# Proteomics Reveals the Molecular Underpinnings of Stronger Learning and Memory in Eastern Compared to Western Bees*[Fn FN2]

**DOI:** 10.1074/mcp.RA117.000159

**Published:** 2017-11-29

**Authors:** Lifeng Meng, Xinmei Huo, Mao Feng, Yu Fang, Bin Han, Han Hu, Fan Wu, Jianke Li

**Affiliations:** From the ‡Institute of Apicultural Research/Key Laboratory of Pollinating Insect Biology, Ministry of Agriculture, Chinese Academy of Agricultural Science, Beijing 100093, China

## Abstract

The eastern (*Apis cerana cerana,* Acc) and western (*Apis mellifera ligustica,* Aml) honeybee are two major honeybee species. Surprisingly, little is known about the fundamental molecular neurobiology of brain suborgans of Acc and Aml. We characterized and compared the proteomes of mushroom bodies (MBs), antennal lobes (ALs) and optical lobes (OLs) in the brain of both species, and biologically validated the functions related to learning and memory. Acc and Aml have evolved similar proteome signatures in MBs and OLs to drive the domain-specific neural activities. In MBs of both species, commonly enriched and enhanced functional groups related to protein metabolism and Ca^2+^ transport relative to ALs and OLs, suggests that proteins and Ca^2+^ are vital for consolidating learning and memory via modulation of synaptic structure and signal transduction. Furthermore, in OLs of both species, the mainly enriched ribonucleoside metabolism suggests its vital role as second messenger in promoting phototransduction. Notably, in ALs of both species, distinct proteome settings have shaped to prime olfactory learning and memory. In ALs of Acc, this is supported by the enriched cytoskeleton organization to sustain olfactory signaling through modulation of plasticity in glomeruli and intracellular transport. In ALs of Aml, however, the enriched functional groups implicated in hydrogen ion transport are indicative of their importance in supporting olfactory processes by regulation of synaptic transmission. The biological confirmation of enhanced activities of protein metabolism and signal transduction in ALs and MBs of Acc relative to in Aml demonstrates that a stronger sense of olfactory learning and memory has evolved in Acc. The reported first in-depth proteome data of honeybee brain suborgans provide a novel insight into the molecular basis of neurobiology, and is potentially useful for further neurological studies in honeybees and other insects.

The eastern and western honeybees are the two most important domesticated honeybee species in the world. *Apis mellifera ligustica* (Aml)^1^, a typical western bee, is maintained across the globe as the most important pollinator and honey and royal jelly producer (1, 2). As a typical representative of the eastern honeybees, *Apis cerana cerana* (Acc) is the second largest bee population in Asia, playing a key role in maintaining ecosystem diversity and economic benefits (3). Western honeybees are believed to have evolved from their Asian counterparts more than 300,000 years ago (4). Because of geographical isolation and long-term evolution by natural selection, they have shaped a wide range of unique behaviors and biological characteristics (5). For instance, Acc has a strong tendency to forage sporadic nectar source flowers in mountain regions and has a stronger capacity to survive in extreme environmental conditions, such as cold and hot weather (6–9). Furthermore, Acc has a stronger ability in learning and memorizing the smell, color, and shape of flowers than Aml (10, 11). In contrast, Aml has a stronger foraging tendency toward large flower patches (6).

The honeybee brain, a very small organ in size comprising 960,000 neurons, is a part of the central nervous system (CNS) that regulates social biology including social behavior, olfactory discrimination, learning and memory (12). However, the brain has its distinct anatomical and functional domains for the regular functioning of the CNS (13). In this context, mushroom bodies (MBs) are the highest-order in the CNS, which integrates massive information streams from various sensory organs to form associated memory (14), whereas antennal lobes (ALs) and optical lobes (OLs) are primary centers where olfactory information detected by the antennae is processed and visual cues are received from the compound eyes (15).

The distinct brain-regions are reported to have specific expression of genes, neuropeptides and proteins to achieve their distinct neural activity for the complex social orders in the honeybee society (16–18). Specifically, the upregulated genes associated with Ca^2+^ signaling pathway (protein kinase C (PKC), Ca^2+^/calmodulin-dependent protein kinase II (CaMK II)) in MBs are necessary for enhancing neuronal plasticity, learning and memory (19–23). In the case of neuropeptides, tachykinin-related peptide is preferentially expressed in MBs and some ALs and OLs neurons, where it is implicated in regulating the activity of neuronal circuits (24, 25). In general, these observations are a manifestation that the honeybee brain has a 'module-functionalization' which is like the tuning of regular neural activity in the human brain (26, 27). Recently, several large-scale proteome works have been performed on honeybee brain to reveal the molecular mechanisms that drive the neural activity in Aml (28–30). However, they were performed only in mushroom body and whole brain tissue. Moreover, there are also genomic works conducted in the brain to explain the behavioral variations among eastern and western honeybees (17). Despite the above-mentioned efforts made to address the regional function of the brain, the results represent only a small fraction of genes, neuropeptides and proteins (31–33). In most case, the measured level of mRNA does not always correlate well with the level of protein, which makes the mRNA level insufficient to predict the corresponding protein abundances. Hence, large scale and comprehensive characterization of the subregional proteome in honeybee brain is vital to gain novel insight into the molecular basis of its neural biology.

Recent advances in mass spectrometry (MS)-based proteomics in terms of enhanced performance in mass accuracy, resolution and sensitivity make it possible to perform a global systems-level investigation on honeybee biology in great depth of knowledge, such as embryogenesis (34, 35), resisting *Varroa* mites (36), development of mandibular glands (37) and hypopharyngeal glands (38). The technological advances in MS offer a state-of-the-art platform to gain better understanding of the molecular basis of neural functionality at the suborgan levels of the honeybee brain. Thus, the aim of this work was to provide an in-depth characterization of the proteome at the suborgan levels (MBs, ALs, and OLs) of Acc and Aml honeybees' brain. Furthermore, a comparison of the differences within and among different species to better understand region-skewed proteome landscapes that underpin the neural biology of both bee species was made. This work, we believe will be used as a potentially important resource and new knowledge for further neuronal activity functional investigations in more specific anatomical regions of honeybee and other insects' brain.

## EXPERIMENTAL PROCEDURES

### 

#### 

##### Chemical Reagents

All chemicals were purchased from Sigma-Aldrich (St. Louis., MO) except modified sequencing grade trypsin that was bought from Promega (Madison, WI). Otherwise, the sources were specified.

##### Sampling MBs, ALs, and OLs from Honeybee Brain

Five uniformly strong colonies of each Aml and Acc species, headed by open mated queens of the same age, were kept at the Institute of Apicultural Research, Chinese Academy of Agricultural Sciences, Beijing, China. From both species, 100 foragers per colony were collected at the hive entrances; only those with a pollen load on their hind legs were sampled as foraging bees. In total, 500 forager bees were sampled from each species. Thereafter, the sampled foragers were anesthetized at 4 °C, followed by brain dissection to collect MBs, ALs, and OLs from the head capsule according to a method described (39). The dissected and collected samples of MBs, ALs, and OLs from brains (a cold PIC, protease inhibitor mixture, was added) were immediately stored at −80 °C until used.

##### Protein Extraction and Digestion

Protein extraction was carried out on the basis of a method previously described (35). Specifically, the collected samples were homogenized with a disposable pestle in a lysis buffer (8 m urea, 2 m thiourea, 4% 3-((3-cholamidopropyl) dimethylammonio)-1-propanesulfonate acid (CHAPS), 20 mm tris-base, 30 mm dithiothreitol (DDT)) on ice for 30 min. The homogenate was centrifuged at 12,000*g* and 4 °C for 15 min, followed by supernatant recovery and protein precipitation by adding 3 volumes of ice-cold acetone for 30 min. The pellet was centrifuged at 13,000 × *g* and 4 °C for 10 min. The supernatant was discarded and the protein pellets were dried at room temperature (RT) and dissolved in 40 mm NH_4_HCO_3_. To prevent reformation of disulfide bonds, the dissolved protein samples were incubated with 100 mm of DDT (DDT/protein (V: V = 1:10)) for 1 h, and then alkylated with 50 mm of iodoacetamide (IAA) (DDT/IAA (V: V = 1:5)) for 1 h in the dark. Finally, the resultant protein was digested with trypsin (enzyme: protein (W: W = 1:62.5)) at 37 °C for 14 h. After digestion, enzymatic reaction was stopped by adding 1 μl of formic acid (FA) into the mixture. The digested peptides were centrifuged at 13,000*g* and 4 °C for 10 min. The supernatant was recovered and extracted using a SpeedVac system (RVC 2–18, Marin Christ, Osterod, Germany) for subsequent LC-MS/MS analysis.

##### LC-MS/MS Analysis

The digested peptide samples were redissolved in 5 μl of 0.1% FA and 3 replicates of each sample were analyzed using an EASY-nLC 1000 (Thermo Fisher Scientific, Bremen, Germany) coupled with LTQ-Orbitrap Elite (Thermo Fisher Scientific, Bremen, Germany) via an ESI ion source (Thermo Fisher Scientific, Bremen, Germany). Before analytical separation, the samples were loaded onto a trap column (2 cm long, 100 μm inner diameter fused silica filling with 5.0 μm Aqua C18 beads, Thermo Fisher Scientific,) in buffer A (0.1% FA in water) for 2 min at a flow rate of 5 μl/min. Then, peptides were separated on an analytical column packed with 3 μm, 100Å, Aqua C18 beads (15 cm long, 75 μm inner diameter, Thermo Fisher Scientific) at a flow rate of 350 nL/min, using a 120-min gradient (from 3 to 8% buffer B (0.1% FA in acetonitrile (ACN) in 5 min, from 8 to 20% buffer B in 80 min, from 20 to 30% buffer B in 20 min, from 30 to 90% buffer B in 5 min, and 90% buffer B in 10 min). The eluting peptides were directly injected into an Orbitrap Elite mass spectrometer (Thermo Fisher Scientific, Bremen, Germany) via ESI. The mass spectrometer was operated in positive ion mode and the data was acquired in a dependent mode. Dynamic exclusion was performed (with a repeated count: 1, exclusion duration: 30 s, charge exclusion: unassigned 1, 8, >8, peptide match: preferred, exclude isotopes: On). The MS1 precursor scan was acquired at a resolution of 60000, at *m*/*z* 400, and scan range: *m*/*z* 300–1800. The top 20 intensity peaks were then fragmented by higher energy collisional dissociation (HCD) in the linear ion trap (resolution: 15,000, isolation window: 2 *m*/*z*, normalized collision energy: 30).

##### Protein Identification and Label-free Quantitation of Abundance Level

The MS/MS raw data were collected using Xcalibur (version 2.2, Thermo Fisher Scientific, Bremen, Germany). Protein identification using in-house PEAKS DB software (version 7.5, Bioinformatics Solutions Inc. Waterloo, Canada) was performed by searching against the sequence database (21,780 protein sequences of *Apis mellifera*, downloaded in February 2015 from NCBI), coupled with a common repository of adventitious proteins database (cRAP, downloaded from The Global Proteome Machine Organization, April, 2012). The search parameters were: parent ion tolerance, 15 ppm; fragment tolerance, 0.05 Da; enzyme, trypsin; maximum missed cleavages, 2; maximum variable PTM per peptide, 3; fixed modification, carbamidomethyl (C,+57.02 Da); and variable modification, oxidation (M,+15.99 Da). A protein was confidently identified only if it contained at least one unique peptide with at least two spectra, applying a threshold of false discovery rate (FDR) ≤ 1.0% by a fusion-decoy database searching strategy, a more conservative FDR estimation than that of target-decoy approach (40). Annotated spectra of single-peptide can be found in supplemental Fig. S1.

Relative protein abundance of the three suborgans within and between the two species was quantified using a label-free quantification performed by PEAKS Q module (version 7.5 Bioinformatics Solutions Inc. Waterloo, Canada). Triplicates of each sample were subjected to the software and one sample was automatically selected as reference. Feature detection was made on each of the samples with an expectation-maximization algorithm. Furthermore, feature alignment of the same peptide from three replicates of each sample was performed with an algorithm for high-performance retention time (41). Normalization was used to calculated sample or group ratios, which was generated by dividing the total ion current (TIC) multiplied by TIC of the reference sample. The mass error tolerance was set according to *m*/*z* shift distribution, whereas the retention time range was set based on RT shift distribution, the feature quality was set based on ratio-quality. Protein expressions in all samples of both bee species were quantified with the sum of three most abundant precursor ion peak intensities of the tryptic peptides. Proteins among different samples were significantly changed in their abundance levels only when they attained the criteria (*p* value <0.05 and a fold change of ≥1.5).

##### Experimental Design and Statistical Rationale

**Fig. 1.**
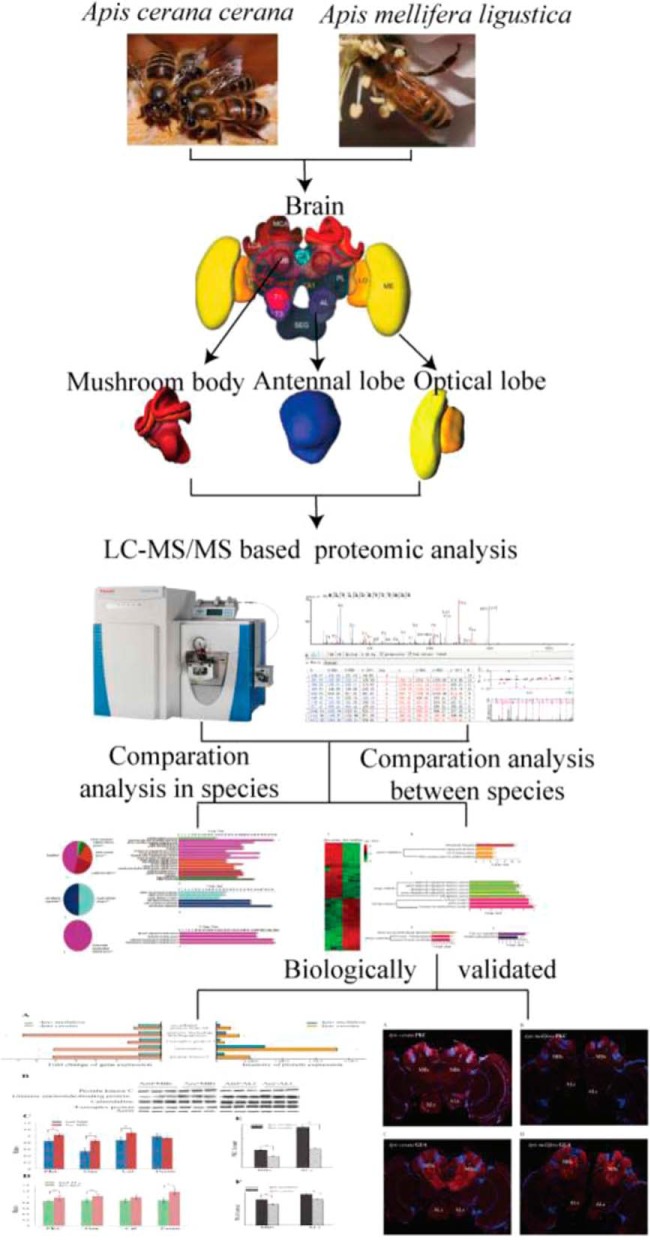
**The workflow chart shows experiment procedure.** Honeybee photos are courteously provided by Dr. Jianke Li. Brain photo is cited from Book: Honeybee-Neurobiology-and-Behavior.

Though, experimental design and statistical rationale for each of the experiments conducted in this work have been described separately and workflow is shown in Fig. 1, 500 foragers were collected from five homogeneous colonies of each species. Protein extraction was made based on produced biological triplicates. Moreover, label-free quantitative comparisons were made using technical triplicates for all samples. Proteins which contain at least one unique peptide and two spectra, with FDR≤1.0% were considered during protein identification. PEAKS Q module was used to assess protein abundance levels in each of the samples during protein quantification, and only *p* value <0.05 and a fold change ≥1.5 were significant. Quantitative real-time PCR (qPCR) was conducted using 5 biological and 3 technological replicates whereas Western blots were performed in 3 biological and technical triplicates. Similarly, the kinase activity assays were performed both in 3 biological and technological replicates. During biological validation (qPCR, Western blots, and Enzyme activity assays), significant differences among the two honeybee species' brain subregions, one-way ANOVA (SPSS version 18.0, SPSS Inc. Chicago, IL) was employed and *p* values <0.05 were significant.

##### Bioinformatics Analysis

The functional gene ontology (GO) term and pathway were assessed using ClueGOv2.1.6, Cytoscape plug-in (http://www.ici.upmc.fr/cluego/) software (42). The analysis was performed by comparing an input data set of identified proteins to all functionally annotated GO categories in the entire genome of *Apis mellifera* from UniProt. The significantly enriched GO terms in biological processes (BPs) and pathways were reported using a right-sided hyper-geometric test and only a *p* value≤0.05 was considered. Then, Bonferroni step-down was used to correct the *p* value to control FDR. Functional grouping of the terms was based on GO hierarchy. The tree level was ranged from 3 to 8, and kappa score level was 0.4. For comparison purpose, sharing 60% of the terms was merged.

To better understand the functional connections among the identified proteins, the protein-protein interaction network (PPI) was created using GeneMANIA, a Cytoscape plug-in (43). The identified honeybee proteins were first blasted against the *Drosophila melanogaster* genome using Blastp by stand-alone. A threshold of *e*-value score < 1e-5 as cutoff was applied to make more reliable alignments. Then, the analogous gene from Drosophila melanogaster with PPI information was subjected to GeneMANIA, and the whole *Drosophila melanogaster* genome was set as background. Coexpression, predicted, genetic, and physical interactions were selected as a network. The top 20 related genes and a maximum of 20 attributes were considered using GO-BPs-based weighting and proteins were grouped by their annotations in GO-BPs.

##### Quantitative Real-Time PCR

Proteins implicated in learning and memory in the MBs and ALs of both species were examined by total RNA extraction (TRLzol reagent, Invitrogen, CA) and quantified by NanoDrop ND91000 spectrophotometer (Thermo Fisher Scientific). Total RNA quality was evaluated by 1.0% denaturing agarose gel electrophoresis based on the visualization of 28s and 18s rRNA bands. PrimeScript RT reagent kit, RR037 (Takara Bio, Kyoto, Japan) was used to generate cDNA. Targeted genes and their primer sequences used in qPCR were listed in supplemental Table S1. The PCR amplification and data retrieval were conducted on iQ5 Multicolor Real-Time PCR Detection System (Bio-Rad, Hercules, CA). The PCR was conducted in a 20 μl reaction system as follows: 95 °C for 30 s; each cycle at 95 °C for 5 s, 60 °C for 30 s, 40 cycles, and melt curve tracking from 55 to 95 °C. Actin was selected as a reference gene and the differences in gene expression were calculated by the 2^−▵▵Ct^ method (44). An error probability *p* < 0.05 was considered as a statistically significant difference in gene expression by one-way ANOVA (SPSS version 18.0, SPSS Inc. Chicago, IL).

##### Western Blotting Analysis

To further verify the differentially expressed proteins associated with learning and memory in the MBs and ALs of both species, PKC, calmodulin, guanine nucleotide-binding protein, and T-complex proteins were selected for Western blotting analysis and each sample was run in 3 replications (45). Polyclonal antibodies of these four proteins were developed in New Zealand female rabbits by Genecreat (Wuhan, China), and were purified by affinity chromatography. Then, the specificity of these antibodies was tested using ELISA (enzyme-linked immunosorbent assays) by Genecreat (Wuhan, China). The primary rabbit polyclonal antibodies were diluted at a ratio of 1:2000, and the secondary antibodies were diluted at a ratio of 1:5000. About 10 μg protein samples were separated by stacking (5%) and separating (15%) SDS-PAGE gels and then transferred to a nitrocellulose membrane (0.2 μm pore size, Invitrogen, Carlsbad, CA) with an iBlot apparatus (iBlot Gel Transfer System, Invitrogen, Eugene, OR). The protein bands were then detected using the ECL Western blotting substrate (Pierce, Rockford, IL) and quantified by densitometry using Quantity-One image analysis system (Bio-Rad, Hercules, CA). Actin was selected as a loading control of the analysis.

##### Activity Assay of PKA (Protein Kinase A) and PKC (Protein Kinase C)

The activity of PKA in MBs and ALs of both species was measured using a PKA activity assay kit (Cat. No. KT-742, Kamiya Biomedical Company, Thousand Oaks). Specifically, ELISA was carried out using 96-well removable microtiter plates (precoated with PKA substrate). Each sample was run in 3 biological and 3 technological replicates. At this point, 10 mg of tissue was homogenized in 200 μl cell lysis buffer containing 1 mm PMSF (Phenylmethanesulfonyl fluoride), 1 μl of PIC per ml of cell lysis buffer, 10 mm phosphatase inhibitor in a 1.5 ml microcentrifuge tube, and incubated for 10 min on ice with occasional vortexing. The supernatants were recovered by centrifuging at 13,000 × *g* and 4 °C for 15 min, followed by diluting 50 times in PKA kinase assay buffer and loaded into plate wells. The enzymatic reaction was started by adding diluted ATP into the plate wells and incubated at 30 °C for 90 min with shaking. Afterward, the solution in the wells was discarded and the wells were washed 4 times. Both goat anti-rabbit IgG: HRP (horseradish peroxidase) conjugate and rabbit phospho PKA substrate antibody were mixed together in the wells for reaction at RT for 60 min with shaking. Finally, the wells were washed 4 times and tetramethylbenzidine substrate was added for color development at RT for 30 min. The reaction was quenched by adding a stop solution. Optical densities of each well were measured with Multiskan GO software (1510 03021, Thermo Fisher scientific, Germany) at a wavelength of 450 nm. The PKA content was calculated according to the calibration curve, which was made with a standard PKA calibrator. A one-way ANOVA (SPSS version 18.0, SPSS Inc. Chicago, IL) was used to detect the differences between Acc and Aml subregions.

The activity of PKC in MBs and ALs of both species was measured using PKC activity assay kit (Cat. No. ADI-EKS-420A, Enzo Life Science, Inc, New York). ELISA and sample preparation was performed as we explained for PKA. Moreover, Enzyme activity, here, was also measured using a similar protocol as for PKA, with modifications in that phosphospecific substrate antibody was added into the wells to bind the phosphorylated peptide substrate in advance, and subsequently bound by anti-rabbit IgG: HRP conjugate. In this case, the relative kinase activity in PKC was calculated as: Average absorbance_sample_ - Average absorbance_blank_/Quantity of crude protein used per assay. Protein concentration was determined using BCA method (BCA Cat. No. P0010, Beyotime Biotechnology Company, China) and bovine serum albumin was used as a standard. A one-way ANOVA (SPSS version 18.0, SPSS Inc. Chicago, IL) was performed to analyze the differences between Acc and Aml brain subregions.

##### Immunohistochemistry (IHC)

Whole mount immunohistochemistry was performed to determine the protein expression in MBs and ALs. Sampled foragers were anesthetized at 4 °C, and the brains were dissected in a drop of cold PBS (phosphate-buffer saline, 6.7 mm) with PIC. The whole brain was fixed in 6.7 mm PBS containing 4% paraformaldehyde at RT. The tissue was dehydrated in ethanol with increasing grades from 50%∼100% and terminated by xylene, then incubated and embedded in paraplast for sectioning. The slides were rehydrated in ethanol by decreasing grades from 100% to 50%. Afterward, the slides were washed 3 times with PBS (0.01 m), and then transferred to hydrogen peroxide solution (3%) and incubated for 10 min in the dark. Subsequently, the slides were transferred to BSA (5%) for 20 min at RT to block nonspecific binding. The diluted primary antibodies in BSA (PKC 1:150; guanine nucleotide-binding protein 1:150) were applied to the slices and incubated overnight at 4 °C. Thereafter, the slices were washed 3 times for 5 min in PBS, then transferred to anti-goat IgG (diluted 1:50 in blocking solution) with Cy3 and finally incubated for 50 min at RT. The washed slices were incubated with DAPI (4′, 6-diamidino-2-phenylindole) for 5 min at RT in the dark. Finally, the slices were washed 3 times for 5 min in PBS, and covered with a drop of DAPI Fluoromount-GTM for fluorescence quenching. Color intensities were then measured in the MicroPublisher 5 RTV (Q-imaging, CA).

## RESULTS

To evaluate the quality of our proteome analysis, protein abundances of 18 raw files across the different suborgans were analyzed. Accordingly, Pearson's correlation coefficients were above 0.94 among replicates of the same samples, and ranged from 0.77 to 0.89 among different samples (supplemental Fig. S2*A*), demonstrating higher technical and biological reproducibility.

### 

#### 

##### A First-time Comprehensive Proteome from MBs, ALs, and OLs of Acc Brain

**Fig. 2.**
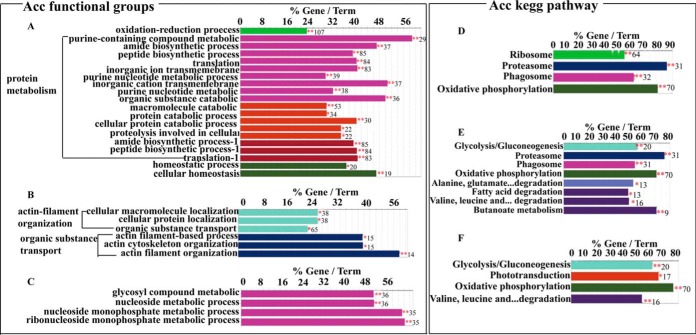
**Comparative analysis of GO terms enriched by identified proteins among mushroom bodies (MBs), antennal lobes (ALs) and optical lobes (OLs) in *Apis cerana ceran* (Acc) brain.** *A*, *B*, and *C*, Histogram representing significantly enriched functional terms in MBs, ALs and OLs of Acc brain respectively. *D*, *E*, and *F*, Histograms representing significantly enriched kegg pathways in MBs, ALs and OLs of Acc brain respectively. The bar represents the number of genes encoding identified proteins and the label displayed on the bar is the percentage of genes encoding the identified proteins comparing to all the genes involved in the specified term. Different color of the bars represents different terms, and the same color indicates same functional group. *, *p* < 0.05; **, *p* < 0.01. See also supplemental Table S5.

To resolve the morphology-based regional protein expression of Acc brain, proteome of MBs, ALs, and OLs were characterized and compared over these 3 regions. A total of 2410 (1134 protein groups), 2264 (1079 protein groups) and 2107 (961 protein groups) proteins were identified from MBs, ALs, and OLs respectively, representing 3186 unique proteins in the Acc brain (supplemental Fig. S2*B*, (supplemental Table S2–S4). In MBs, protein metabolism was mainly enriched in both functional groups and pathways (Fig. 2*A* and *D*, supplemental Table S5). Whereas, 2 functional groups (actin filament organization and organic substance transport) and 2 pathways (energy and amino acid metabolism) were the most enriched GO terms in ALs (Fig. 2*B* and 2*E*, supplemental Table S5). Although ribonucleoside monophosphate metabolic process and 4 pathways associated with phototransduction and energy metabolism were mainly enriched in OLs (Fig. 2*C* and 2*F*, supplemental Table S5).

##### Quantitative Proteome Differences among Acc Brain SubRegions

In this case, when we see the regionally changed protein abundance levels and their functions, from a total of 3186 identified proteins in the 3 regions, 266 of them were differentially expressed (141, 78 and 47 from MBs, ALs and OLs respectively) (supplemental Table S6). More specifically, upregulated proteins in MBs were enriched in protein complex assembly, calcium ion transport, intracellular signal transduction, and pathways implicated in protein synthesis (supplemental Fig. S3*A* and S3*D*, supplemental Table S7). Further, upregulated proteins in ALs were enriched in microtubule-based process and cell redox homeostasis, and pathways associated with amino acid metabolism (supplemental Fig. S3*B* and S3*E*, supplemental Table S7). However, those upregulated proteins in OLs were mainly enriched in molecule transport (such as anion and endocytosis) and pathways implicated in phototransduction and energy metabolism (supplemental Fig. S3*C* and S3*F*, supplemental Table S7).

**Fig. 3.**
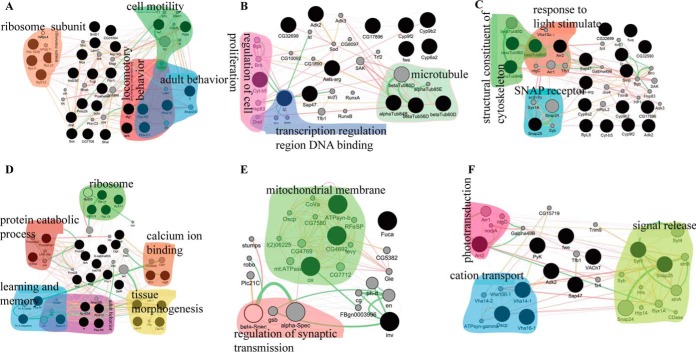
**Protein-protein interaction (PPI) network of upregulated proteins identified in mushroom bodies (MBs), antennal lobes (ALs) and optical lobes (OLs) of *Apis cerana cerana* (Acc) and *Apis mellifera ligustica* (Aml) brain.** *A*, *B*, and *C*, PPI networks of upregulated proteins from MBs, ALs and OLs of Acc brain respectively. *D*, *E*, and *F*, PPI networks of upregulated proteins from MBs, ALs and OLs of Aml brain respectively. The interaction among upregulated proteins is mapped using GeneMANIA, a cytoscape plug-in. The identified proteins are first blasted to *Drosophila melanogaster* genome using stand-alone Blastp program. A threshold of *e*-value score < 1e-5 as cutoff was applied to make more reliable alignments. Then, genes from *Drosophila melanogaster* are subjected to GeneMANIA, and its whole genome is set as background. Coexpression, predicted, genetic, and physical interaction networks are selected. Top 20 related genes and a maximum of 20 attributes are exhibited using GO biological process-based weighting. Proteins are grouped by their annotations in GO biological processes.

PPI analysis in GeneMANIA for upregulated proteins in MBs, ALs and OLs was performed based on Coexpression, Predicted, Genetic and Physical interaction as parameters and interactions were selected among the available data for *Drosophila melanogaster*, and protein interactions were based on biological processes. Consequently, we found that the ribosomal subunit proteins were linked with many other proteins as a hub in the MBs. For instance, ribosome protein S12 was found to be interacted with other proteins in facilitating the adult behaviors and locomotory behaviors. Furthermore, ribosome protein L23 was associated with twinfilin protein to promote cell motility (Fig. 3*A*). On the other hand, microtubule proteins were found to be interacted with cell proliferation regulation and transcription regulatory region DNA binding in the ALs (Fig. 3*B*). Moreover, snap receptor proteins and microtubule proteins were the ones found to interact with each other in response to light stimulus in the OLs (Fig. 3*C*).

##### In-depth Proteome Profiles of Aml Brain Subregions

**Fig. 4.**
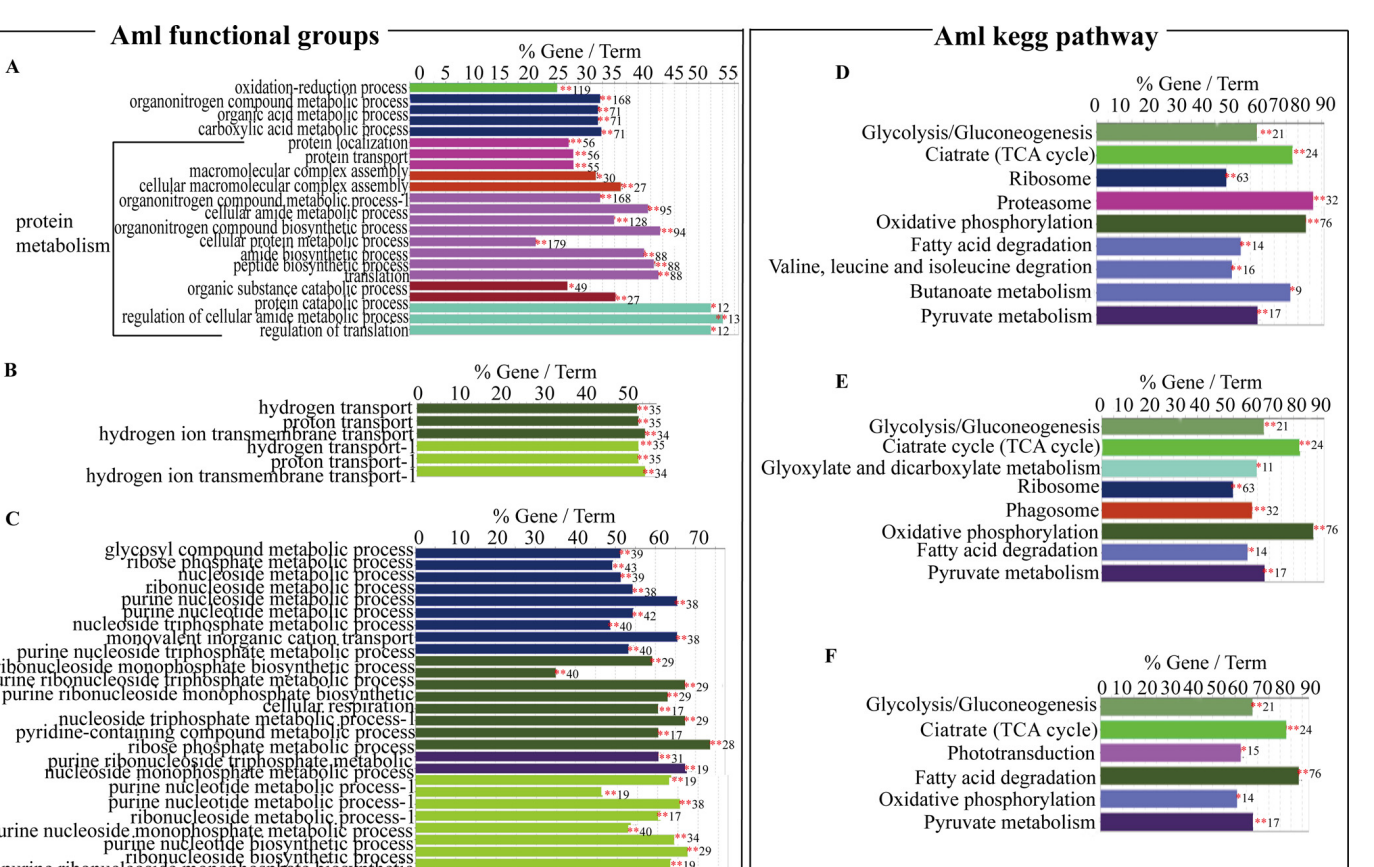
**Comparative analysis of GO terms enriched in mushroom bodies (MBs), antennal lobes (ALs) and optical lobes (OLs) in *Apis mellifera ligustica* (Aml) brain.** *A*, *B*, and *C*, Histogram represents enriched functional groups in MBs, in ALs and OLs in Aml brain respectively. *D*, *E*, and *F*, Histogram representing enriched kegg pathway in MBs, ALs and OLs in Aml brain respectively. The bar represents the number of gene encoding the identified protein and the label displayed on the bar means the percentage of gene encoding the identified protein compared with all the genes involved in that term. Different color bar represents different term, and the same color means the same functional group. *, *p* < 0.05; **, *p* < 0.01. See also supplemental Table S12.

Generally, a total of 3281 proteins have been identified from the 3 subregions of Aml brain, in which, 2516 (1246 protein groups), 2204 (1075 protein groups), and 2212 (1087 protein groups) proteins were from MBs, ALs, and OLs, respectively (supplemental Fig. S2*C*, supplemental Table S8–S10). Regarding the functional groups and pathways, enriched protein groups related to protein metabolism and pathways implicated in energy metabolism were the most abundant ones among a total of 7 functional groups and 7 pathways identified in MBs (Fig. 4*A* and 4*D*, supplemental Table S11). In case of ALs, 2 functional groups (hydrogen transport and hydrogen ion transmembrane transports) and 8 pathways mainly involved in protein metabolism and transport were enriched (Fig. 4*B*and 4*E*, supplemental Table S11). Whereas, purine metabolic process, phototransduction and energy metabolism were the major enriched GO terms in OLs (Fig. 4*C* and 4*F*, supplemental Table S11).

##### Quantitative Proteome Differences among Aml Brain Subregions

Among a total of identified proteins (3281) in 3 subregions of Aml brain, 281 proteins were differentially regulated being, 152, 31, and 98 proteins upregulated in MBs, ALs, and OLs, respectively (supplemental Table S12). According to the downstream analysis results, we have observed that the highly abundant proteins in MBs were enriched in protein metabolism, calcium ion transmembrane transport, and intracellular signal transduction functional groups, and phosphatidylinositol signaling system, and protein metabolism pathways (supplemental Fig. S4*A* and S4*D*, supplemental Table S13). Those upregulated proteins in ALs were mainly enriched in the hydrogen transport and hydrogen ion transport functional groups; and energy and amino acid metabolism pathways (supplemental Fig. S4*B* and S4*E*, supplemental Table S13). Although, upregulated proteins in OLs were found to be enriched only in energy metabolism GO terms (supplemental Fig. S4*C* and S4*F*, supplemental Table S13).

Regarding the upregulated proteins in MBs linked to the PPI network, proteasome proteins and ribosome proteins were associated with 14–3-3 protein. In addition, calcium-binding proteins were found to be interacted with PKA base on genetic interaction analysis (Fig. 3*D*). In ALs, mitochondrial membrane proteins were the ones found to be interacted with spectrin proteins (Fig. 3*E*). Whereas, cation transport proteins were found to be the physically interacted group with signal release proteins in OLs (Fig. 3*F*).

##### Proteome Comparisons Between the Acc and Aml Brain Subregions

To understand the functional differences driven by protein expression, proteomes of the MBs, ALs and OLs were compared between Acc and Aml brains. In MBs, 2516 and 2410 proteins were identified in Acc and Aml, respectively, representing 2939 unique proteins. Similar functional groups and pathways were enriched in both bee species, in which purine nucleoside monophosphate metabolic processes, protein metabolism, and energy metabolism were the majorly enriched functional classes and pathways (supplemental Fig. S5*A* and S5*B*, supplemental Table S14). However, of the 186 differentially expressed proteins in MBs of both bee species, 95 and 91 were highly abundant in Acc and Aml, respectively (supplemental Table S15). In Acc, the upregulated proteins were enriched in protein biosynthesis (supplemental Fig. S6*A* and S6*B*, supplemental Table S16), whereas, in Aml, the upregulated proteins were enriched in substance transport and energy metabolism (supplemental Fig. S6*C* and S6*D*, supplemental Table S16).

In case of ALs, 2264 and 2204 proteins were identified in Acc and Aml respectively, representing a total of 2708 individual proteins. Specifically, purine nucleoside monophosphate metabolic process, regulation of actin cytoskeleton organization function groups, and protein metabolism pathway were the main enriched in Acc (supplemental Fig. S7*A*, supplemental Table S14). Whereas, hydrogen ion transmembrane transport and energy metabolism were the most abundant enriched function group and pathway respectively in Aml (supplemental Fig. S7*B*, supplemental Table S14).

Quantitatively, among a total of 95 differentially expressed proteins in the ALs of Acc and Aml brain (supplemental Table S15), the 45 upregulated proteins in Acc were enriched in functional groups implicated in protein synthesis and amino acid metabolism pathway (supplemental Fig. S6*E* and S6*F*, supplemental Table S16). On the other hand, the 50 upregulated proteins in the ALs of Aml were enriched in 2 functional groups and 2 pathways in which both are associated with energy metabolism (supplemental Fig. S6*G* and S6*H*, supplemental Tables S16).

Regarding the OLs, 2107 and 2212 proteins were identified in Acc and Aml respectively, represented 2716 of the exclusive proteins. Qualitatively, purine nucleoside metabolic process, oxidative phosphorylation and protein metabolism were mainly enriched in both bee species (supplemental Fig. S8*A* and S8*B*, supplemental Table S14). On the other hand, based on the quantitative analysis, we found that among a total of 167 proteins that have changed their expression levels in the OLs, 59 and 108 proteins were upregulated in Acc and Aml respectively (supplemental Table S15). The upregulated proteins in the OLs of Acc were enriched in function groups implicated in microtubule cytoskeleton organization and cellular homeostasis, and pathways associated with energy metabolism (supplemental Fig. S9*A* and S9*B*, supplemental Table S16). Similarly, upregulated proteins in the OLs of Aml brain, metabolism processes and energy production were the mainly enriched functional groups and pathways (supplemental Fig. S9*C* and S9*D*, supplemental Table S16).

##### Biological Verification of Stronger Olfactory Learning and Memory in Acc than that in Aml

**Fig. 5.**
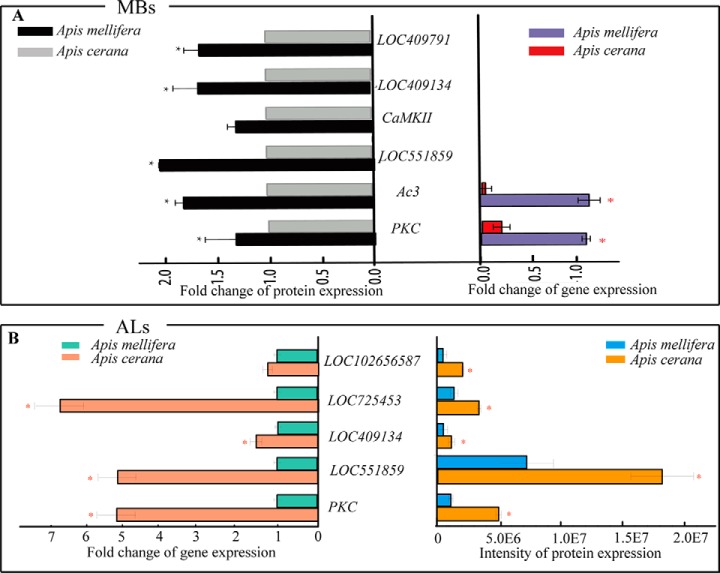
**Quantitative Real-Time PCR Verification of different expressed proteins associated with learning and memory in antennal lobes (ALs) and mushroom bodies (MBs) between *Apis cerana cerana* (Acc) and *Apis mellifera ligustica* (Aml).** *A*, Comparison of expression trends between mRNA and abundance level of differential proteins (fold change ≥1.5 and *p* < 0. 05) related to learning and memory in MBs of Acc and Aml brains. *B*, mRNA and protein expression levels of differential proteins (fold change ≥1.5 and *p* < 0. 05) associated to learning and memory in ALs of Acc and Aml bees. Error bars are standard deviations. *, *p* < 0.05.

**Fig. 6.**
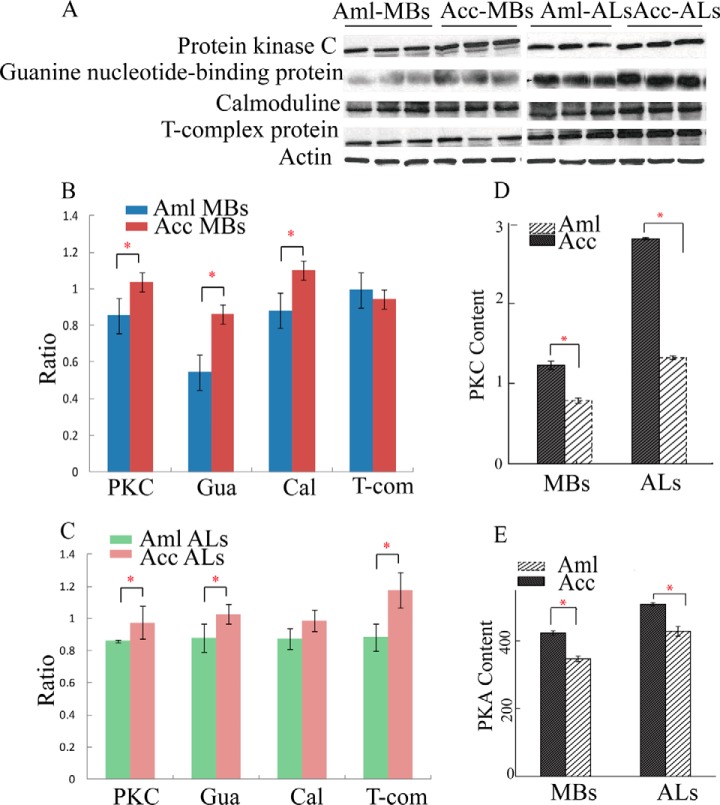
**Verification of different proteins implicated in learning and memory in antennal lobes (ALs) and mushroom bodies (MBs) between *Apis cerana cerana* (Acc) and *Apis mellifera ligustica* (Aml).** *A*, The Western-Blotting bands of protein kinase A, guanine nucleotide-binding protein, calmoduline, T-complex protein, and actin is used as reference. *B*, *C*, The relative expression values of protein kinae A, guanine nucleotide-binding protein, calmoduline, T-complex protein in MBs and ALs in Acc and Aml brain, normalized by actin. The error bar is standard deviation. The asterisks denote significant differences (*p* < 0.05). *D*, *E*, The enzymatic activity of PKA and PKC detected in ALs and MBs in Acc and Aml brains. The error bar is standard deviation. The asterisks show significant differences (*p* < 0.05).

**Fig. 7.**
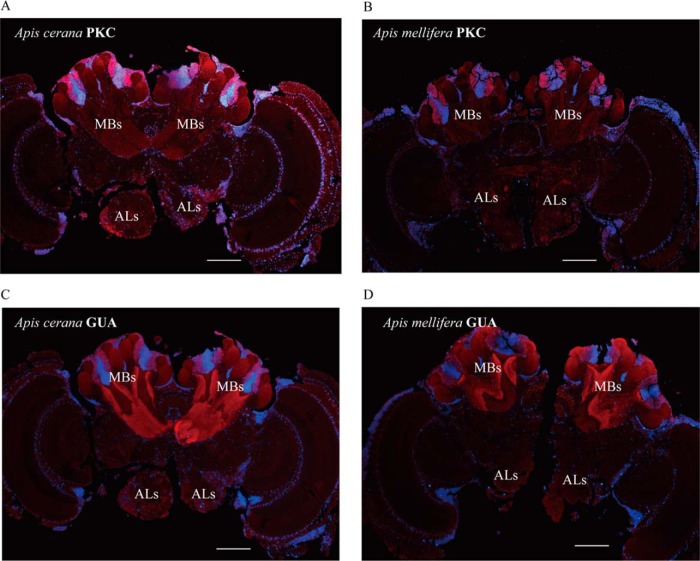
**Brain sections were immunostained with antibodies of protein kinase C (PKC) and guanine nucleotide-binding protein (GUA).** The blue was stained with 4′, 6-diamidino-2-phenylindole. The red fluorescence was stained with Cy3-conjugated antibodies. *A*, Immunofluorescence of PKC in *Apis cerana cerana* (Acc) brain sections. *B*, Immunofluorescence of PKC in *Apis mellifera ligustica* (Aml) brain sections. *C*, Immunofluorescence of GUA in Acc brain sections. *D*, Immunofluorescence of GUA in Aml brain sections. Scale bars represent 250 μm.

Based on the above analyses, the stronger olfactory learning and memory abilities expressed in Acc than in Aml has attracted our attention. Accordingly, those differentially regulated proteins involved in the functionality of learning and memory were confirmed through qPCR, Western blot, IHC and kinase activity assays. For this purpose, nine proteins were selected from Acc and Aml brain subregions to evaluate their expression trends at transcriptional level. Accordingly, six of them showed similar expression tendency both at the protein and transcriptional levels. However, the abundance level of adenylate cyclase 3 isoform X3 and PKC was greater in the MBs of Acc than in Aml (Fig. 5*A*). Furthermore, the expression levels of calmodulin, PKC, T-complex protein, guanine nucleotide-binding protein and ras-related protein rab-10 were higher in the ALs of Acc than in those of Aml (Fig. 5*B*). In our western-blotting analysis, calmodulin, PKC, guanine nucleotide-binding protein and T-complex protein expression was again higher in both ALs and MBs of Acc than in those of Aml (Fig. 6*A*, 6*B*, and 6*C*). This was also verified by IHC analysis, but only two of them, PKC and guanine nucleotide-binding protein were observed highly expressed in Acc than that of Aml (Fig. 7*A*, 7*B*, 7*C*, and 7*D*). Furthermore, enzymatic activity of PKC and PKA, both essential enzymes in insect learning and memory, was also found to be higher in MBs and ALs of Acc than in those of Aml (Fig. 6*D* and 6*E*).

## DISCUSSION

At this point, it is wise to notify that this work comprises results of a comprehensive region-specific proteome of the honeybee brain for the first time than its kind which gives us a chance to look at in-depth molecular portraits of the neural biology in Acc and Aml. Generally, these are the two different honeybee species developed through natural selection for thousands of years, and have evolved a wide repertoire of distinct biology including foraging behavior, and learning and memory (5, 10). Hence, targeting to understand the molecular underpinnings of the neural activity at subregion levels, the proteomes of MBs, ALs and OLs of Acc and Aml brain were defined and compared between each of the subregions and across species. Here, the cerebrum functions of MBs and OLs of both species employ similar proteome settings to achieve distinct neural functions in each of the subregions. This is manifested by the fact that a plethora of enriched functional classes implicated in protein metabolism and enhanced activity of calcium ion transmembrane transport in MBs relative to those in ALs and OLs. These enriched and enhanced functional classes are vital in driving learning and memory by regulating synaptic plasticity and signal transduction facilitation in MBs. In OLs, the enriched ribonucleoside metabolism relative to those in MBs and ALs is suggestive of their vital roles in priming the visual system functioning through maintaining the potentials of electrical charges and G-protein signaling transduction. However, distinct proteome signatures have been shaped in the ALs of the two species to support the functionality of olfaction. The significantly enriched actin filament organization and substance transport in ALs of Acc, compared with those in MBs and OLs, are indicative of their key roles in the olfactory signaling process by modulating the plasticity of glomeruli and sustaining intracellular transports. In ALs of Aml, however, the enriched hydrogen transport and hydrogen ion transport, over those in MBs and OLs, indicate their vital importance in driving olfactory learning and memory (OLM) process via regulation of synaptic transmission. Notably, the exclusively enriched purine nucleoside monophosphate involved in signal transduction, and the enhanced function of protein metabolism in ALs of Acc, as compared with those in Aml, indicates that Acc has shaped a better olfactory sense than Aml via synaptic plasticity regulation for the formation of memory. This is further supported by the enhanced protein biosynthesis and ribosome pathway in the MBs of Acc, as compared with those in Aml, and is validated through different biological confirmatory analyses.

### 

#### 

##### Protein Metabolism and Ca^2+^ Mediated Activity of Signal Pathways has Driven Learning and Memory in MBs

Long-term memory is a stable memory process, which needs synthesis of new proteins to change the structure of synapses, thereby stabilizing and consolidating specific memory traces (46). This is reflected in our data from protein synthesis targeted enriched peptide metabolic processes in the MBs of both bee species, and by the establishment of protein localization in MBs of Aml. It is also further supported by the enriched ribosome pathway in both bee species, post-transcriptional regulation of gene expression in Aml, and the translational processes in Acc. All these functional classes and pathways are vital for new protein synthesis in translation machinery, suggesting that protein synthesis is essential for the formation of learning and memory in MBs of honeybee brain. This is consistent with the knowledge that volume of MBs is remarkably increased after foraging or odor treatments (47, 48), and this morphology change needs synthesis of new proteins to stabilize and consolidate learning and memorization of flowers' characteristics and location of food sources (49, 50).

On the other hand, protein catabolic and proteasome pathways are important in maintaining protein homeostasis in cells via degradation, and they play key roles in the update and reorganization of early and late long-term memory in honeybees (51). Here, the enriched pathways associated with protein degradation in both Acc and Aml may indicate their roles in maintaining learning and memory of honeybees. This is further supported by our PPI network analysis, in which proteasome proteins interacted with 14–3-3 protein to promote learning or memory (52). Moreover, proteasome also influences signaling molecules implicated in Ca^2+^ mediated signal transduction, such as Ca^2+^/CaMK II (53). In addition, CaMK II is known to catalyze phosphorylation of many neuronal proteins, thereby affecting both short and long-term memories (54). Here, the upregulated CaMK II in Acc is a manifestation of its crucial role in structural plasticity of neurons and memory formation (55).

Signaling molecules are fundamentally important in learning and memory as they regulate gene transcription, translation and protein degradation in honeybees (56). For instance, Ca^2+^, an important second messenger, can activate a wide range of Ca^2+^-dependent enzymes, which further activate the related signal transduction to promote learning and memory in MBs (57). A typical example is the Ca^2+^/phospholipid-dependent PKC/PKA cascade pathway, which controls the transcription processes during long-term memory formation and long-lasting neuronal plasticity in honeybees (58). Here, upregulated proteins involved in calcium ion transmembrane transport in MBs of both bee species, as compared with those in ALs and OLs, suggest that elevation of Ca^2+^ concentration is critical for learning and memory (59). Furthermore, the enriched phosphatidylinositol signaling system in Aml suggests its role in elevating intracellular Ca^2+^ concentration to boost long term memory (60). Overall, we understood that protein synthesis and catabolism are vital for the establishment of the honeybee memory.

##### Actin Cytoskeleton Organization in ALs is Vital for Honeybee Olfaction

Actin-filament and microtubule cytoskeleton are critical for maintaining the plasticity of neuropil and intracellular signal molecule transport in the olfactory system. The enriched actin-filament and enhanced activity in microtubule-based processes in ALs of Acc suggest that cytoskeleton functions in modulating plasticity of glomeruli and intracellular signal molecule transport in honeybee olfactory processes (61, 62). Actin, a major cytoskeletal element, is vital in proper axon guidance, and microtubules are necessary in axon structure and elongation (63). Hence, the enriched actin filament organization in ALs of Acc is supposed to function in modulating plasticity of glomeruli. This is similar to the actin-based cytoskeleton organization in the axonal growth of olfactory nerve layers during olfactory development in mice (64). Furthermore, in the PPI network, the elevated levels of tubulin alpha-2/alpha-4 chain-like isoform X4 and tubulin beta-3 chain-like isoform 2 in ALs of Acc suggest their enhanced roles to prime the glomeruli plasticity after odor-exposures by interacting with cell proliferation regulation and transcription regulatory region of DNA binding (65, 66). Actin filaments and microtubules are also essential in the neural system intracellular transport. Microtubule cooperating with kinesis motors is one of the most important cargo transportation mechanisms in the nervous system (67). Hence, the enriched microtubule-based processes by highly abundant proteins in ALs of Acc is indicative of the fact that its sensitive olfaction is likely to be driven by stronger activity of synaptic transmission and cell motility in the olfactory system (63).

Searching and collecting nectar and pollen is a process that demands an intensive energy supply to signal food source information back to colony members and recruit foragers to initiate foraging activity (68). To this effect, the enriched pathways related to energy metabolism in the ALs of both bees, such as oxidative phosphorylation, glycolysis/gluconeogensis and the citrate cycle, are believed to be involved in providing energy in the ALs for better neural performance during field foraging. The enriched hydrogen and hydrogen ion transmembrane transports for the synthesis of ATP in Aml may serve as the same purpose as energy production. Moreover, in the PPI network, we found that ATP synthase interacted with spectrin proteins to regulate synaptic transmission via genetic interaction, suggests that ATP synthase plays an important role in olfactory signal transmission in ALs of honeybees.

##### Metabolizing Ribonucleoside Monophosphate in OLs Promotes Phototransduction via G-Protein Signaling and Energy Turnover

Photoreceptors require a substantial amount of molecules that can generate energy to phosphorylate rhodopsin in daylight and maintain ion gradients in the dark (69). At this point, the enriched ribonucleoside monophosphate metabolic processes in Acc, purine ribonucleoside metabolic processes and purine nucleoside metabolic processes in Aml are suggestive of their roles in providing energy molecules such as ATP, GTP and GMP. Furthermore, phototransduction is a G protein-mediated process (70), where GTP and GMP cycles are vital in visual recovery and phototransduction. Thus, the enriched purine ribonucleoside metabolic process in OLs of Acc implies its central role in phototransduction. Moreover, the enriched ATP metabolic processes in OLs of Aml and the enriched oxidative phosphorylation, glyoxylate and arboxylate metabolic processes in OLs of both bee species may play key roles in the phototransduction process through increasing ATP supply. Similarly, the phototransduction cascade demands an electrical charge potential across the cell membrane (71). To this end, upregulated proteins in Aml which are enriched in hydrogen ion transmembrane indicates that hydrogen ions are also involved in influencing membrane potential and mediating depolarization (72), which are vital in regulating phototransduction in the honeybee brain (73). Furthermore, in the PPI network, cation transport interacted with signal release proteins influence phototransduction process. This suggests that cation transport and hydrogen ion transmembrane play key roles in phototransduction for signal release and maintaining transmembrane potential.

##### Acc Evolved a Better Sense in OLM than Aml

Different olfactory strategies have been shaped between Acc and Aml by natural evolution (74). Interestingly, the olfactory signaling processes in ALs of Acc are supported by enriched actin filament organization that is crucial for plasticity of glomeruli and intracellular molecular transport (29). In contrast, in ALs of Aml, the olfactory processes are supported by enriched hydrogen transport and hydrogen ion transport via regulation of synaptic transmission (72). This difference is also consistent with the knowledge that odorant gene expression is different in these species (5). Furthermore, Acc antennae have a higher number of olfactory sensilla and olfactory coreceptors than that of Aml (74, 75). In this context, the enriched functional terms implicated in signal transduction and morphology plasticity of olfactory neuropil in ALs of Acc, suggest their function in cementing a strong capacity to deal with olfaction and odor in neurons. Specifically, the abundant enriched purine nucleoside monophosphates (such as AMP and GMP, important second messengers) are suggestive of an enhanced signal transduction in memory activities of ALs of Acc (76, 77). The enriched cytoskeleton organization in the ALs of Acc can possibly boost learning and memory processes by elevating signal transduction and glomeruli morphogenesis after learning (78). Remarkably, enhanced expression of proteins related to protein synthesis, which provides new proteins for the increased changes in synaptic morphology after odor learning, further indicates the stronger ability of learning and memory in ALs of Acc. Furthermore, the strong expression of PKC, guanine nucleotide-binding protein and calmodulin in Acc also are further evidences for elevated function in signal transduction in memory processes (79). This agrees with the upregulated encoding genes of the three proteins, and is further supported by Western blotting and immunohistochemistry analyses. PKC and PKA are both important molecules in mediating learning and memory in honeybees (53). They also show a higher enzymatic activity in ALs of Acc, compared with those in Aml. These observations can possibly be explained by the fact that Acc has experienced a strong selection for adaptation to its current ecological habitat in mountainous regions, where the major nectar resources are sporadically available (80). Moreover, an enhanced sensitivity in smell acquisition by Acc is also an evolutionary strategy to respond smartly to various floral volatile compounds during foraging.

The integrative neuropils of the honeybee olfactory pathway comprises ALs and MBs, where the olfactory information must transmit from ALs into MBs to form long-term memory (81). In MBs of Acc, compared with those in Aml, the strongly expressed proteins implicated in protein synthesis, are likely involved in boosting learning and memory performances (82). Furthermore, some proteins involved in learning and memory activities are highly abundant in the MBs of Acc. This further strengthens the fact that Acc has adapted a better learning and memory ability than Aml. For instance, adenylate cyclase 3 isoform X3 and PKC, which were strongly expressed in Acc compared with in Aml, are important in memory formation through phosphorylation of target proteins (83). These findings are in line with the higher levels of PKC mRNA and enzymatic activity in Acc than Aml. The above discussed proteins are also known to be vital for Acc in achieving a stronger ability in learning dance language, color, and grating patterns (10, 11). However, enhanced hydrogen transport functional groups and energy metabolism pathways in Aml are also found to provide the same role in substance transport during learning and memory processes.

## CONCLUSIONS

To our knowledge, our data defines the first time comprehensive proteome of MBs, ALs and OLs in the eastern and western honeybees' brain. Similar region-specific proteomic settings of MBs and OLs have been developed in both honeybee species to achieve their distinct roles in driving neuronal biology. Learning and memory formation in the MBs of both bees are underpinned by a wide range of functional classes implicated in protein metabolism and calcium ion transport that modulate synaptic structure and signal transduction, to consolidate memory traces. In OLs, phototransduction activity is achieved by the enriched functional groups in purine ribonucleoside metabolism and energy production via signal release and maintenance of transmembrane potential. To be noted, the ALs of both bees has evolved distinct proteome programs to prime OLM. In ALs of Acc, olfactory signaling pathway is consolidated by enriched cytoskeleton organization via regulation of glomeruli plasticity and intracellular transport. On the other hand, in ALs of Aml, olfactory processes are accomplished by enriched hydrogen transport and hydrogen ion transport via regulation of synaptic transmission. Remarkably, Acc has adapted a stronger sense of olfactory learning and memory than Aml as a result of enhanced activity of signal transduction and protein metabolism in both ALs and MBs. This is further supported by supplemental qPCR, Western-blotting, immunohistochemistry and enzymatic activity analyses. Finally, it is wise to remind that the novelty and significance of this work to future honeybee neurobiology studies at the suborgan level is immense important.

## Data Availability

The LC-MS/MS data have been deposited to the Proteome Xchange Consortium (http://proteomecentral.proteomexchange.org) via the PRIDE partner repository with the data set identifier: PXD007091.

## Supplementary Material

Supplemental Data

## Footnotes

^1^ The abbreviations used are:

Aml: *Apis mellifera ligustica*
Acc: *Apis cerana cerana*
ACN: acetonitrile
ALs: antennal lobes
BPs: biological processes
CaMK II: Ca2+/calmodulin-dependent protein kinase II
CNS: central nervous system
cRAP: common repository of adventitious proteins database
DAPI 4′: 6-diamidino-2-phenylindole
FA: formic acid
FDR: false discovery rate
GO: gene ontology
IAA: iodoacetamide
IHC: immunohistochemistry
MBs: mushroom bodies
OLs: optical lobes
OLM: olfactory learning and memory
PBS: phosphate-buffer saline
PCA: principal components analysis
PMSF: Phenylmethanesulfonyl
PIC: protease inhibitor cocktail
PKA: protein kinase A
PKC: protein kinase C
TIC: total ion current.
