# Supplementary material for: Proteomics Reveals the Molecular Underpinnings of Stronger Learning and Memory in Eastern Compared to Western Bees
Source: Mol Cell Proteomics. 2017 Nov 29;17(2):255–69. doi: 10.1074/mcp.RA117.000159 (PMC5795390; doi:10.1074/mcp.RA117.000159)
Supplement: Supplemental Data [file supp_17_2_255__index.html]

Proteomics Reveals the Molecular Underpinnings of Stronger Learning and Memory in Eastern Compared to Western Beess — Sub-organ Proteome of Honeybee Brain — Proteomics Reveals the Molecular Underpinnings of Stronger Learning and Memory in Eastern Compared to Western Bees — Subregion Proteome of Honeybee Brain — Supplemental Data 

# Proteomics Reveals the Molecular Underpinnings of Stronger Learning and Memory in Eastern Compared to Western Bees

## Supplemental Data

- supplemental tables - supplemental tables for protein identified and biological function analysis
- Supplemental figure - Supplemetal figure for manuscript
- Annotated spectra of single-peptide - Annotated spectra of single-peptide identifications of proteins in mushroom bodies,antennal lobes and optical lobes of Apis cerana and Apis mellifera
