# Supplementary material for: Proteomics Reveals the Molecular Underpinnings of Stronger Learning and Memory in Eastern Compared to Western Bees
Source: Mol Cell Proteomics. 2017 Nov 29;17(2):255–69. doi: 10.1074/mcp.RA117.000159 (PMC5795390; doi:10.1074/mcp.RA117.000159)
Supplement: Supplemental Data [file supp_RA117.000159_4755_1_supp_19944_2yx65z.pdf]

Supplemental figure legends

Supplemental Figure 1. Annotated spectra of single-peptide from mushroom bodies (MBs), antennal lobes (ALs) and optical lobes (OLs) in *Apis cerana cerana* (Acc) and *Apis mellifera ligustica* (Aml).

Supplemental Figure 2. Data evaluation and comparative proteomic analysis of mushroom bodies (MBs), antennal lobes (ALs) and optical lobes (OLs). (A) The matrix of 18 correlation plots of protein abundance level in three replications. The numbers are the value of Pearson correlation coefficient. (B, C) Venn diagrams indicating the numbers of common and unique proteins identified in the MBs, ALs and OLs in *Apis cerana cerana* (Acc) and *Apis mellifera ligustica* (Aml) brain respectively.

Supplemental Figure 3. Functional groups and biological pathways enriched by up-regulated proteins in mushroom bodies (MBs), antennal lobes (ALs) and optical lobes (OLs) of *Apis cerana cerana* (Acc) brain. (A, B and C) Histograms indicating enriched functional groups of up-regulated proteins in MBs, ALs and OLs of Acc brain respectively. (D, E and F) Histograms indicating enriched biological pathways of up-regulated proteins in MBs, ALs and OLs of the Acc brain respectively. The bars represent numbers of genes encoding identified proteins and the labels, displayed on the bars, explained percentages of genes encoding identified proteins compared to all the genes involved in that specified term. Different colored bars represent different terms, and same color means the same pathway. *, $P < 0.05$; **, $P < 0.01$. See also Table S7.

Supplemental Figure 4. Functional group and pathway enriched by up-regulated proteins in mushroom bodies (MBs), antennal lobes (ALs), optical lobes (OLs) in brain of *Apis mellifera ligustica* (Aml). (A, B and C) Functional group enriched by up-regulated proteins in MBs, ALs

and OLs in Aml brain respectively. (D, E and F) Pathway enriched by up-regulated proteins in MBs, ALs and OLs in brain of Aml respectively. The bar represents the number of gene encoding the identified protein and the label displayed on the bar means the percentage of gene encoding the identified protein compared to all the genes involved in that term. Different color bar represents different term, and the same color means the same pathway. *, $P < 0.05$; **, $P < 0.01$.

Supplemental Figure 5. Comparative analysis of functional group and biological pathway enriched by identified proteins in mushroom bodies (MBs) between *Apis cerana cerana* (Acc) and *Apis mellifera ligustica* (Aml). (A and B) Histogram indicates specific term in MBs of Acc and Aml brain respectively. The bar represents the number of gene encoding the identified protein and the label displayed on the bar means the percentage of gene encoding the identified protein compared to all the genes involved in that term. Different color bar represents different term, and the same color means the same pathway. *, $P < 0.05$; **, $P < 0.01$. See also Table S14.

Supplemental Figure 6. Comparative analysis of functional groups and biological pathways enriched by up-regulated proteins in mushroom bodies (MBs) and antennal lobes (ALs) of *Apis cerana cerana* (Acc) and *Apis mellifera ligustica* (Aml) brains. (A, B) Functional groups and biological pathways enriched by up-regulated proteins in MBs of Acc brain respectively. (C and D) Functional groups and biological pathways enriched by up-regulated proteins in MBs of Aml brain espectively. (E, F) Functional groups and biological pathways enriched by up-regulated proteins in ALs of Acc brains respectively. (G, H) Functional groups and biological pathways enriched by up-regulated proteins in ALs of Aml brain. The bar and its labels showing numbers

and percentages of genes encoding the identified proteins from all genes involved in those specific terms. Different colored bars represents the different terms, and the same colors show same groups. *, $P < 0.05$; **, $P < 0.01$. See also Table S15-16.

Supplemental Figure 7. Comparative analysis of functional groups and biological pathways enriched by identified proteins in antennal lobes (ALs) of *Apis cerana cerana* (Acc) and *Apis mellifera ligustica* (Aml) brains. (A) A Histogram representing enriched GO terms in ALs of Acc brain. (B) A Histogram indicating GO terms in ALs of Aml brain. The bars and its labels show numbers and percentages of genes encoding identified proteins from all genes involved in those specific terms. Different colored bars represents different terms, and same colors show same groups. *, $P < 0.05$; **, $P < 0.01$. See also Table S14.

Supplemental Figure 8. Comparative analysis of functional group and biological pathway enriched by the identified proteins in optical lobes (OLs) between *Apis cerana cerana* (Acc) and *Apis mellifera ligustica* (Aml). (A and B) Histogram chart shows functional group and biological pathway enriched in OLs of Acc and Aml brain respectively. The bar represents the number of gene encoding the identified protein and the label displayed on the bar means the percentage of gene encoding the identified protein compared to all the genes involved in that term. Different color bar represents different term, and the same color means the same pathway. *, $P < 0.05$; **, $P < 0.01$. See also Table S14.

Supplemental Figure 9. Comparative analysis of functional group and biological pathway enriched by the up-regulated proteins in optical lobes (OLs) between *Apis cerana cerana* (Acc) and *Apis mellifera ligustica* (Aml). (A, B) Functional group and biological pathway enriched by the up-regulated proteins in OLs of Acc brain. (C, D) Histogram indicates functional term and

biological pathway enriched by up-regulated proteins in OLs of Aml brain. The bar represents the number of gene encoding the identified protein and the label displayed on the bar means the percentage of gene encoding the identified protein compared to all the genes involved in that term. Different color bar represents different term, and the same color means the same pathway. *, $P < 0.05$; **, $P < 0.01$. See also Table S15-16.

Supplemental figures

https://mcponline.msubmit.net. Supplementary_Figure_S1.pdf

Supplemental figure 1

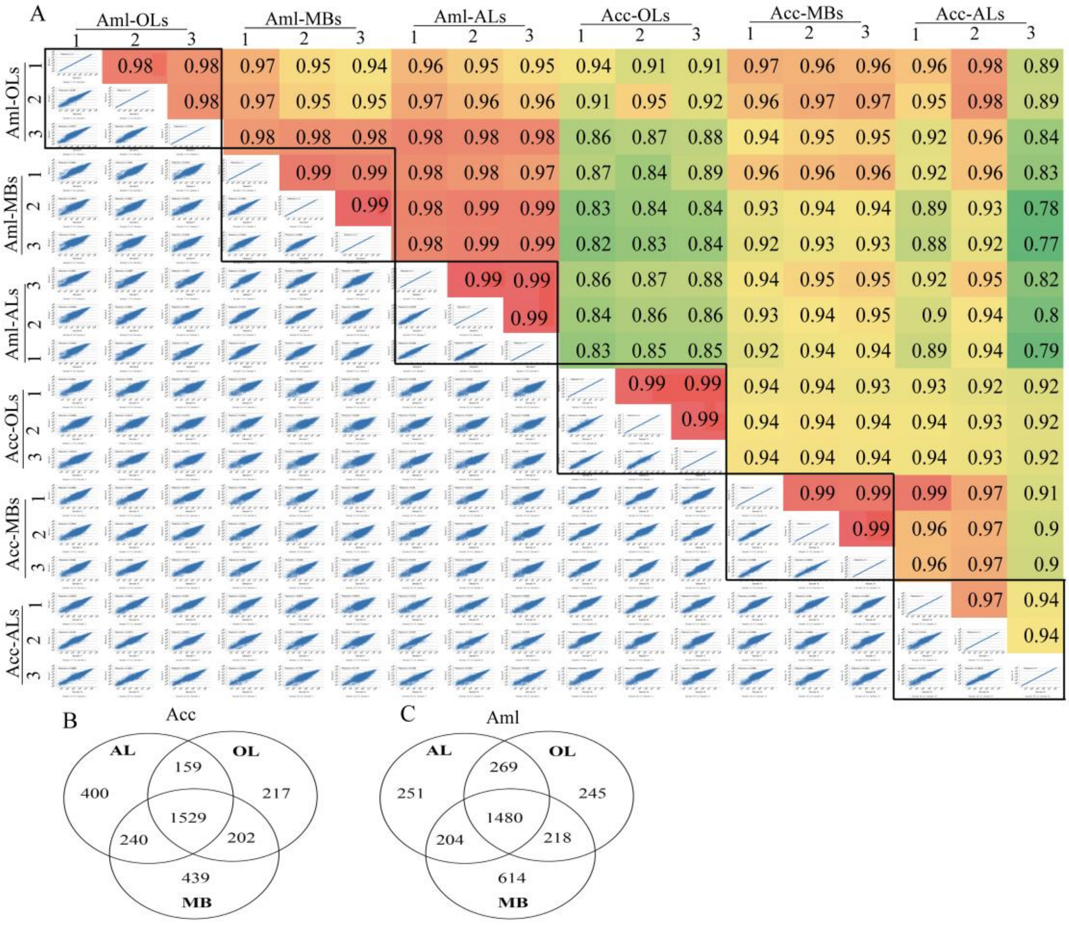

Supplemental figure 2

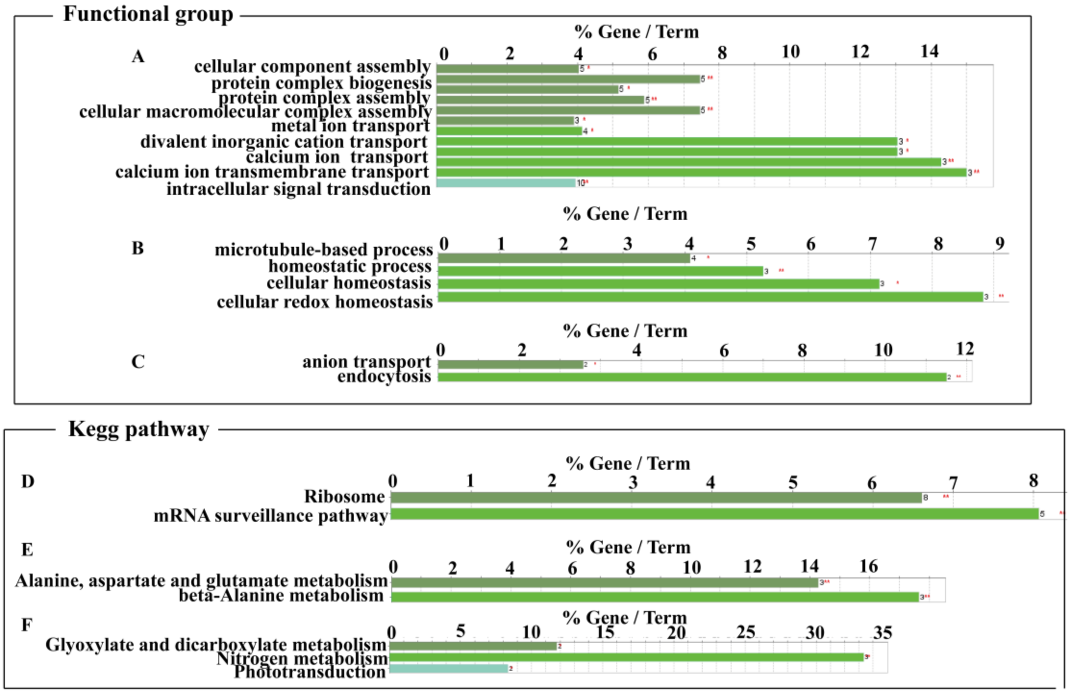

Supplemental figure 3

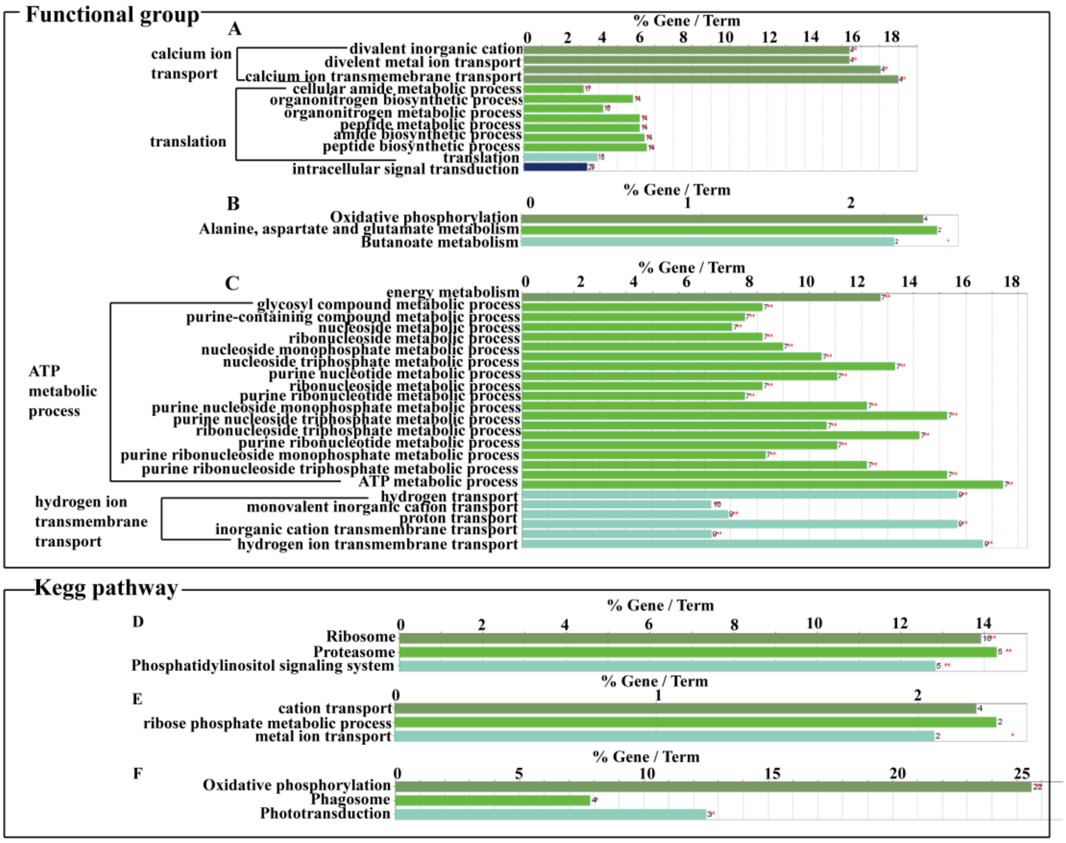

Supplemental figure 4

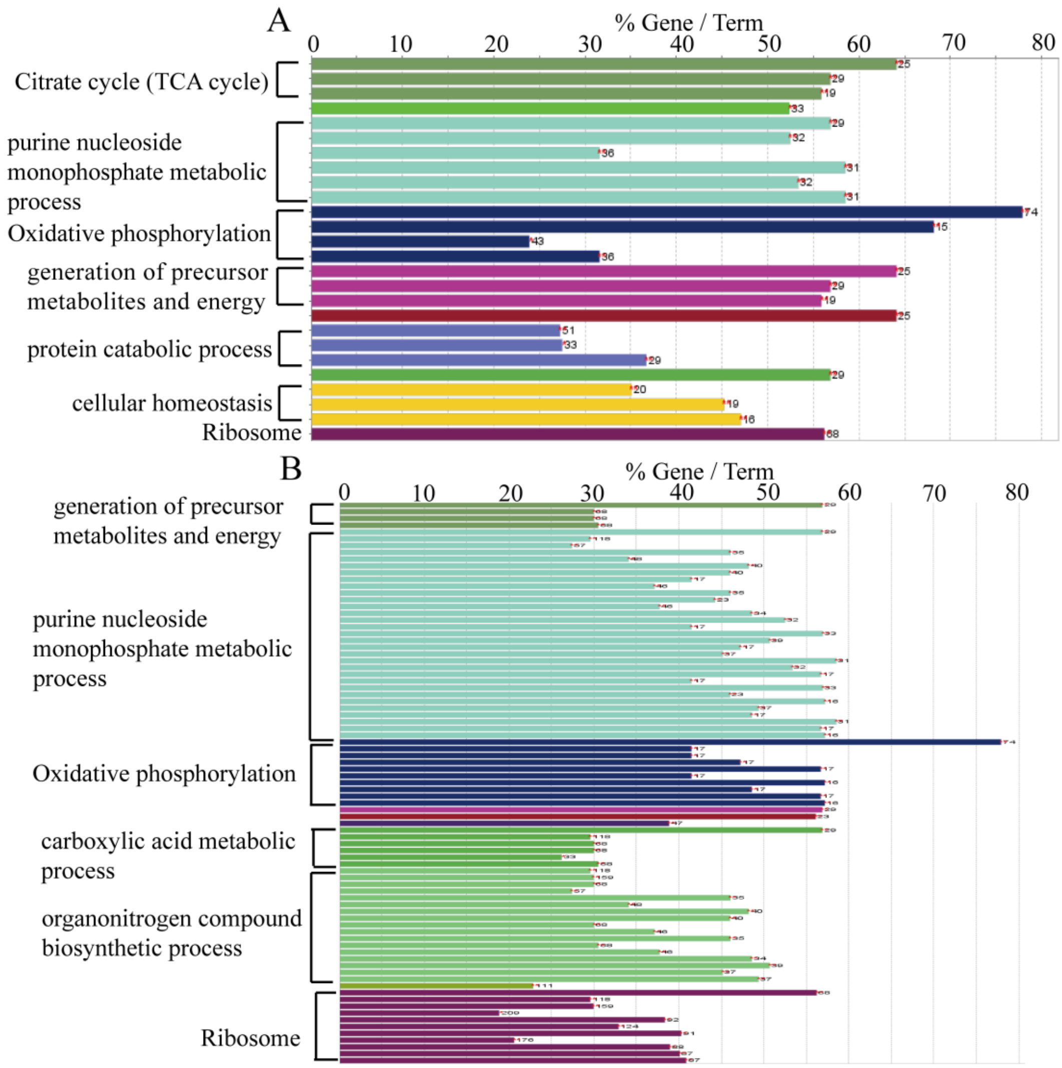

Supplemental figure 5

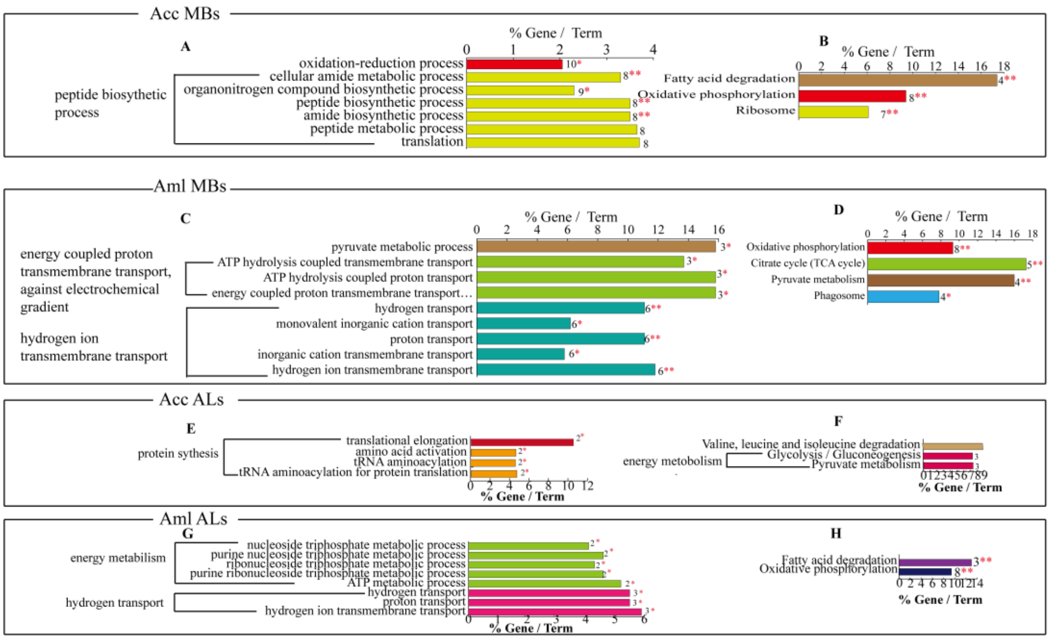

Supplemental figure 6

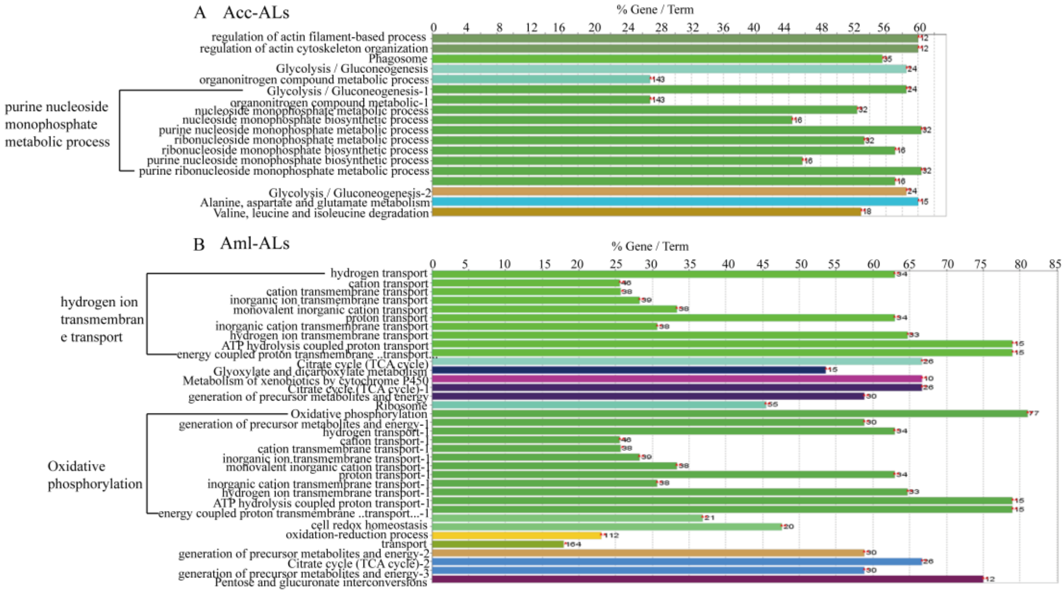

Supplemental figure 7

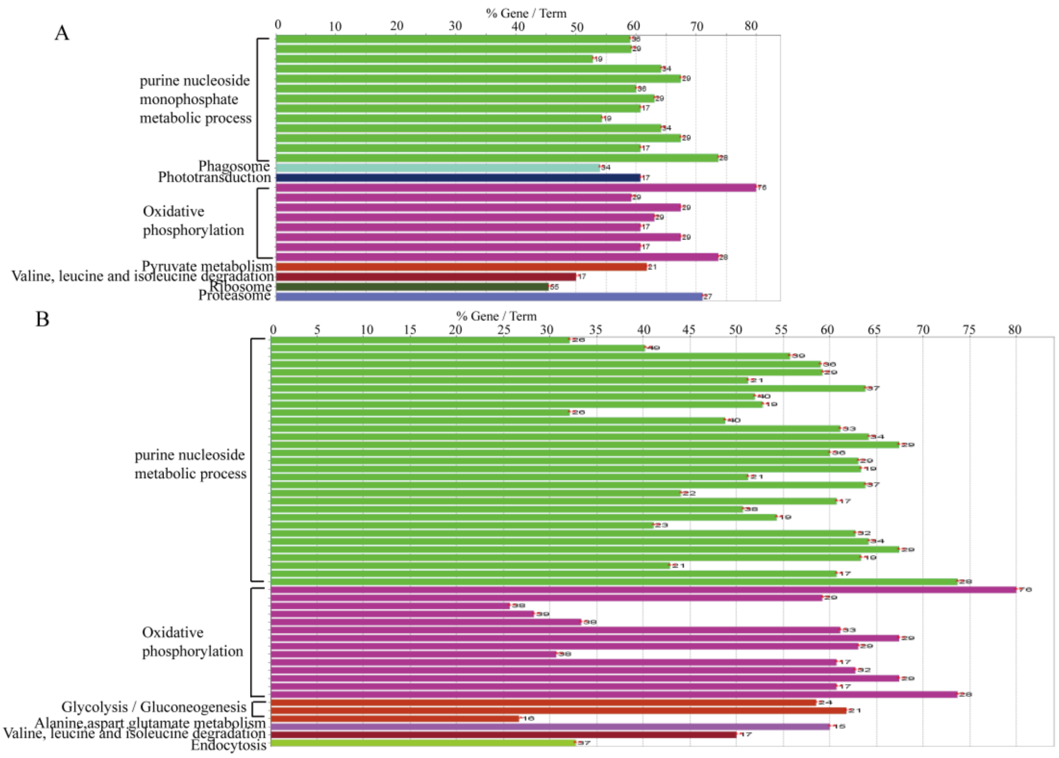

Supplemental figure 8

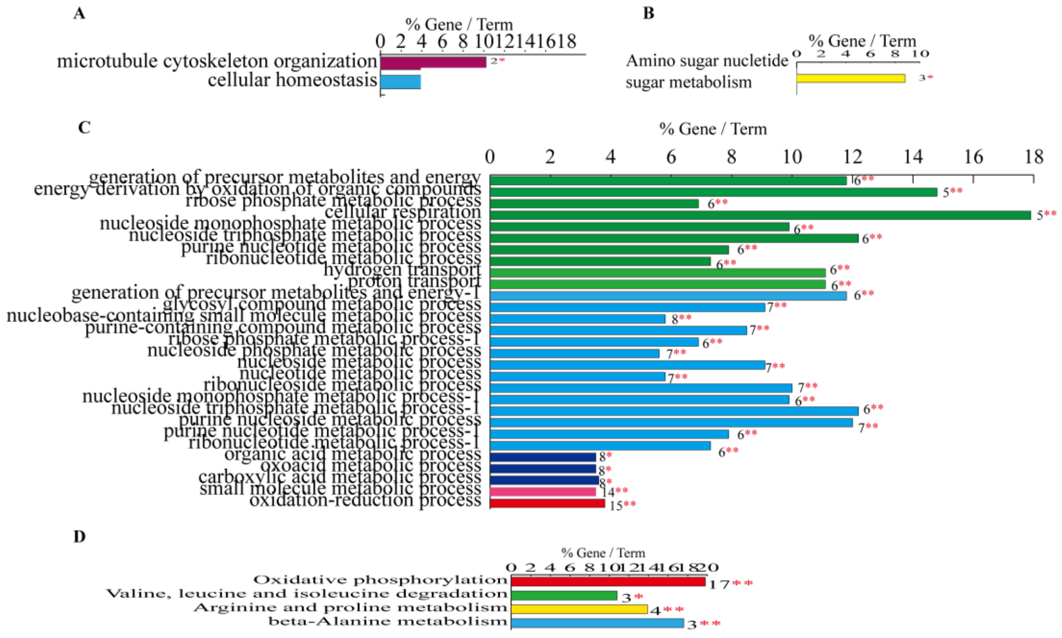

Supplemental figure 9
