# Supplementary material for: Proteomics Reveals the Molecular Underpinnings of Stronger Learning and Memory in Eastern Compared to Western Bees
Source: Mol Cell Proteomics. 2017 Nov 29;17(2):255–69. doi: 10.1074/mcp.RA117.000159 (PMC5795390; doi:10.1074/mcp.RA117.000159)

Annotated spectra of single-peptide identifications of proteins in mushroom bodies of *Apis cerana*

gi|571523207

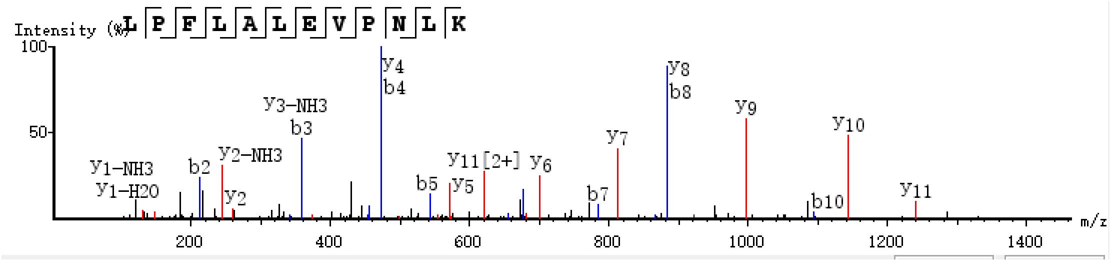

gi|110756942

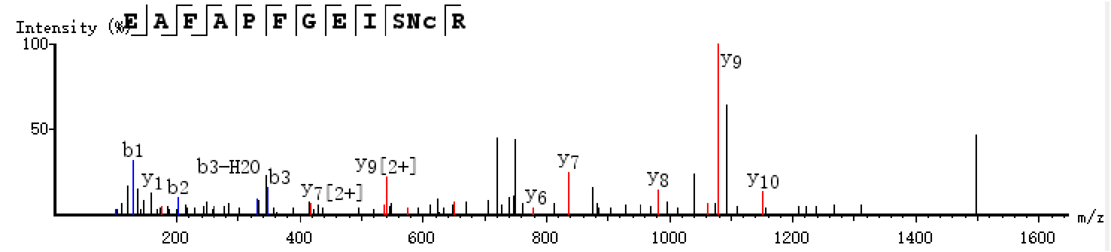

gi|48097576

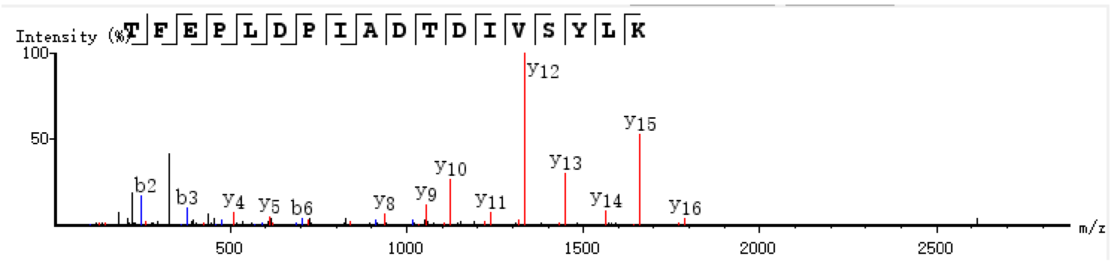

gi|571541806

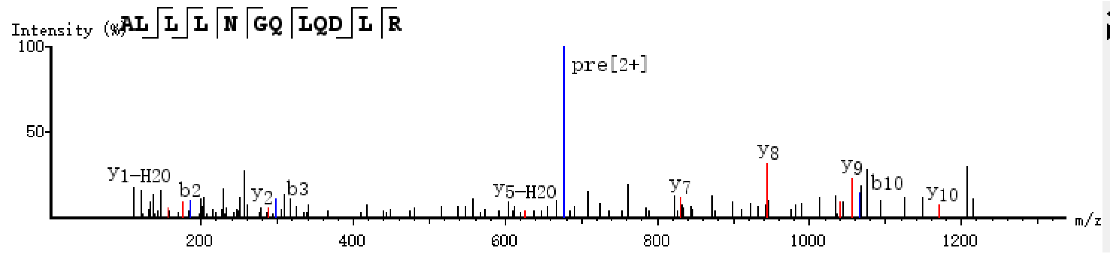

gi|571541813

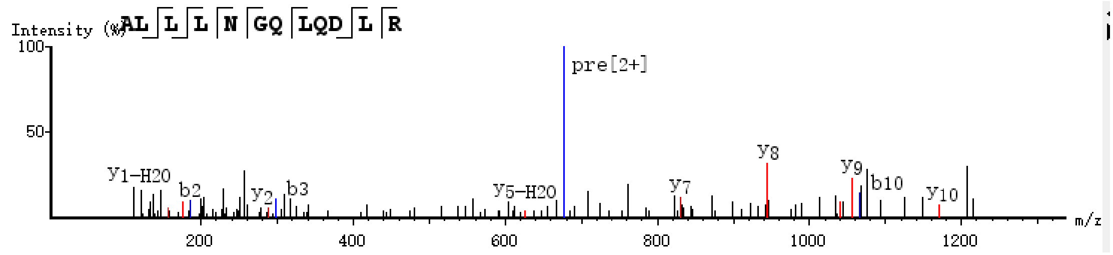

gi|571541810

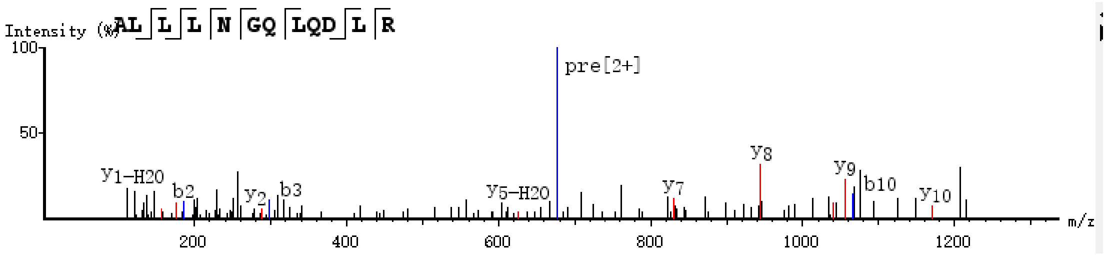

gi|571499584

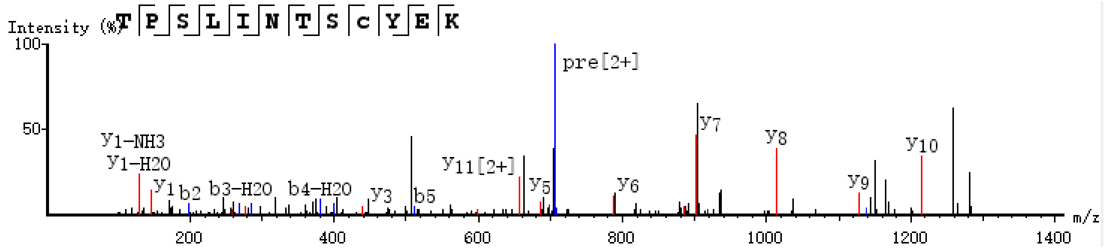

gi|571576925

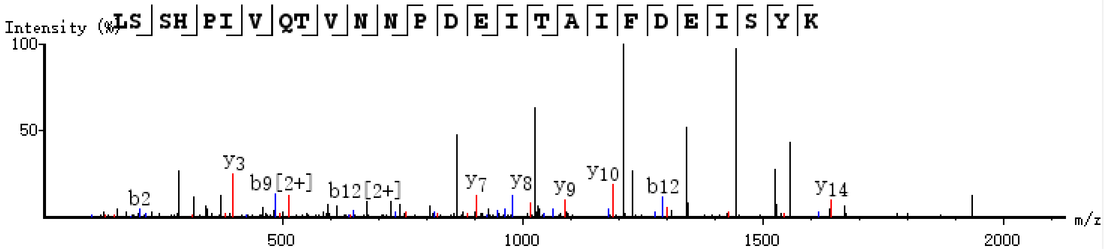

gi|66551883

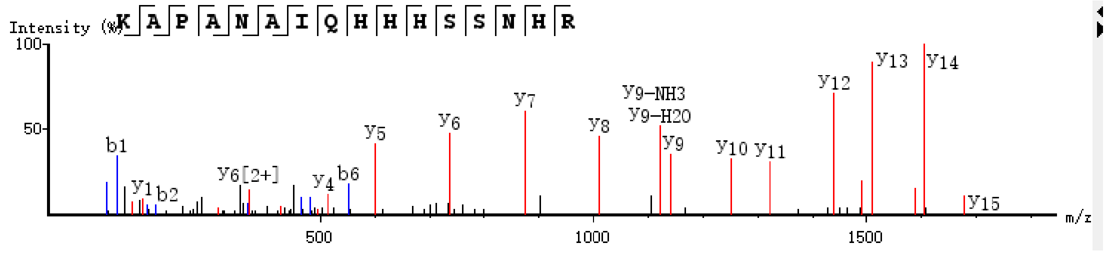

gi|571507129

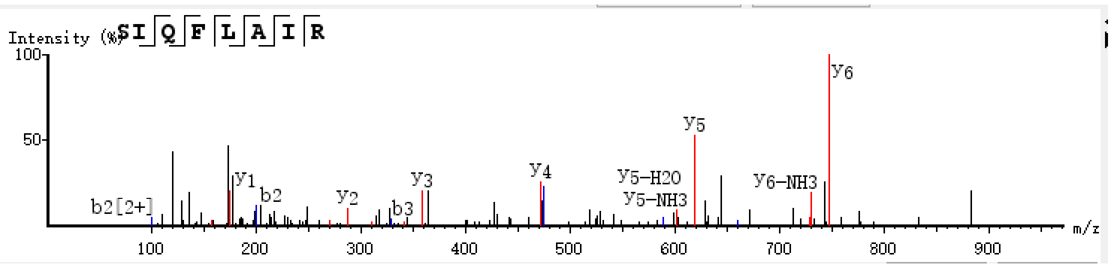

gi|571507133

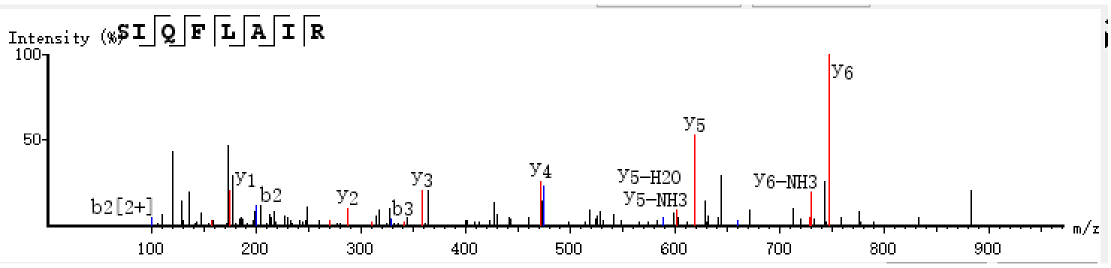

gi|571500982

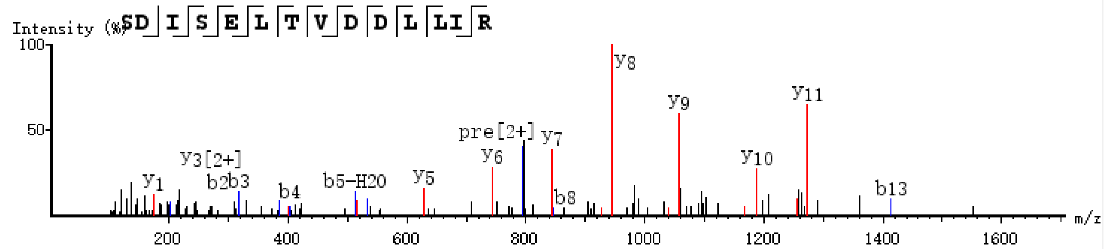

gi|328781481

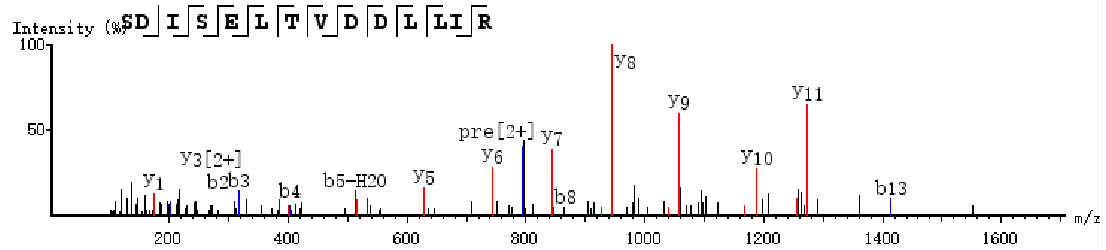

gi|571573002

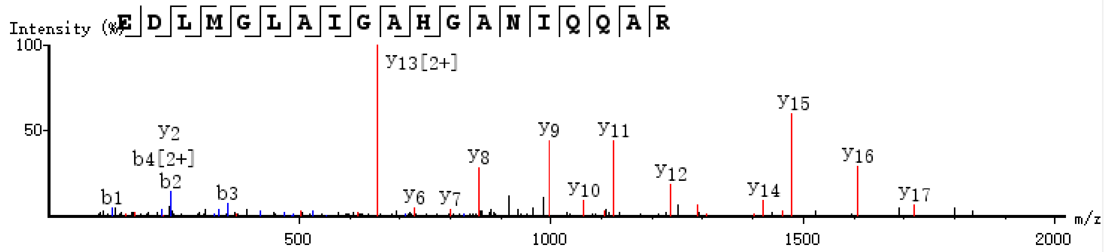

gi|110762954

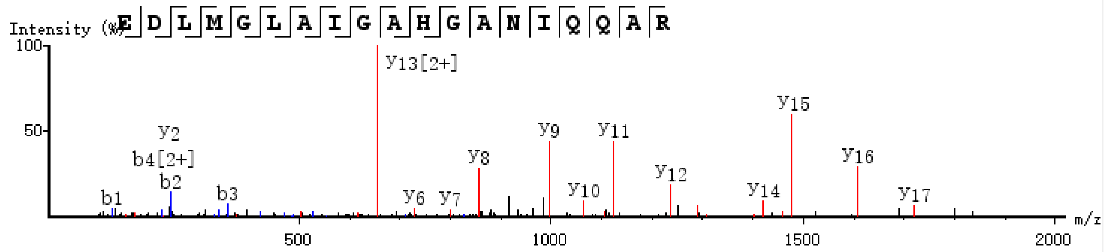

gi|66517407

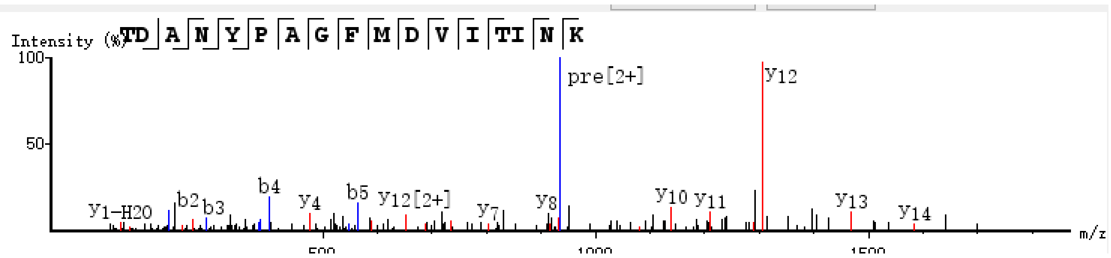

gi|571566140

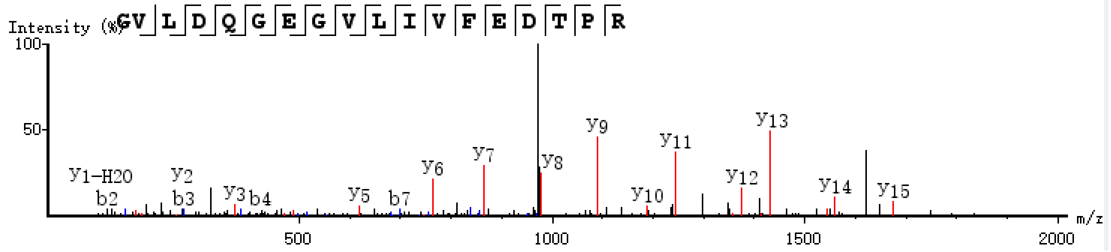

gi|334883192

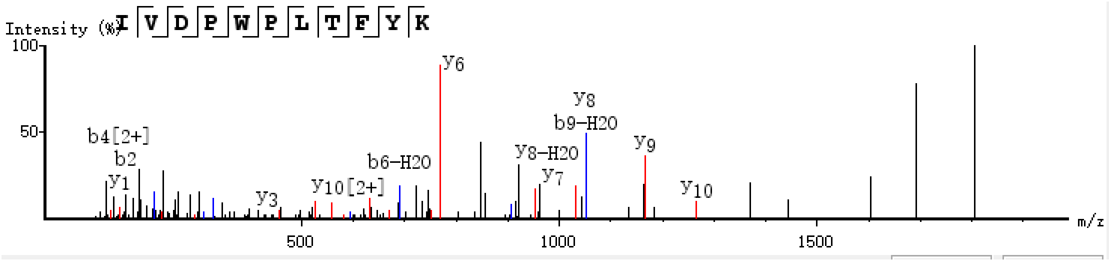

gi|66516052

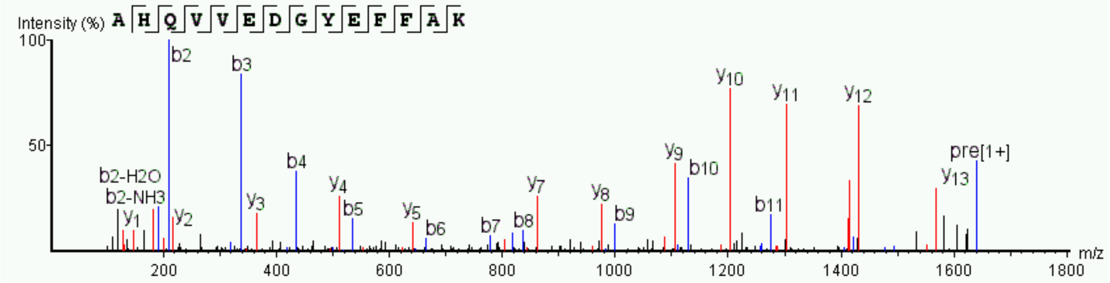

gi|328791406

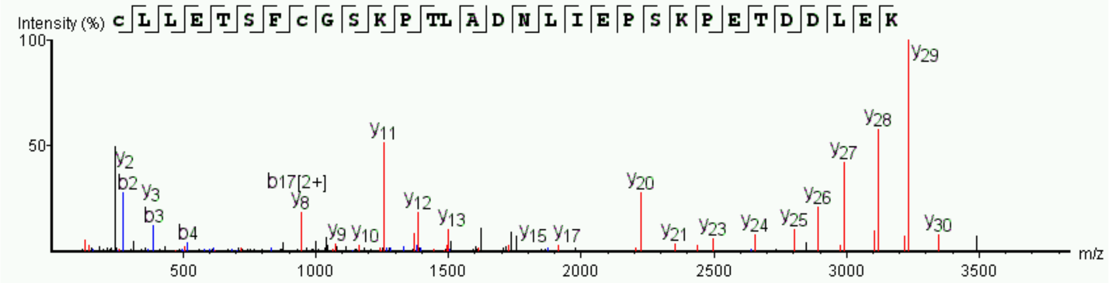

gi|571541221

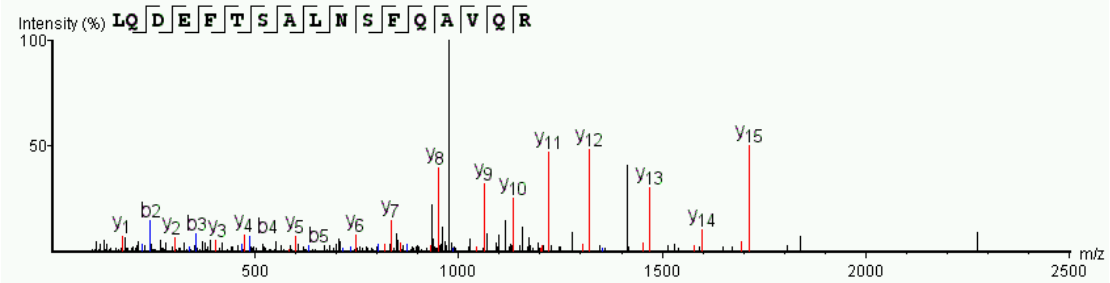

gi|571541223

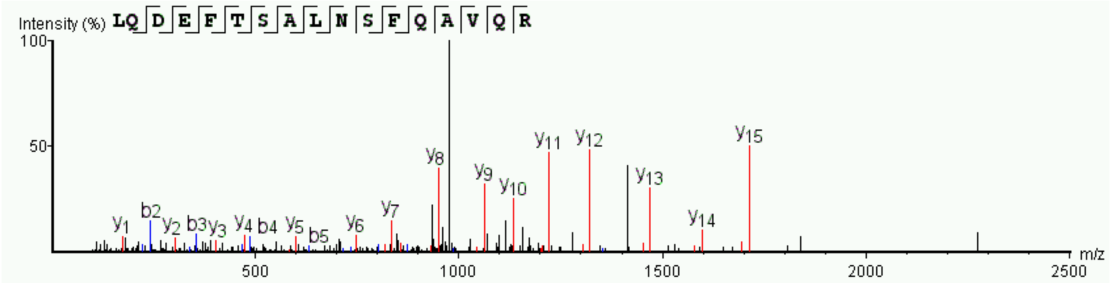

gi|571541219

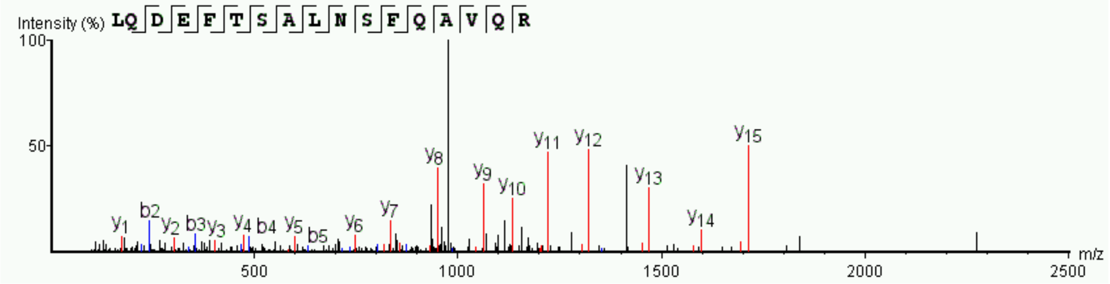

gi|571552022

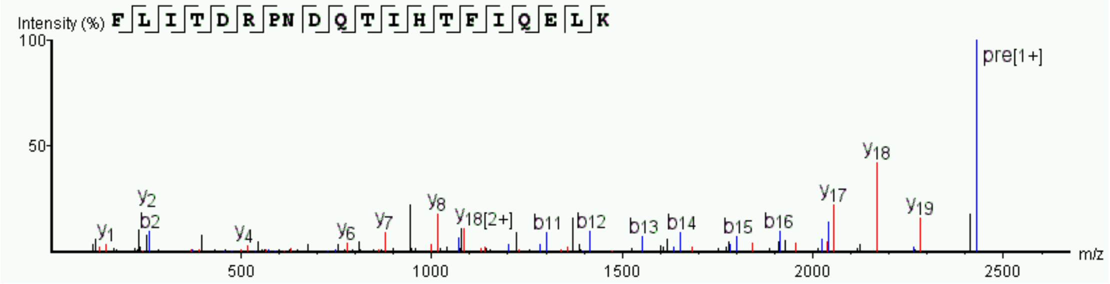

gi|328779321

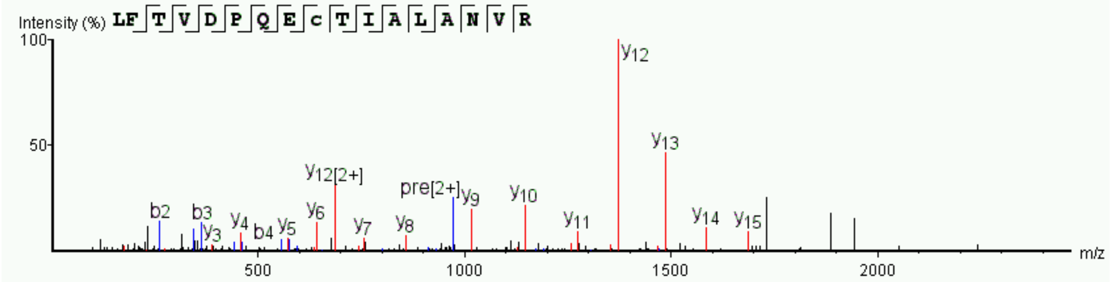

328786253

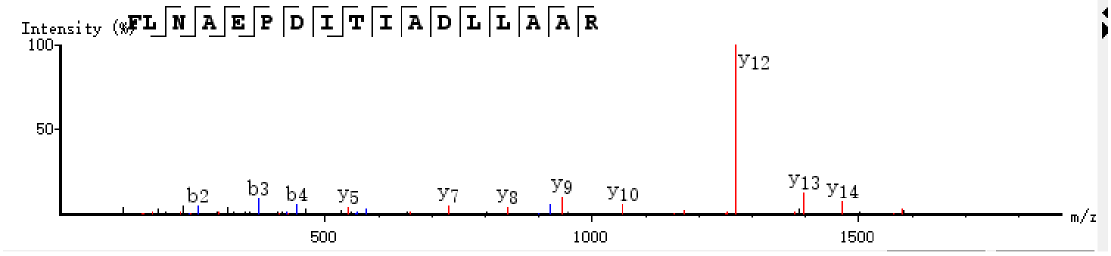

gi|571551784

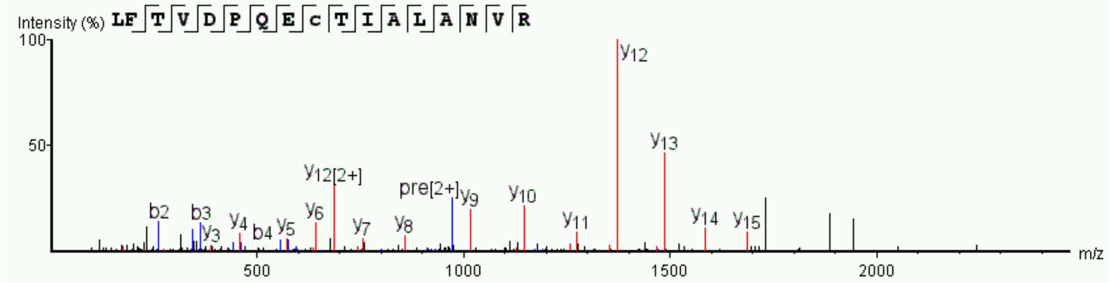

gi|571568560

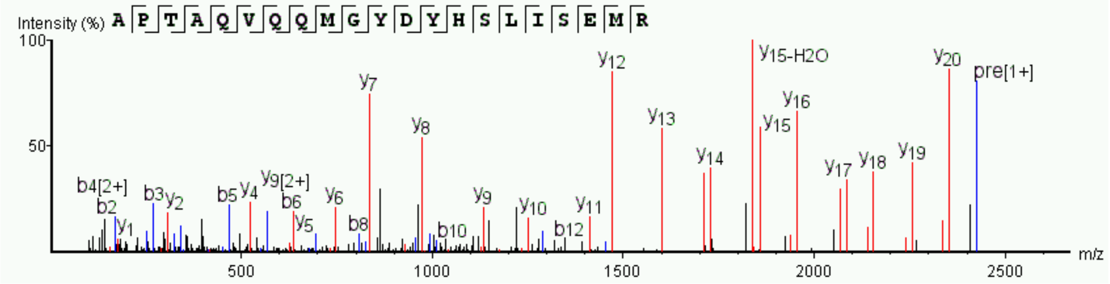

gi|328786065

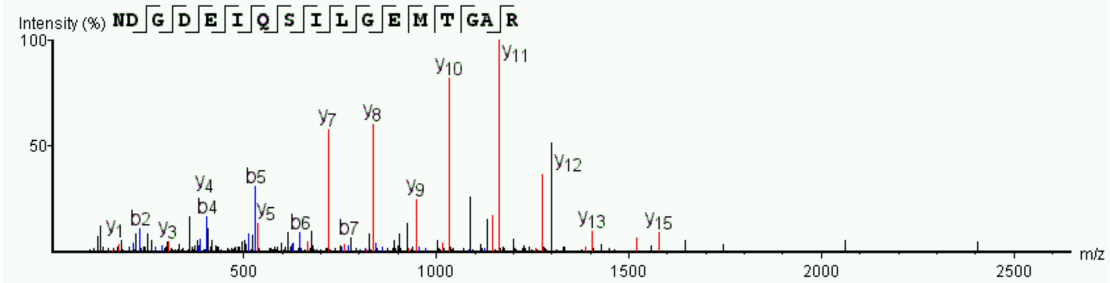

gi|110764555

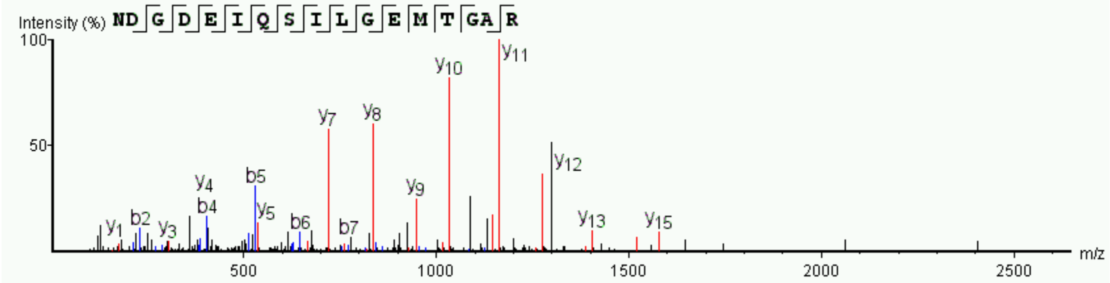

gi|328786067

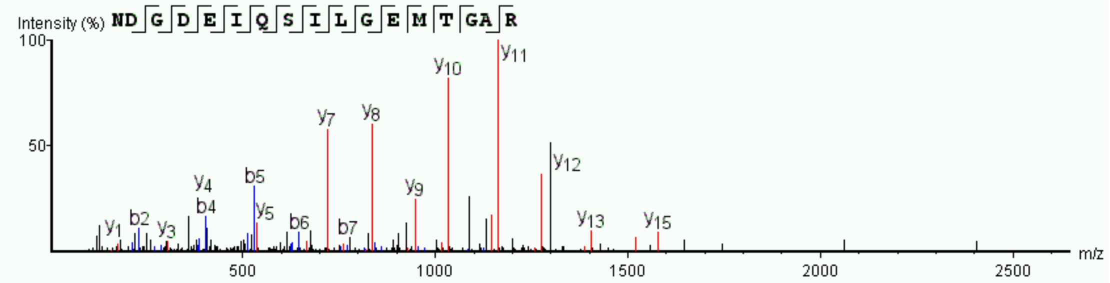

gi|571530735

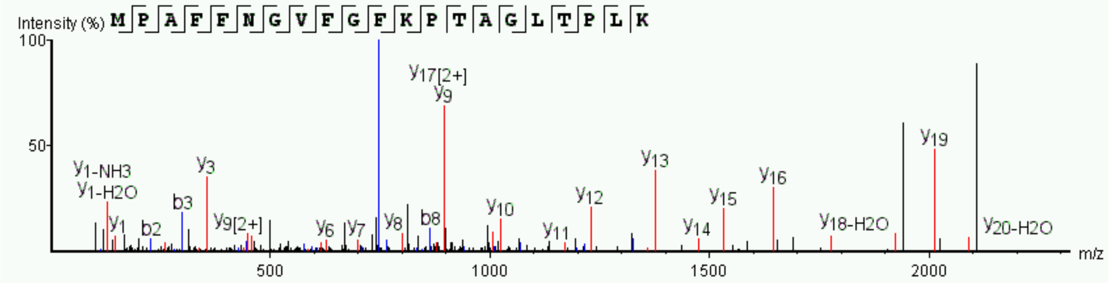

gi|66565444

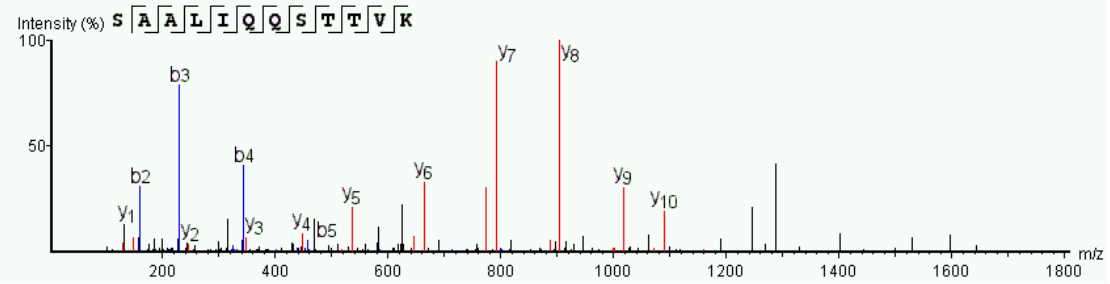

gi|66526748

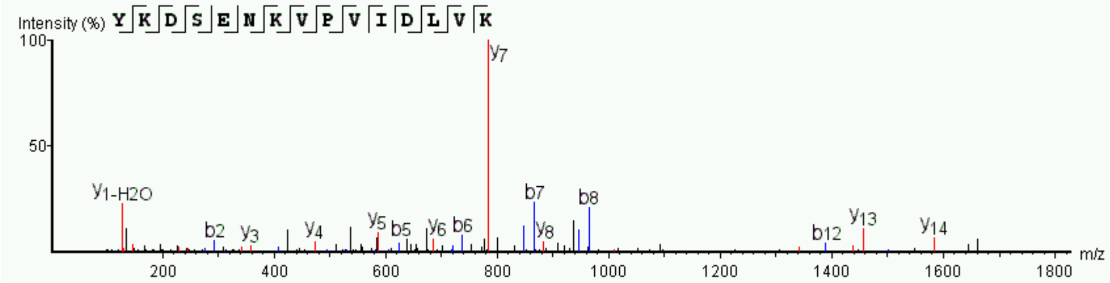

gi|571576016

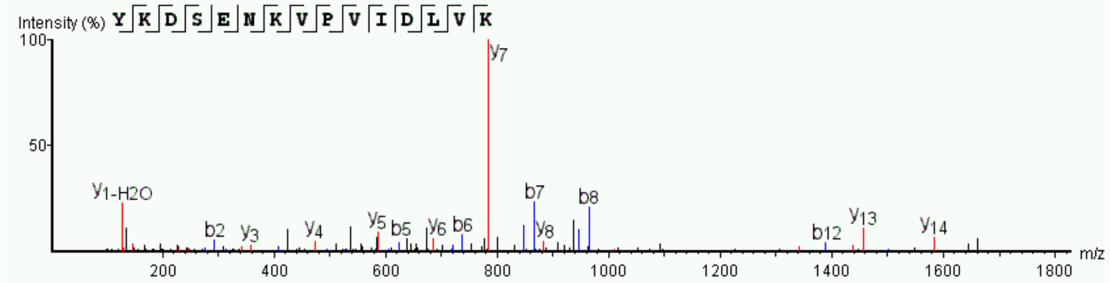

gi|571524333

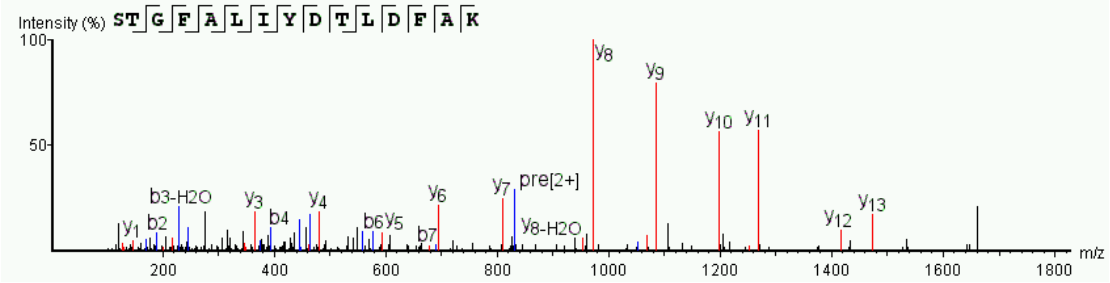

gi|48095625

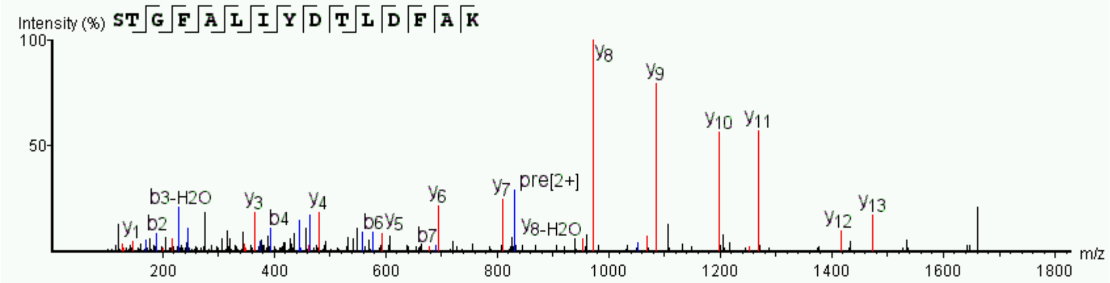

gi|571524328

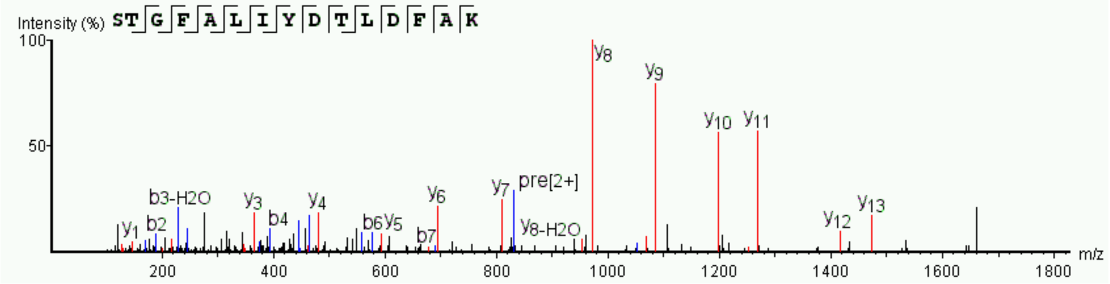

gi|571509628

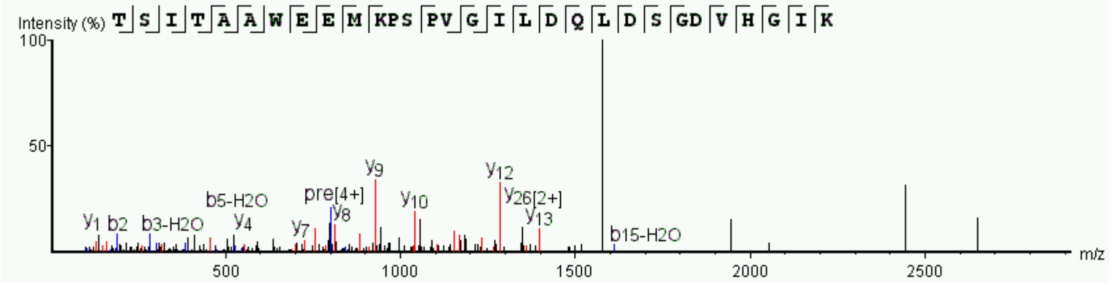

gi|571499378

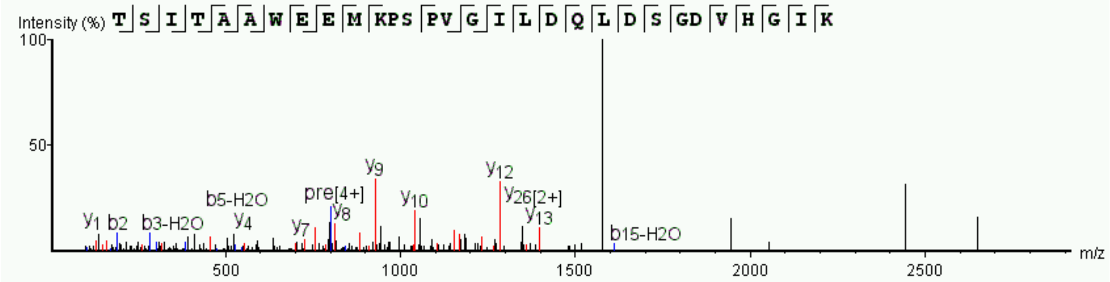

gi|571499384

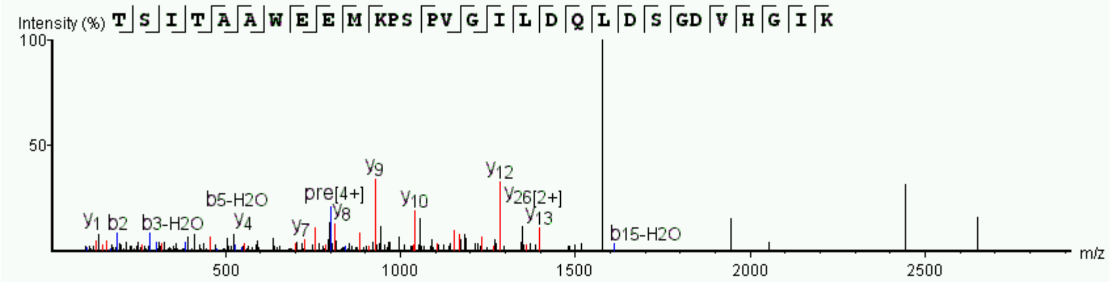

gi|571499391

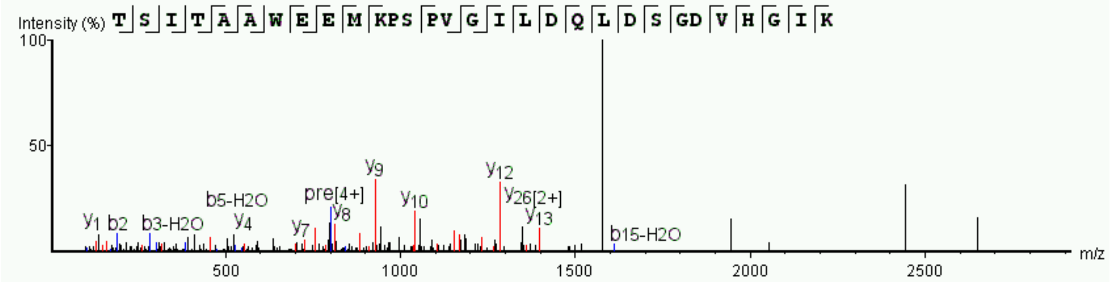

gi|571499394

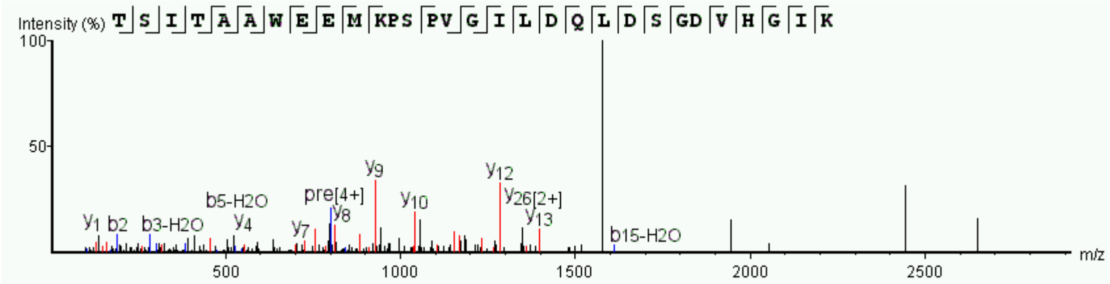

gi|571499398

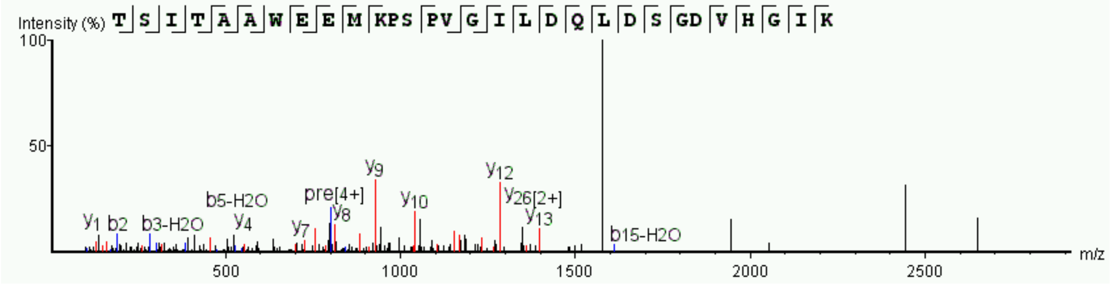

gi|328776065

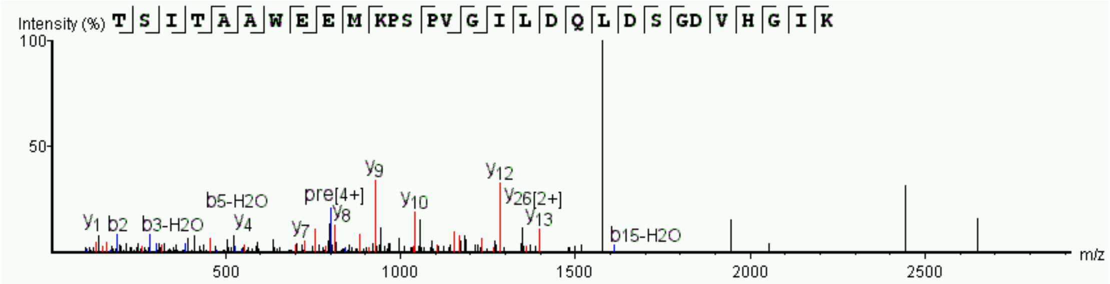

gi|571509645

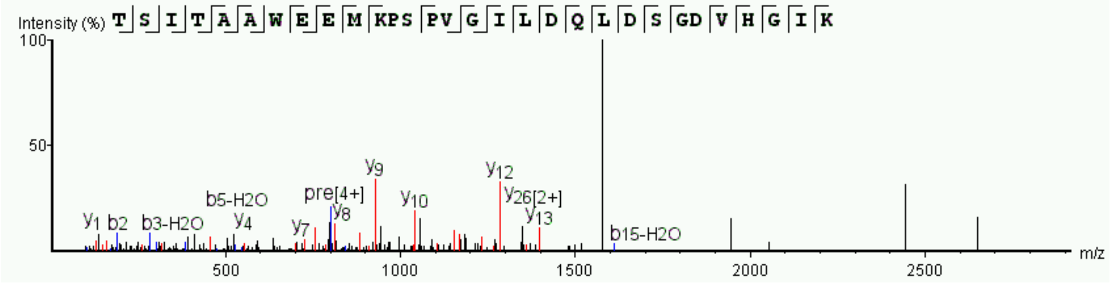

gi|571509648

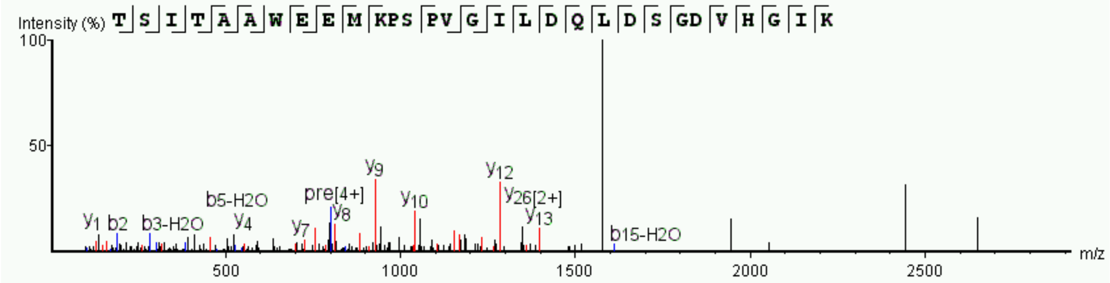

gi|571509634

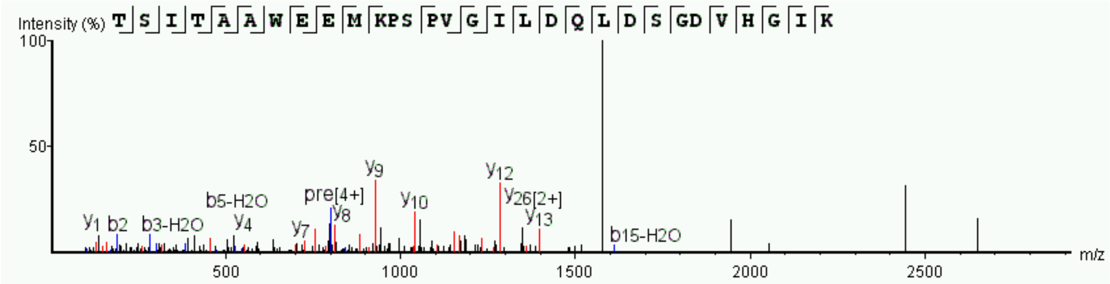

gi|571509637

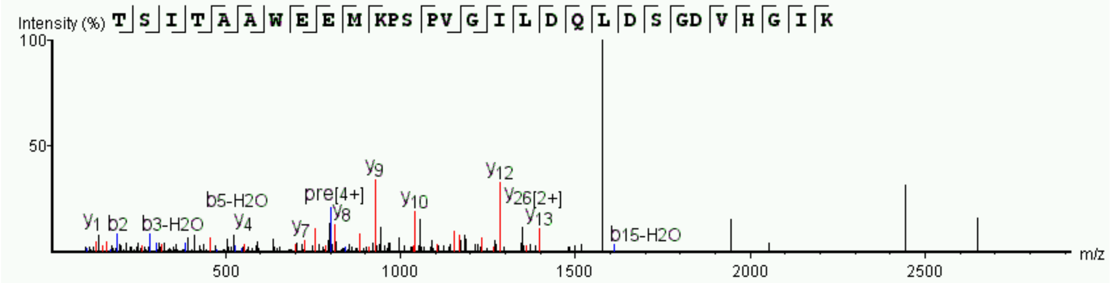

gi|571509641

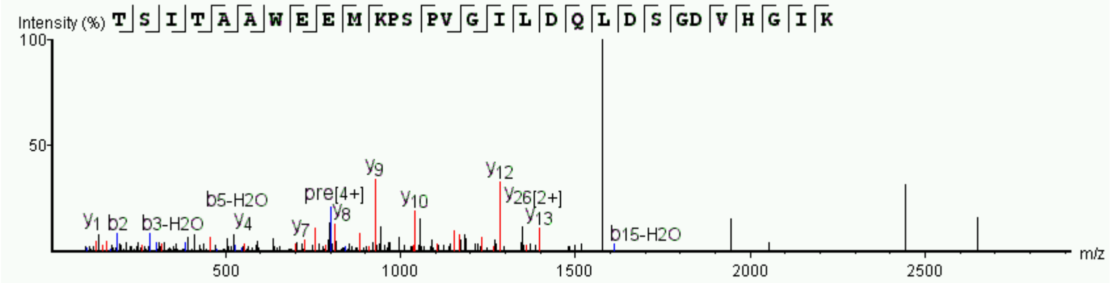

gi|571509657

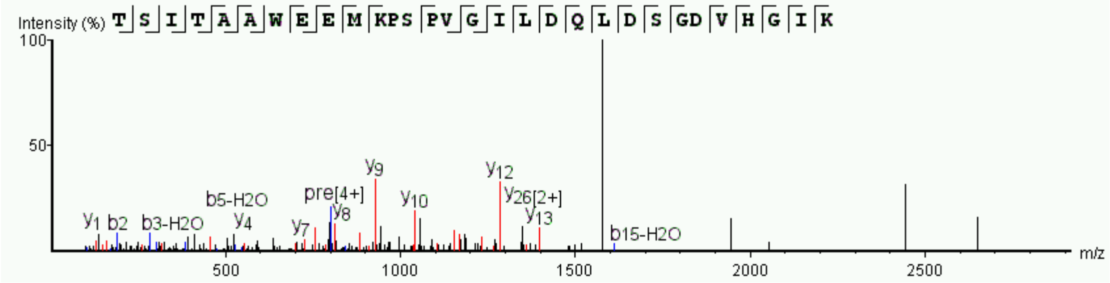

gi|571509659

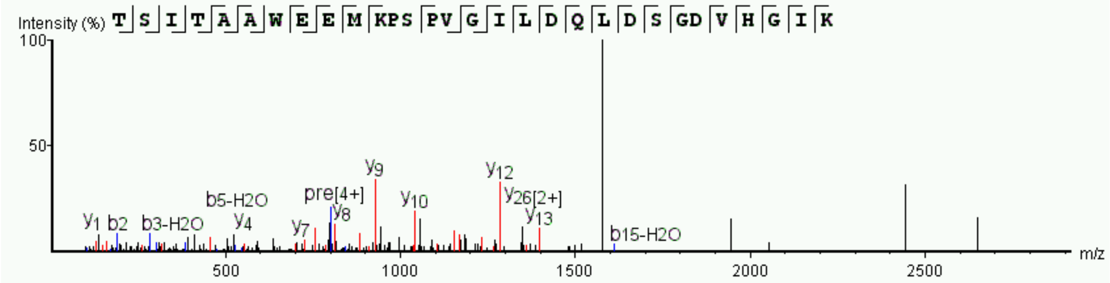

gi|571509654

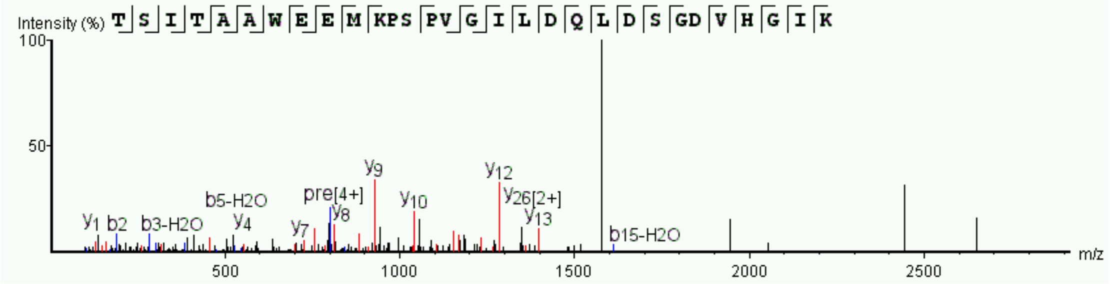

gi|571573525

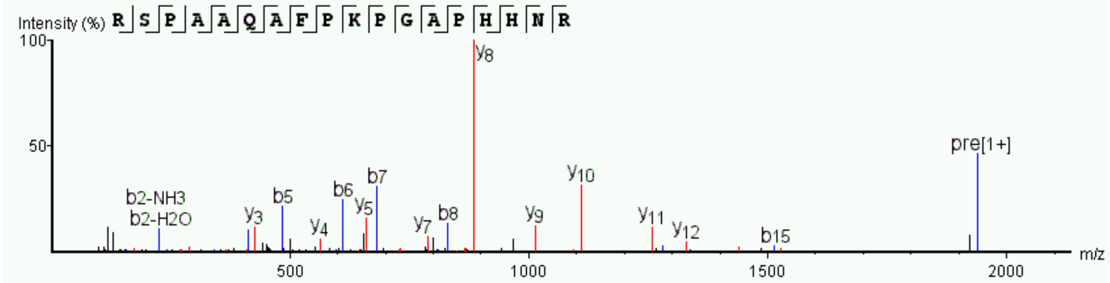

gi|571573523

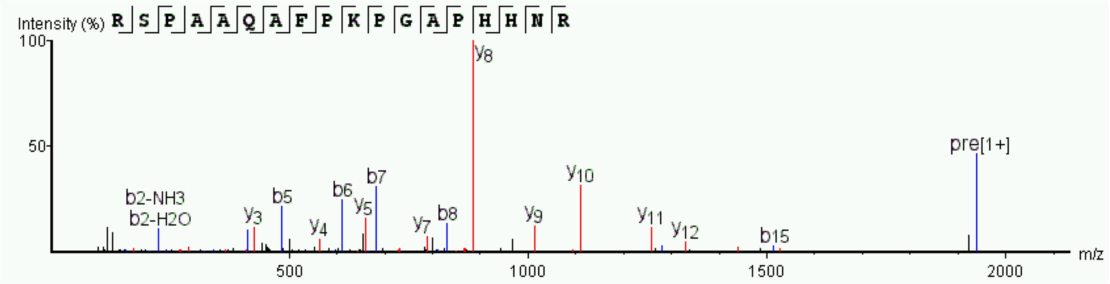

gi|571560041

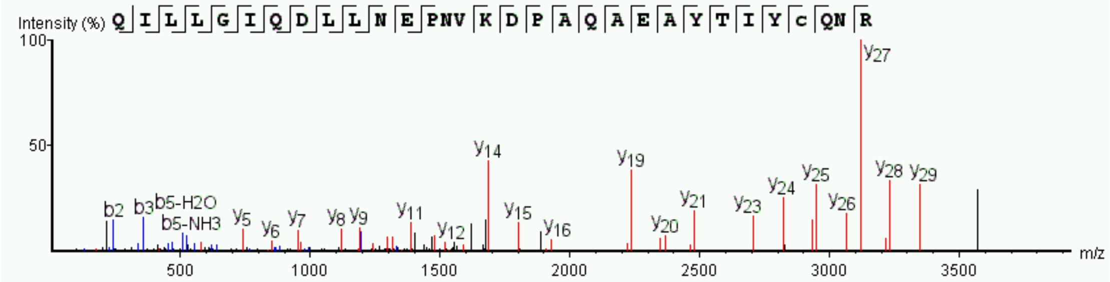

gi|571560038

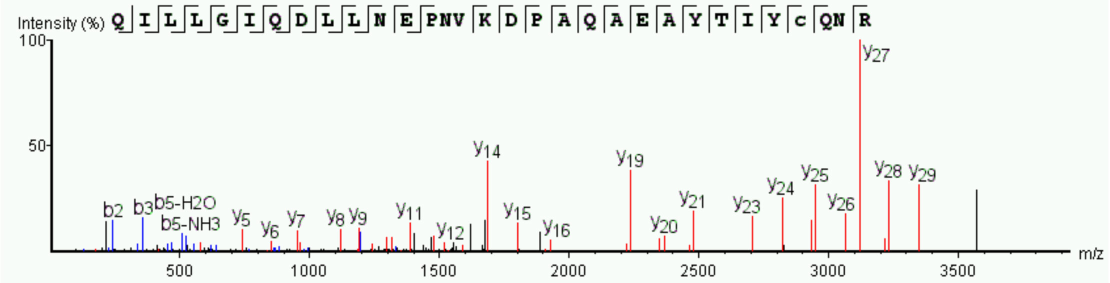

gi|328789575

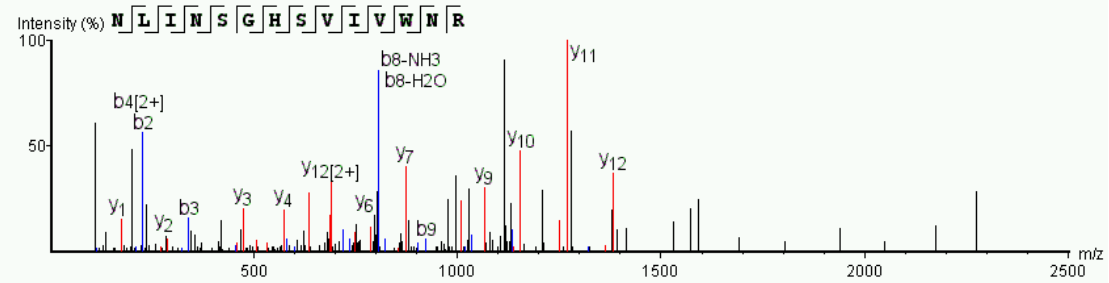

gi|48096769

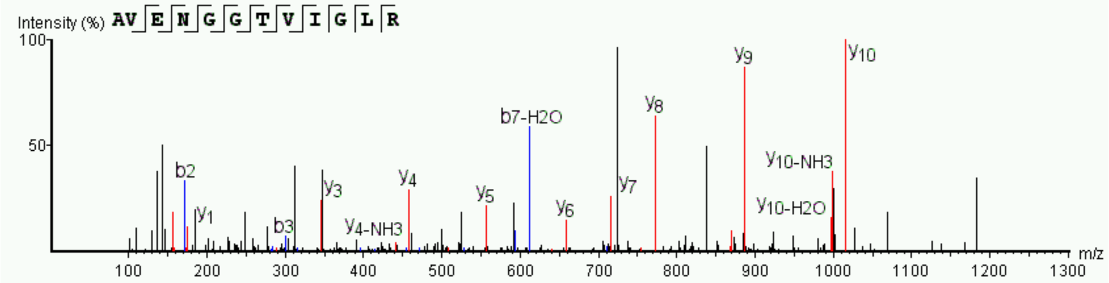

gi|48140497

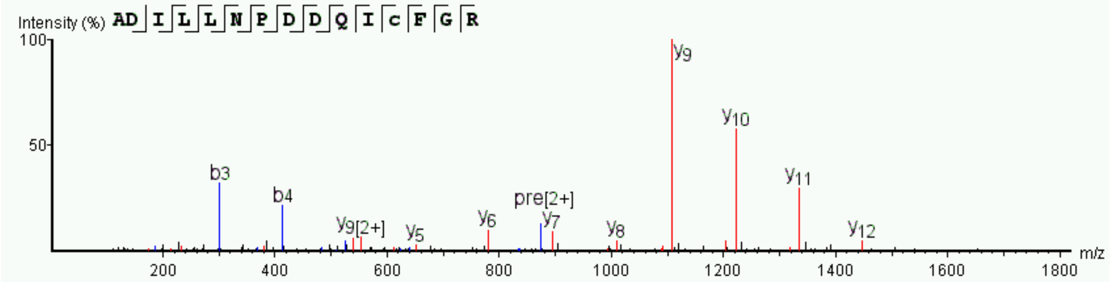

gi|571553257

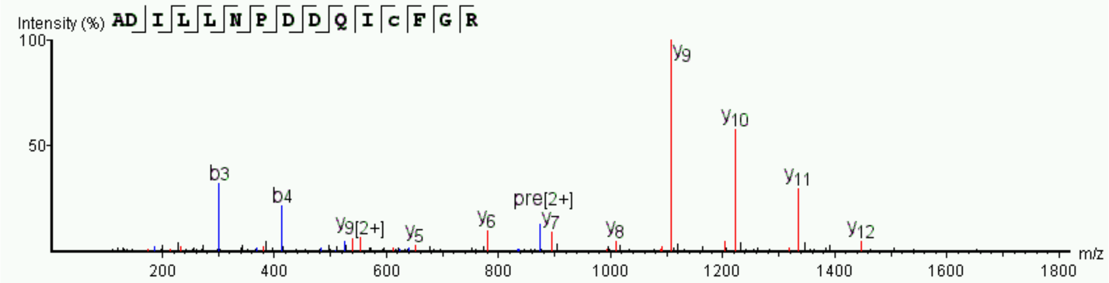

gi|66511337

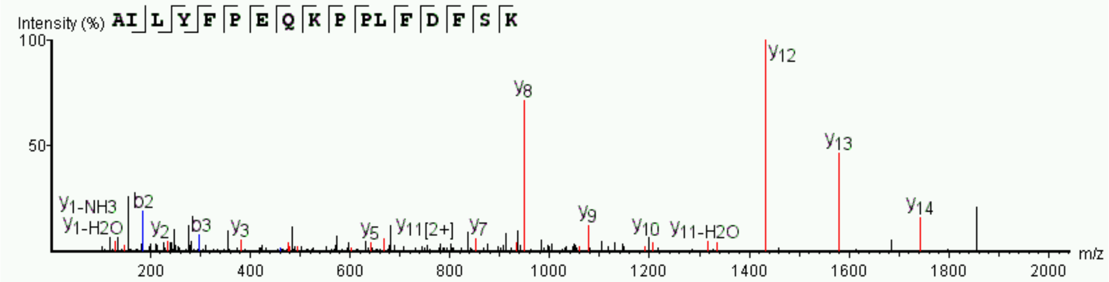

gi|328793729

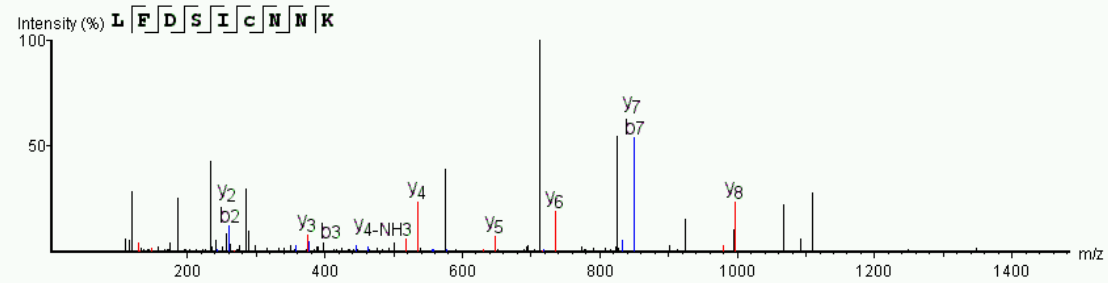

gi|328789473

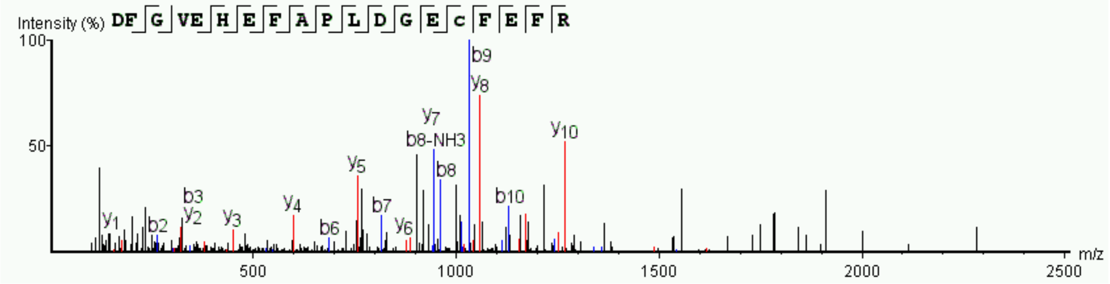

gi|571575189

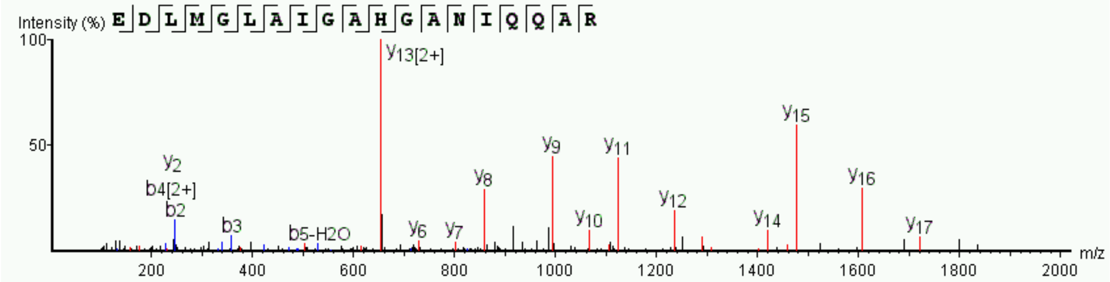

gi|571575193

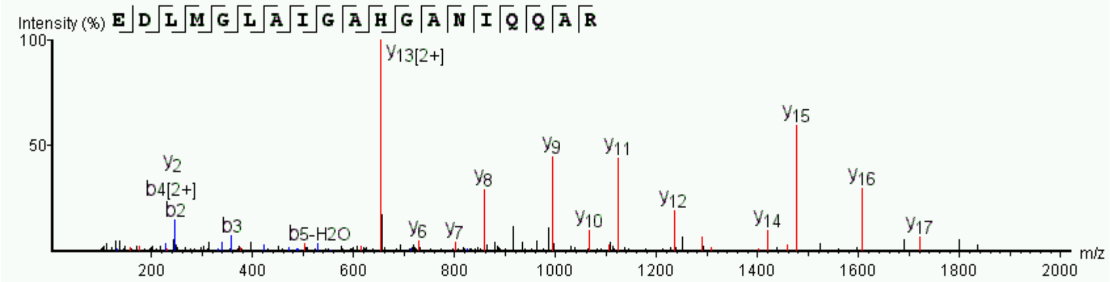

gi|571575195

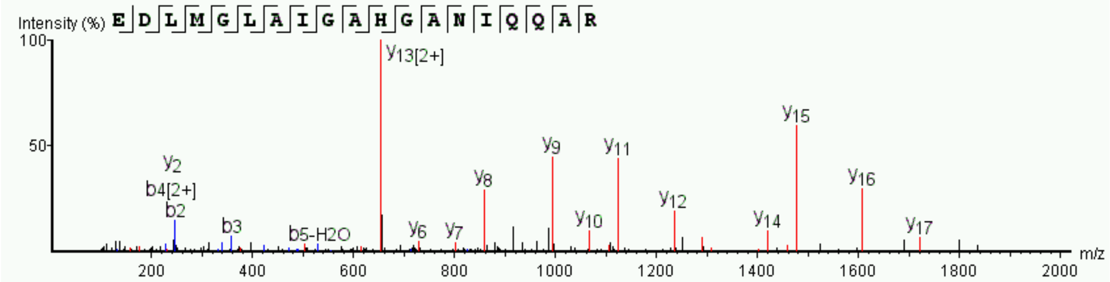

gi|571575198

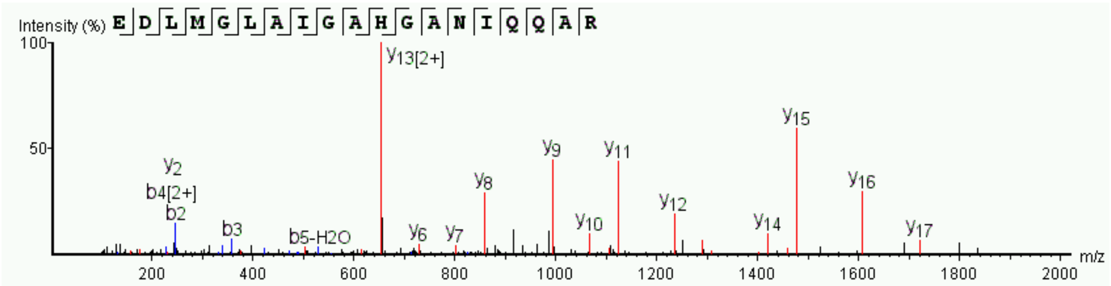

gi|328783508

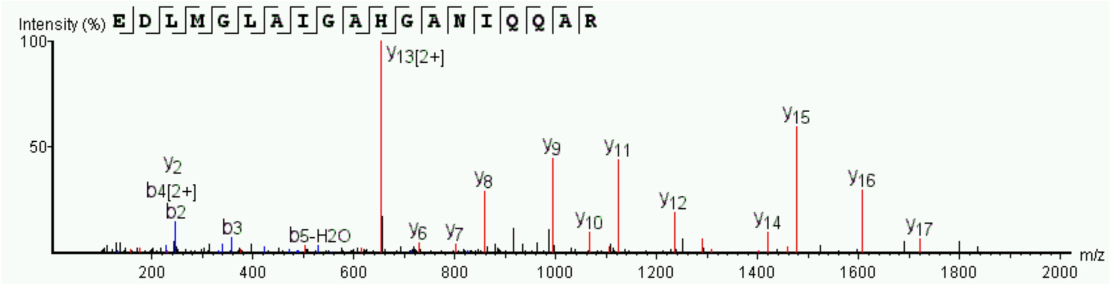

gi|571575191

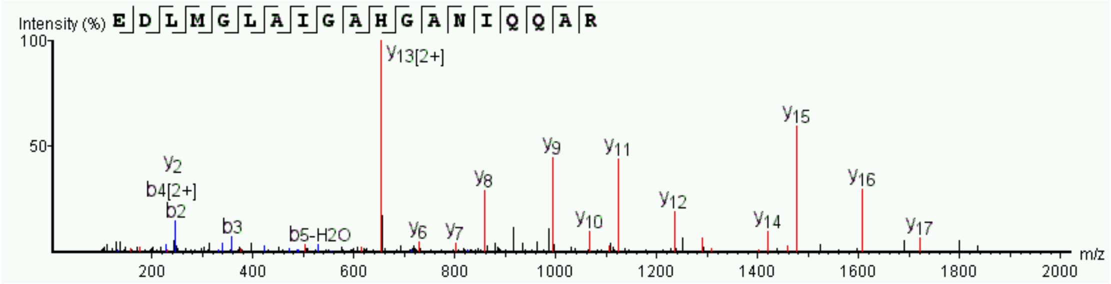

gi|571569633

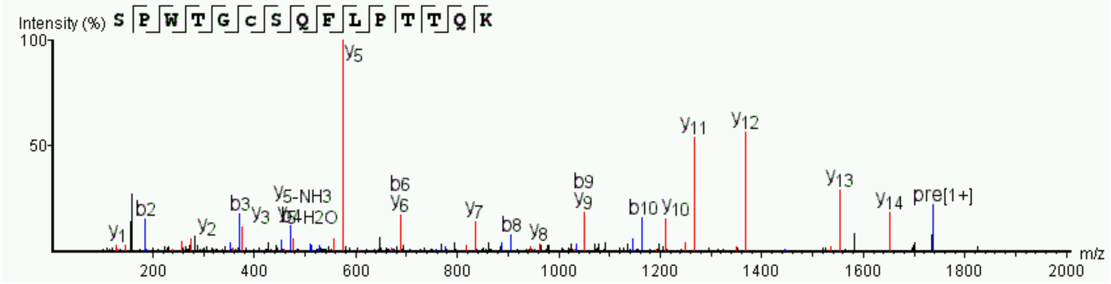

gi|66543768

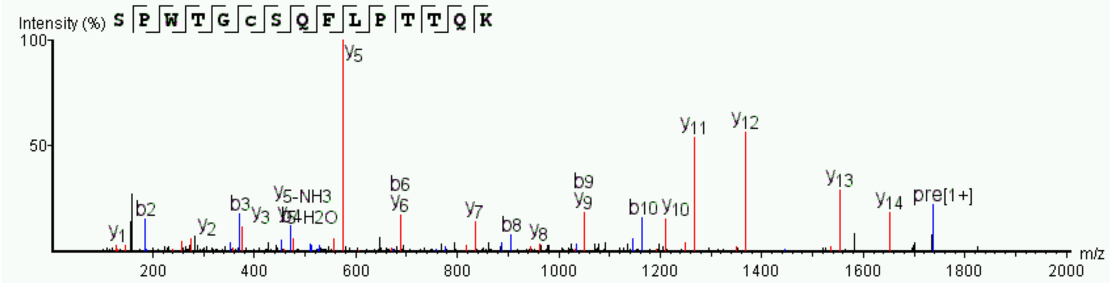

gi|571569639

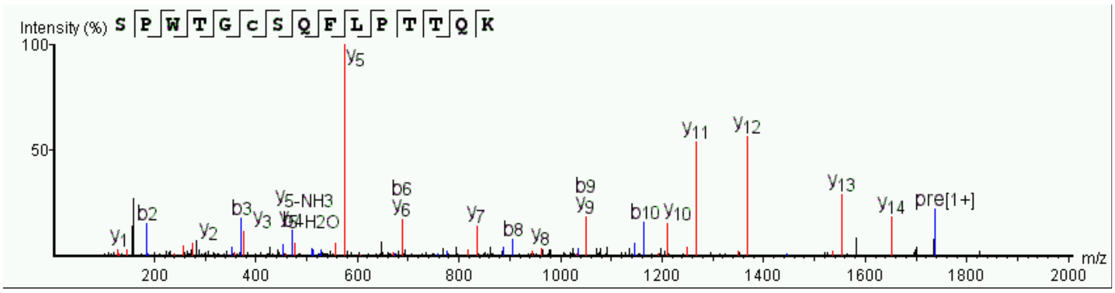

gi|571548900

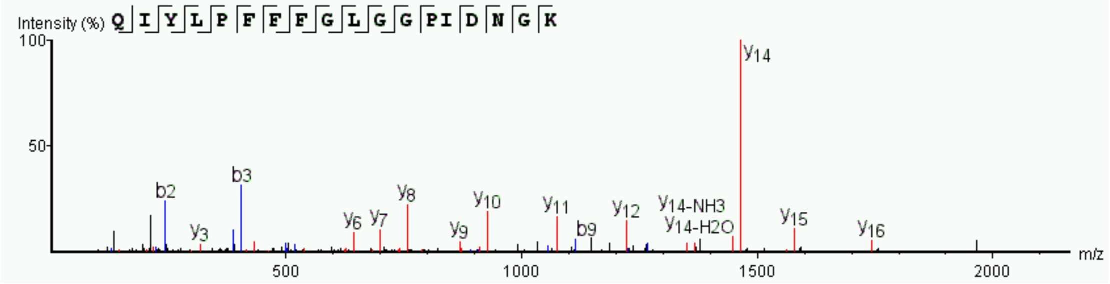

gi|66550870

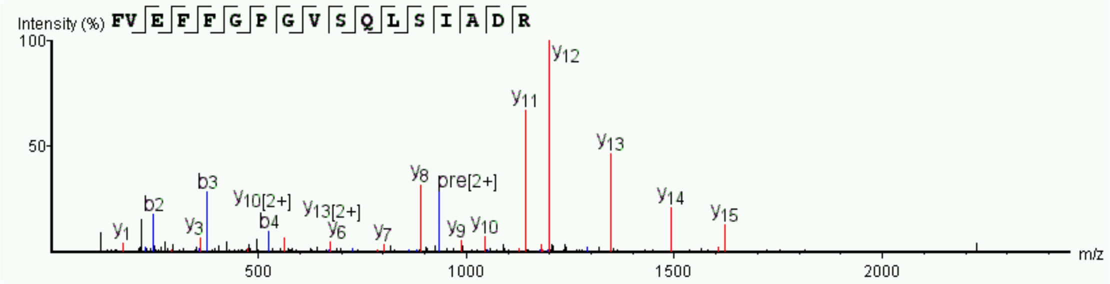

gi|571575903

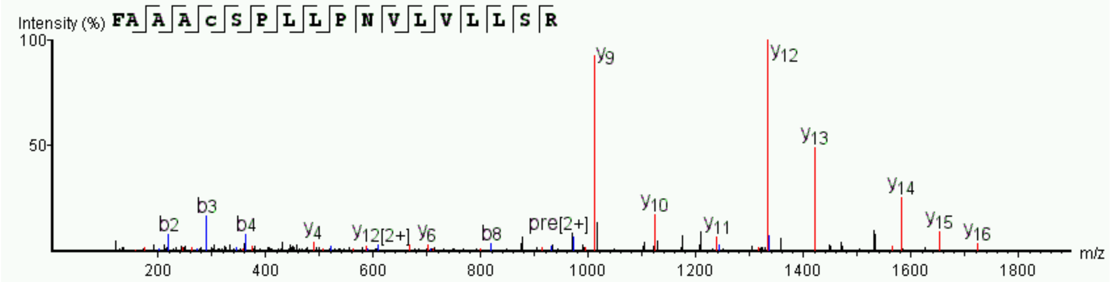

gi|571575905

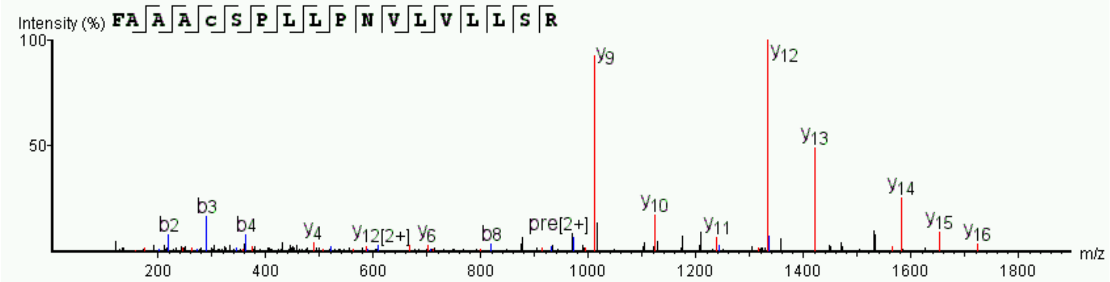

gi|571575901

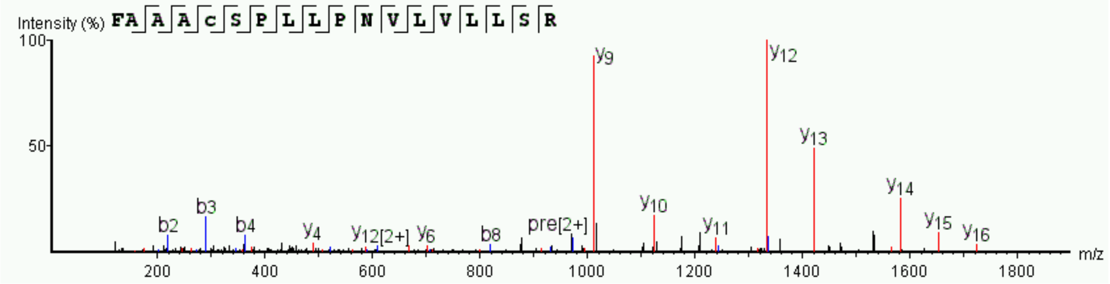

gi|328780974

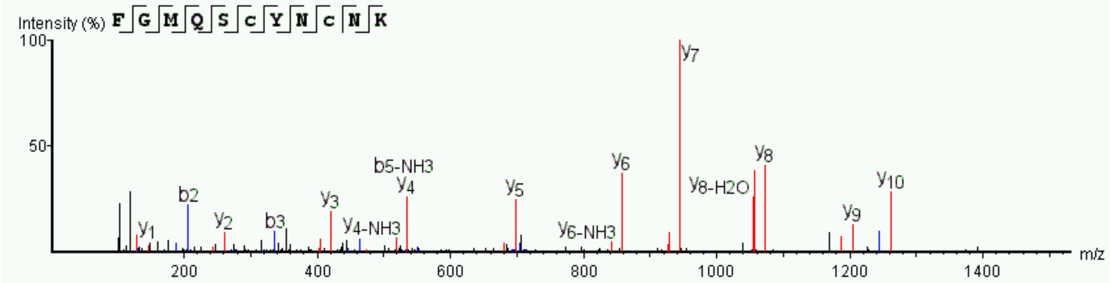

gi|571548542

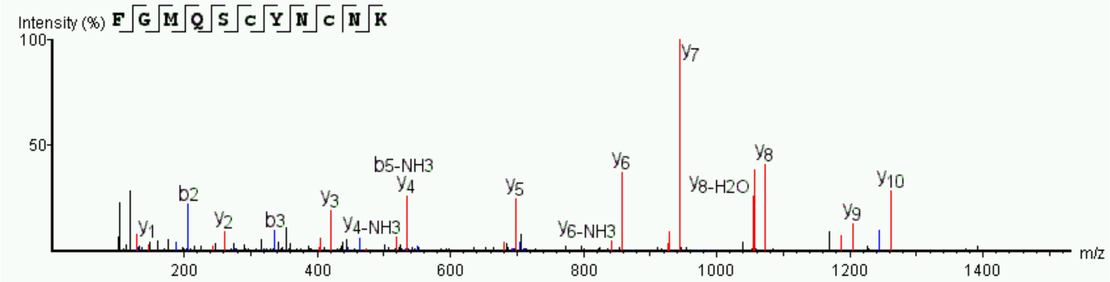

gi|110759374

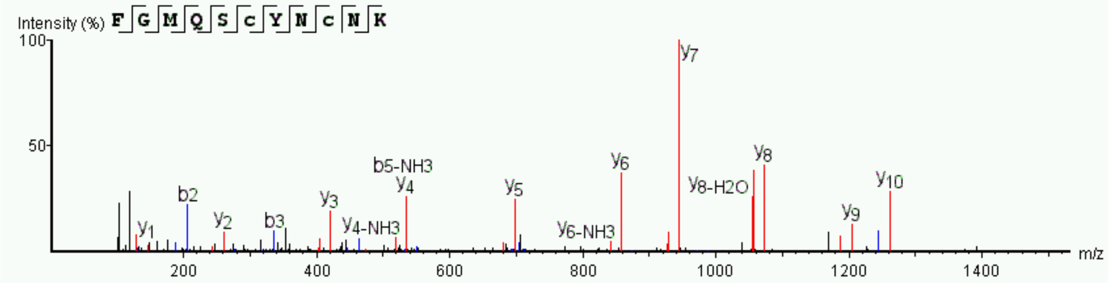

gi|66549799

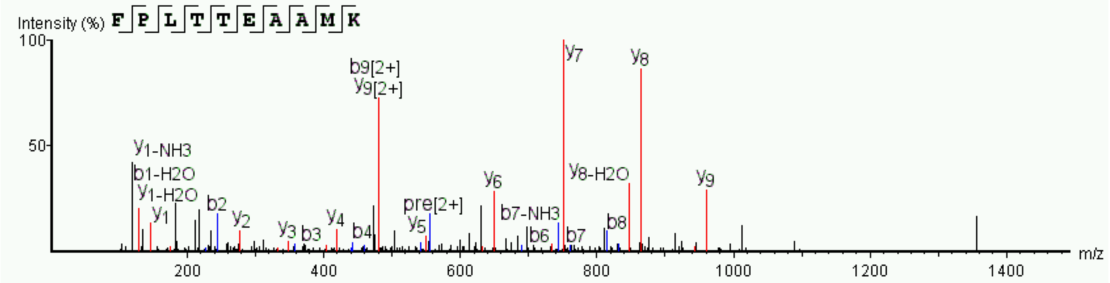

gi|571576123

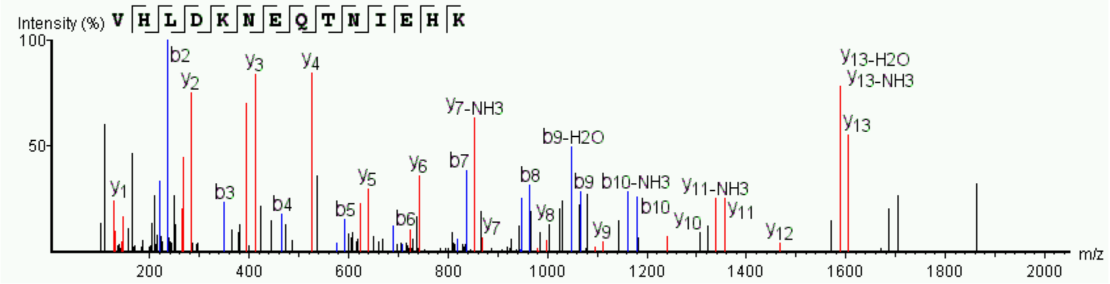

gi|571575119

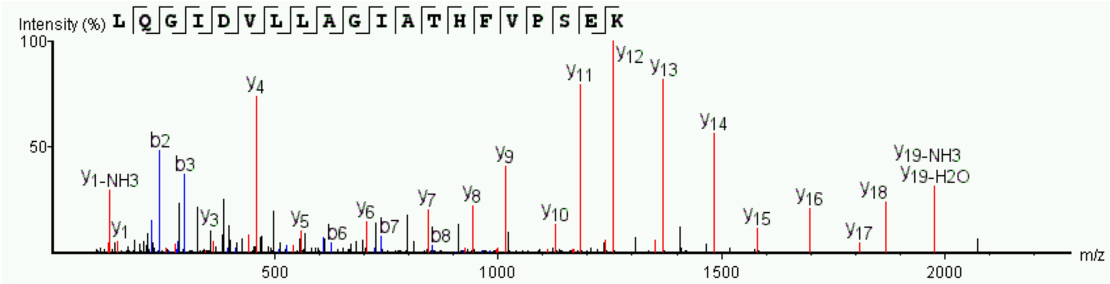

gi|328783479

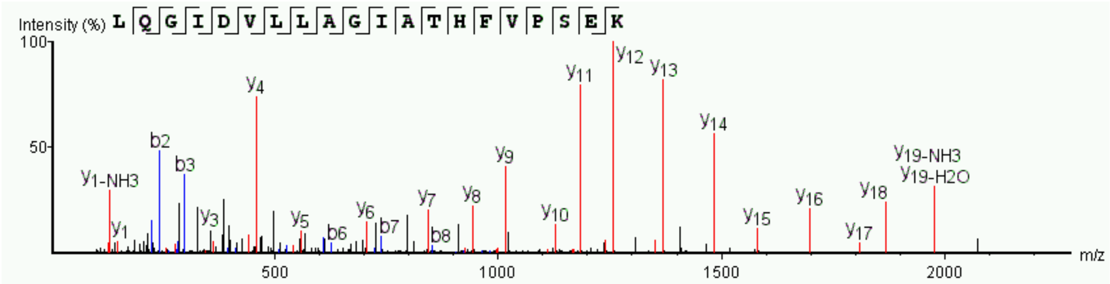

gi|571575116

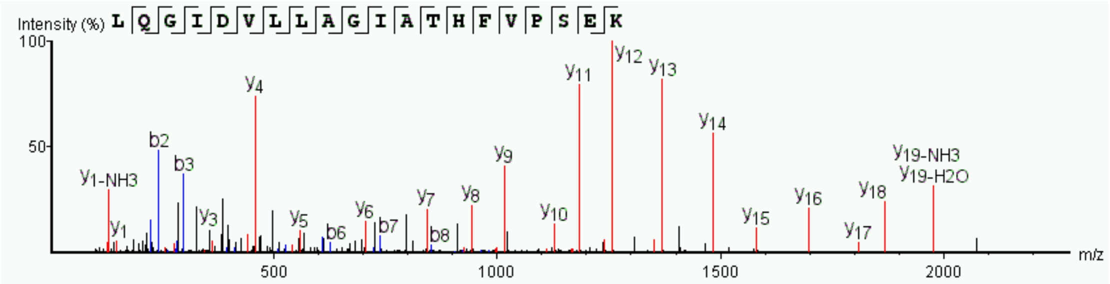

gi328780510

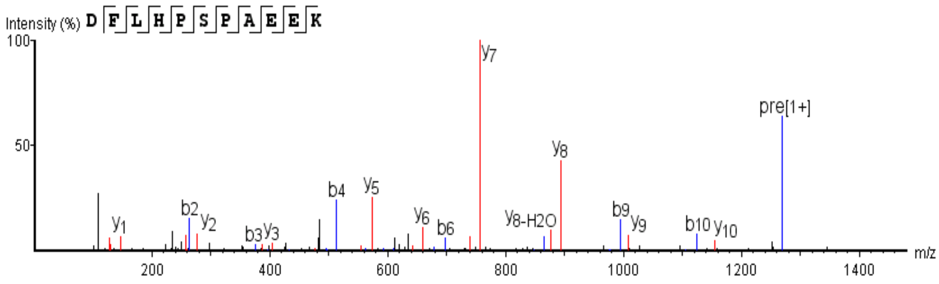

gi328781115

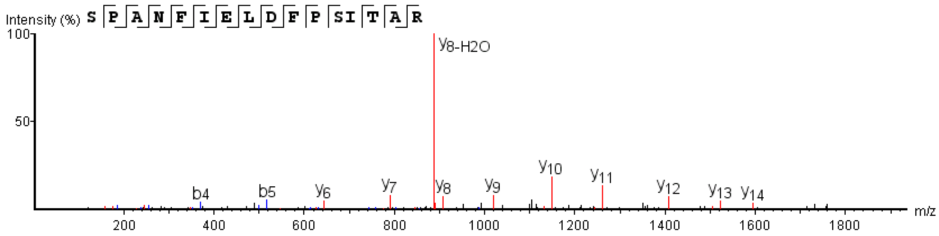

gi328781218

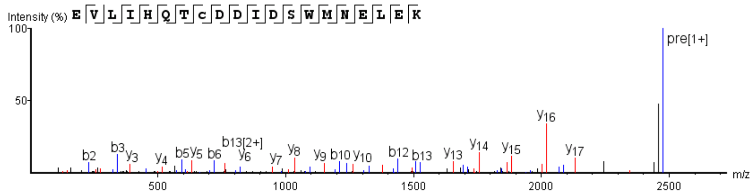

gi328782156

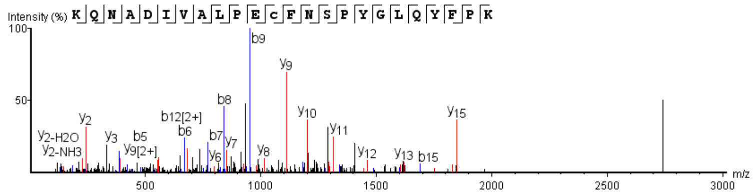

gi328782283

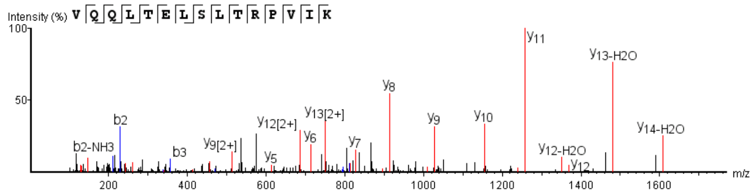

gi|571538909

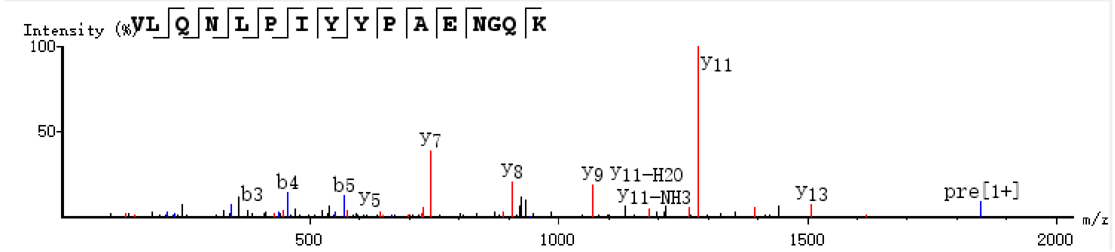

gi328782334

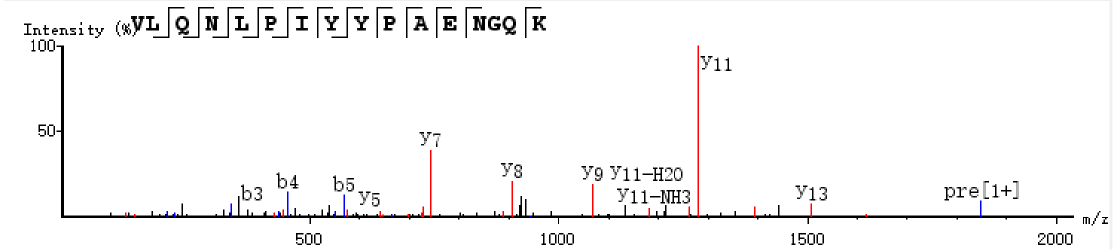

gi|571523743

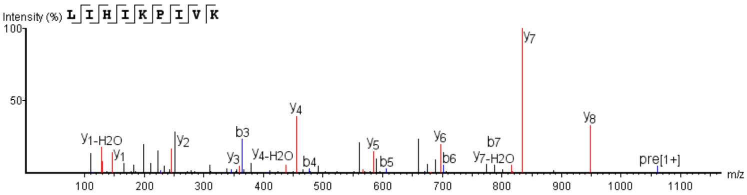

gi|571523747

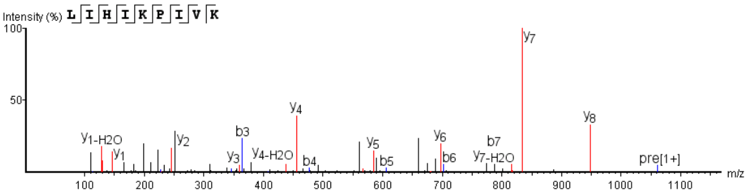

gi328782499

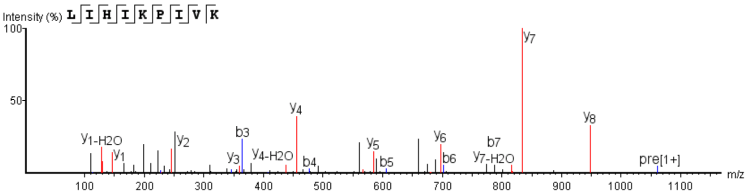

i328782520

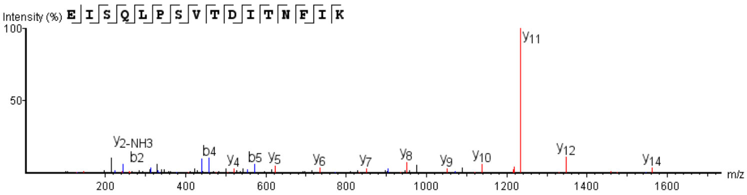

gi|571571071

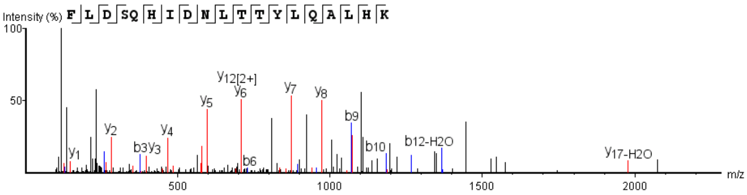

gi328782752

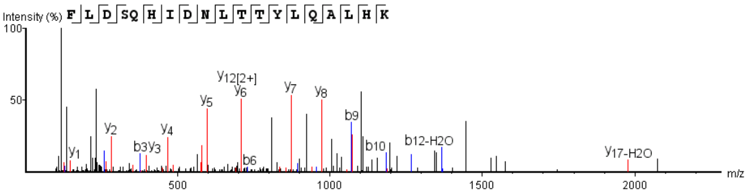

gi328782906

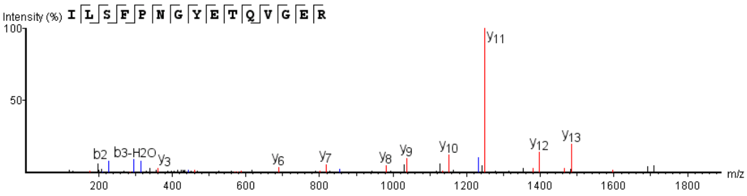

gi328783026

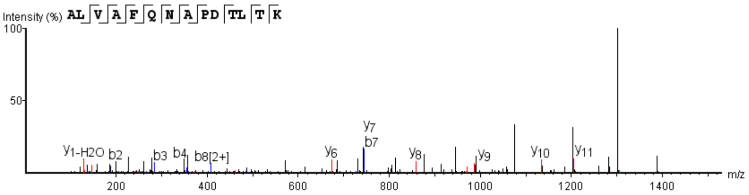

gi328784195

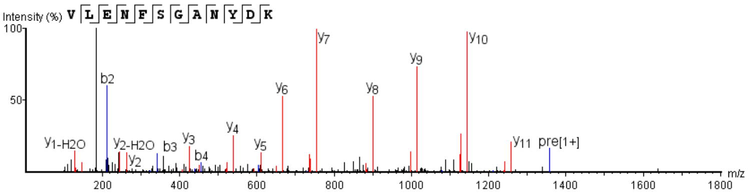

gi328784410

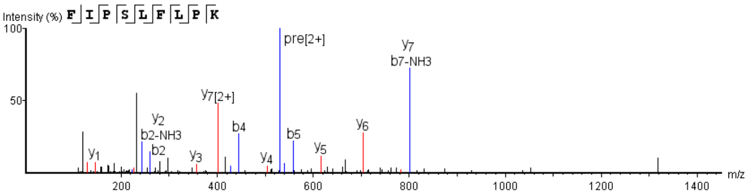

gi|571553681

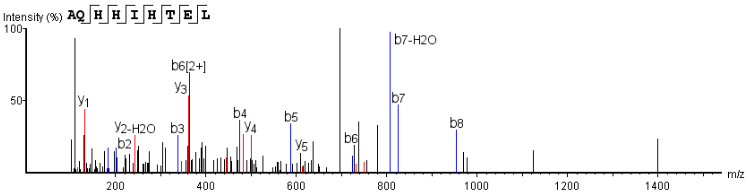

gi328784622

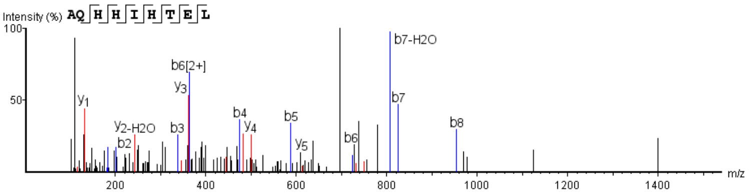

gi328785087

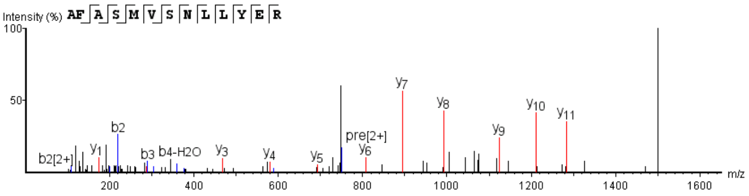

gi|48128110

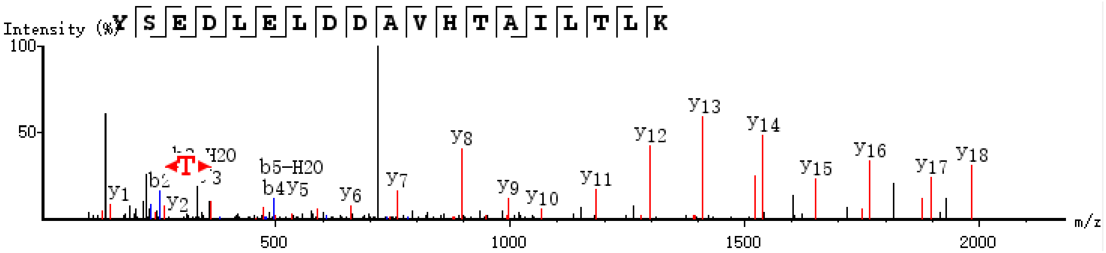

gi328785946

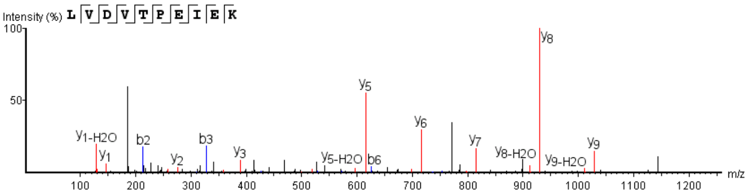

gi328786568

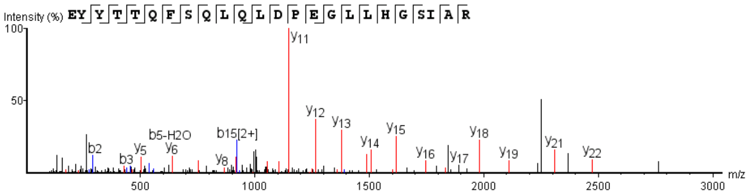

gi328786971

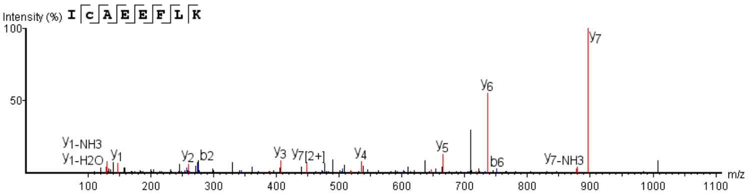

gi328787488

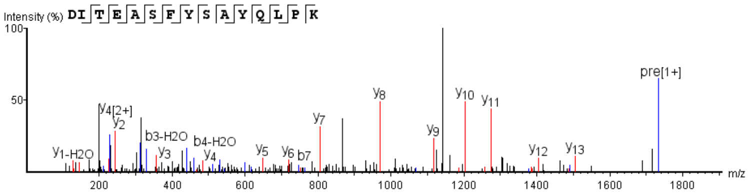

gi328787541

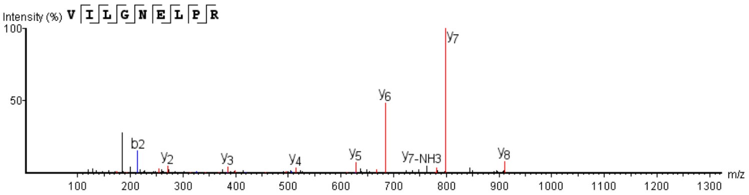

gi328788017

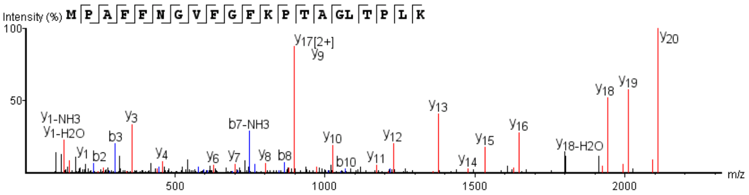

gi|571550221

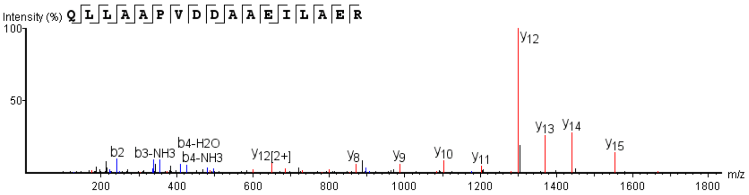

gi328788157

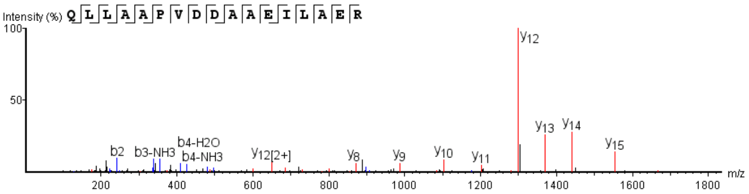

gi328788528

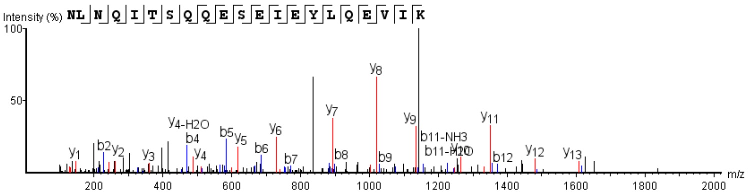

gi328788813

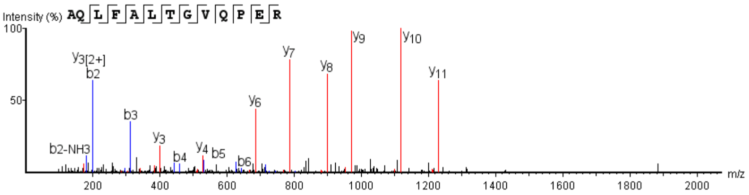

gi328788838

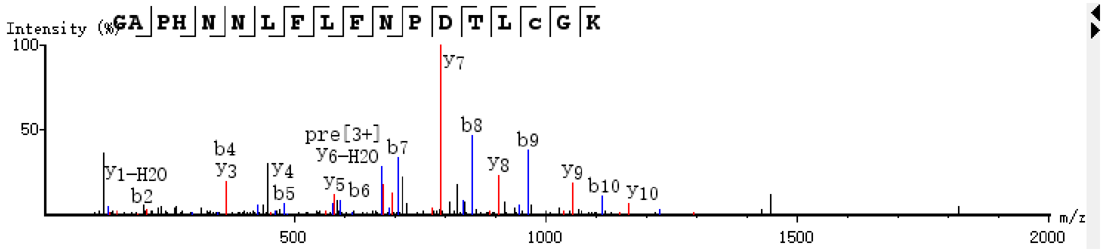

gi328789347

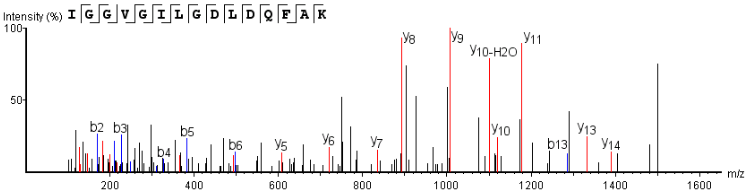

gi328789366

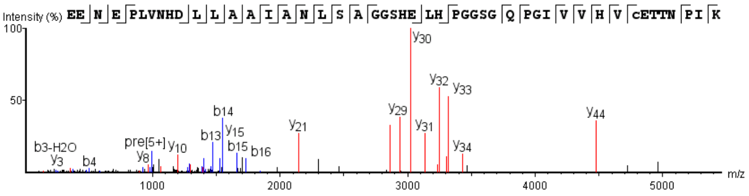

gi328789409

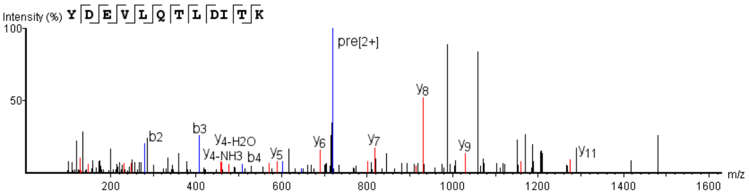

gi|571556448

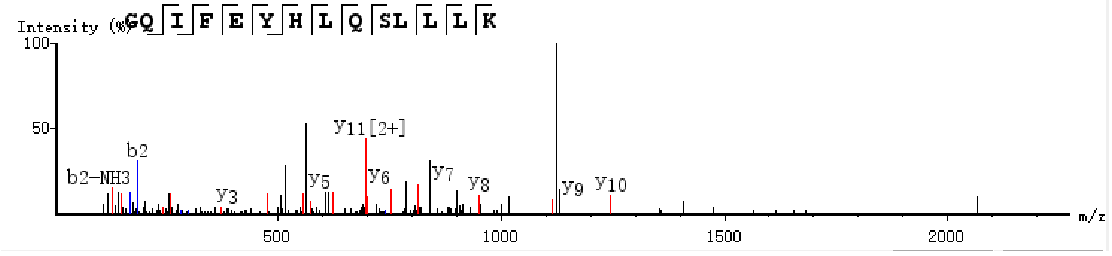

gi328789542

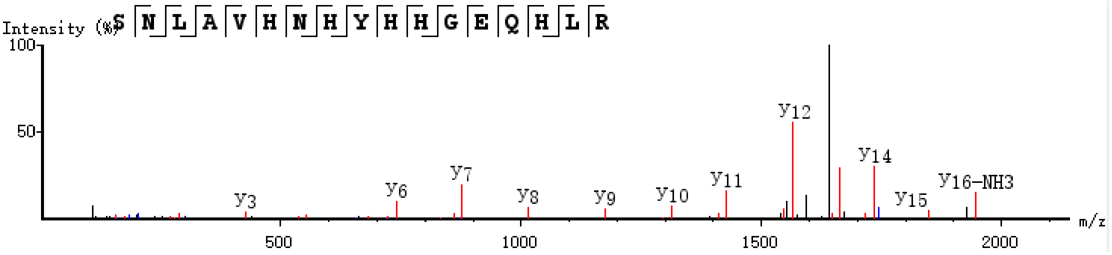

gi328790880

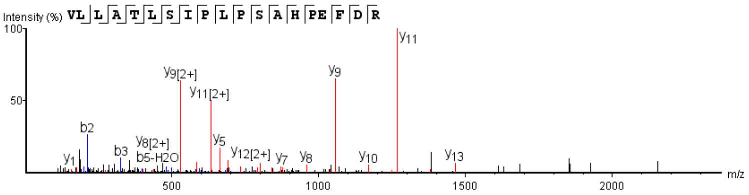

gi328791588

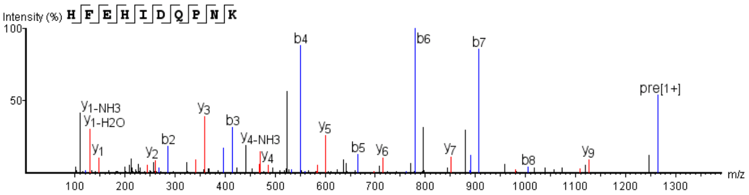

gi328792314

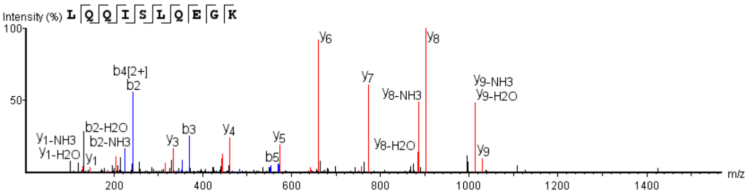

gi328792316

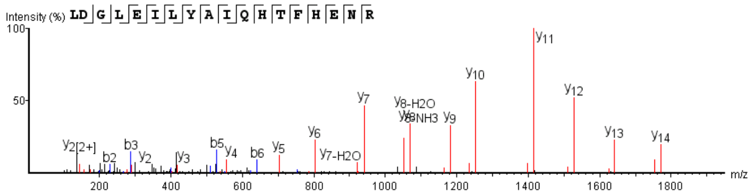

gi|571577410

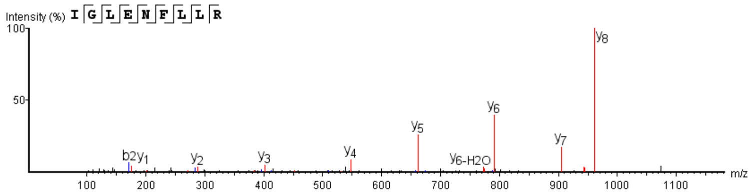

gi328793251

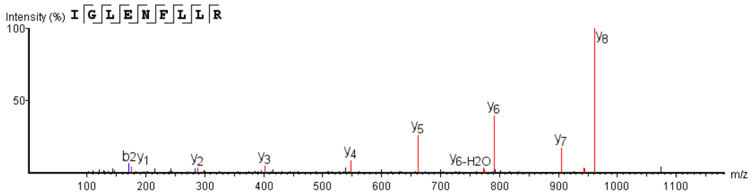

gi|571577853

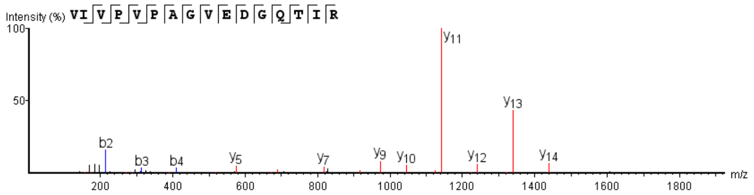

gi328793508

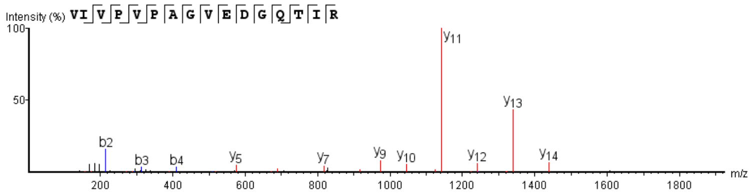

gi328793540

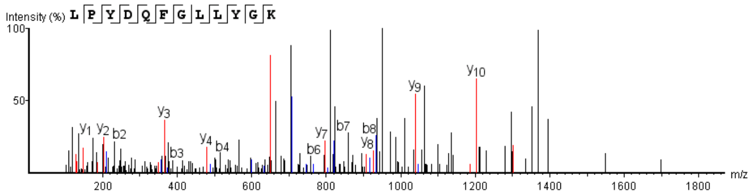

gi|58585248

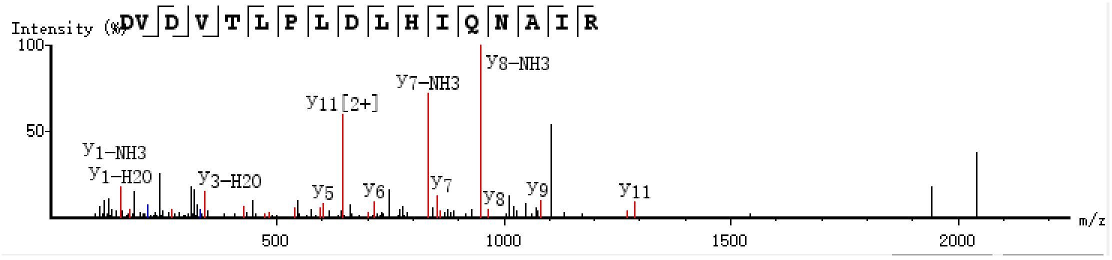

gi335892839

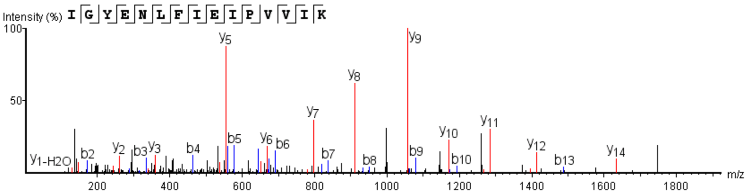

gi336391185

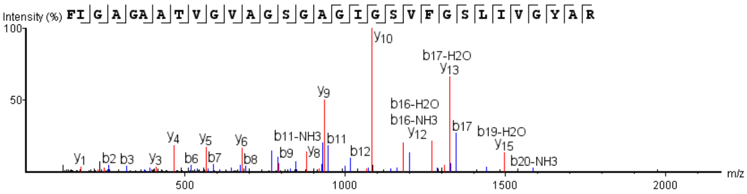

gi343790874

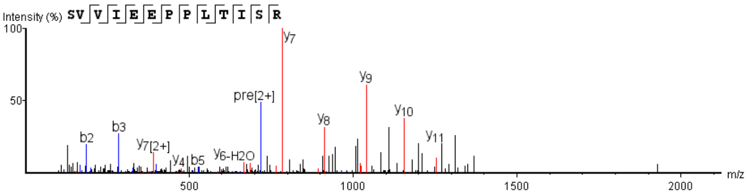

gi571499378

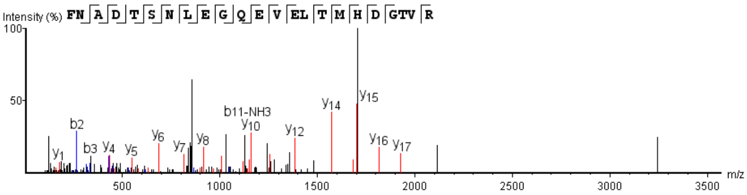

gi571499384

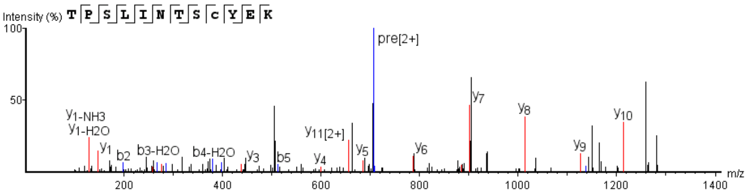

gi|328779522

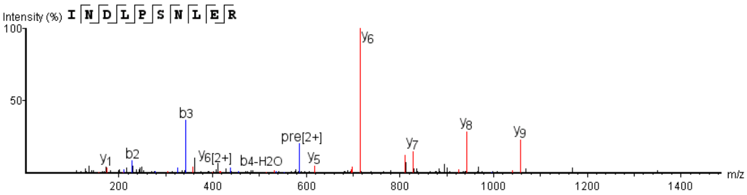

gi571500503

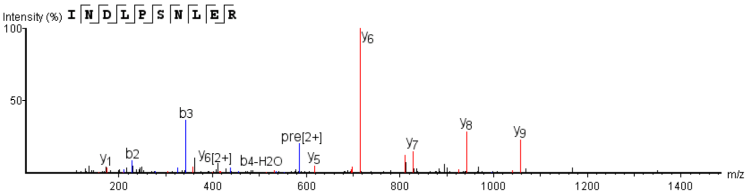

gi 571500507

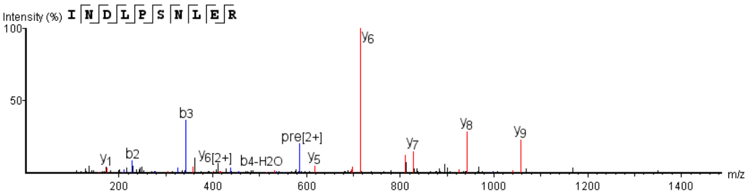

gi571500512

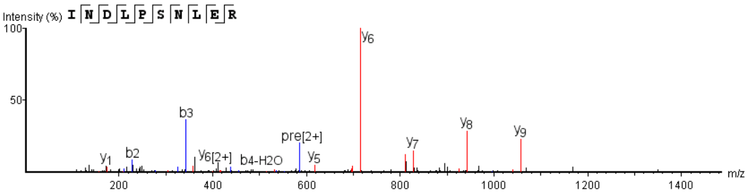

gi571501059

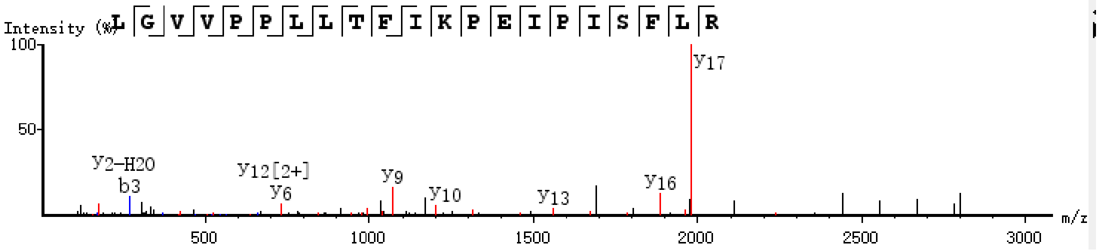

gi571501620

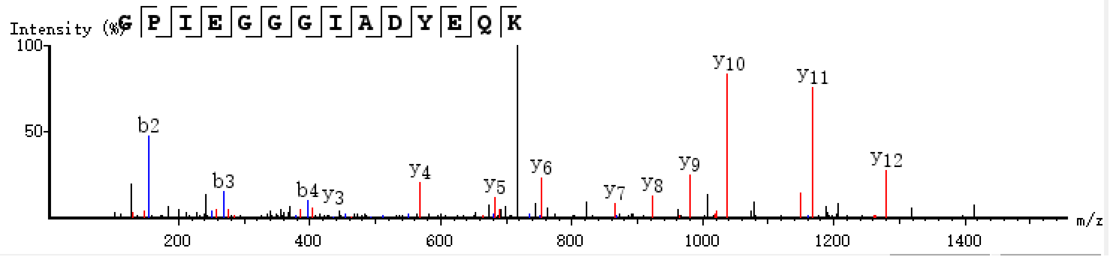

gi571501696

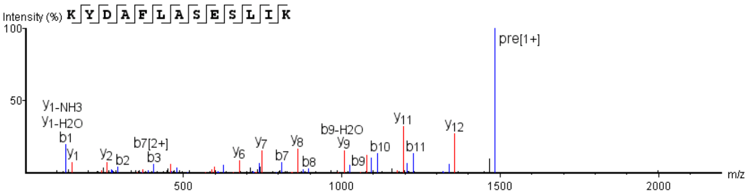

gi571502990

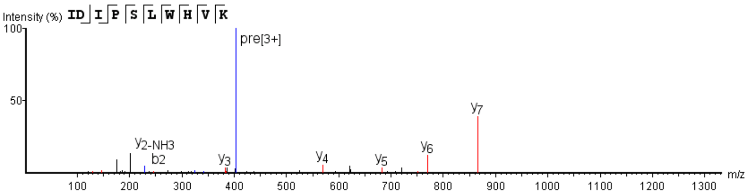

gi571503152

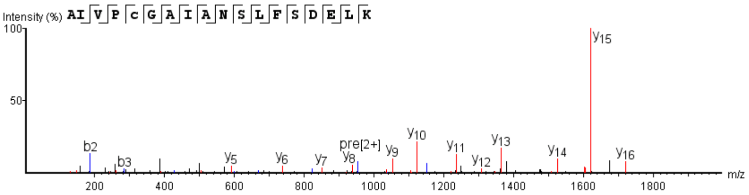

gi571503331

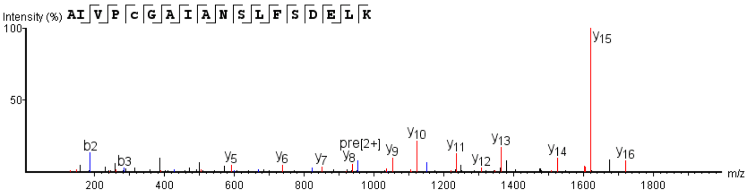

gi571503334

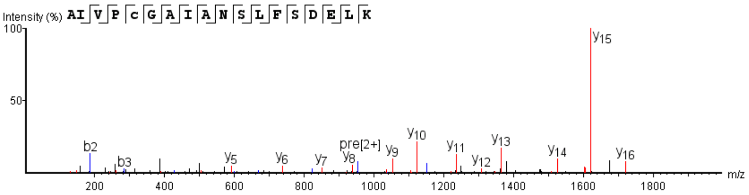

gi|66544564

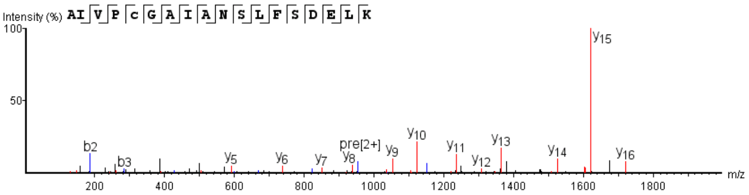

gi571503337

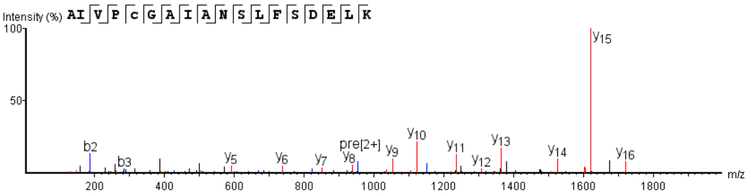

gi571503340

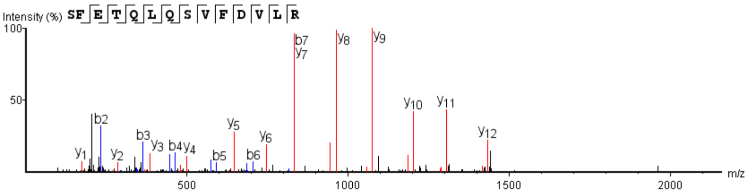

gi571503655

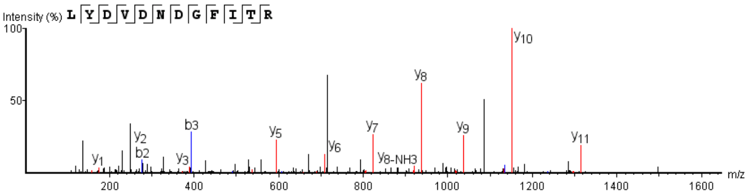

gi571503659

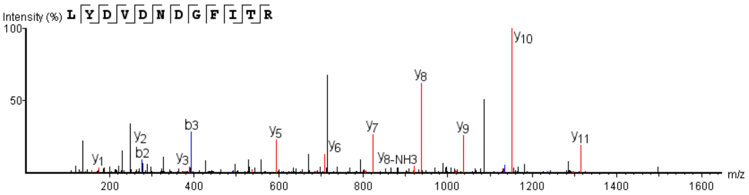

gi571503749

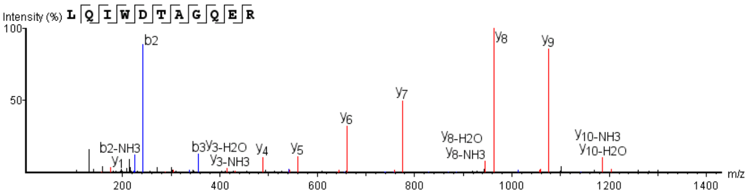

gi571503850

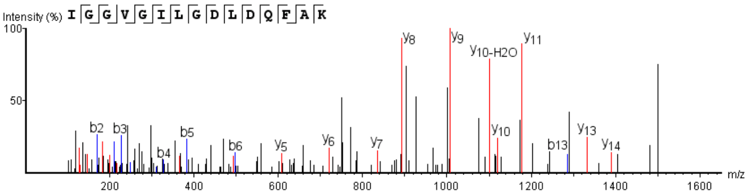

gi571503986

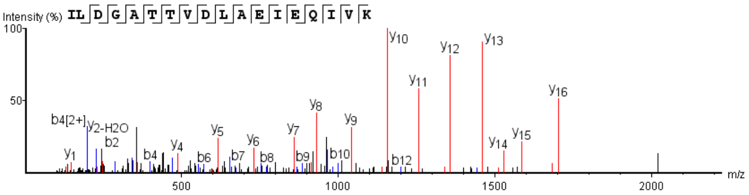

gi571503989

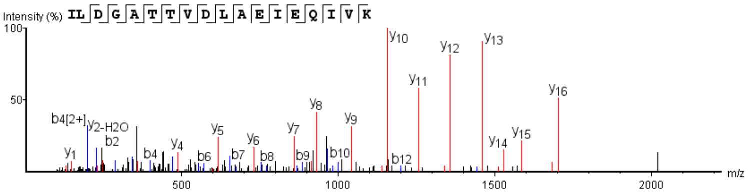

gi571503993

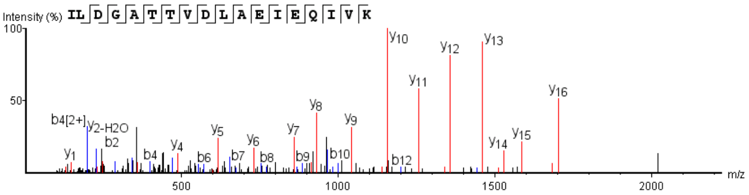

gi571503997

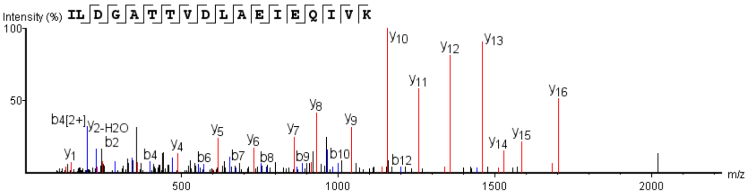

gi571504000

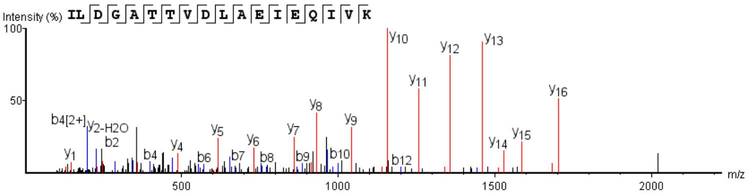

gi571504003

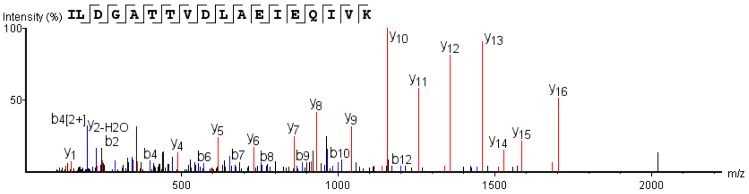

gi571504006

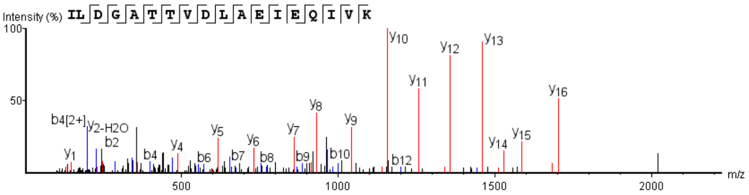

gi571504009

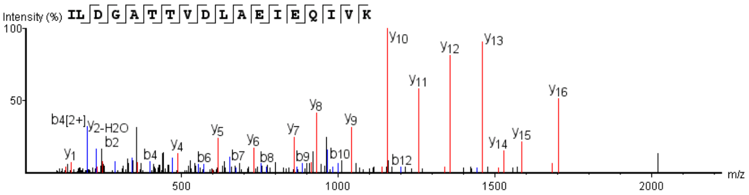

gi|328790379

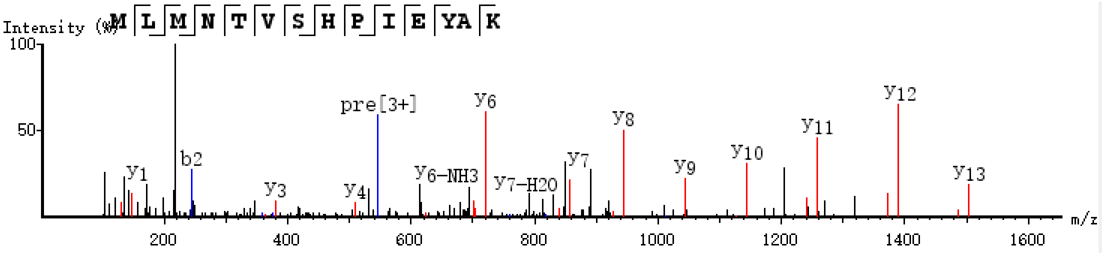

gi|571504099

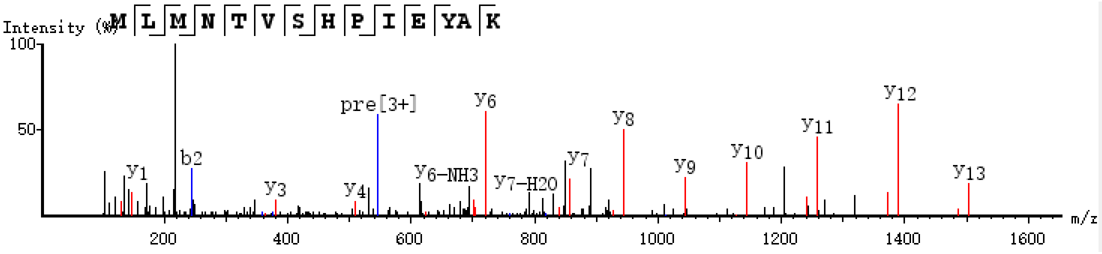

gi571504104

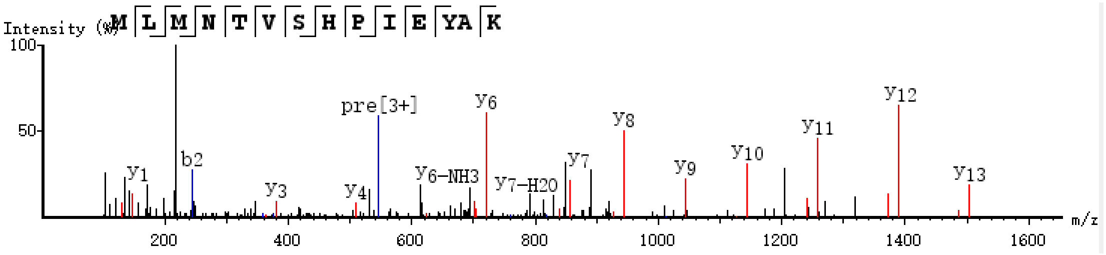

gi|66542091

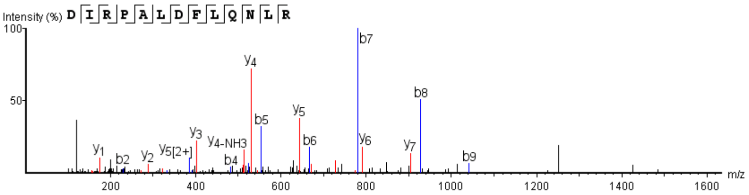

gi571504286

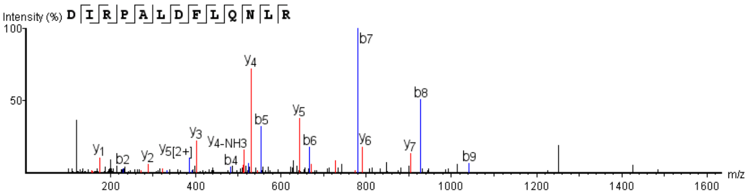

gi571504293

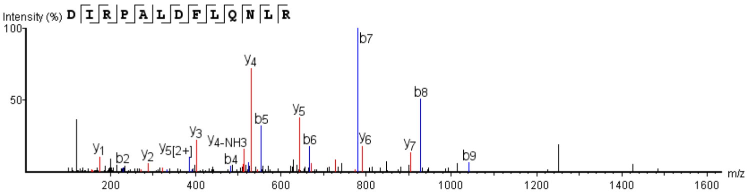

gi571504295

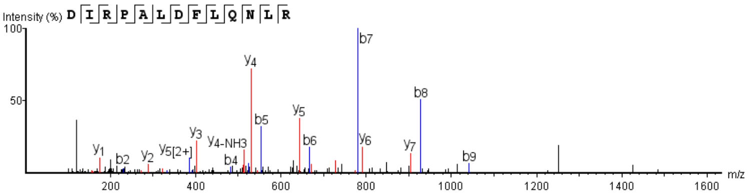

gi571504299

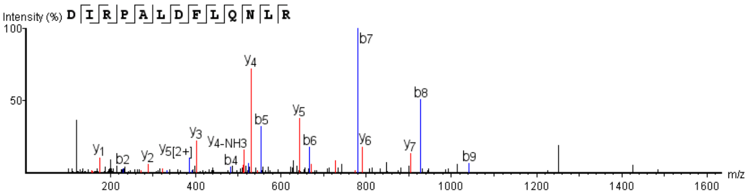

gi571505277

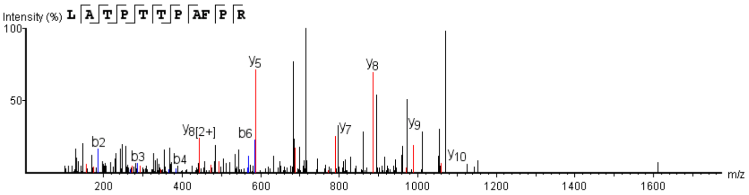

gi571505803

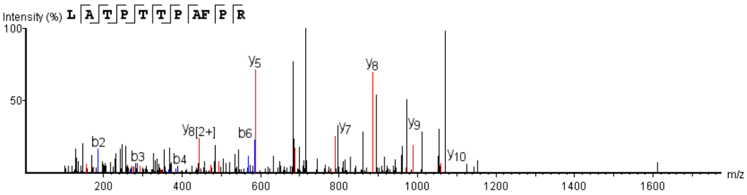

gi|571505865

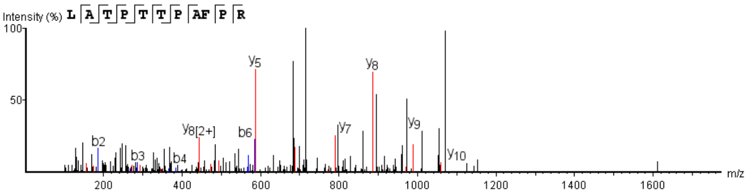

gi571506170

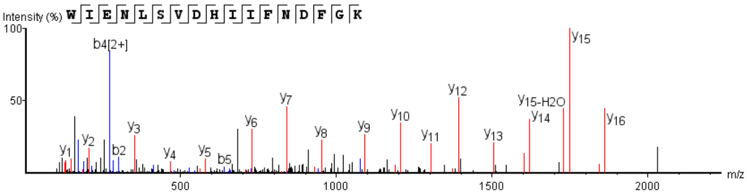

gi571506973

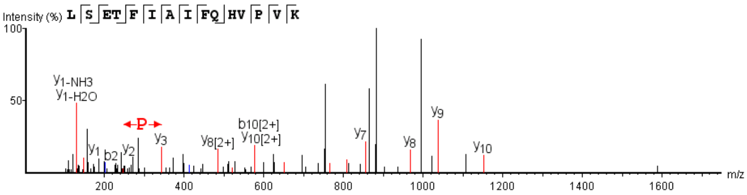

gi571506977

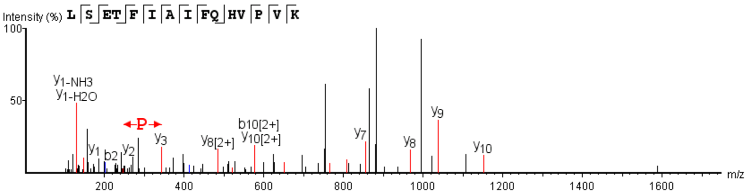

gi571507129

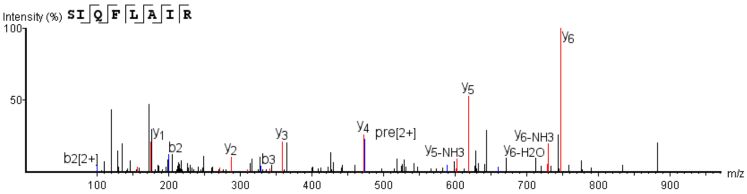

gi|328792171

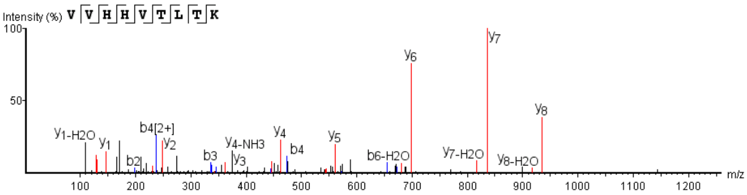

gi571507133

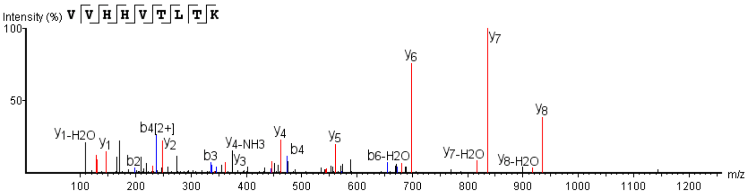

gi571507387

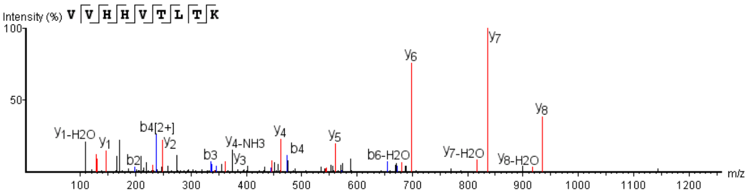

gi571507392

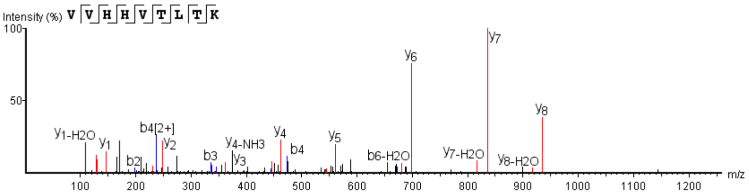

gi571507395

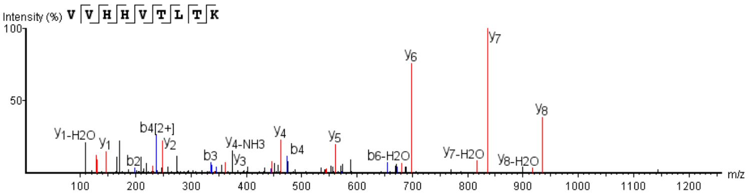

gi571507399

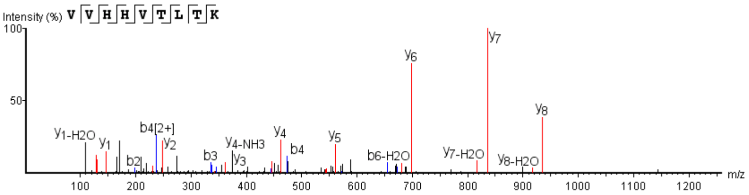

gi571507402

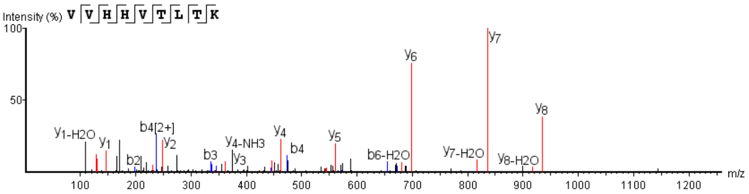

gi571507406

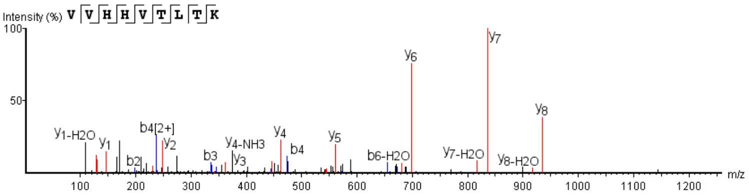

gi571507410

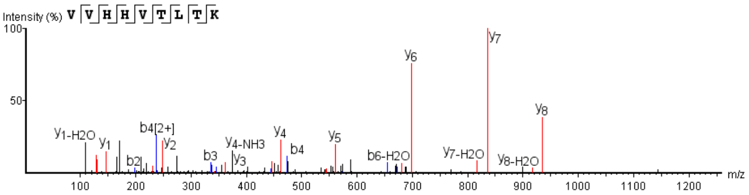

gi571507413

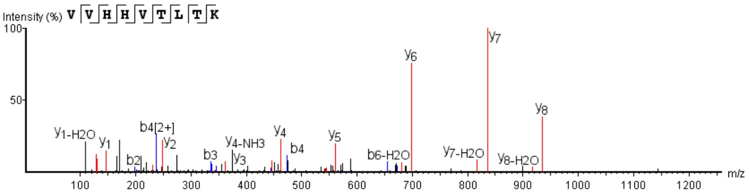

gi571507416

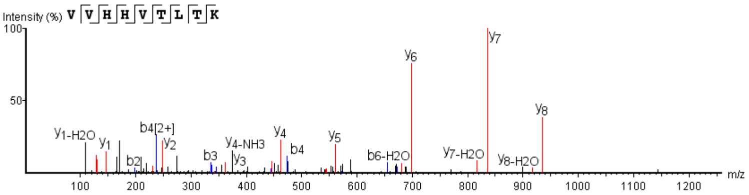

gi571507420

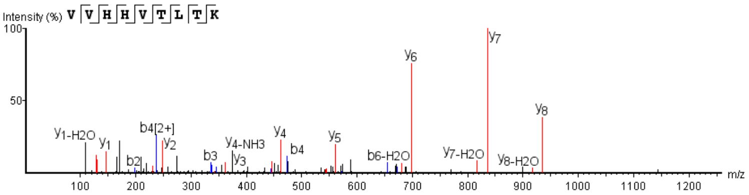

gi571507424

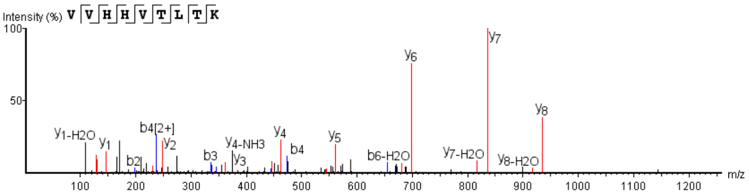

gi571507427

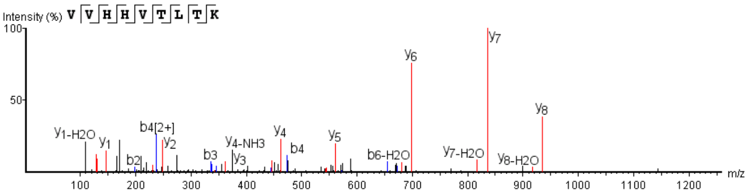

gi571507430

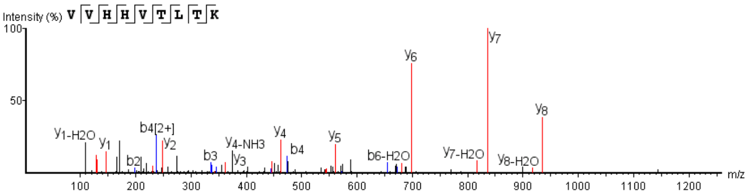

gi571507434

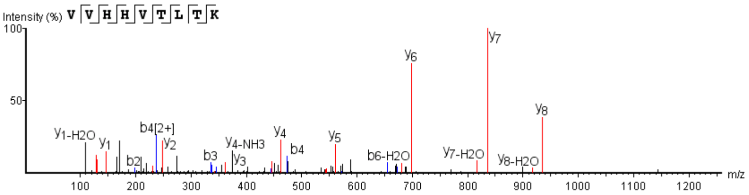

gi571508050

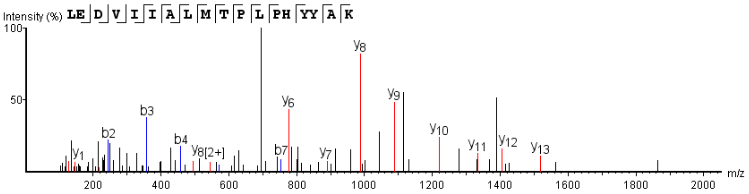

gi571508183

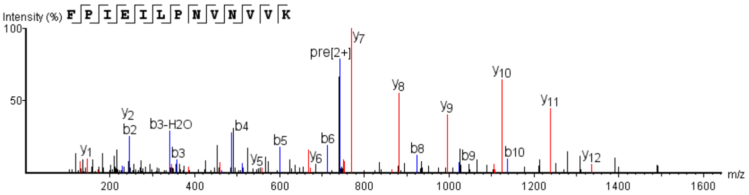

gi571508187

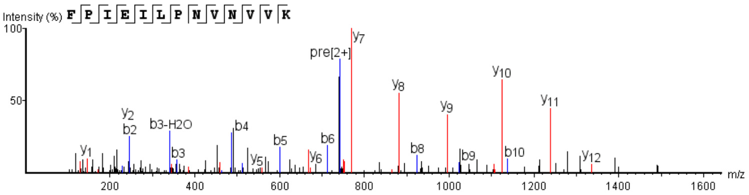

gi571508425

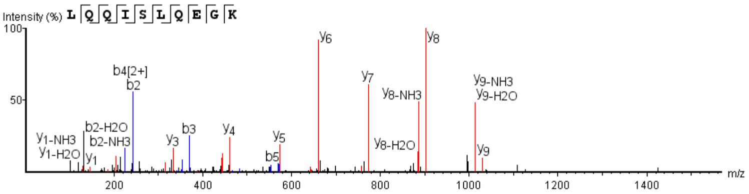

gi571508429

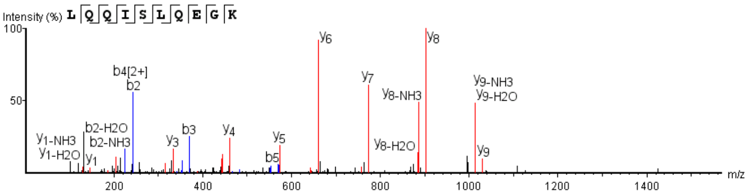

gi571508435

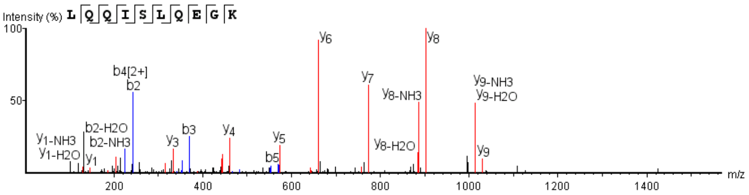

gi571508695

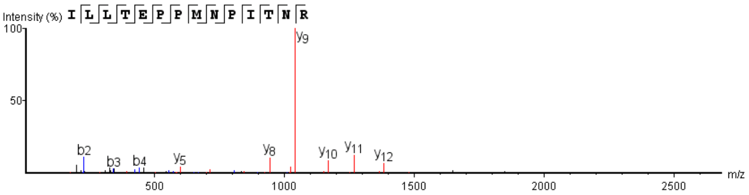

gi571508747

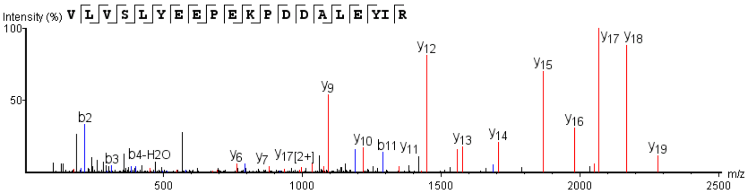

gi571509803

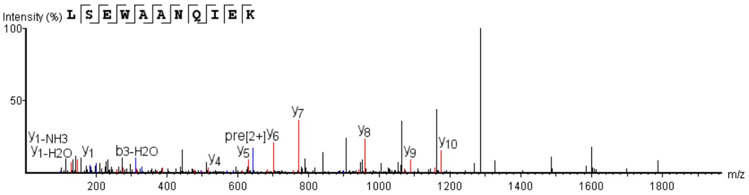

gigi571510158

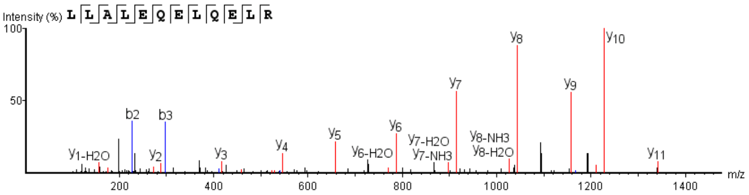

gi|110767181

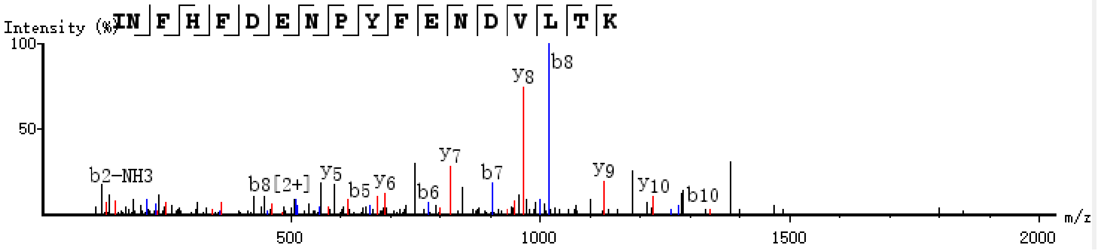

gi571510978

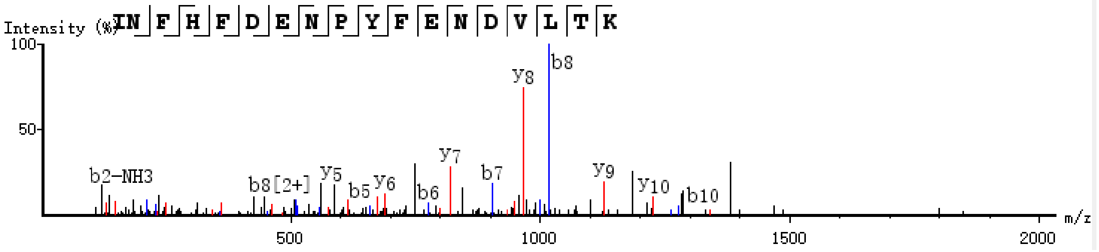

gi571511040

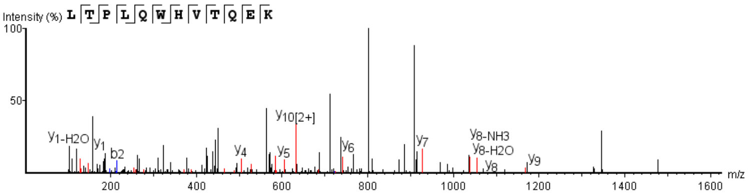

gi571511327

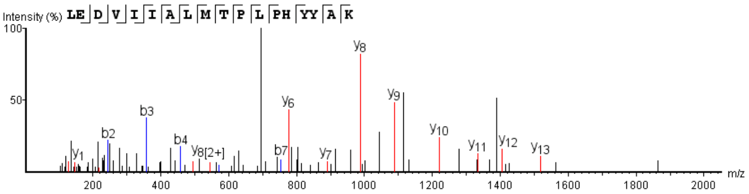

gi571512347

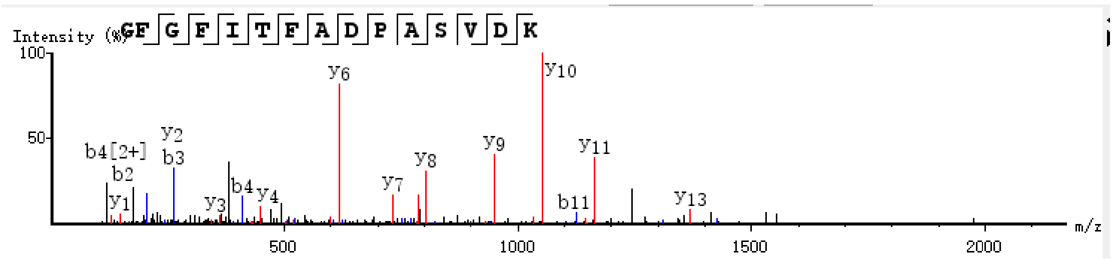

gi571513237

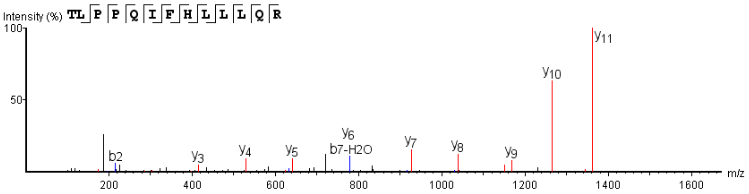

gi|48105316

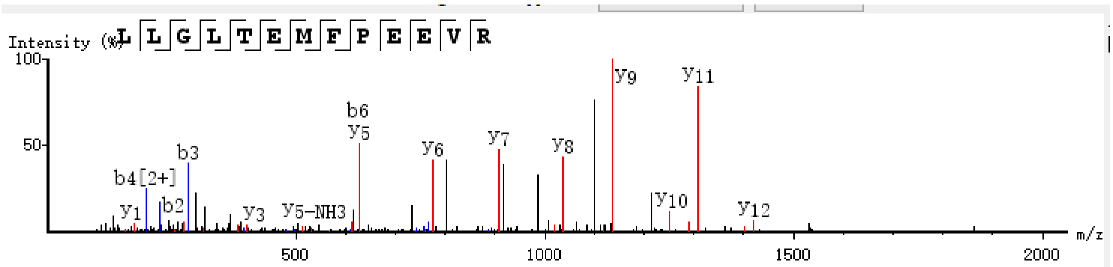

gi571514456

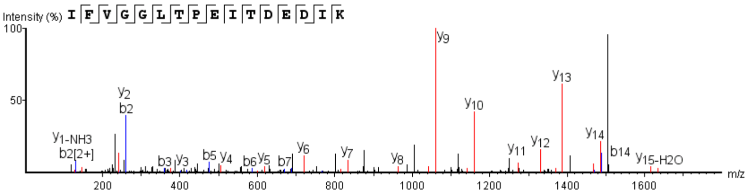

gi|571515763

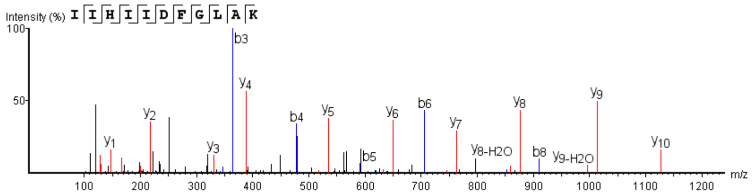

gi571515726

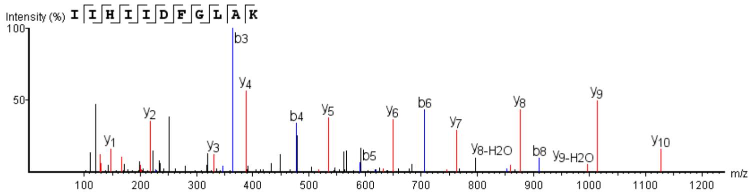

gi571515732

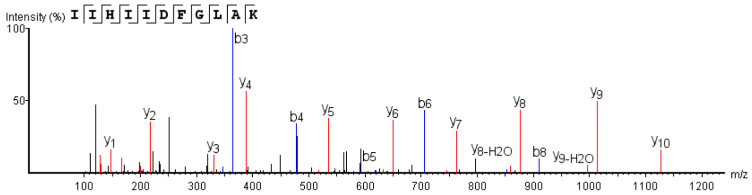

gi571515736

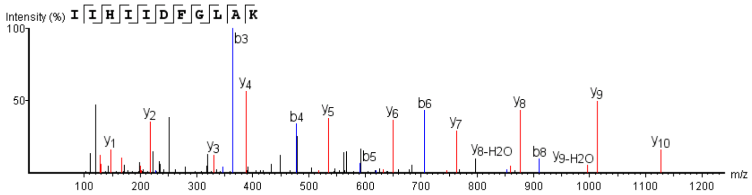

gi571515740

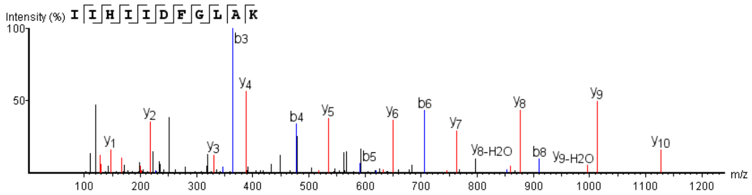

gi571515743

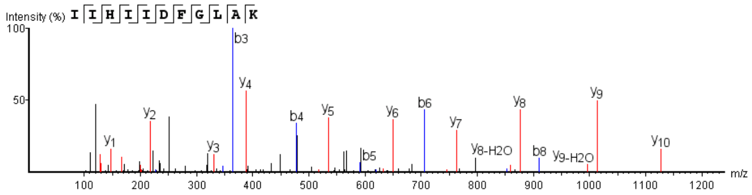

gi571515747

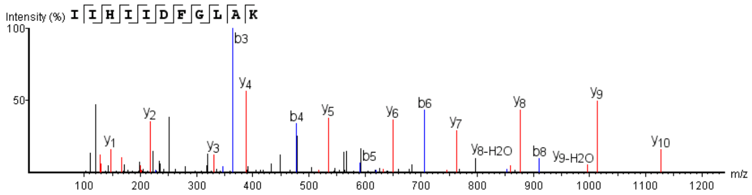

gi571515751

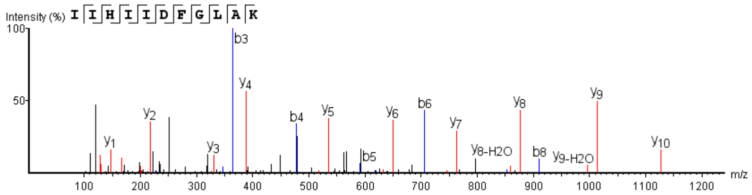

gi571515755

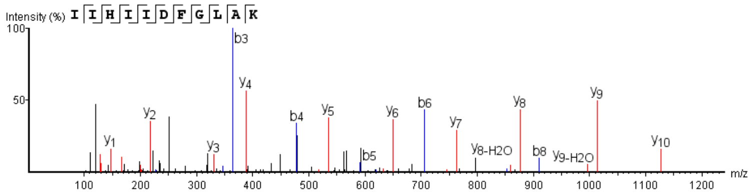

gi571515759

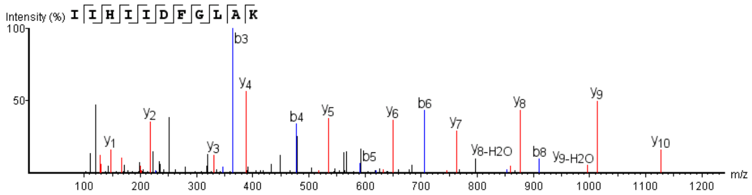

gi571516333

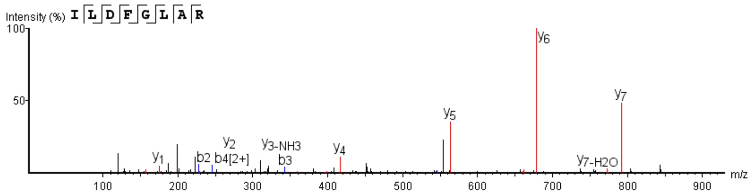

gi571516301

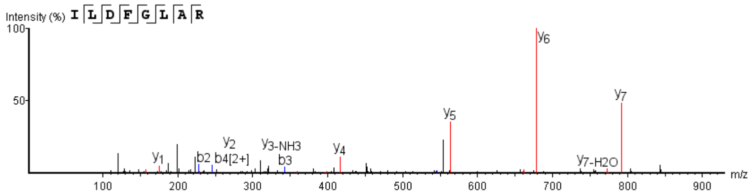

gi571516304

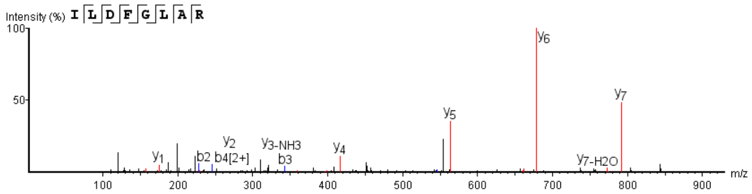

gi571516307

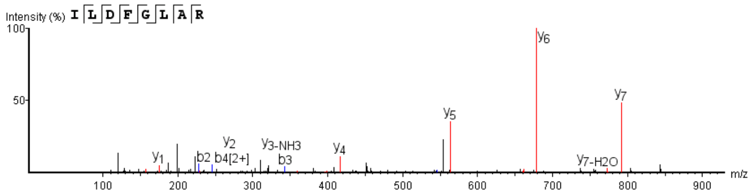

gi571516310

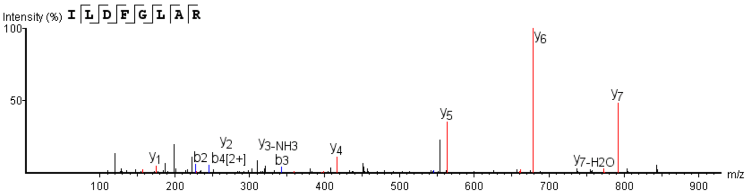

gi571516313

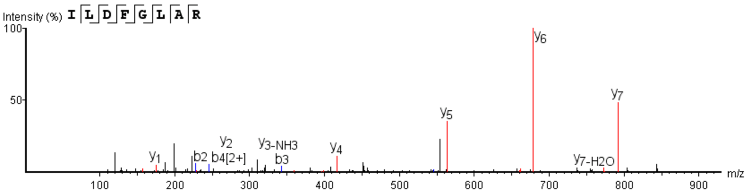

gi571516317

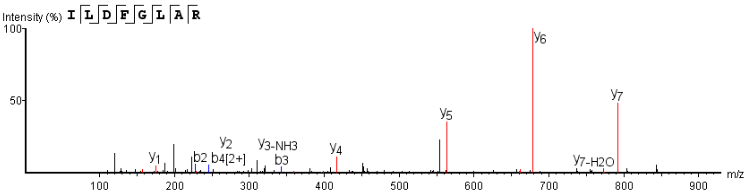

gi571516320

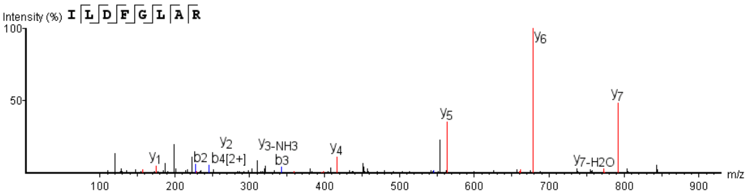

gi571516326

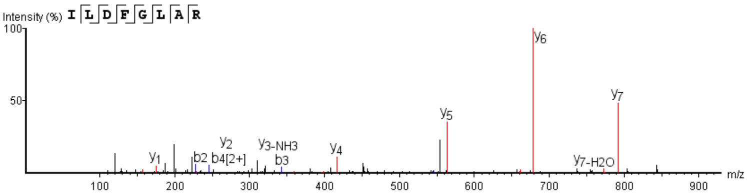

gi571516330

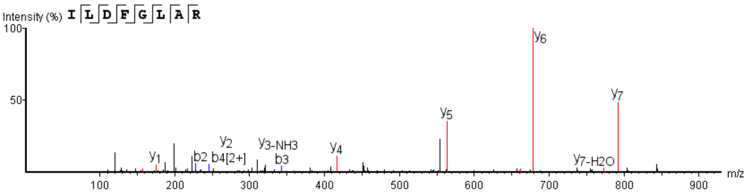

gi571517007

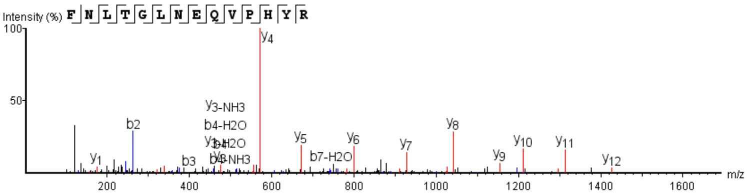

gi571517011

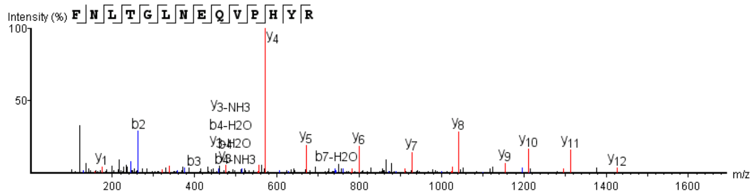

gi571517016

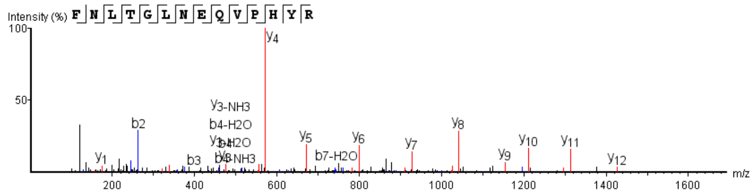

gi|328777239

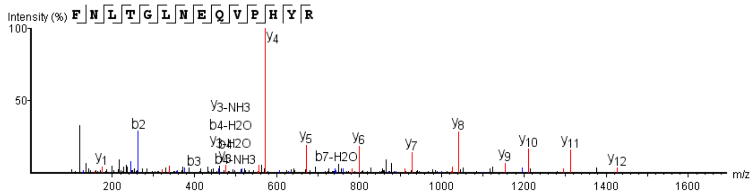

gi571517101

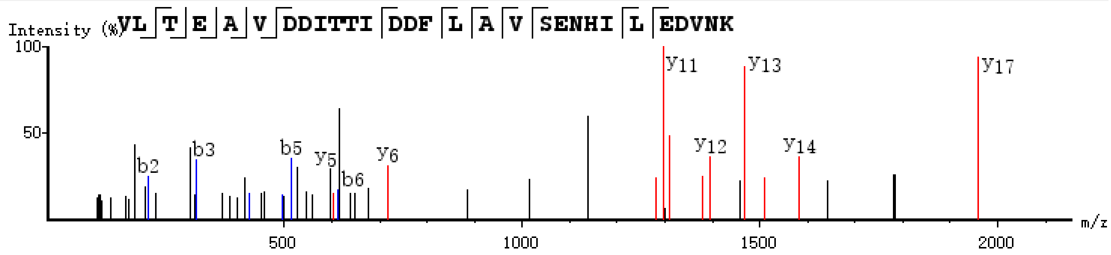

gi571517115

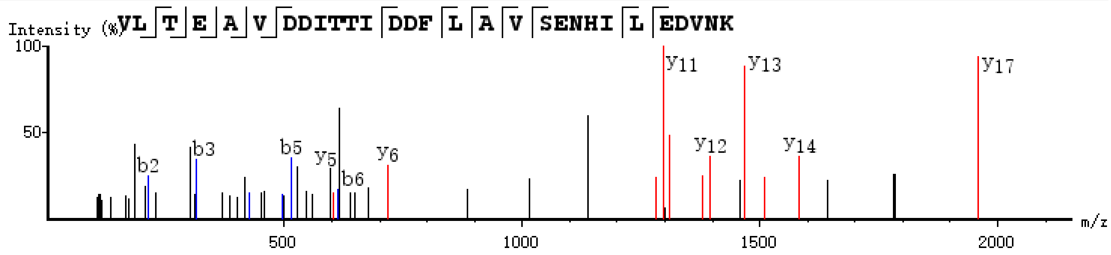

gi571517543

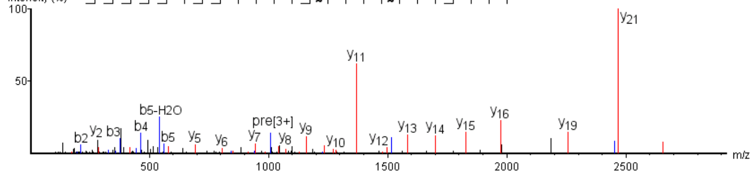

gi|328778864

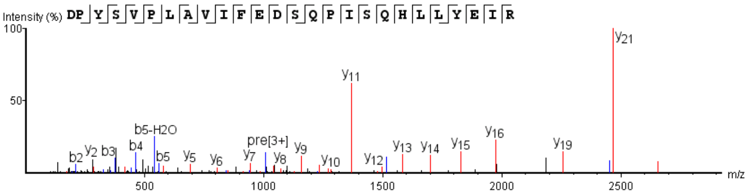

gi571518466

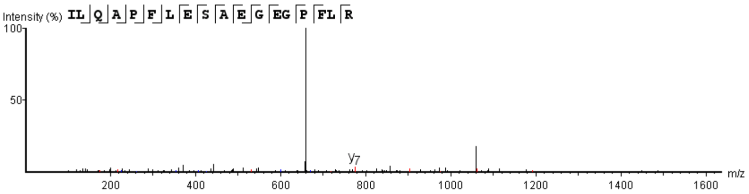

gi571521158

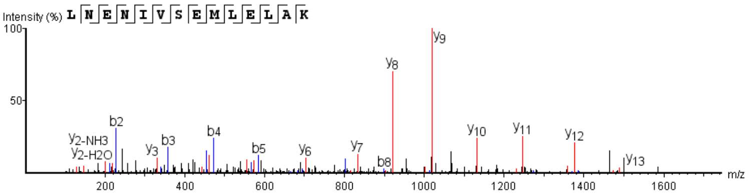

gi571521696

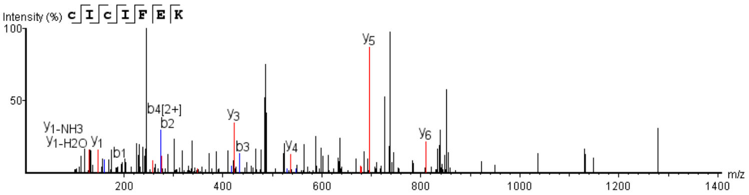

gi571522078

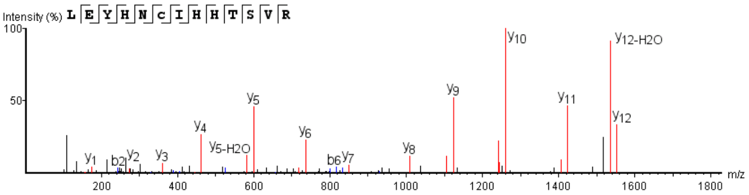

gi571522669

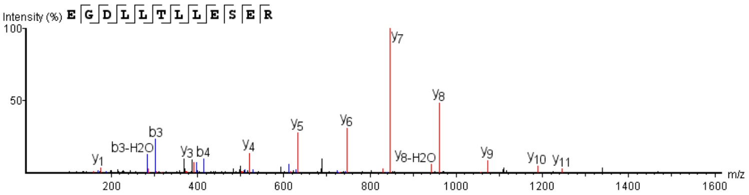

gi571522855

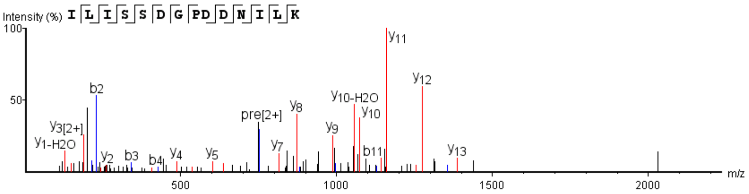

gi571522976

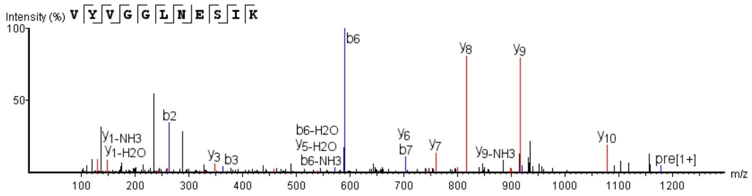

gi571523648

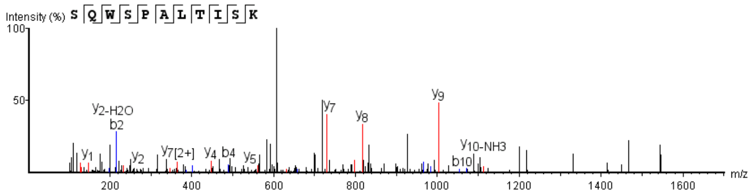

gi571523949

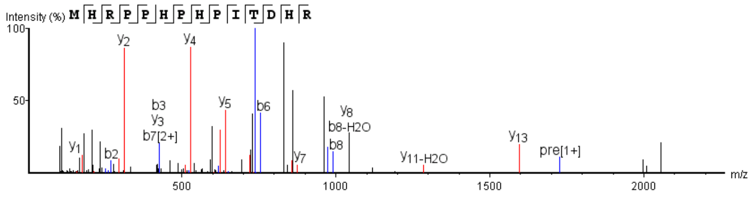

gi571523953

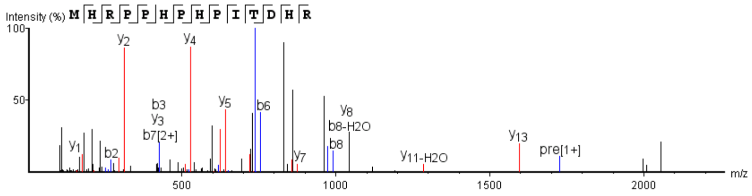

gi571523957

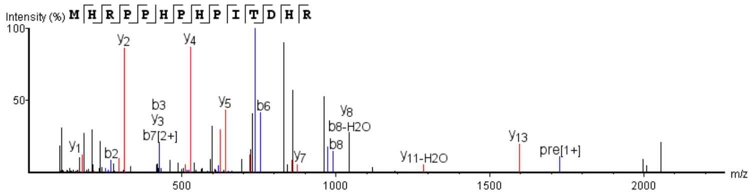

gi571523961

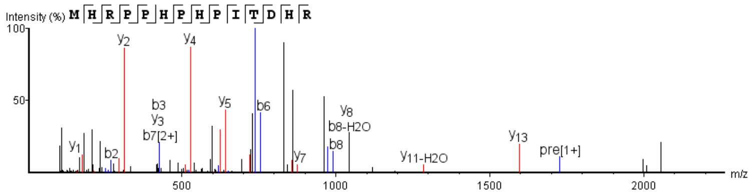

gi571523965

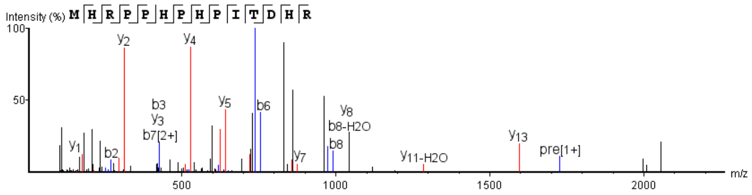

gi571523968

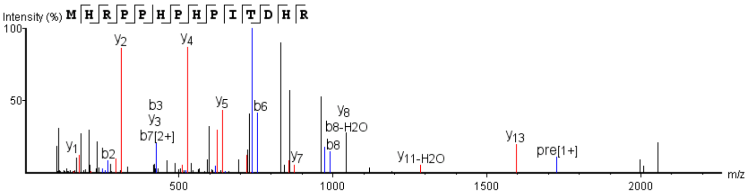

gi571523972

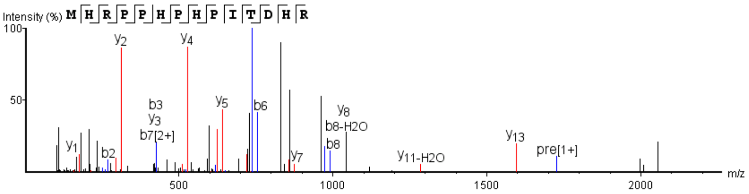

gi571523976

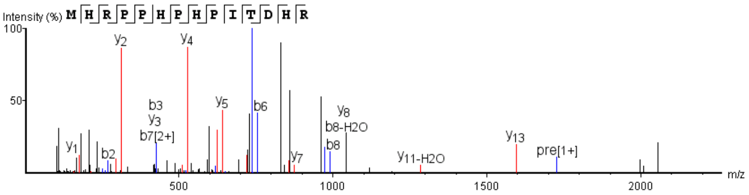

gi571523978

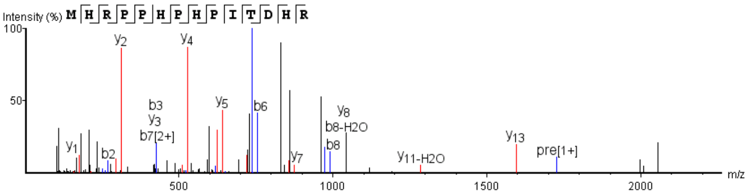

gi571523984

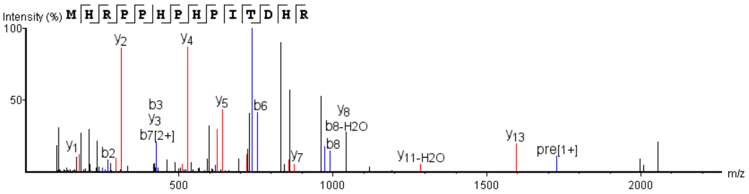

gi571524209

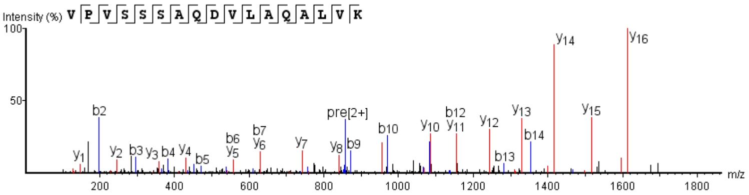

gi571524250

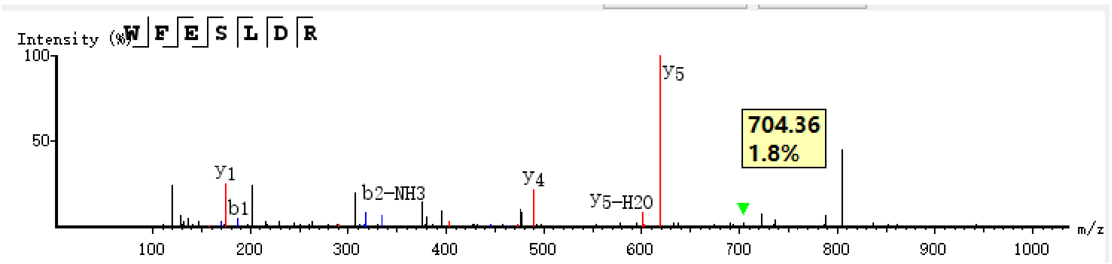

gi571524852

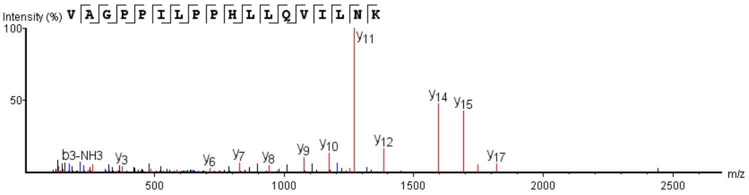

gi|571524852

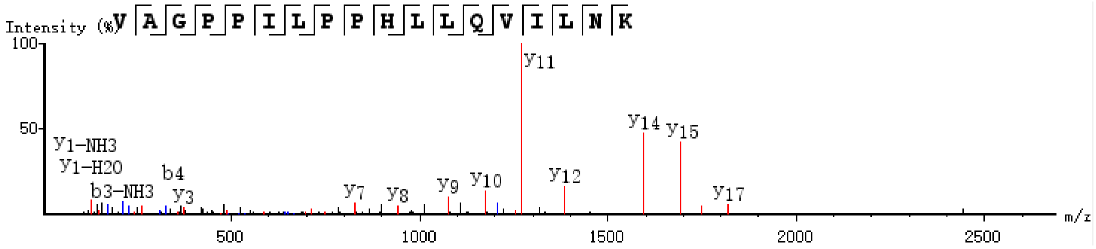

gi|571524858

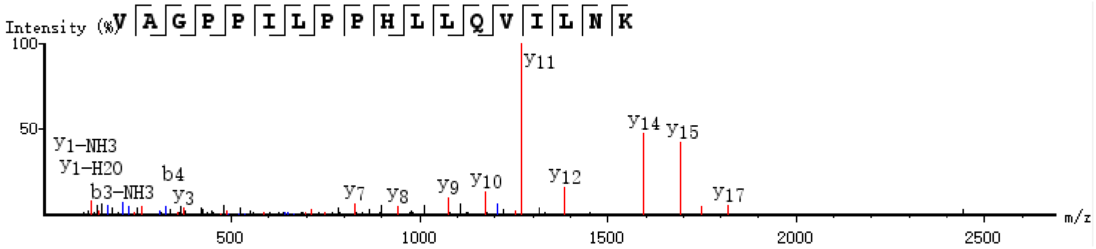

gi|48113496

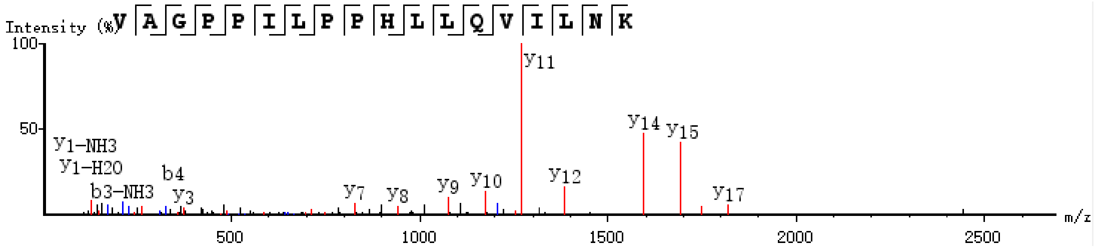

gi571524916

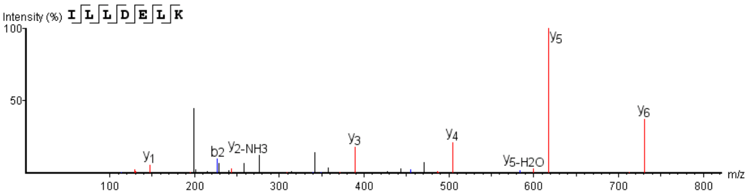

gi571525189

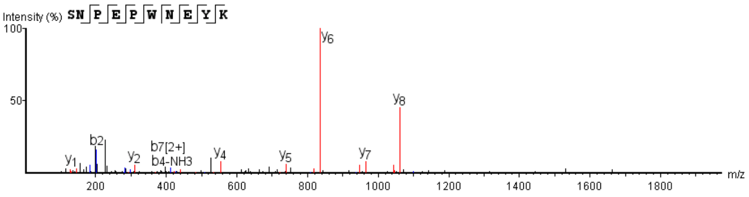

gi|48102172

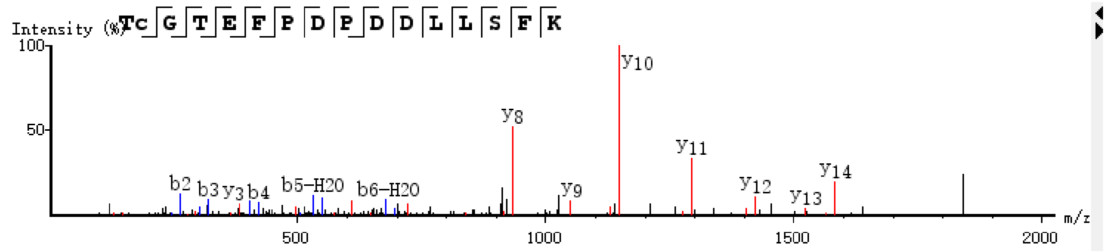

gi571527109

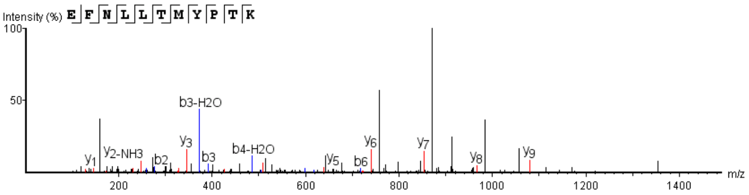

gi|571513678

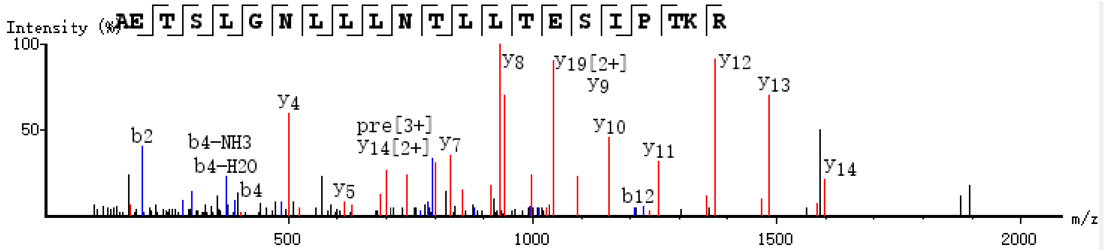

gi|571513682

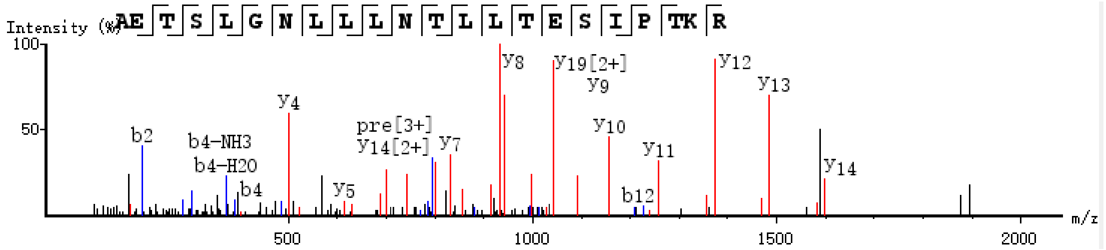

gi|571513674

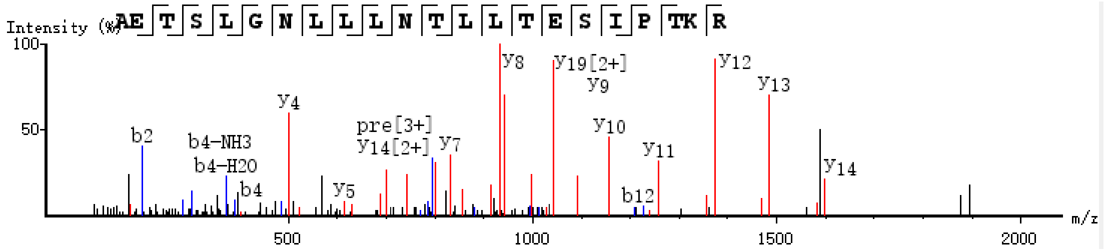

gi571527843

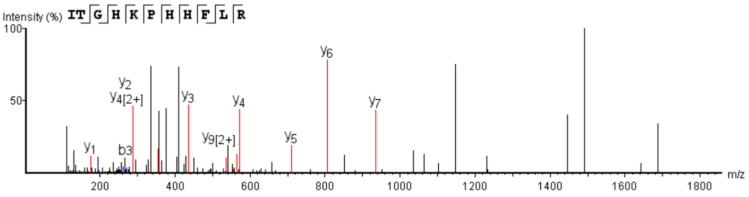

gi571528227

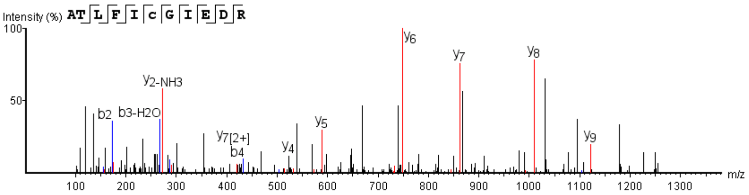

gi|571528291

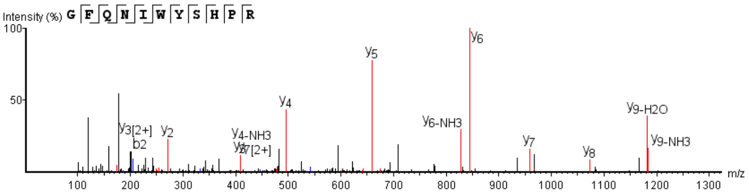

gi571528288

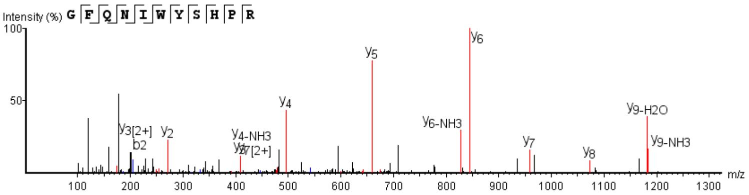

gi571528797

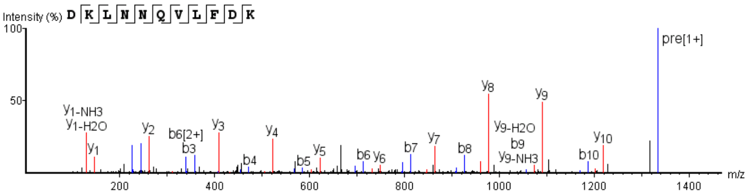

gi571529116

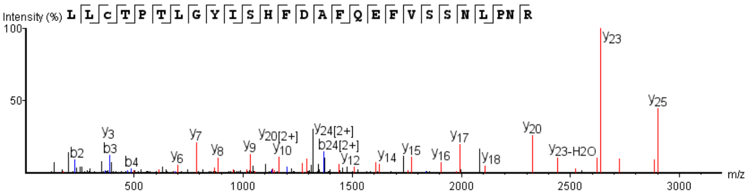

gi571530529

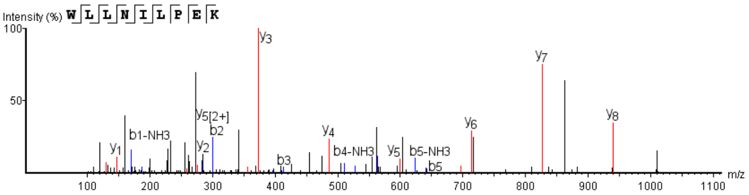

gi571530533

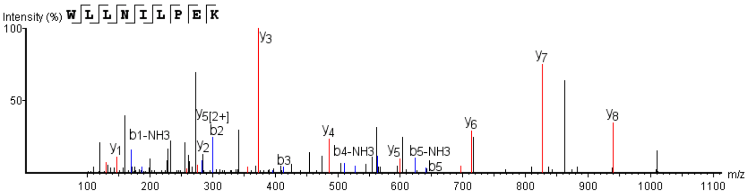

gi571530959

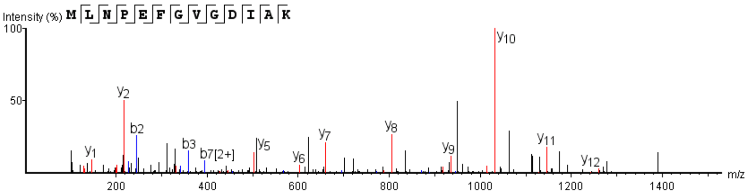

gi571532244

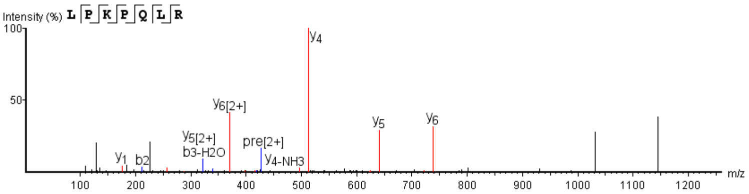

gi|571532252

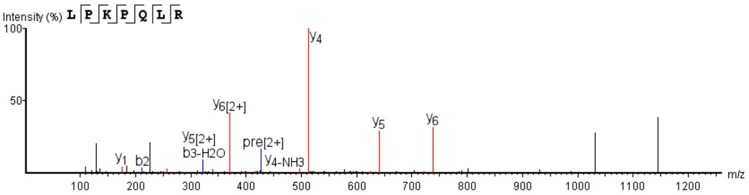

gi571532248

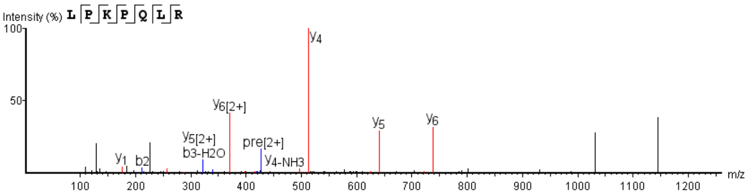

gi571532256

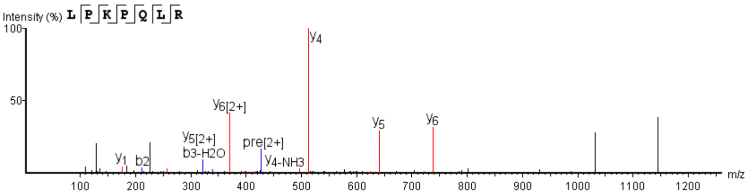

gi571532260

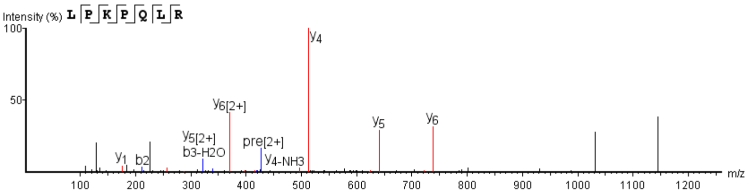

gi571532438

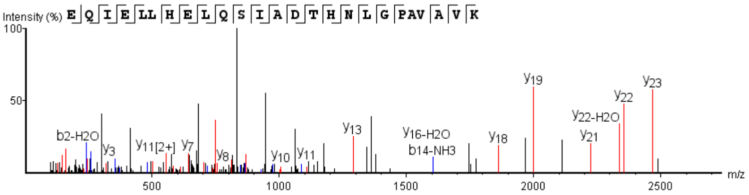

gi571532679

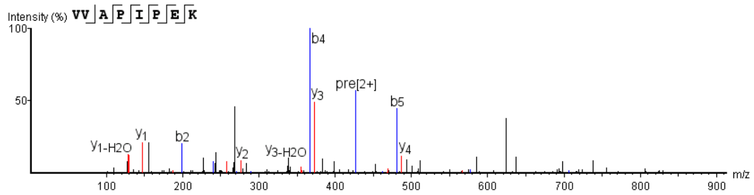

gi571532691

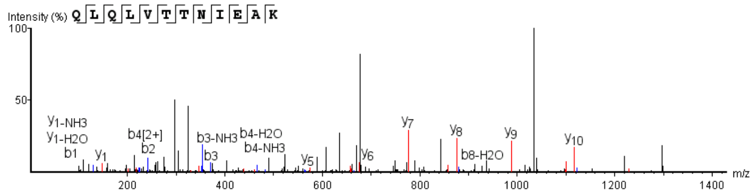

gi571533174

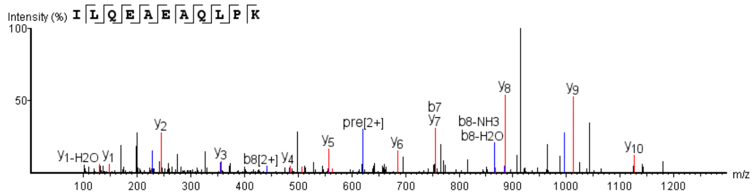

gi571533533

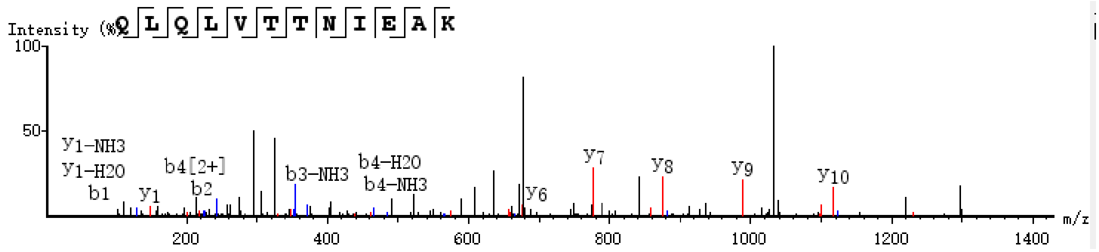

gi|571507709

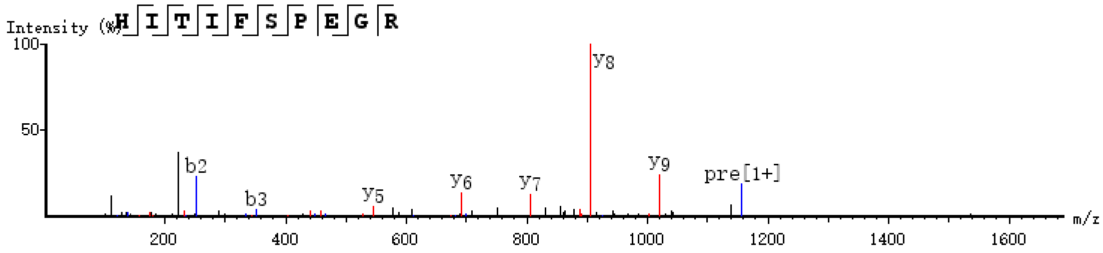

gi|571534127

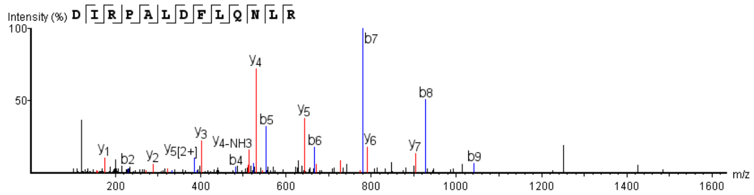

gi328789379

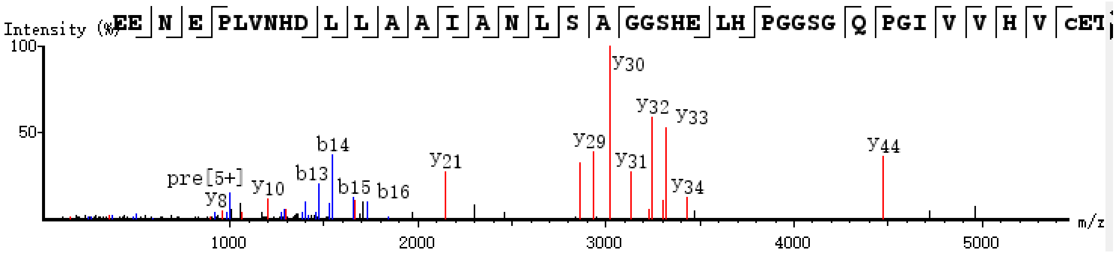

gi|571534130

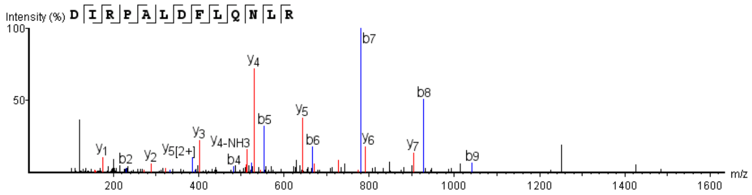

gi571534133

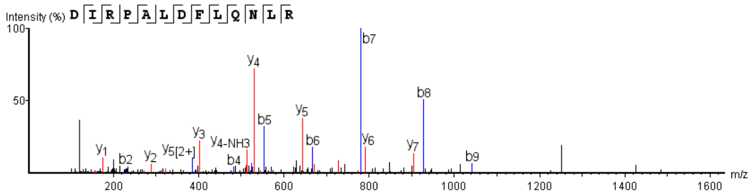

gi|328785653

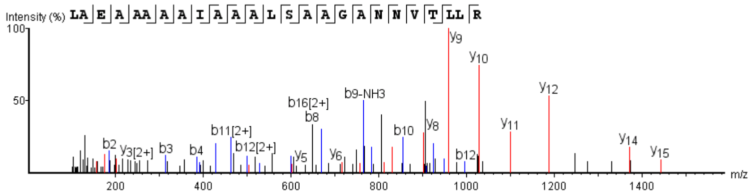

gi|571534940

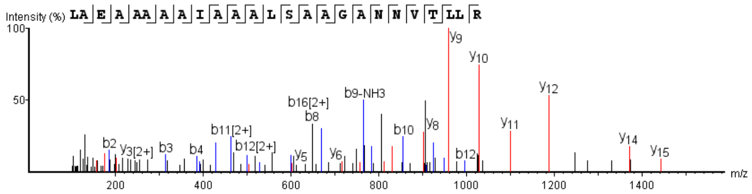

gi|571534943

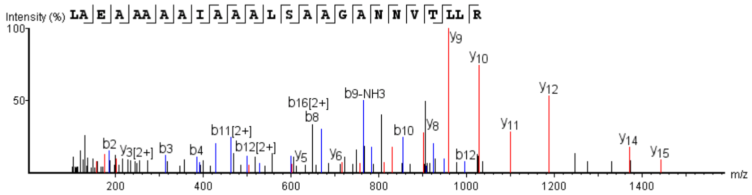

gi|571534947

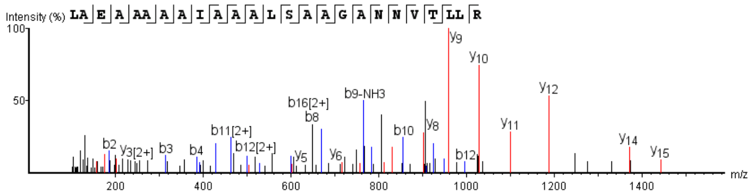

gi|57153495

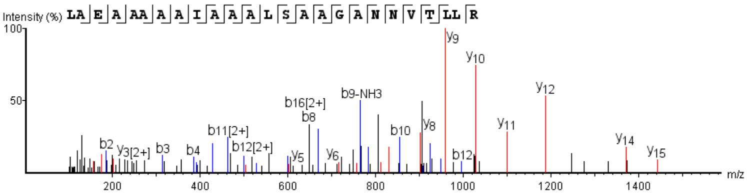

gi1571534954

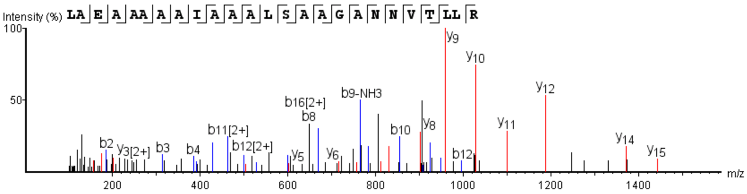

gi571534961

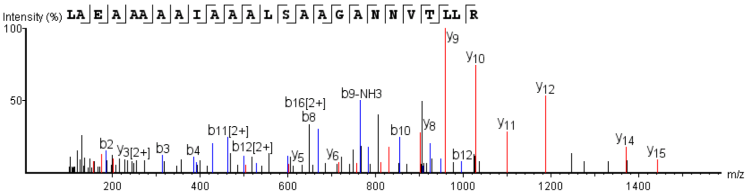

gi571535113

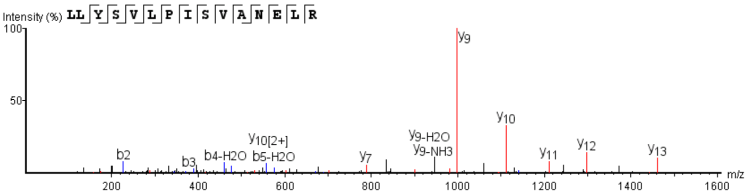

gi571561389

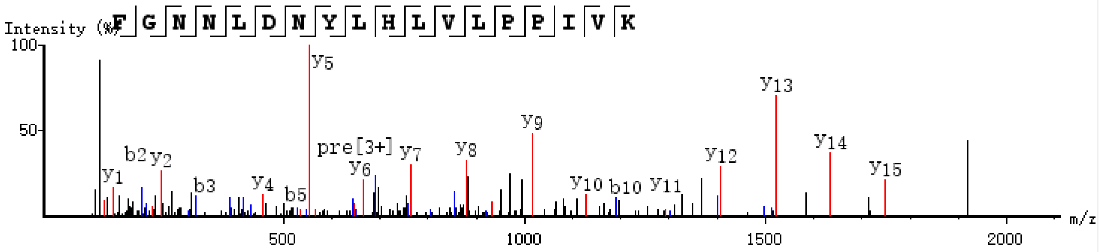

gi|328791460

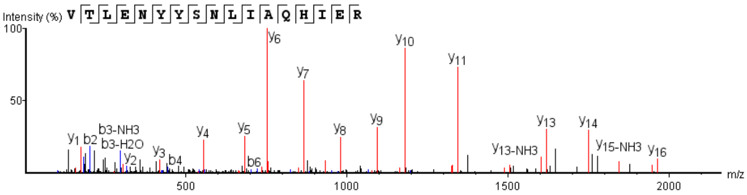

gi571535995

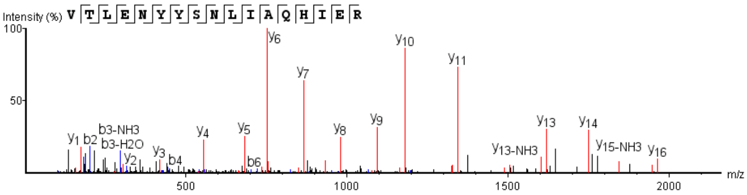

gi571536003

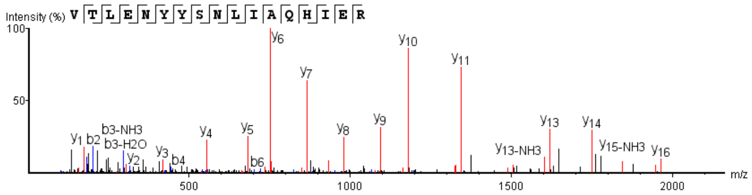

gi571536006

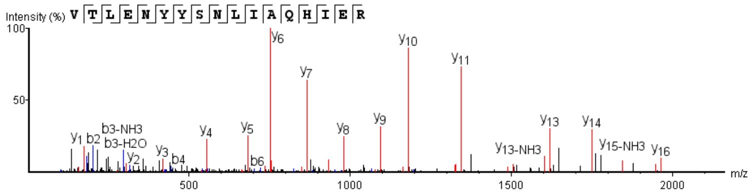

gi571536010

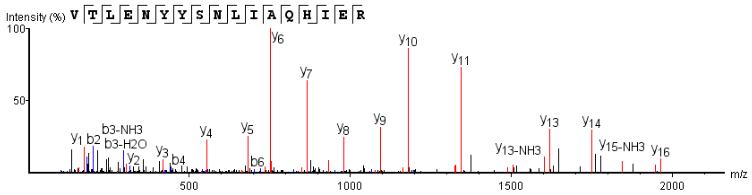

gi571536013

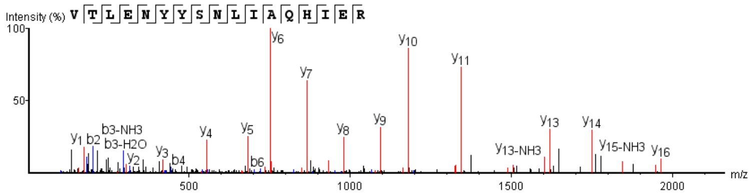

gi571536016

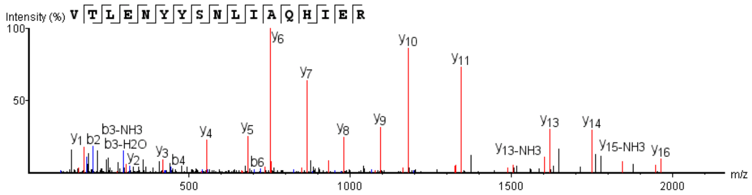

gi571536261

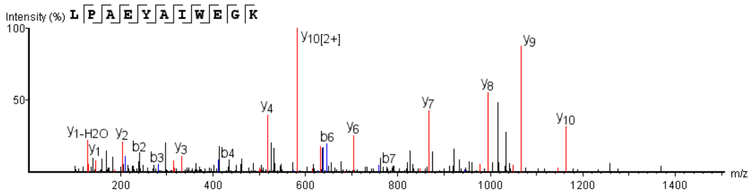

gi|571537408

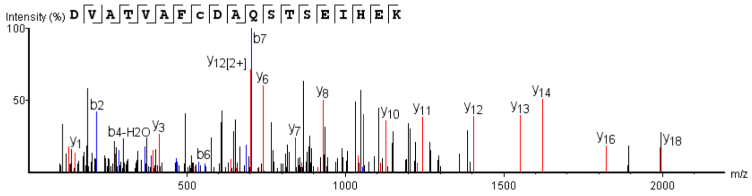

gi|328776329

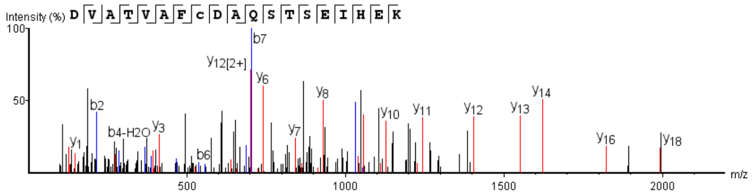

gi571537384

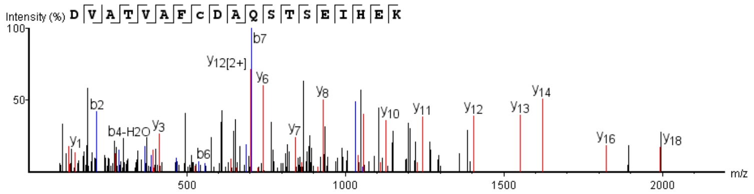

gi571537387

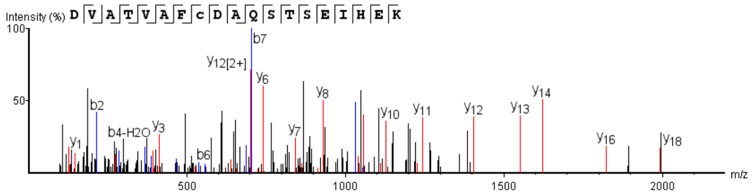

gi571537391

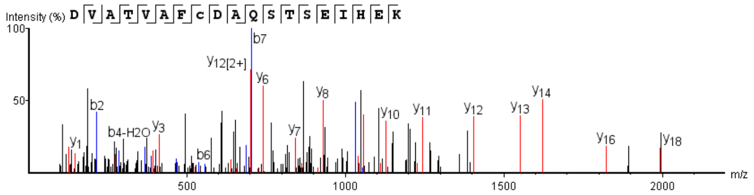

gi571537395

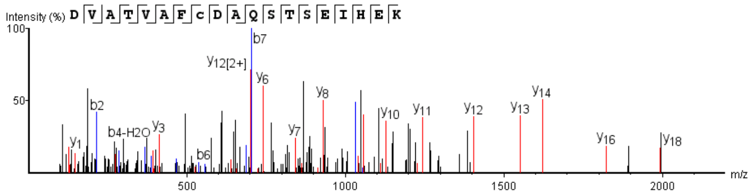

gi571537398

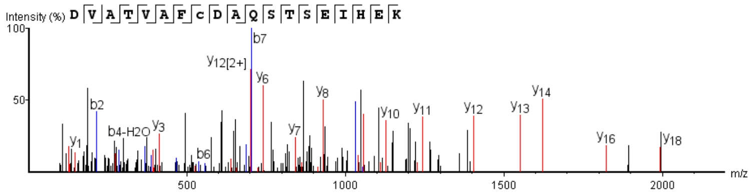

gi571537401

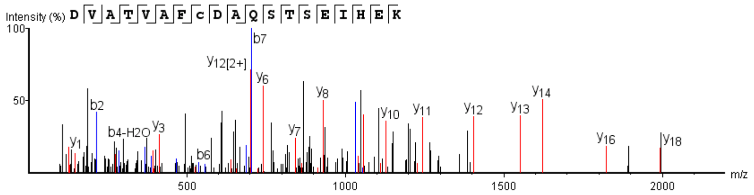

gi571537405

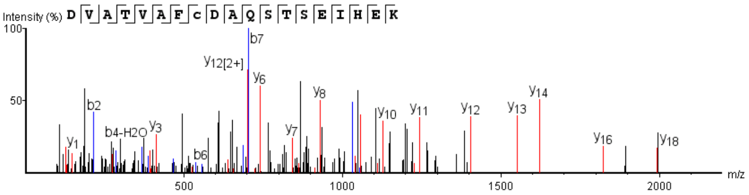

gi|110765112

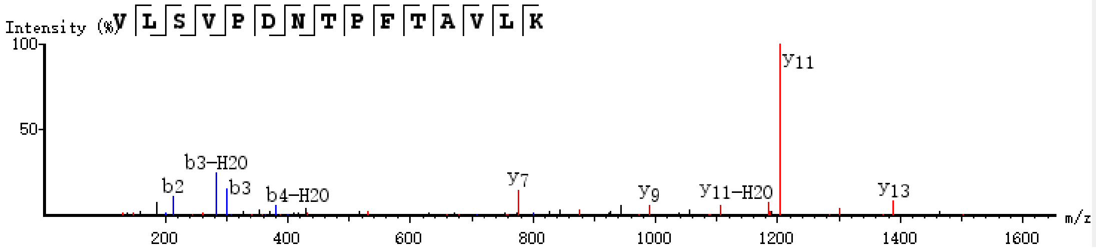

gi571538017

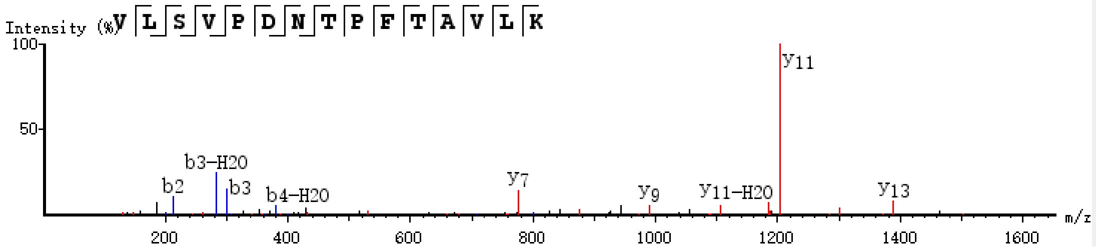

gi571538660

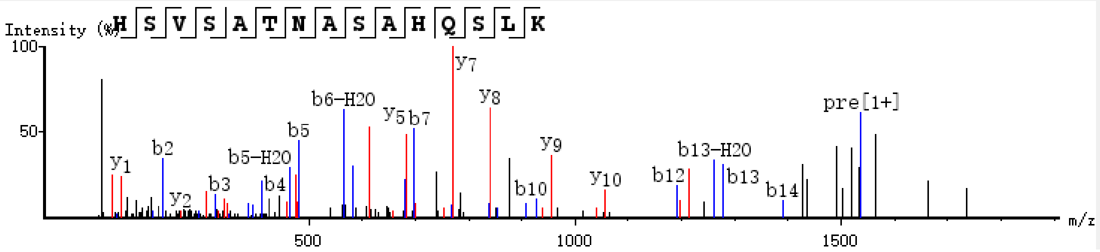

gi571547498

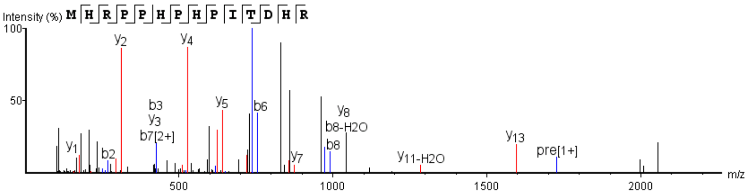

gi|328782357

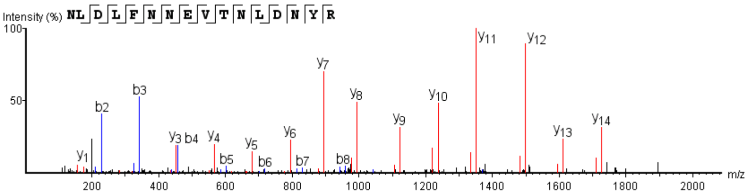

gi571539002

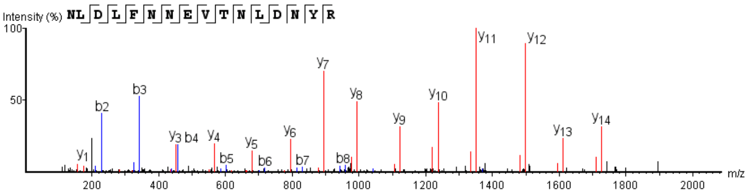

gi571539230

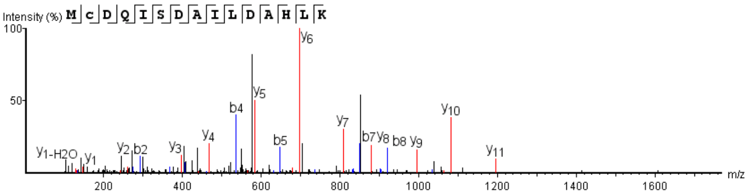

gi571539233

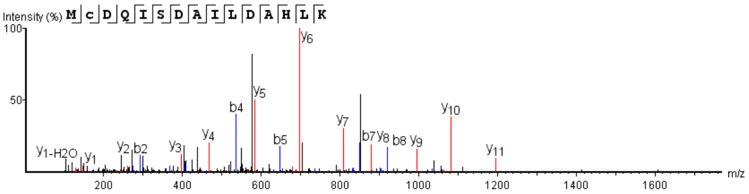

gi571540738

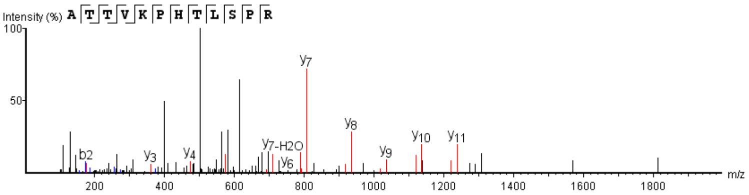

gi571540960

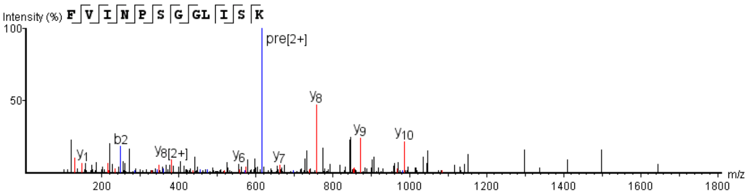

gi571540964

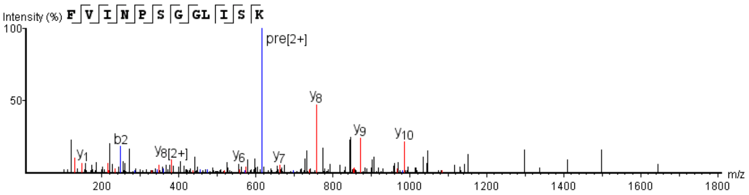

gi|110758613

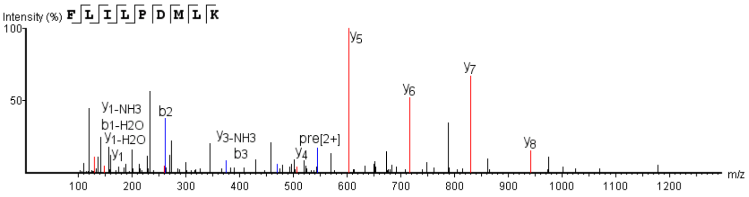

gi571542313

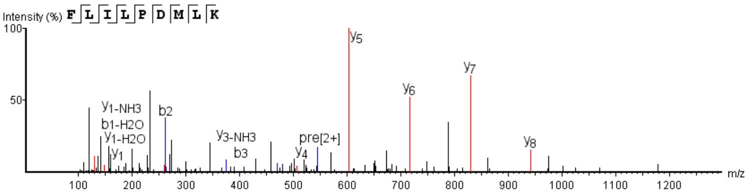

gi|571542631

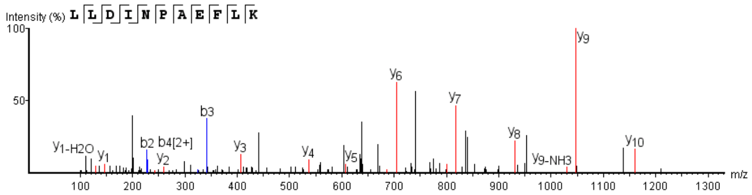

gi|571542645

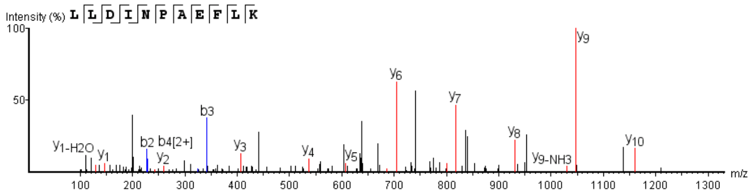

gi571542574

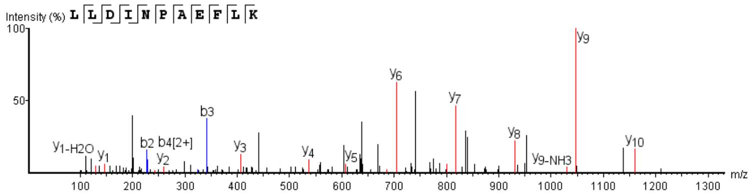

gi571542577

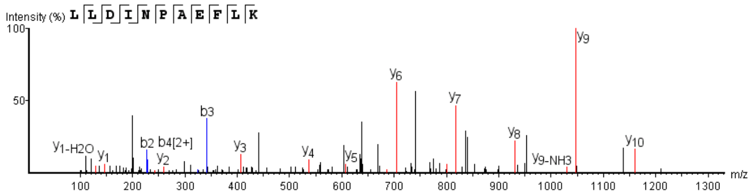

gi571542581

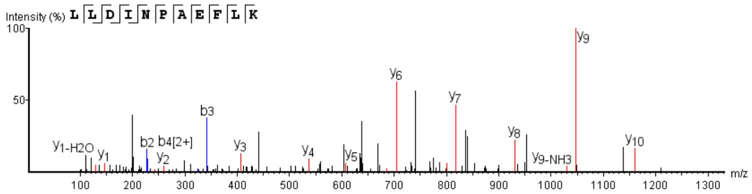

gi571542585

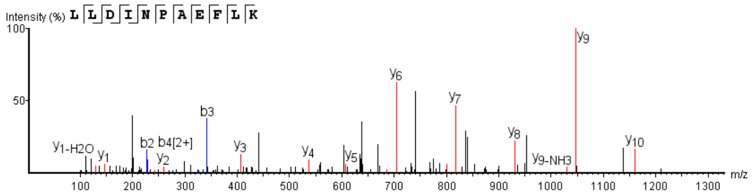

gi571542589

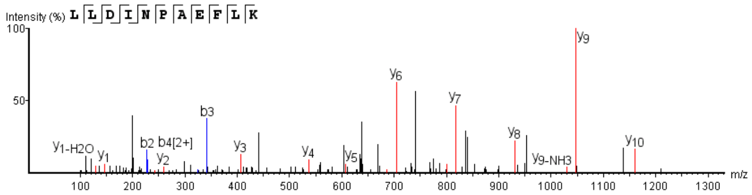

gi571542593

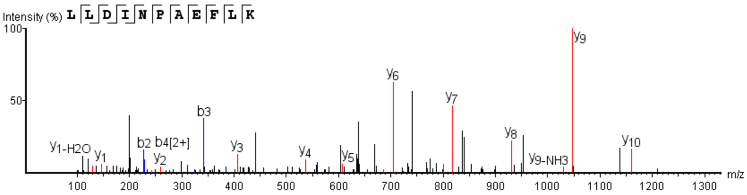

gi571542597

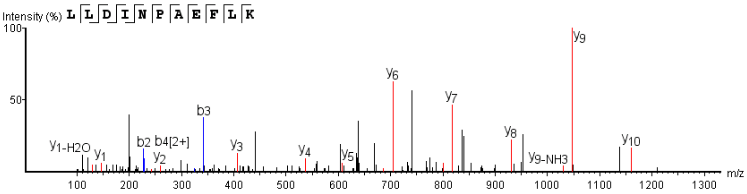

gi571542600

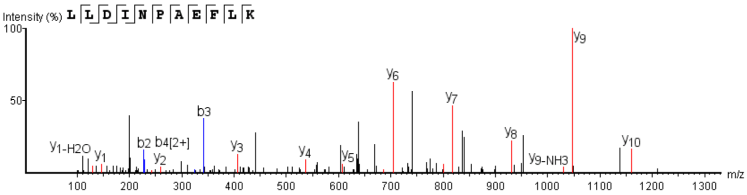

gi571542604

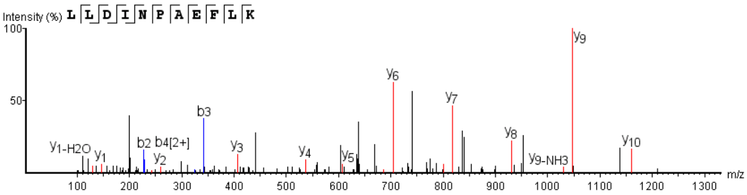

gi571542608

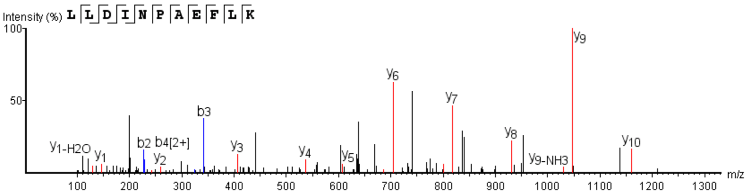

gi571542611

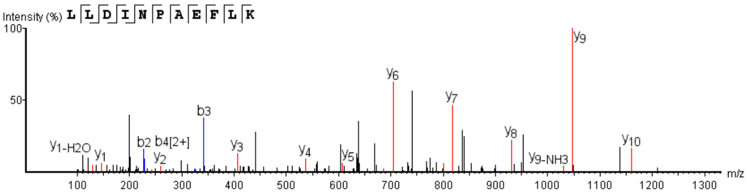

gi571542614

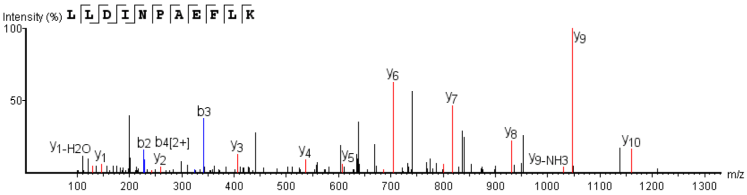

gi571542618

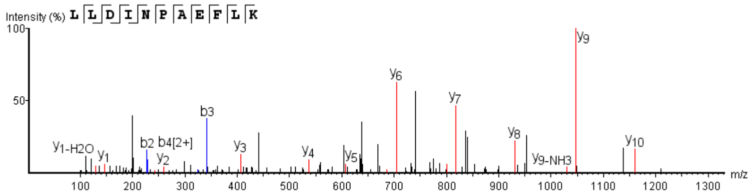

gi571542624

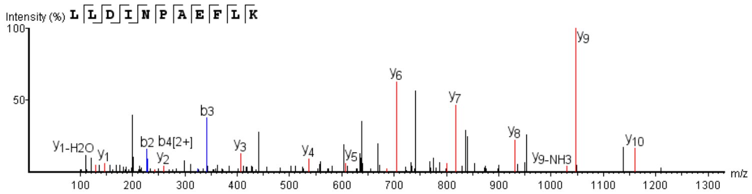

gi571542628

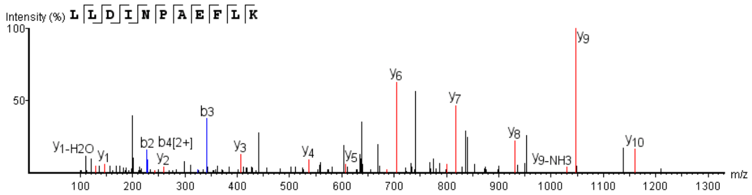

gi571542635

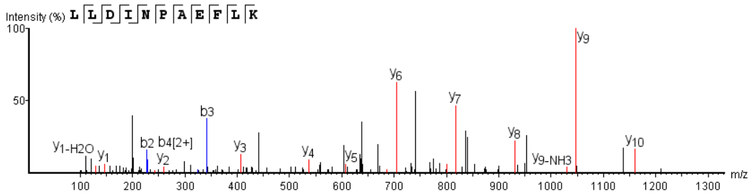

gi571542638

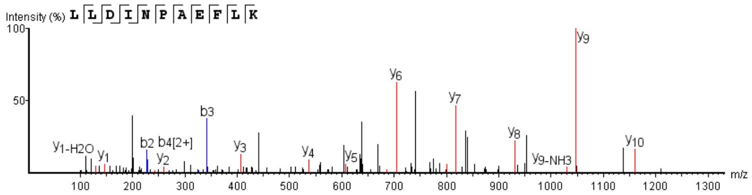

gi571542641

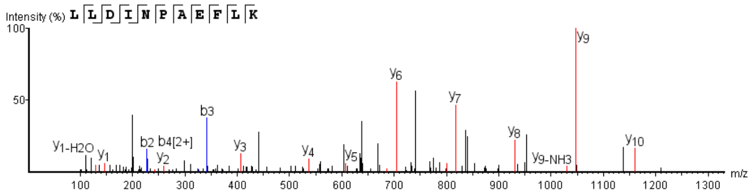

gi|571503152

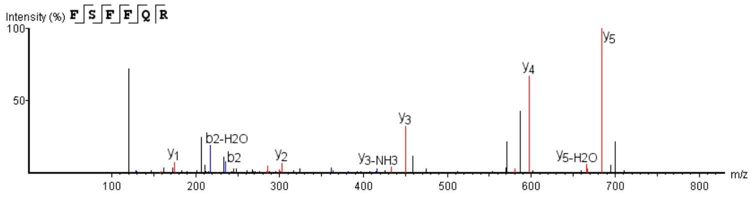

gi571542705

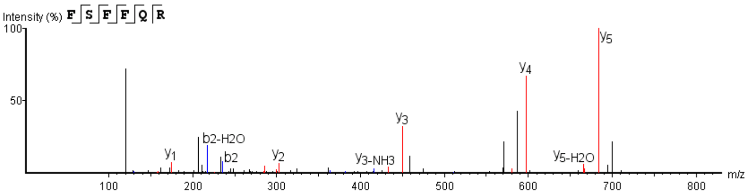

gi571543246

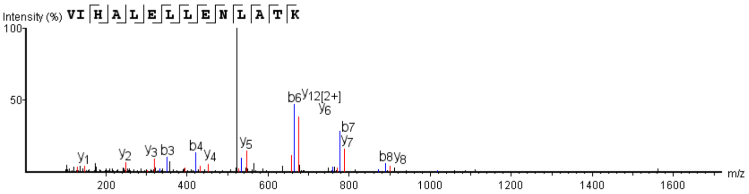

gi571543249

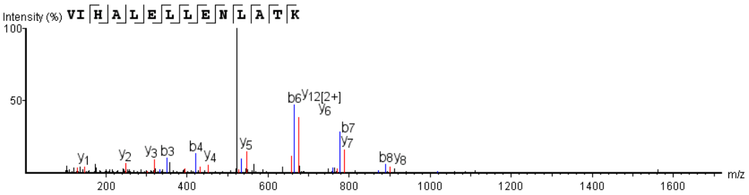

gi571543561

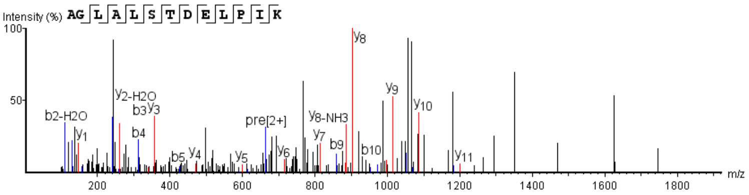

gi571543706

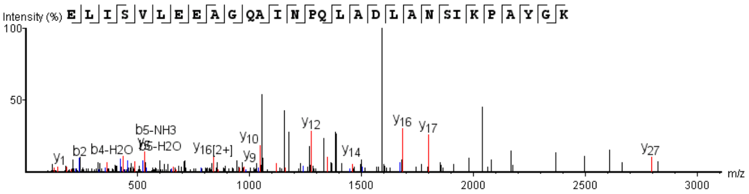

gi571544763

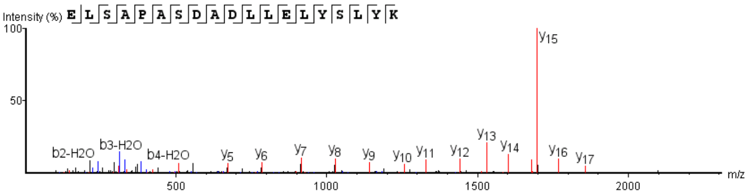

gi571545068

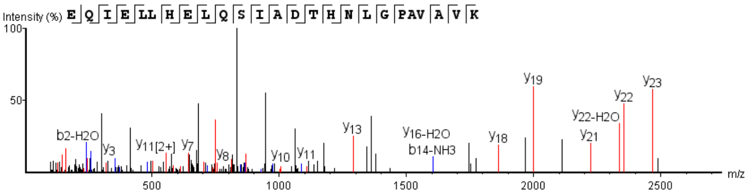

gi571545167

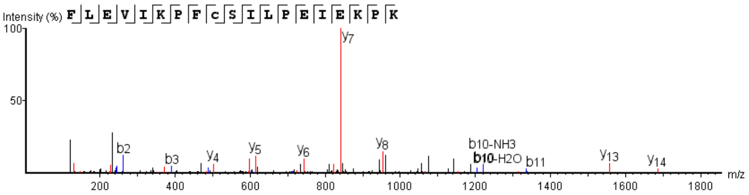

gi571546054

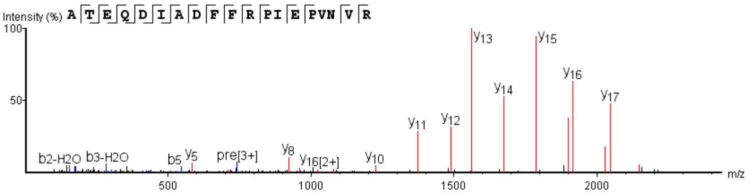

gi571546058

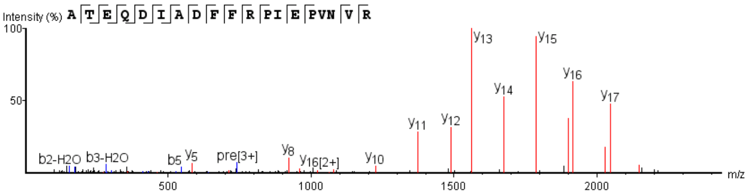

gi571546934

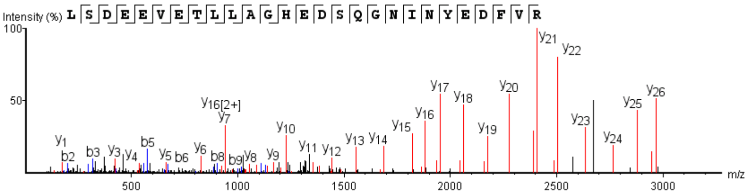

gi571546944

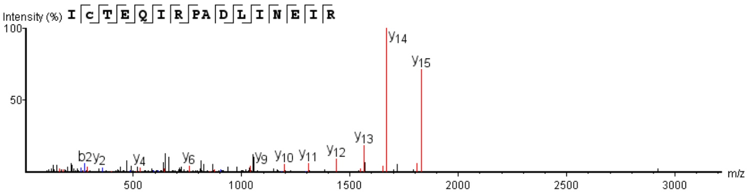

gi|66516304

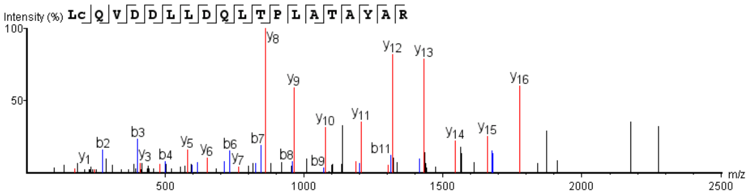

gi571547954

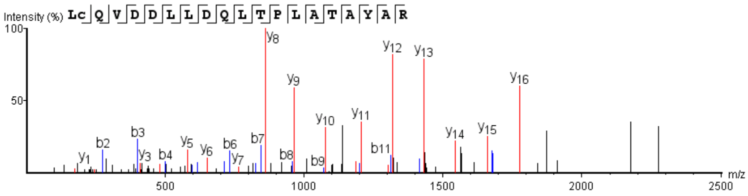

gi571548456

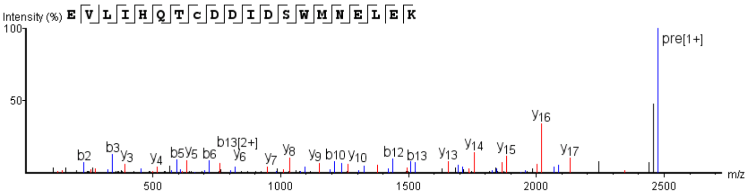

gi|328781423

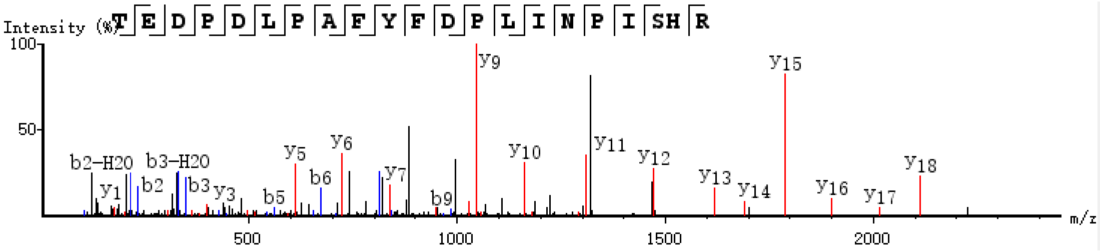

gi571548748

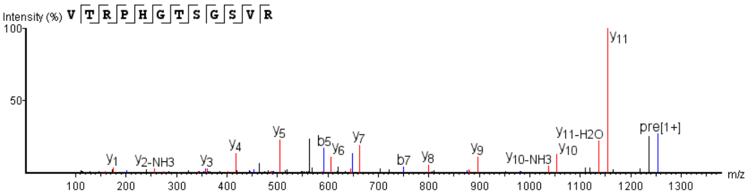

gi|57154875

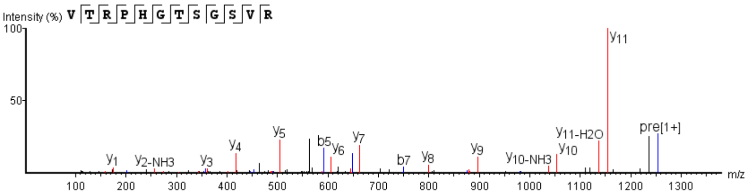

gi|571548754

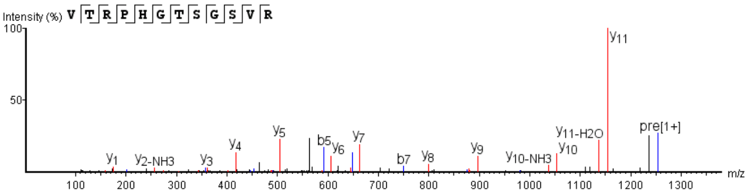

gi|328781218

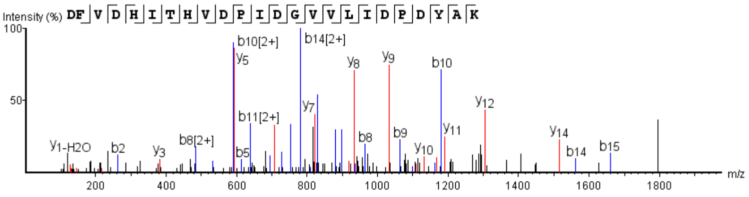

gi571549591

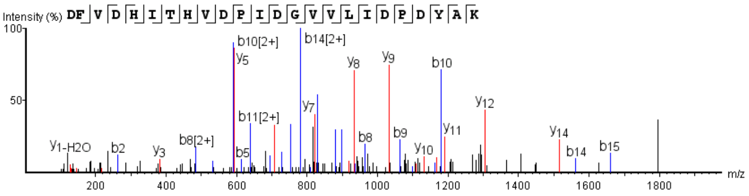

gi571549598

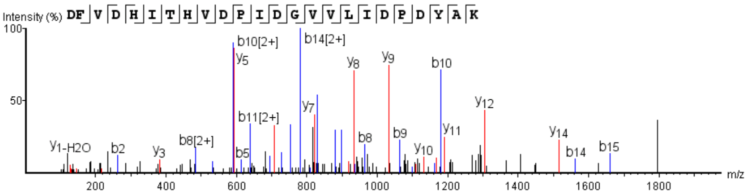

gi571549603

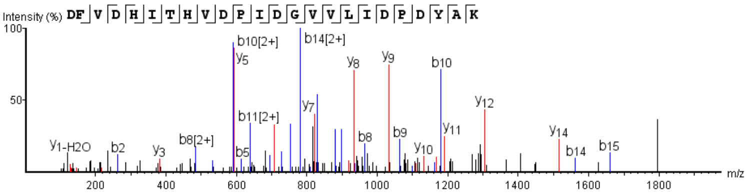

gi571550223

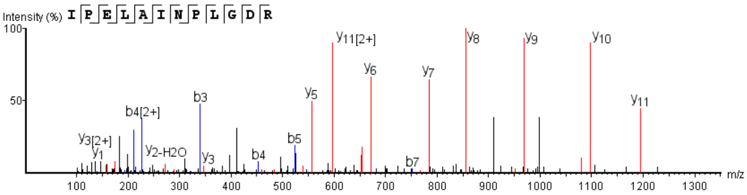

gi571551208

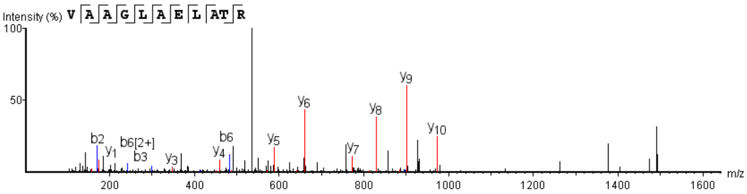

gi 571551834

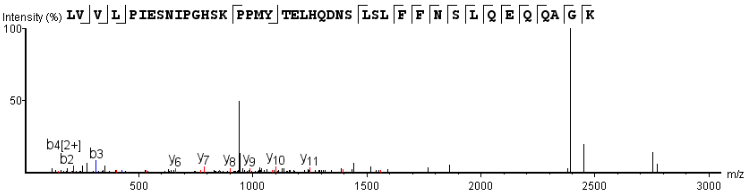

gi571554164

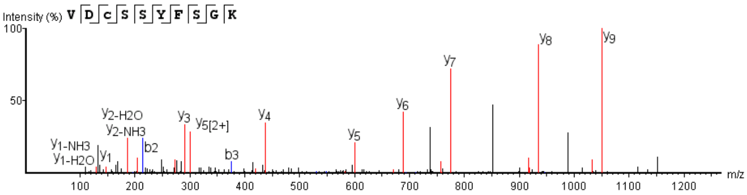

gi|66517577

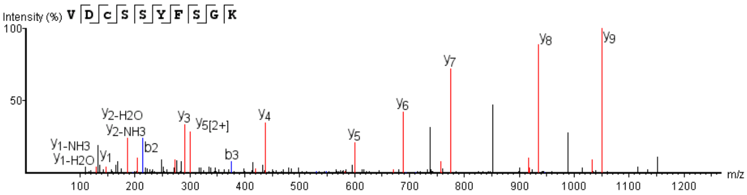

gi|571554820

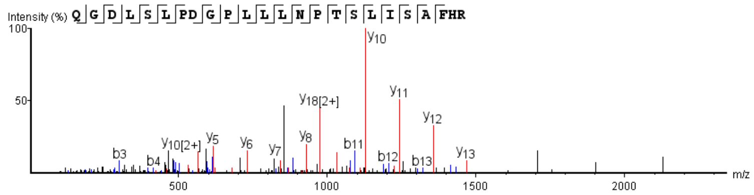

gi|328786485

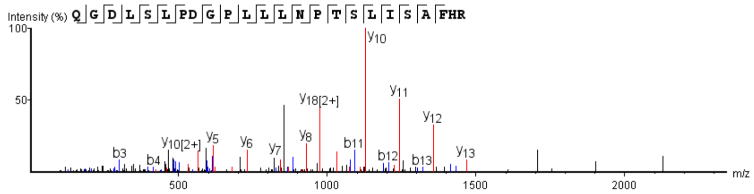

gi571554817

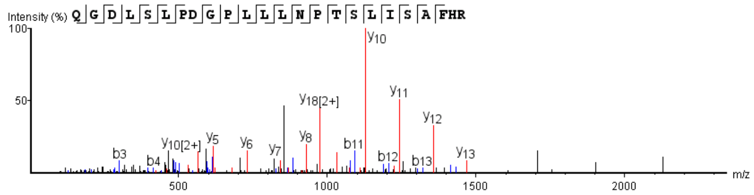

gi66514137

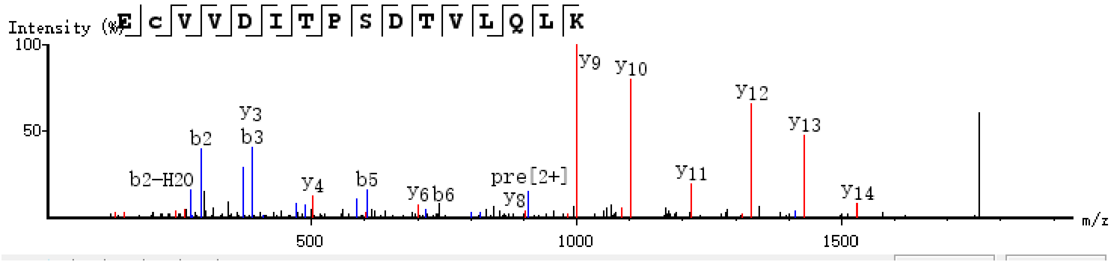

gi571555562

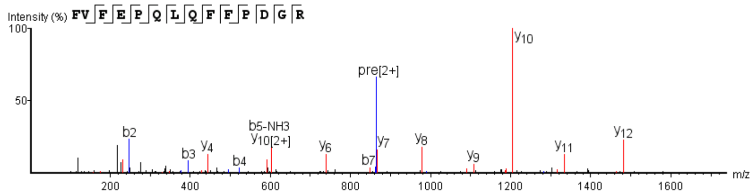

gi571555576

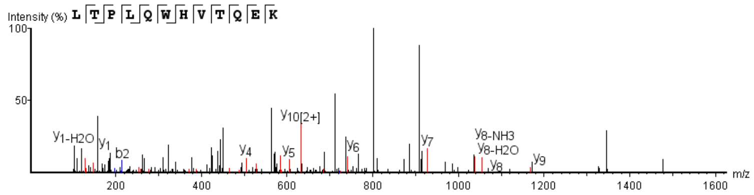

gi571555973

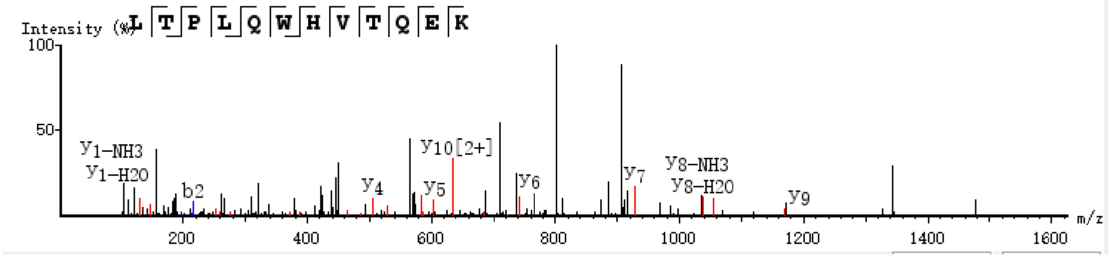

gi571555981

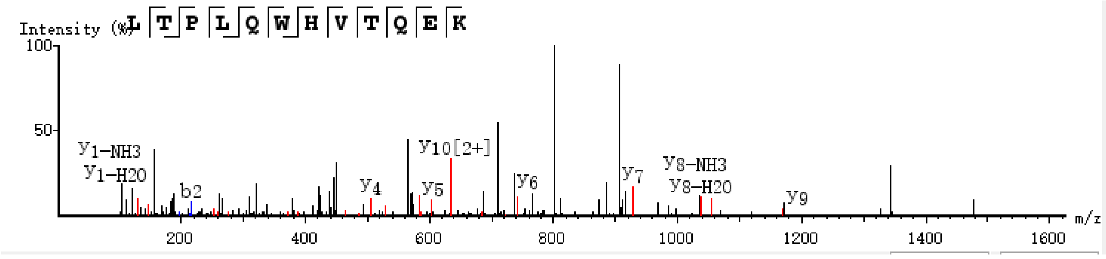

gi571555985

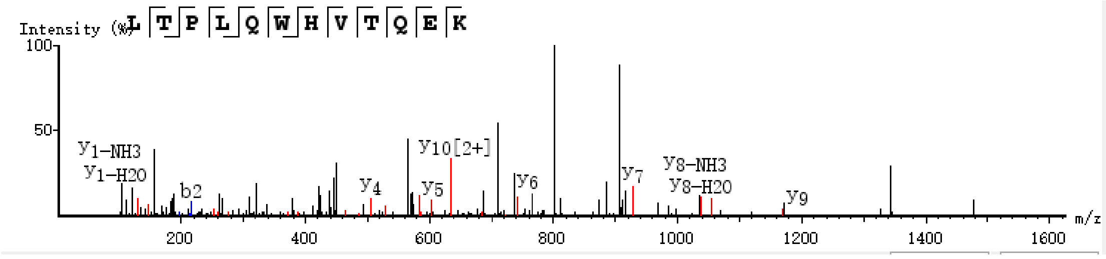

gi|328789842

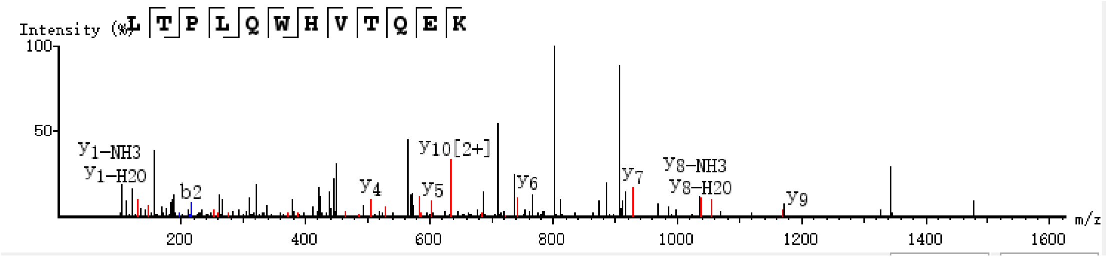

gi|328789844

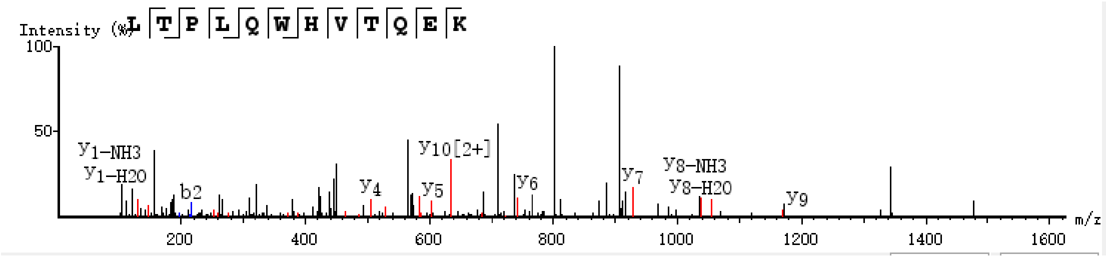

gi571556092

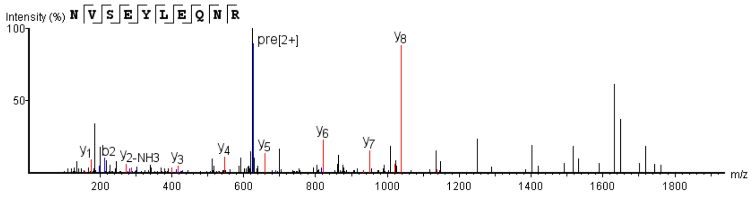

gi|571557529

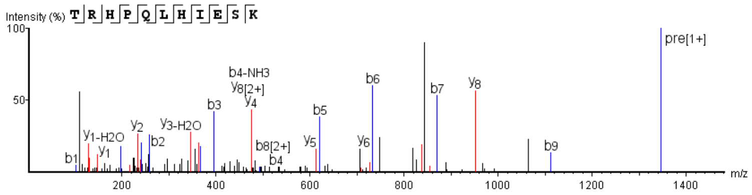

gi571557508

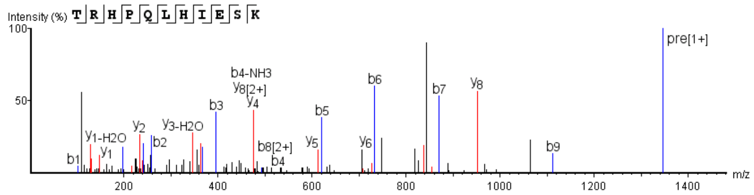

gi571557511

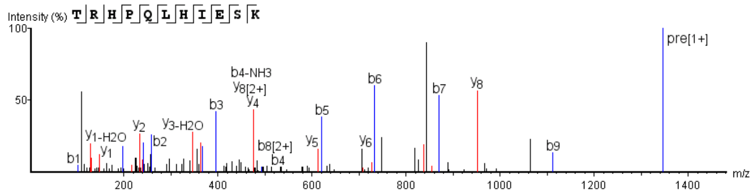

gi571557515

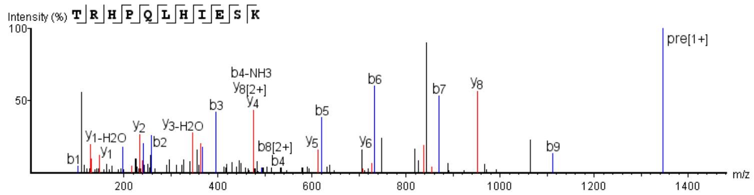

gi571557519

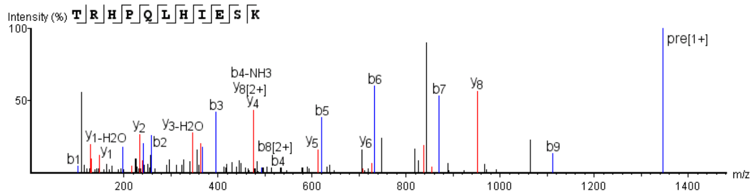

gi571557523

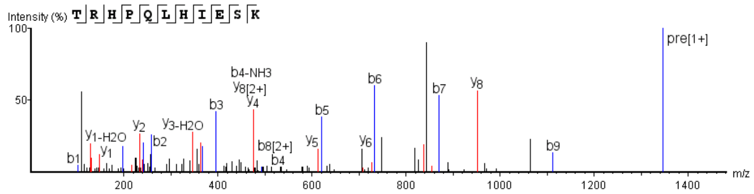

gi571558139

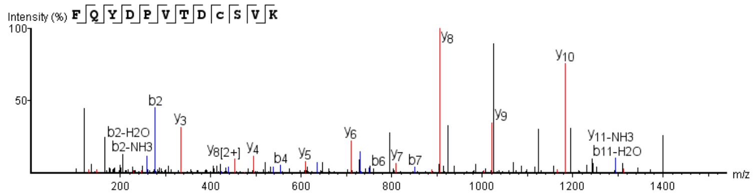

gi|571567131

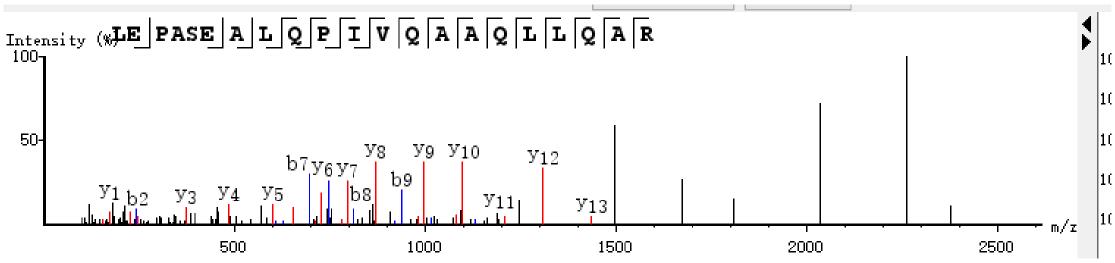

gi571558256

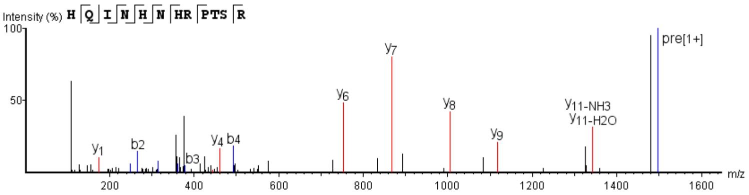

gi571558849

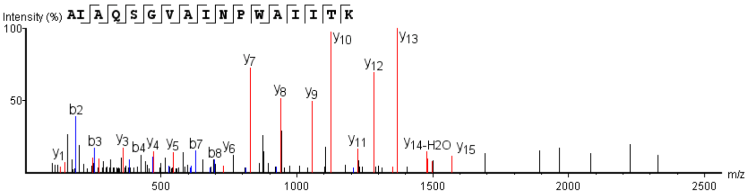

gi571558853

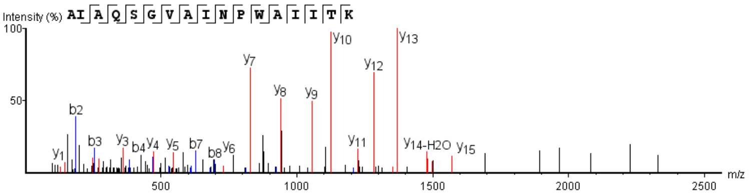

gi571558857

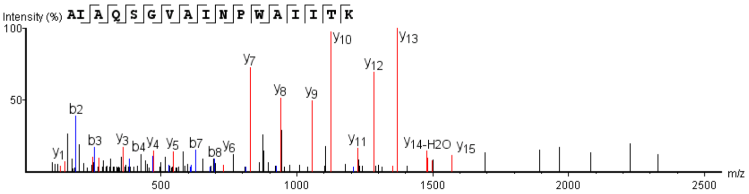

gi571558902

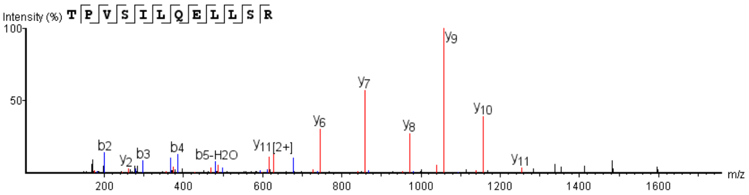

gi571558905

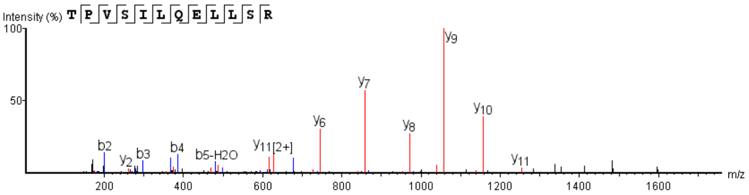

gi571558909

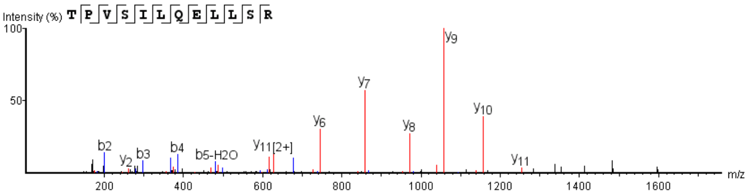

gi571558912

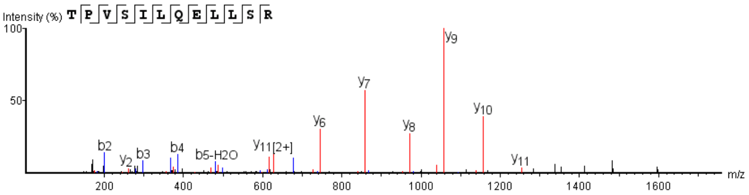

gi571558915

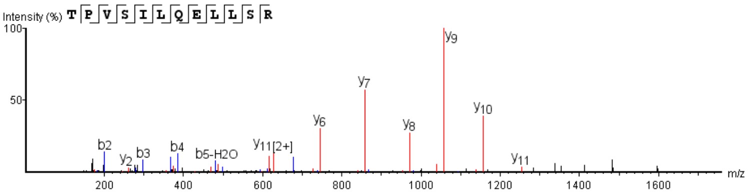

gi 571559072

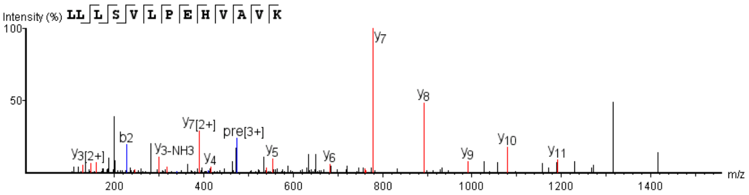

gi|110757619

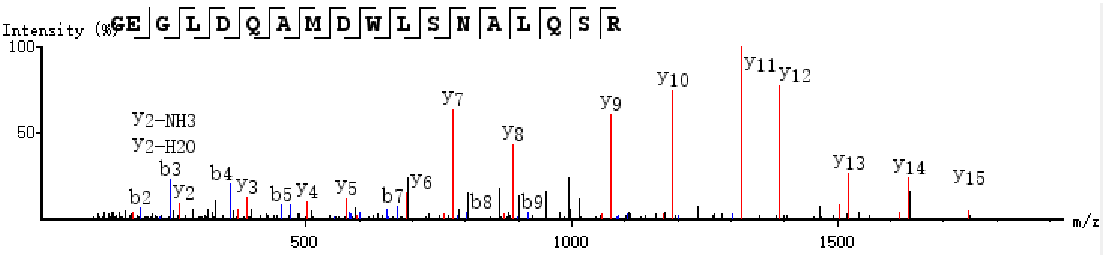

gi571559065

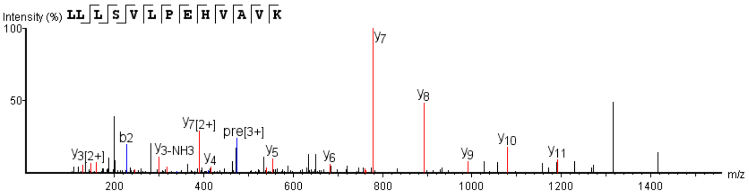

gi571559068

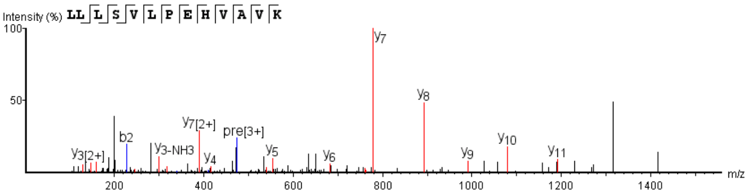

gi571559277

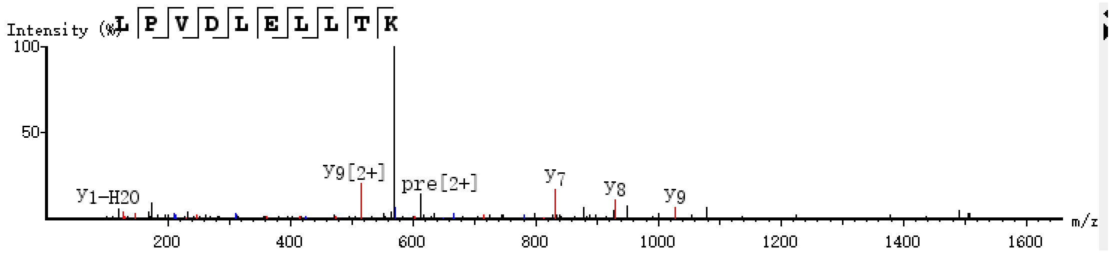

gi571559721

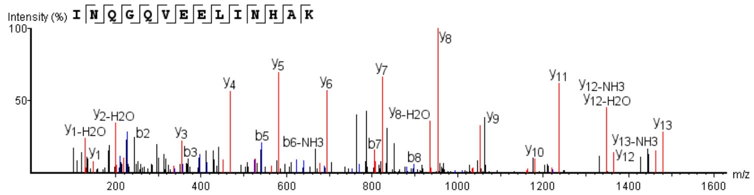

gi|571561371

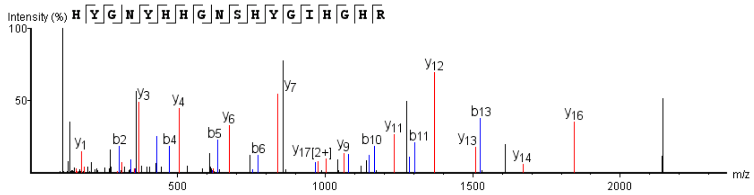

gi|571546900

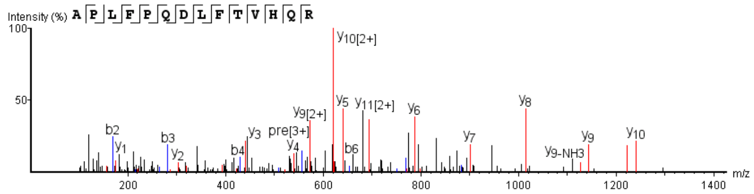

gi|571546934

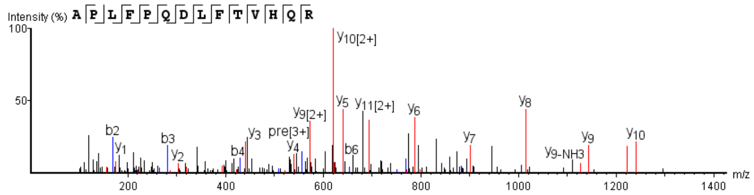

gi|571546944

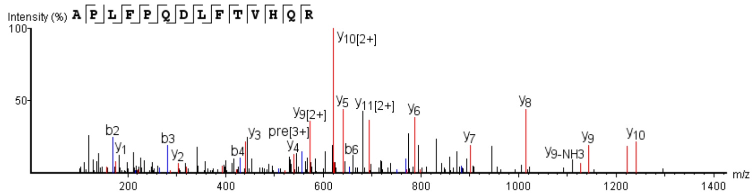

gi|571546904

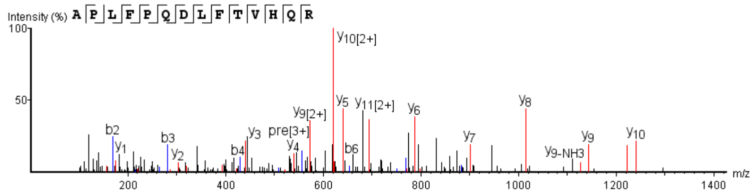

gi|571546911

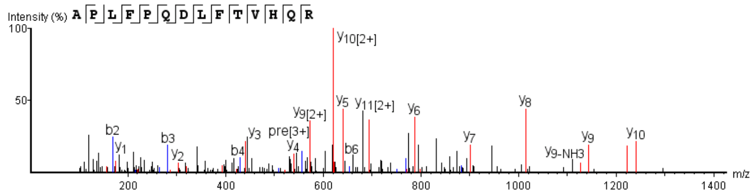

gi|571546915

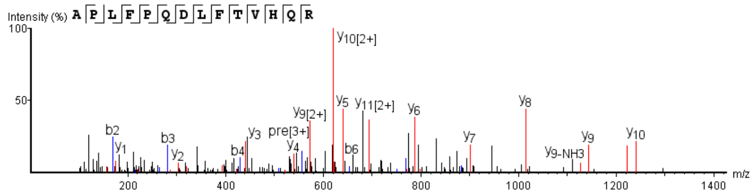

gi|110764759

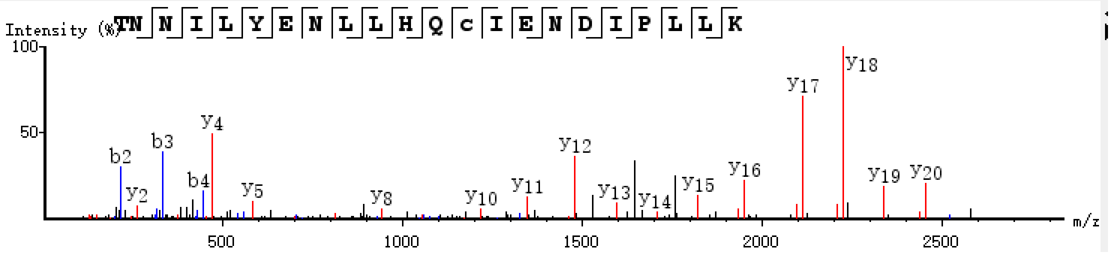

gi|571561359

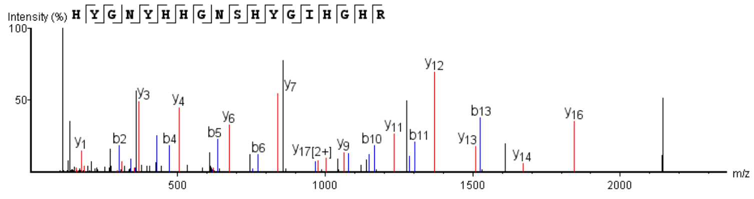

gi571561363

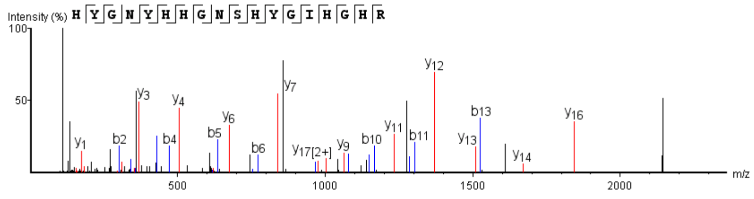

gi571561367

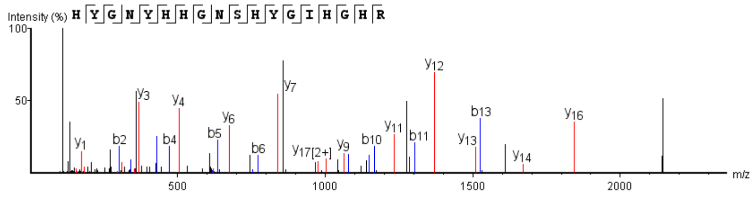

gi571561371

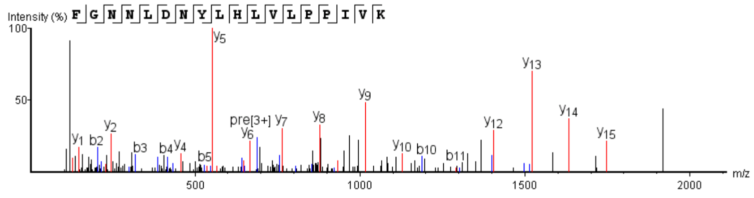

gi571561698

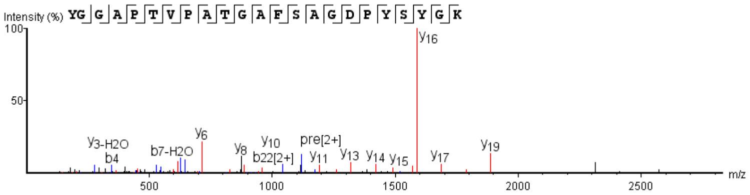

gi571561705

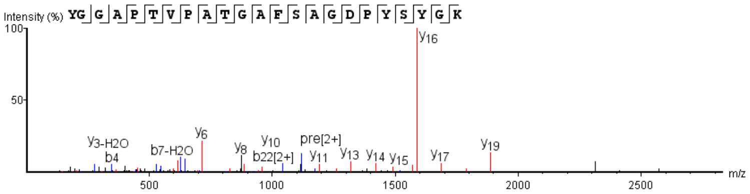

gi571561709

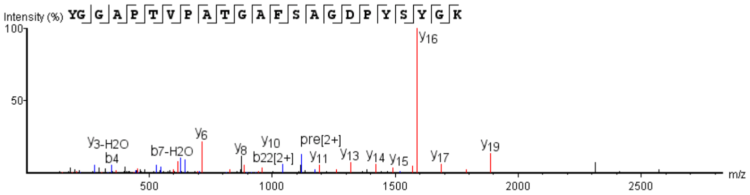

gi571561713

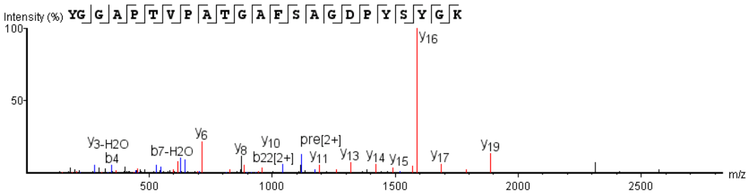

gi571561717

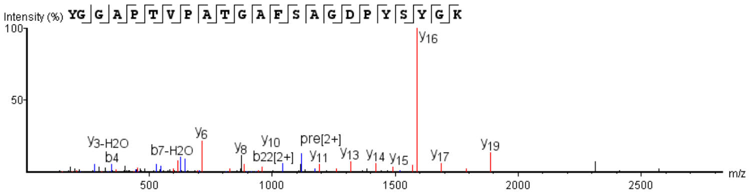

gi571561721

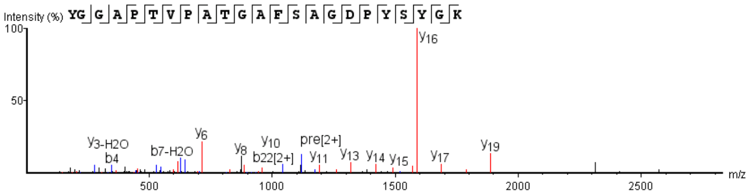

gi571561725

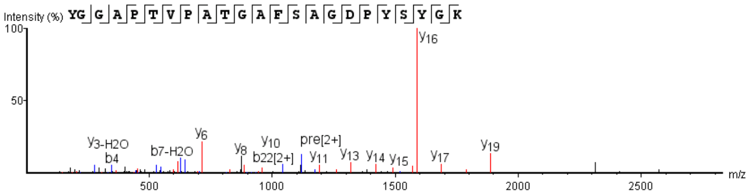

gi|328787142

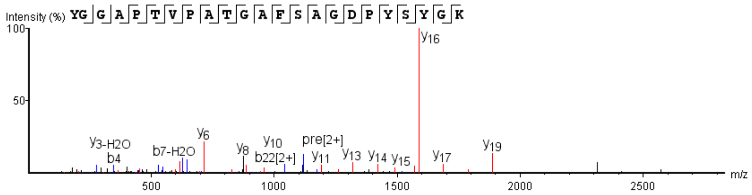

gi571561759

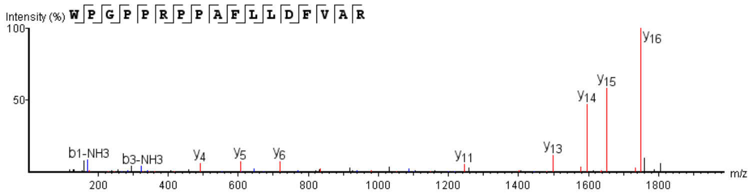

gi571562027

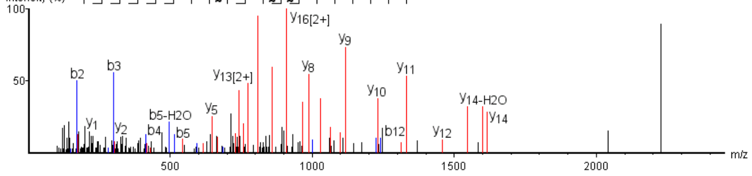

gi571562031

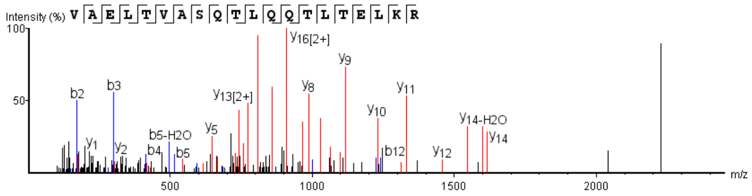

gi571563070

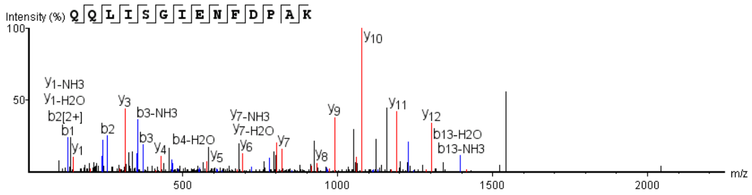

gi|571563985

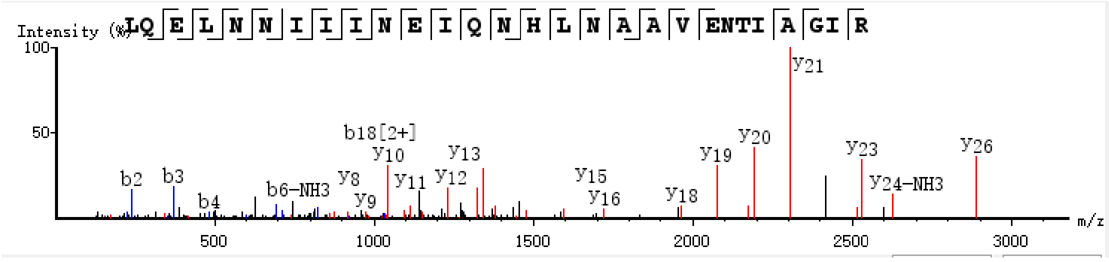

gi571563982

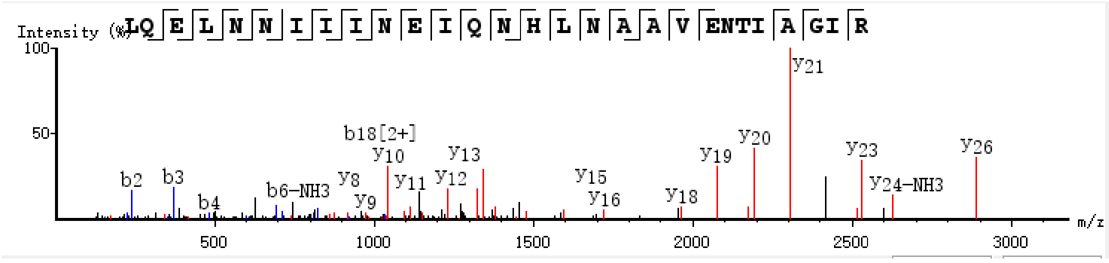

gi571563988

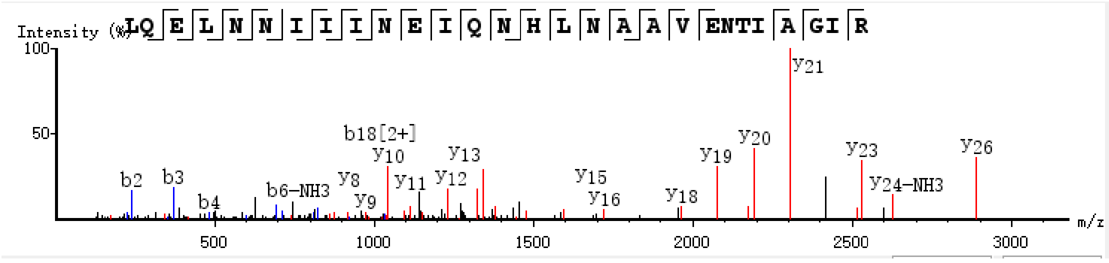

gi571563990

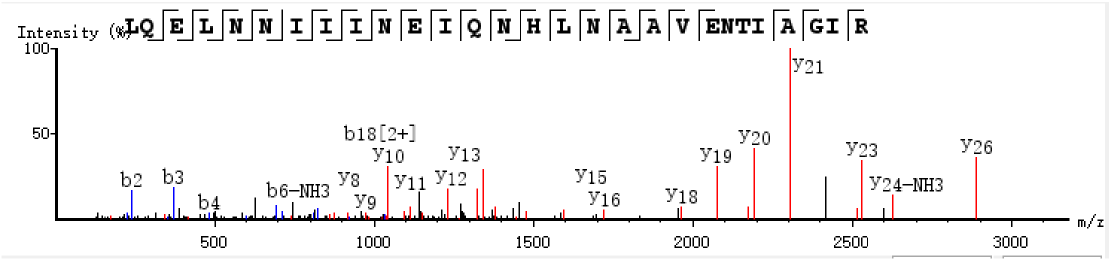

gi571564004

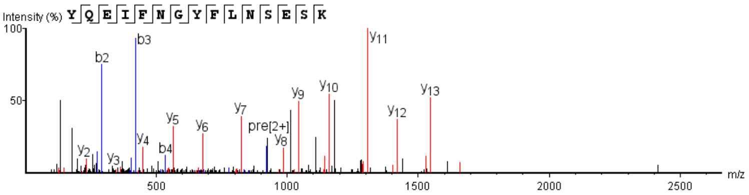

gi571564503

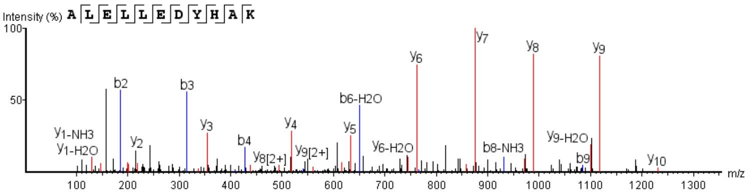

gi571564978

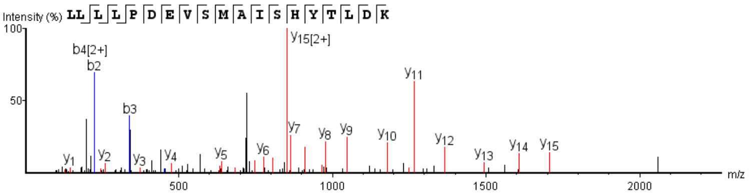

gi571564981

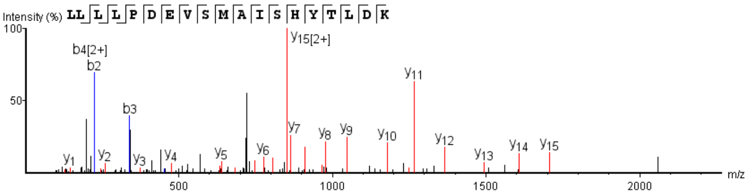

gi|328789160

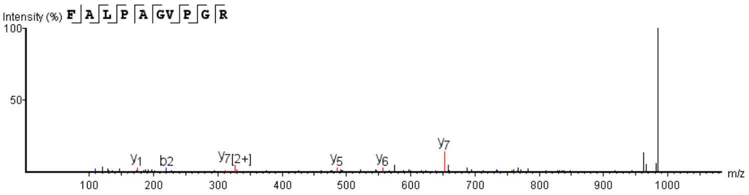

gi|571503499

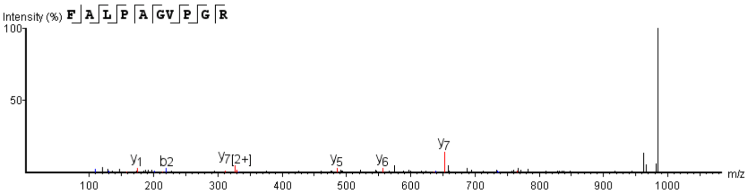

gi|48103761

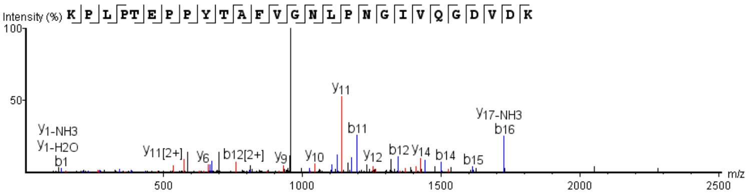

gi571565430

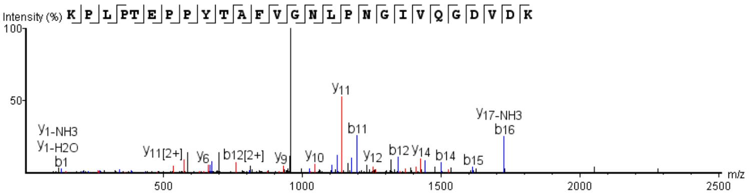

gi|328777360

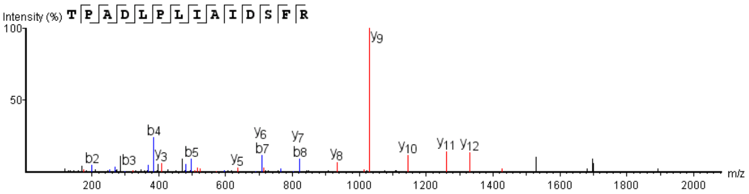

gi571566909

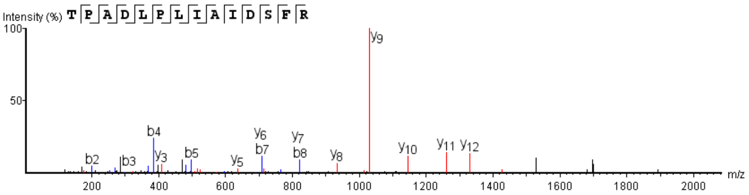

gi571567903

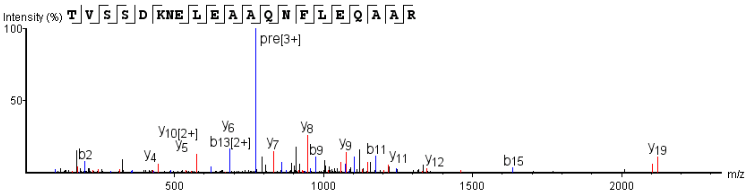

gi571567906

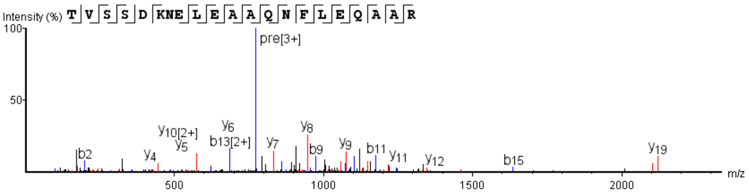

gi571569633

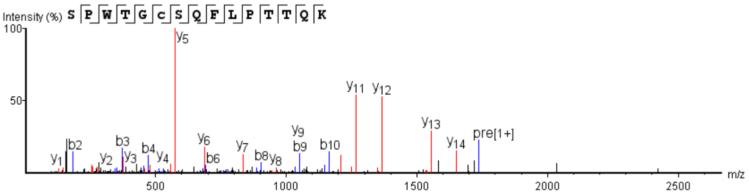

gi571570081

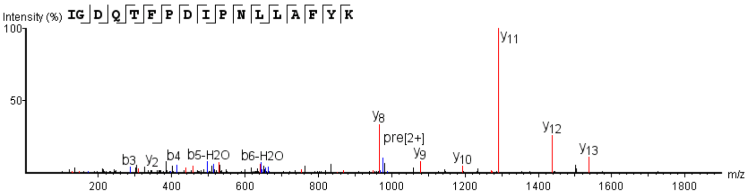

gi|571570085

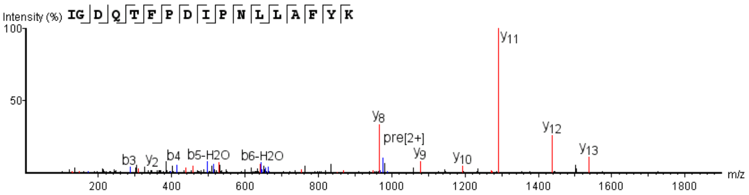

gi|571570083

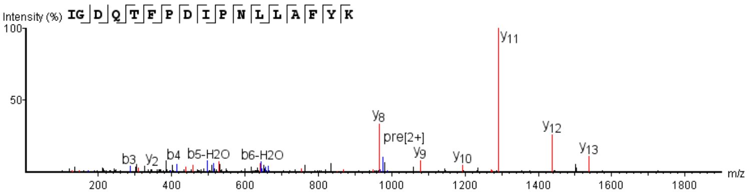

gi571570113

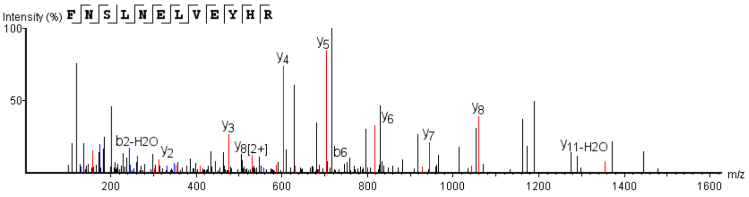

gi571570476

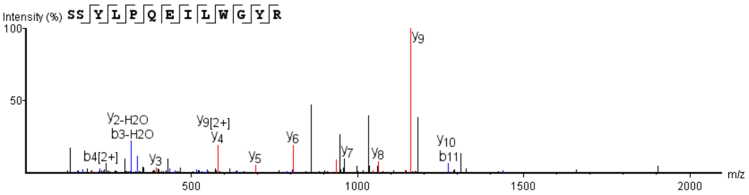

gi571570487

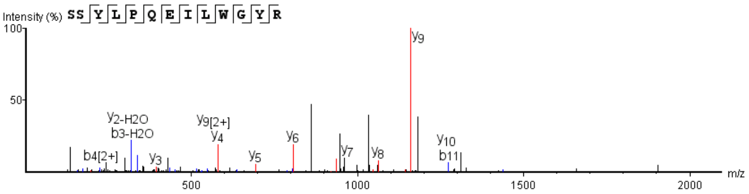

gi571570493

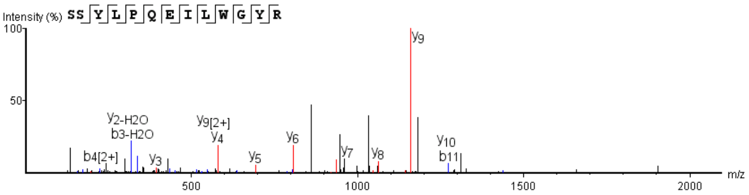

gi571570496

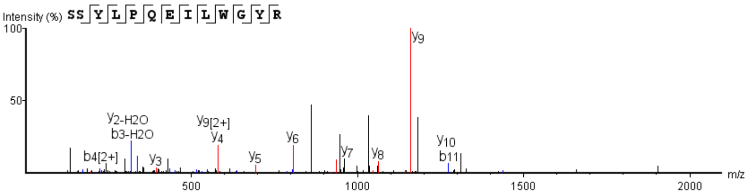

gi571570501

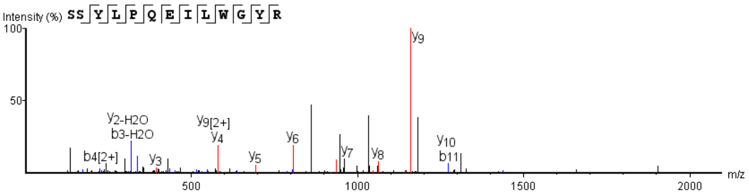

gi571570507

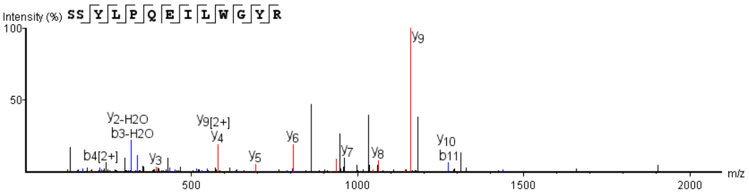

gi571570515

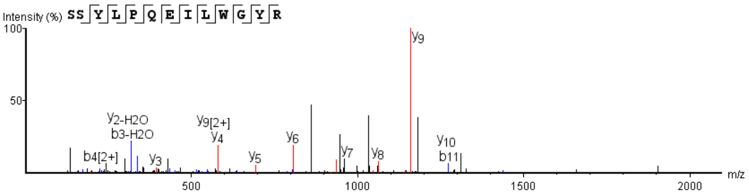

gi571570521

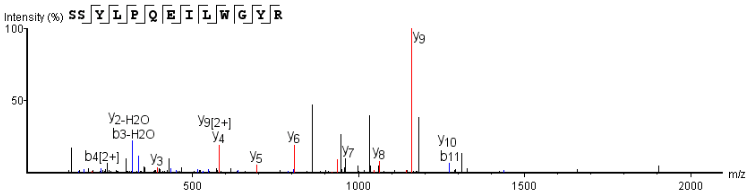

gi571570525

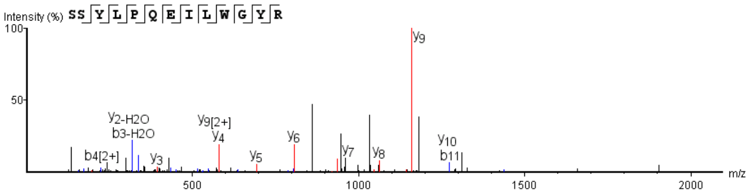

gi571570530

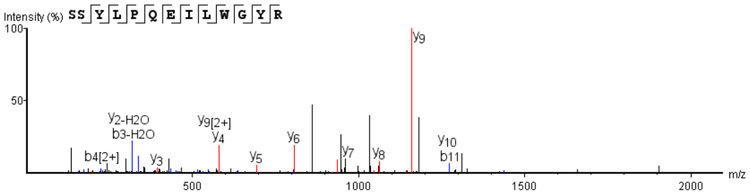

gi|571570511

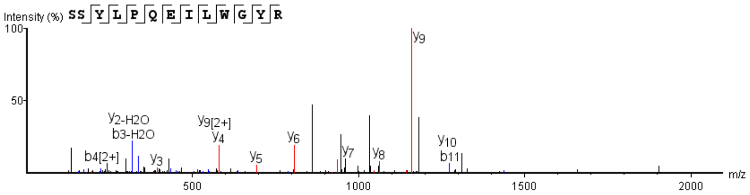

gi|571573722

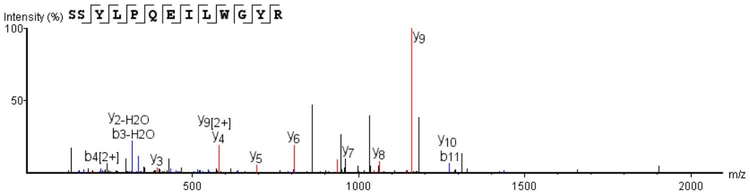

gi|328782779

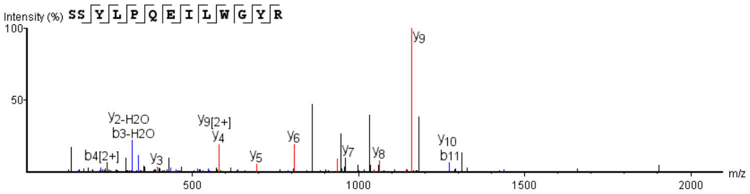

gi571570534

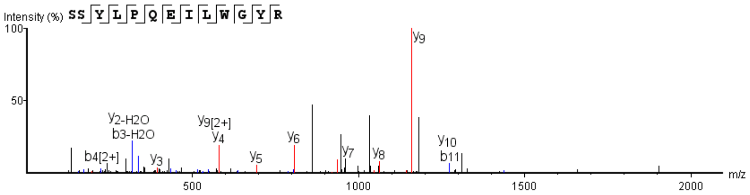

gi571570586

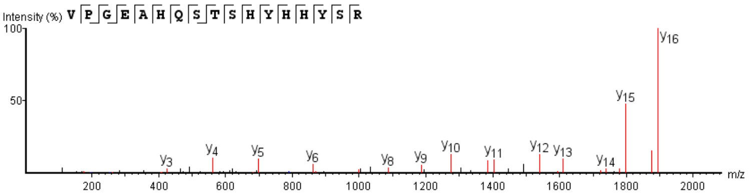

gi571570594

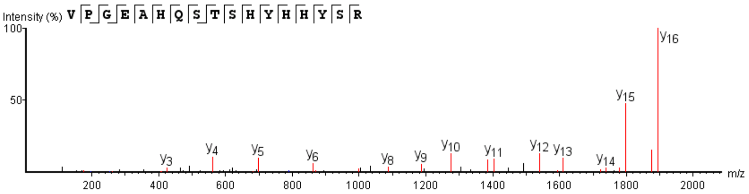

gi571570602

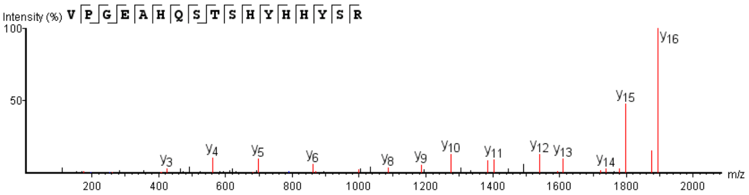

gi571570610

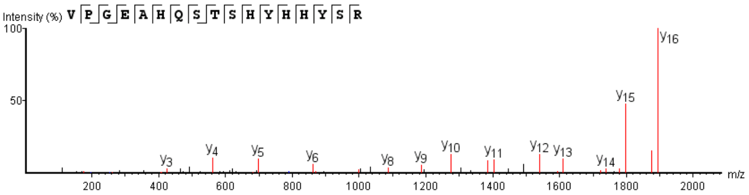

gi571570616

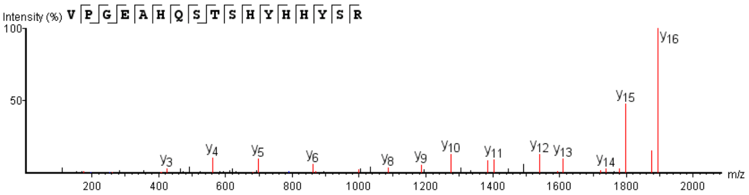

gi571570622

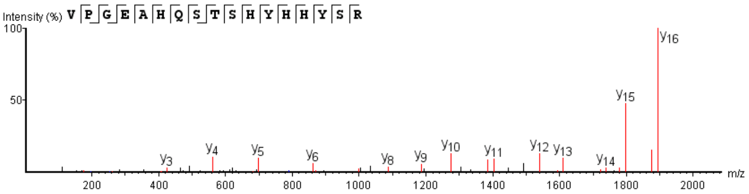

gi571570635

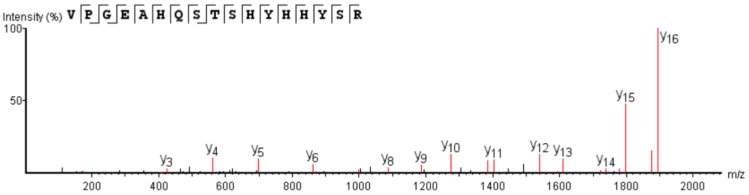

gi571570641

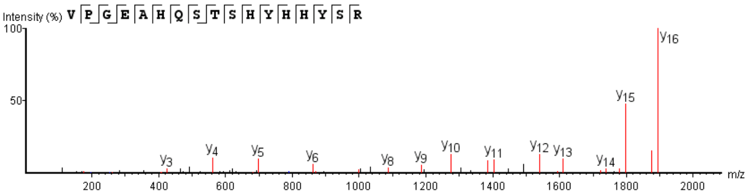

gi|571571279

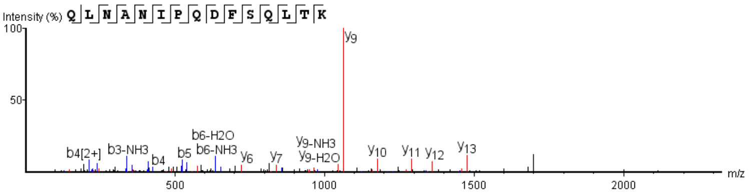

gi571571223

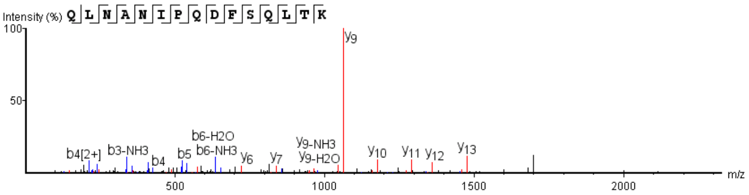

gi571571231

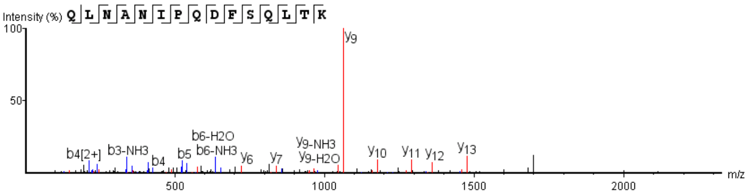

gi571571243

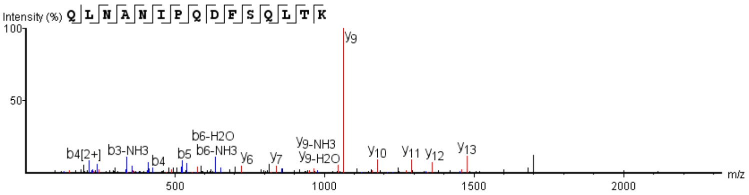

gi571571253

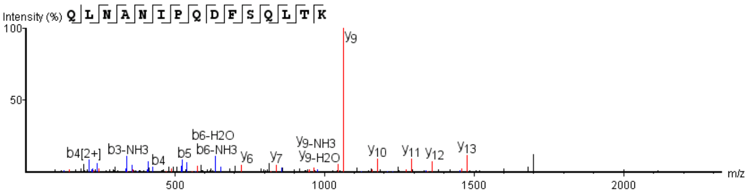

gi571571261

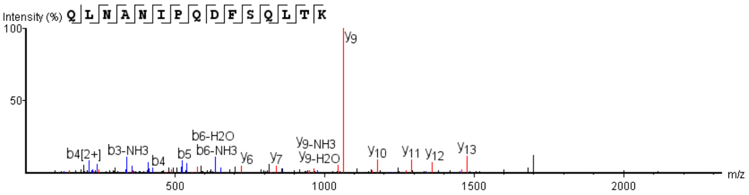

gi571571878

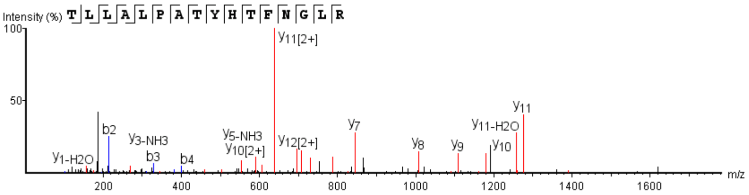

gi571572247

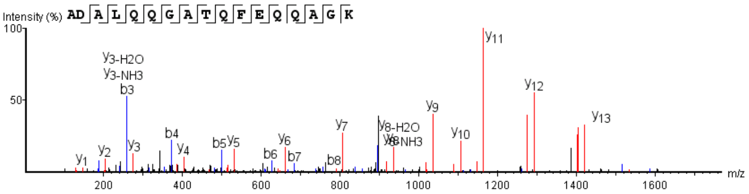

gi571572819

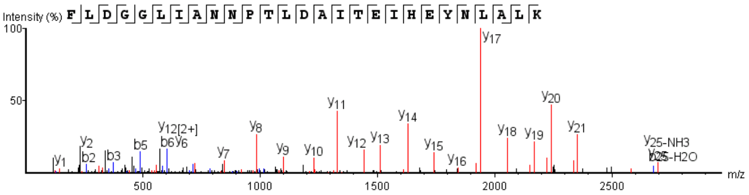

gi|571573316

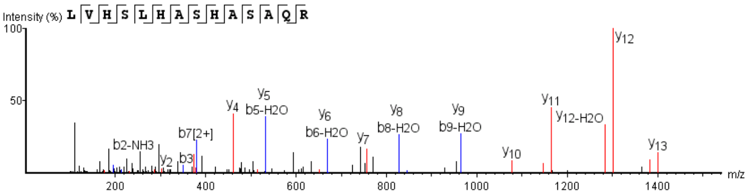

gi|571573318

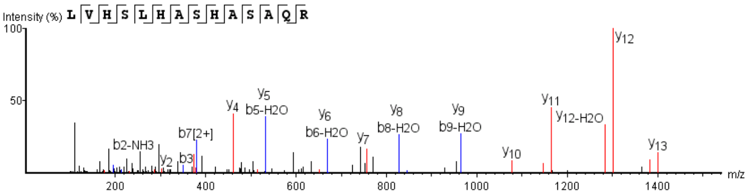

gi|571573320

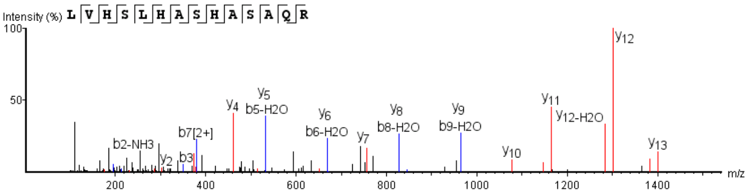

gi|571573322

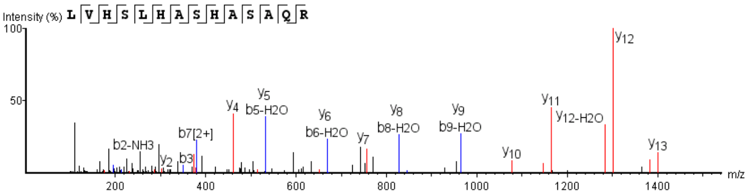

gi|571573324

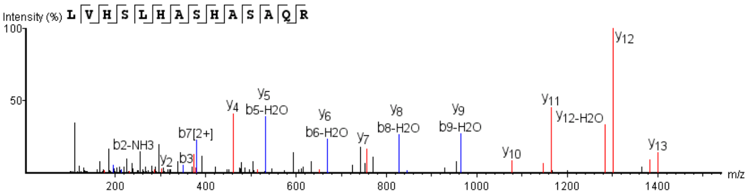

gi571573326

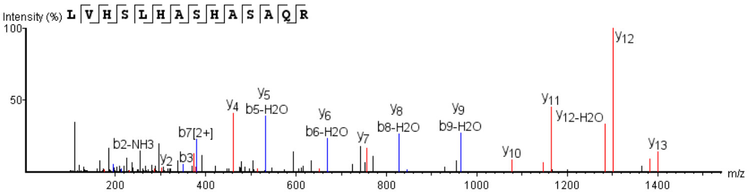

gi571573328

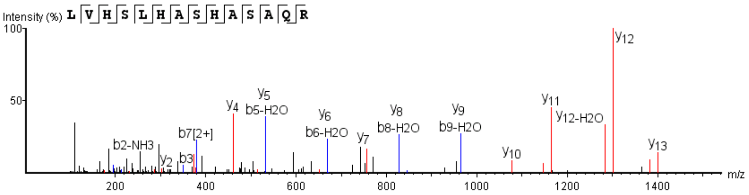

gi571573330

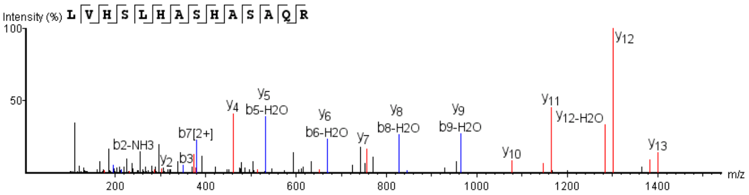

gi571573590

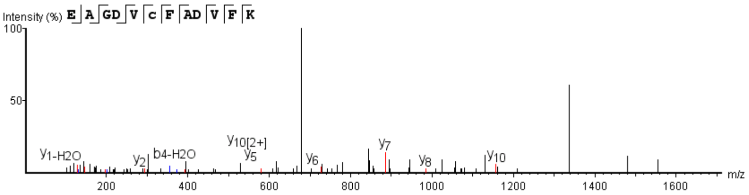

gi571573648

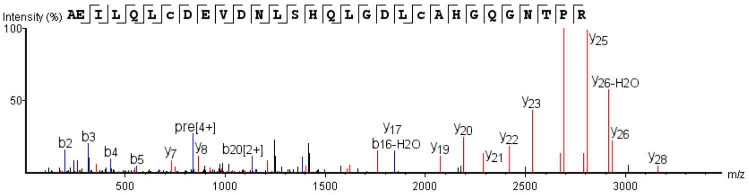

gi|328788617

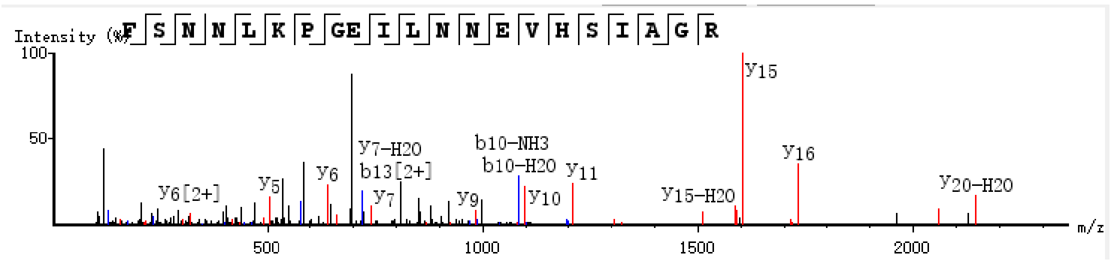

gi|328788617

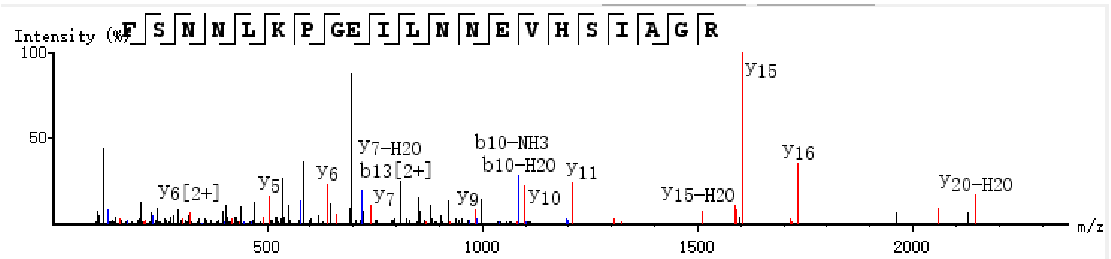

gi571573722

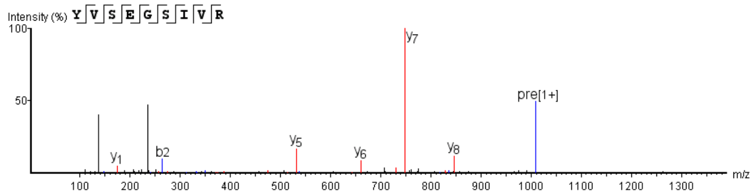

gi|571573888

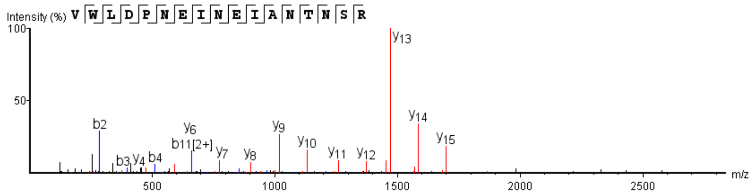

gi571573884

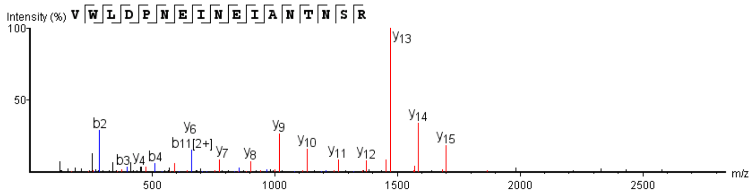

gi571573886

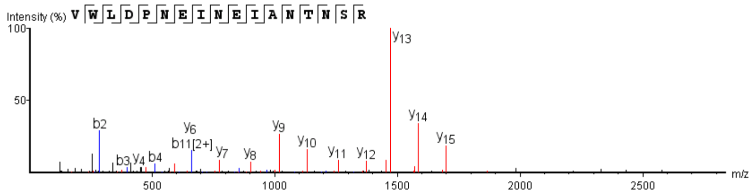

gi5834932

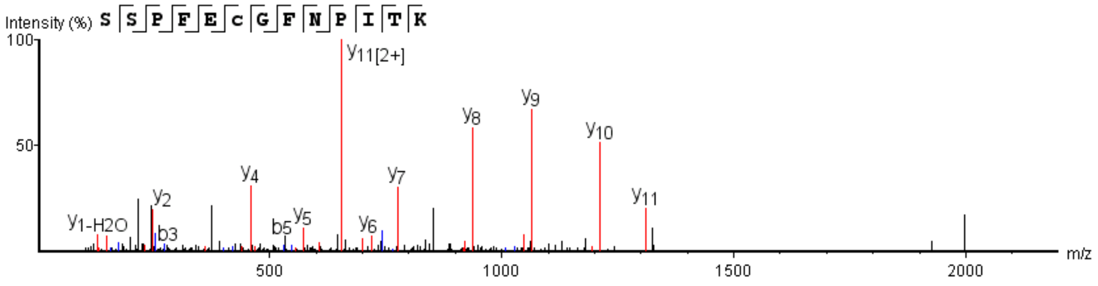

gi48094341

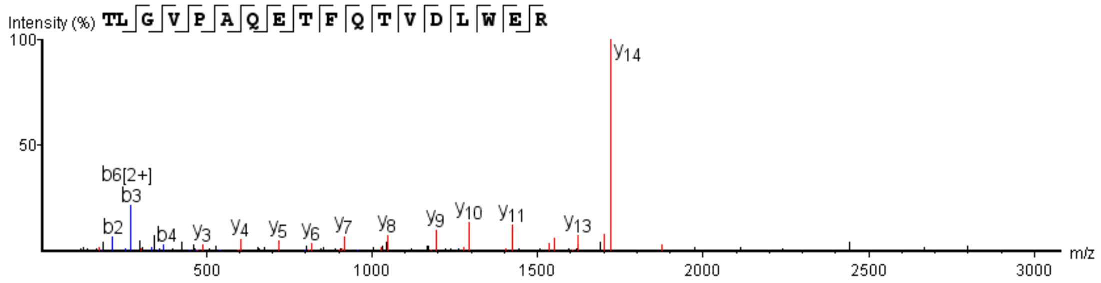

gi48095935

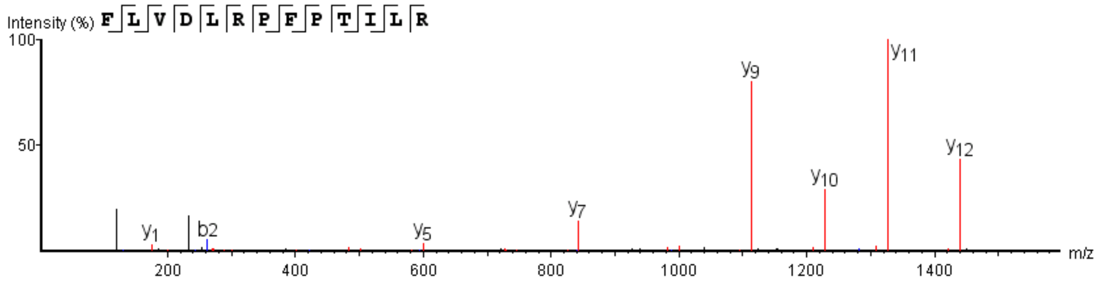

gi|571541890

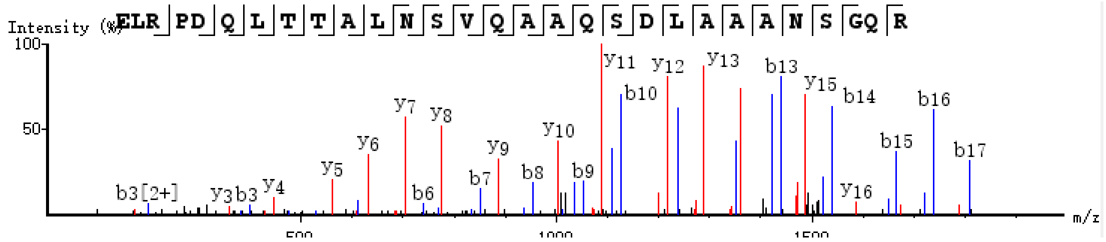

gi|328777798

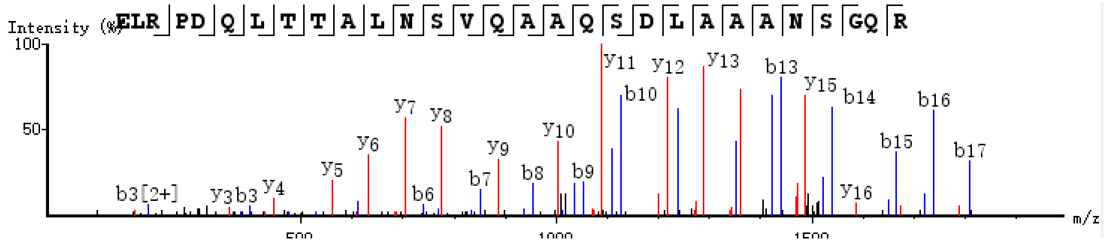

gi48097144

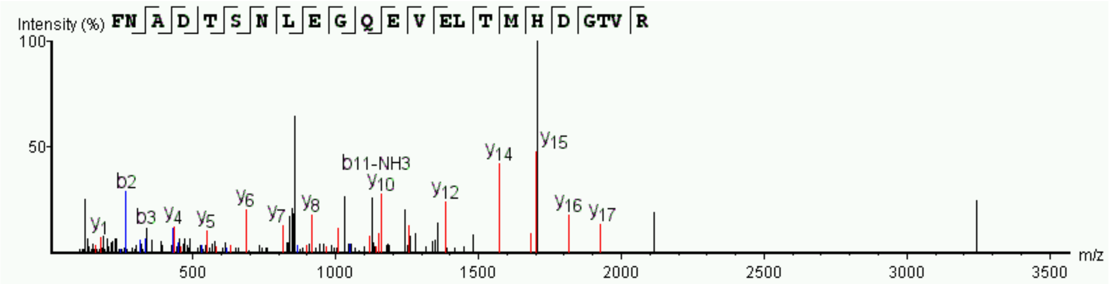

gi|571536261

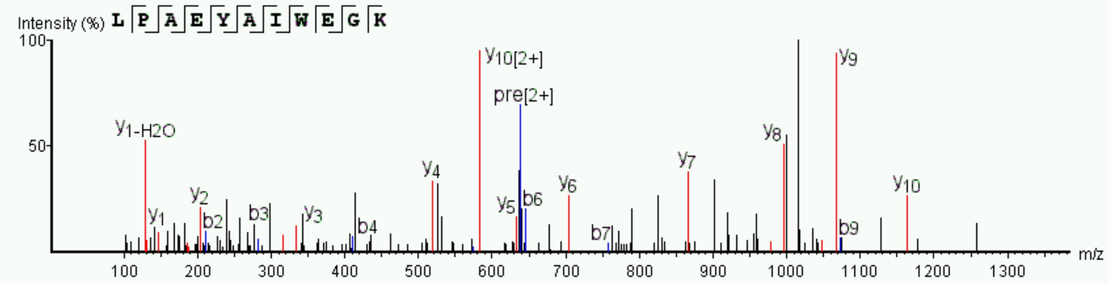

gi|571552411

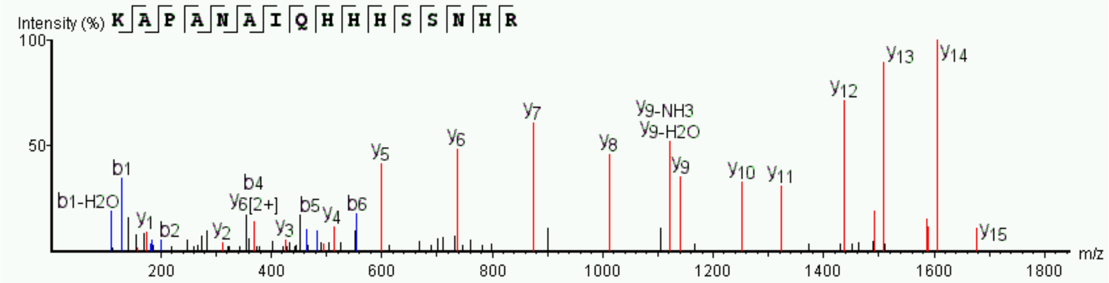

gi|571539223

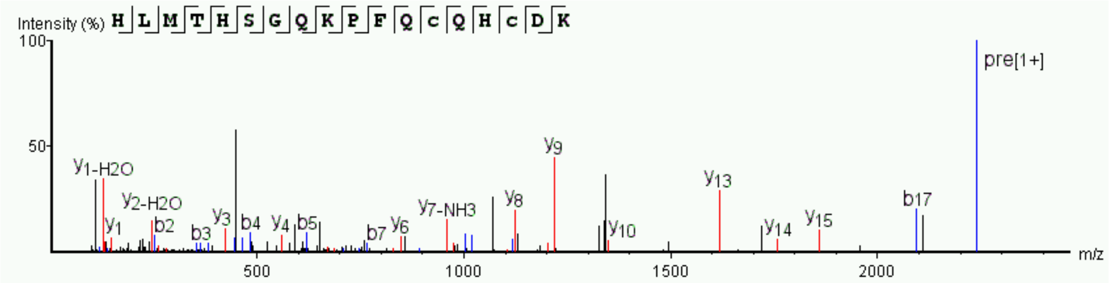

gi66499475

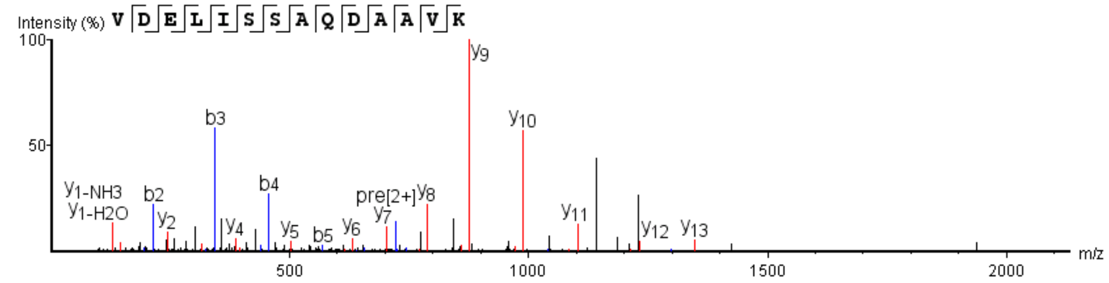

gi571542631

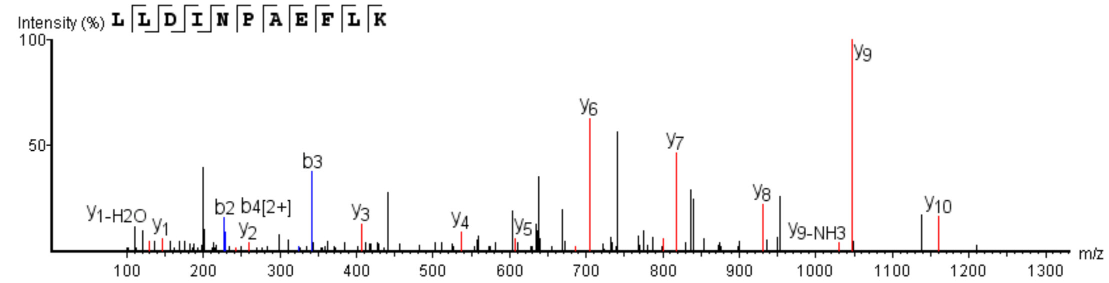

gi|66500934

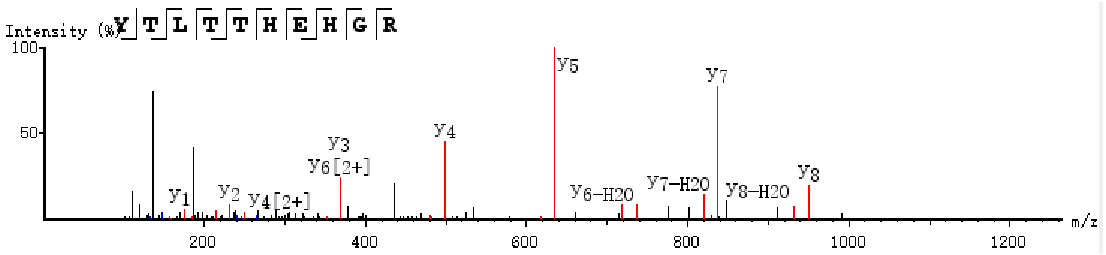

gi328788820

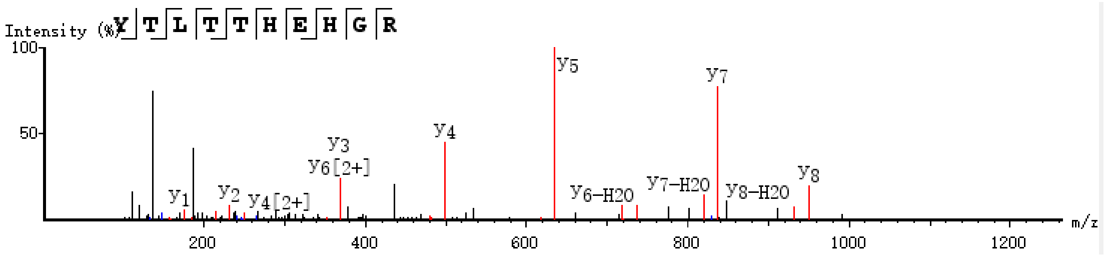

gi48098905

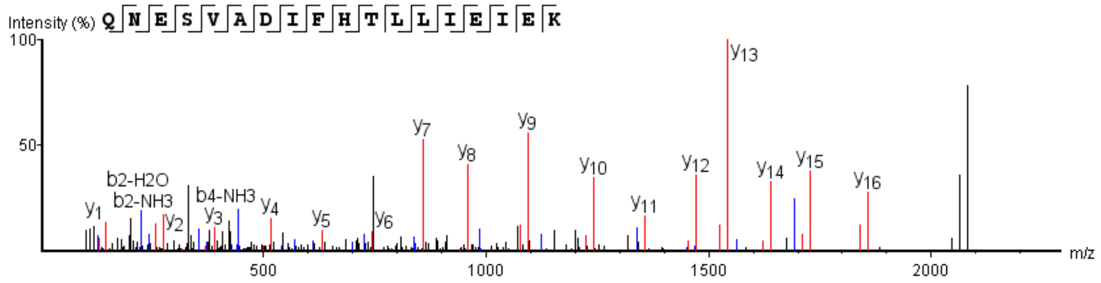

gi48102403

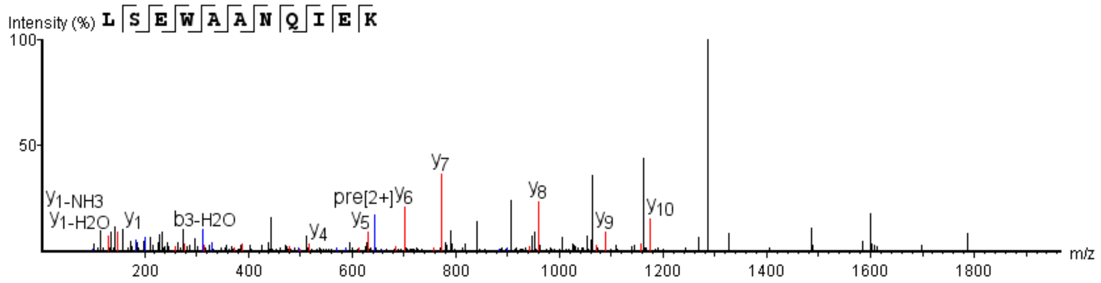

gi48103761

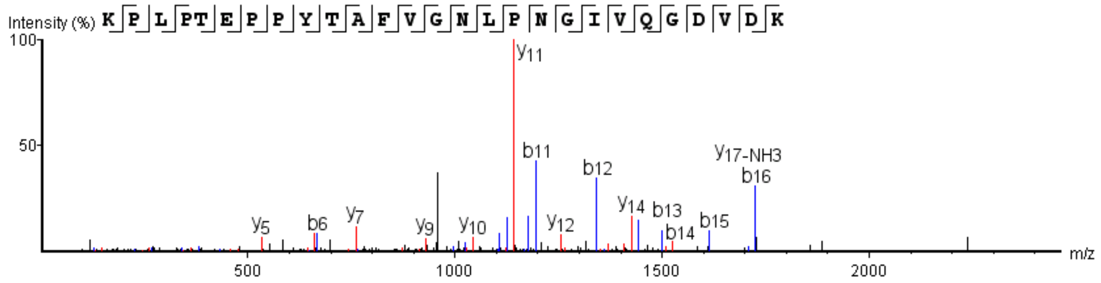

gi|66546725

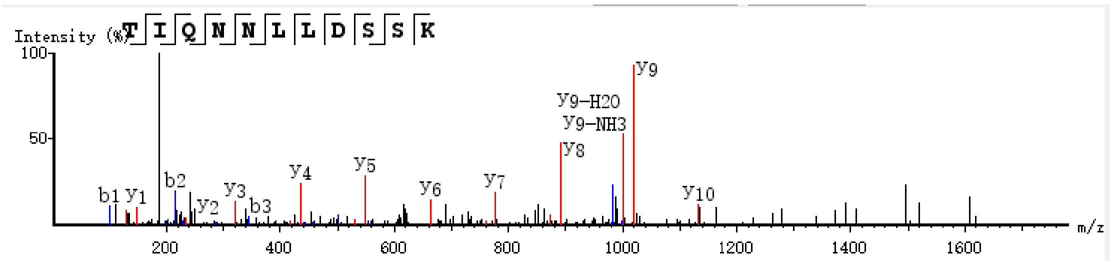

gi48104864

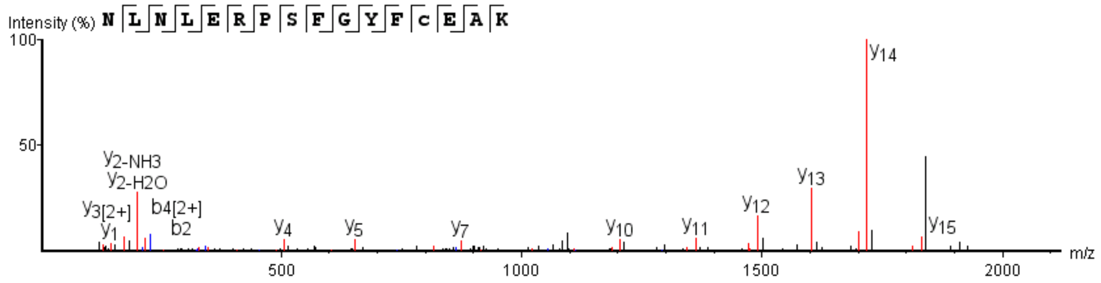

gi 48139270

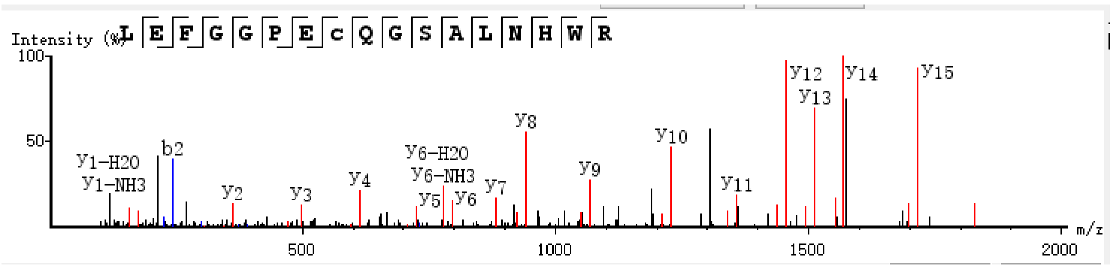

gi|571517348

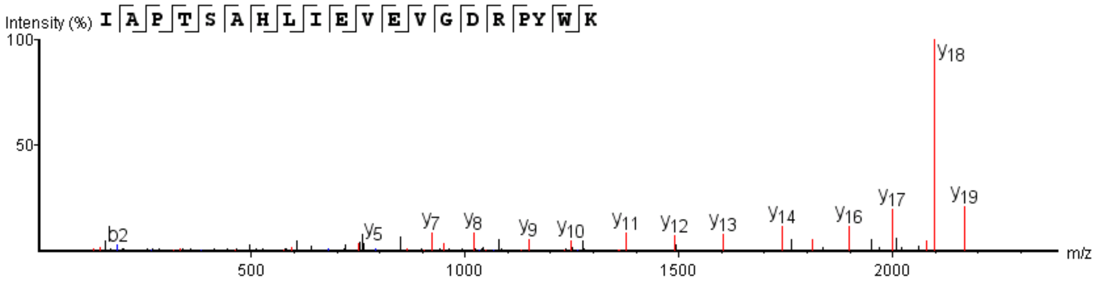

gi48104995

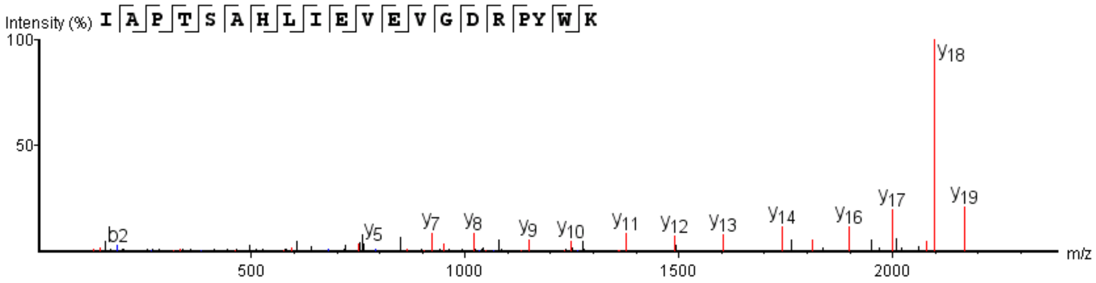

gi48139270

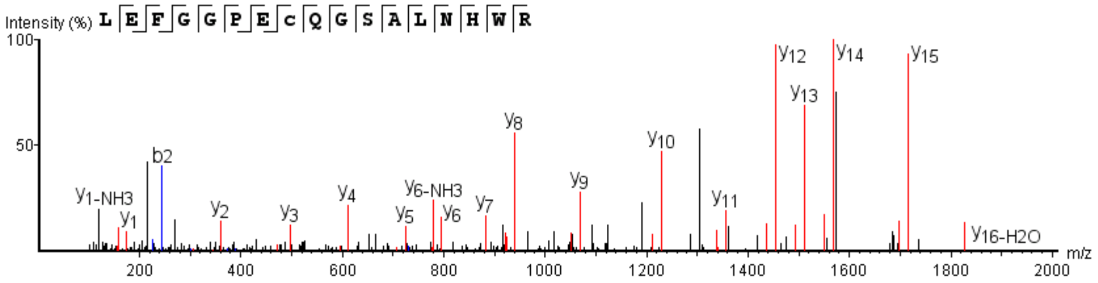

gi57153412

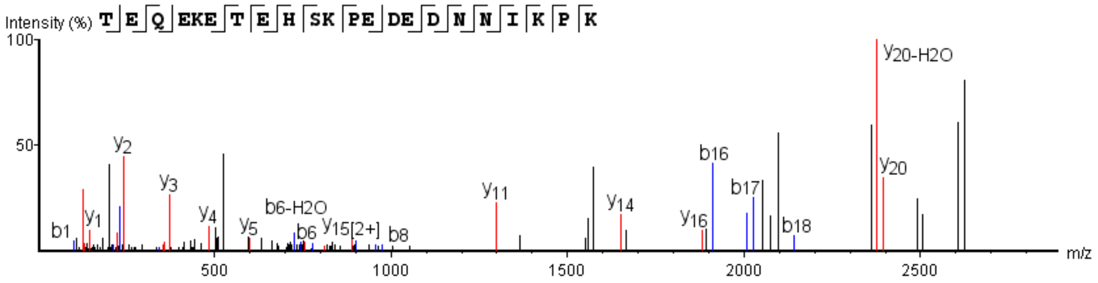

gi58585132

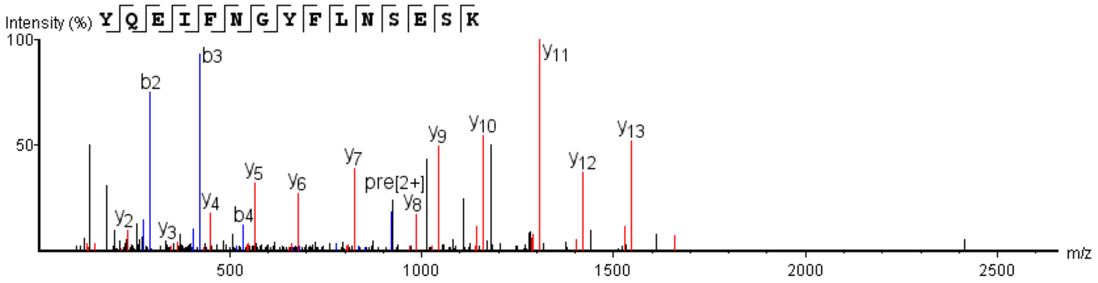

gi58585206

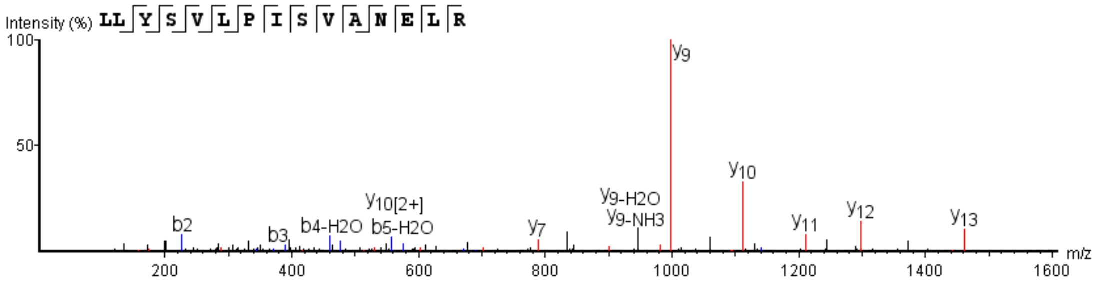

gi66499475

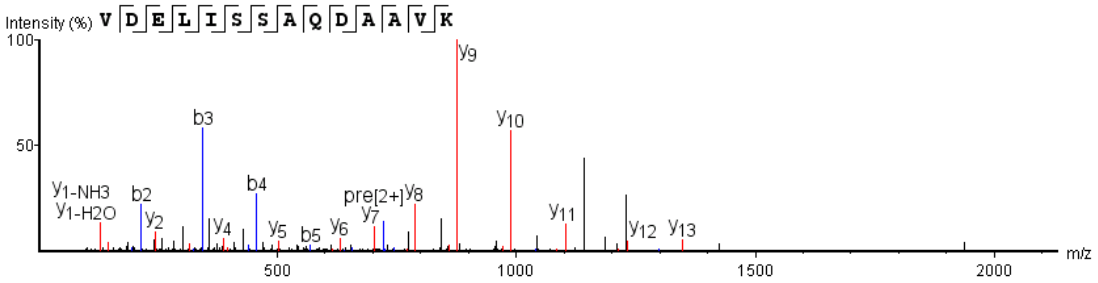

gi|571549102

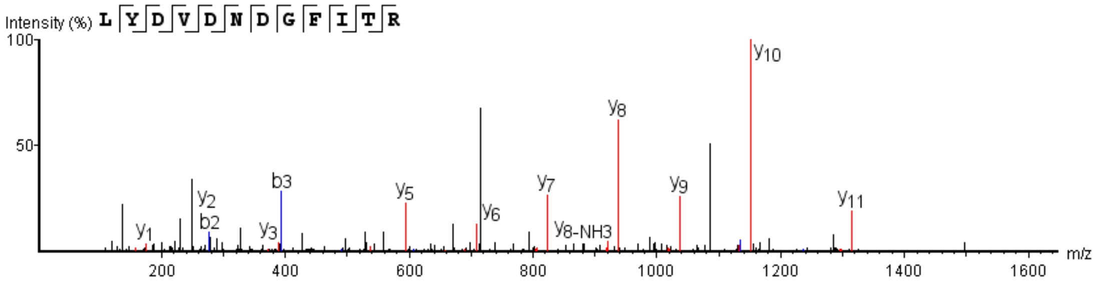

gi|571549099

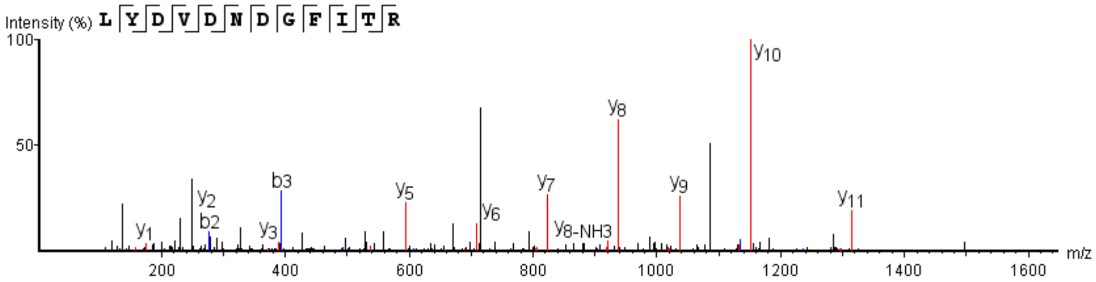

gi66499717

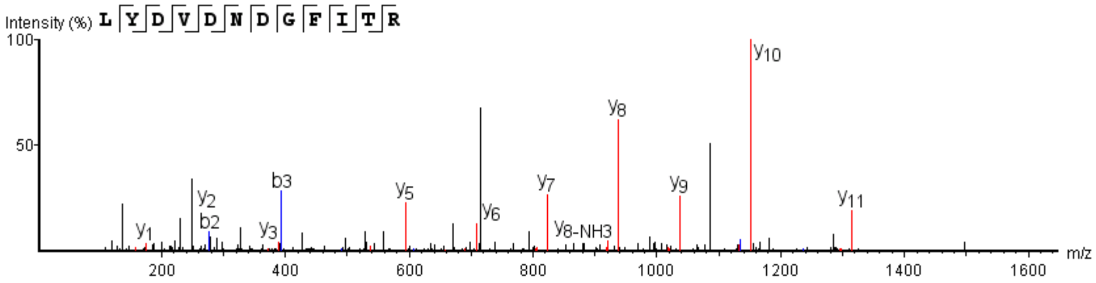

gi66500455

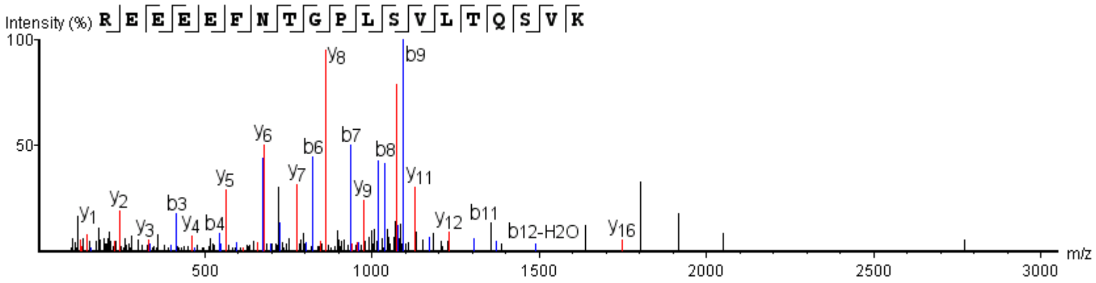

gi66504030

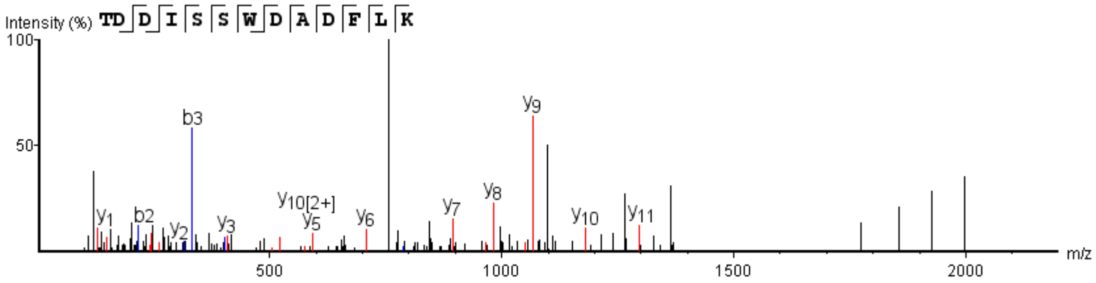

gi66504263

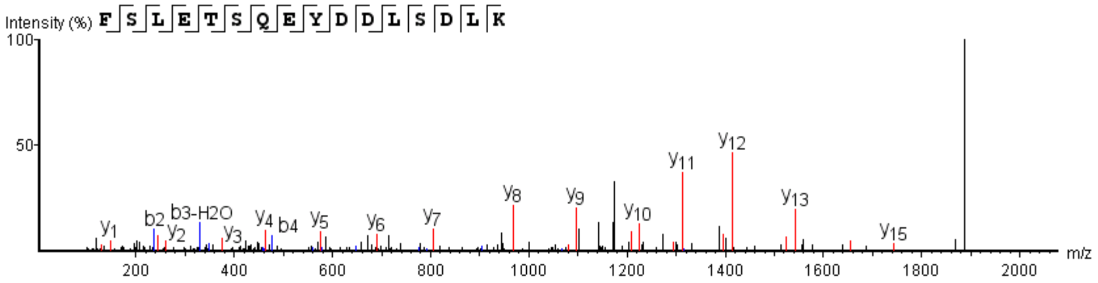

gi66505264

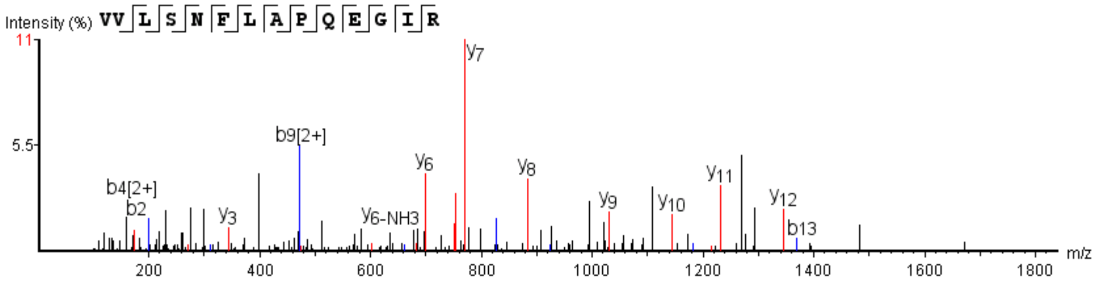

gi66506759

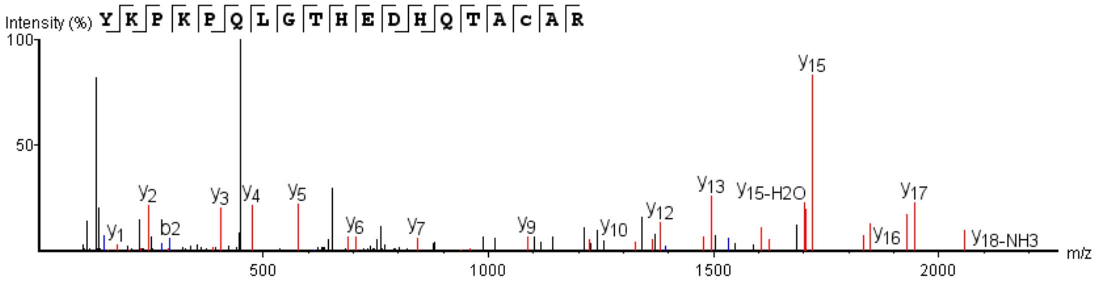

gi66508242

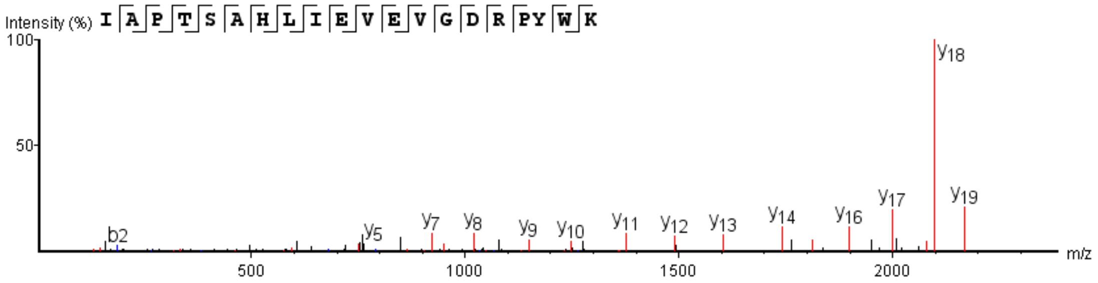

gi|571545333

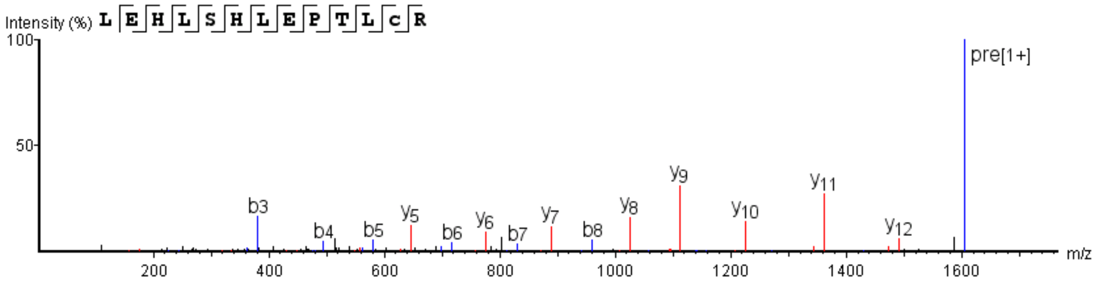

gi|571545340

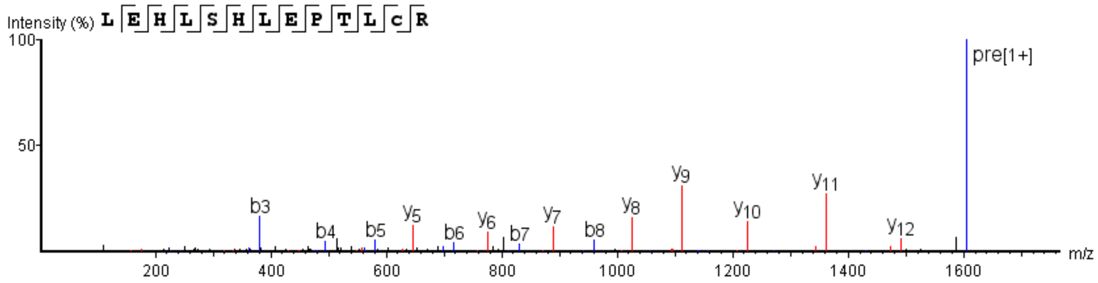

gi|571545346

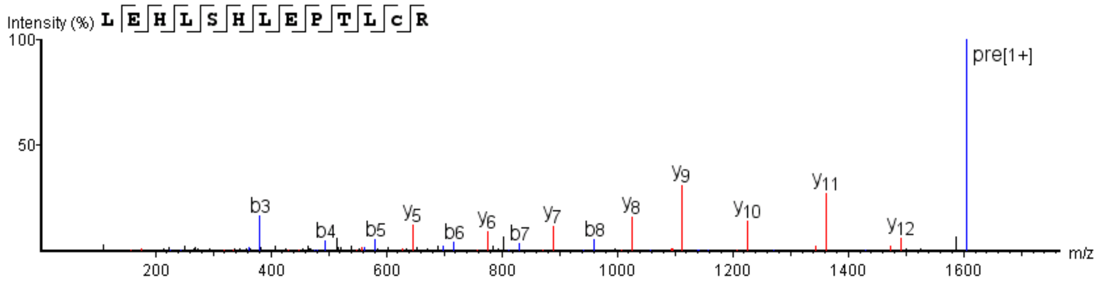

gi|571545349

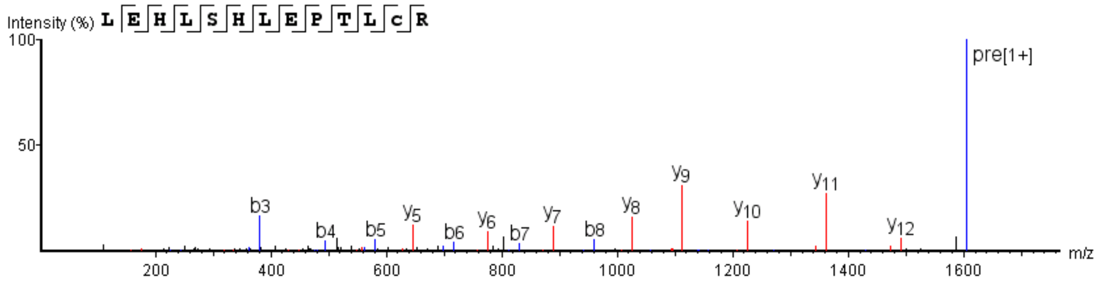

gi|571575410

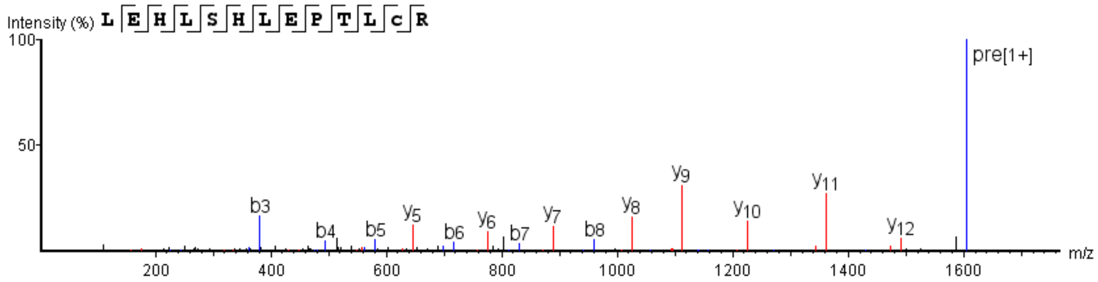

gi|571531426

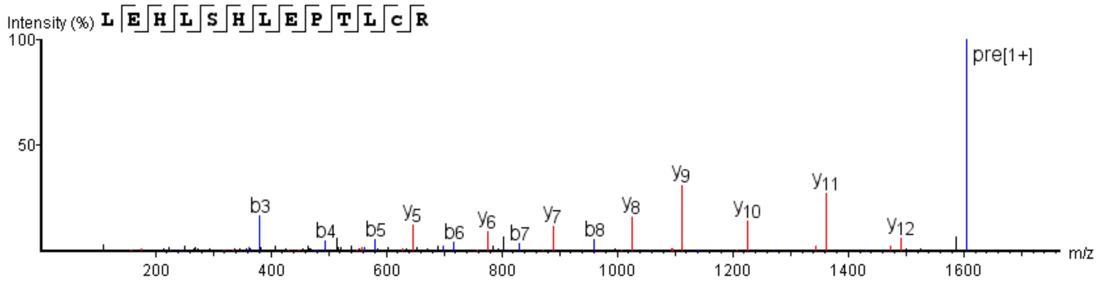

gi66509585

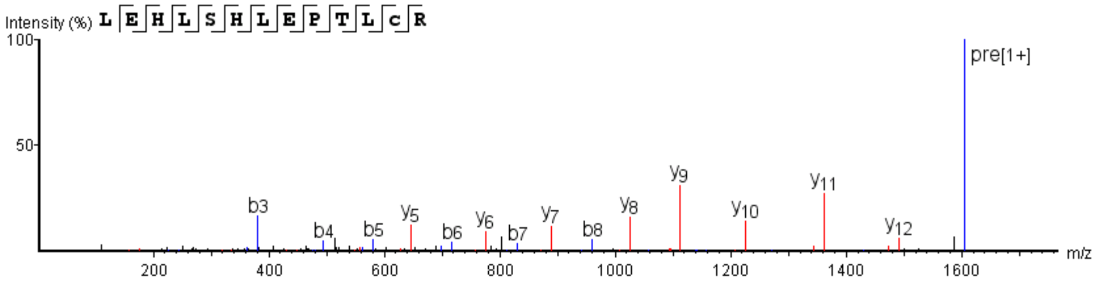

gi|571541144

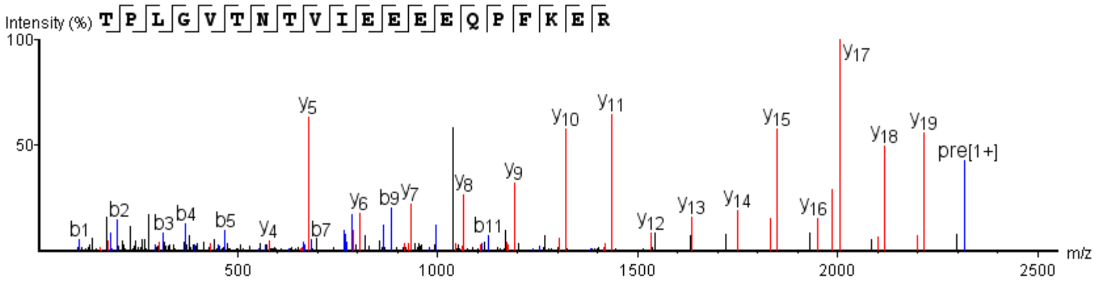

gi66512104

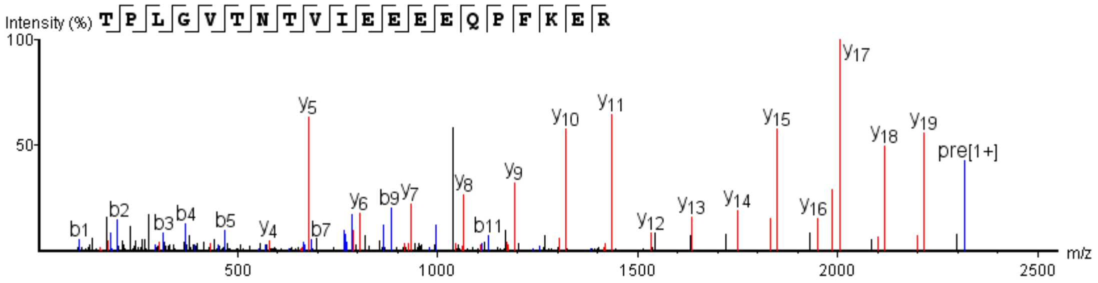

gi66515508

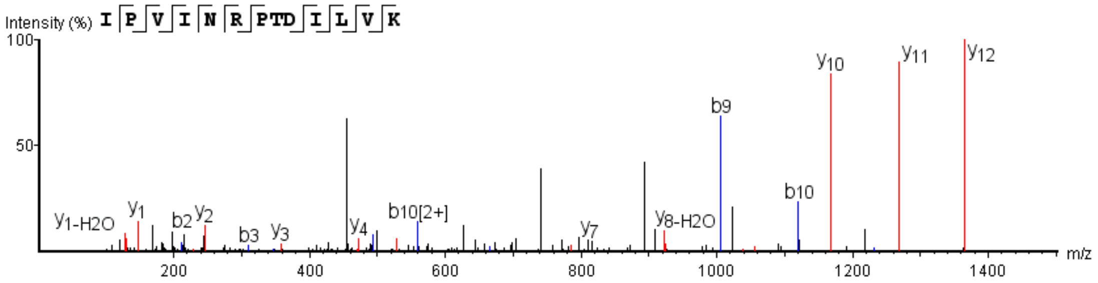

gi66516093

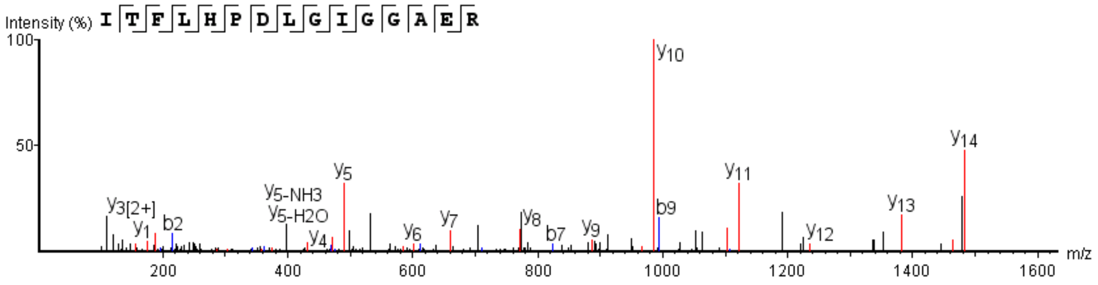

gi66516304

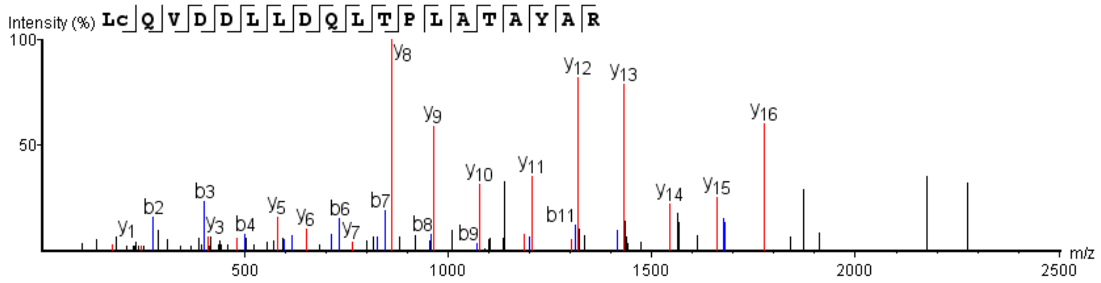

gi66517185

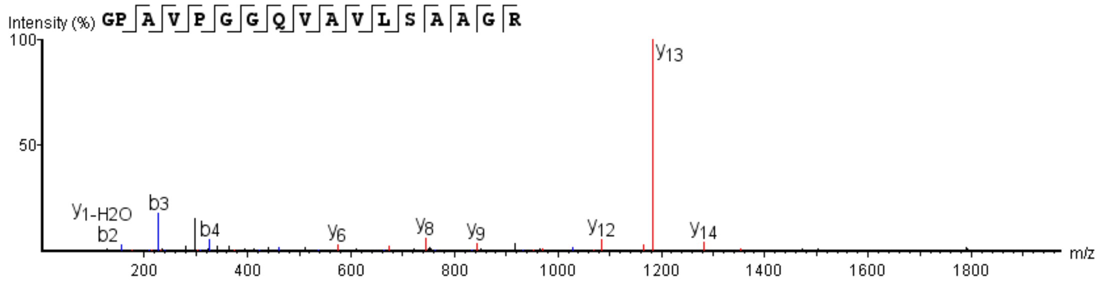

gi66517238

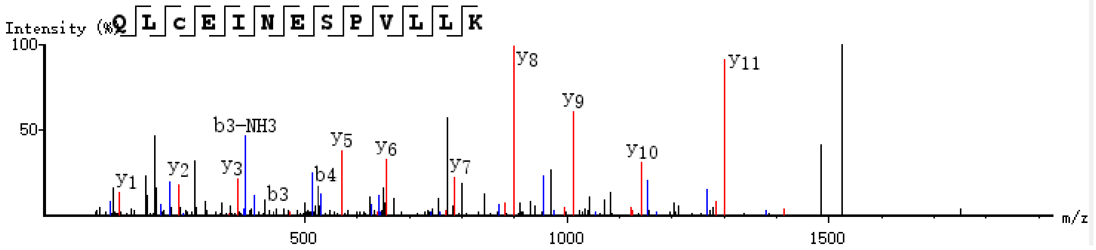

gi|571554726

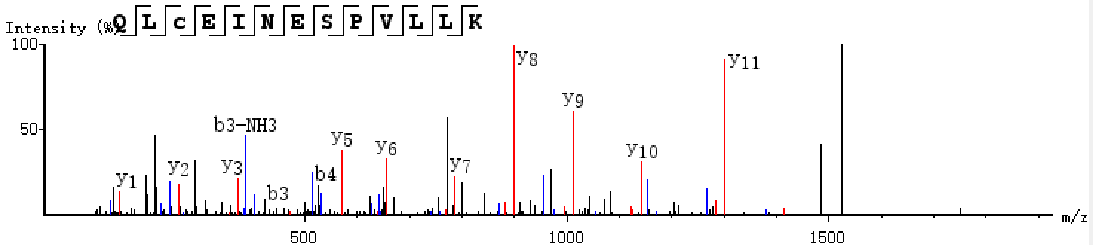

gi|571554730

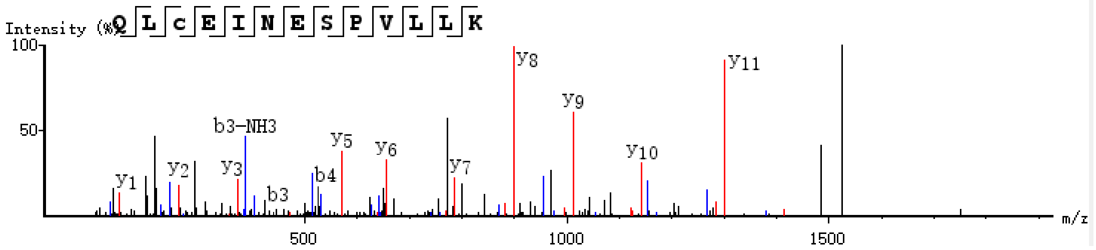

gi66519683

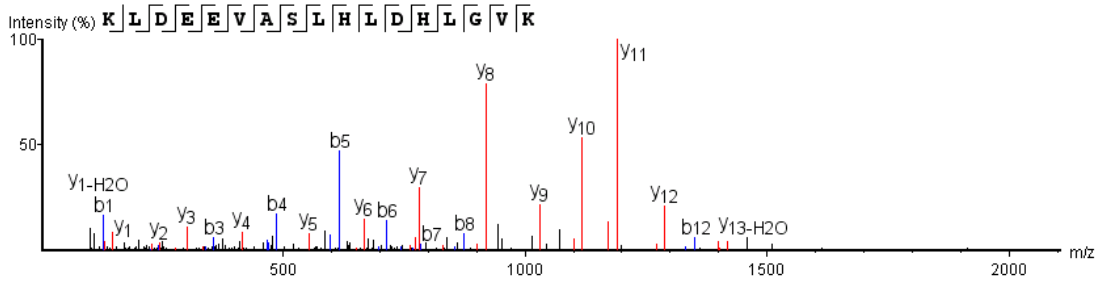

gi|571566593

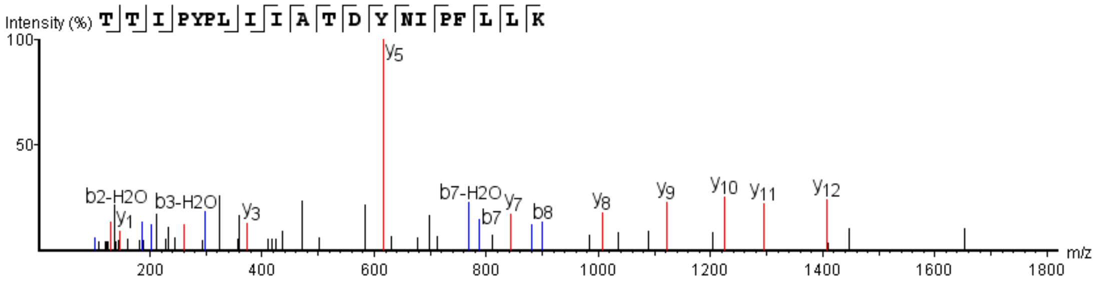

gi|571566596

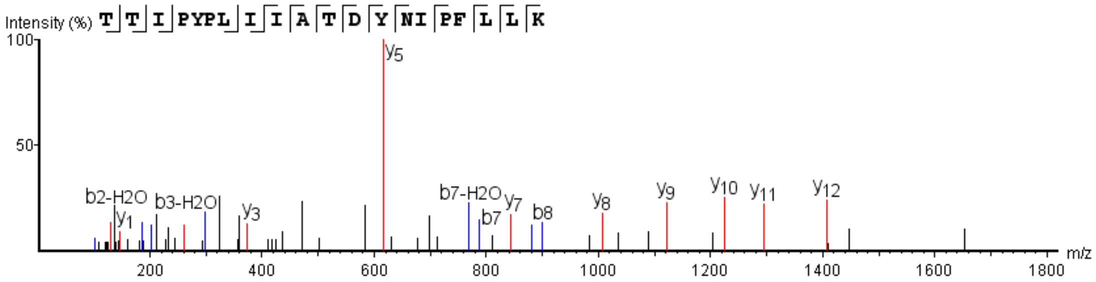

gi|571566599

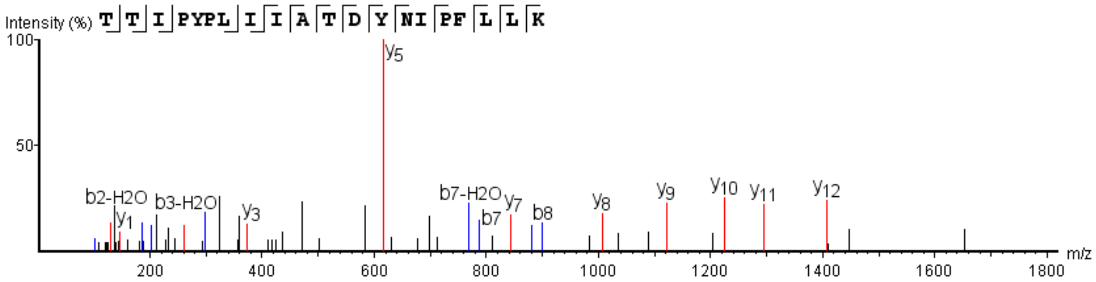

gi66522157

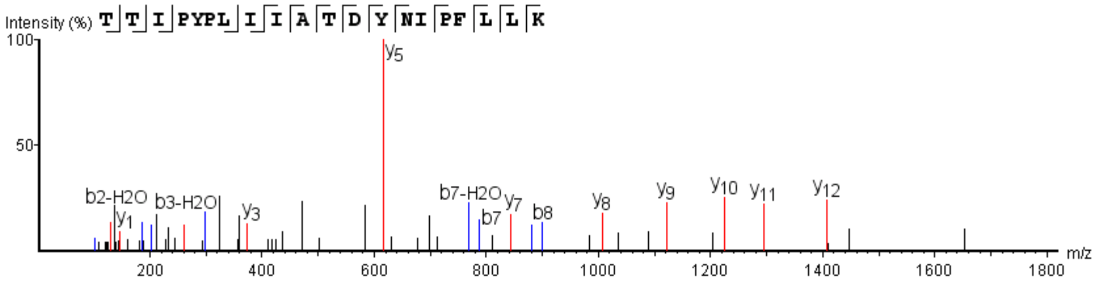

gi66525345

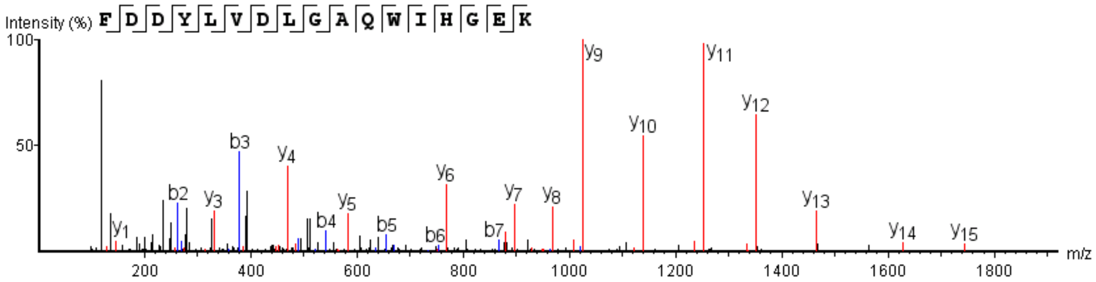

gi66525427

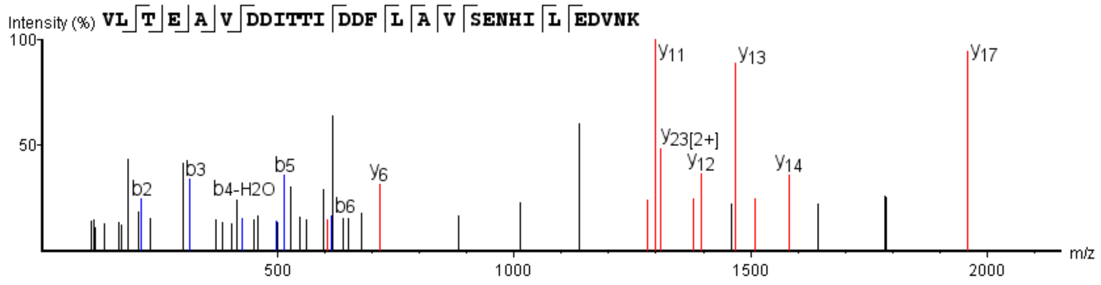

gi66525633

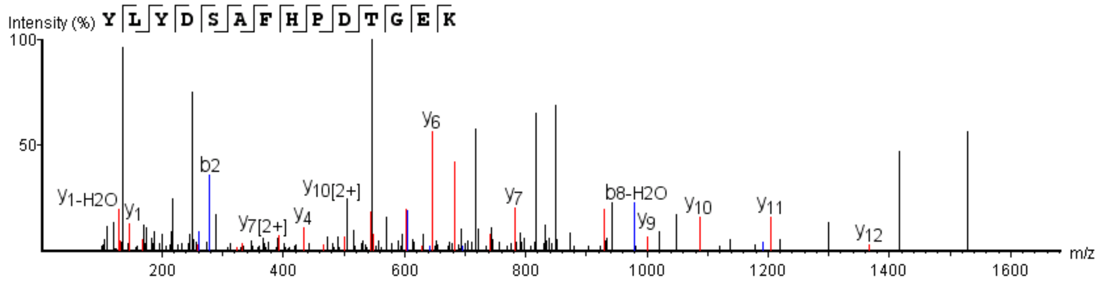

gi66529630

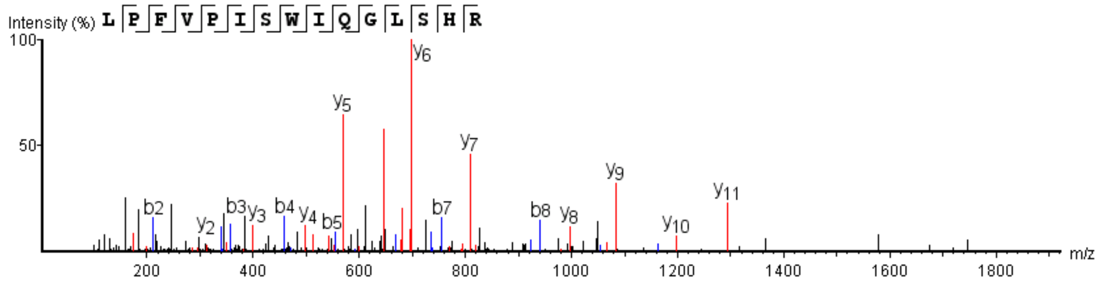

gi66529797

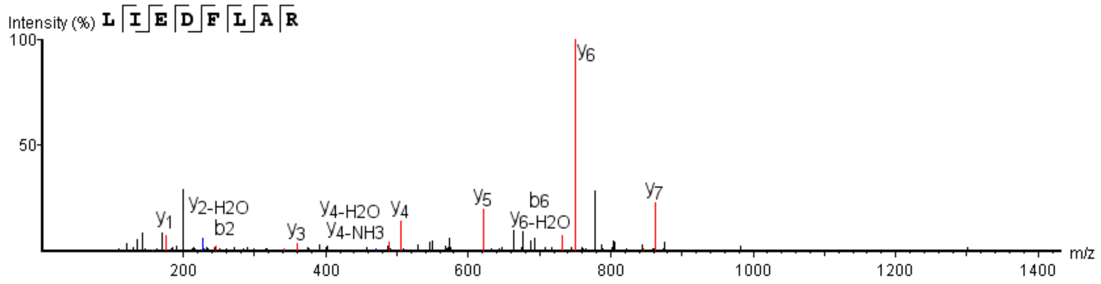

gi66531704

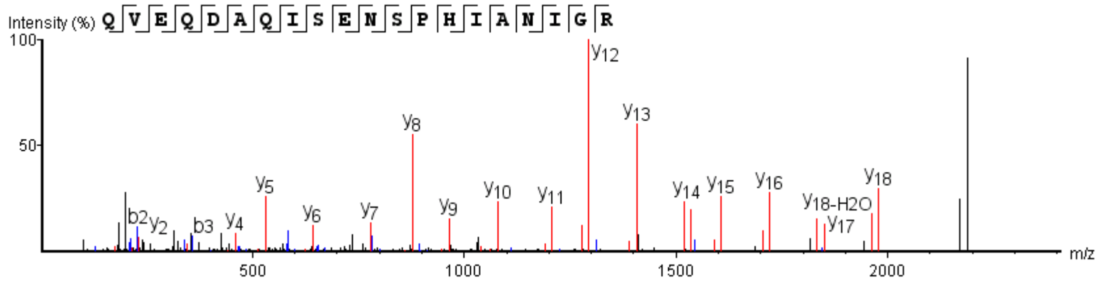

gi66532731

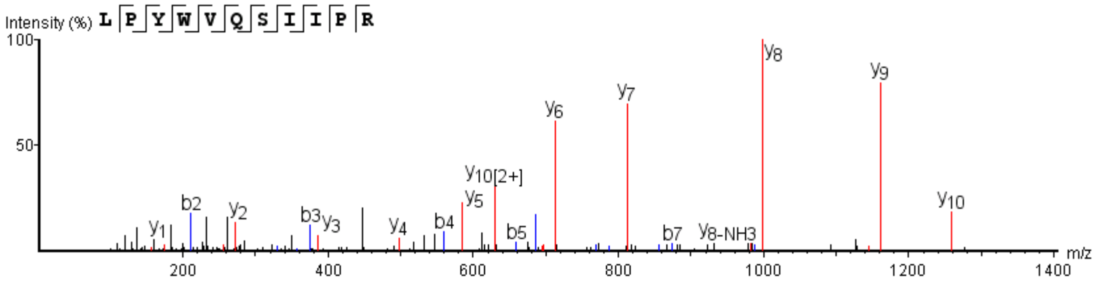

gi66540209

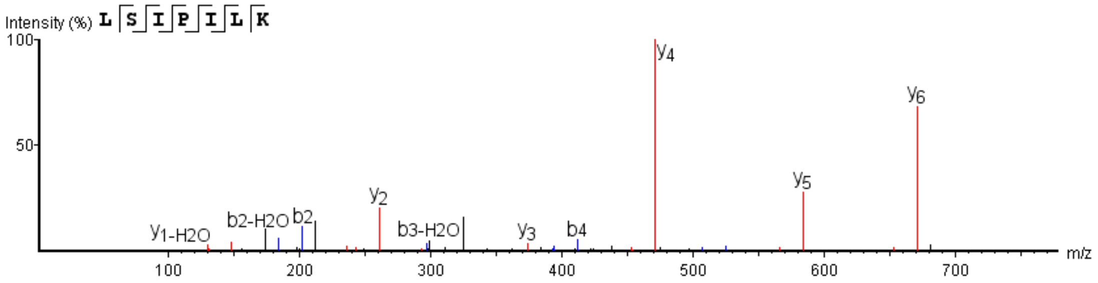

gi66555143

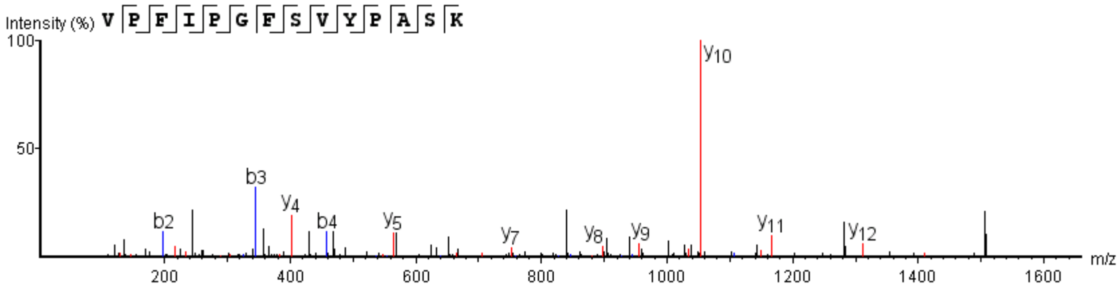

gi66555021

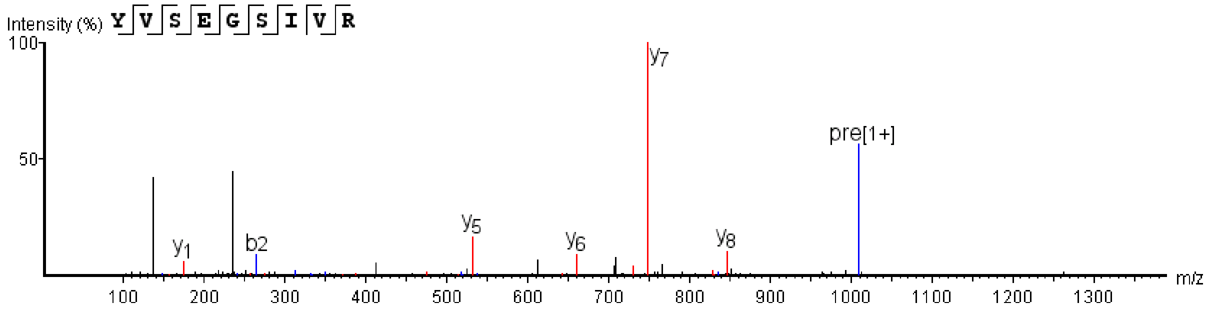

gi66554764

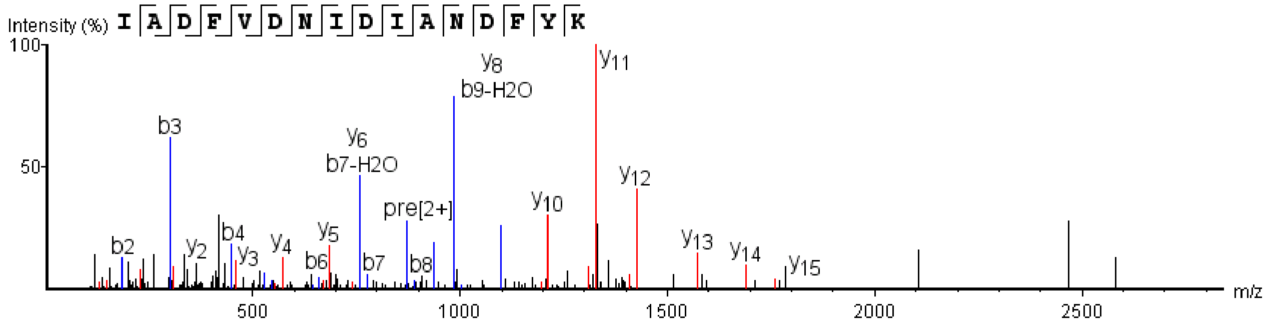

gi66553455

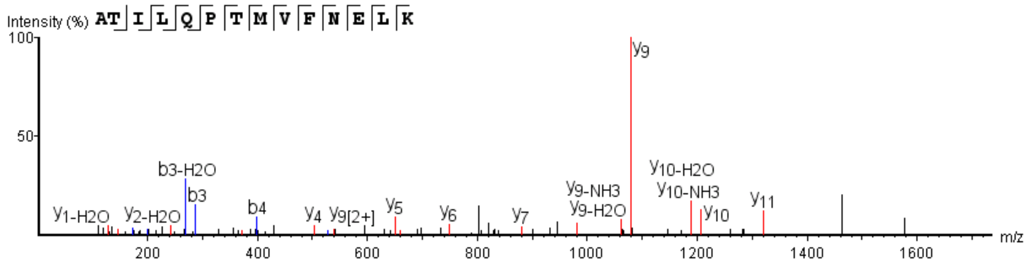

gi66552169

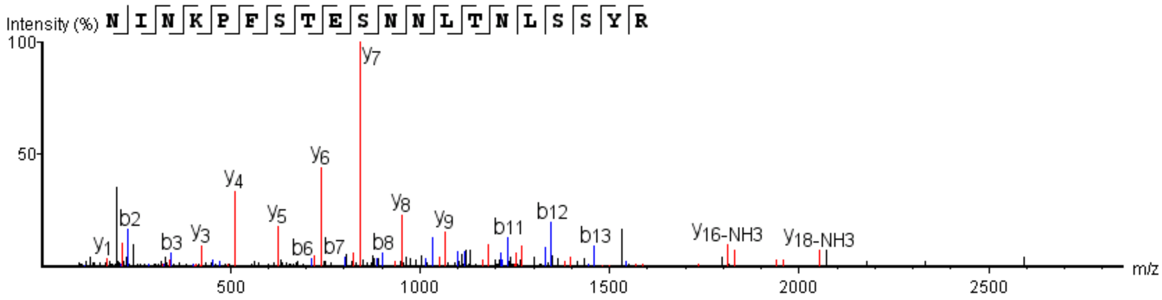

gi66551959

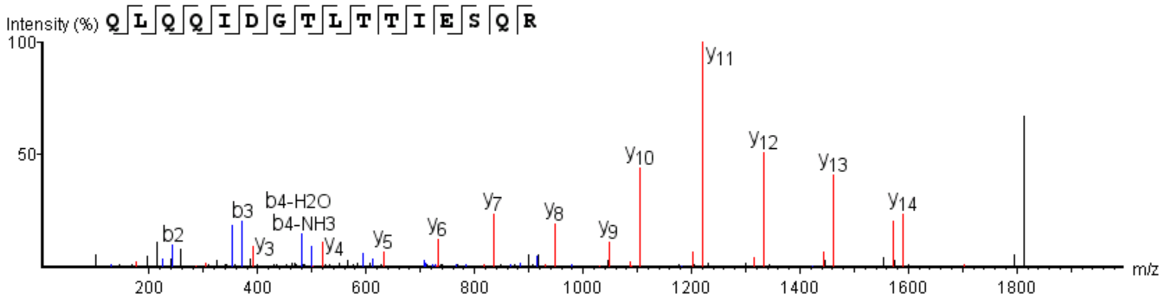

gi66551653

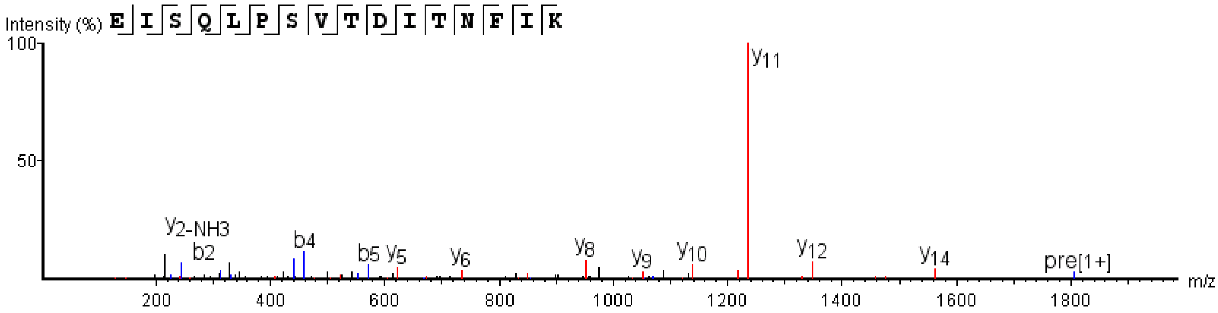

gi66549806

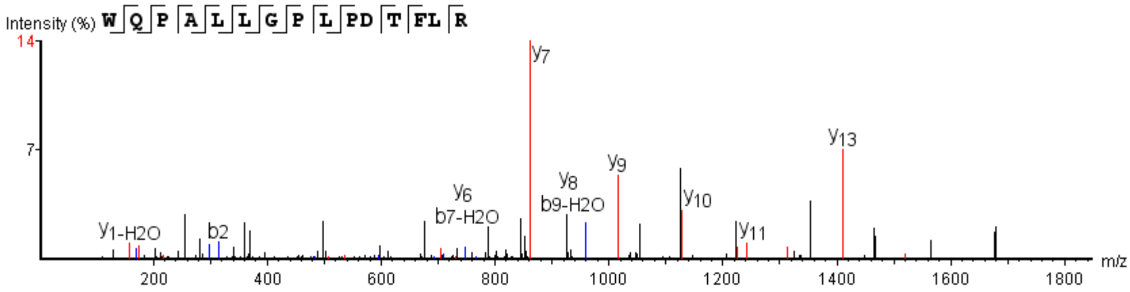

gi66549336

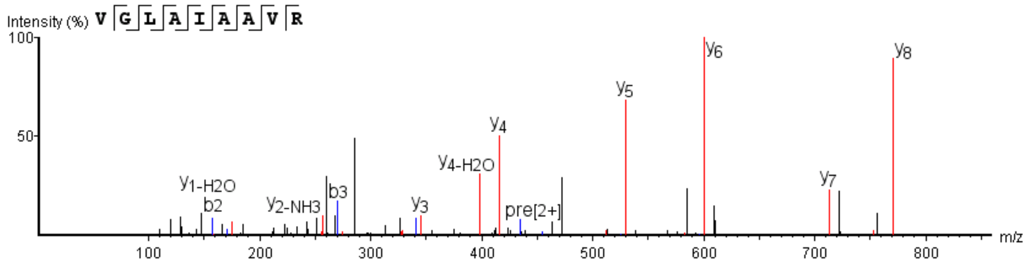

gi66544564

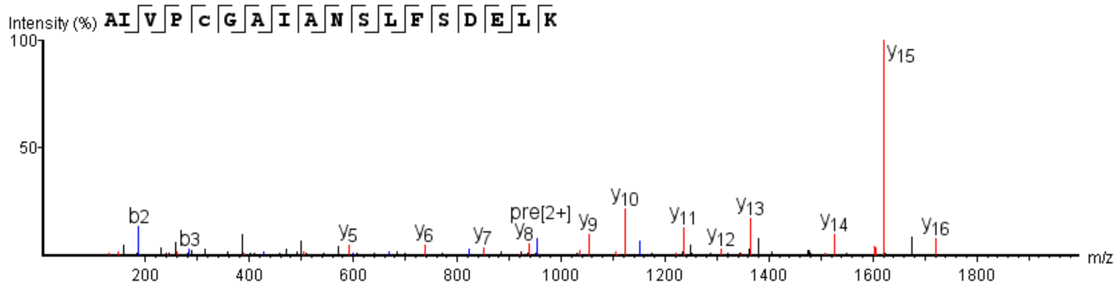

gi66542158

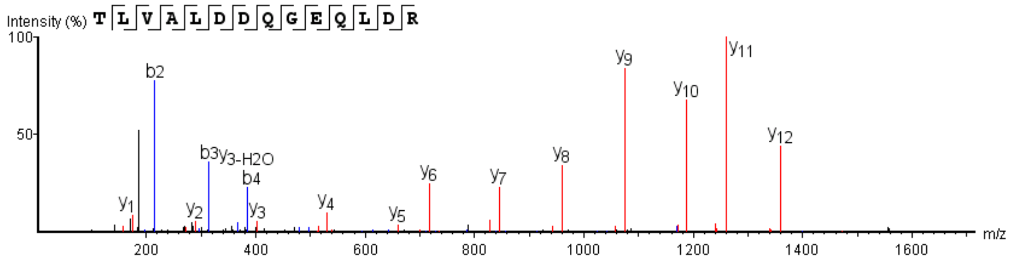

gi66542091

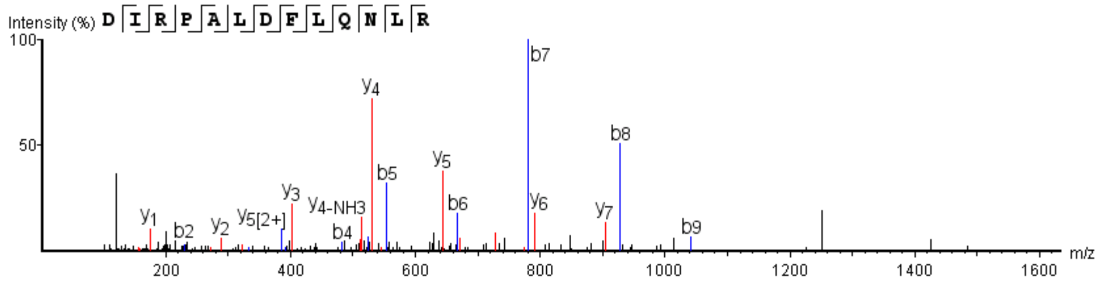

gi110755033

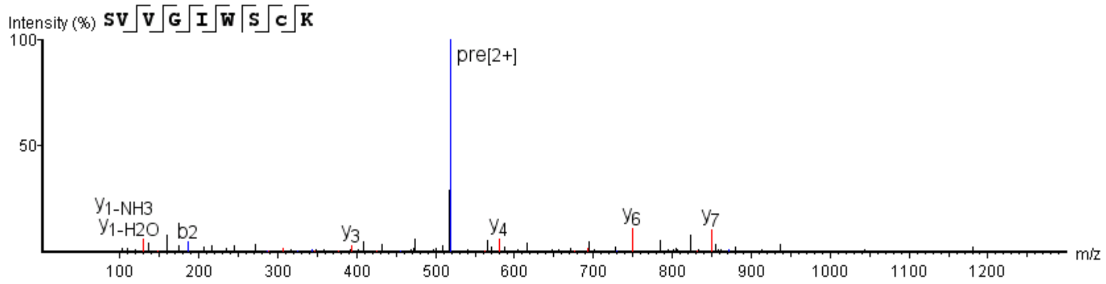

gi110749393

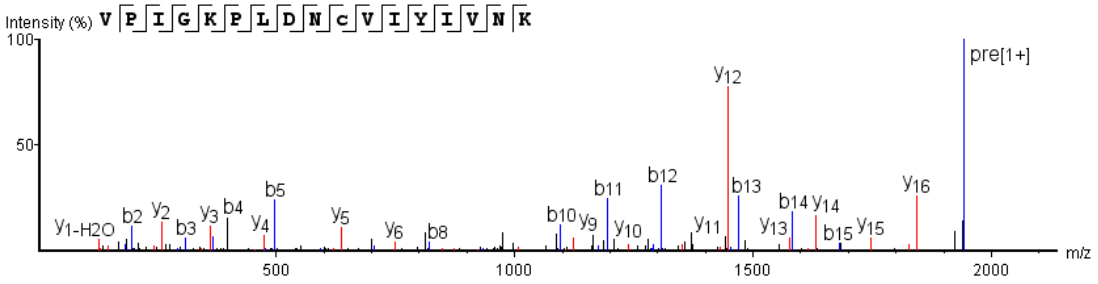

gi110749234

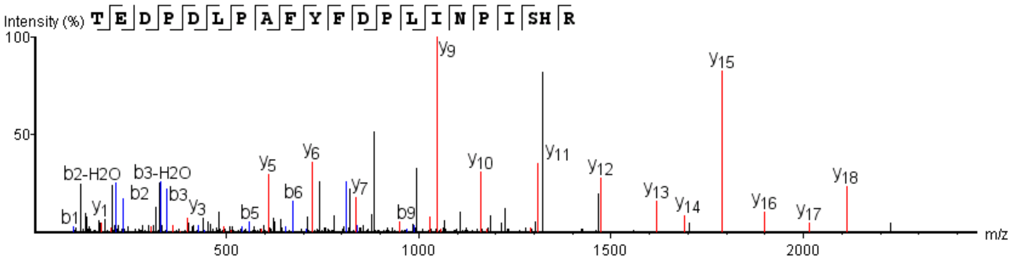

gi110748994

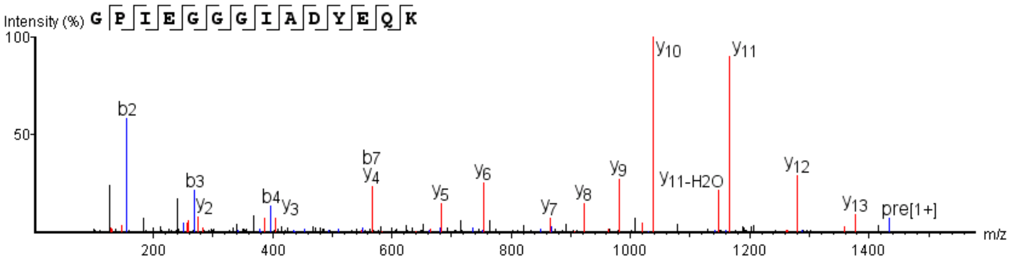

gi71573316

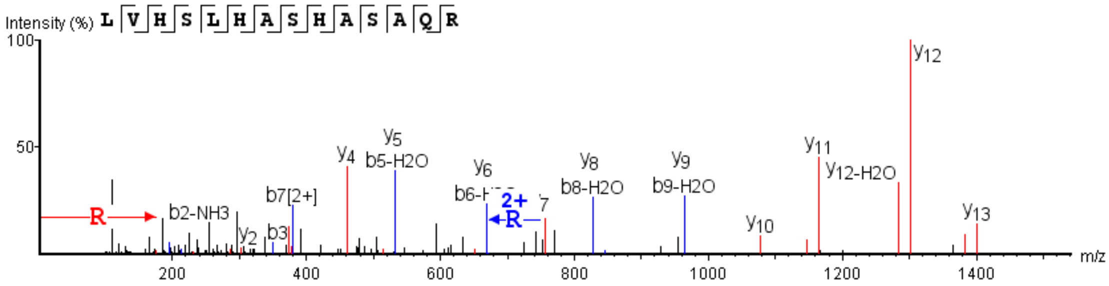

gi|571535386

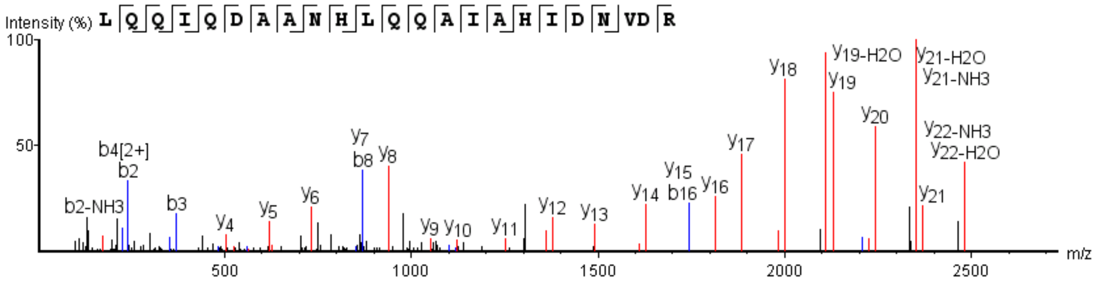

gi66564441

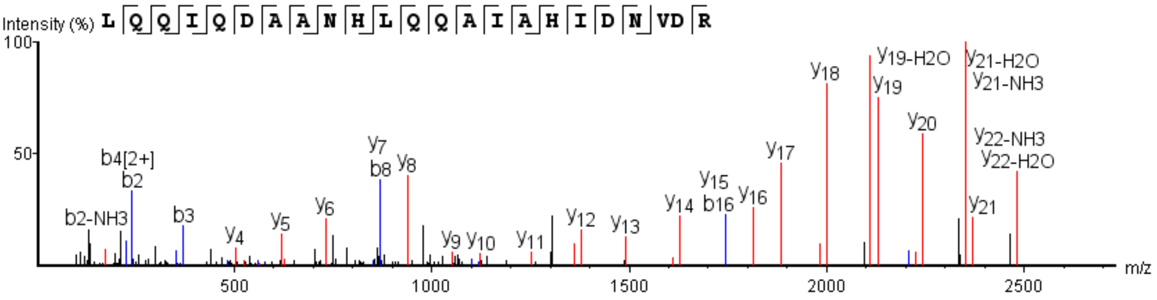

gi66562243

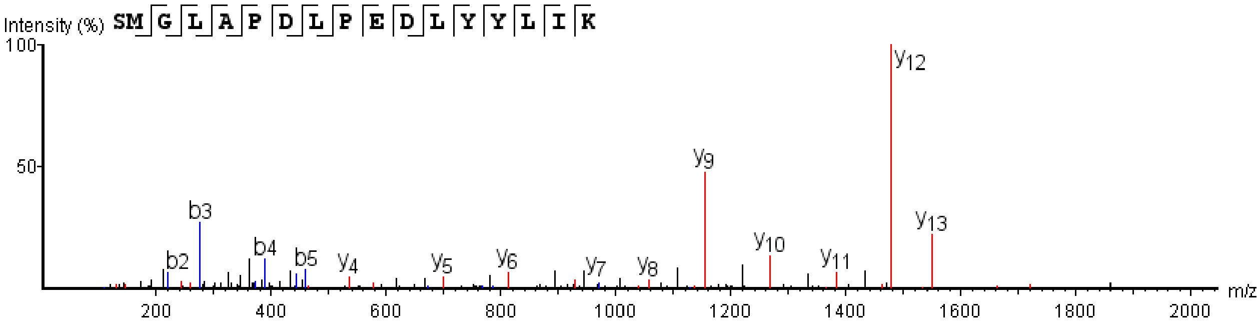

gi66561731

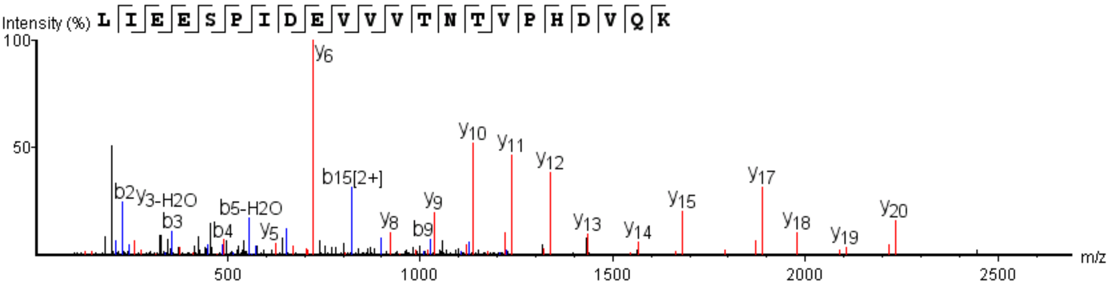

gi66560294

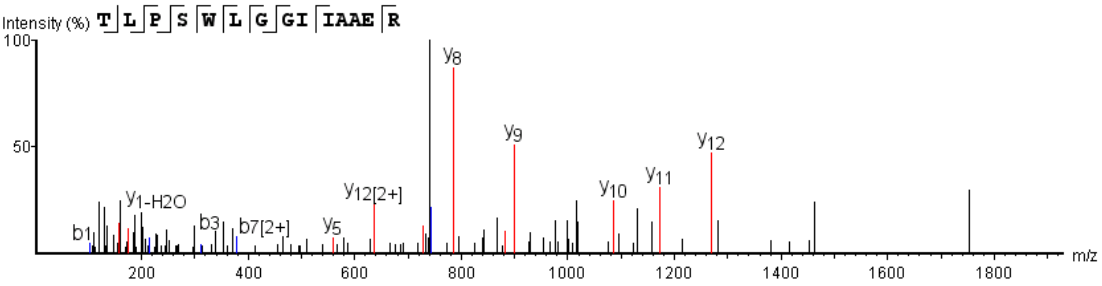

gi66558726

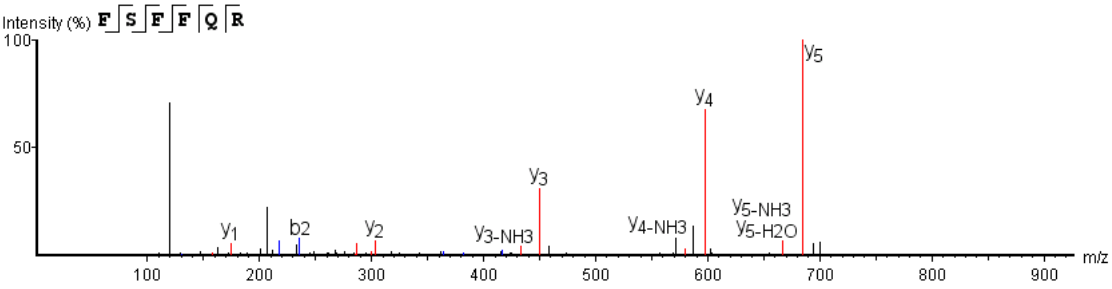

gi66556835

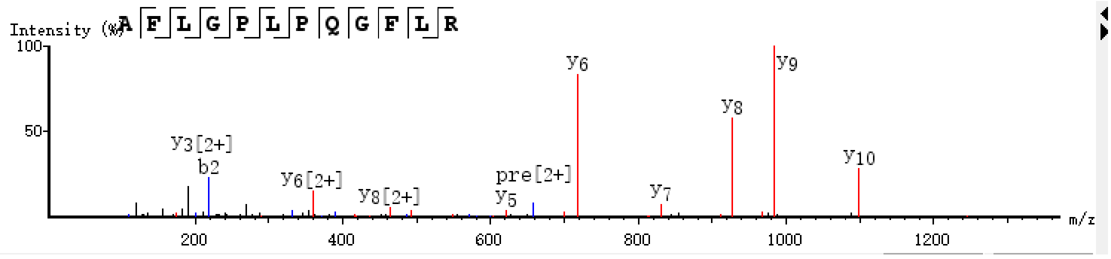

gi110761627

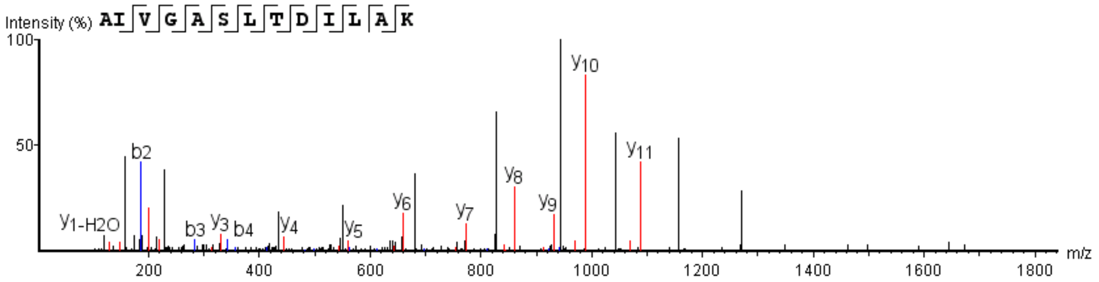

gi|571560975

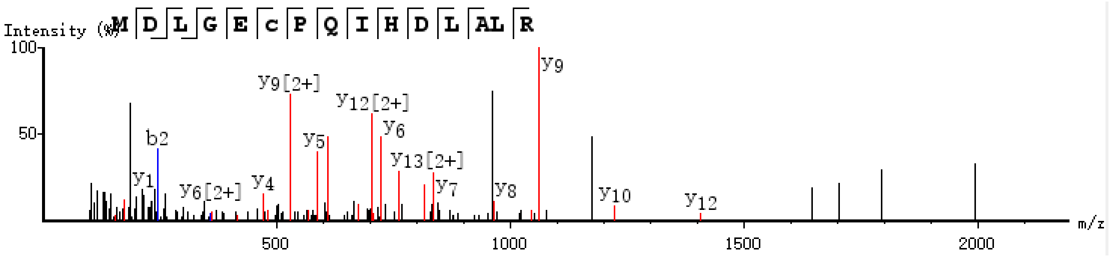

gi|571560979

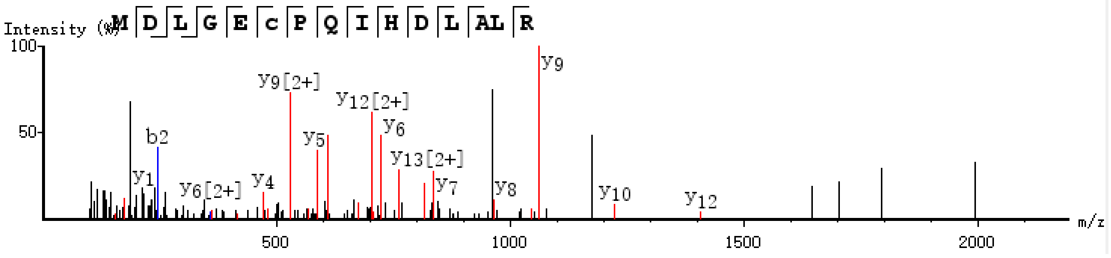

gi|571560983

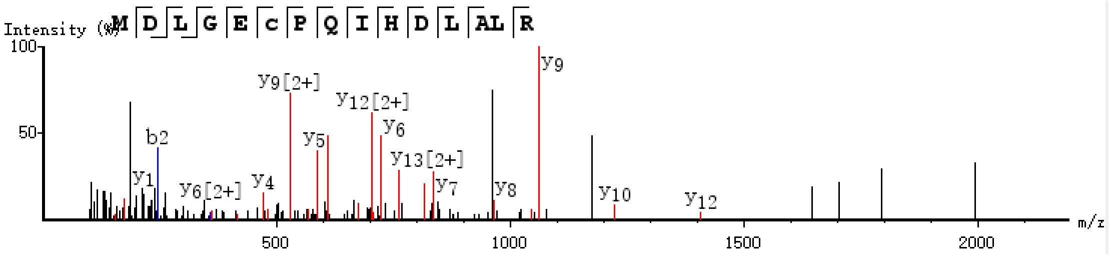

gi|571560987

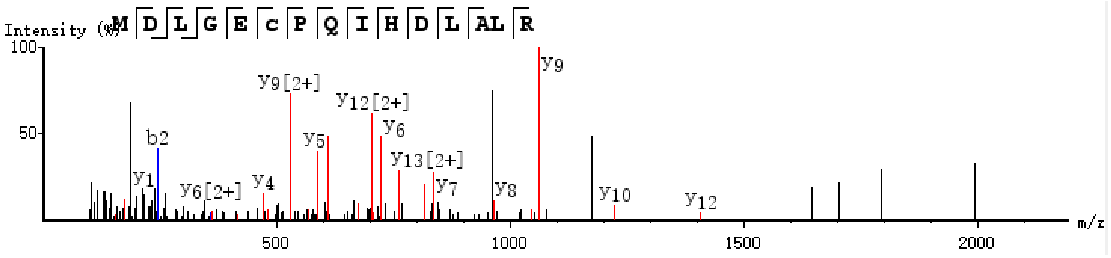

gi|571560991

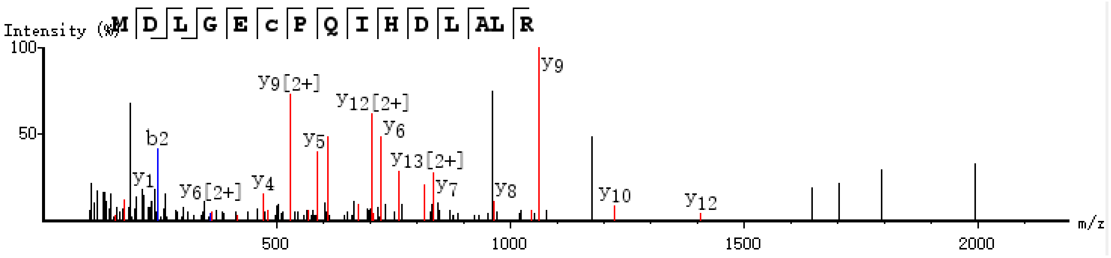

gi|571560995

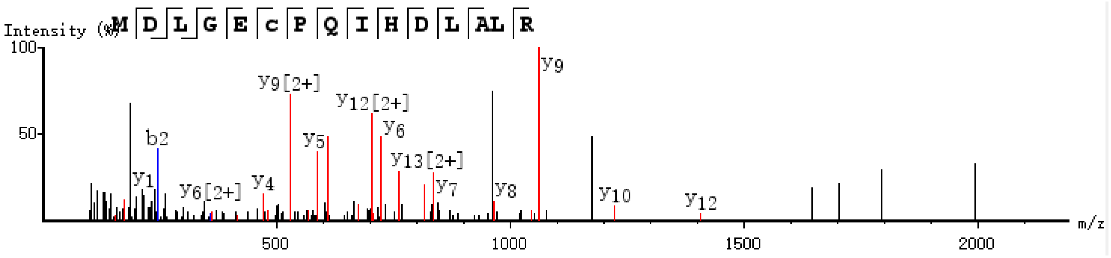

gi|571560999

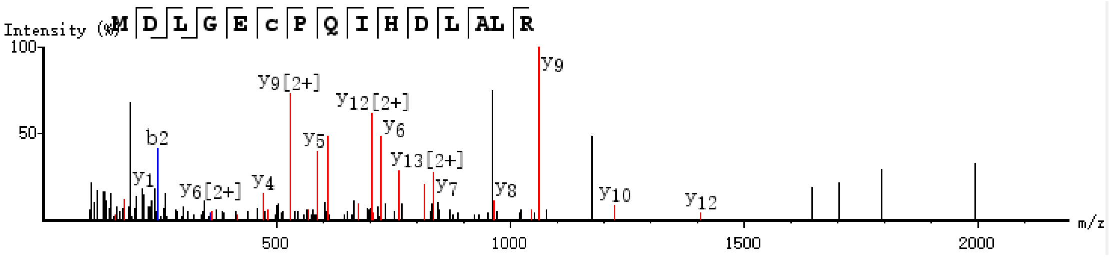

gi110761115

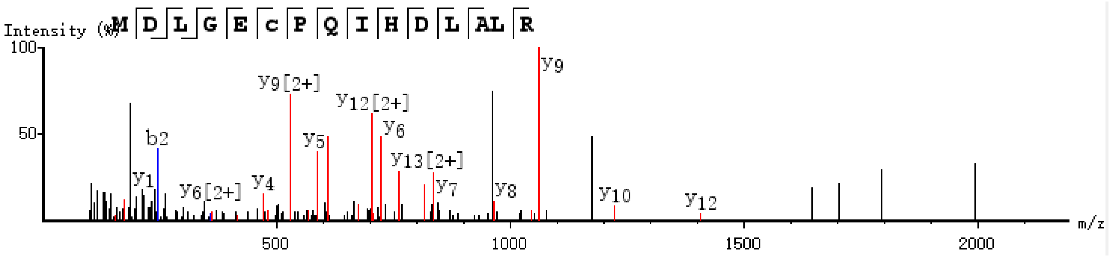

gi|328777929

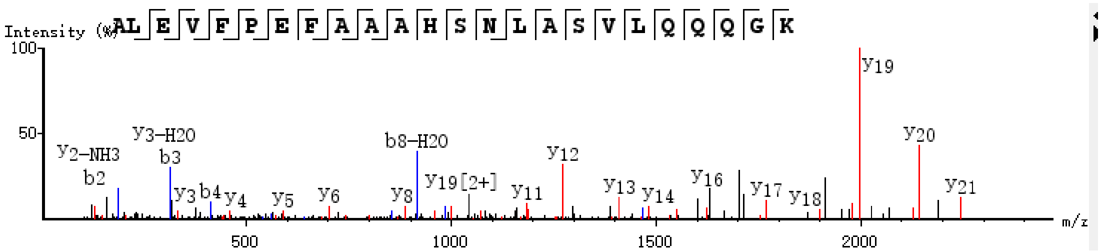

110760723

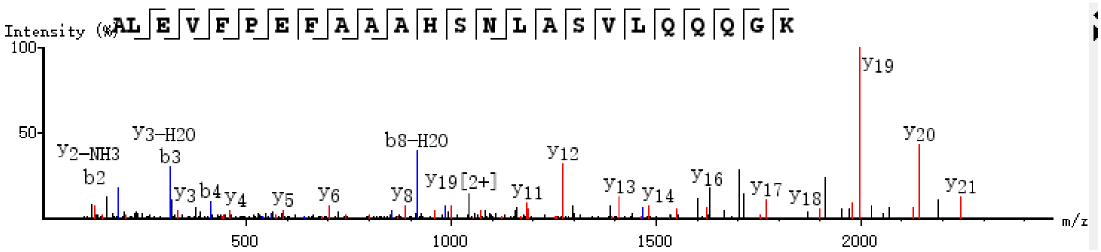

gi|571553163

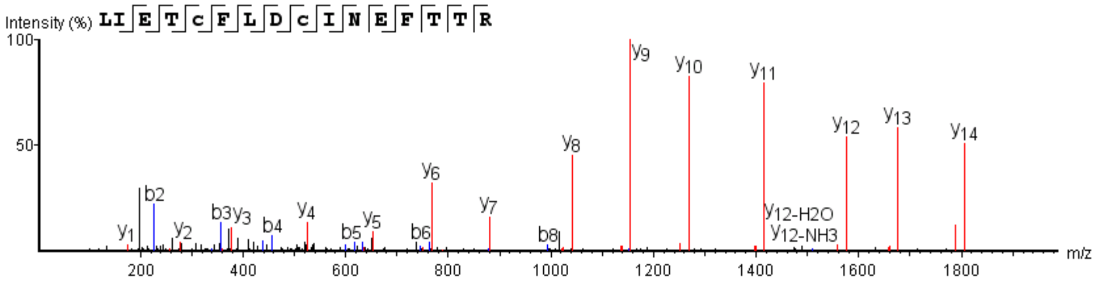

gi110760068

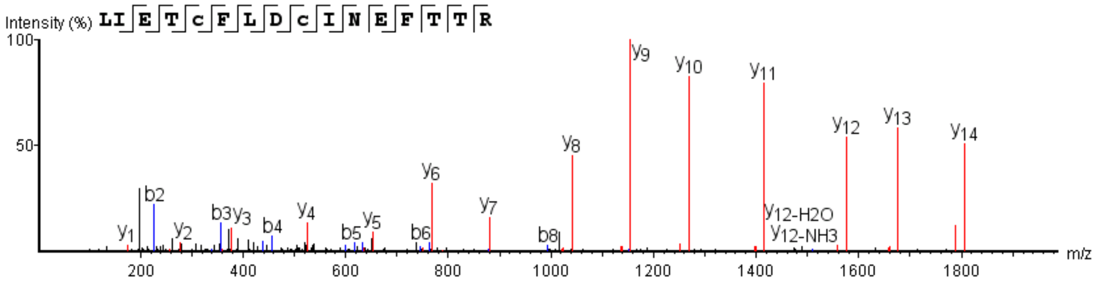

gi110759426

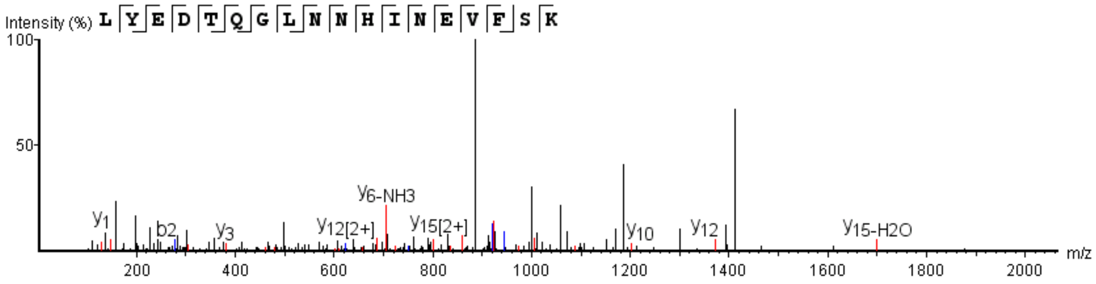

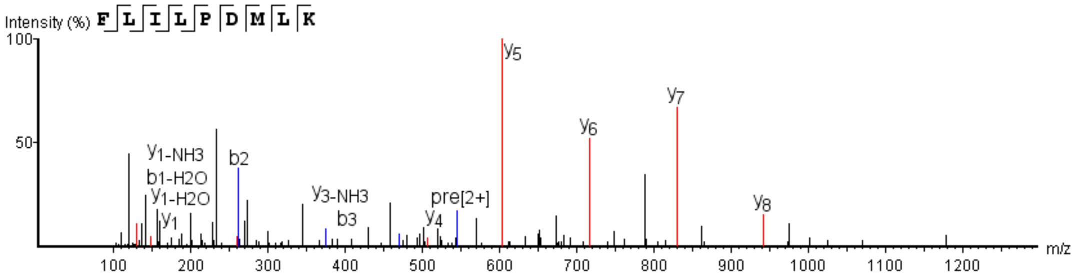

gi110756641

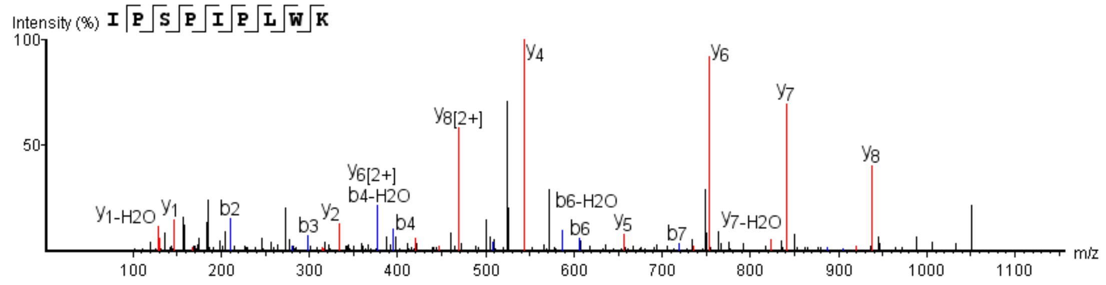

gi|571571431

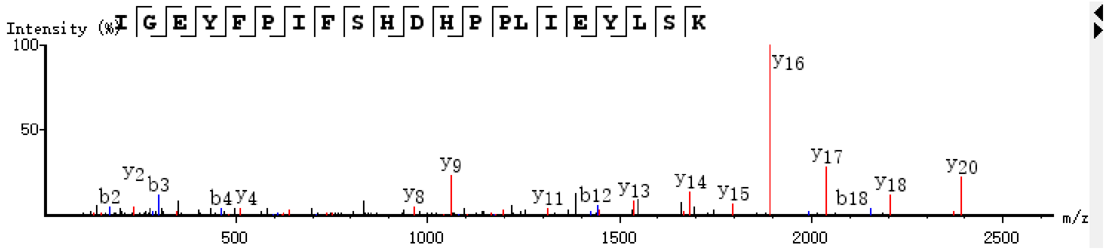

gi|571571434

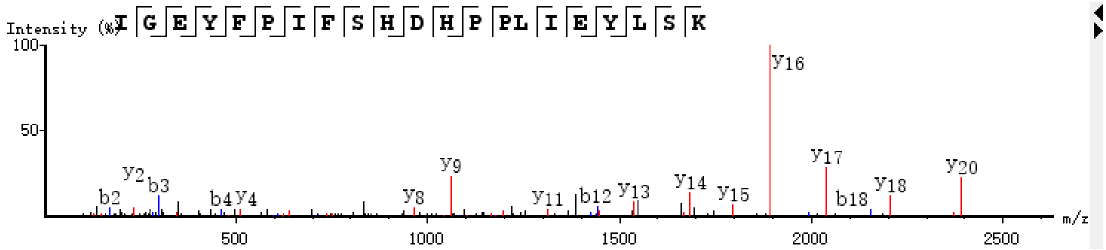

gi|328790105

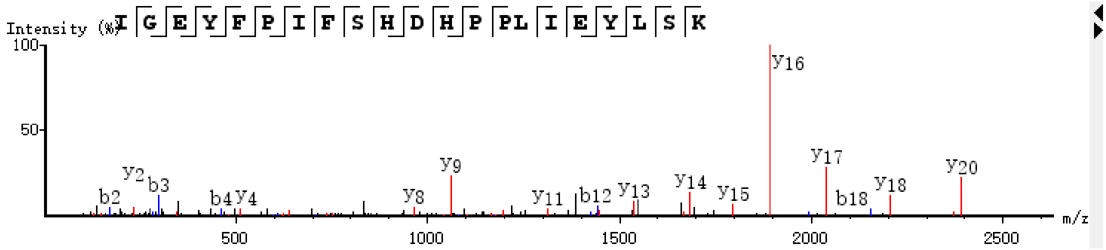

gi110756571

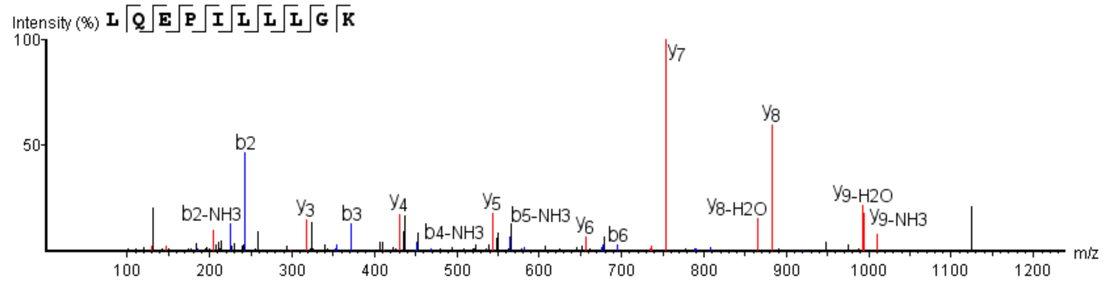

gi110755421

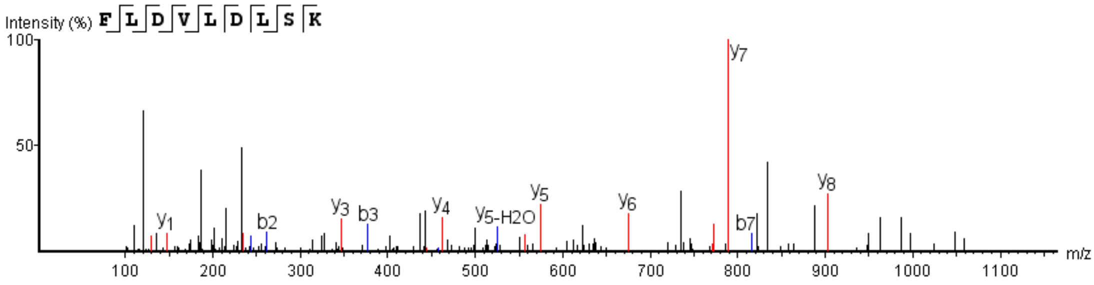

gi110755171

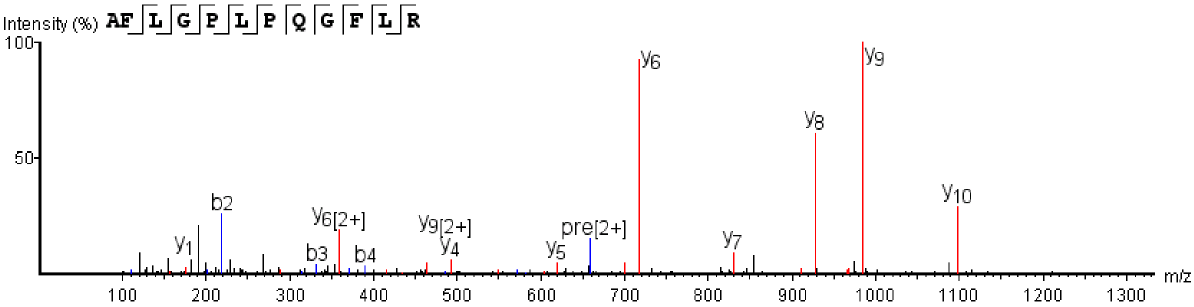

gi118150480

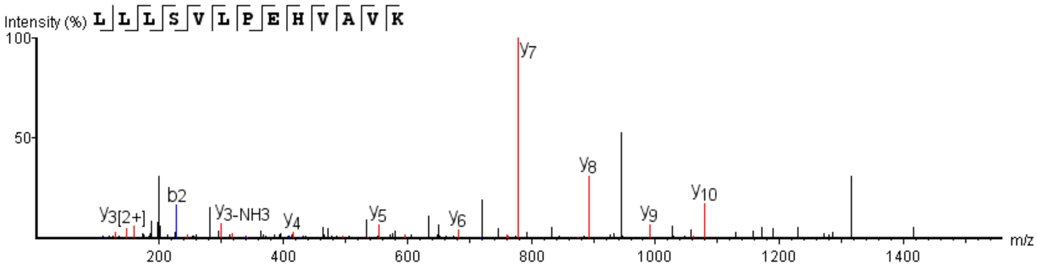

gi110772198

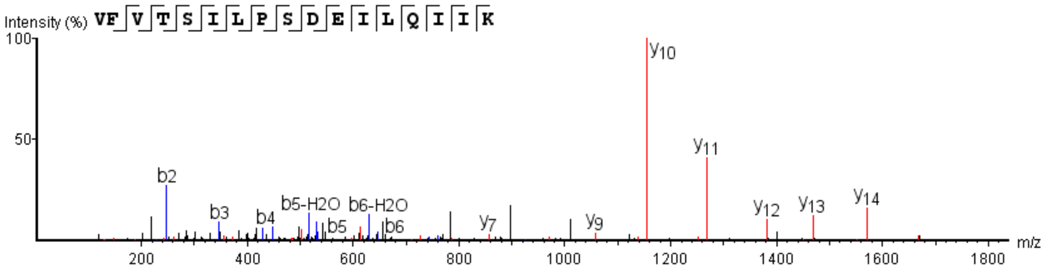

gi110771708

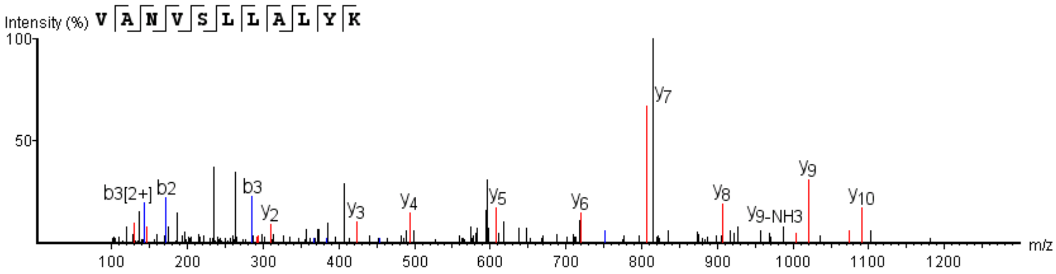

gi110767181

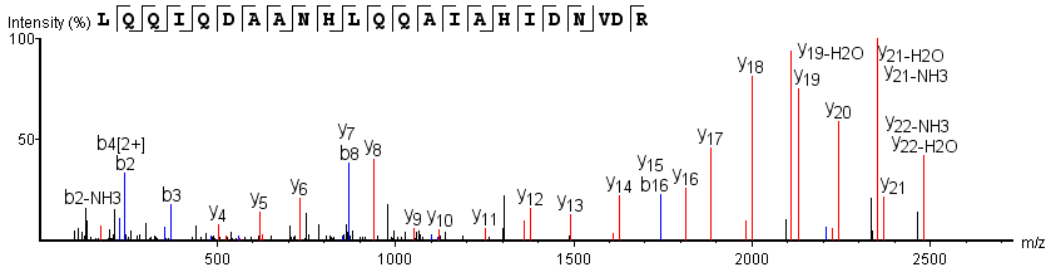

gi110766823

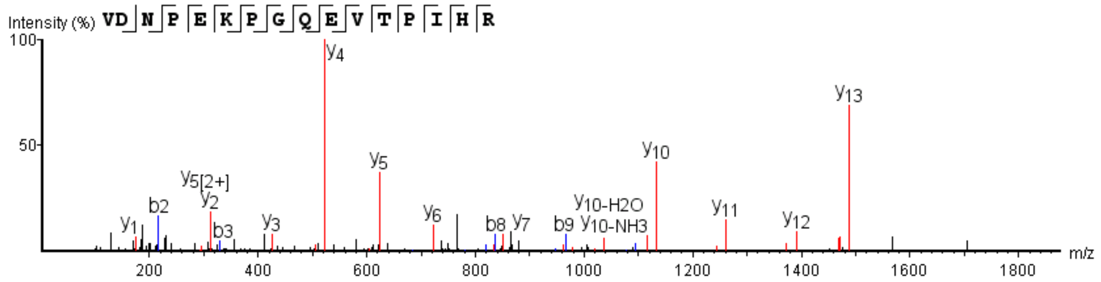

gi|328779841

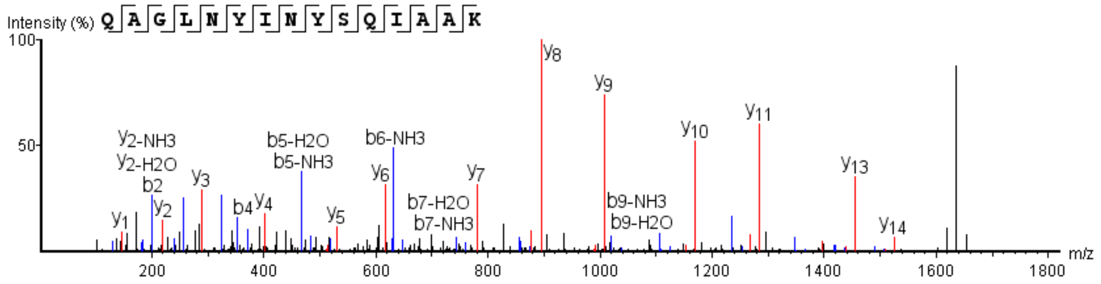

gi110764763

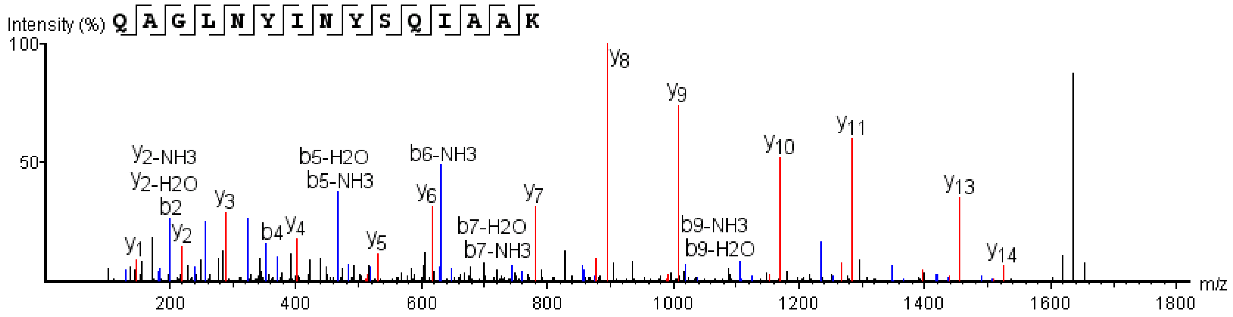

gi110764754

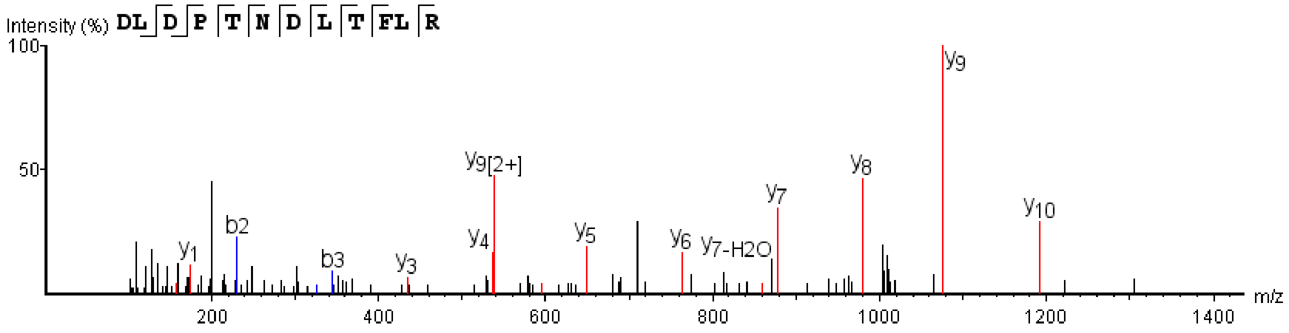

gi110764733

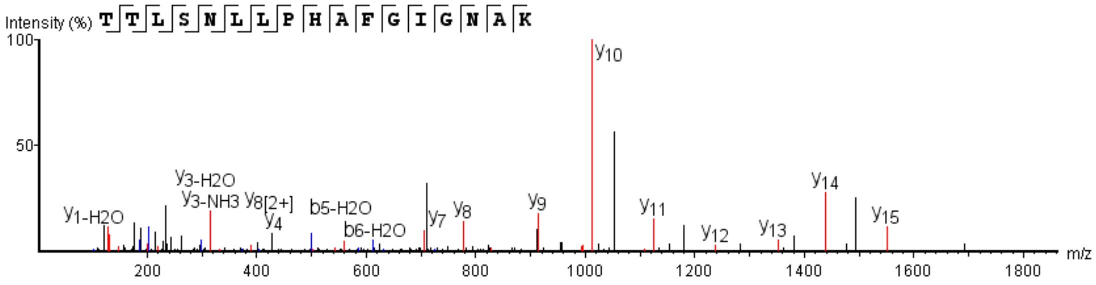

gi|571561398

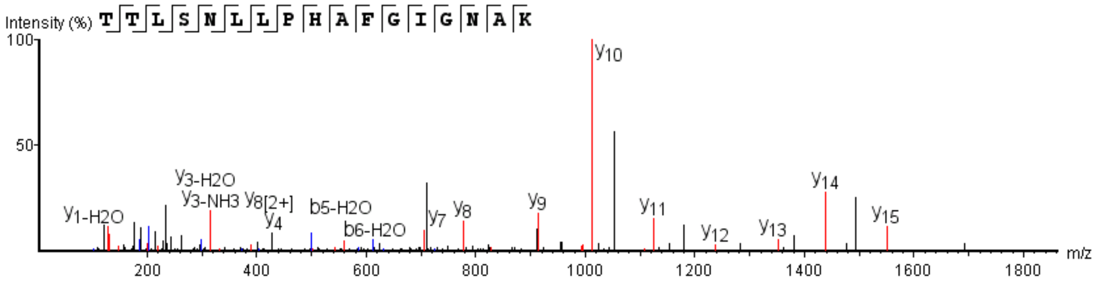

gi|571561402

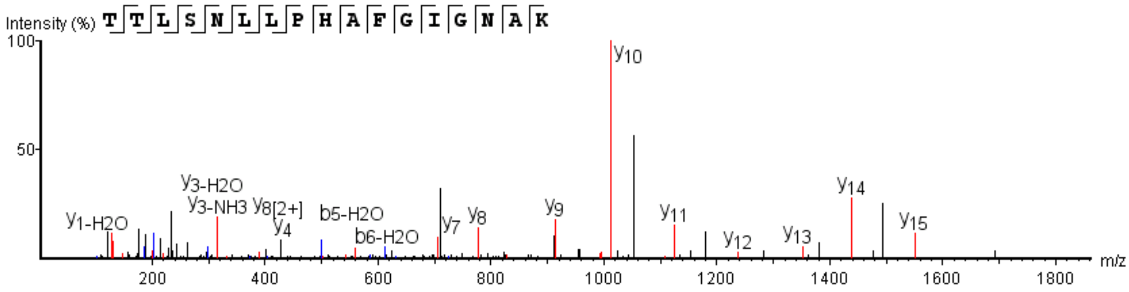

gi110763495

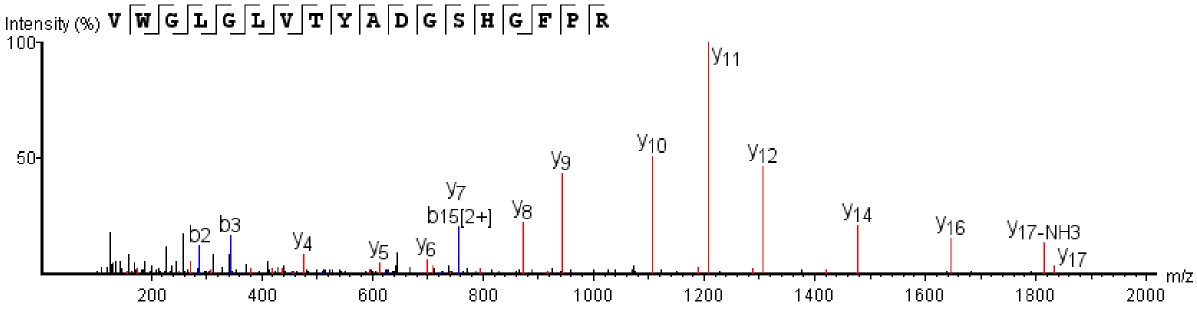

gi110762818

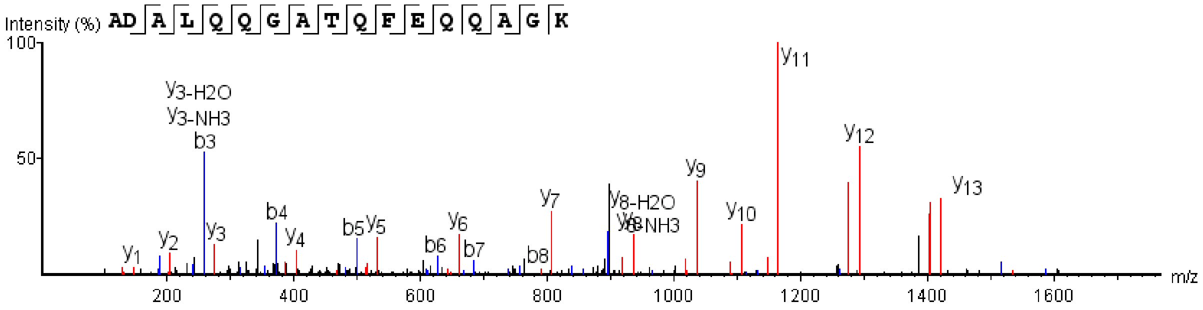

gi571579618

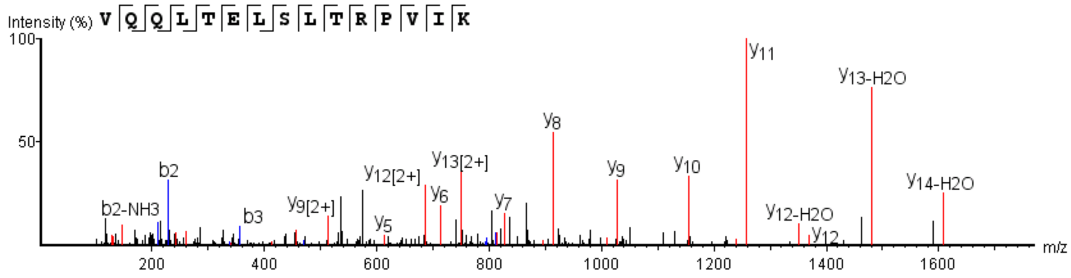

gi571579593

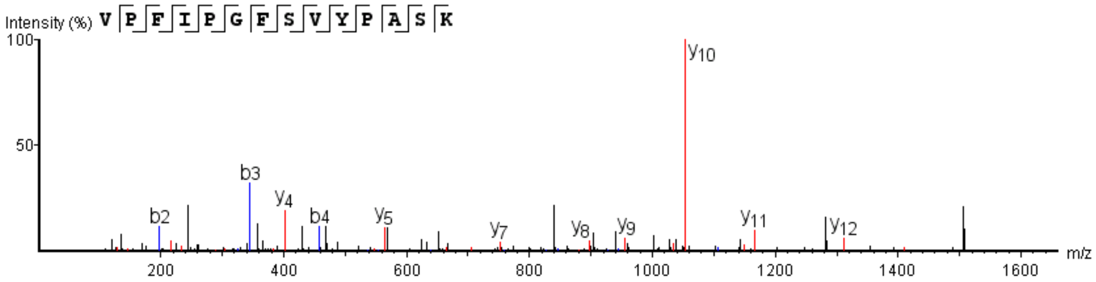

gi|66555143

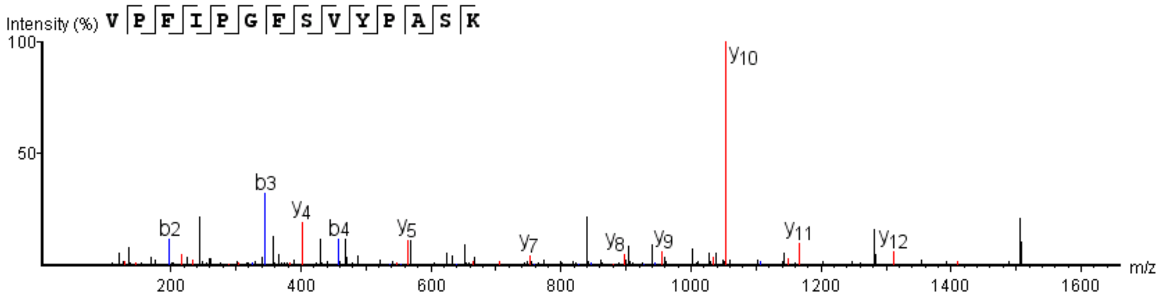

gi571579428

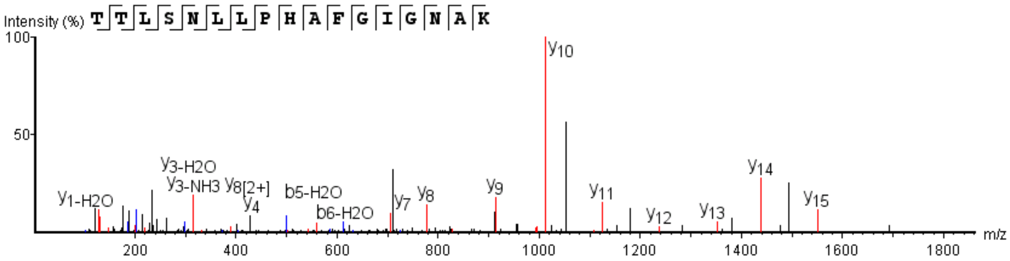

gi571579183

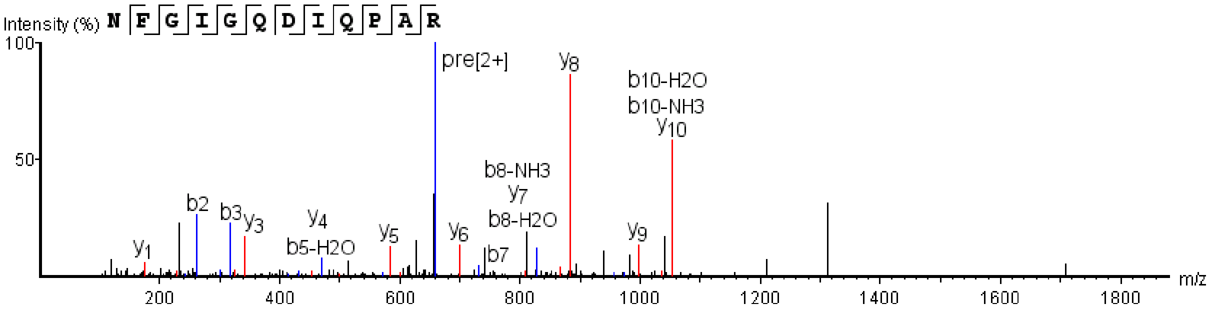

gi571578335

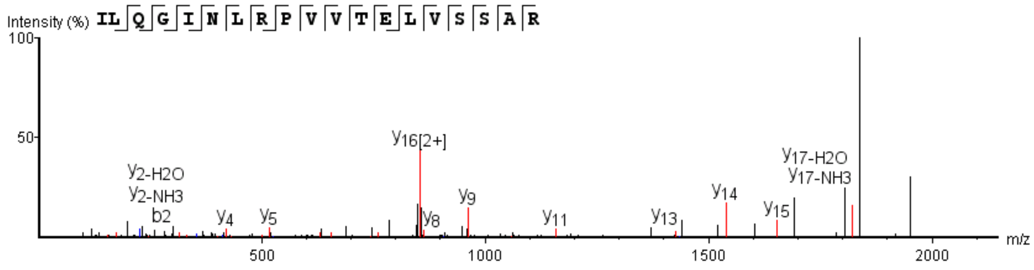

gi571577995

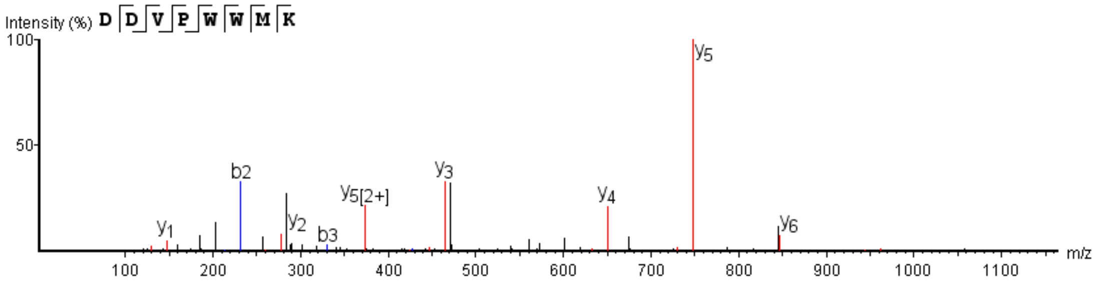

gi|66509518

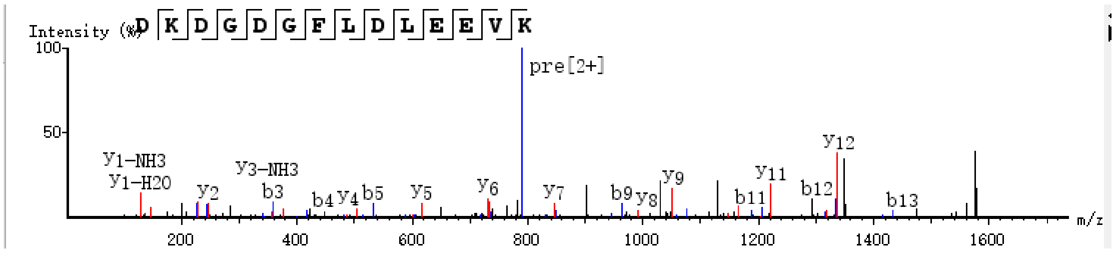

gi571577993

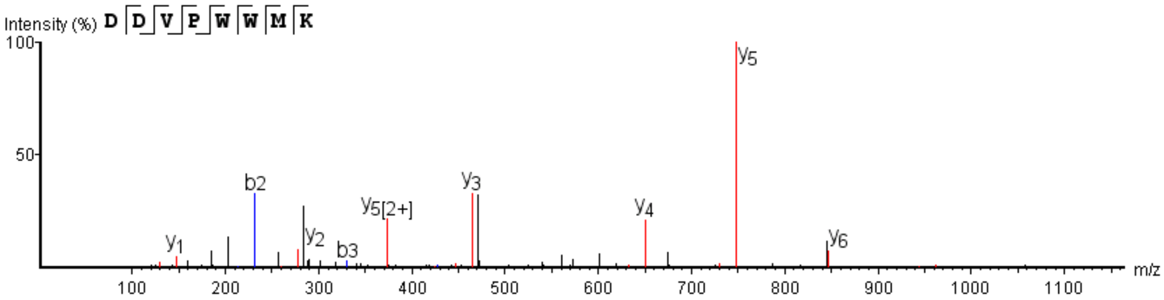

gi571577853

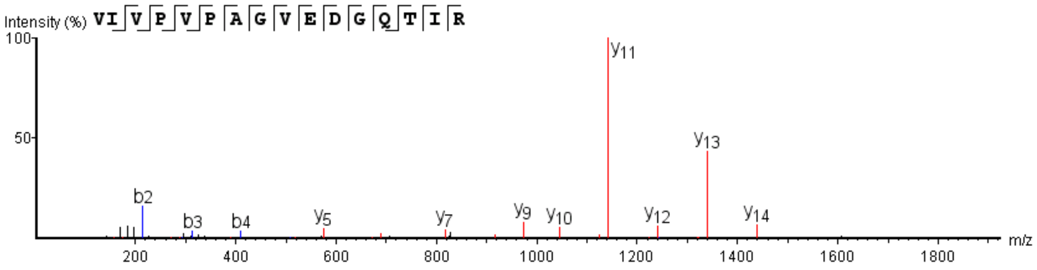

gi|66540209

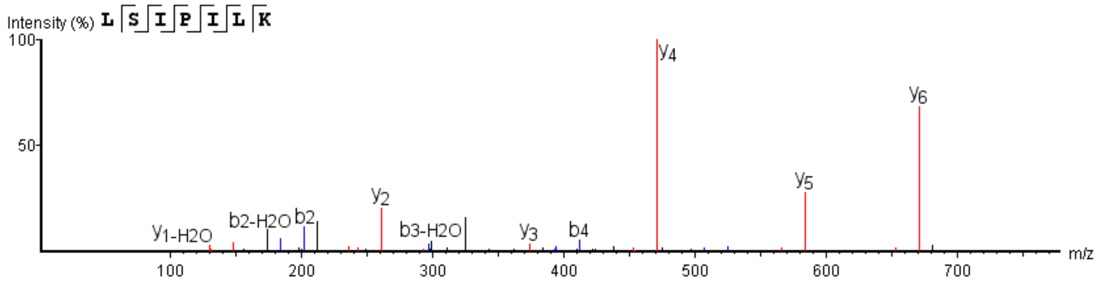

gi571577798

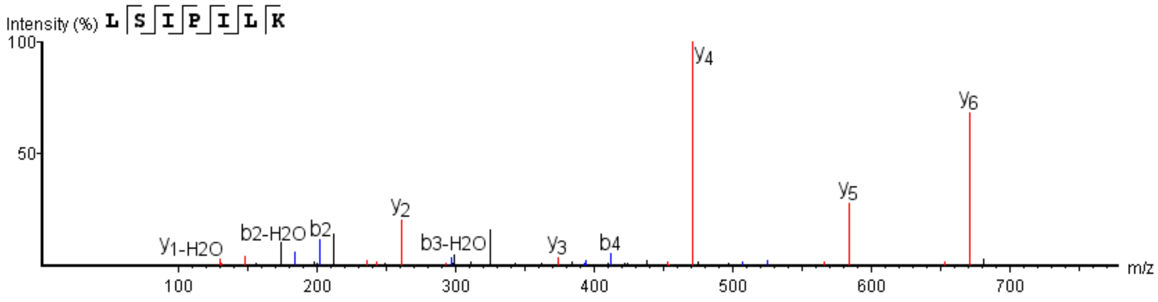

gi571577744

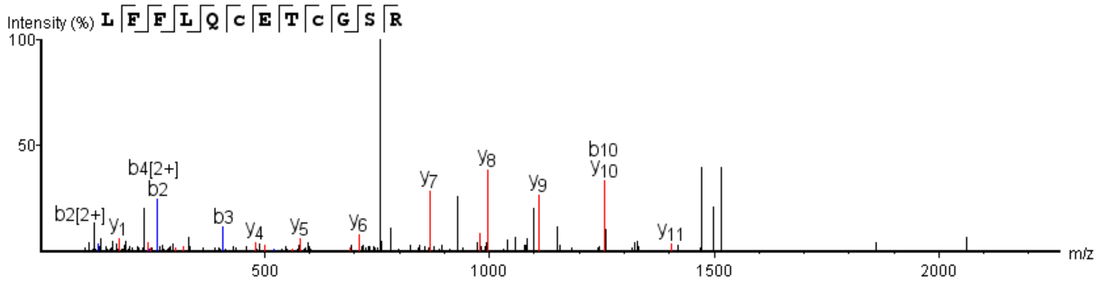

gi571577742

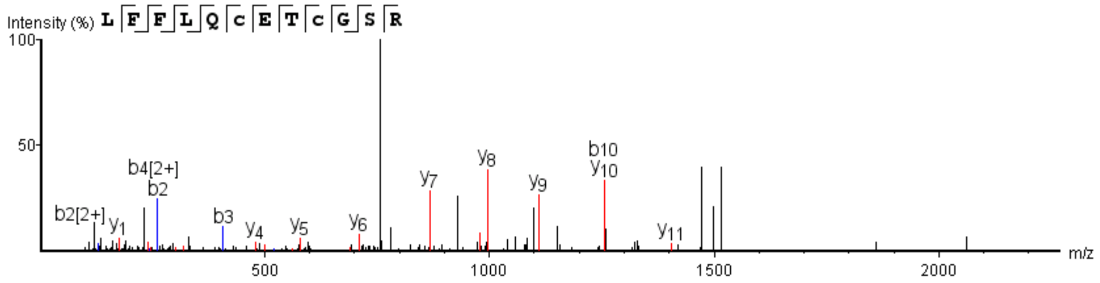

gi571577740

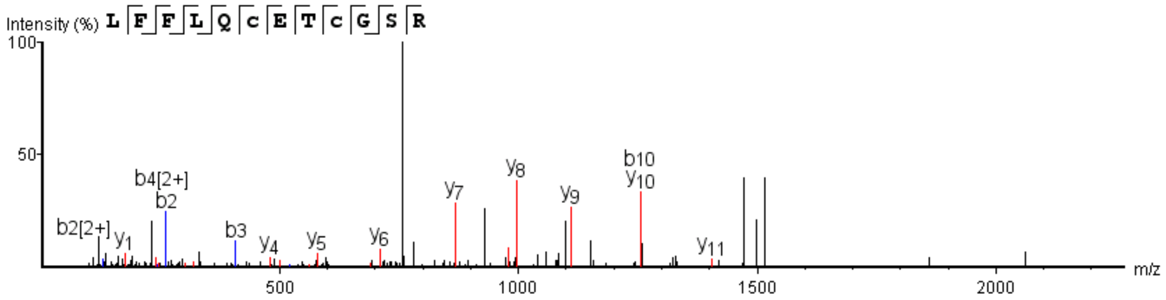

gi571577410

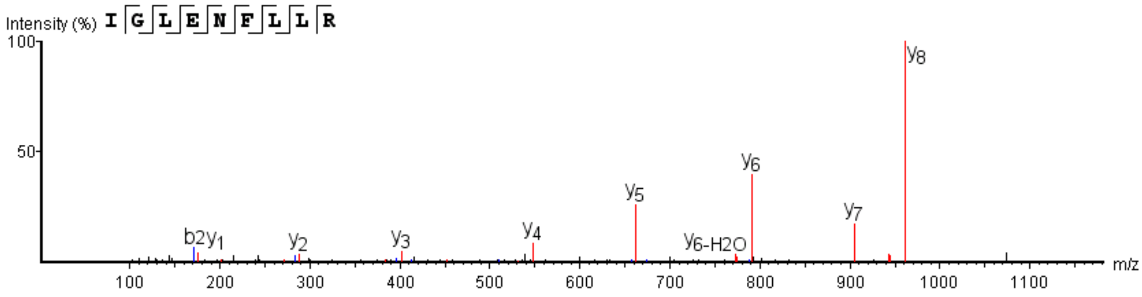

gi571577379

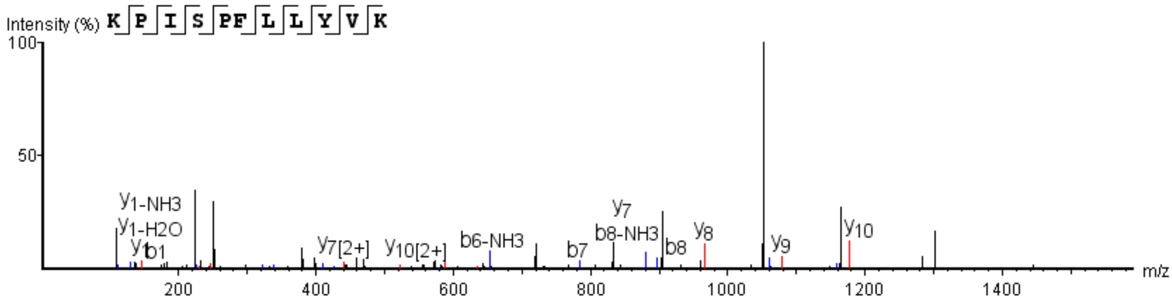

gi571577319

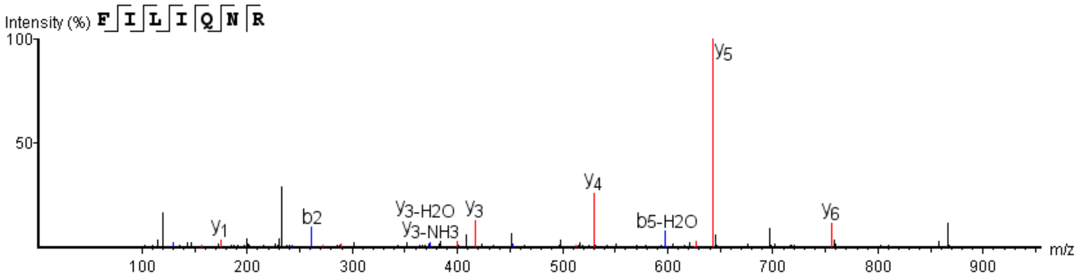

gi571577109

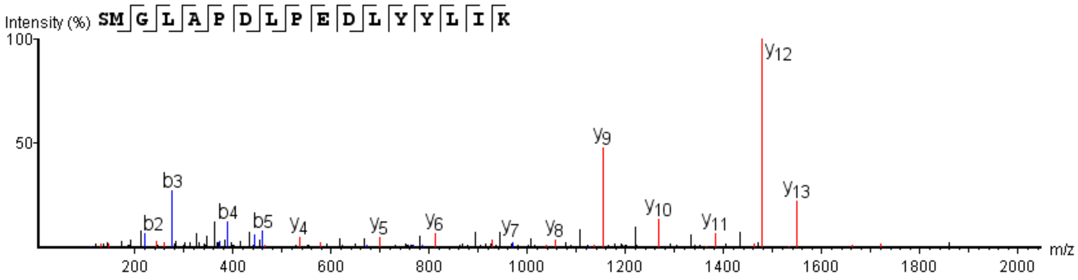

gi|328792927

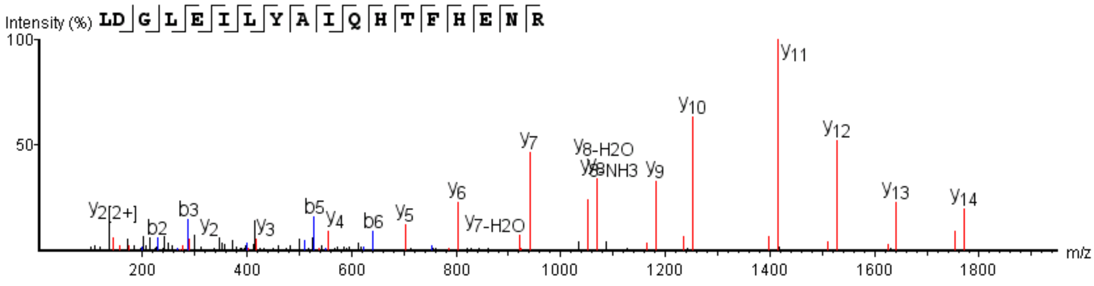

gi571576771

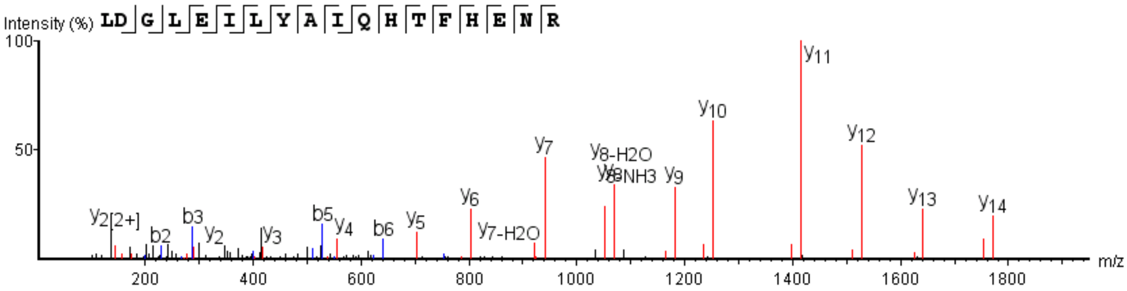

gi571576665

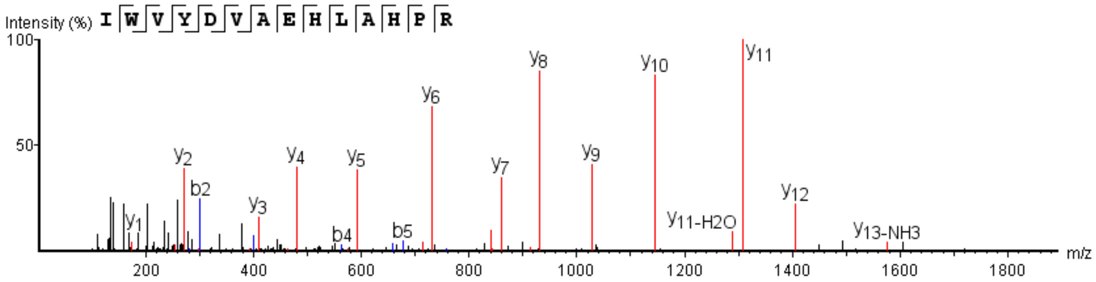

gi571576661

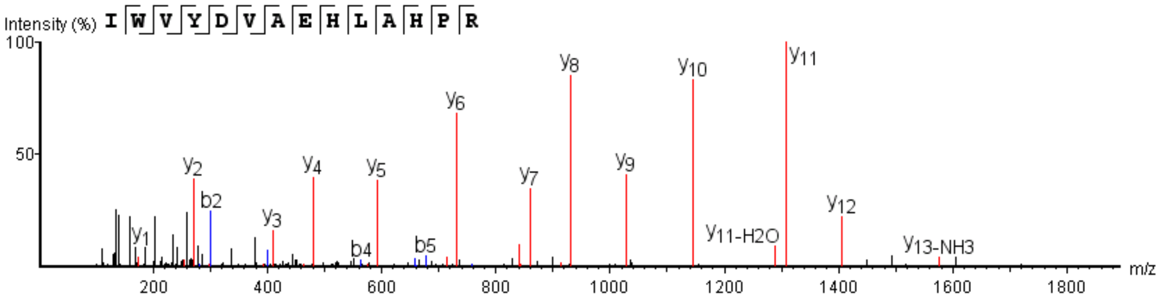

gi571576557

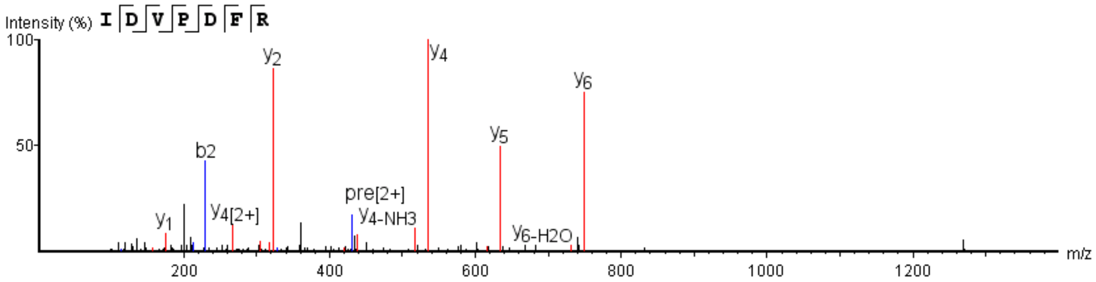

gi571576555

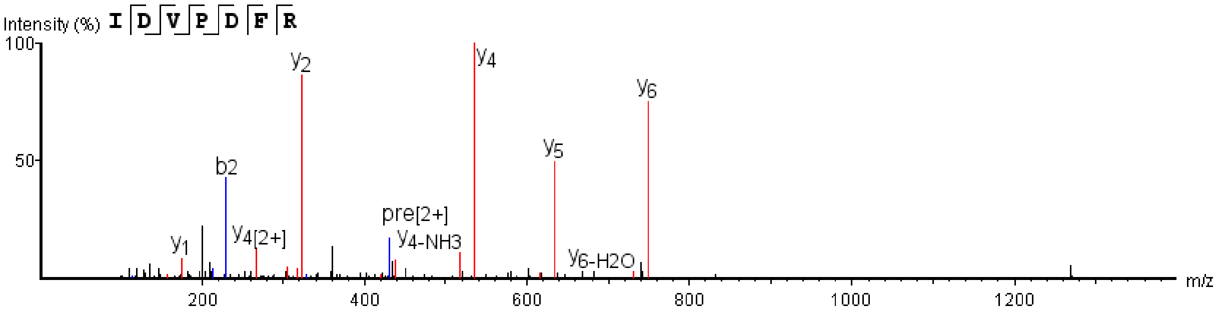

gi571576460

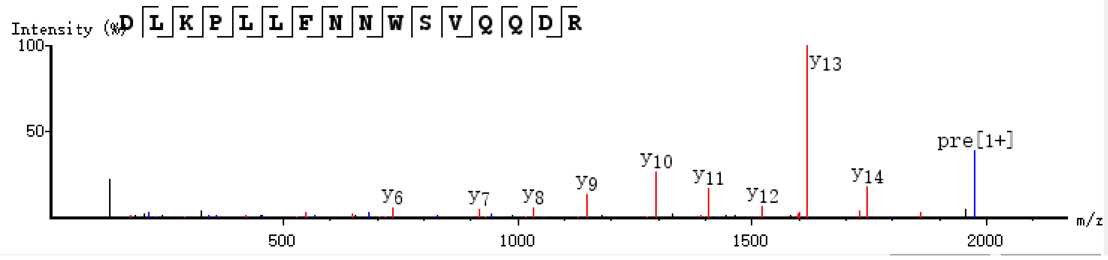

gi571576251

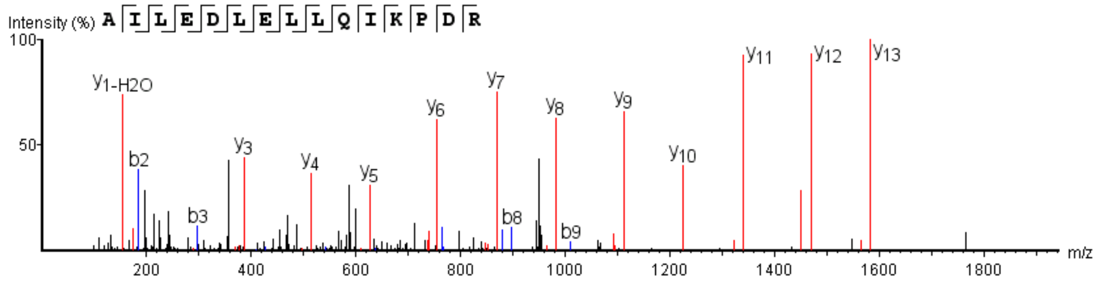

gi571576183

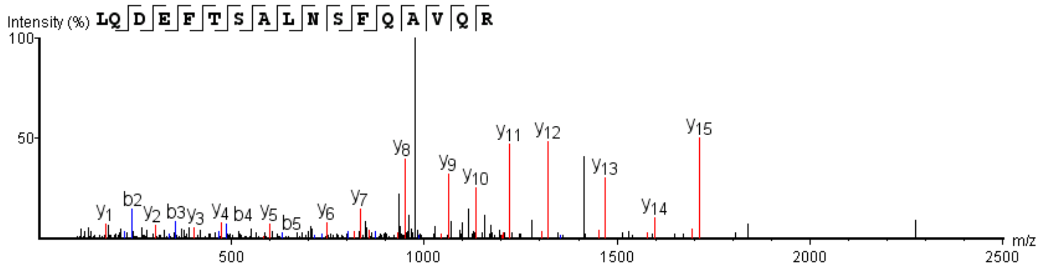

gi571575549

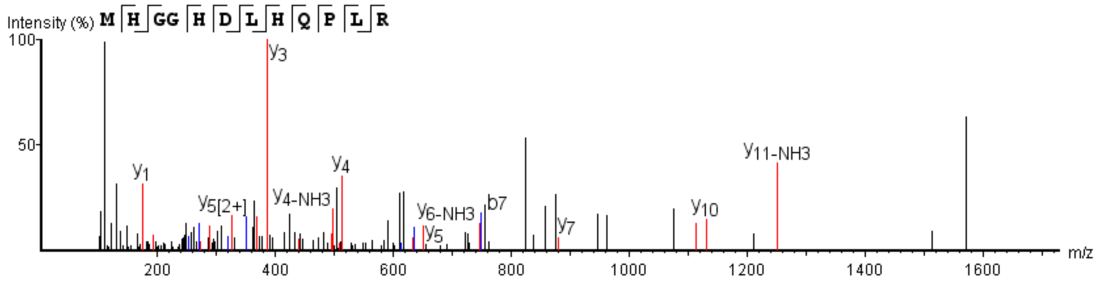

gi571566017

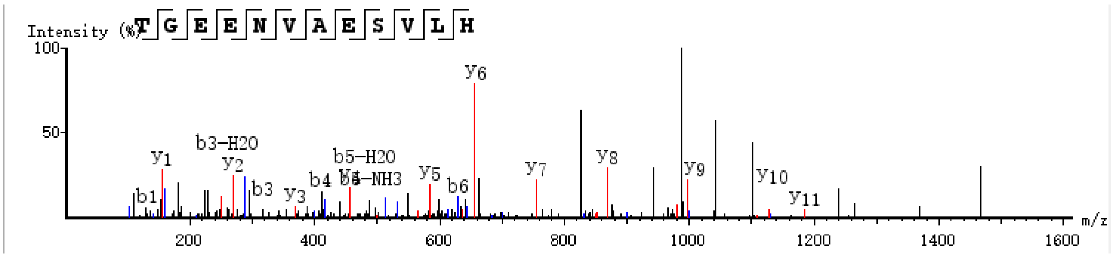

gi571575531

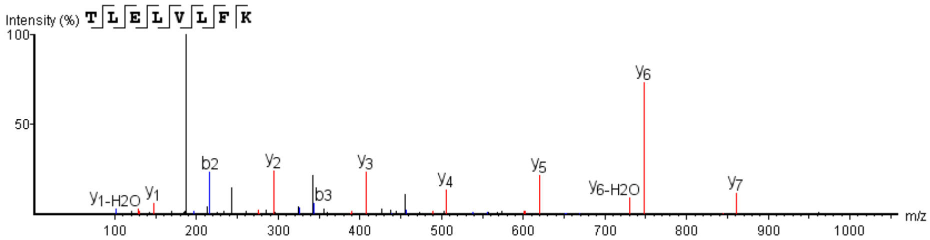

gi571575529

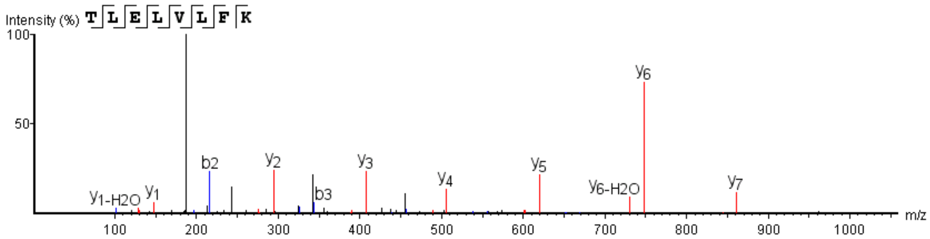

gi571575329

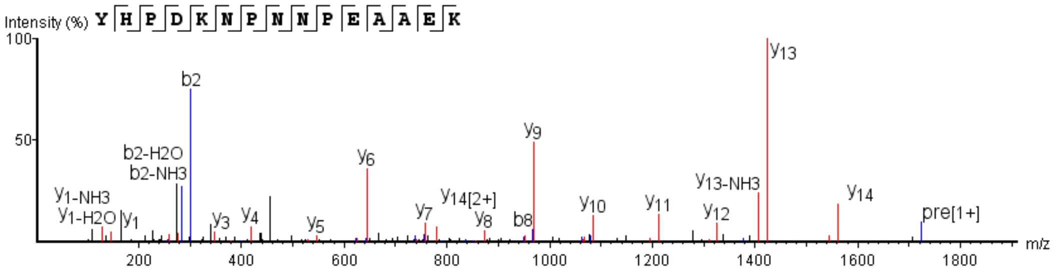

gi571575043

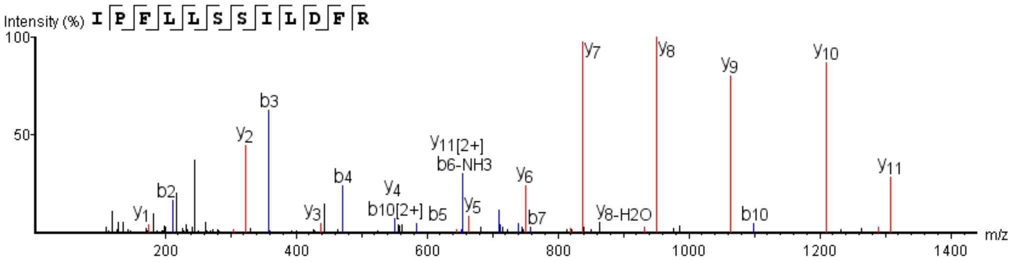

gi571574994

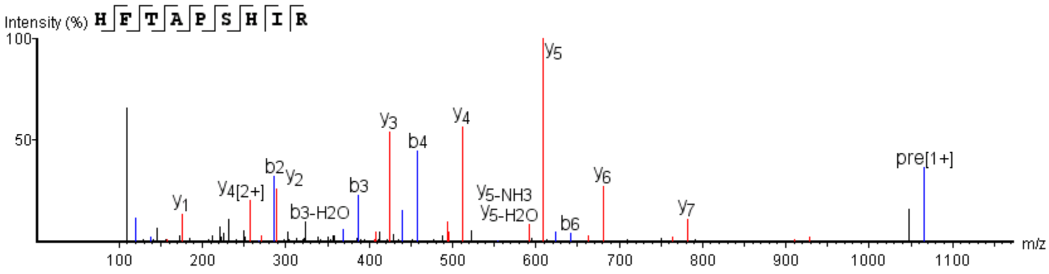

gi571574918

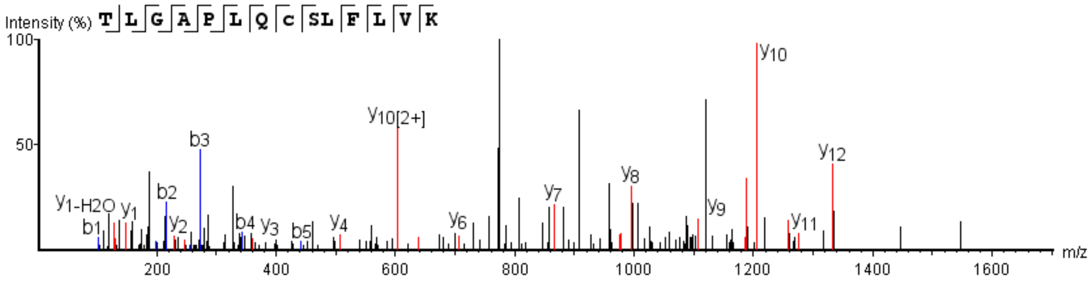

gi571574741

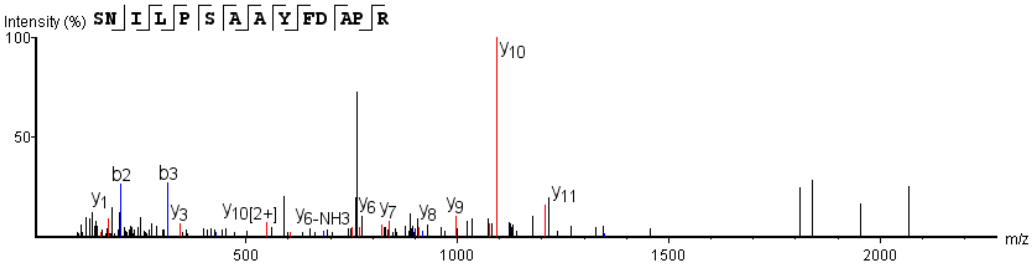

gi571574521

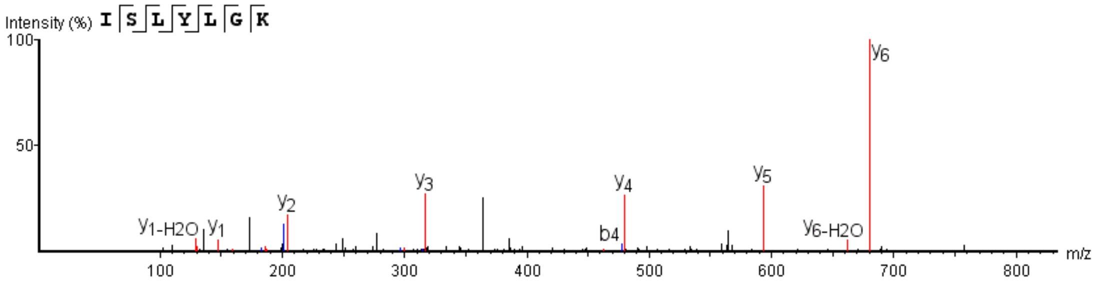

gi571574519

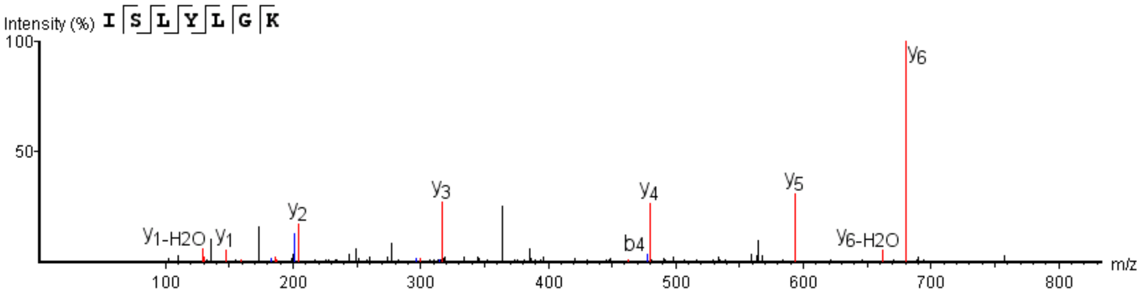

gi|66525427

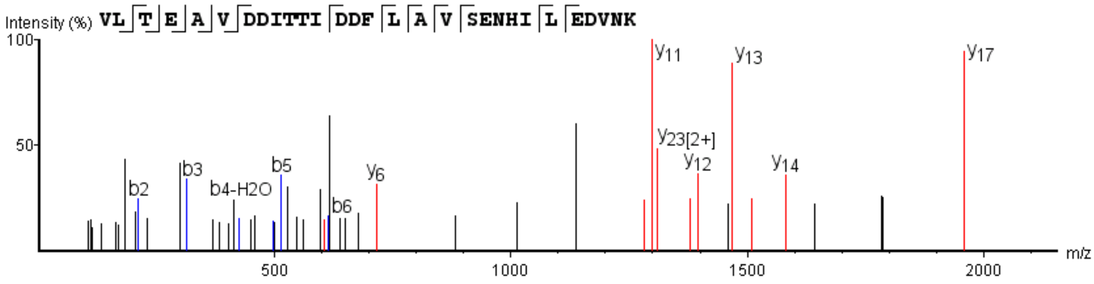

gi571574449

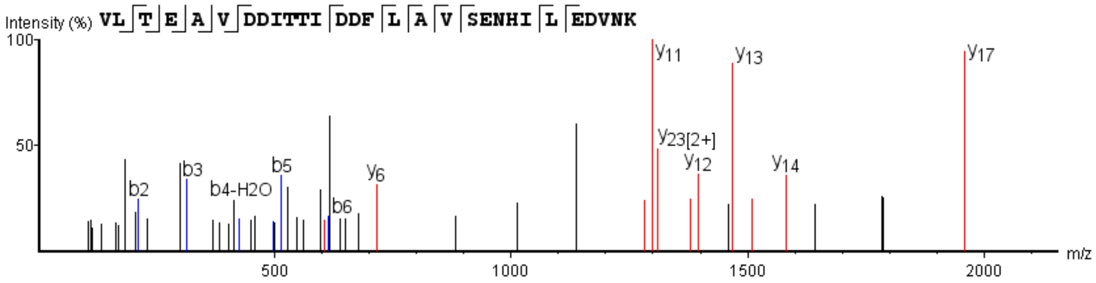

gi571574451

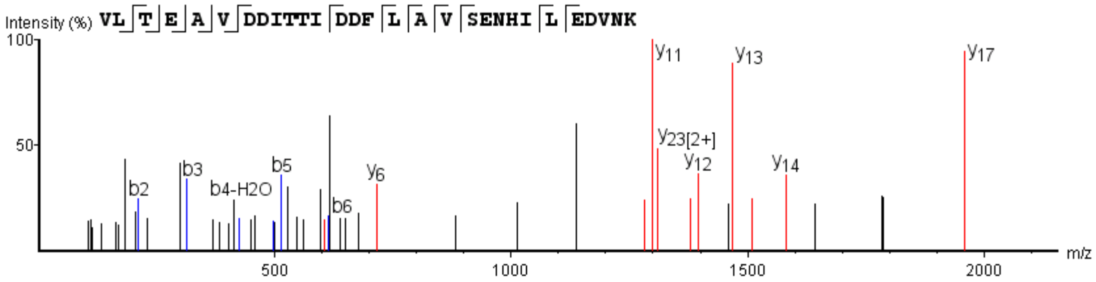

gi571574447

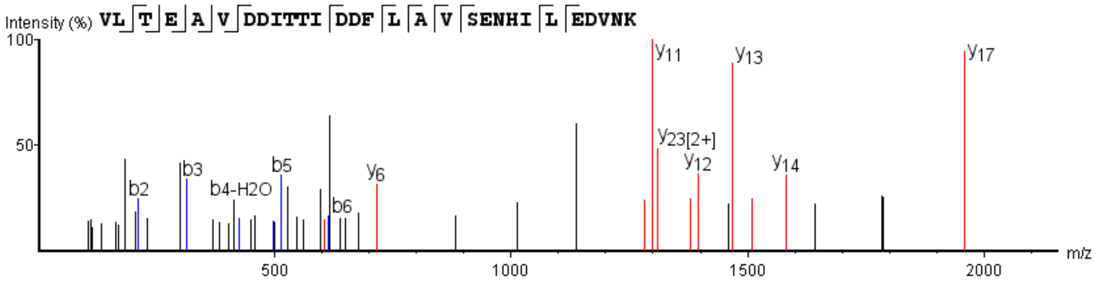

gi571574432

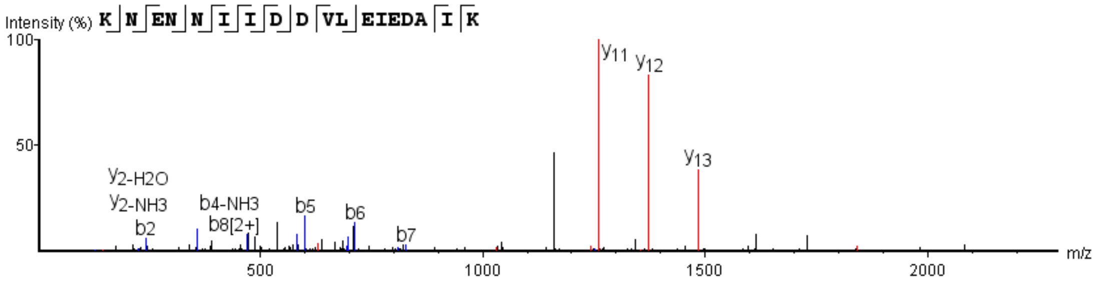

gi571574430

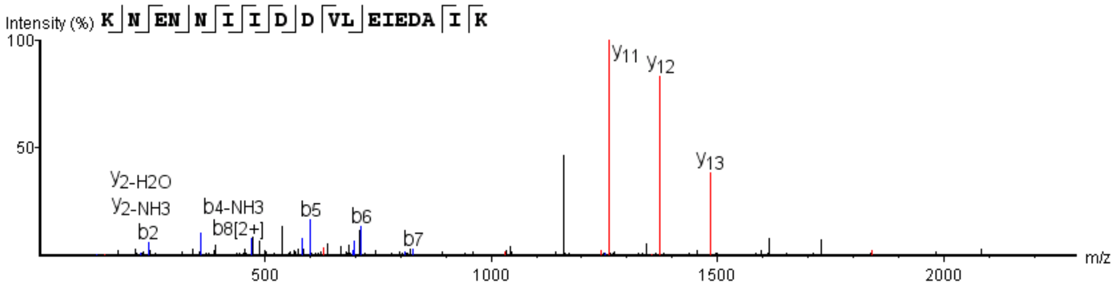

gi|328783491

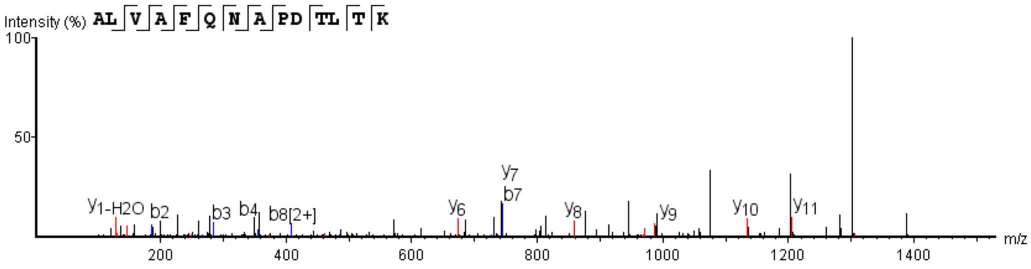

gi571574442

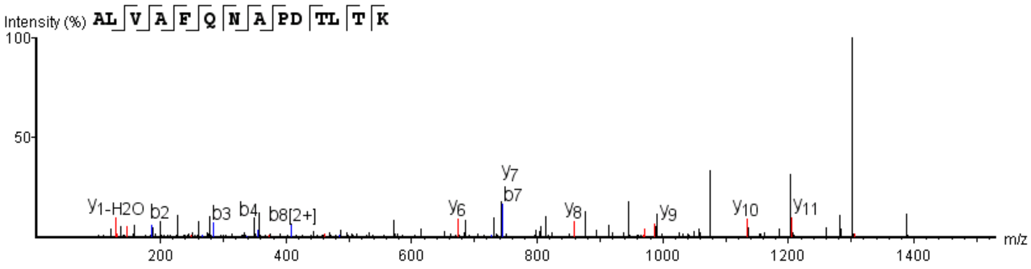

gi 571574200

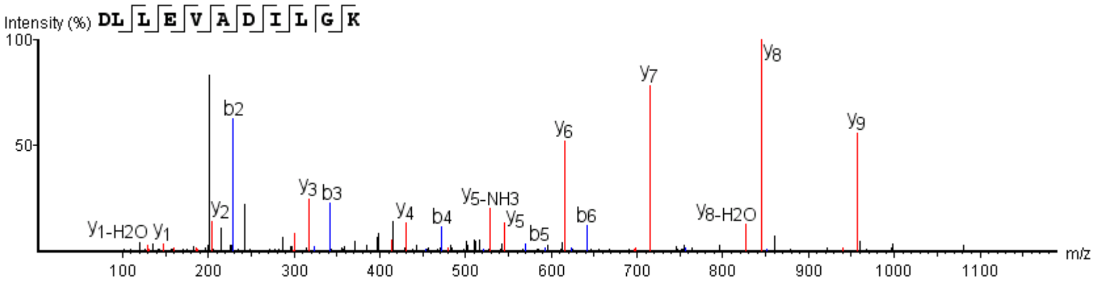

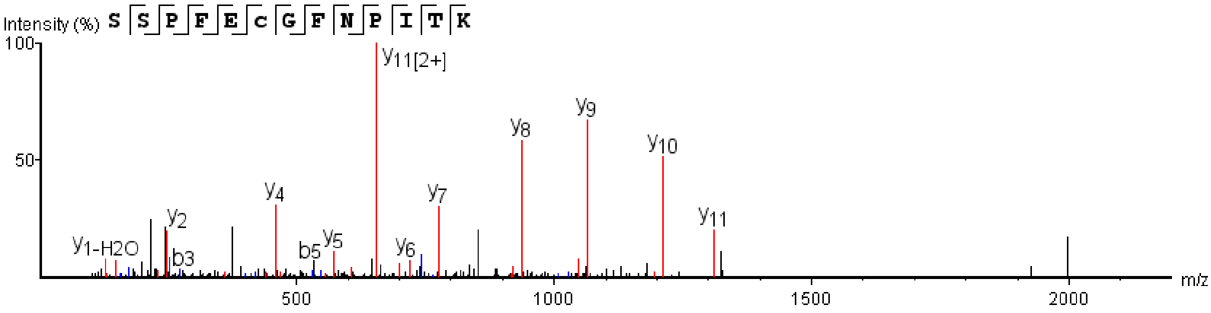

gi571573892

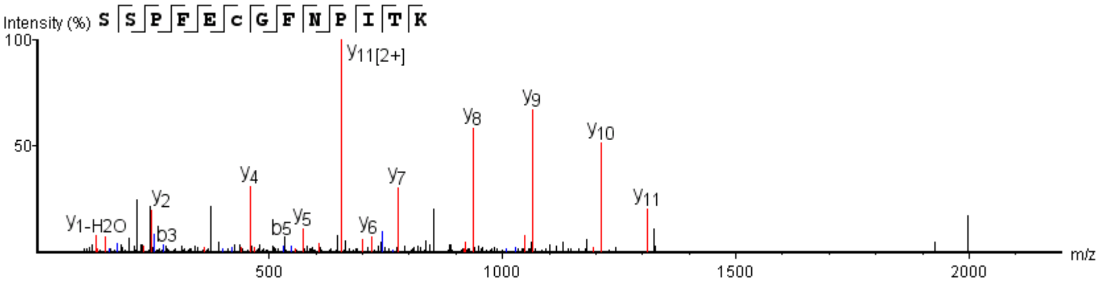

gi571573890

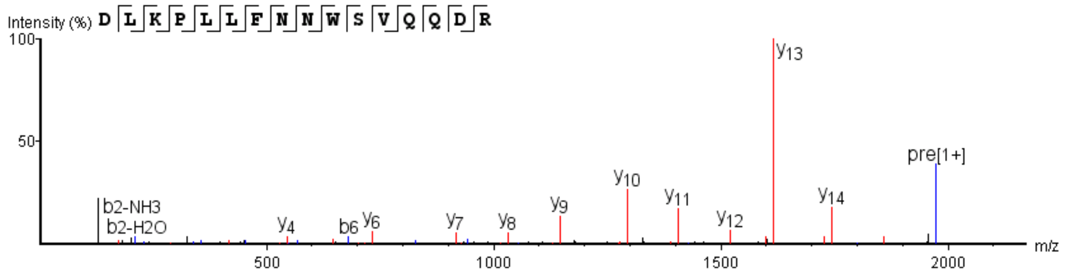

gi328777360

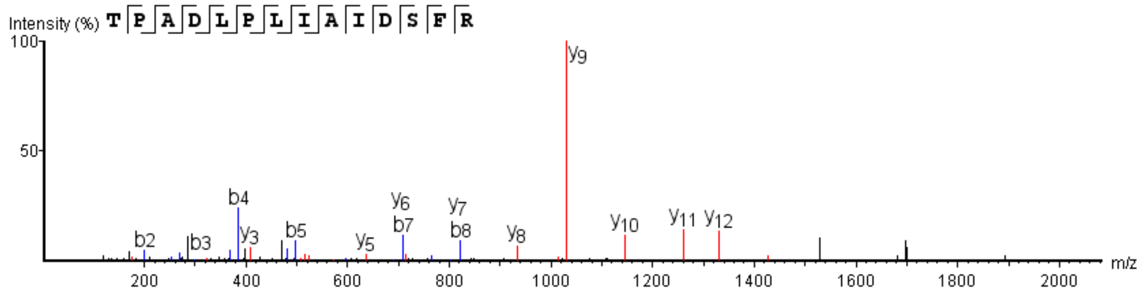

gi328777239

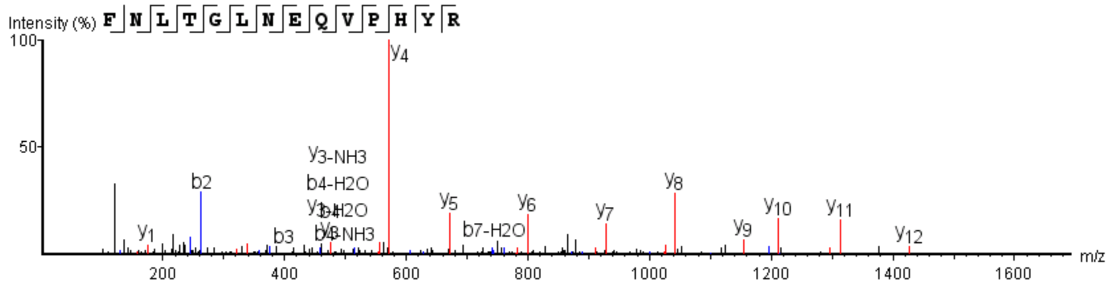

gi328776594

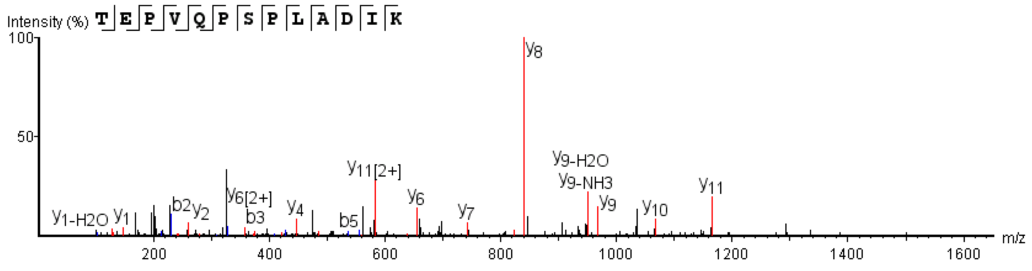

gi328776555

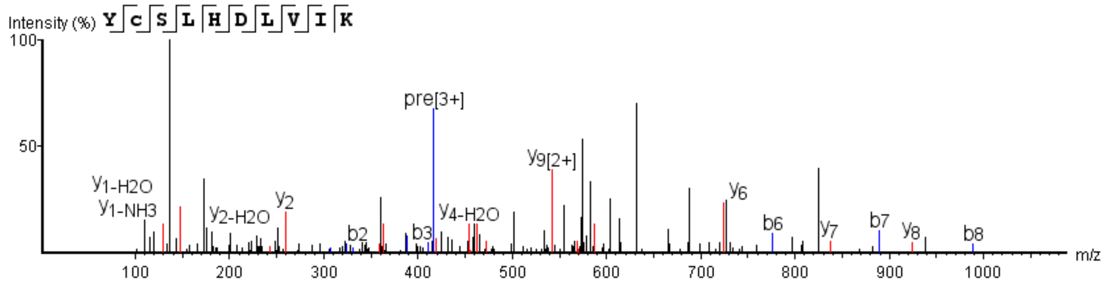

gi328776329

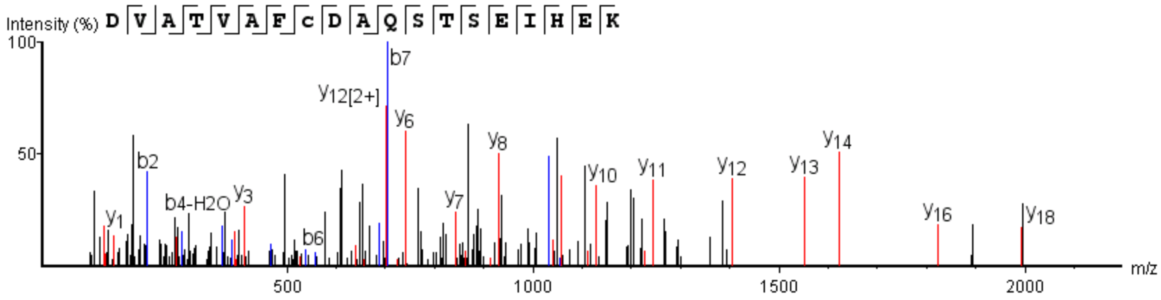

gi|571529309

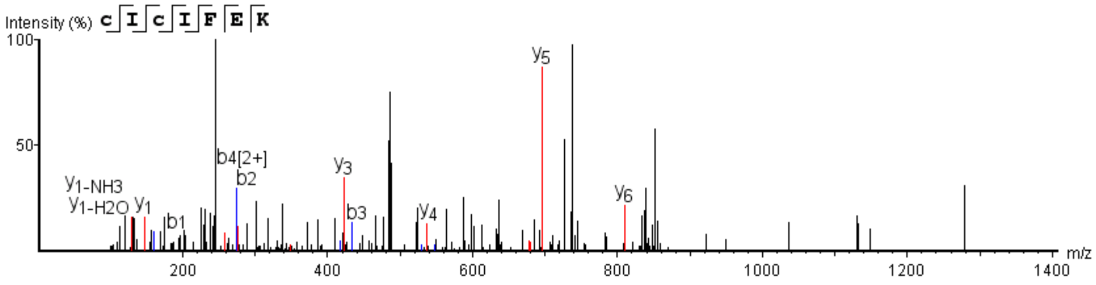

gi|571529312

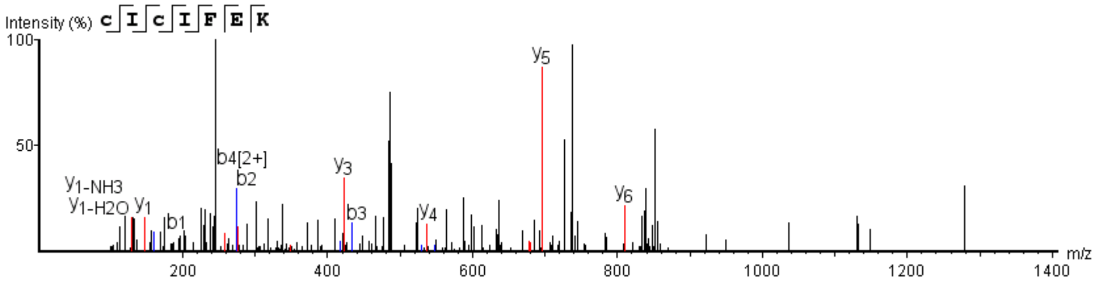

gi|571521696

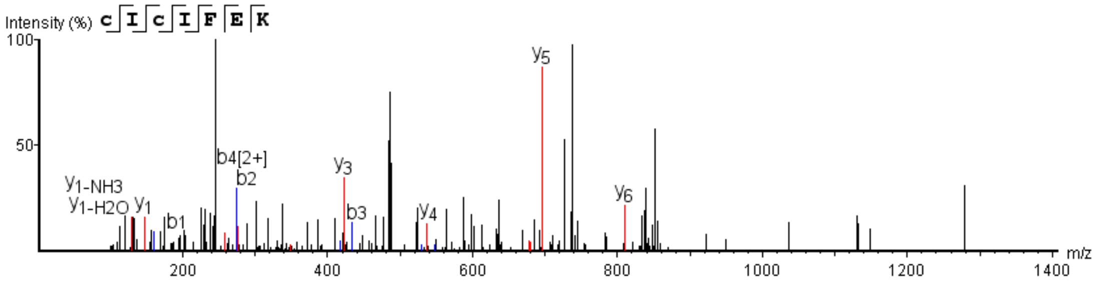

gi|571521709

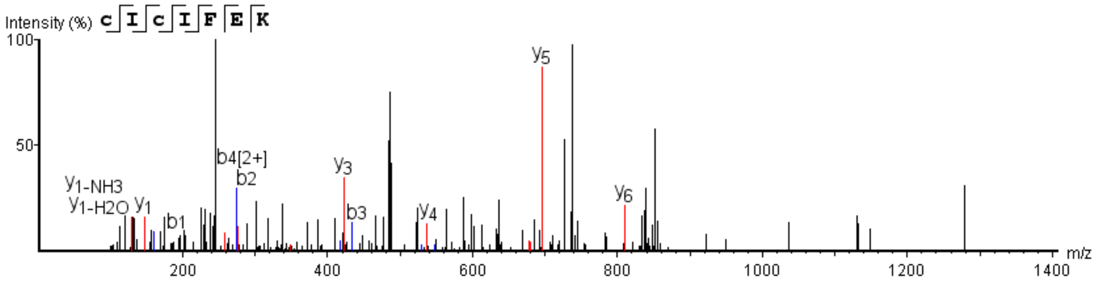

gi328775985

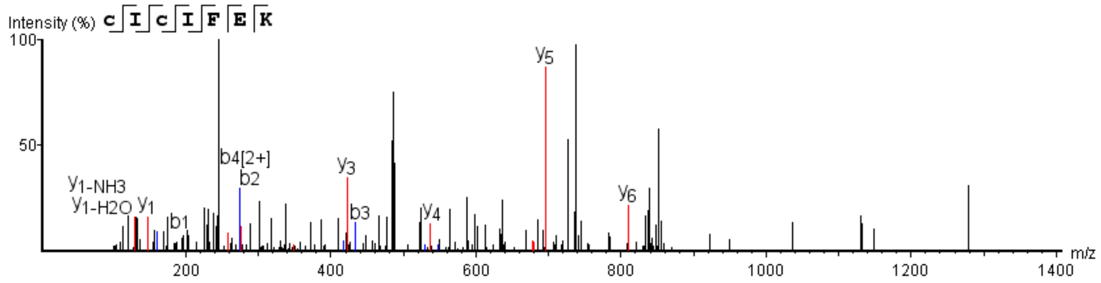

gi 32877590

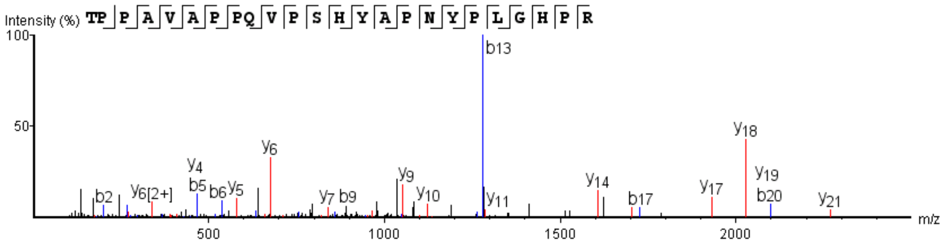

0

gi327315378

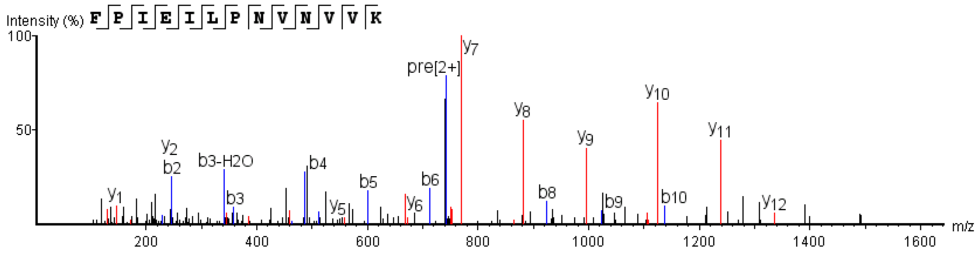

gi288872189

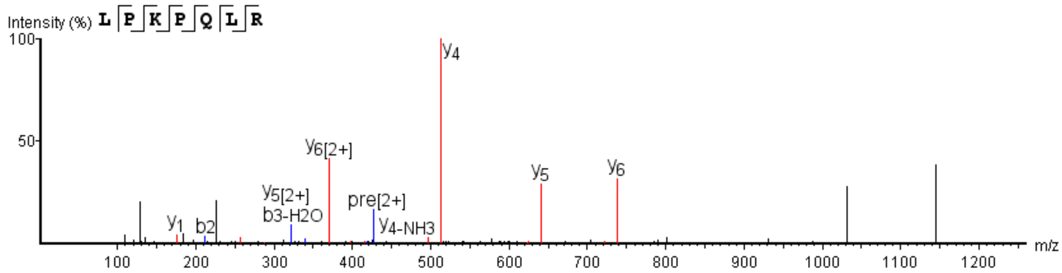

gi219842191

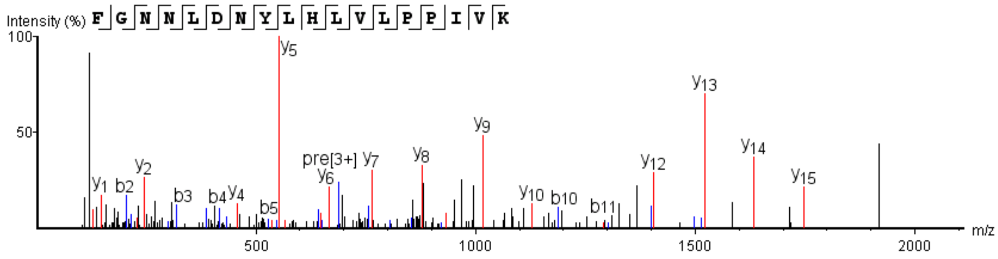

gi118150510

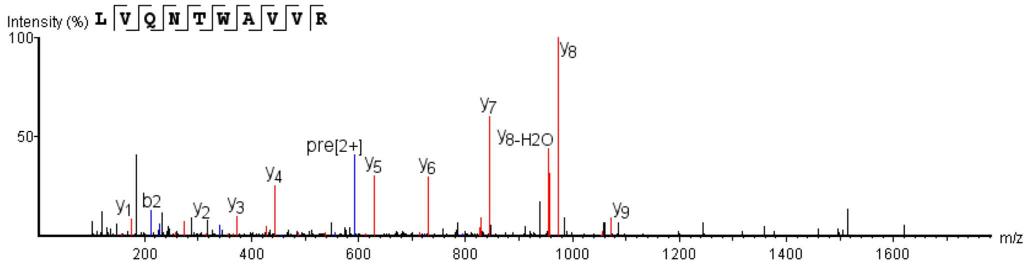

gi|571563867

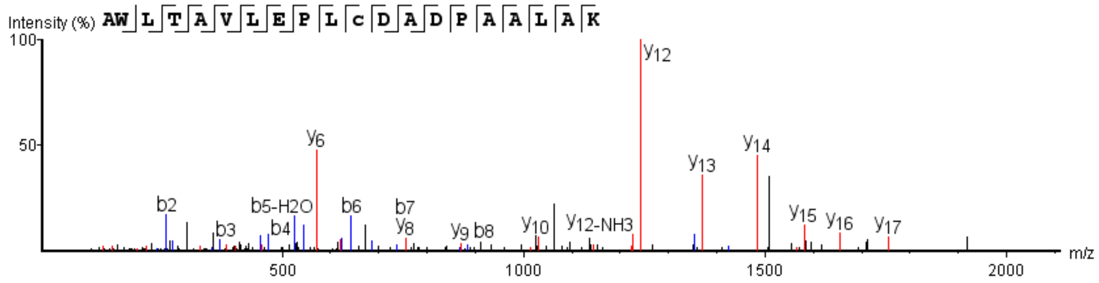

gi328780081

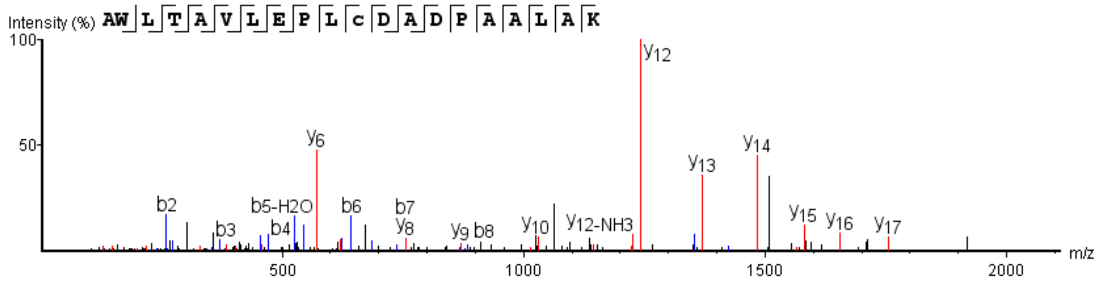

gi328779841

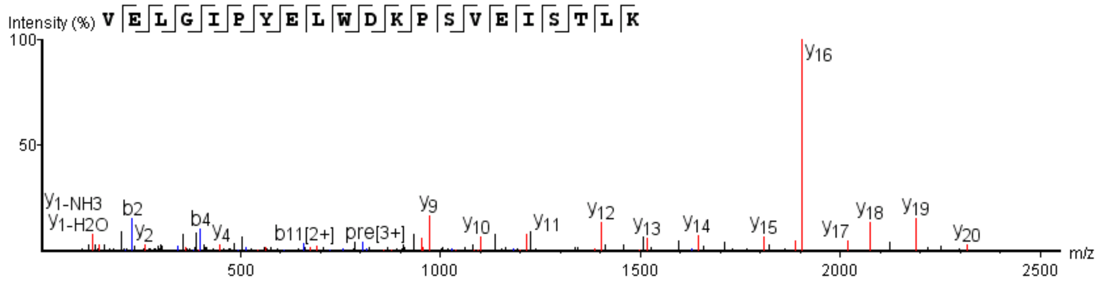

gi|571568001

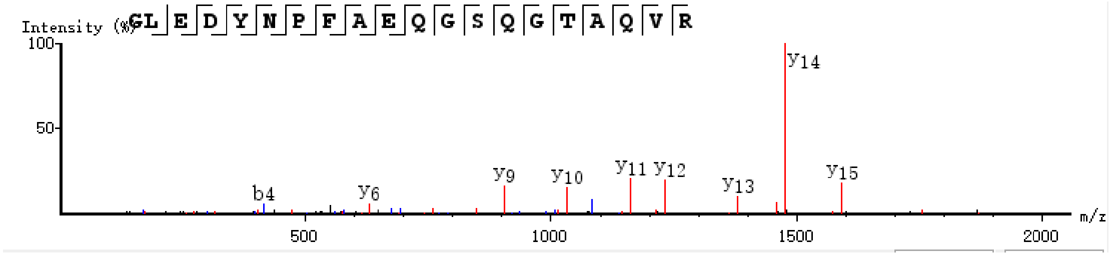

gi328779563

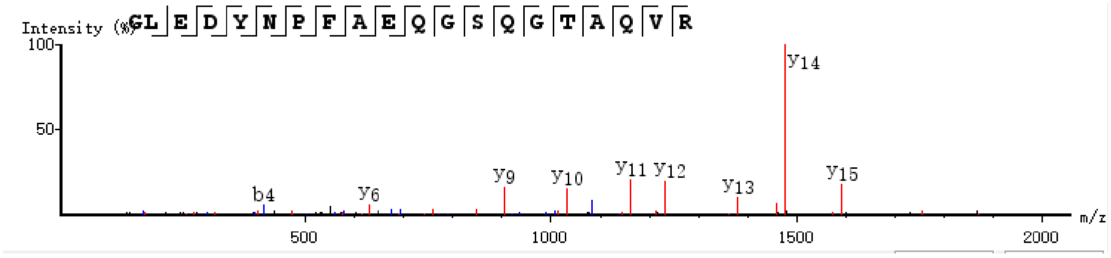

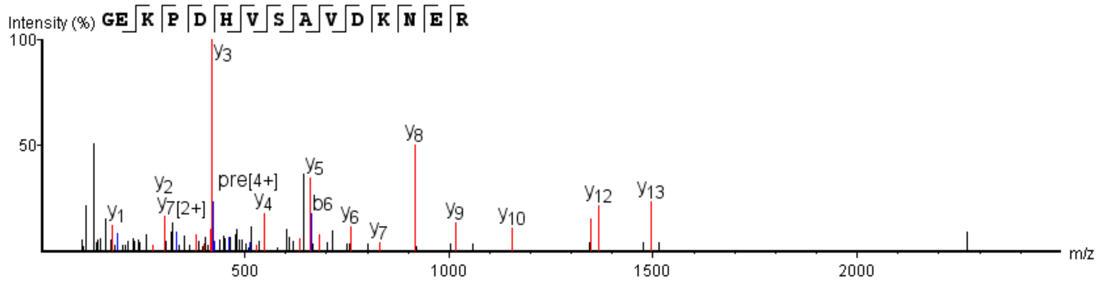

gi328778902

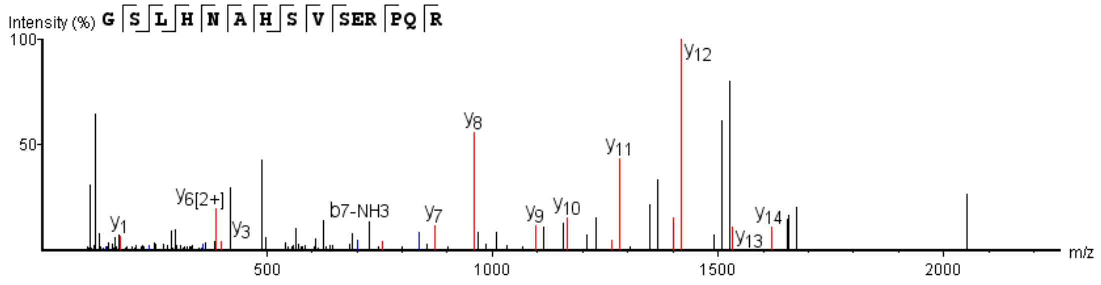

gi328778851

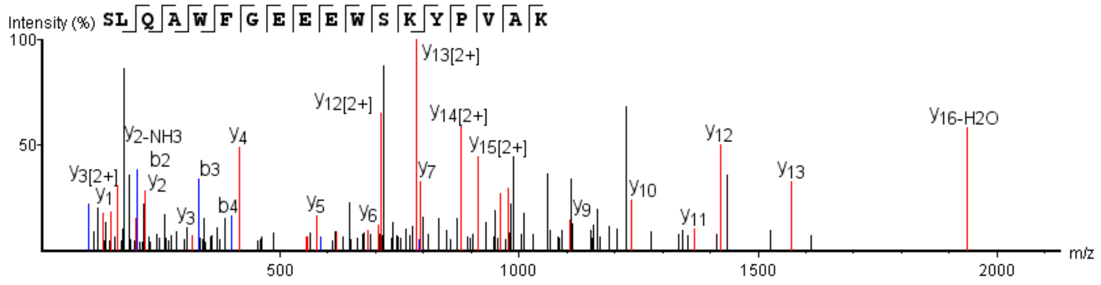

gi|571577919

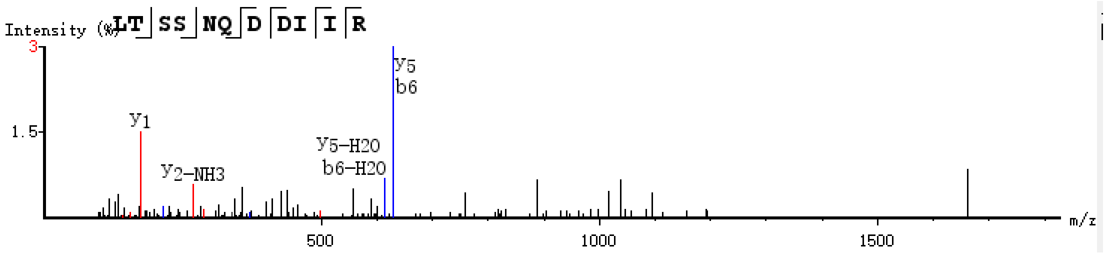

gi|571577921

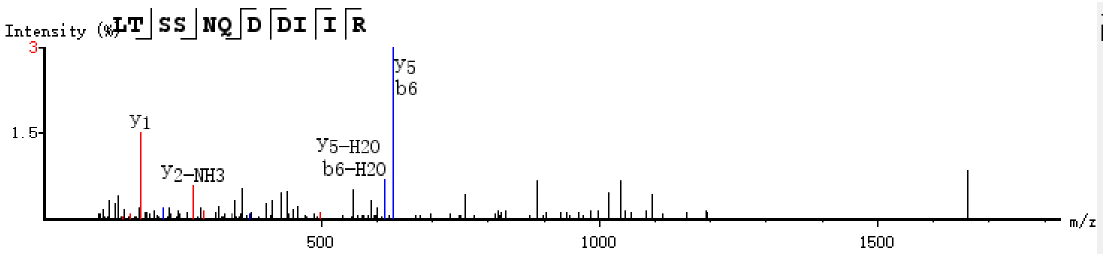

gi|571577923

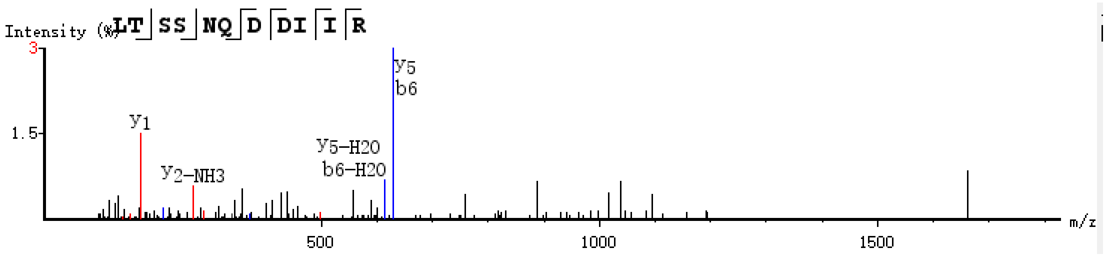

gi|571577925

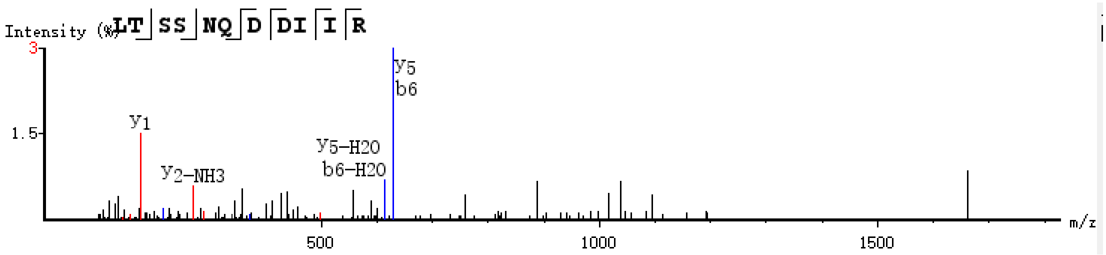

gi|571577927

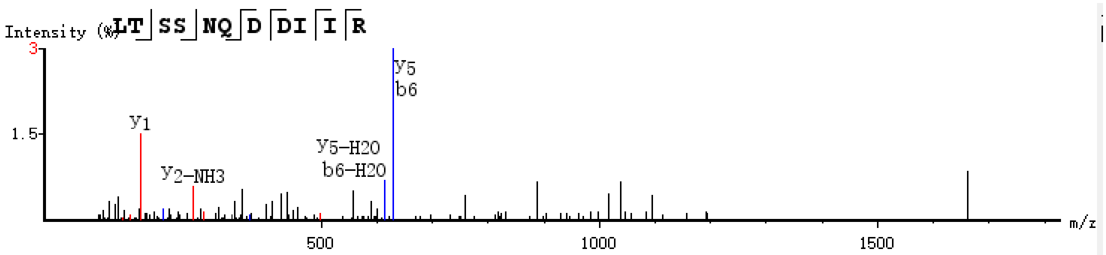

gi|571577929

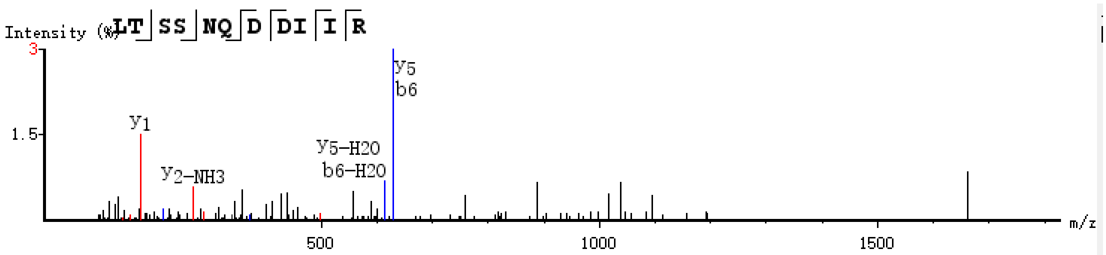

gi|571577931

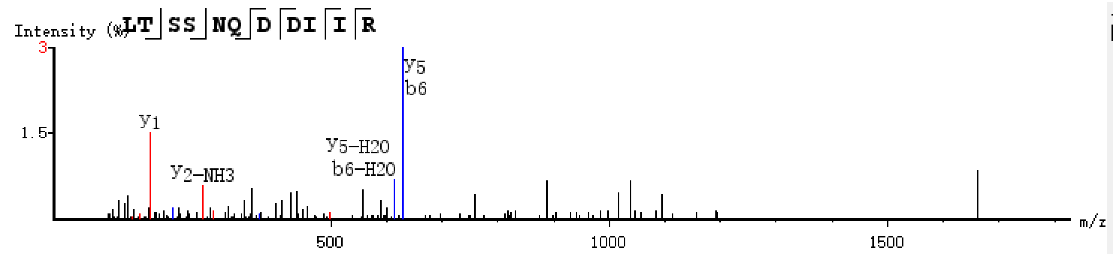

gi|571577933

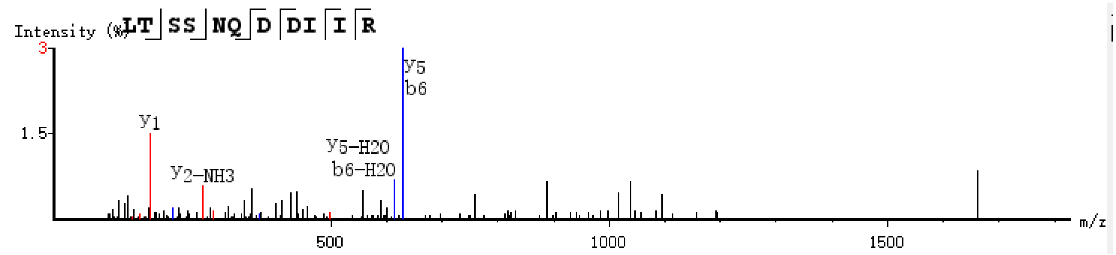

gi|571543209

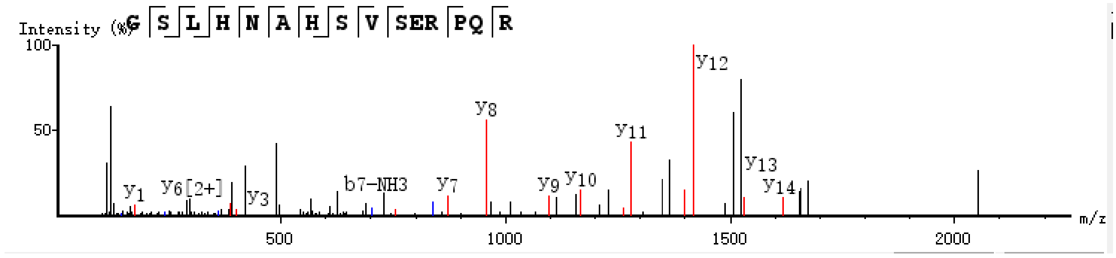

gi|571543214

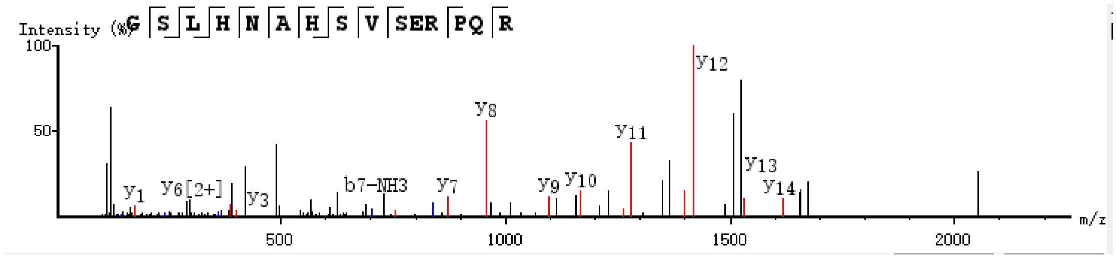

gi|571543217

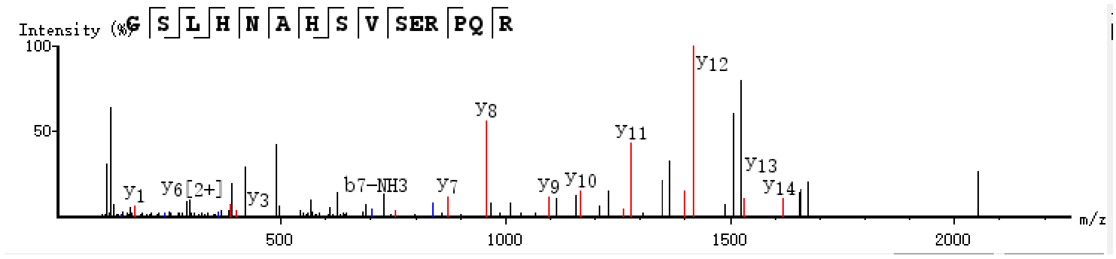

gi328778597

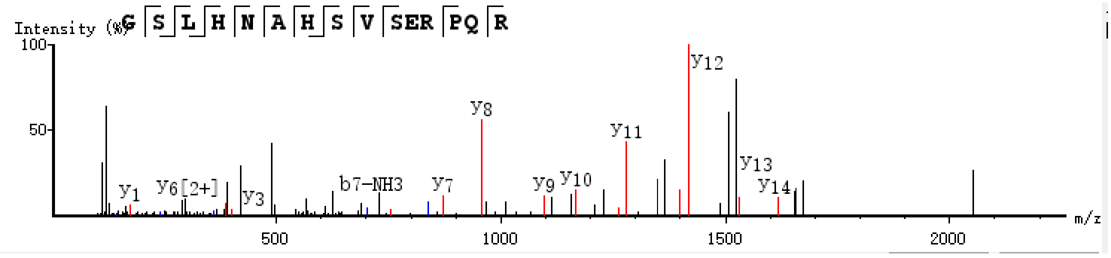

gi328778195

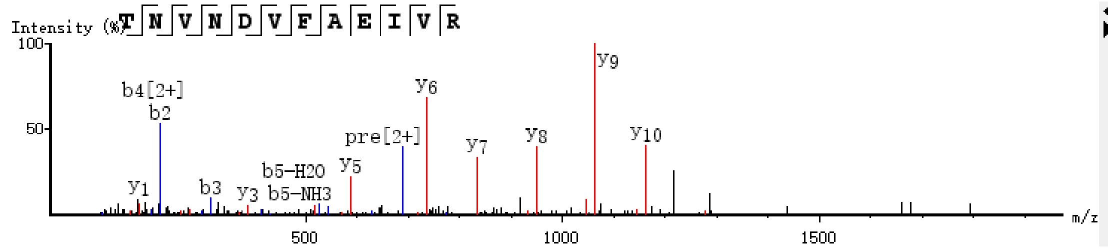

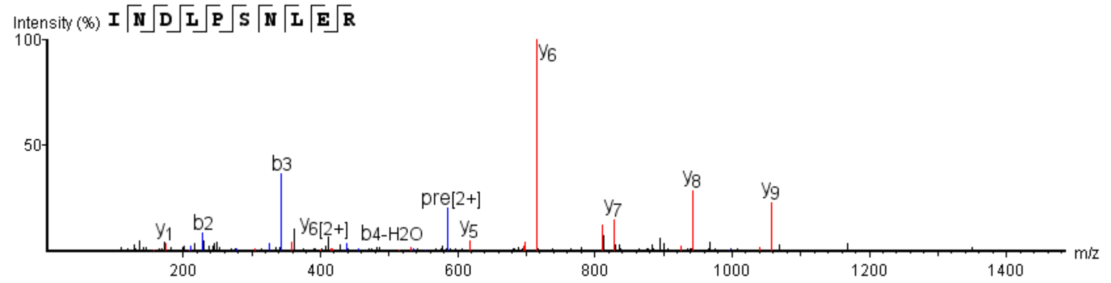

gi 328777695

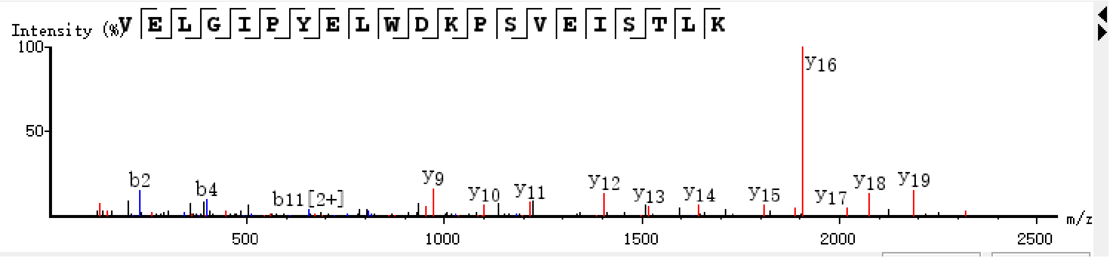

gi|571539226

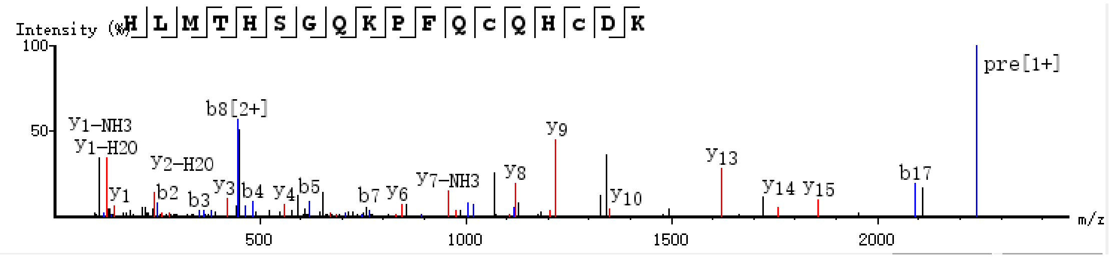

Annotated spectra of single-peptide identifications of proteins in antennal bodies of *Apis cerana*

gi328788820

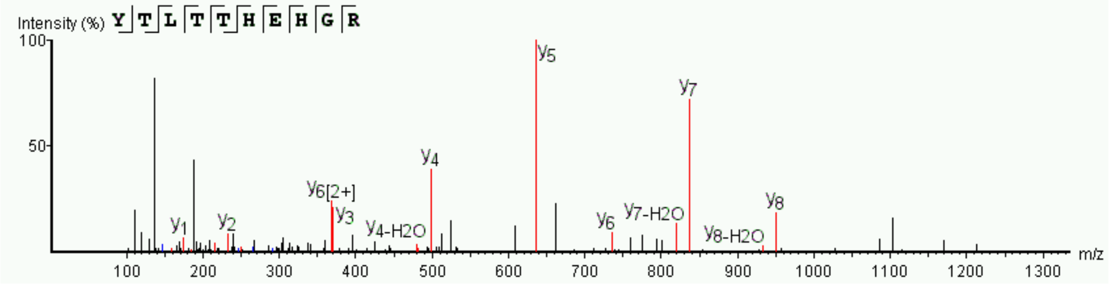

gi66540209

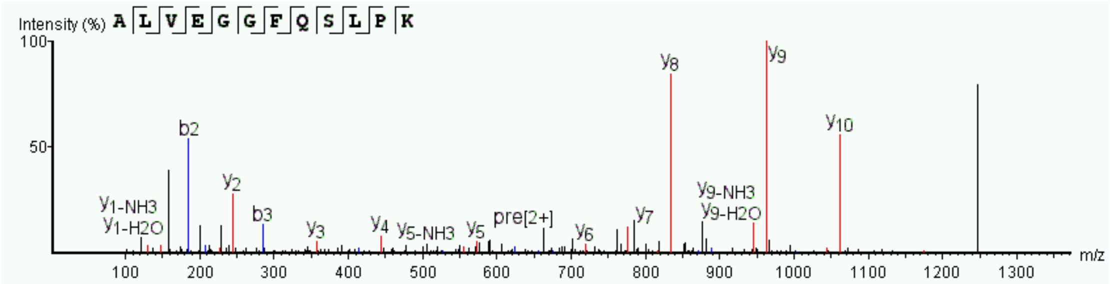

gi571577798

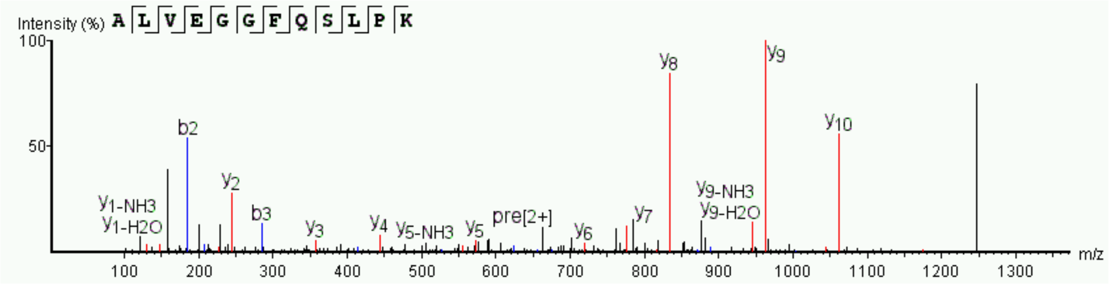

gi571559515

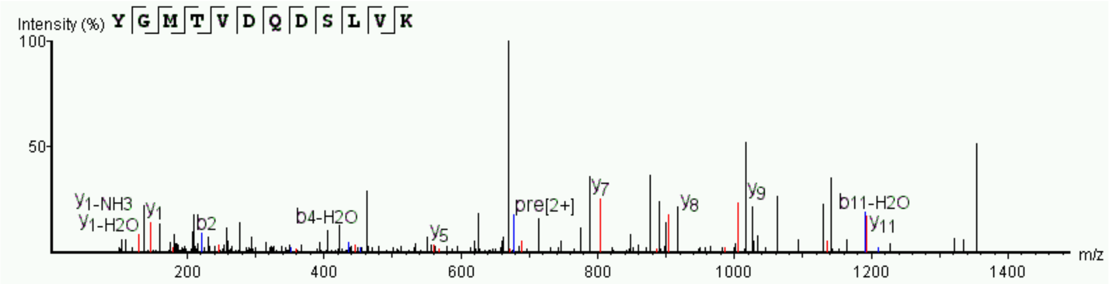

gi48103761

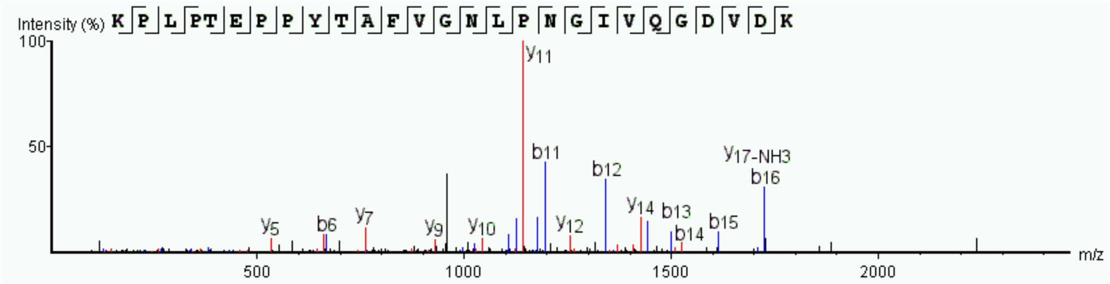

gi66531704

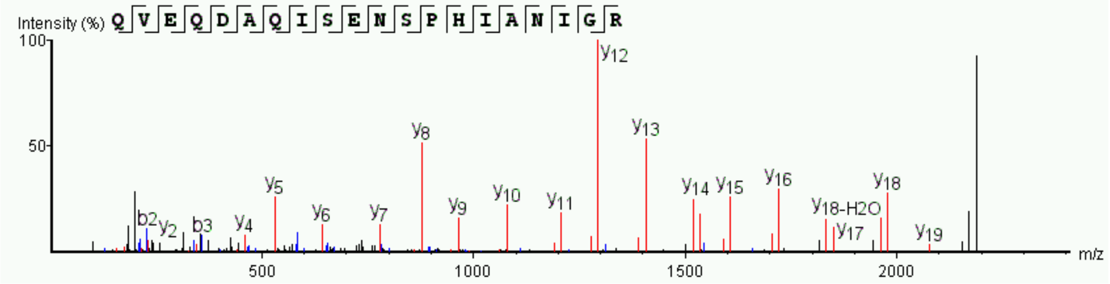

gi571501205

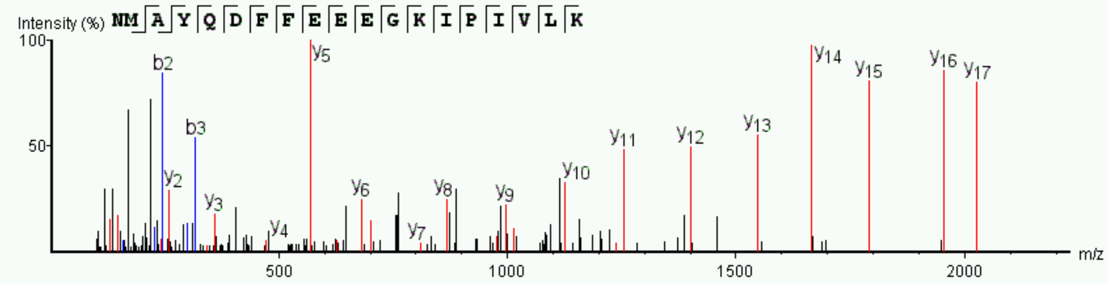

gi328790105

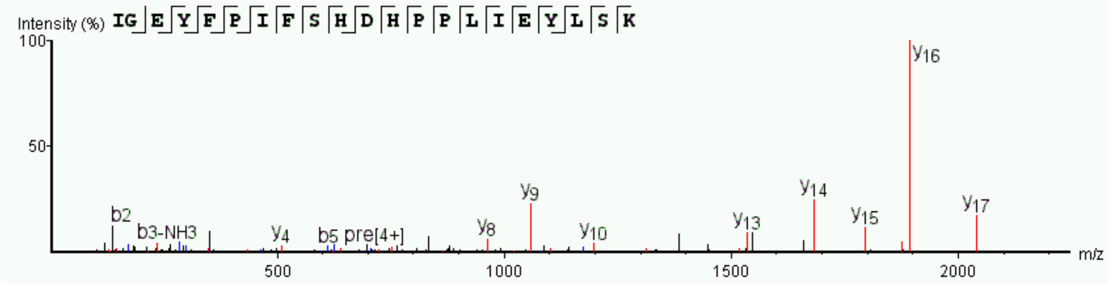

gi571515342

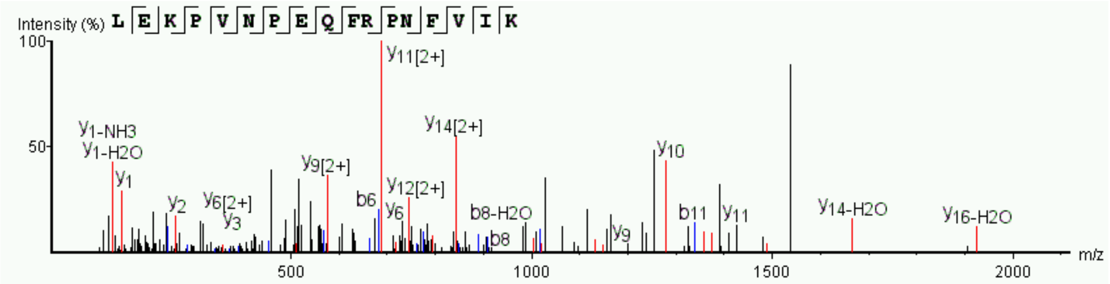

gi571517372

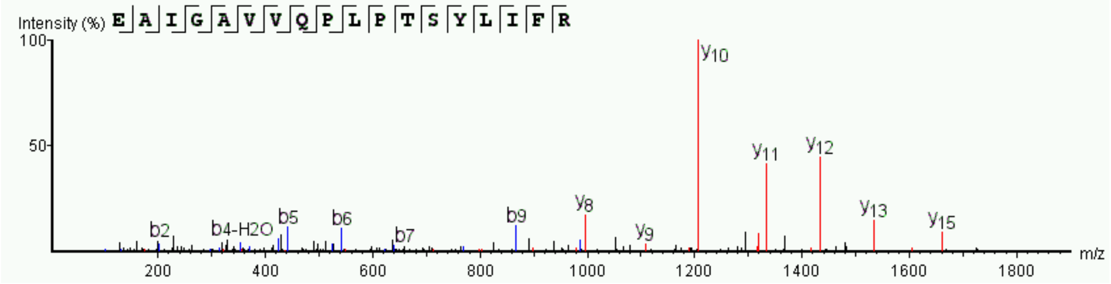

gi571517403

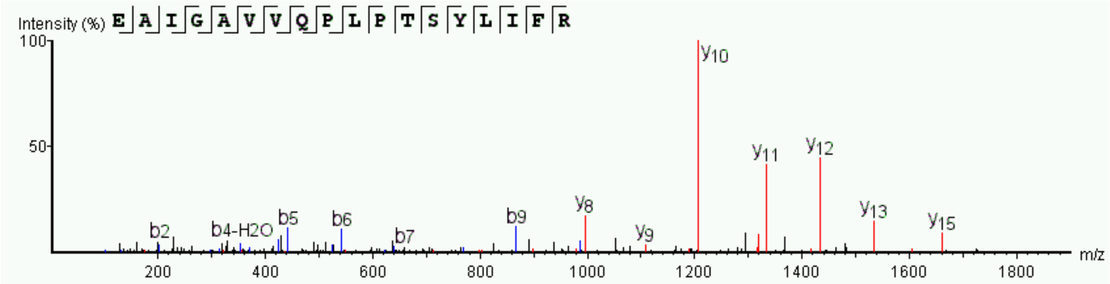

gi571517376

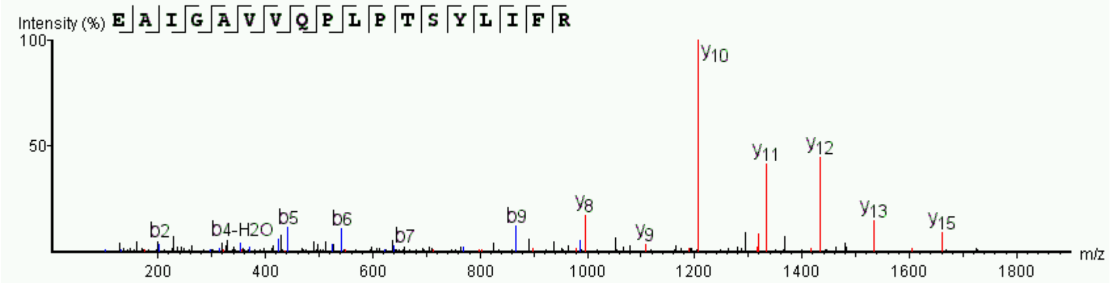

gi571517379

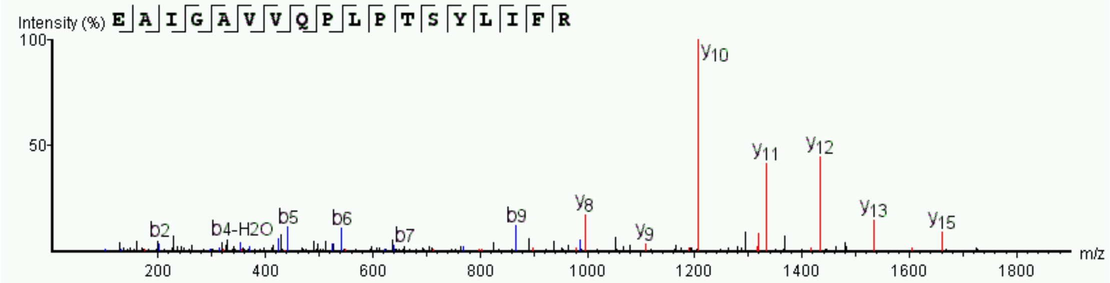

gi571517382

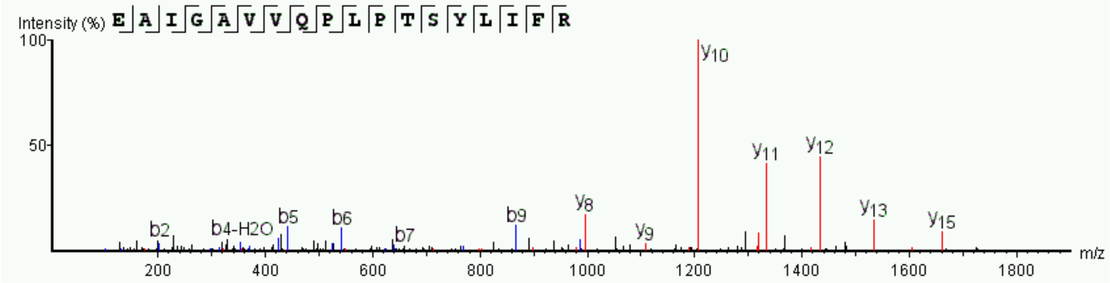

gi571517385

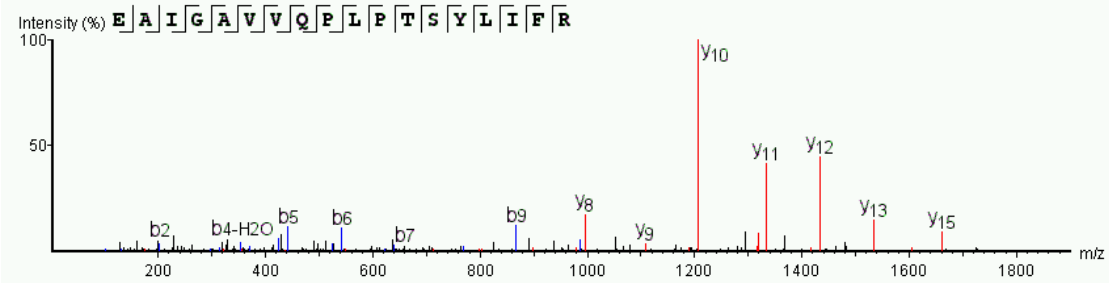

gi571517389

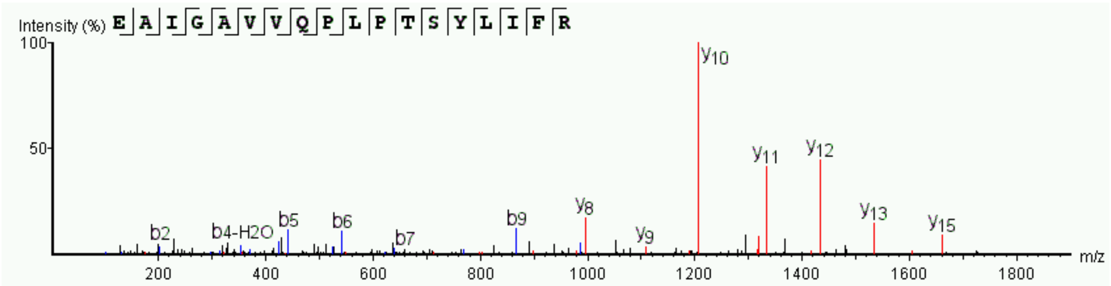

gi571517392

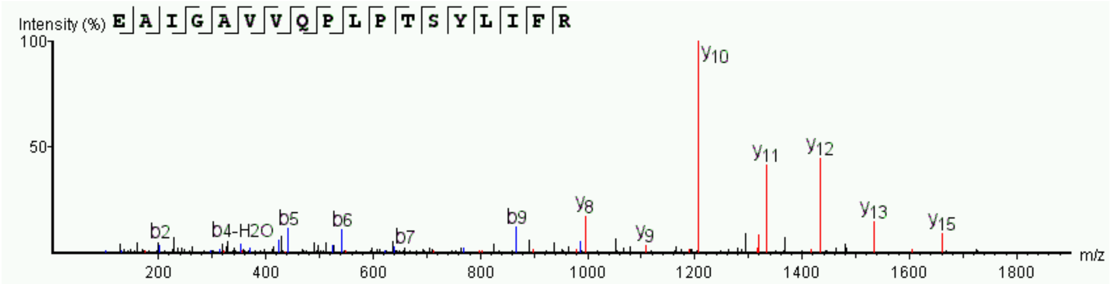

gi571517396

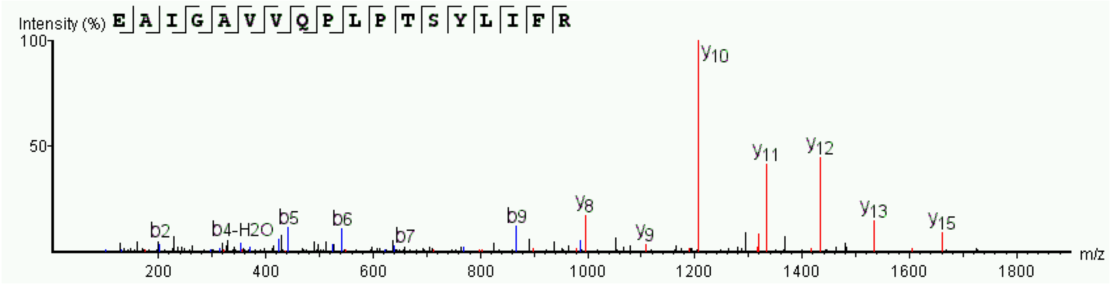

gi571541340

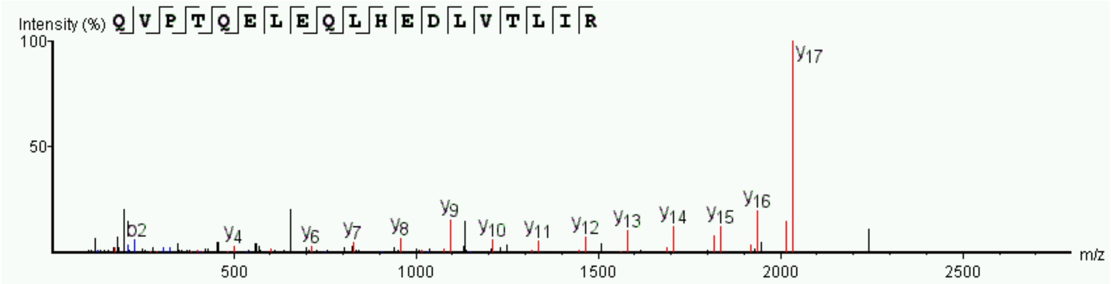

gi571528435

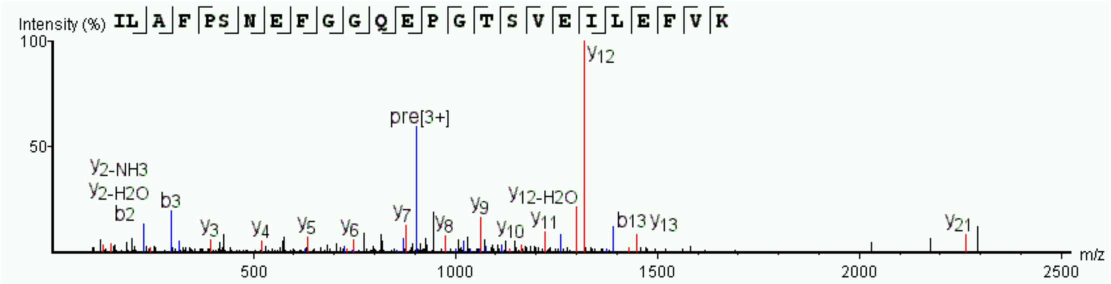

gi571565660

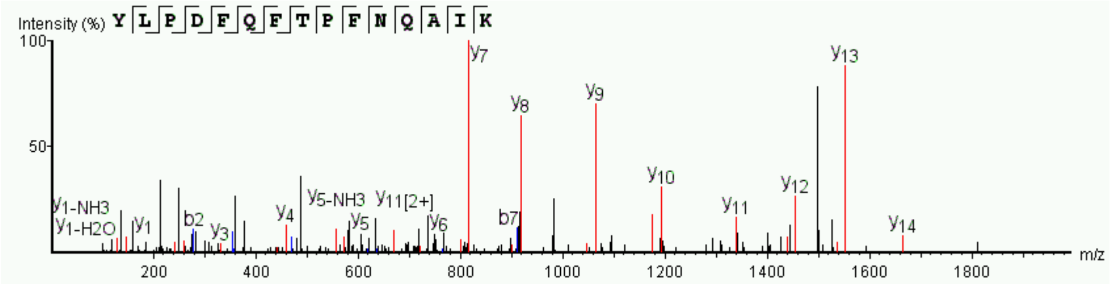

gi571565656

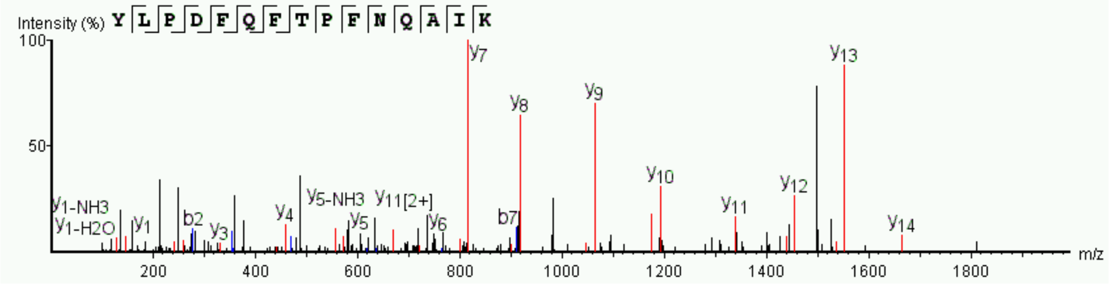

gi571523743

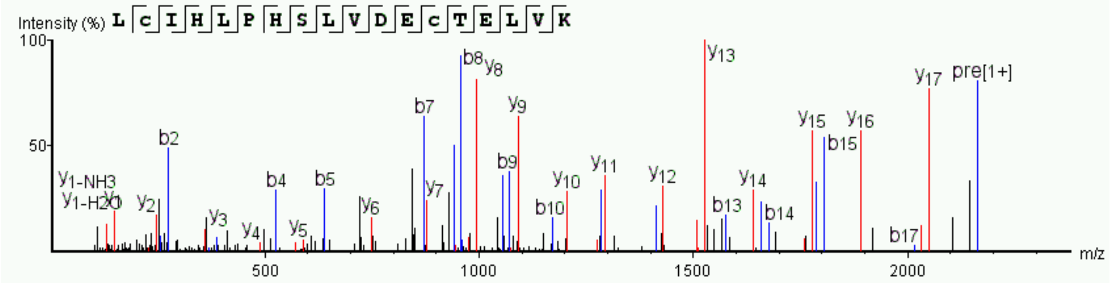

gi571523747

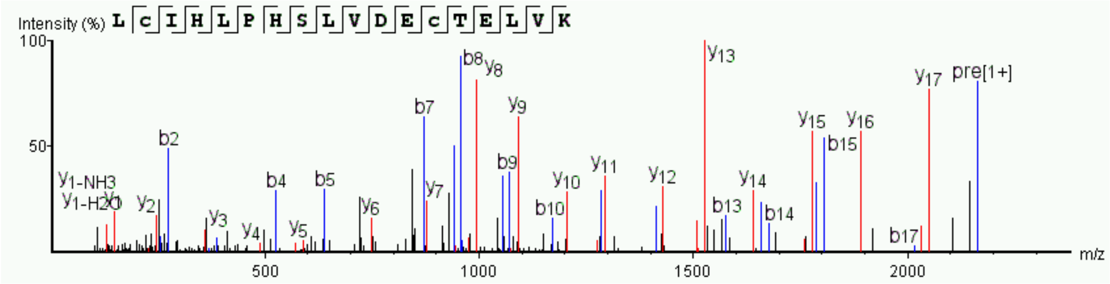

gi328782499

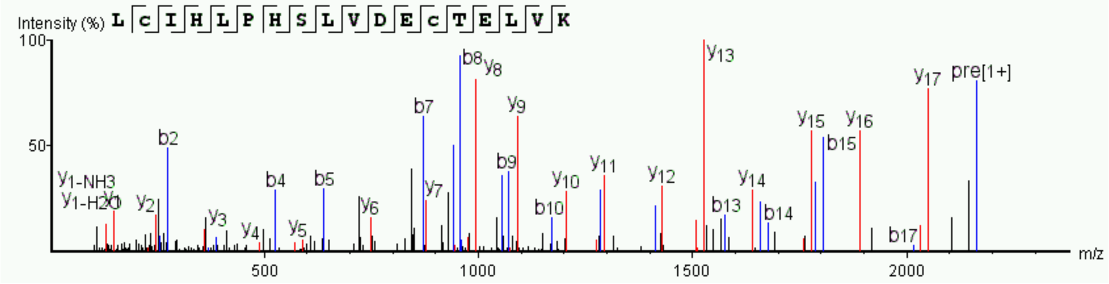

gi48128110

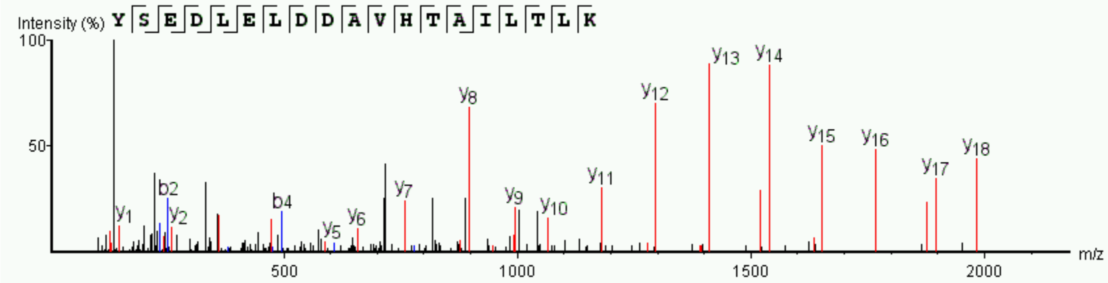

gi48103834

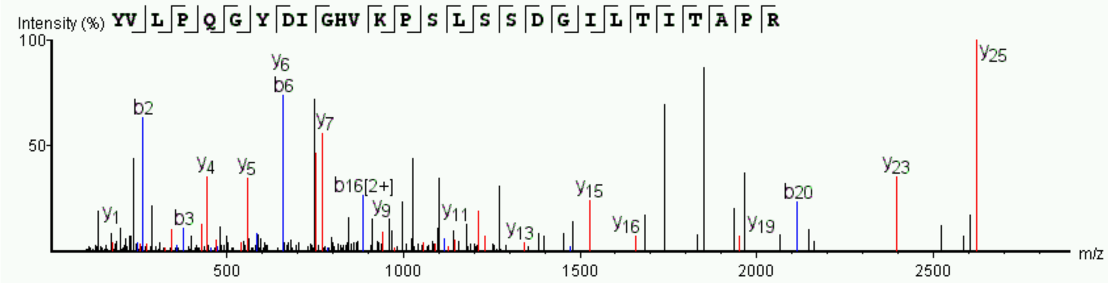

gi571537013

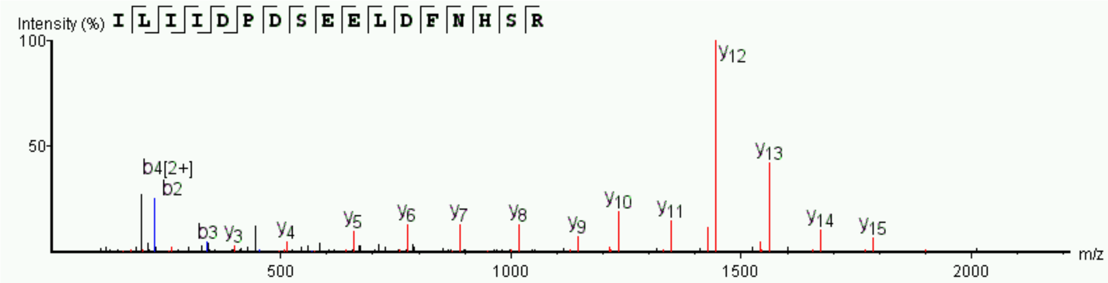

gi571508050

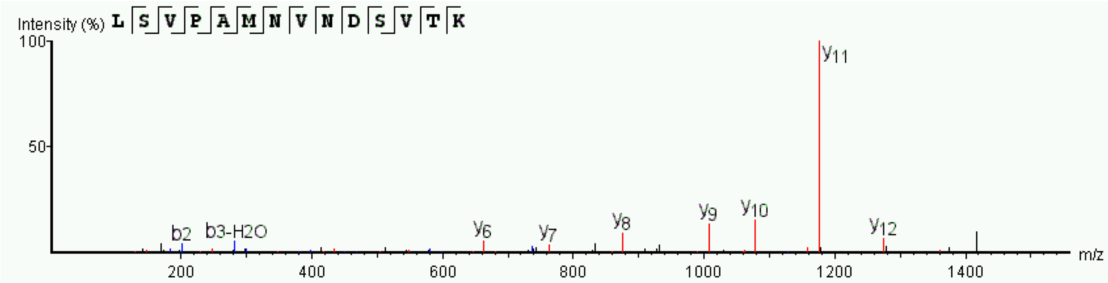

gi110766219

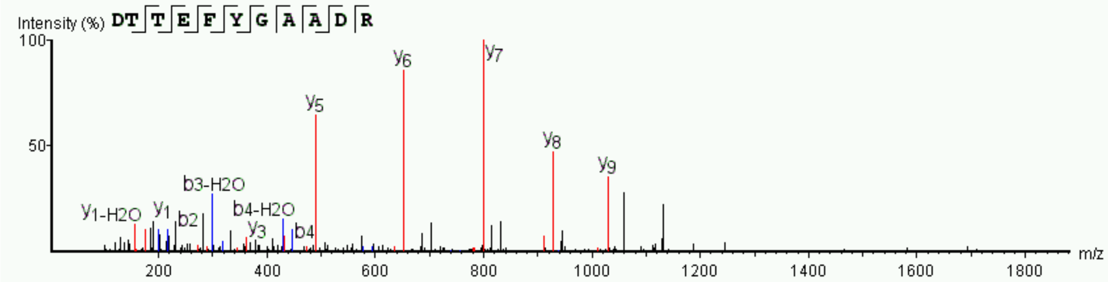

gi571516301

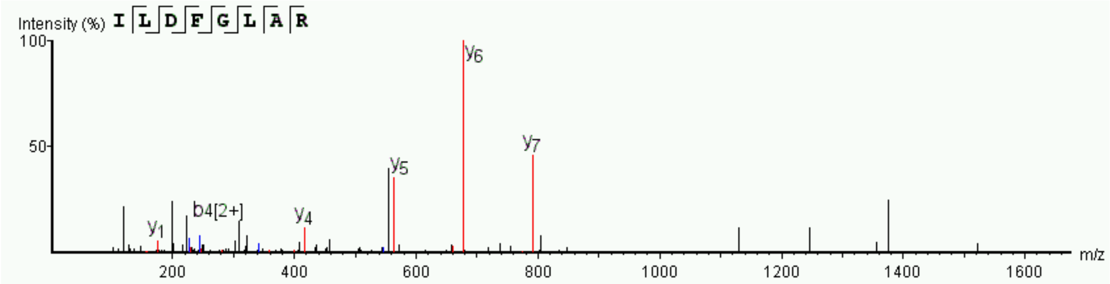

gi571516333

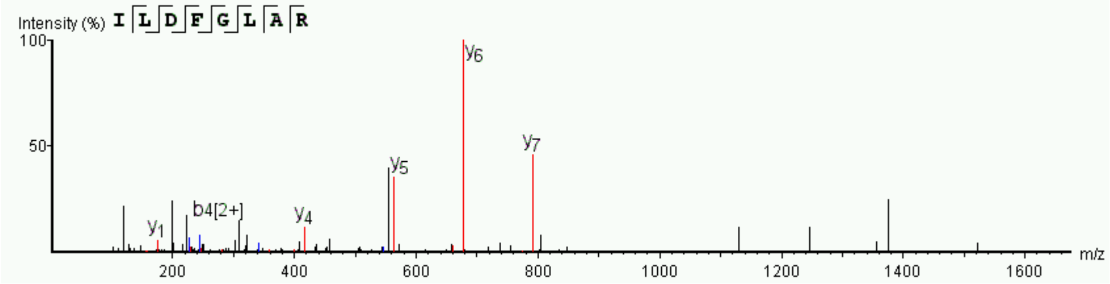

gi571516304

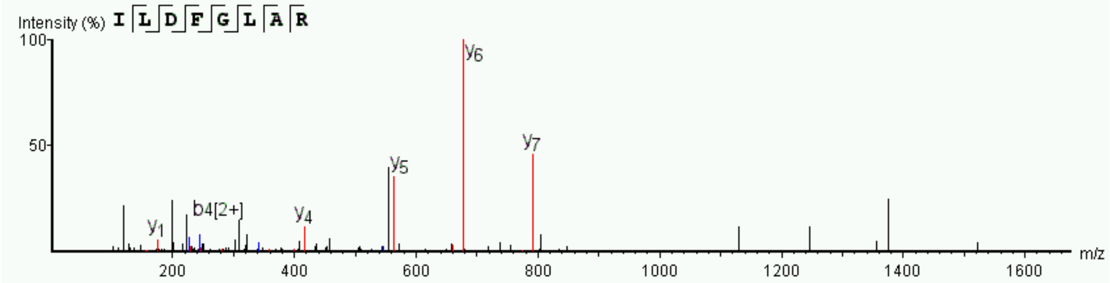

gi571516307

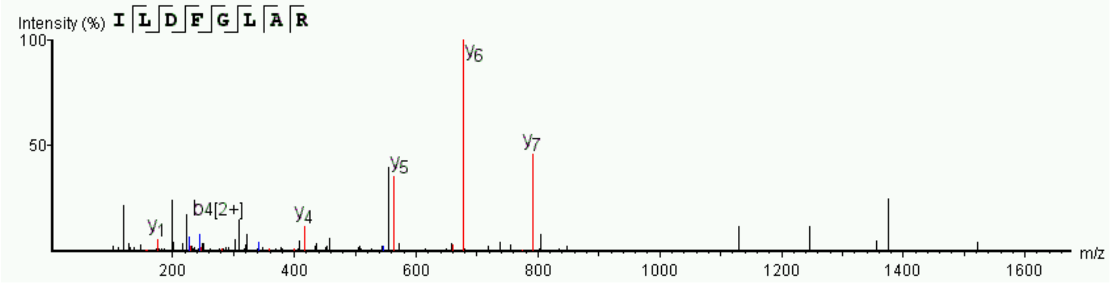

gi571516310

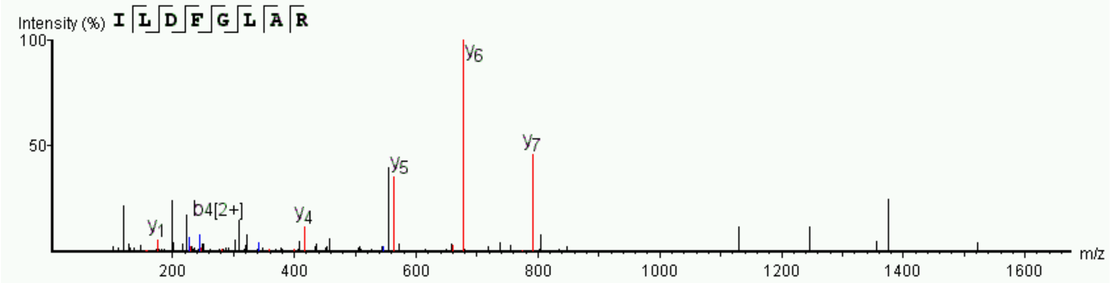

gi571516313

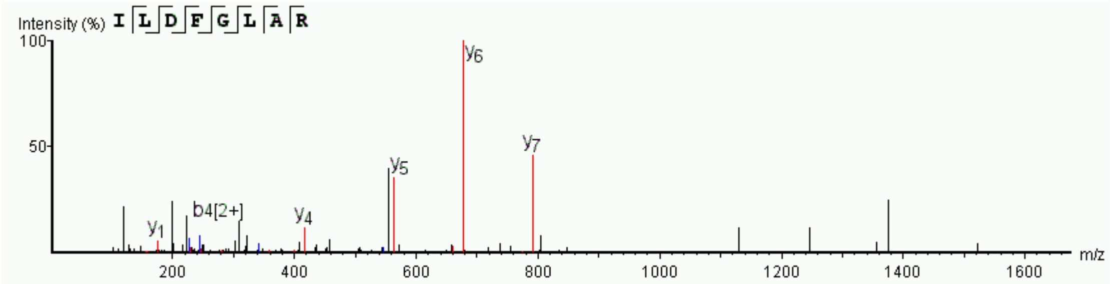

gi571516317

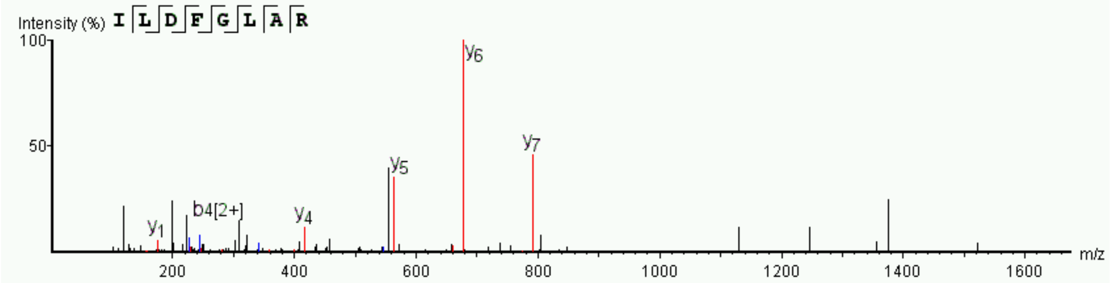

gi571516320

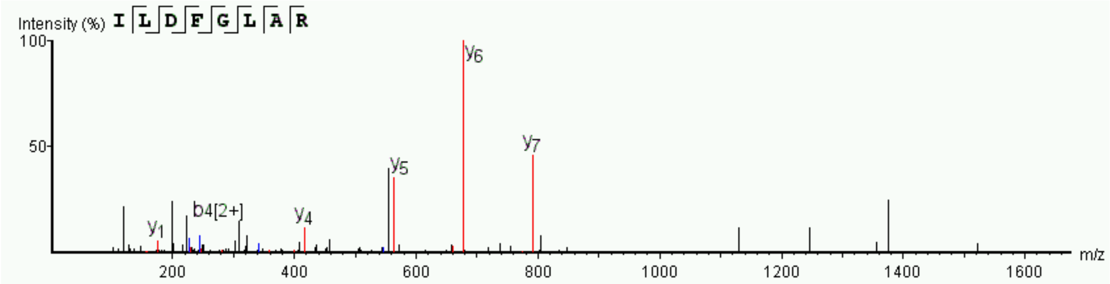

gi571516326

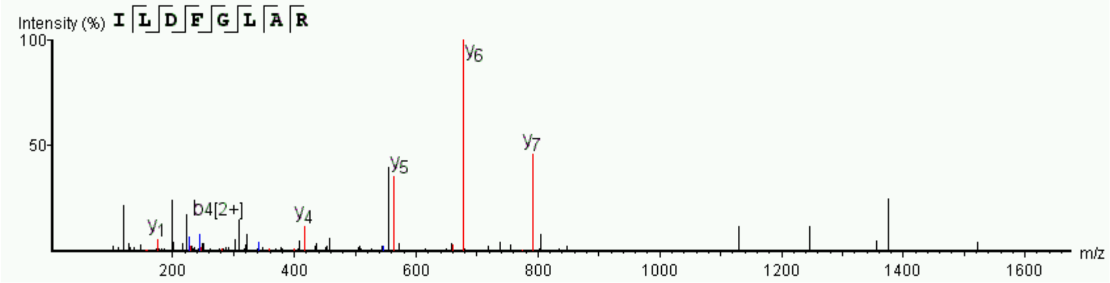

gi571516330

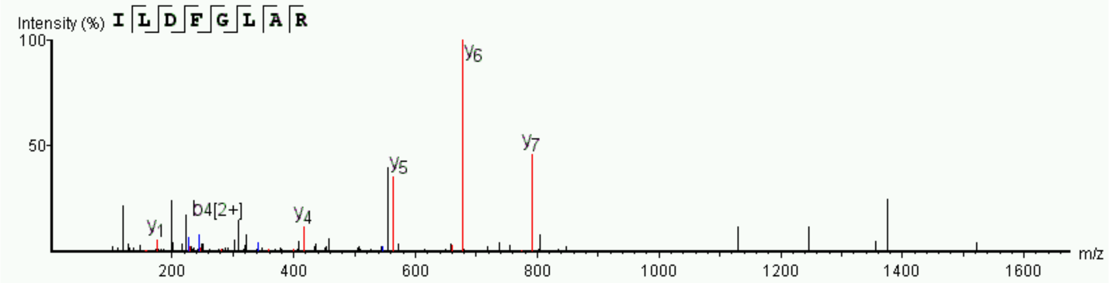

gi571568775

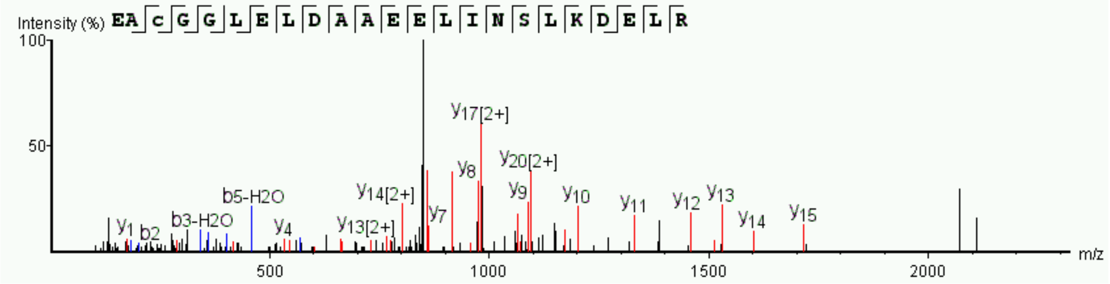

gi571523648

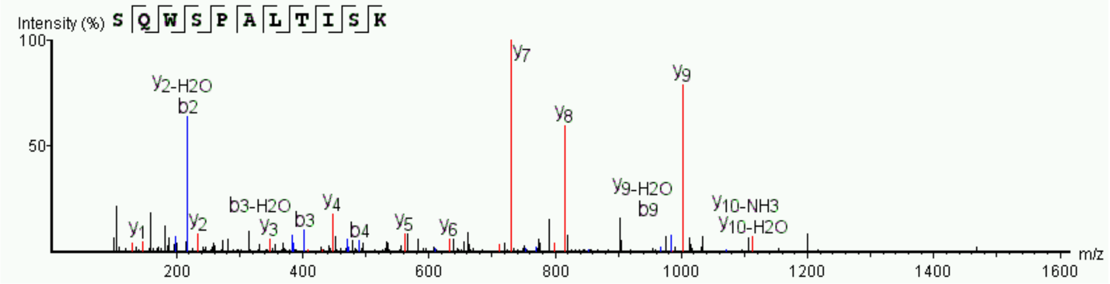

gi571543658

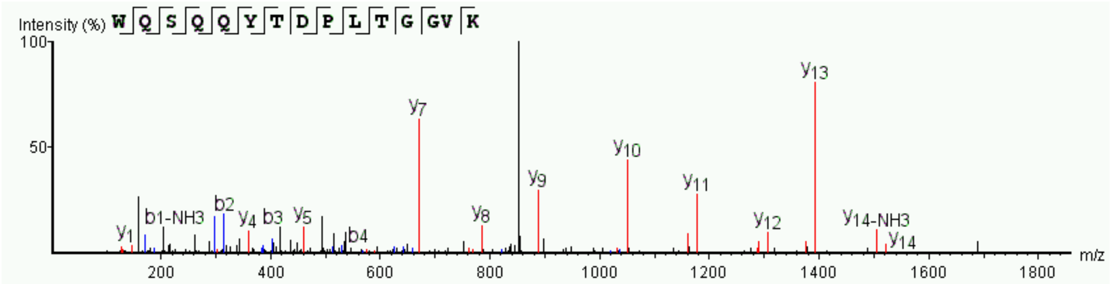

gi571543688

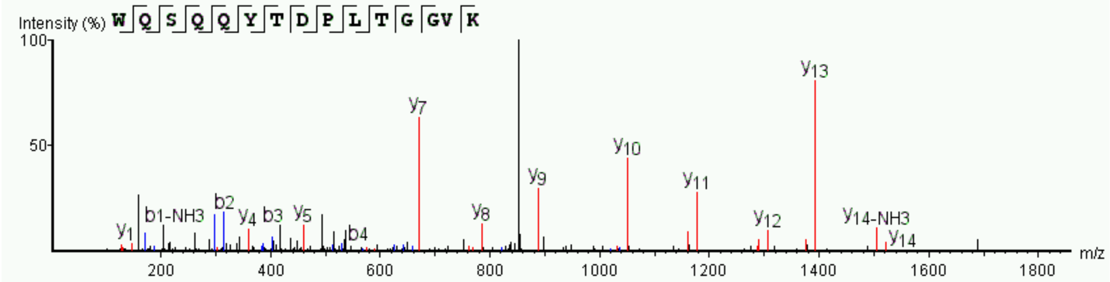

gi571543693

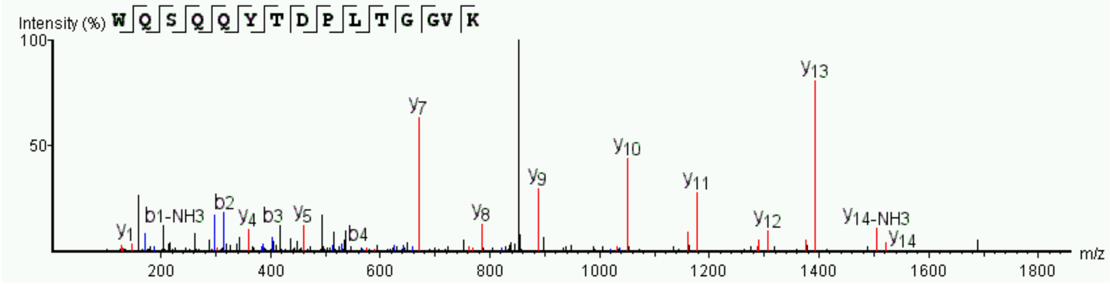

gi571543661

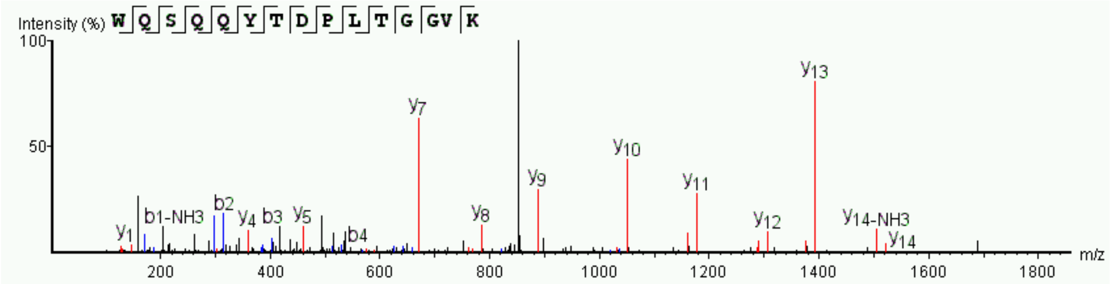

gi571543664

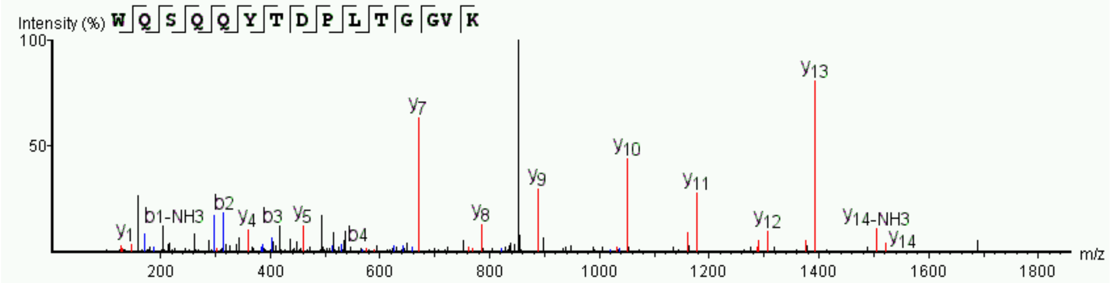

gi571543668

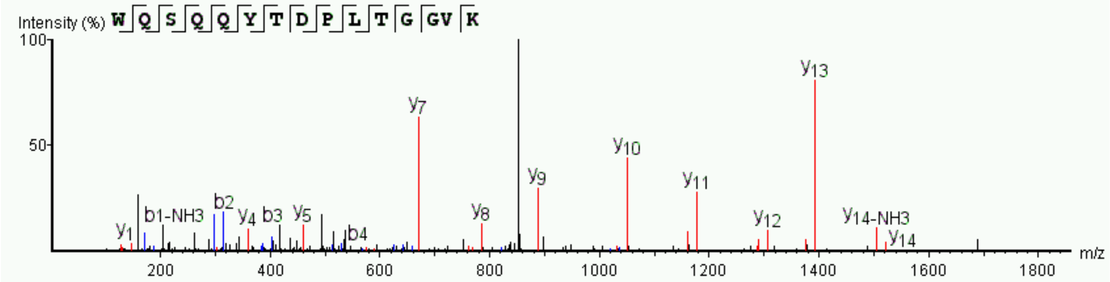

gi571543672

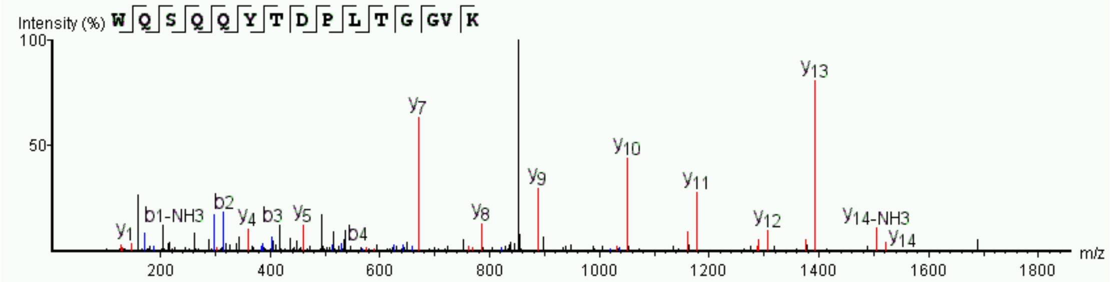

gi571543676

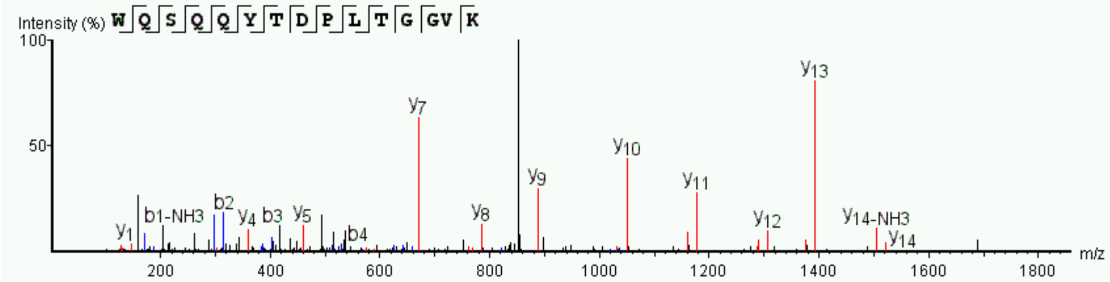

gi571543679

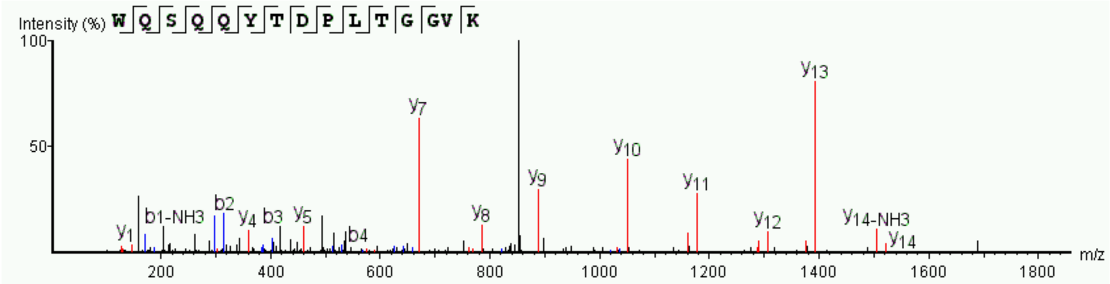

gi571543682

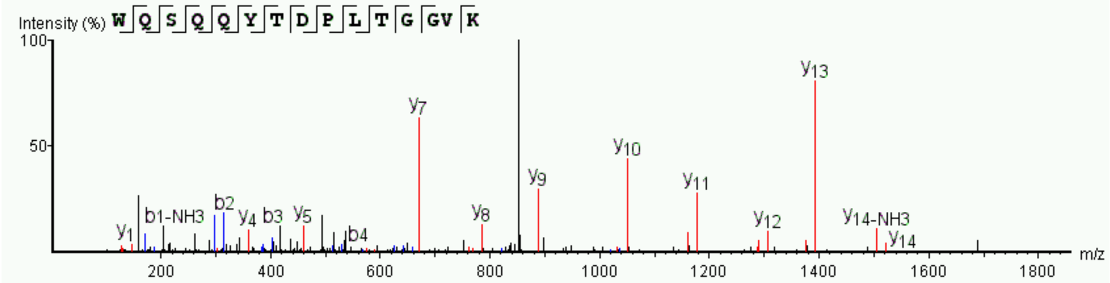

gi571543685

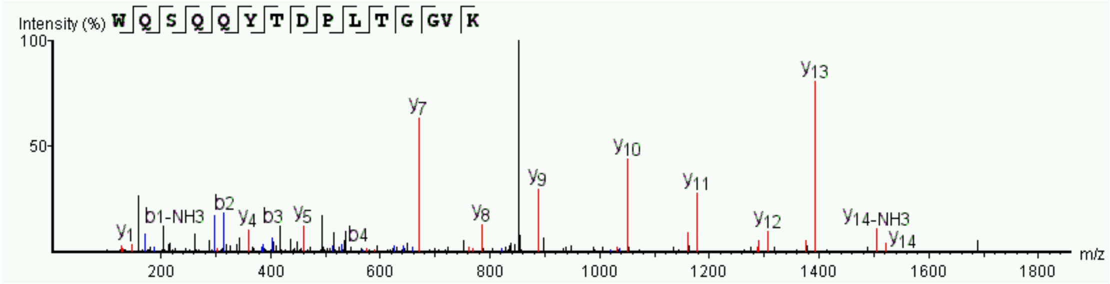

gi571570118

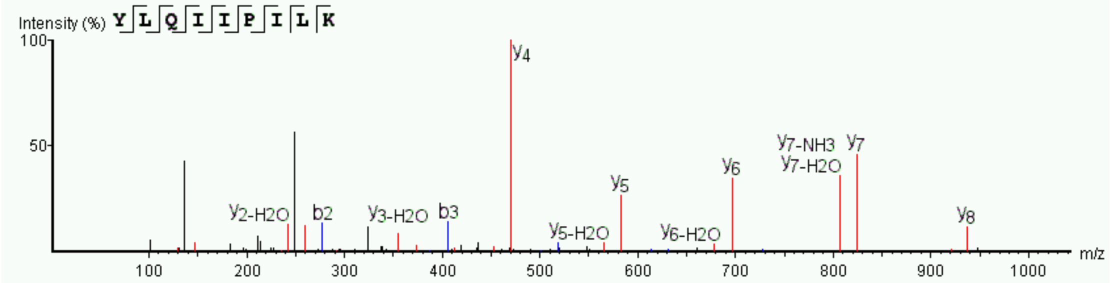

gi328792943

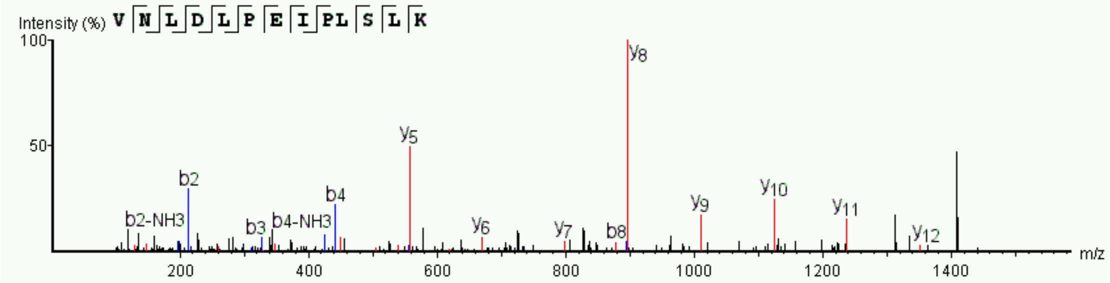

gi288872189

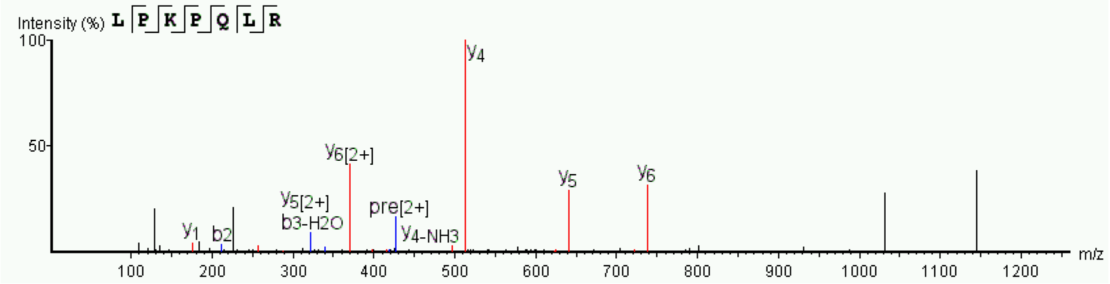

gi66524128

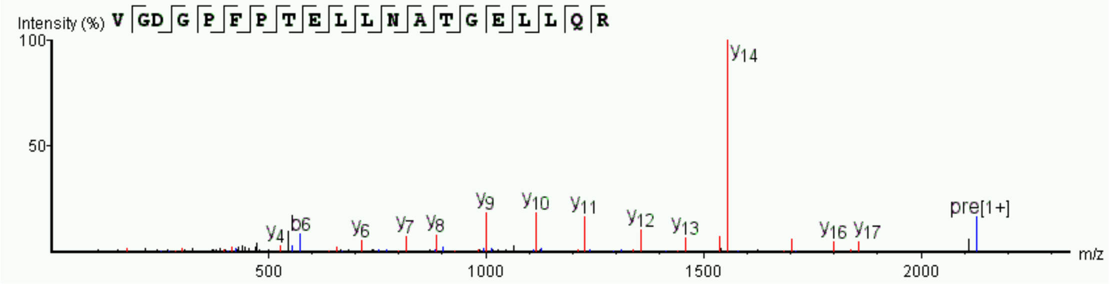

gi571540738

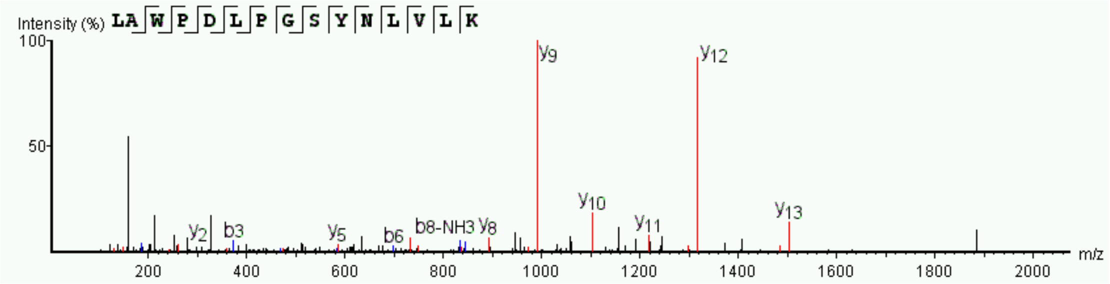

gi571512479

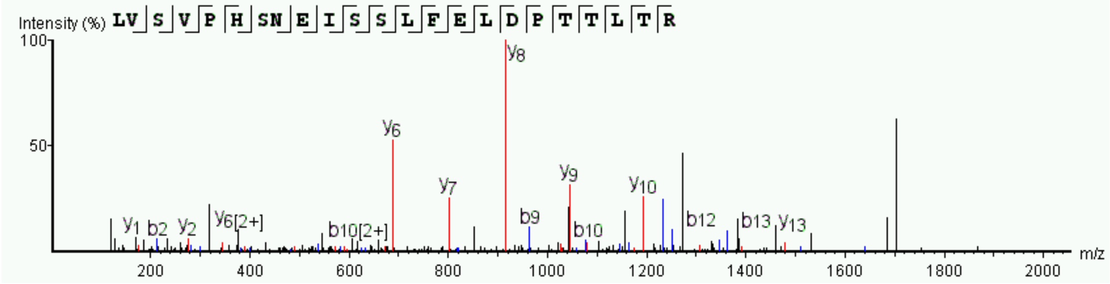

gi571512509

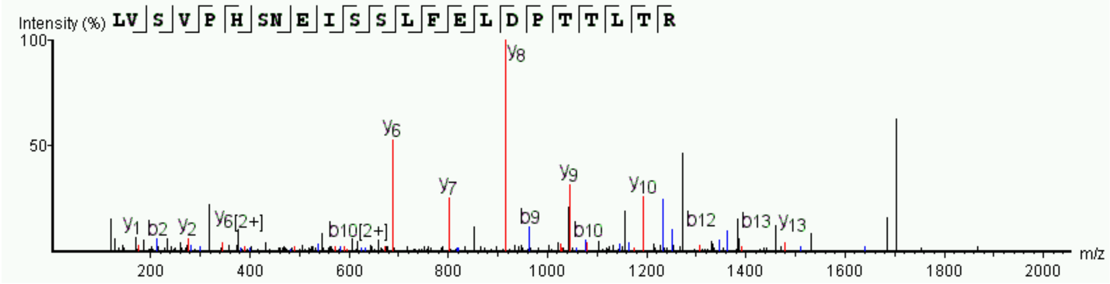

gi571512481

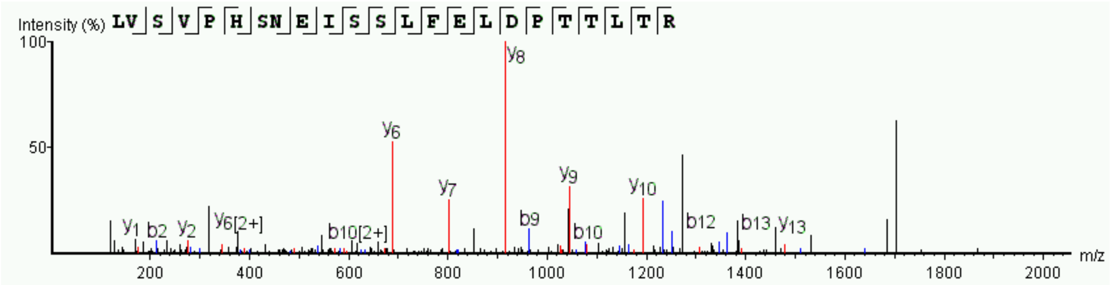

gi571512485

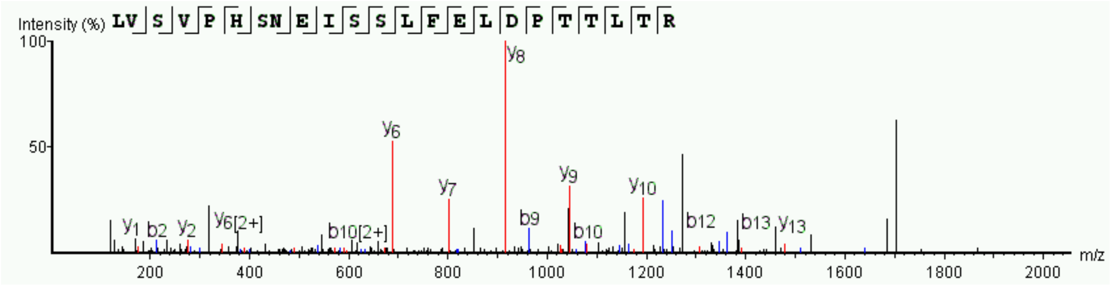

gi571512492

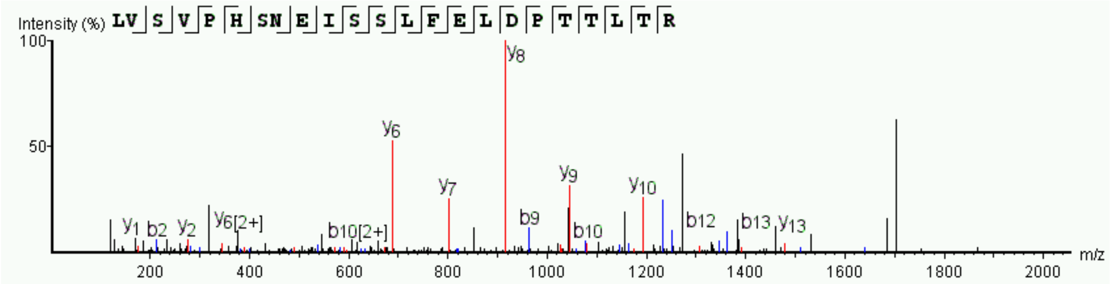

gi571512495

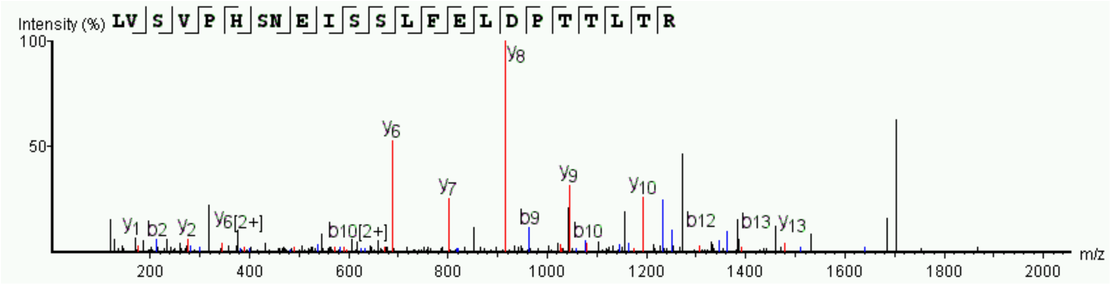

gi571512499

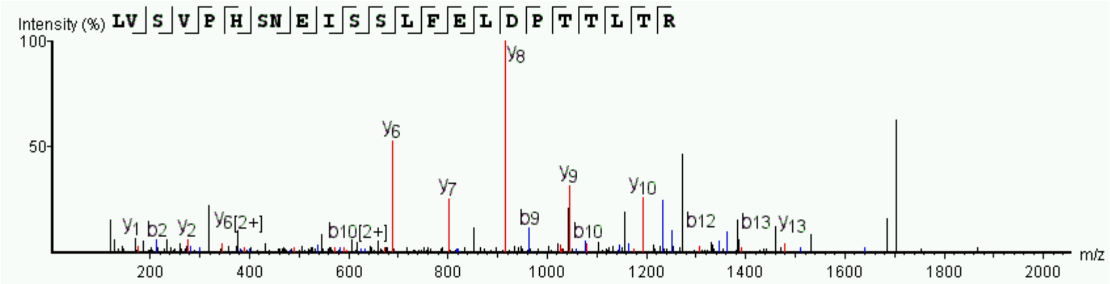

gi571512503

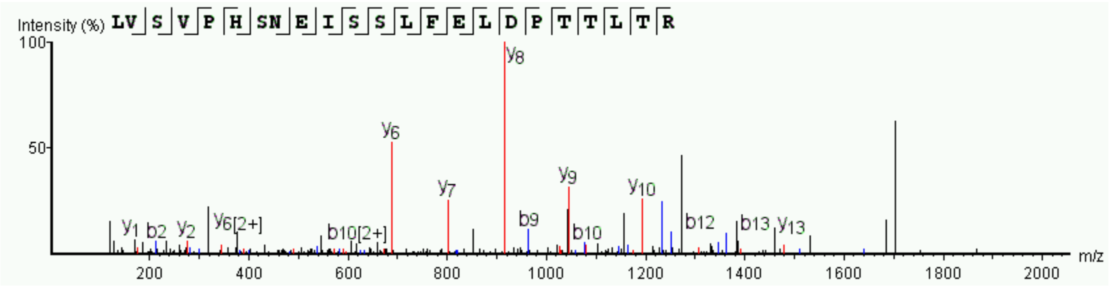

gi328778689

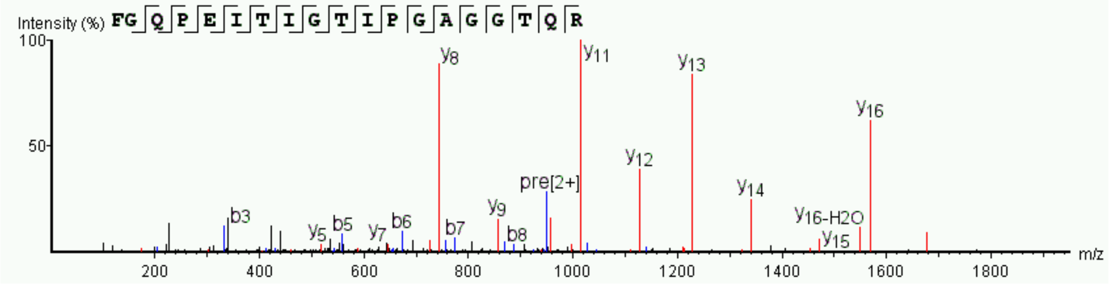

gi571566593

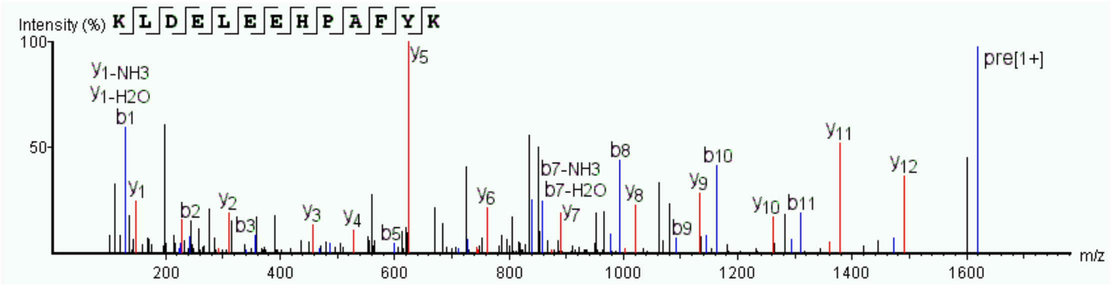

gi571566596

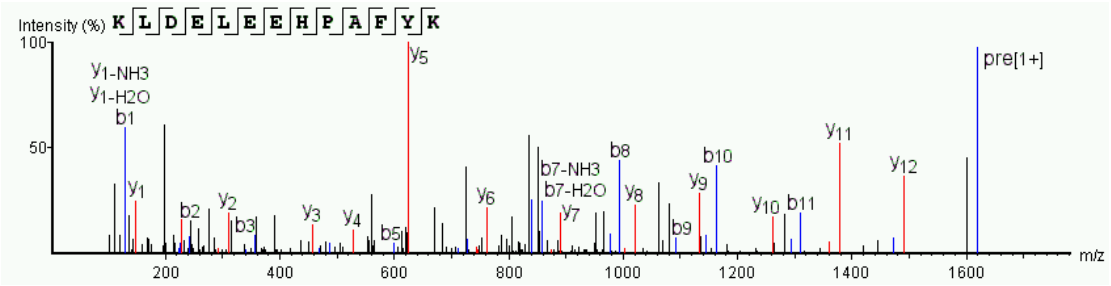

gi571566599

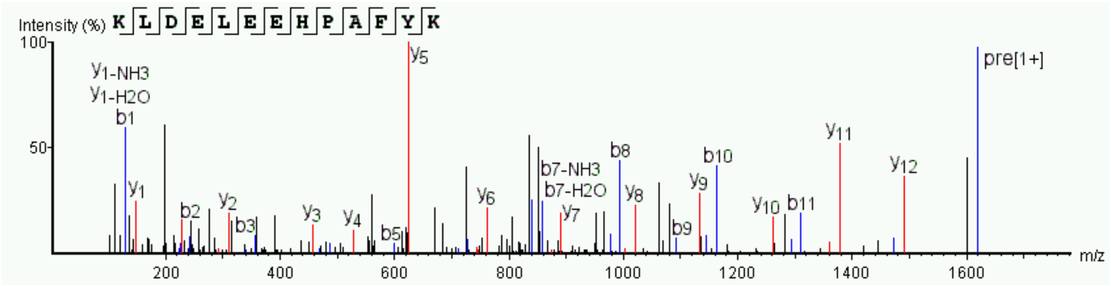

gi66522157

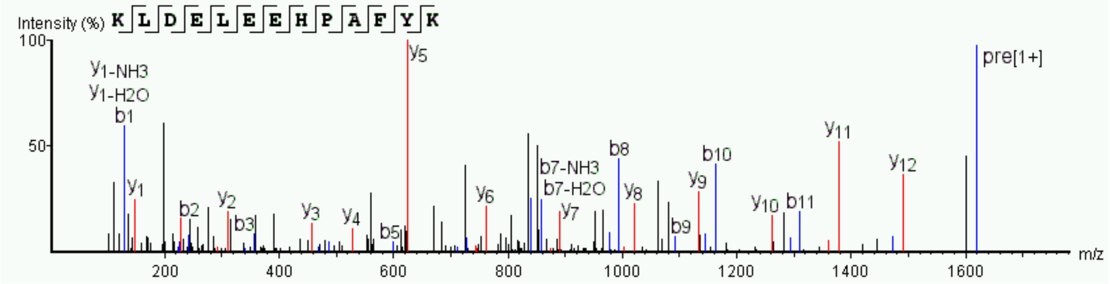

gi328784314

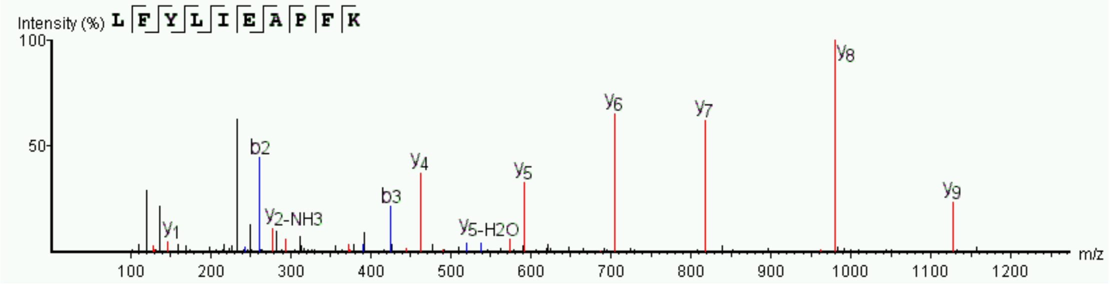

gi328784994

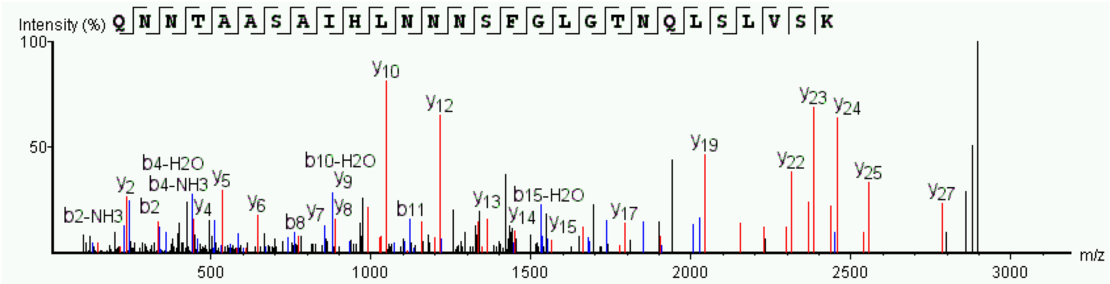

gi571528578

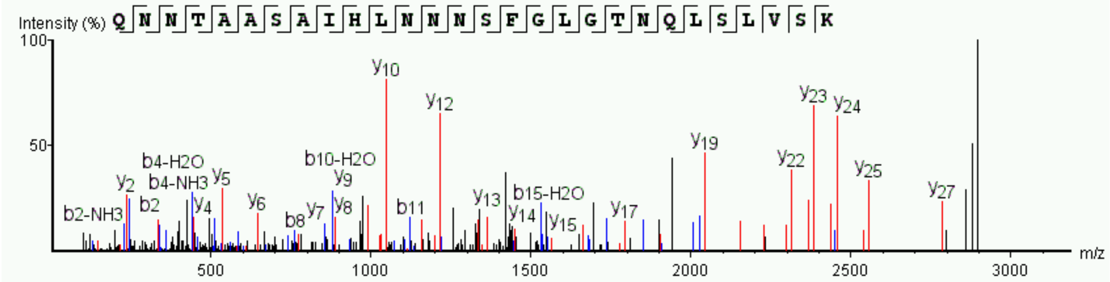

gi571528581

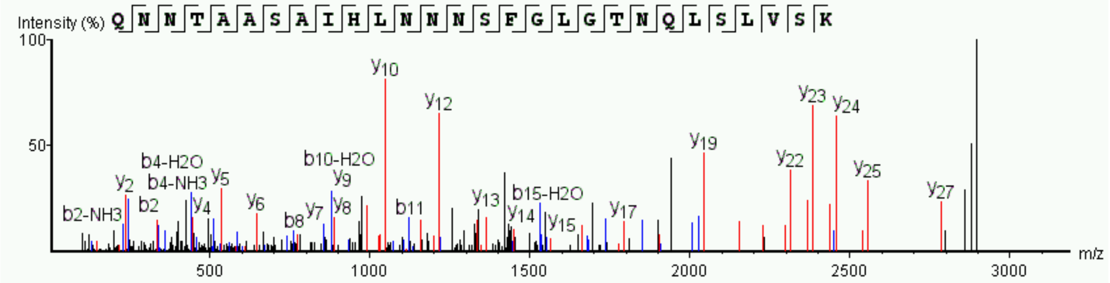

gi571506076

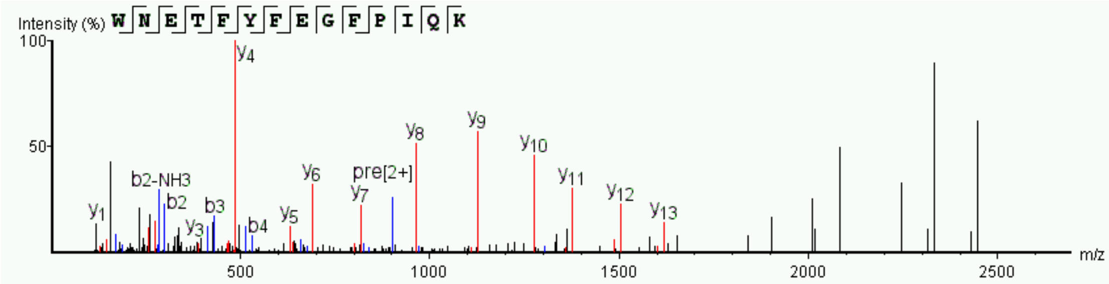

gi571506106

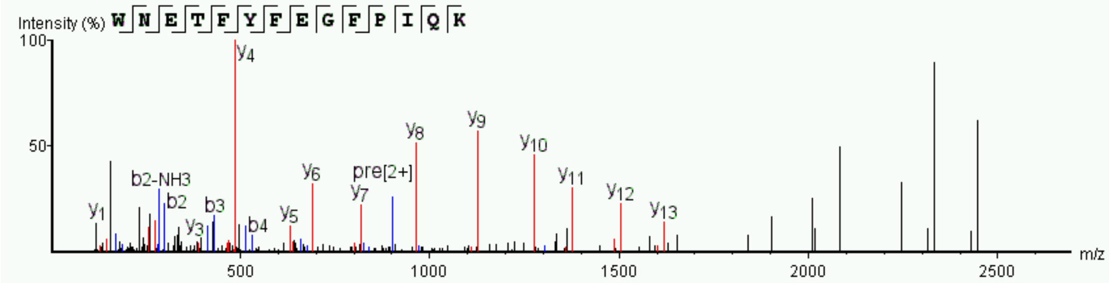

gi571506110

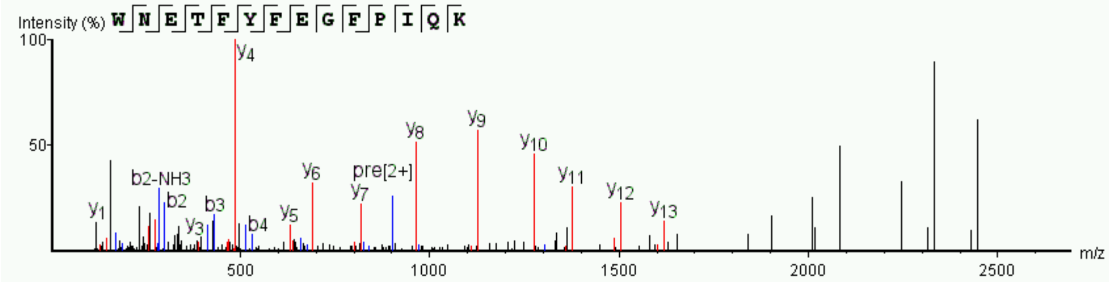

gi571506113

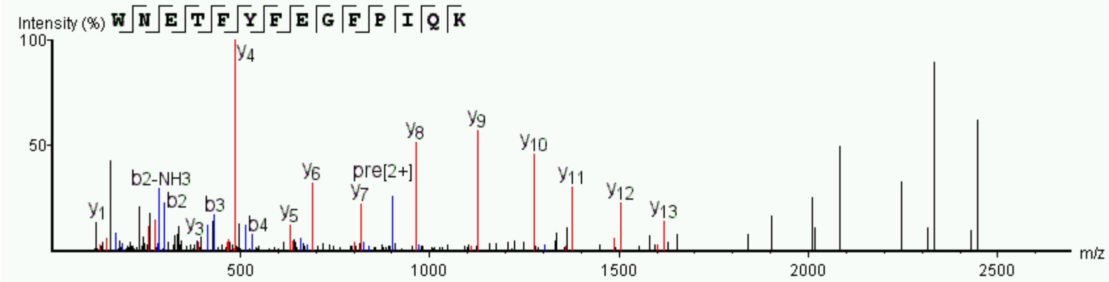

gi571506079

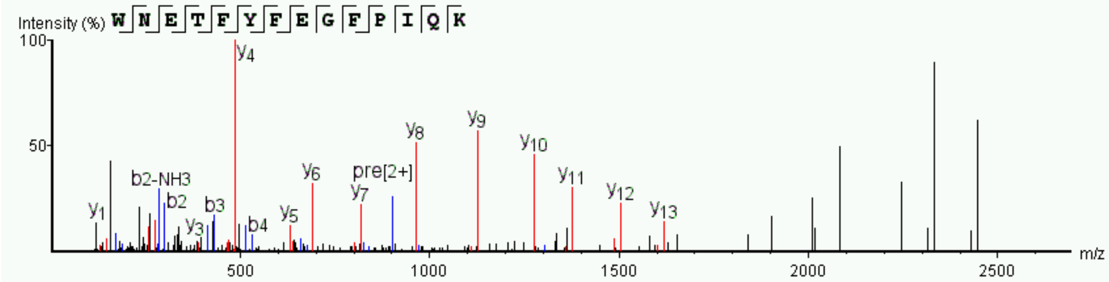

gi571506083

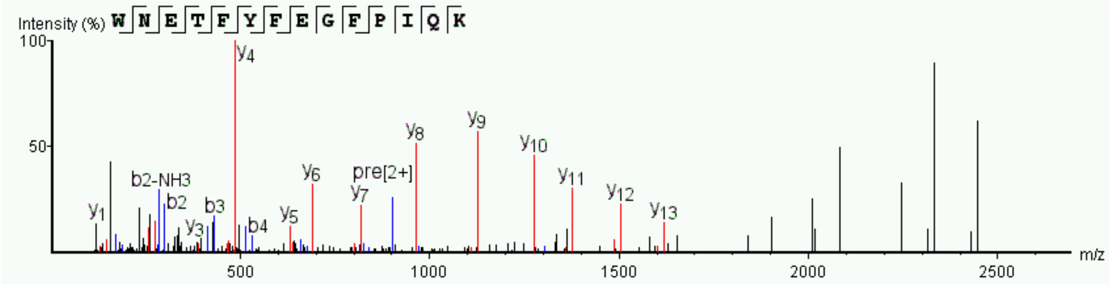

gi571506086

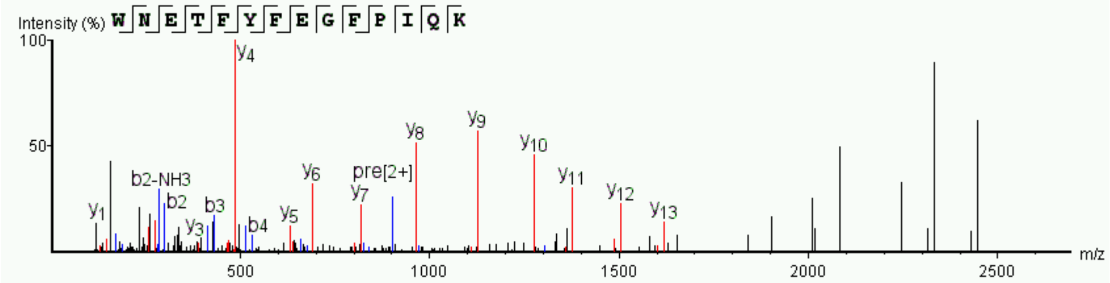

gi571506090

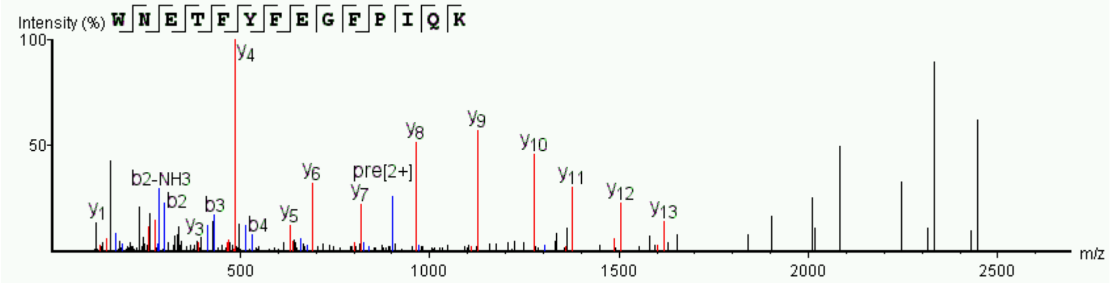

gi571506093

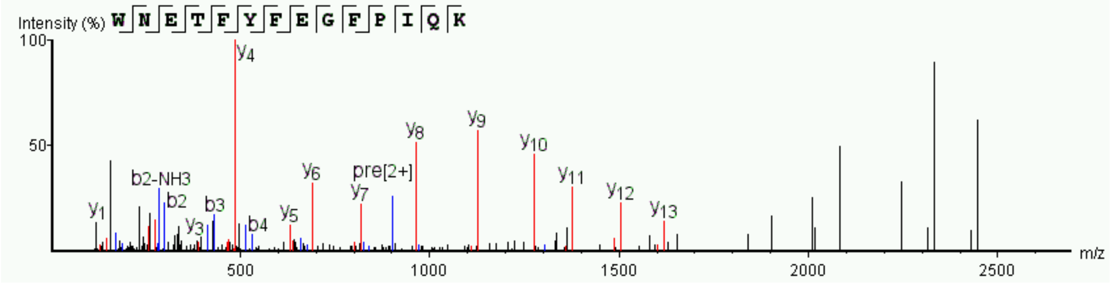

gi571506096

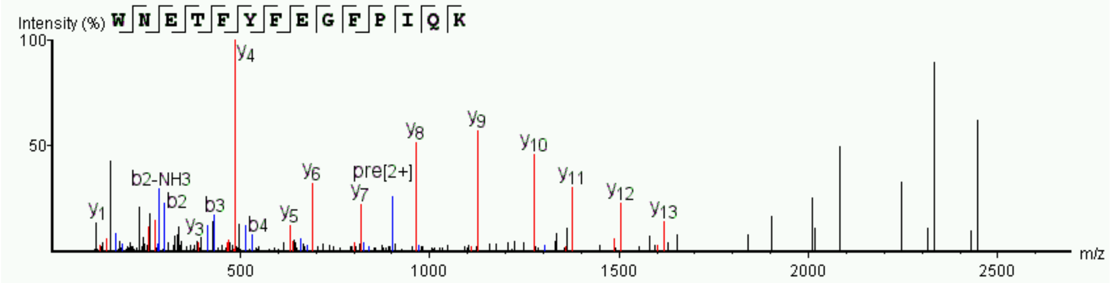

gi571506100

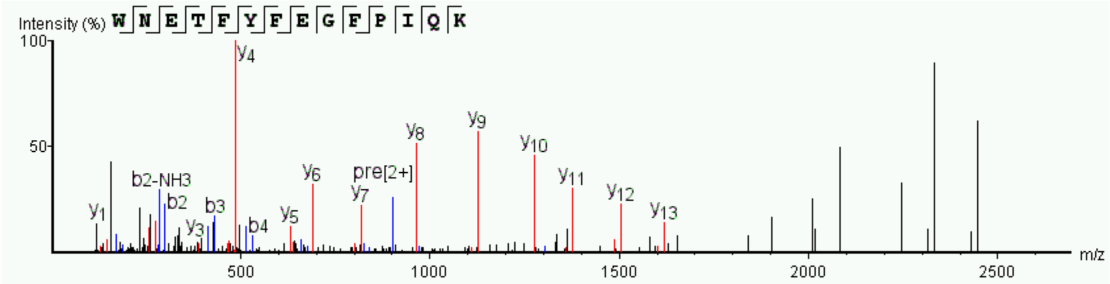

gi571506103

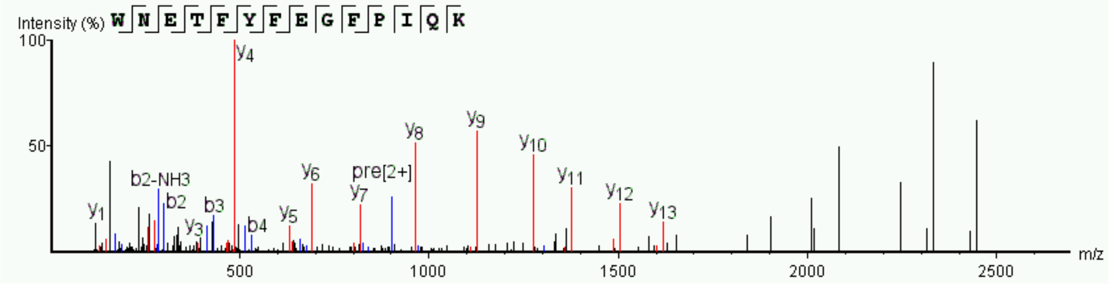

gi571549591

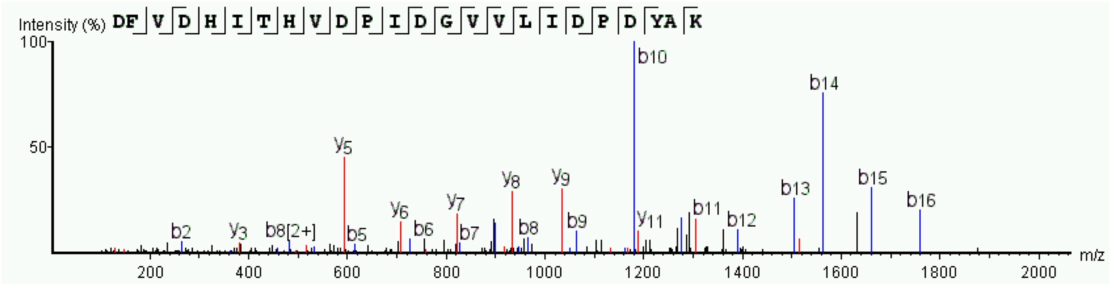

gi571549598

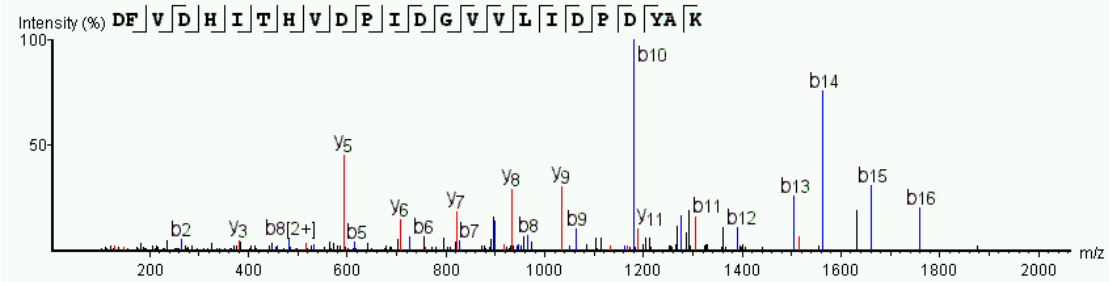

gi571549603

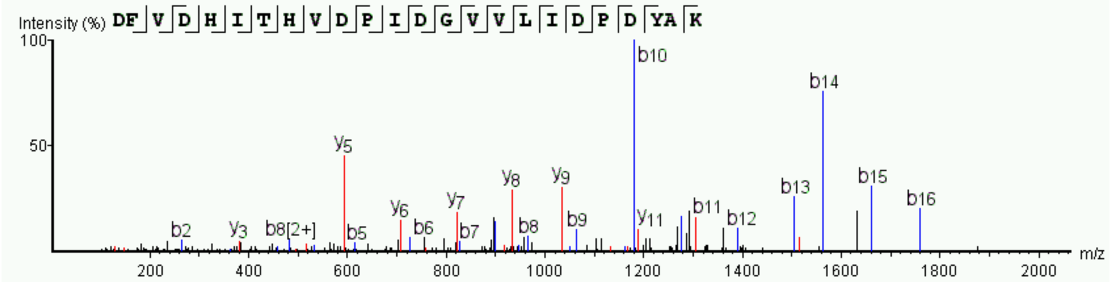

gi328781218

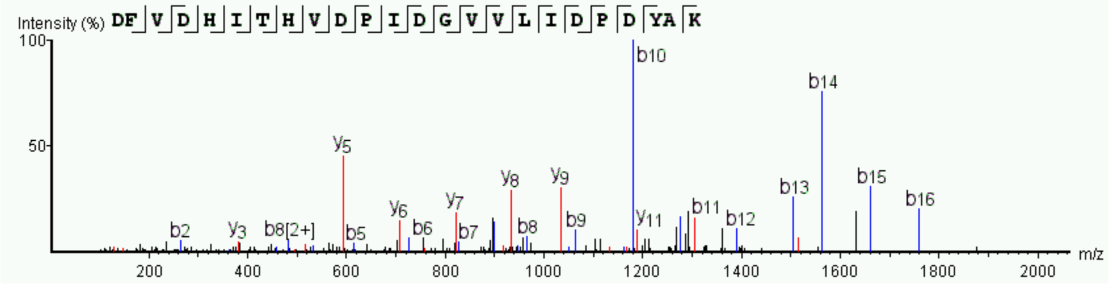

gi571521696

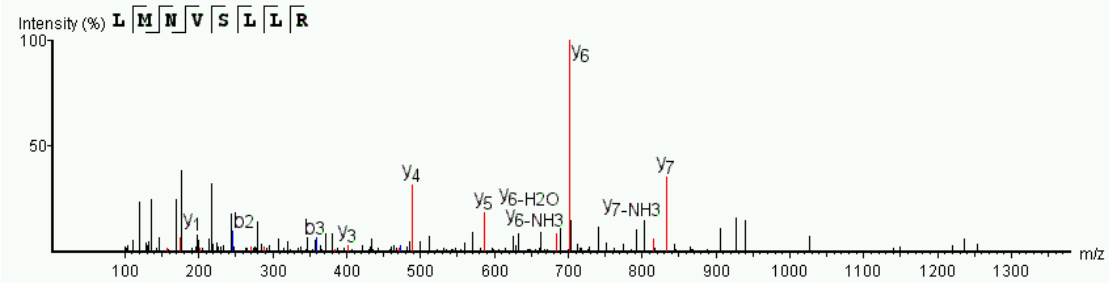

gi571521709

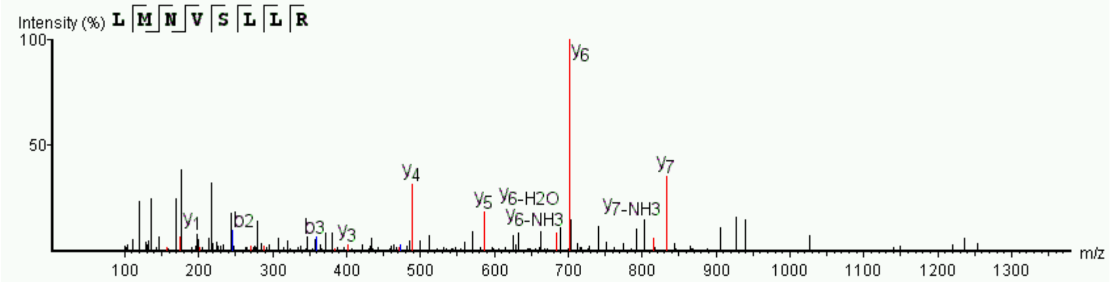

gi328775985

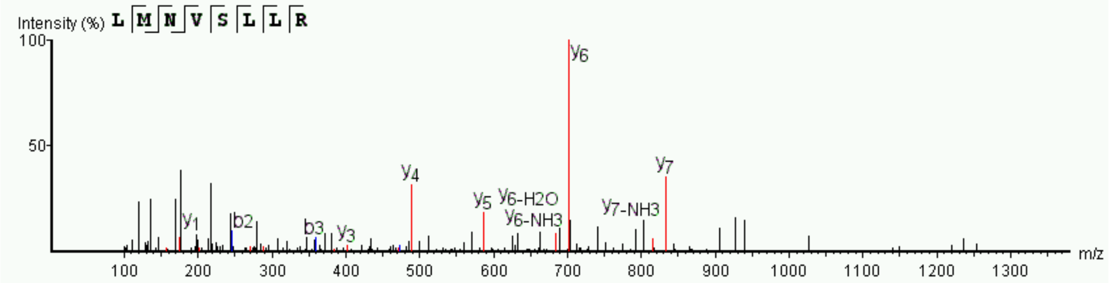

gi66512785

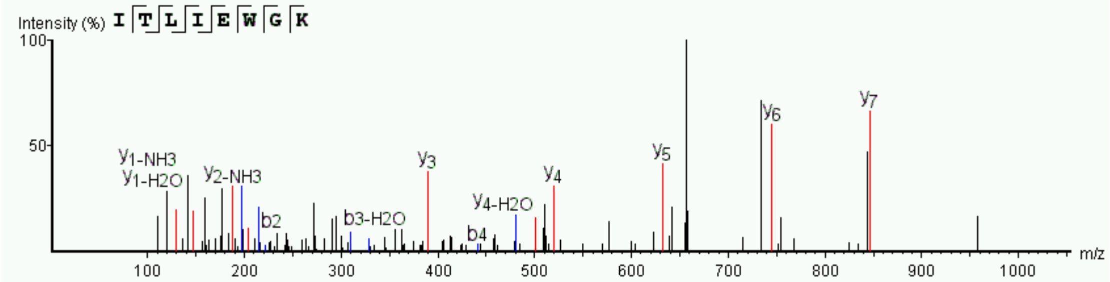

gi571538912

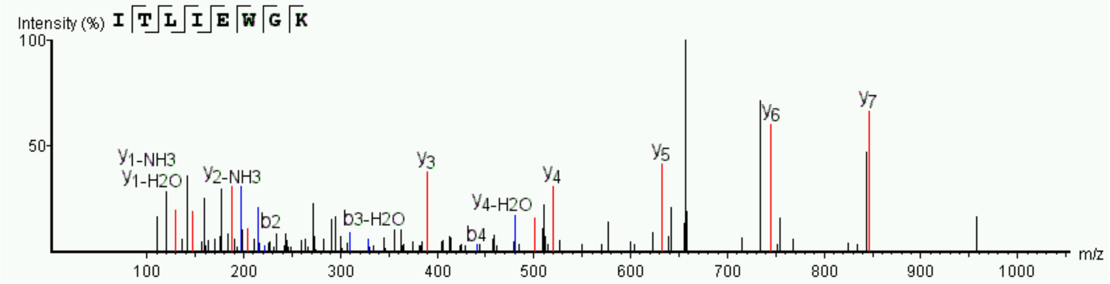

gi328791545

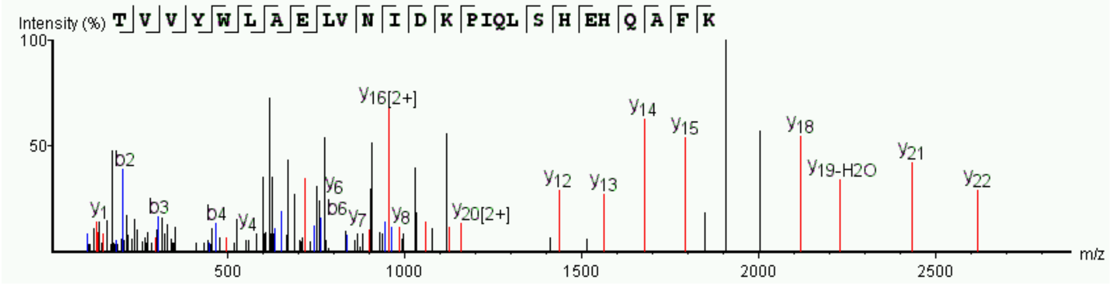

gi571505889

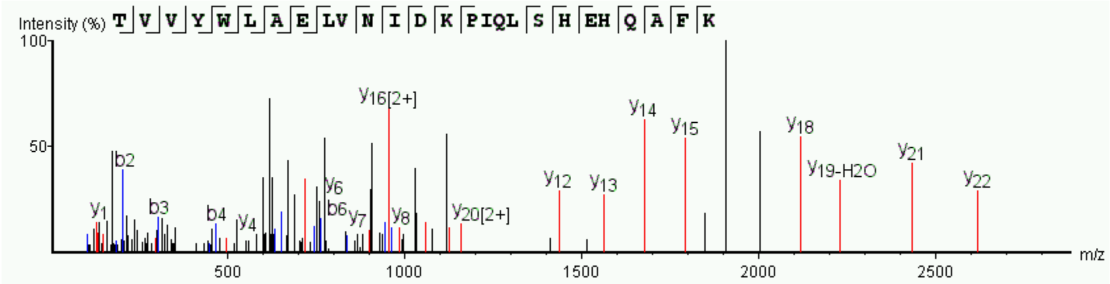

gi571505892

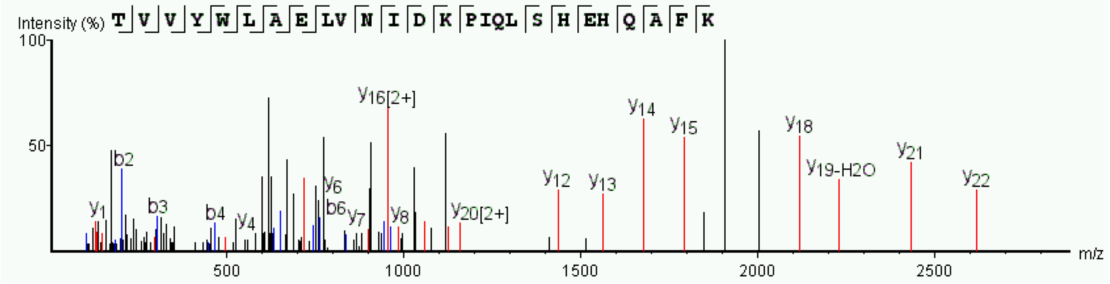

gi571544412

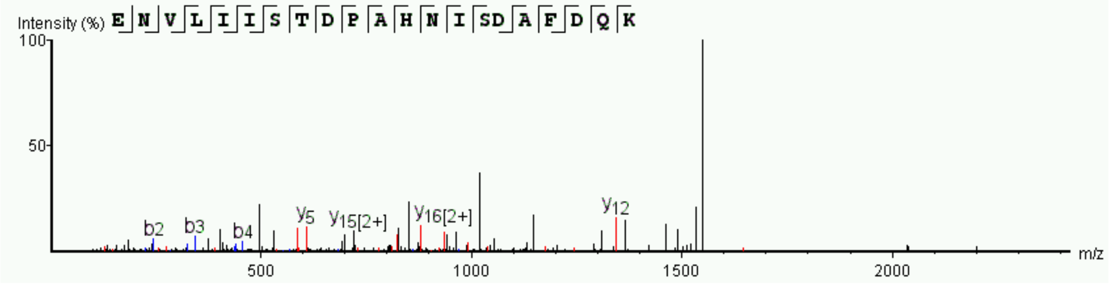

gi328786971

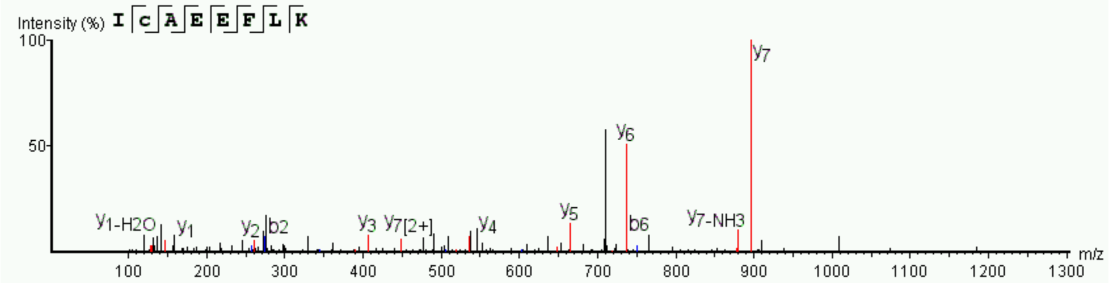

gi571544412

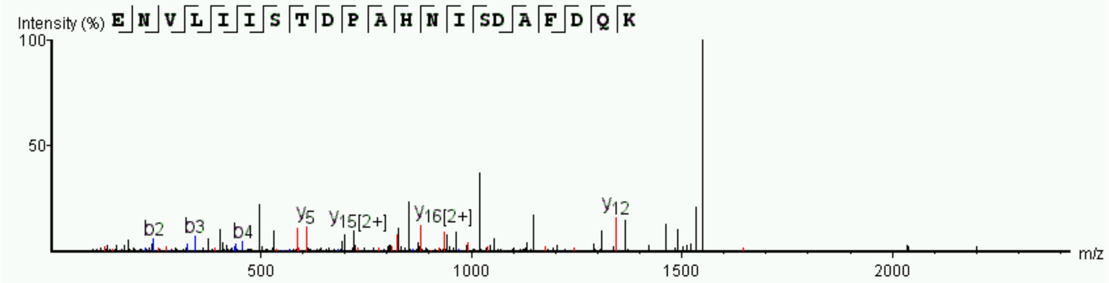

gi328786971

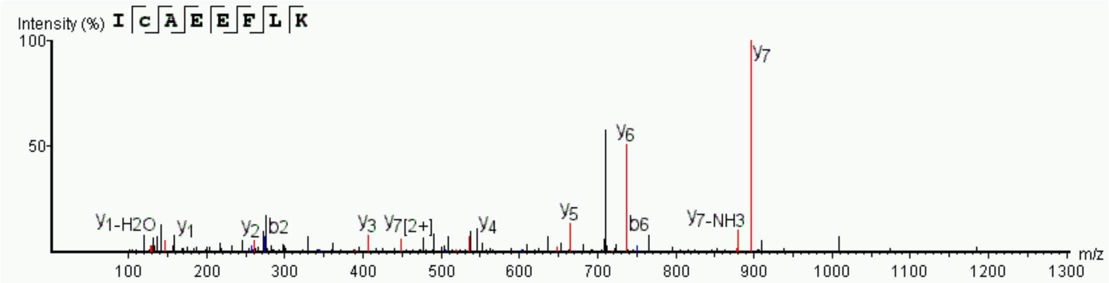

gi571532025

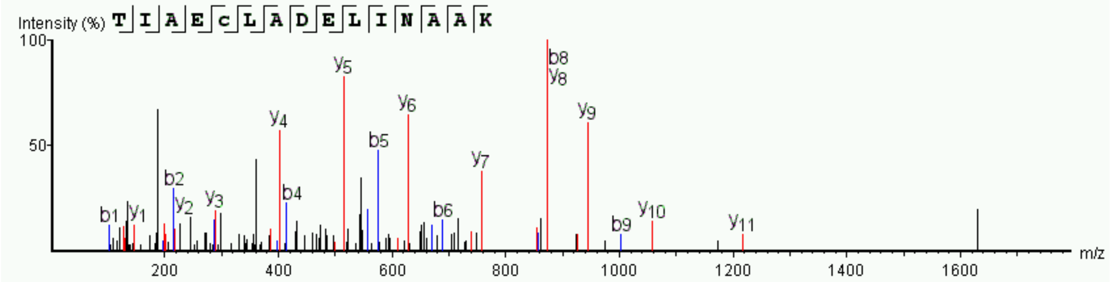

gi571532029

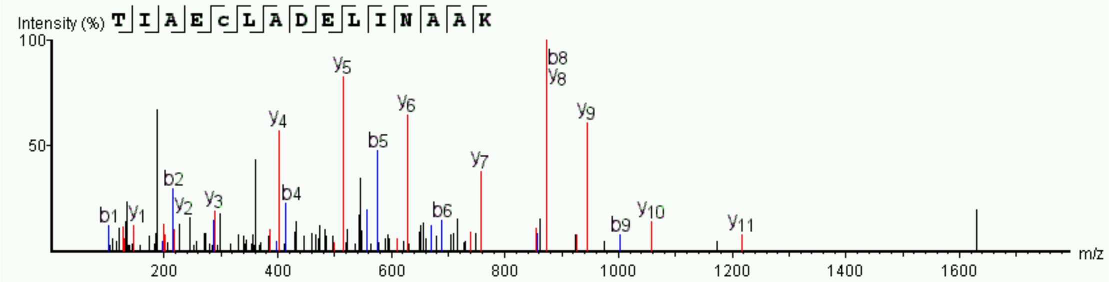

gi328789366

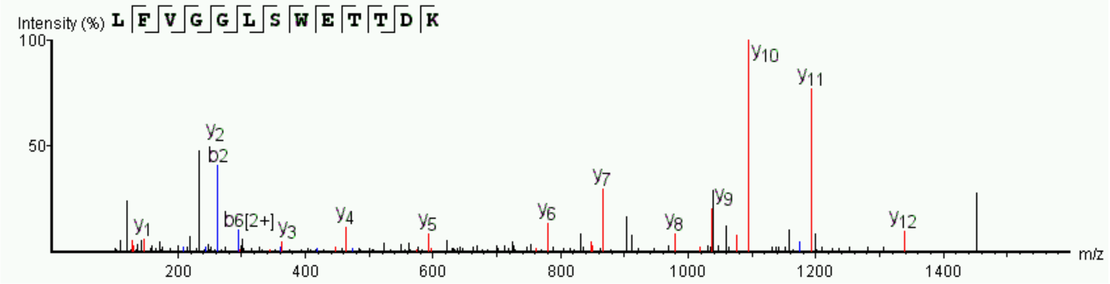

gi571565991

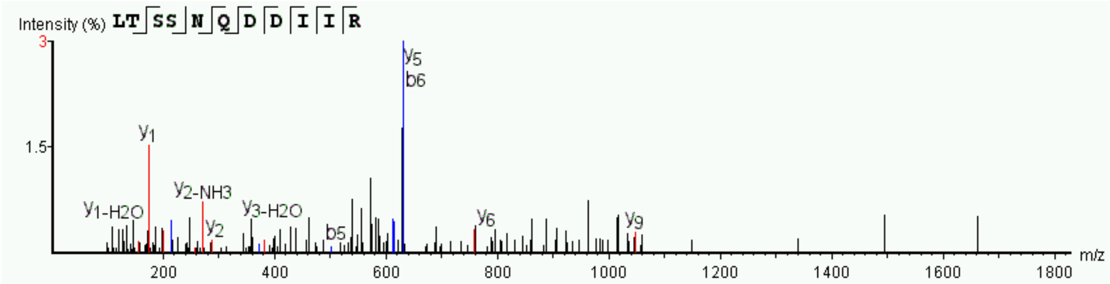

gi571577919

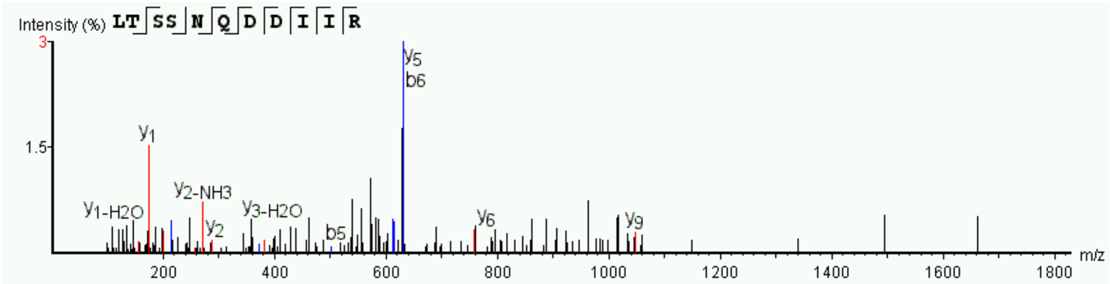

gi571577921

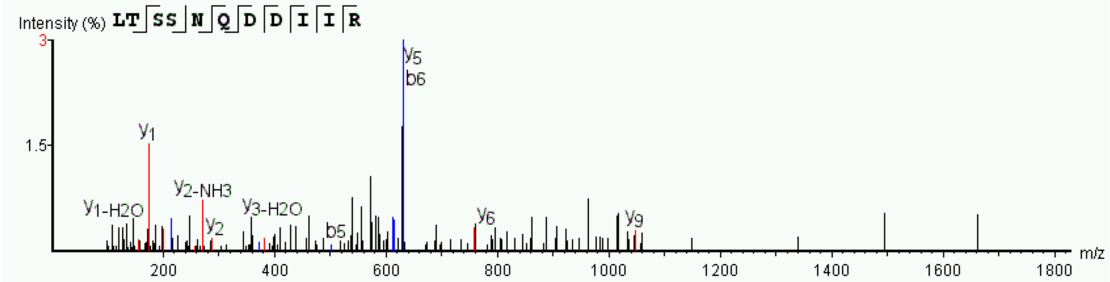

gi571577923

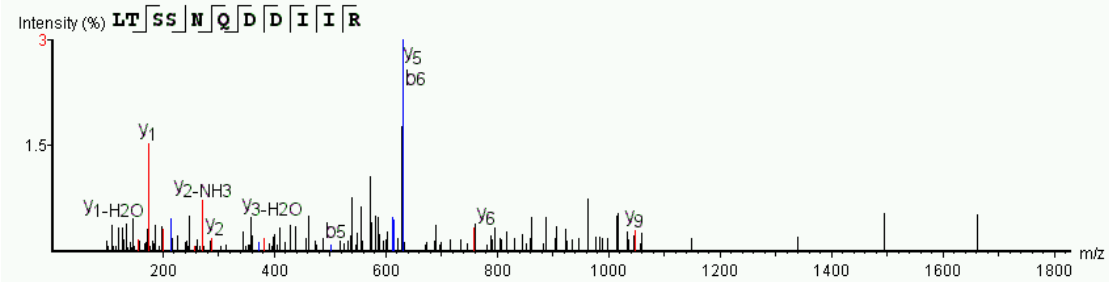

gi571577925

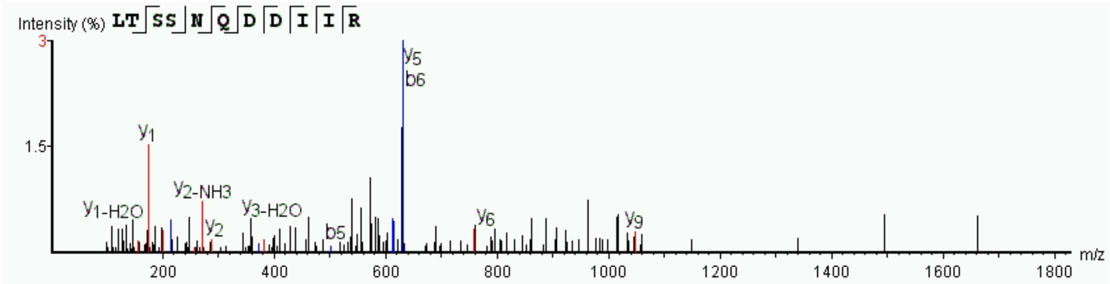

gi571577927

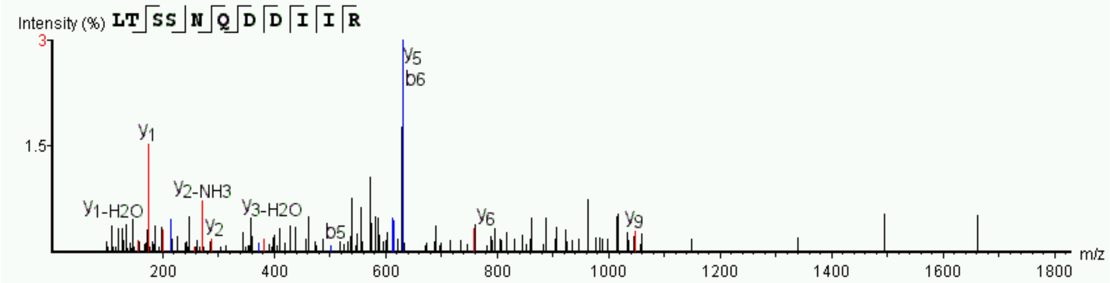

gi571577929

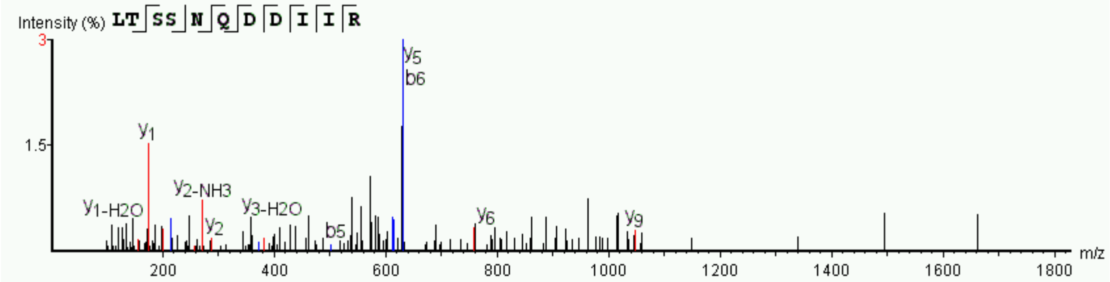

gi571577931

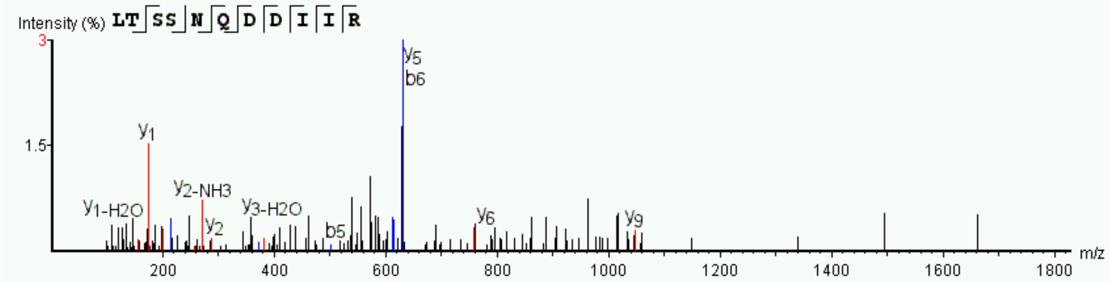

gi571577933

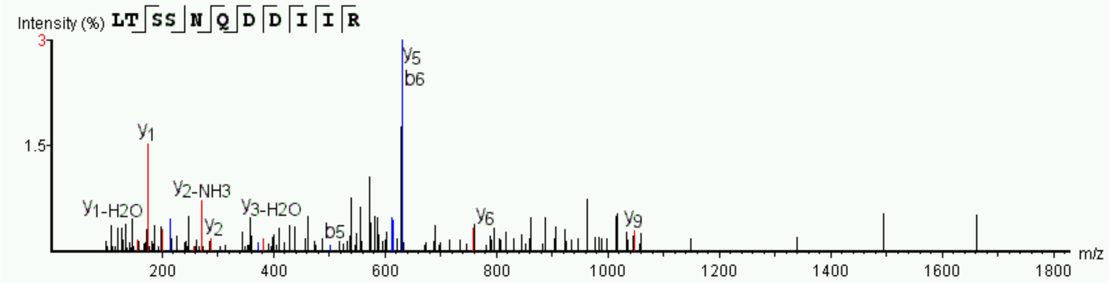

gi571559017

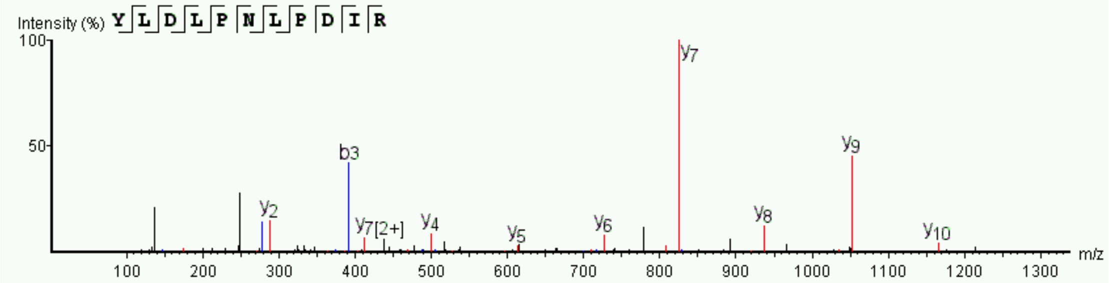

gi571516617

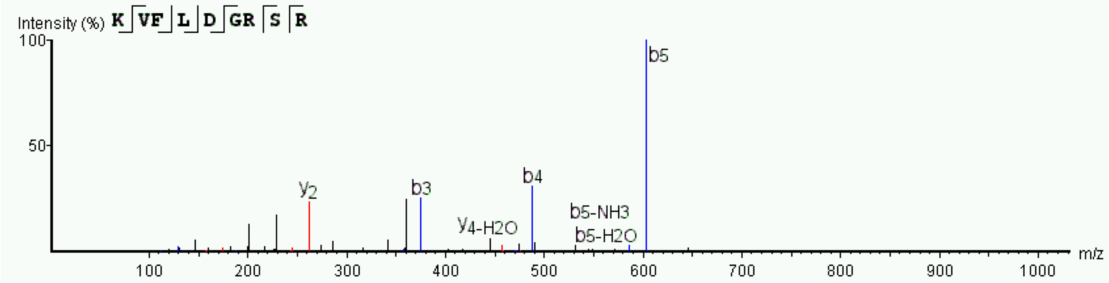

gi571566027

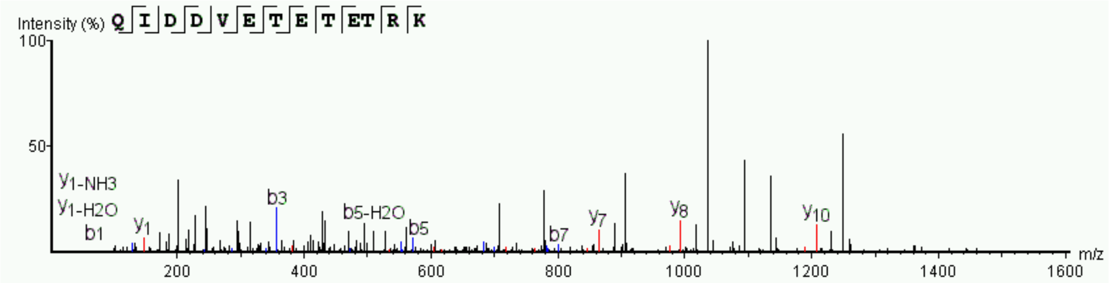

gi270289764

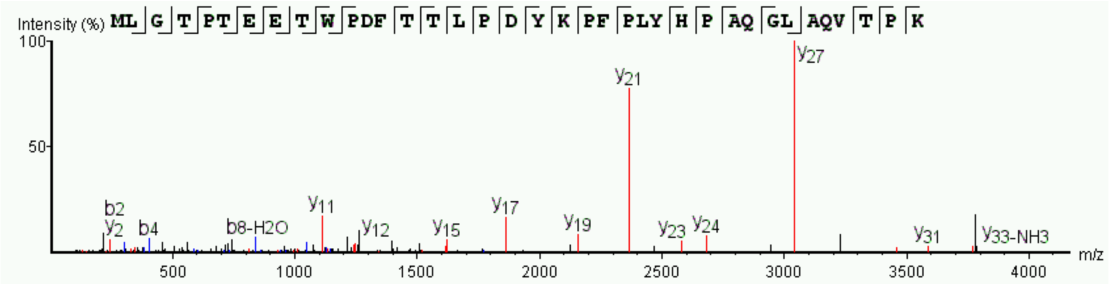

gi571519398

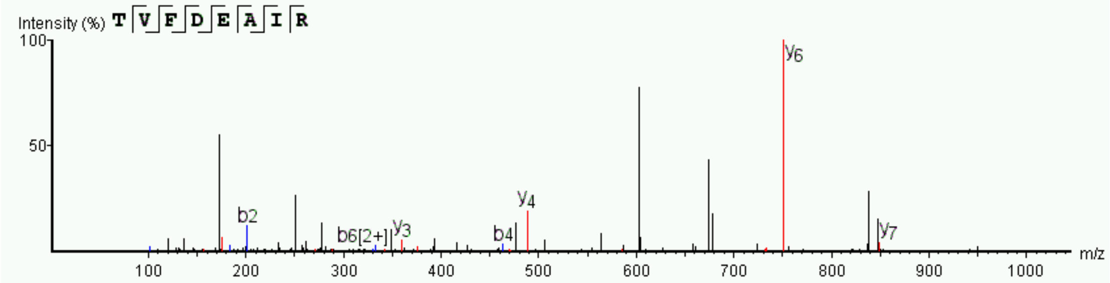

gi571519402

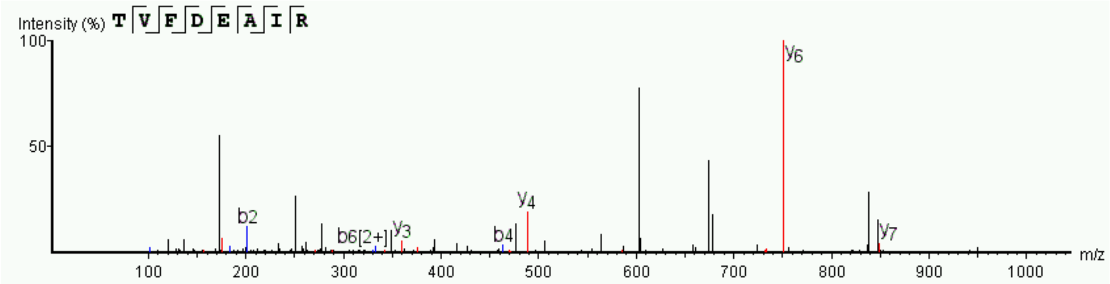

gi571519406

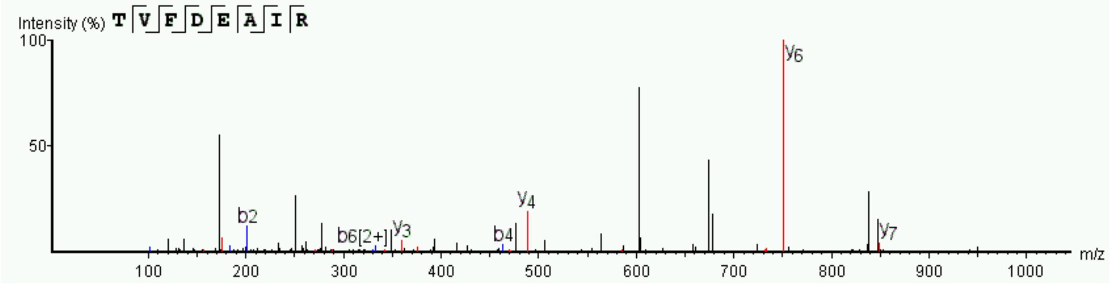

gi571519411

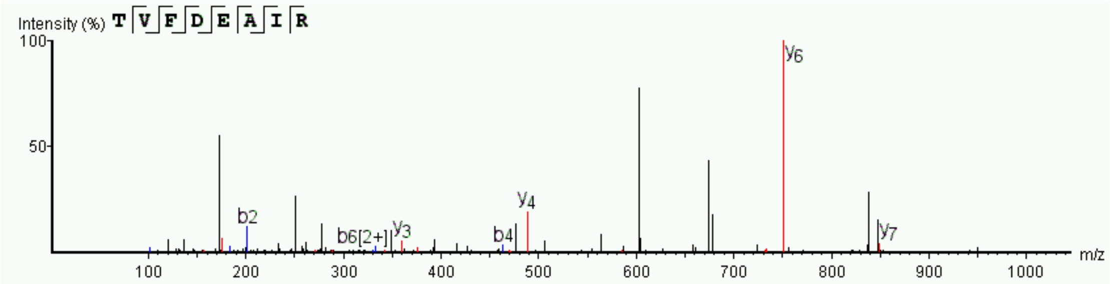

gi571540528

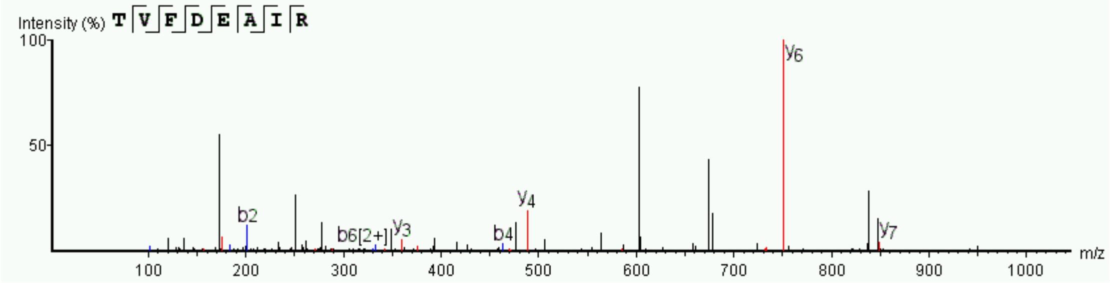

gi571540535

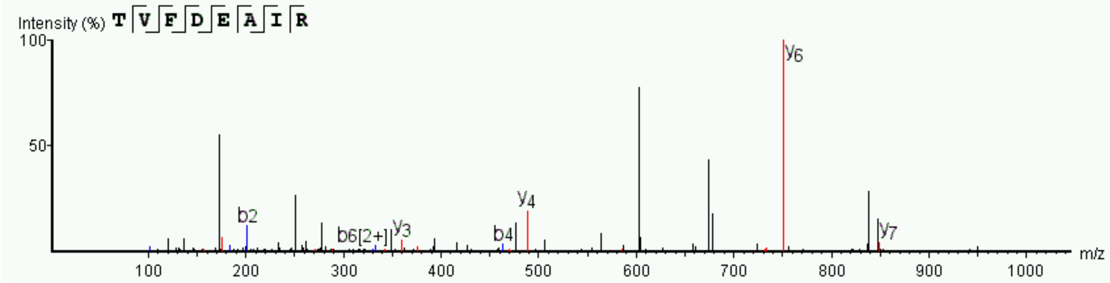

gi571540539

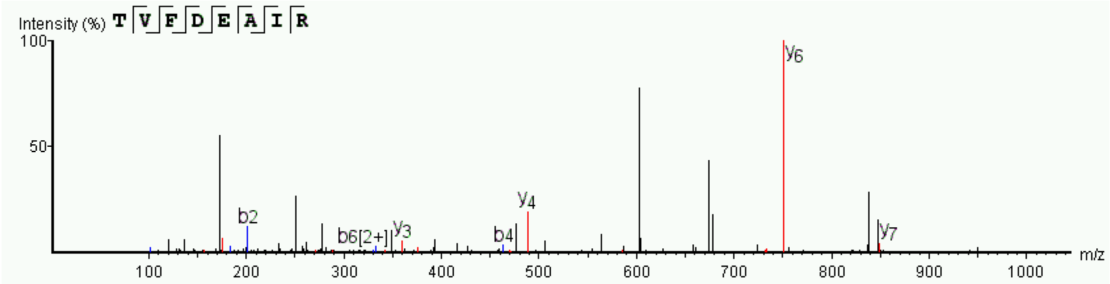

gi571540531

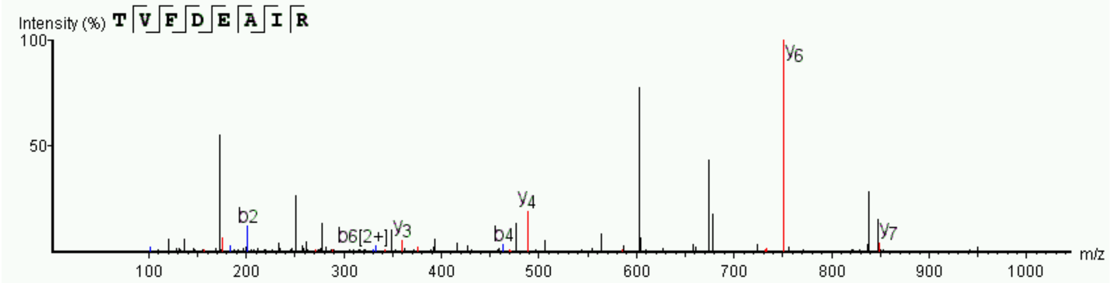

gi110761543

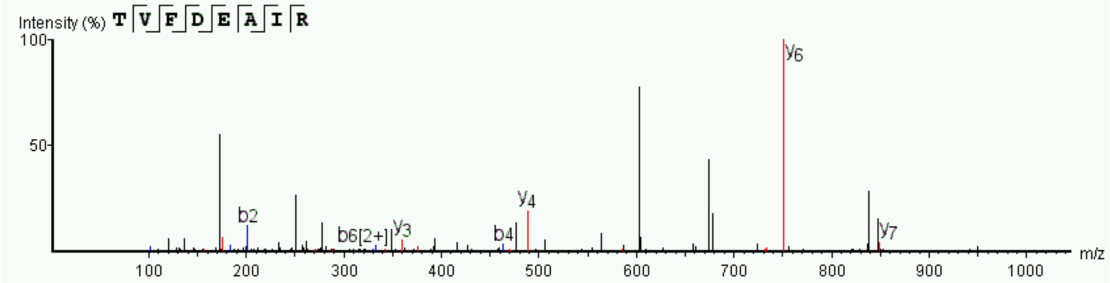

gi571562181

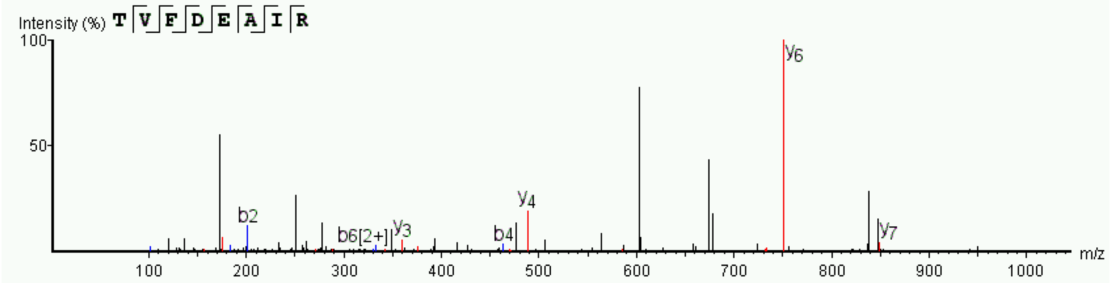

gi66550153

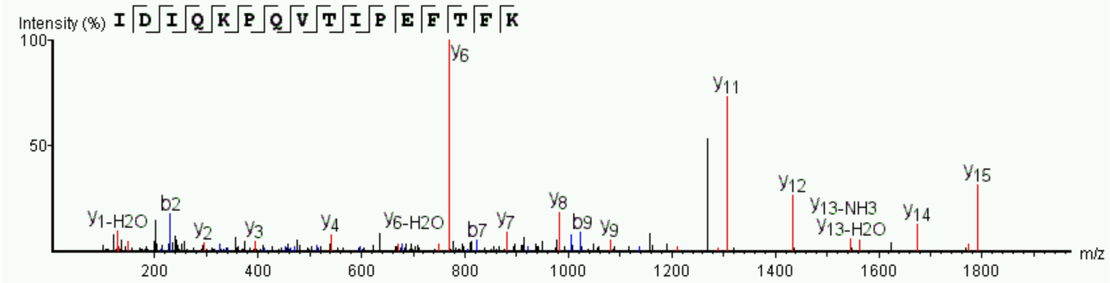

gi328792585

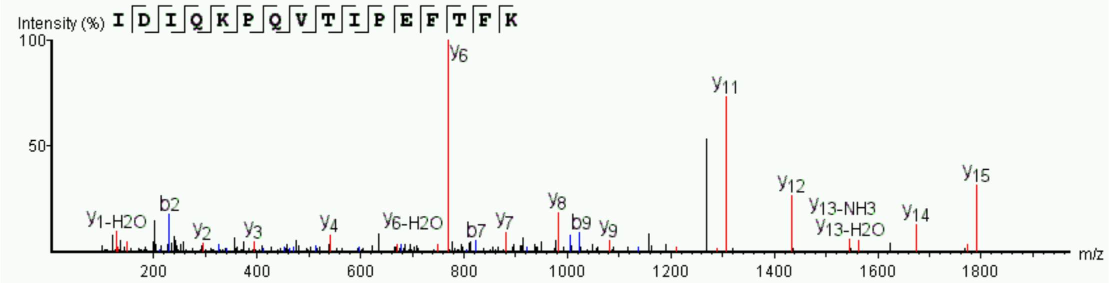

gi110763426

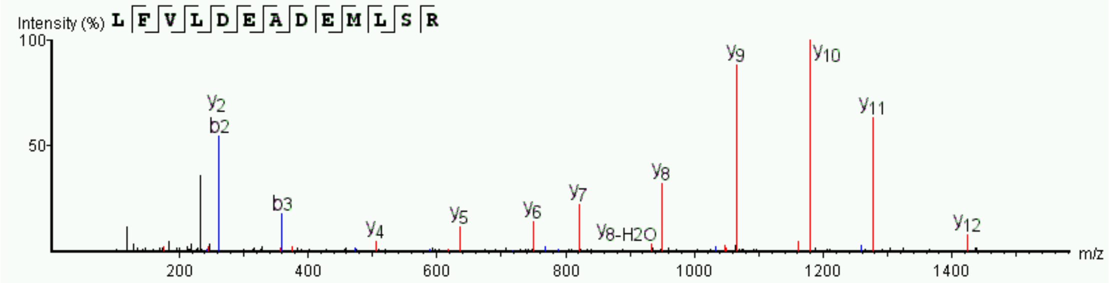

gi571523319

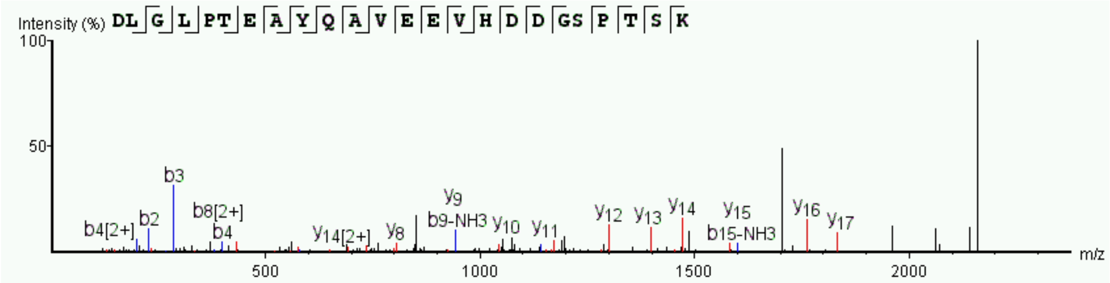

gi571523323

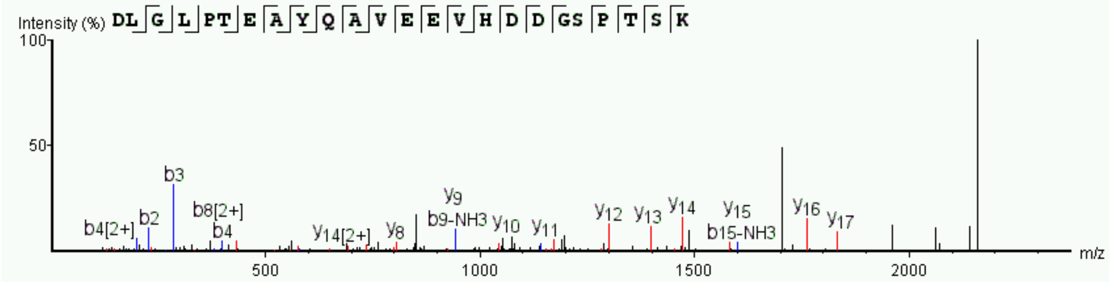

gi66547724

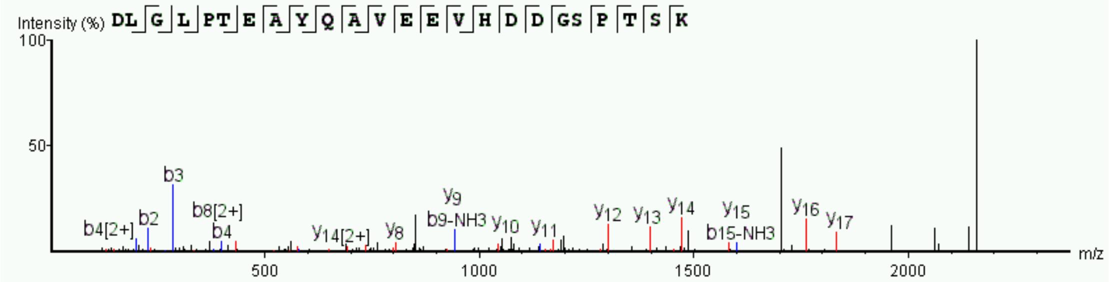

gi571544563

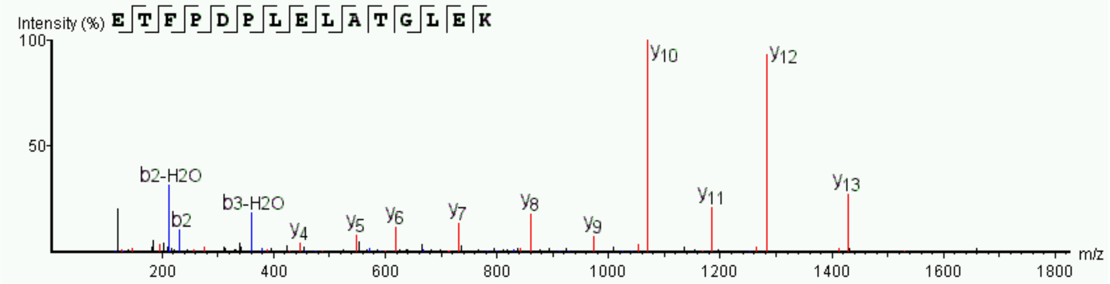

gi571520919

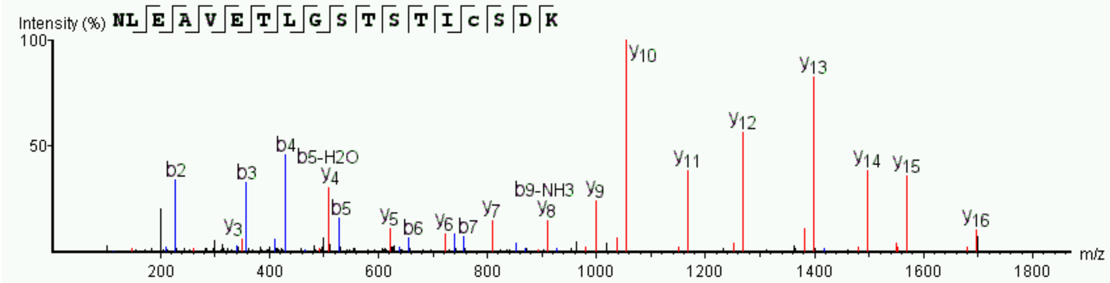

gi571520923

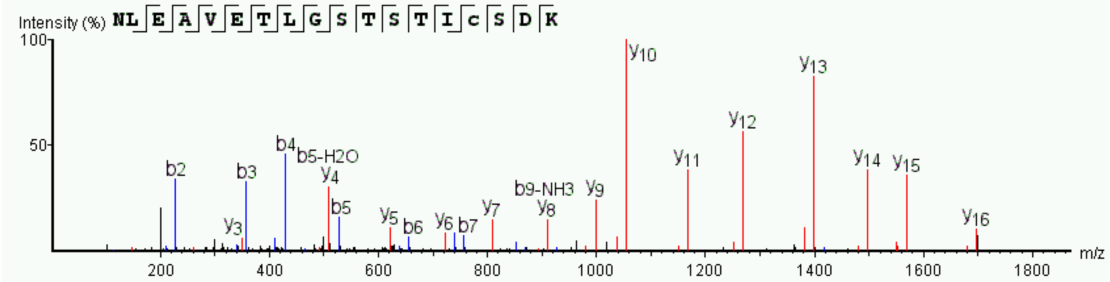

gi571520927

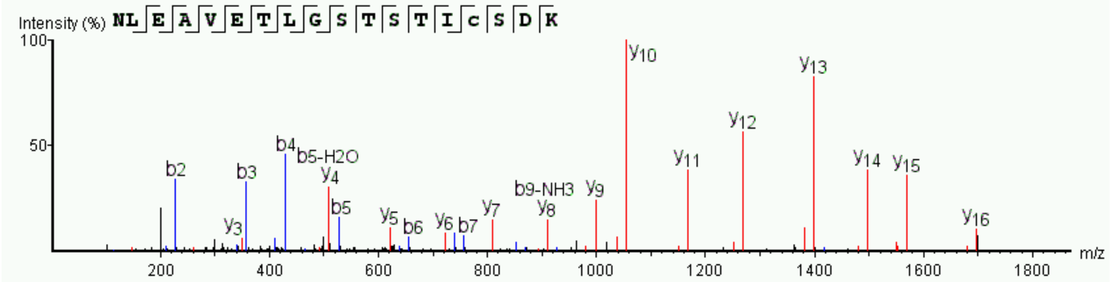

gi48095246

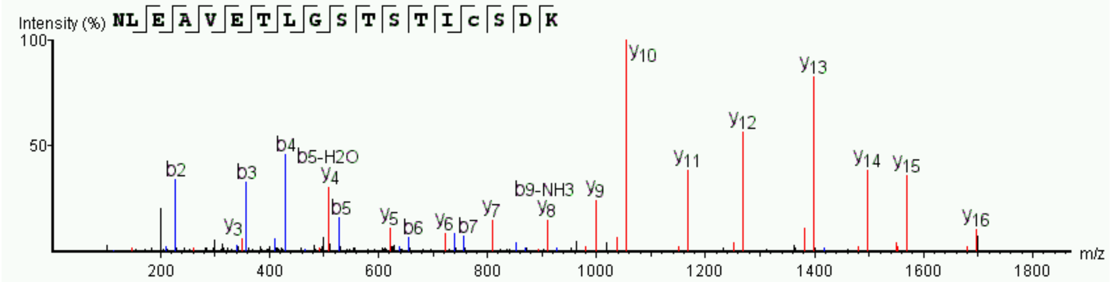

gi328781481

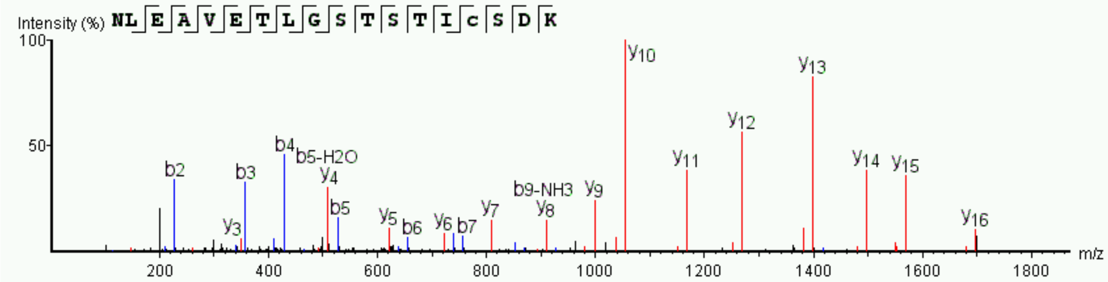

gi571500982

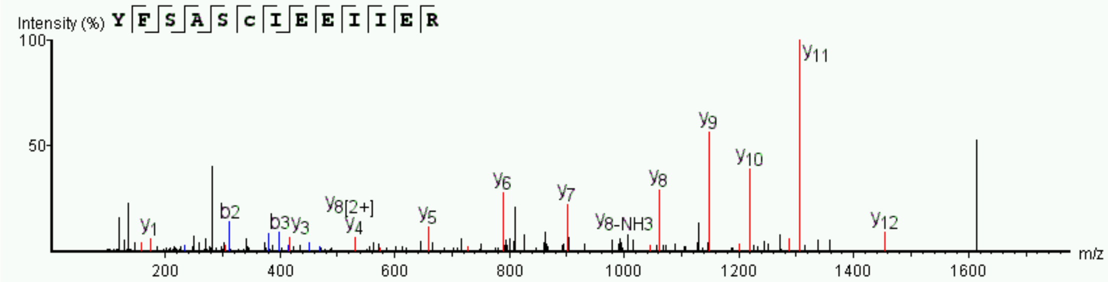

gi224809502

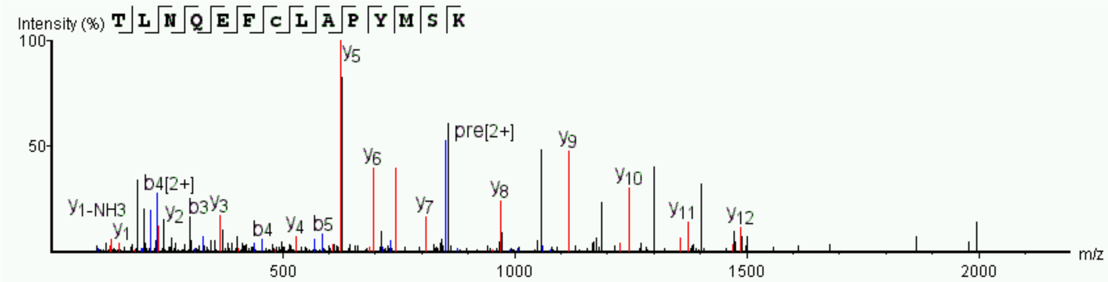

gi571566027

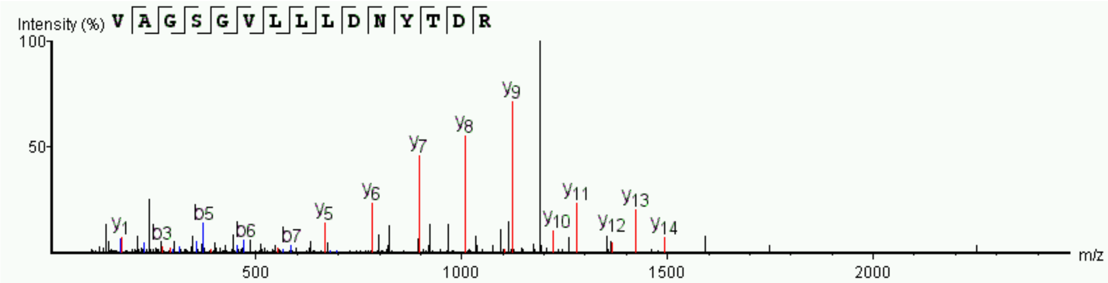

gi224809502

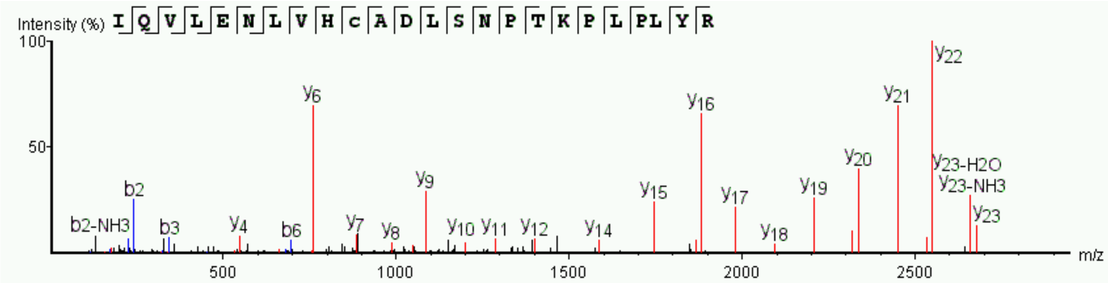

gi66559873

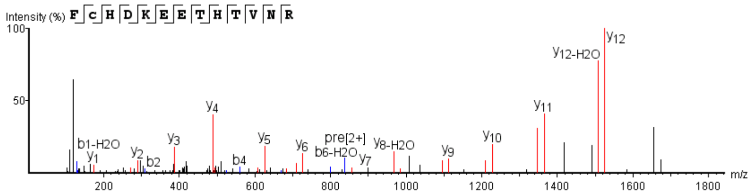

gi48094508

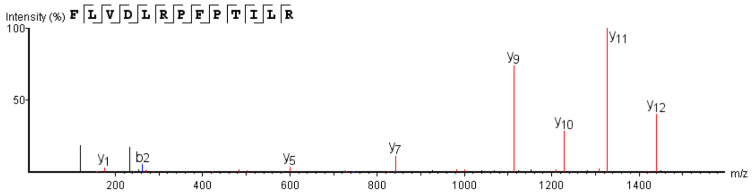

gi48095935

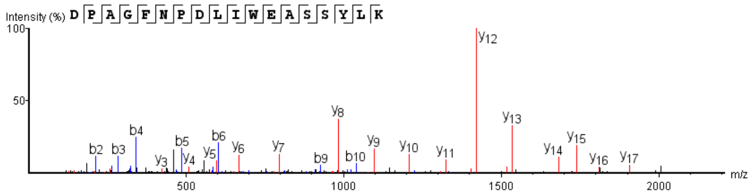

gi48097144

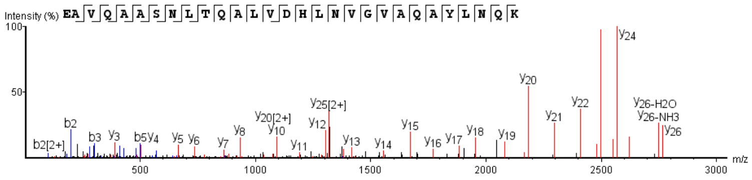

gi48097870

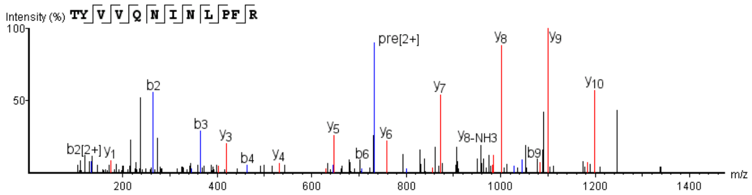

gi48097928

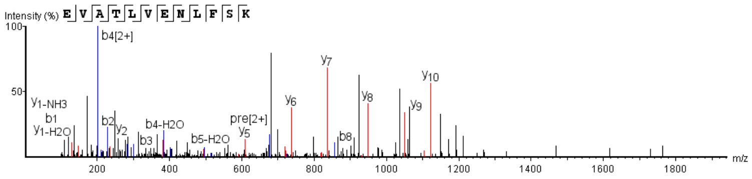

gi48098249

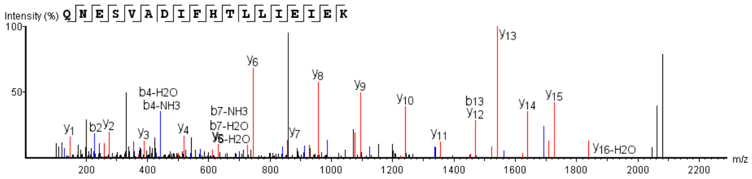

gi48098905

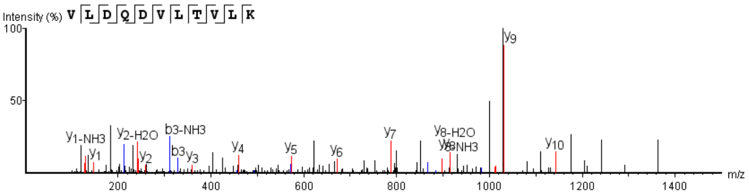

gi48103887

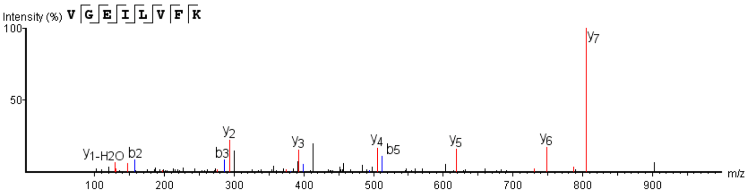

gi48104021

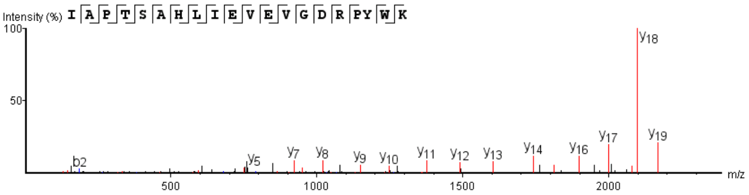

gi48104995

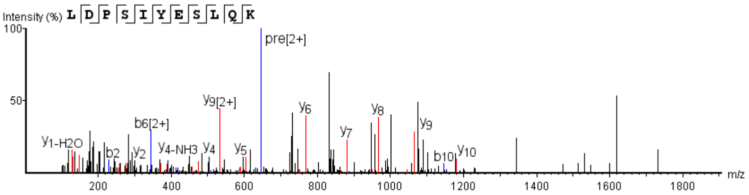

gi48106137

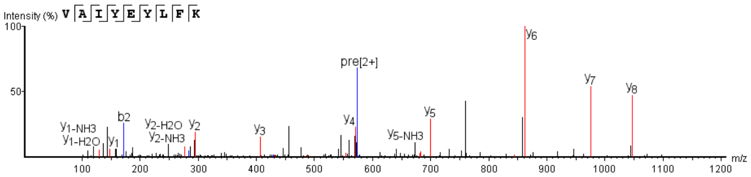

gi48106363

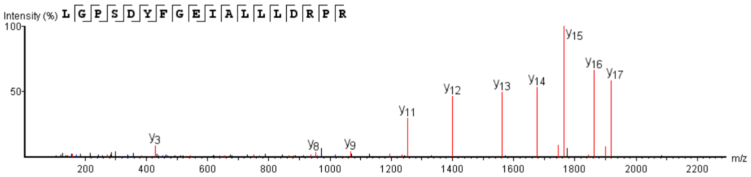

gi48106841

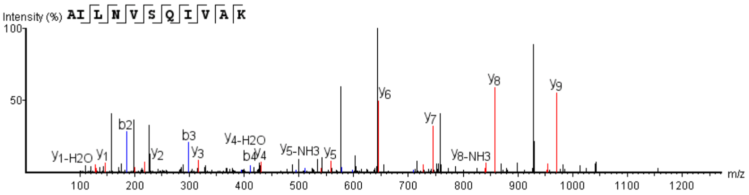

gi48130977

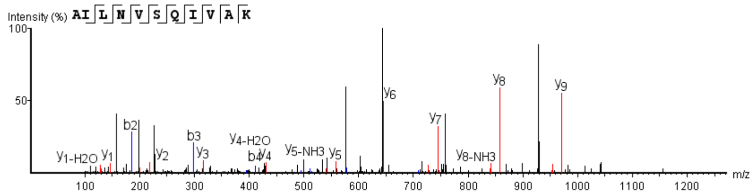

gi48139441

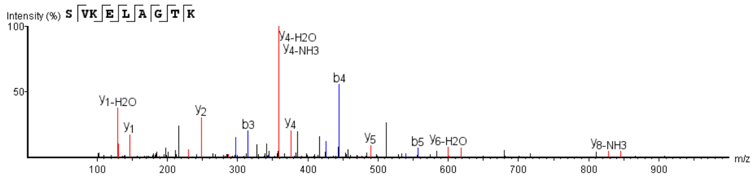

gi48141273

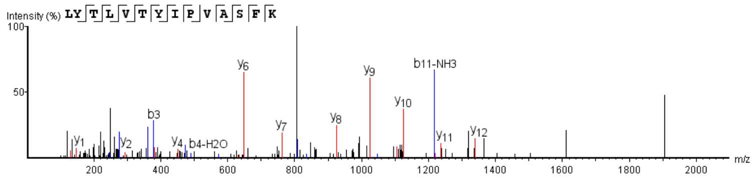

gi48142237

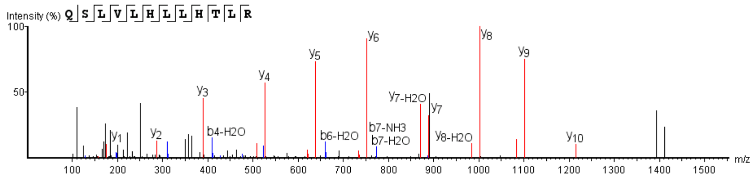

gi57154098

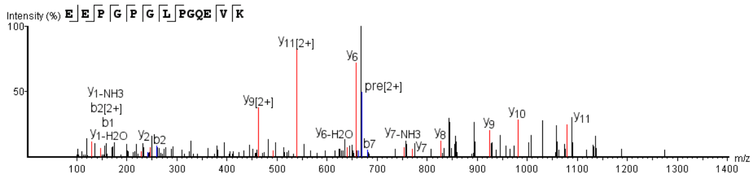

gi58585078

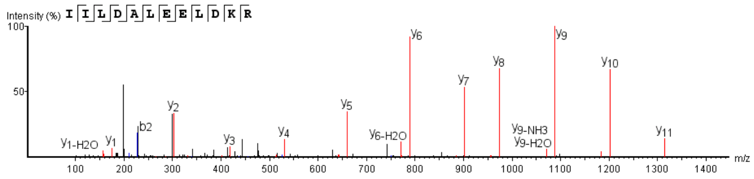

gi58585094

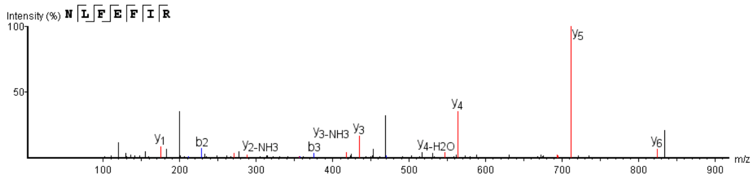

gi58585096

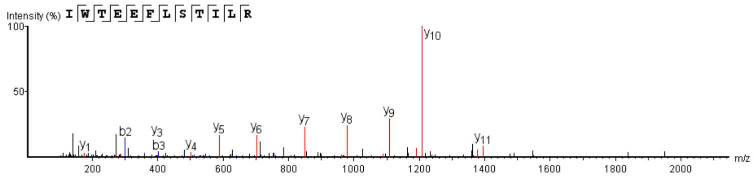

gi58585110

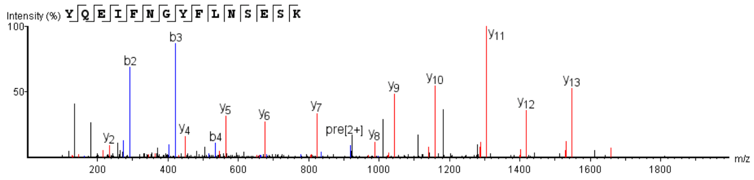

gi58585132

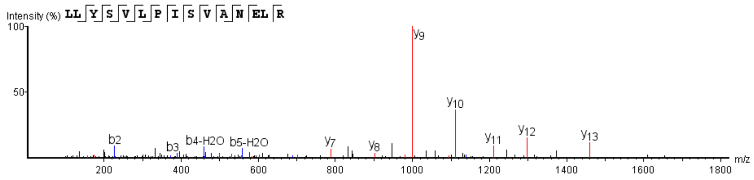

gi58585206

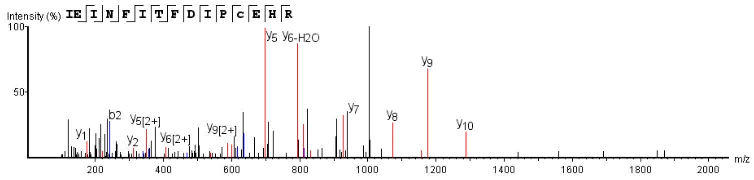

gi60460899

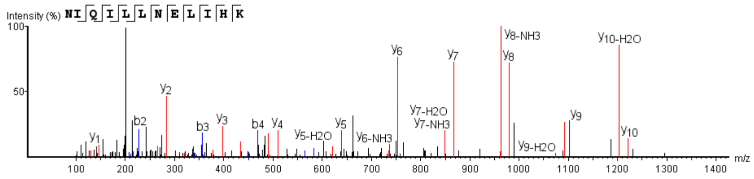

gi66499175

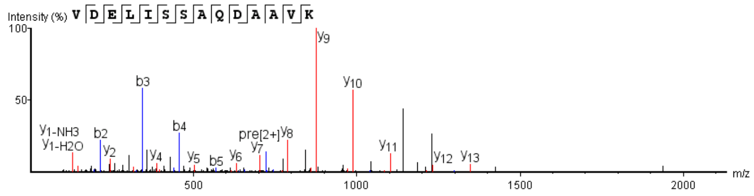

gi66499475

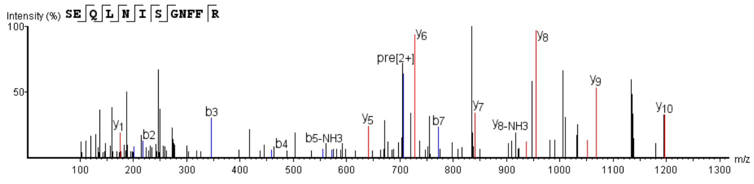

gi66500885

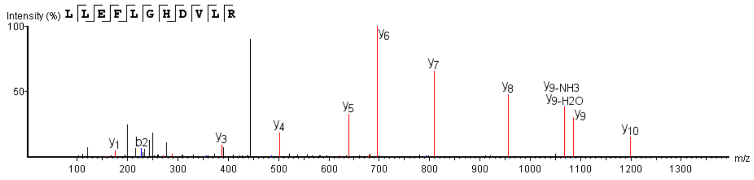

gi66501636

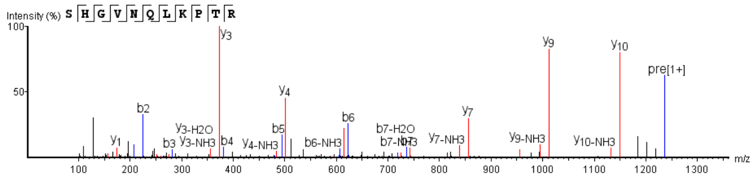

gi66506623

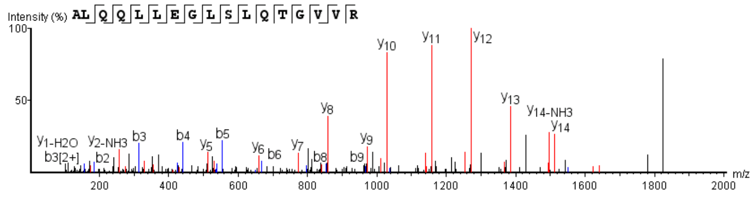

gi66509969

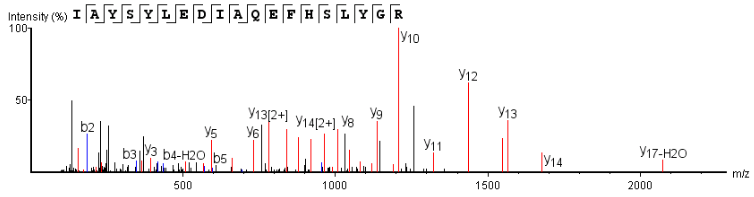

gi66513815

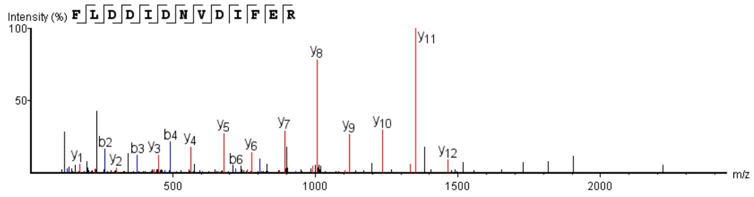

gi66514558

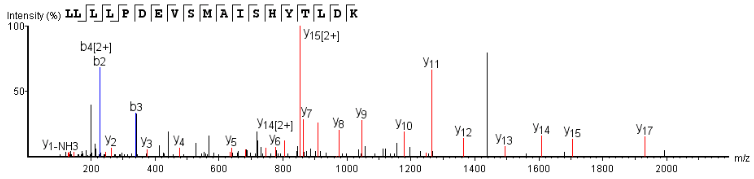

gi66517238

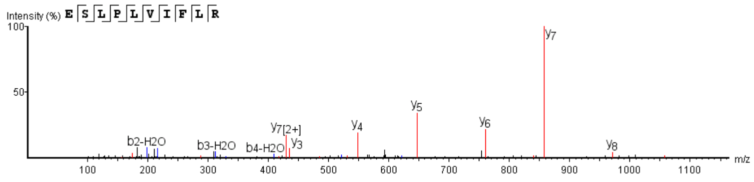

gi66517407

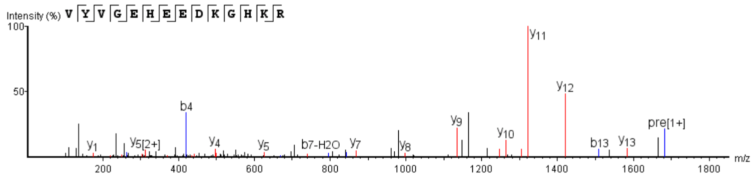

gi66517414

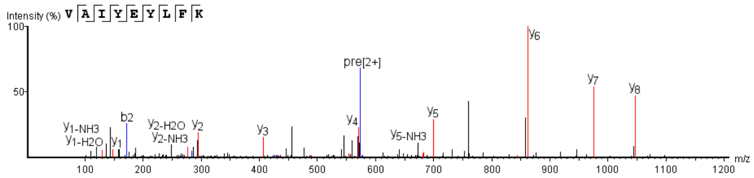

gi66517421

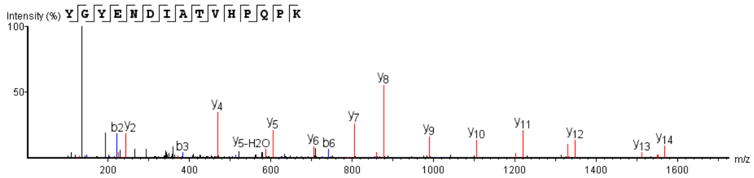

gi66519149

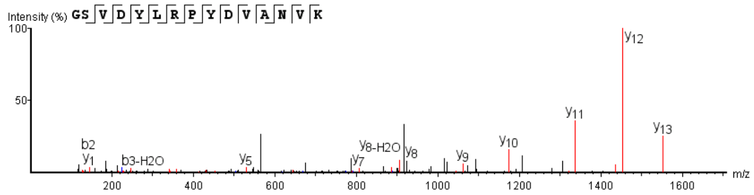

gi66519842

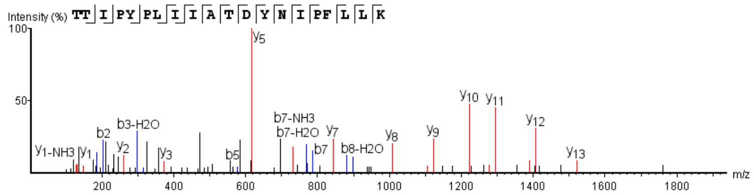

gi66522157

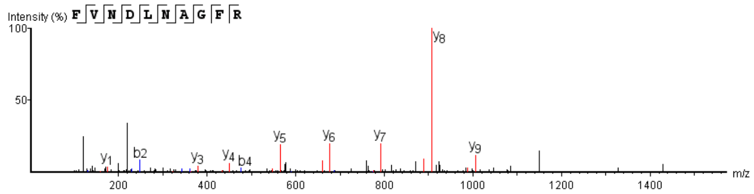

gi66523789

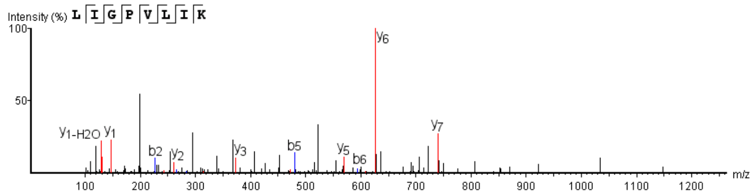

gi66525117

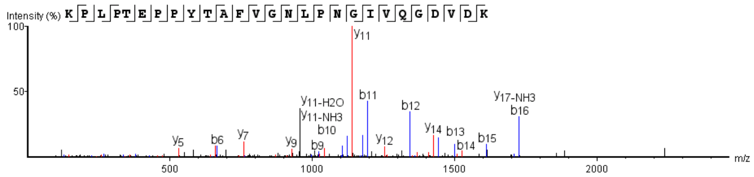

gi66532125

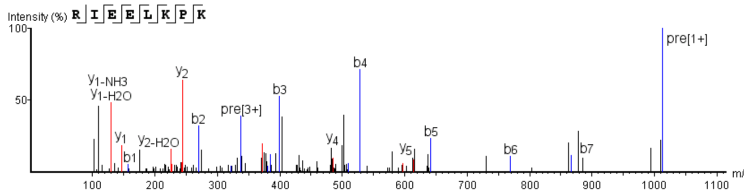

gi66533332

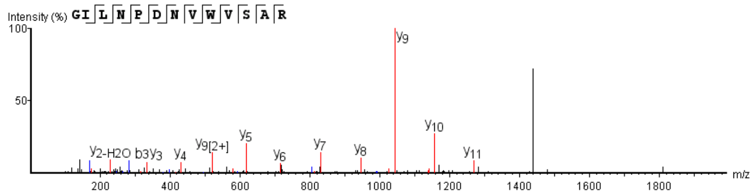

gi66534900

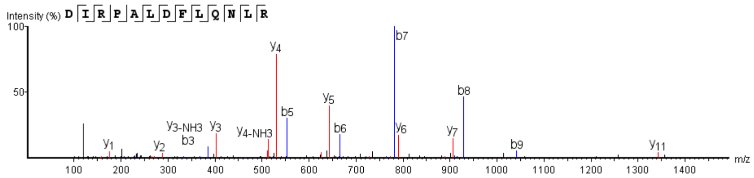

gi66542091

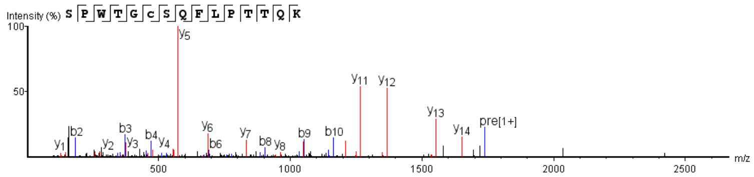

gi66543768

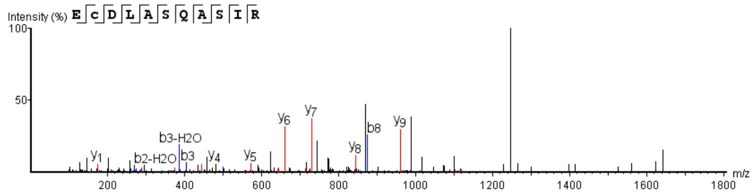

gi66549683

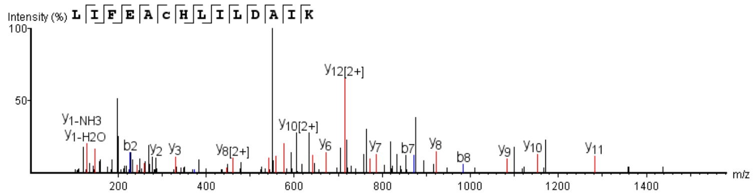

gi66549686

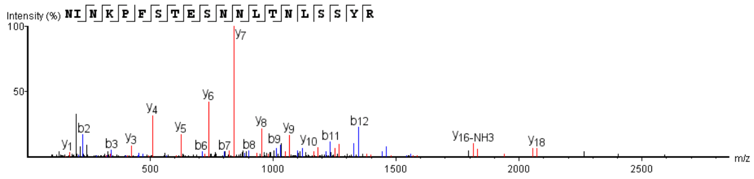

gi66552169

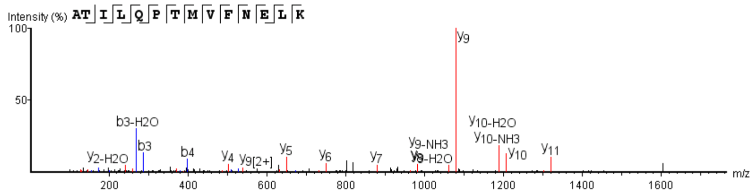

gi66553455

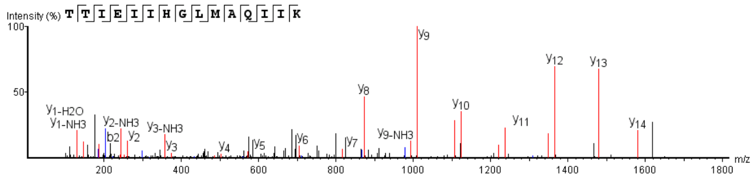

gi66554150

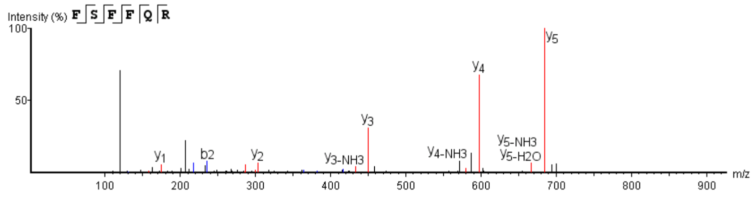

gi571532244

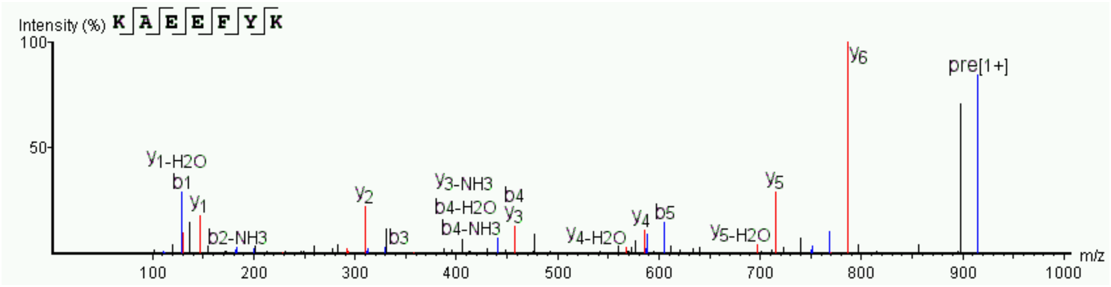

gi571532248

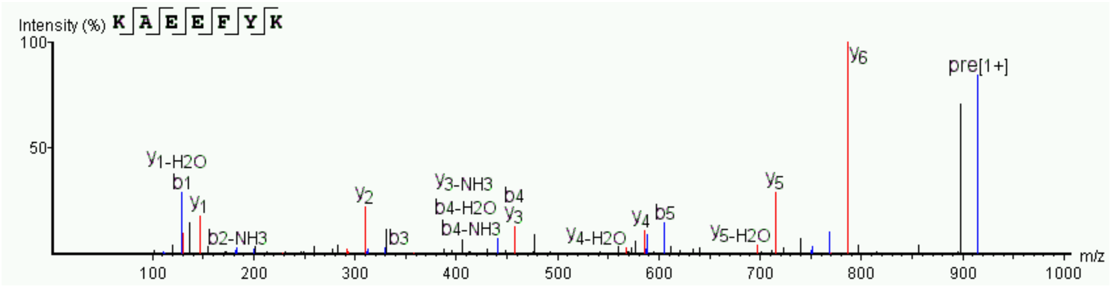

gi571532252

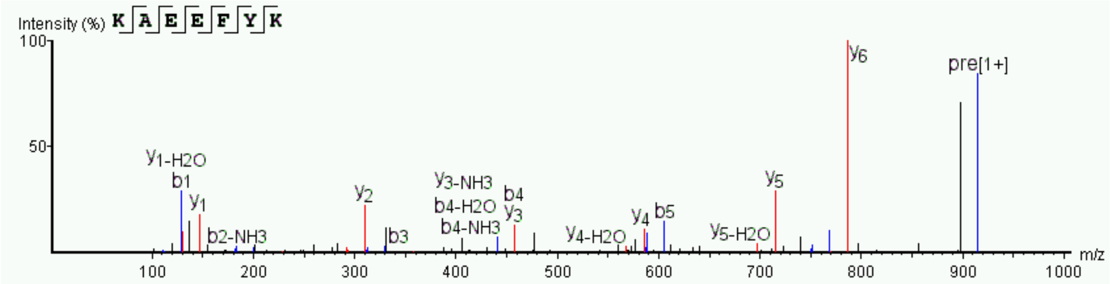

gi571532256

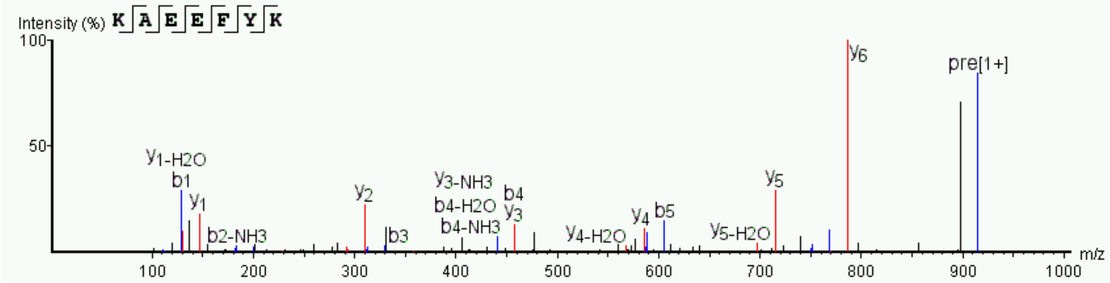

gi571532260

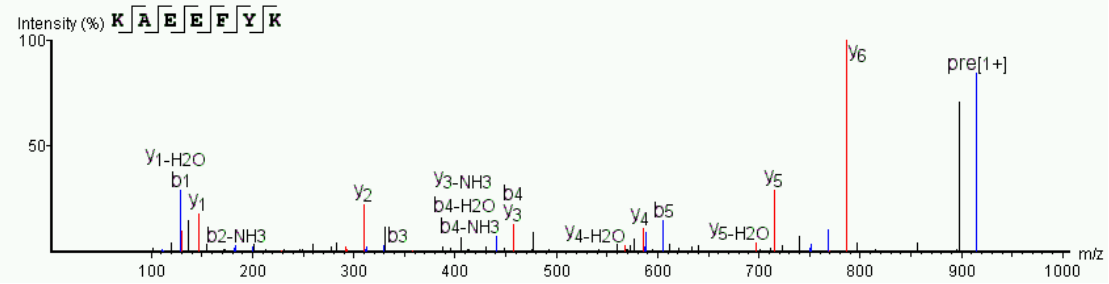

gi66558726

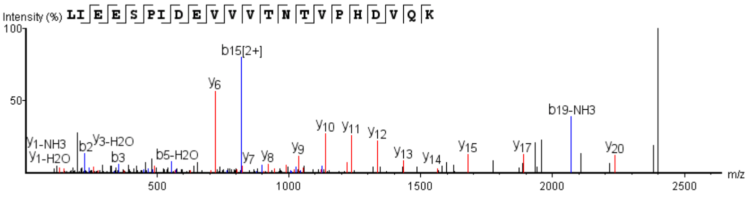

gi571513618

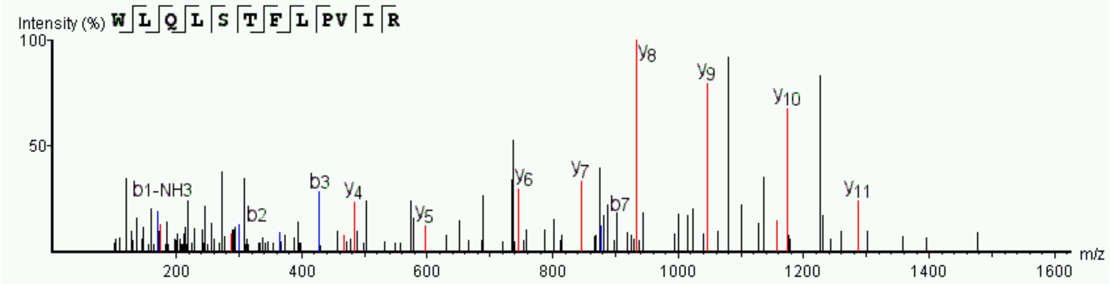

gi571513622

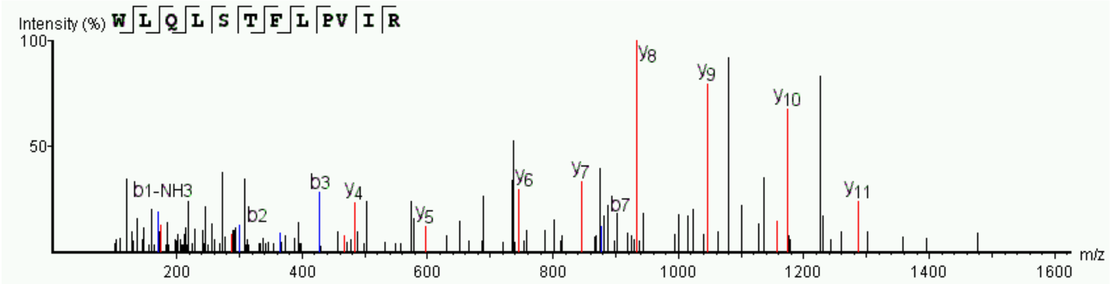

gi571513626

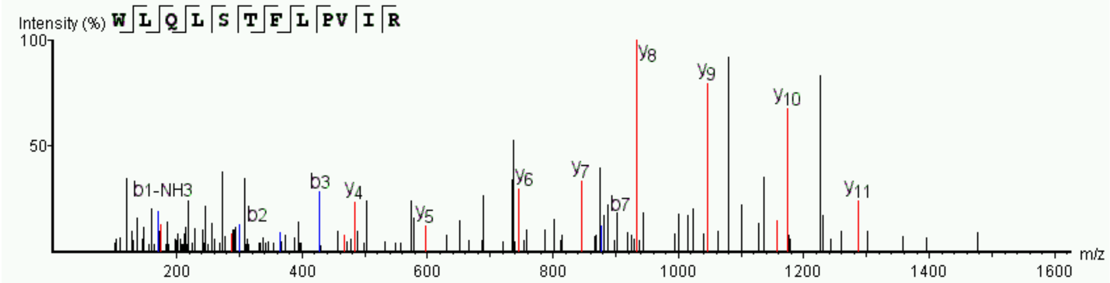

gi571513630

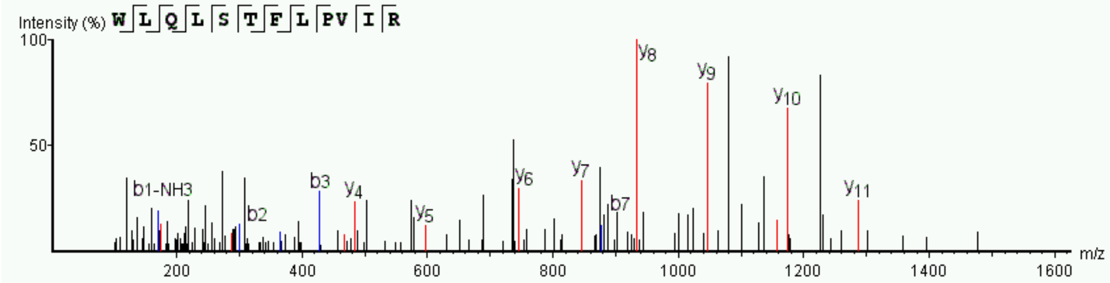

gi571513632

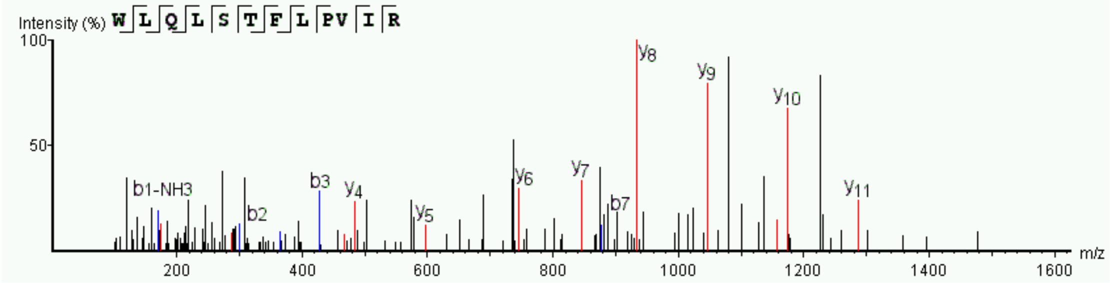

gi571513636

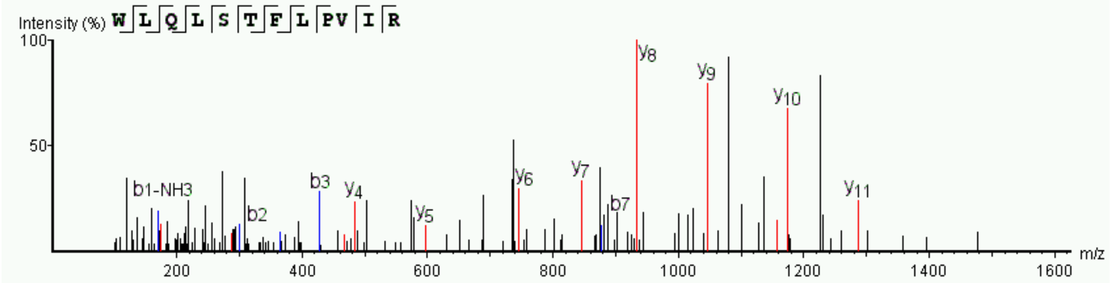

gi571541144

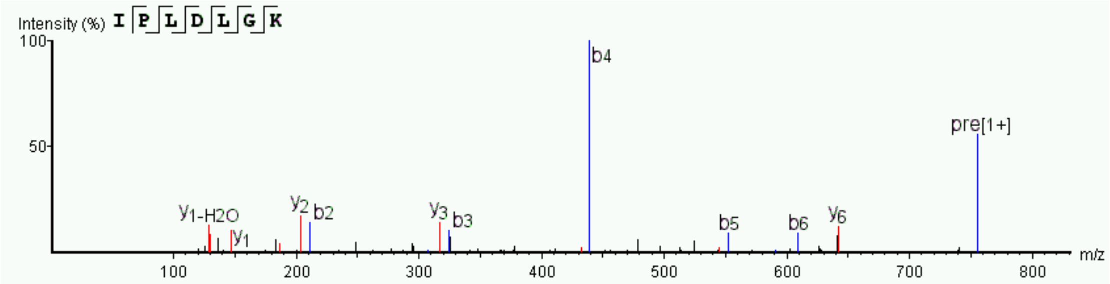

gi66512104

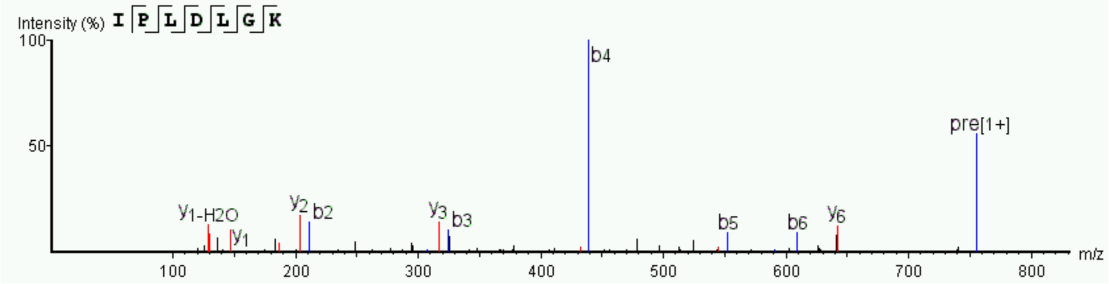

gi571551826

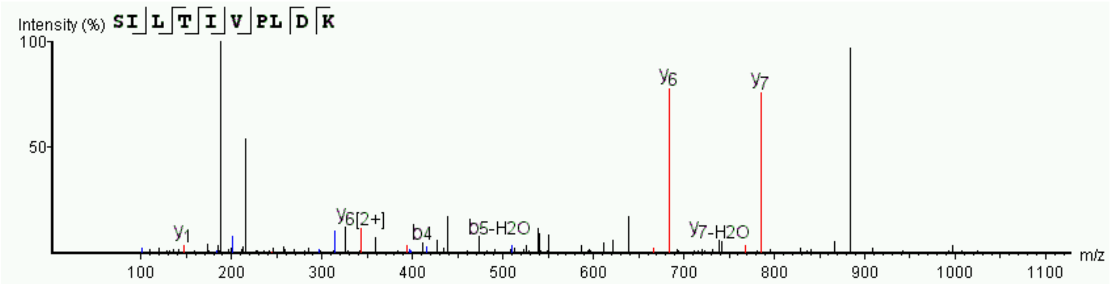

gi571551830

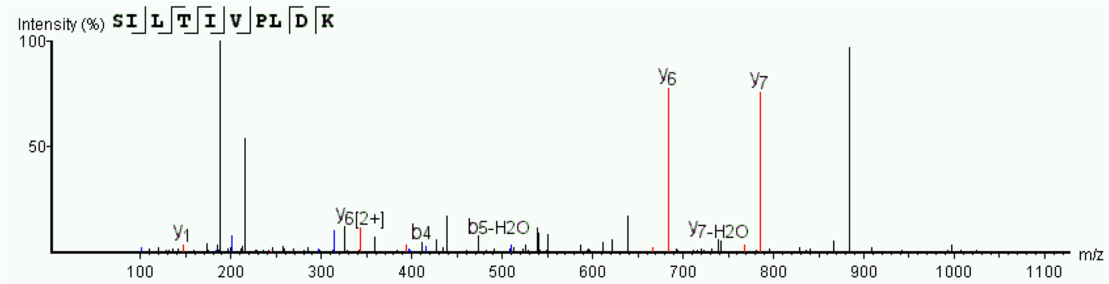

gi66561731

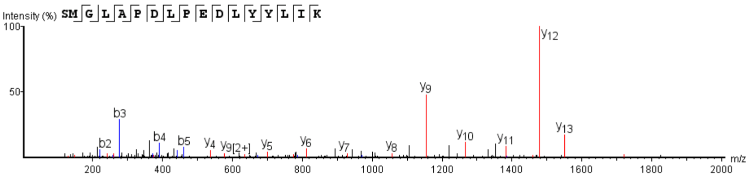

gi66562243

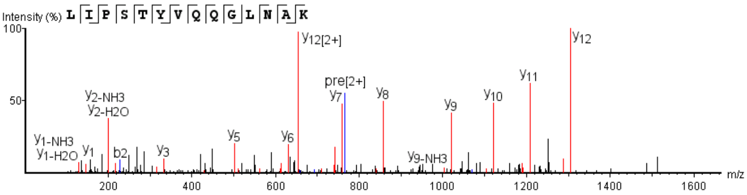

gi66565384

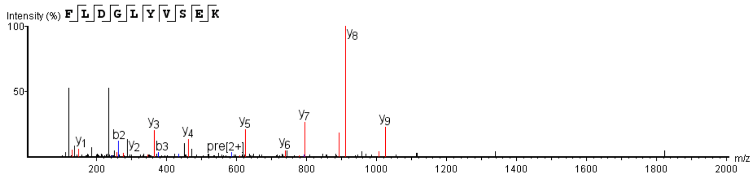

gi66565444

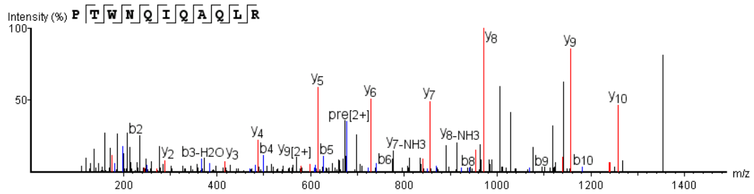

gi110755171

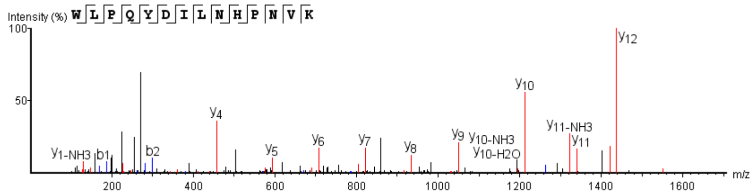

gi110756134

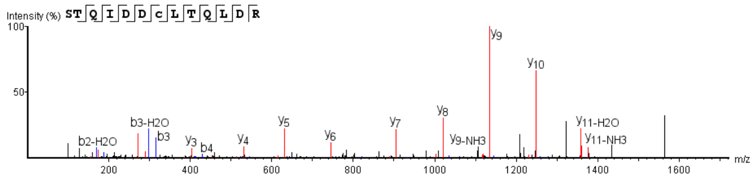

gi110756153

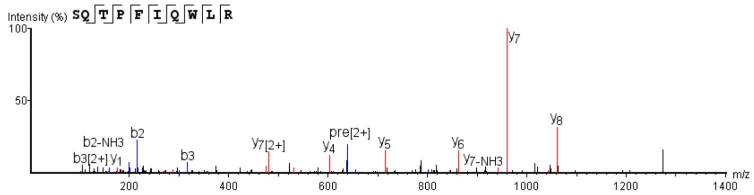

gi110758517

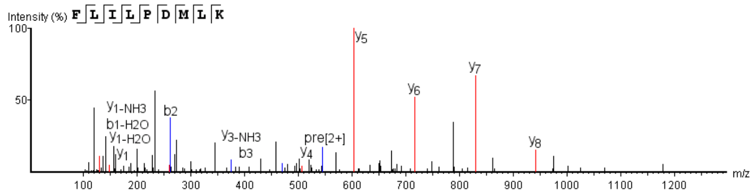

gi110758613

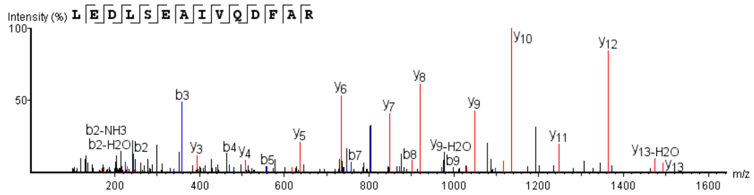

gi110758628

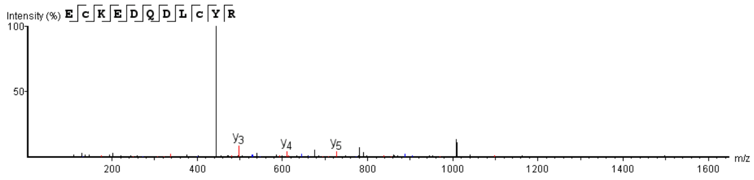

gi110759374

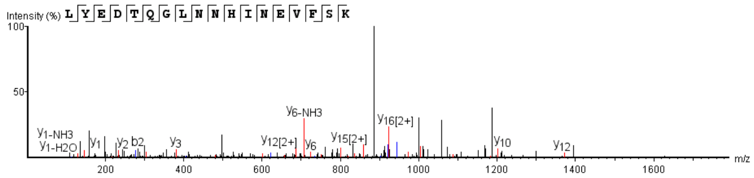

gi110759426

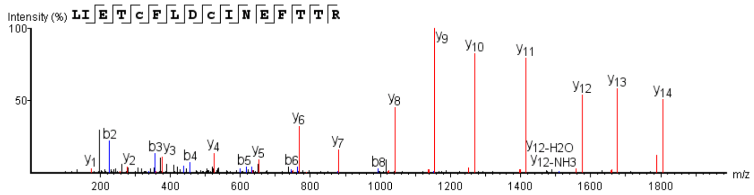

gi110760068

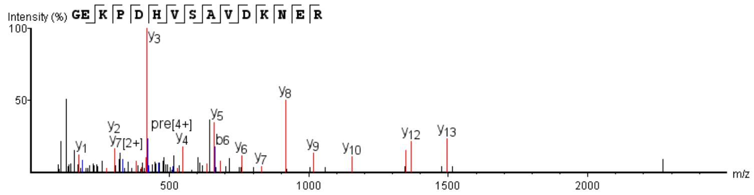

gi110760723

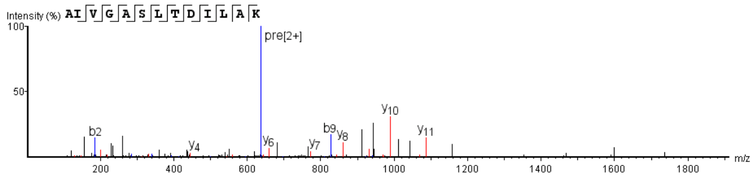

gi110761627

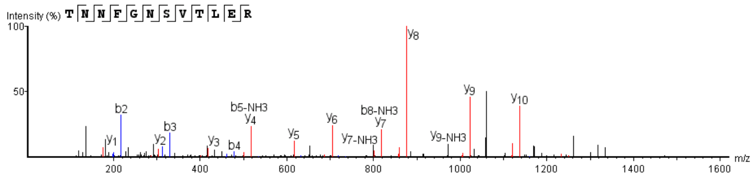

gi110762954

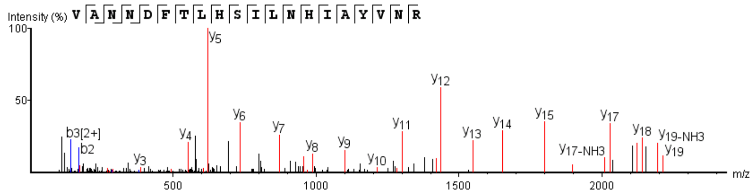

gi110763875

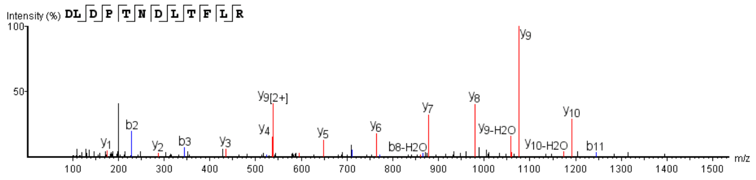

gi110764754

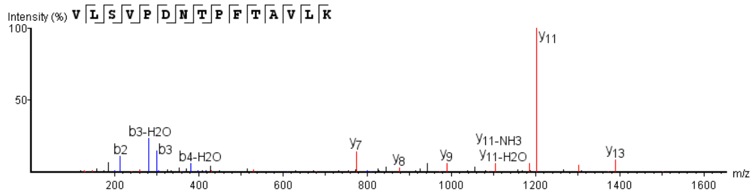

gi110765112

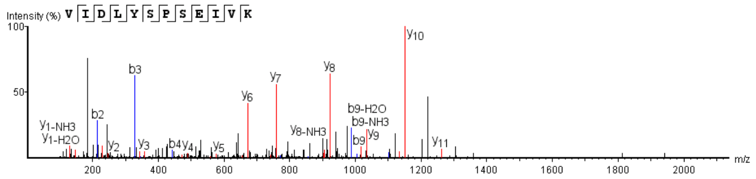

gi110766823

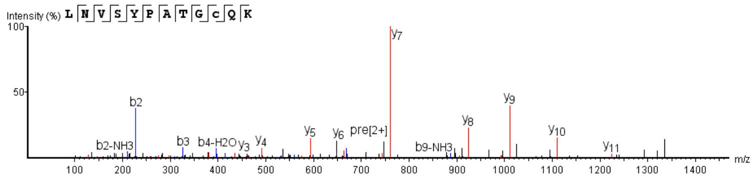

gi110767080

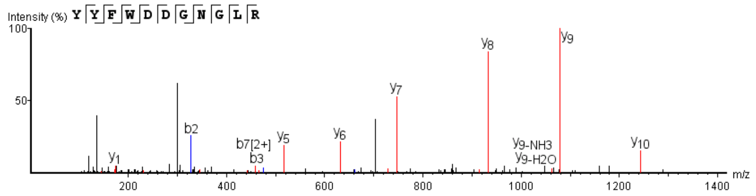

gi110767674

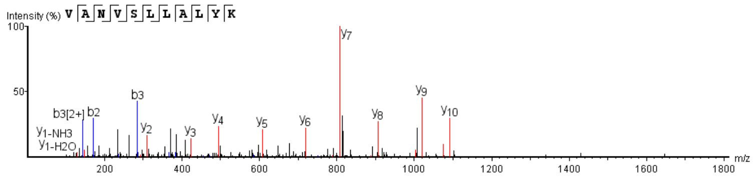

gi110771708

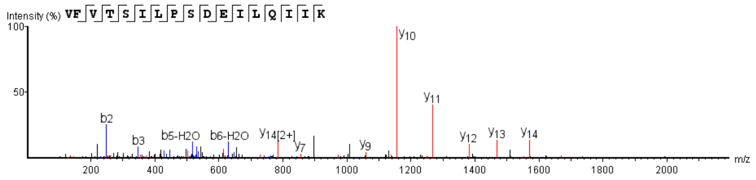

gi110772198

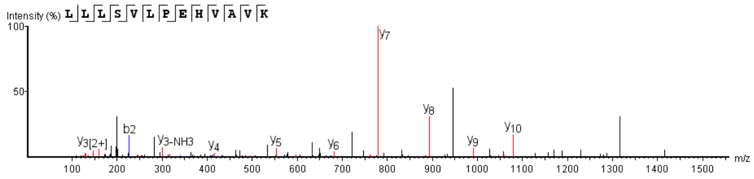

gi118150480

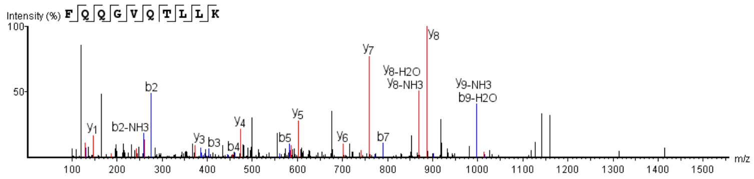

gi166795901

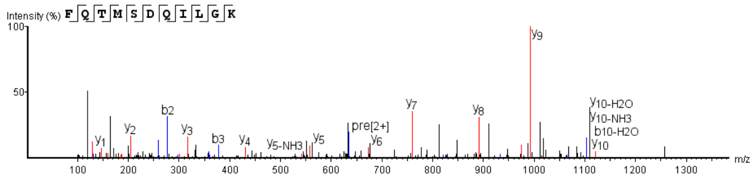

gi209969778

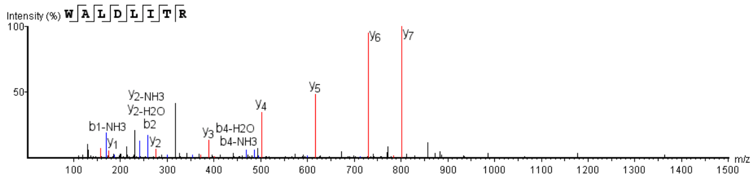

gi226533687

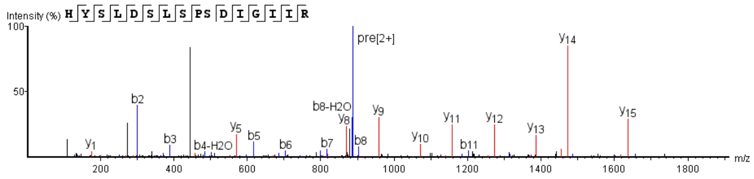

gi268370271

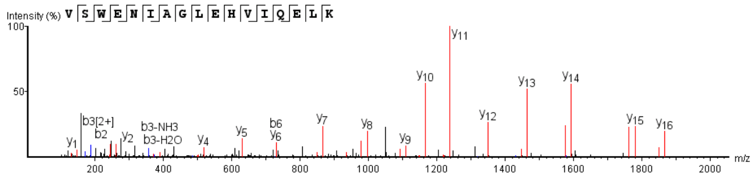

gi328775871

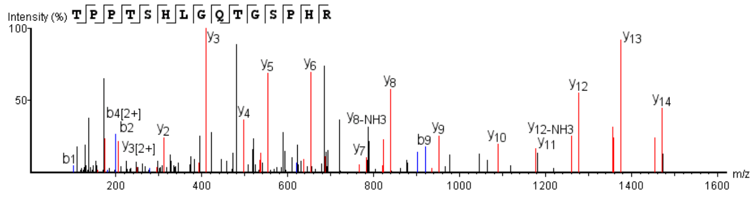

gi328776379

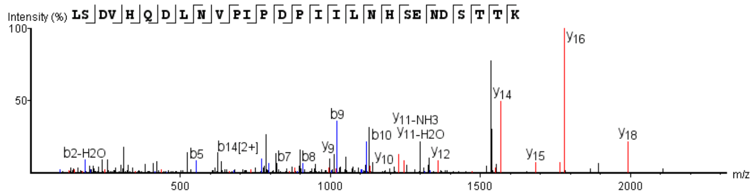

gi328776555

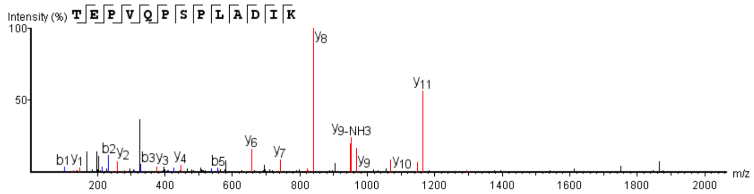

gi328776594

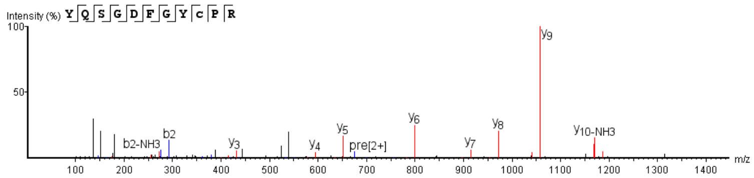

gi328777239

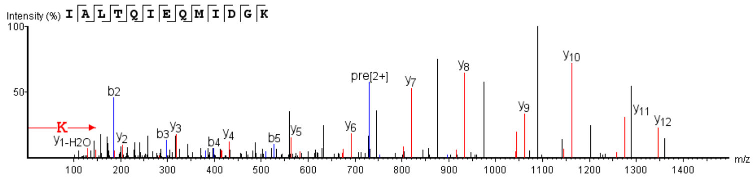

gi328782247

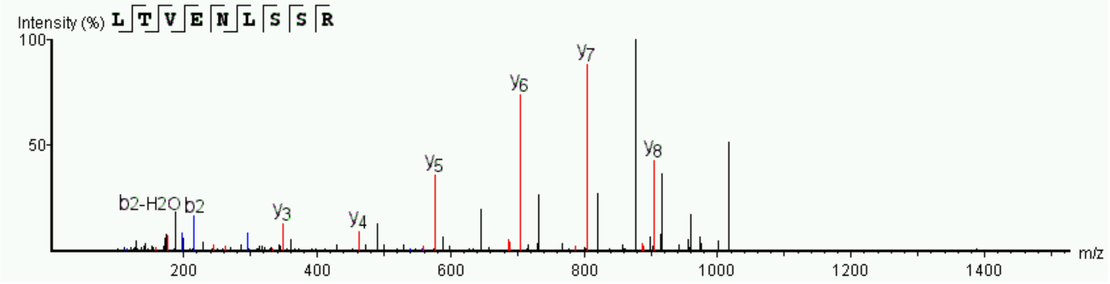

gi66506397

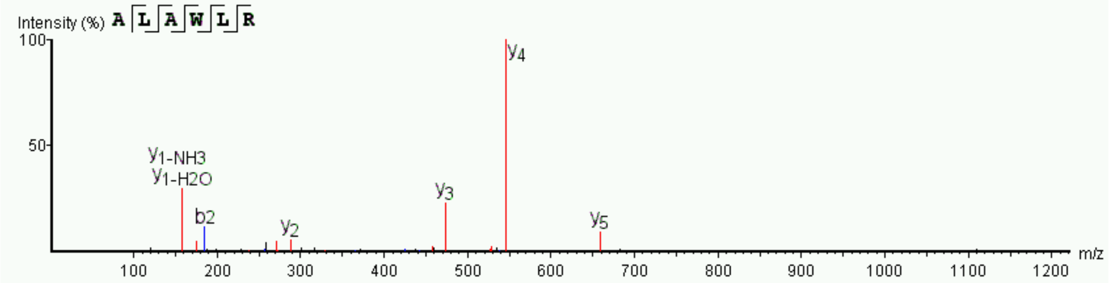

gi571523784

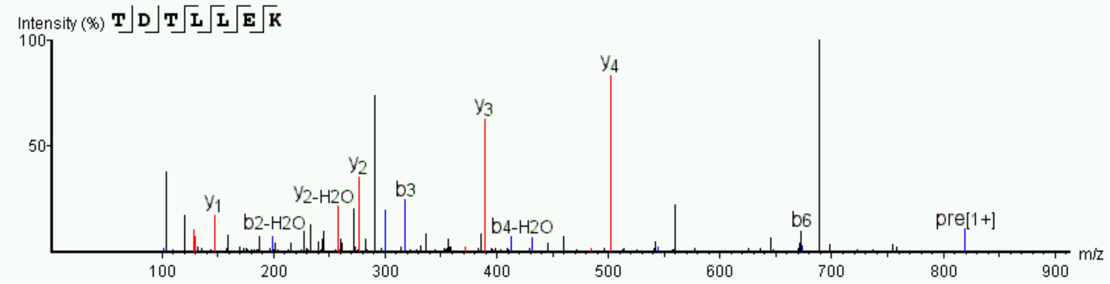

gi571522017

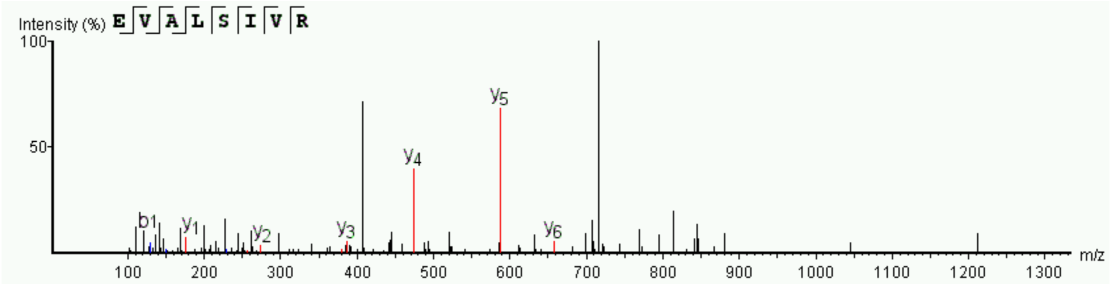

gi571522020

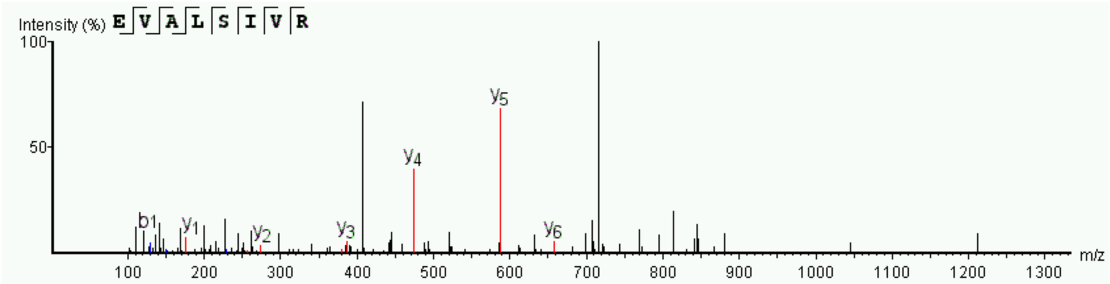

gi571522031

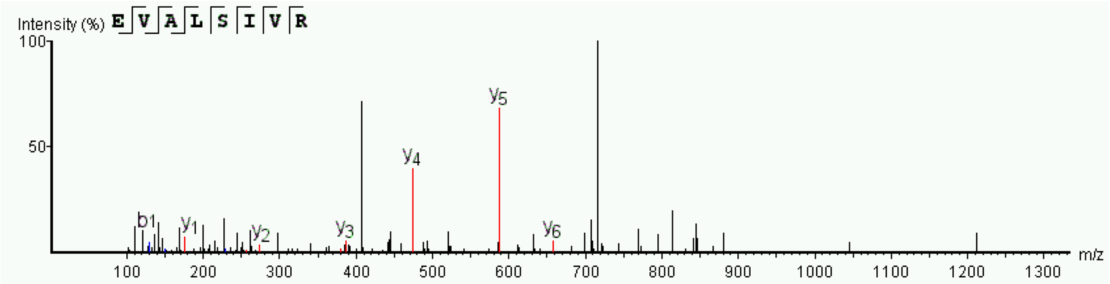

gi571550350

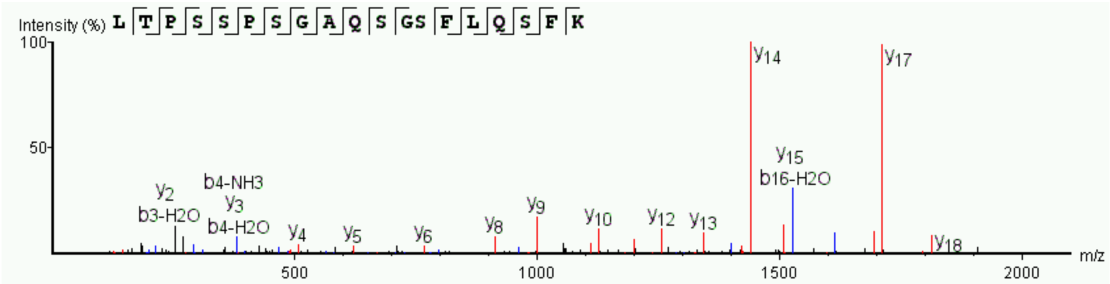

gi571550353

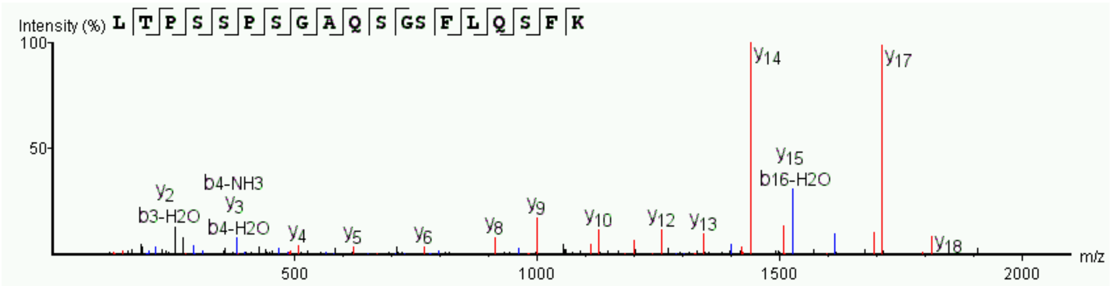

gi571550357

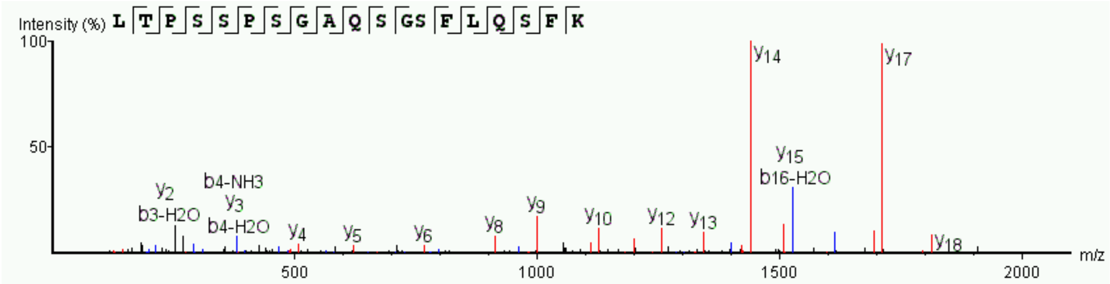

gi571550346

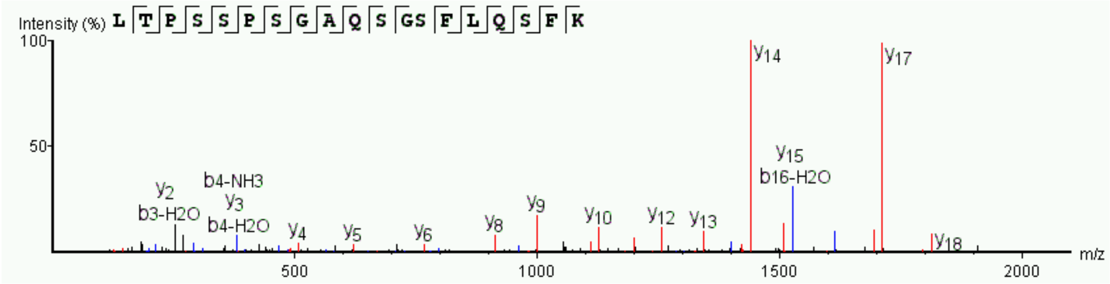

gi571548074

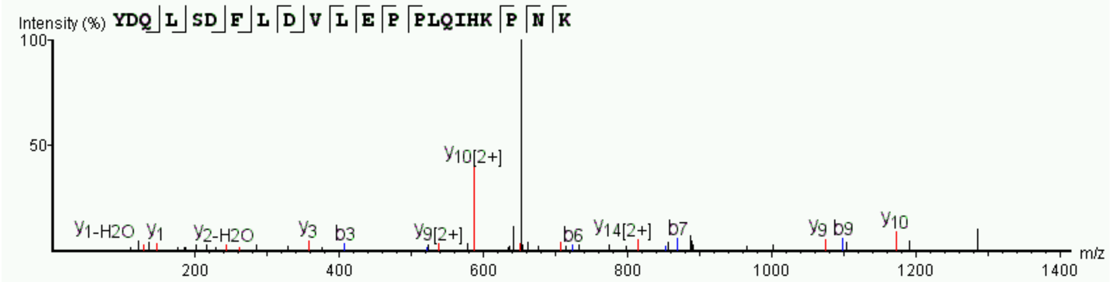

gi571548084

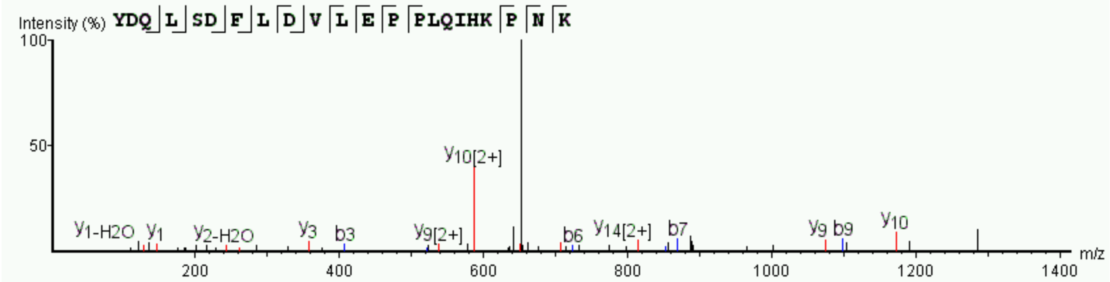

gi571548012

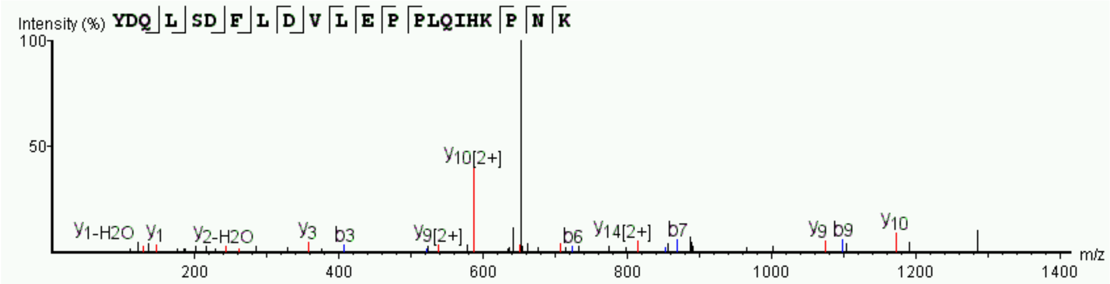

gi571548094

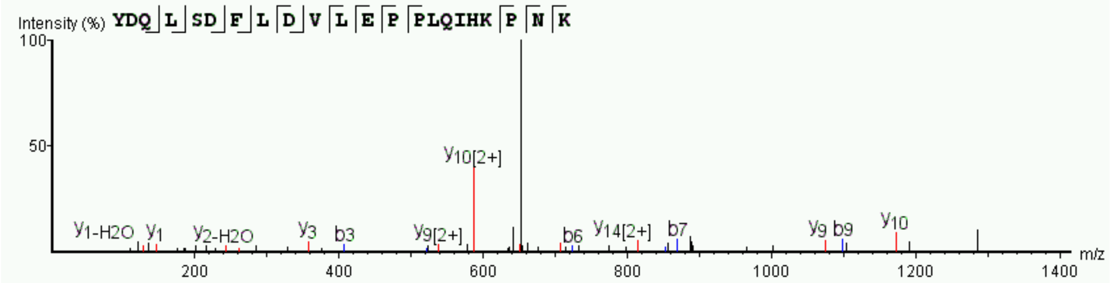

gi571548106

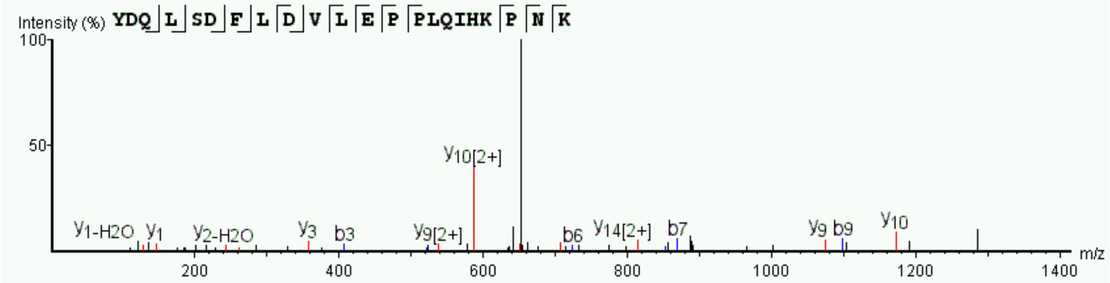

gi571548116

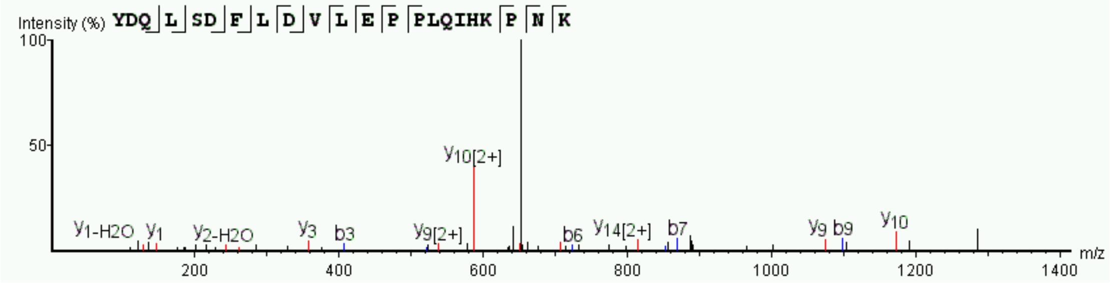

gi571548120

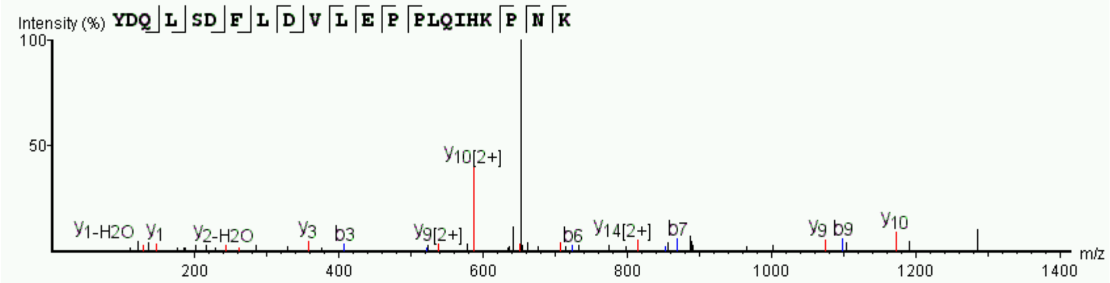

gi571548126

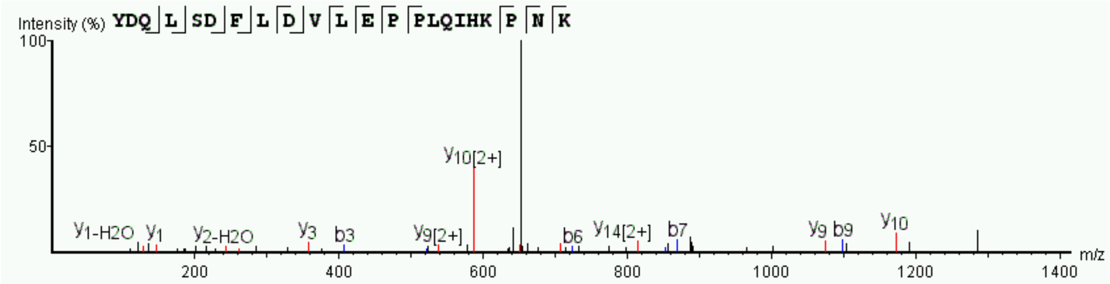

gi571548129

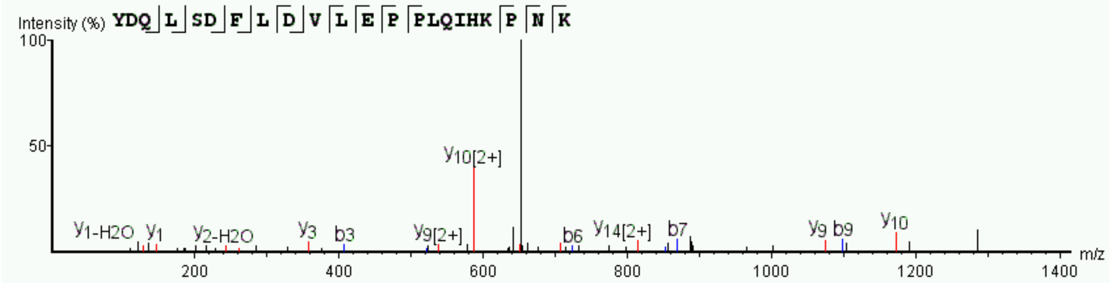

gi571548024

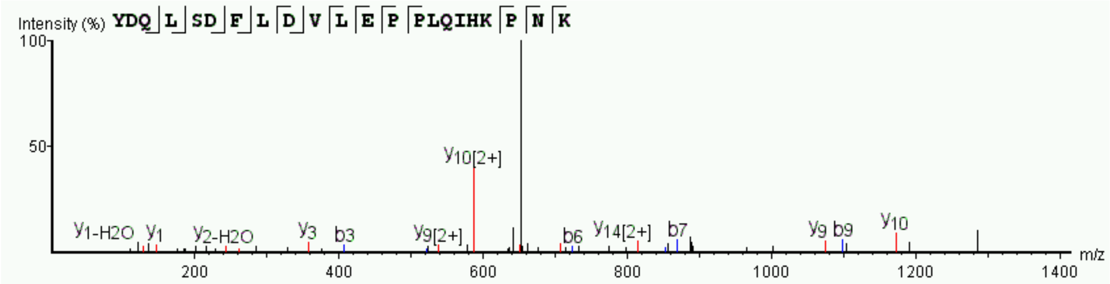

gi571548057

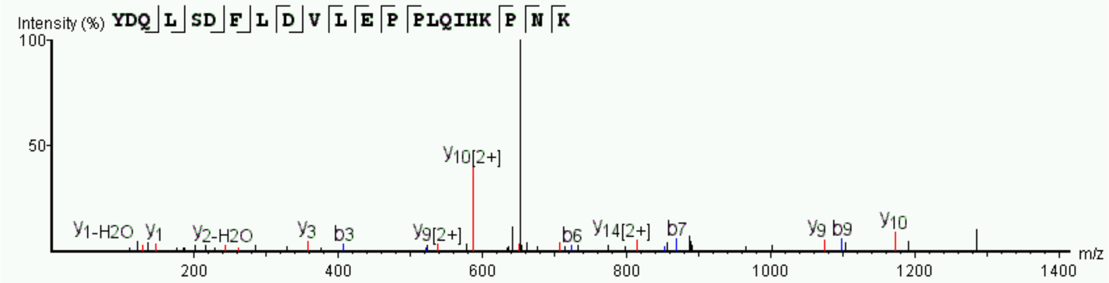

gi571576952

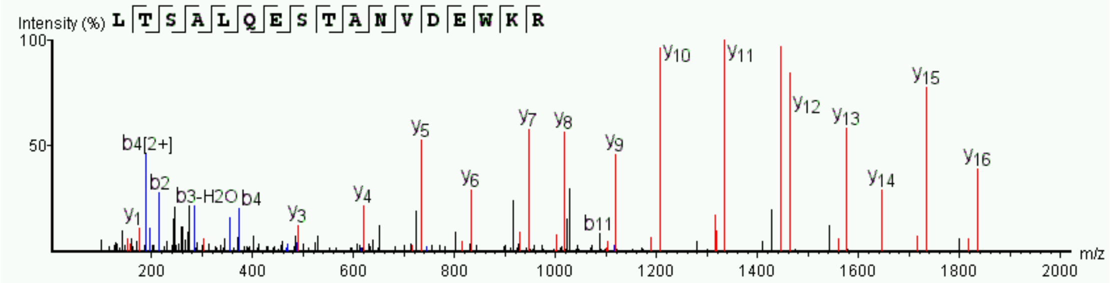

gi571576954

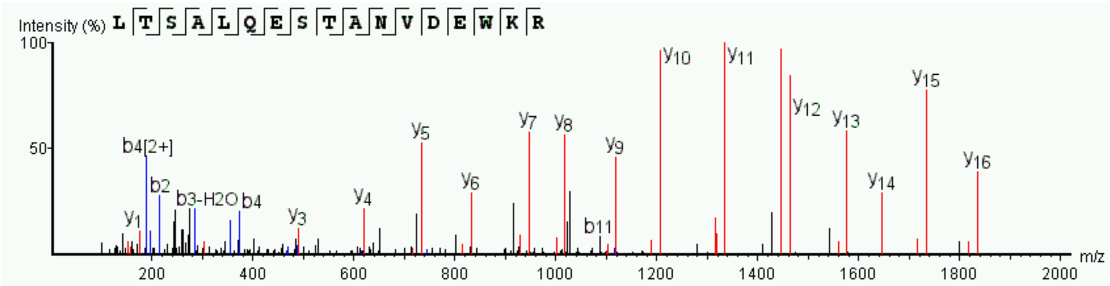

gi571576958

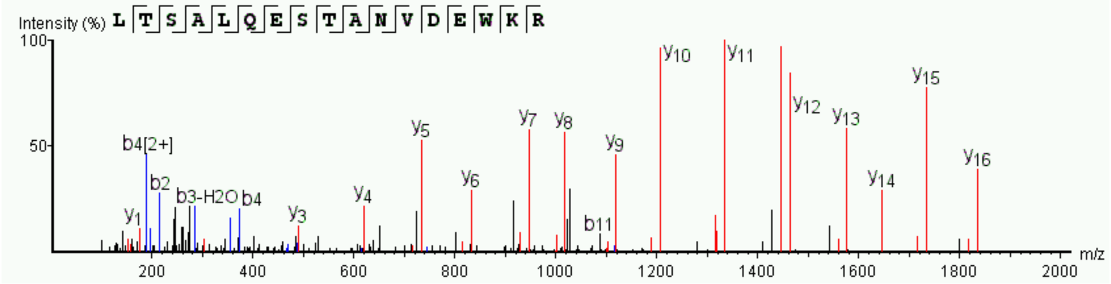

gi328790266

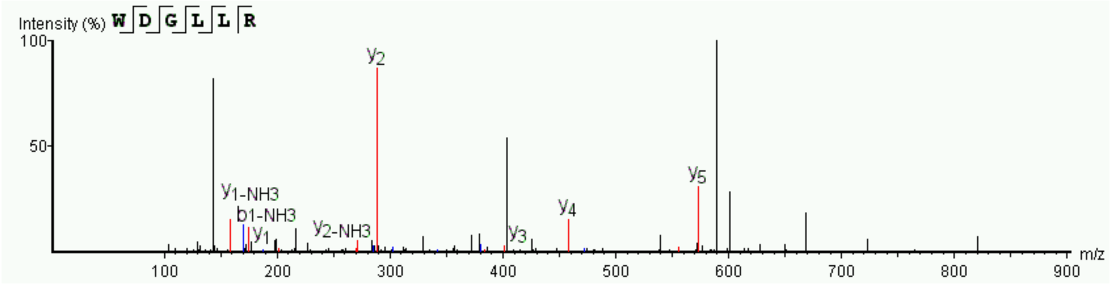

gi571508747

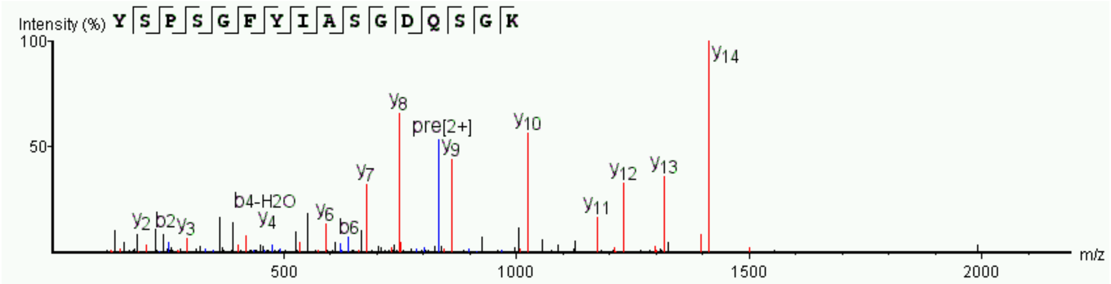

gi328777263

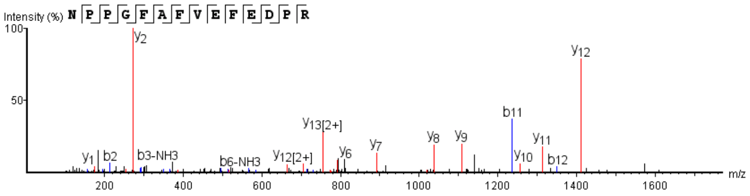

gi328777412

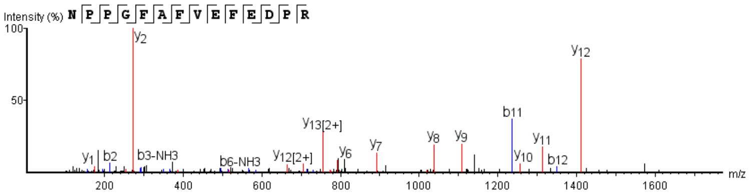

gi328777414

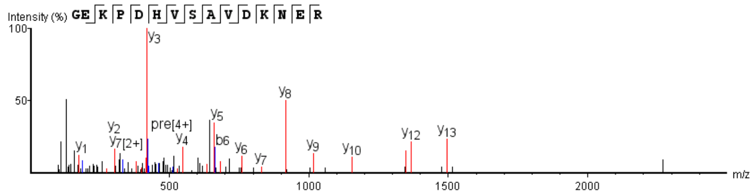

gi328777929

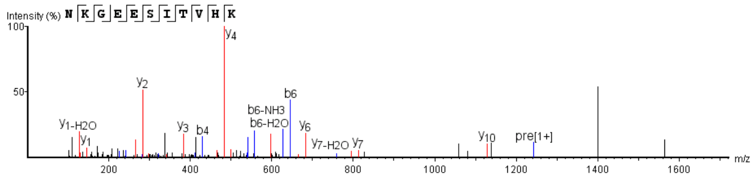

gi328778154

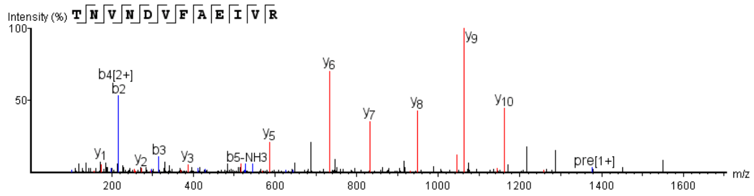

gi328778195

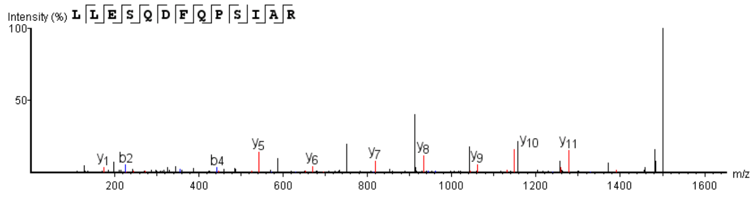

gi328778947

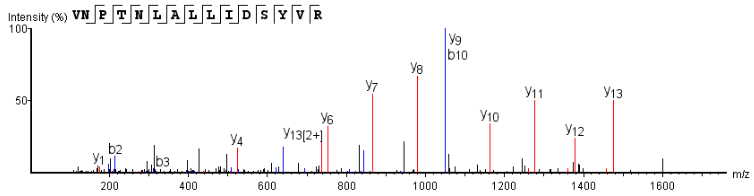

gi328779207

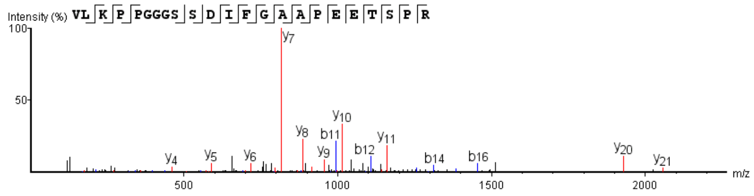

gi328779806

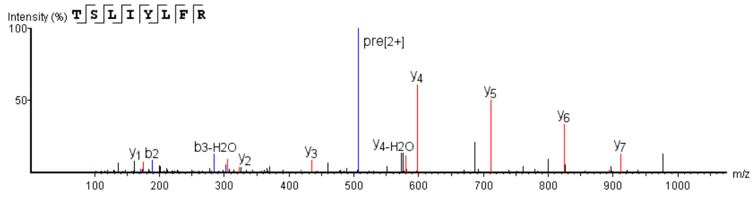

gi328780475

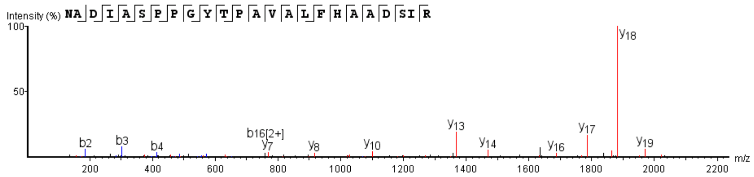

gi328780829

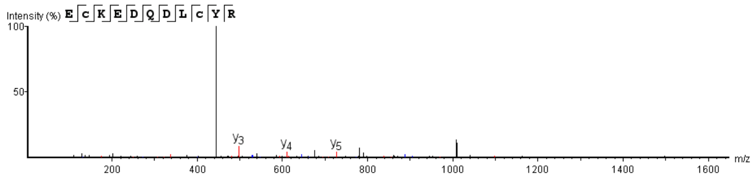

gi328780974

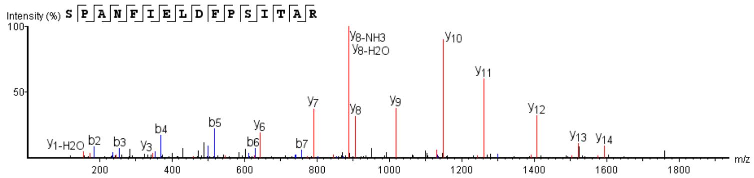

gi328781115

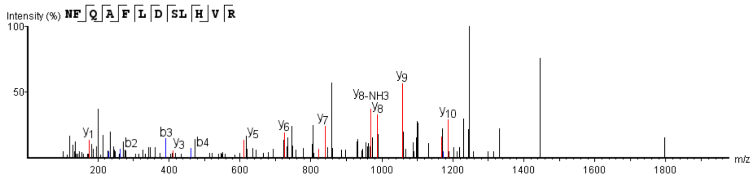

gi328781520

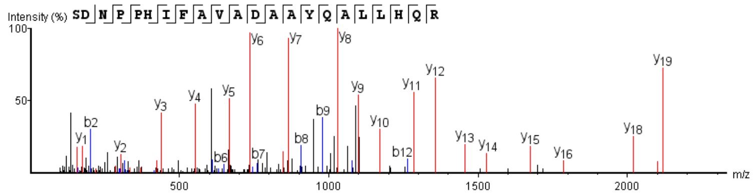

gi328781964

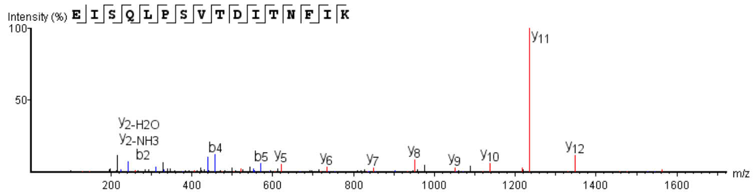

gi328782520

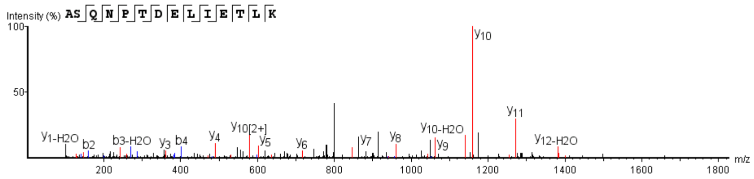

gi328782553

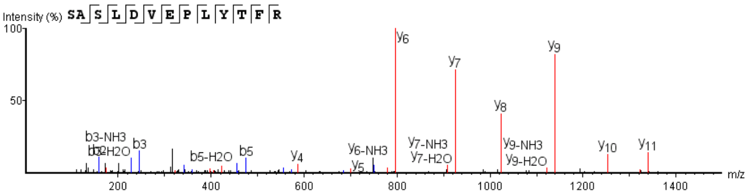

gi328782716

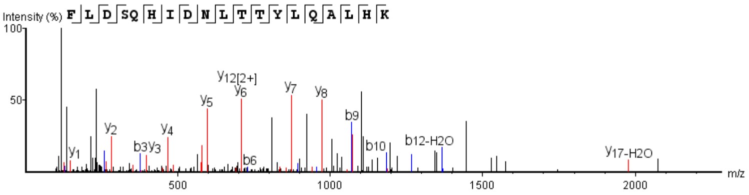

gi328782752

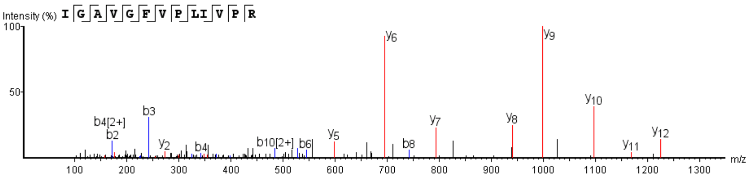

gi328782992

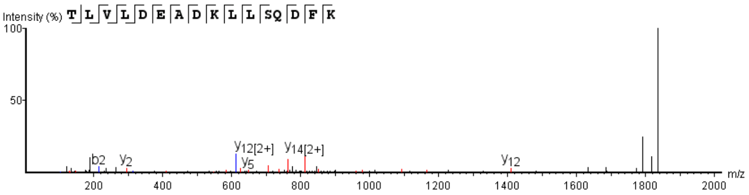

gi328783051

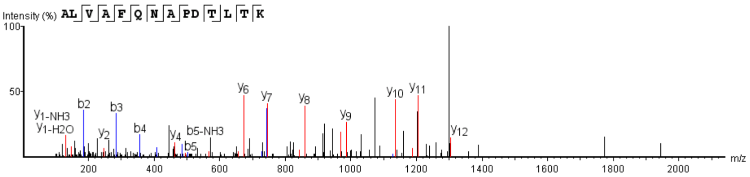

gi328783491

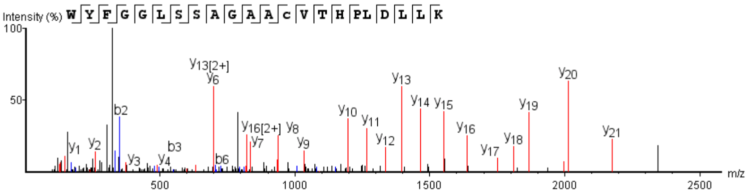

gi328783561

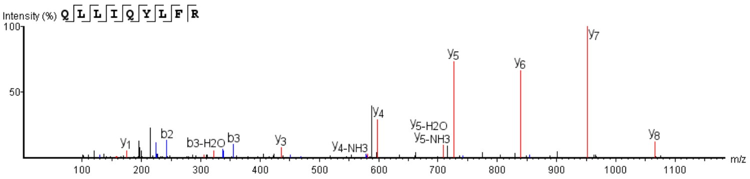

gi328783748

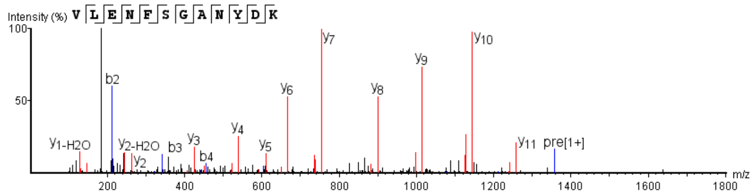

gi328784195

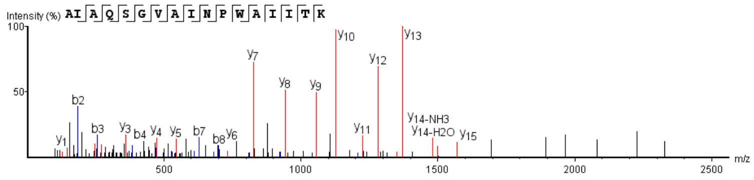

gi328784556

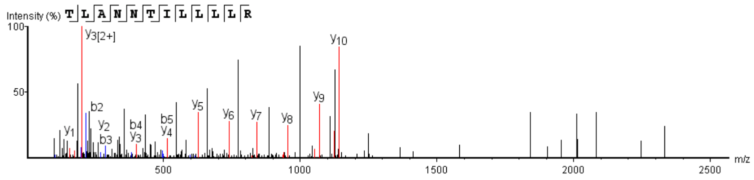

gi328784729

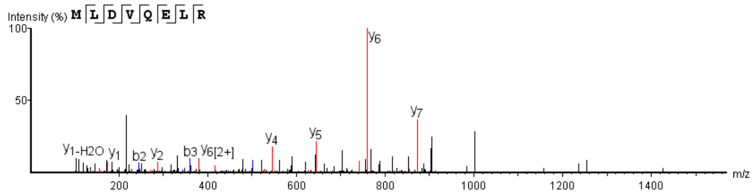

gi328784942

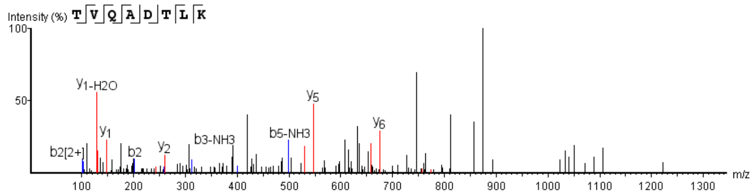

gi328785302

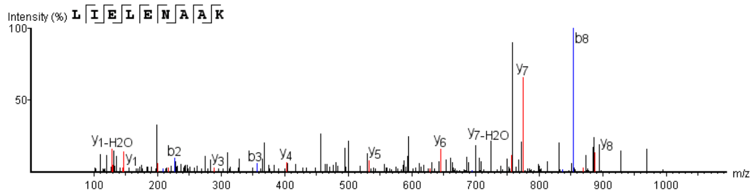

gi328785330

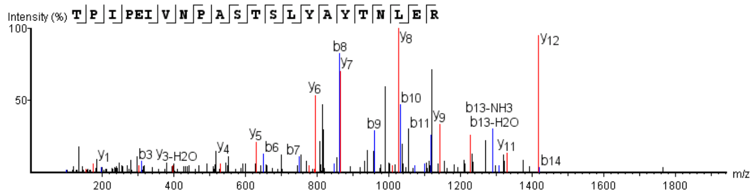

gi328786356

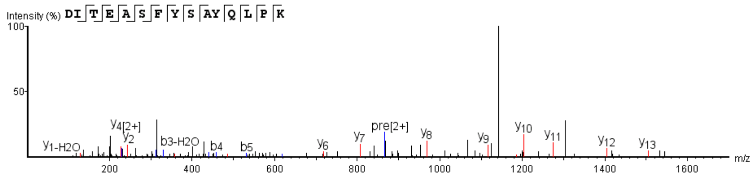

gi328787488

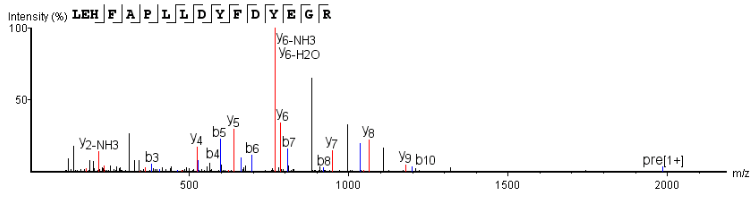

gi328787733

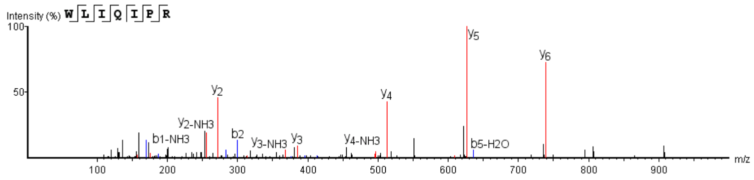

gi328788208

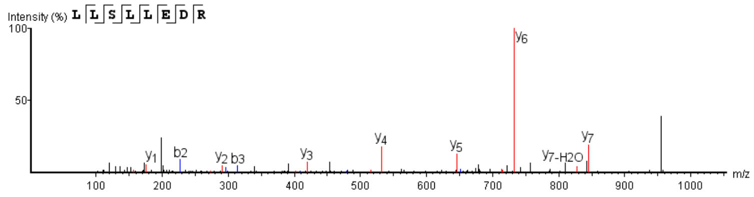

gi328788274

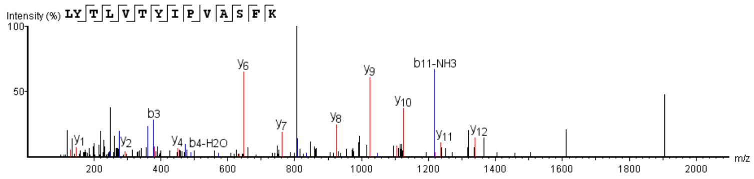

gi328788691

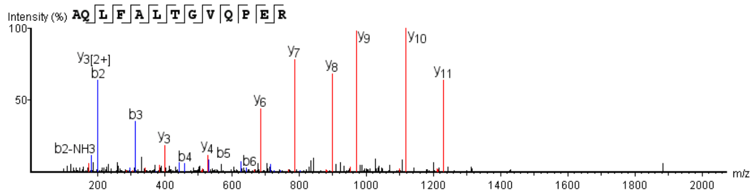

gi328788813

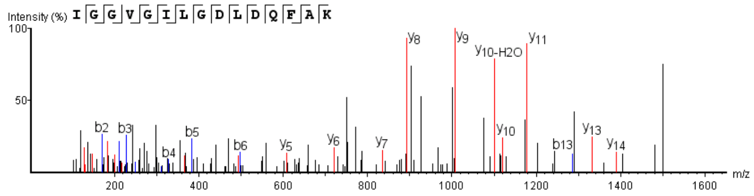

gi328789347

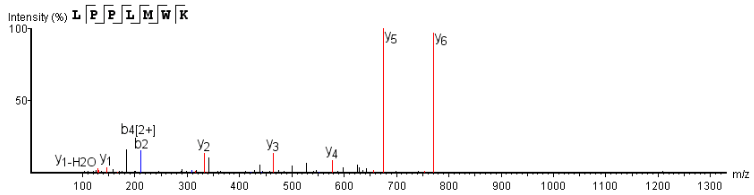

gi328789486

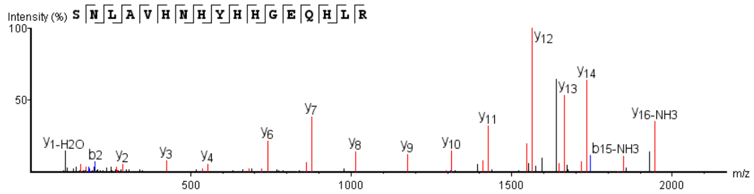

gi328789542

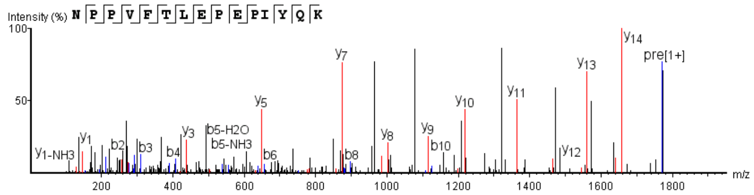

gi328790053

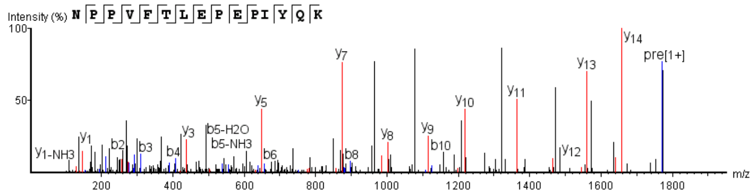

gi328790055

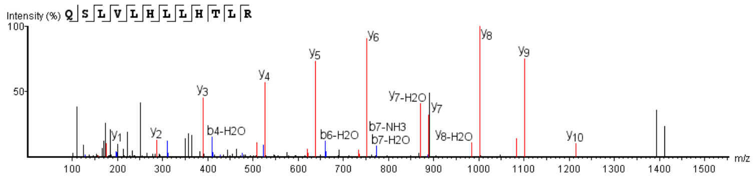

gi328790235

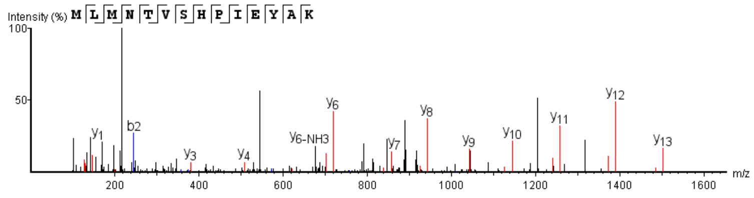

gi328790379

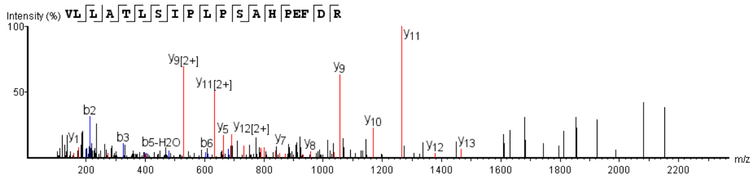

gi328790880

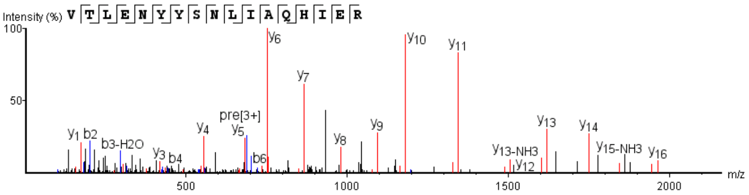

gi328791460

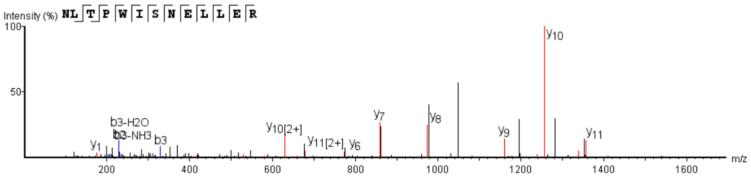

gi328792621

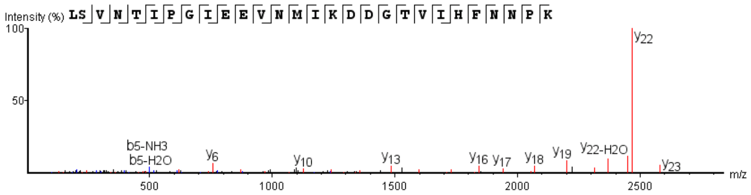

gi328792910

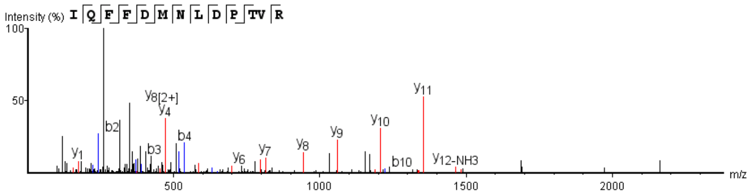

gi328793534

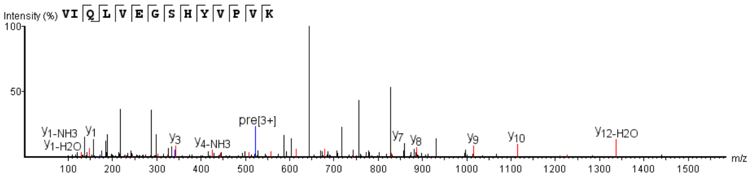

gi335057529

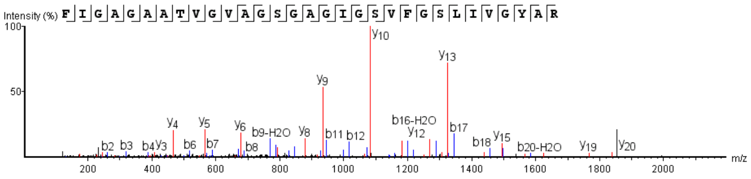

gi336391185

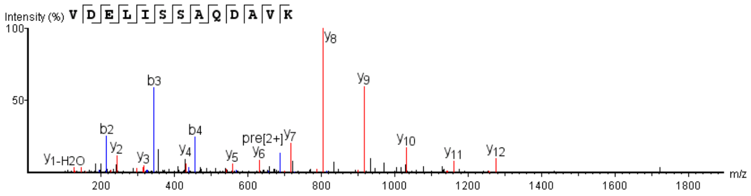

gi571499266

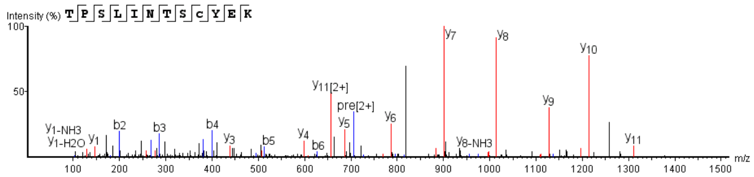

gi571499584

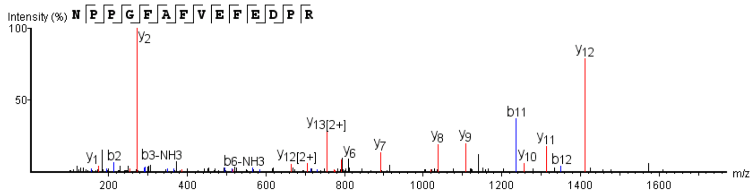

gi571500029

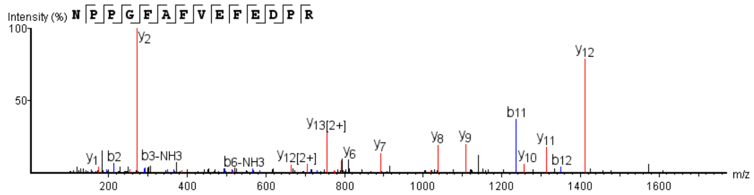

gi571500035gi571500039

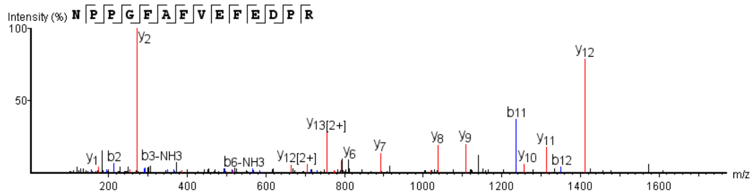

gi571500044

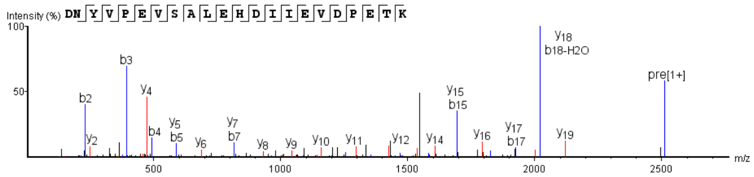

gi571500813

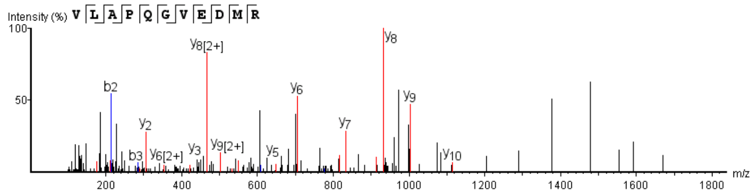

gi571502355

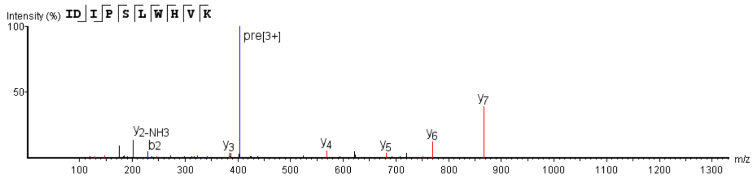

gi571502990

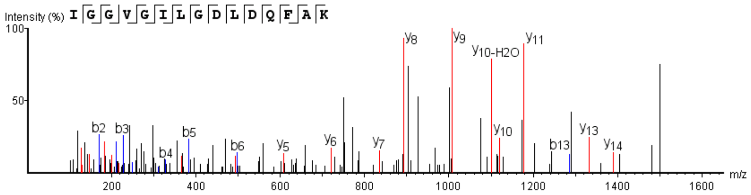

gi571503850

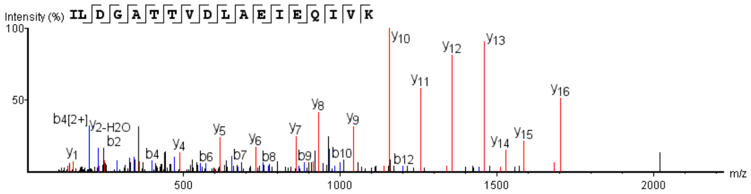

gi571503997

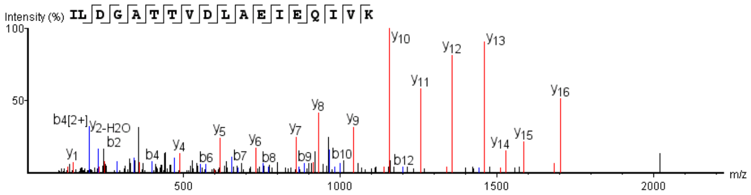

gi571504000

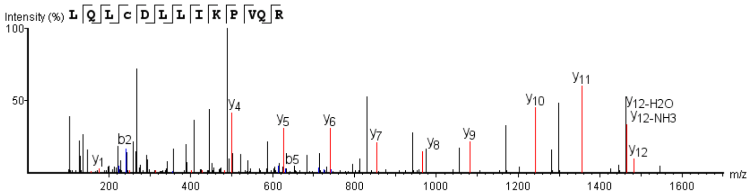

gi571504009

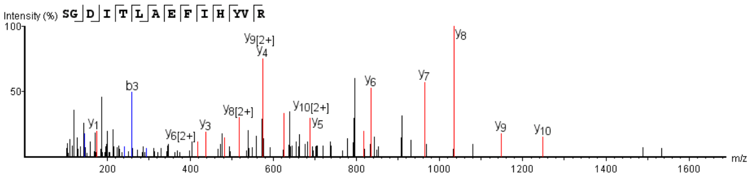

gi571504092

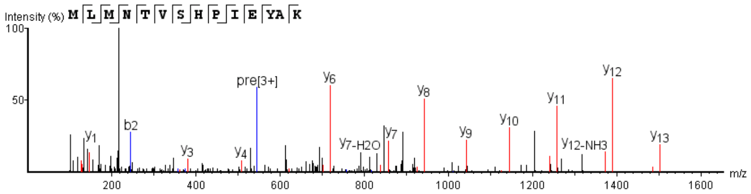

gi571504099

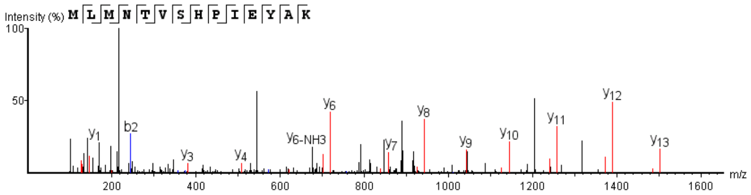

gi571504104

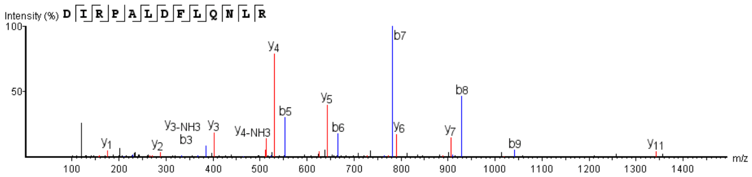

gi571504286

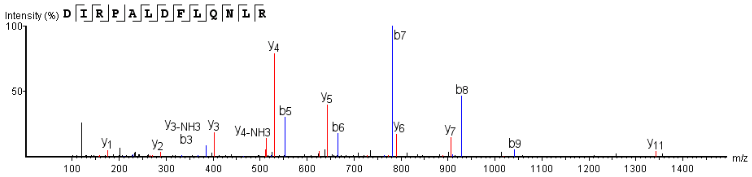

gi571504293

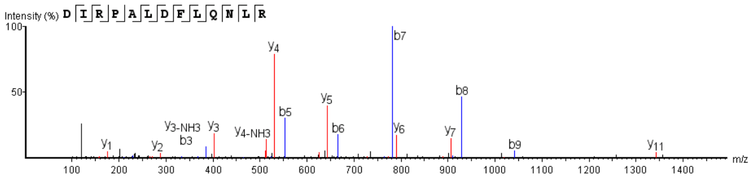

gi571504295

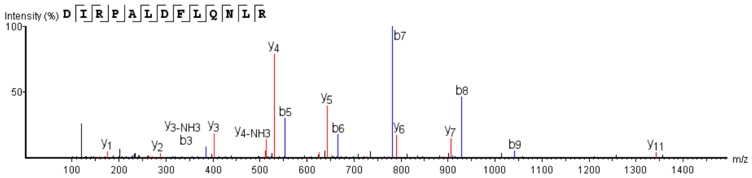

gi571504299

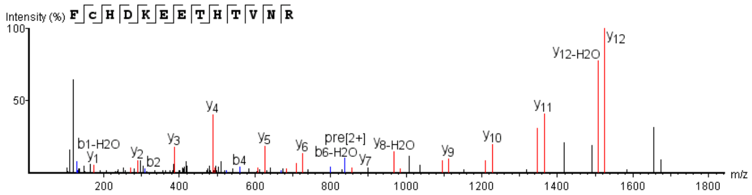

gi571504485

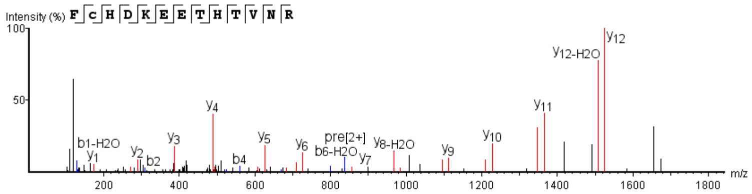

gi571504489

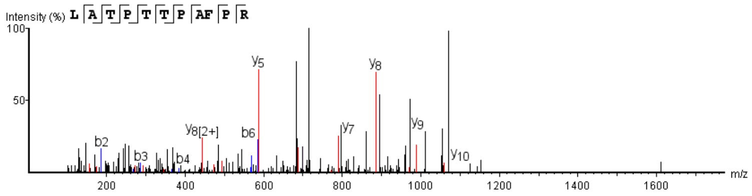

gi571505799

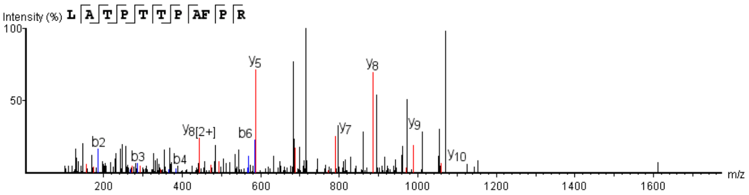

gi571505803

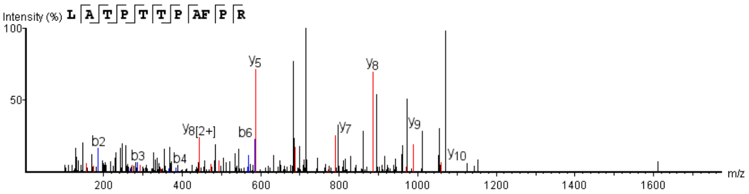

gi571505843

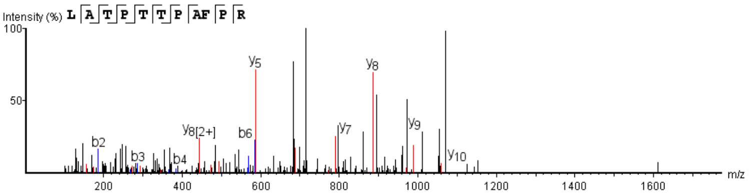

gi571505846

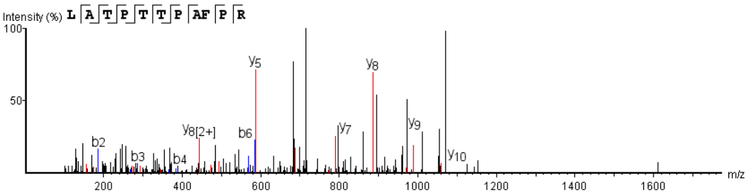

gi571505849

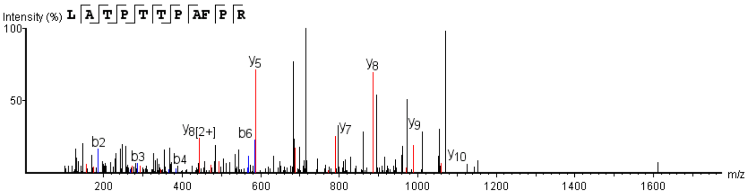

gi571505852

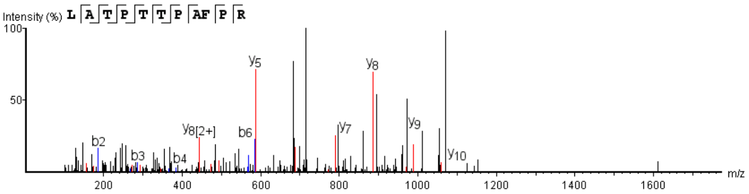

gi571505855

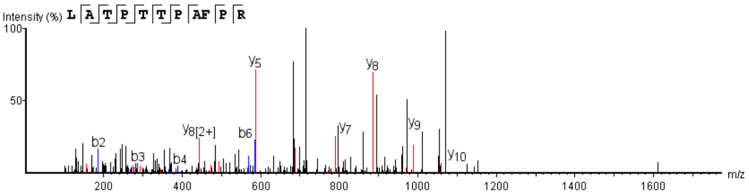

gi571505858

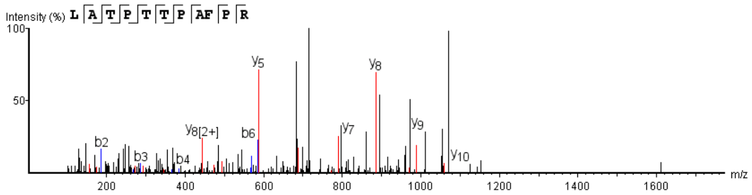

gi571505861

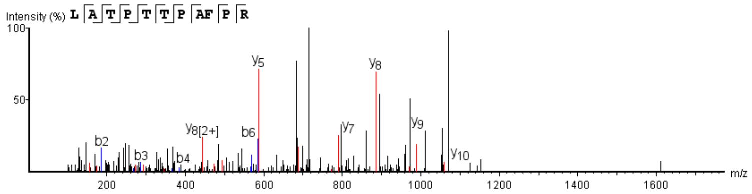

gi571505865

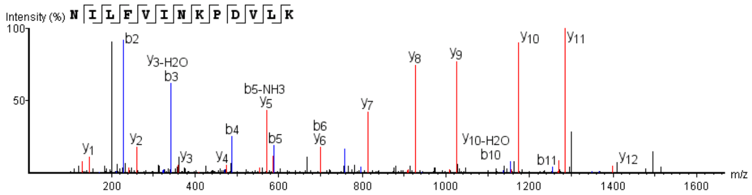

gi571507494

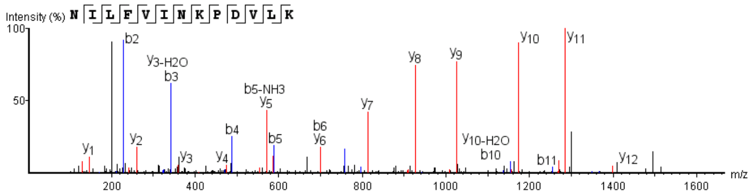

gi571507498

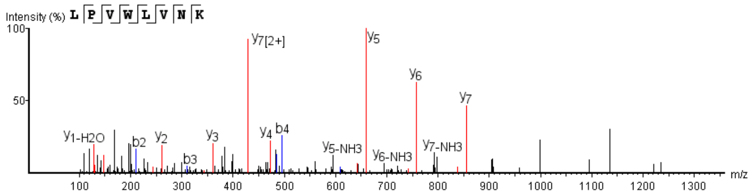

gi571509114

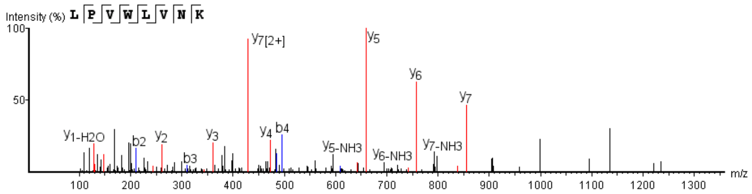

gi571509118

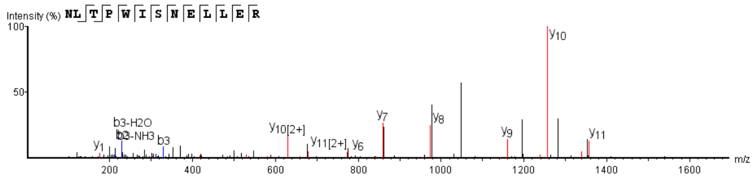

gi571509569

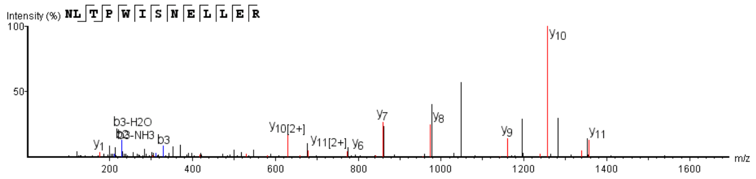

gi571509575

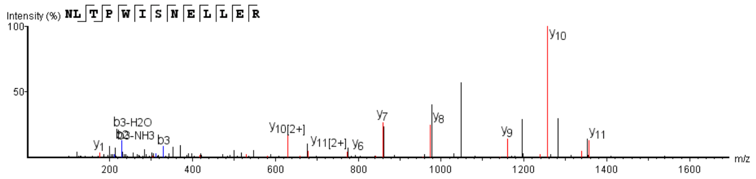

gi571509578

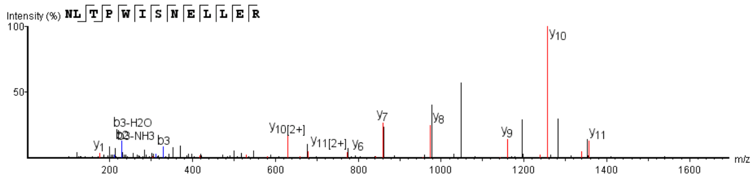

gi571509582

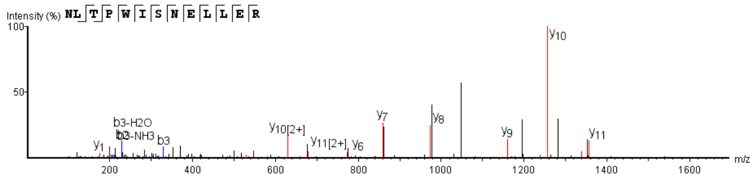

gi571509585

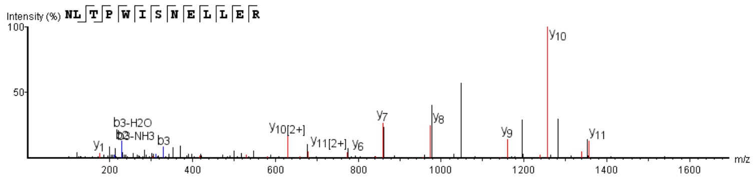

gi571509589

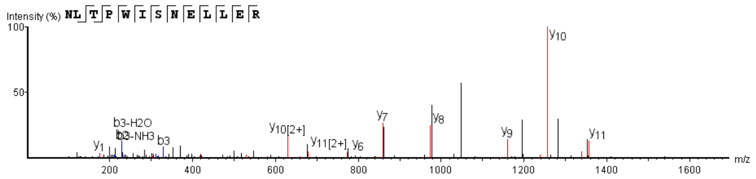

gi571509593

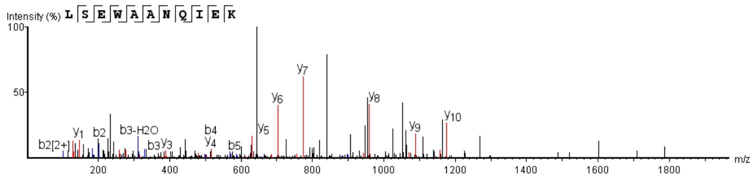

gi571509803

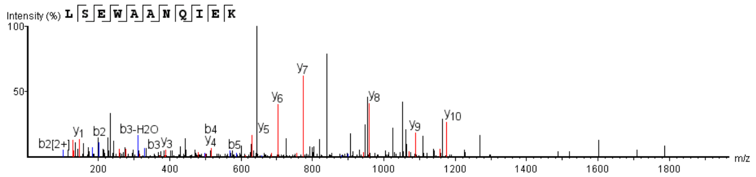

gi571509806

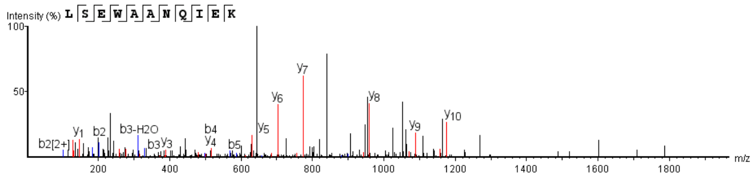

gi571509810

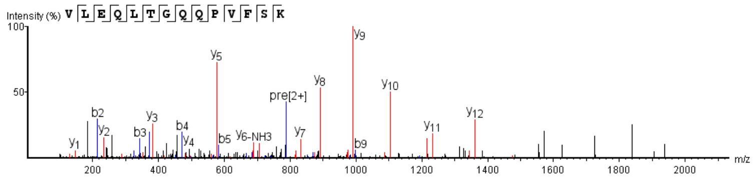

gi571510850

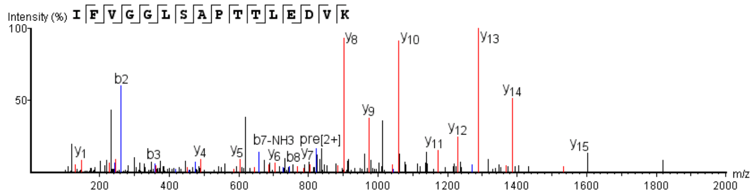

gi571512336

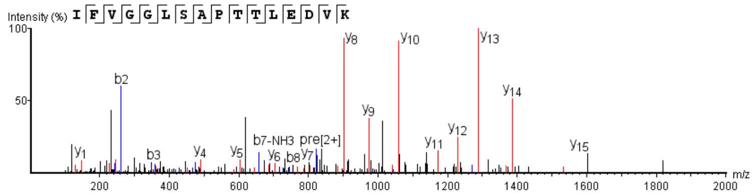

gi571512339

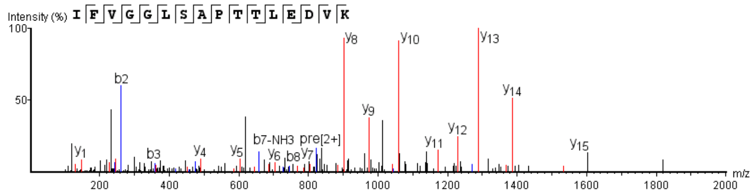

gi571512342

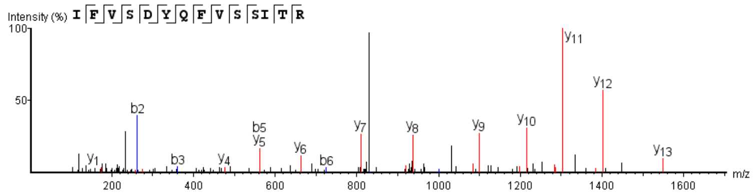

gi571512898

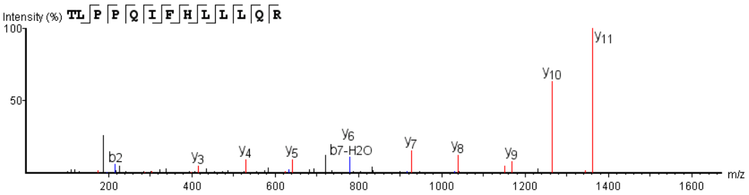

gi571513237

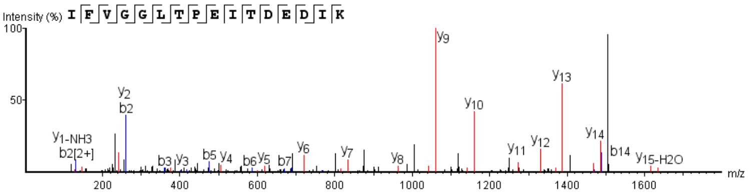

gi571514456

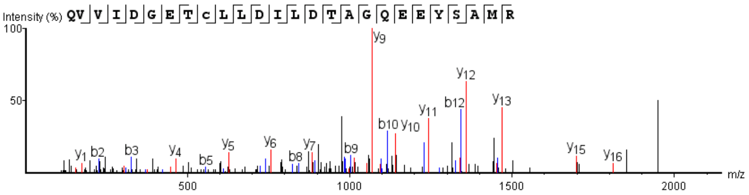

gi571514556

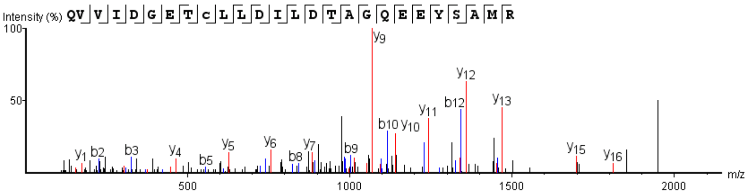

gi571514559

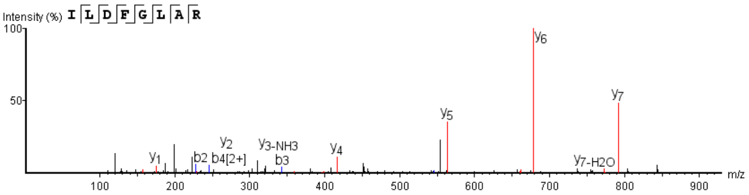

gi571516304

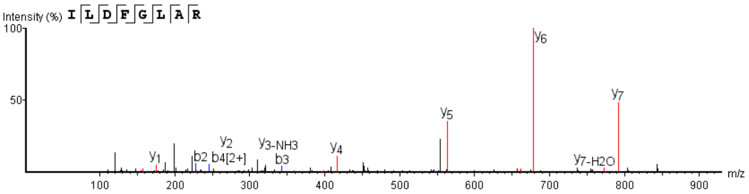

gi571516310

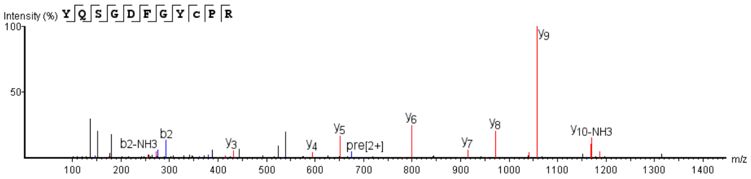

gi571517007

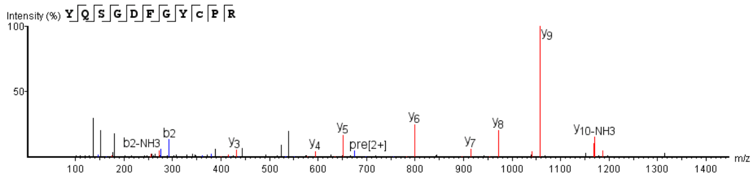

gi571517011

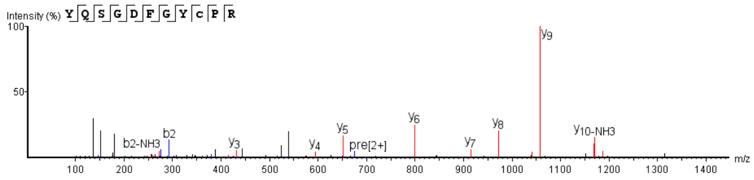

gi571517016

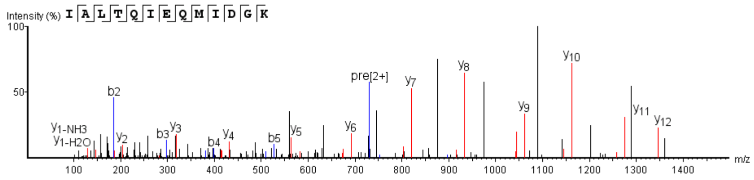

gi571517147

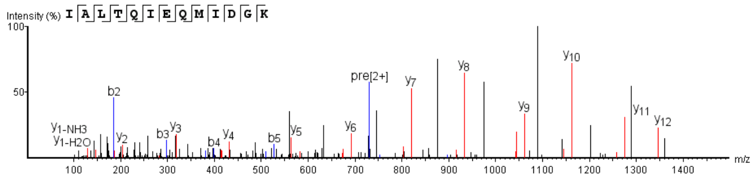

gi571517153

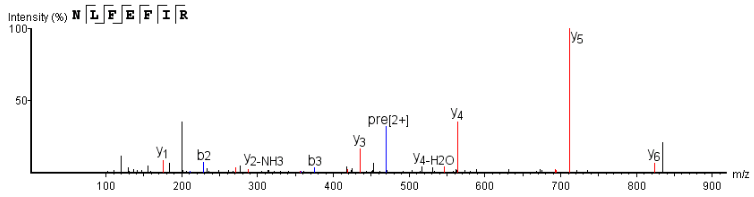

gi571517320

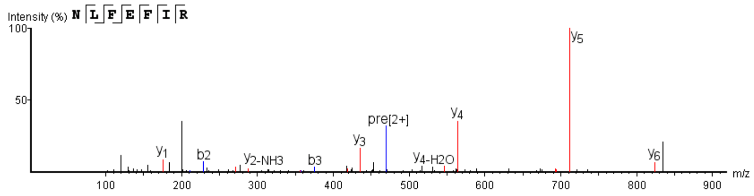

gi571517323

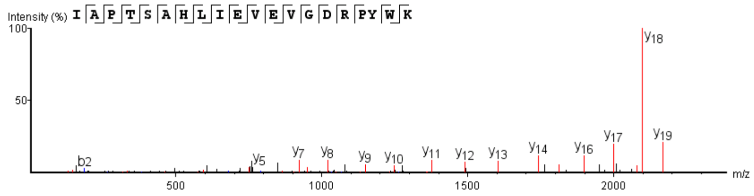

gi571517348

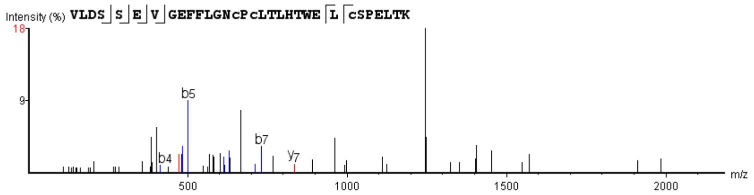

gi571520564

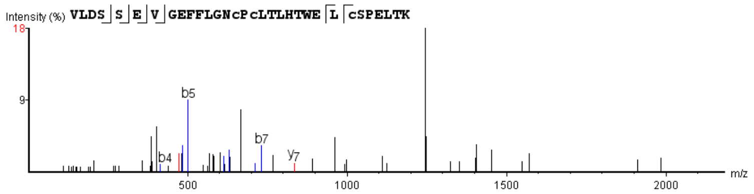

gi571520568

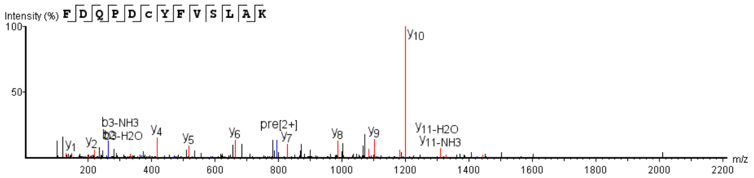

gi571520775

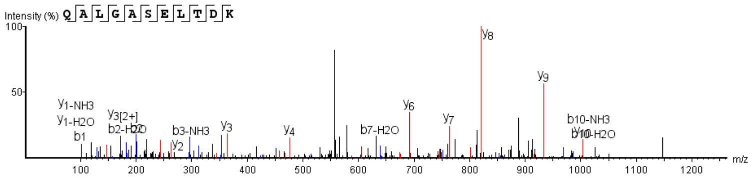

gi571520919

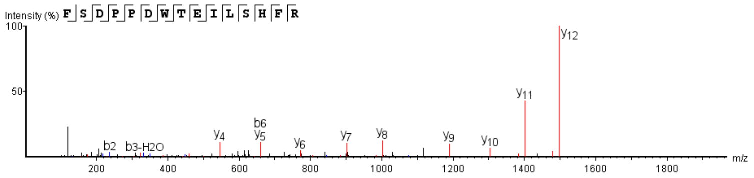

gi571521115

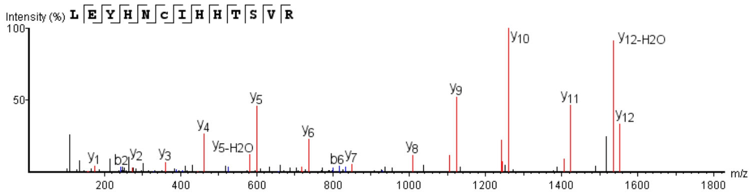

gi571522078

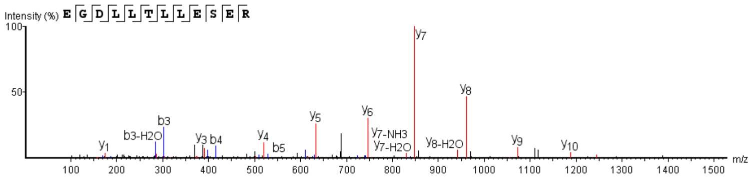

gi571522669

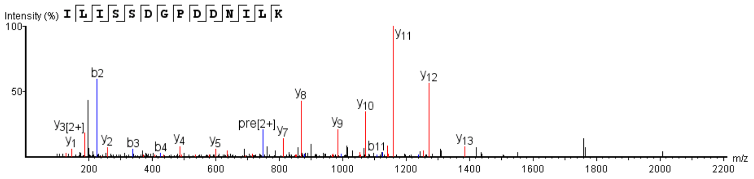

gi571522855

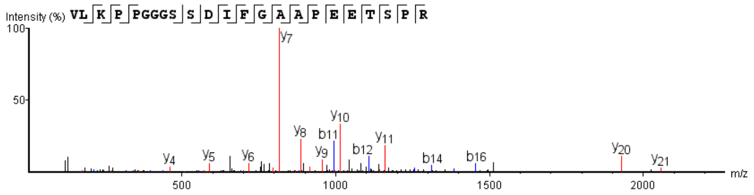

gi571522890

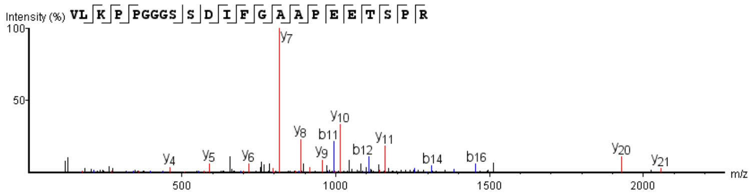

gi571522894

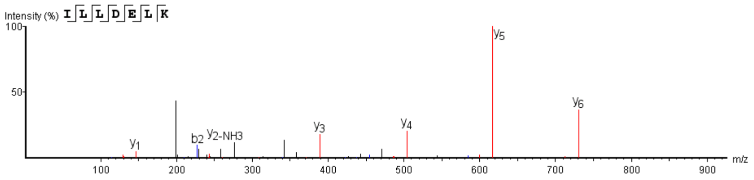

gi571524916

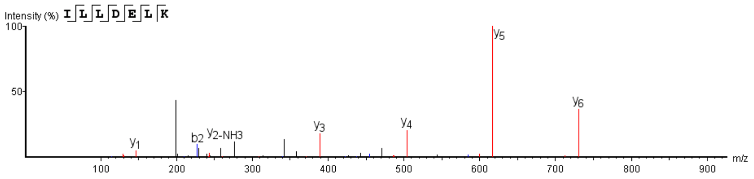

gi571524919

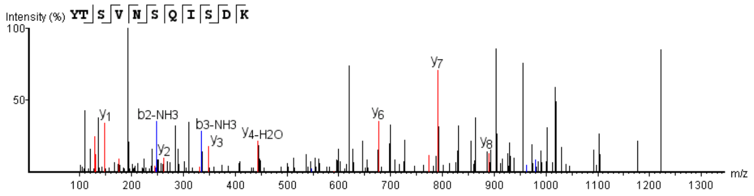

gi571525006

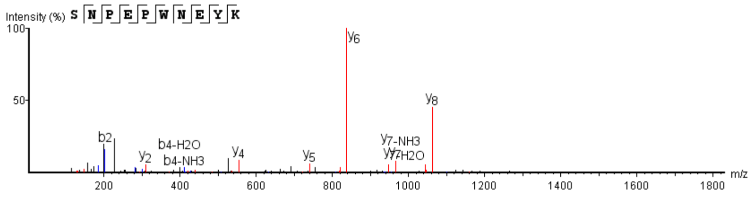

gi571525189

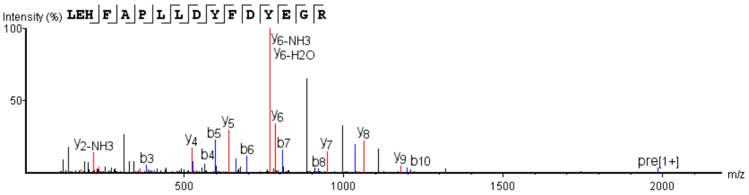

gi571525515

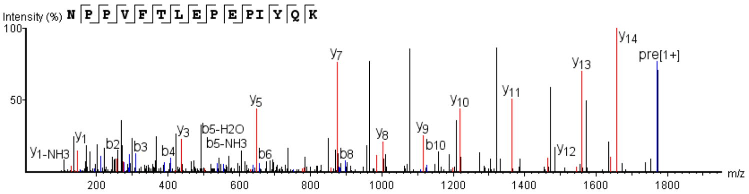

gi571525957

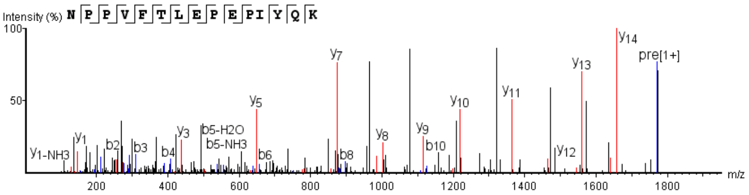

gi571525961

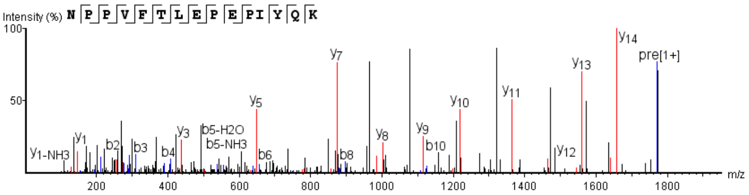

gi571525968

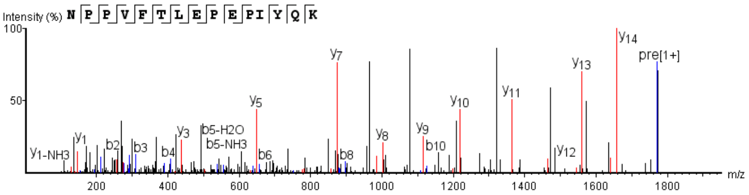

gi571525972

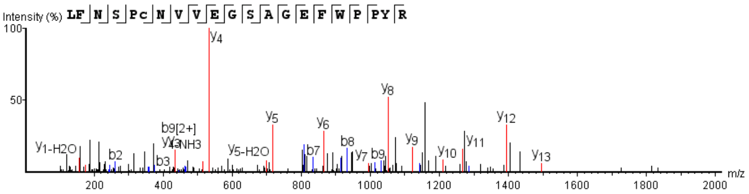

gi571525993

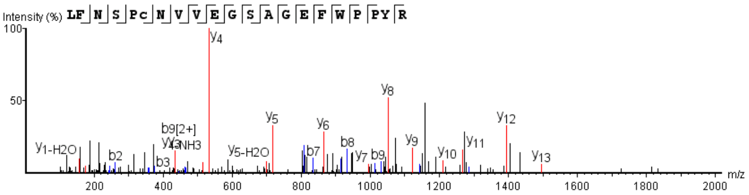

gi571525997

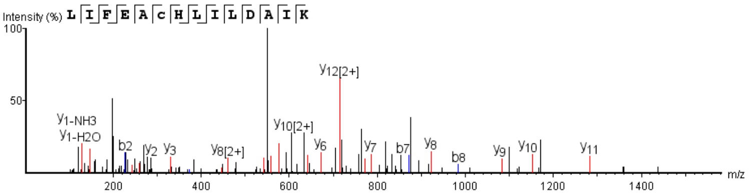

gi571526495

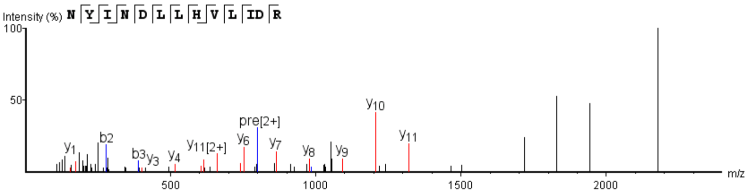

gi571527870

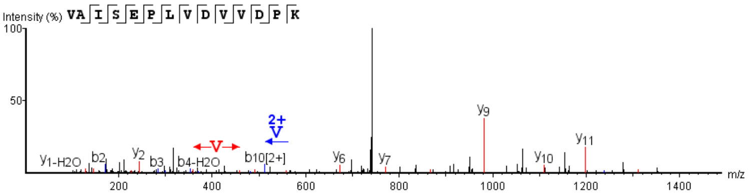

gi571528227

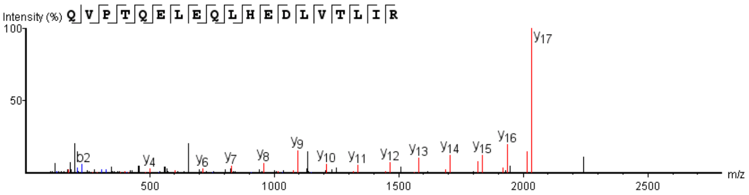

gi571528435

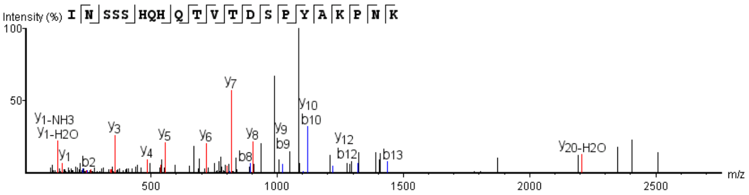

gi571529205

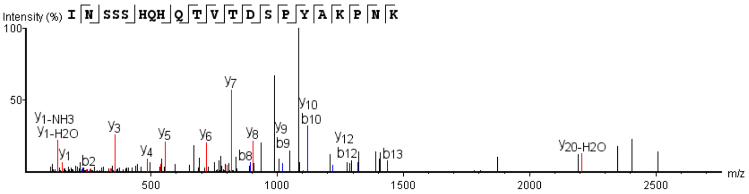

gi571529209

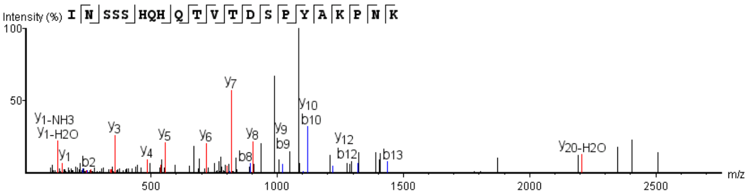

gi571529213

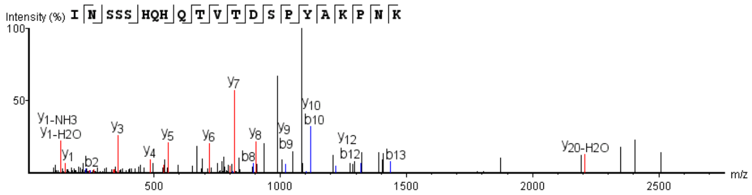

gi571529216

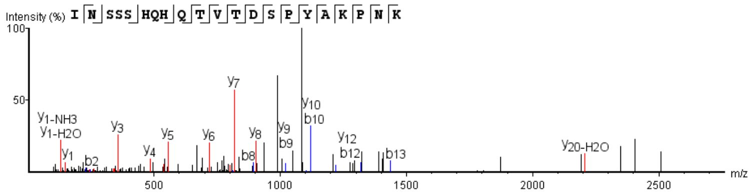

gi571529220

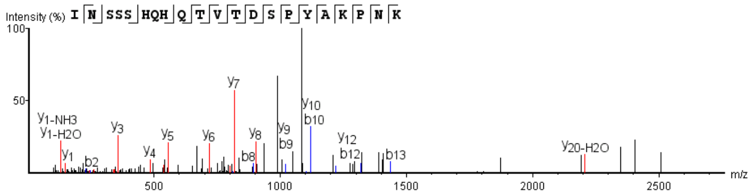

gi571529224

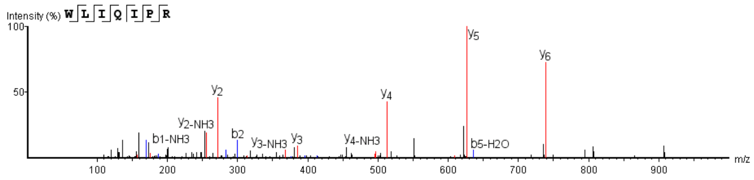

gi571529406

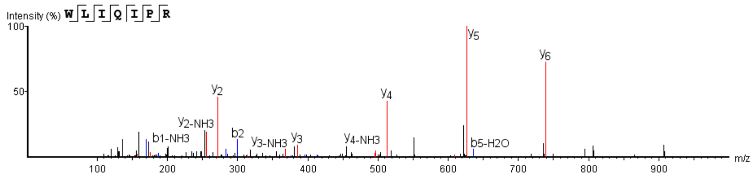

gi571529409

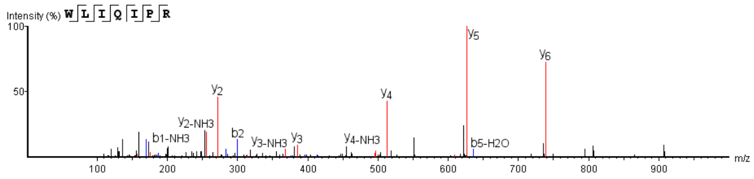

gi571529412

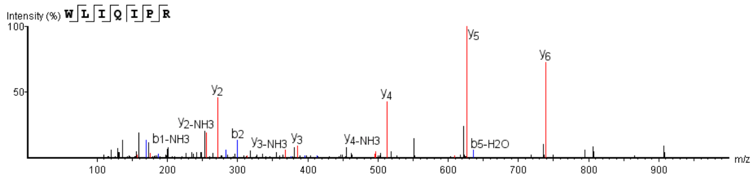

gi571529417

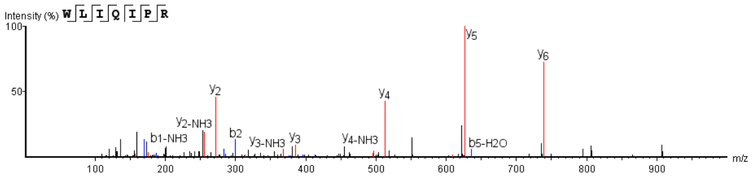

gi571529421

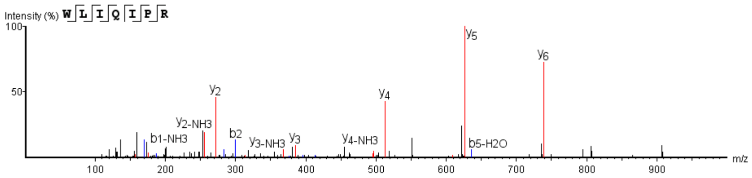

gi571529425

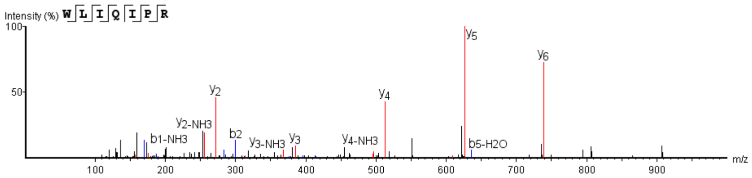

gi571529429

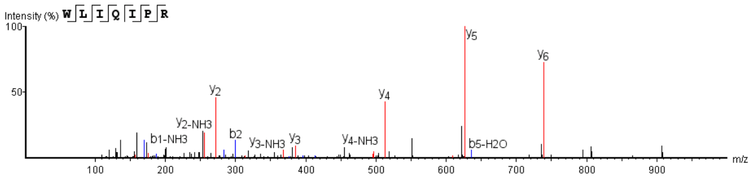

gi571529433

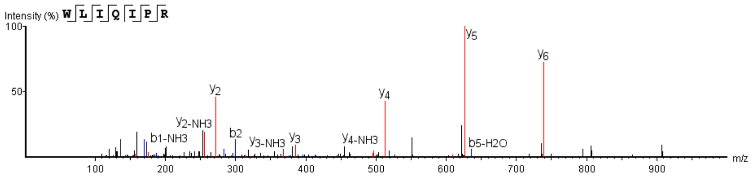

gi571529436

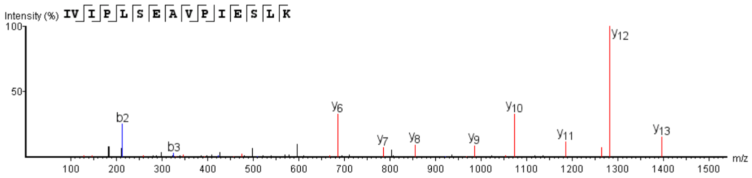

gi571529541

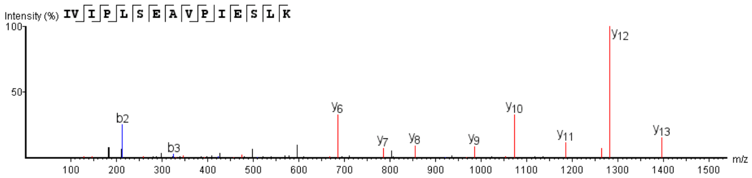

gi571529545

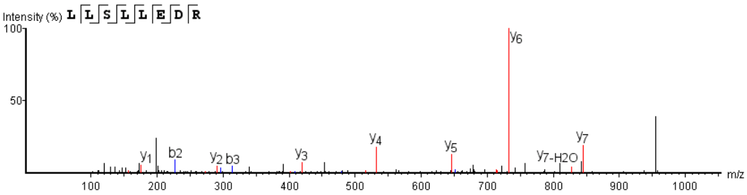

gi571529763

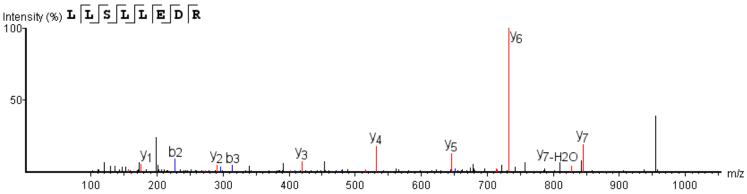

gi571529767

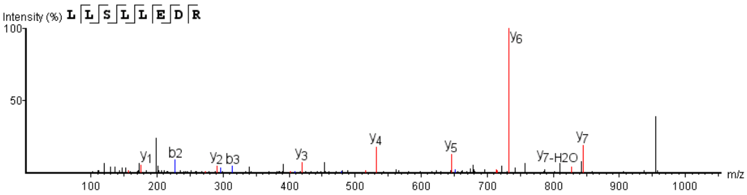

gi571529770

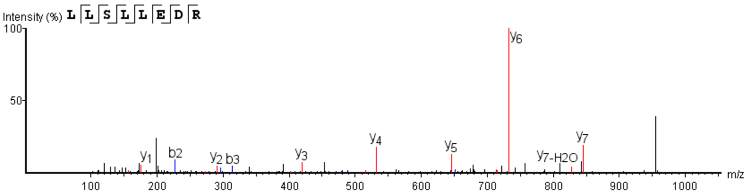

gi571529773

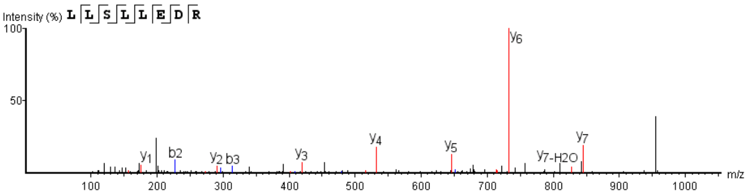

gi571529779

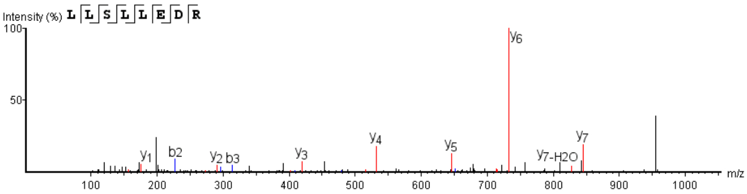

gi571529782

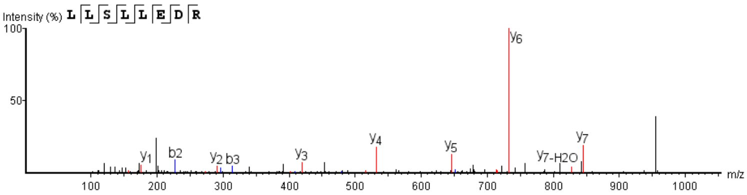

gi571529785

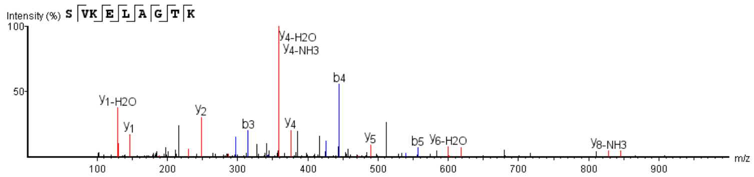

gi571530481

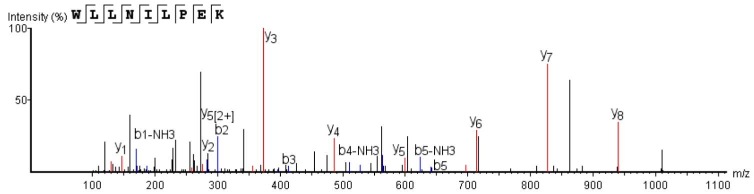

gi571530529

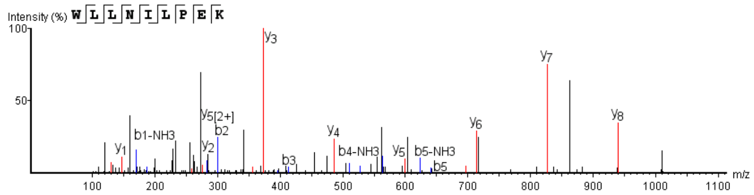

gi571530533

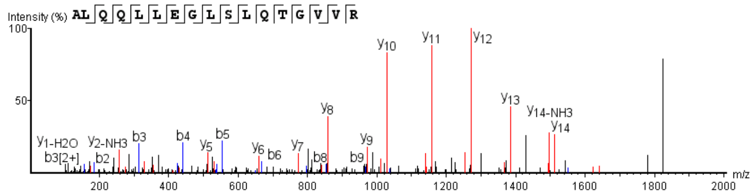

gi571531847

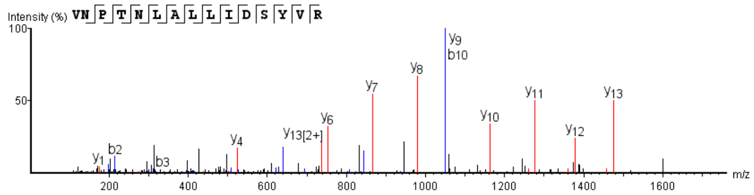

gi571532722

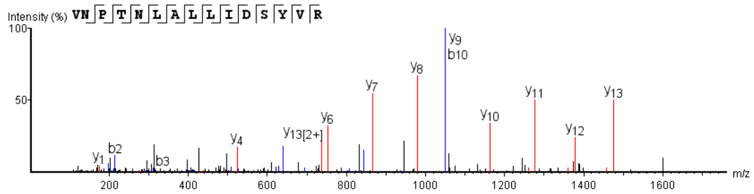

gi571532725

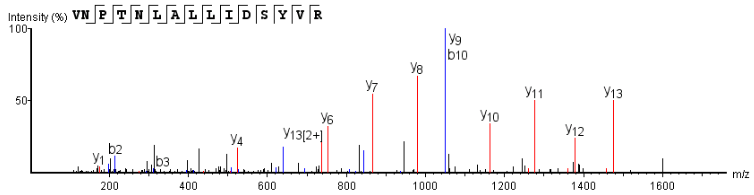

gi571532729

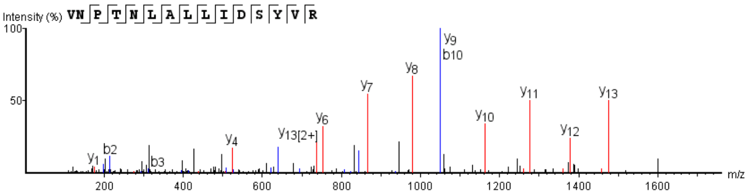

gi571532734

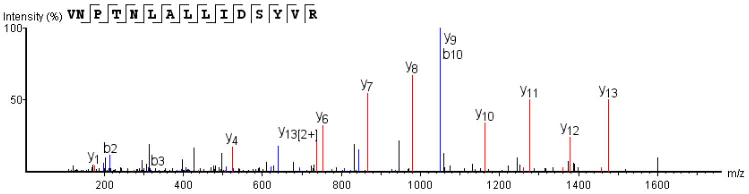

gi571532738

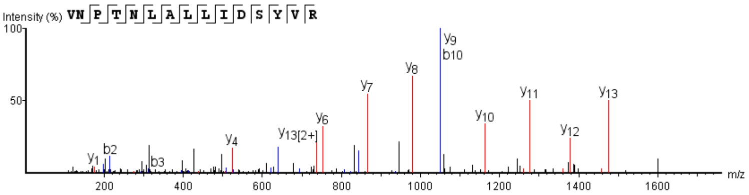

gi571532741

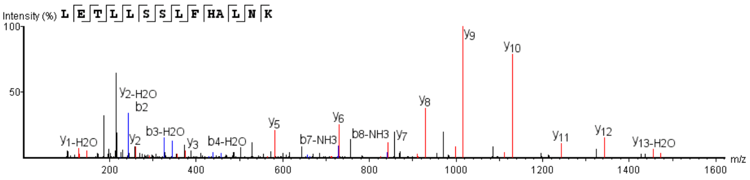

gi571533068

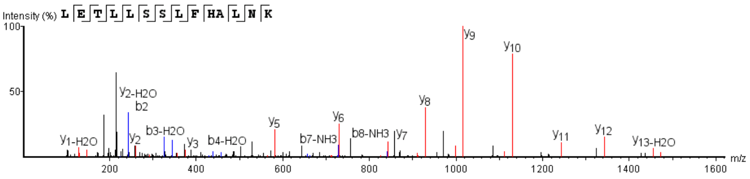

gi571533072

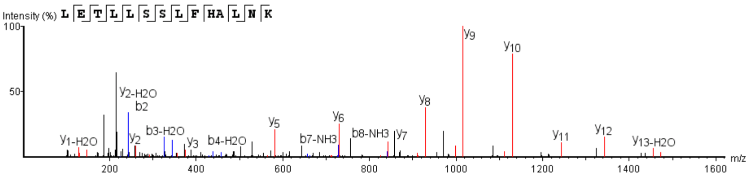

gi571533076

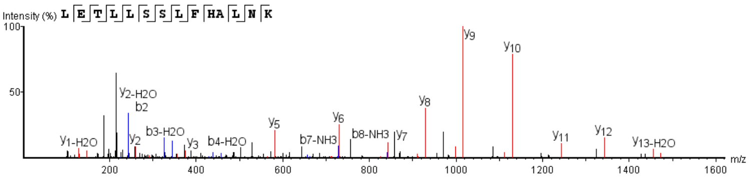

gi571533080

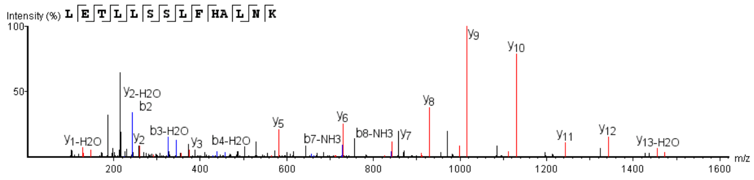

gi571533083

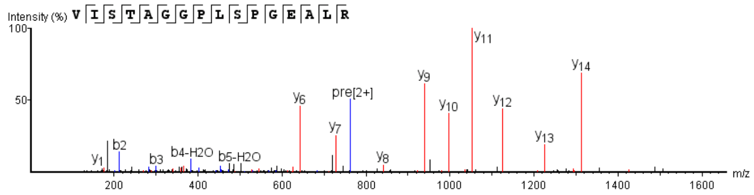

gi571533194

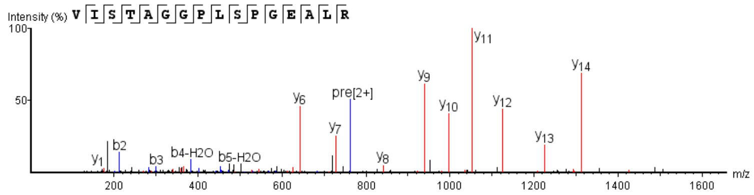

gi571533197

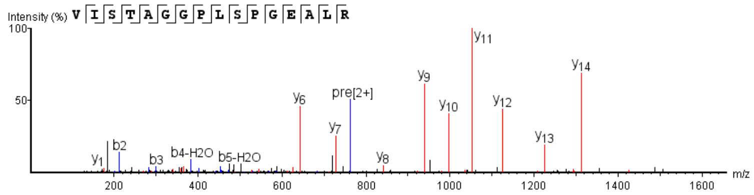

gi571533201

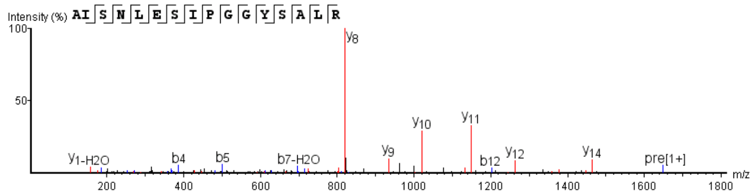

gi571533610

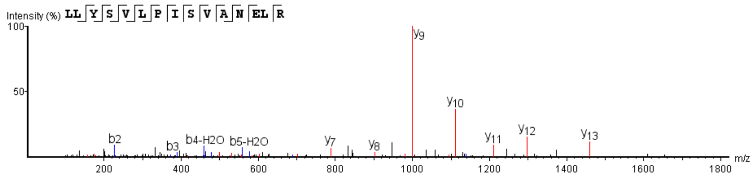

gi571535113

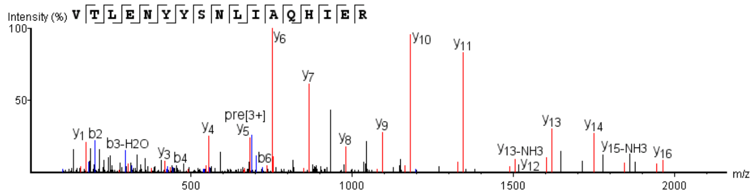

gi571535995

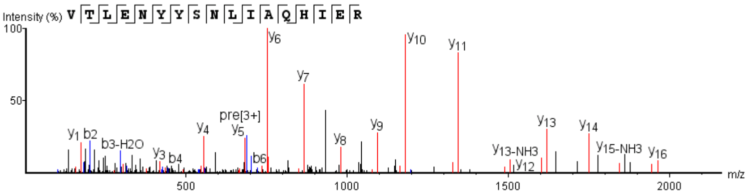

gi571536003

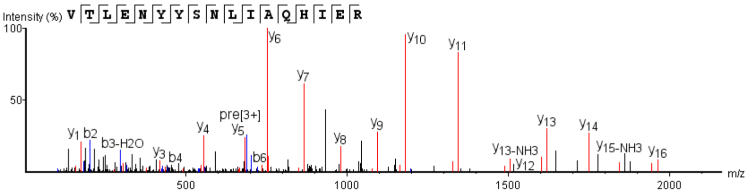

gi571536006

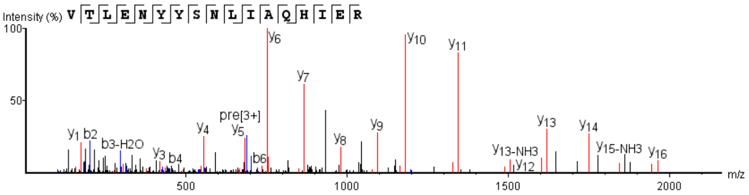

gi571536010

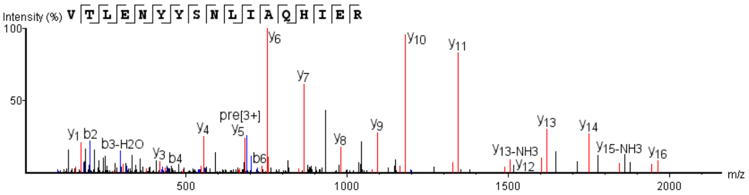

gi571536013

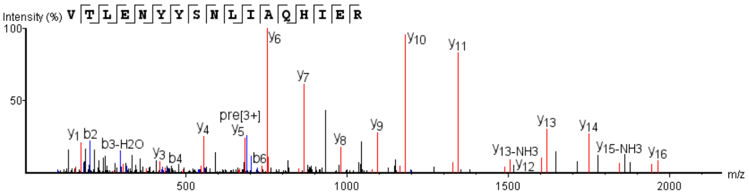

gi571536016

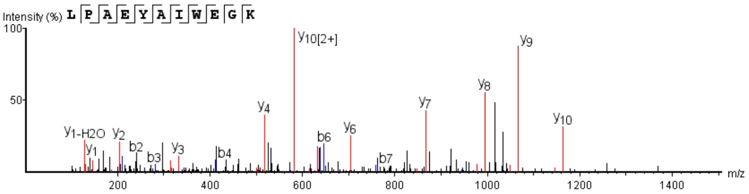

gi571536261

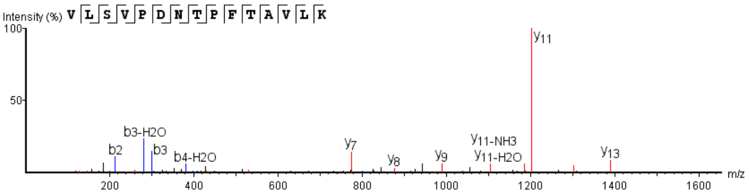

gi571538017

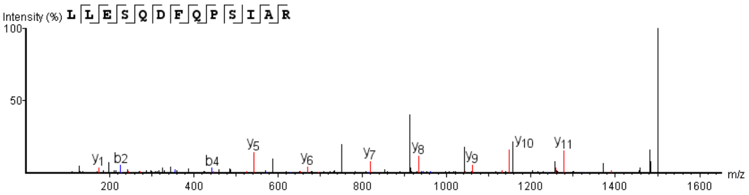

gi571538301

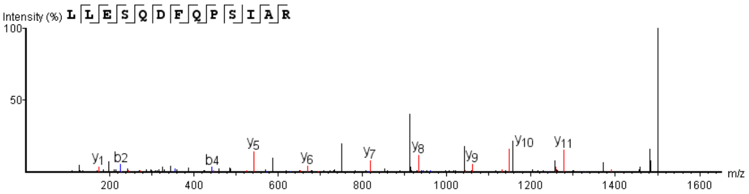

gi571538305

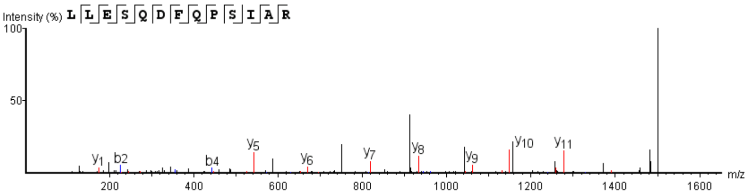

gi571538309

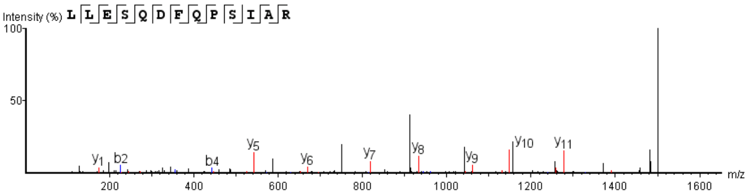

gi571538313

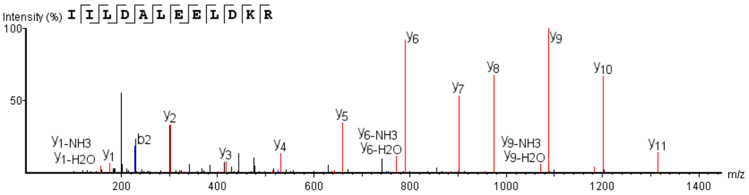

gi571538622

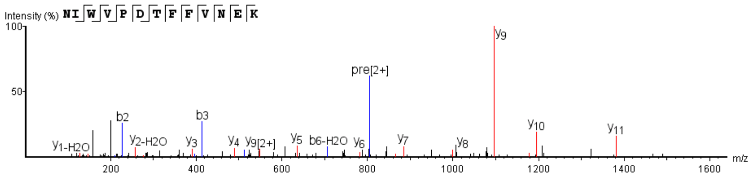

gi571539510

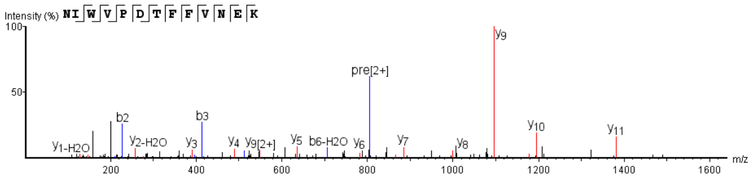

gi571539513

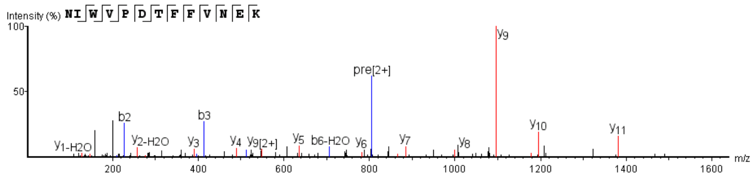

gi571539517

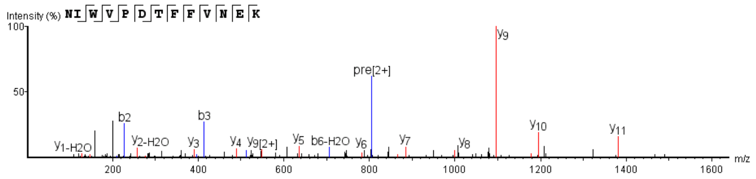

gi571539527

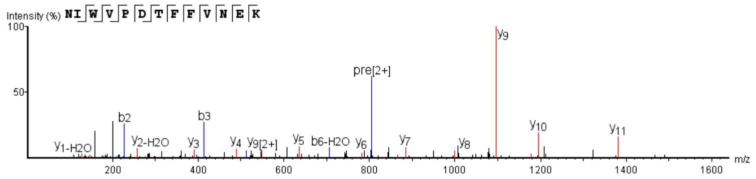

gi571539551

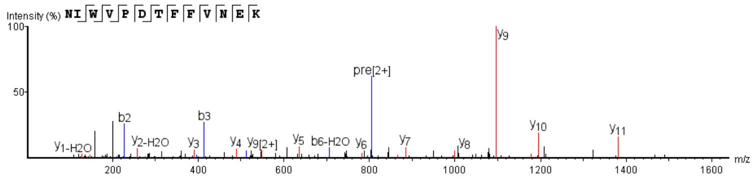

gi571539554

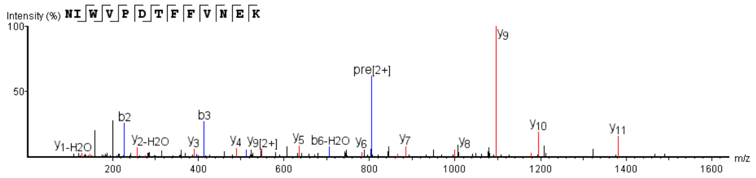

gi571539557

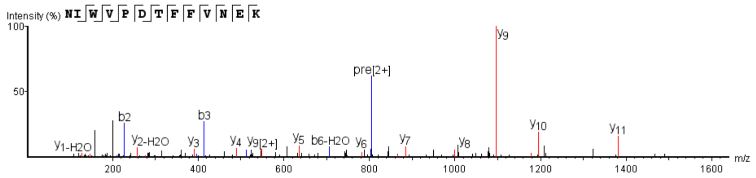

gi571539560

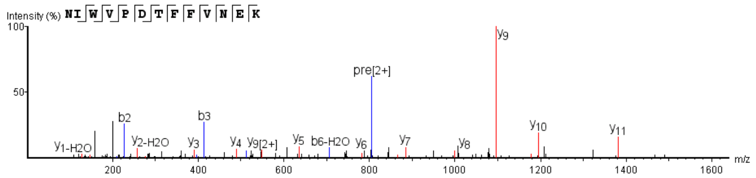

gi571539564

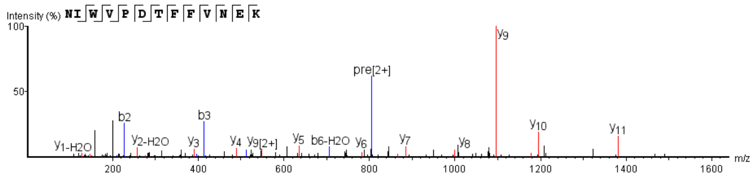

gi571539567

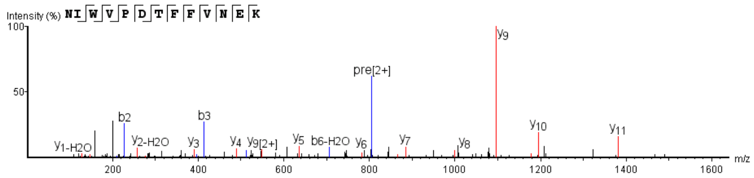

gi571539570

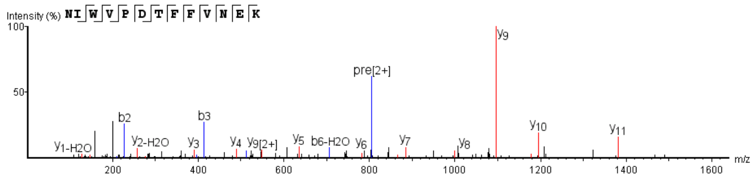

gi571539574

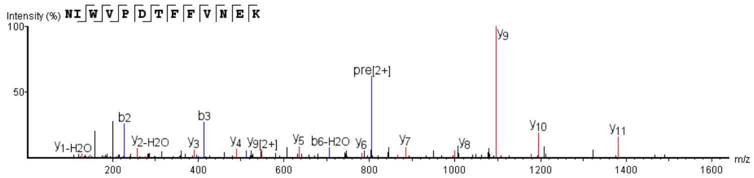

gi571539578

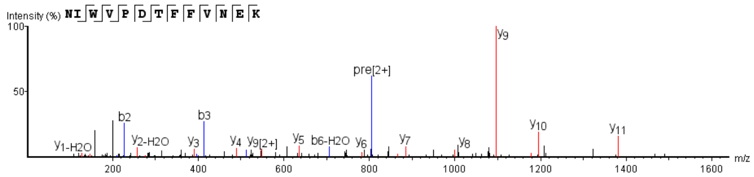

gi571539581

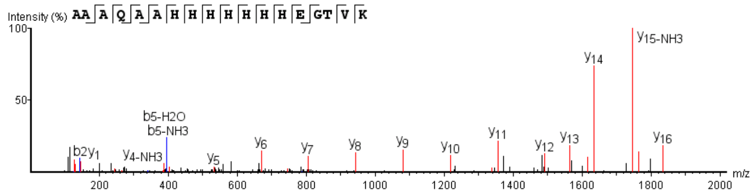

gi571540063

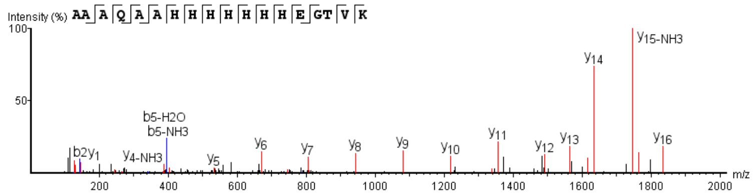

gi571540067

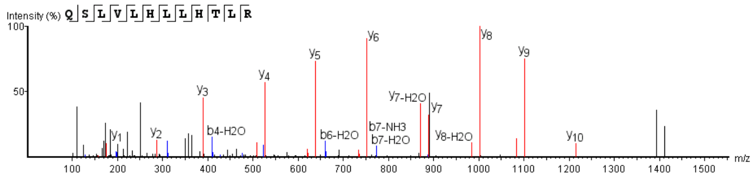

gi571540977

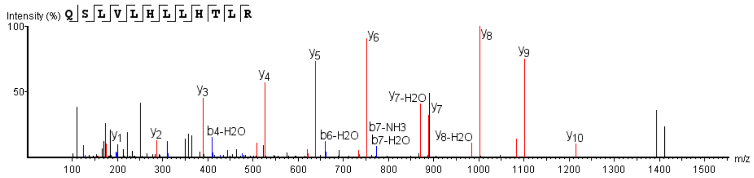

gi571540985

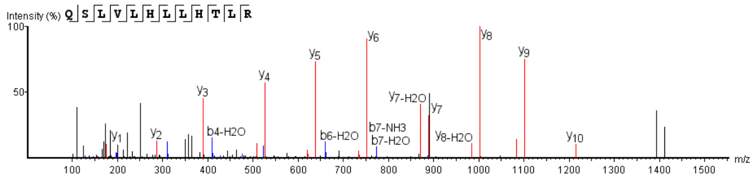

gi571540987

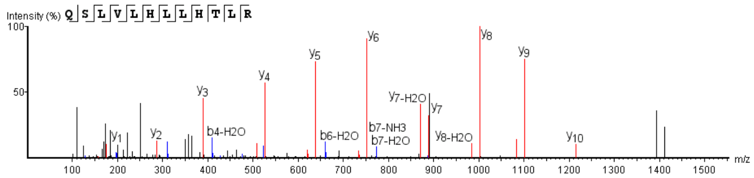

gi571540991

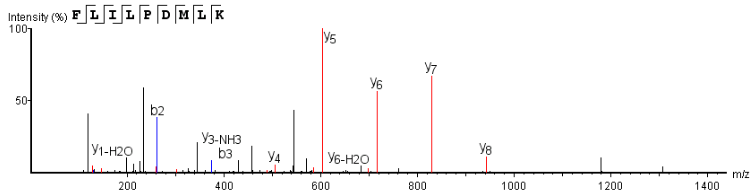

gi571542313

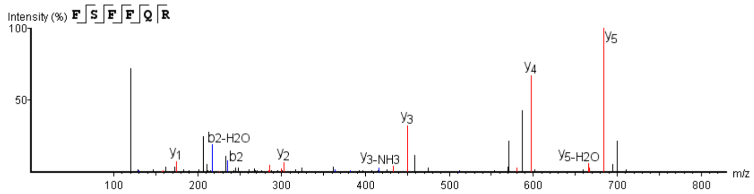

gi571542705

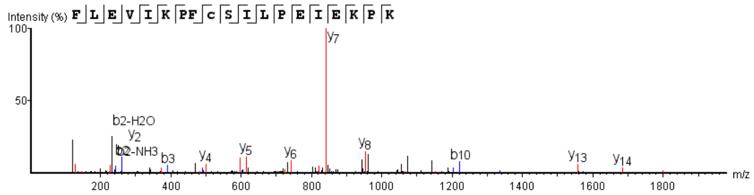

gi571545167

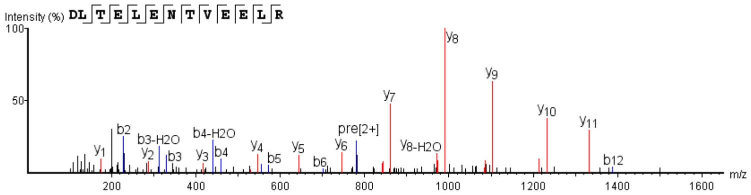

gi571545333

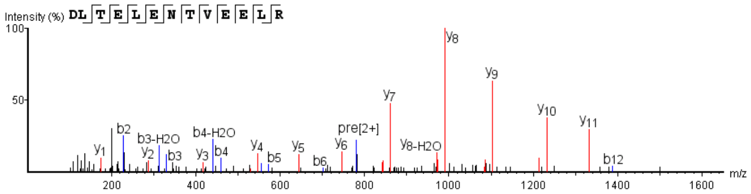

gi571545337

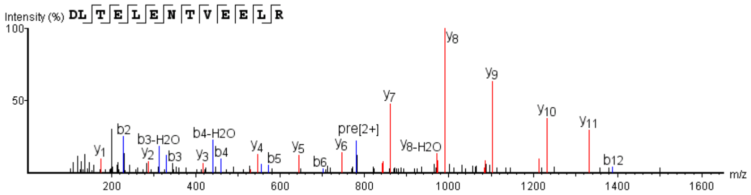

gi571545340

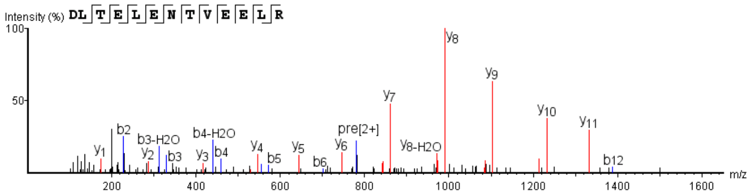

gi571545343

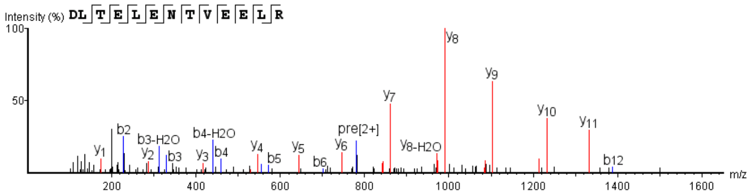

gi571545346

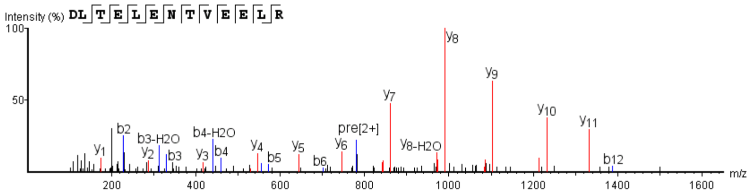

gi571545349

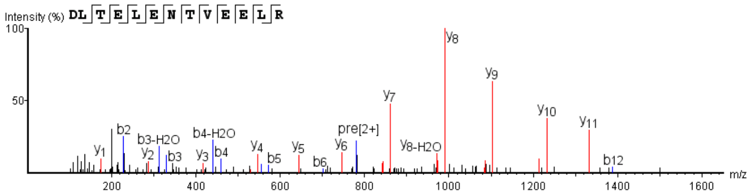

gi571545352

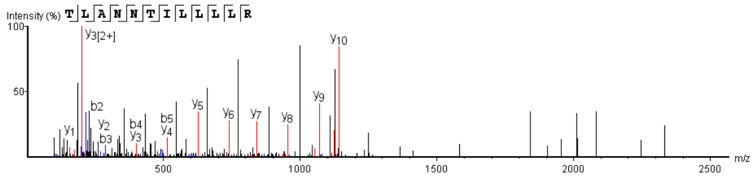

gi571545383

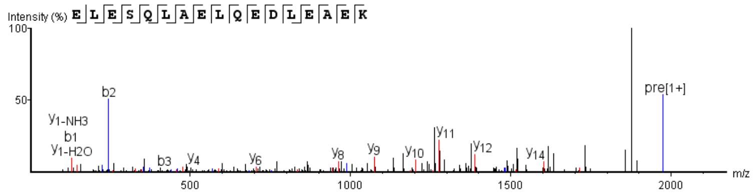

gi571546709

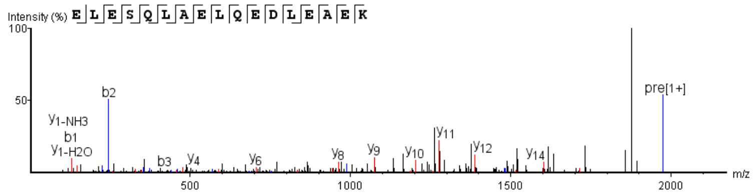

gi571546713

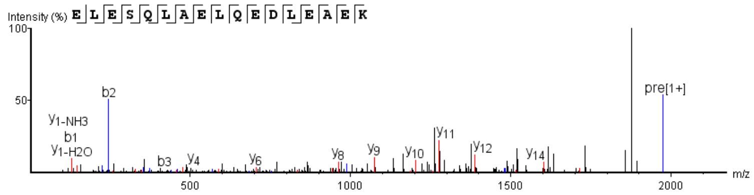

gi571546716

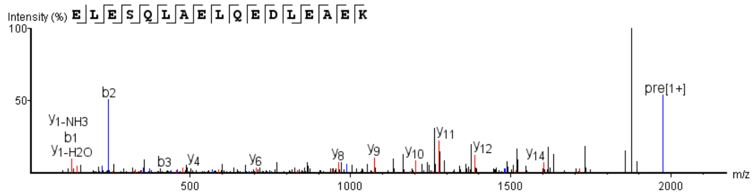

gi571546720

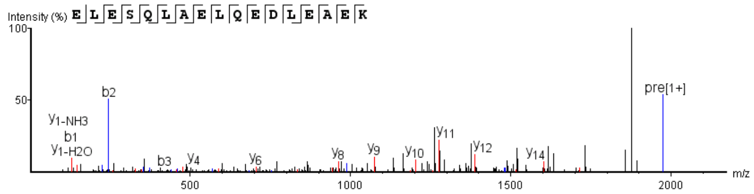

gi571546724

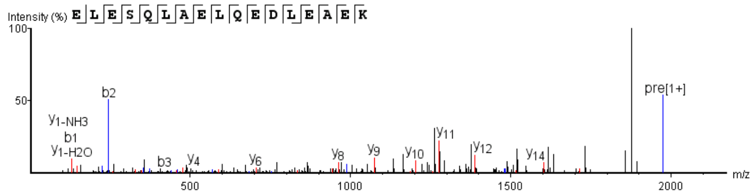

gi571546728

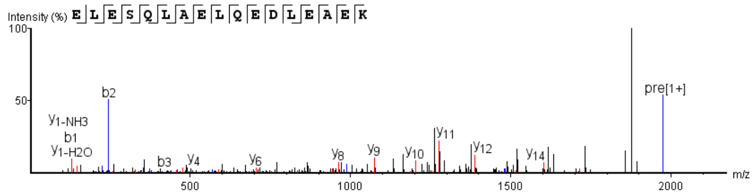

gi571546732

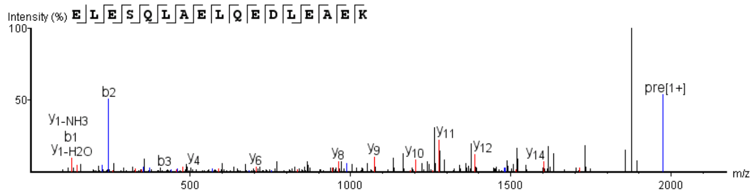

gi571546736

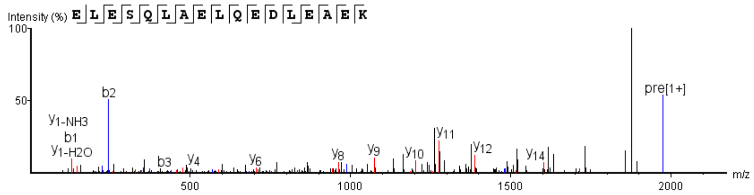

gi571546740

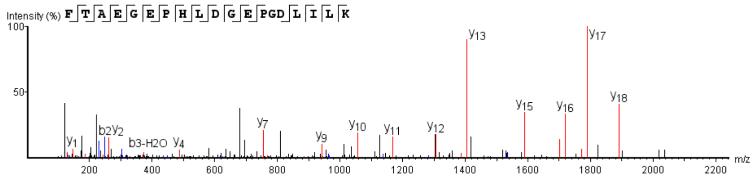

gi571547079

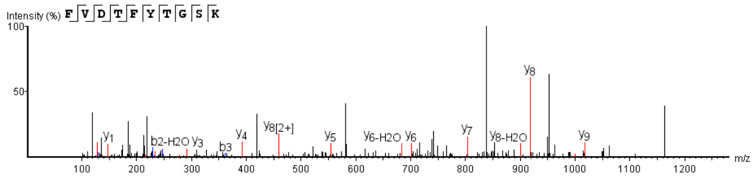

gi571547498

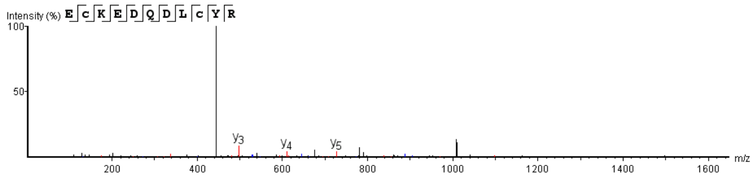

gi571548542

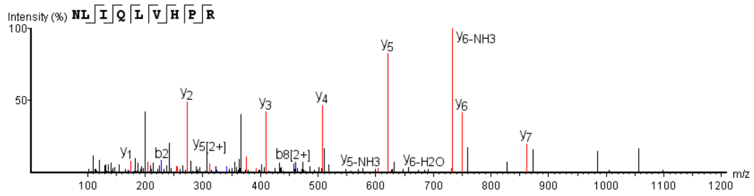

gi571549109

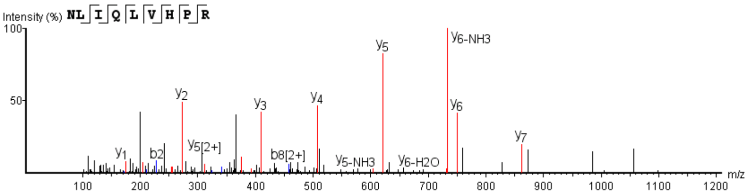

gi571549112

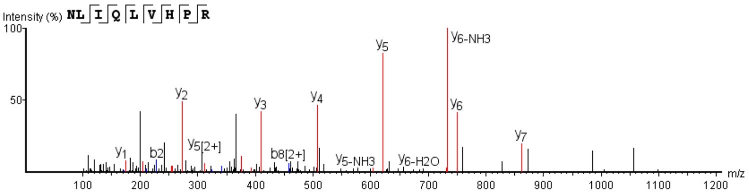

gi571549115

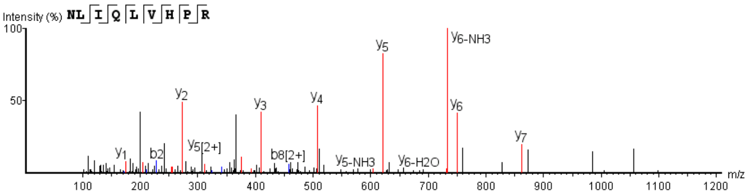

gi571549118

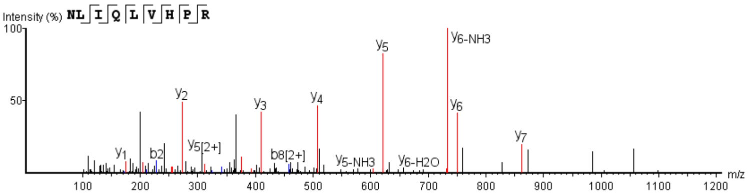

gi571549122

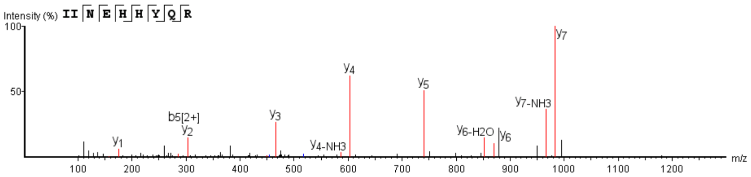

gi571550579

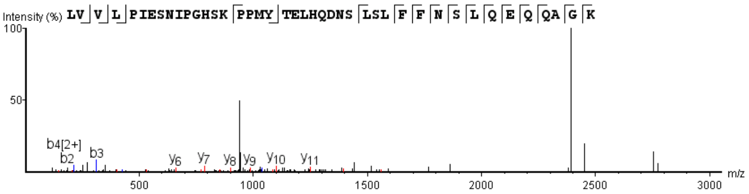

gi571551826

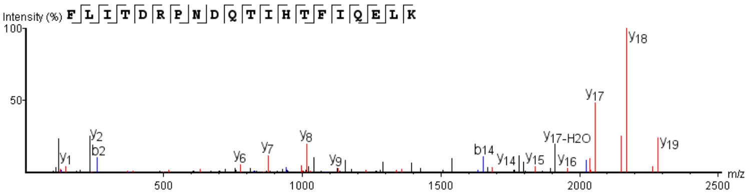

gi571571951

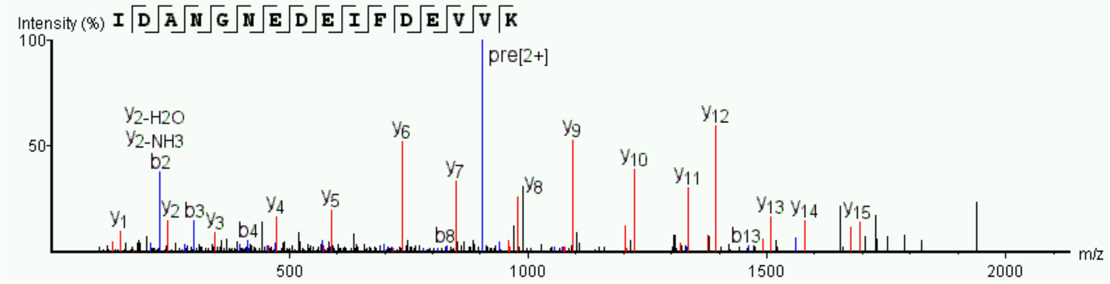

gi328777341

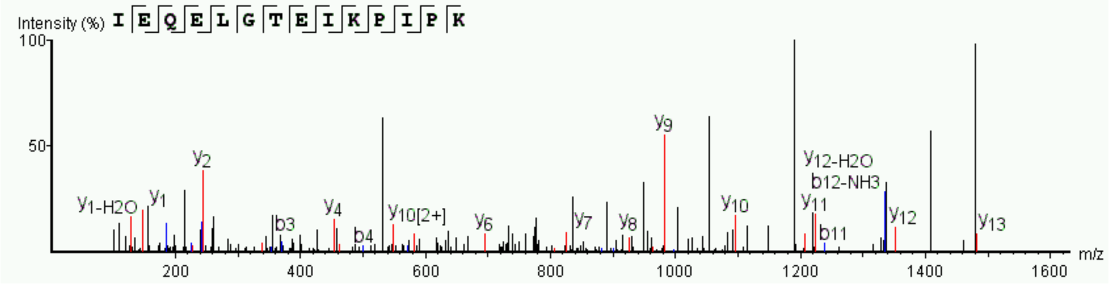

gi571573449

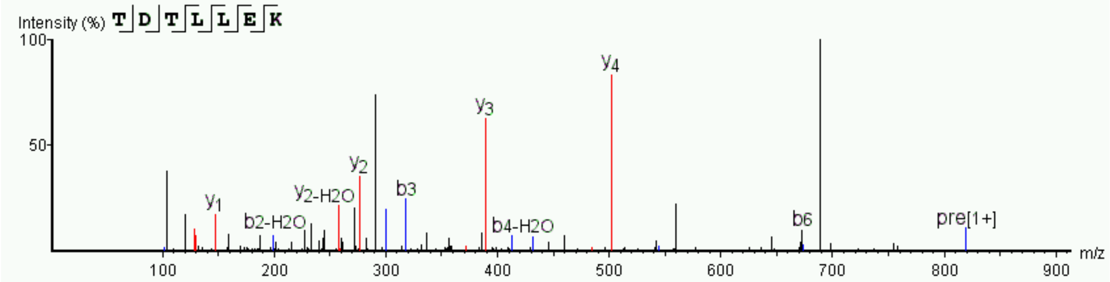

gi328776965

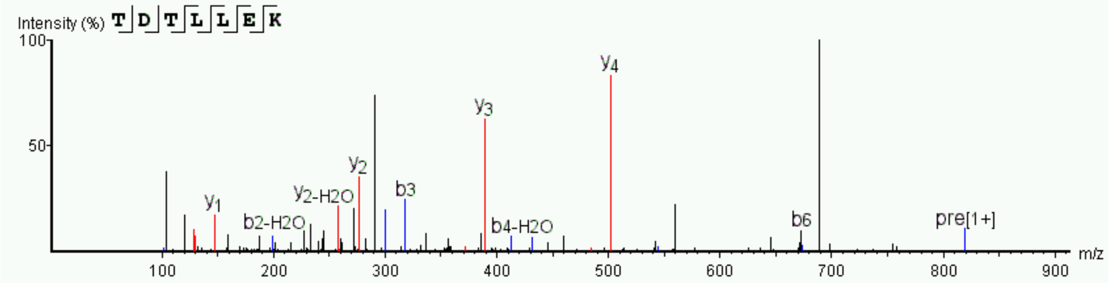

gi571501733

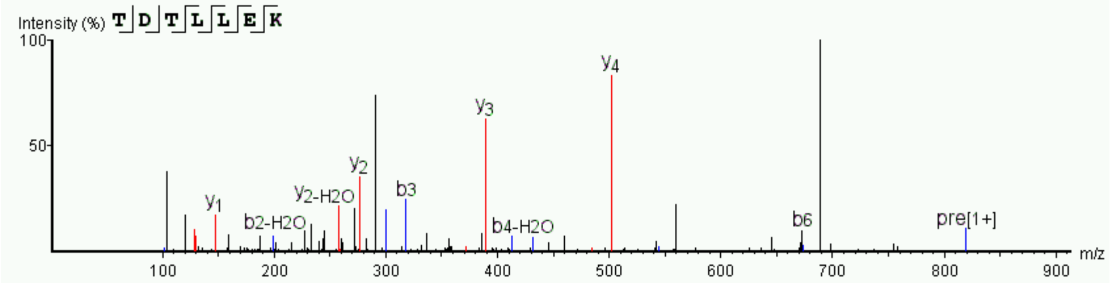

gi66547108

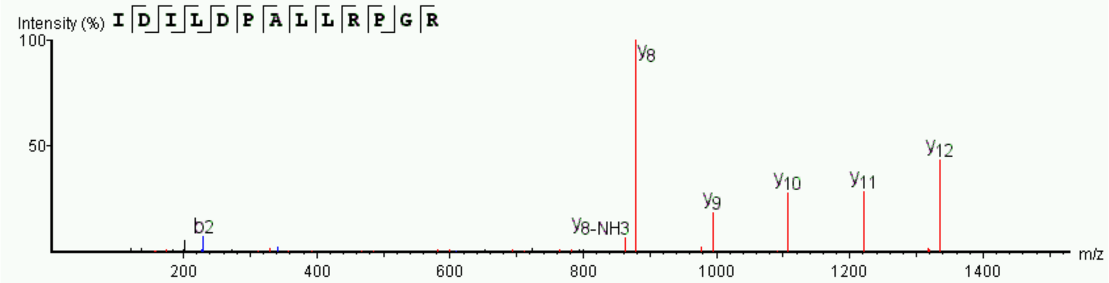

gi571509128

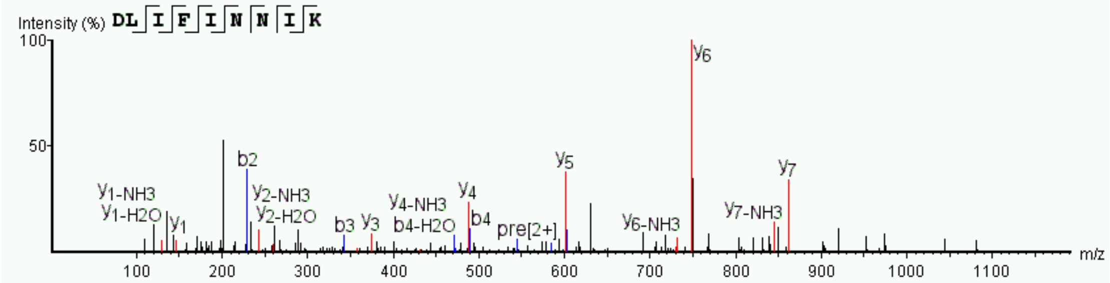

gi328792443

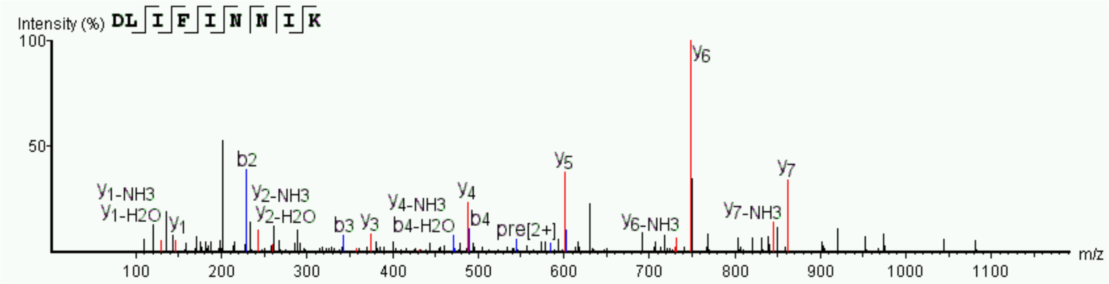

gi571534149

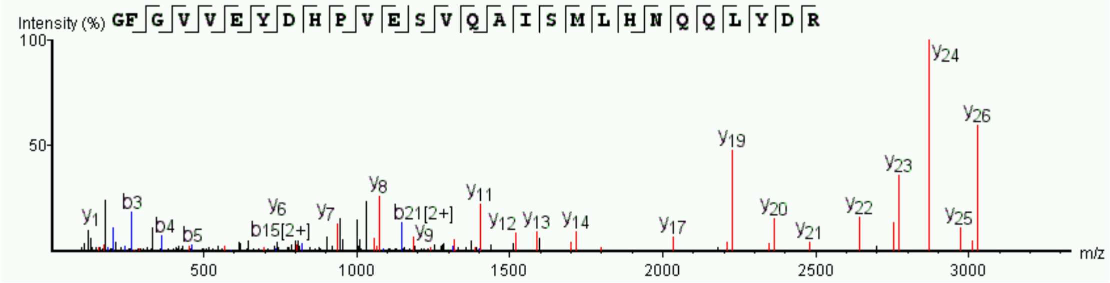

gi571534155

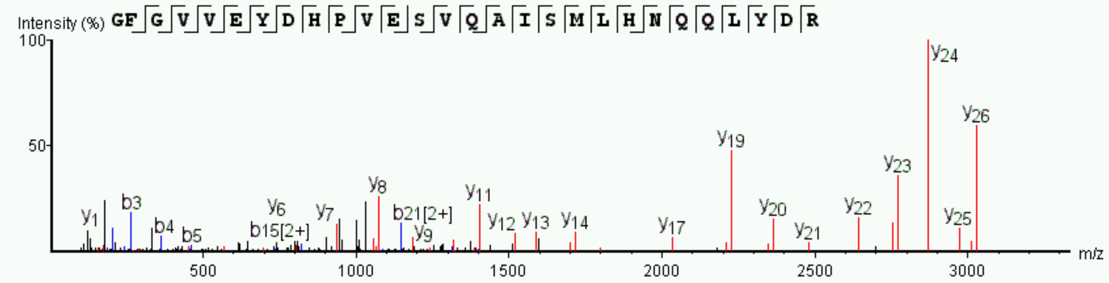

gi328785653

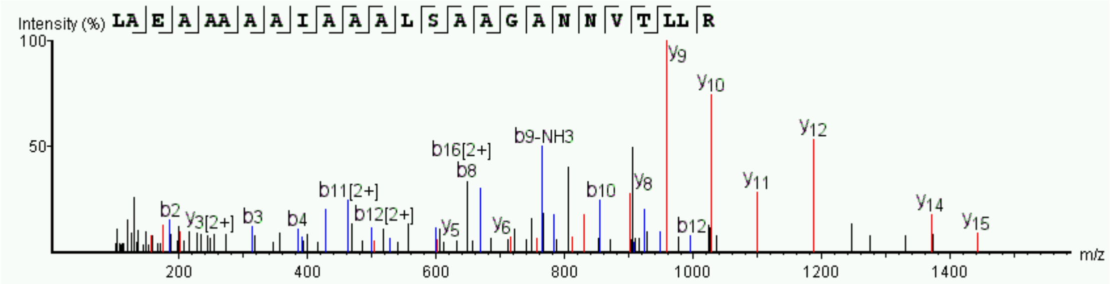

gi571534943

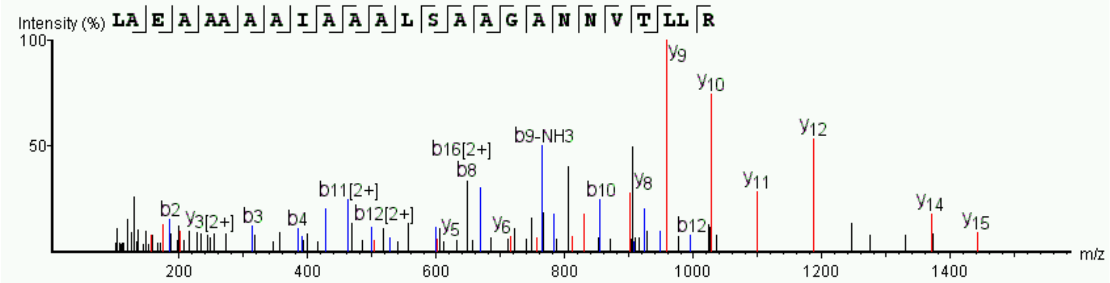

gi571534951

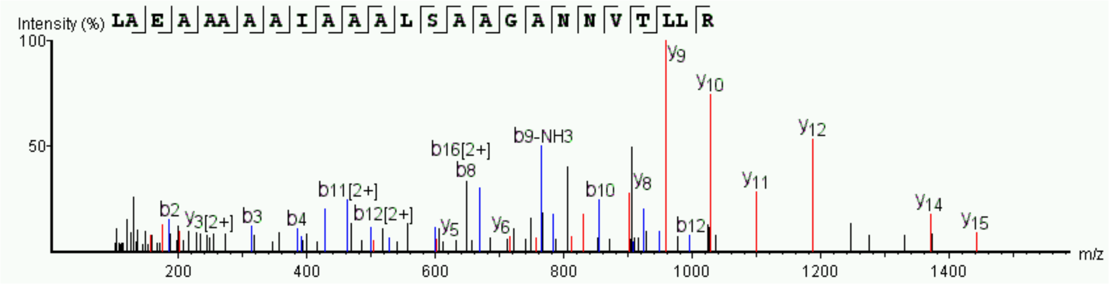

gi571525611

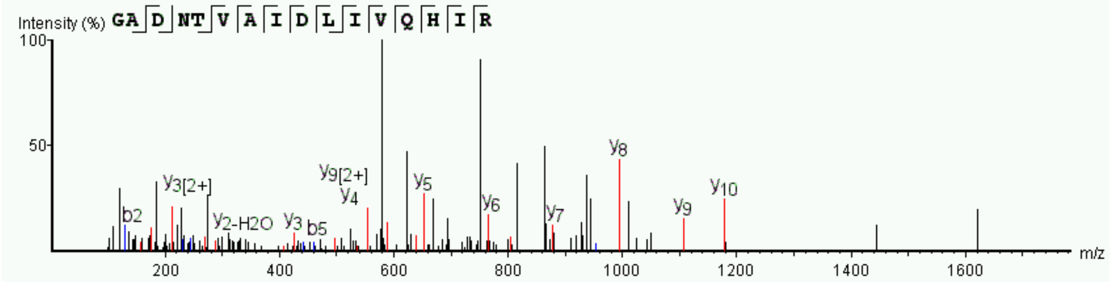

gi110756046

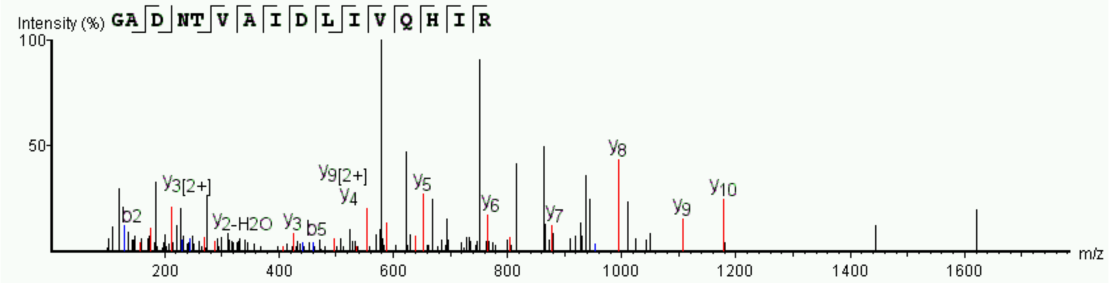

gi571526019

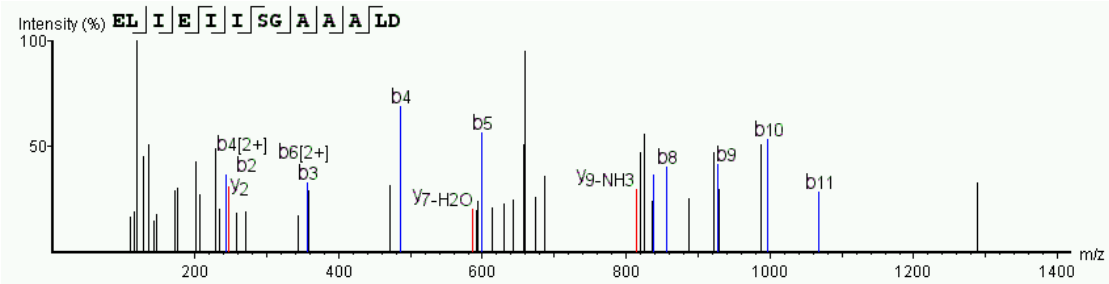

gi571526021

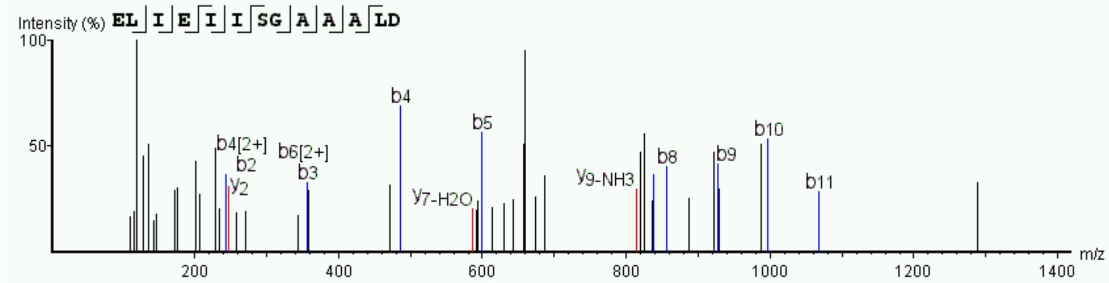

gi571526047

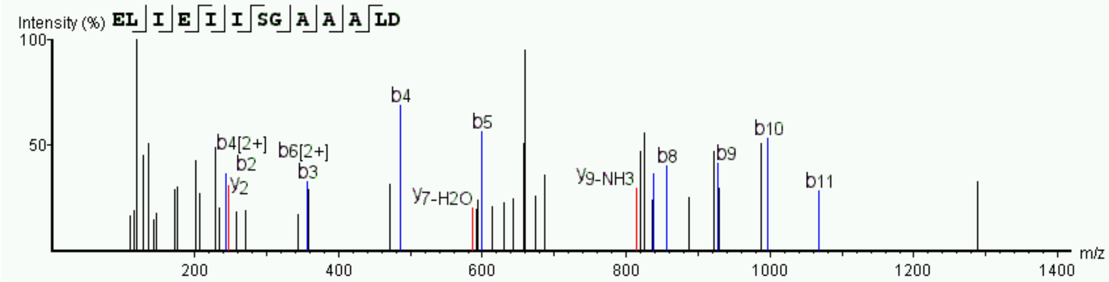

gi571526051

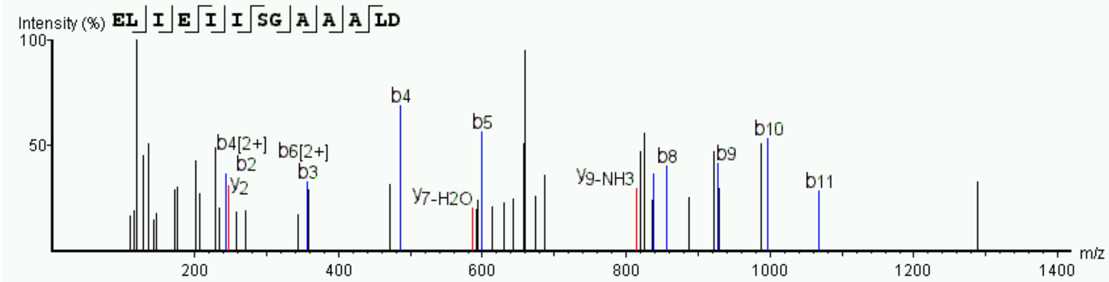

gi571526055

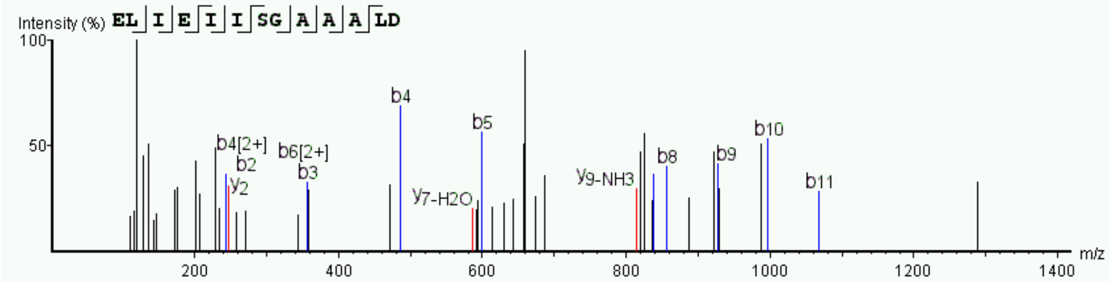

gi328790061

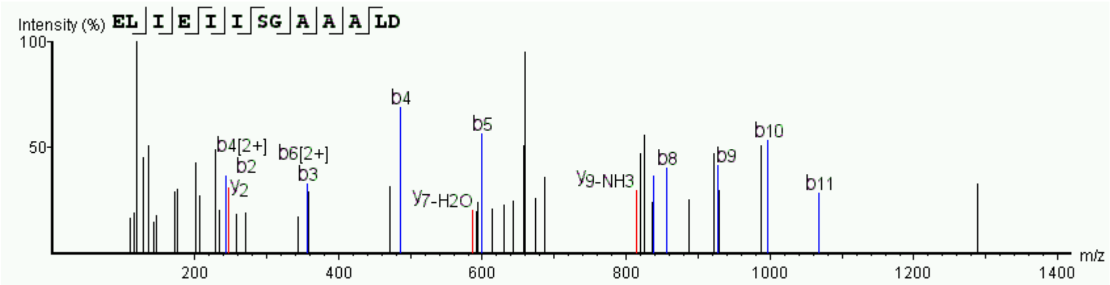

gi571548186

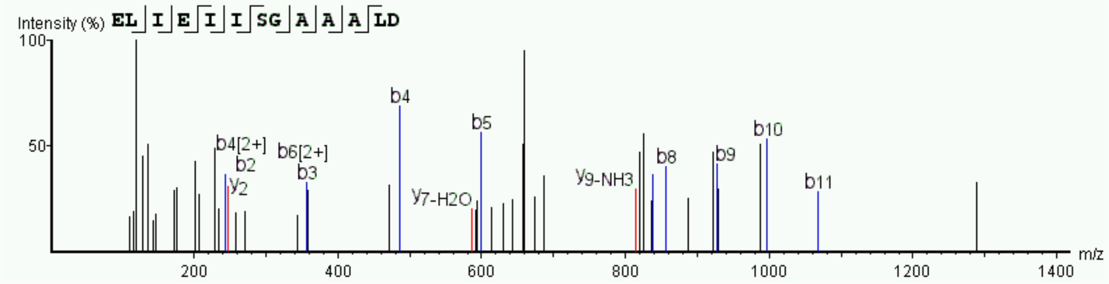

gi571548456

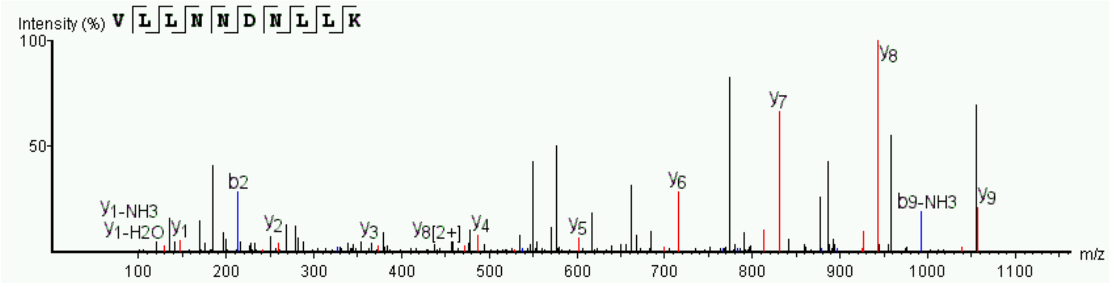

gi328781423

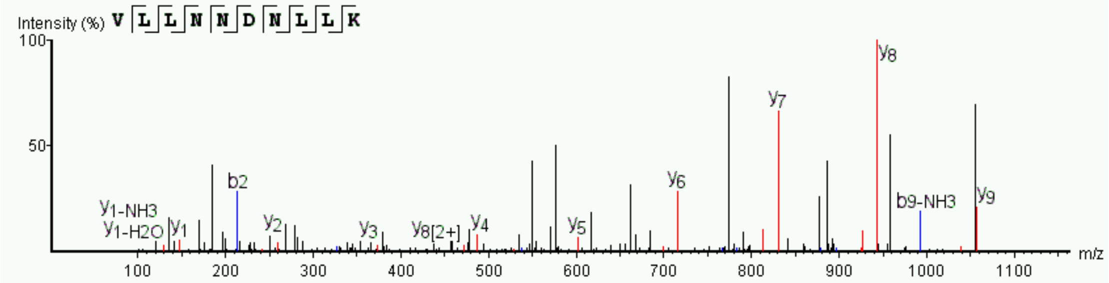

gi571541887

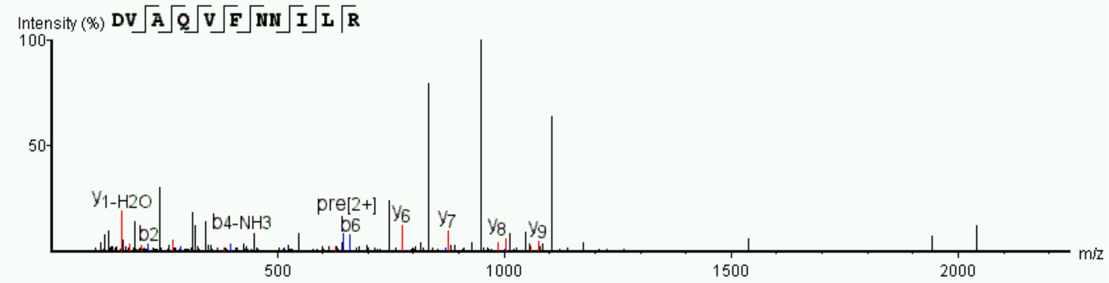

gi571570671

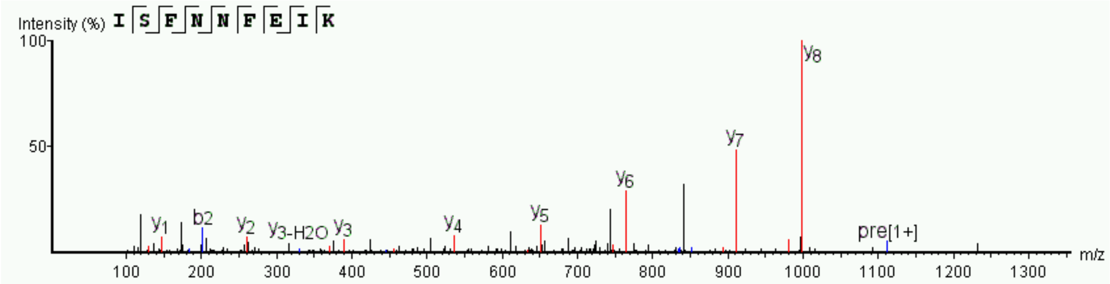

gi66523953

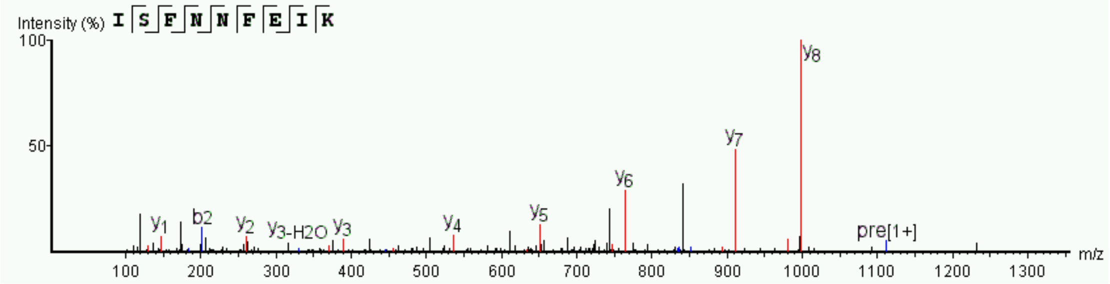

gi571525869

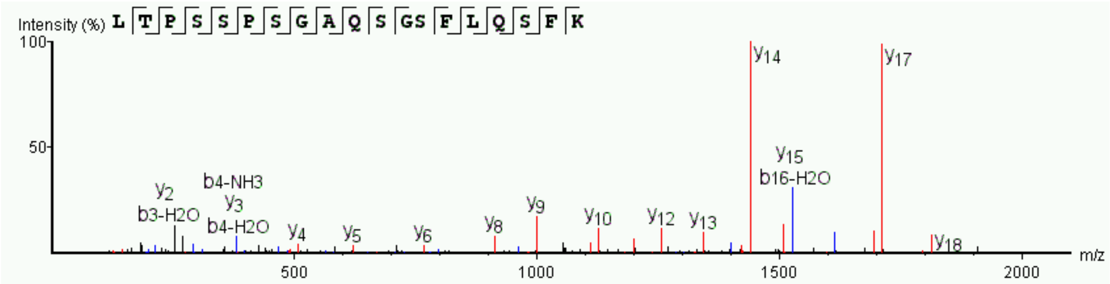

gi571525842

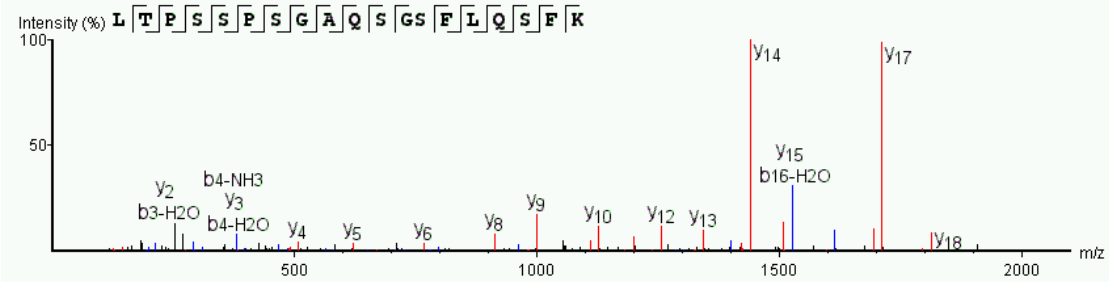

gi571525846

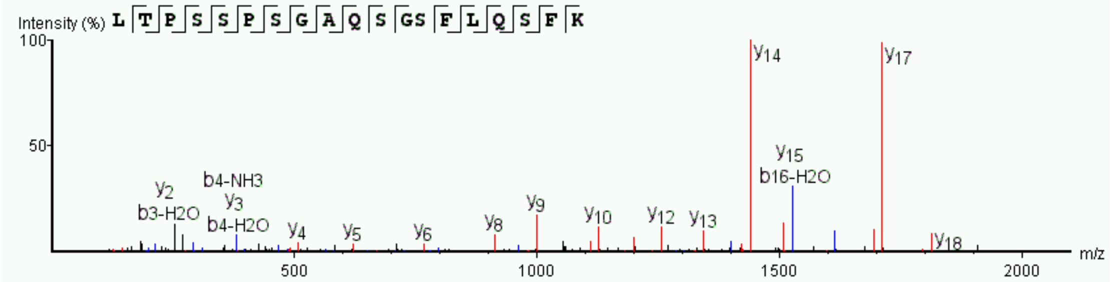

gi571525849

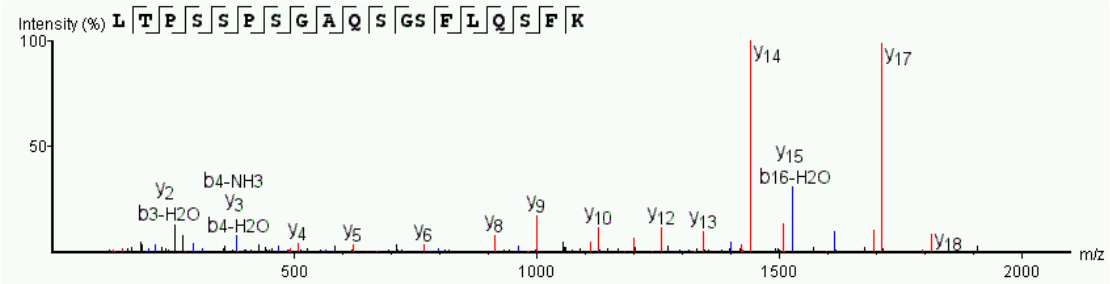

gi571525852

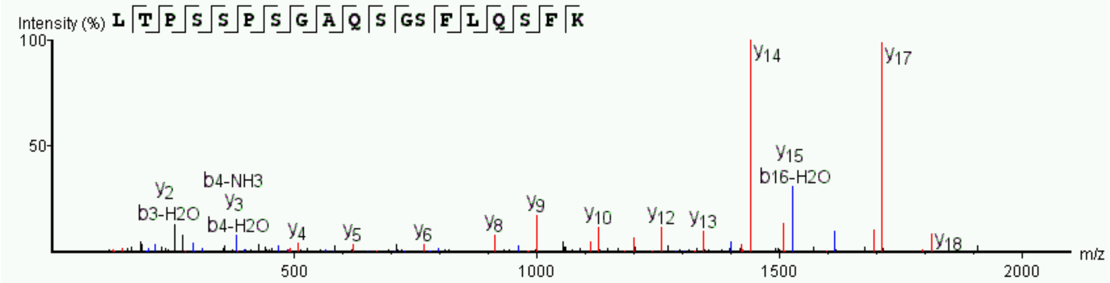

gi571525855

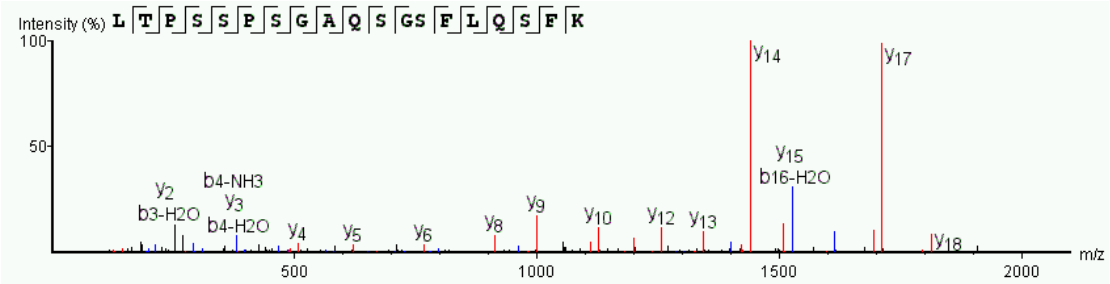

gi571525858

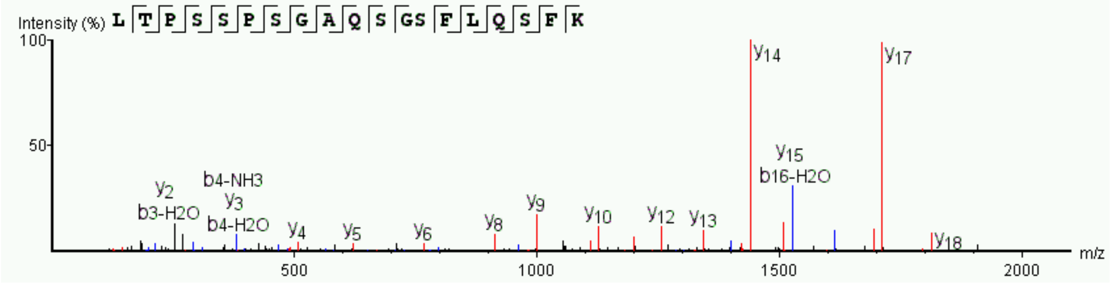

gi571525861

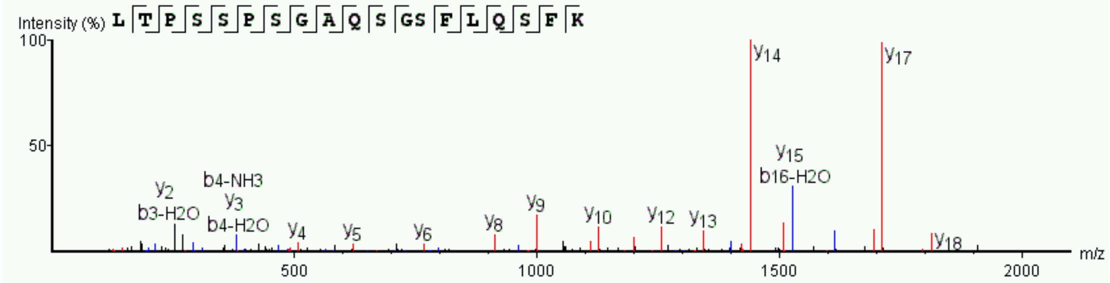

gi571525866

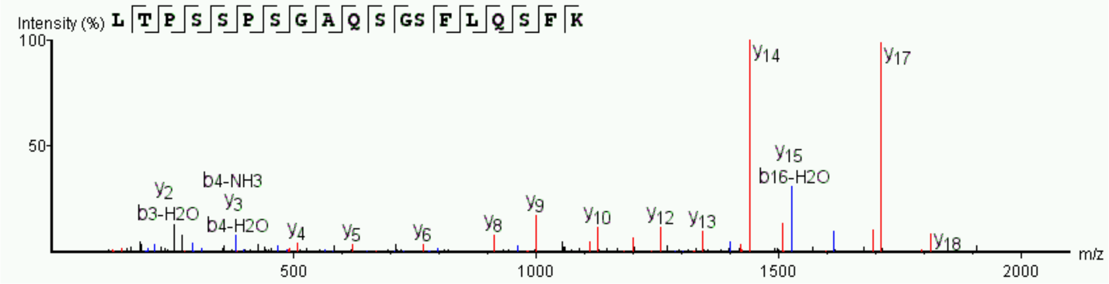

gi571508663

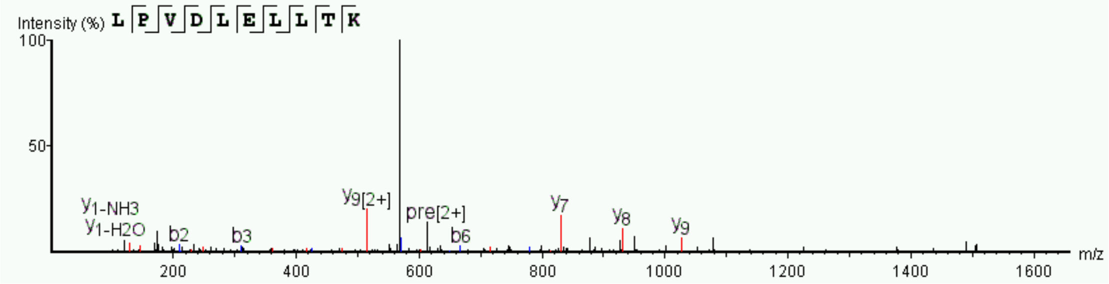

gi571552022

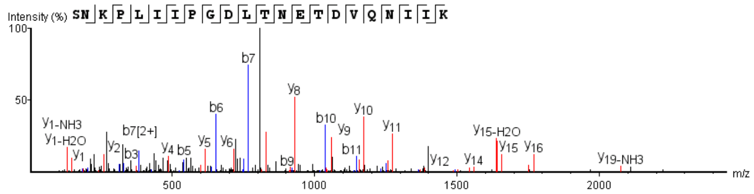

gi571552435

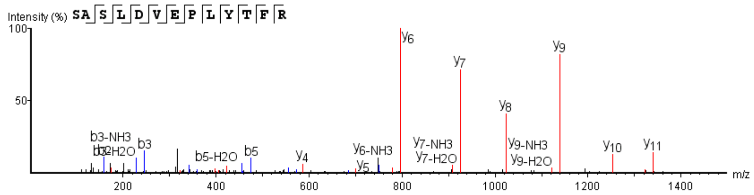

gi571552849

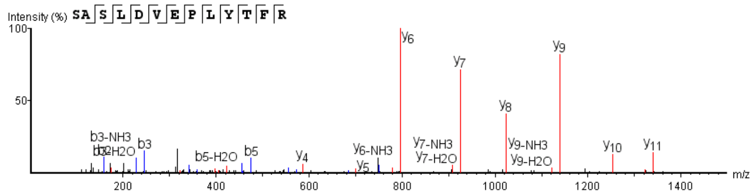

gi571552867

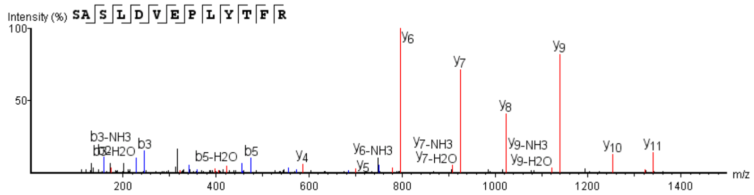

gi571552871

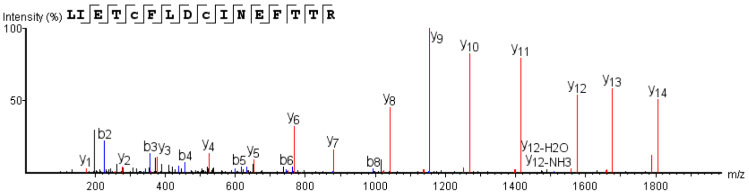

gi571553163

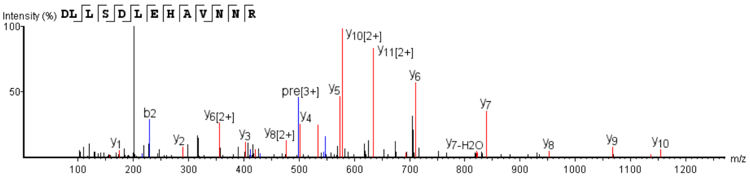

gi571553208

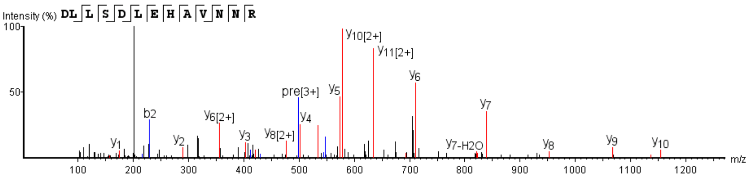

gi571553212

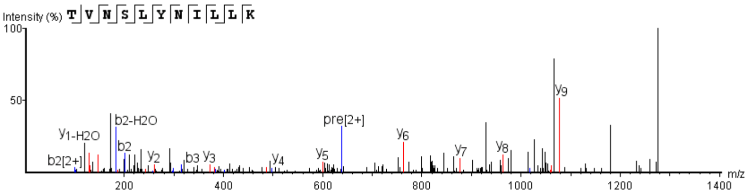

gi571554543

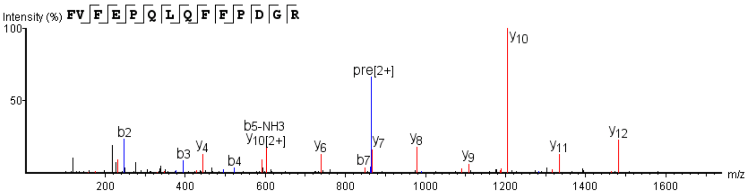

gi571555562

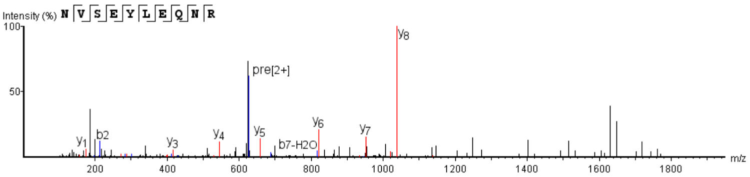

gi571556092

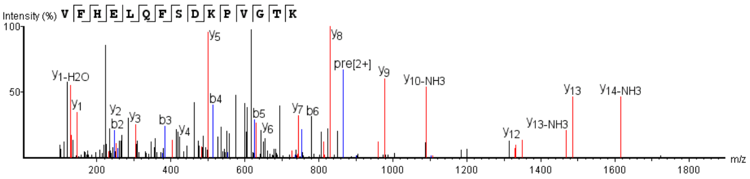

gi571558139

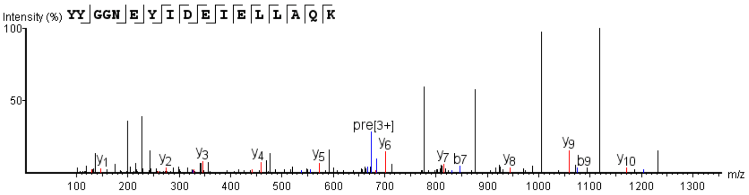

gi571558147

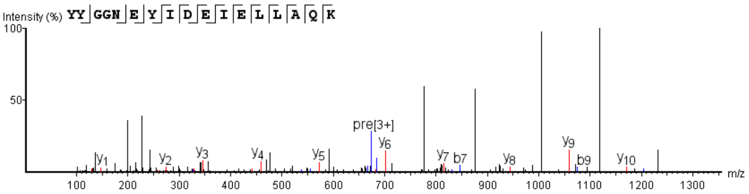

gi571558150

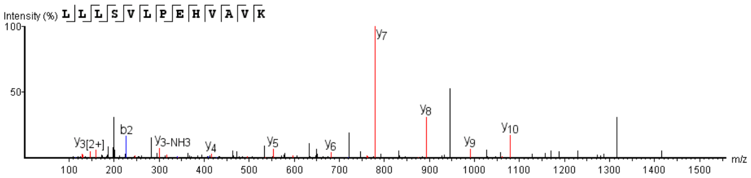

gi571559065

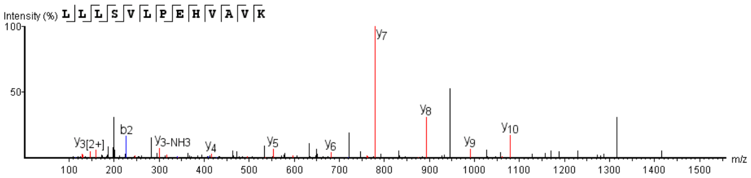

gi571559068

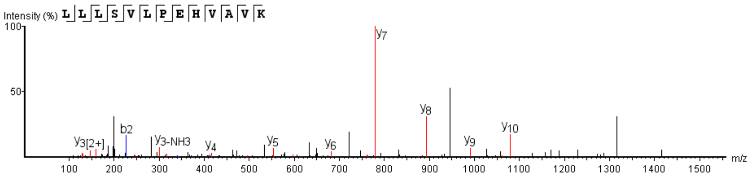

gi571559072

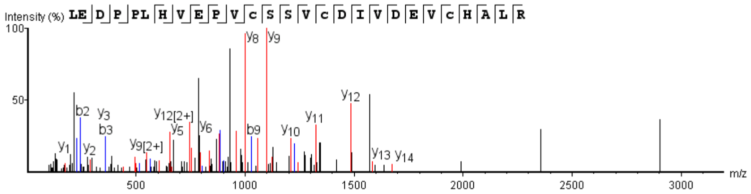

gi571559774

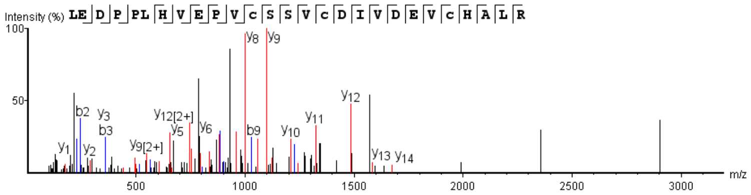

gi571559781

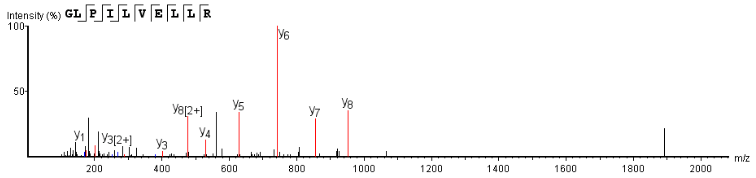

gi571560469

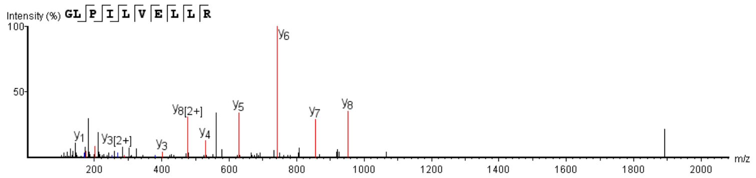

gi571560472

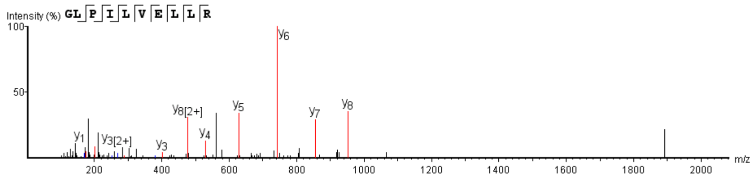

gi571560476

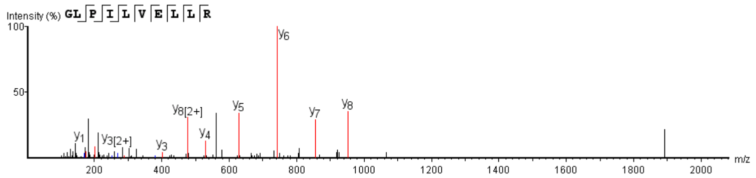

gi571560479

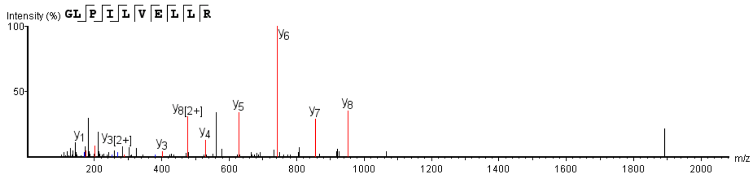

gi571560482

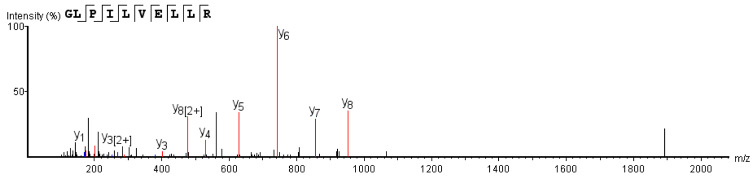

gi571560485

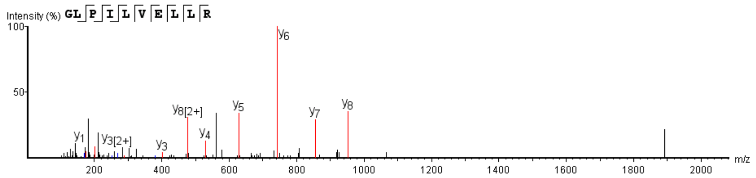

gi571560489

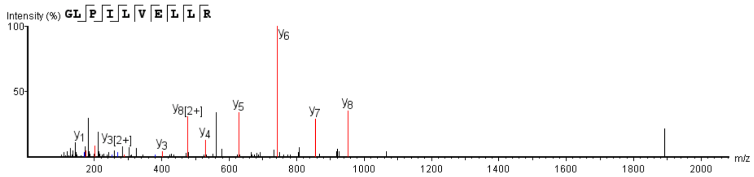

gi571560493

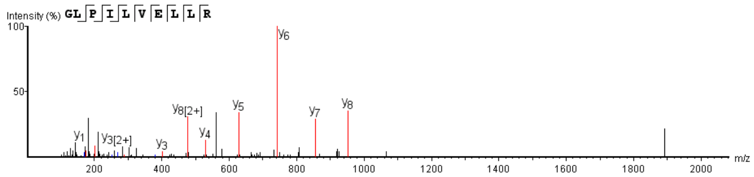

gi571560498

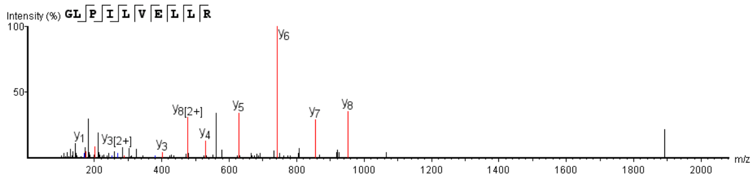

gi571560501

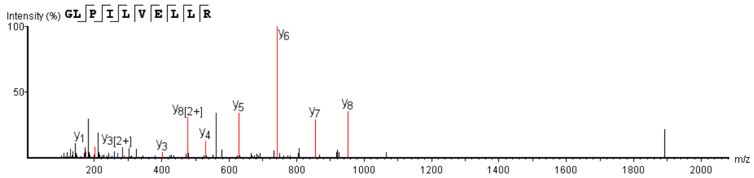

gi571560504

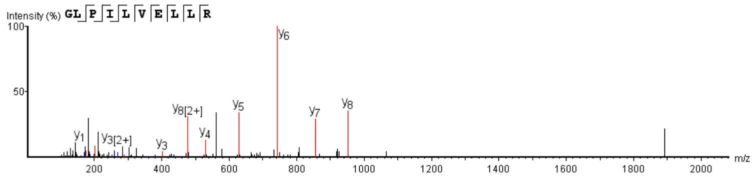

gi571560507

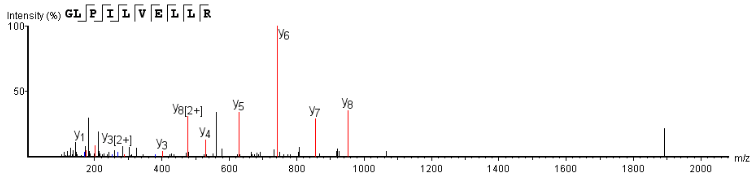

gi571560511

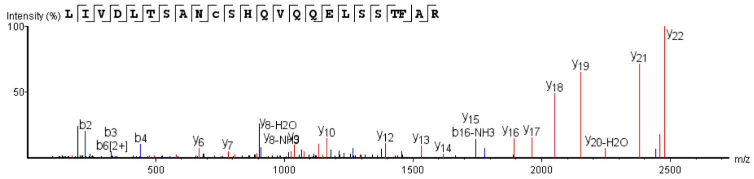

gi571560773

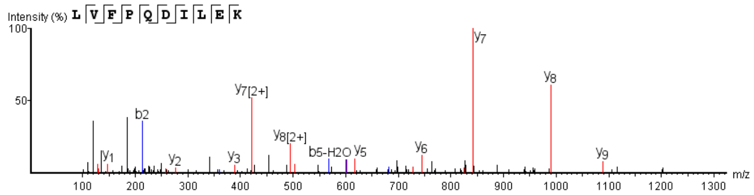

gi571562354

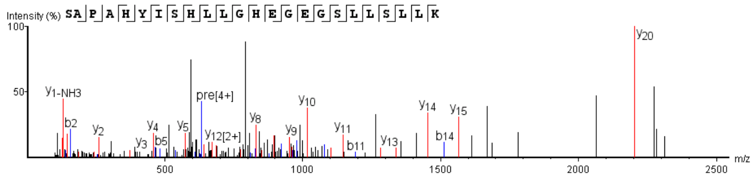

gi571563394

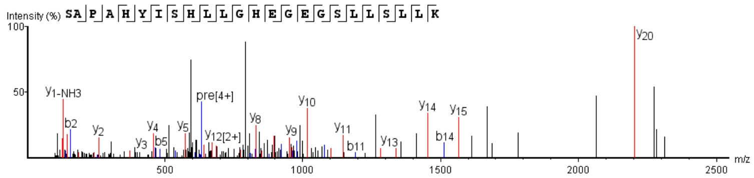

gi571563397

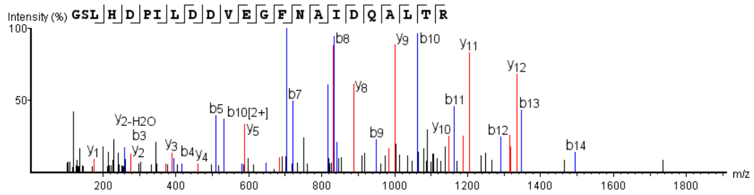

gi571563508

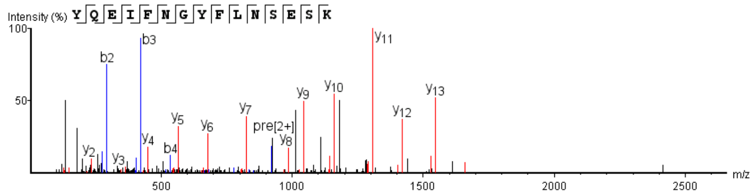

gi571564004

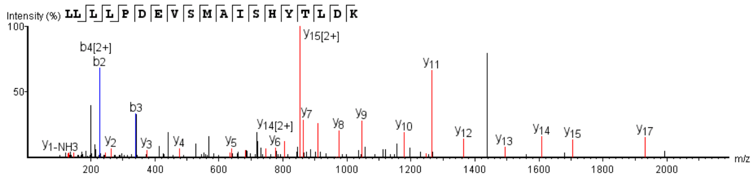

gi571564978

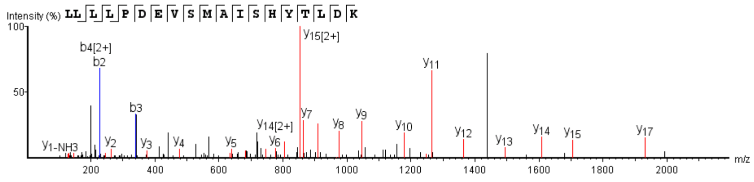

gi571564981

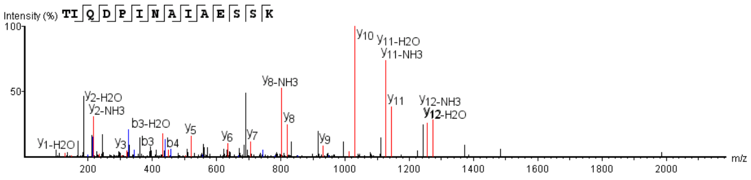

gi571565123

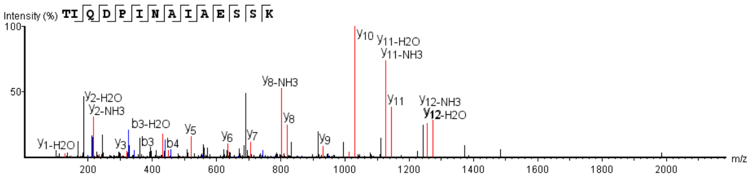

gi571565126

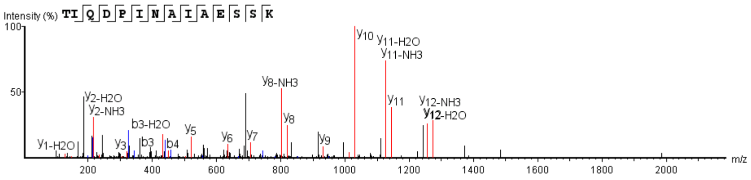

gi571565430

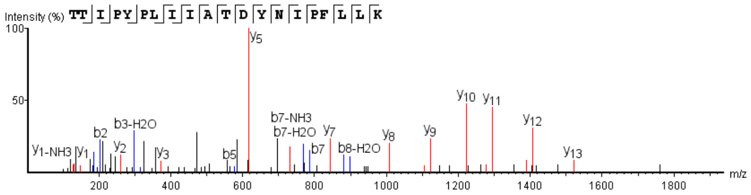

gi571566599

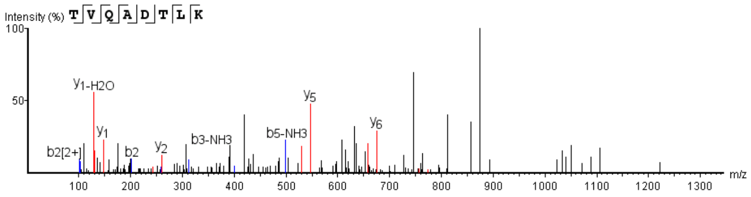

gi571568566

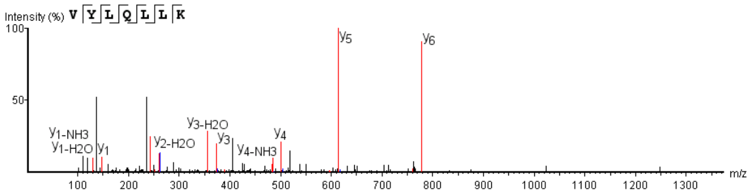

gi571569497

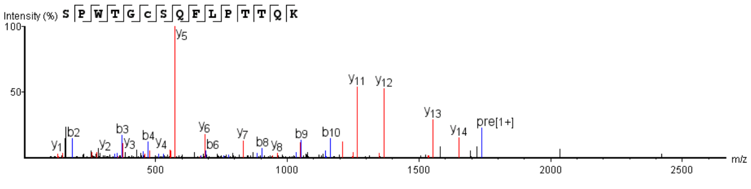

gi571569633

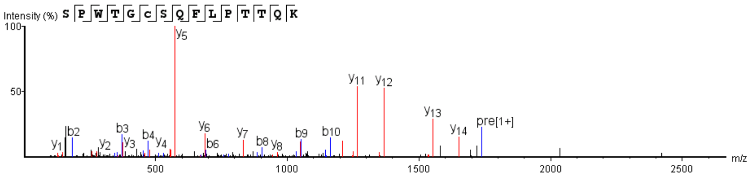

gi571569639

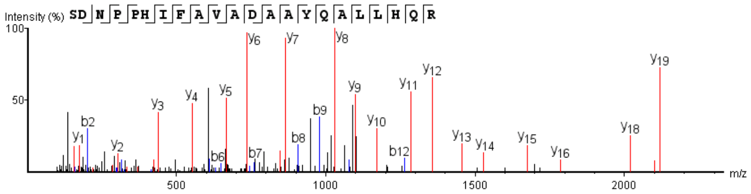

gi571570187

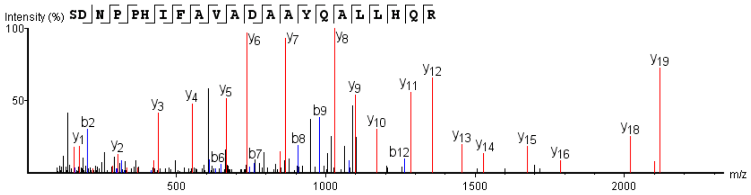

gi571570190

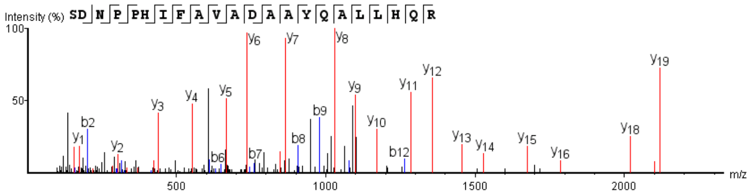

gi571570192

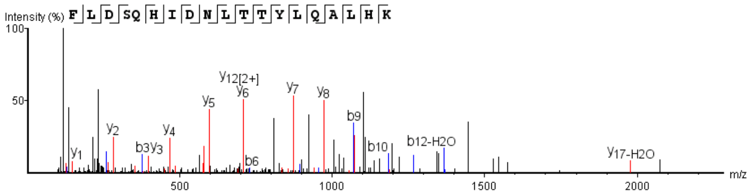

gi571571071

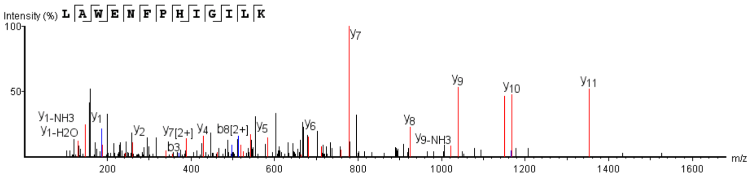

gi571572159

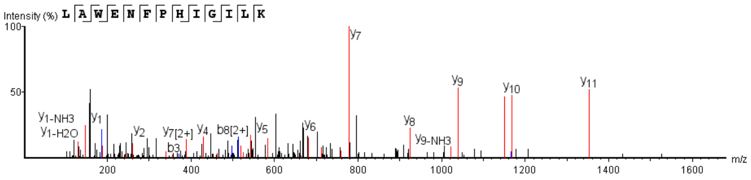

gi571572163

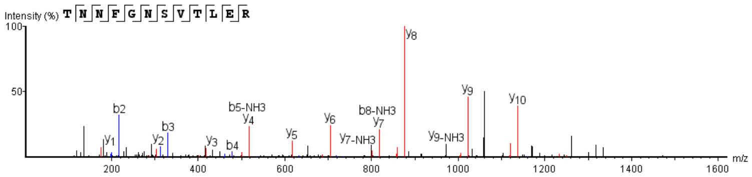

gi571573002

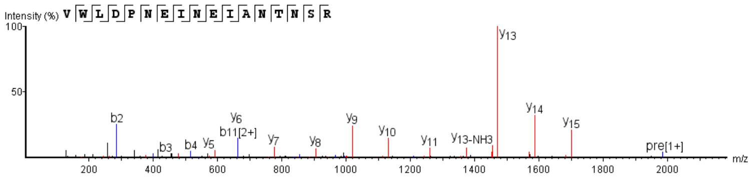

gi571573543

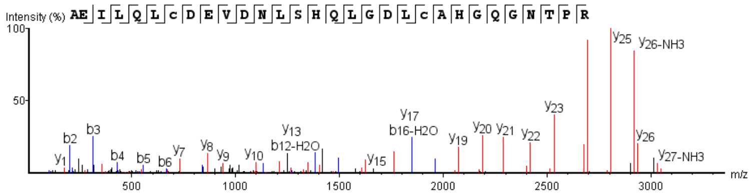

gi571573648

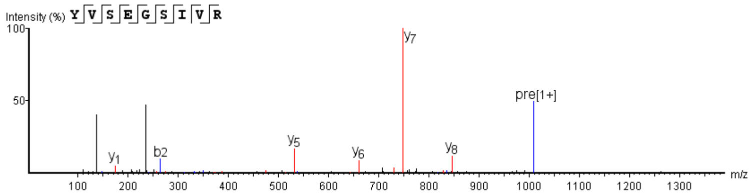

gi571573722

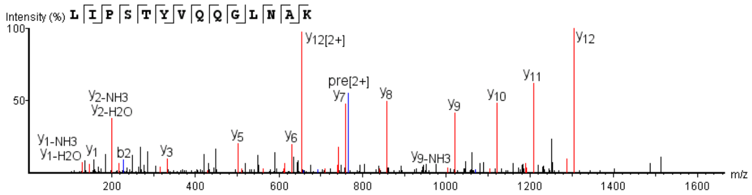

gi571573896

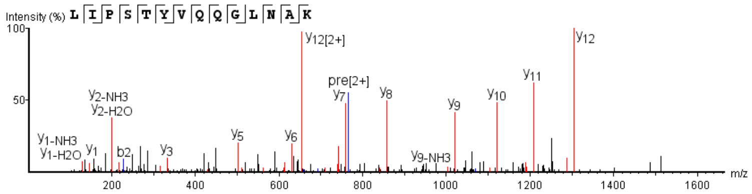

gi571573898

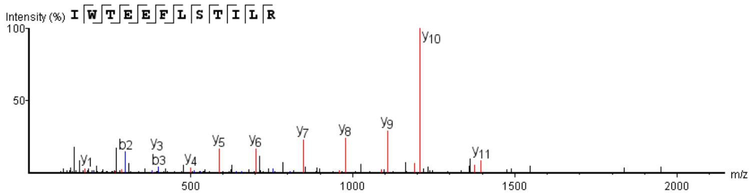

gi571574298

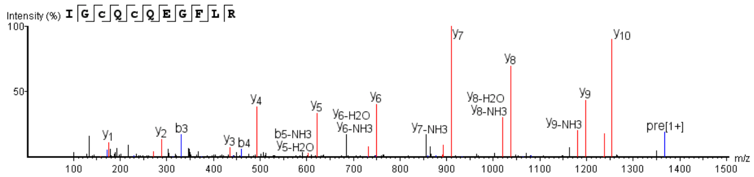

gi571574384

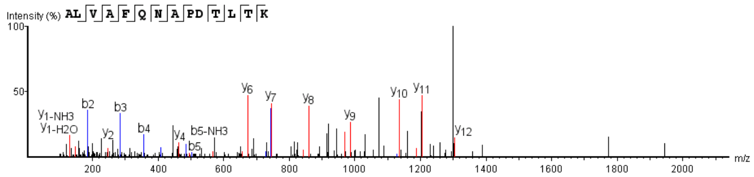

gi571574442

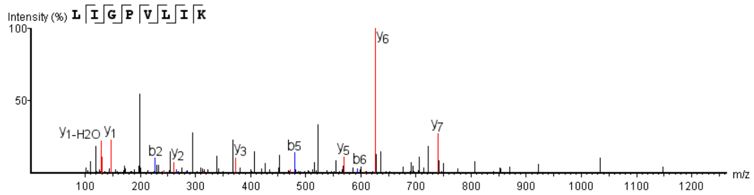

gi571574771

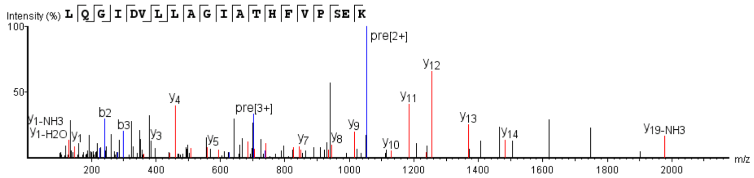

gi571575116

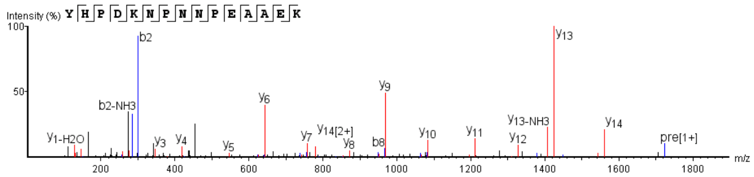

gi571575329

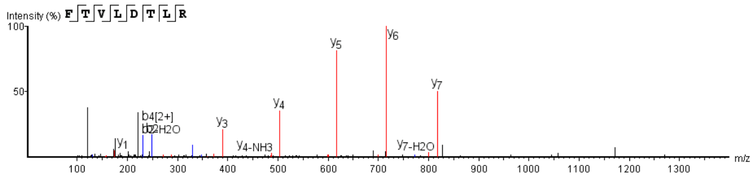

gi571575497

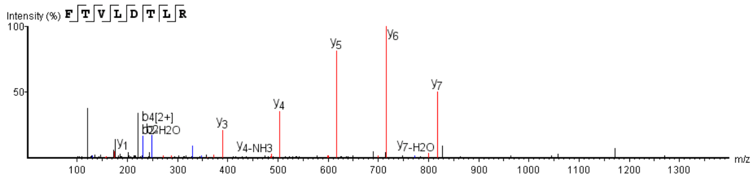

gi571575499

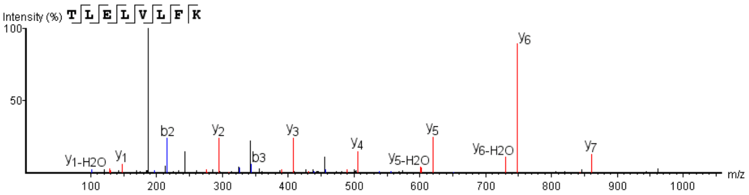

gi571575529

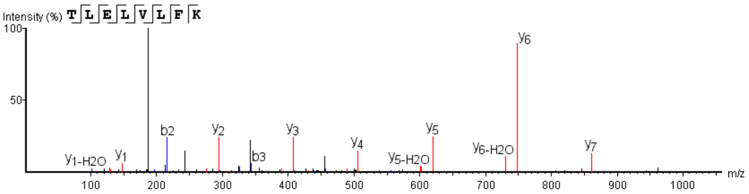

gi571575531

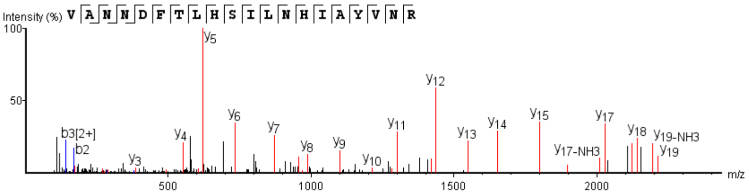

gi571575582

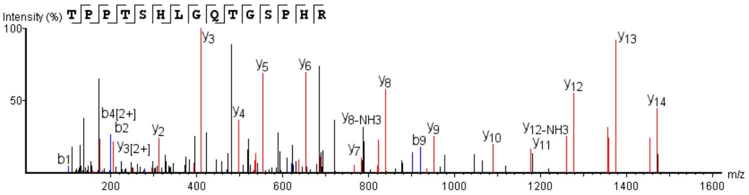

gi571575635

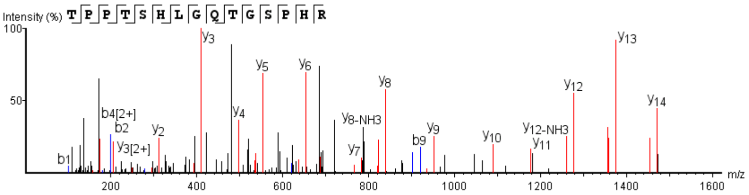

gi571575637

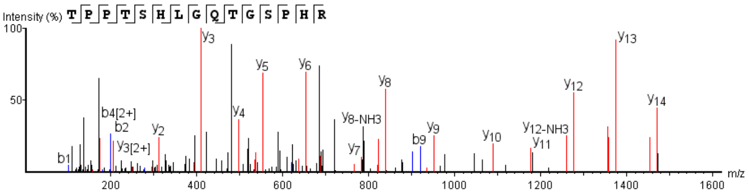

gi571575639

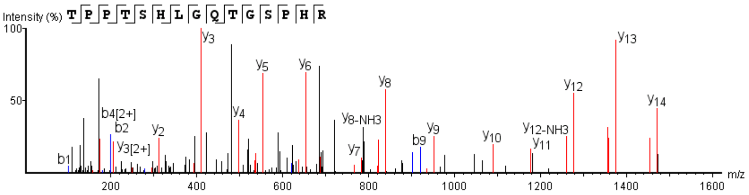

gi571575641

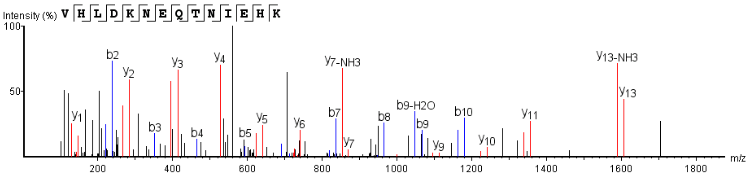

gi571576123

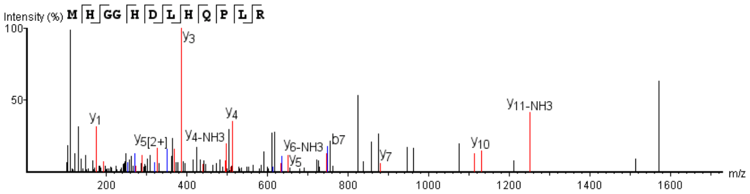

gi571576165

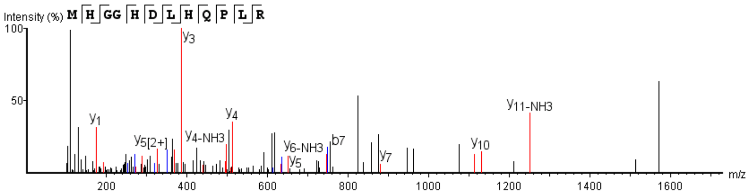

gi571576167

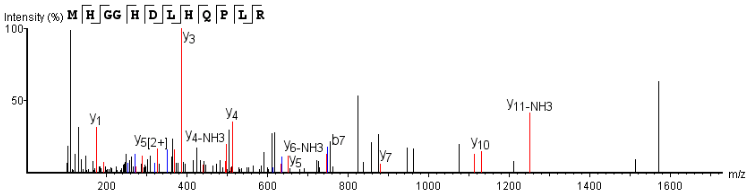

gi571576169

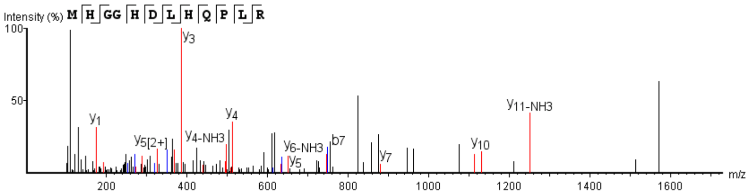

gi571576171

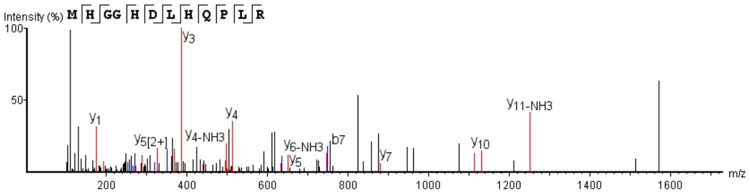

gi571576173

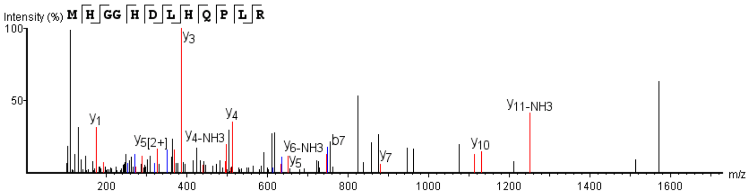

gi571576175

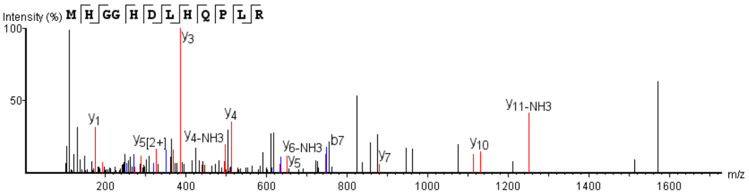

gi571576177

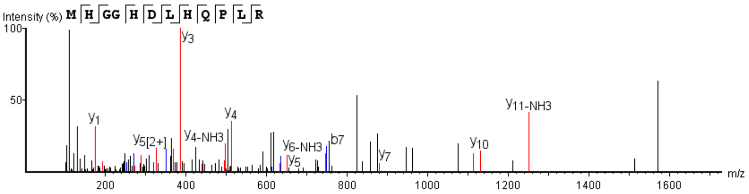

gi571576179

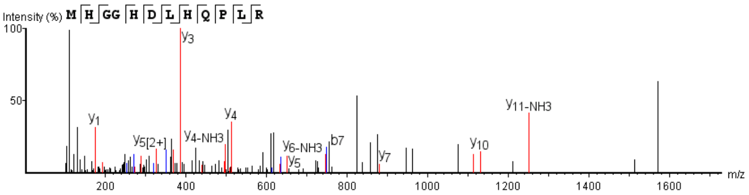

gi571576181

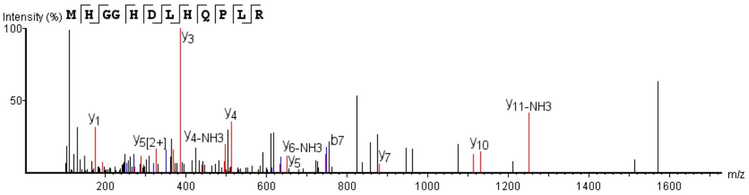

gi571576183

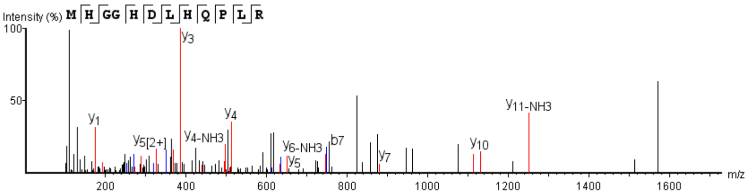

gi571576185

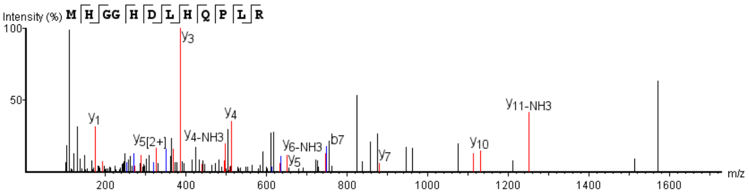

gi571576187

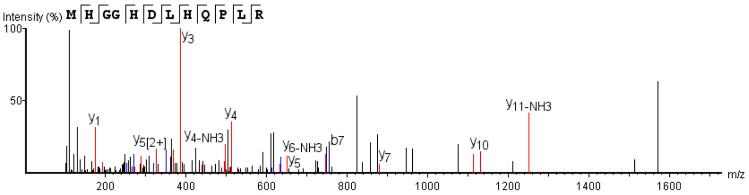

gi571576189

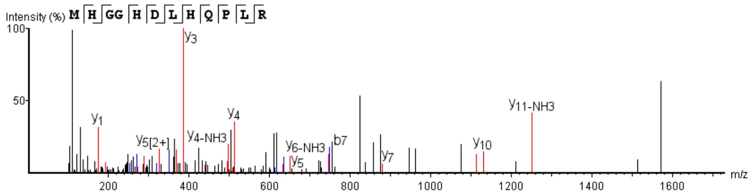

gi571576191

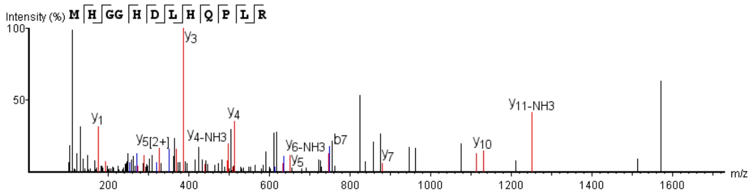

gi571576193

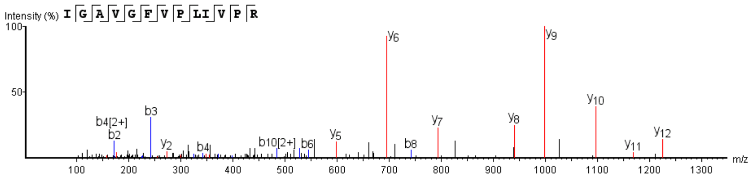

gi571576551

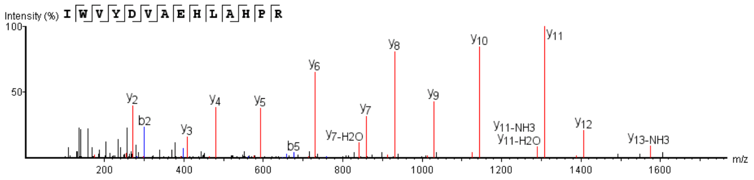

gi571576661

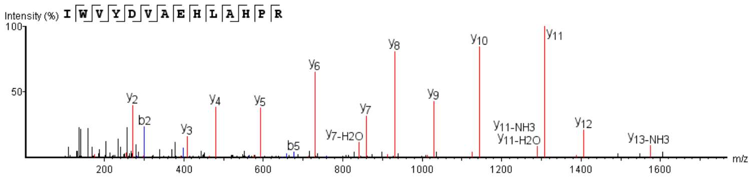

gi571576665

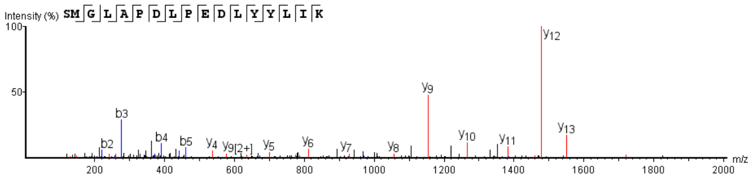

gi571577109

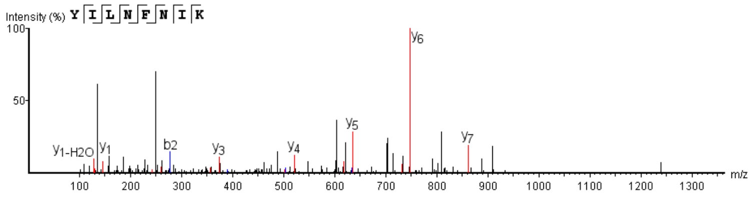

gi571577865

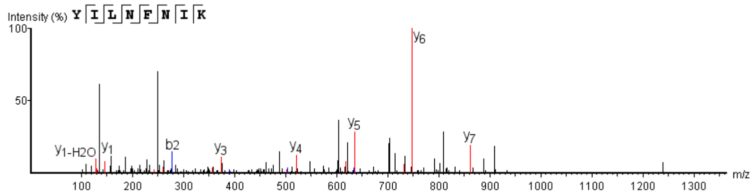

gi571577867

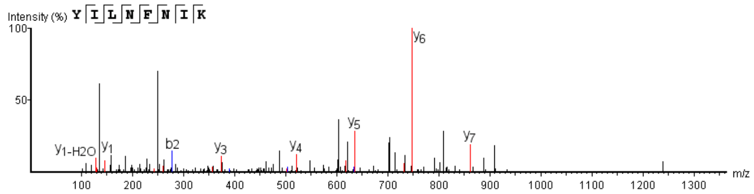

gi571577869

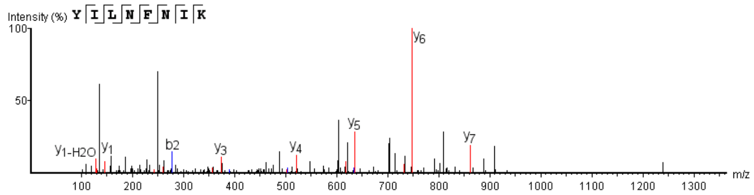

gi571577871

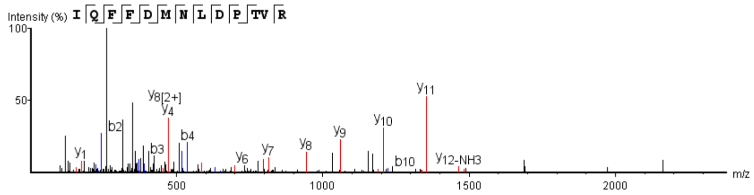

gi571577901

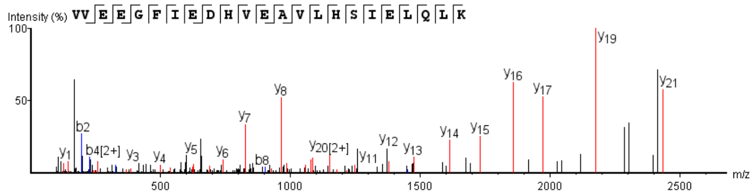

gi571577978

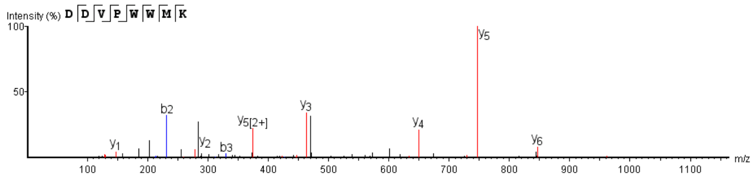

gi571577993

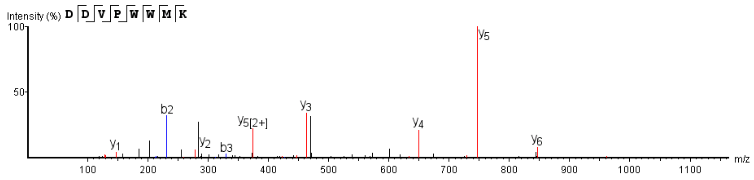

gi571577995

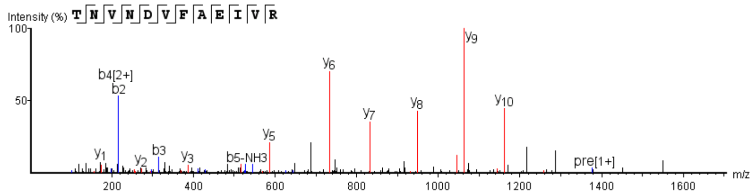

gi571578152

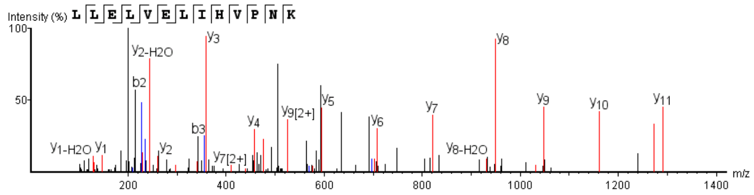

gi571578527

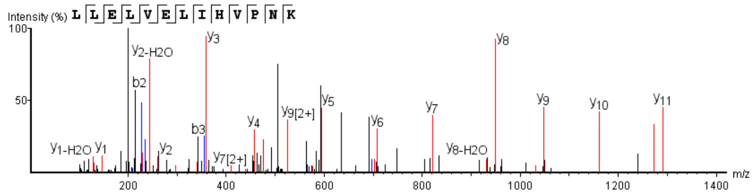

gi571578529

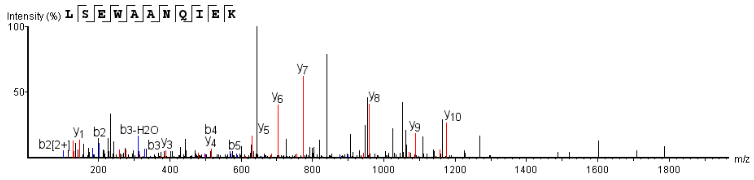

gi48102403

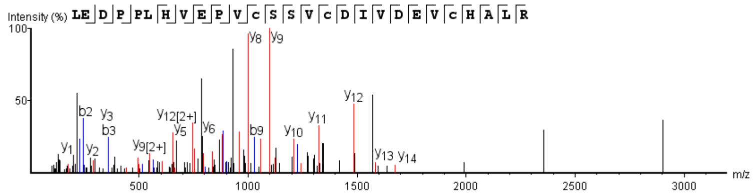

gi328786818

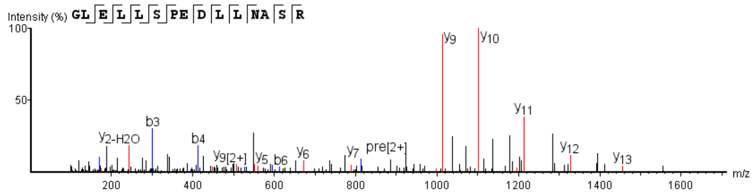

Annotated spectra of single-peptide identifications of proteins in optical bodies of *Apis cerana*

gi|571575116

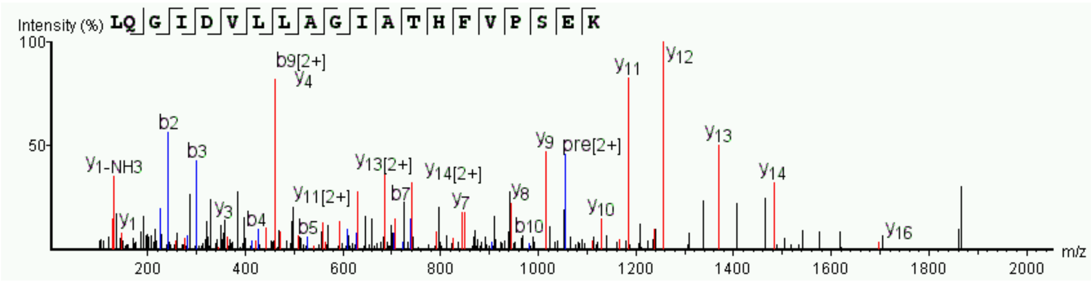

gi|571575119

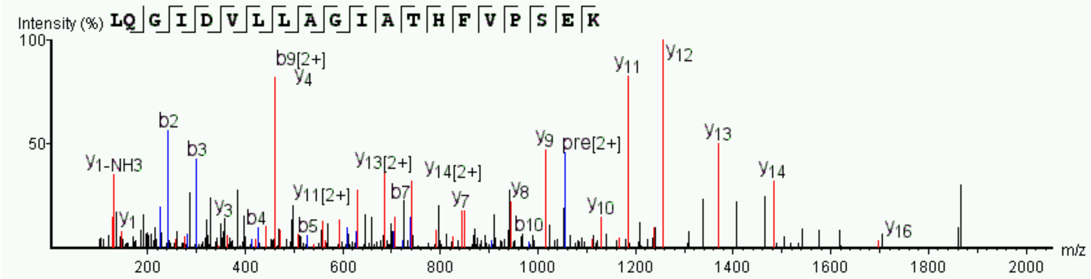

gi|328783479

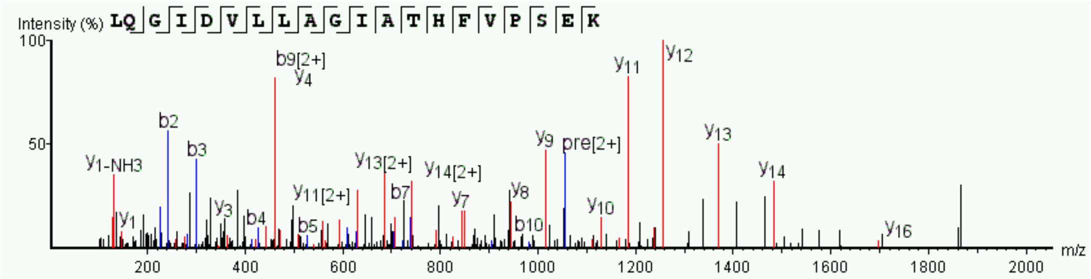

gi|571541951

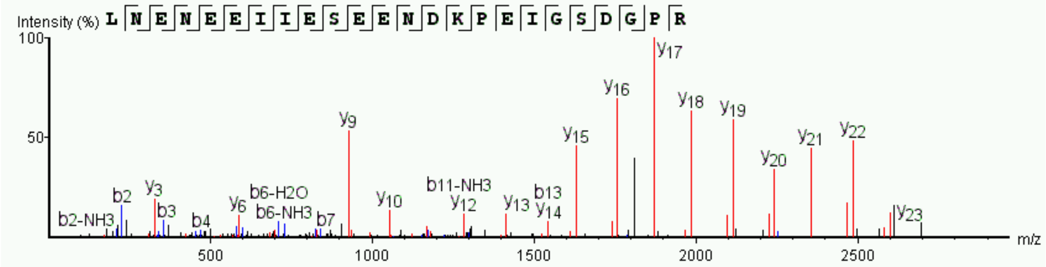

gi|571541954

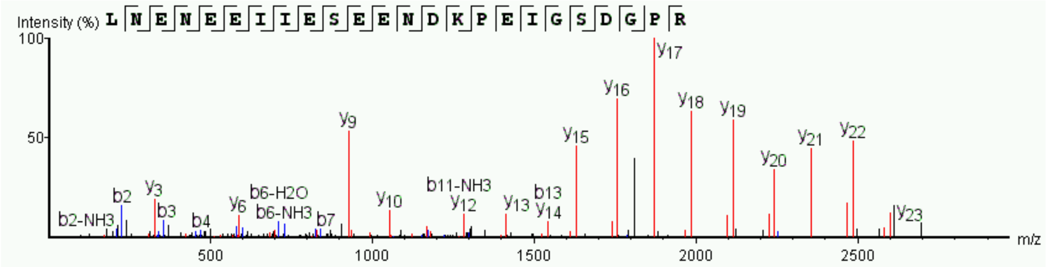

gi|571541958

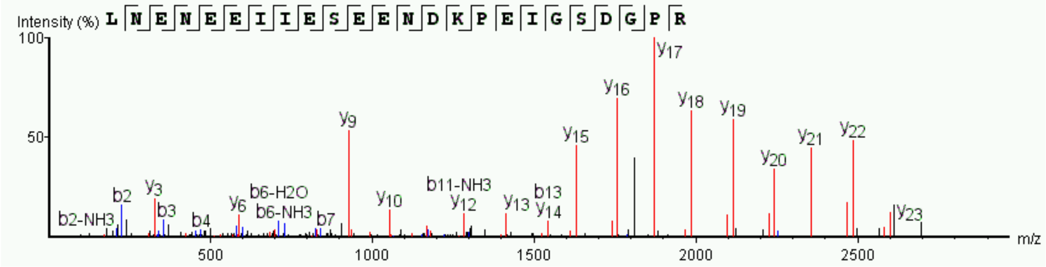

gi|571575329

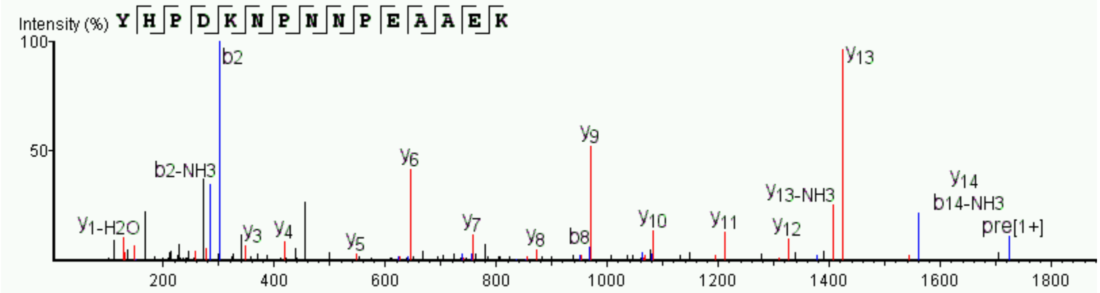

gi|571576551

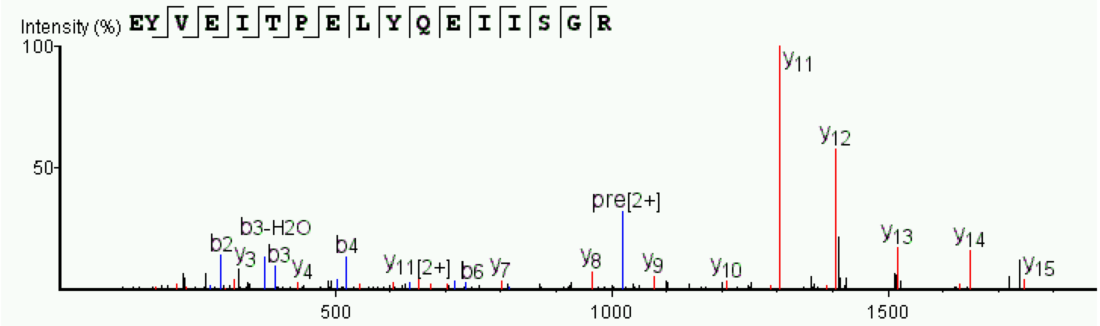

gi|328782992

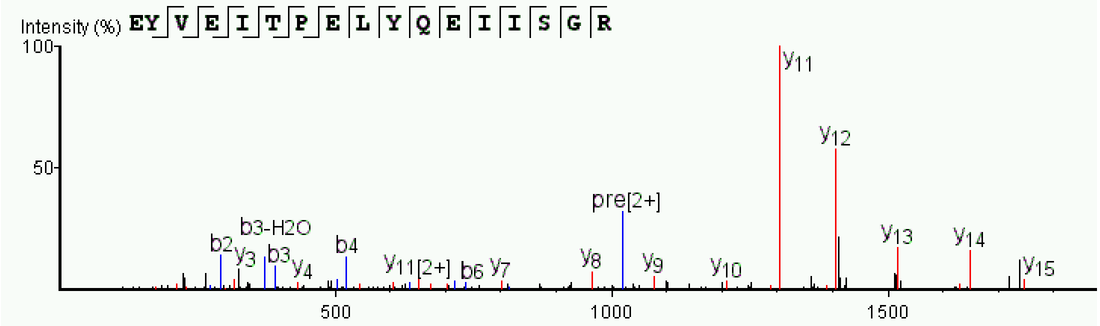

gi|571513398

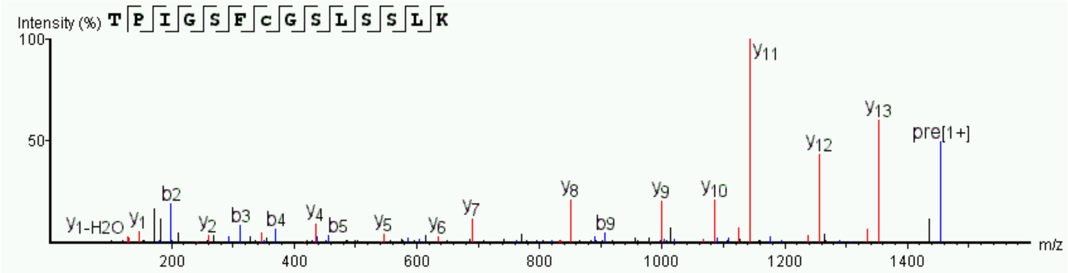

gi|571517320

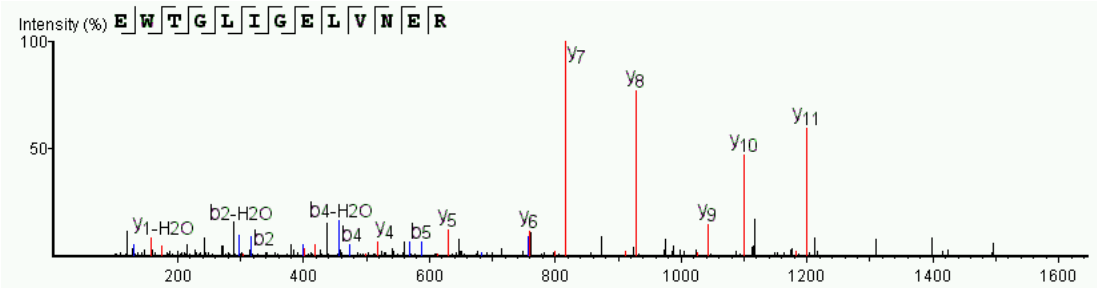

gi|571517323

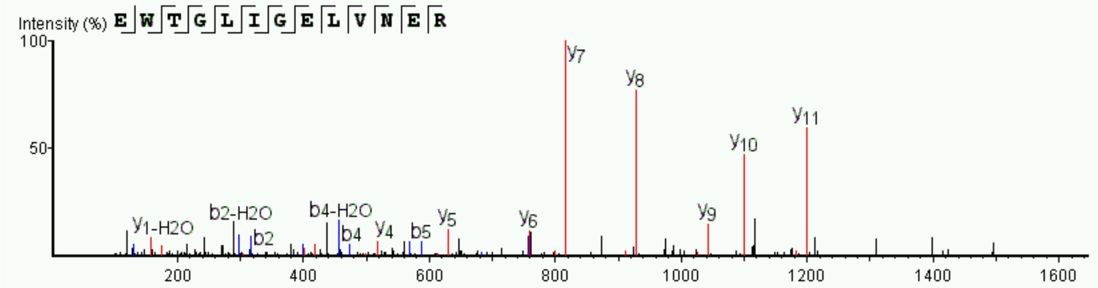

gi|571563582

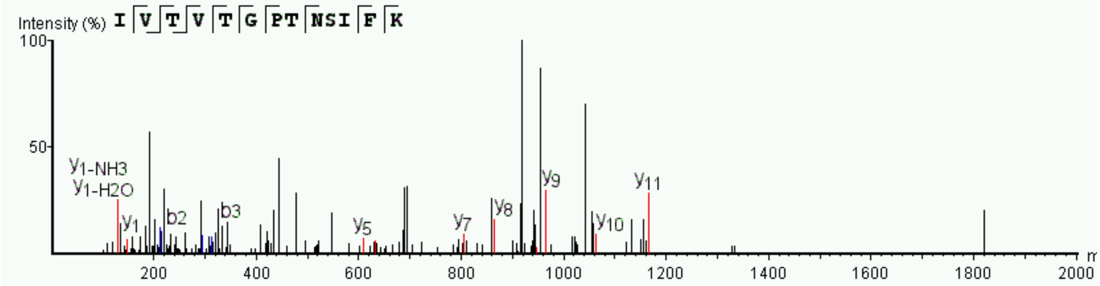

gi|571563586

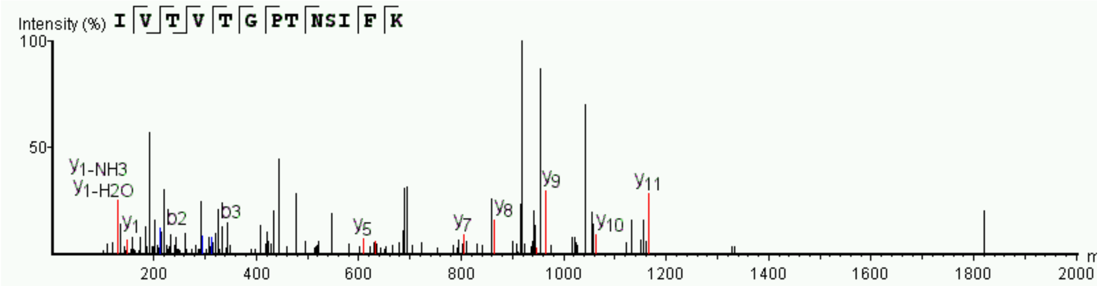

gi|571563590

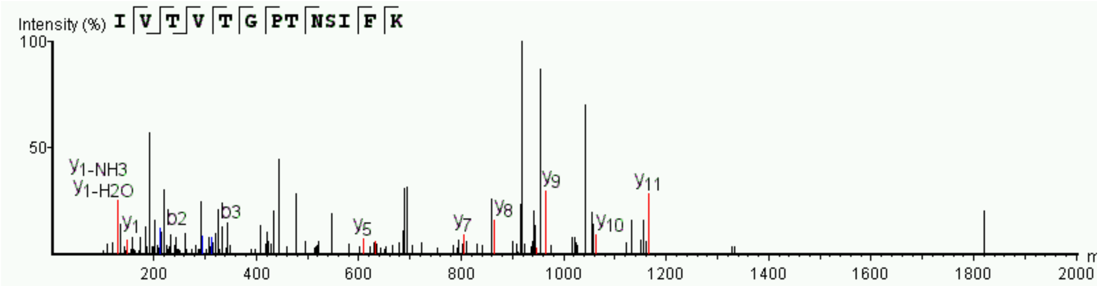

gi|571563594

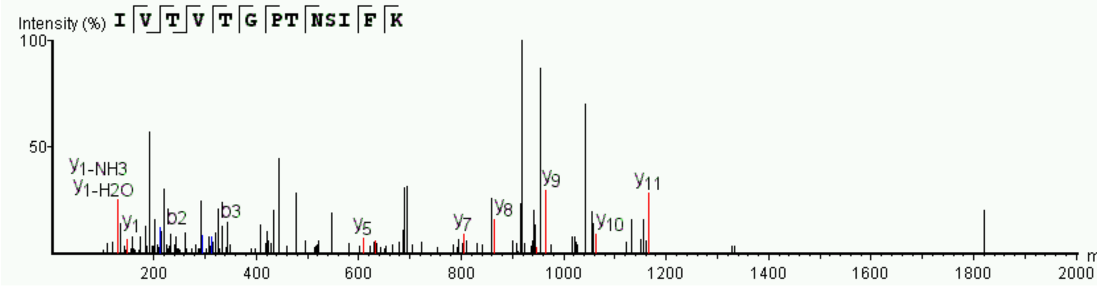

gi|571537253

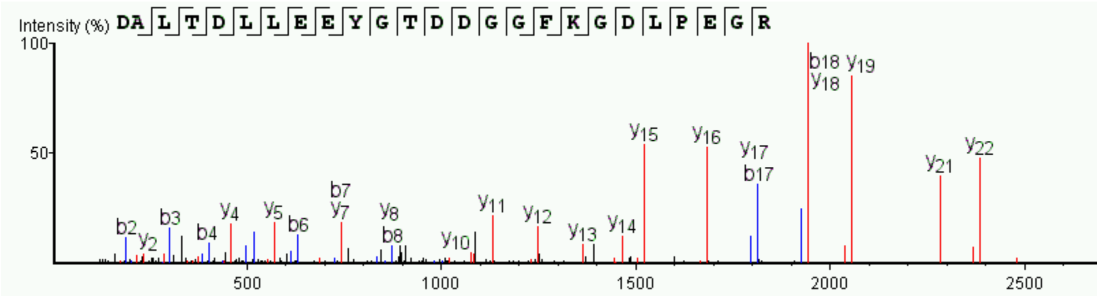

gi|571537256

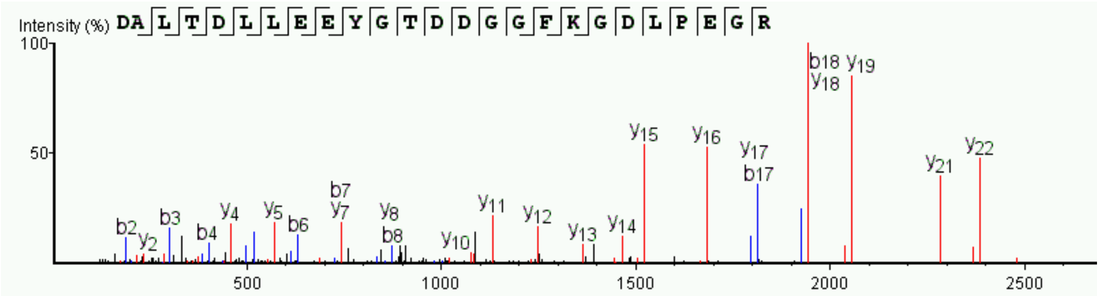

gi|571537262

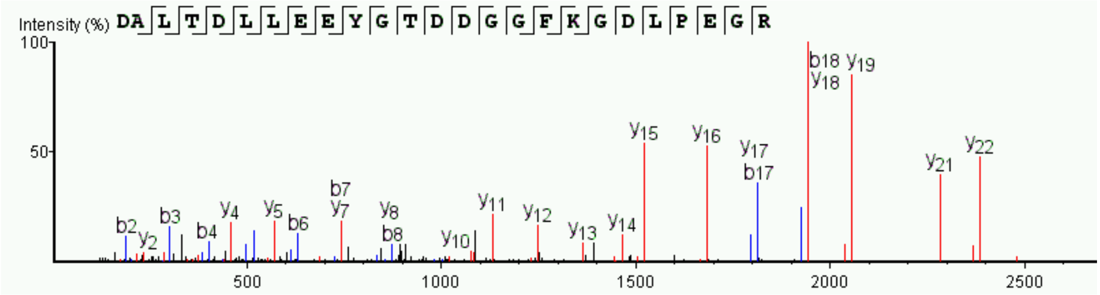

gi|66551967

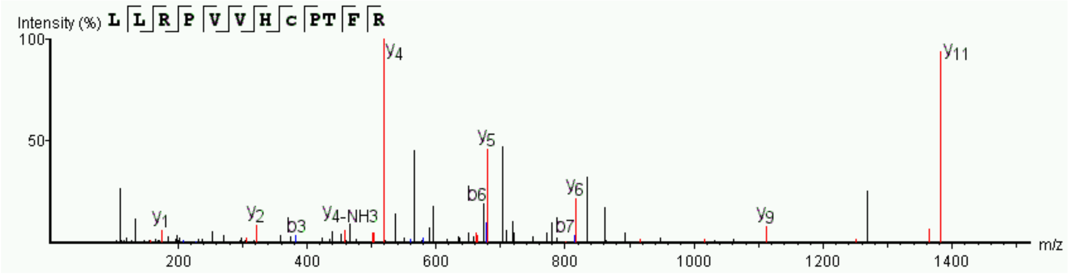

gi|571506703

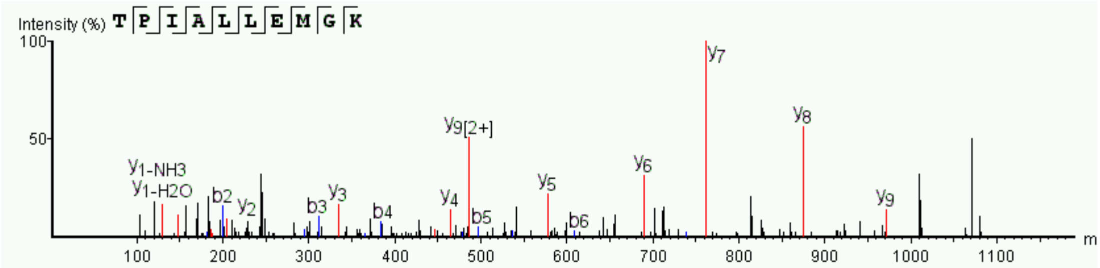

gi|571506707

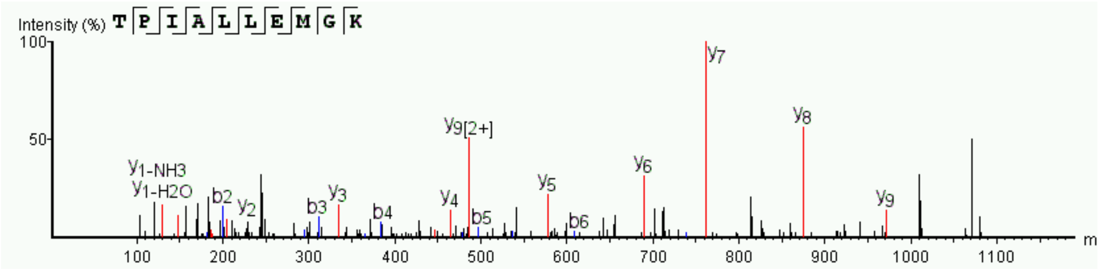

gi|571506711

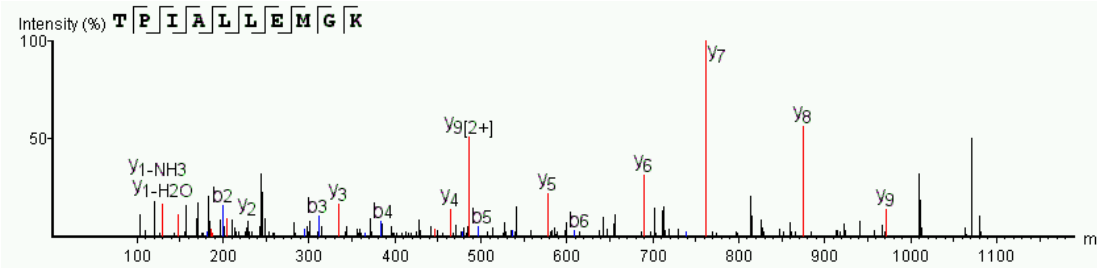

gi|571506700

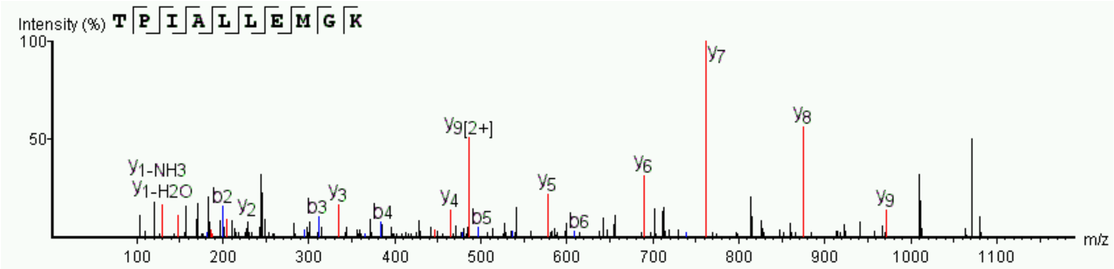

gi|66562741

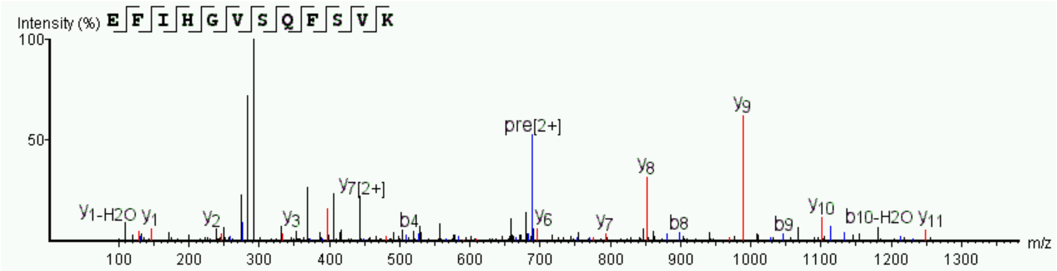

gi|110756942

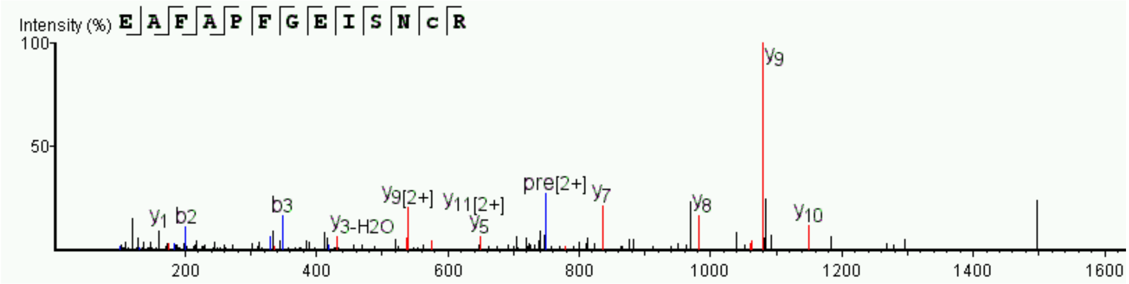

gi|48097576

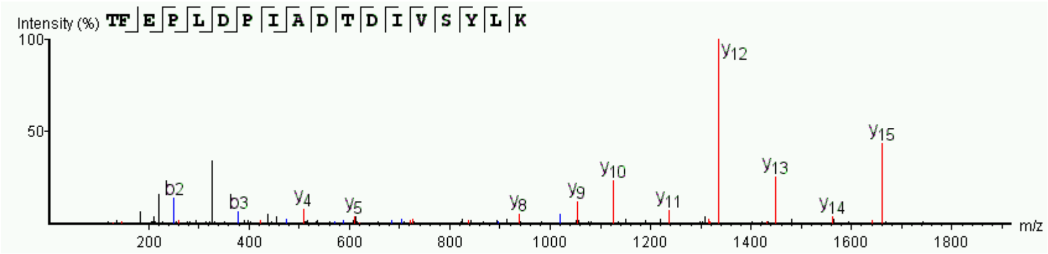

gi|571513678

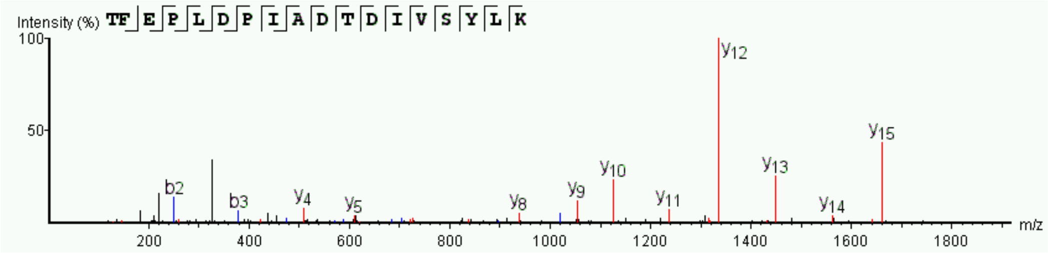

gi|571513682

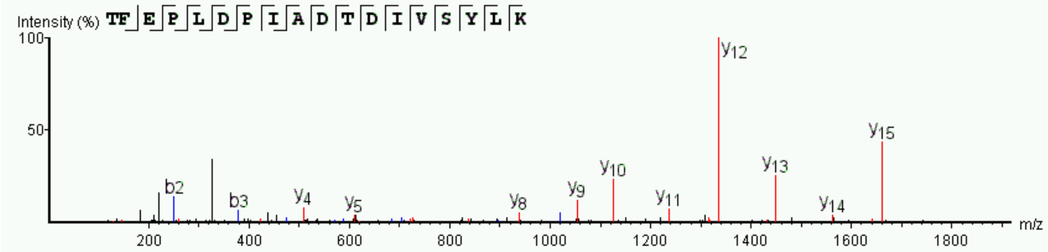

gi|571513674

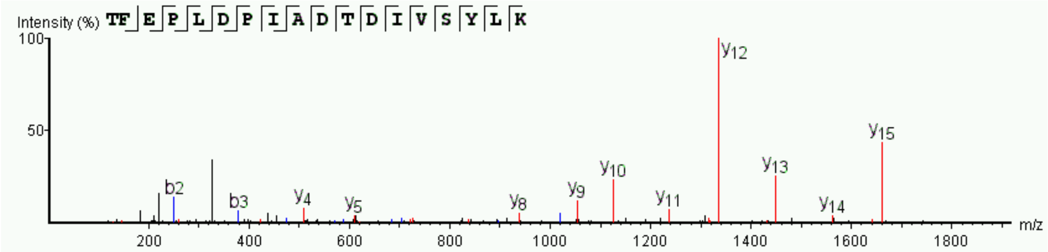

gi|66518451

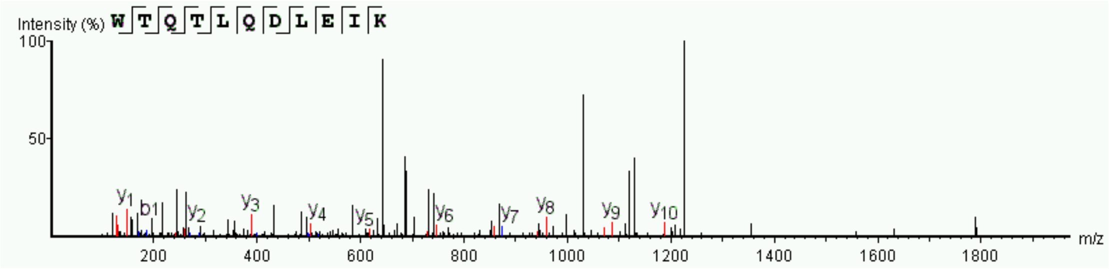

gi|571528288

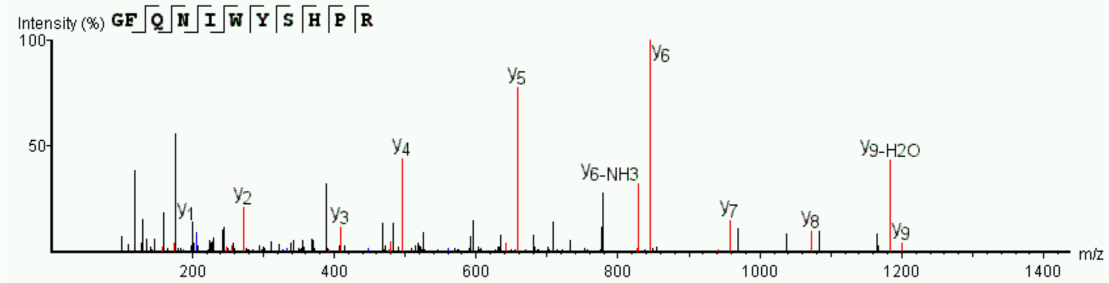

gi|571553690

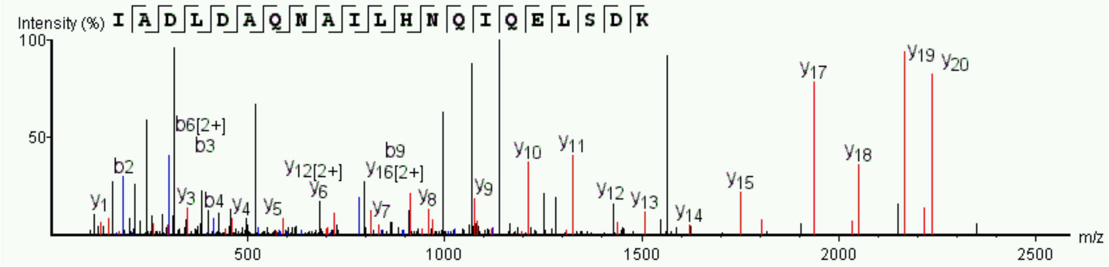

gi|571566413

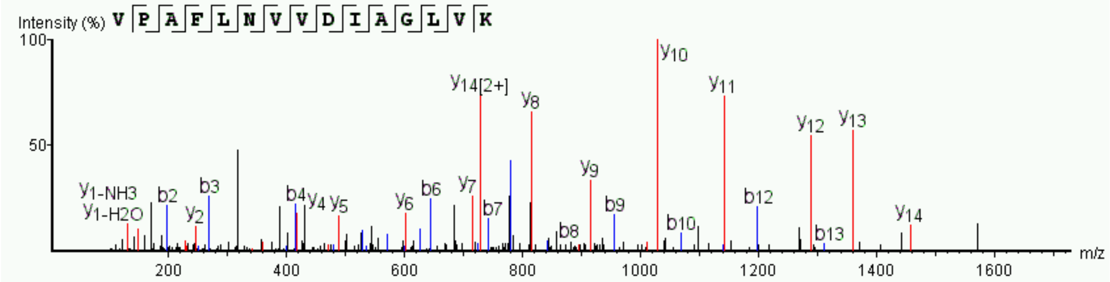

gi|110762023

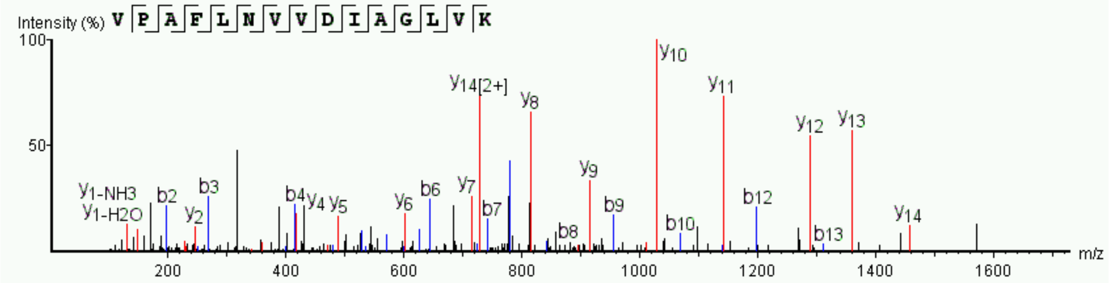

gi|571523207

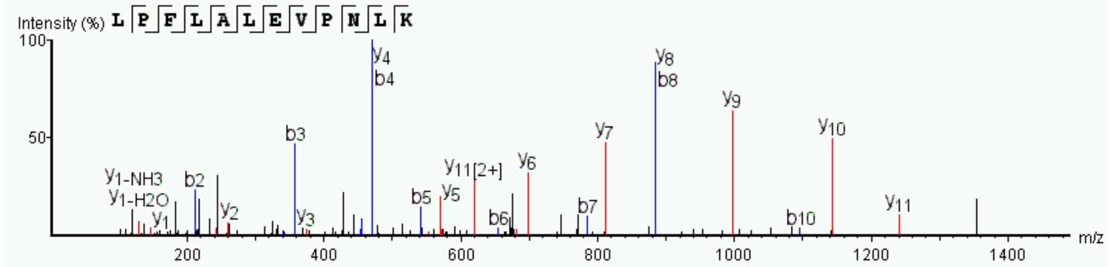

gi|328789542

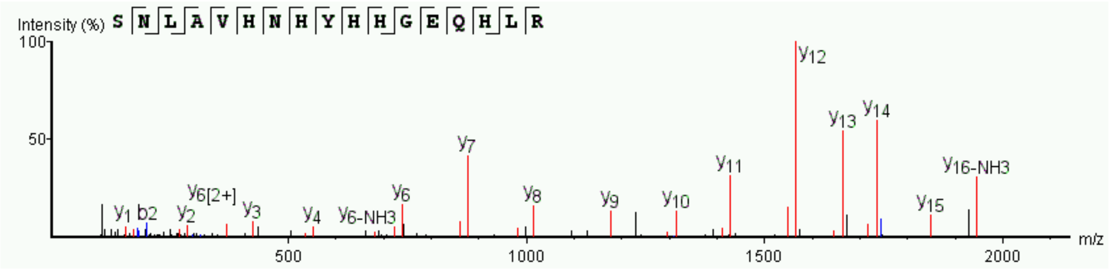

gi|328782599

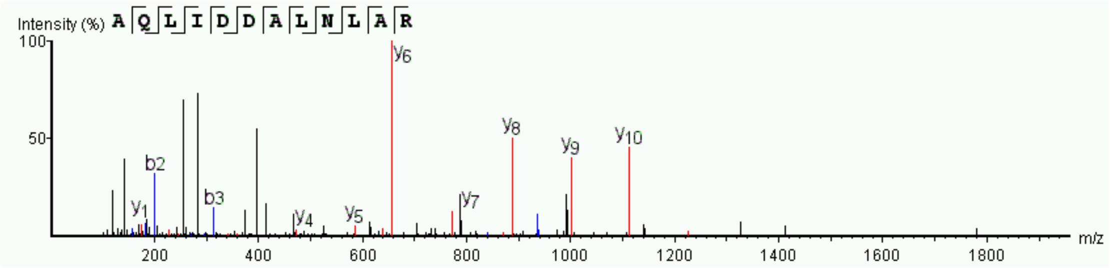

gi|328776594

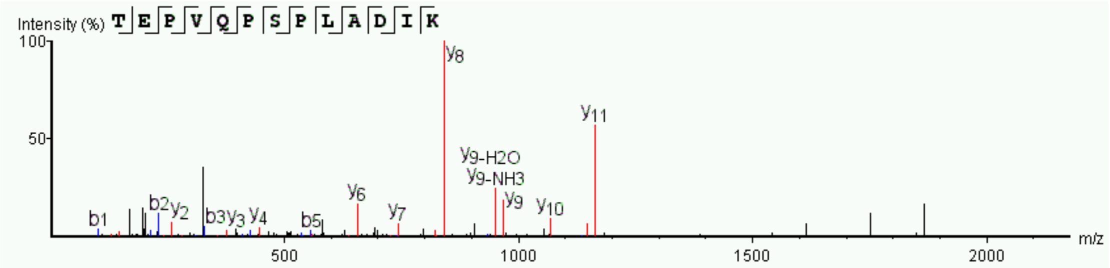

gi|571552397

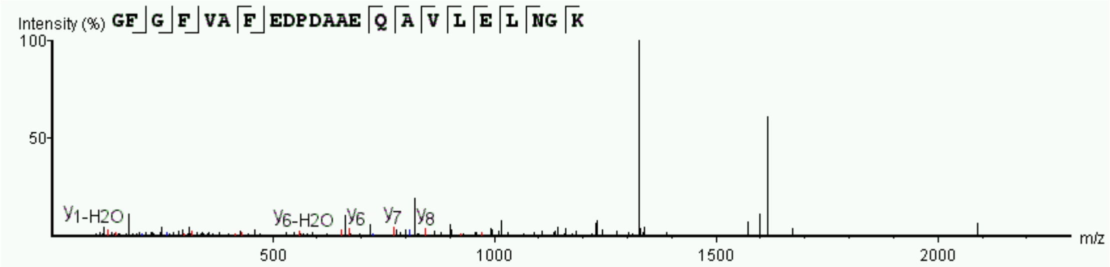

gi|571552393

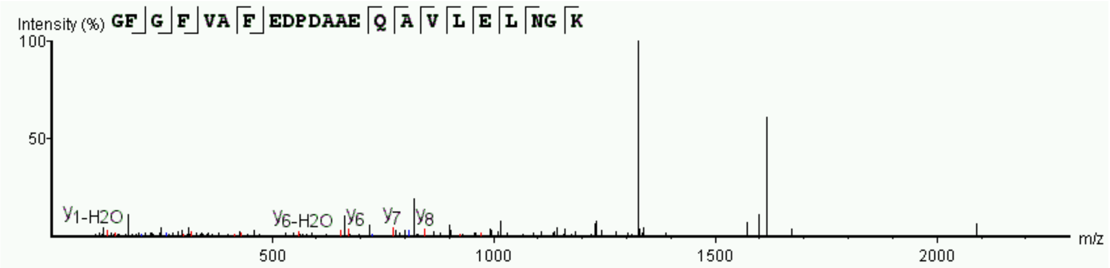

gi|571540698

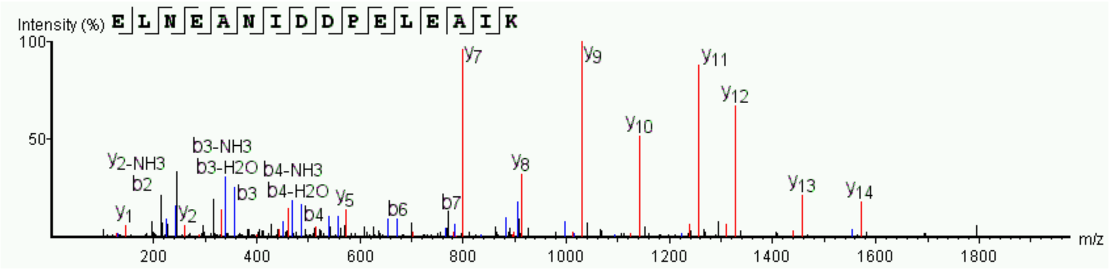

gi|571508080

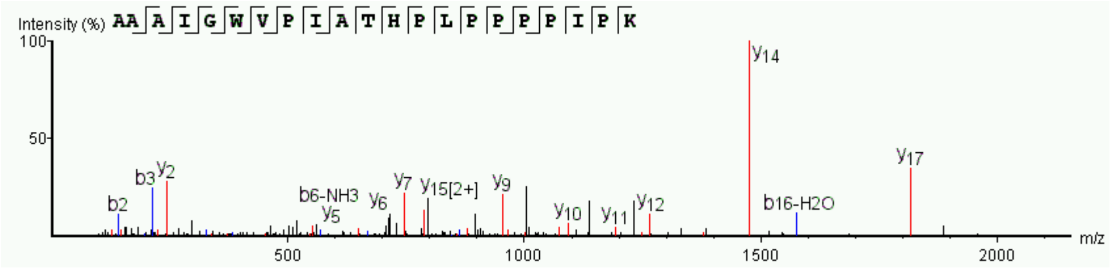

gi|571508083

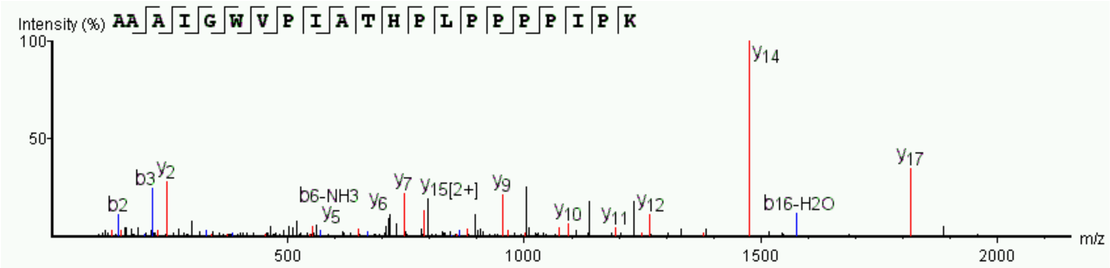

gi|571508087

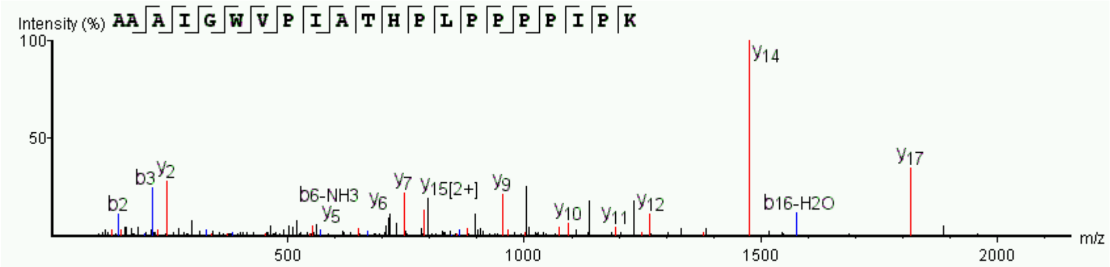

gi|571508090

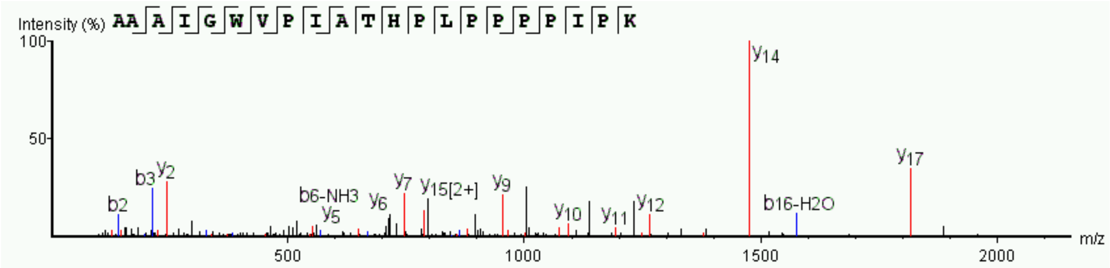

gi|571508093

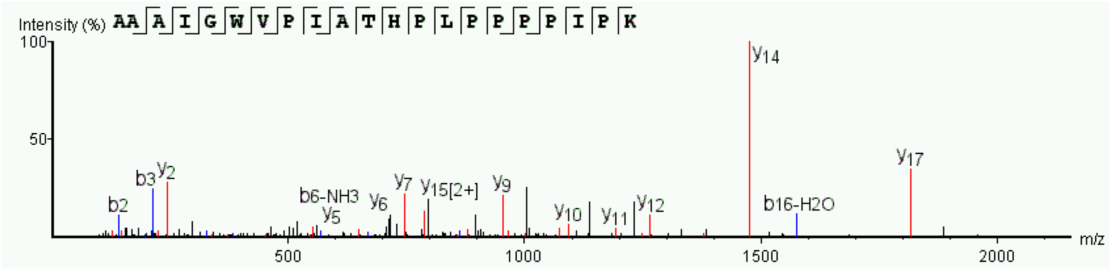

gi|571508096

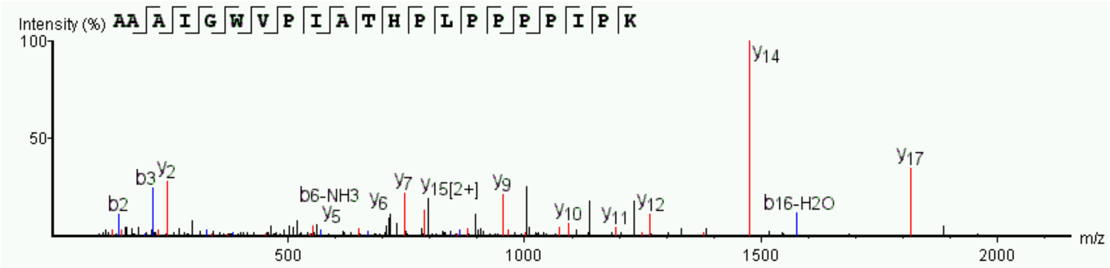

gi|571508102

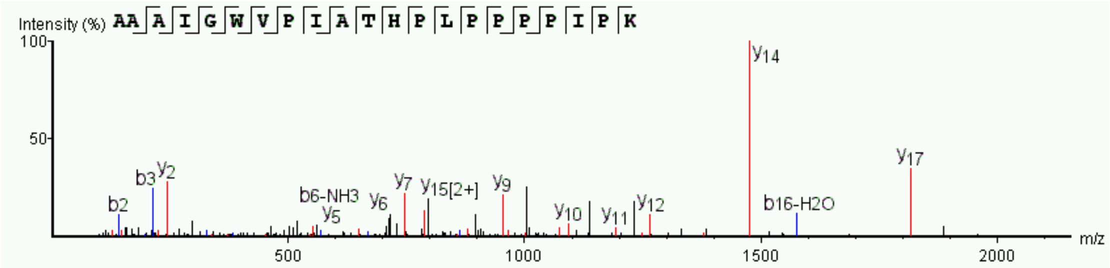

gi|328792267

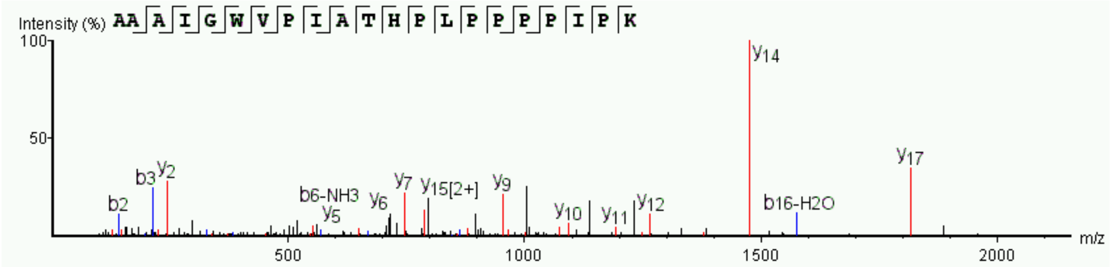

gi|571508077

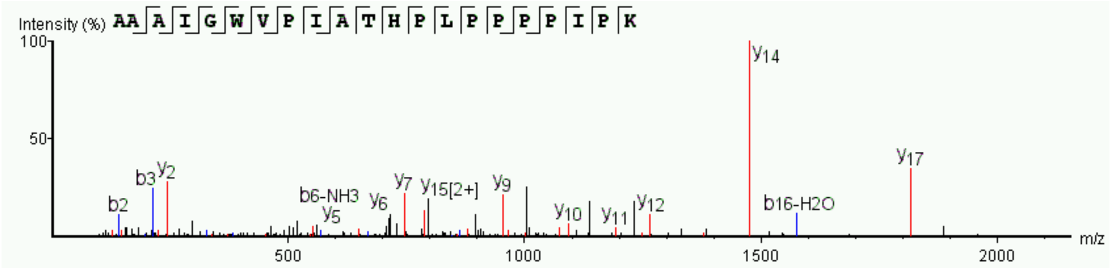

gi|571529116

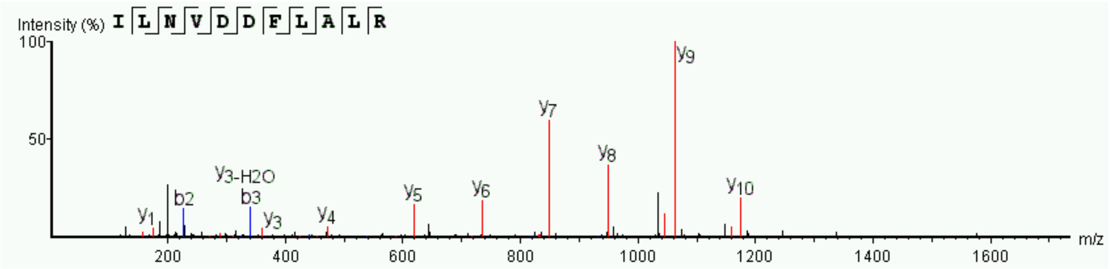

gi|571565660

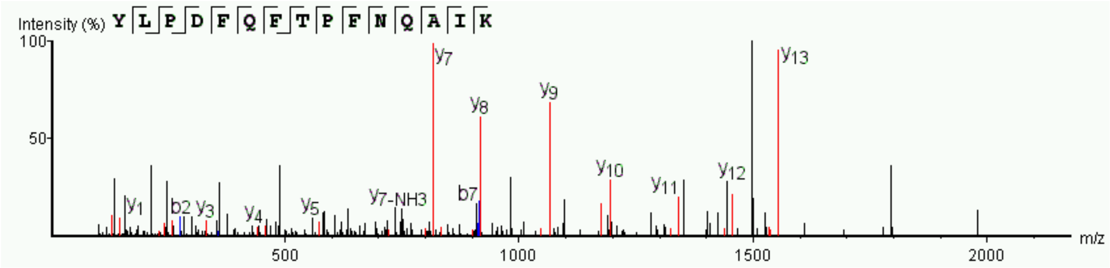

gi|571565656

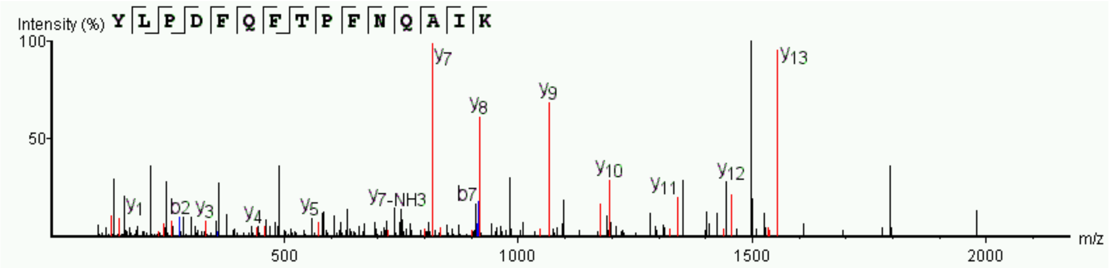

gi|48095935

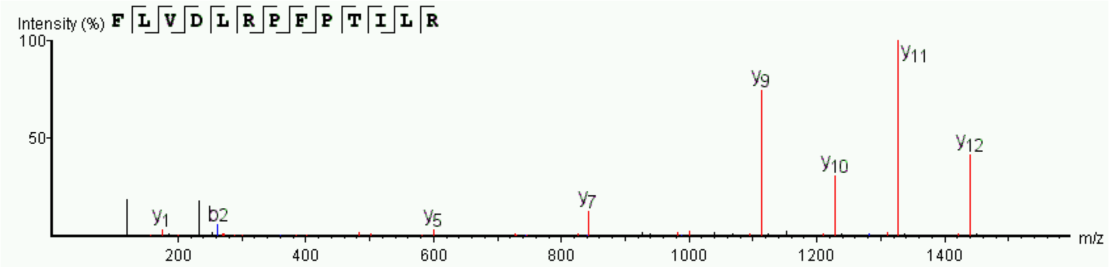

gi|48097144

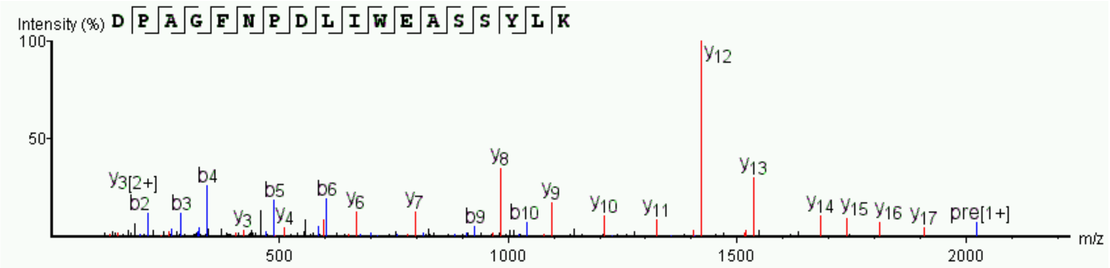

gi|571516991

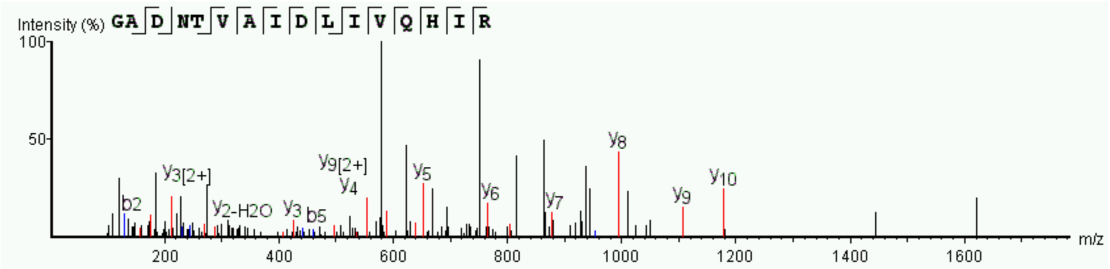

gi|48094929

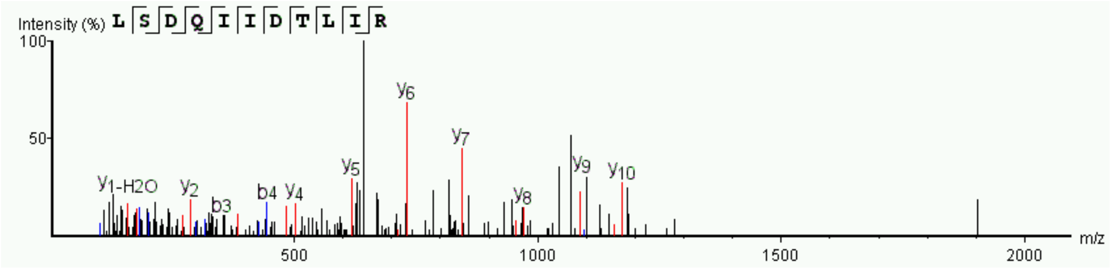

gi|328789503

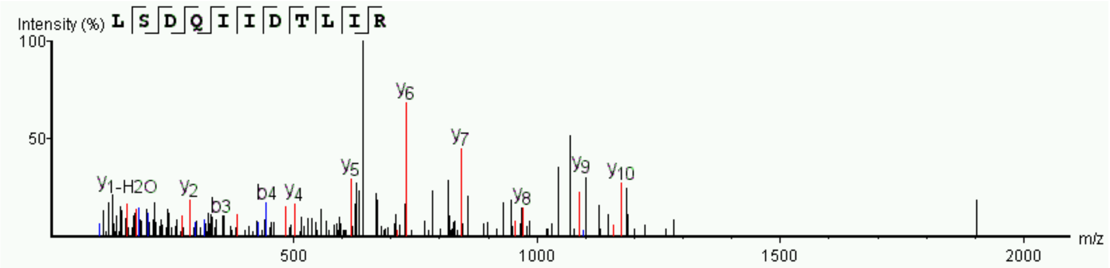

gi|571522941

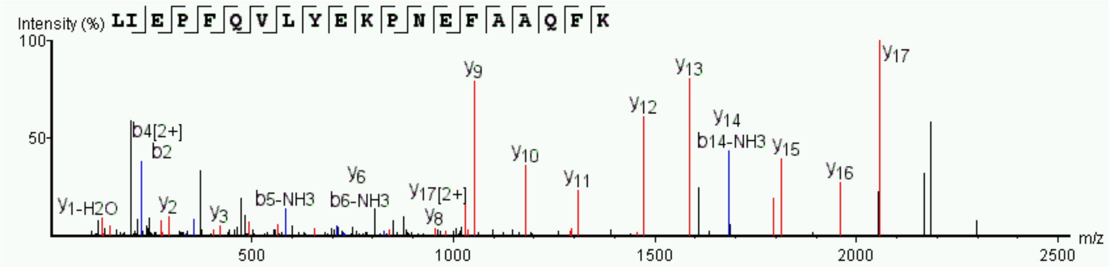

gi|66524764

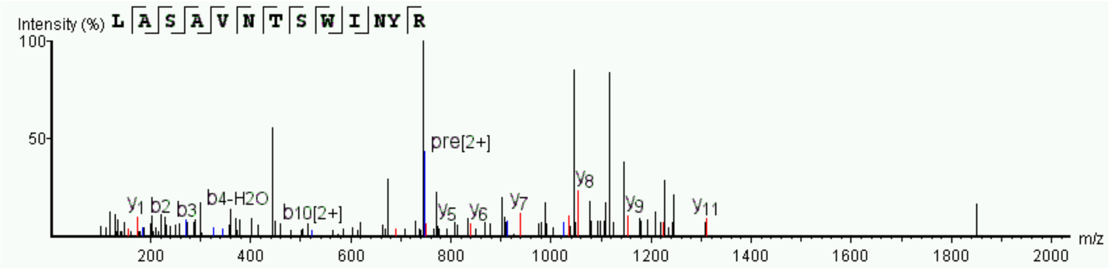

gi|571570586

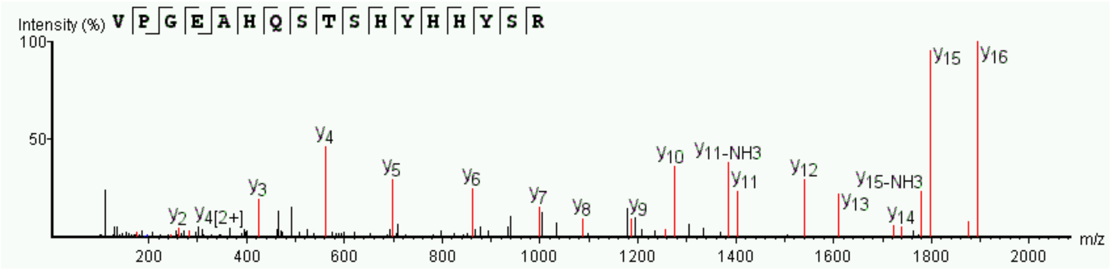

gi|571570594

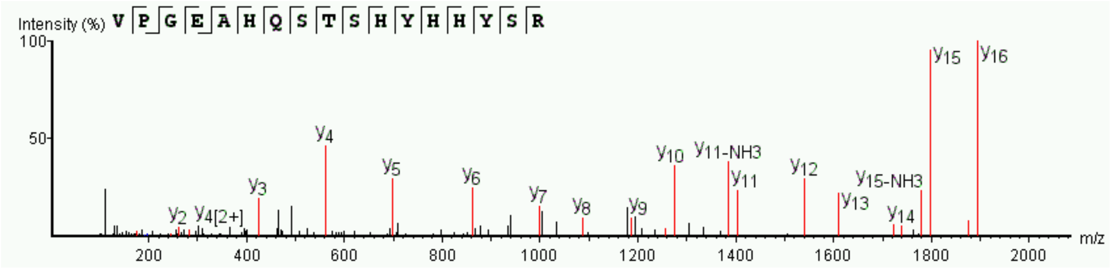

gi|571570602

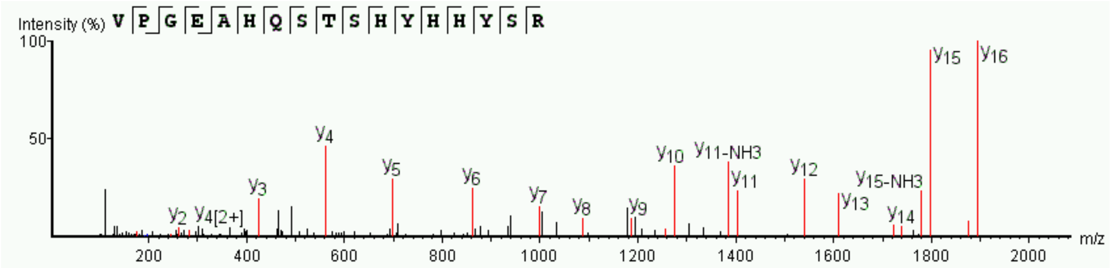

gi|571570616

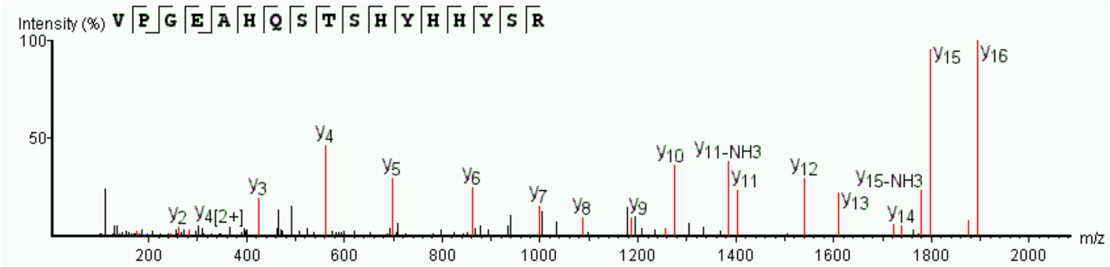

gi|571570622

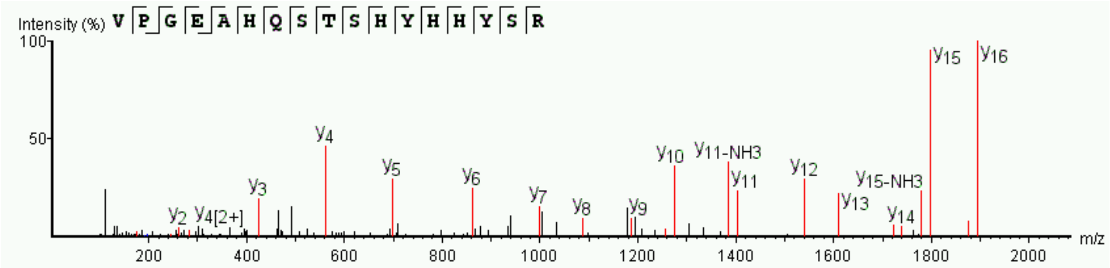

gi|571570635

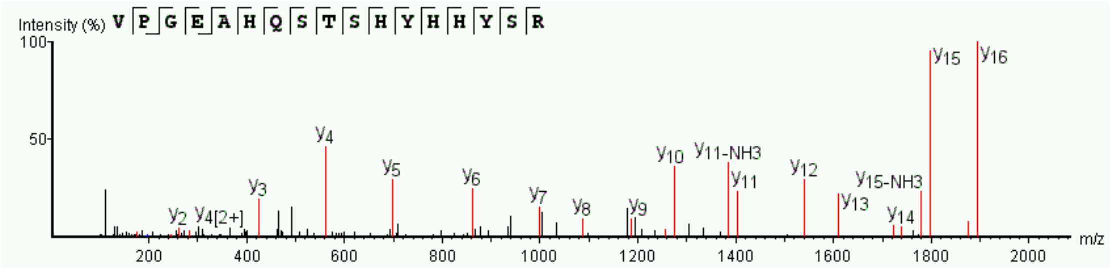

gi|571570641

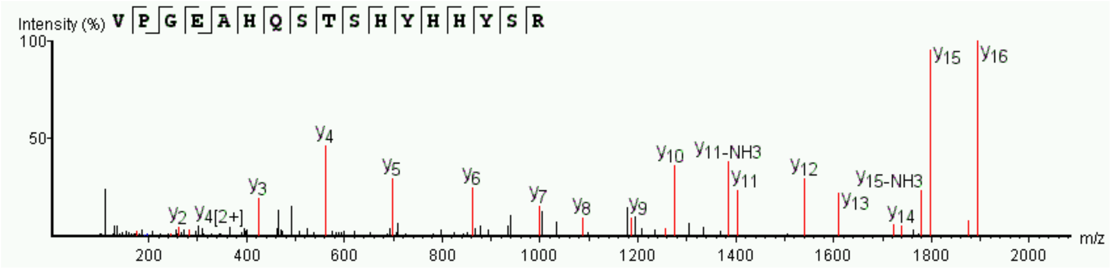

gi|571570610

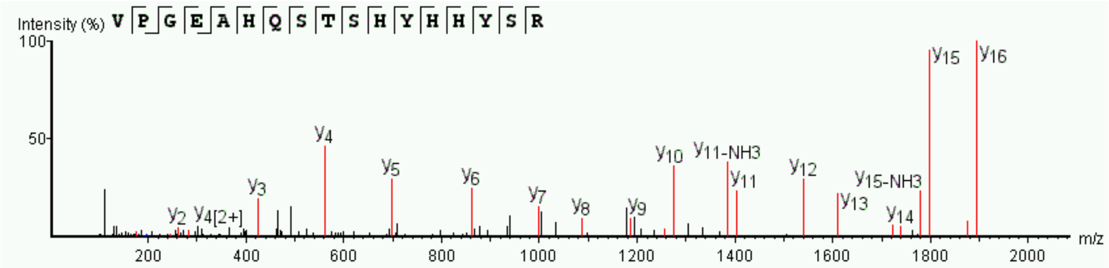

gi|571575529

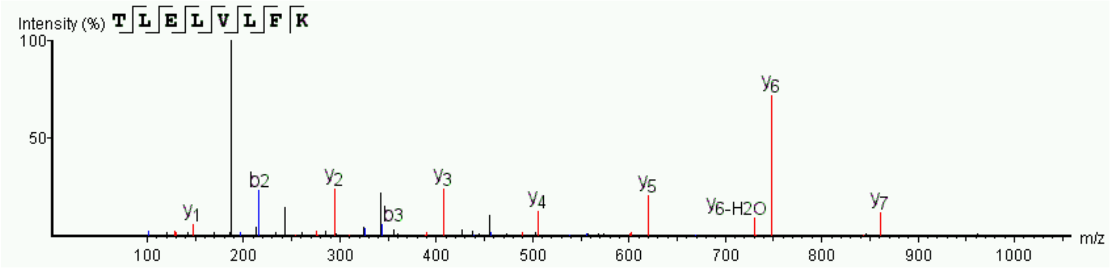

gi|571575531

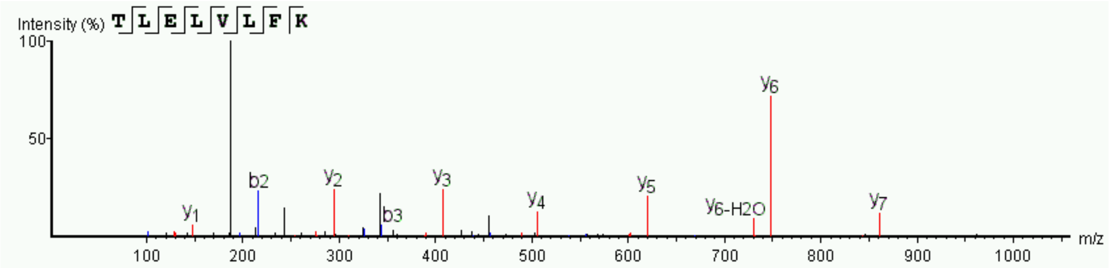

gi|48128110

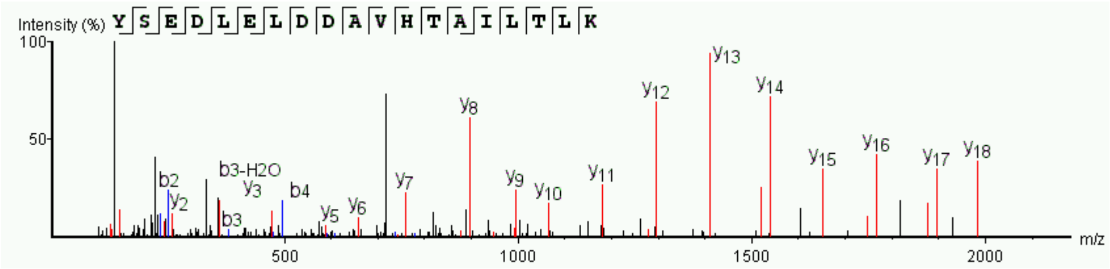

gi|48096769

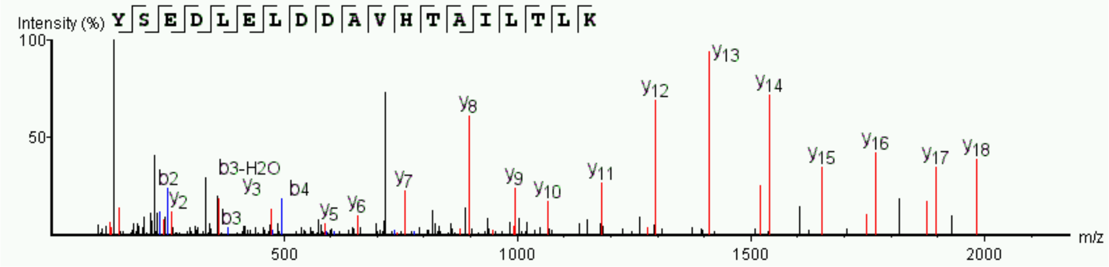

gi|328787541

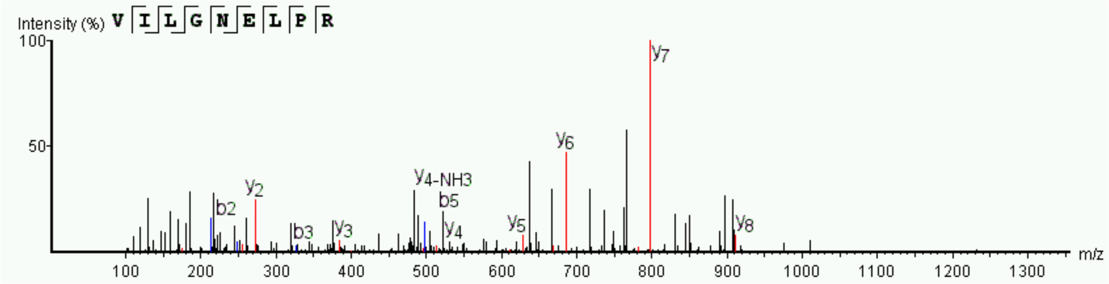

gi|571539727

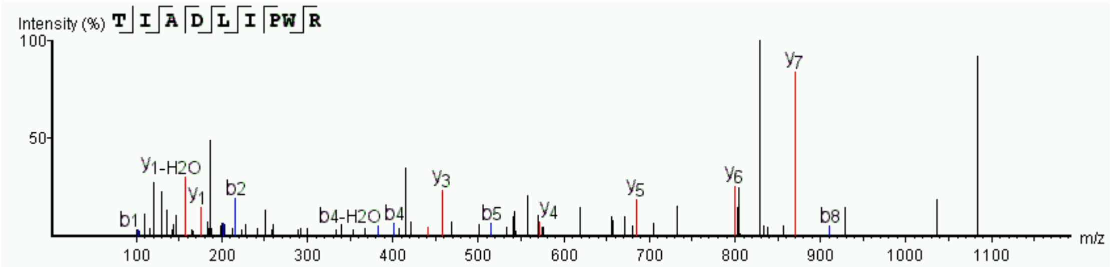

gi|571539731

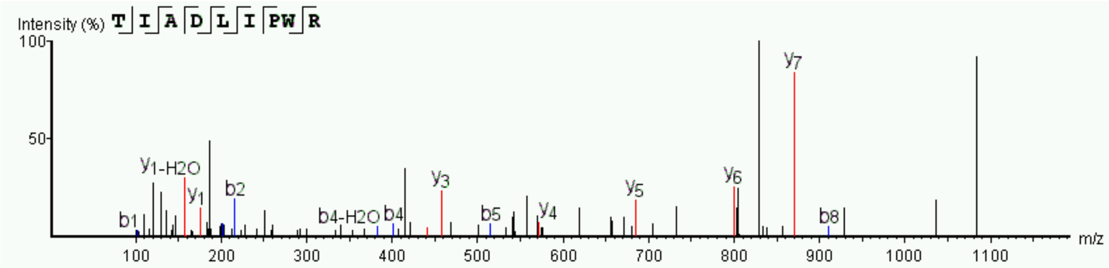

gi|571528227

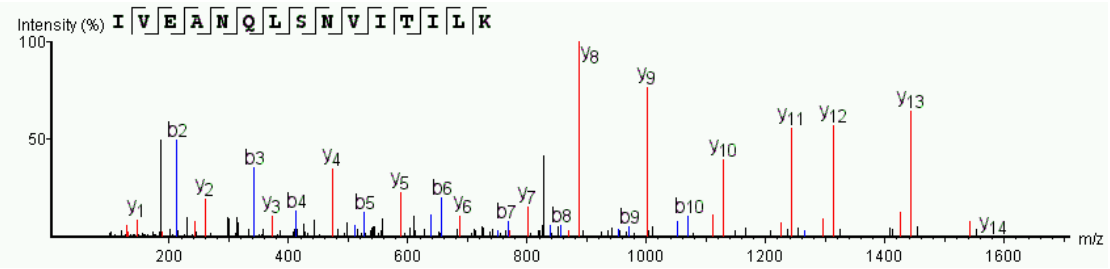

gi|571576759

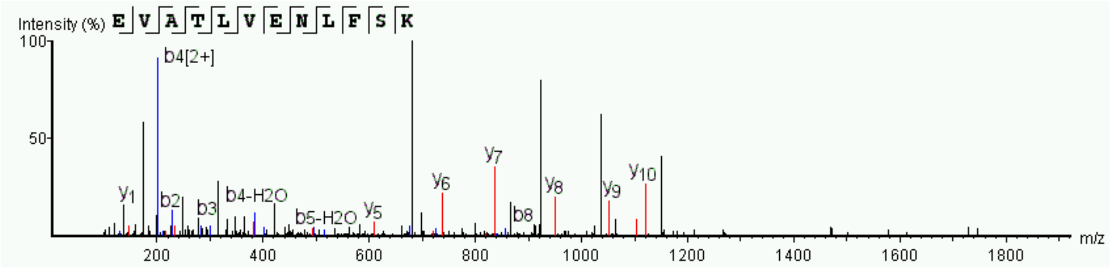

gi|571576761

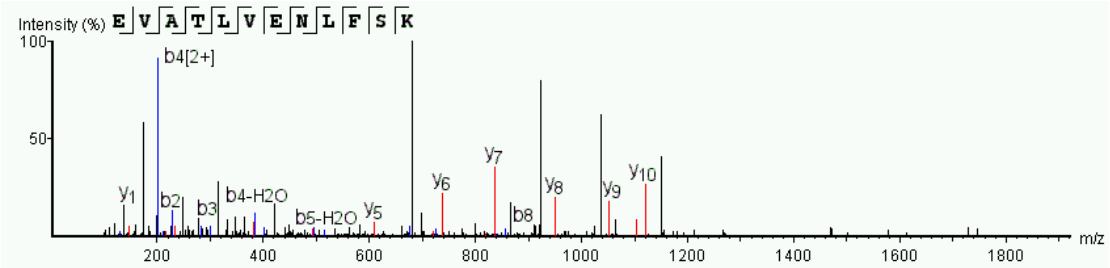

gi|571576763

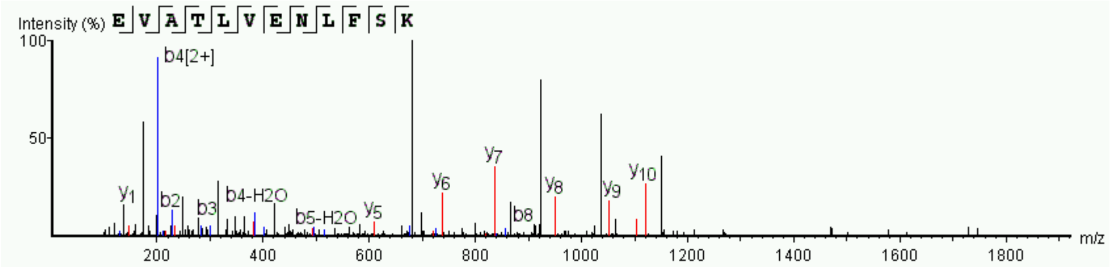

gi|328792920

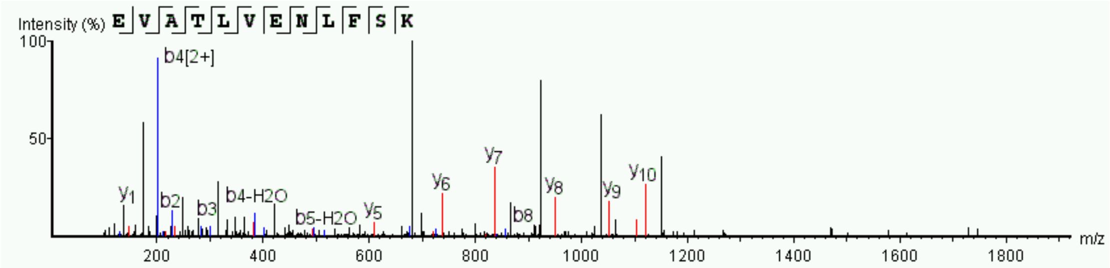

gi|571576756

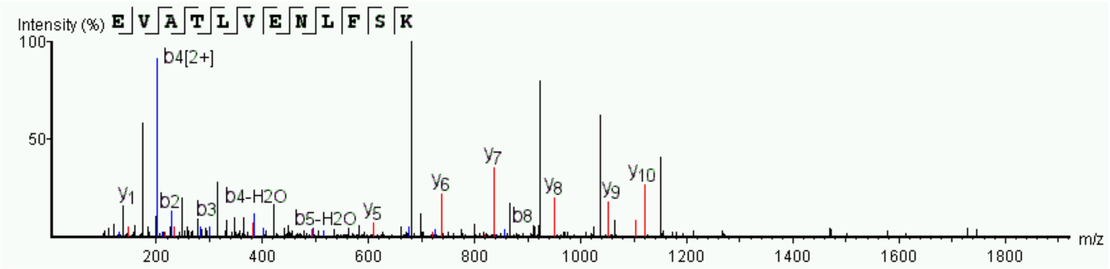

gi|571538169

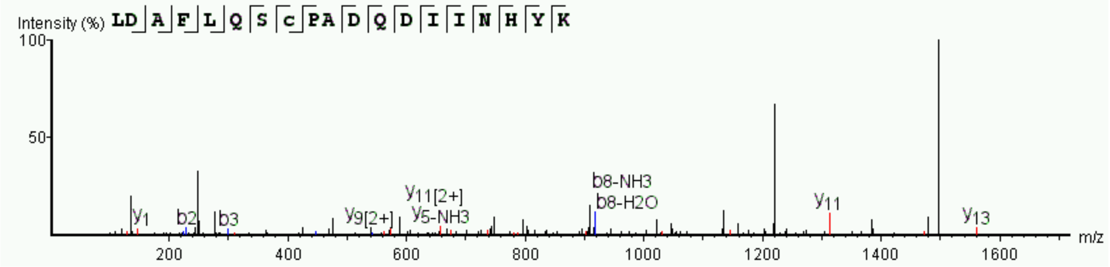

gi|328777457

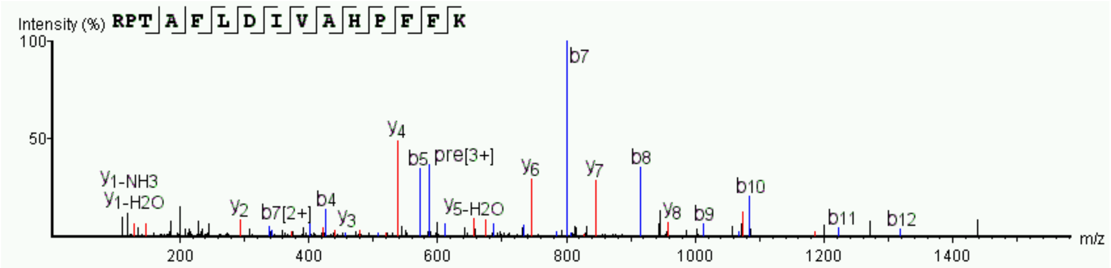

gi|571500050

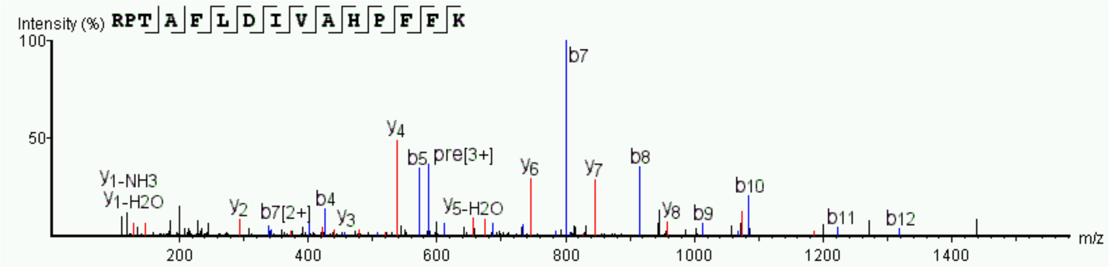

gi|48103834

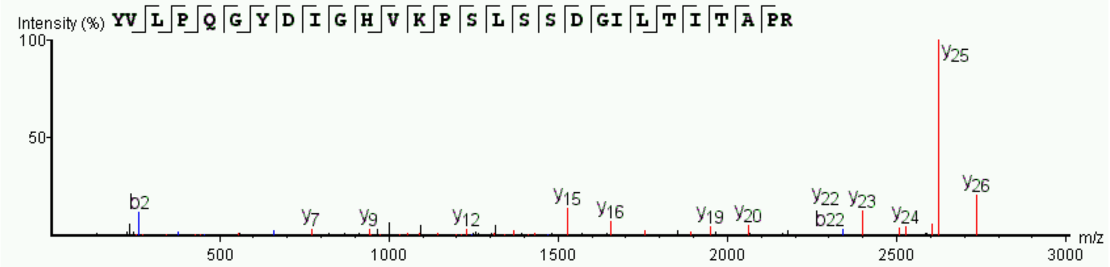

gi|571566701

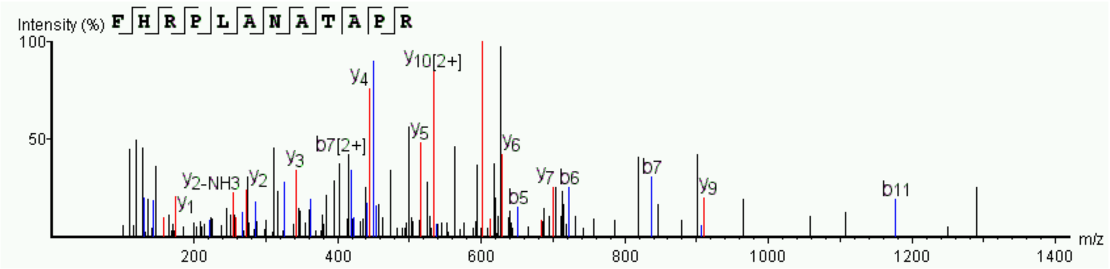

gi|328782520

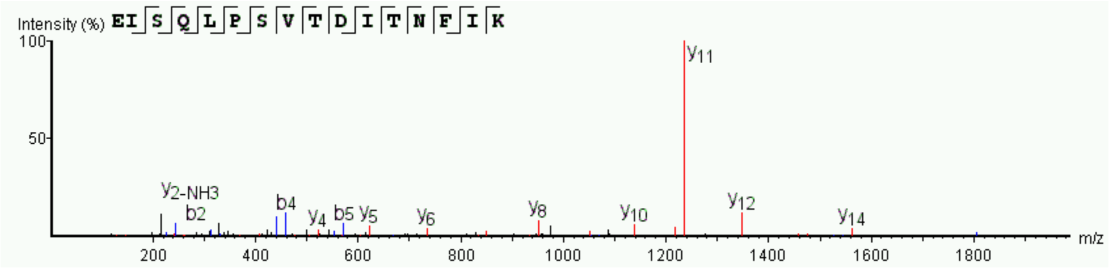

gi|571510978

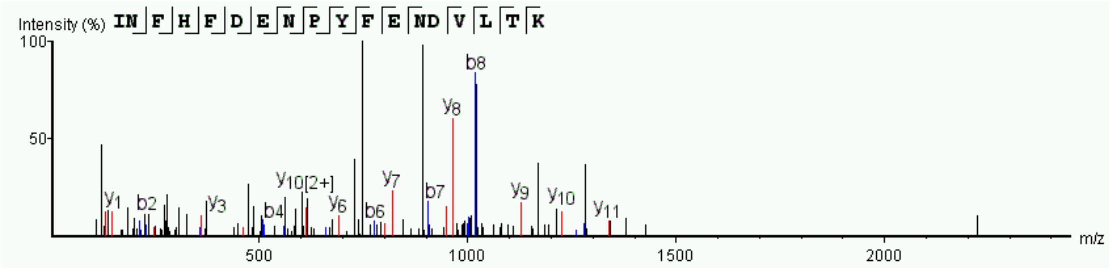

gi|110767181

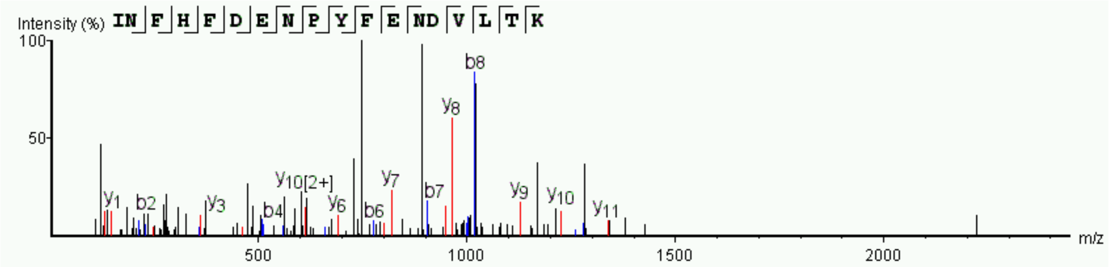

gi|328788157

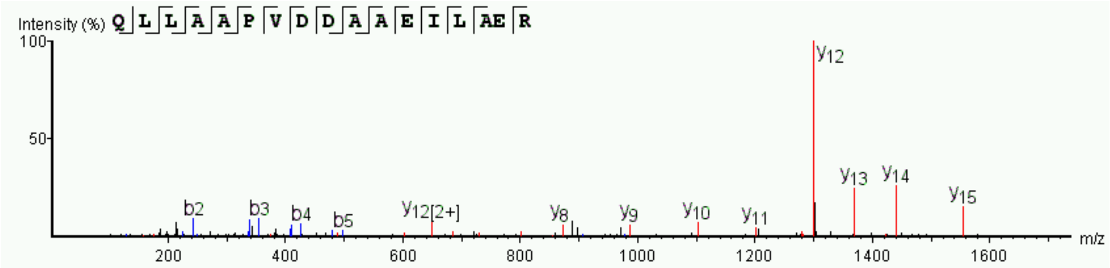

gi|571550221

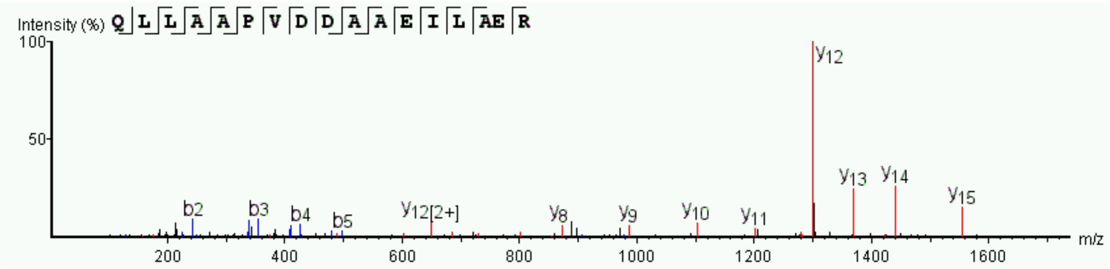

gi|328793508

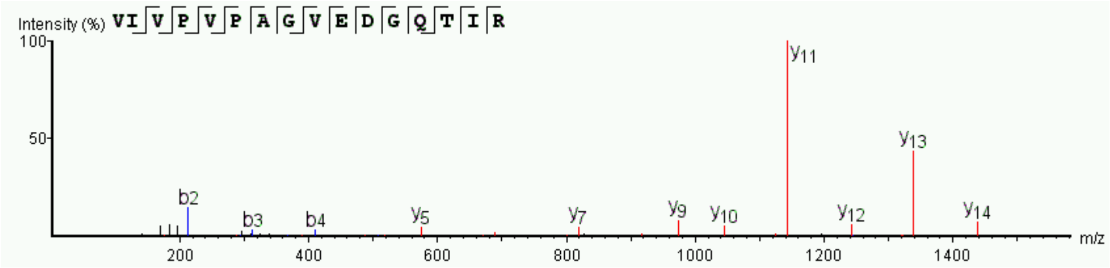

gi|571577853

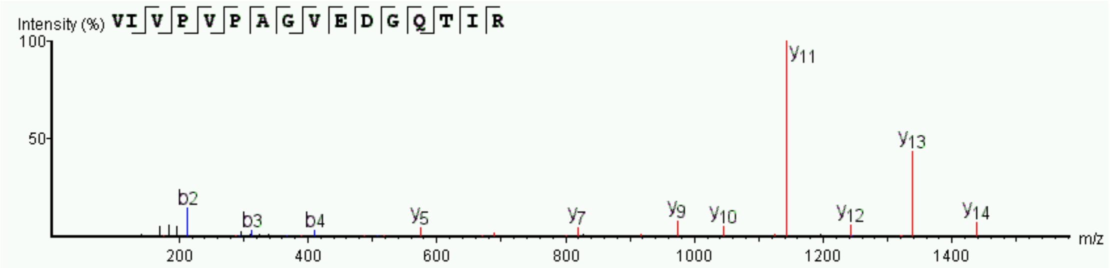

gi|571525961

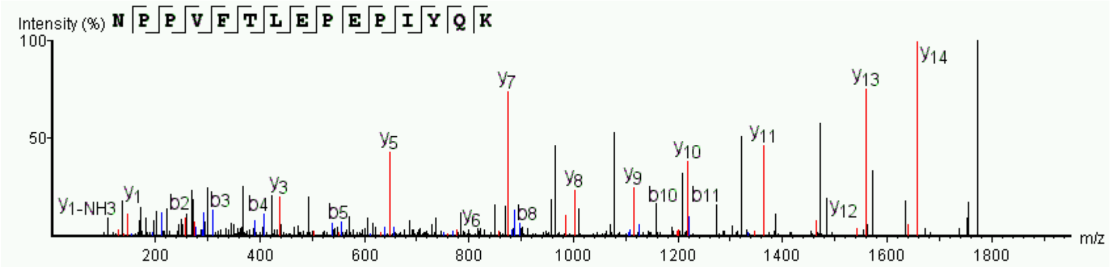

gi|571525968

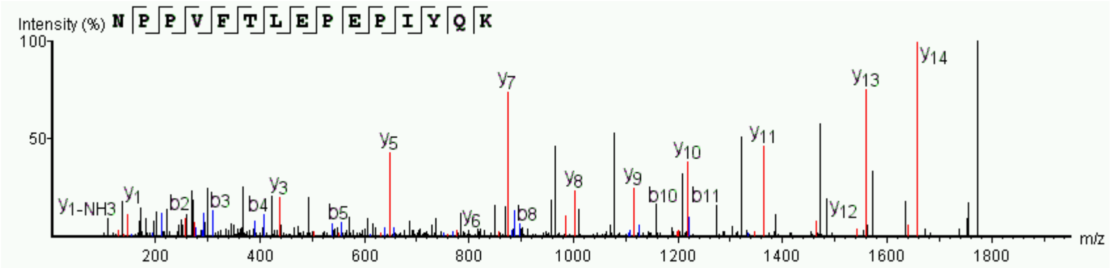

gi|571525972

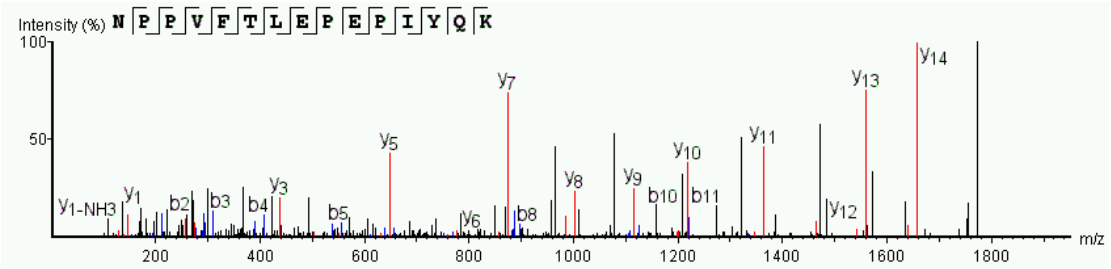

gi|328790055

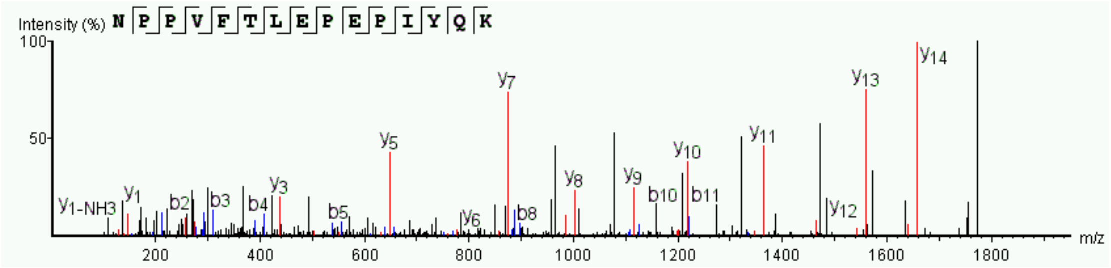

gi|328790053

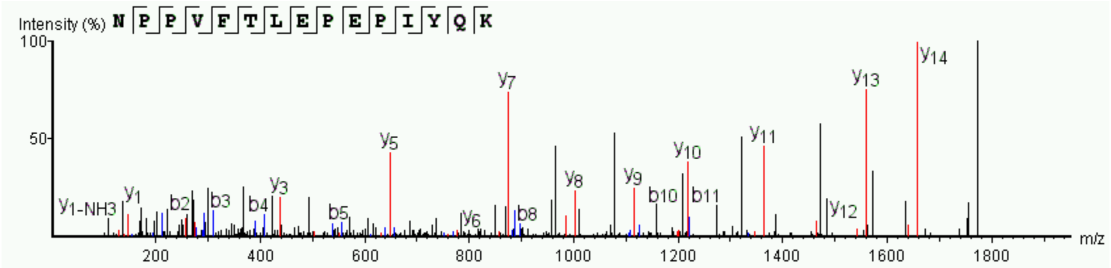

gi|571525957

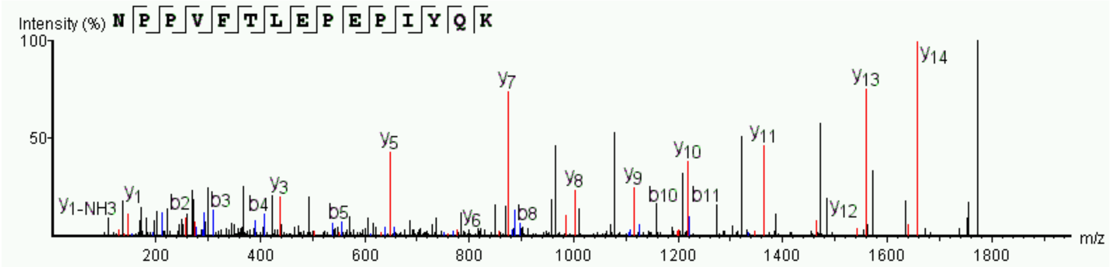

gi|571552022

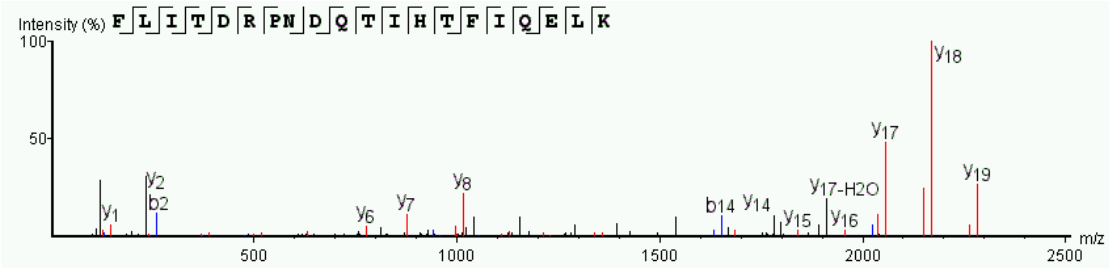

gi|328777886

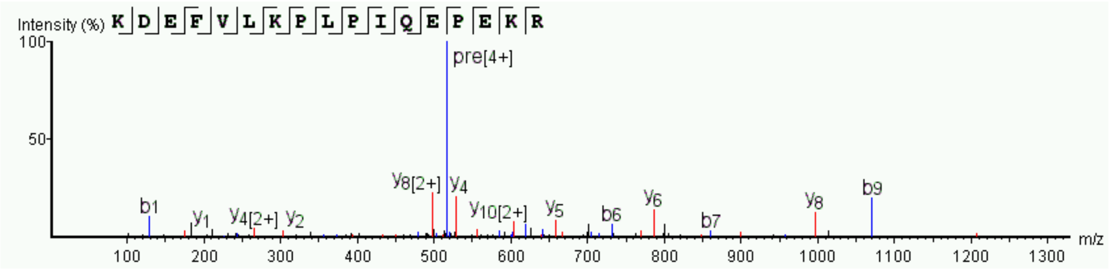

gi|571576460

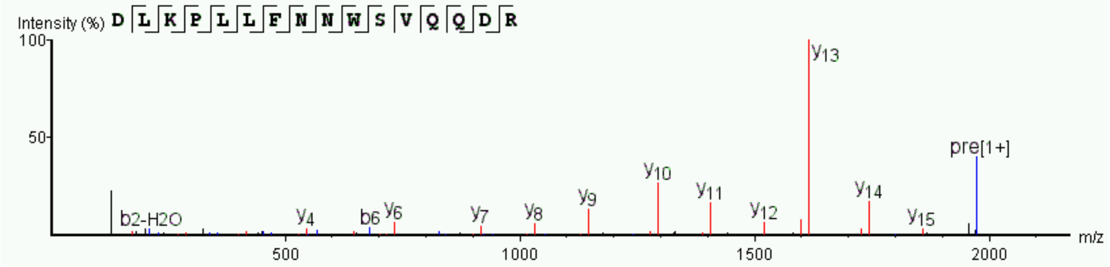

gi|571546273

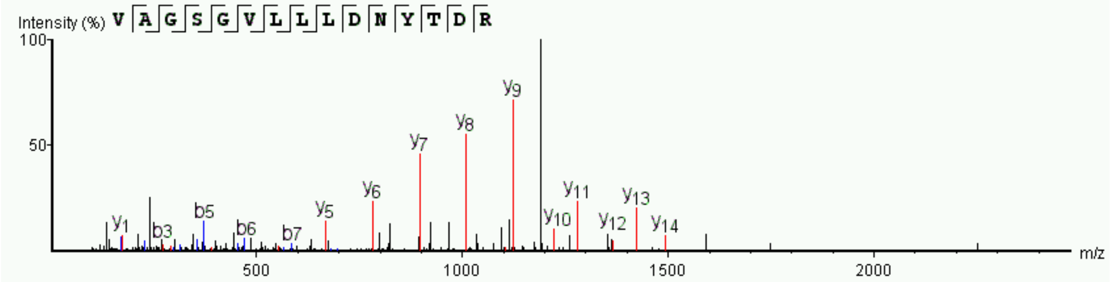

gi|571546308

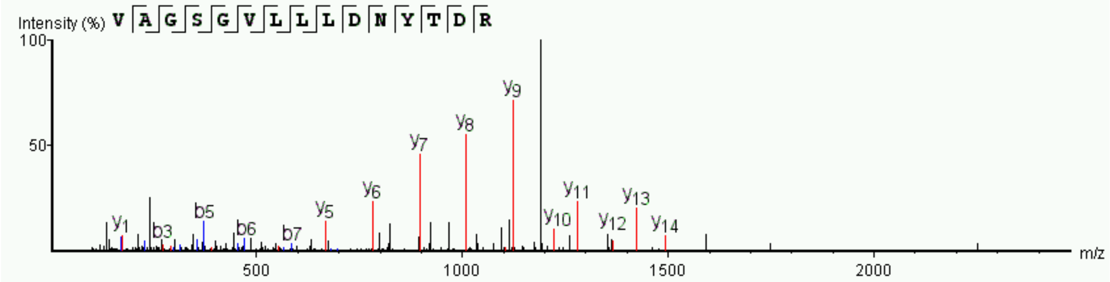

gi|571546312

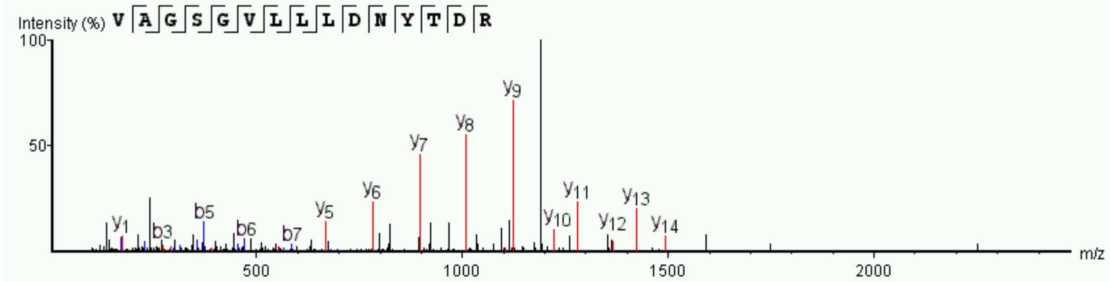

gi|571546316

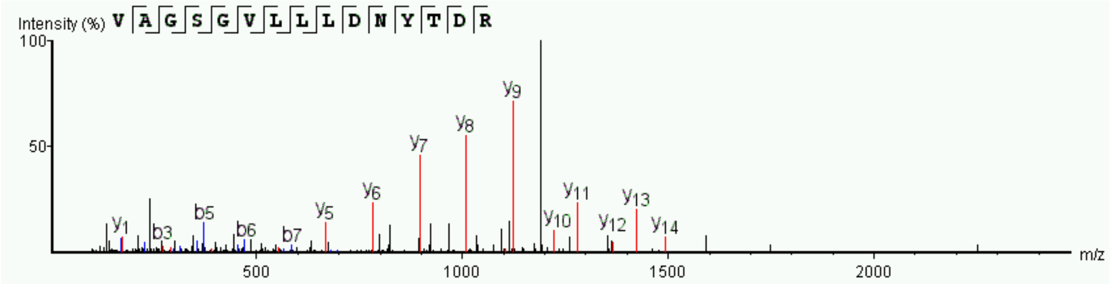

gi|571546320

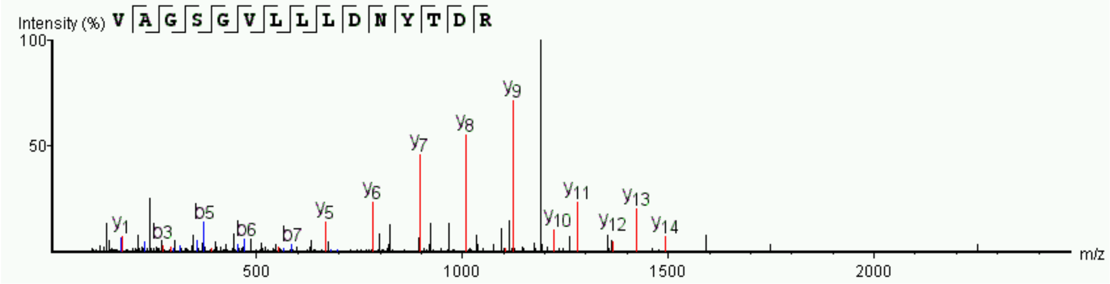

gi|571546323

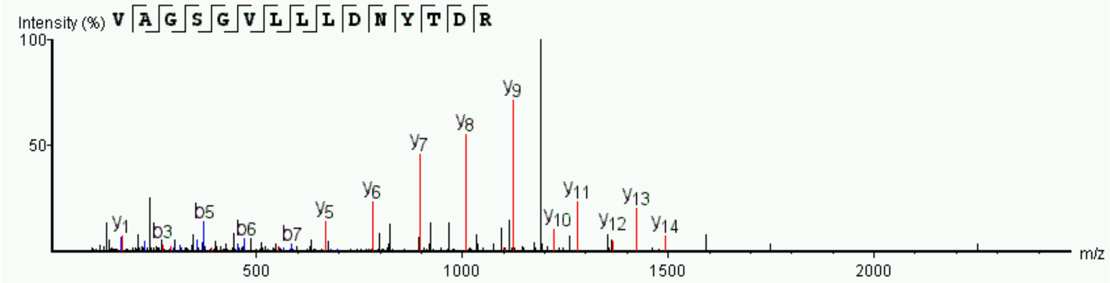

gi|571546326

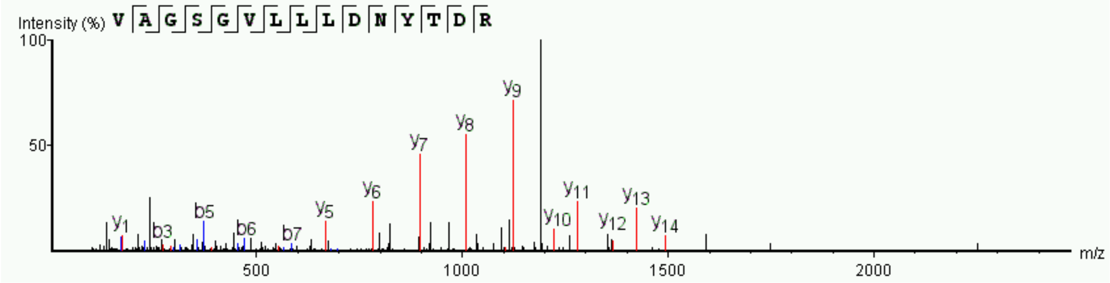

gi|571546330

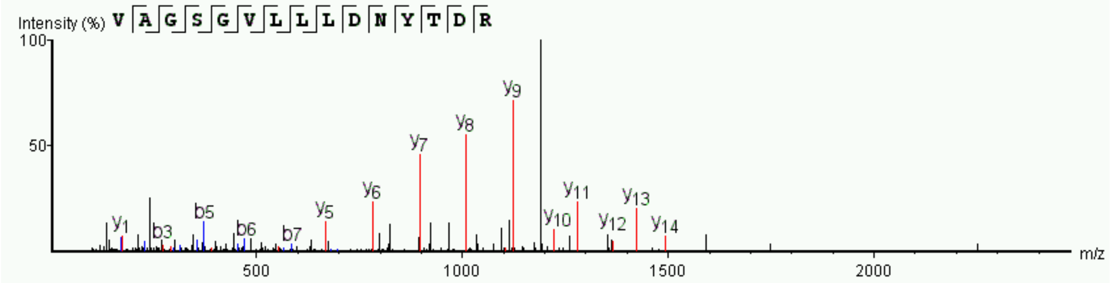

gi|571546334

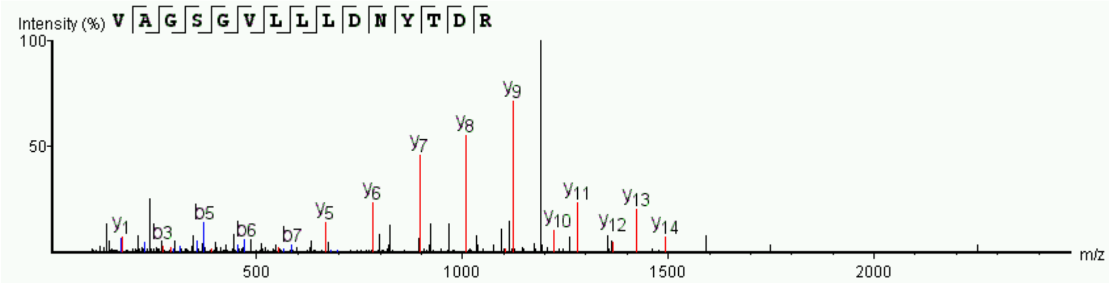

gi|571546338

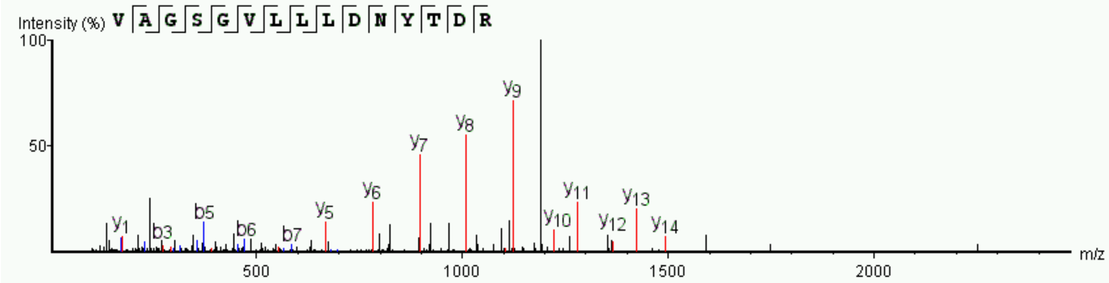

gi|571546341

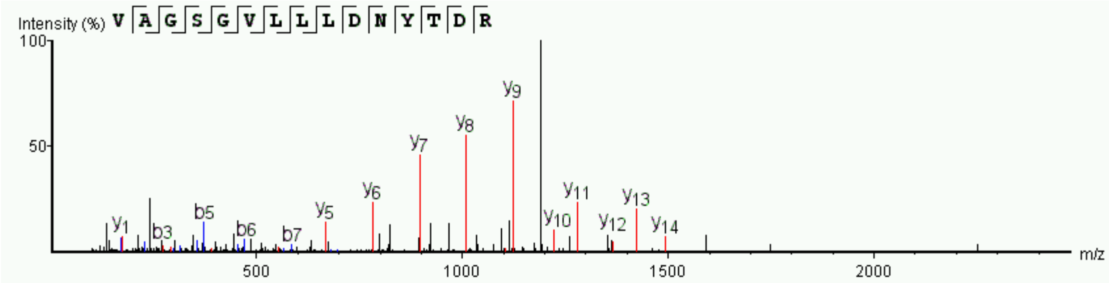

gi|571546277

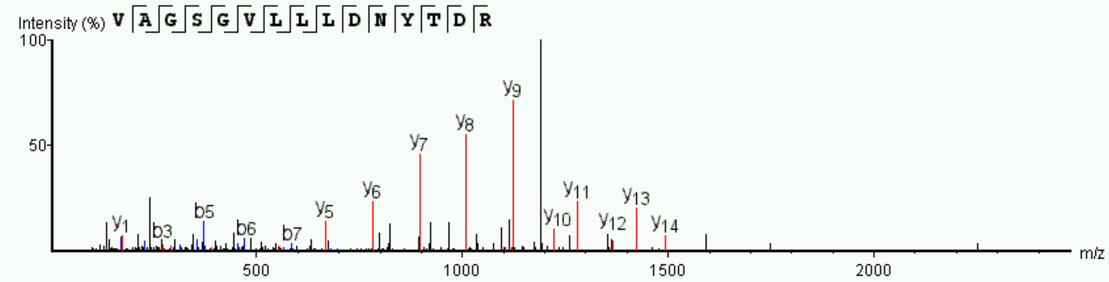

gi|328789199

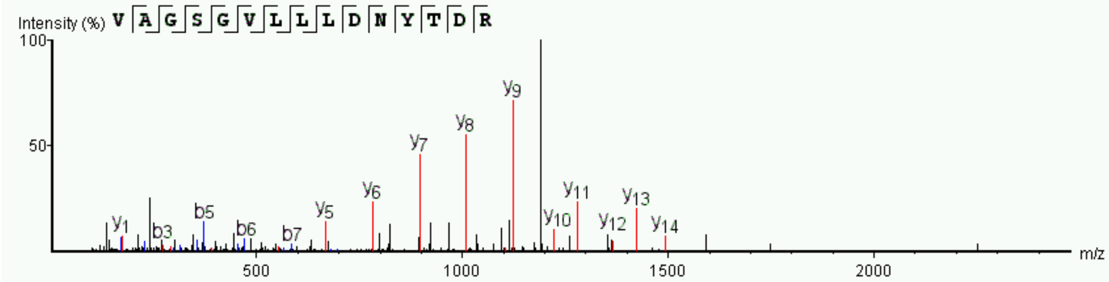

gi|571546281

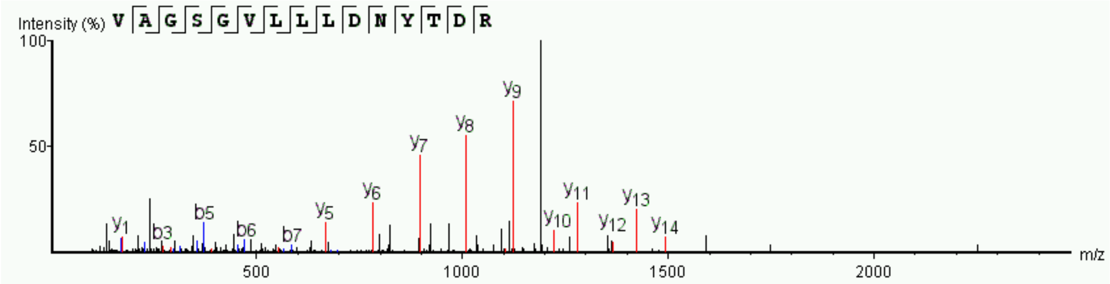

gi|571546285

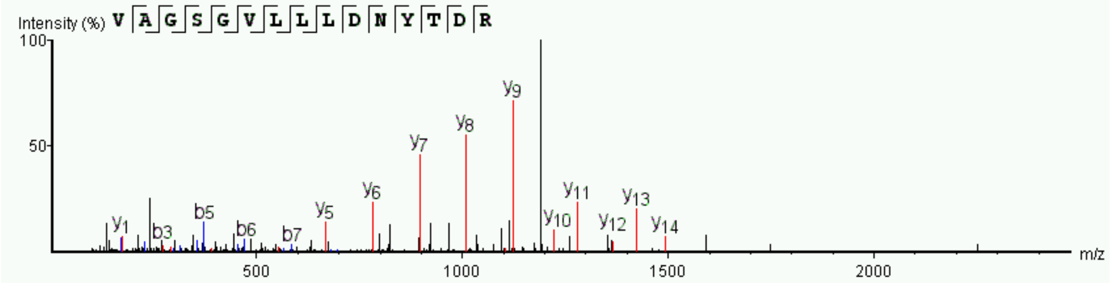

gi|571546289

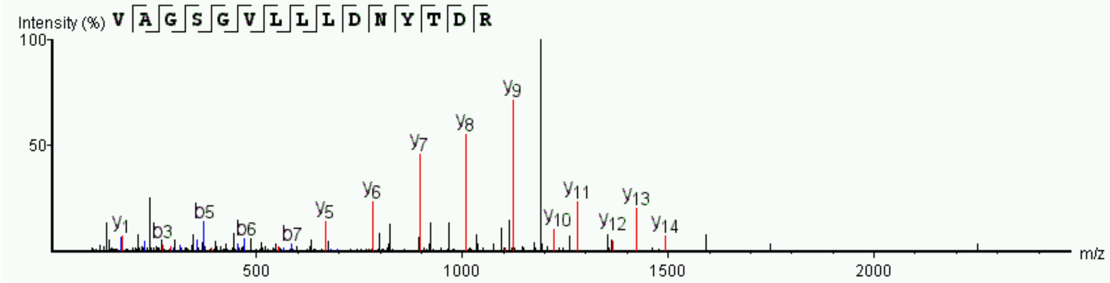

gi|571546293

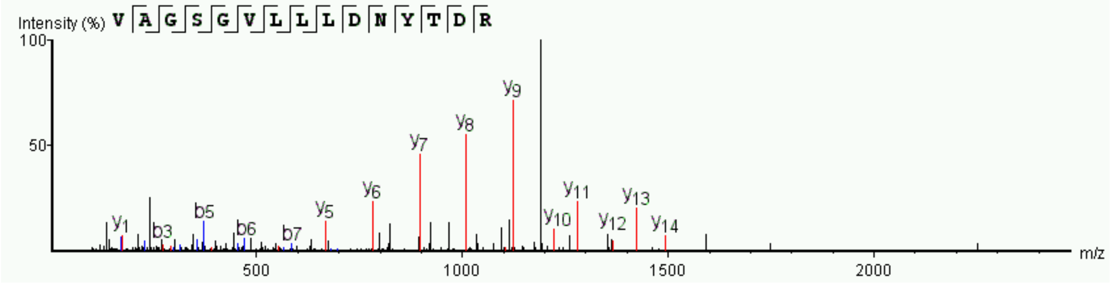

gi|571546296

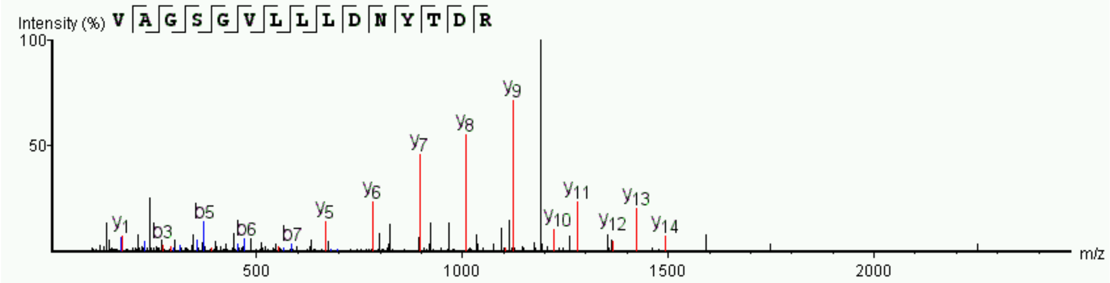

gi|571546300

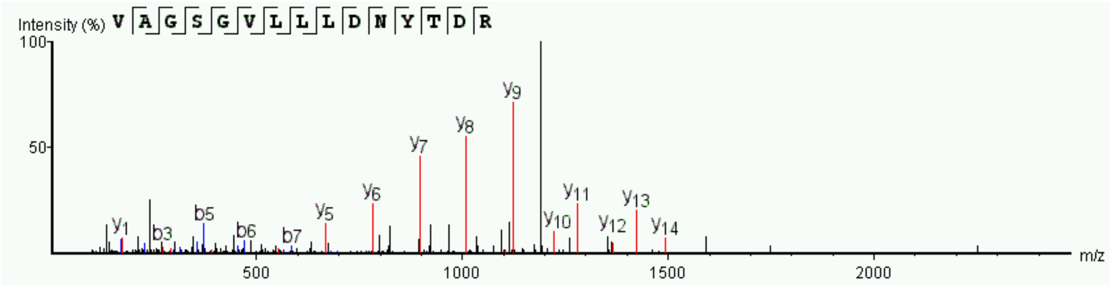

gi|571546304

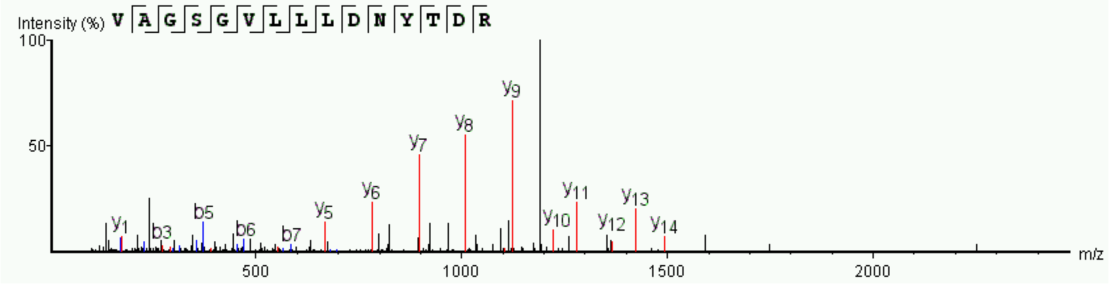

gi|571544763

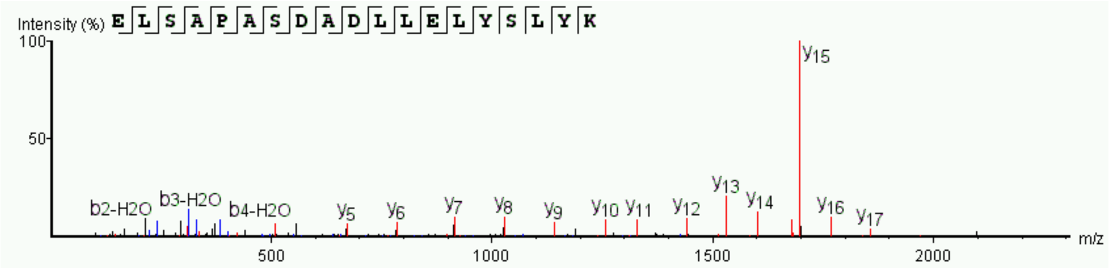

gi|328786659

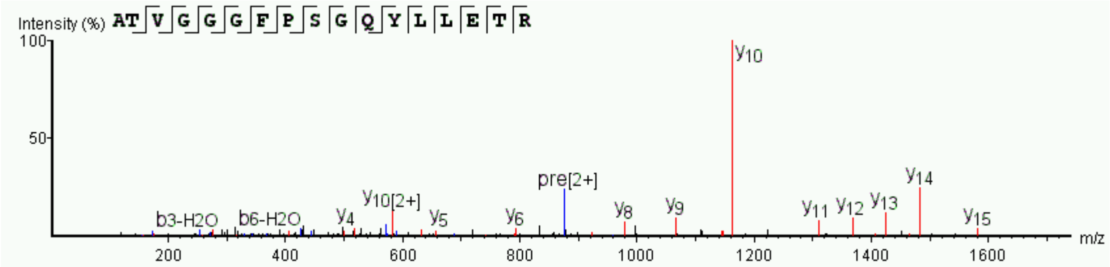

gi|571554418

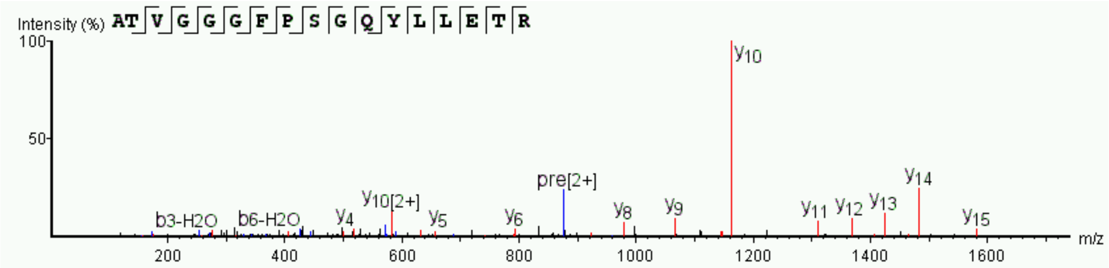

gi|571504340

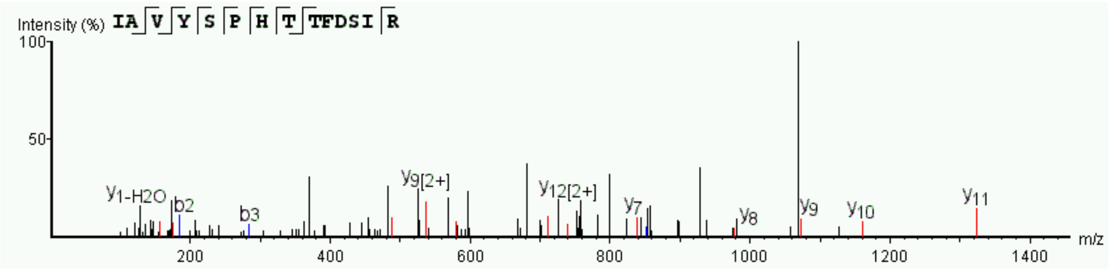

gi|571577493

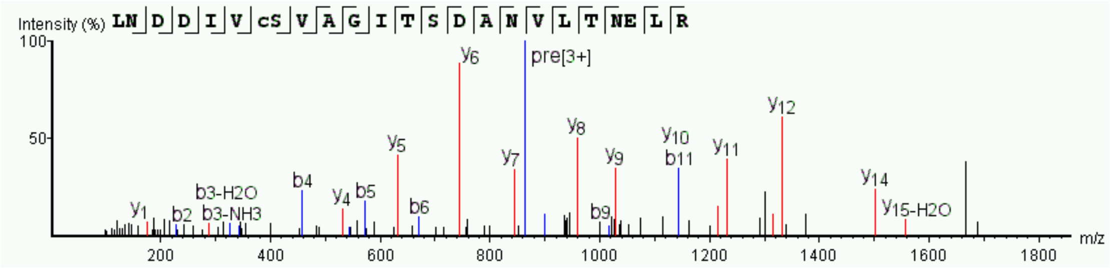

gi|328793299

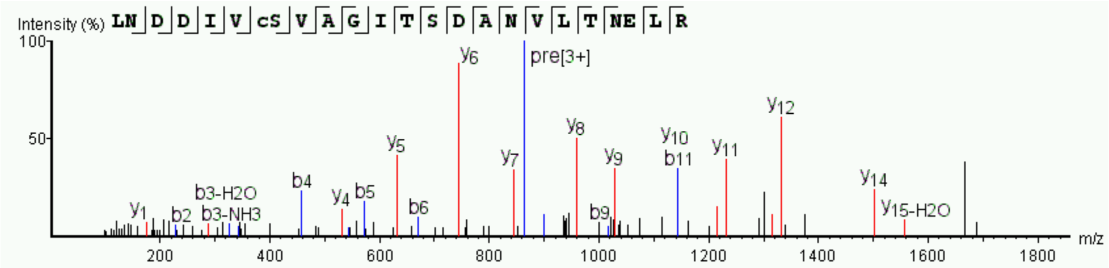

gi|571532691

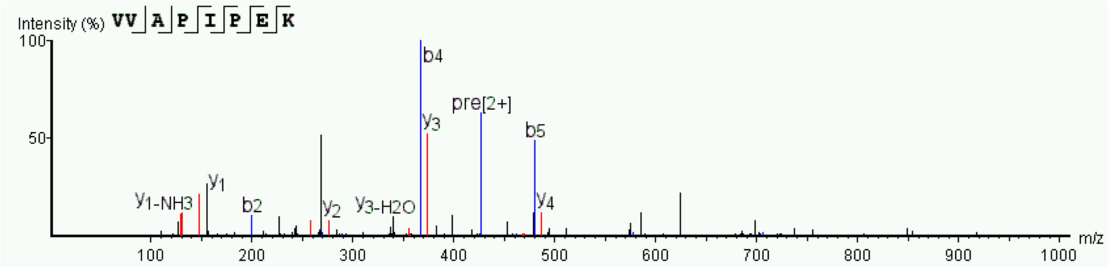

gi|328788528

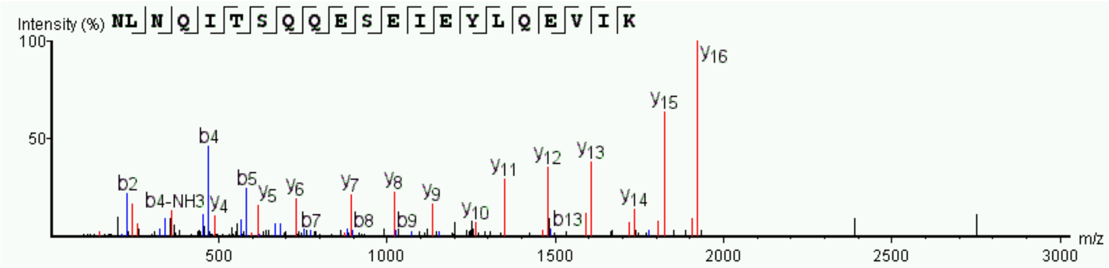

gi|328789575

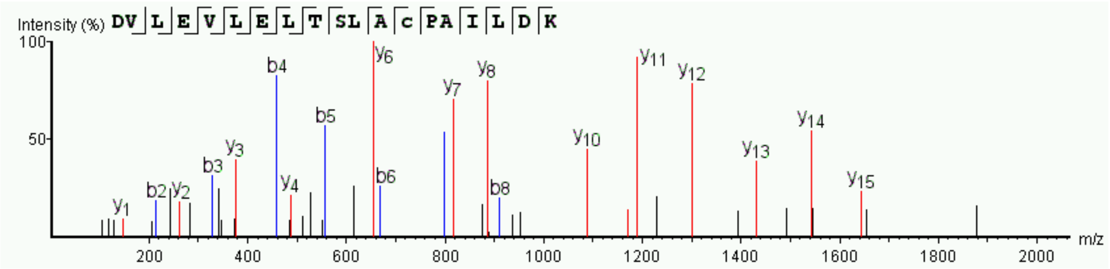

gi|328786568

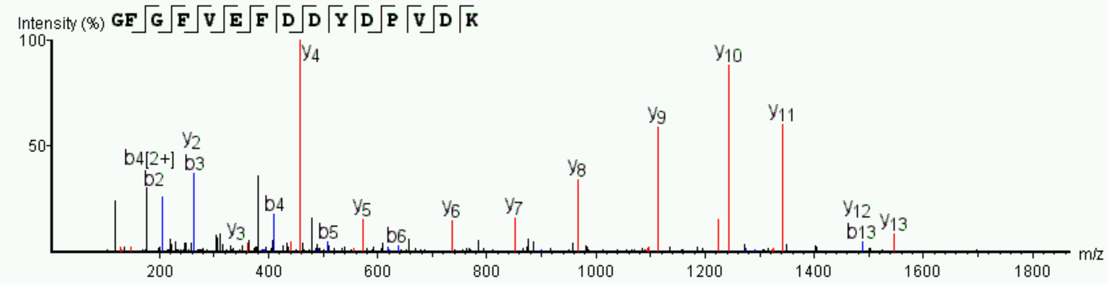

gi|571499266

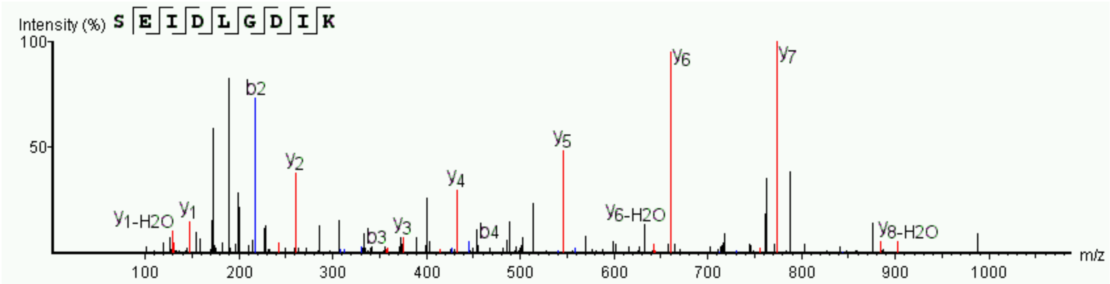

gi|571518074

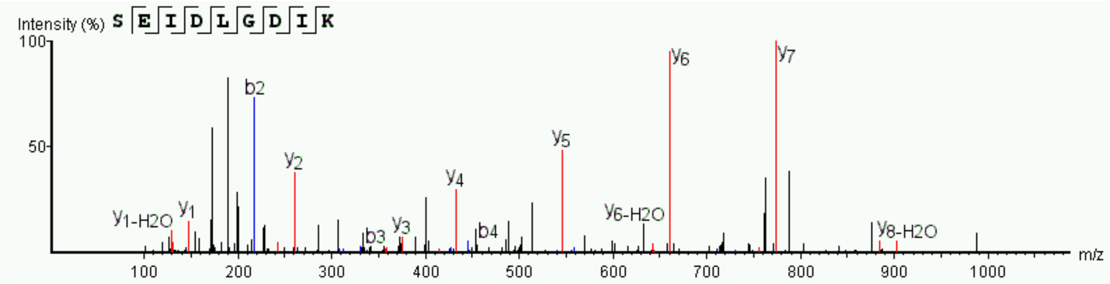

gi|571508187

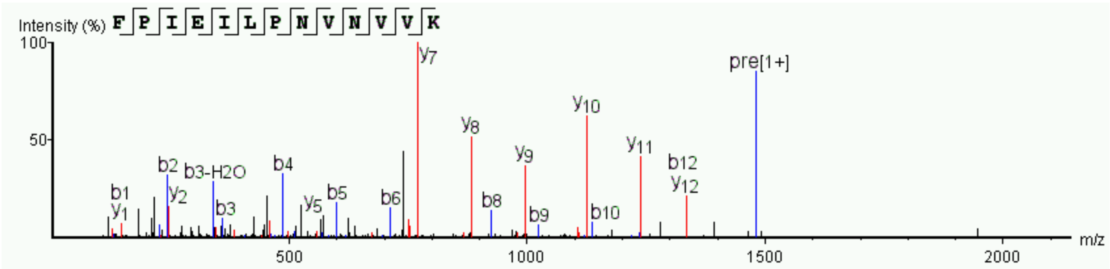

gi|571508183

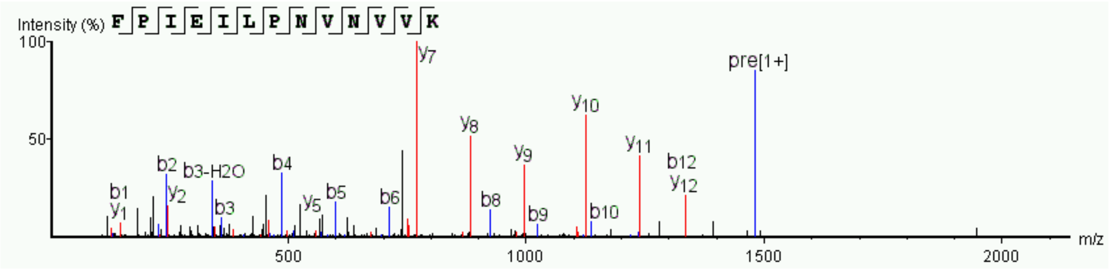

gi|328781494

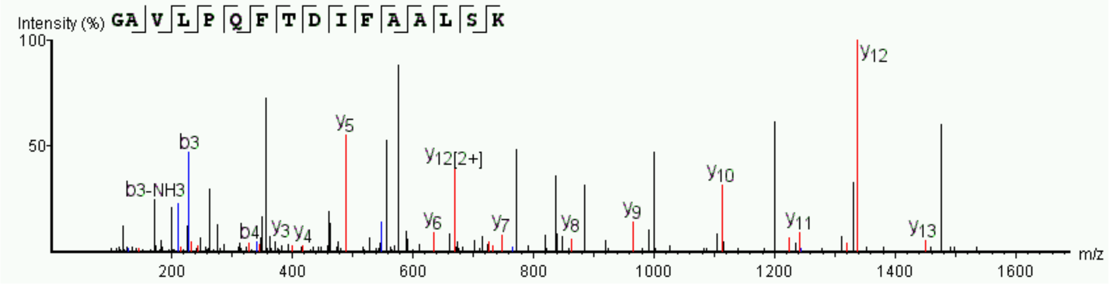

gi|571560291

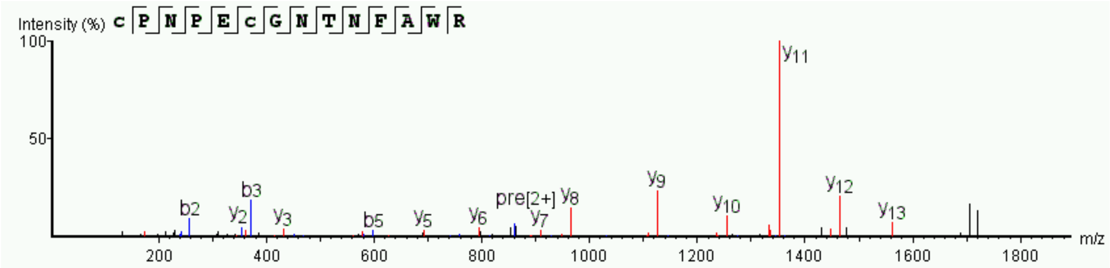

gi|571560294

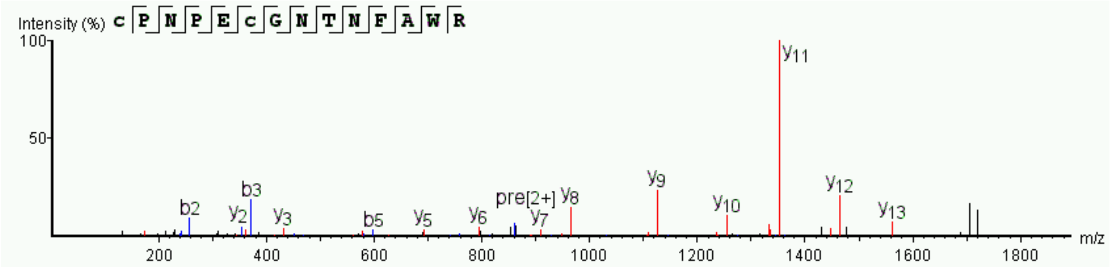

gi|571560288

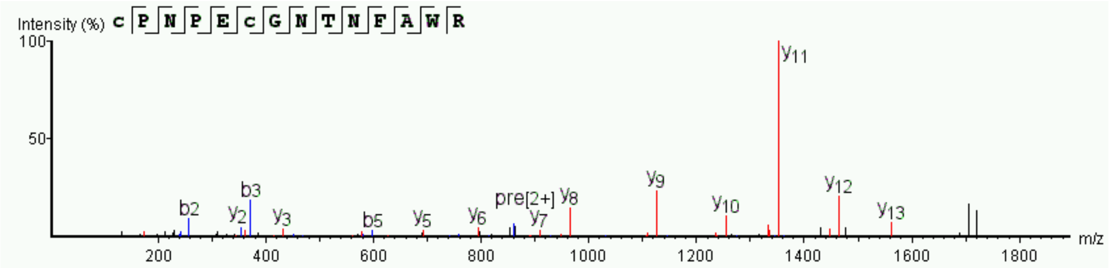

gi|571514456

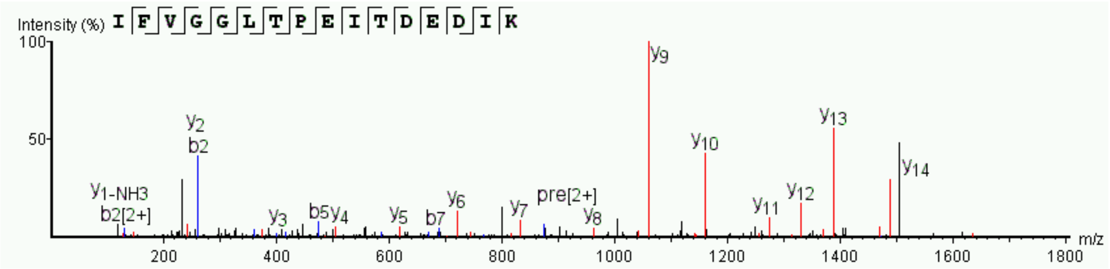

gi|571539233

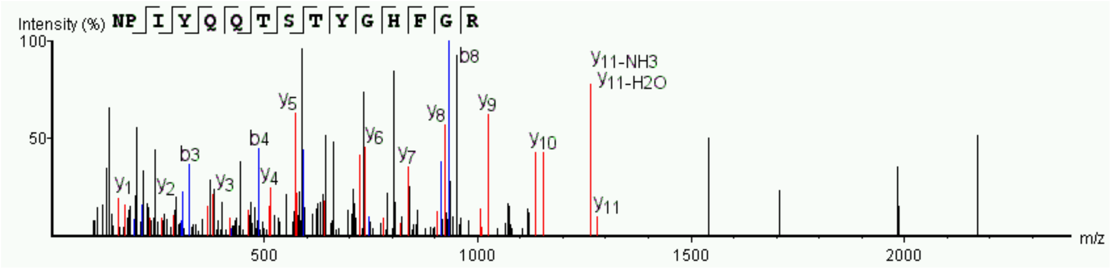

gi|571568001

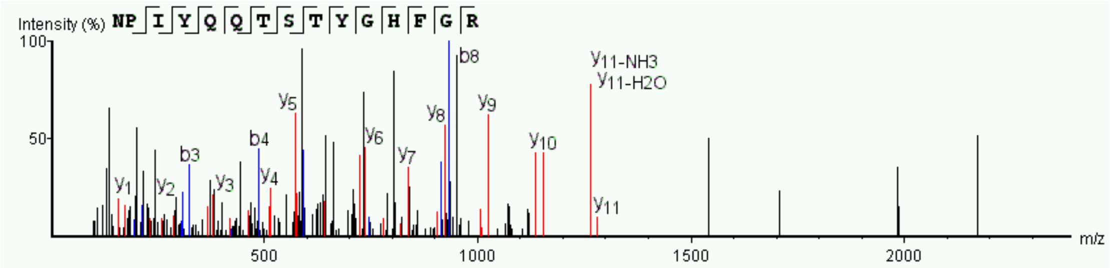

gi|571539230

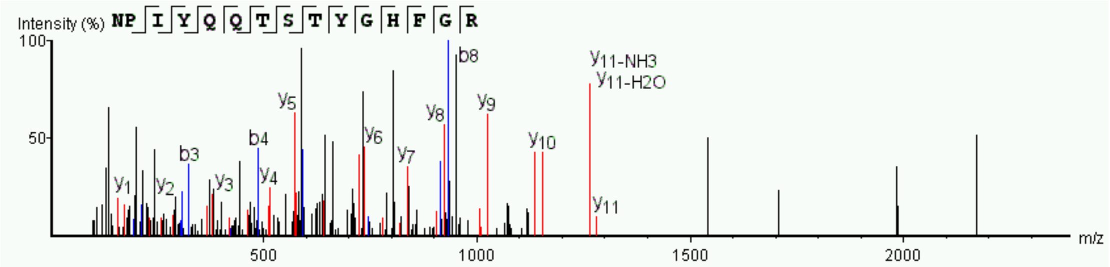

gi|328779563

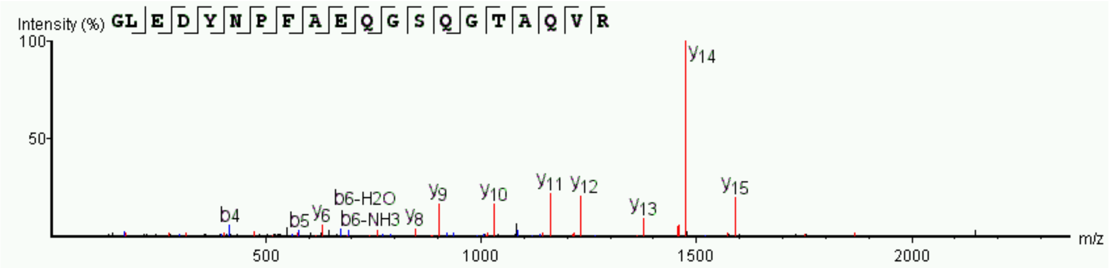

gi|328791460

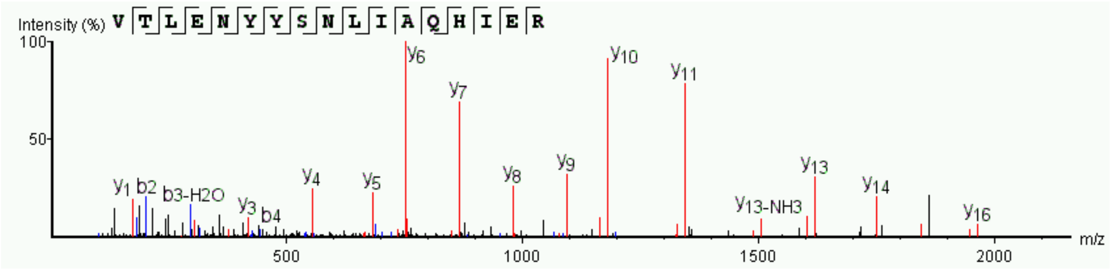

gi|571535995

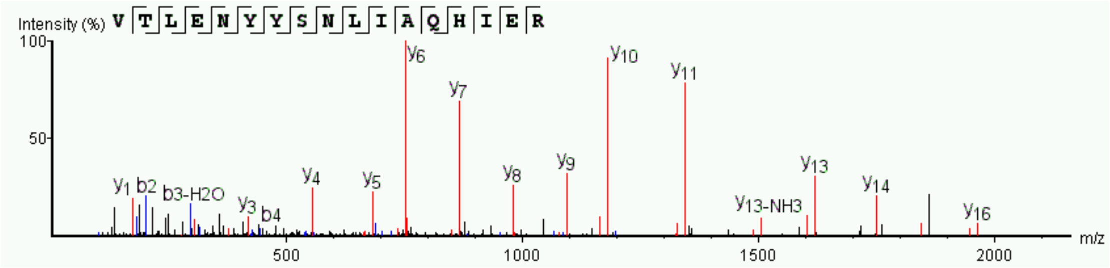

gi|571536003

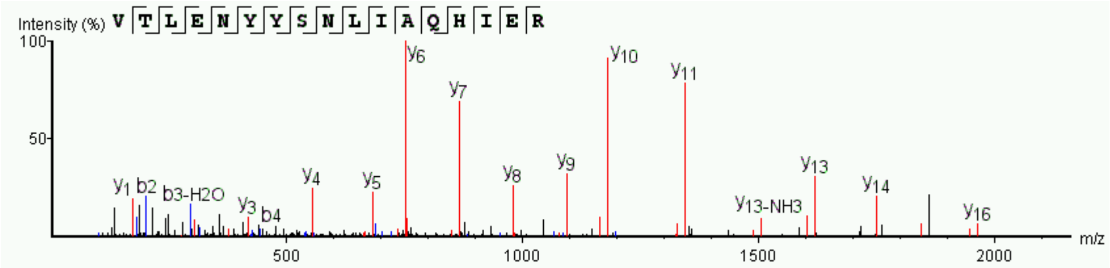

gi|571536006

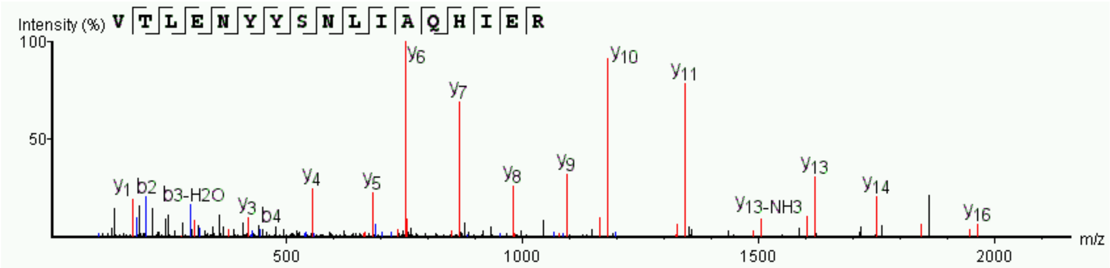

gi|571536010

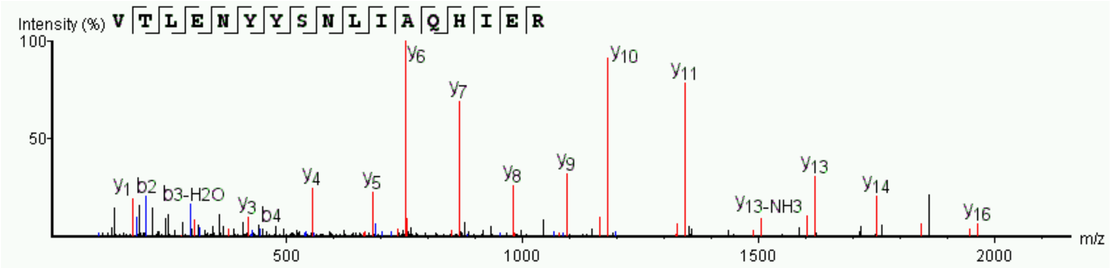

gi|571536013

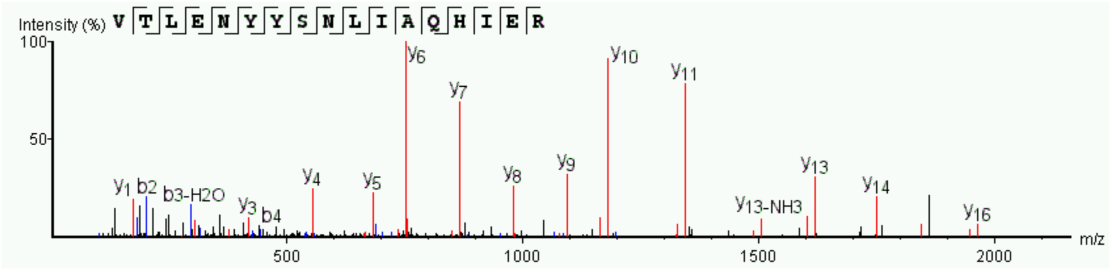

gi|571536016

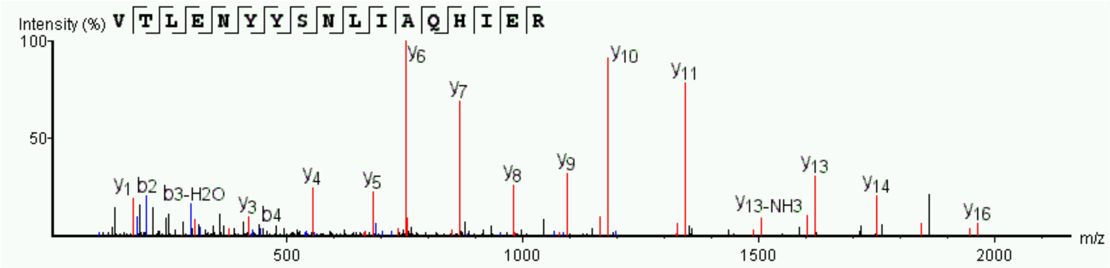

gi|571506283

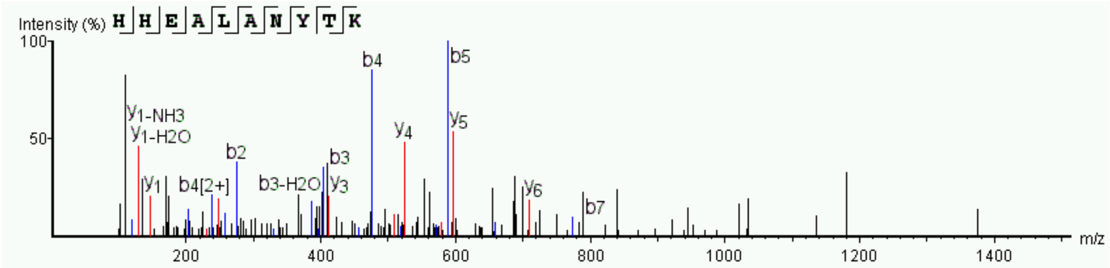

gi|571506280

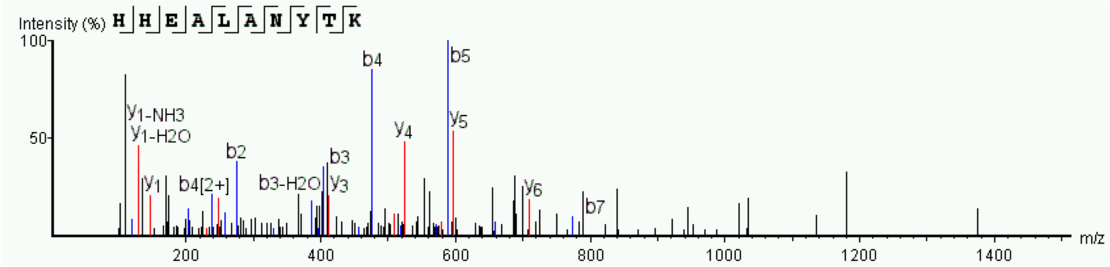

gi|66549336

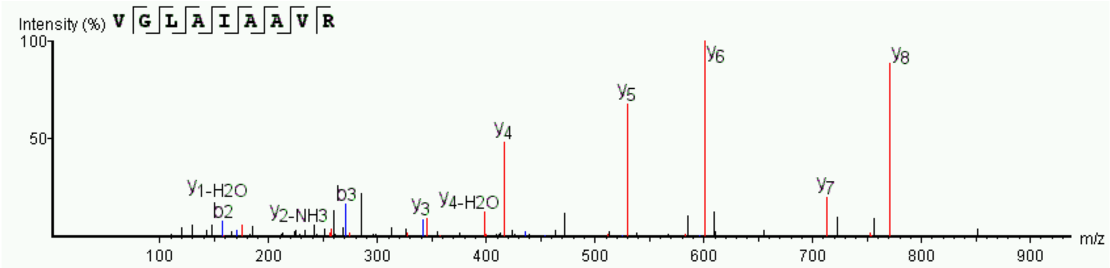

gi|571507731

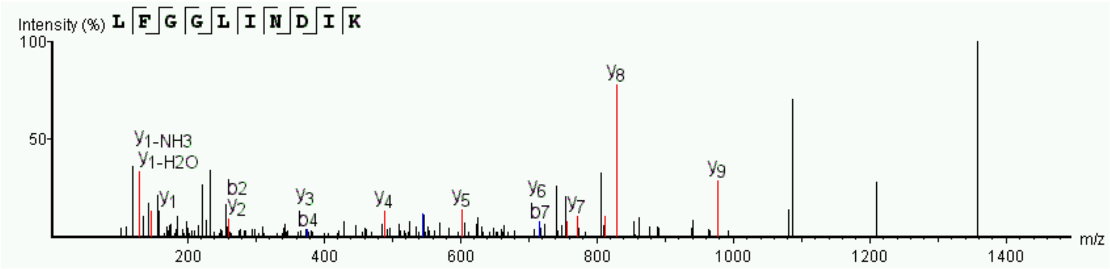

gi|571507770

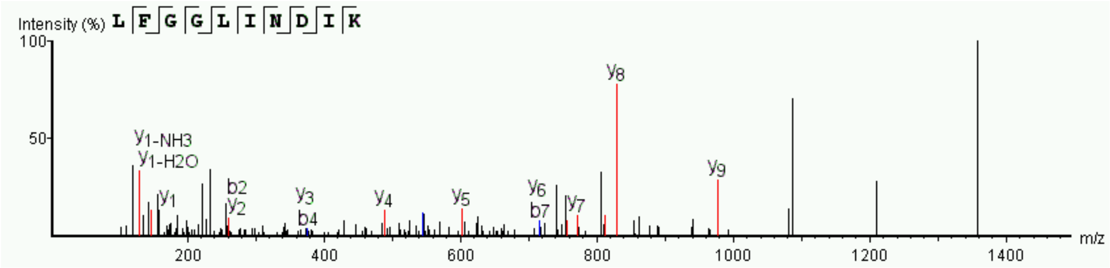

gi|571507774

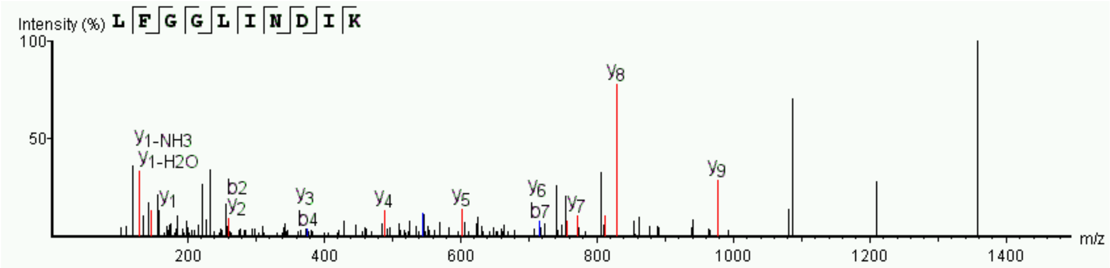

gi|571507777

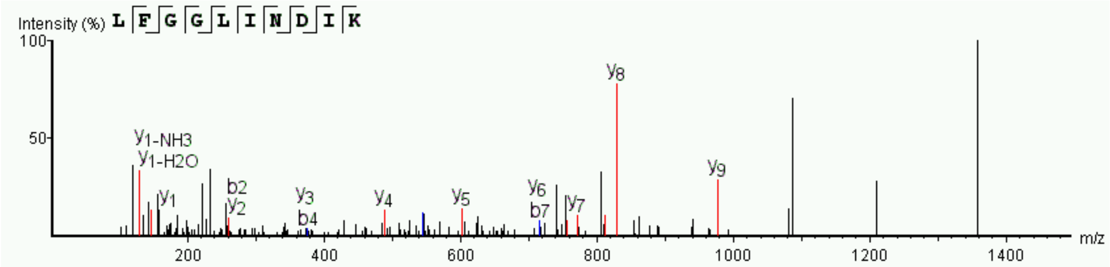

gi|571507781

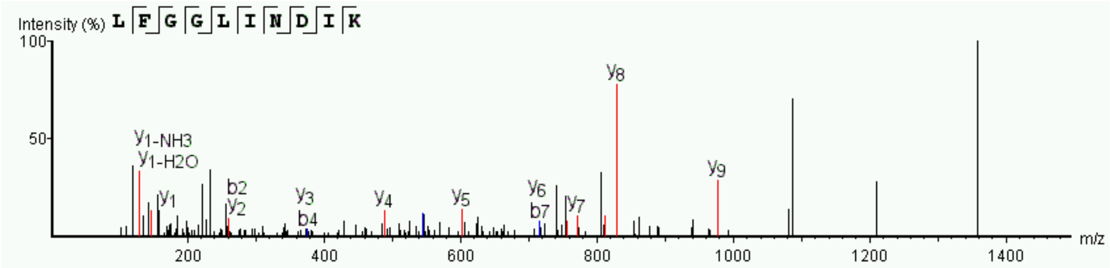

gi|571507785

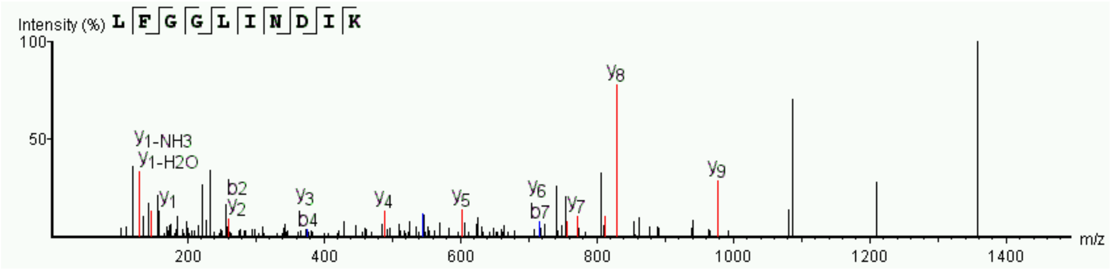

gi|571507789

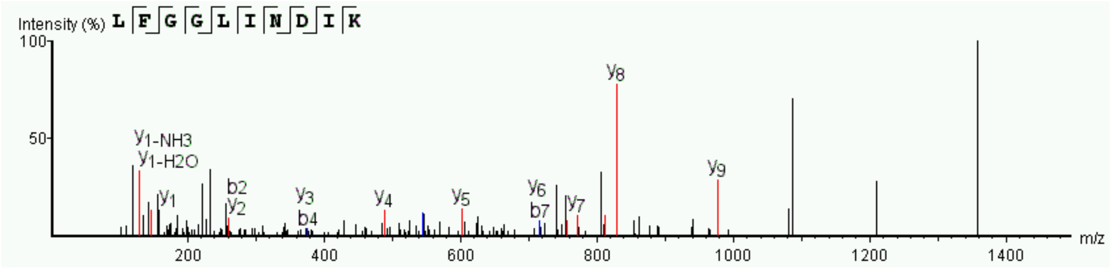

gi|571507793

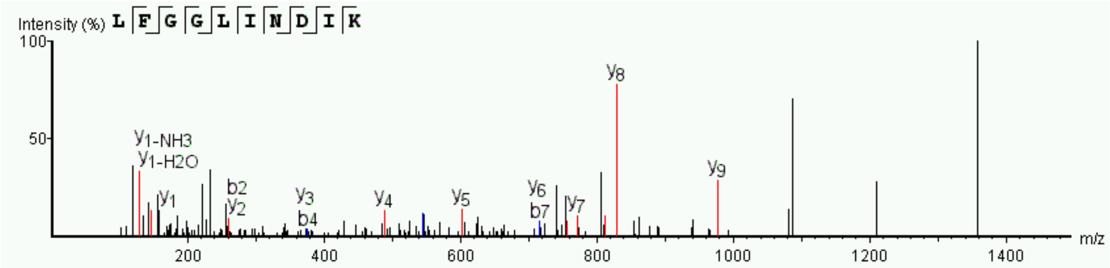

gi|571507797

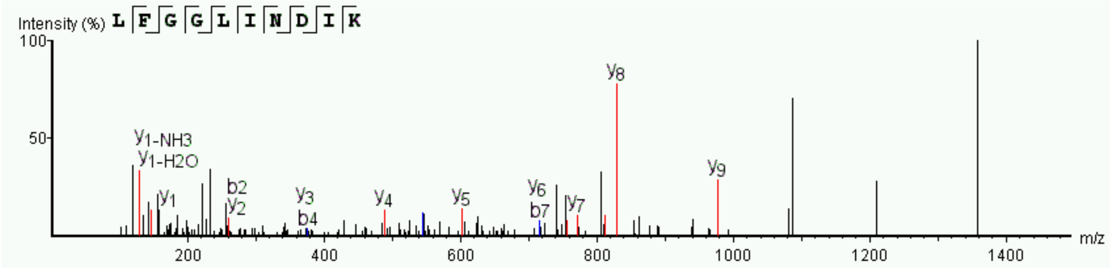

gi|571507800

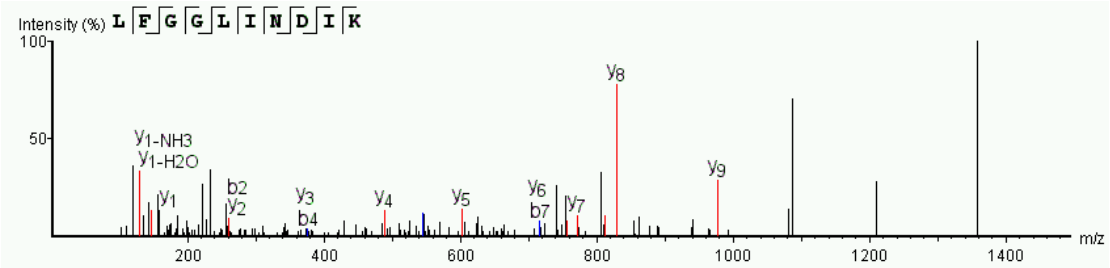

gi|571507735

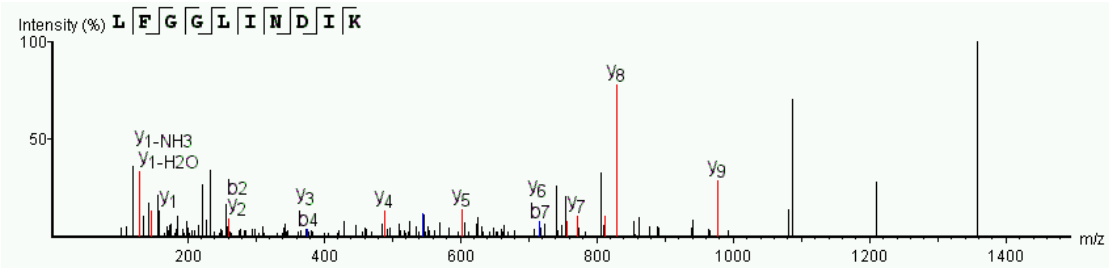

gi|571507738

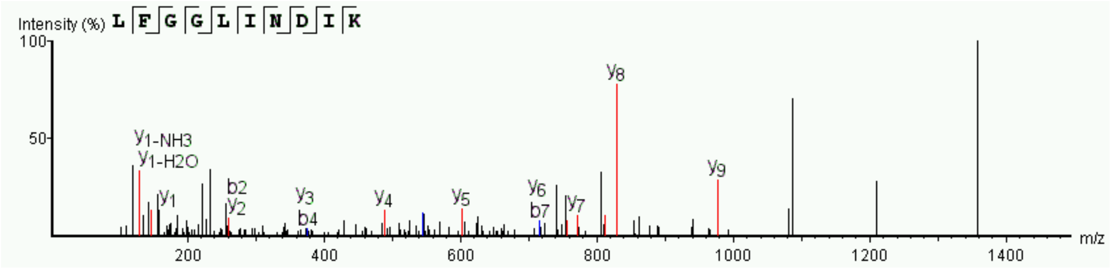

gi|571507742

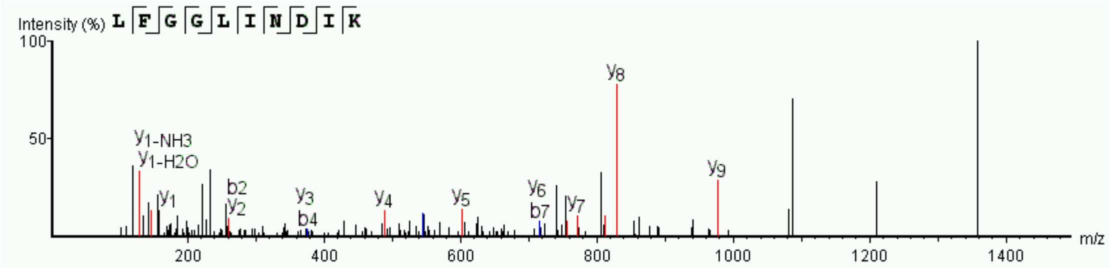

gi|571507754

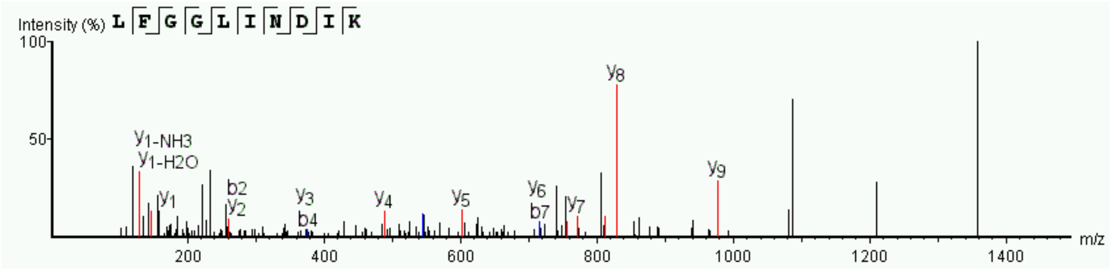

gi|571507757

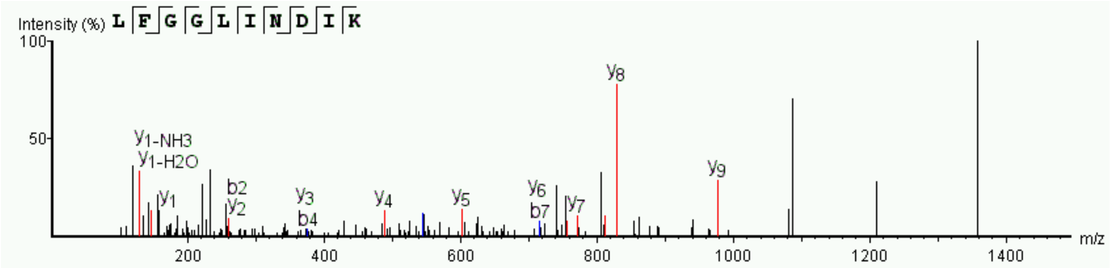

gi|571507760

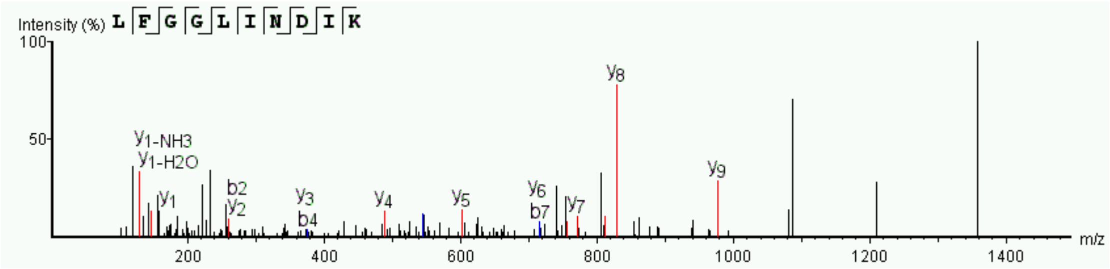

gi|571507763

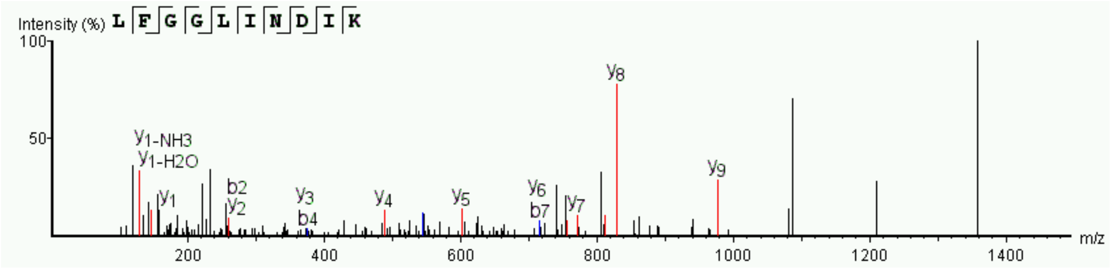

gi|571507767

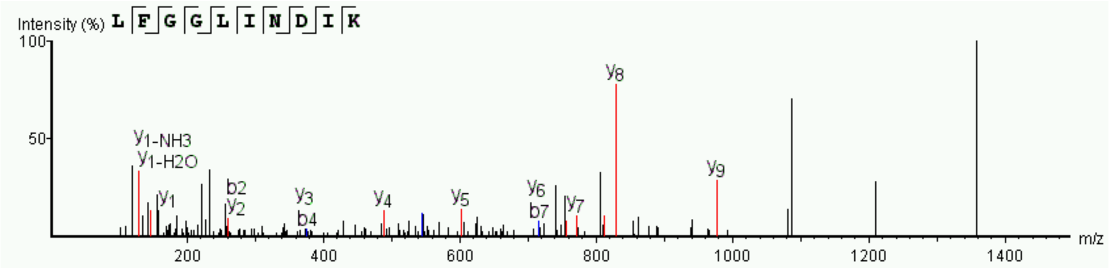

gi|66525345

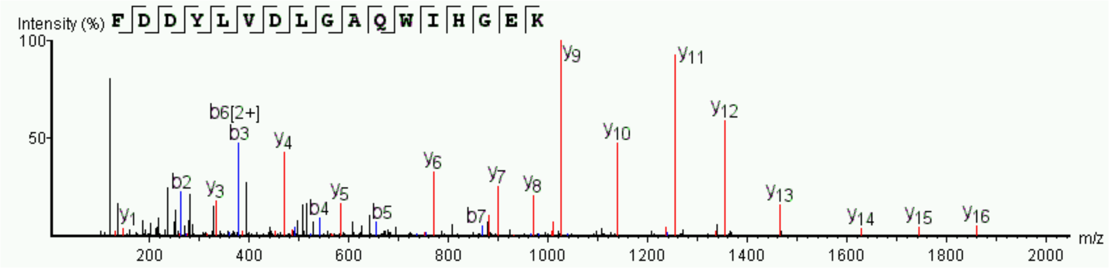

gi|571556649

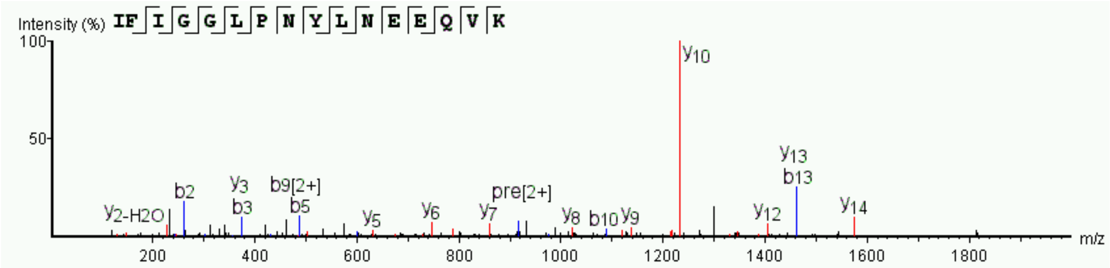

gi|66520699

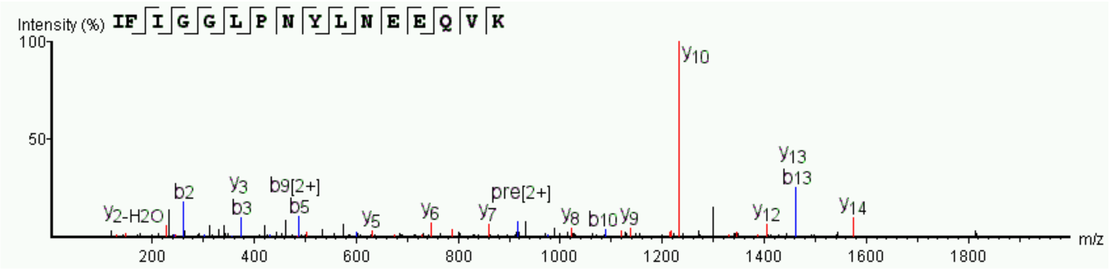

gi|110762818

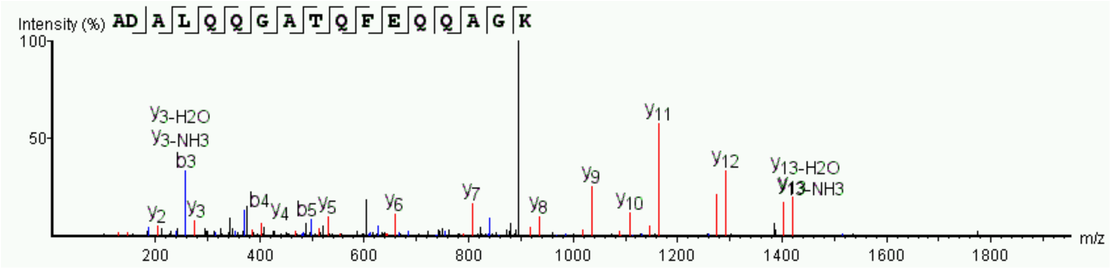

gi|571572247

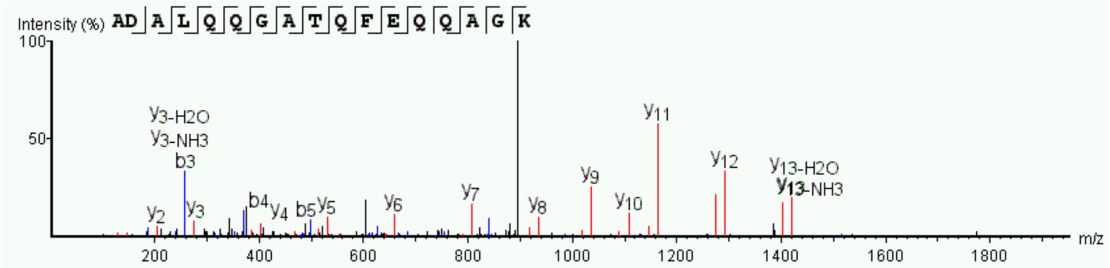

gi|571541223

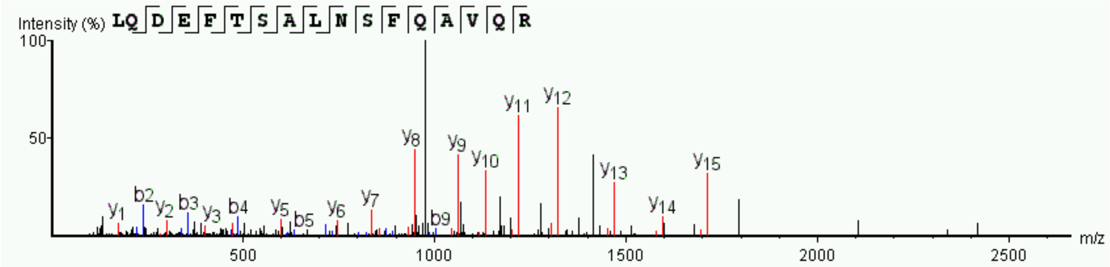

gi|571541219

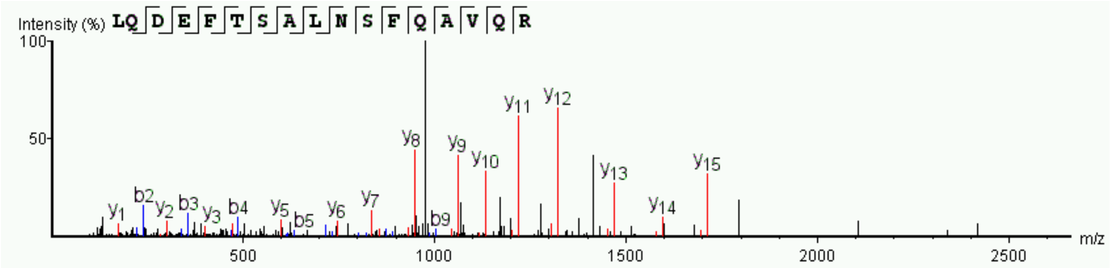

gi|571541221

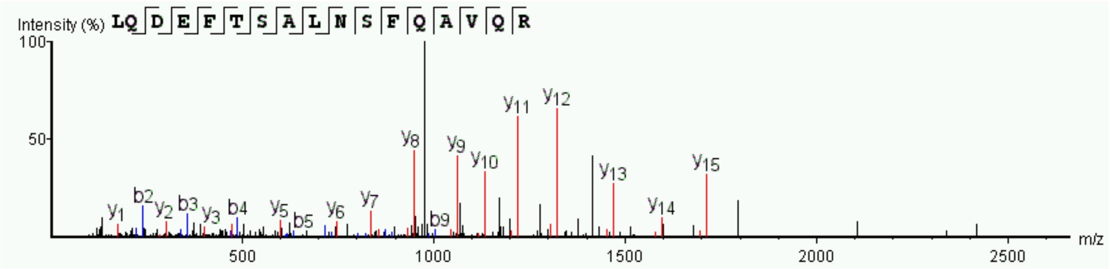

gi|571520114

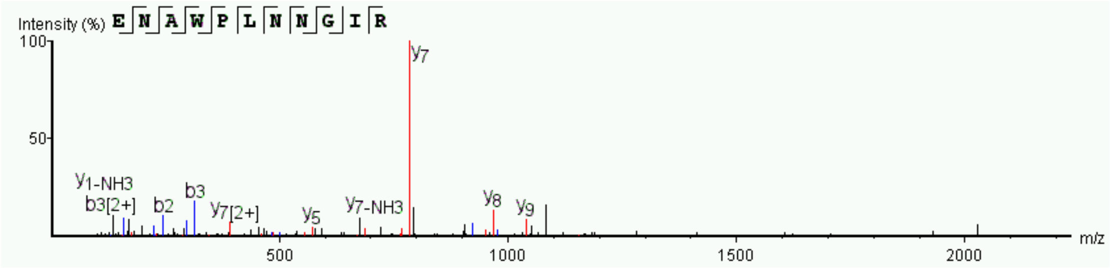

gi|571520119

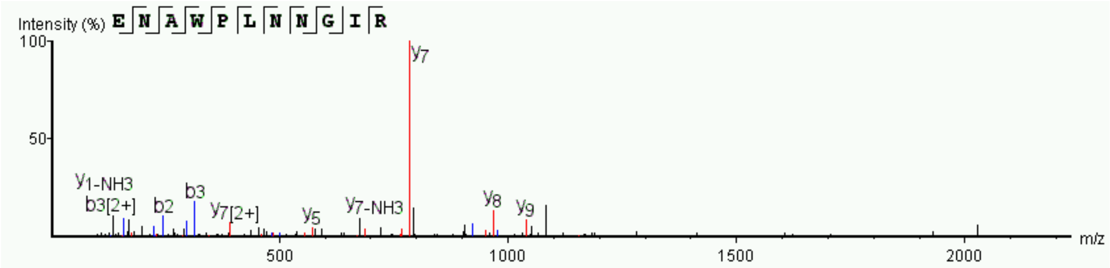

gi|328788626

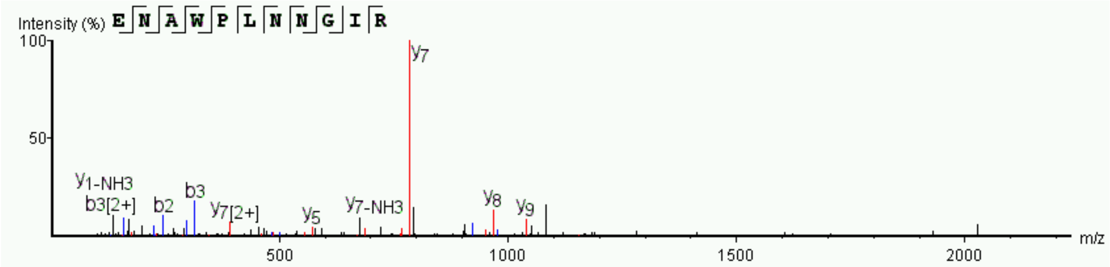

gi|571520111

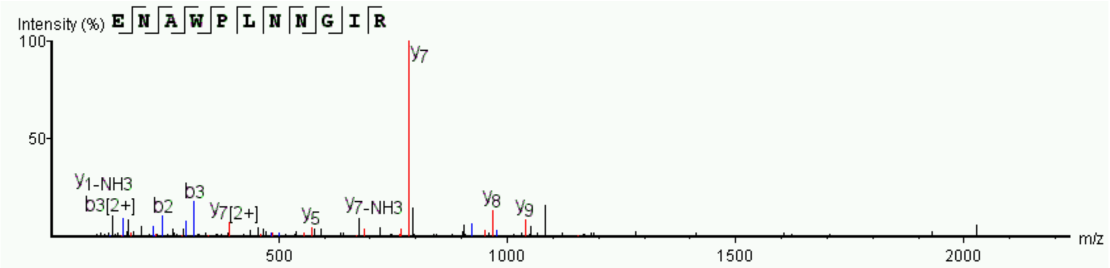

gi|571522078

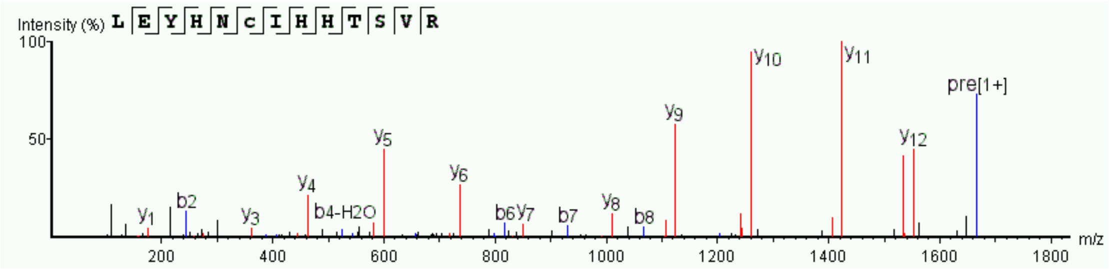

gi|571569154

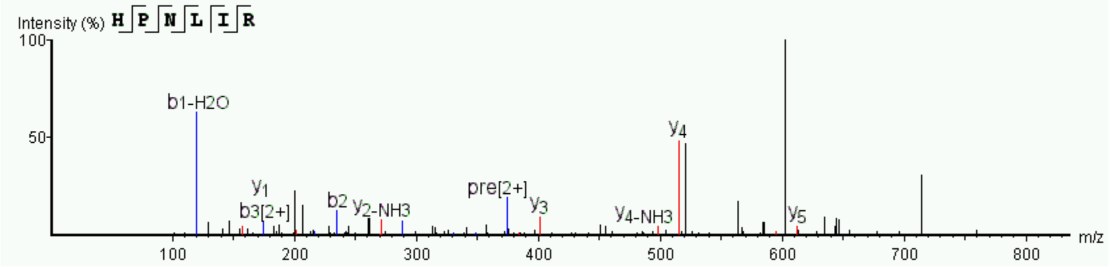

gi|48104995

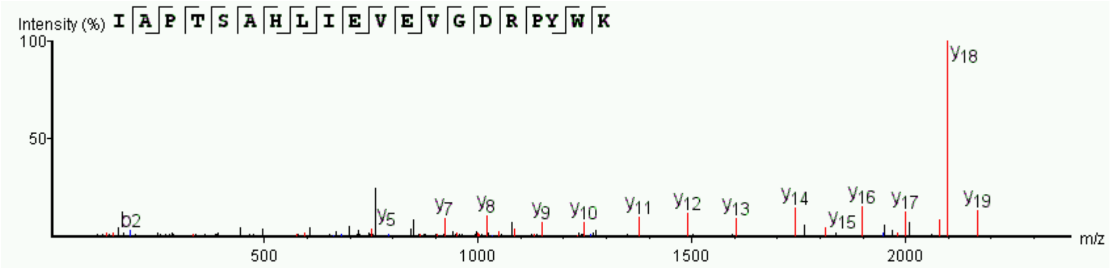

gi|571517348

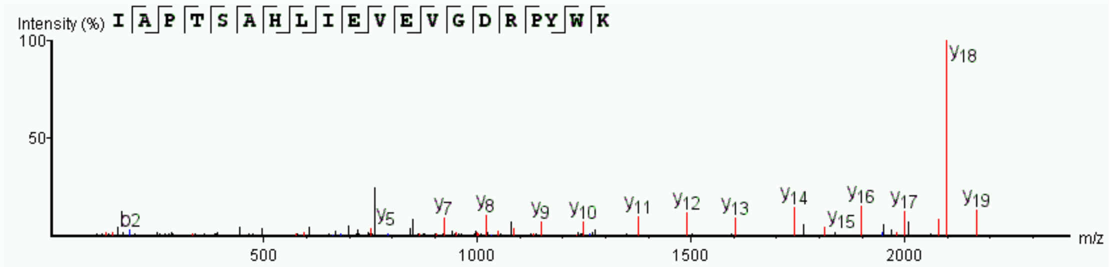

gi|48096617

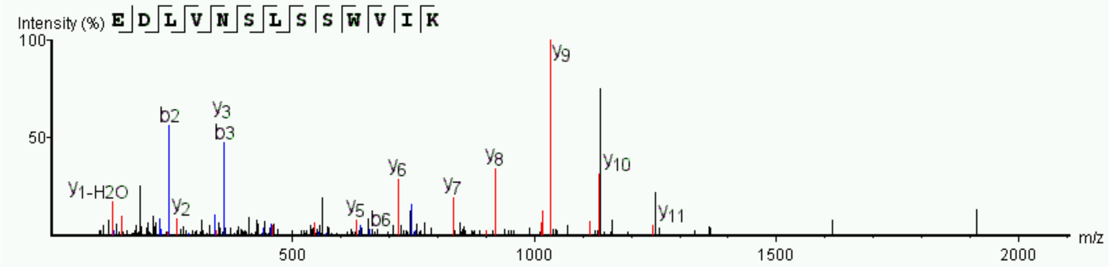

gi|66556835

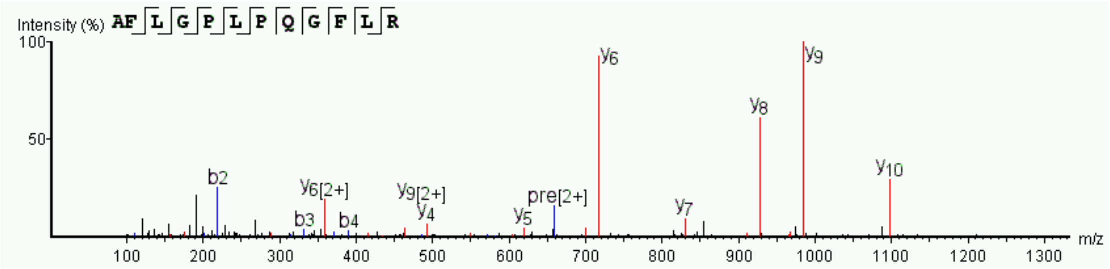

gi|328775950

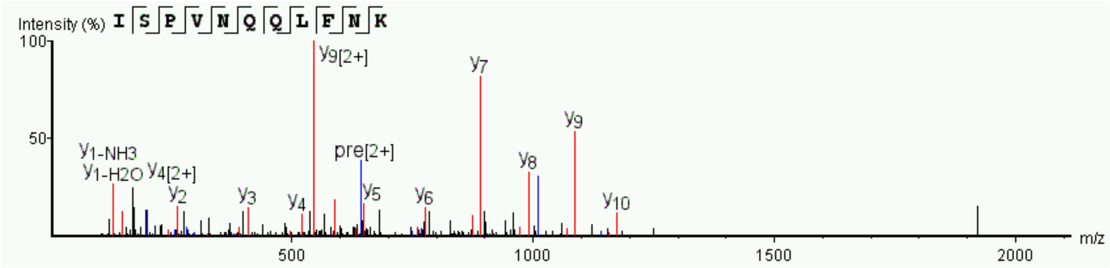

gi|66507736

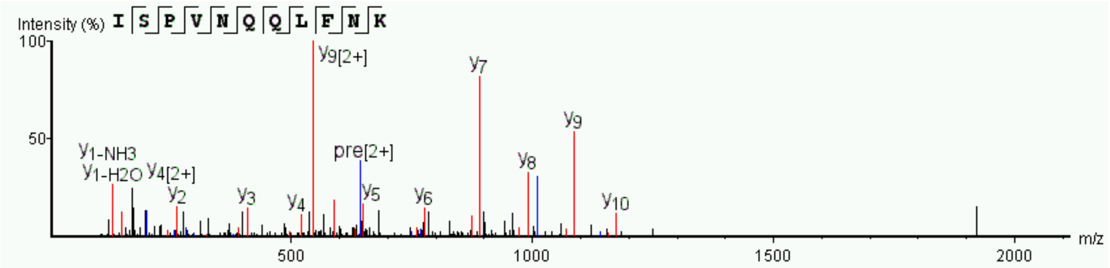

gi|571537241

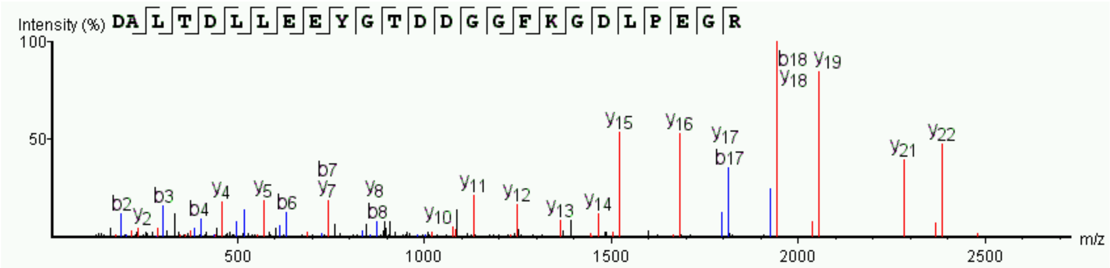

gi|571537249

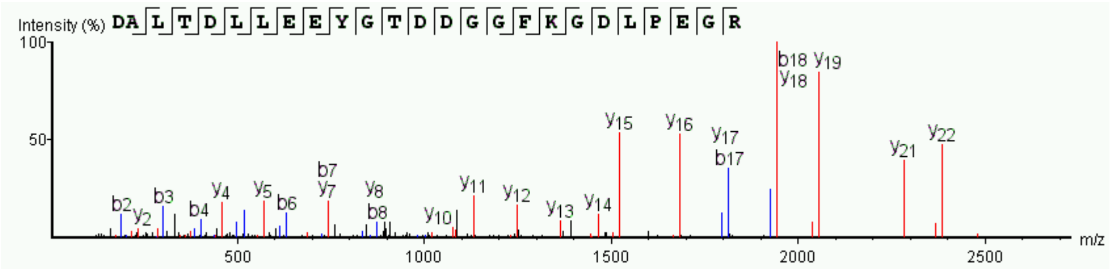

gi|571537256

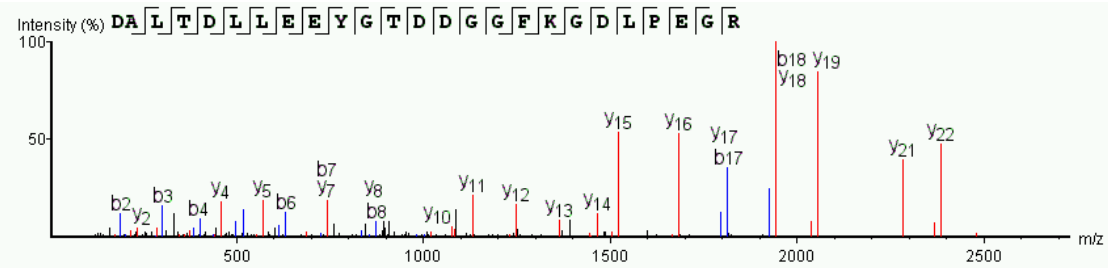

gi|66529630

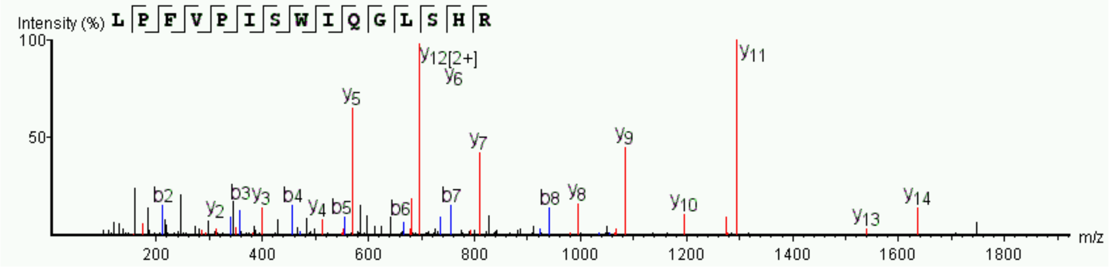

gi|110756641

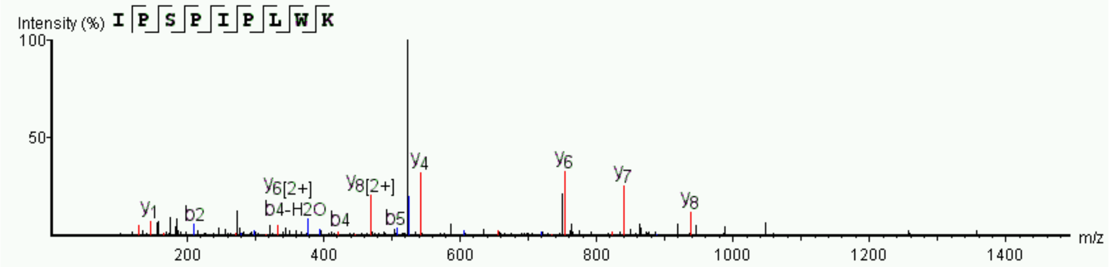

gi|328789935

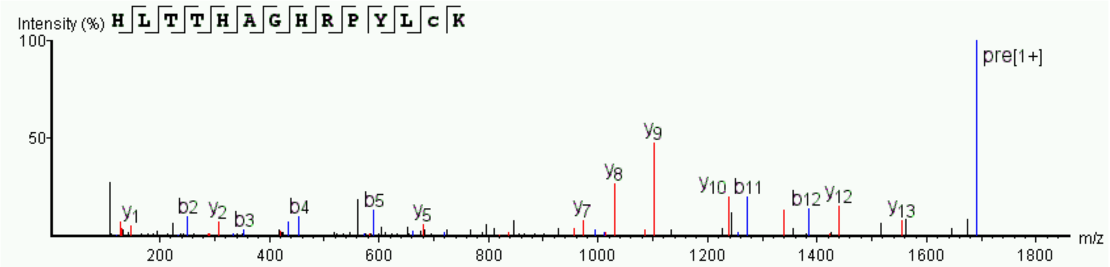

gi|571552435

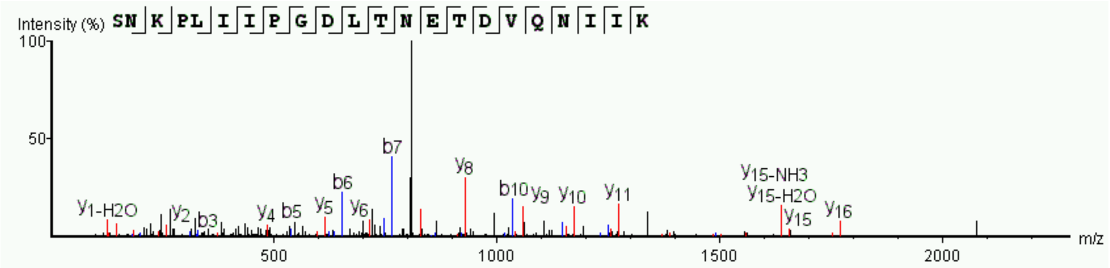

gi|571533174

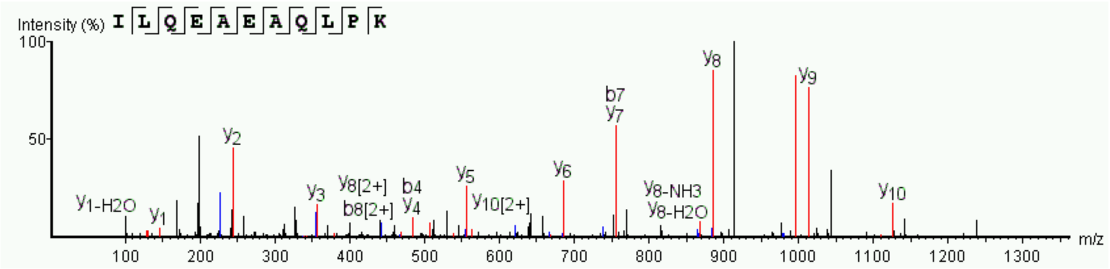

gi|328782269

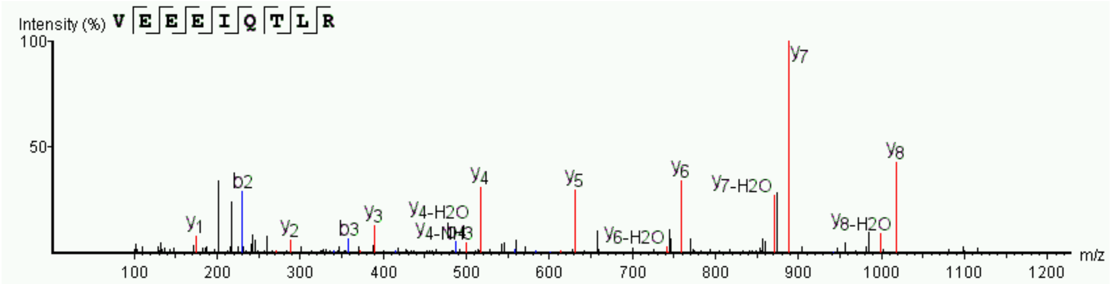

gi|571552571

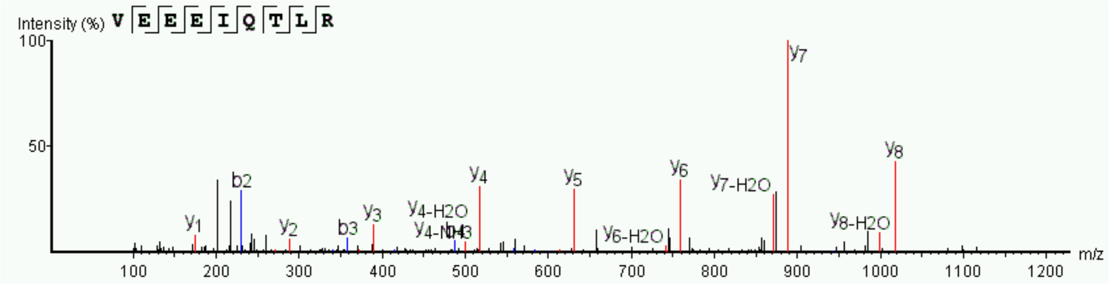

gi|571541896

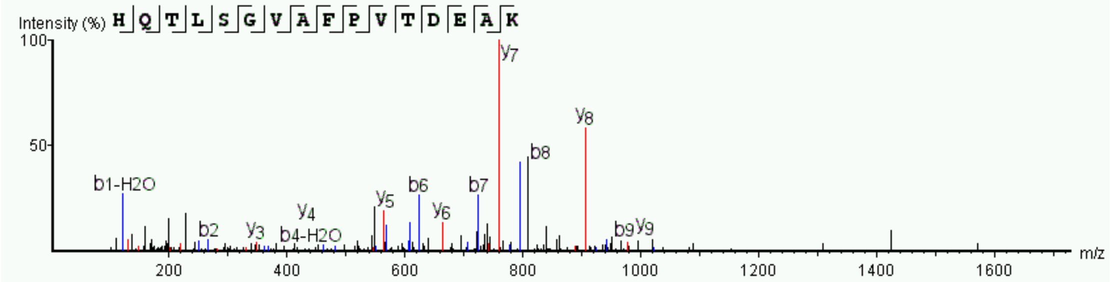

gi|571541899

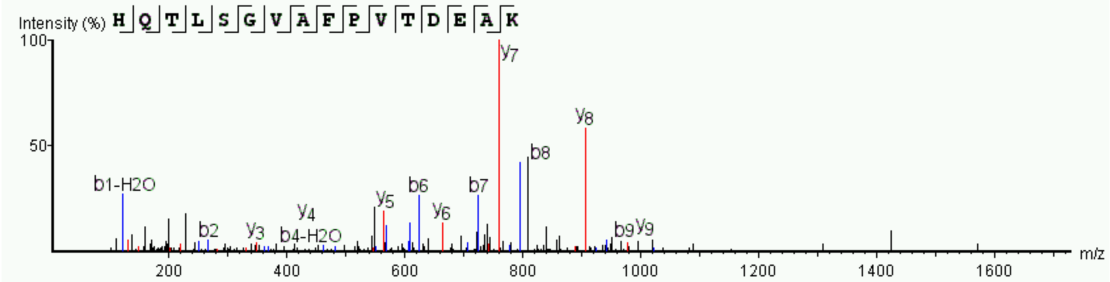

gi|571541903

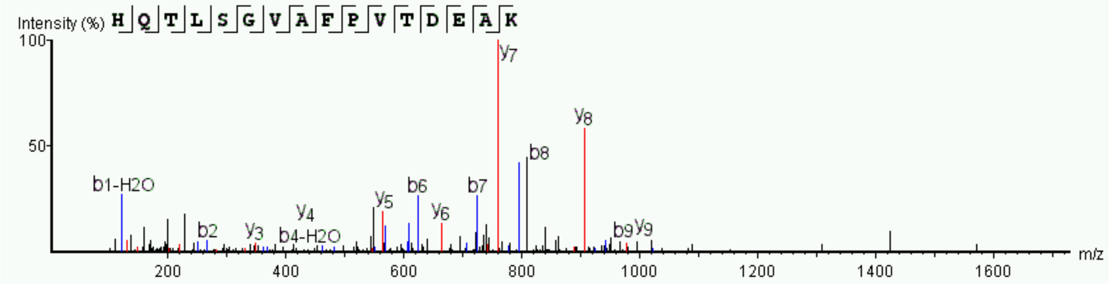

gi|571503359

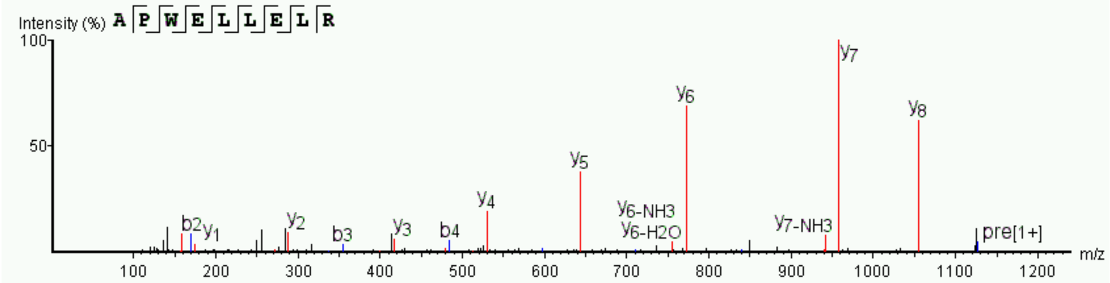

gi|571503362

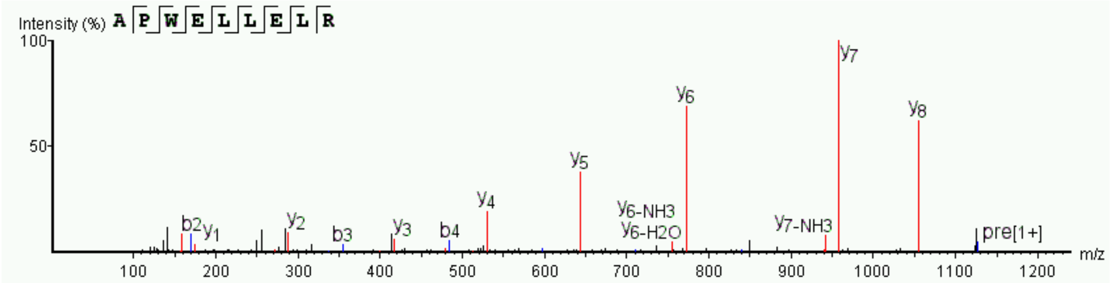

gi|571503366

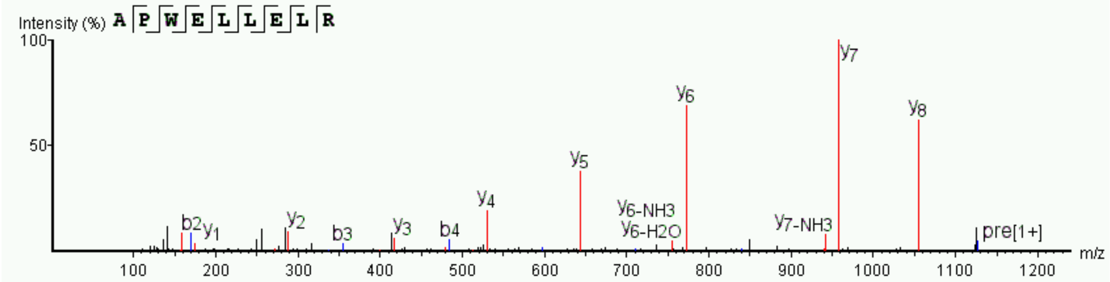

gi|571503369

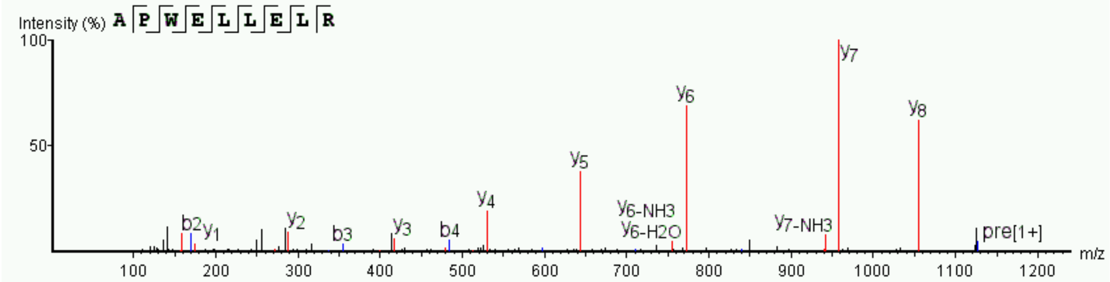

gi|571503372

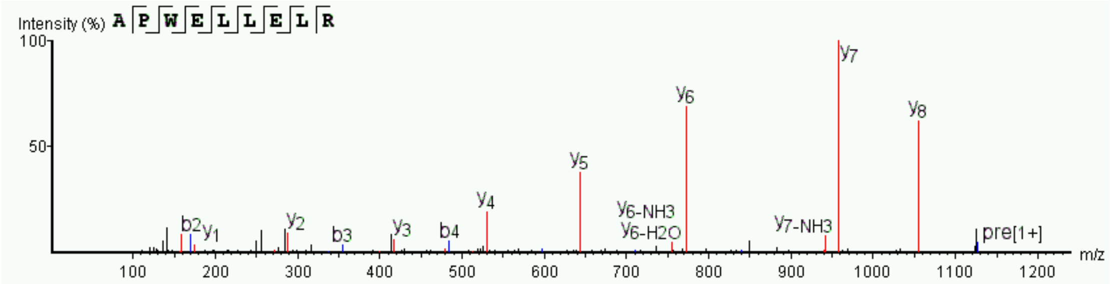

gi|571503376

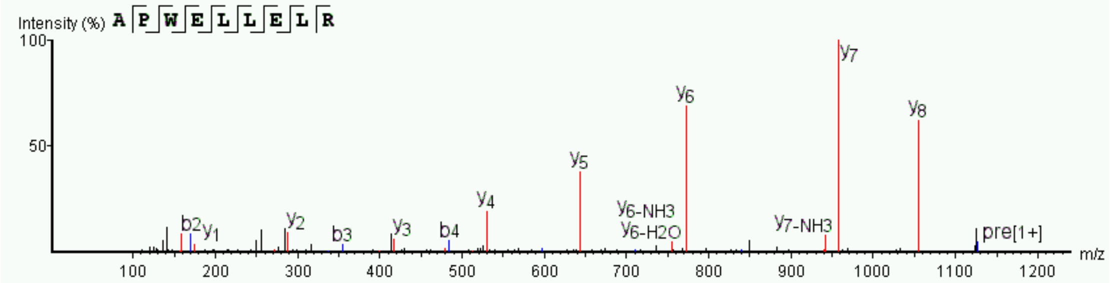

gi|571503379

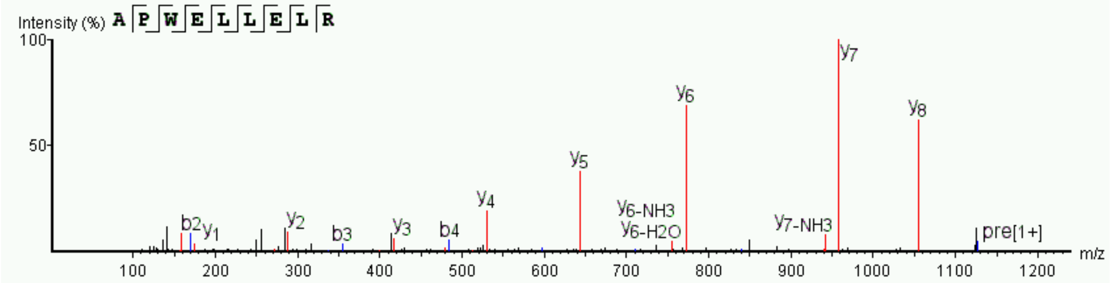

gi|328788805

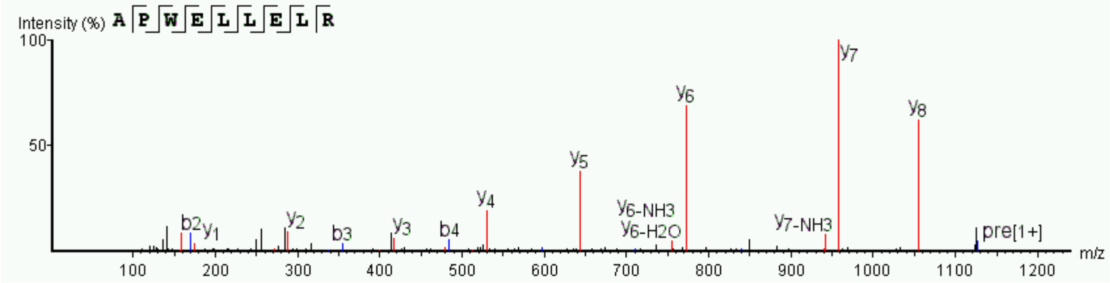

gi|571571820

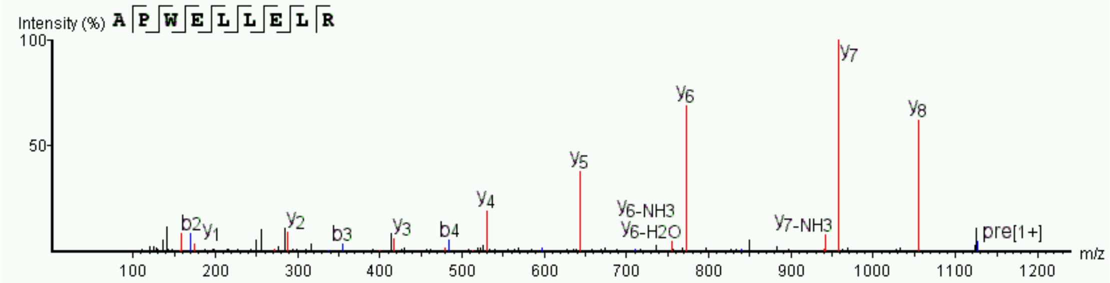

gi|571571824

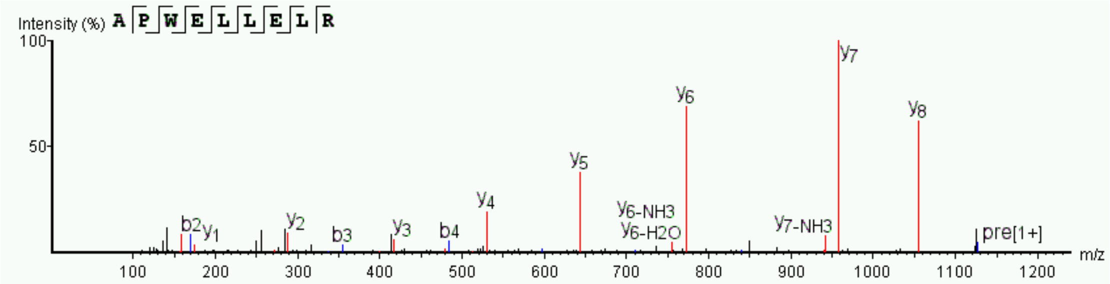

gi|571571828

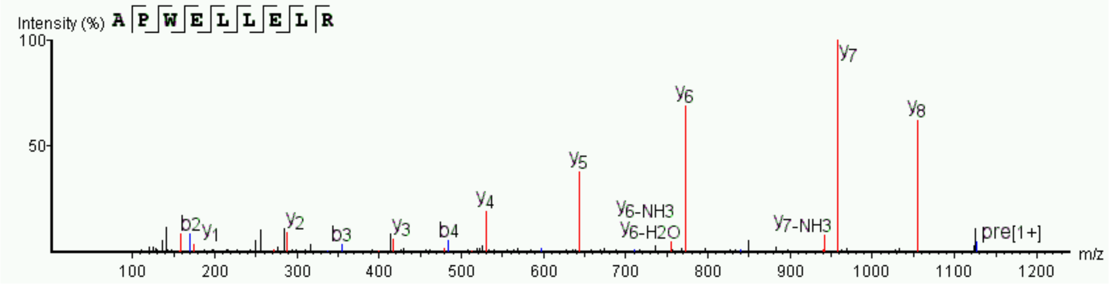

gi|571577446

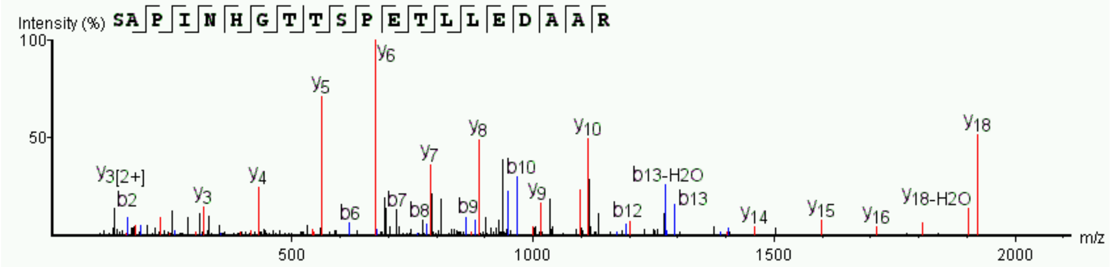

gi|571577448

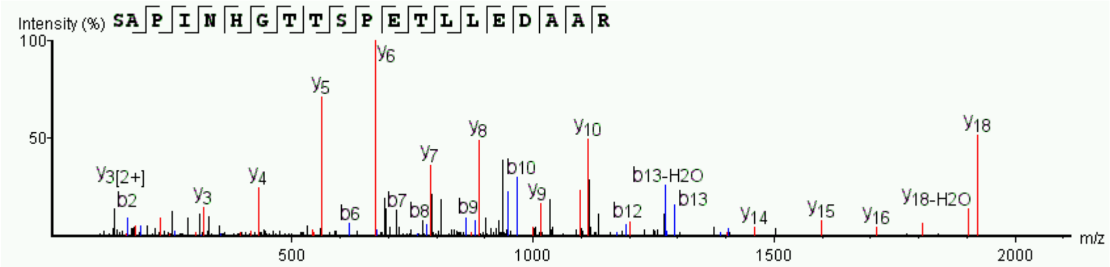

gi|48103883

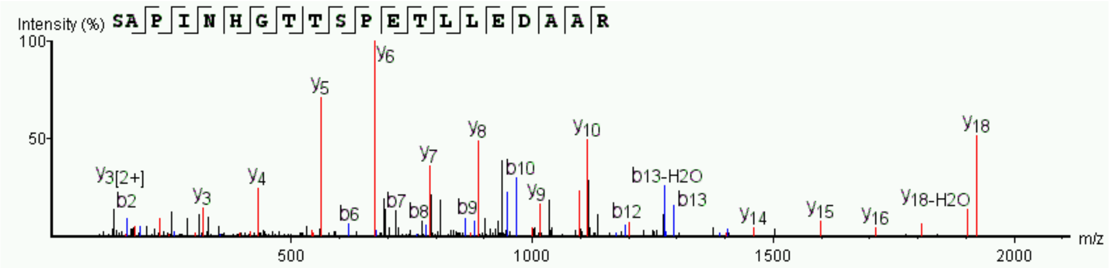

gi|48138568

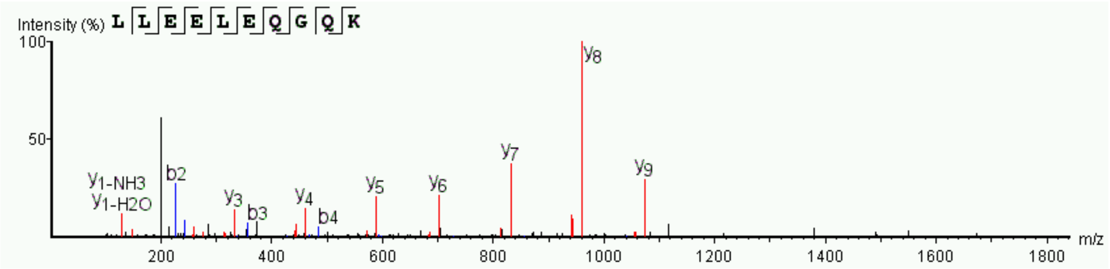

gi|571538017

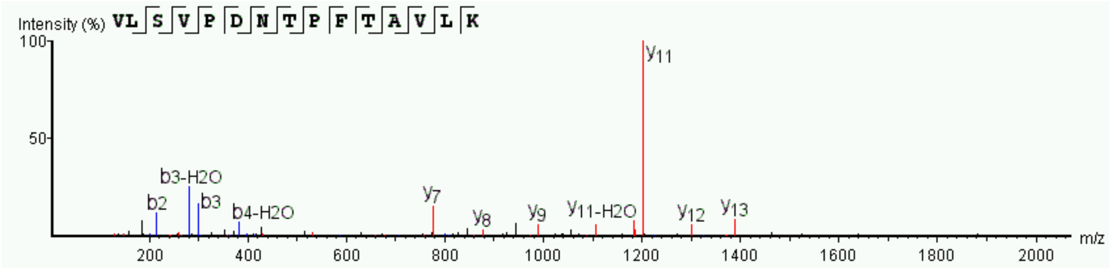

gi|110765112

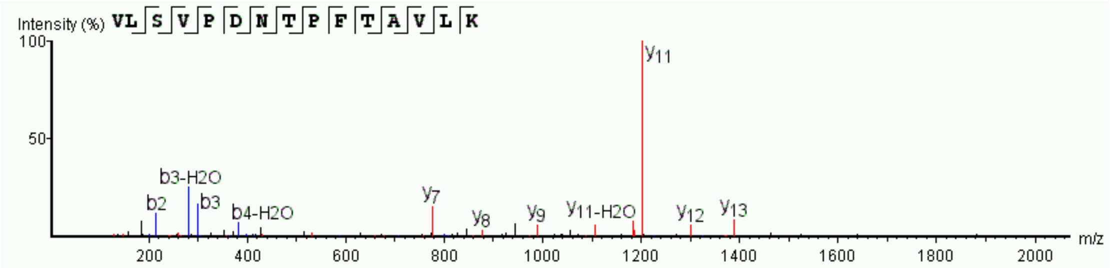

gi|66514137

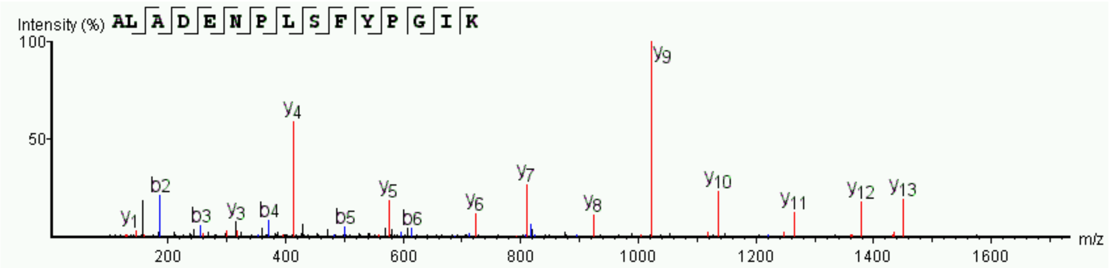

gi|66534585

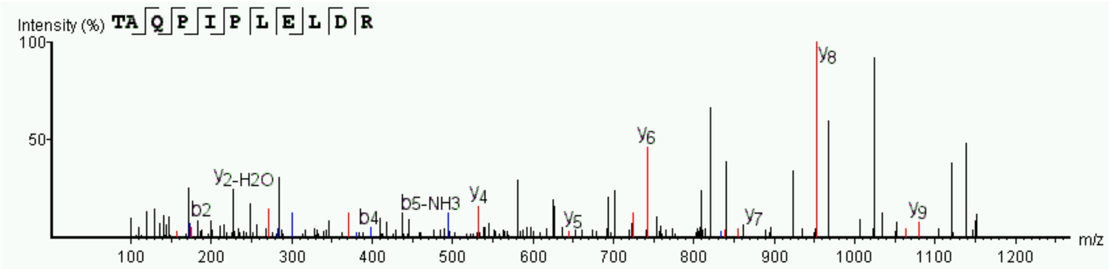

gi|571574478

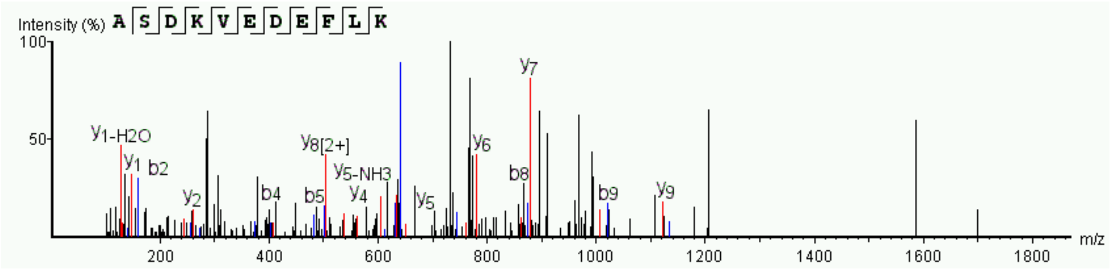

gi|571574496

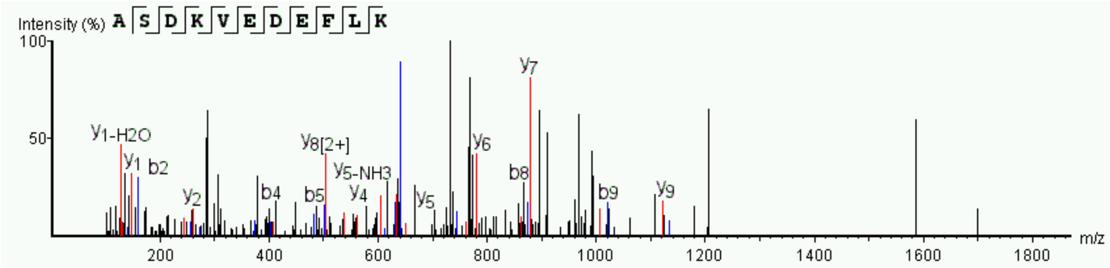

gi|571574502

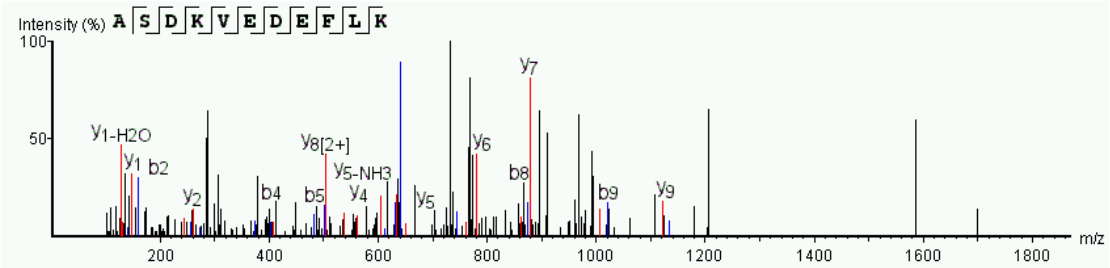

gi|571574486

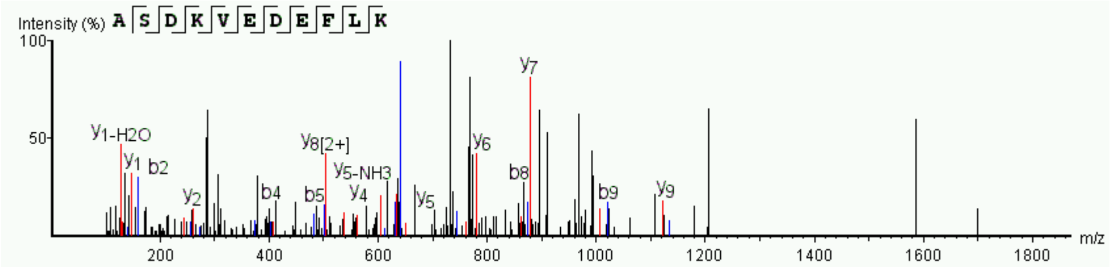

gi|571574488

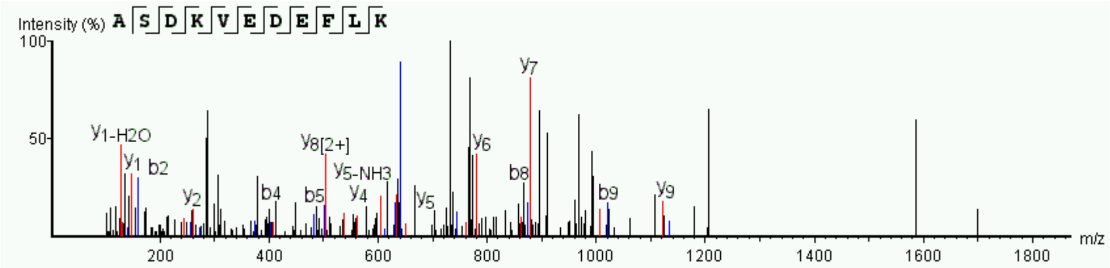

gi|571574490

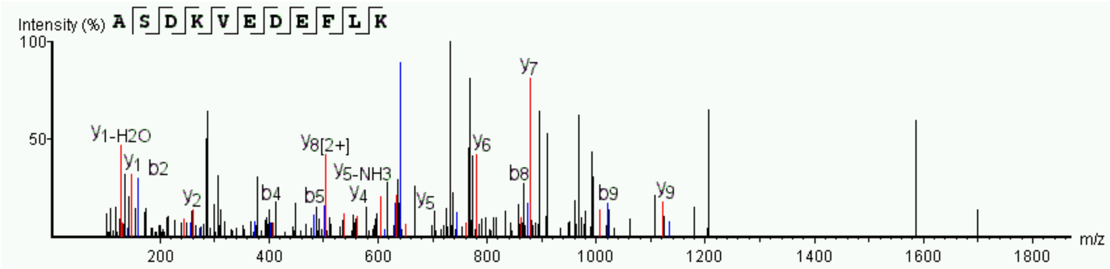

gi|571574494

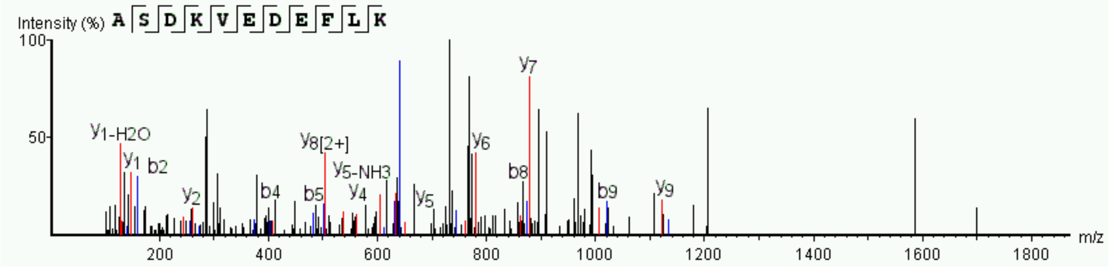

gi|328777351

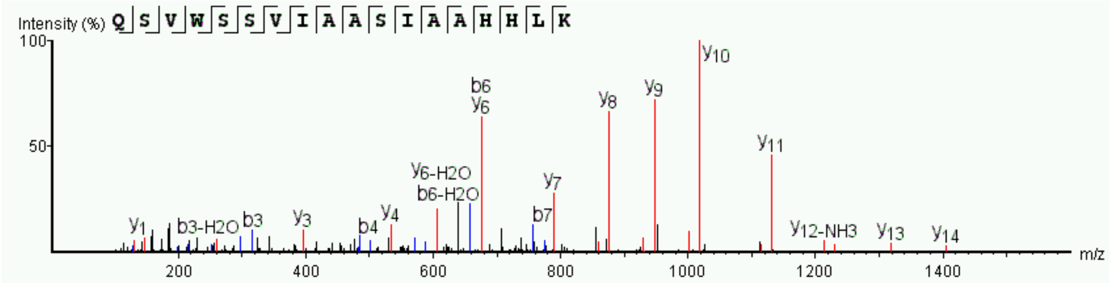

gi|571566895

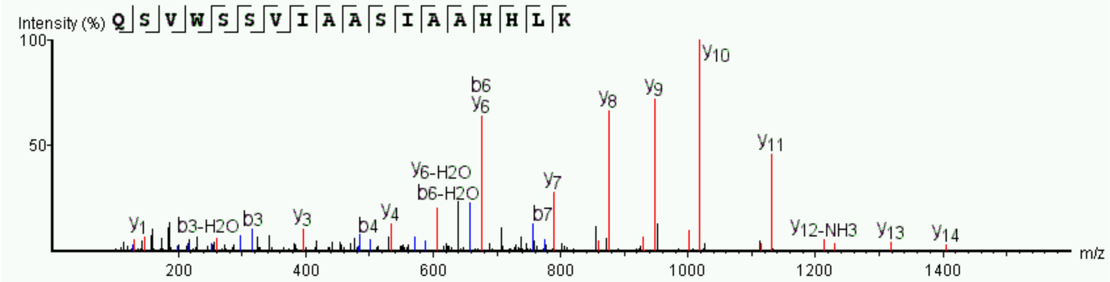

gi|571566901

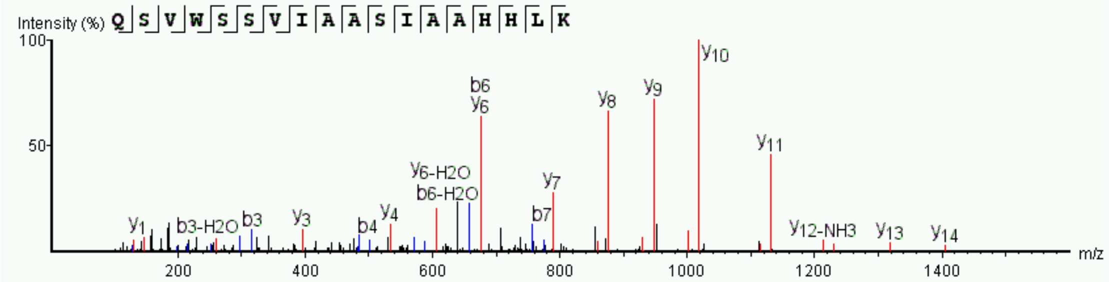

gi|571534306

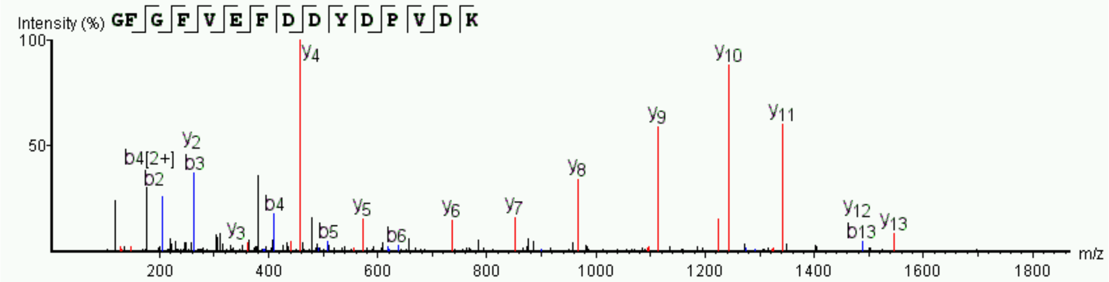

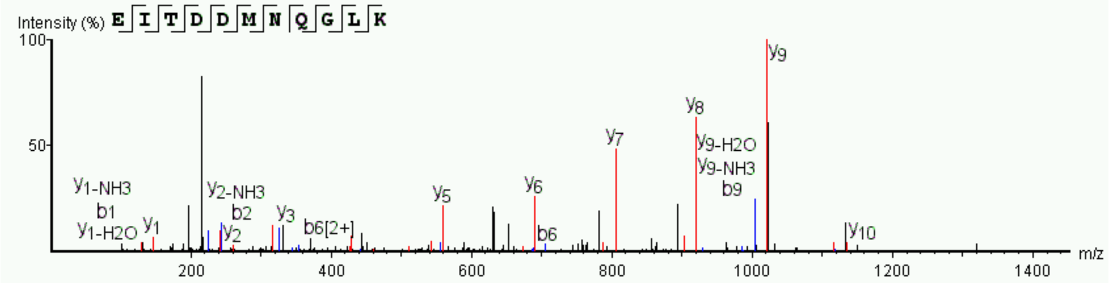

gi|571512347

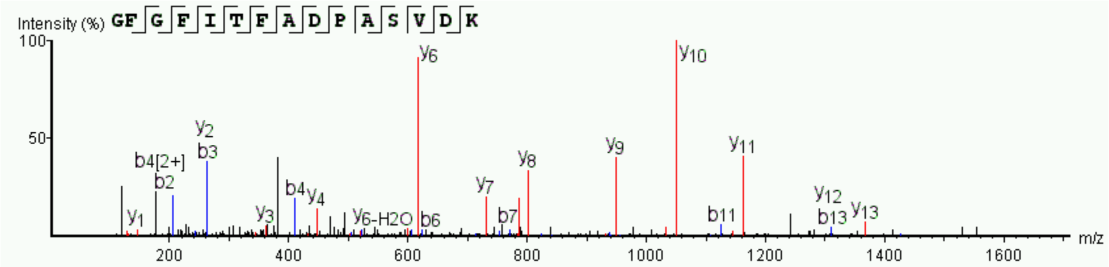

gi|571512898

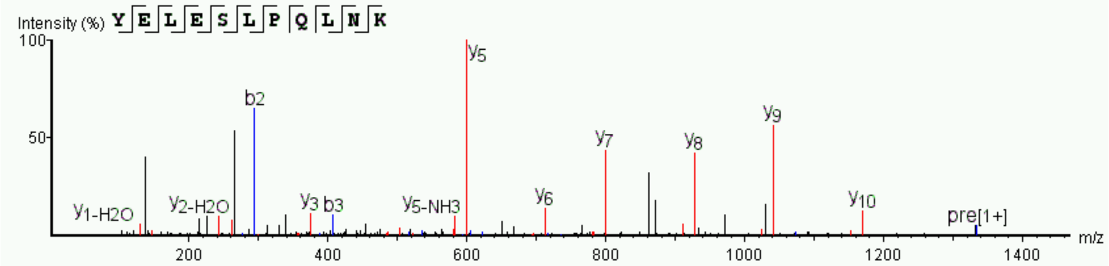

gi|571551216

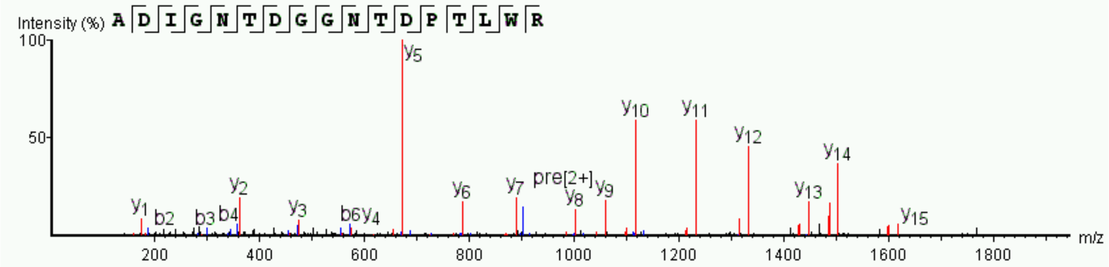

gi|571561556

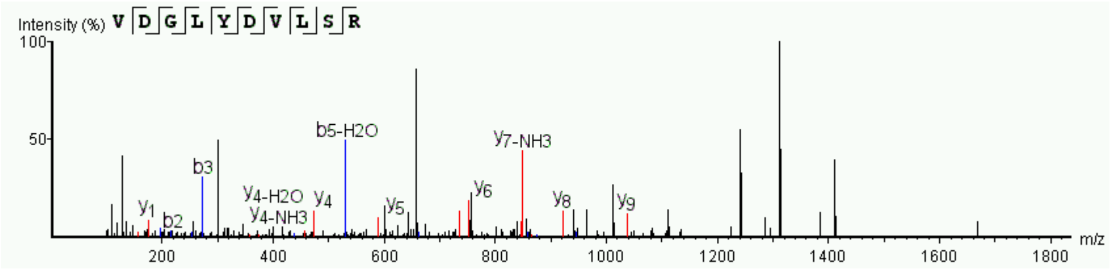

gi|571561560

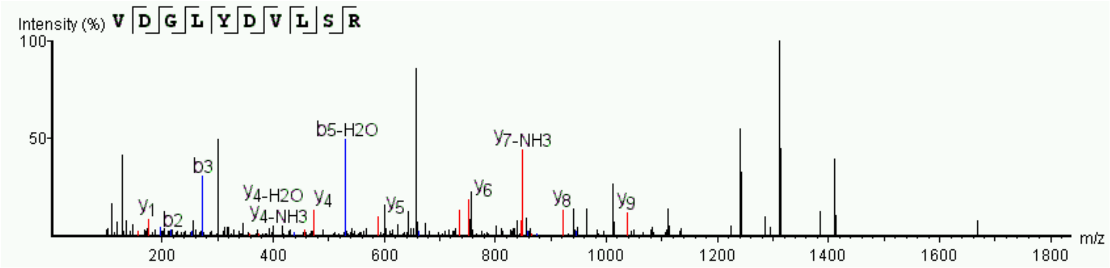

gi|571561564

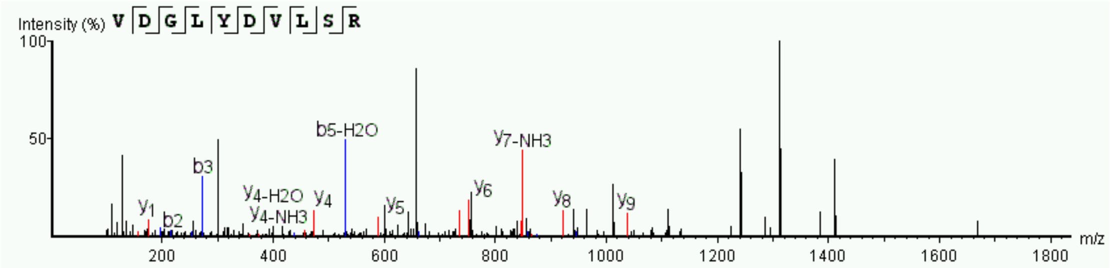

gi|571578780

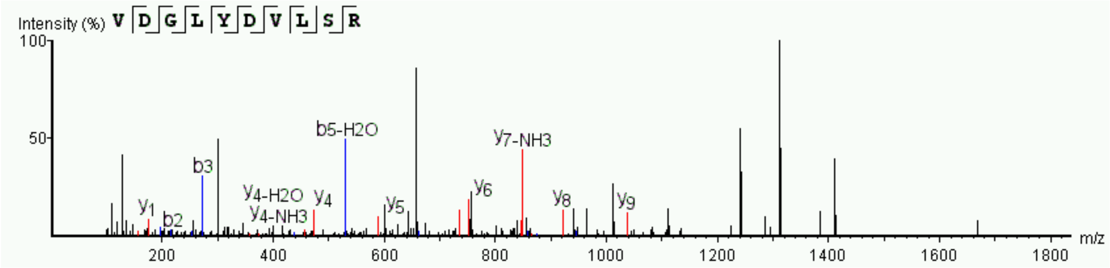

gi|571578782

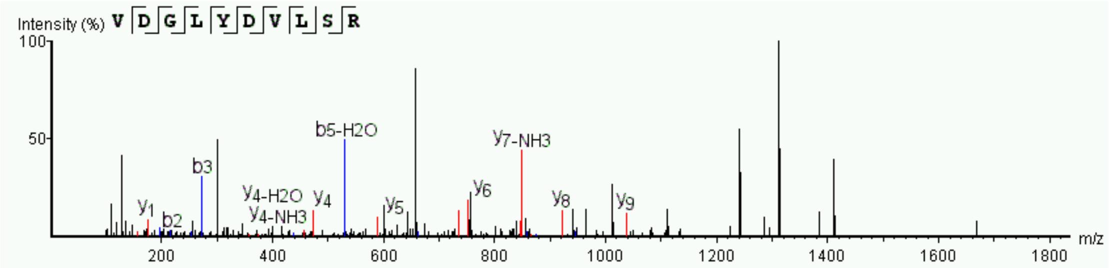

gi|571545149

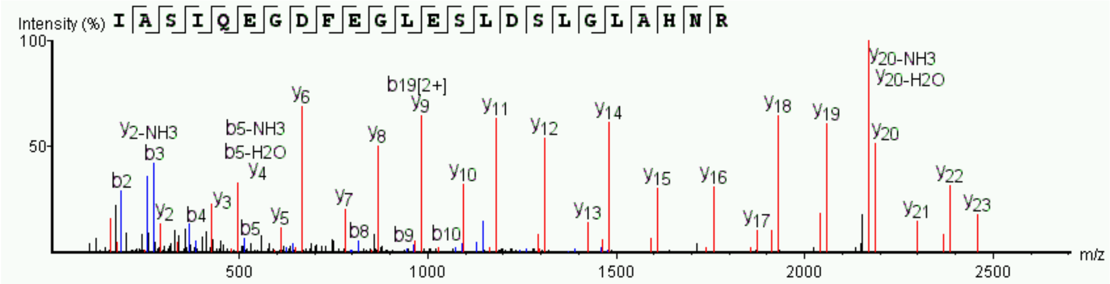

gi|571545145

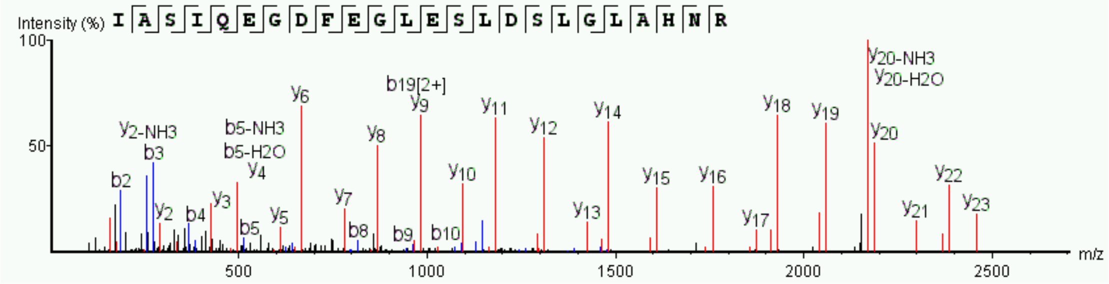

gi|66565869

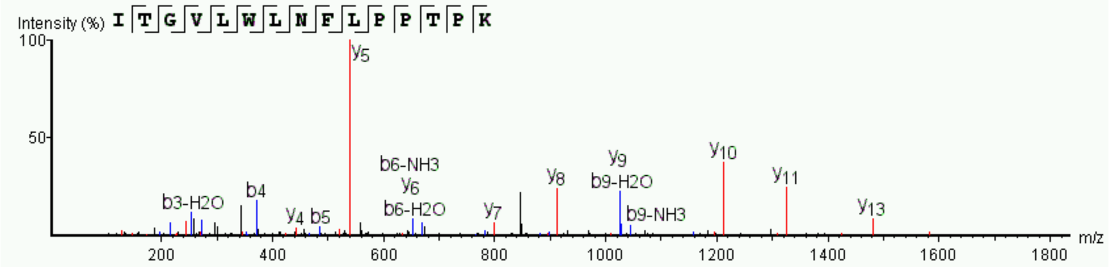

gi|571563070

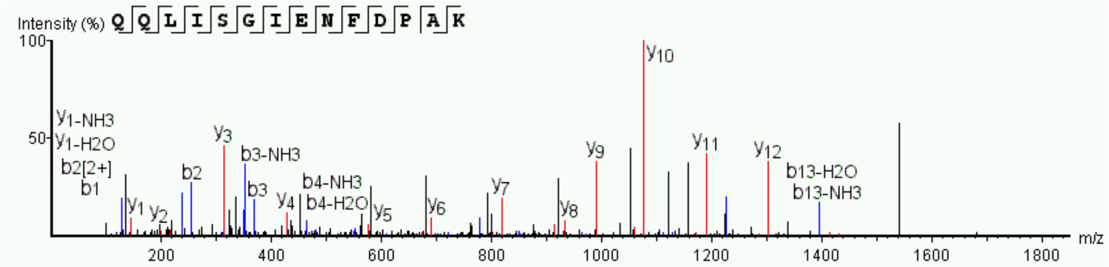

gi|328776397

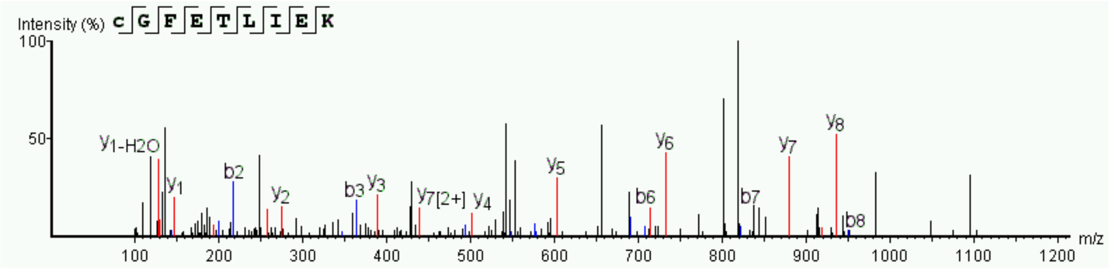

gi|328786574

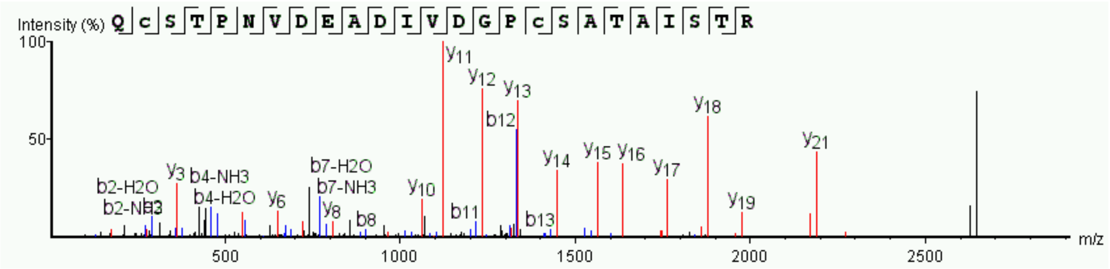

gi|571572915

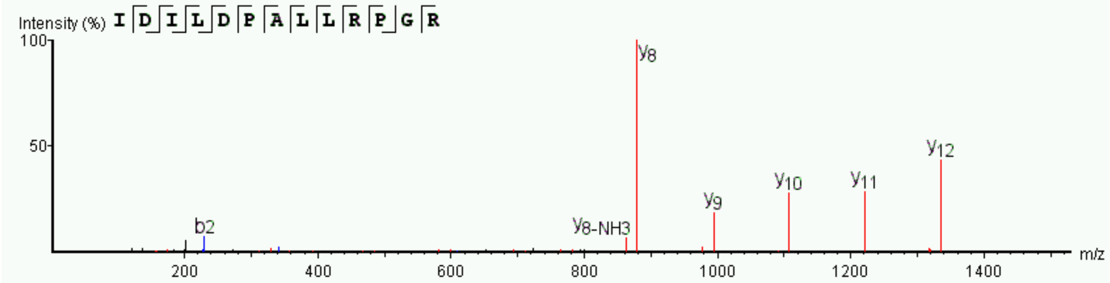

gi|571572933

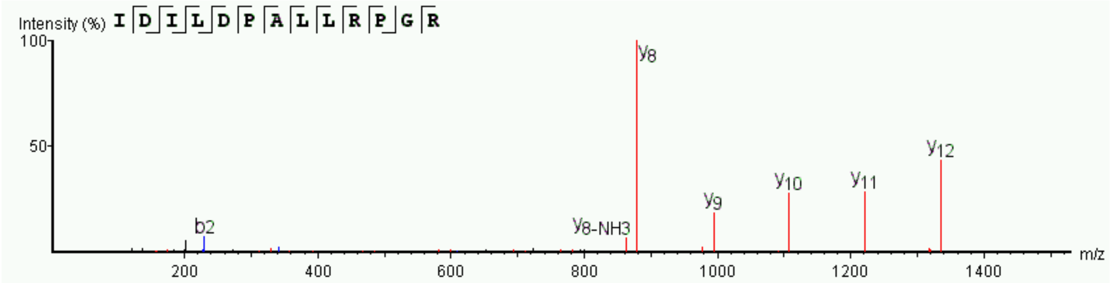

gi|571572941

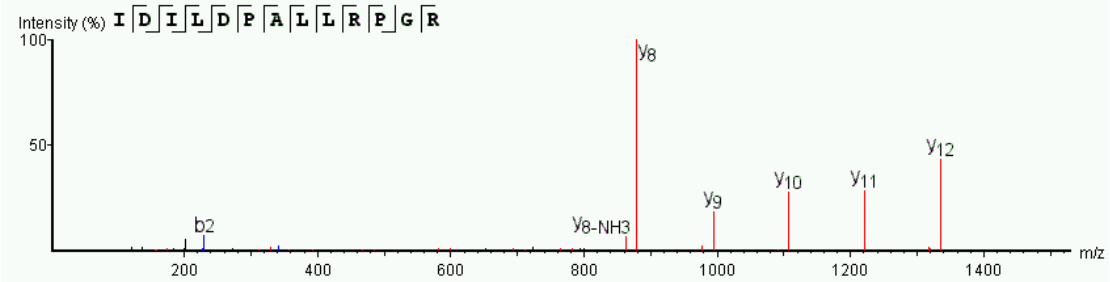

gi|571572949

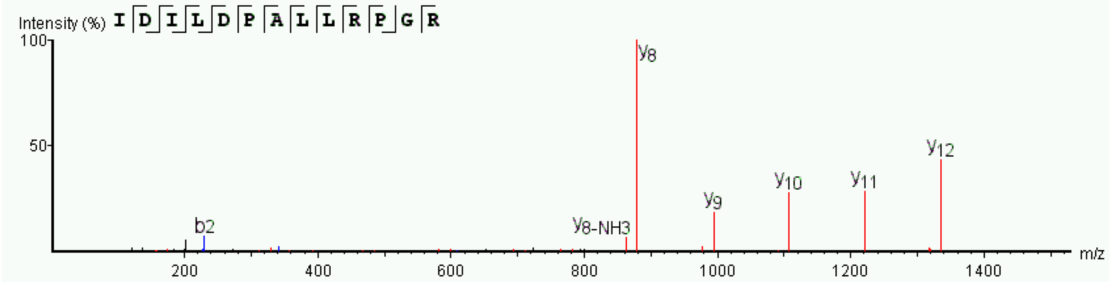

gi|571572929

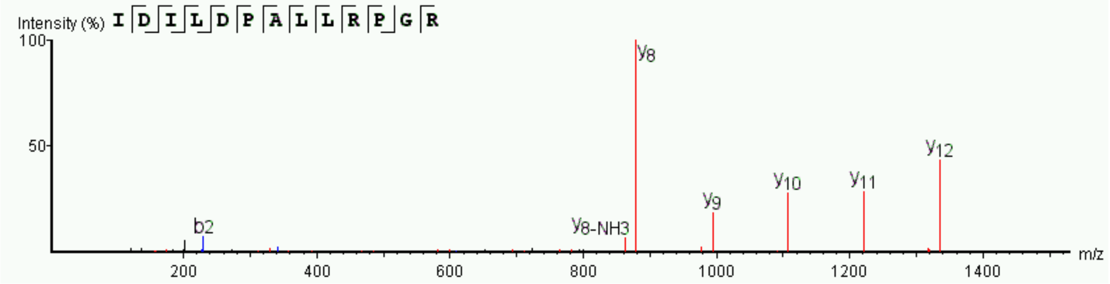

gi|571503771

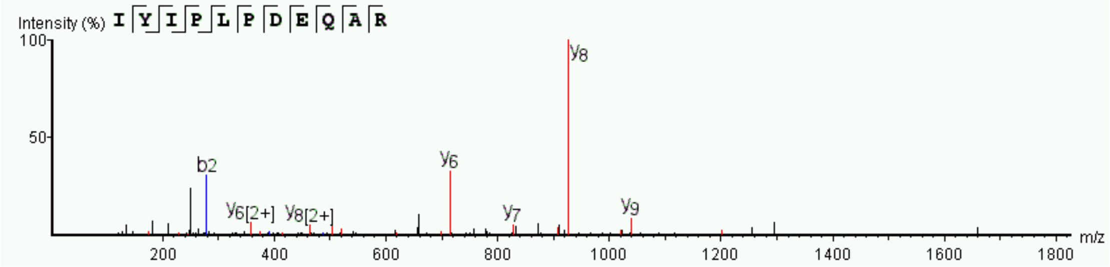

gi|66564933

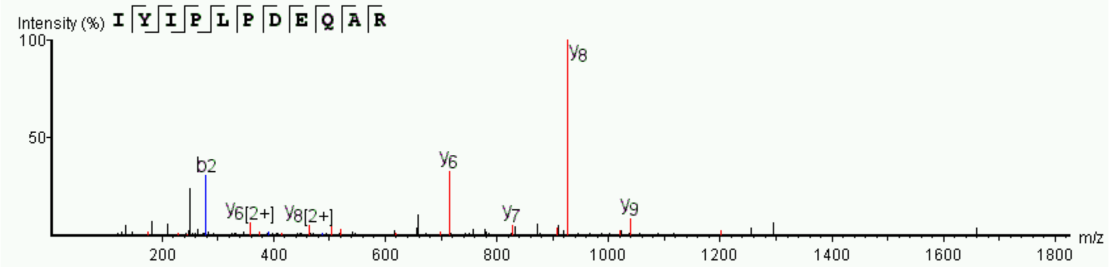

gi|571501581

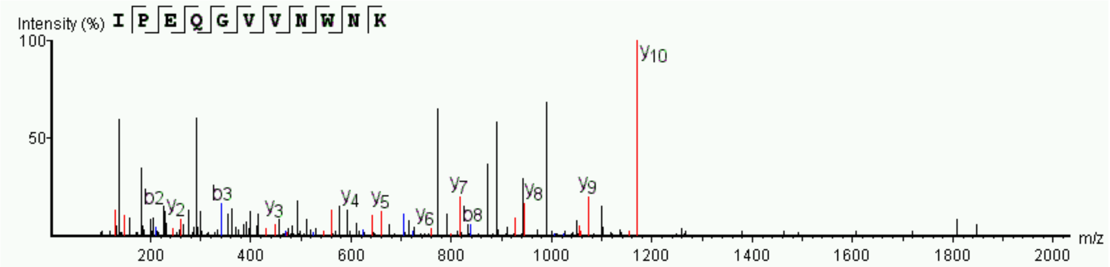

gi|66513815

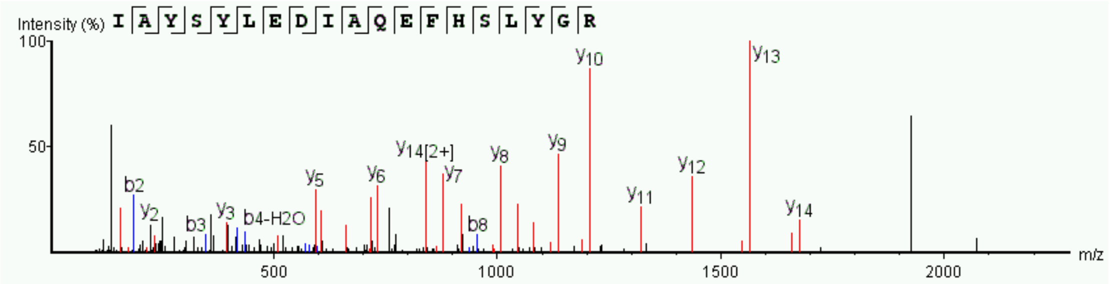

gi|571566027

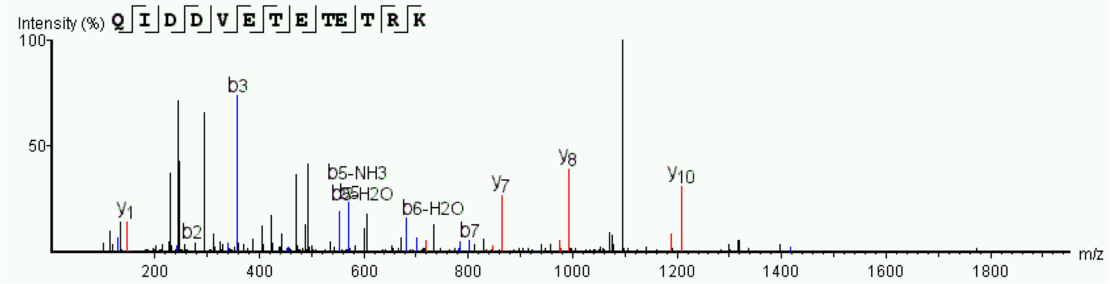

gi|571552411

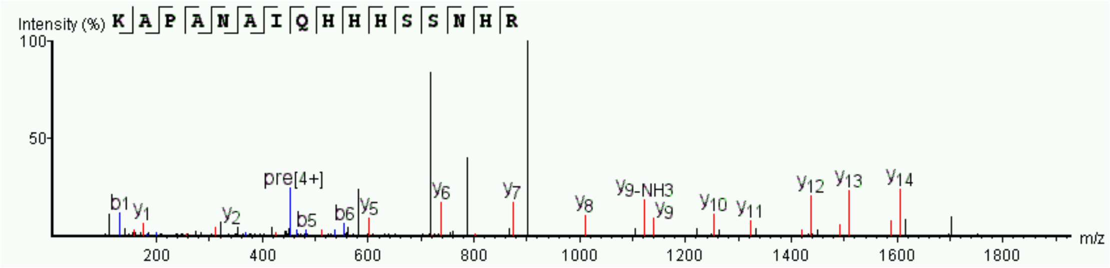

gi|66551883

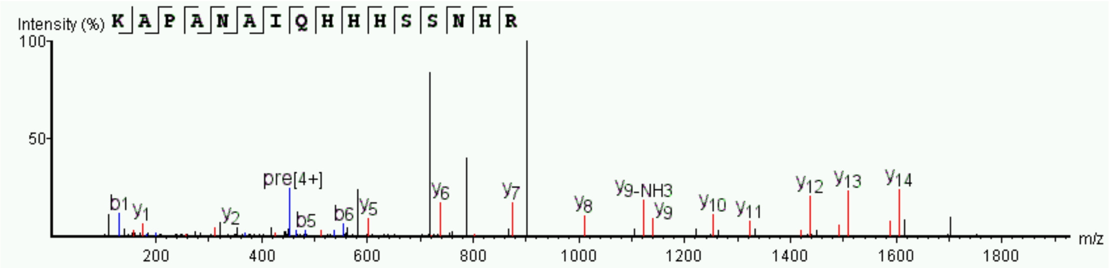

gi|571562837

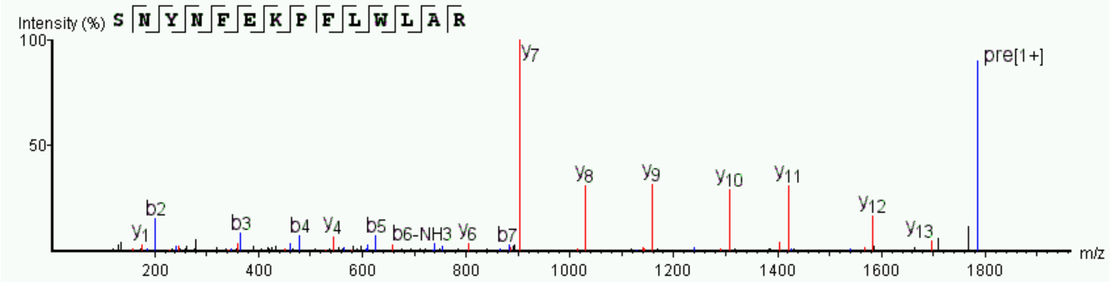

gi|48097366

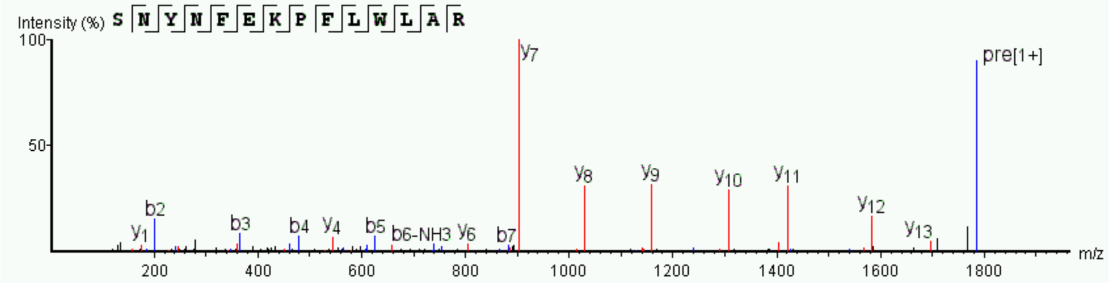

gi|571578042

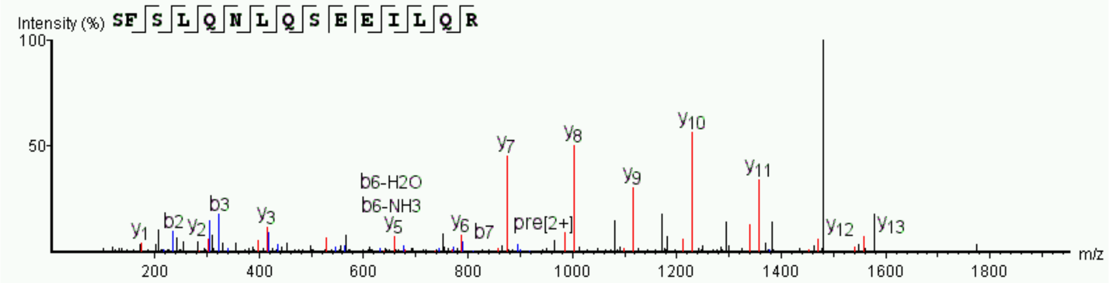

gi|571525698

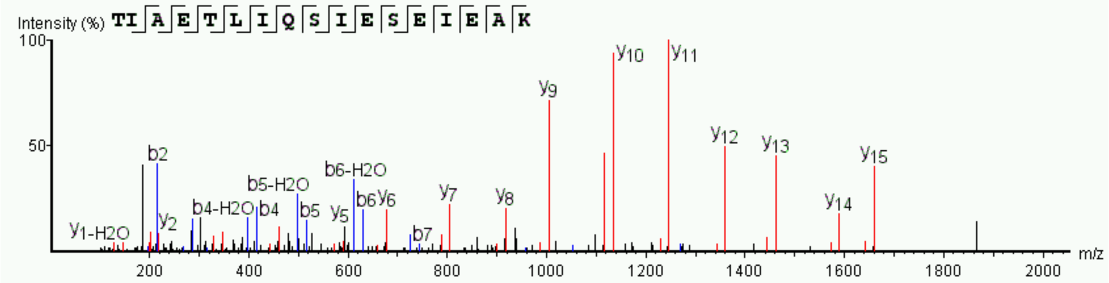

gi|571574918

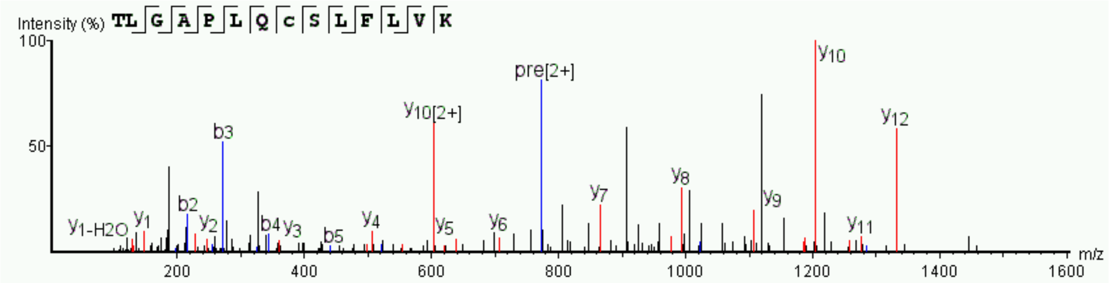

gi|110772198

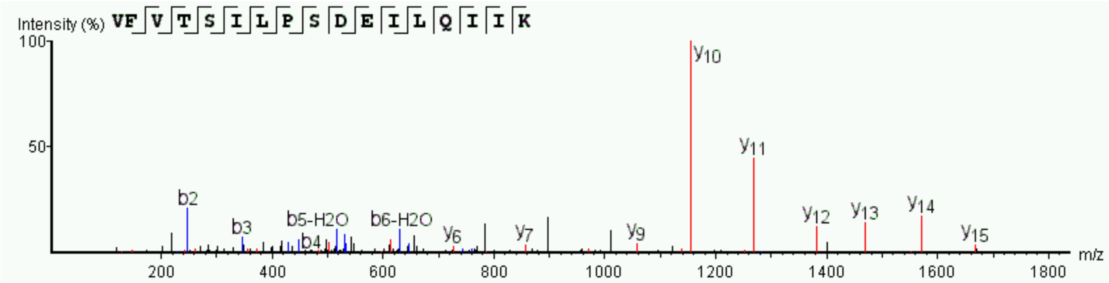

gi|571575119

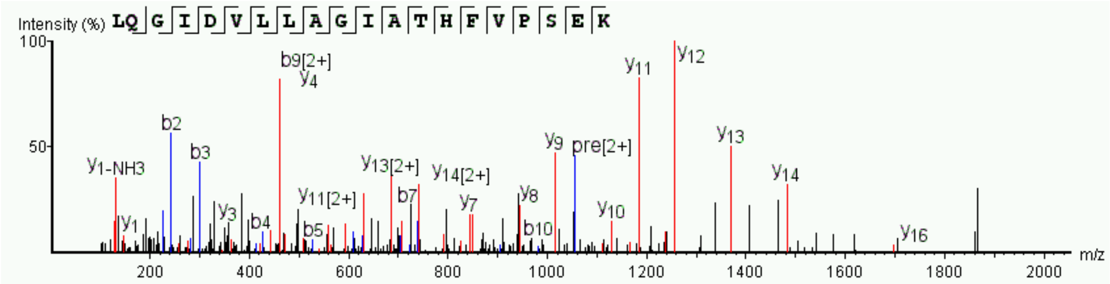

gi|328783479

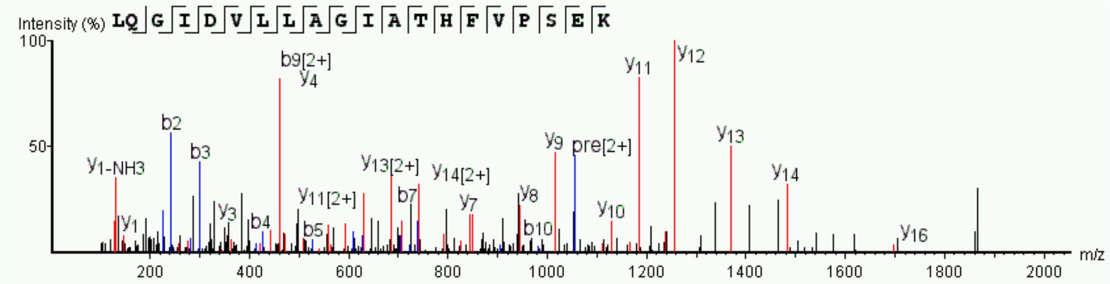

gi|571575116

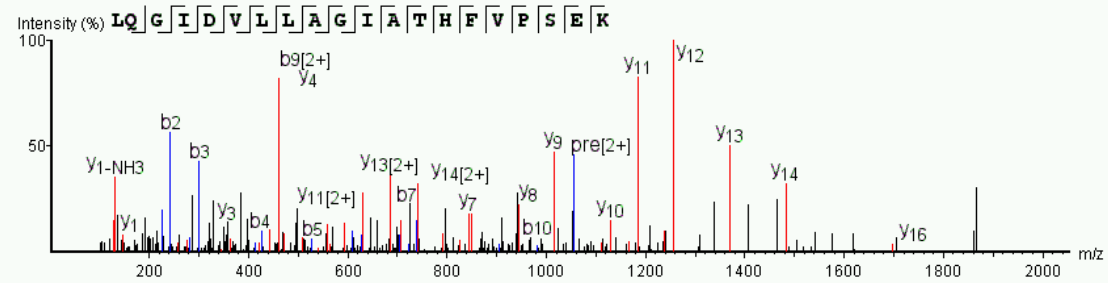

gi|571528291

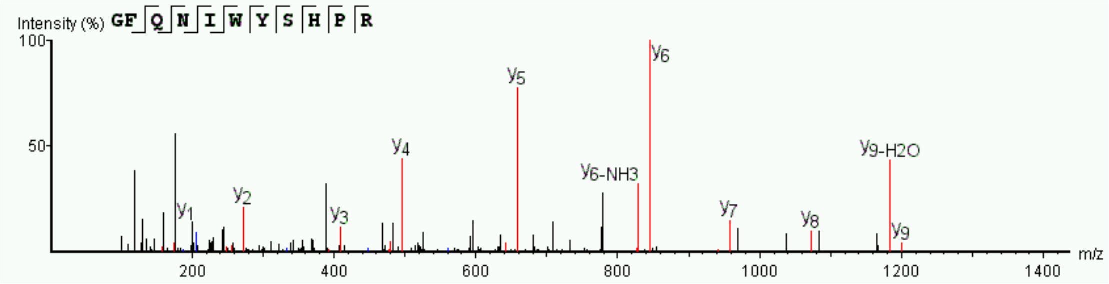

gi|66517407

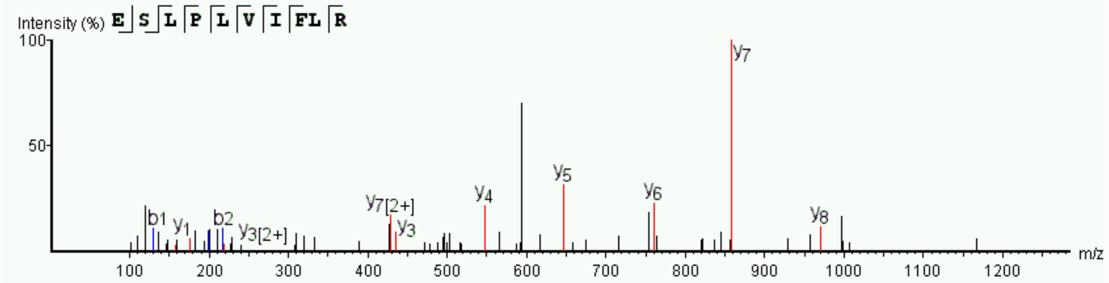

gi|571576123

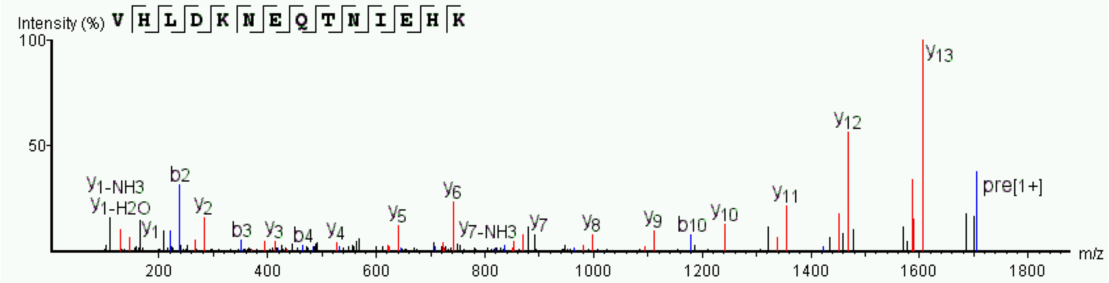

gi|571524852

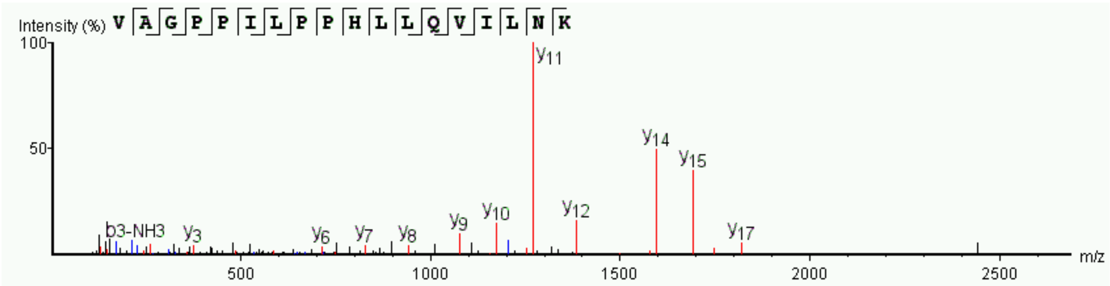

gi|571524858

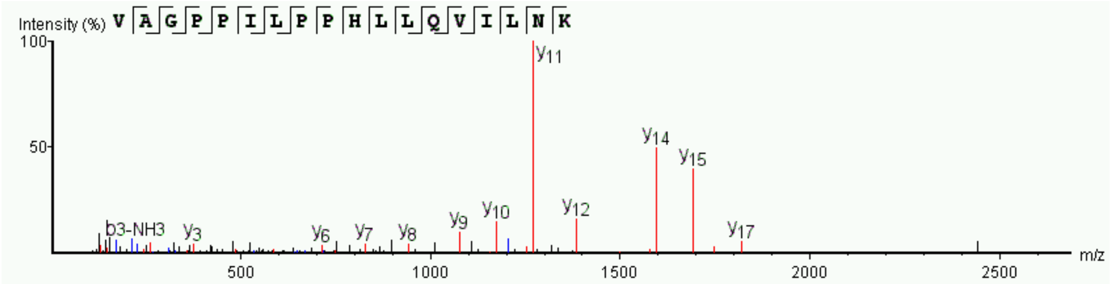

gi|48113496

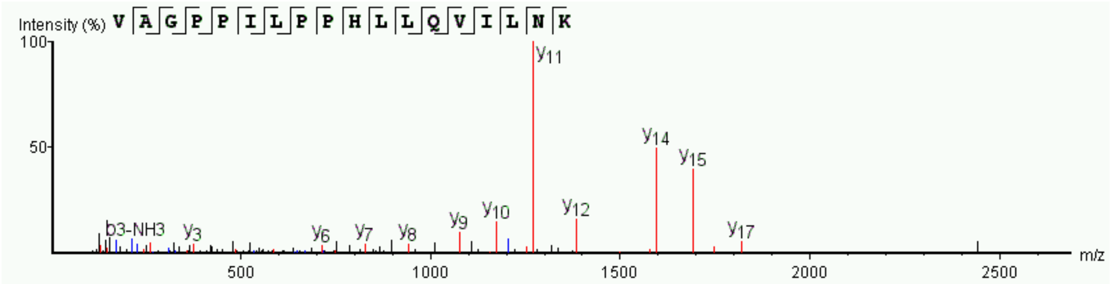

gi|48113496

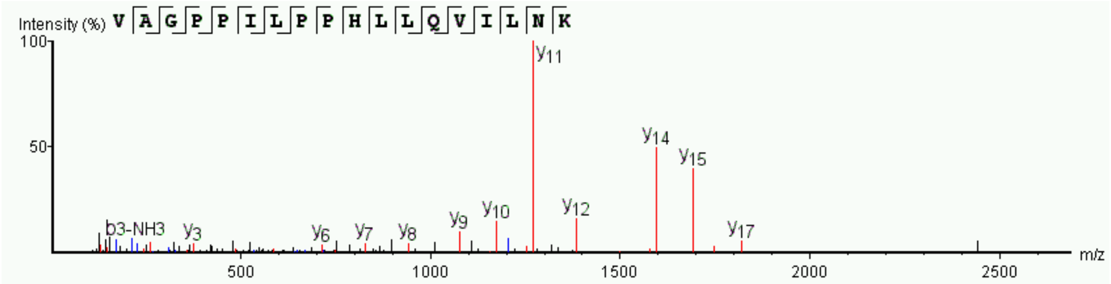

gi|571501696

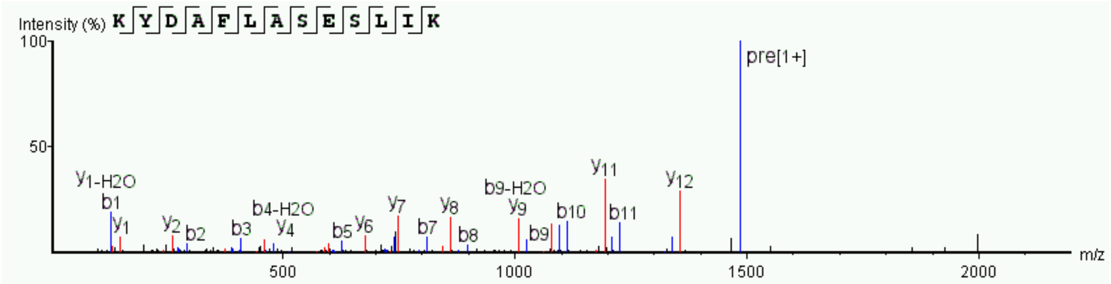

gi|571510850

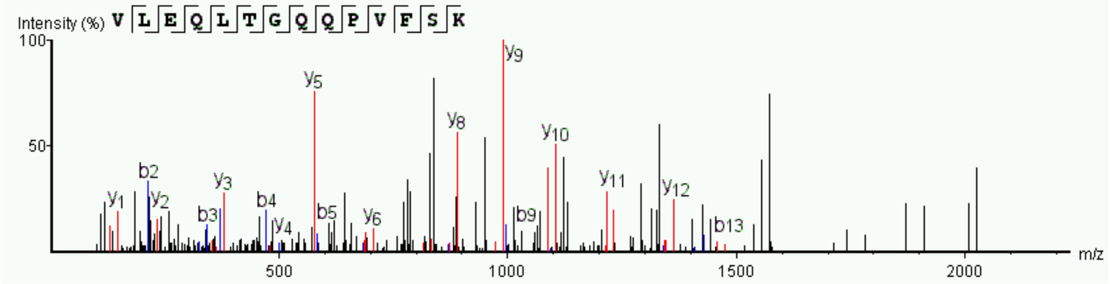

gi|48130977

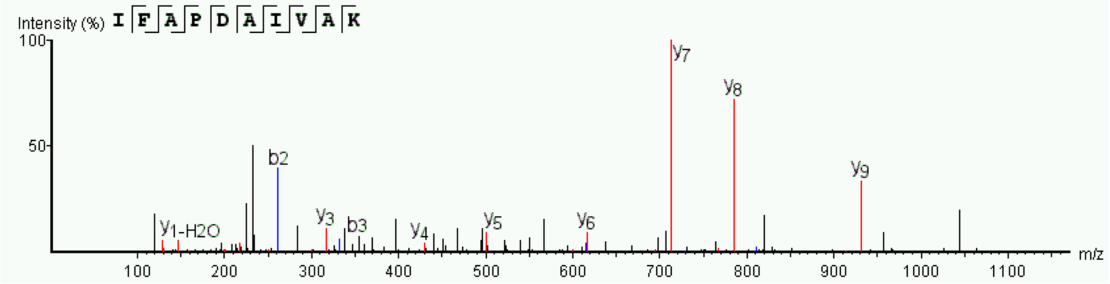

gi|571573543

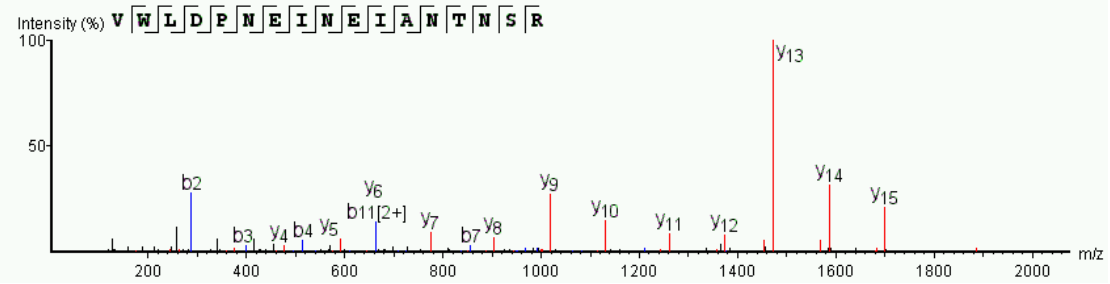

gi|110762813

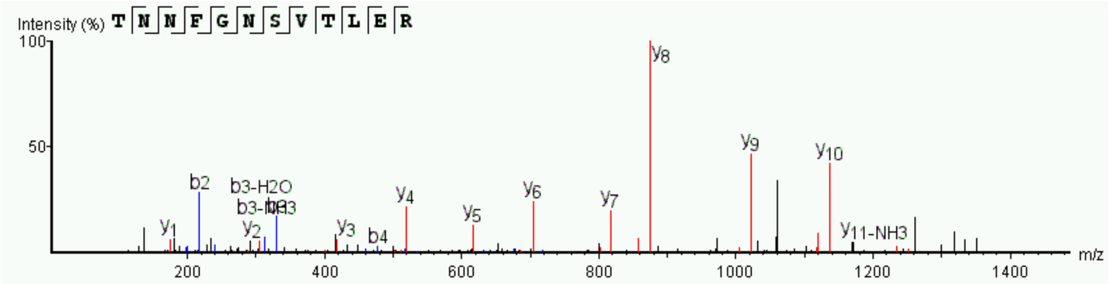

gi|66558956

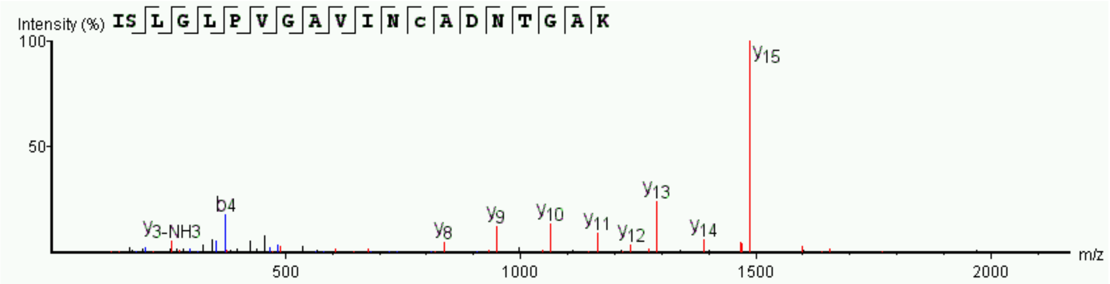

gi|110761627

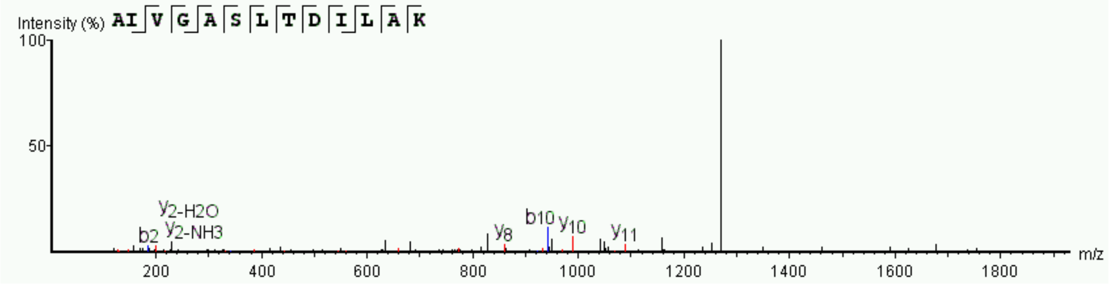

gi|571574994

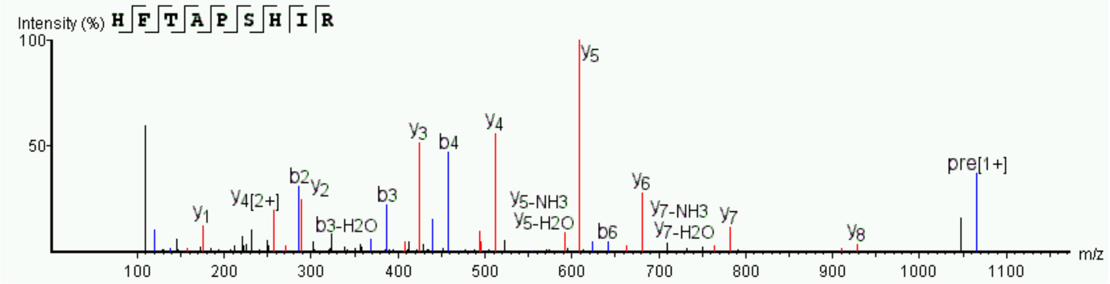

gi|66552169

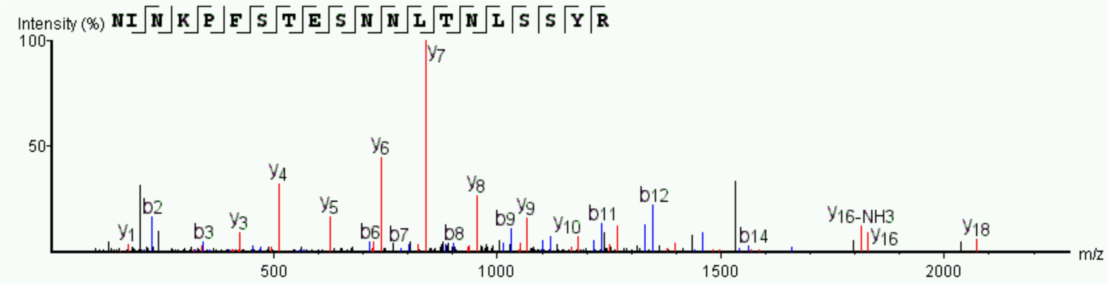

gi|571548748

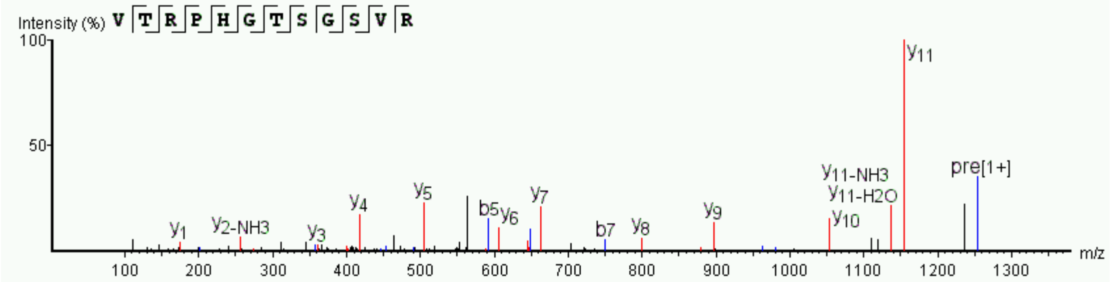

gi|571548751

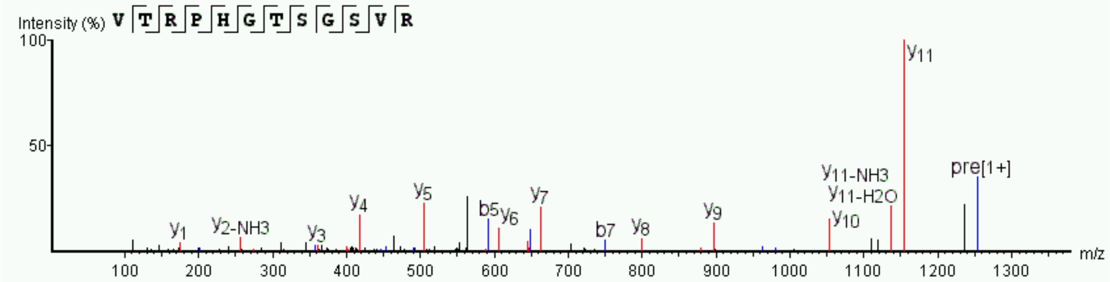

gi|571548754

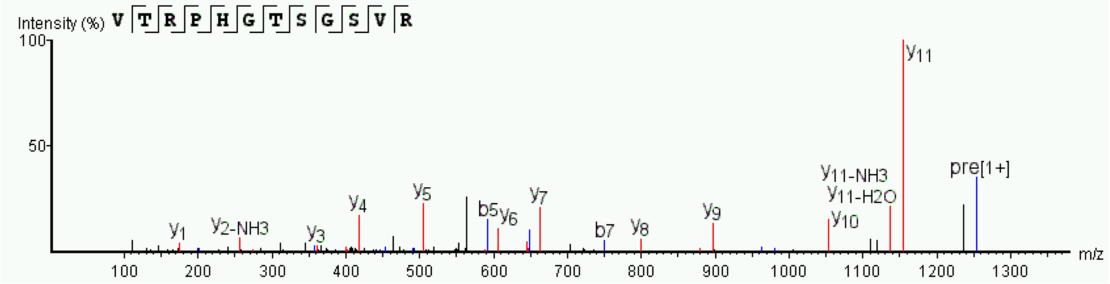

gi|48105889

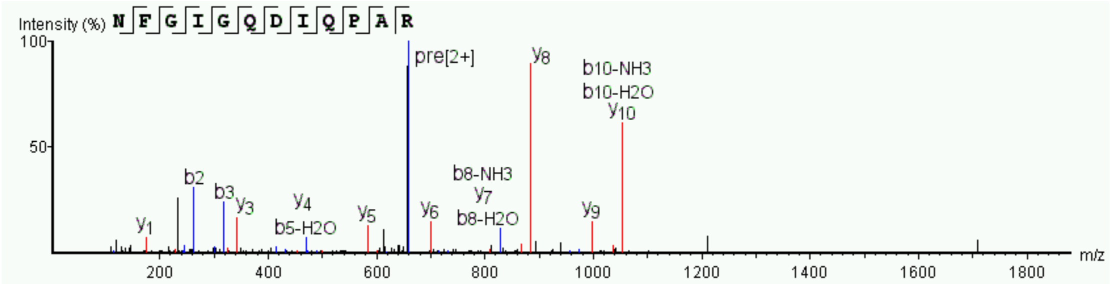

gi|571579183

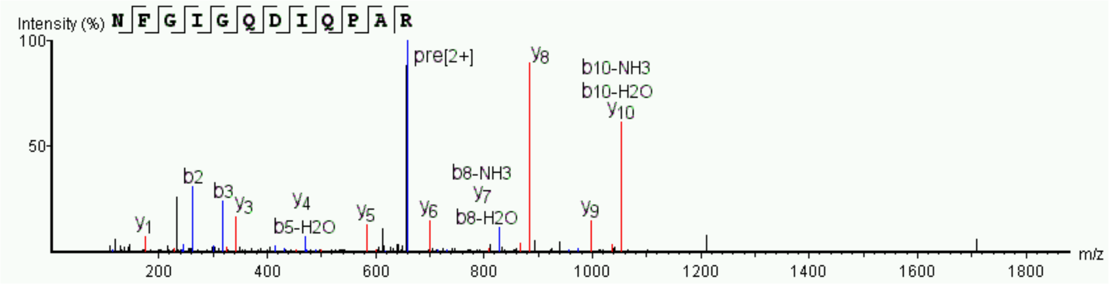

gi|66516926

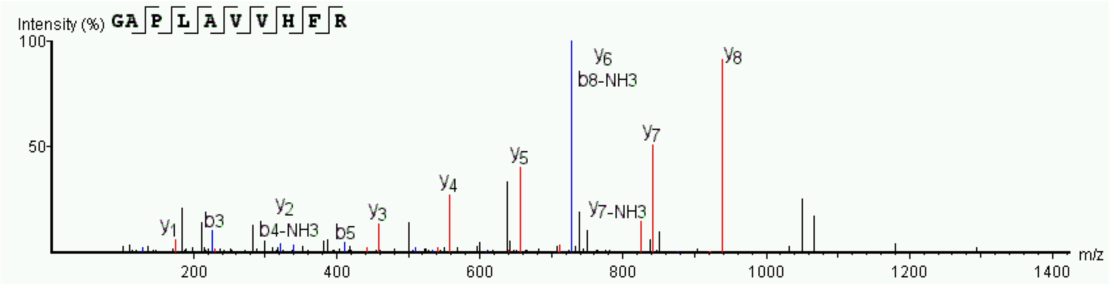

gi|571555366

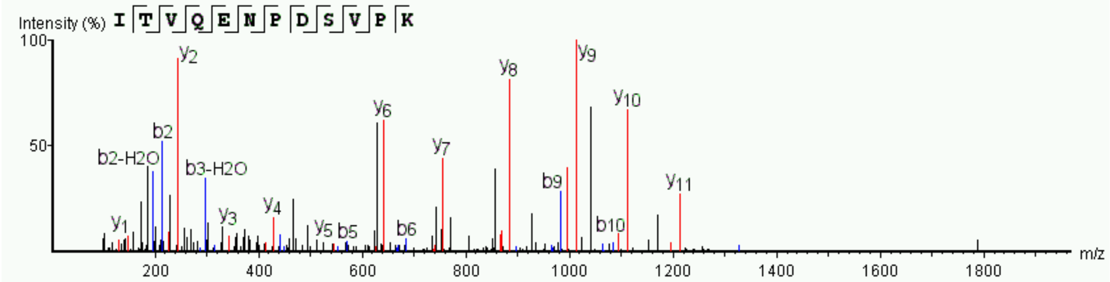

gi|571555369

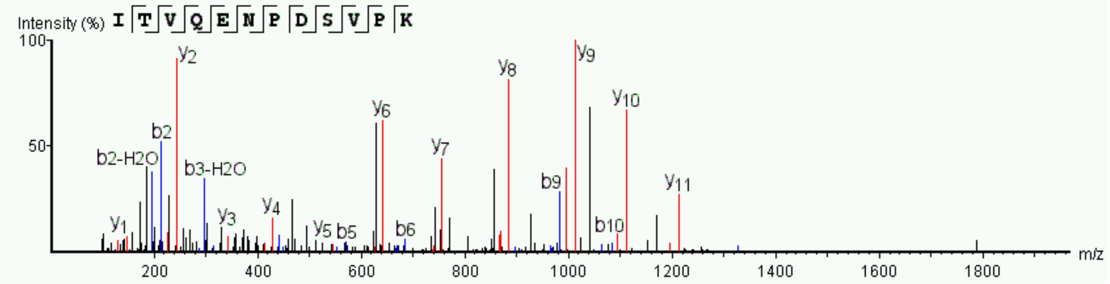

gi|571539002

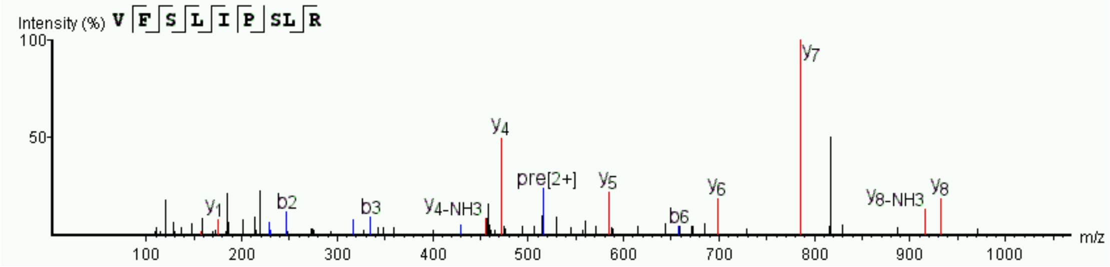

gi|328782357

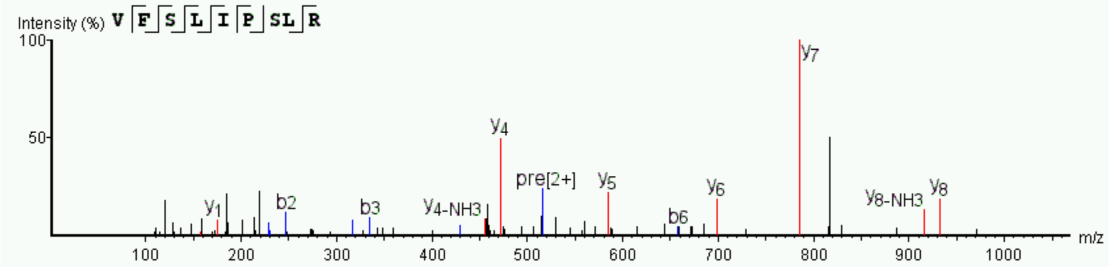

gi|110756153

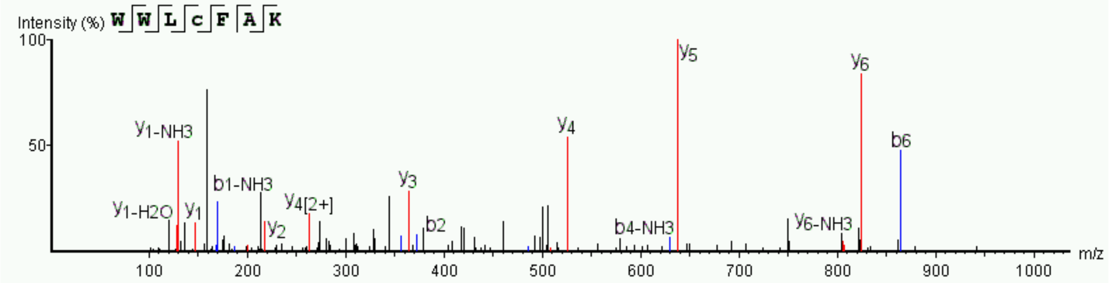

gi|571570081

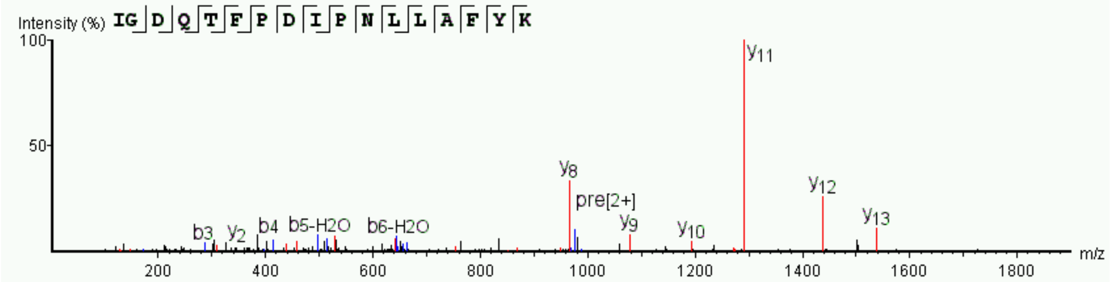

gi|571570085

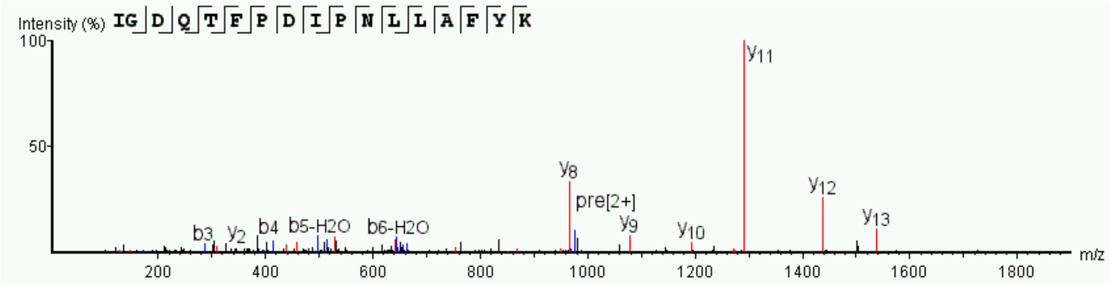

gi|571570083

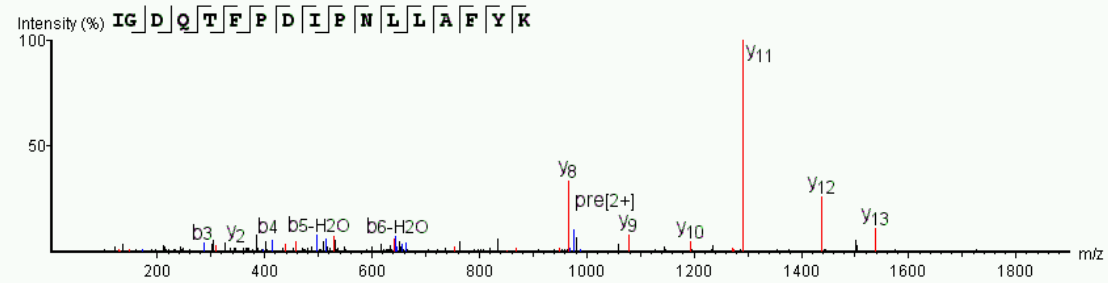

gi|66519683

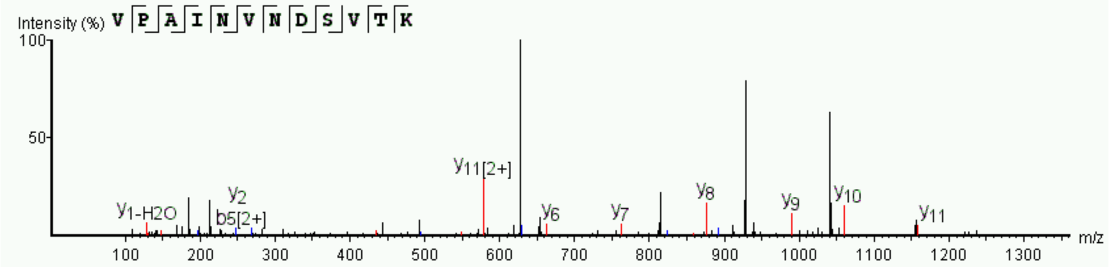

gi|110758776

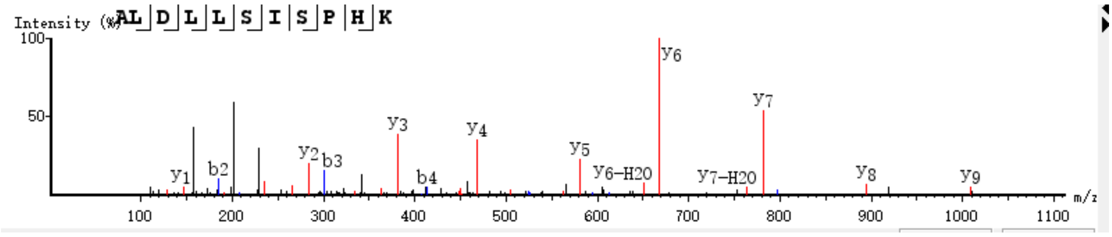

gi|571522855

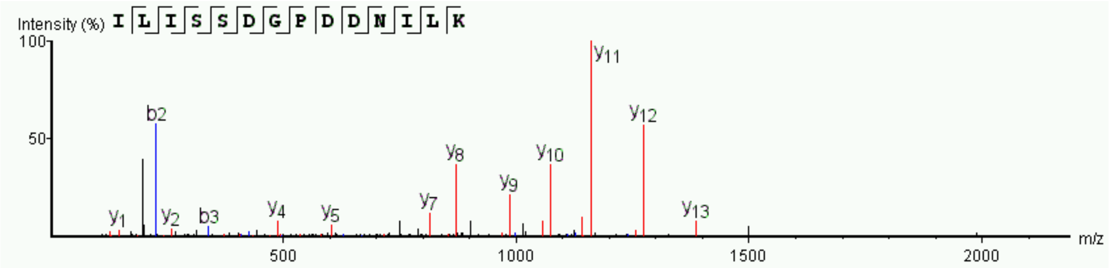

gi|571572159

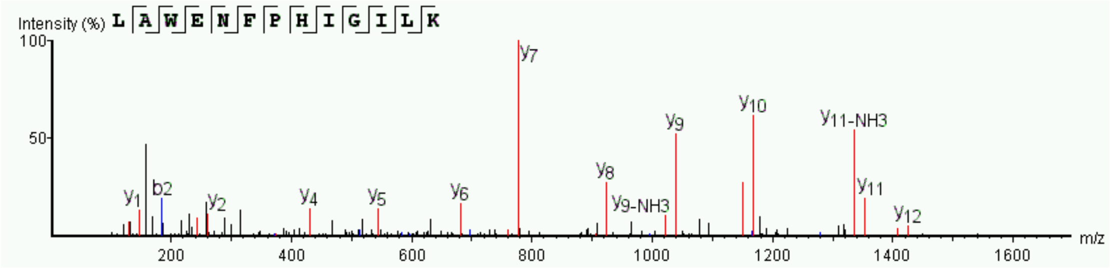

gi|571572163

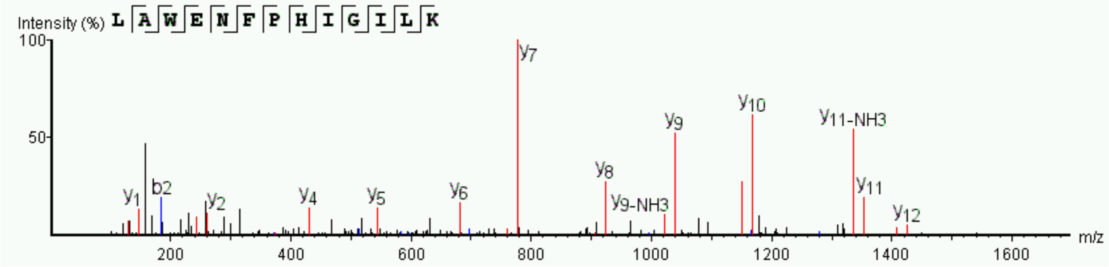

gi|571529433

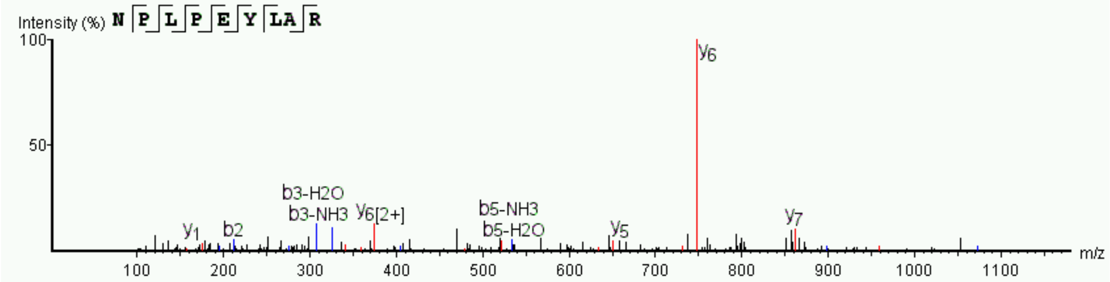

gi|571529436

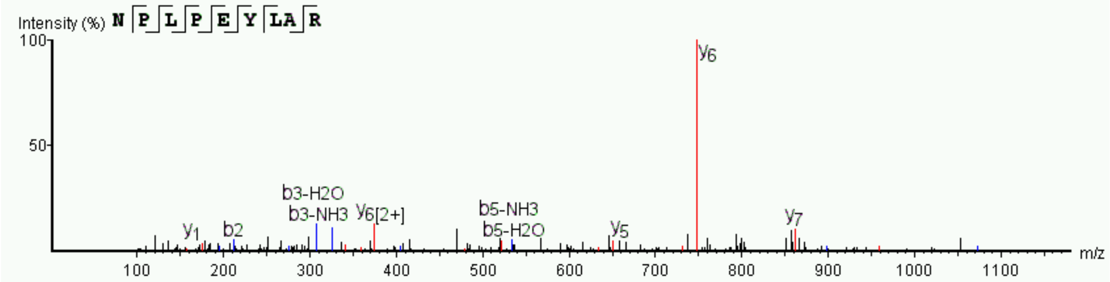

gi|571529406

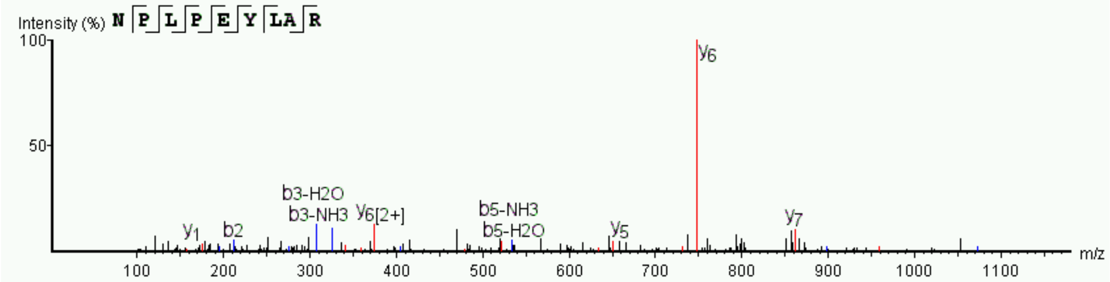

gi|571529409

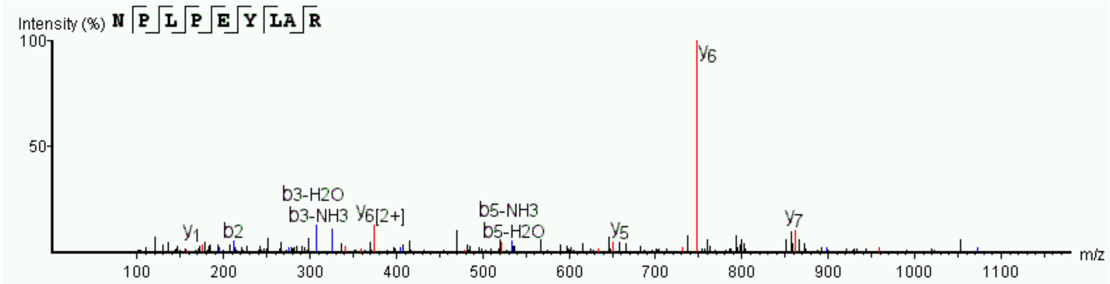

gi|571529412

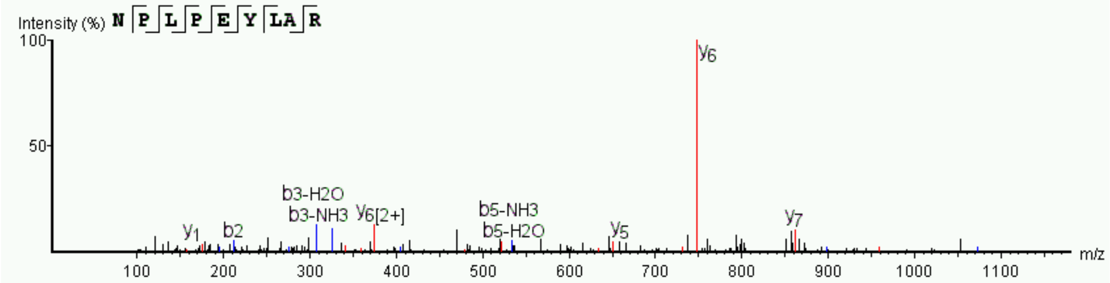

gi|571529417

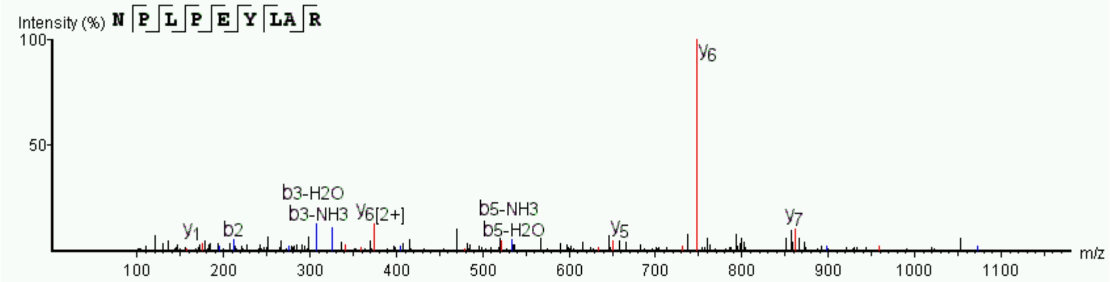

gi|571529421

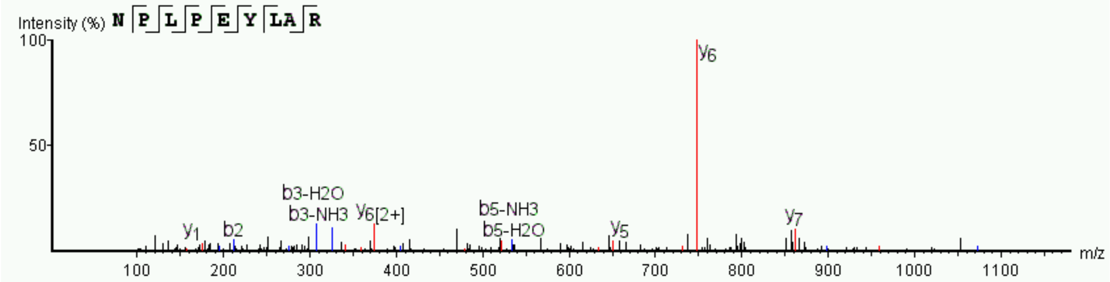

gi|571529425

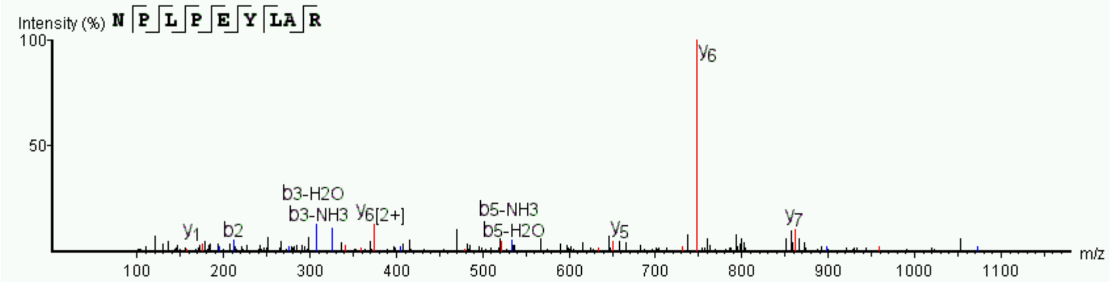

gi|571529429

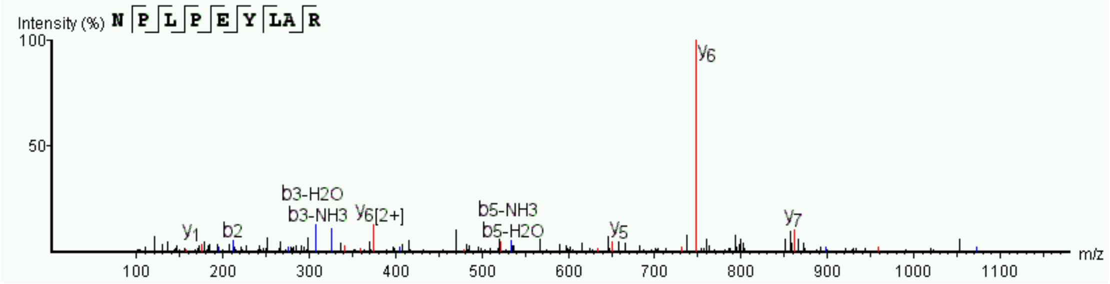

gi|328788208

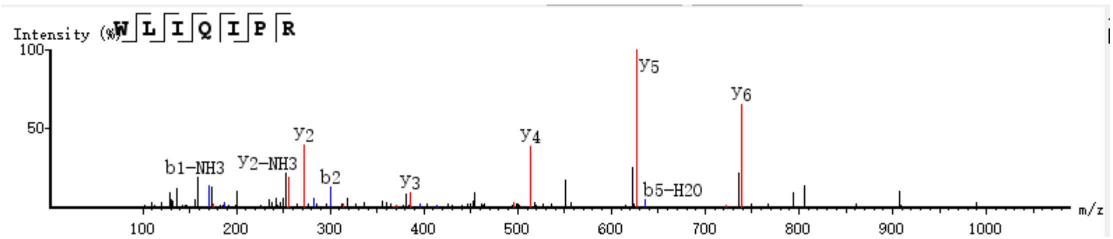

gi|571531564

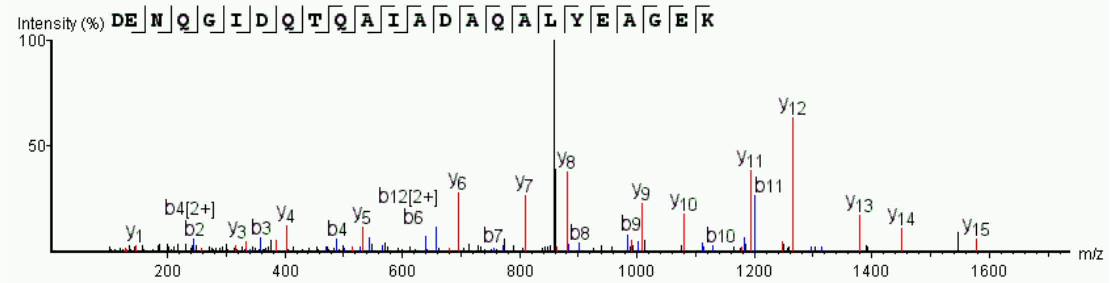

gi|66555021

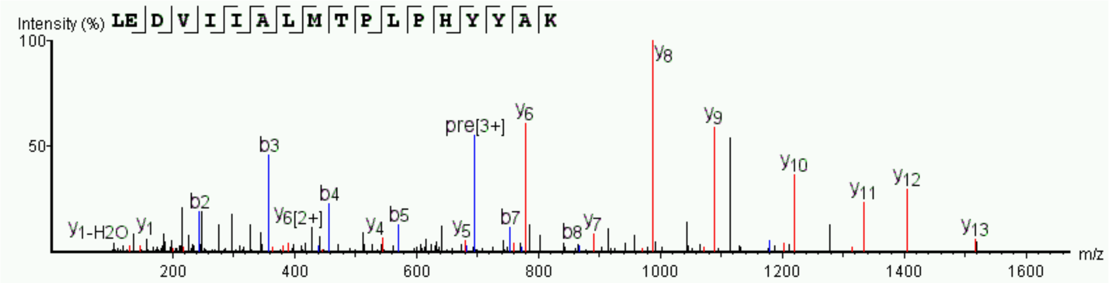

gi|66526174

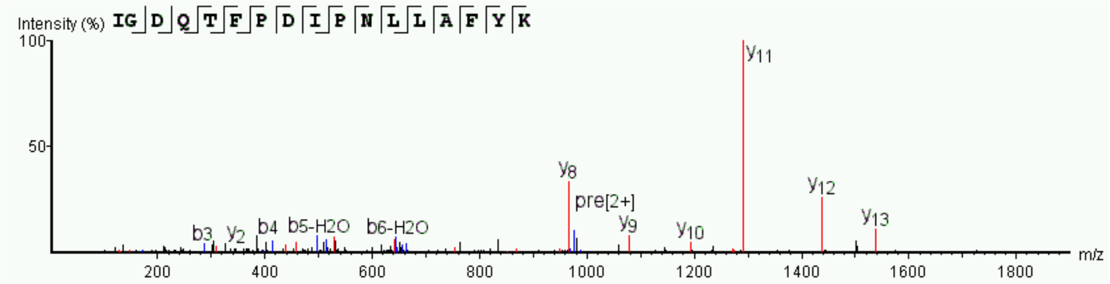

gi|110748994

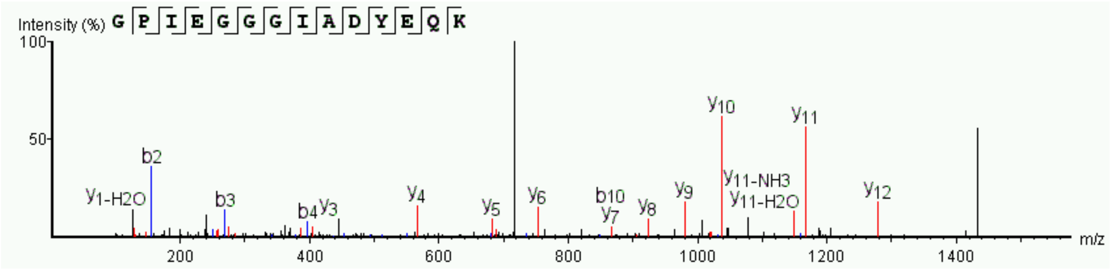

gi|571501620

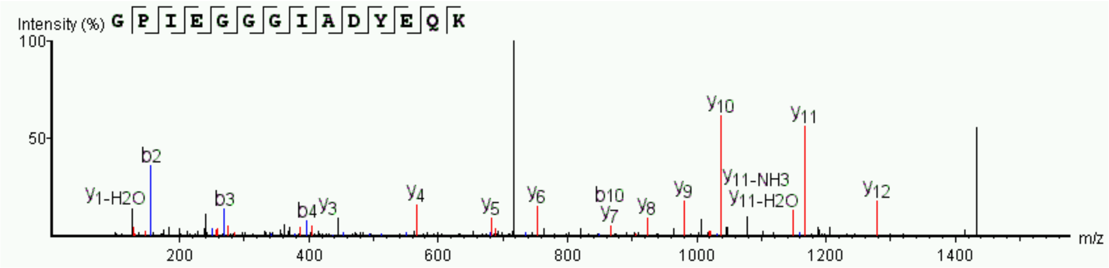

gi|571575497

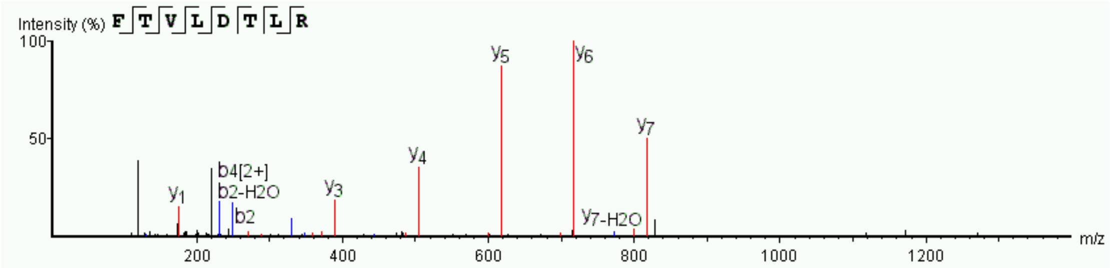

gi|571575499

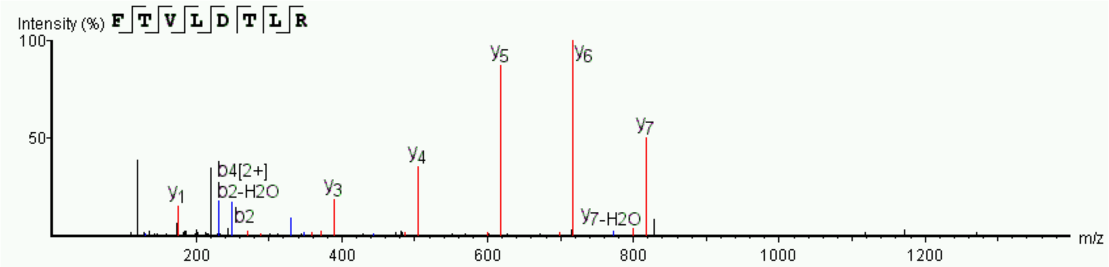

gi|571517543

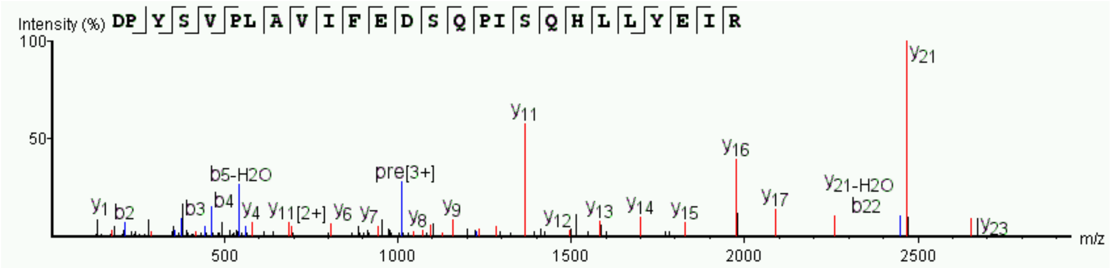

gi|328778864

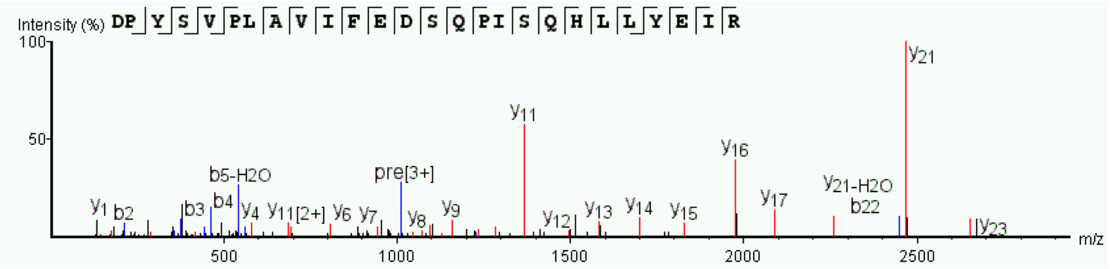

gi|571576251

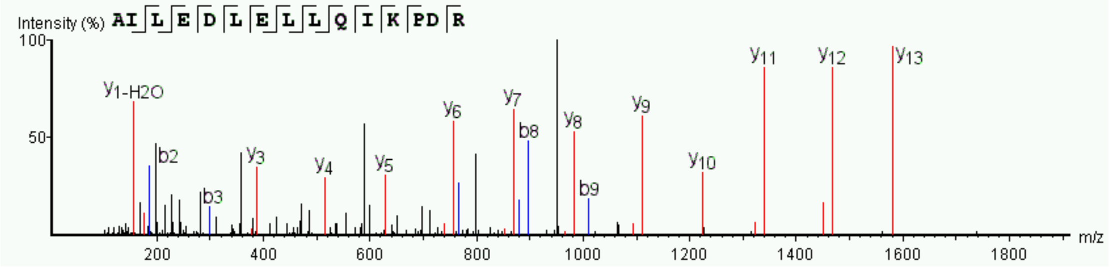

gi|571558849

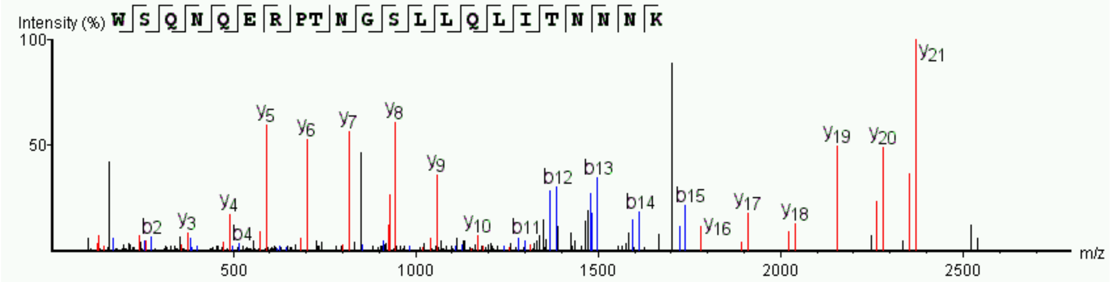

gi|571558853

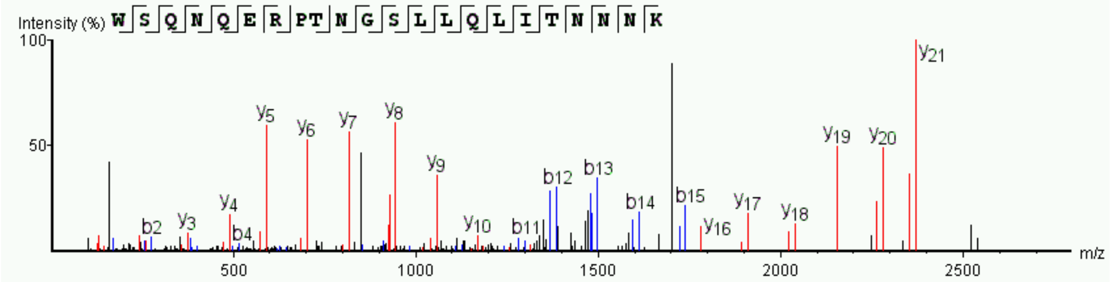

gi|571558857

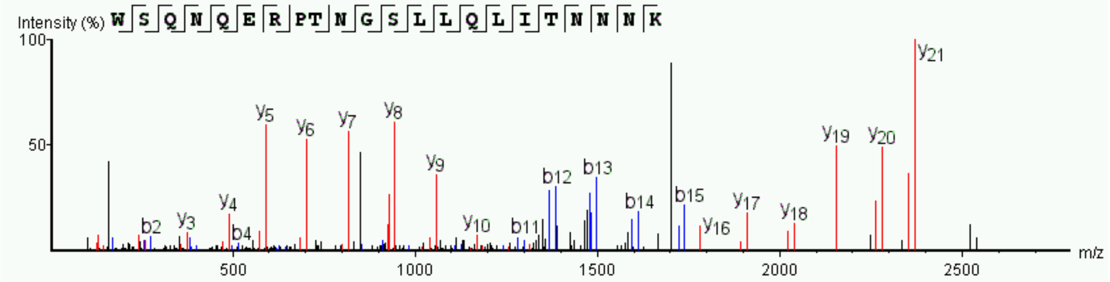

gi|571564381

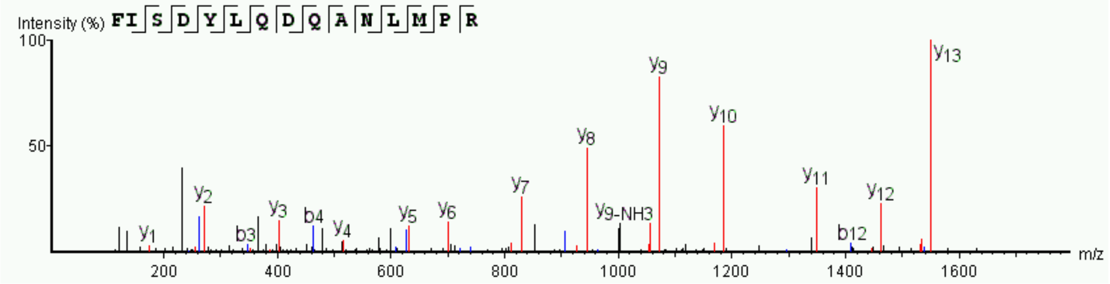

gi|571550223

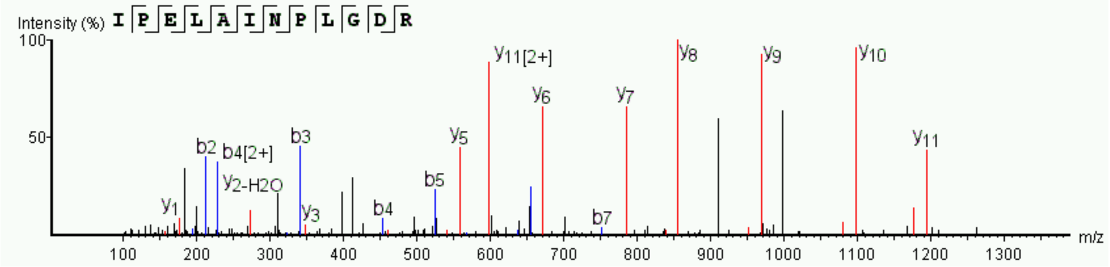

gi|571577993

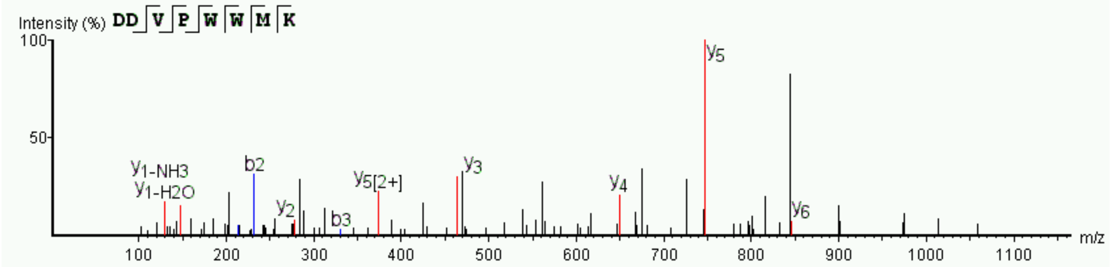

gi|571577995

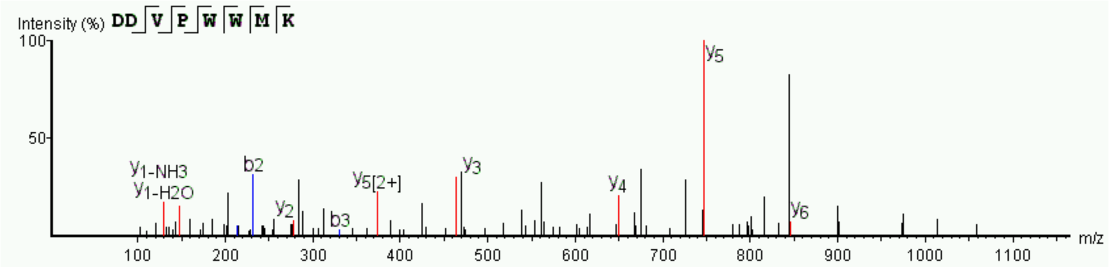

gi|66564402

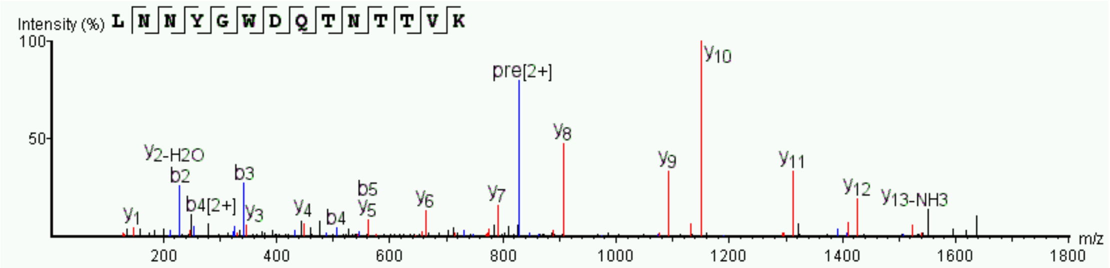

gi|571506170

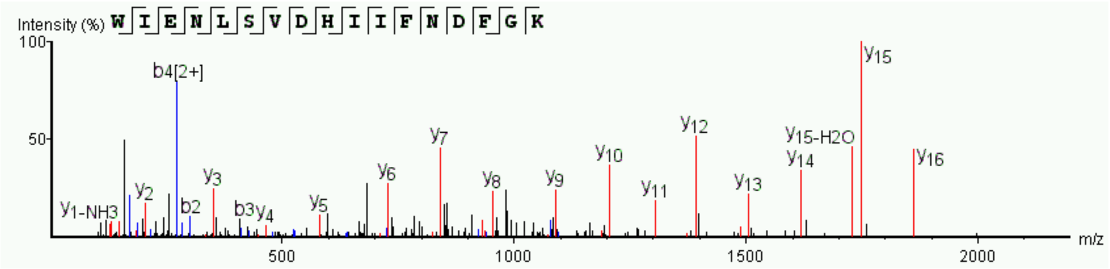

gi|571574447

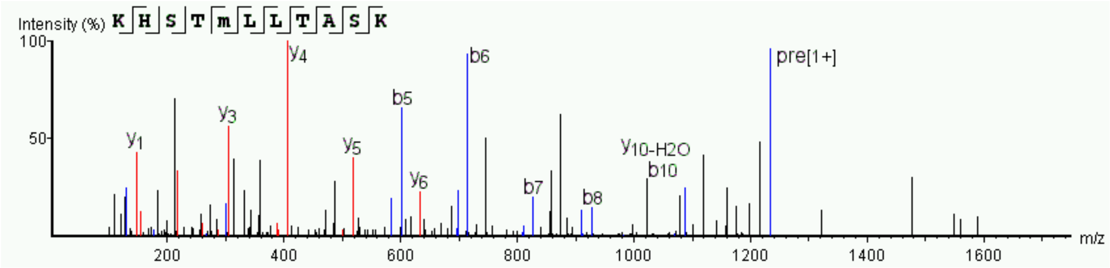

gi|571574449

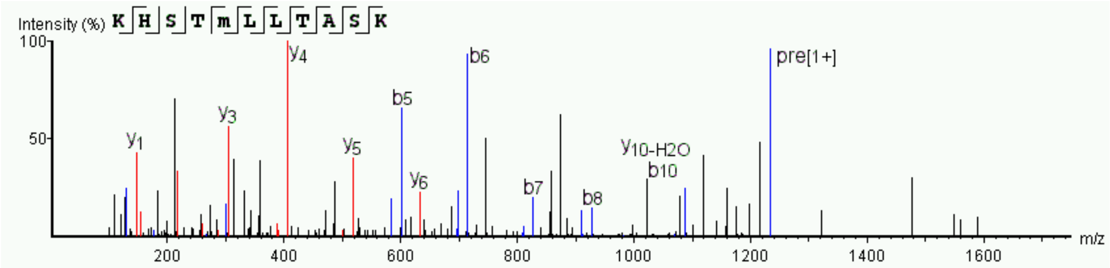

gi|571574451

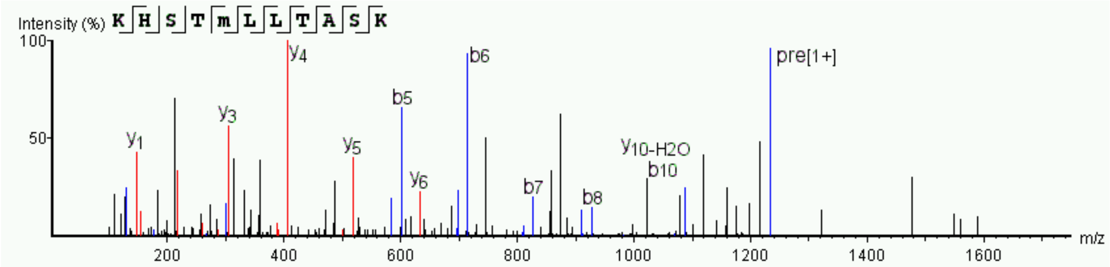

gi|66525427

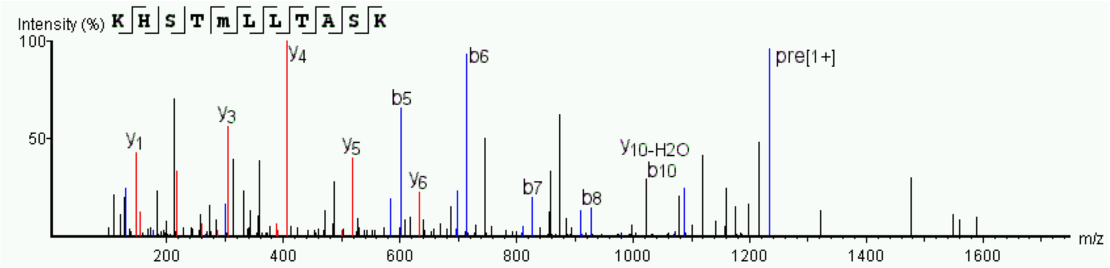

gi|571524916

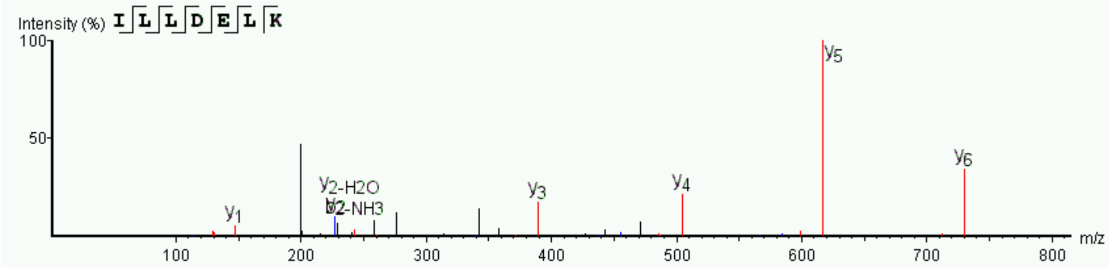

gi|571524919

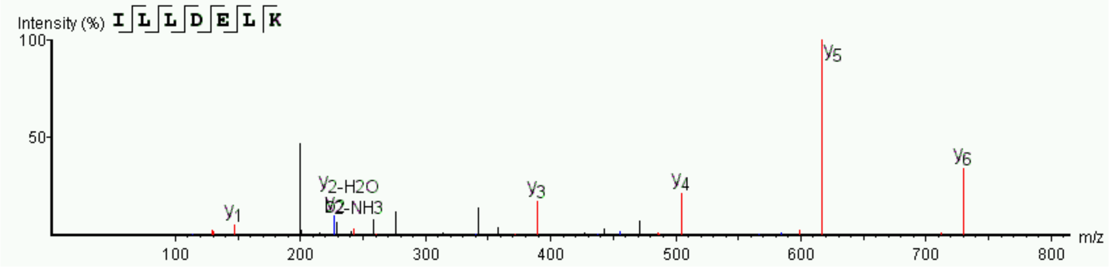

gi|66551959

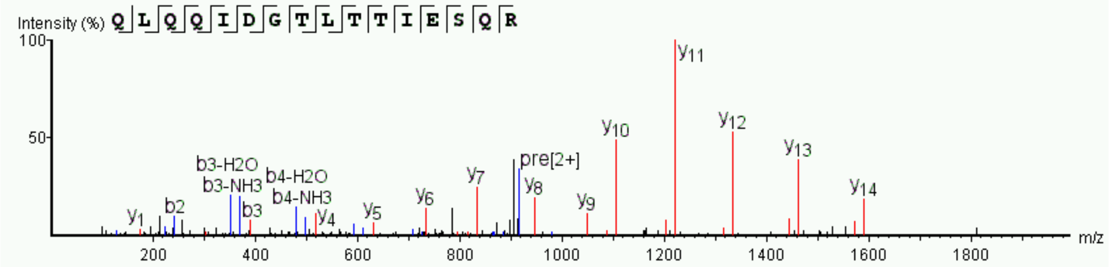

gi|571574384

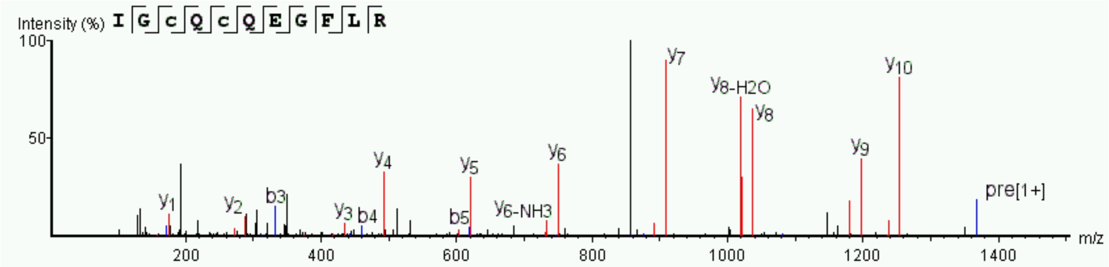

gi|66499810

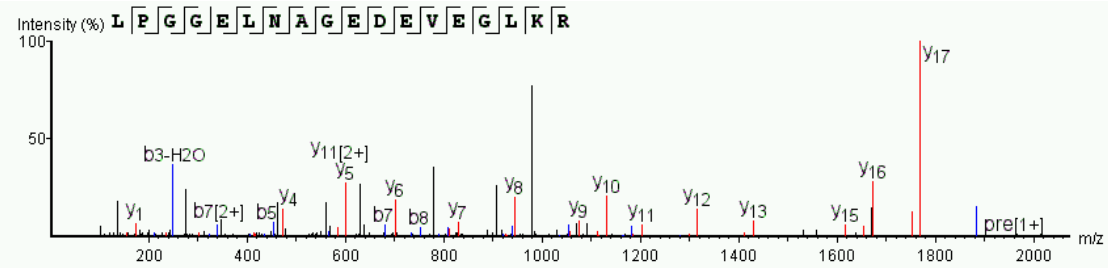

gi|571537384

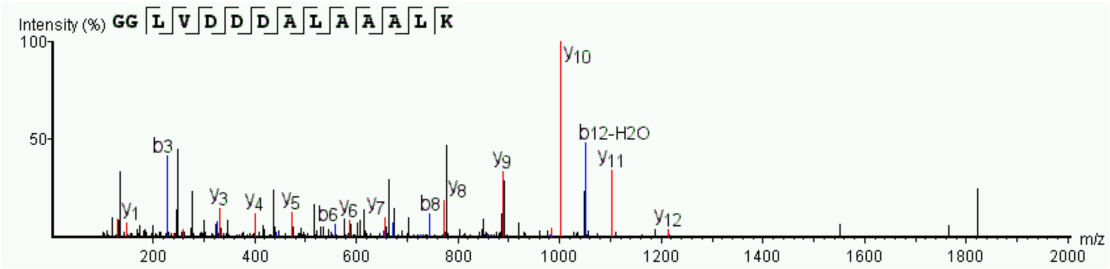

gi|571537387

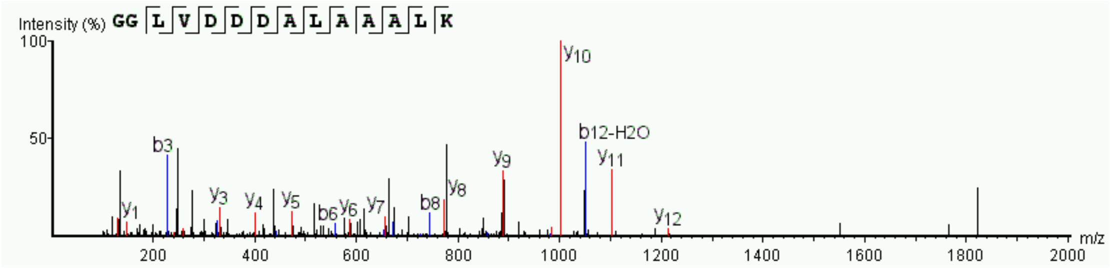

gi|571537391

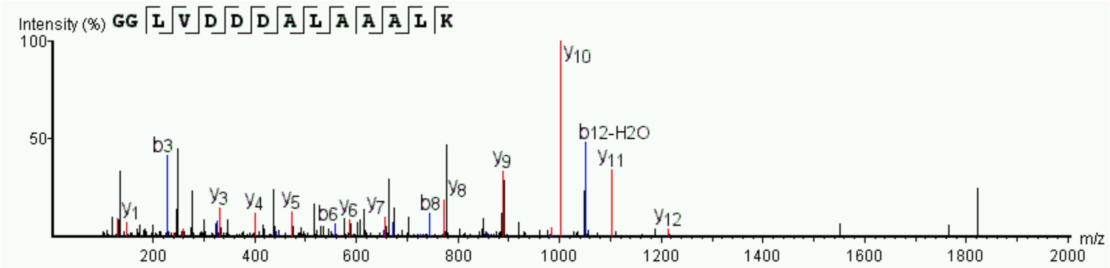

gi|571537395

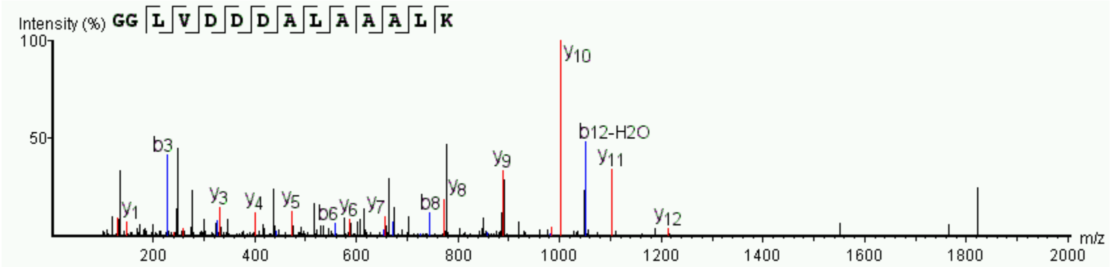

gi|571537398

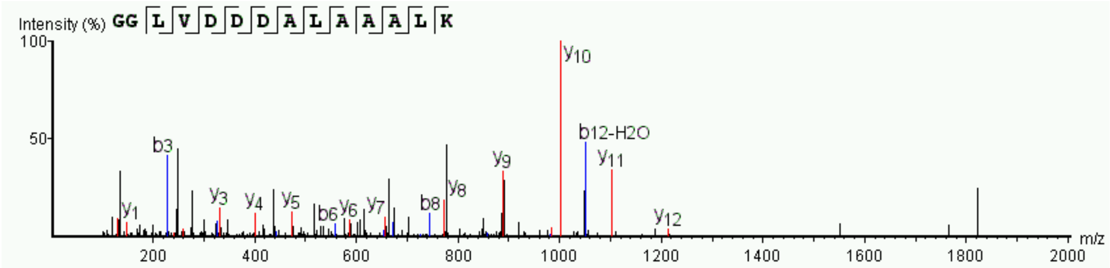

gi|571537401

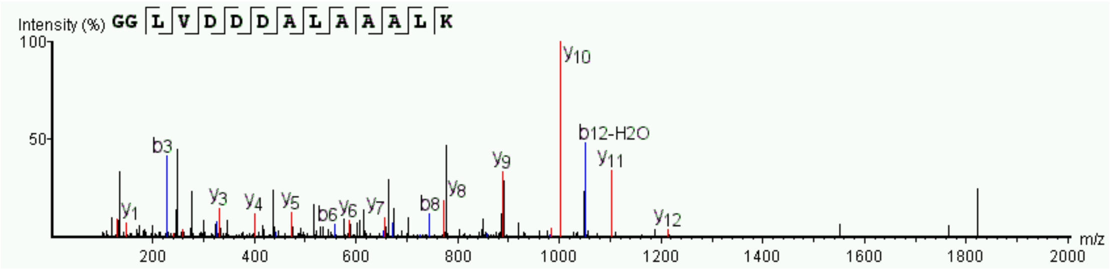

gi|571537405

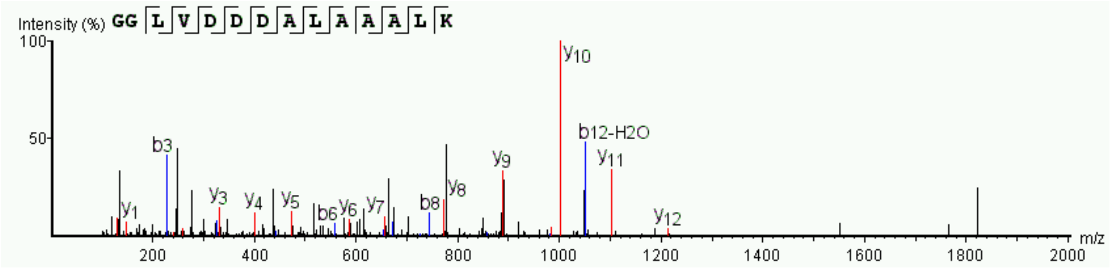

gi|571537408

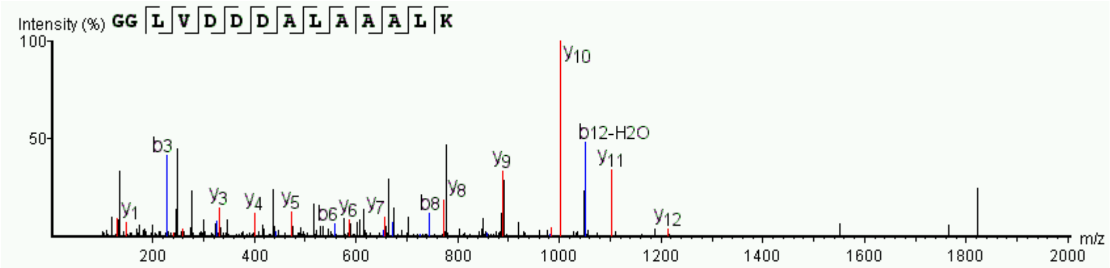

gi|328776329

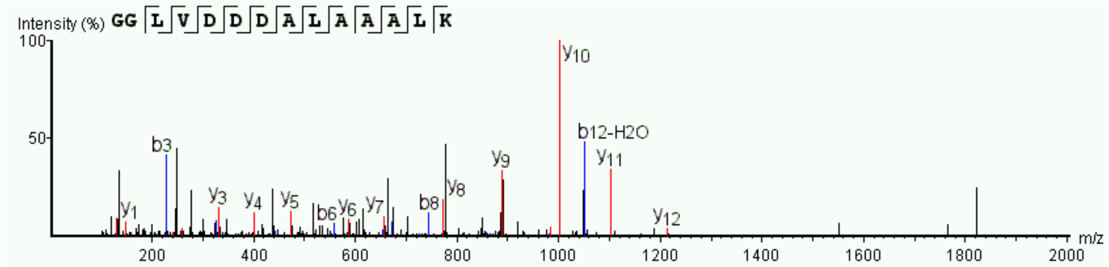

gi|571561468

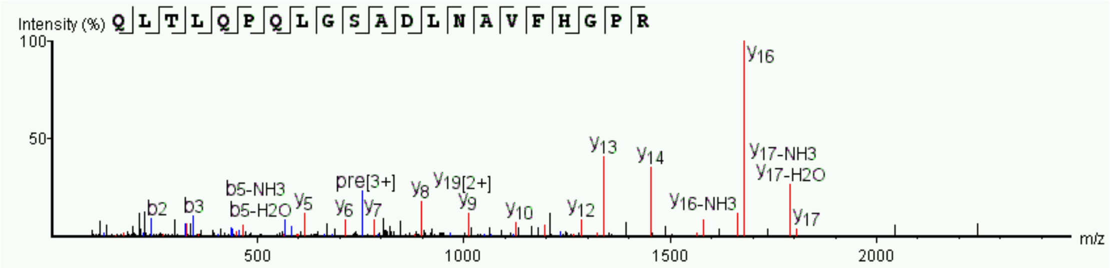

gi|571561464

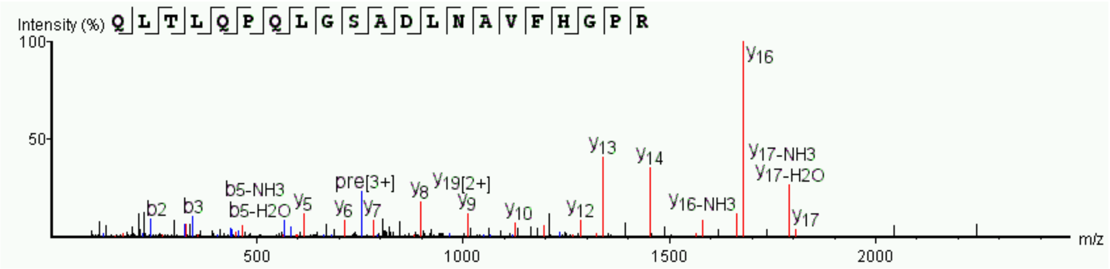

gi|571532248

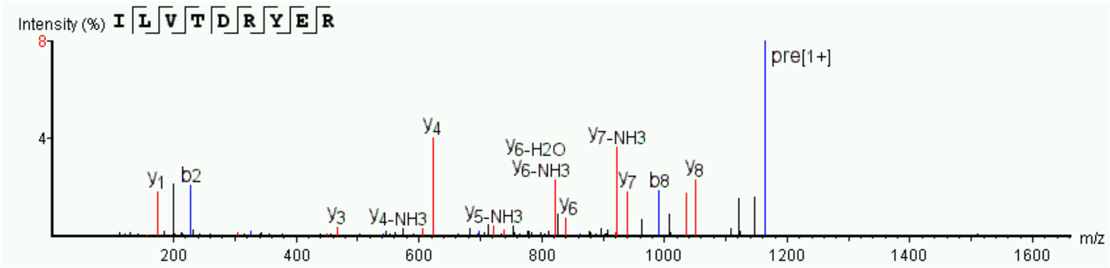

gi|571532252

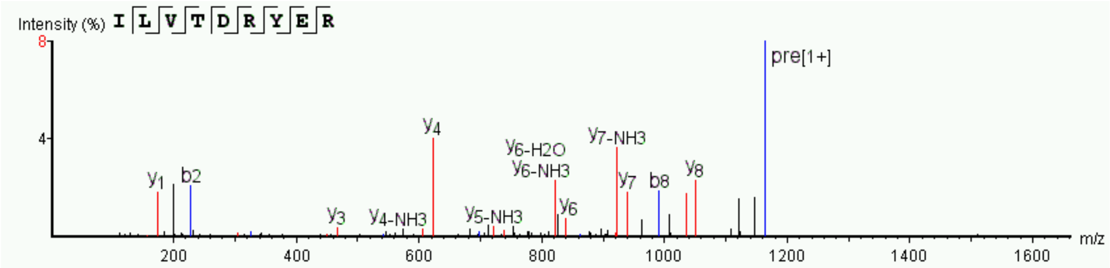

gi|571532256

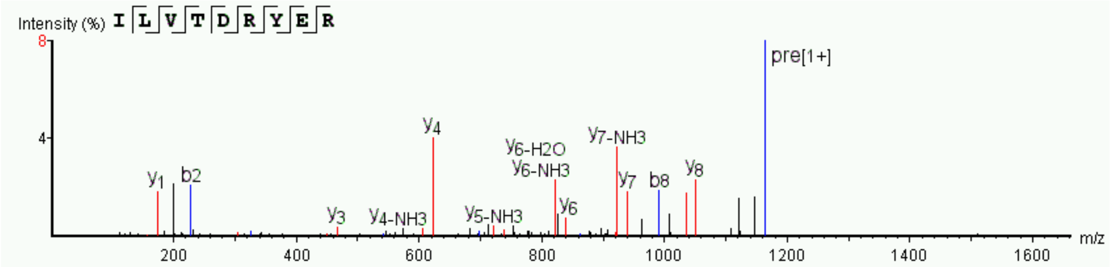

gi|571532260

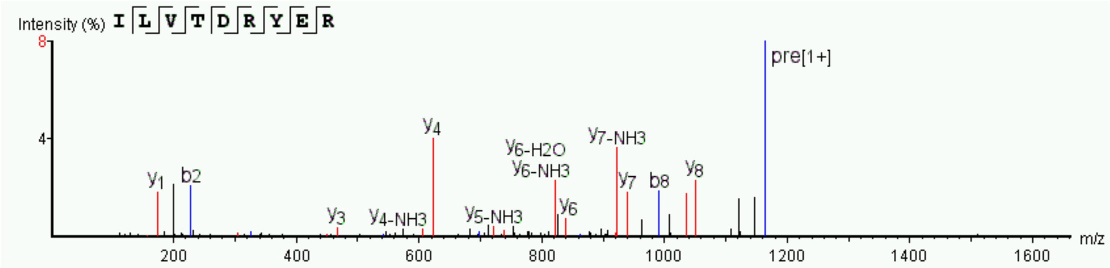

gi|571532244

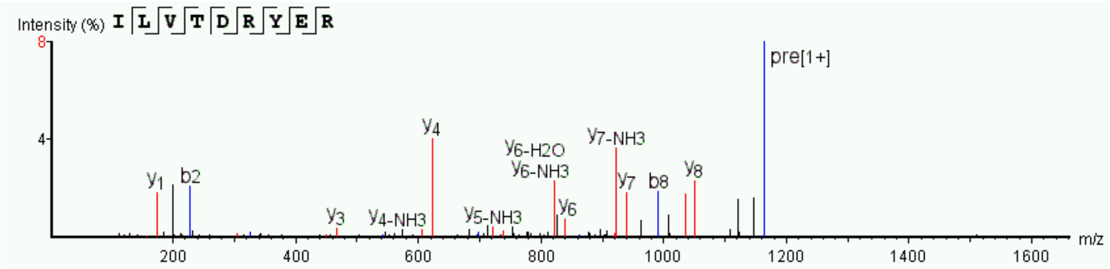

gi|66532731

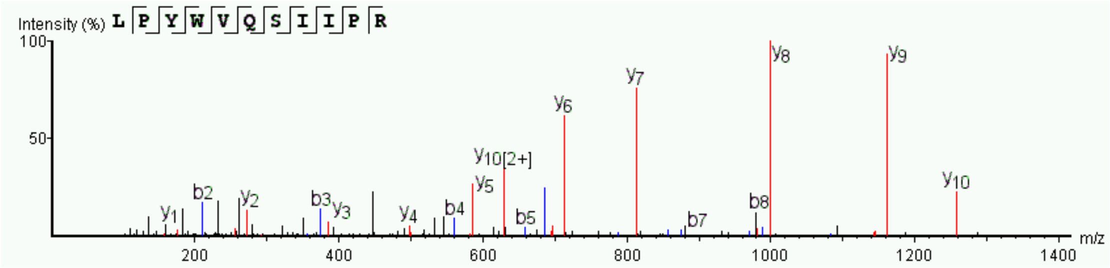

gi|48098155

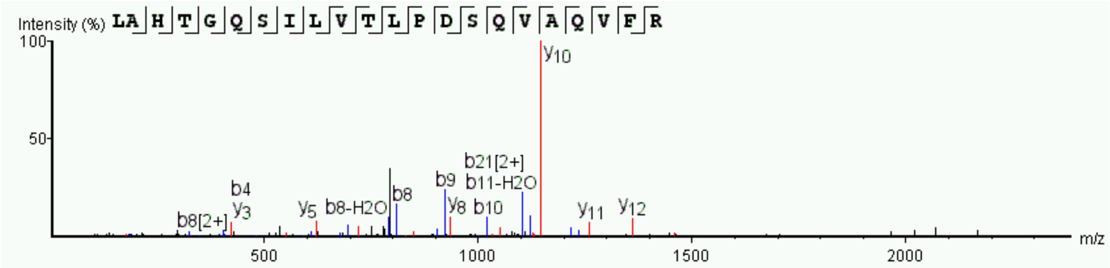

gi|328783311

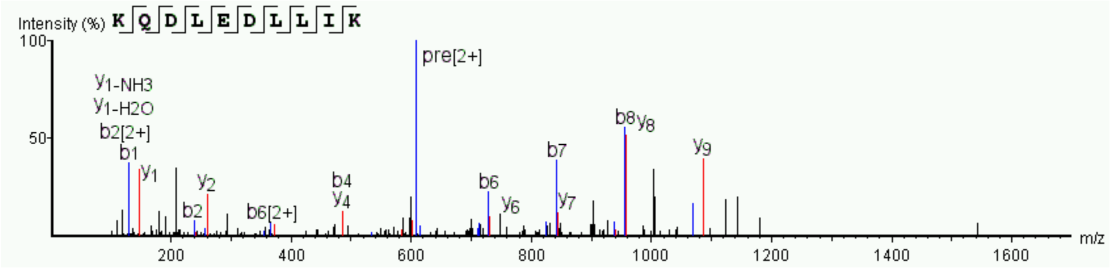

gi|328783748

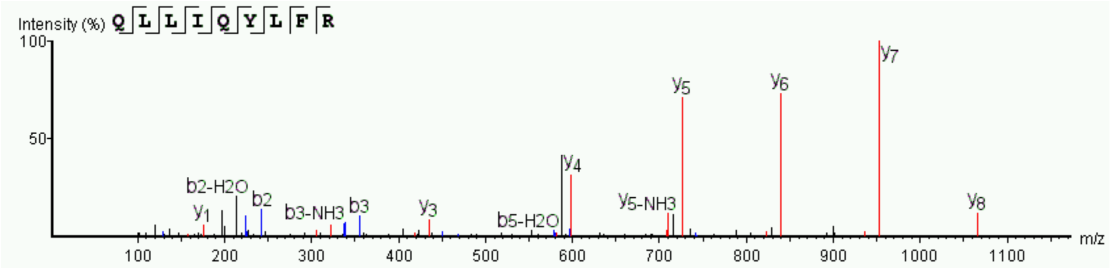

gi|571527600

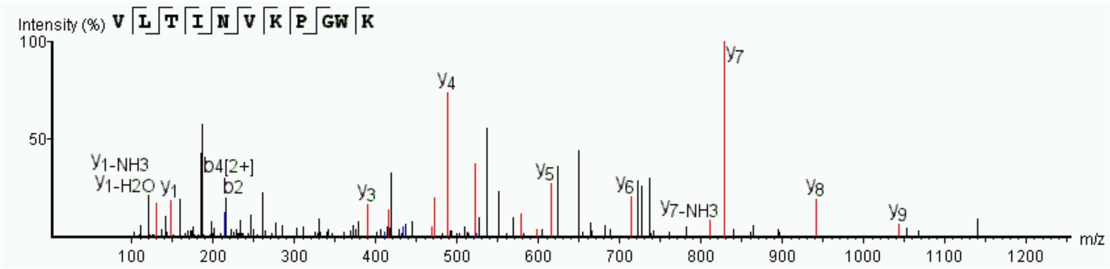

gi|571550208

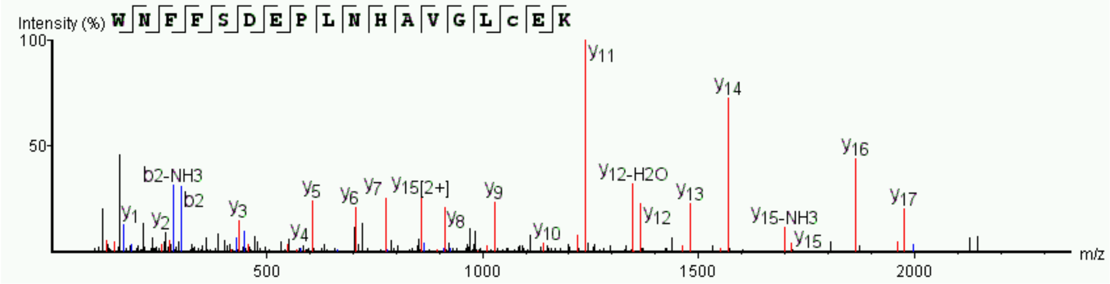

gi|571513636

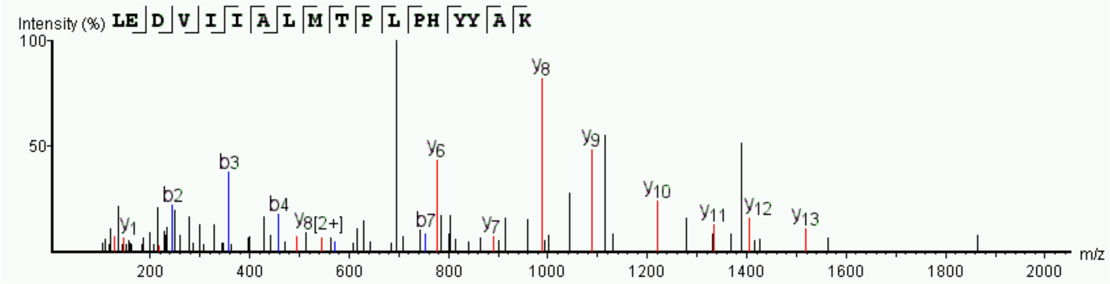

gi|571513622

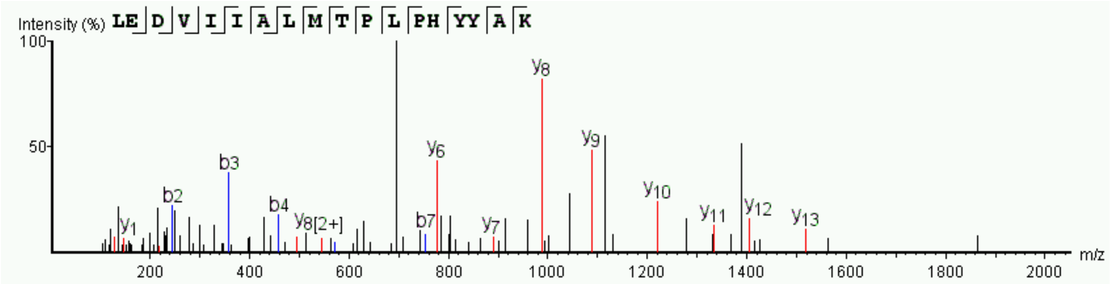

gi|571513626

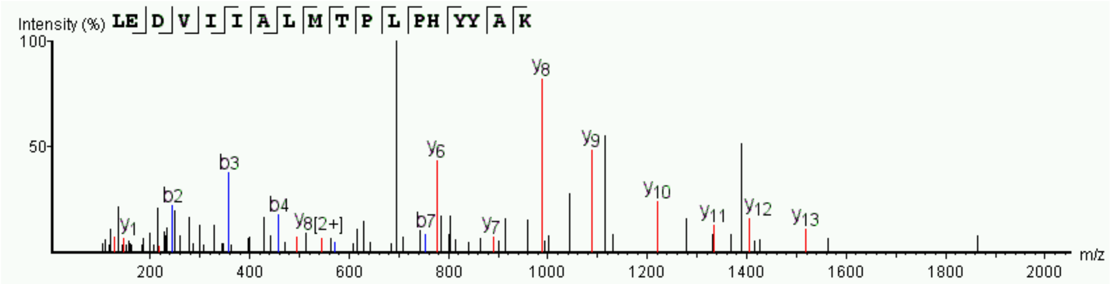

gi|571513630

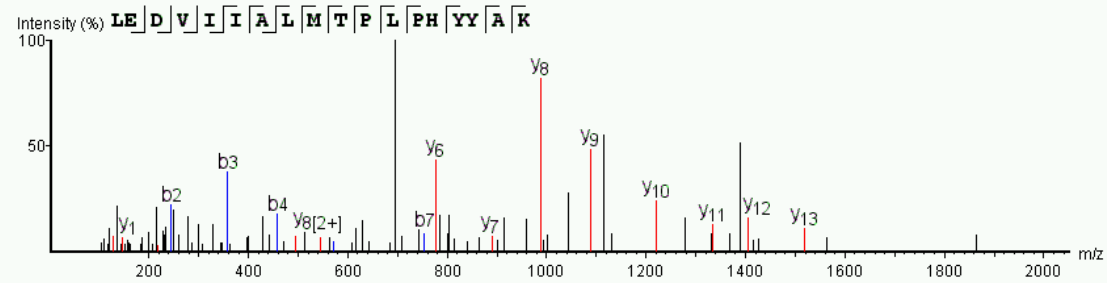

gi|571513618

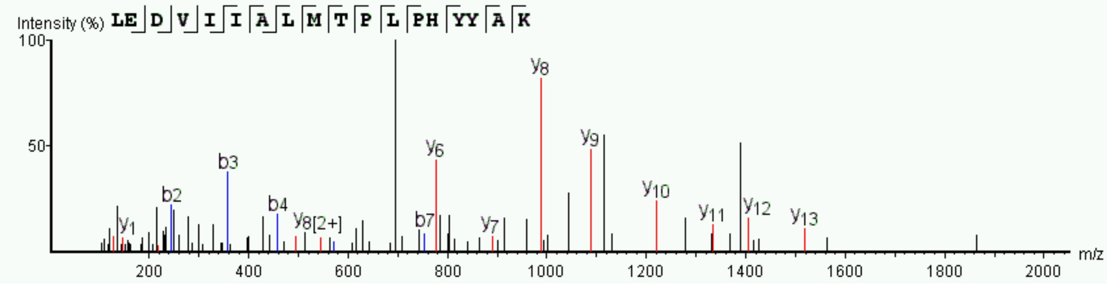

gi|571513632

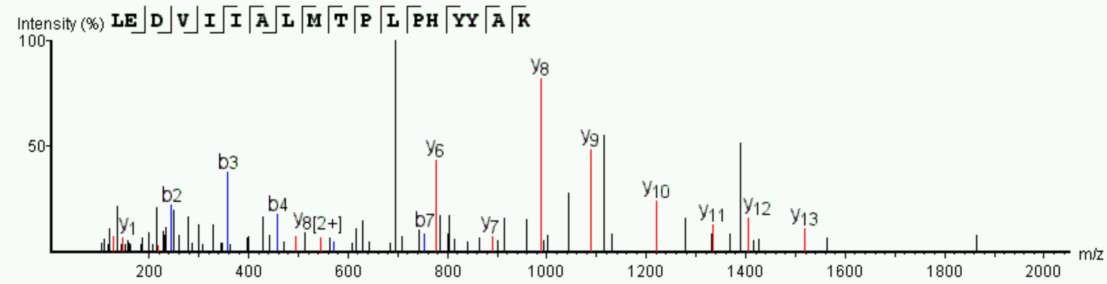

gi|328783818

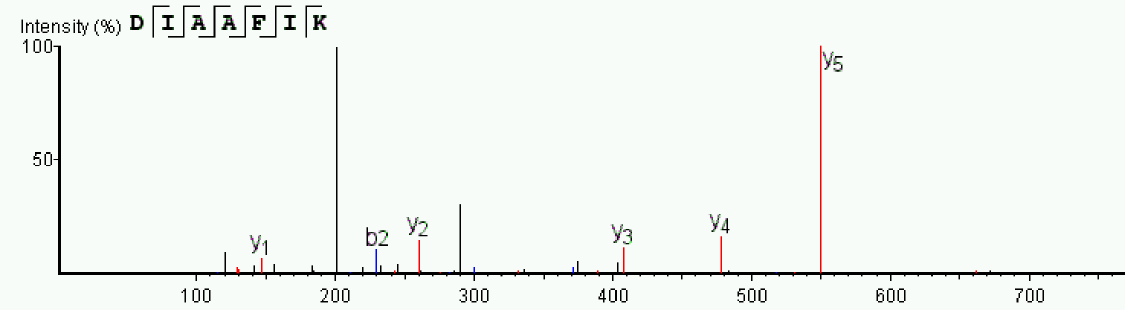

gi|328780820

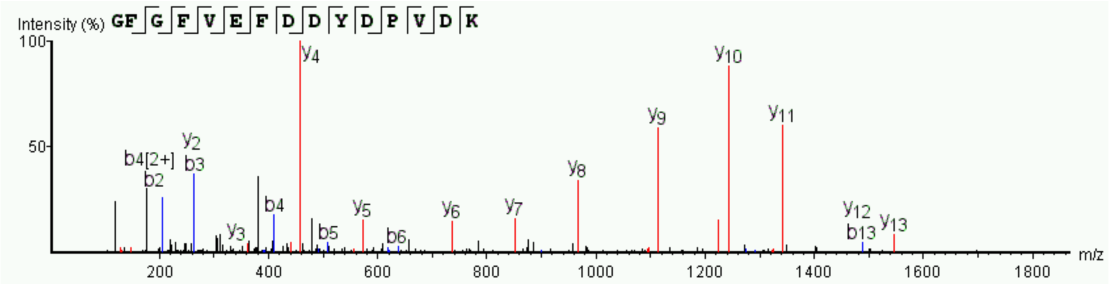

gi|571566909

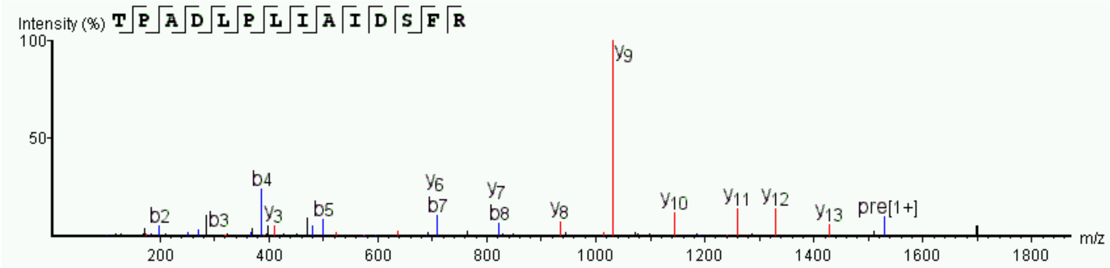

gi|328777360

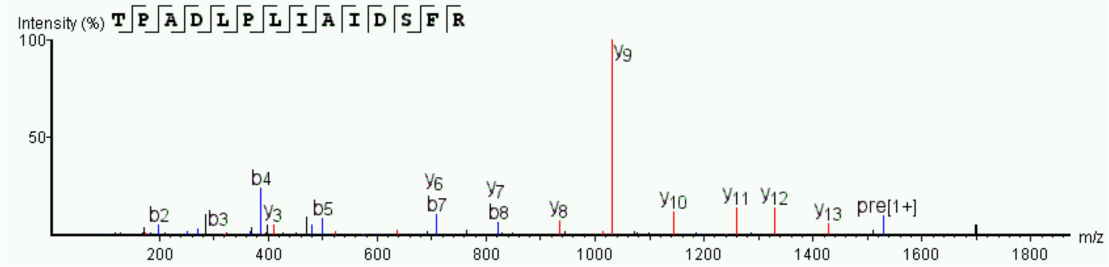

gi|328788967

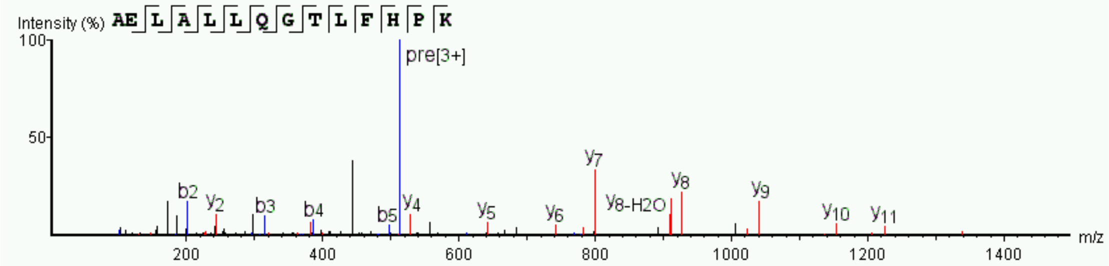

gi|328792599

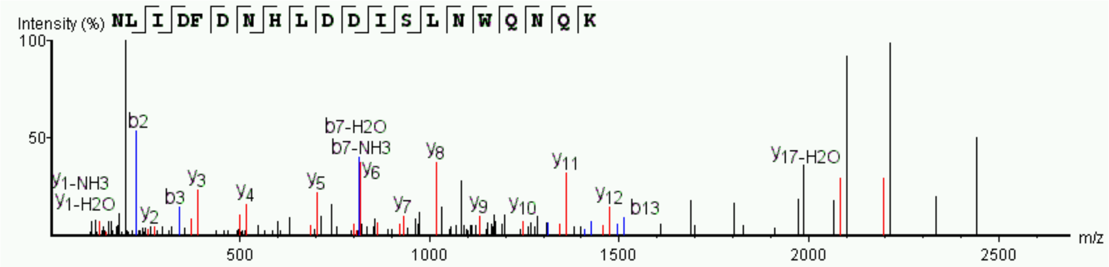

gi|48099074

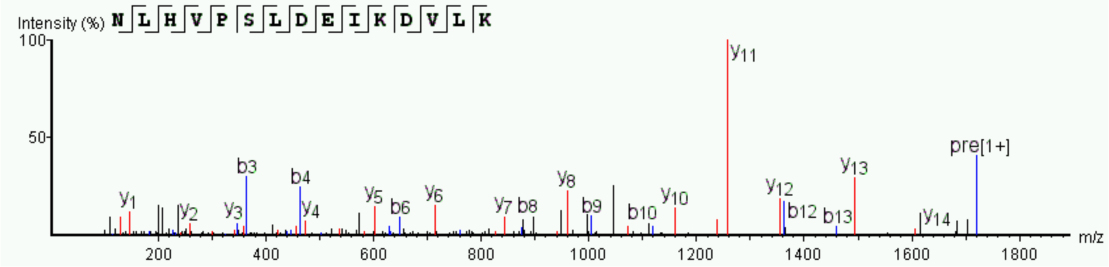

gi|328790880

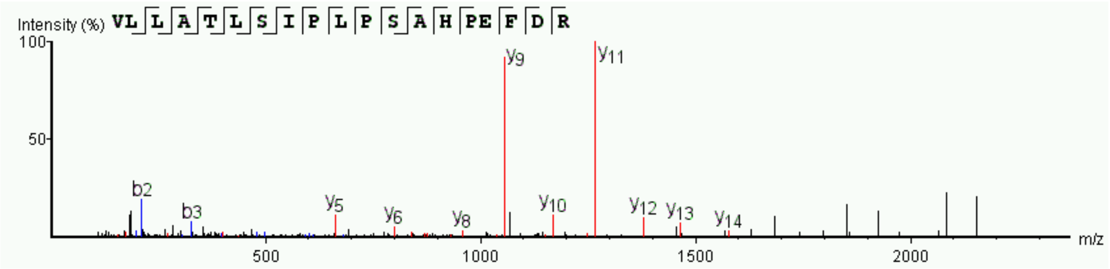

gi|571577643

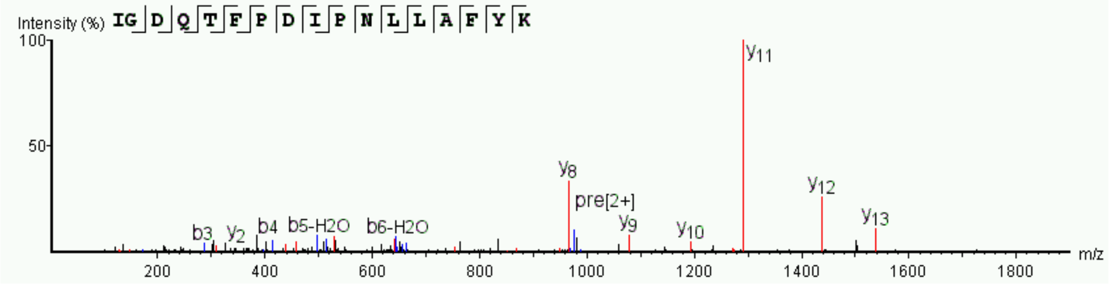

gi|110767655

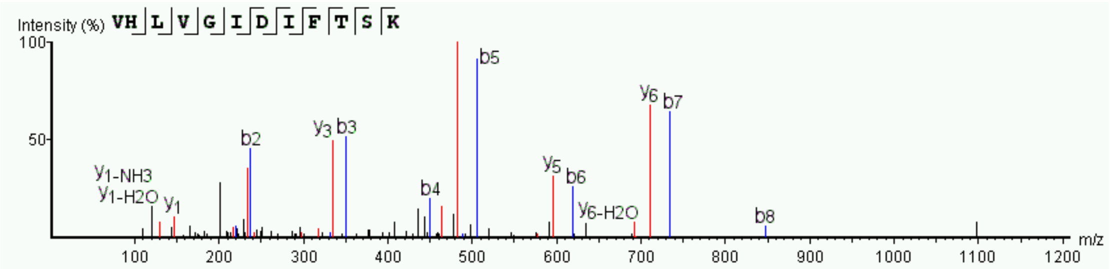

gi|571575749

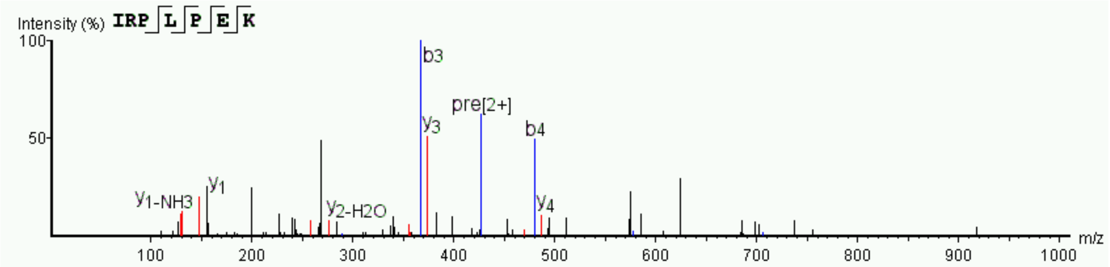

gi|66531704

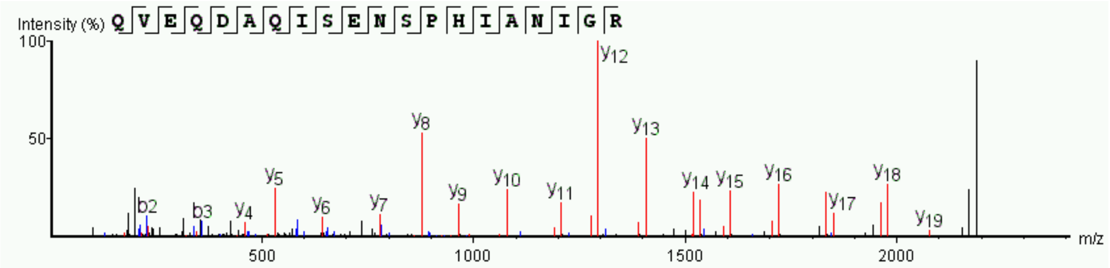

gi|571560348

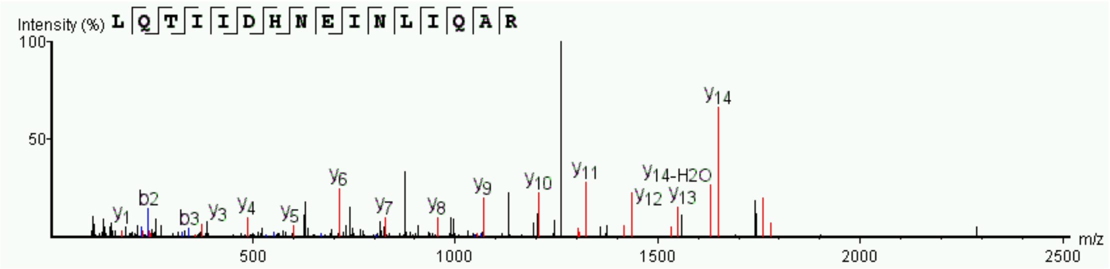

gi|571564796

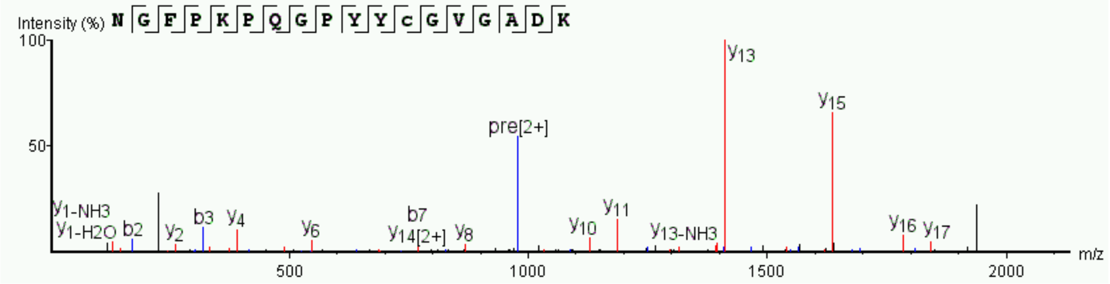

gi|571503655

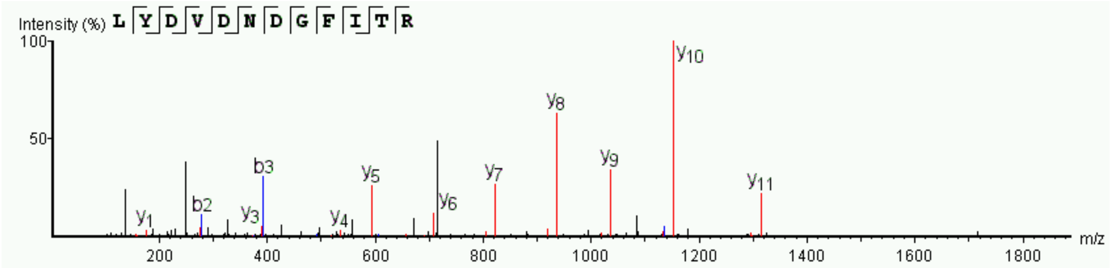

gi|66499717

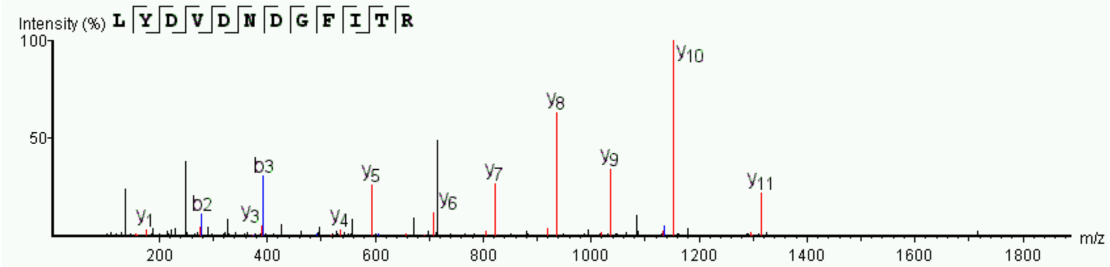

gi|571503659

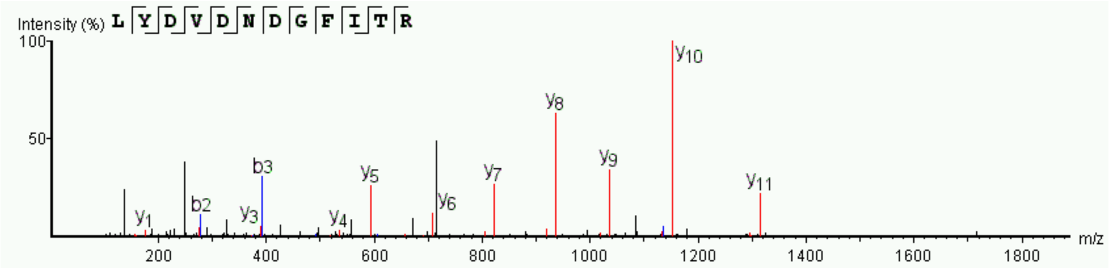

gi|571552254

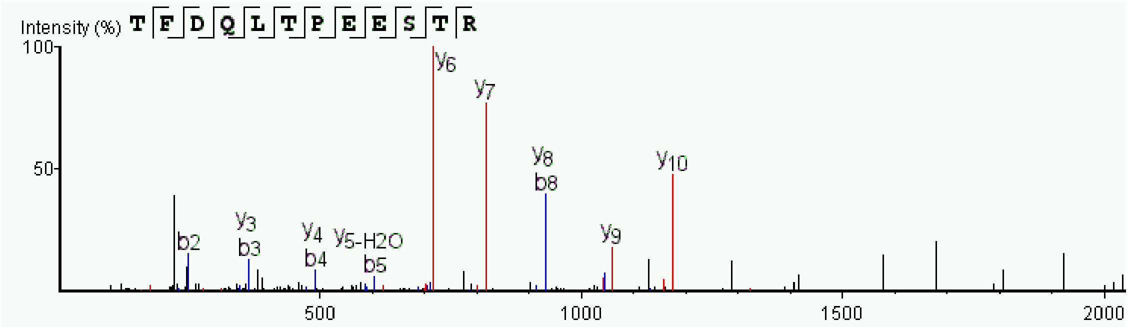

gi|328781627

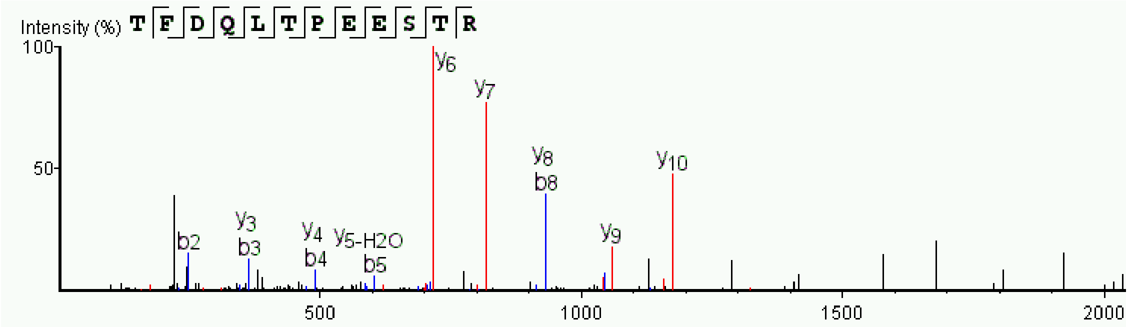

gi|328781625

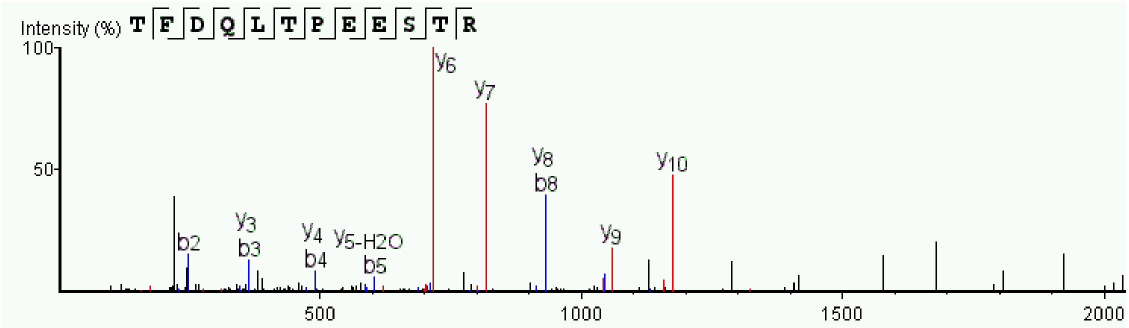

gi|571549099

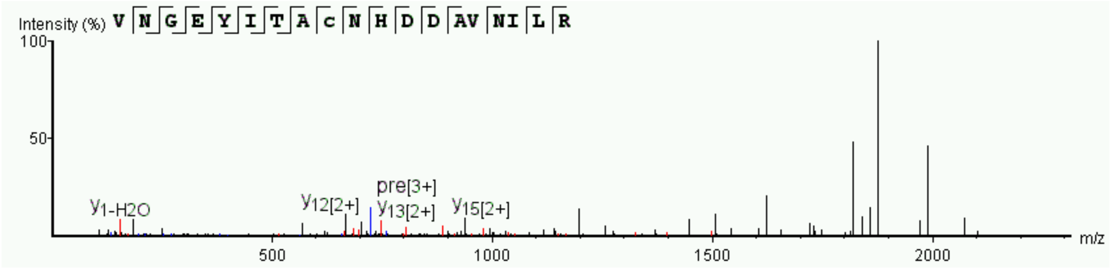

gi|571549102

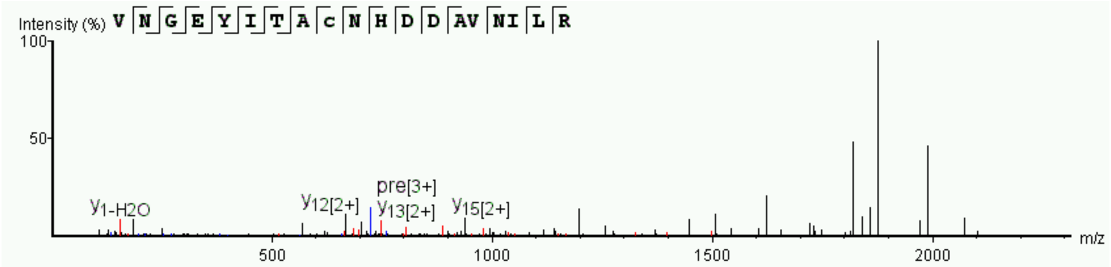

gi|571539527

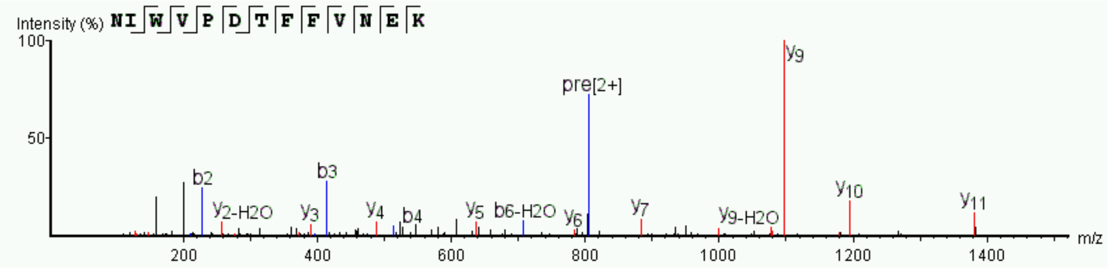

gi|571539567

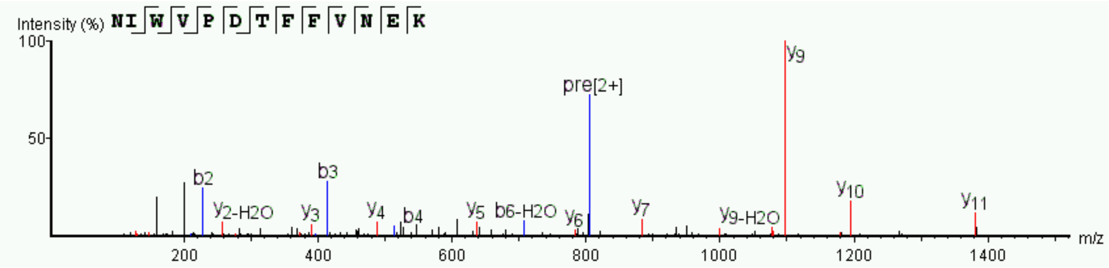

gi|571539570

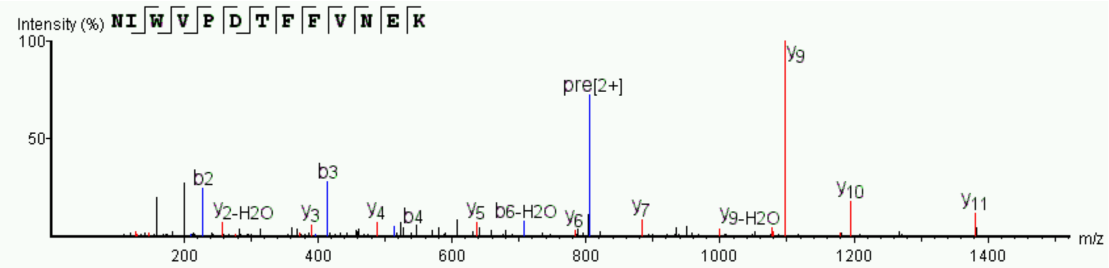

gi|571539574

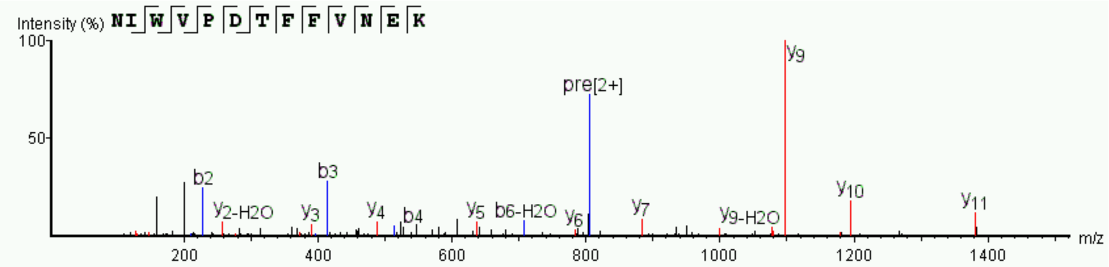

gi|571539578

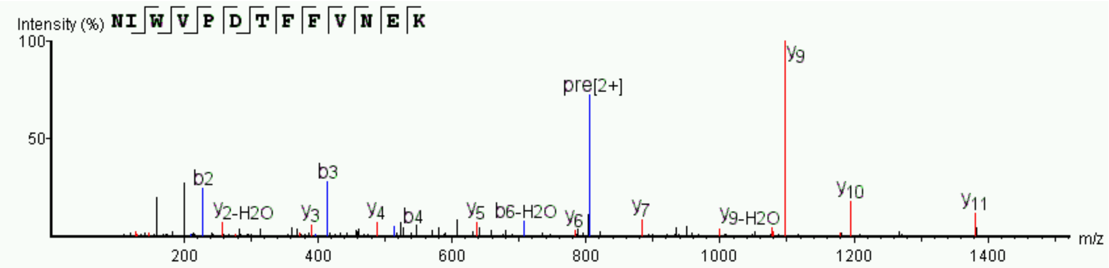

gi|571539581

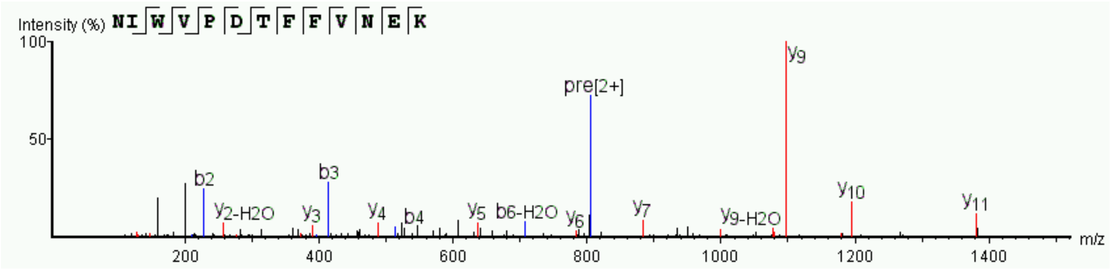

gi|571539510

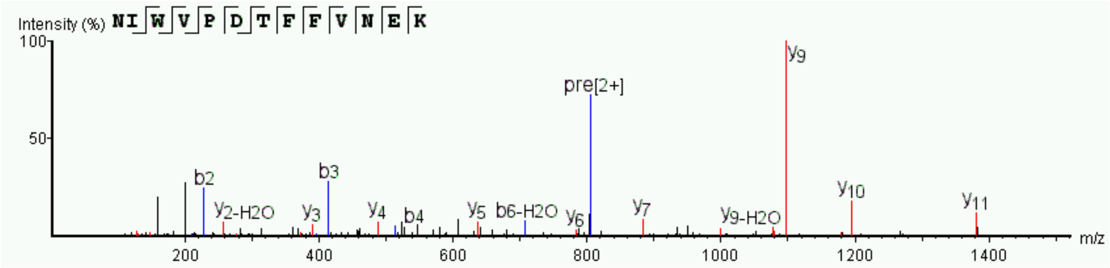

gi|571539513

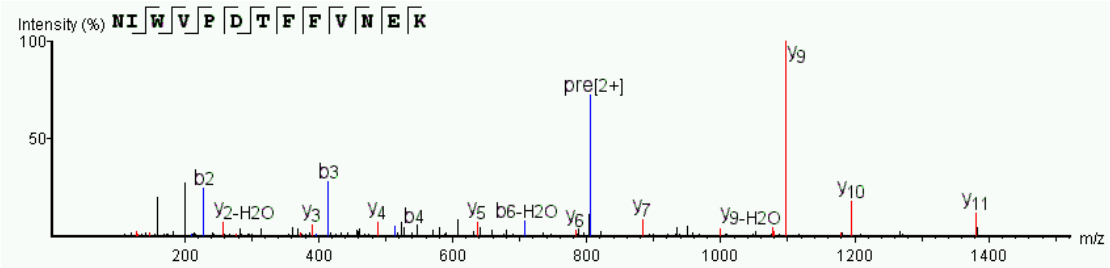

gi|571539517

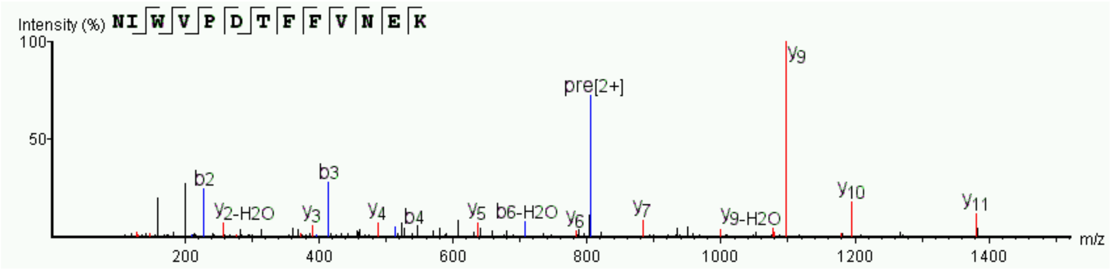

gi|571539551

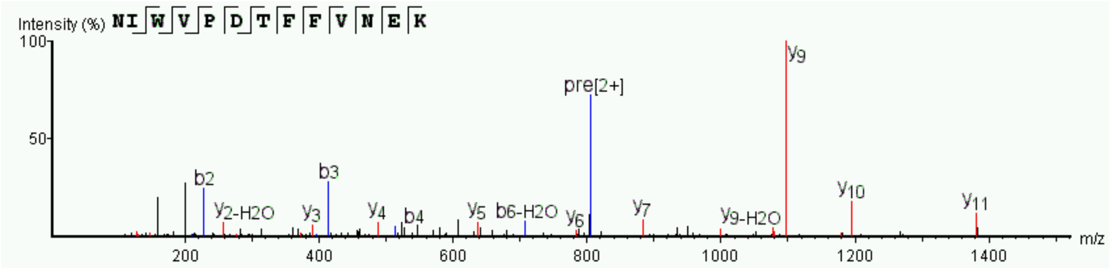

gi|571539554

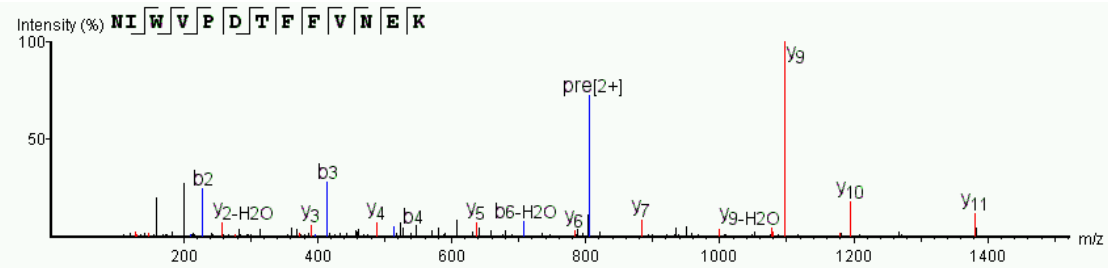

gi|571539557

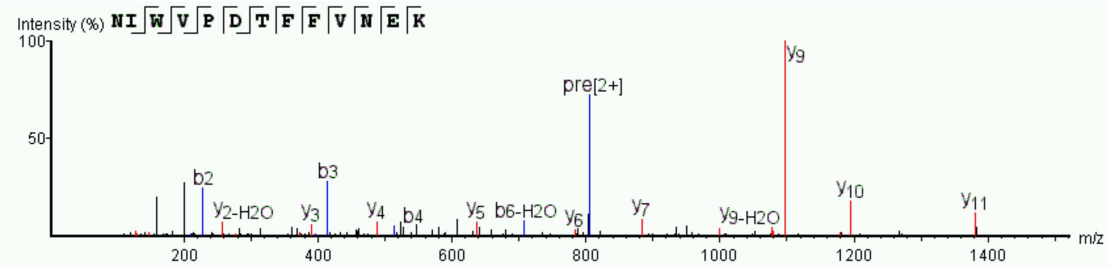

gi|571539560

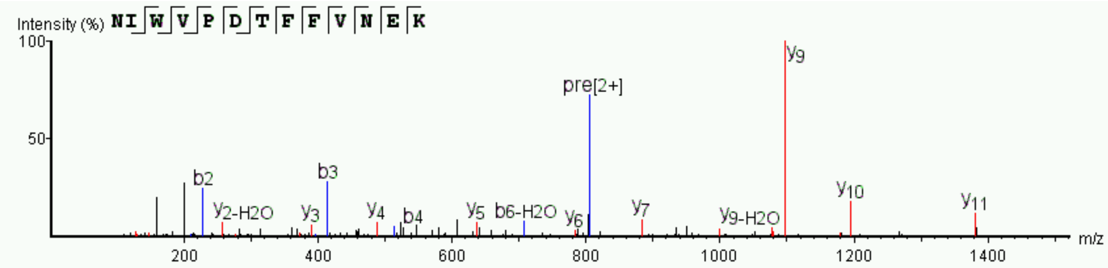

gi|571539564

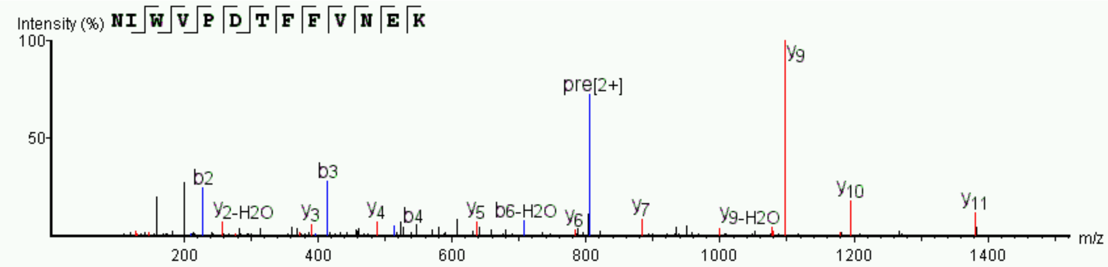

gi|571538660

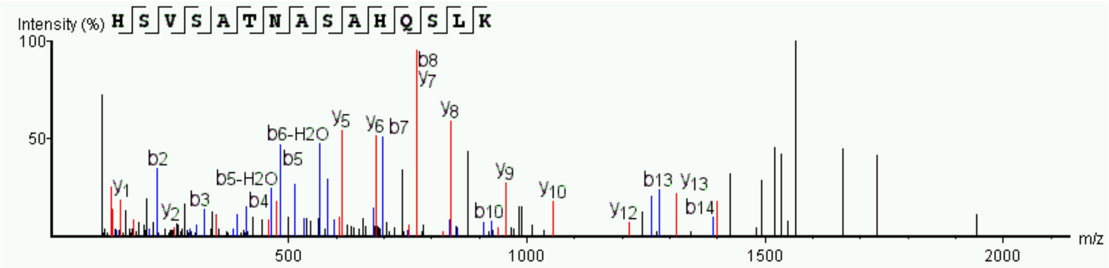

gi|571519668

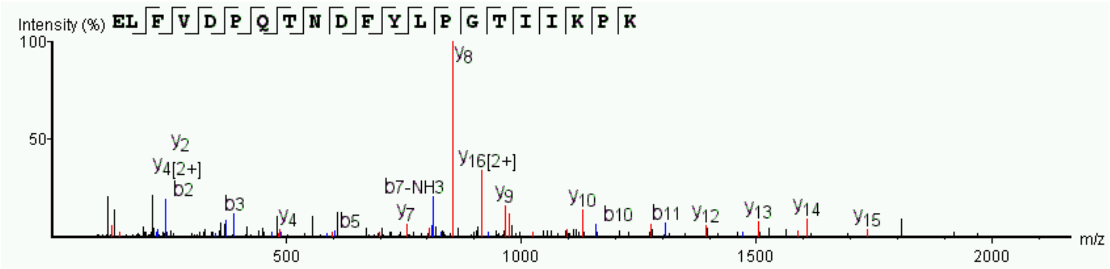

gi|110755339

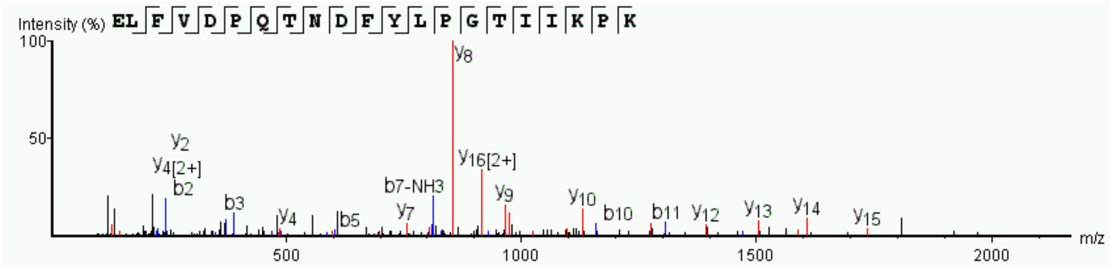

gi|571519665

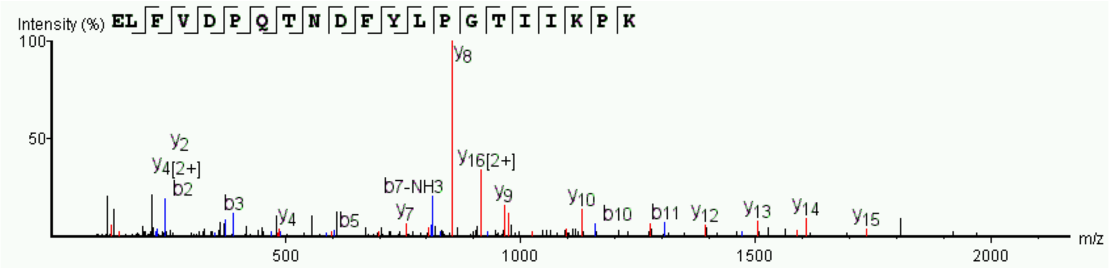

gi|571569736

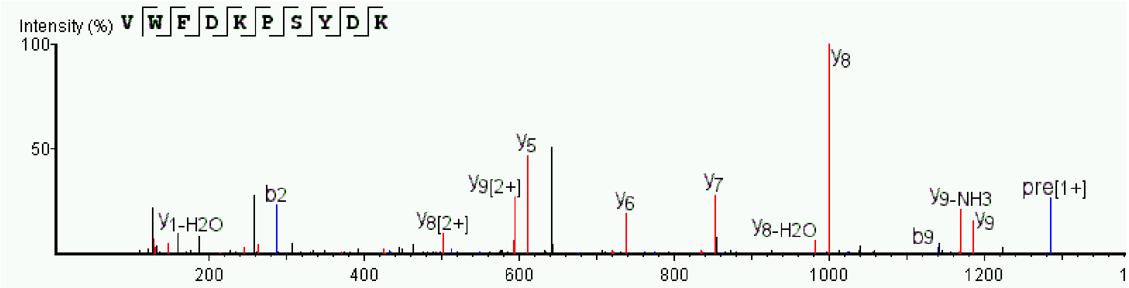

gi|571561918

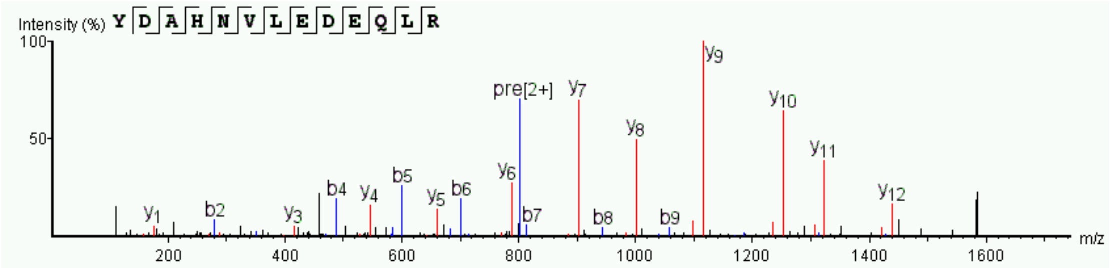

gi|571561922

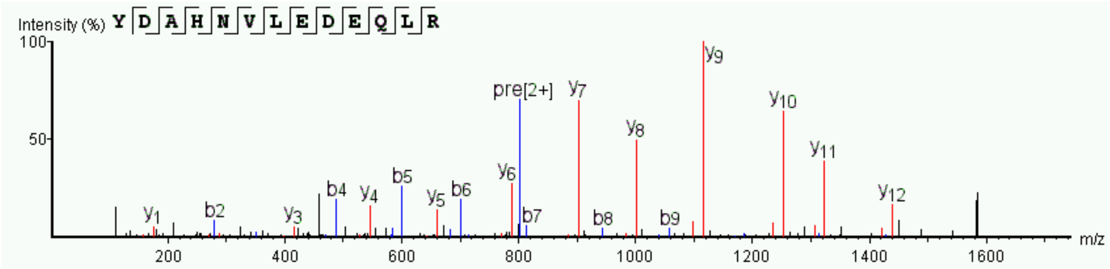

gi|66519264

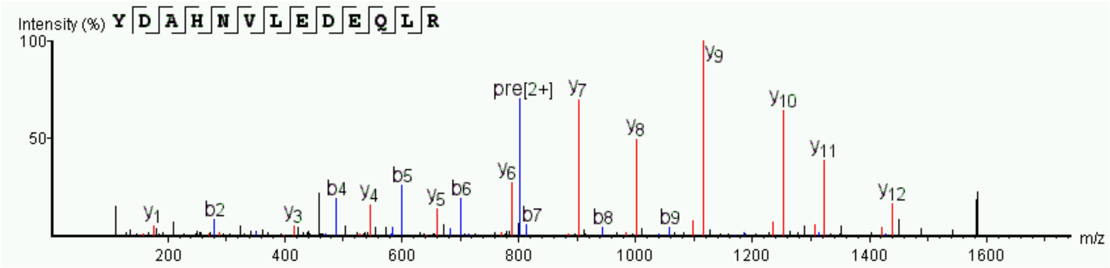

gi|571561914

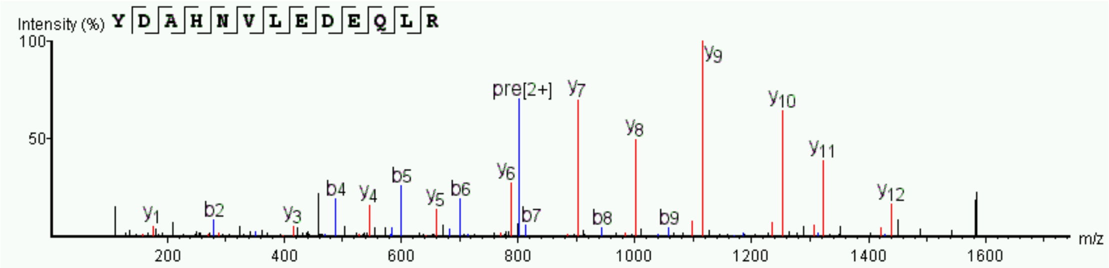

gi|66522082

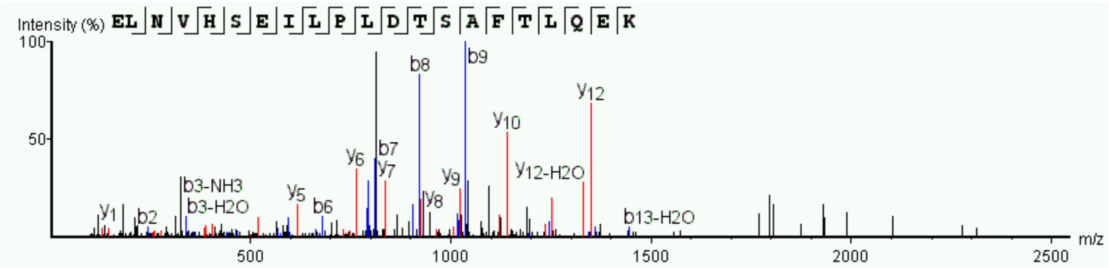

gi|66554764

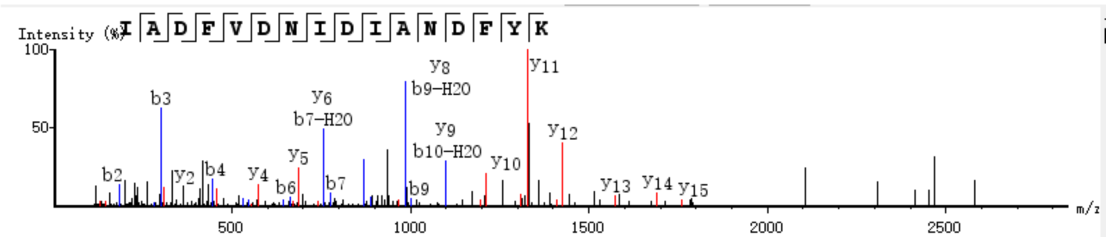

|66546729

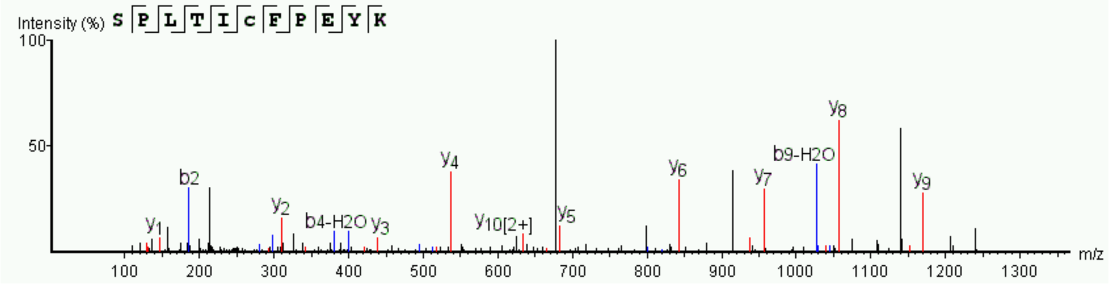

|48095594

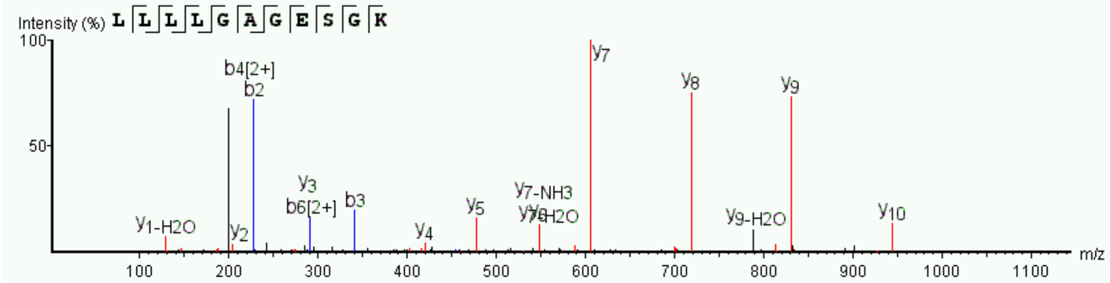

|110771519

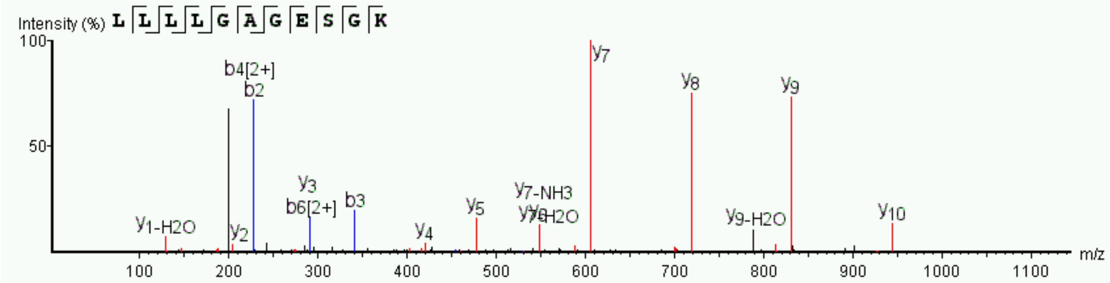

|66513952

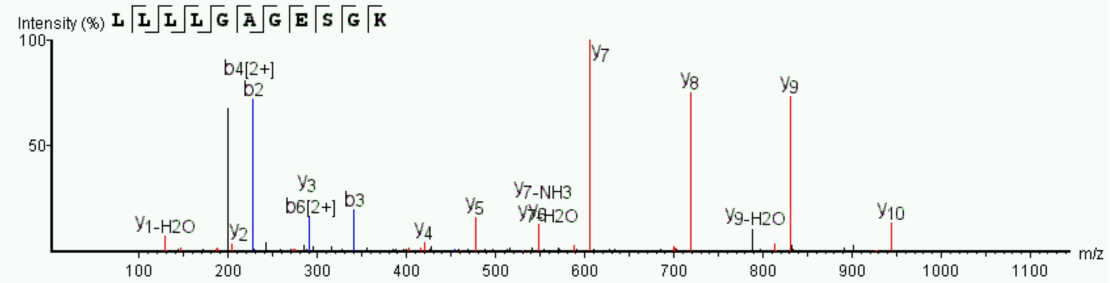

|328777691

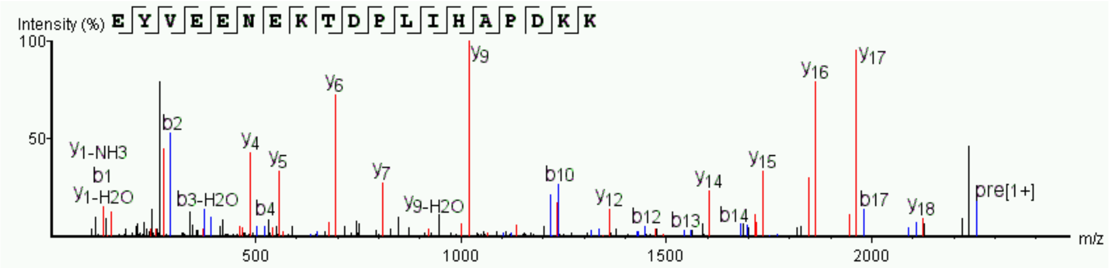

|66509537

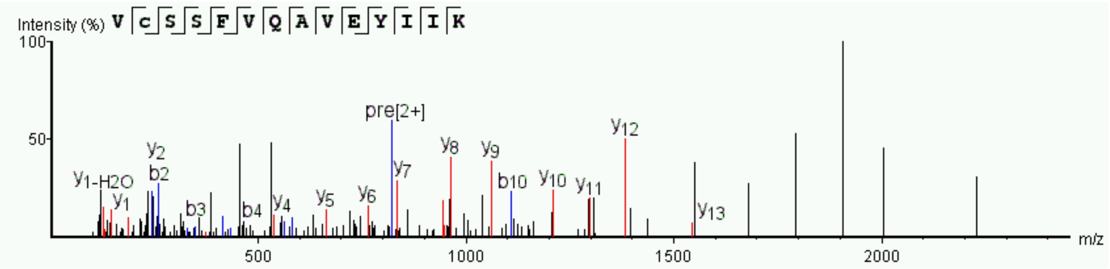

|571511606

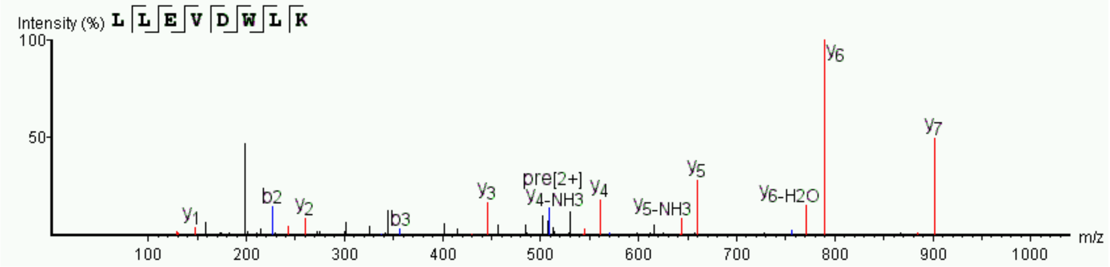

|571534598

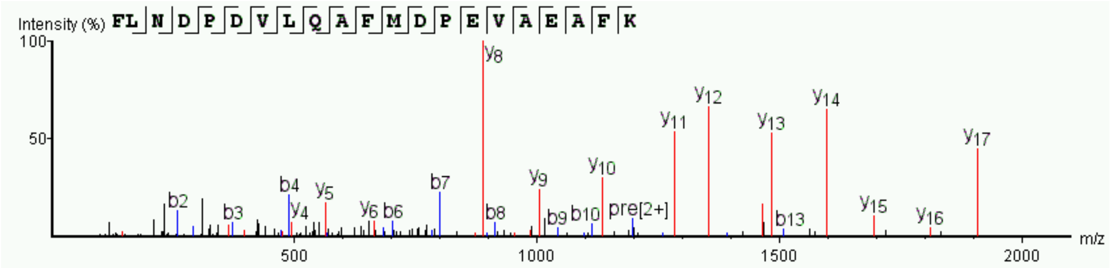

|571534601

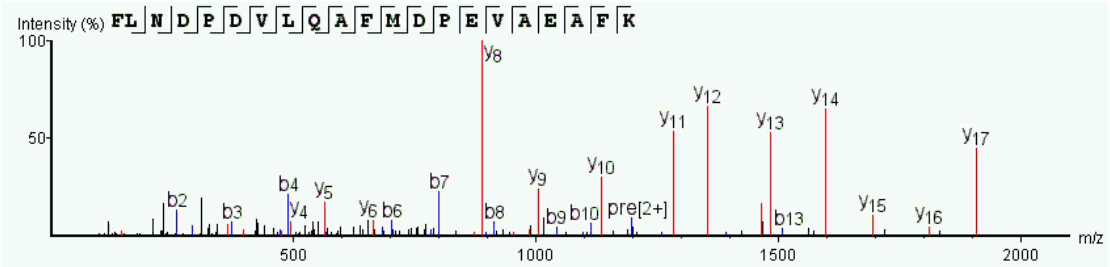

|571554856

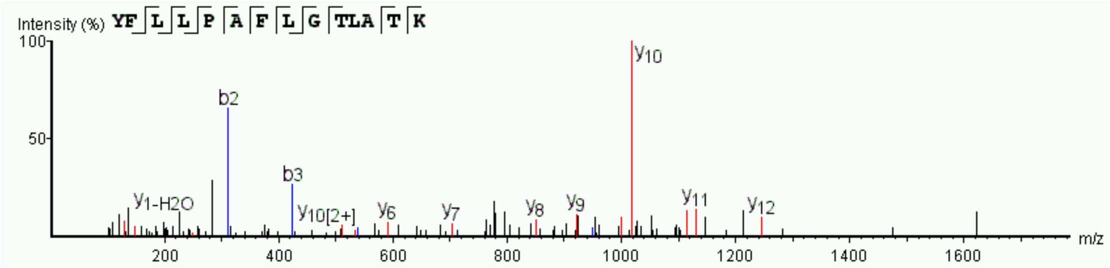

|571567906

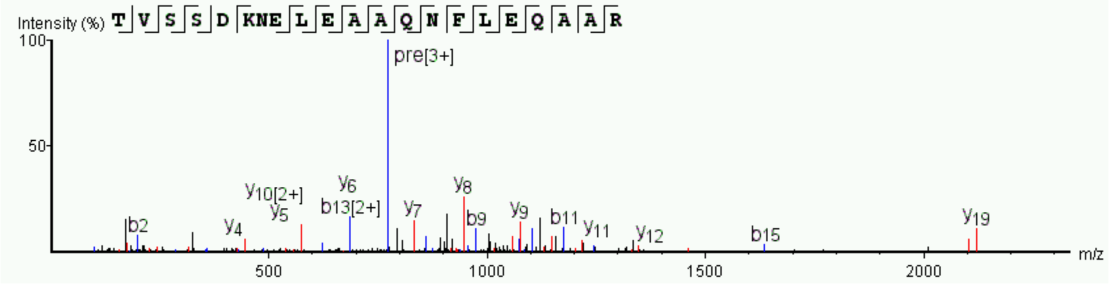

|571512505

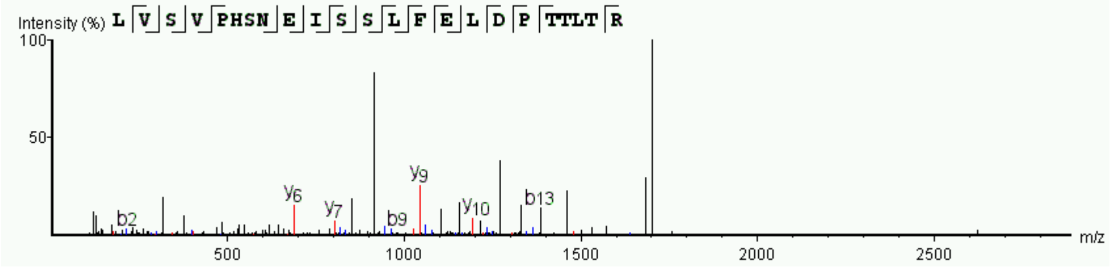

|571512479

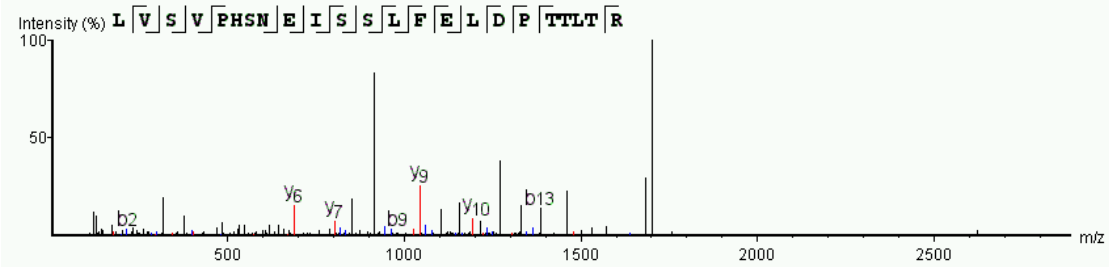

|571512509

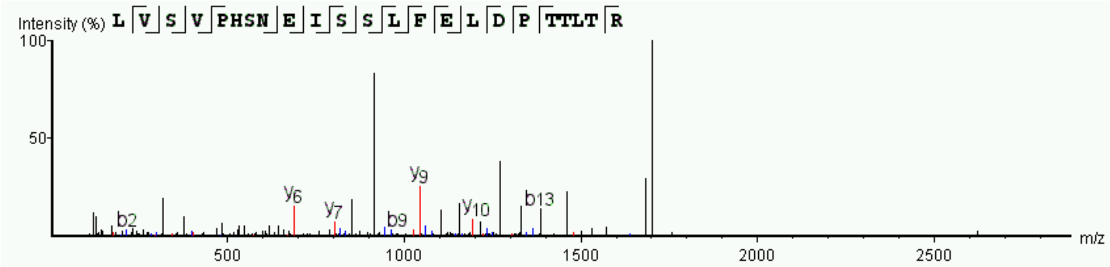

|571512481

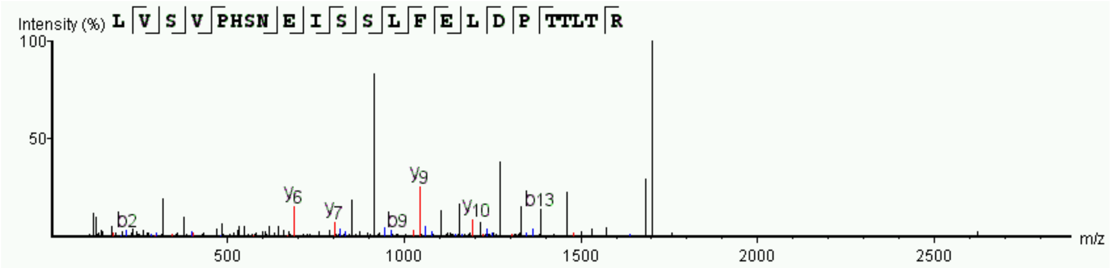

|571512485

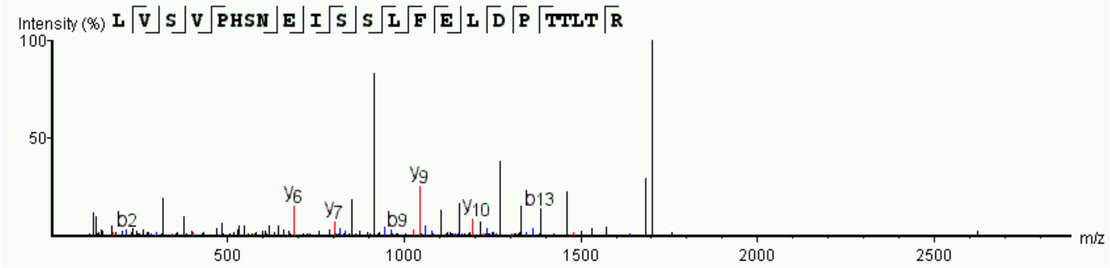

|571512488

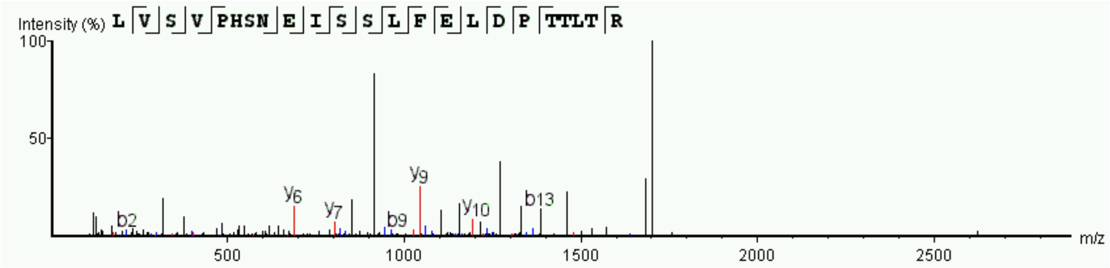

|571512492

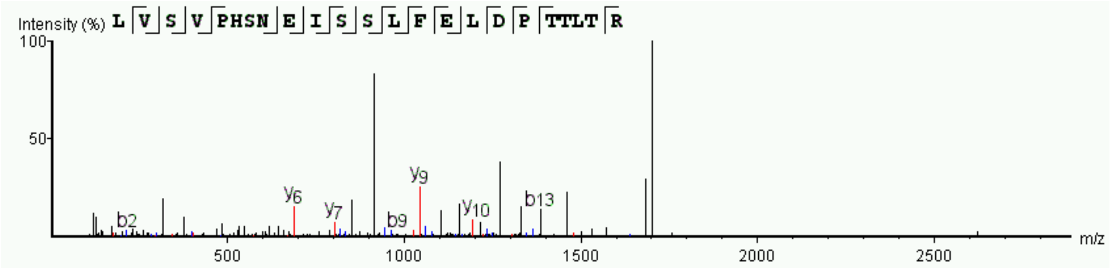

|571512495

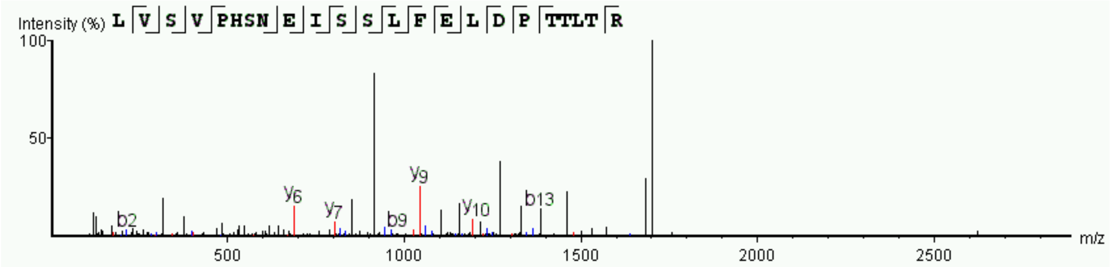

|571512499

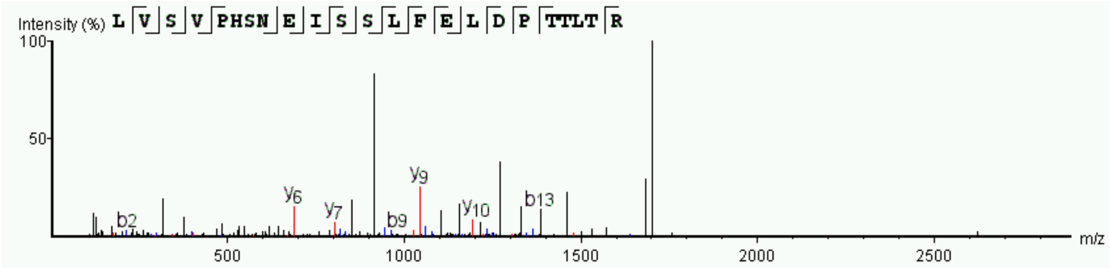

|571512503

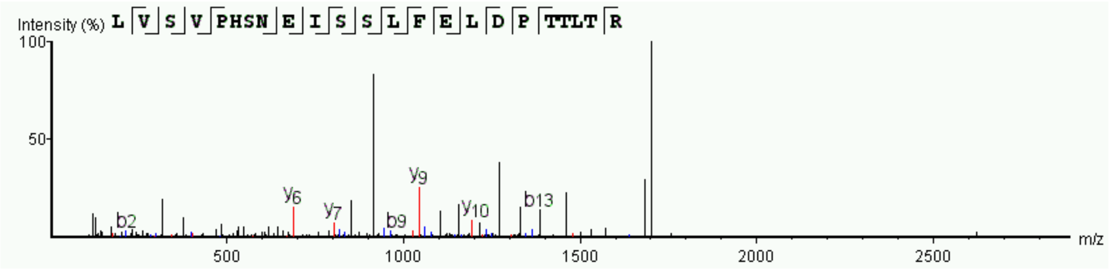

|66551651

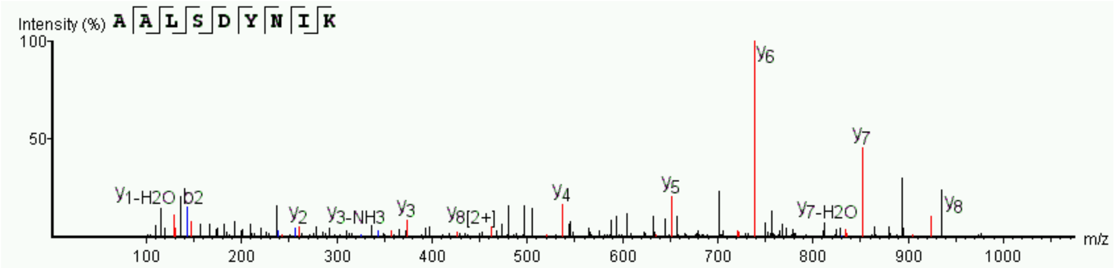

|66522232

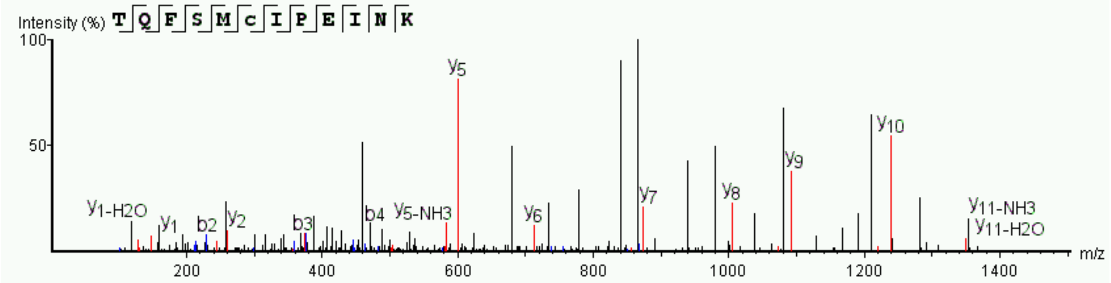

| 571566693

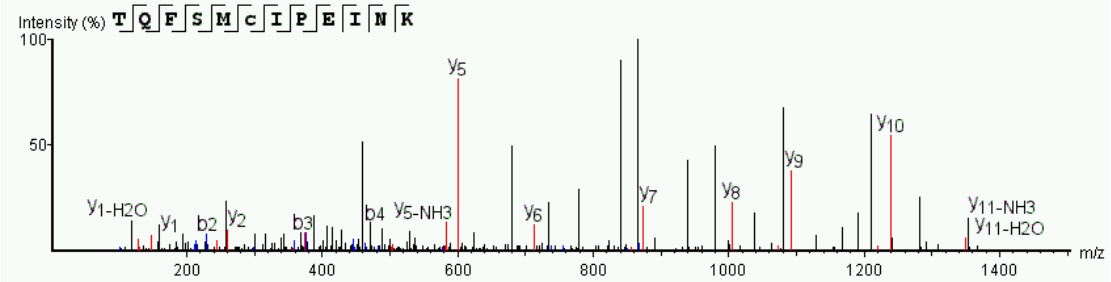

|571566697

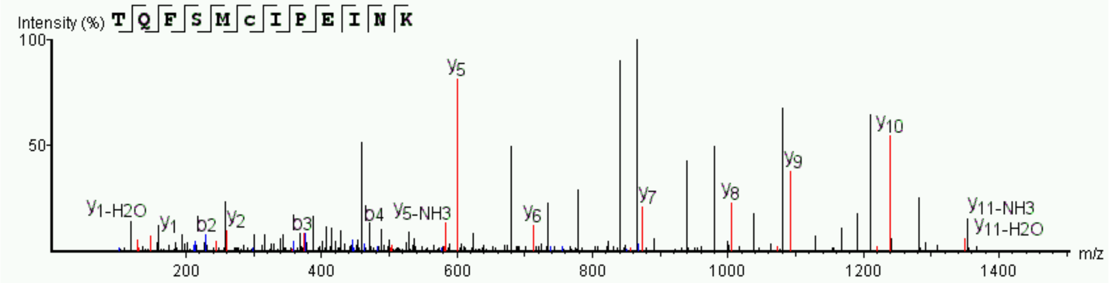

|110755692

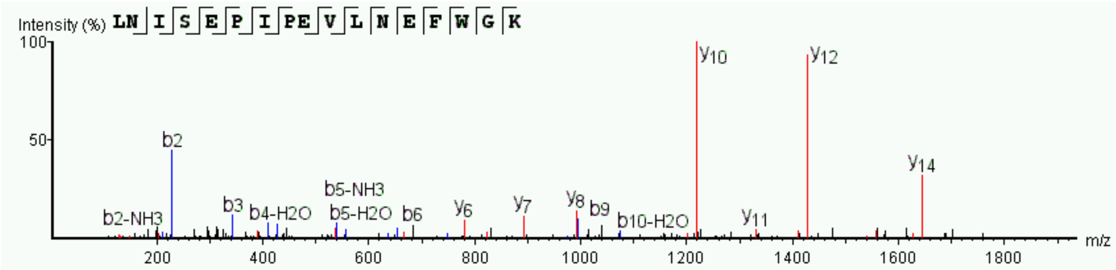

|571574430

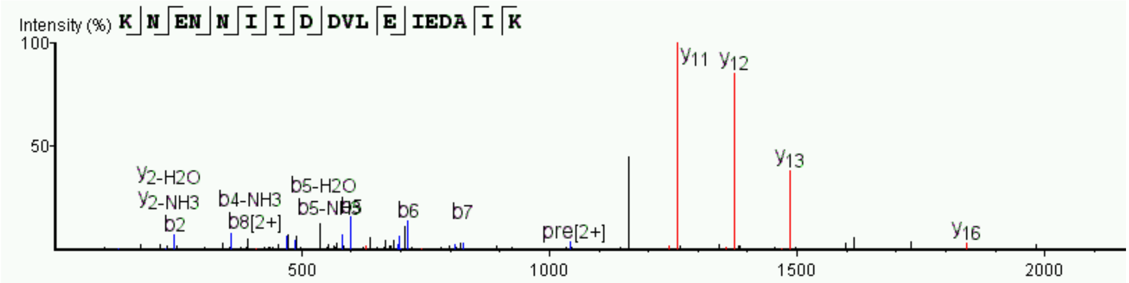

|571574432

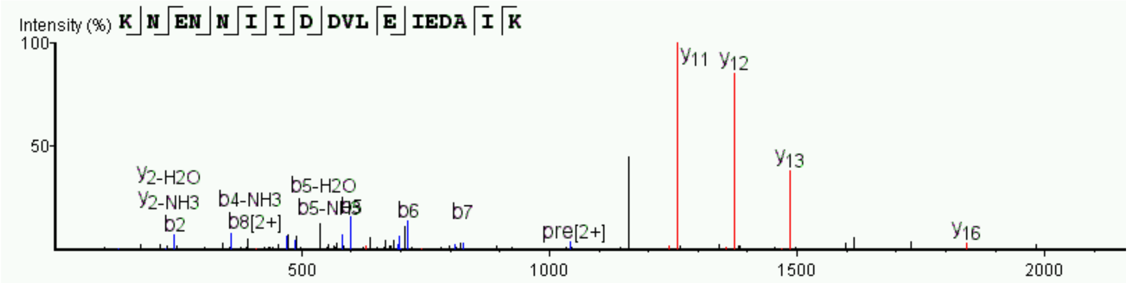

|328782625

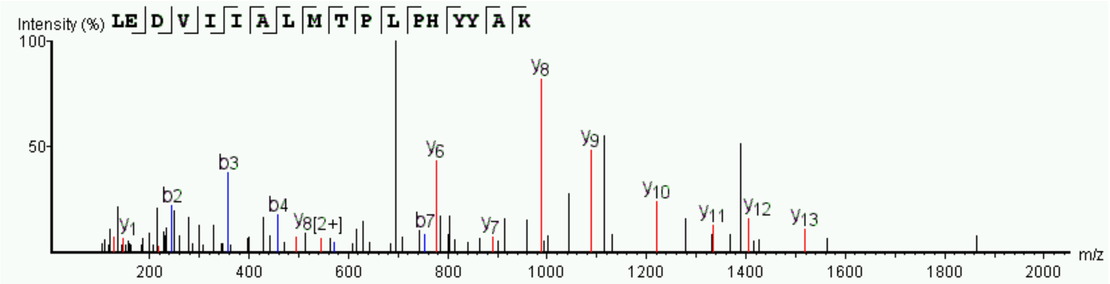

|328780564

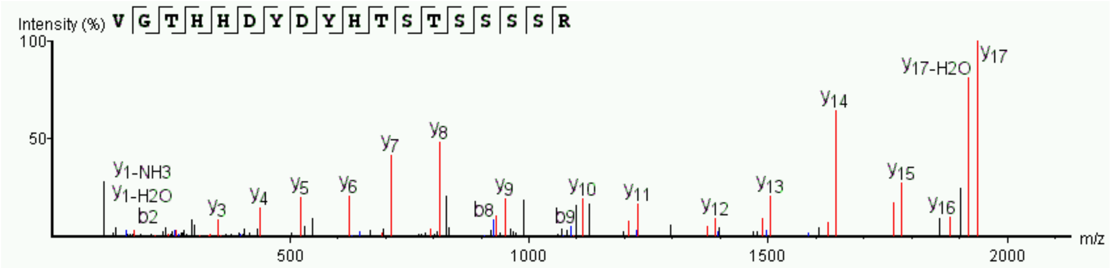

|571511719

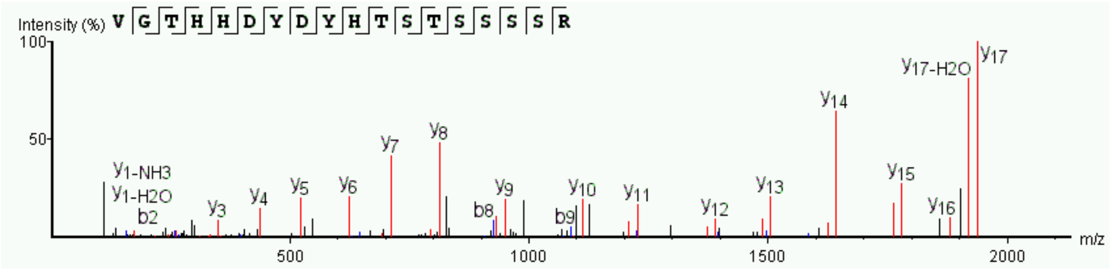

|571511728

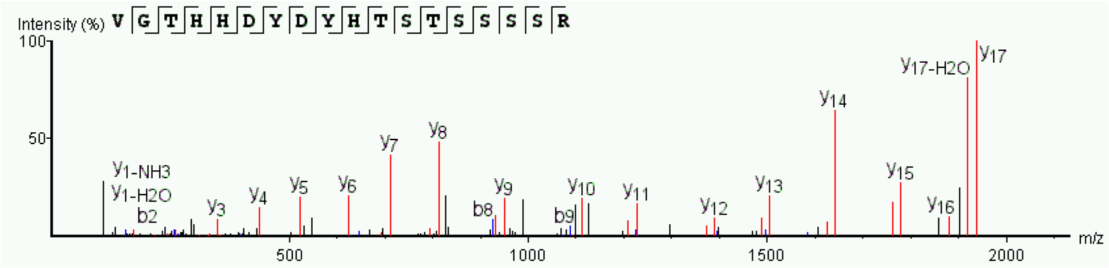

|571504286

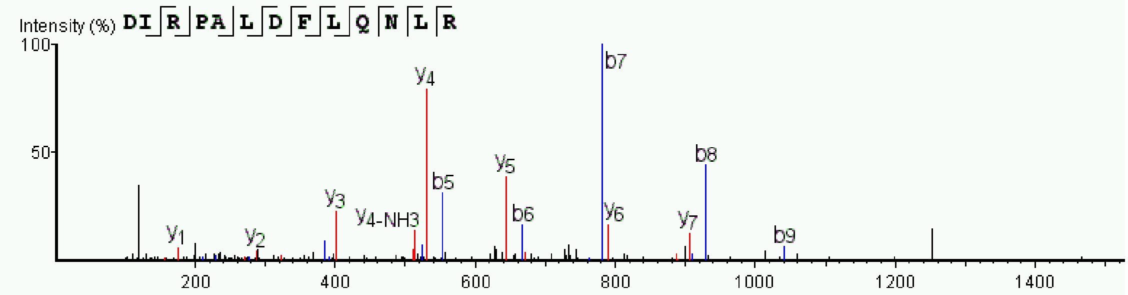

|571504293

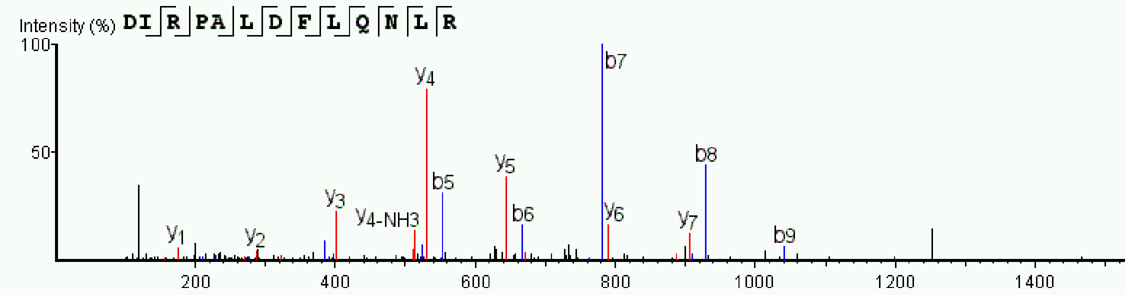

|571504295

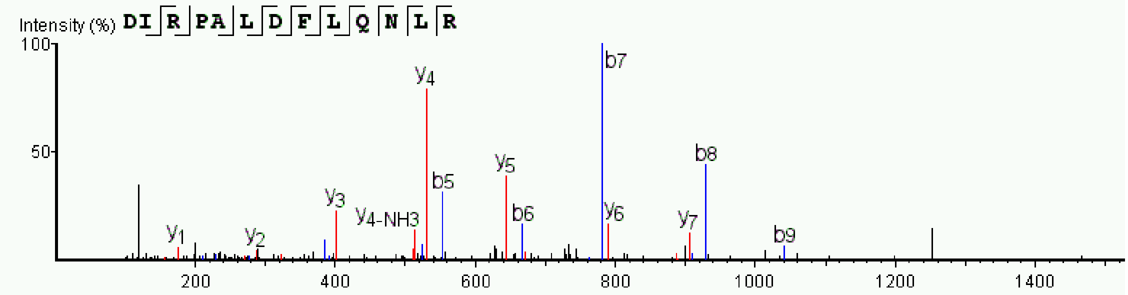

|571504299

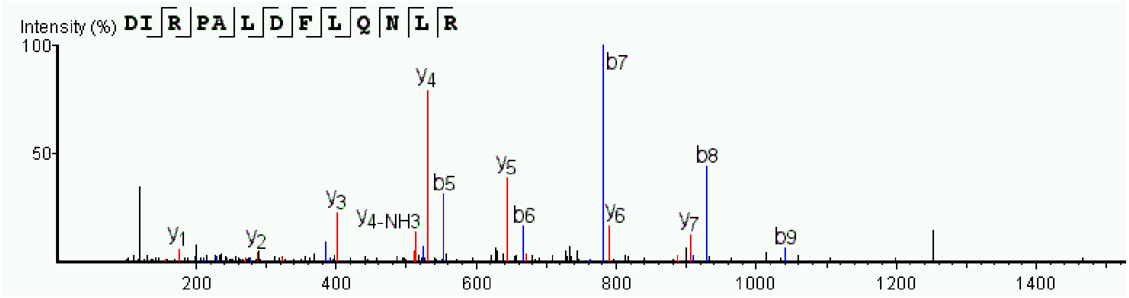

|66542091

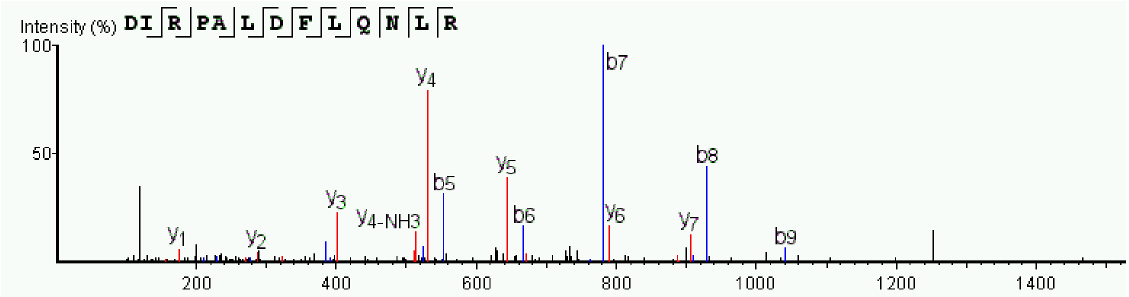

|571534940

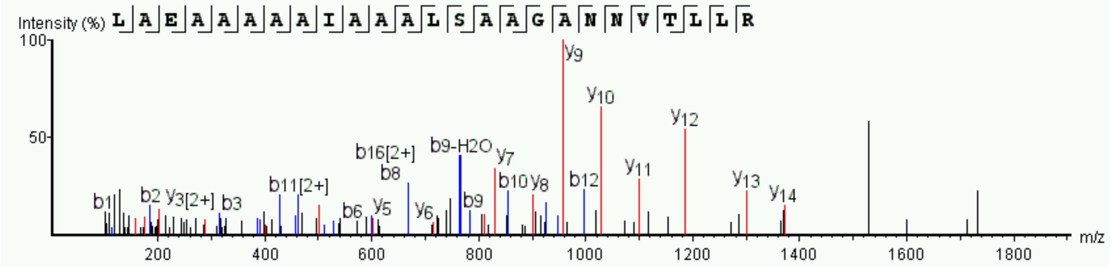

|571534943

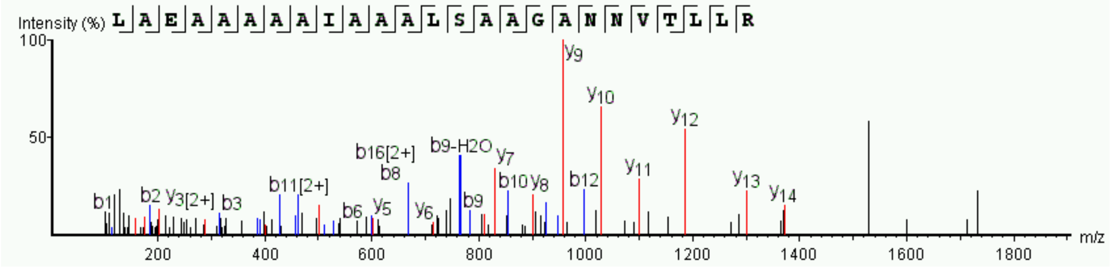

|571534947

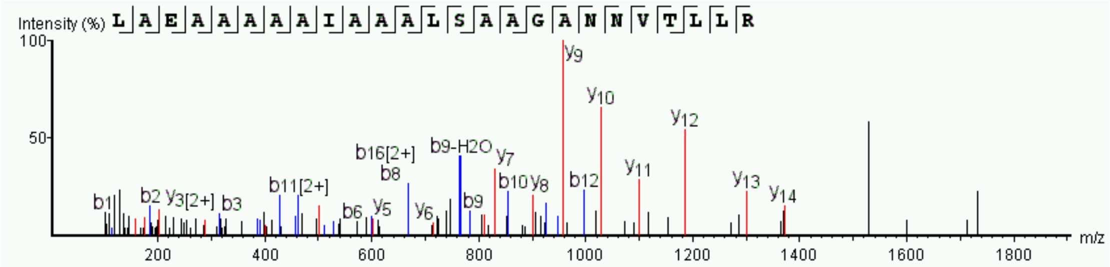

|571534951

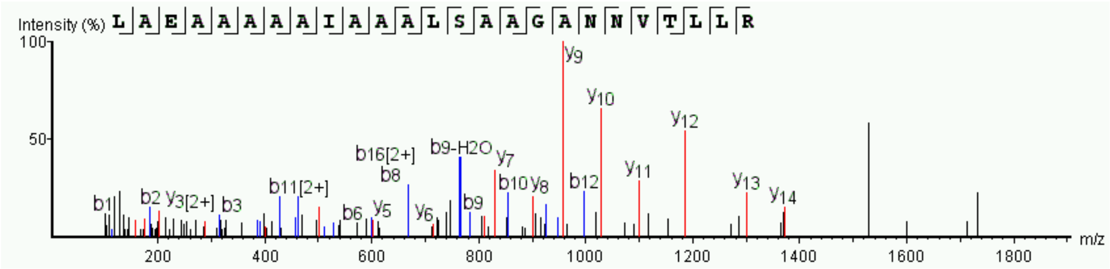

|571534954

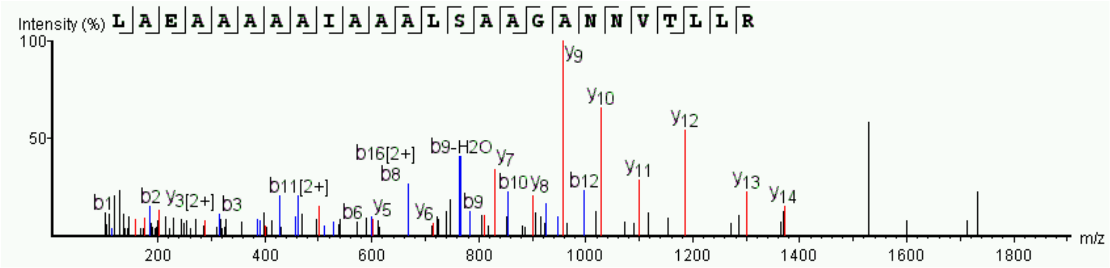

|571534961

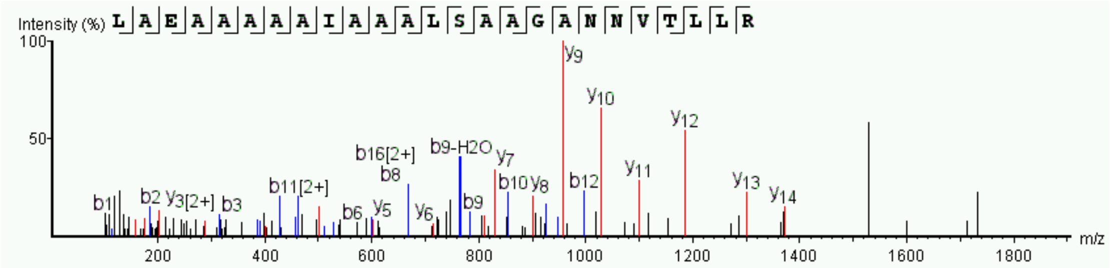

|328785653

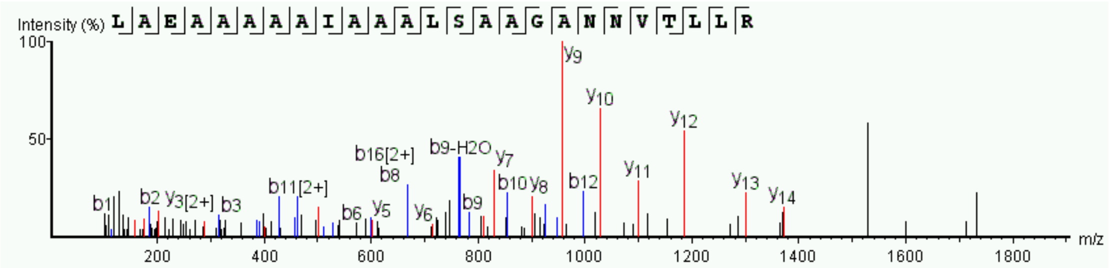

|328781115

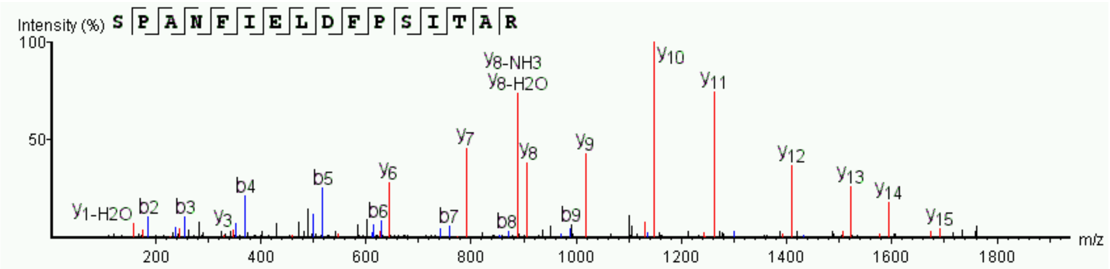

|328790105

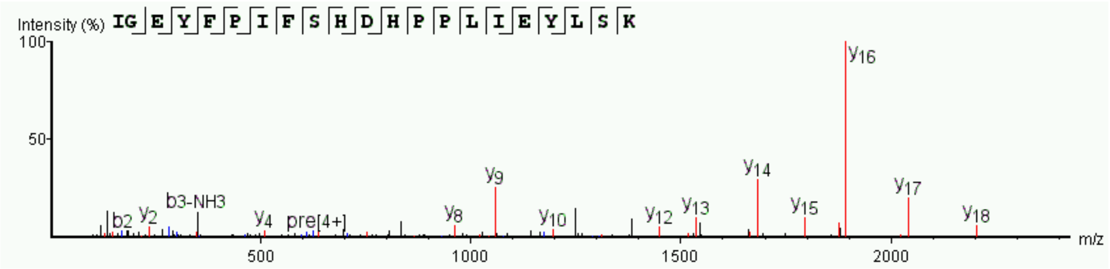

|571571261

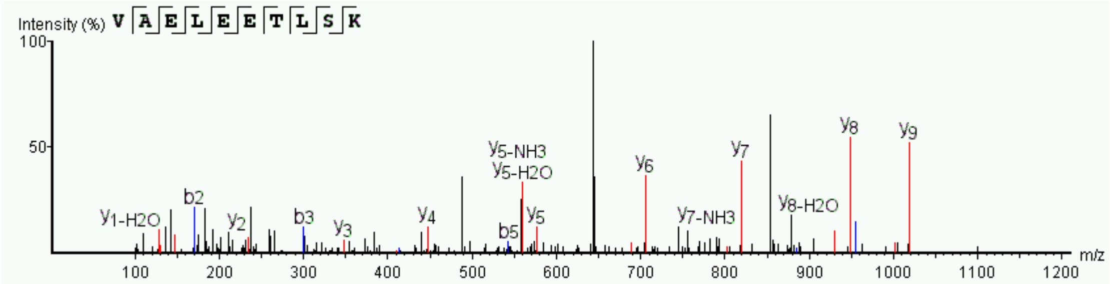

|571571265

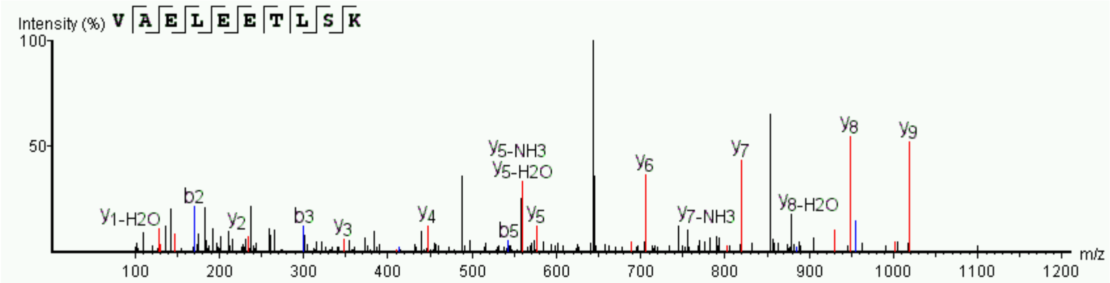

|571571269

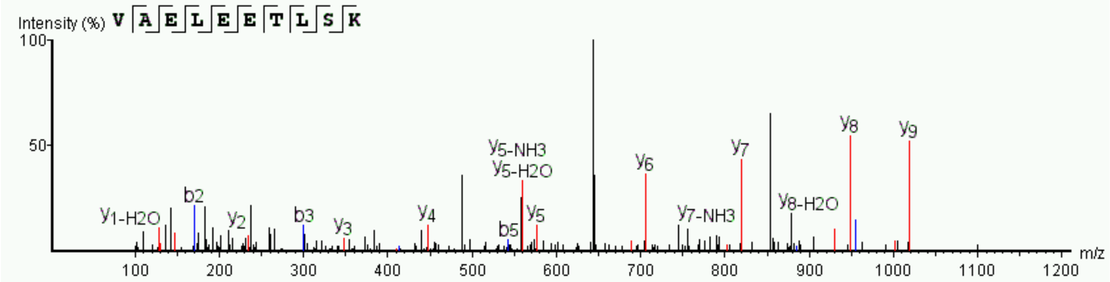

|571571279

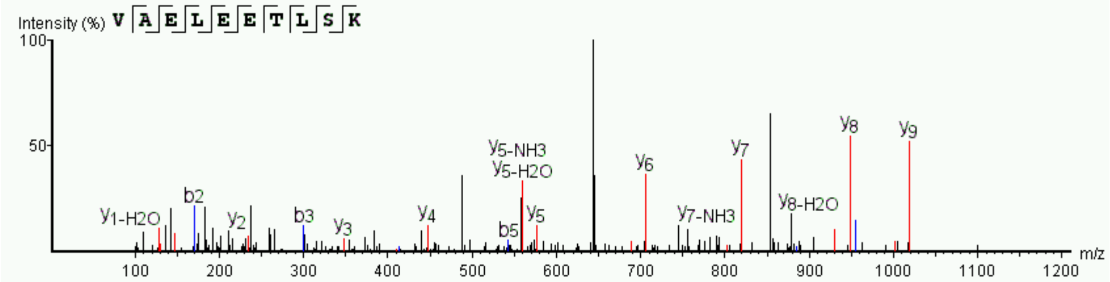

|571571227

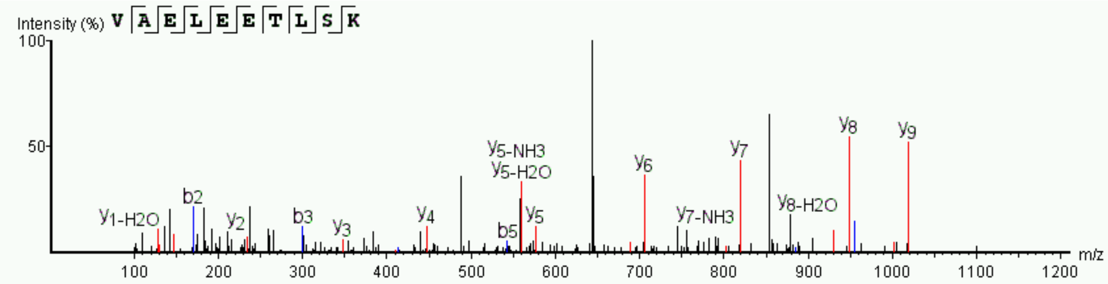

|571571231

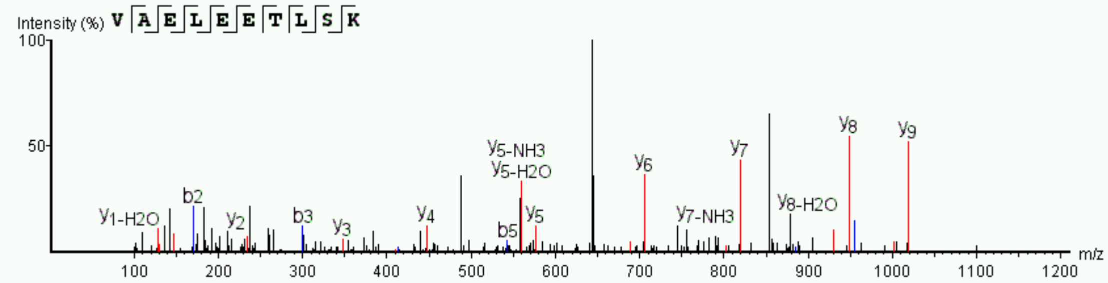

|571571235

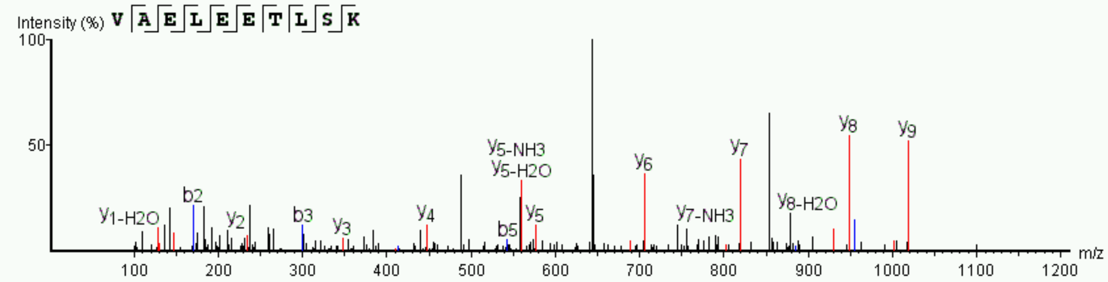

|571571239

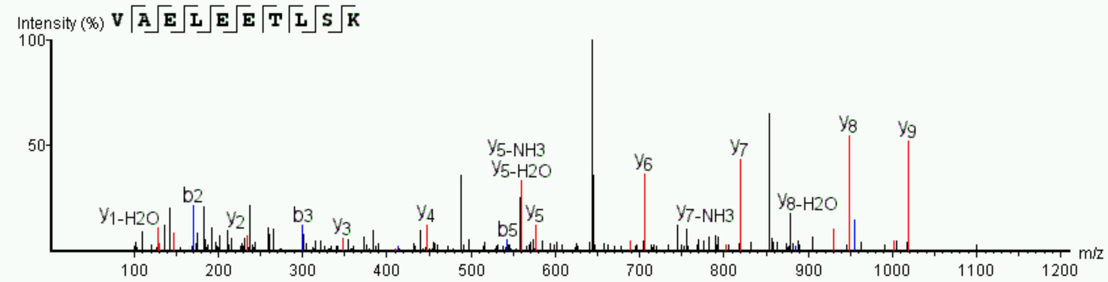

|571571243

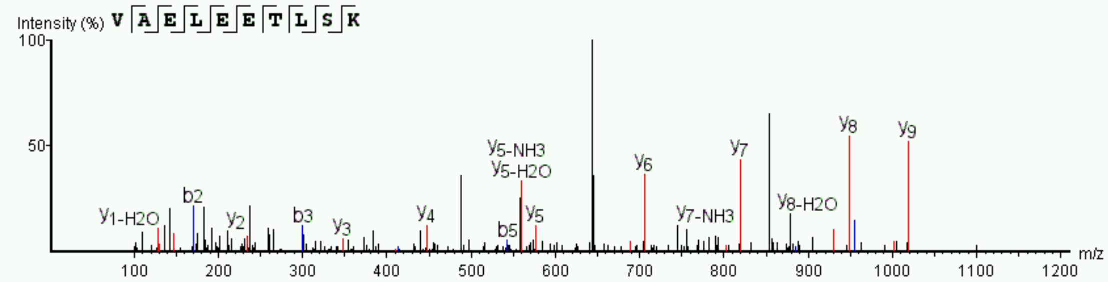

|571571249

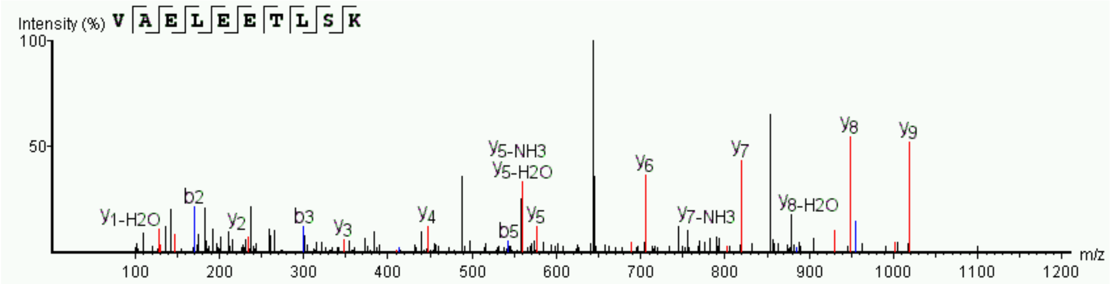

|571571253

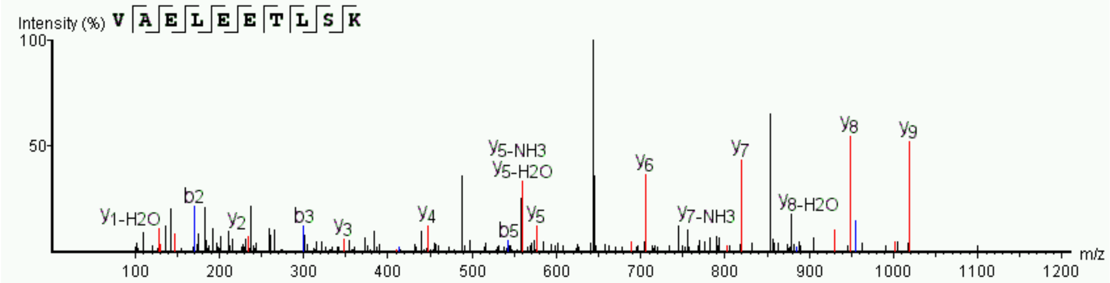

|571571257

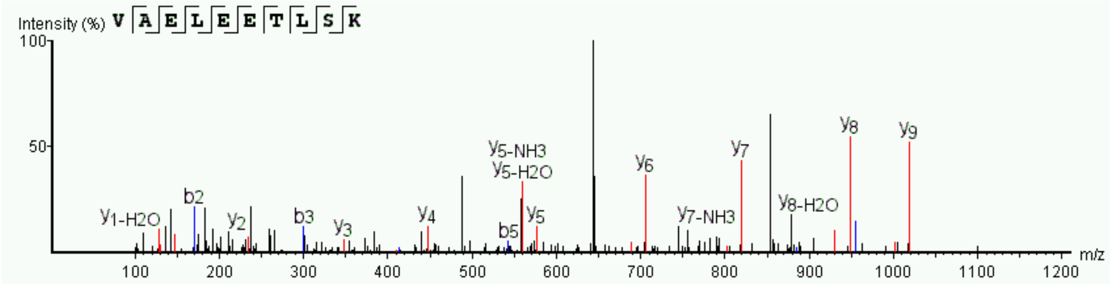

|571571223

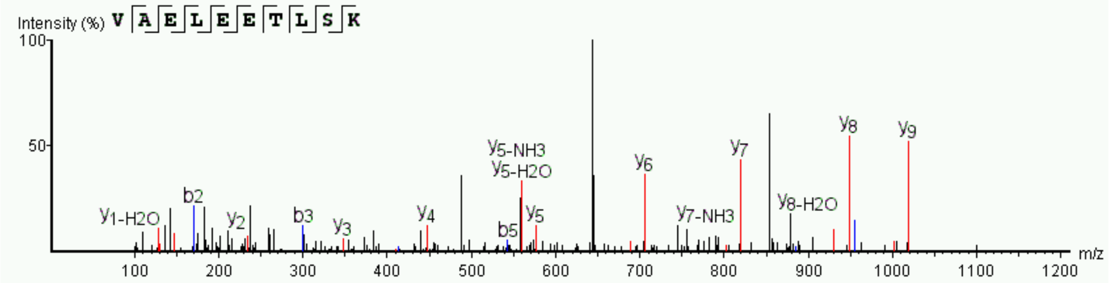

|571575591

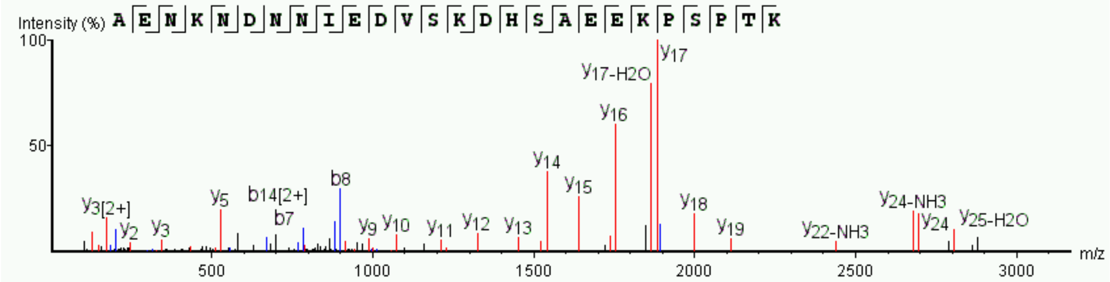

|571575589

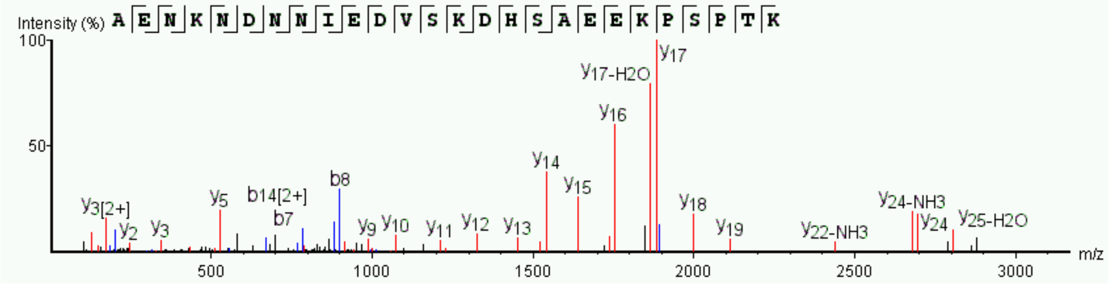

|571501325

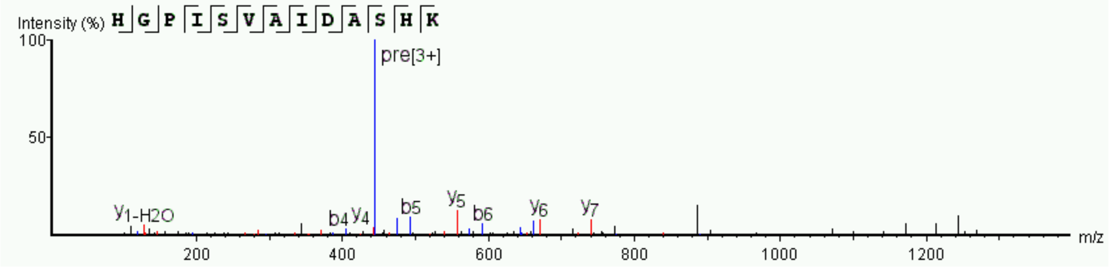

|571521603

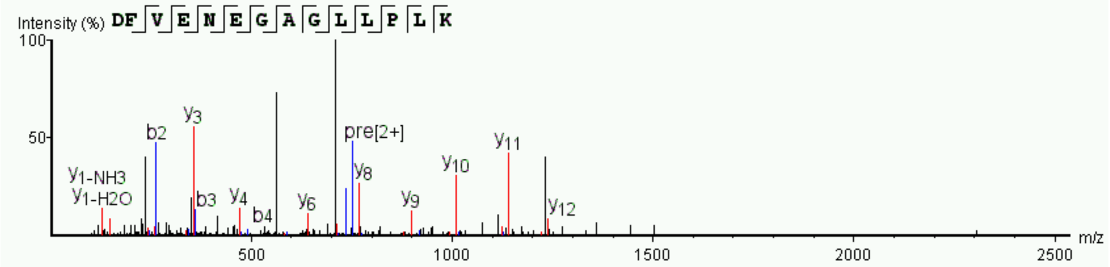

|571533533

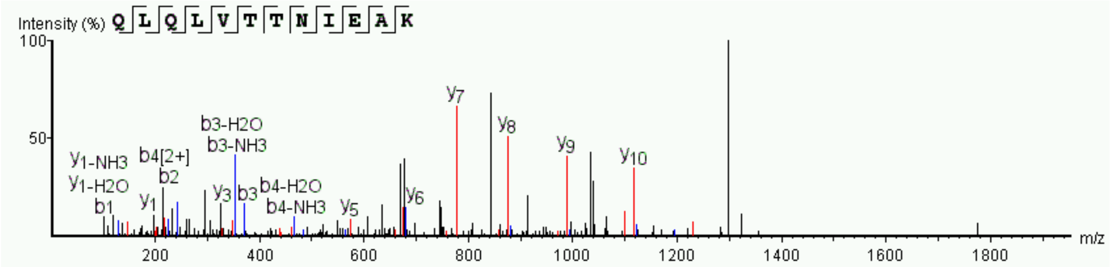

|571533533

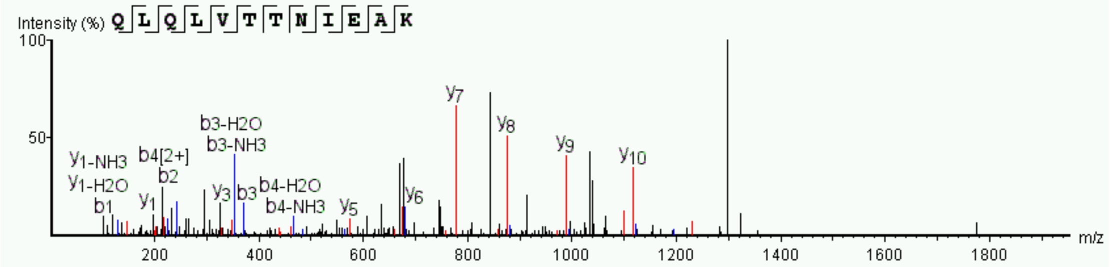

|571522190

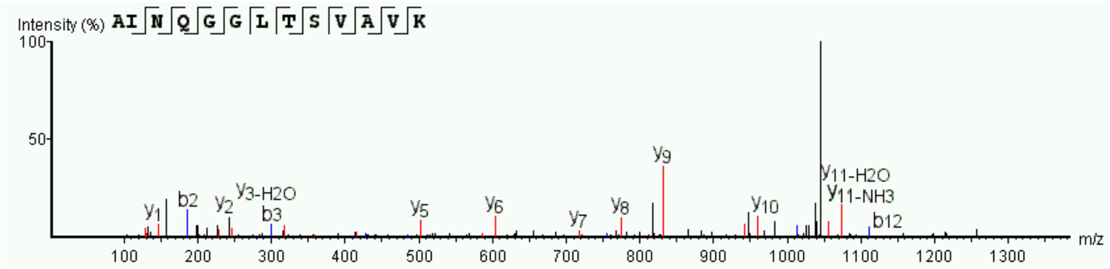

|571573162

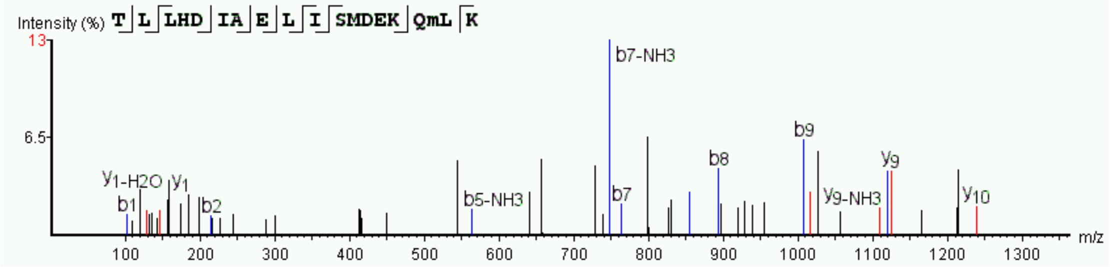

|571573648

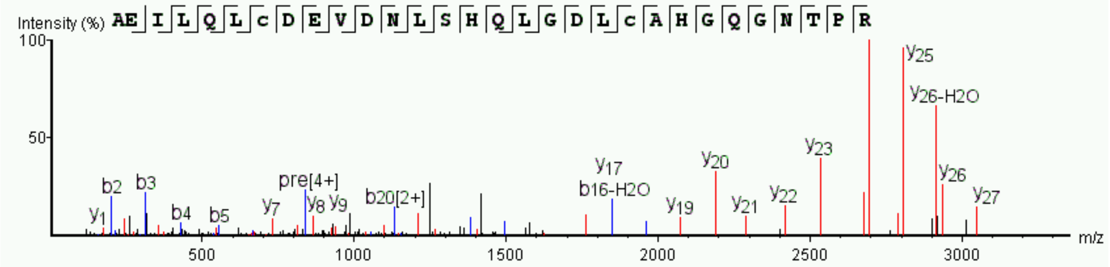

|571573606

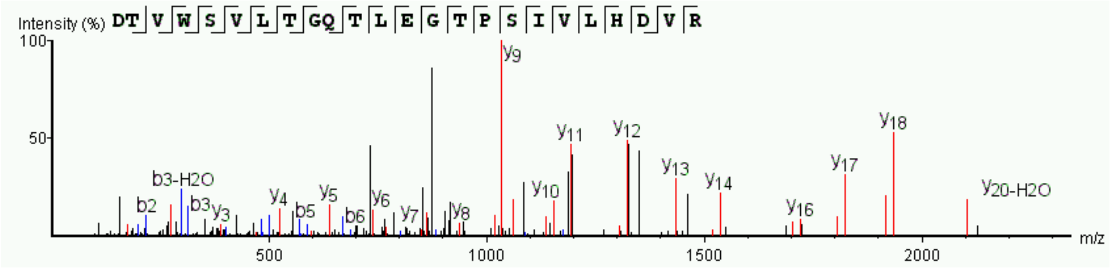

|571573608

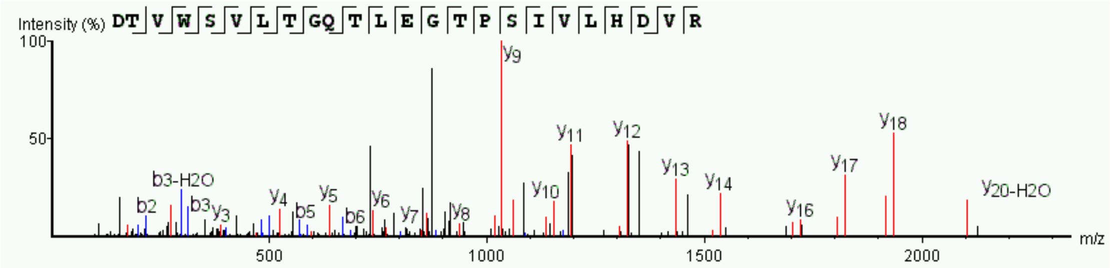

|571573610

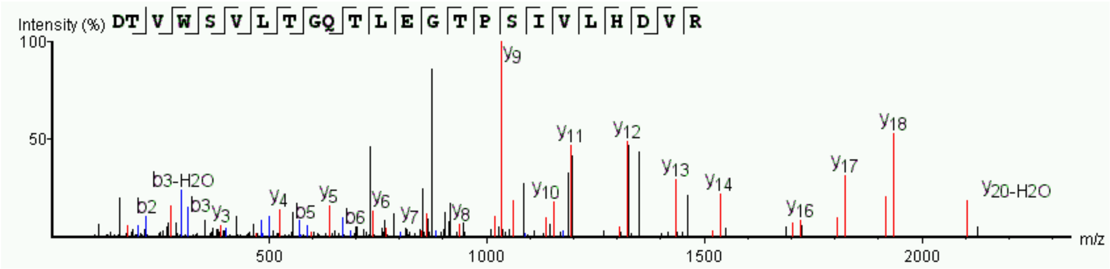

|571573612

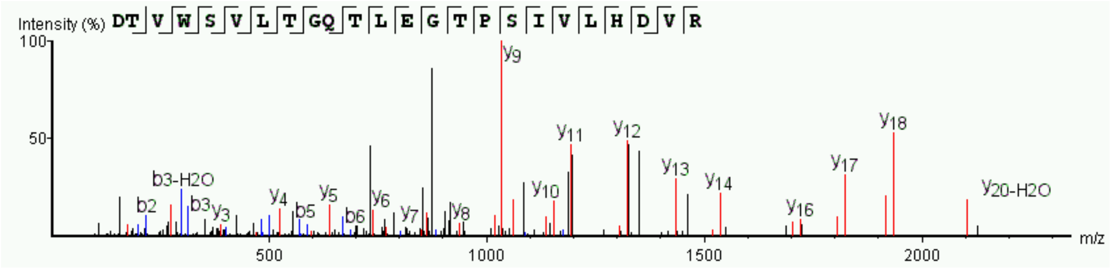

|571573614

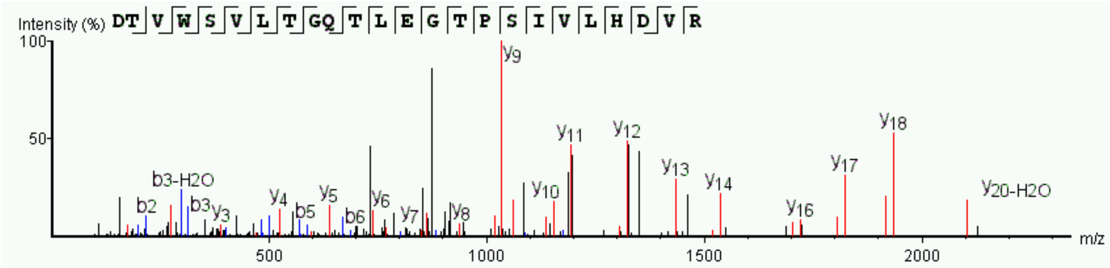

|571573616

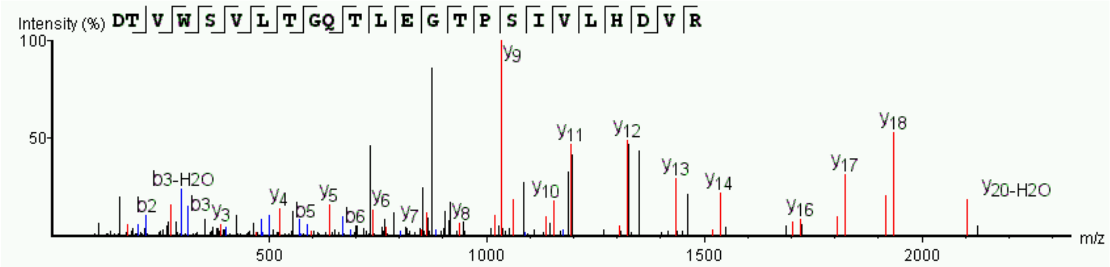

|571573618

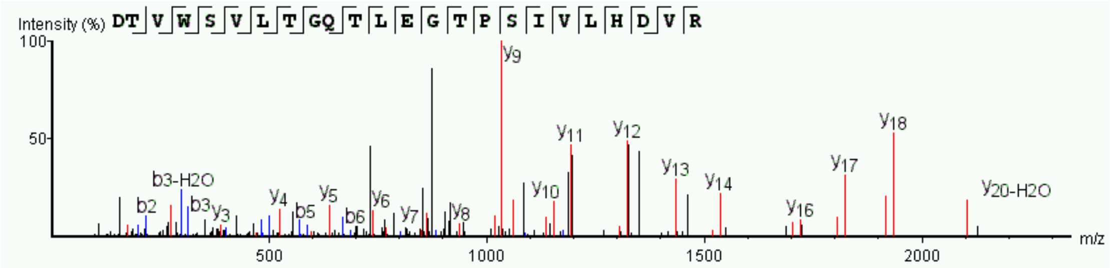

|571573620

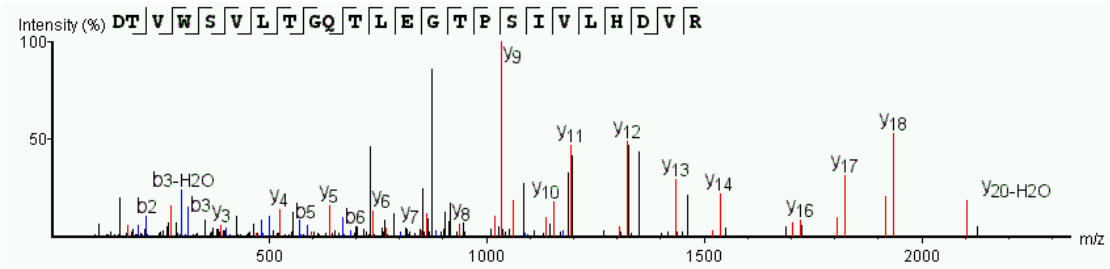

|571573604

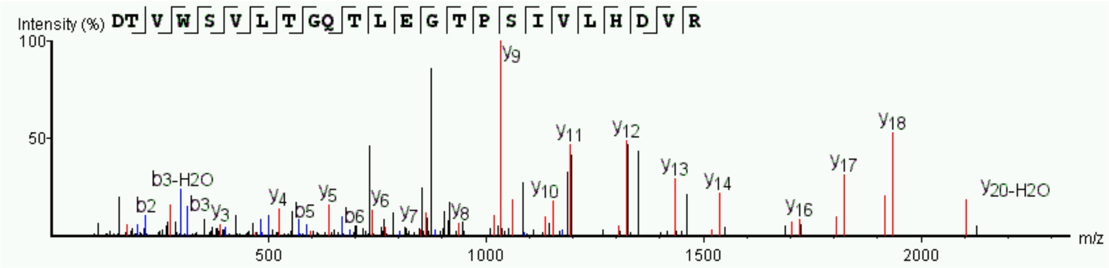

|571508626

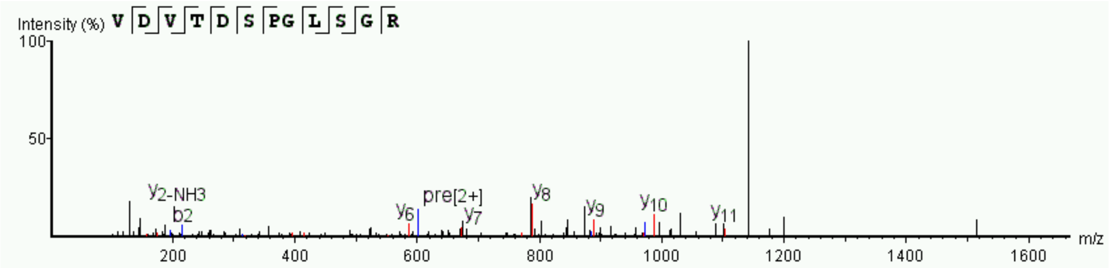

|571575043

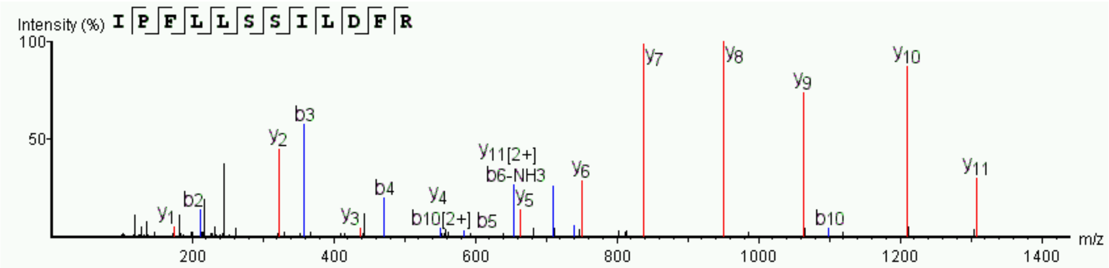

|571555981

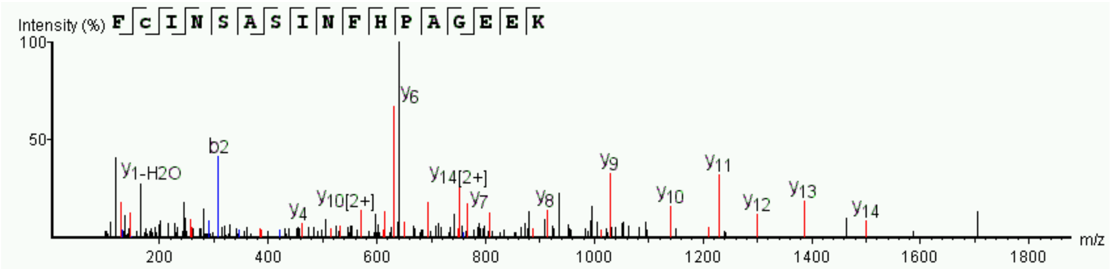

|571555985

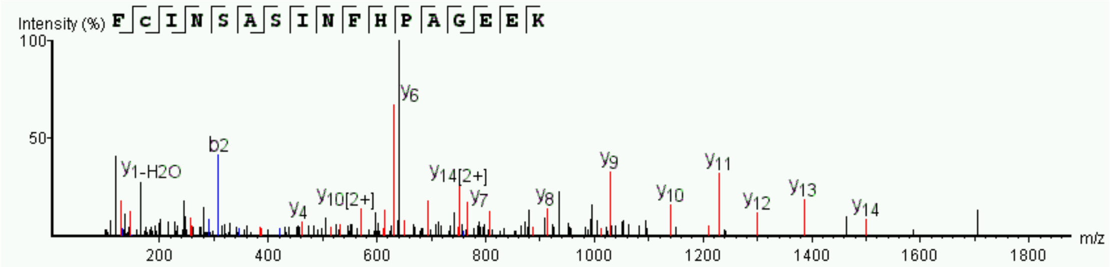

|328789842

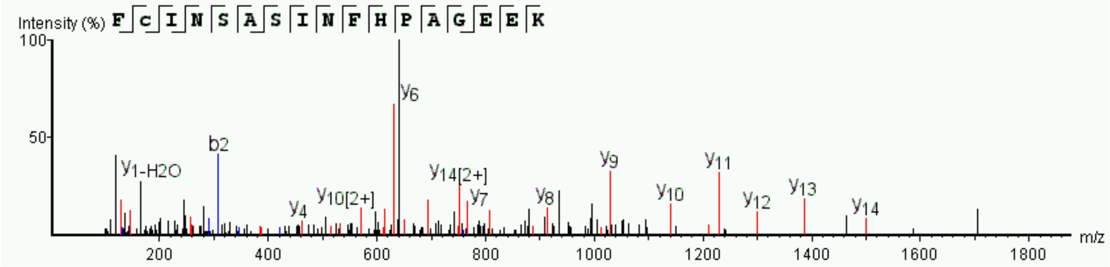

|328789844

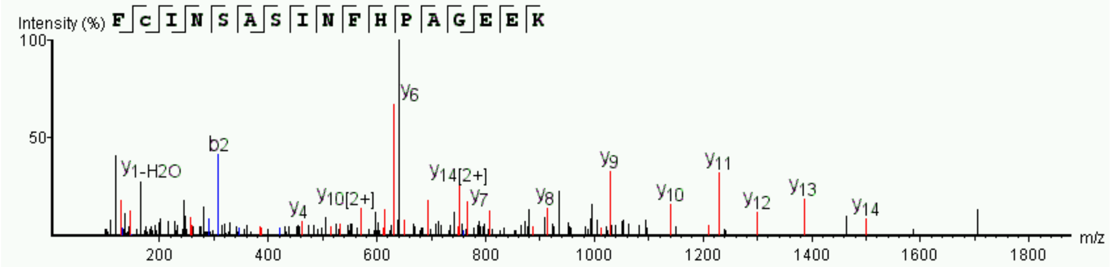

|571555973

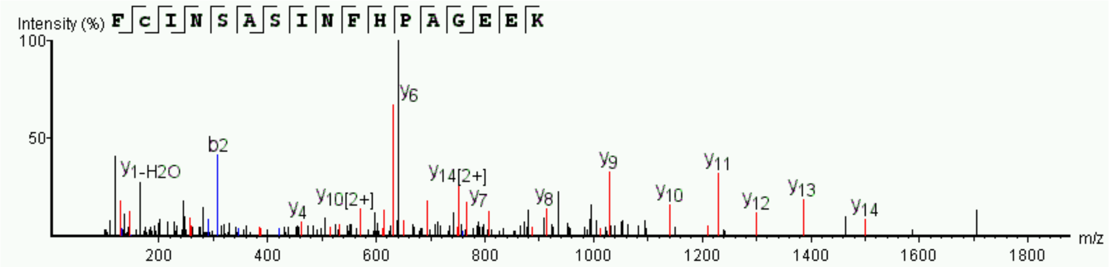

|328776866

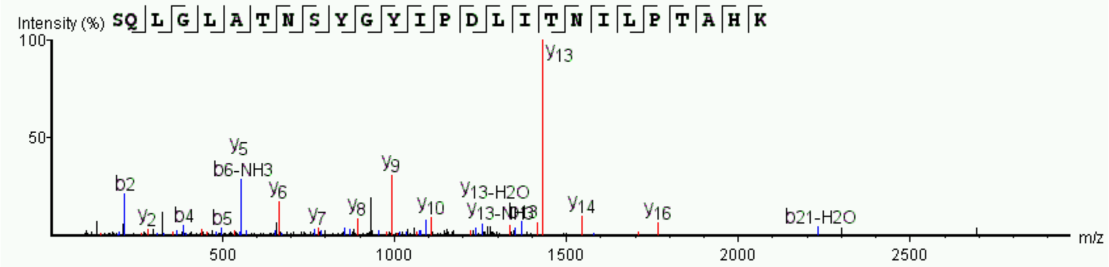

|66505264

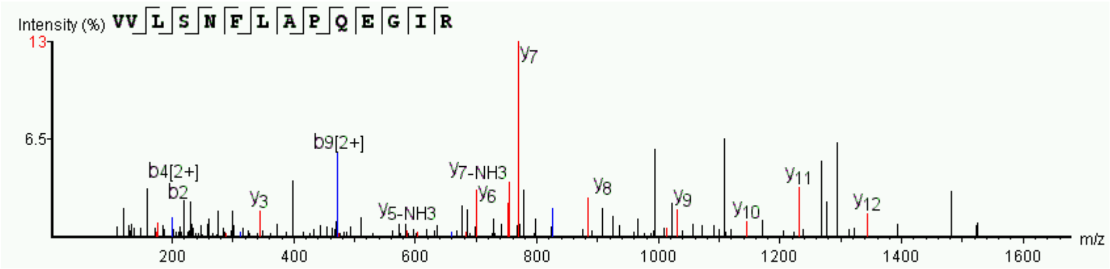

|571511327

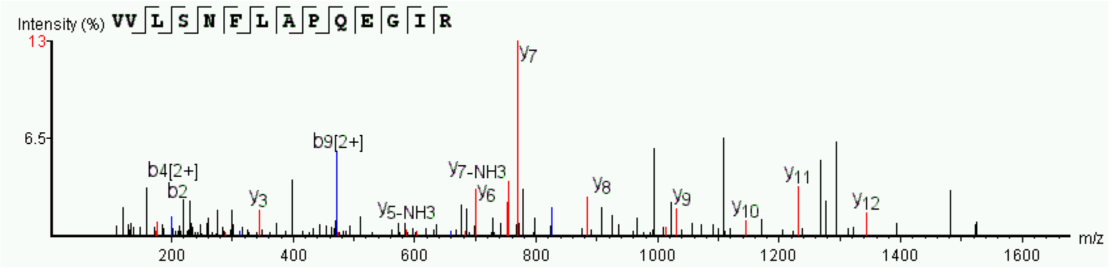

|571510158

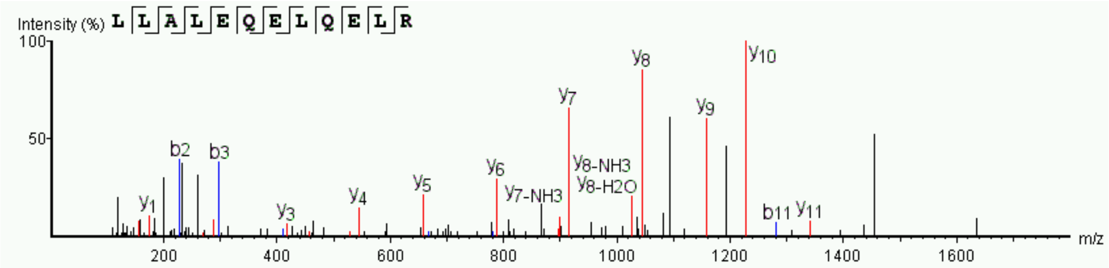

|571508425

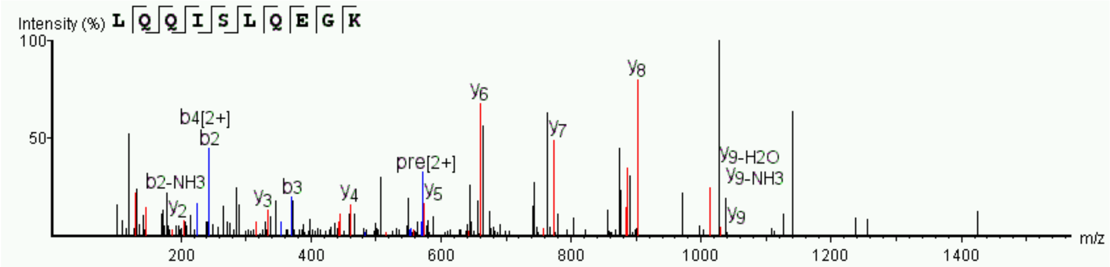

|571508429

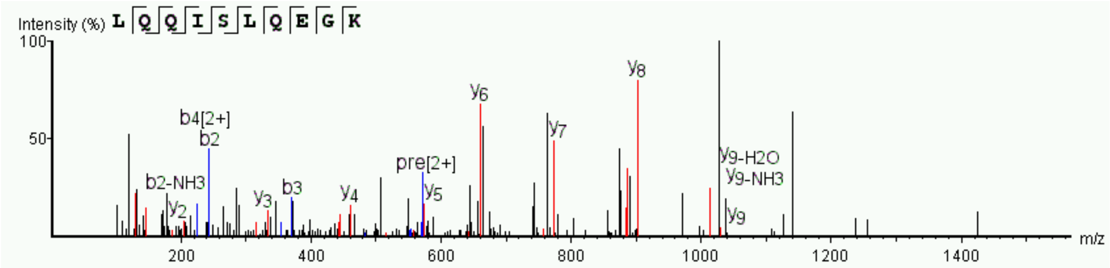

|571508435

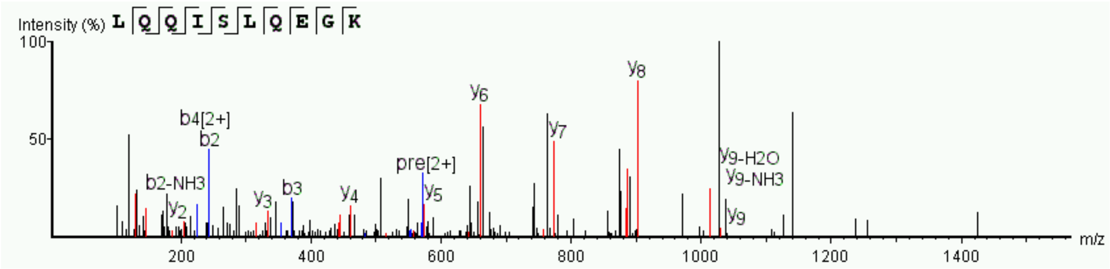

|328792314

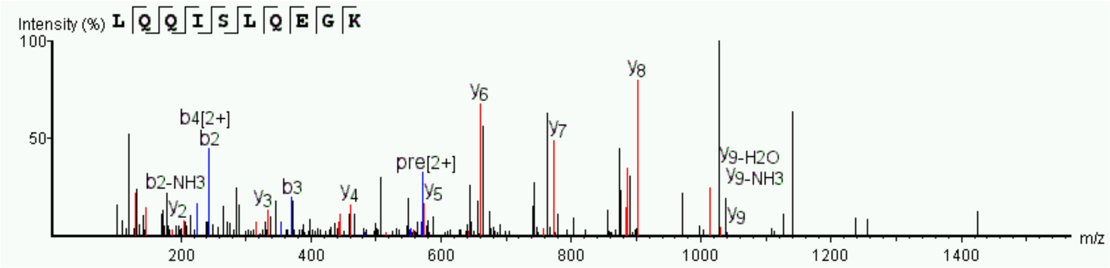

|328792316

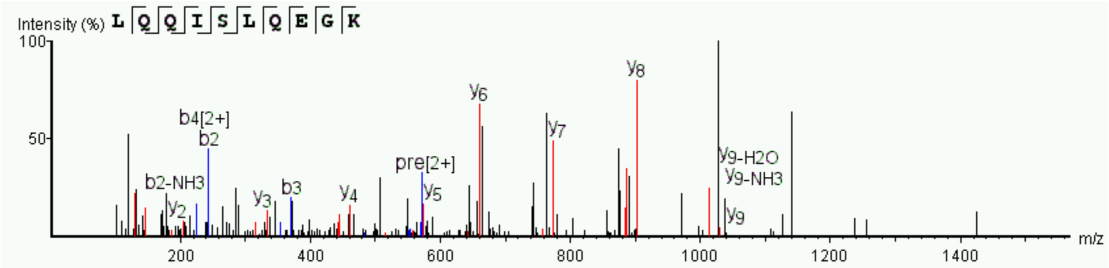

|571527239

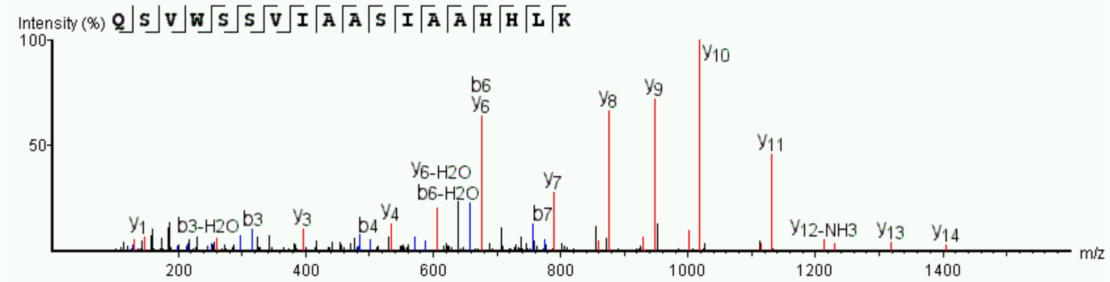

|328781720

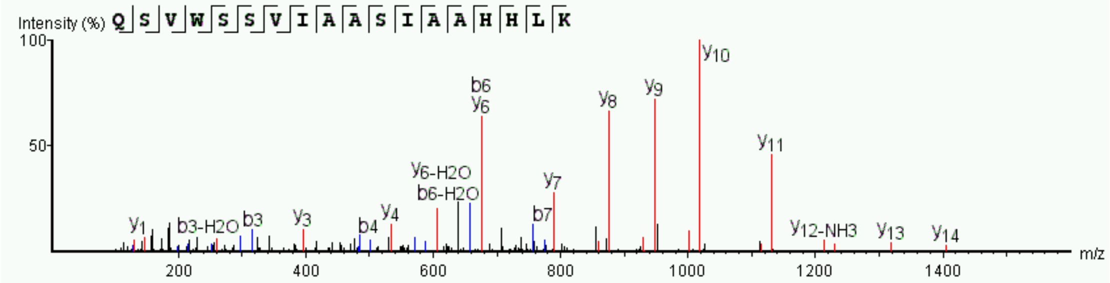

|571527236

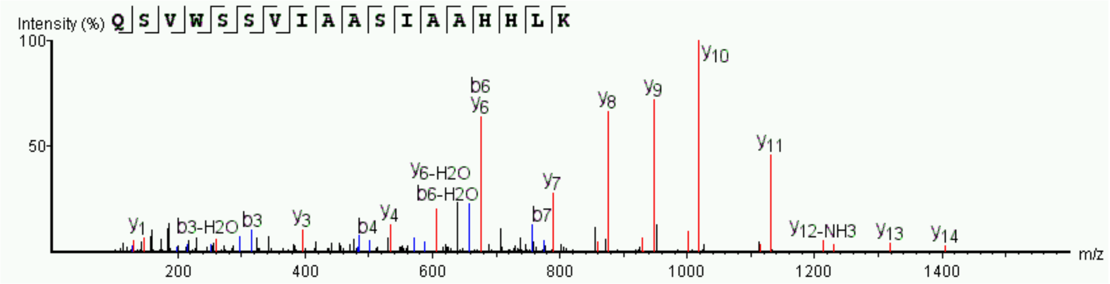

|66519233

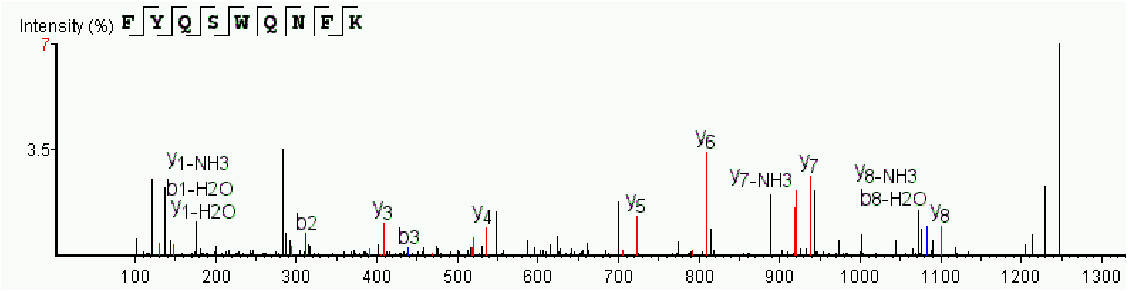

|110760068

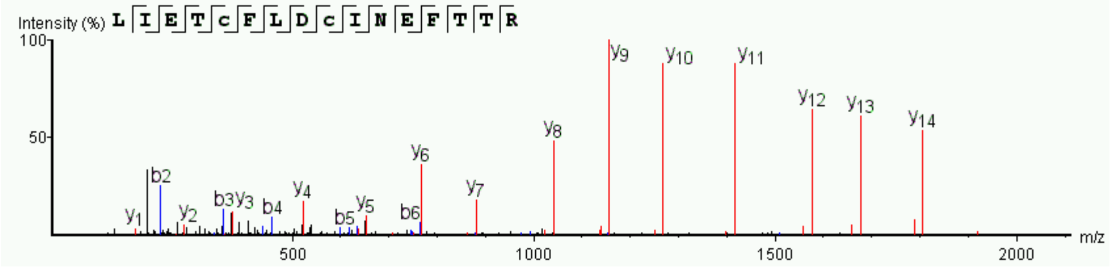

|571553163

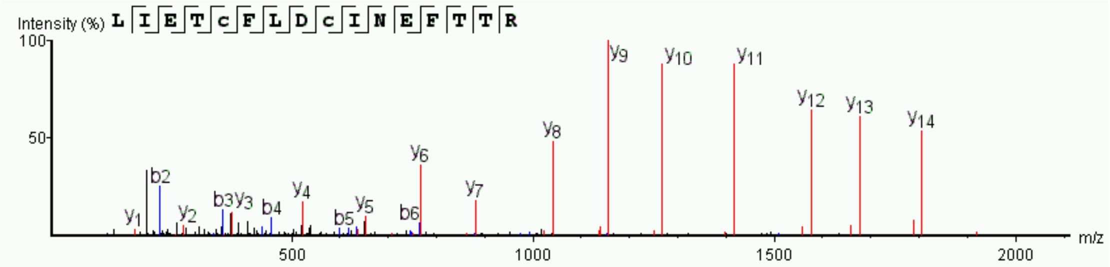

|571513237

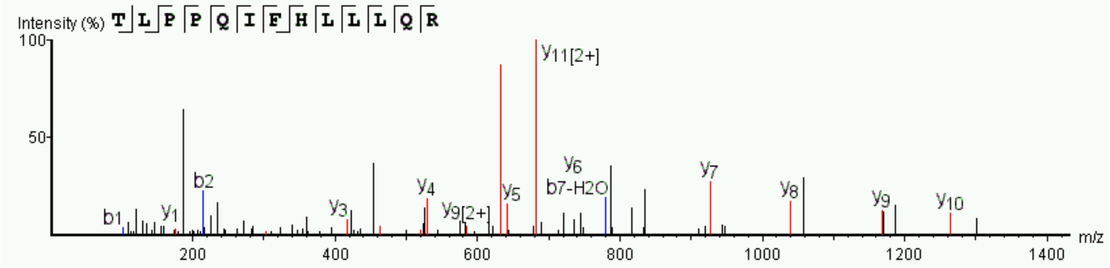

|48105316

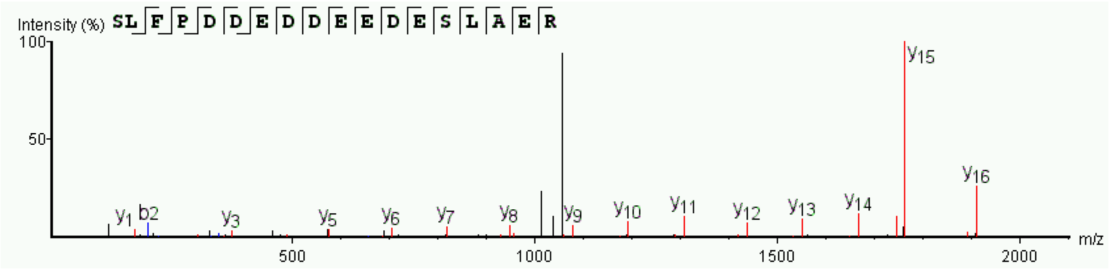

|66517185

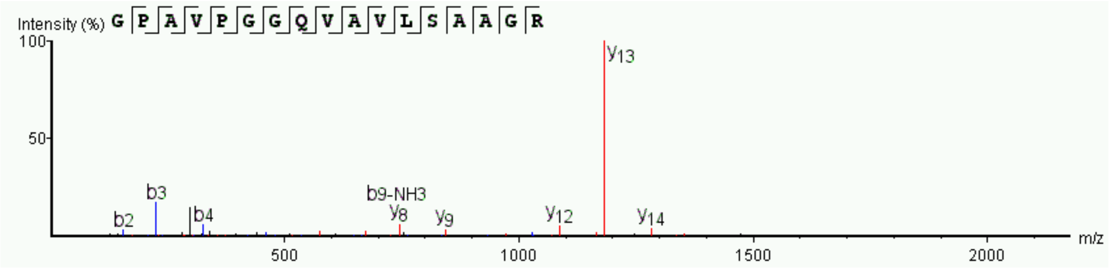

|328783268

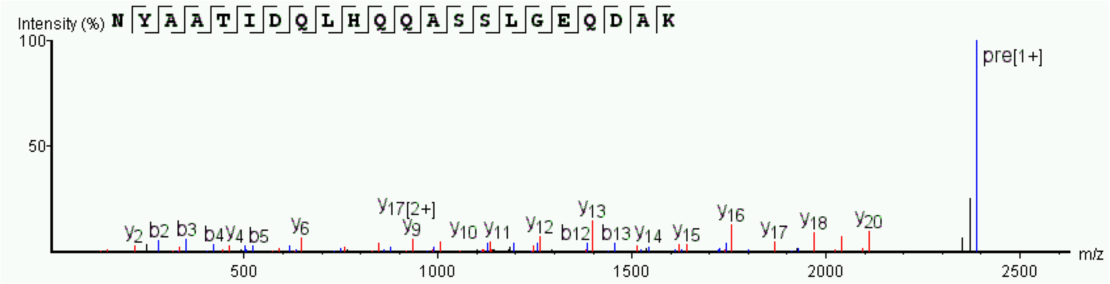

gi|328794153

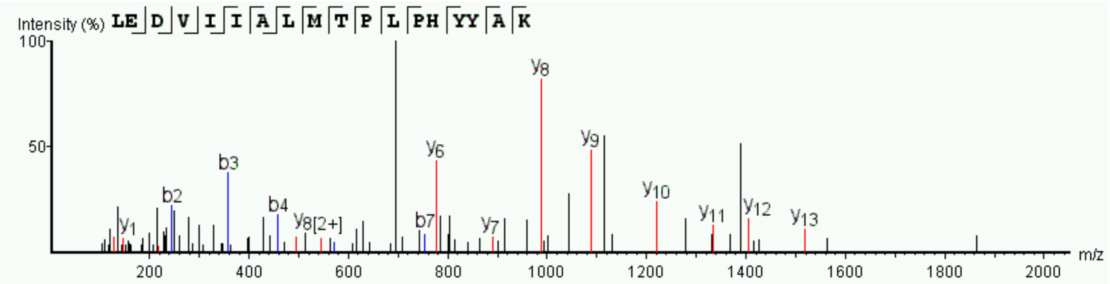

gi|571522038

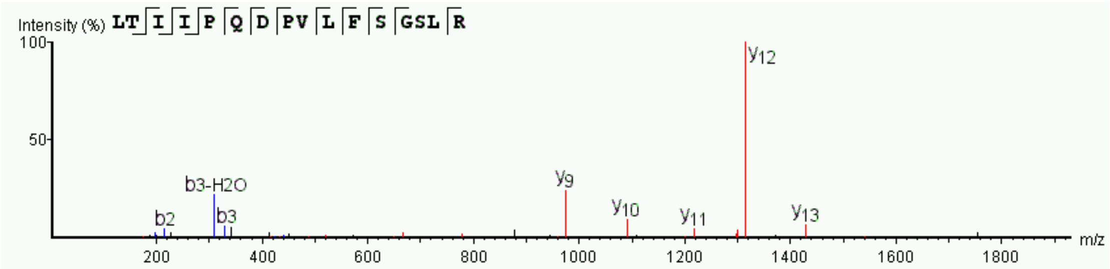

gi|571522042

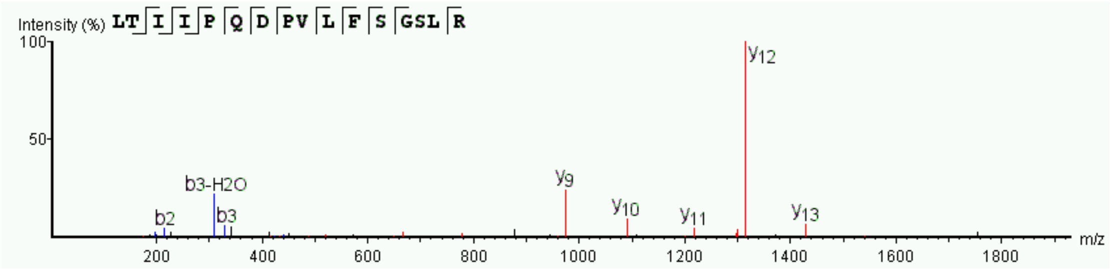

gi|571522046

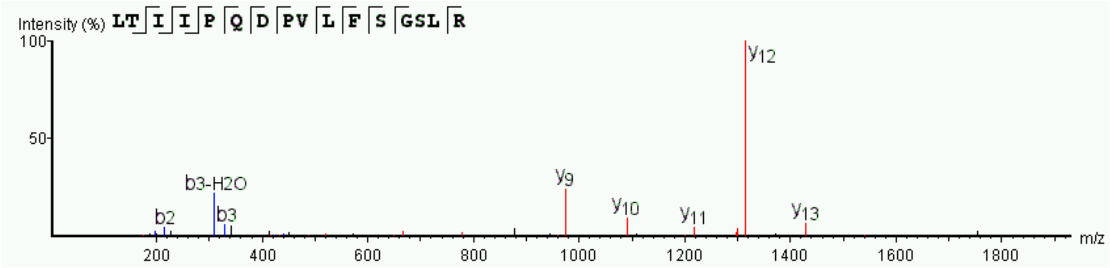

gi|571522050

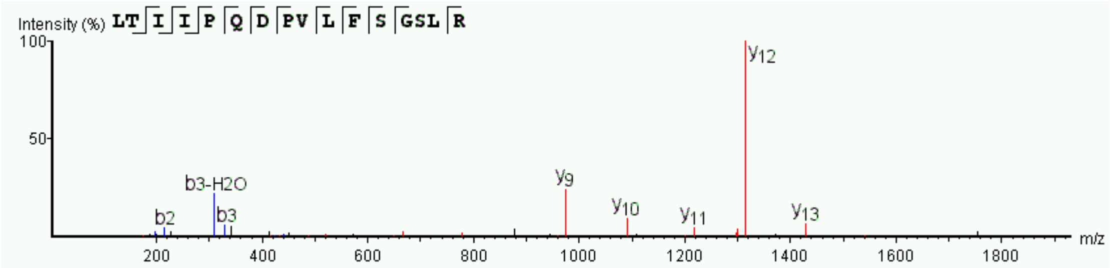

gi|571522053

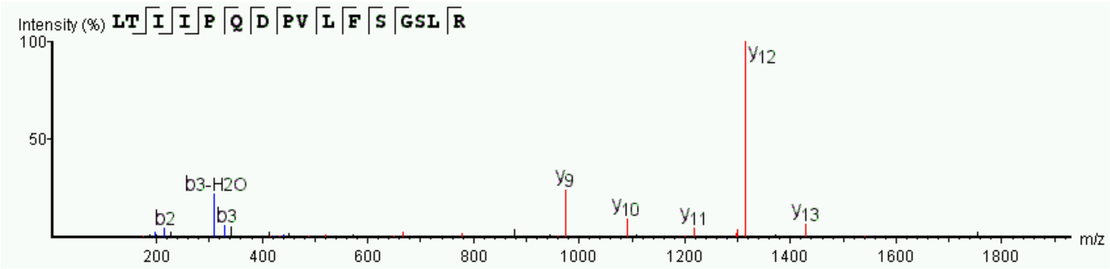

gi|571522057

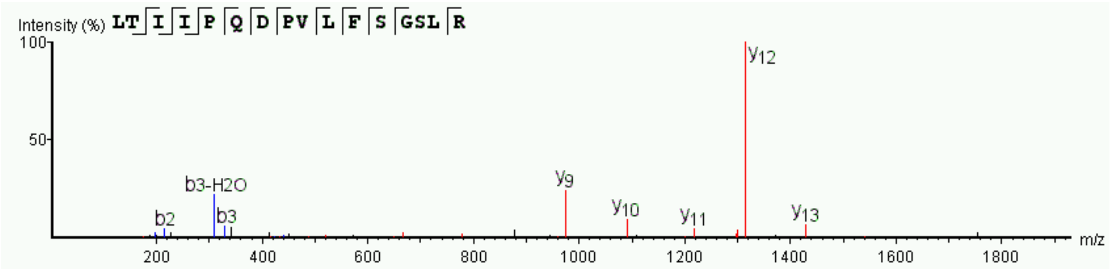

gi|571522034

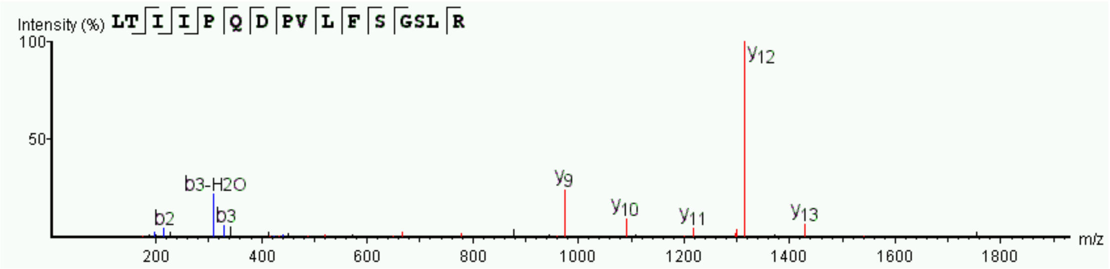

gi|571576771

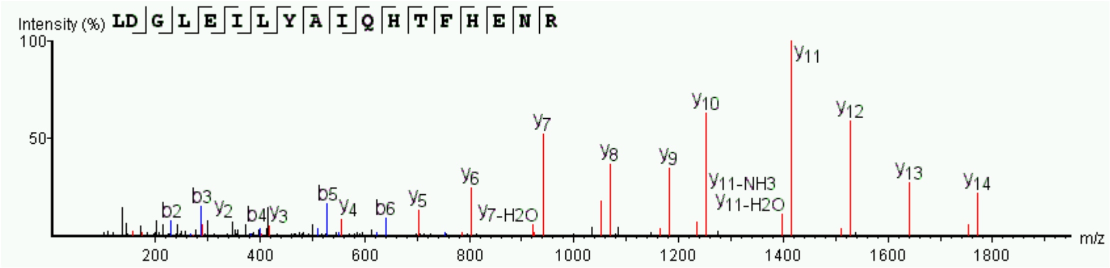

gi|328792927

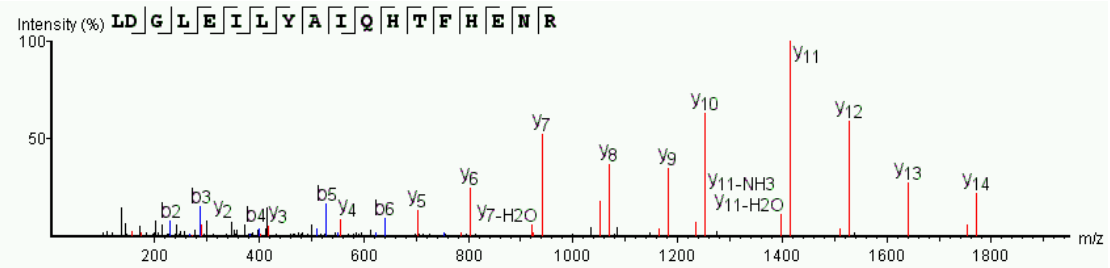

gi|571561363

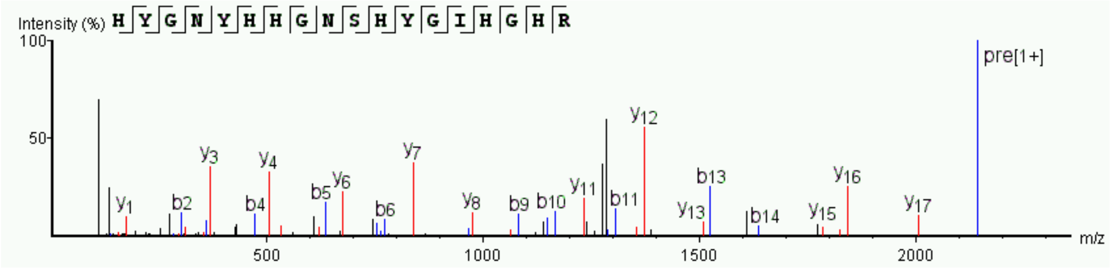

gi|571561367

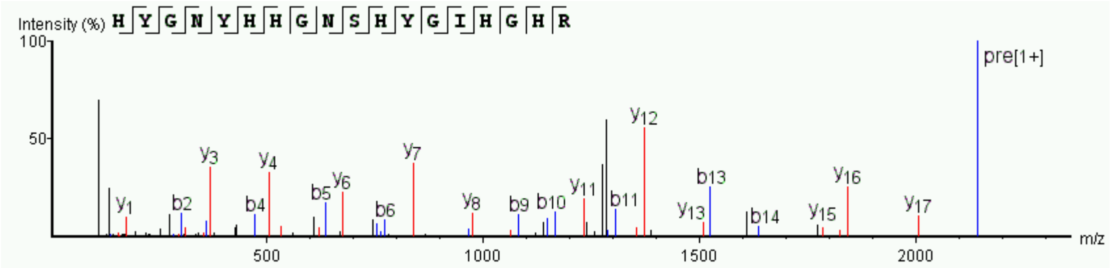

gi|571561371

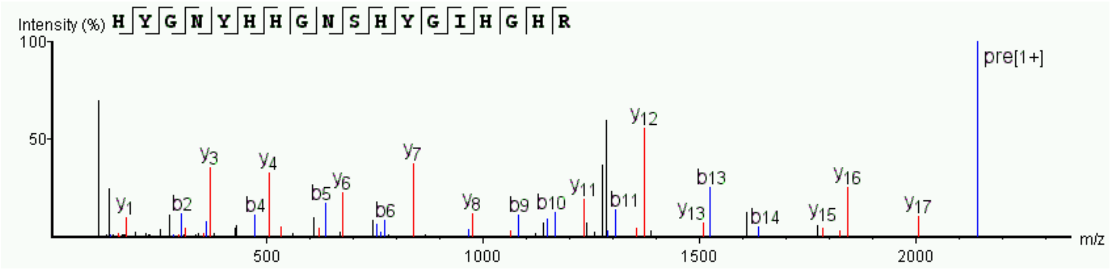

gi|571561359

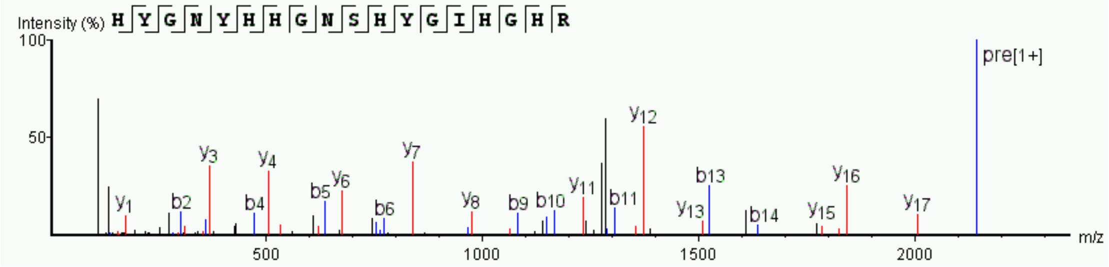

gi|48094341

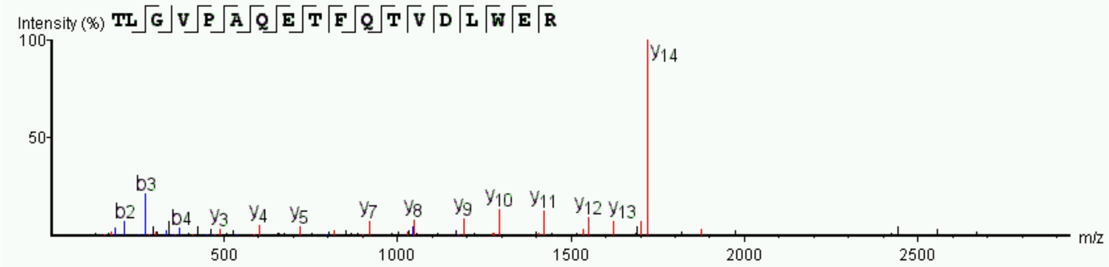

gi|571563508

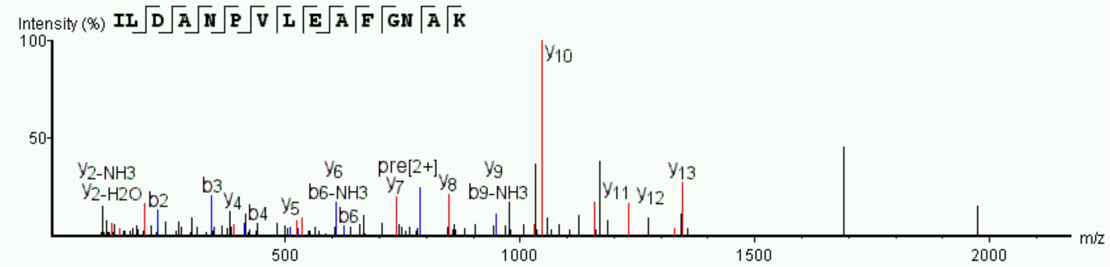

gi|328777790

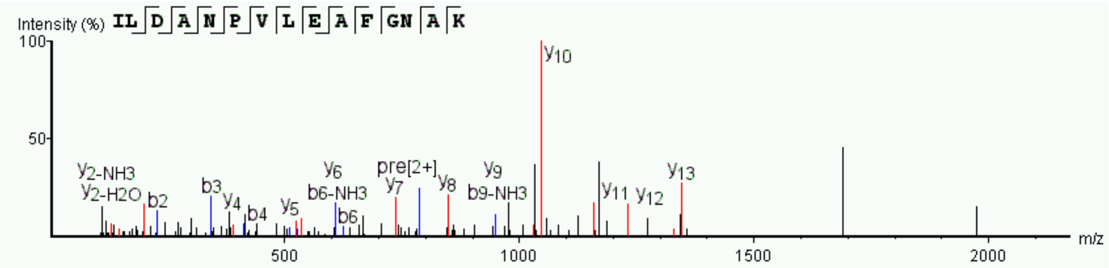

gi|571525189

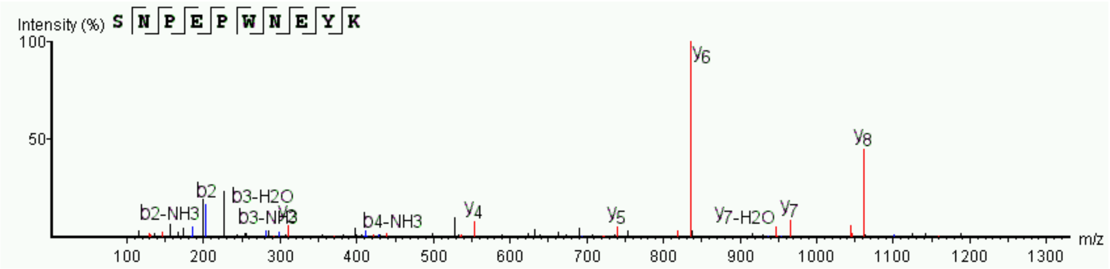

gi|571507494

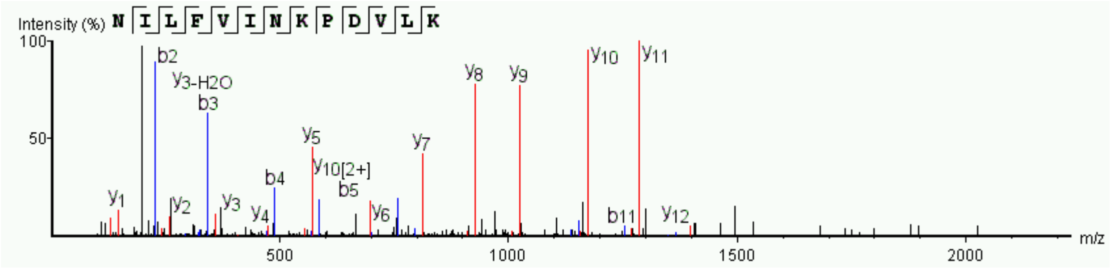

gi|571507498

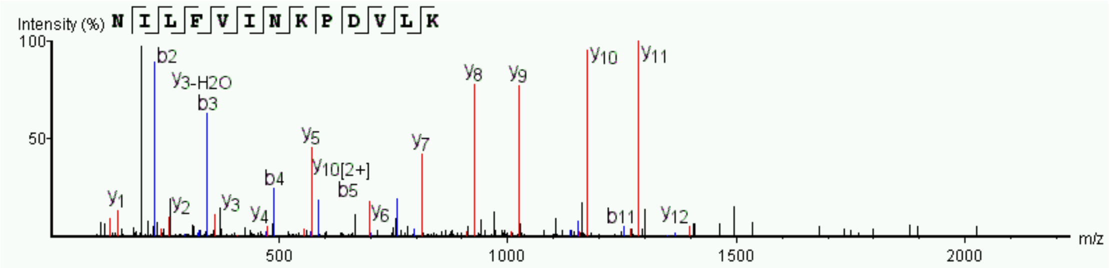

gi|48102172

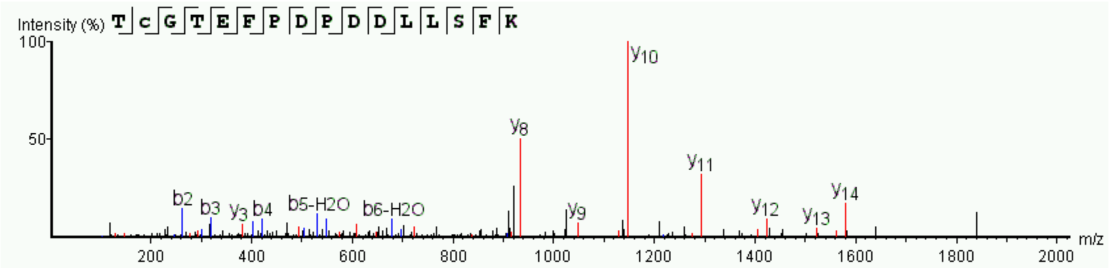

gi|571541806

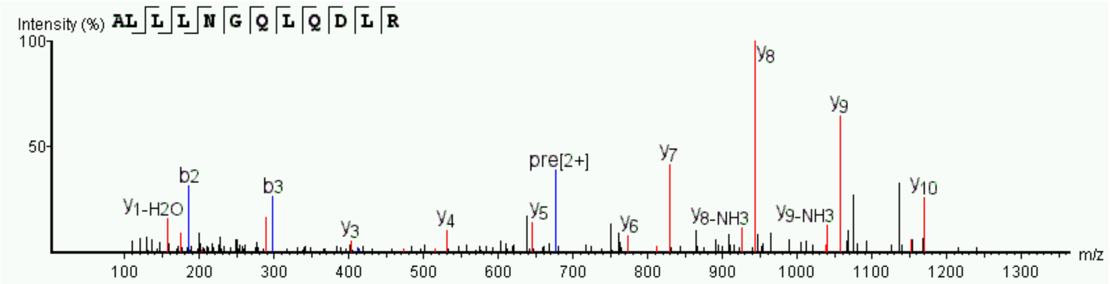

-

gi|335057540

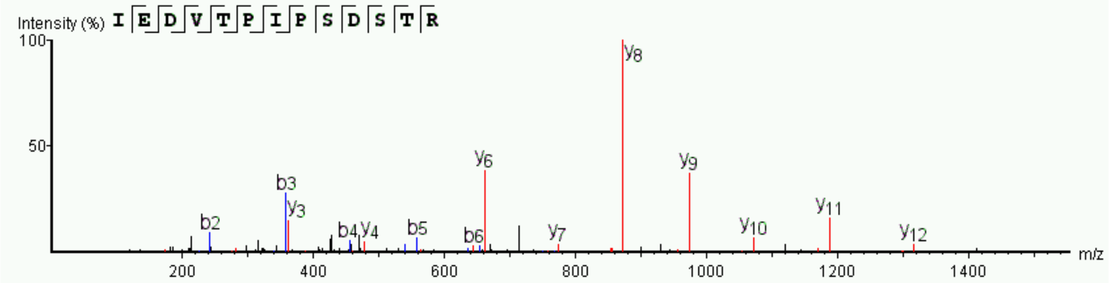

gi|336391185

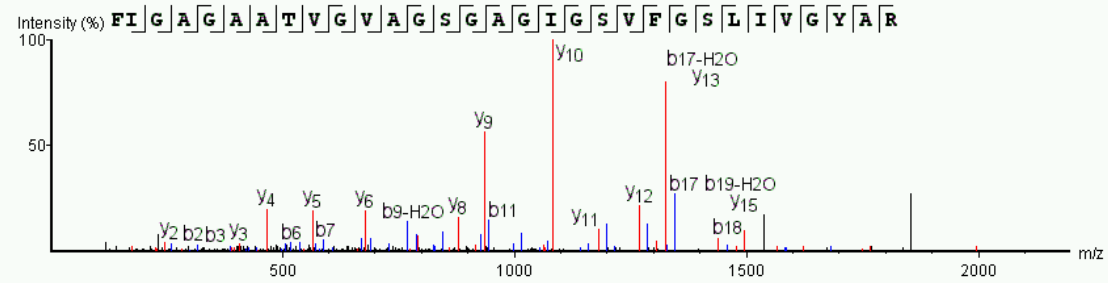

gi|288872189

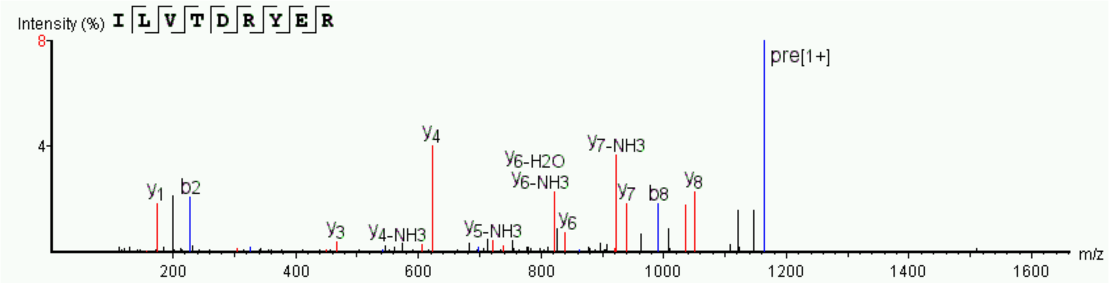

gi|118150510

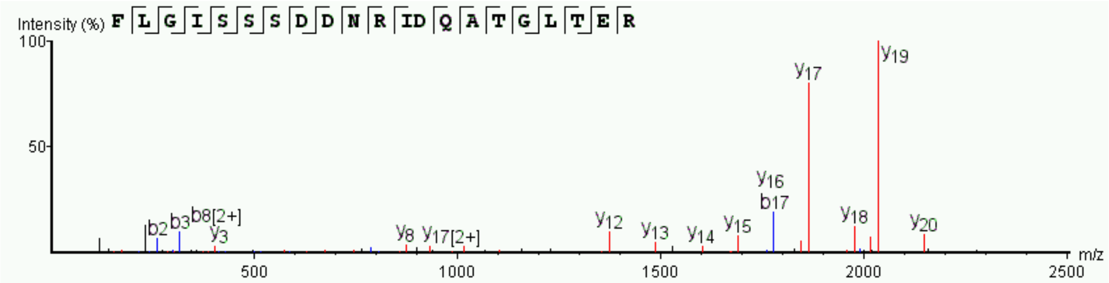

gi|118150484

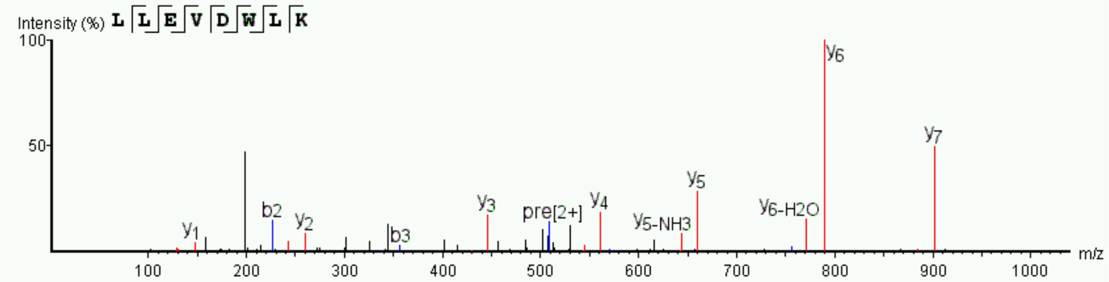

gi|58585078

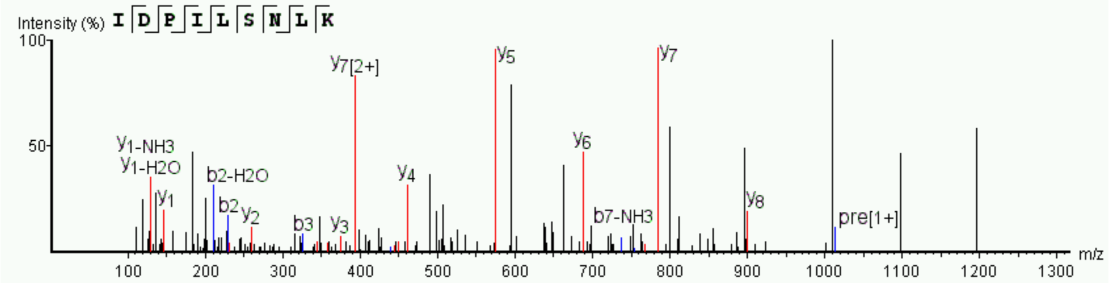

gi|60115688

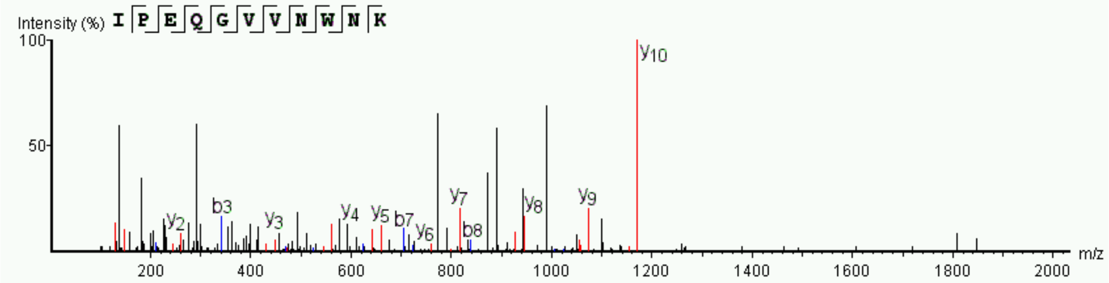

gi|58585096

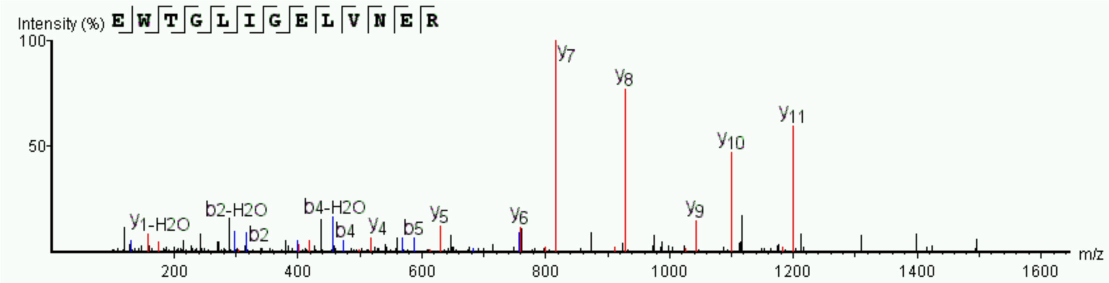

gi|268370271

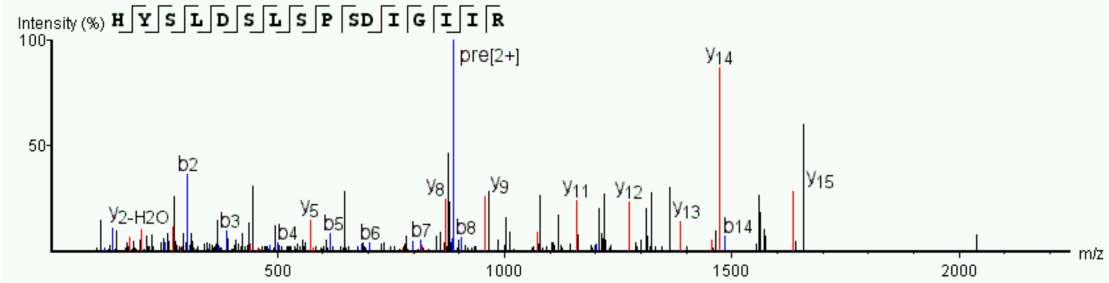

gi|335892852

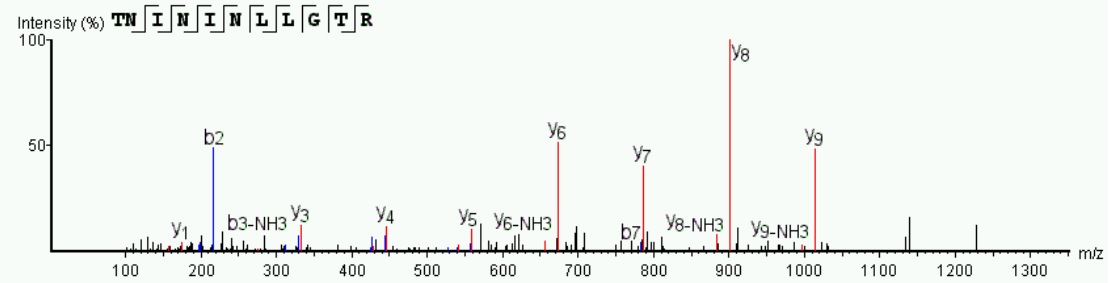

gi|334883143

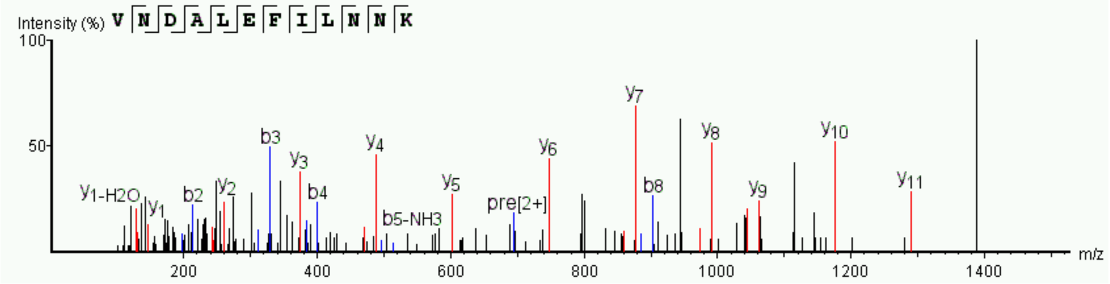

gi|327315378

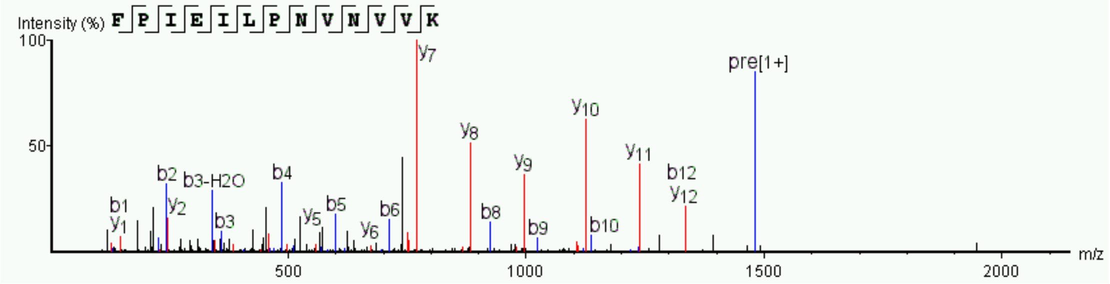

gi|571525362

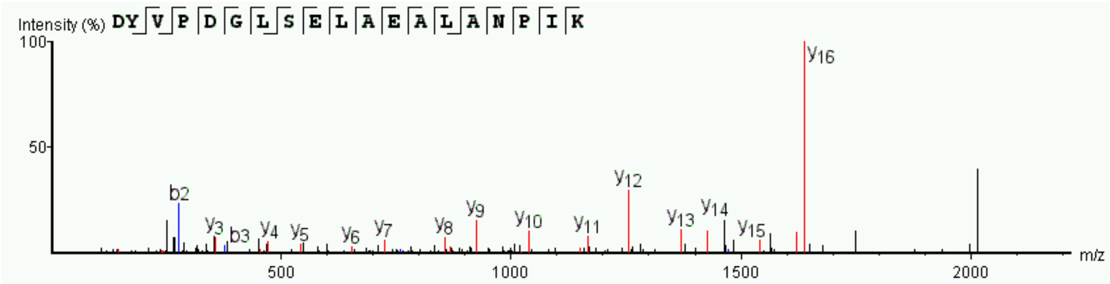

gi|571525366

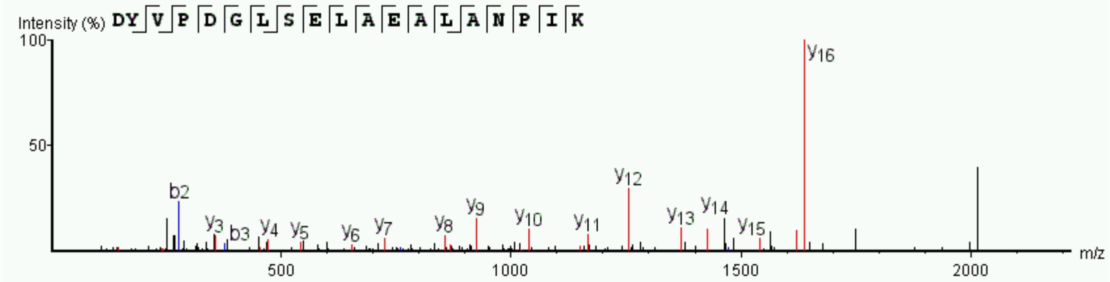

gi|328787709

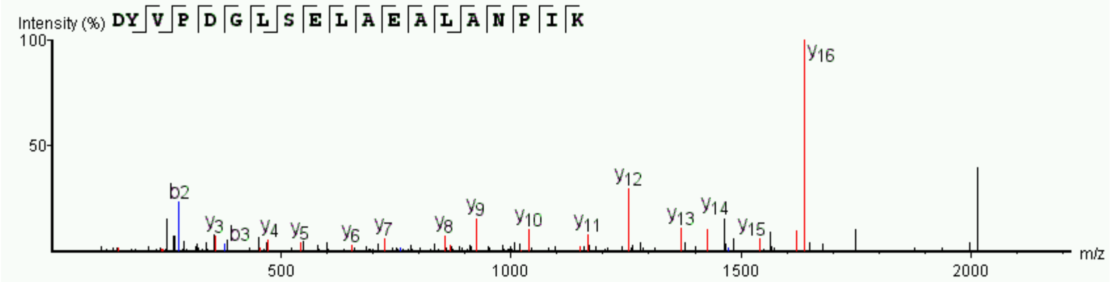

gi|328787711

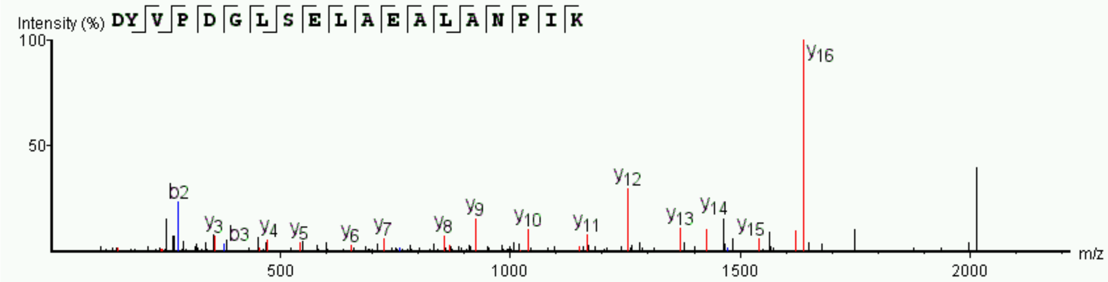

gi|571508698

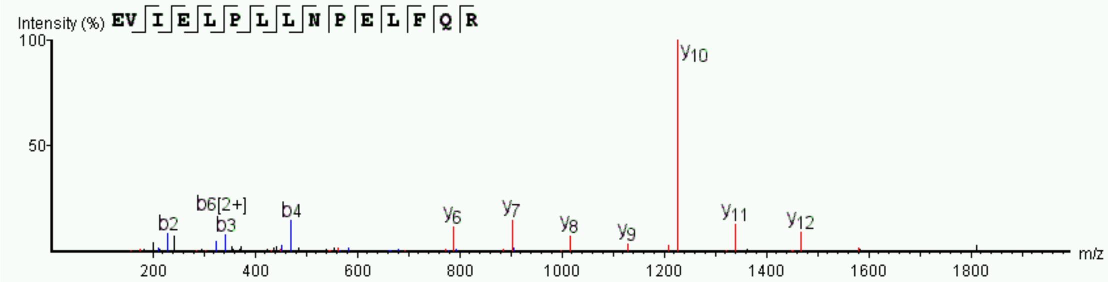

gi|328790710

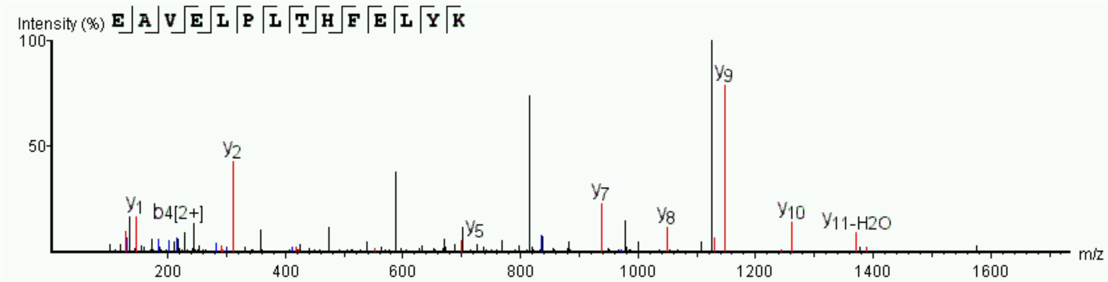

gi|571576907

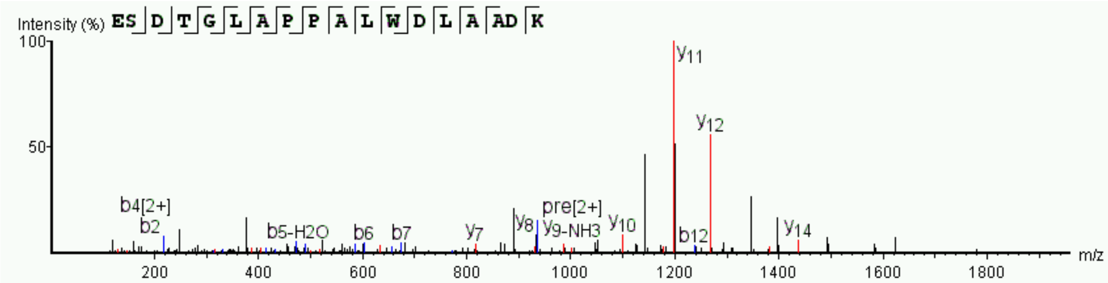

gi|110762783

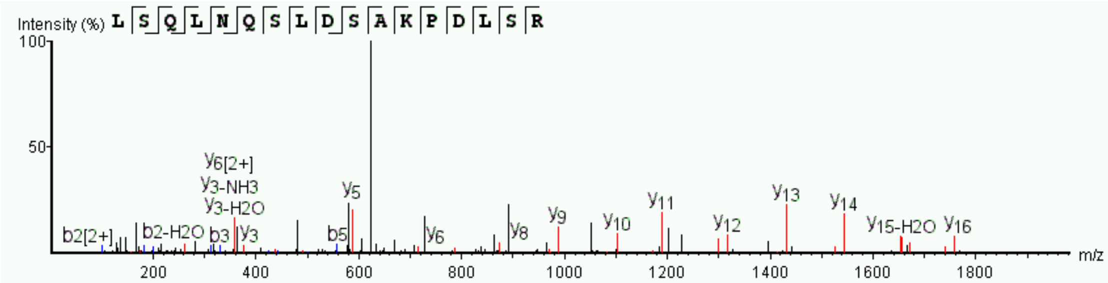

gi|328792301

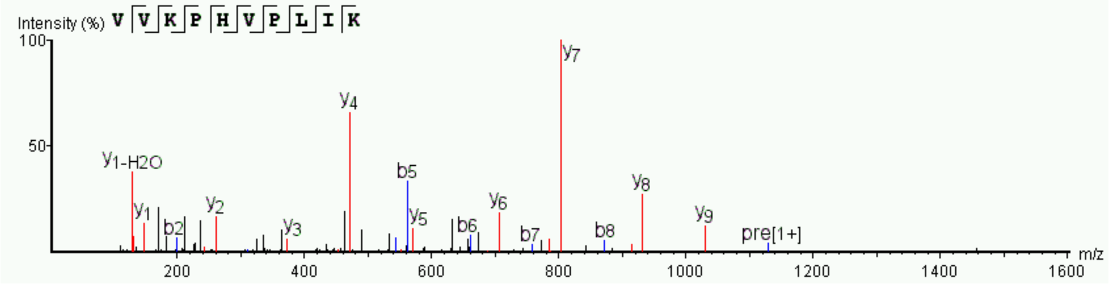

gi|571521798

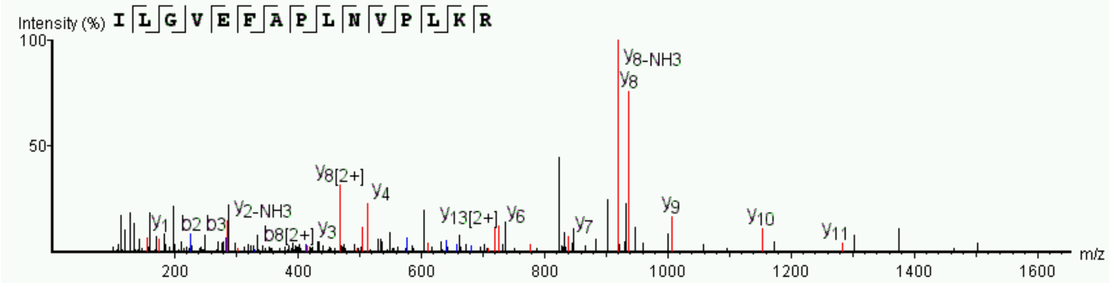

gi|66531974

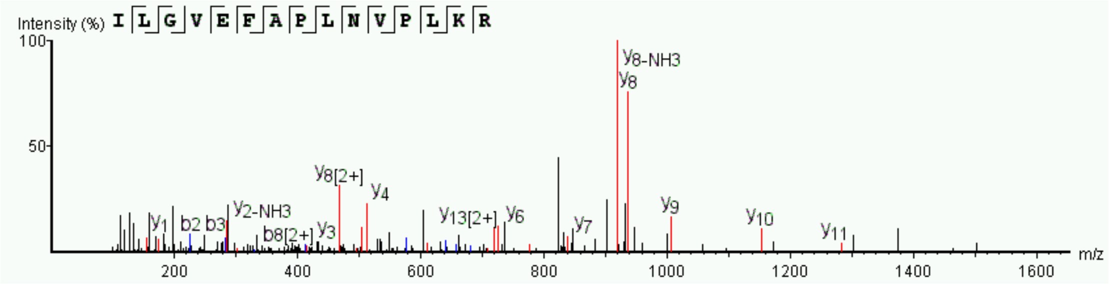

gi|571504413

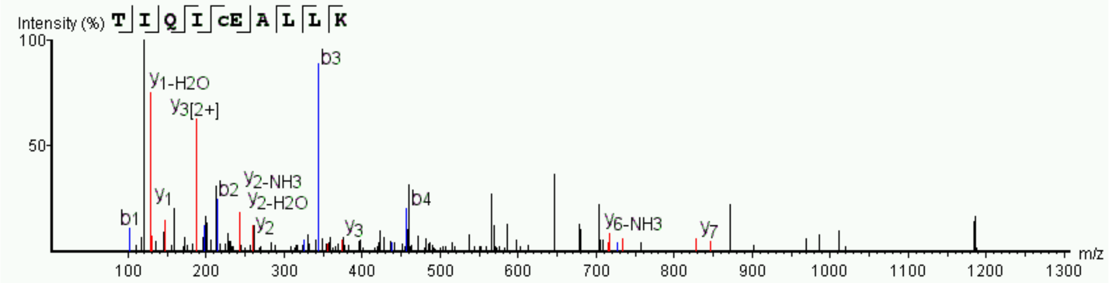

gi|48097100

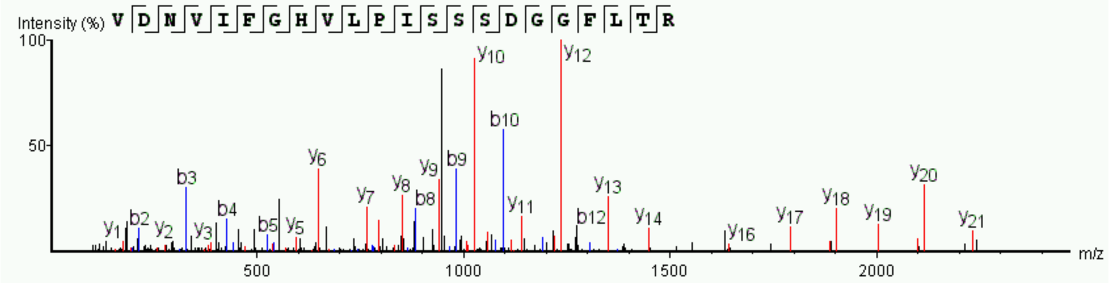

gi|110757985

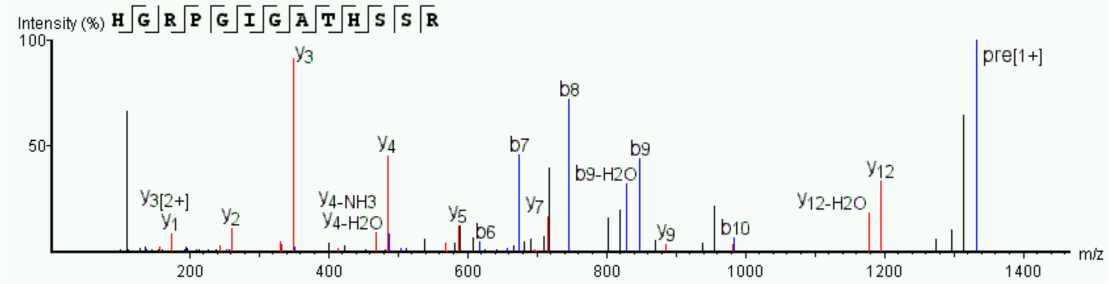

gi|48095625

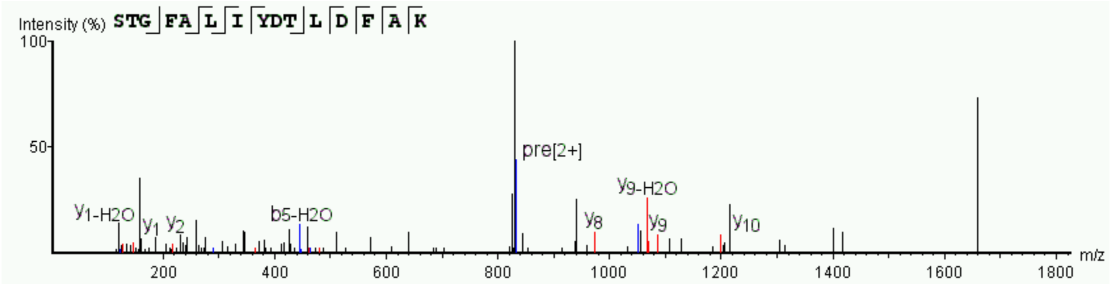

gi|571524333

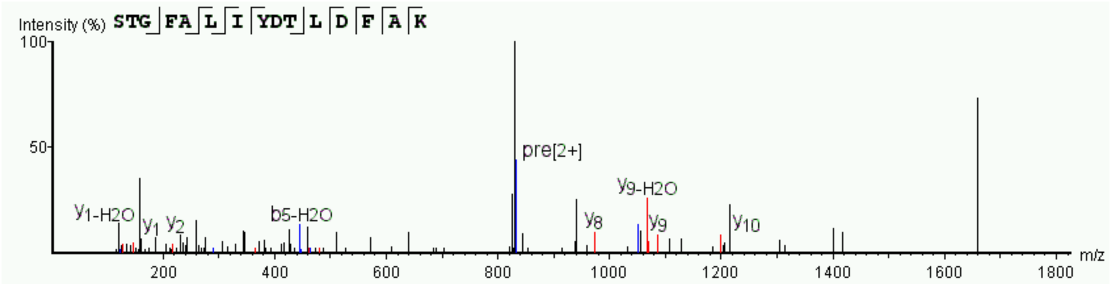

gi|571524328

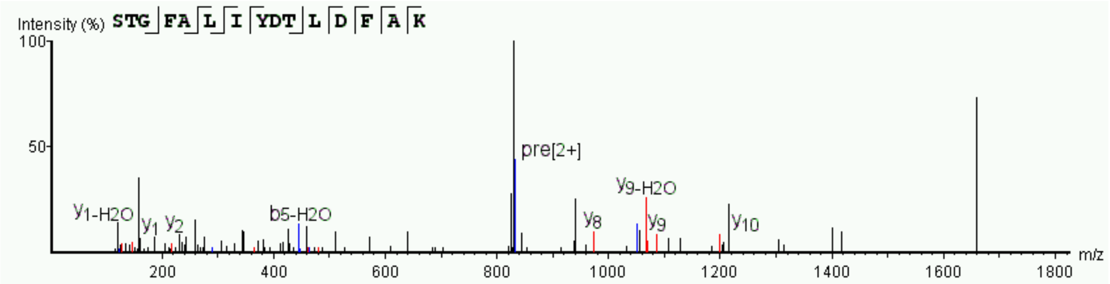

gi|571528797

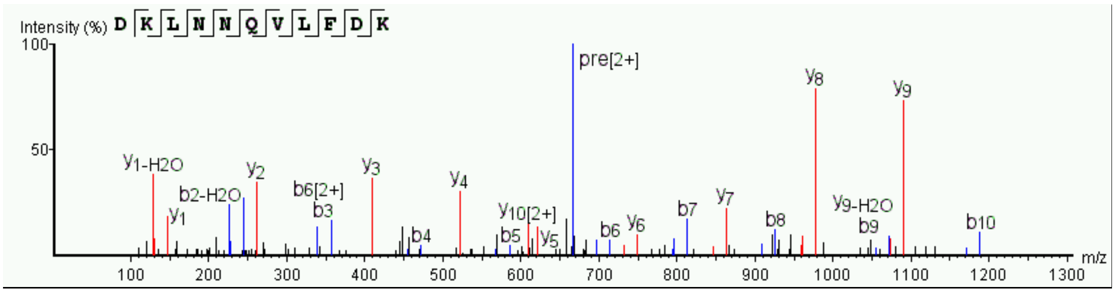

gi|571522669

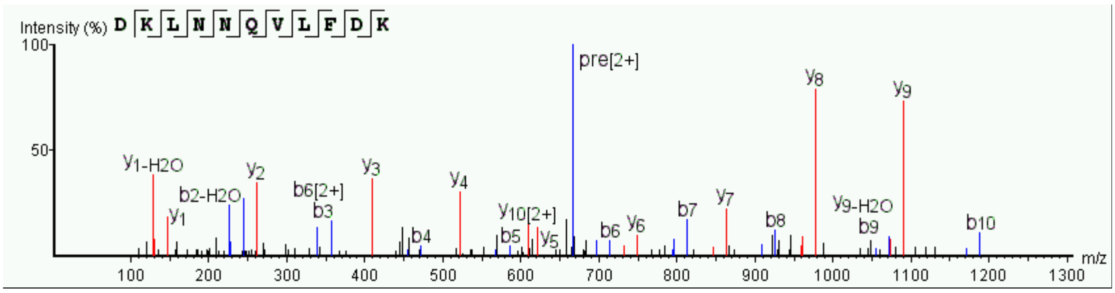

Annotated spectra of single-peptide identifications of proteins in mushroom bodies of *Apis mellifera*

gi|571571828

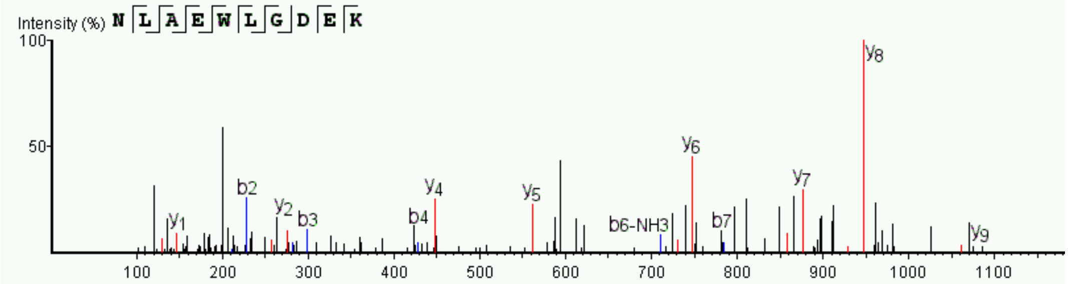

gi|571531847

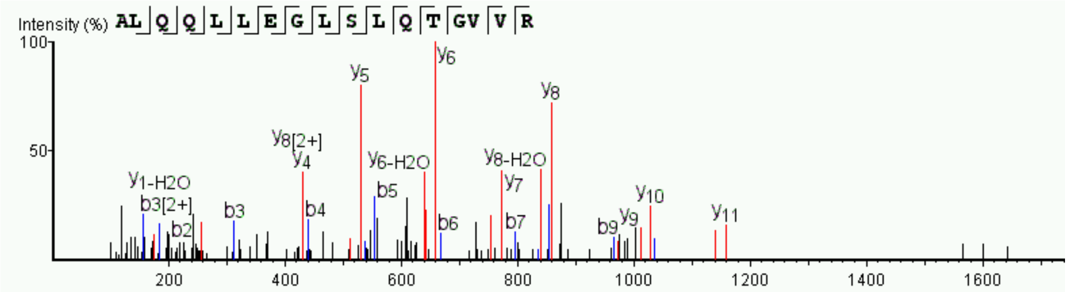

gi|66509969

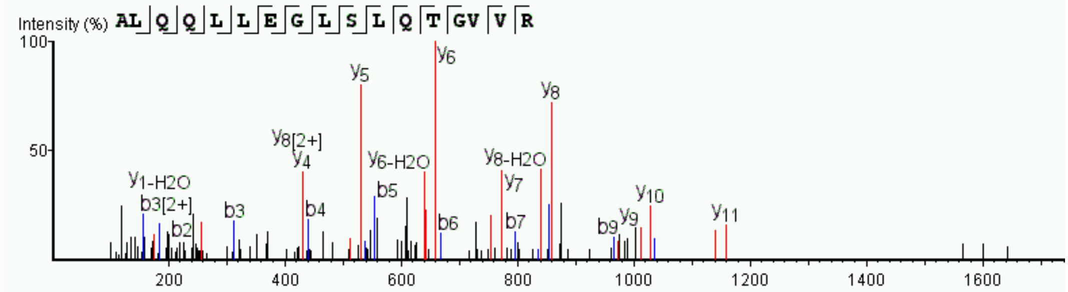

gi|571510609

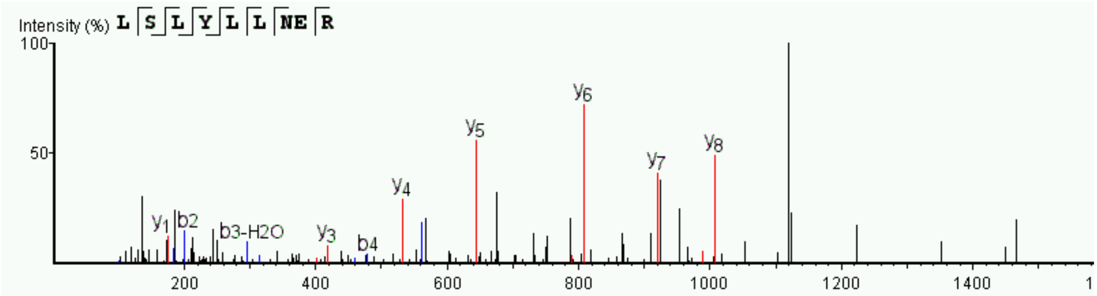

gi|571510614

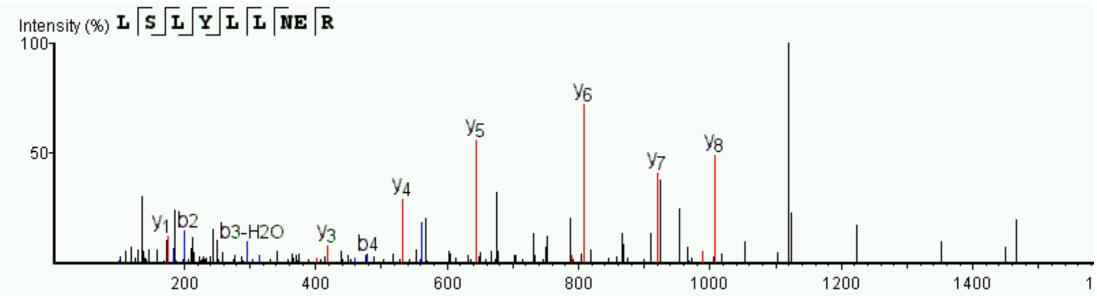

gi|571510617

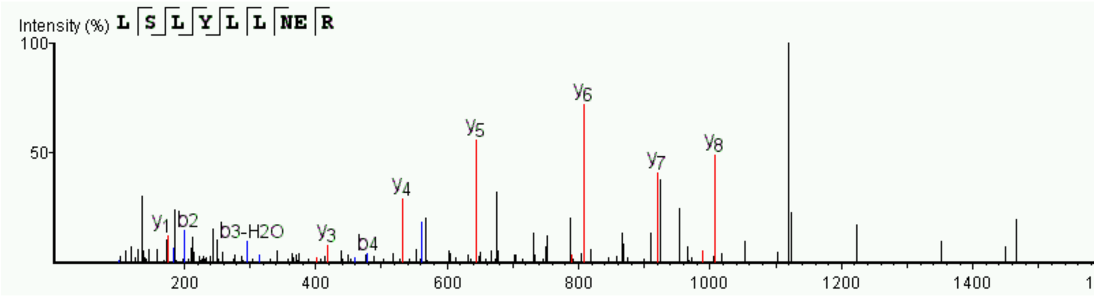

gi|328792853

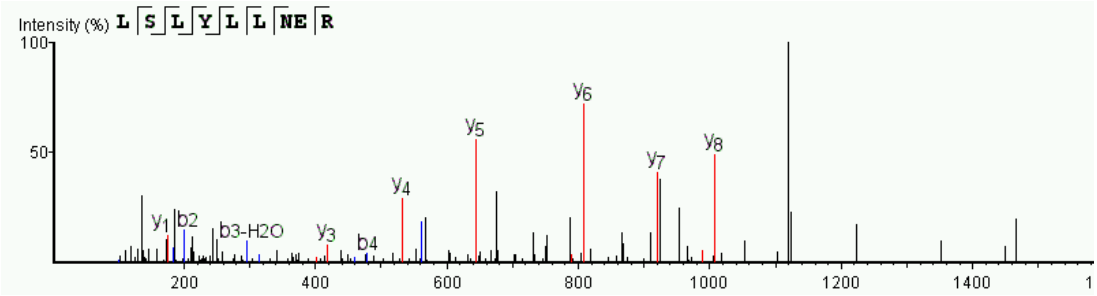

gi|571567574

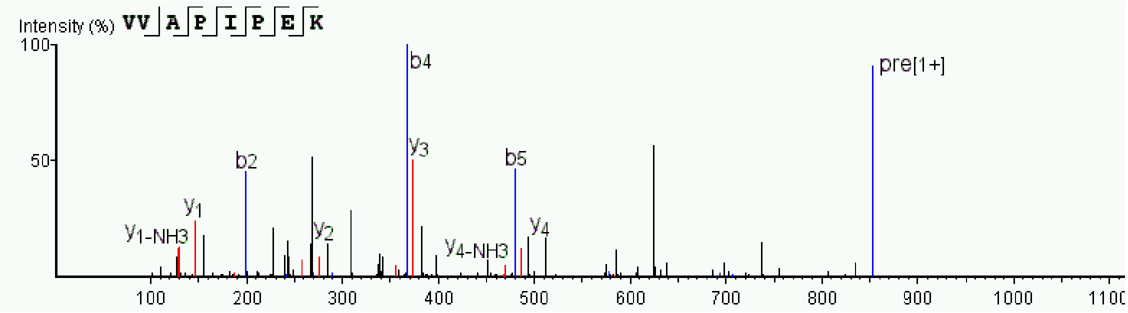

gi|571502282

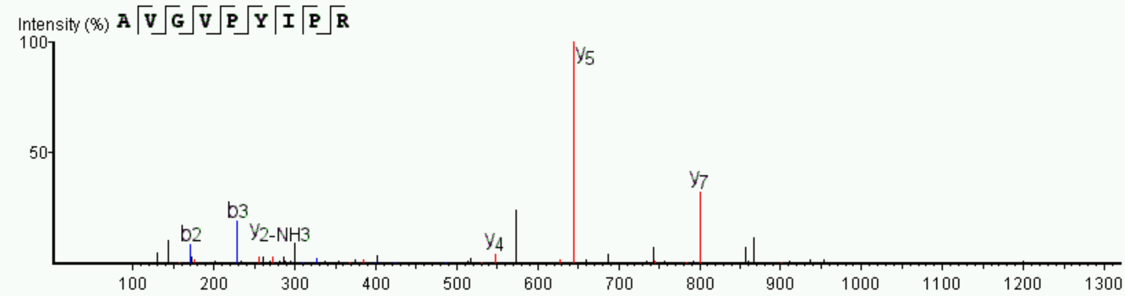

gi|66531704

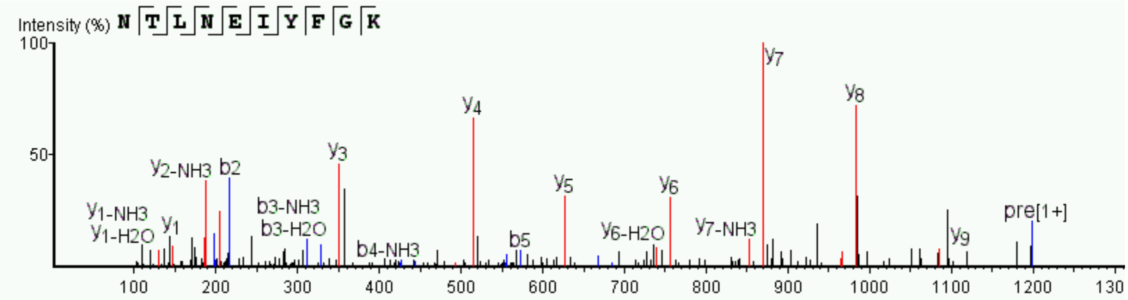

gi|571532679

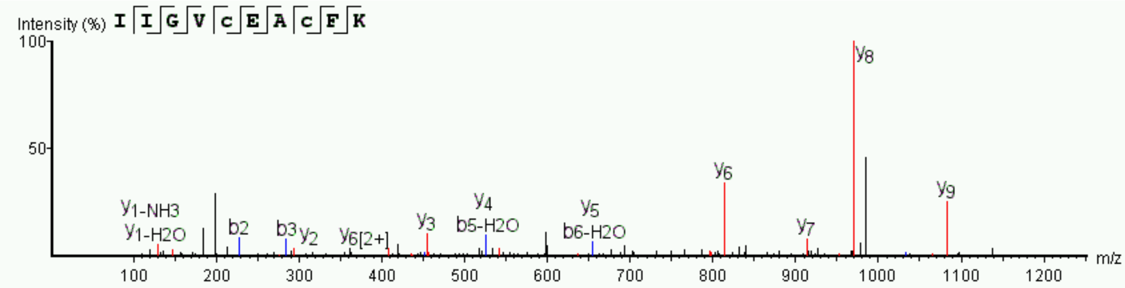

gi|571569633

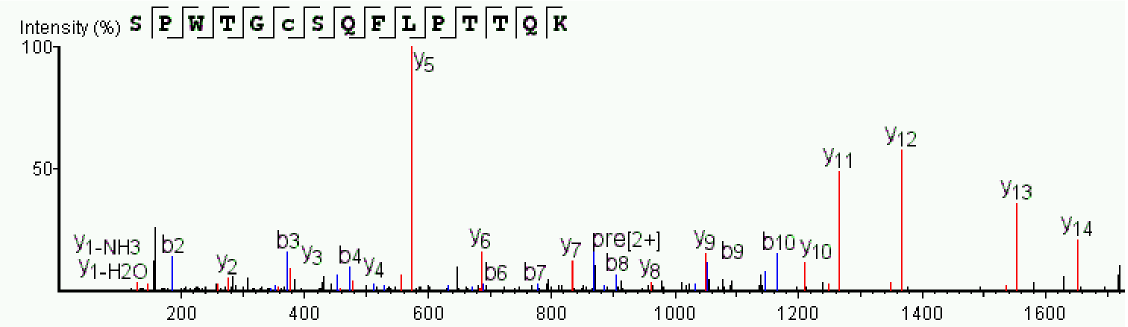

gi|571569639

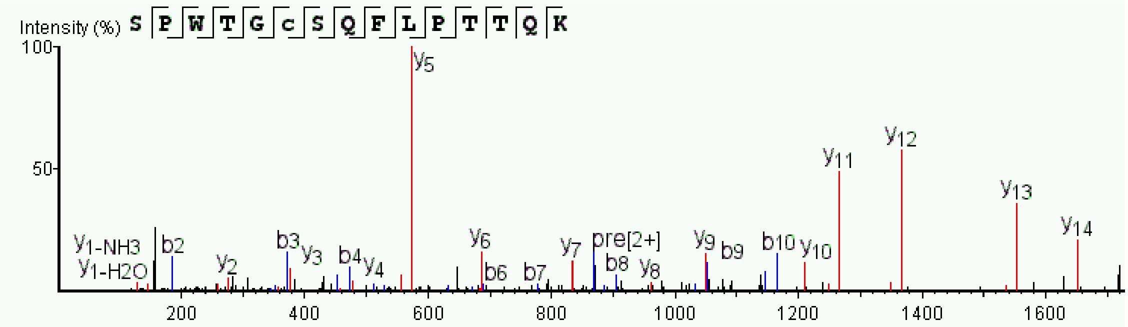

gi|66543768

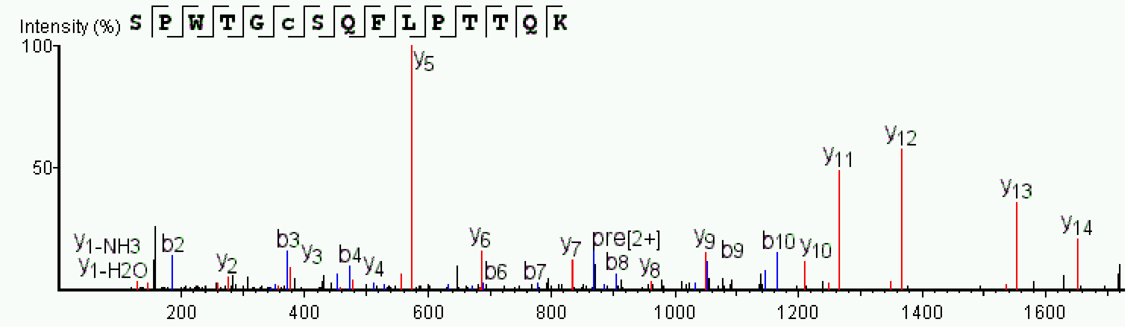

gi|571560836

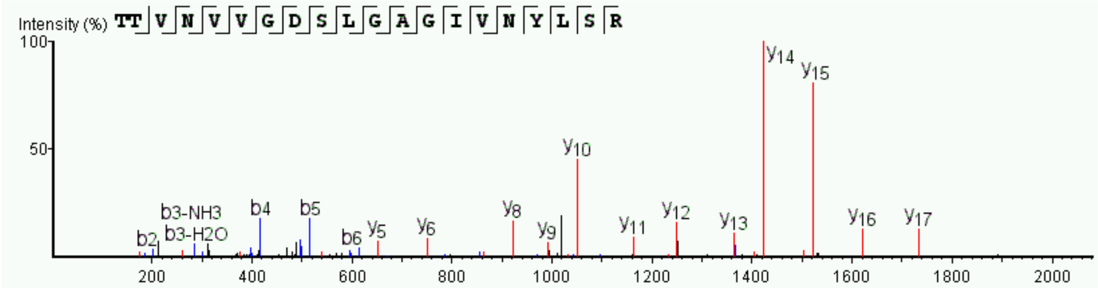

gi|571560840

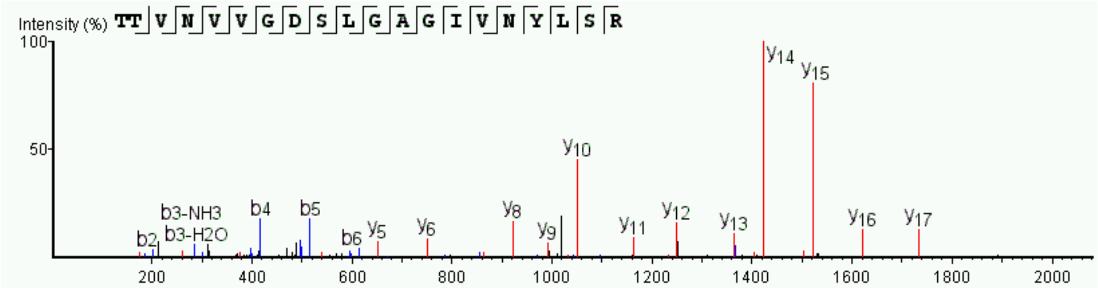

gi|571560844

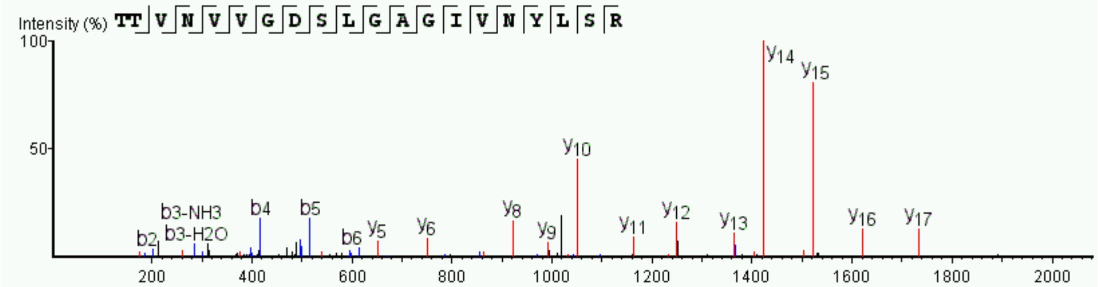

gi|571560848

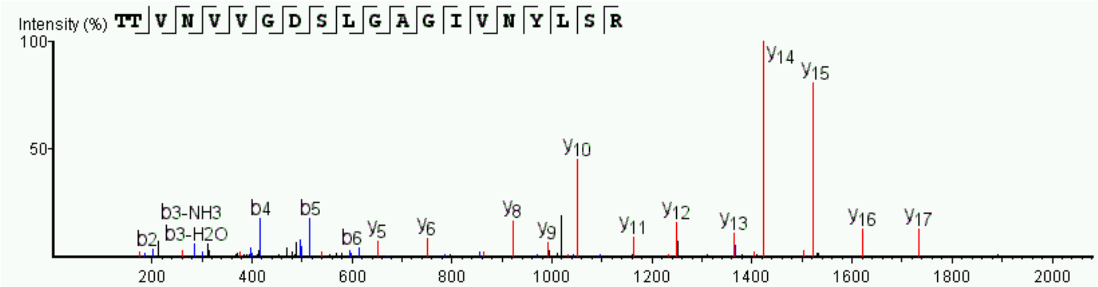

gi|571577740

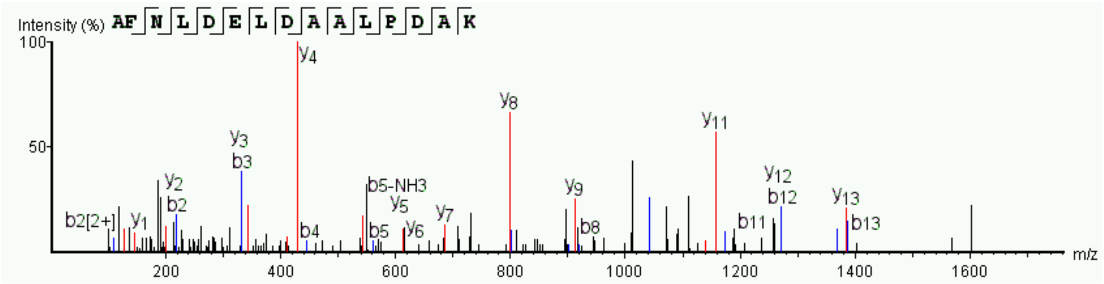

gi|571577742

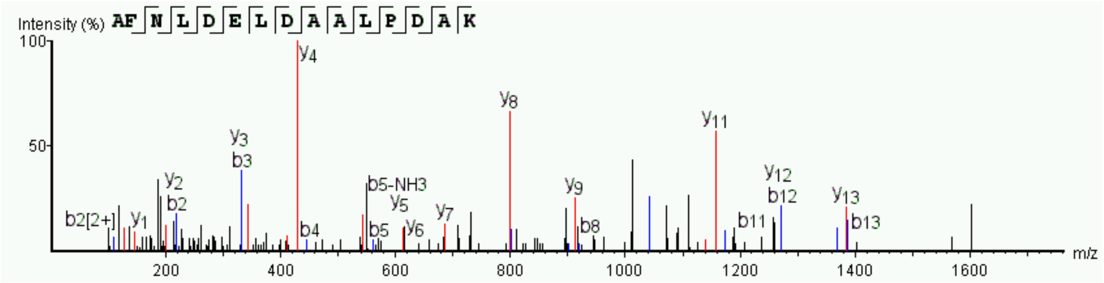

gi|571577744

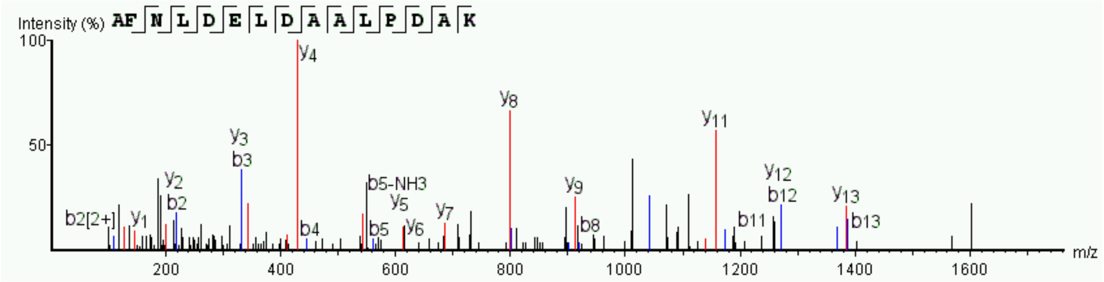

gi|571545068

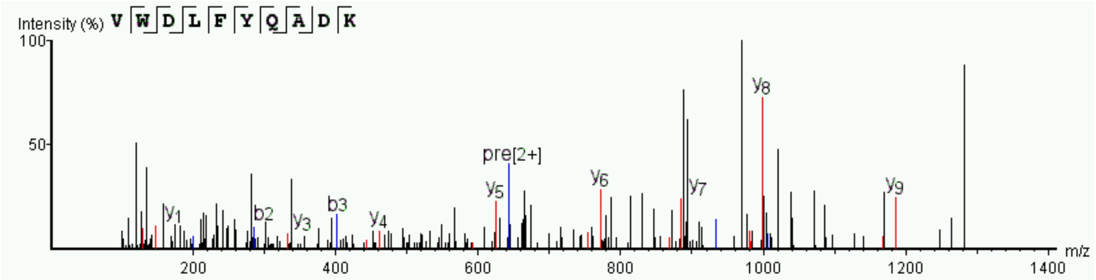

gi|328789473

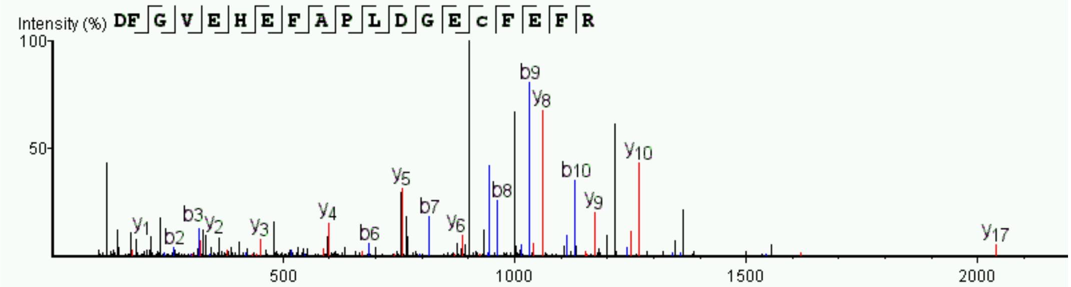

gi|571531971

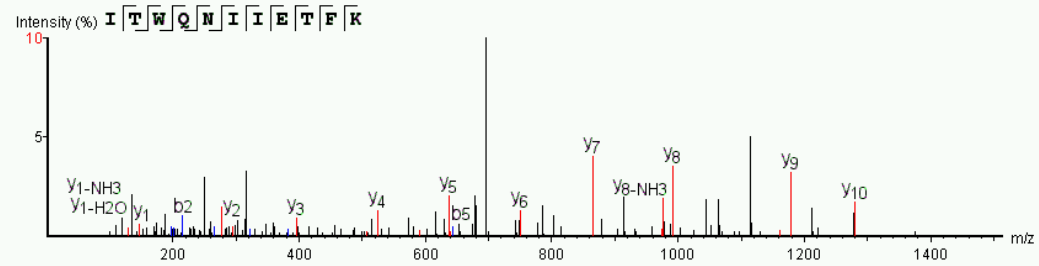

gi|571531975

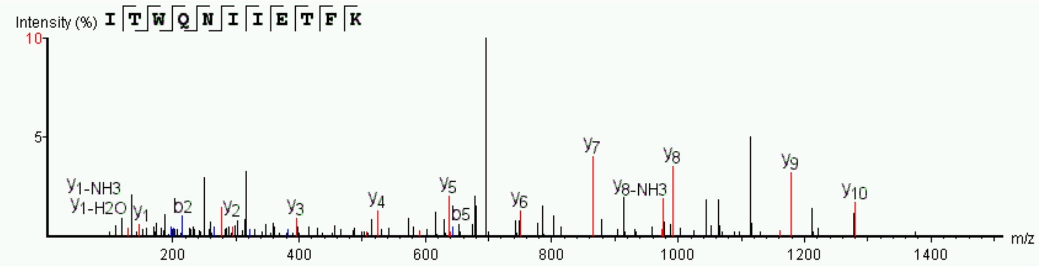

gi|571574918

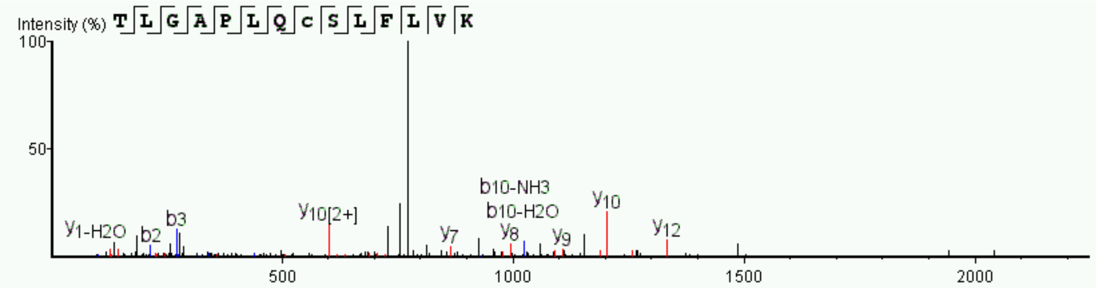

gi|571547726

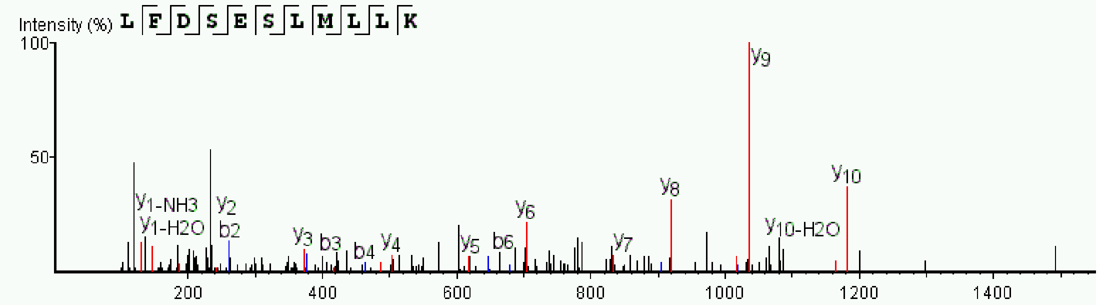

gi|571547729

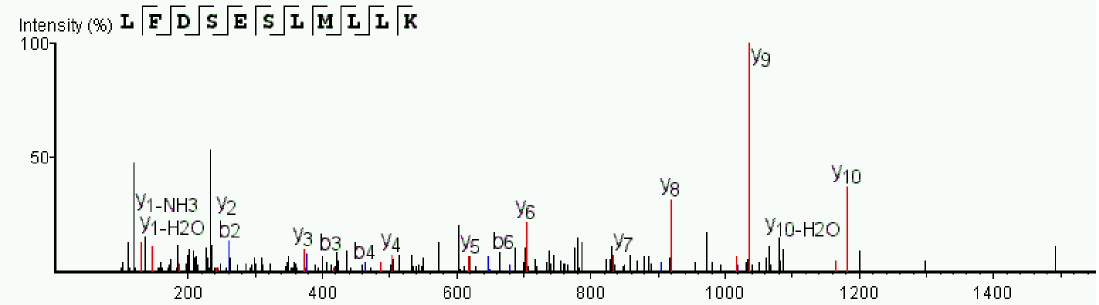

gi|571505416

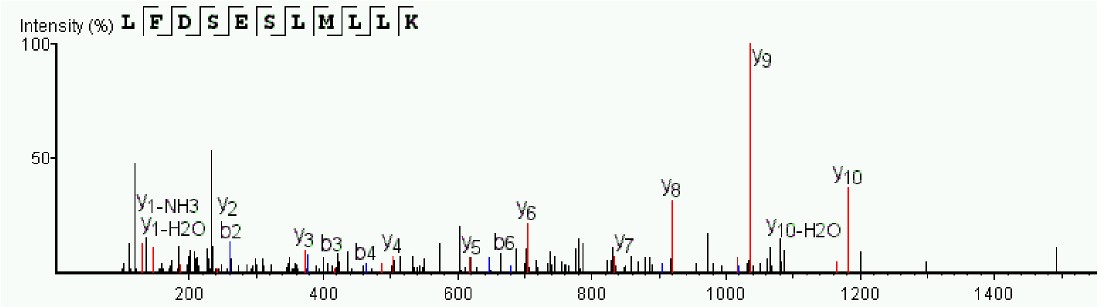

gi|66500045

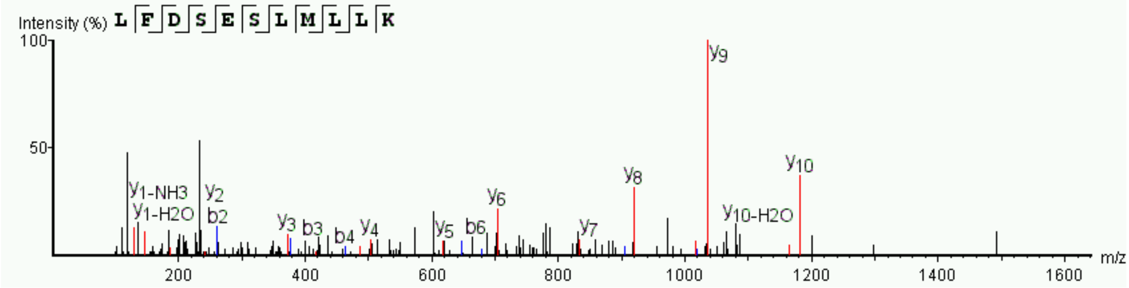

gi|110764555

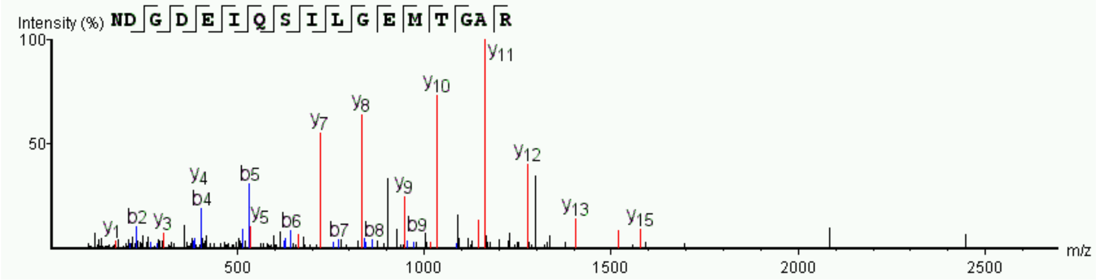

gi|328786065

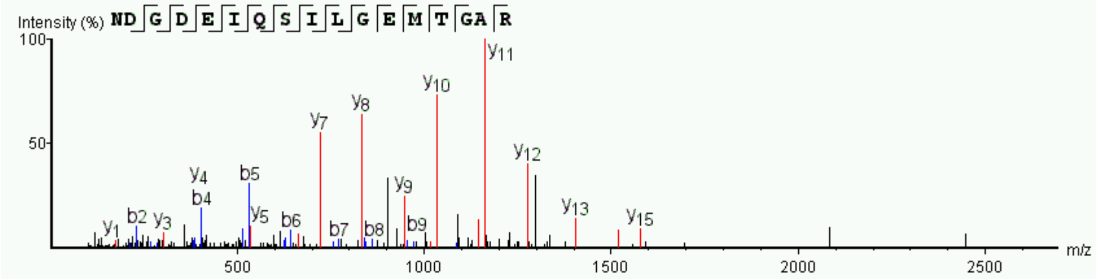

gi|328786067

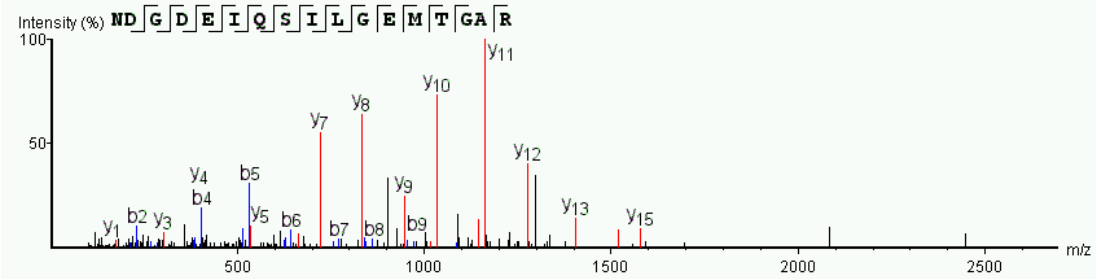

gi|571501987

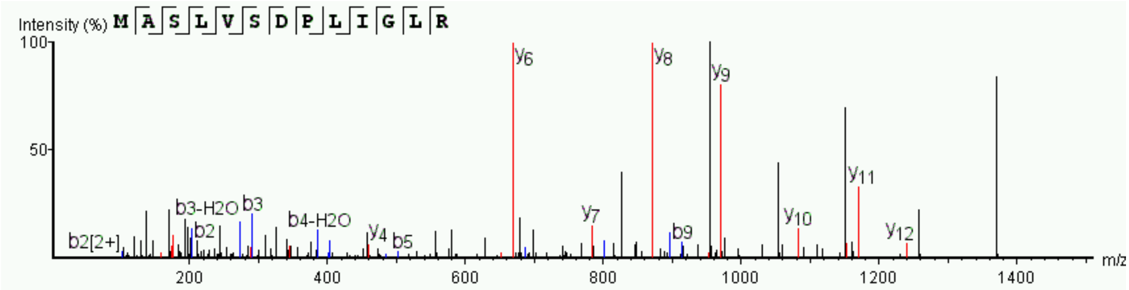

gi|328783034

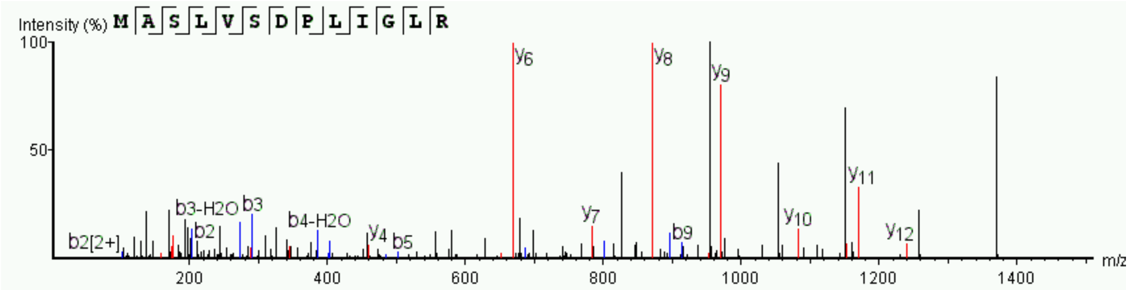

gi|328784463

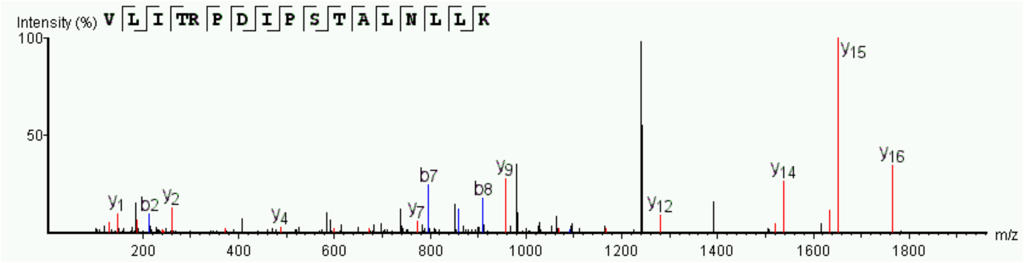

gi|571558990

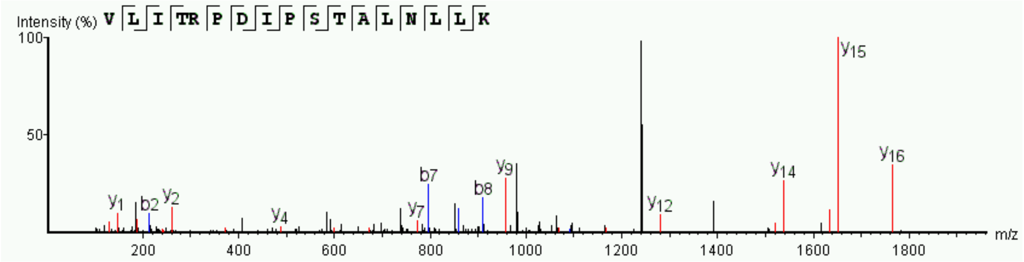

gi|571558993

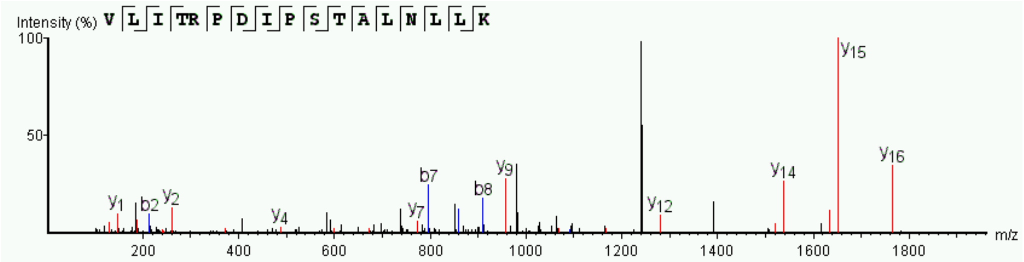

gi|571558997

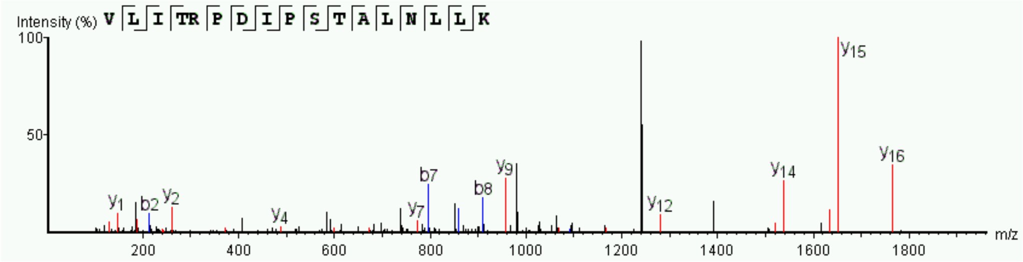

gi|571559000

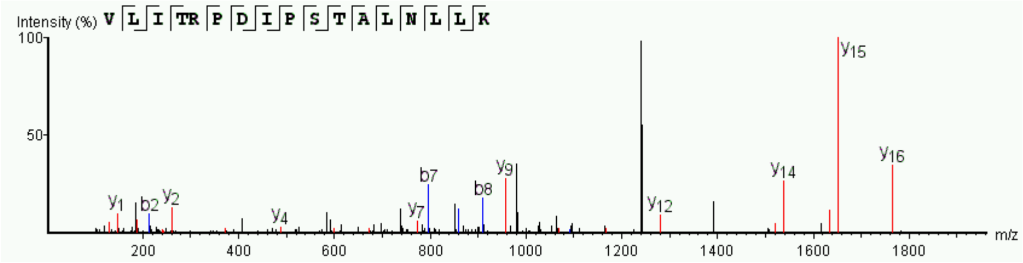

gi|66499182

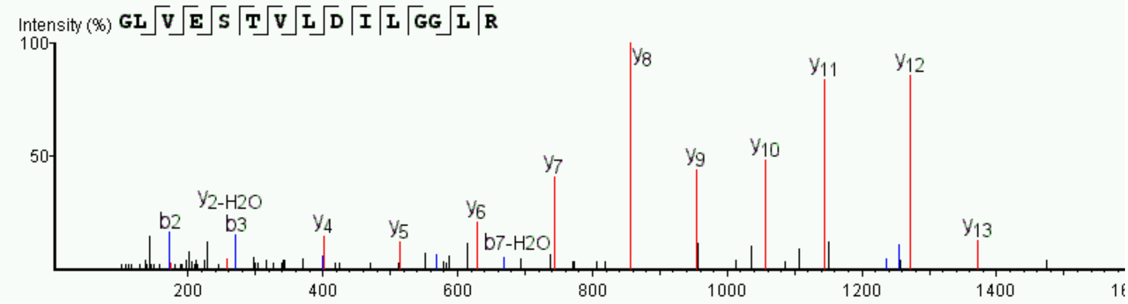

gi|571560348

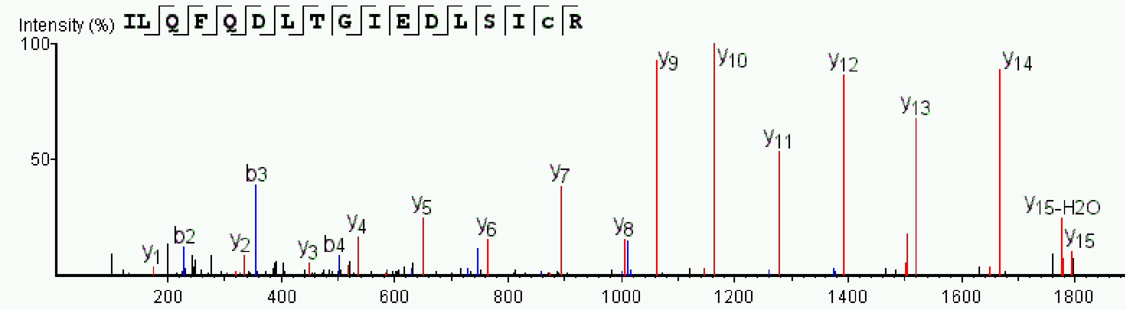

gi|571558404

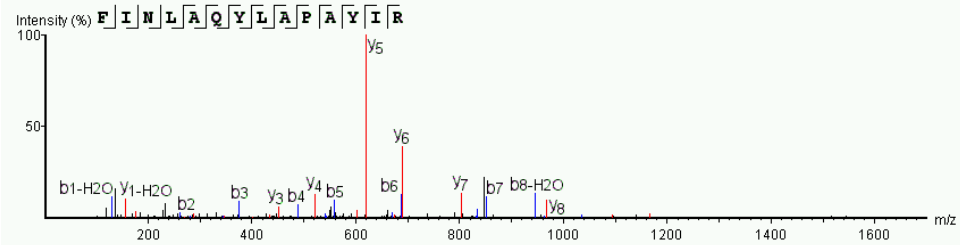

gi|571558407

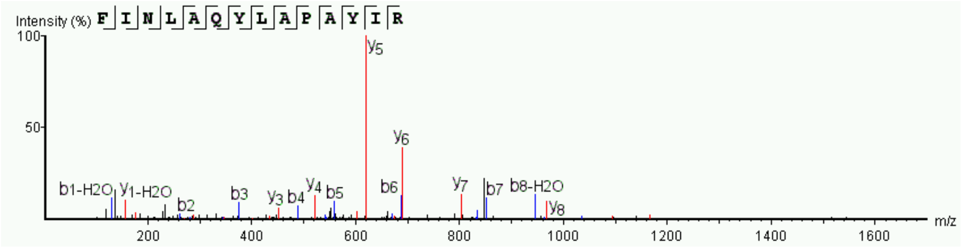

gi|110751164

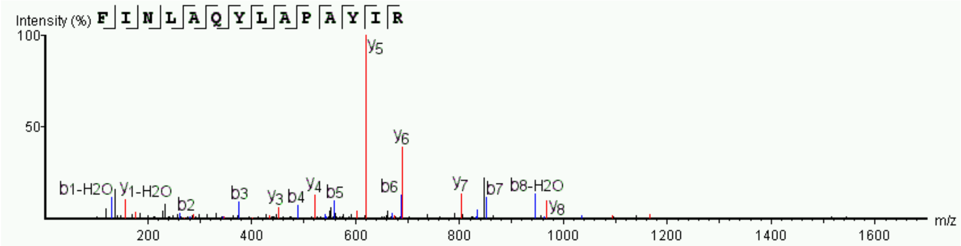

gi|571574413

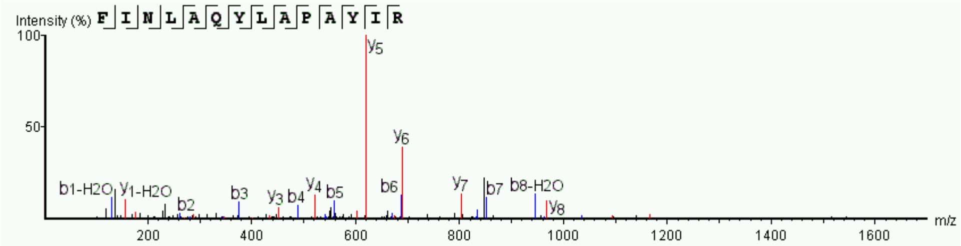

gi|571574415

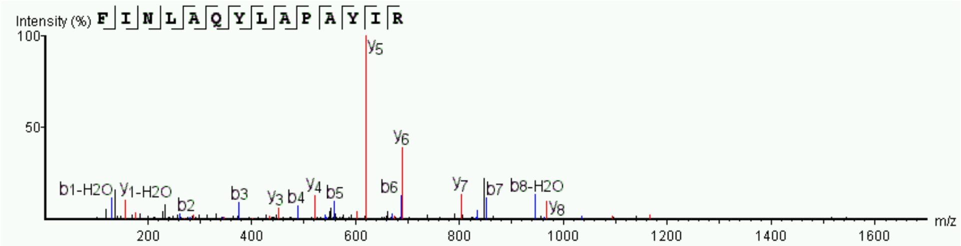

gi|571546054

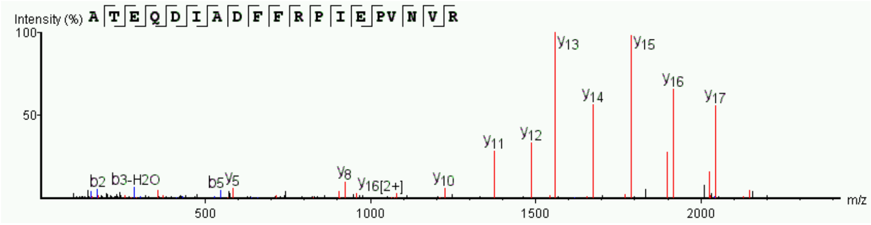

gi|571546058

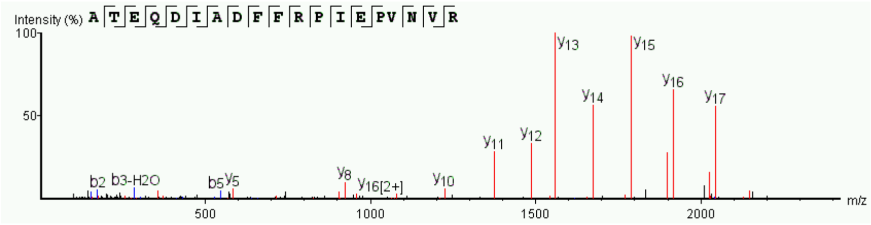

gi|571533174

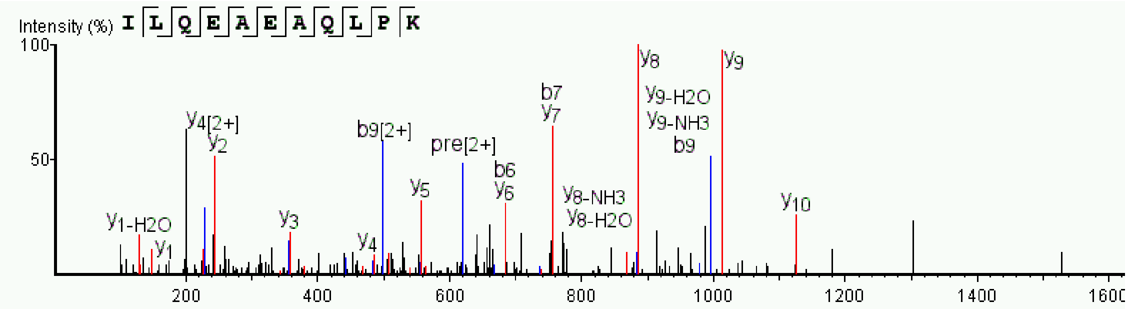

gi|571510150

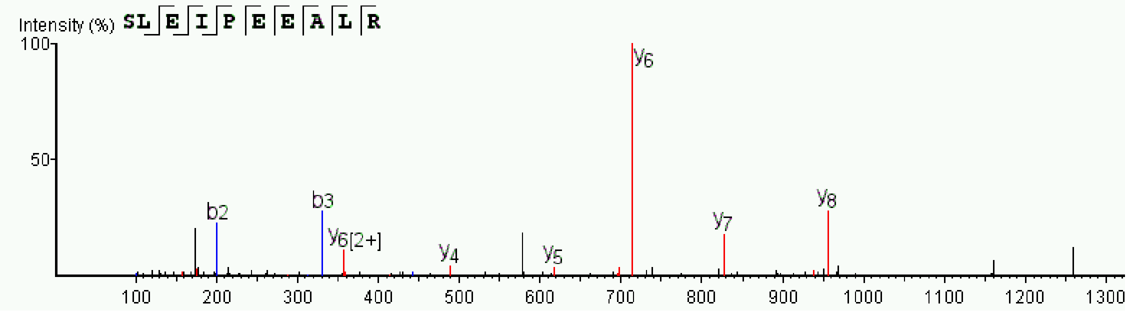

gi|571574200

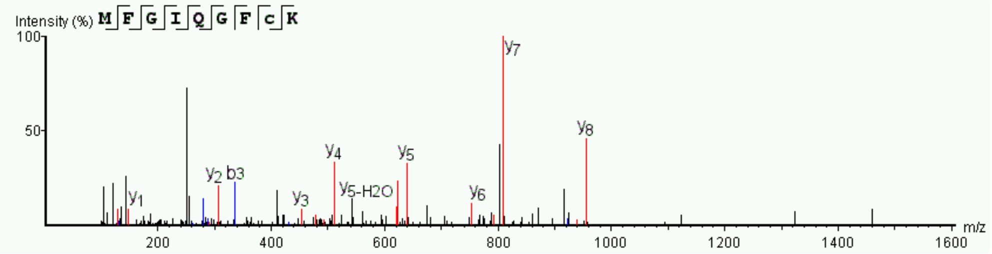

gi|571503655

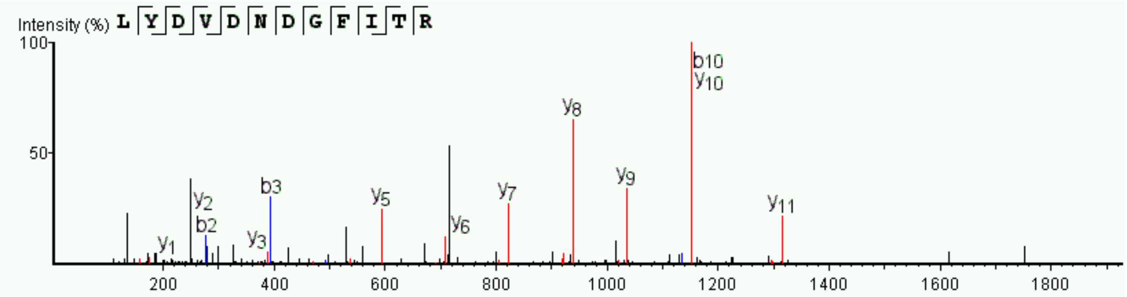

gi|571503659

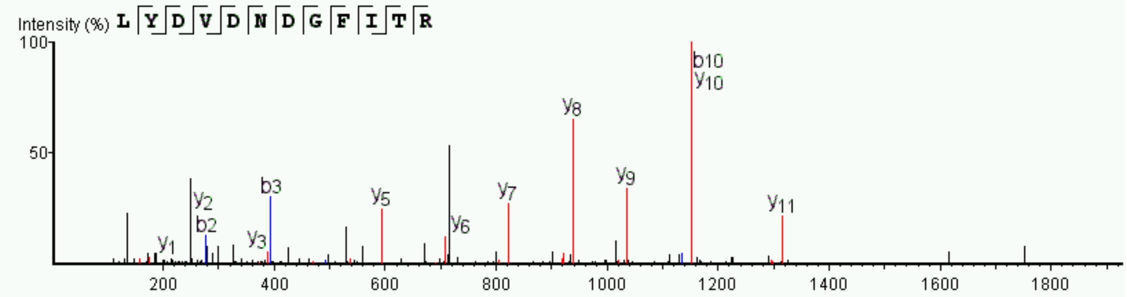

gi|66499717

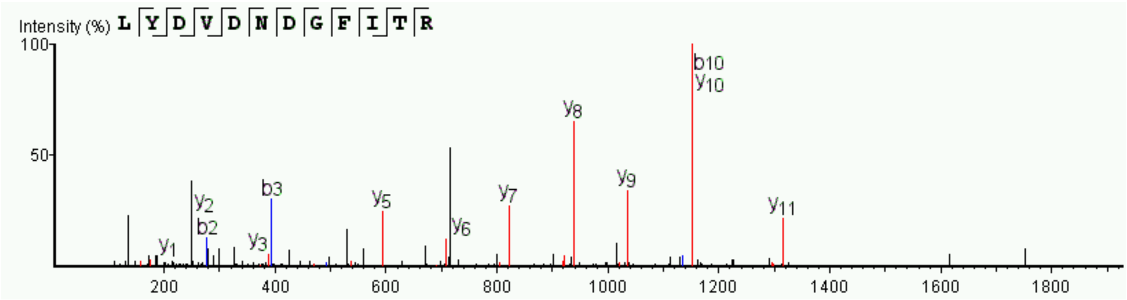

gi|66516817gi|571556252

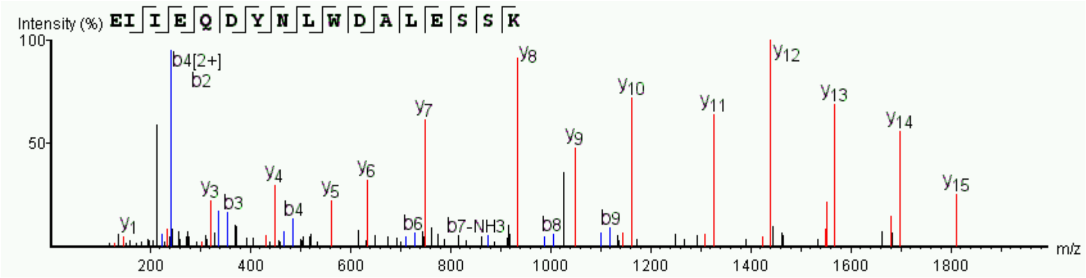

gi|571530481

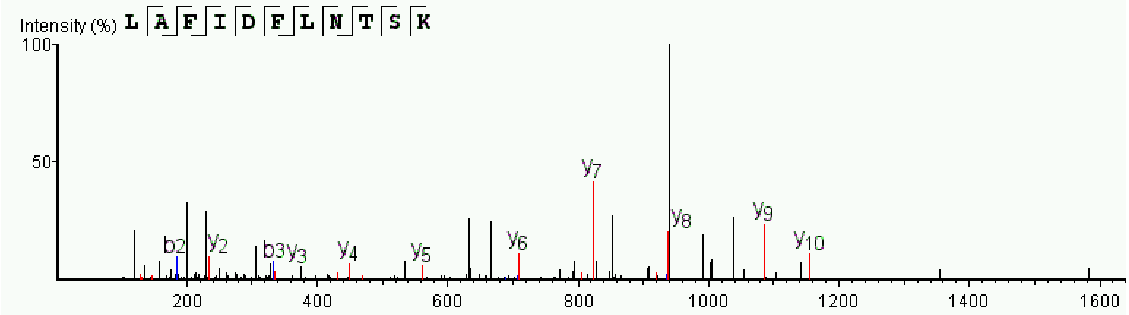

gi|48141273

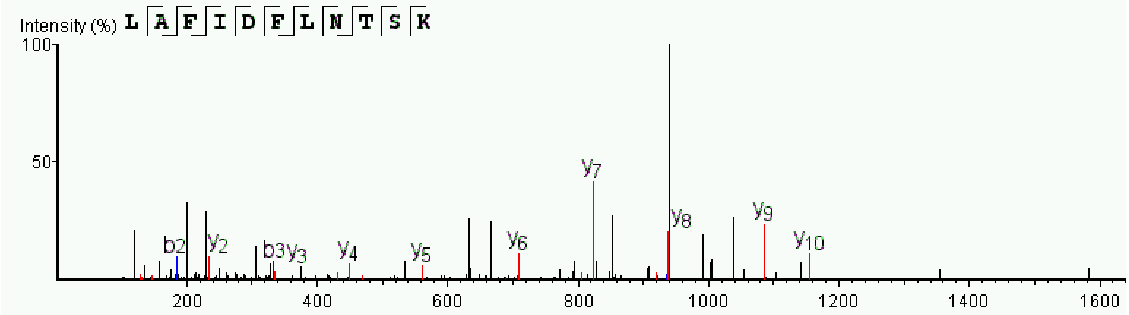

gi|571567903

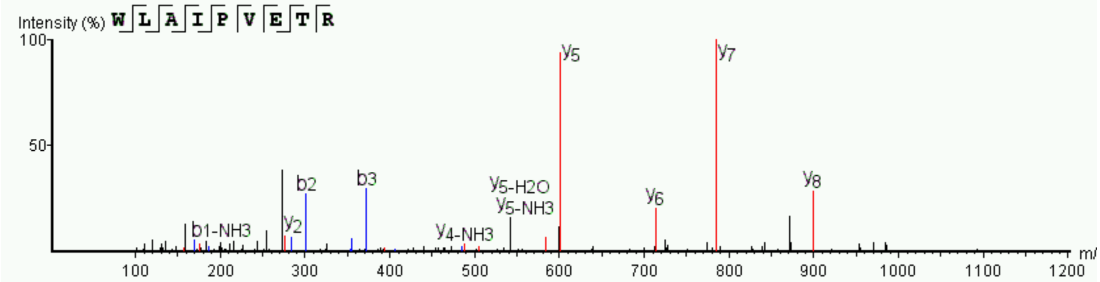

gi|571567906

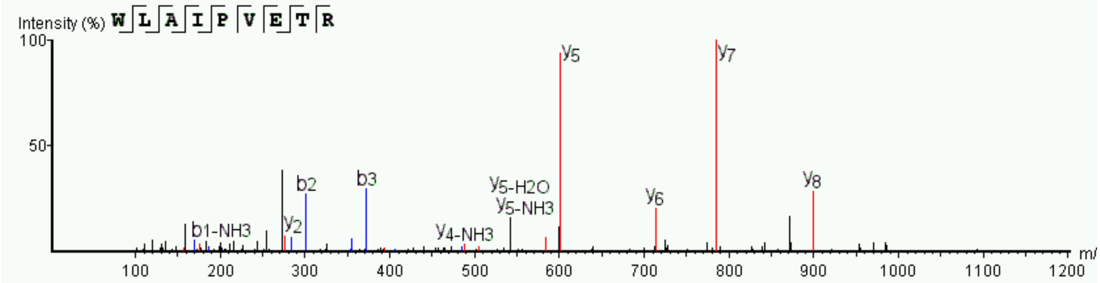

gi|328792738

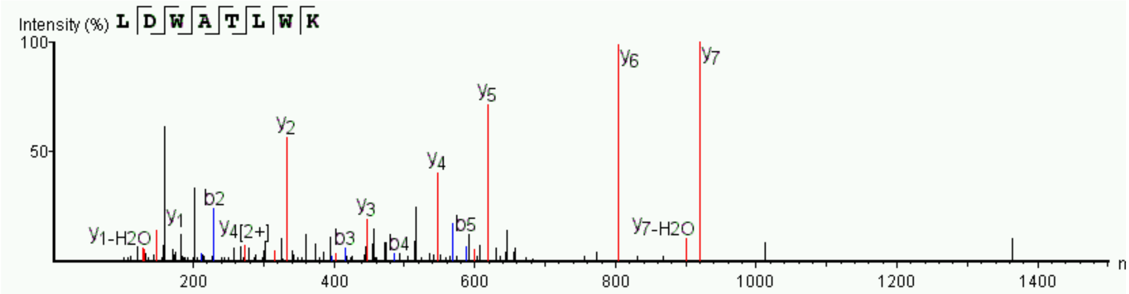

gi|571529052

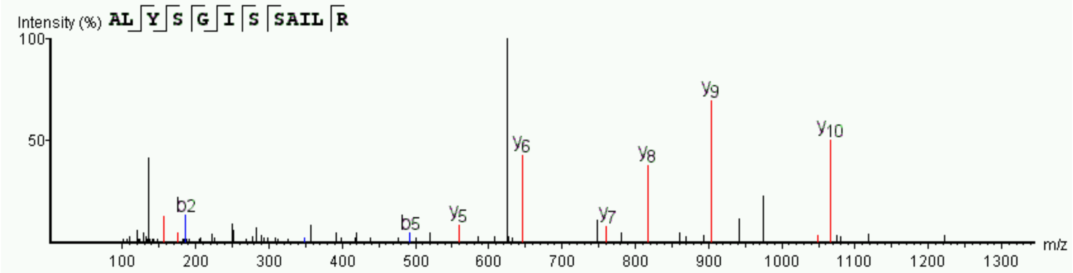

gi|571529058

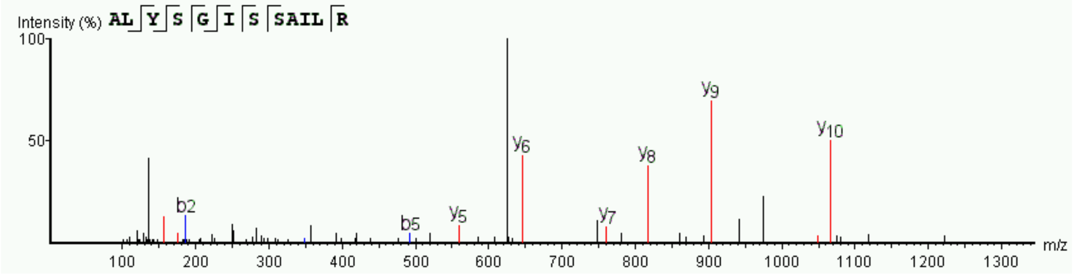

gi|571503986

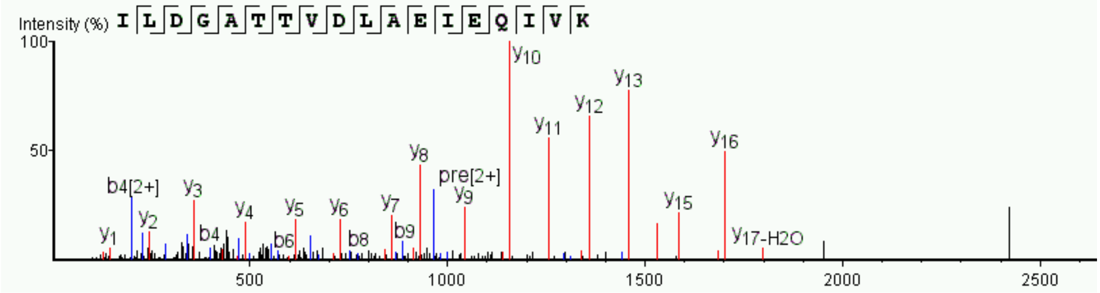

gi|571503989

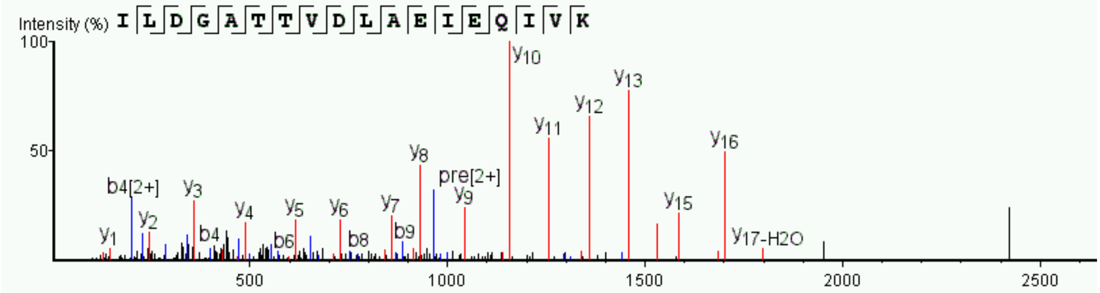

gi|571503993

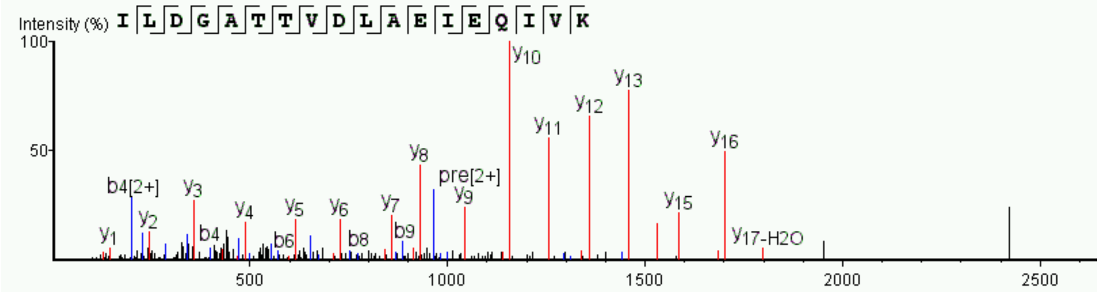

gi|571503997

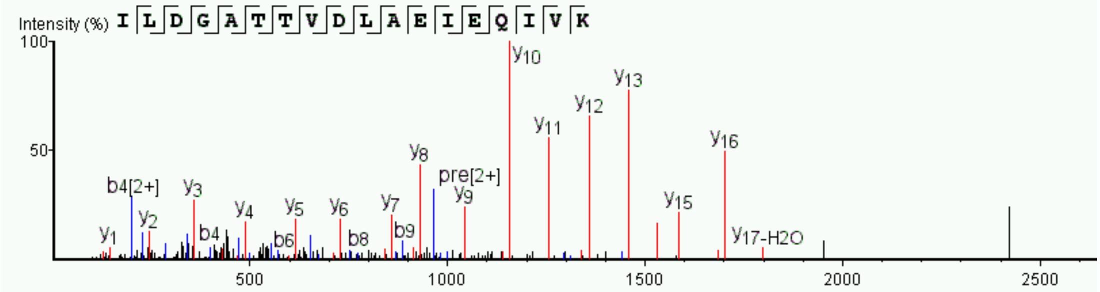

gi|571504000

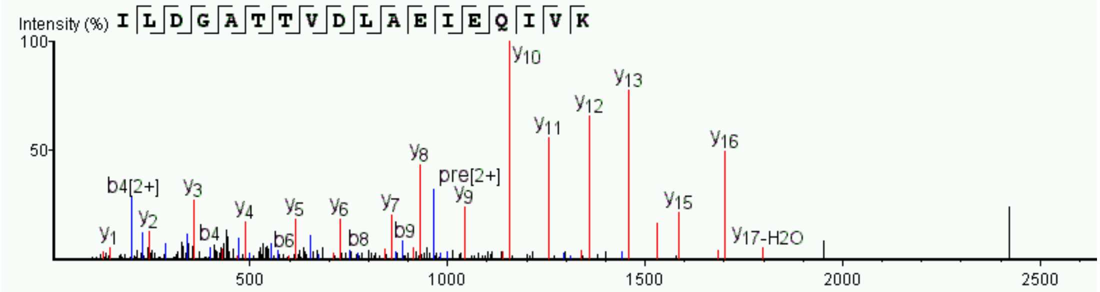

gi|571504003

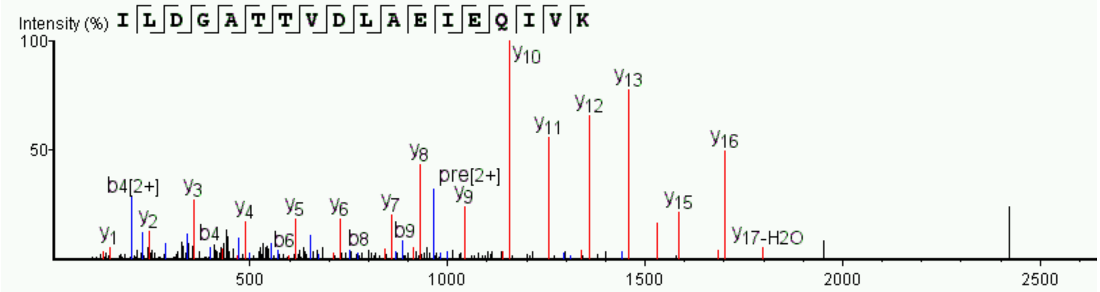

gi|571504006

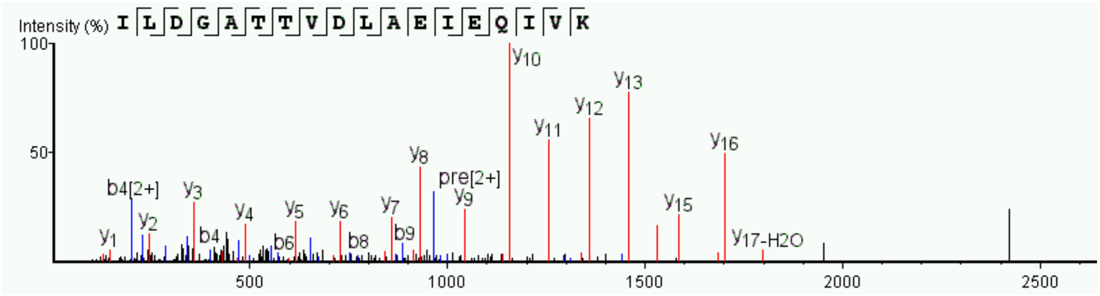

gi|571504009

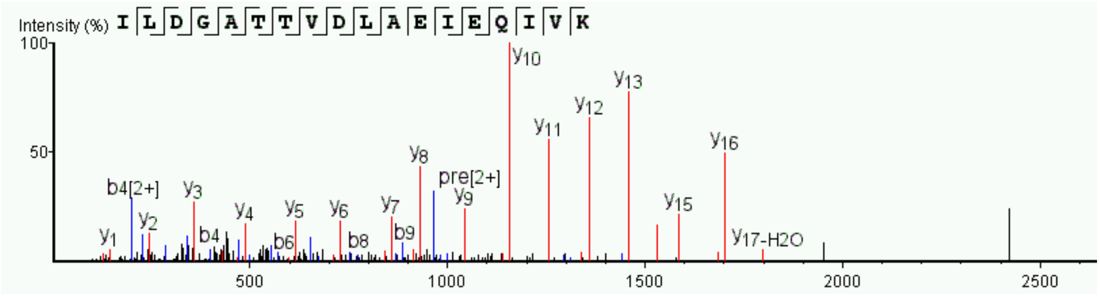

gi|571553455

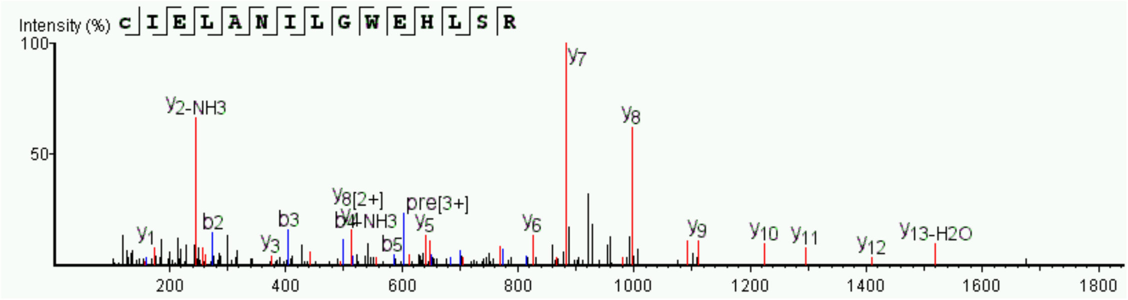

gi|571575589

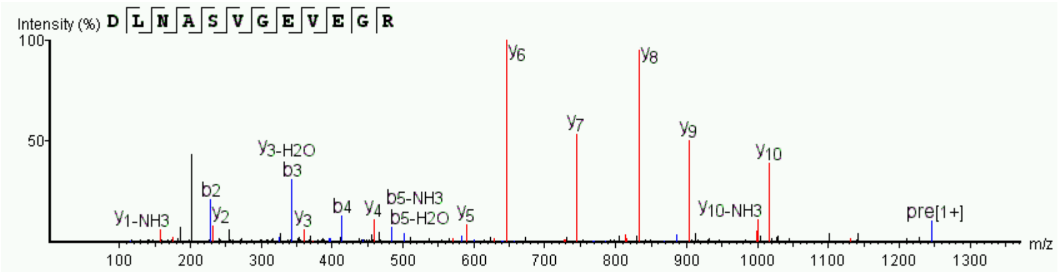

gi|571575591

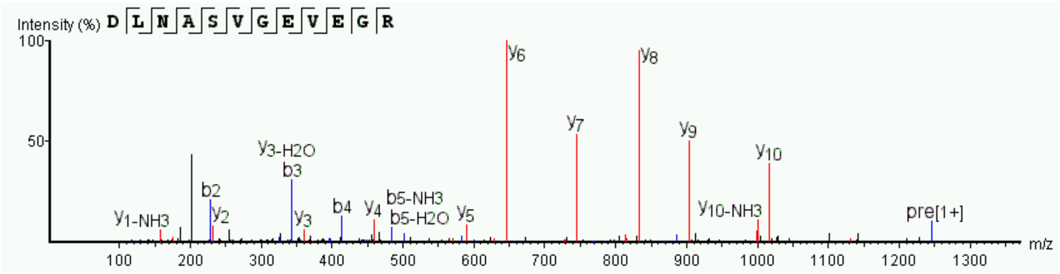

gi|110763879

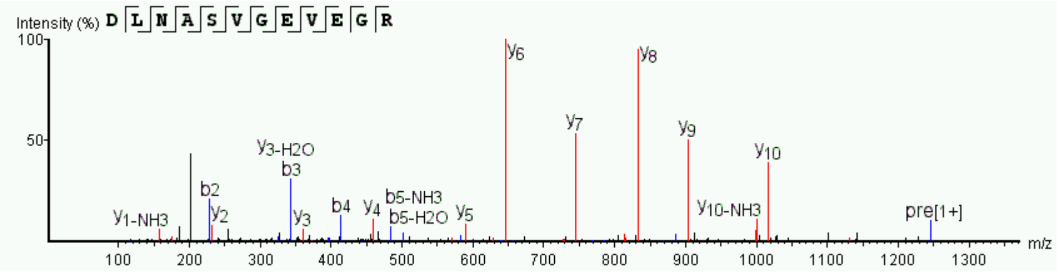

gi|328789361

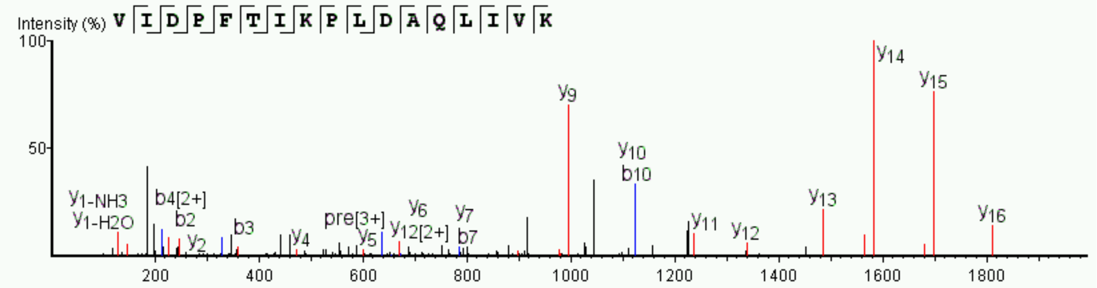

gi|66503776

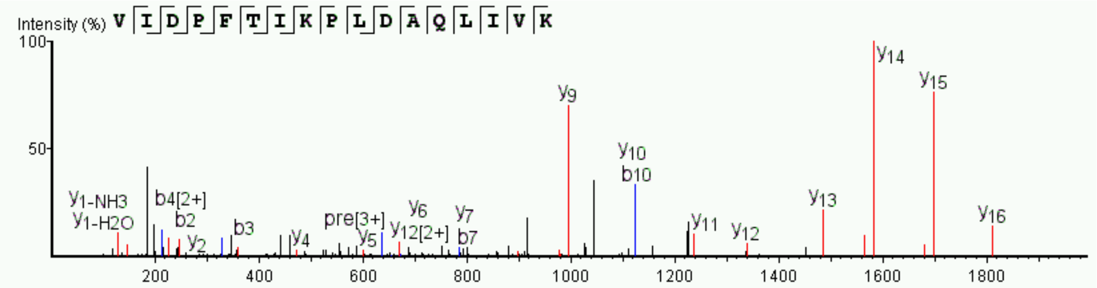

gi|48096617

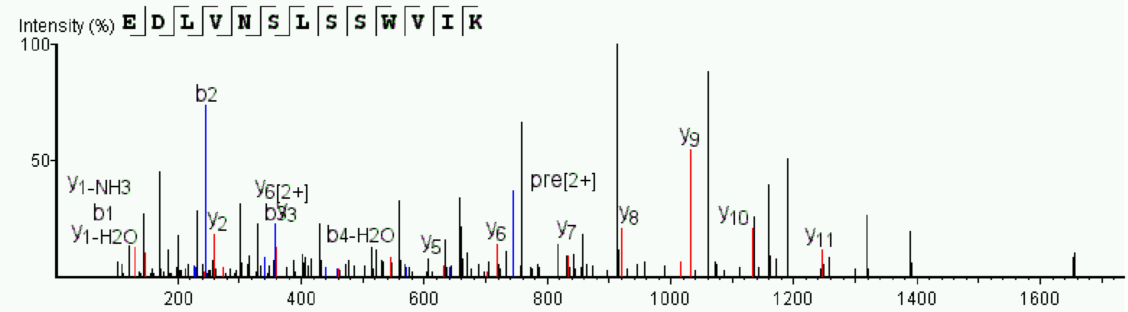

gi|328784195

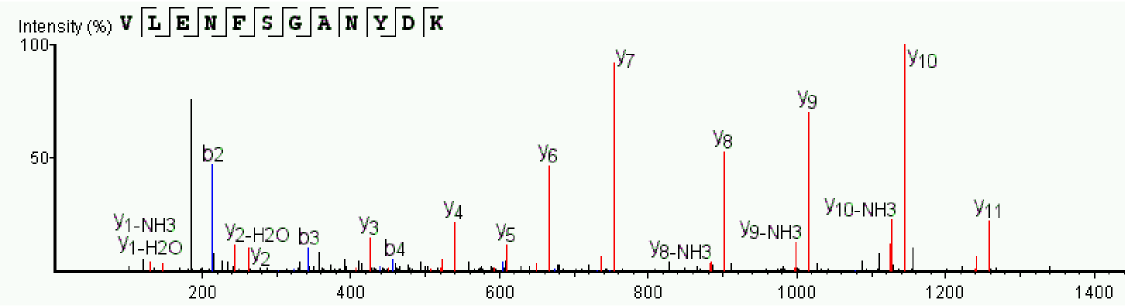

gi|328783026

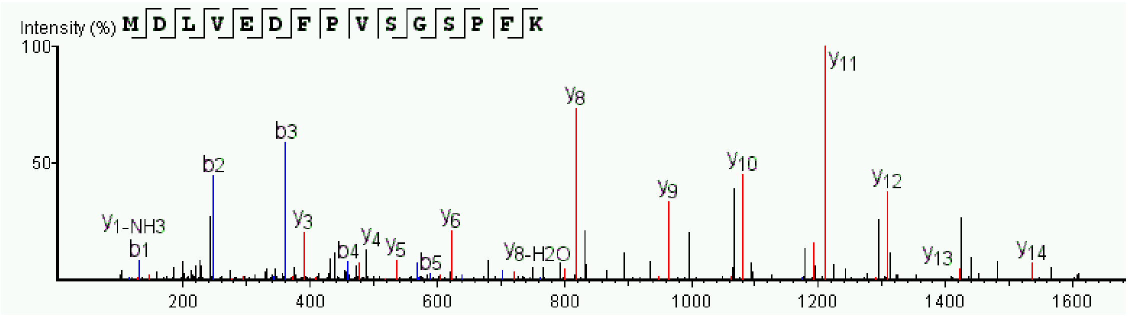

gi|66556835

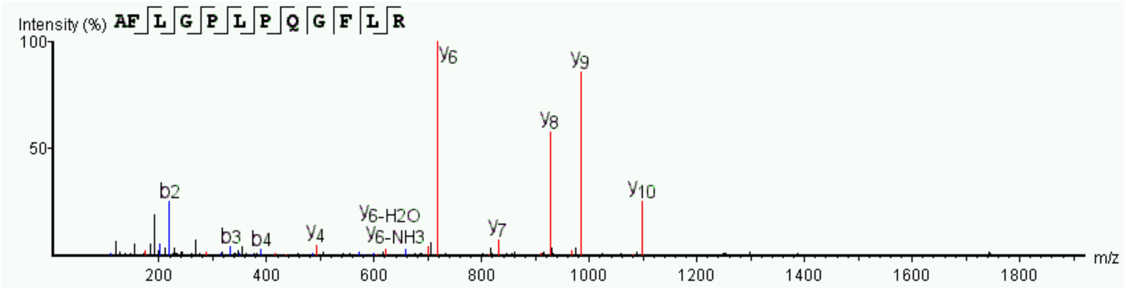

gi|571559277

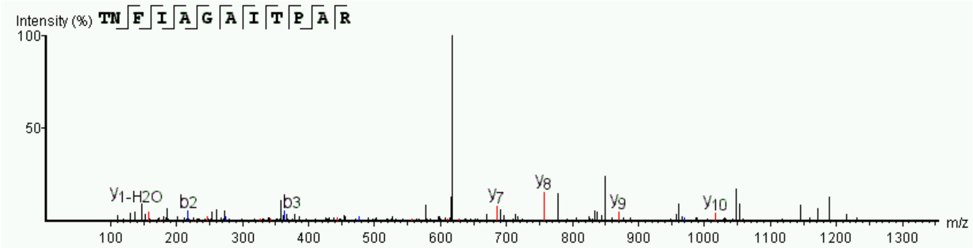

gi|328777308

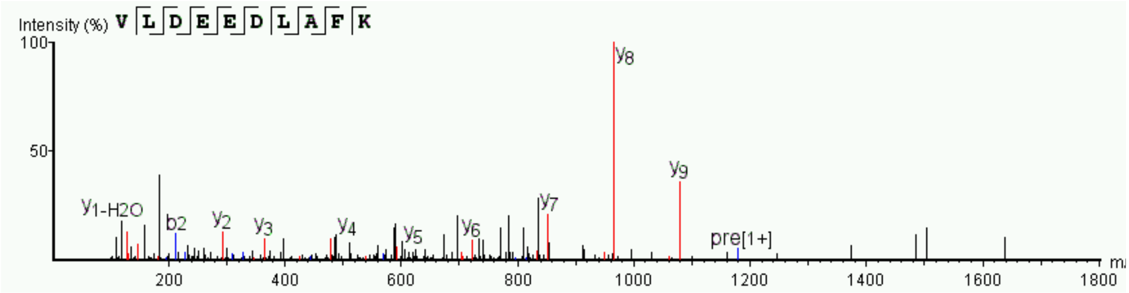

gi|571568741

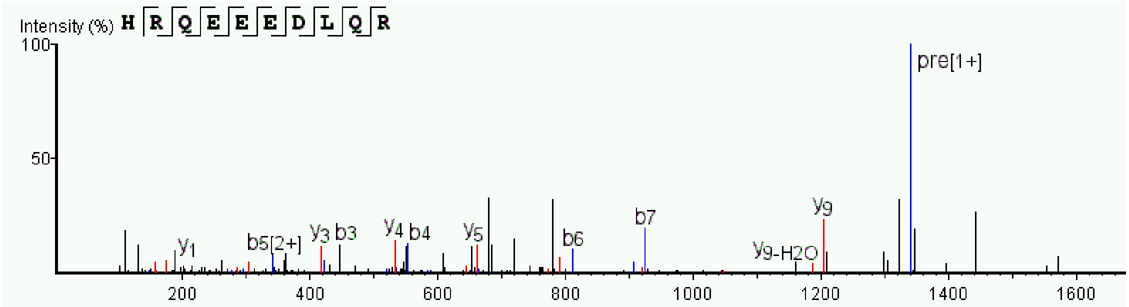

gi|571578492

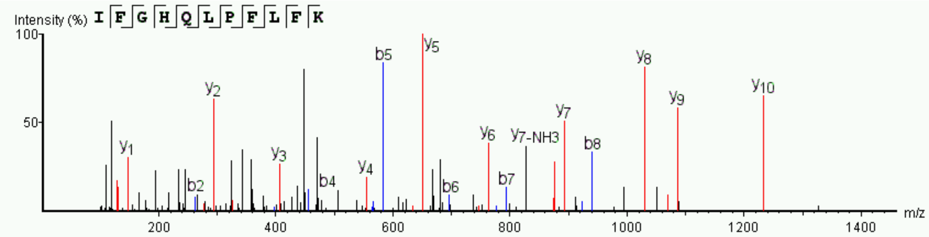

gi|571578495

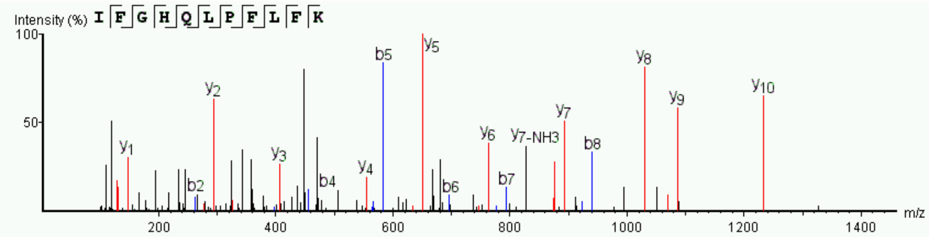

gi|66564896

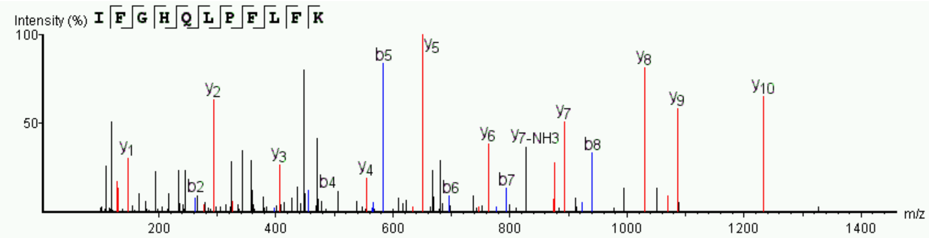

gi|66519946

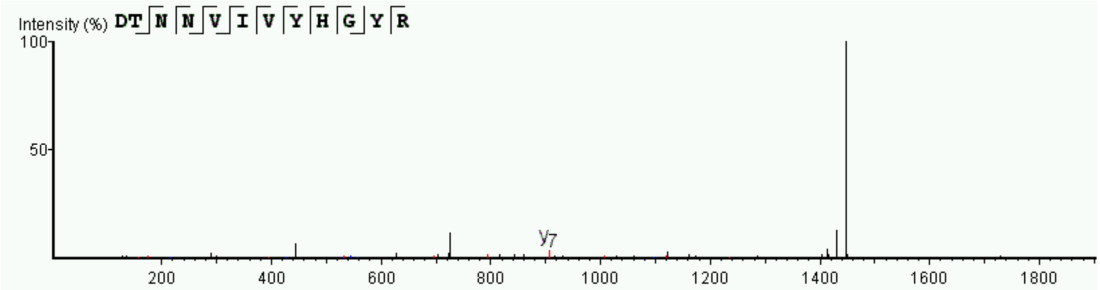

gi|571513874

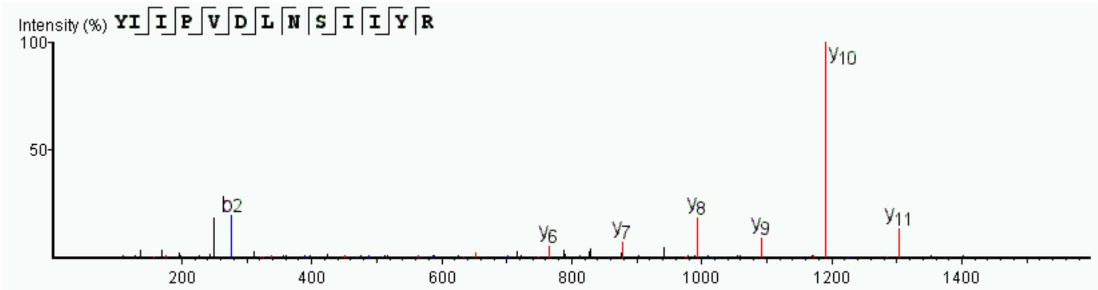

gi|571573604

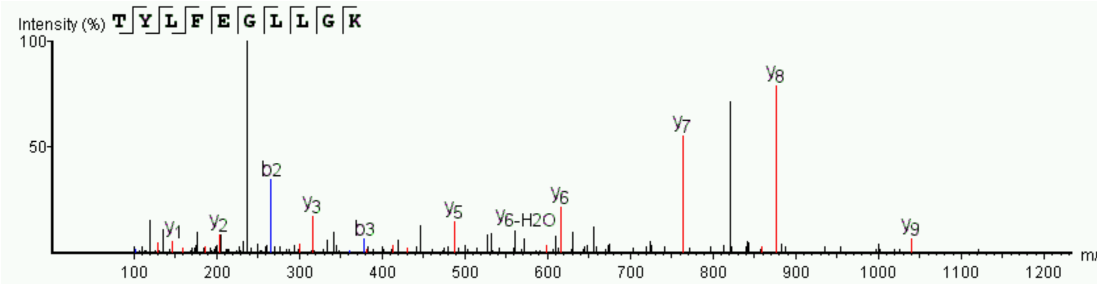

gi|571573606

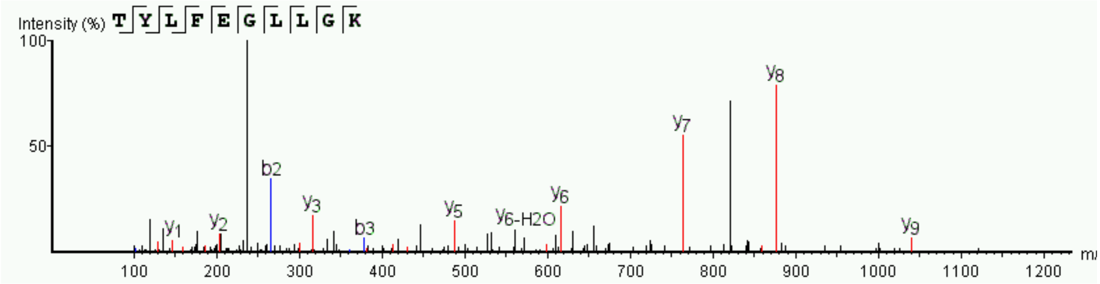

gi|571573608

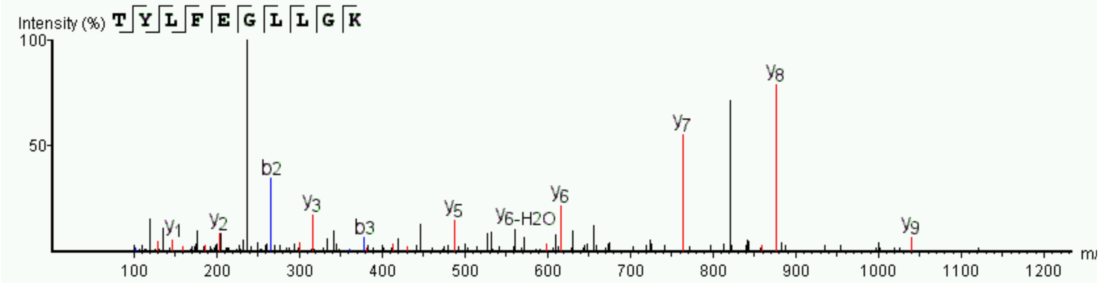

gi|571573610

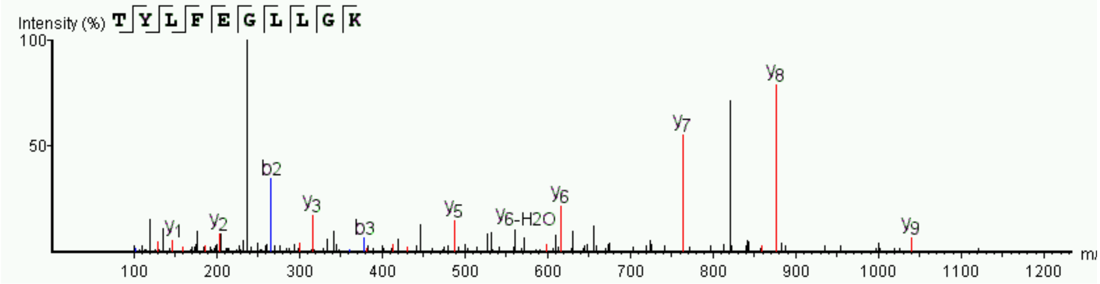

gi|571573612

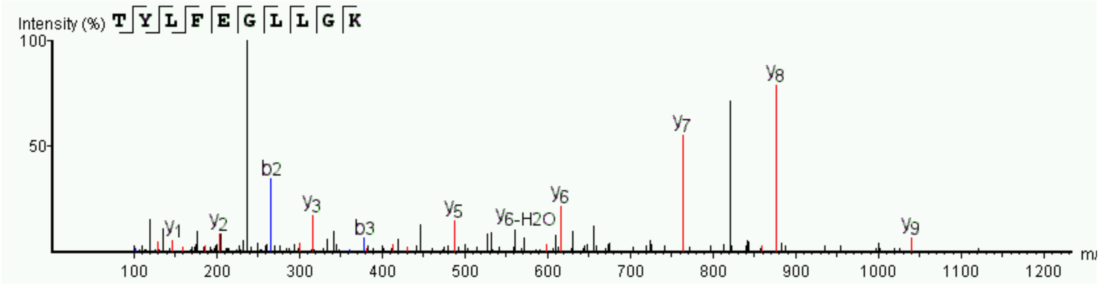

gi|571573614

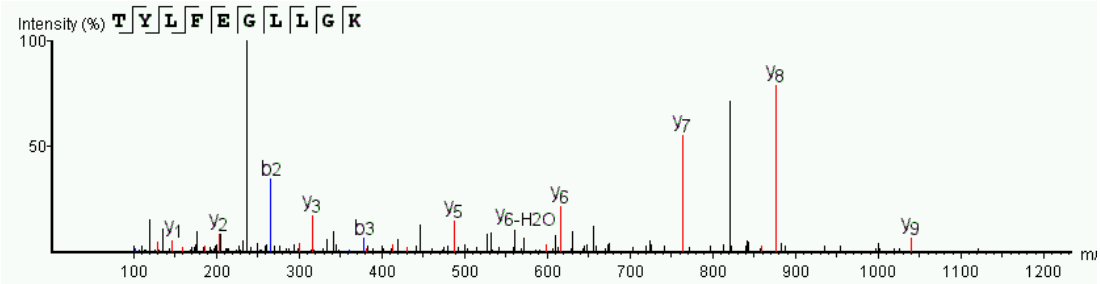

gi|571573616

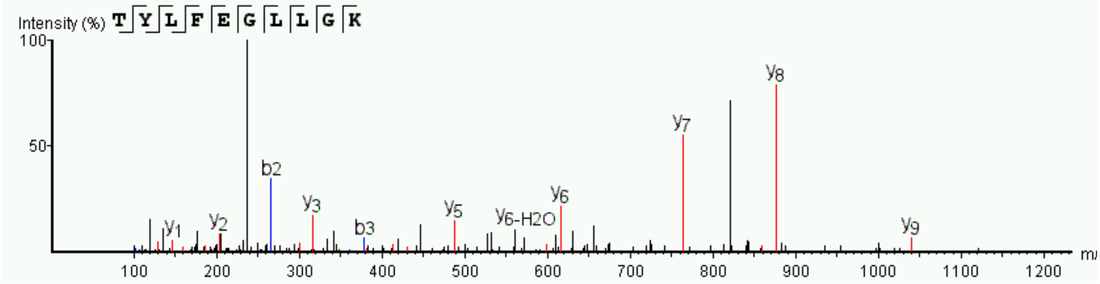

gi|571573618

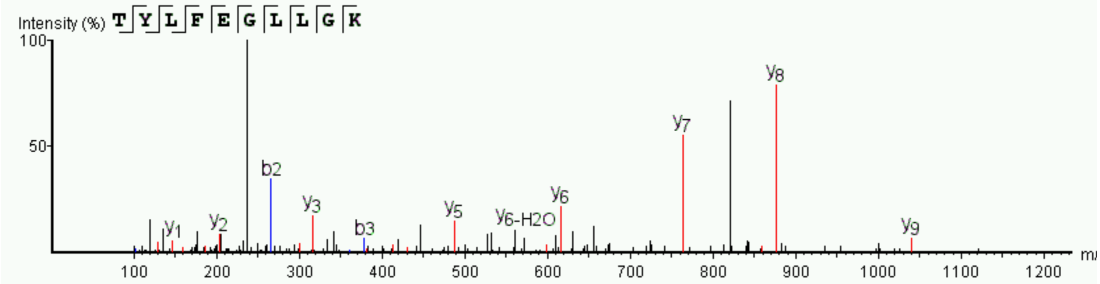

gi|571573620

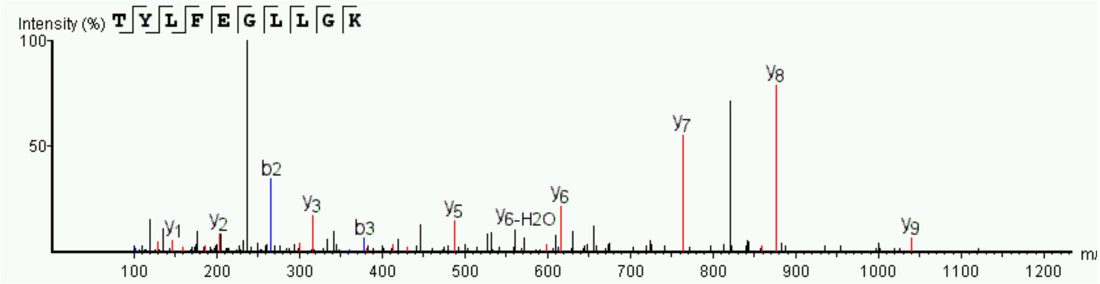

gi|48104536

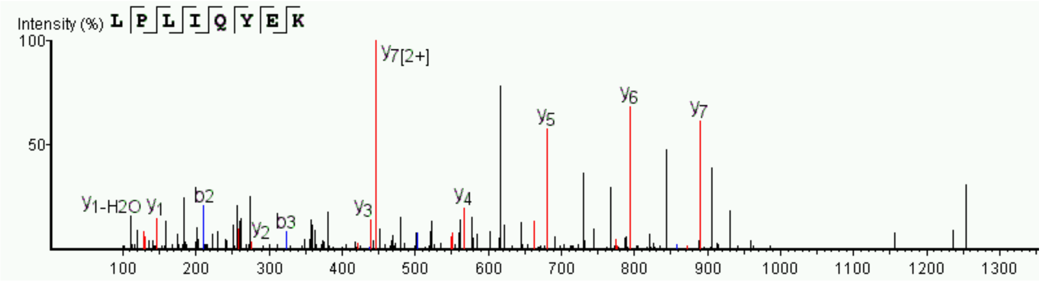

gi|571514786

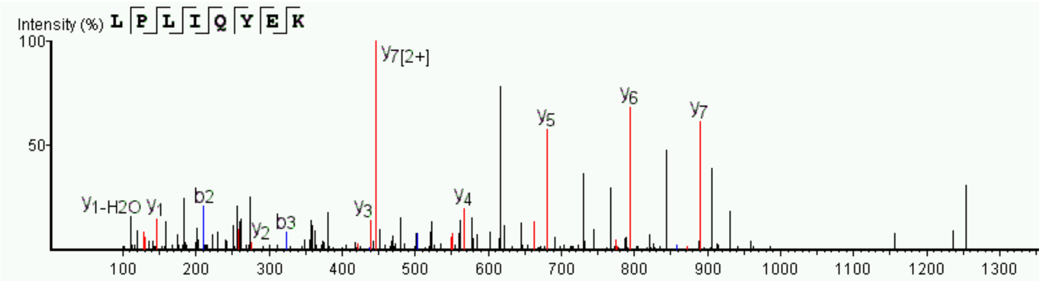

gi|571514793

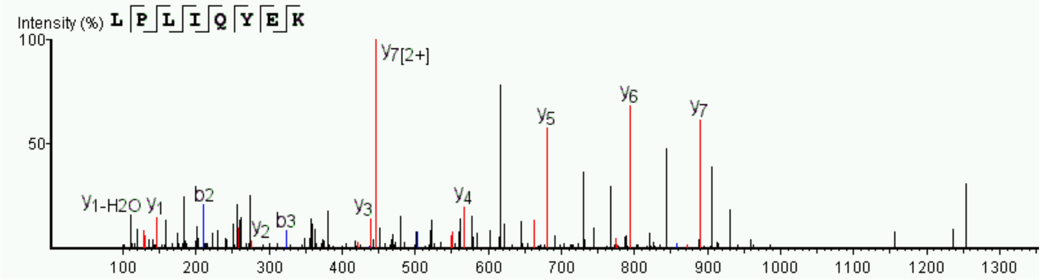

gi|66529961

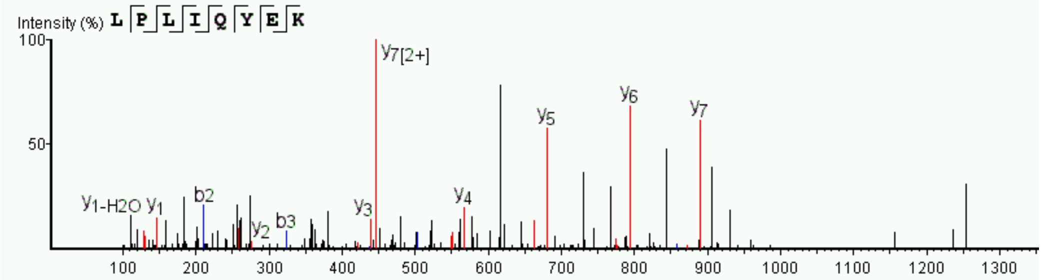

gi|110758628

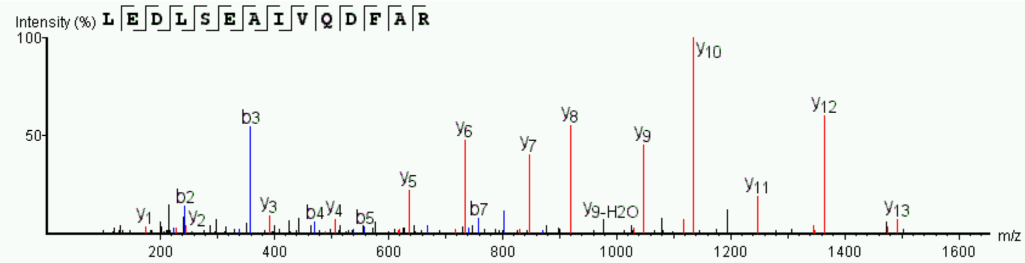

gi|571505799

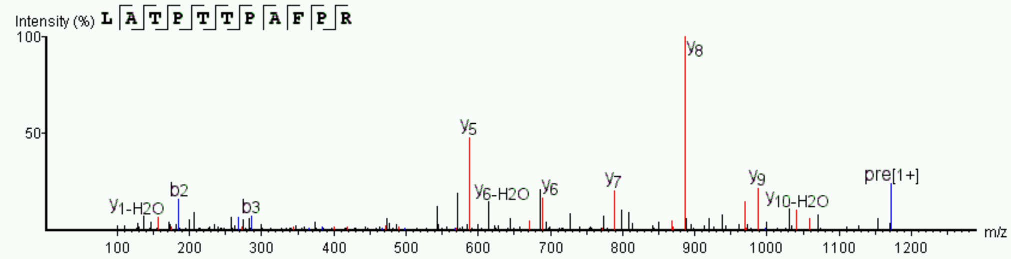

gi|571505835

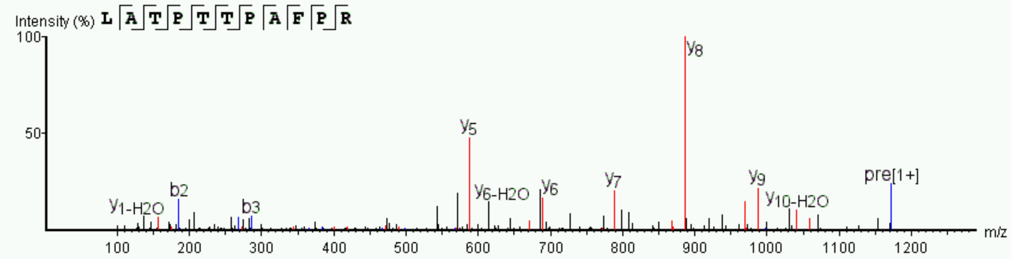

gi|571505843

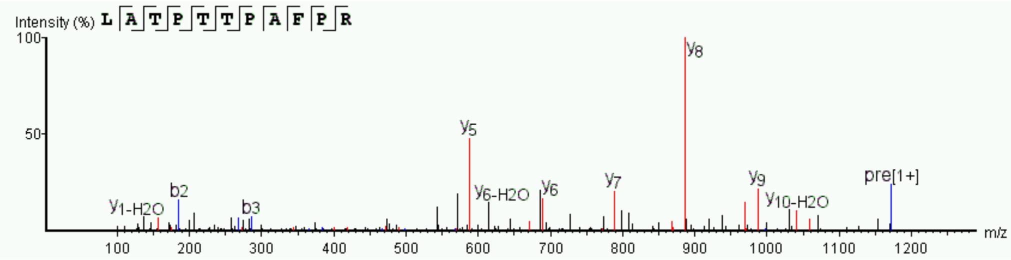

gi|571505846

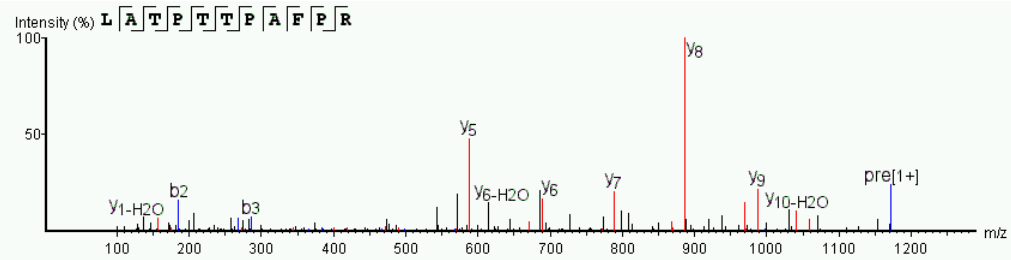

gi|571505865

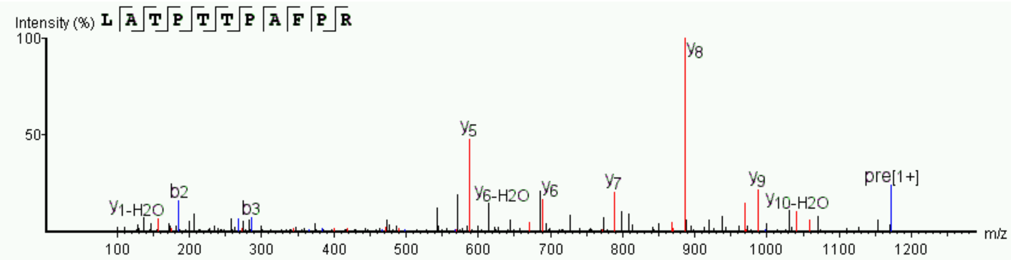

gi|571505803

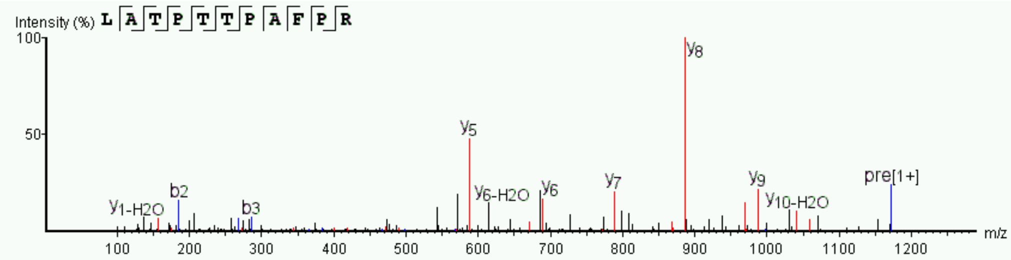

gi|571505811

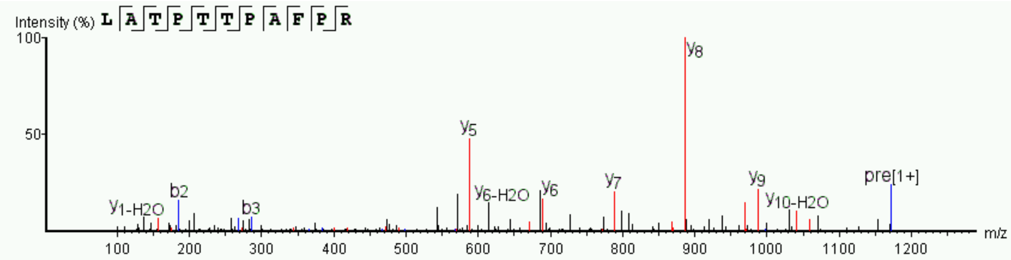

gi|571547459

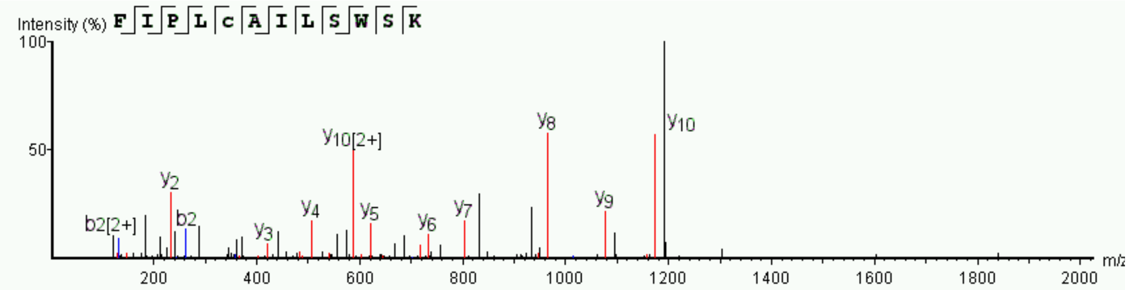

gi|328777174

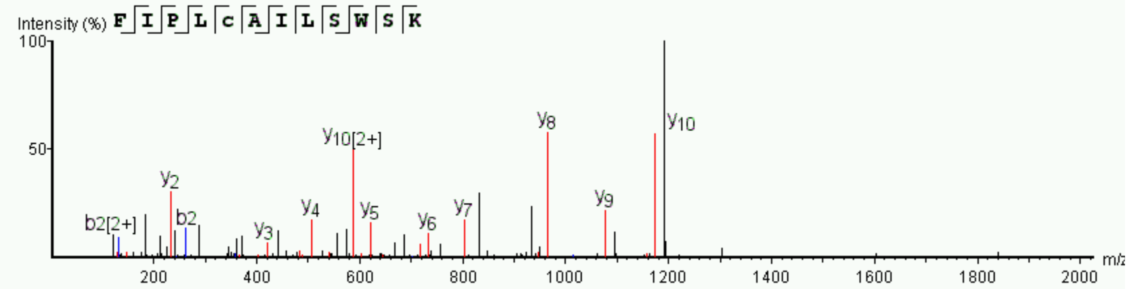

gi|571558657

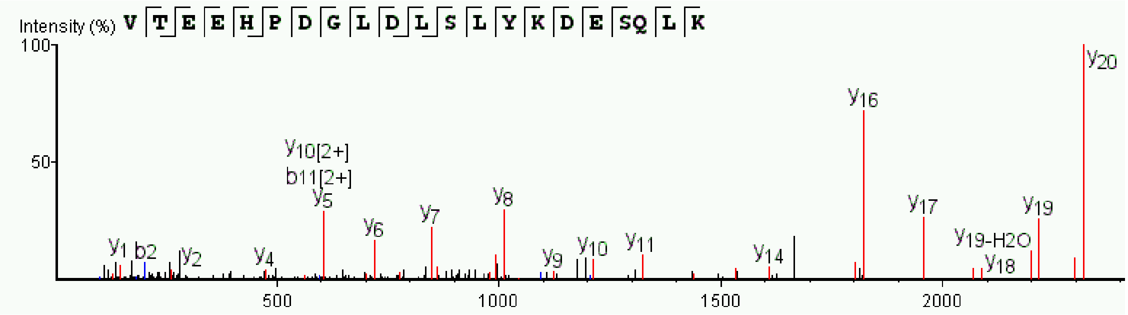

gi|571558660

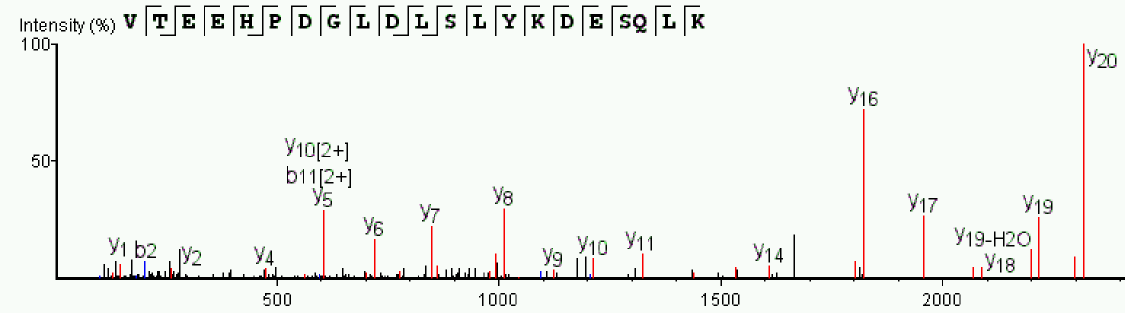

gi|328784509

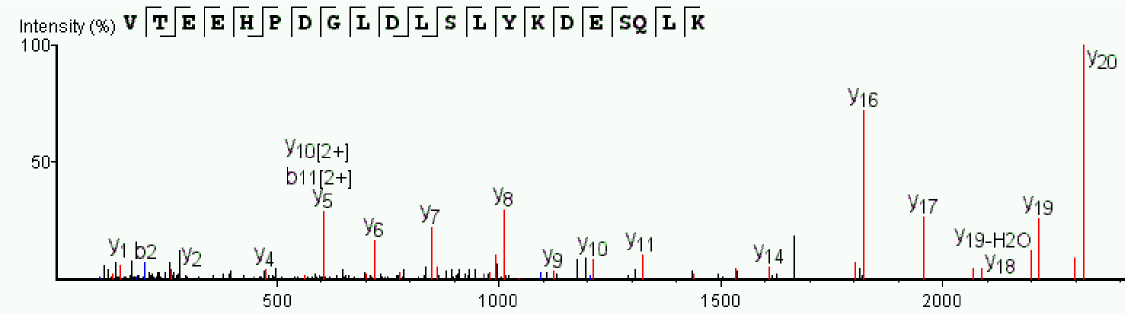

gi|328790379

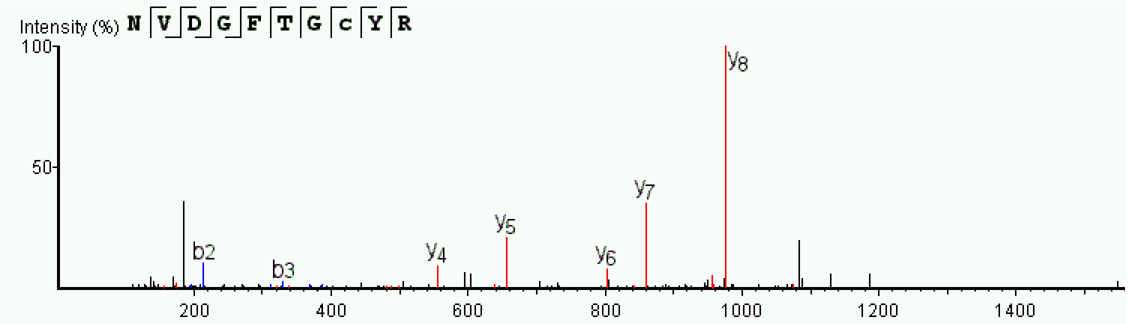

gi|571504099

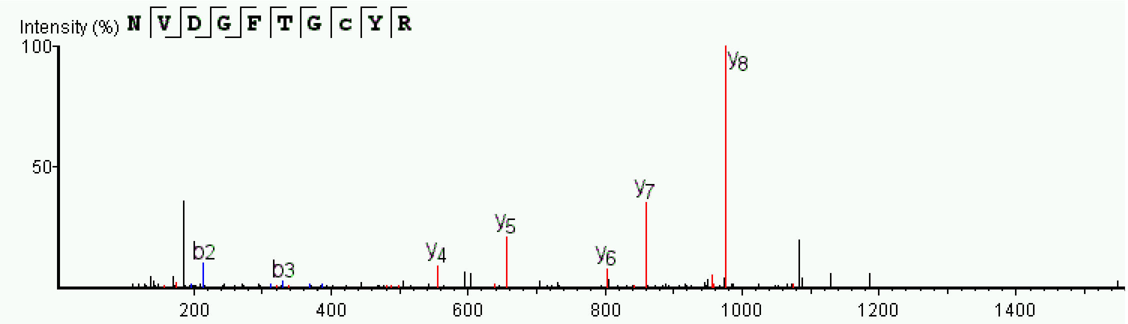

gi|571504104

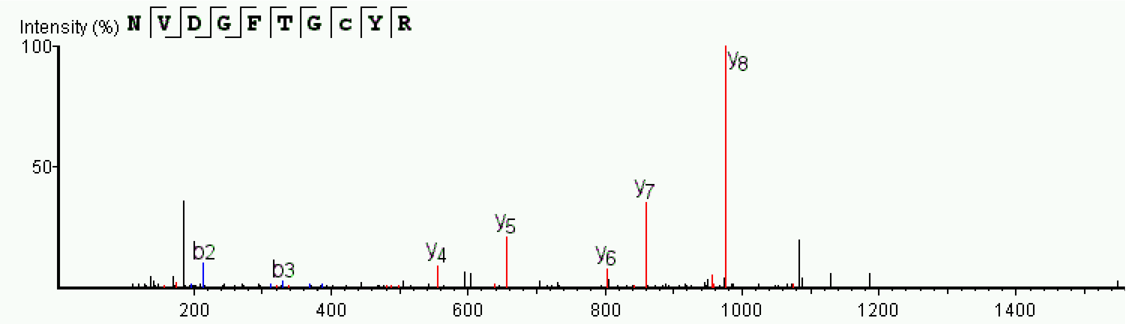

gi|110760068

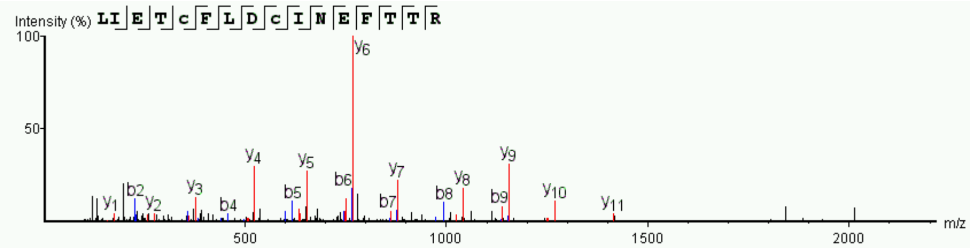

gi|571553163

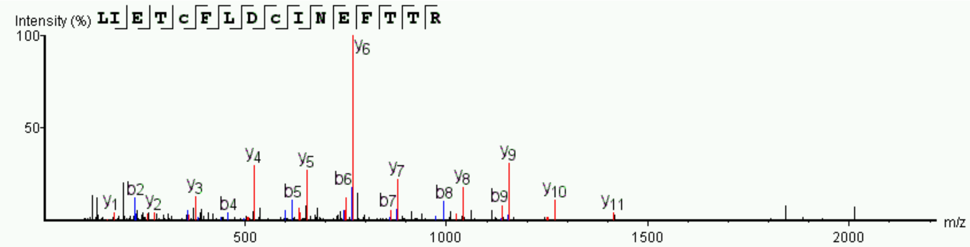

gi|571518510

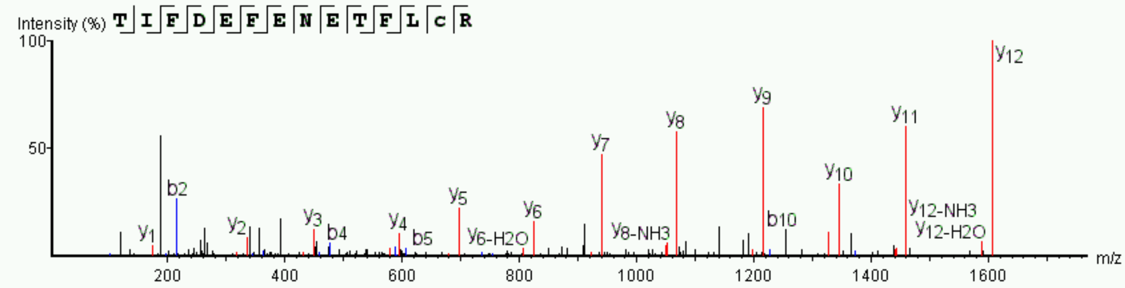

gi|328780159

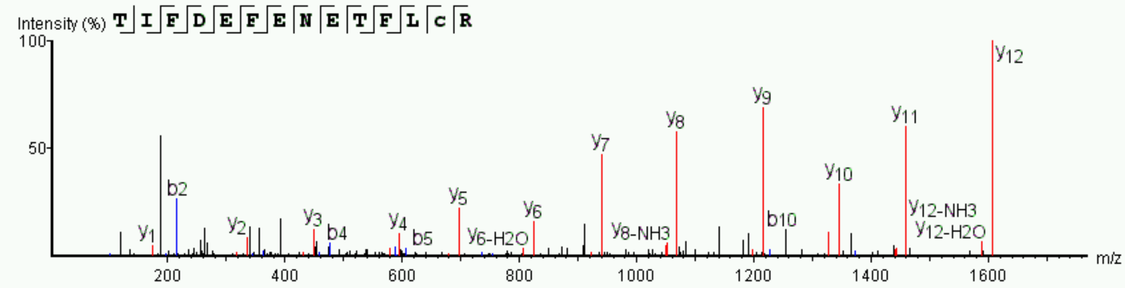

gi|66500091

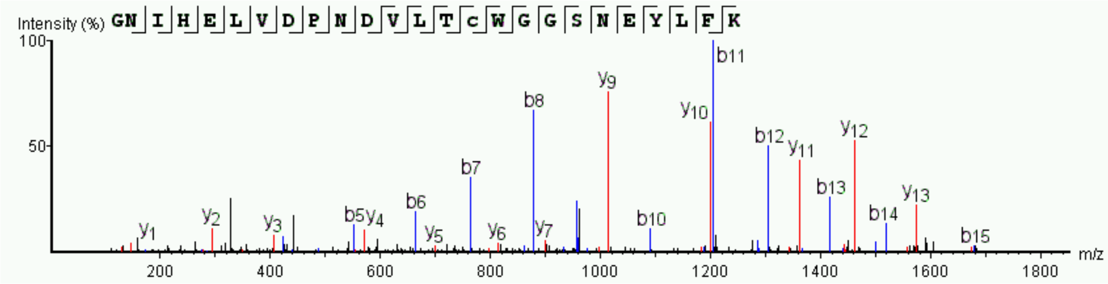

gi|571541013

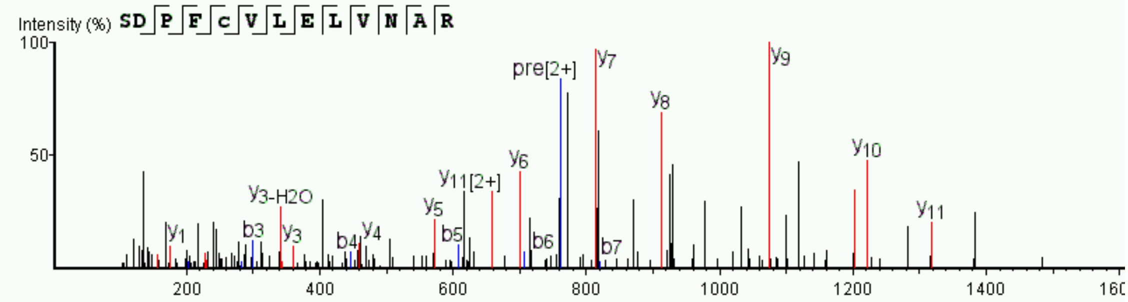

gi|571541015

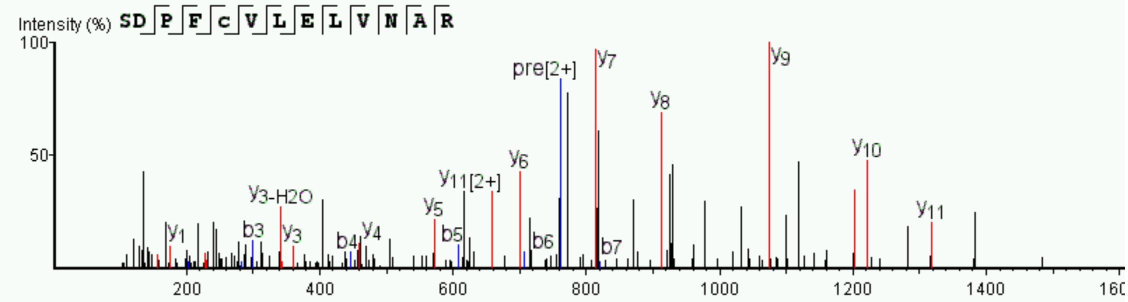

gi|110758517

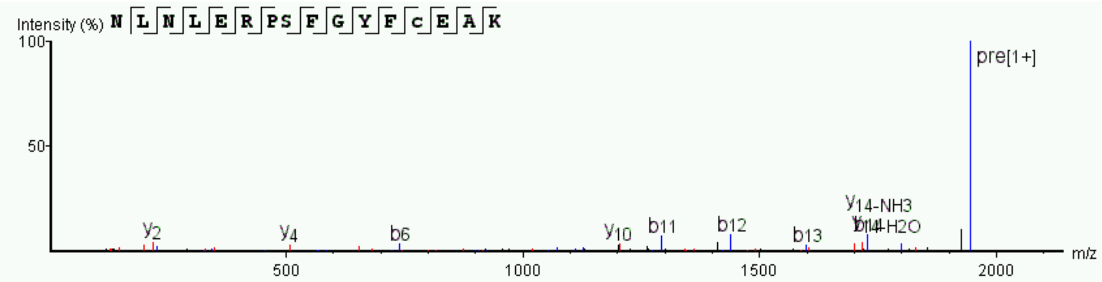

gi|48104864

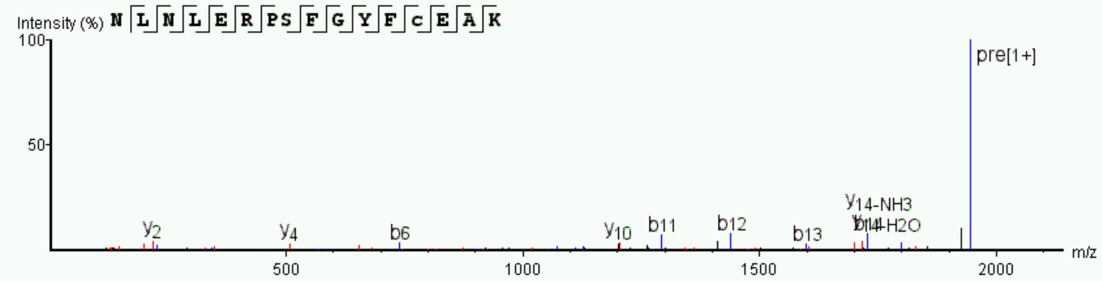

gi|571576518

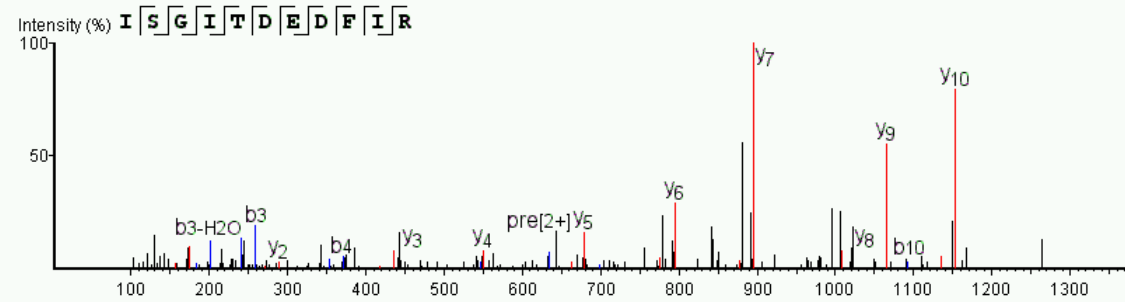

gi|571576520

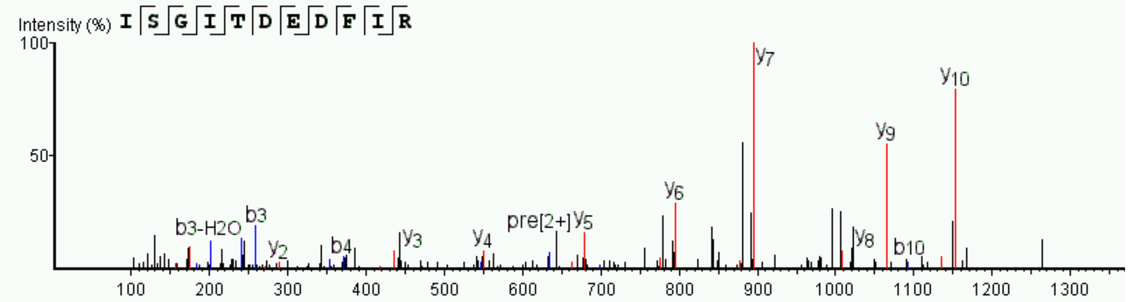

gi|571513674

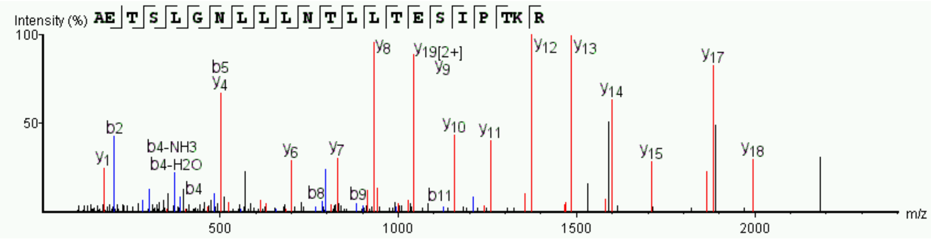

gi|571513678

gi|571513682

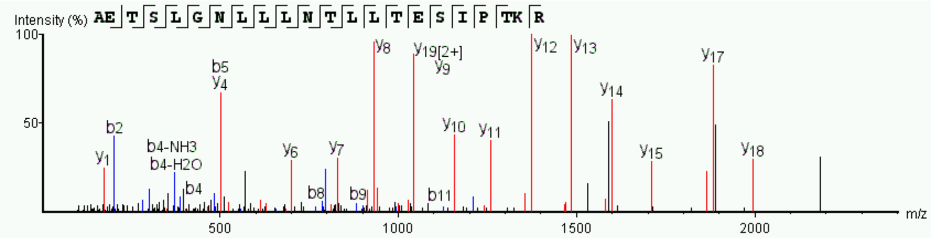

gi|571553257

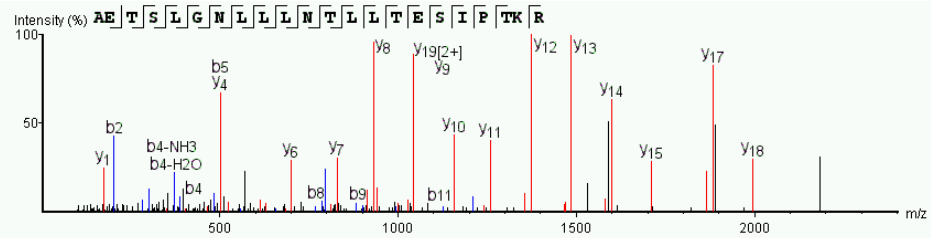

gi|48140497

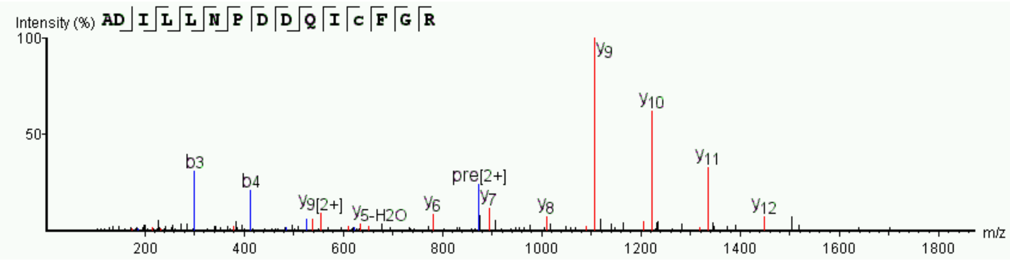

gi|571528435

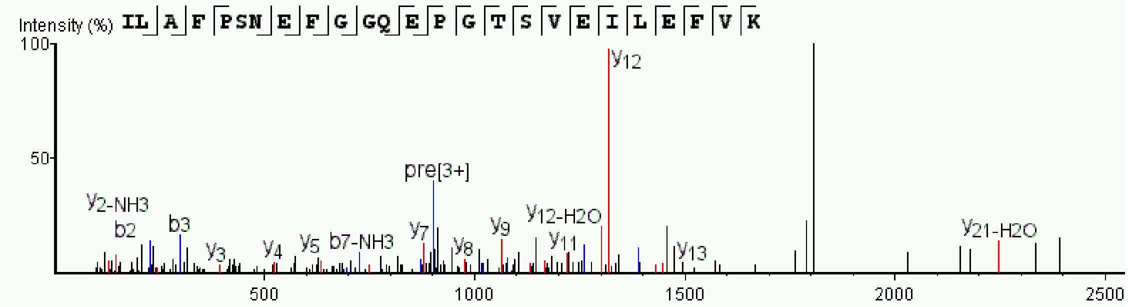

gi|328784953

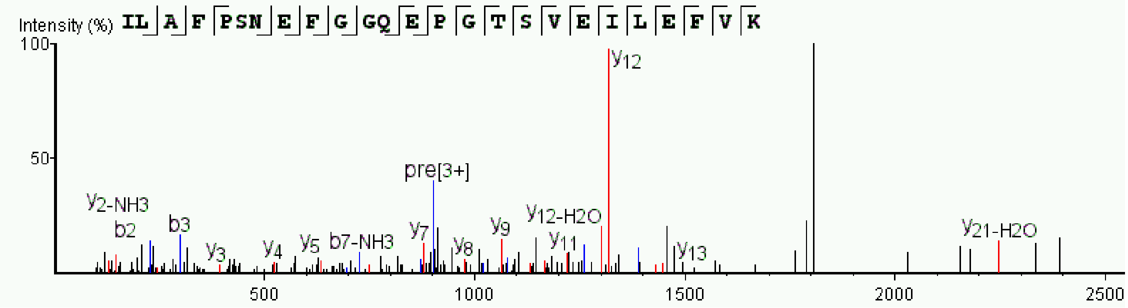

gi|571502210

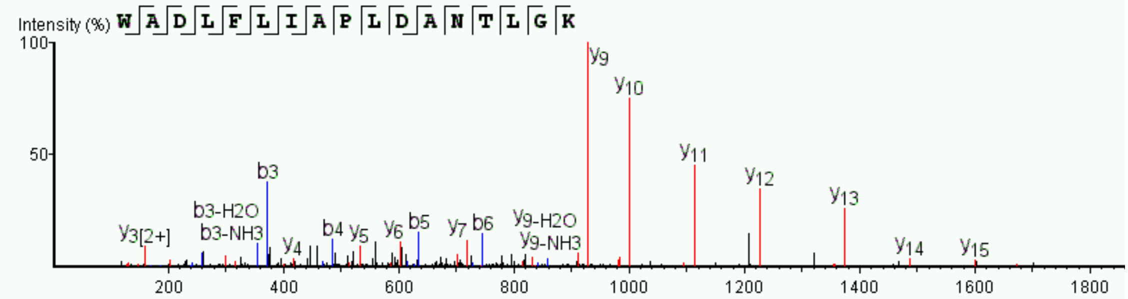

gi|48103807

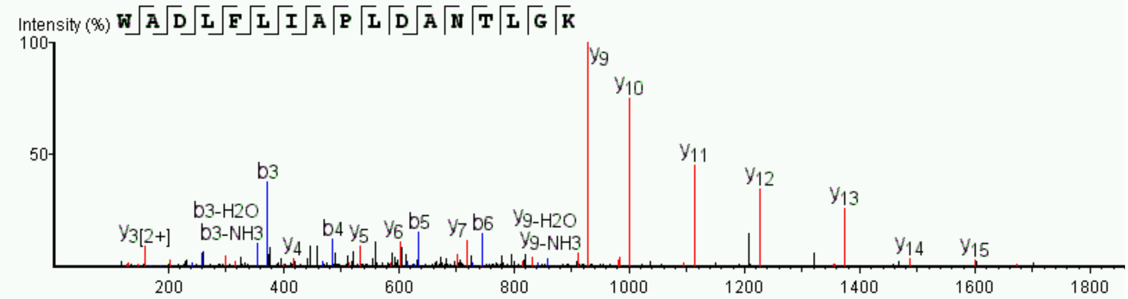

gi|571547176

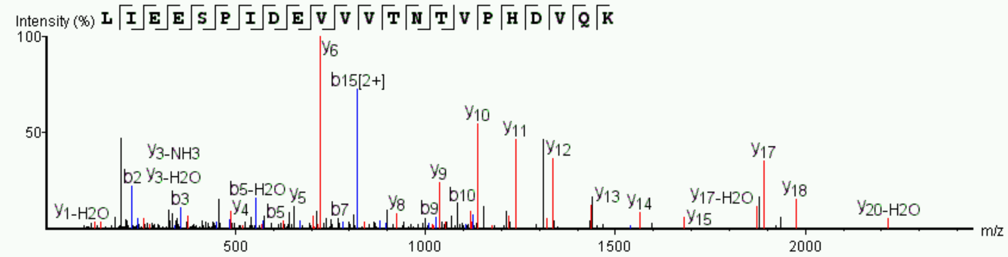

gi|66561731

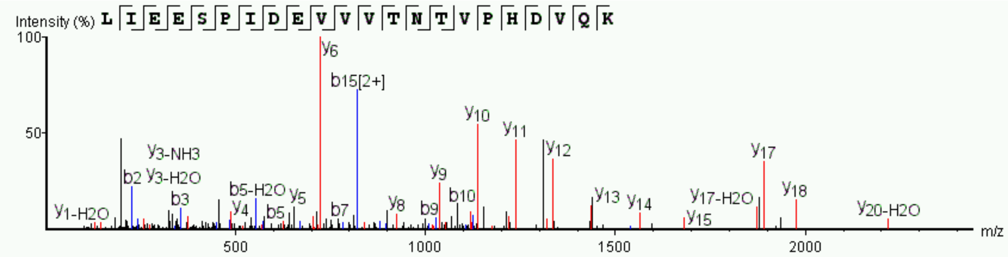

gi|571523743

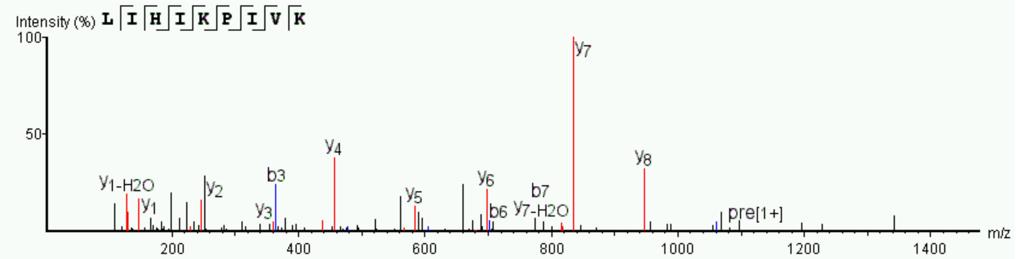

gi|571523747

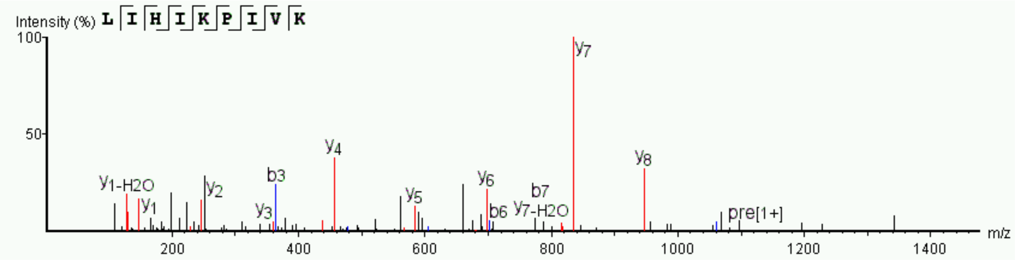

gi|328782499

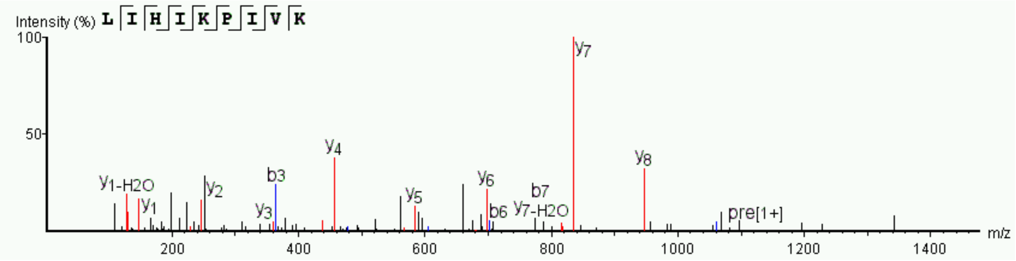

gi|571575531

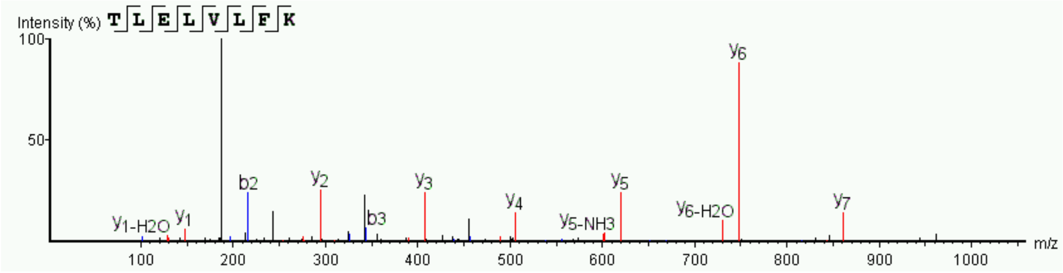

gi|571575529

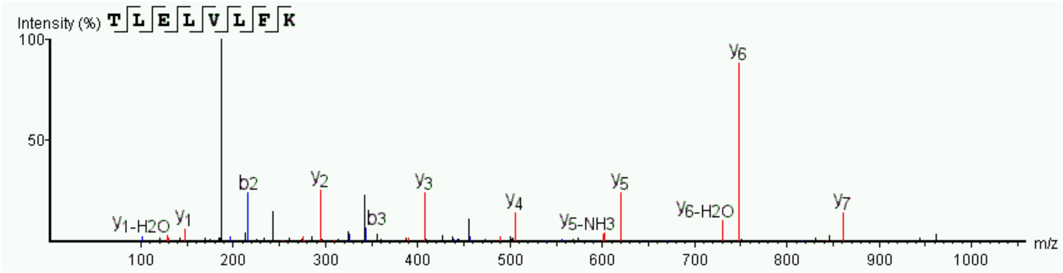

gi|571552029

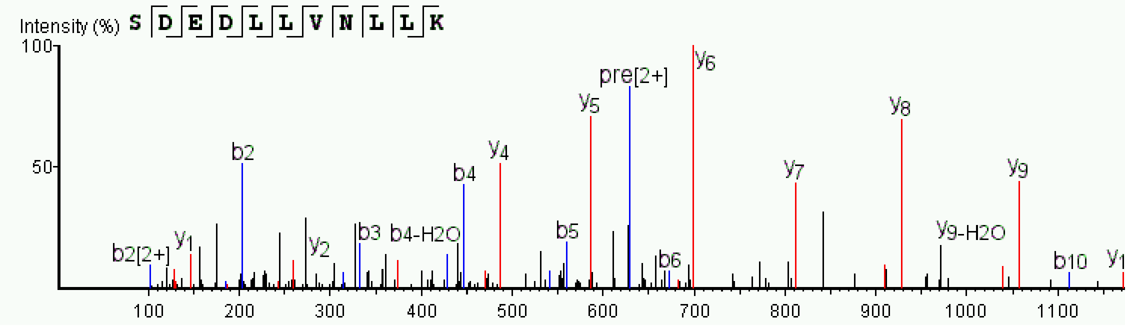

gi|66518140

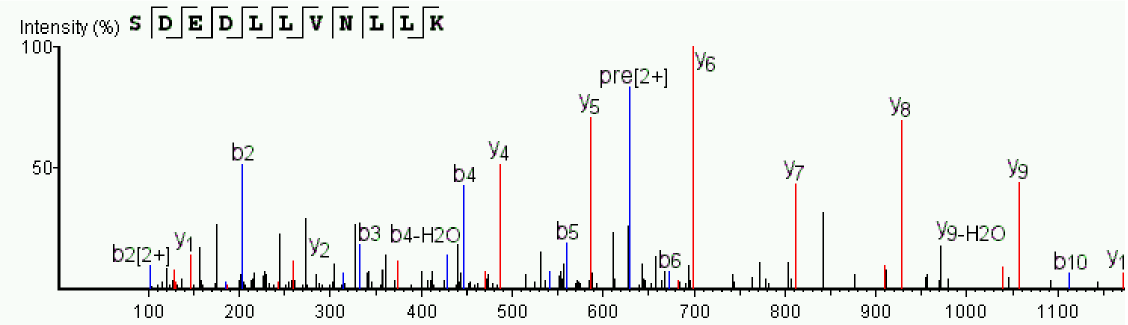

gi|66534585

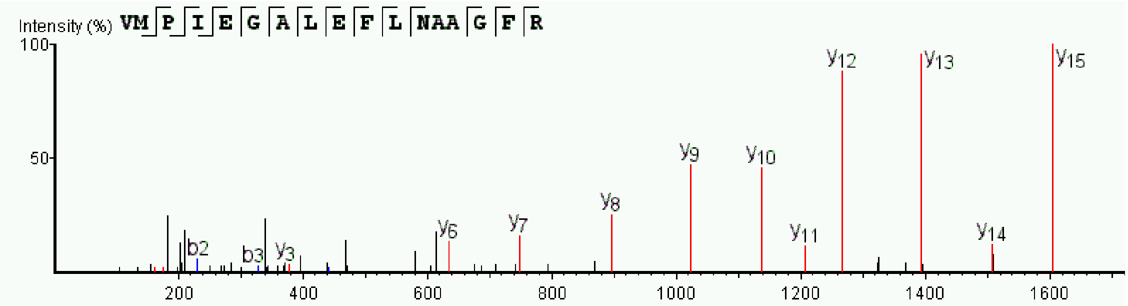

gi|571519324

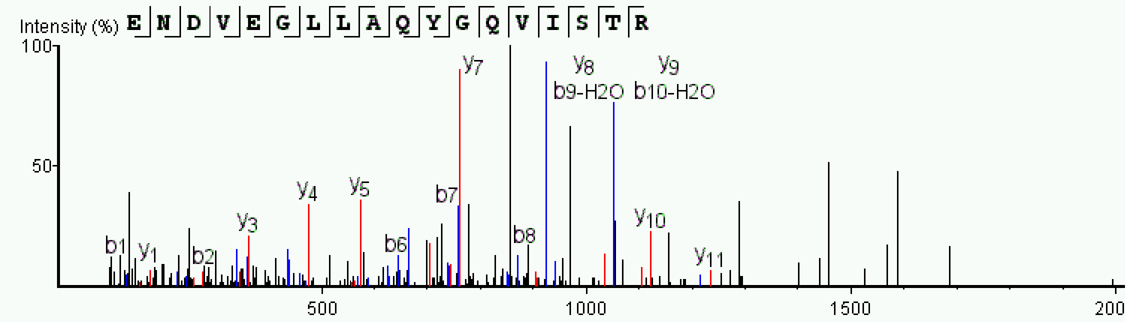

gi|571519356

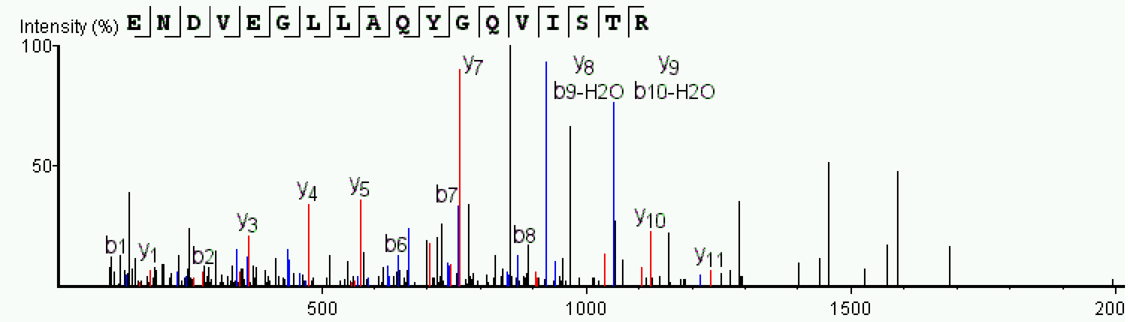

gi|571519360

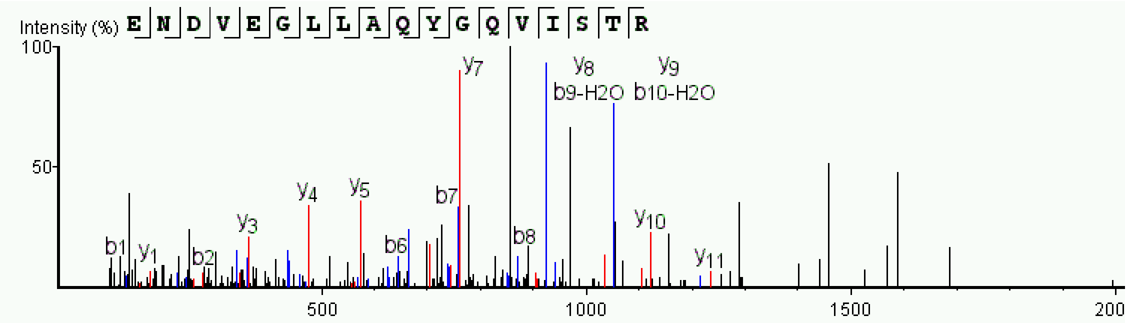

gi|571519364

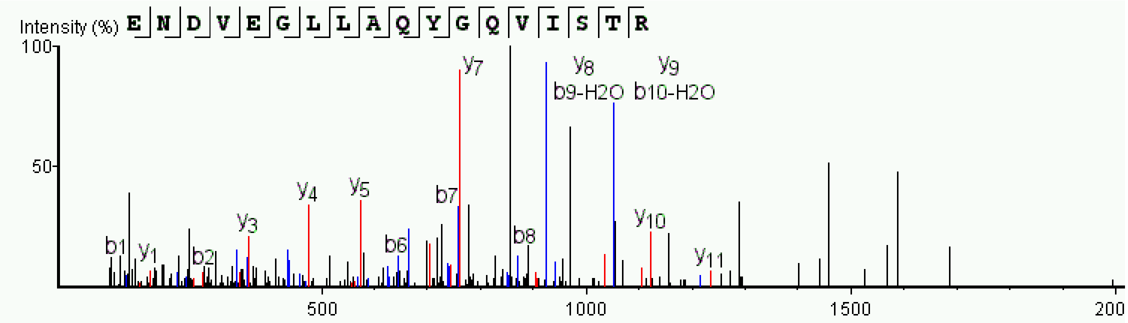

gi|571519327

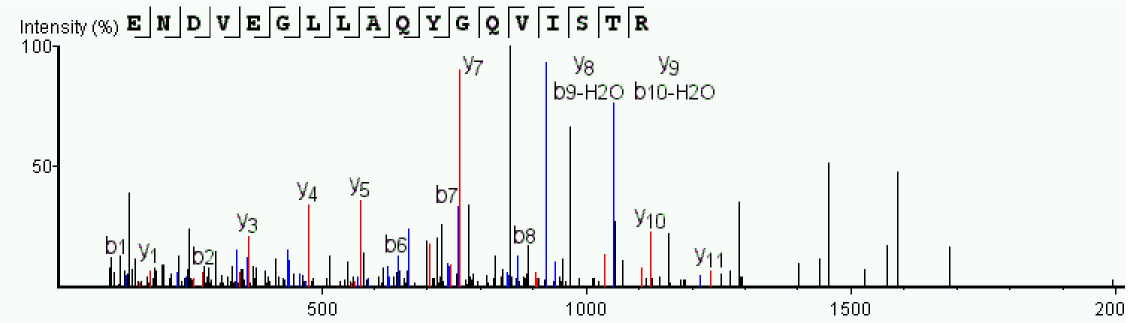

gi|571519331

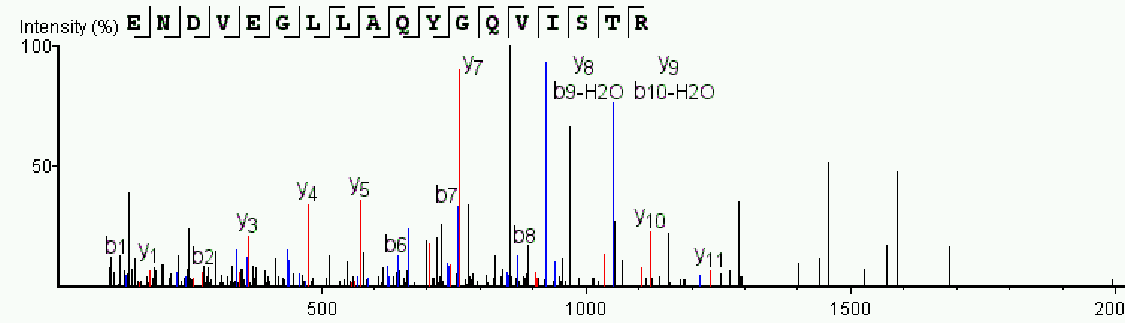

gi|571519334

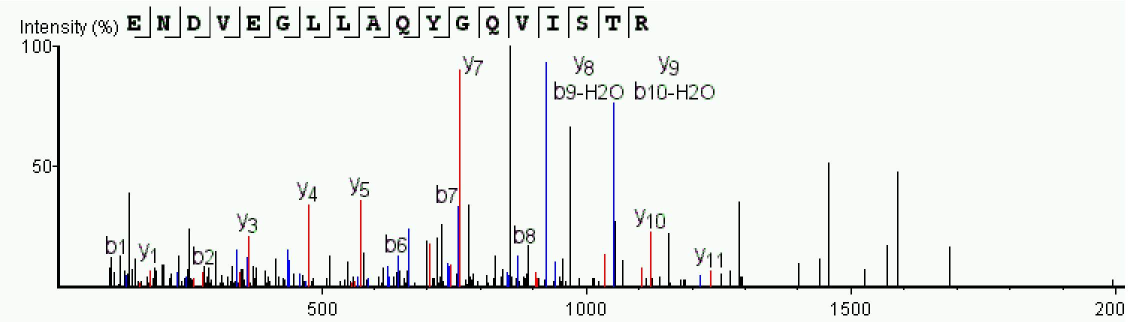

gi|571519338

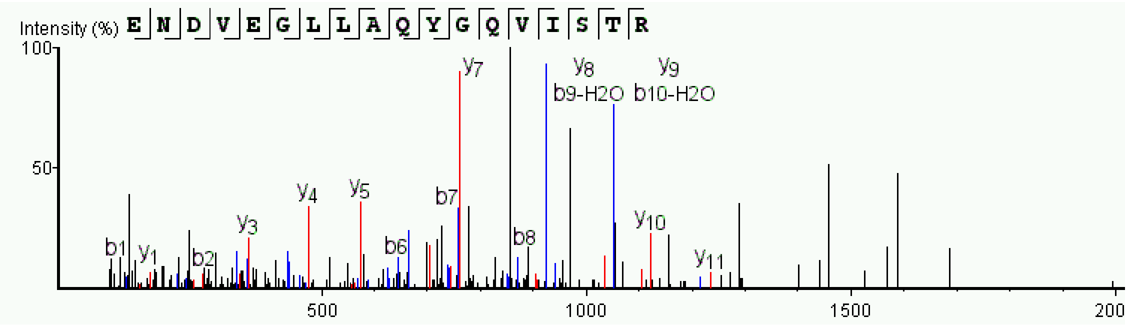

gi|571519342

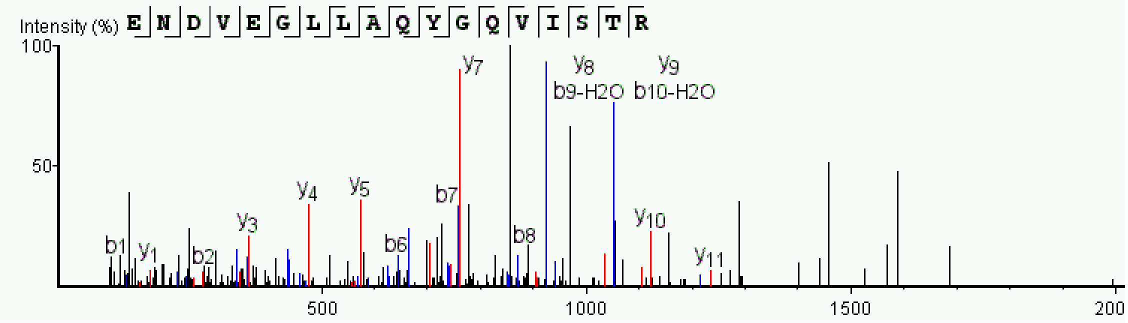

gi|571519345

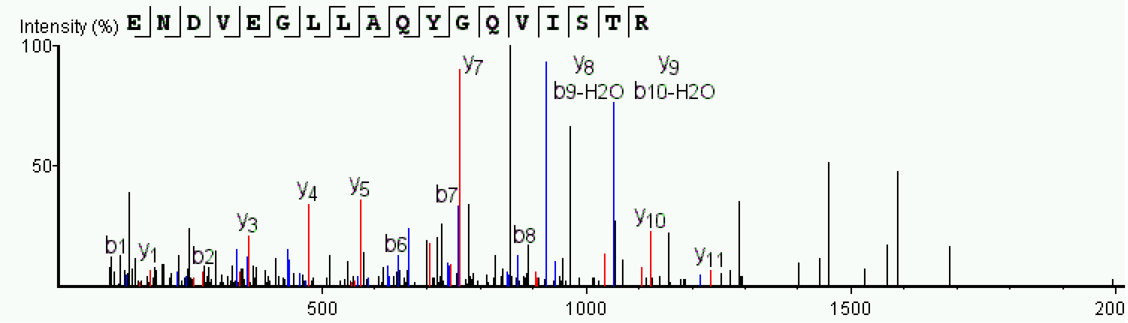

gi|571519348

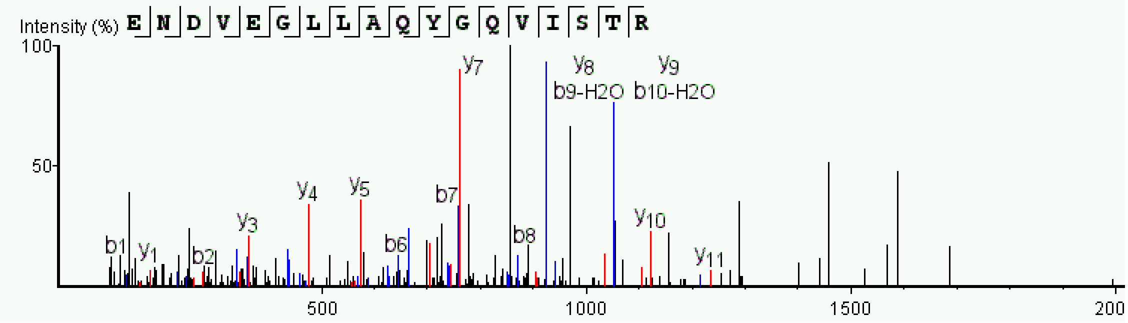

gi|571519352

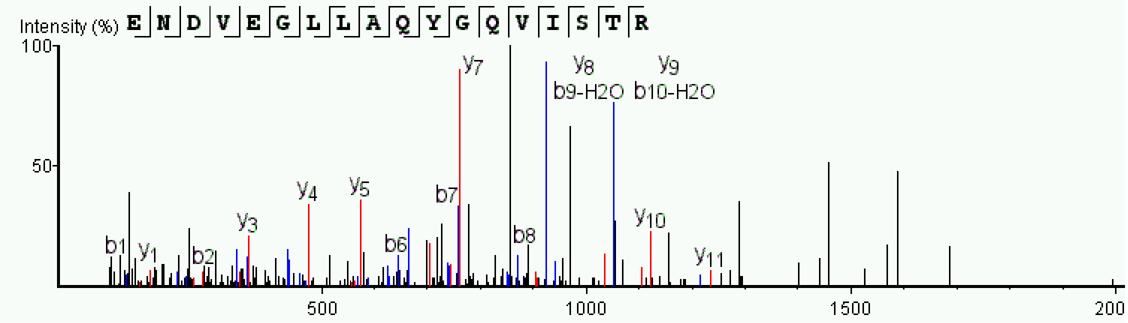

gi|66561608

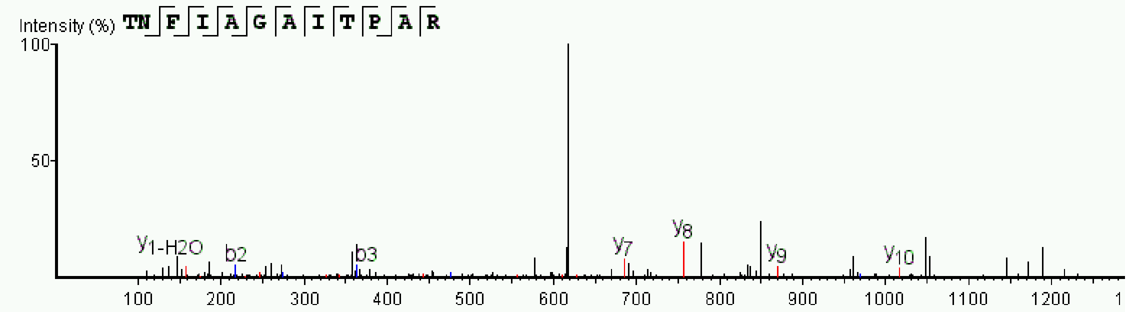

gi|571532722

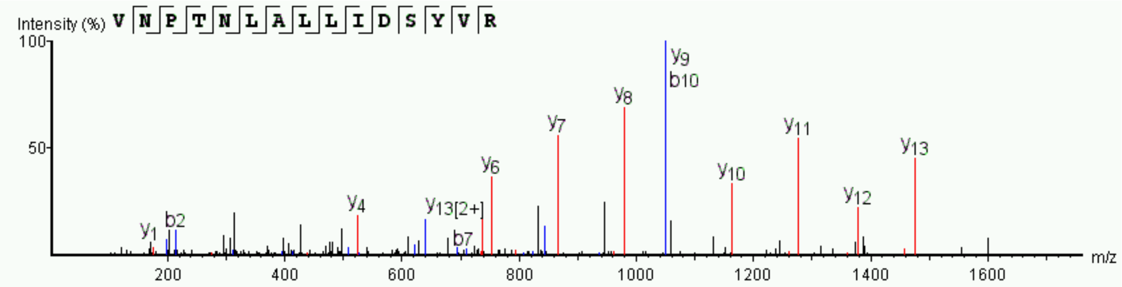

gi|571532725

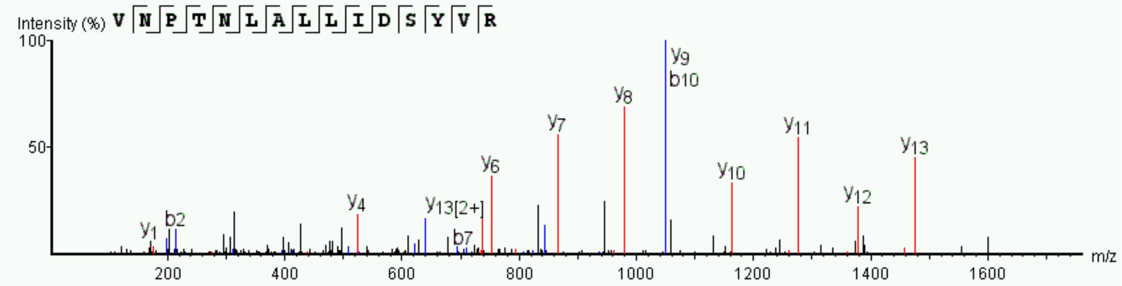

gi|571532729

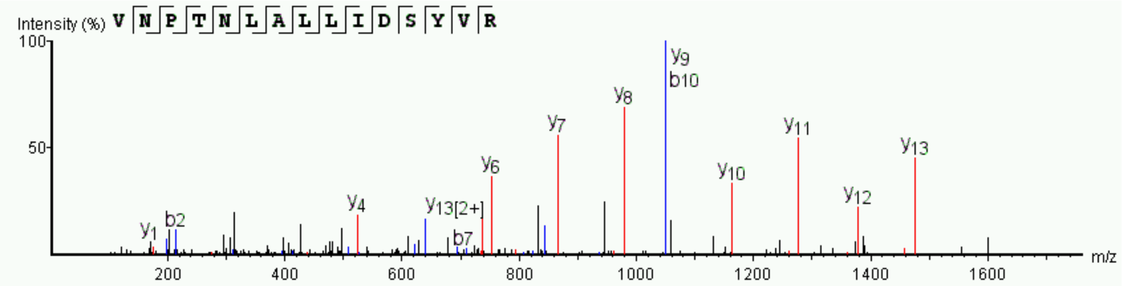

gi|571532734

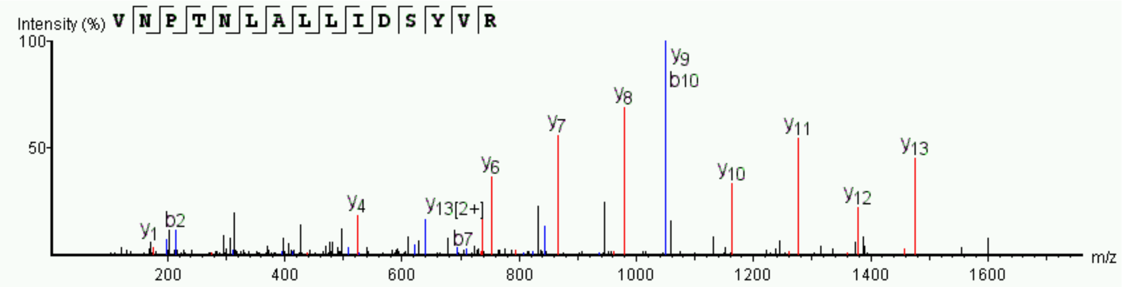

gi|571532738

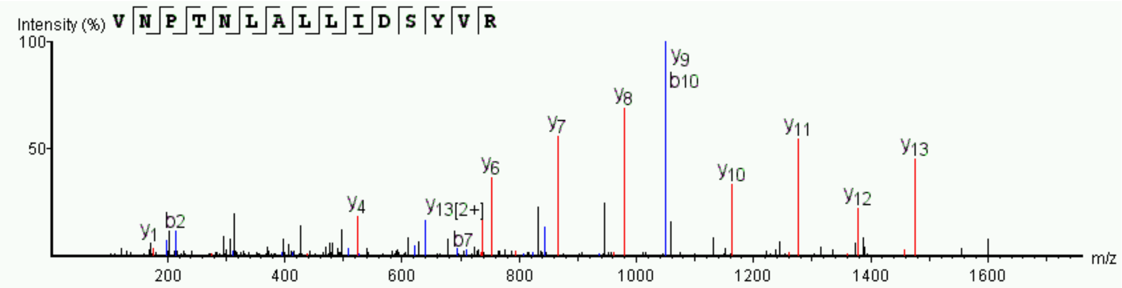

gi|571532741

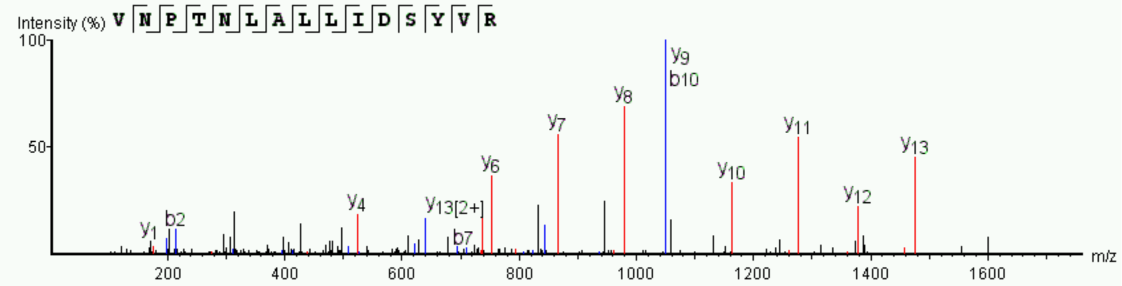

gi|328779207

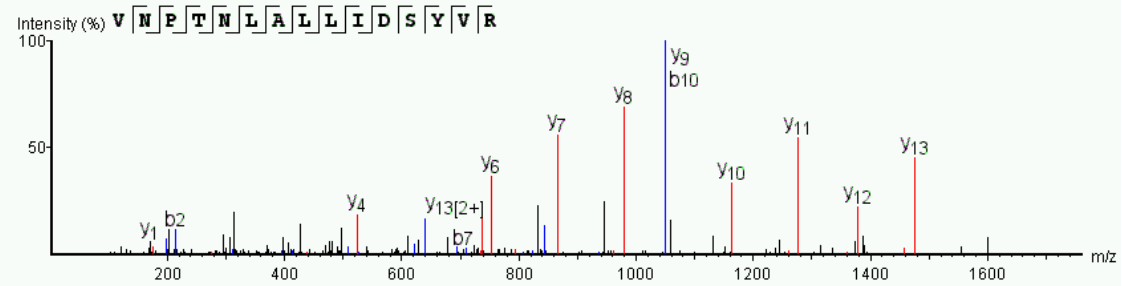

gi|571574486

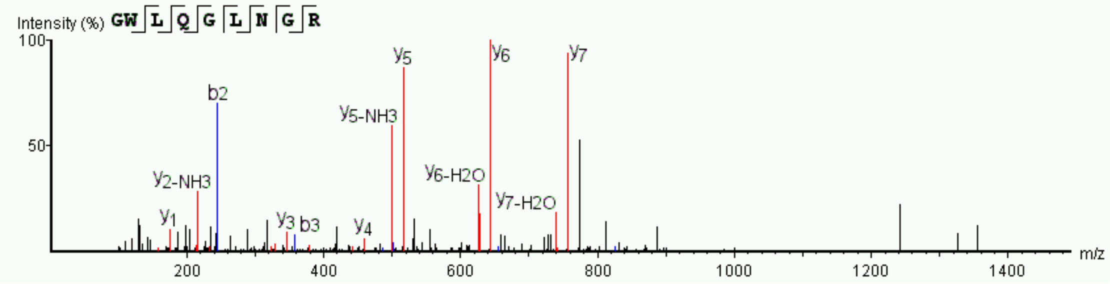

gi|571523160

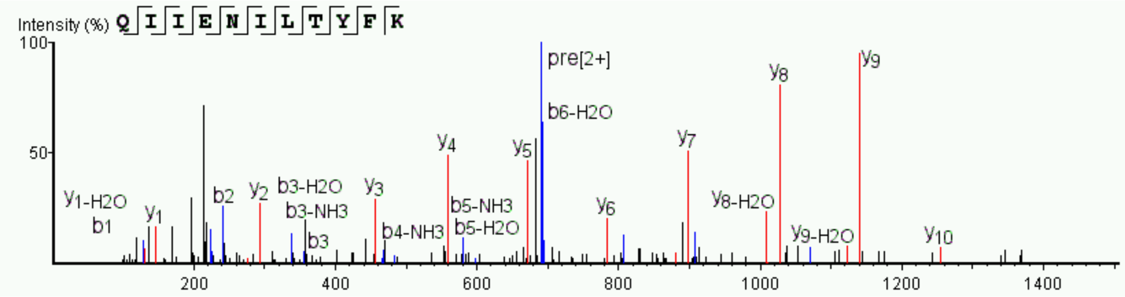

gi|571523164

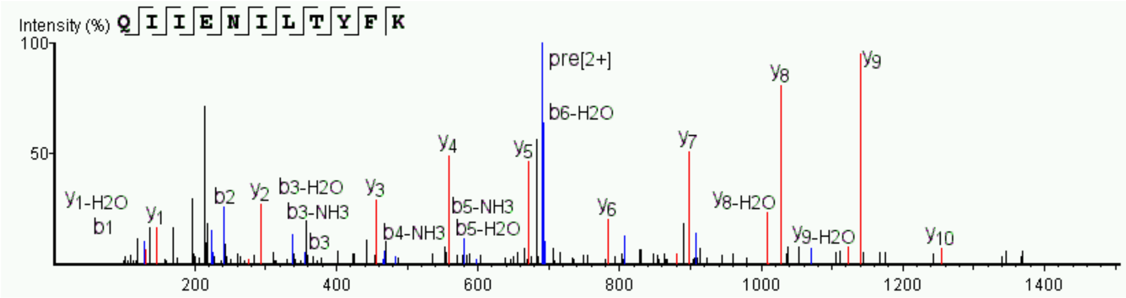

gi|571523168

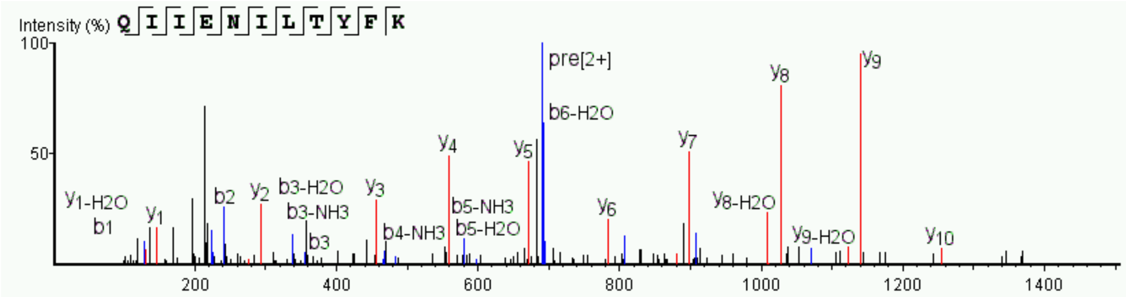

gi|571523172

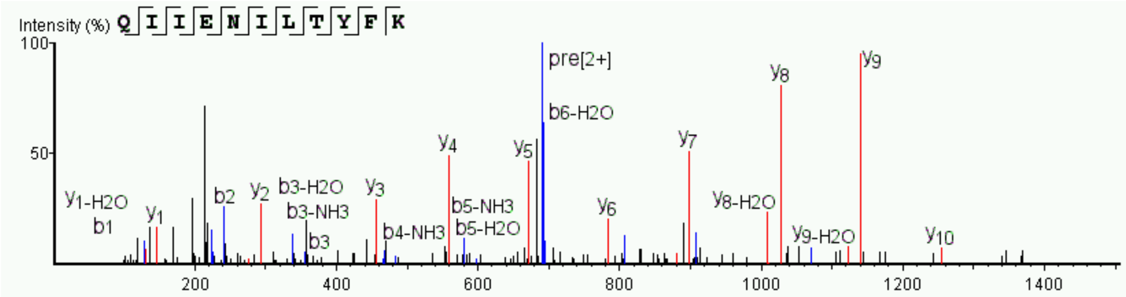

gi|328778208

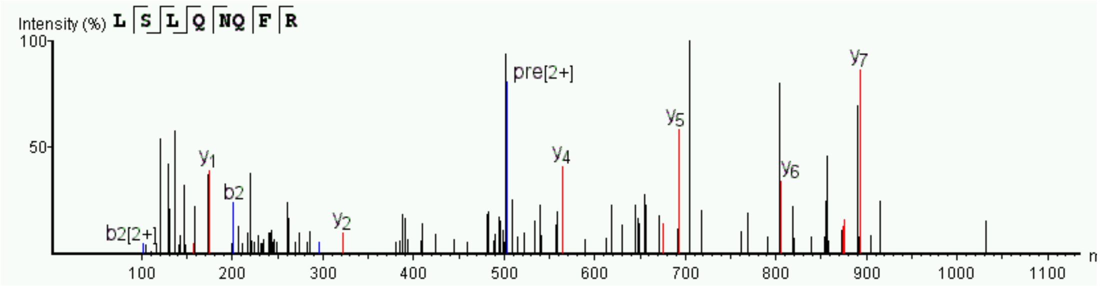

gi|571545167

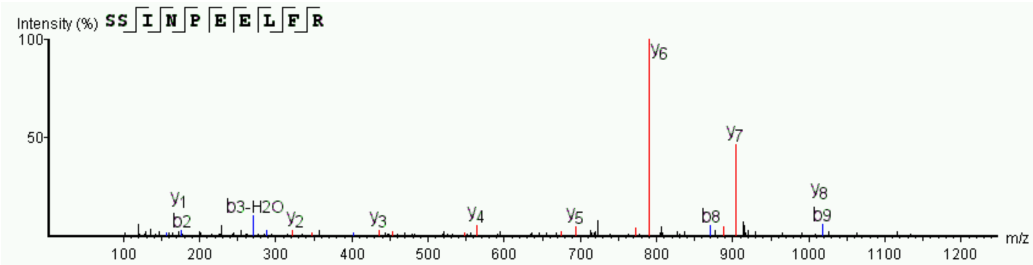

gi|571577853

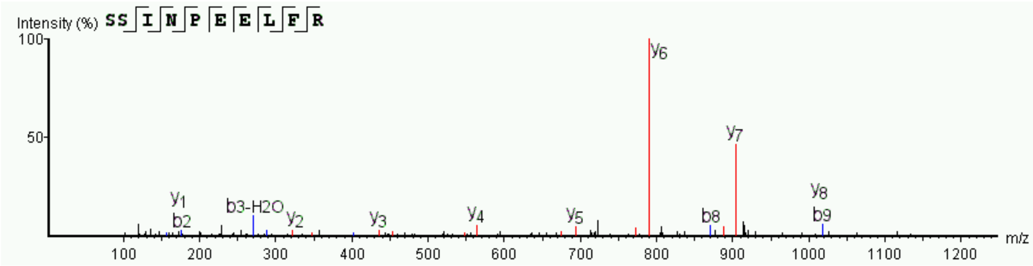

gi|328777351

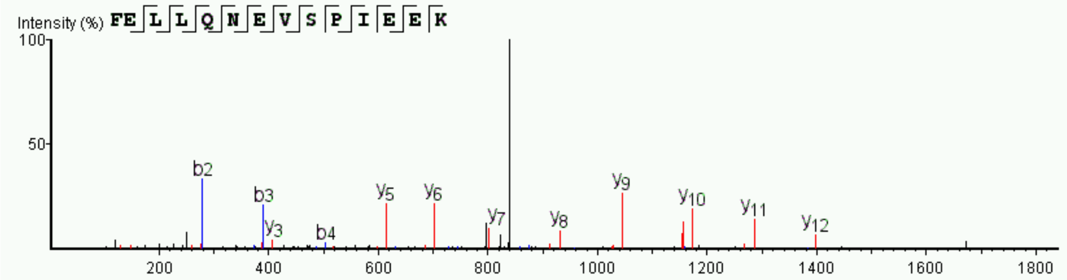

gi|571566895

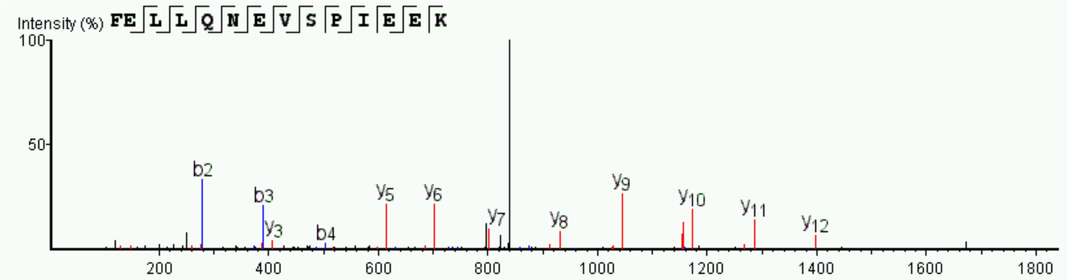

gi|571566901

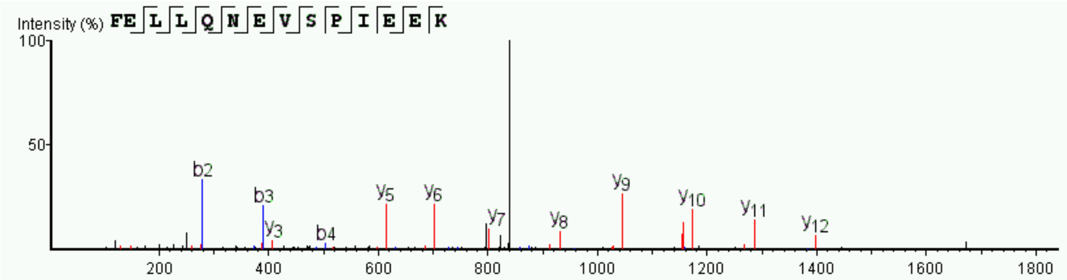

gi|571545145

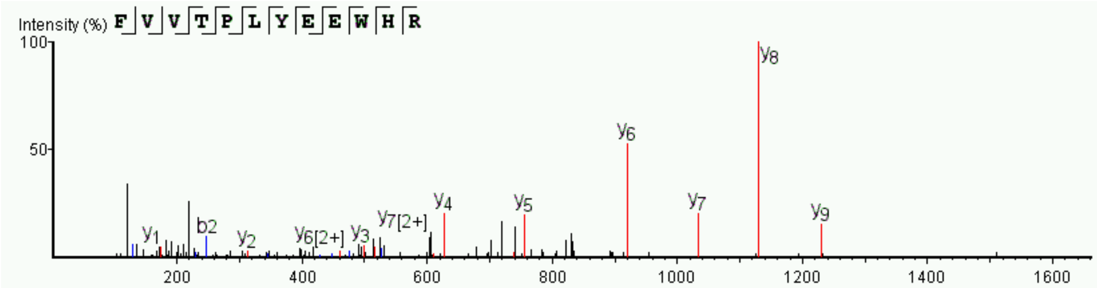

gi|571545149

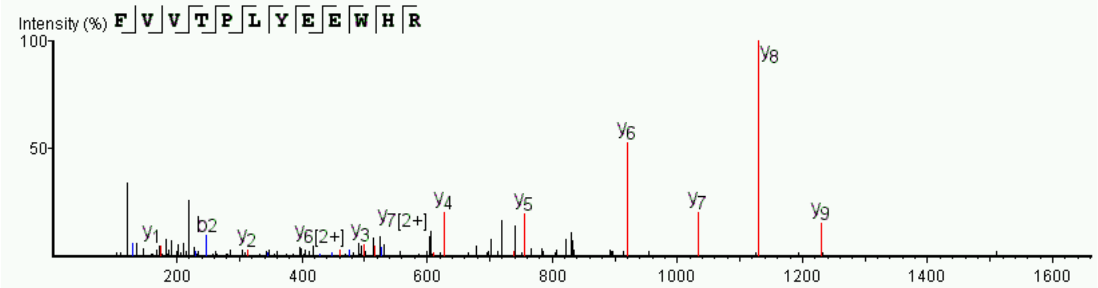

gi|571501855

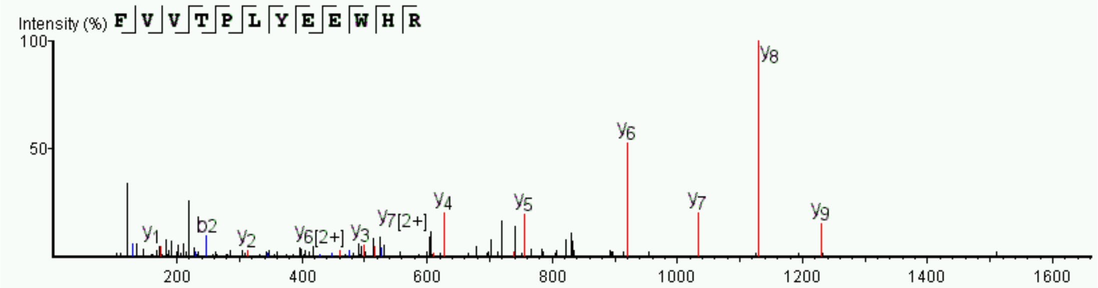

gi|571501858

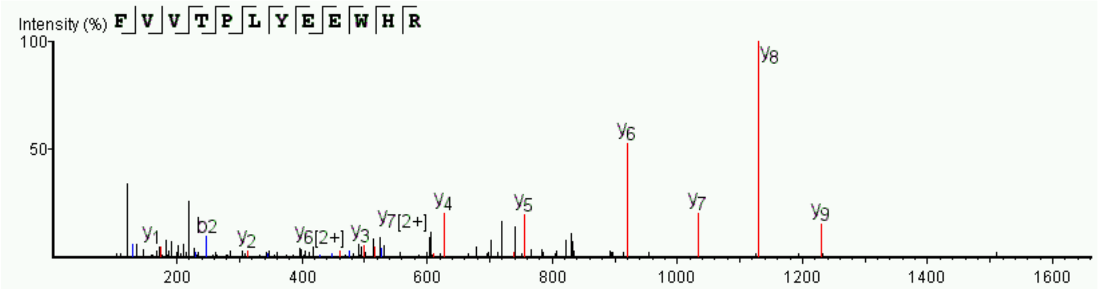

gi|571501862

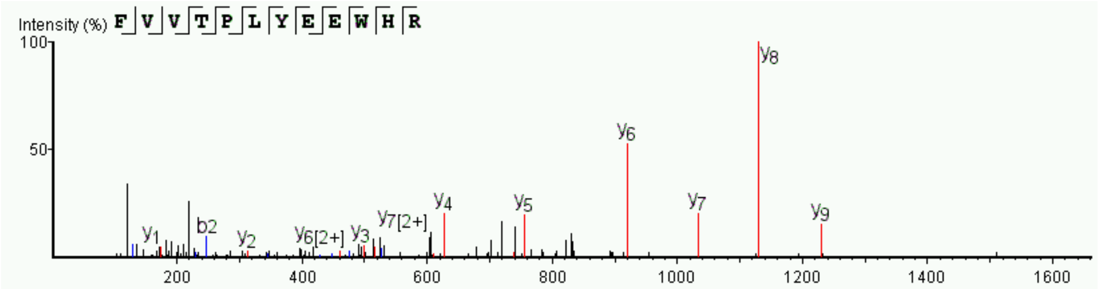

gi|571501869

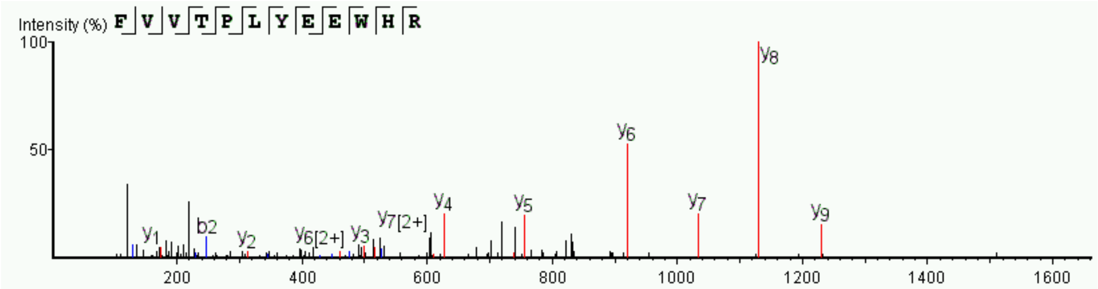

gi|571501872

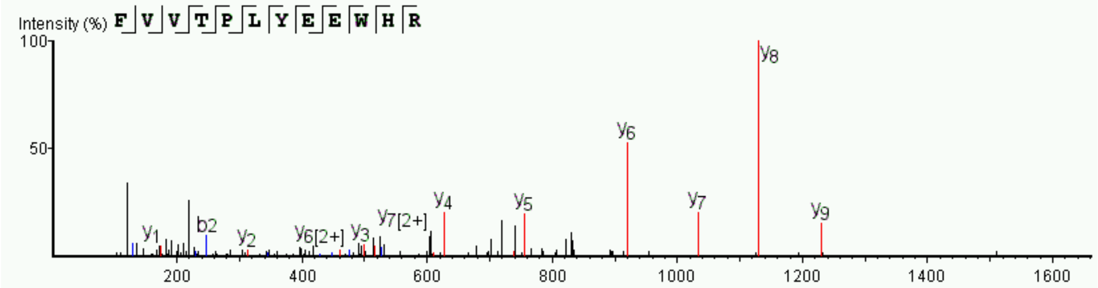

gi|571501876

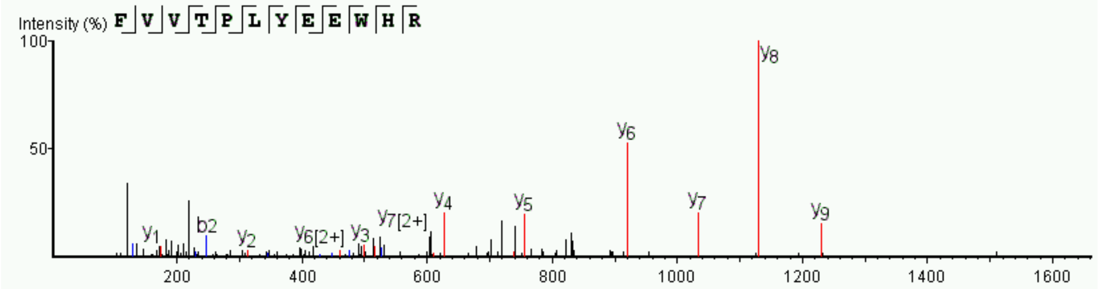

gi|571501896

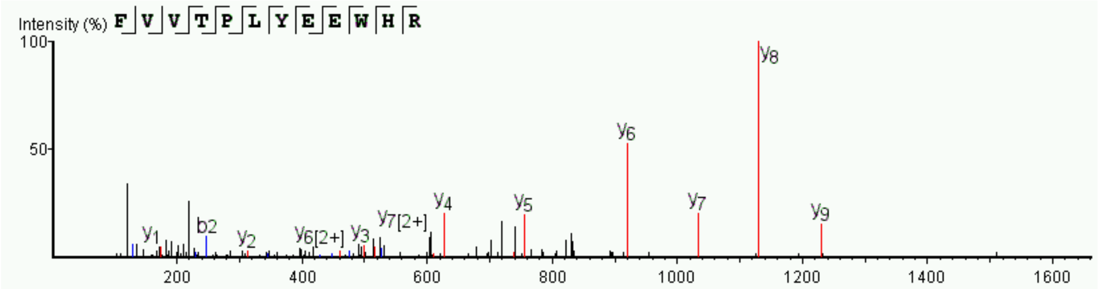

gi|571501852

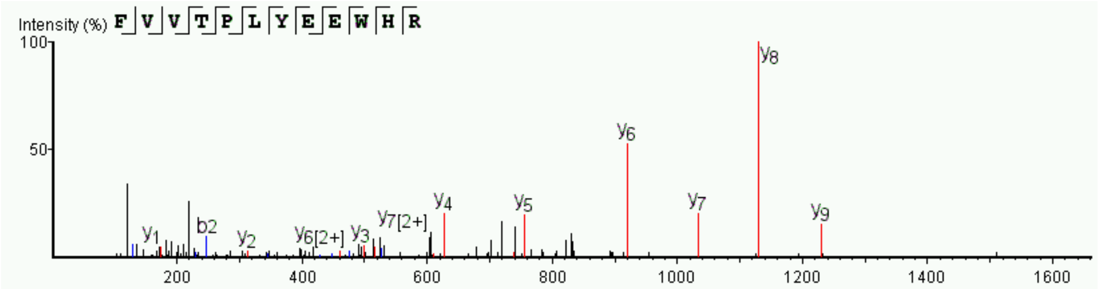

gi|571524250

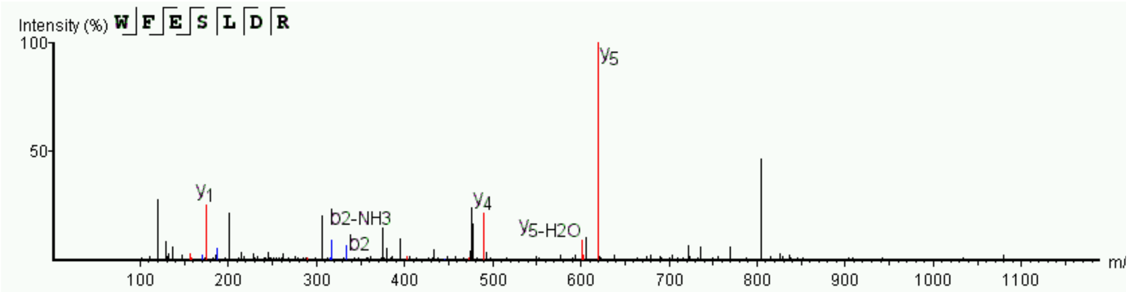

gi|571551895

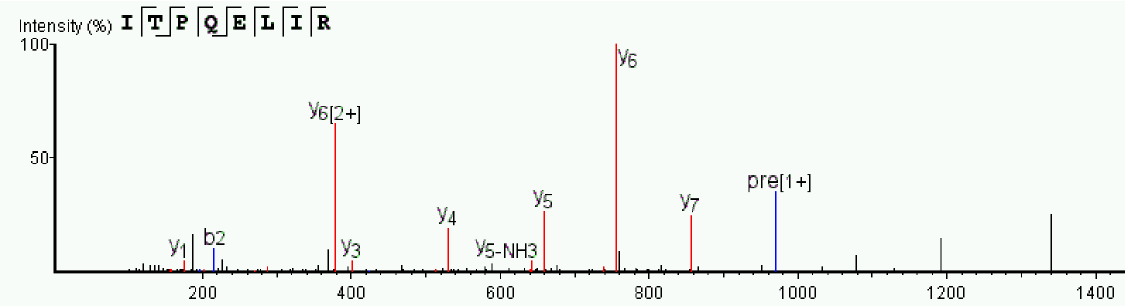

gi|571529309

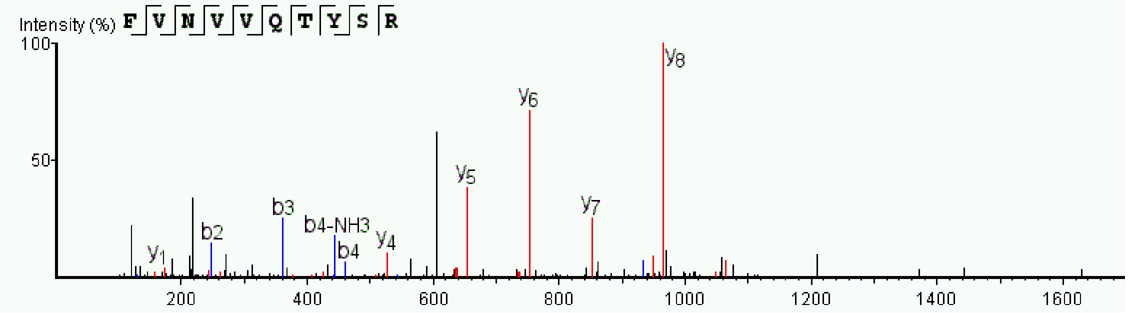

gi|571529312

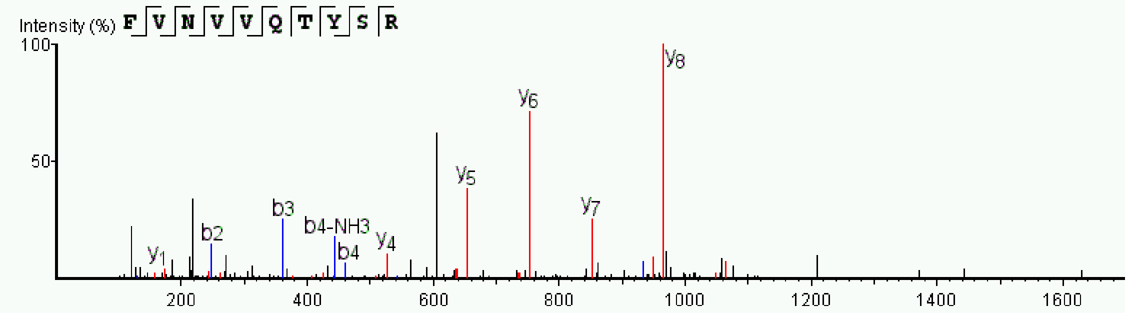

gi|571552022

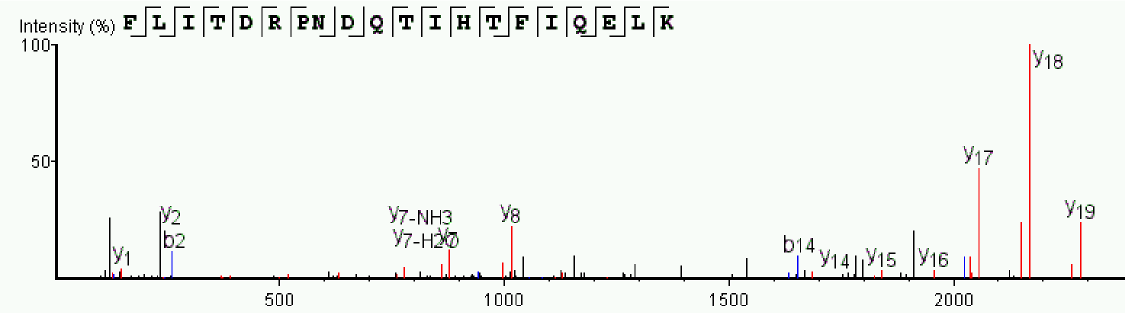

gi|571535386

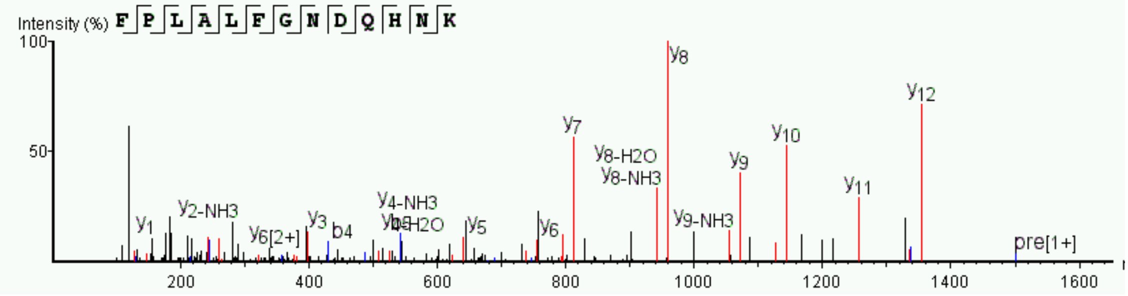

gi|66564441

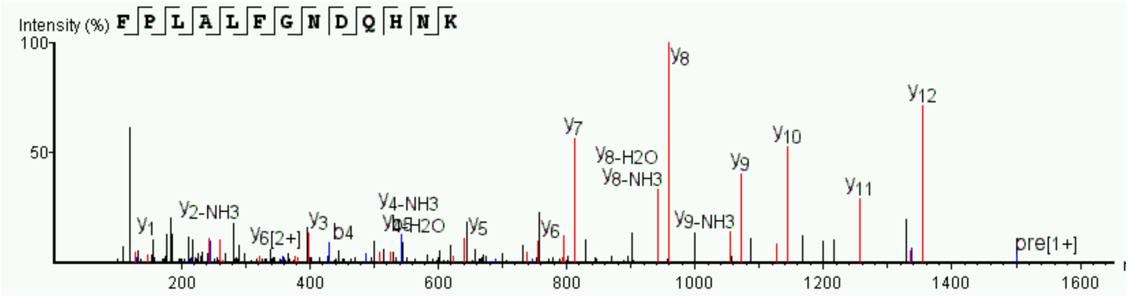

gi|571570530

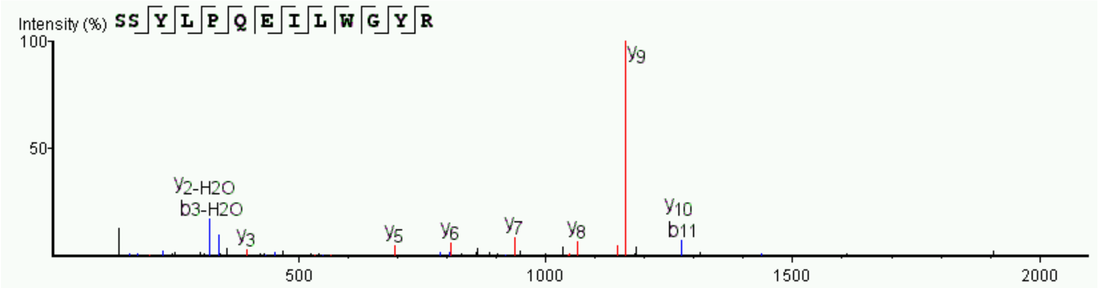

gi|571570534

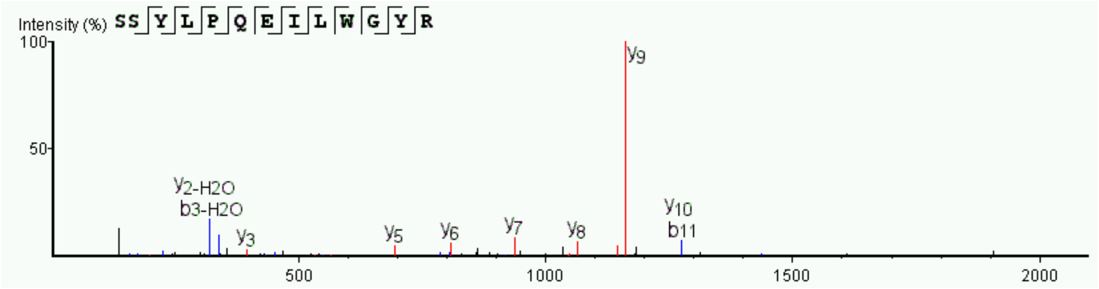

gi|571502919

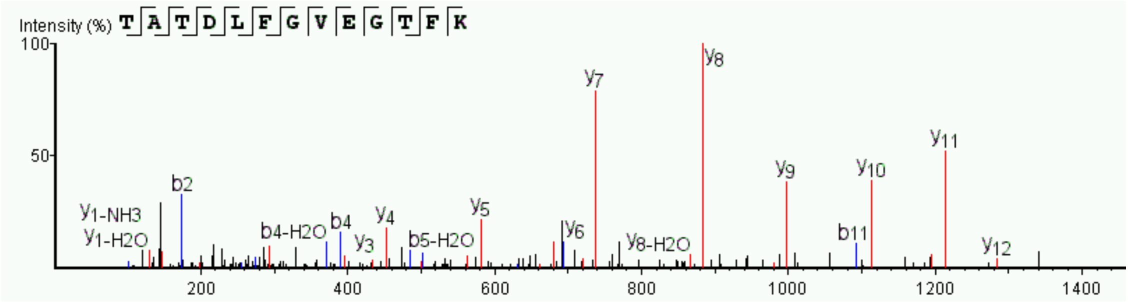

gi|571566593

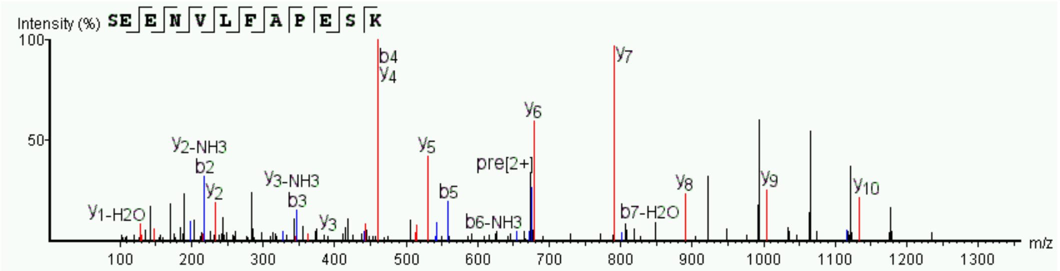

gi|571566596

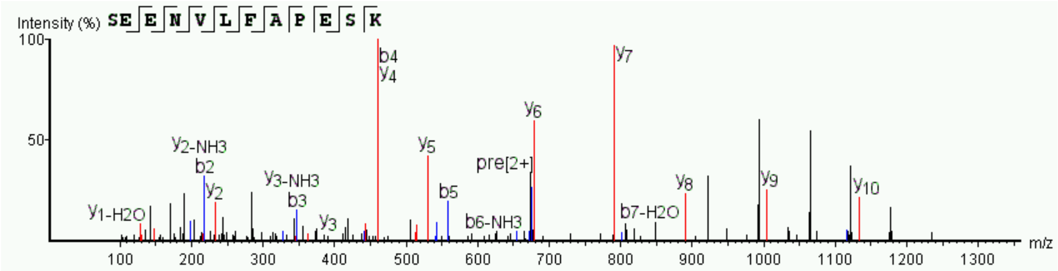

gi|571566599

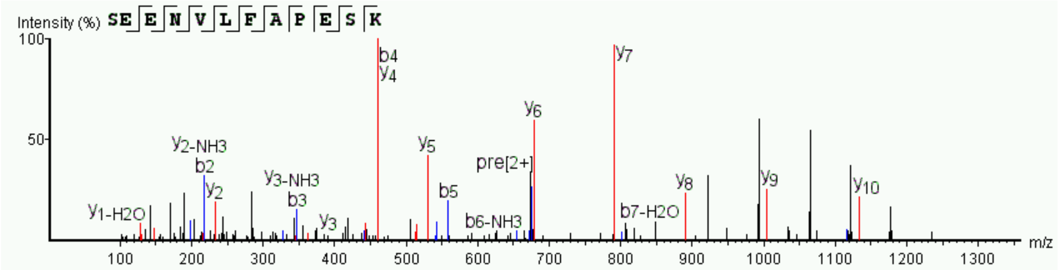

gi|66522157

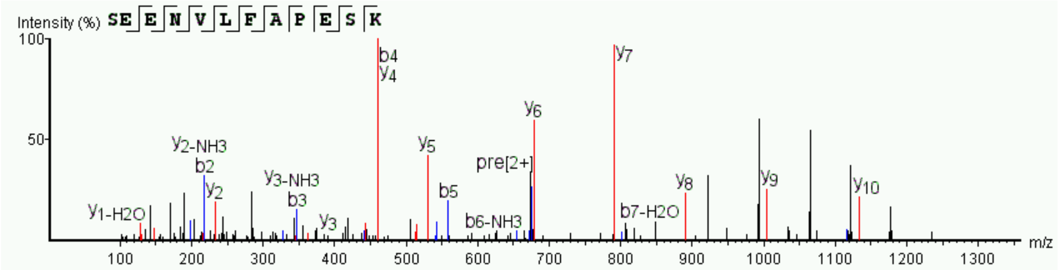

gi|571549269

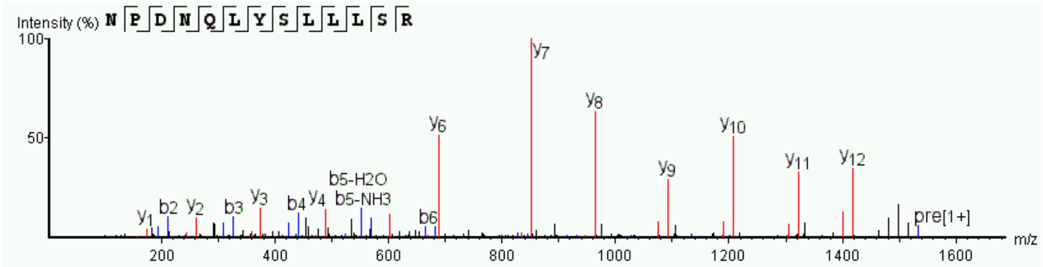

gi|328781169

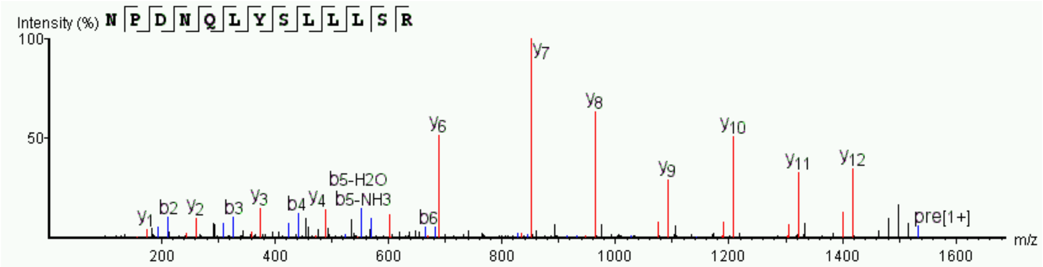

gi|571510045

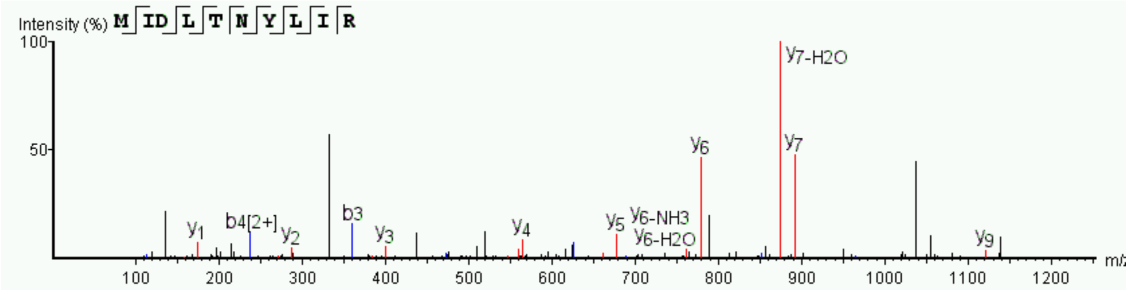

gi|571510049

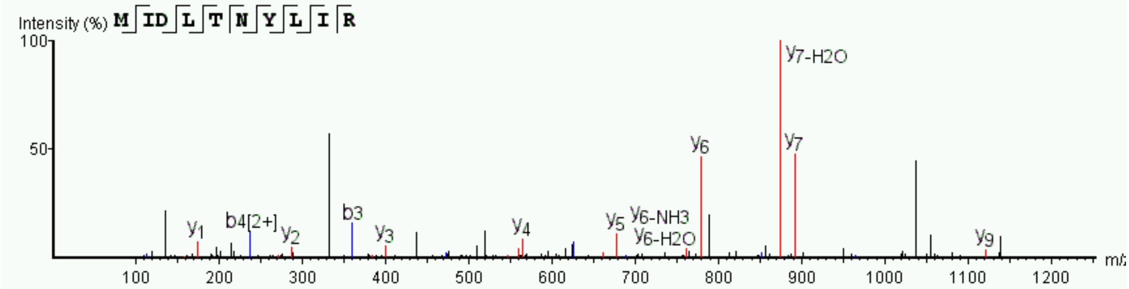

gi|571530780

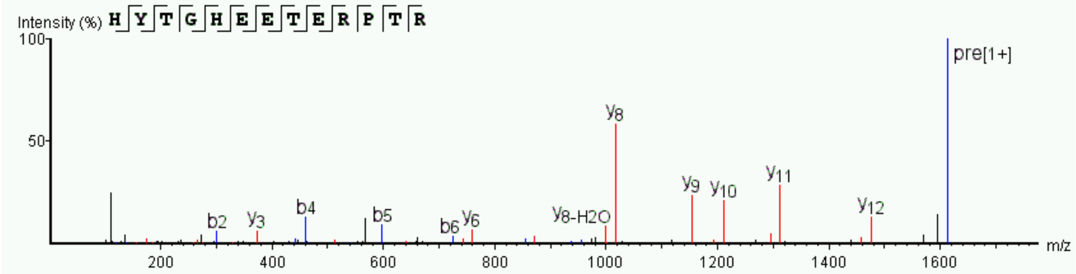

gi|571530782

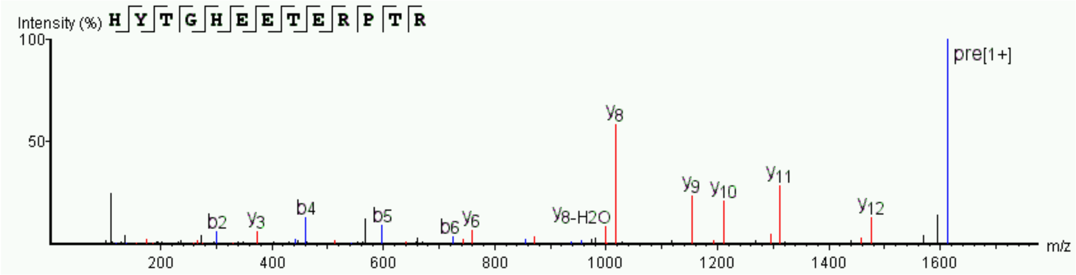

gi|328790254

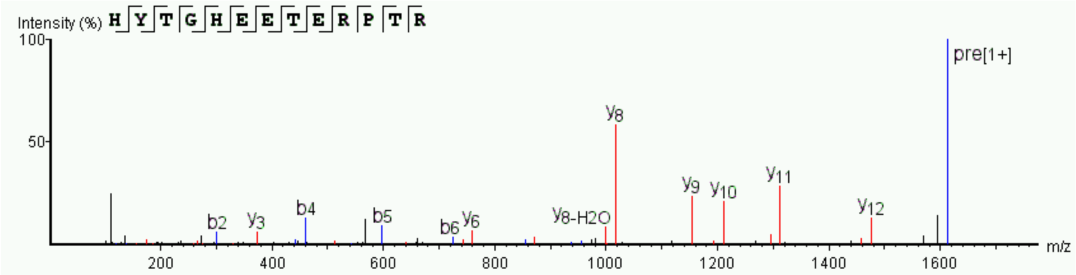

gi|571567914

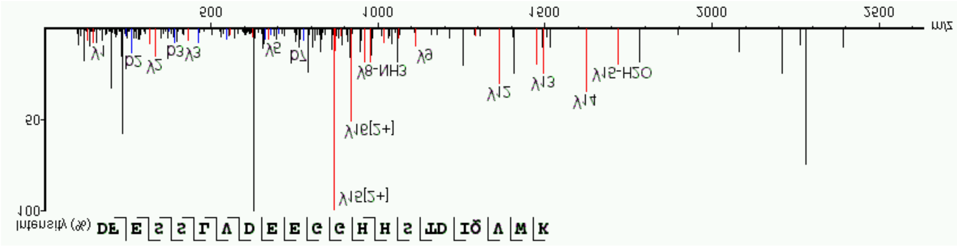

gi|66522619

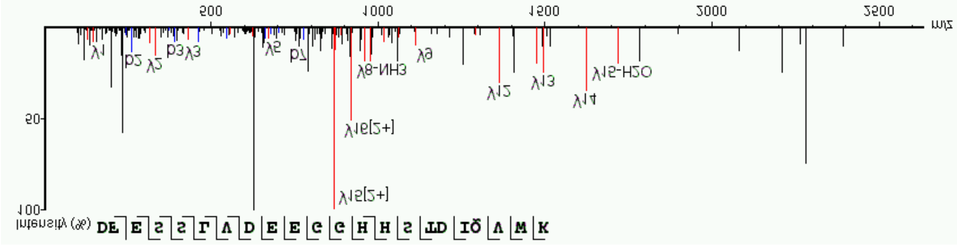

gi|571547162

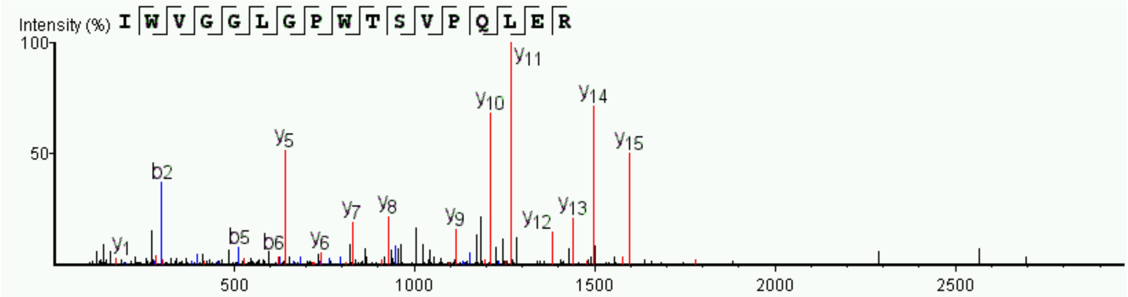

gi|328775847

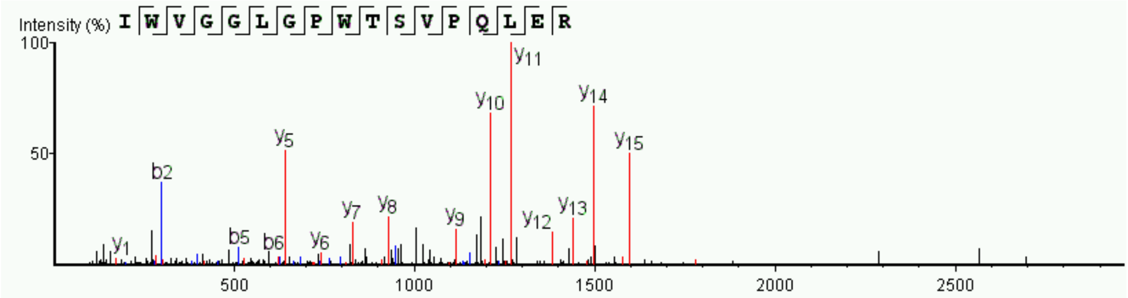

gi|571578780

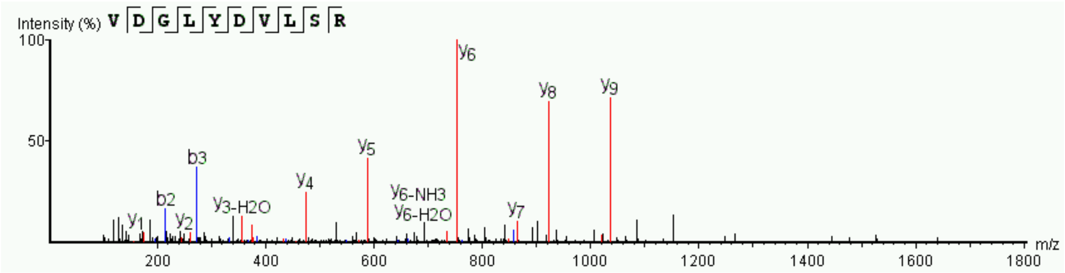

gi|571578782

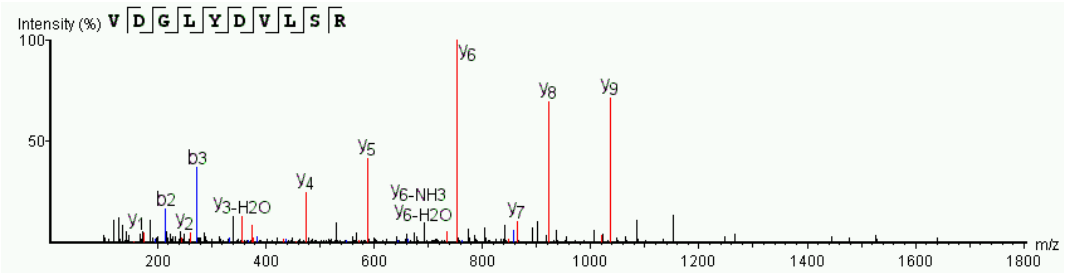

gi|571560975

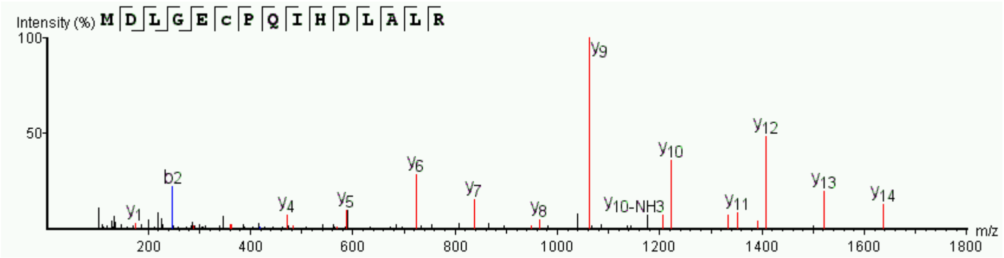

gi|571560979

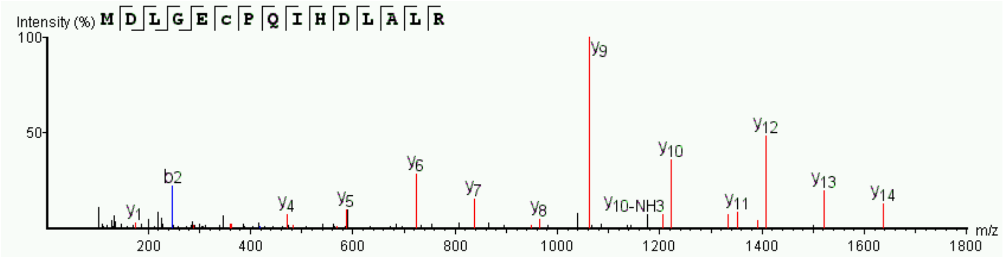

gi|571560983

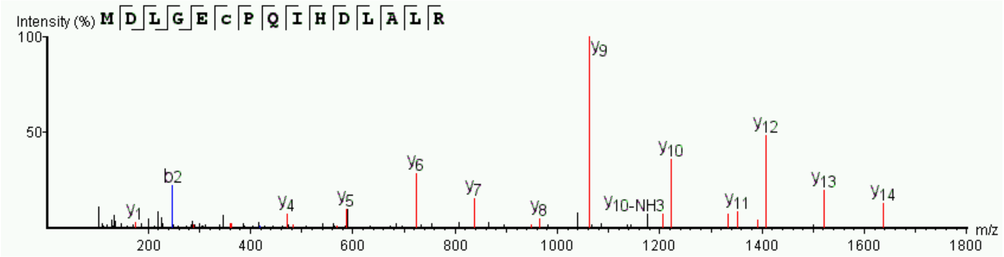

gi|571560987

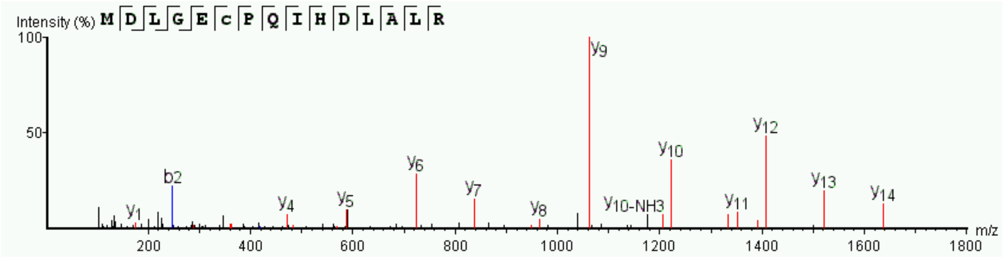

gi|571560991

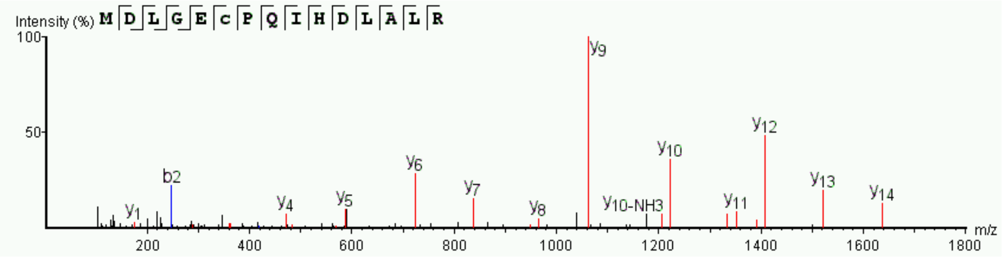

gi|571560995

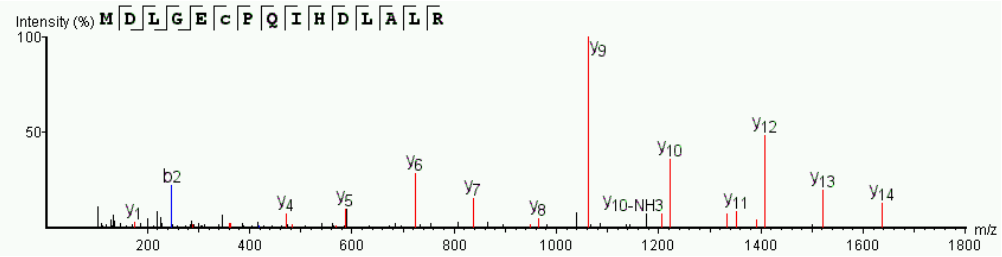

gi|571560999

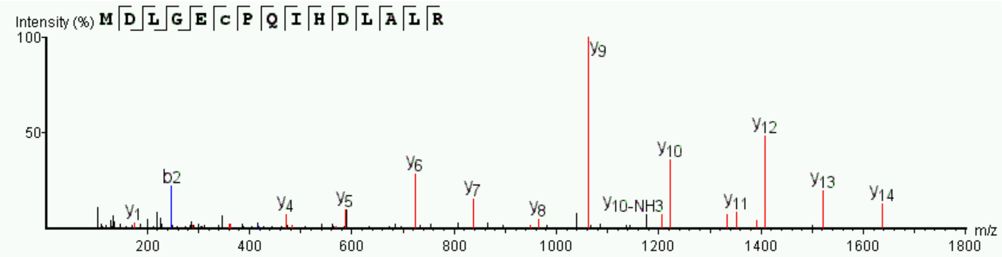

gi|110761115

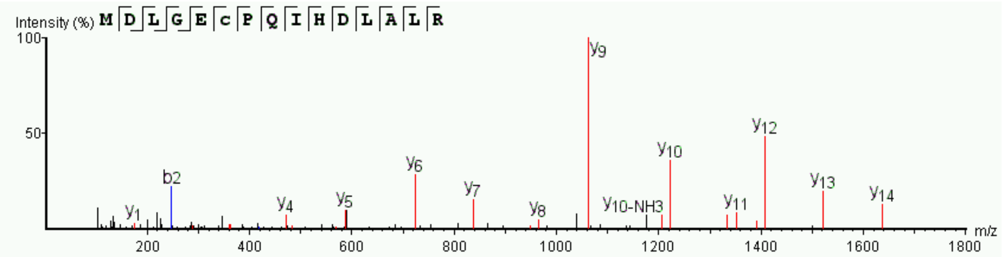

gi|571542426

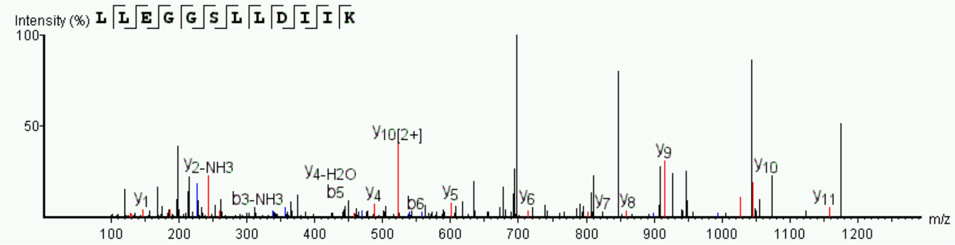

gi|571542429

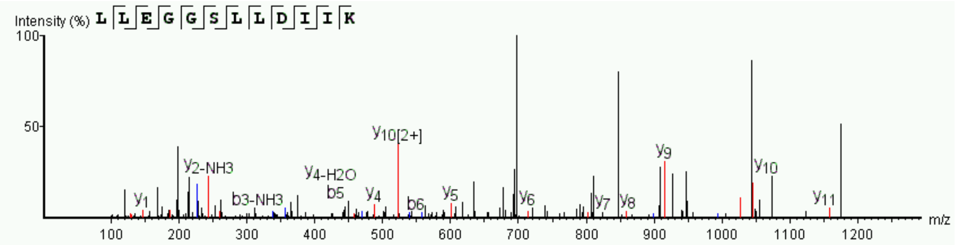

gi|571542432

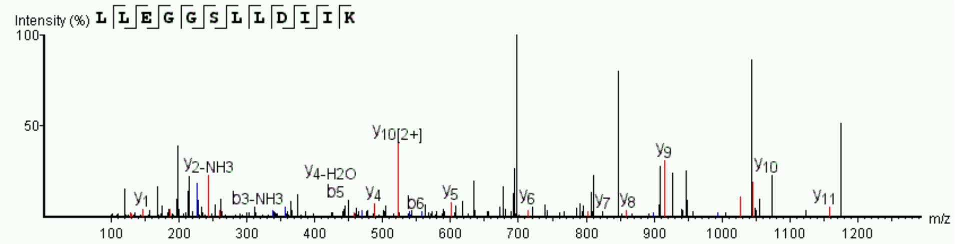

gi|571542435

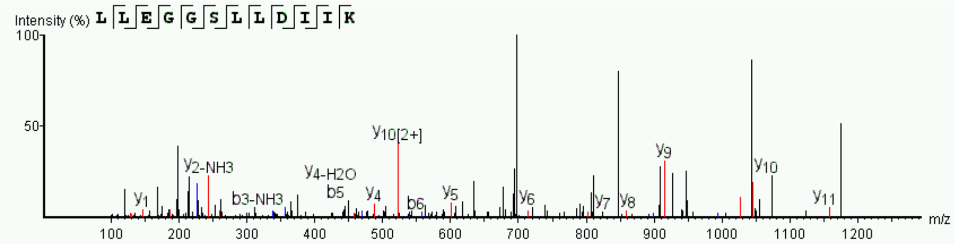

gi|571542438

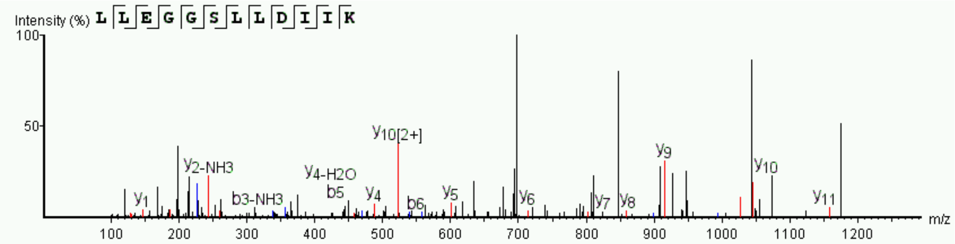

gi|571542441

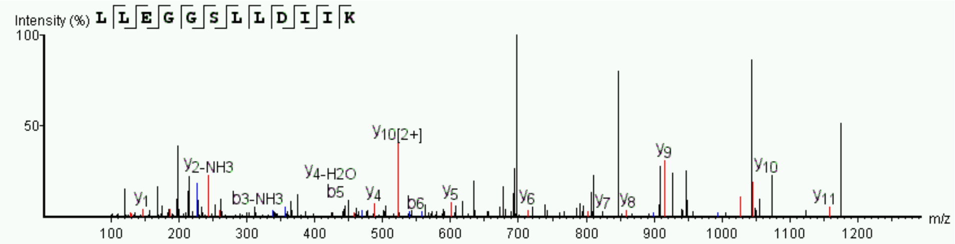

gi|571499523

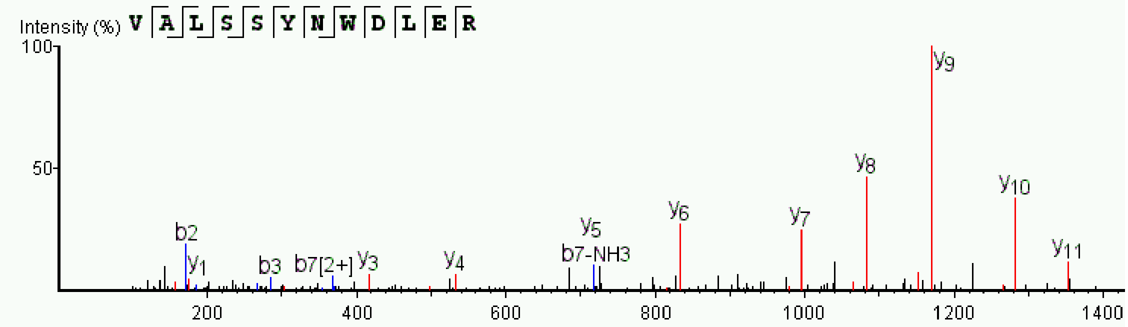

gi|571568731

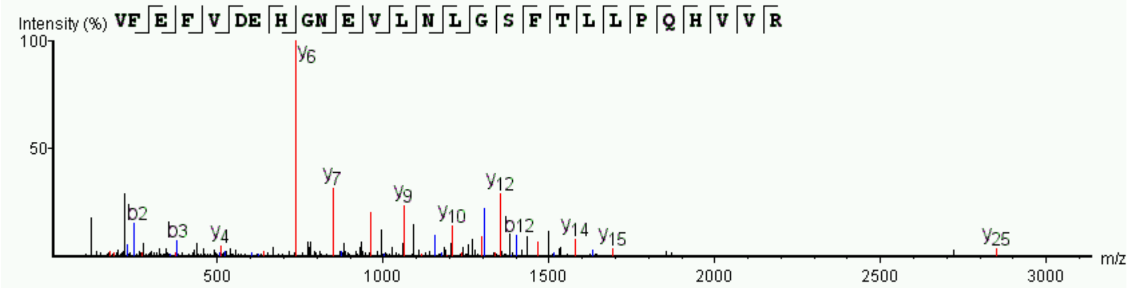

gi|571568734

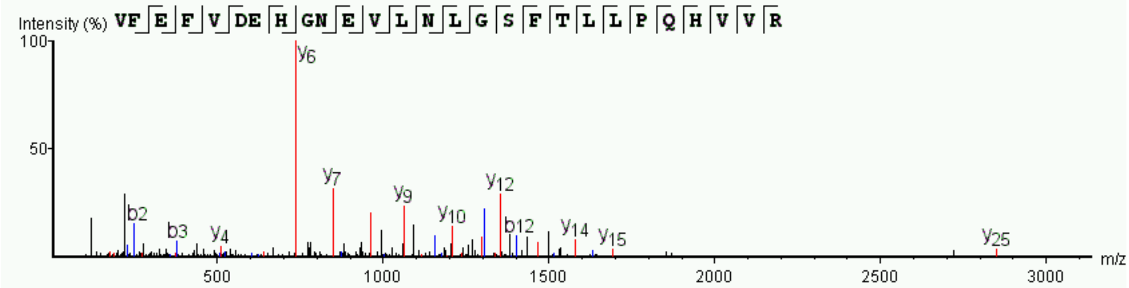

gi|571568738

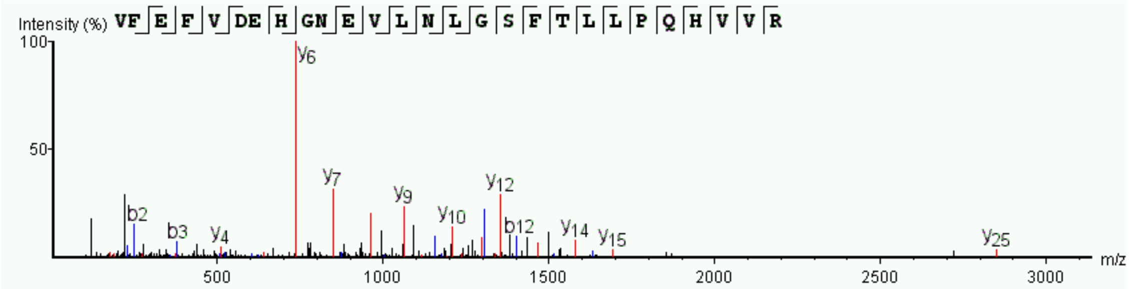

gi|110765112

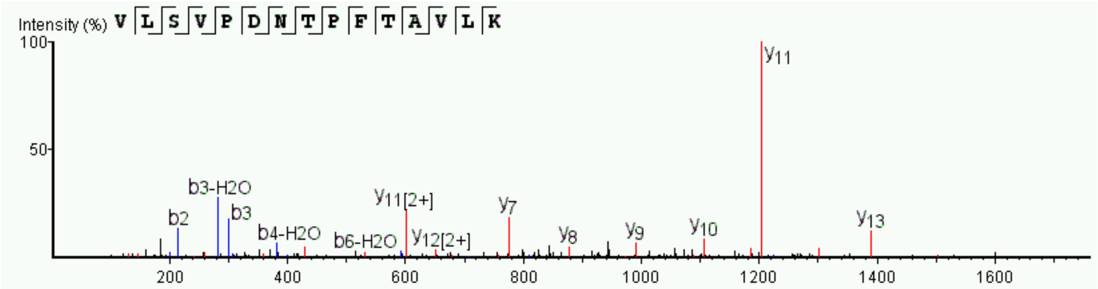

gi|571538017

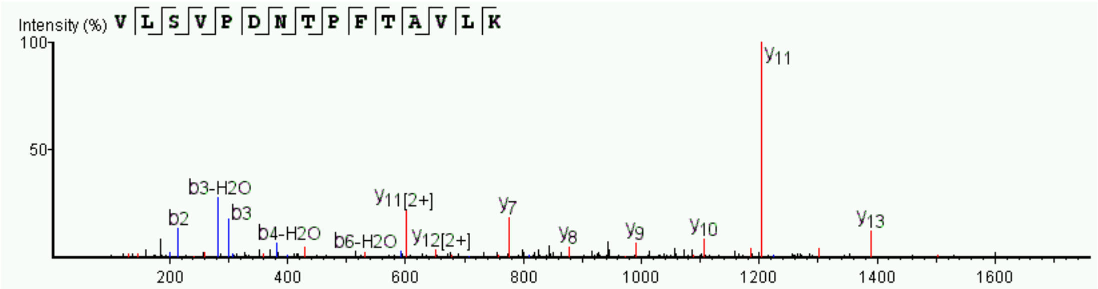

gi|571534107

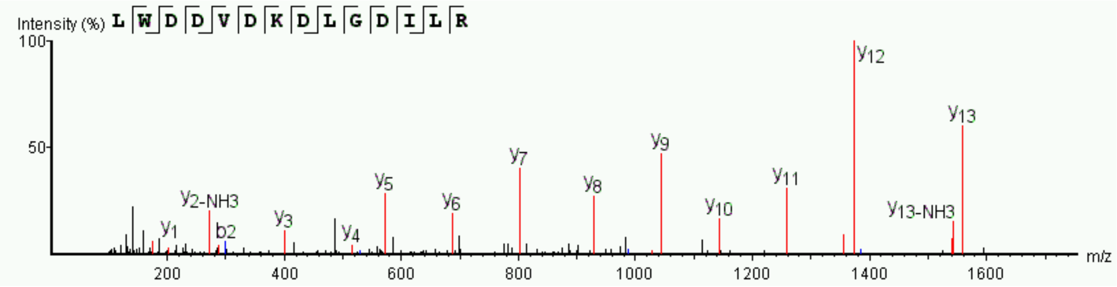

gi|571534110

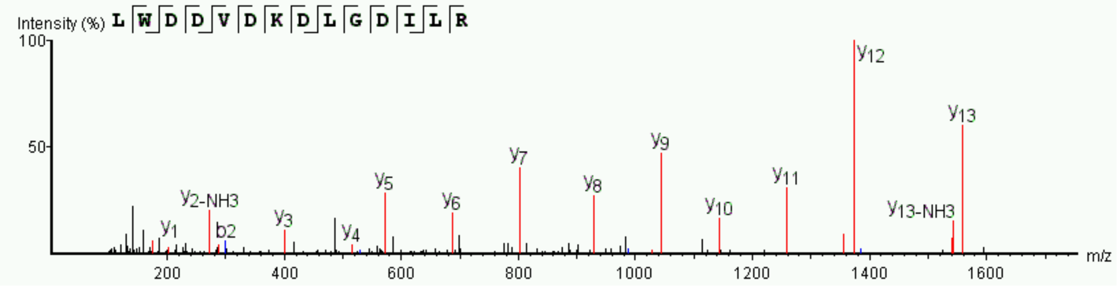

gi|571534113

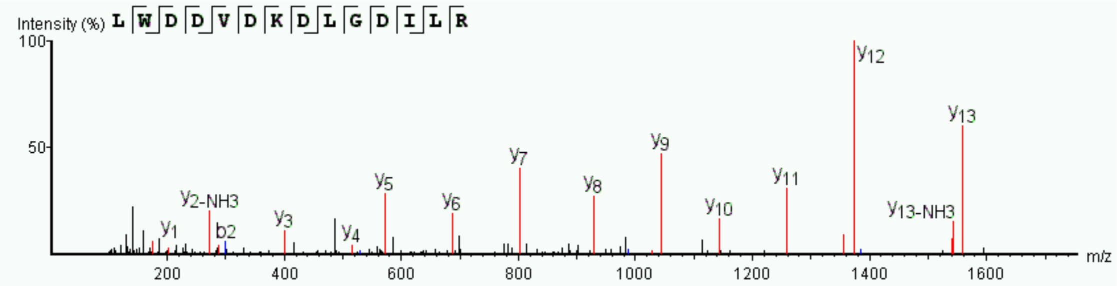

gi|571534116

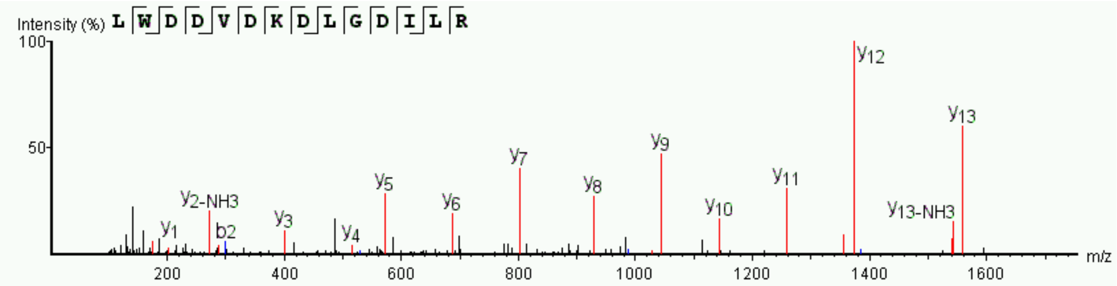

gi|571534120

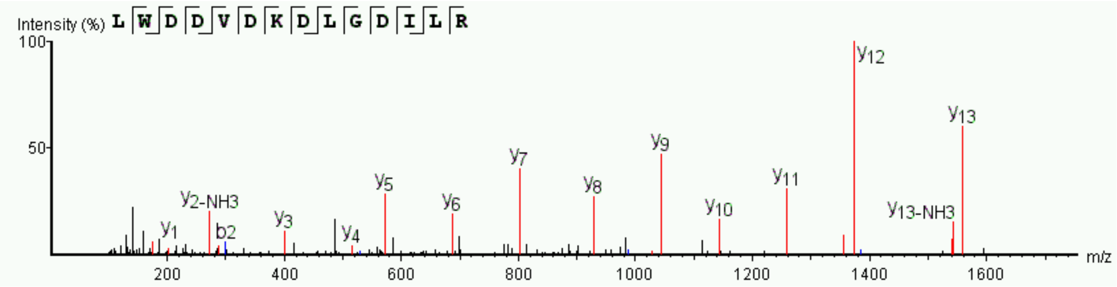

gi|48097928

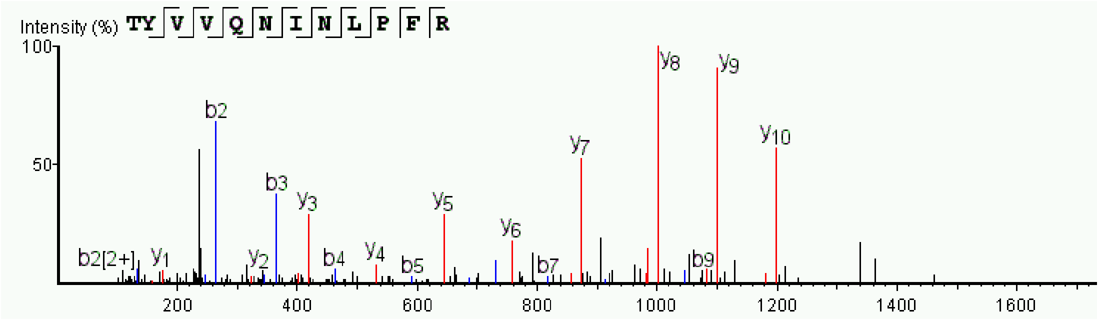

gi|66531593

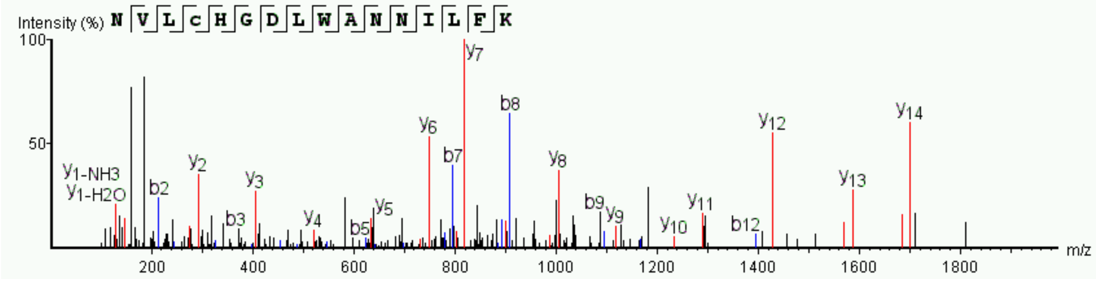

gi|328787676

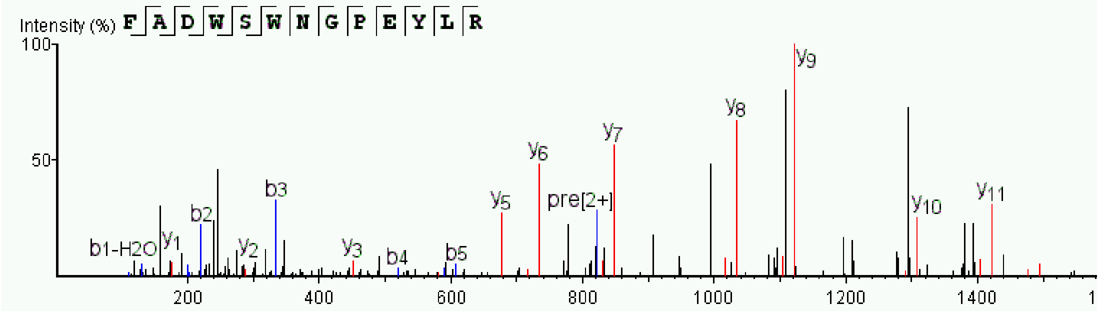

gi|571565027

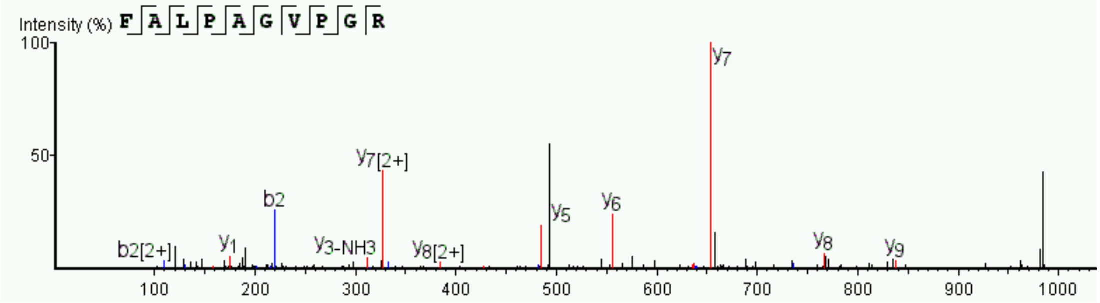

gi|571565030

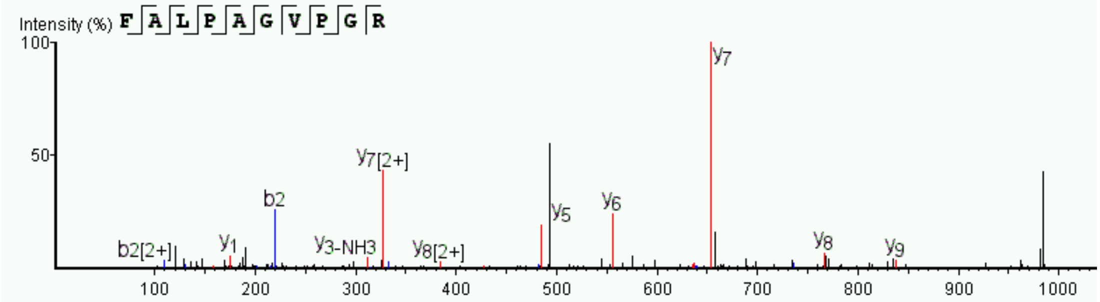

gi|571565033

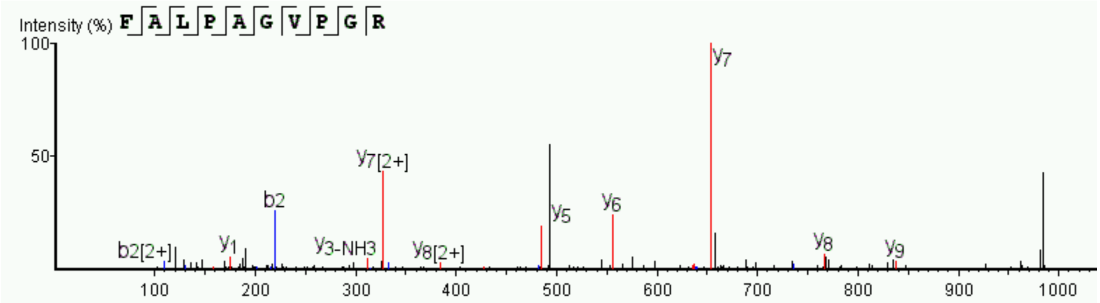

gi|110749038

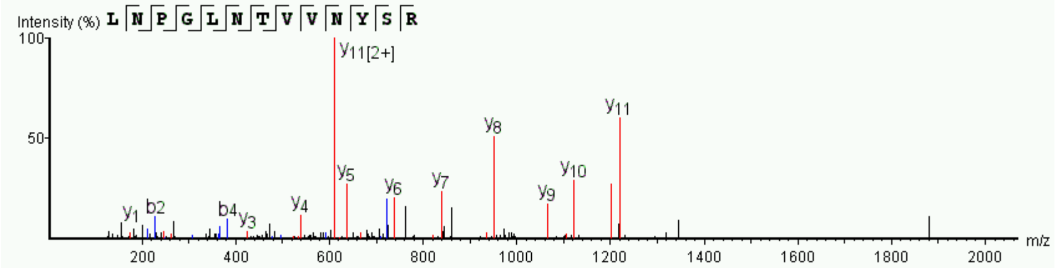

gi|571509610

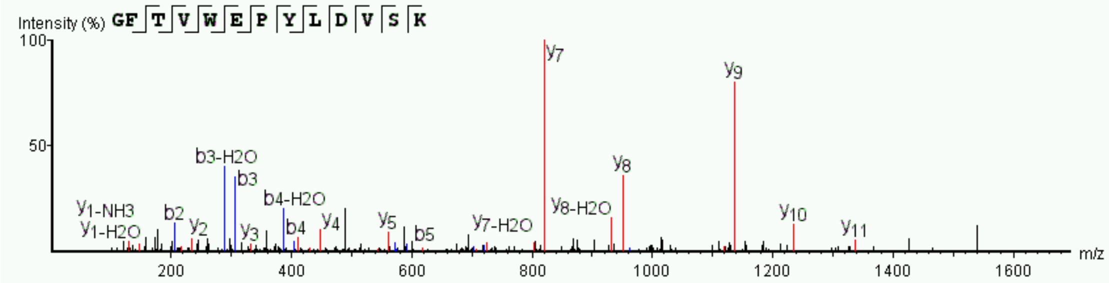

gi|571509645

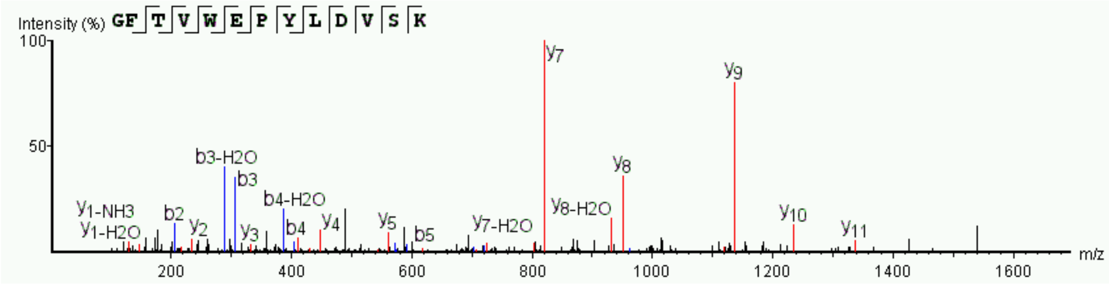

gi|571509648

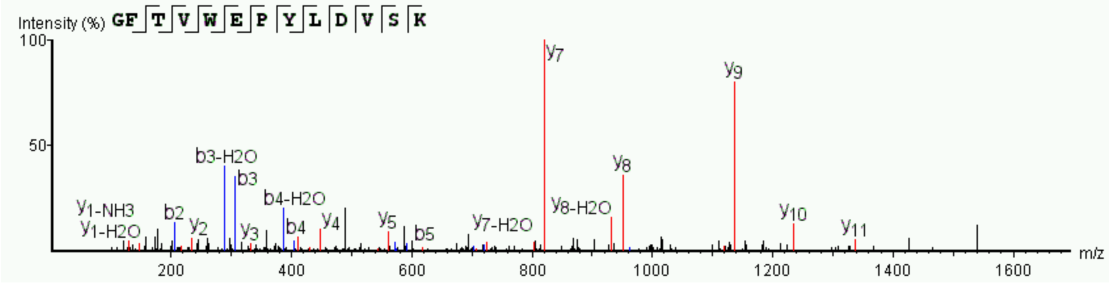

gi|571509651

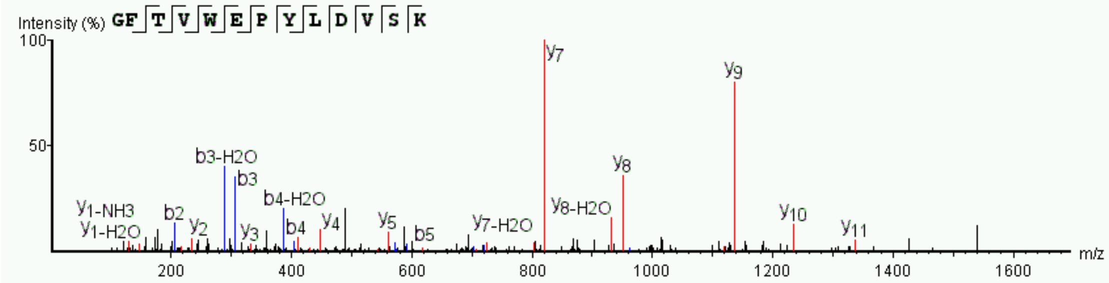

gi|571509654

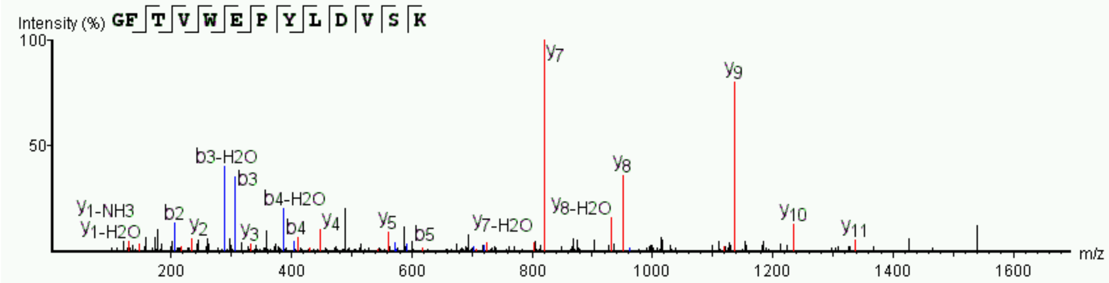

gi|571509657

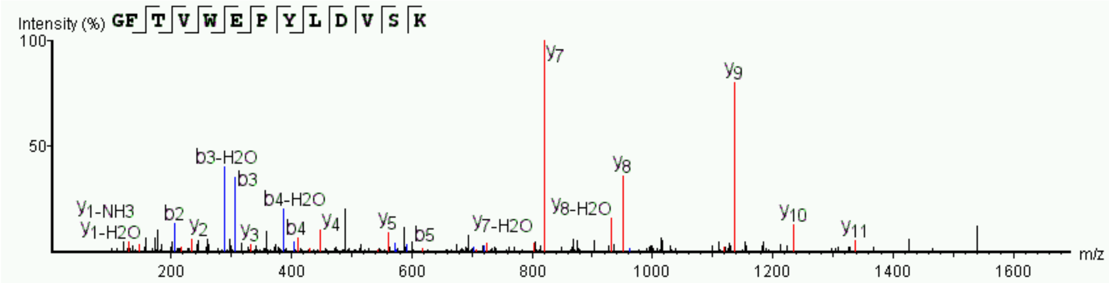

gi|571509659

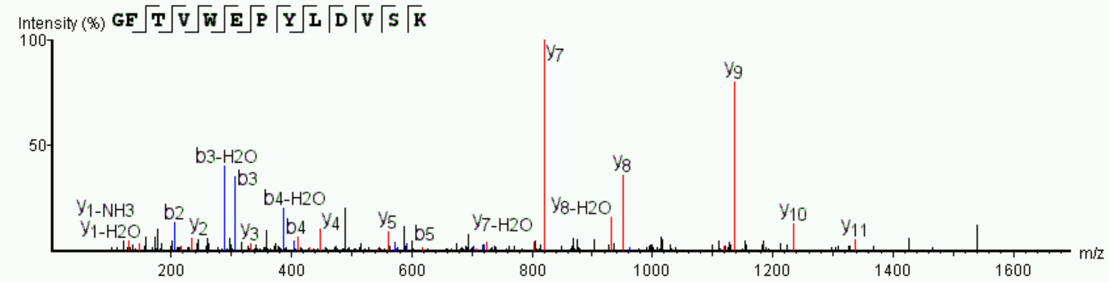

gi|571509614

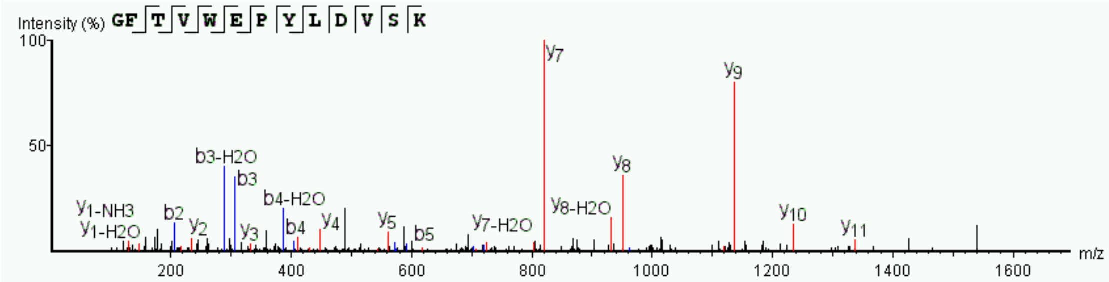

gi|571509618

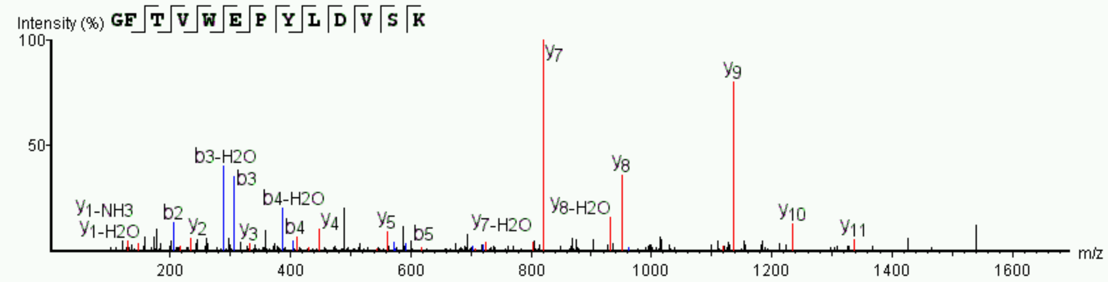

gi|571509621

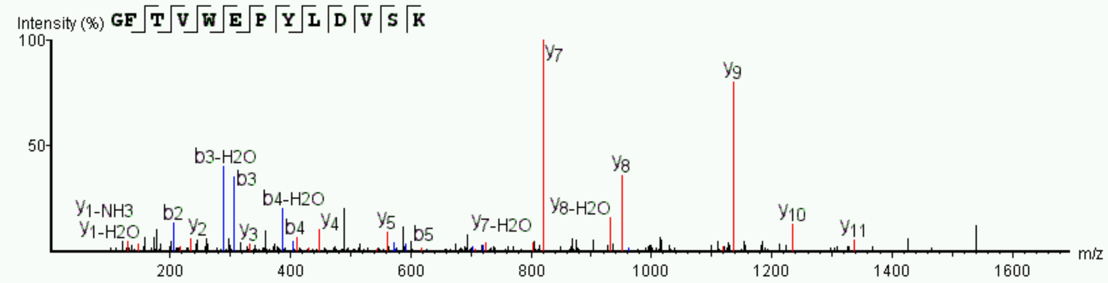

gi|571509624

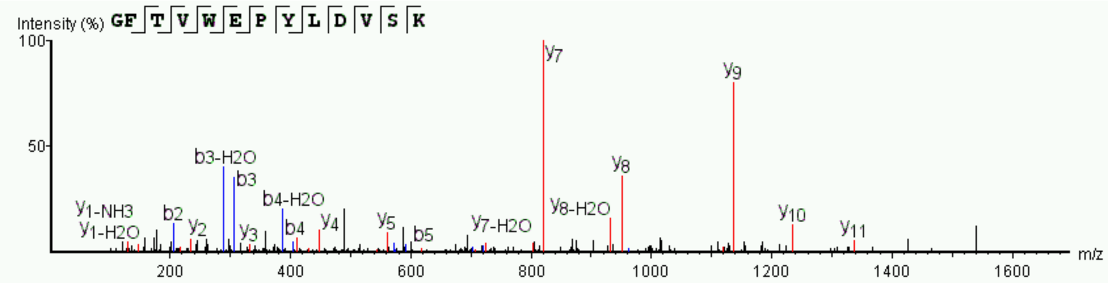

gi|571509628

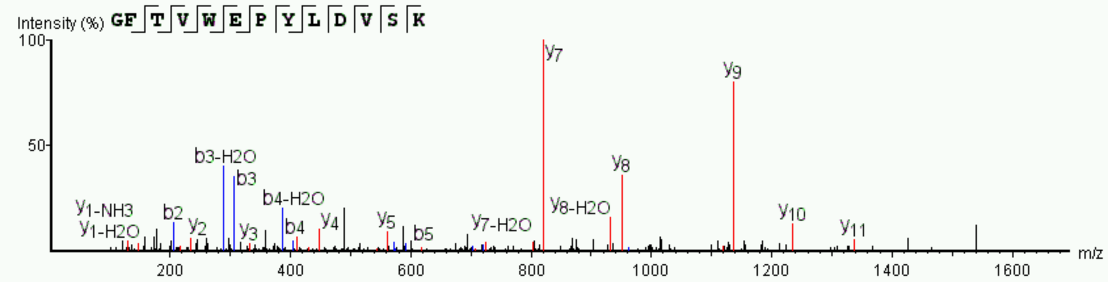

gi|571509634

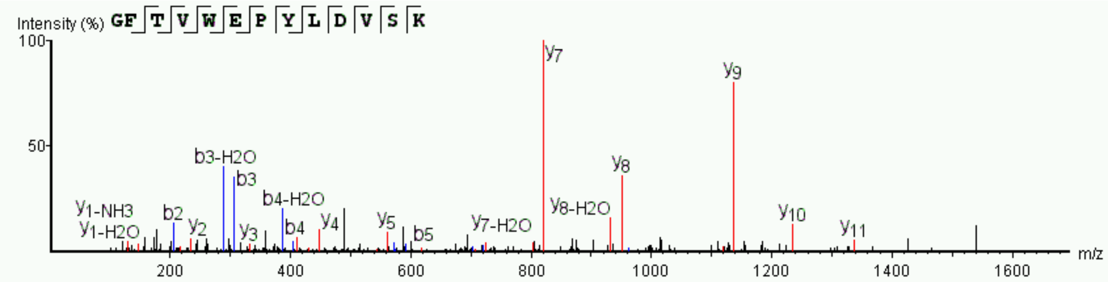

gi|571509637

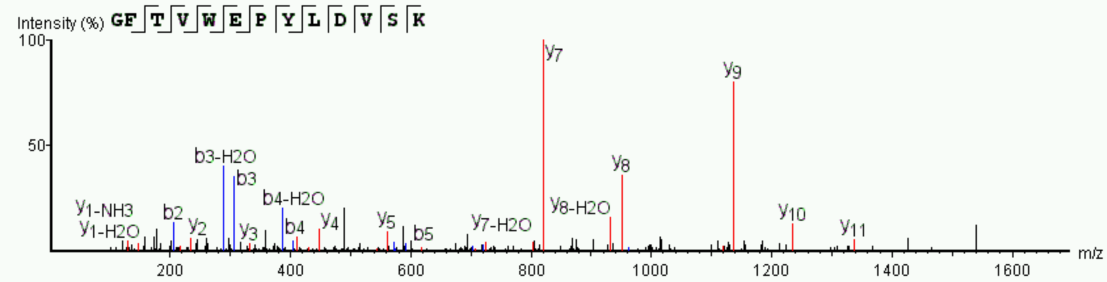

gi|571509641

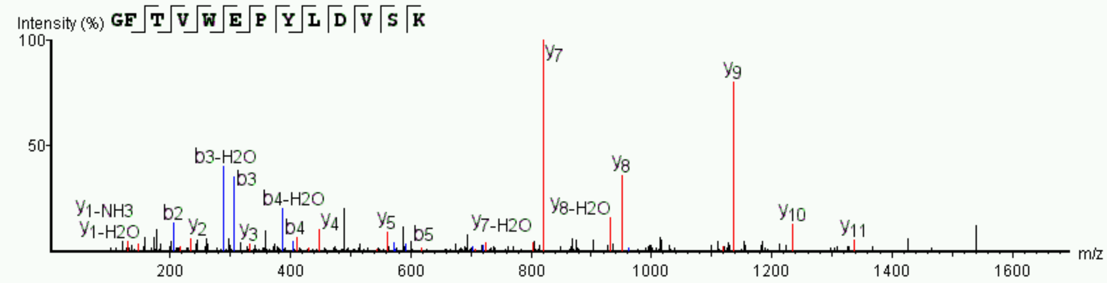

gi|571567623

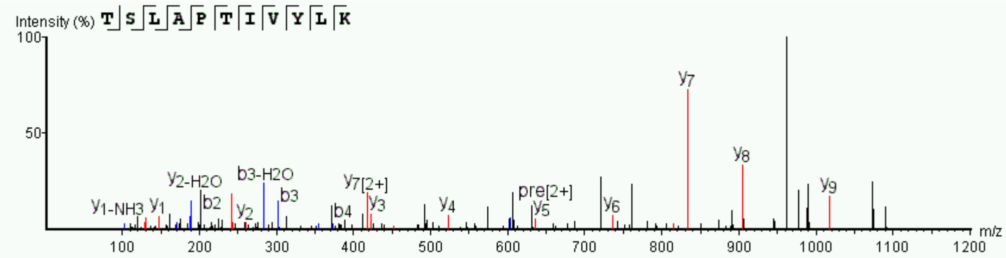

gi|571567660

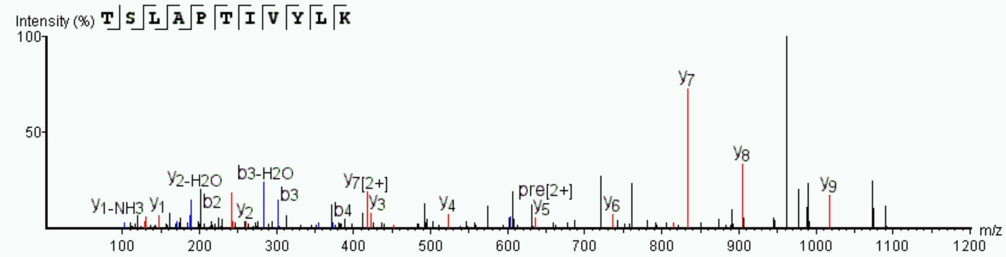

gi|571567664

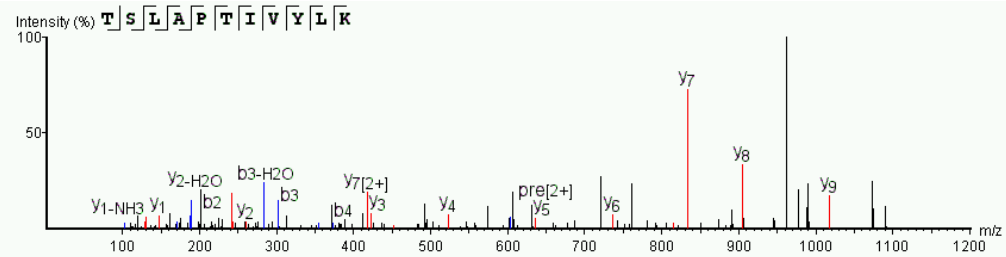

gi|571567668

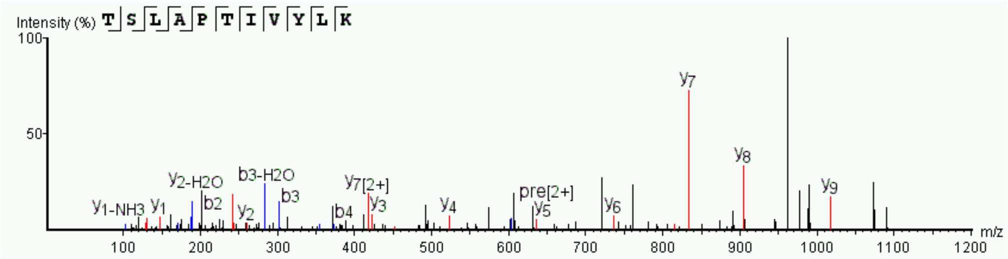

gi|571567674

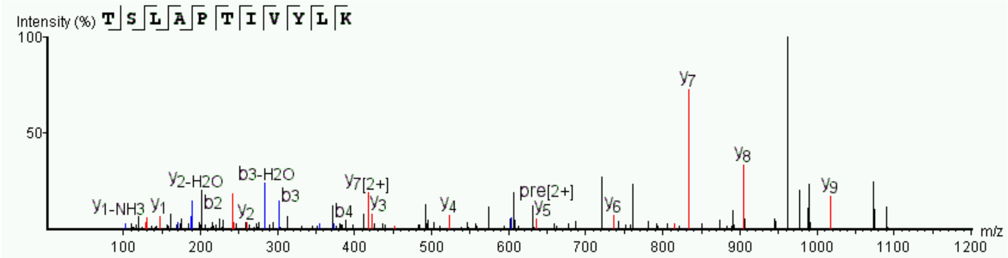

gi|571567677

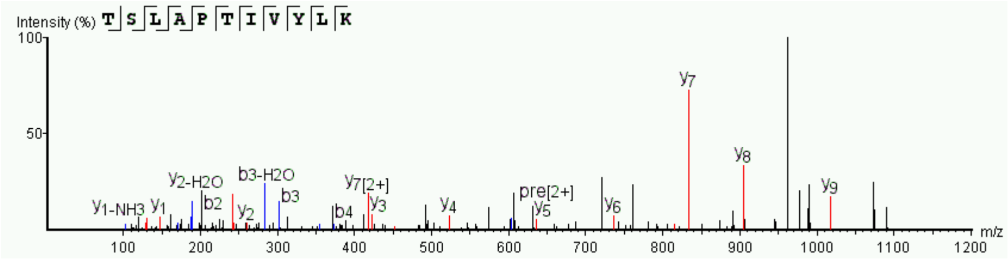

gi|571567680

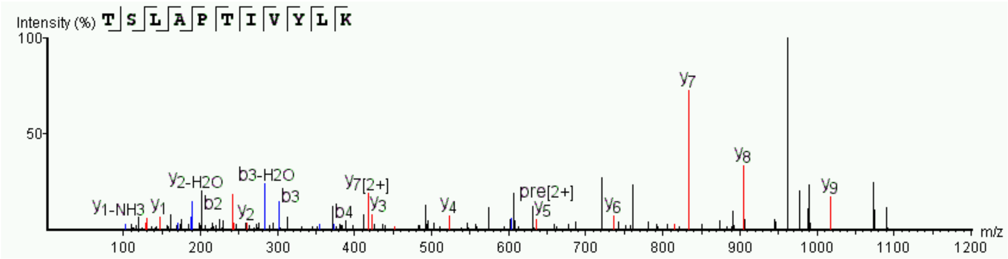

gi|571567683

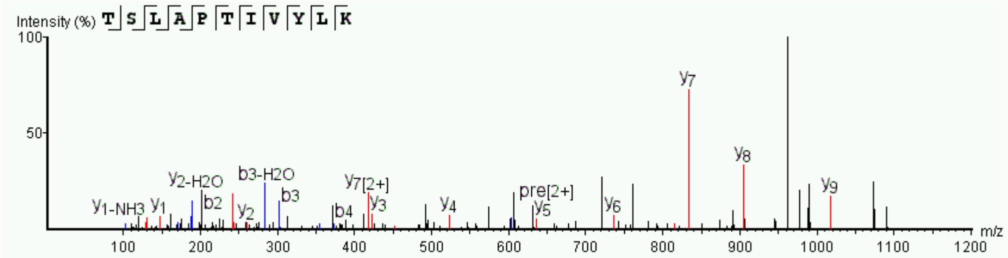

gi|571567687

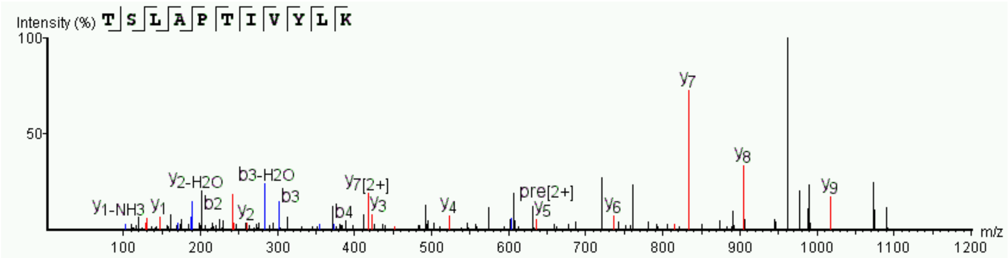

gi|571567626

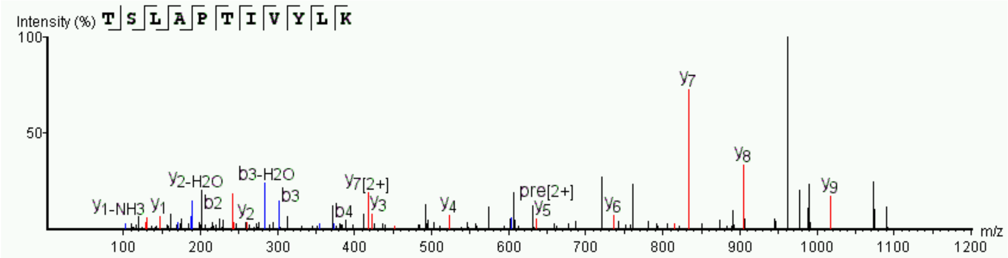

gi|571567629

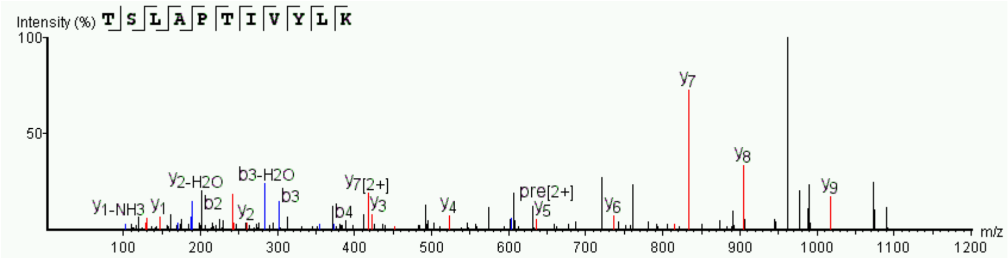

gi|571567632

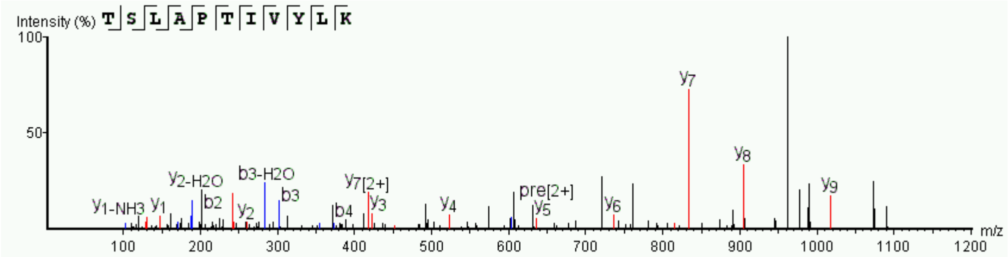

gi|571567637

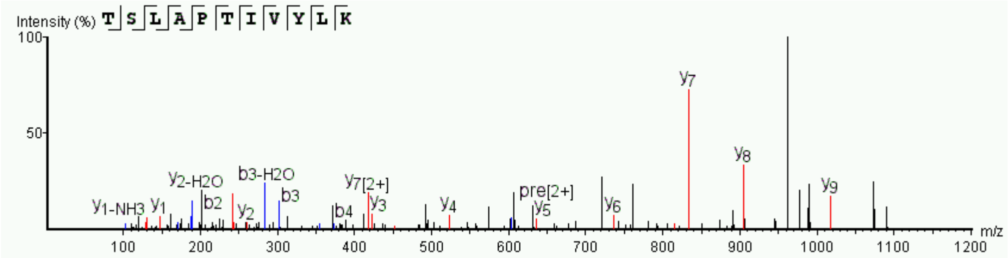

gi|571567640

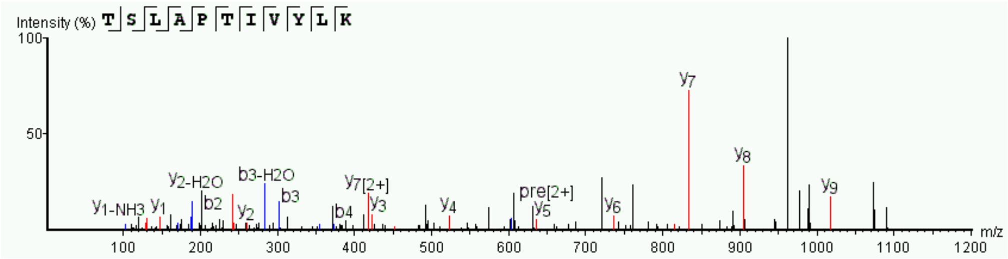

gi|571567643

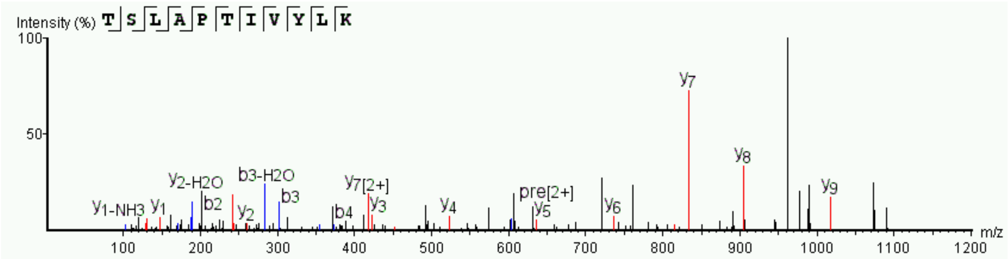

gi|571567650

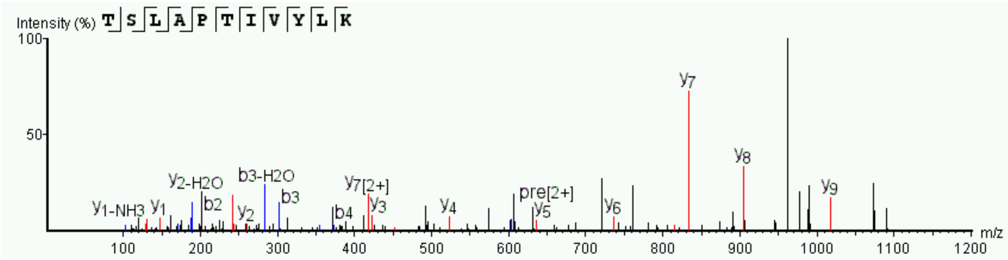

gi|571577919

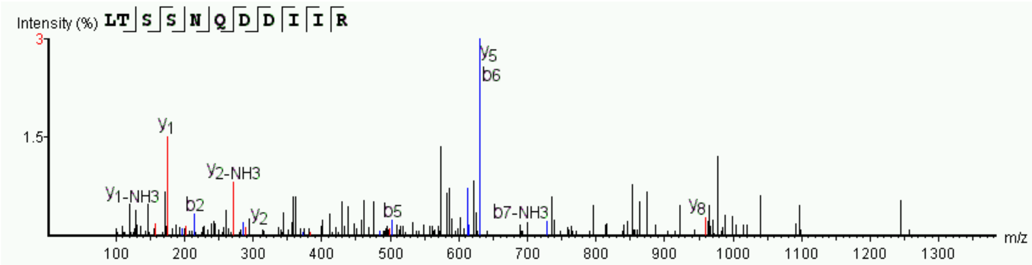

gi|571577921

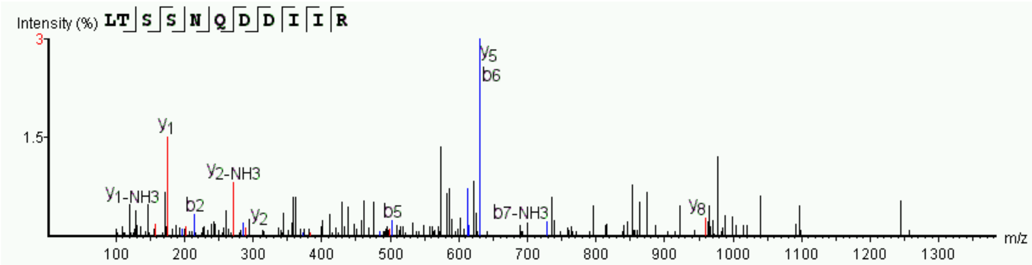

gi|571577923

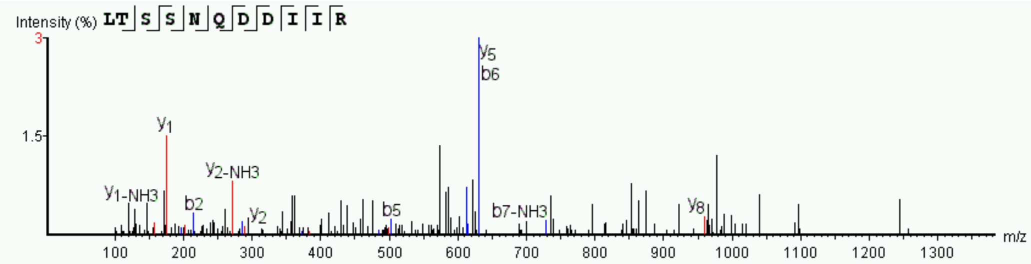

gi|571577925

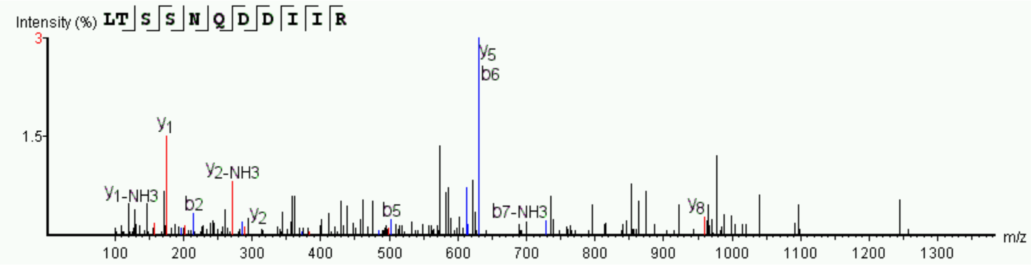

gi|571577927

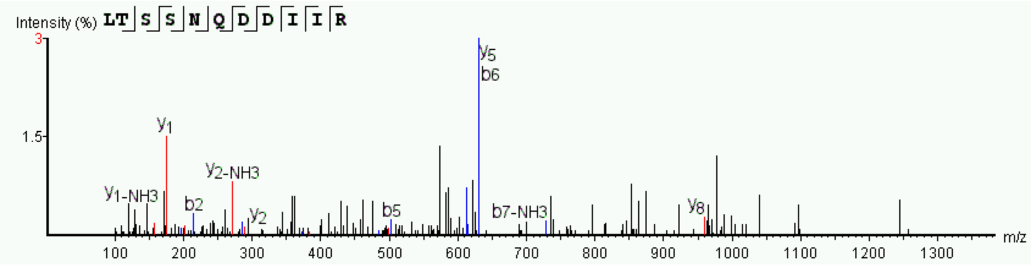

gi|571577929

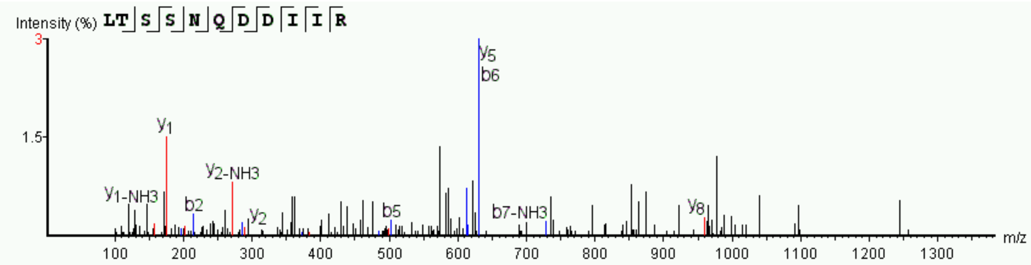

gi|571577931

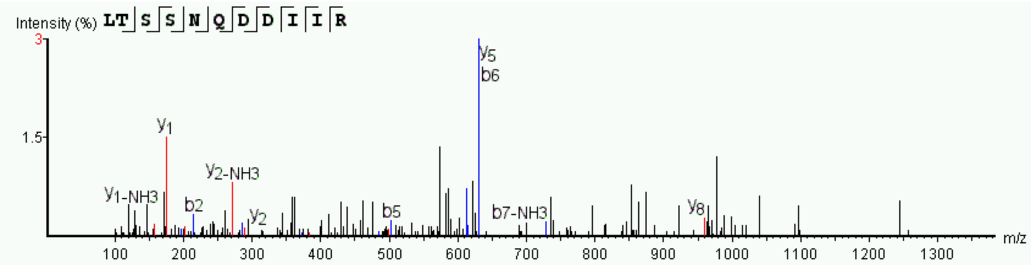

gi|571577933

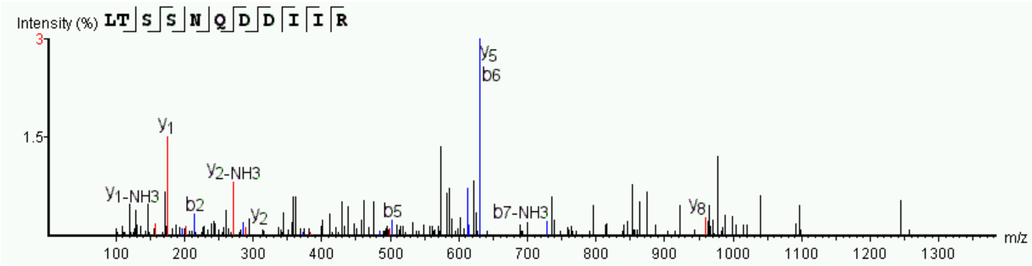

gi|328793799

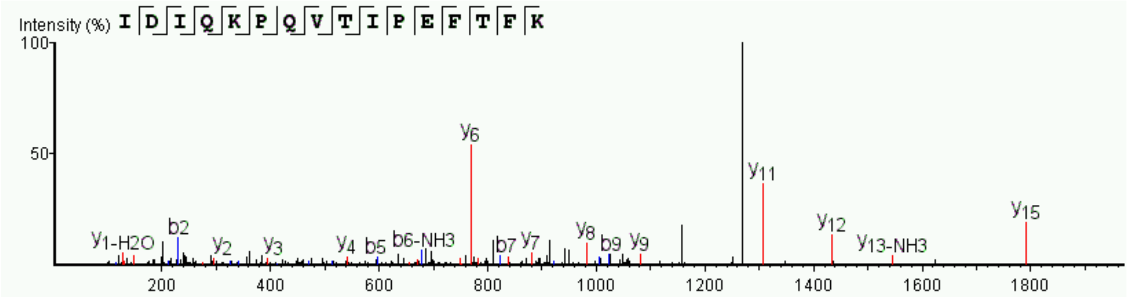

gi|571567654

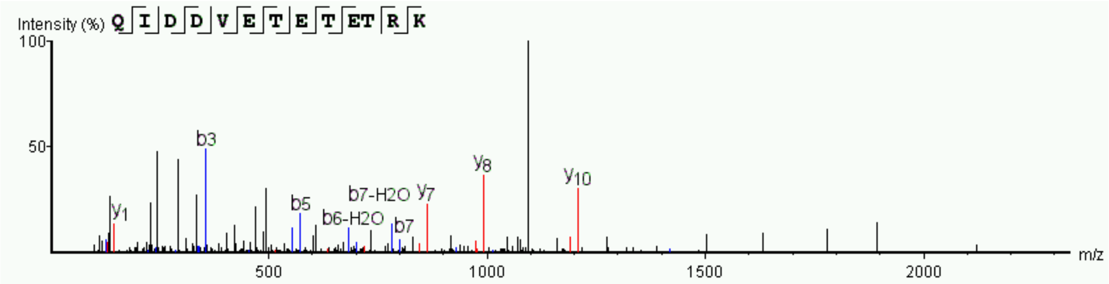

gi|571559017

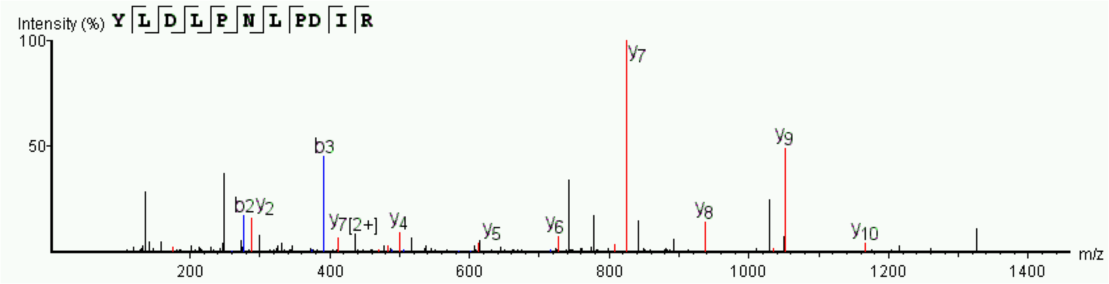

gi|328786521

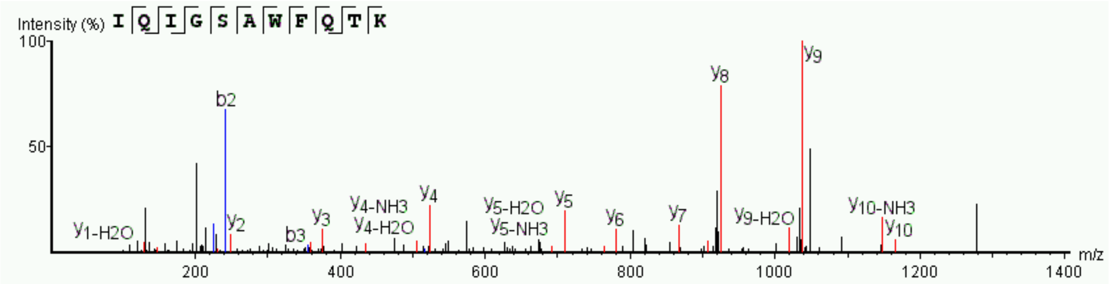

gi|571578311

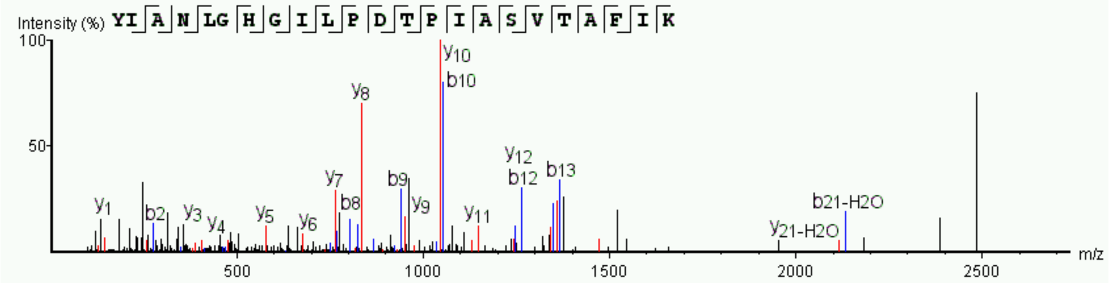

gi|66559873

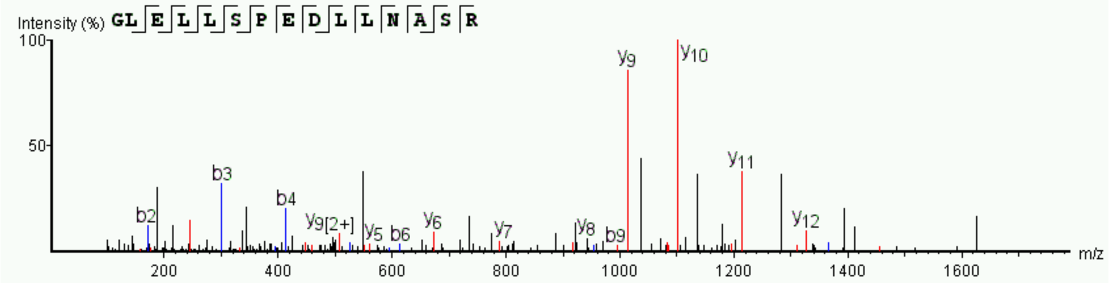

gi|48142179

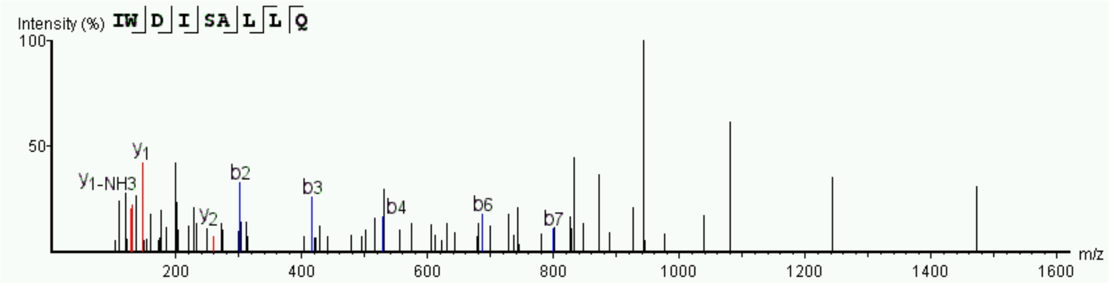

gi|571539226

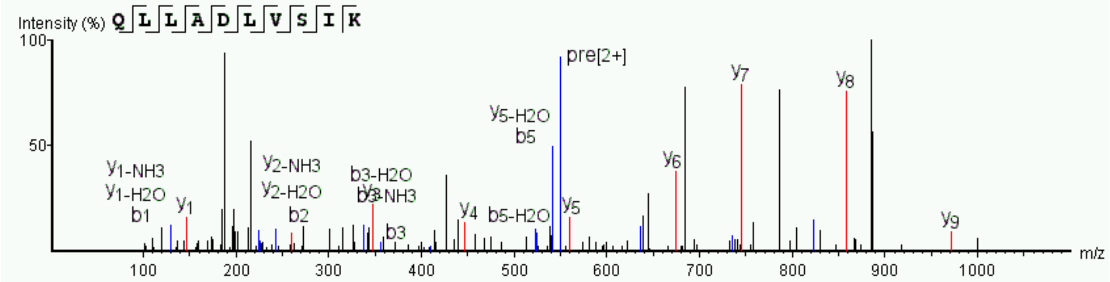

gi|571539223

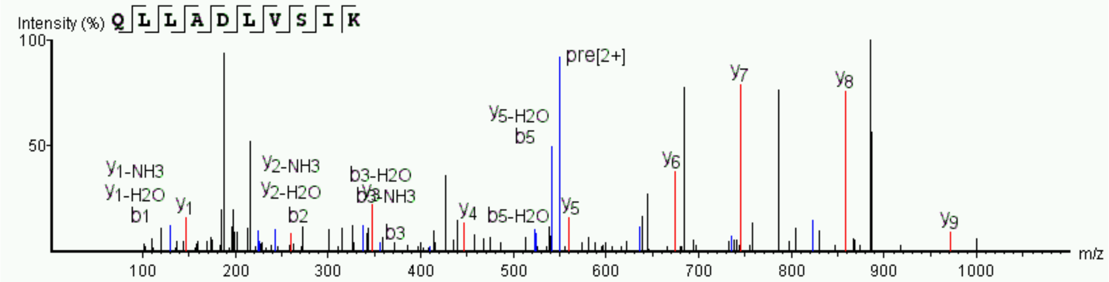

gi|48104995

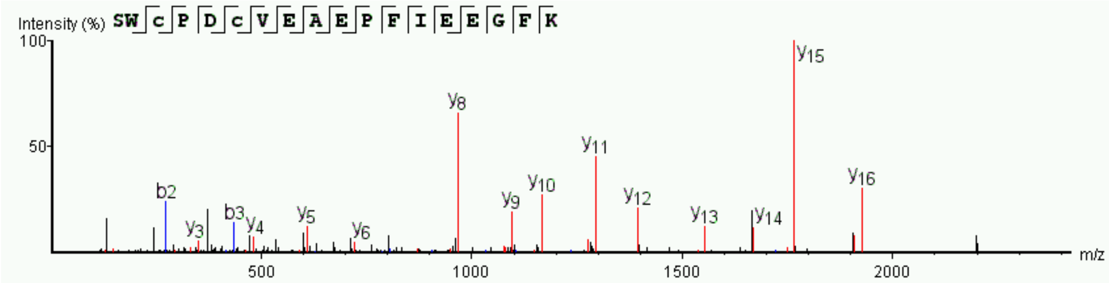

gi|571517348

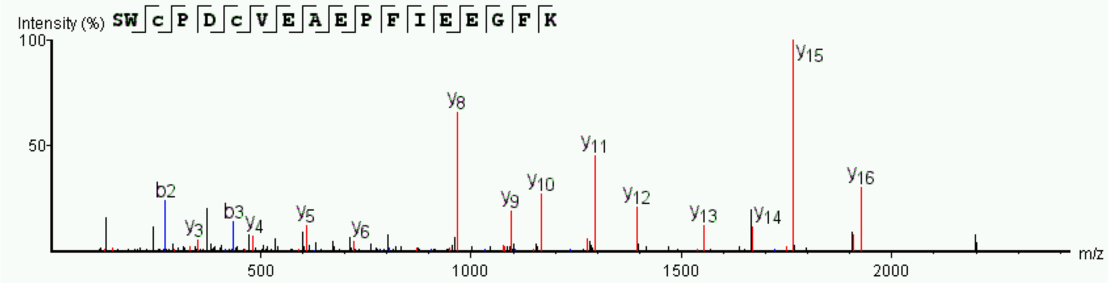

gi|571562624

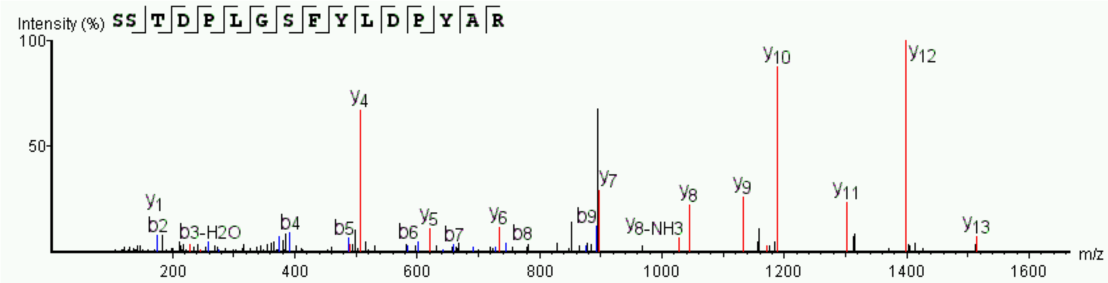

gi|571564004

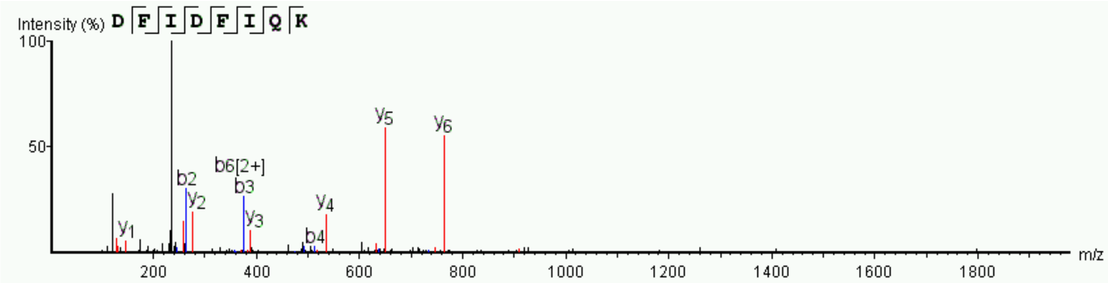

gi|328783180

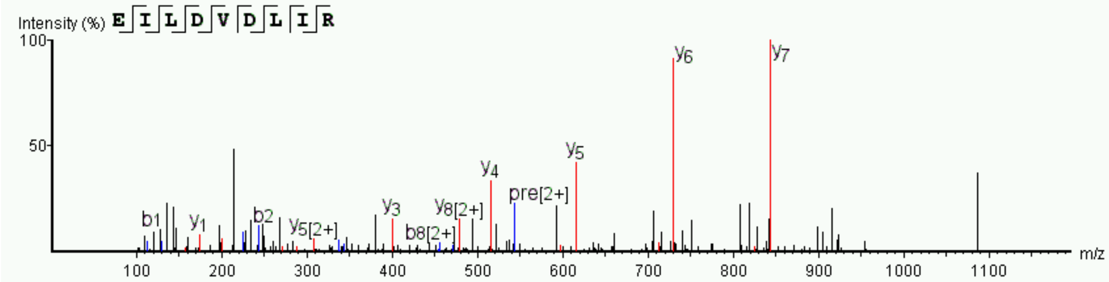

gi|571501719

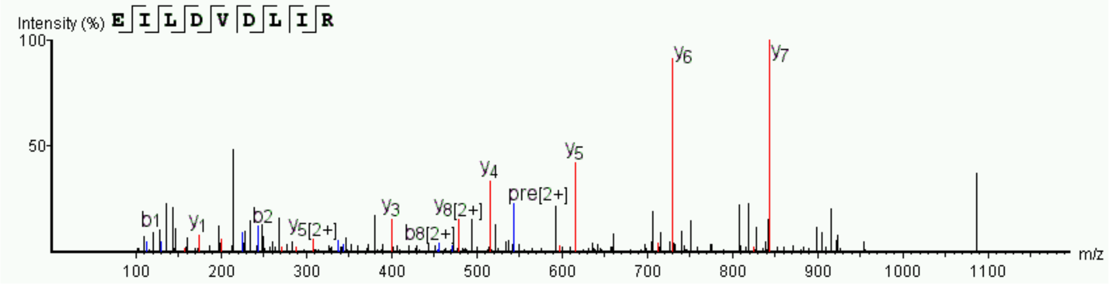

gi|66549382

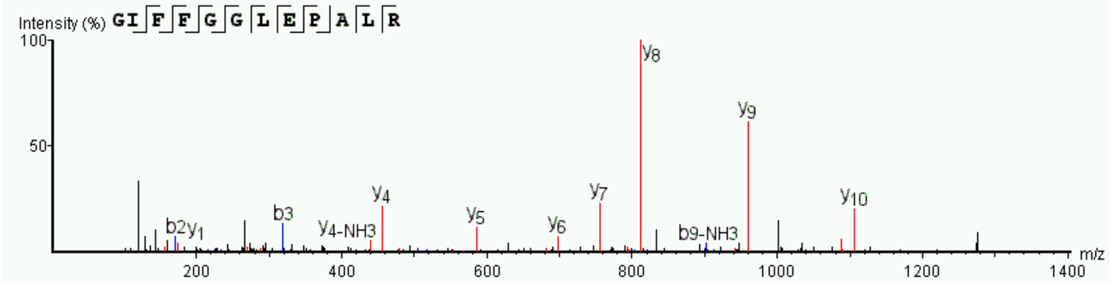

gi|571540223

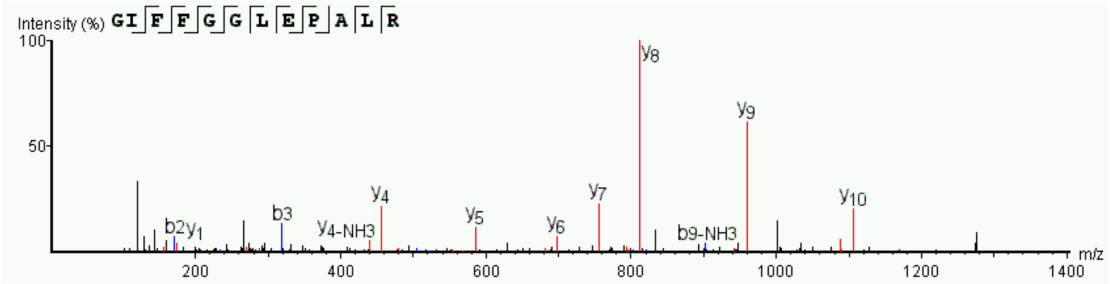

gi|66558726

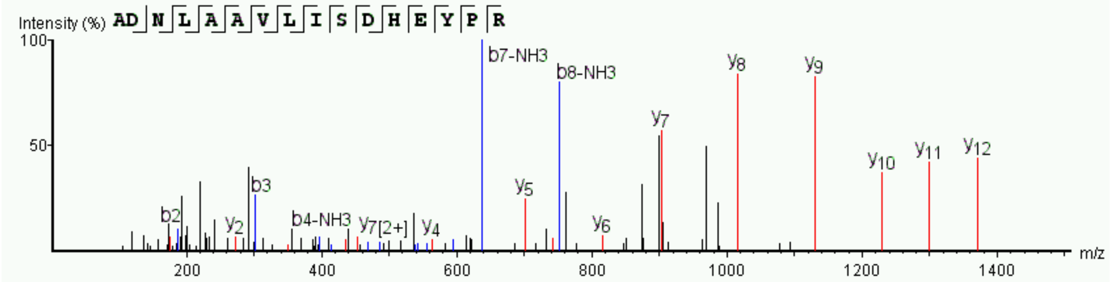

gi|571542705

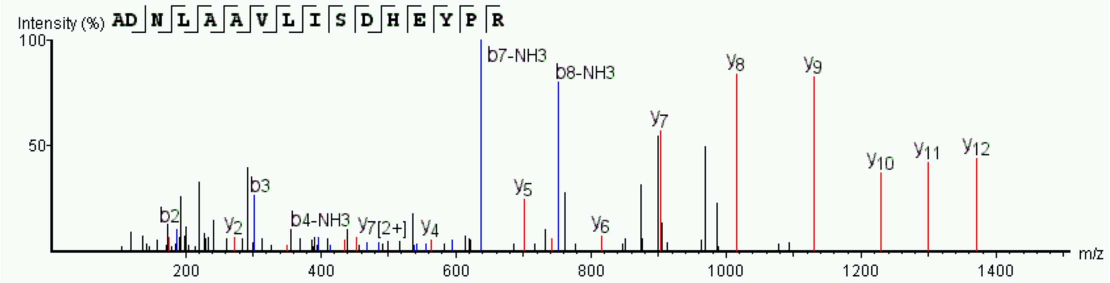

gi|571574853

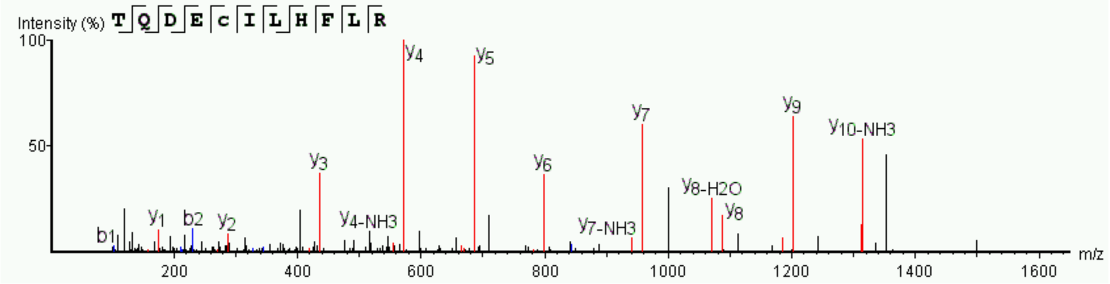

gi|328788838

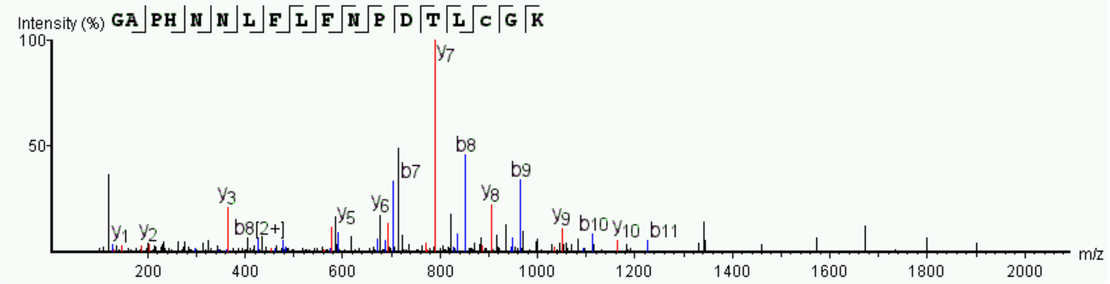

gi|66520699

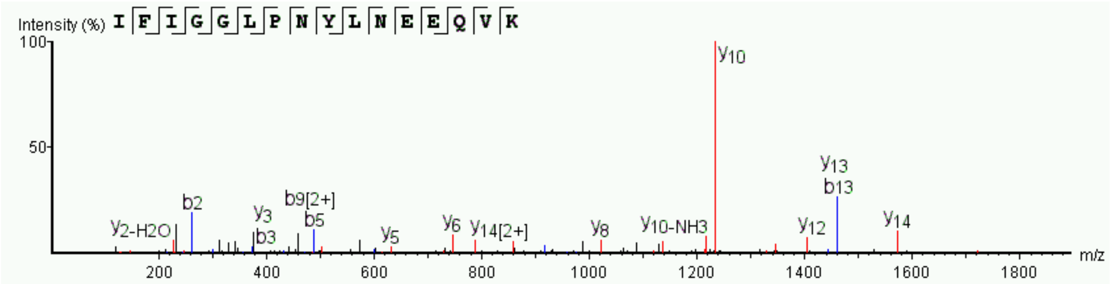

gi|571556649

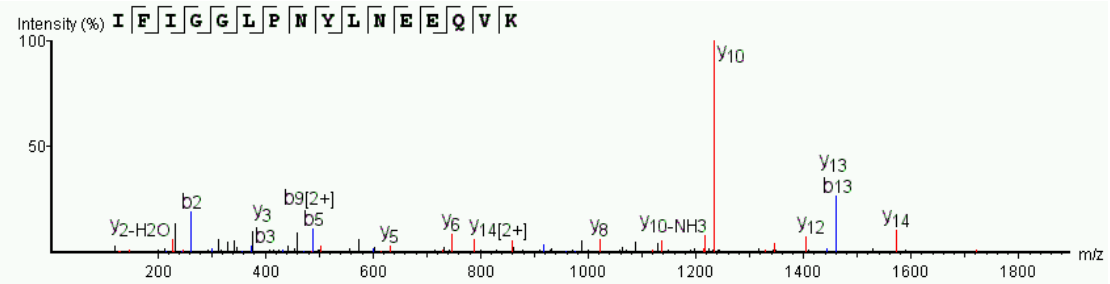

gi|110756801

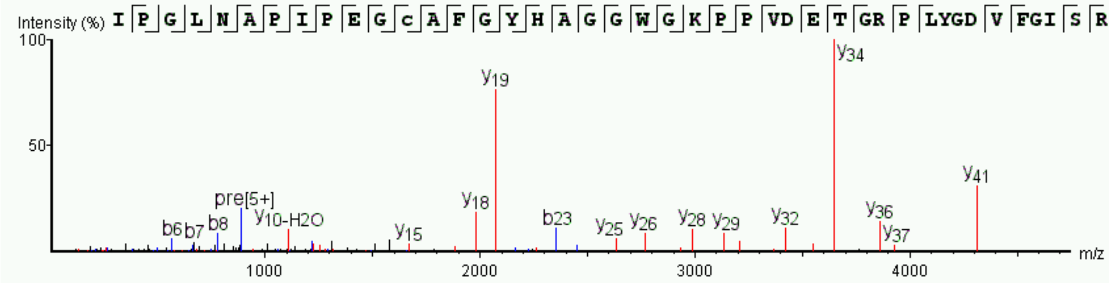

gi|571575127

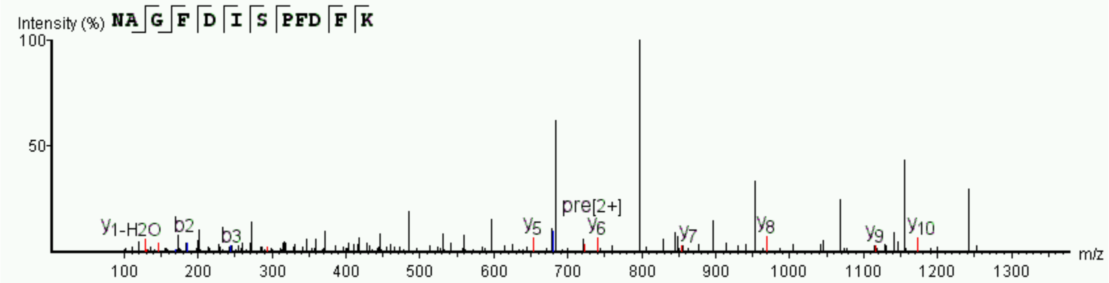

gi|66504030

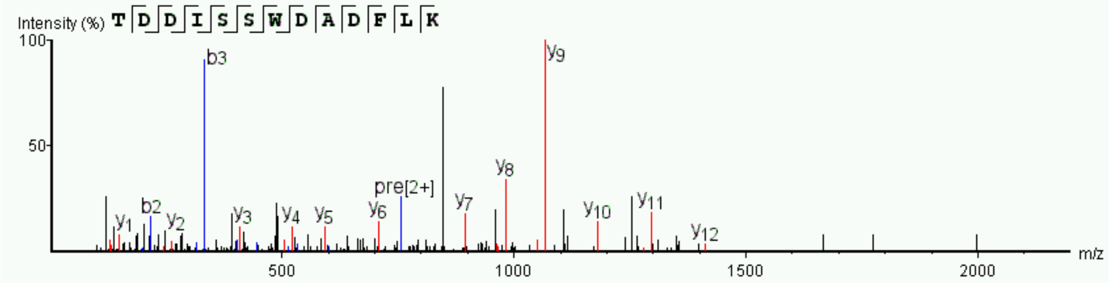

gi|66525345

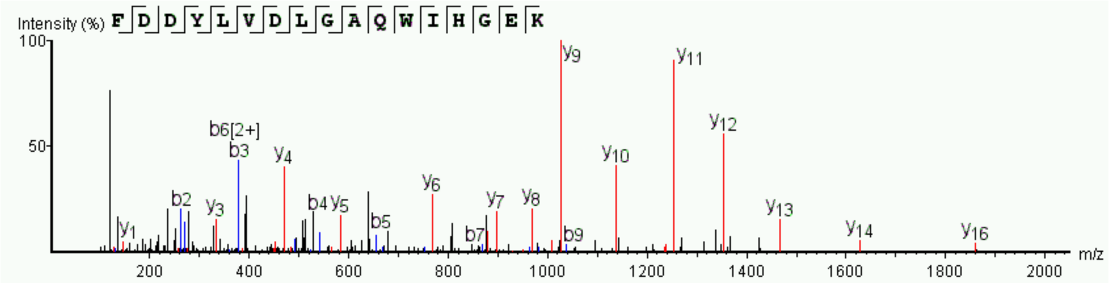

gi|571575549

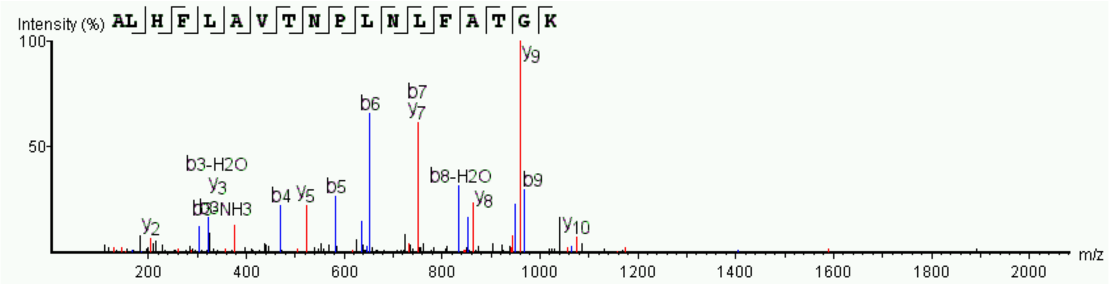

gi|571575571

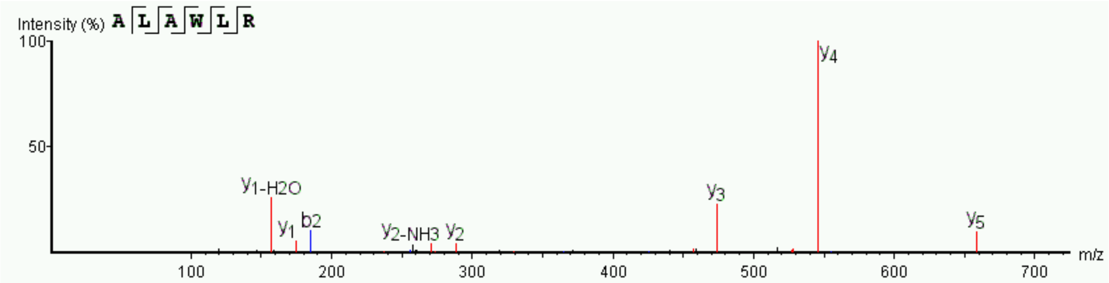

gi|571542313

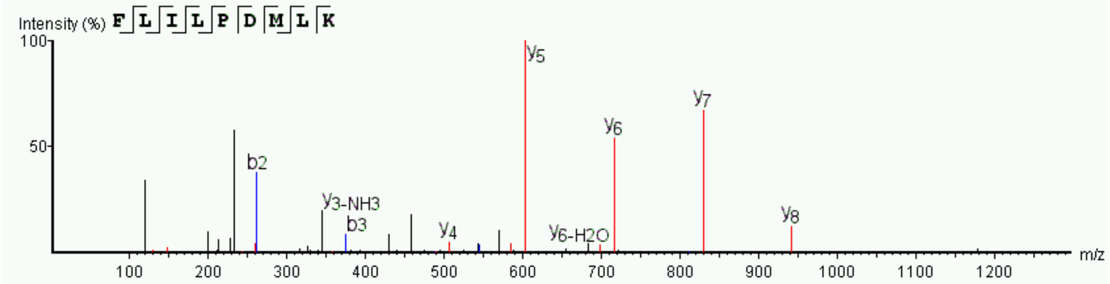

gi|110758613

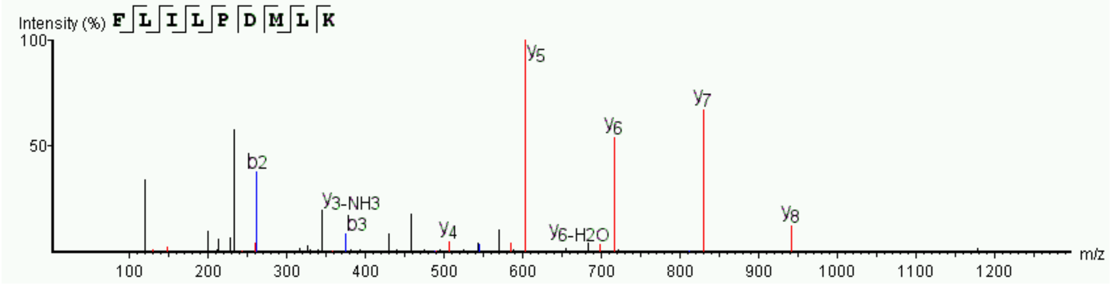

gi|110767674

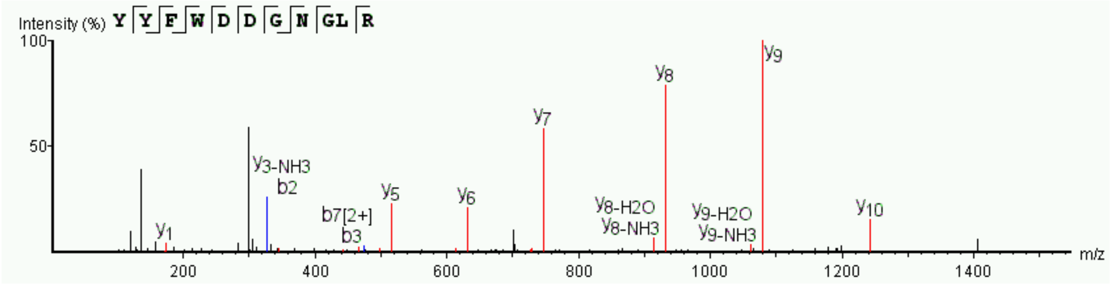

gi|66525633

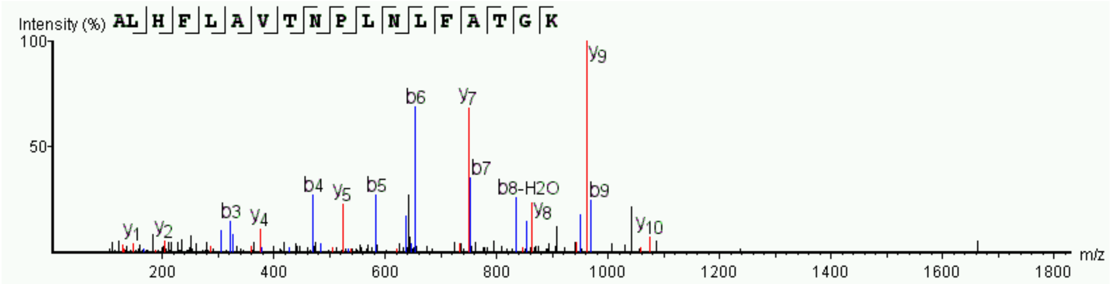

gi|66557634

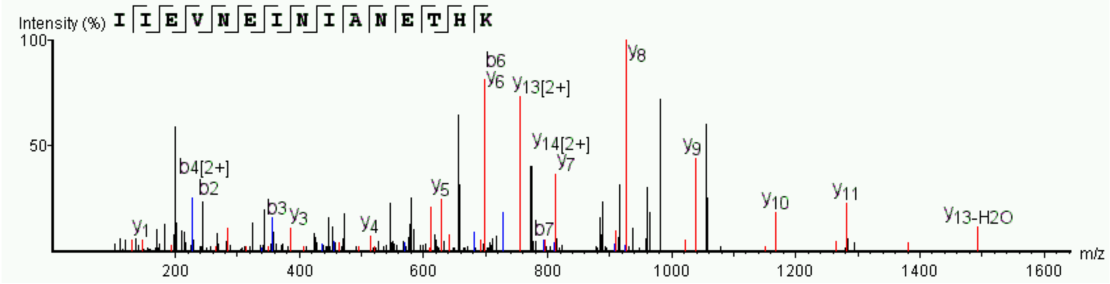

gi|571514770

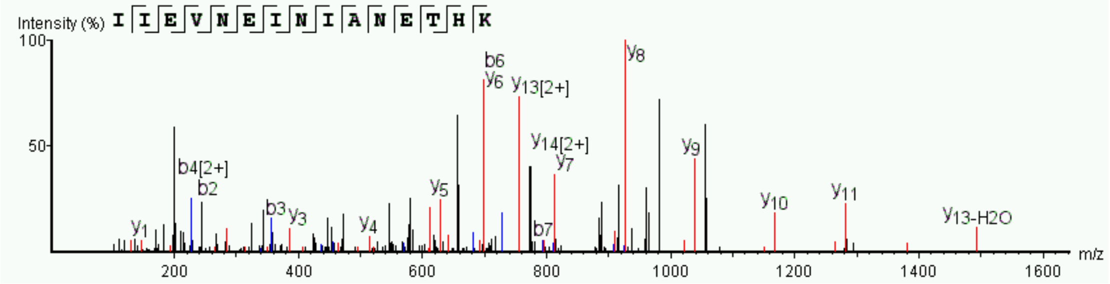

gi|328778804

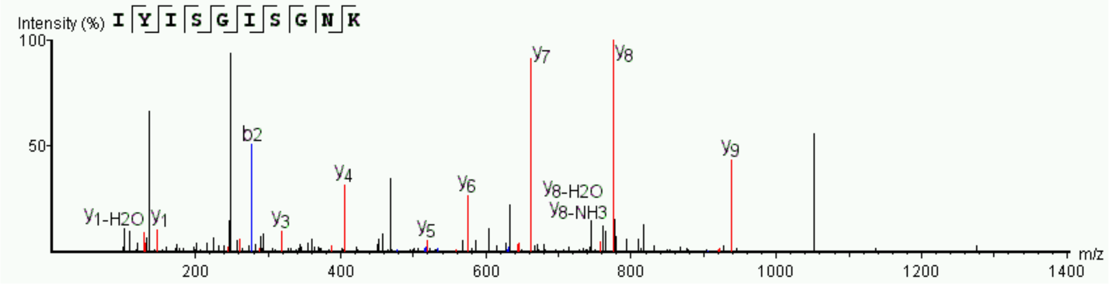

gi|66560999

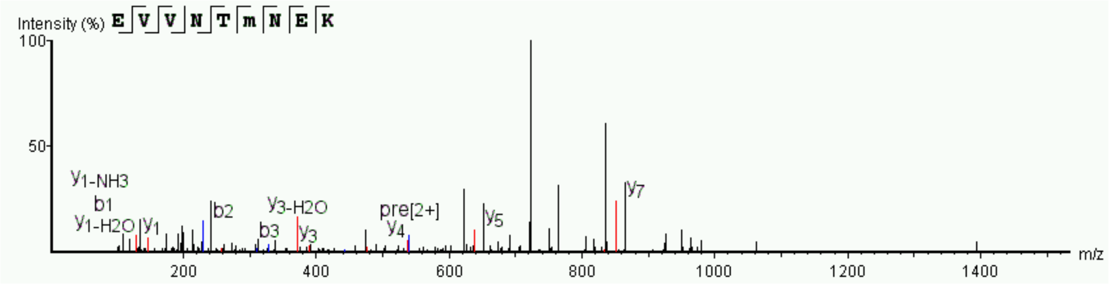

gi|571578209

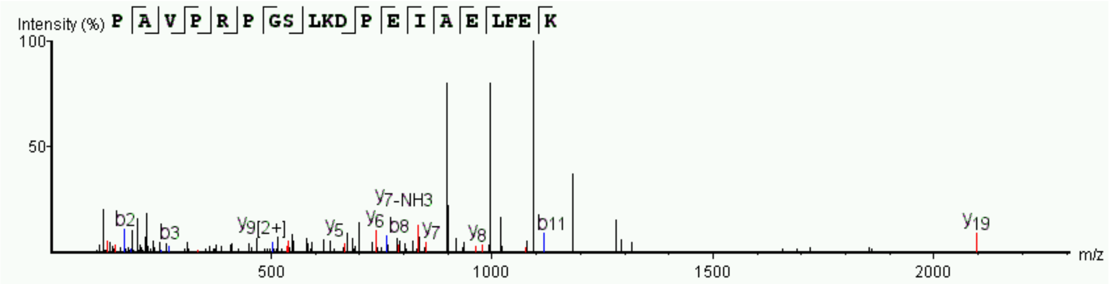

gi|571534388

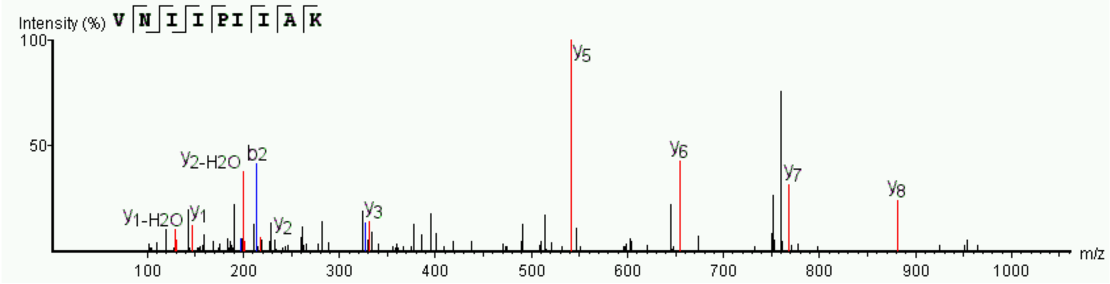

gi|571534385

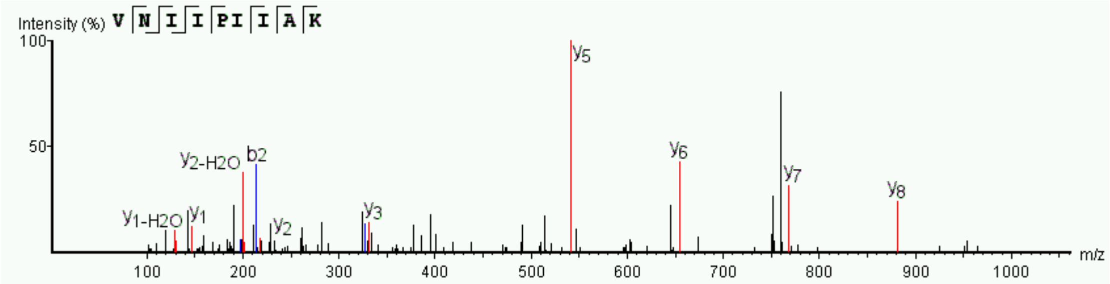

gi|571534382

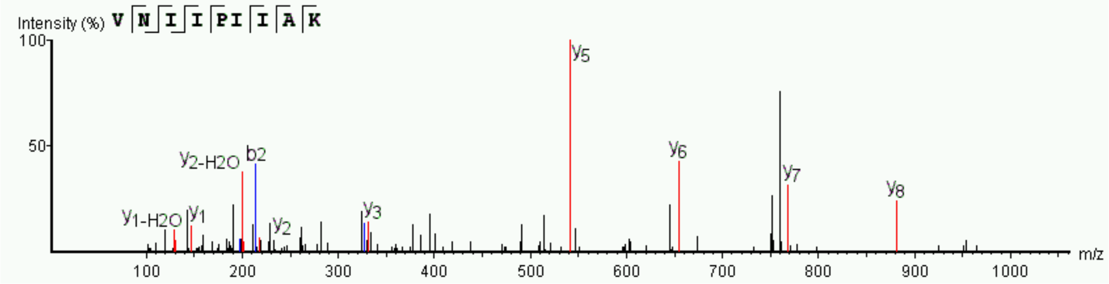

gi|328779563

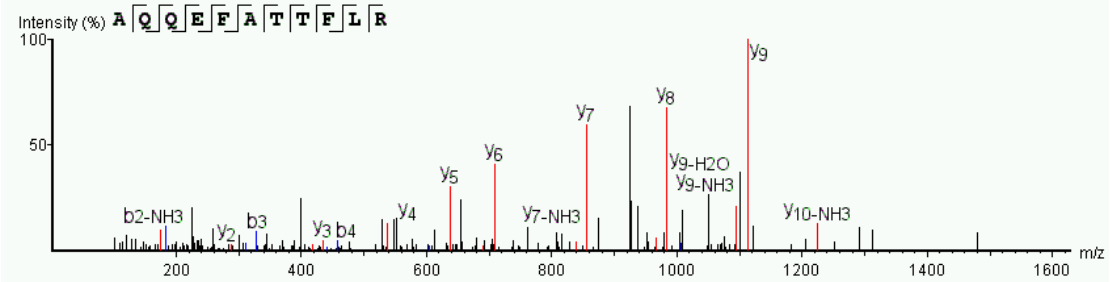

gi|571568001

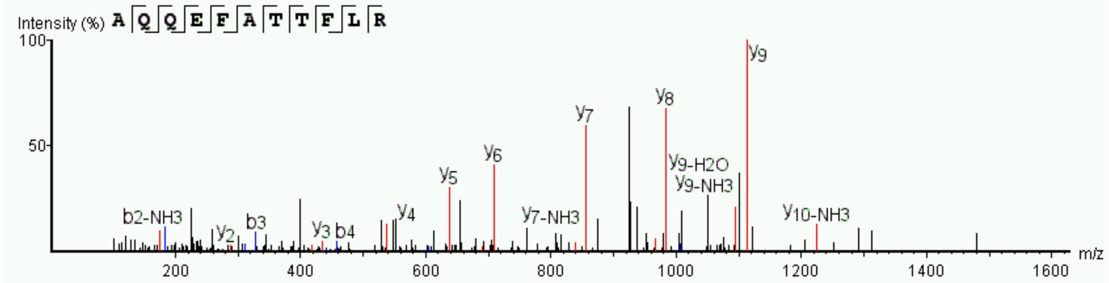

gi|571522976

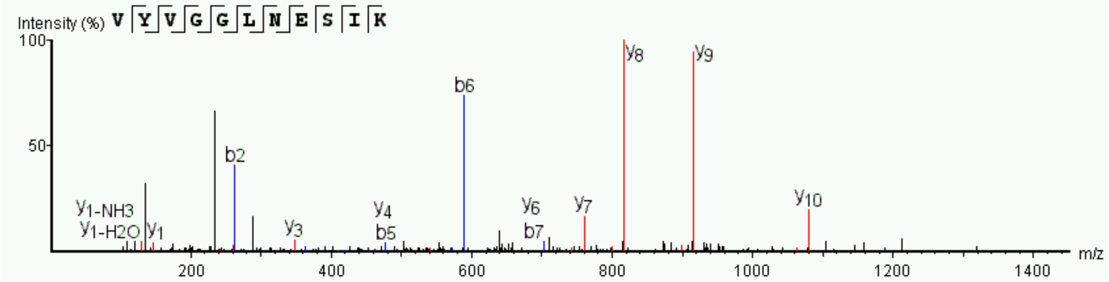

gi|571565646

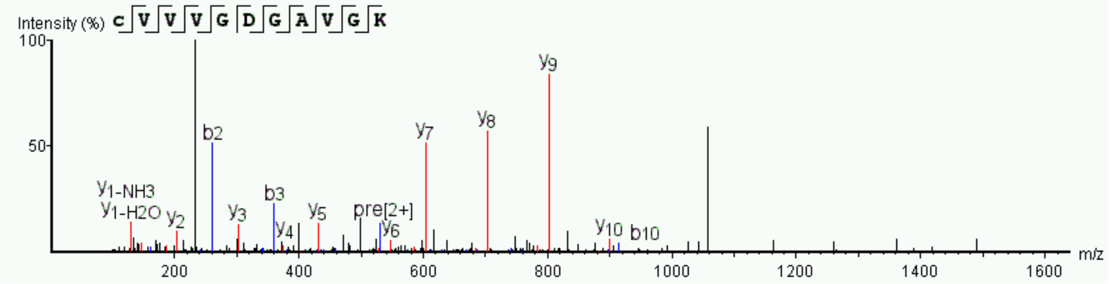

gi|328780081

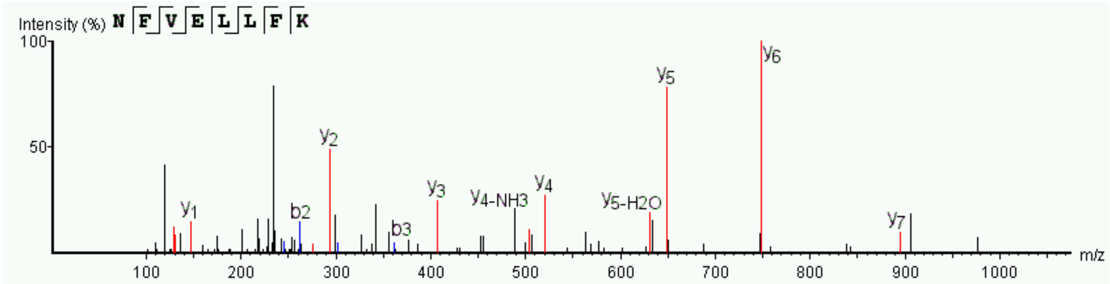

gi|571563867

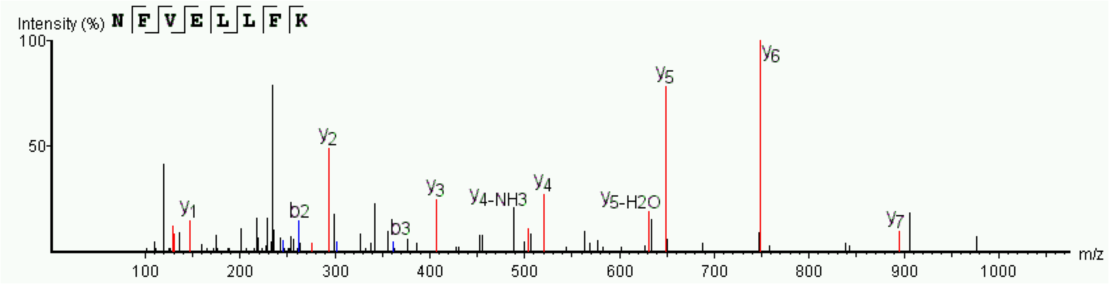

gi|571558915

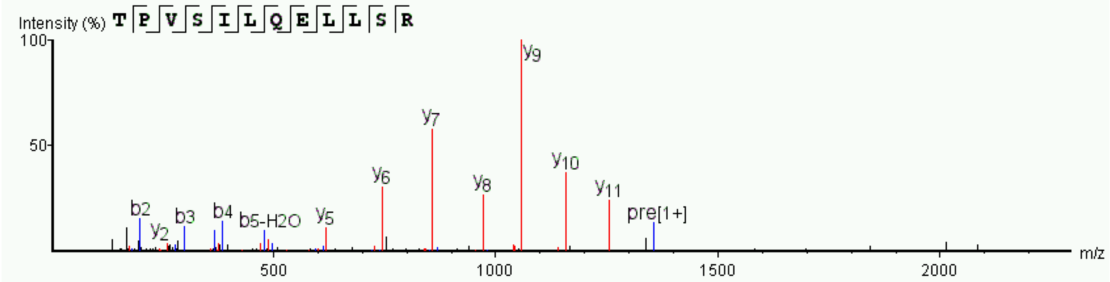

gi|571558912

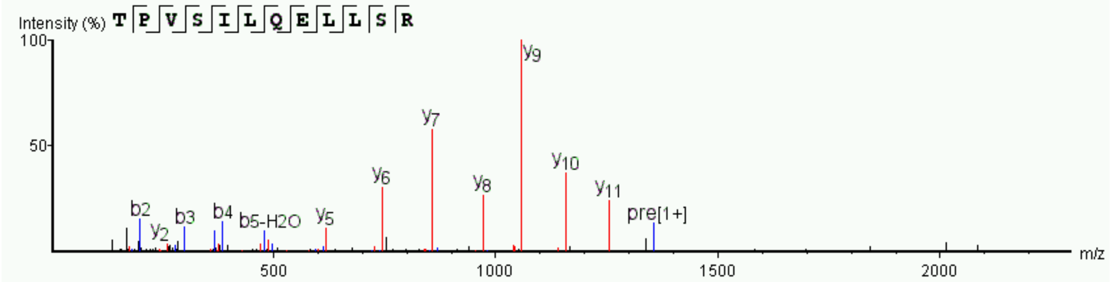

gi|571558905

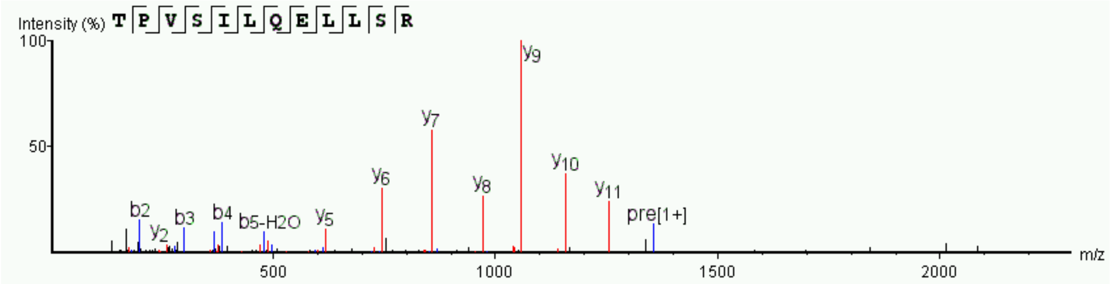

gi|571558909

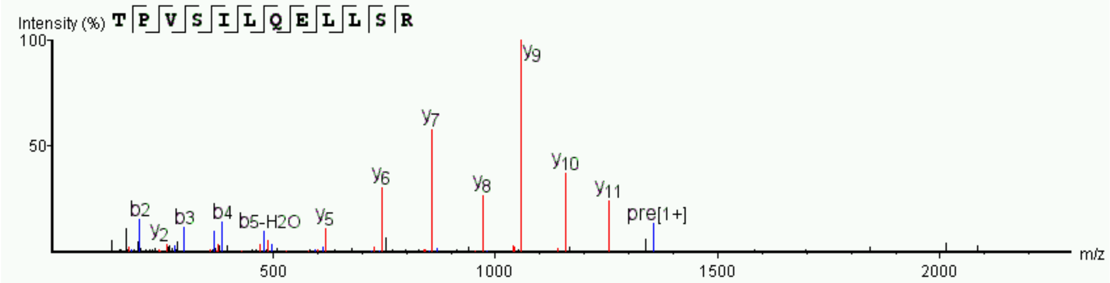

gi|571558902

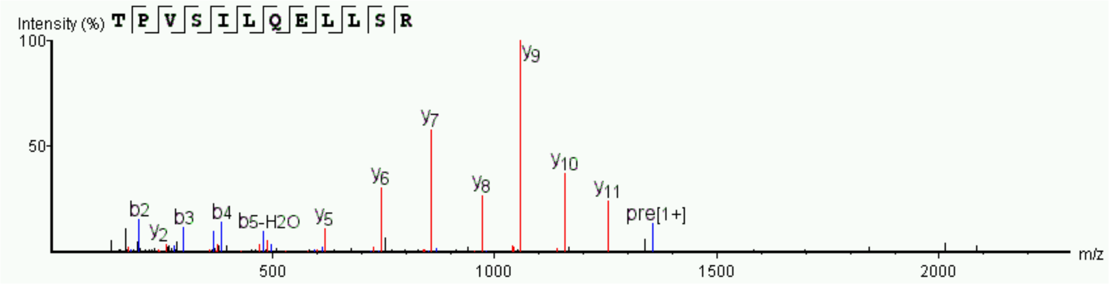

gi|571543246

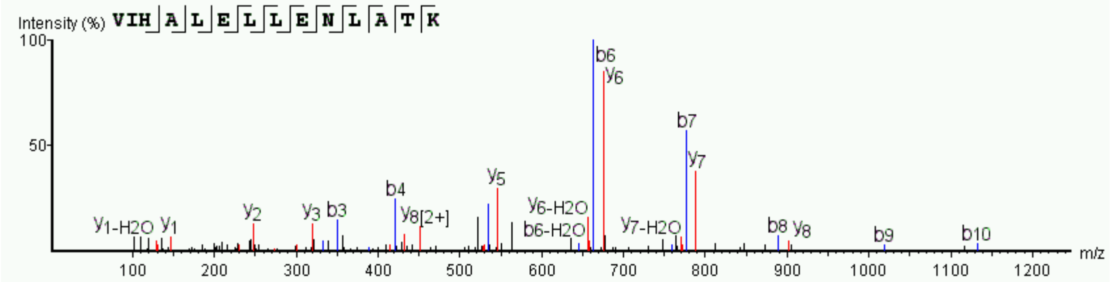

gi|571543249

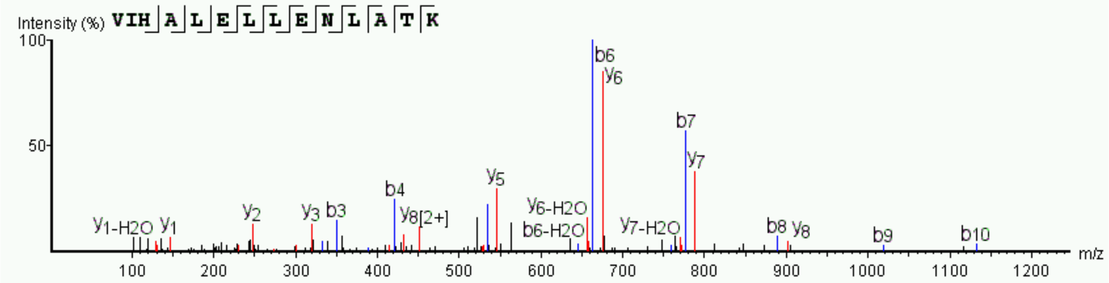

gi|571526744

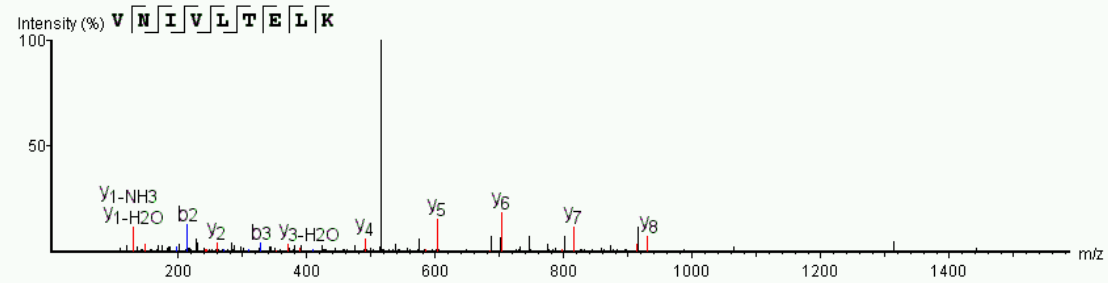

gi|48142169

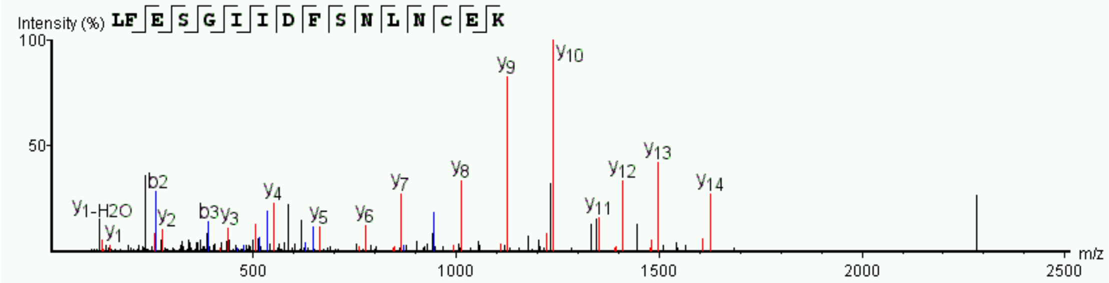

gi|571508187

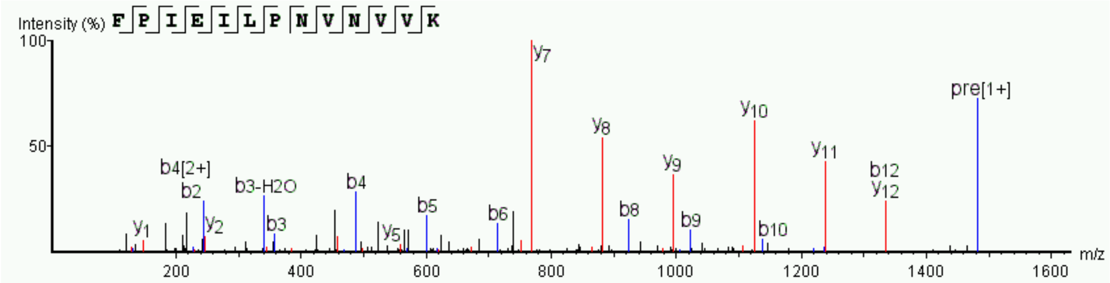

gi|571508183

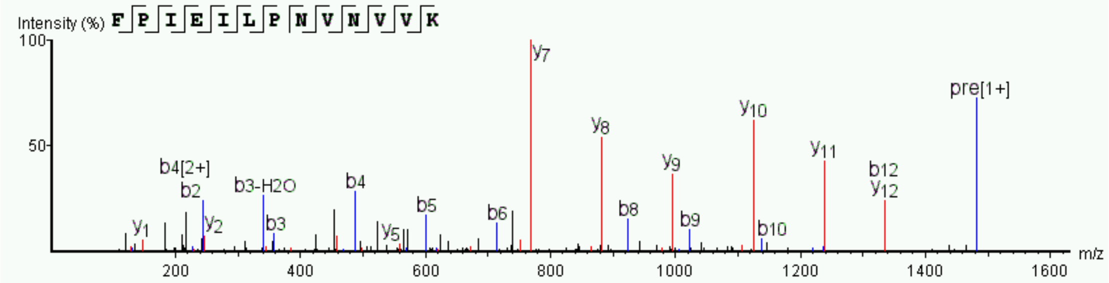

gi|66499475

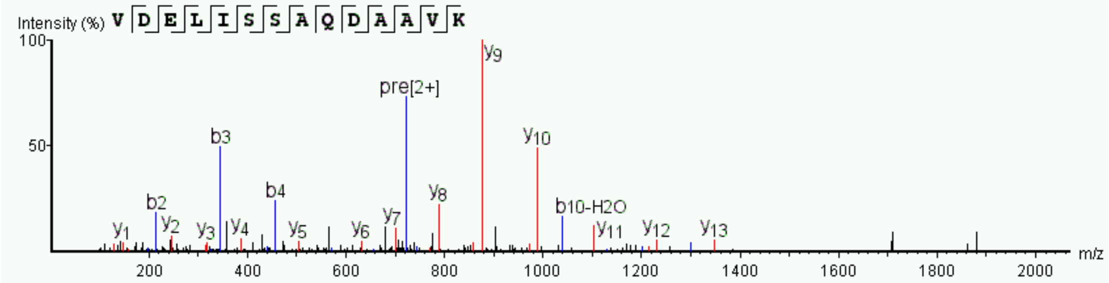

gi|328778195

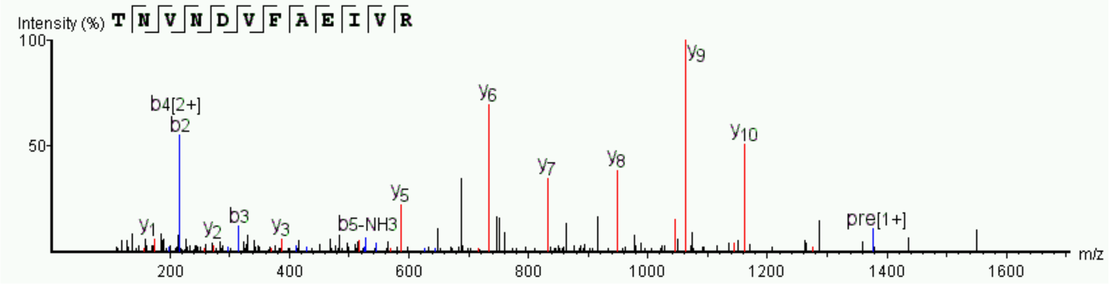

gi|66513978

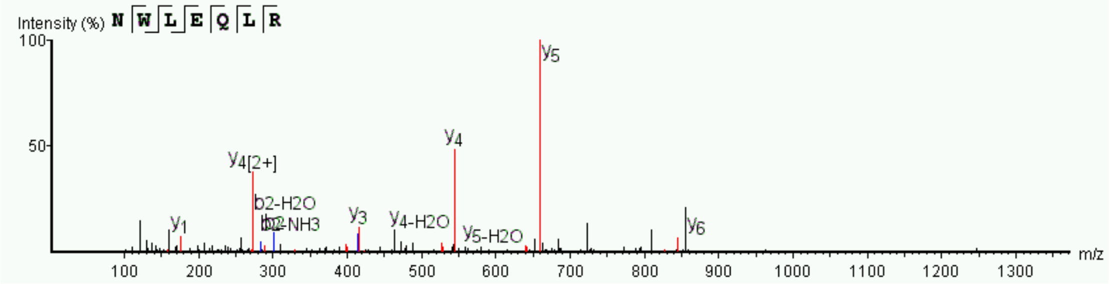

gi|328782334

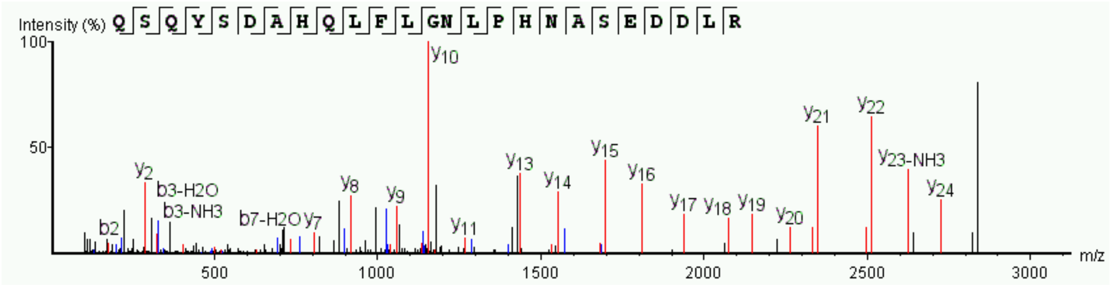

gi|571538909

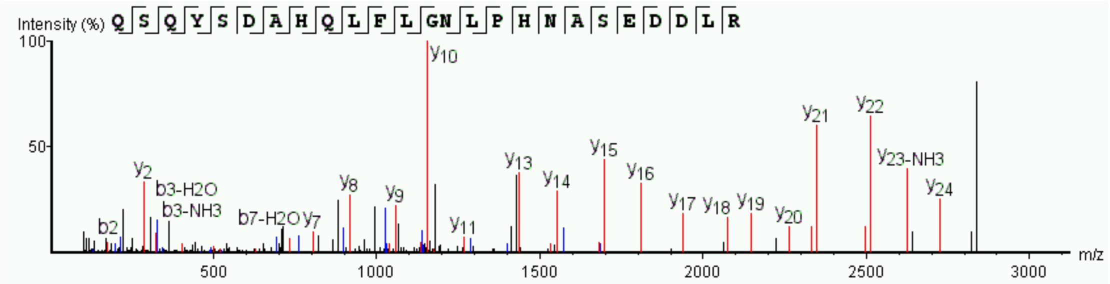

gi|571541137

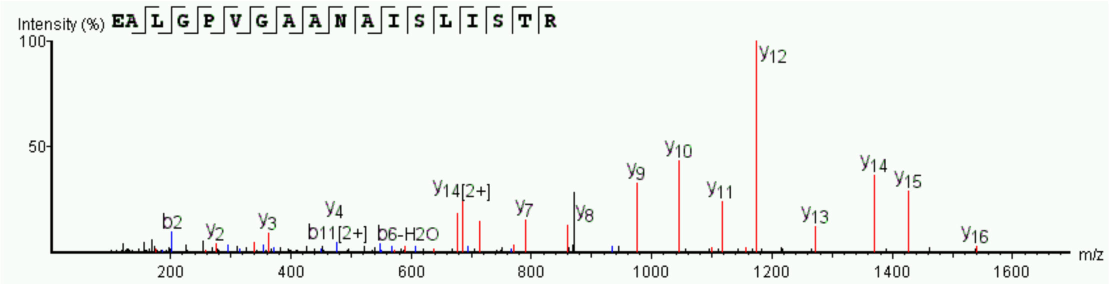

gi|110761748

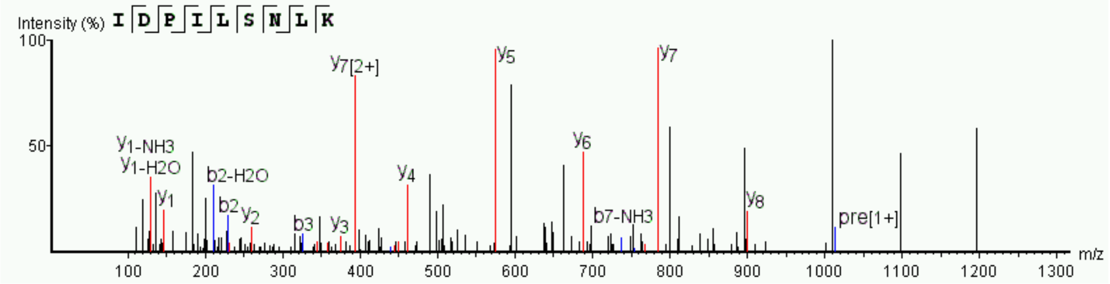

gi|571530920

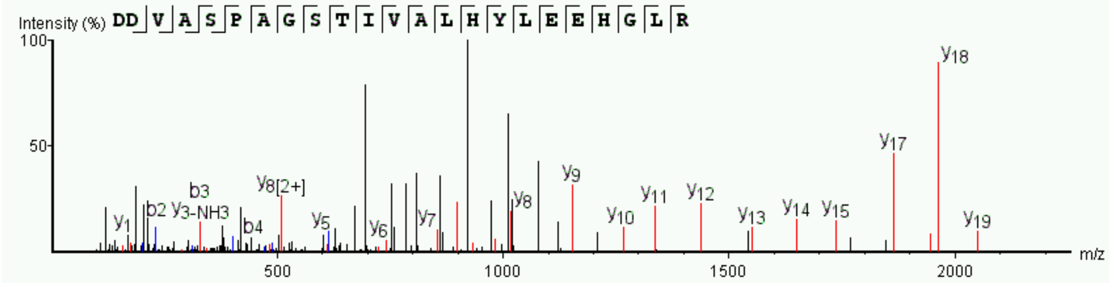

gi|48096057

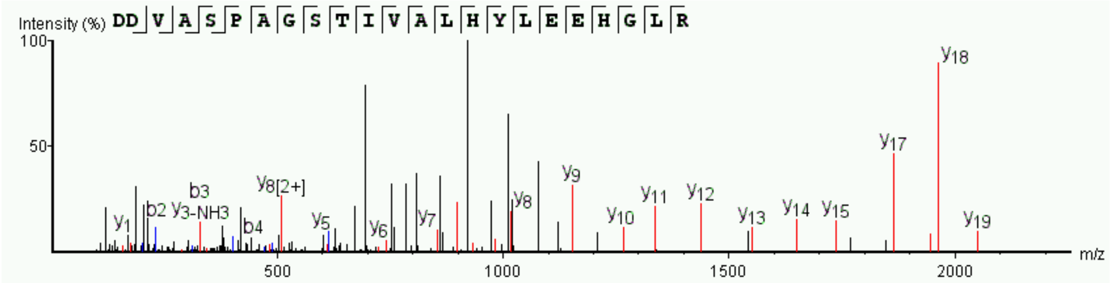

gi|571530913

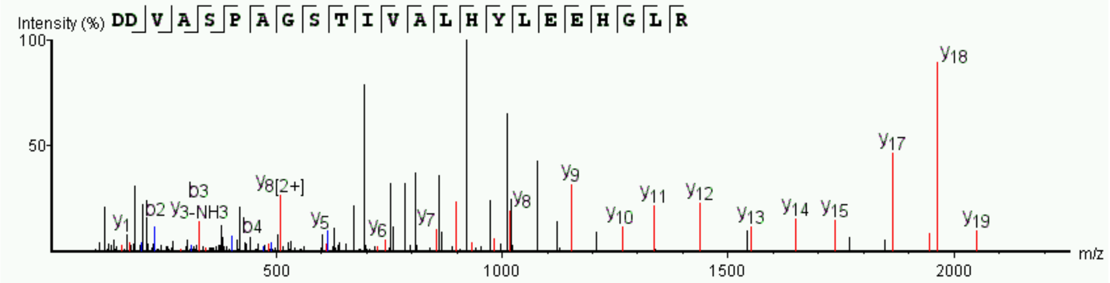

gi|66534900

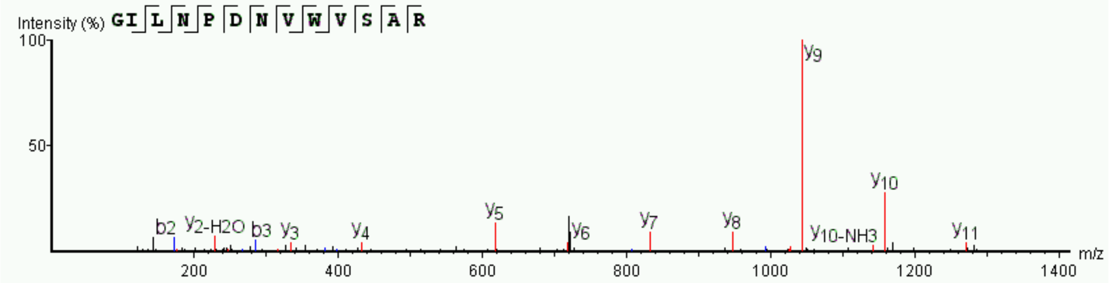

gi|328792791

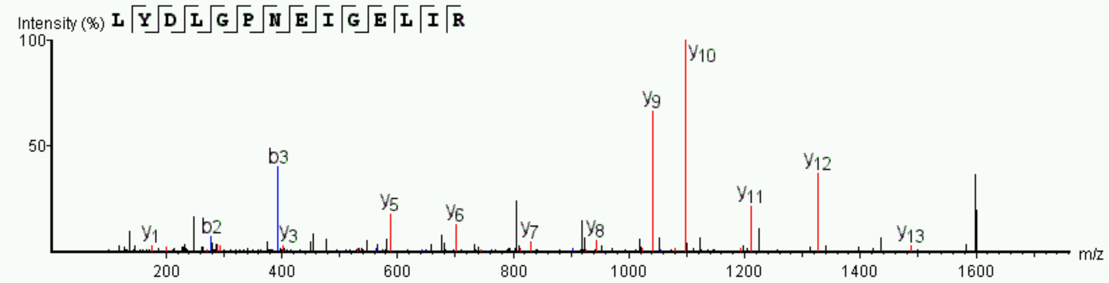

gi|328788528

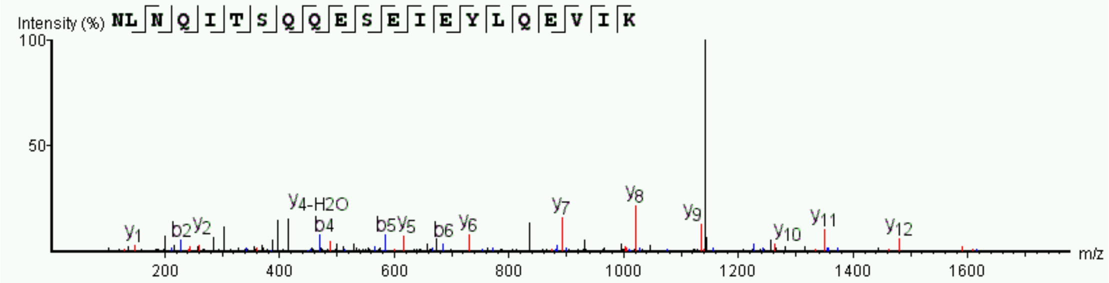

gi|328782255

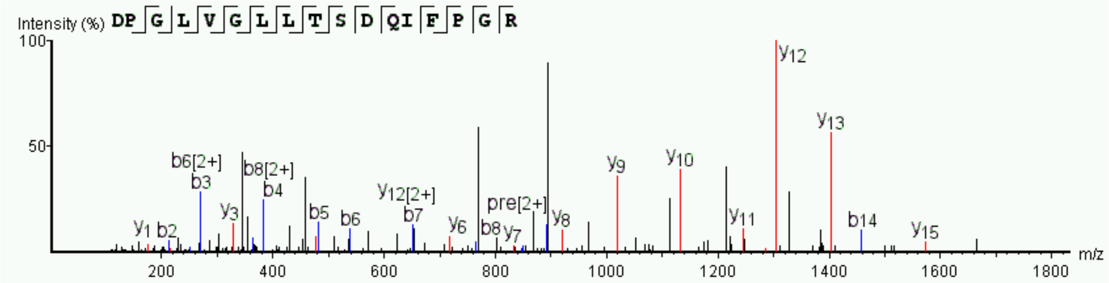

gi|571532691

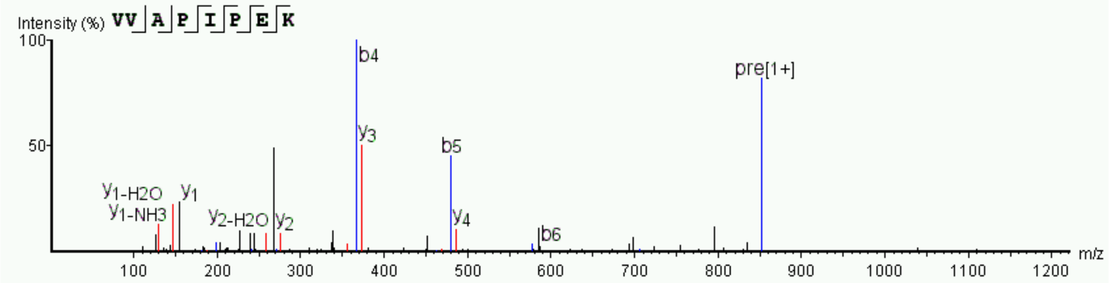

gi|328785850

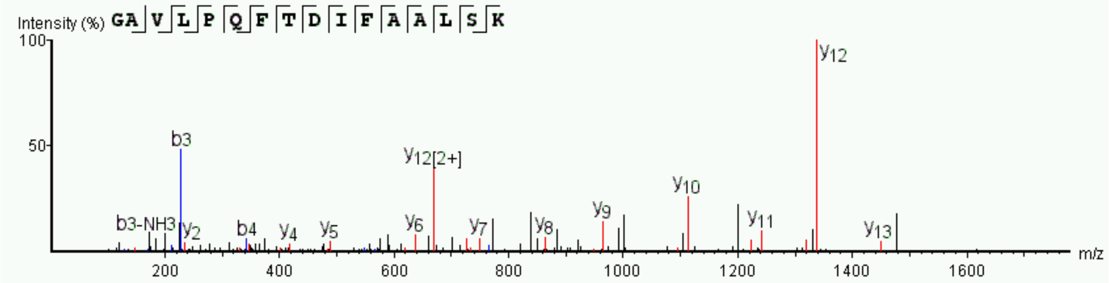

gi|328782520

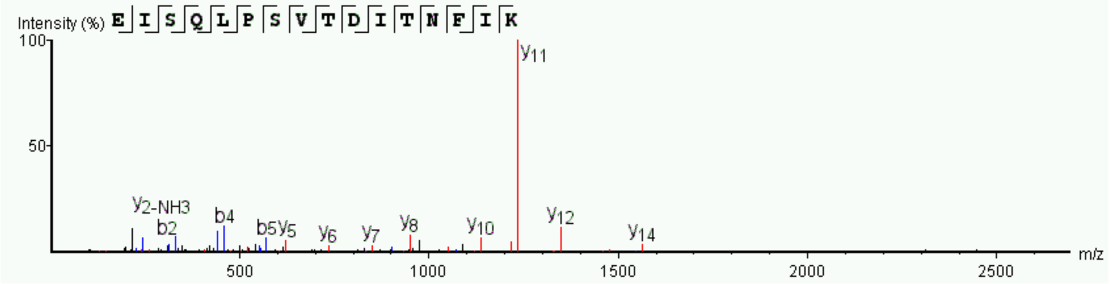

gi|110750012

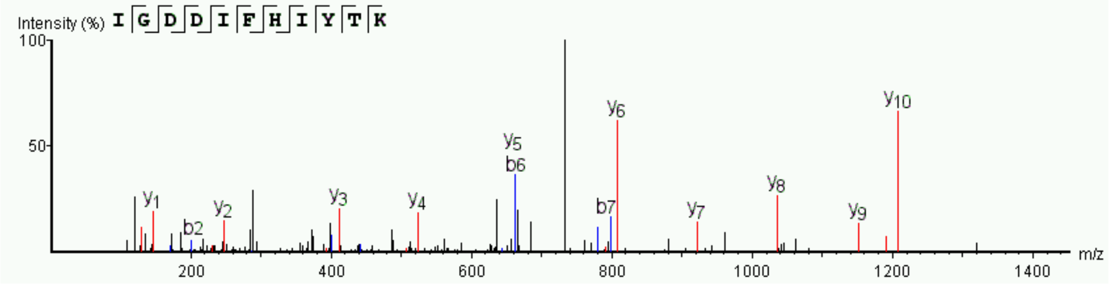

gi|328778377

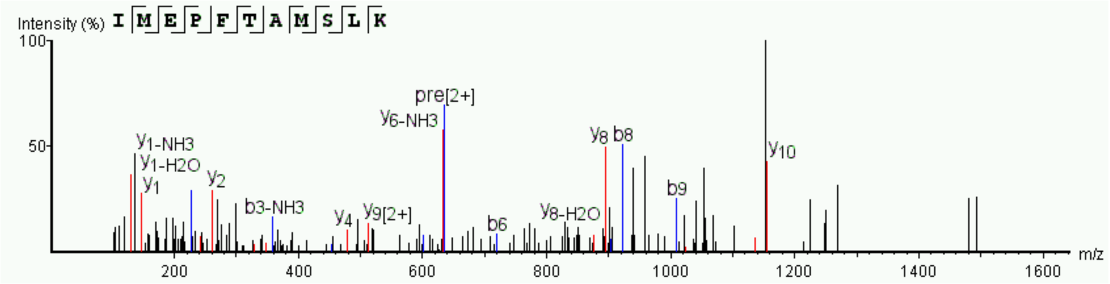

gi|571542915

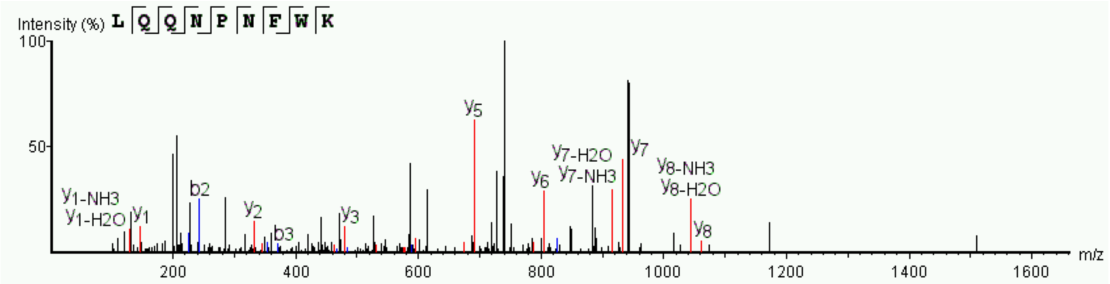

gi|66548732

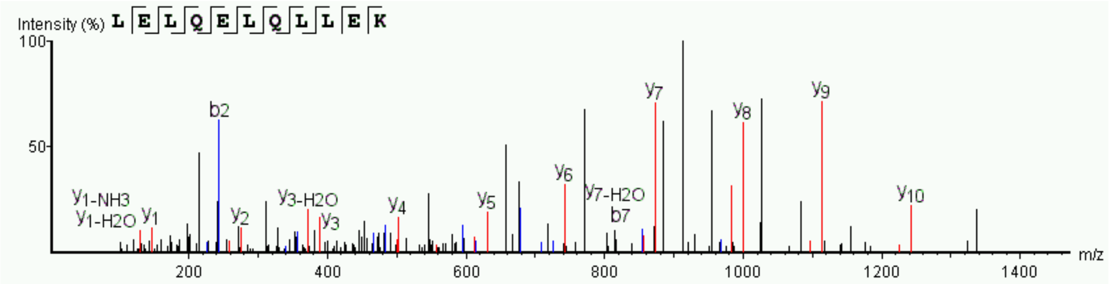

gi|48103834

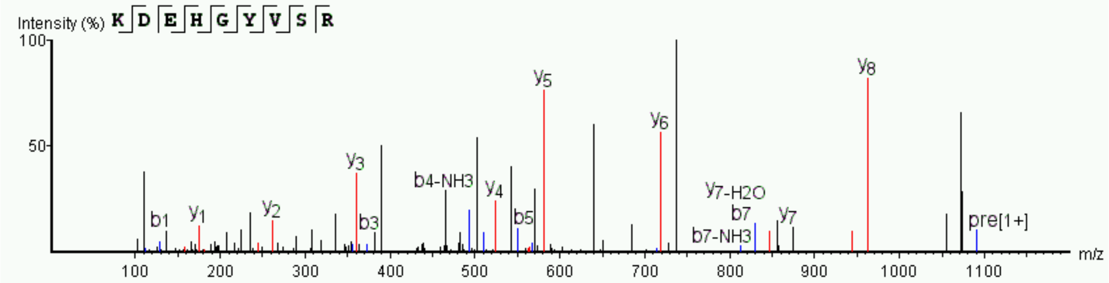

gi|571568560

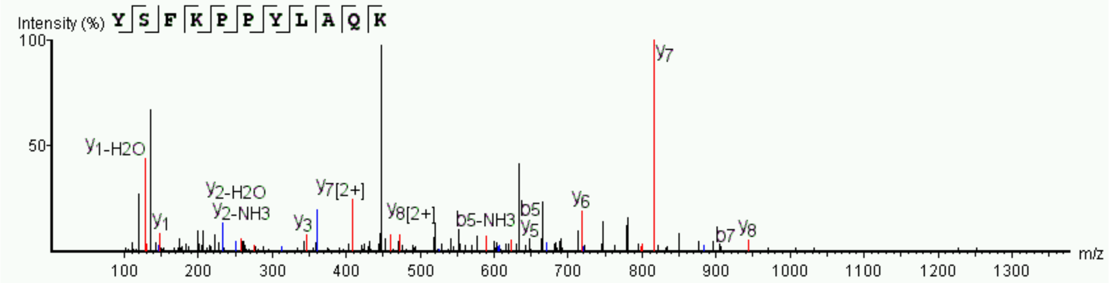

gi|571525107

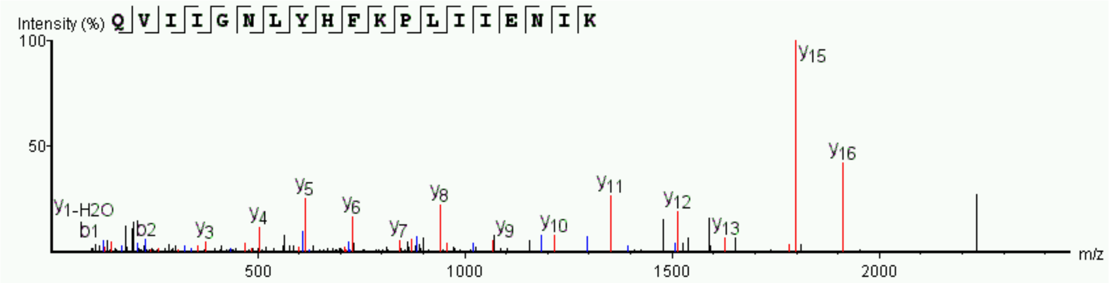

gi|66519842

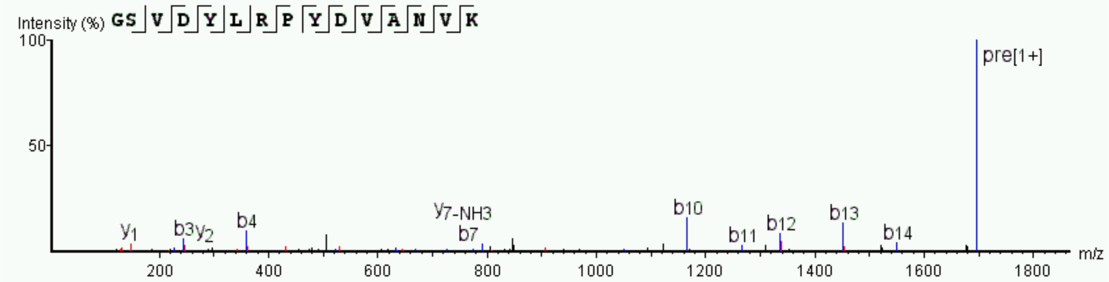

gi|66524764

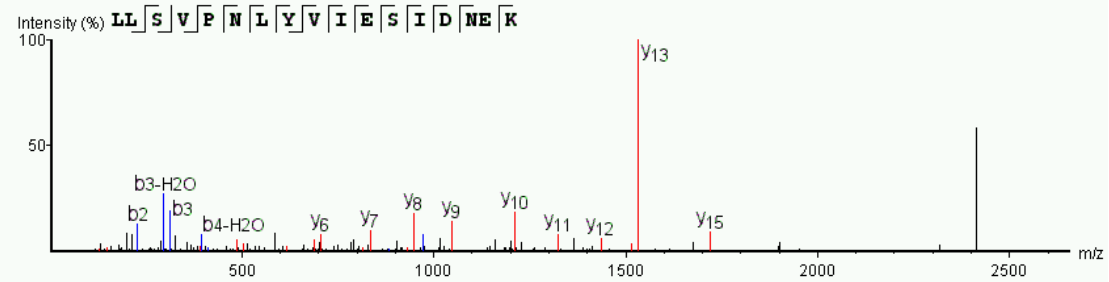

gi|66500455

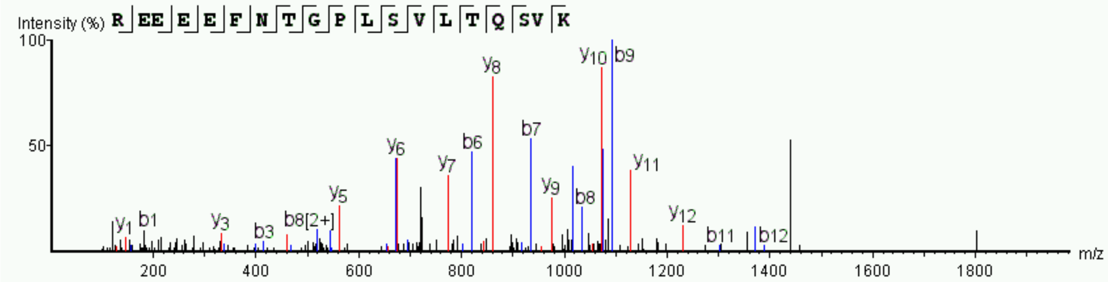

gi|66509459

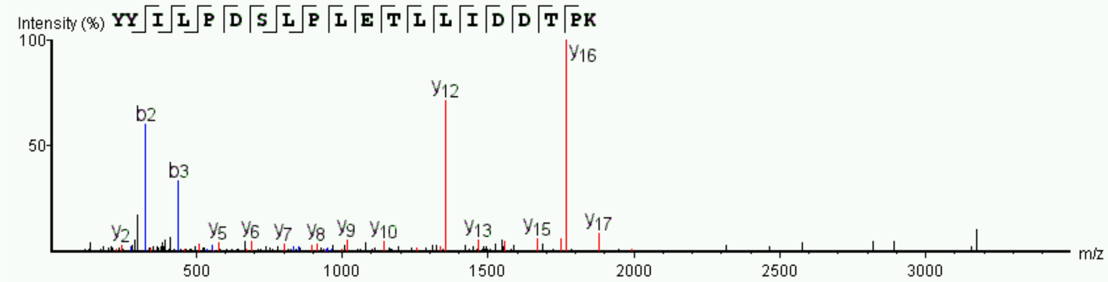

gi|66525153

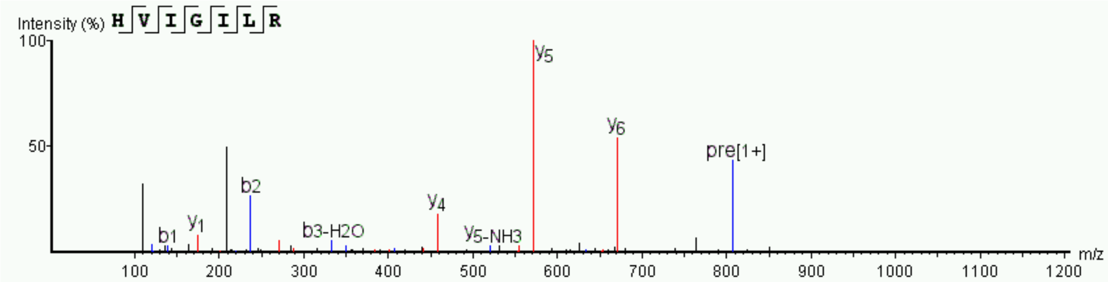

gi|48097144

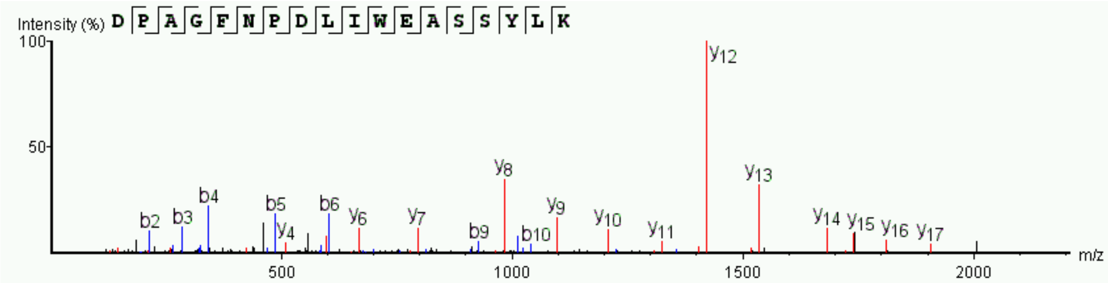

gi|571536610

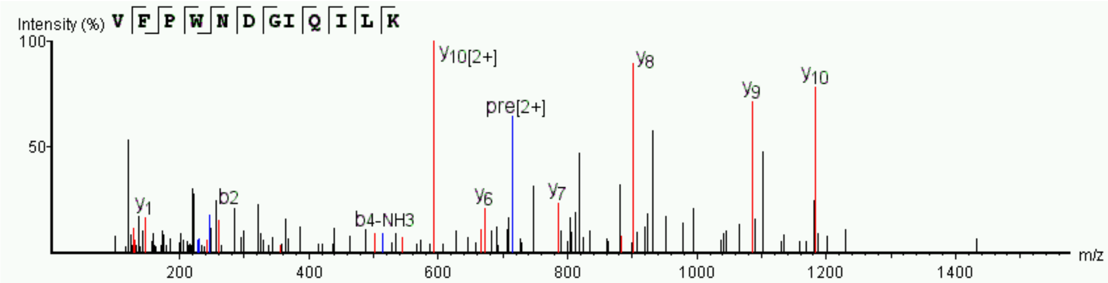

gi|571510083

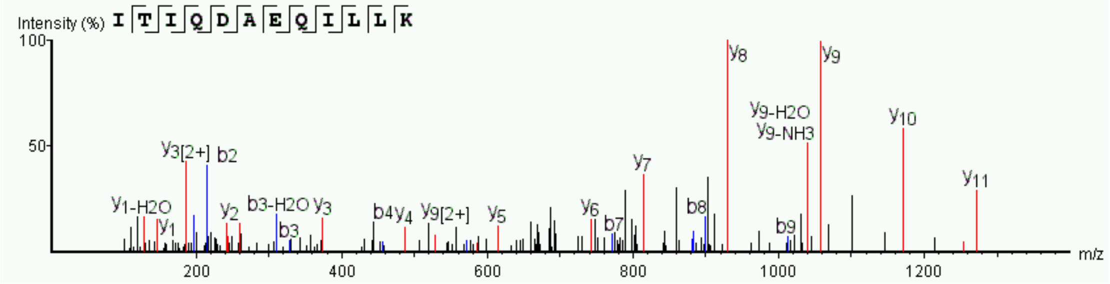

gi|48095935

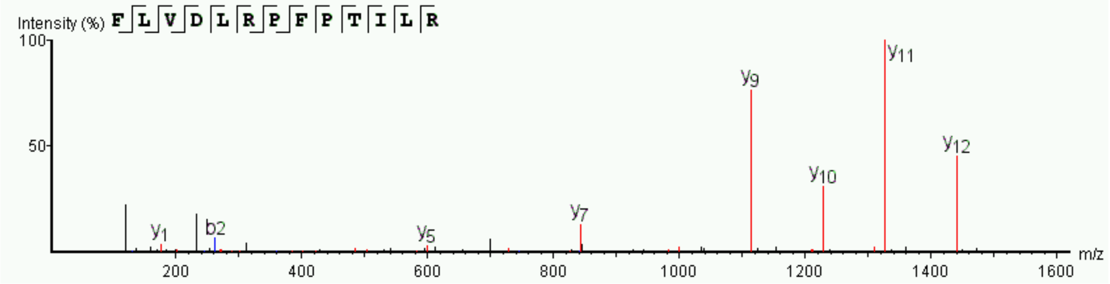

gi|48106137

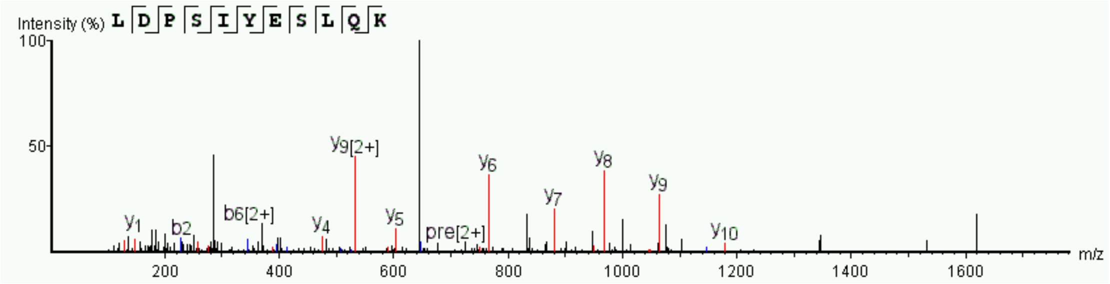

gi|571540698

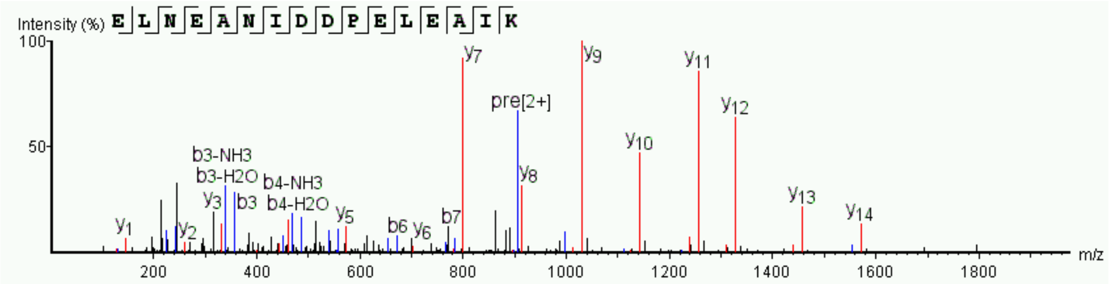

gi|571541867

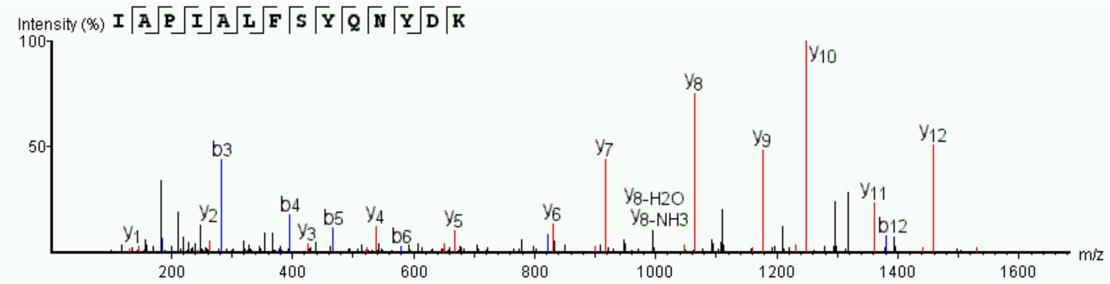

gi|328776594

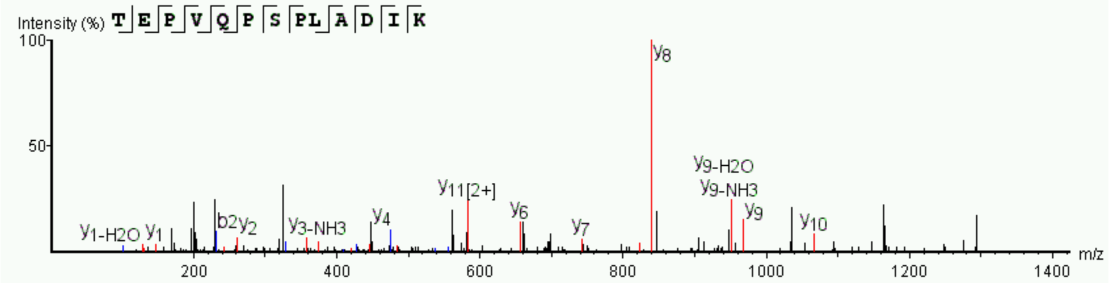

gi|571541340

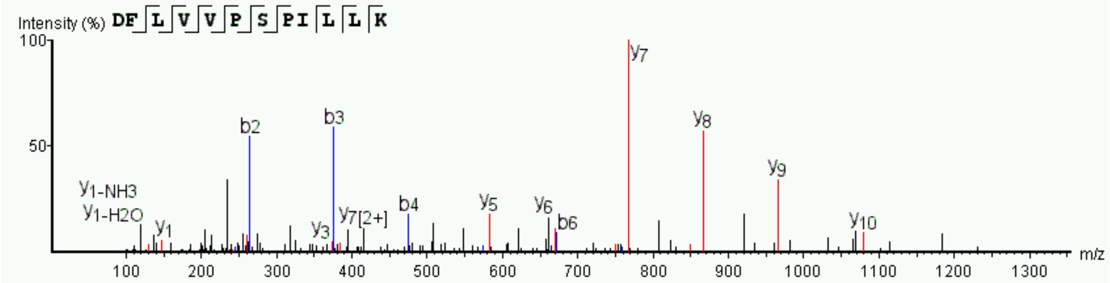

gi|571579311

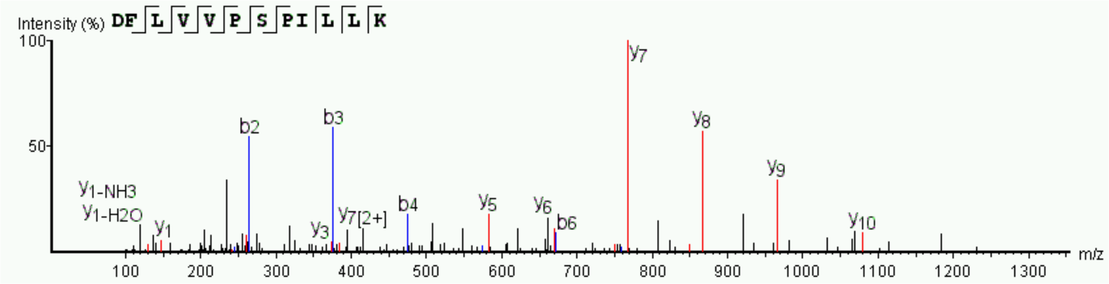

gi|110755171

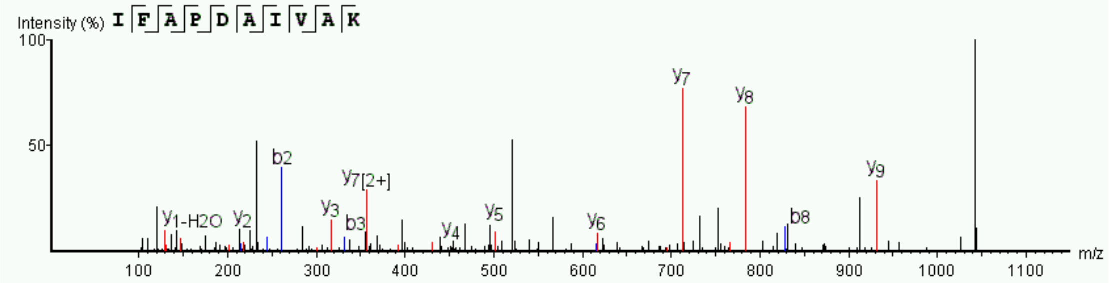

gi|571523207

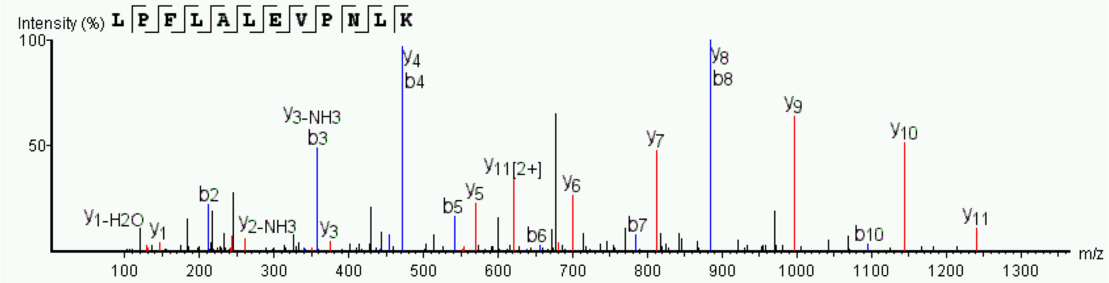

gi|110756942

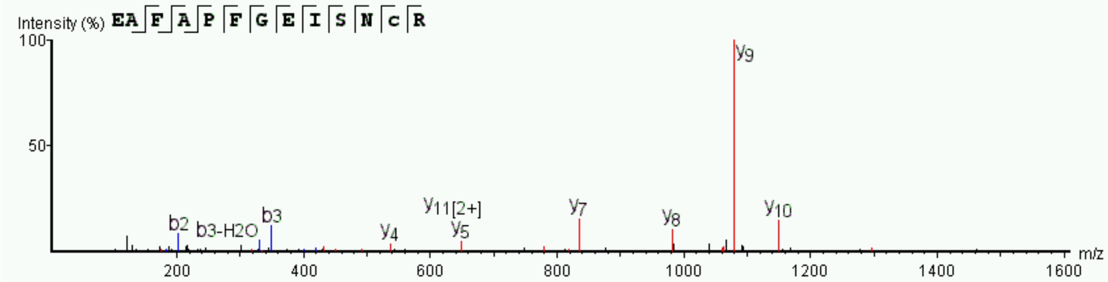

gi|571504735

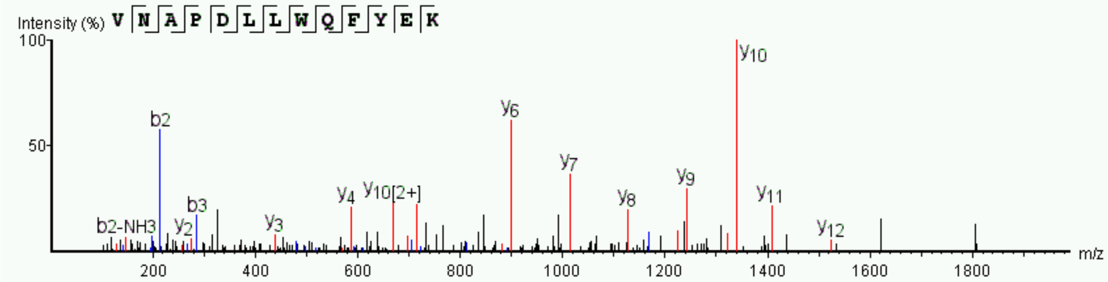

gi|66543732

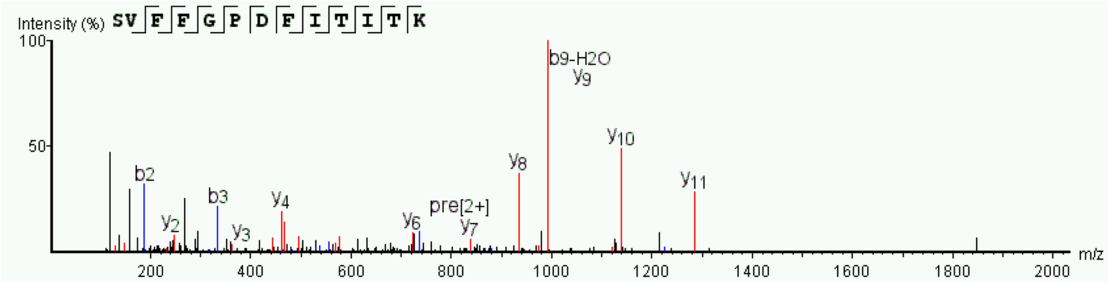

gi|571527109

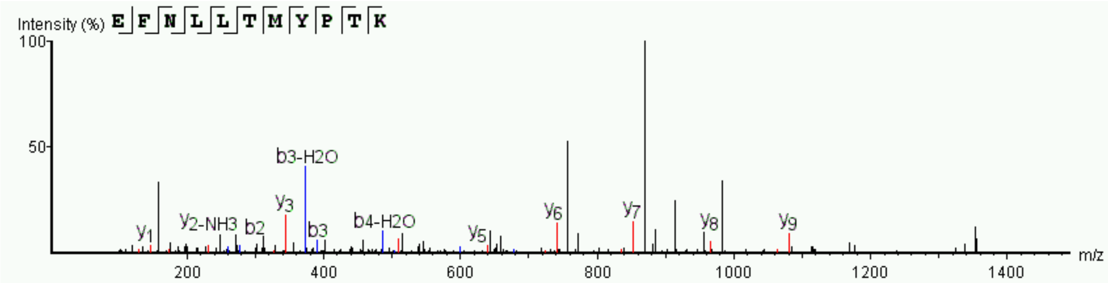

gi|571525796

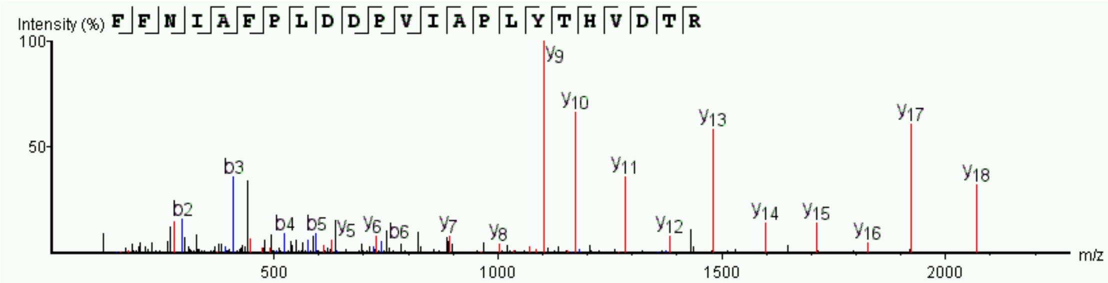

gi|571544898

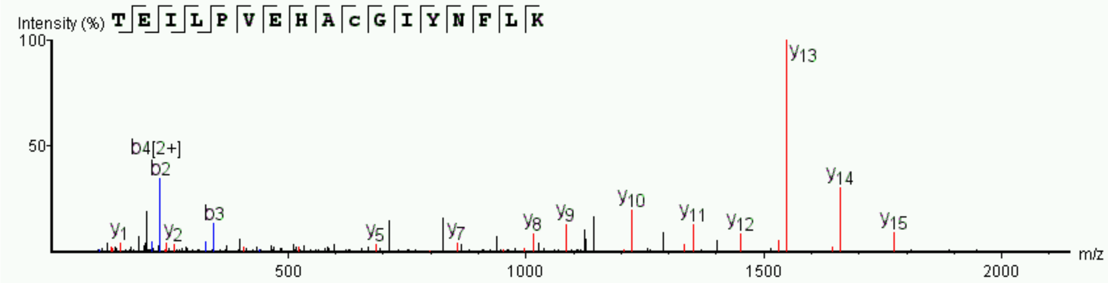

gi|571546530

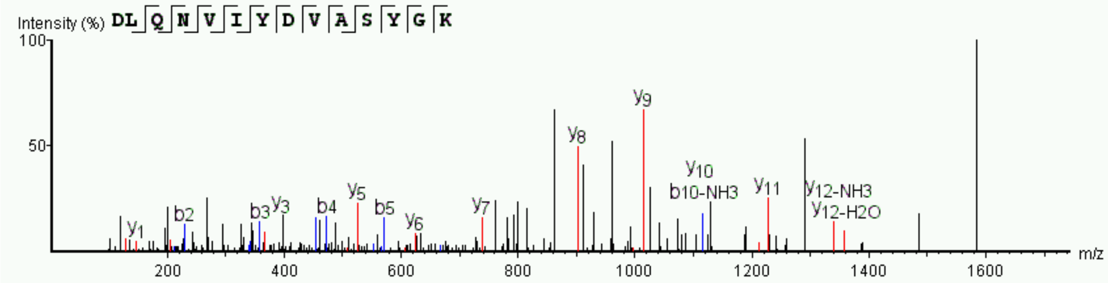

gi|571515342

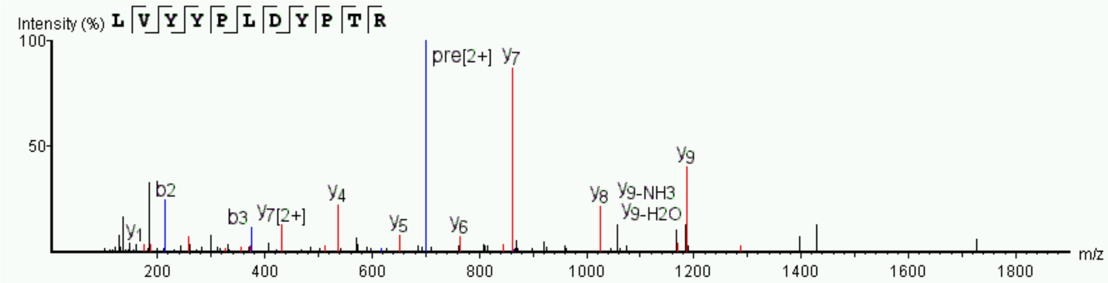

gi|571560259

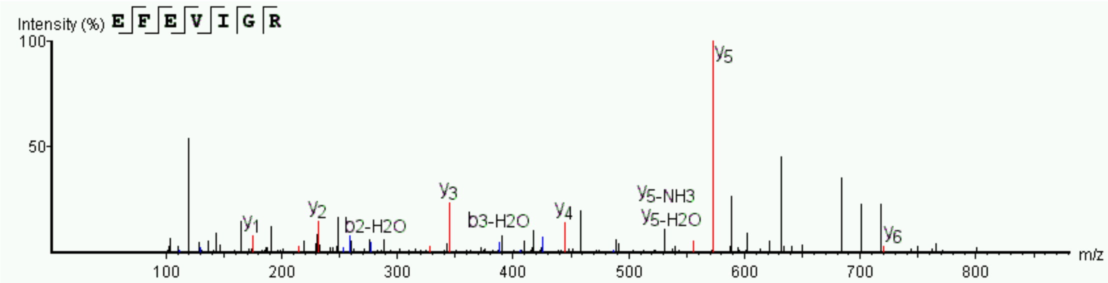

gi|48105316

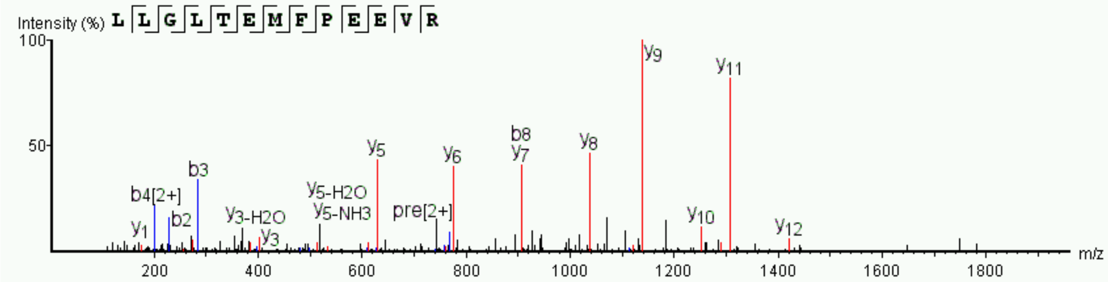

gi|571510158

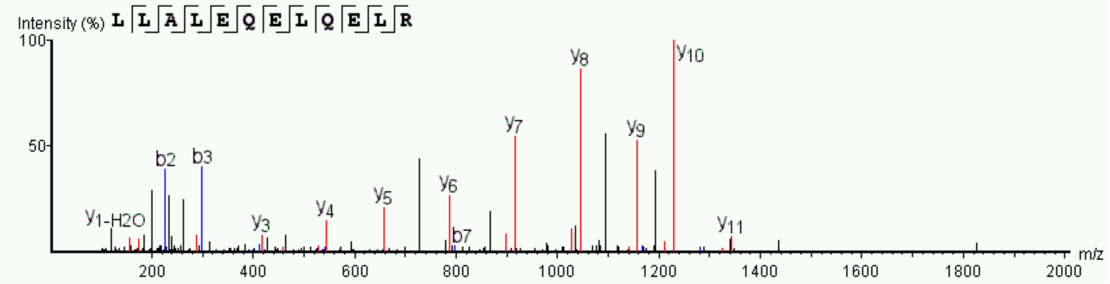

gi|328783561

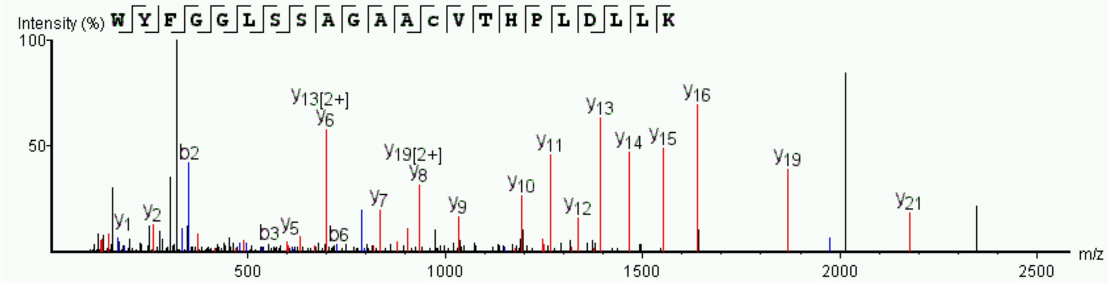

gi|571562354

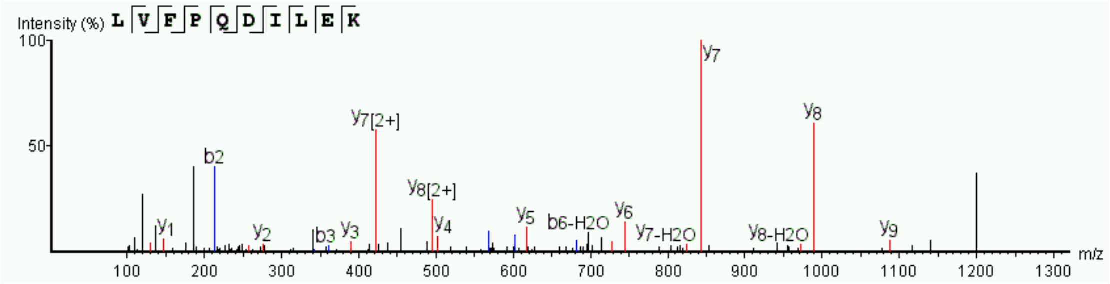

gi|328776866

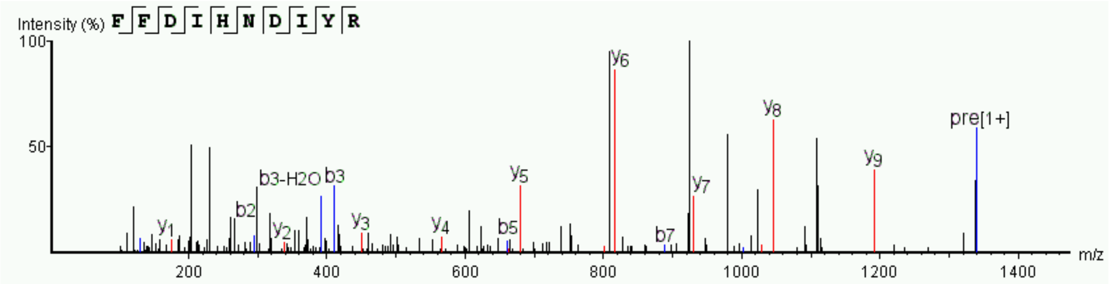

gi|66526670

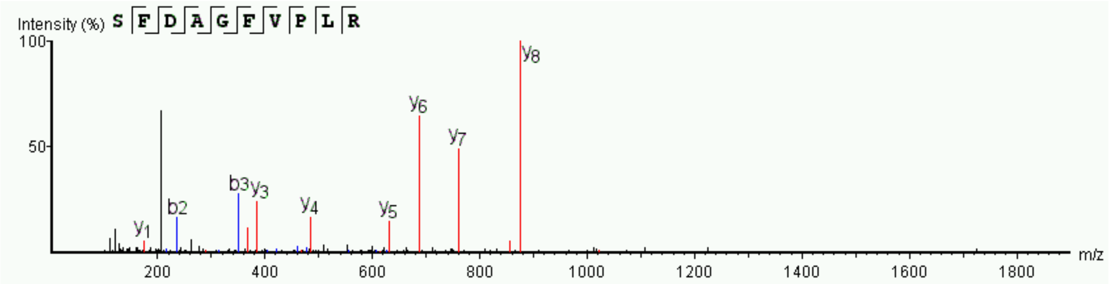

gi|571575043

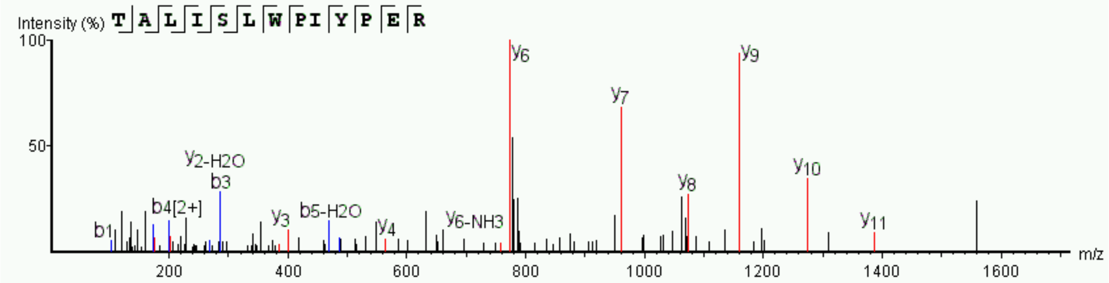

gi|328787116

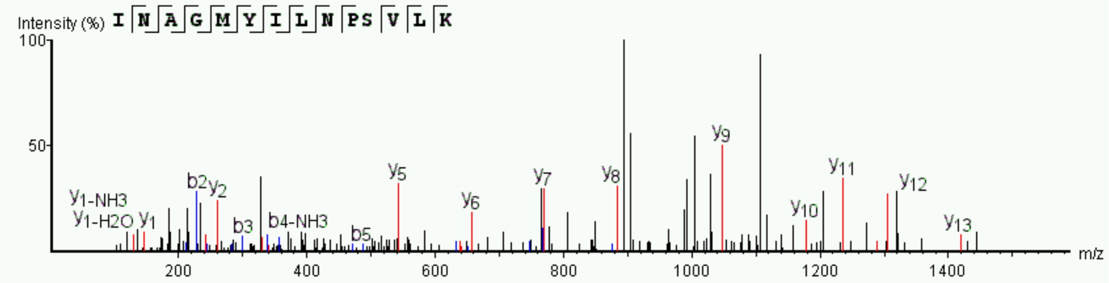

gi|110762605

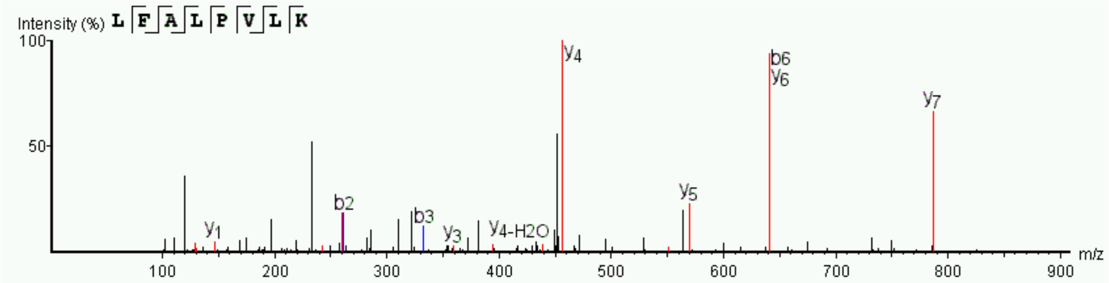

gi|110750888

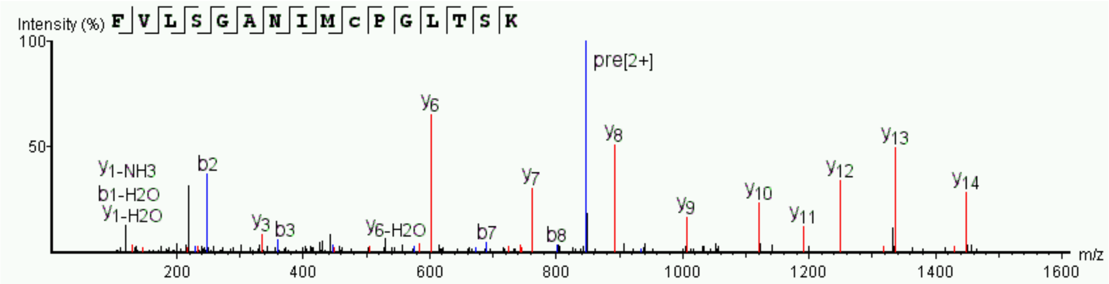

gi|571502292

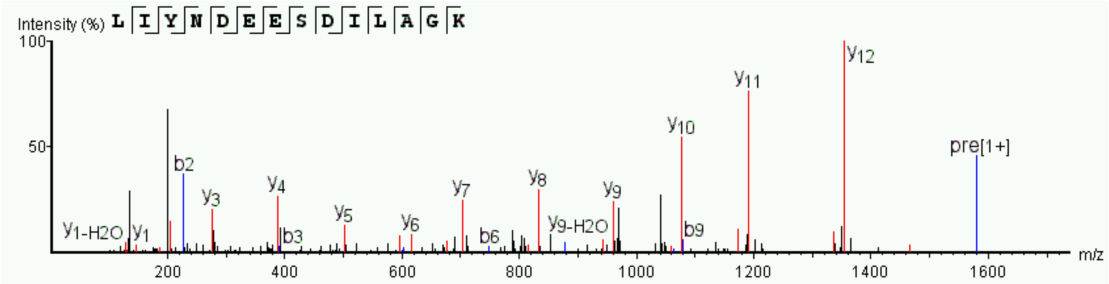

gi|328783171

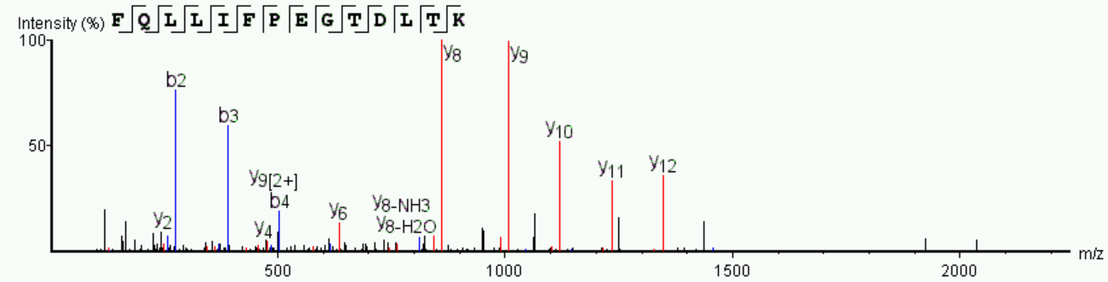

gi|571573648

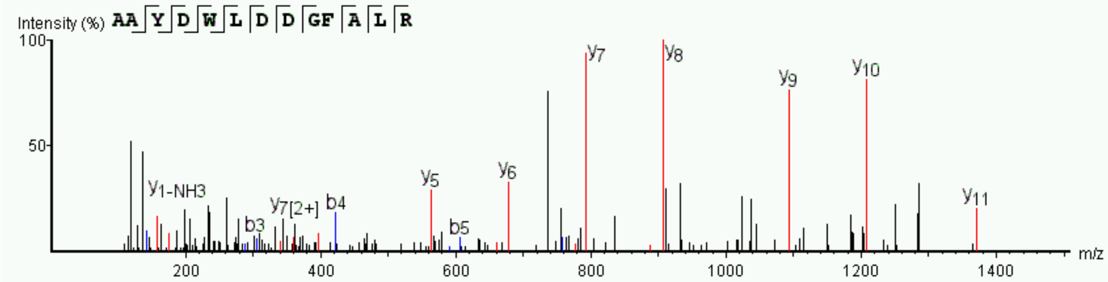

gi|571534241

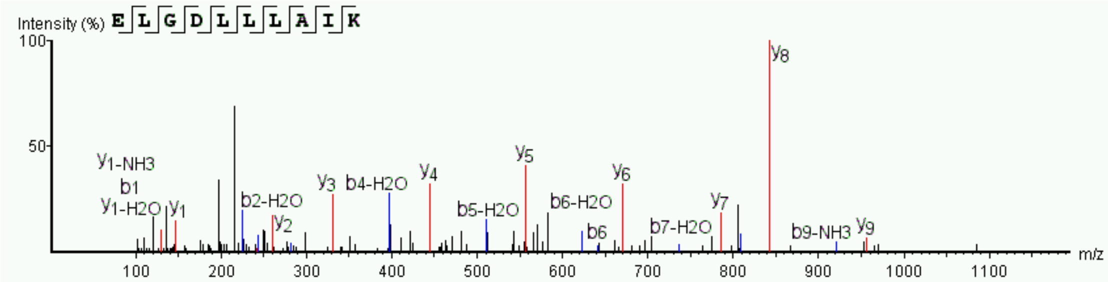

gi|571578350

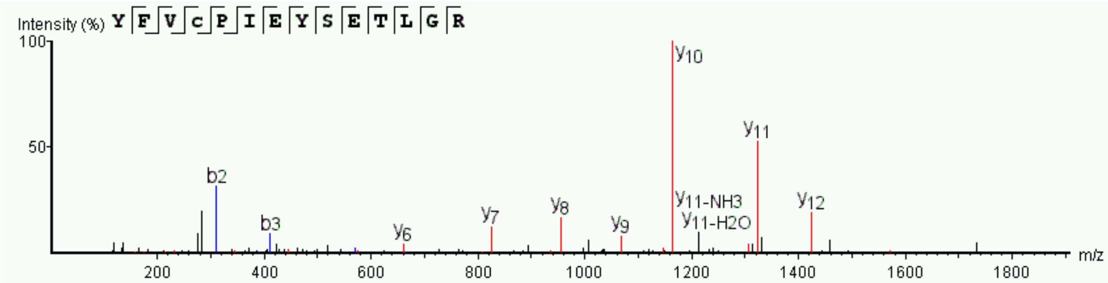

gi|571507579

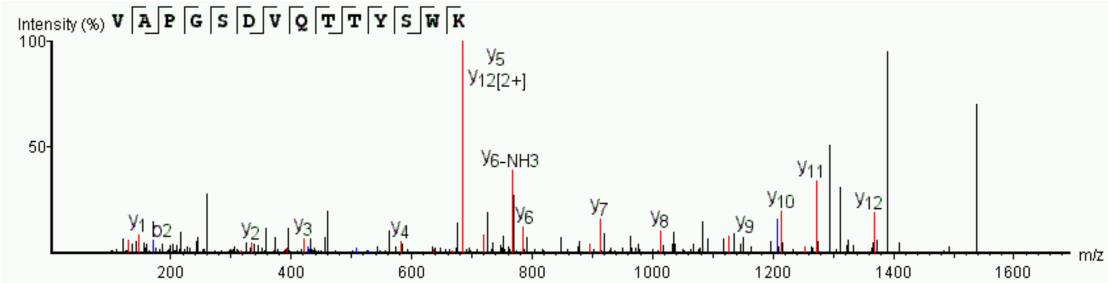

gi|571576375

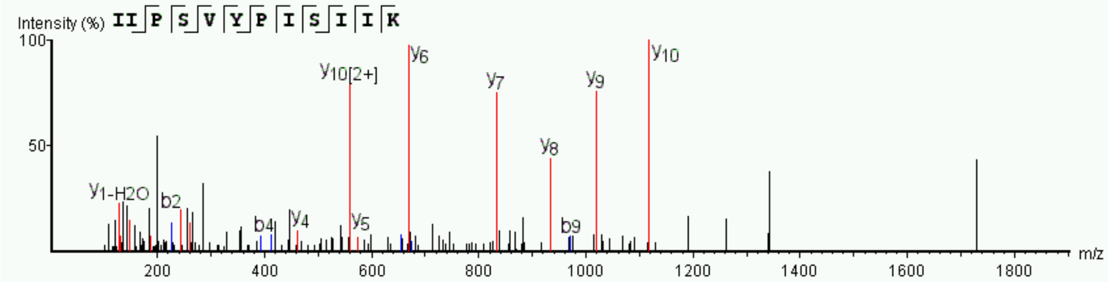

gi|328781115

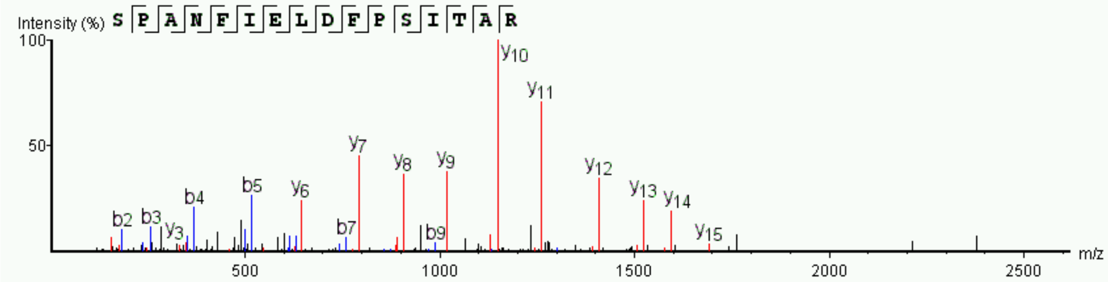

gi|571559721

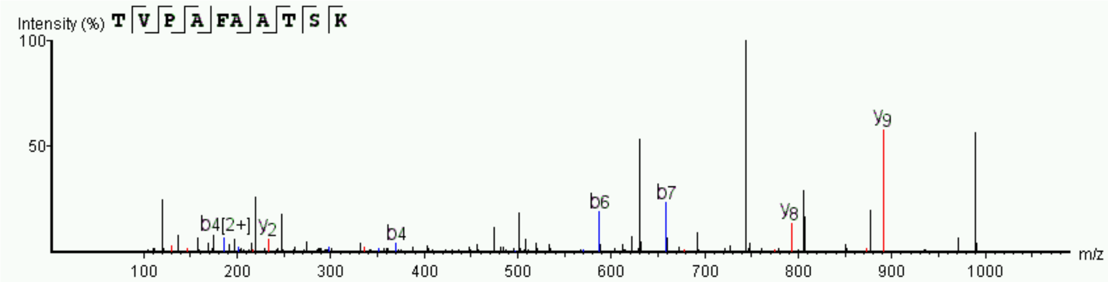

gi|110755692

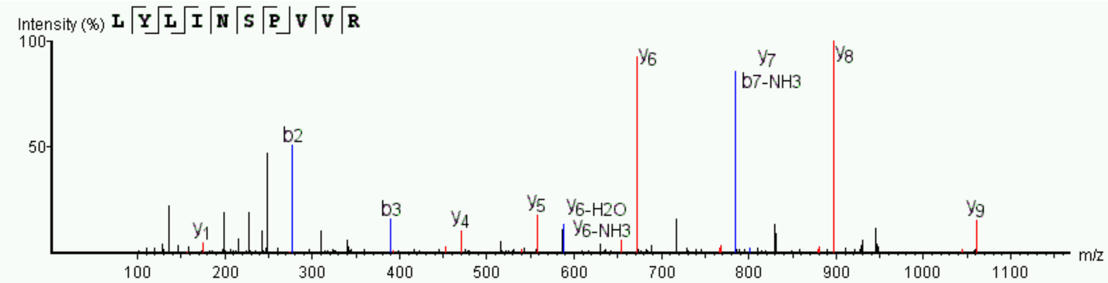

gi|66554764

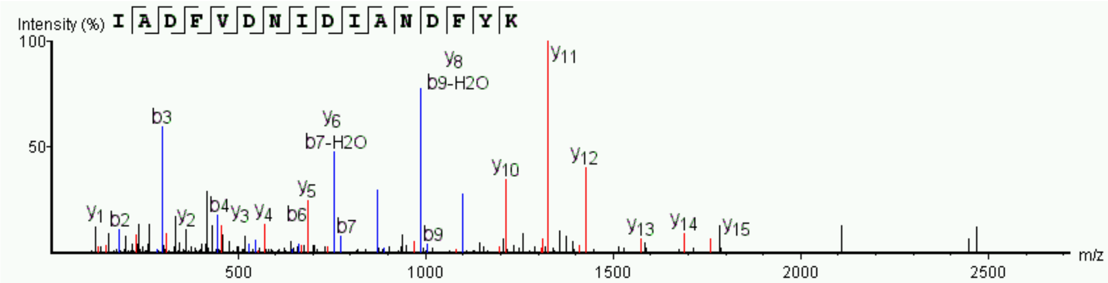

gi|66509537

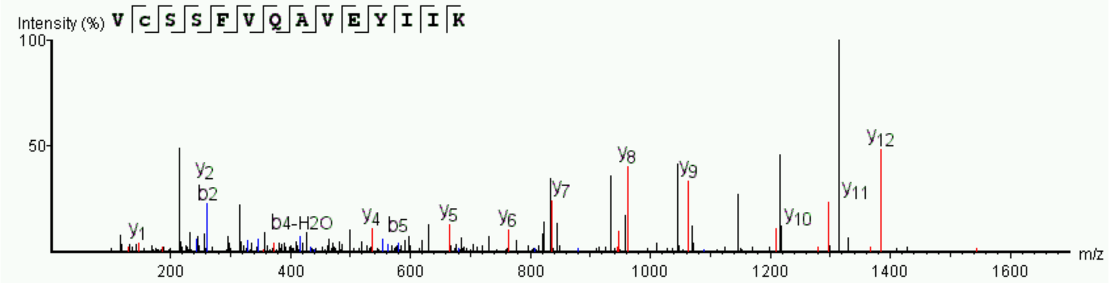

gi|48126476

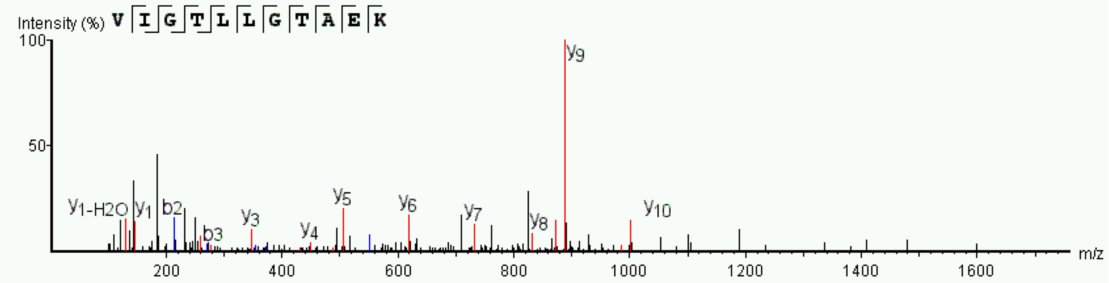

gi|571543561

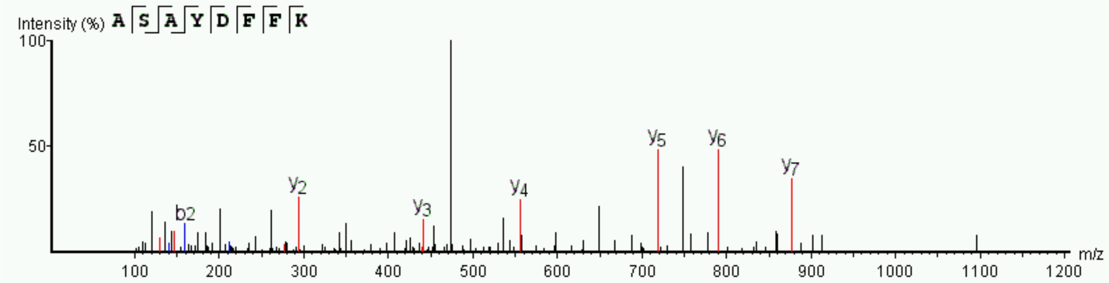

gi|66545512

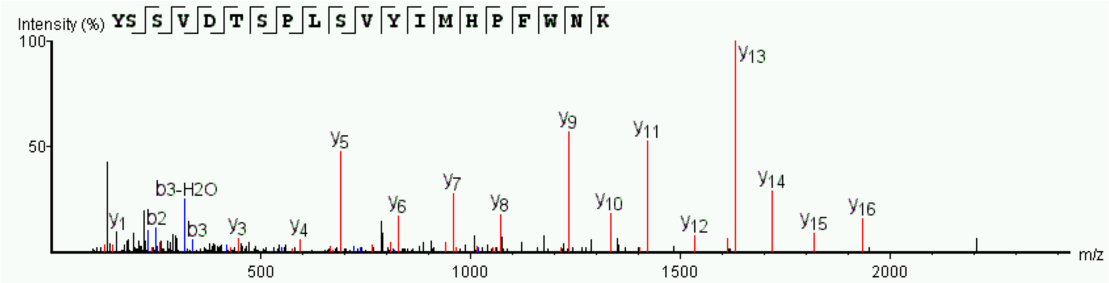

gi|571556068

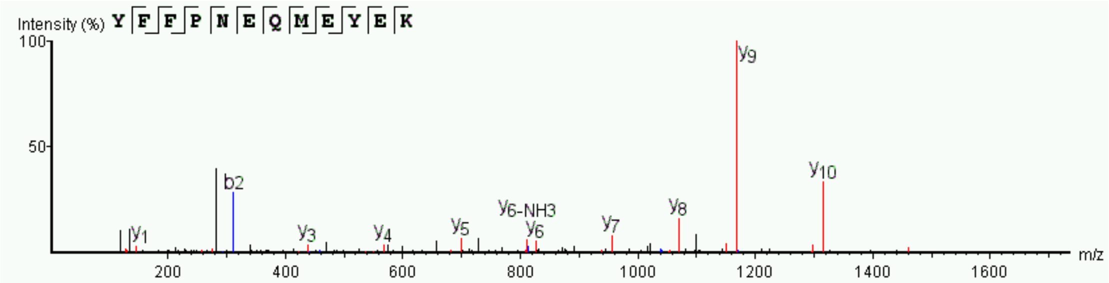

gi|571524328;

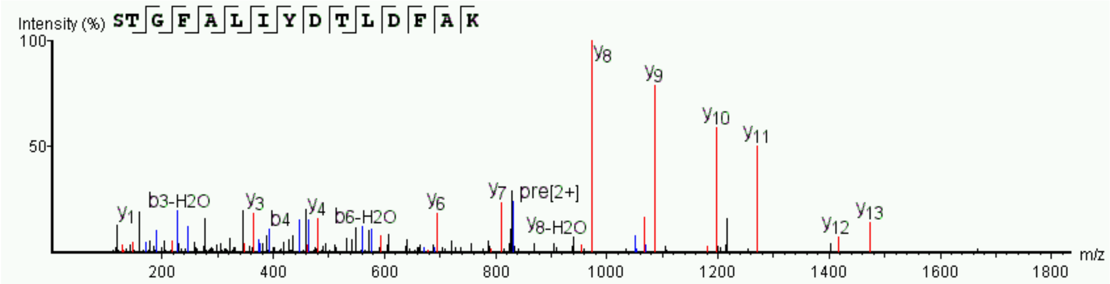

gi|571524333;

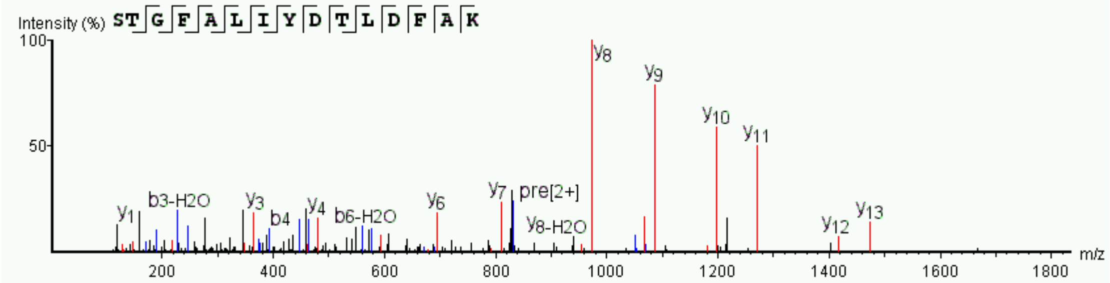

gi|48095625

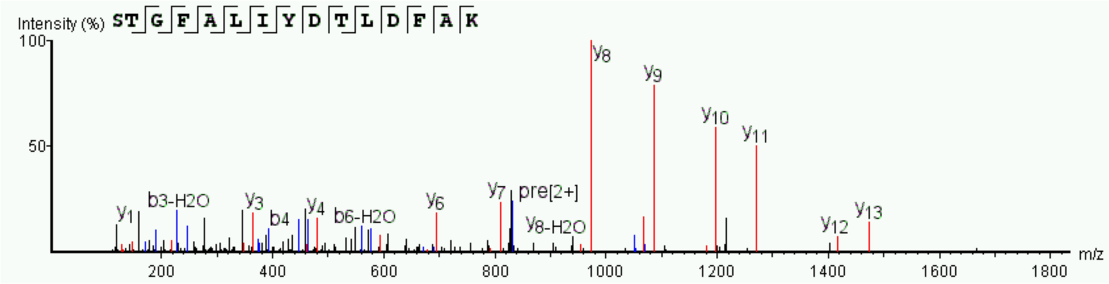

gi|328787488

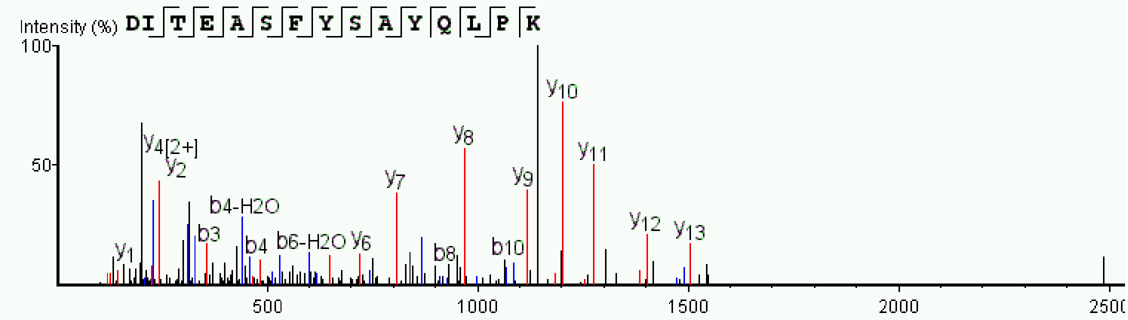

gi|571528288;

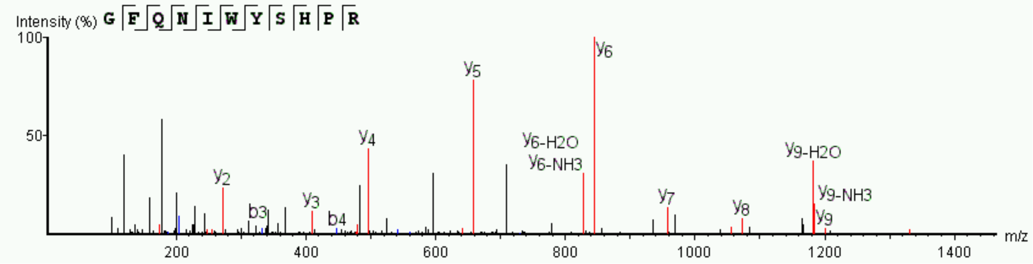

gi|571528291

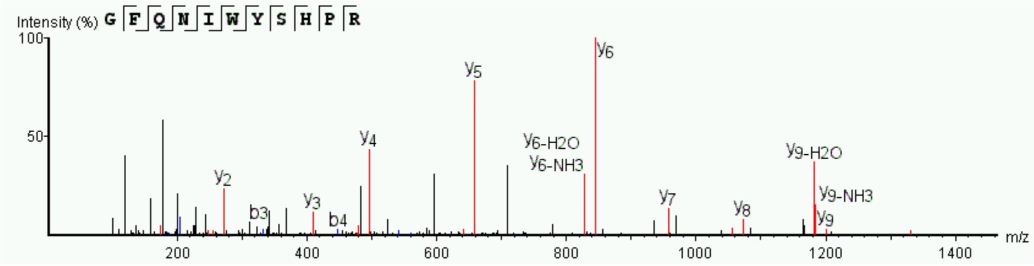

gi|110765226

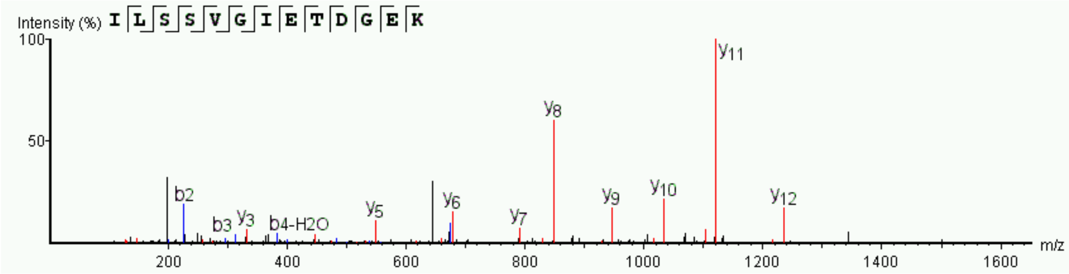

gi|66524917

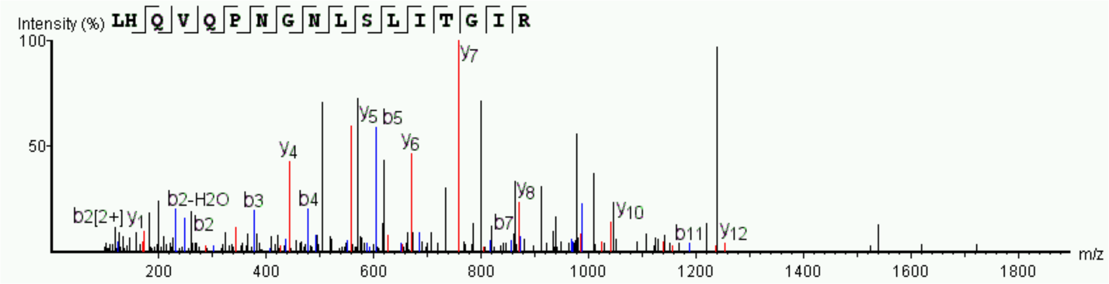

gi|58585114

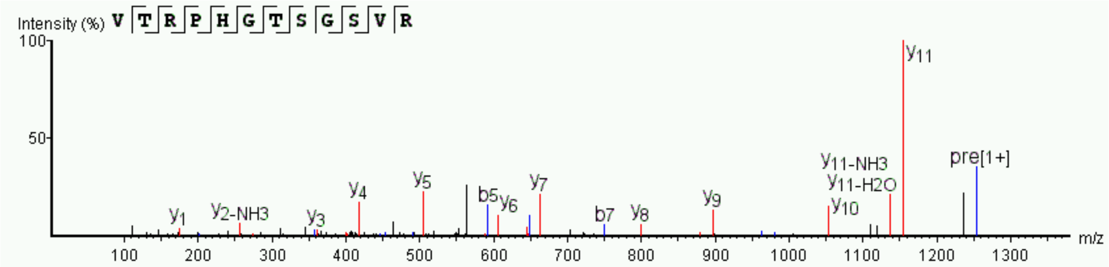

gi|66564768

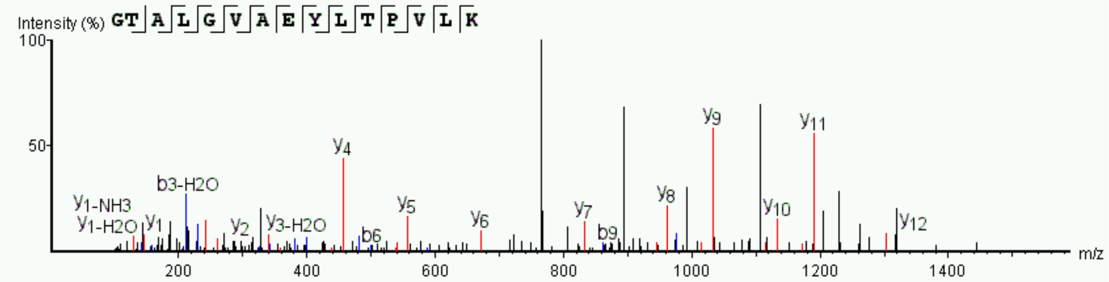

gi|571521115;

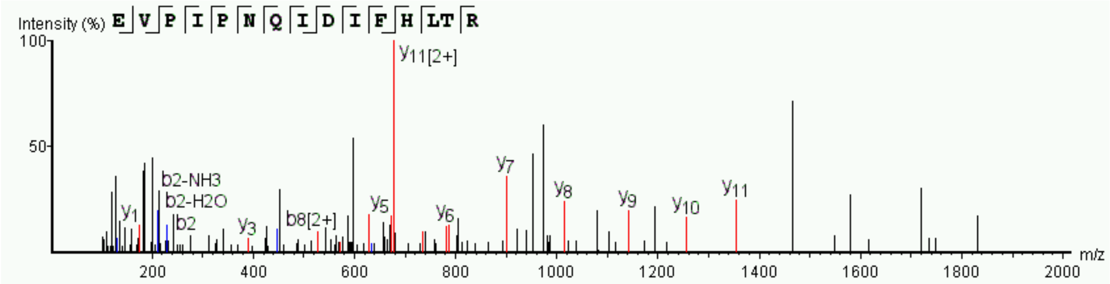

gi|571579446;

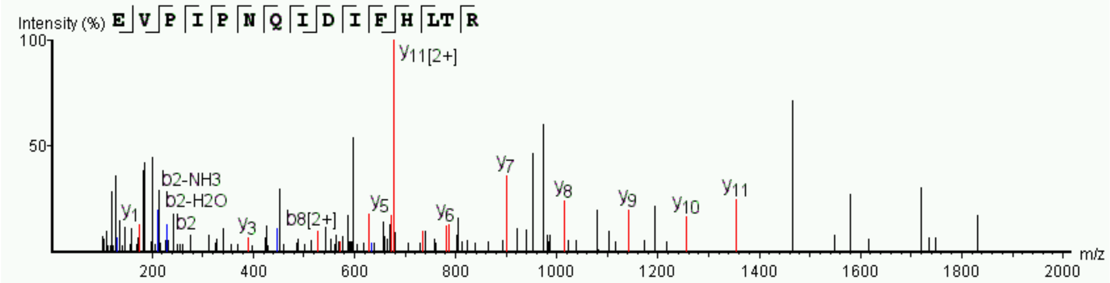

gi|571542548;

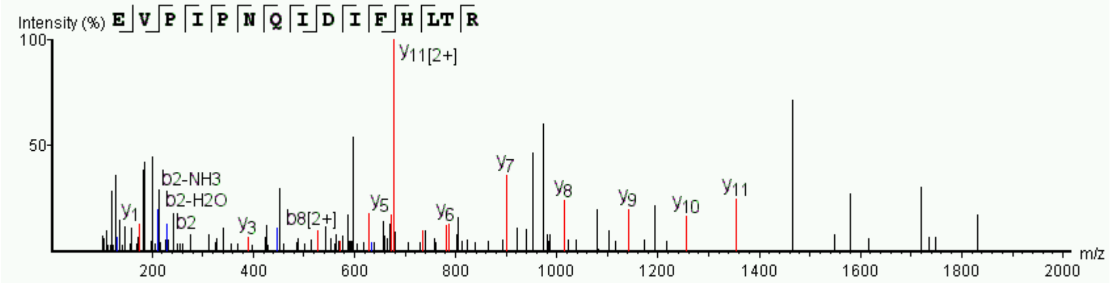

gi|571542551

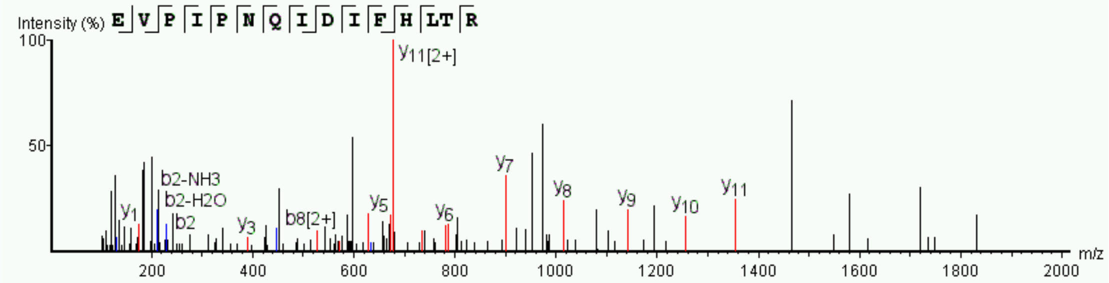

gi|571573543

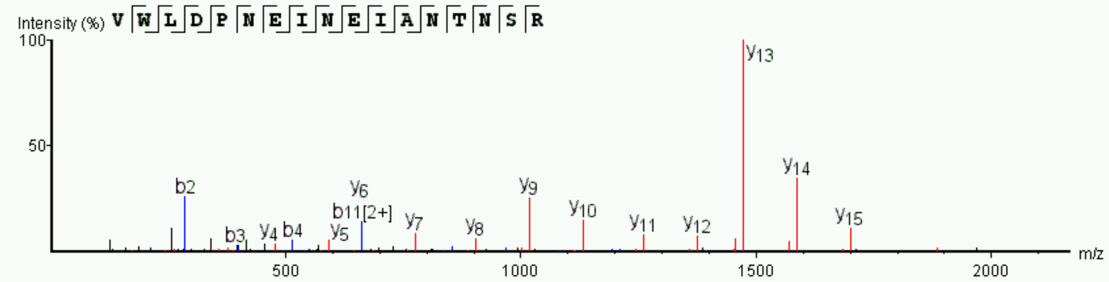

gi|58585164

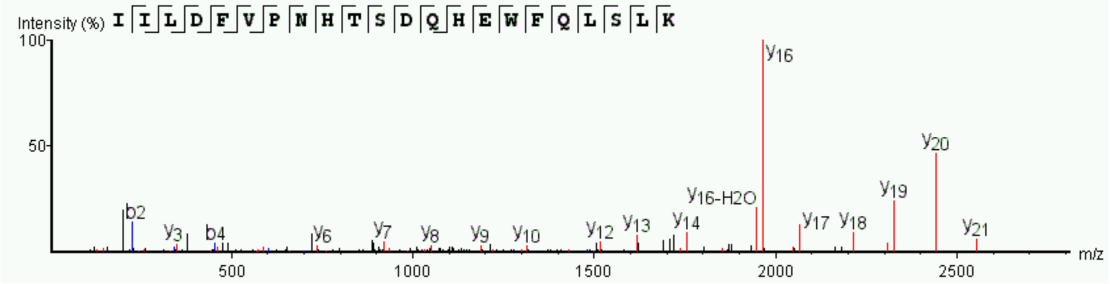

gi|571547079

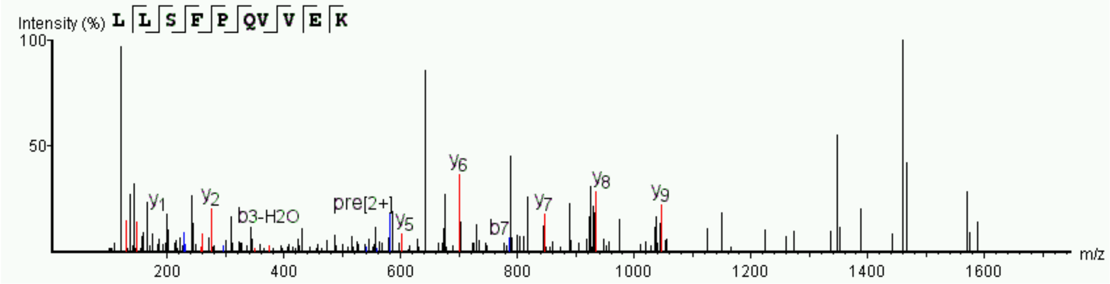

gi|328783748

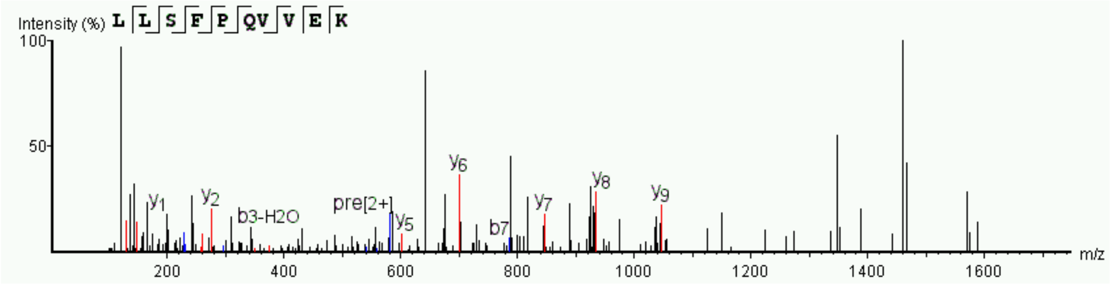

gi|571544412

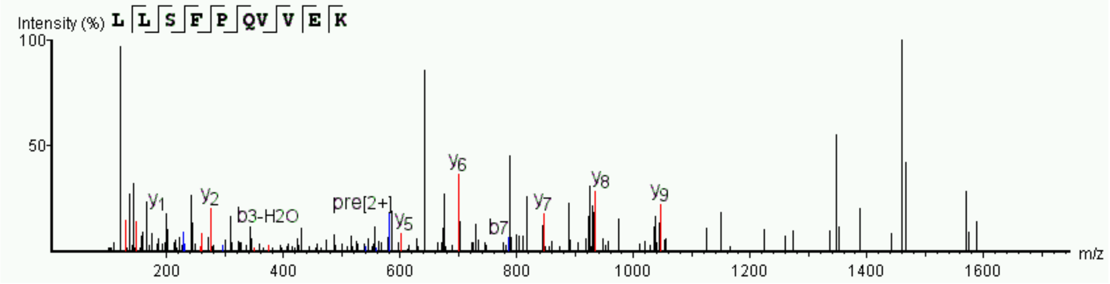

gi|571554717

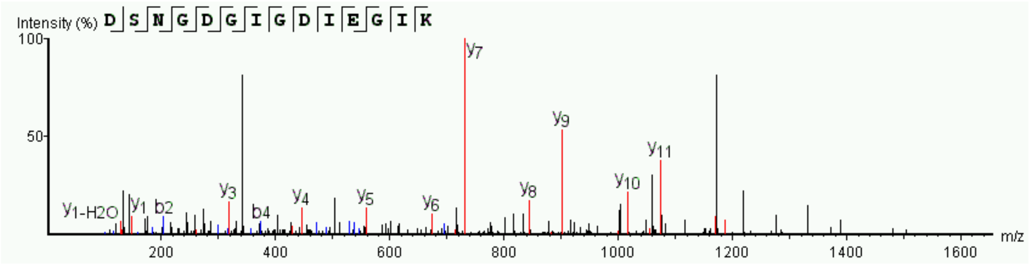

gi|571554721

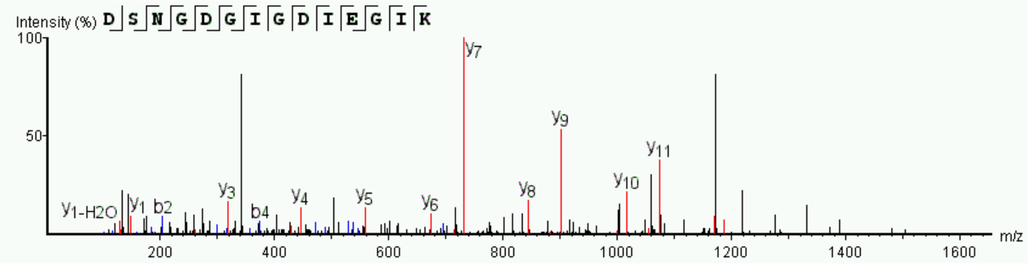

gi|328786465

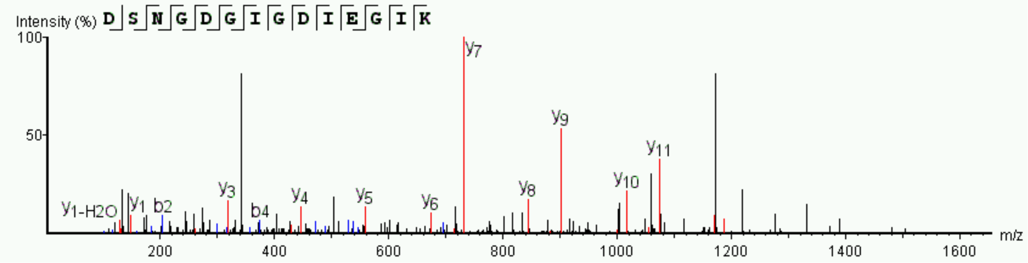

gi|571554030

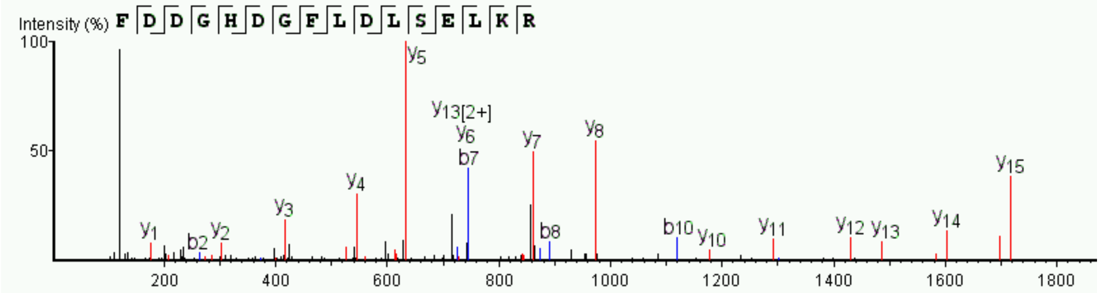

gi|571554034

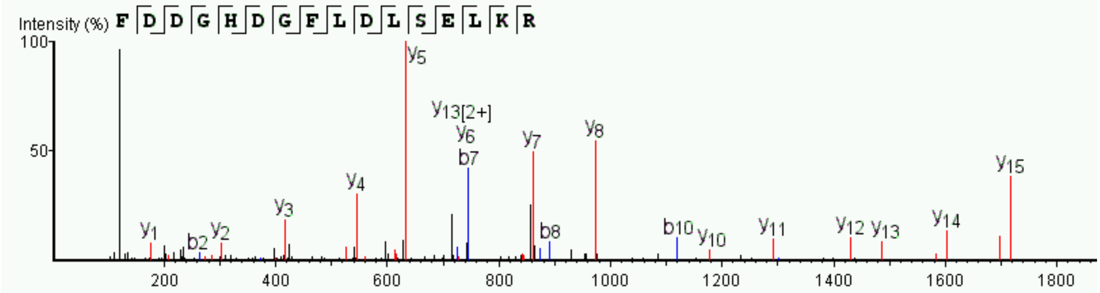

gi|66530680

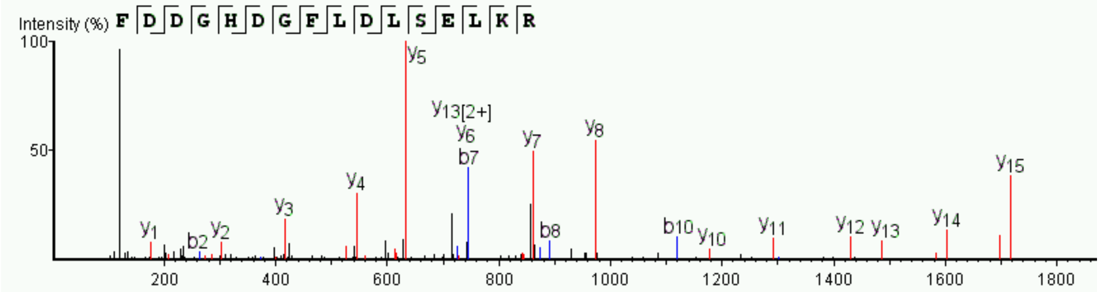

gi|571517547

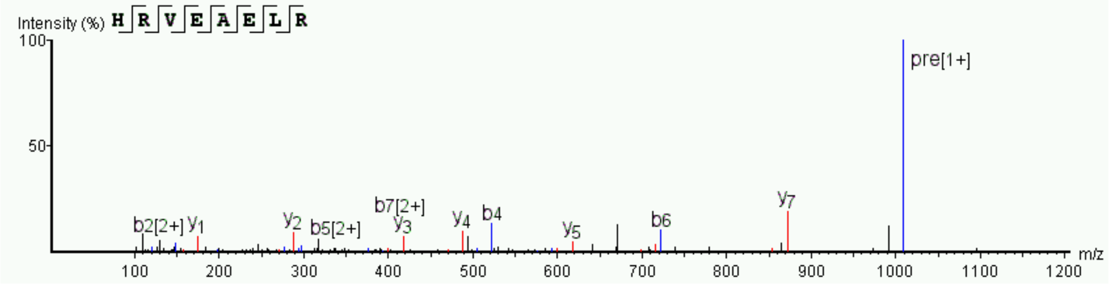

gi|110759426

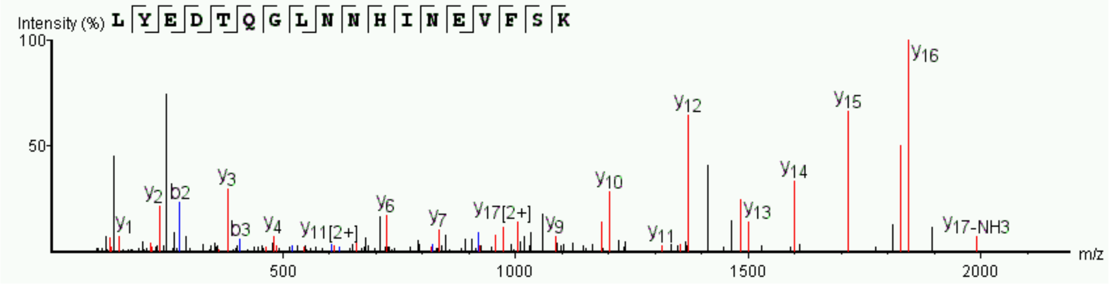

gi|571576863

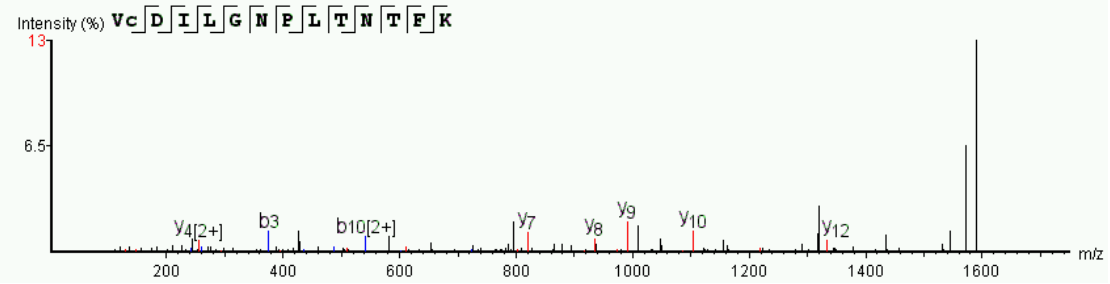

gi|328784835

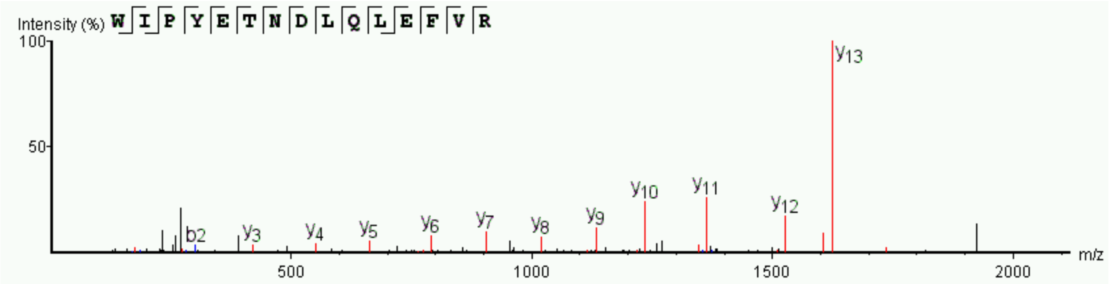

gi|110764763;

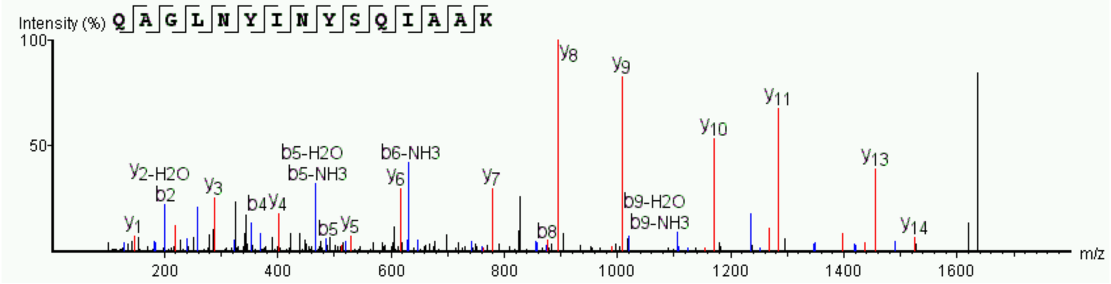

gi|328779841

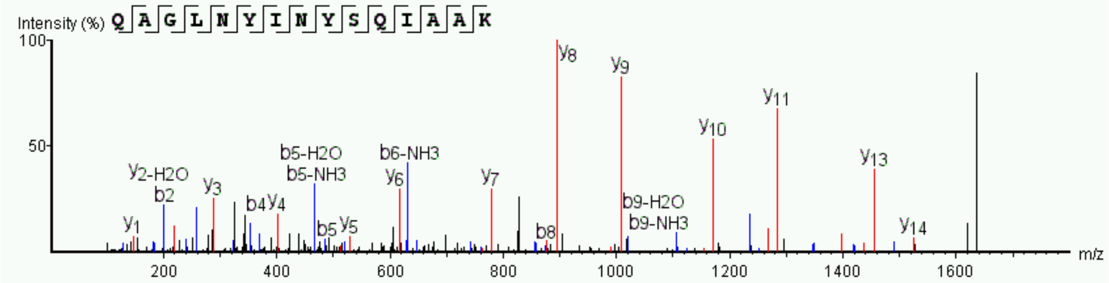

gi|66547758

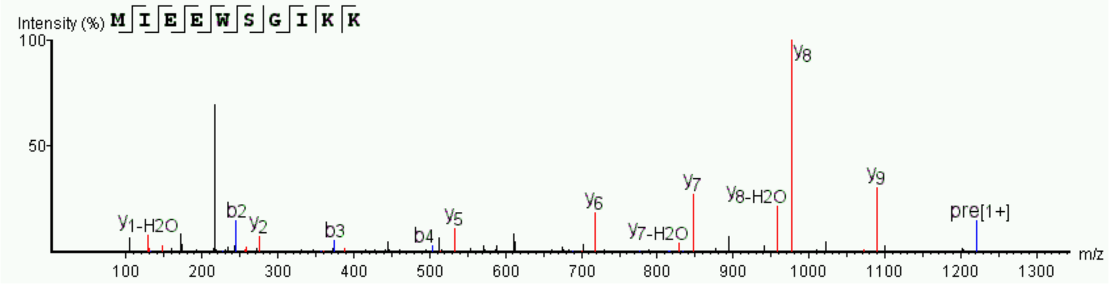

gi|571574994

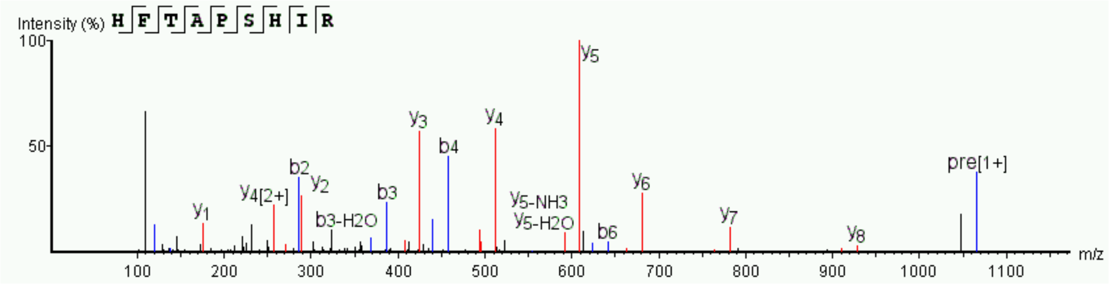

gi|336391185

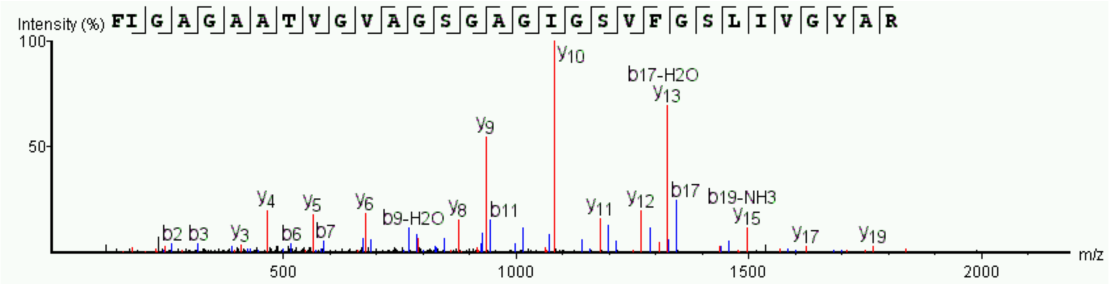

gi|571575755

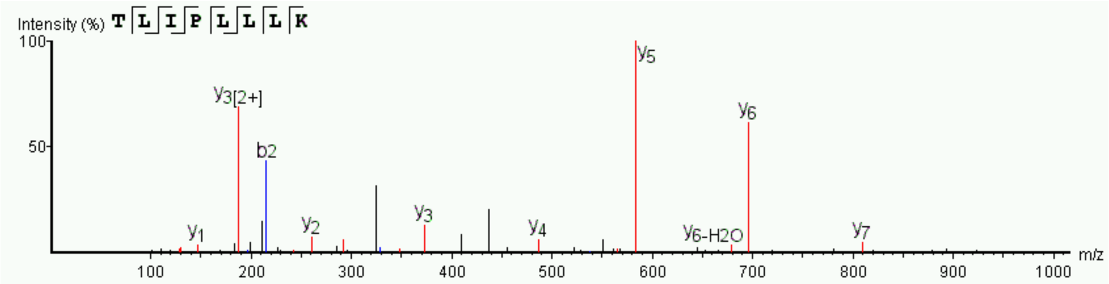

gi|58585106

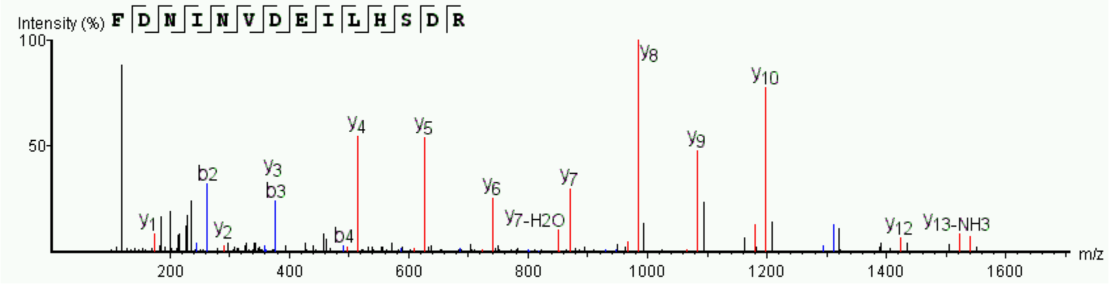

gi|571574750

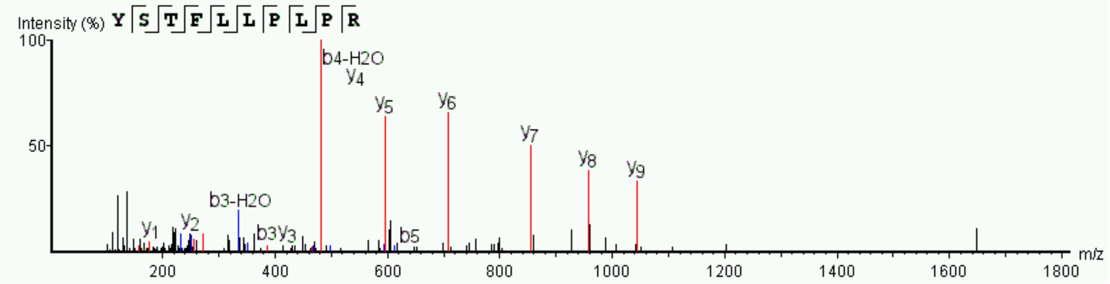

gi|571502990

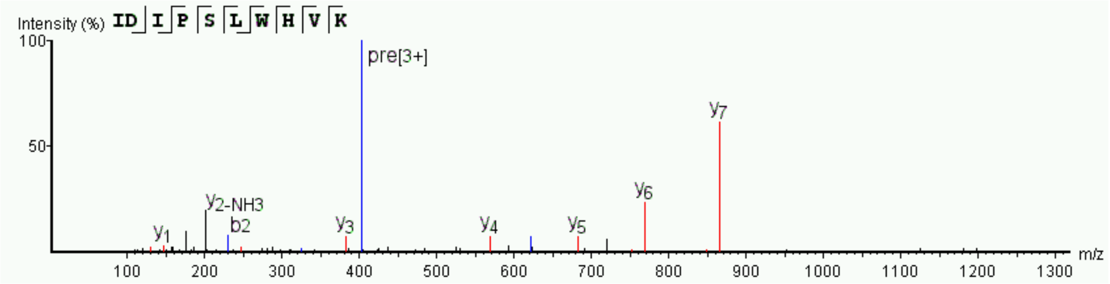

gi|571532899;

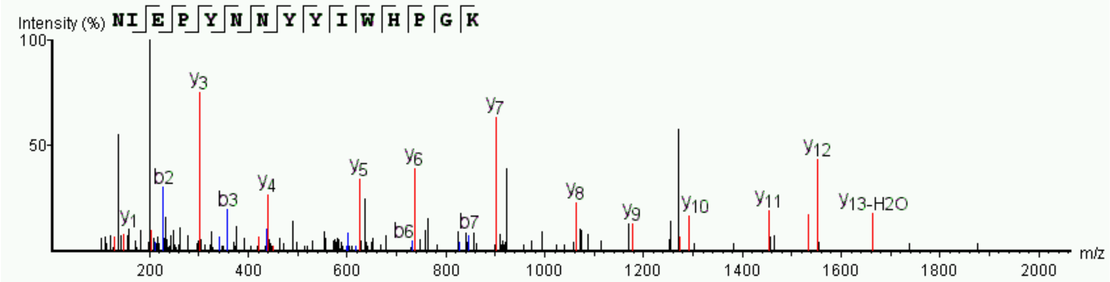

gi|571532902;

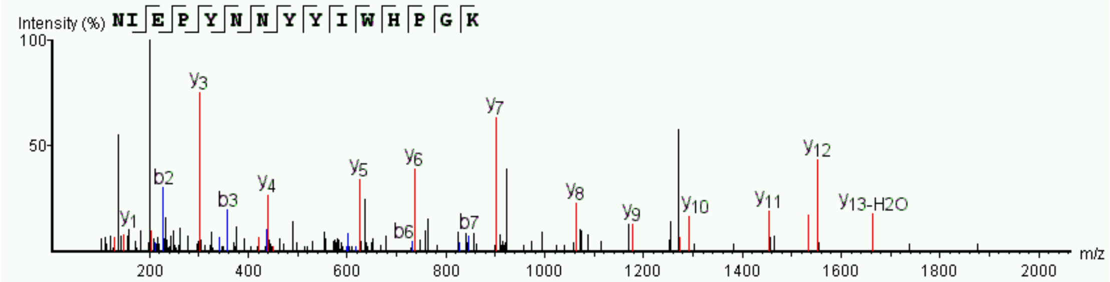

gi|571532906

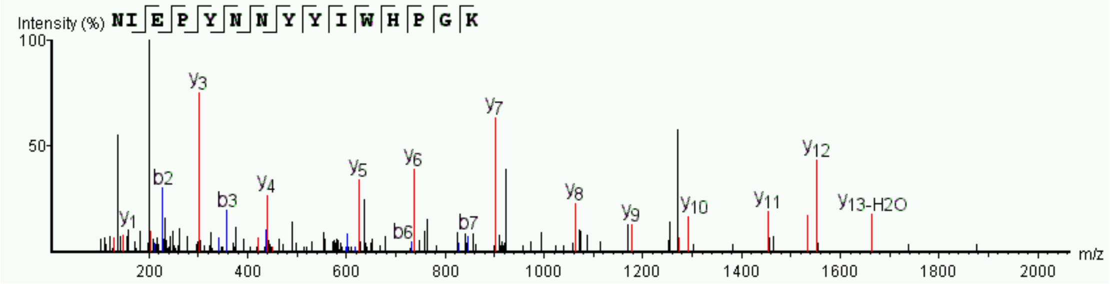

gi|571532910

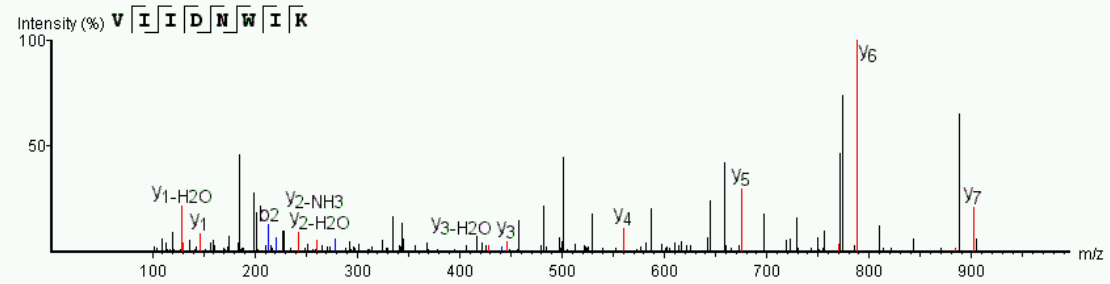

gi|328786429;

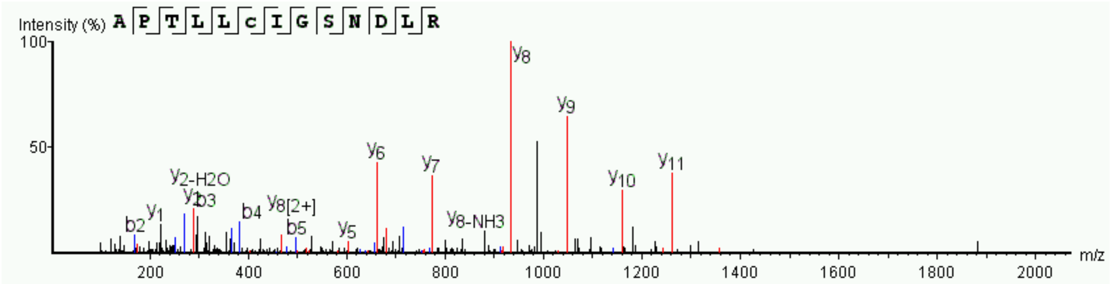

gi|571553992

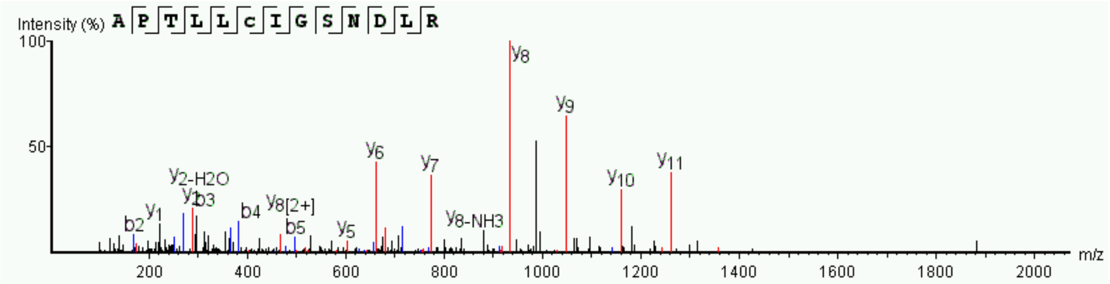

gi|328786971

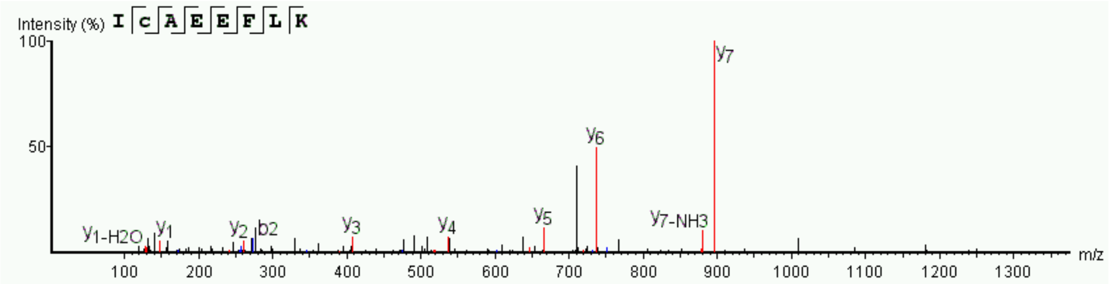

gi|571561861;

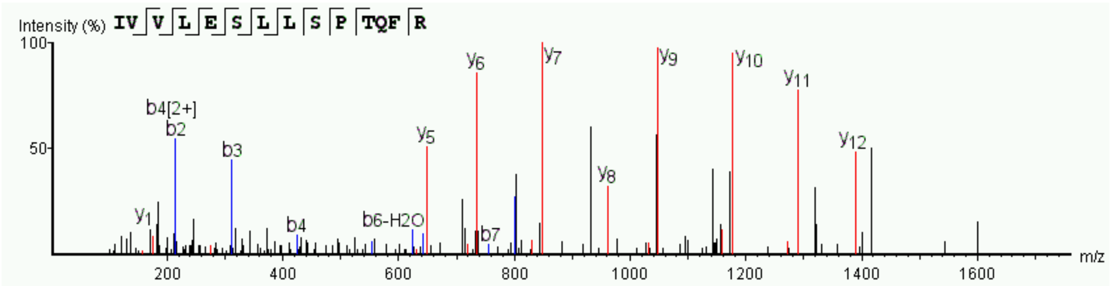

gi|328787167

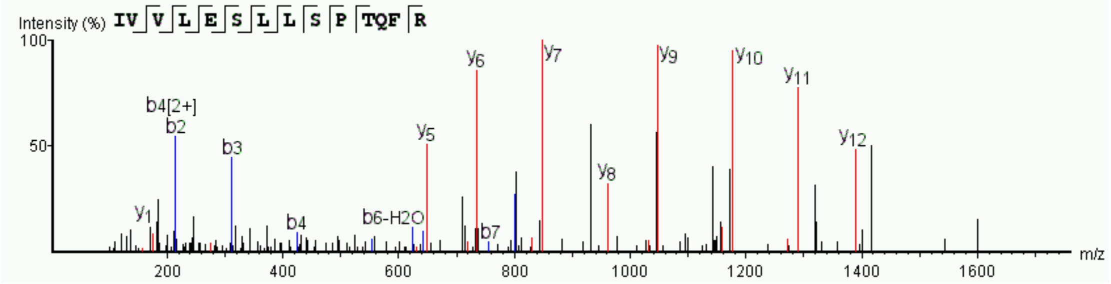

gi|571504170;

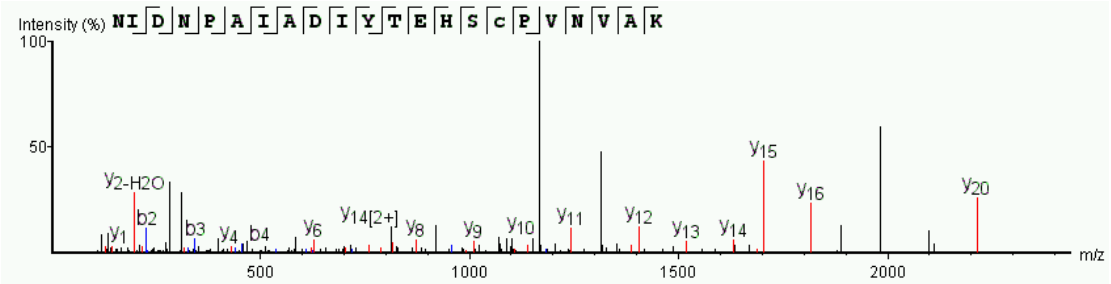

gi|328790339

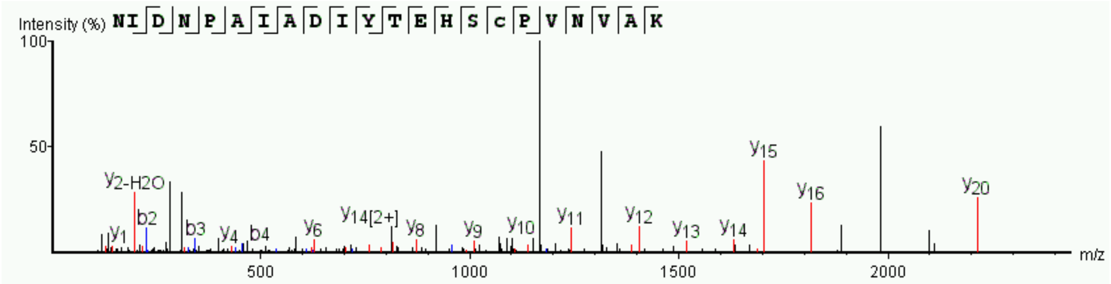

gi|571519817;

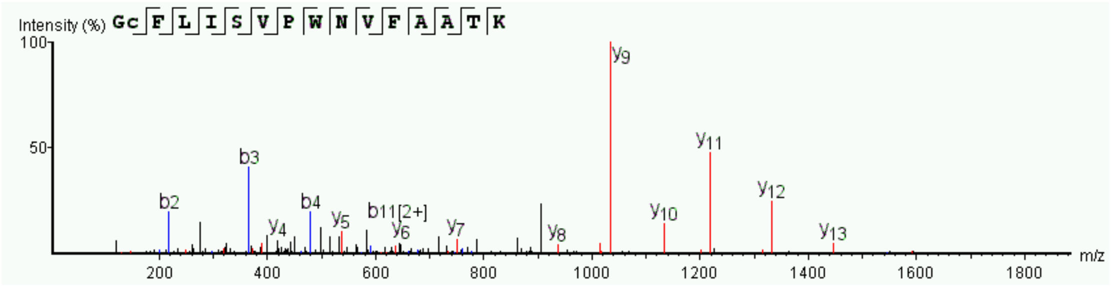

gi|66505792

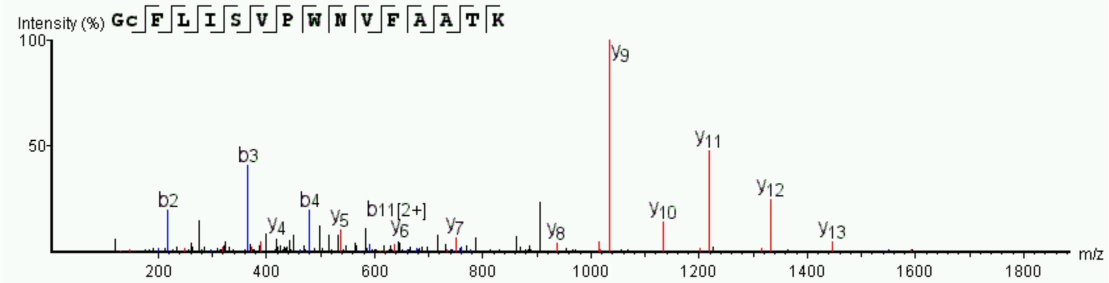

gi|328793818

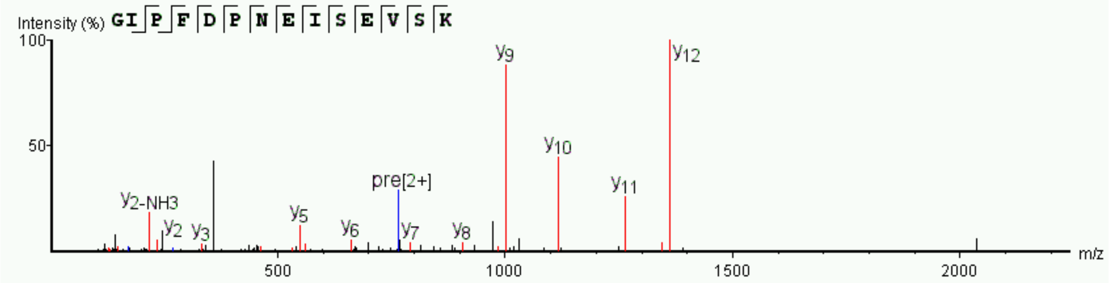

gi|571558444;

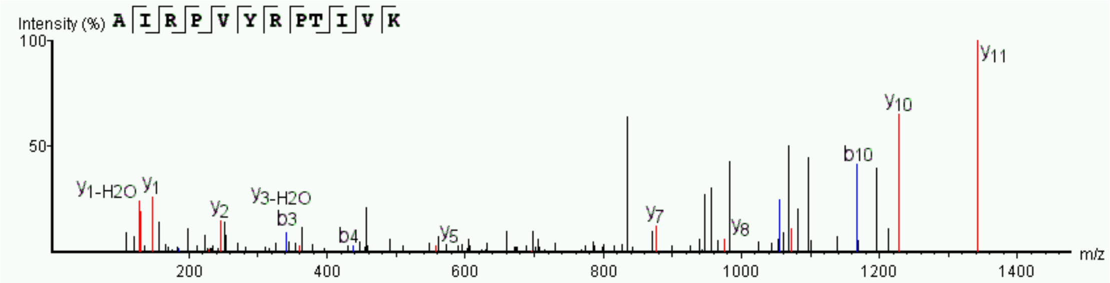

gi|571558447

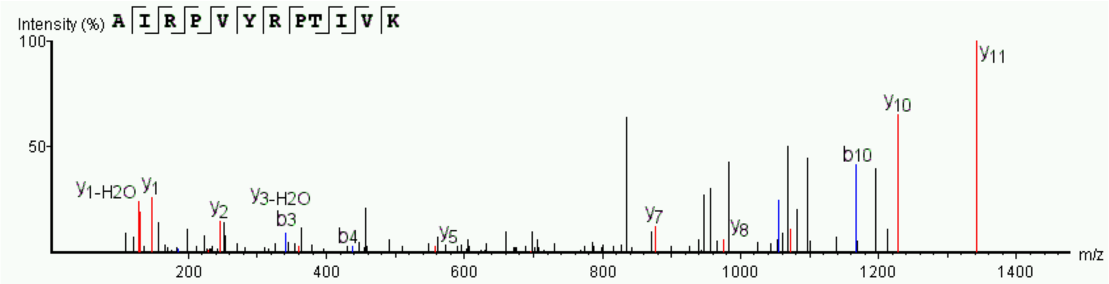

gi|328788691;

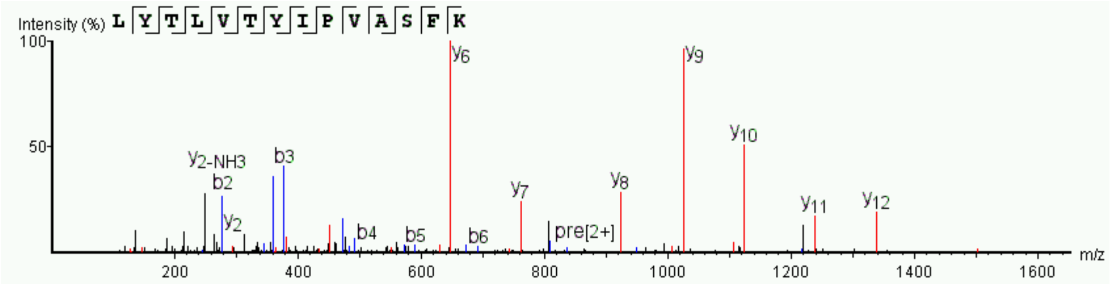

gi|48142237

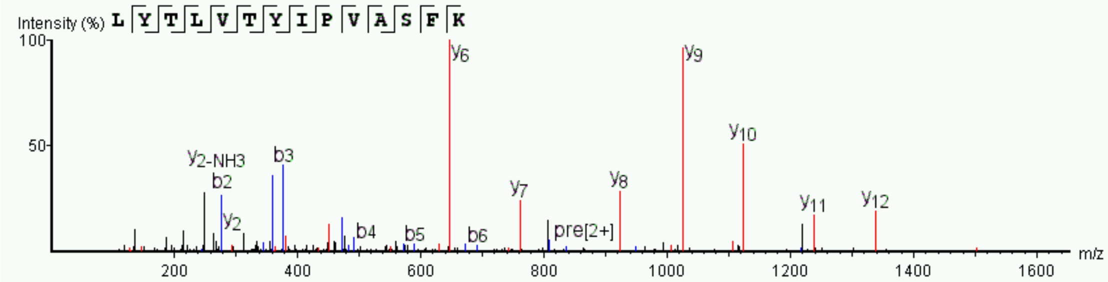

gi|335057536

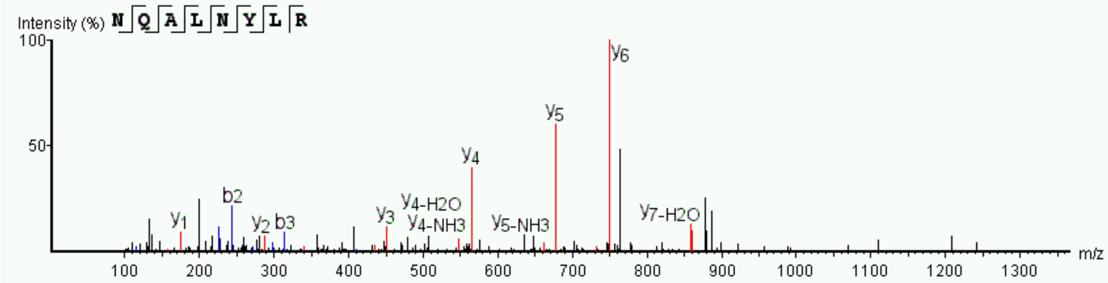

gi|5834937

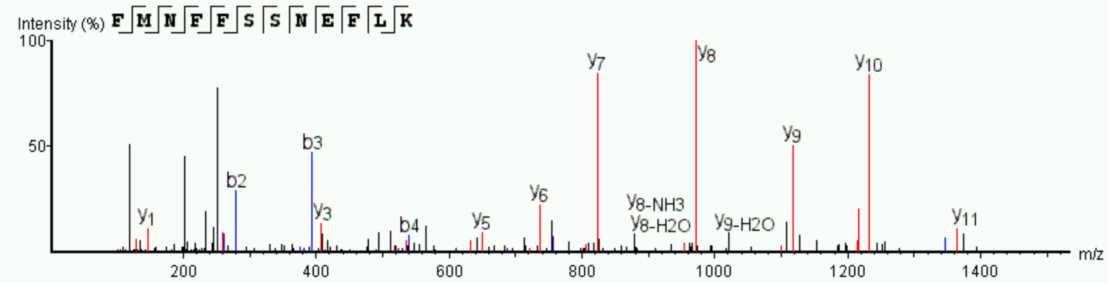

gi|288872189

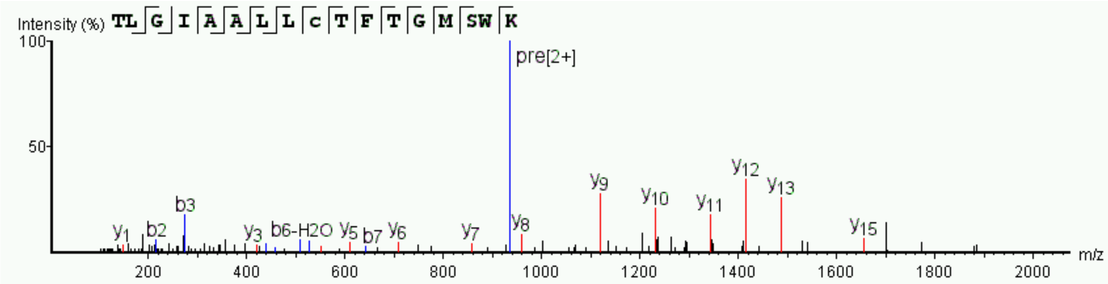

gi|118150510

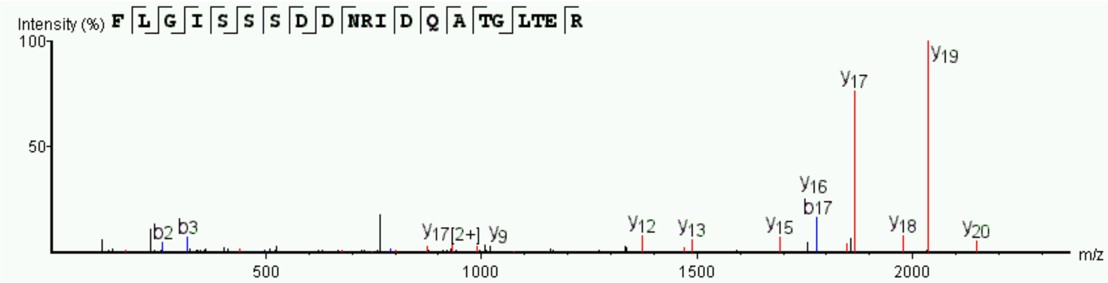

gi|209969778

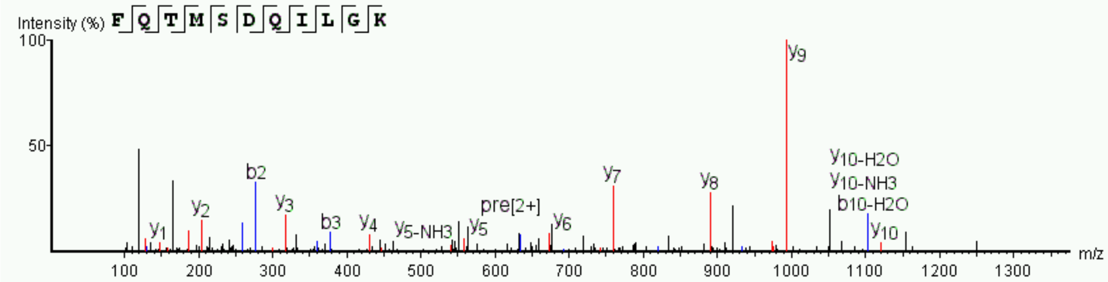

gi|66566113

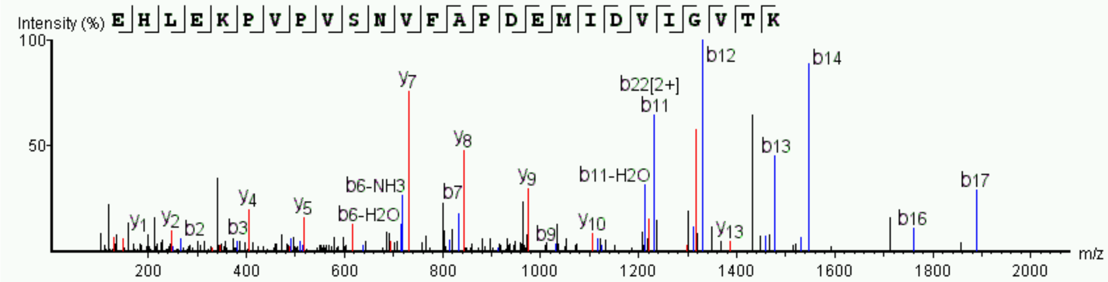

gi|335892823

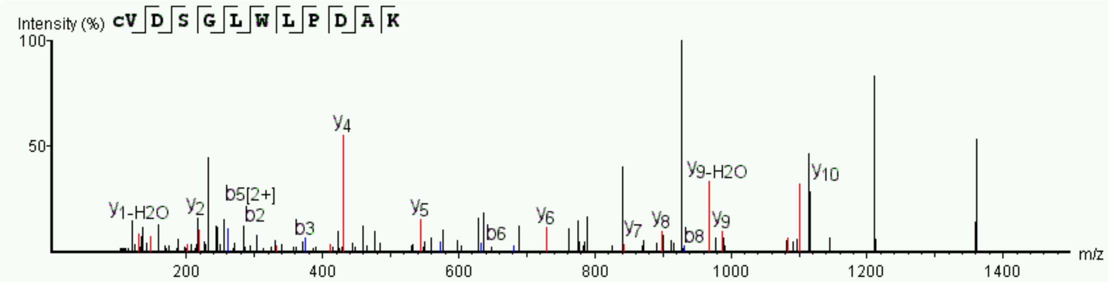

gi|571532244

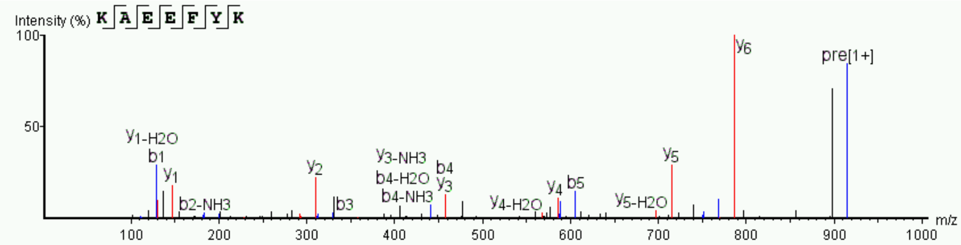

gi|571532248

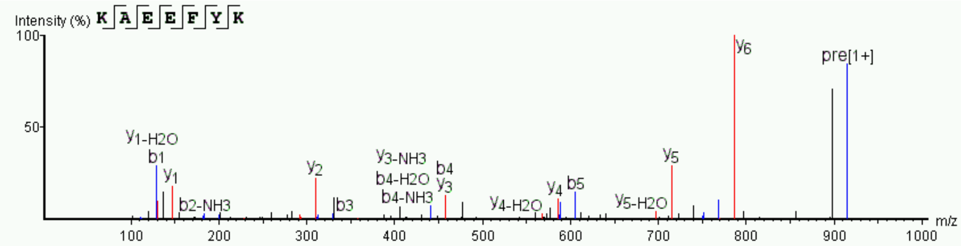

gi|571532252

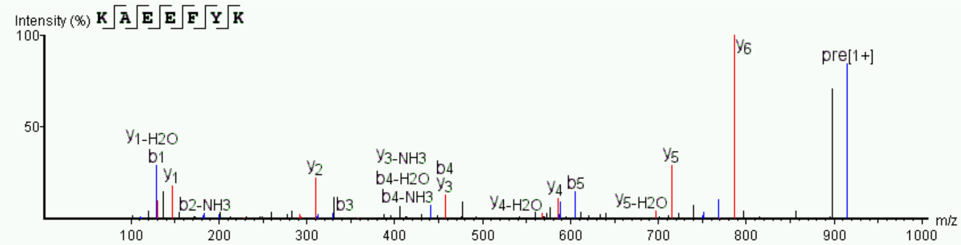

gi|571532256

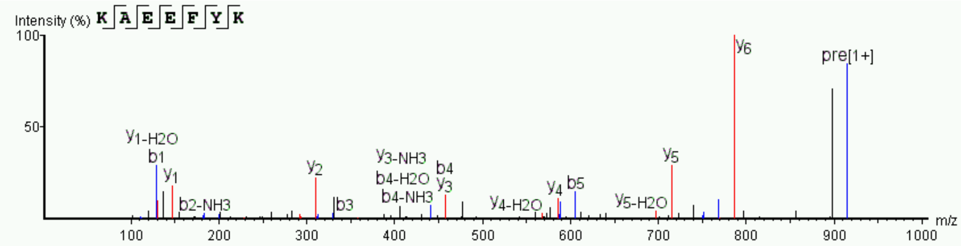

gi|571532260

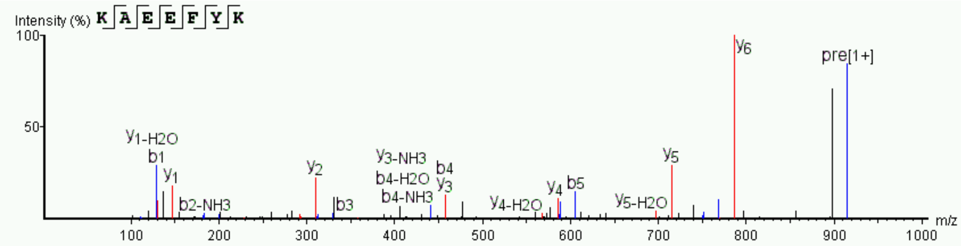

gi|328783193

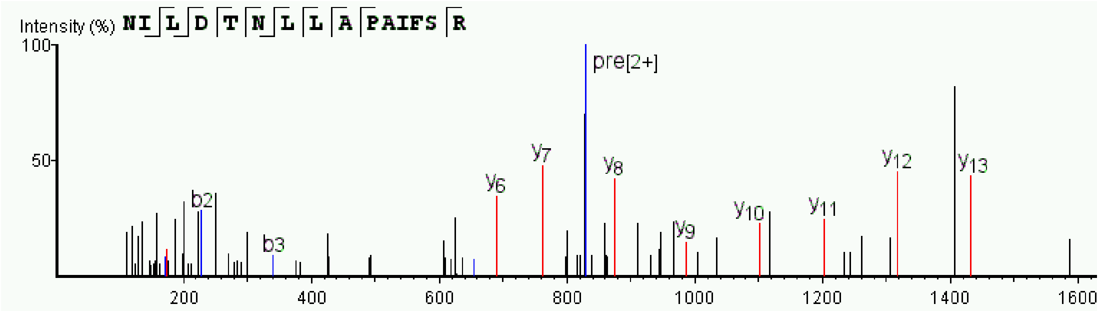

gi|571501760

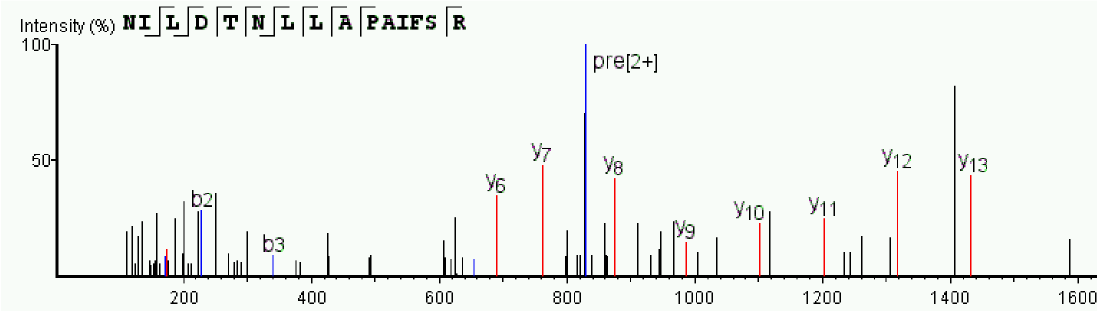

gi|48095369

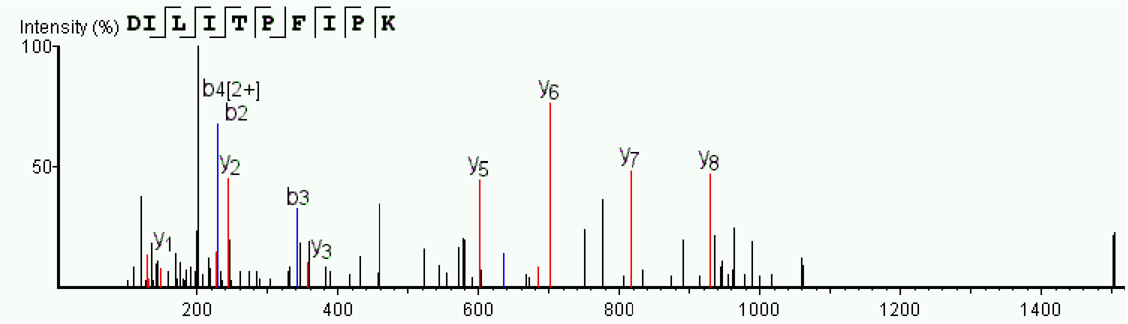

gi|110751008

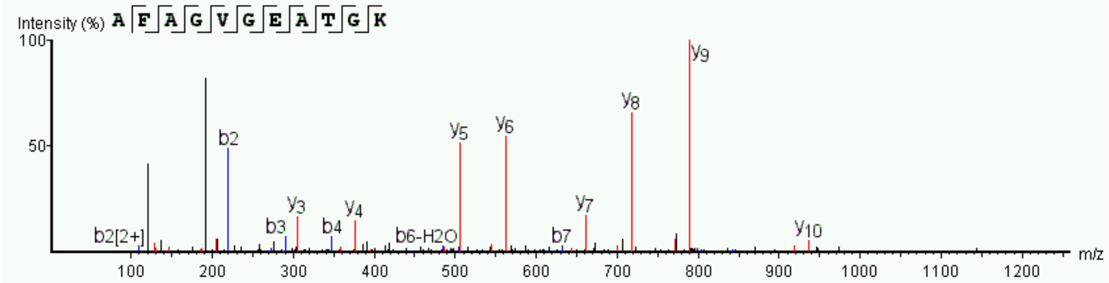

gi|240255421

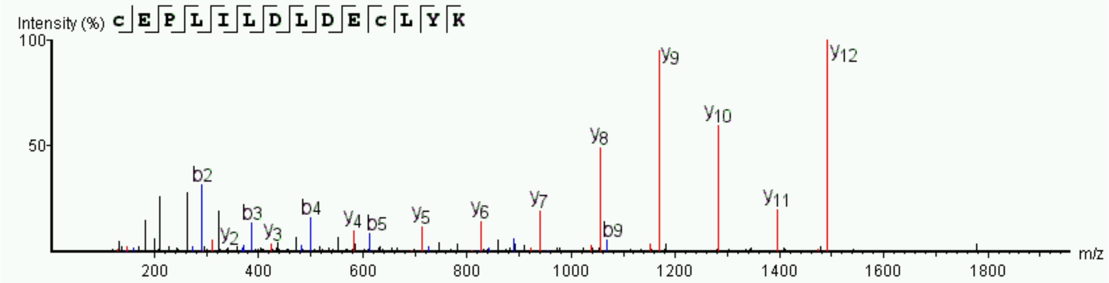

gi|5834932

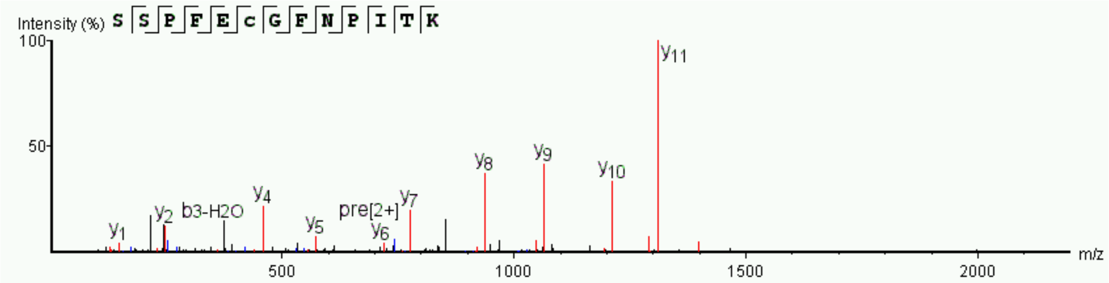

gi|571576016;

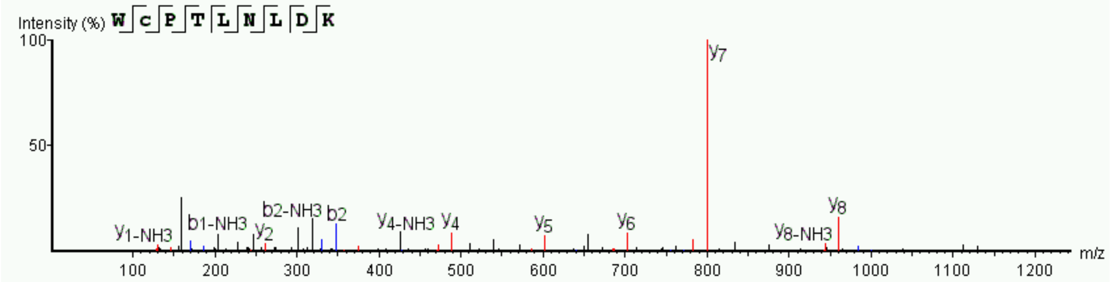

gi|66526748gi

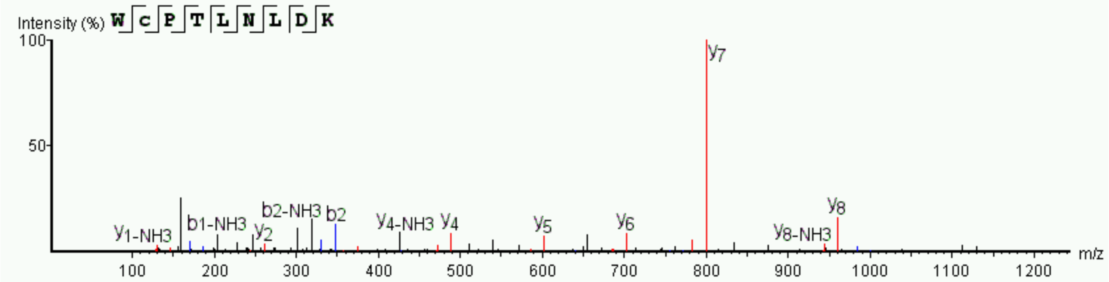

||268370271

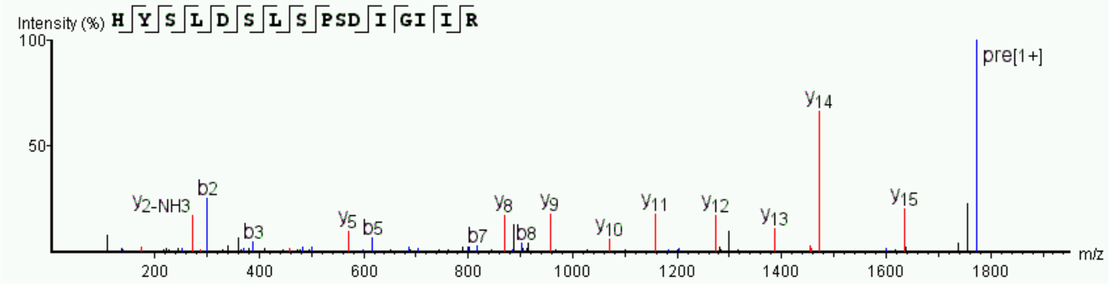

gi|48140499

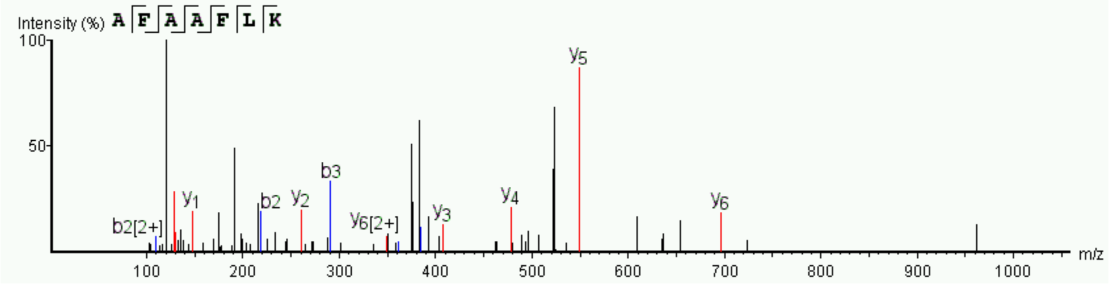

gi|327315378

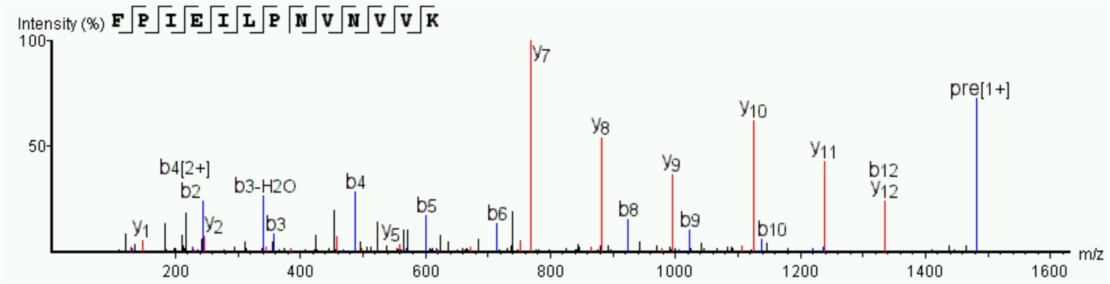

gi|110771708

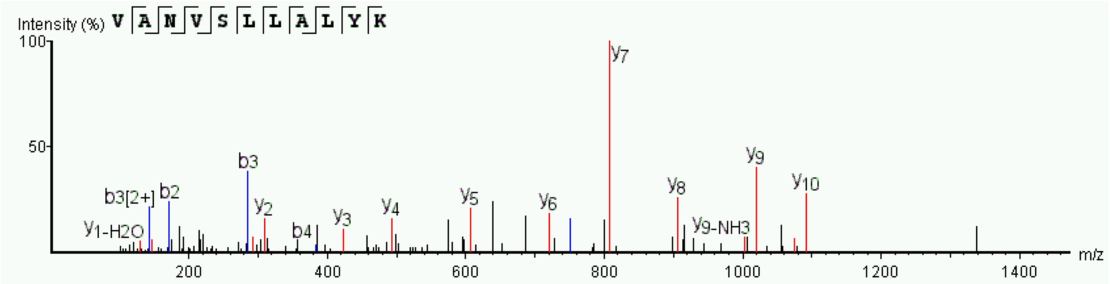

gi|110766823

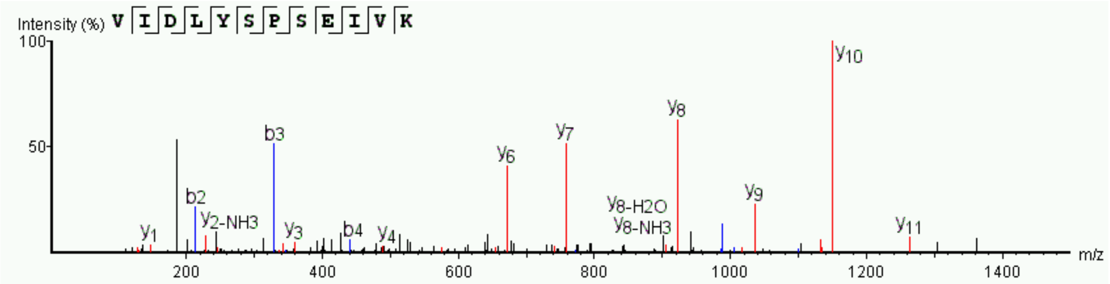

gi|239787841

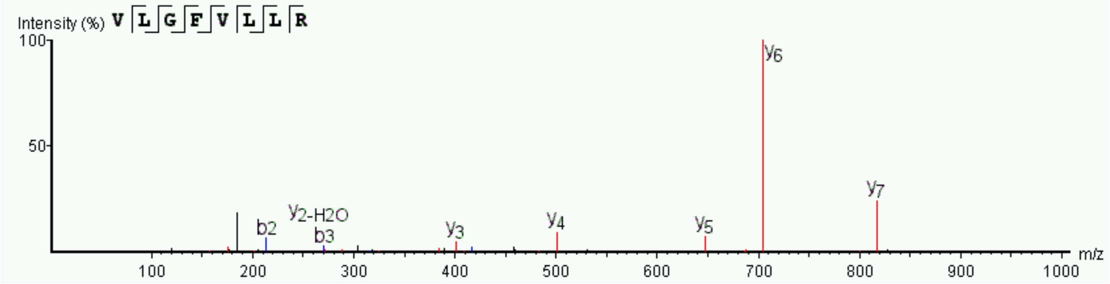

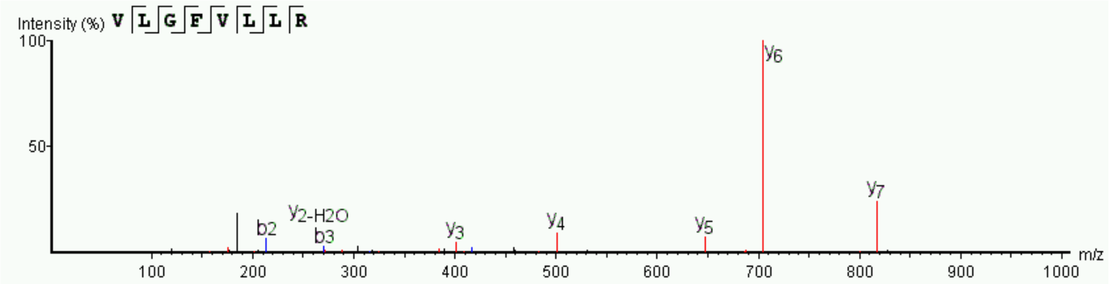

gi|571545002

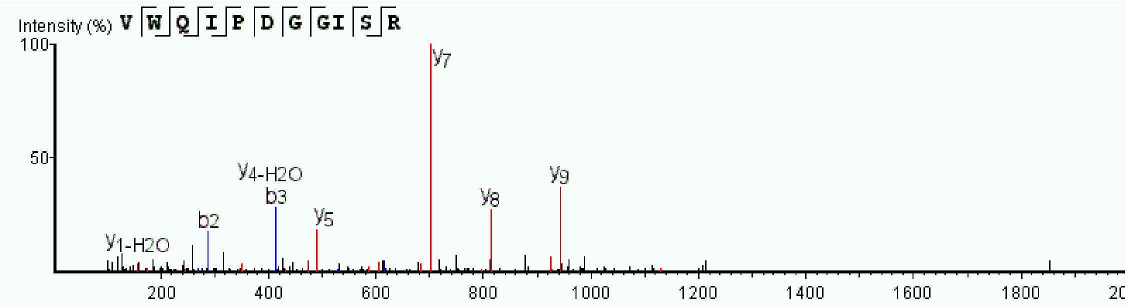

gi|571545005

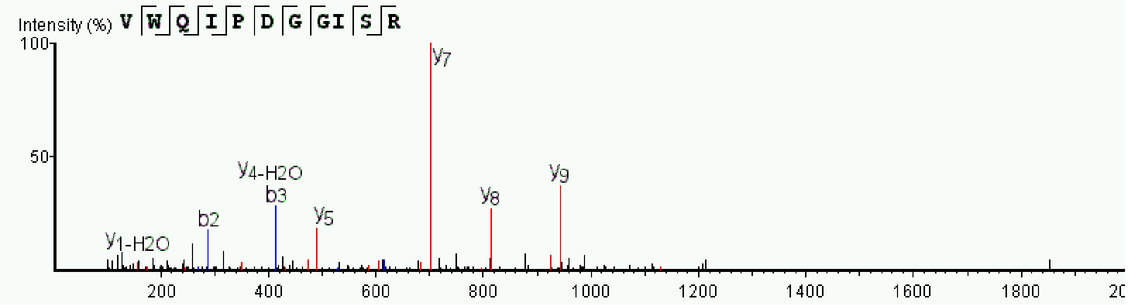

gi|571545009

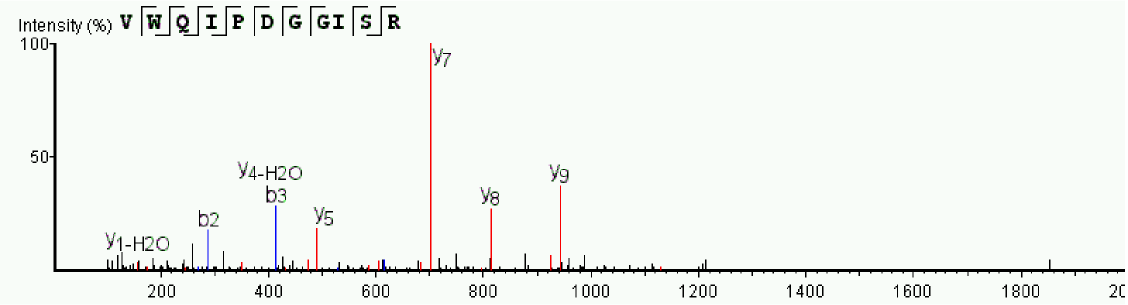

gi|571545012

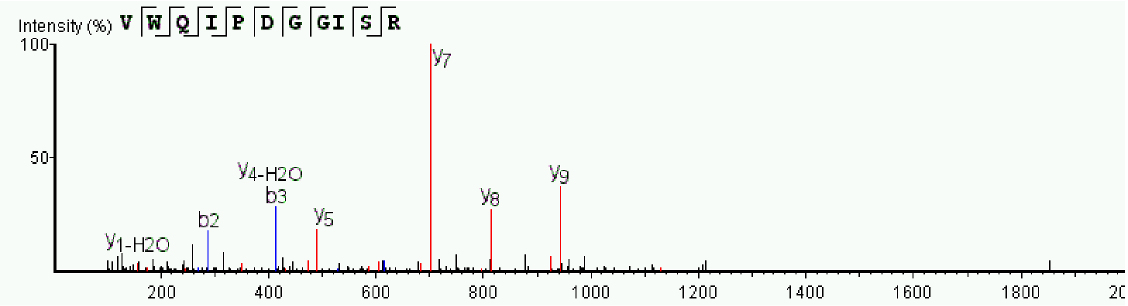

gi|571545015

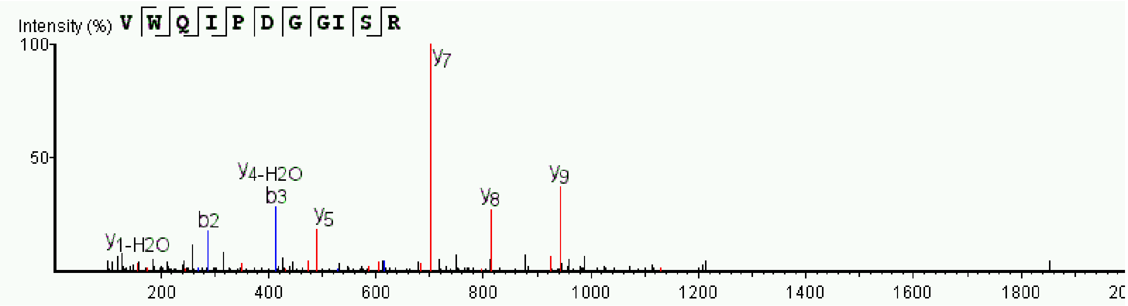

gi|571522953

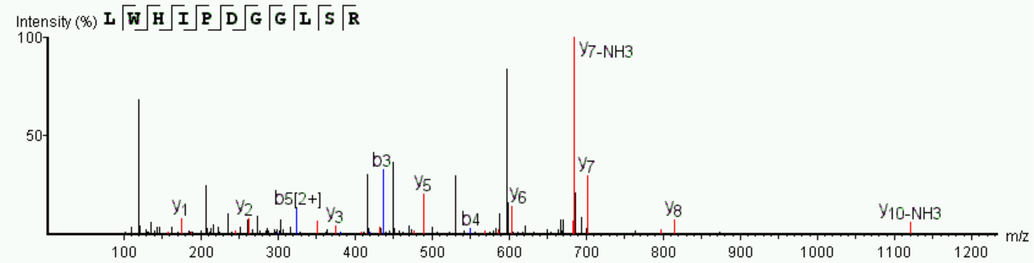

gi|571522957

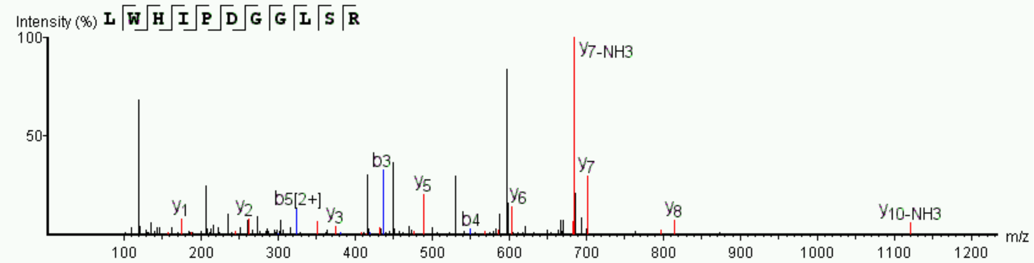

gi|571522961

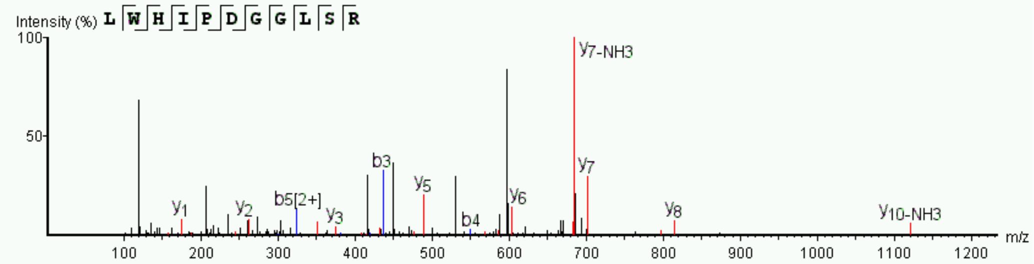

gi|571522949

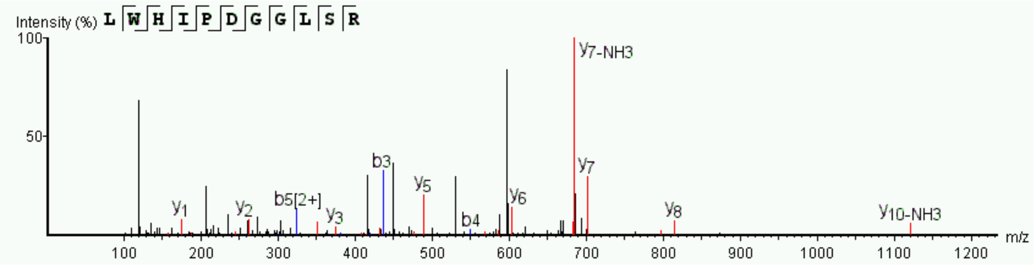

gi|571567945

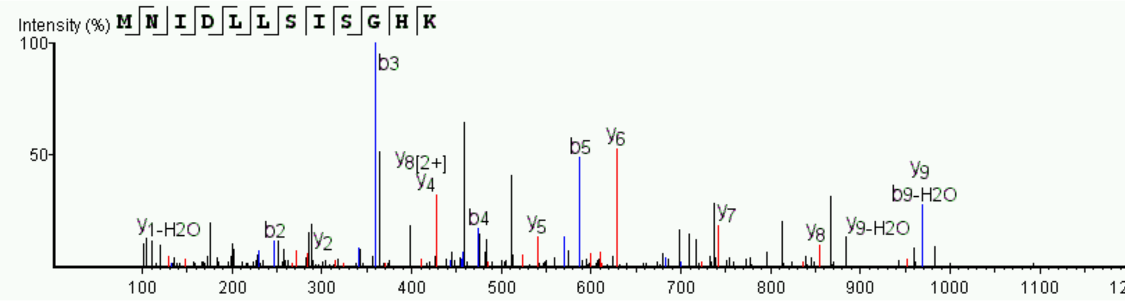

gi|571567948

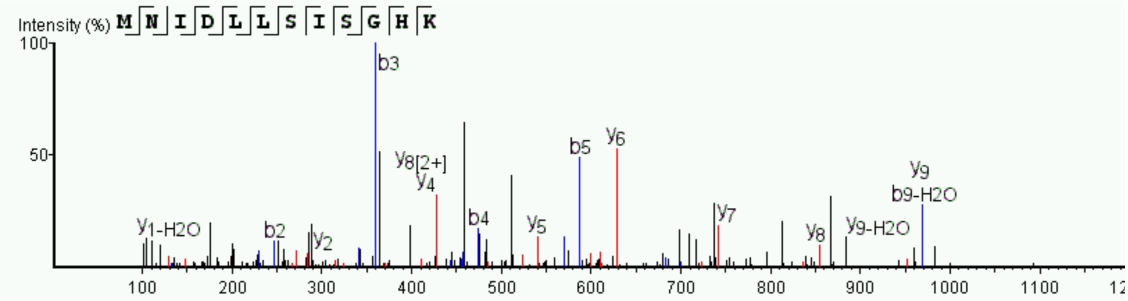

gi|328779544

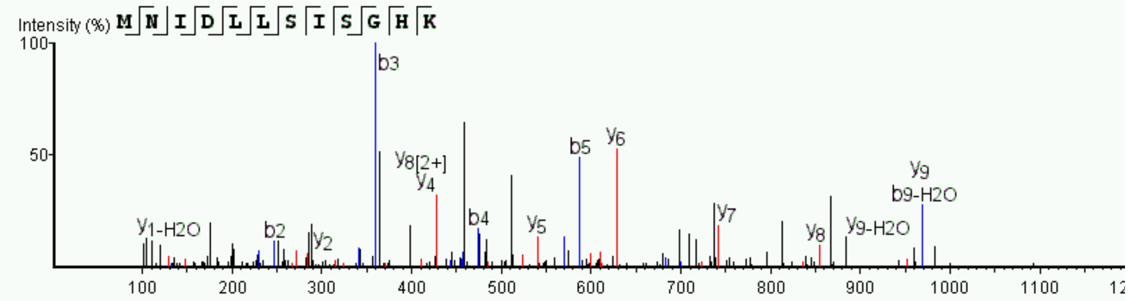

gi|571575329

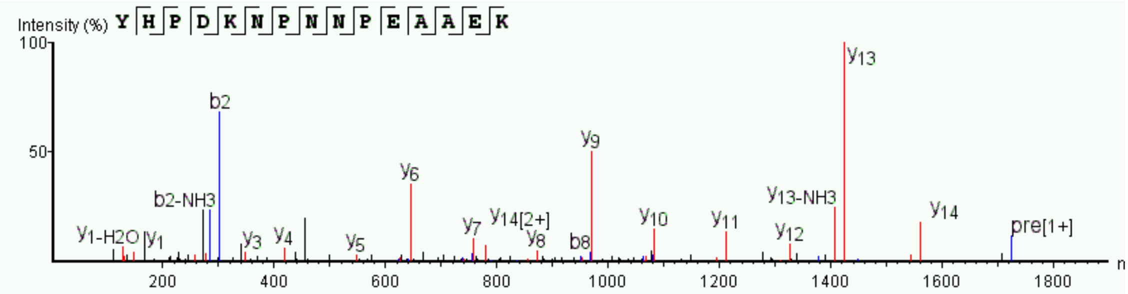

gi|110757574

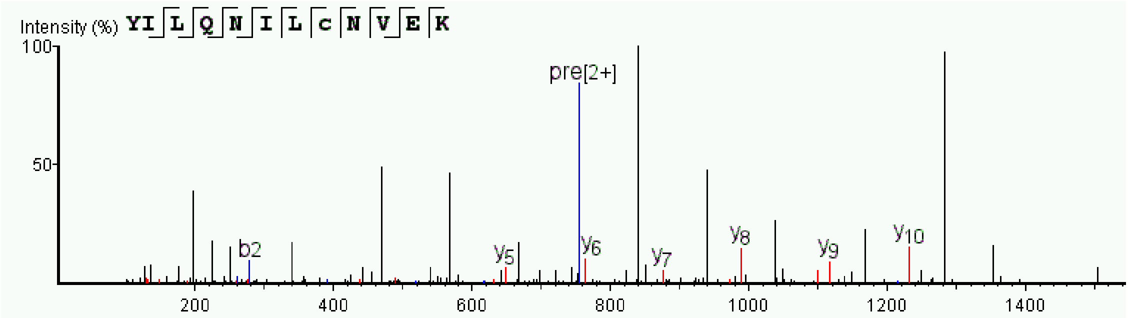

gi|328778851

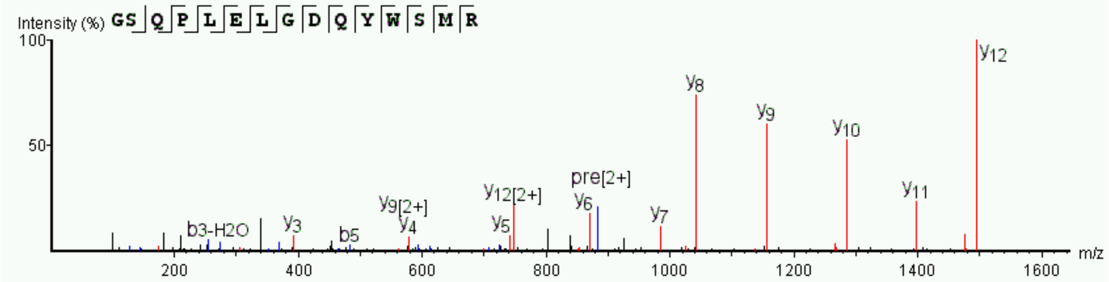

gi|110759577

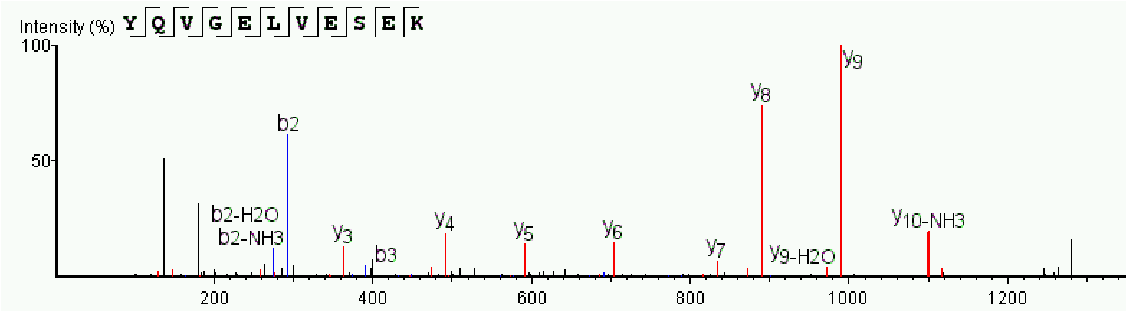

gi|328781214

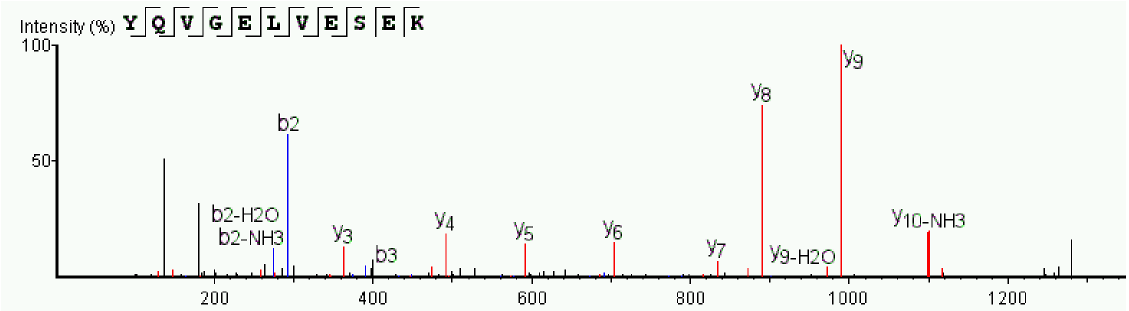

gi|110772198

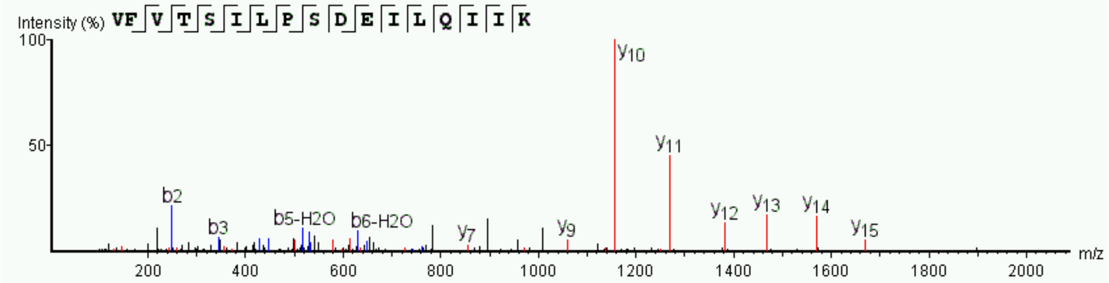

gi|66524513

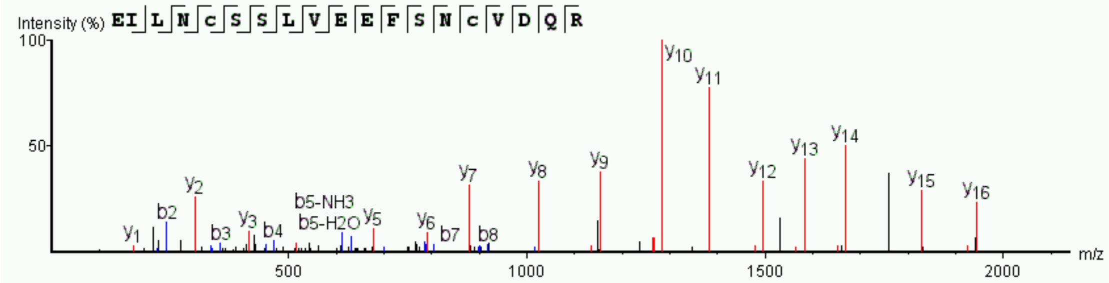

gi|334852197

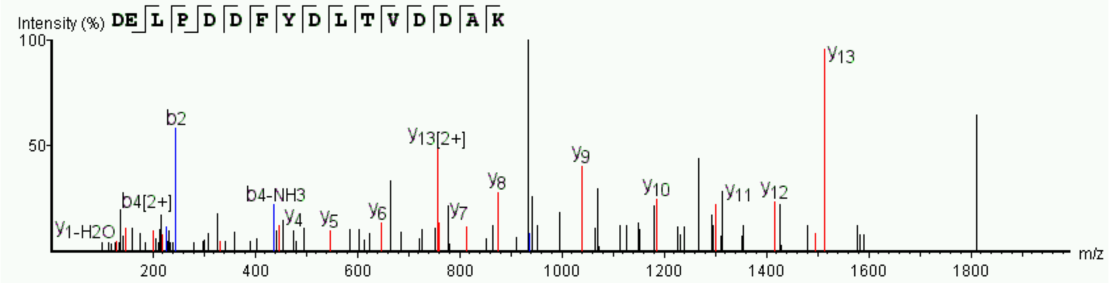

gi|58585132

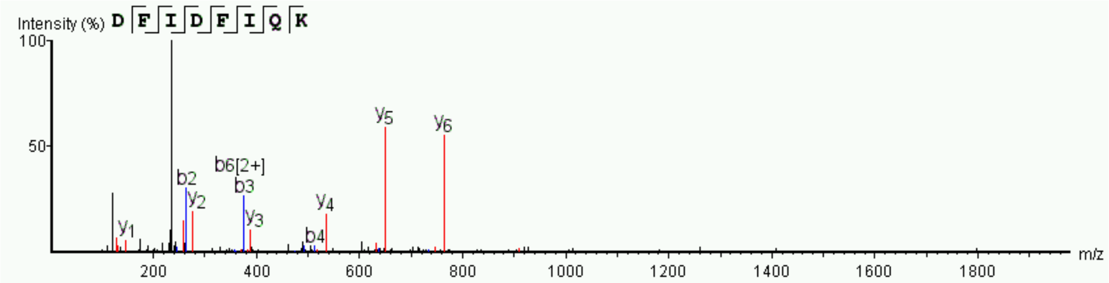

gi|162952028

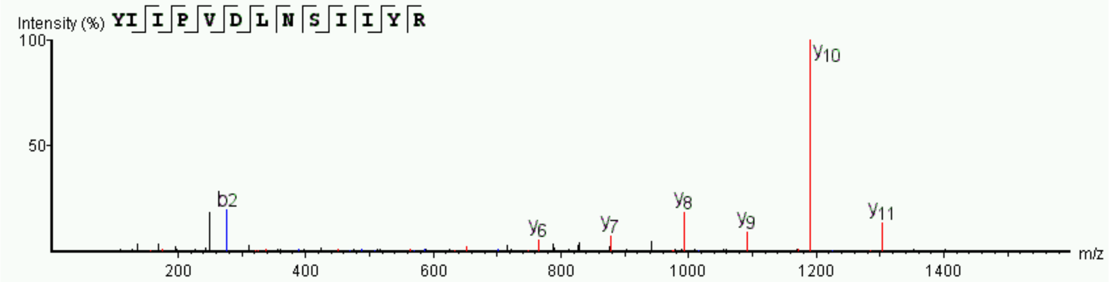

gi|571540469;

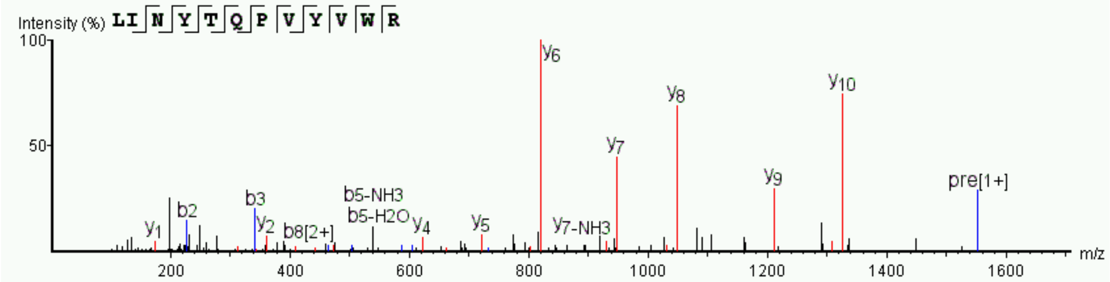

gi|571540475;

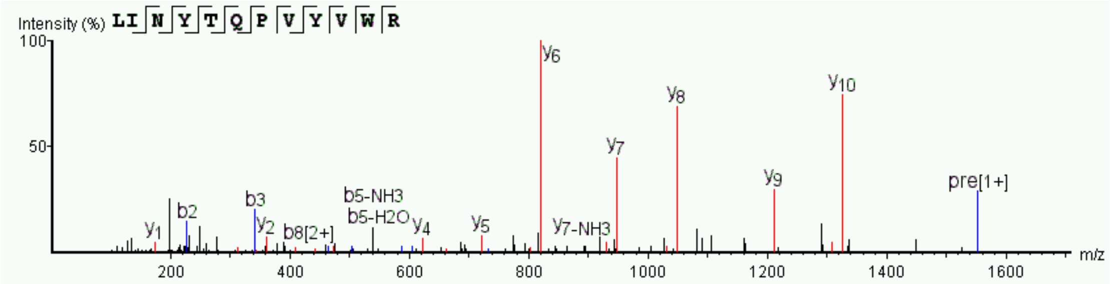

gi|571540483;

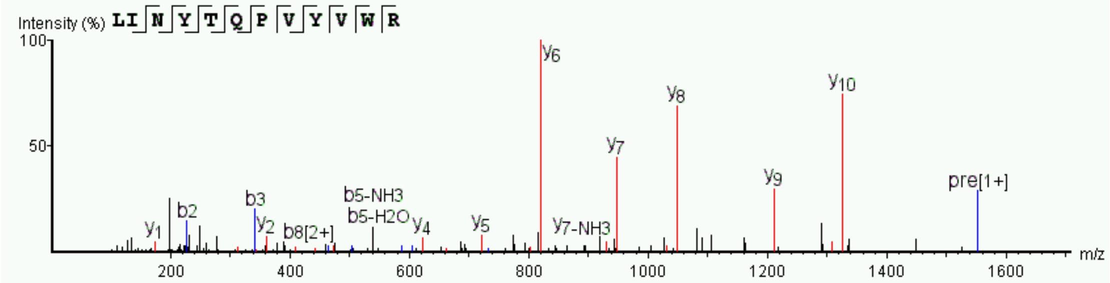

gi|571540493

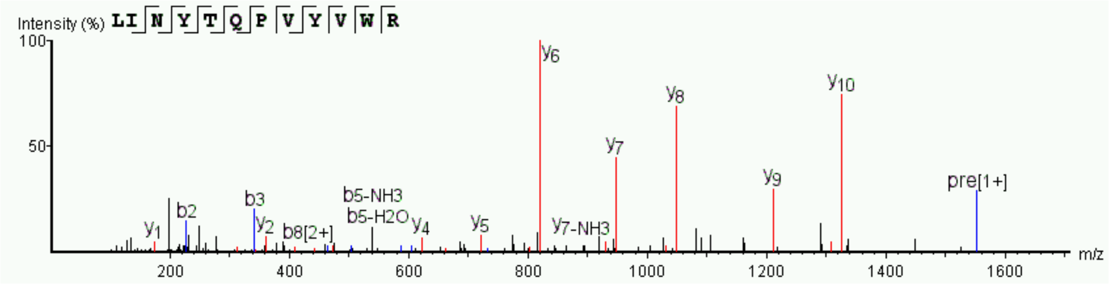

gi|571558762 ;

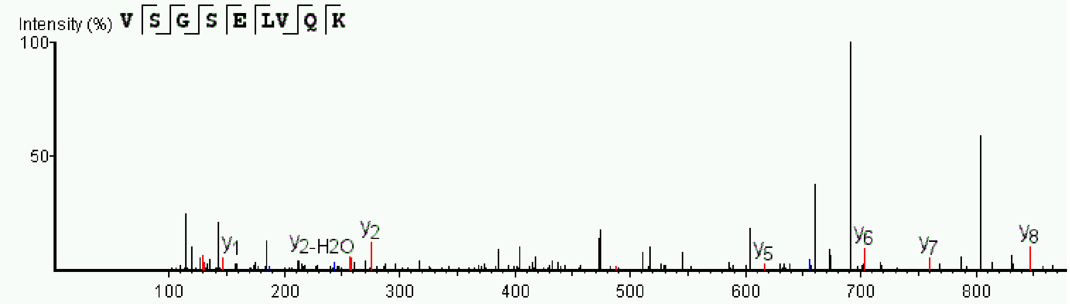

gi|571558766;

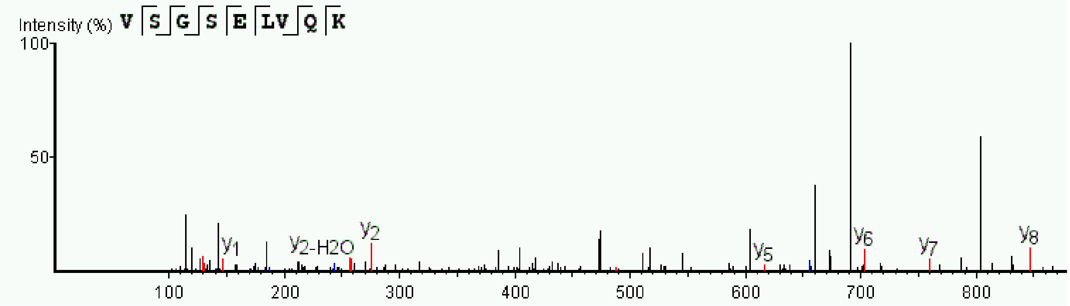

gi|571558772 ;

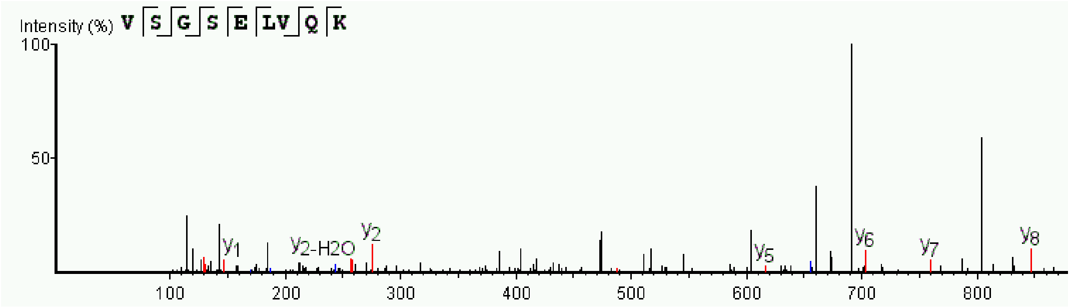

gi|571558776

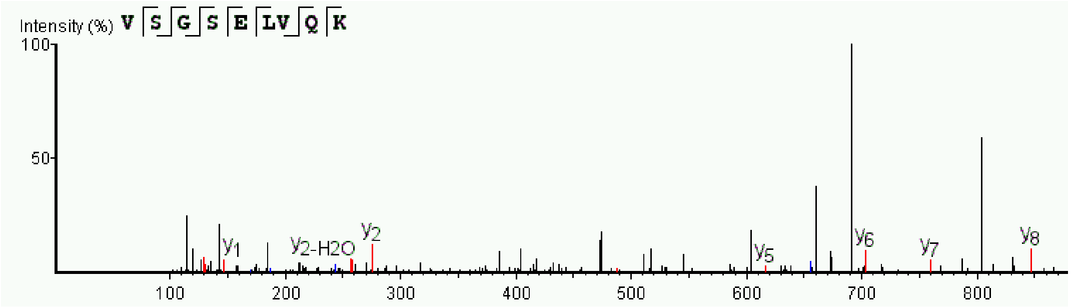

gi|571566140

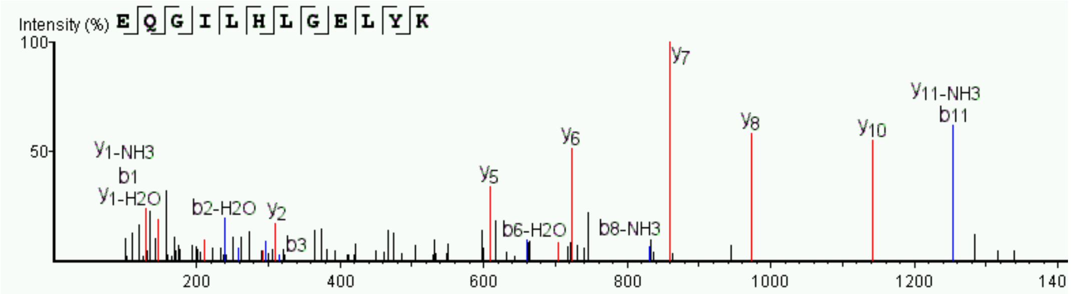

gi|571578878

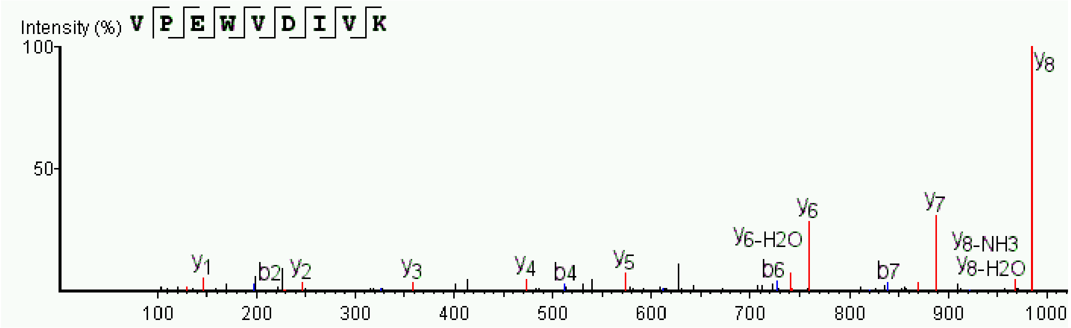

gi|571521158

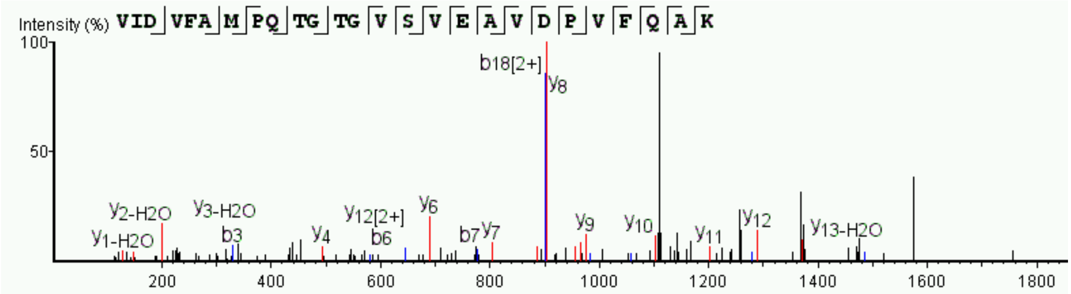

gi|571561875

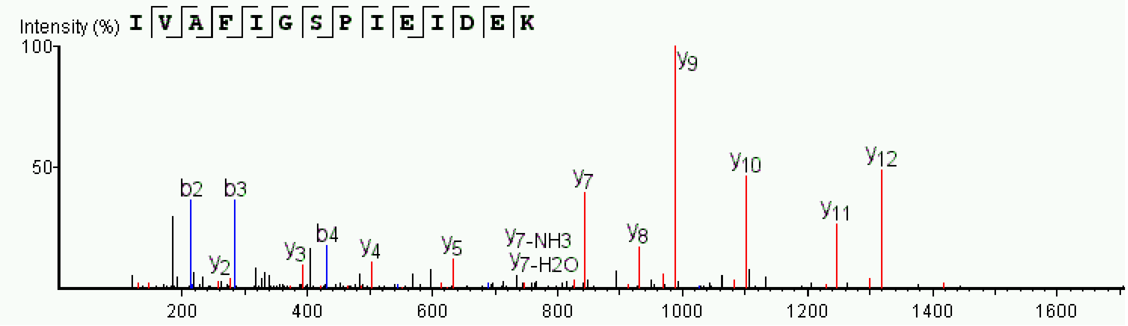

gi|110762783

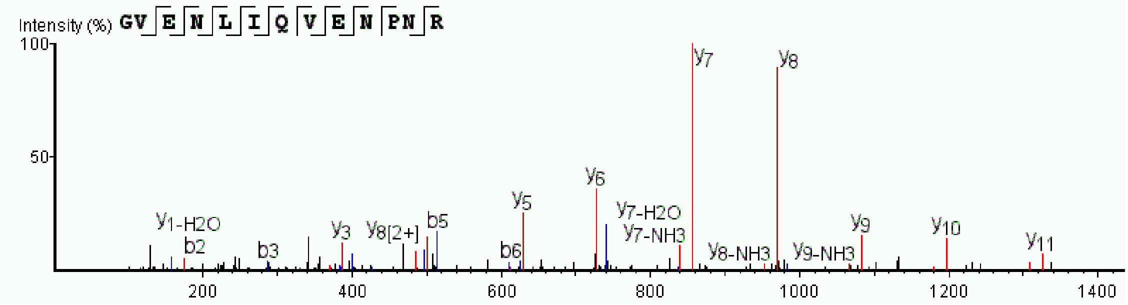

gi|110763030

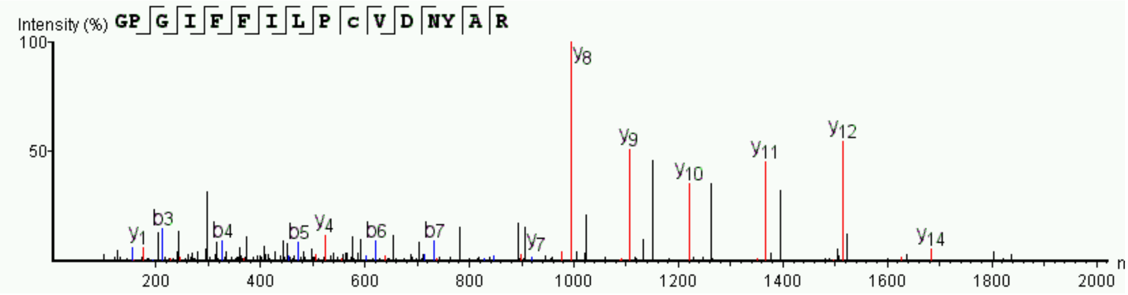

gi|571572787

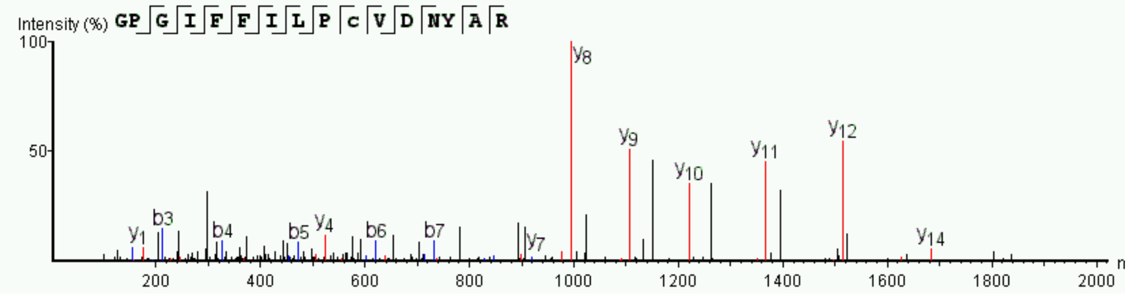

gi|571572789

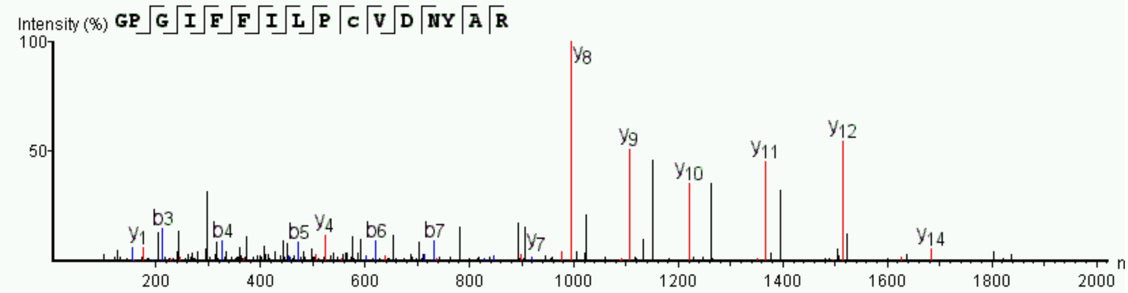

gi|571572791

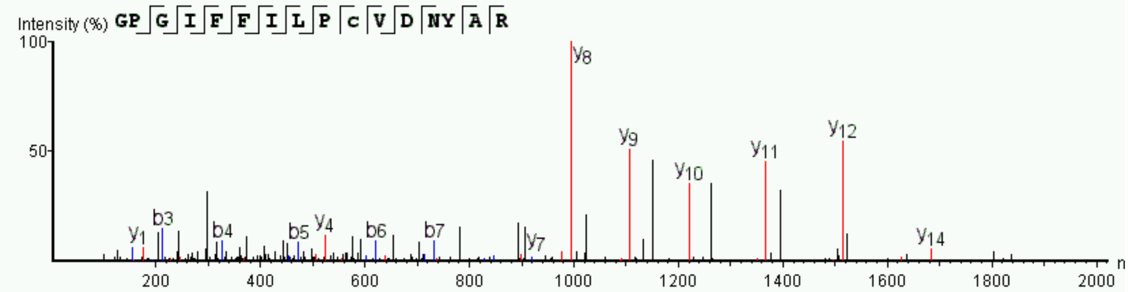

gi|571572793

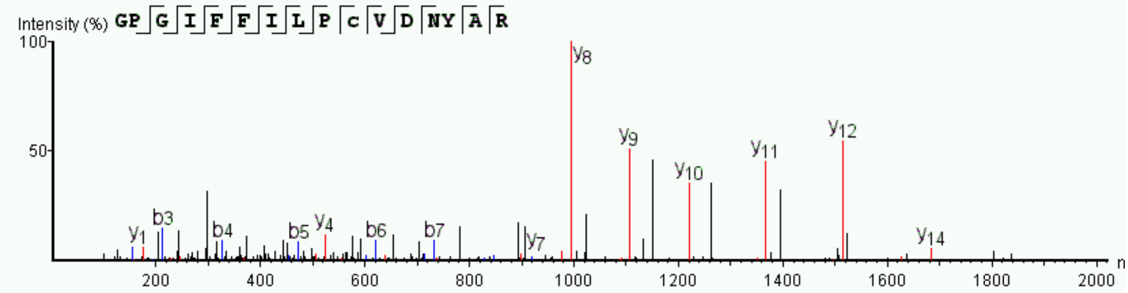

gi|571572796

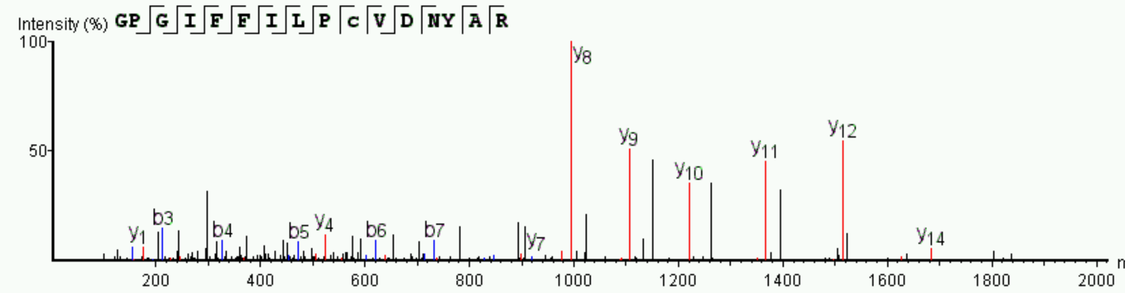

gi|571572798

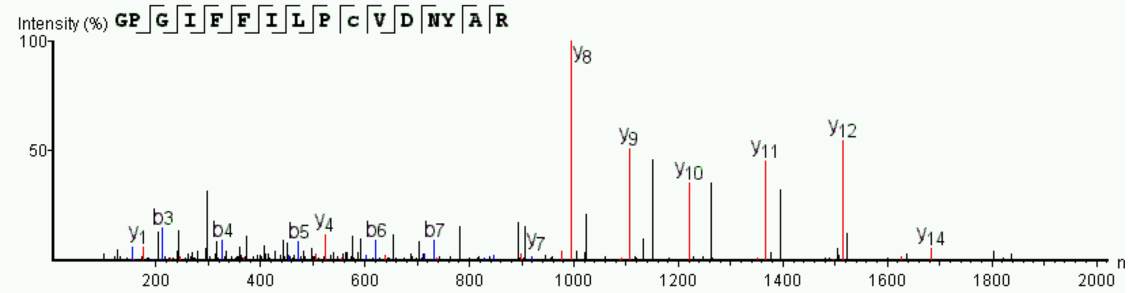

gi|571572801

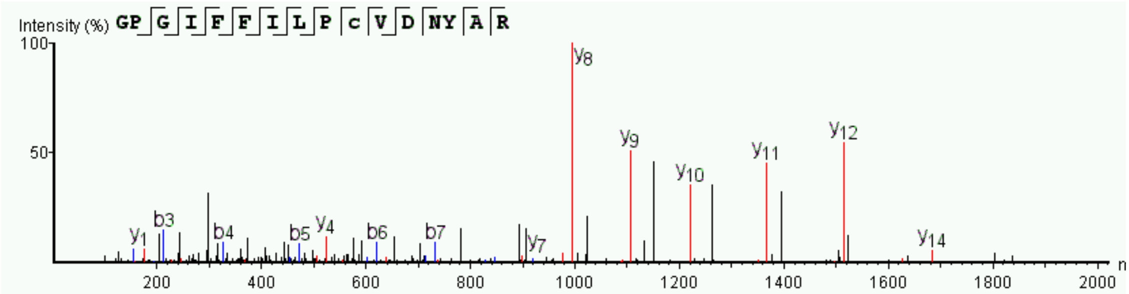

gi|571572803

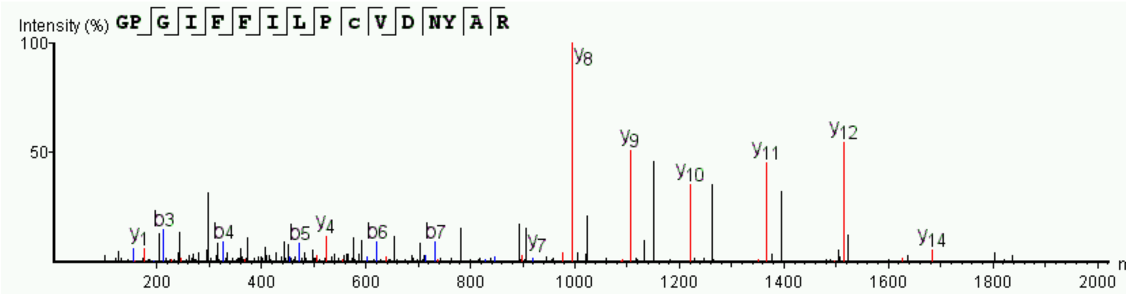

gi|571516024

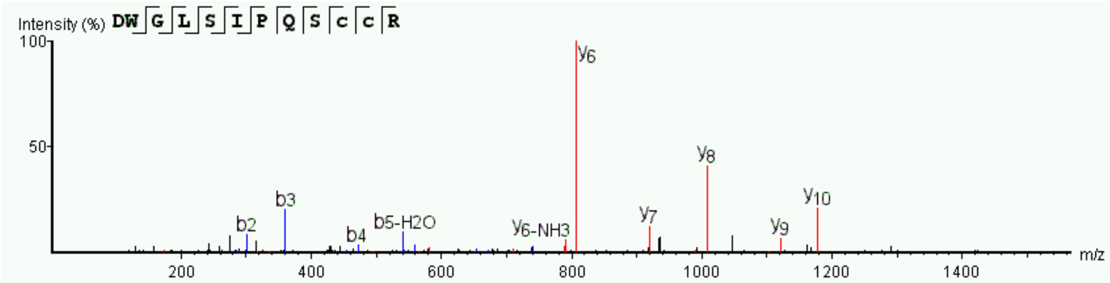

gi|571516027

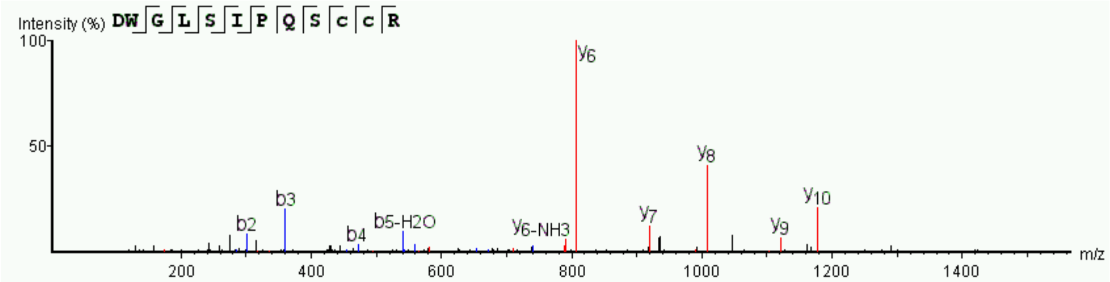

gi|571516030

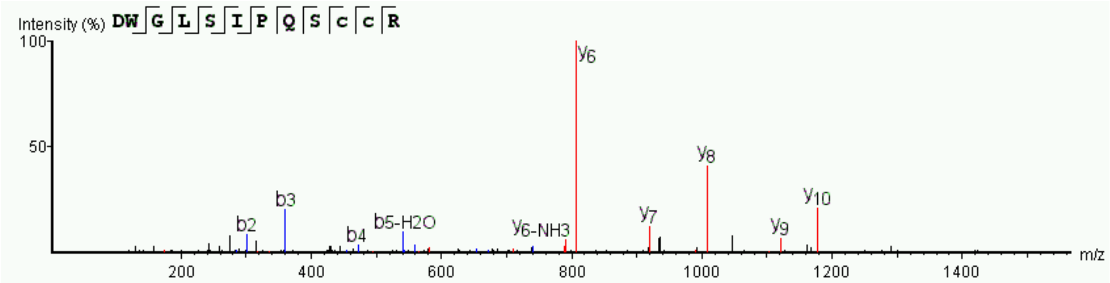

gi|66504429

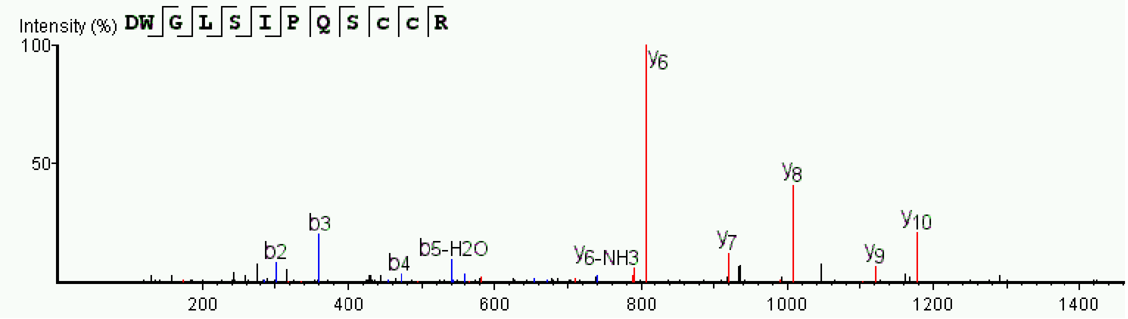

gi|571503331

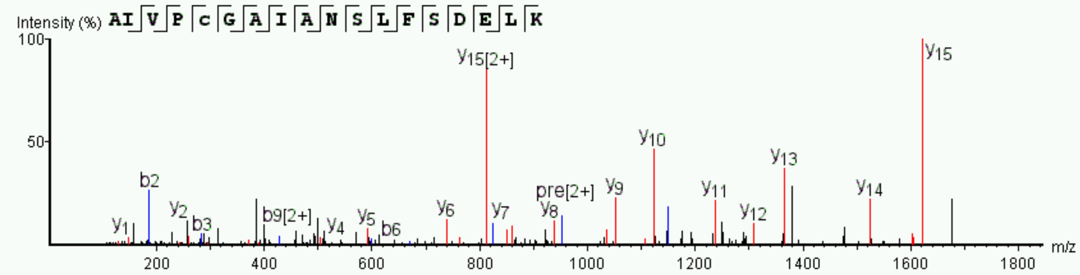

gi|571503334

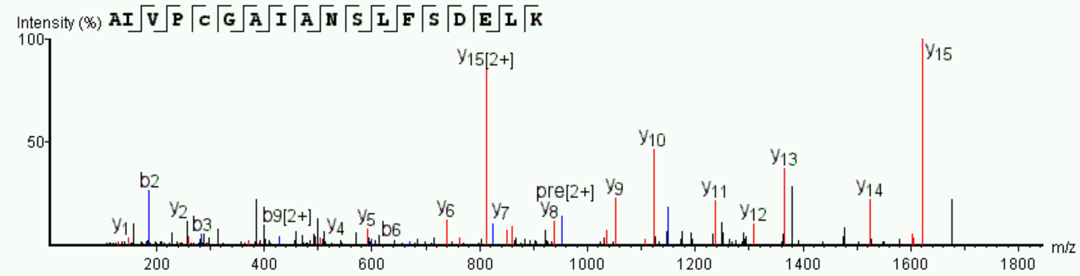

gi|571503337

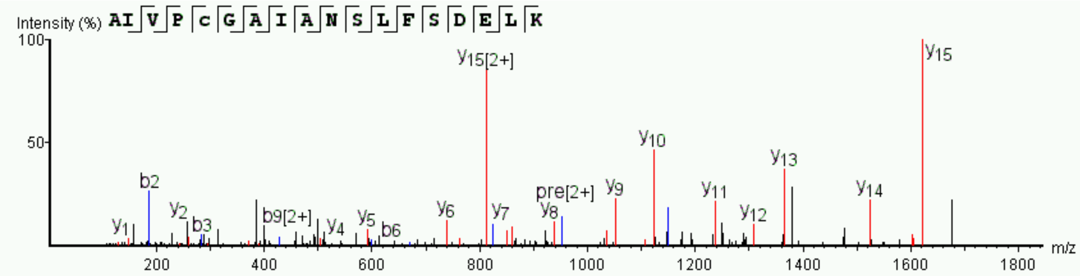

gi|571503340

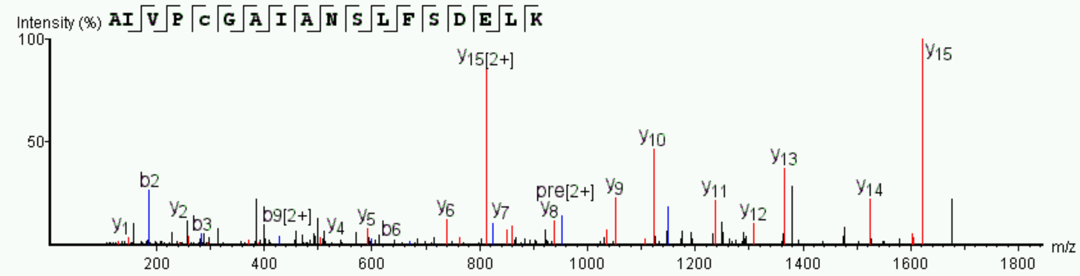

gi|66544564

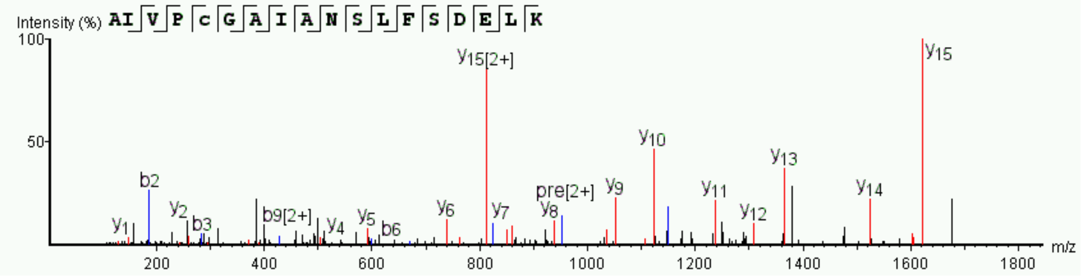

gi|571566909

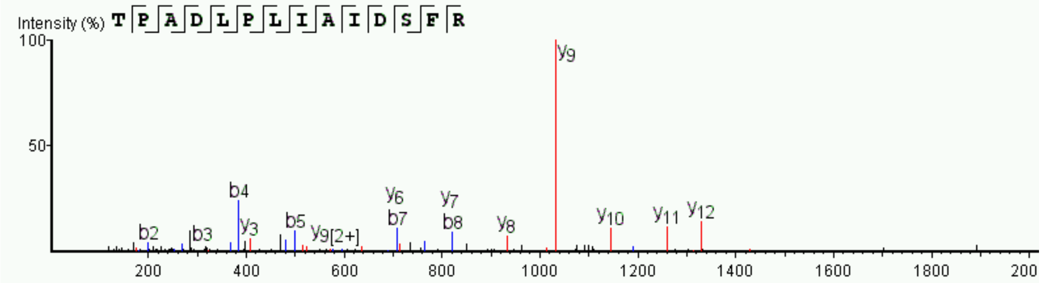

gi|328777360

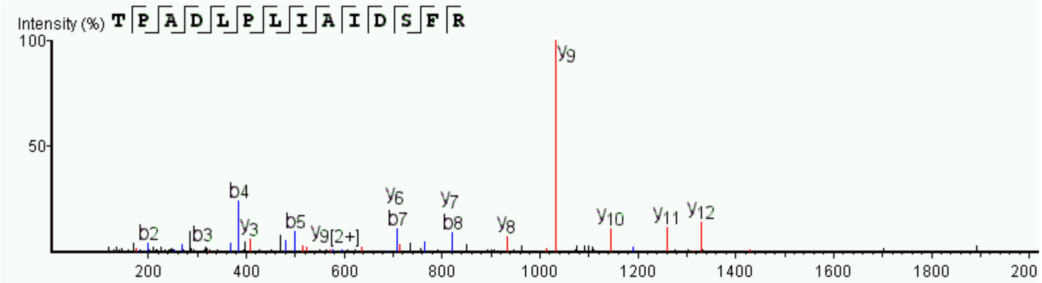

gi|571533507

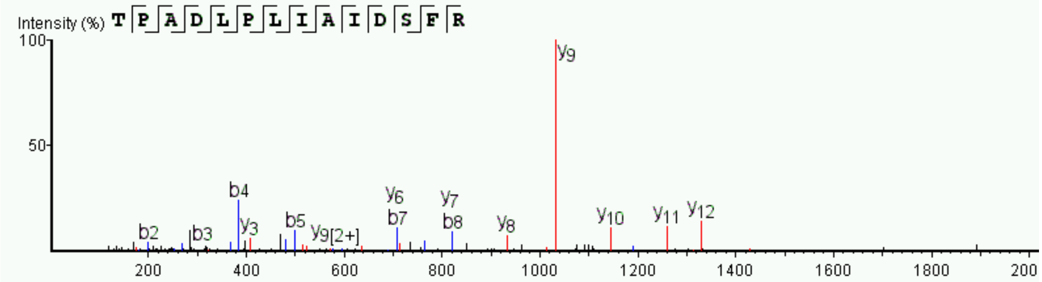

gi|571541144

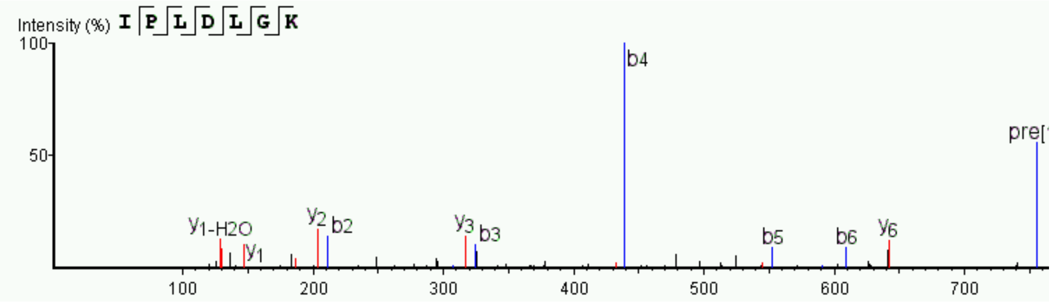

gi|66512104

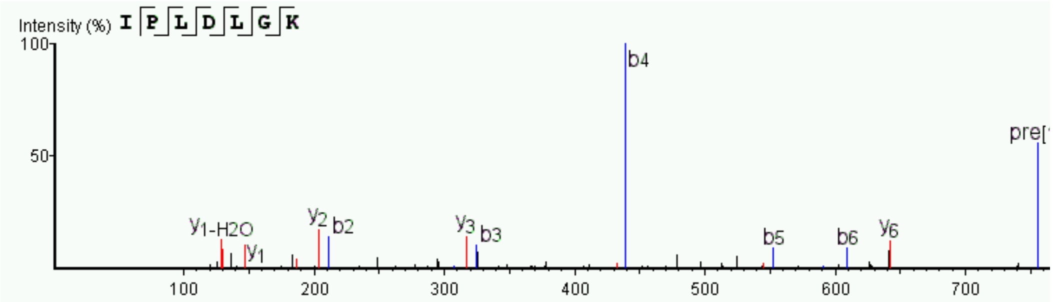

gi|328780829

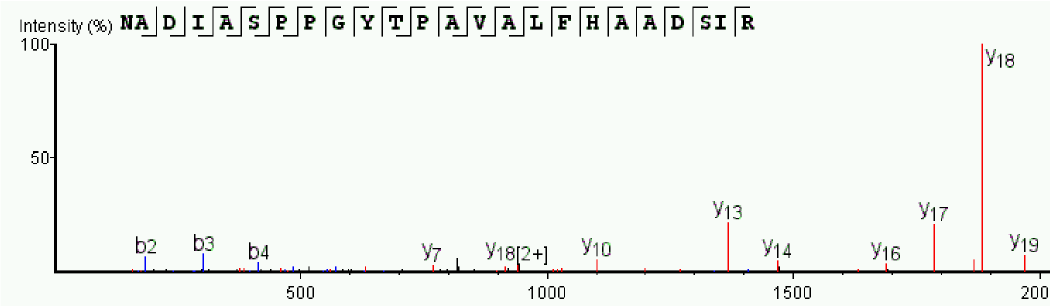

gi|571533515

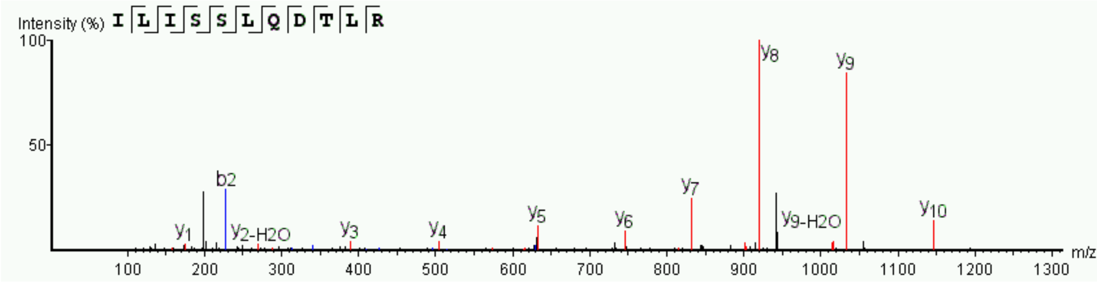

gi|571559515

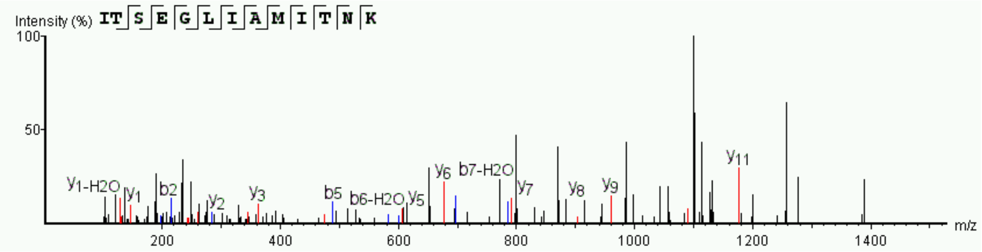

gi|571565418

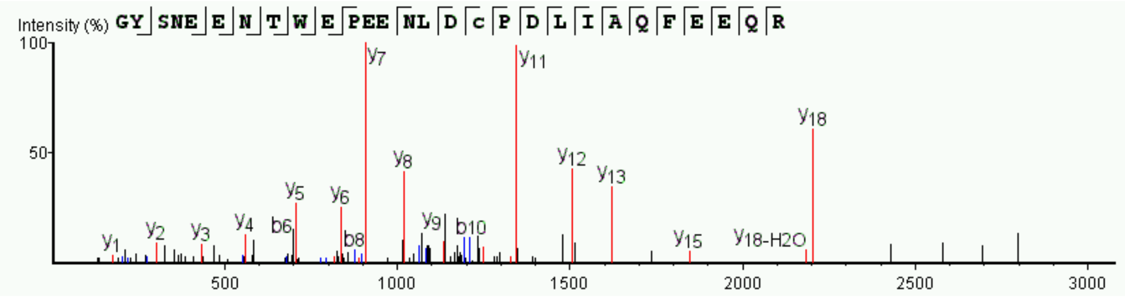

gi|48097746

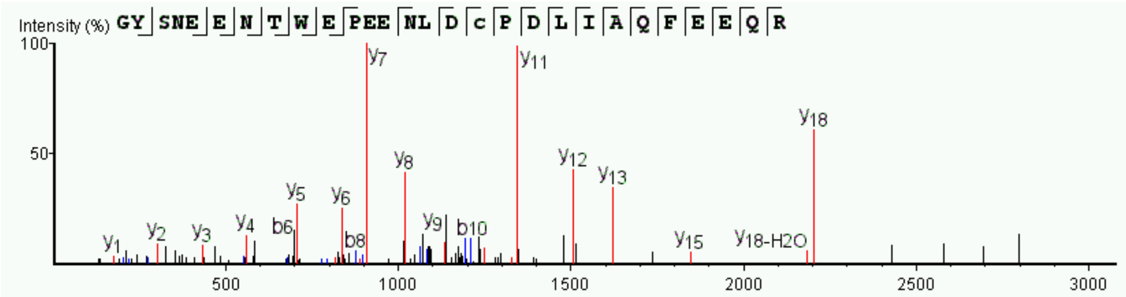

gi|48098249

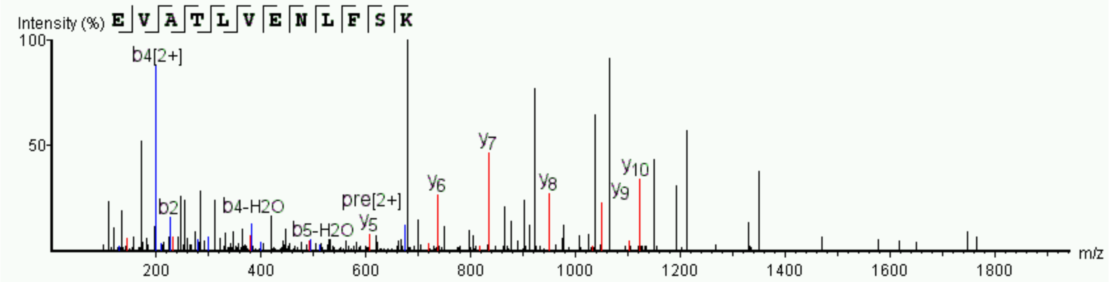

gi|328784410

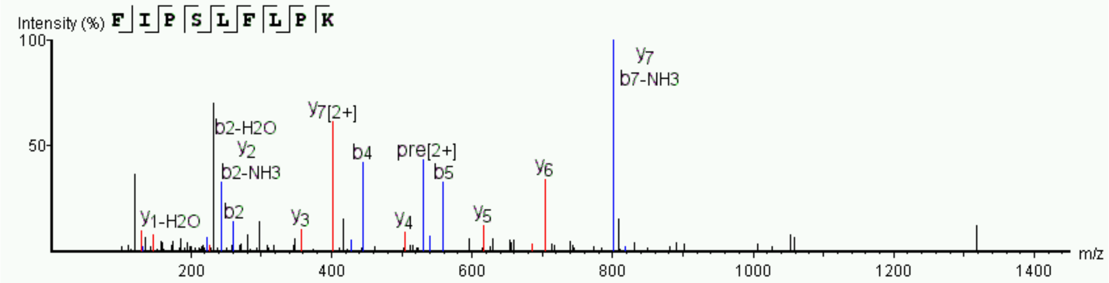

gi|328789409

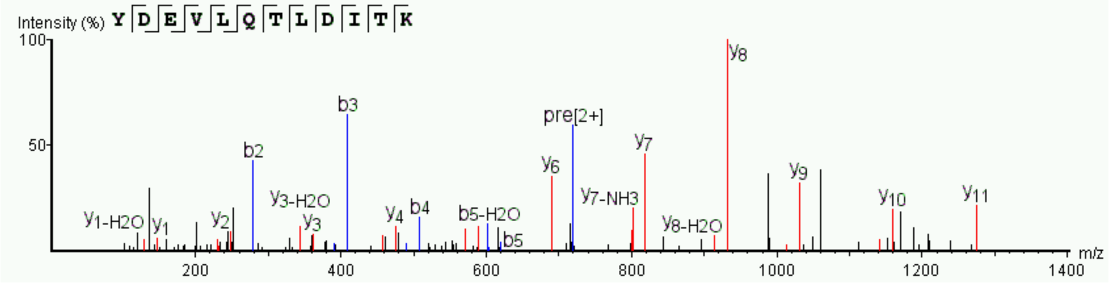

gi|571504763

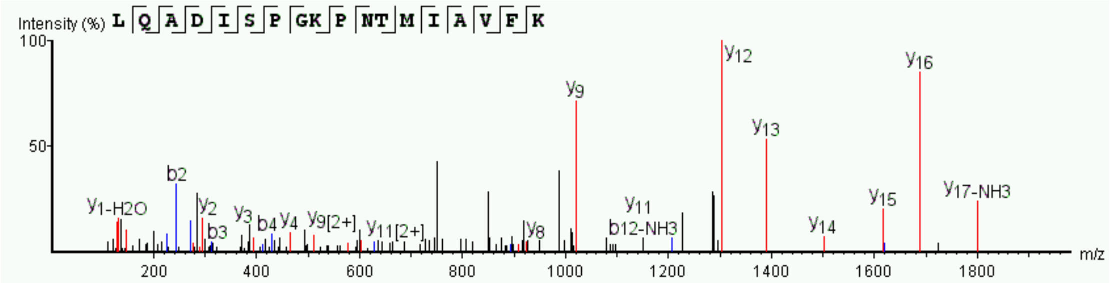

gi|571504766

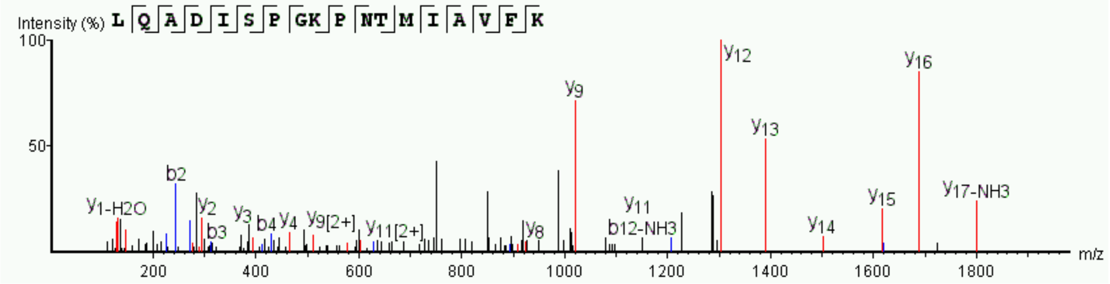

gi|571504092

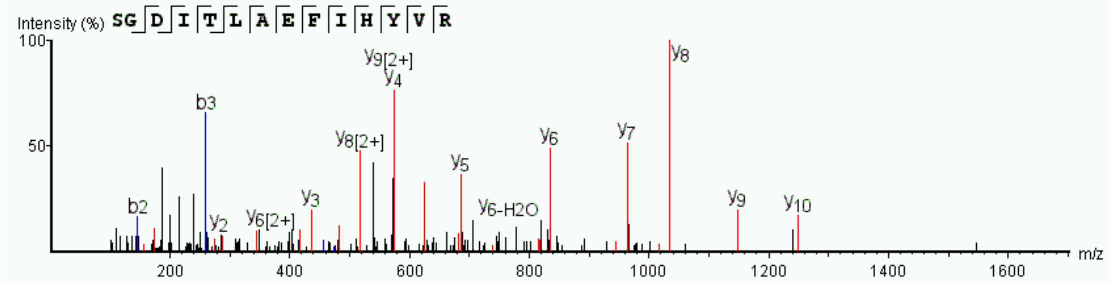

gi|571577993;

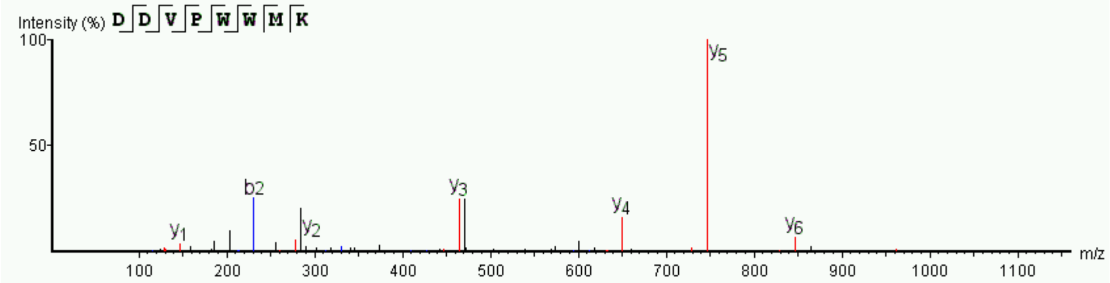

gi|571577995

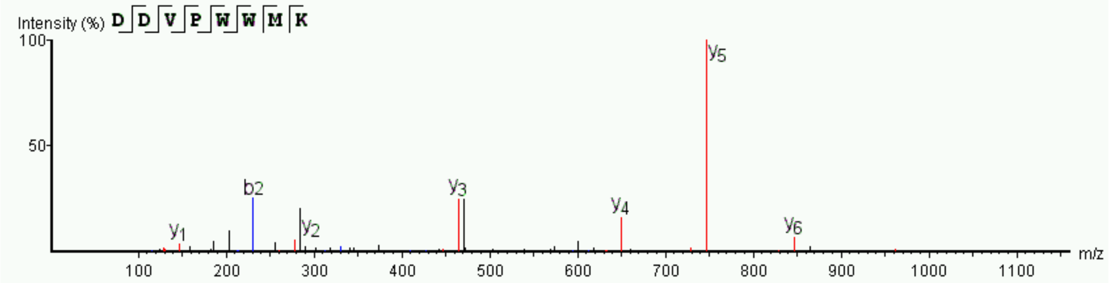

gi|571509742;

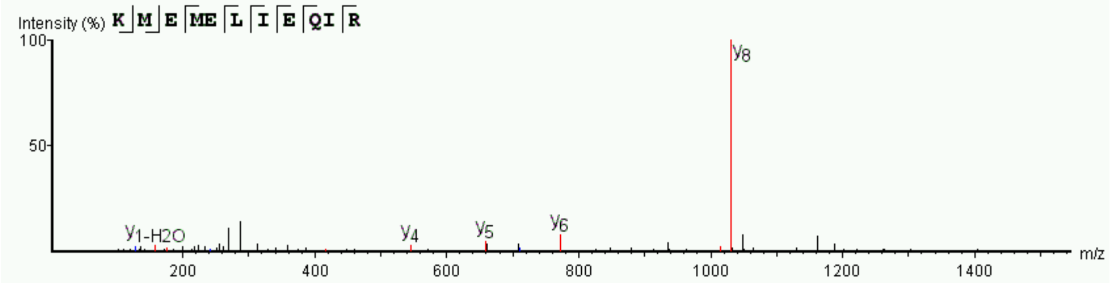

gi|571509745

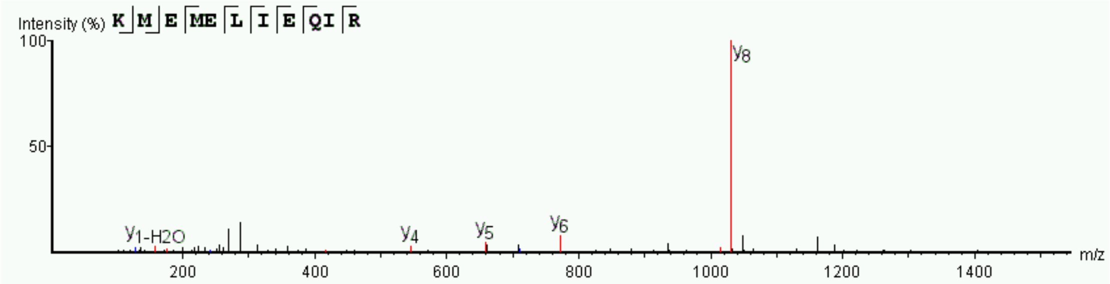

gi|571509749

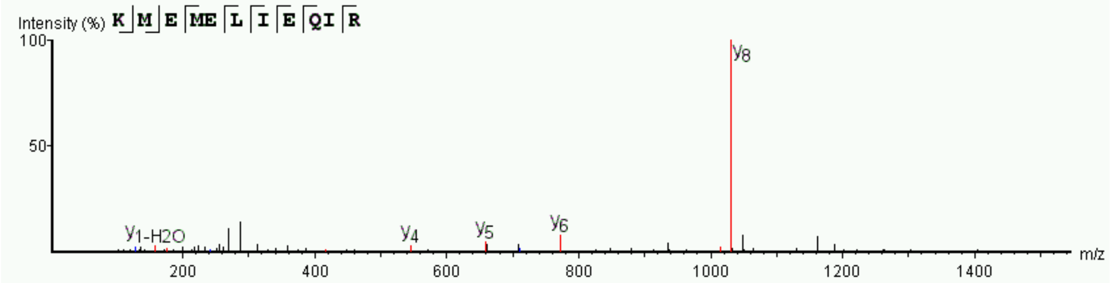

gi|571509753

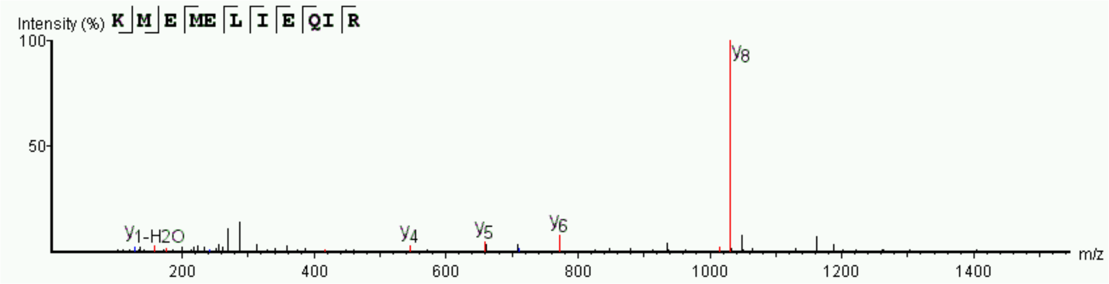

gi|571558853;

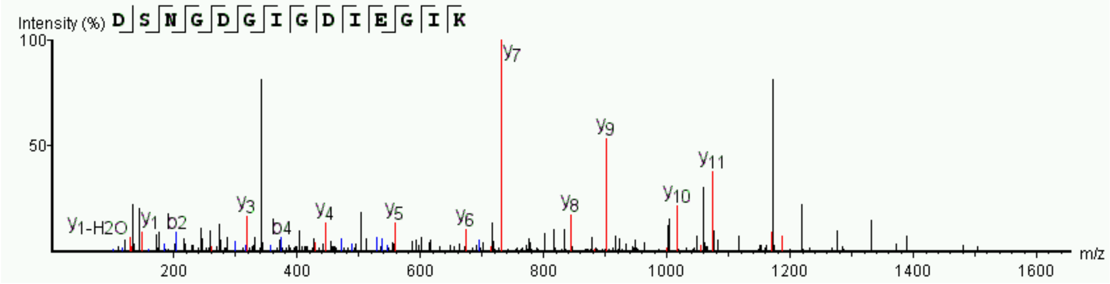

gi|571558857;

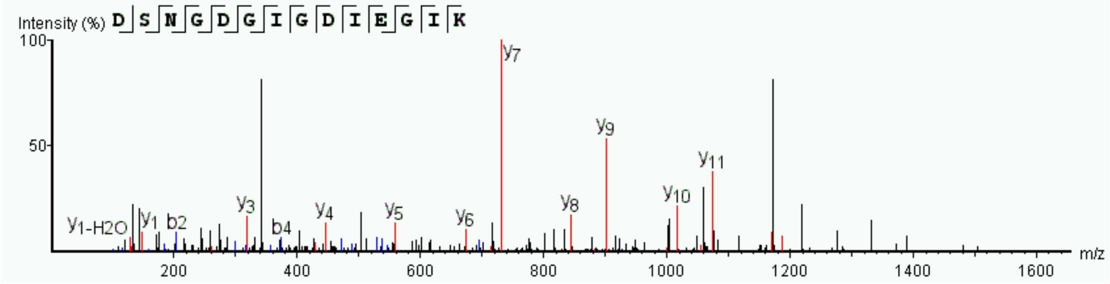

gi|328784556;

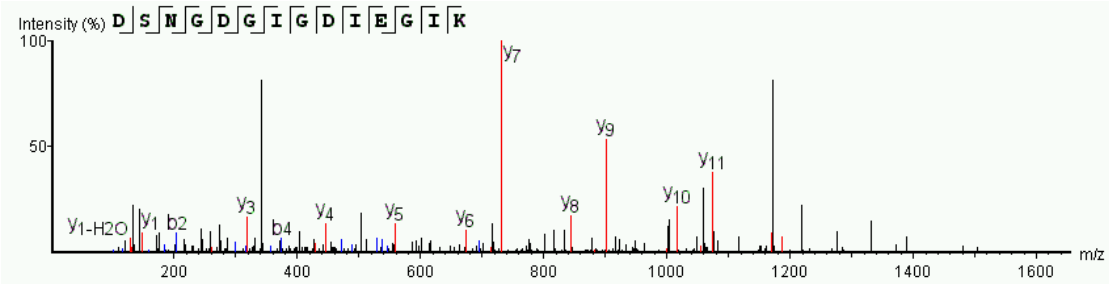

gi|571558849

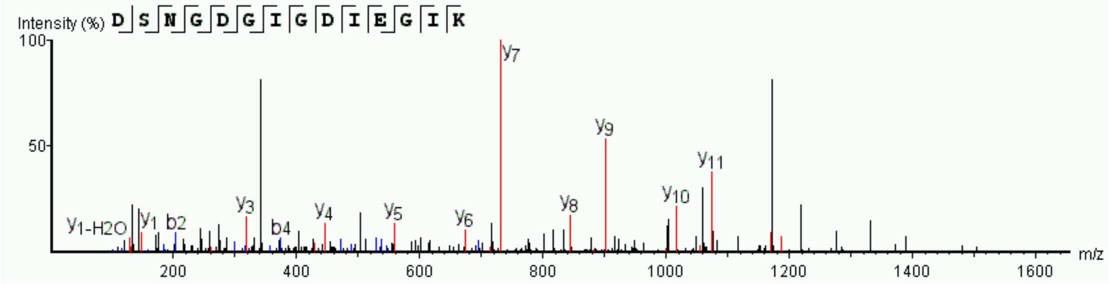

gi|66516304

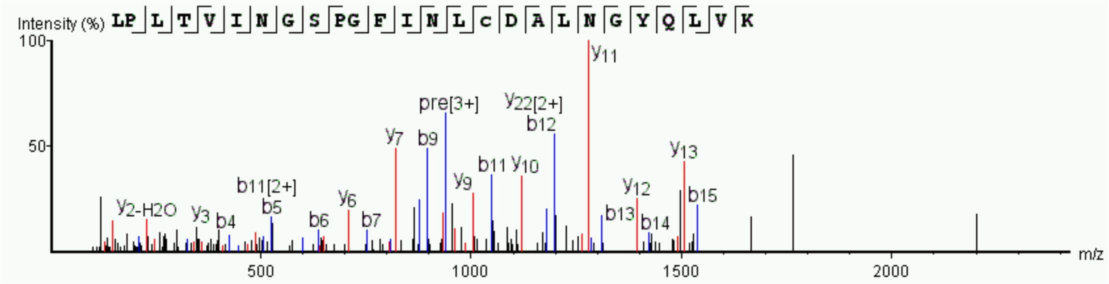

gi|571546866

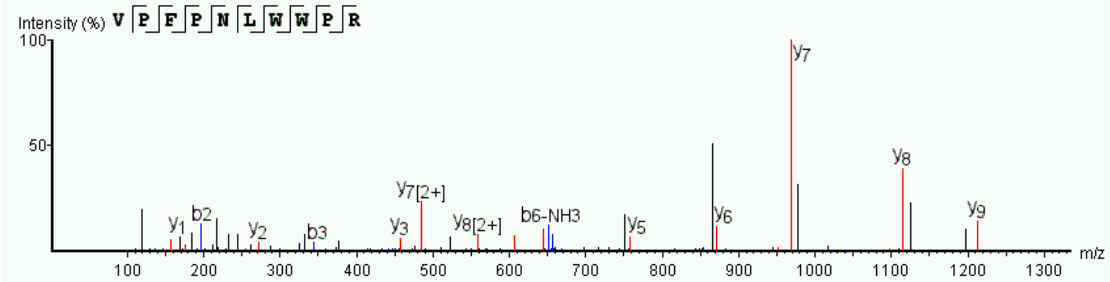

gi|66549206

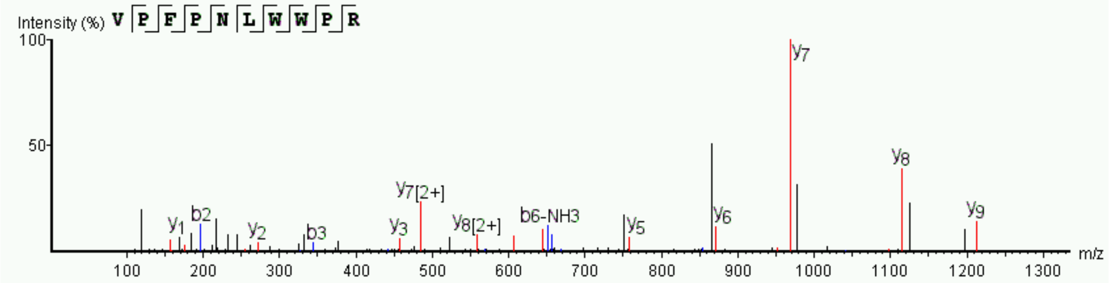

gi|571553856

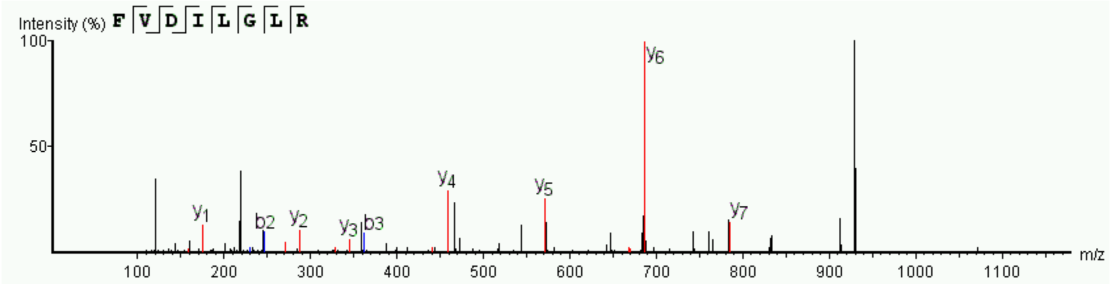

gi|571553859

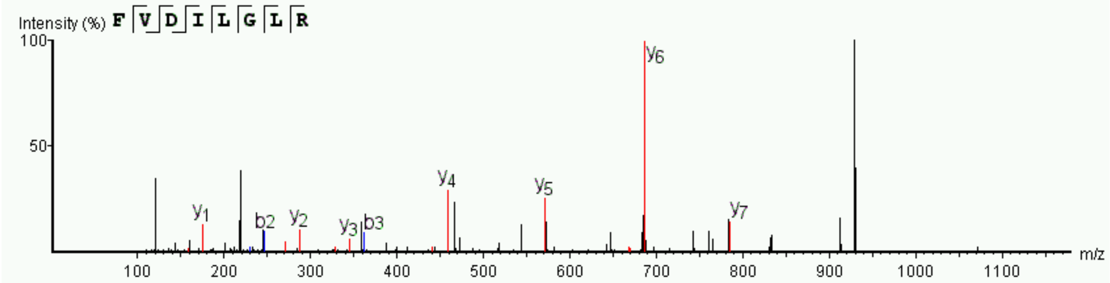

gi|48114117

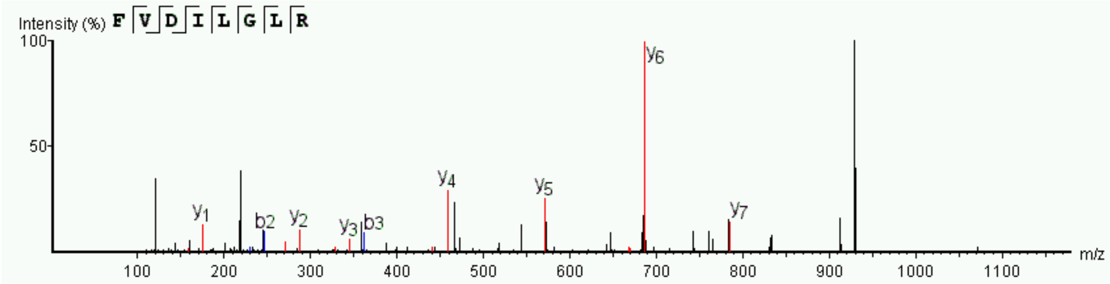

gi|571569183;

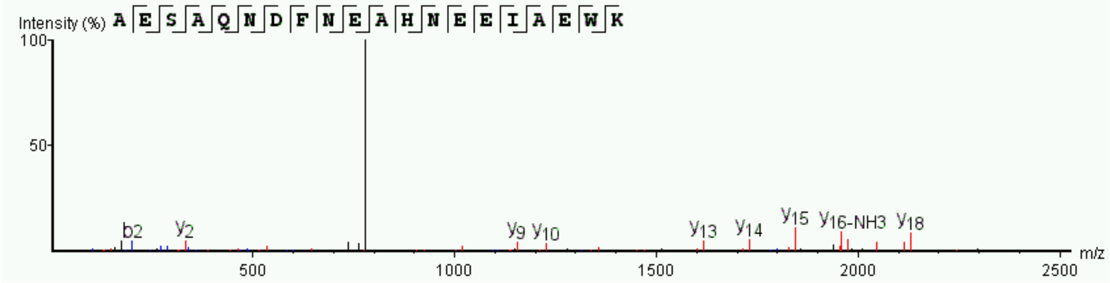

gi|571569186

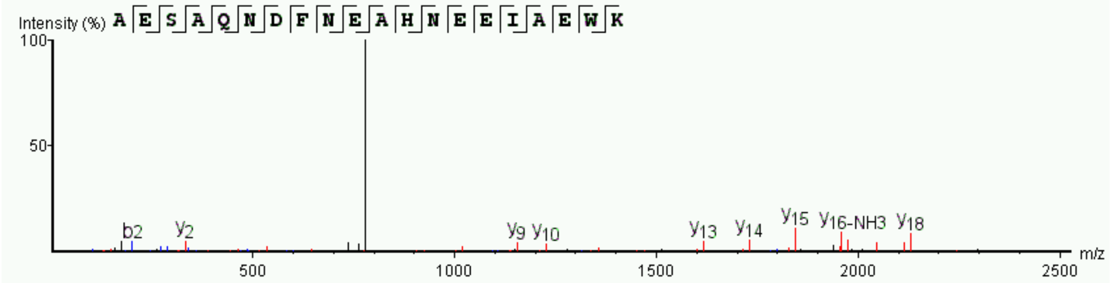

gi|110757985

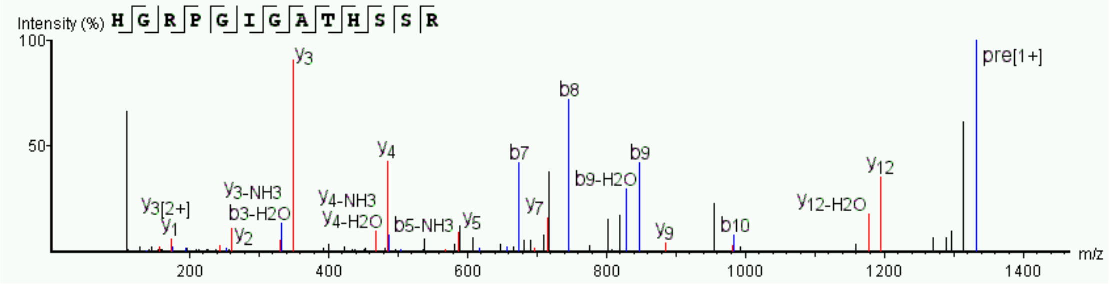

gi|571577109;

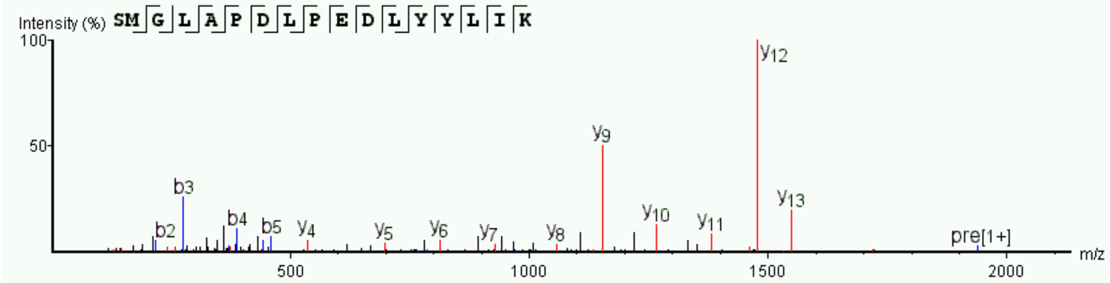

gi|66562243

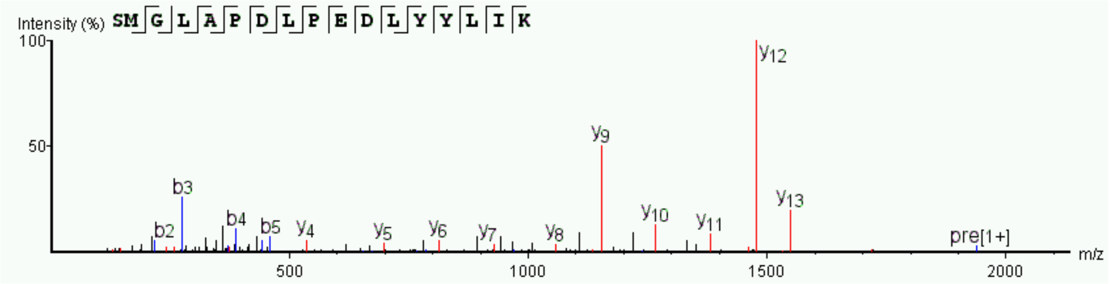

gi|48106363;

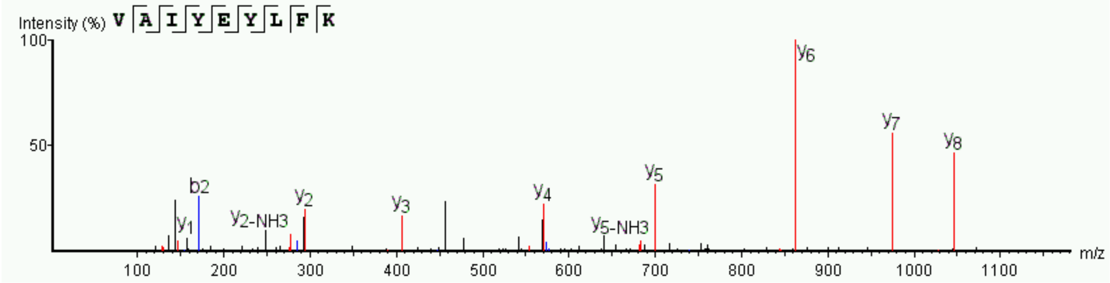

gi|66517421

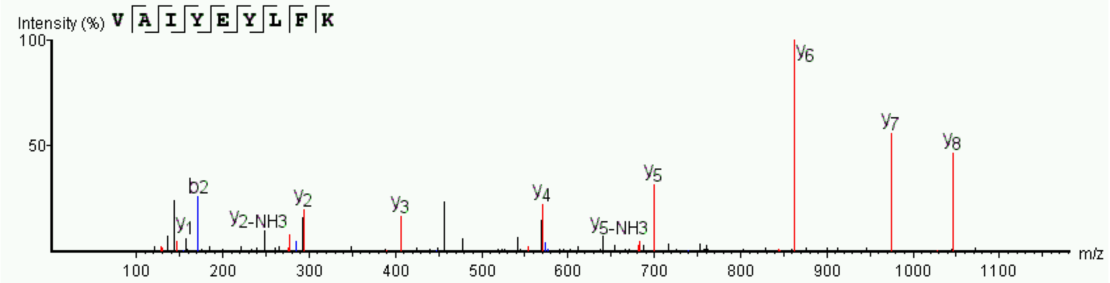

gi|571543571

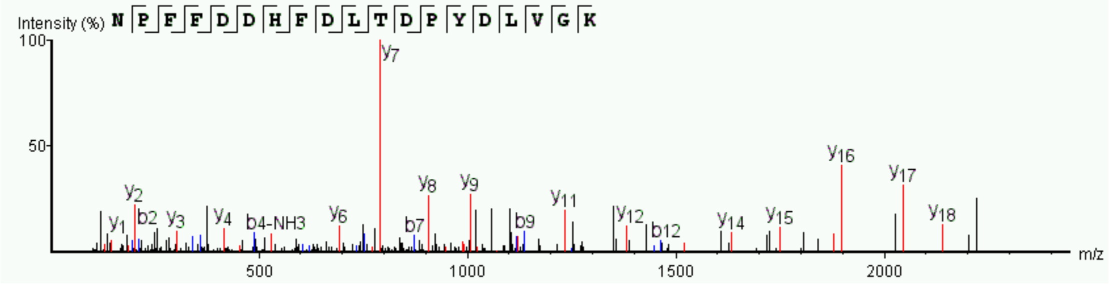

Annotated spectra of single-peptide identifications of proteins in antennal bodies of *Apis mellifera*

gi|328785120

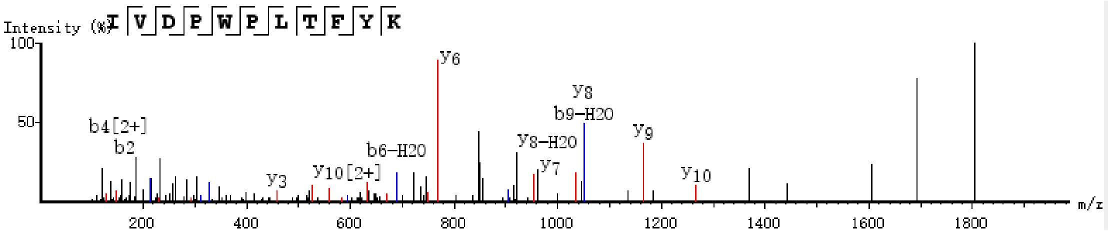

gi|328783319

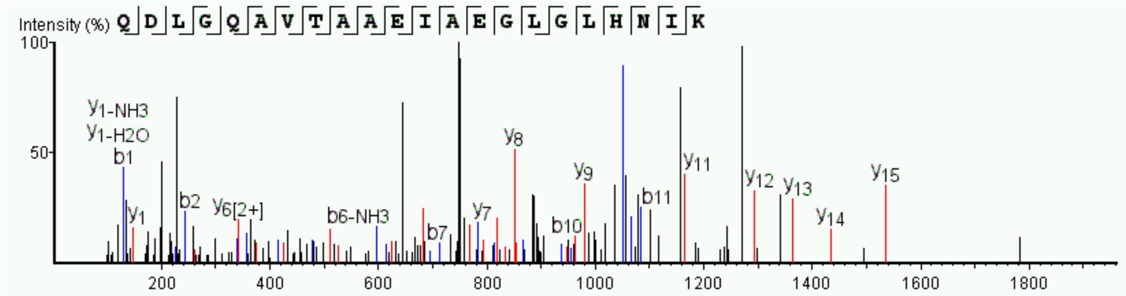

gi|571539622

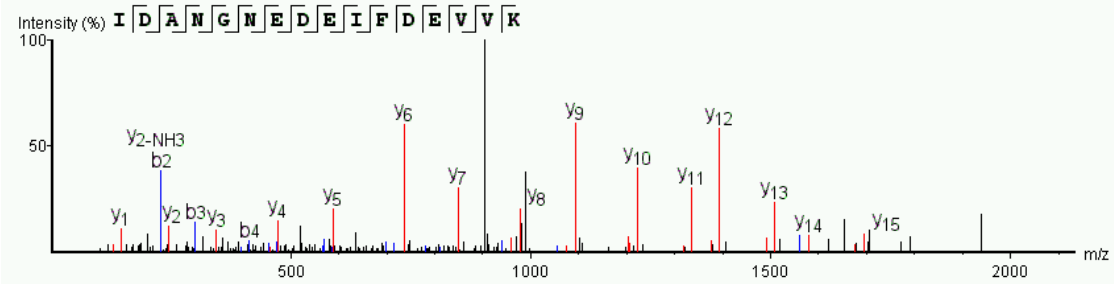

gi|571539625

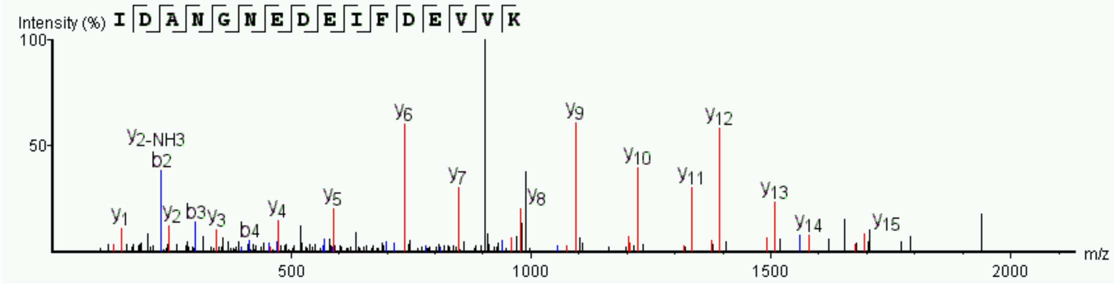

gi|571520923

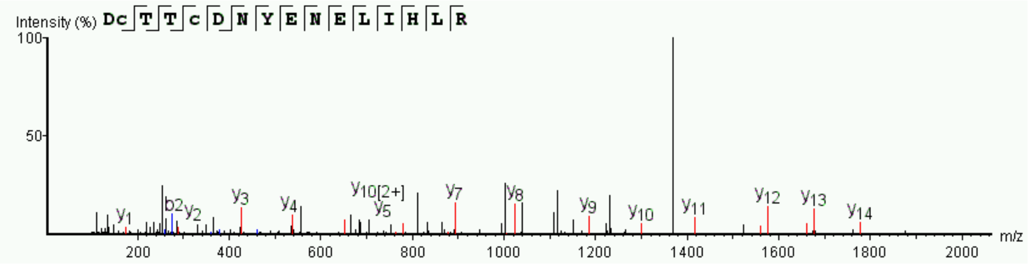

gi|571520927

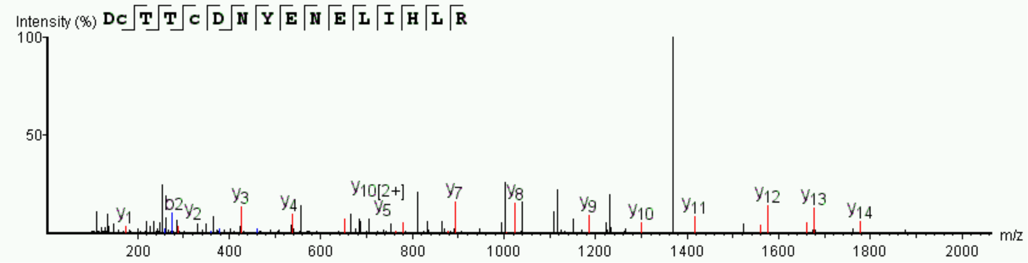

gi|48095246

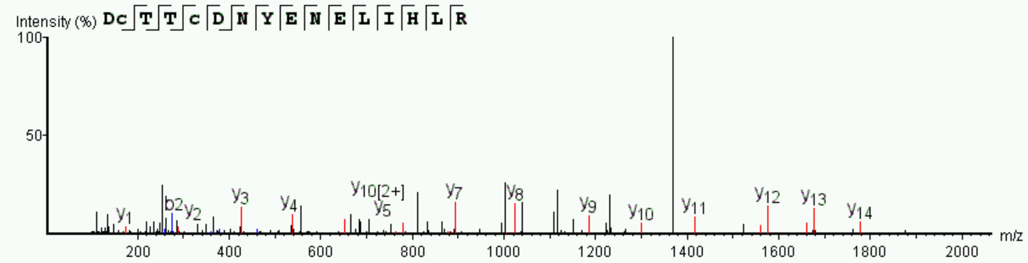

gi|110764763

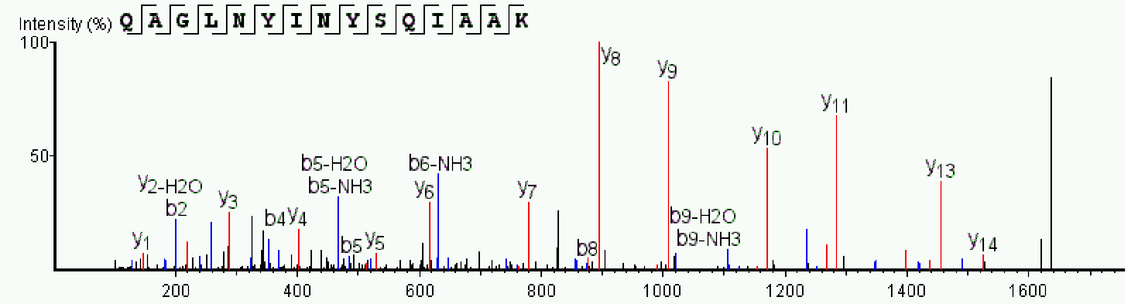

gi|328779841

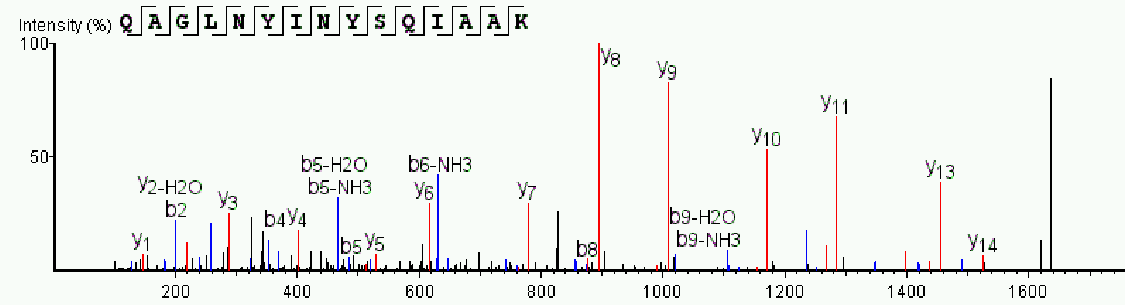

gi|571549591

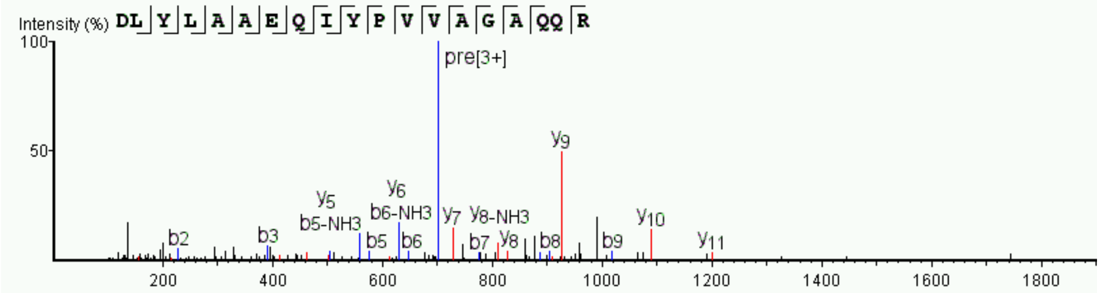

gi|571549598

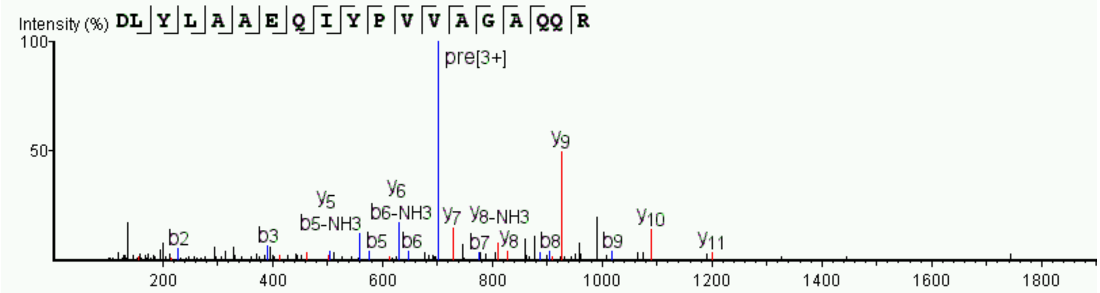

gi|571549603

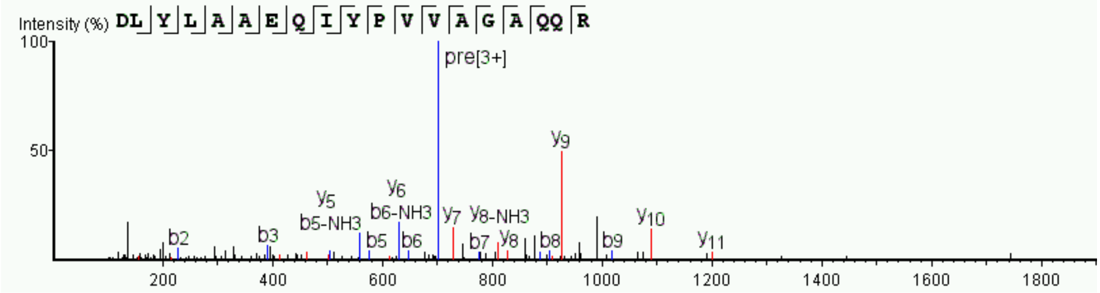

gi|328781218

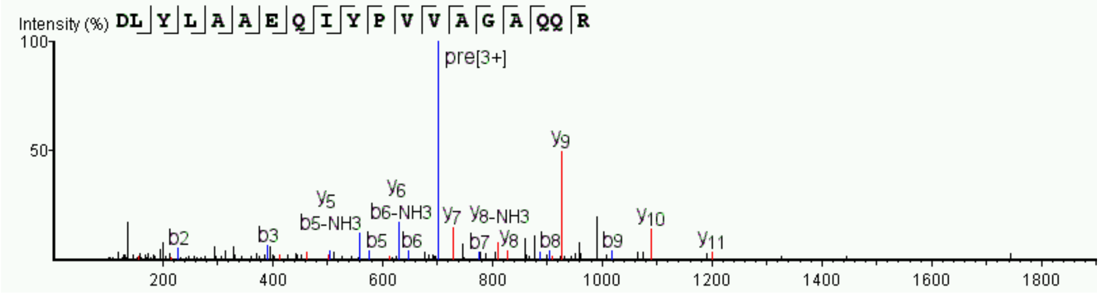

gi|571576251

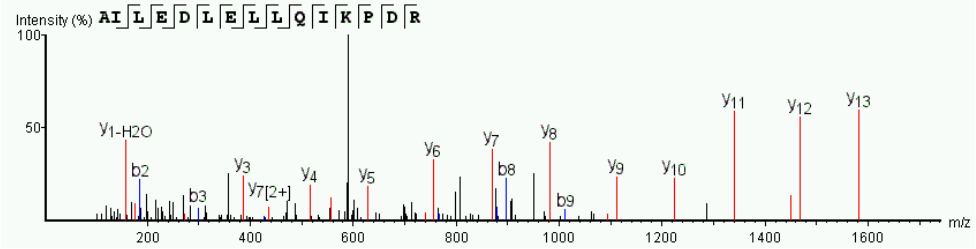

gi|571517543

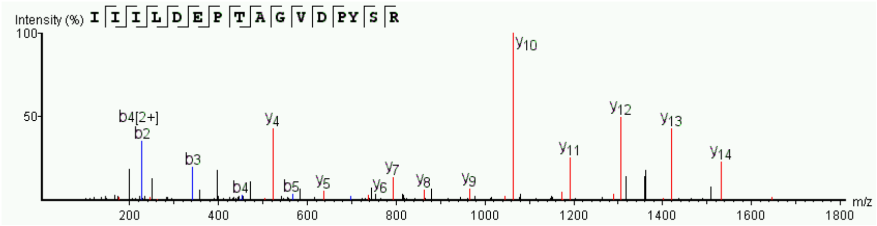

gi|328778864

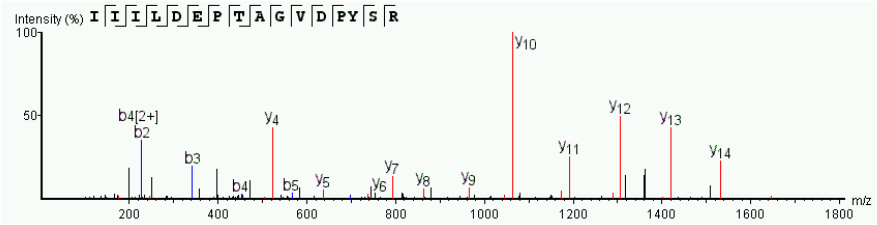

gi|571572791

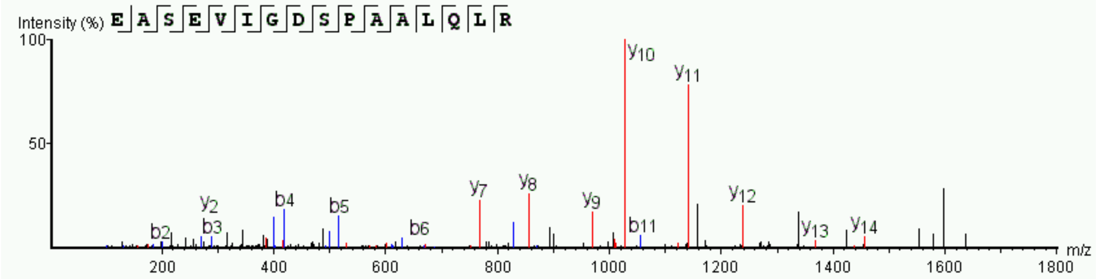

gi|571572798

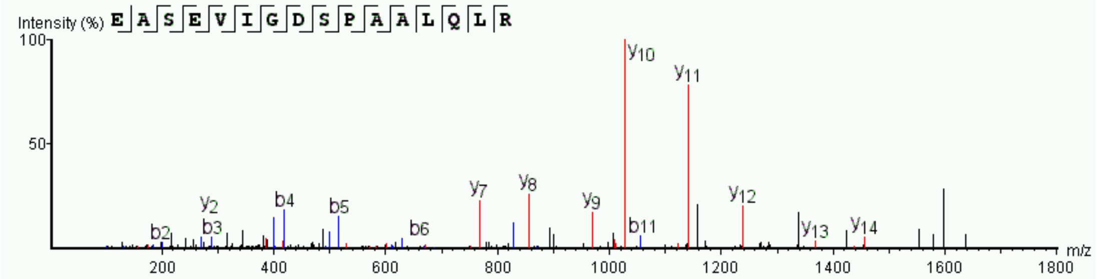

gi|571547954

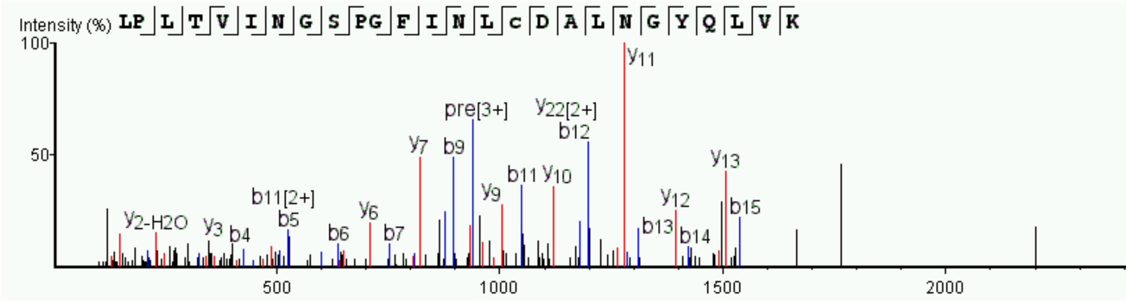

gi|66516304

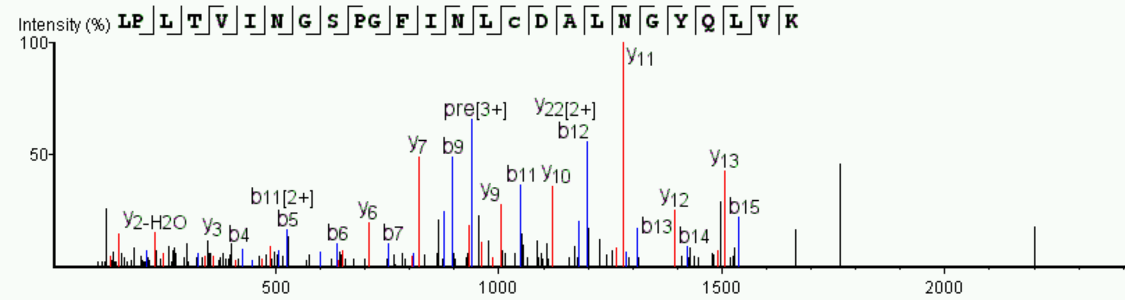

gi|571577993

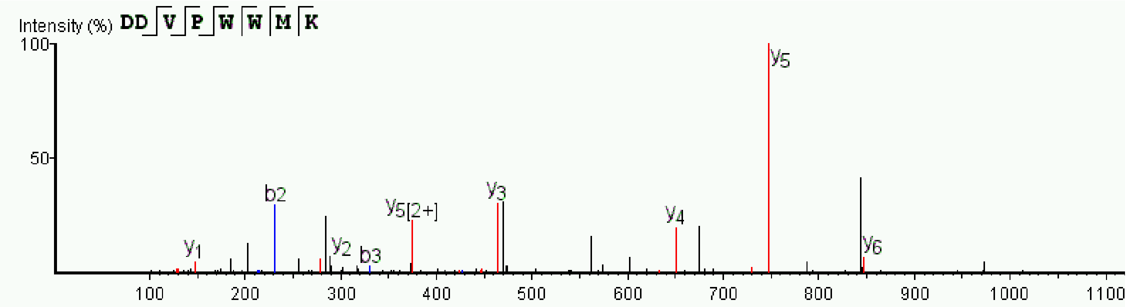

gi|571577995

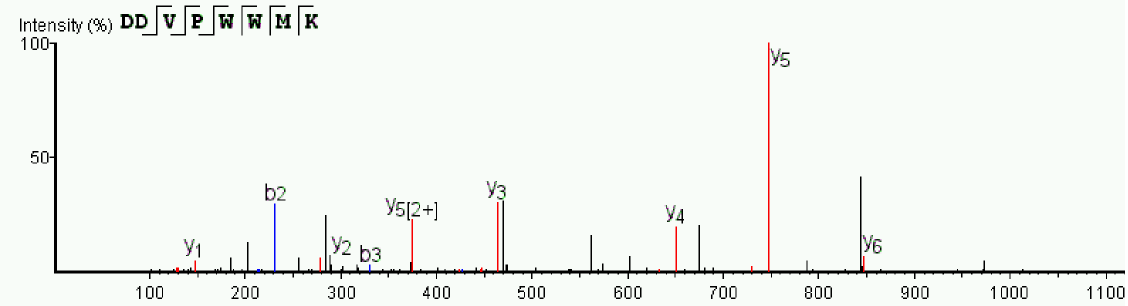

gi|328781034

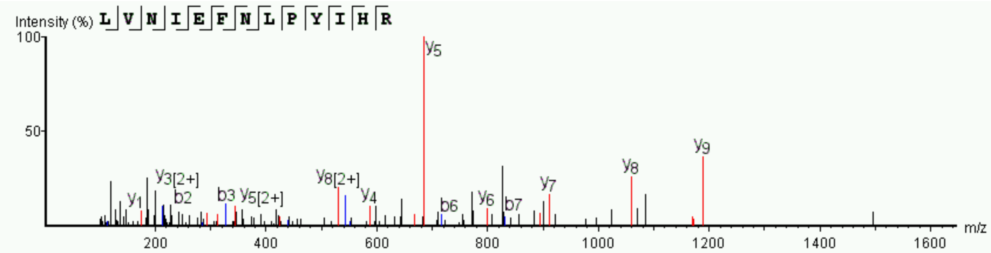

gi|571548834

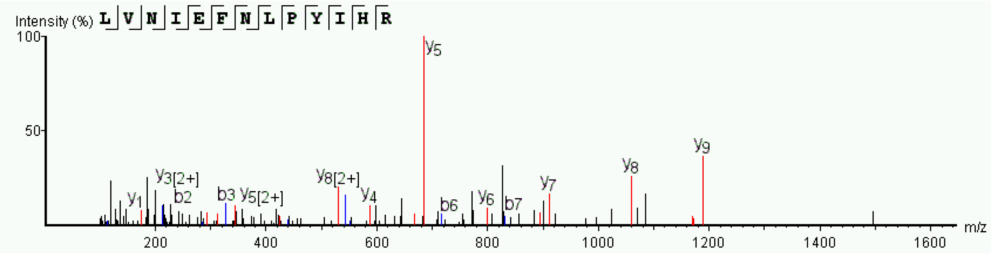

gi|571523319

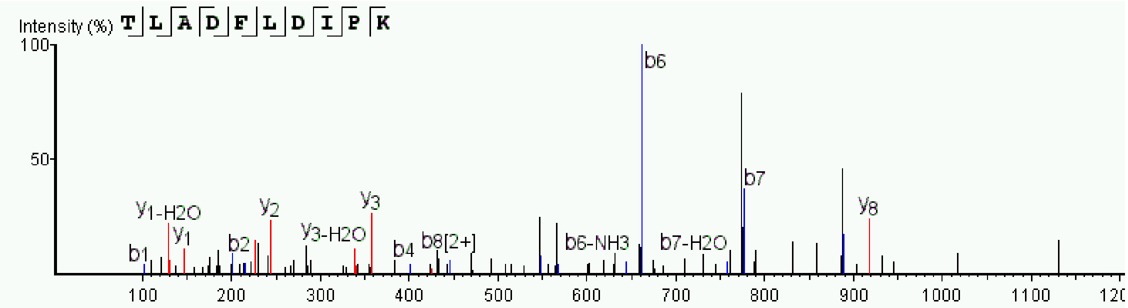

gi|66547724

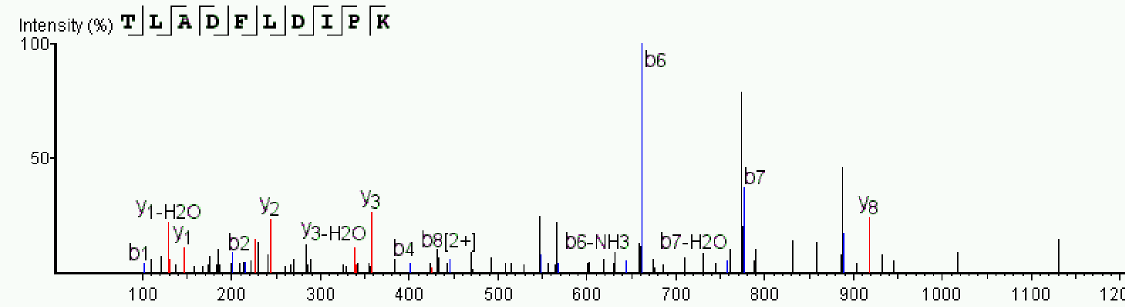

gi|571549109

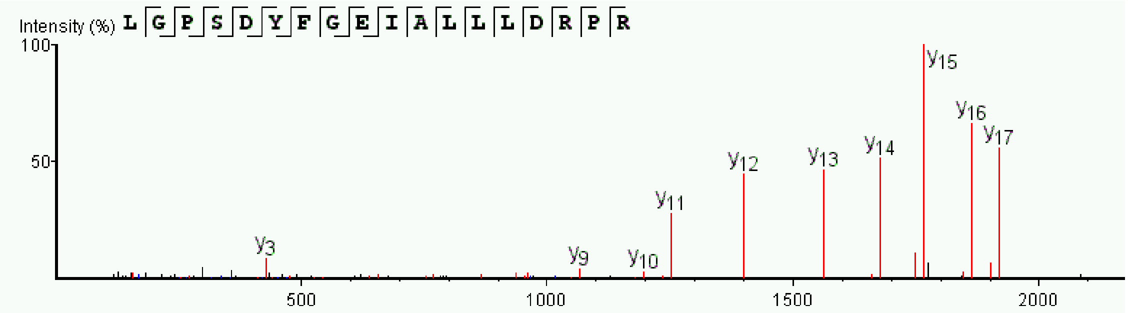

gi|571549112

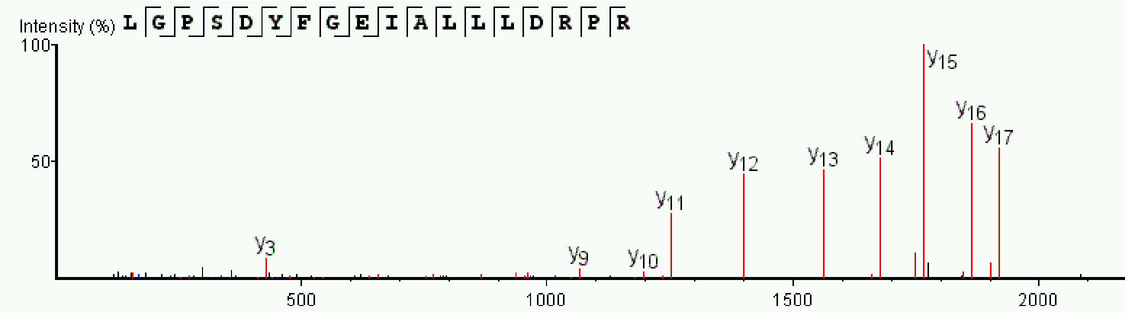

gi|571549115

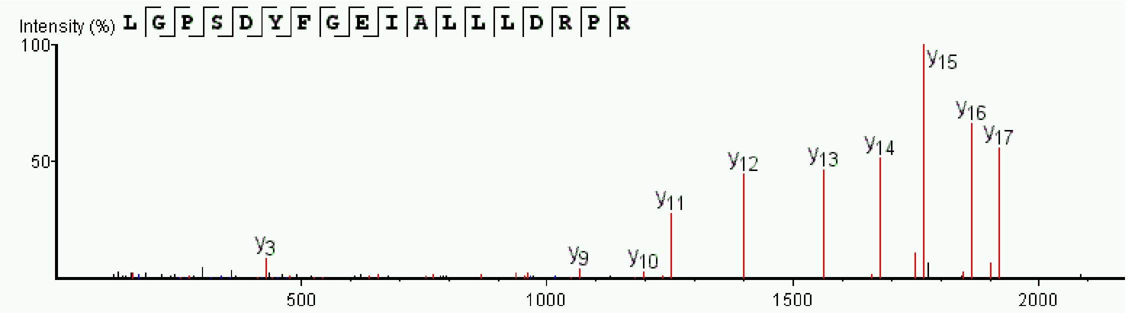

gi|571549118

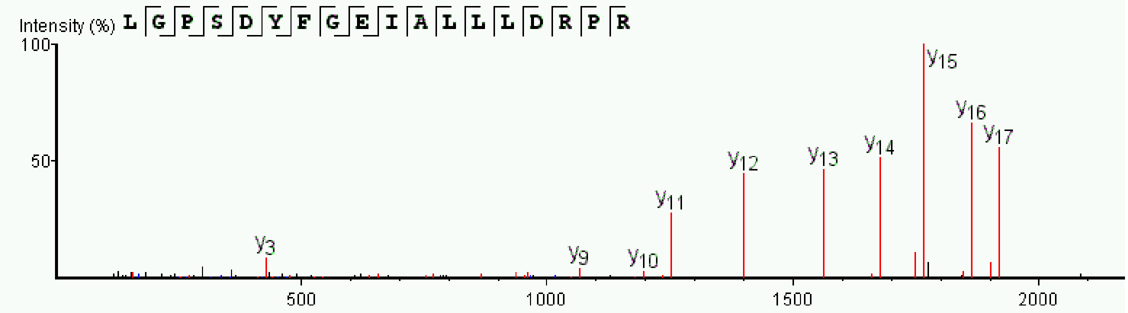

gi|48106841

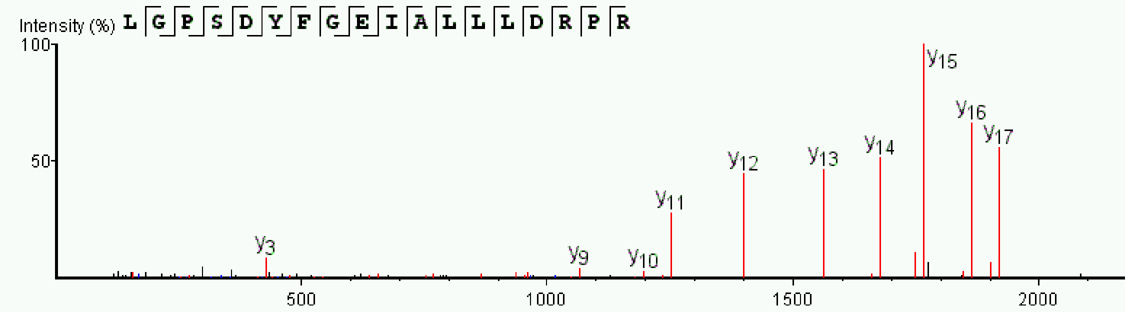

gi|571517007

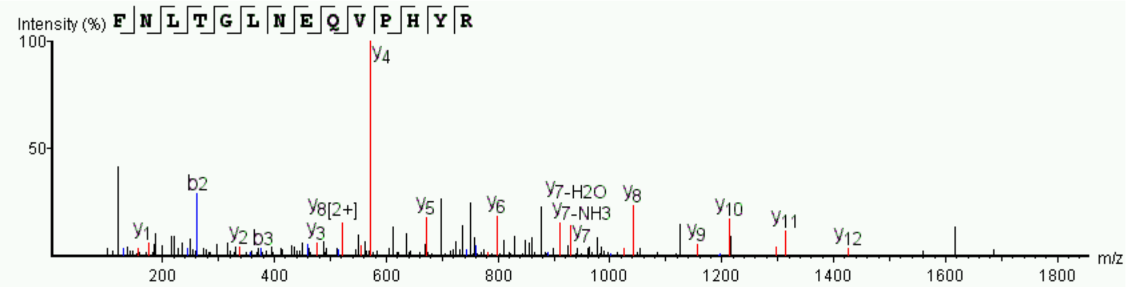

gi|571517011

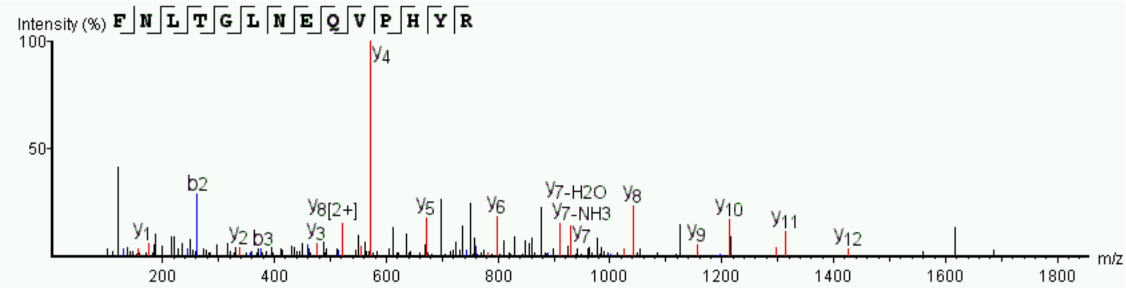

gi|571517016

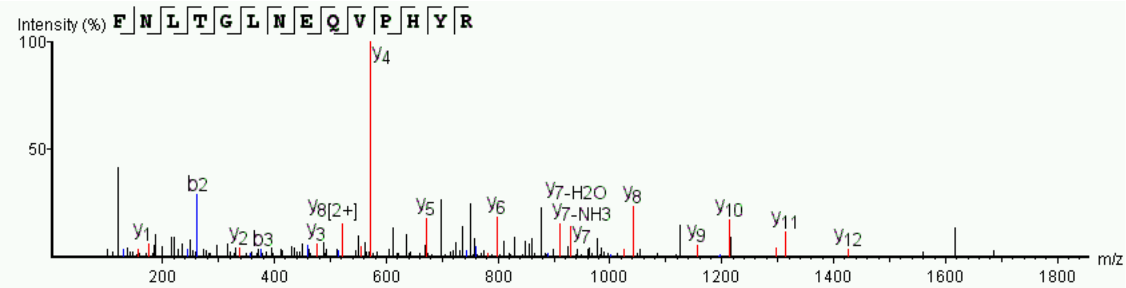

gi|328777239

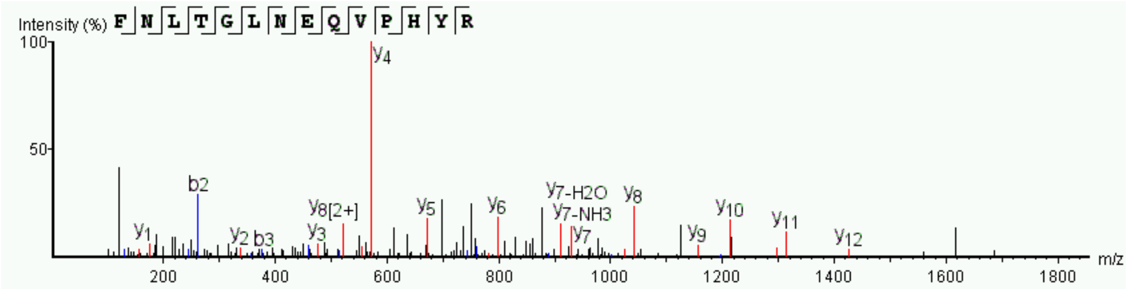

gi|571574447

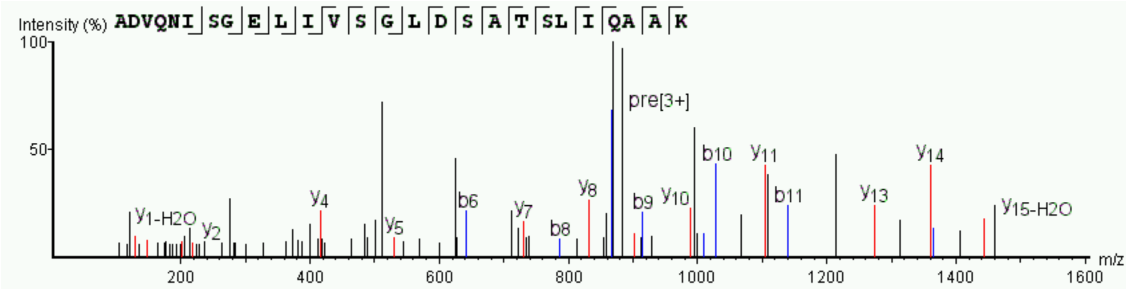

gi|571574449

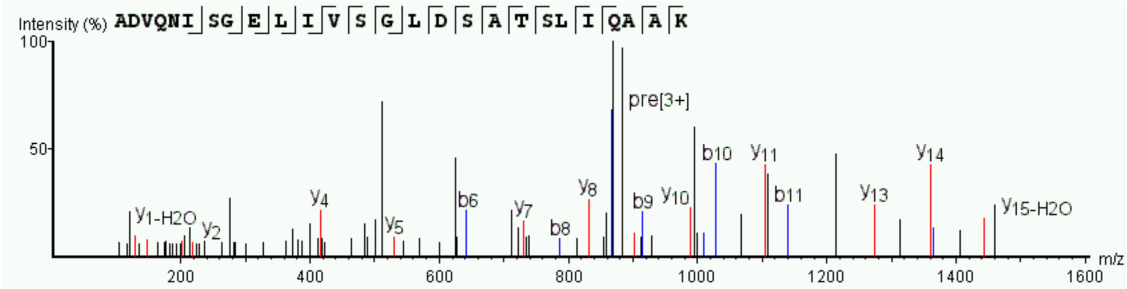

gi|571574451

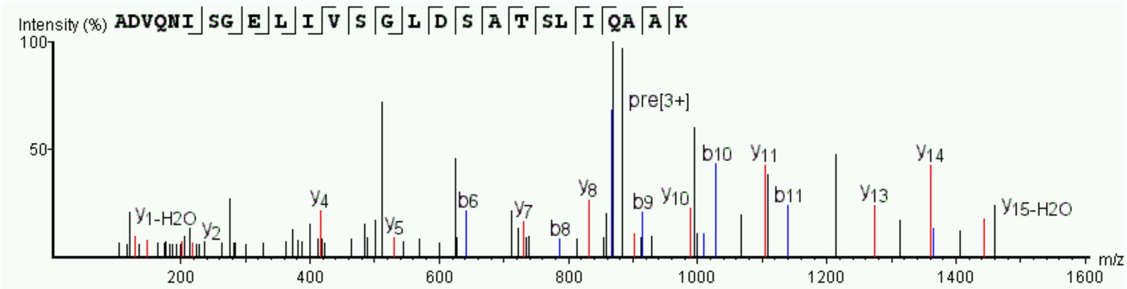

gi|66525427

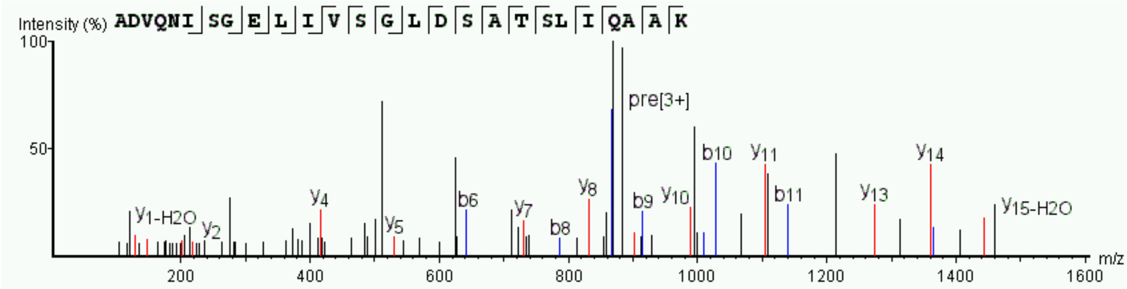

gi|571522061

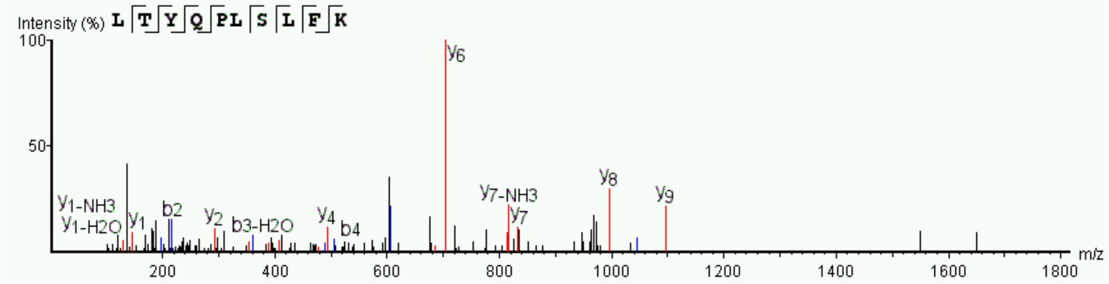

gi|328788820

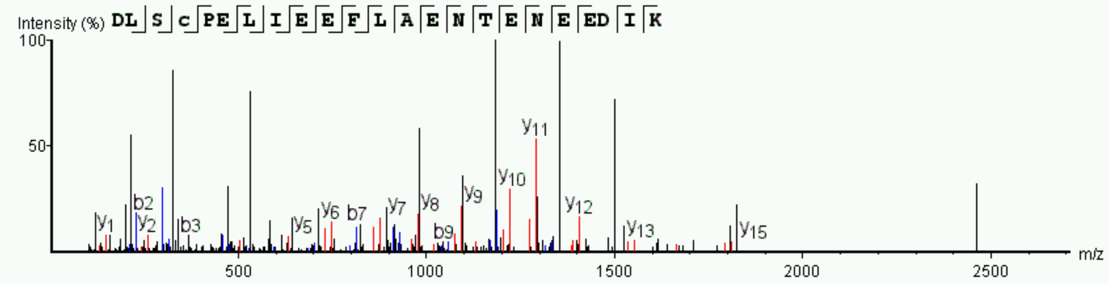

gi|66551959

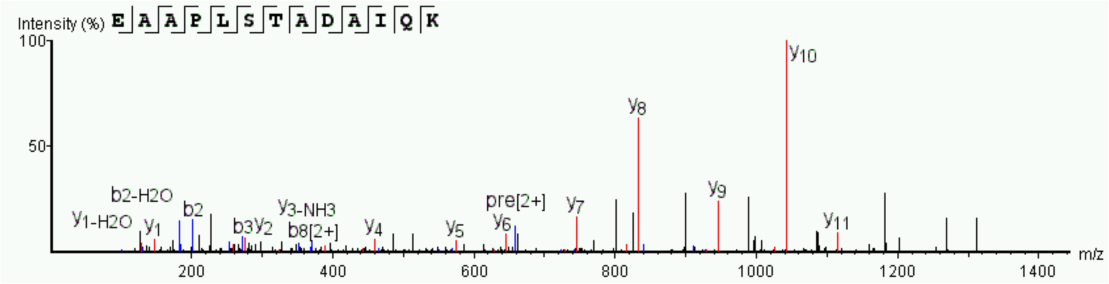

gi|328790504

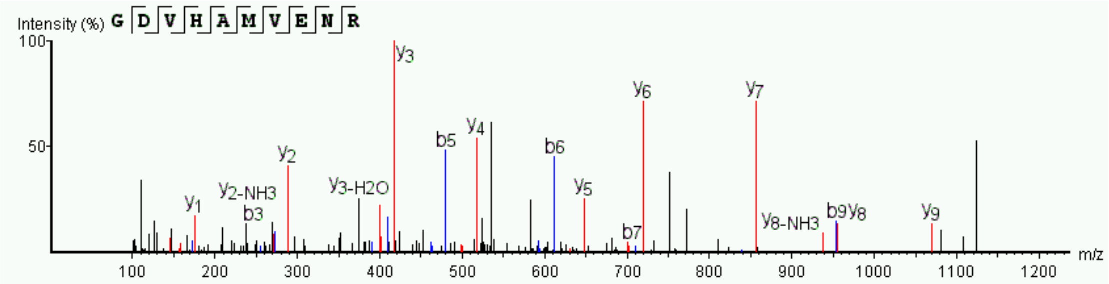

gi|571504092

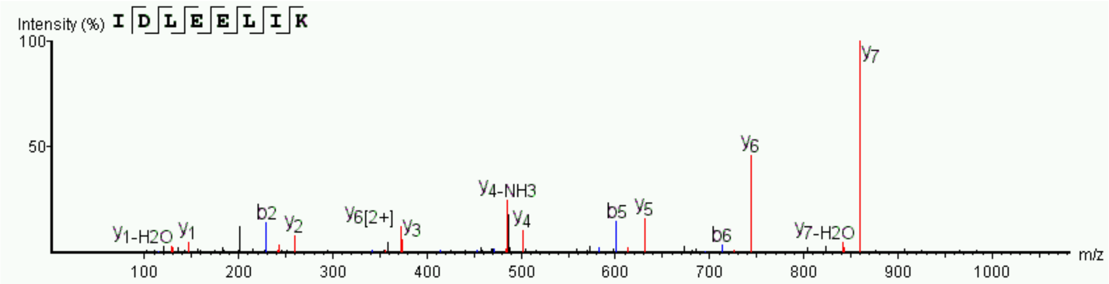

gi|571540738

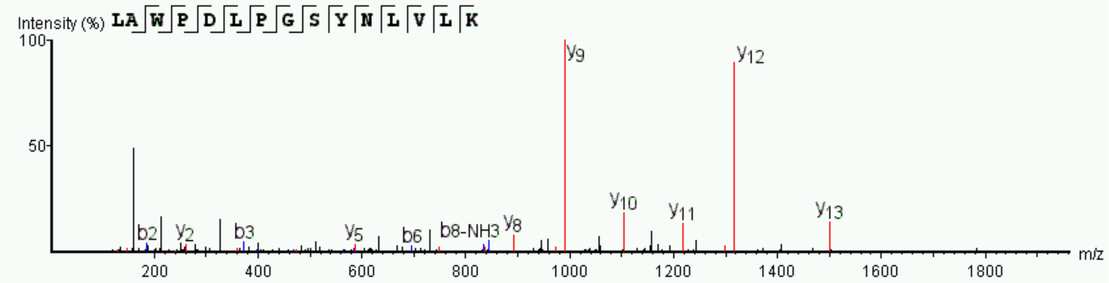

gi|328782832

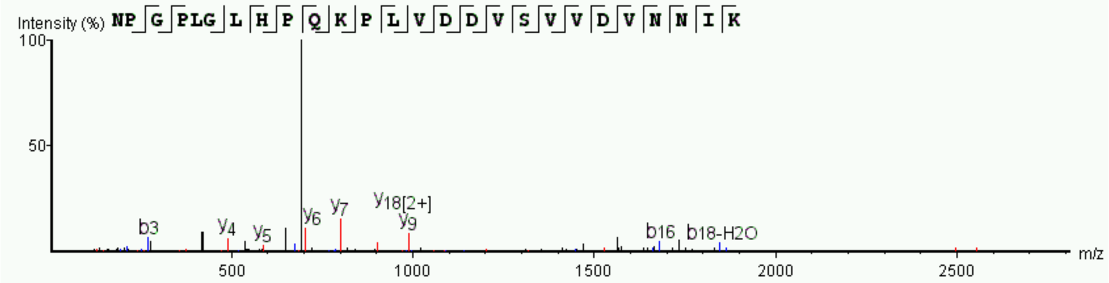

gi|66564768

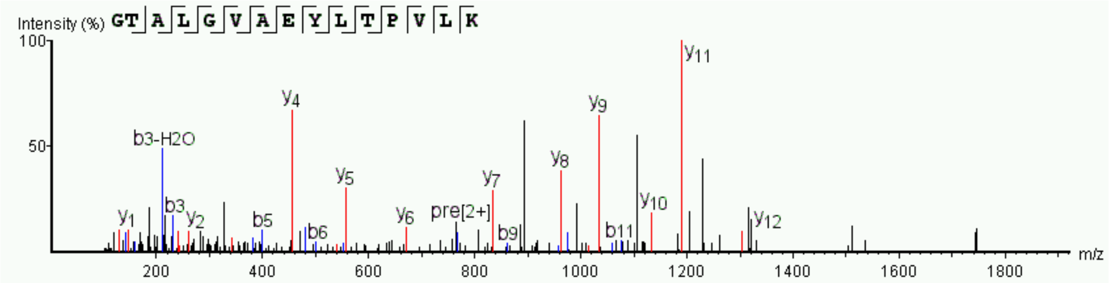

gi|328775871

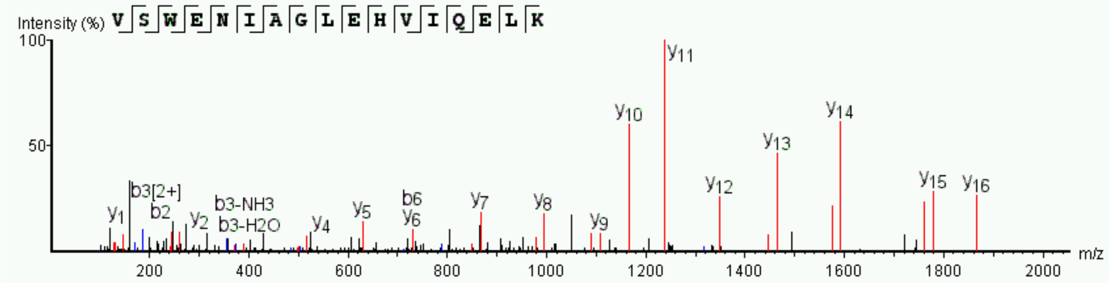

gi|571544412

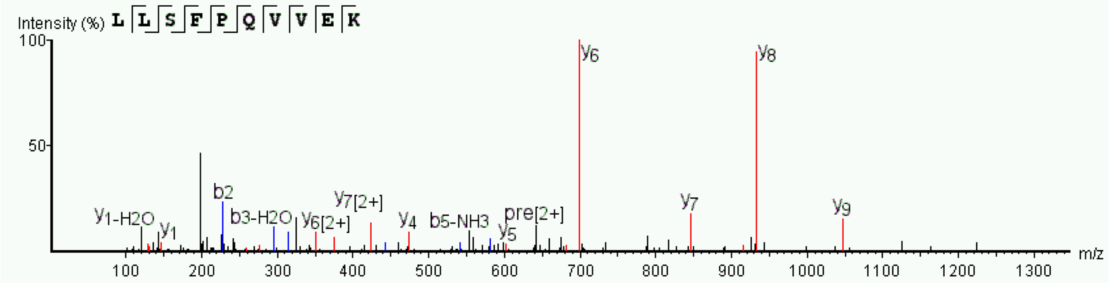

gi|110760120

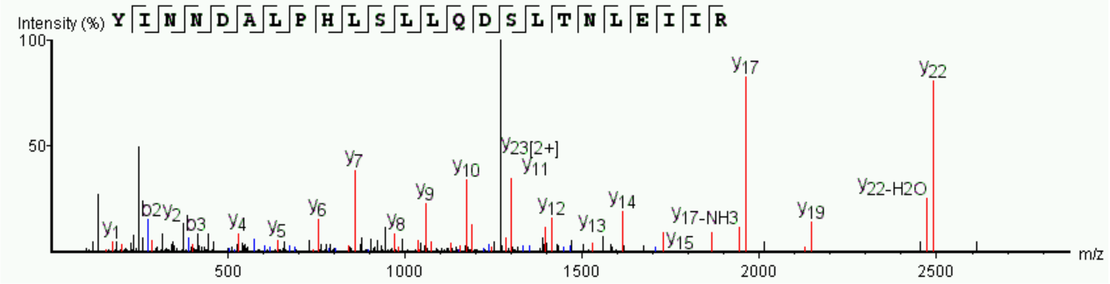

gi|571520912

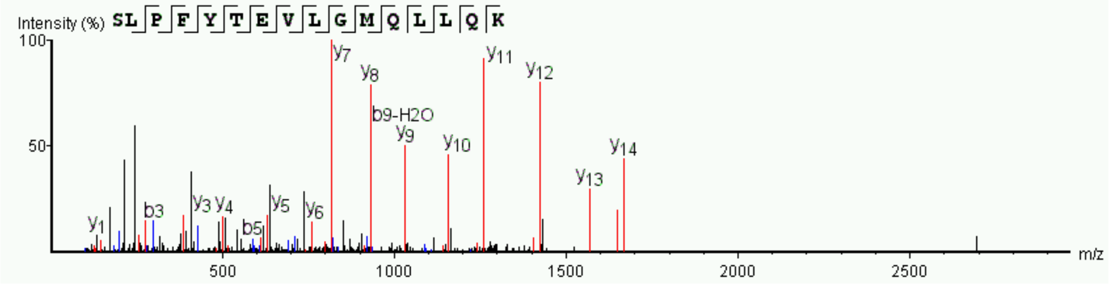

gi|66536388

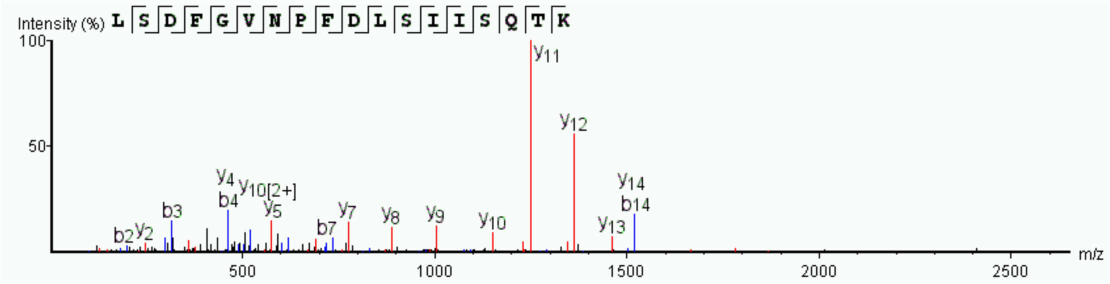

gi|571563087

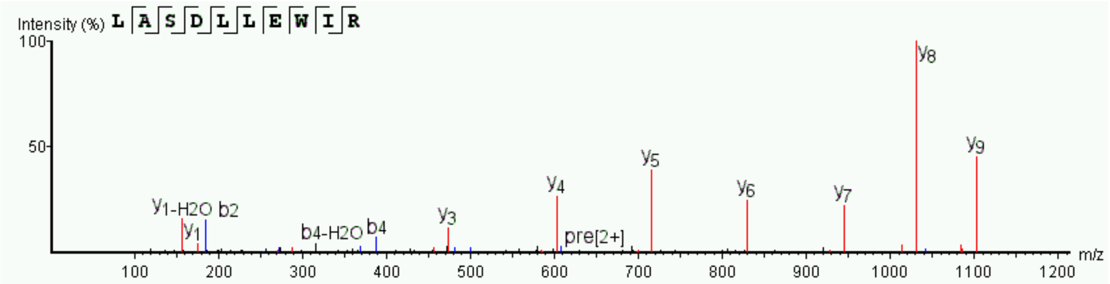

gi|66519683

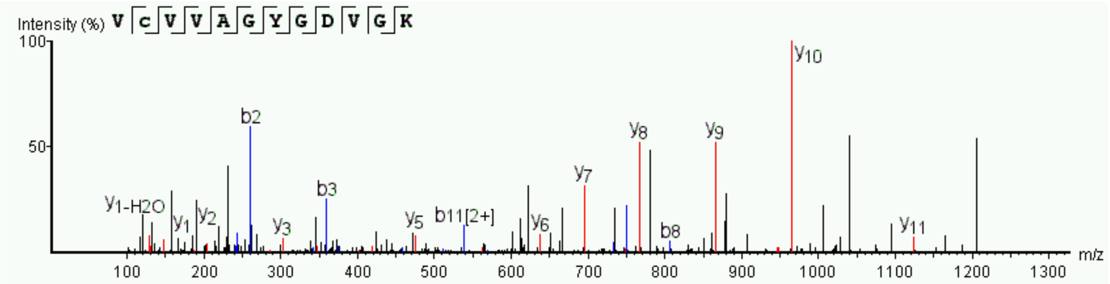

gi|66524917

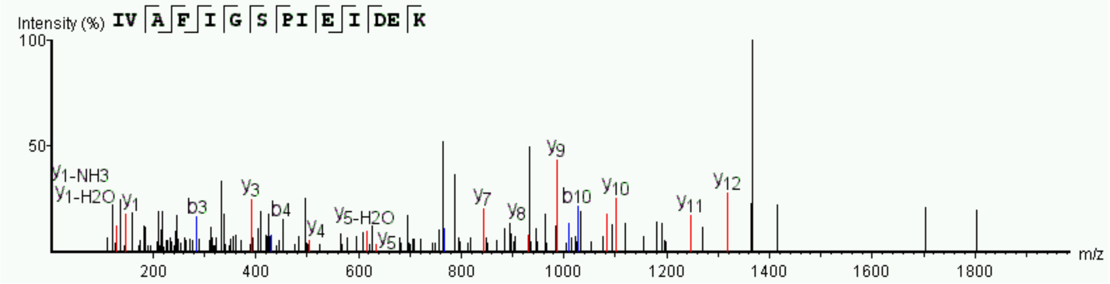

gi|283436150

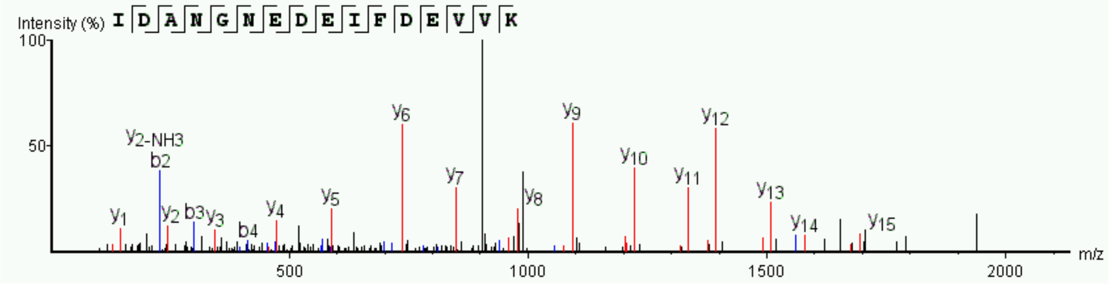

gi|571575901

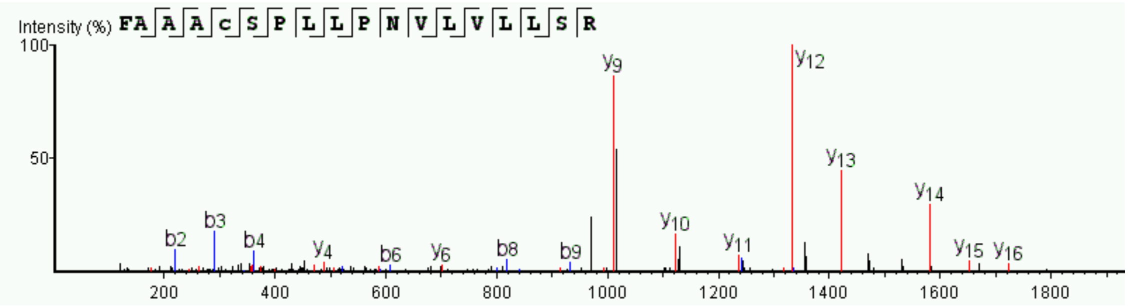

gi|571575903

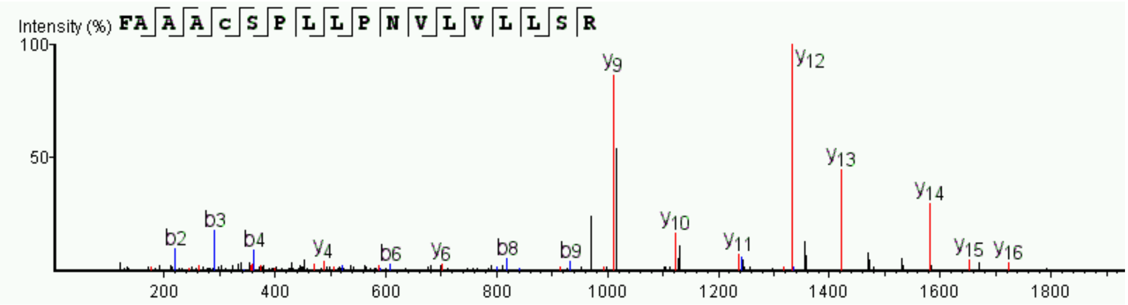

gi|571575905

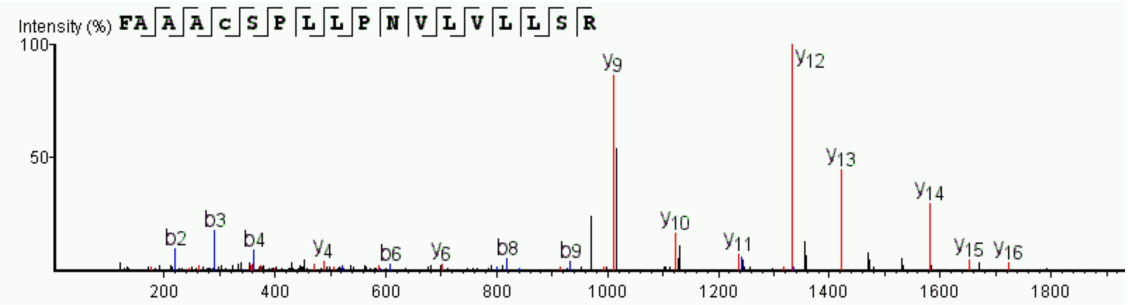

gi|571519398

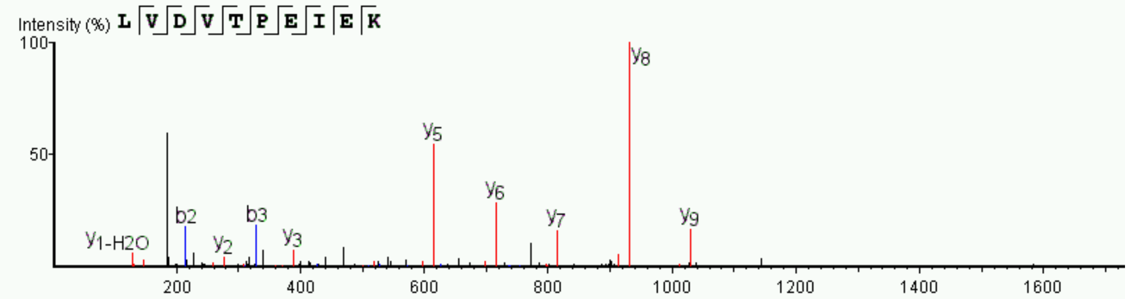

gi|571540528

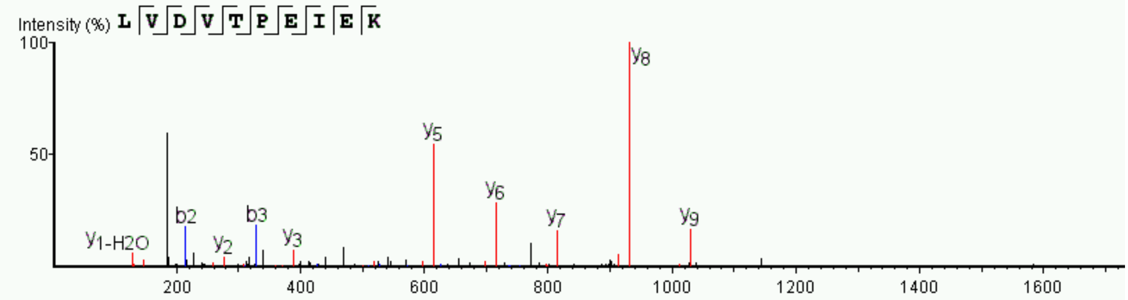

gi|571540539

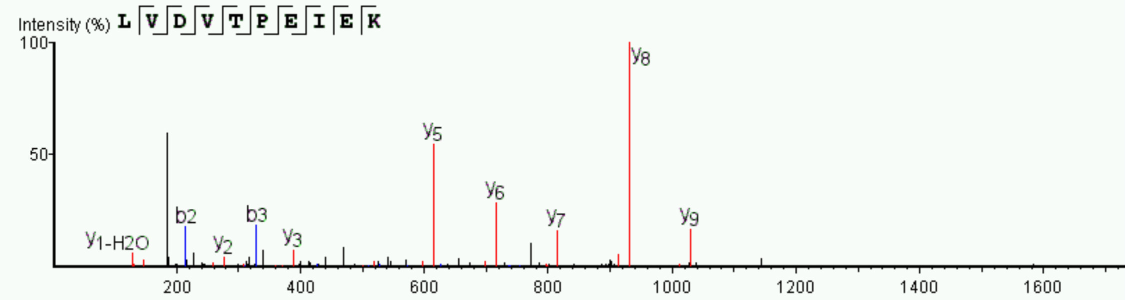

gi|571540531

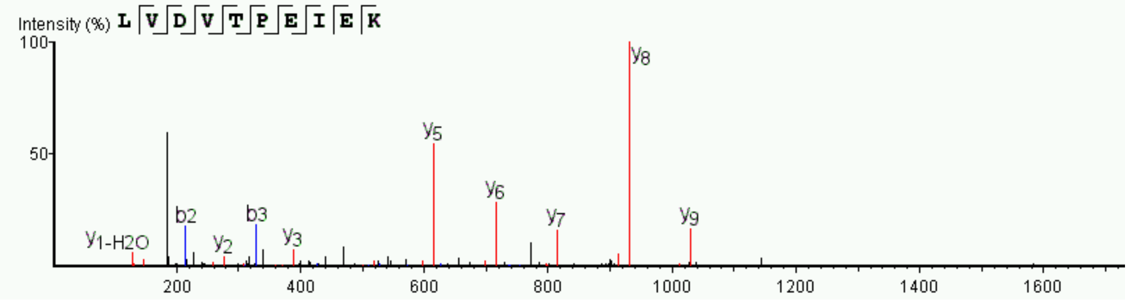

gi|110761543

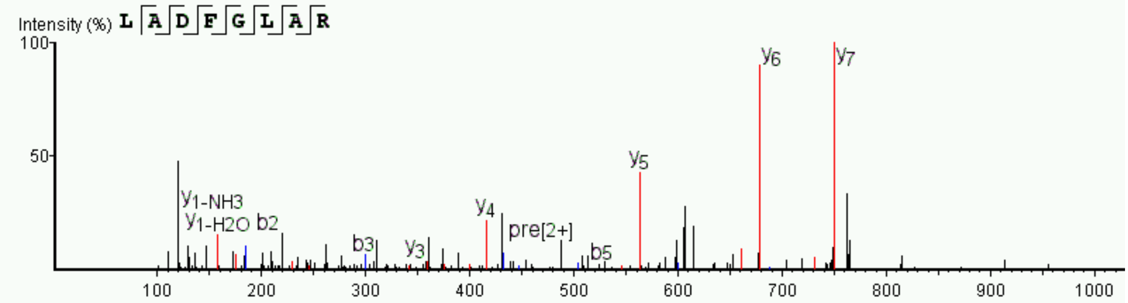

gi|571562181

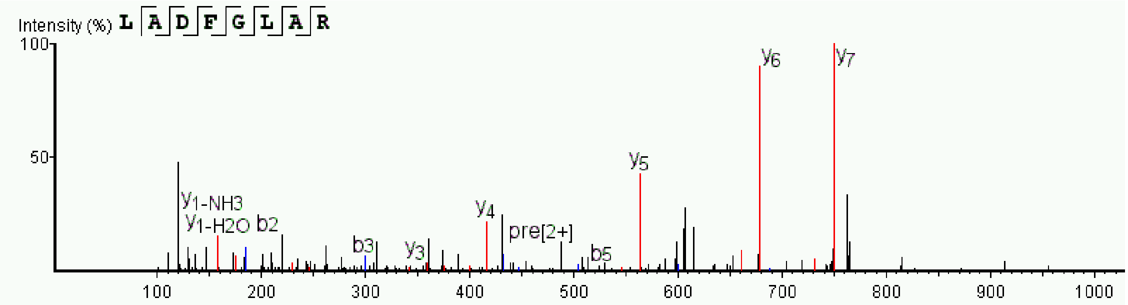

gi|571526930

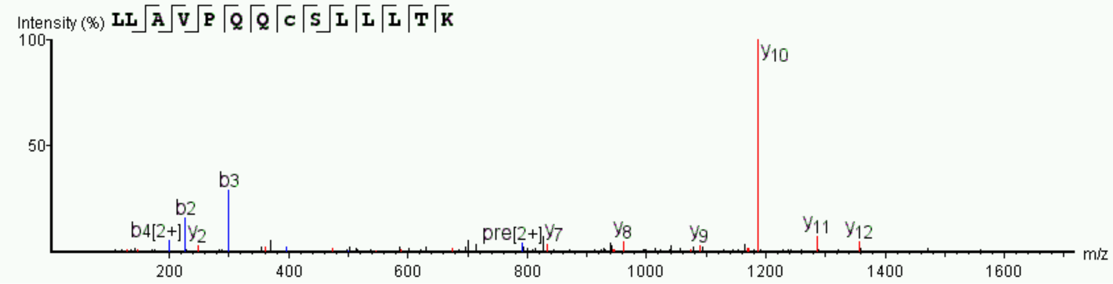

gi|571526934

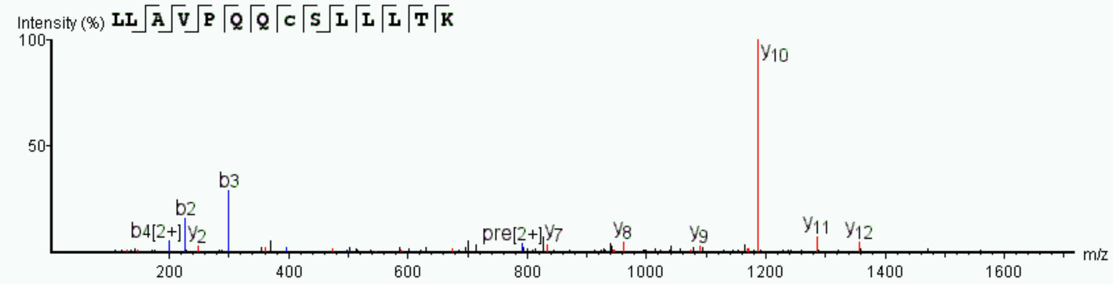

gi|66508242

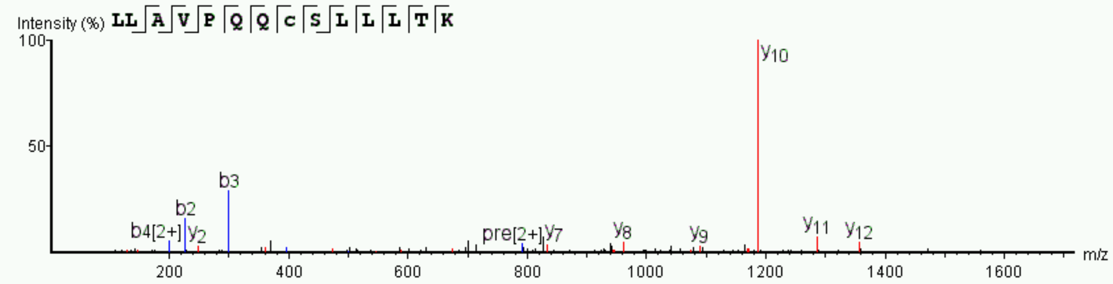

gi|571577495

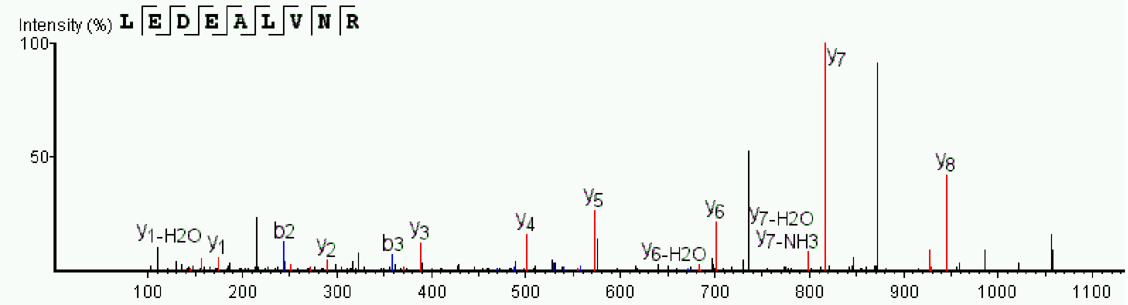

gi|571577497

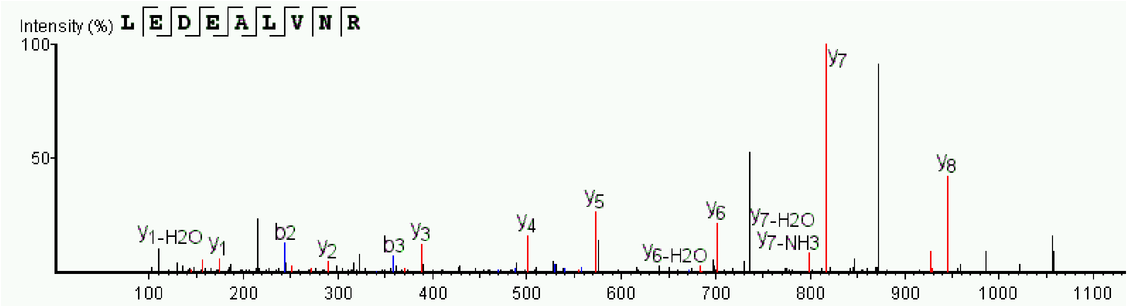

gi|571532260

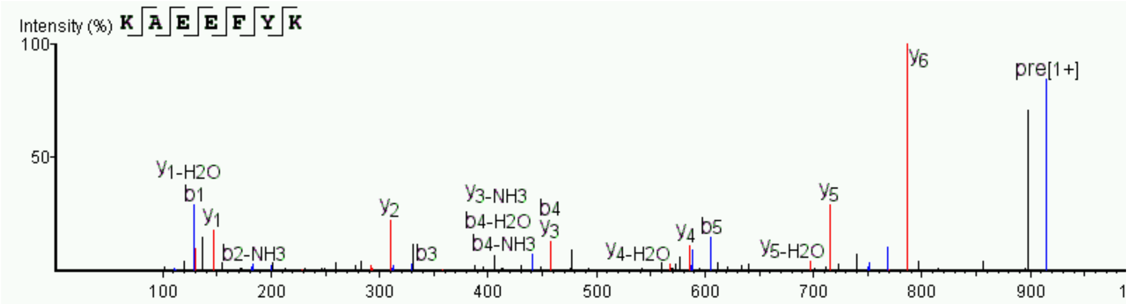

gi|571532244

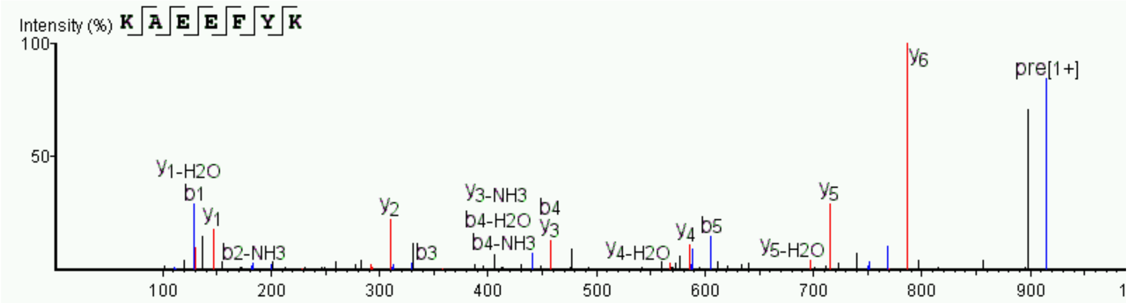

gi|571532248

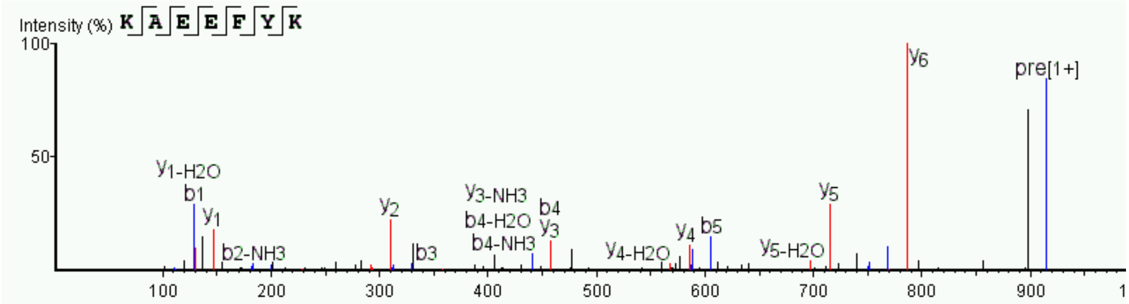

gi|571532252

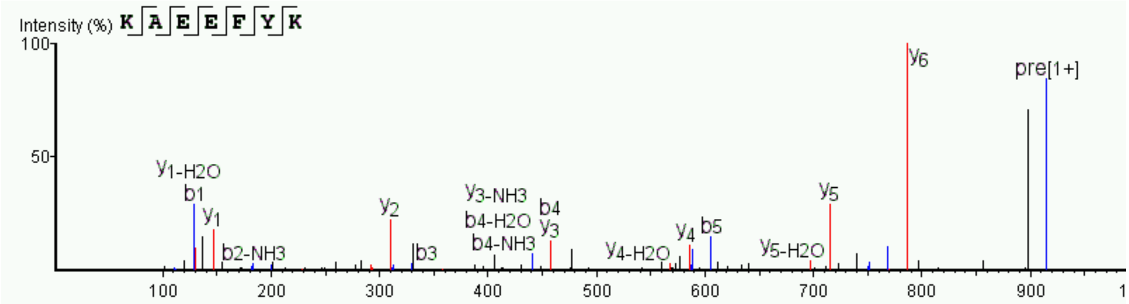

gi|571532256

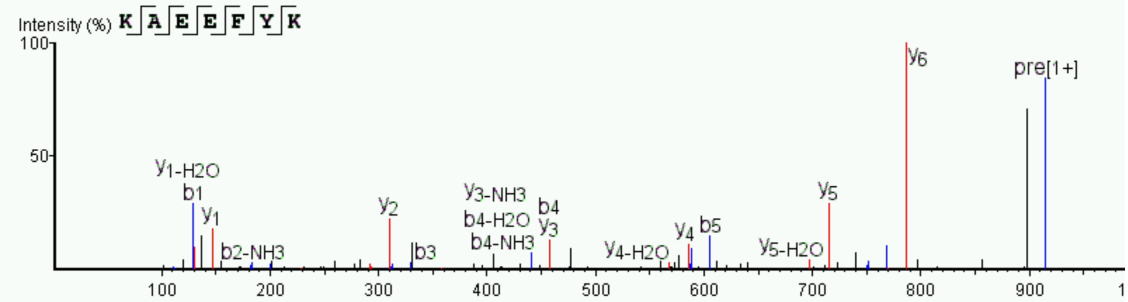

gi|571573882

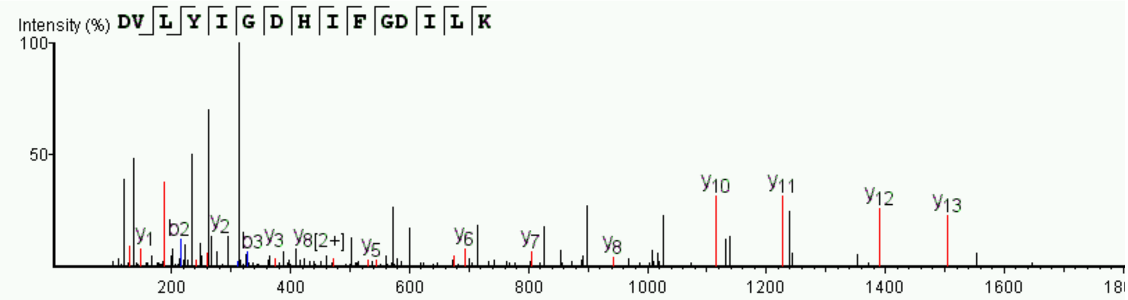

gi|571573884

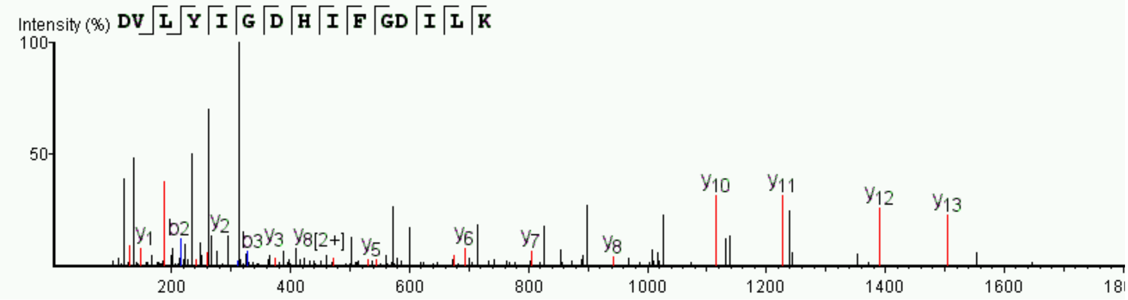

gi|571573886

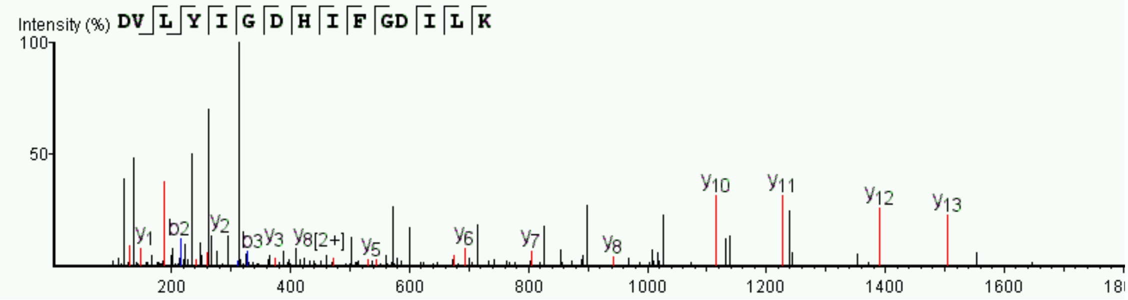

gi|571573888

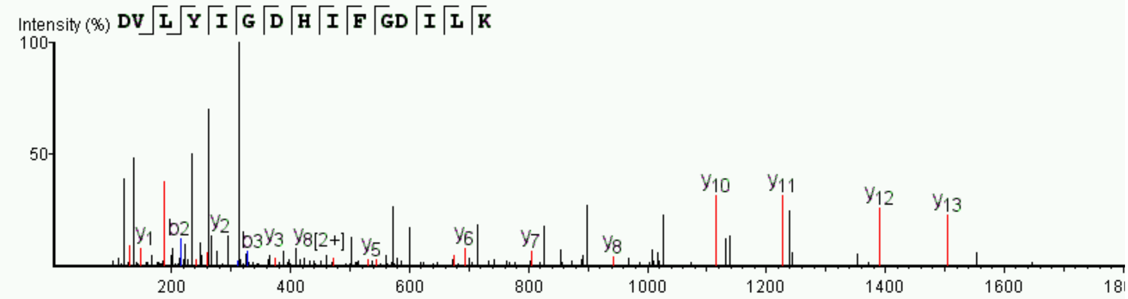

gi|571573890

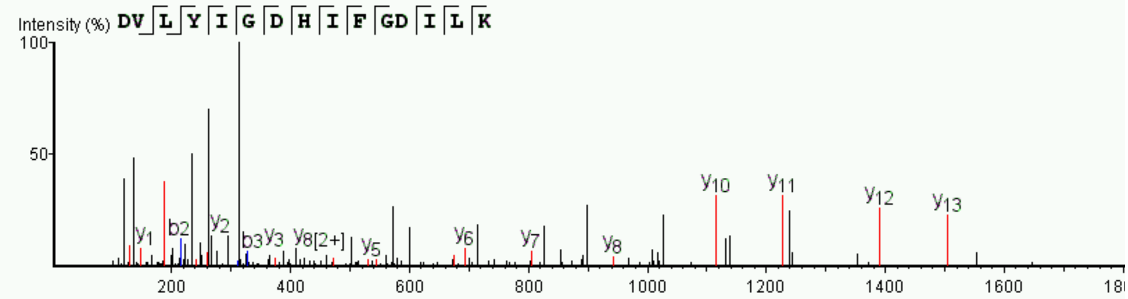

gi|571573892

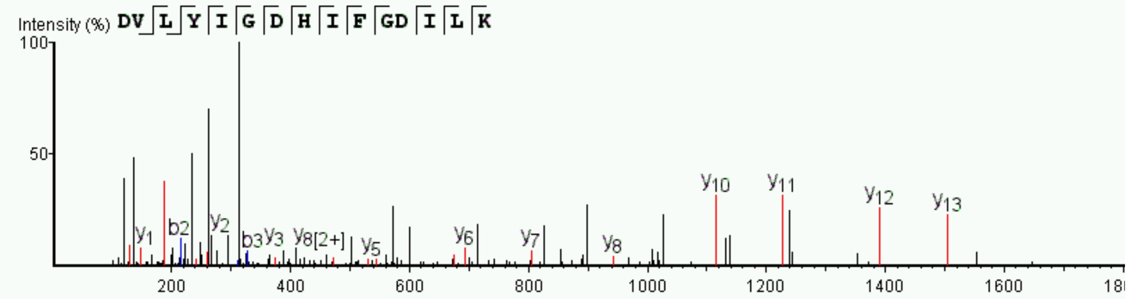

gi|571573894

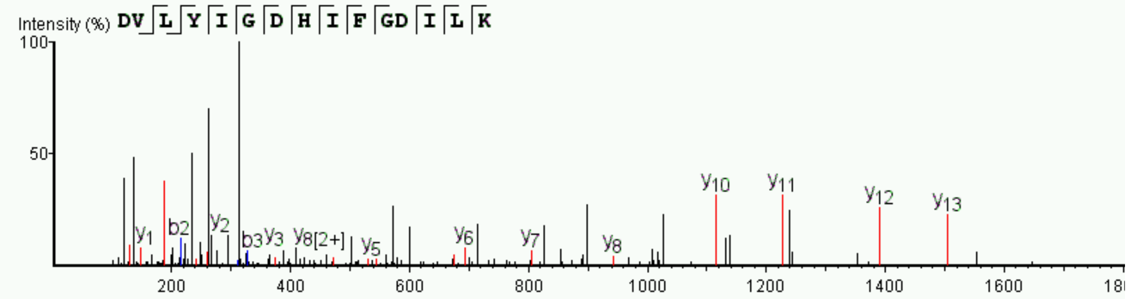

gi|571506816

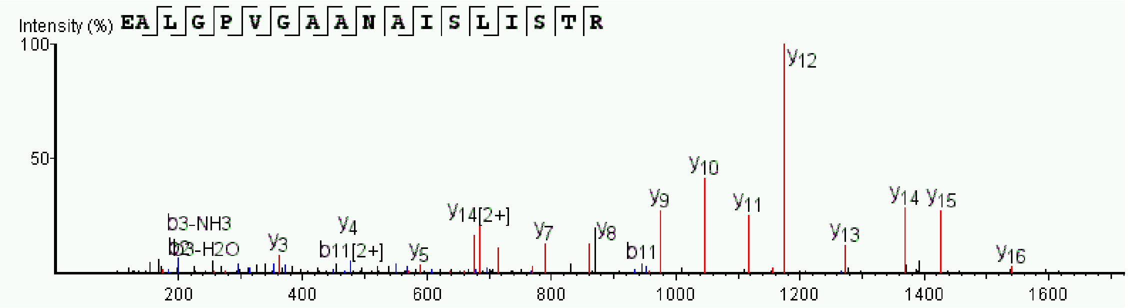

gi|571506819

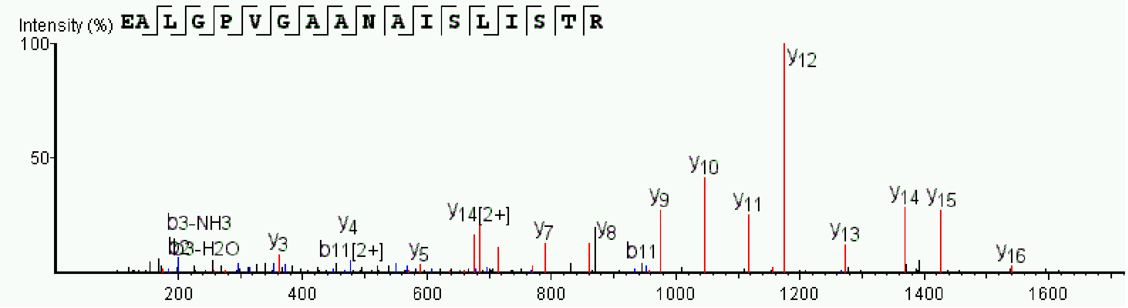

gi|328791738

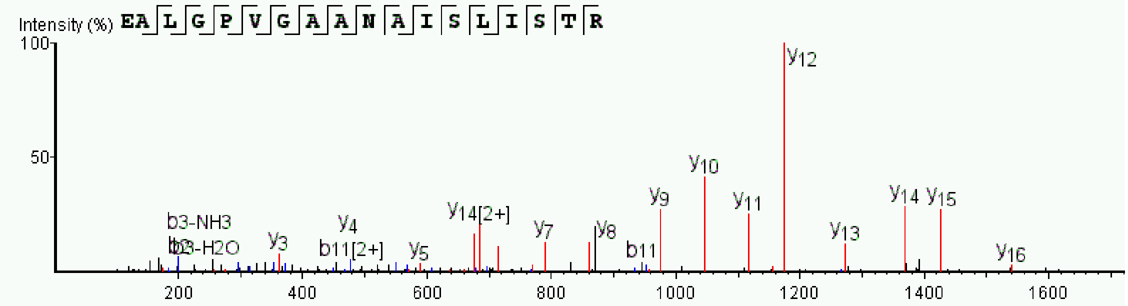

gi|571569372

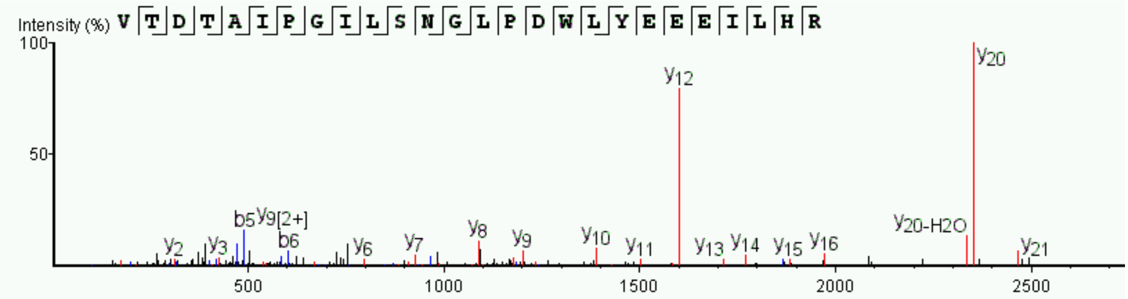

gi|328777707

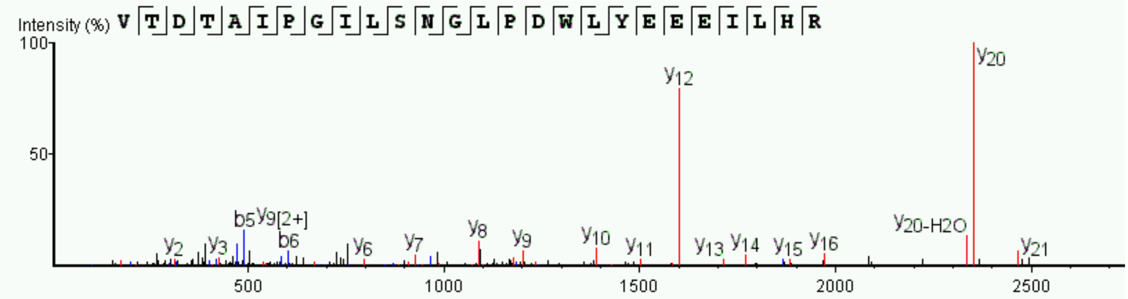

gi|571554160

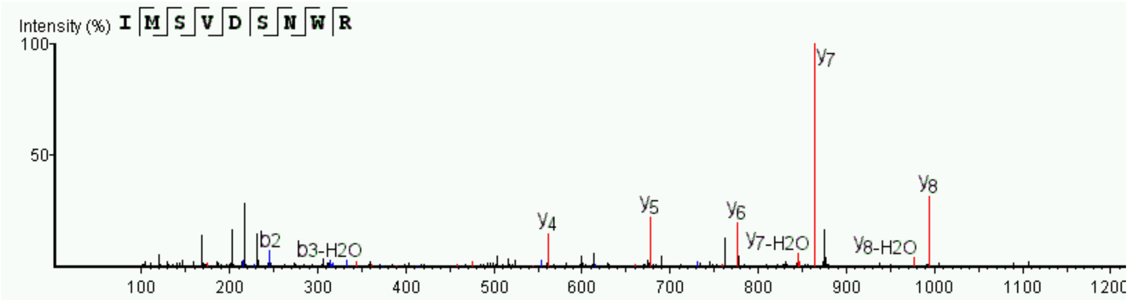

gi|571554164

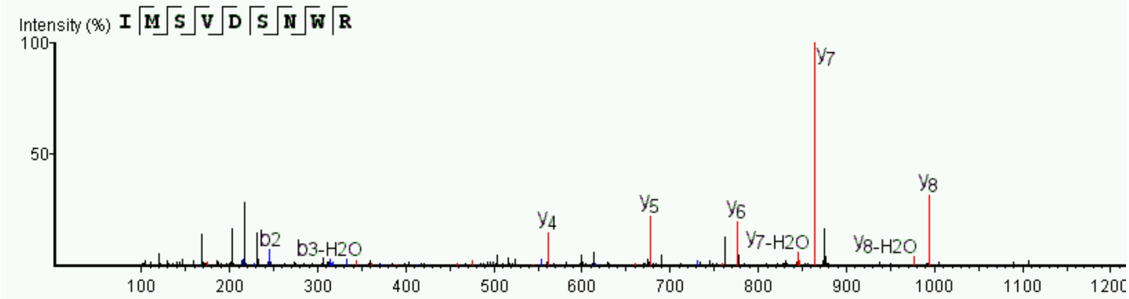

gi|66517577

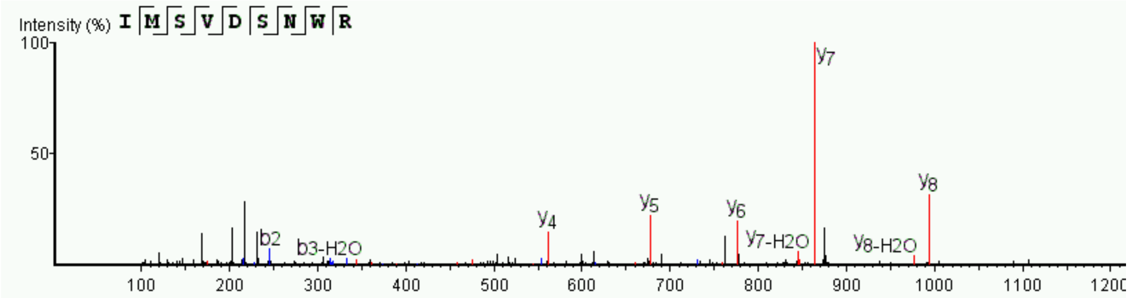

gi|571577798

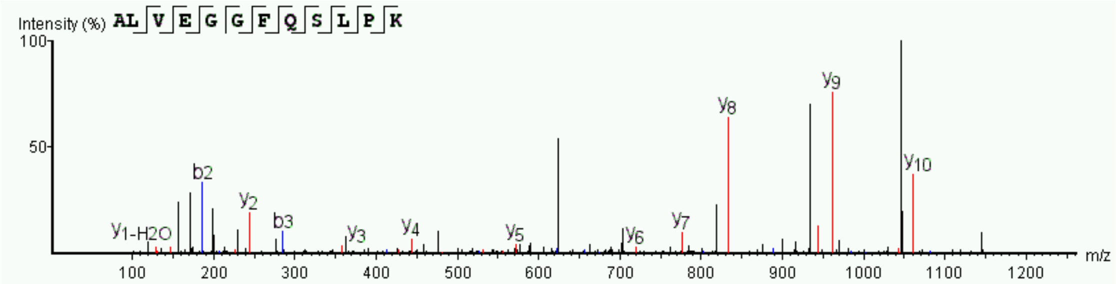

gi|66540209

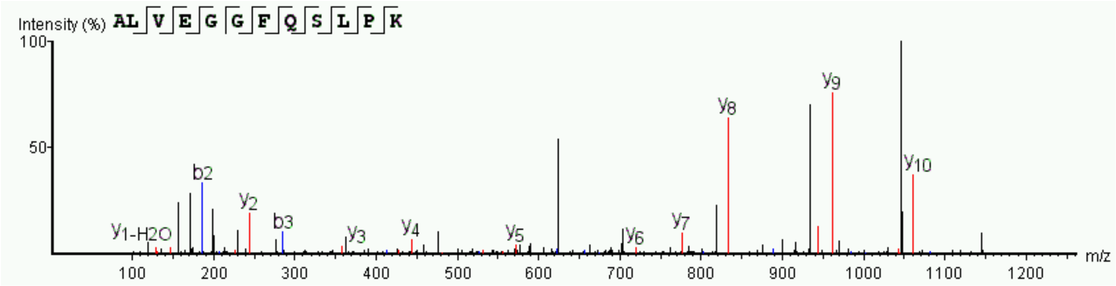

gi|571566909

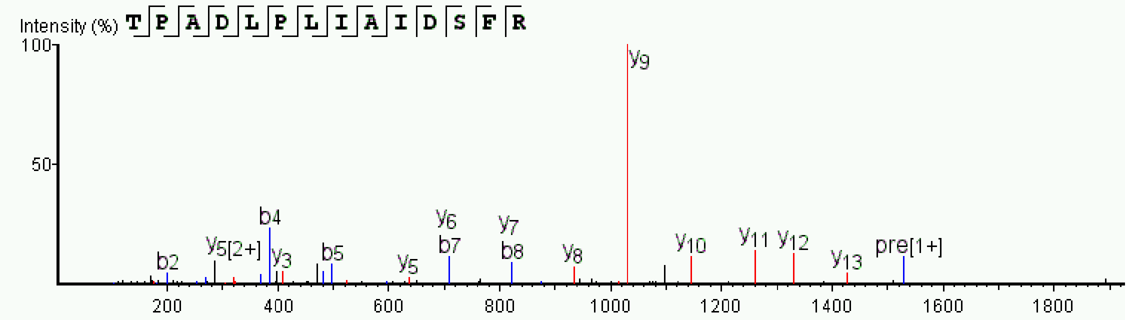

gi|328777360

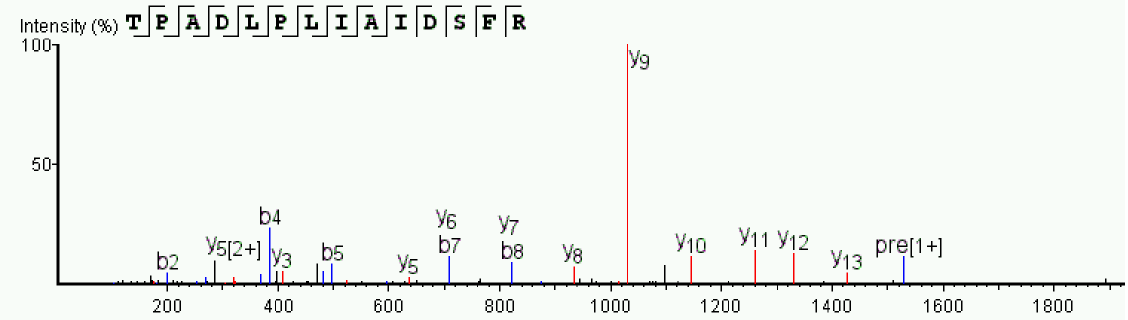

gi|571509803

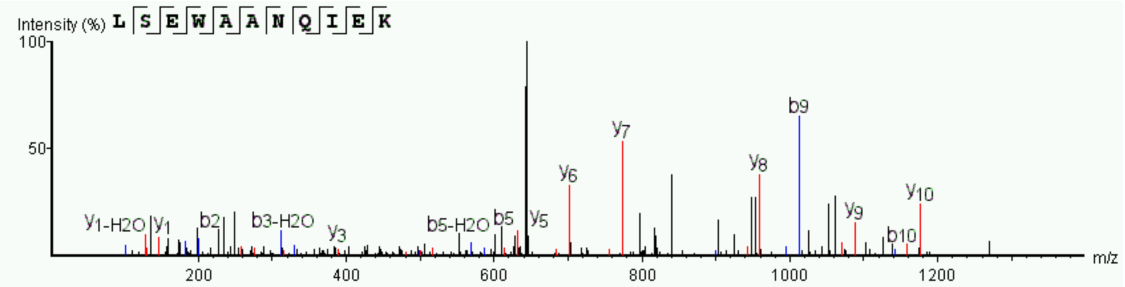

gi|571509806

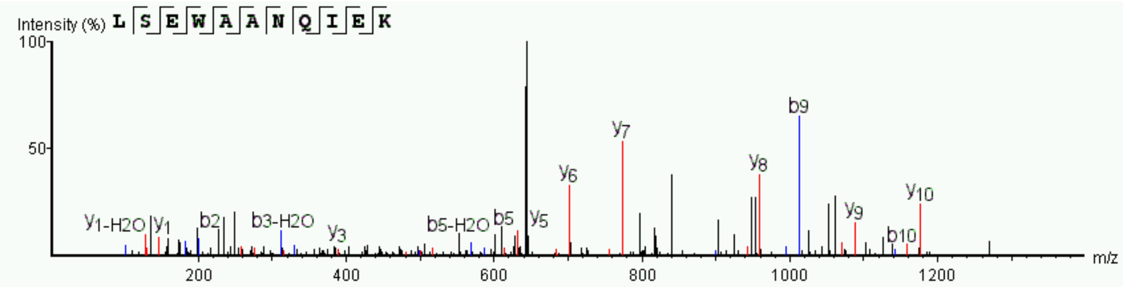

gi|571509810

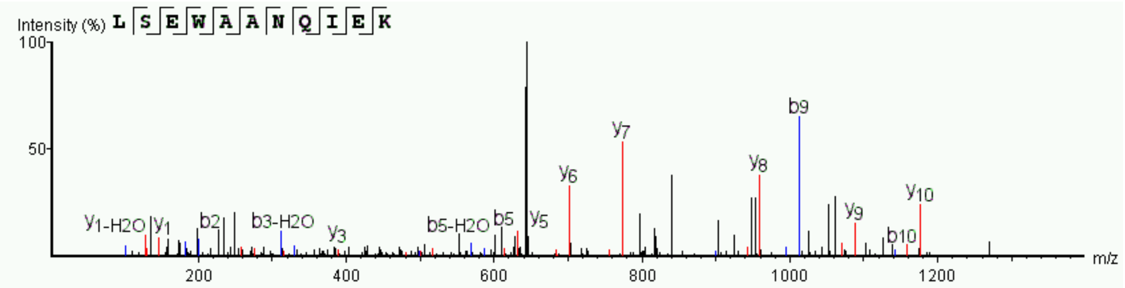

gi|48102403

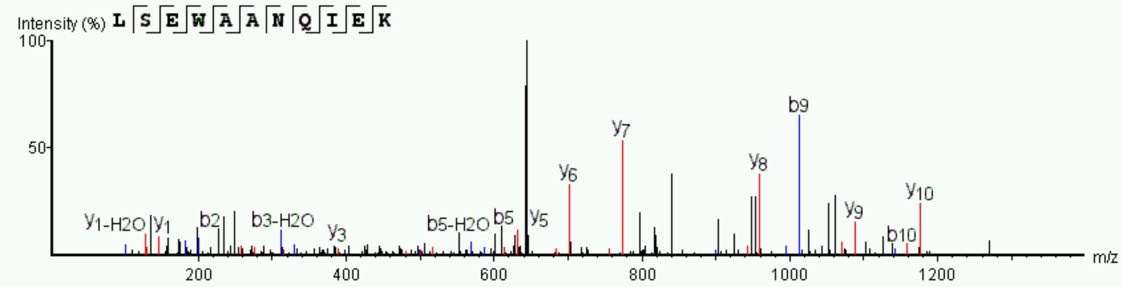

gi|328792599

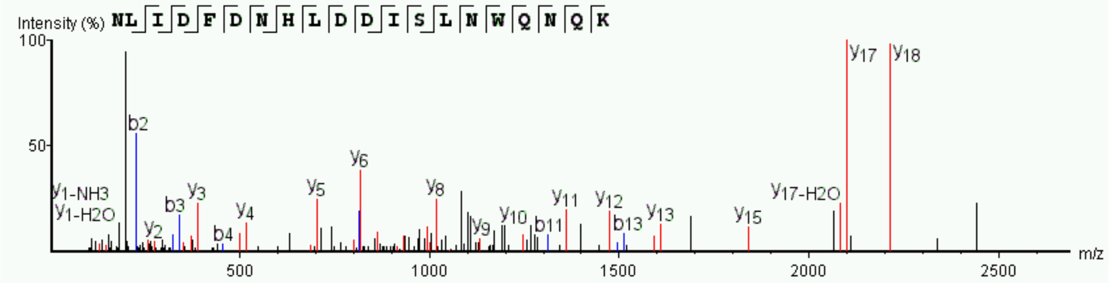

gi|571577740

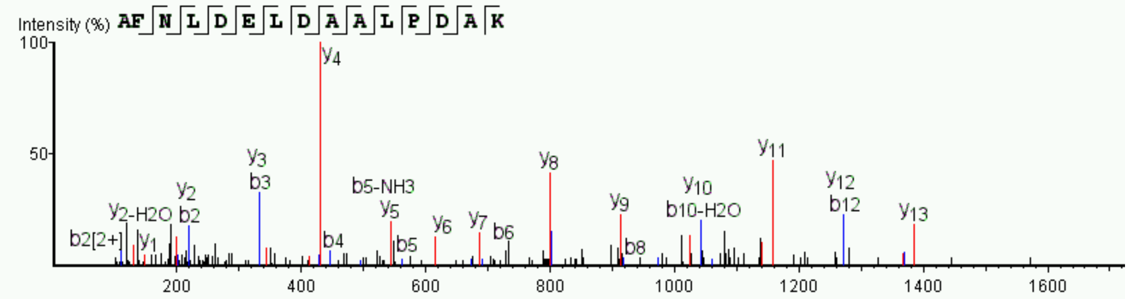

gi|571577742

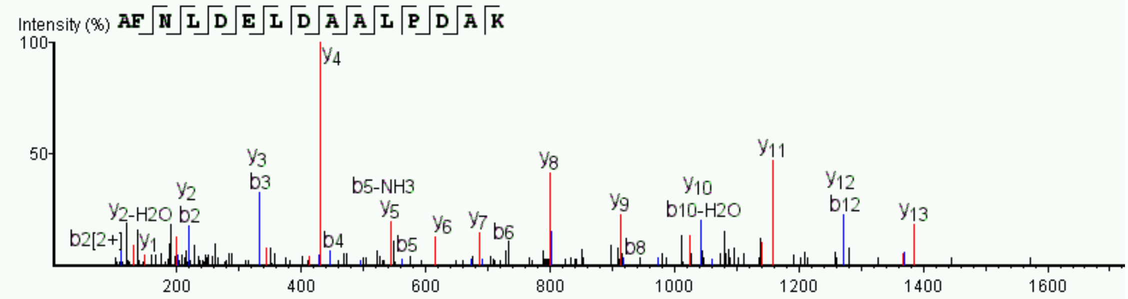

gi|571577744

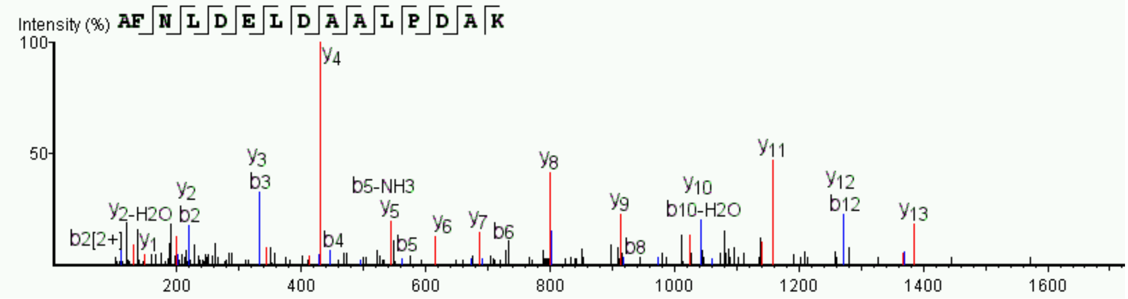

gi|571577901

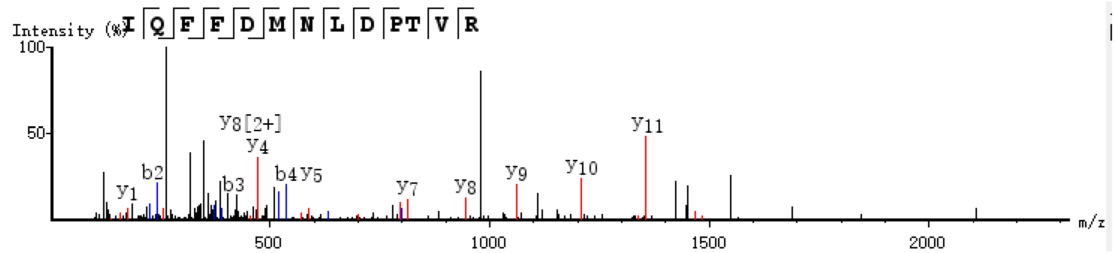

gi|328793534

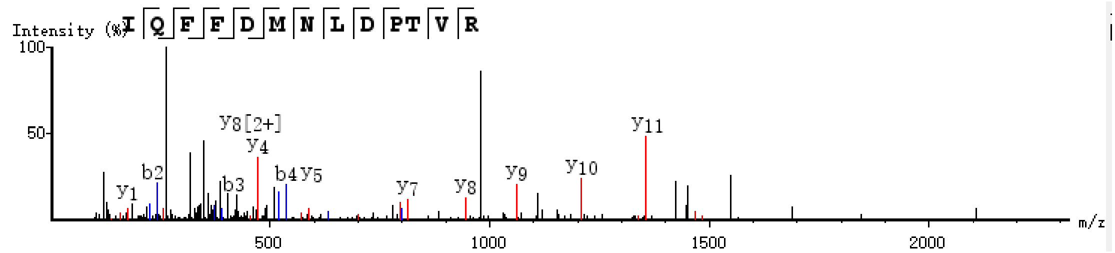

gi|571507731

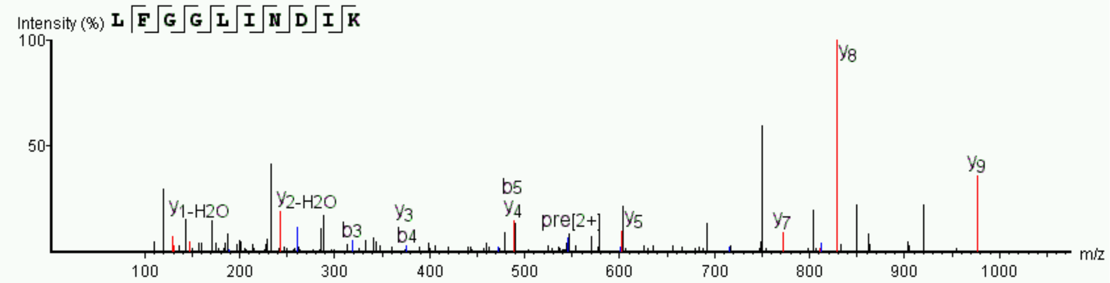

gi|571507770

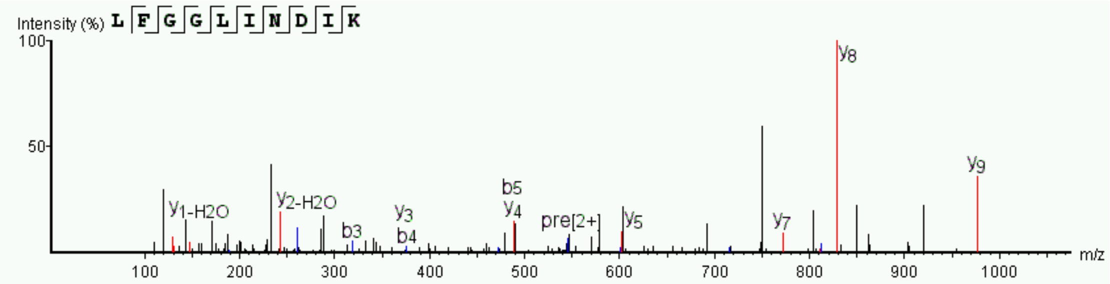

gi|571507774

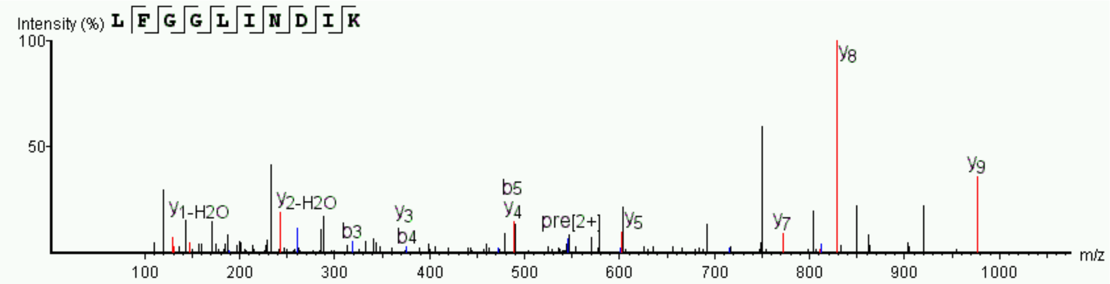

gi|571507777

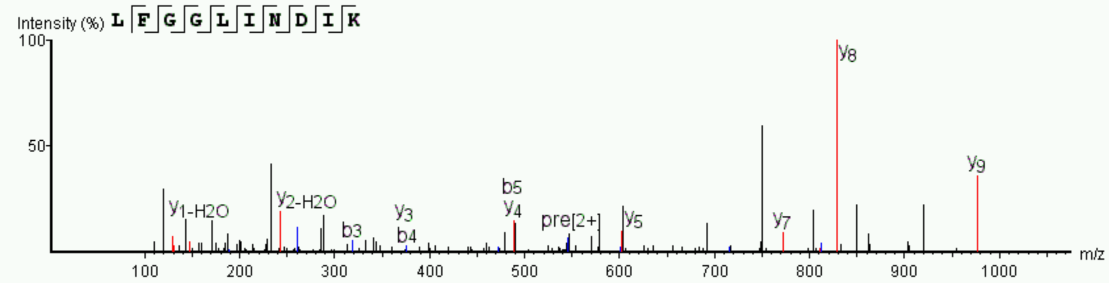

gi|571507781

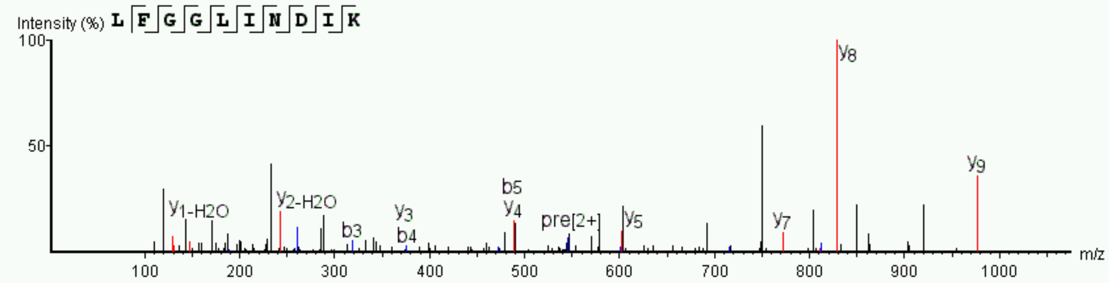

gi|571507785

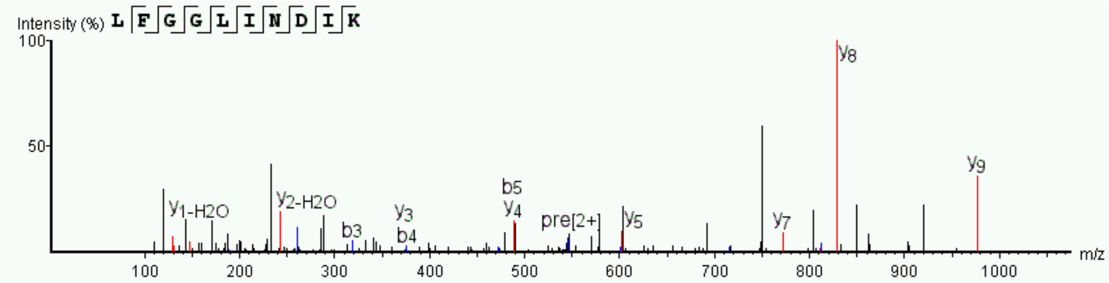

gi|571507789

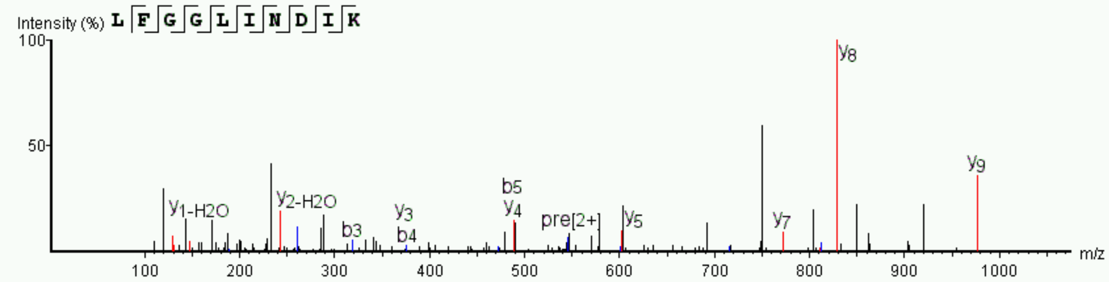

gi|571507793

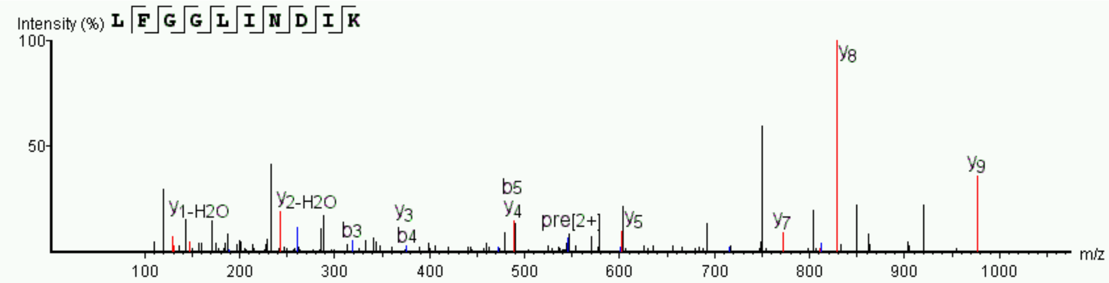

gi|571507797

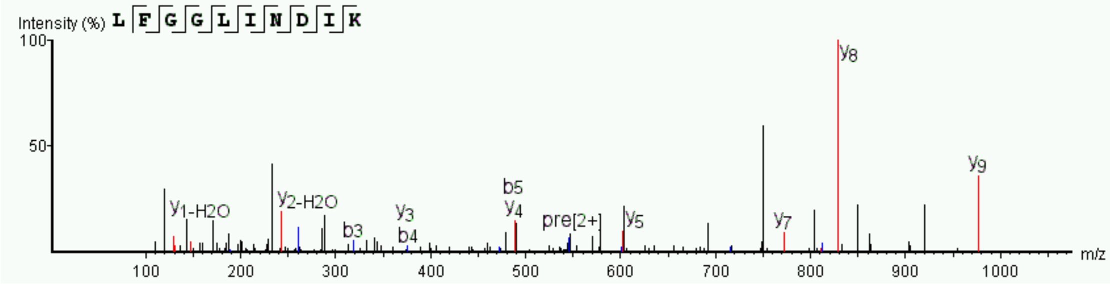

gi|571507800

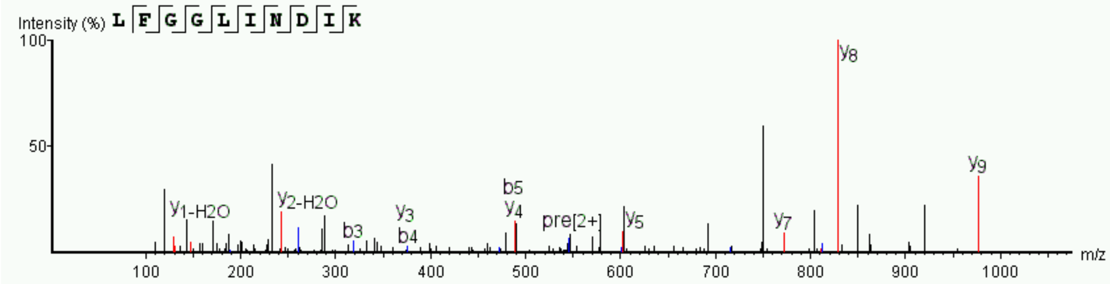

gi|571507735

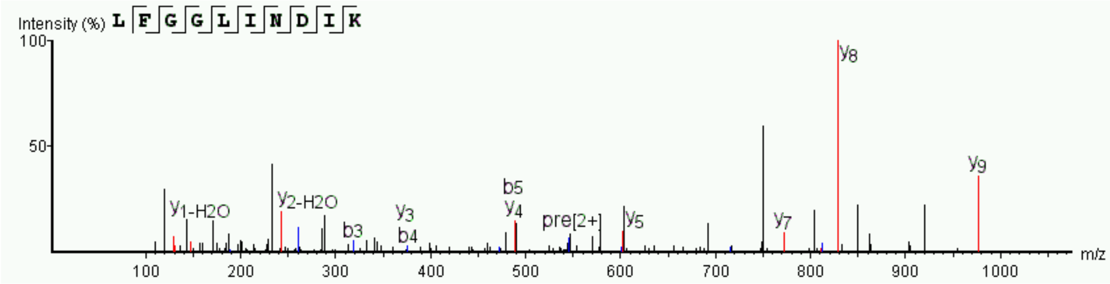

gi|571507738

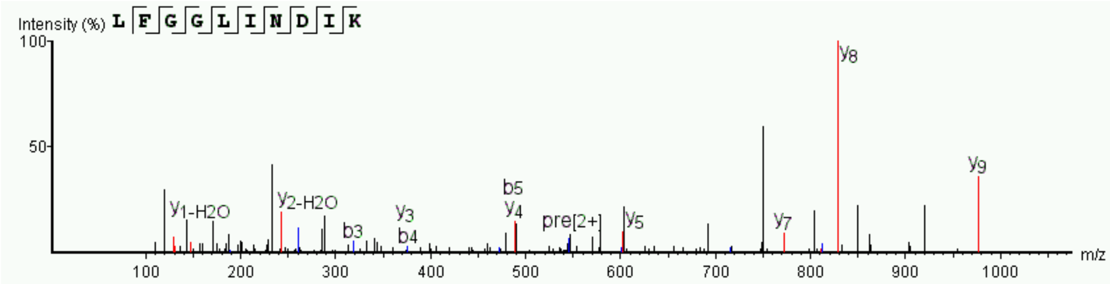

gi|571507742

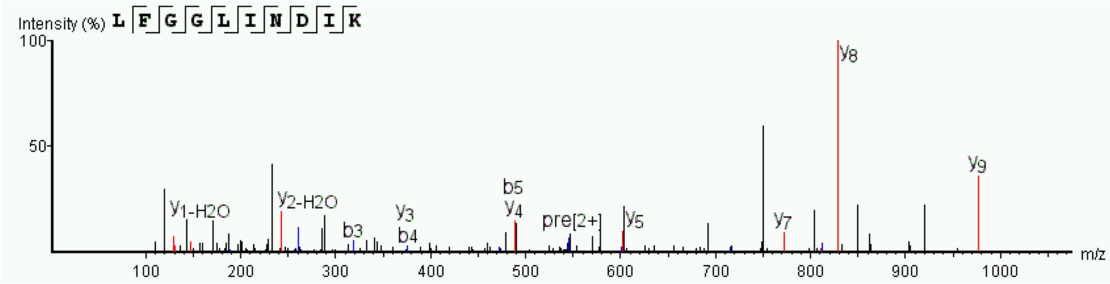

gi|571507754

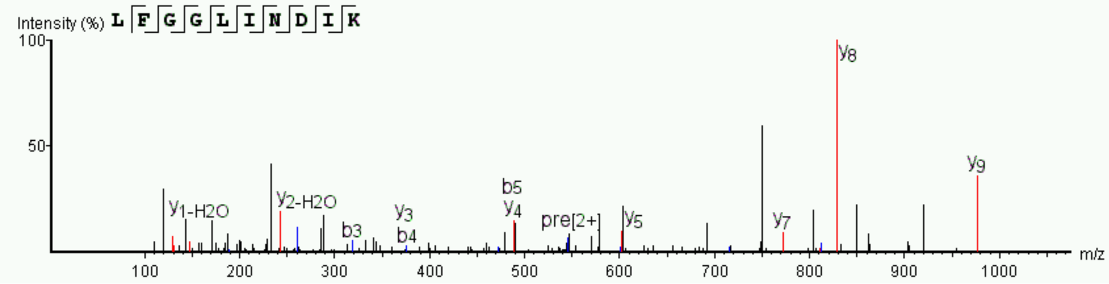

gi|571507757

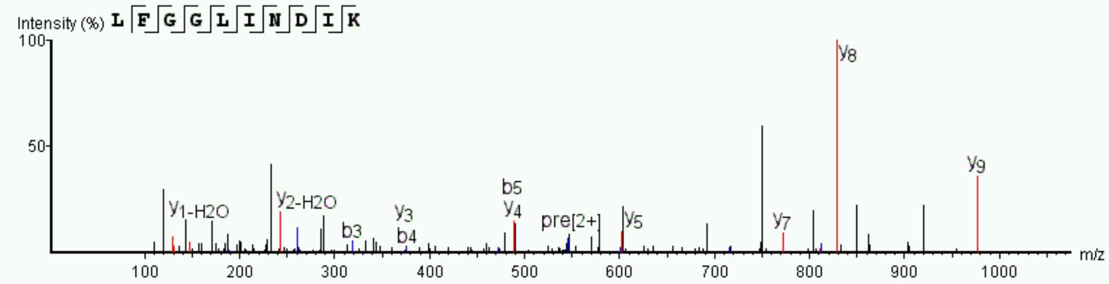

gi|571507760

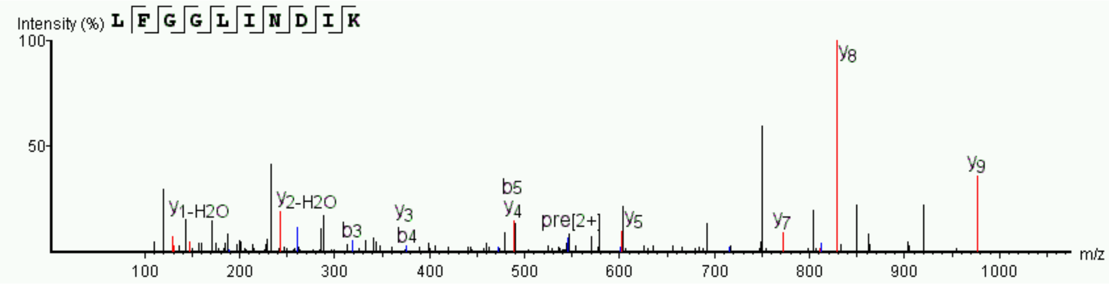

gi|571507763

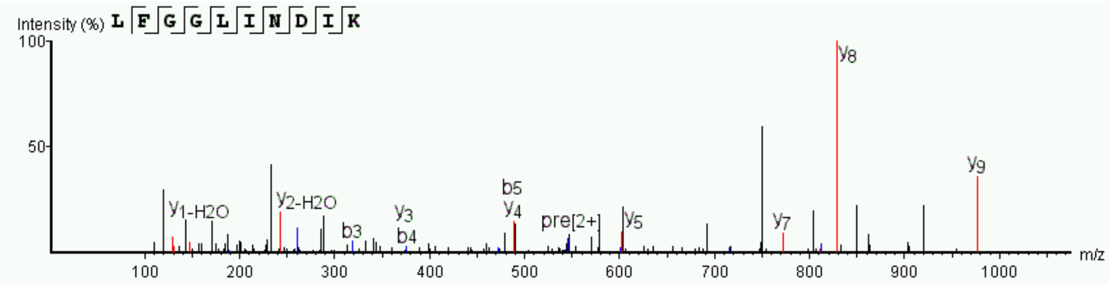

gi|571507767

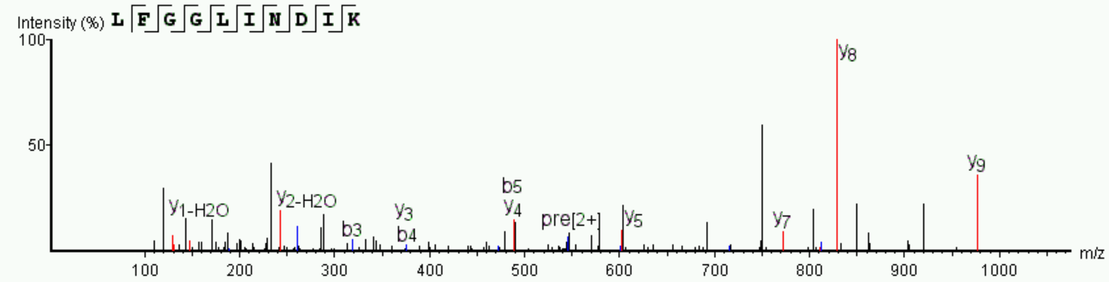

gi|571576165

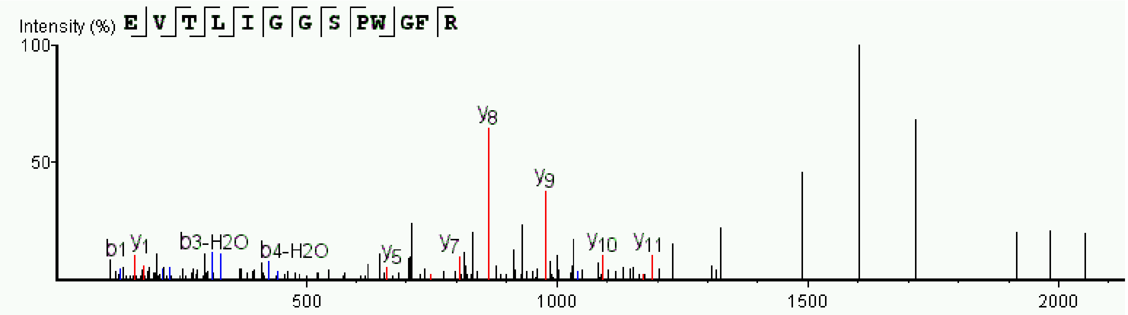

gi|571576183

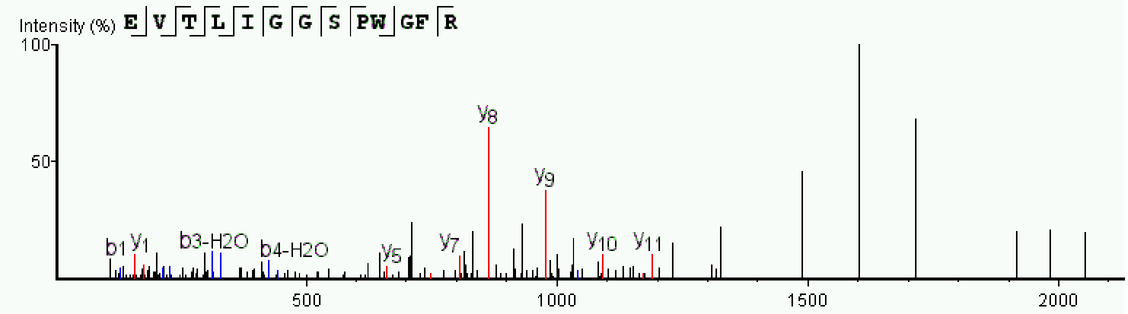

gi|571576185

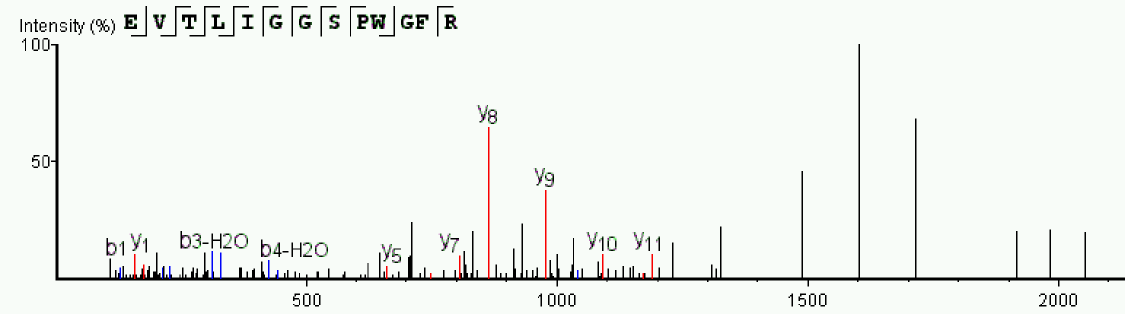

gi|571576187

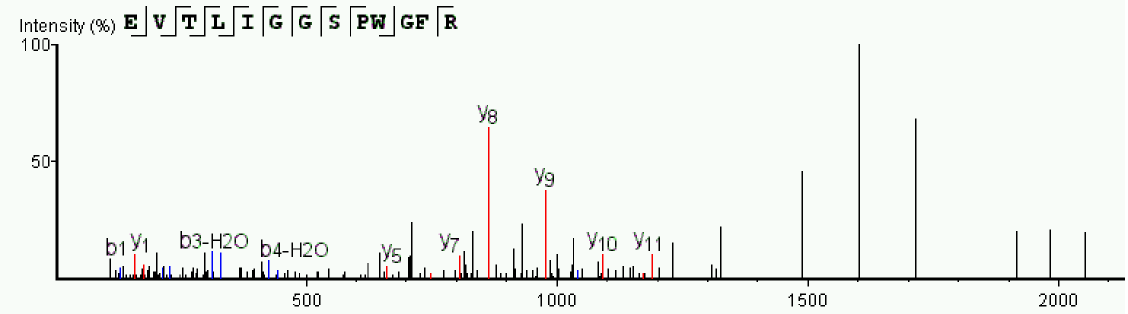

gi|571576189

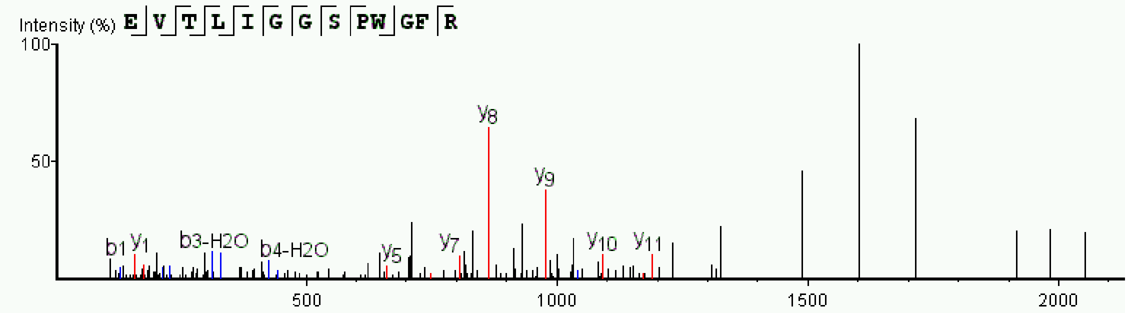

gi|571576191

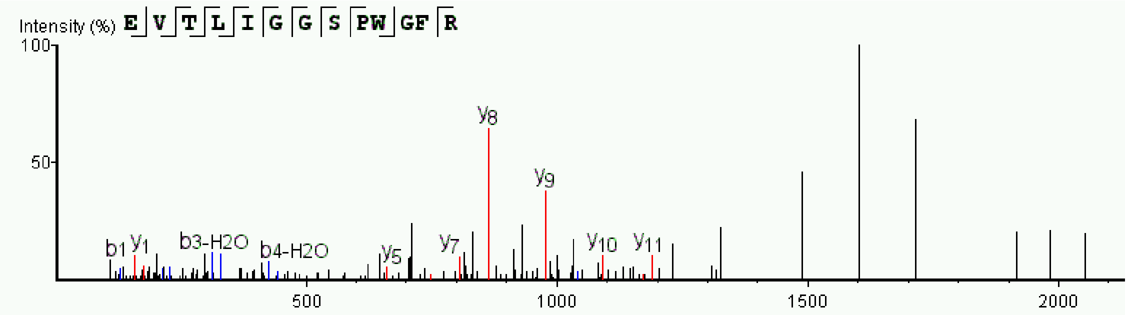

gi|571576193

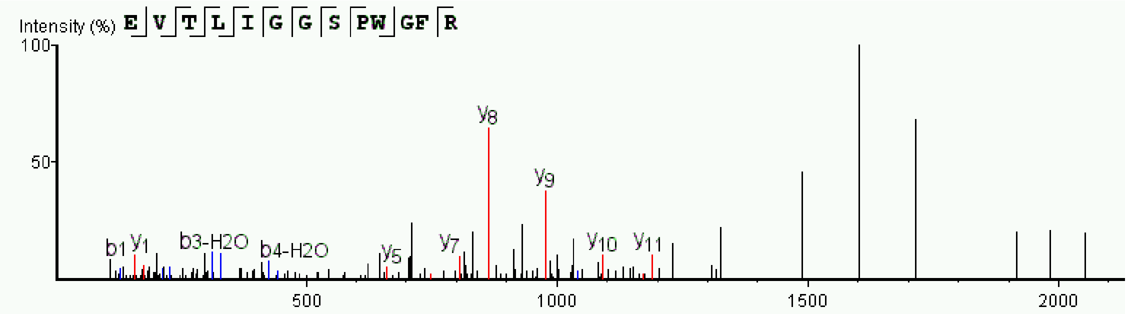

gi|571576167

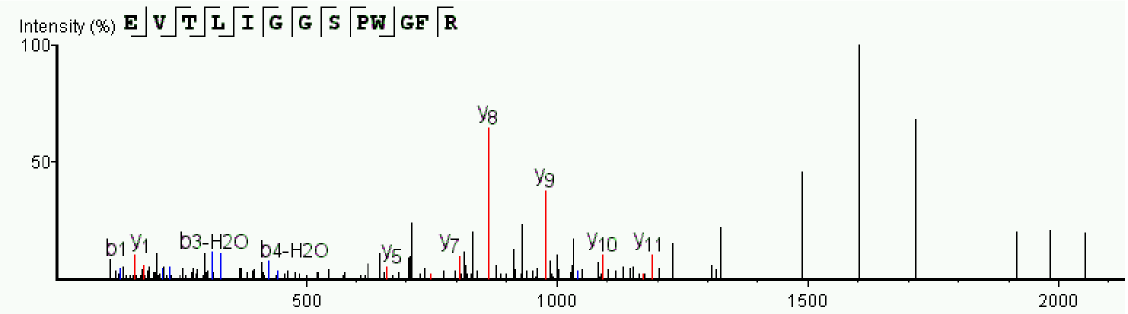

gi|571576169

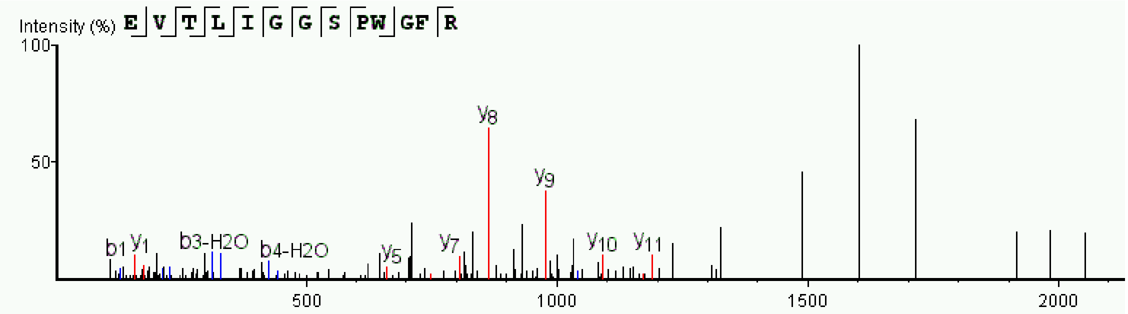

gi|571576171

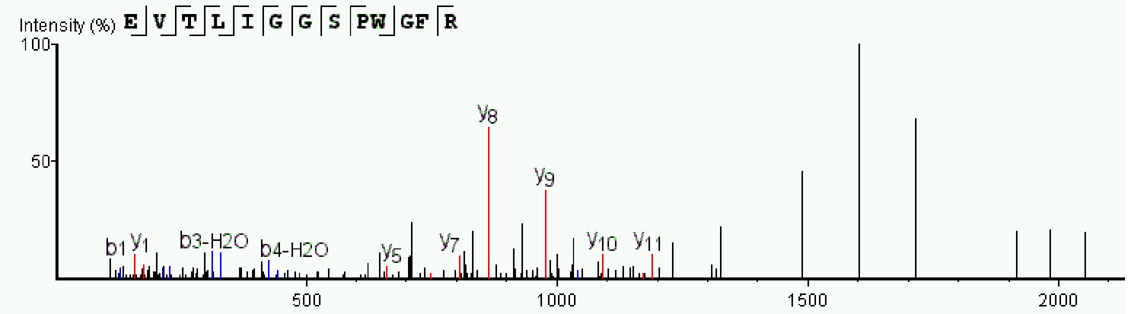

gi|571576173

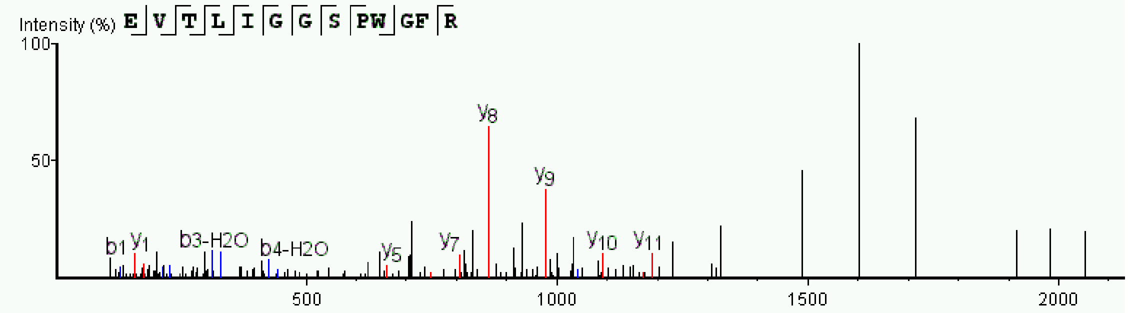

gi|571576175

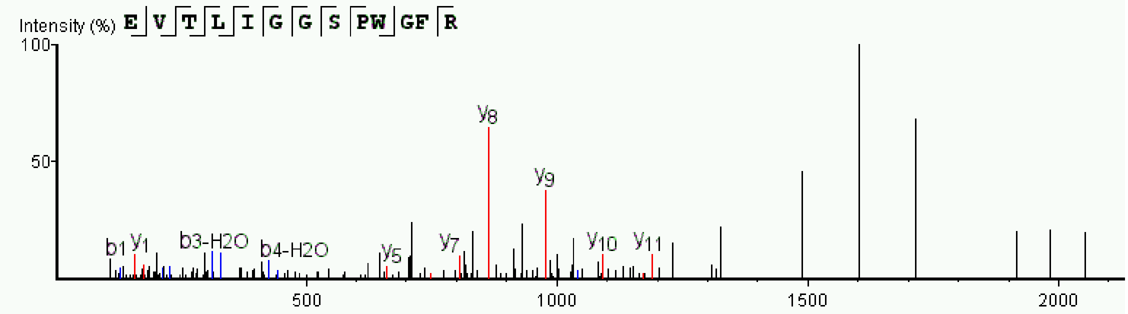

gi|571576177

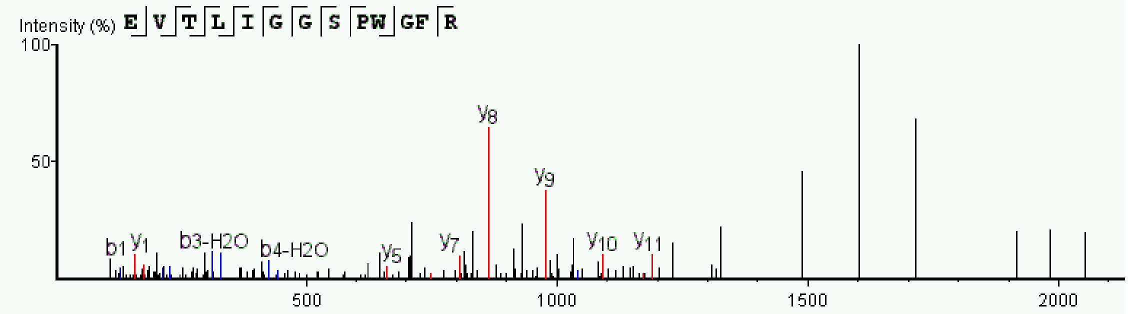

gi|571576179

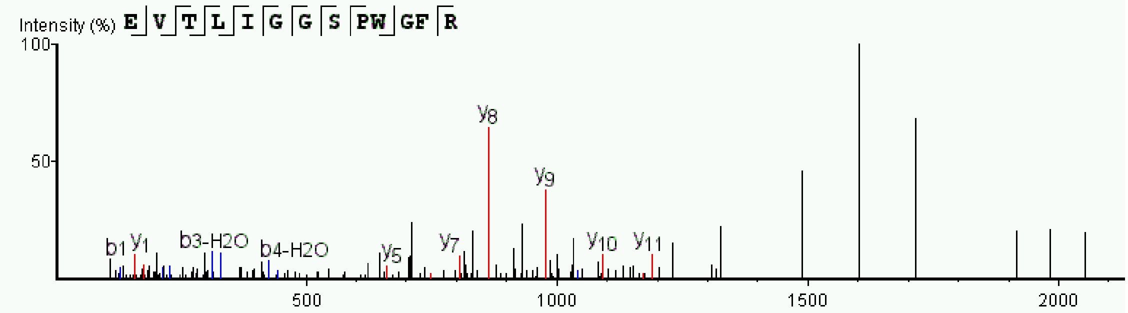

gi|571576181

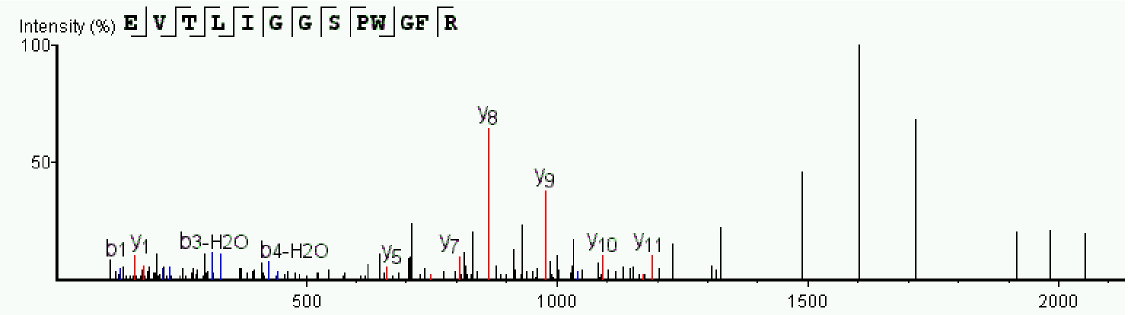

gi|571520111

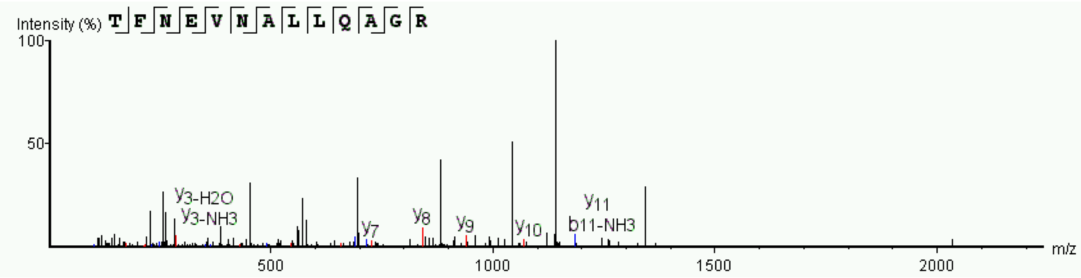

gi|571520119

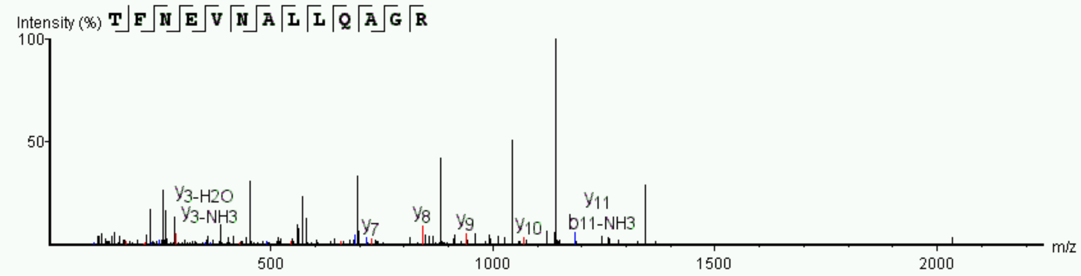

gi|328788626

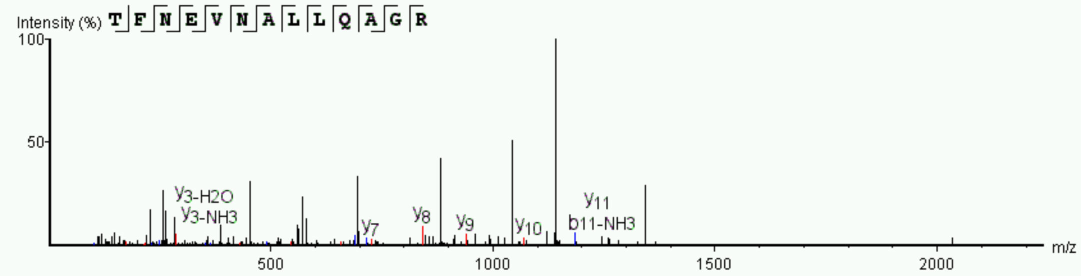

gi|328783062

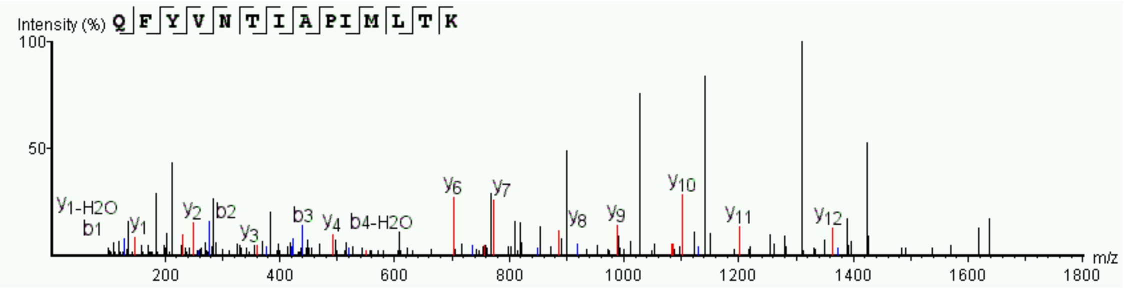

gi|571502248

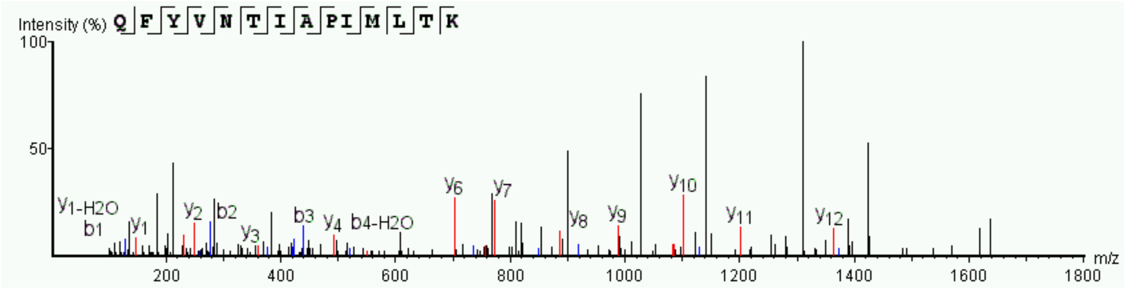

gi|571502252

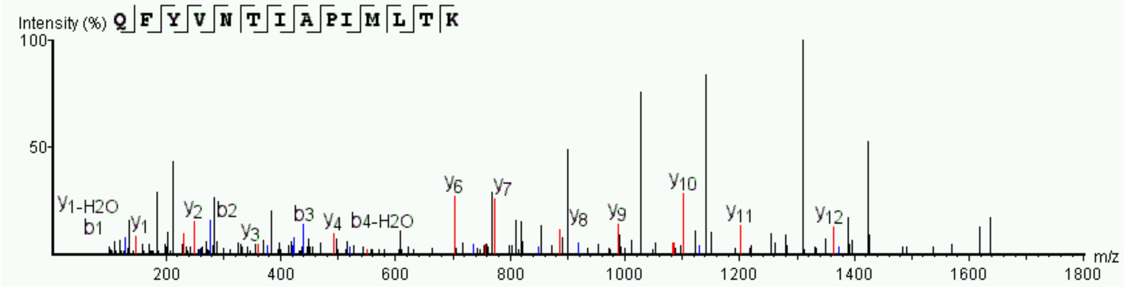

gi|571570476

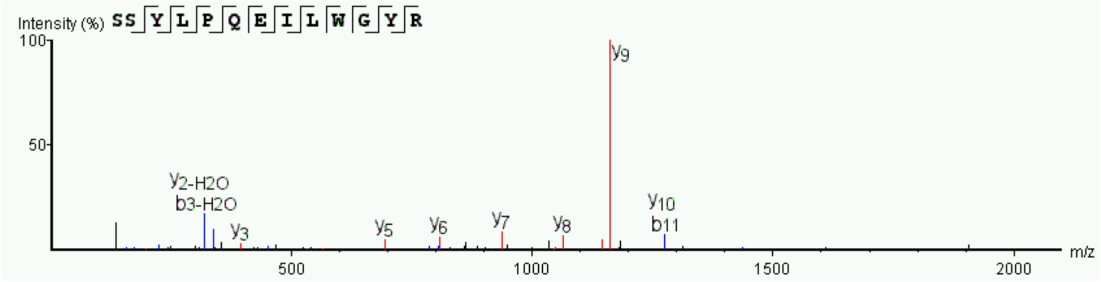

gi|571570525

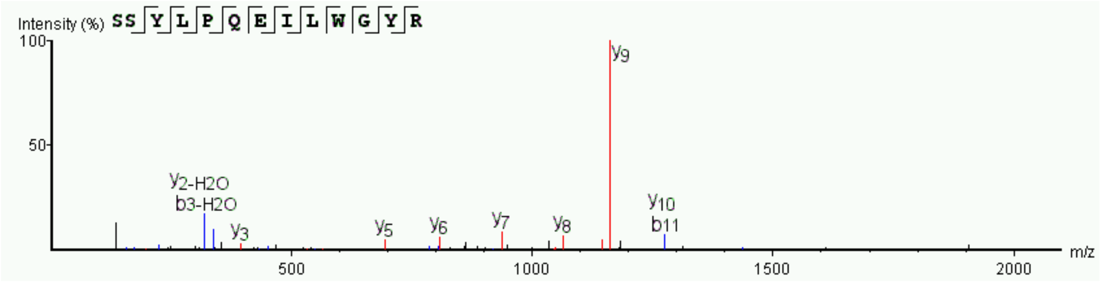

gi|571570530

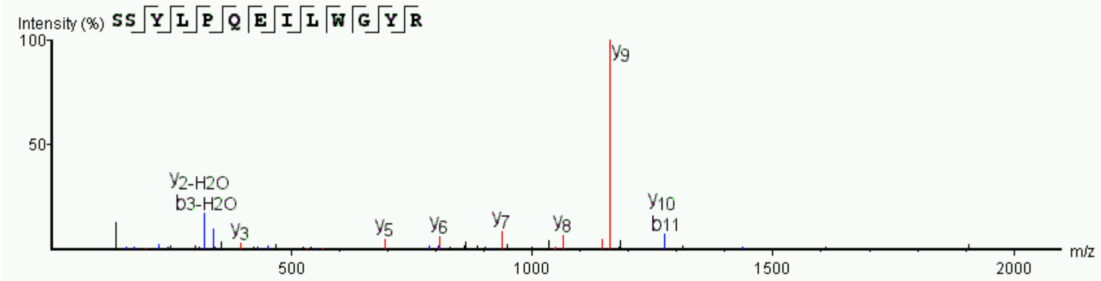

gi|571570534

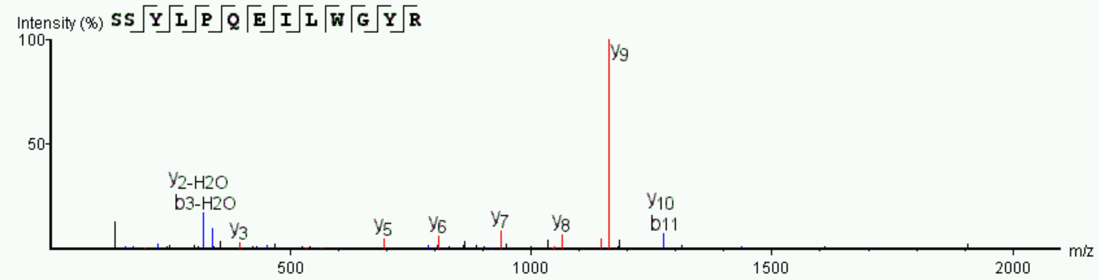

gi|328782779

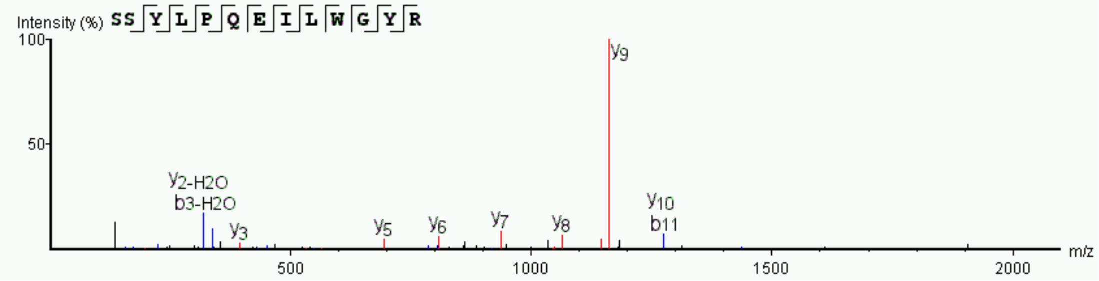

gi|571570487

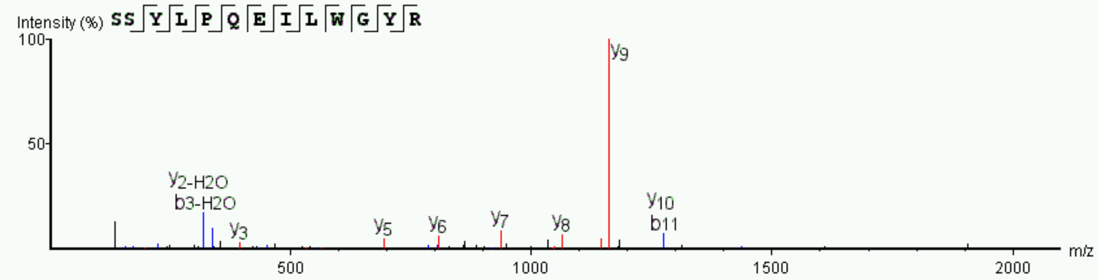

gi|571570493

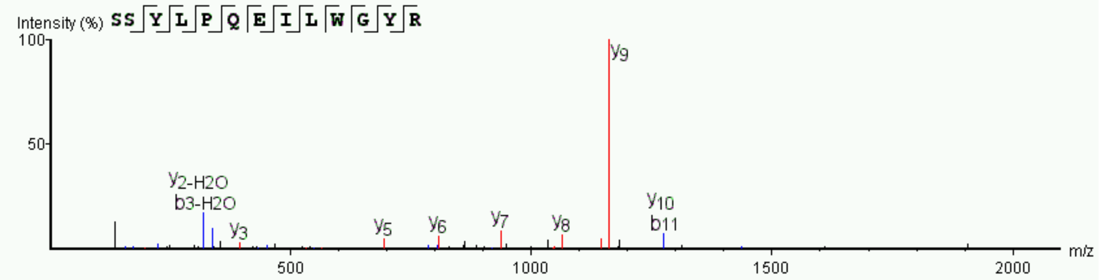

gi|571570496

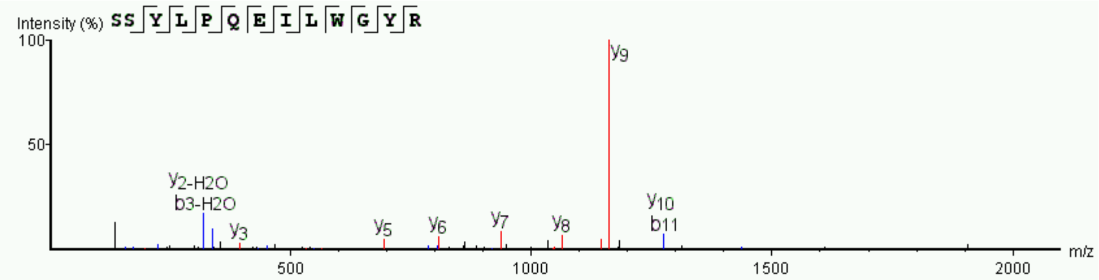

gi|571570501

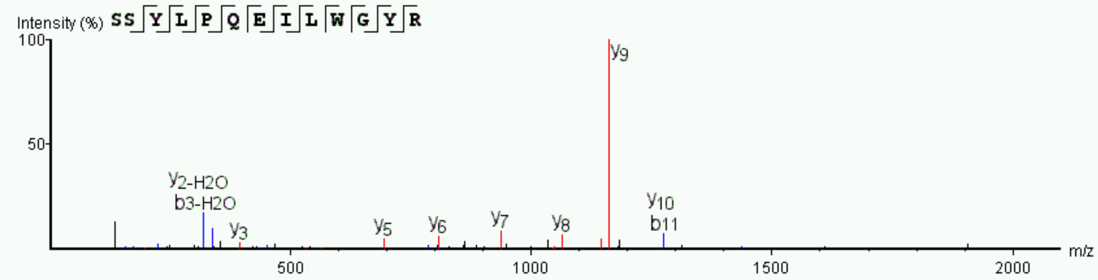

gi|571570507

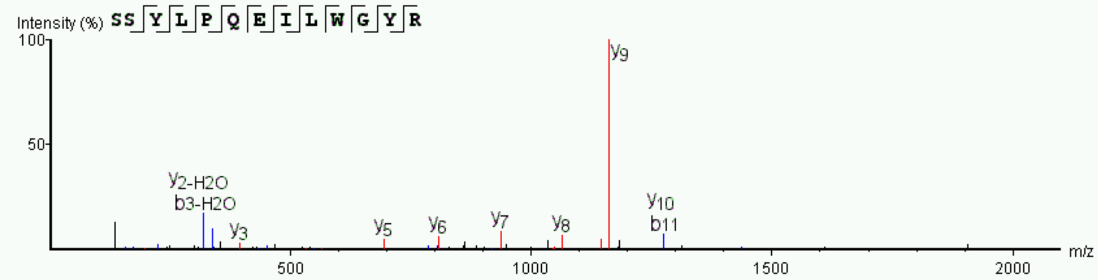

gi|571570511

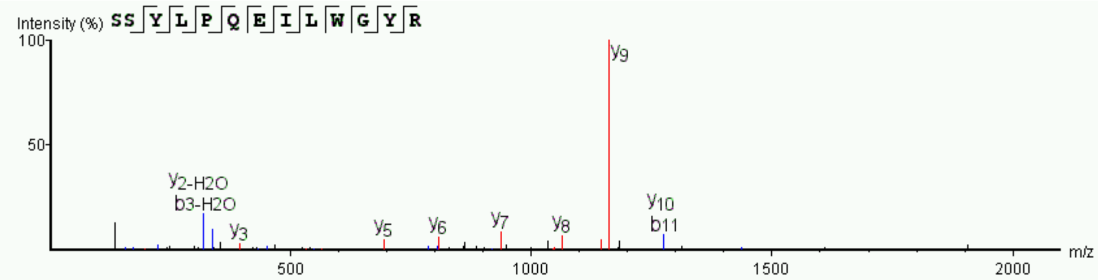

gi|571570515

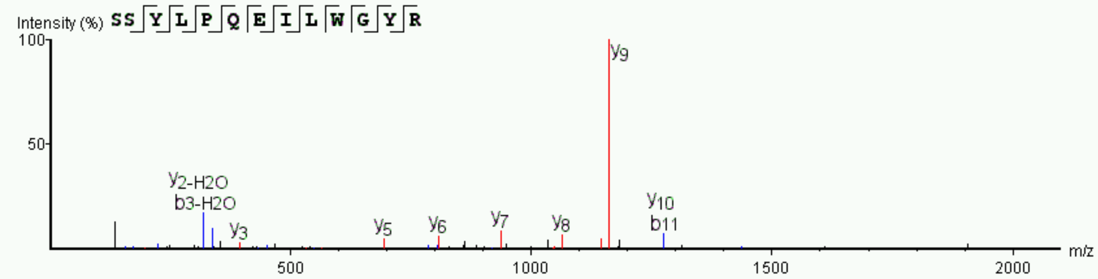

gi|571570521

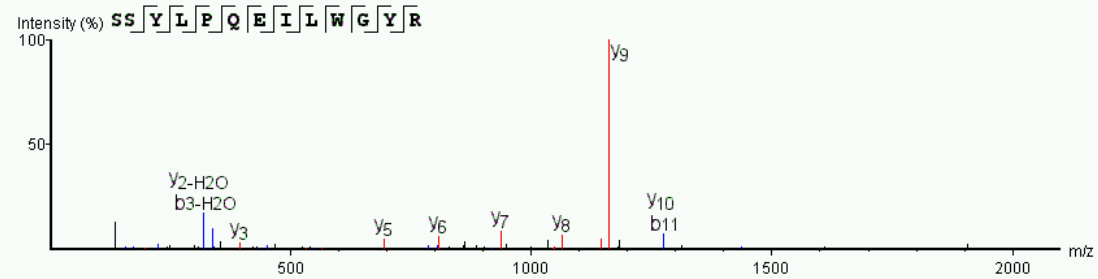

gi|571549269 gi|328781169

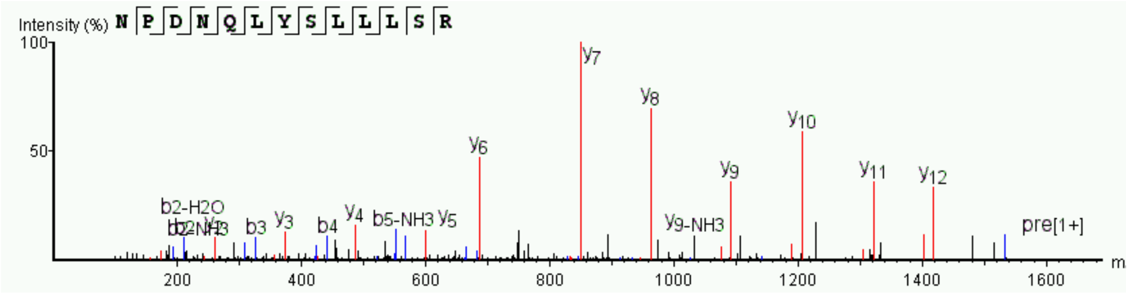

gi|571566049

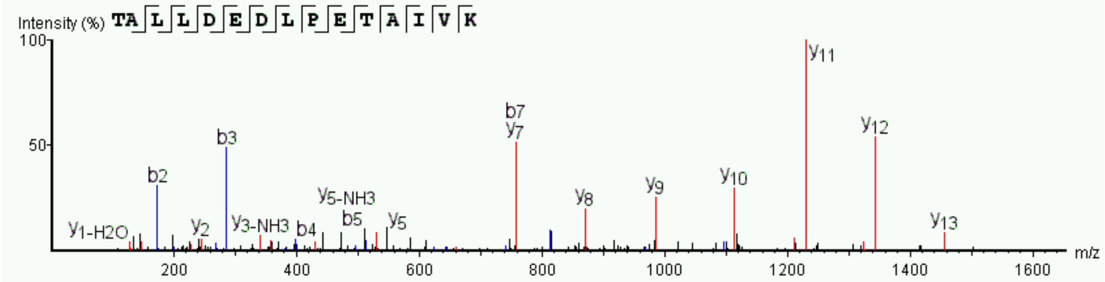

gi|328776180

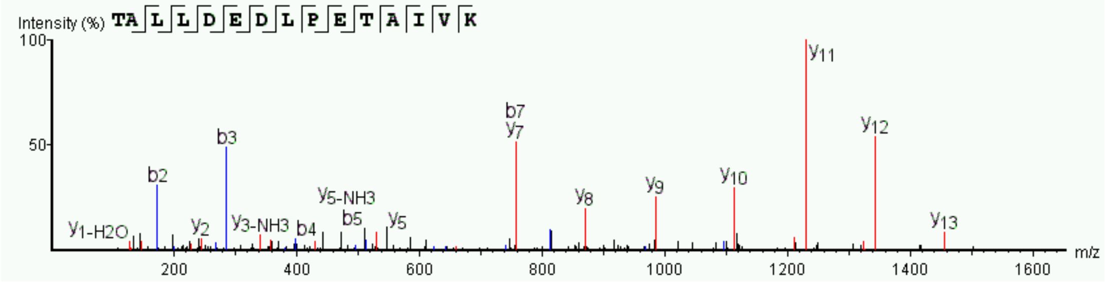

gi|571538040

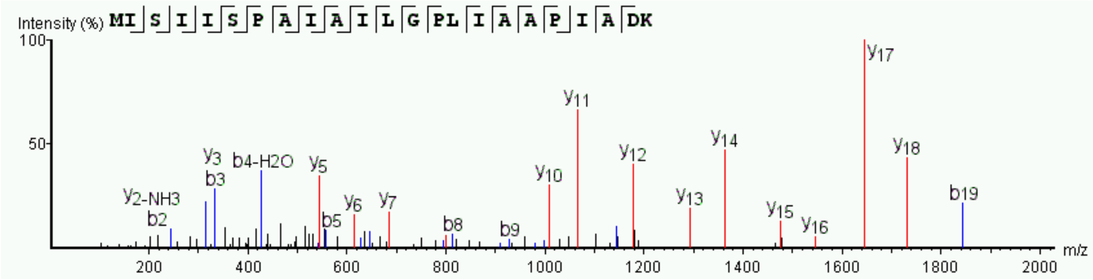

gi|571538043

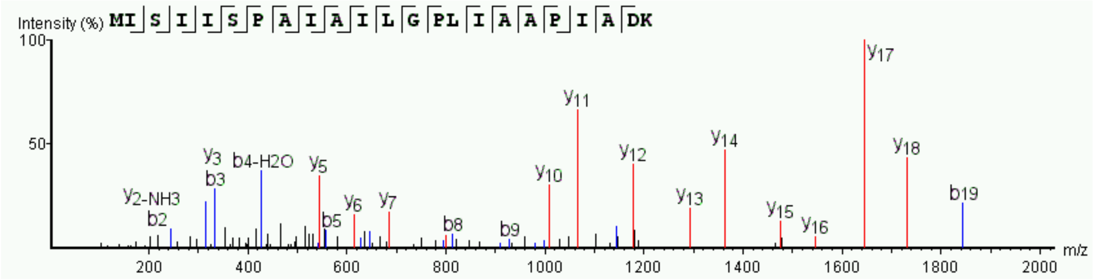

gi|571538046

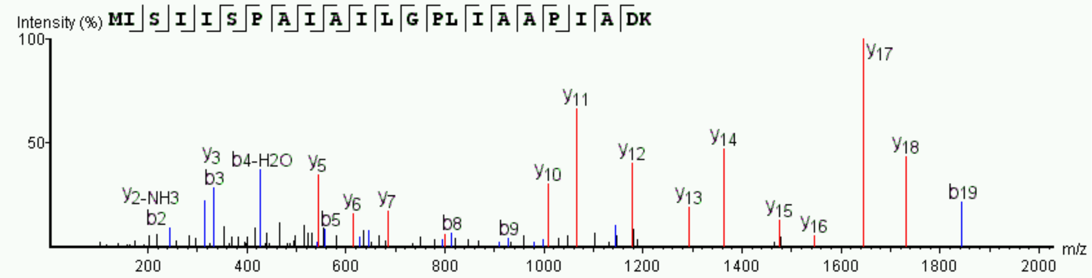

gi|571538049

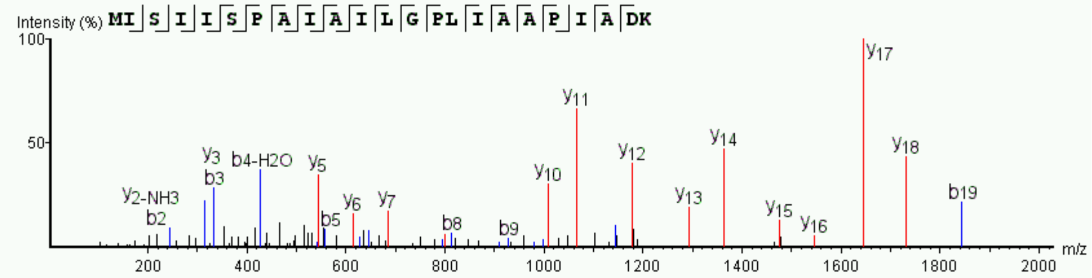

gi|571538052

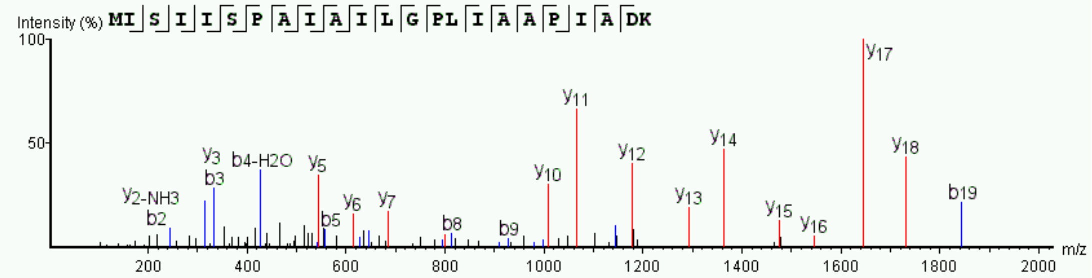

gi|571538056

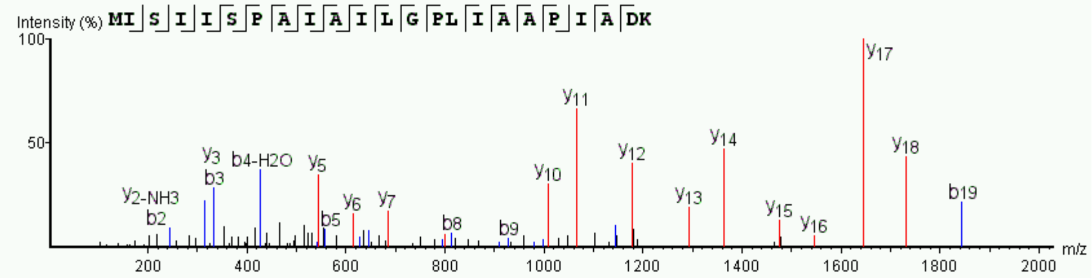

gi|571575408

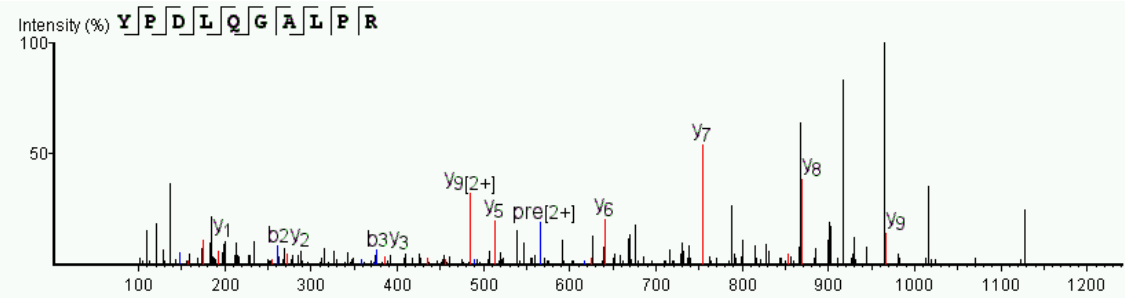

gi|328775931

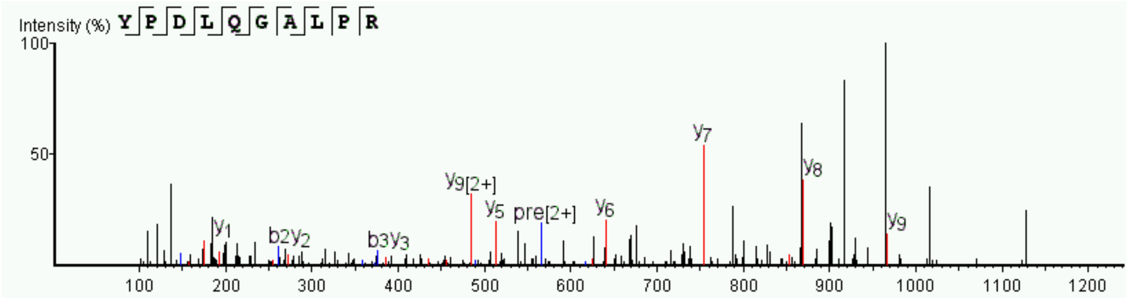

gi|571531426

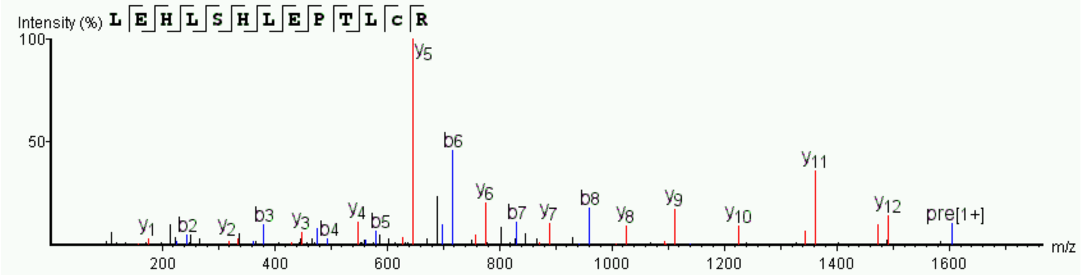

gi|66509585

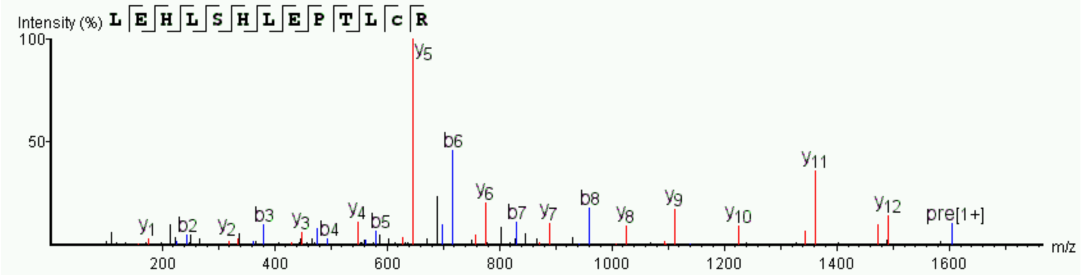

gi|571520632

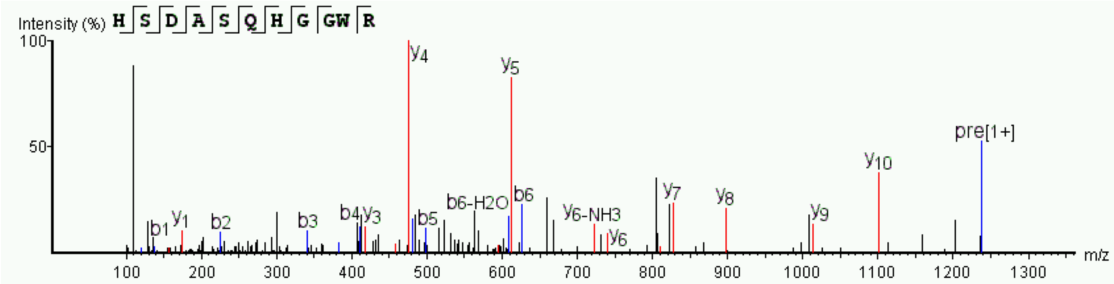

gi|571520635

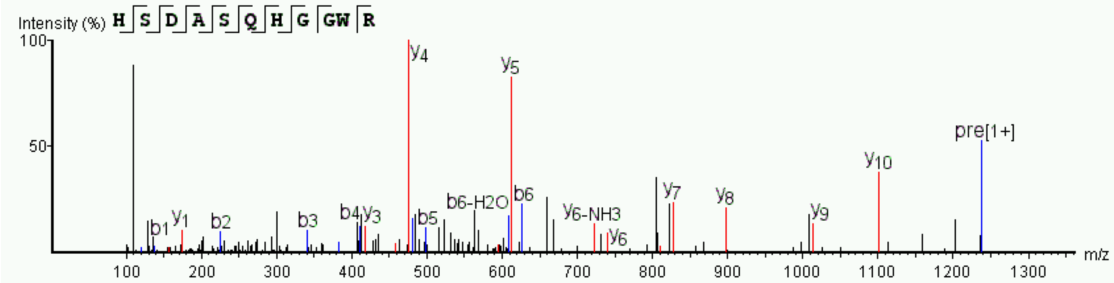

gi|571520638

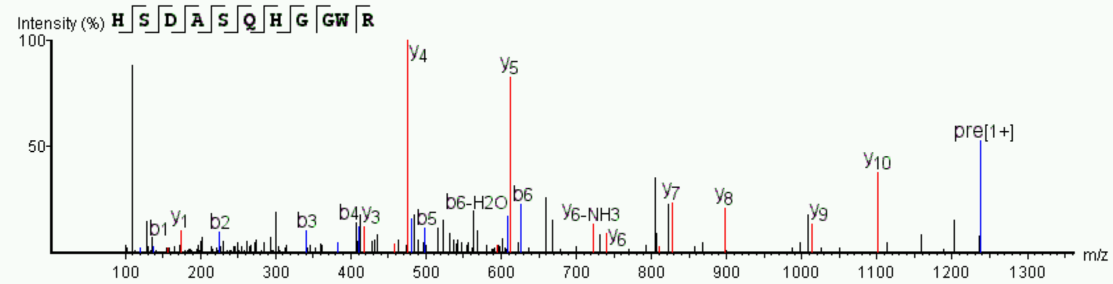

gi|571520624

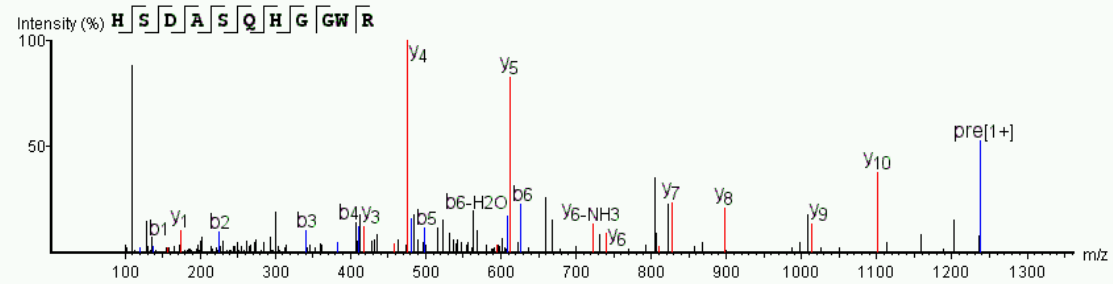

gi|571520628

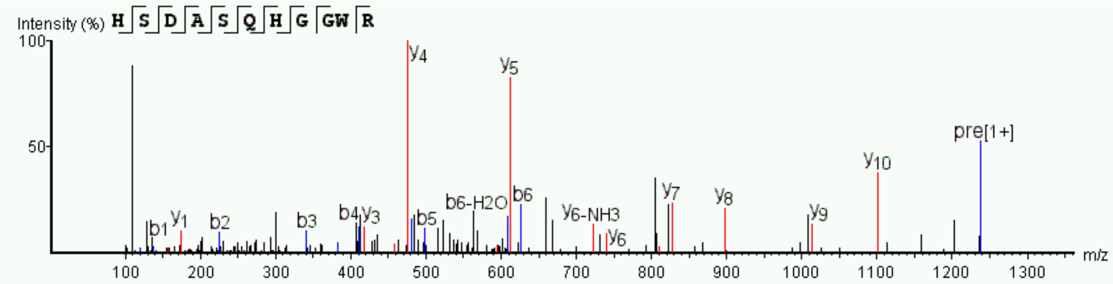

gi|571525511

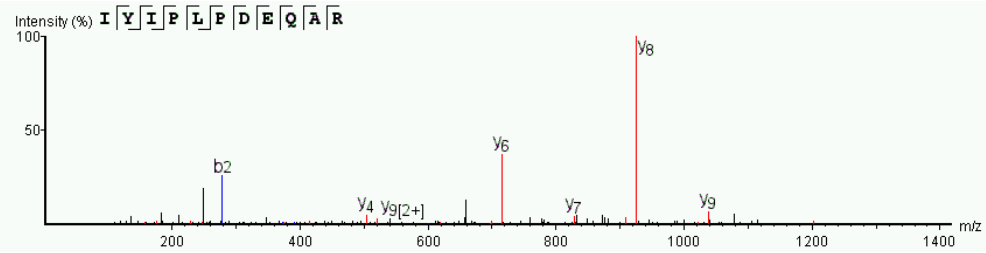

gi|571525515

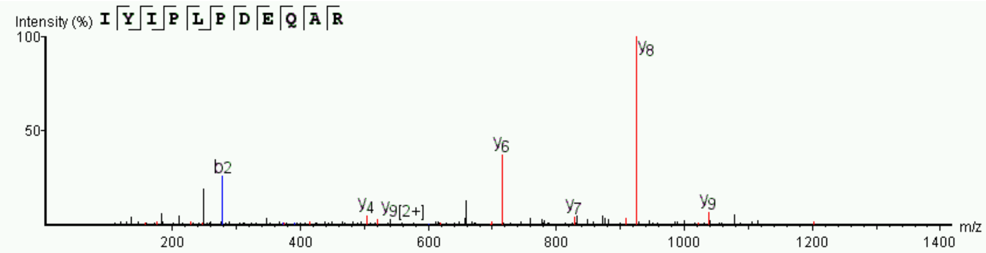

gi|328787733

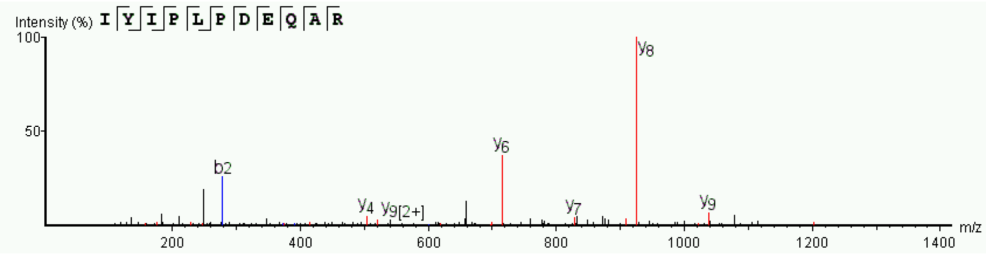

gi|571503771

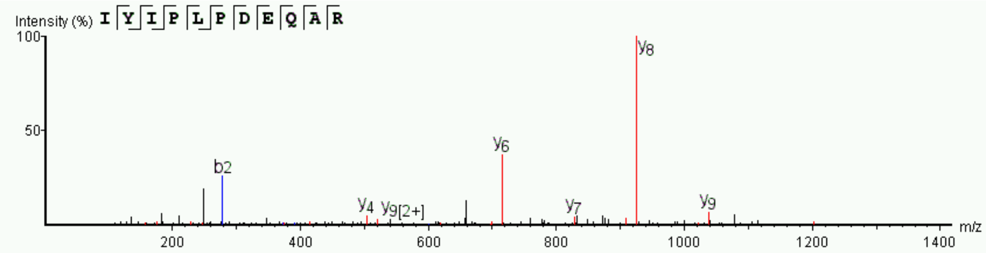

gi|66564933

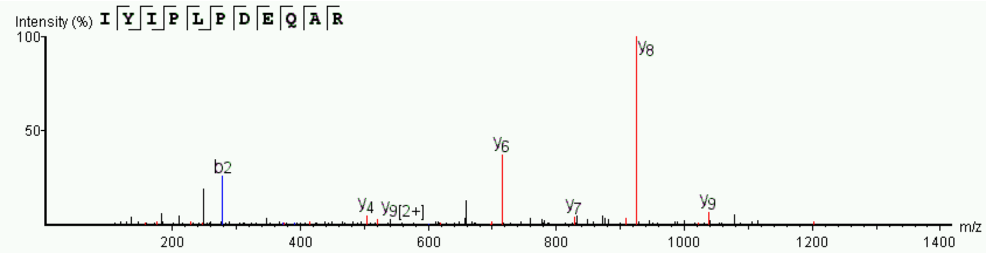

gi|571533194

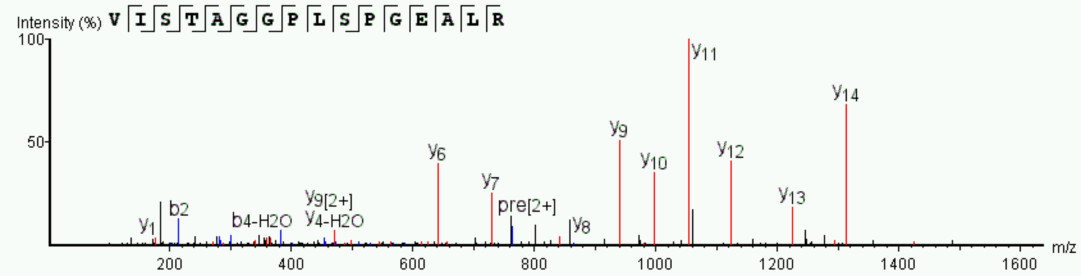

gi|571533197

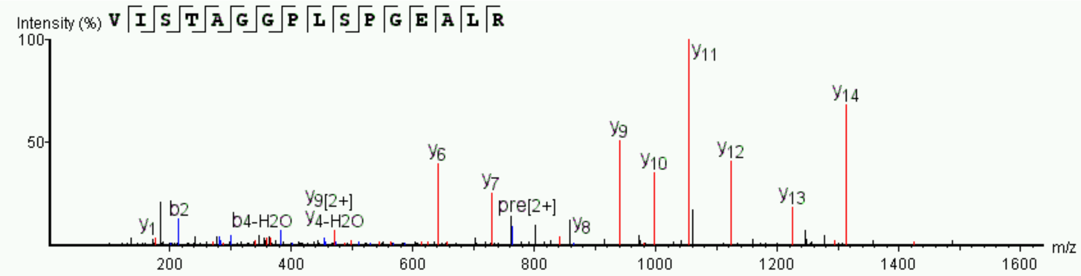

gi|571533201

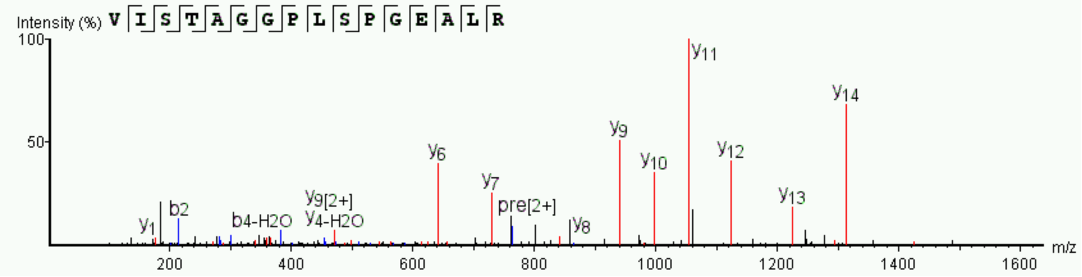

gi|58585110

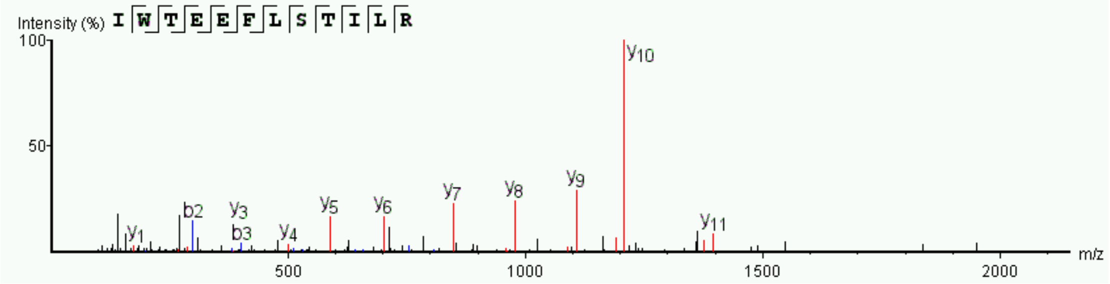

gi|48099854

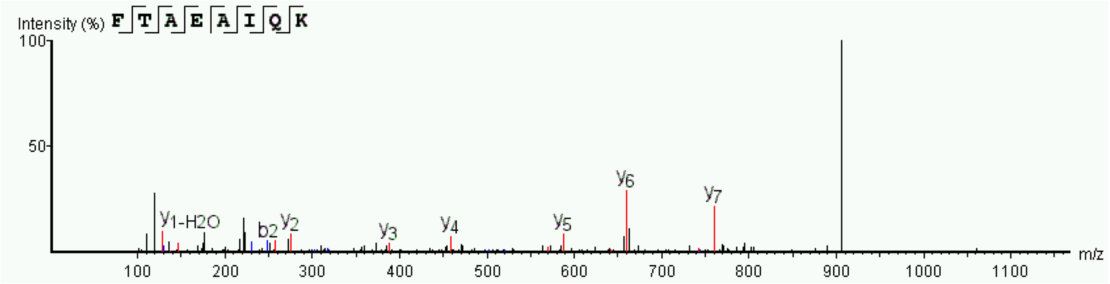

gi|328786356

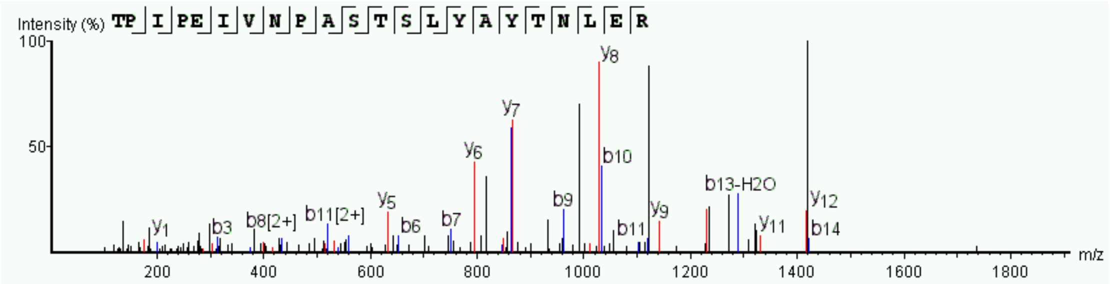

gi|571501581

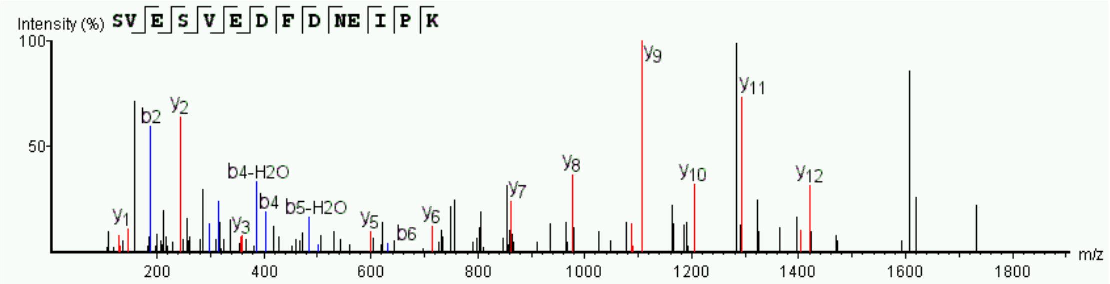

gi|328792943

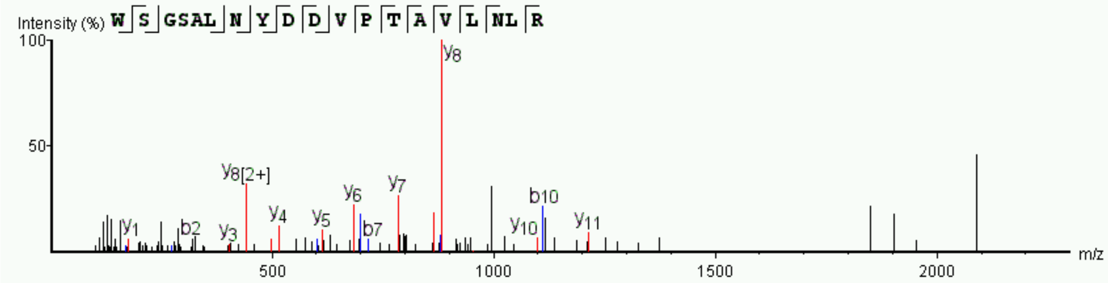

gi|571559017

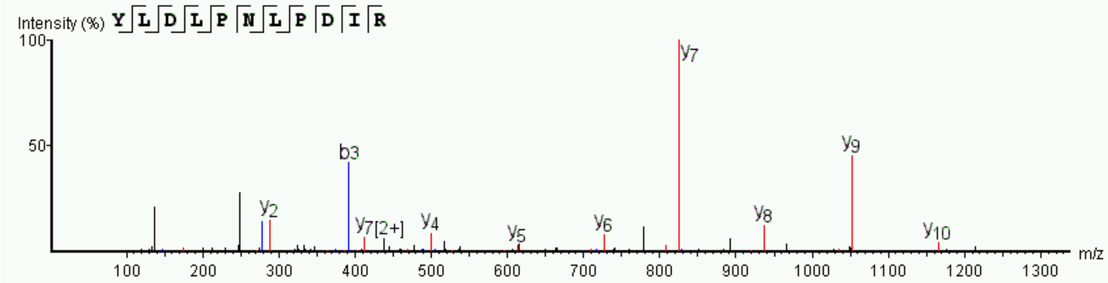

gi|571529256

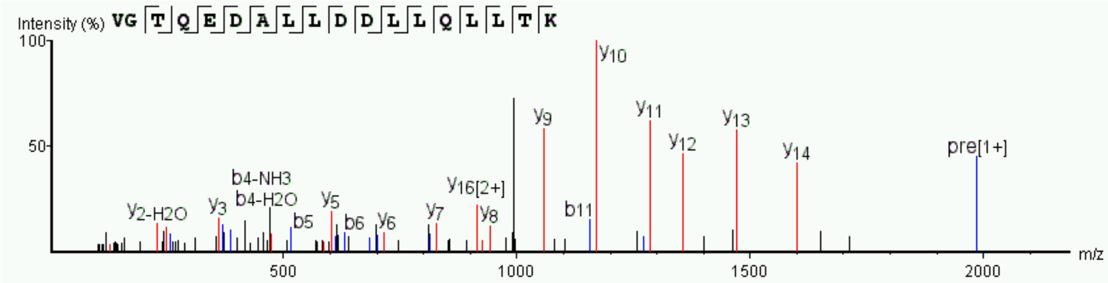

gi|571520744

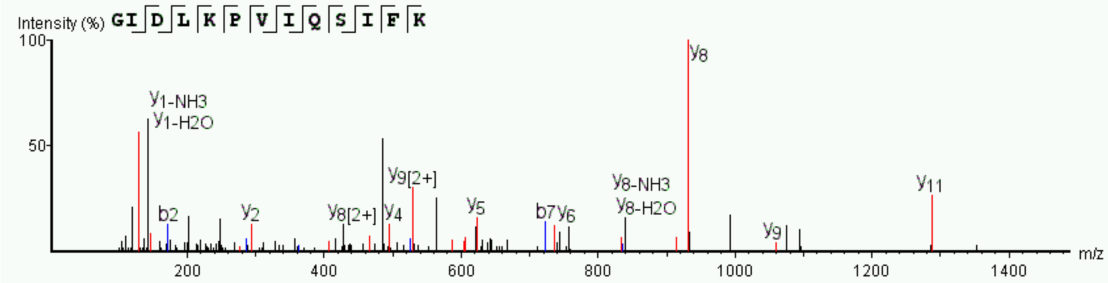

gi|328784535

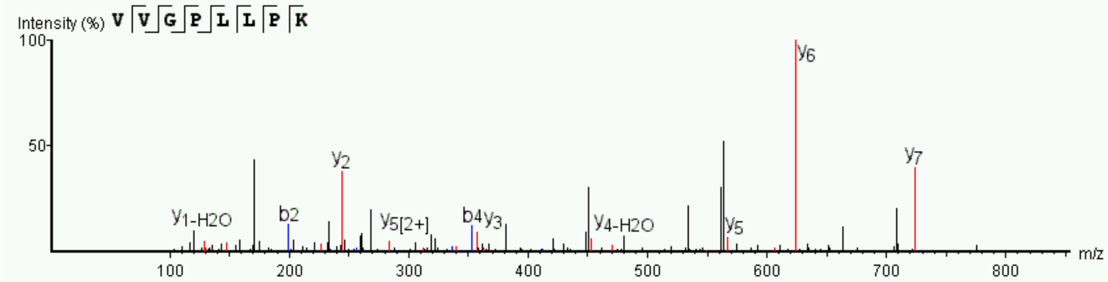

gi|328780847

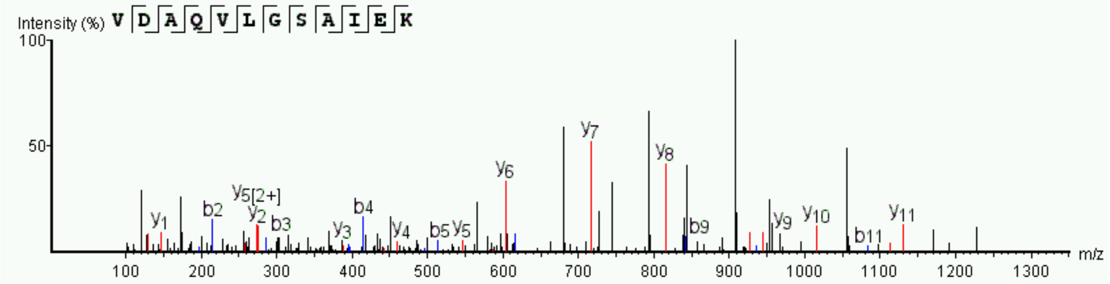

gi|571529312

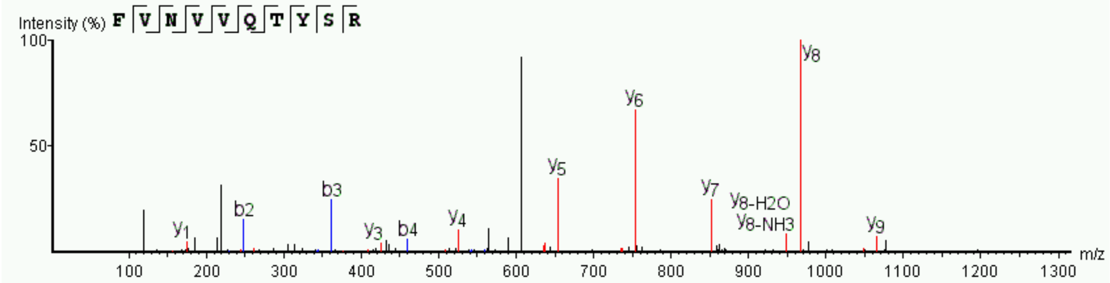

gi|571529309

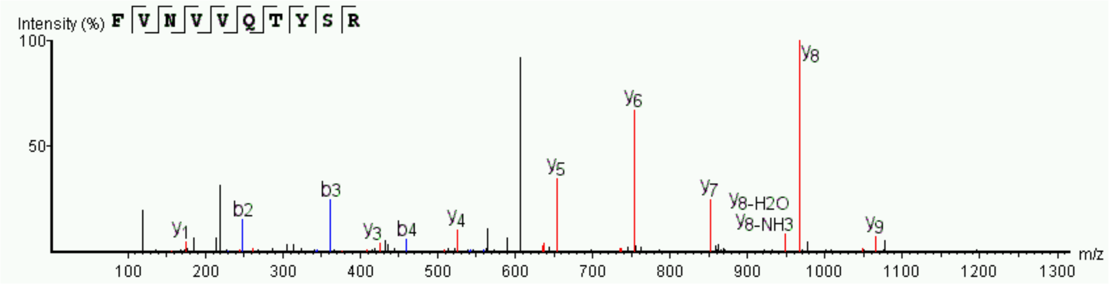

gi|328789317

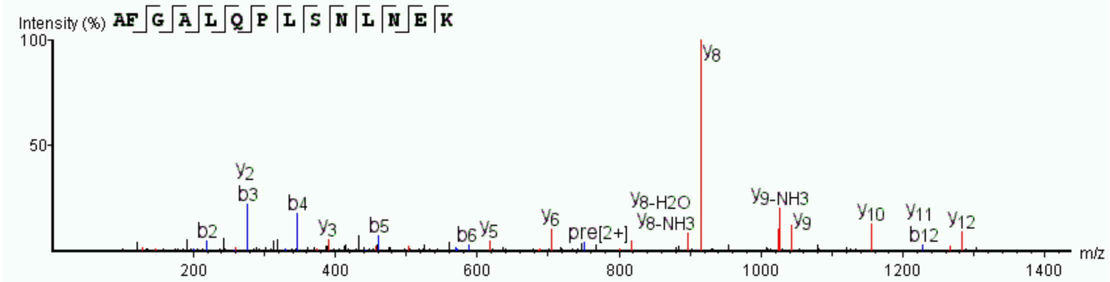

gi|571503693

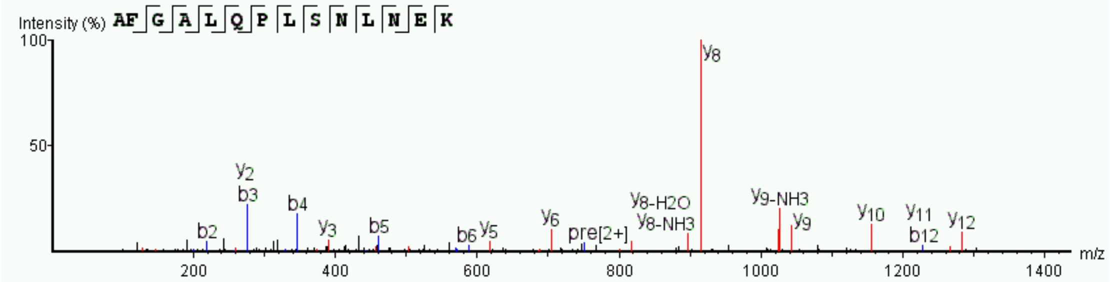

gi|571503690

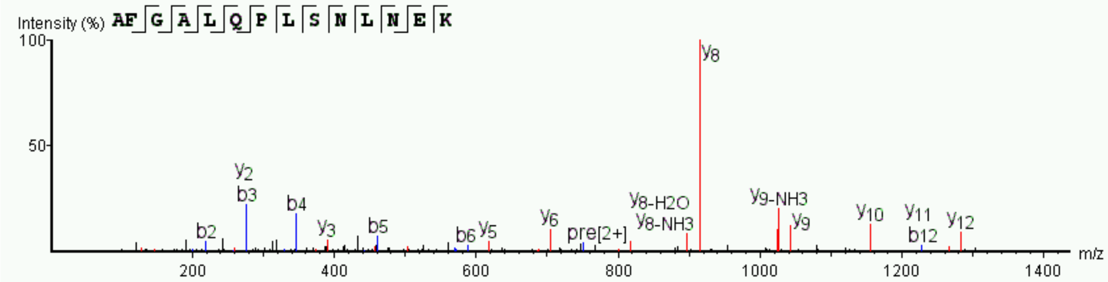

gi|571563070

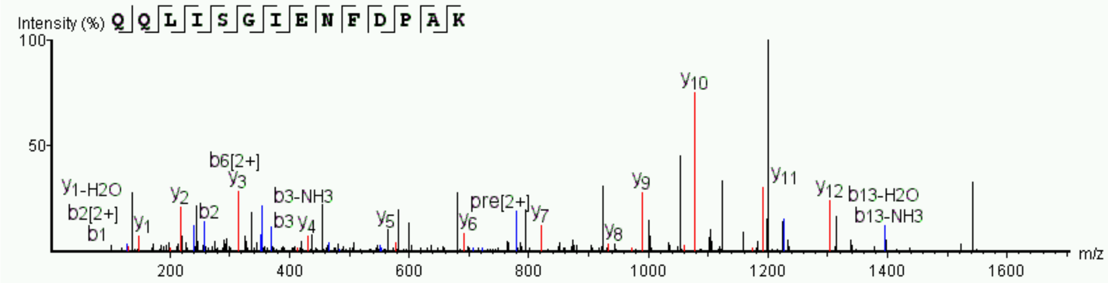

gi|571551216

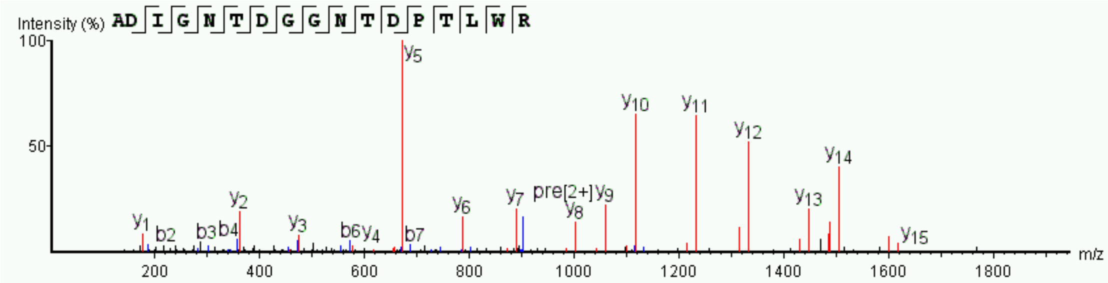

gi|571566031

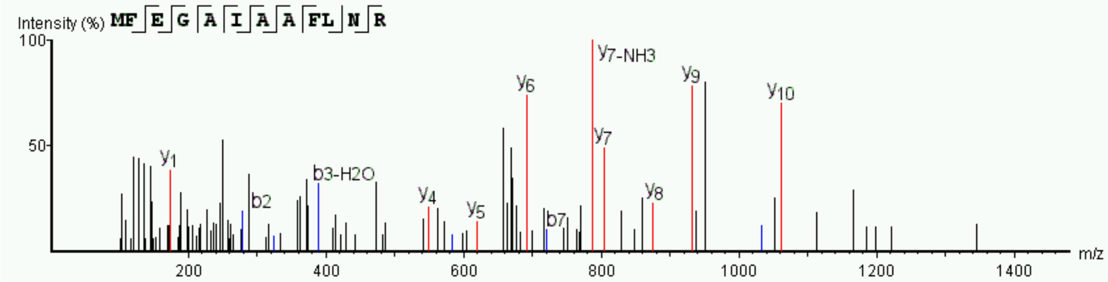

gi|571512347

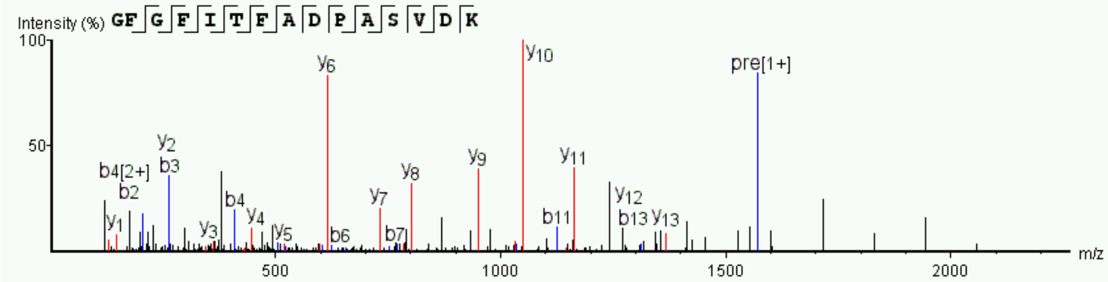

gi|571579097

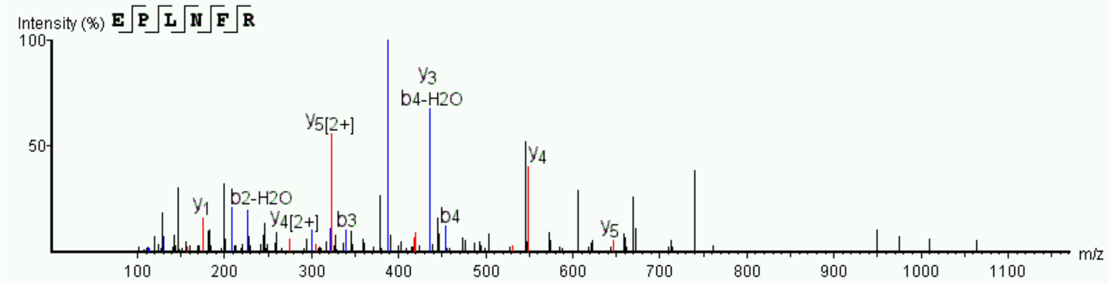

gi|328789366

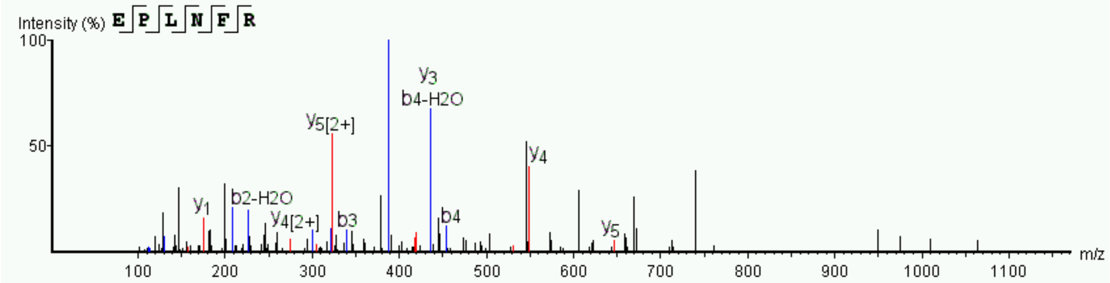

gi|571535244

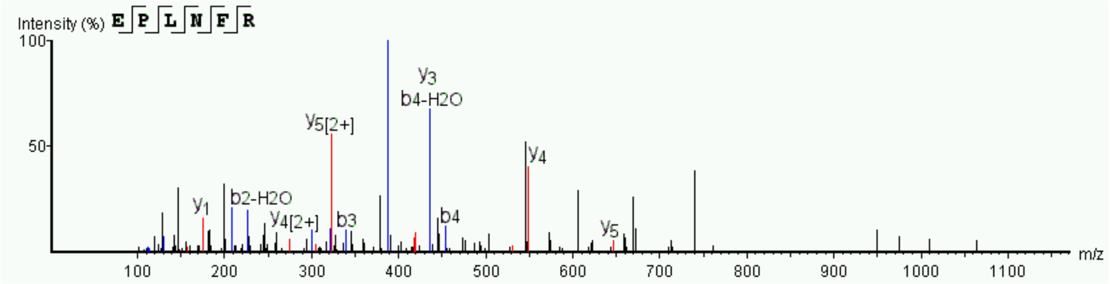

gi|571538420

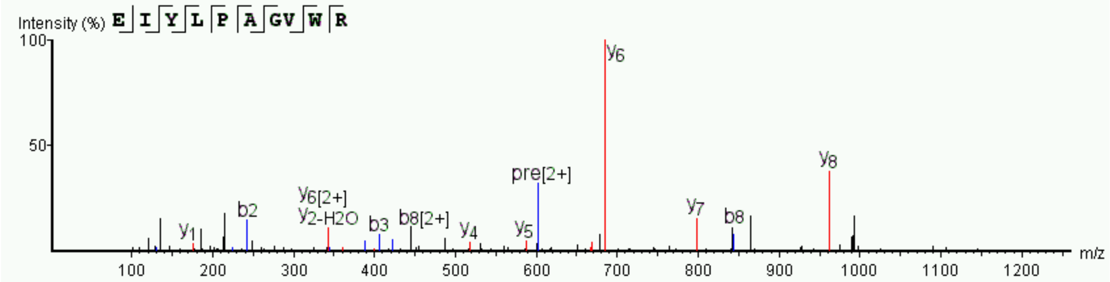

gi|571538417

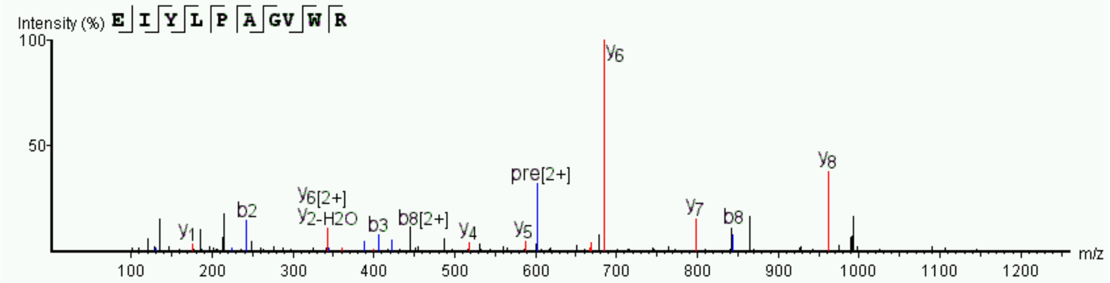

gi|110756134

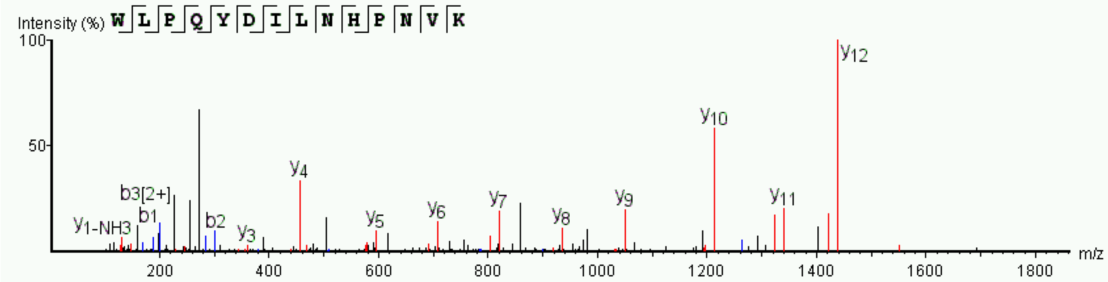

gi|48097928

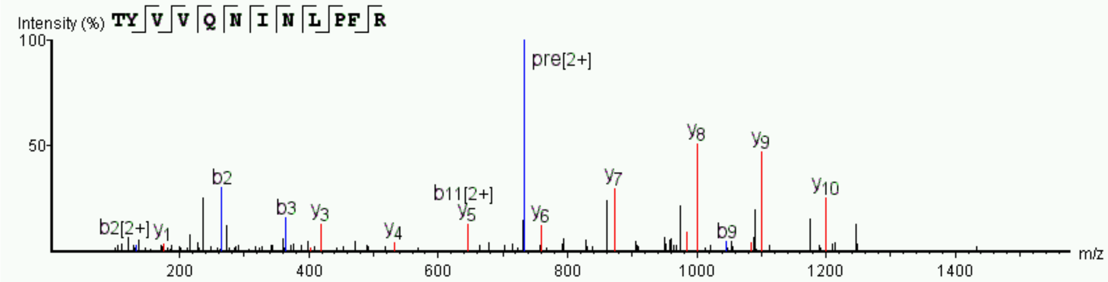

gi|571523648

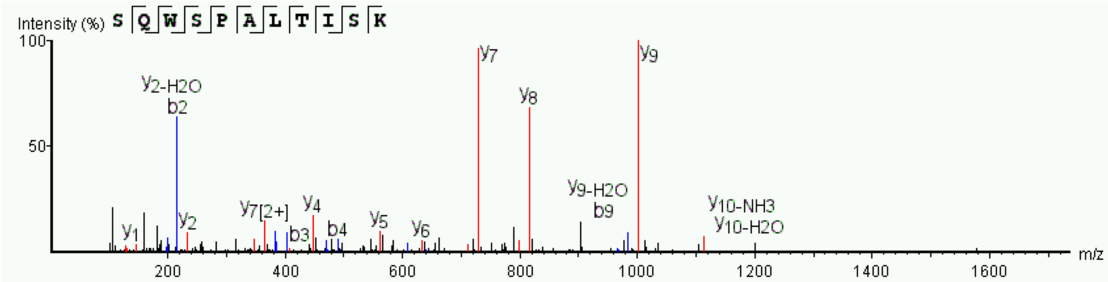

gi|328788813

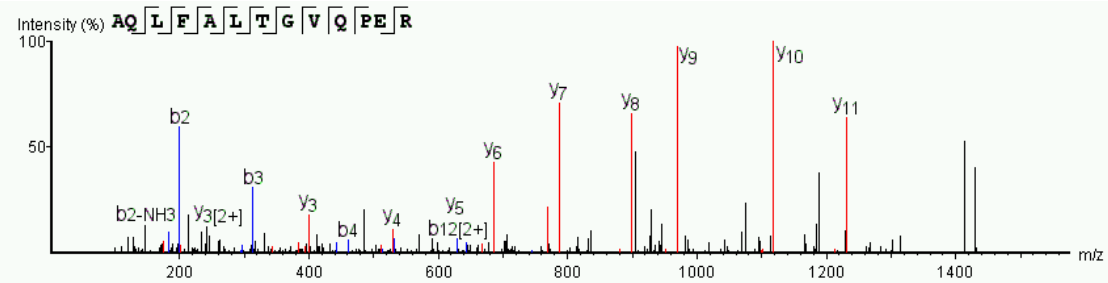

gi|571533610

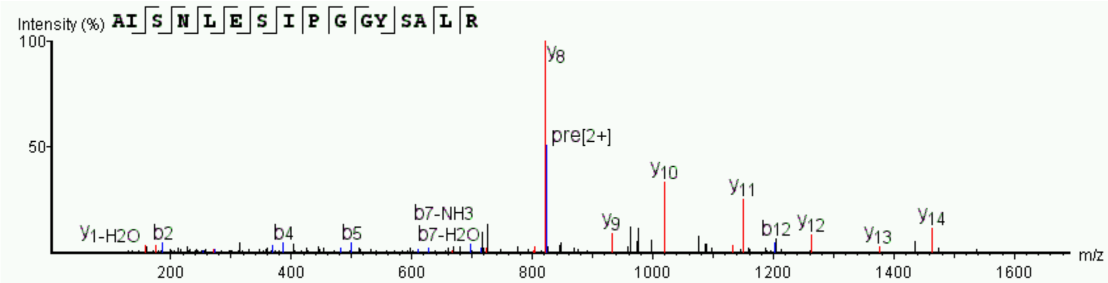

gi|571556157

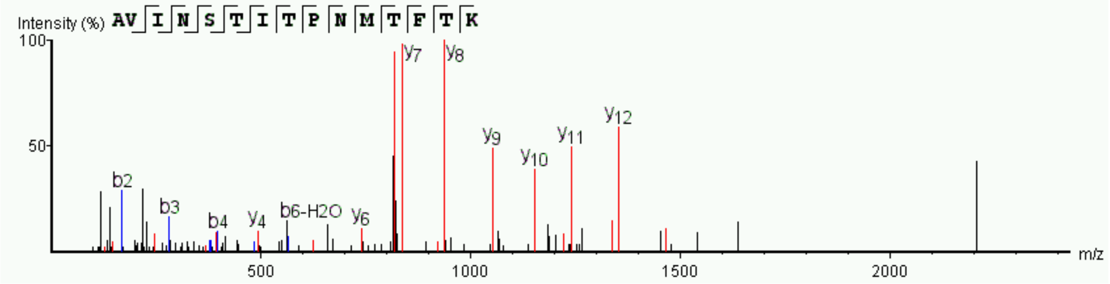

gi|66518218

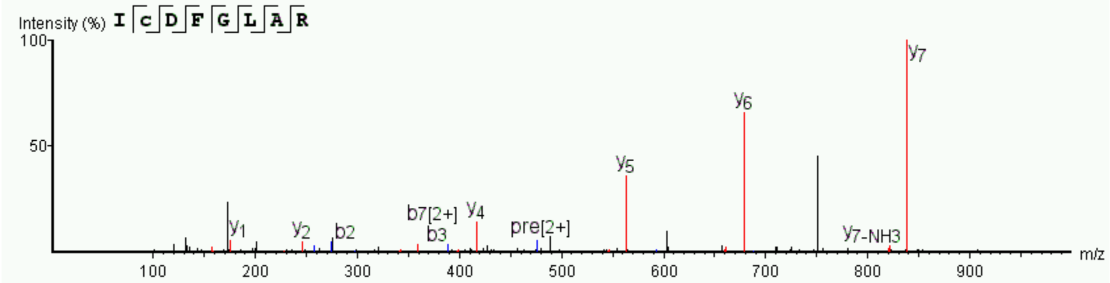

gi|571551750

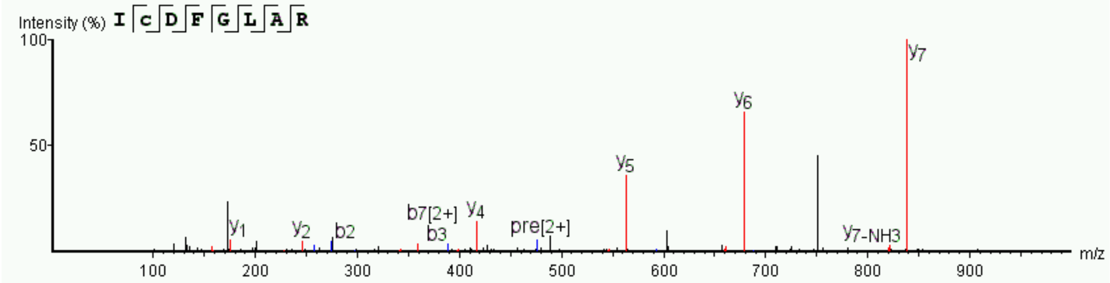

gi|571551746

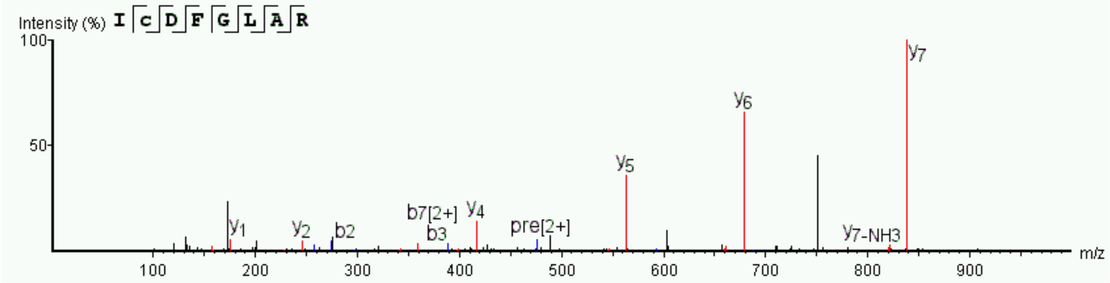

gi|571551742

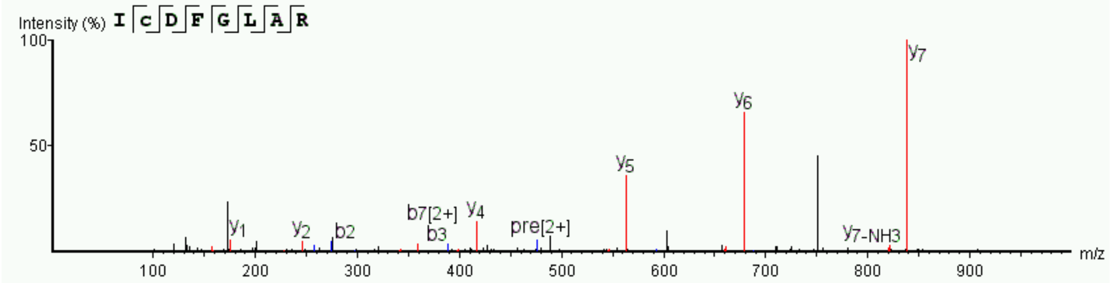

gi|571503372

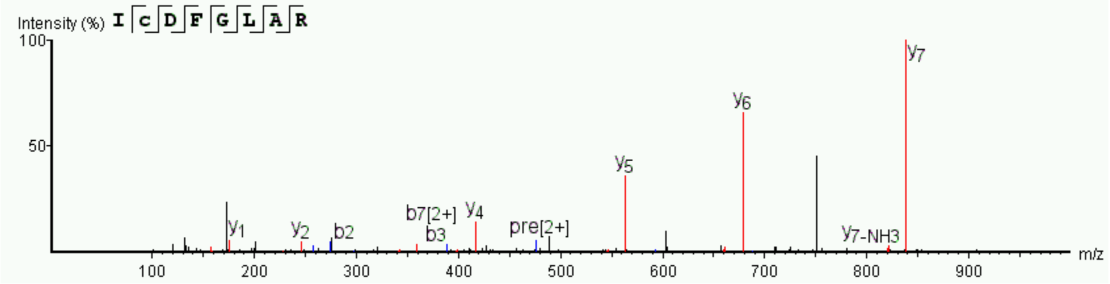

gi|571574298

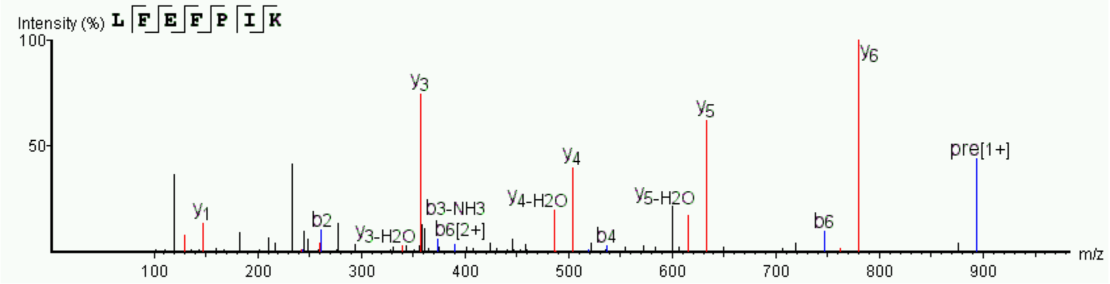

gi|571544693

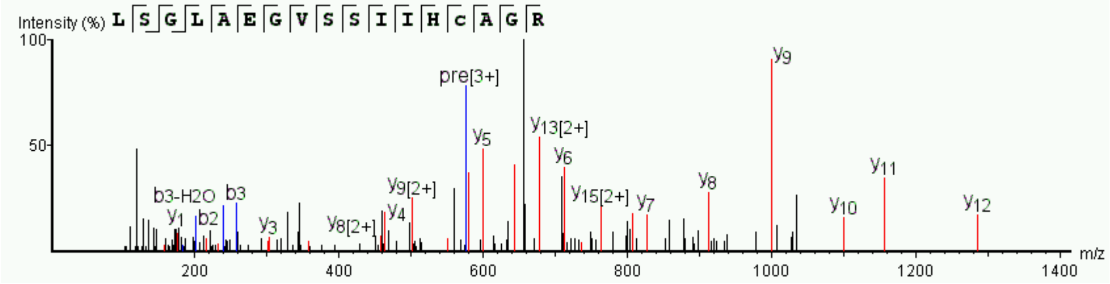

gi|571544690

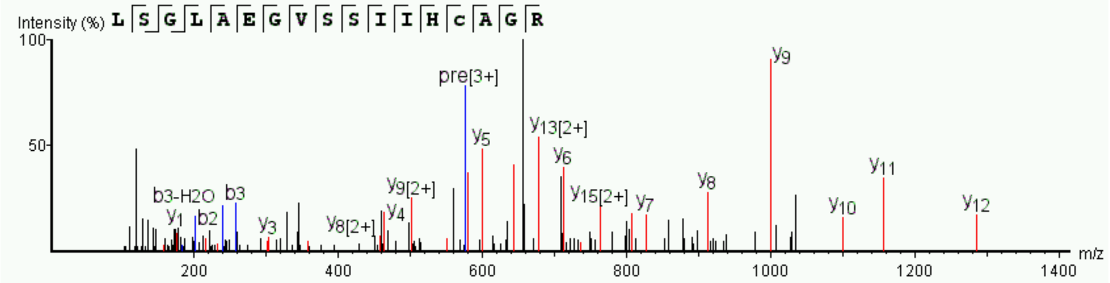

gi|571552571

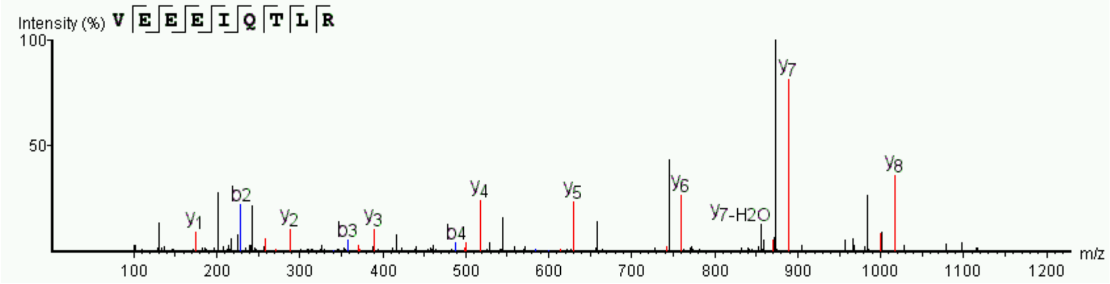

gi|571552567

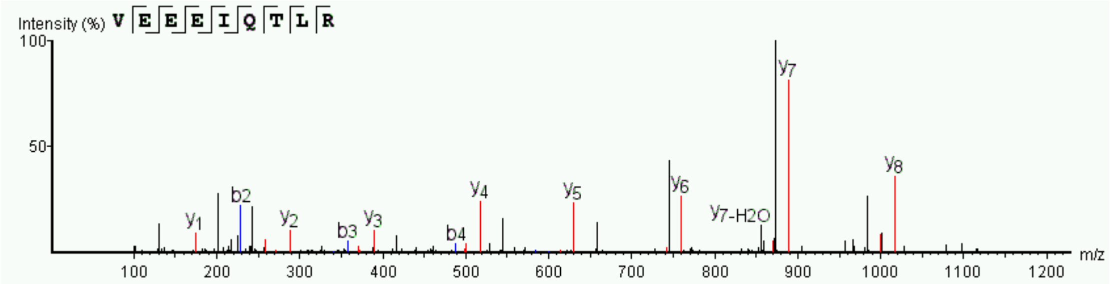

gi|328782269

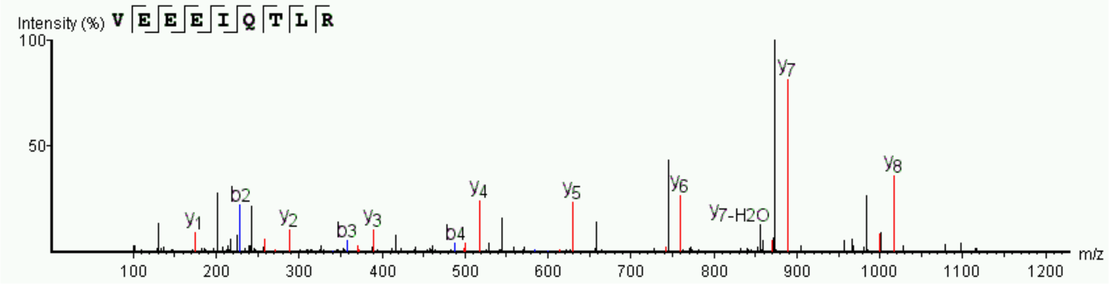

gi|571552435

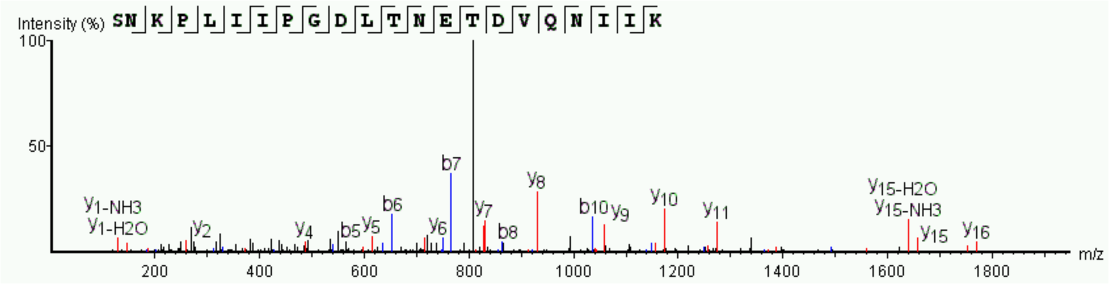

gi|571501487

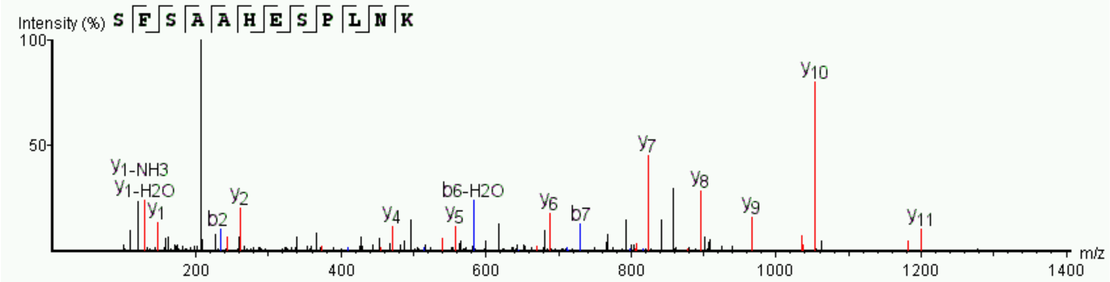

gi|110748900

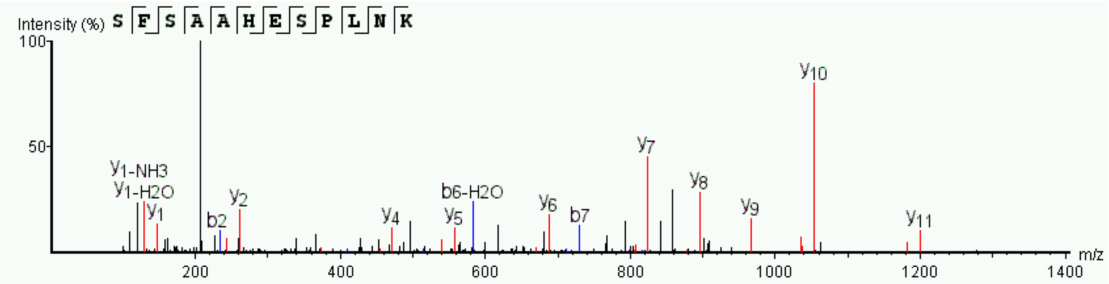

gi|66523789

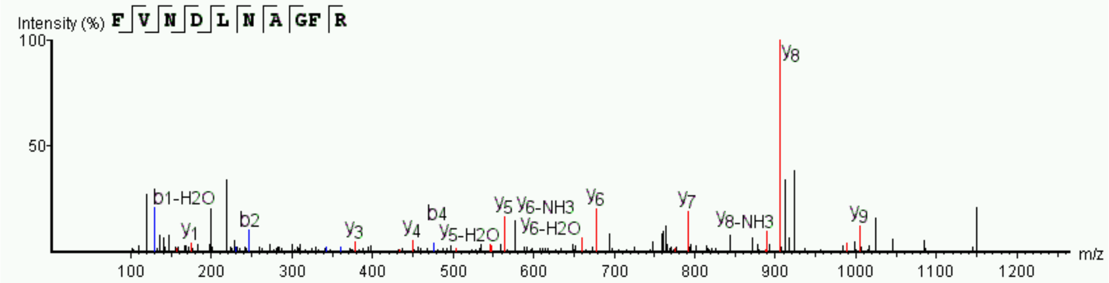

gi|66515987

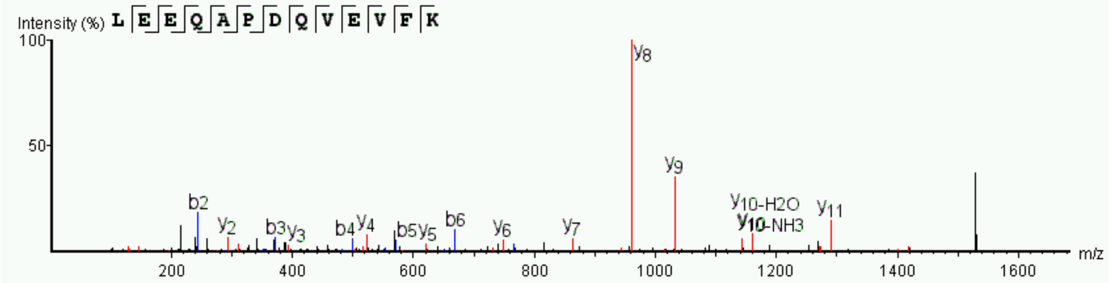

gi|571533551

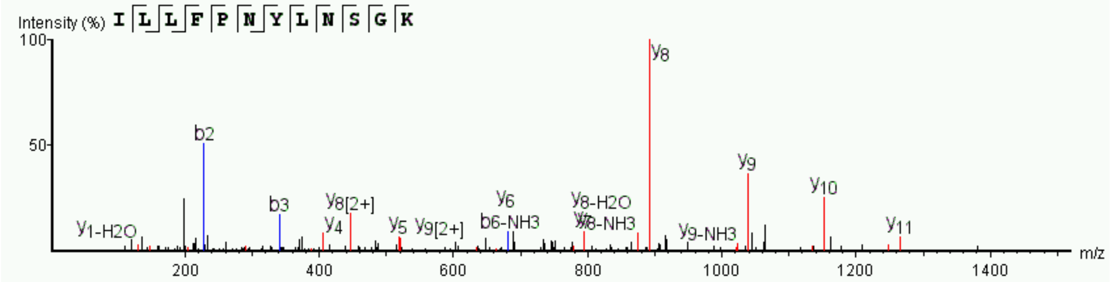

gi|328782257

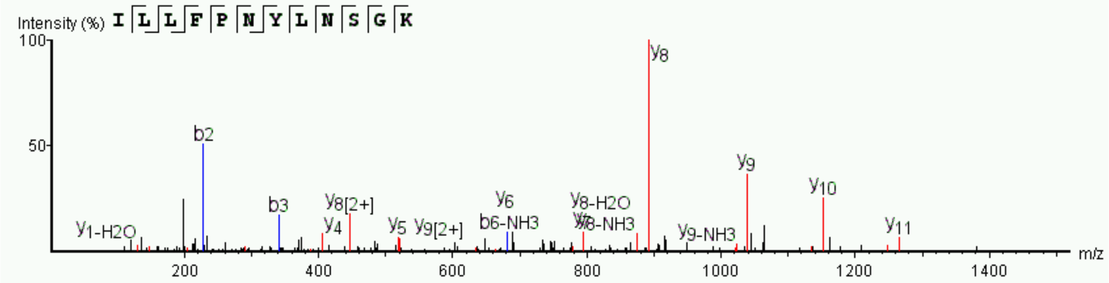

gi|571537266

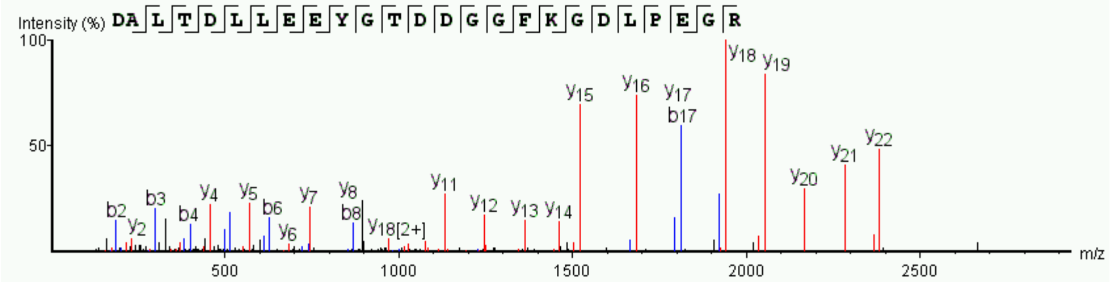

gi|571537262

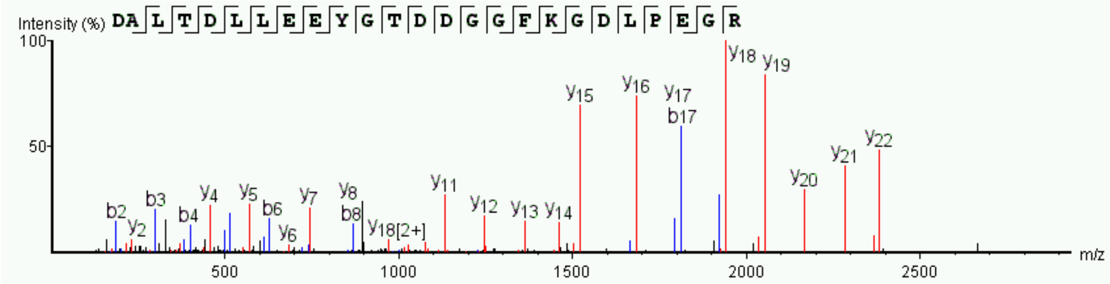

gi|571537256

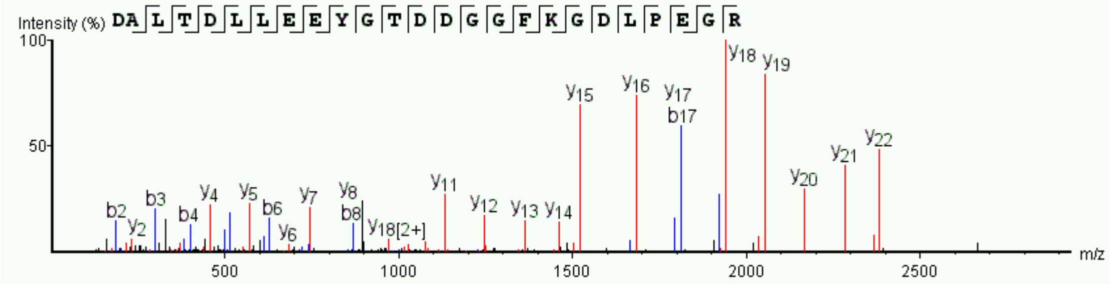

gi|571537253

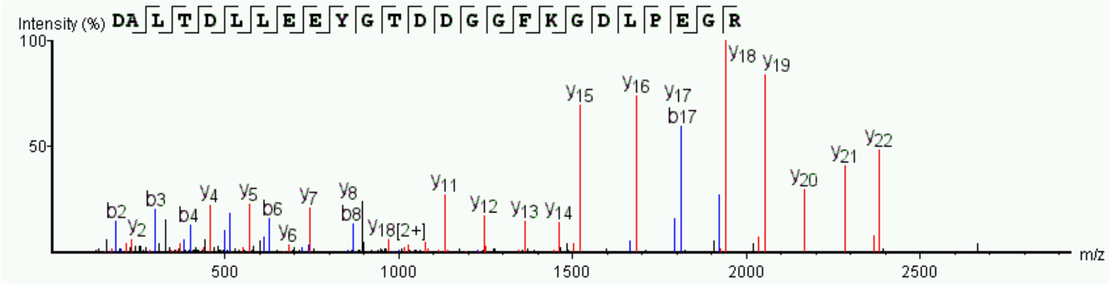

gi|571537249

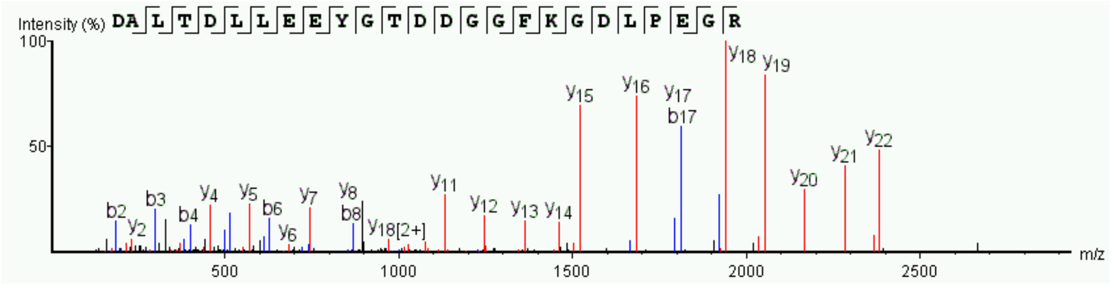

gi|571537245

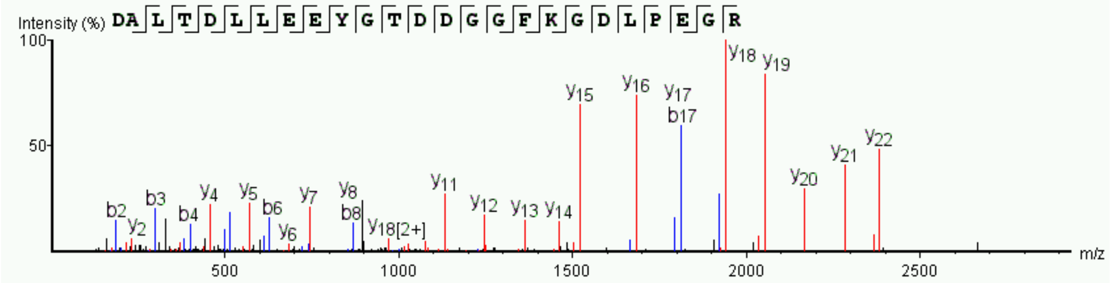

gi|571537241

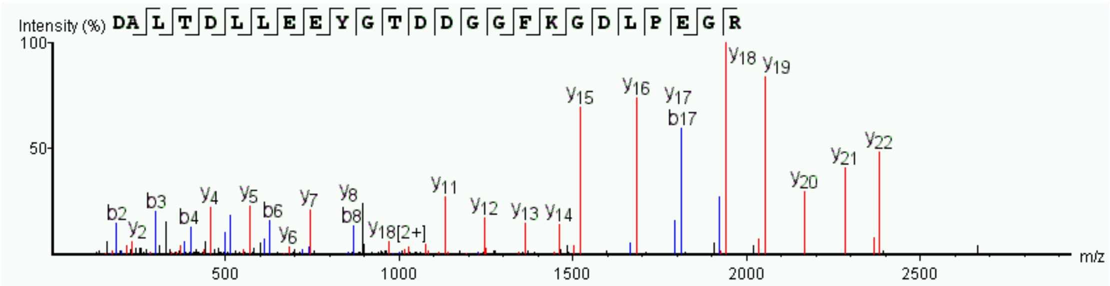

gi|328775950

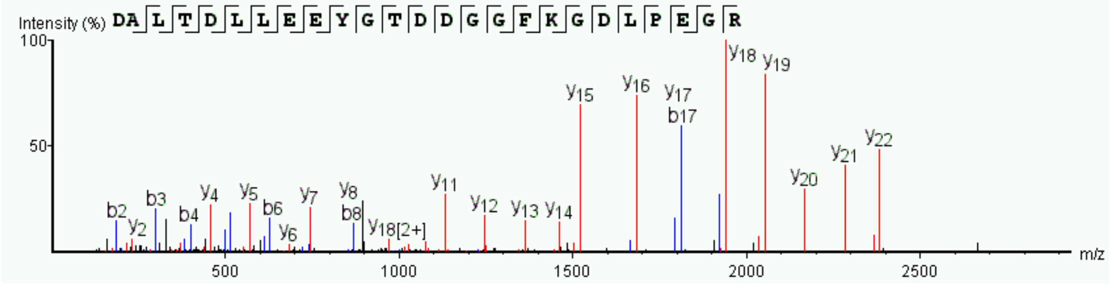

gi|571562620

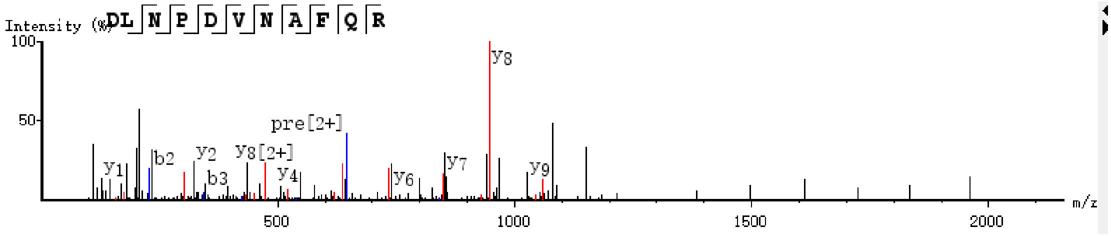

gi|66507736

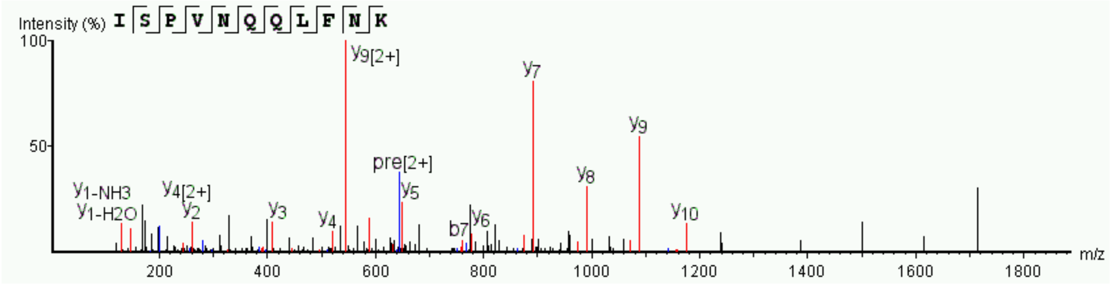

gi|66556835

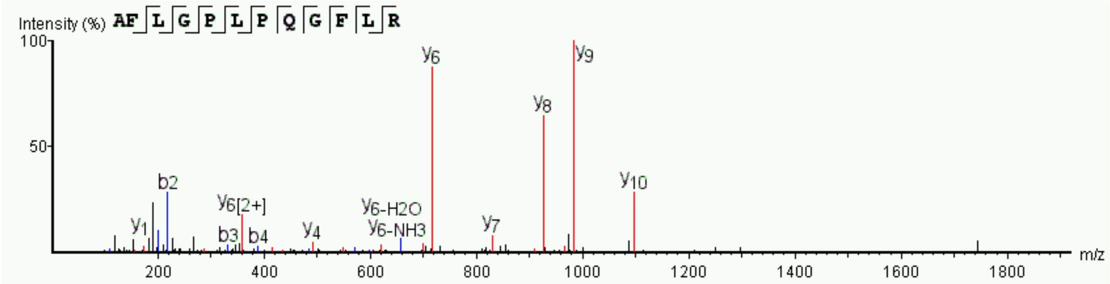

gi|328784195

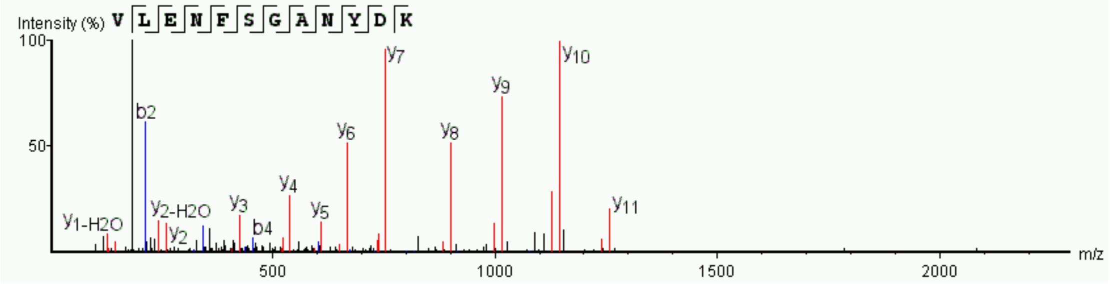

gi|328778481

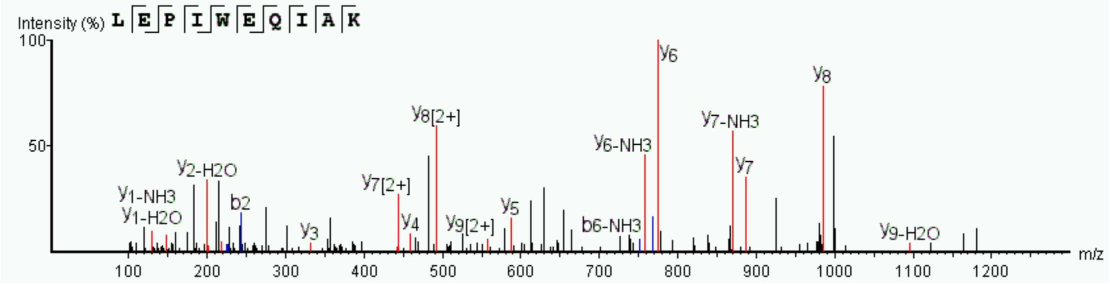

gi|571574967

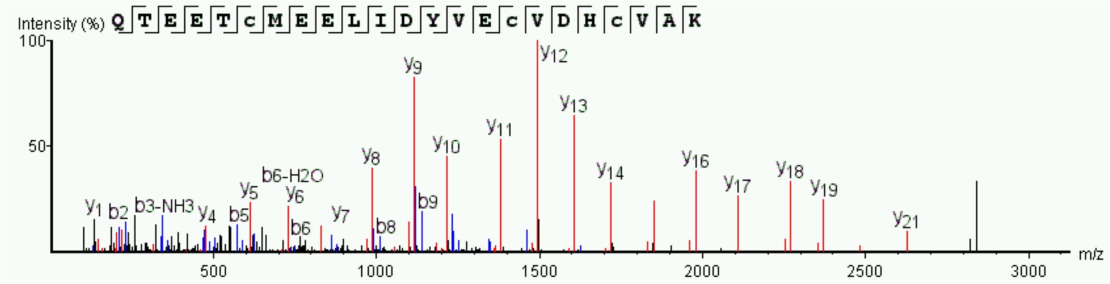

gi|328785093

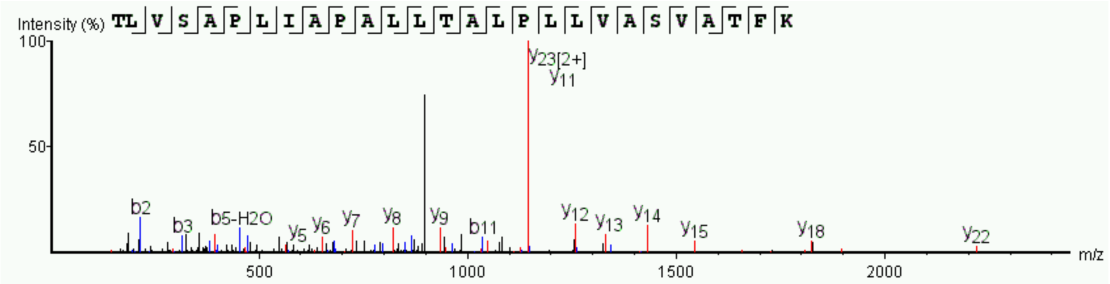

gi|66524811

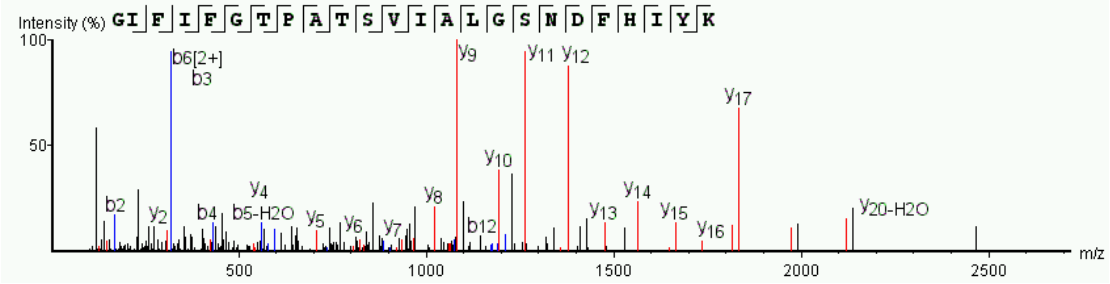

gi|571575127

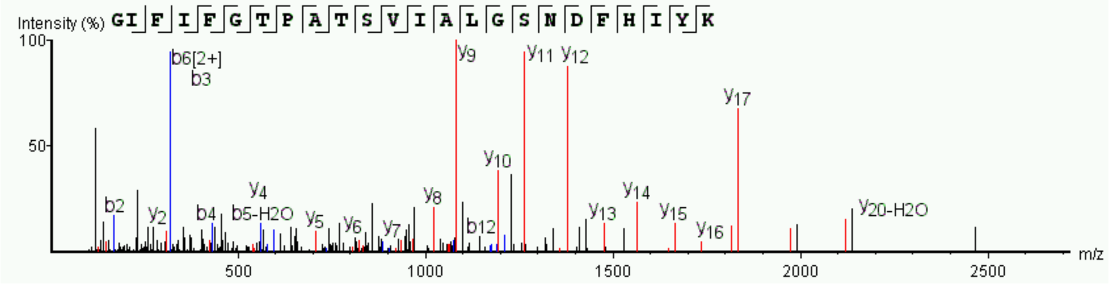

gi|66504030

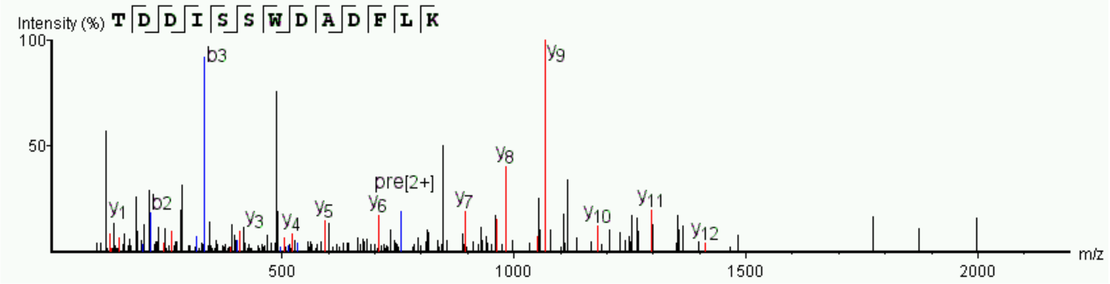

gi|571526645

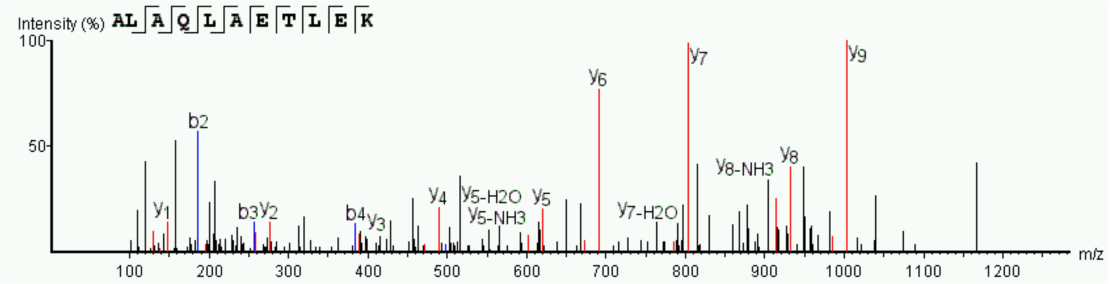

gi|571515131

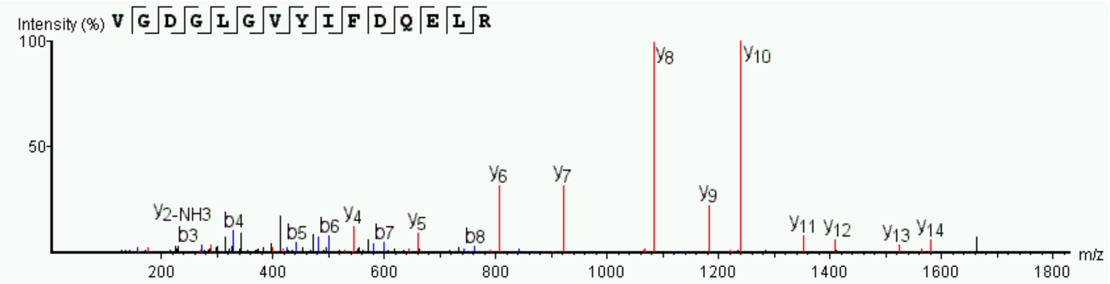

gi|571575549

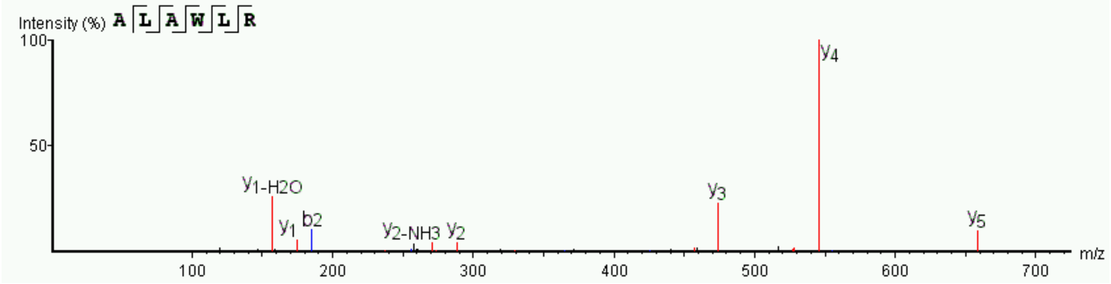

gi|571575573

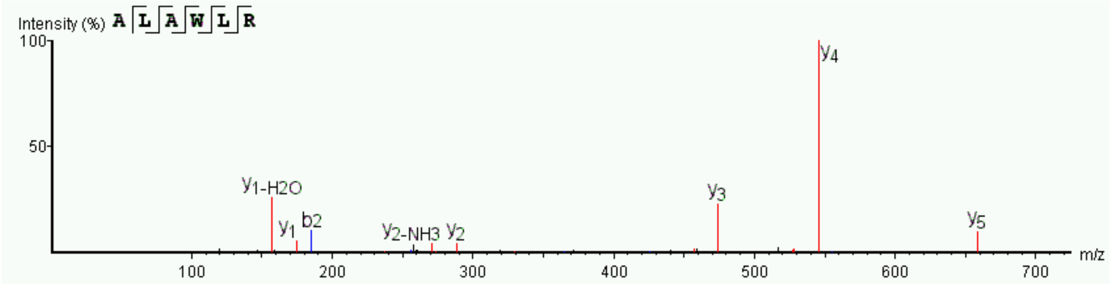

gi|571575571

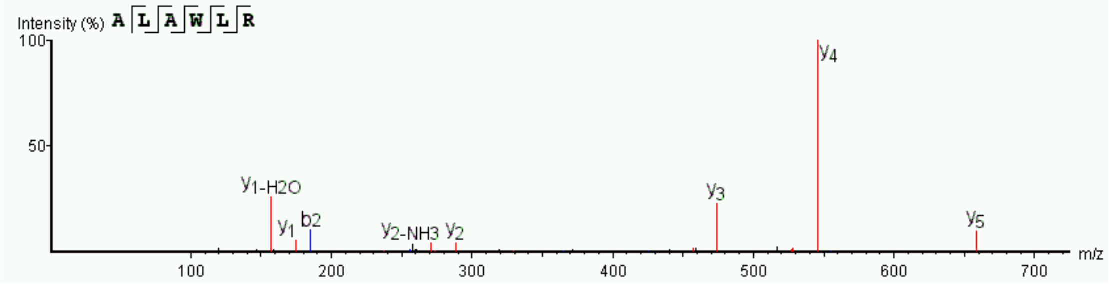

gi|571560348

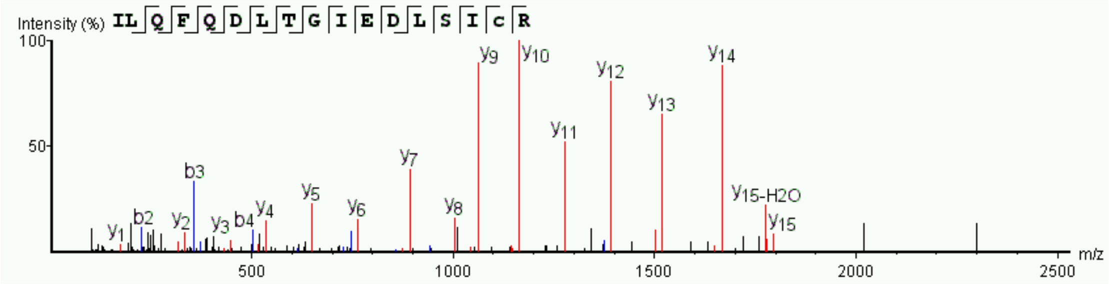

gi|571547726

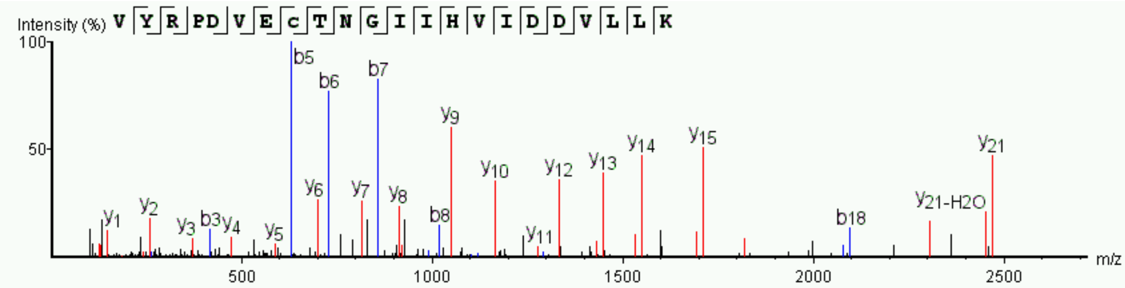

gi|571547729

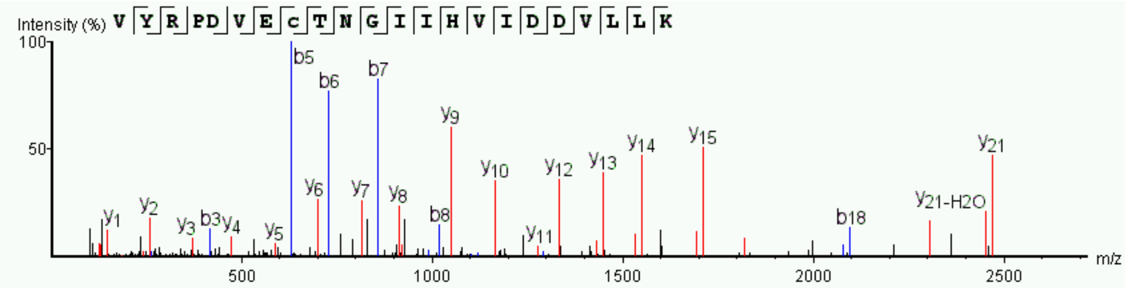

gi|571532722

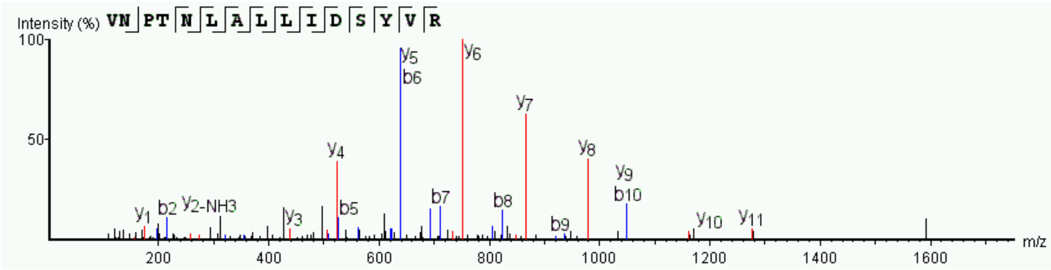

gi|571532725

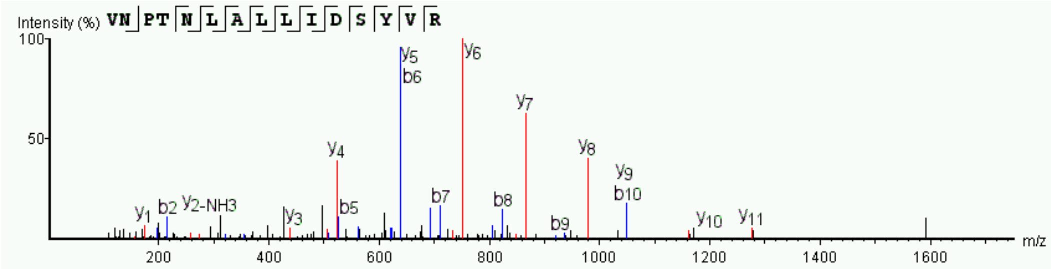

gi|571532729

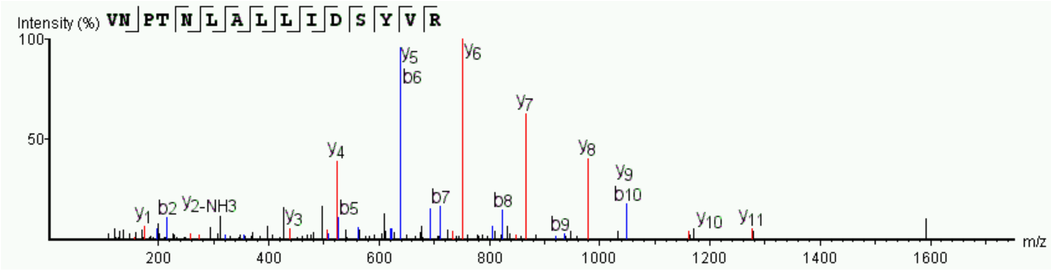

gi|571532734

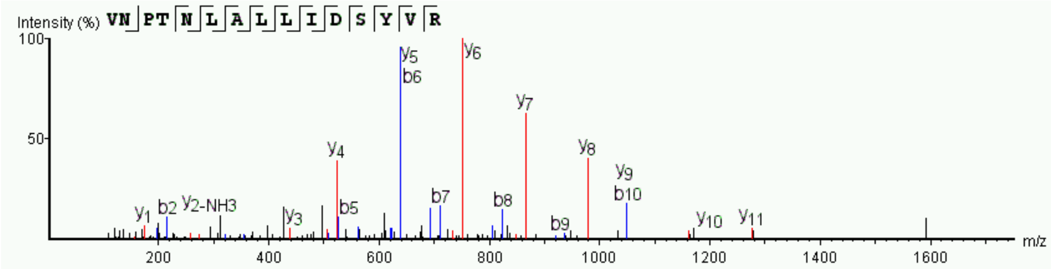

gi|571532738

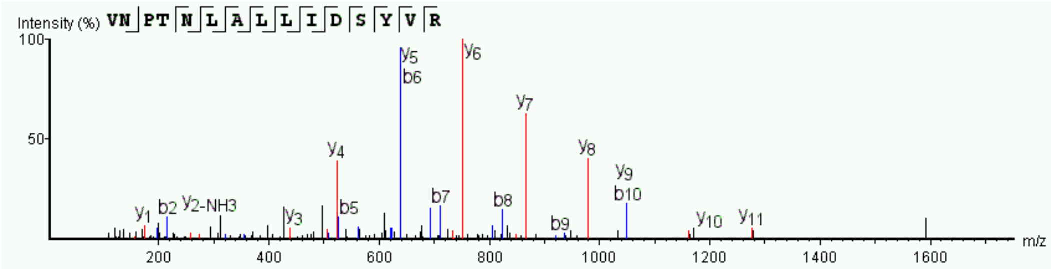

gi|571532741

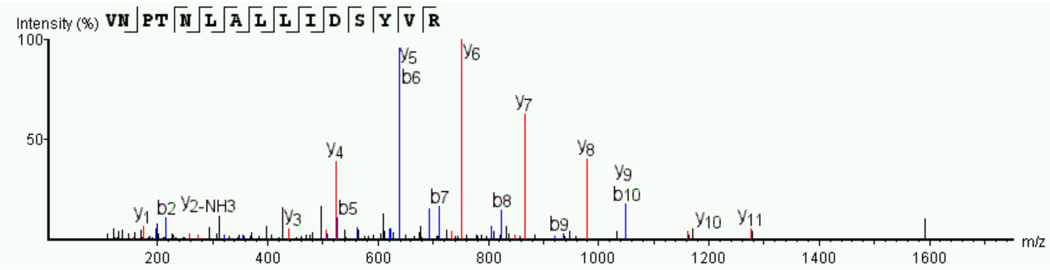

gi|328779207

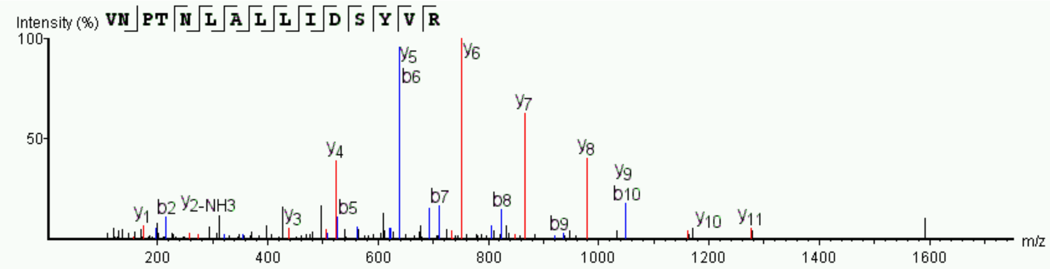

gi|571510489

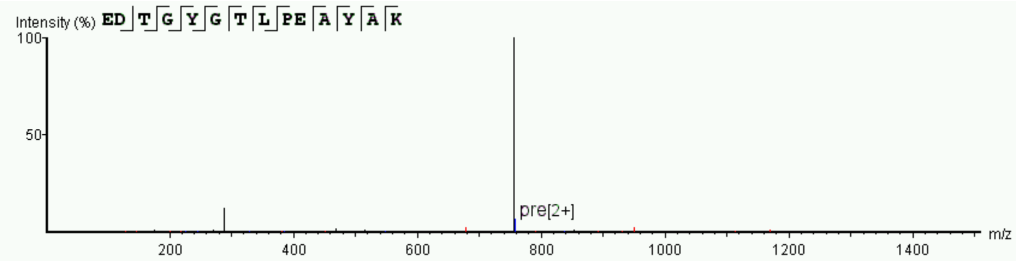

gi|571510492

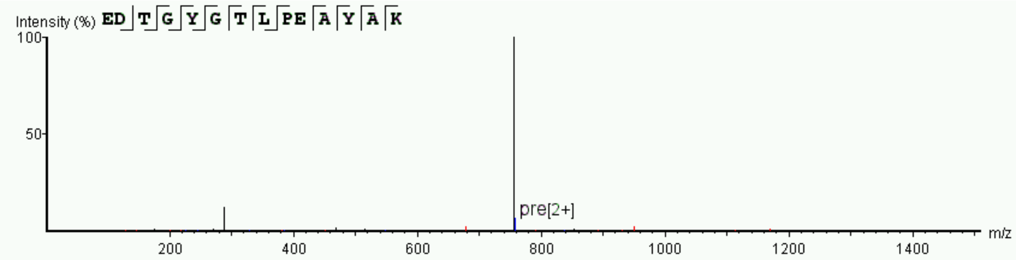

gi|571510495

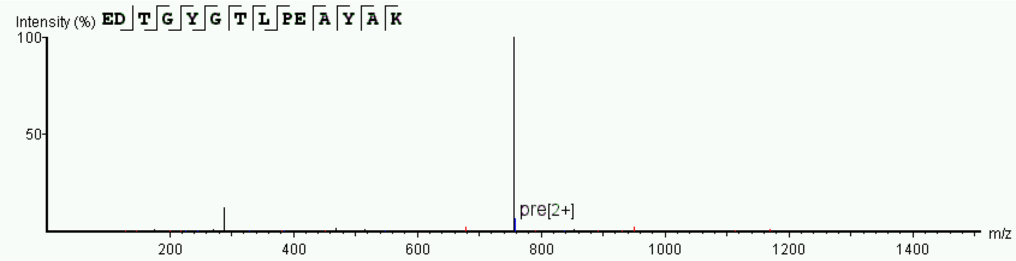

gi|571510498

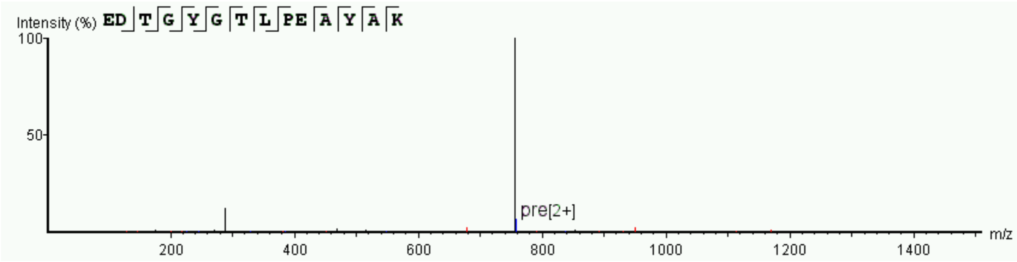

gi|571510502

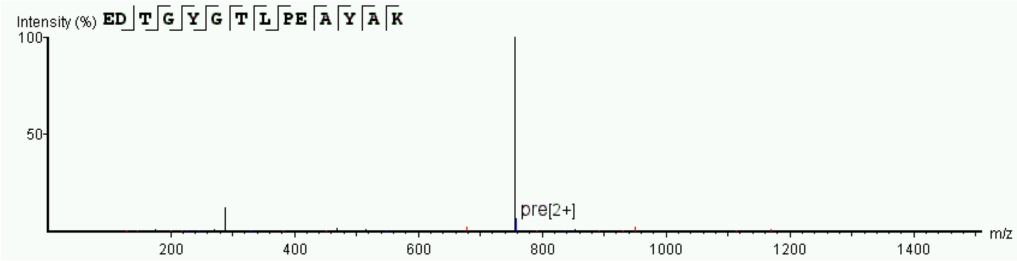

gi|571510506

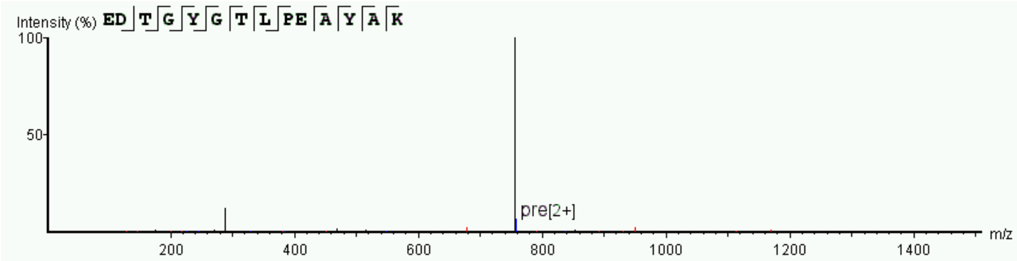

gi|571510510

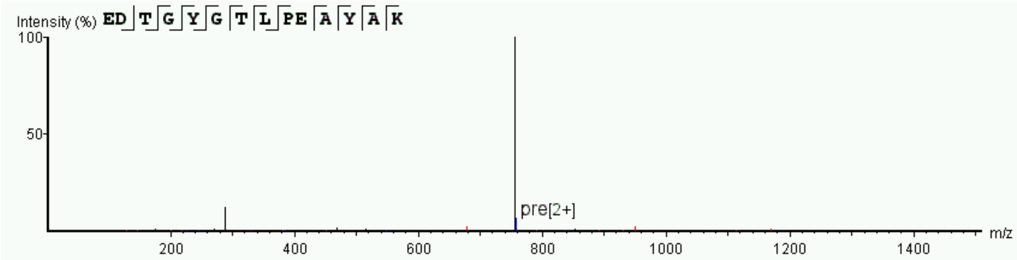

gi|328781812

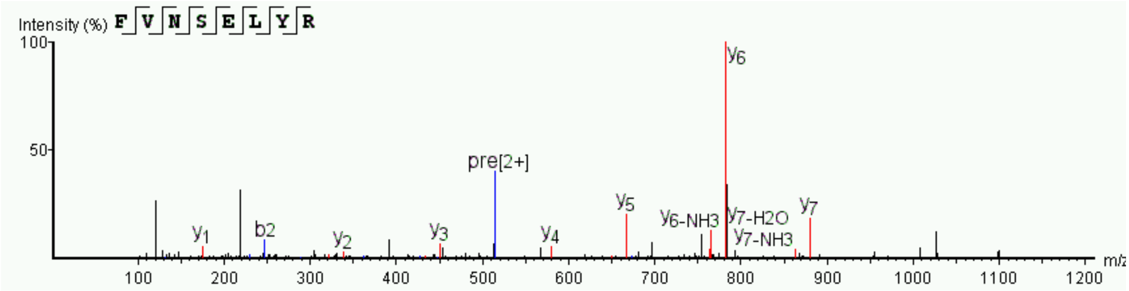

gi|571527387

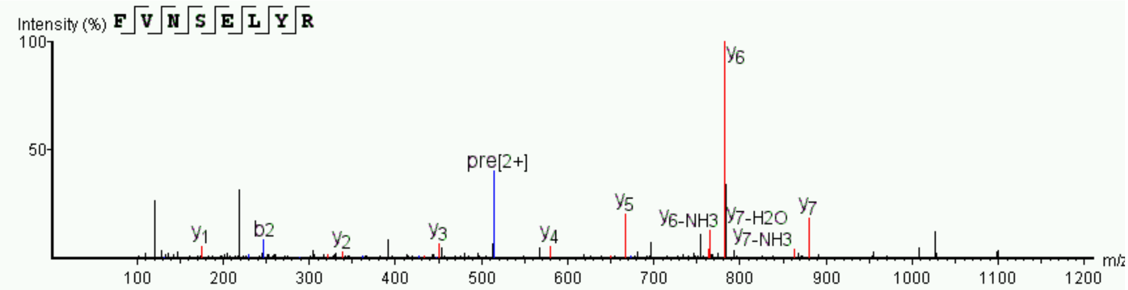

gi|571527394

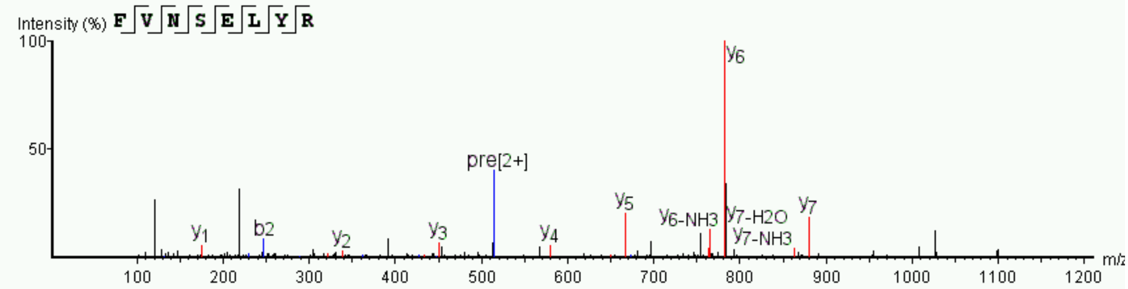

gi|571527397

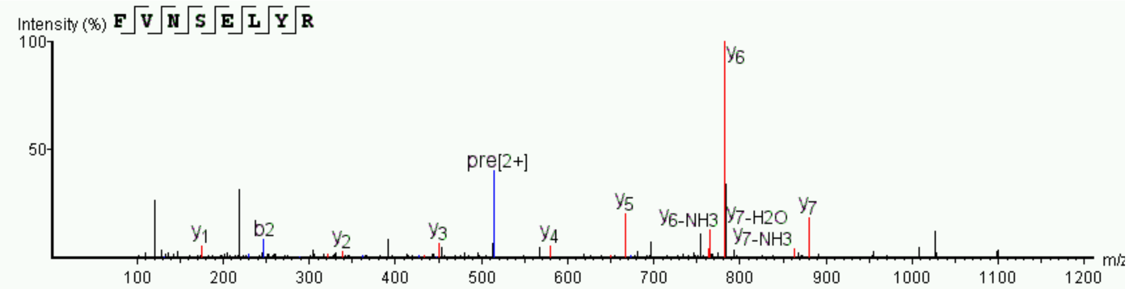

gi|571511272

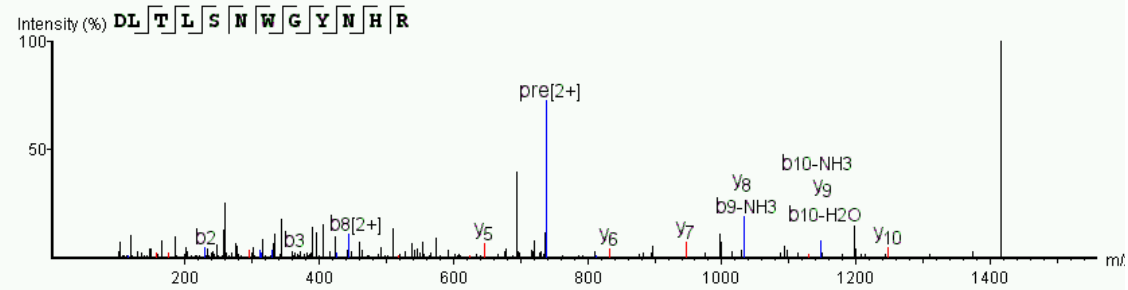

gi|571511275

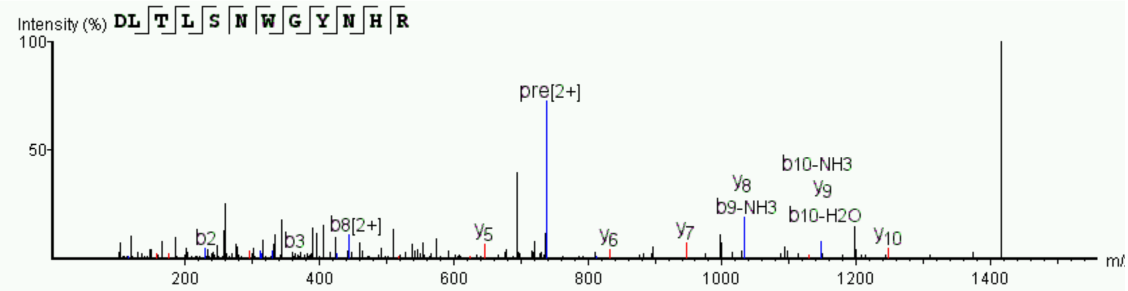

gi|571511278

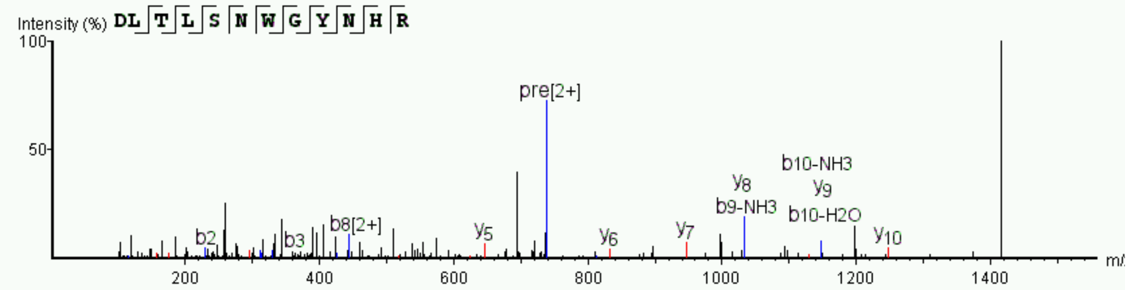

gi|571511281

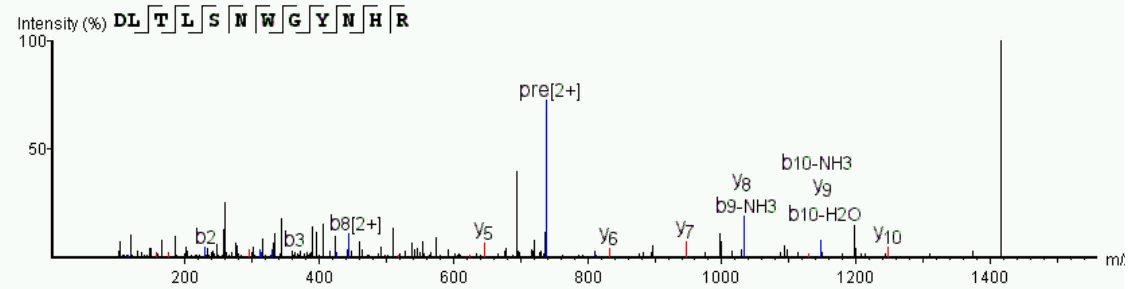

gi|571511284

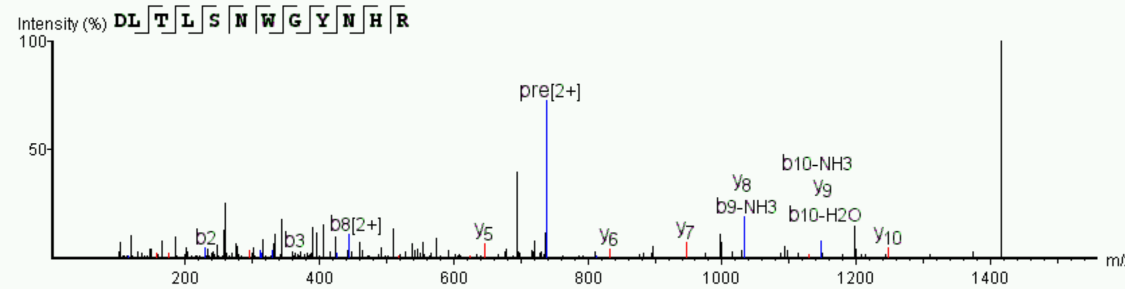

gi|571511287

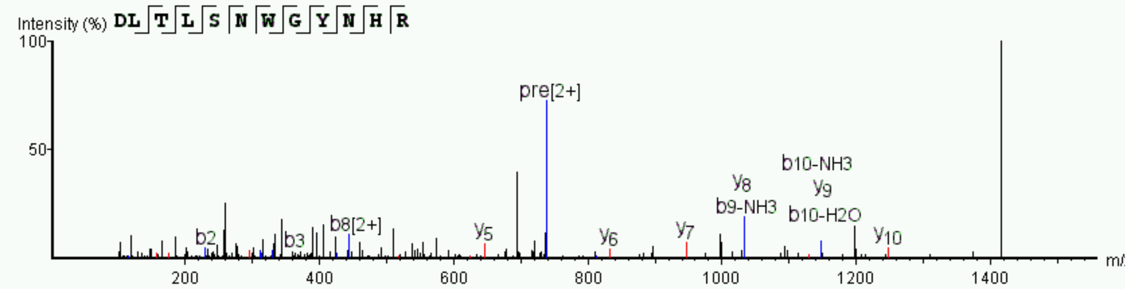

gi|571511290

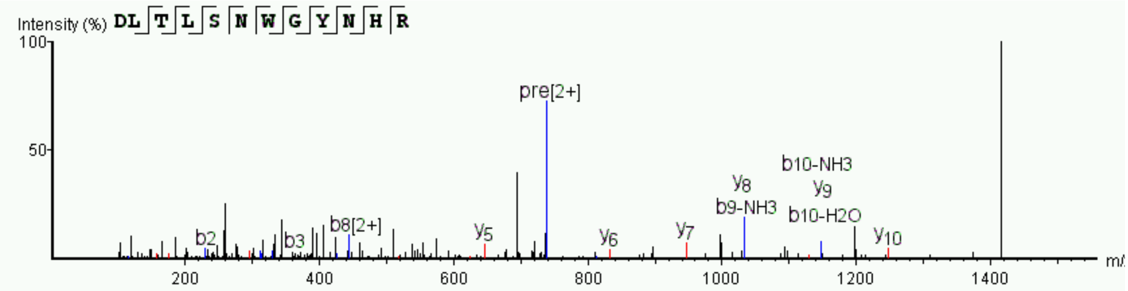

gi|328778104

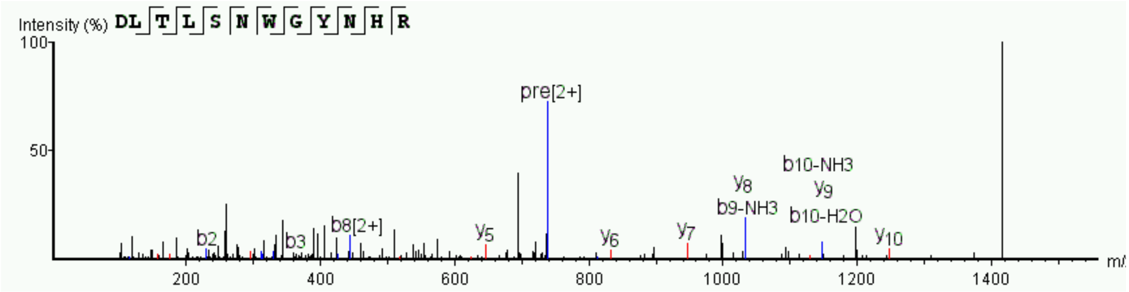

gi|571527707

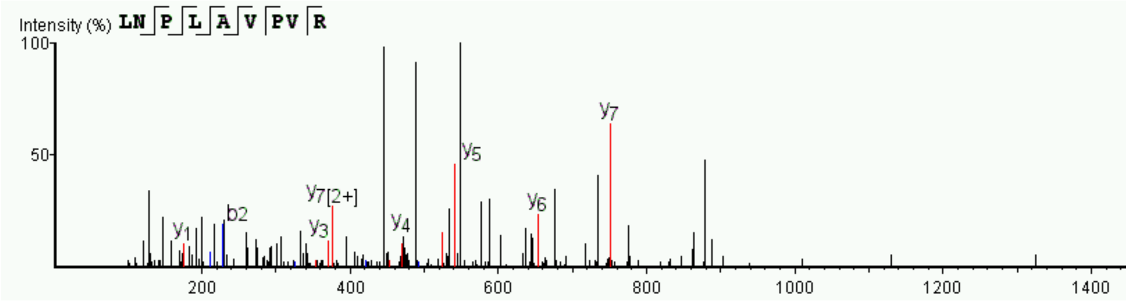

gi|328781704

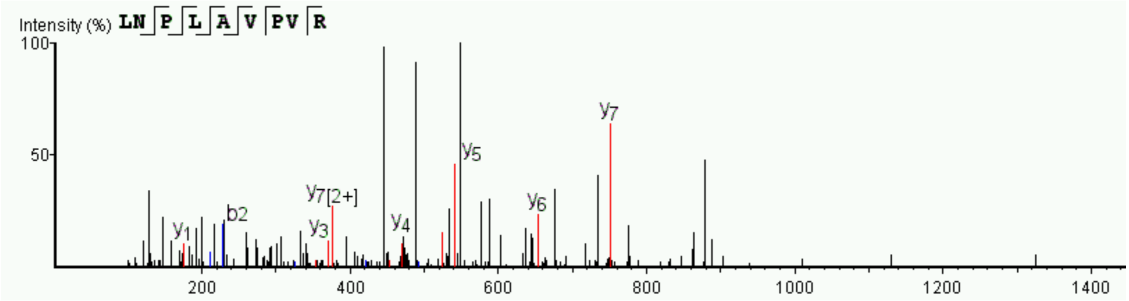

gi|328777414

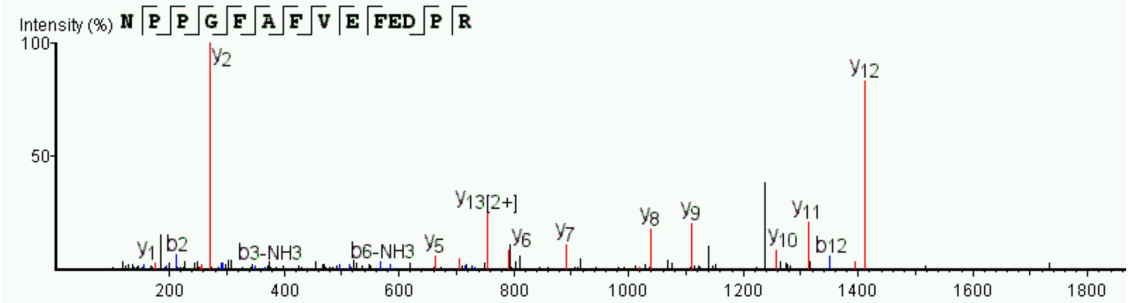

gi|571500029

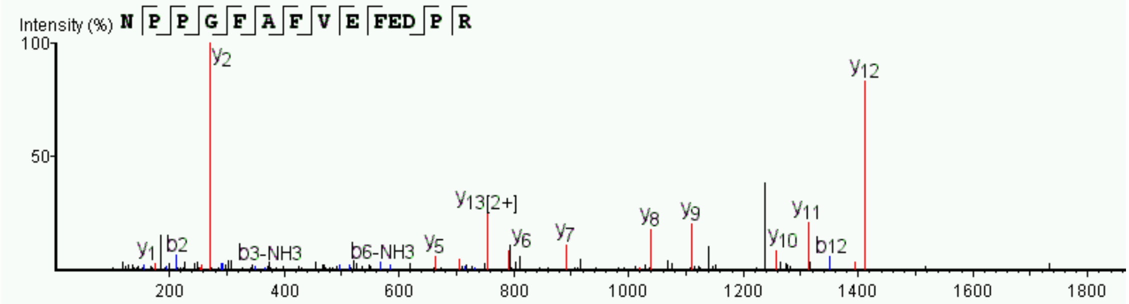

gi|571500035

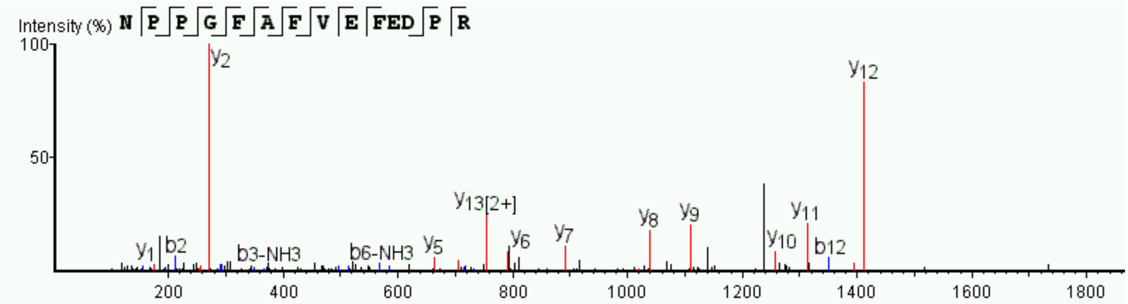

gi|571500039

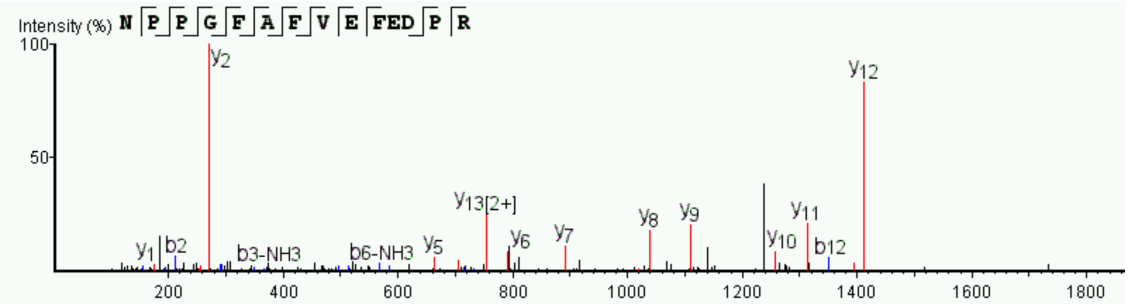

gi|571500044

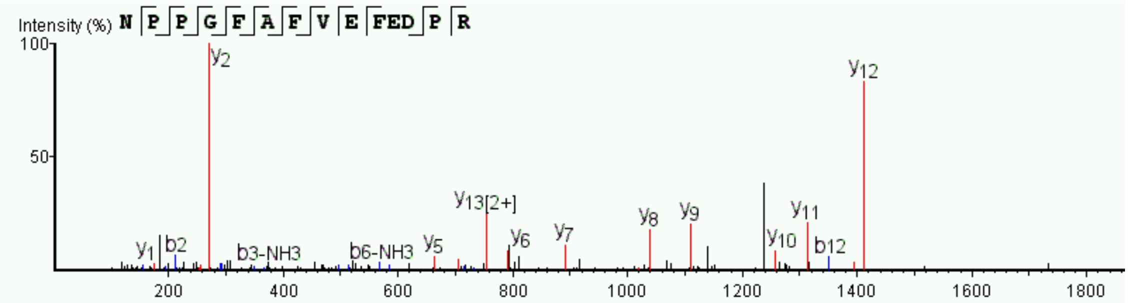

gi|328777412

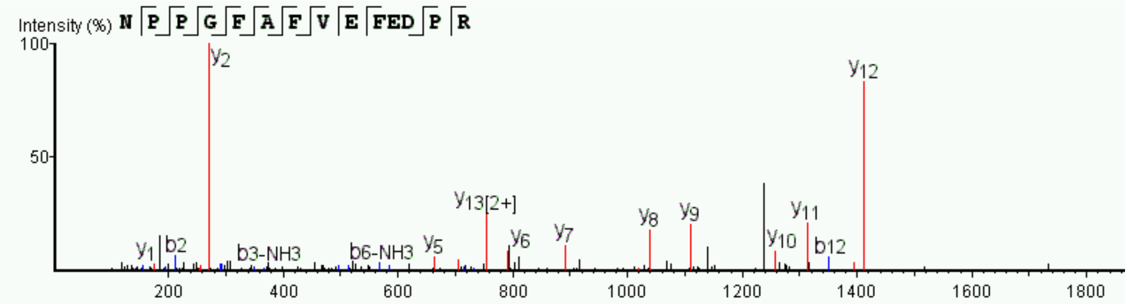

gi|571512336

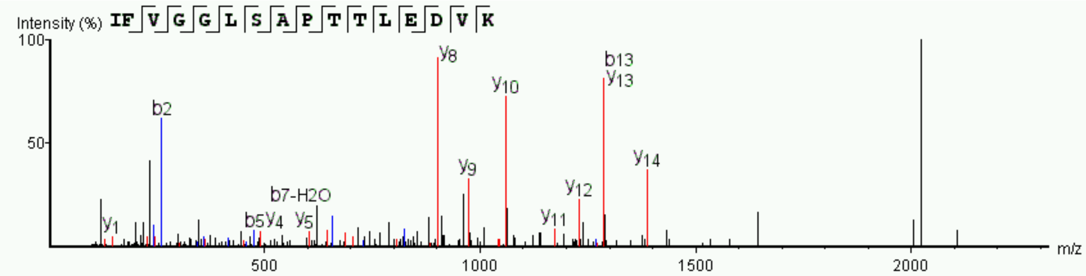

gi|571512339

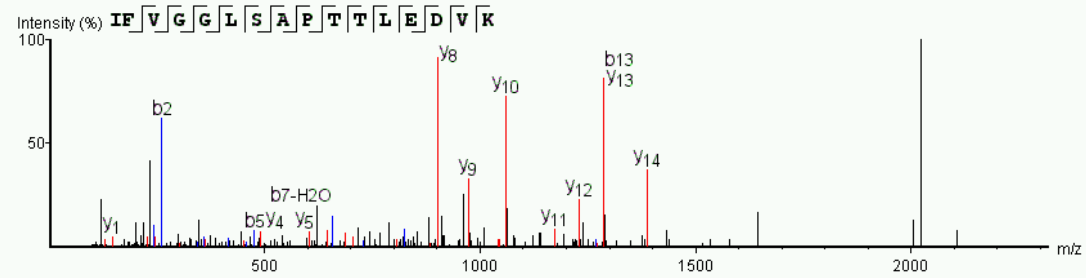

gi|571512342

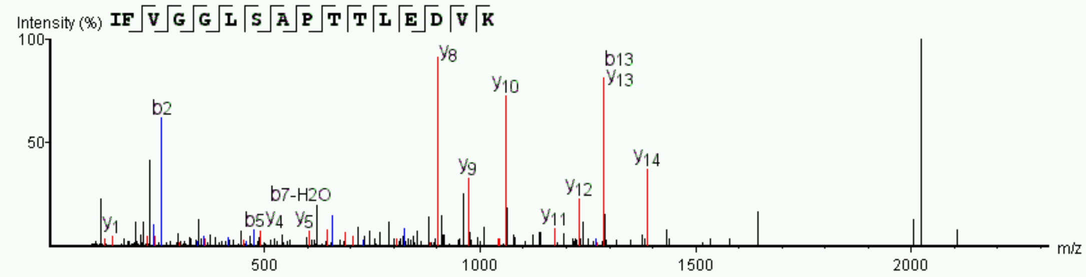

gi|571575626

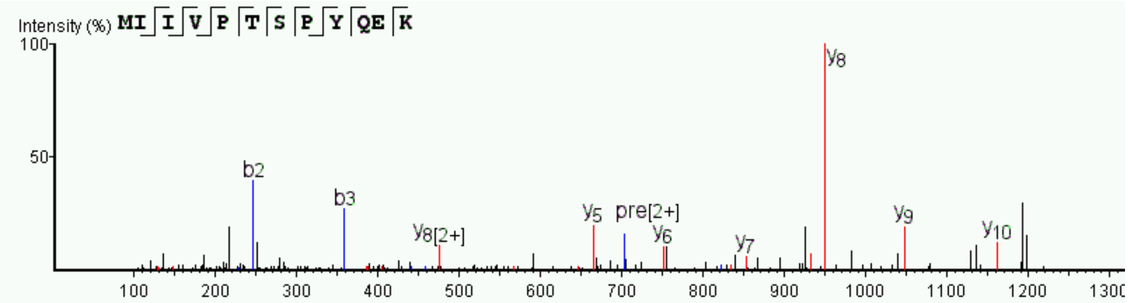

gi|571575628

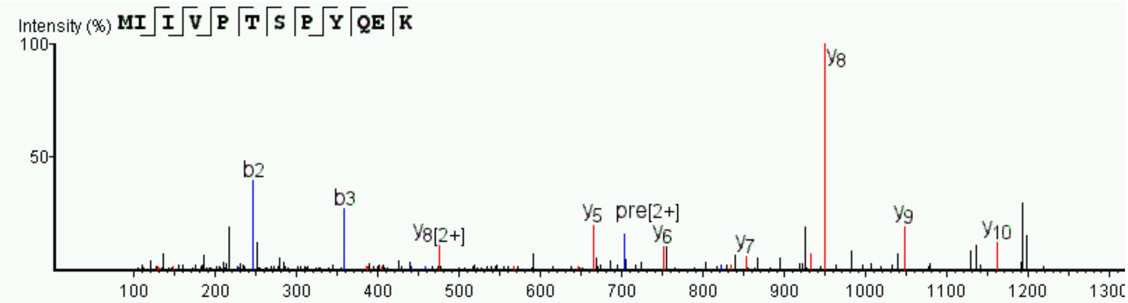

gi|571575630

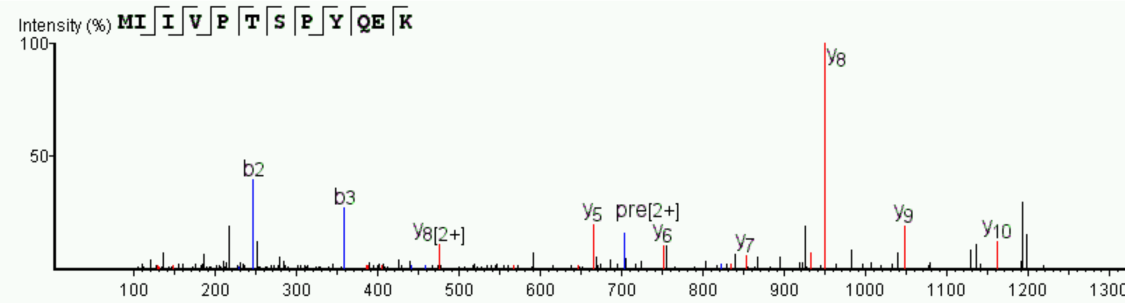

gi|571558143

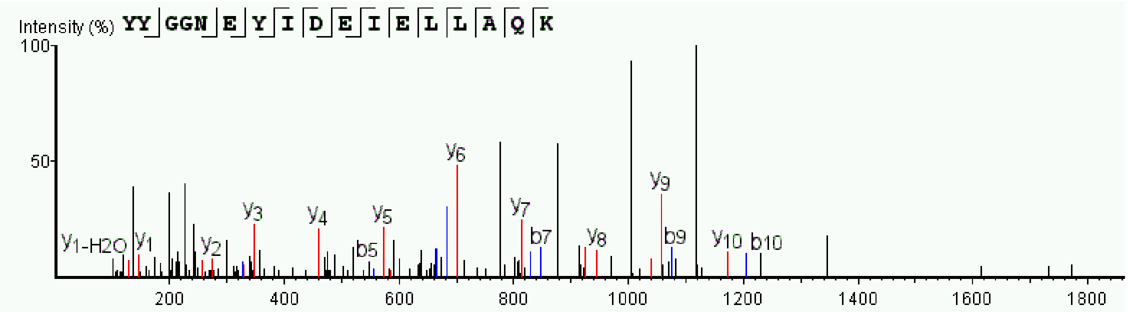

gi|571558147

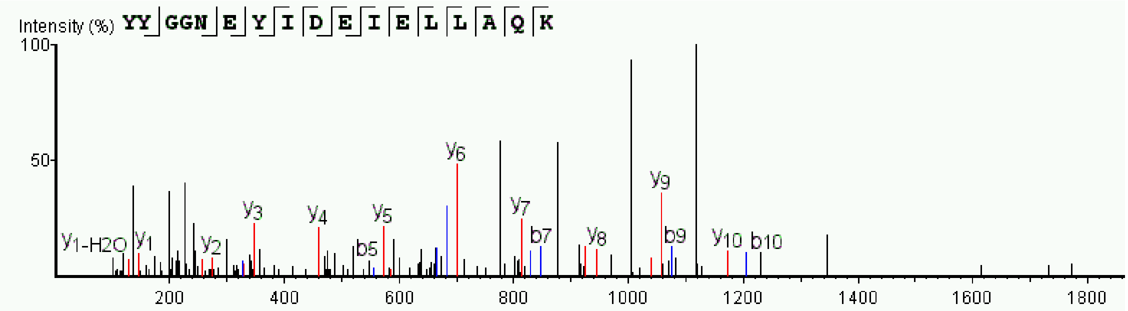

gi|571558150

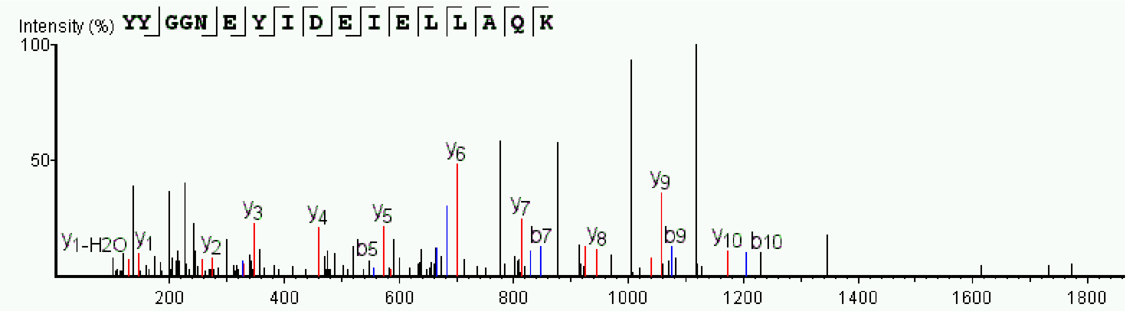

gi|571506280

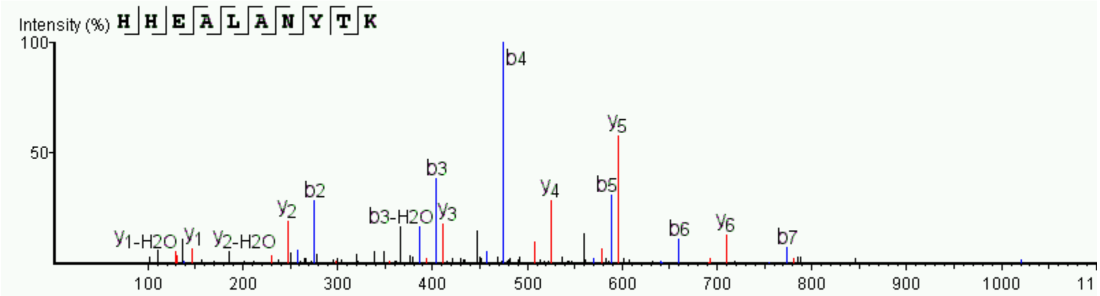

gi|571506283

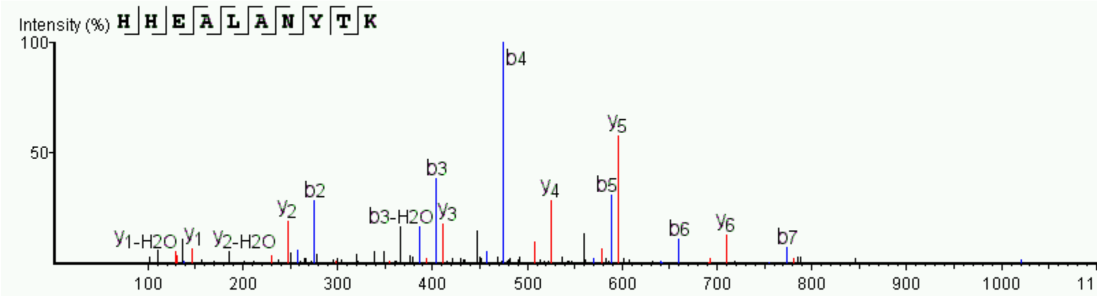

gi|571568731

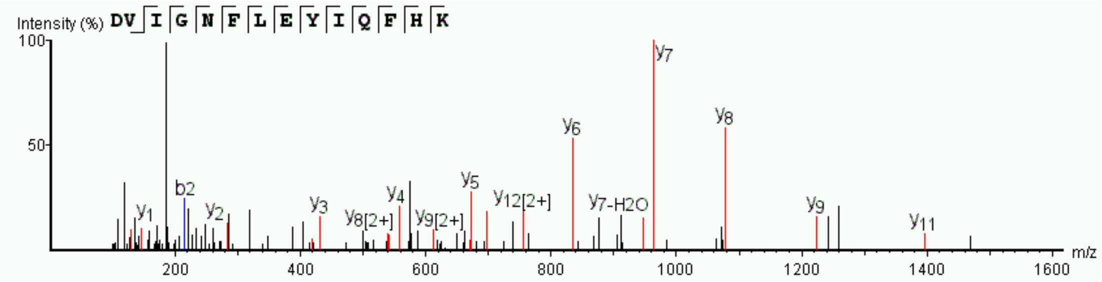

gi|571568734

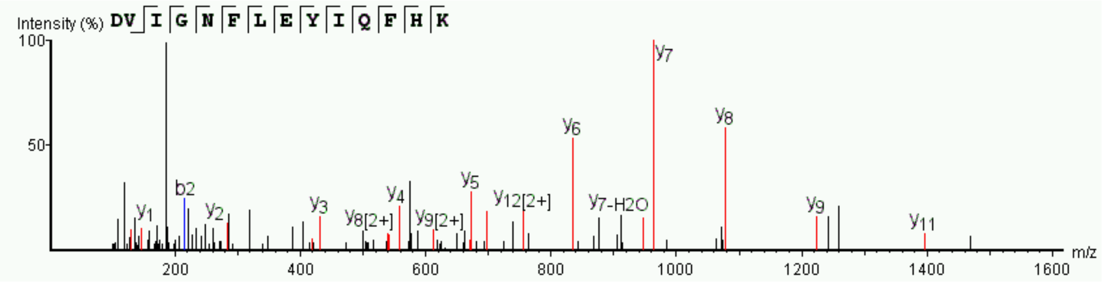

gi|571568738

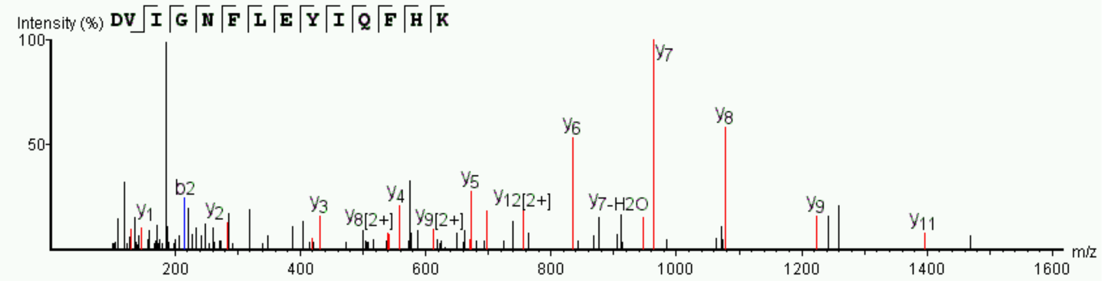

gi|66509916

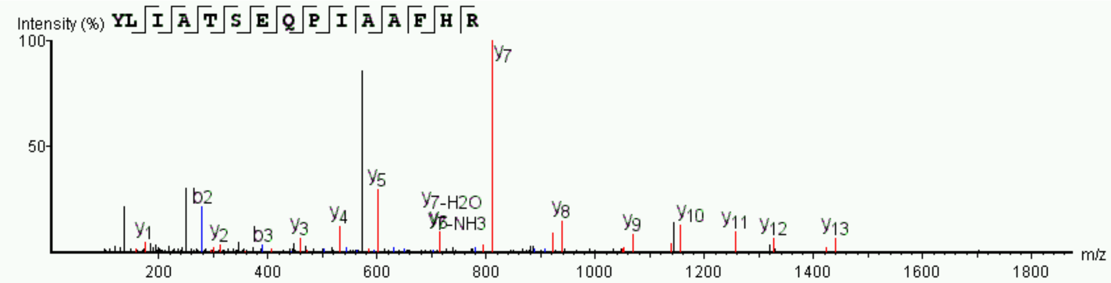

gi|571577865

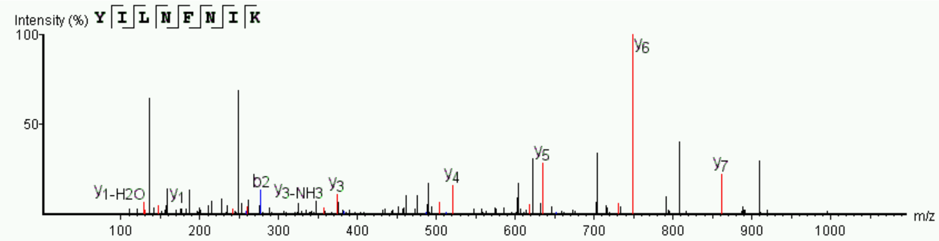

gi|571577867

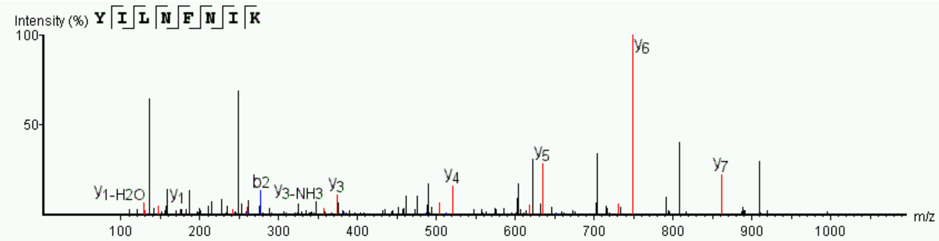

gi|571577869

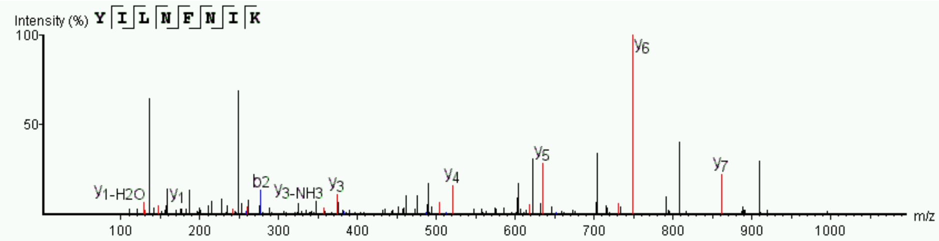

gi|571577871

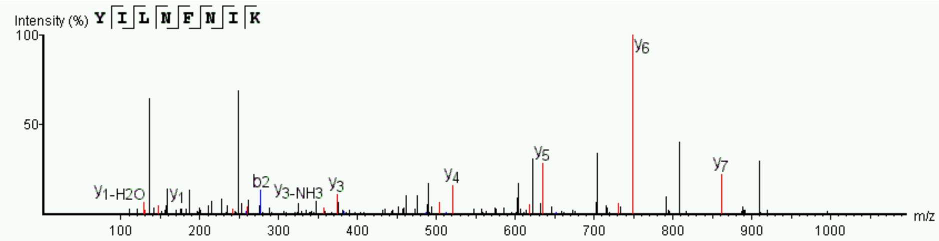

gi|571508459

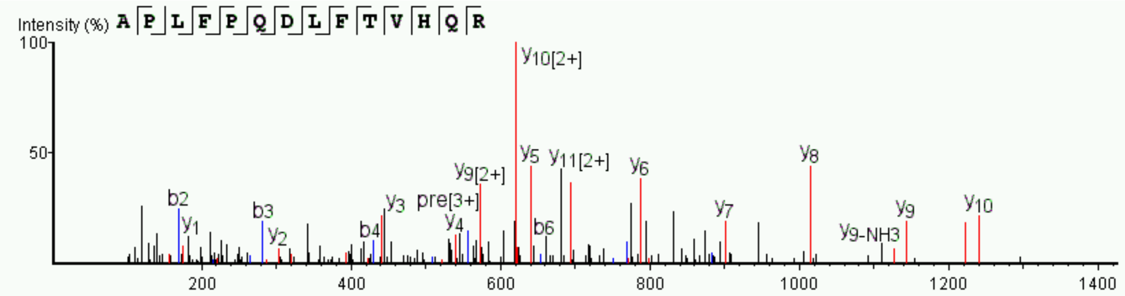

gi|571508466

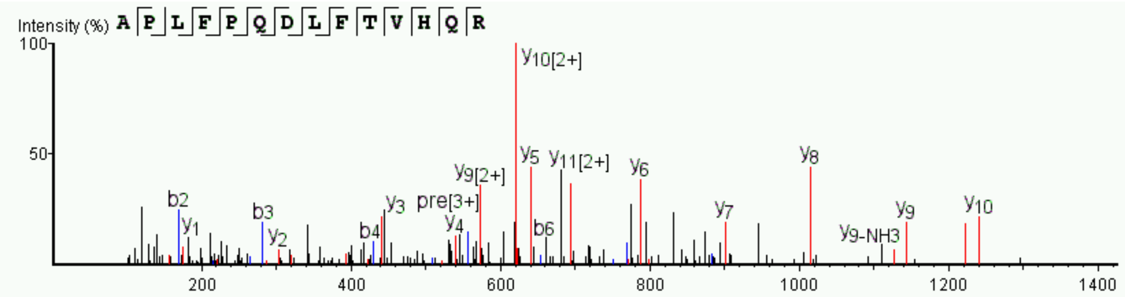

gi|571508469

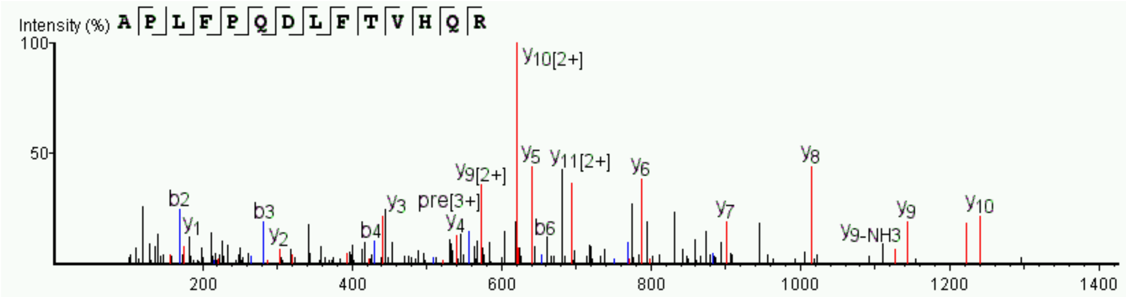

gi|571508472

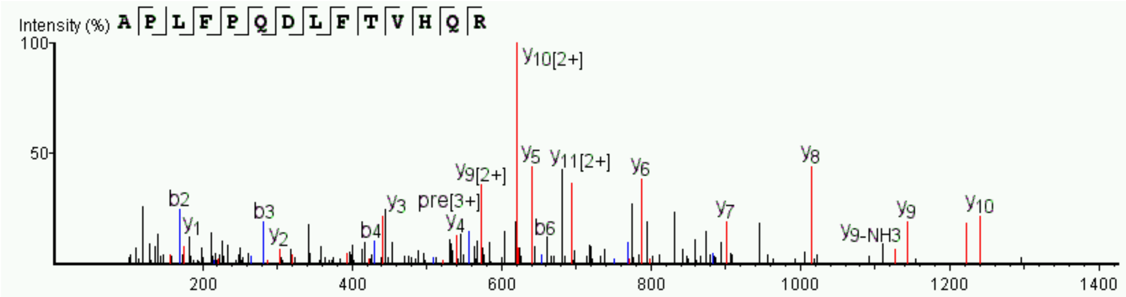

gi|328792320

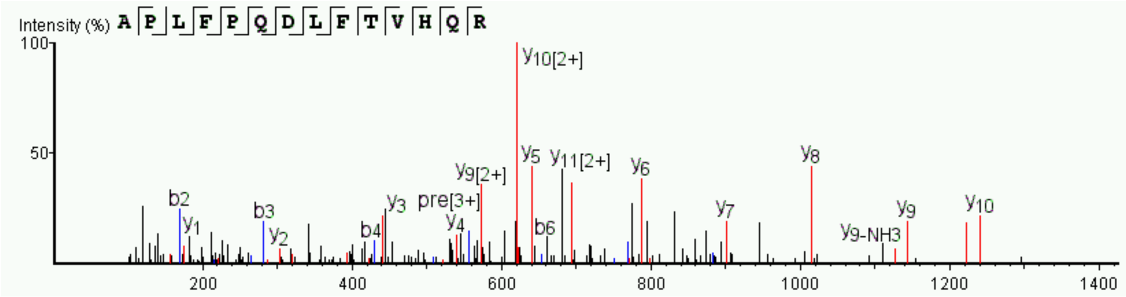

gi|110767674

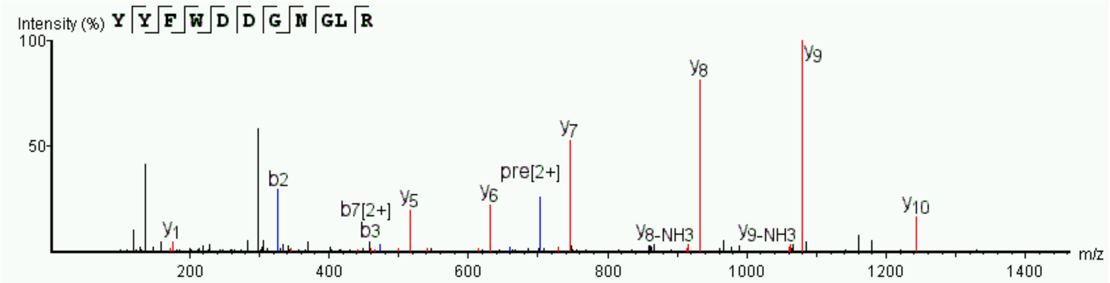

gi|110755131

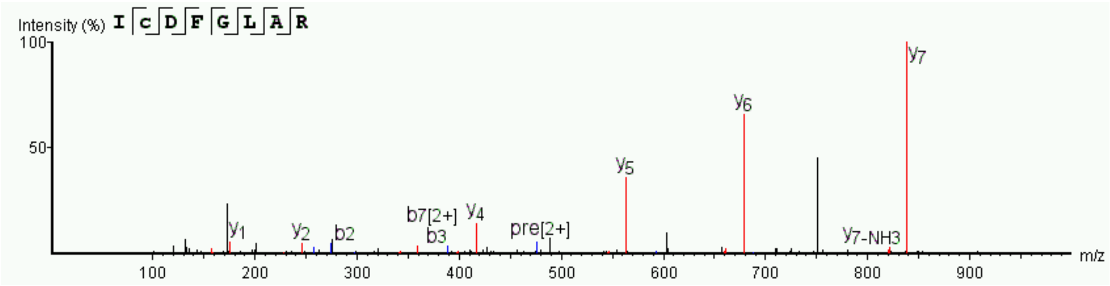

gi|571518891

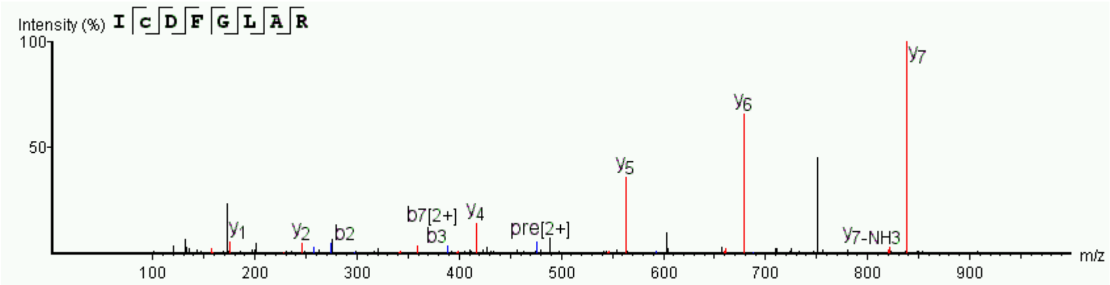

gi|571518888

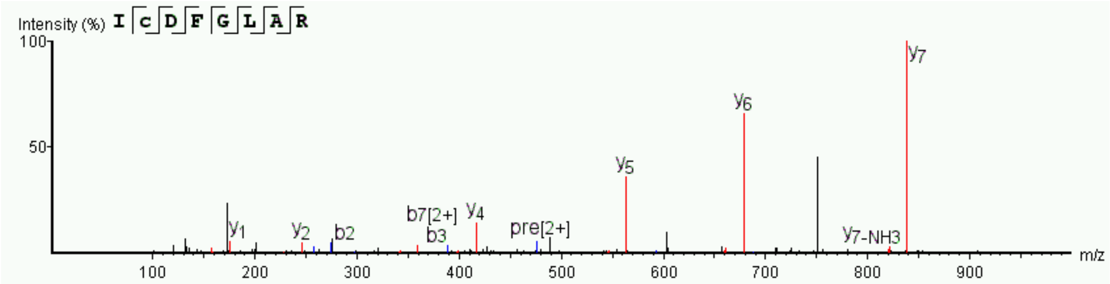

gi|571518885

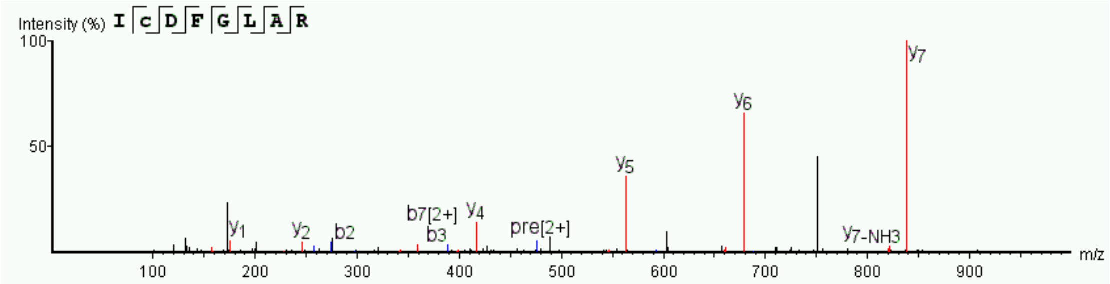

gi|571550357

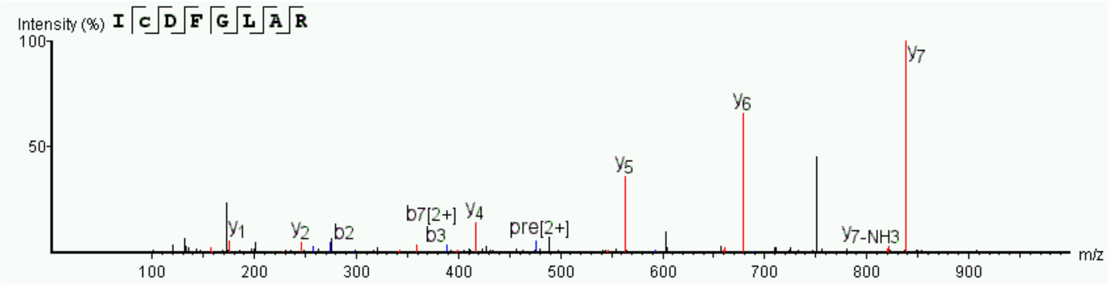

gi|571514456

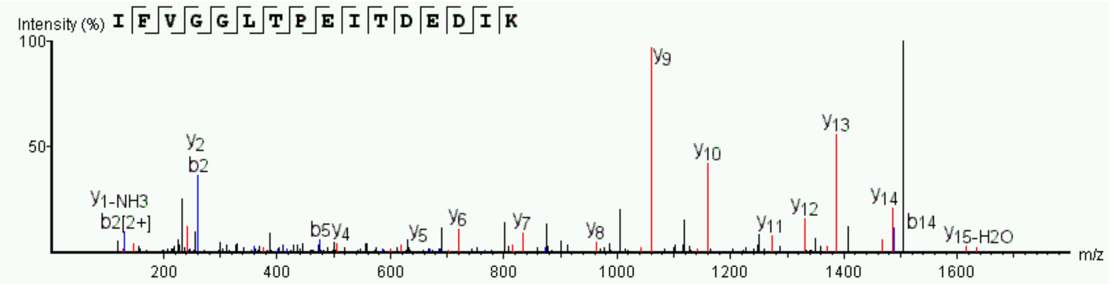

gi|48142169

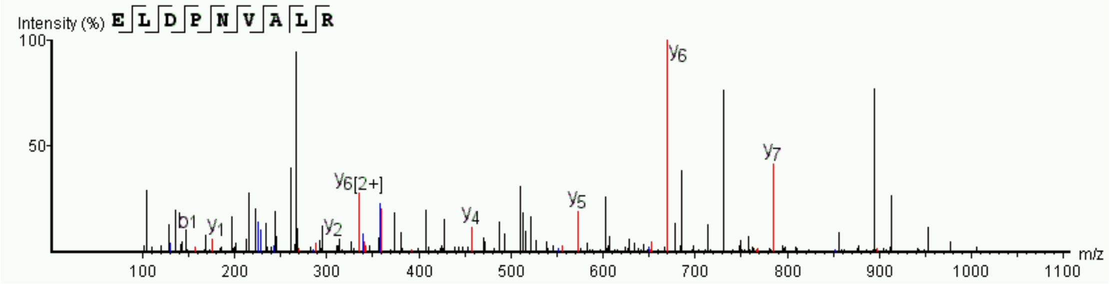

gi|66499475

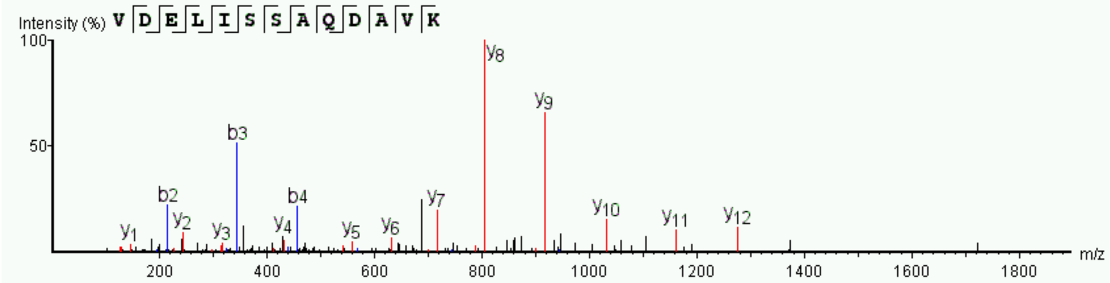

gi|571499266

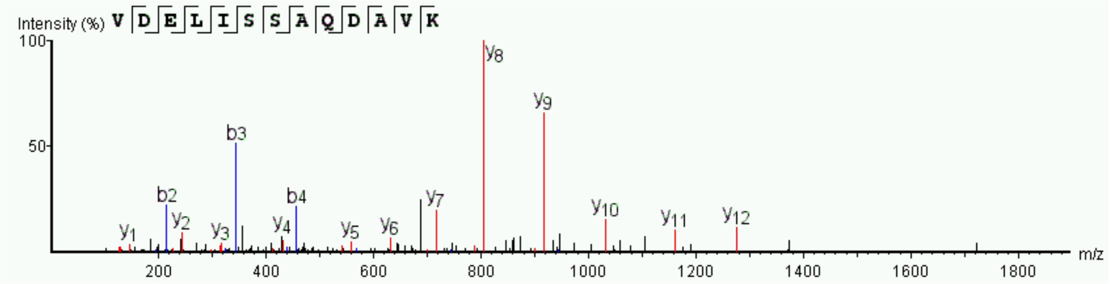

gi|48103887

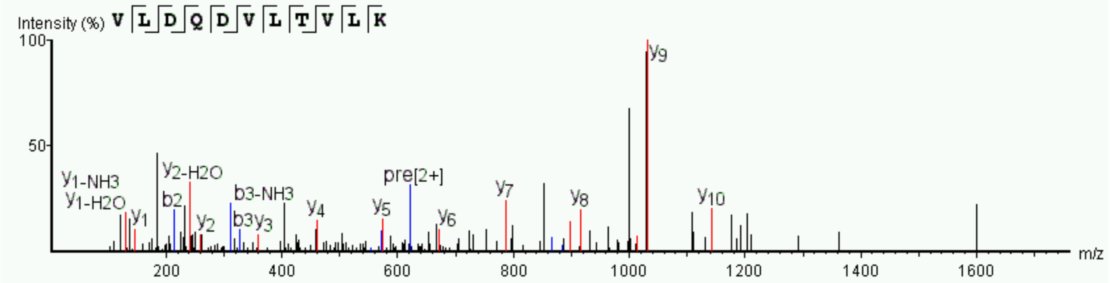

gi|66560175

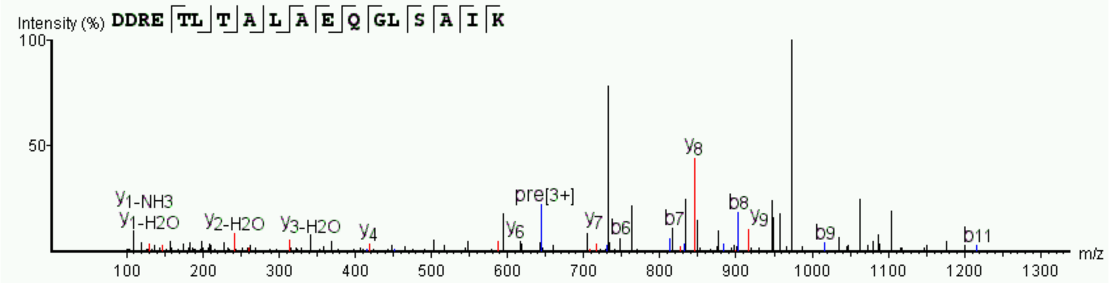

gi|48105901

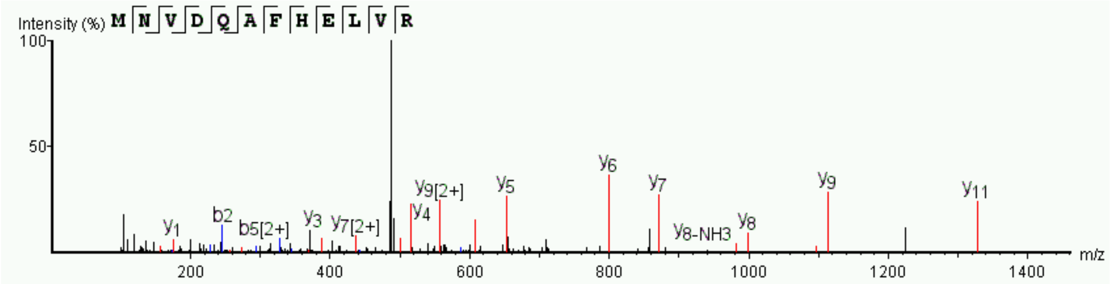

gi|571546226

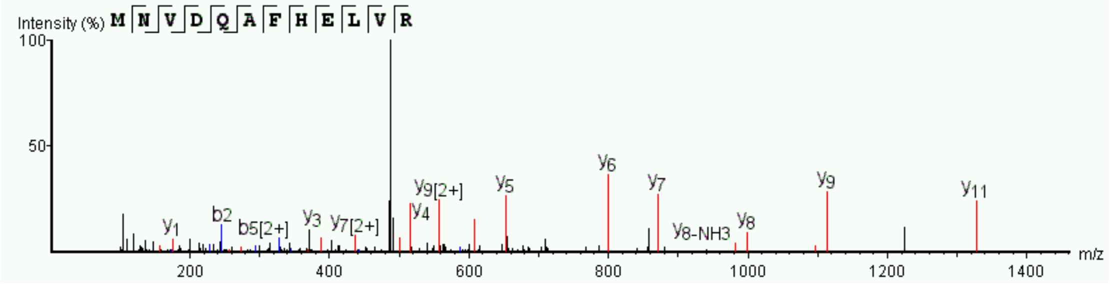

gi|571522020

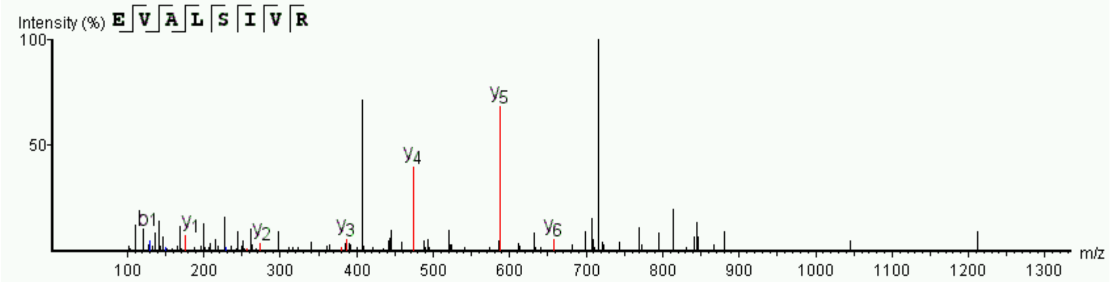

gi|571522010

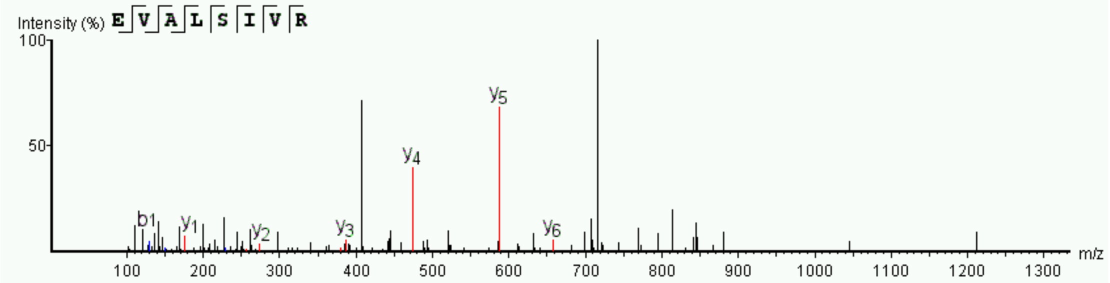

gi|571554543

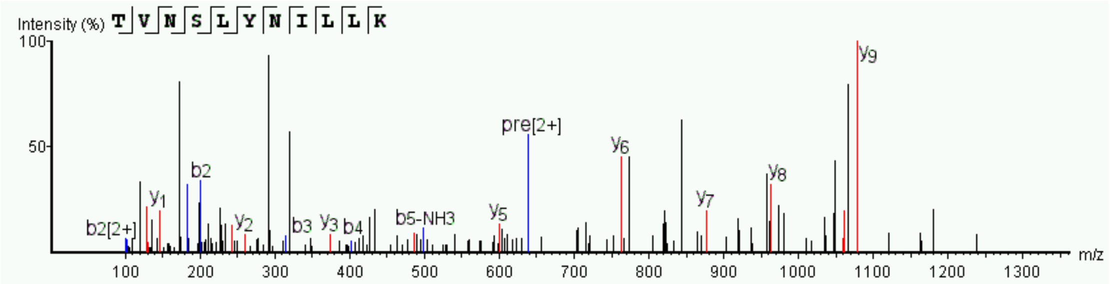

gi|328788528

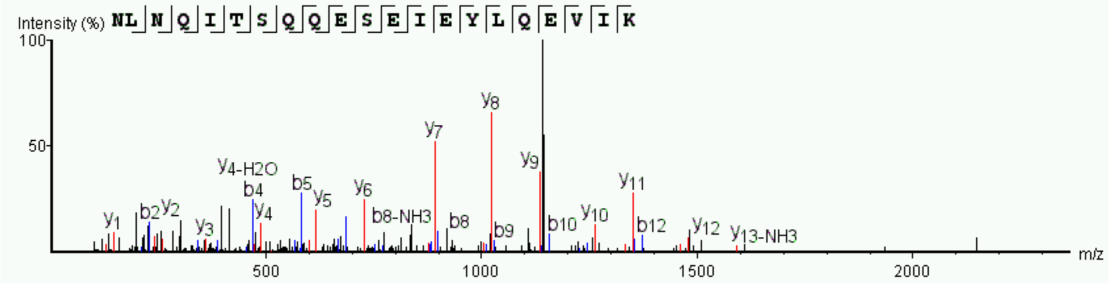

gi|66522157

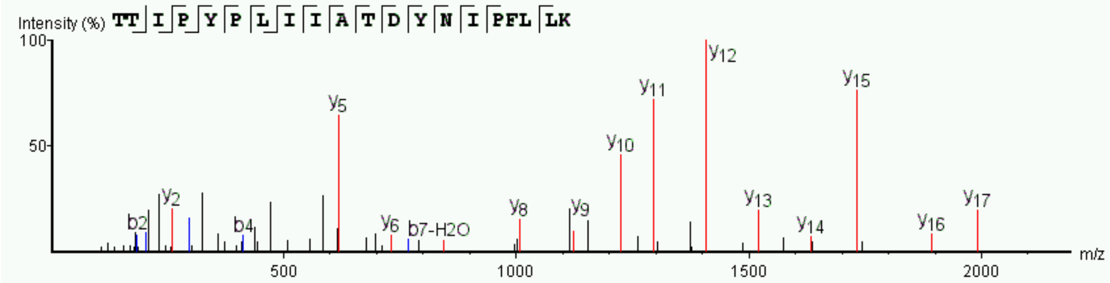

gi|571566599

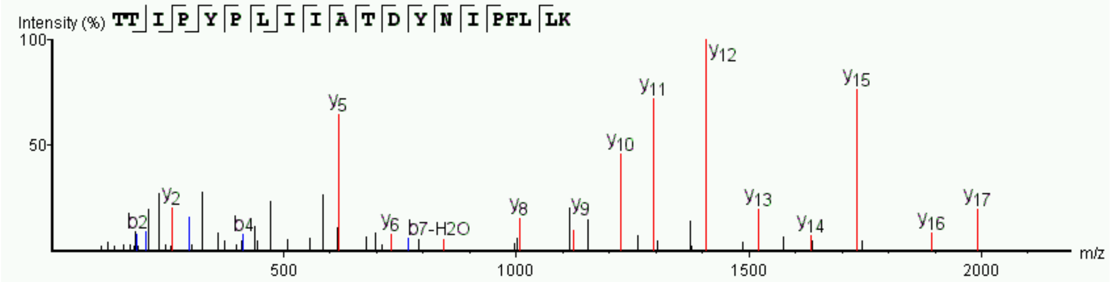

gi|571566596

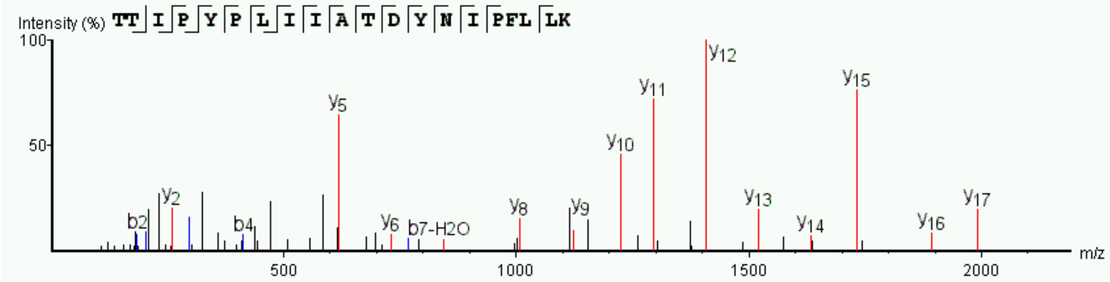

gi|571566593

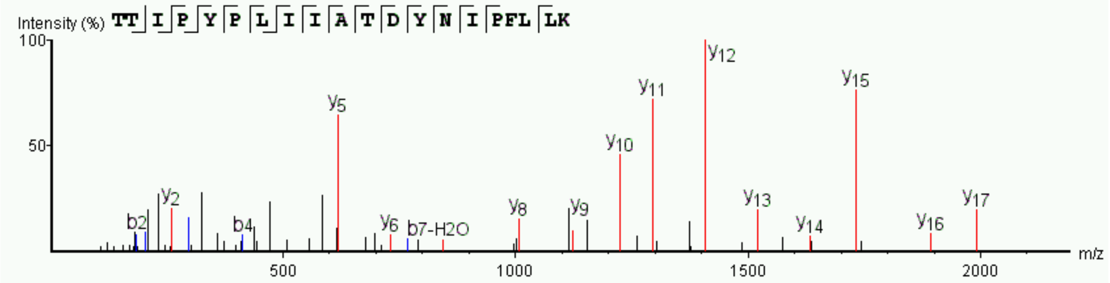

gi|66530260

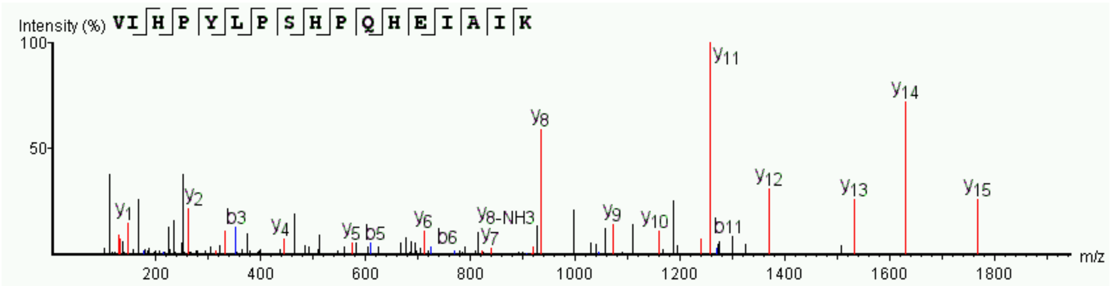

gi|571564565

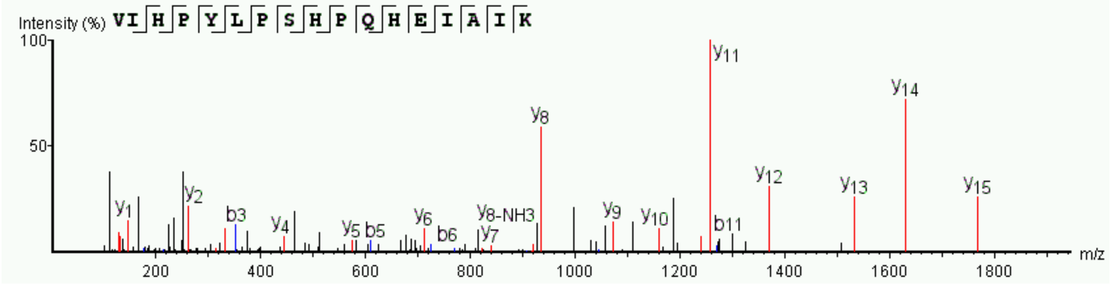

gi|571514744

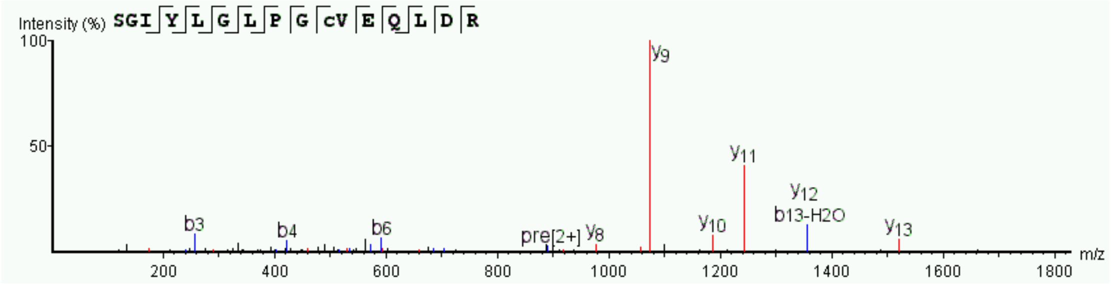

gi|328785850

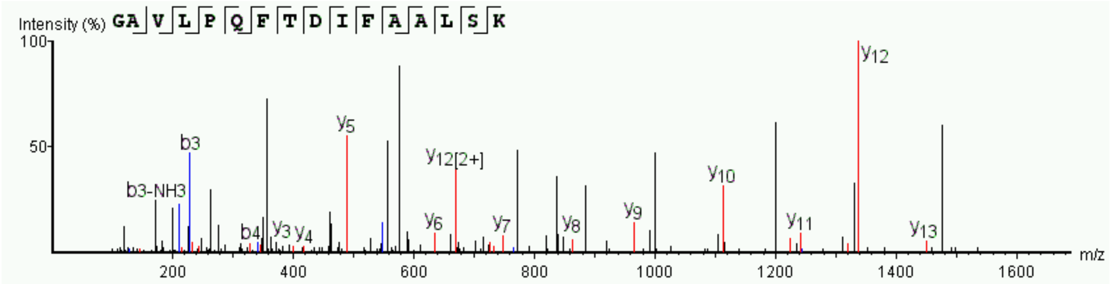

gi|571577853

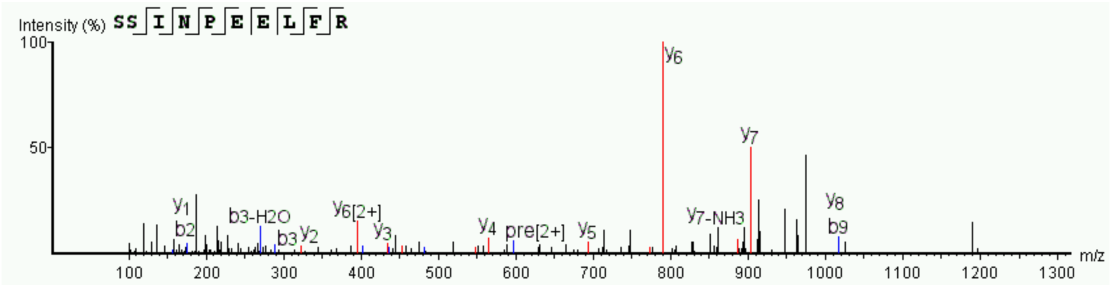

gi|328793508

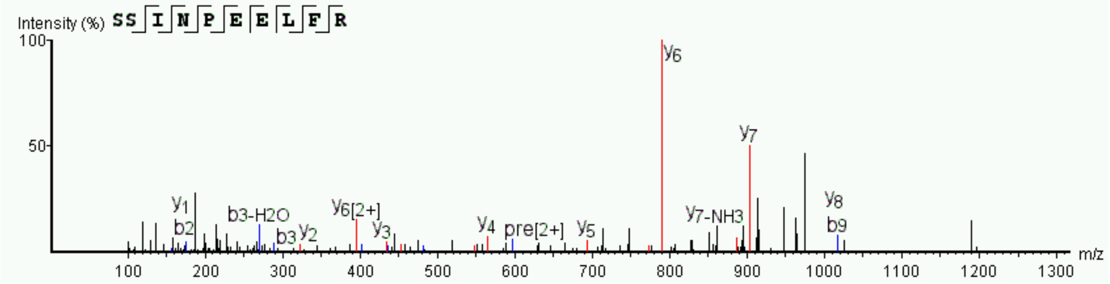

gi|66564441

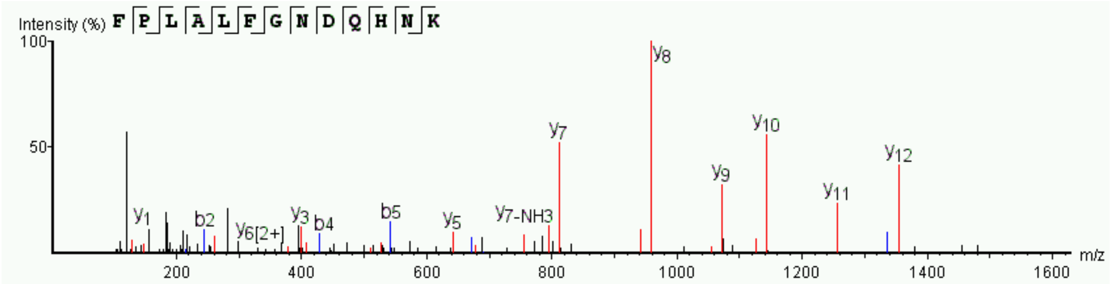

gi|571535386

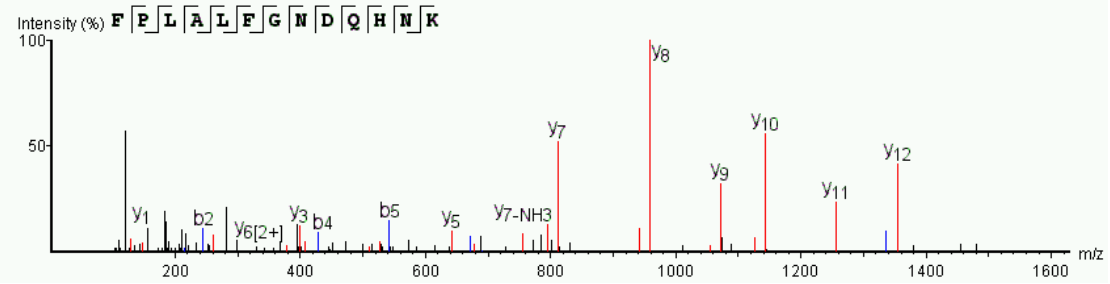

gi|328782520

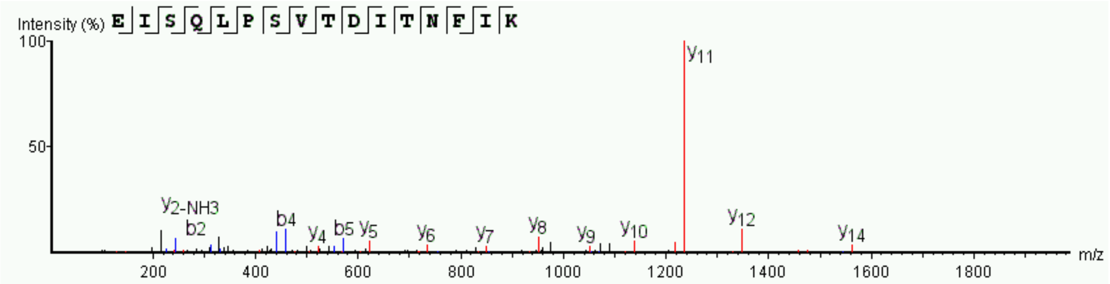

gi|571537013

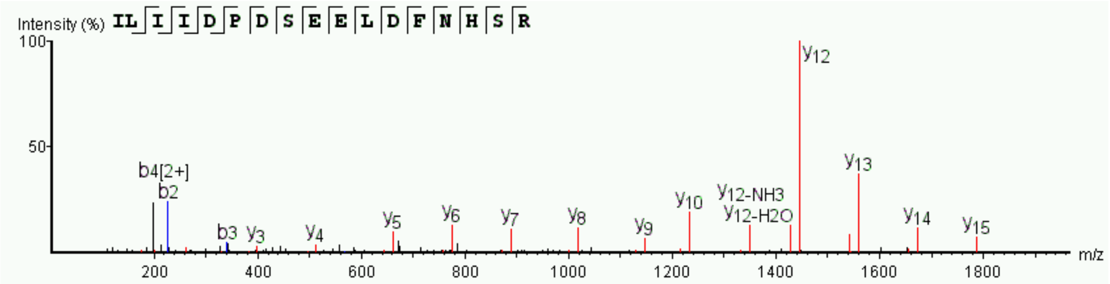

gi|571537200

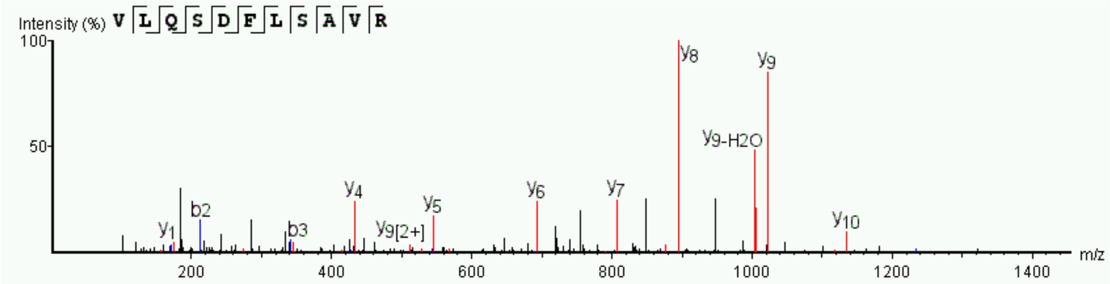

gi|571537196

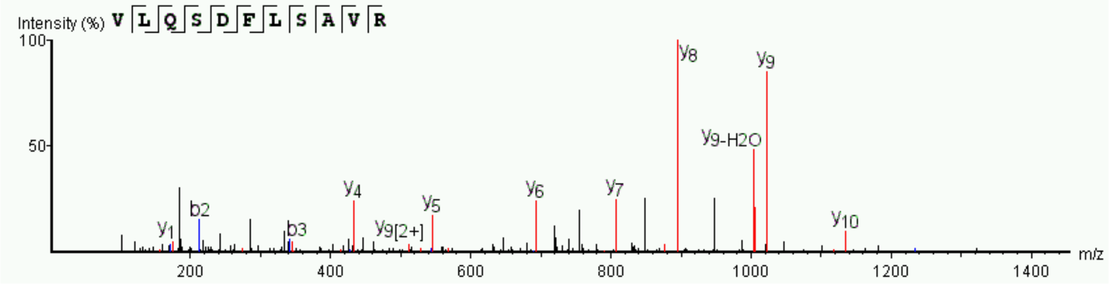

gi|48103834

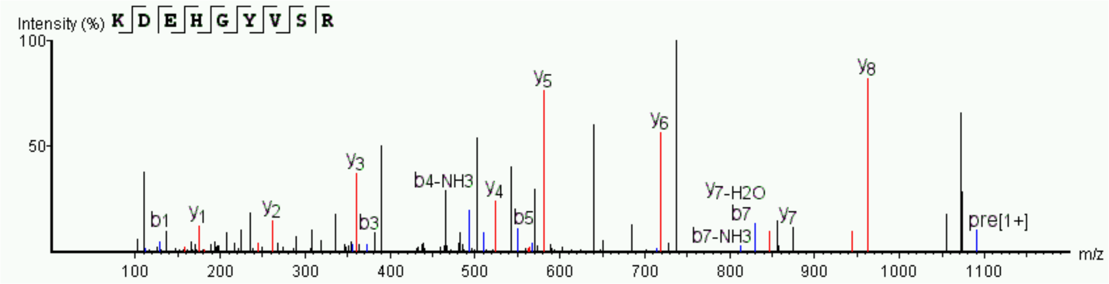

gi|571568560

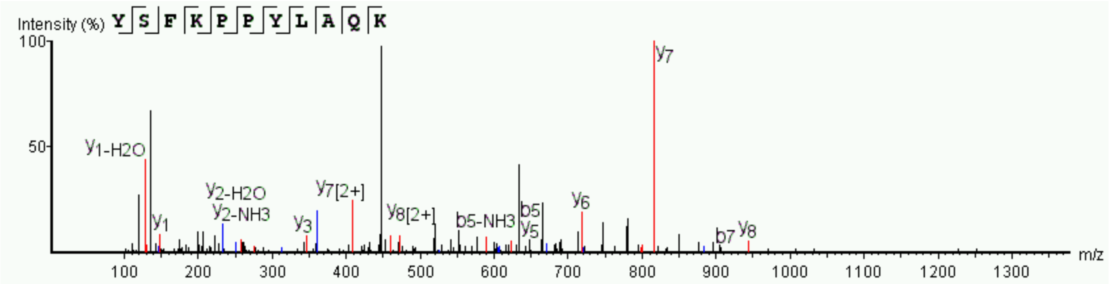

gi|571570113

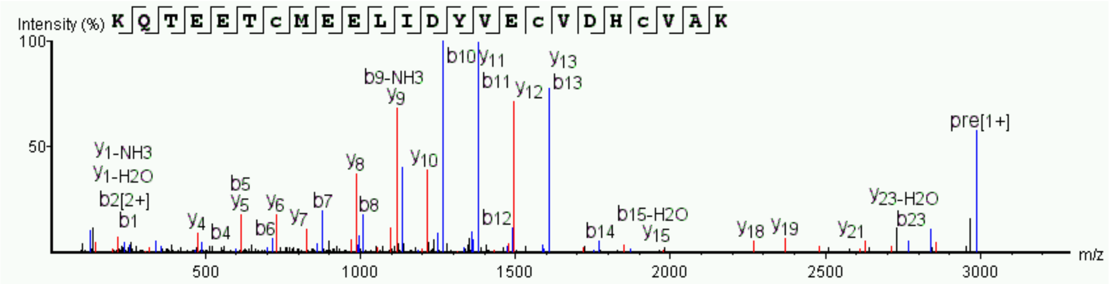

gi|571570671

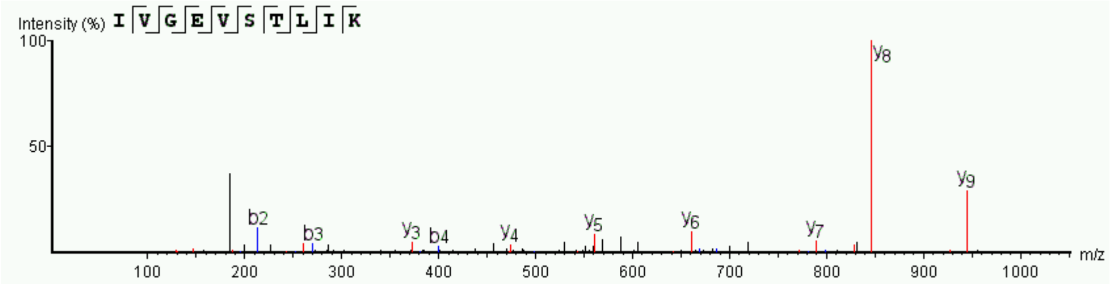

gi|571570655

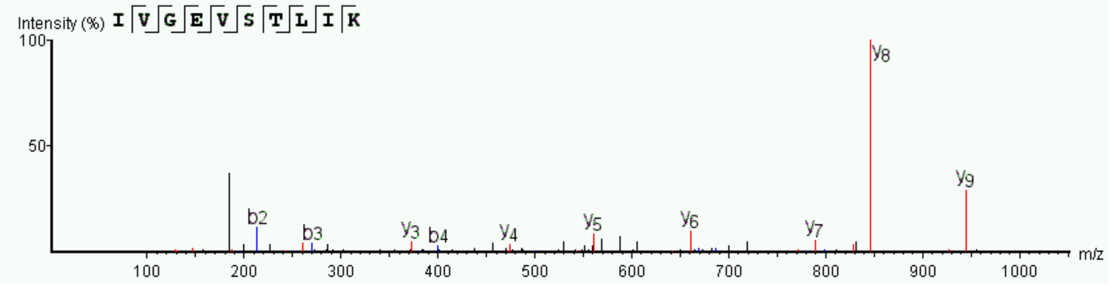

gi|571528227

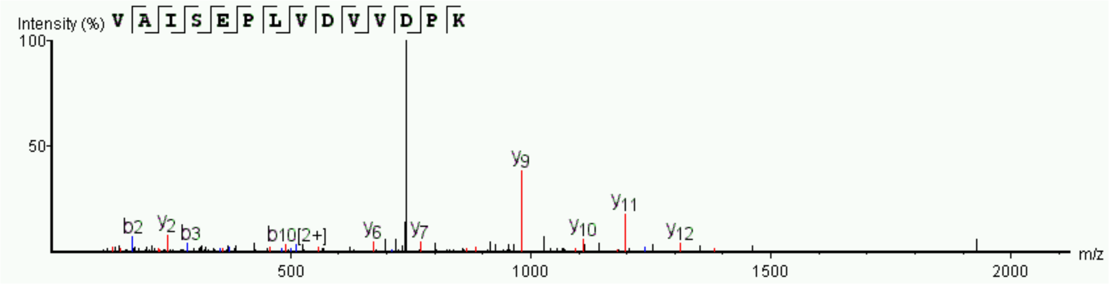

gi|328787761

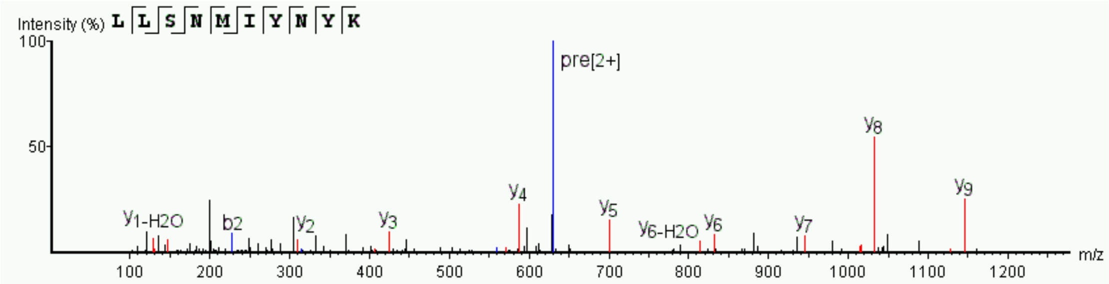

gi|328785087

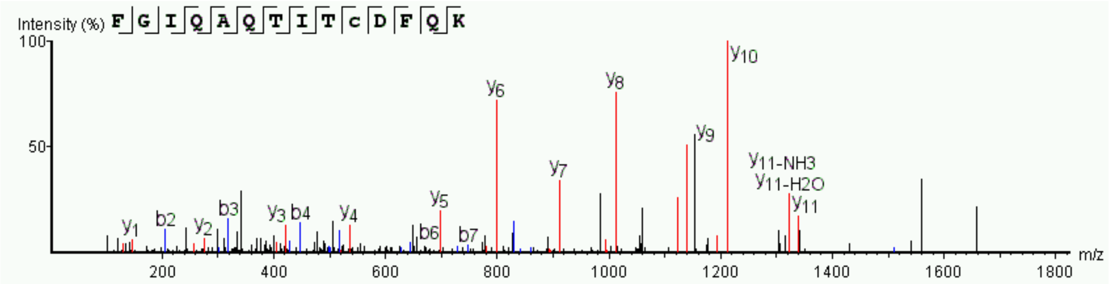

gi|66519157

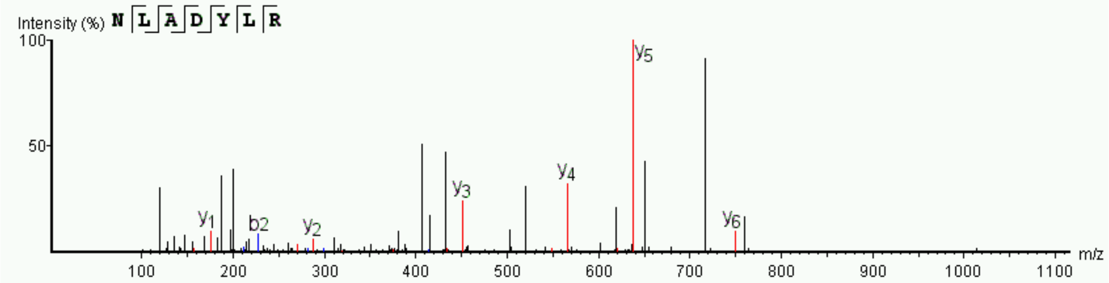

gi|571509446

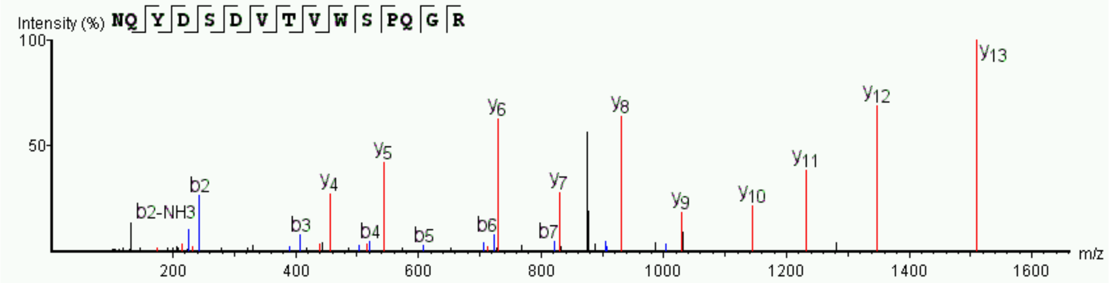

gi|66518140

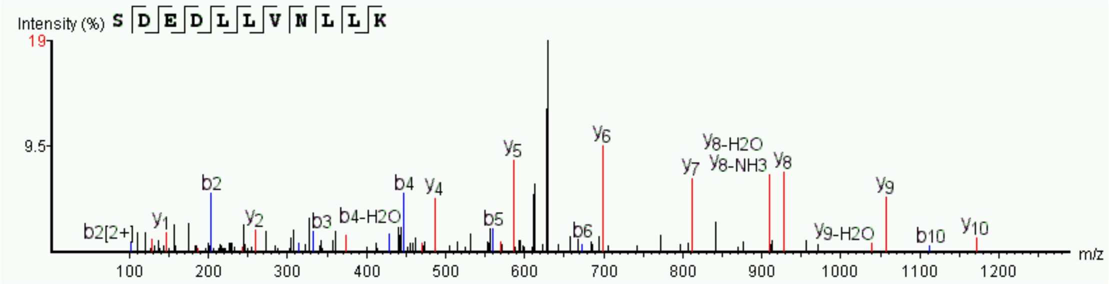

gi|328776555

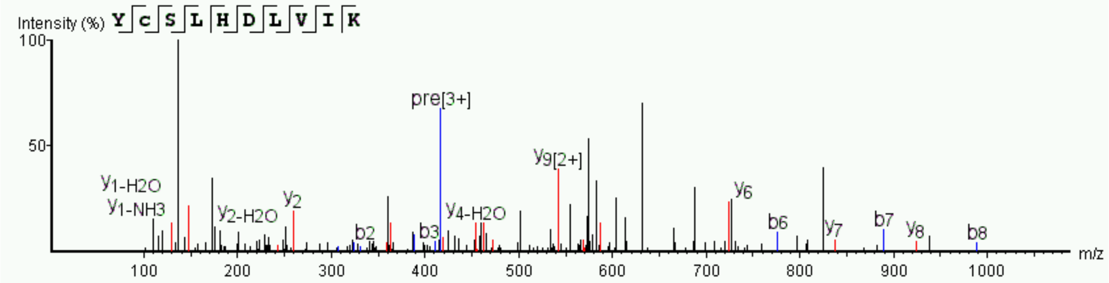

gi|66553455

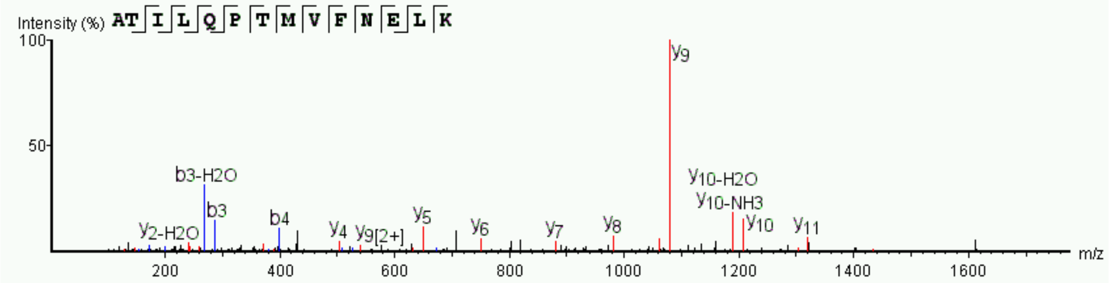

gi|571575529

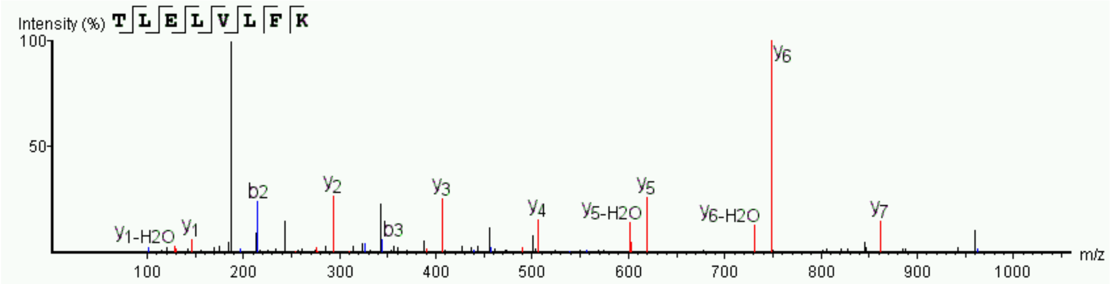

gi|571575531

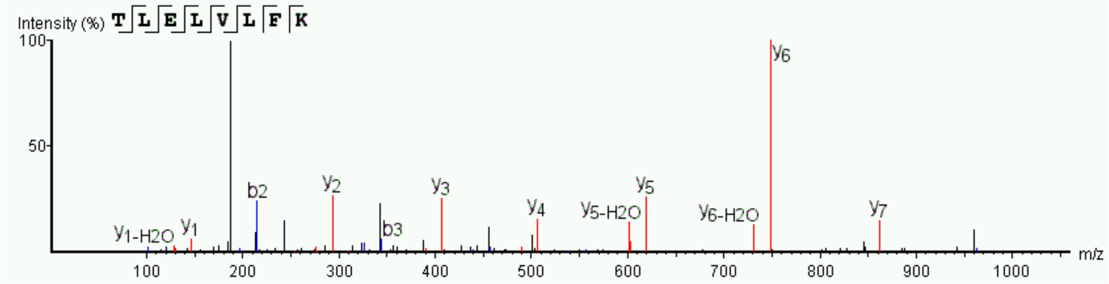

gi|328782499

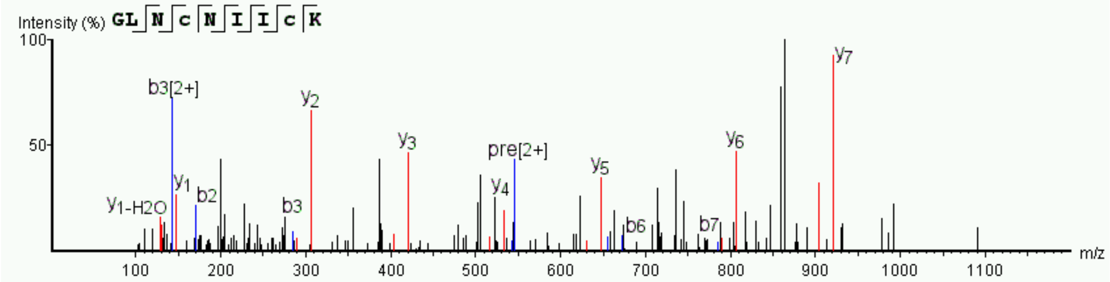

gi|571523747

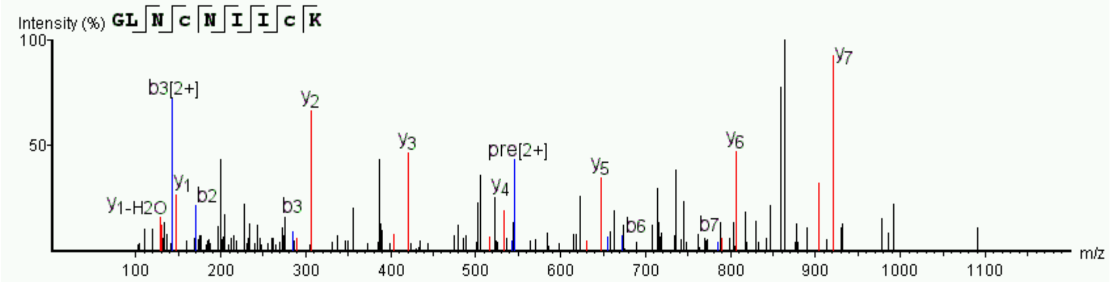

gi|571523743

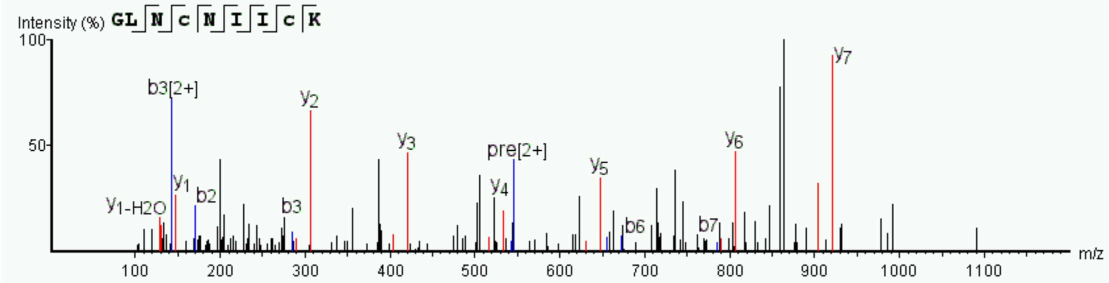

gi|571522941

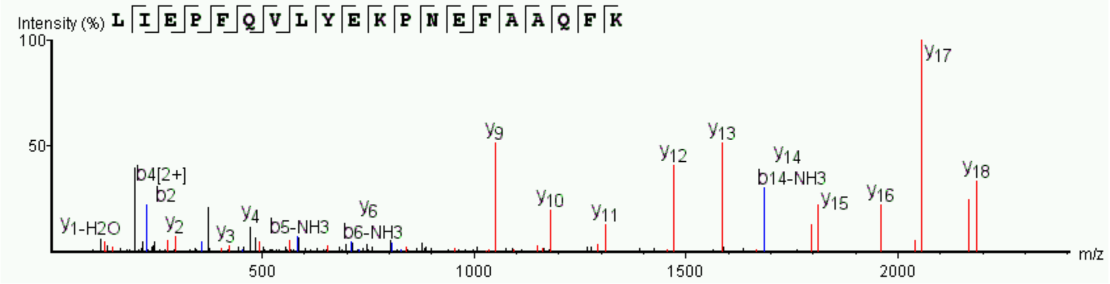

gi|48094929

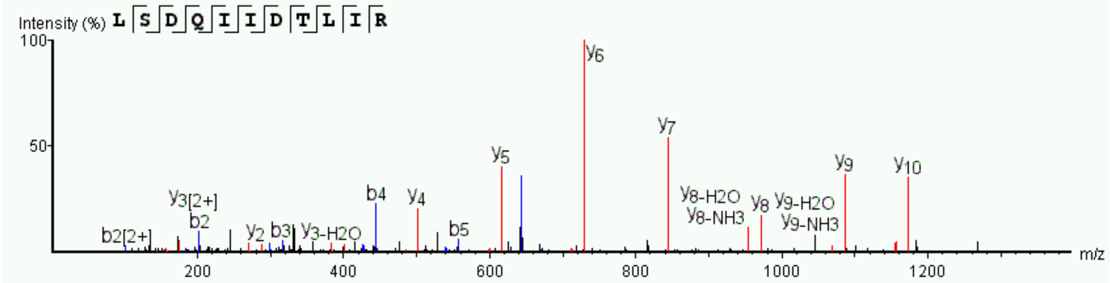

gi|328789503

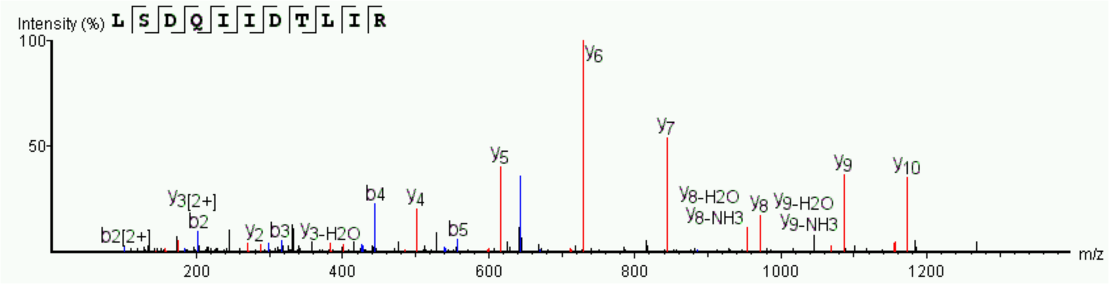

gi|48097144

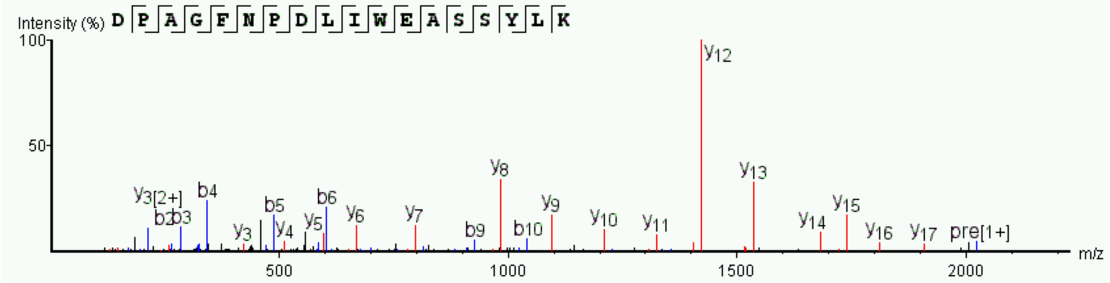

gi|571573864

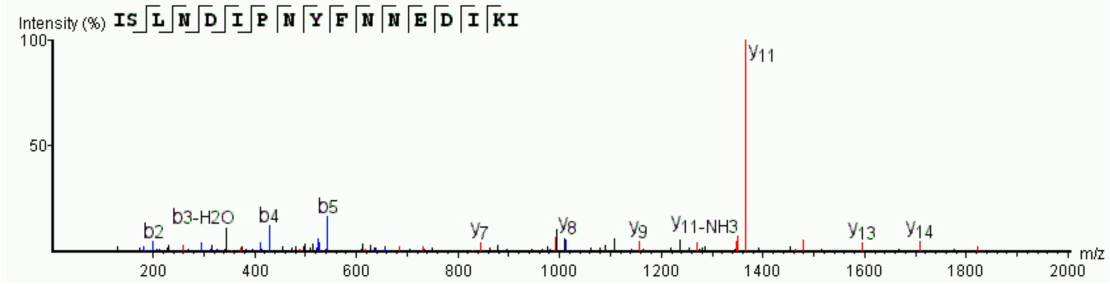

gi|571573862

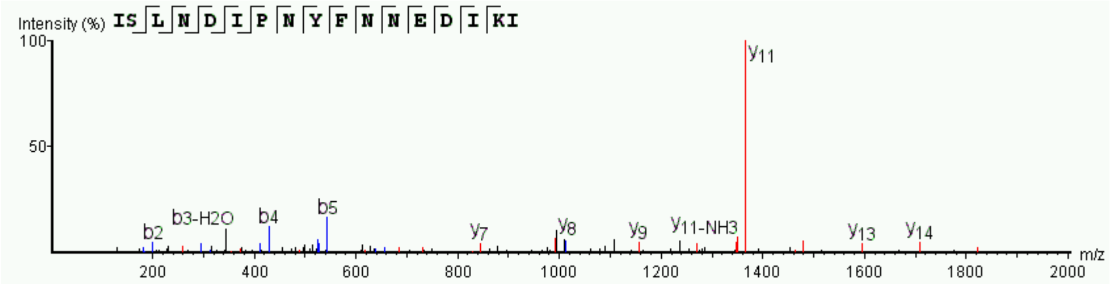

gi|571573860

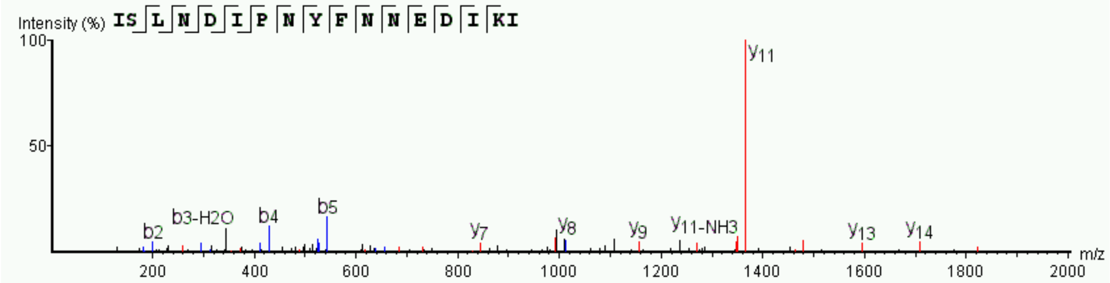

gi|571573858

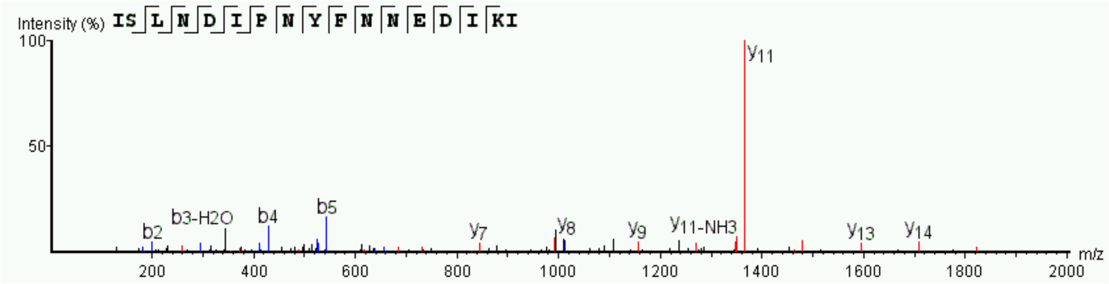

gi|571516016

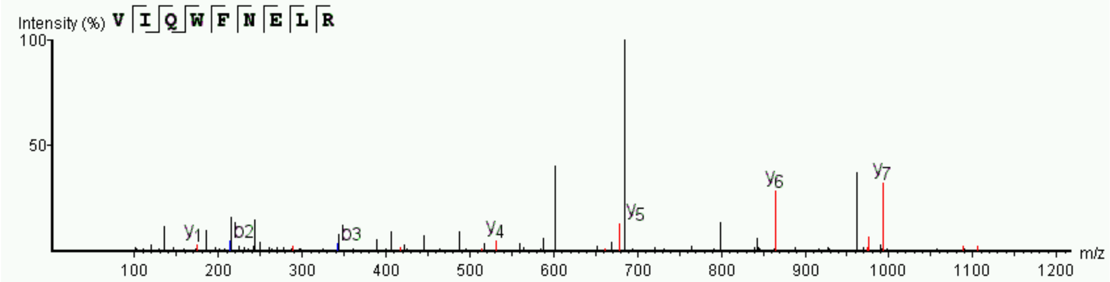

gi|571516012

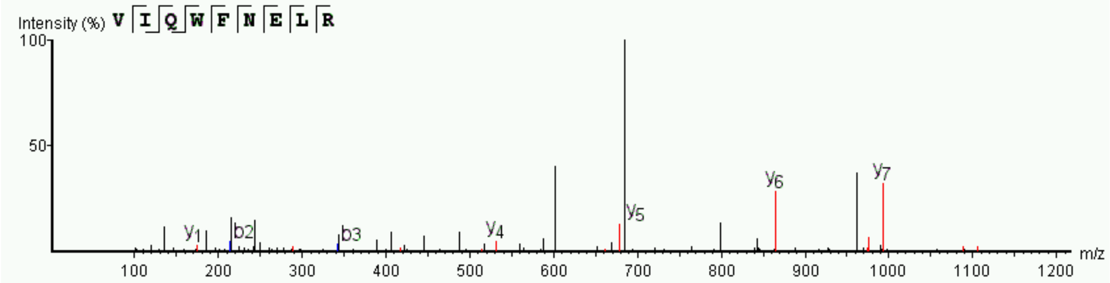

gi|571516009

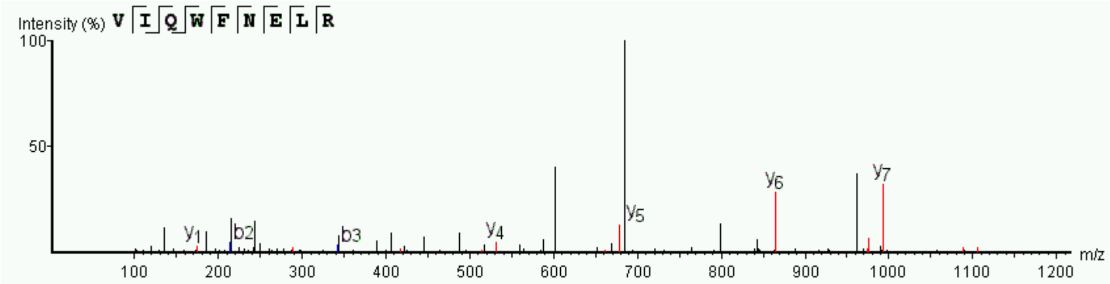

gi|571516005

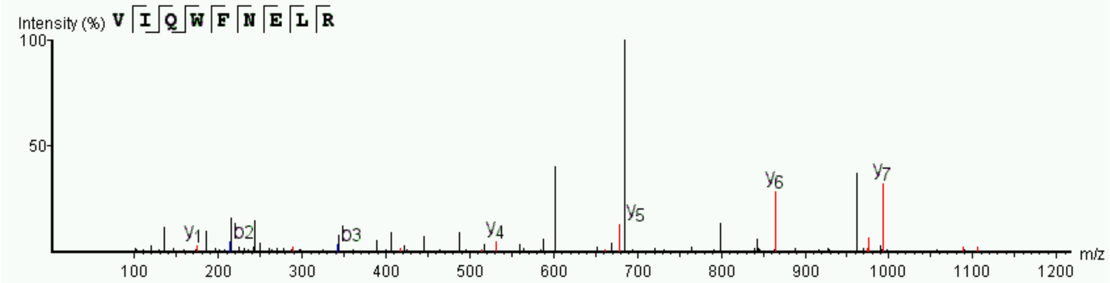

gi|571516001

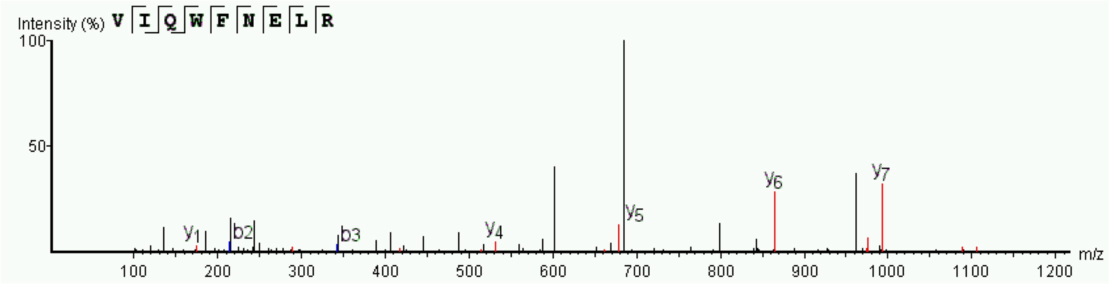

gi|48104167

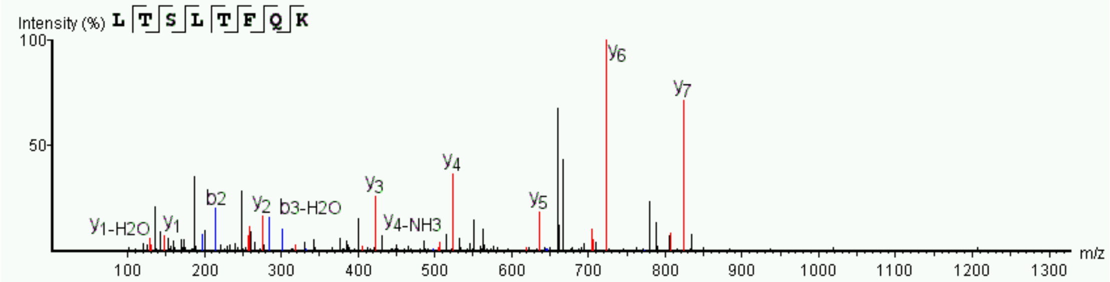

gi|328788603

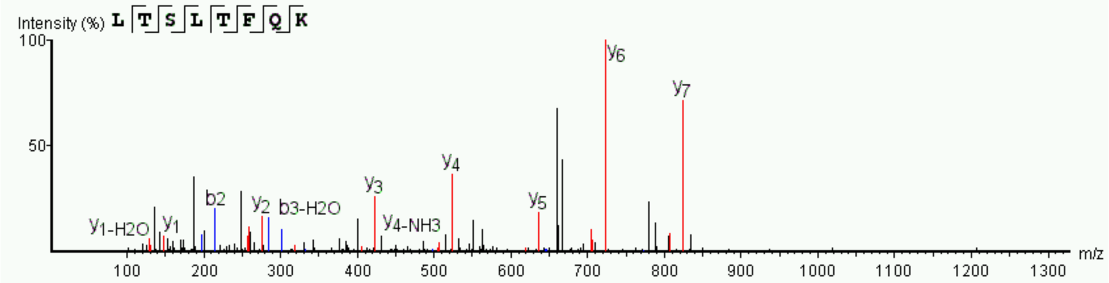

gi|48095935

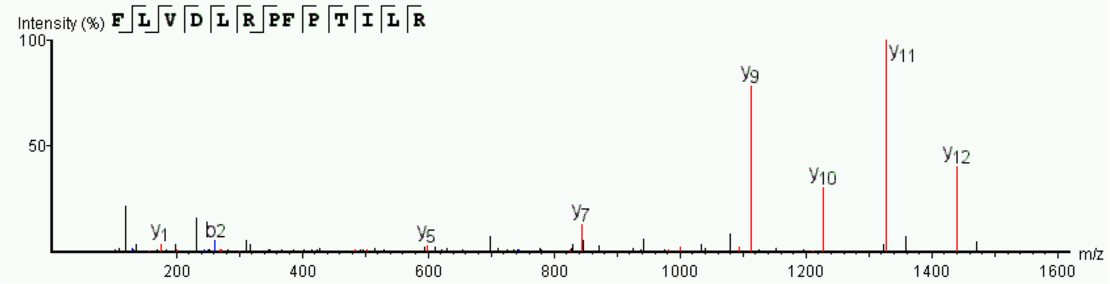

gi|571571176

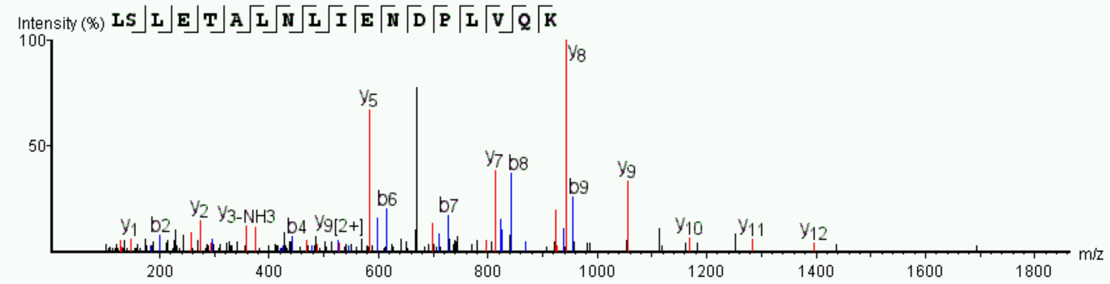

gi|571549351

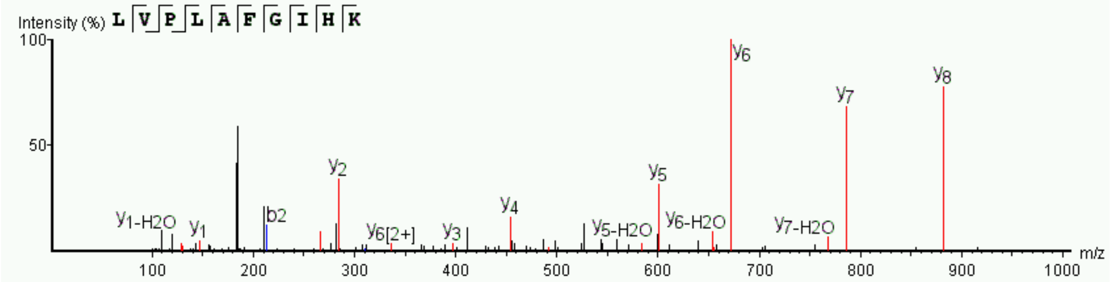

gi|571549348

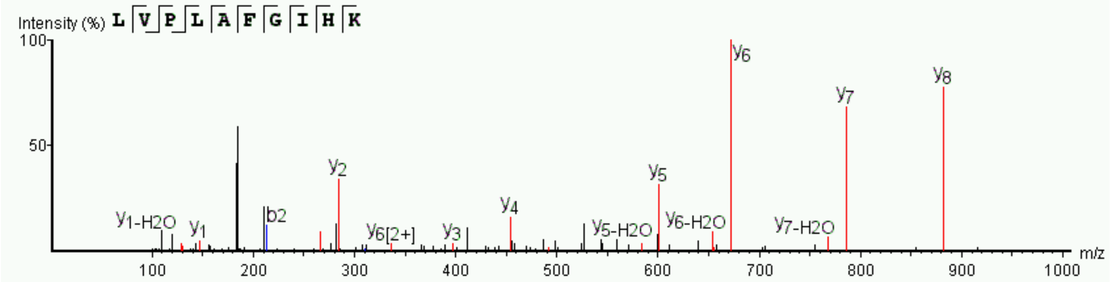

gi|571549345

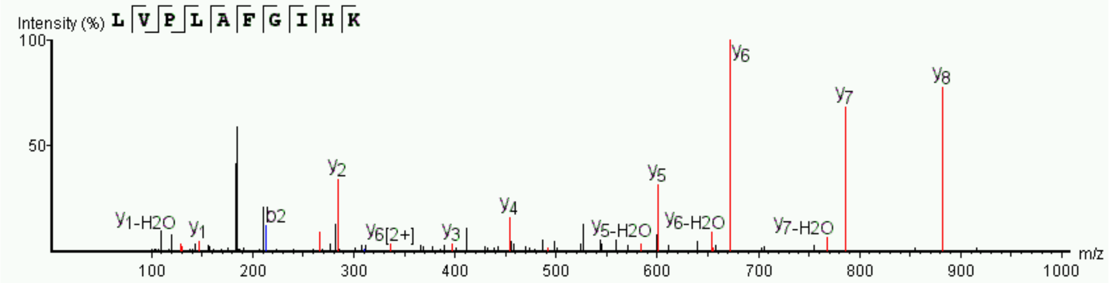

gi|48101013

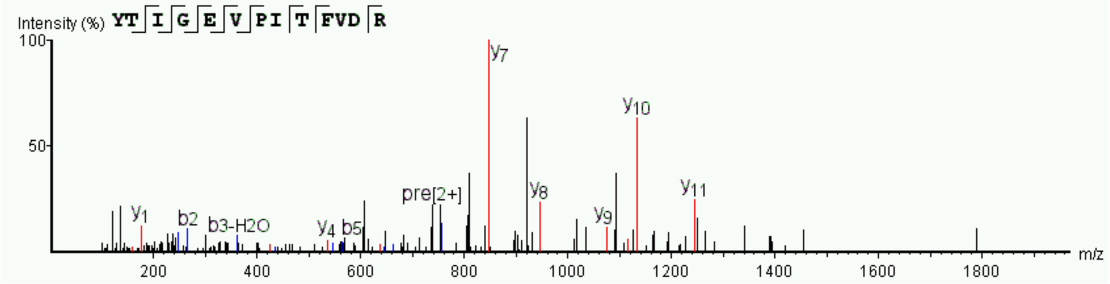

gi|66546020

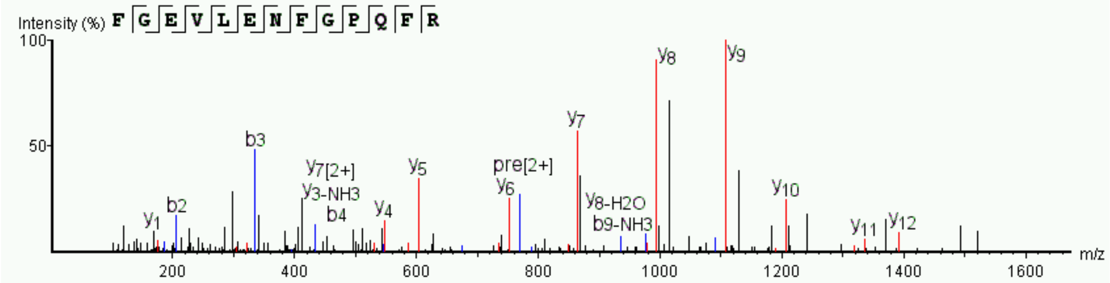

gi|571577775

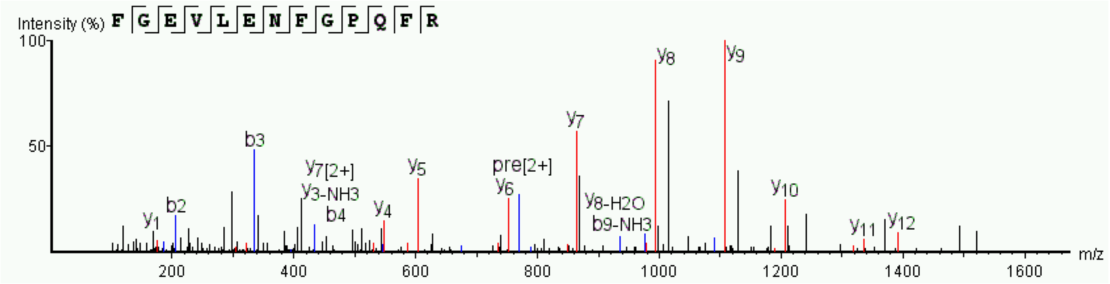

gi|571570405

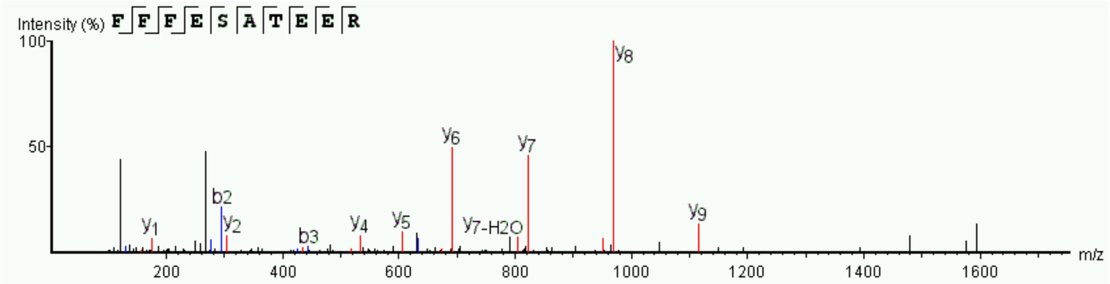

gi|571510578

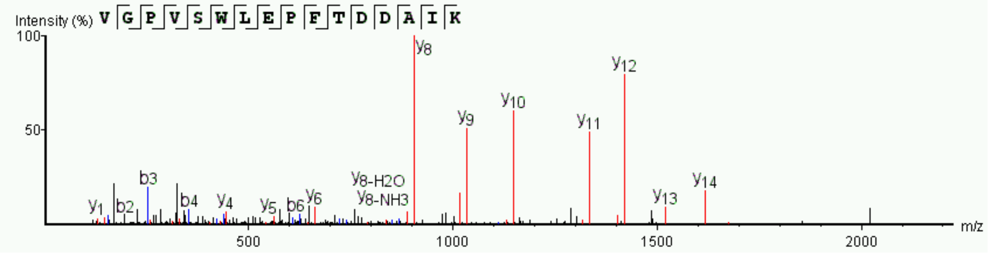

gi|571510581

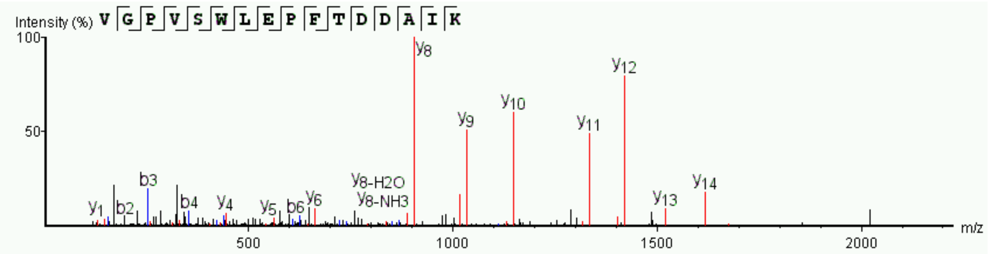

gi|571510585

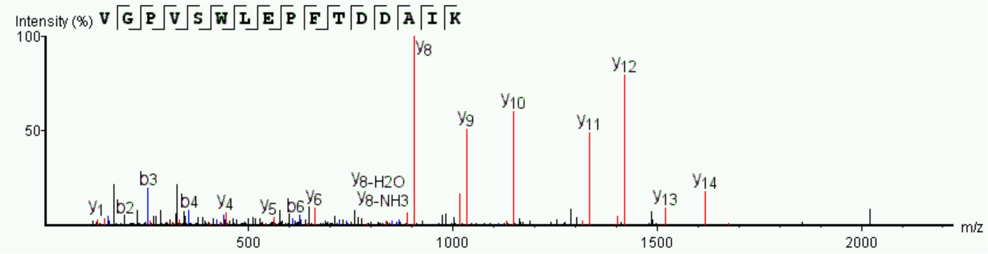

gi|571554302

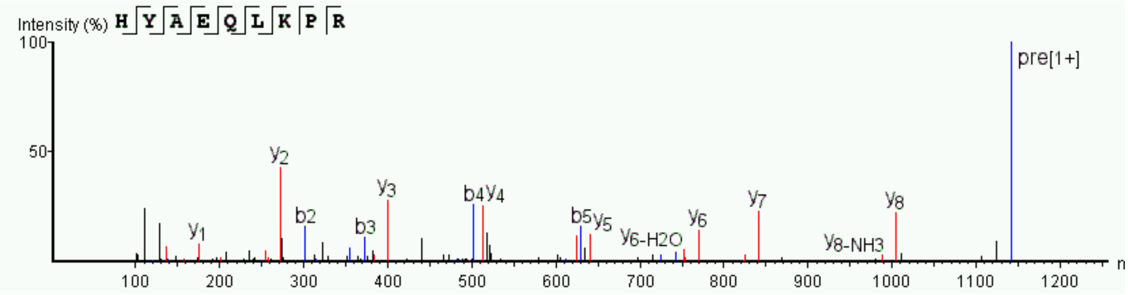

gi|571554305

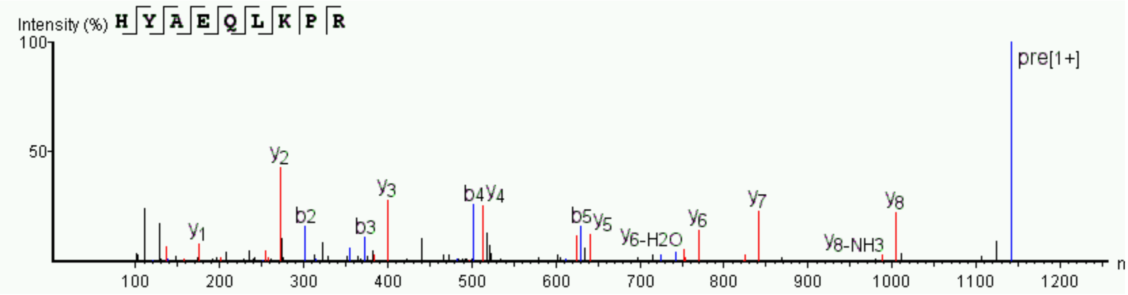

gi|571554308

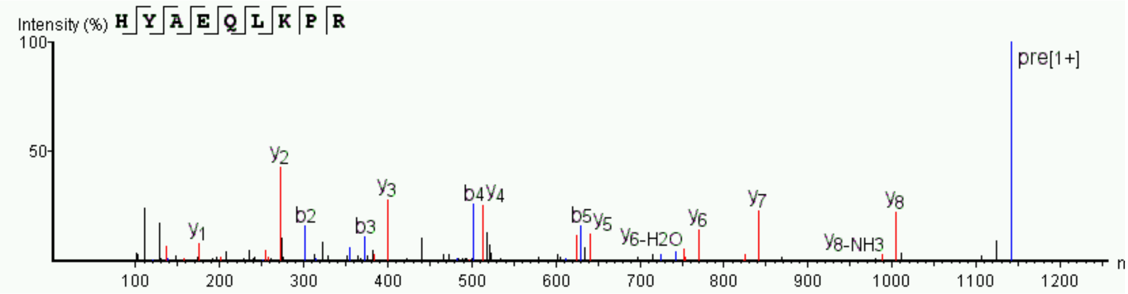

gi|571554312

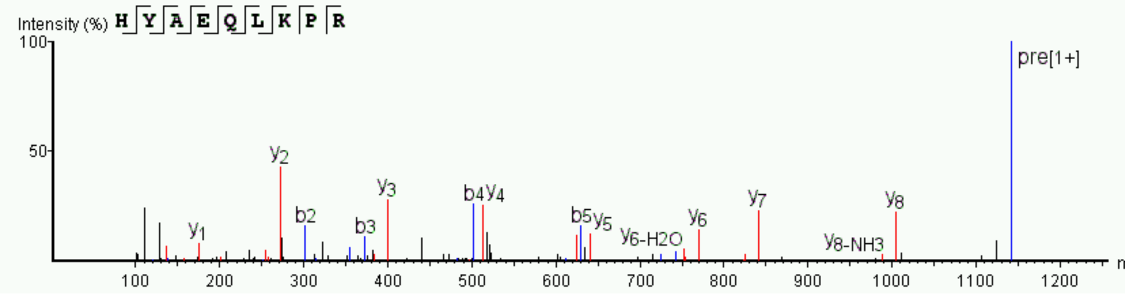

gi|571554316

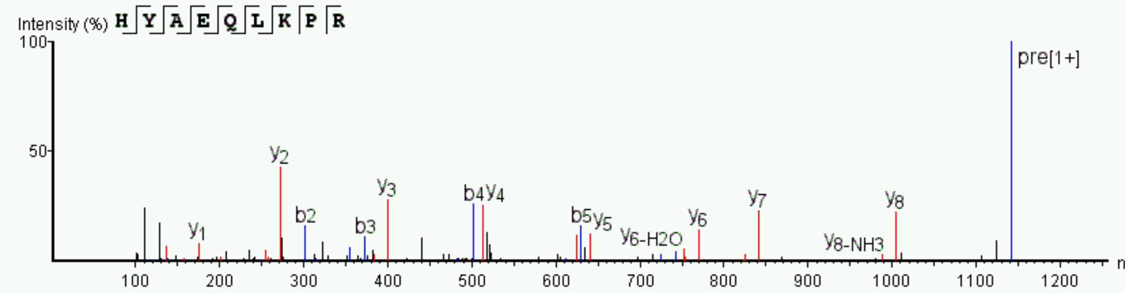

gi|571554319

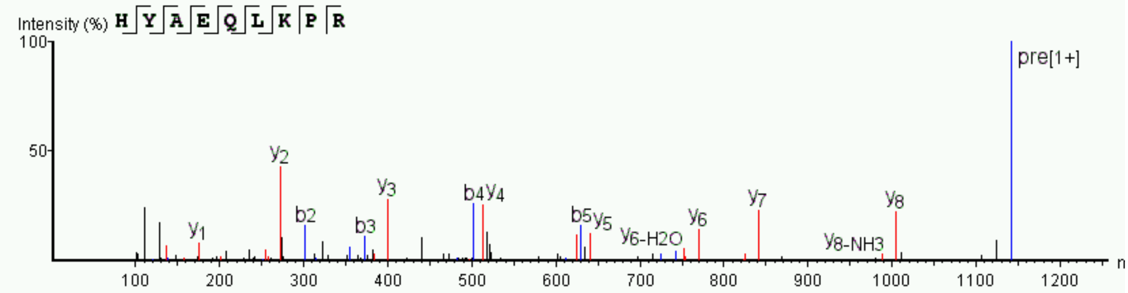

gi|571554322

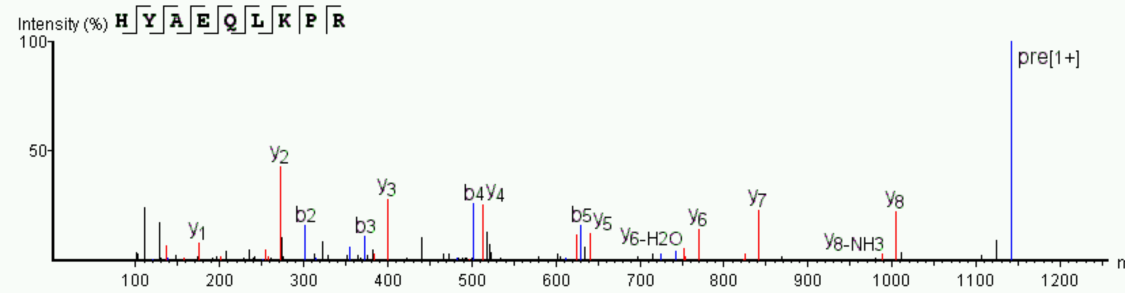

gi|571549099

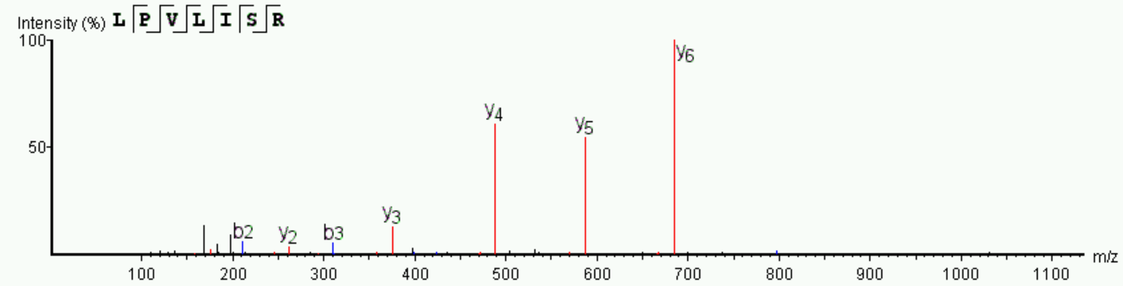

gi|571549102

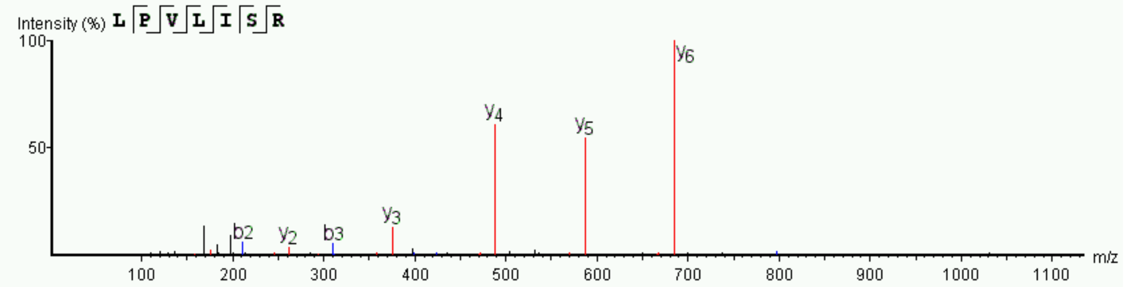

gi|571509128

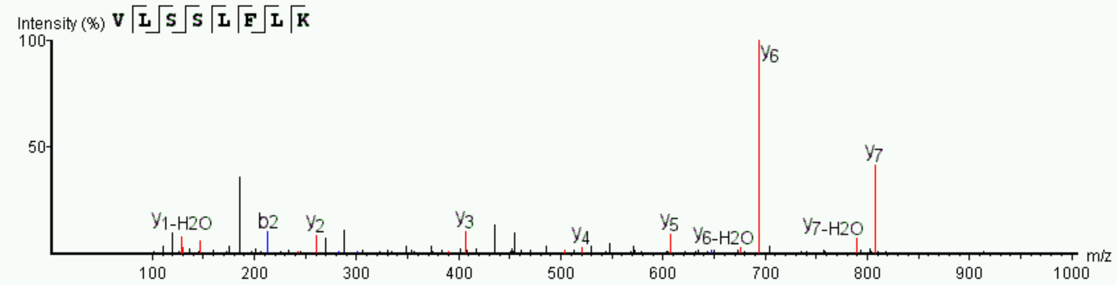

gi|328792443

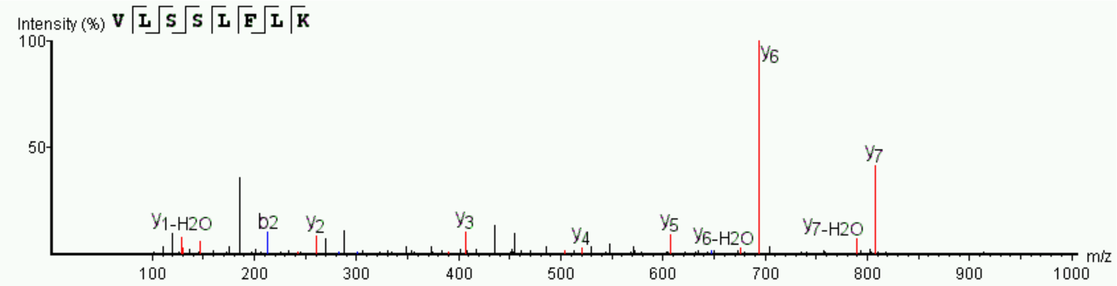

gi|571556287

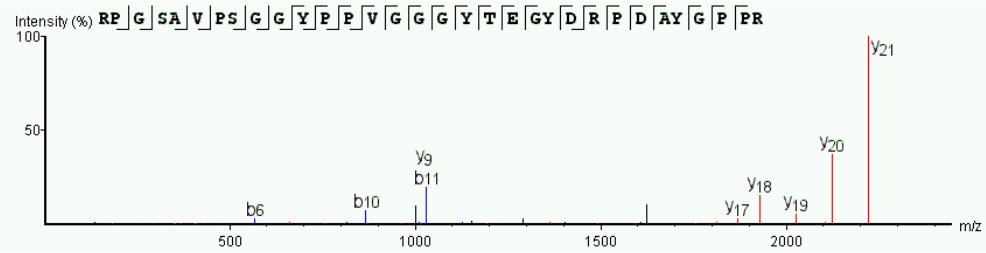

gi|66516797

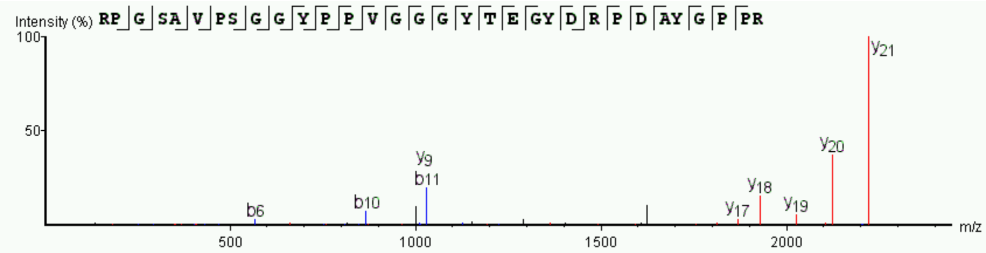

gi|571556291

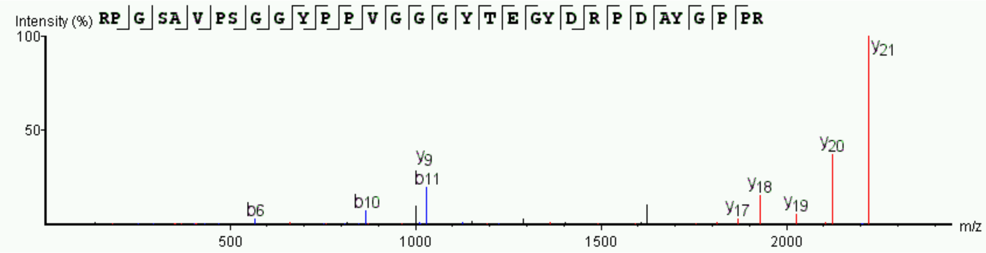

gi|571556295

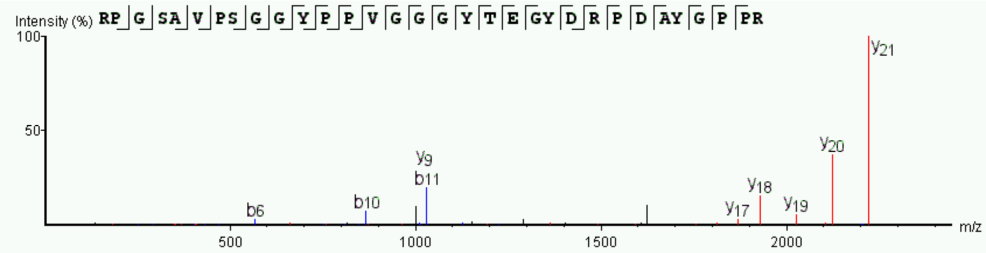

gi|571556299

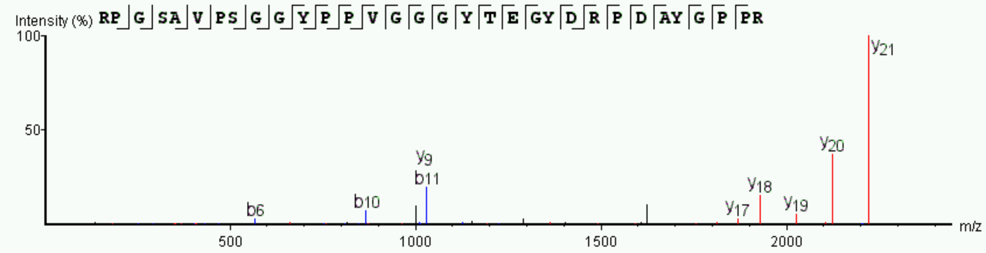

gi|571556305

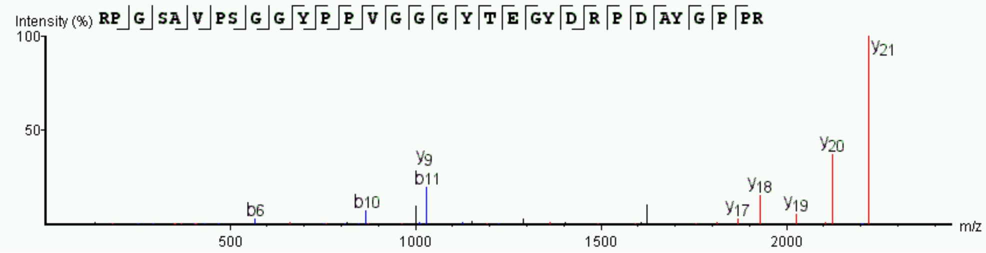

gi|571556309

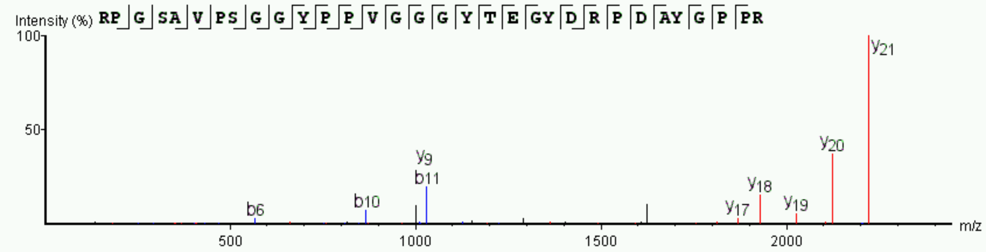

gi|571556316

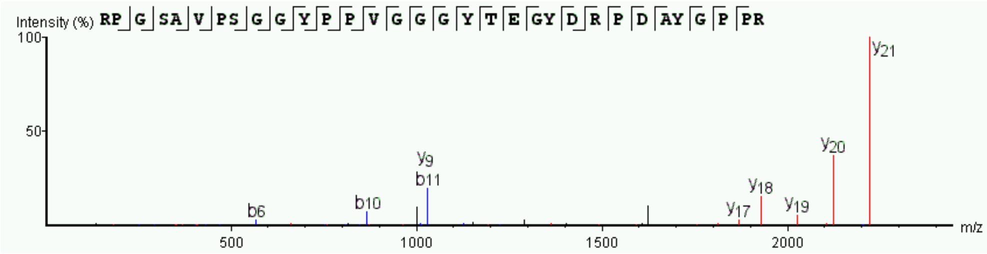

gi|571563982

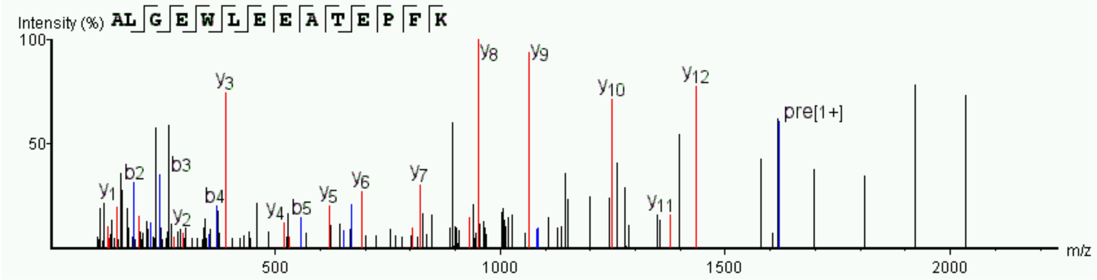

gi|571563985

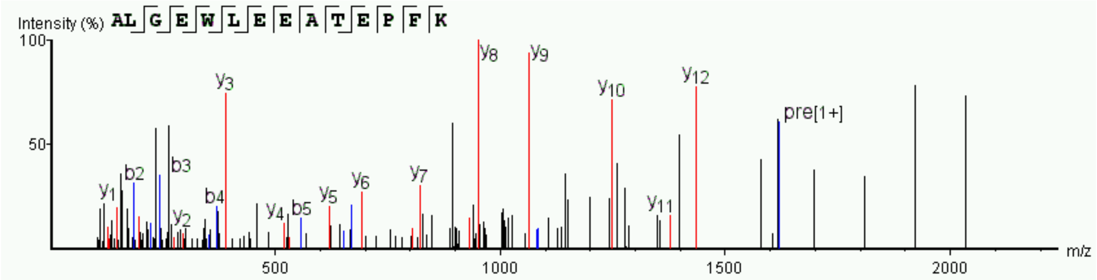

gi|571563988

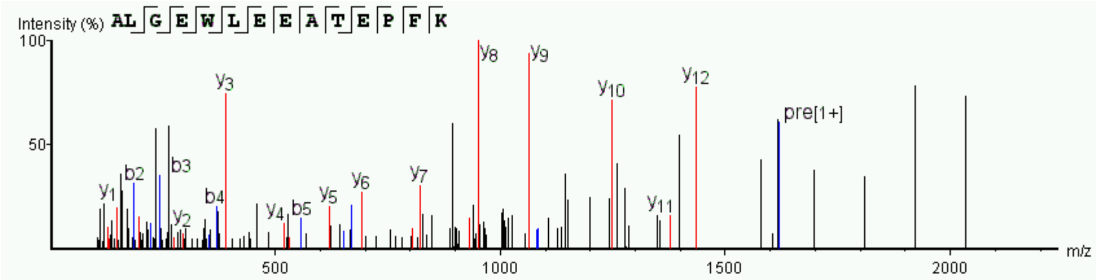

gi|571563990

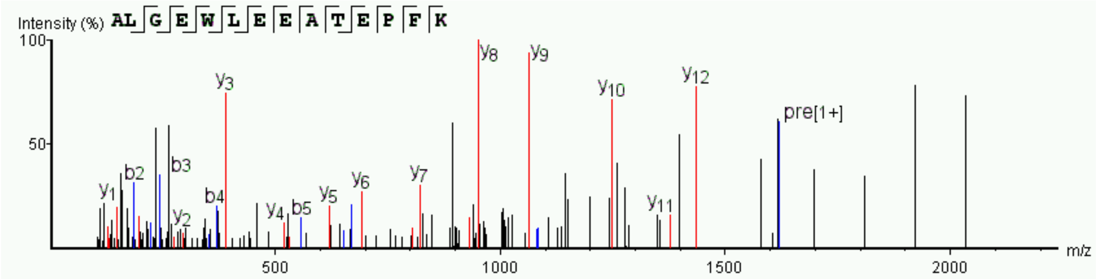

gi|571574413

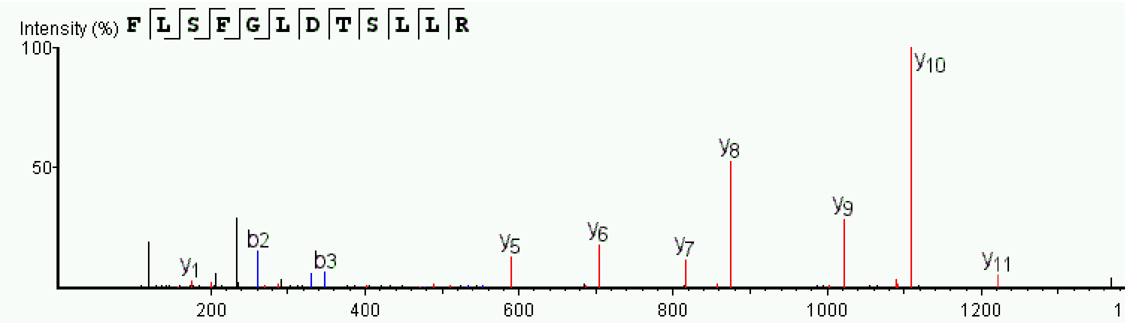

gi|571574415

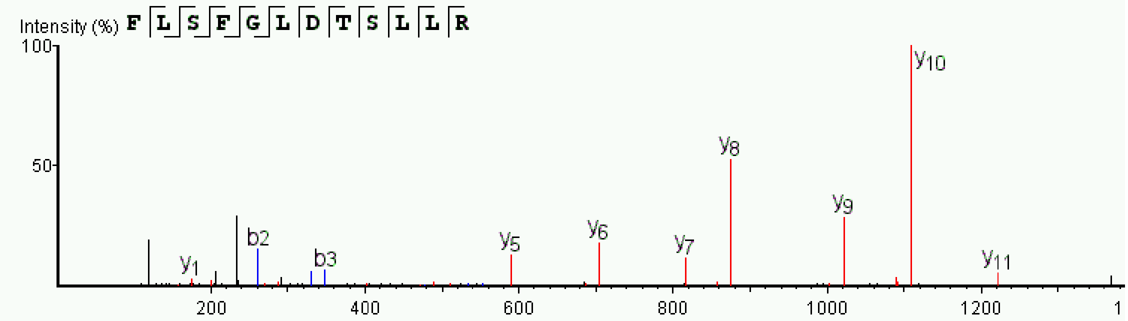

gi|571534149

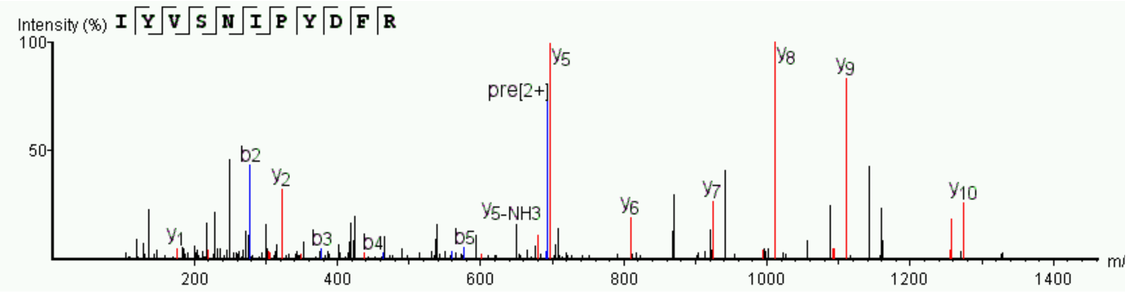

gi|571534152

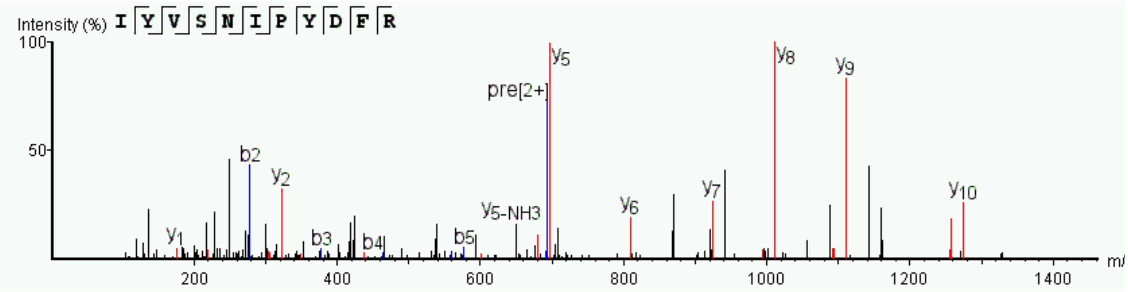

gi|571534155

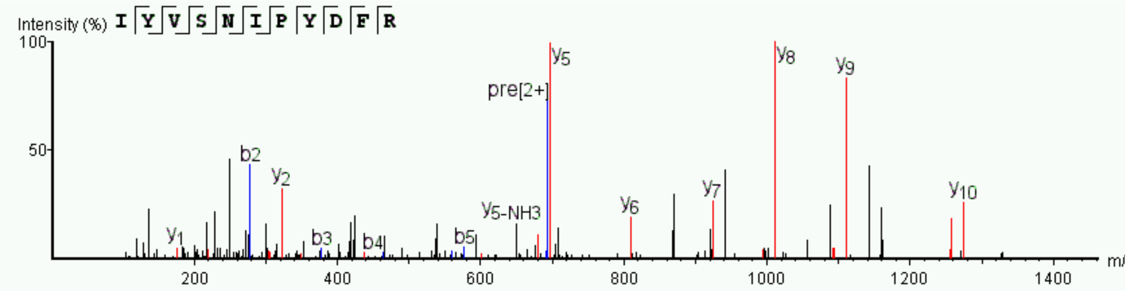

gi|571534158

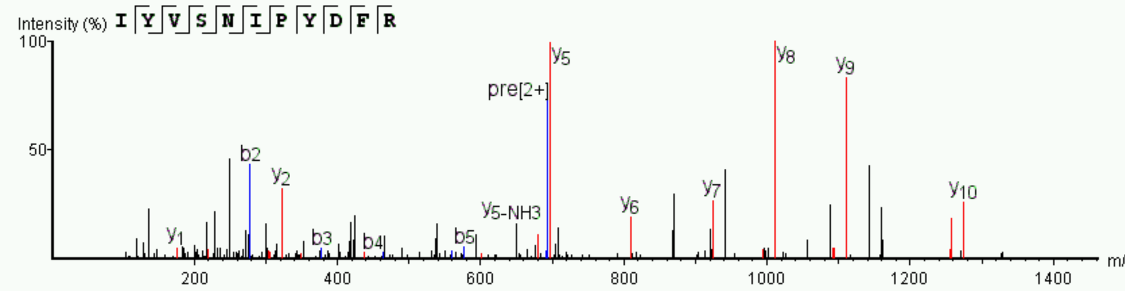

gi|571534161

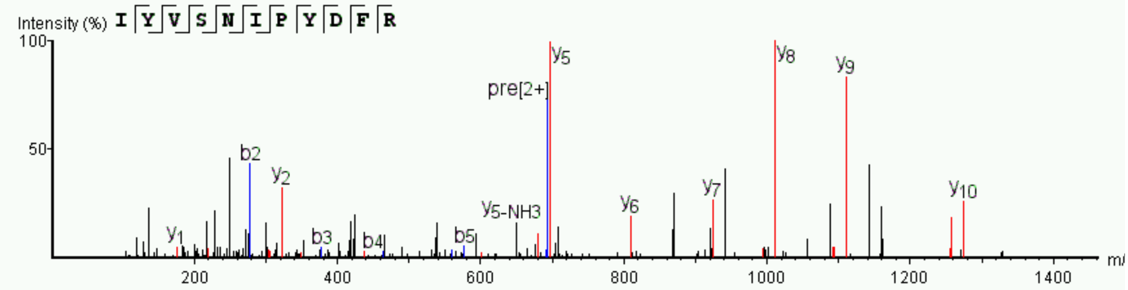

gi|571506852

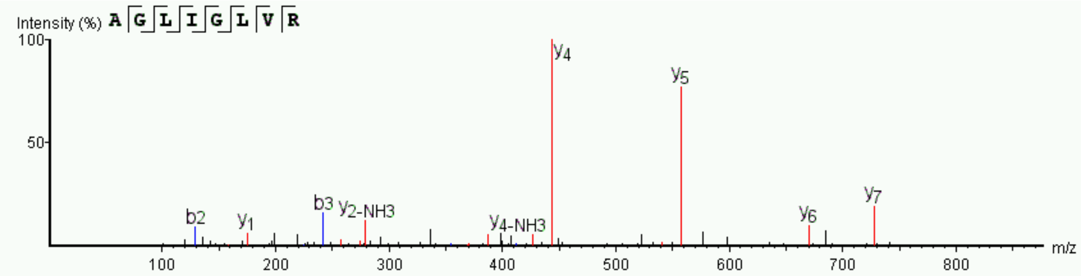

gi|571506855

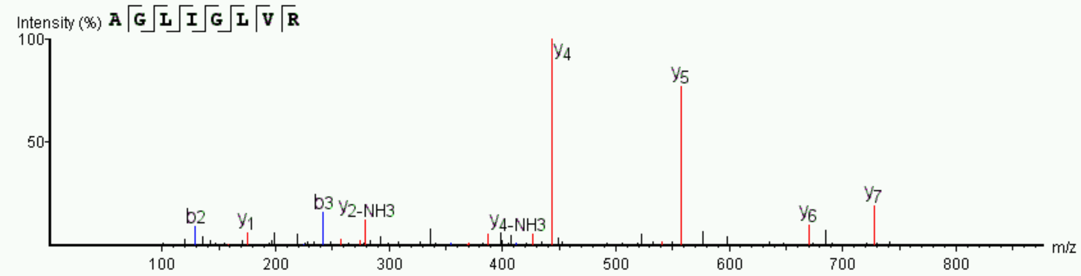

gi|571506858

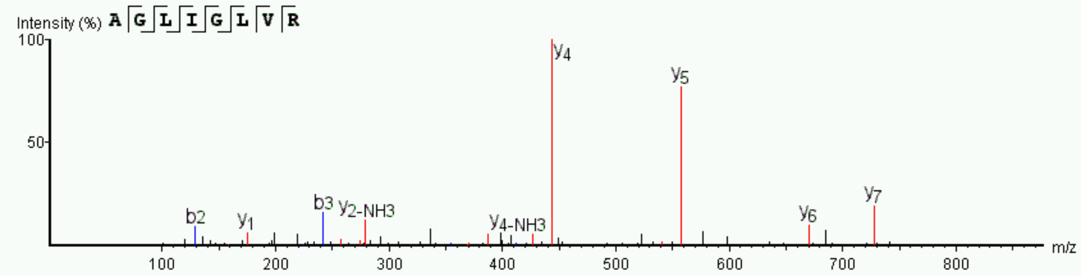

gi|66500257

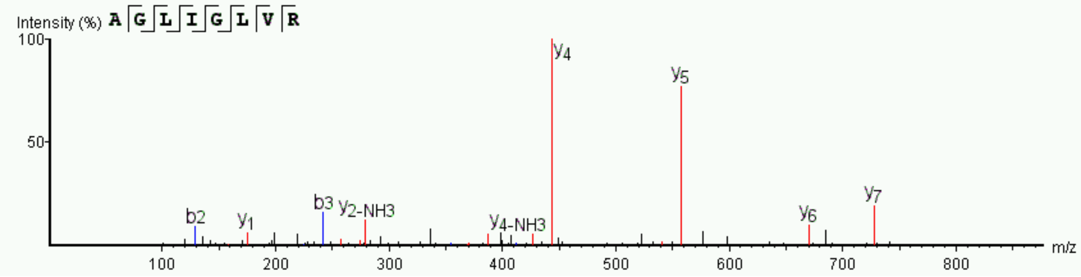

gi|571511334

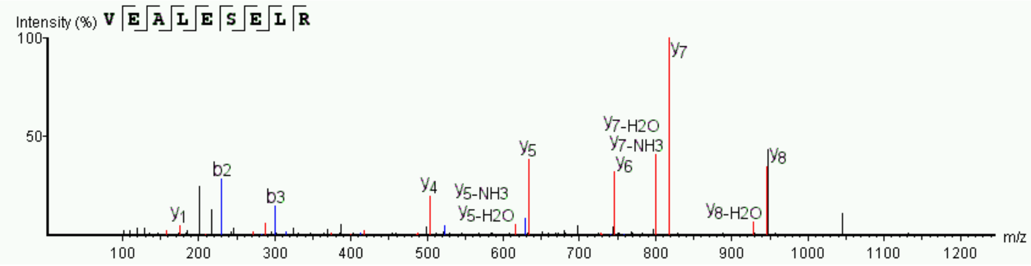

gi|328778117

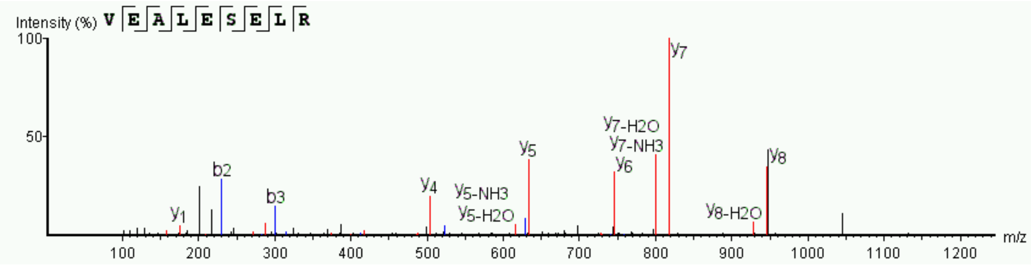

gi|328792621

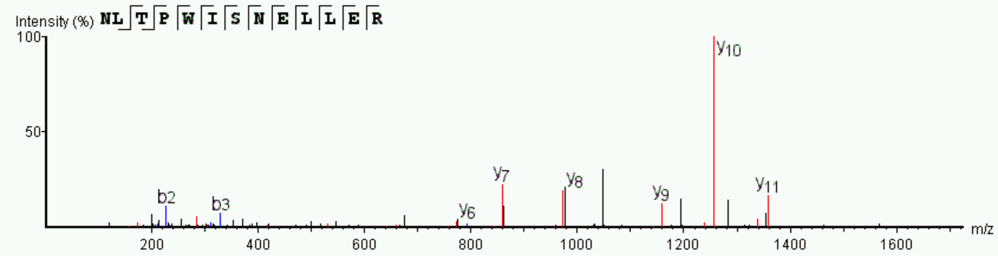

gi|571509569

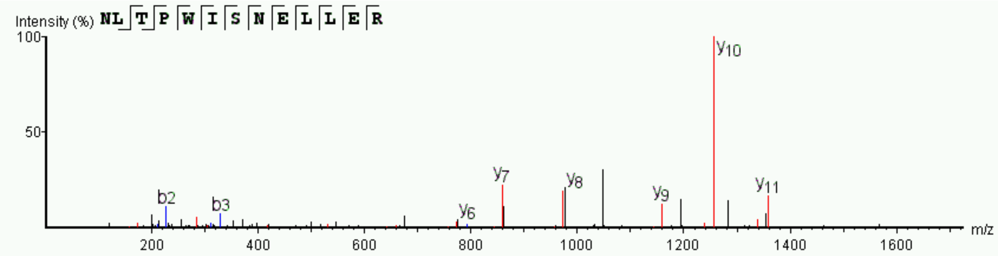

gi|571509575

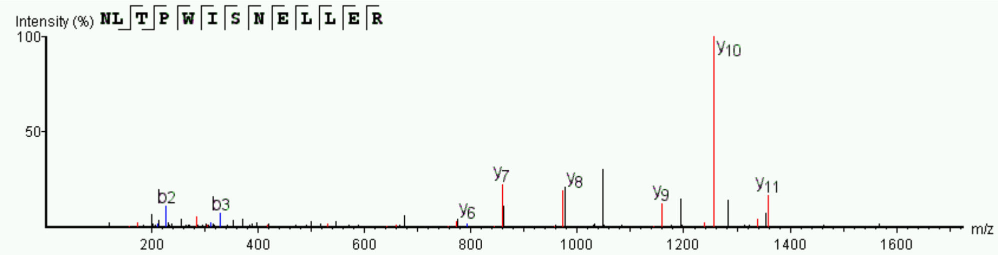

gi|571509578

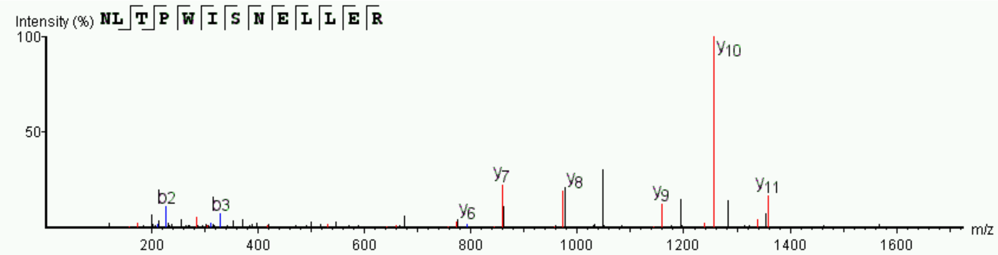

gi|571509582

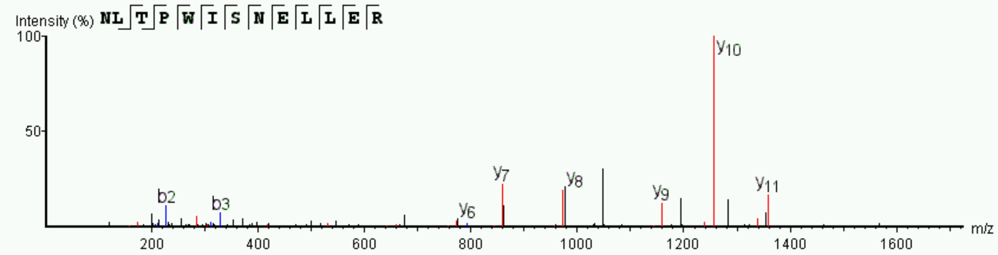

gi|571509585

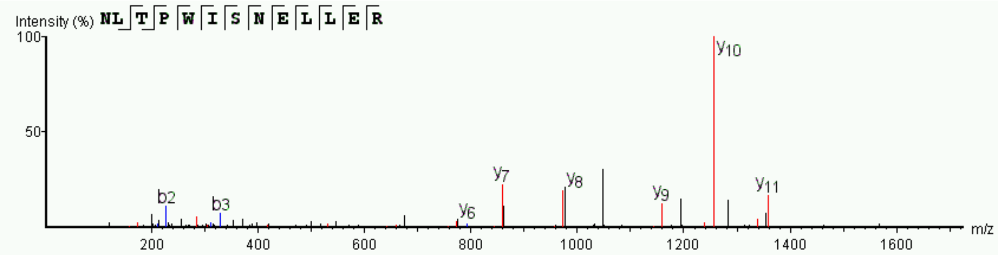

gi|571509589

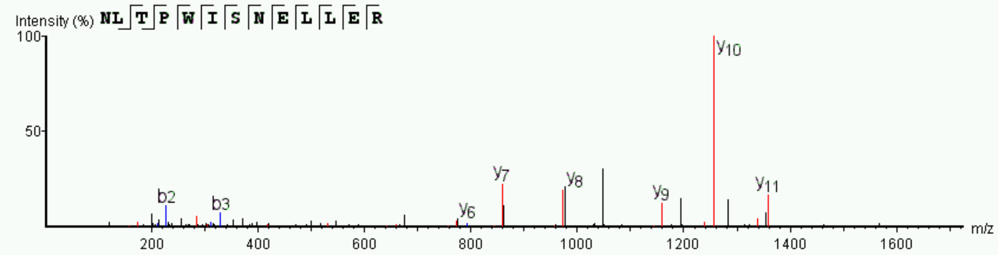

gi|571509593

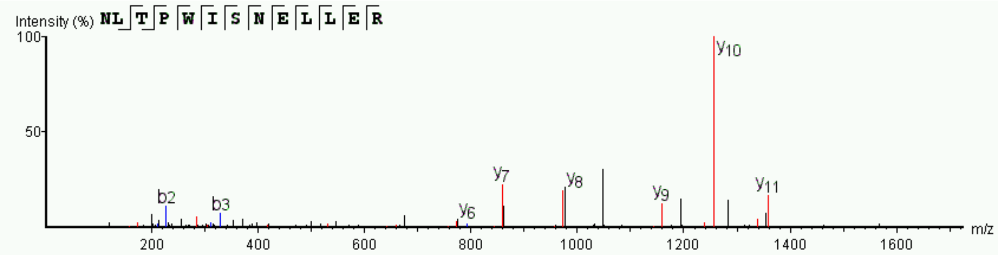

gi|571534598

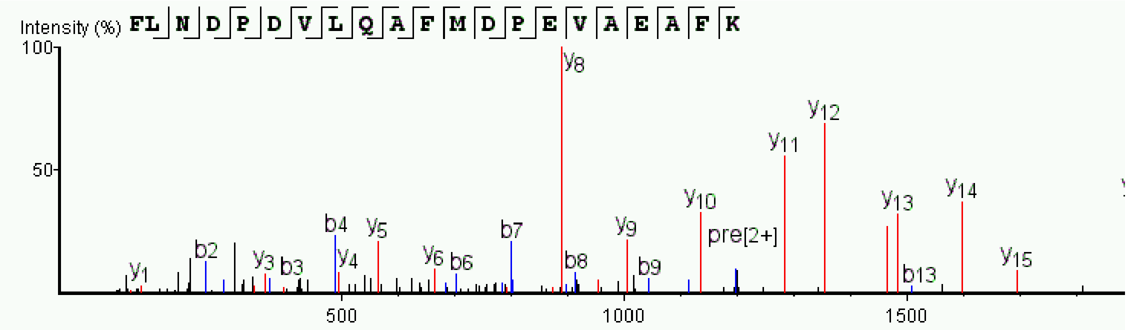

gi|571534601

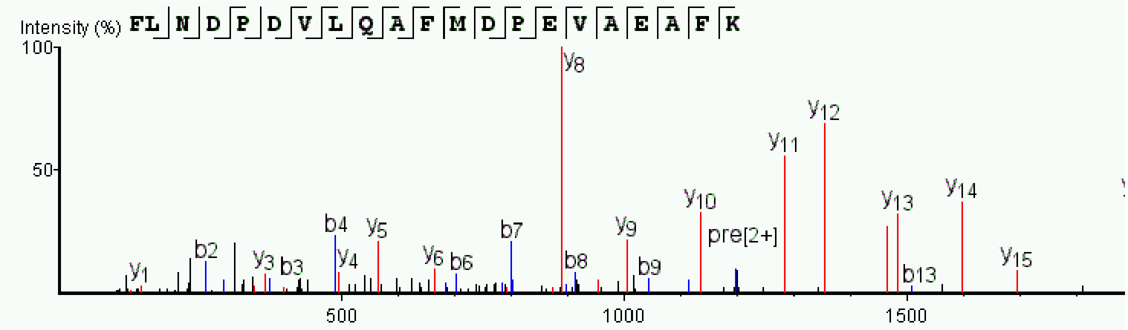

gi|571534840

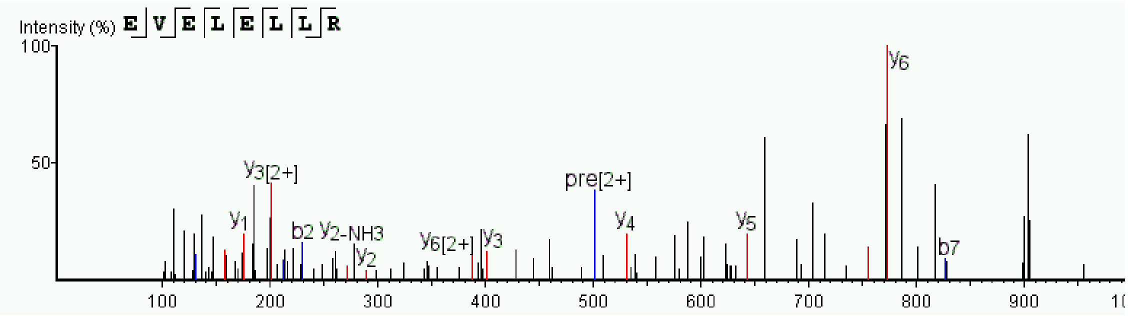

gi|571534843

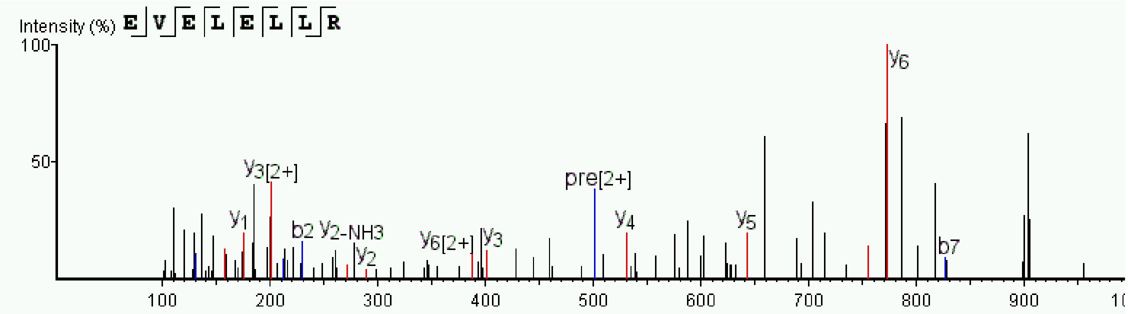

gi|571534846

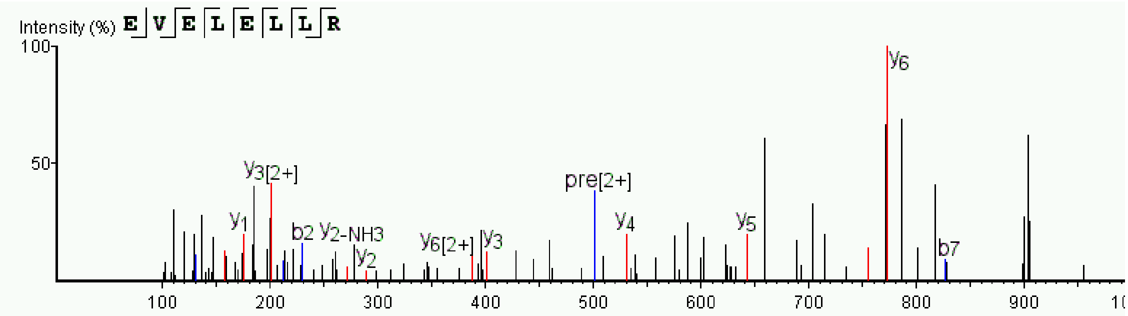

gi|571534849

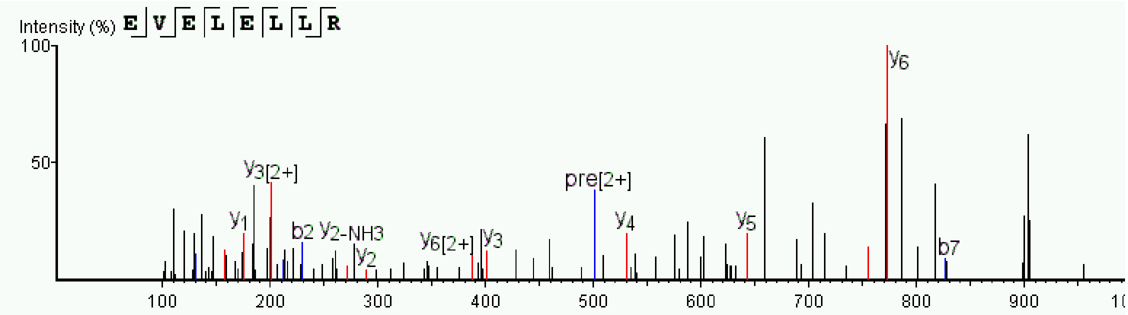

gi|571534853

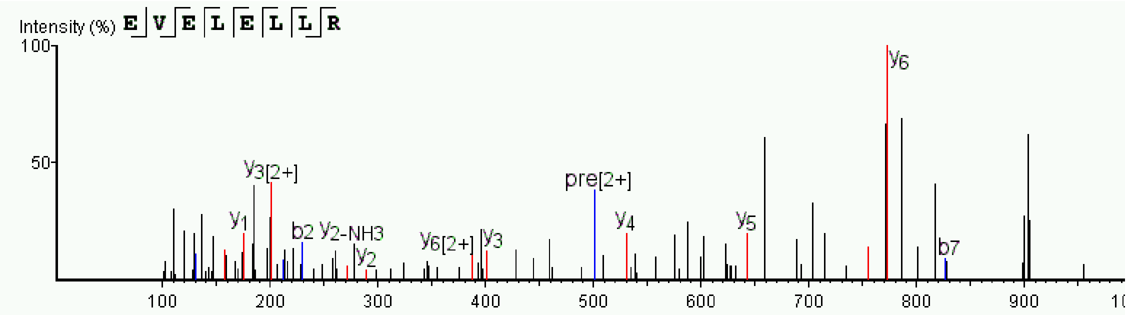

gi|571504286

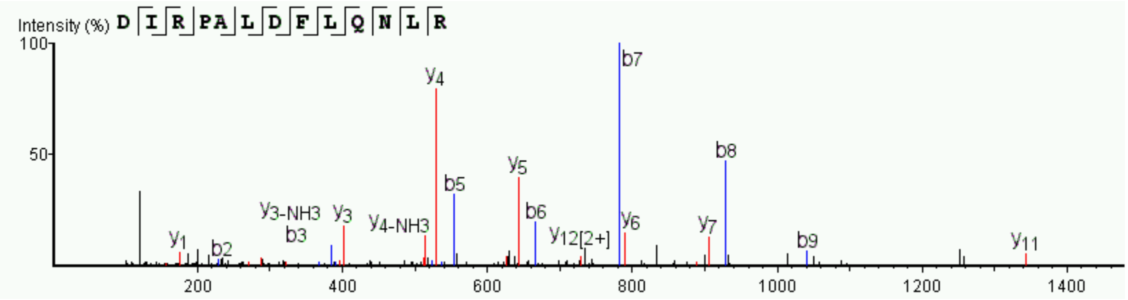

gi|571504293

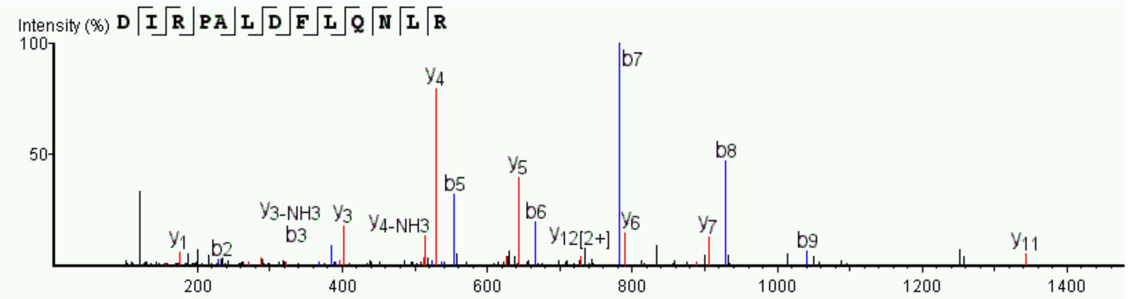

gi|571504295

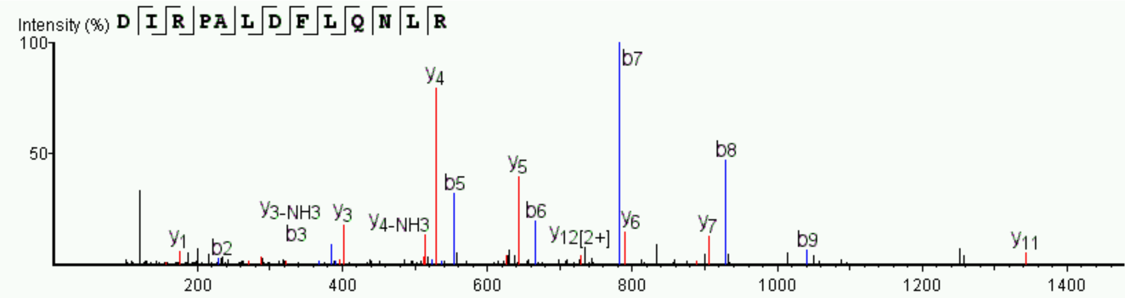

gi|571504299

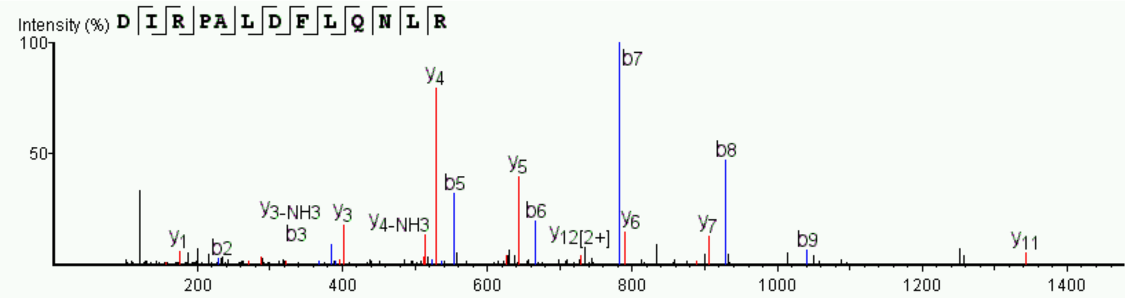

gi|66542091

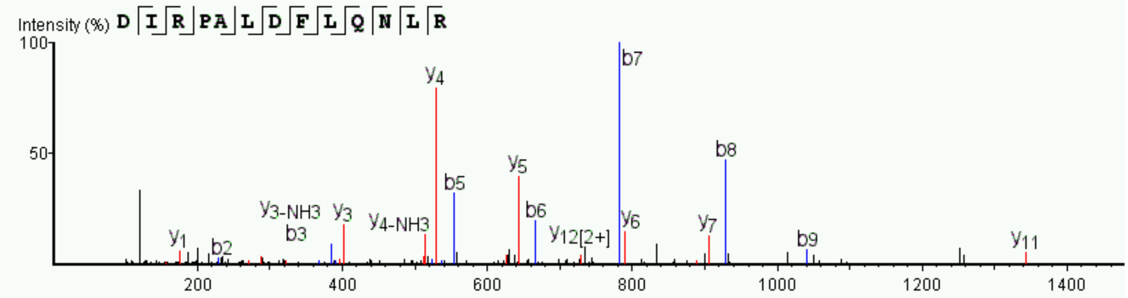

gi|571571431

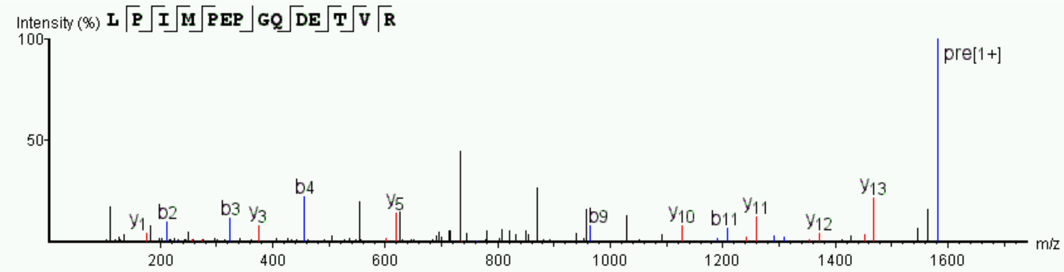

gi|571571434

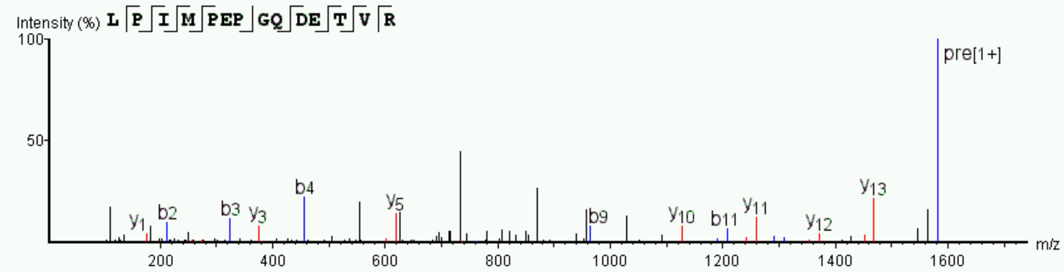

gi|571571438

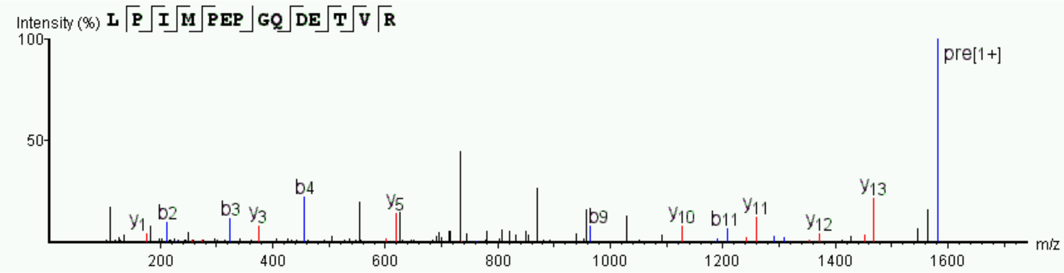

gi|571571223

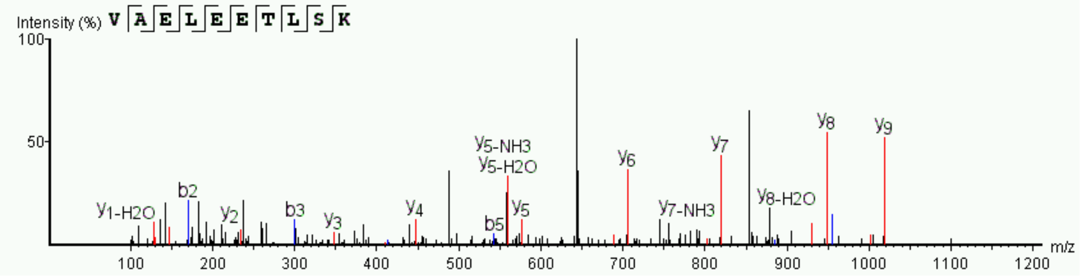

gi|571571261

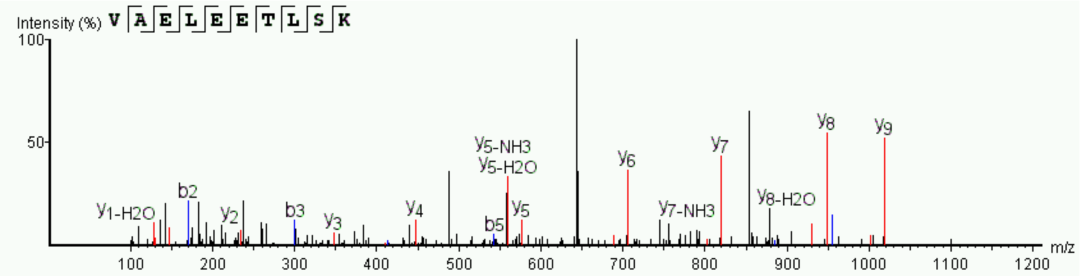

gi|571571265

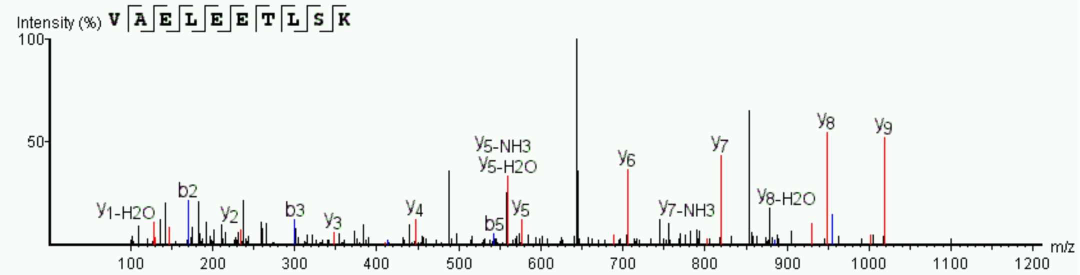

gi|571571269

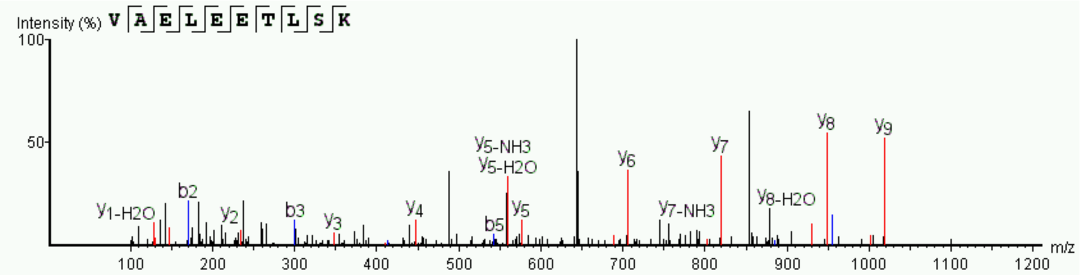

gi|571571275

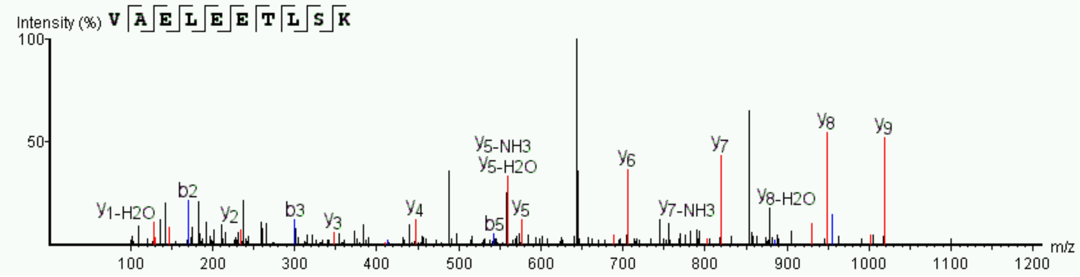

gi|571571279

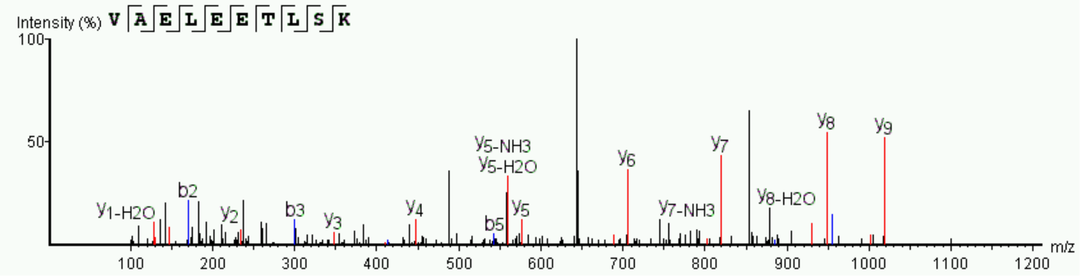

gi|571571227

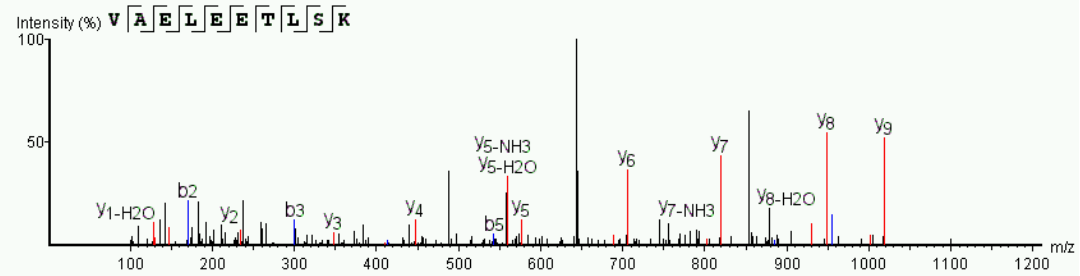

gi|571571231

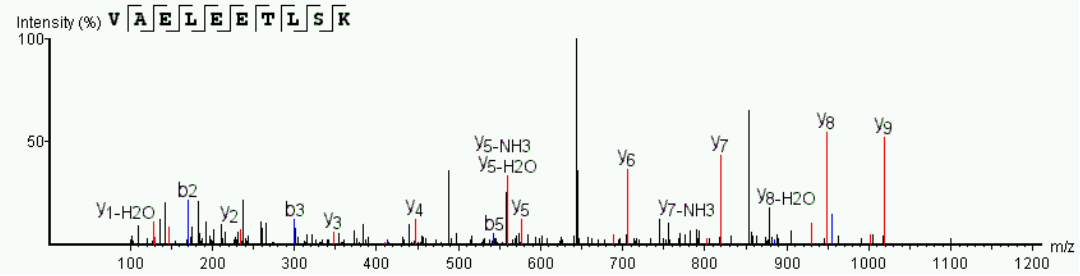

gi|571571235

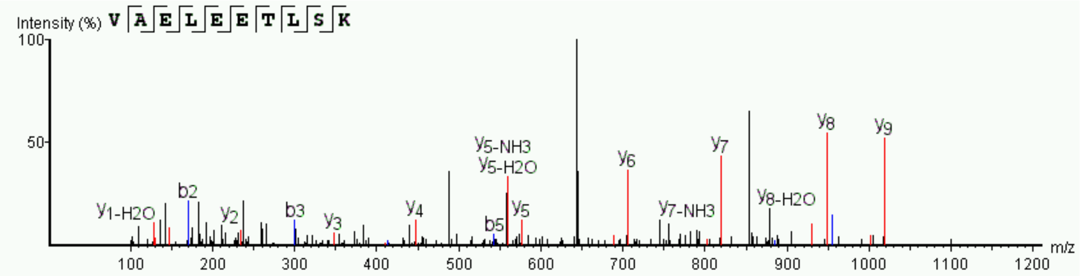

gi|571571239

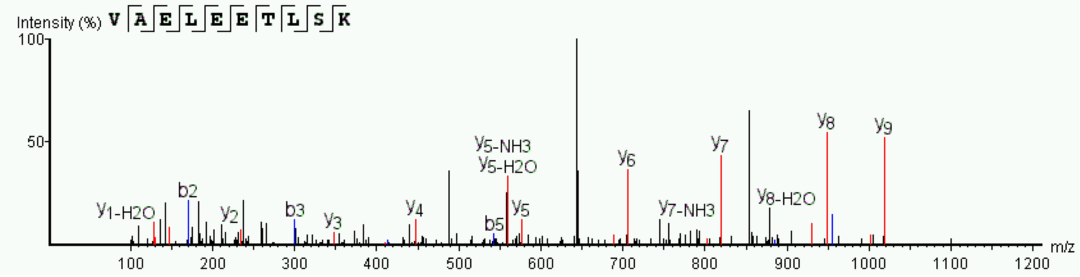

gi|571571243

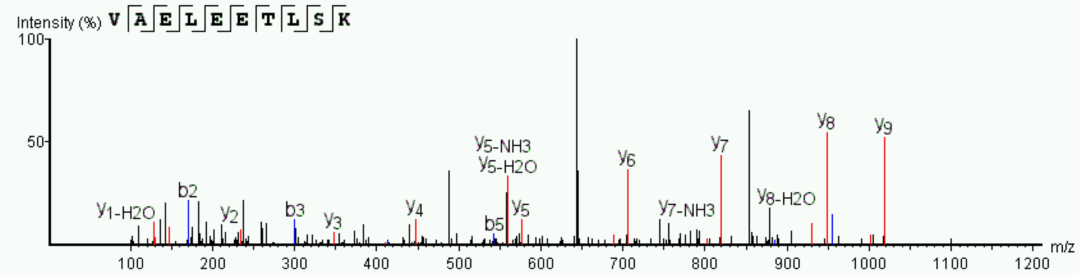

gi|571571249

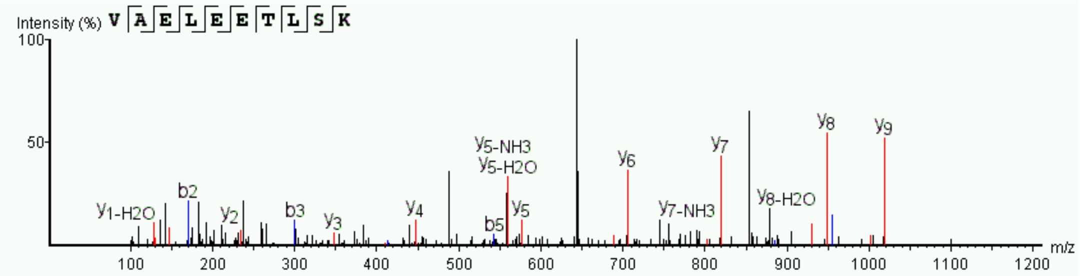

gi|571571253

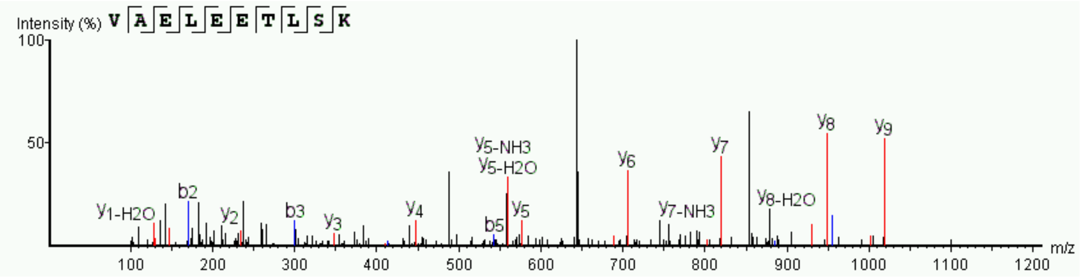

gi|571571257

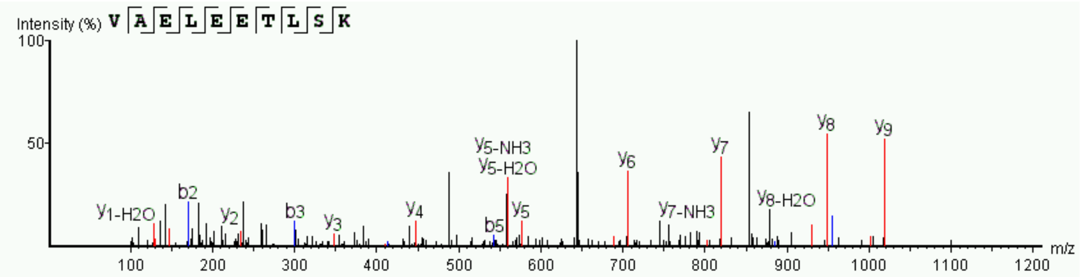

gi|328788274

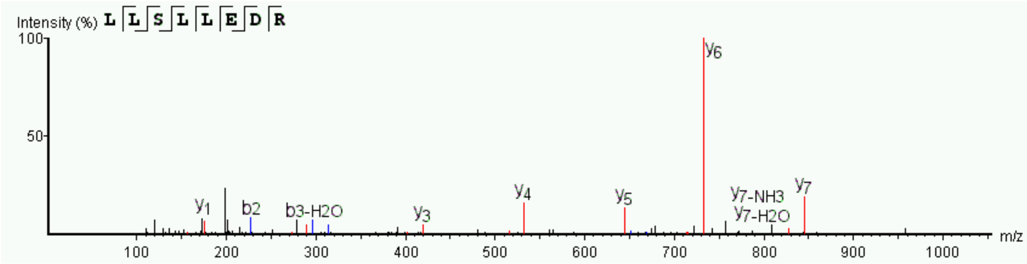

gi|571529763

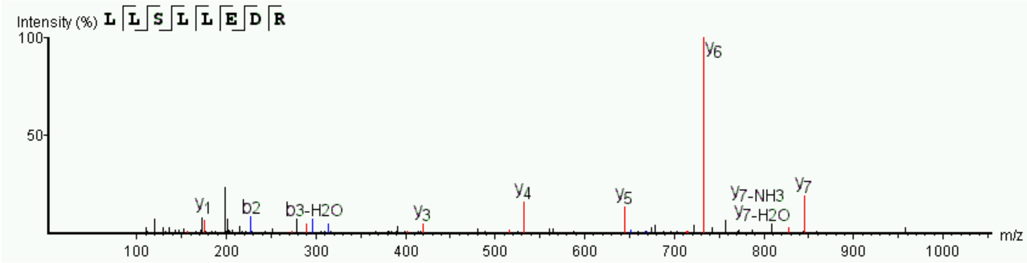

gi|571529767

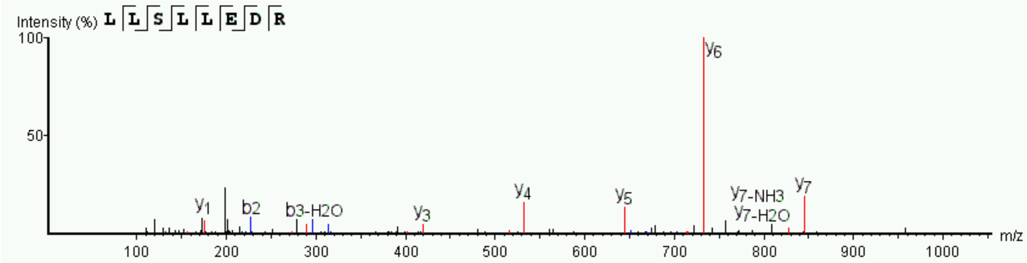

gi|571529770

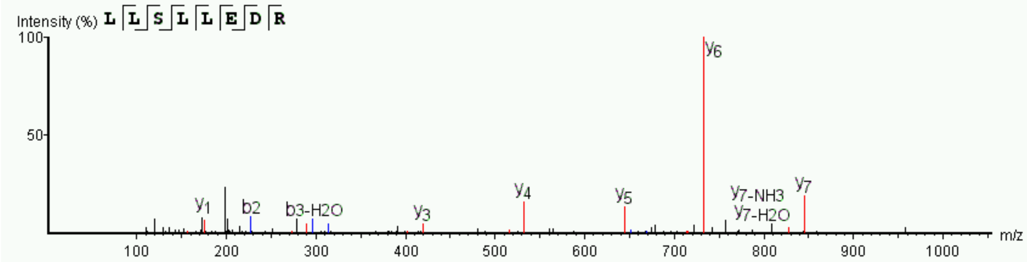

gi|571529773

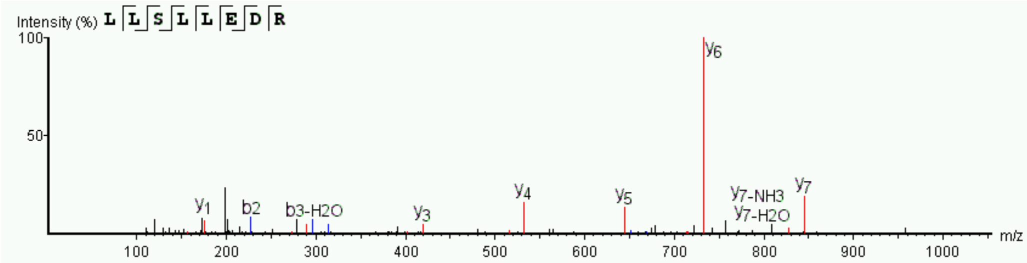

gi|571529779

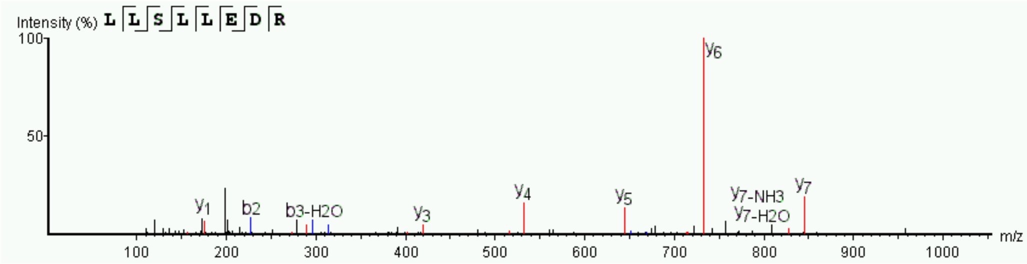

gi|571529782

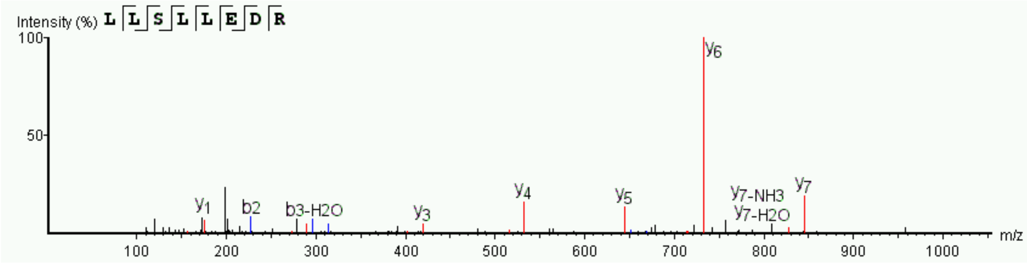

gi|571529785

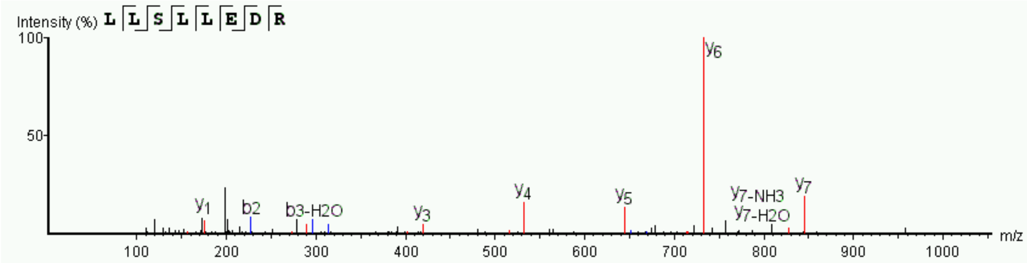

gi|110760068

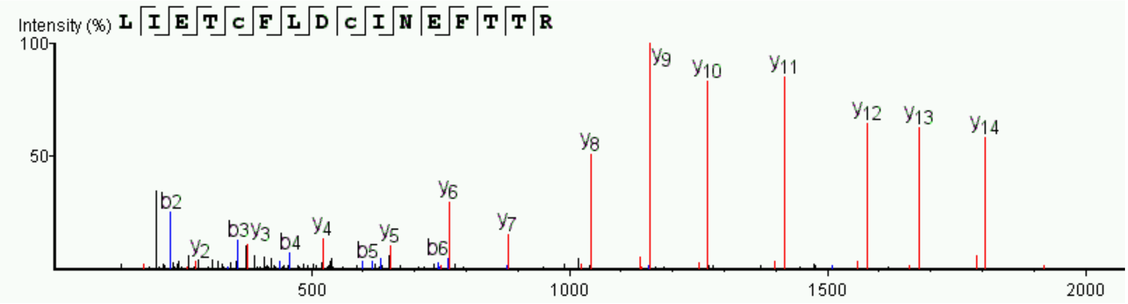

gi|571553163

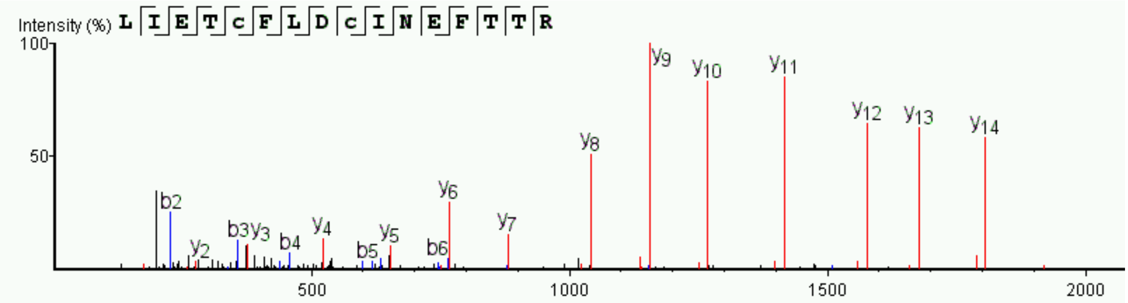

gi|328777790

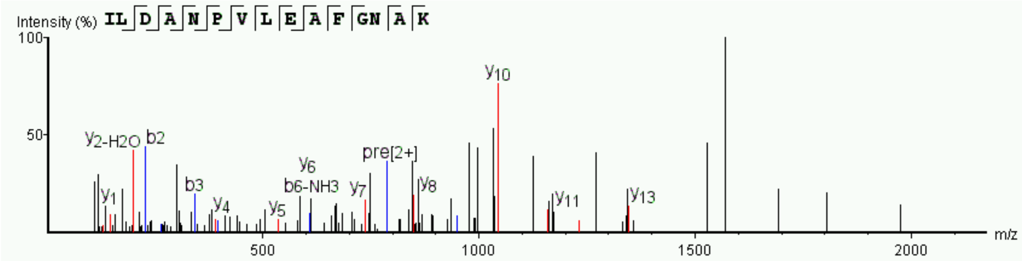

gi|571563508

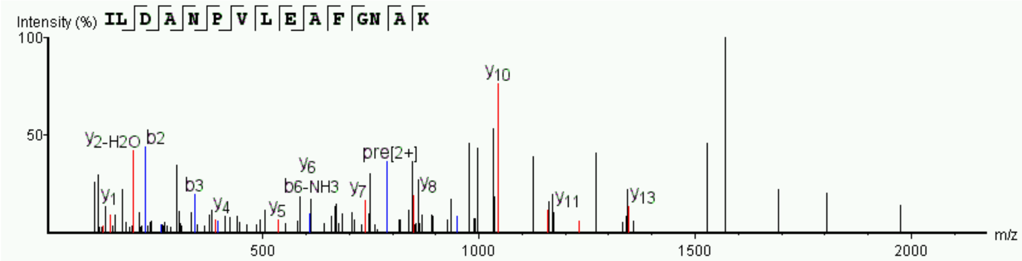

gi|571546713

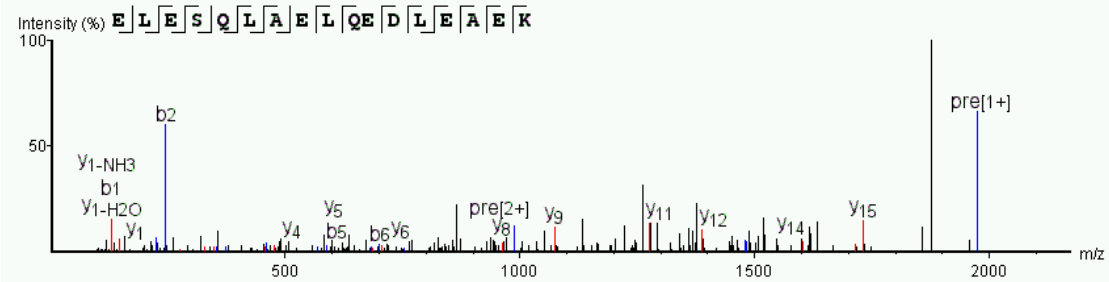

gi|571546716

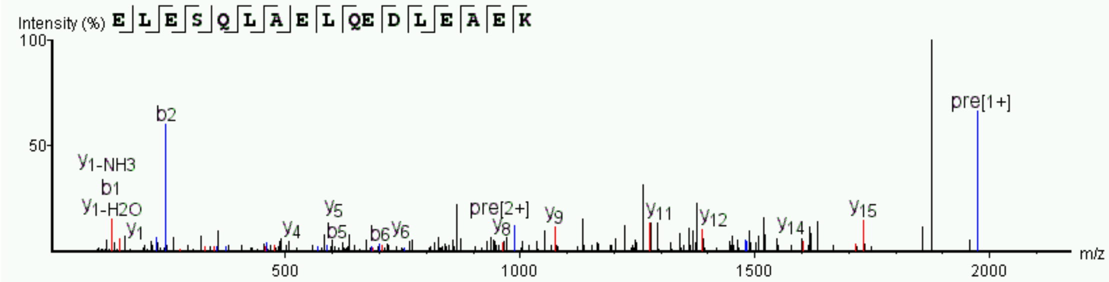

gi|571546720

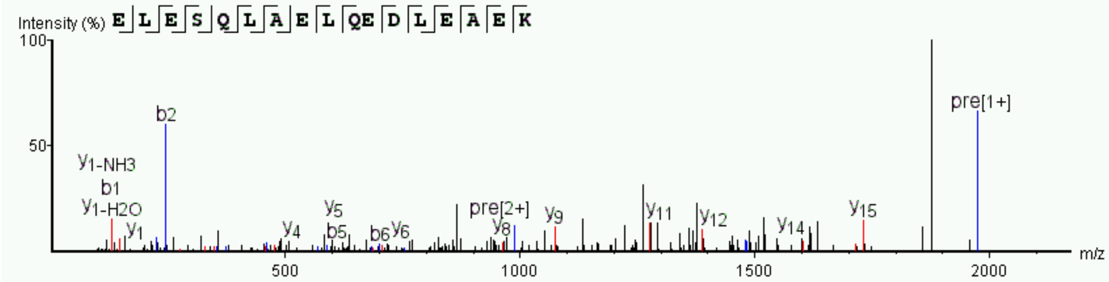

gi|571546724

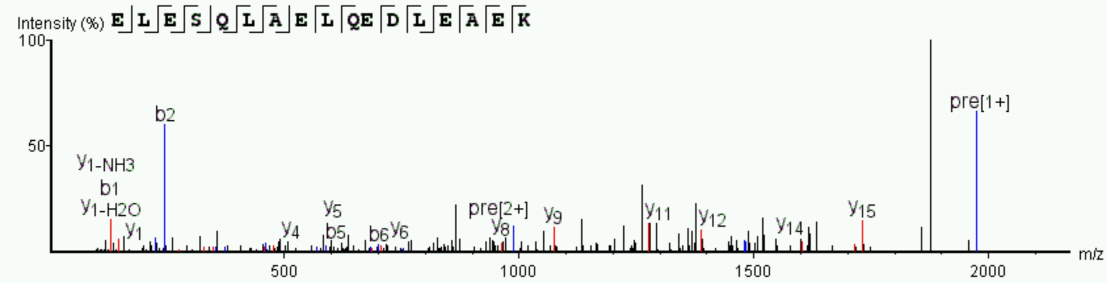

gi|571546728

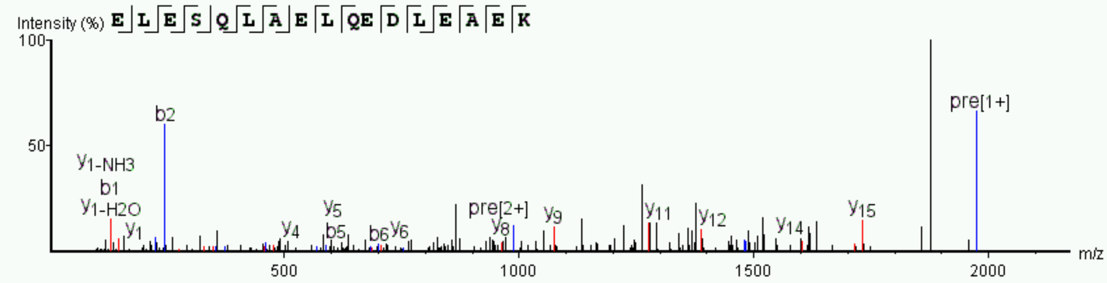

gi|571546732

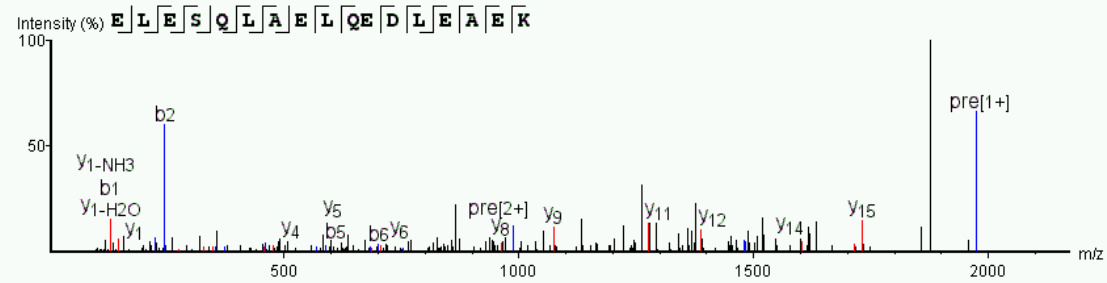

gi|571546736

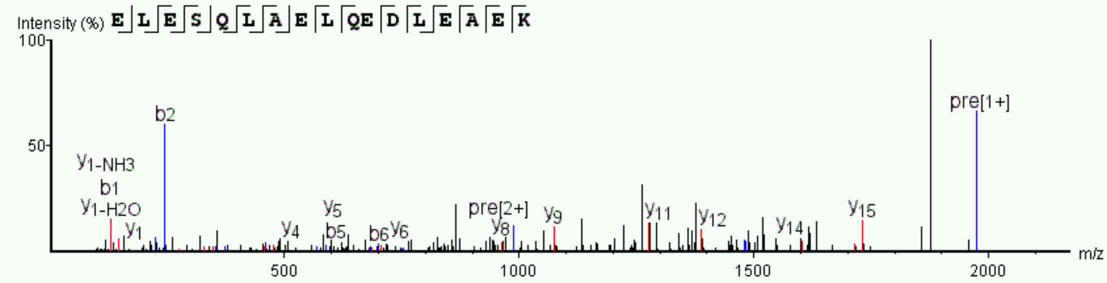

gi|571546740

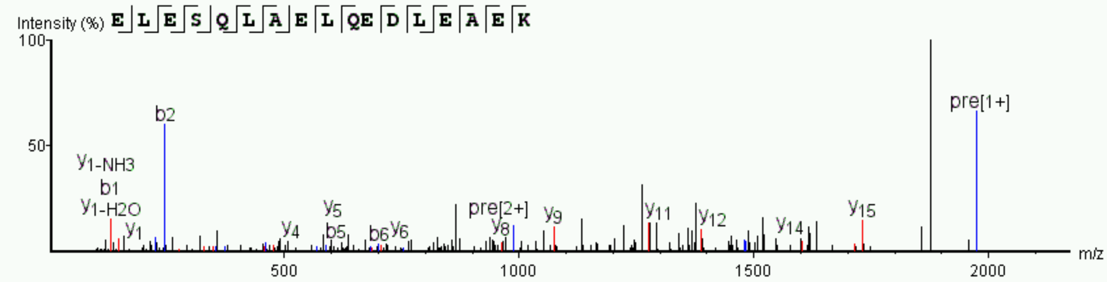

gi|571546709

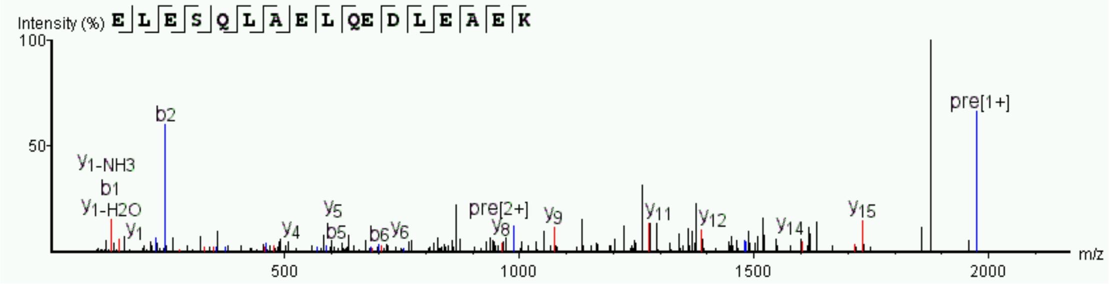

gi|571552397

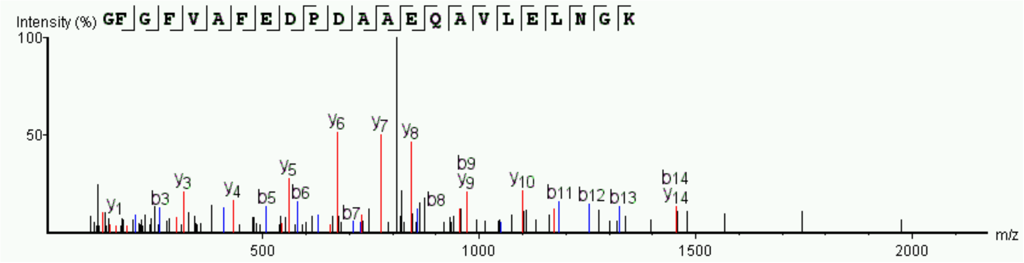

gi|571552393

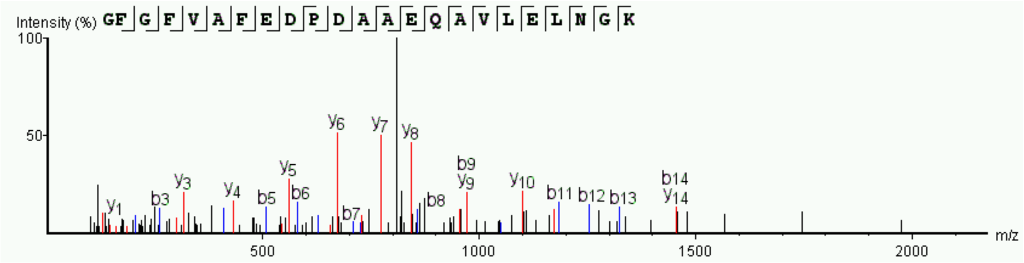

gi|571510037

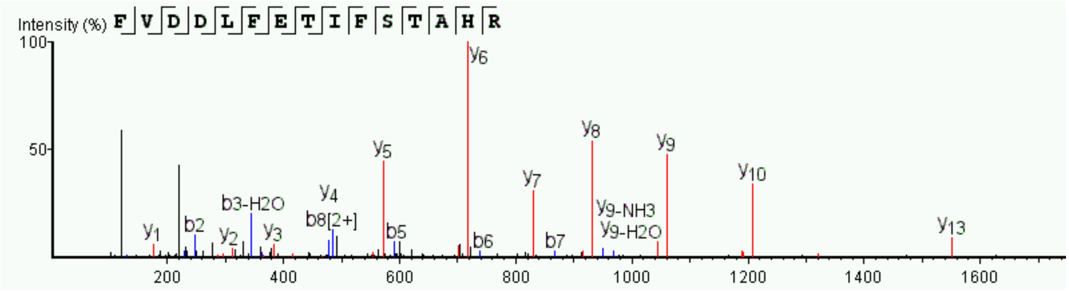

gi|571510040

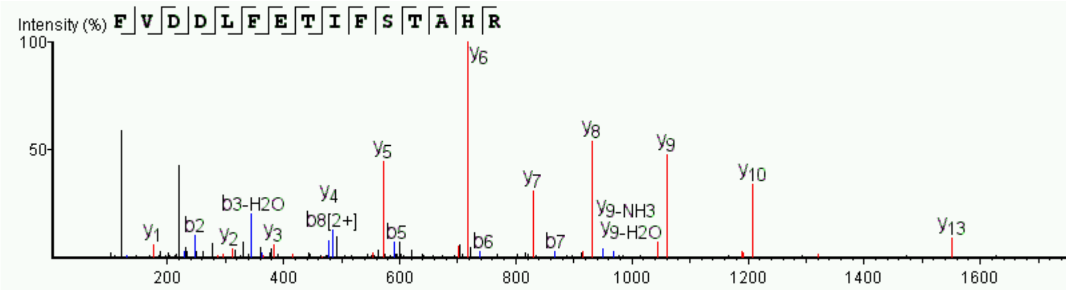

gi|110750043

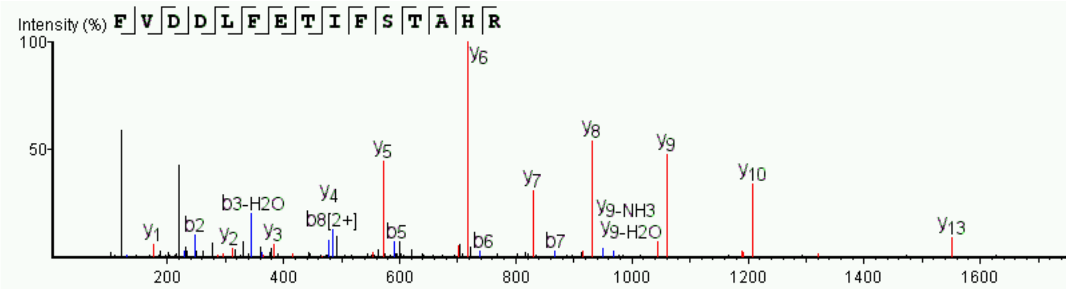

gi|571578229

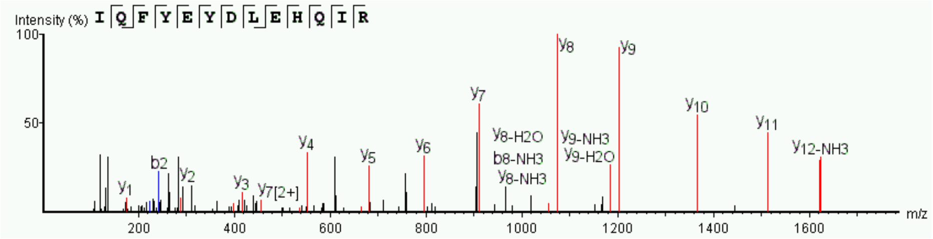

gi|571578231

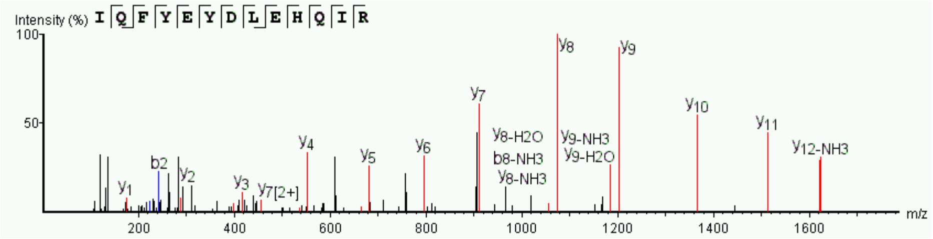

gi|571578233

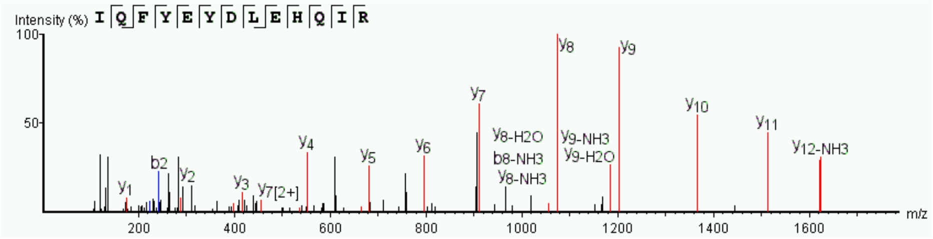

gi|66533867

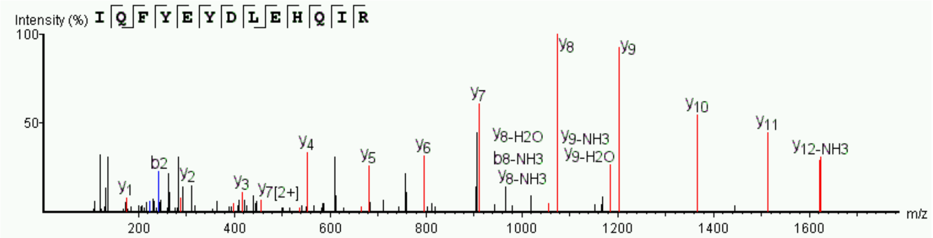

gi|571502210

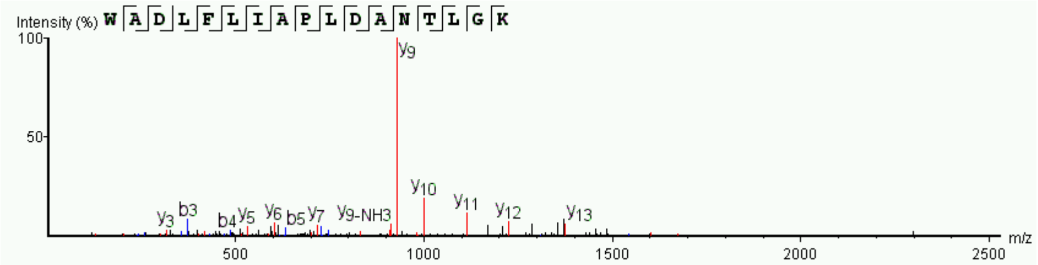

gi|48103807

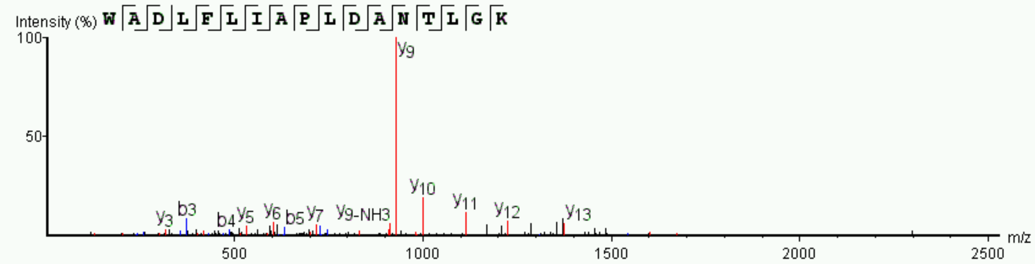

gi|328776594

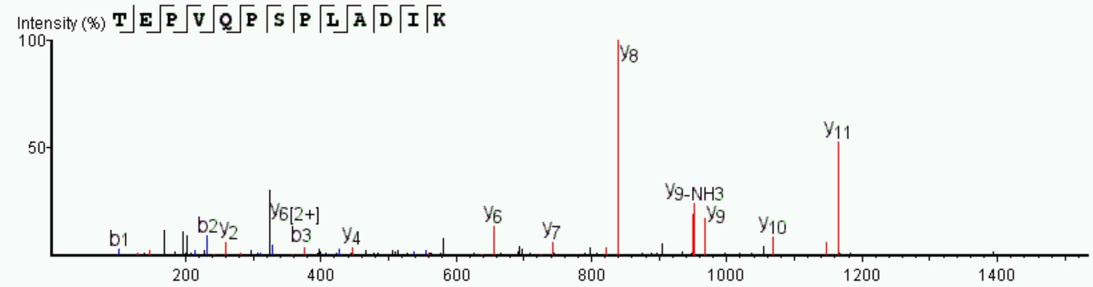

gi|328792171

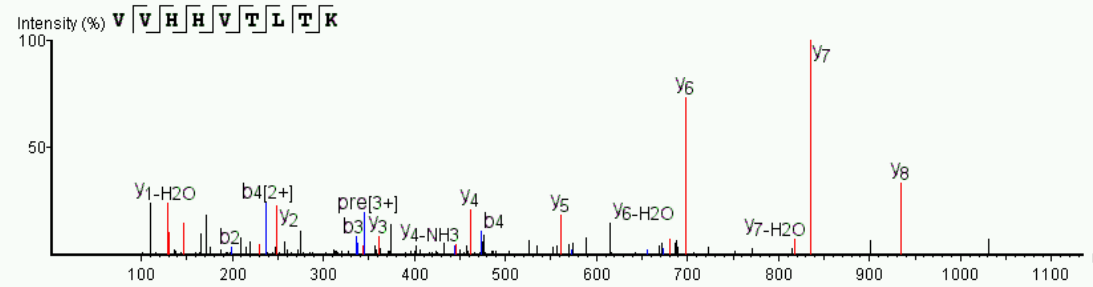

gi|571507416

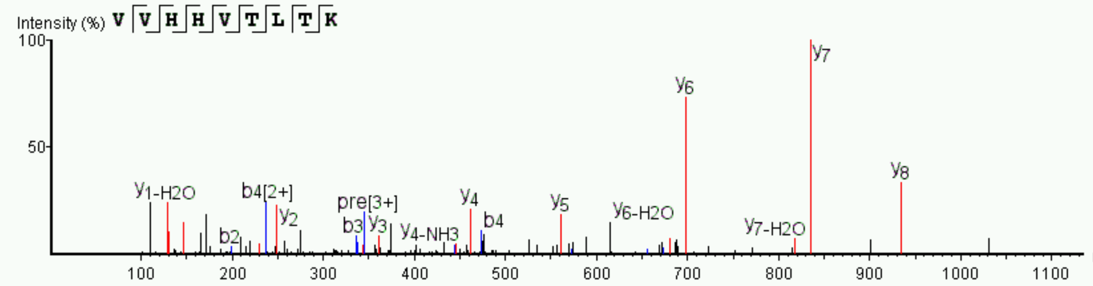

gi|571507420

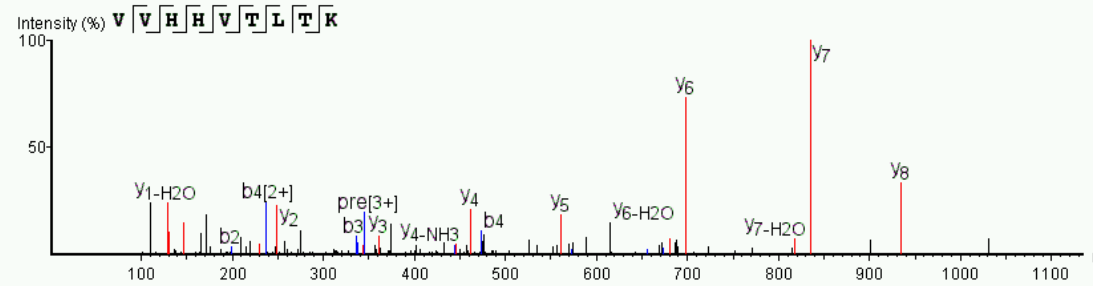

gi|571507424

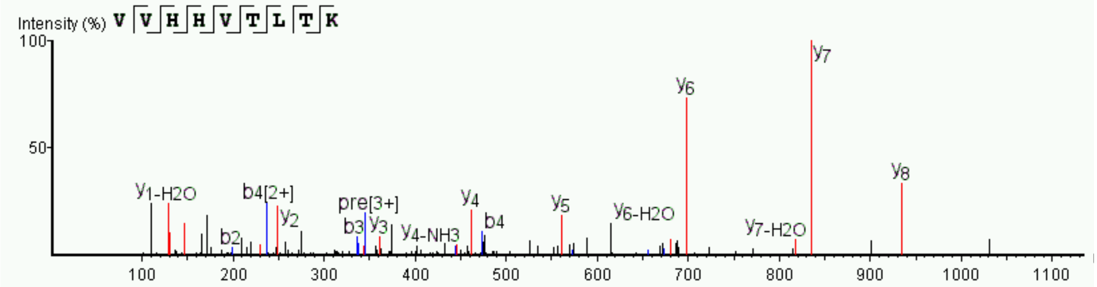

gi|571507427

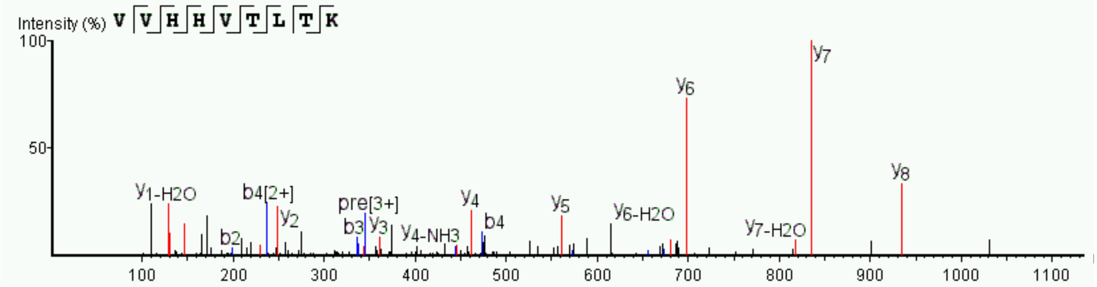

gi|571507430

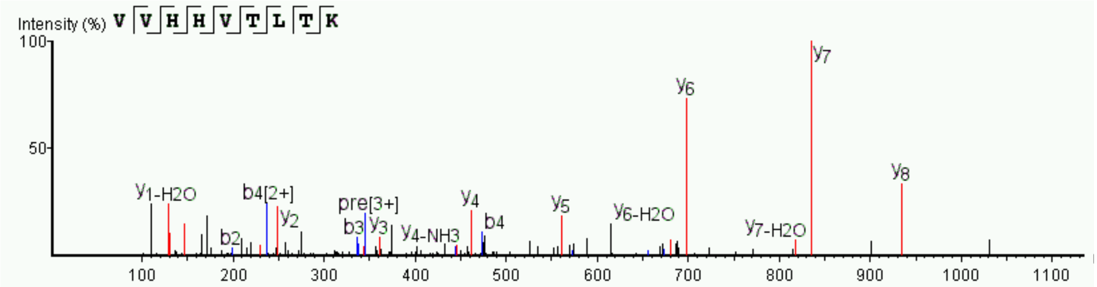

gi|571507387

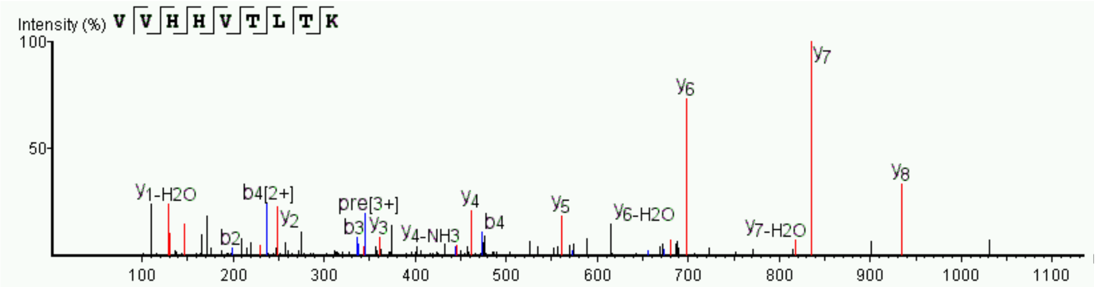

gi|571507434

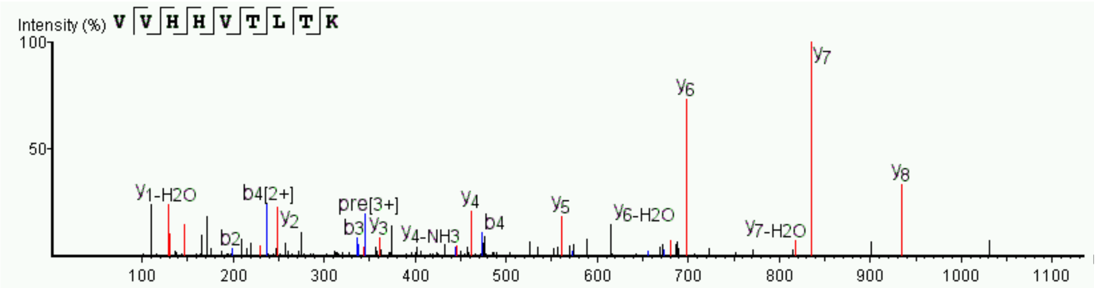

gi|571507392

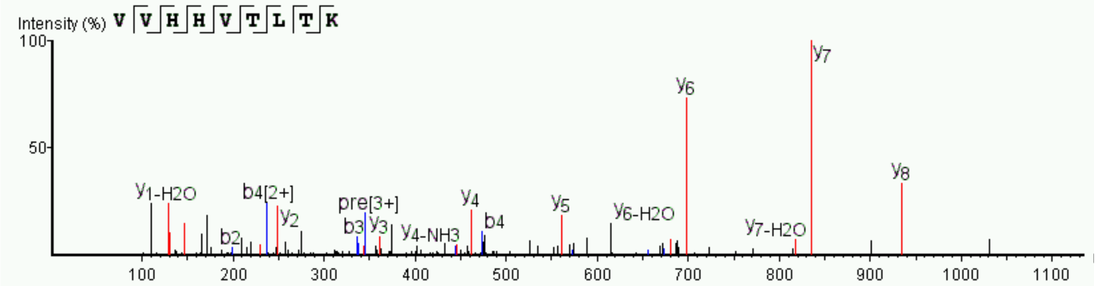

gi|571507395

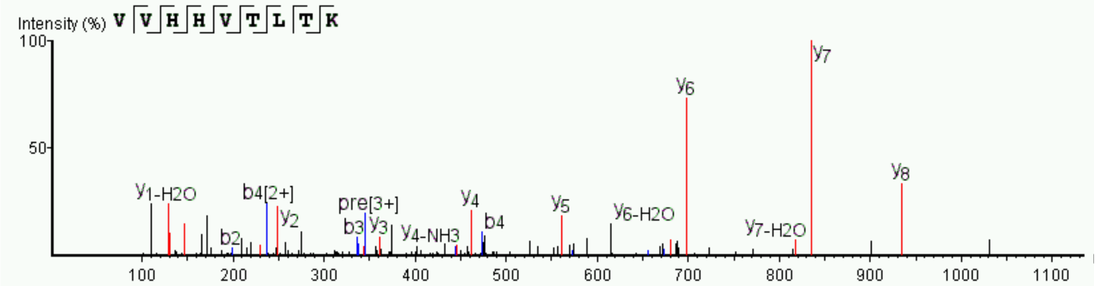

gi|571507399

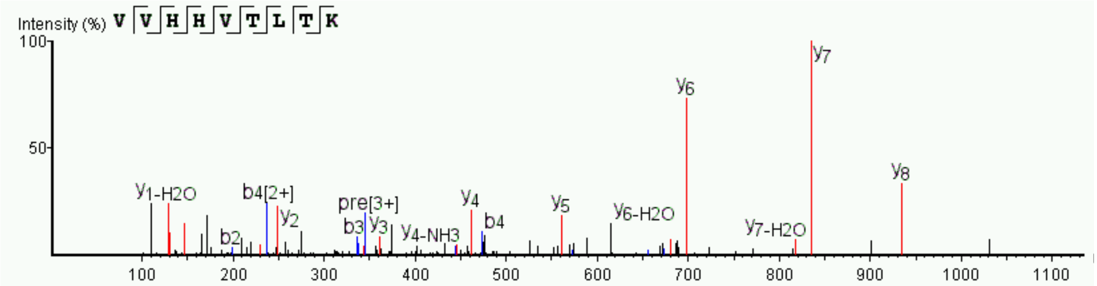

gi|571507402

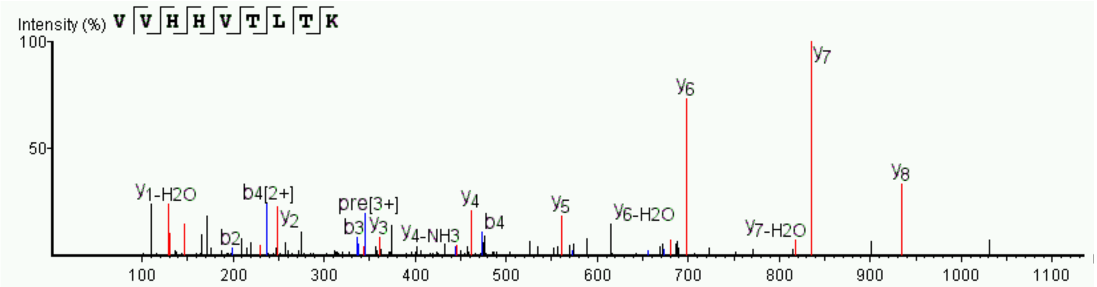

gi|571507406

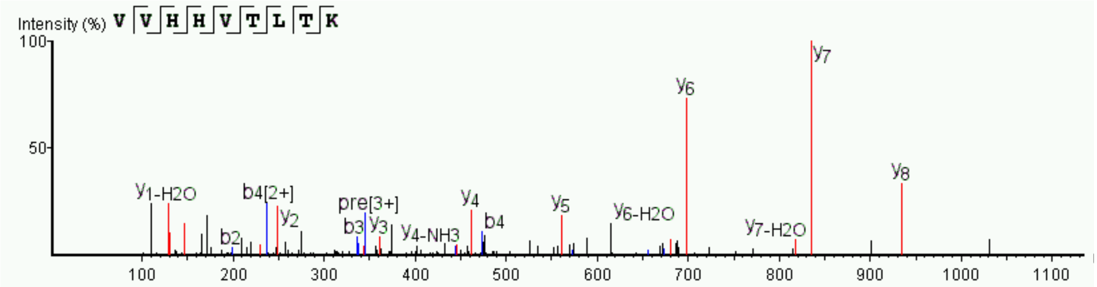

gi|571507410

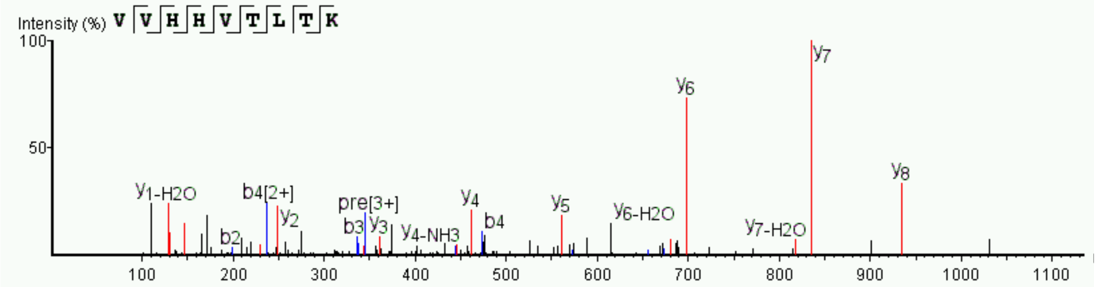

gi|571507413

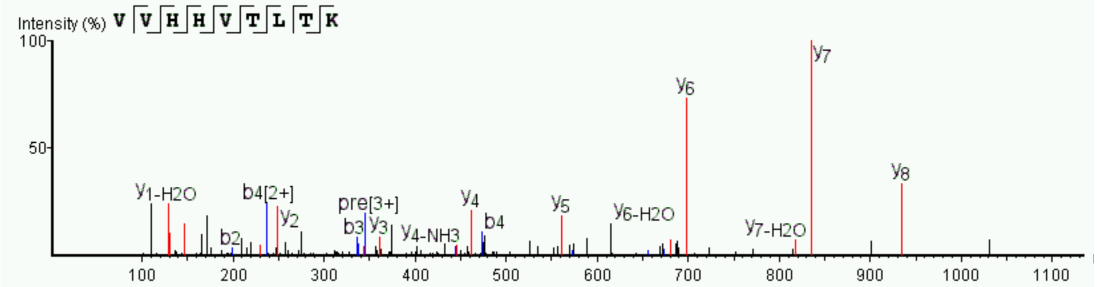

gi|328784953

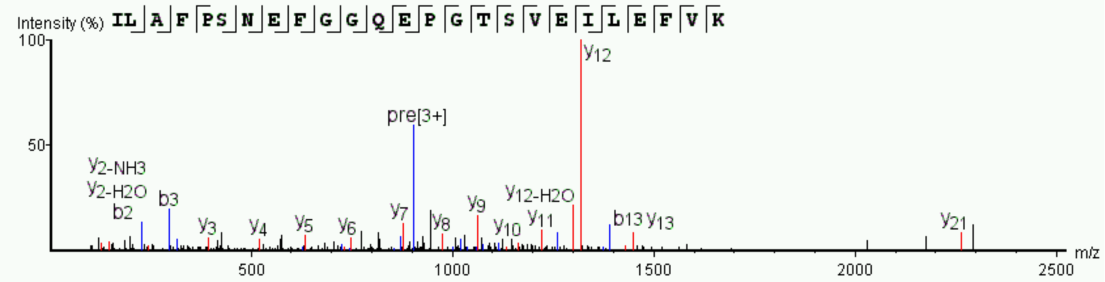

gi|571528435

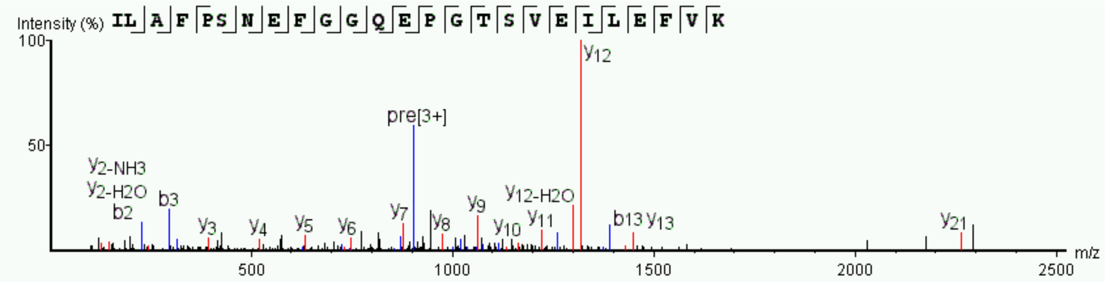

gi|328787622

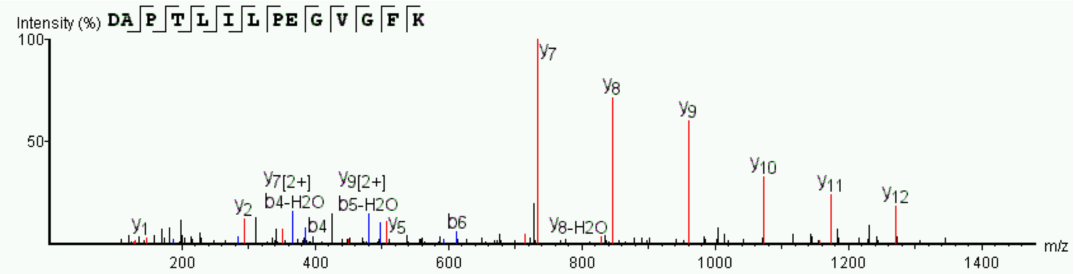

gi|571541806

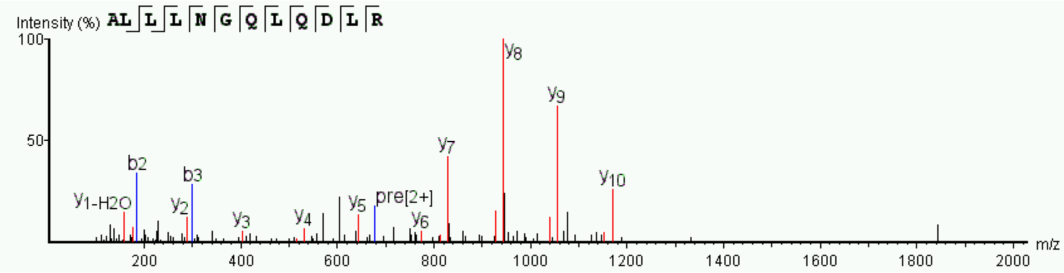

gi|571541802

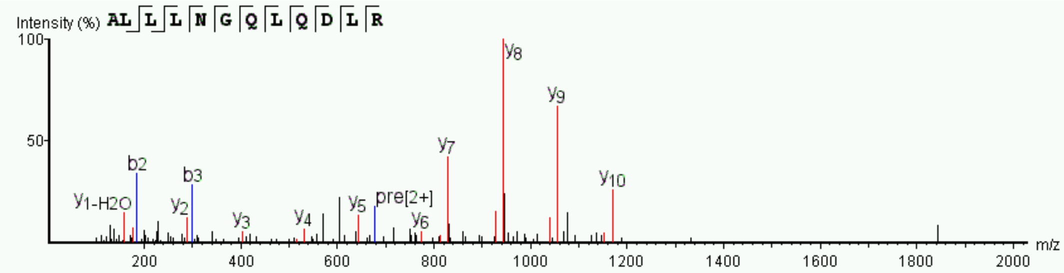

gi|571541813

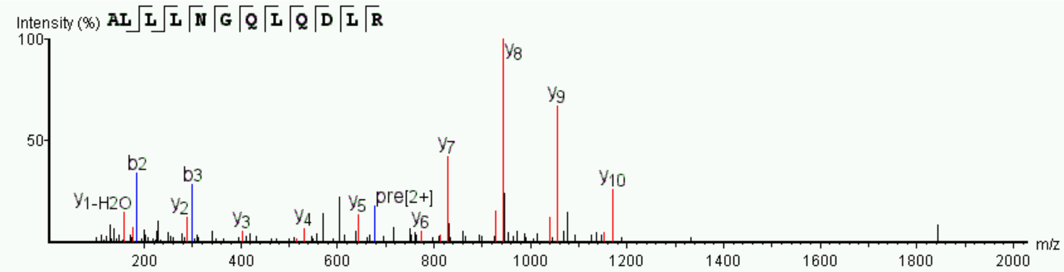

gi|571541816

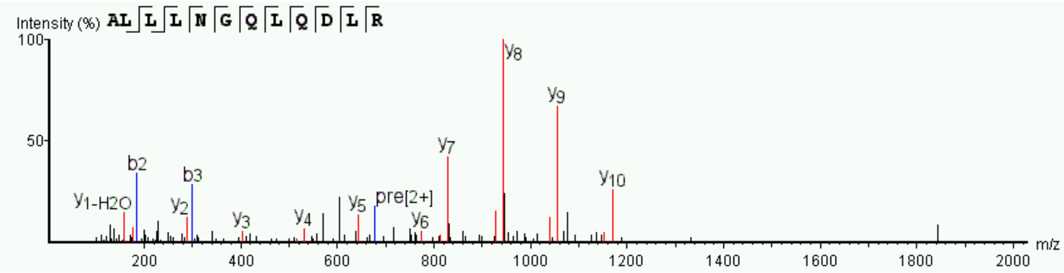

gi|571541819

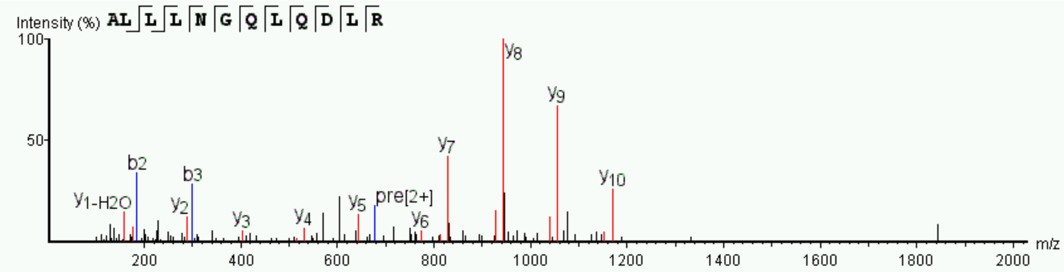

gi|571541810

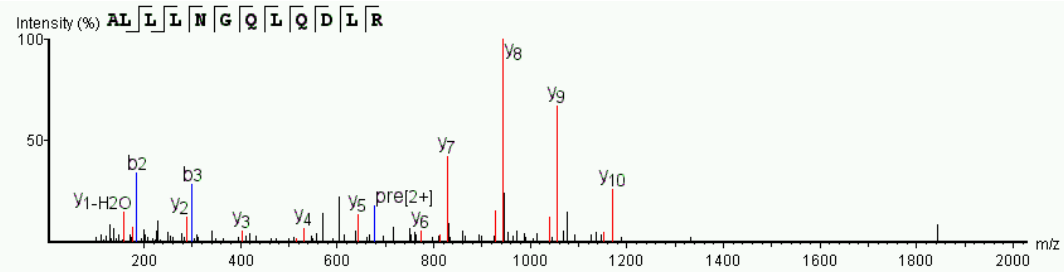

gi|571549289

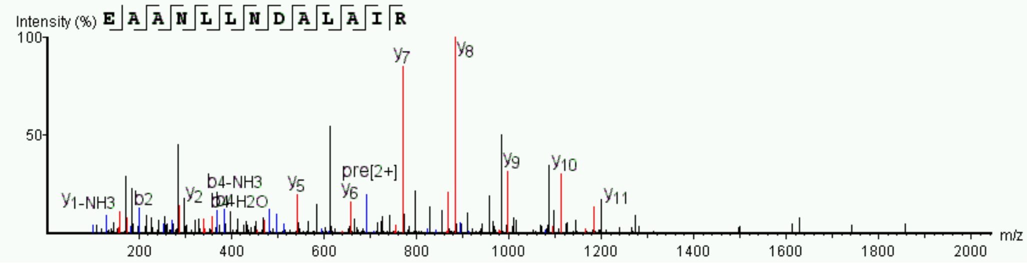

gi|571517320

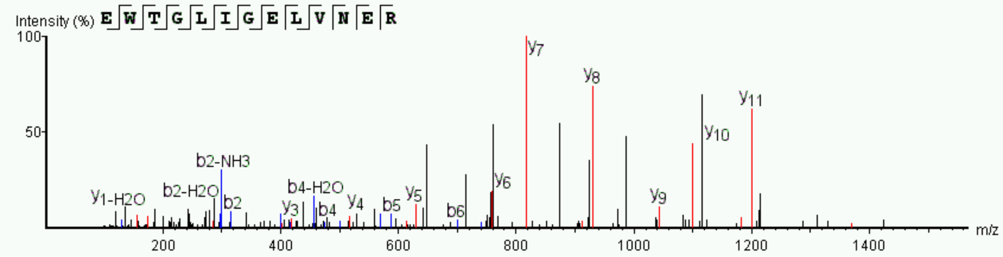

gi|571517323

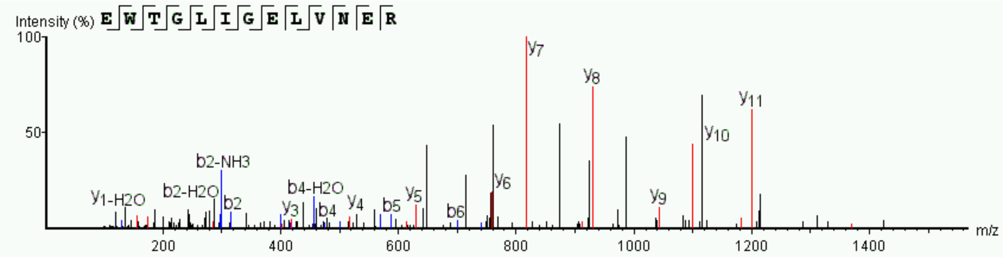

gi|571556448

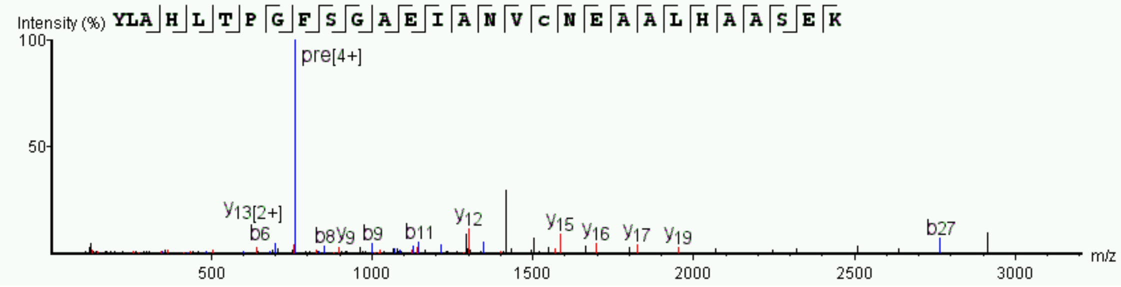

gi|571568205

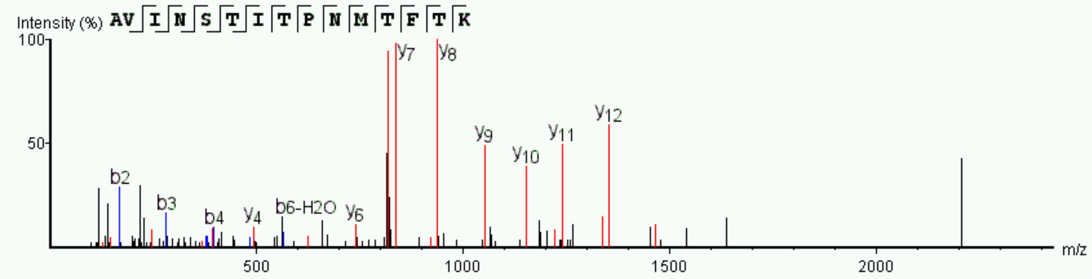

gi|328782542

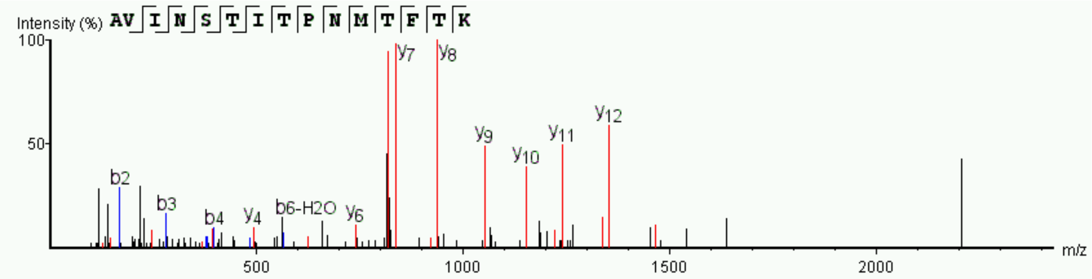

gi|571514498

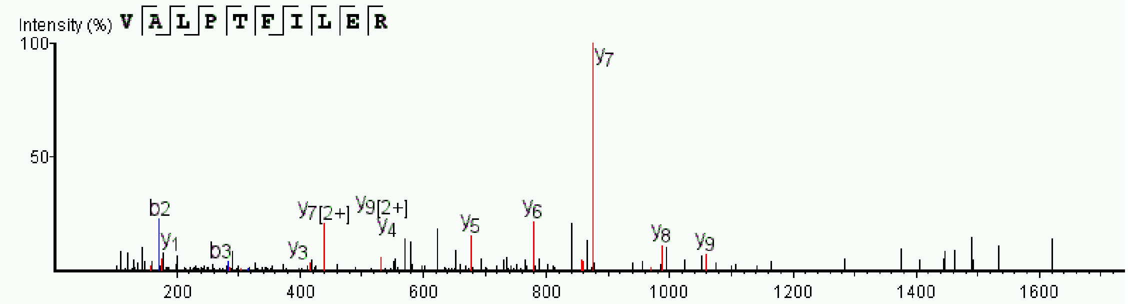

gi|571514510

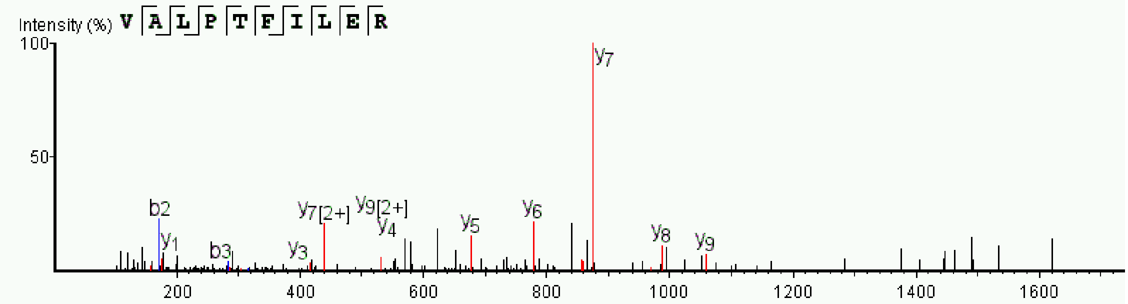

gi|571514506

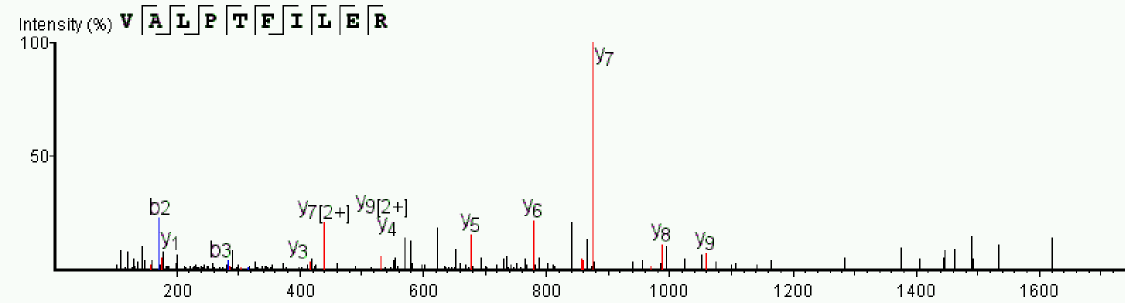

gi|571514502

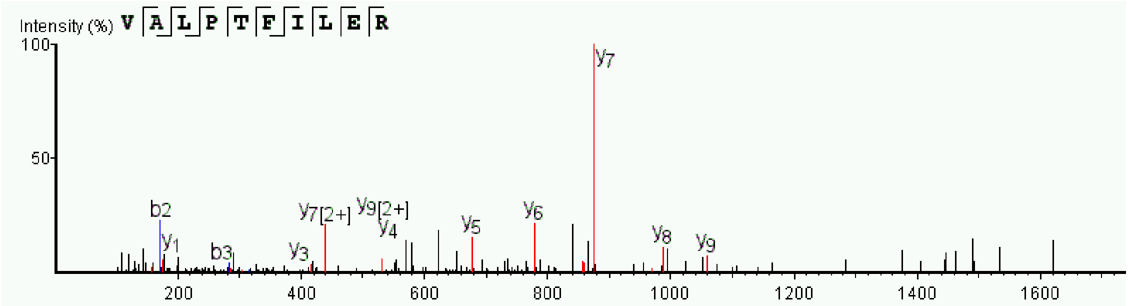

gi|66565384

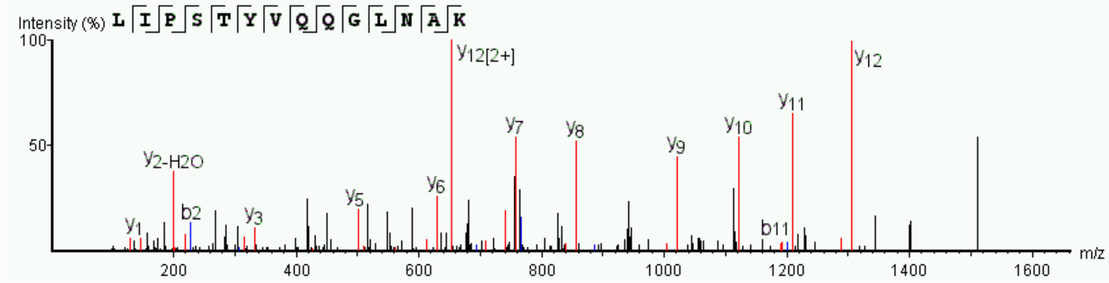

gi|571573898

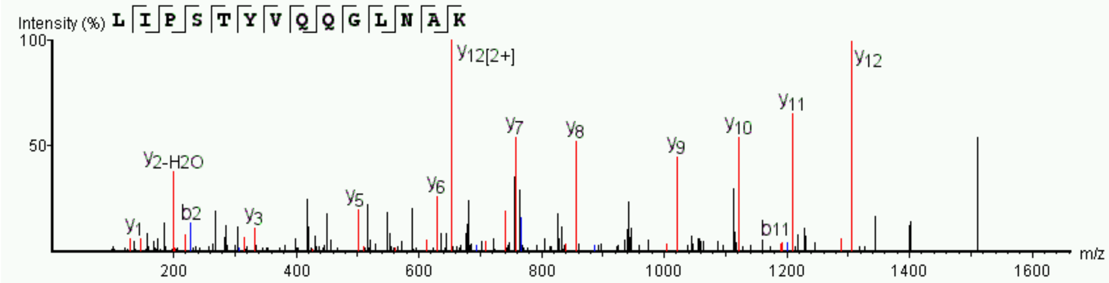

gi|571573896

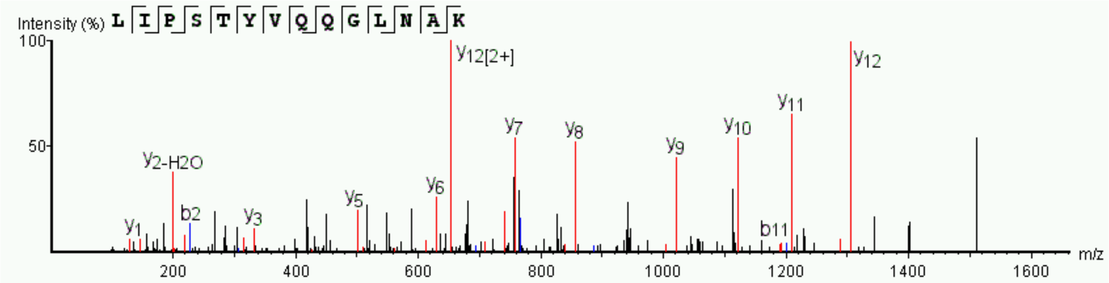

gi|571540964

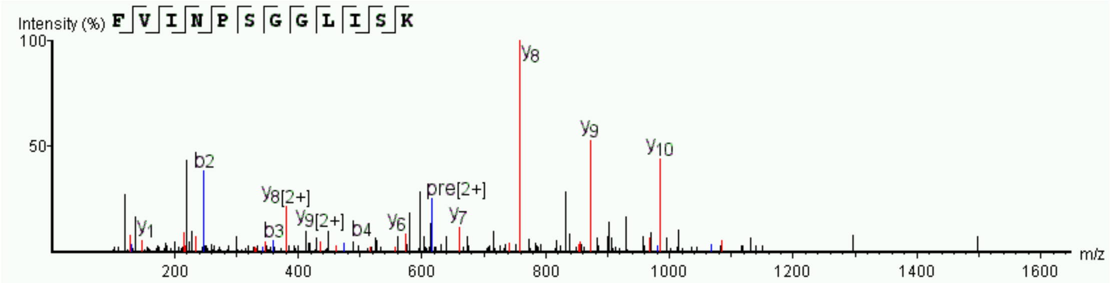

gi|571540960

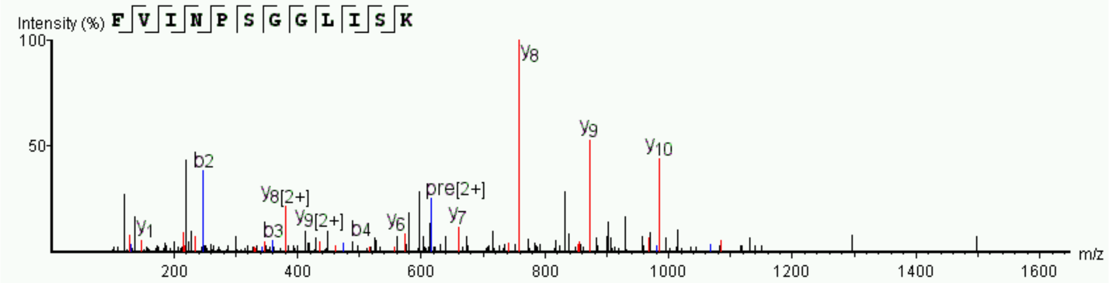

gi|66543732

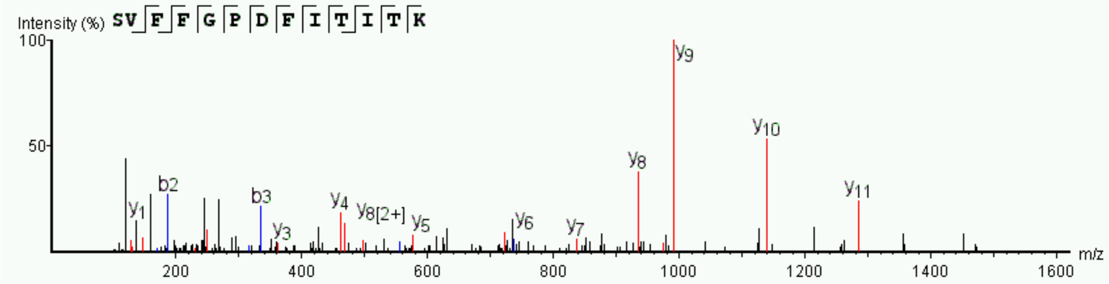

gi|66500170

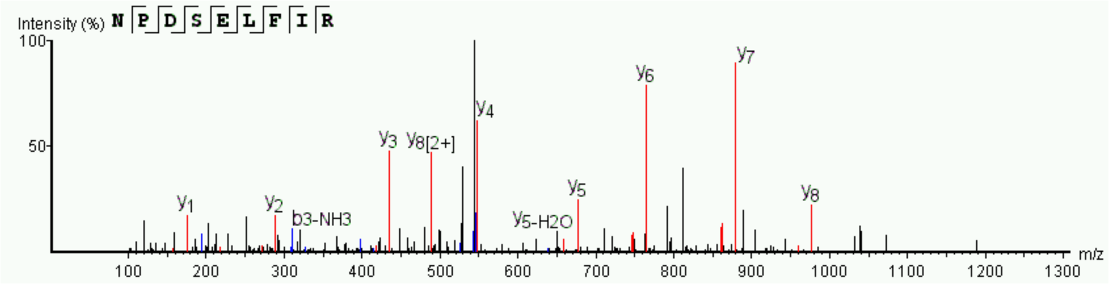

gi|48102172

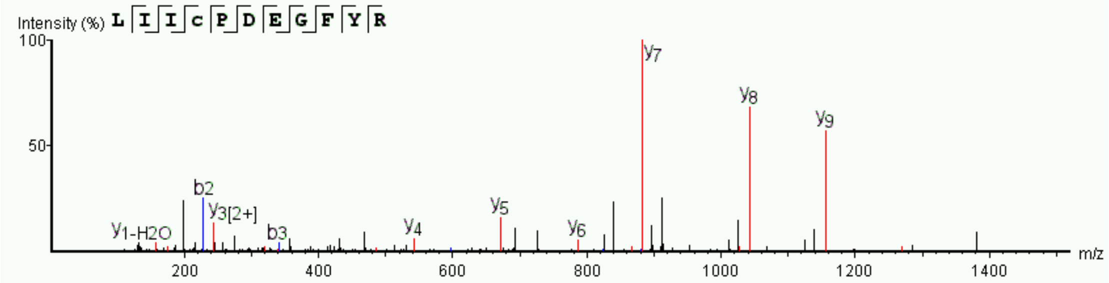

gi|571507498

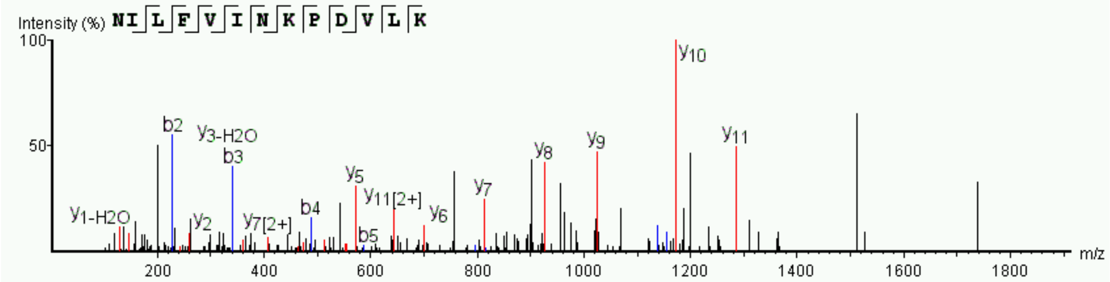

gi|571507494

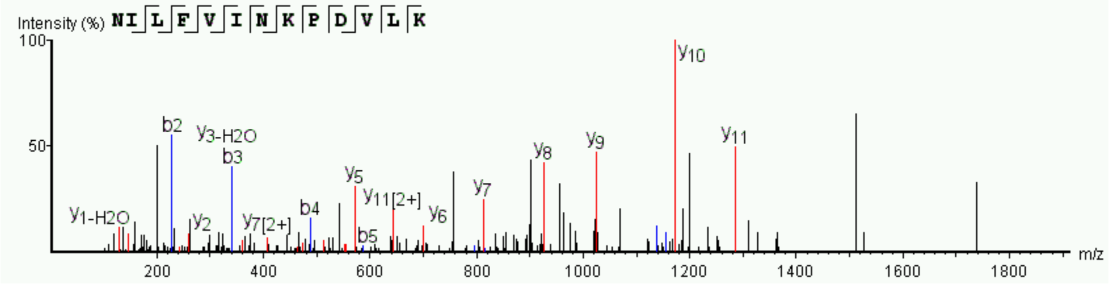

gi|48098241

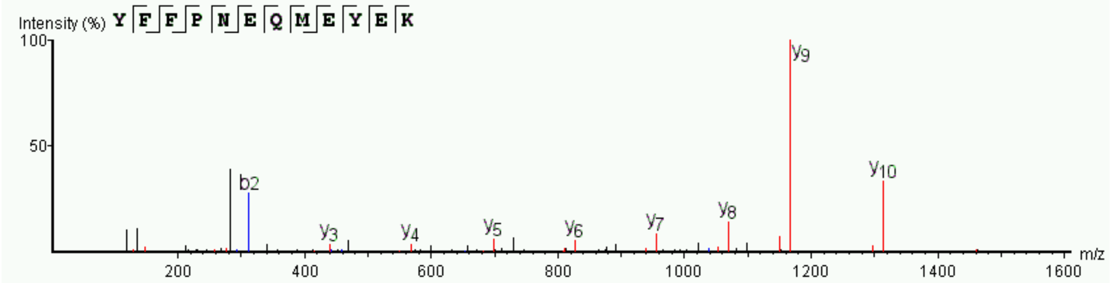

gi|571573138

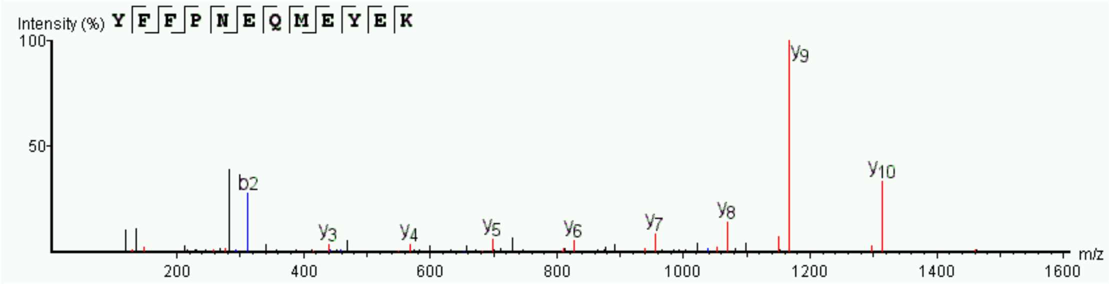

gi|571505185

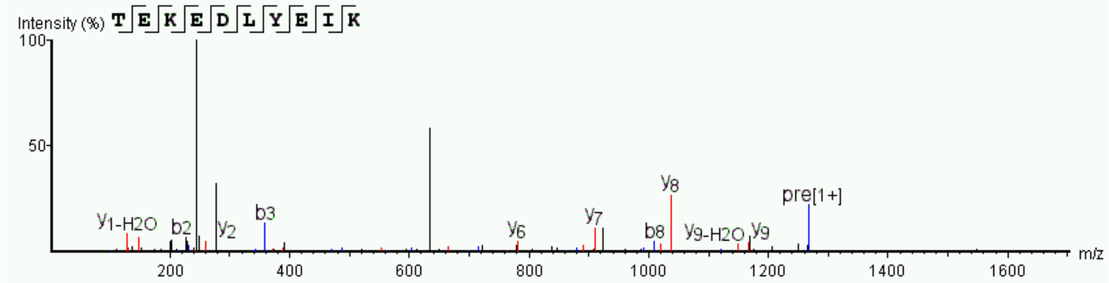

gi|571544898

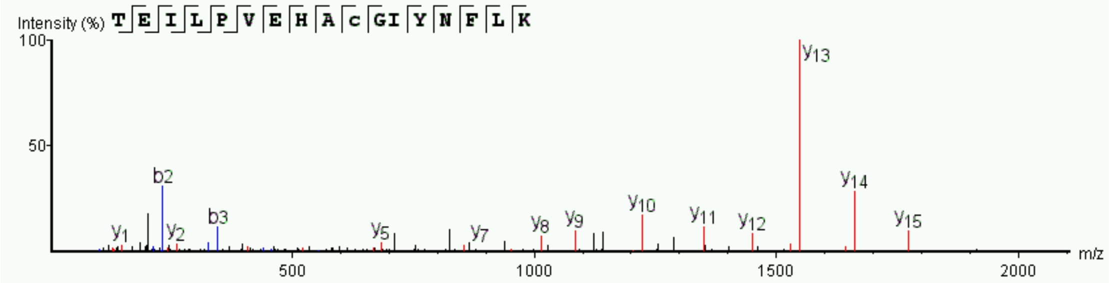

gi|48094341

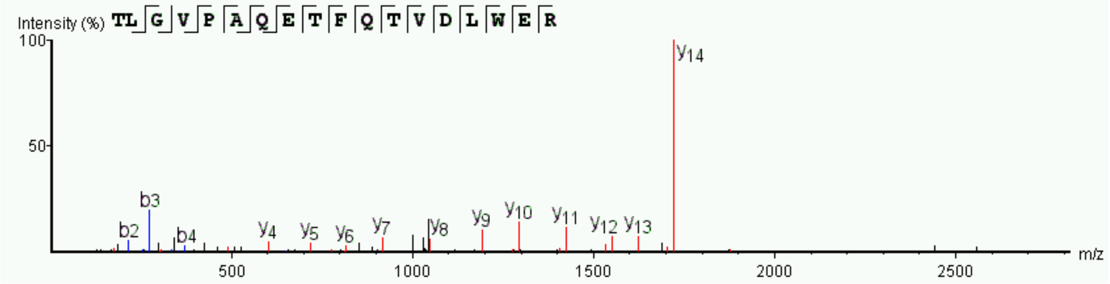

gi|571522057

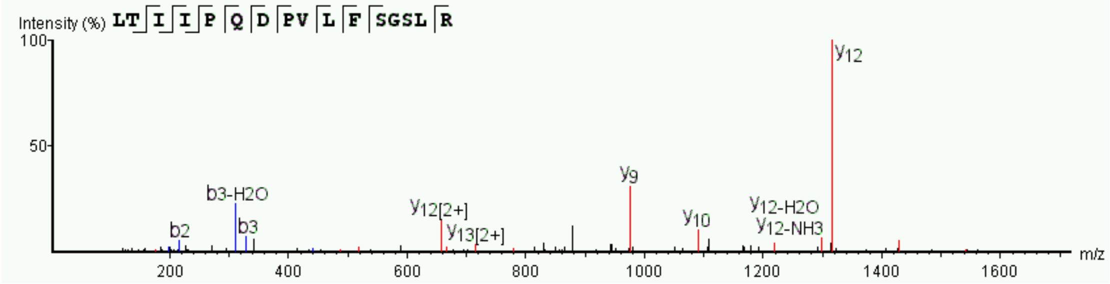

gi|571522053

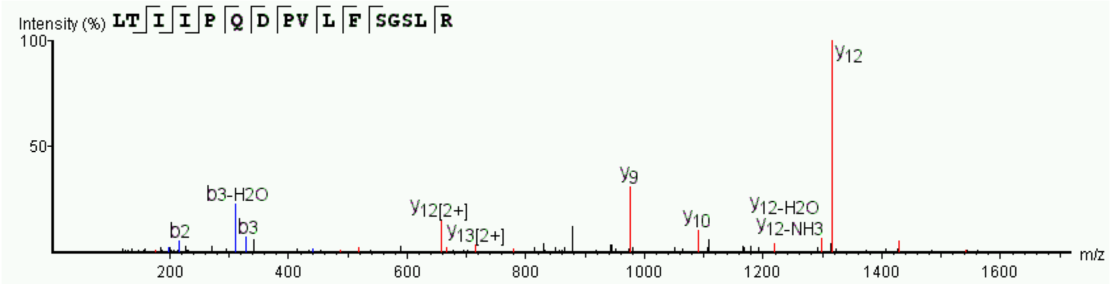

gi|571522050

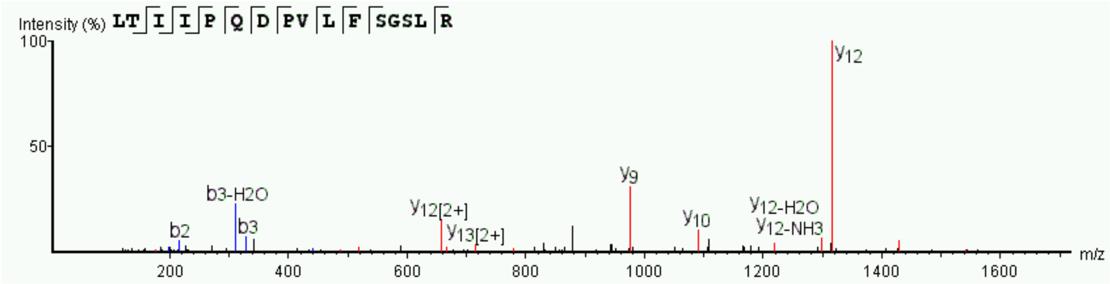

gi|571522046

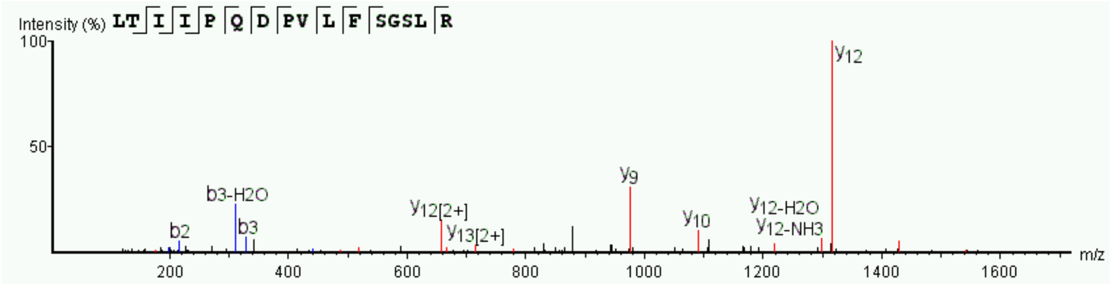

gi|571522042

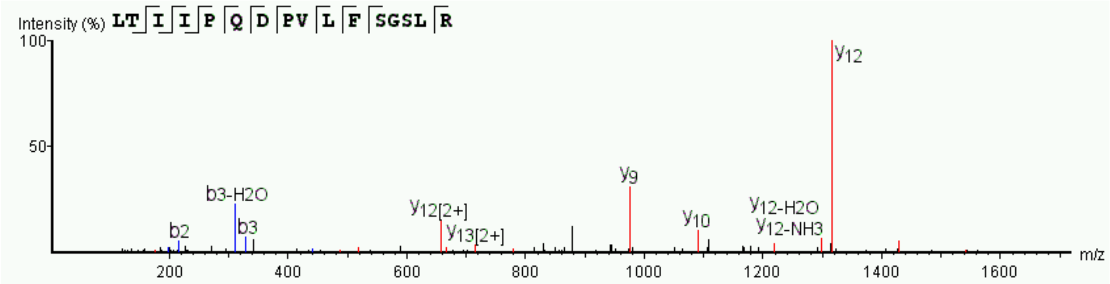

gi|571522038

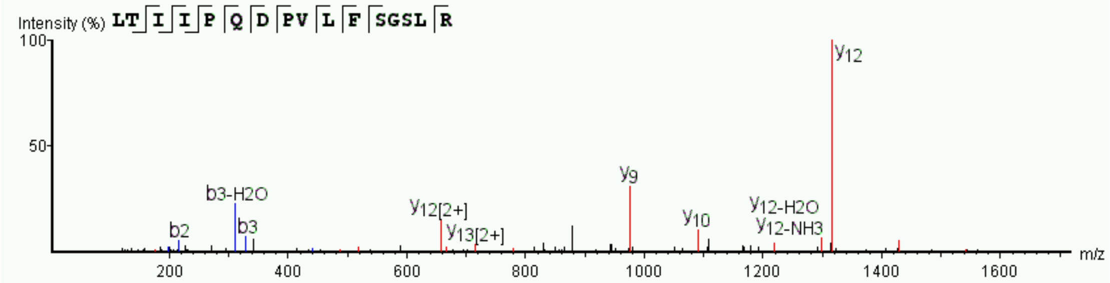

gi|571522034

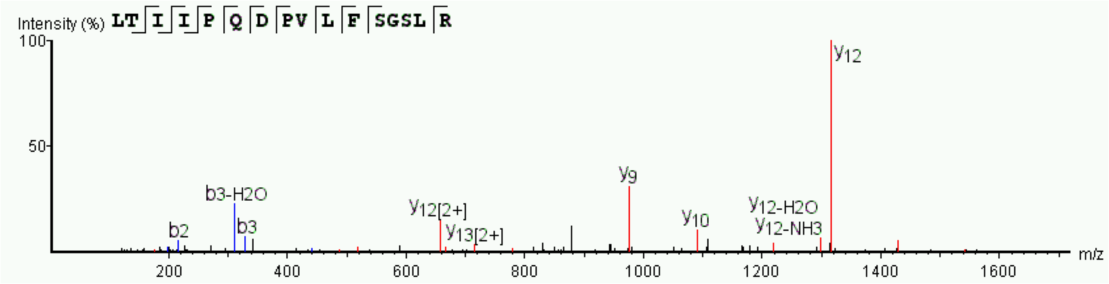

gi|328780159

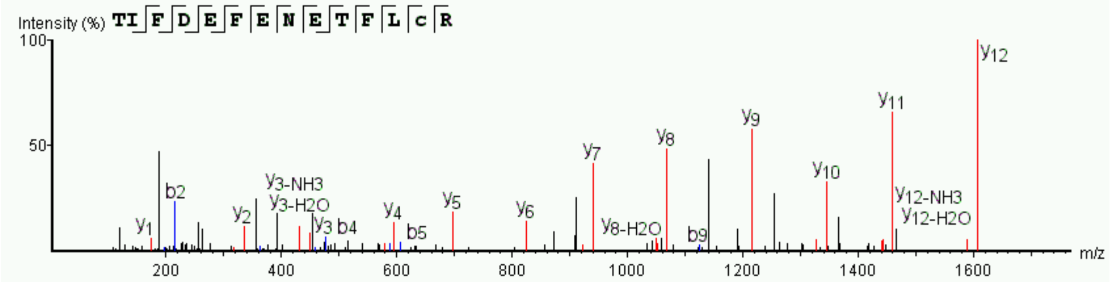

gi|571518510

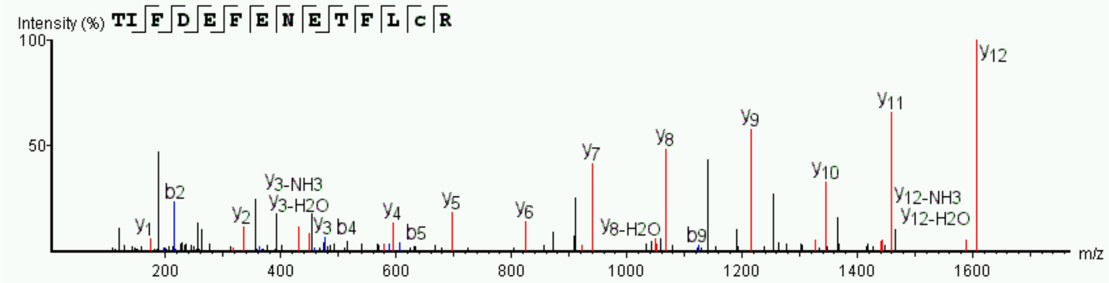

gi|328779694

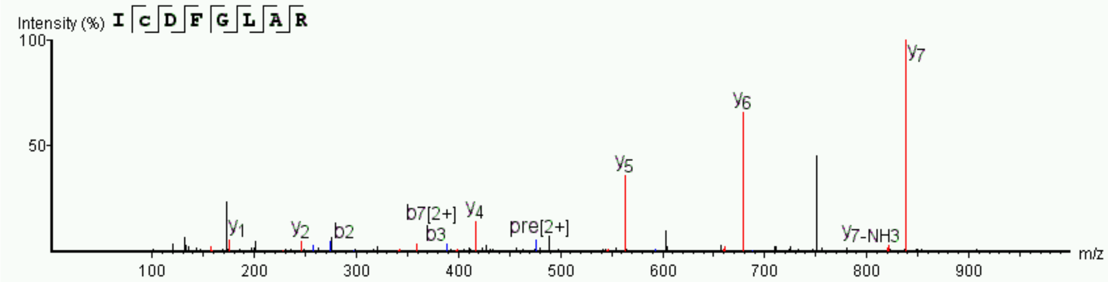

gi|110748745

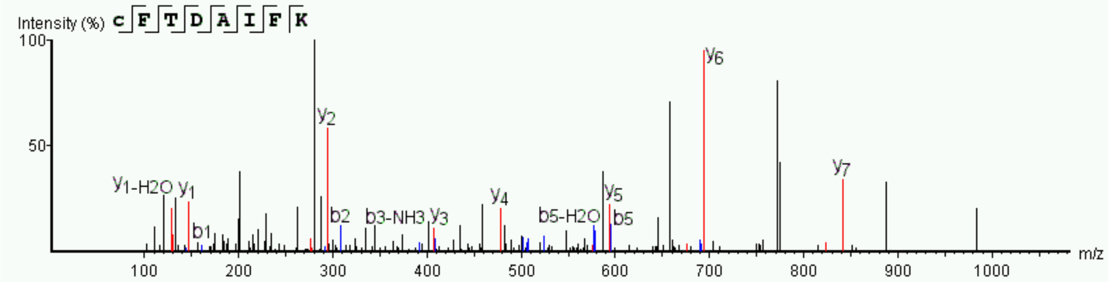

gi|328782218

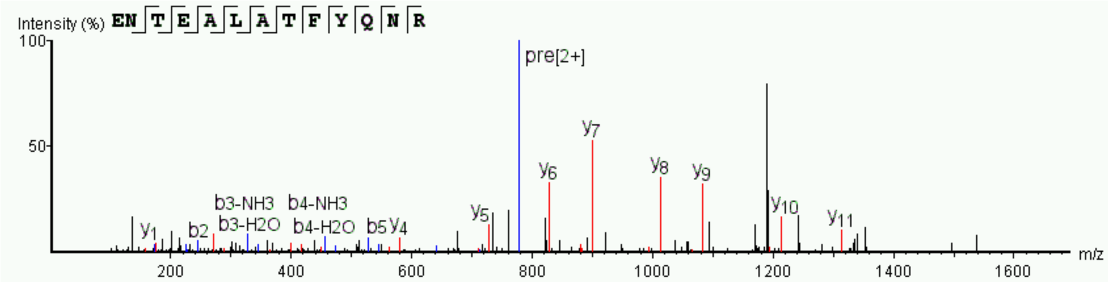

gi|48105316

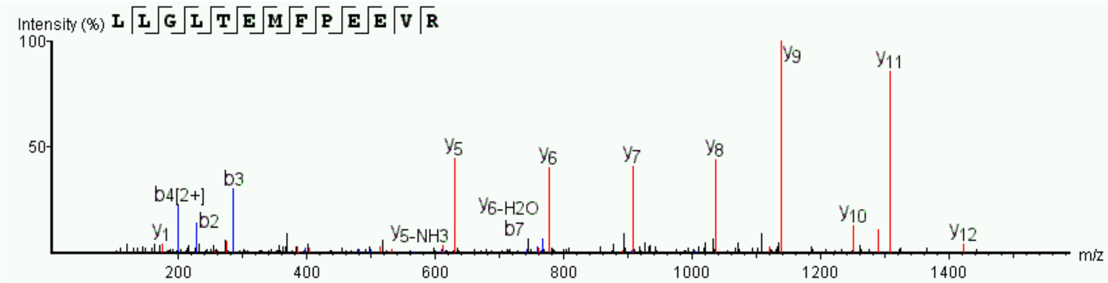

gi|110749921

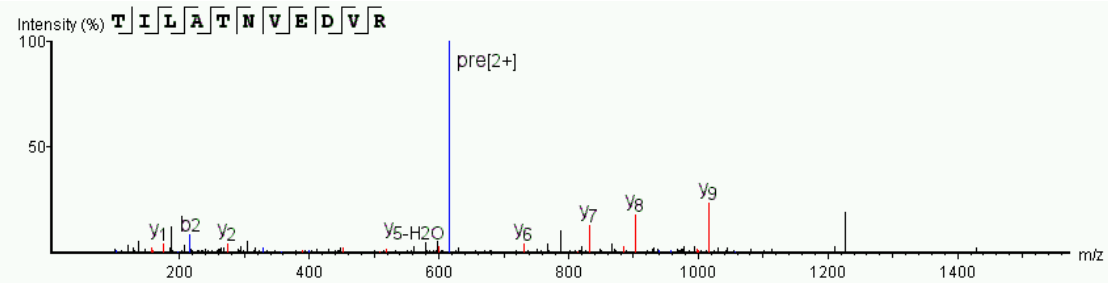

gi|66519233

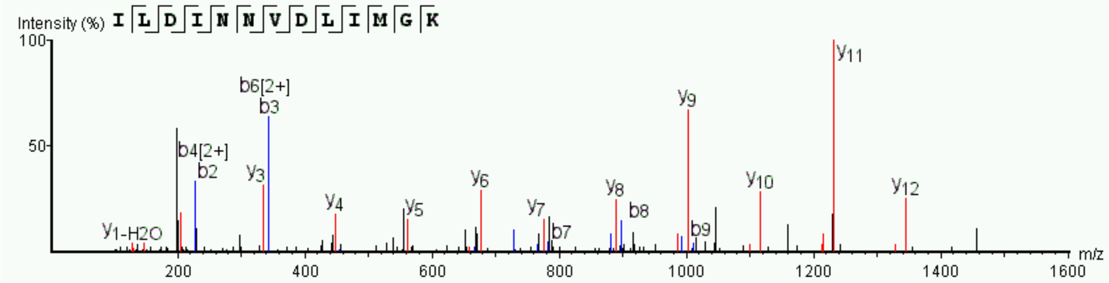

gi|571510158

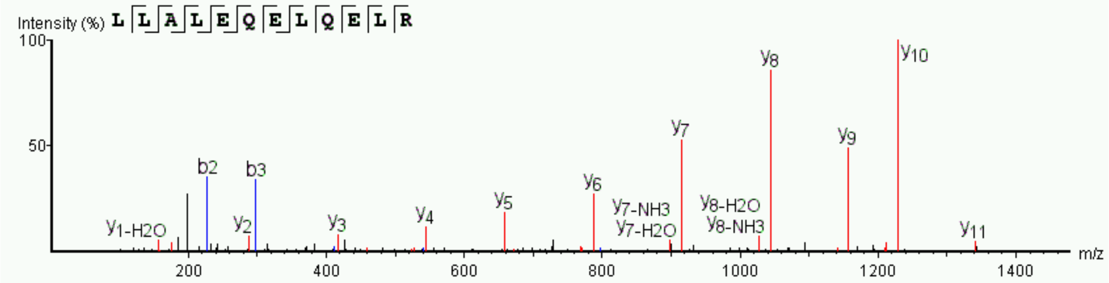

gi|328783561

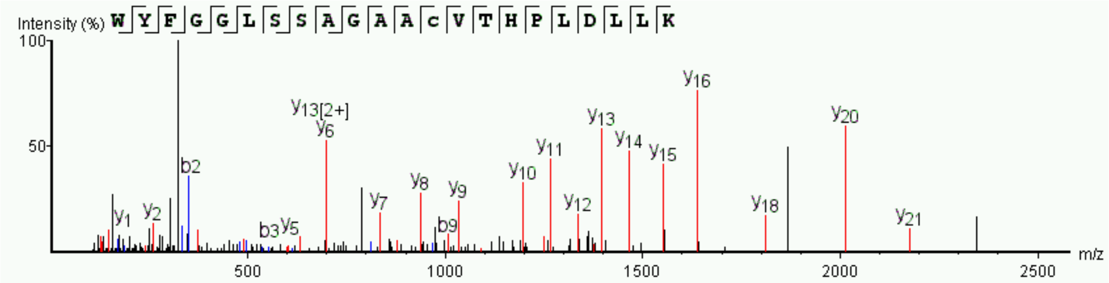

gi|571562354

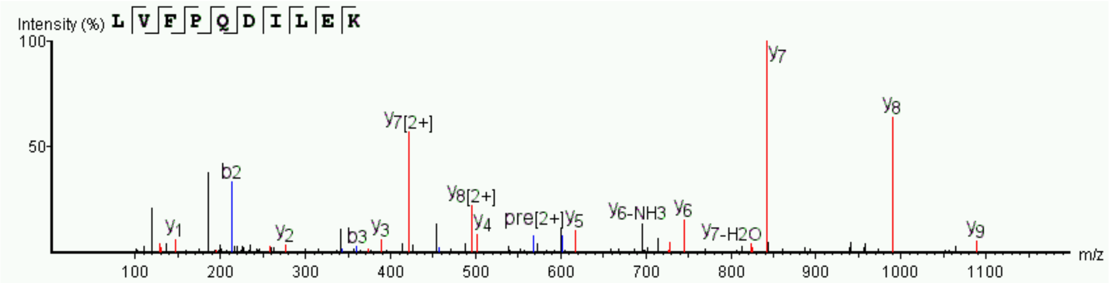

gi|48094957

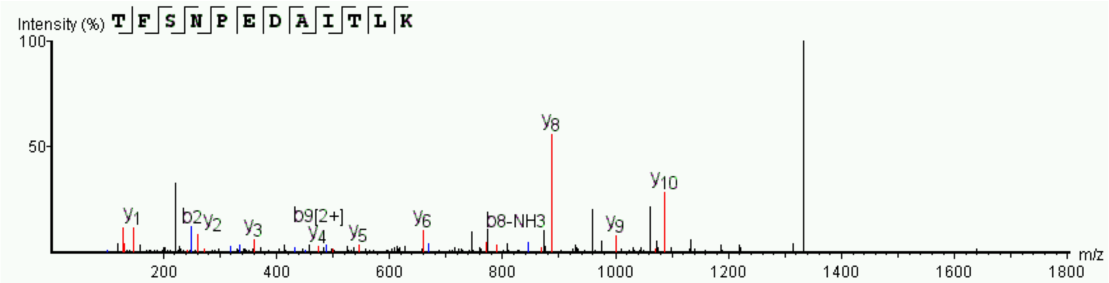

gi|328786562

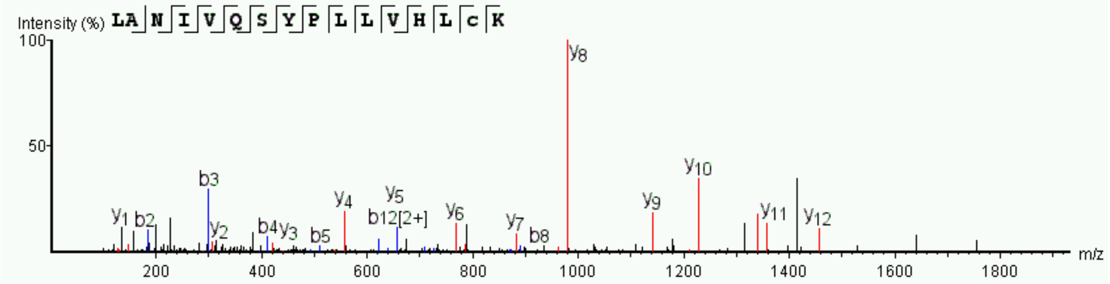

gi|66533332

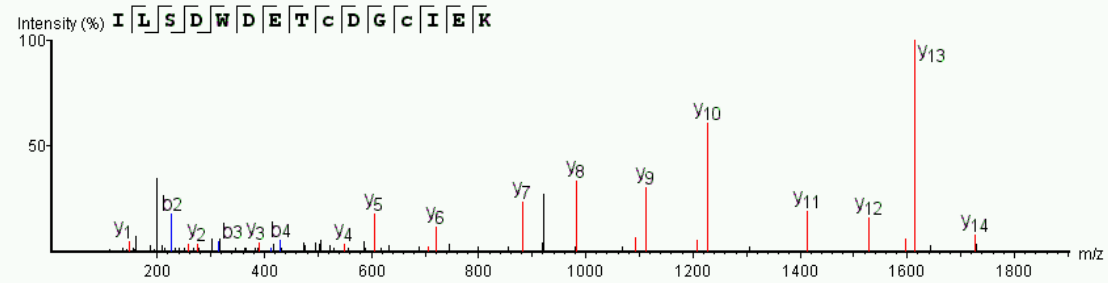

gi|571503615

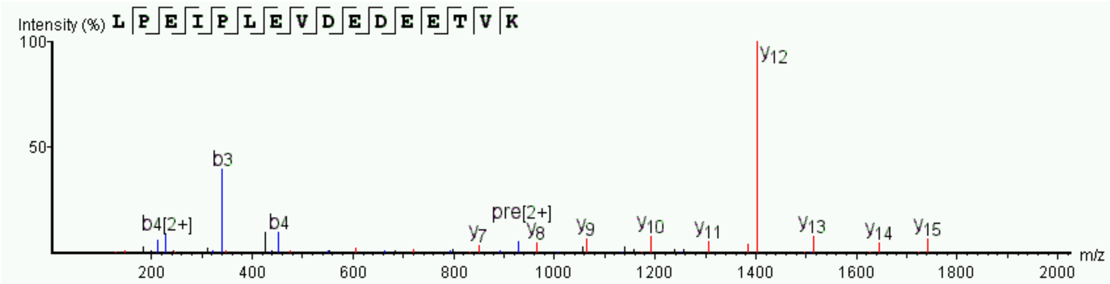

gi|571503611

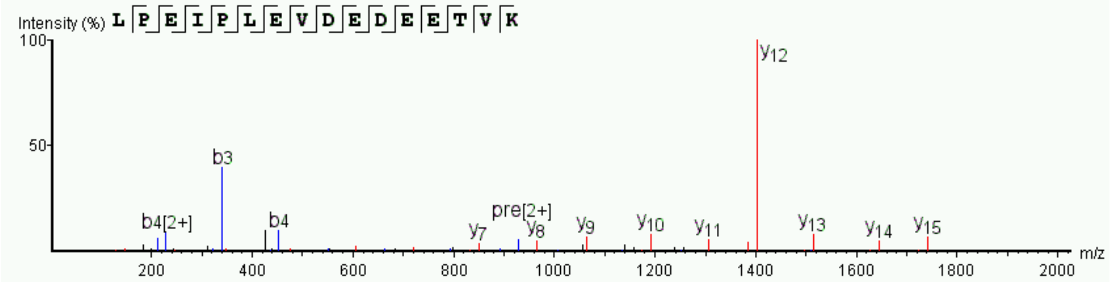

gi|571503608

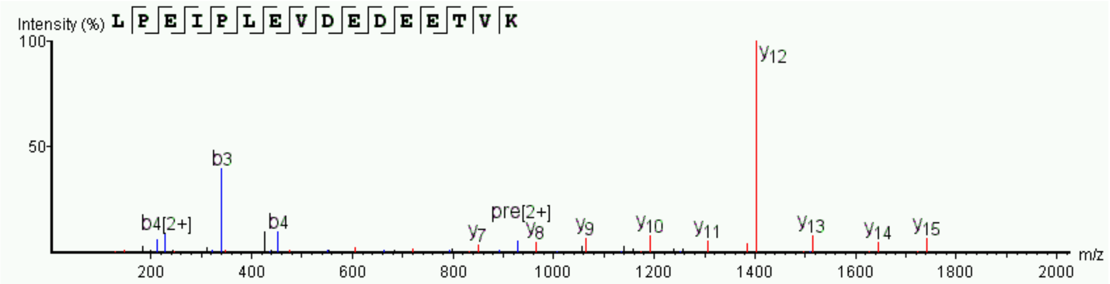

gi|110759417

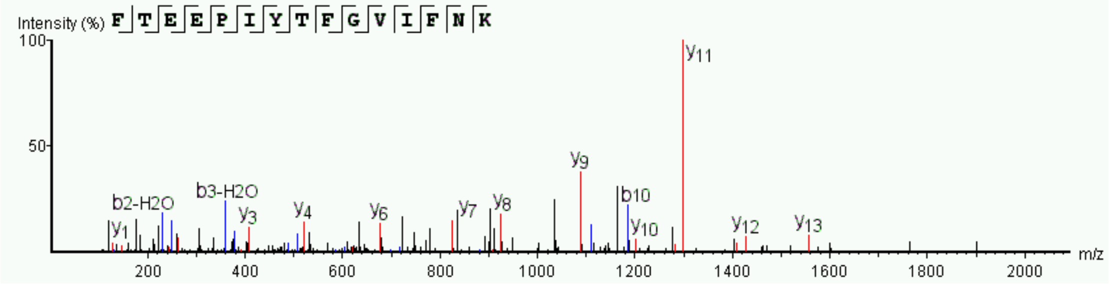

gi|571530533

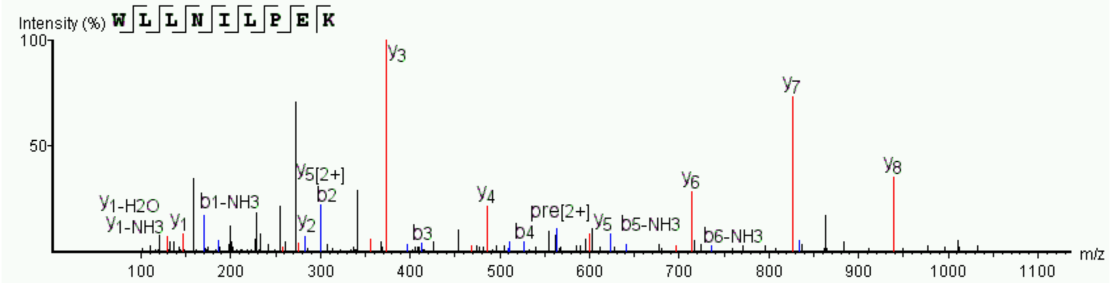

gi|571530529

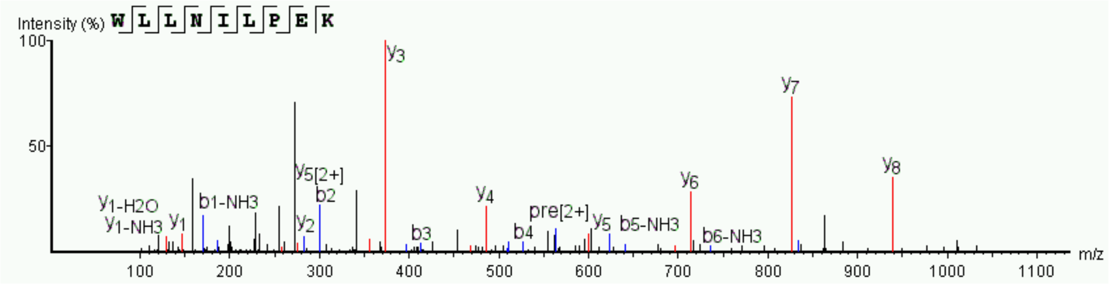

gi|48139441

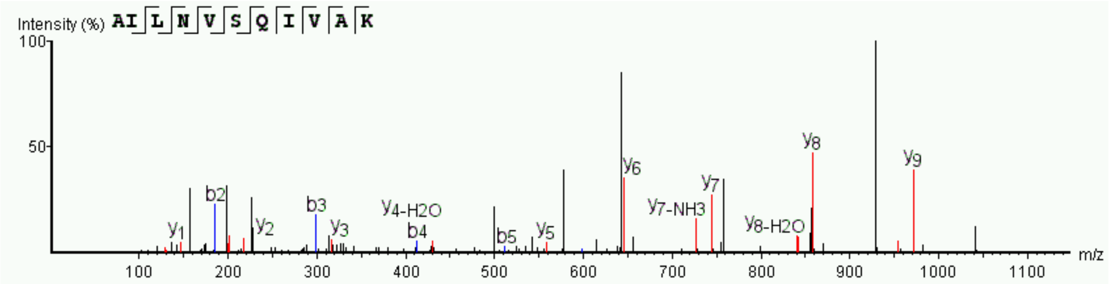

gi|571558139

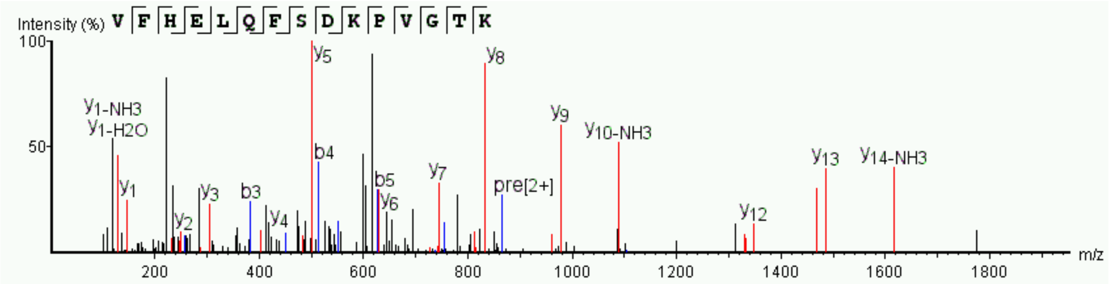

gi|571547030

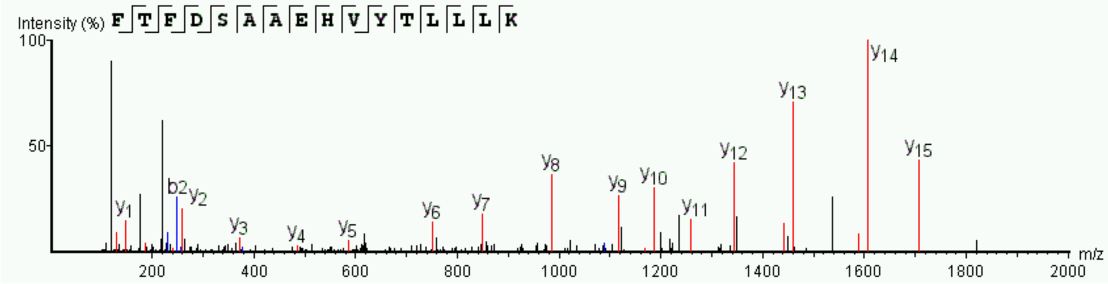

gi|571526489

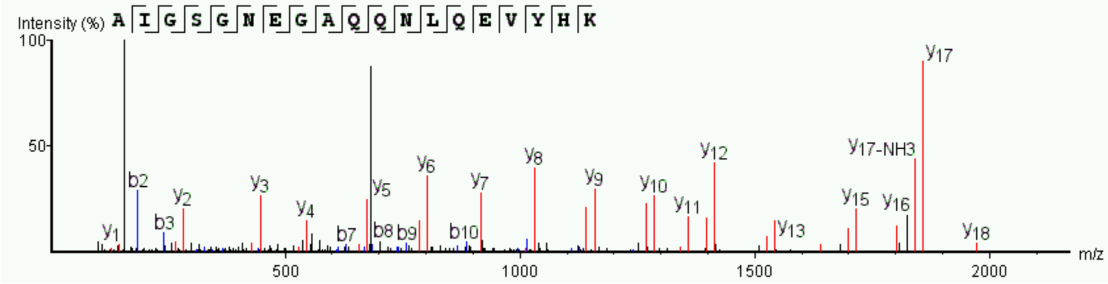

gi|571540042

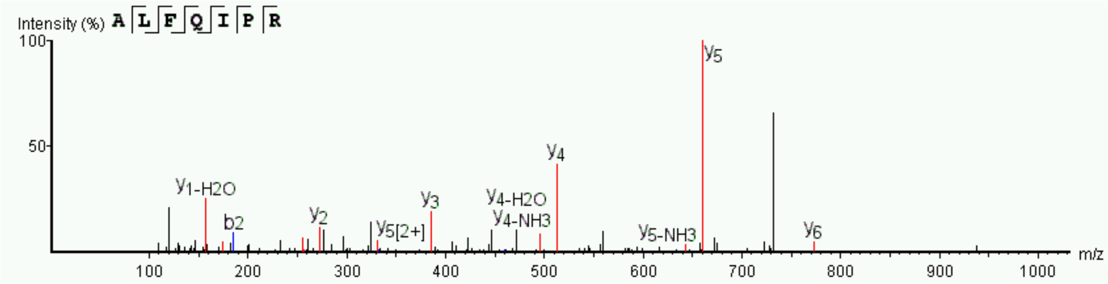

gi|571550579

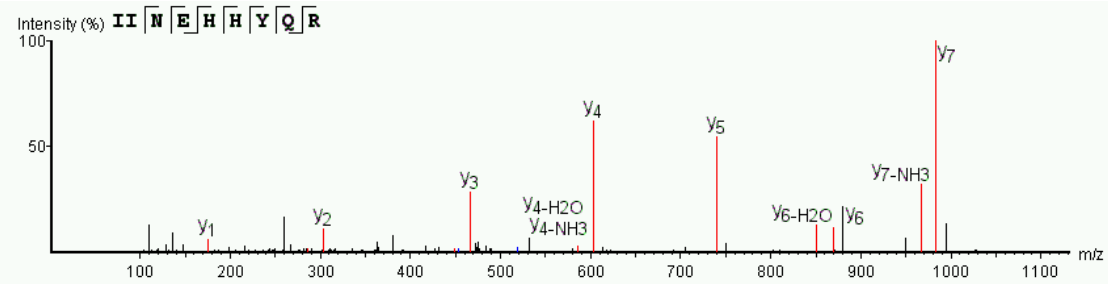

gi|571513398

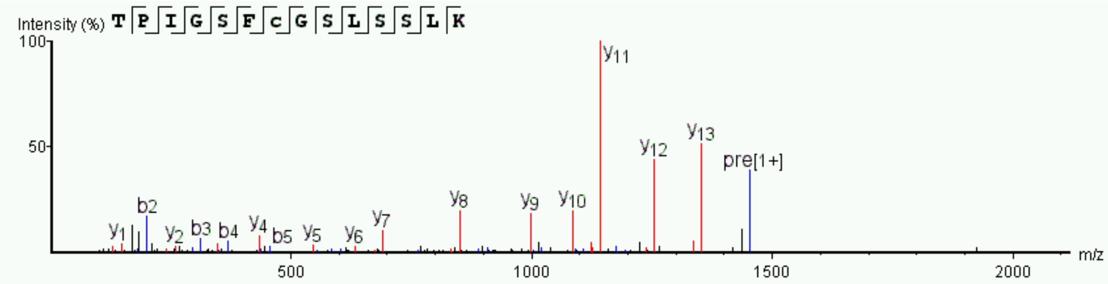

gi|328780510

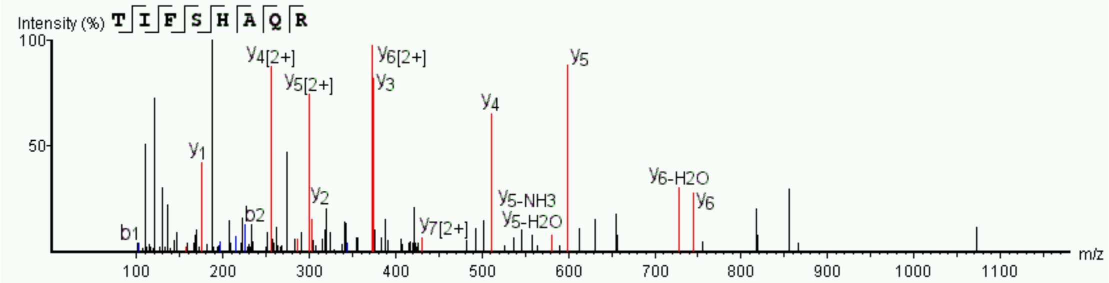

gi|328782992

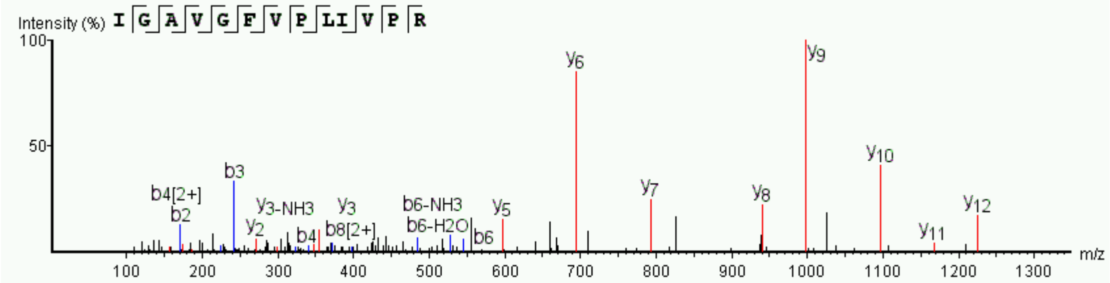

gi|571576551

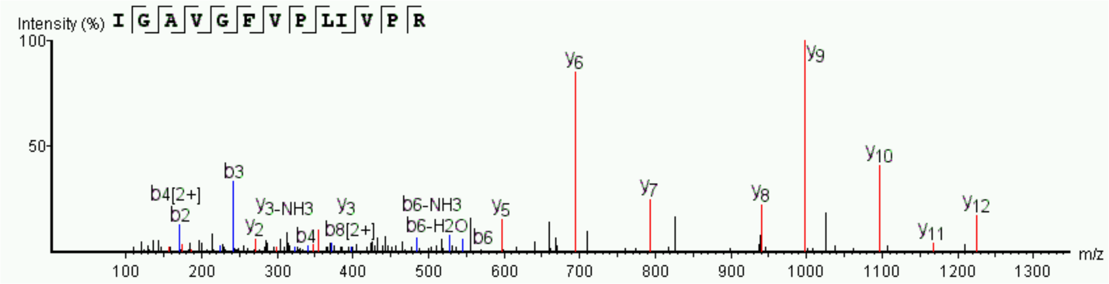

gi|328790105

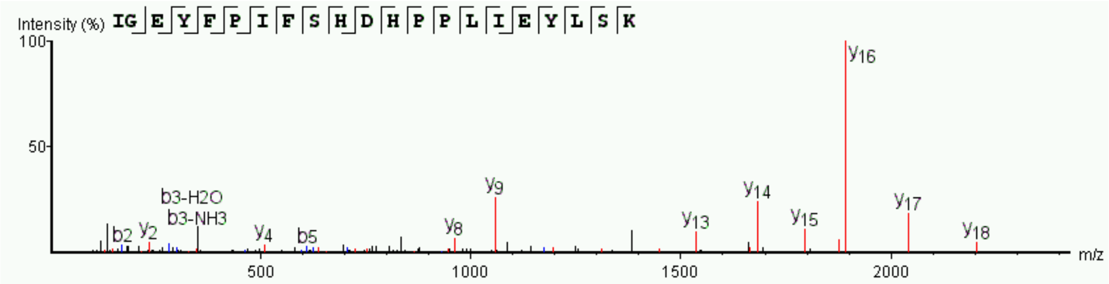

gi|571523088

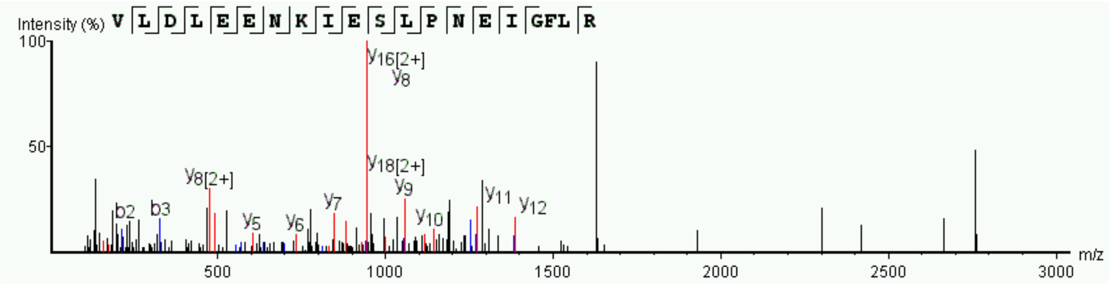

gi|328781115

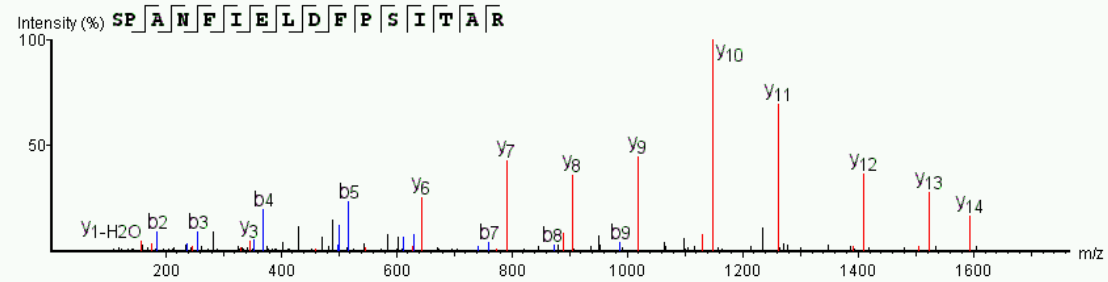

gi|571557555

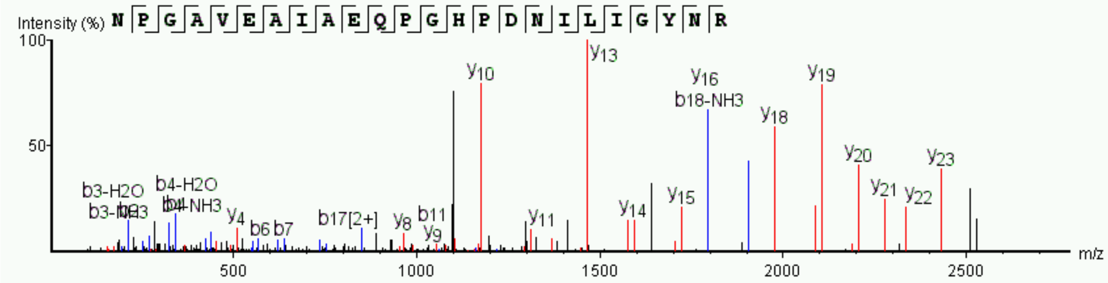

gi|66551651

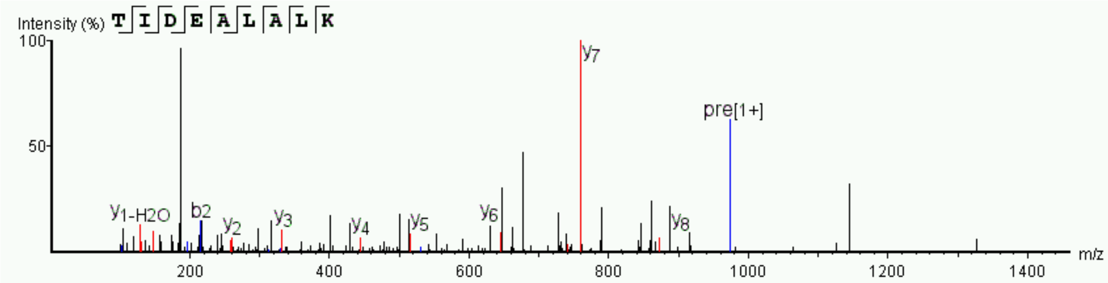

gi|66512887

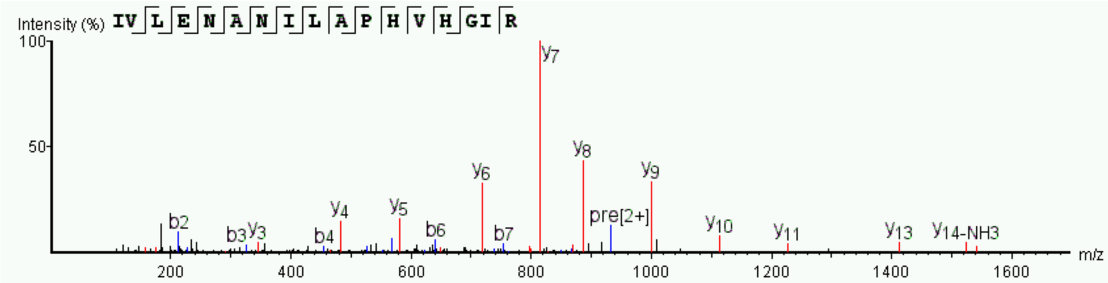

gi|66554764

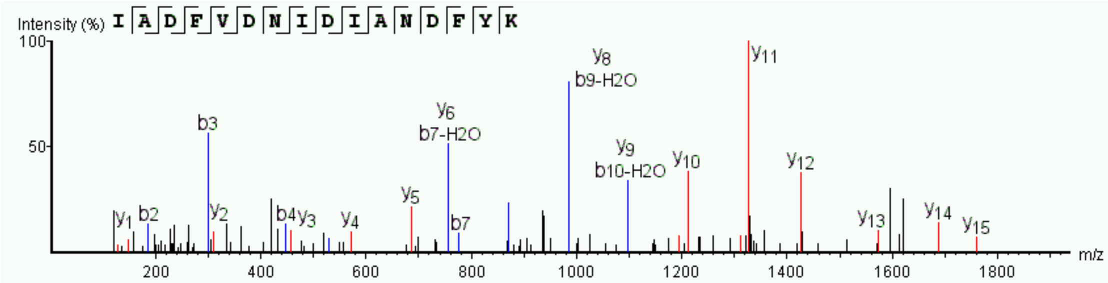

gi|328792179

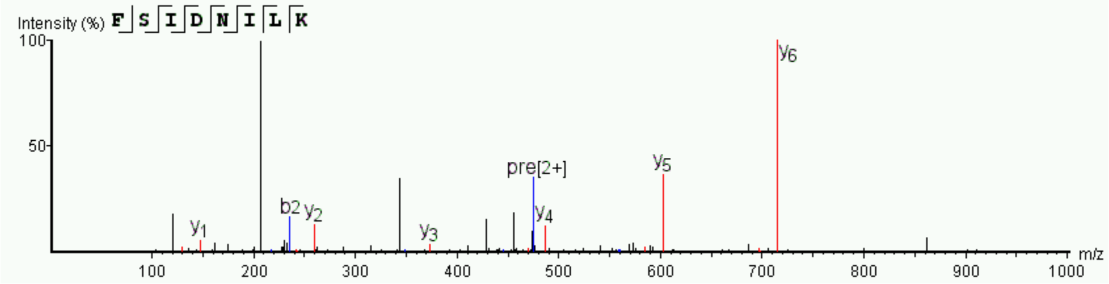

gi|571510150

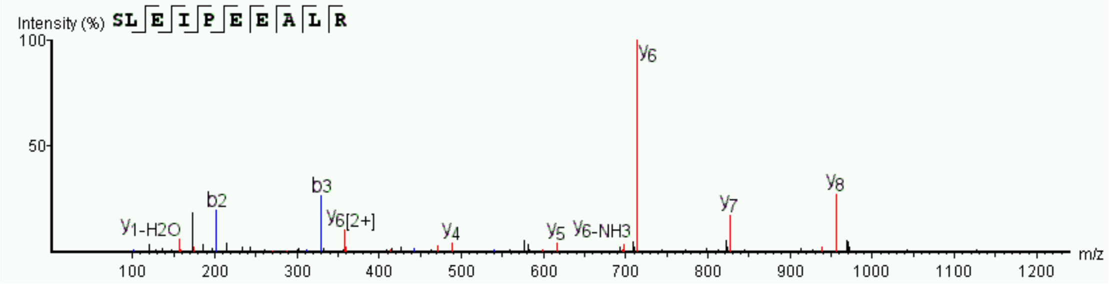

gi|66511337

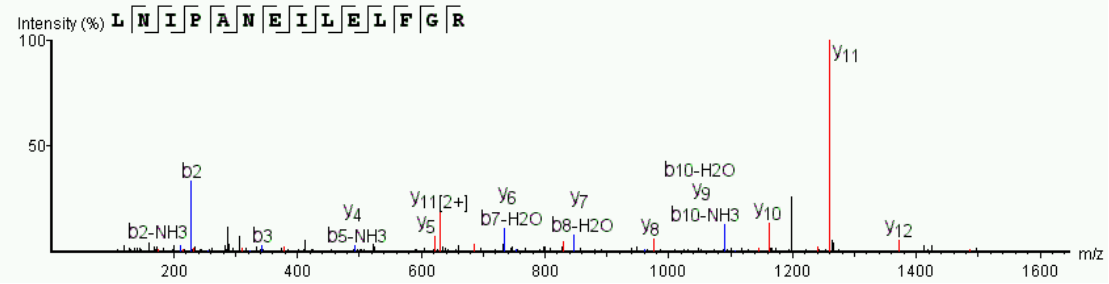

gi|571535113

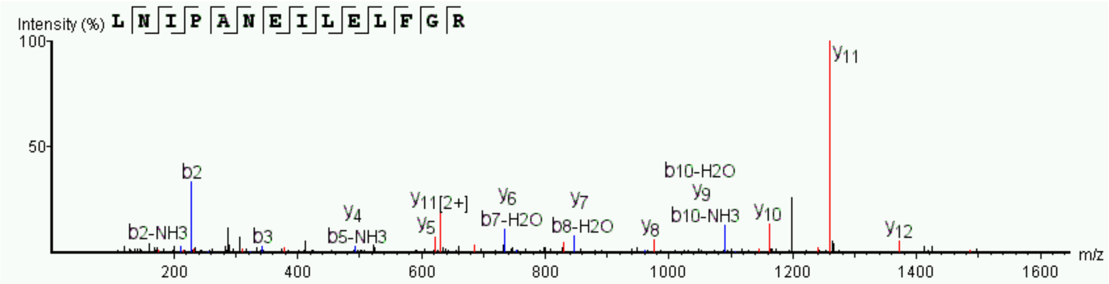

gi|571499584

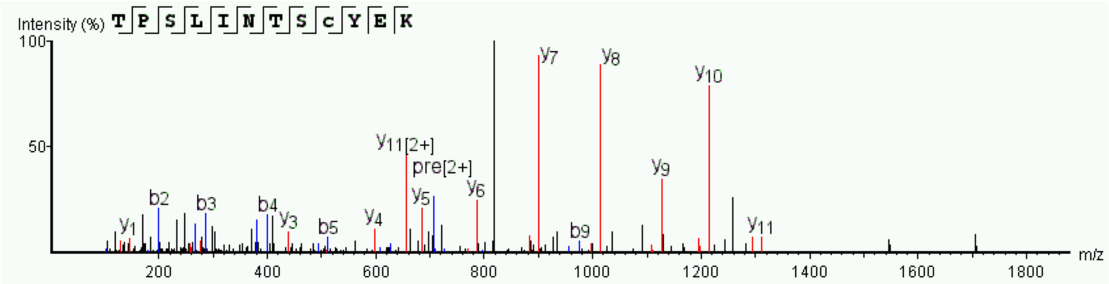

gi|571573797

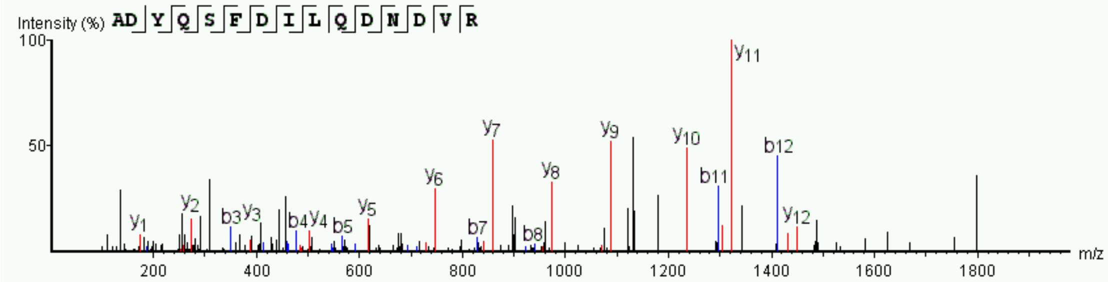

gi|328789473

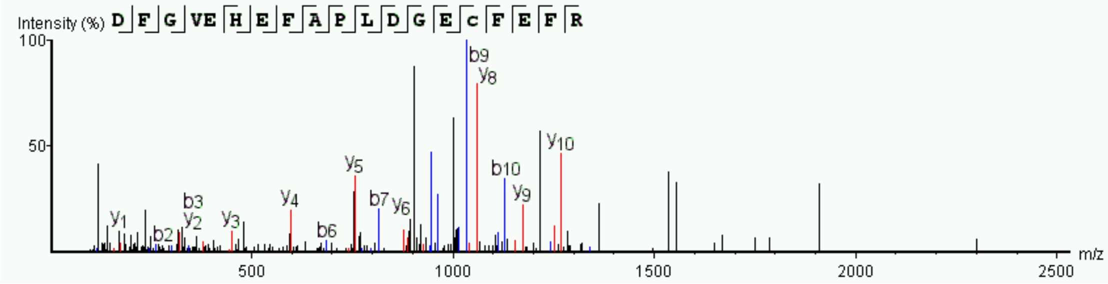

gi|571538660

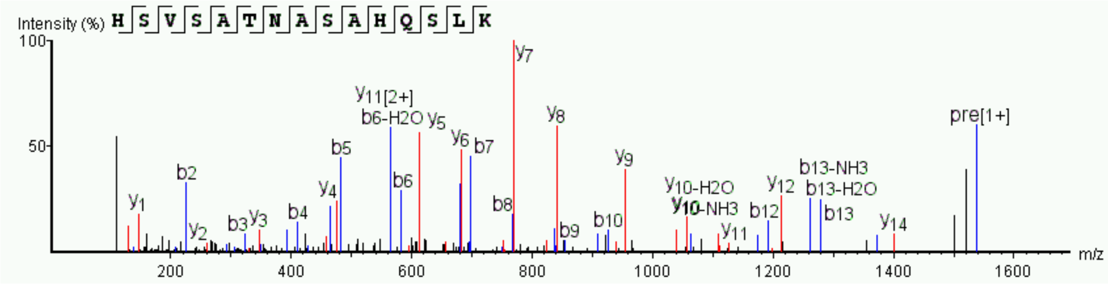

gi|328791134

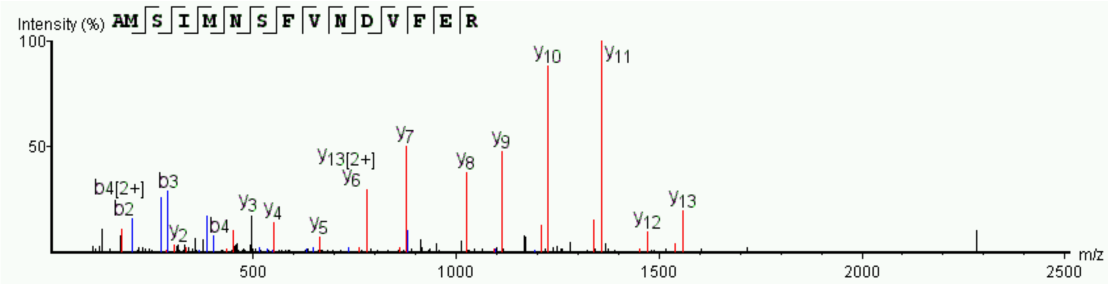

gi|328793605

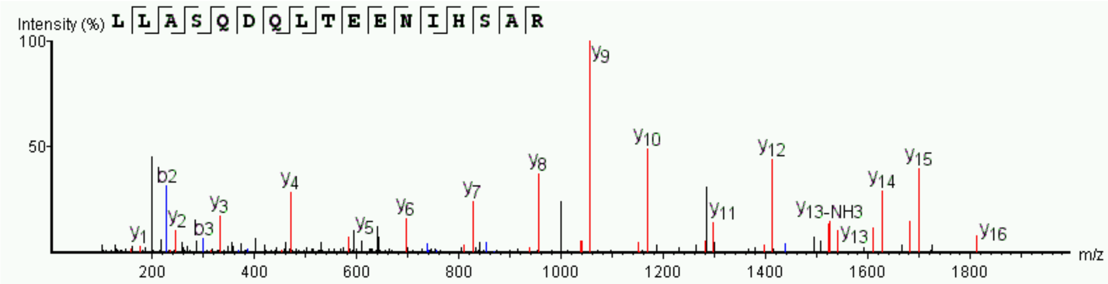

gi|571503163

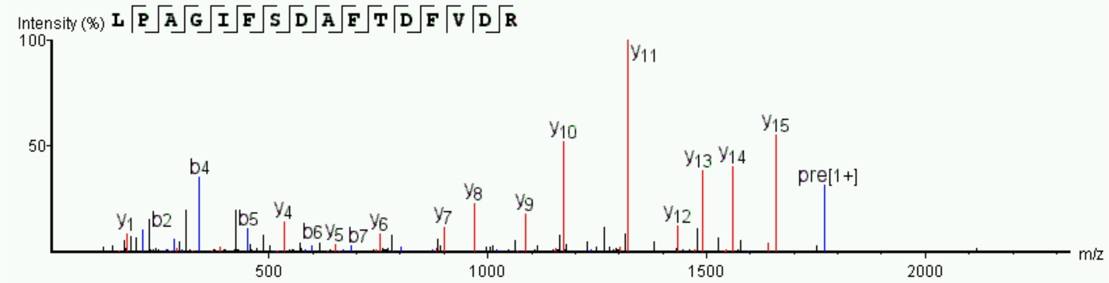

gi|571576863

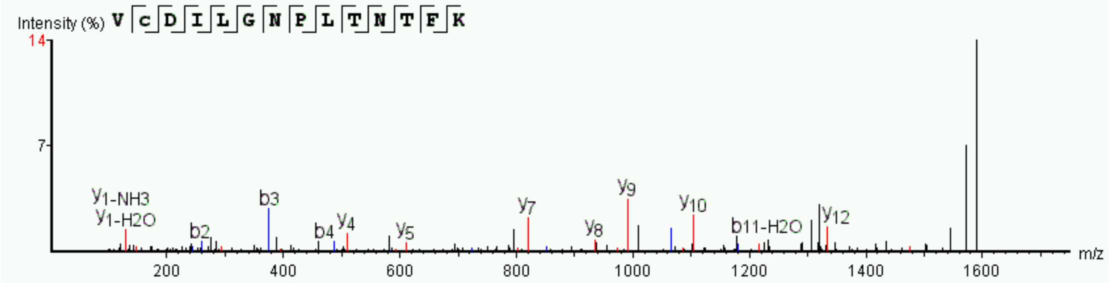

gi|571527600

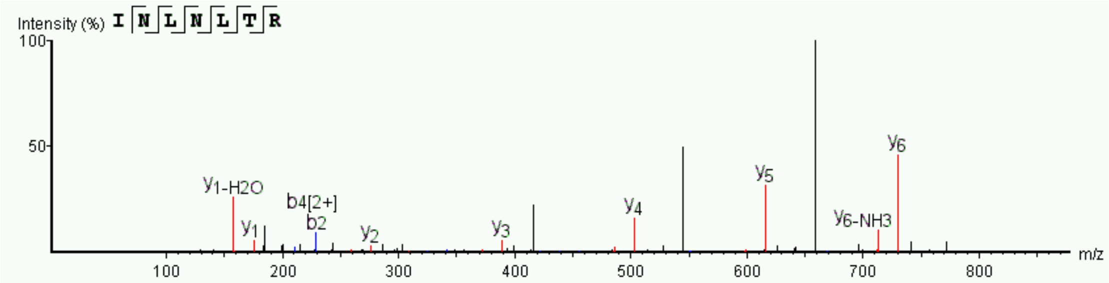

gi|571547079

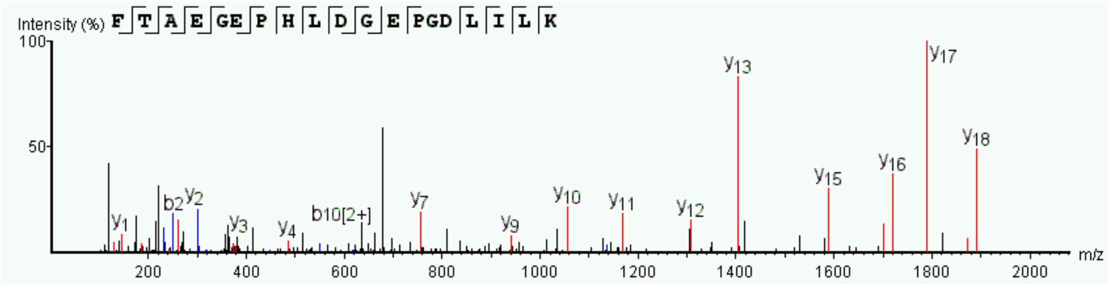

gi|66509442

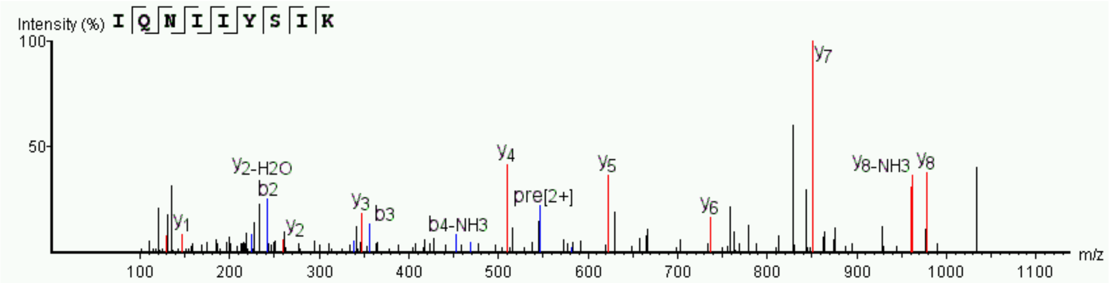

gi|571536590

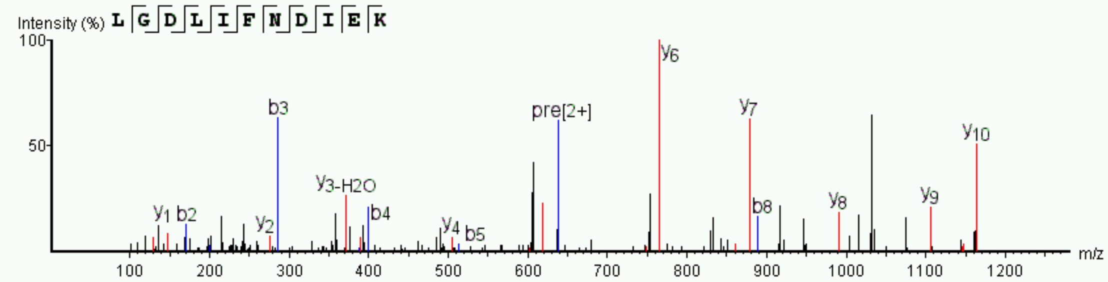

gi|66532731

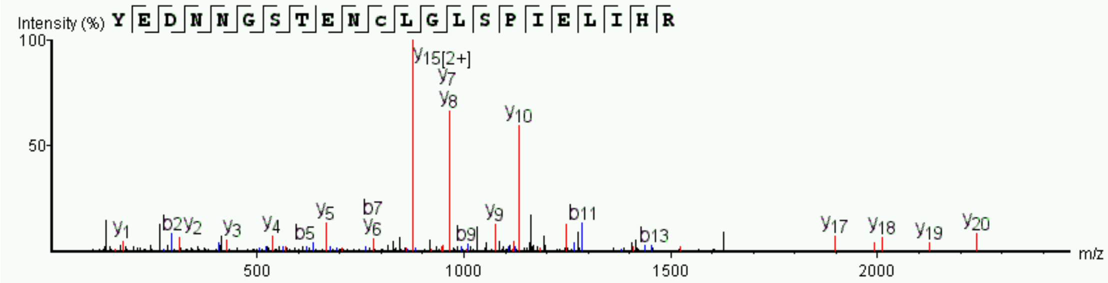

gi|571575329

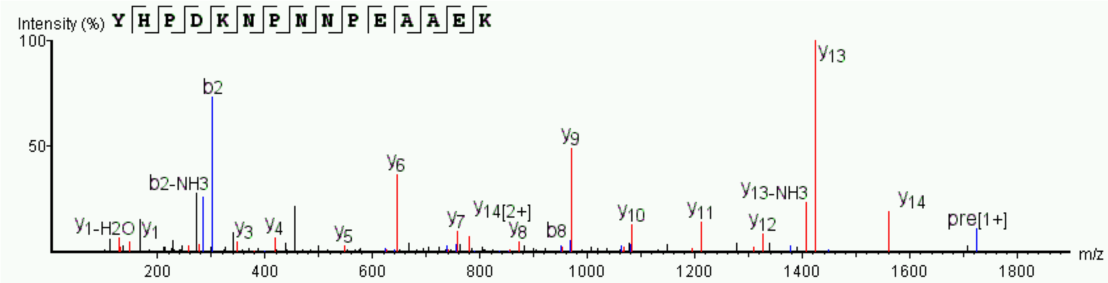

gi|571576935

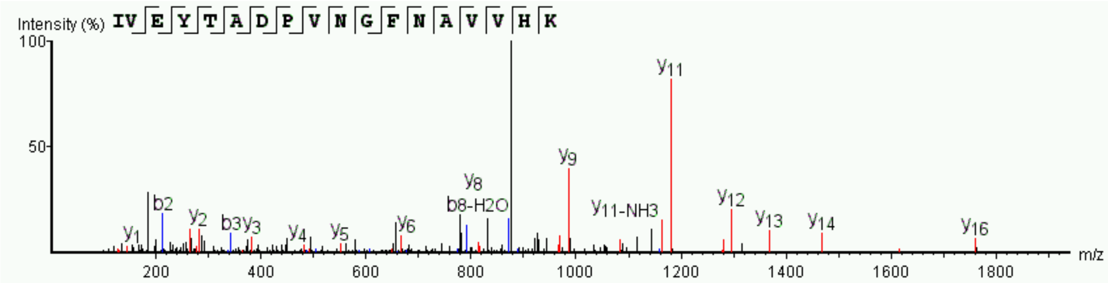

gi|110772198

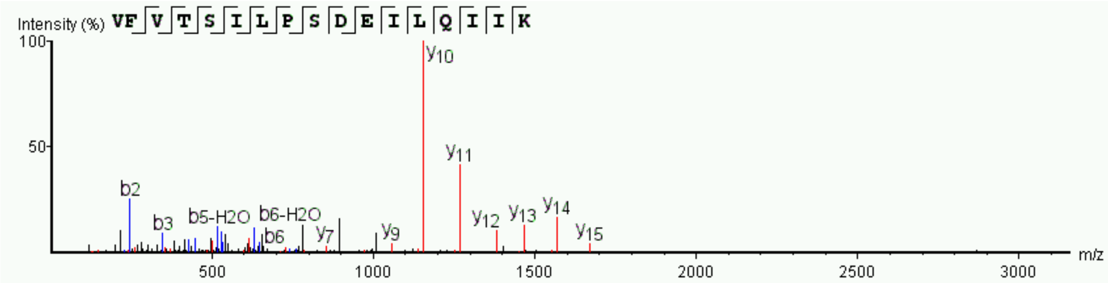

gi|571530426

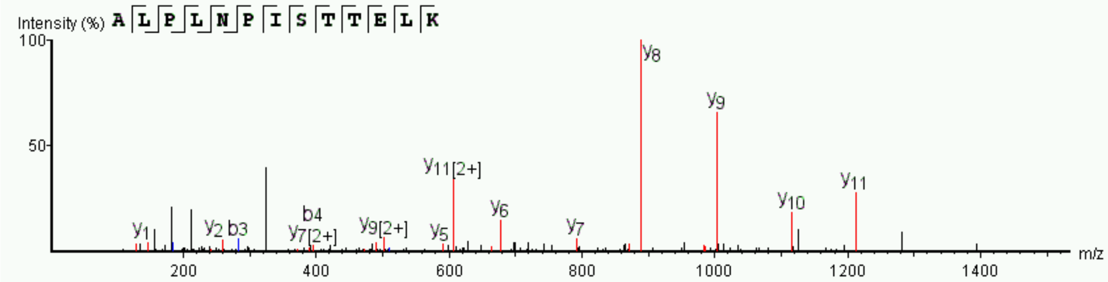

gi|336391185

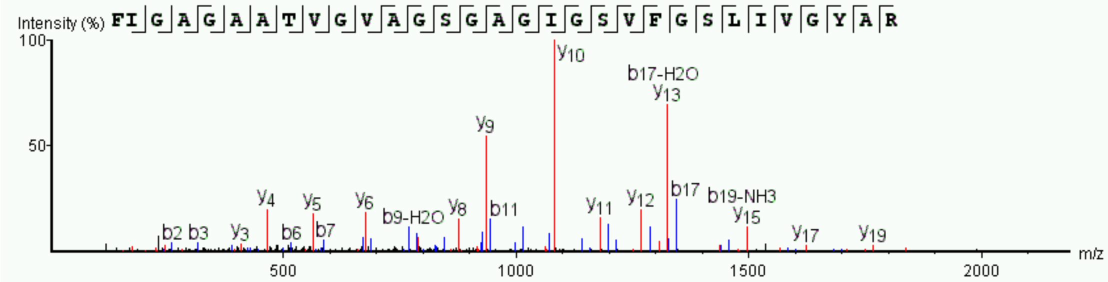

gi|58585106

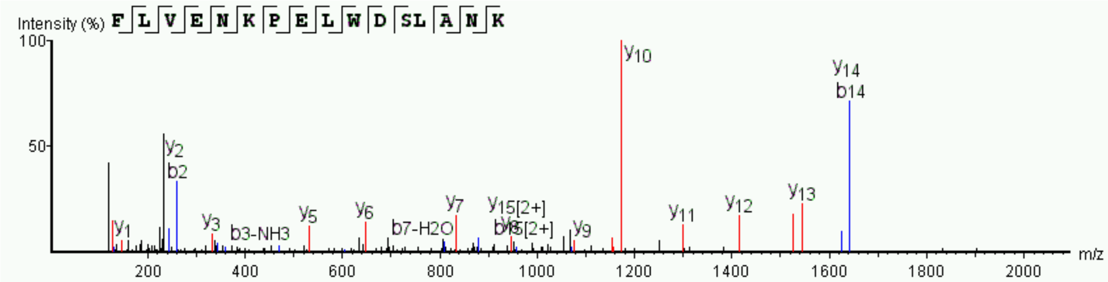

gi|571569183

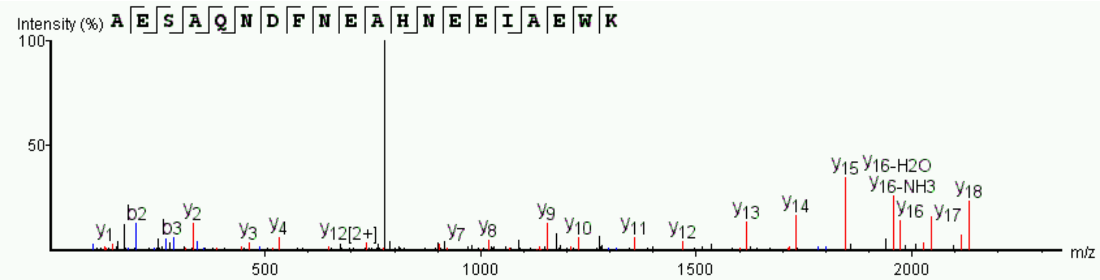

gi|571569186

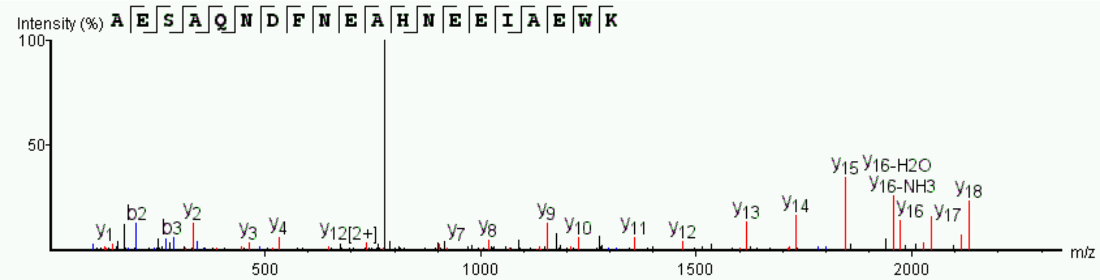

gi|399220300

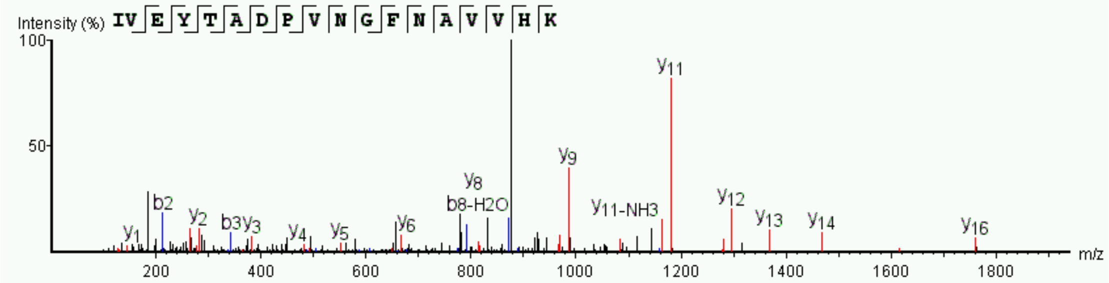

gi|270289764

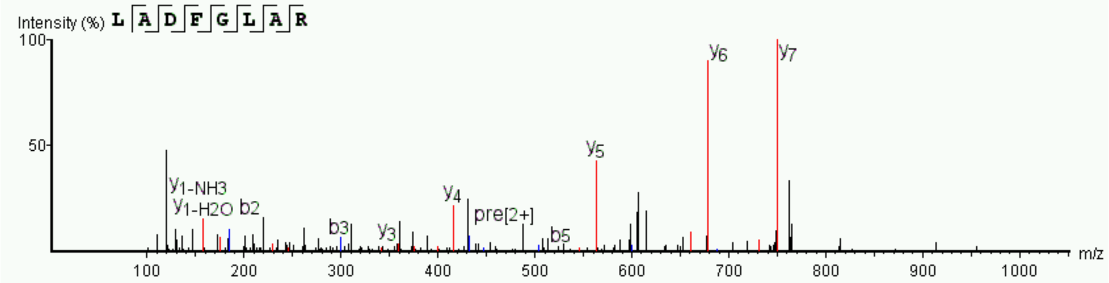

gi|5834937

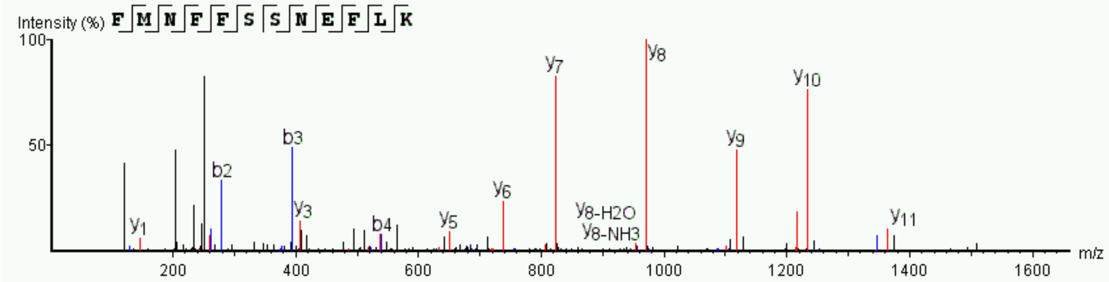

gi|288872189

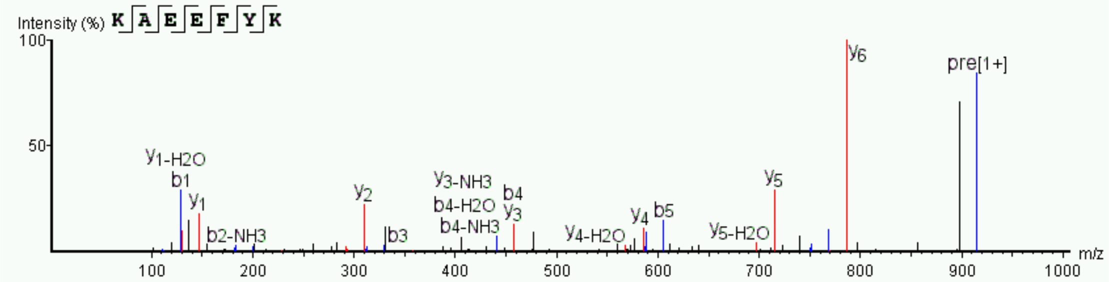

gi|58585206

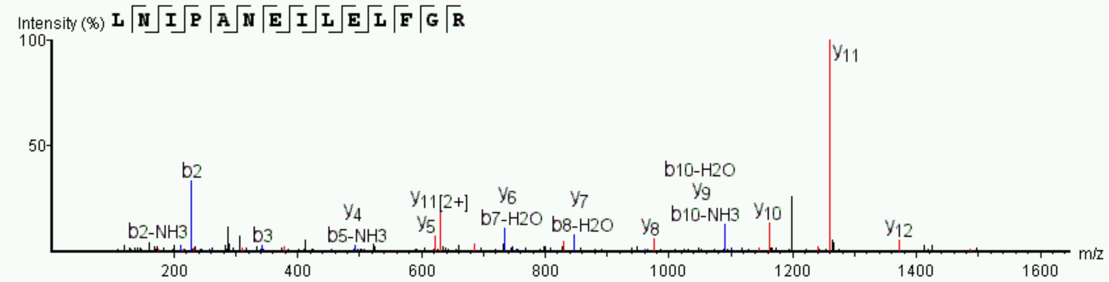

gi|58585078

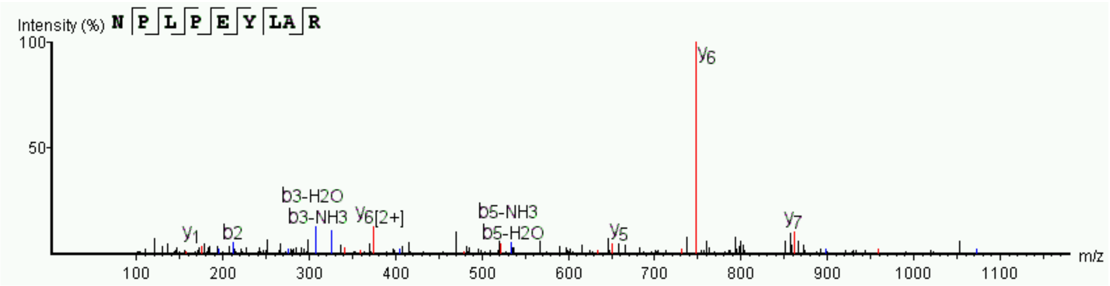

gi|571552606

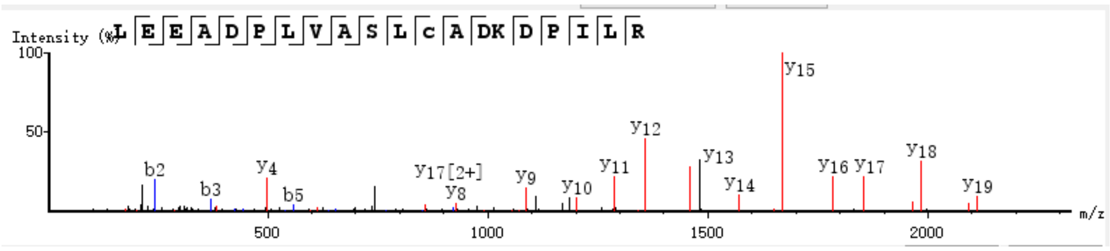

gi|60115688

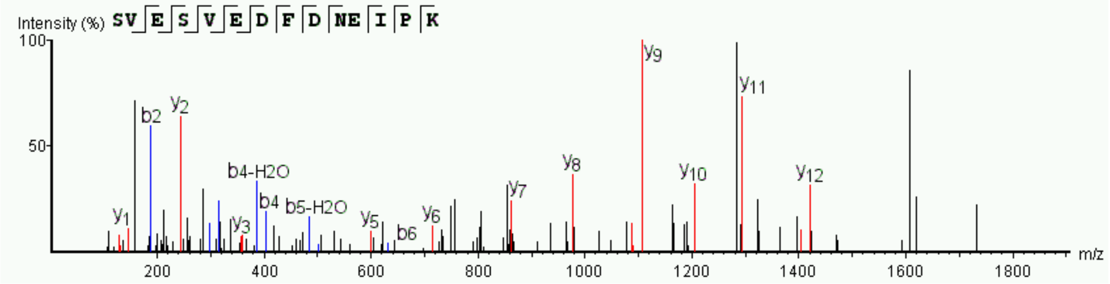

gi|209180477

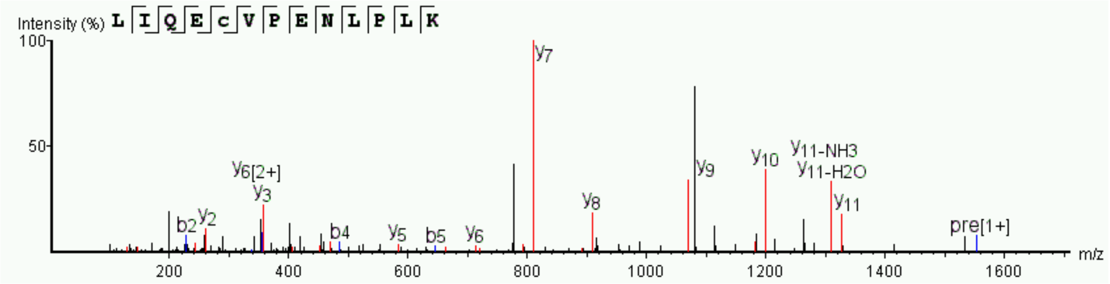

gi|58585154

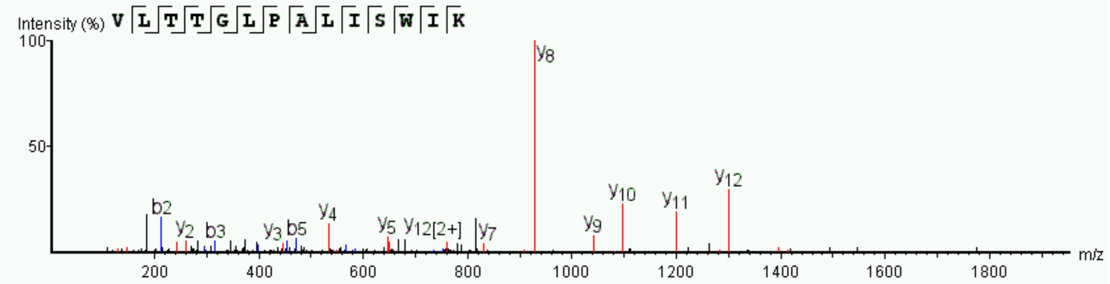

gi|5834932

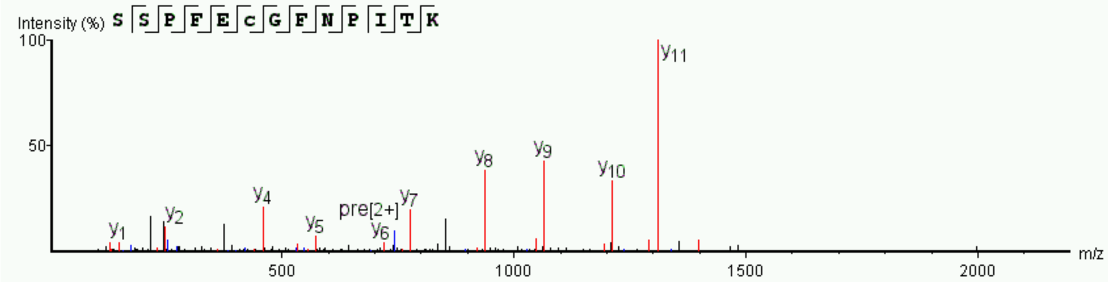

gi|58585096

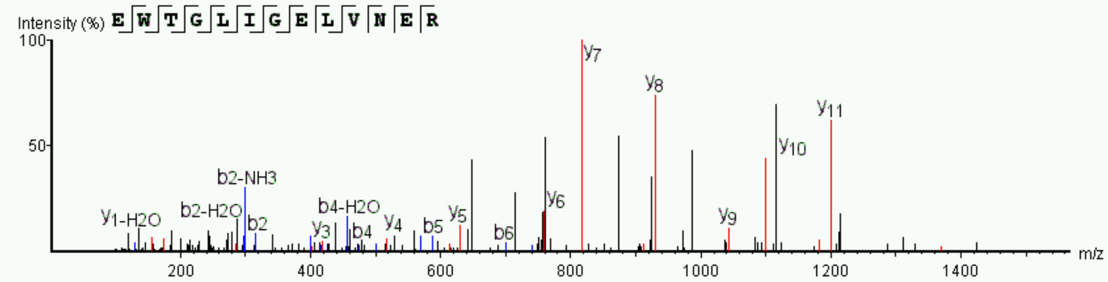

gi|66547365

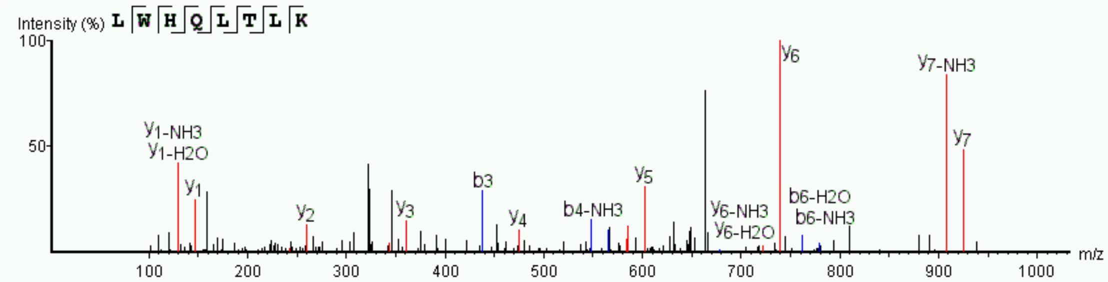

gi|571578355

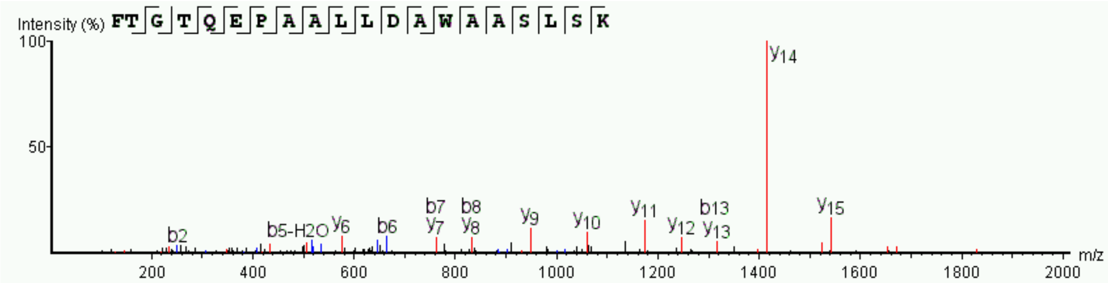

gi|328781273

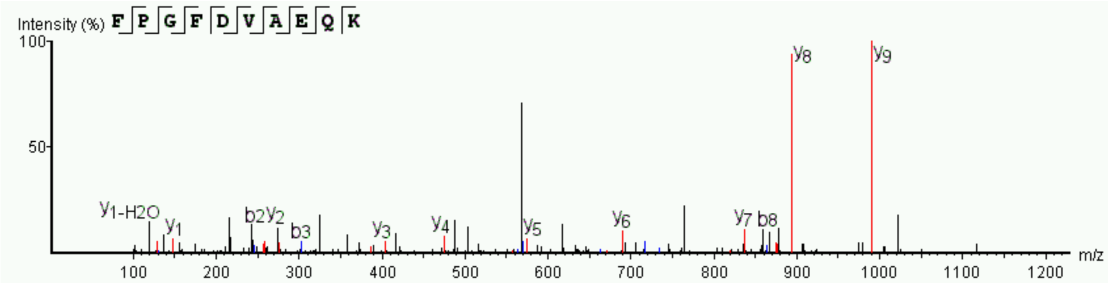

gi|571558762

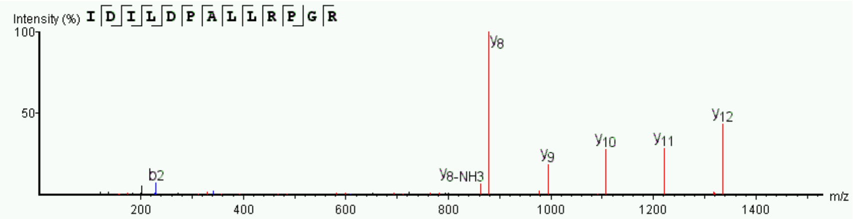

gi|571558766

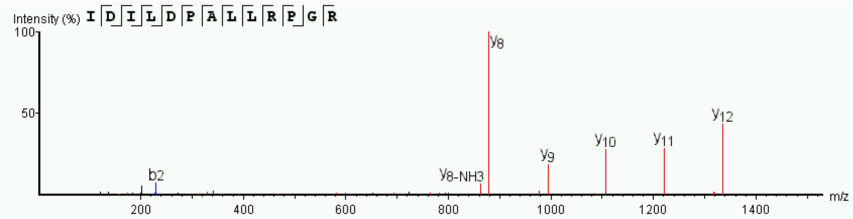

gi|571558772

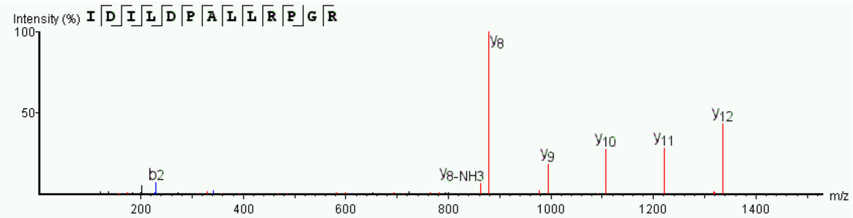

gi|571558776

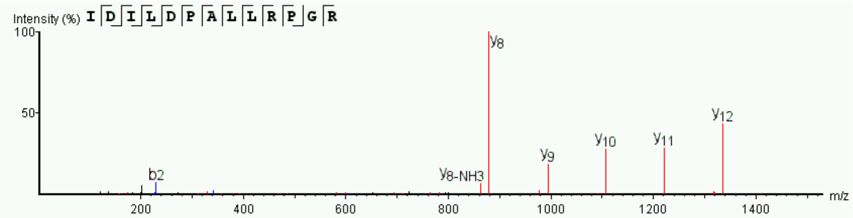

gi|571524852

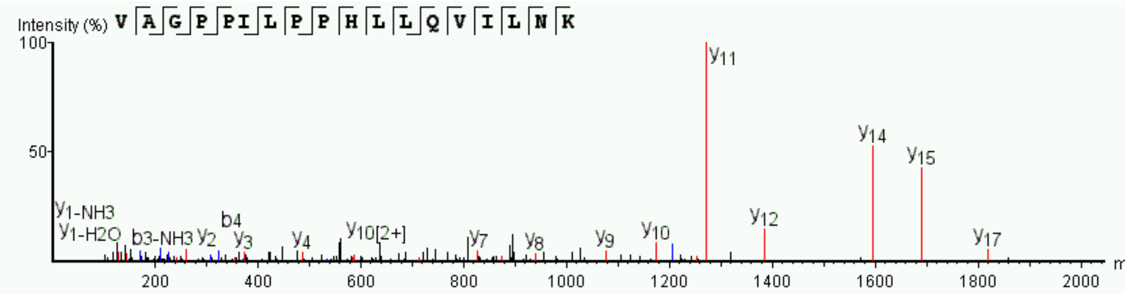

gi|571524858

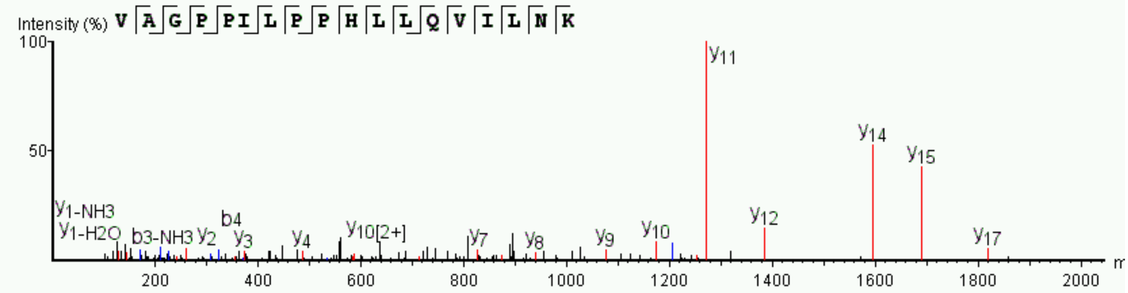

gi|48113496

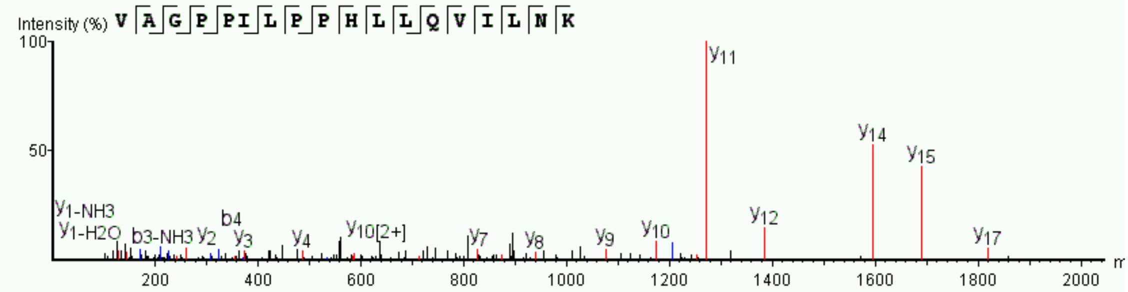

gi|571573002

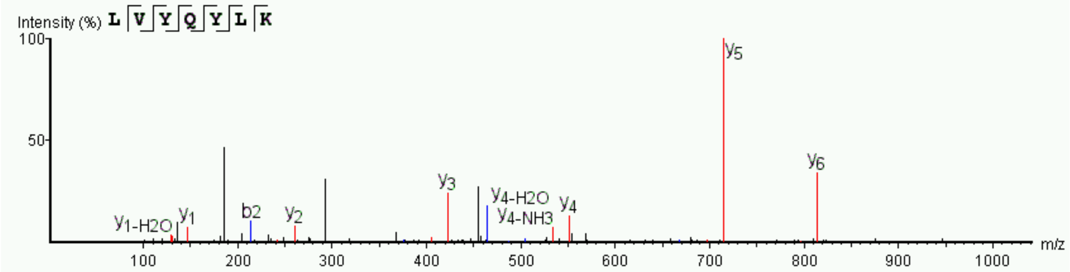

gi|110762954

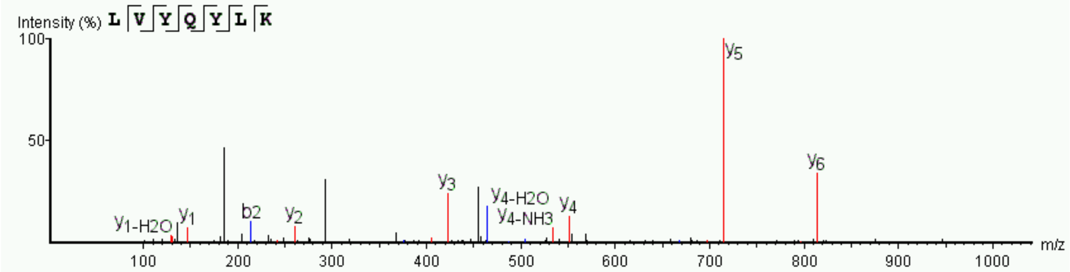

gi|48096310

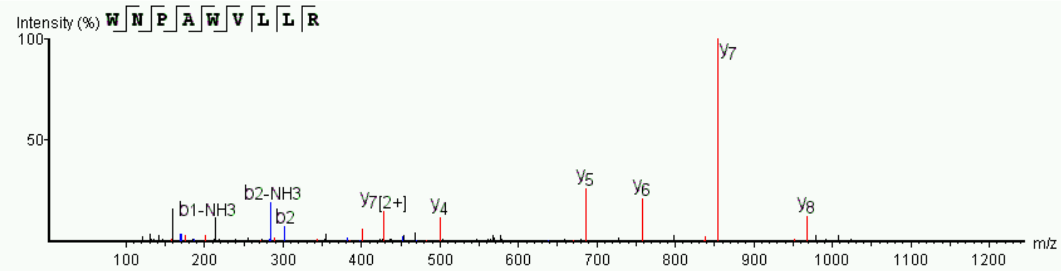

gi|328793058

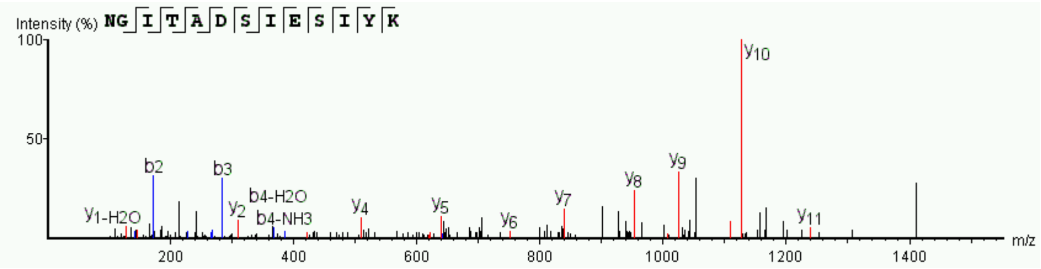

gi|328788691

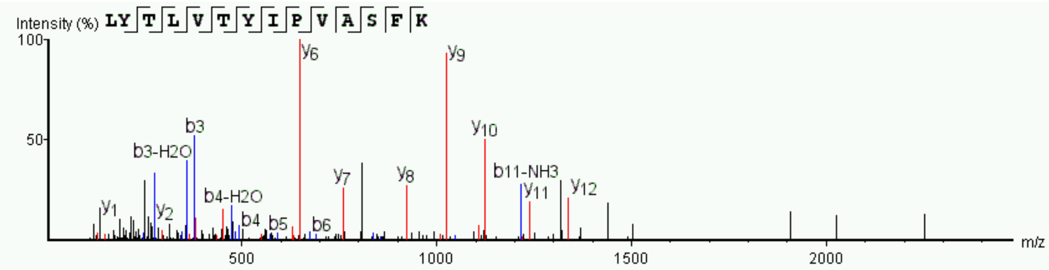

gi|48142237

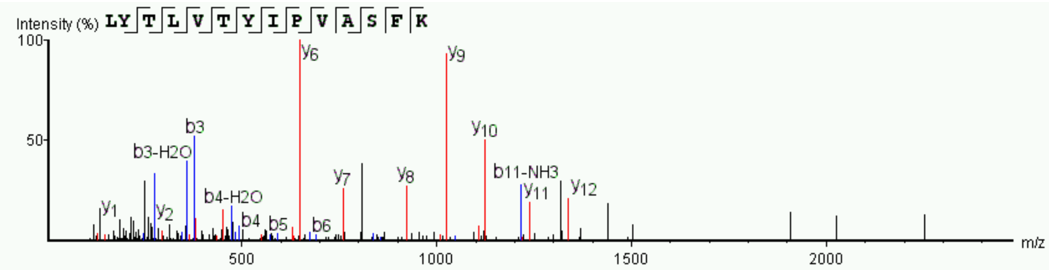

gi|571555366

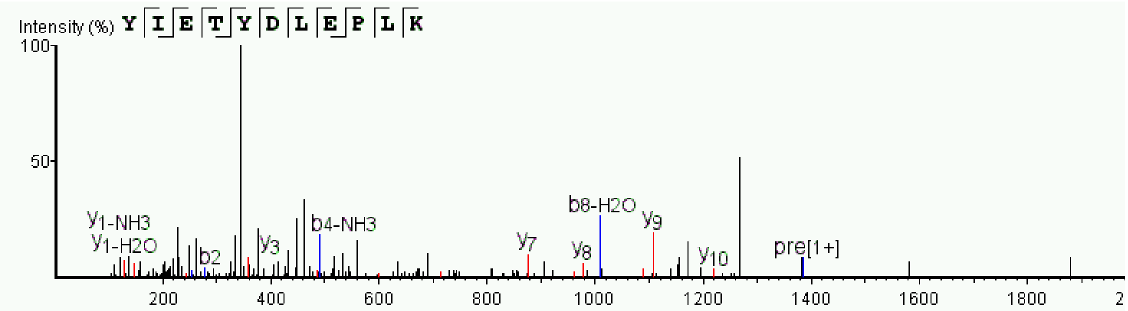

gi|571555369

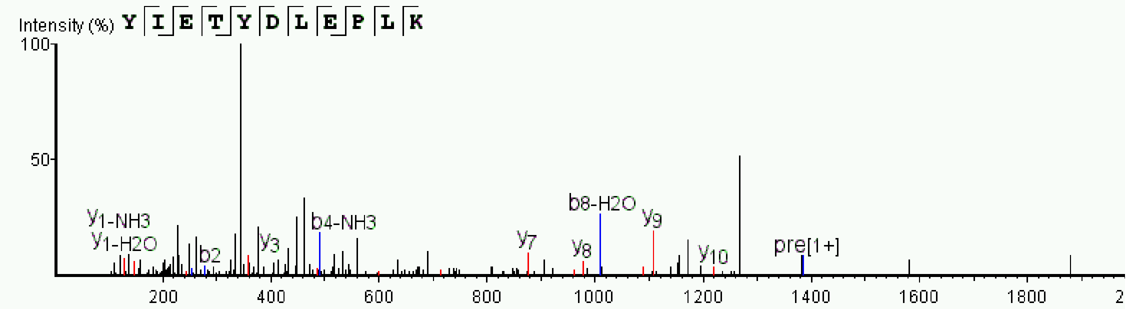

gi|571572819

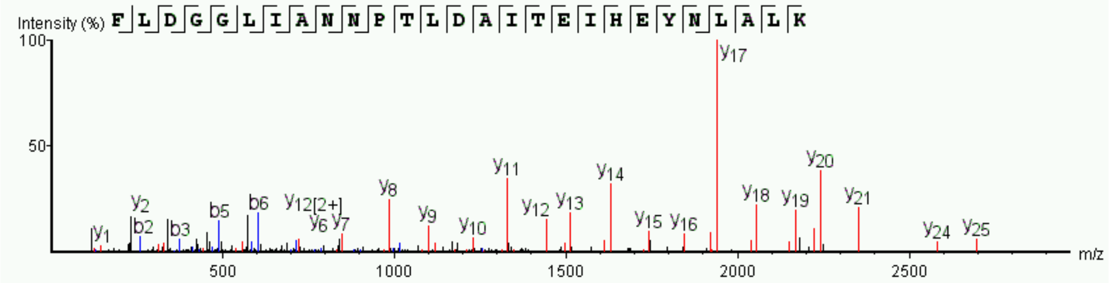

gi|66552169

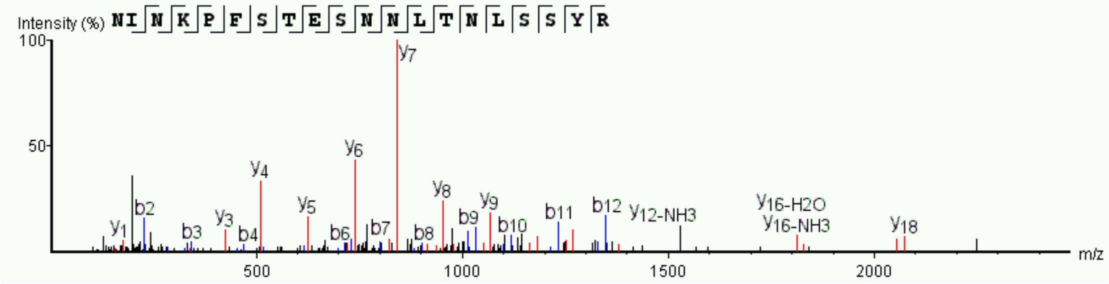

gi|66526748

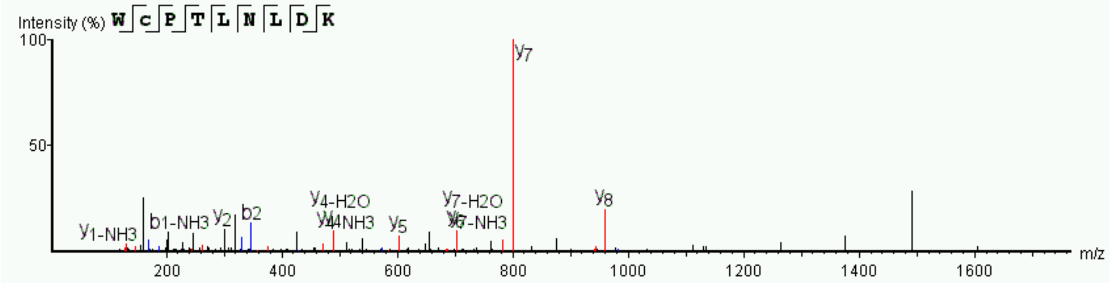

gi|571576016

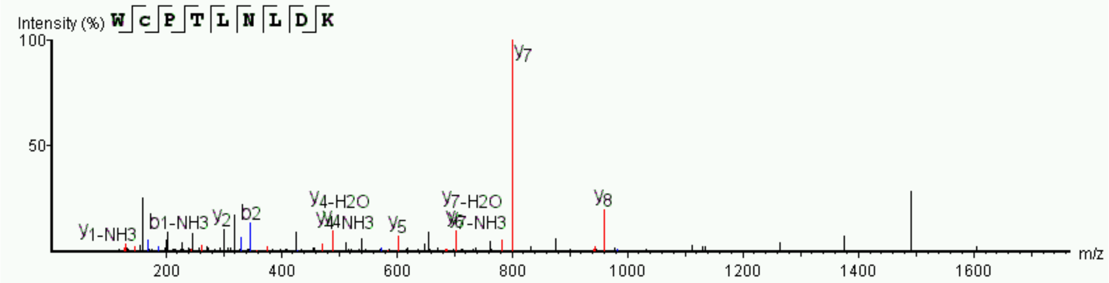

gi|571574994

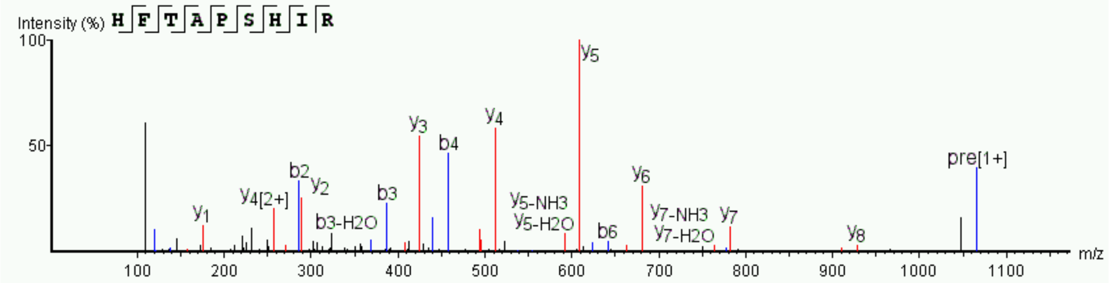

gi|110761627

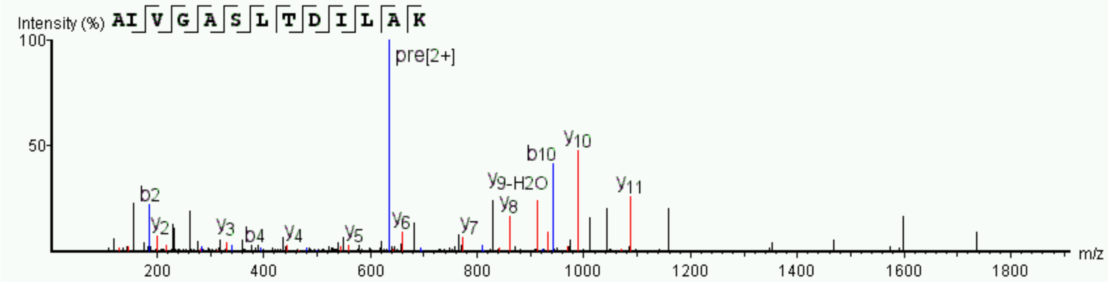

gi|110762813

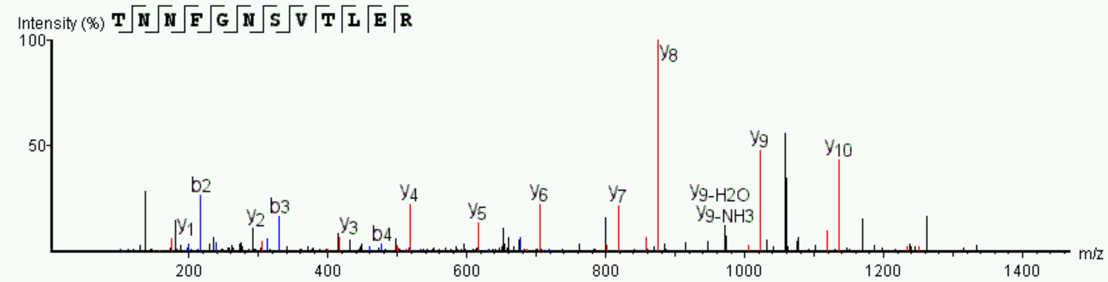

gi|571573543

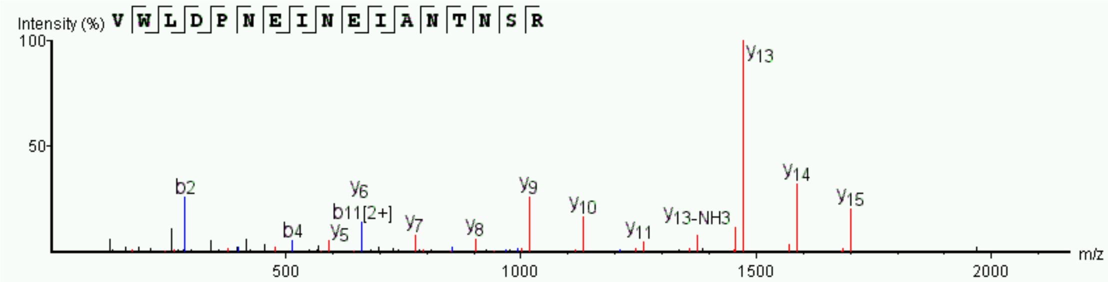

gi|571522669

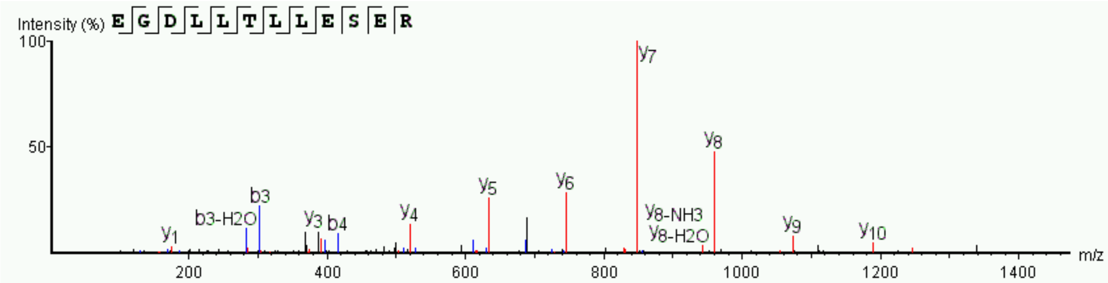

gi|328787488

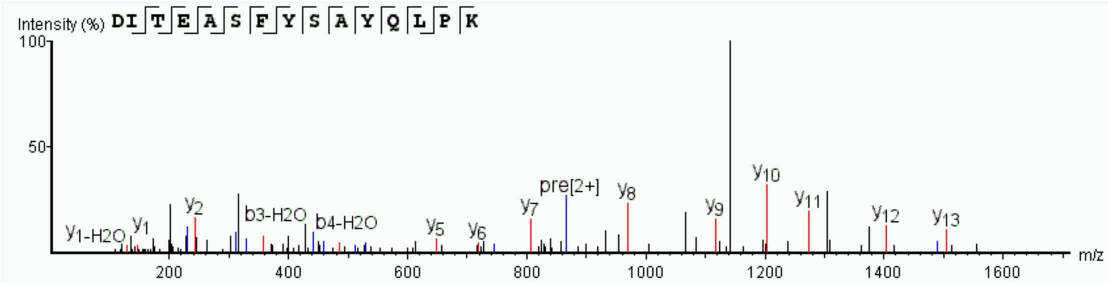

gi|110771708

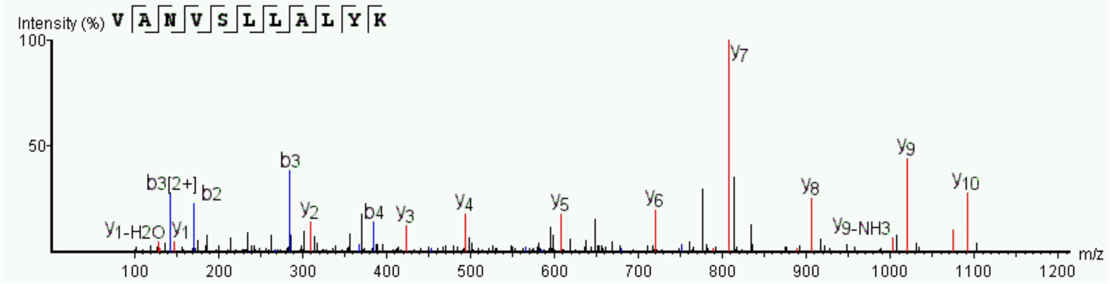

gi|110766823

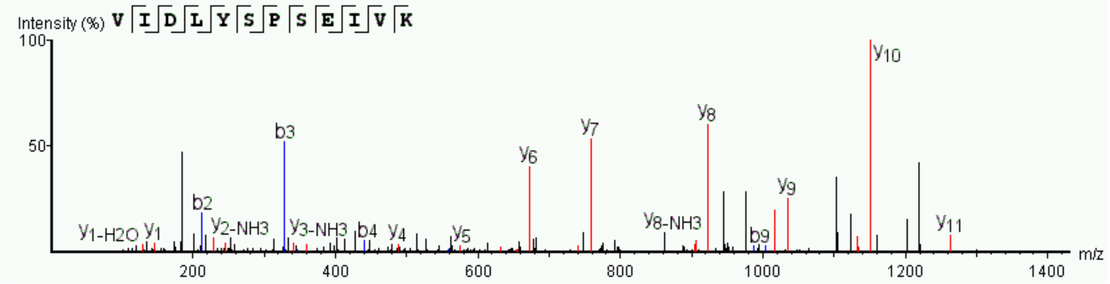

gi|328788587

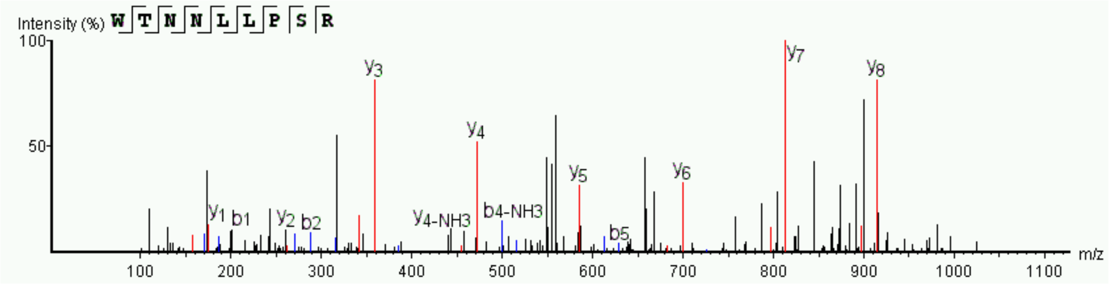

gi|110757985

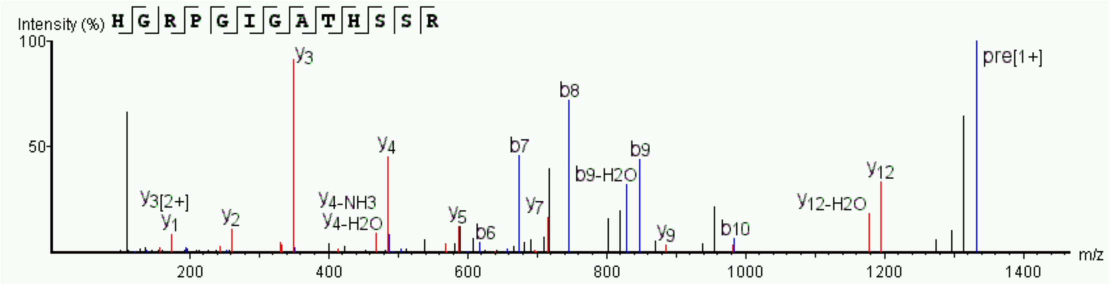

gi|66562243

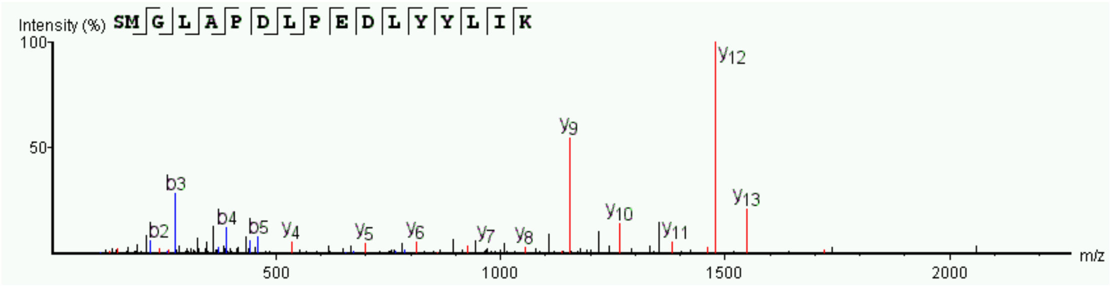

gi|571577109

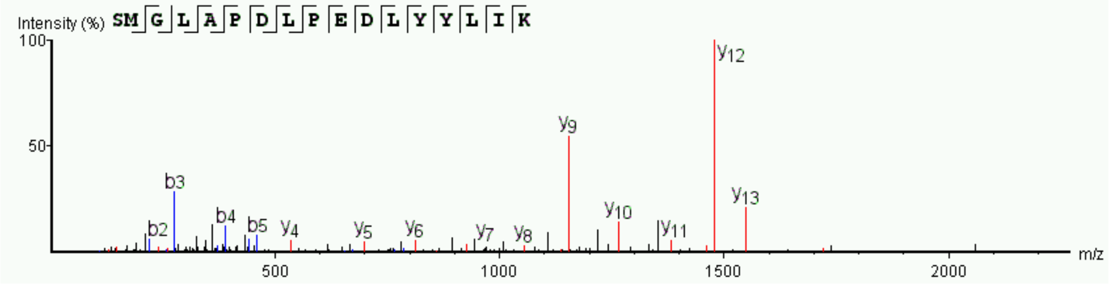

gi|66517421

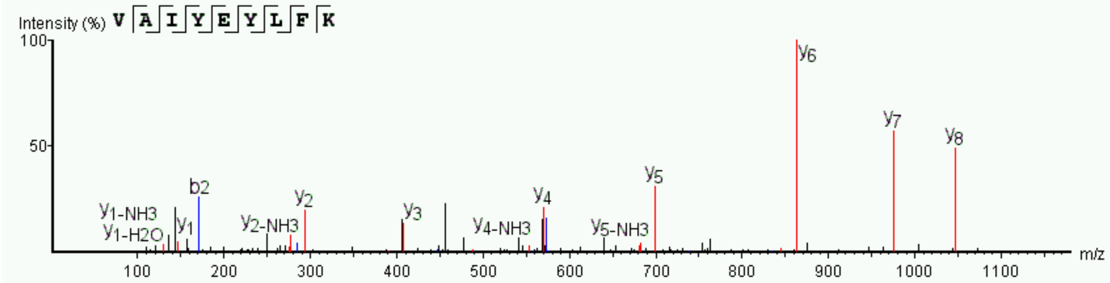

gi|48106363

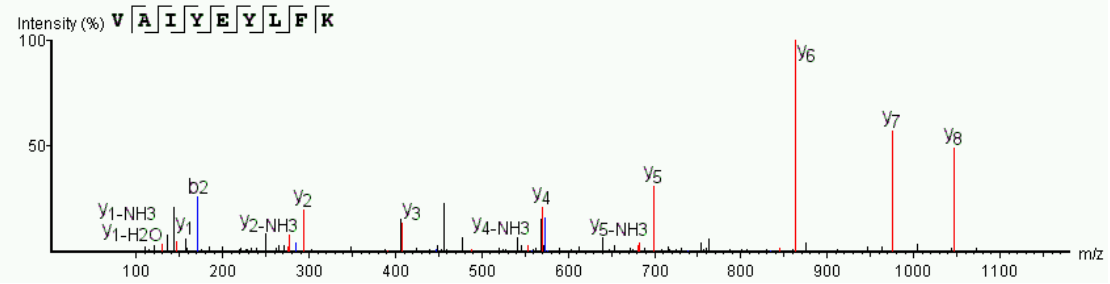

gi|571514405

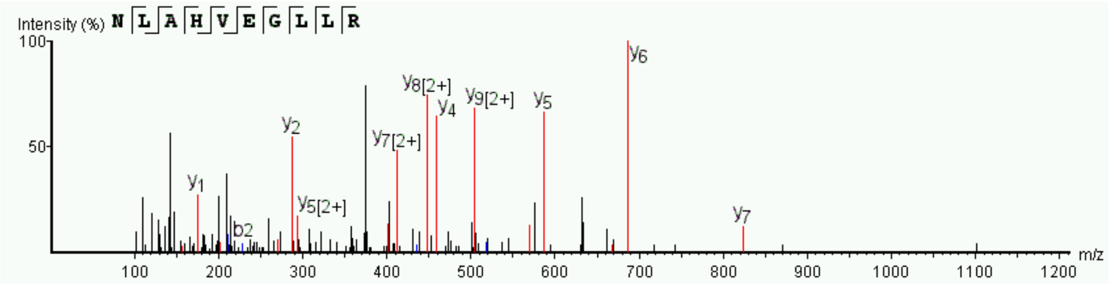

Annotated spectra of single-peptide identifications of proteins in optical bodies of *Apis mellifera*

gi|297591983

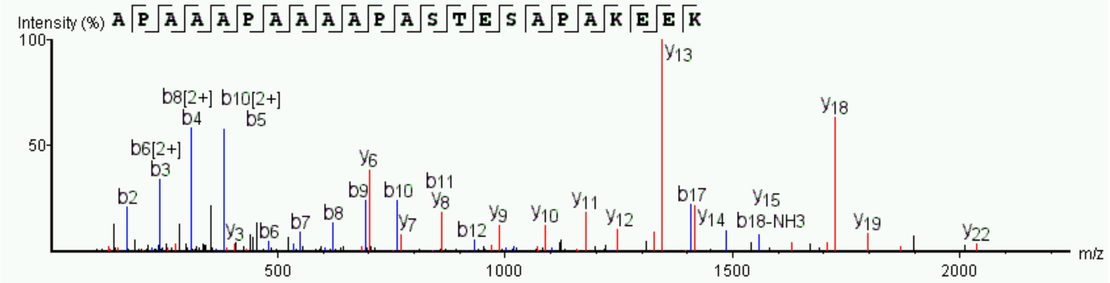

gi|118150480

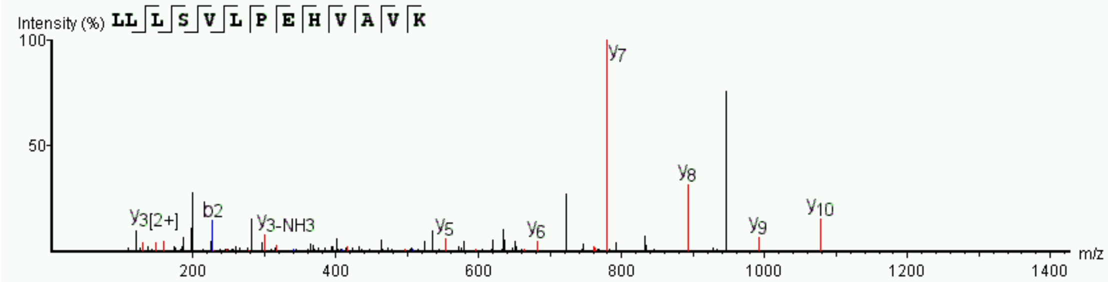

gi|94158854

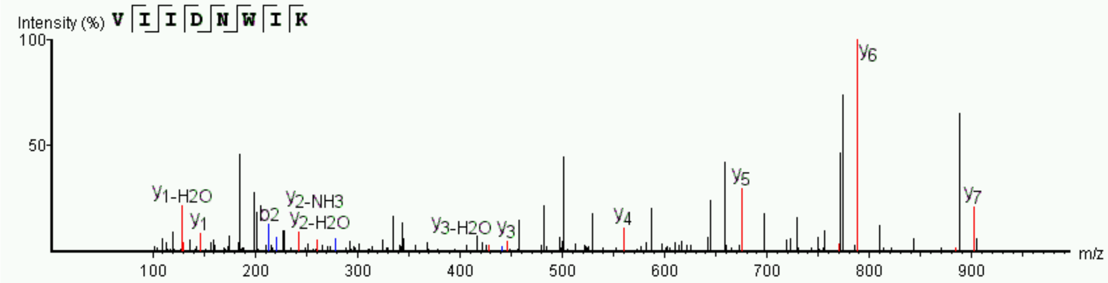

gi|166795901

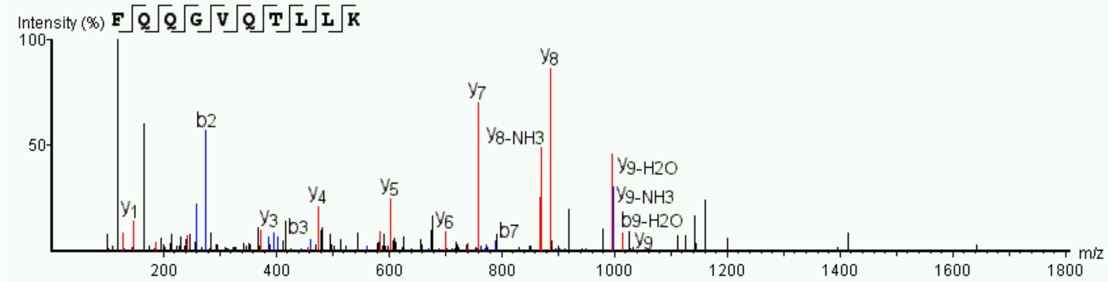

gi|336391185

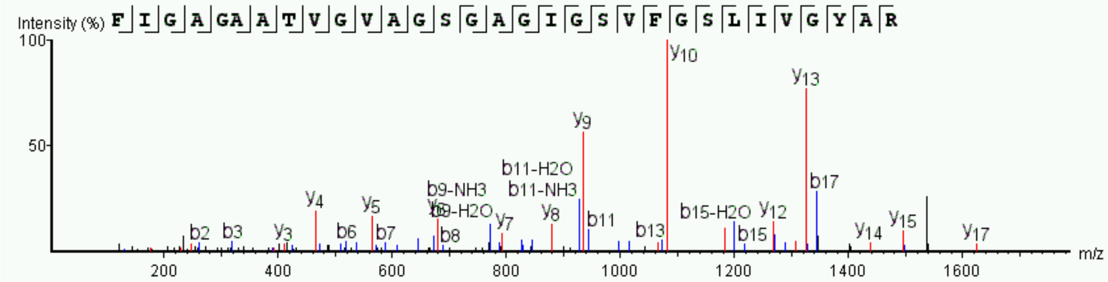

gi|270289764

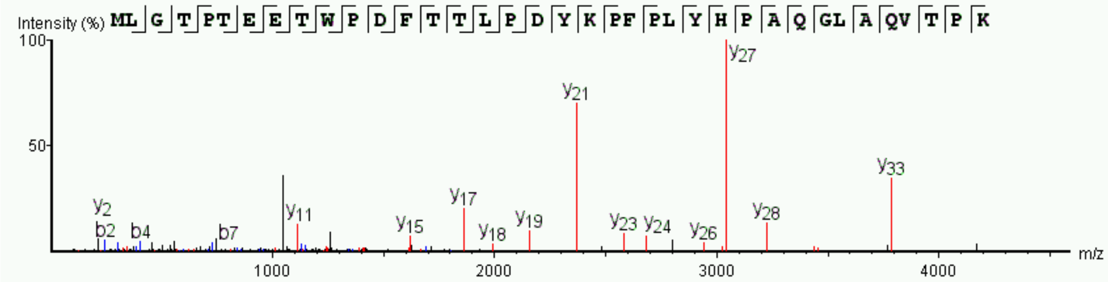

gi|5834937

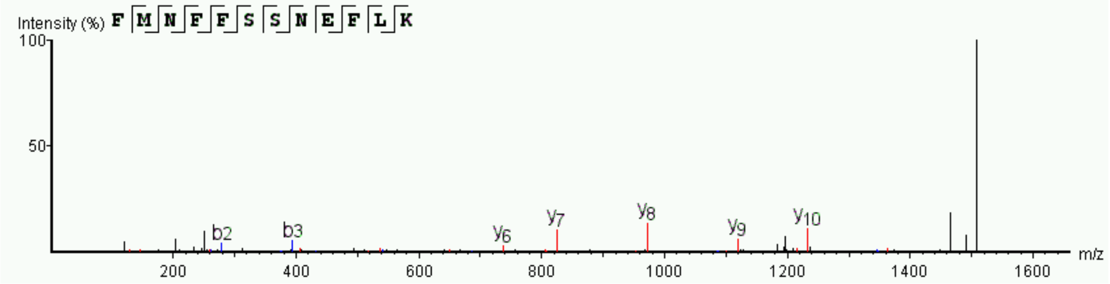

gi|254910938

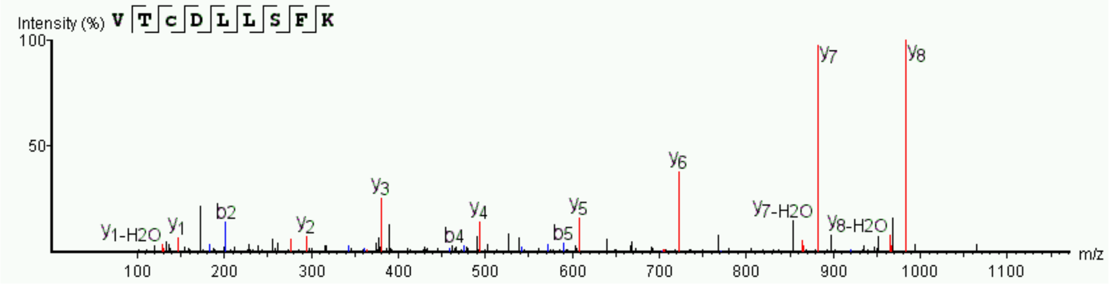

gi|335892843

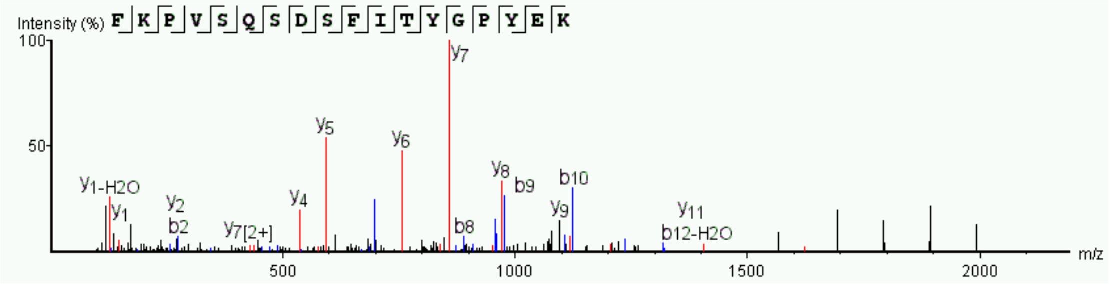

gi|58585206

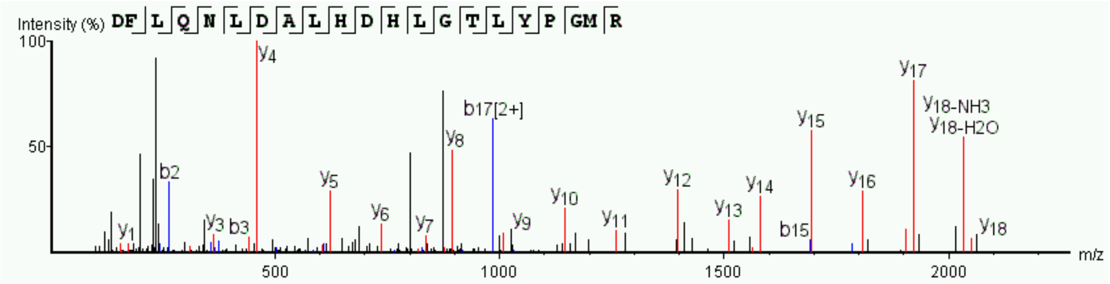

gi|209969778

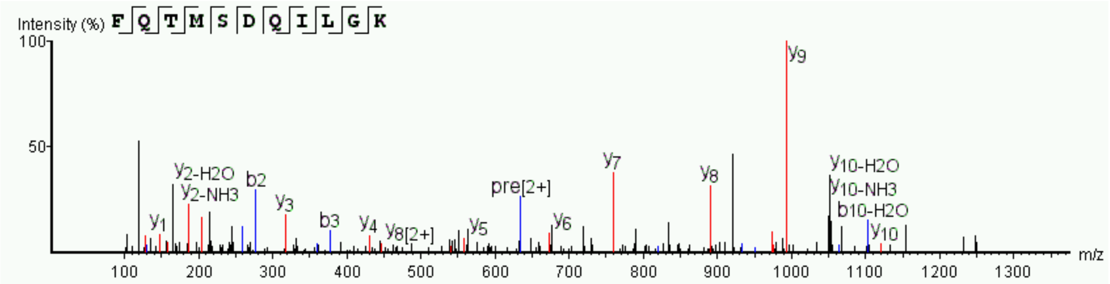

gi|60115688

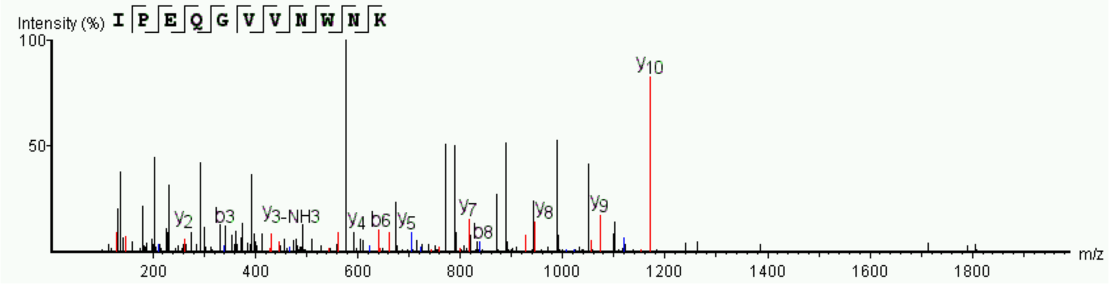

gi|209180477

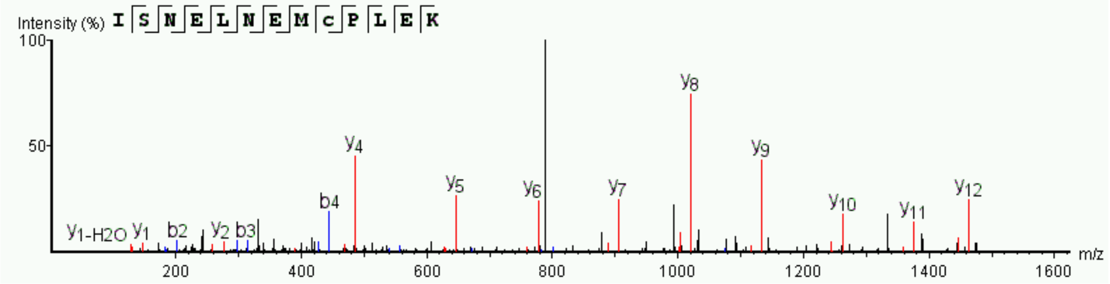

gi|58585098

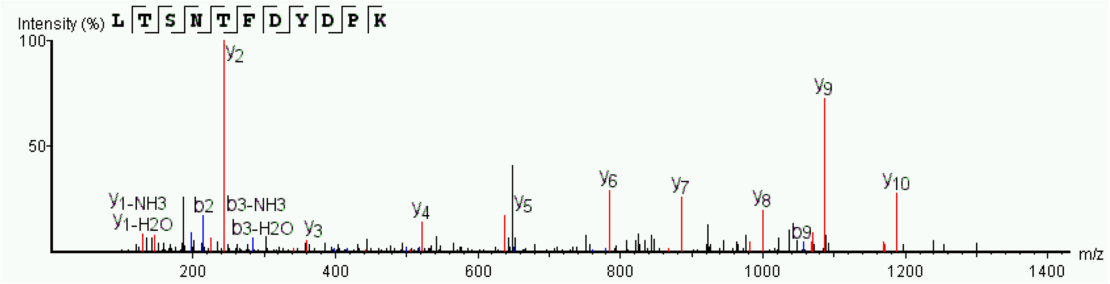

gi|58585138

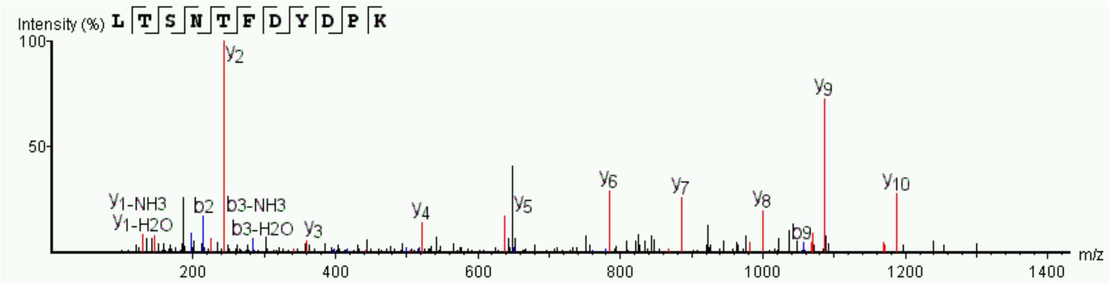

gi|5834932

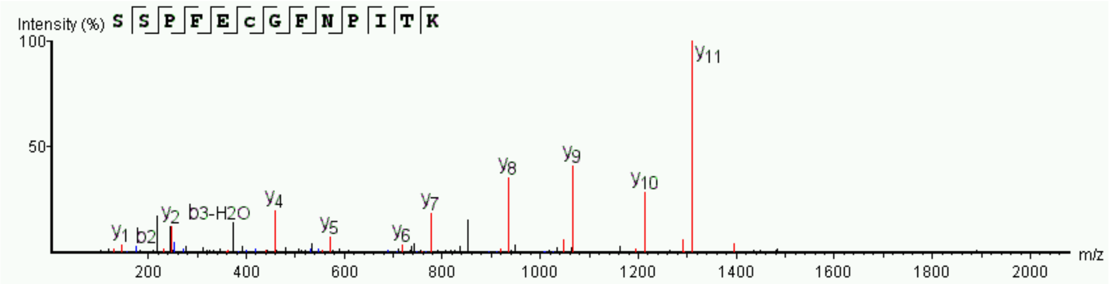

gi|328781273

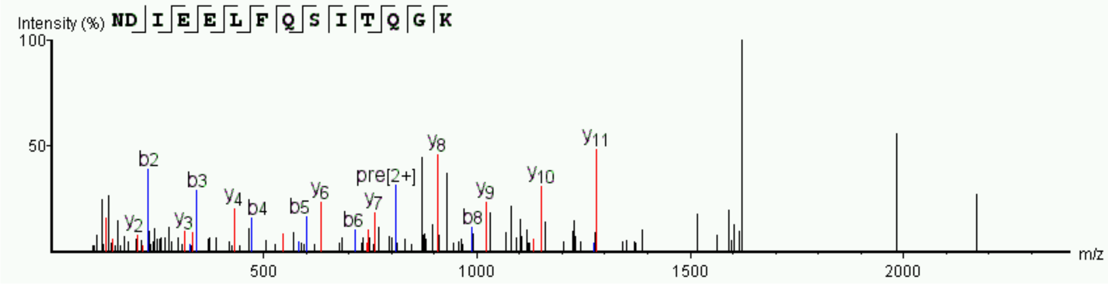

gi|571521798

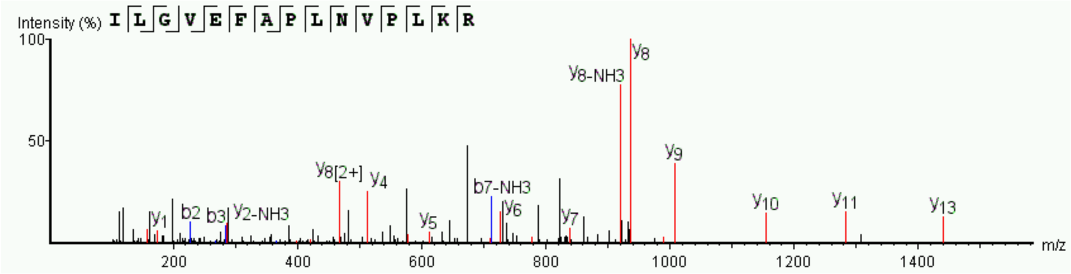

gi|66531974

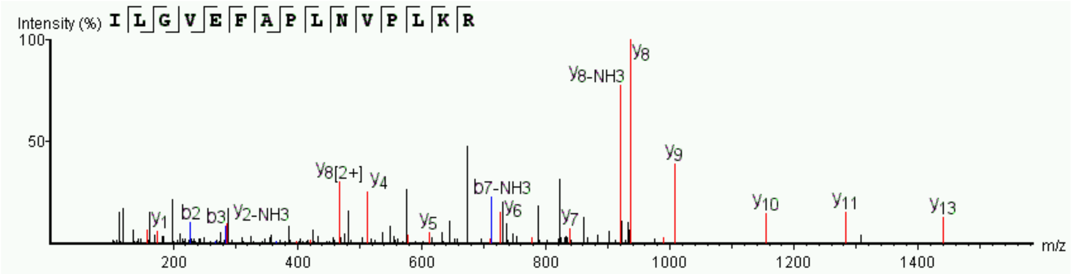

gi|571543571

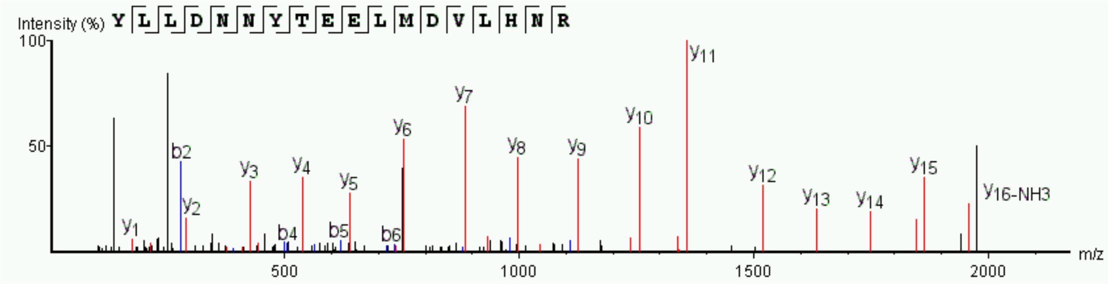

gi|571569110

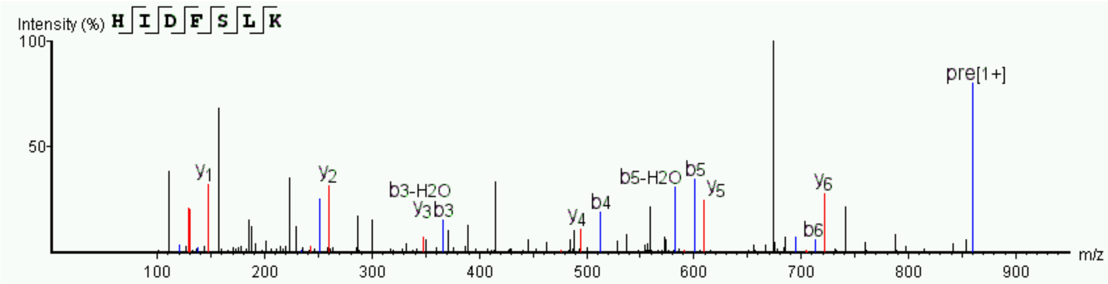

gi|110767080

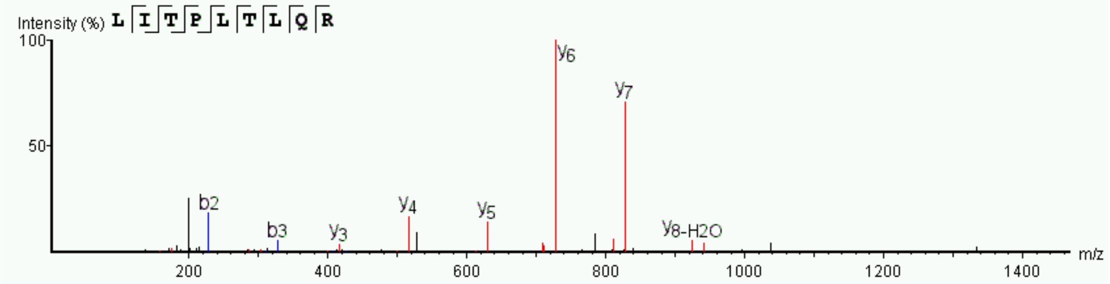

gi|571532029

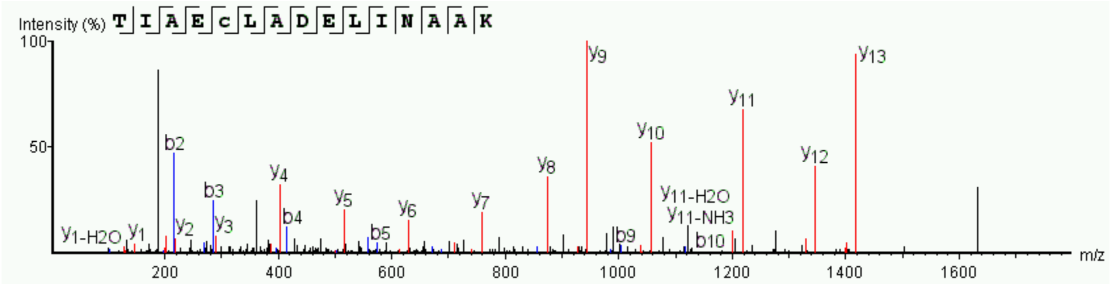

gi|571532025

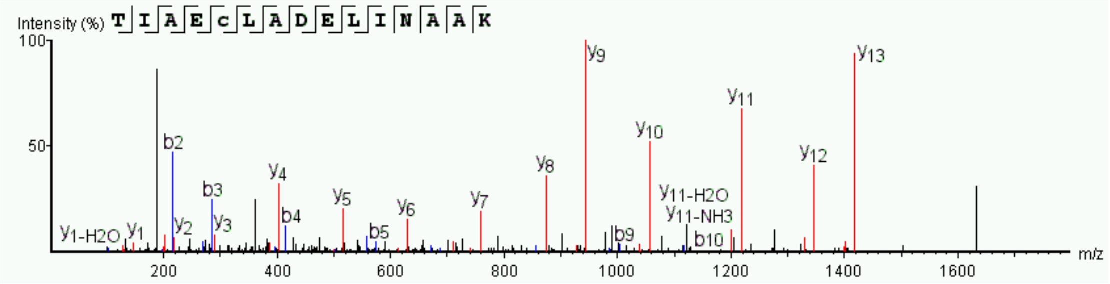

gi|571528291

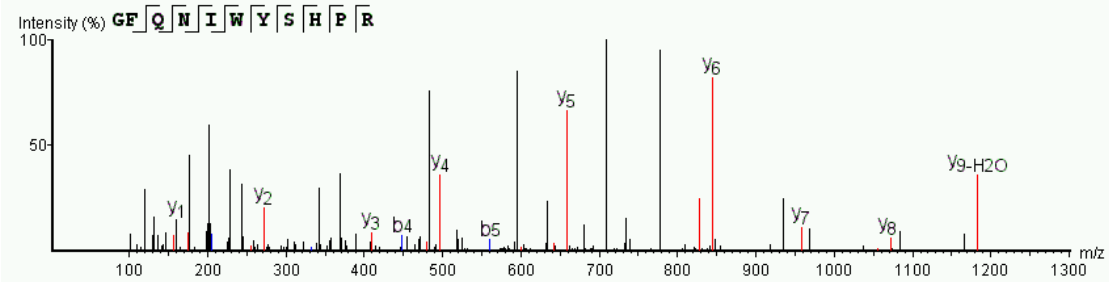

gi|571528288

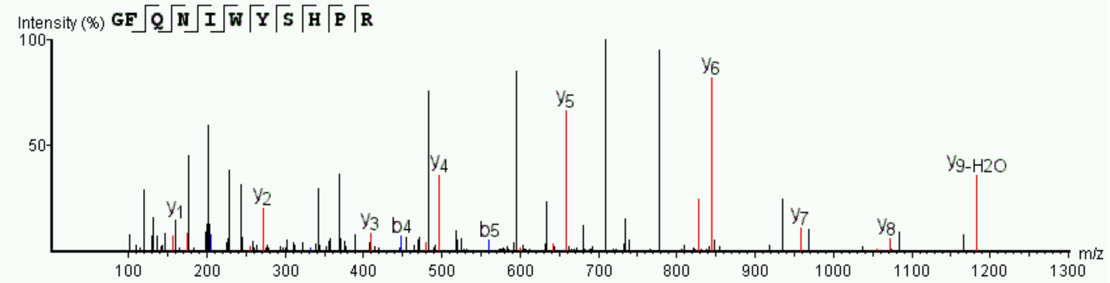

gi|110771708

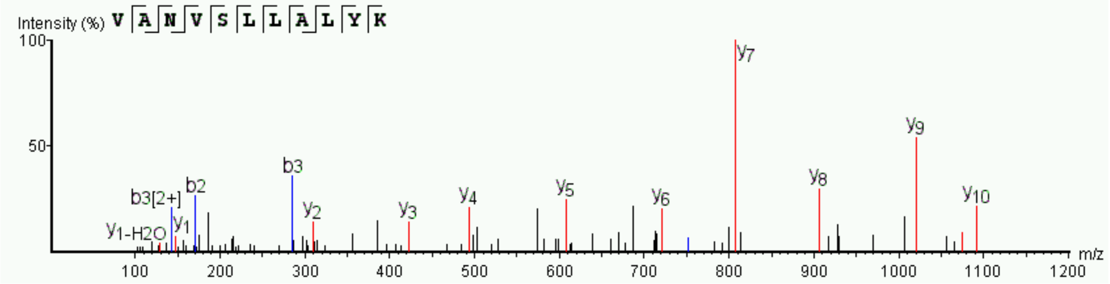

gi|110766823

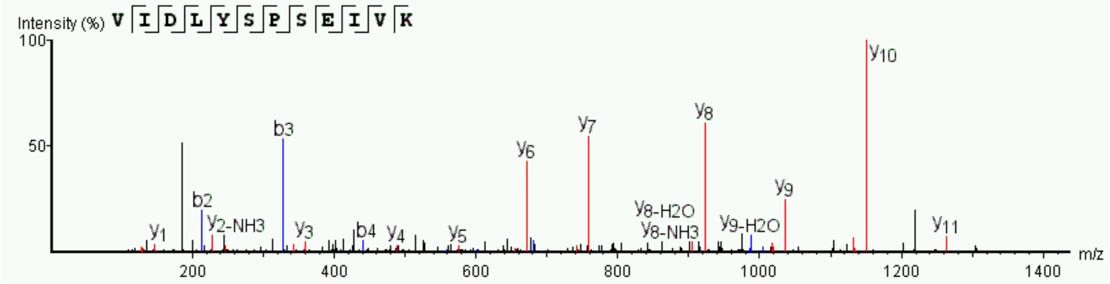

gi|48140499

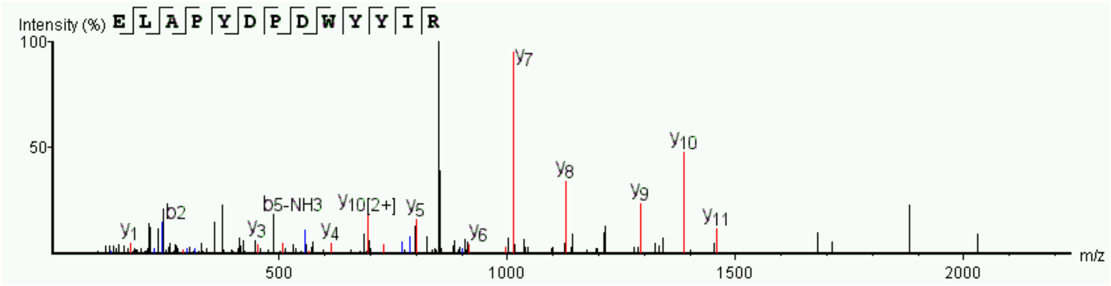

gi|571578878

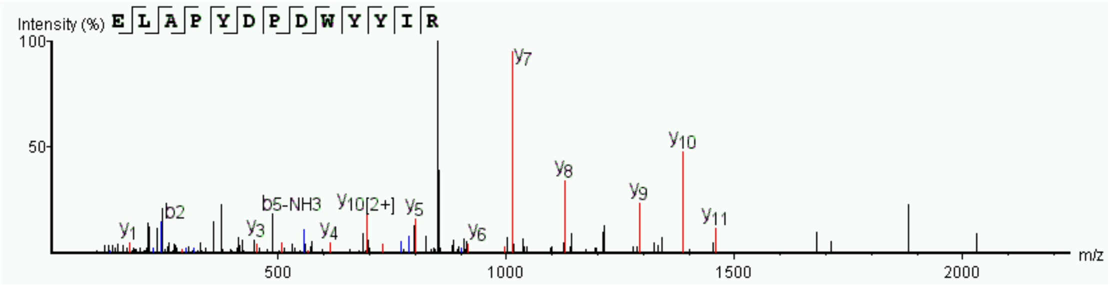

gi|110756571

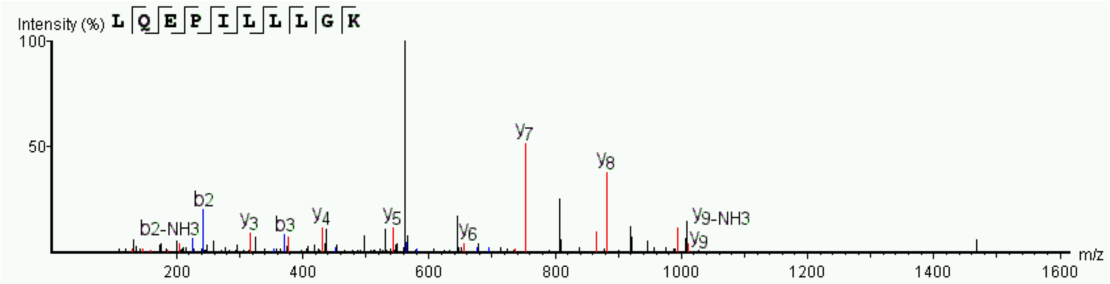

gi|328788587

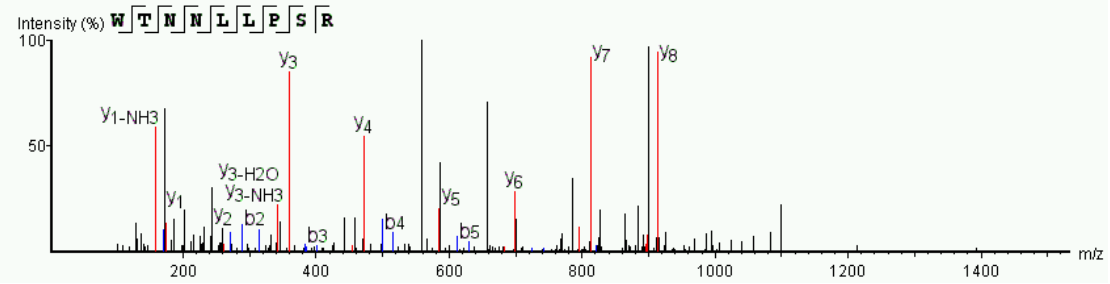

gi|66562243

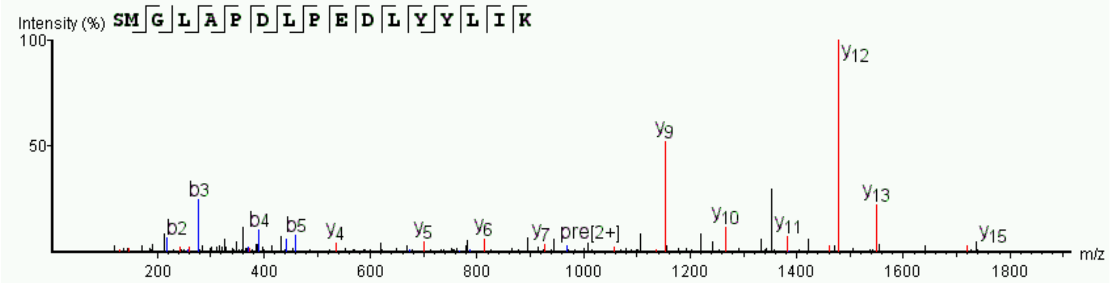

gi|571577109

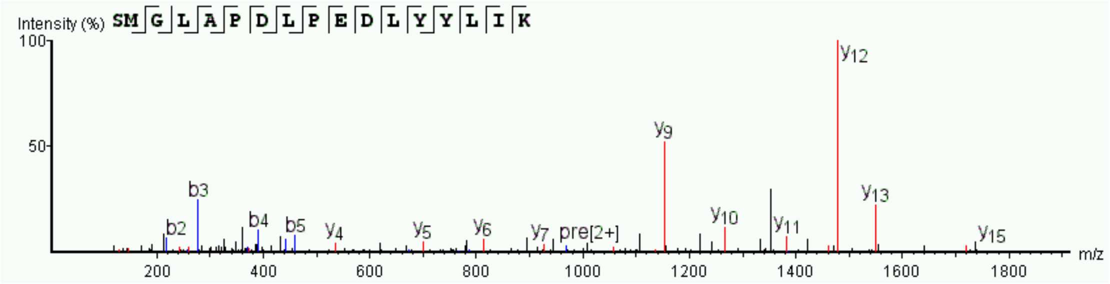

gi|571527606

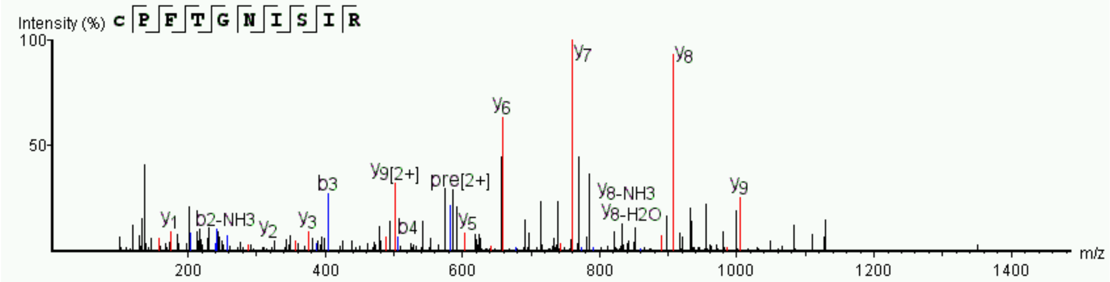

gi|571527608

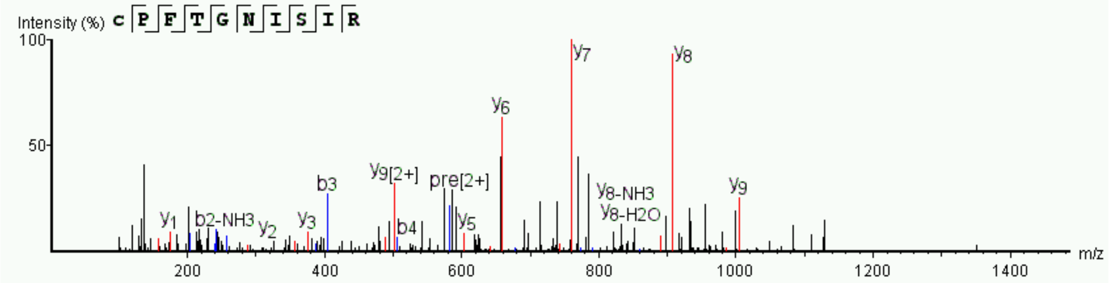

gi|66517421

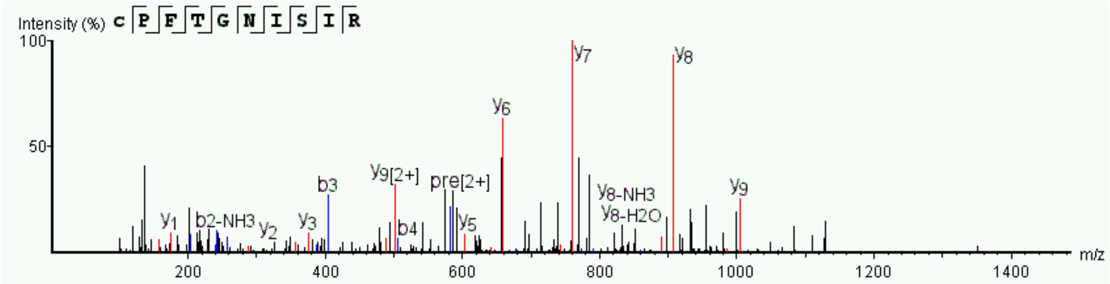

gi|48106363

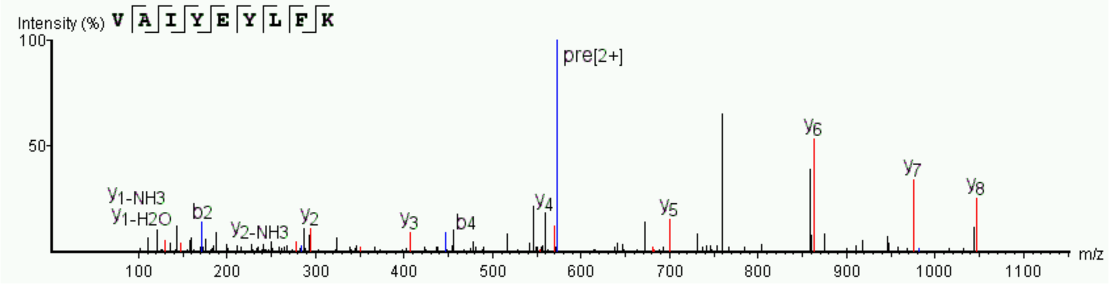

gi|66522335

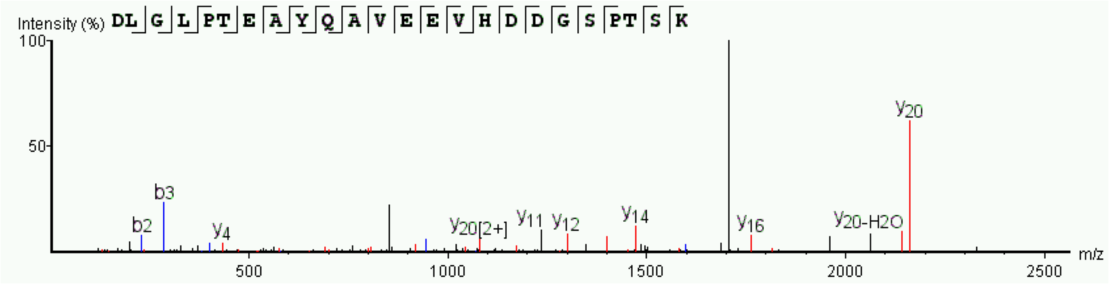

gi|571508698

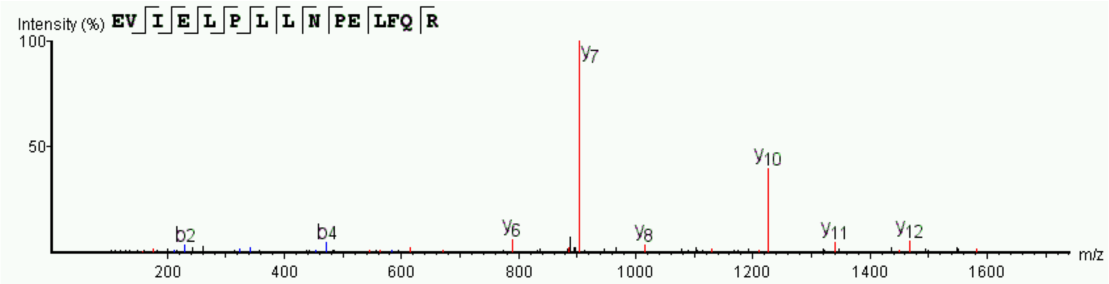

gi|66547365

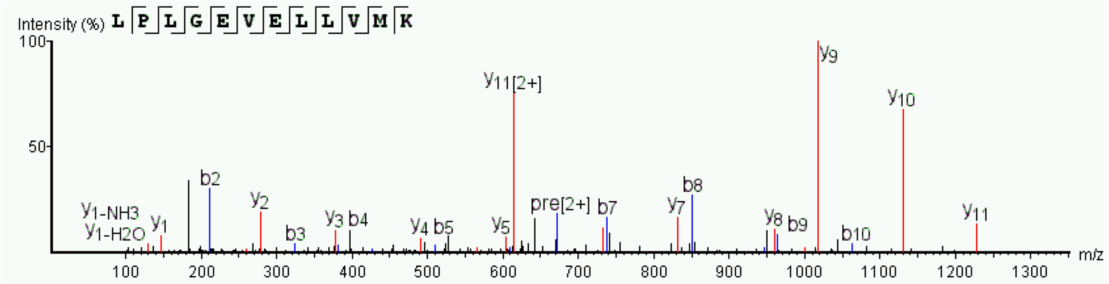

gi|571578355

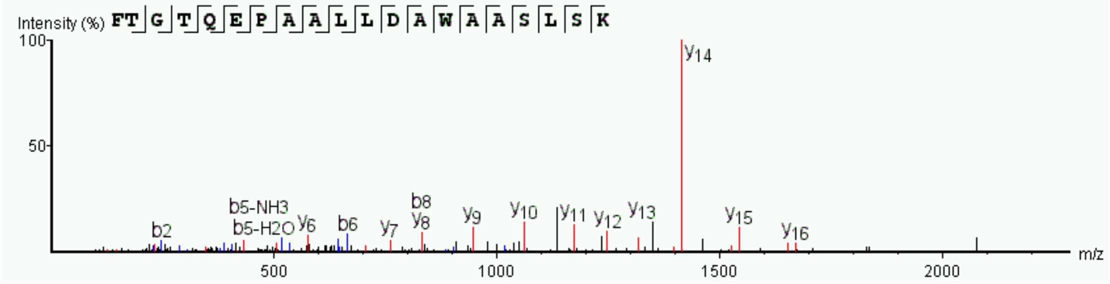

gi|66509032

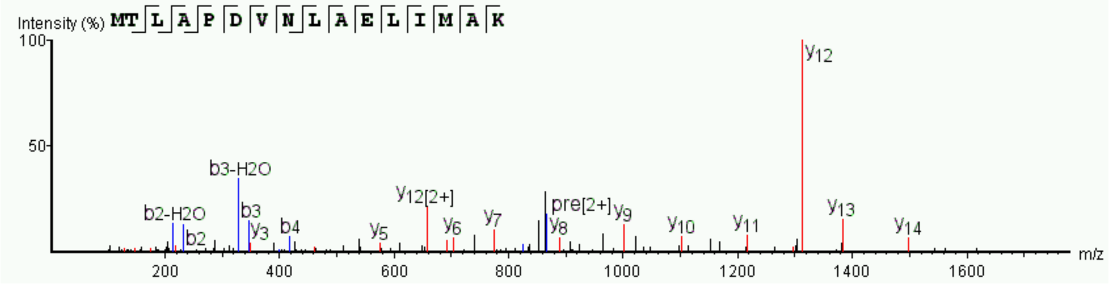

gi|571558766

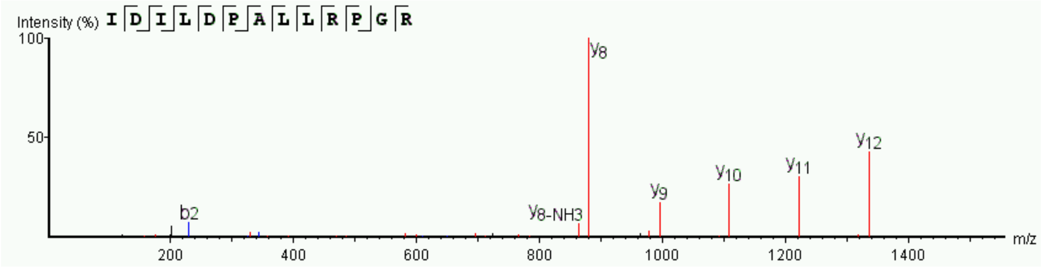

gi|571558762

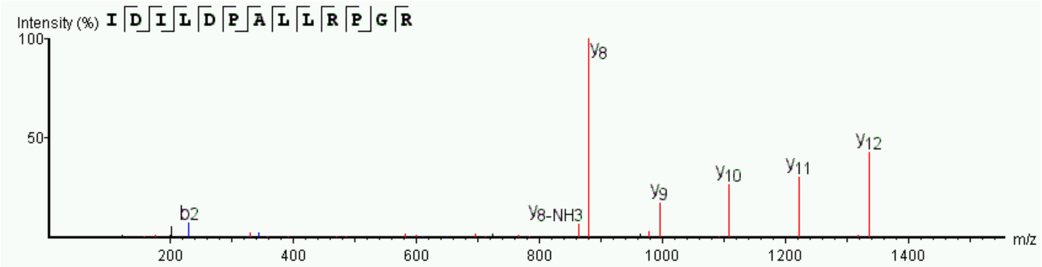

gi|571558772

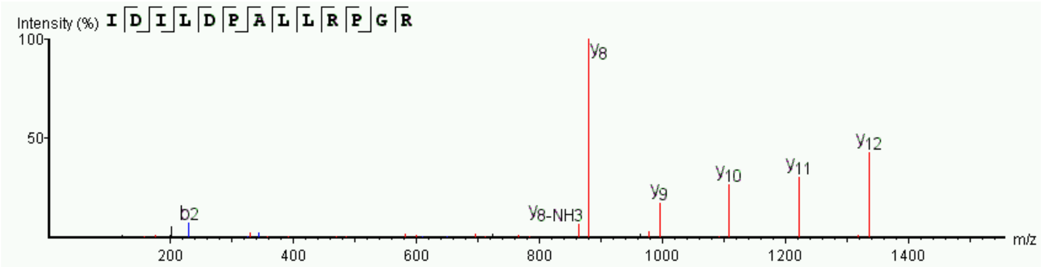

gi|571558776

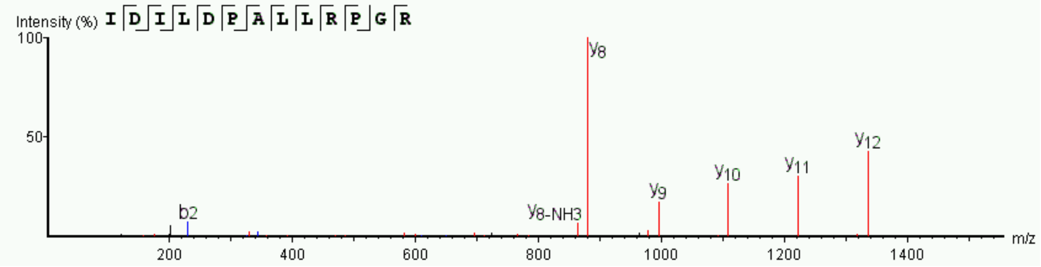

gi|571542461

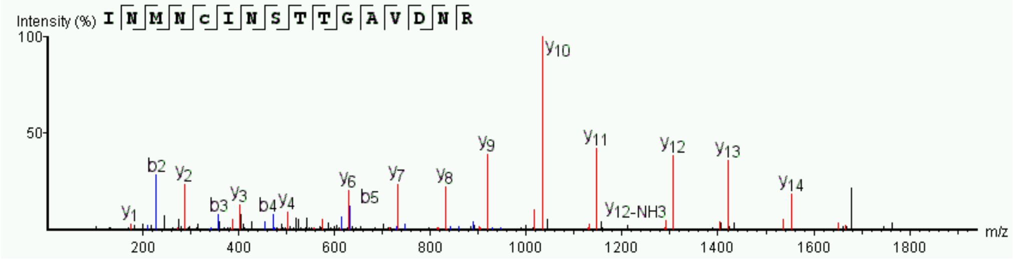

gi|328778492

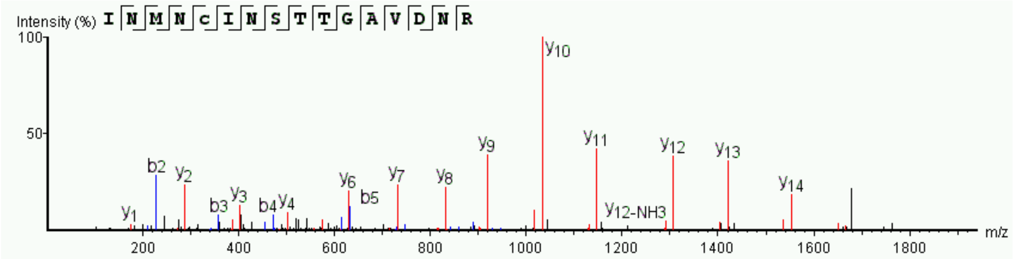

gi|571524852

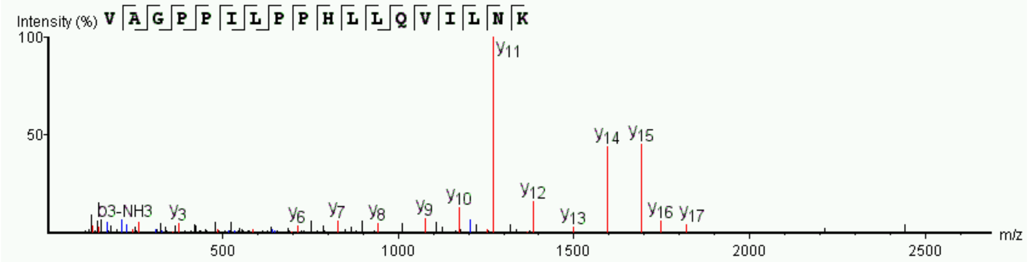

gi|571524858

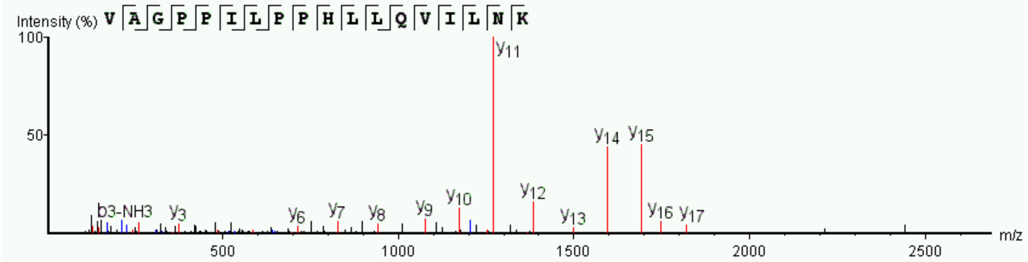

gi|48113496

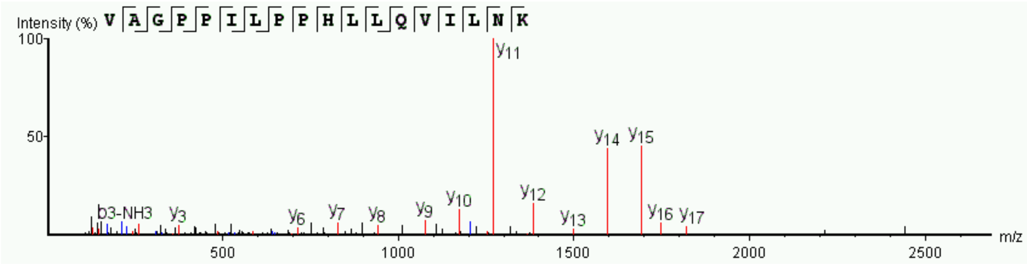

gi|571556092

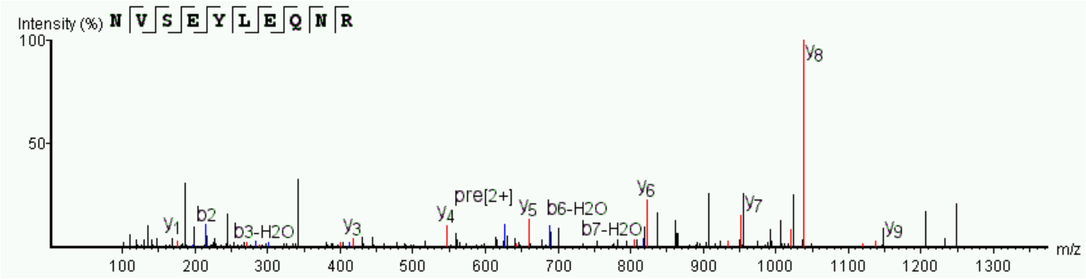

gi|571552343

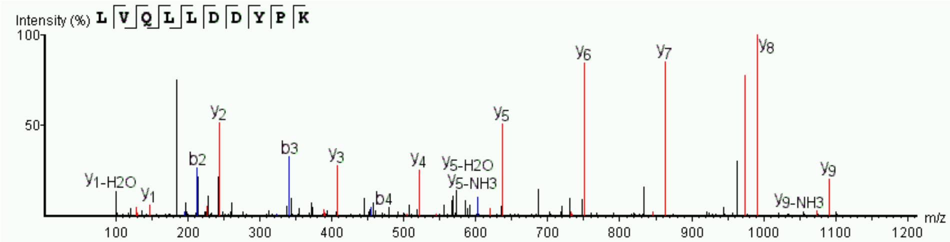

gi|66559310

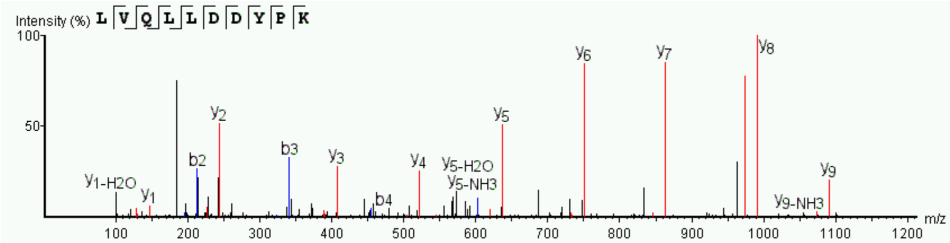

gi|66506623

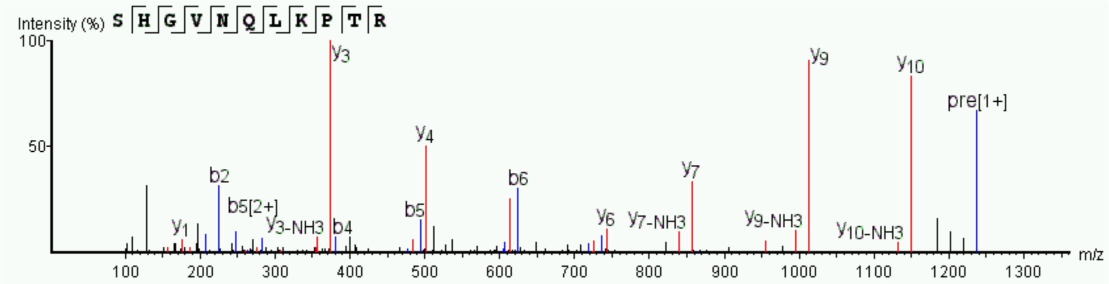

gi|66551967

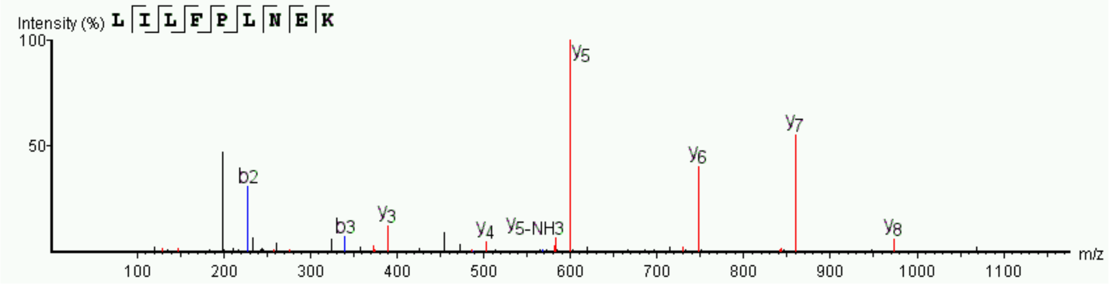

gi|66499585

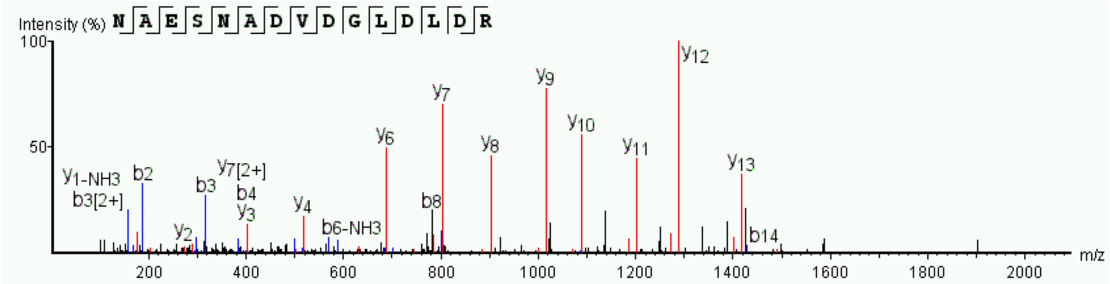

gi|328788782

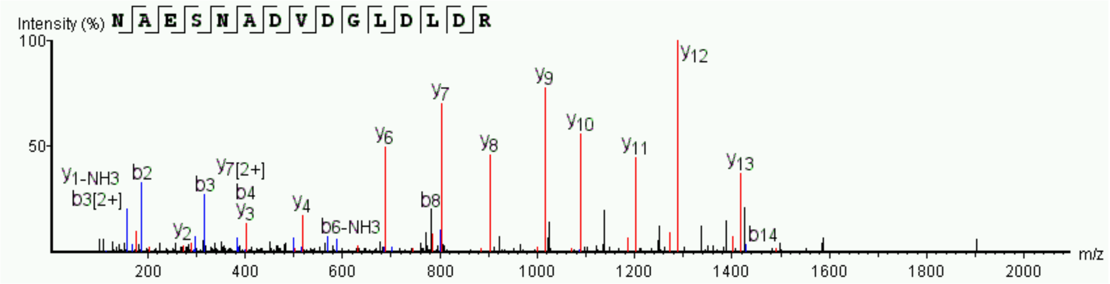

gi|48139903

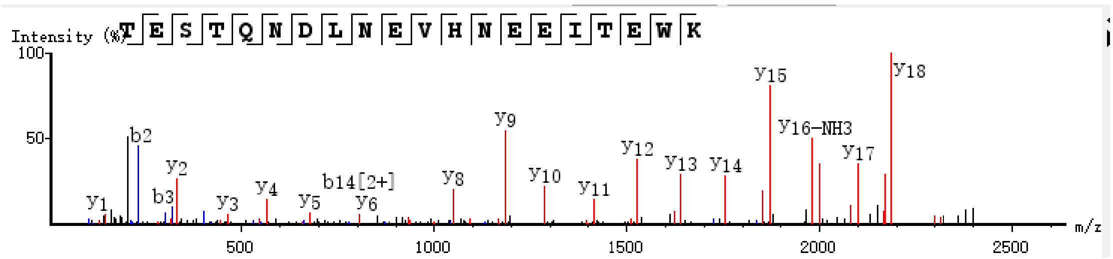

gi|571569183 gi|571569186

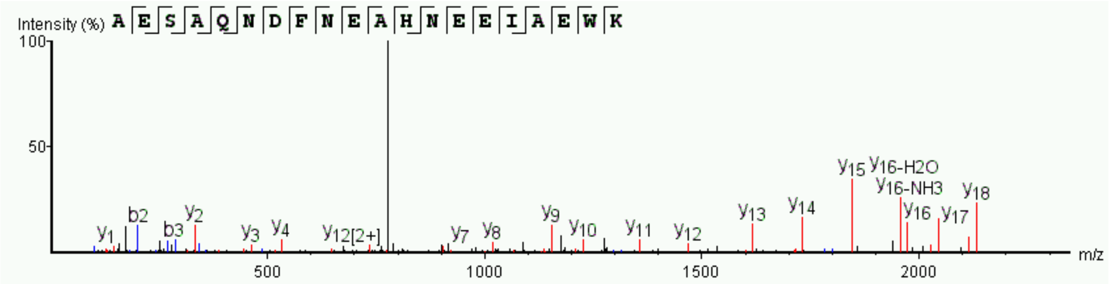

gi|328784556

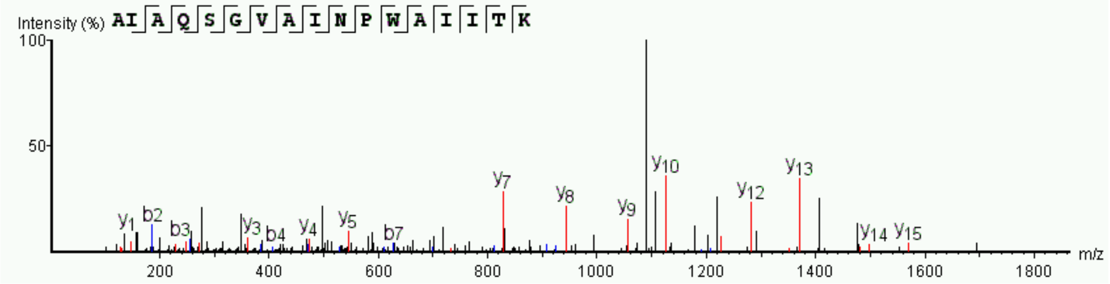

gi|571577127

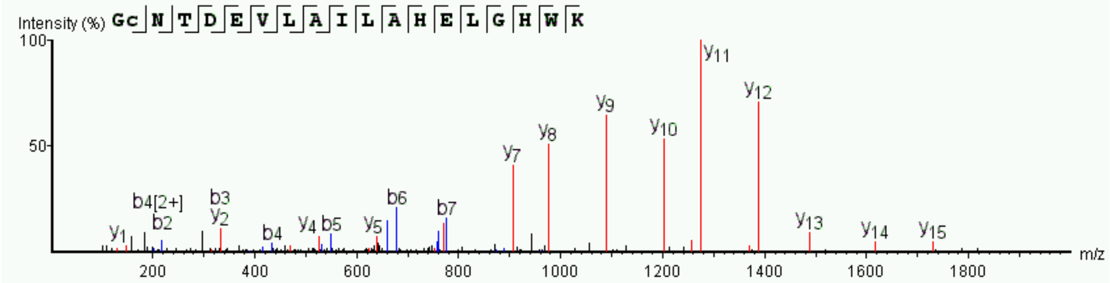

gi|571577129

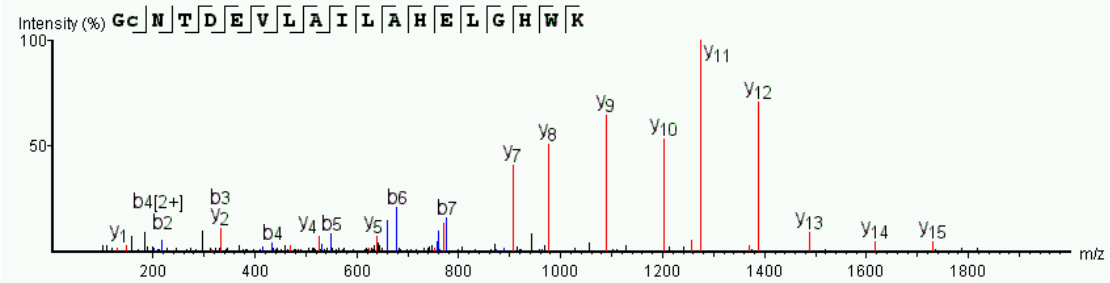

gi|328793120

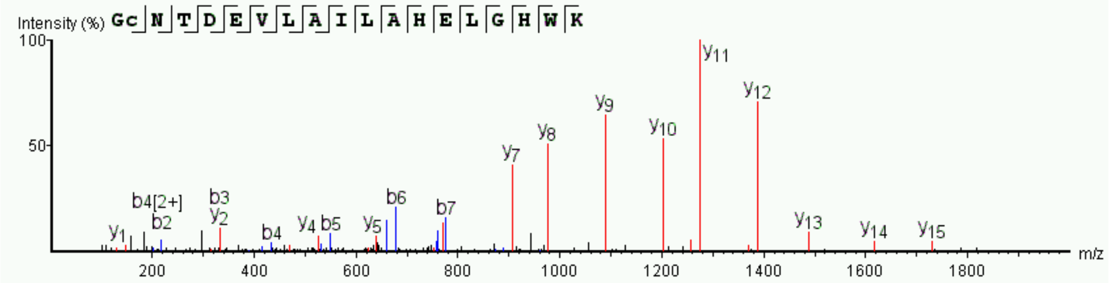

gi|571532925

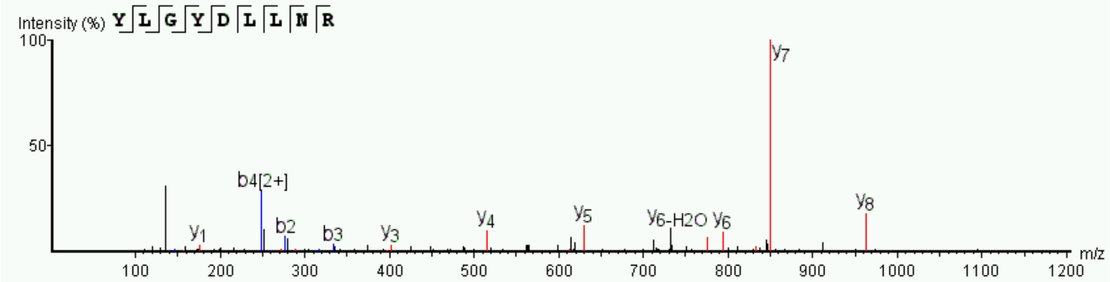

gi|571532928

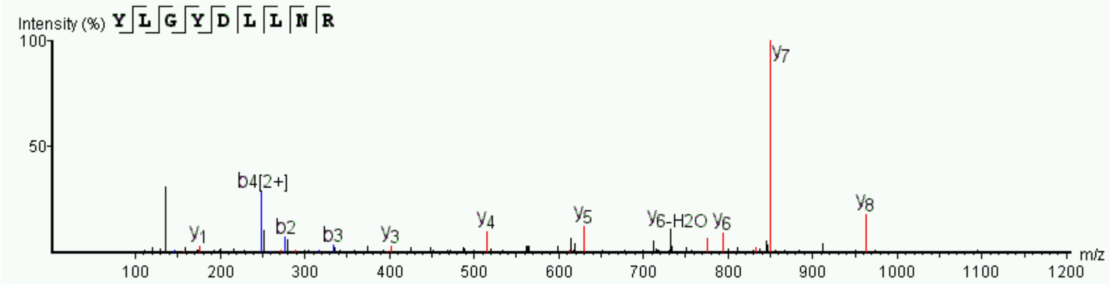

gi|571532931

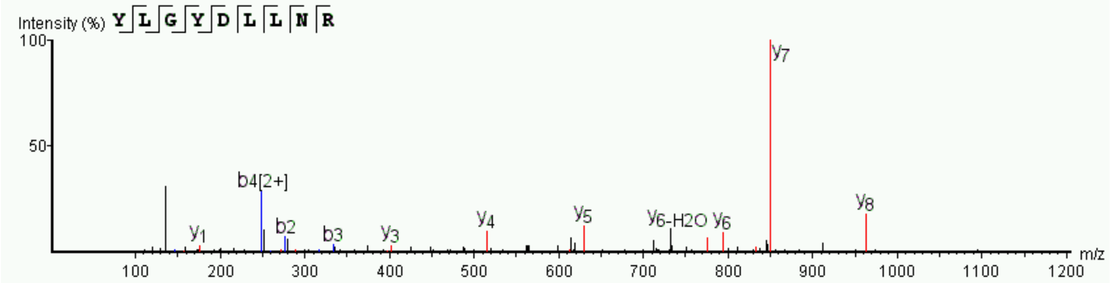

gi|571532935

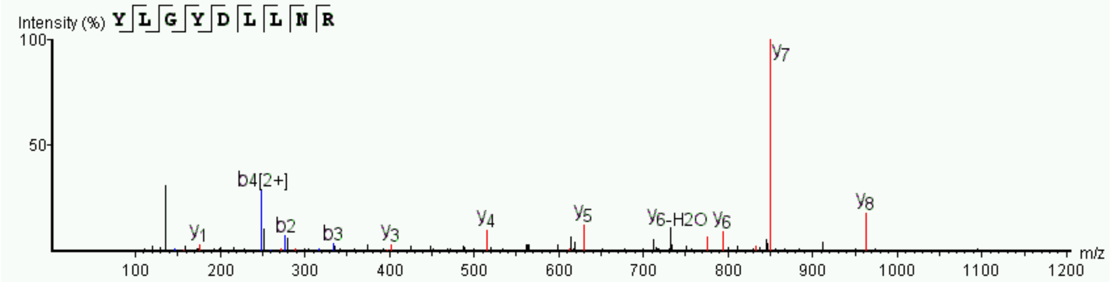

gi|571532938

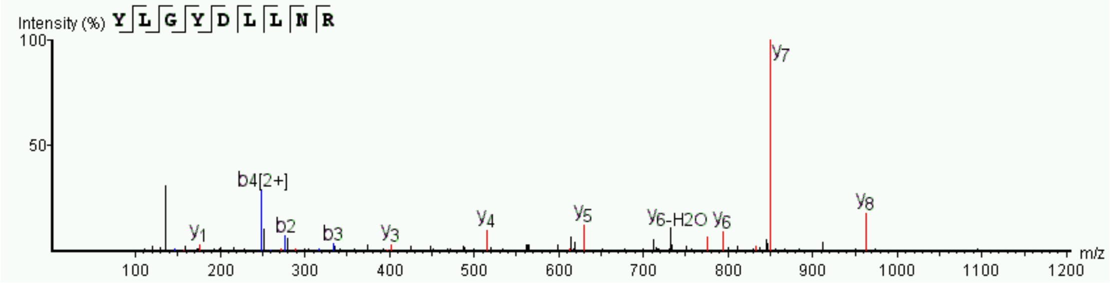

gi|571532941

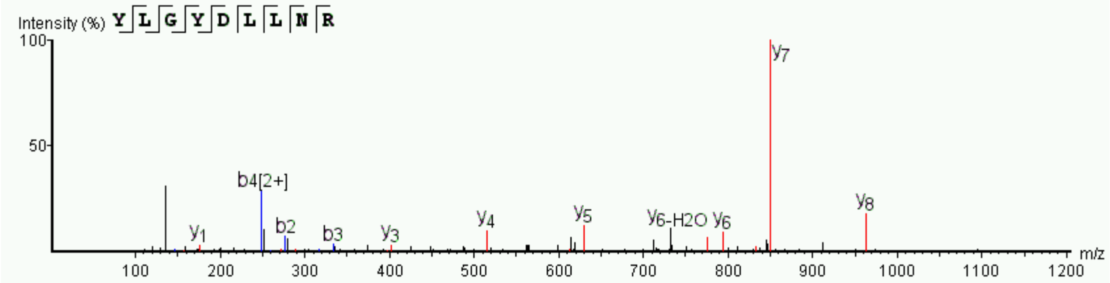

gi|571532945

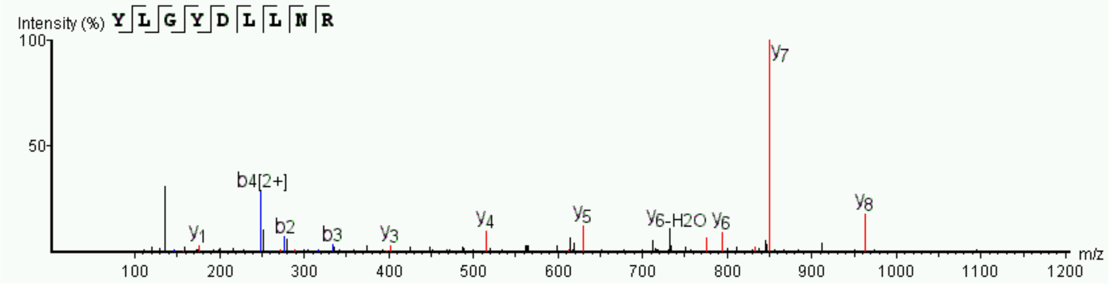

gi|571541951

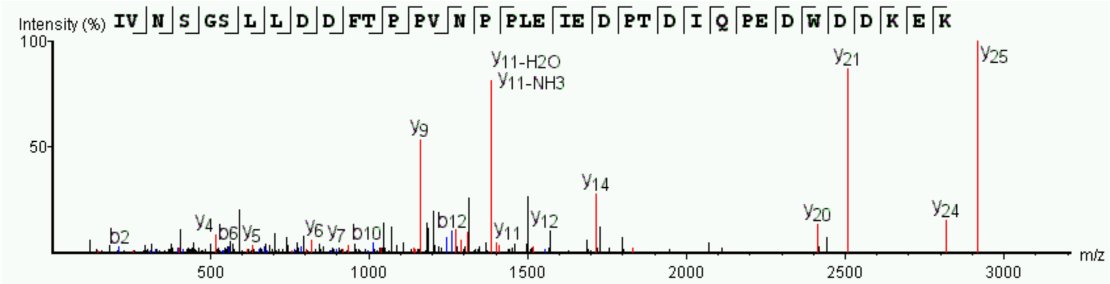

gi|571541954

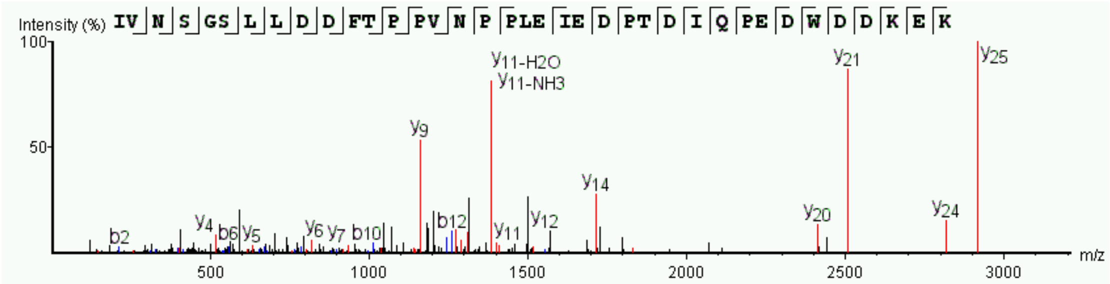

gi|571541958

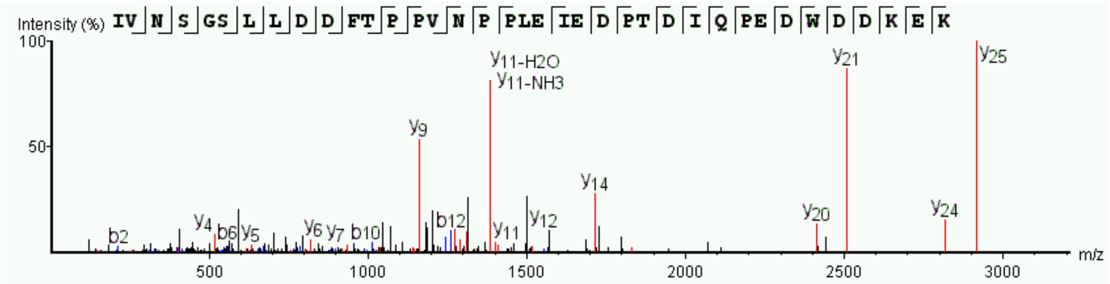

gi|571523319

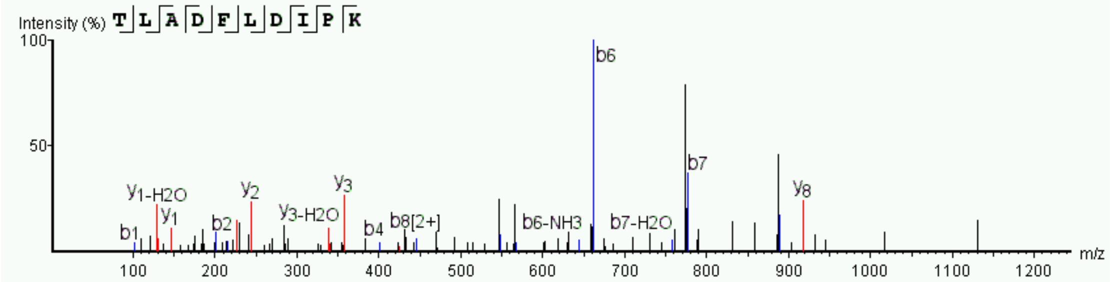

gi|571523323

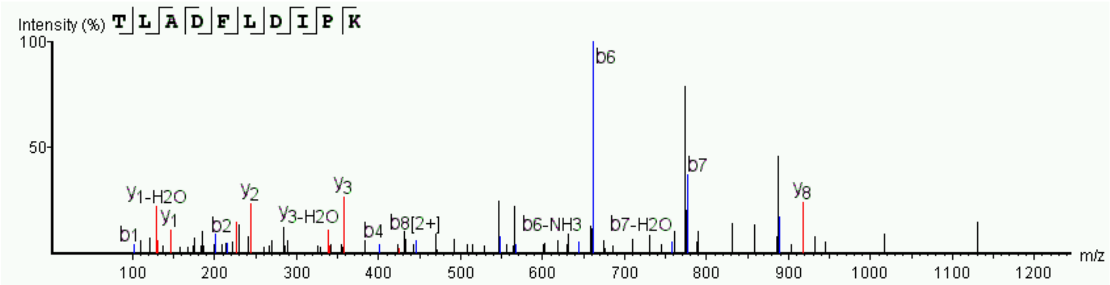

gi|66547724

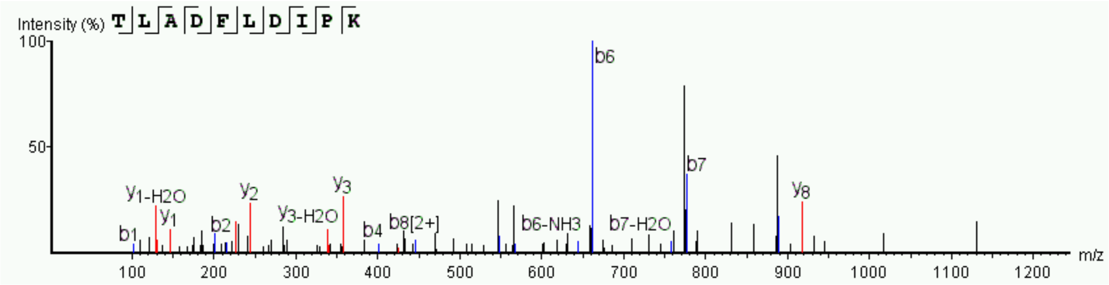

gi|571554160

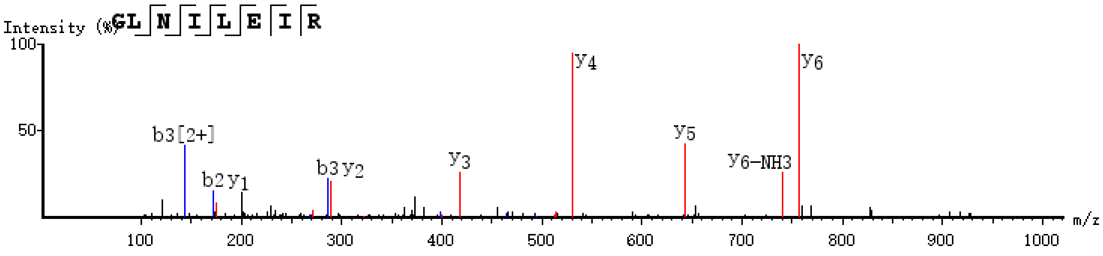

gi|571517007

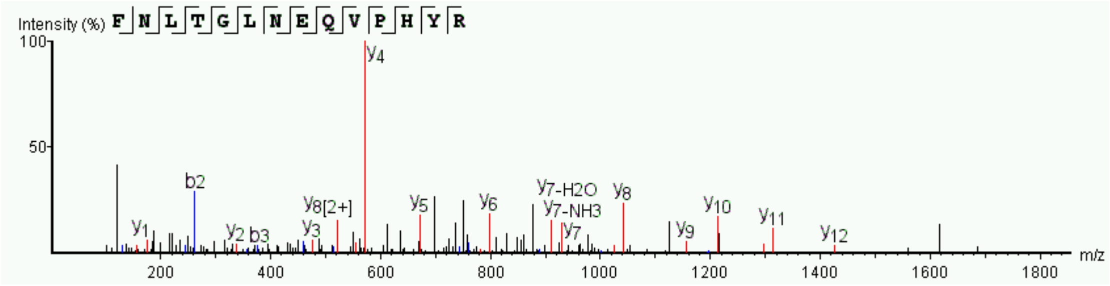

gi|571517011

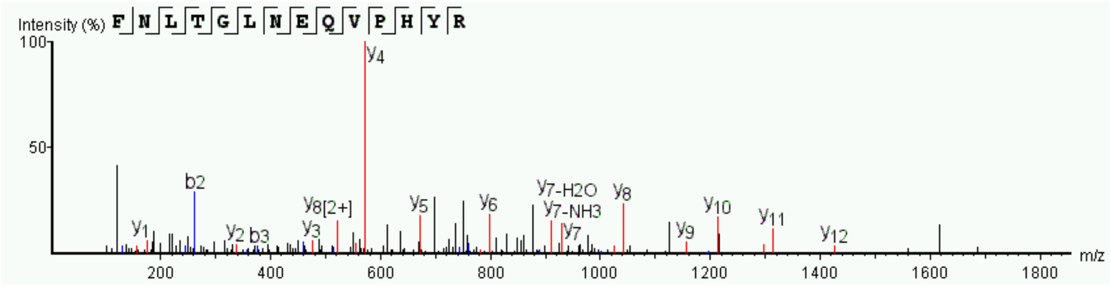

gi|571517016

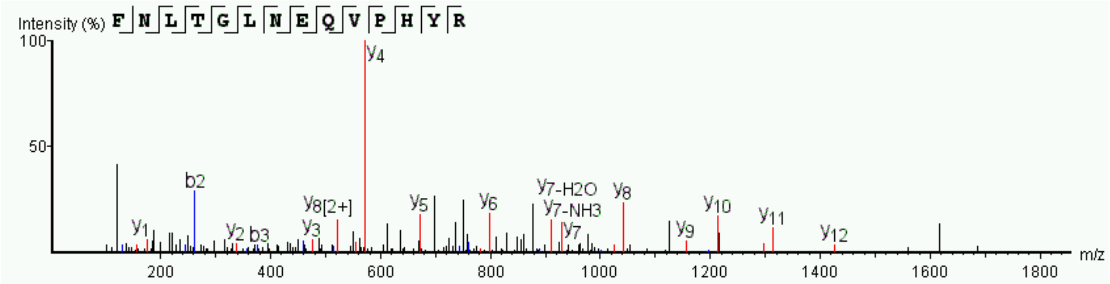

gi|328777239

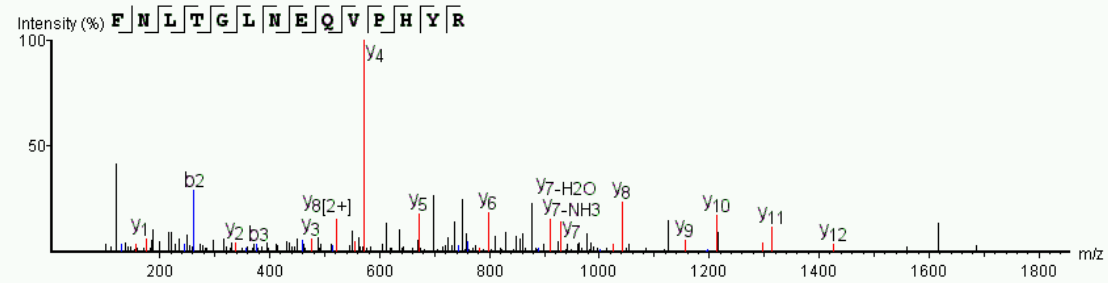

gi|571560469

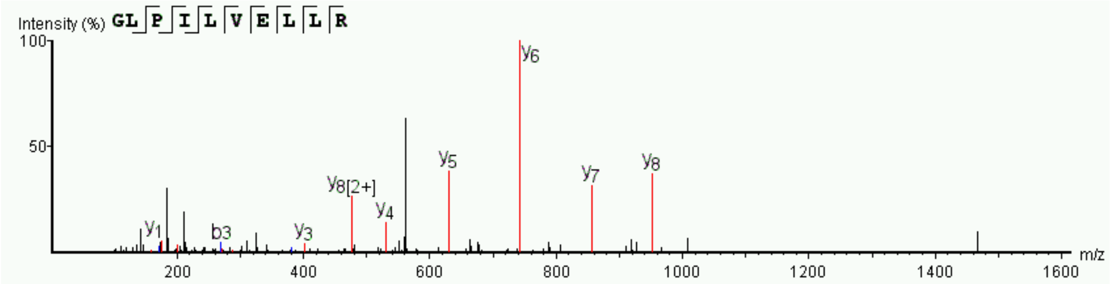

gi|571560501

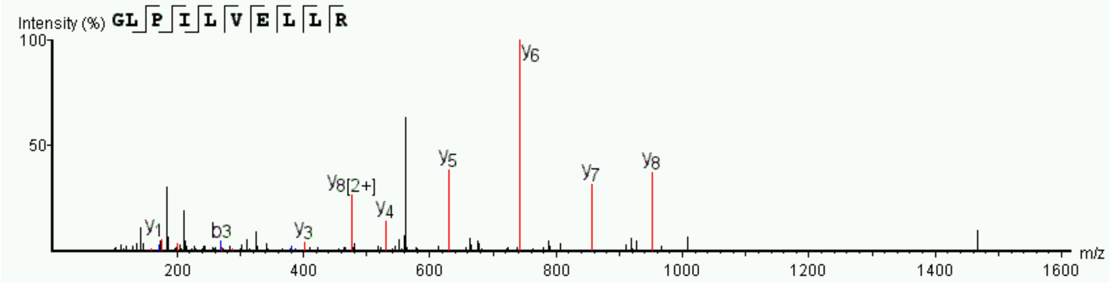

gi|571560504

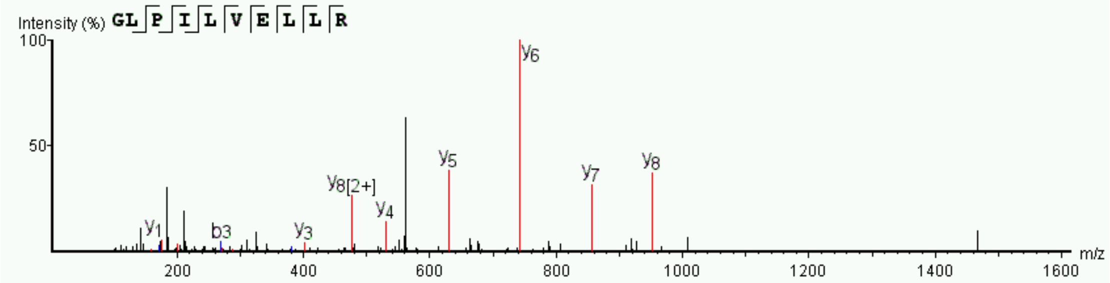

gi|571560507

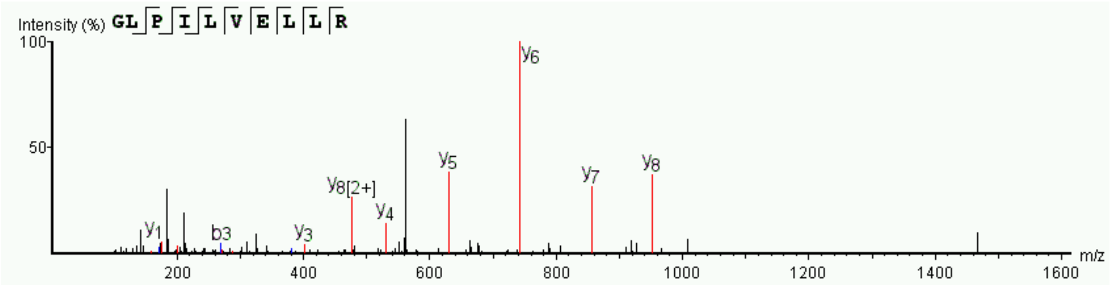

gi|571560511

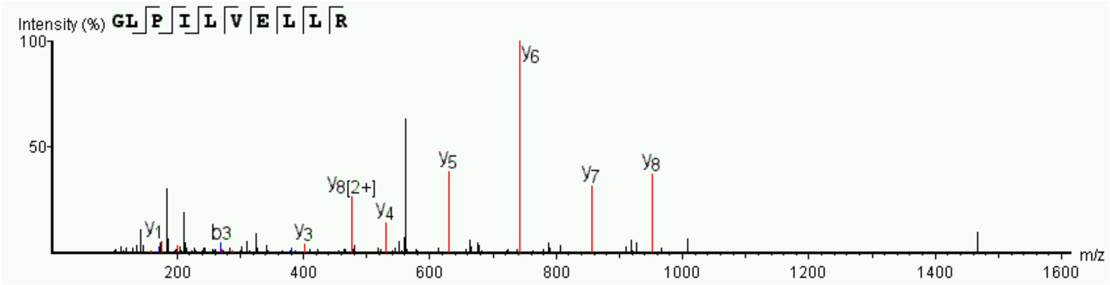

gi|571560472

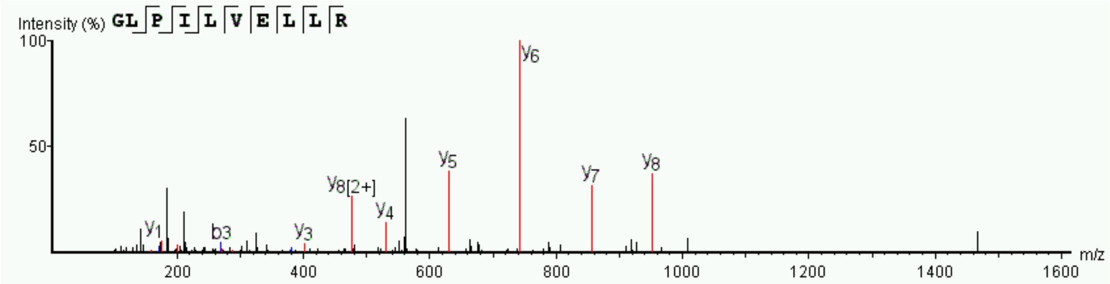

gi|571560476

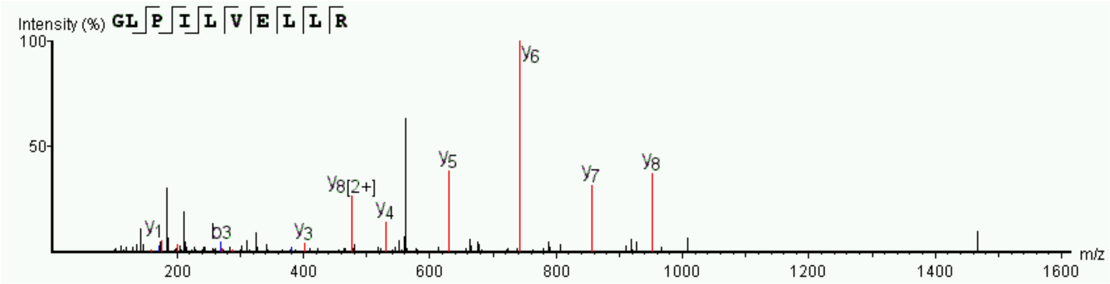

gi|571560479

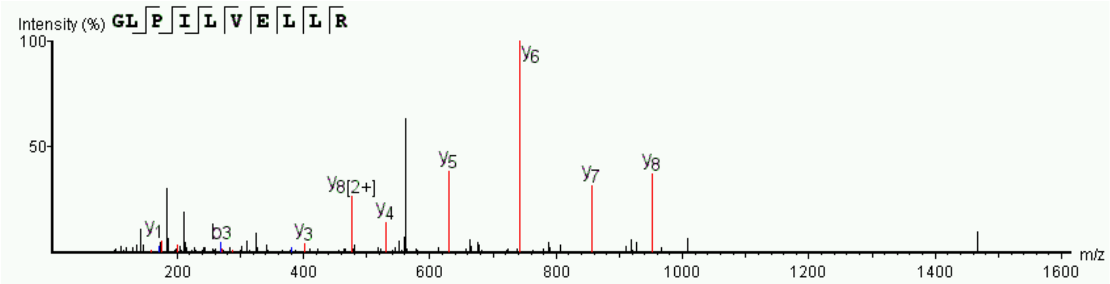

gi|571560482

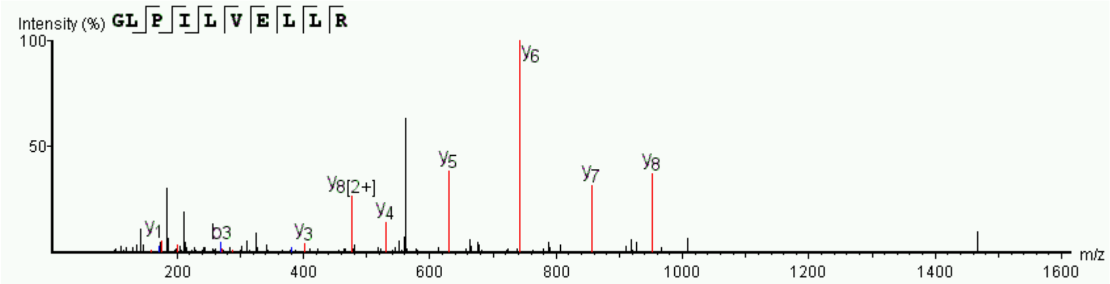

gi|571560485

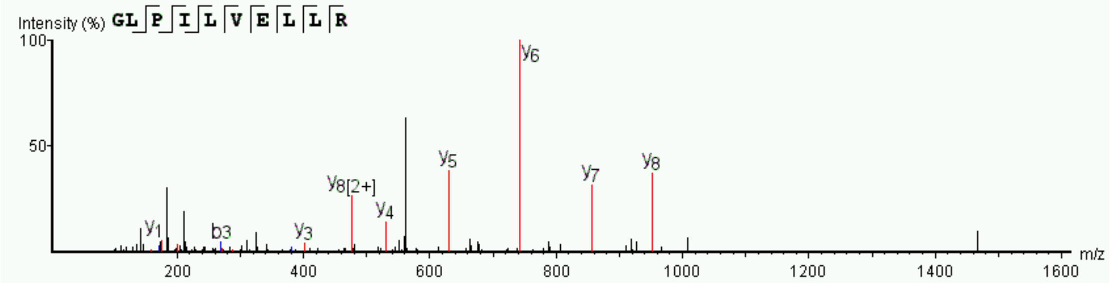

gi|571560489

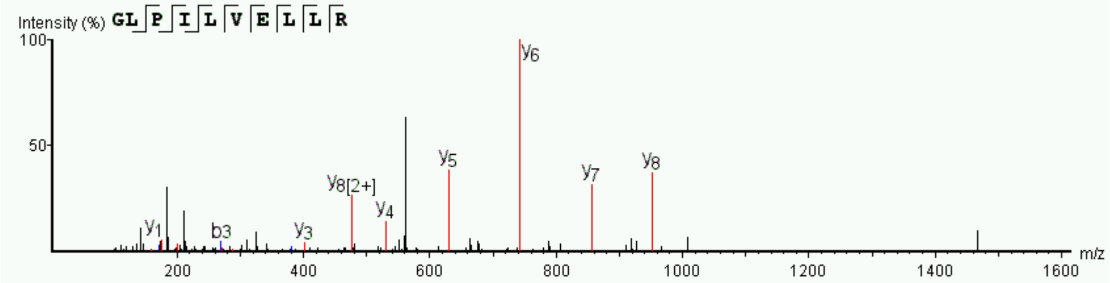

gi|571560493

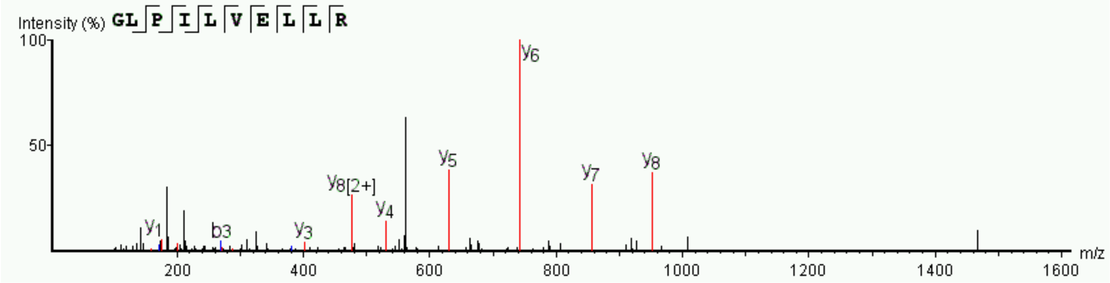

gi|571560498

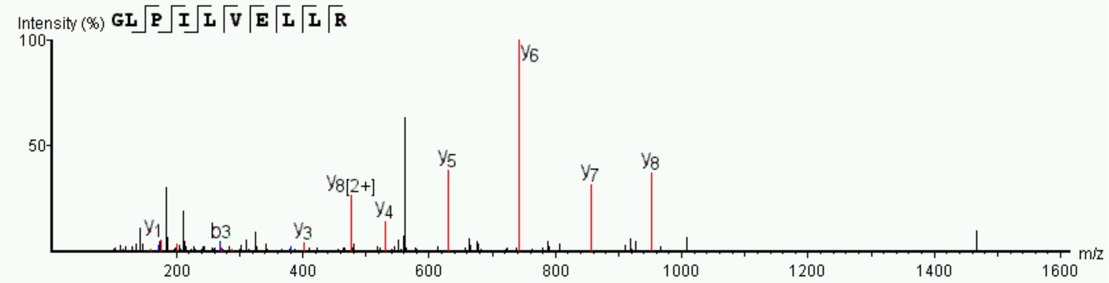

gi|110759374

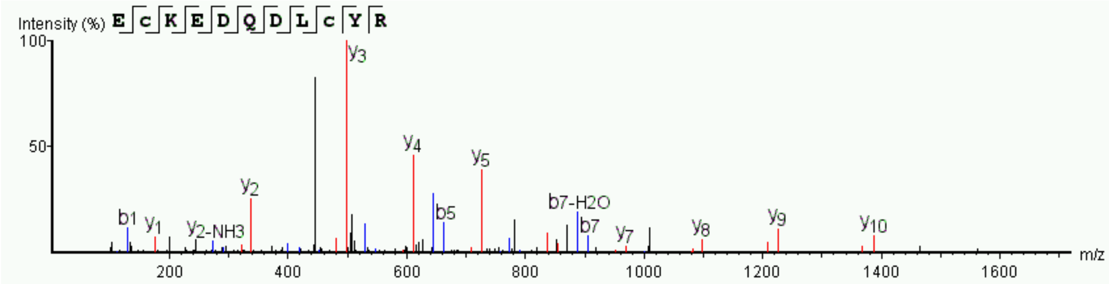

gi|328780974

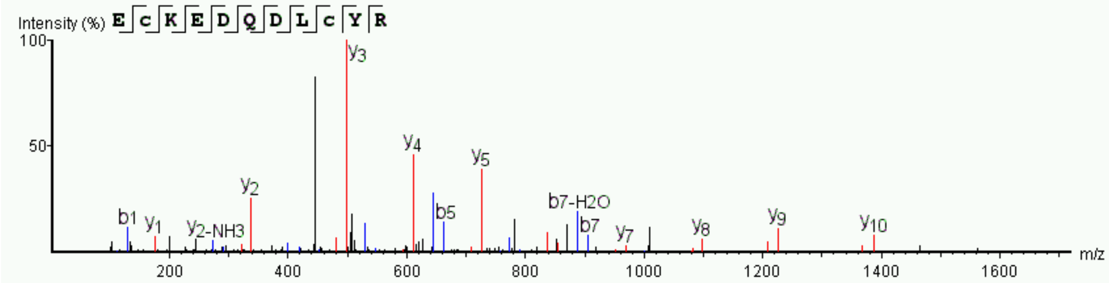

gi|571548542

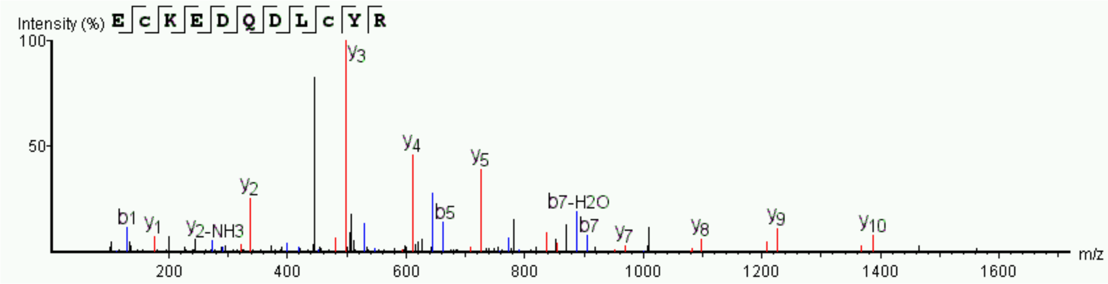

gi|571525362

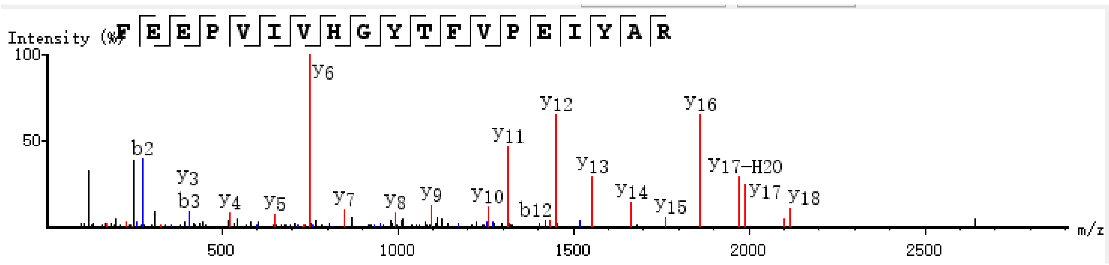

gi|328788820

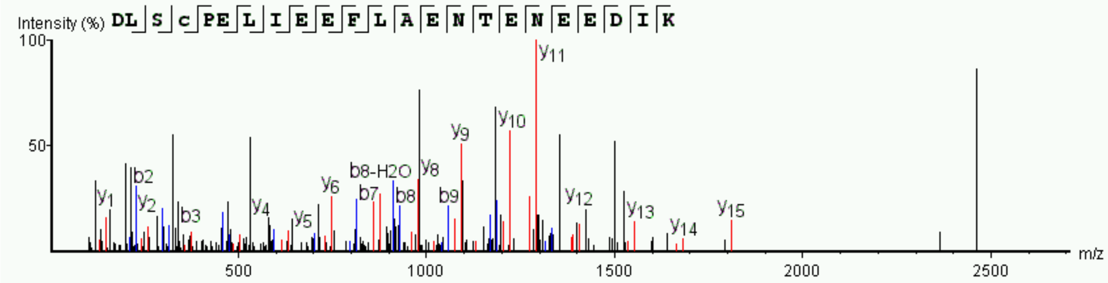

gi|571564826

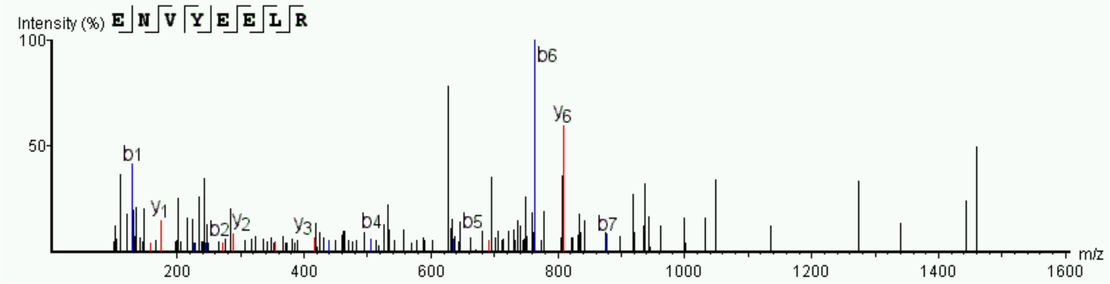

gi|571516024

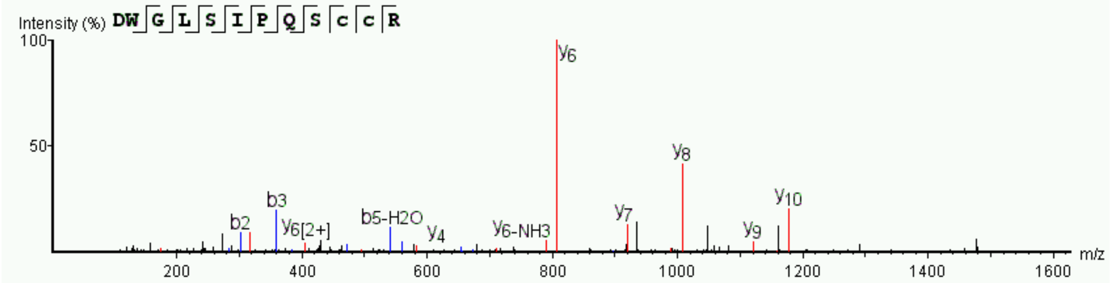

gi|571516027

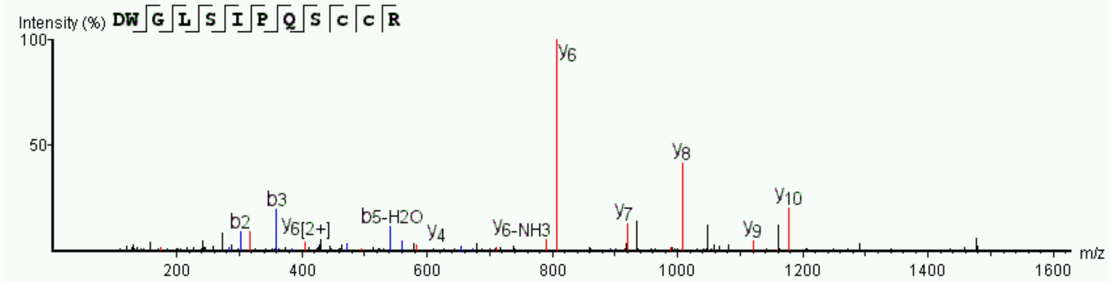

gi|571516030

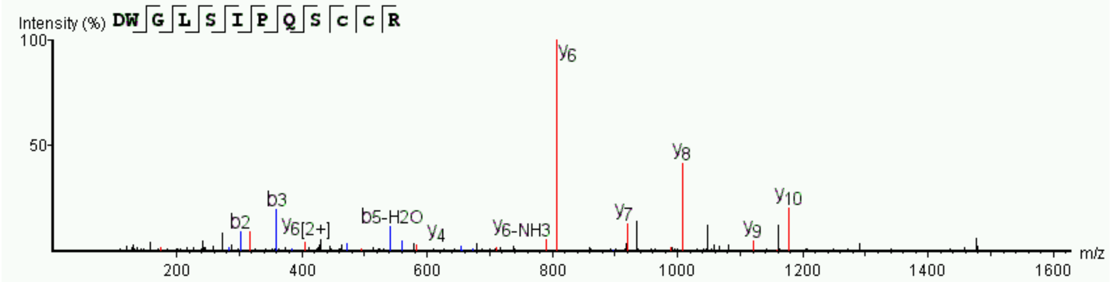

gi|66504429

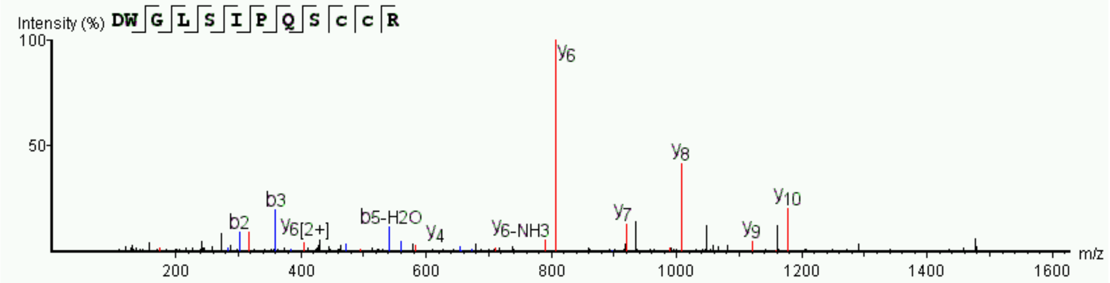

gi|571574382

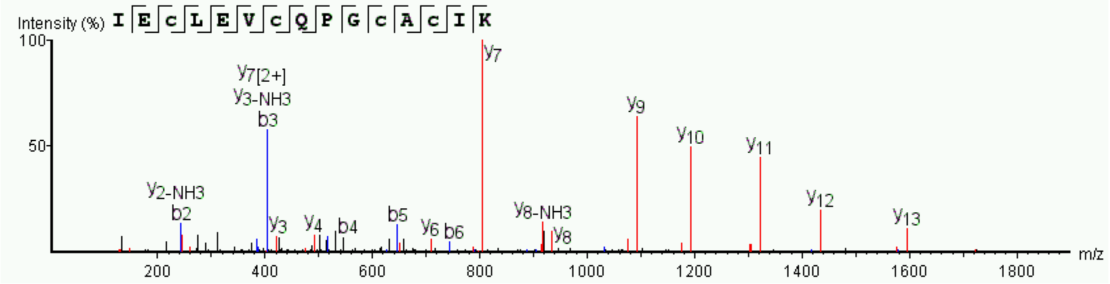

gi|66499068

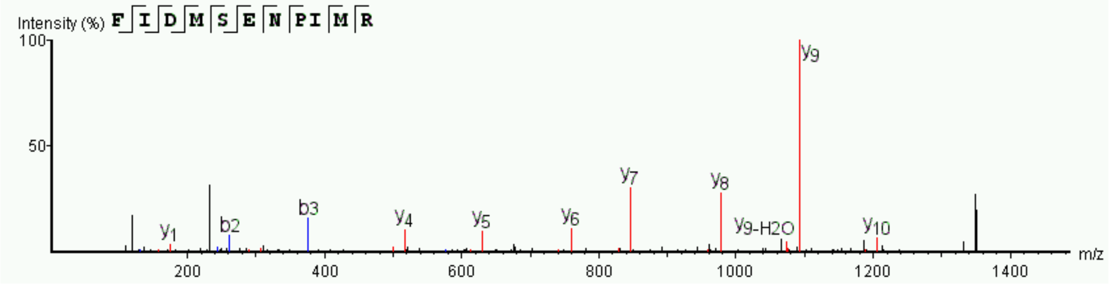

gi|571522061

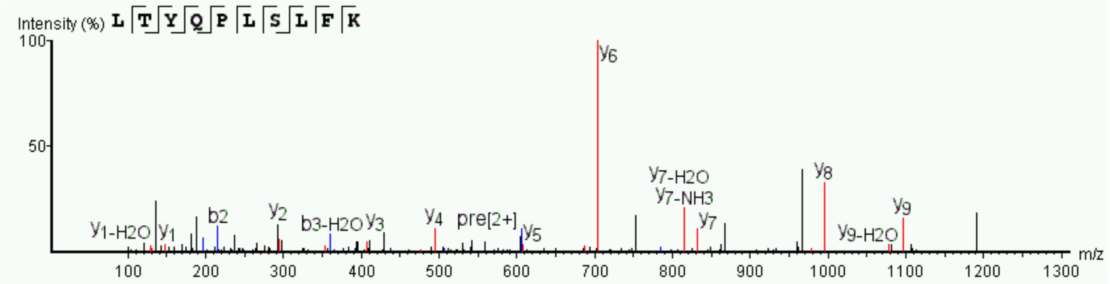

gi|110765036

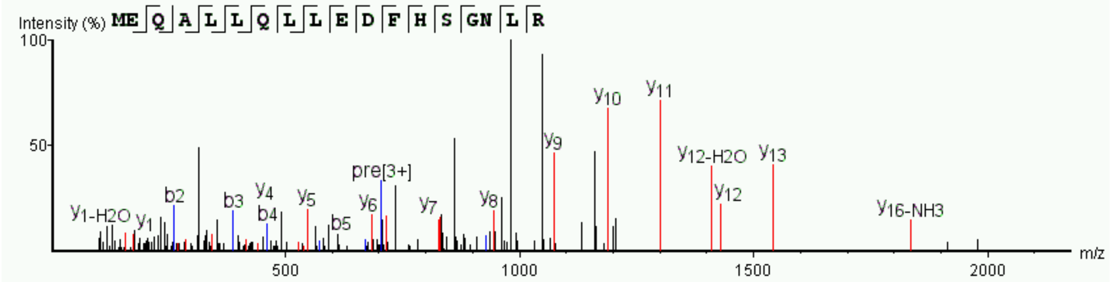

gi|571577966

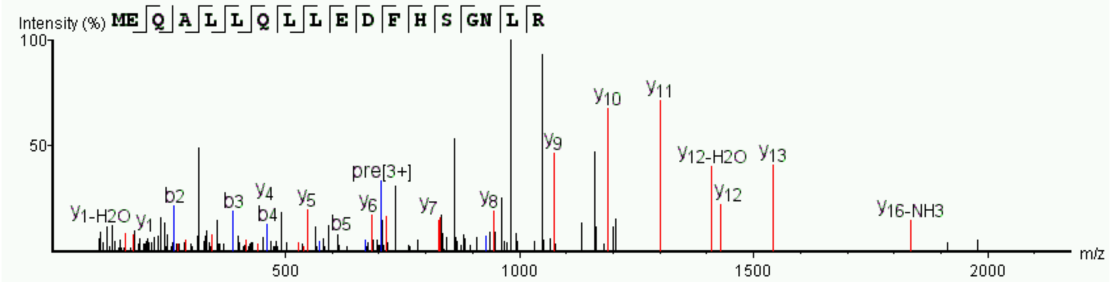

gi|571577968

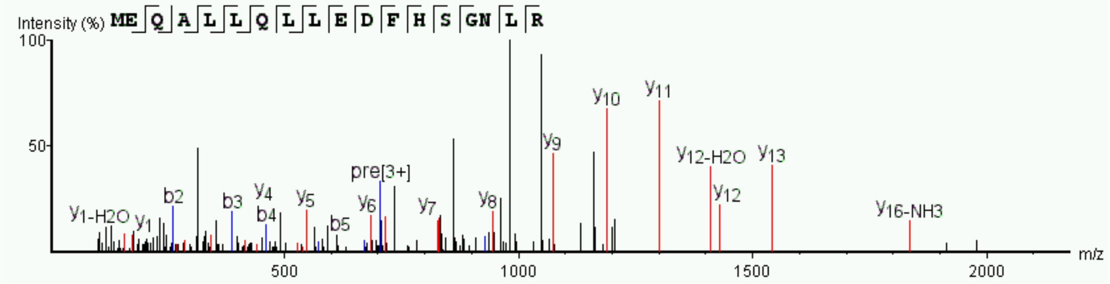

gi|571564978

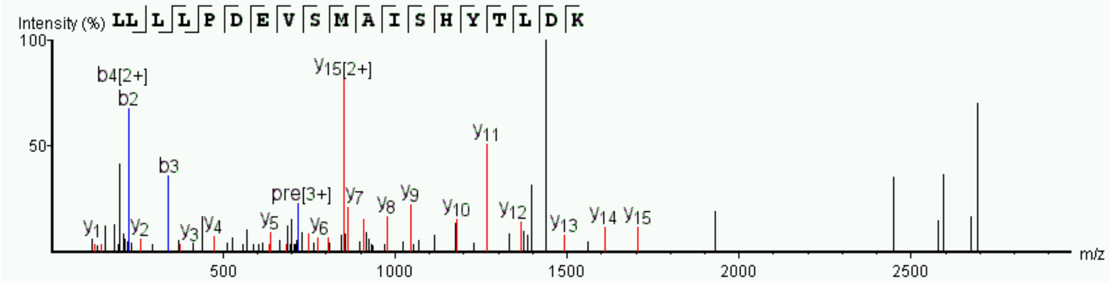

gi|571564981

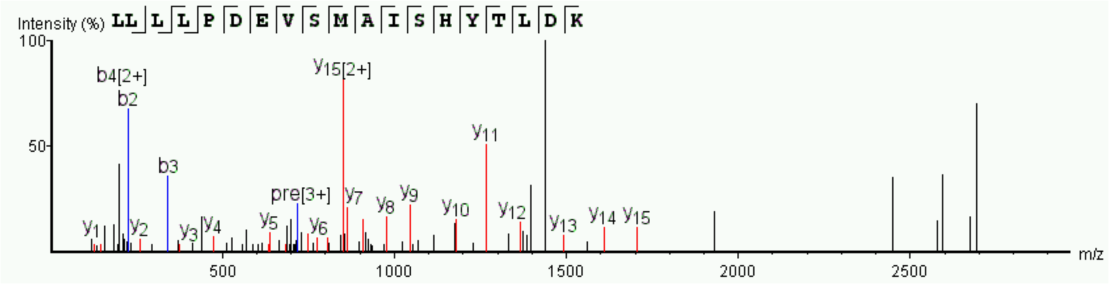

gi|66549806

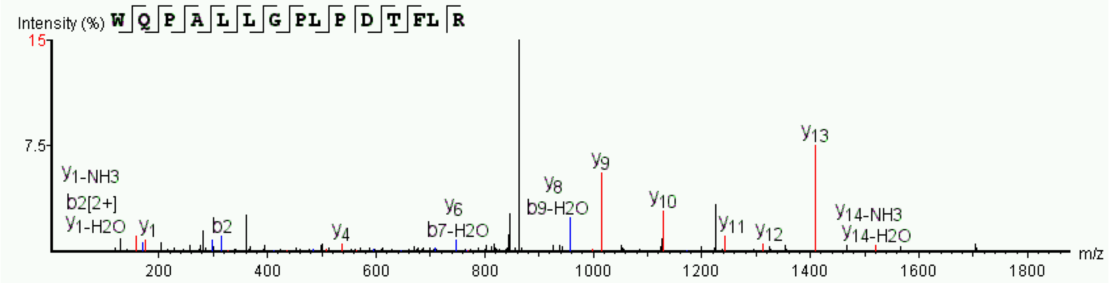

gi|571545012

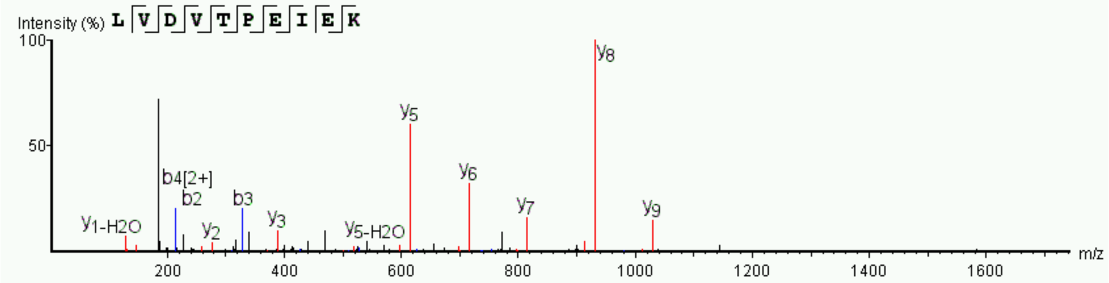

gi|110772198

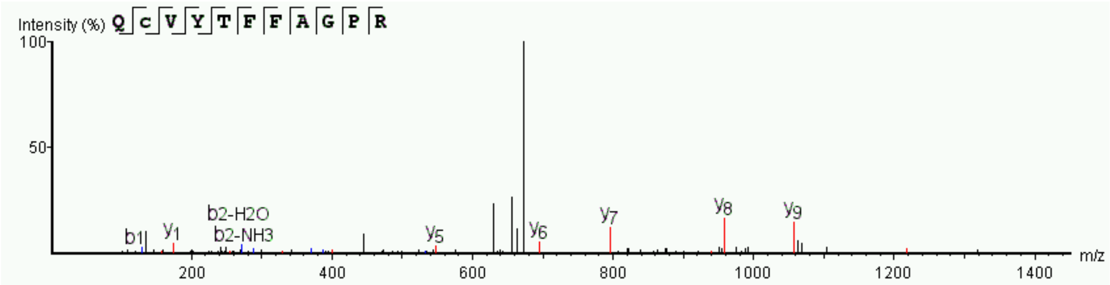

gi|571526265

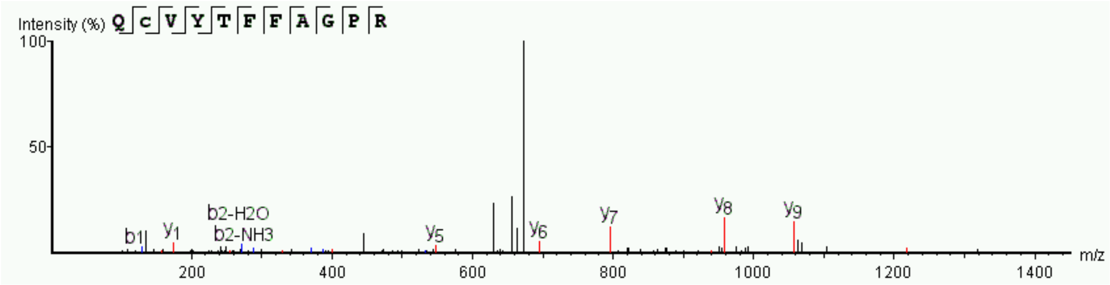

gi|571526271

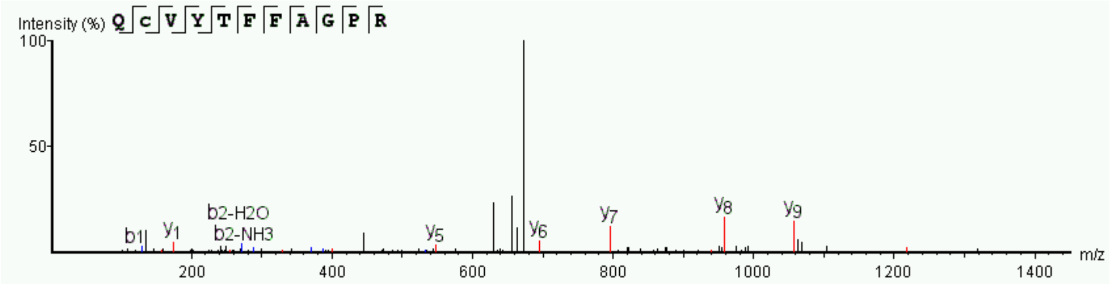

gi|571526275

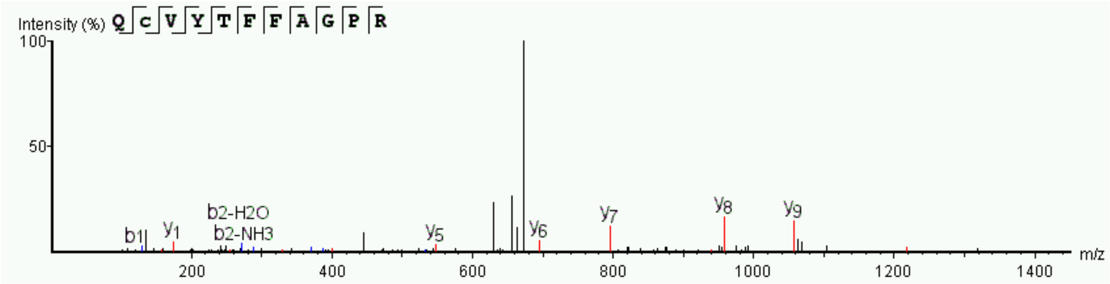

gi|571526279

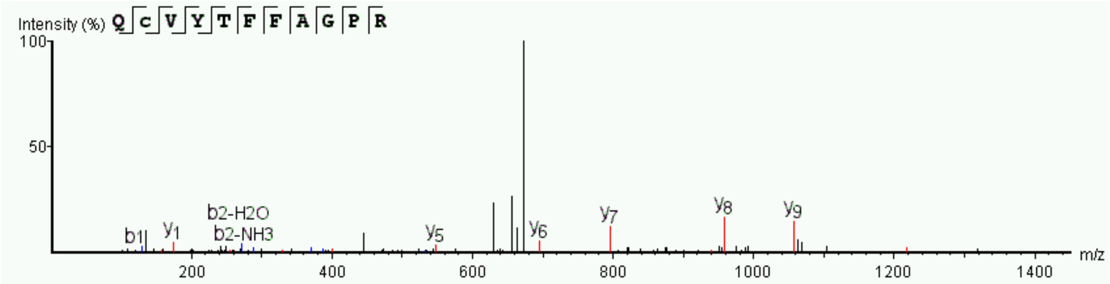

gi|571526283

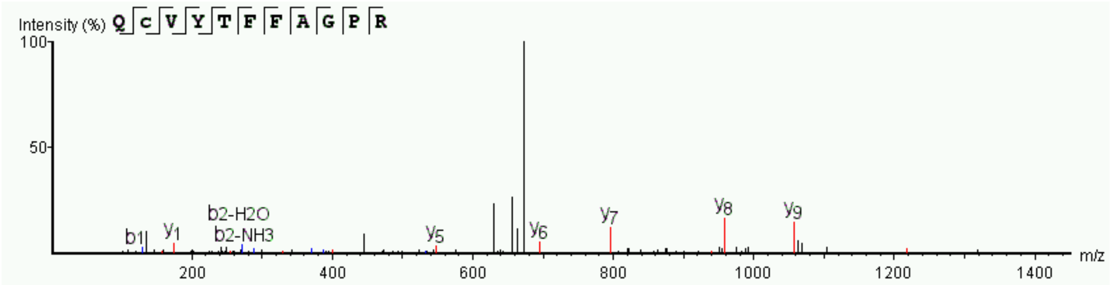

gi|571526286

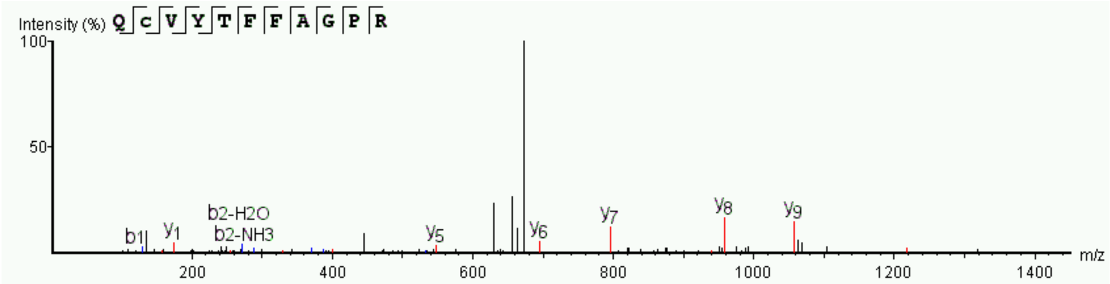

gi|571526289

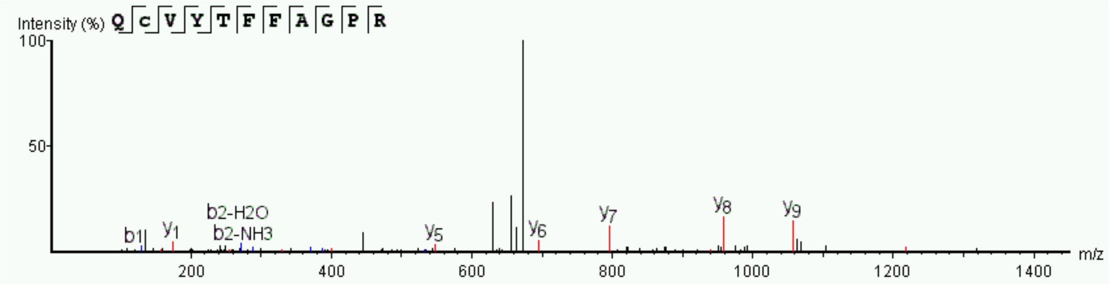

gi|110772198

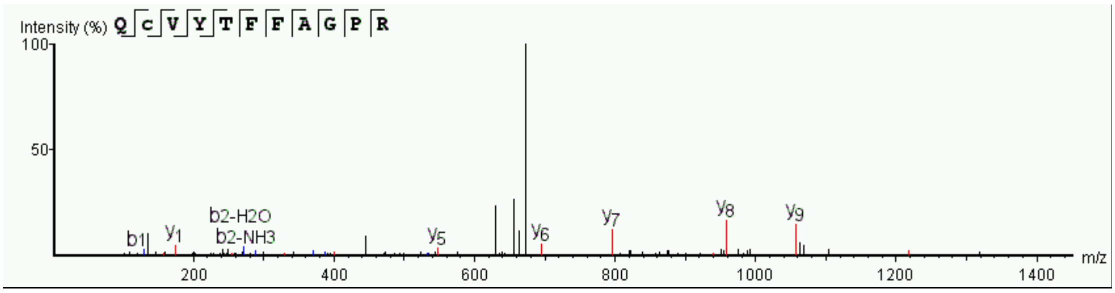

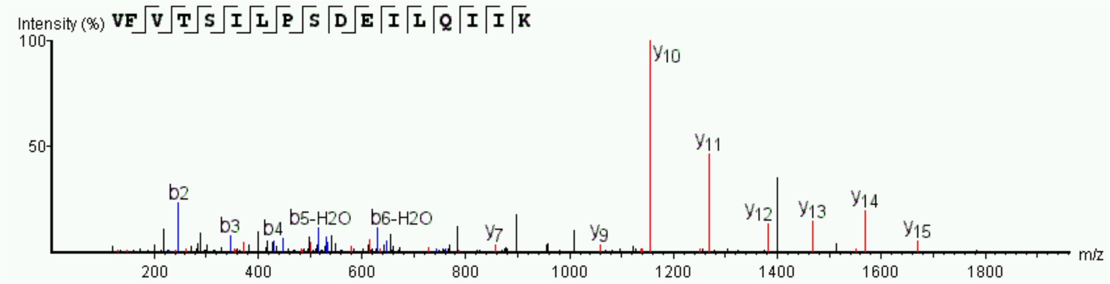

gi|571512237

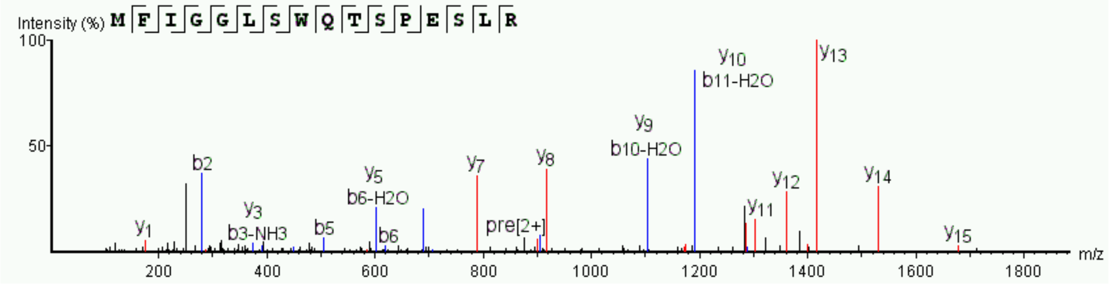

gi|571567945

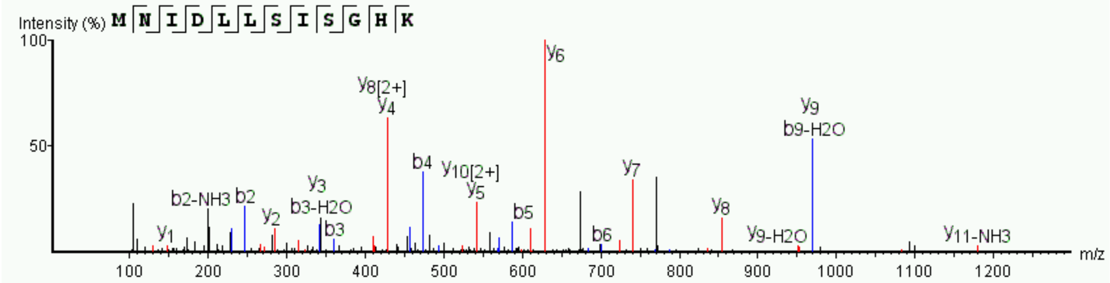

gi|571567948

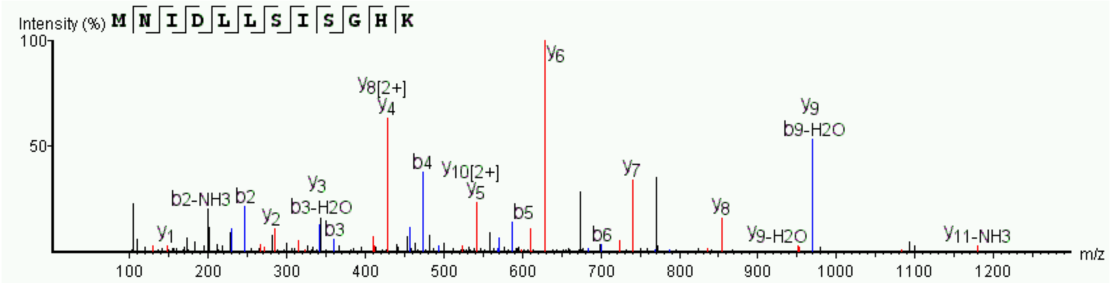

gi|328779544

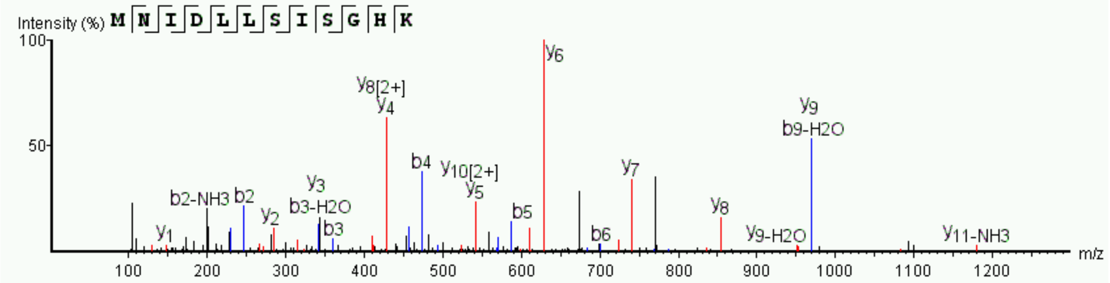

gi|66550870

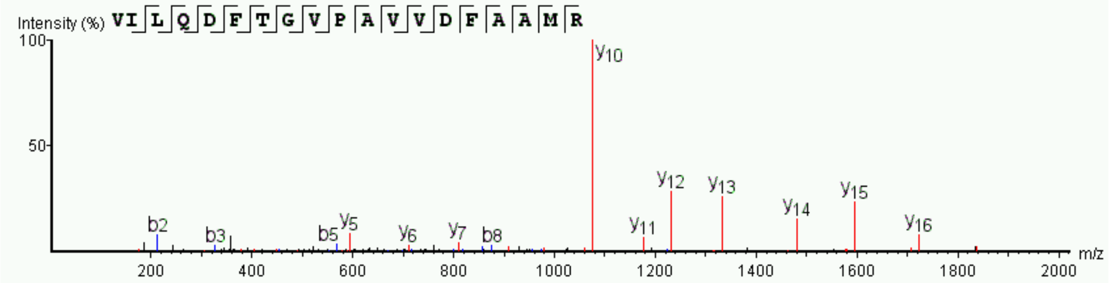

gi|571506816

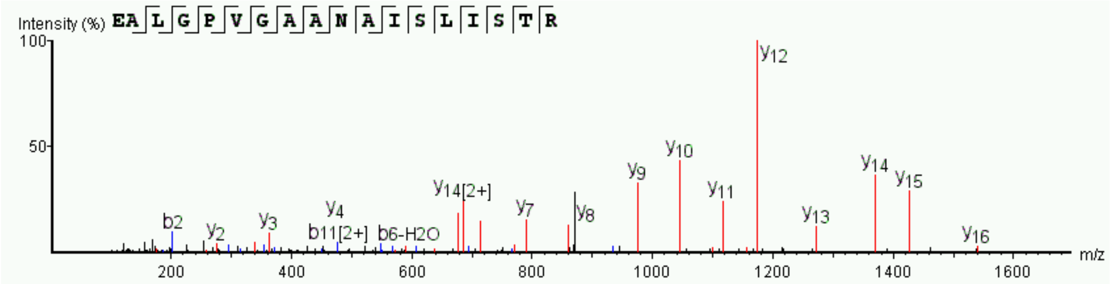

gi|571506819

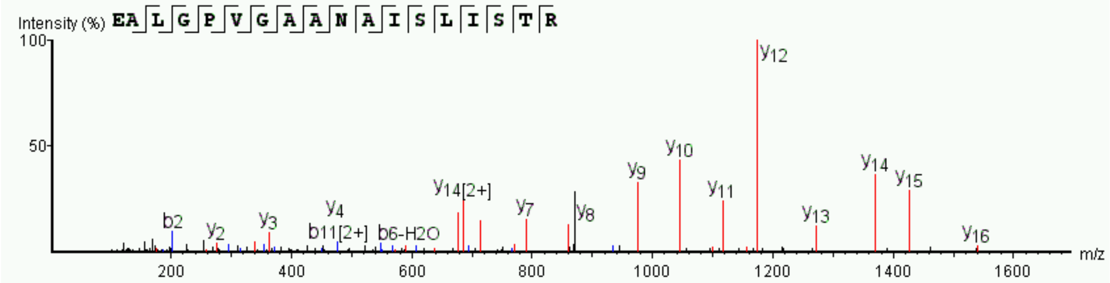

gi|328791738

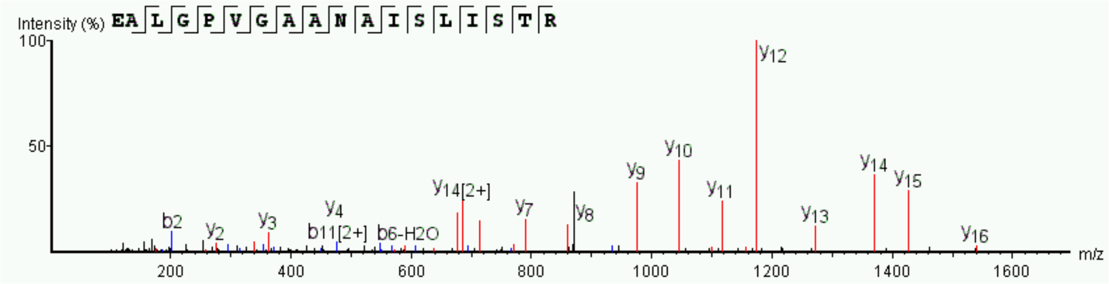

gi|66509442

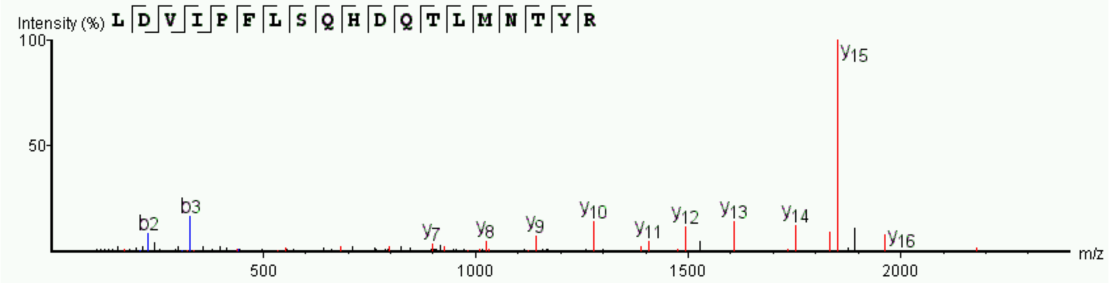

gi|571545429

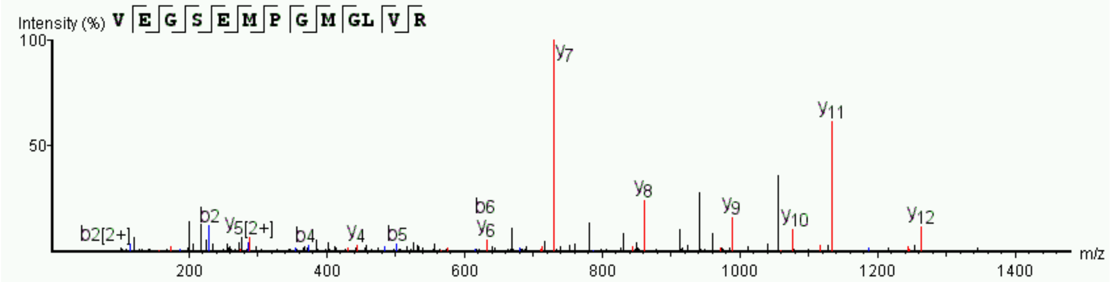

gi|571545459

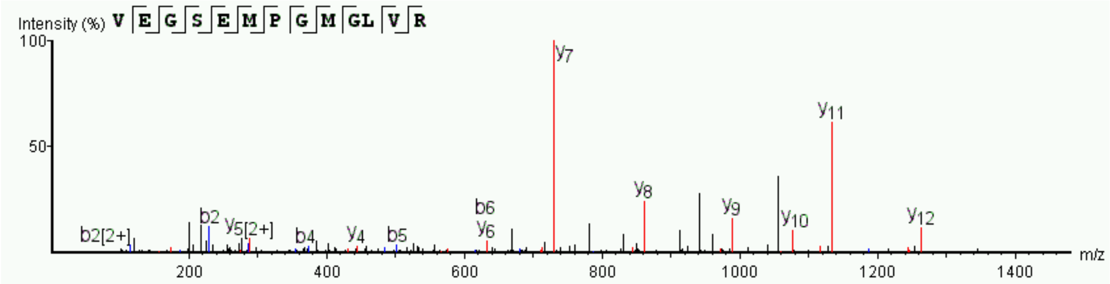

gi|571545463

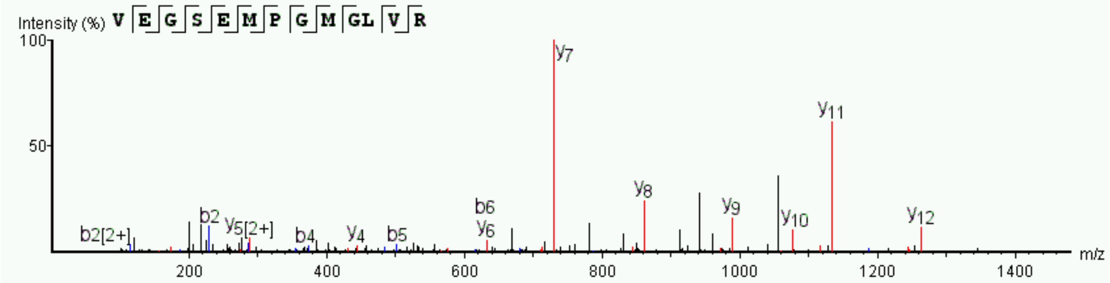

gi|571545467

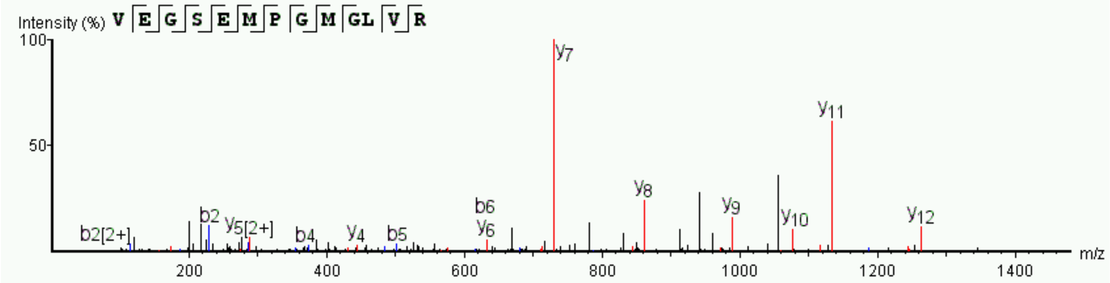

gi|571545471

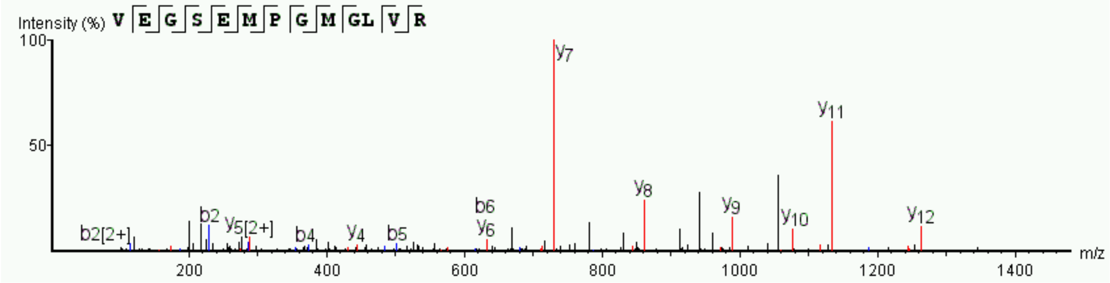

gi|571545432

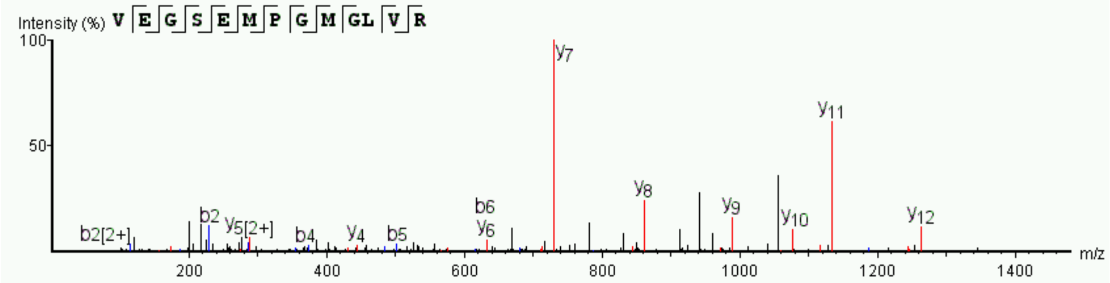

gi|571545436

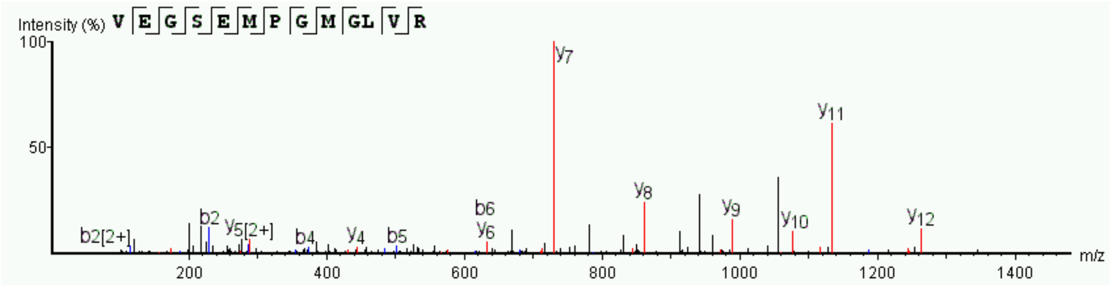

gi|571545439

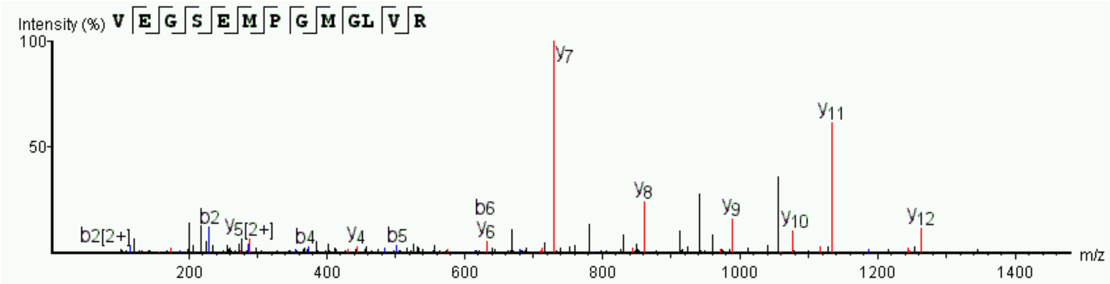

gi|571545442

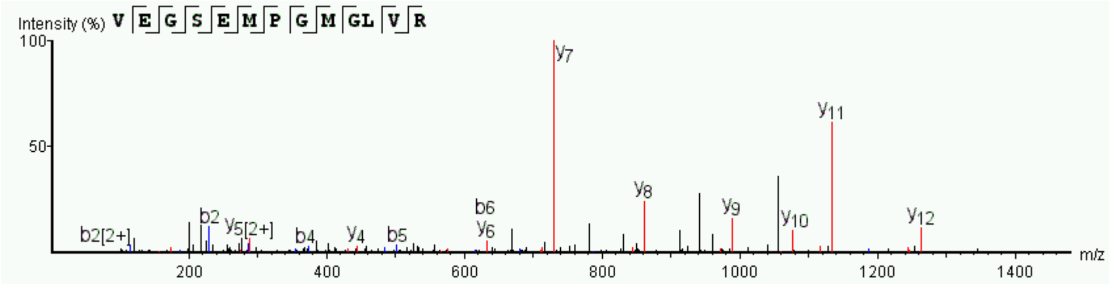

gi|571545446

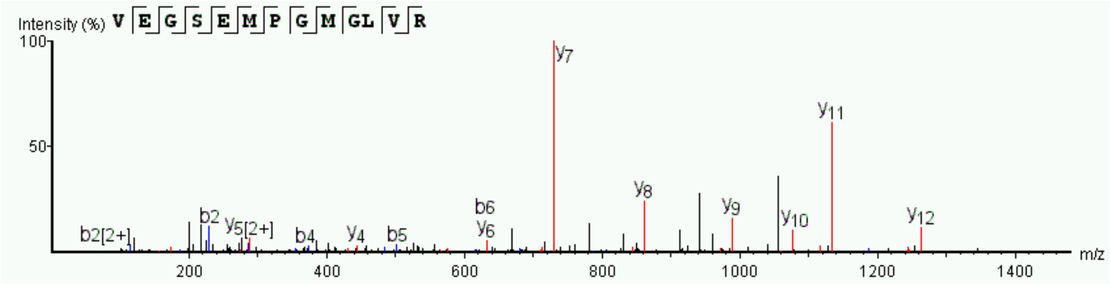

gi|571545449

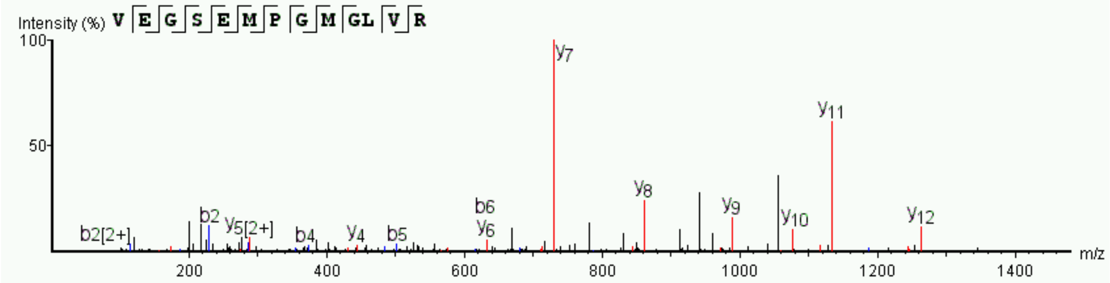

gi|571545452

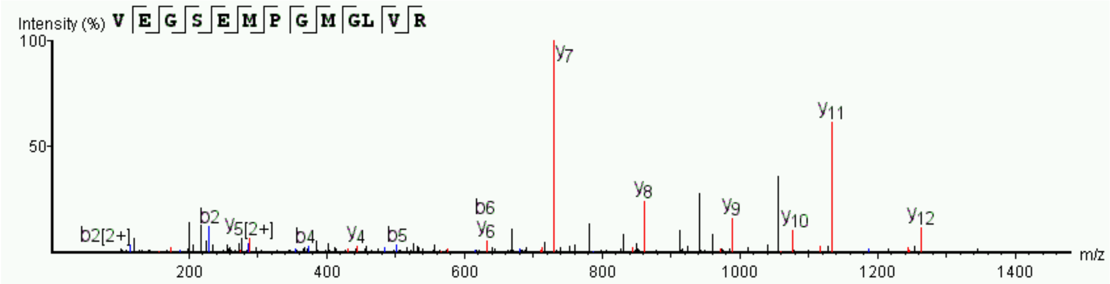

gi|571545455

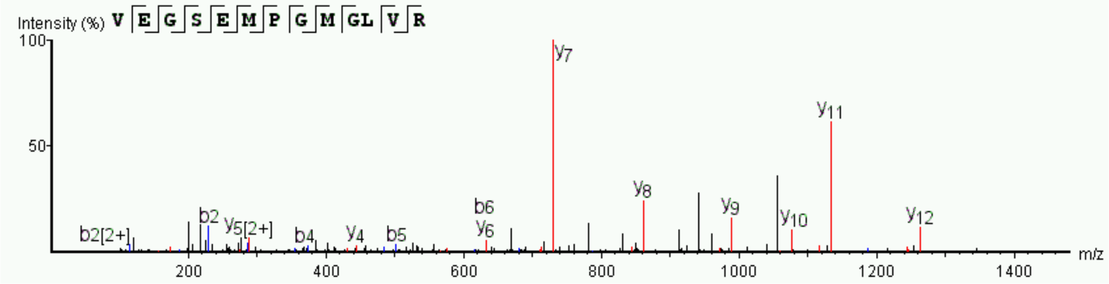

gi|571567987

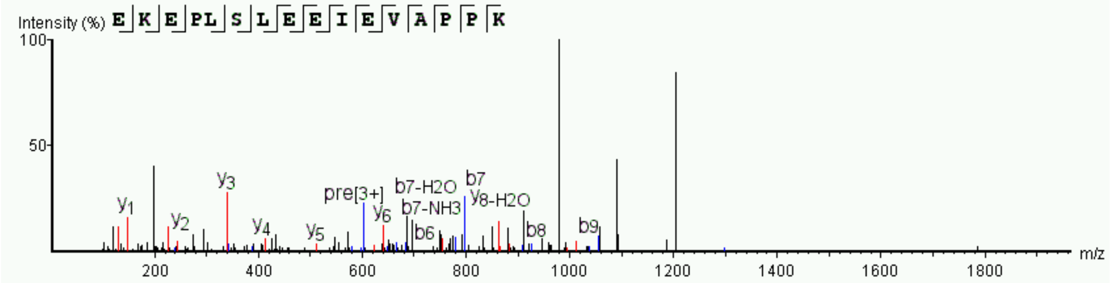

gi|571567990

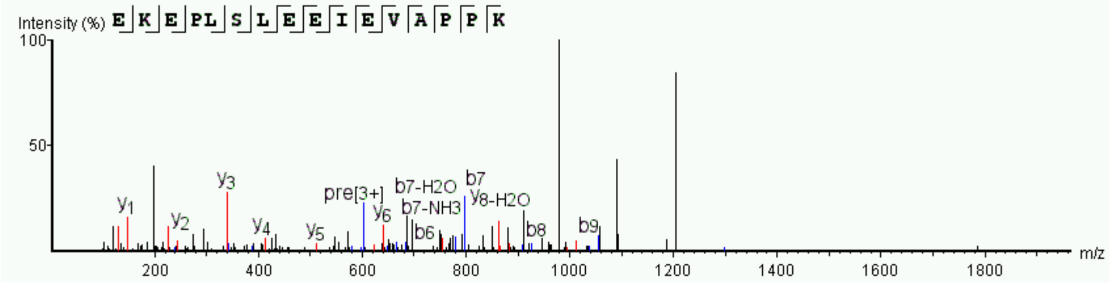

gi|571567994

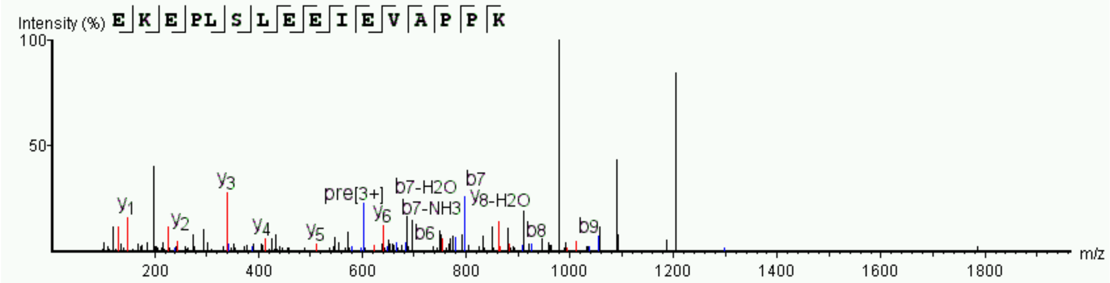

gi|571567998

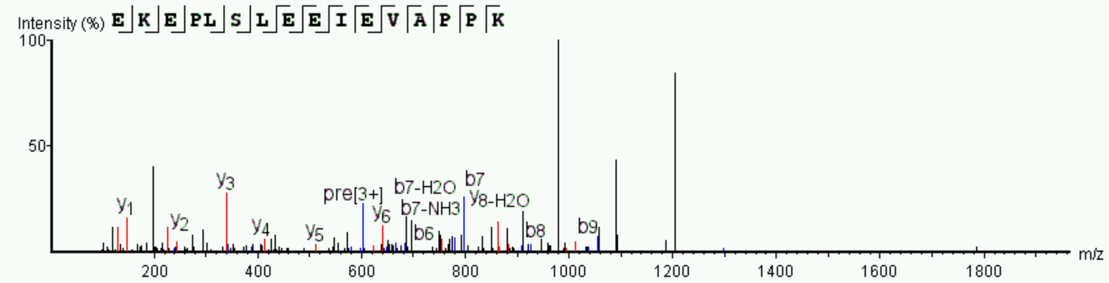

gi|571531961

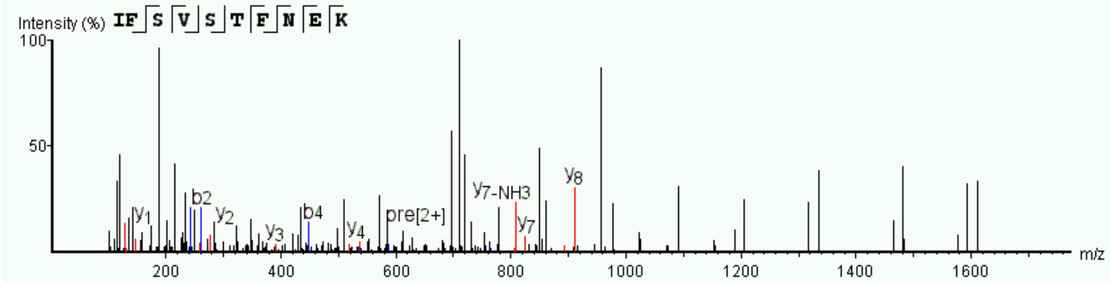

gi|571531965

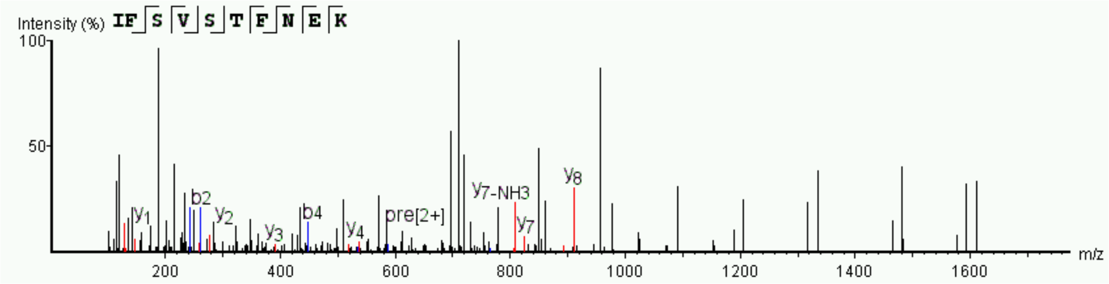

gi|571544767

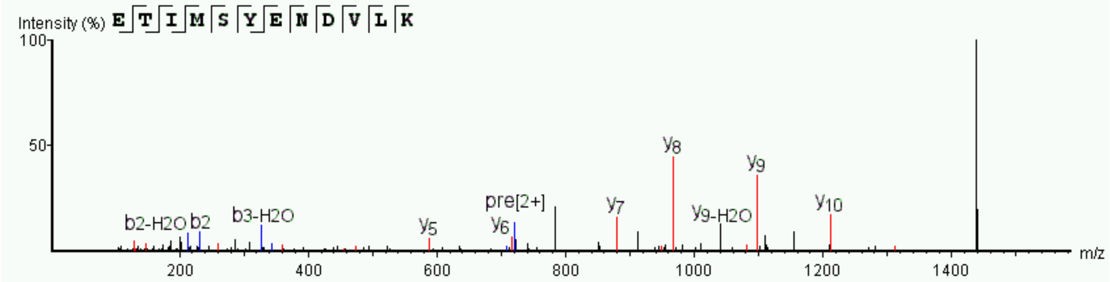

gi|571544771

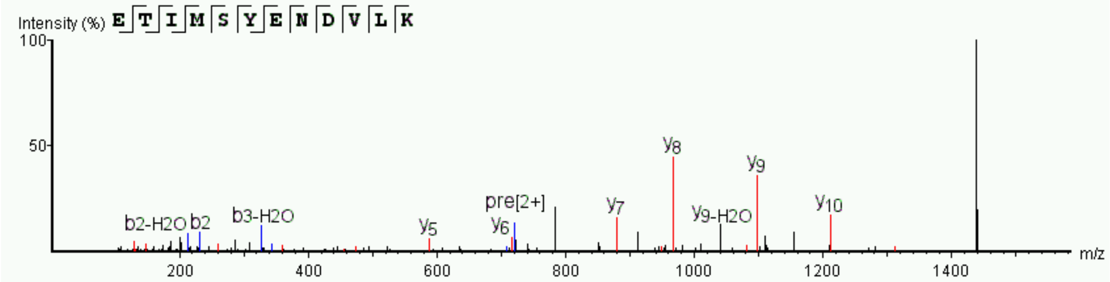

gi|571544775

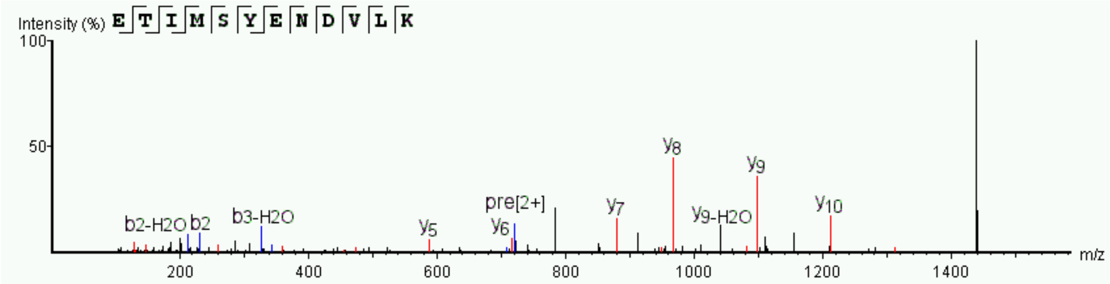

gi|571544778

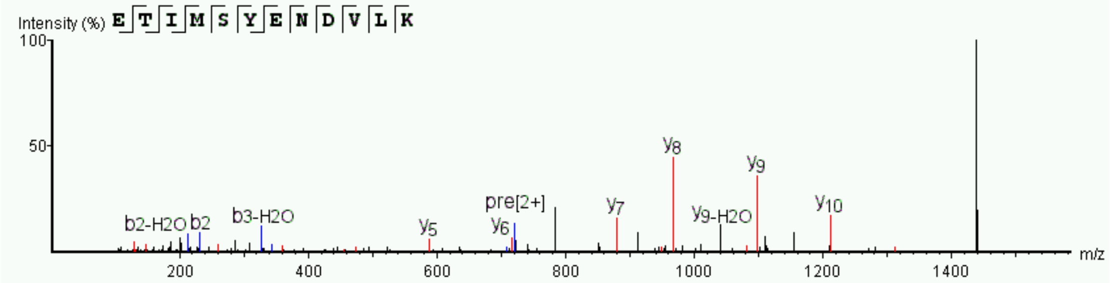

gi|571544781

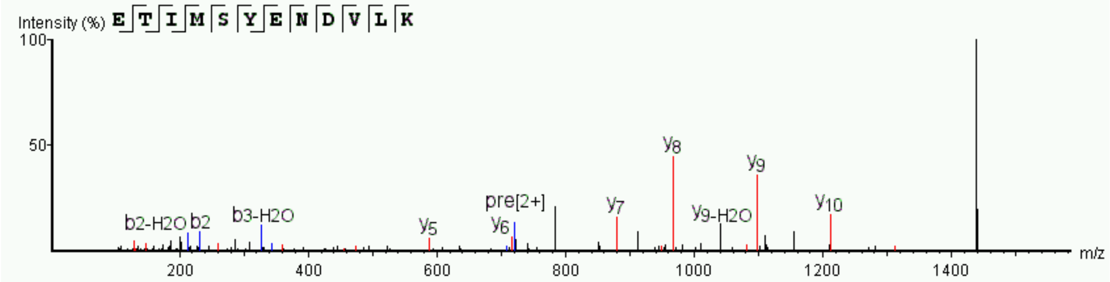

gi|571544784

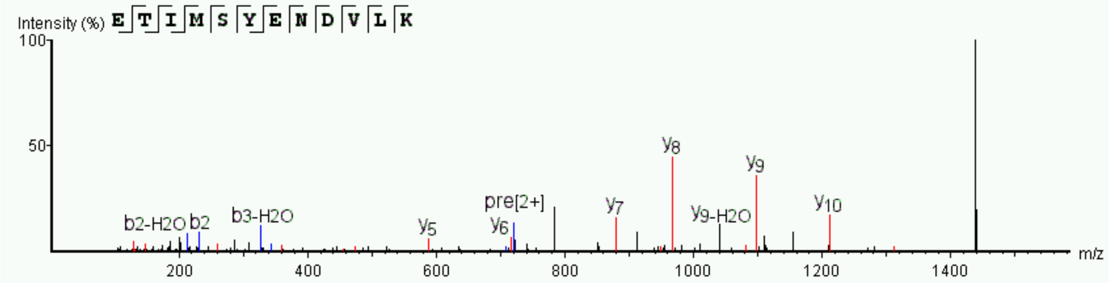

gi|328783748

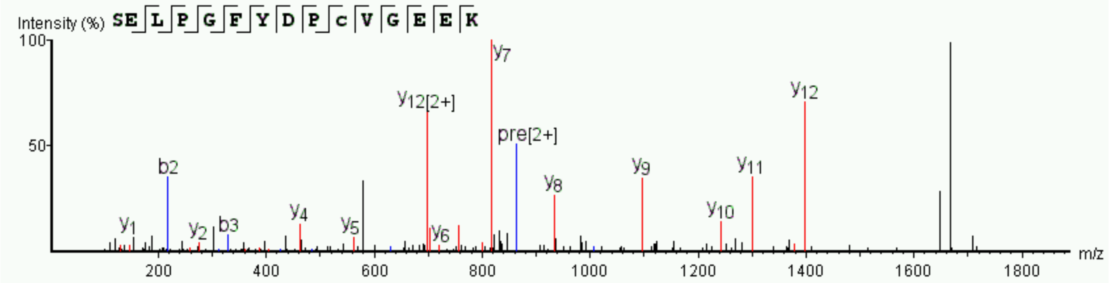

gi|571527600

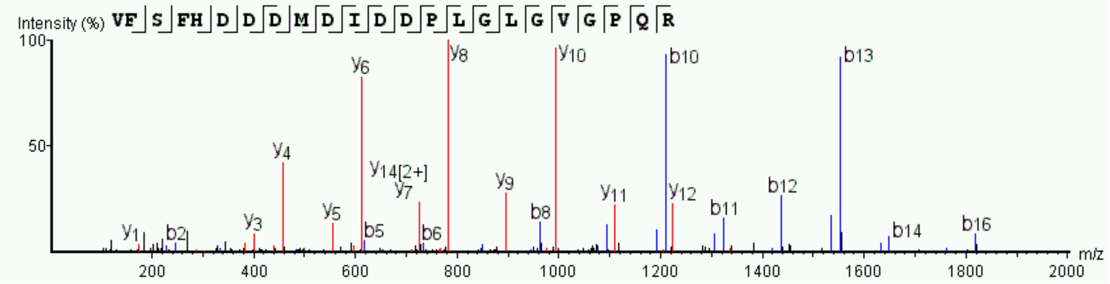

gi|571503163

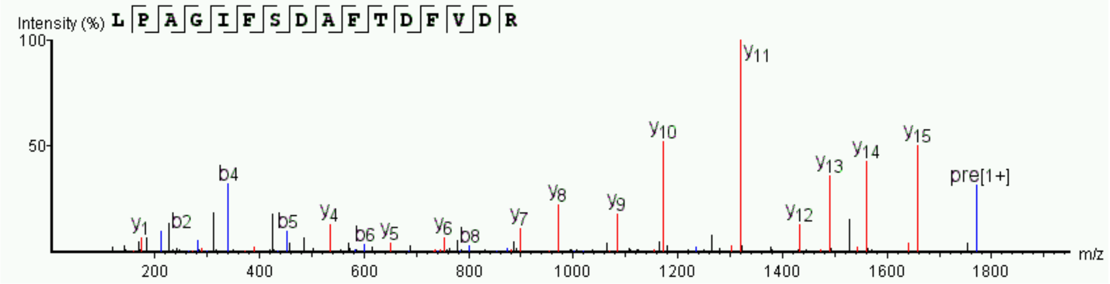

gi|328783650

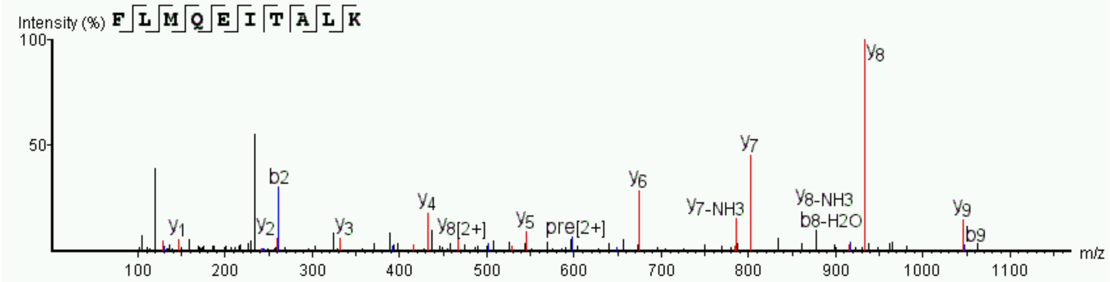

gi|571574746

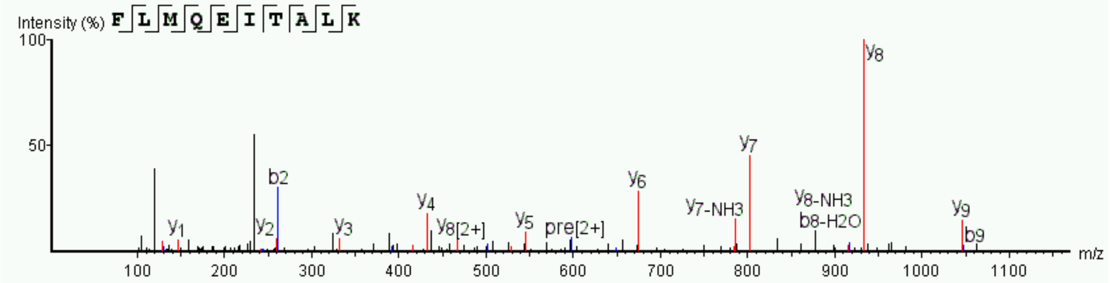

gi|110759426

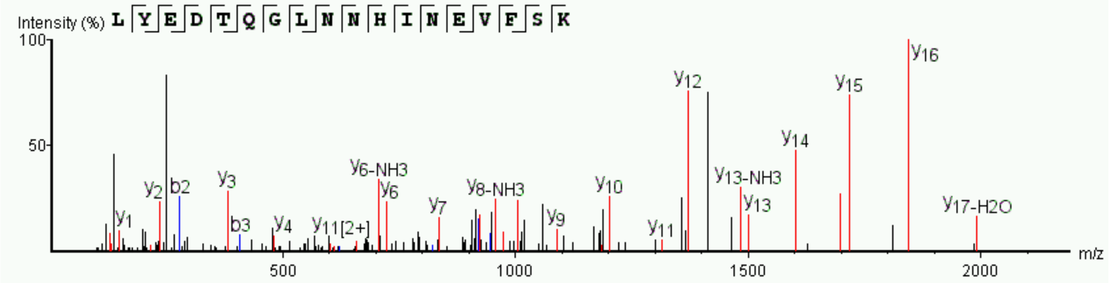

gi|571568885

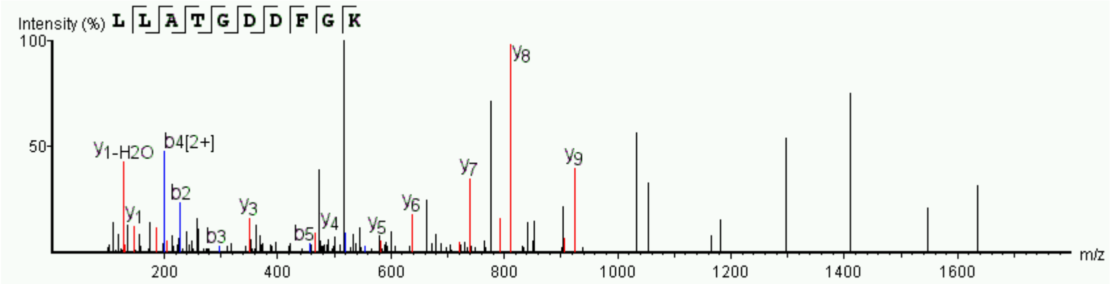

gi|571568893

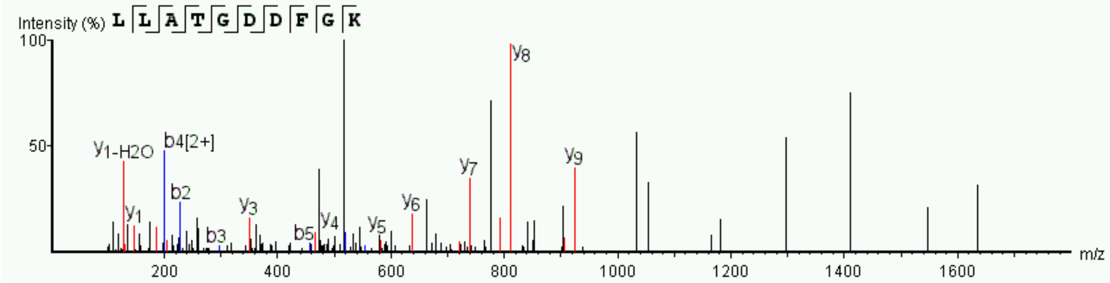

gi|571568897

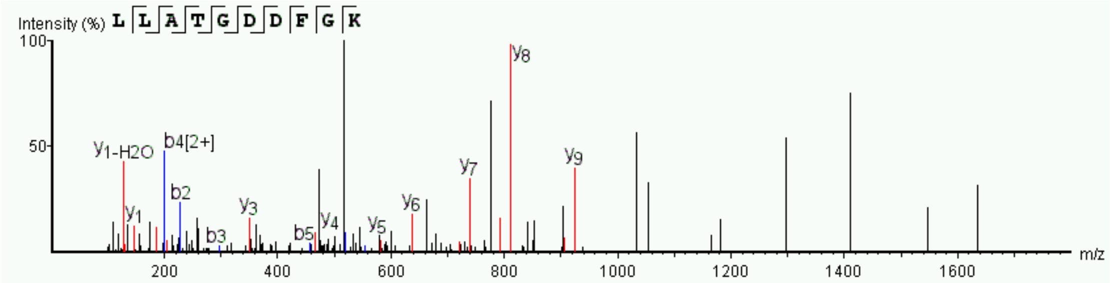

gi|571568901

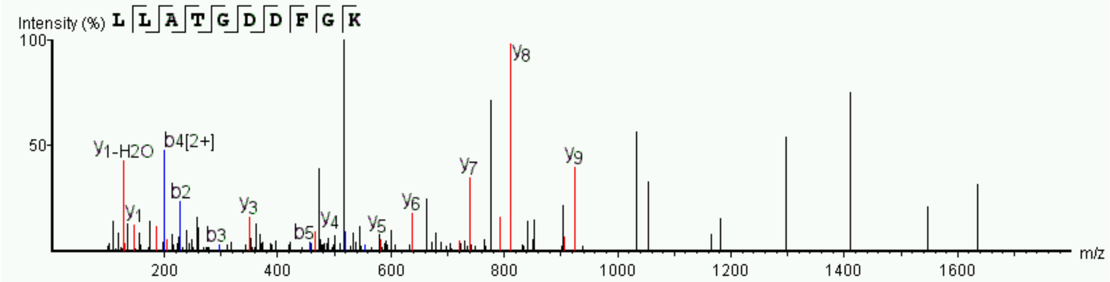

gi|571568907

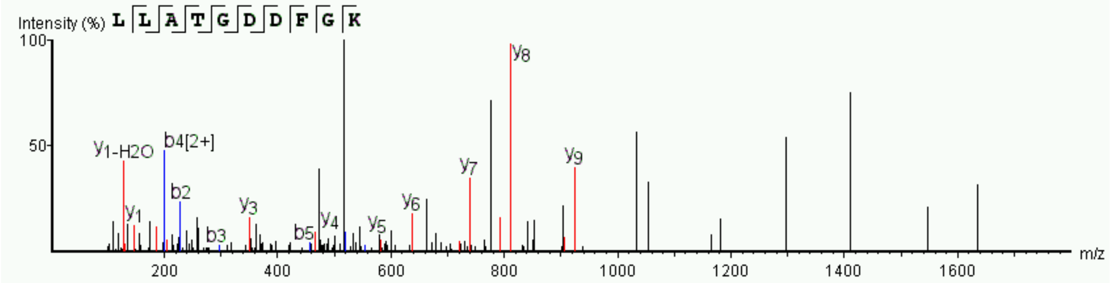

gi|571568910

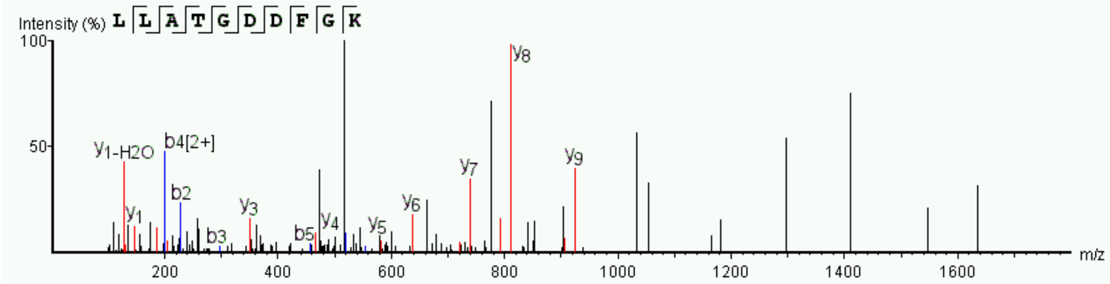

gi|571568913

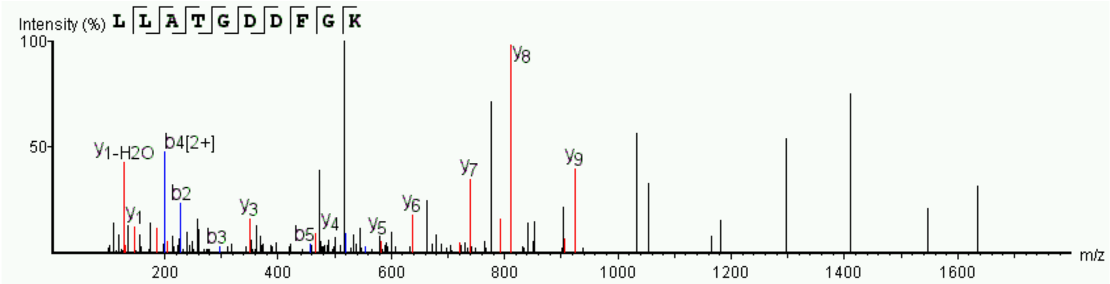

gi|328788758

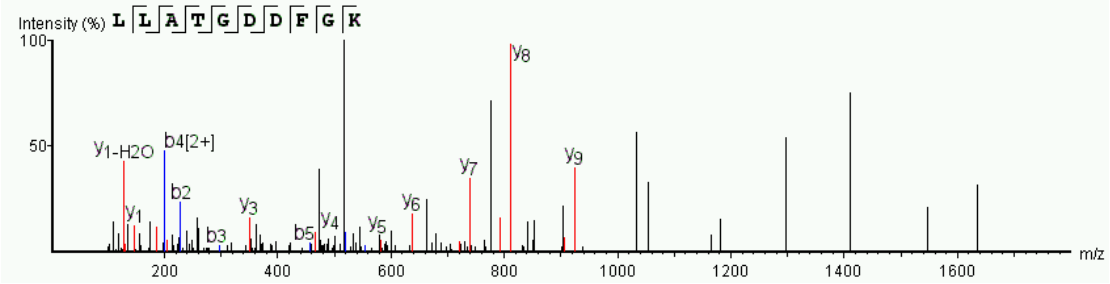

gi|571554030

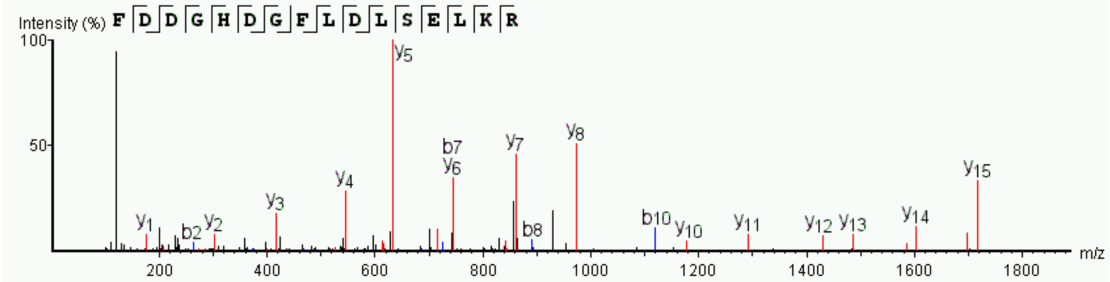

gi|66530680

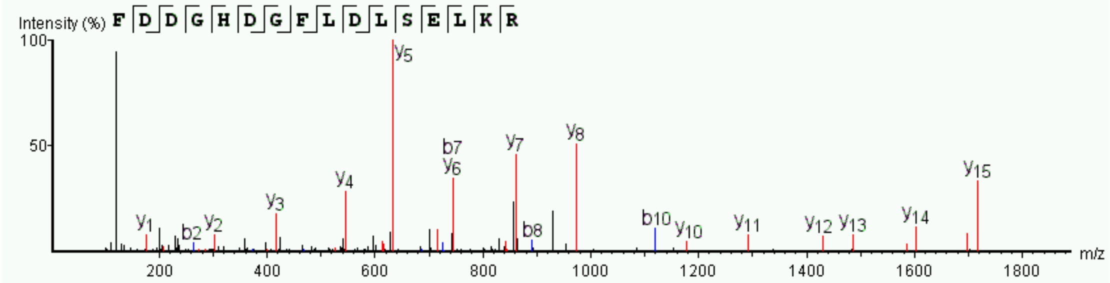

gi|328788967

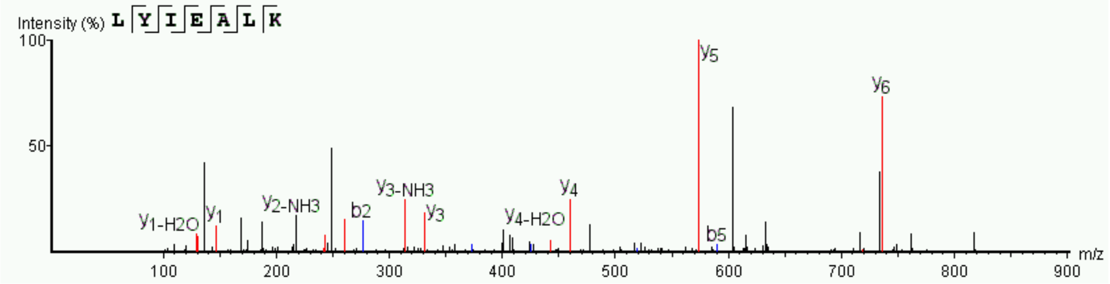

gi|571541144

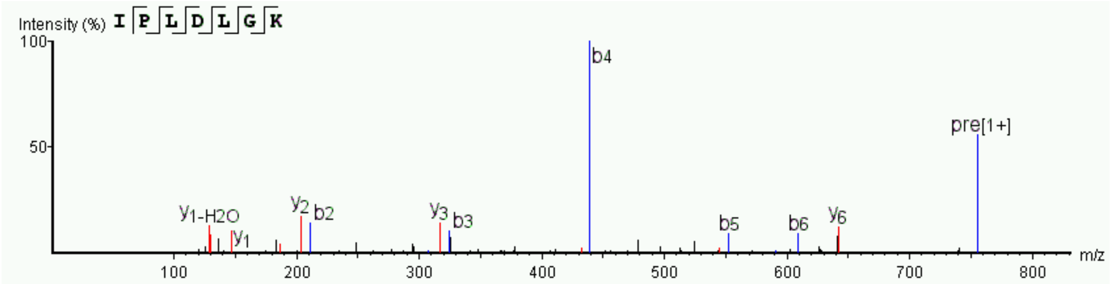

gi|66512104

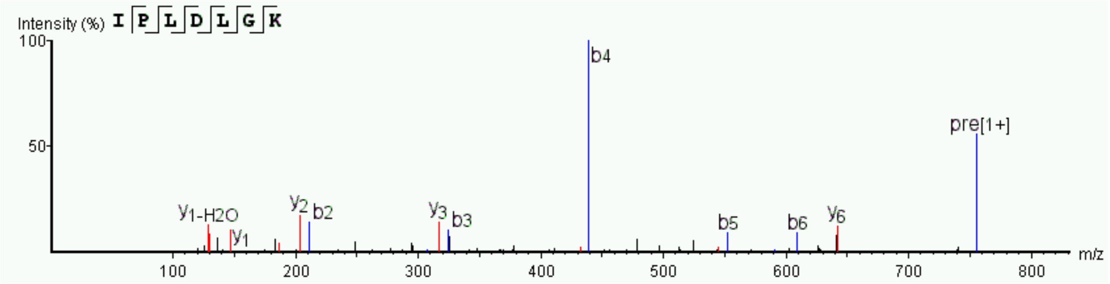

gi|571569633

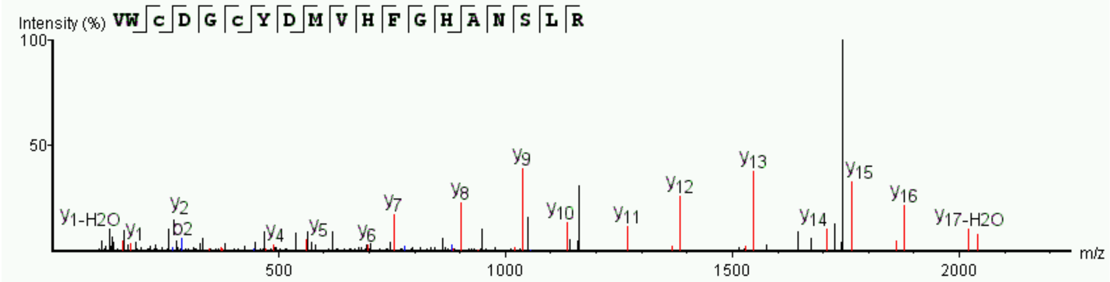

gi|571569639

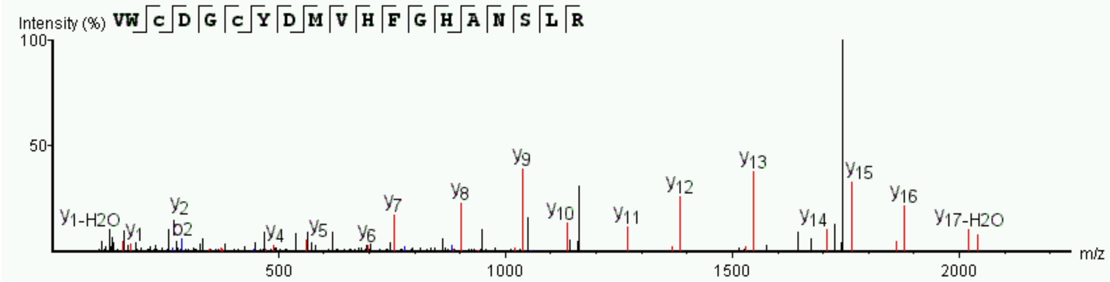

gi|66543768

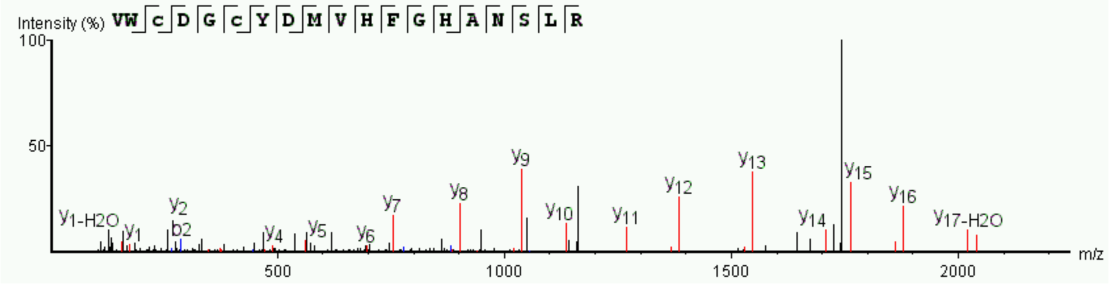

gi|66551115

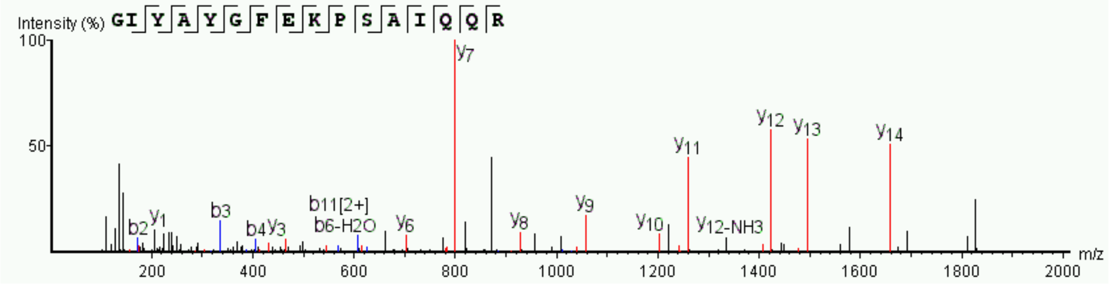

gi|571577740

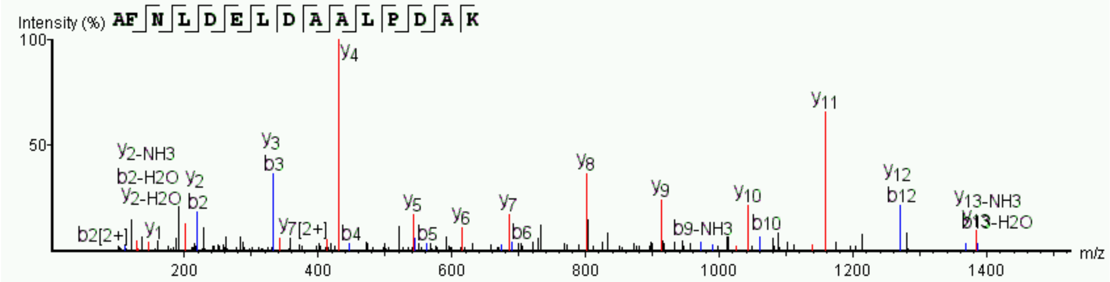

gi|571577742

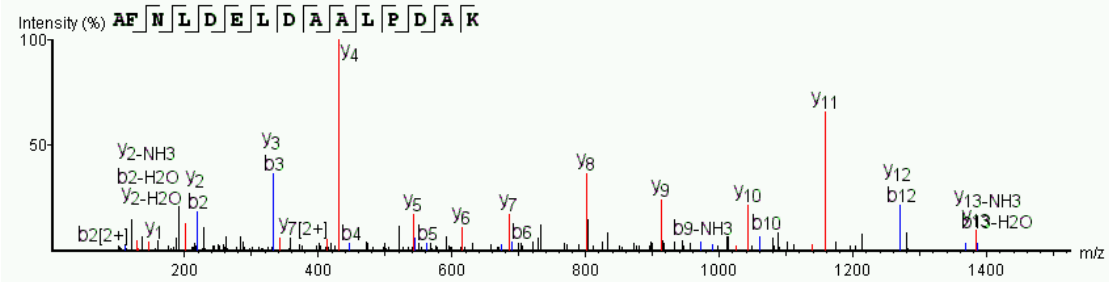

gi|571577744

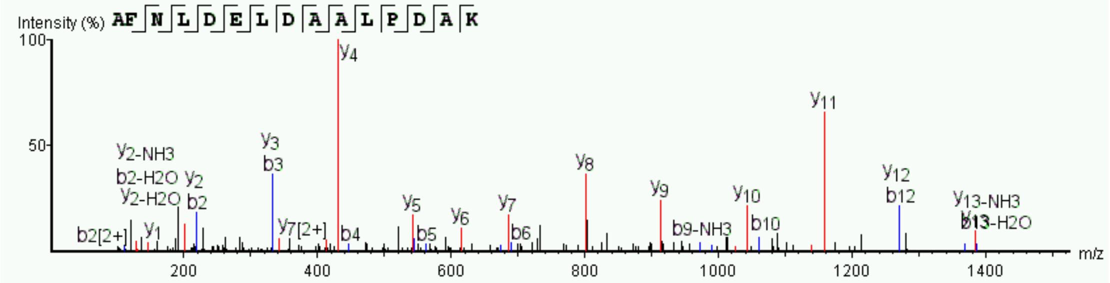

gi|110755833

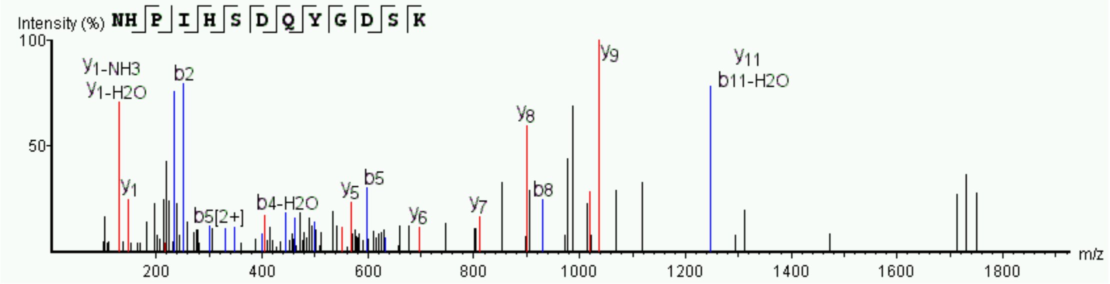

gi|328793605

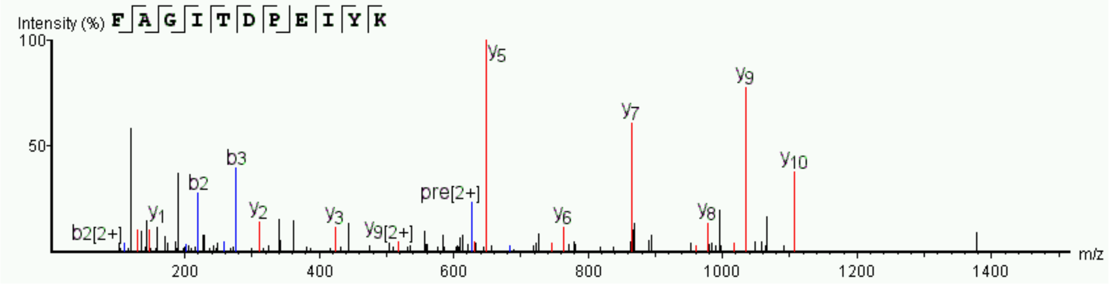

gi|571547726

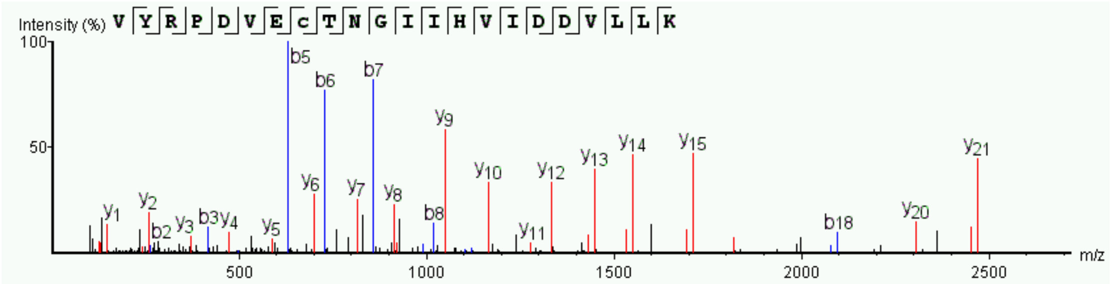

gi|571547729

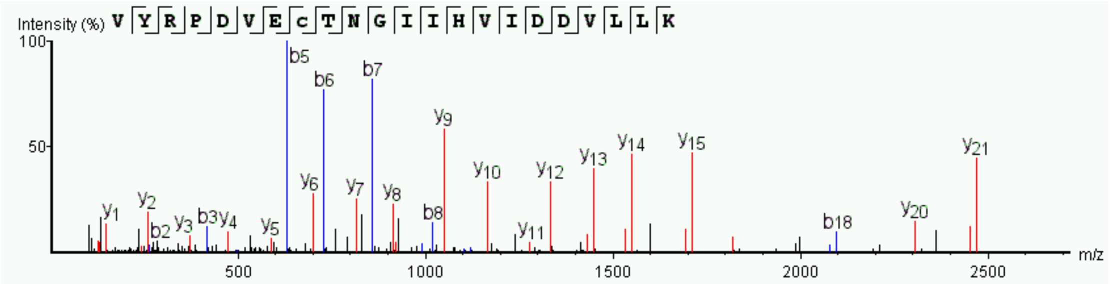

gi|571530735

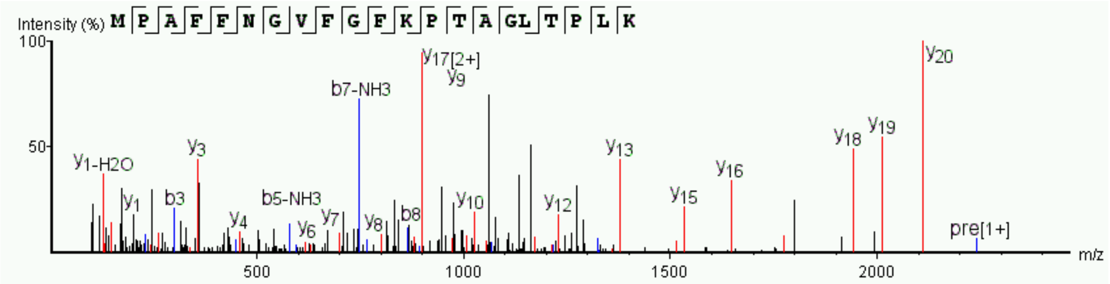

gi|571511387

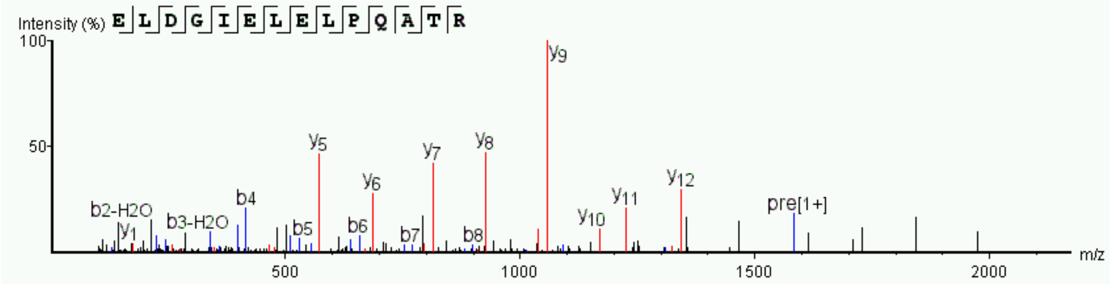

gi|66529483

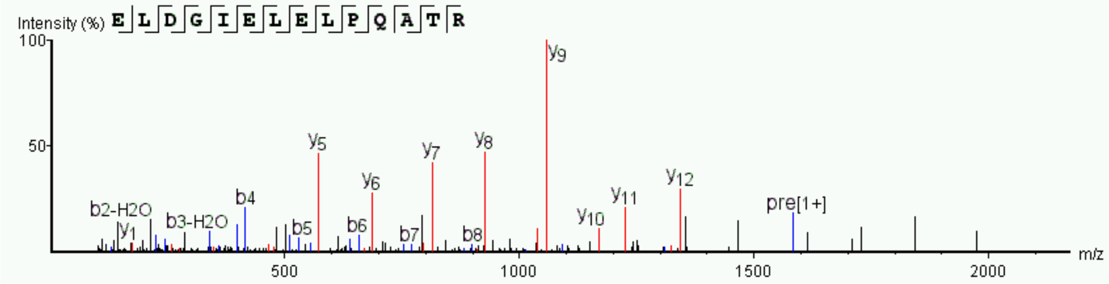

gi|571510578

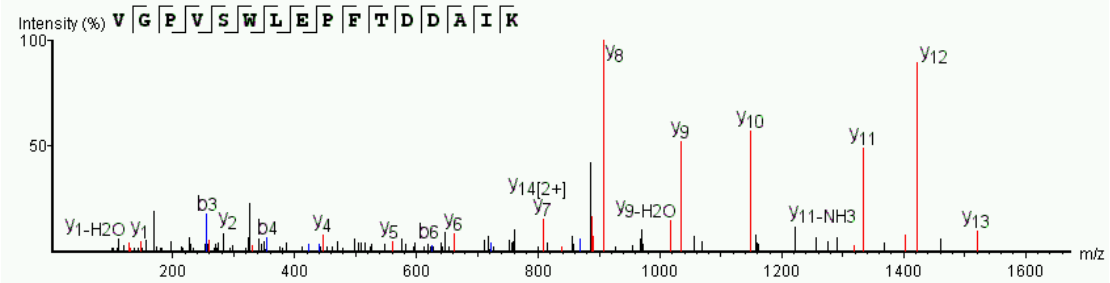

gi|571510585

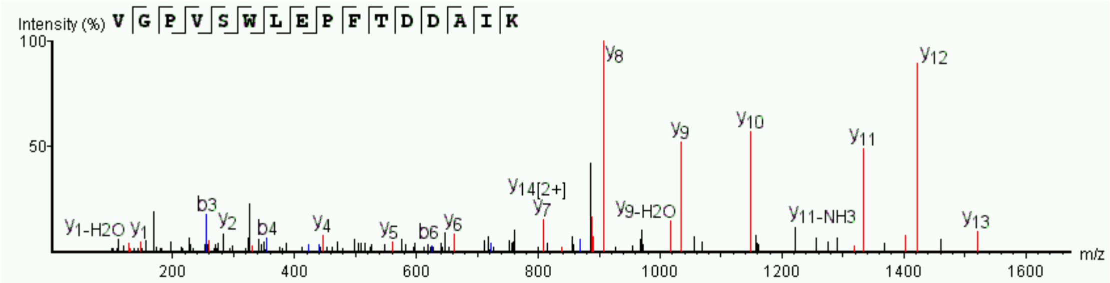

gi|571510581

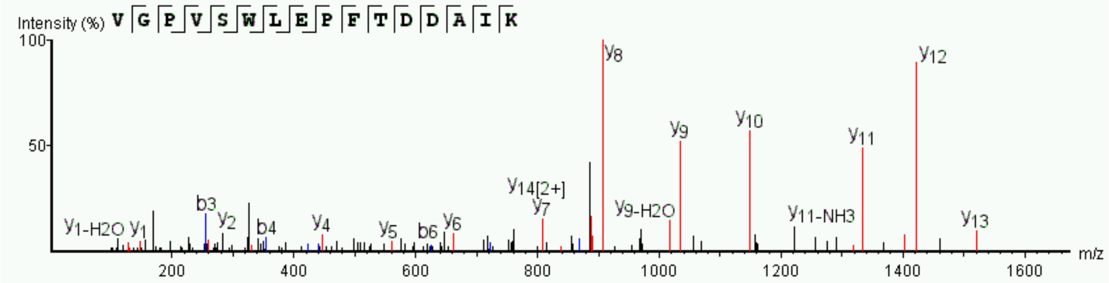

gi|571538660

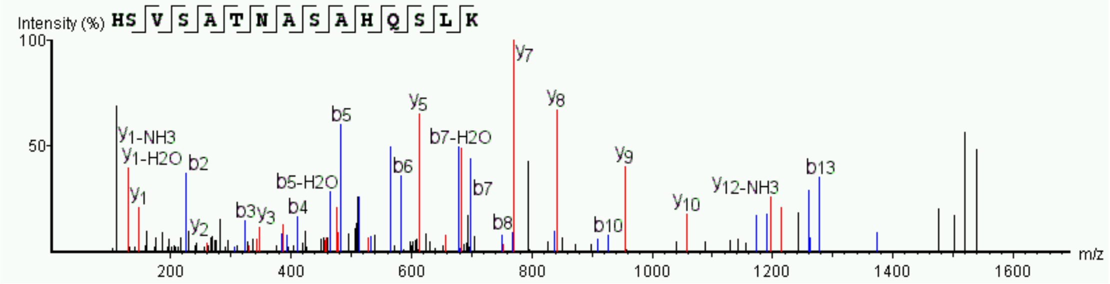

gi|110755339

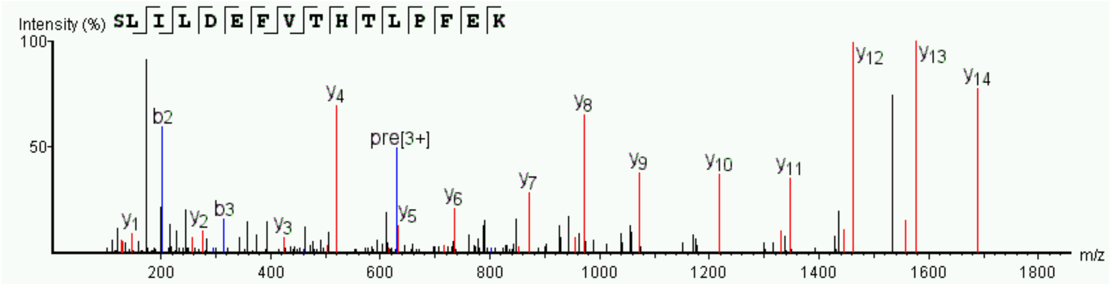

gi|571519665

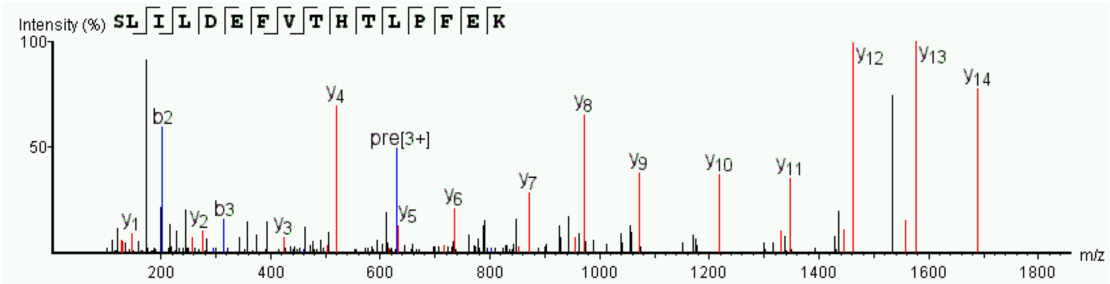

gi|571519668

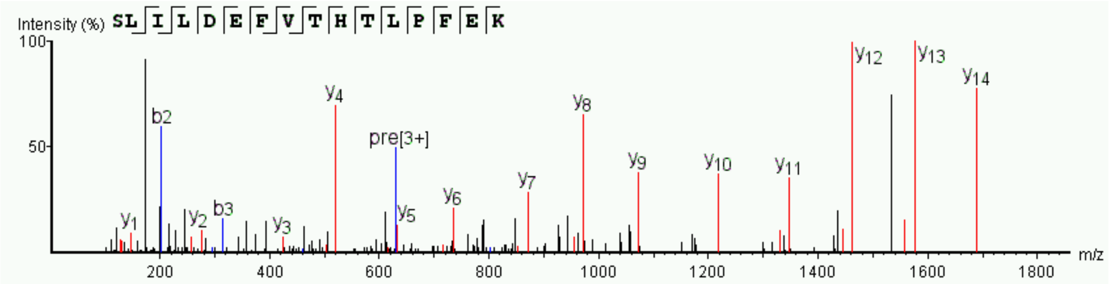

gi|571509128

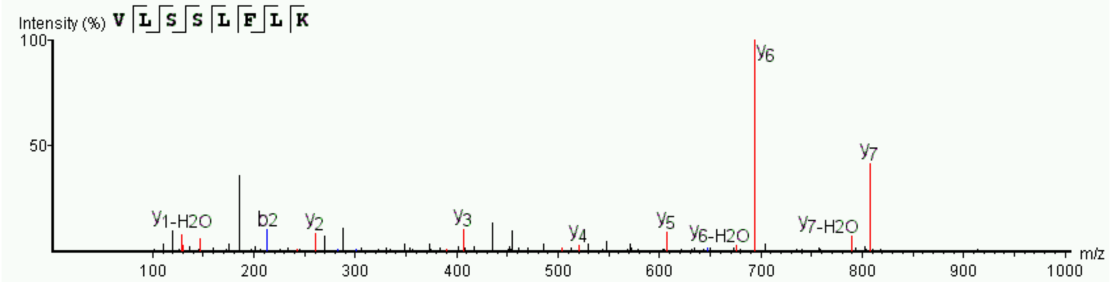

gi|328792443

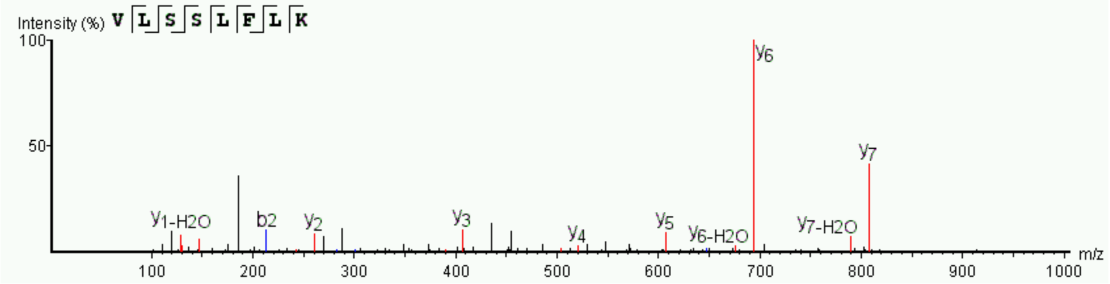

gi|571574918

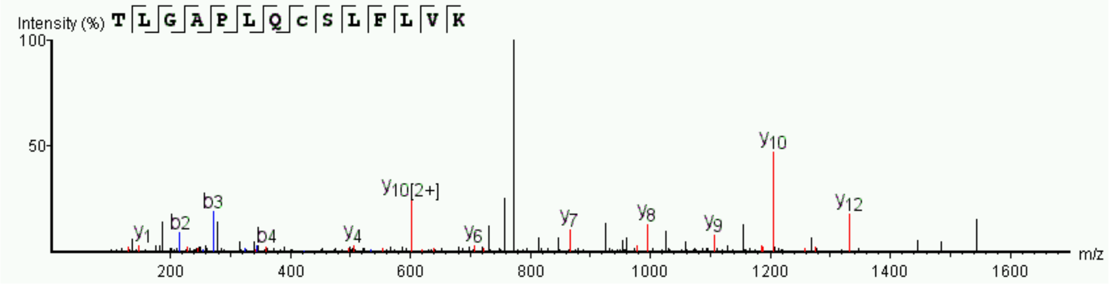

gi|110764555

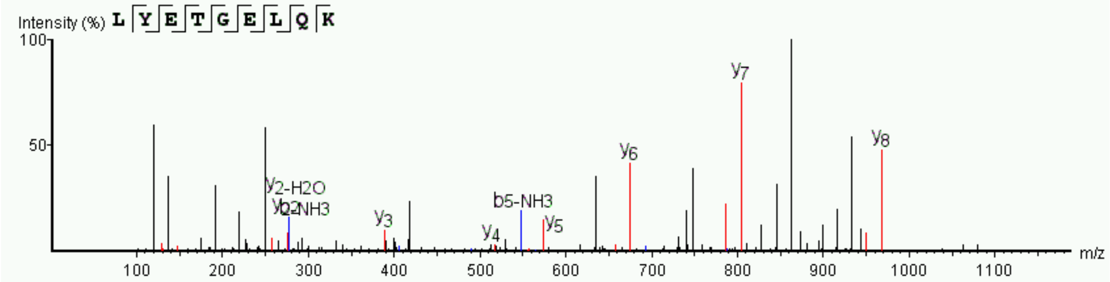

gi|328786065

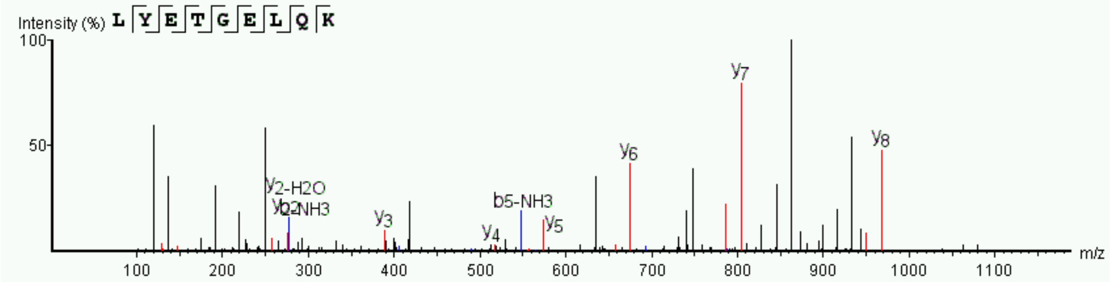

gi|328786067

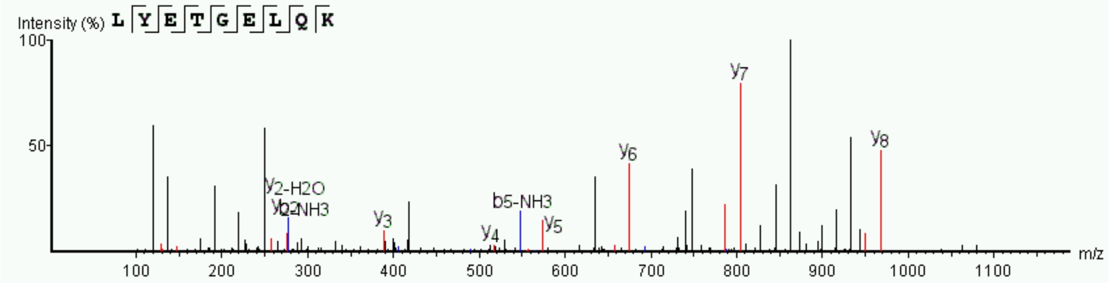

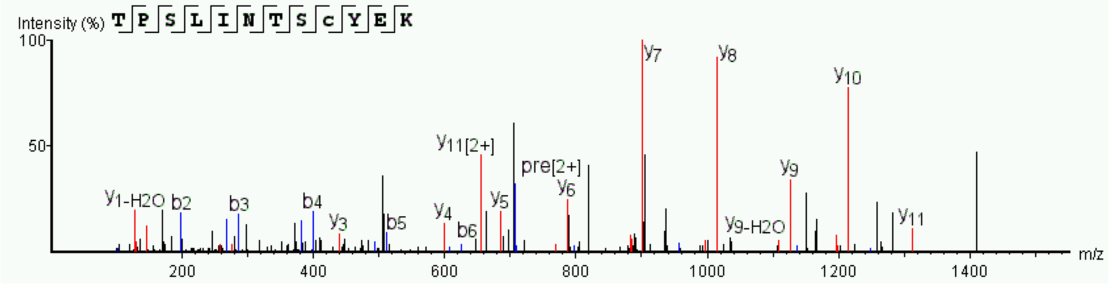

gi|571558993

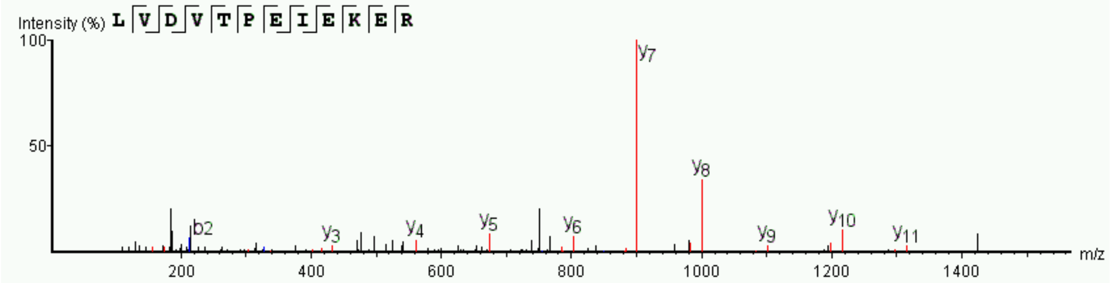

gi|571559000

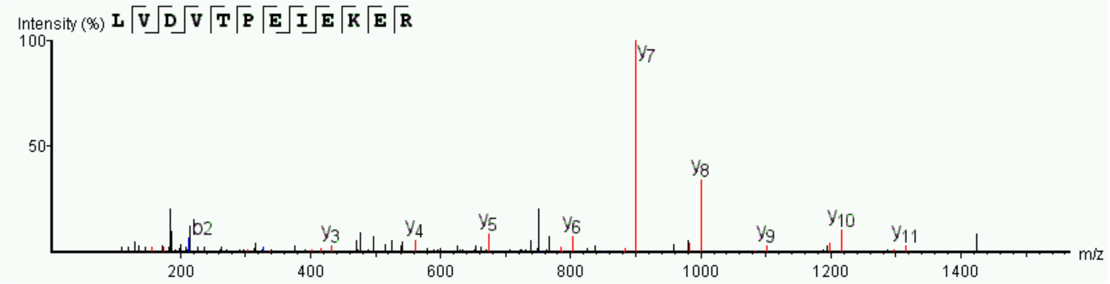

gi|66522082

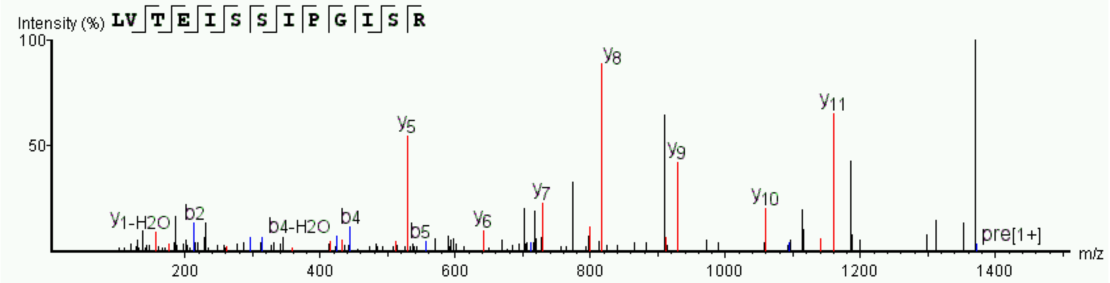

gi|328793467

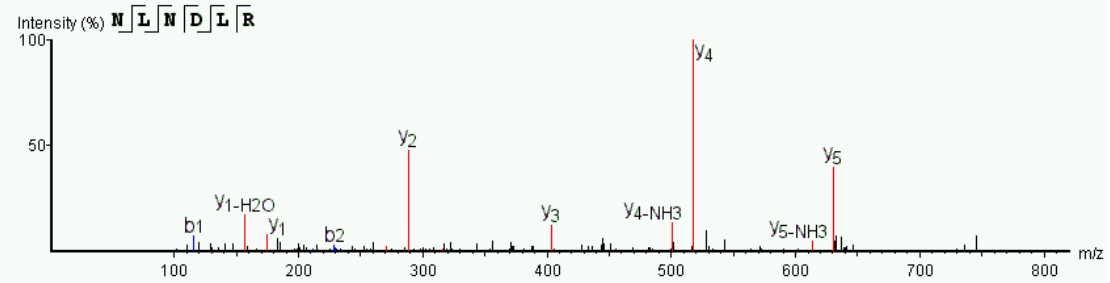

gi|328793729

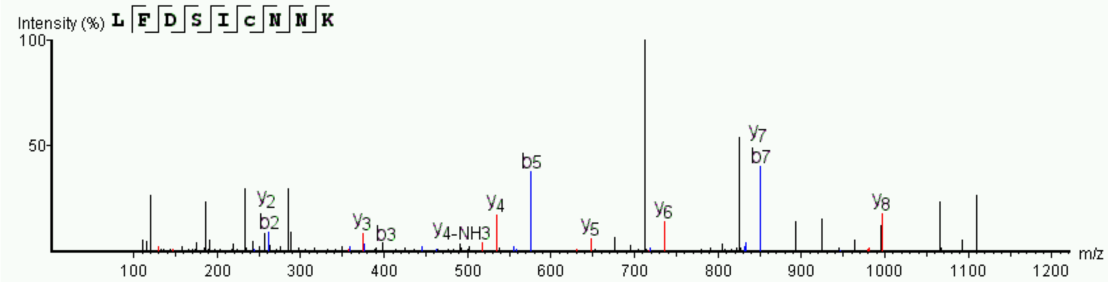

gi|571535113

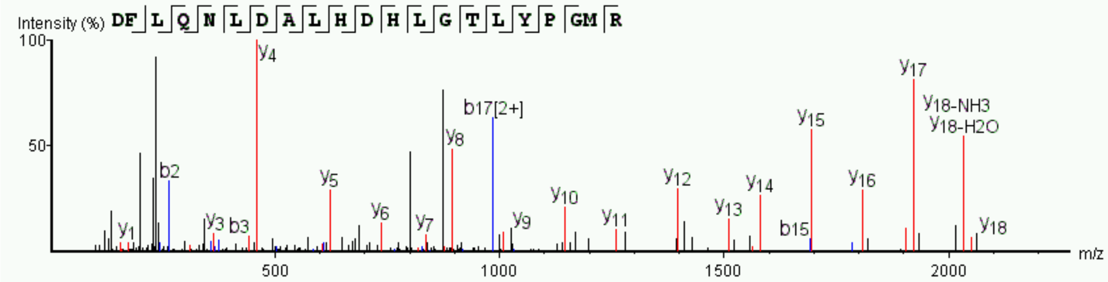

gi|66509537

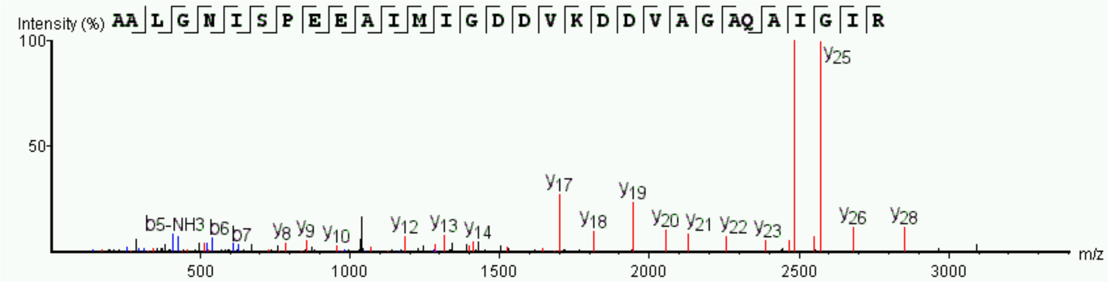

gi|571547190

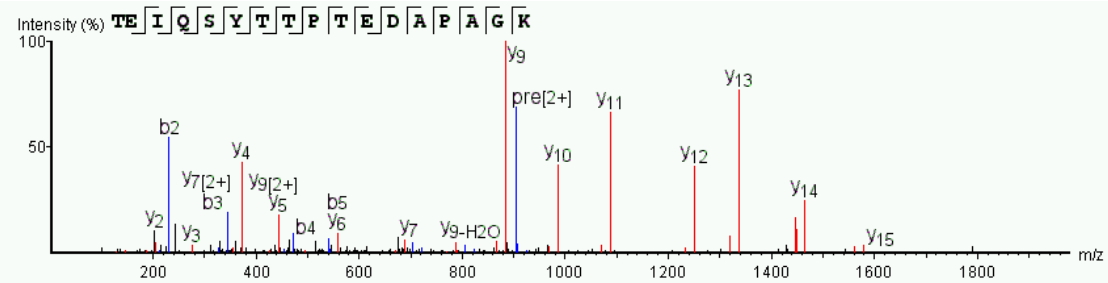

gi|66511337

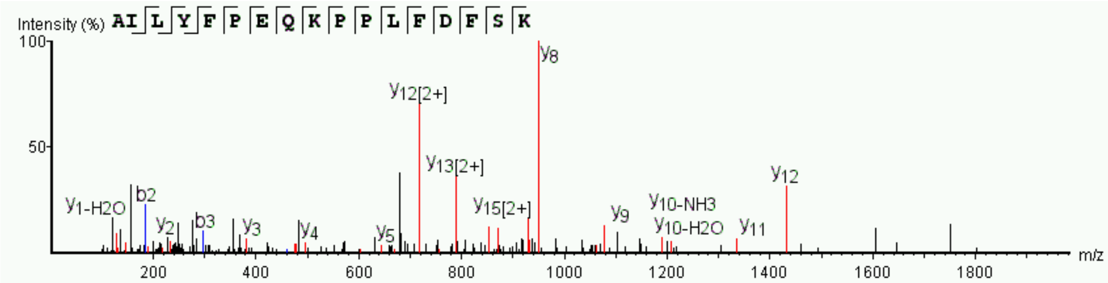

gi|571523225

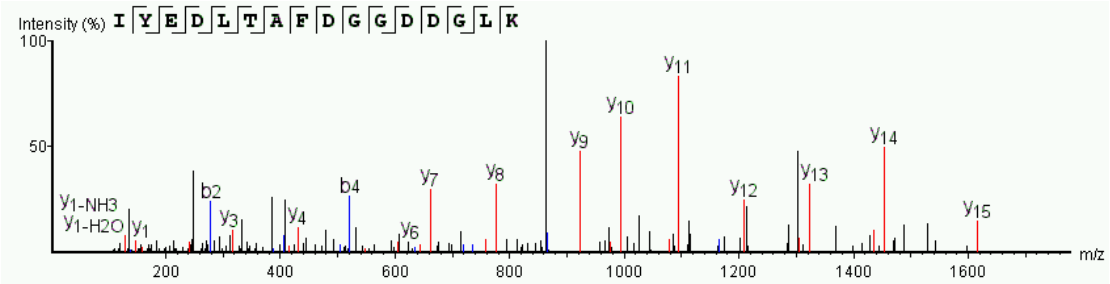

gi|66547731

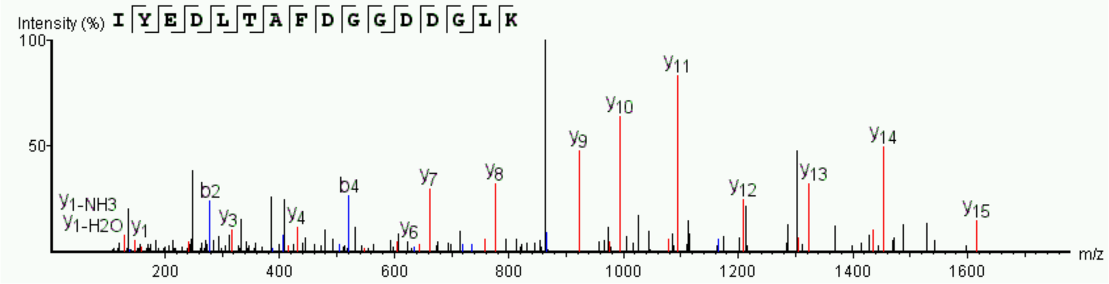

gi|328786150

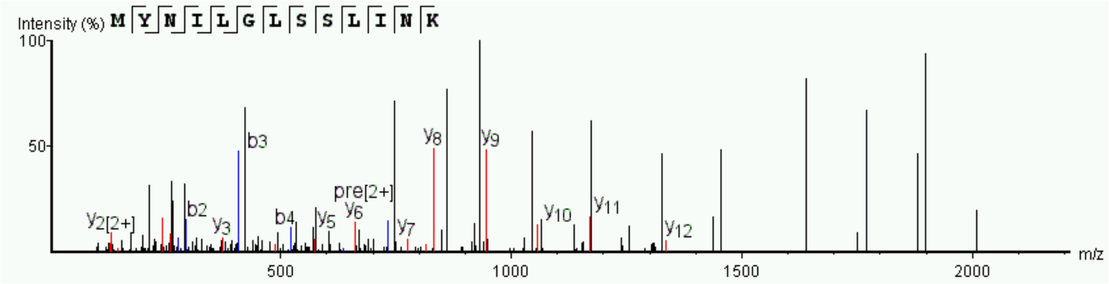

gi|328792179

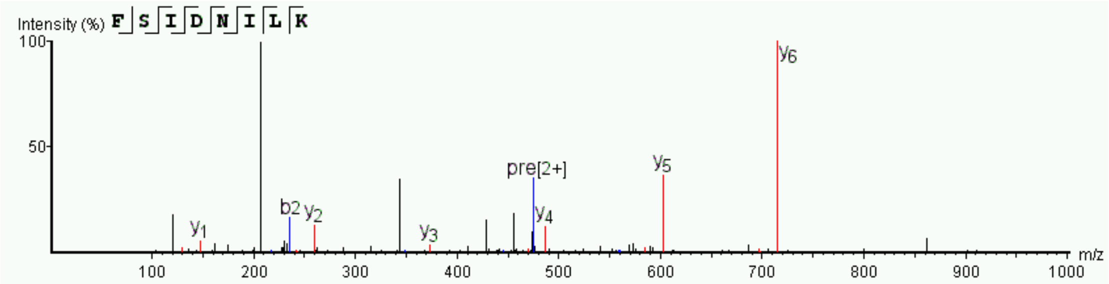

gi|328792621

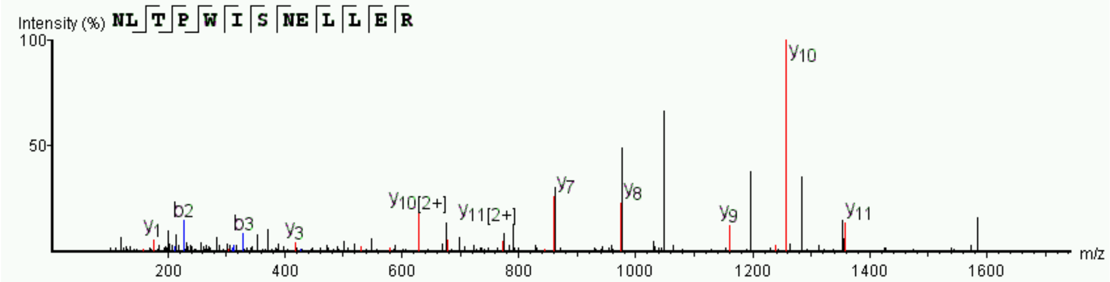

gi|571509569

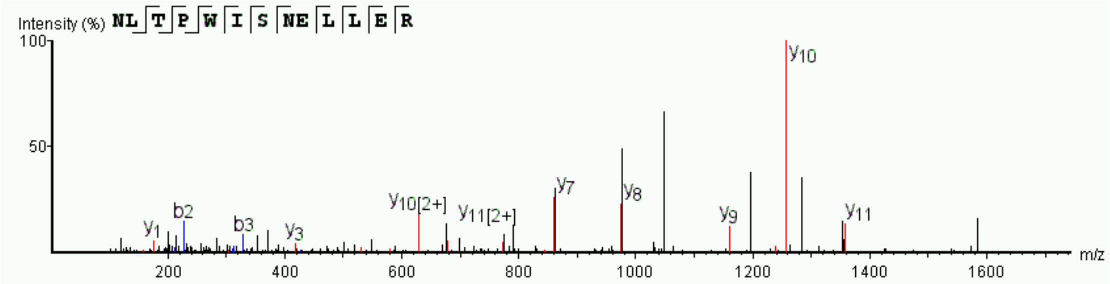

gi|571509575

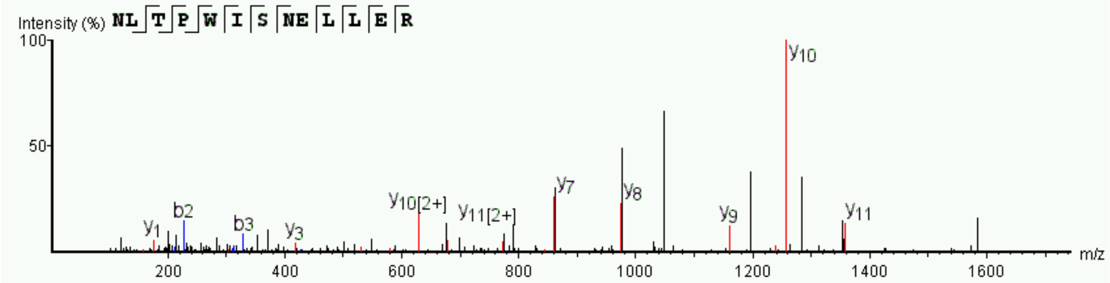

gi|571509578

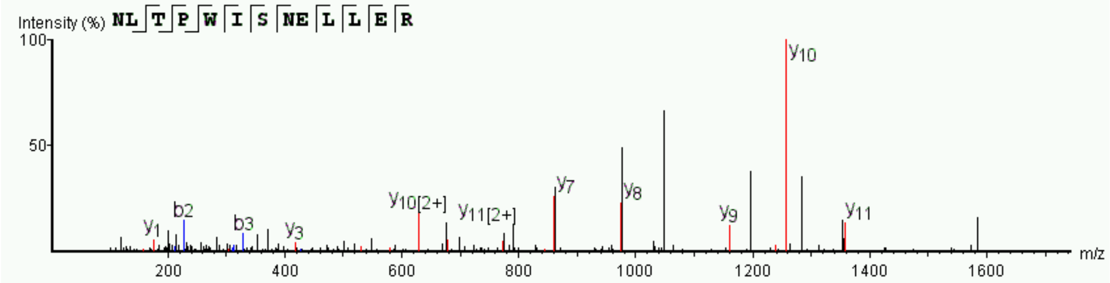

gi|571509582

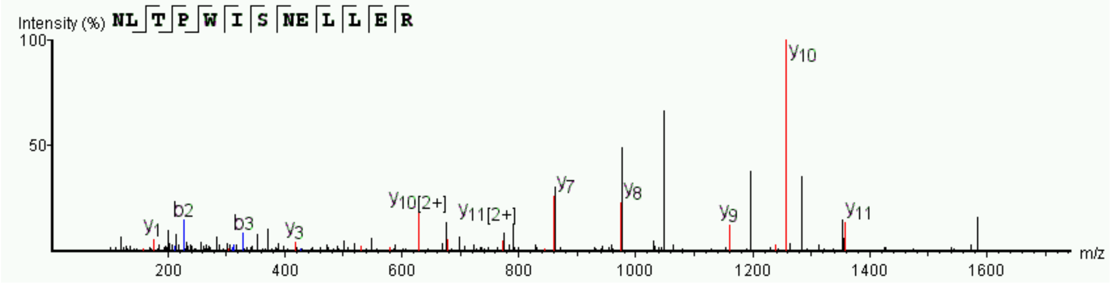

gi|571509585

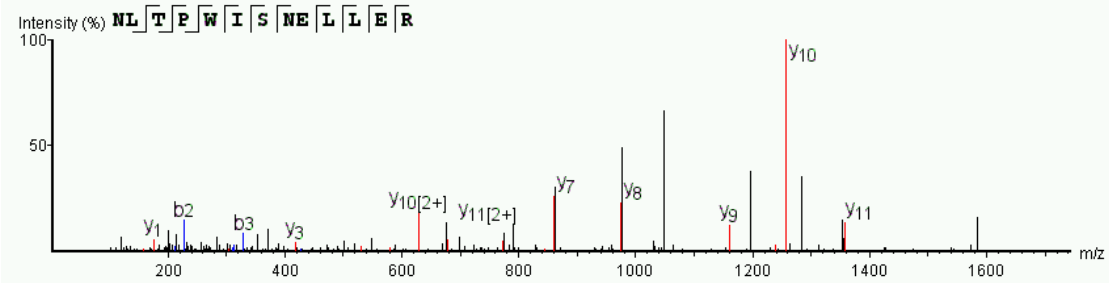

gi|571509589

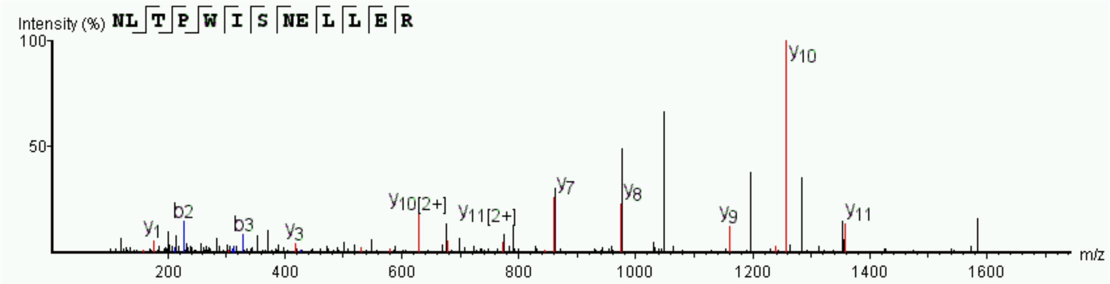

gi|571509593

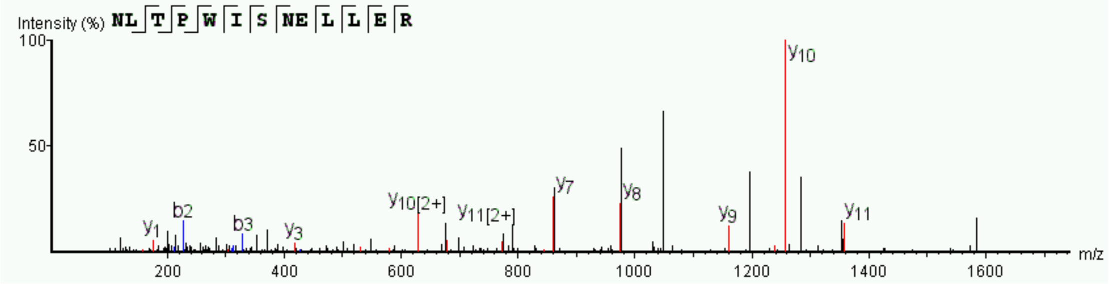

gi|571541861

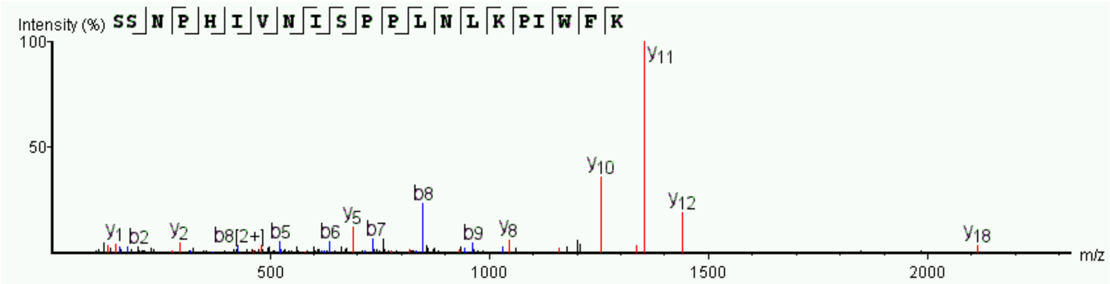

gi|66530010

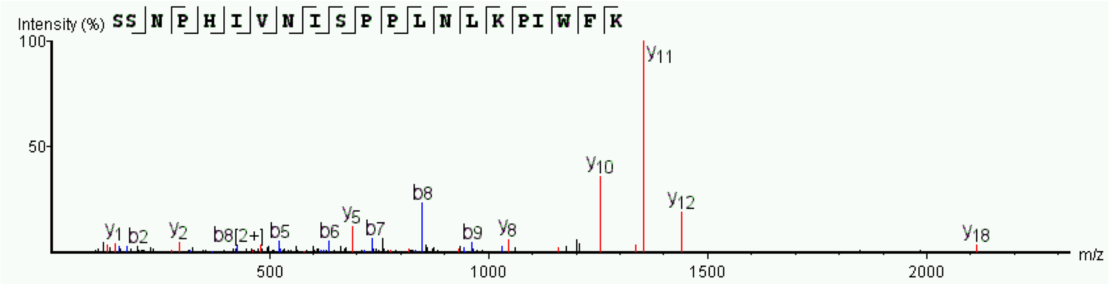

gi|571553669

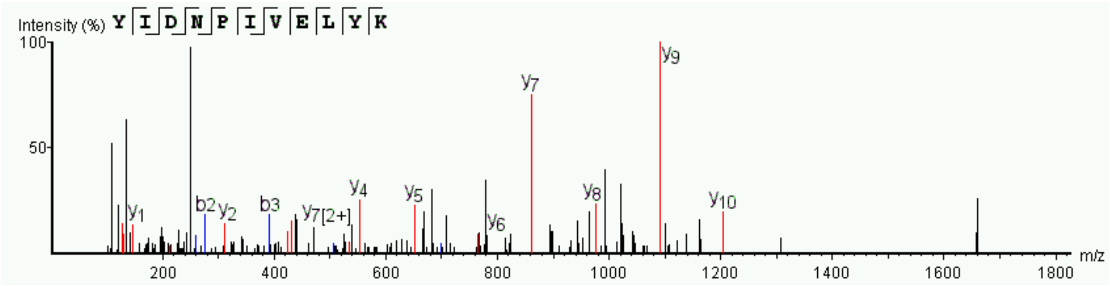

gi|328784616

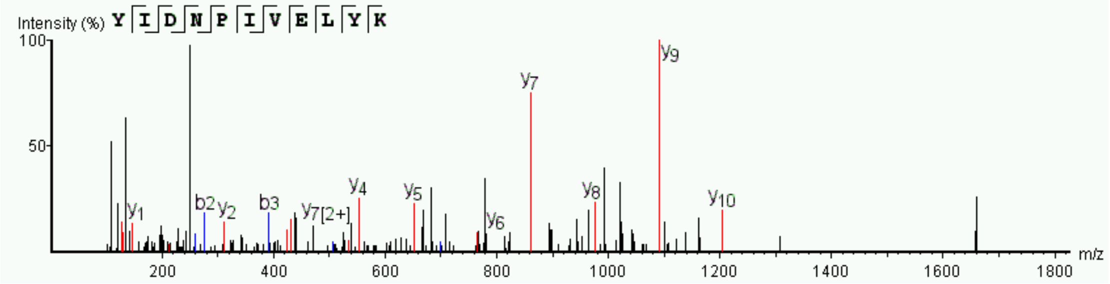

gi|571512479

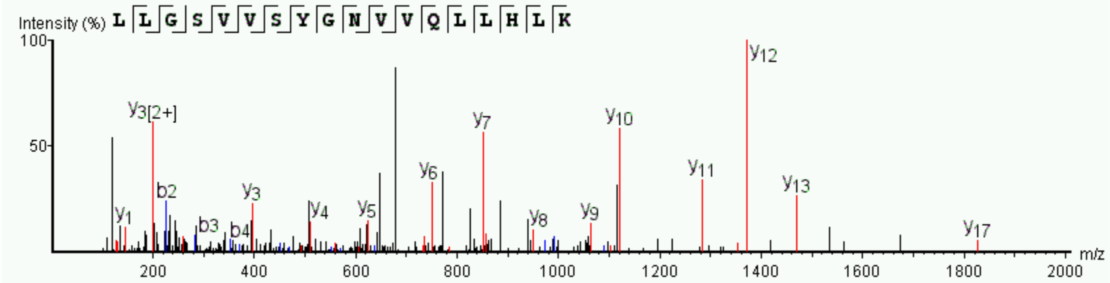

gi|571512509

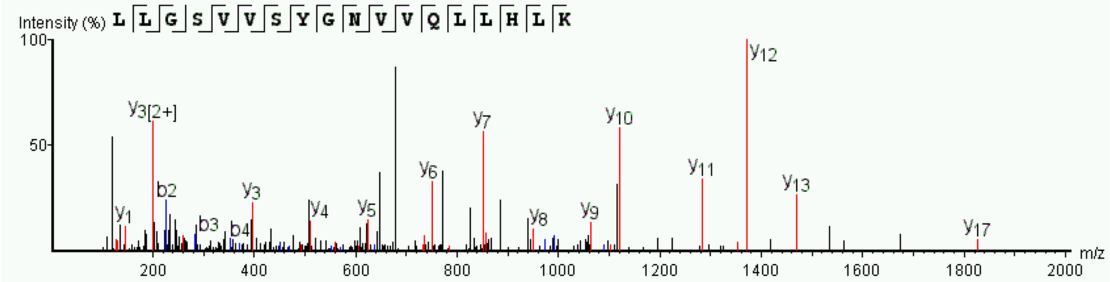

gi|571512481

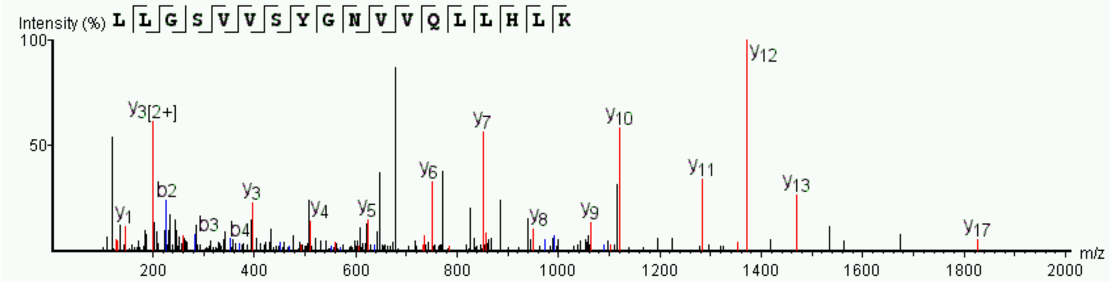

gi|571512485

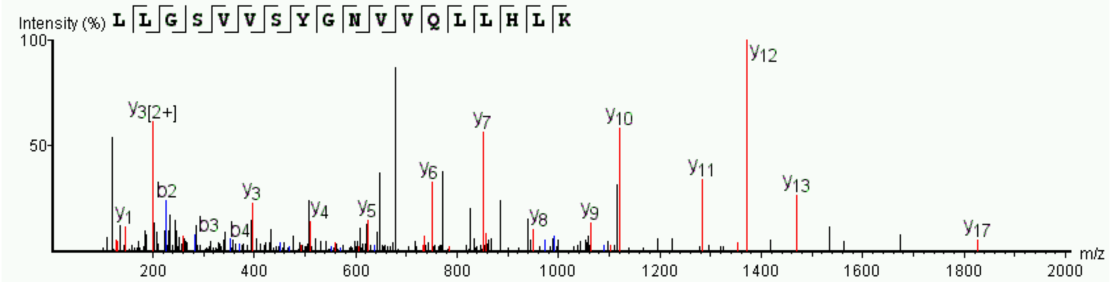

gi|571512488

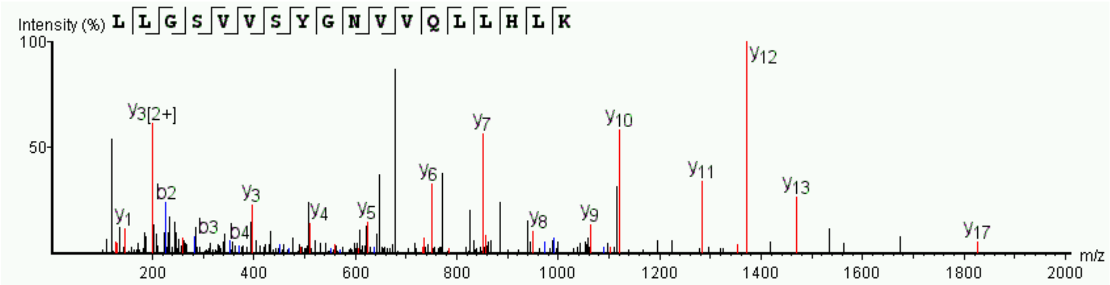

gi|571512492

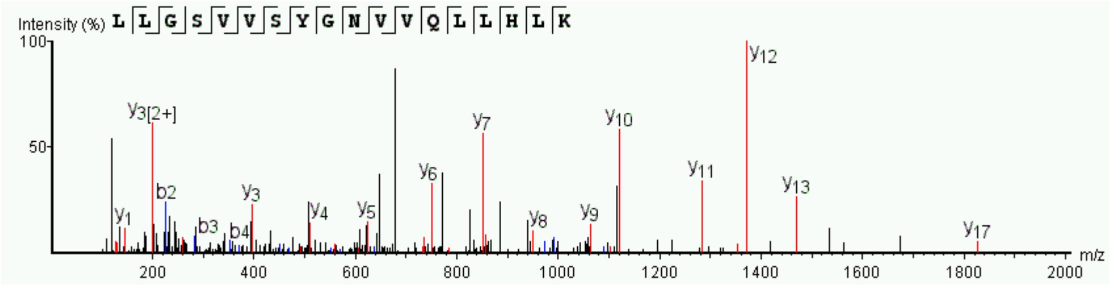

gi|571512495

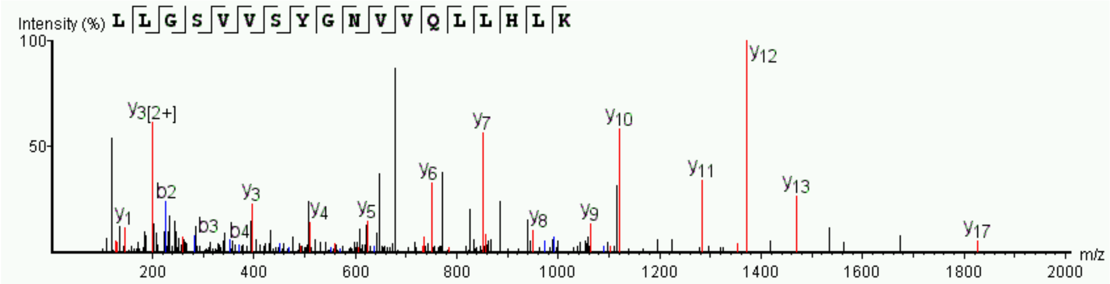

gi|571512499

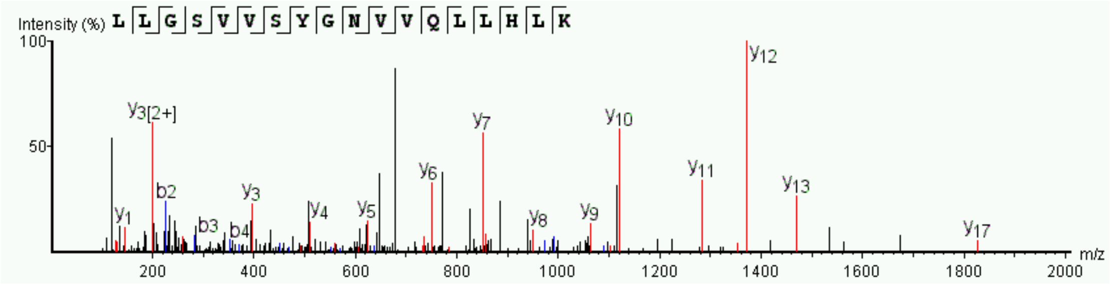

gi|571512503

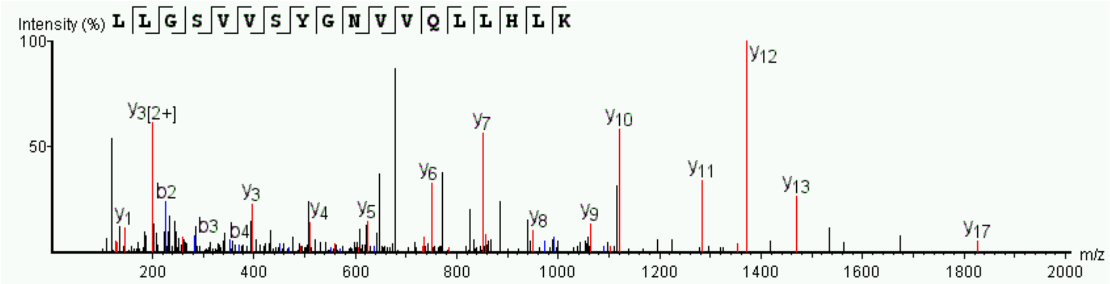

gi|571512505

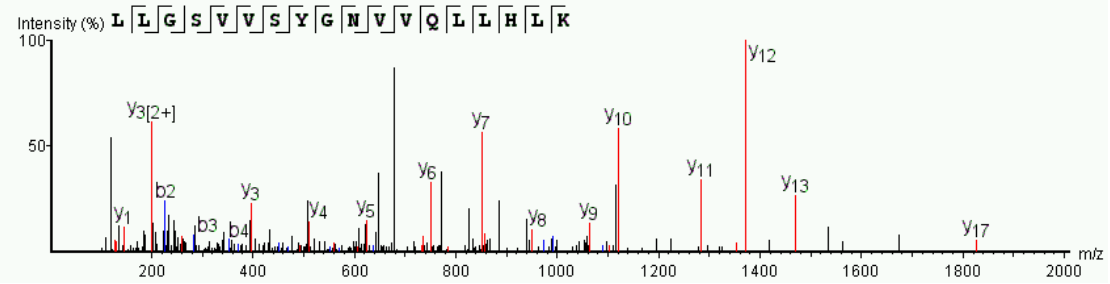

gi|328781115

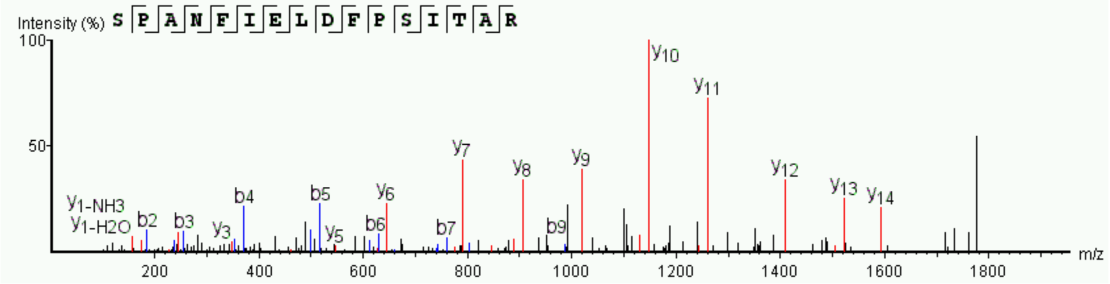

gi|571523088

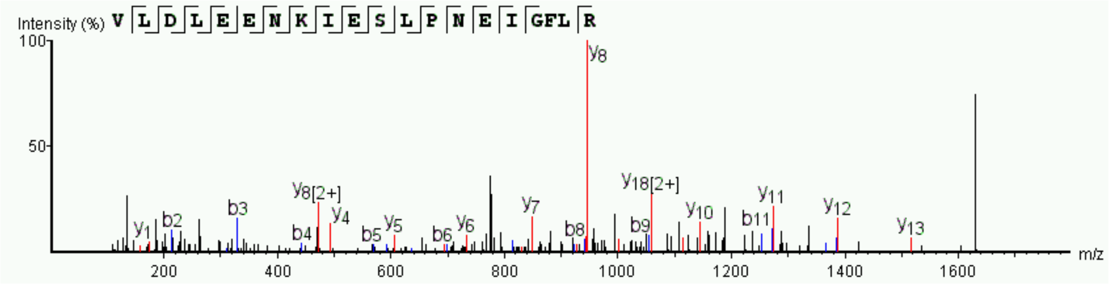

gi|328790105

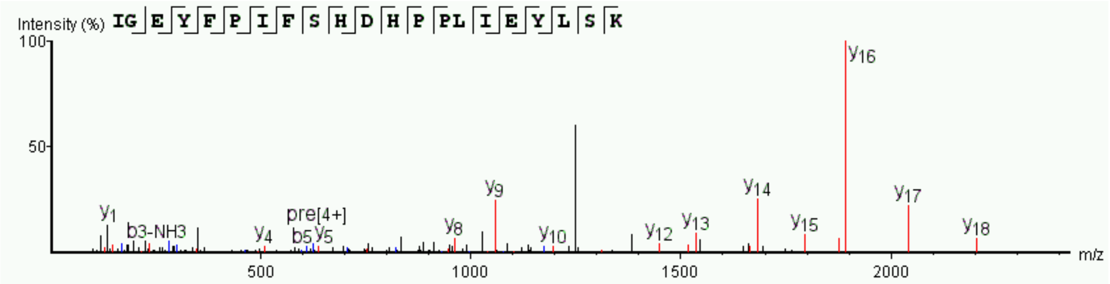

gi|571571431

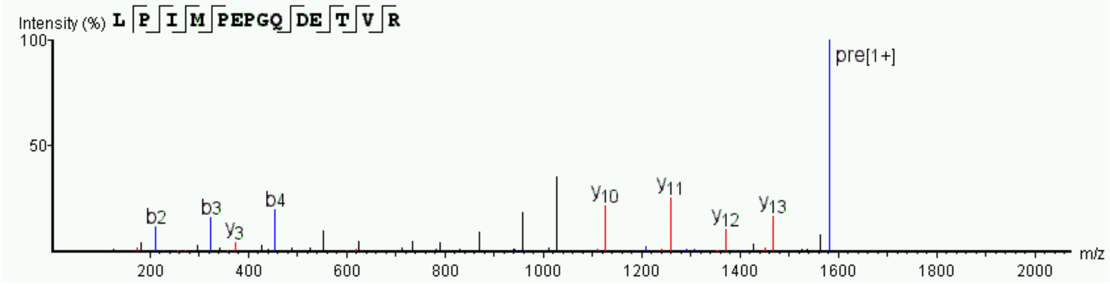

gi|571571434g

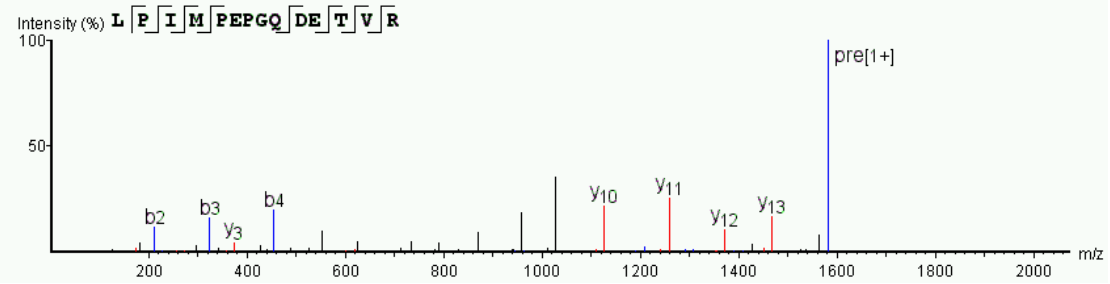

gi|571571223

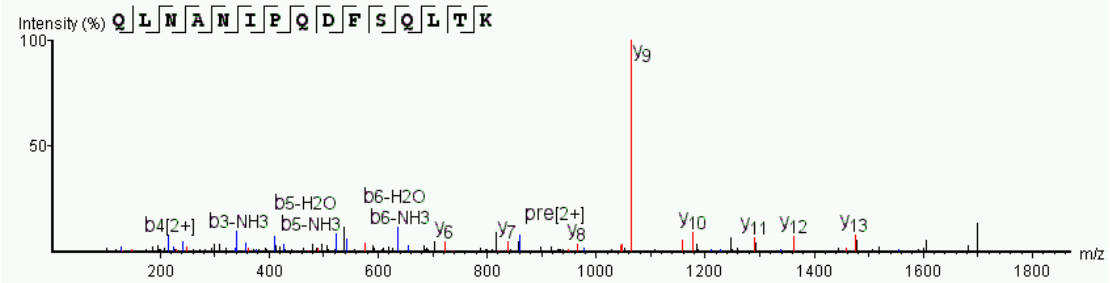

gi|571571261

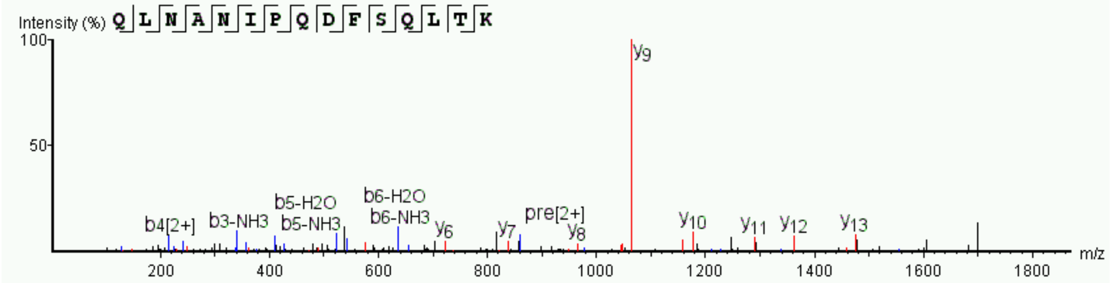

gi|571571265

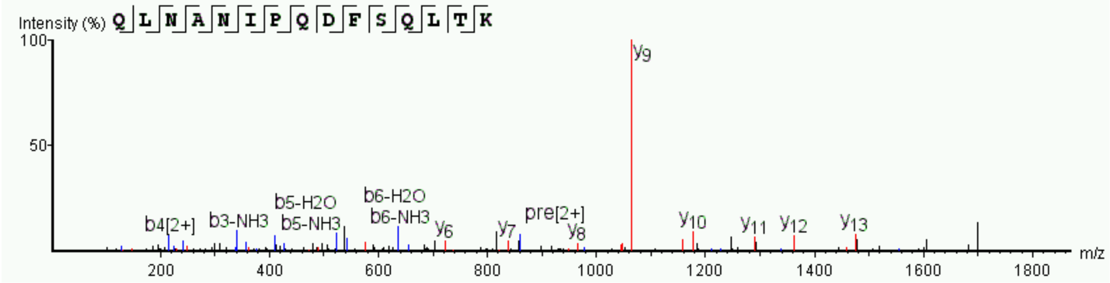

gi|571571269

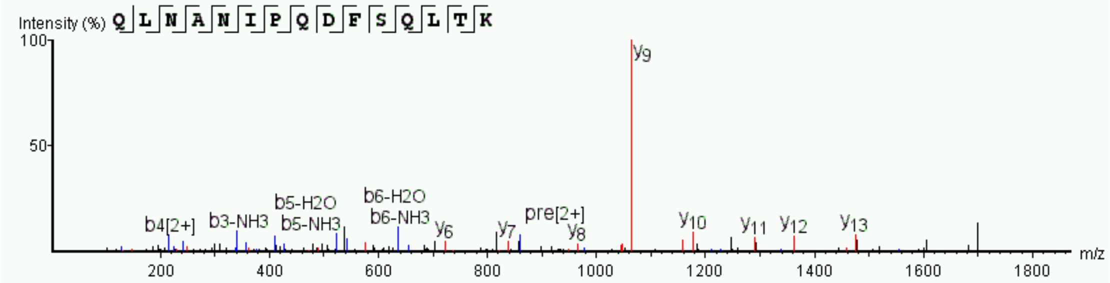

gi|571571279

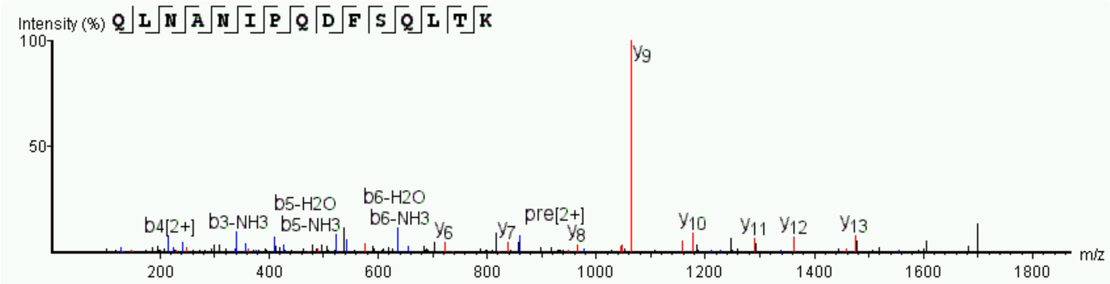

gi|571571227

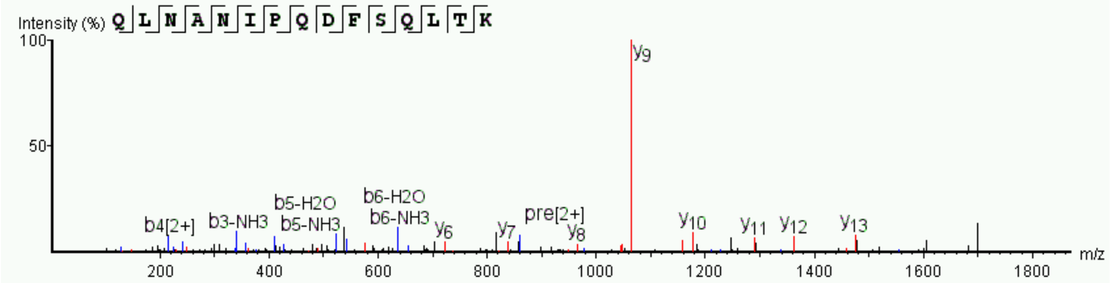

gi|571571231

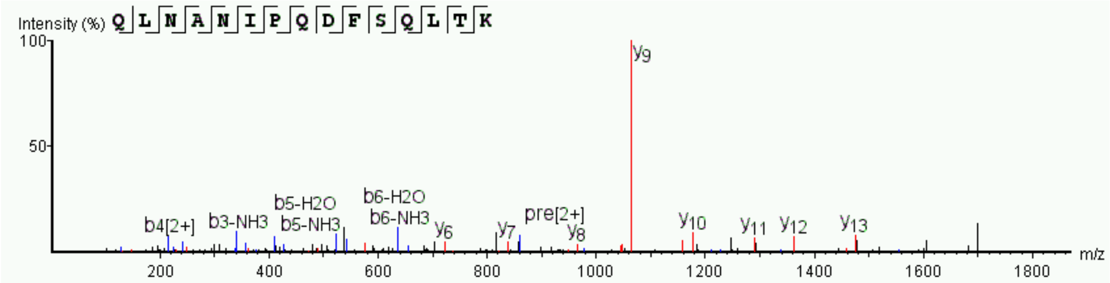

gi|571571235

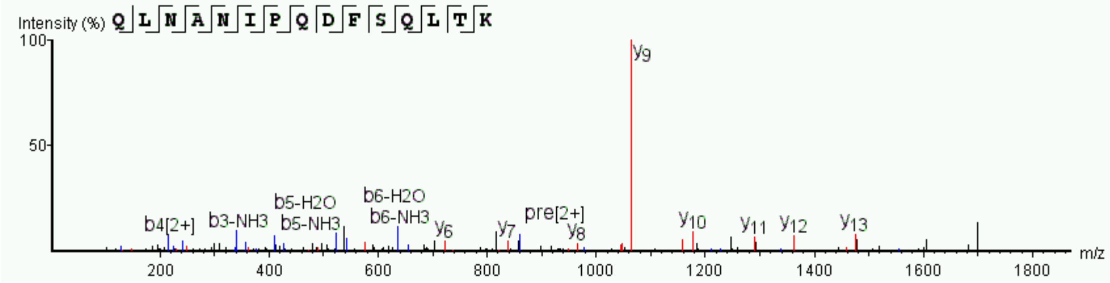

gi|571571239

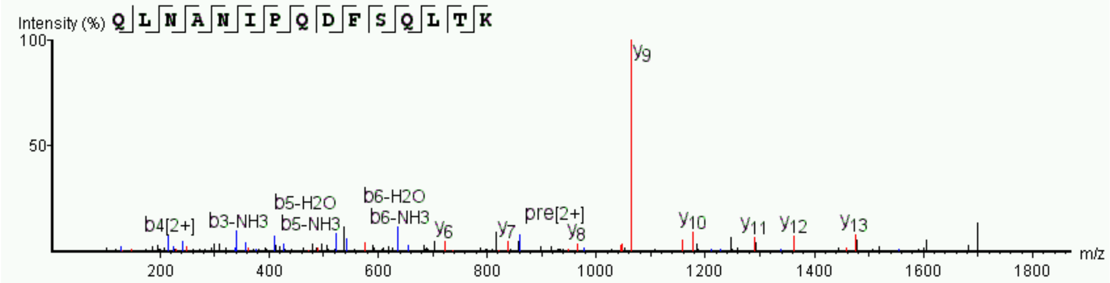

gi|571571243

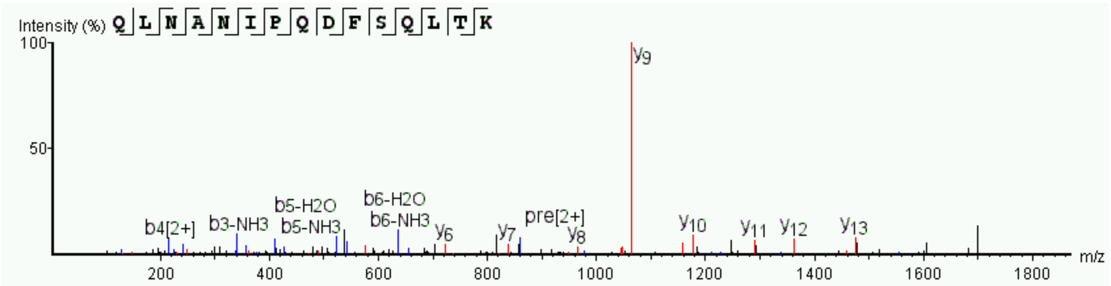

gi|571571249

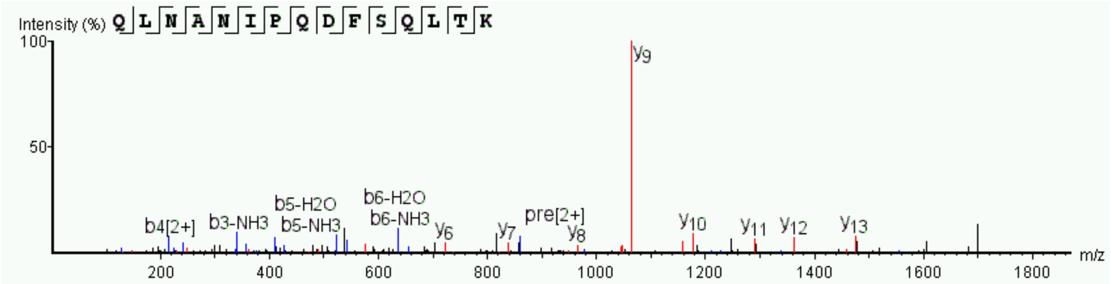

gi|571571253

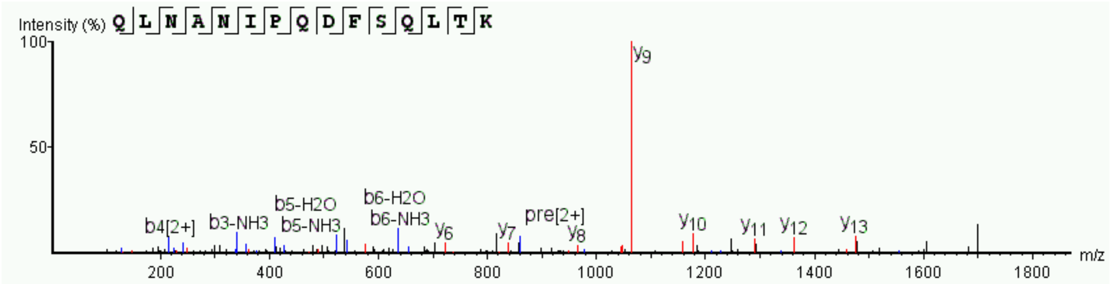

gi|571571257

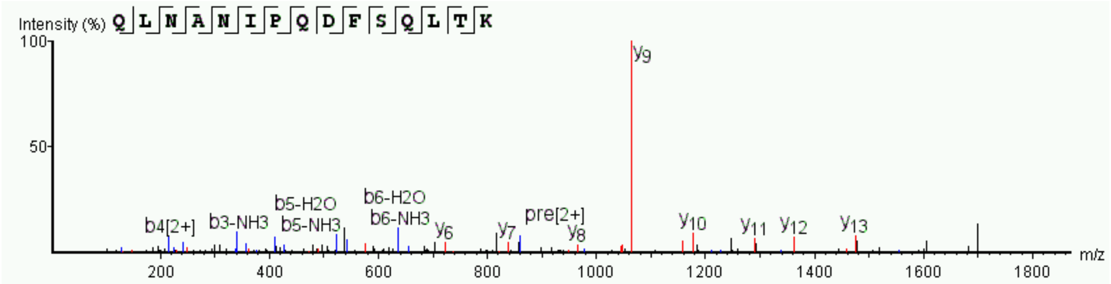

gi|571525572

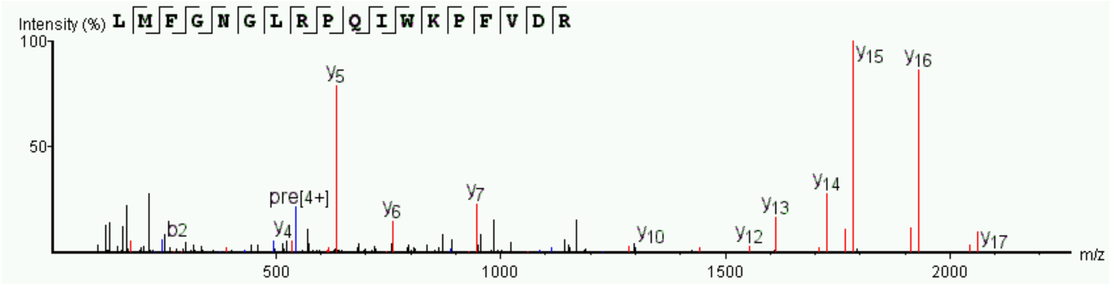

gi|571533533

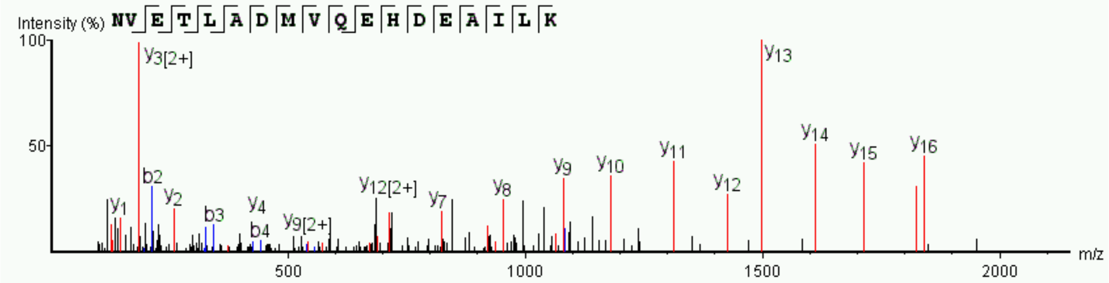

gi|571526489

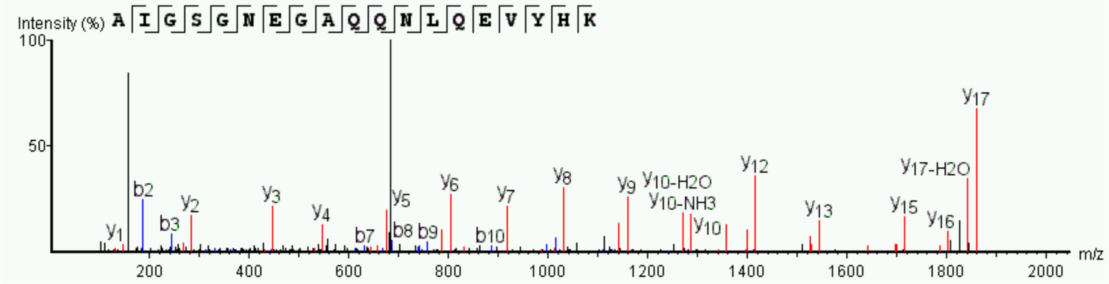

gi|571507709

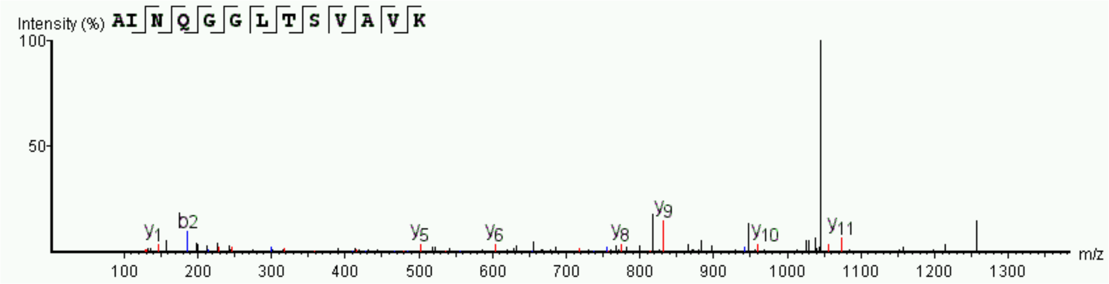

gi|571558139

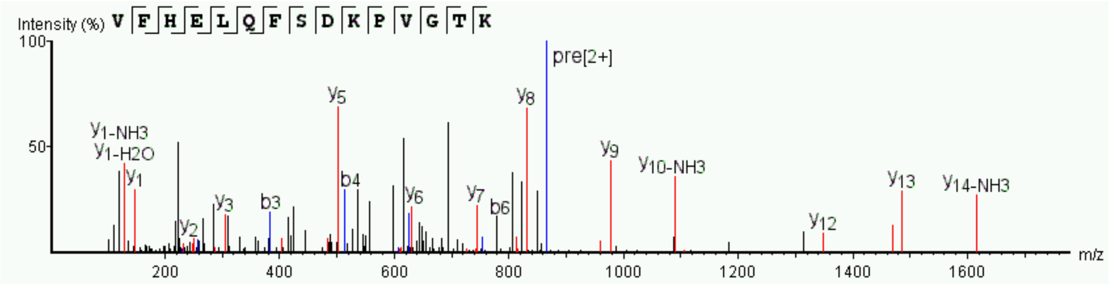

gi|571530529

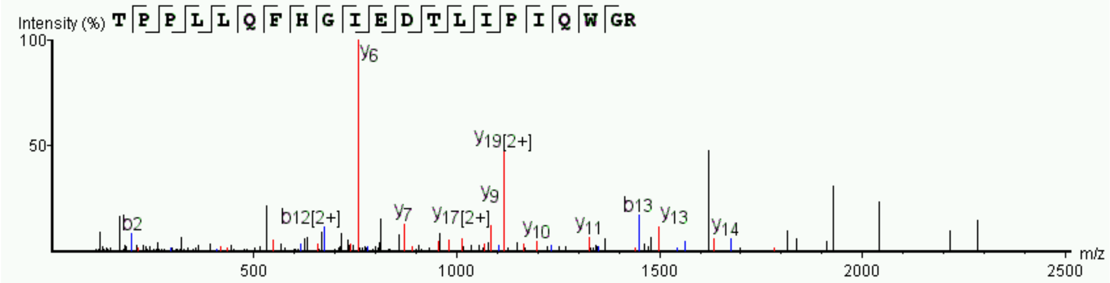

gi|571530533

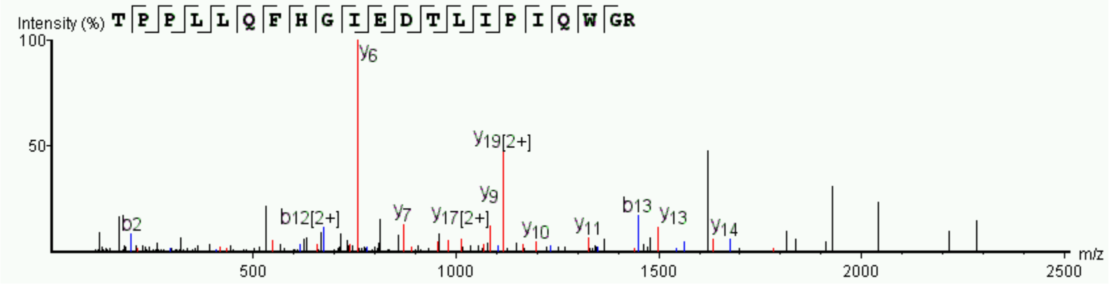

gi|571557461

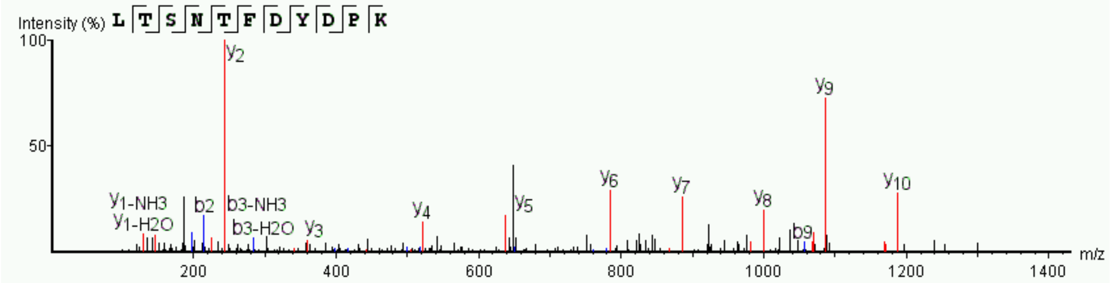

gi|571557464

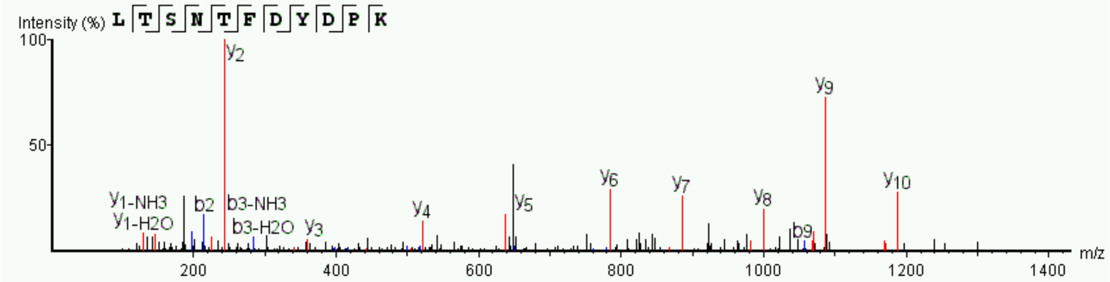

gi|571557467

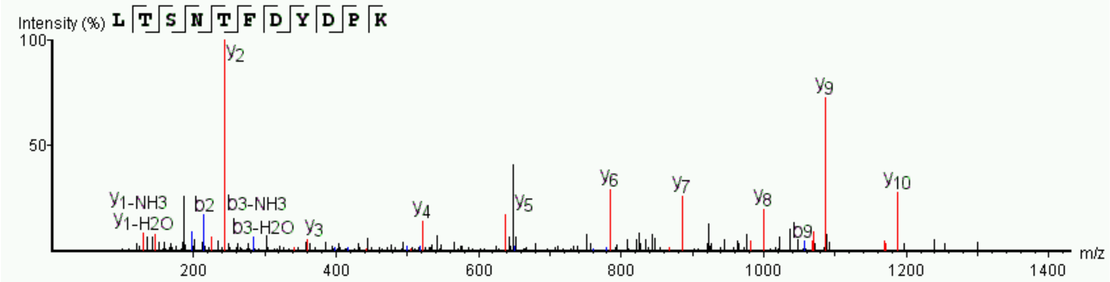

gi|571557471

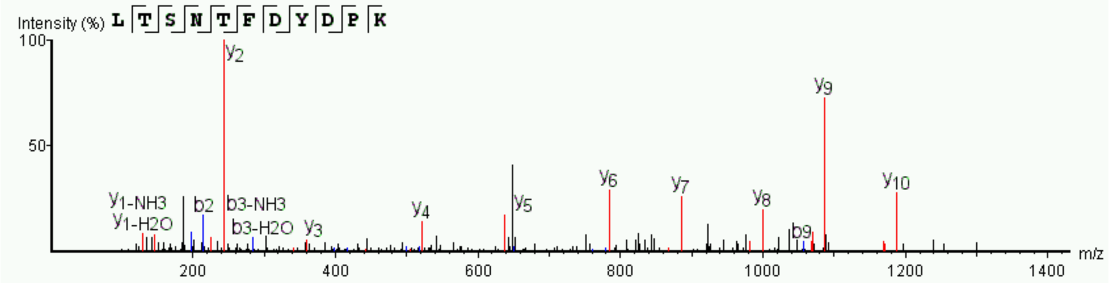

gi|328790379

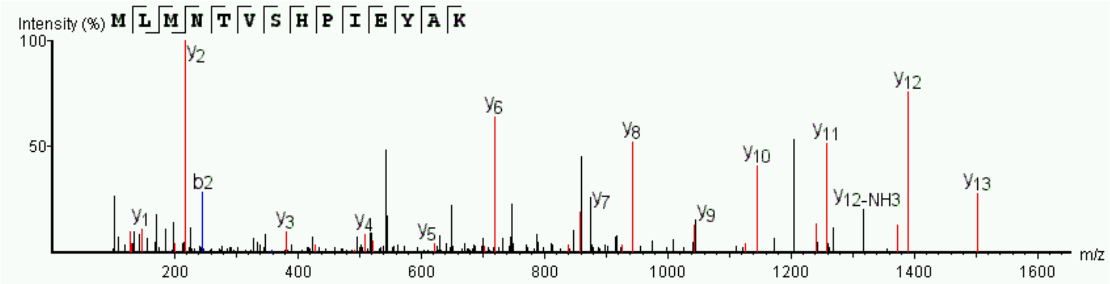

gi|571504099

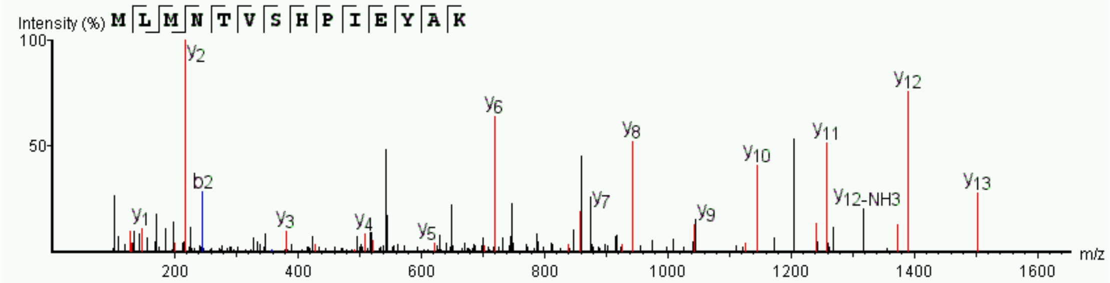

gi|571504104

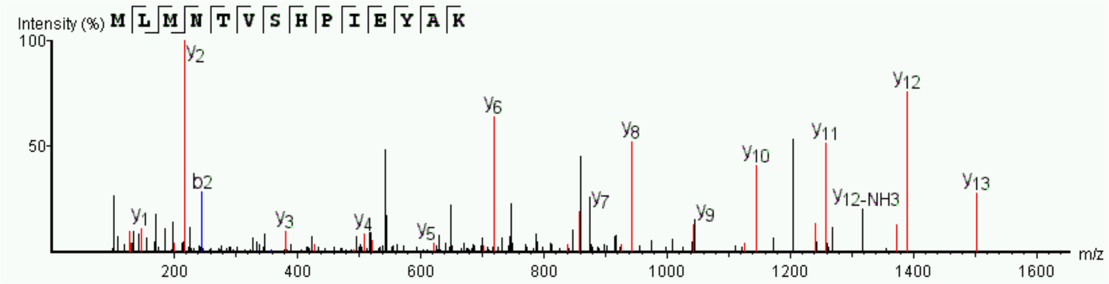

gi|110749921

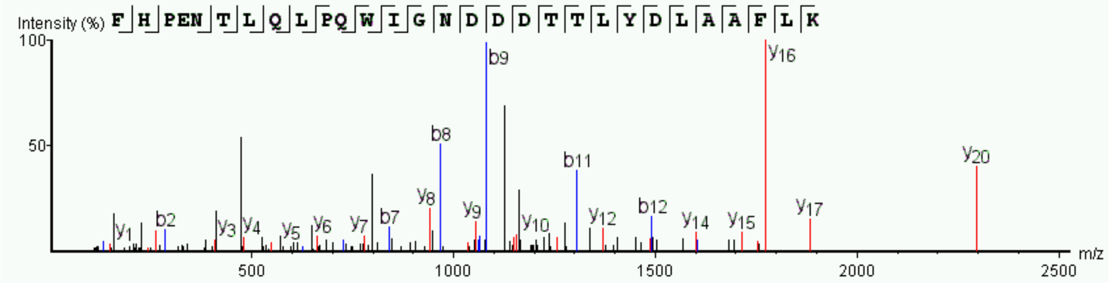

gi|110760068

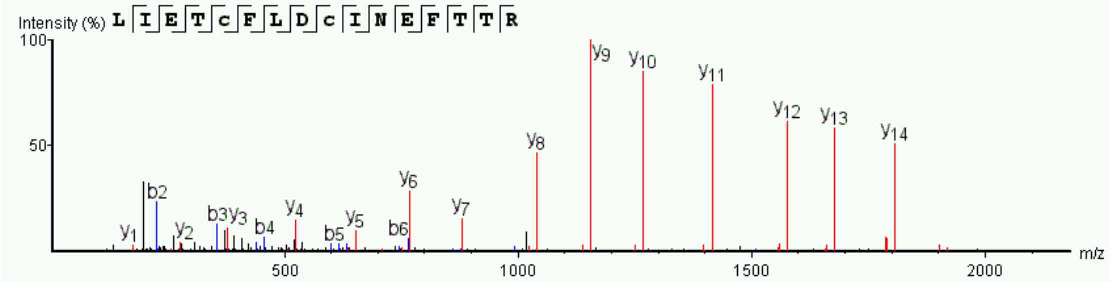

gi|571553163

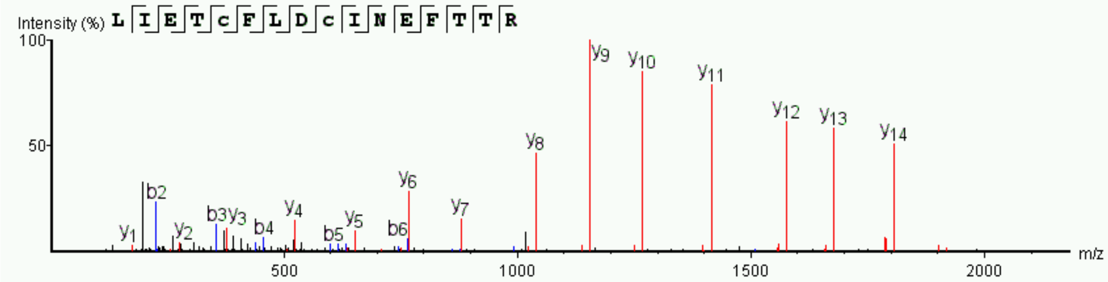

gi|328779694

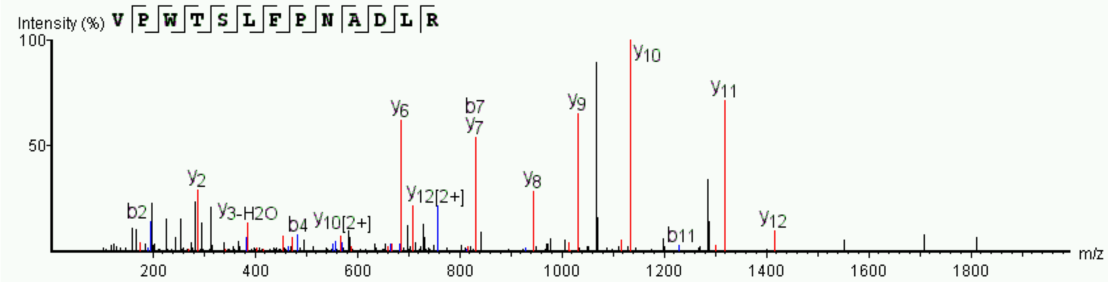

gi|110748745

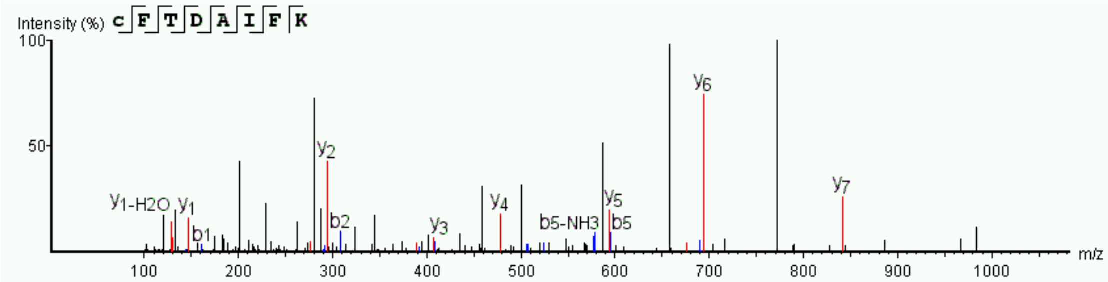

gi|328782218

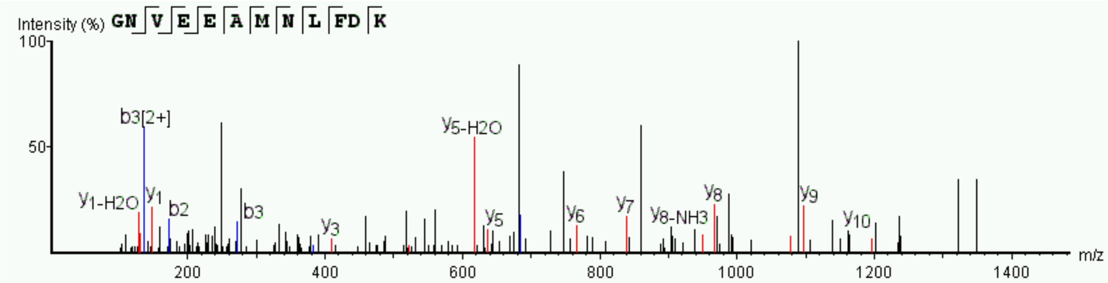

gi|48105316

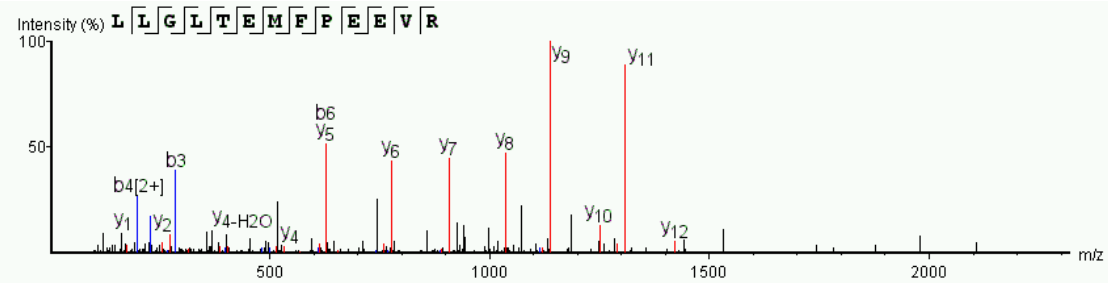

gi|571510158

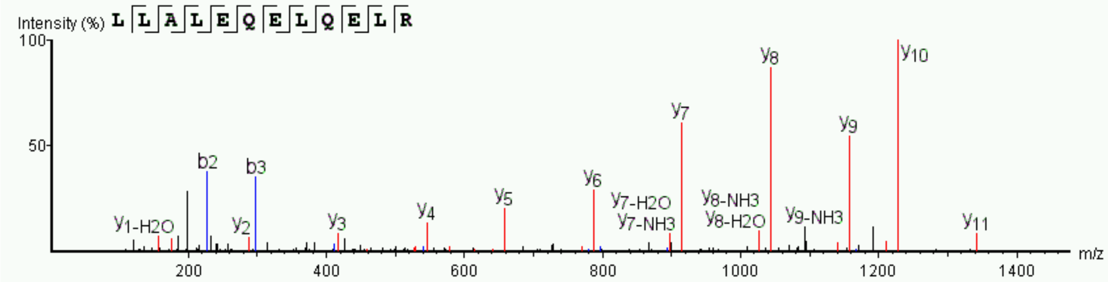

gi|571574786

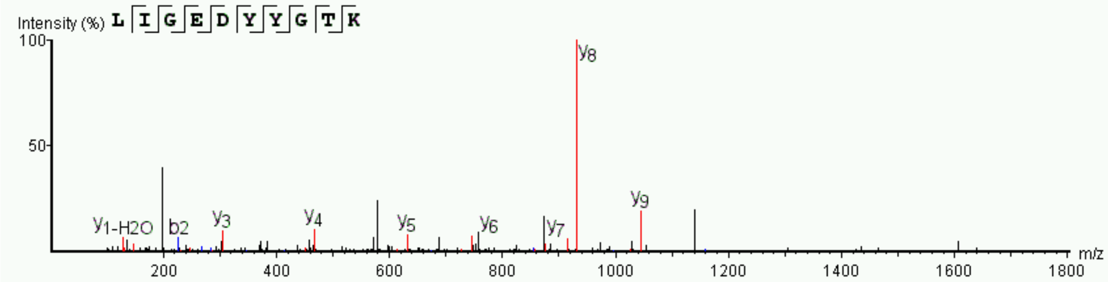

gi|571568566

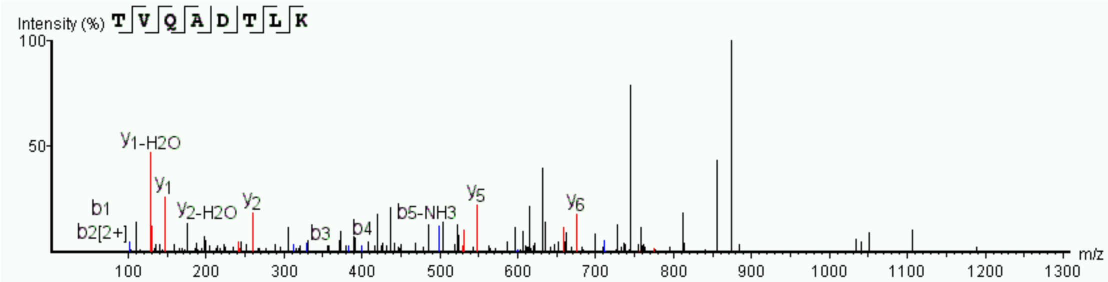

gi|328778643

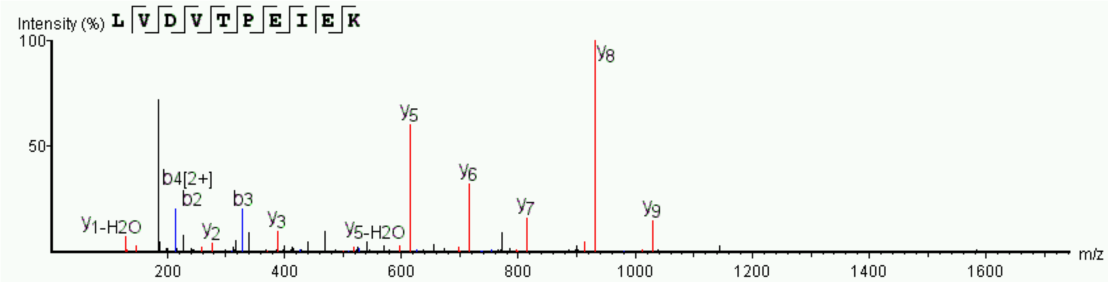

gi|571508626

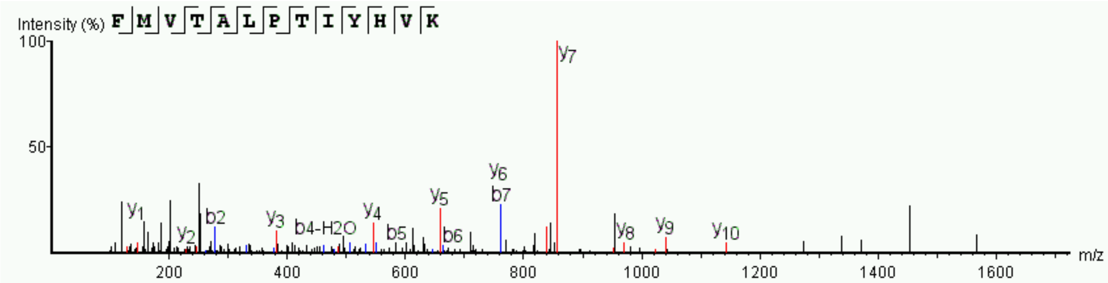

gi|328787116

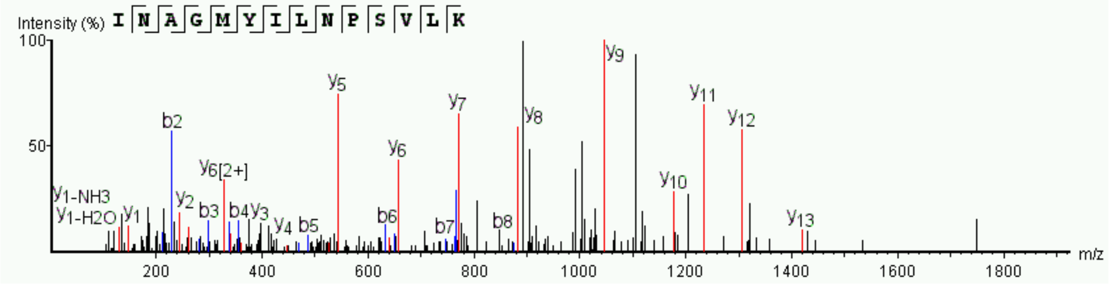

gi|110750888

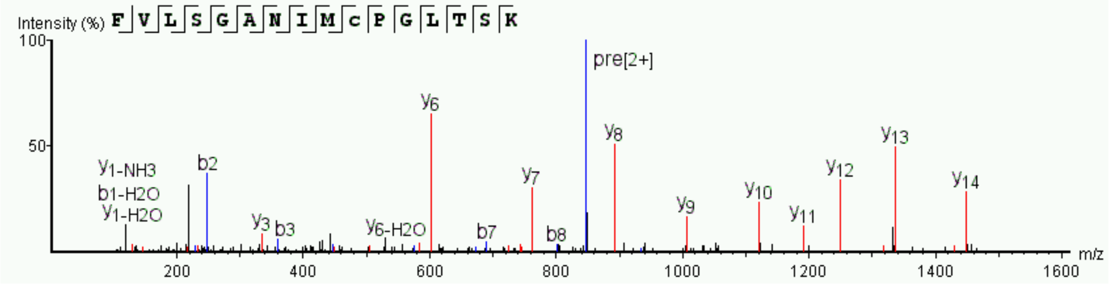

gi|571503608

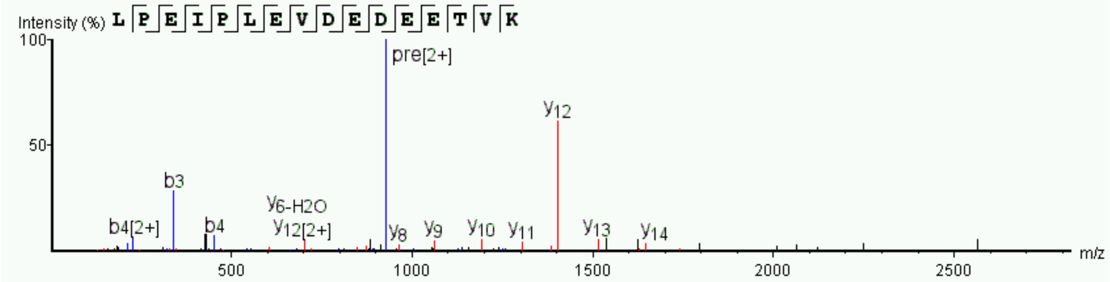

gi|571503611

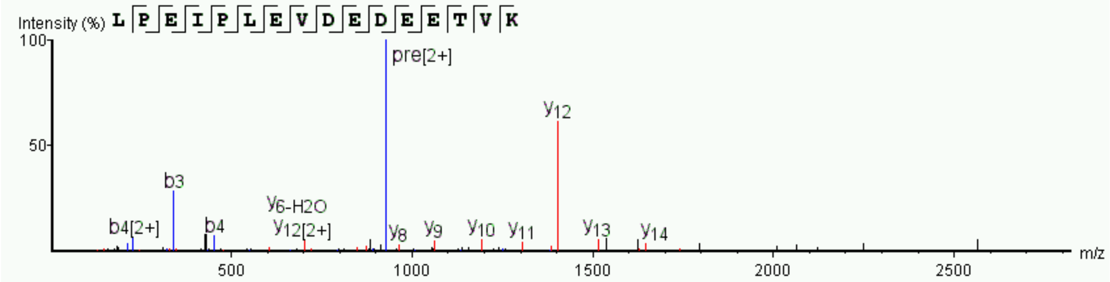

gi|571503615

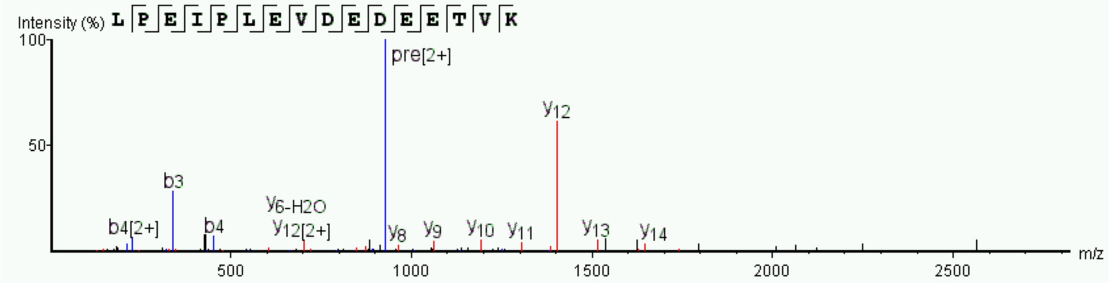

gi|48139441

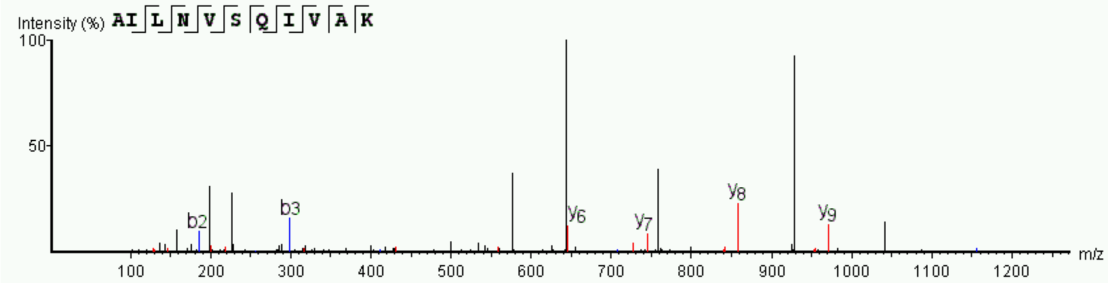

gi|571573648

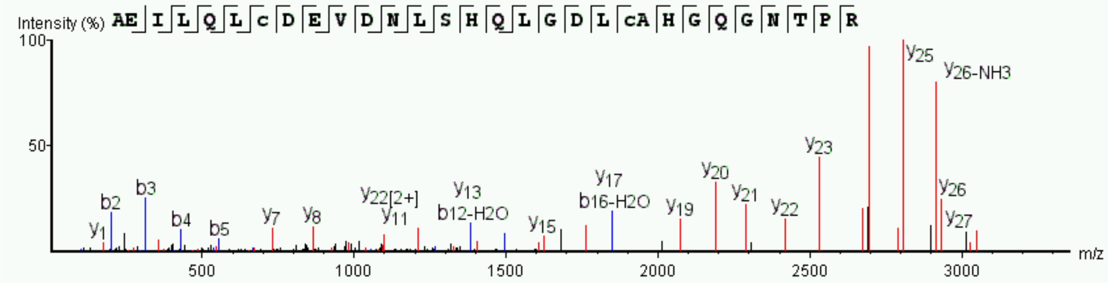

gi|571520775

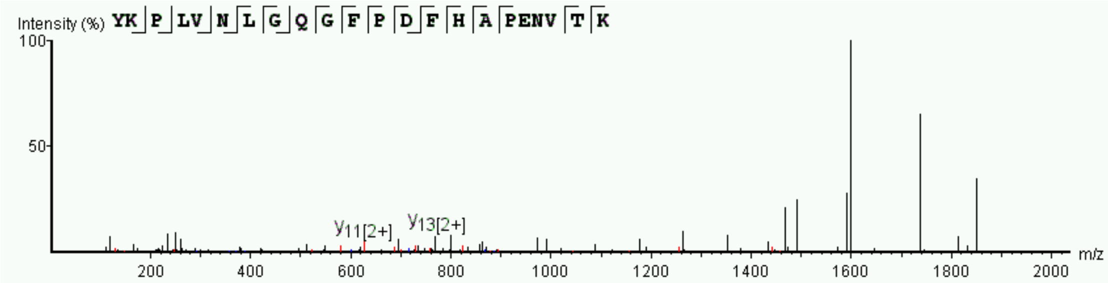

gi|571507579

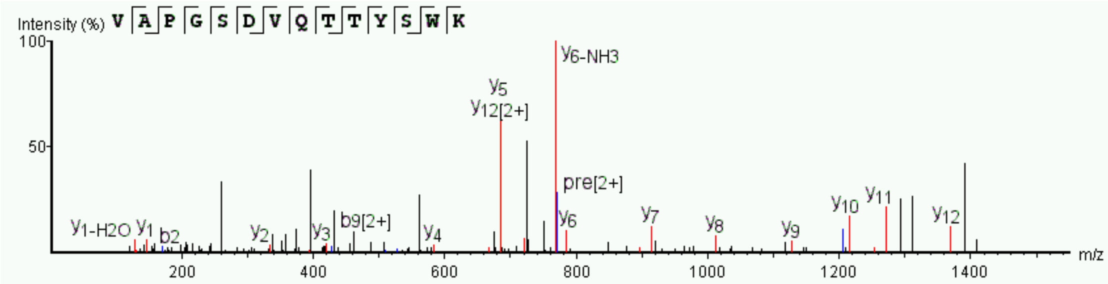

gi|571569364

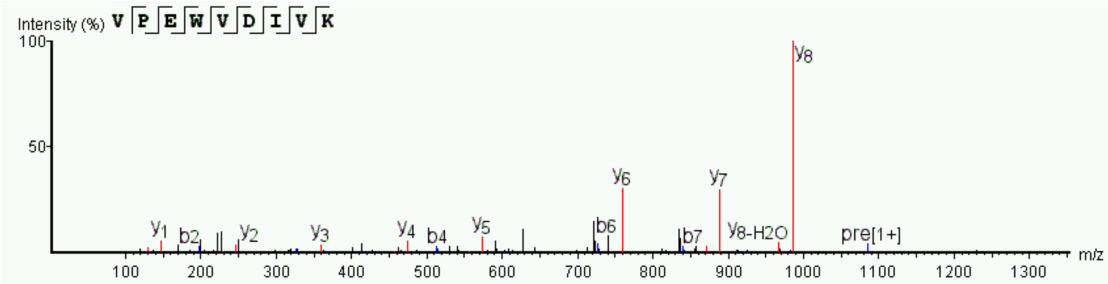

gi|66508366

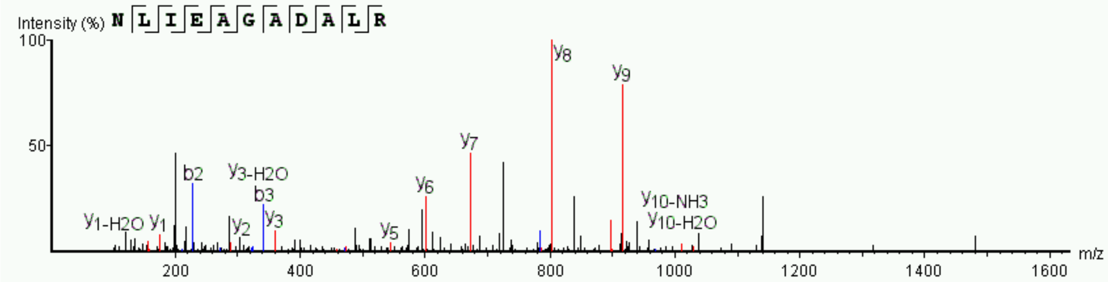

gi|571522614

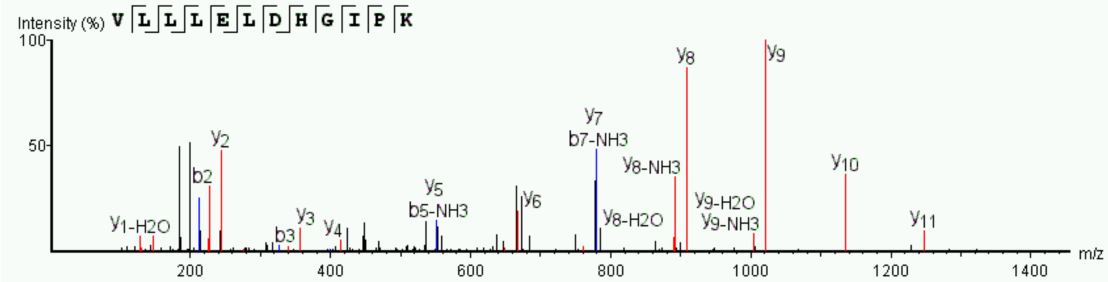

gi|66560294

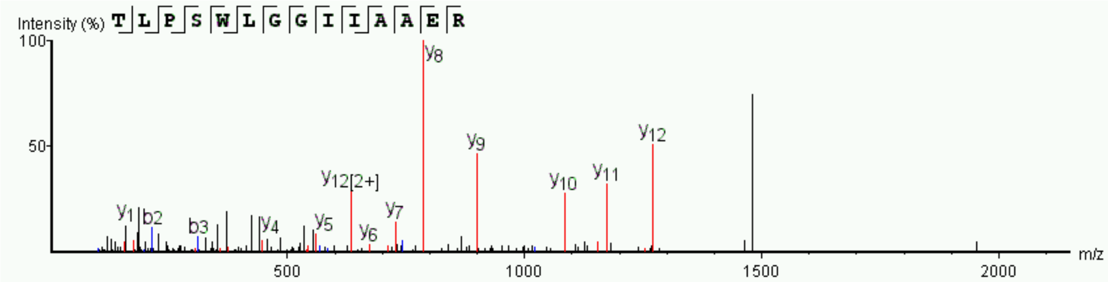

gi|571534155

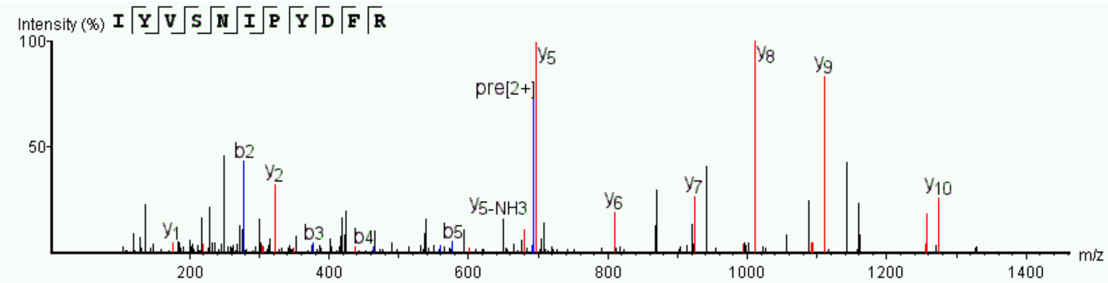

gi|571577935

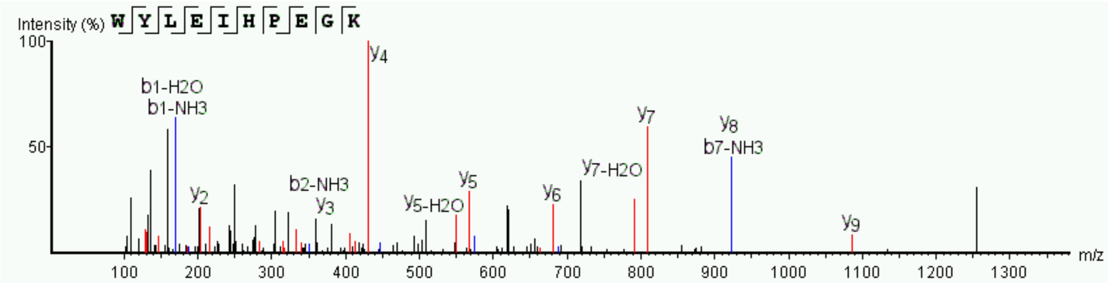

gi|571568097

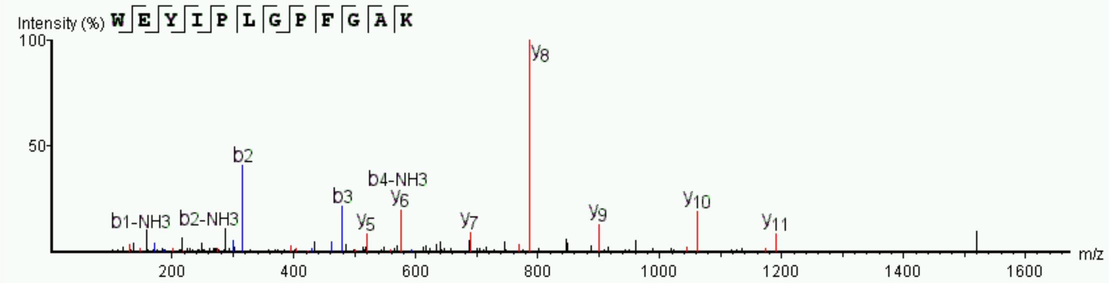

gi|571576209

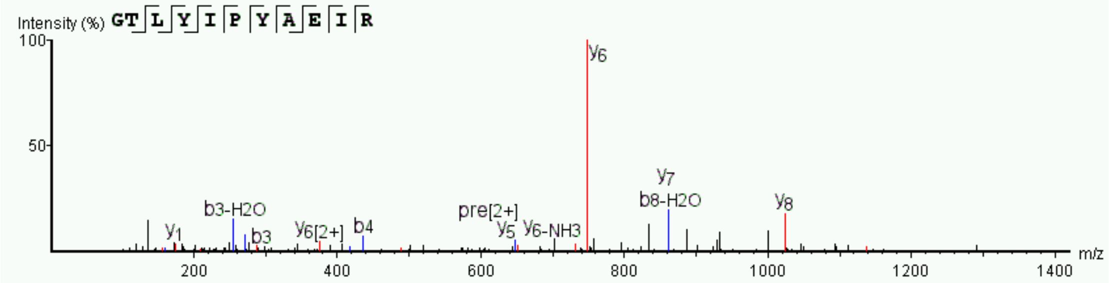

gi|571539574

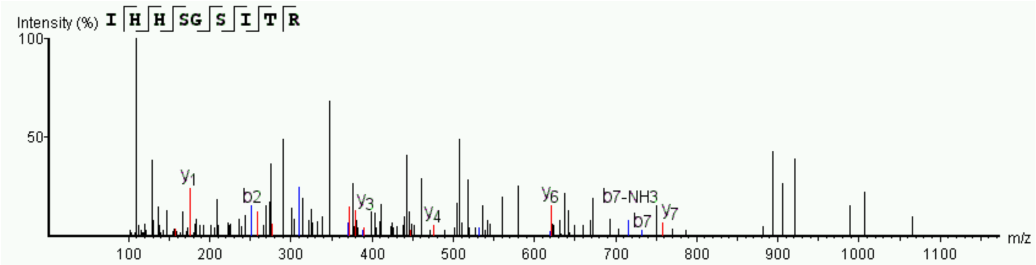

gi|571539517

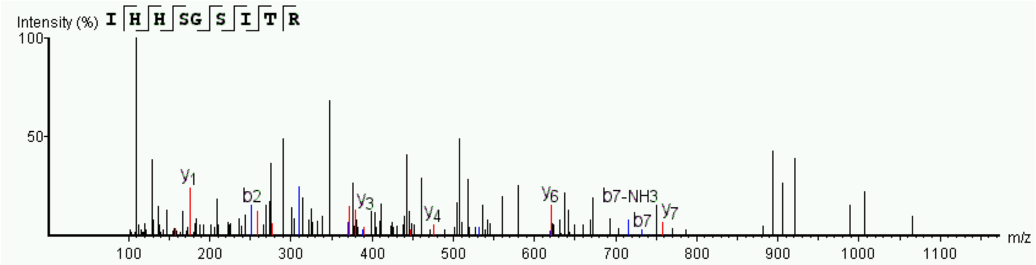

gi|66515288

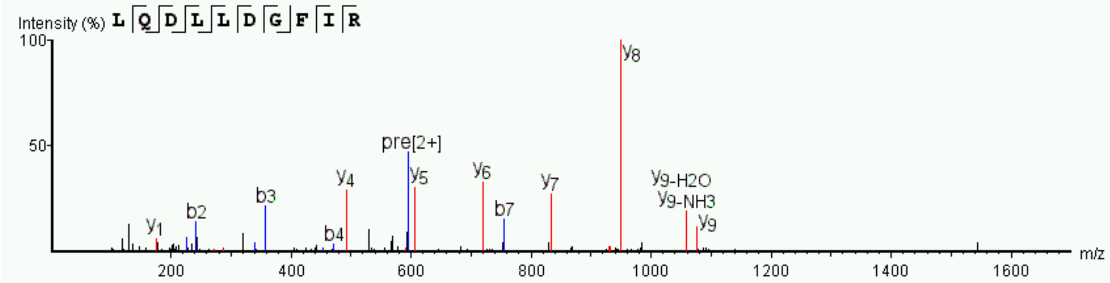

gi|571550203

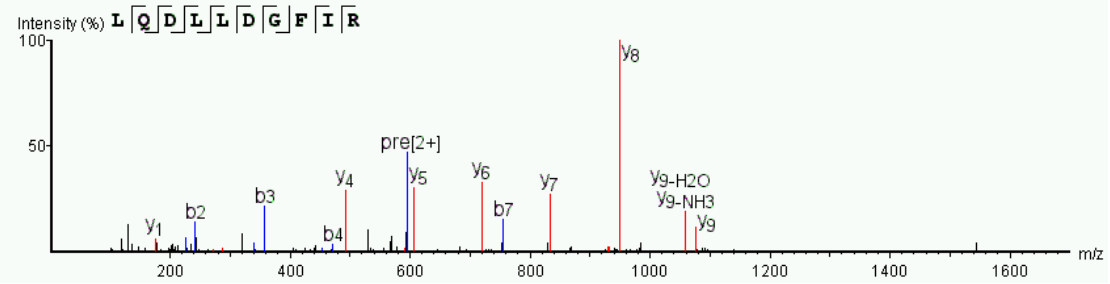

gi|66545512

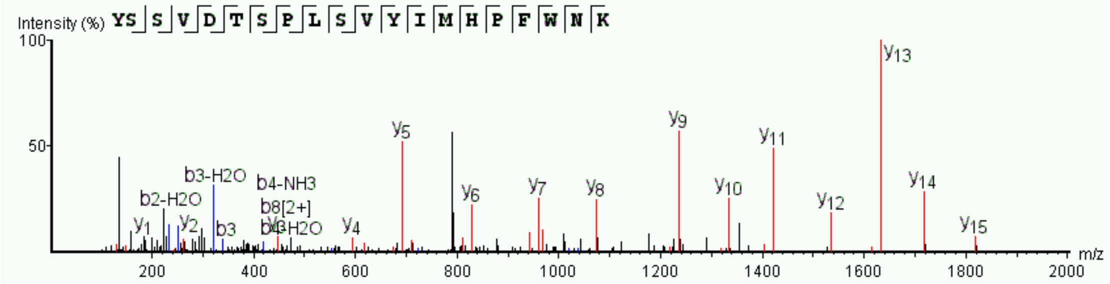

gi|571533068

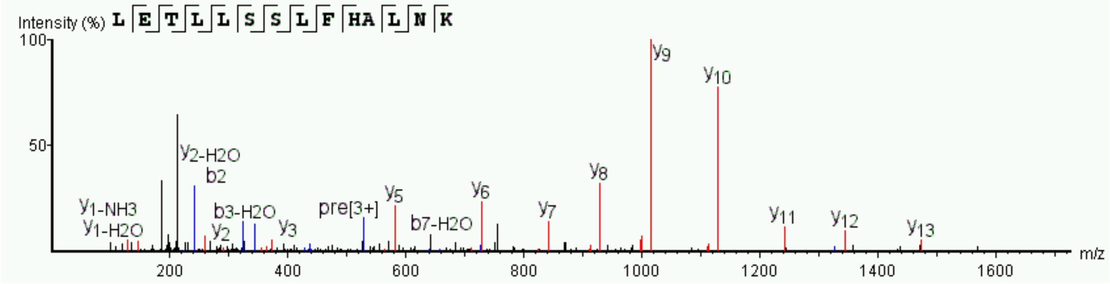

gi|571533072

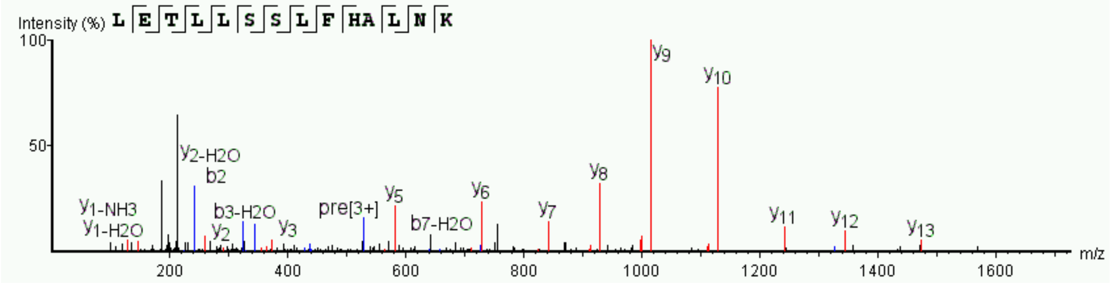

gi|571533076

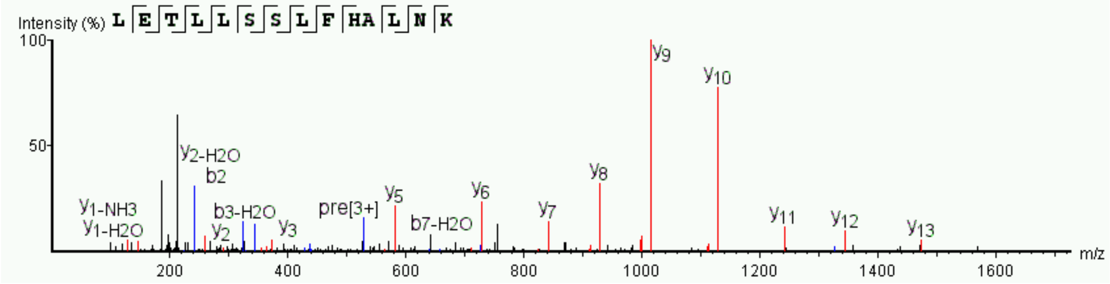

gi|571533080

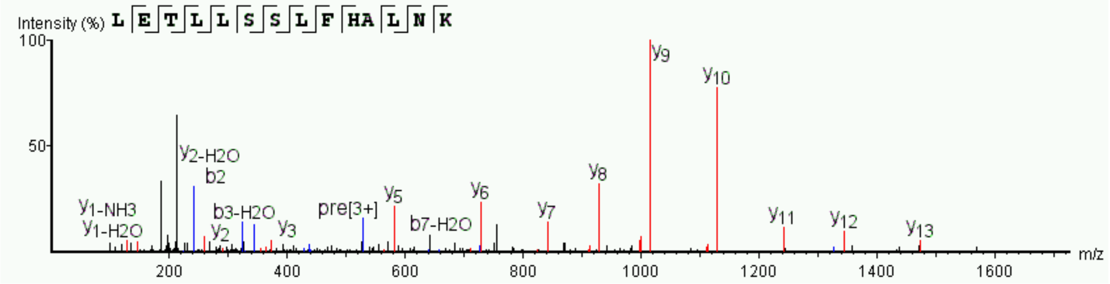

gi|571575755

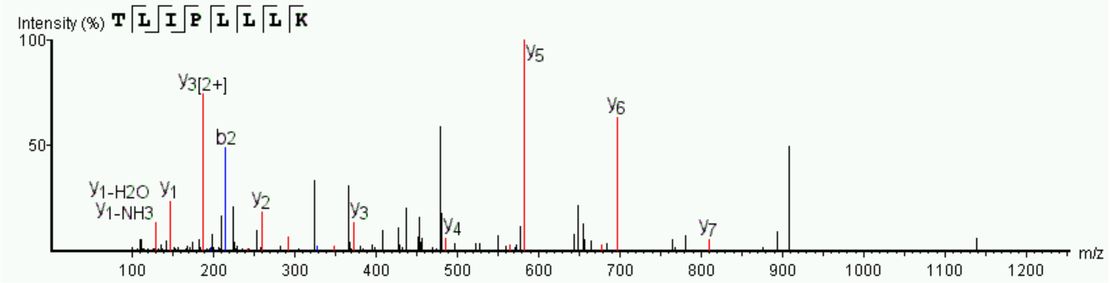

gi|328777707

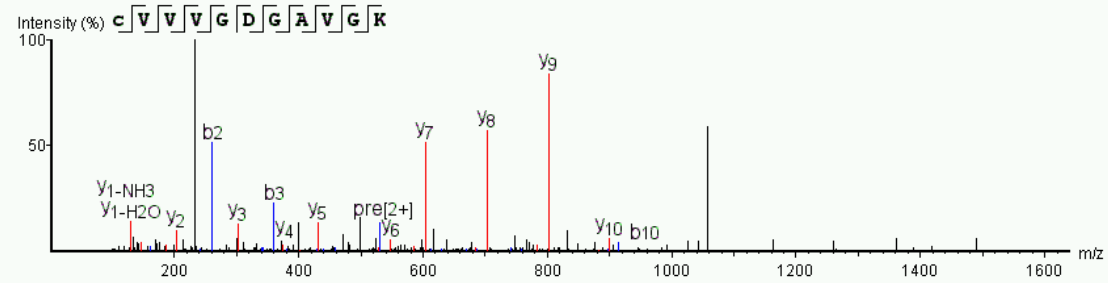

gi|571575329

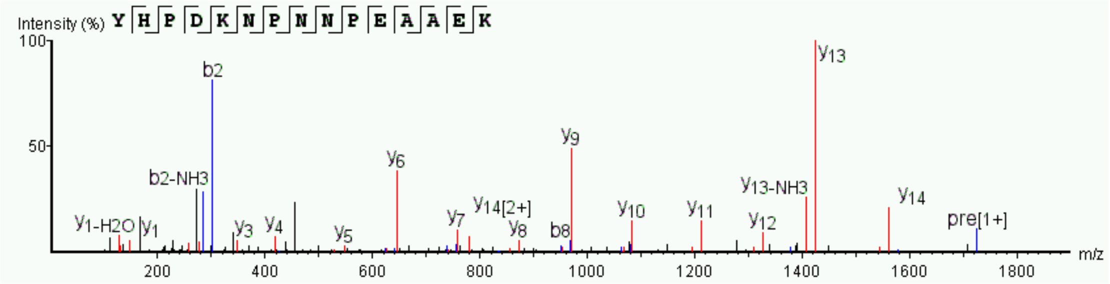

gi|571562181

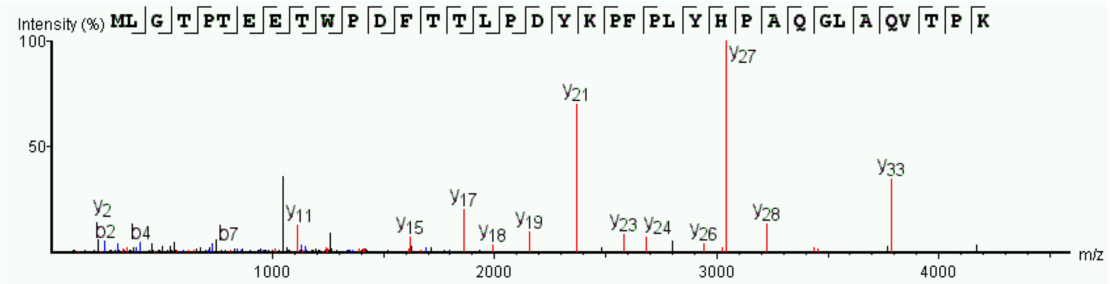

gi|571537005

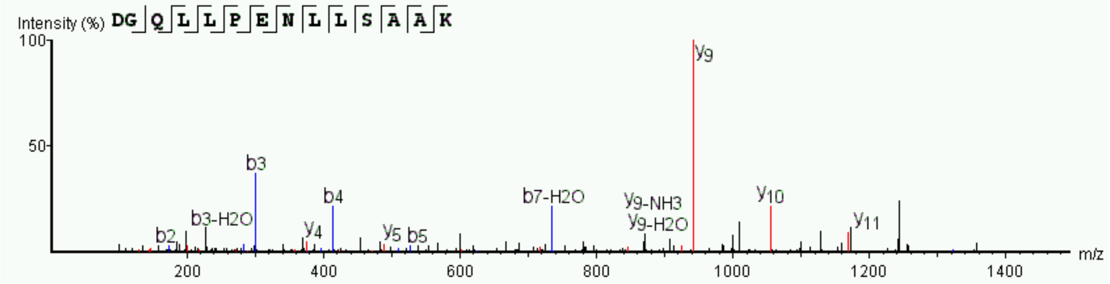

gi|48106841

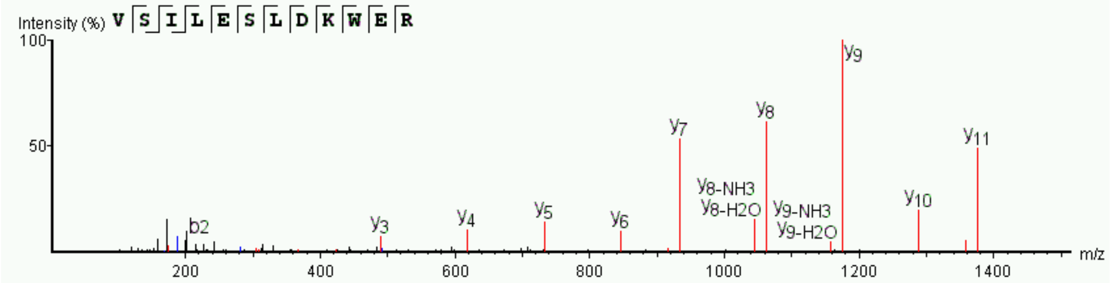

gi|571549109

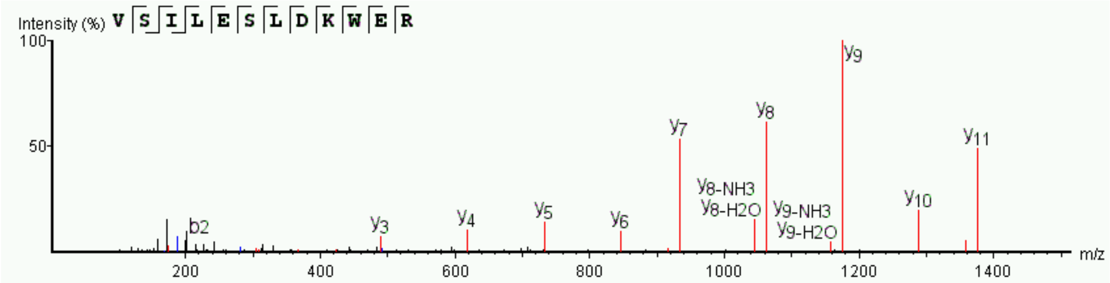

gi|571549112

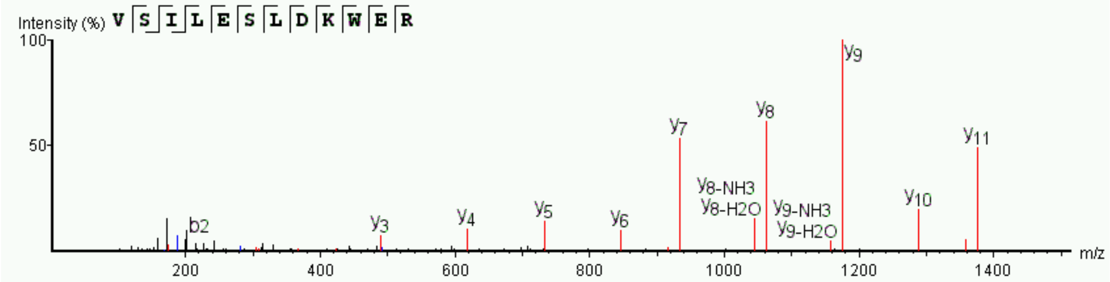

gi|571549115

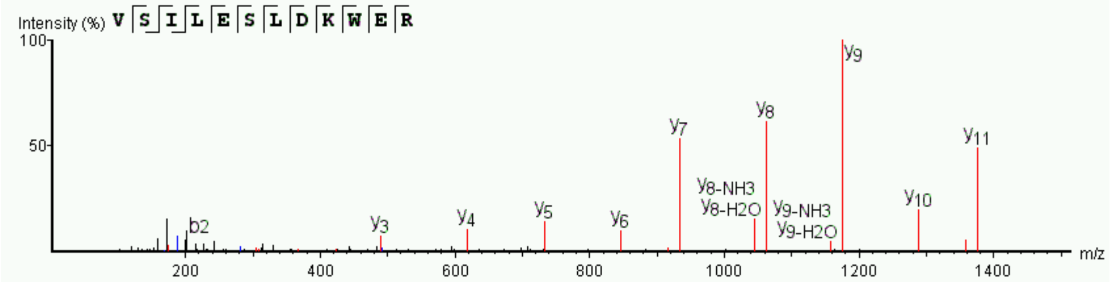

gi|571549118

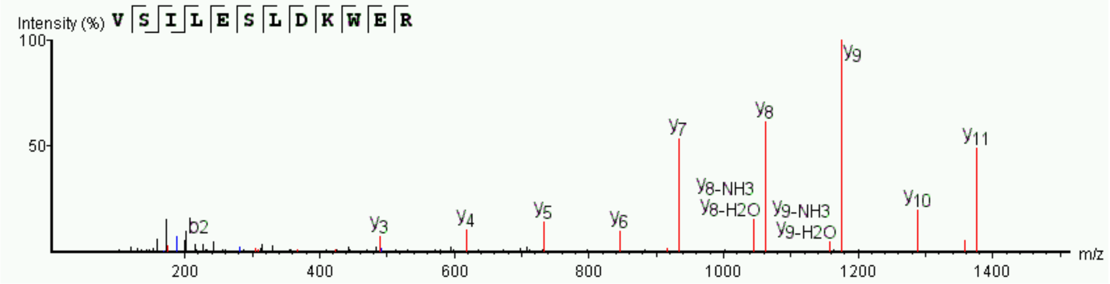

gi|571546273

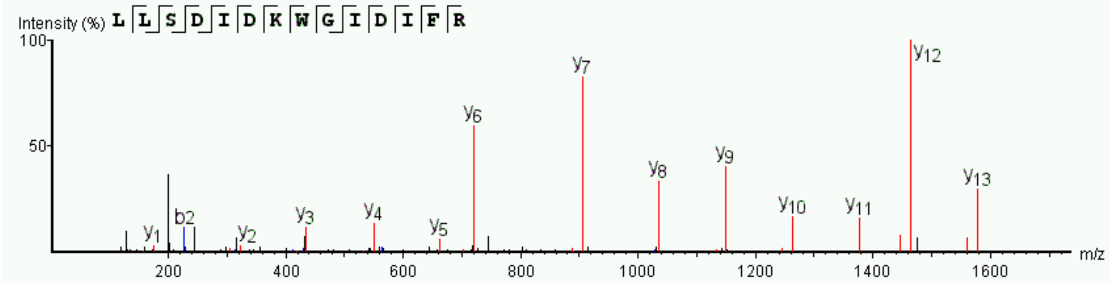

gi|571546308

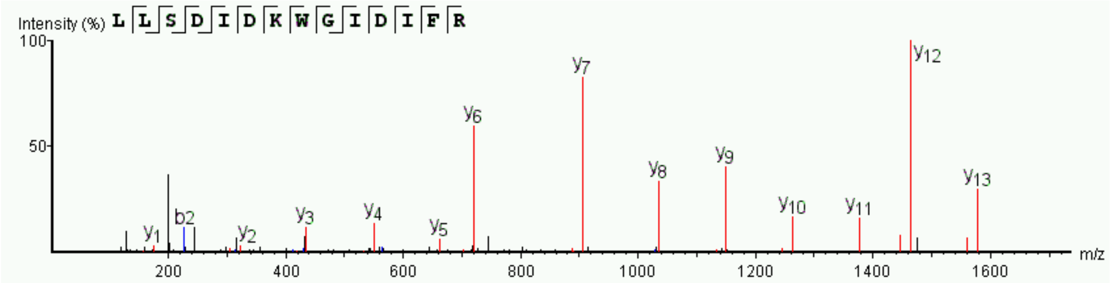

gi|571546312

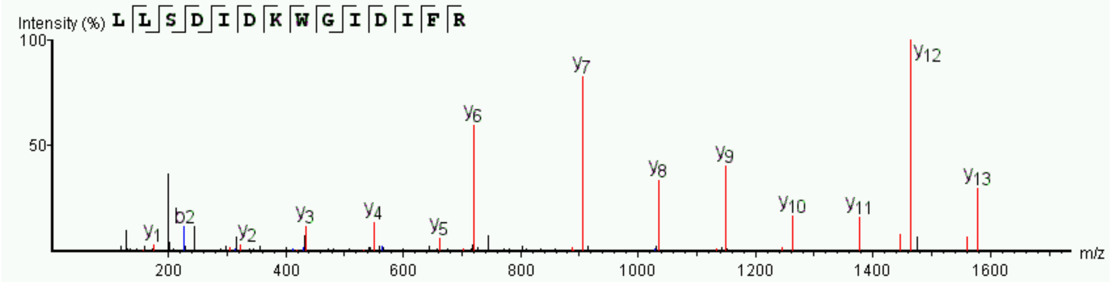

gi|571546316

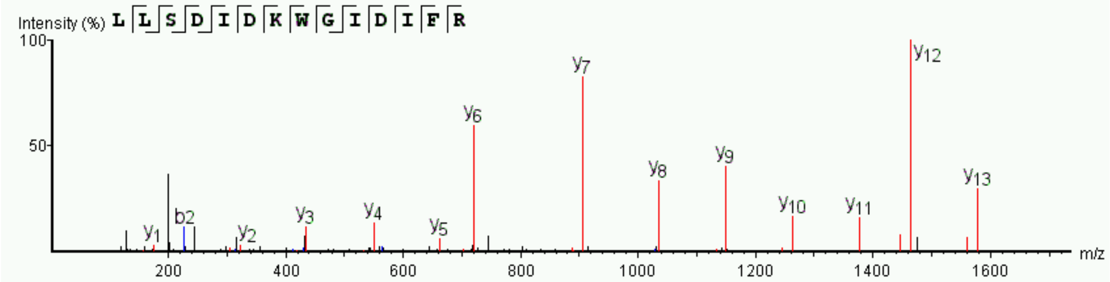

gi|571546320

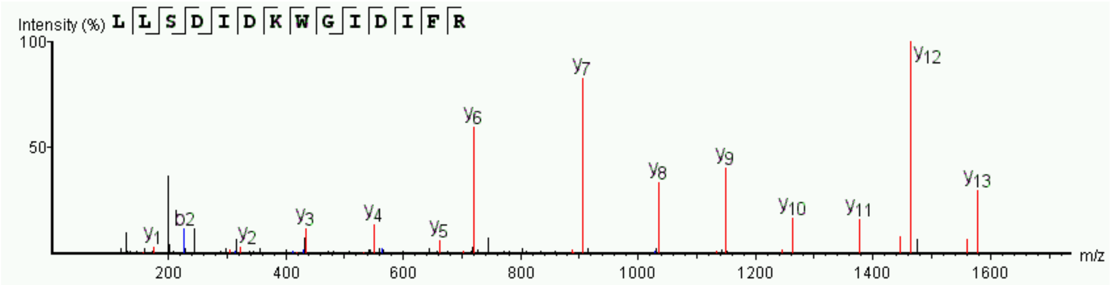

gi|571546323

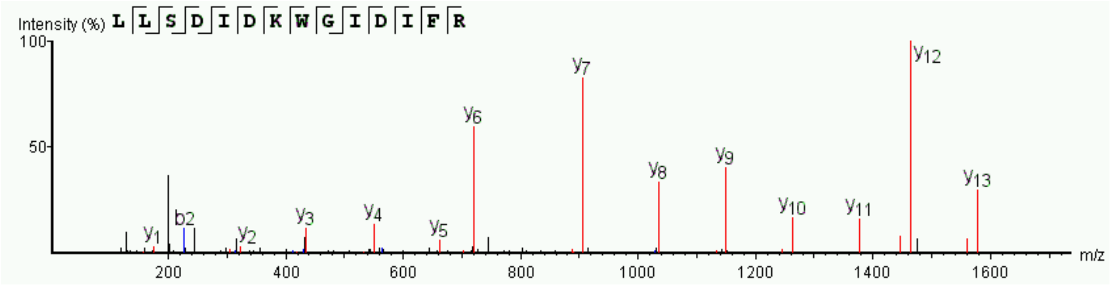

gi|571546326

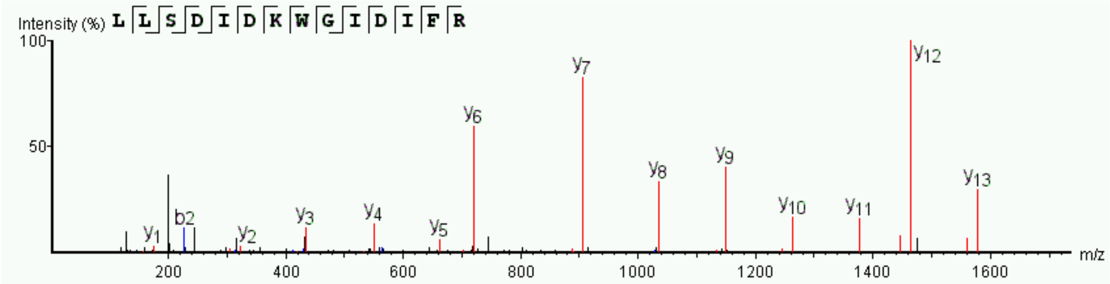

gi|571546330

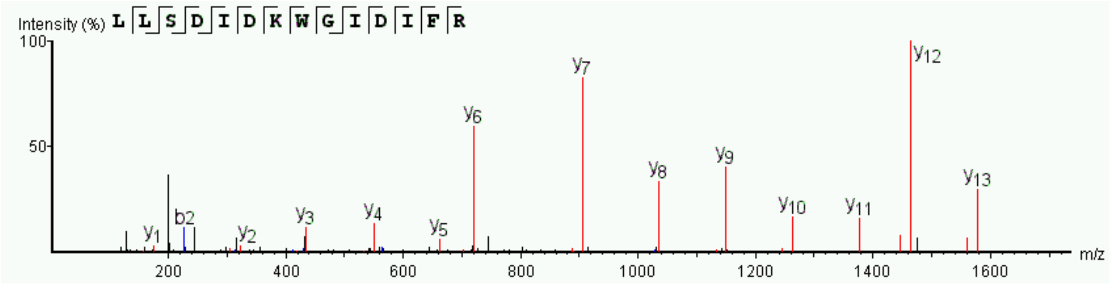

gi|571546334

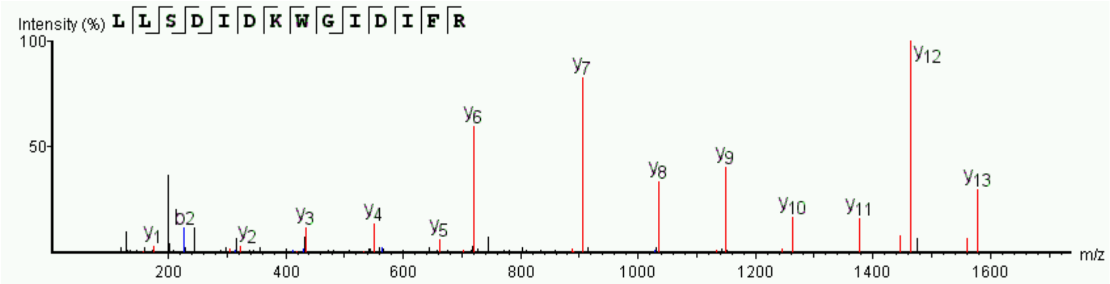

gi|571546338

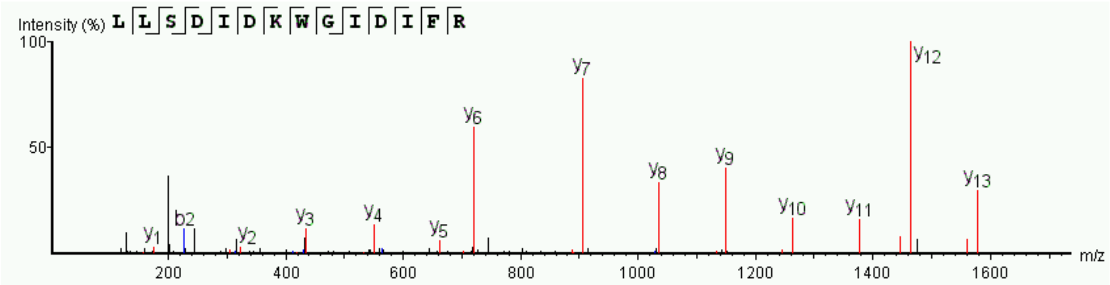

gi|571546341

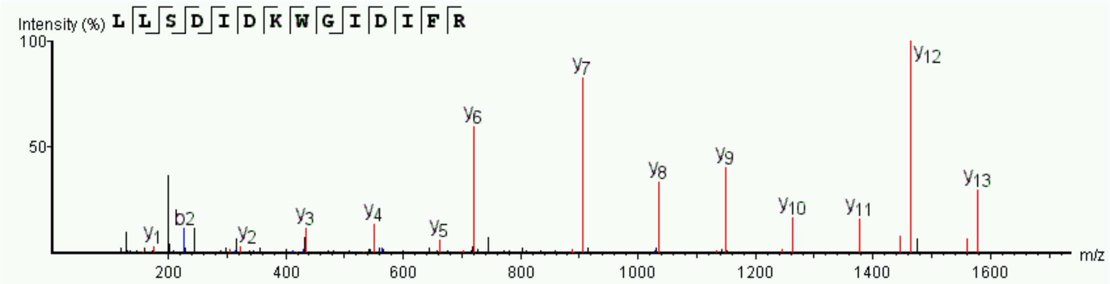

gi|571546277

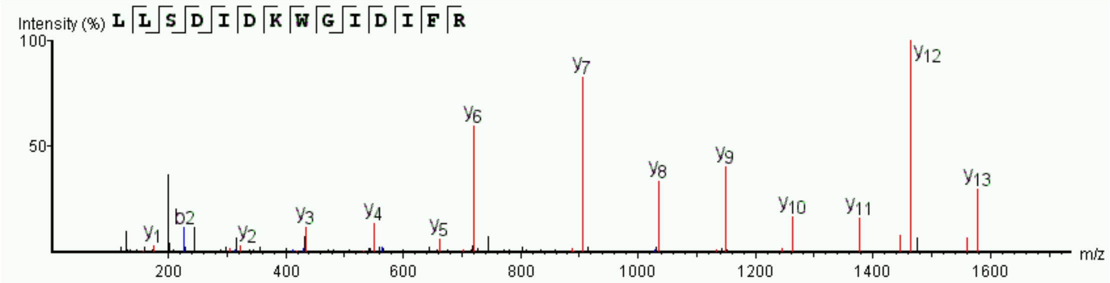

gi|328789199

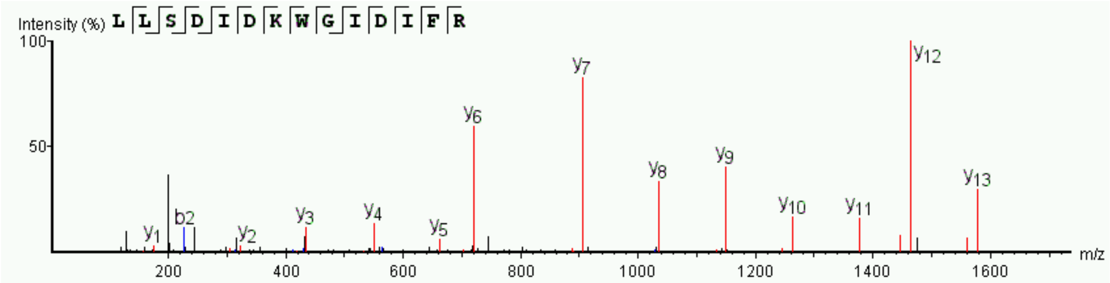

gi|571546281

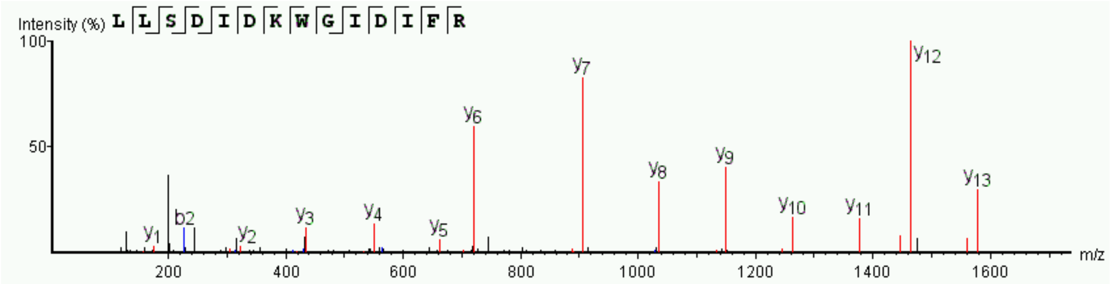

gi|571546285

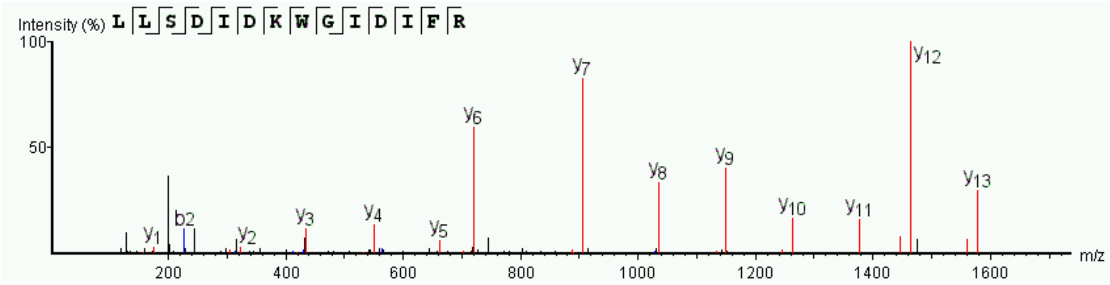

gi|571546289

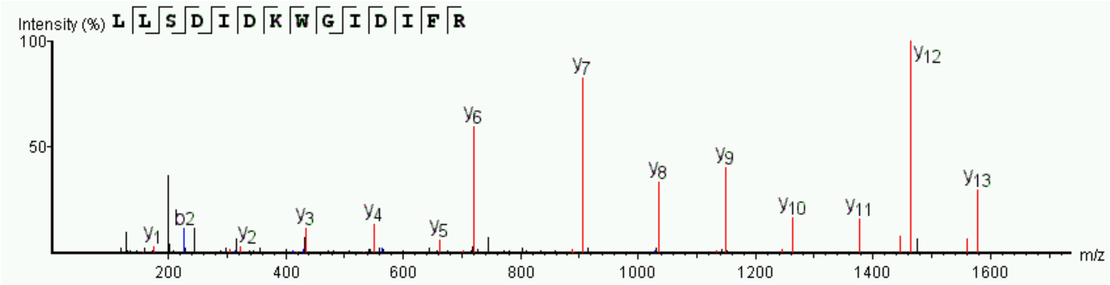

gi|571546293

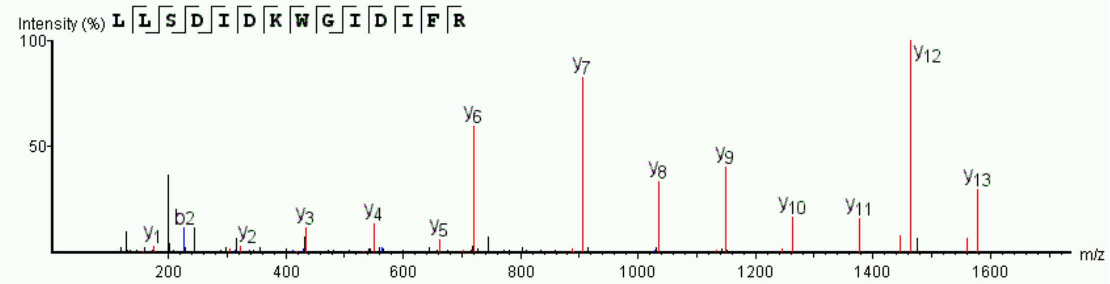

gi|571546296

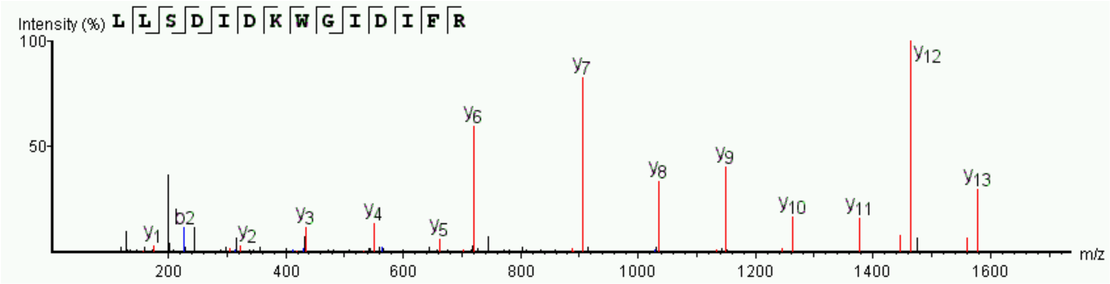

gi|571546300

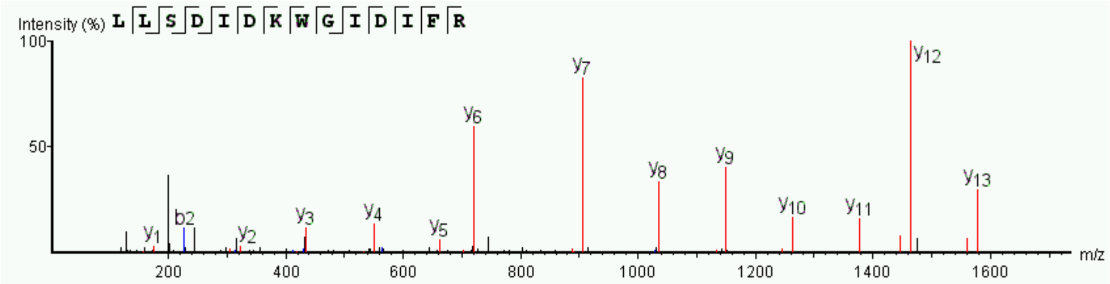

gi|571546304

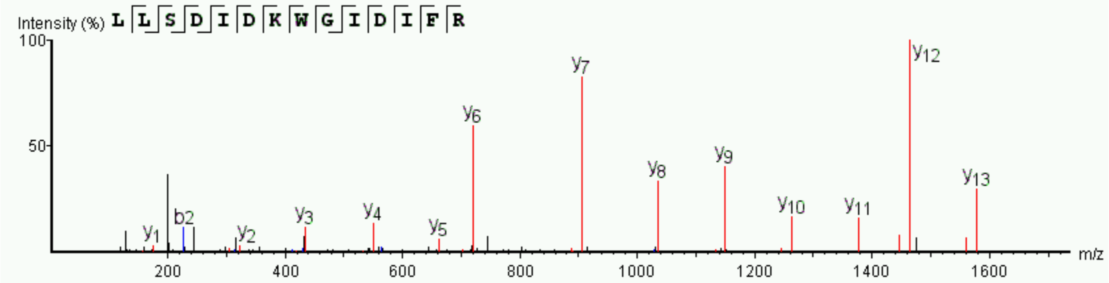

gi|328784410

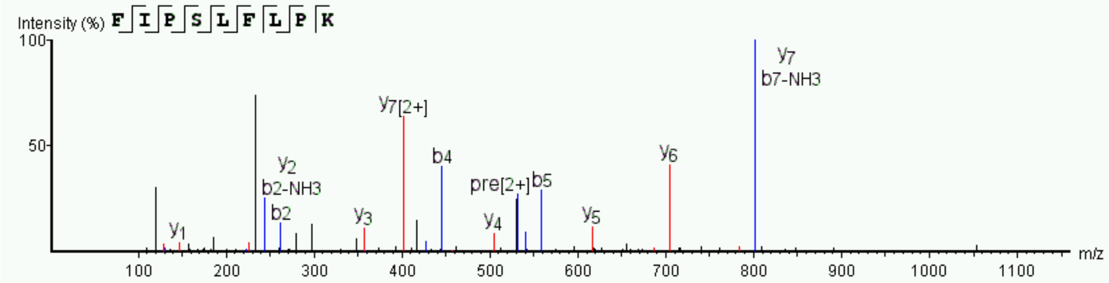

gi|571504763

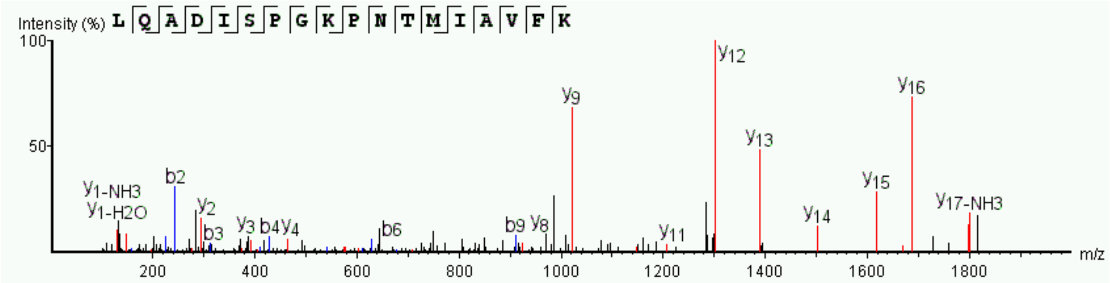

gi|571504766

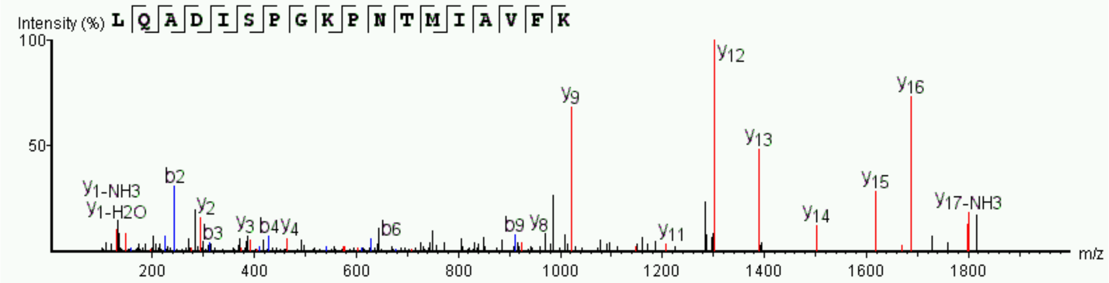

gi|66564402

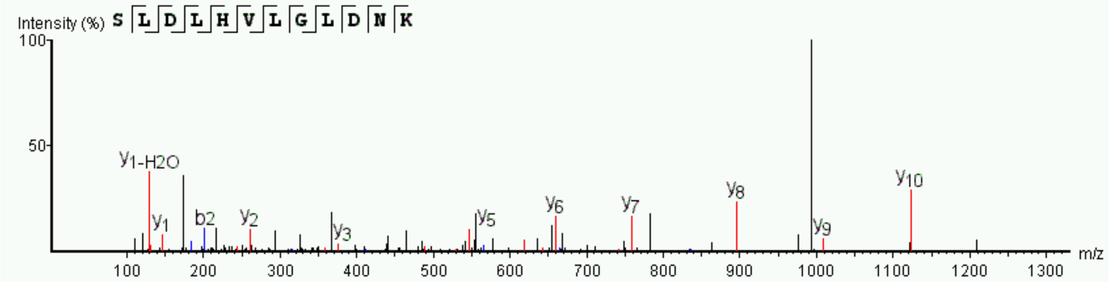

gi|328781034

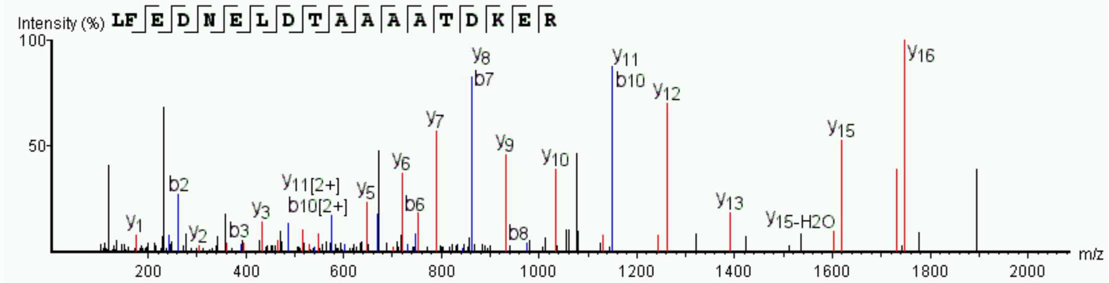

gi|110763495

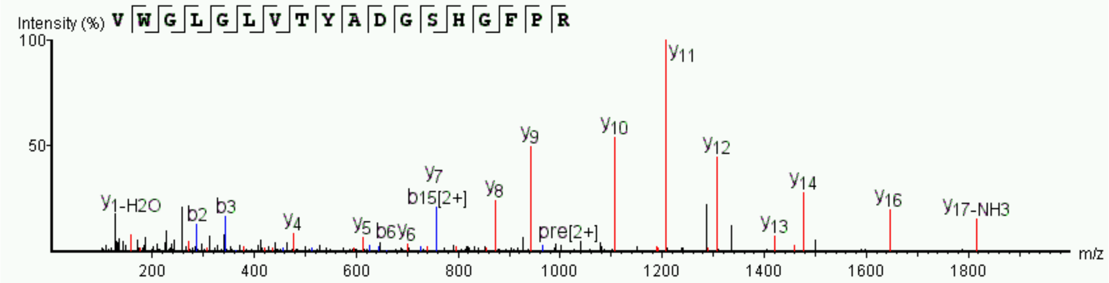

gi|571515342

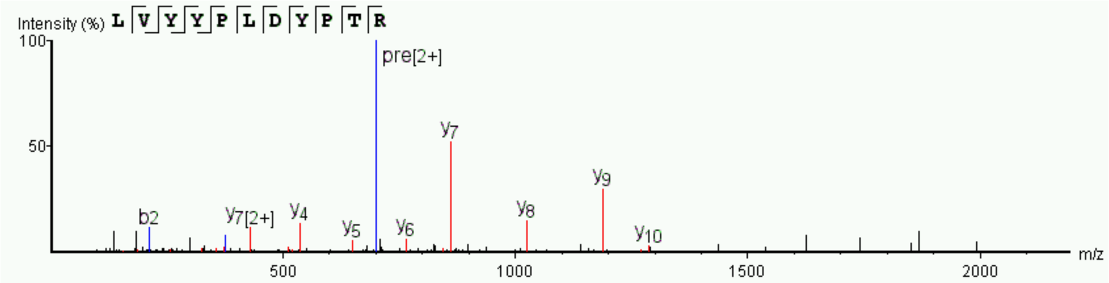

gi|571573693

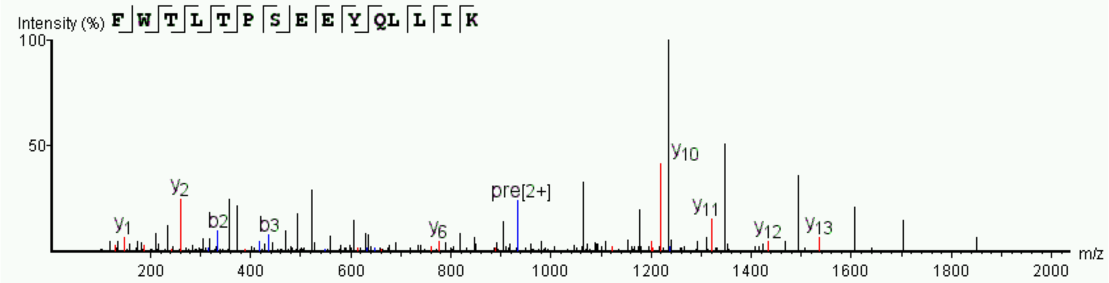

gi|571545933

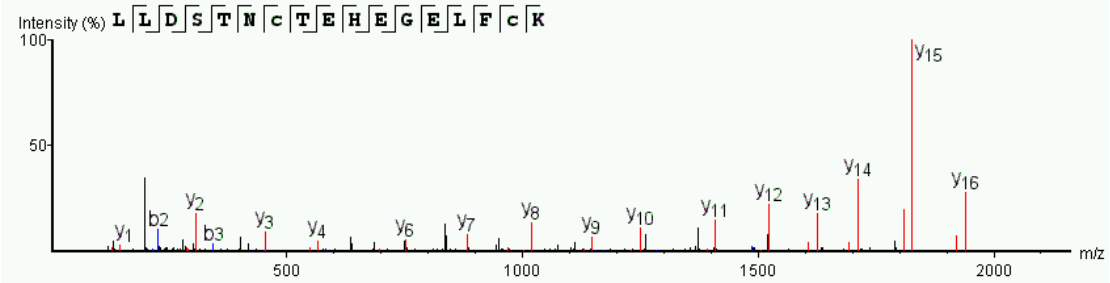

gi|571545937

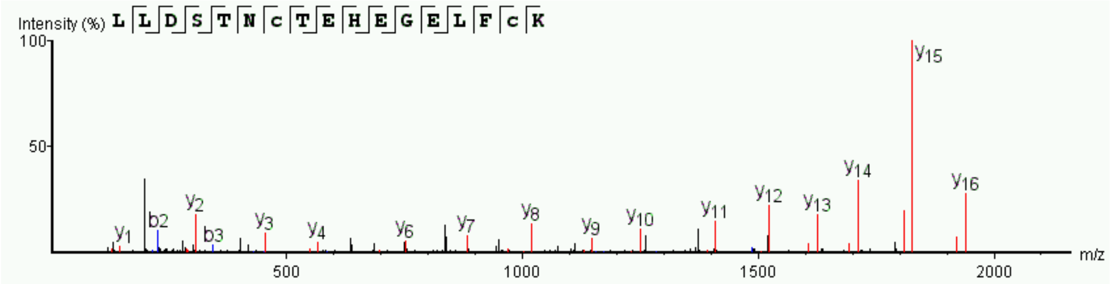

gi|328788697

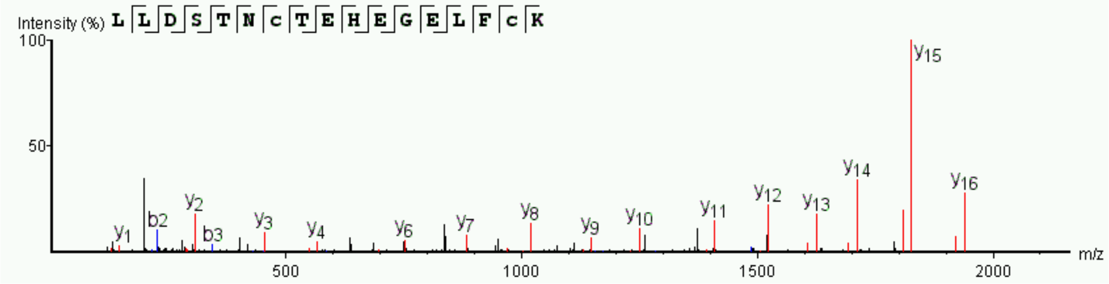

gi|571563508

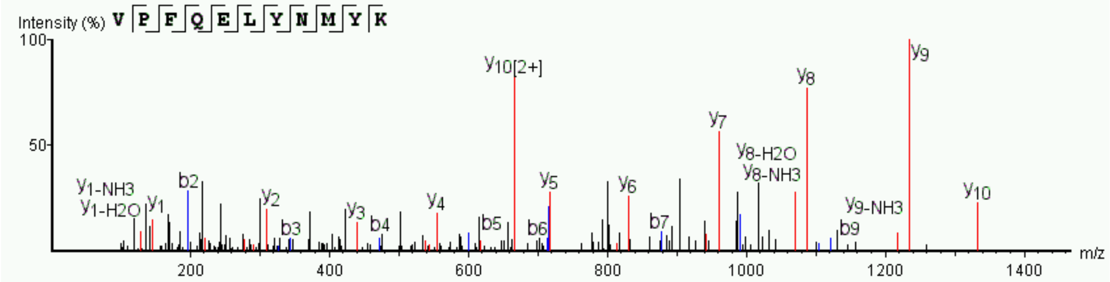

gi|328777790

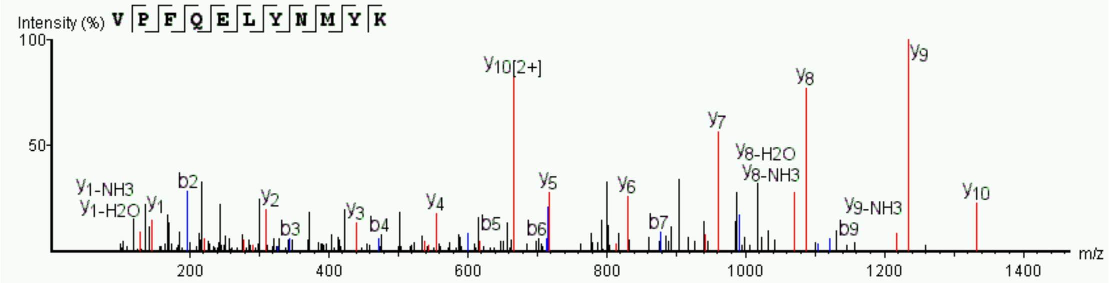

gi|571507494

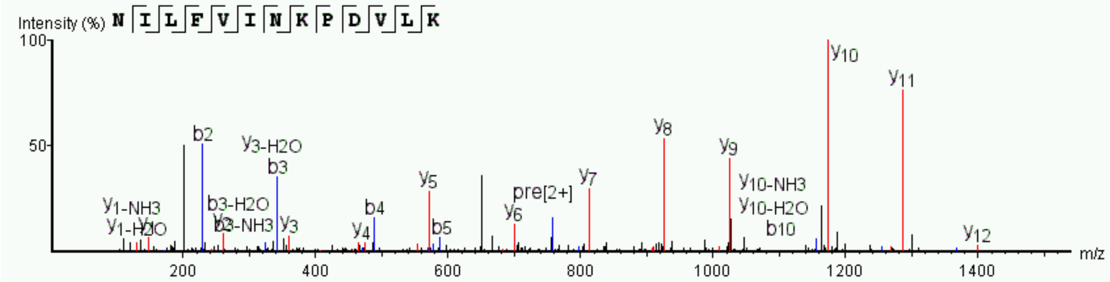

gi|571507498

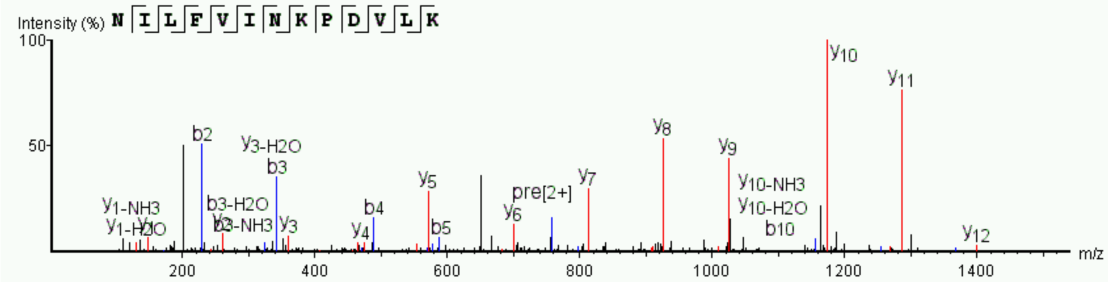

gi|48102172

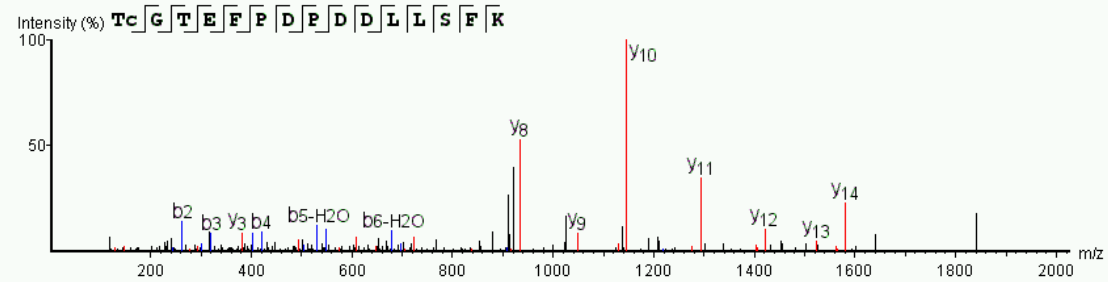

gi|571541806

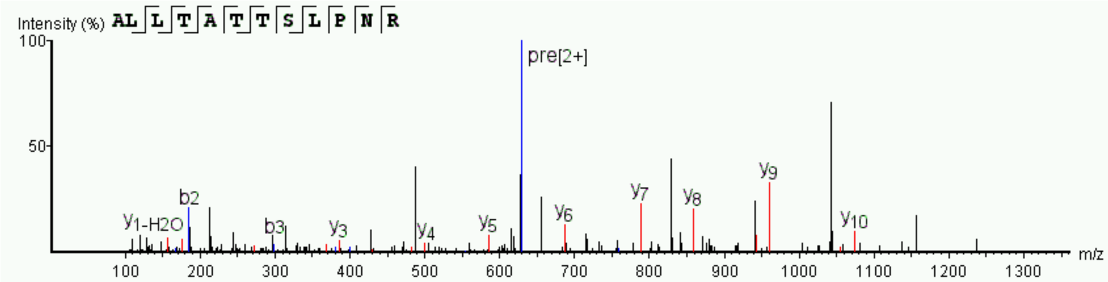

gi|571566413

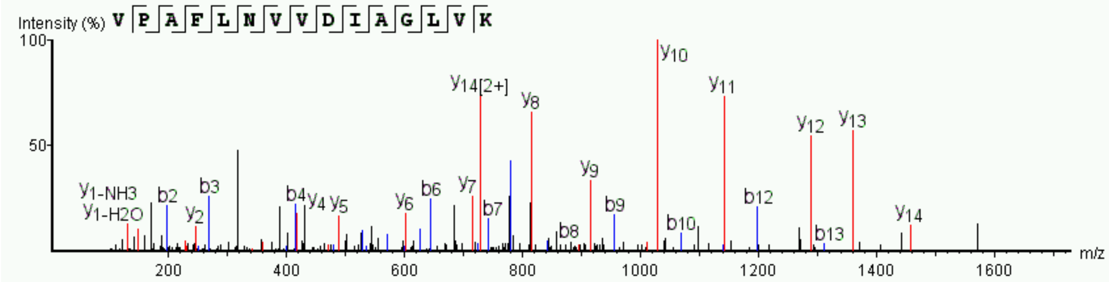

gi|110762023

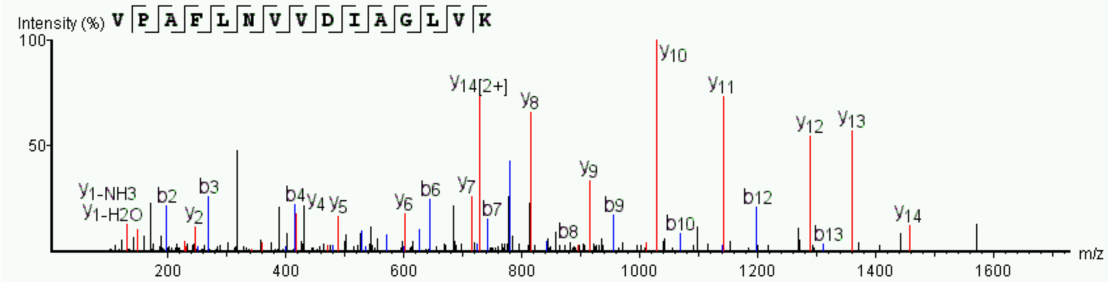

gi|571552590

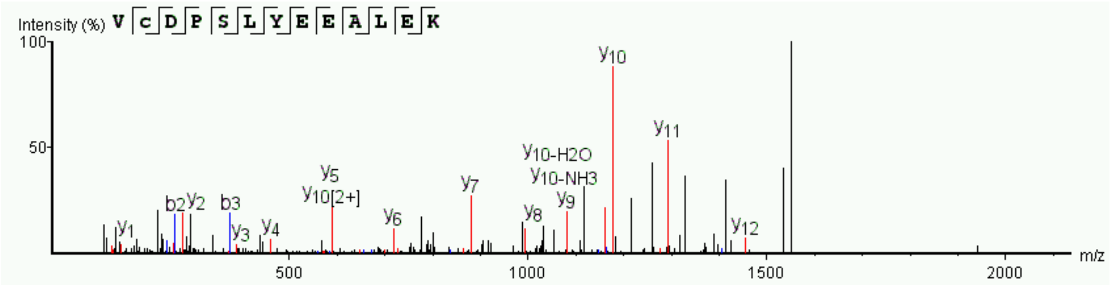

gi|328782271

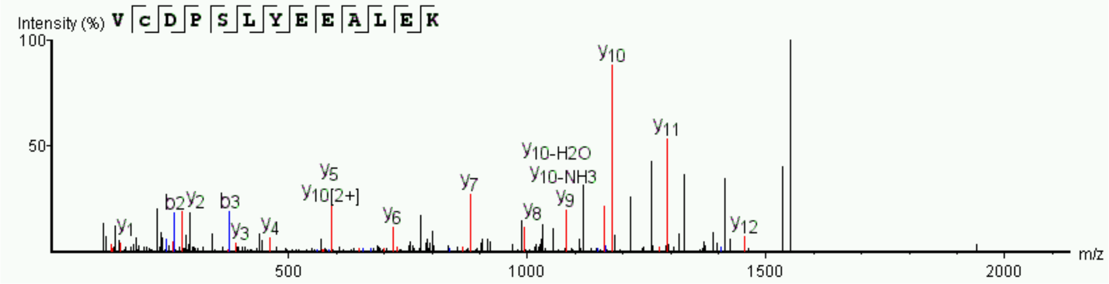

gi|571517372

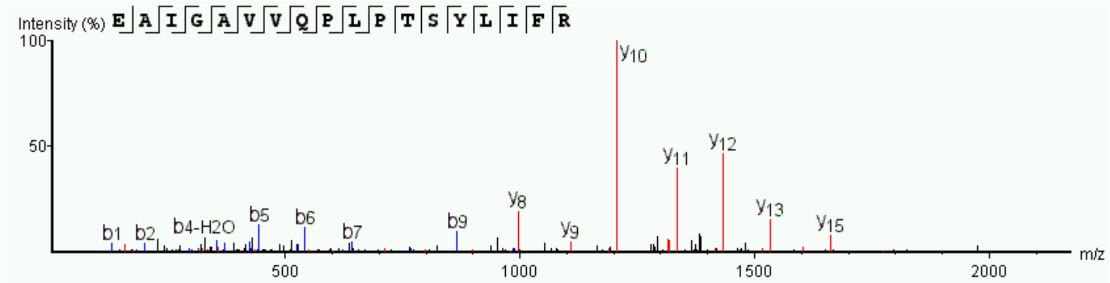

gi|571517403

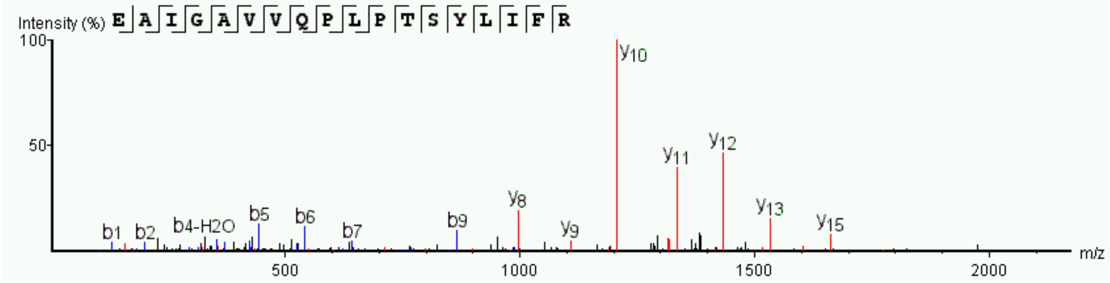

gi|571517376

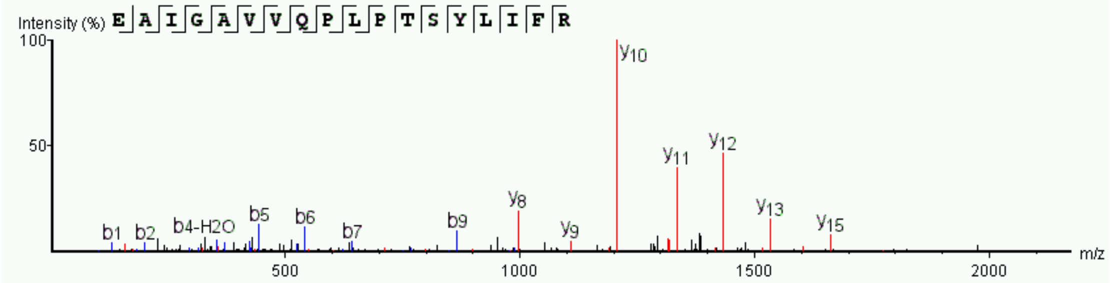

gi|571517379

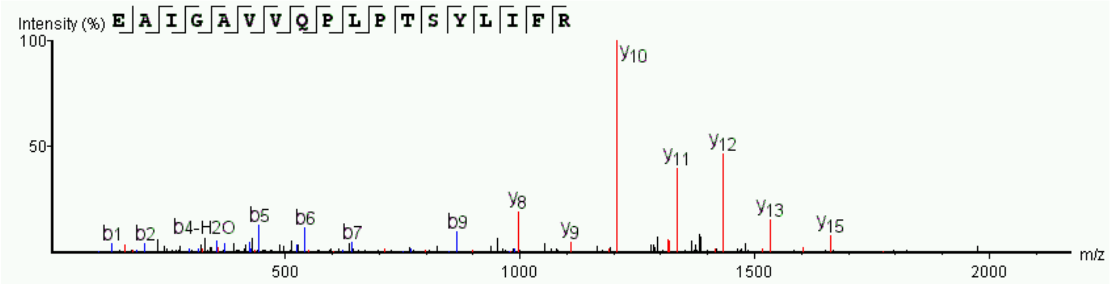

gi|571517382

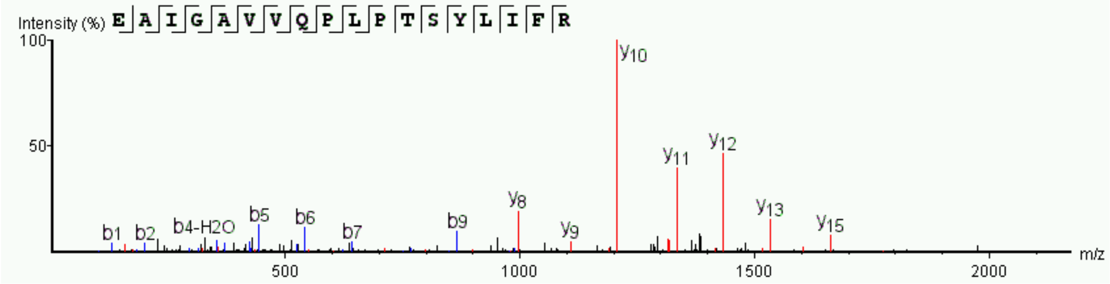

gi|571517385

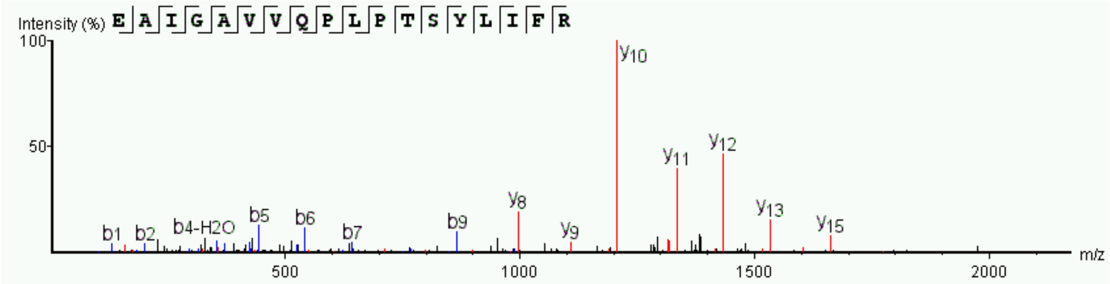

gi|571517389

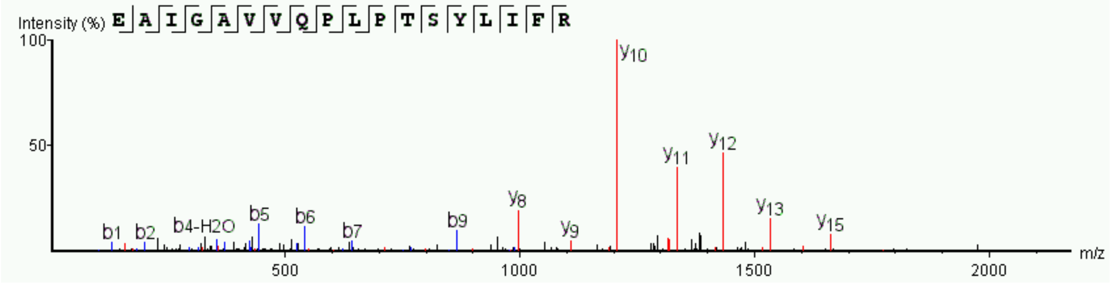

gi|571517392

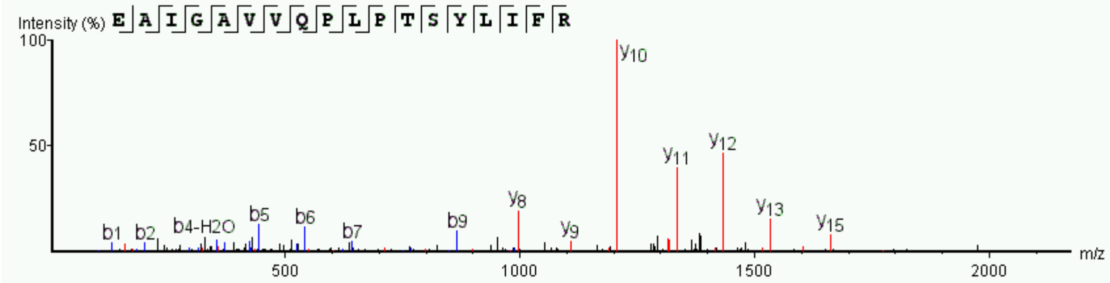

gi|571517396

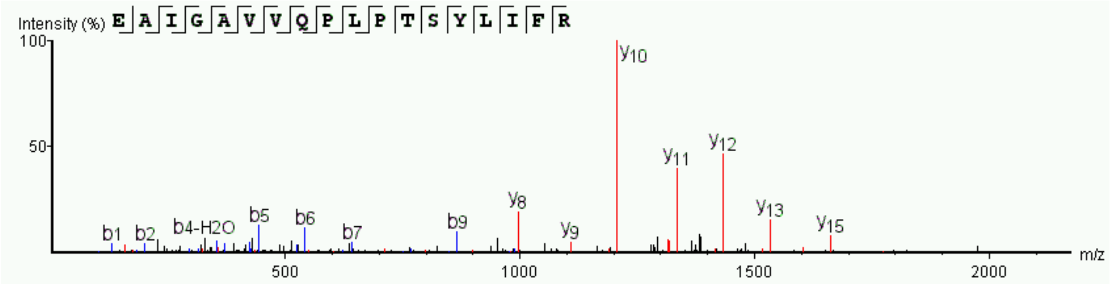

gi|571517400

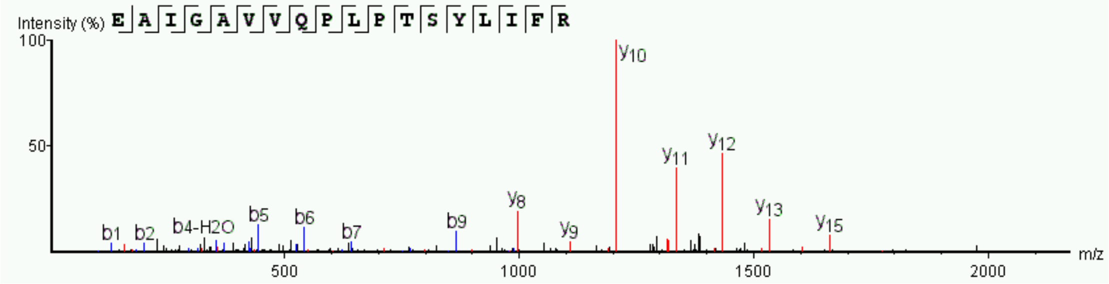

gi|571501998

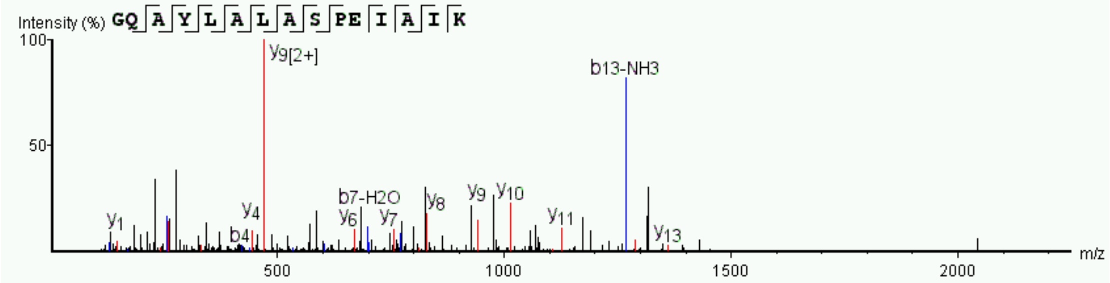

gi|66499186

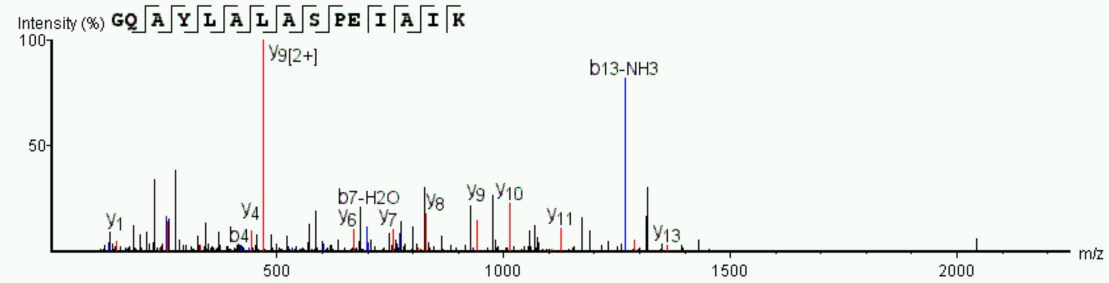

gi|571553257

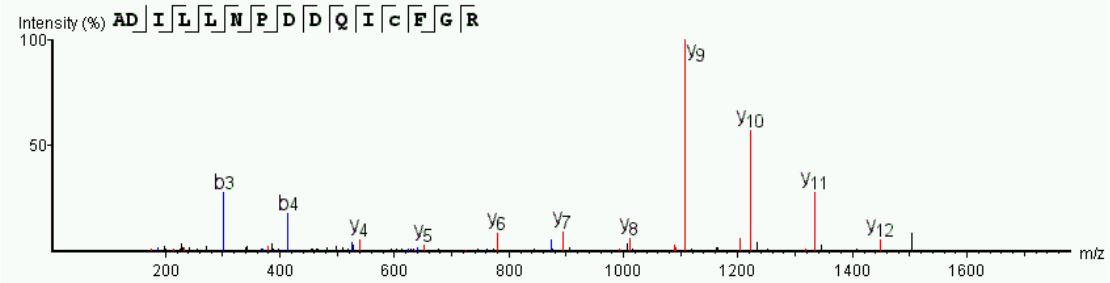

gi|48140497

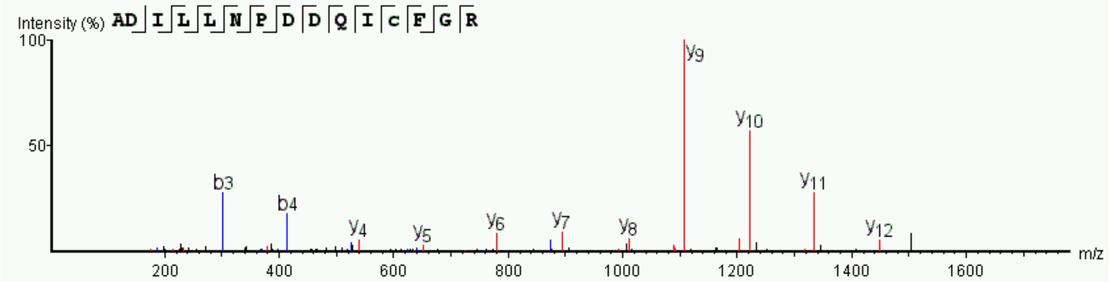

gi|571573752

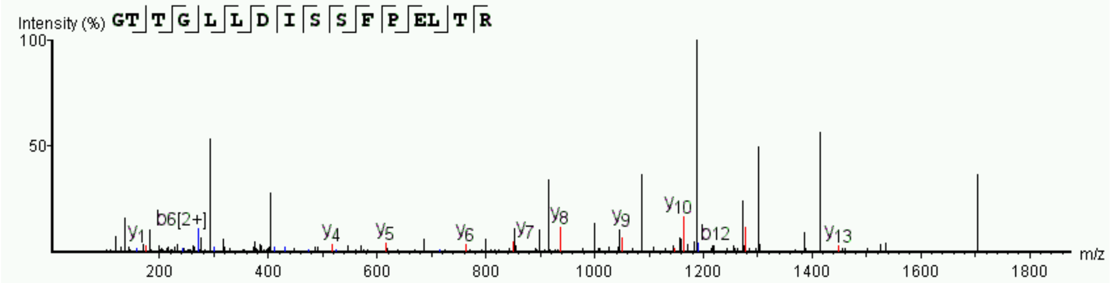

gi|571573754

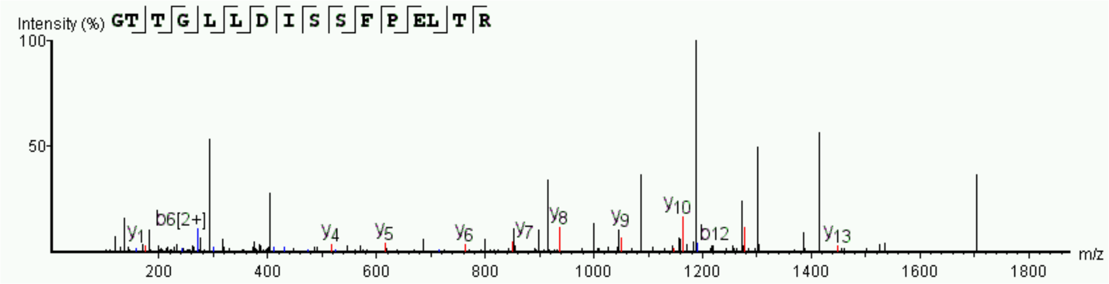

gi|571541340

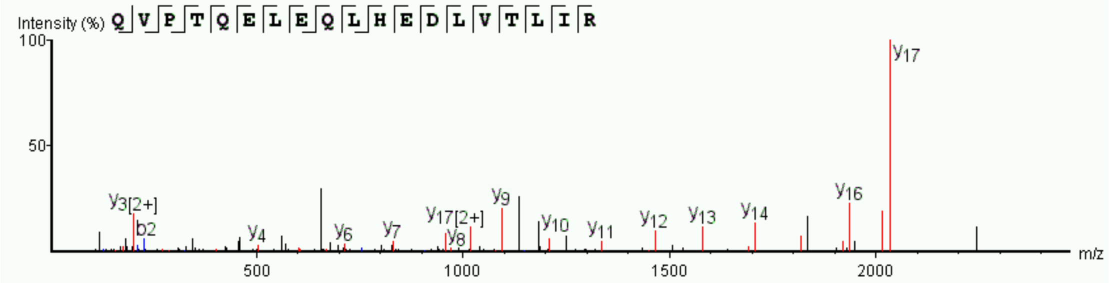

gi|328792171

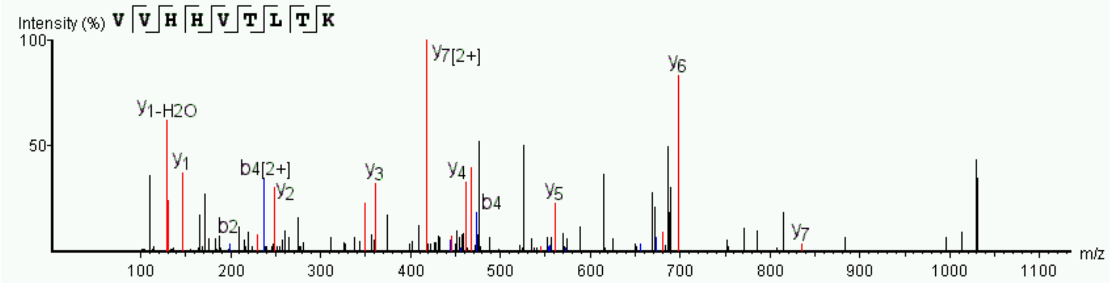

gi|571507416

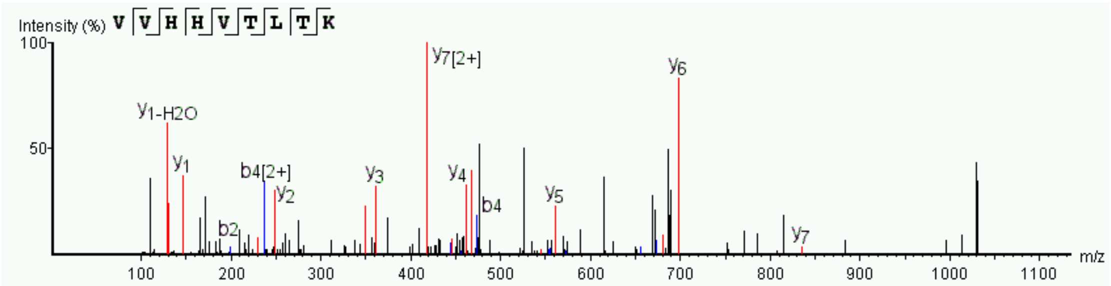

gi|571507420

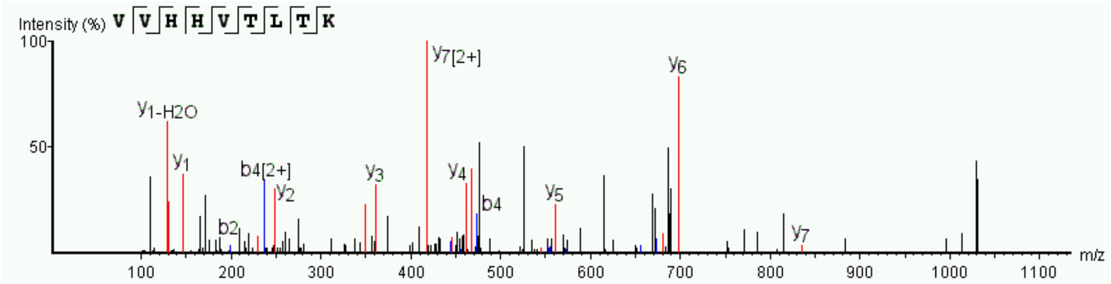

gi|571507424

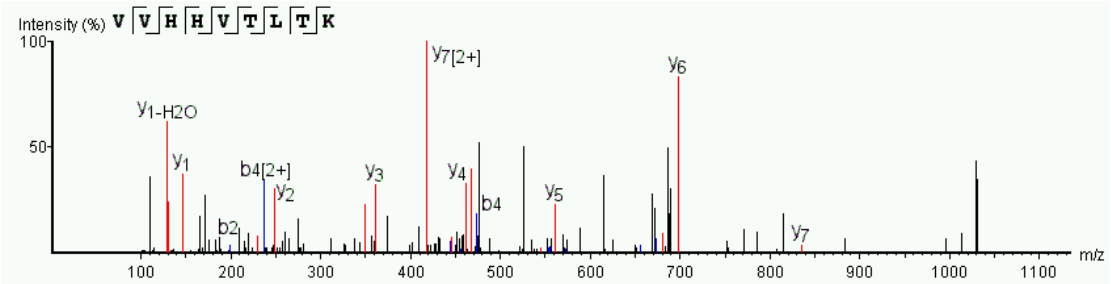

gi|571507427

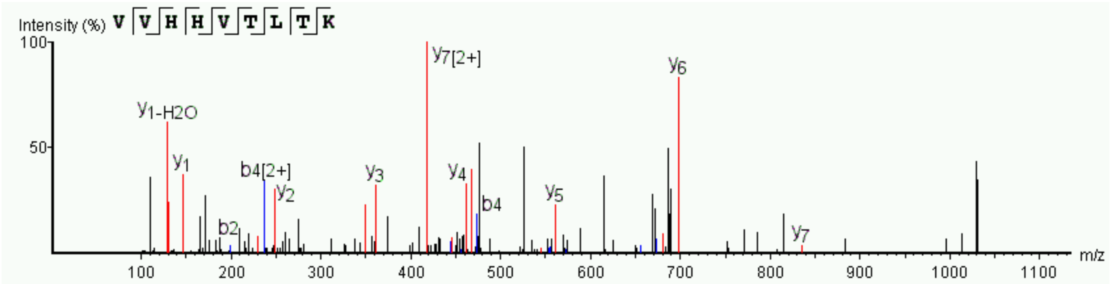

gi|571507430

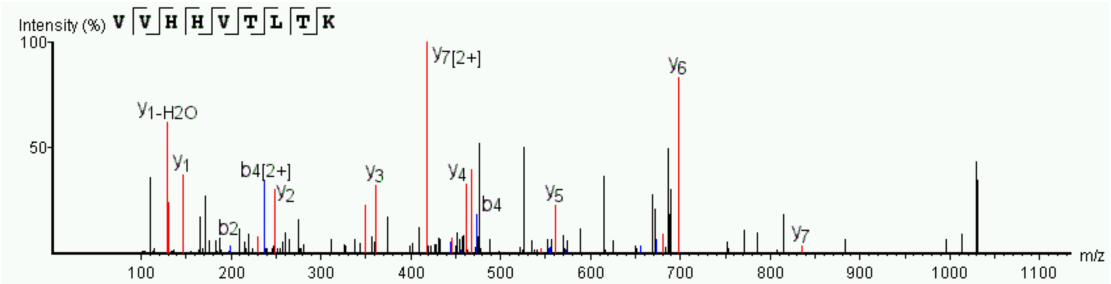

gi|571507434

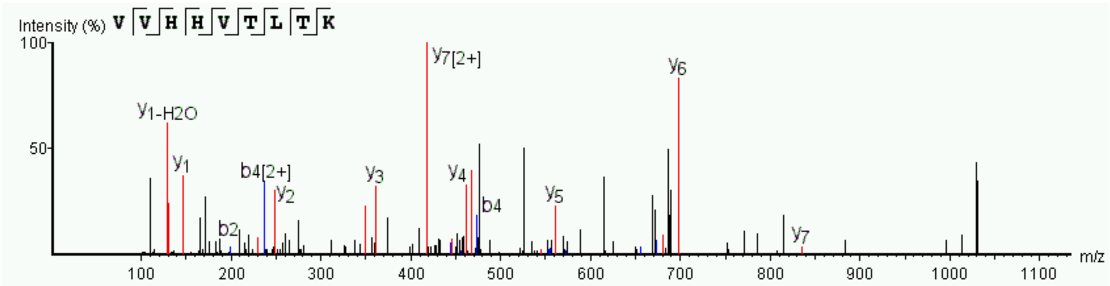

gi|571507387

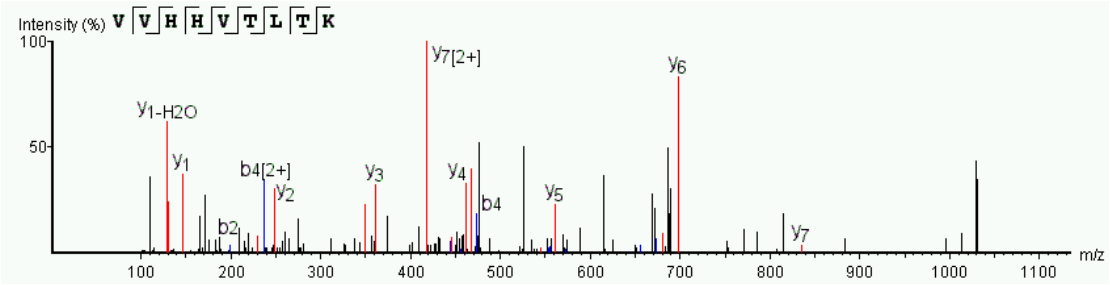

gi|571507392

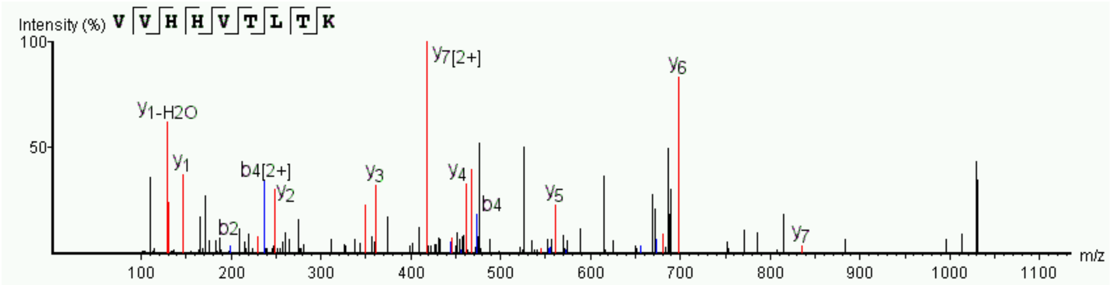

gi|571507395

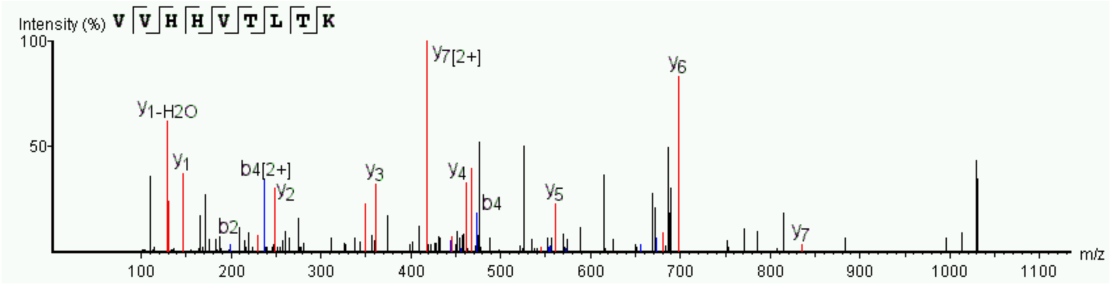

gi|571507399

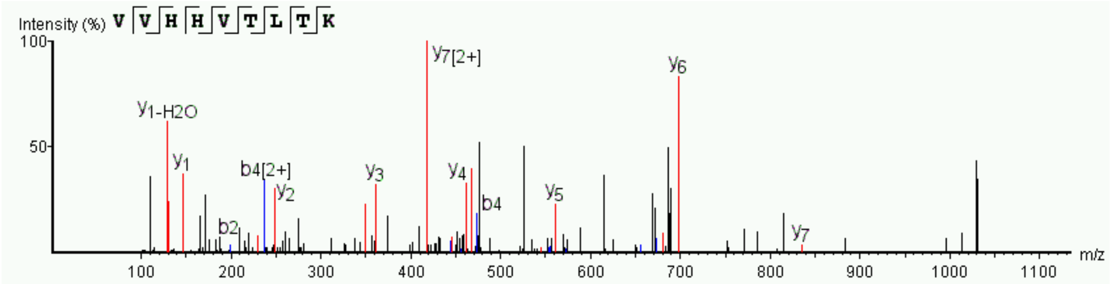

gi|571507402

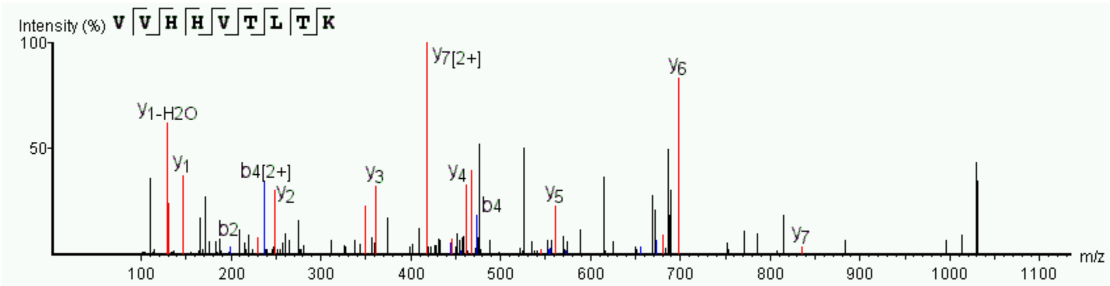

gi|571507406

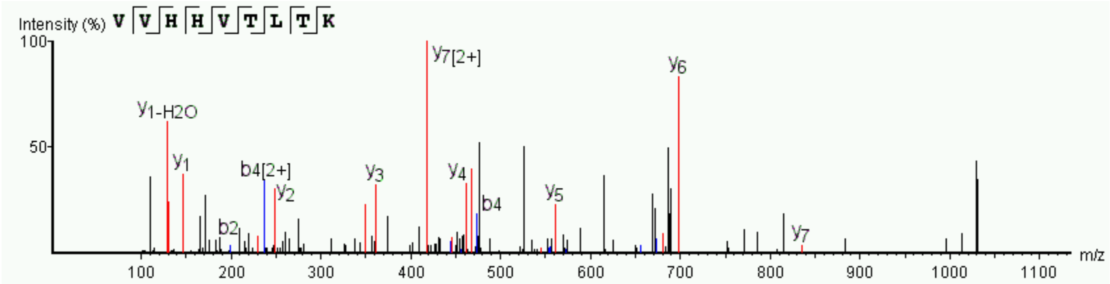

gi|571507410

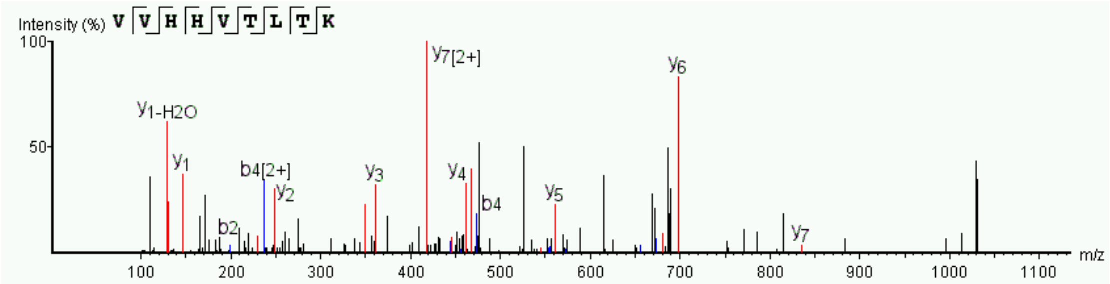

gi|571507413

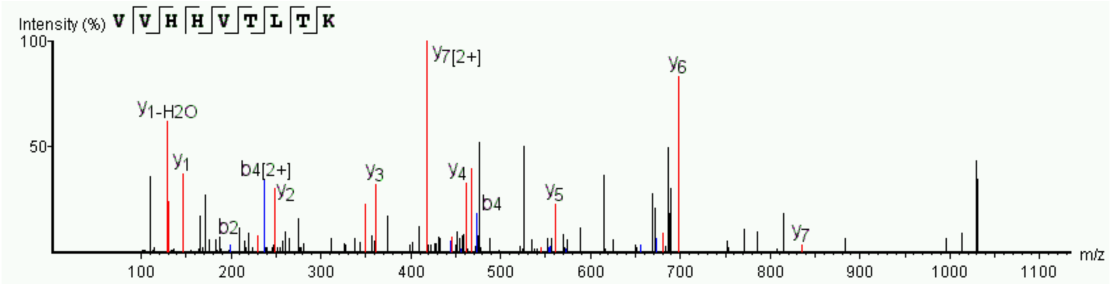

gi|571578229

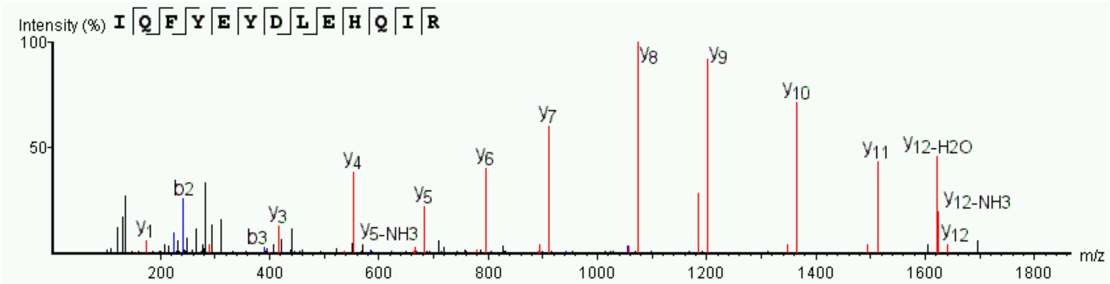

gi|571578231

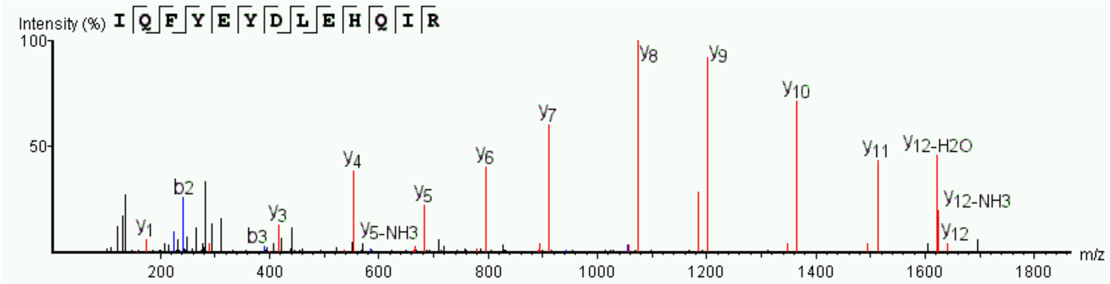

gi|571578233

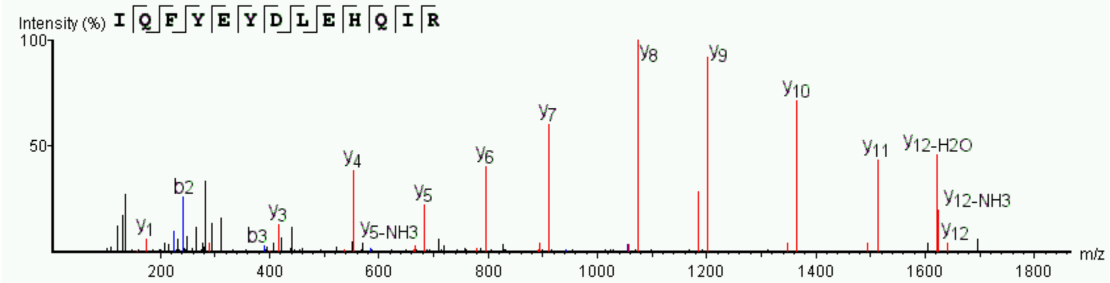

gi|66533867

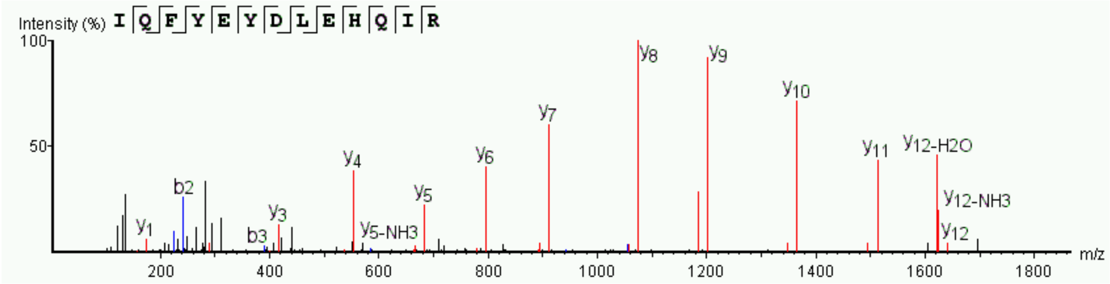

gi|328778152

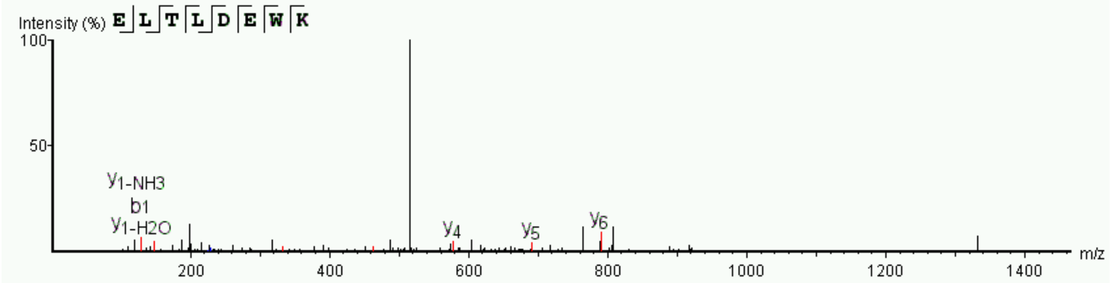

gi|571510037

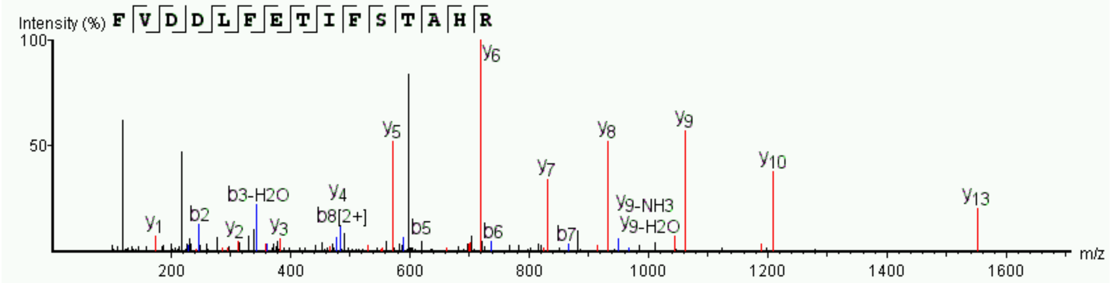

gi|571510040

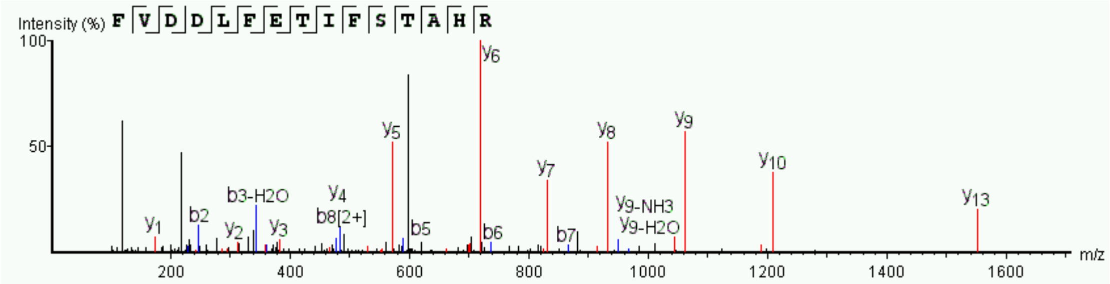

gi|110750043

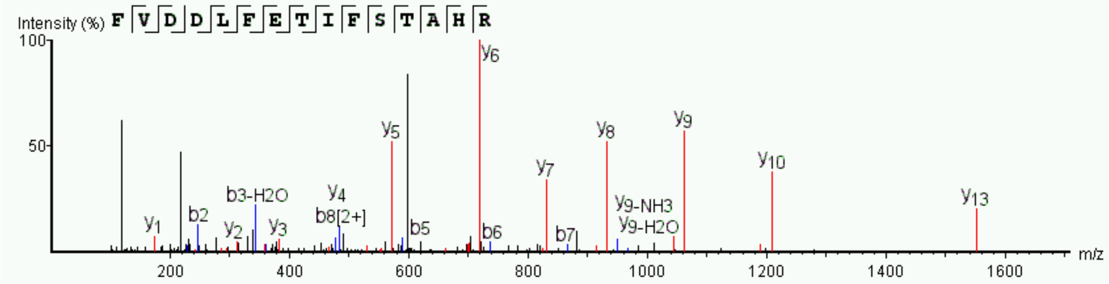

gi|571552393

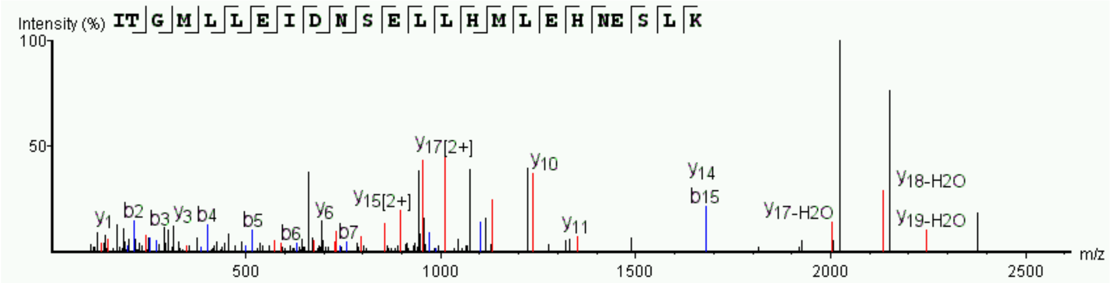

gi|571552397

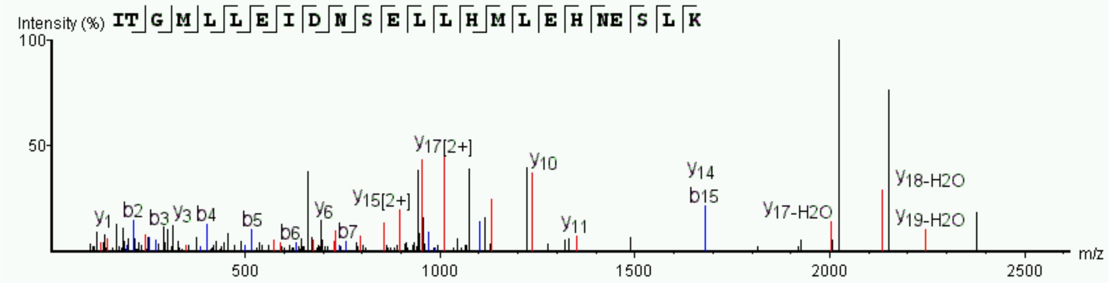

gi|571540698

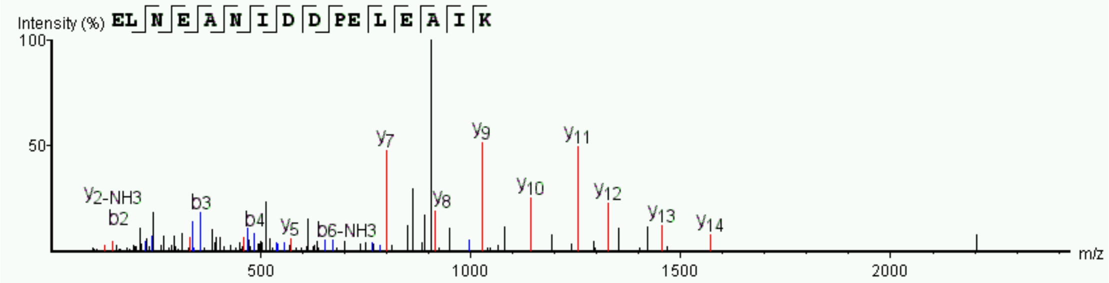

gi|571508087

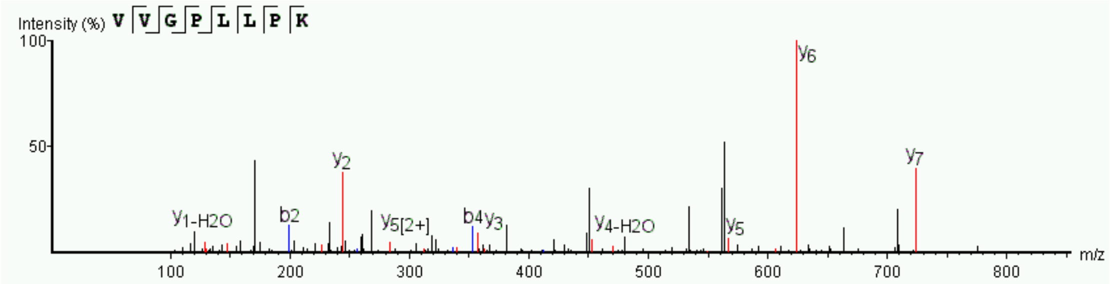

gi|571508090

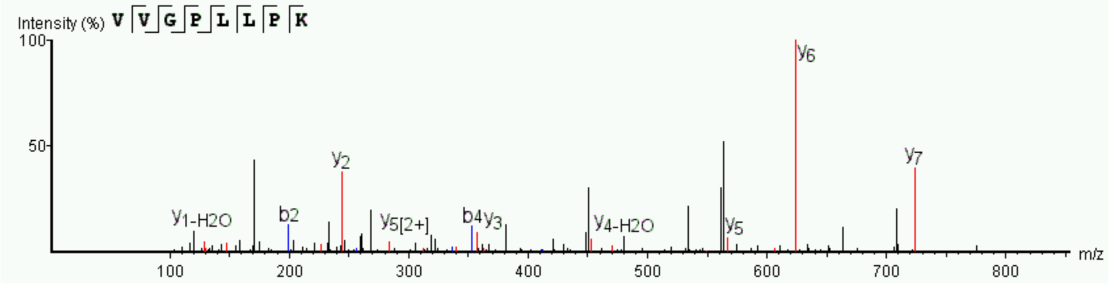

gi|571529116

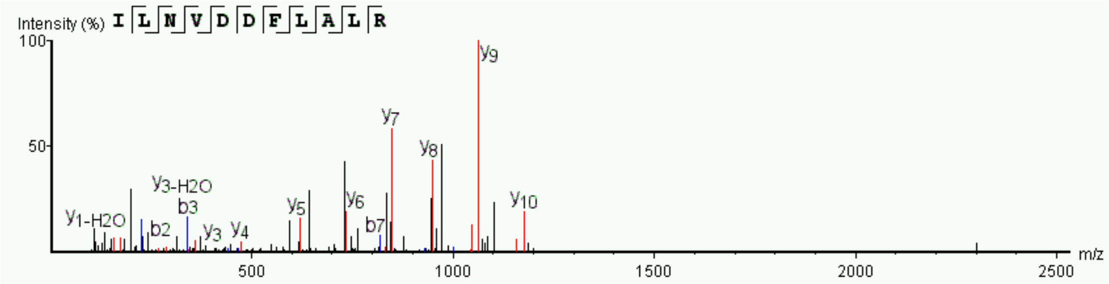

gi|571559086

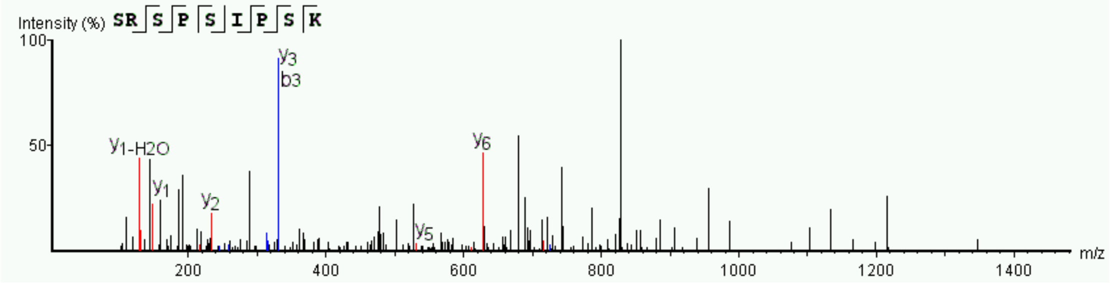

gi|571559093

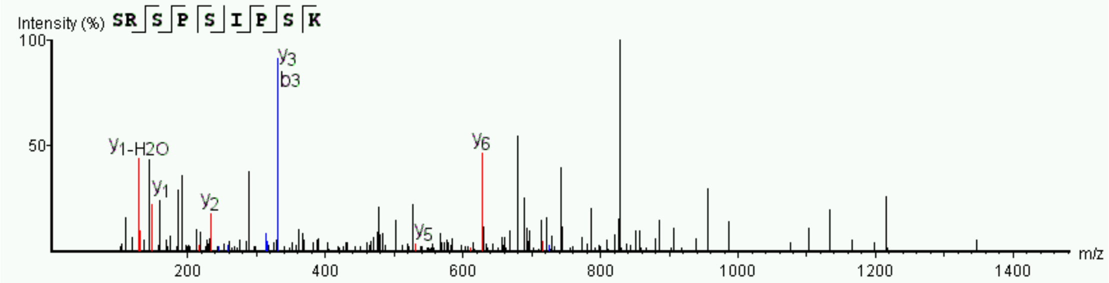

gi|328788274

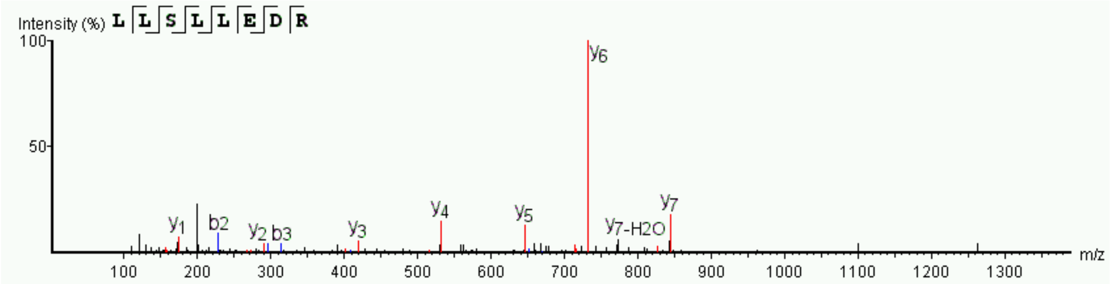

gi|571529763

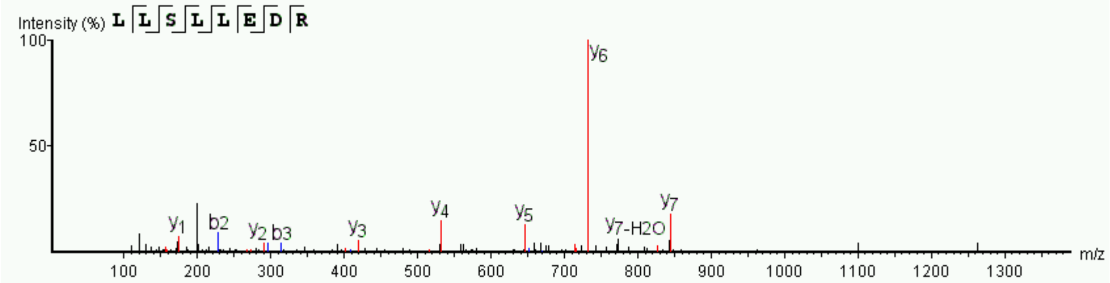

gi|571529767

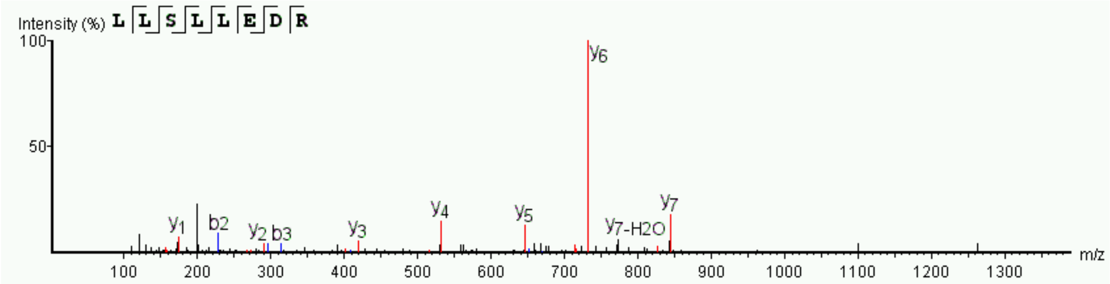

gi|571529770

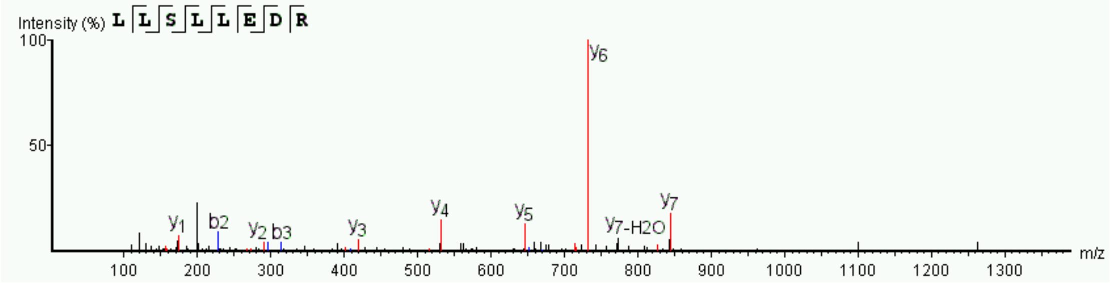

gi|571529773

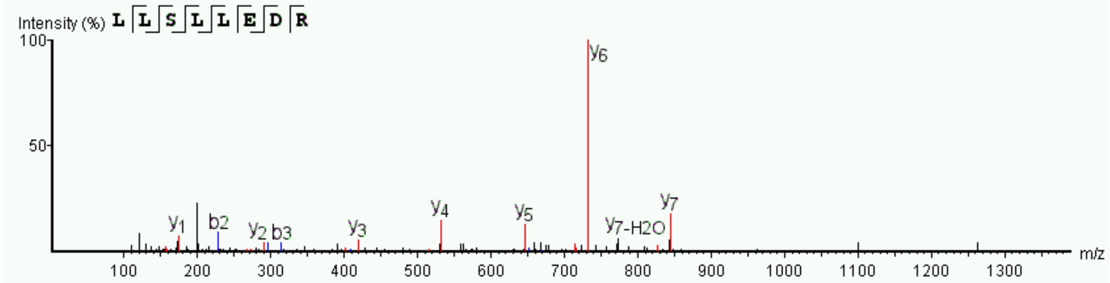

gi|571529779

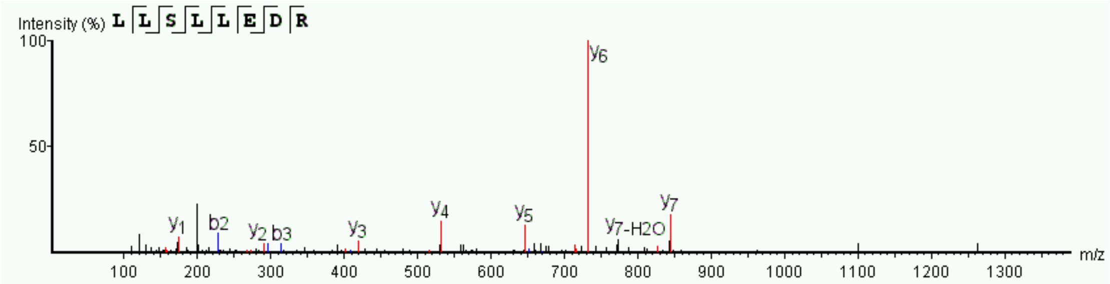

gi|571529782

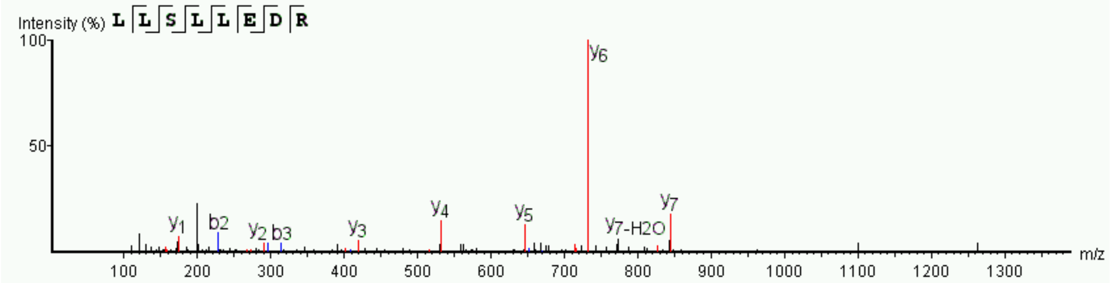

gi|571529785

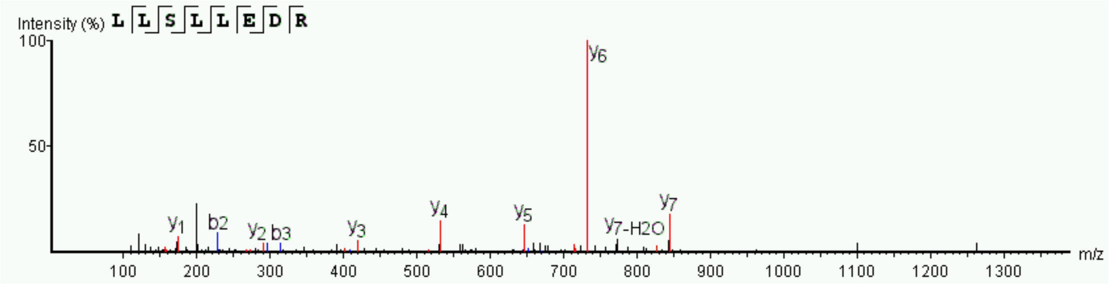

gi|571565660

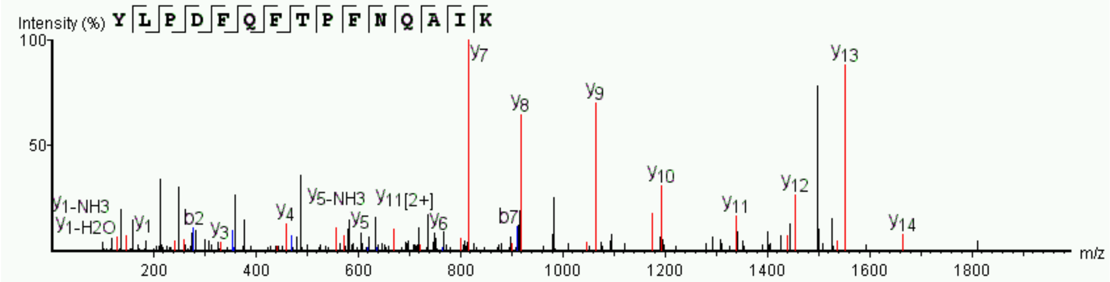

gi|571565656

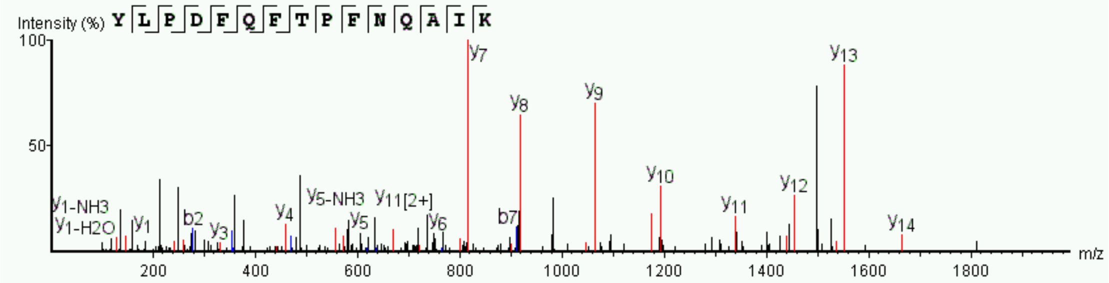

gi|66500946

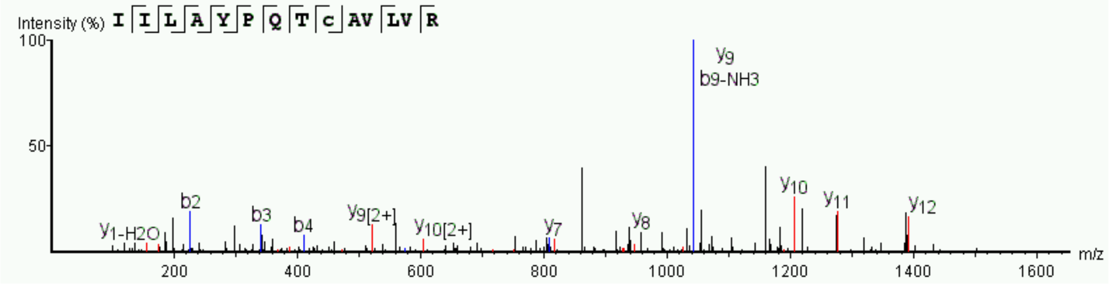

gi|328788603

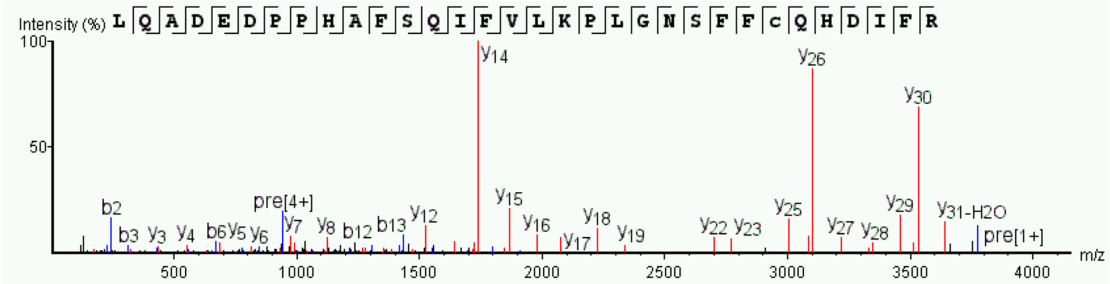

gi|48104167

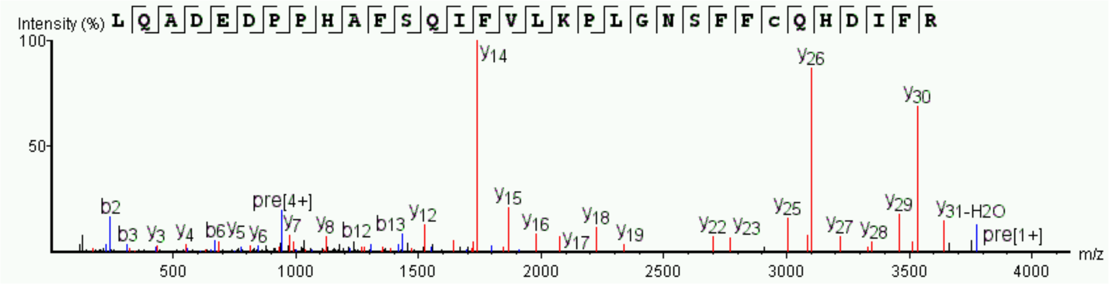

gi|571515962

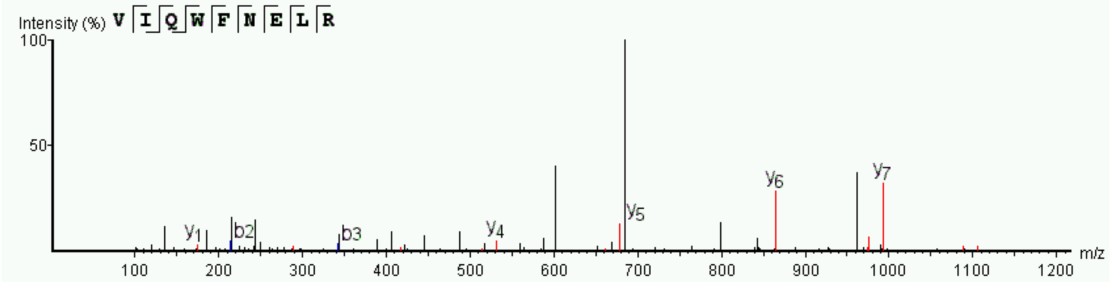

gi|571516001

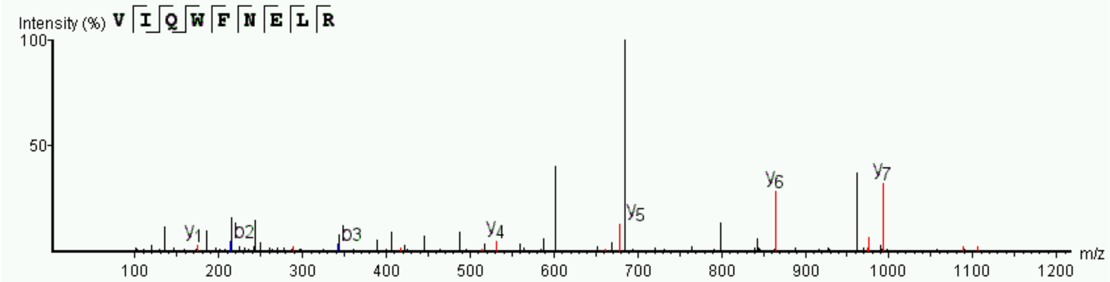

gi|571516016

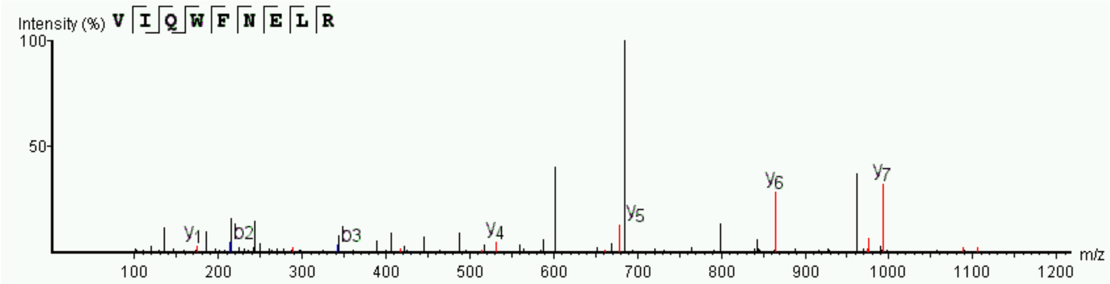

gi|328789642

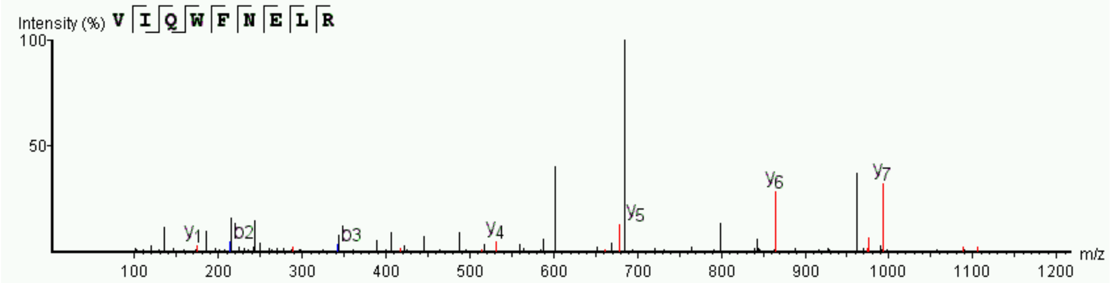

gi|571515966

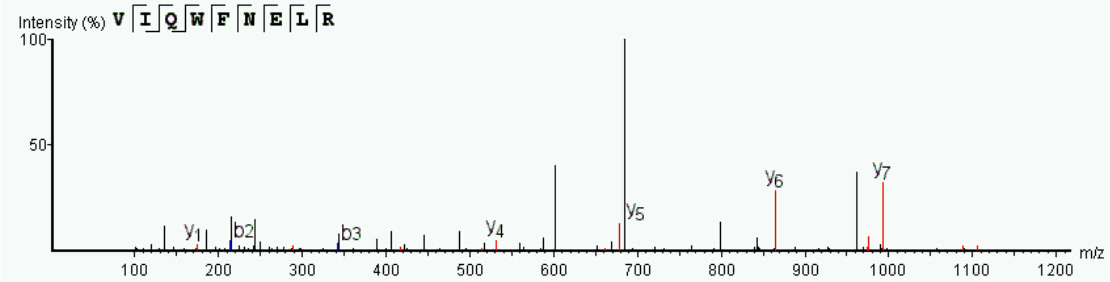

gi|571515974

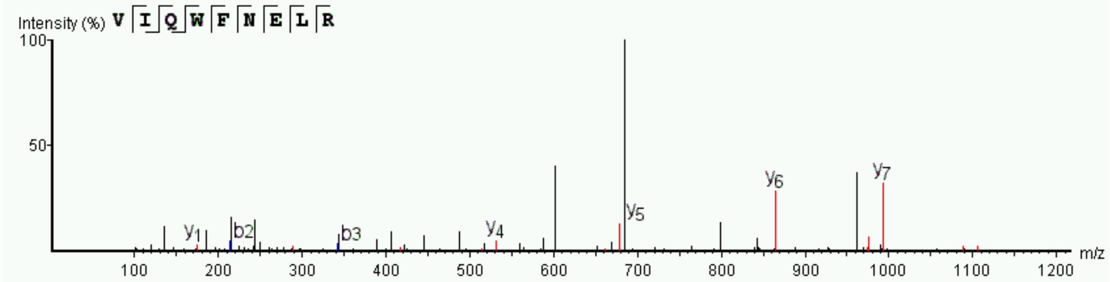

gi|571515981

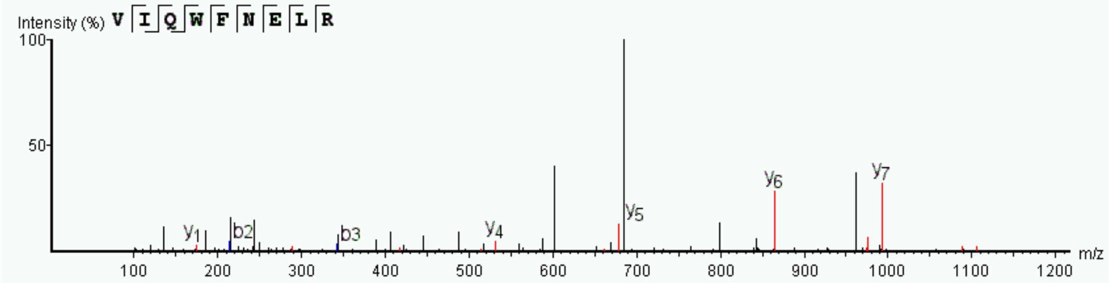

gi|48097144

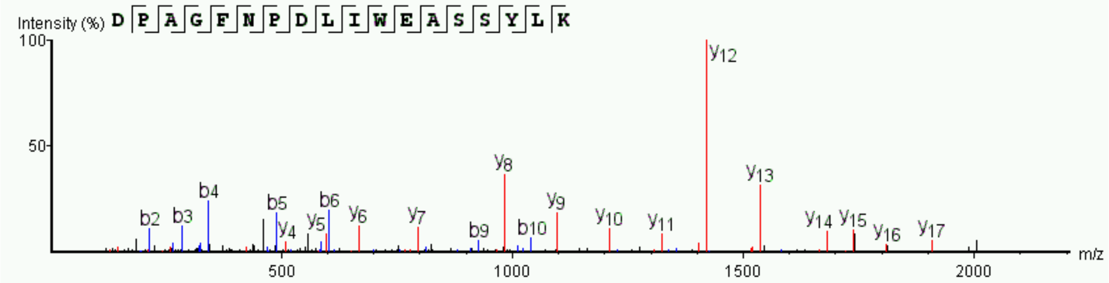

gi|328778339

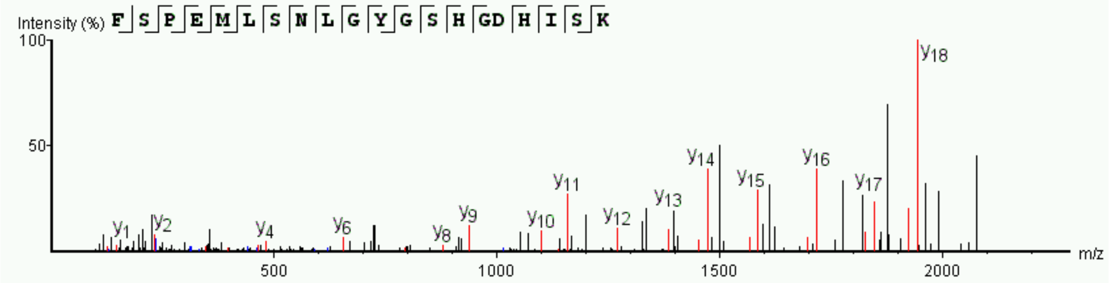

gi|571575531

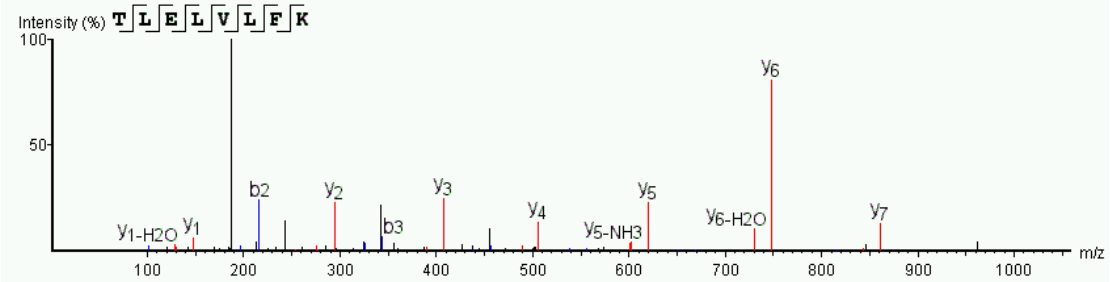

gi|571575529

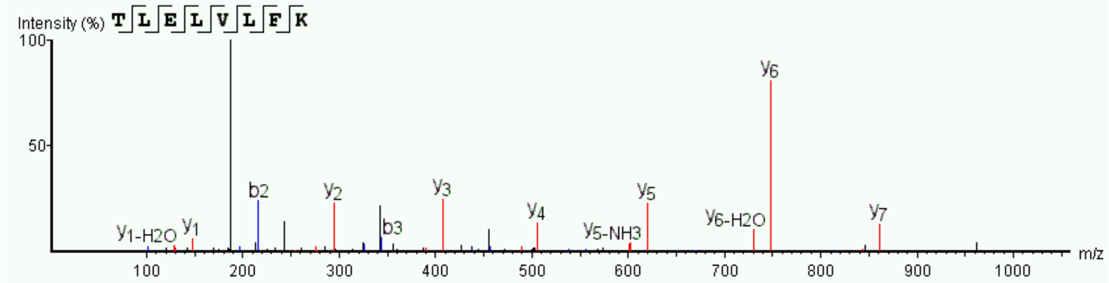

gi|48128110

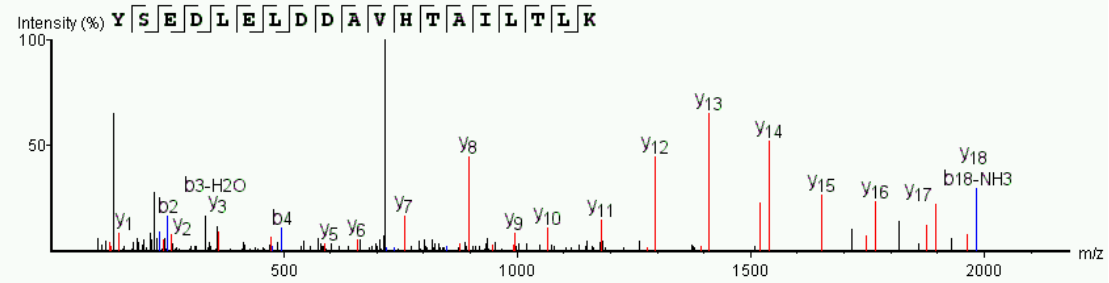

gi|48096769

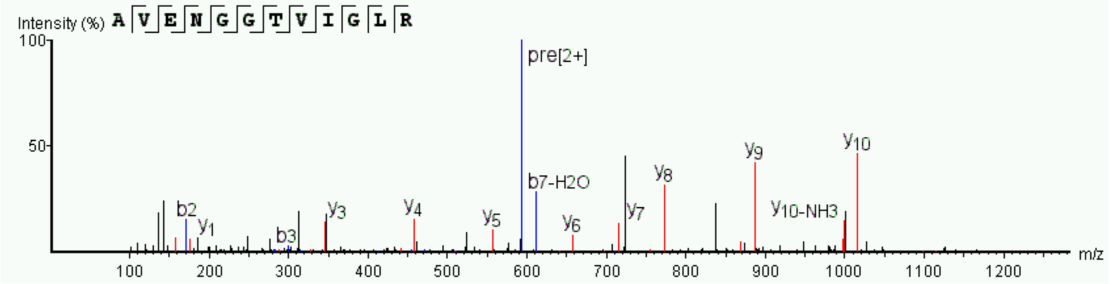

gi|571531985

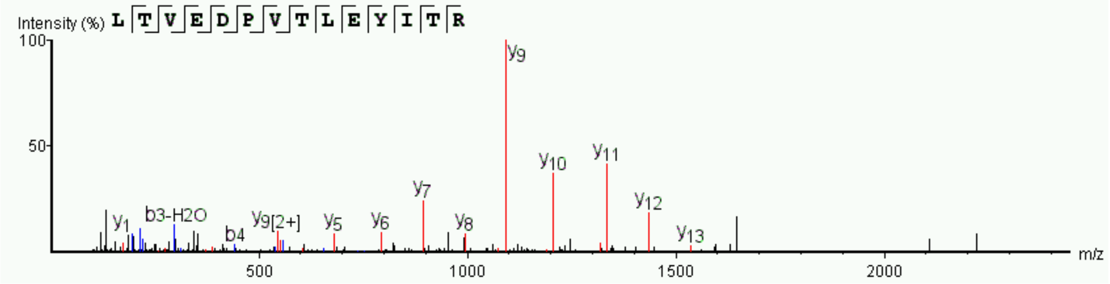

gi|571509446

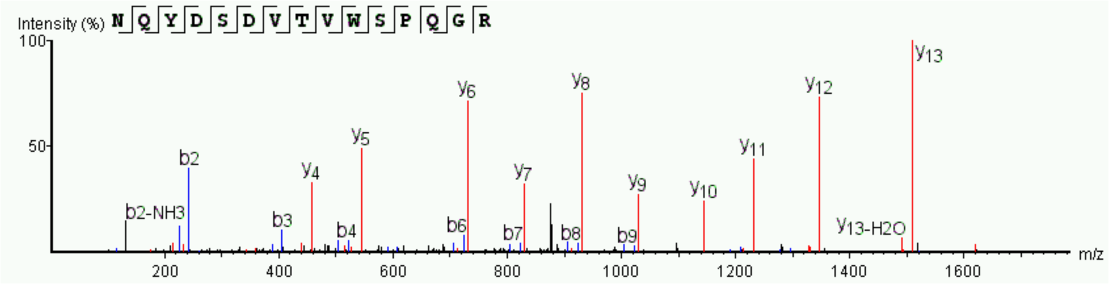

gi|66553455

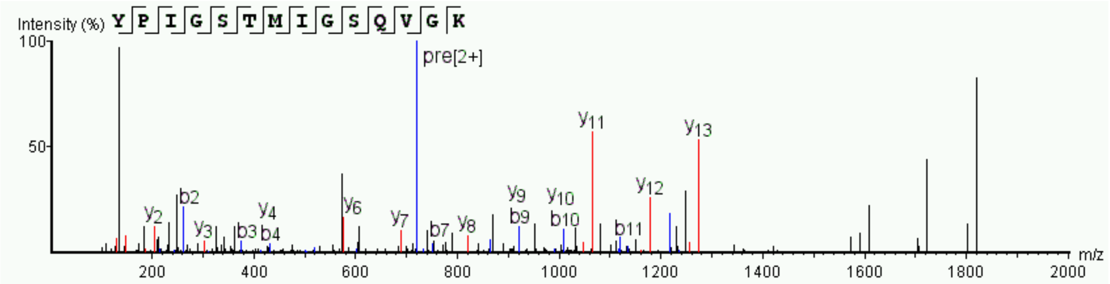

gi|66519157

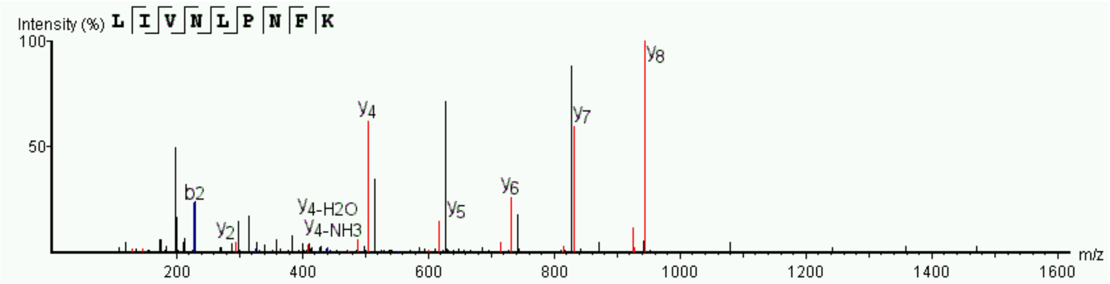

gi|328787761

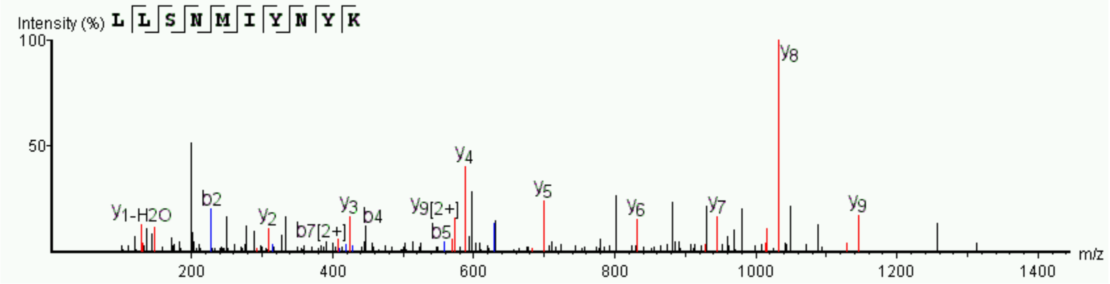

gi|571570655

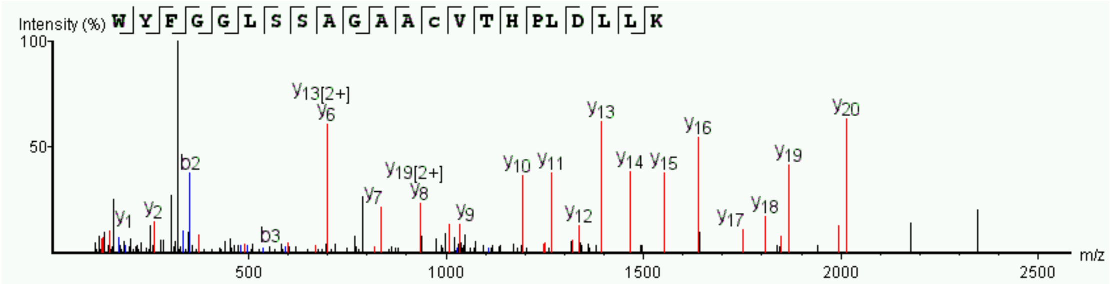

gi|571570671

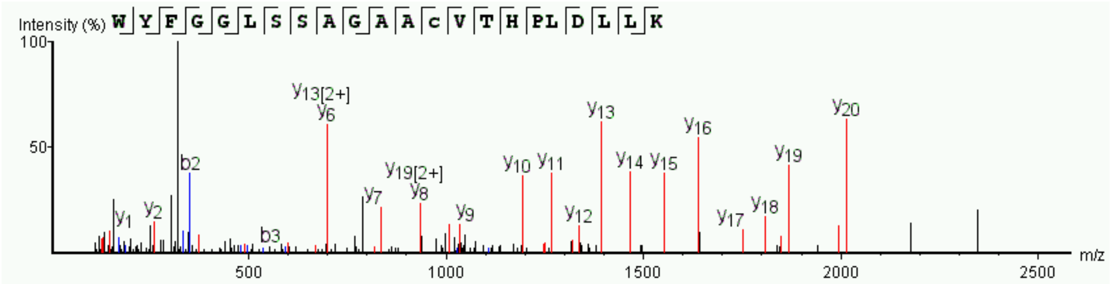

gi|571570685

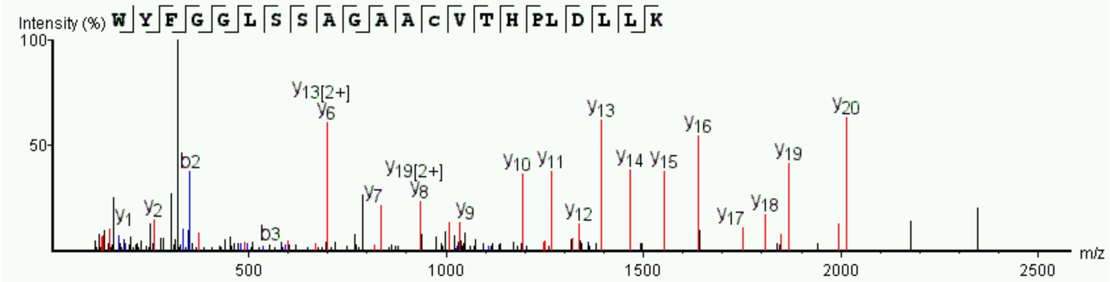

gi|66523953

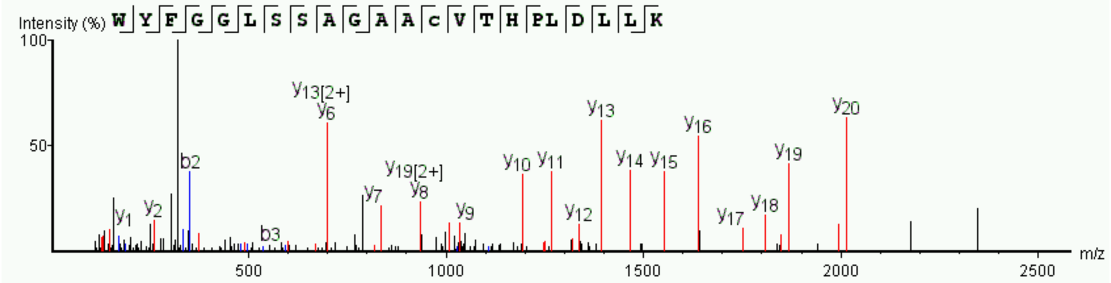

gi|571509939

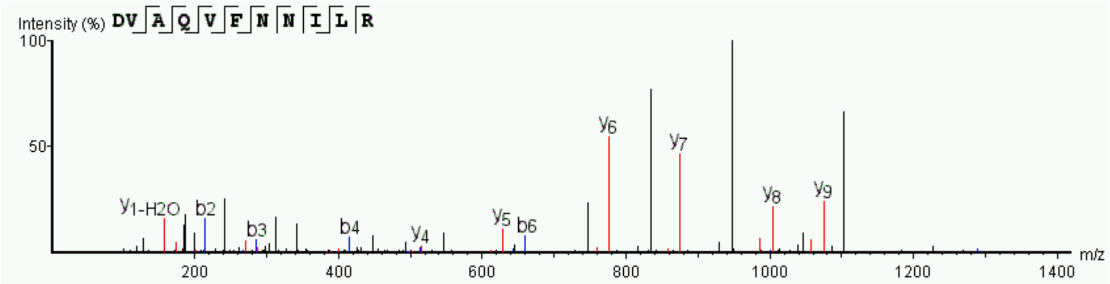

gi|571509942

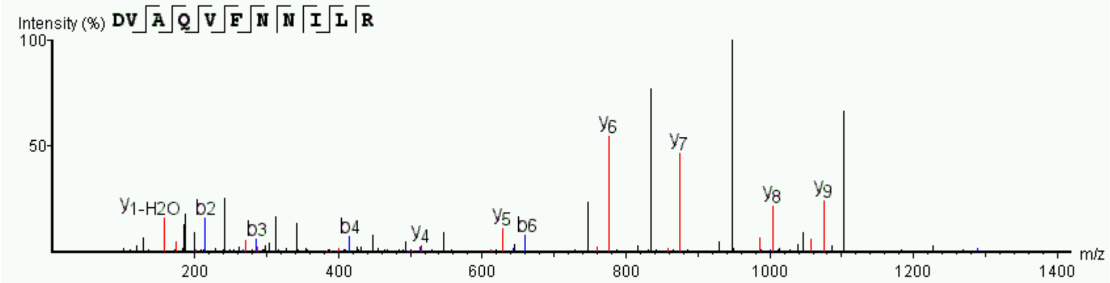

gi|571509949

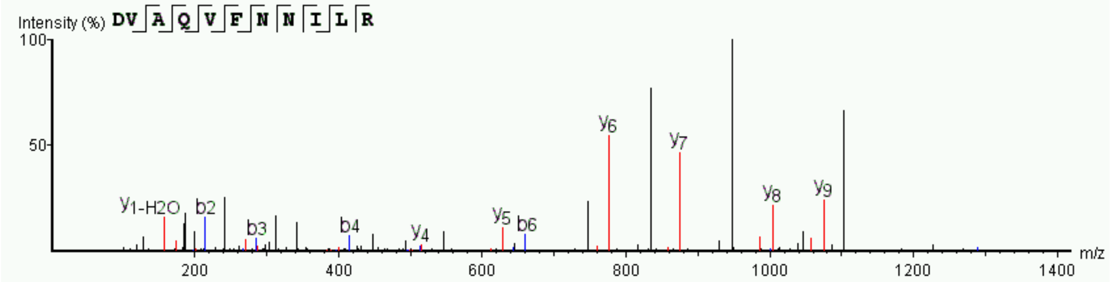

gi|571509953

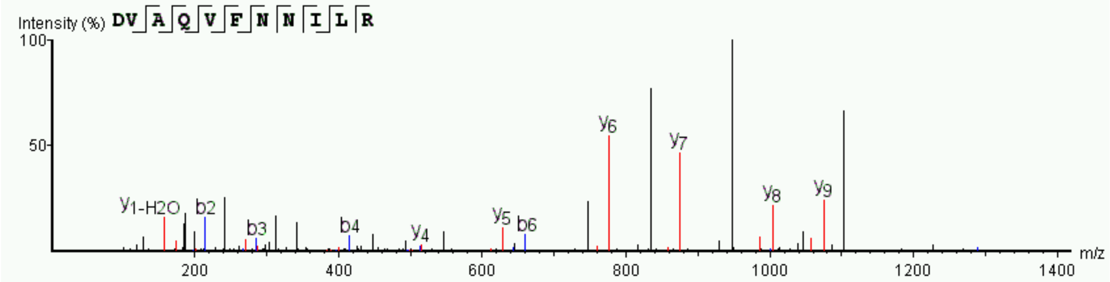

gi|66501185

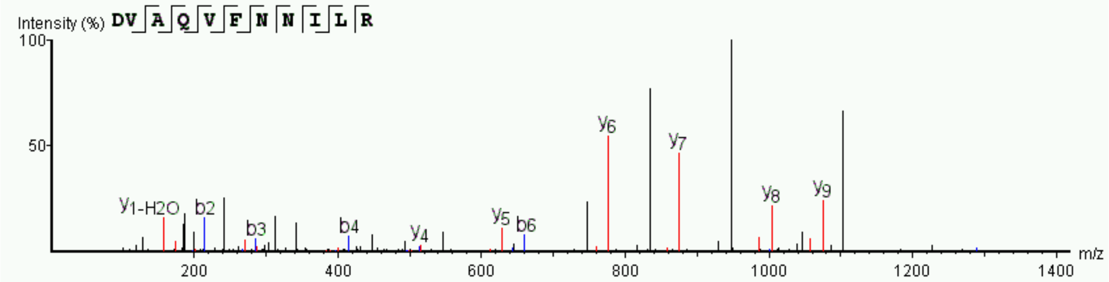

gi|571532722

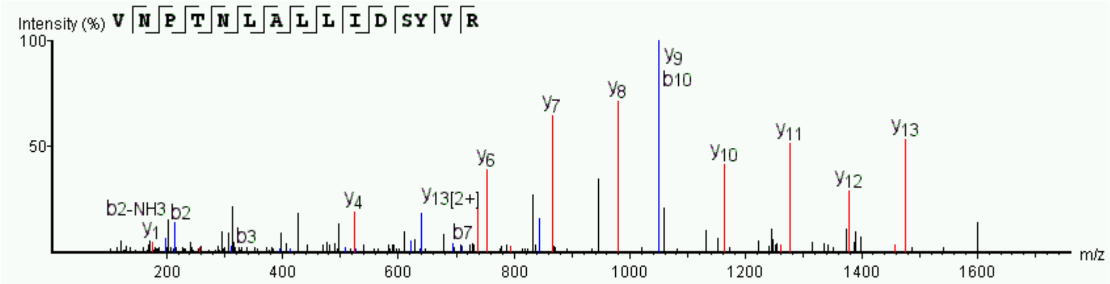

gi|571532725

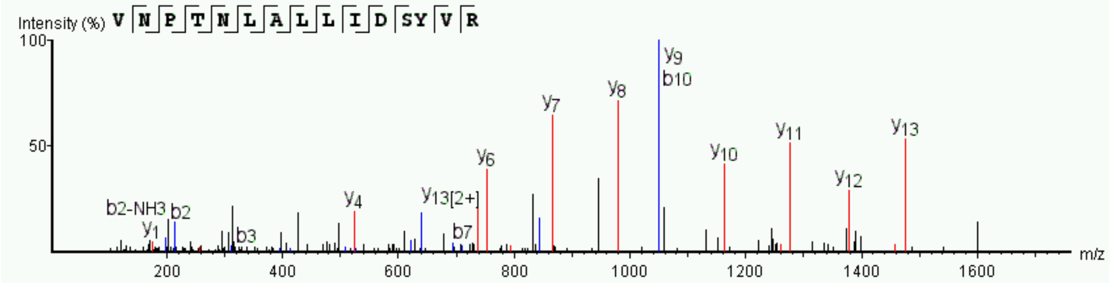

gi|571532729

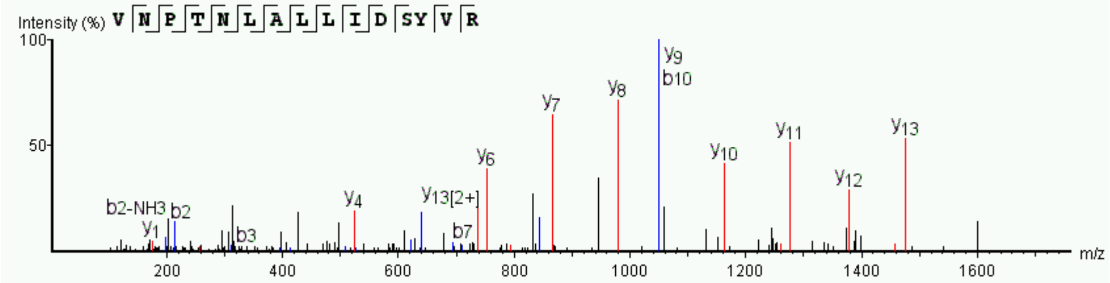

gi|571532734

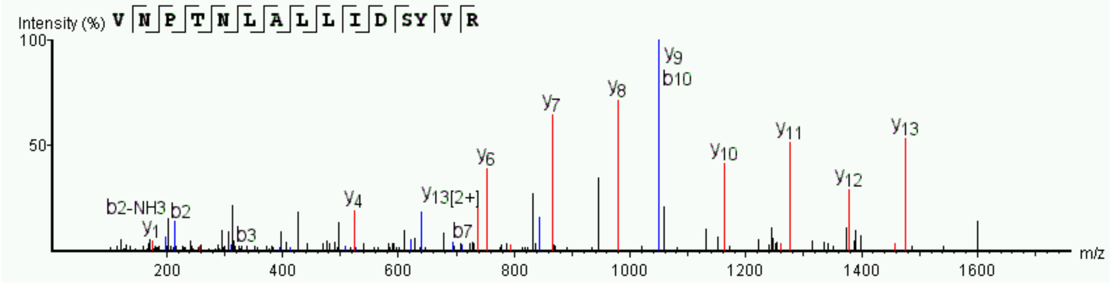

gi|571532738

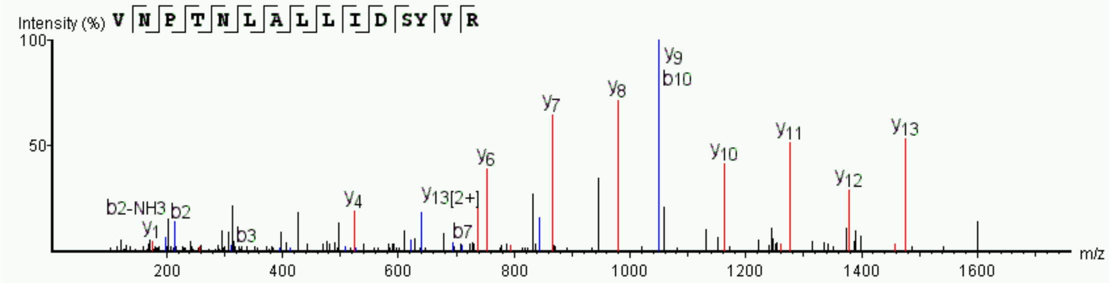

gi|571532741

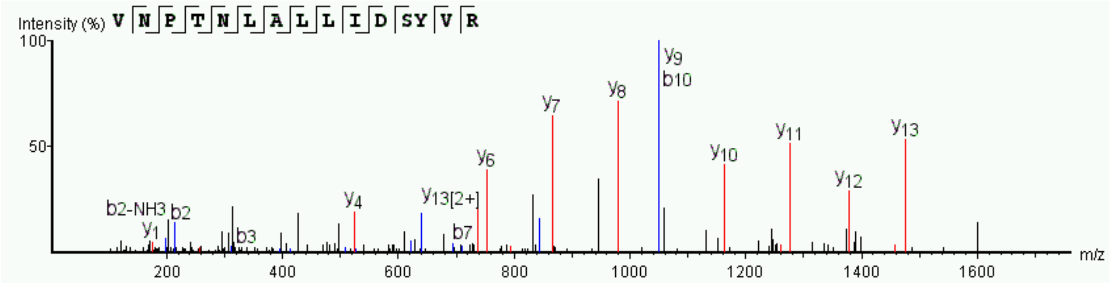

gi|328779207

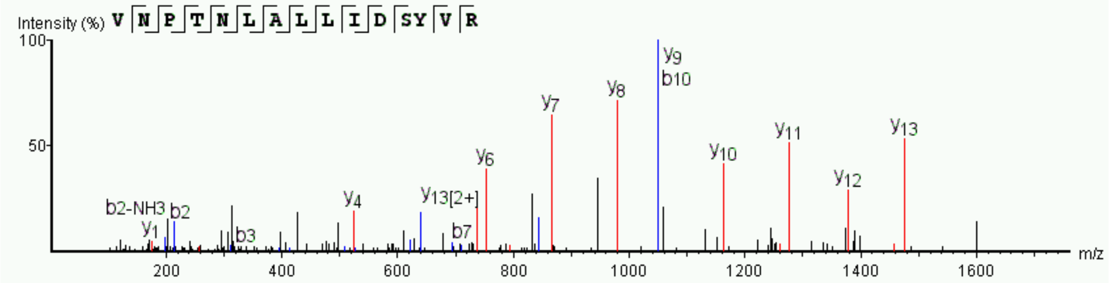

gi|571566701

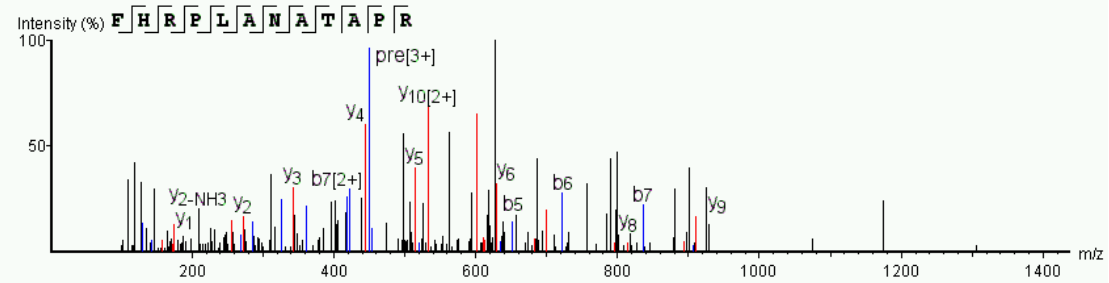

gi|328788421

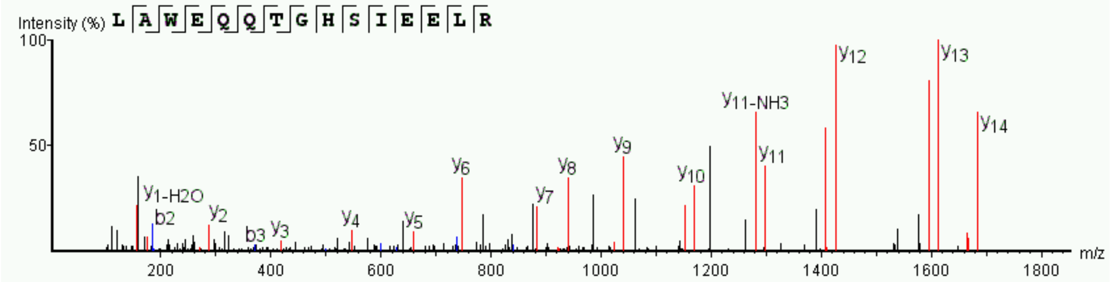

gi|571530521

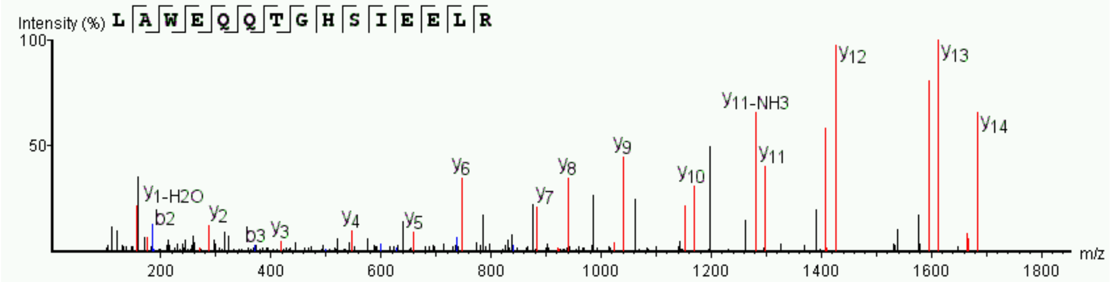

gi|571530525

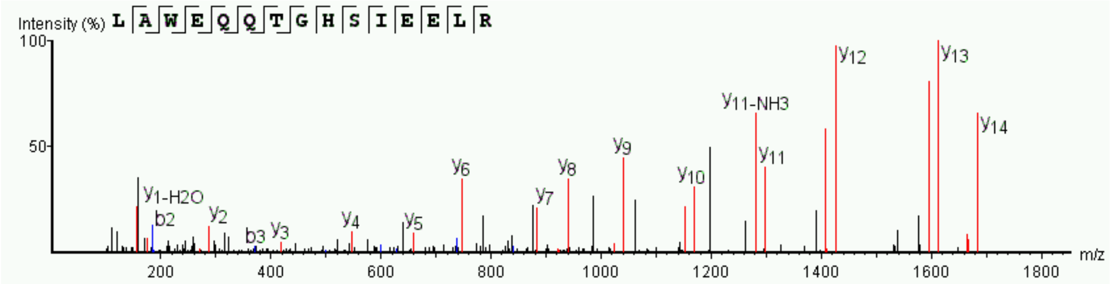

gi|571530489

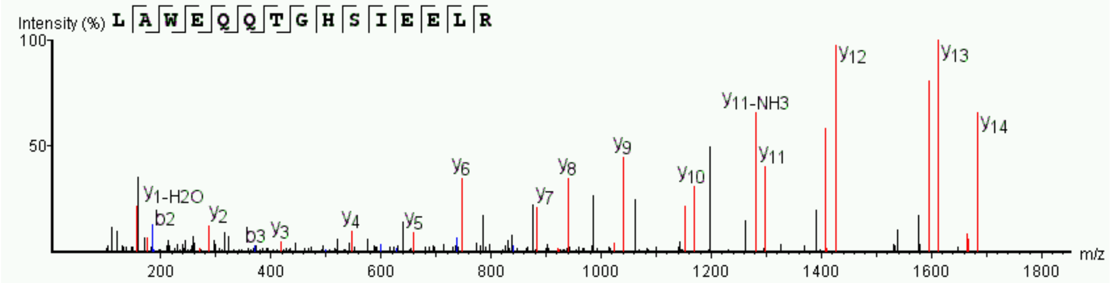

gi|571530492

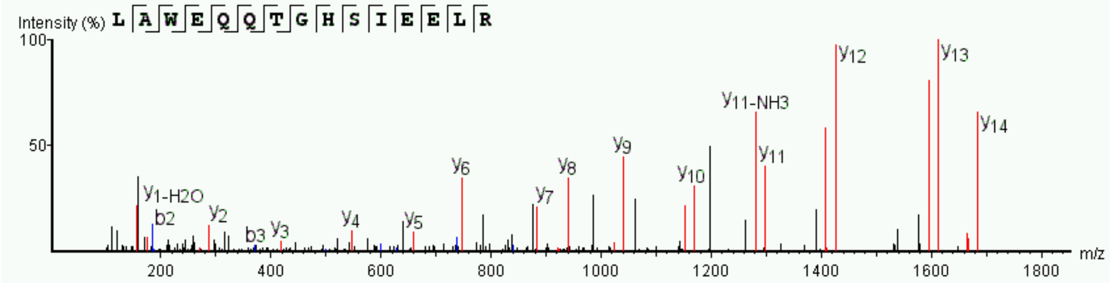

gi|571530496

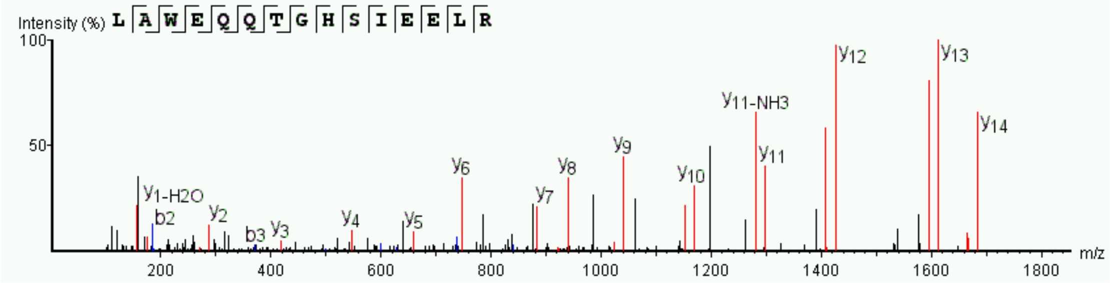

gi|571530502

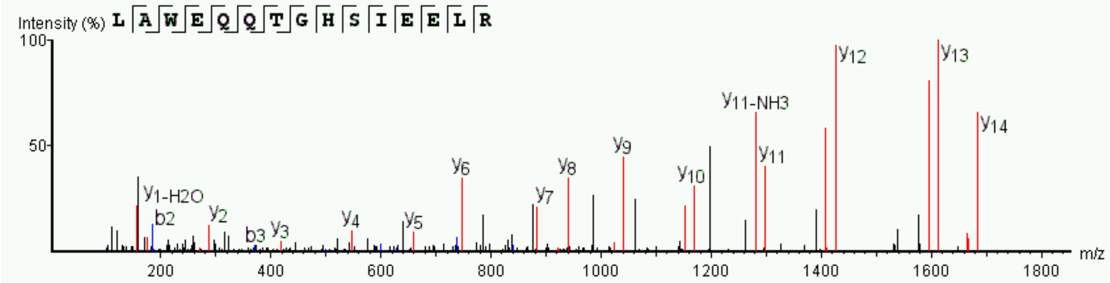

gi|571530506

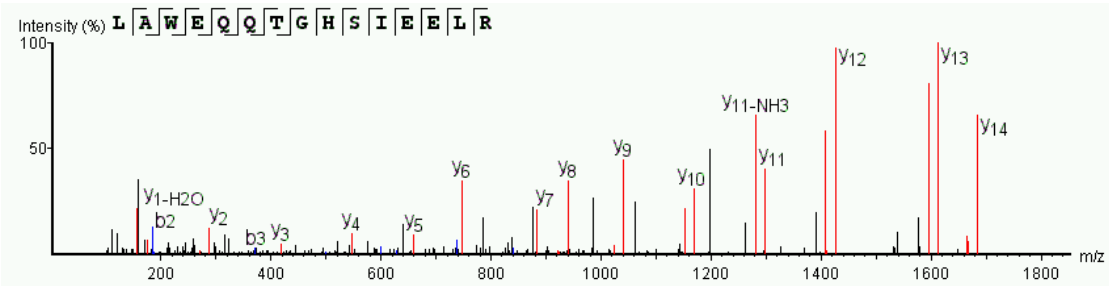

gi|571530511

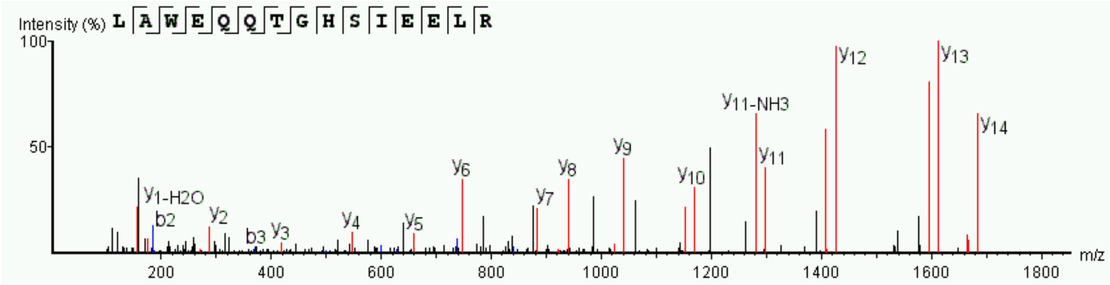

gi|571530515

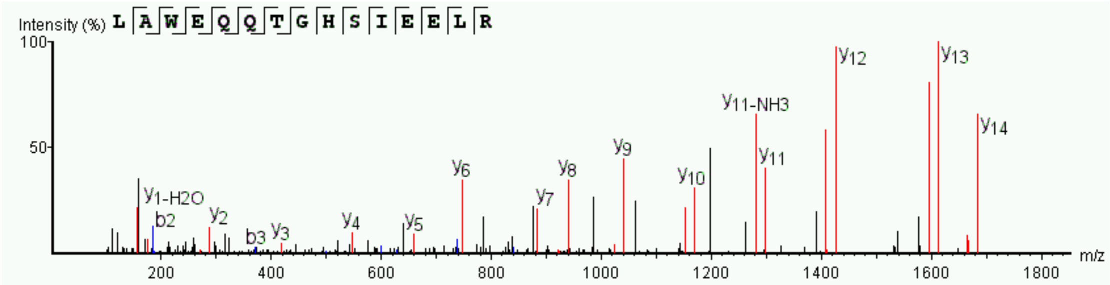

gi|571530518

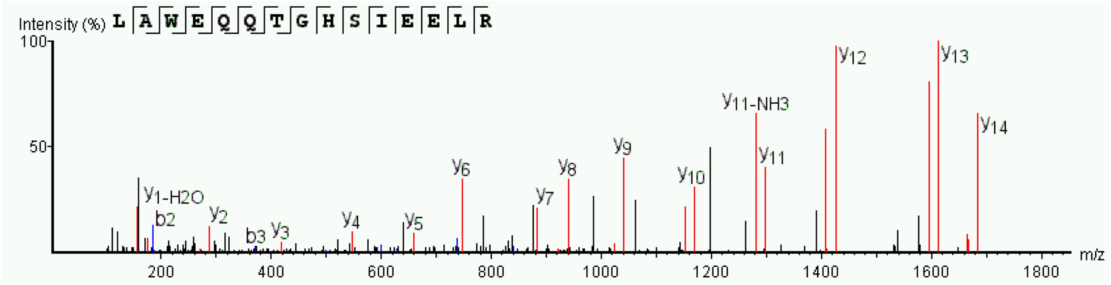

gi|110750012

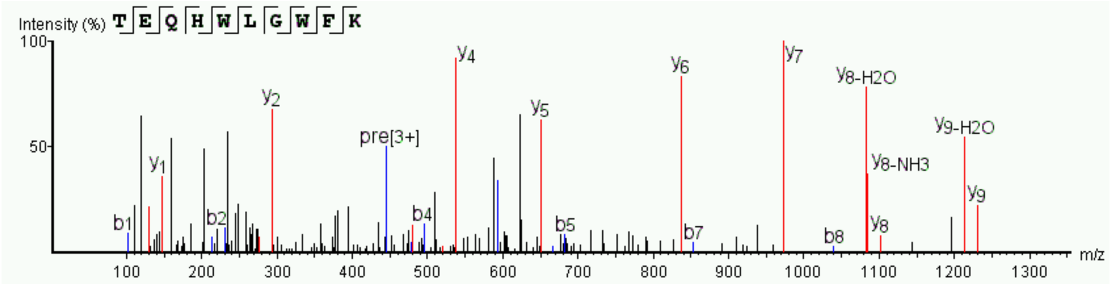

gi|328782520

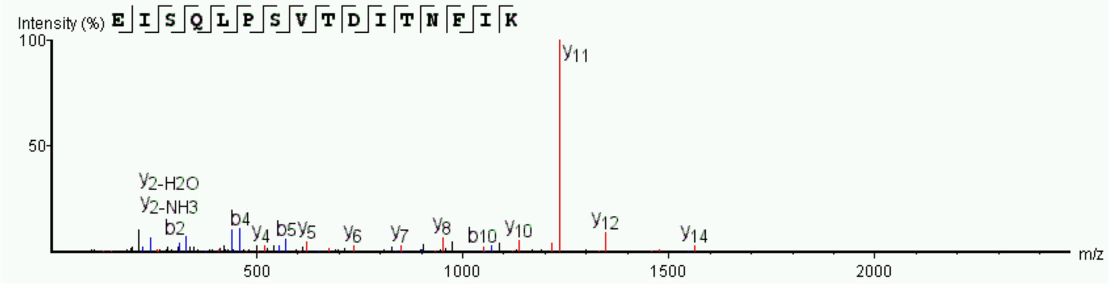

gi|571506842

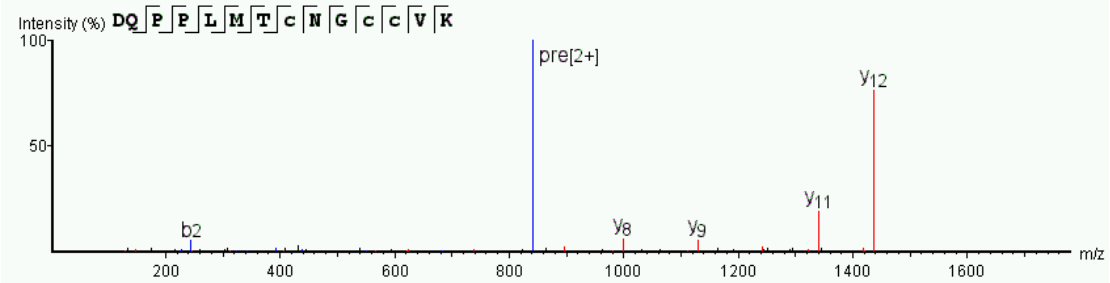

gi|571506845

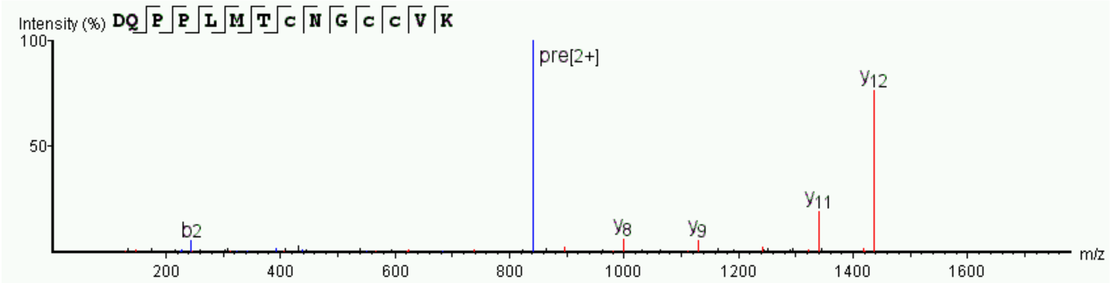

gi|571506848

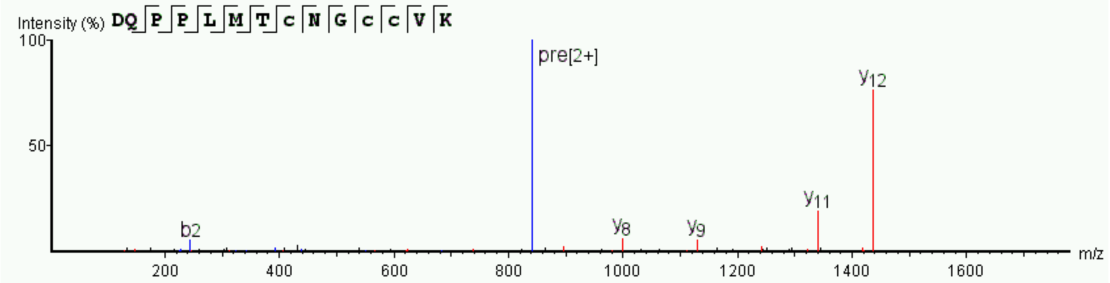

gi|571550221

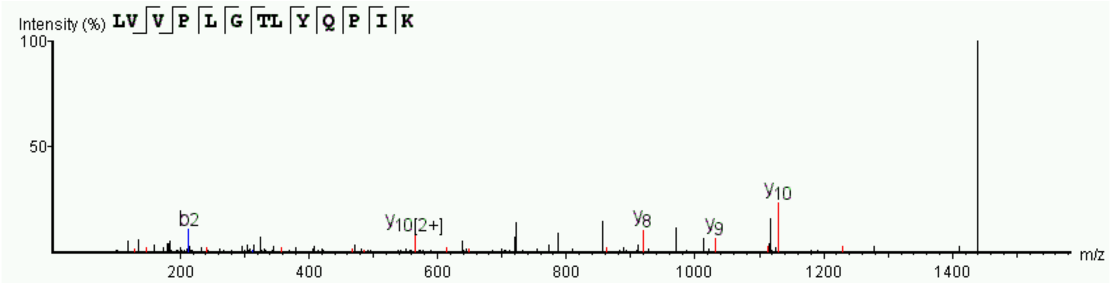

gi|328788157

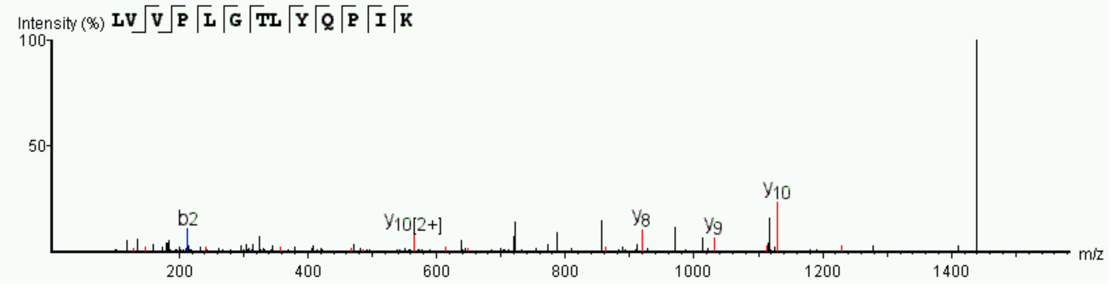

gi|571525946

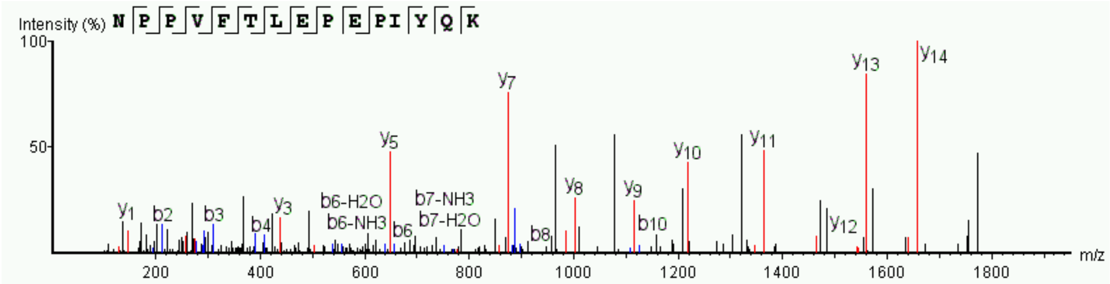

gi|571525950

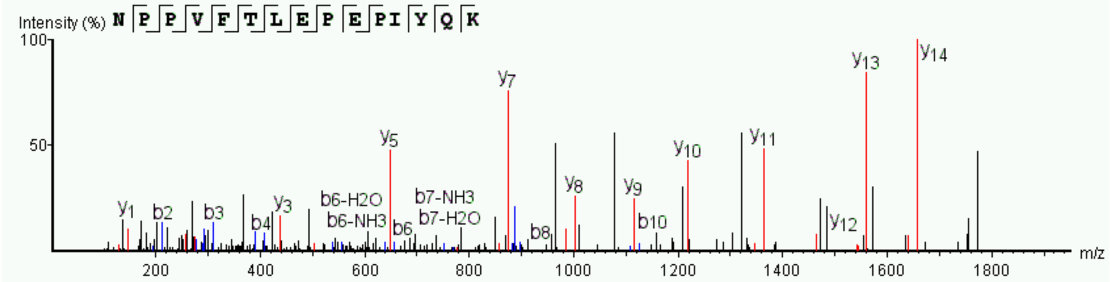

gi|571525957

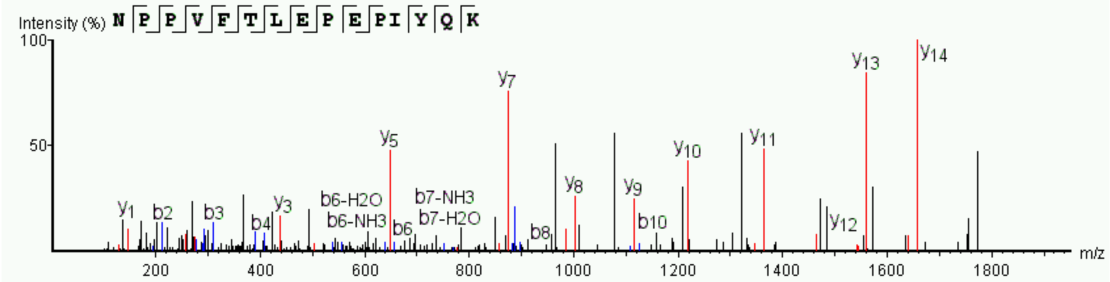

gi|571525961

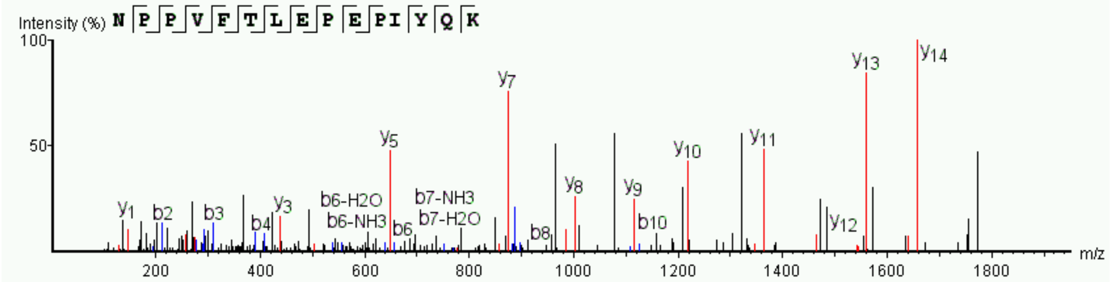

gi|571525968

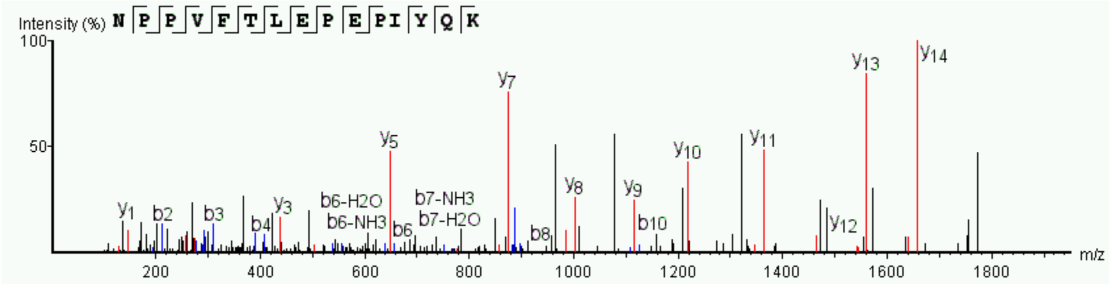

gi|571525972

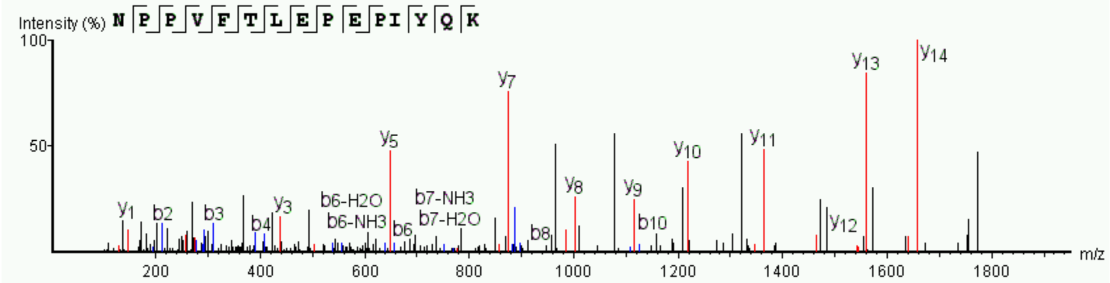

gi|328790055

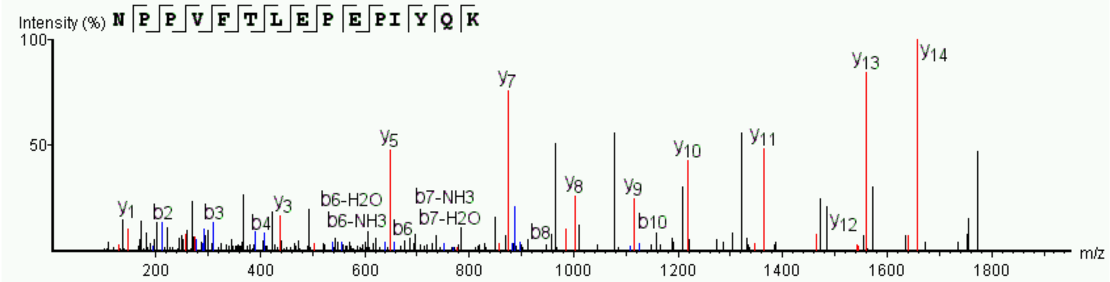

gi|328790053

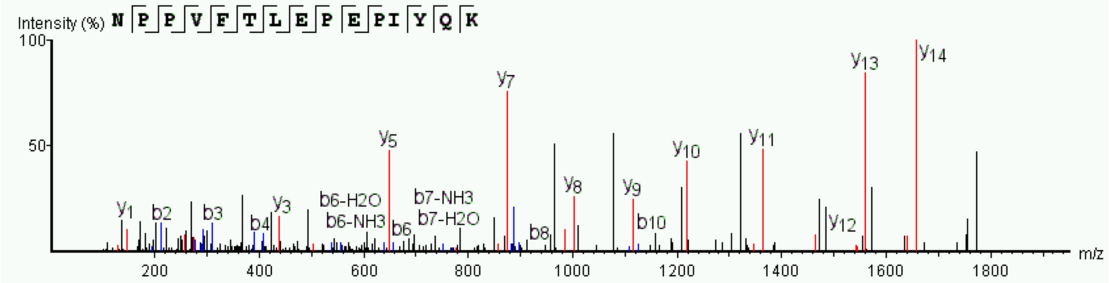

gi|571552419

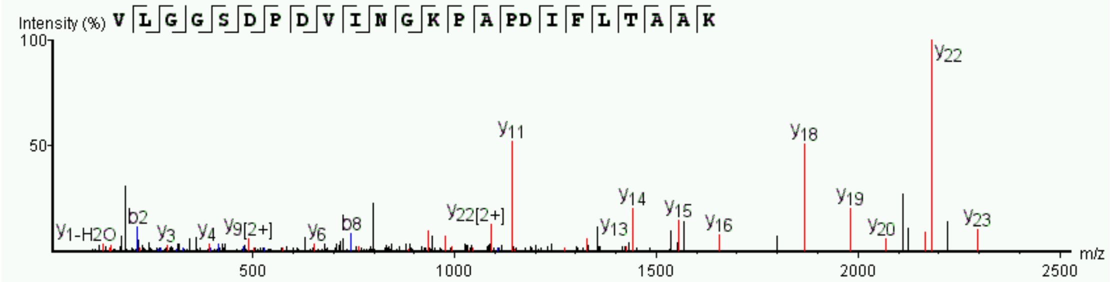

gi|571552423

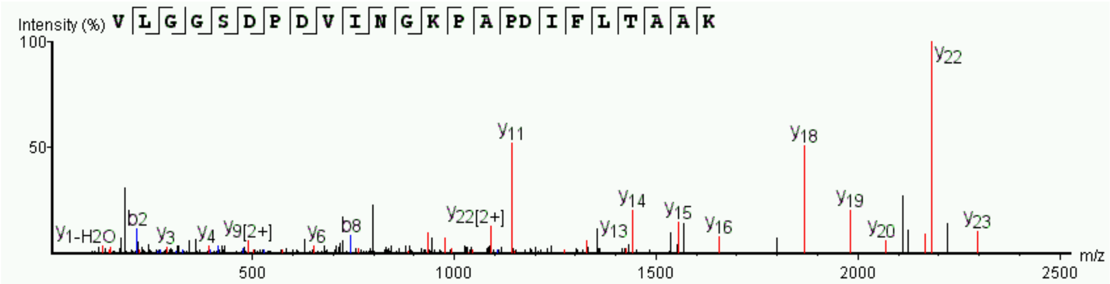

gi|328782042

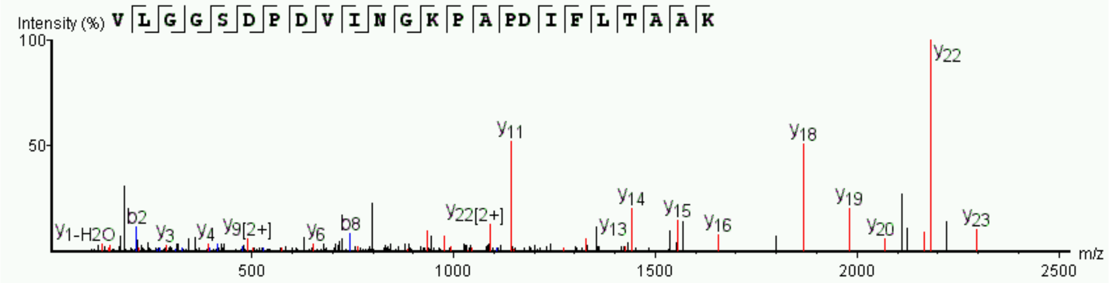

gi|571508047

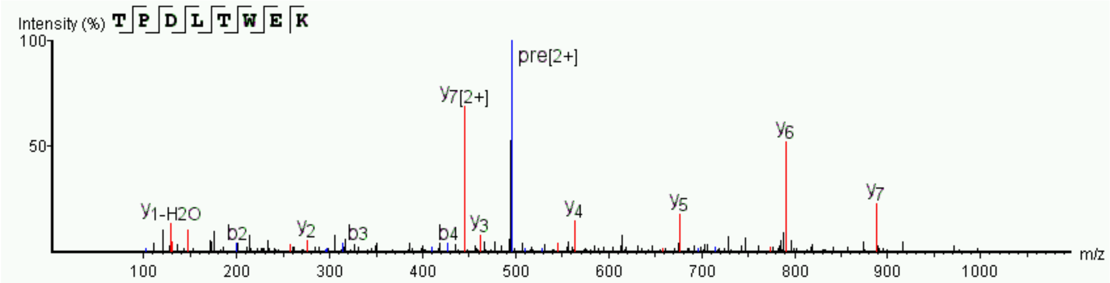

gi|571508050

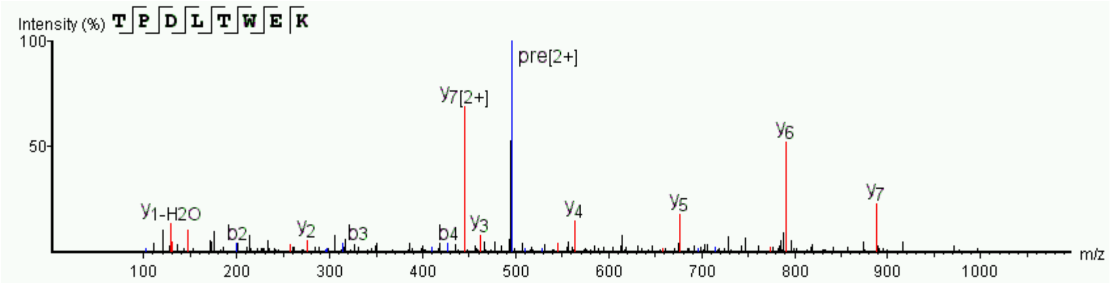

gi|110749750

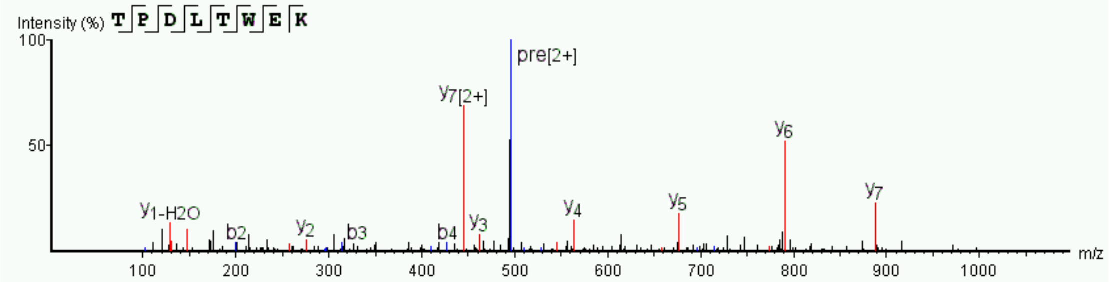

gi|571566593

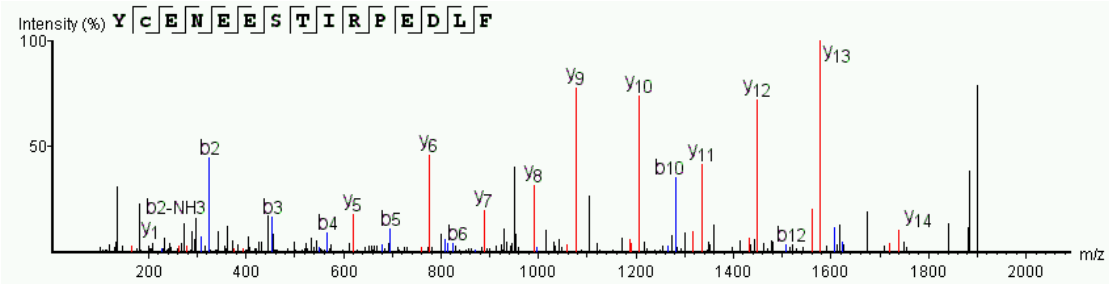

gi|571566596

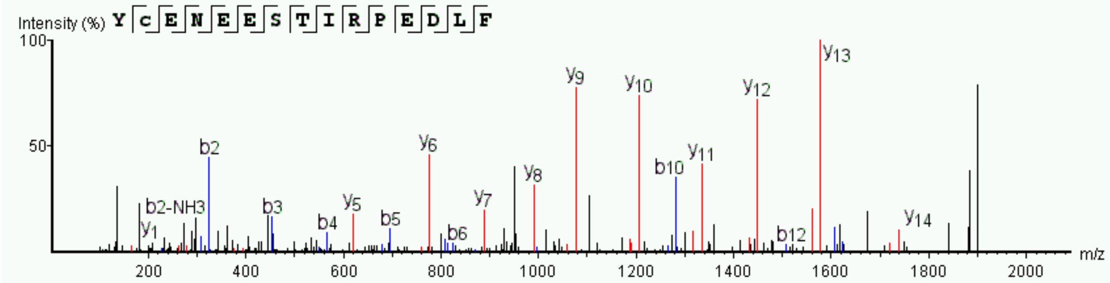

gi|571566599

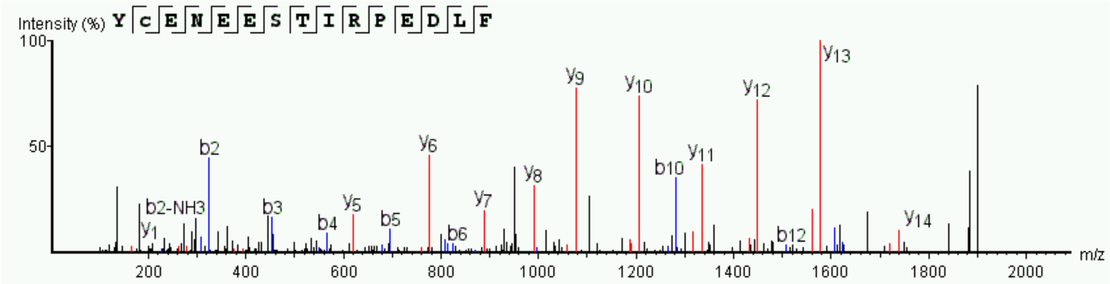

gi|66522157

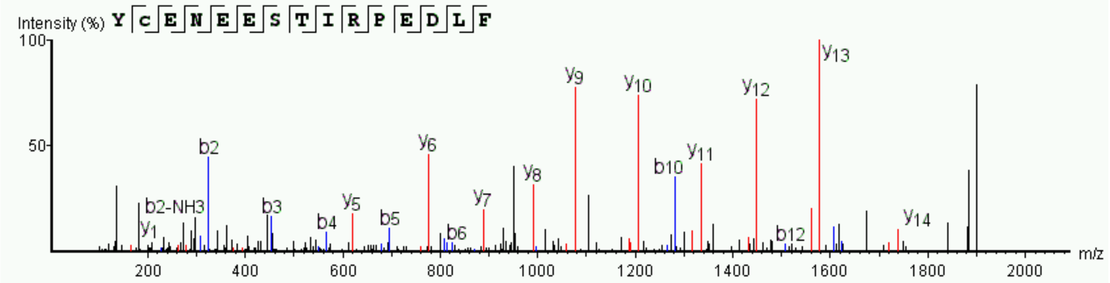

gi|571577493

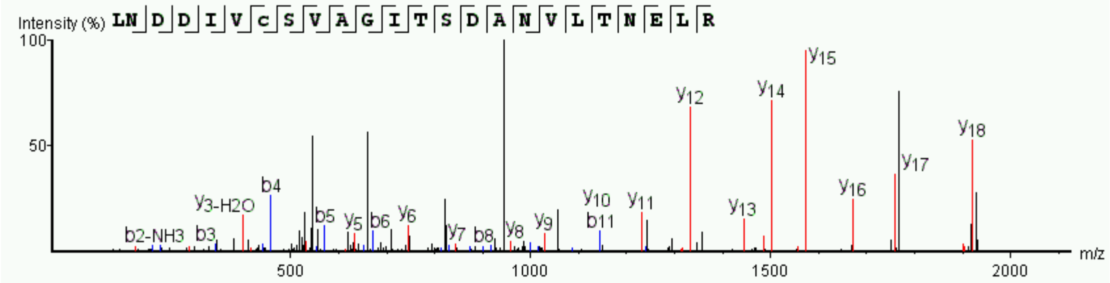

gi|328793299

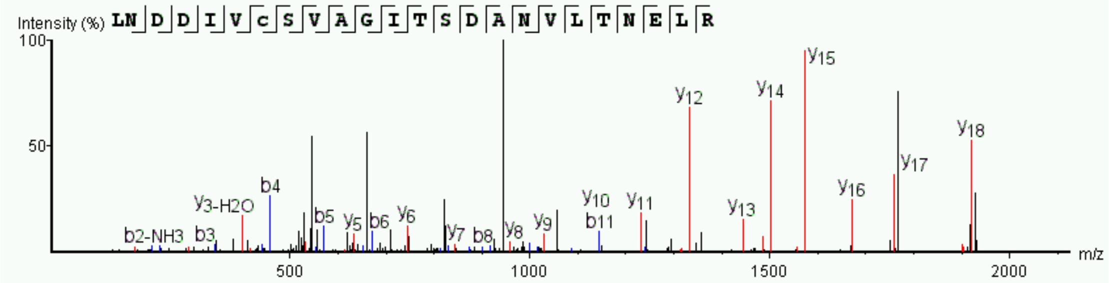

gi|66504967

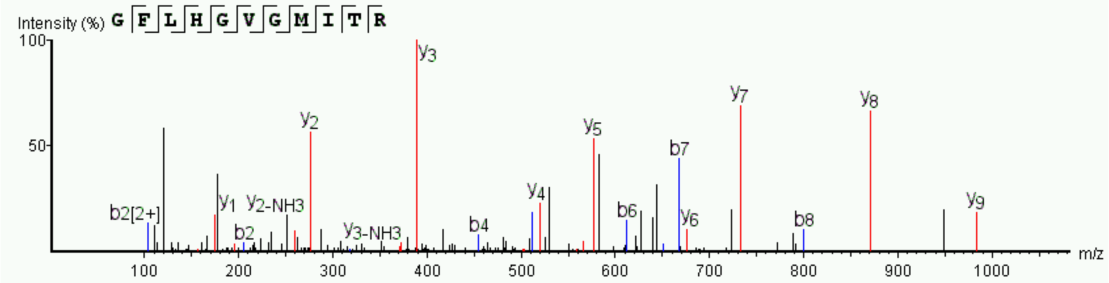

gi|571554543

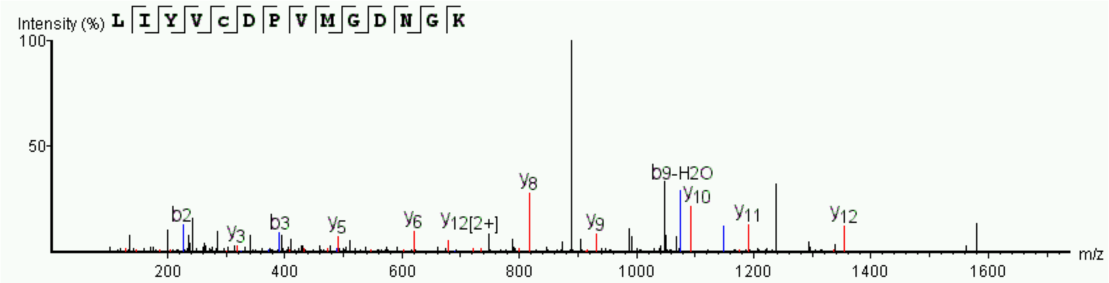

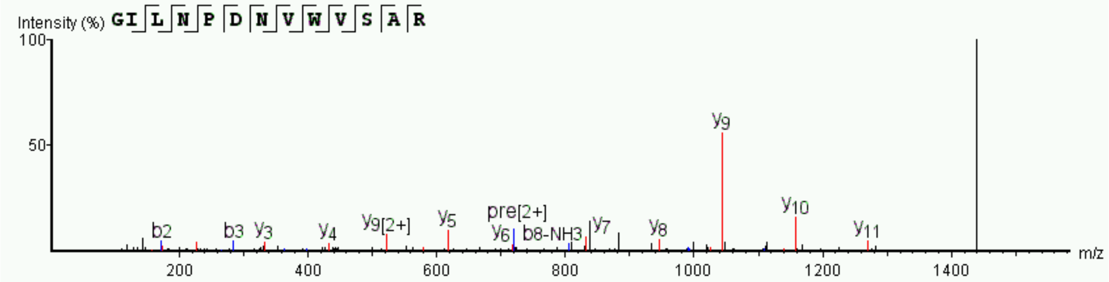

gi|571514556

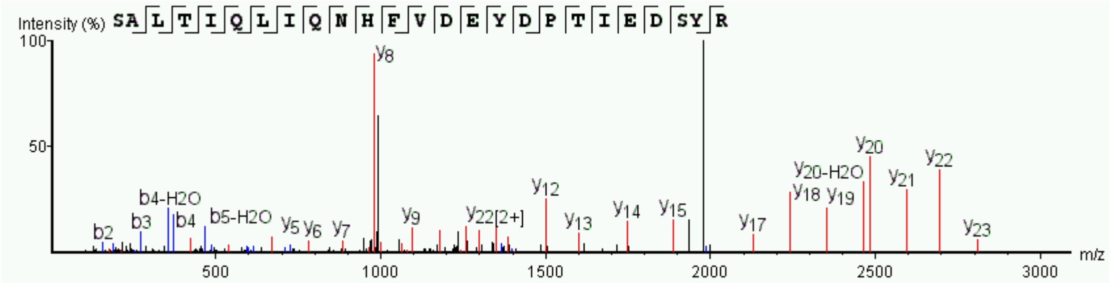

gi|571514559

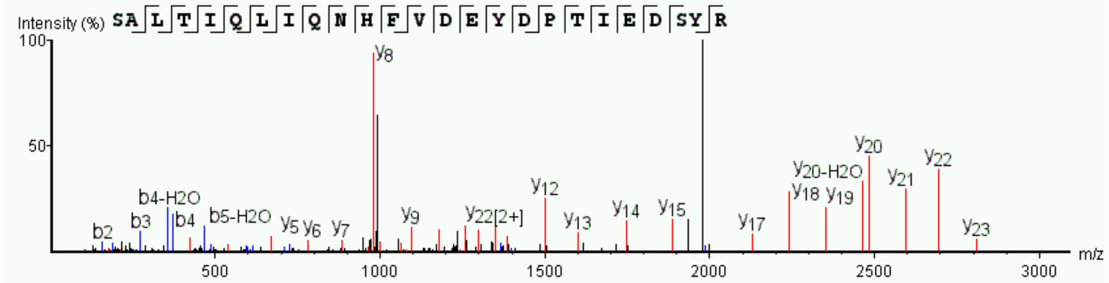

gi|48103887

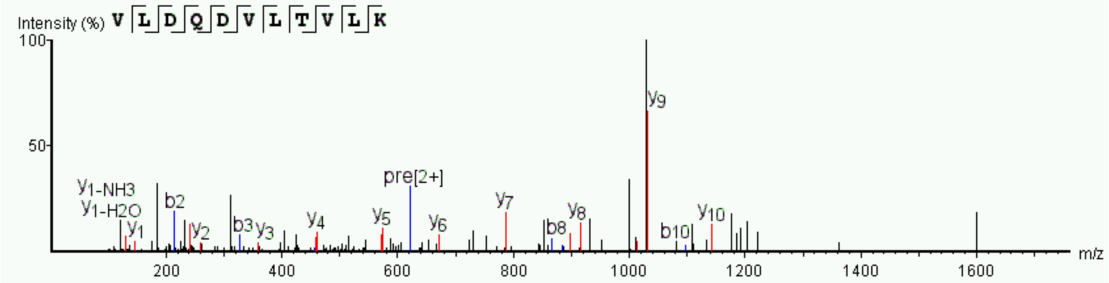

gi|571510495

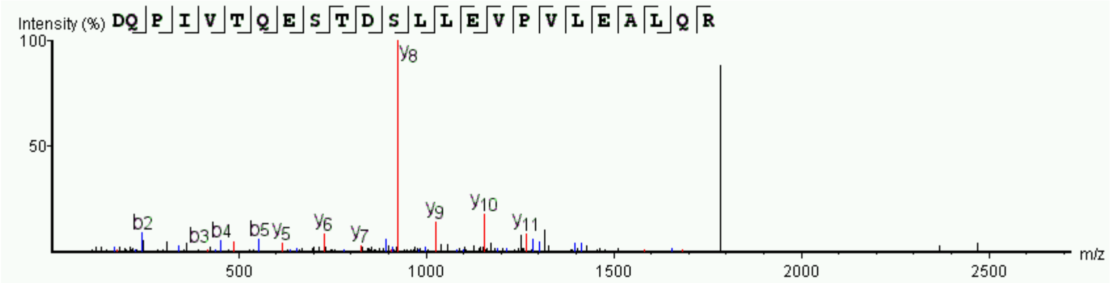

gi|571510498

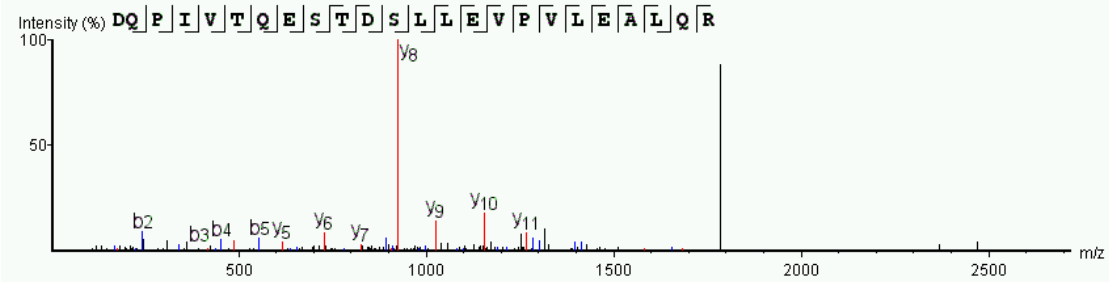

gi|571510510

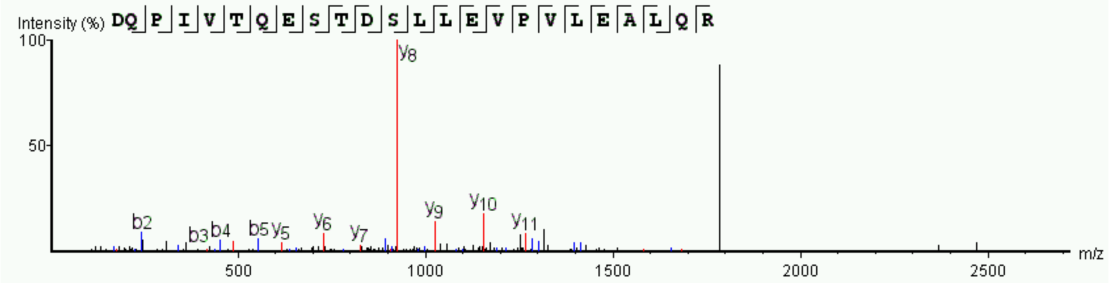

gi|571547398

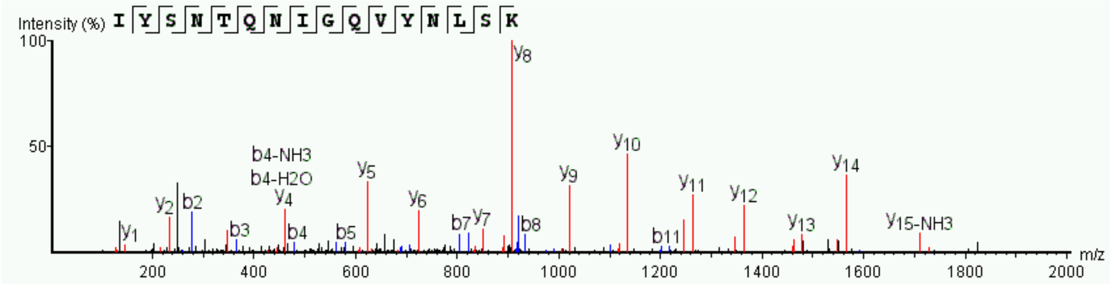

gi|328776891

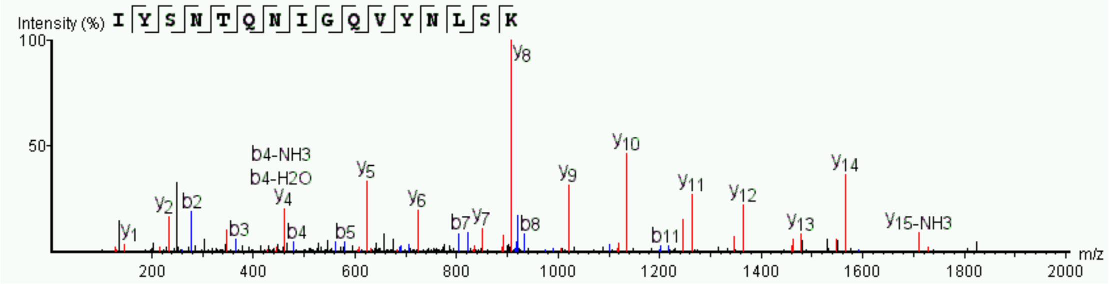

gi|571542574

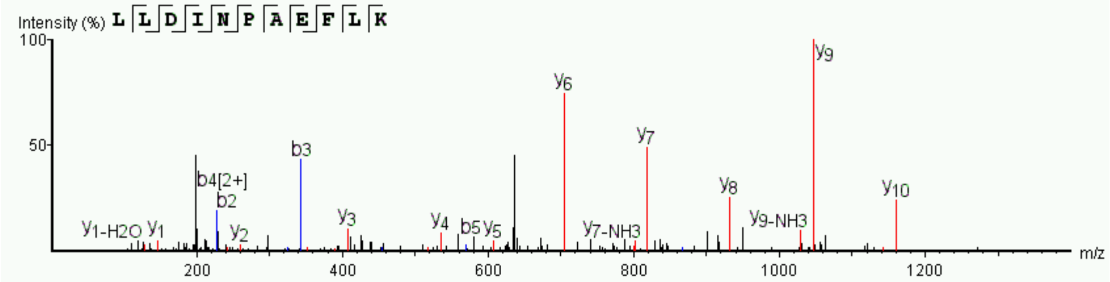

gi|571542608

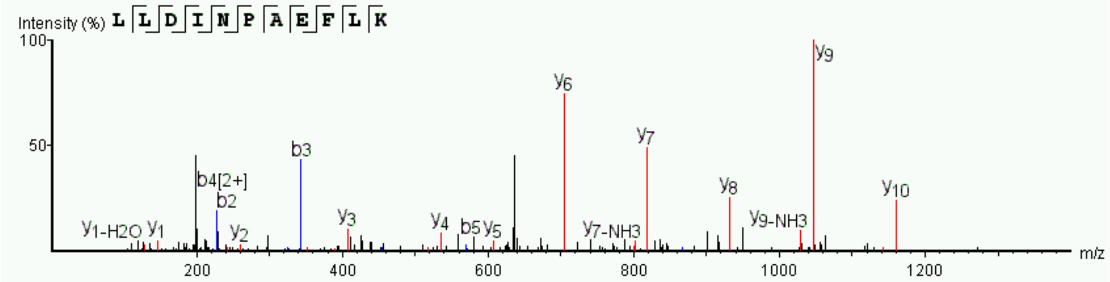

gi|571542611

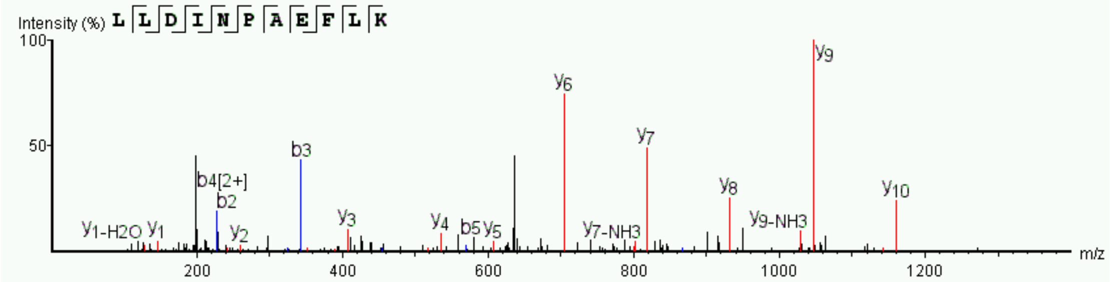

gi|571542614

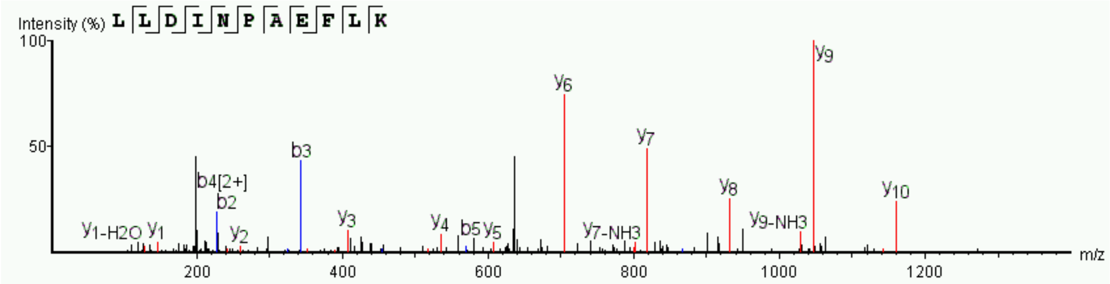

gi|571542618

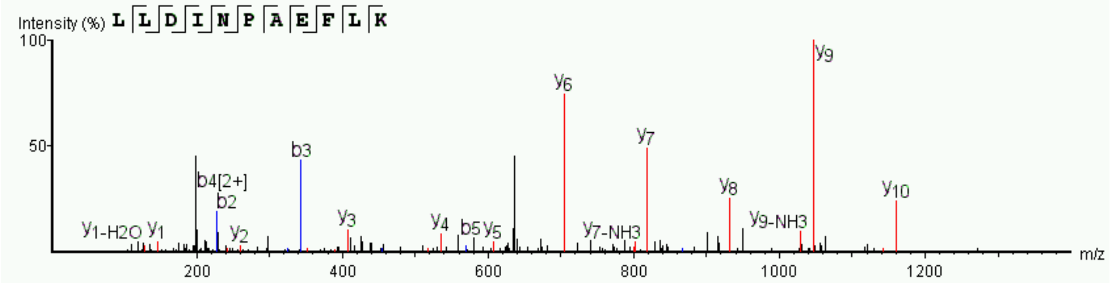

gi|571542624

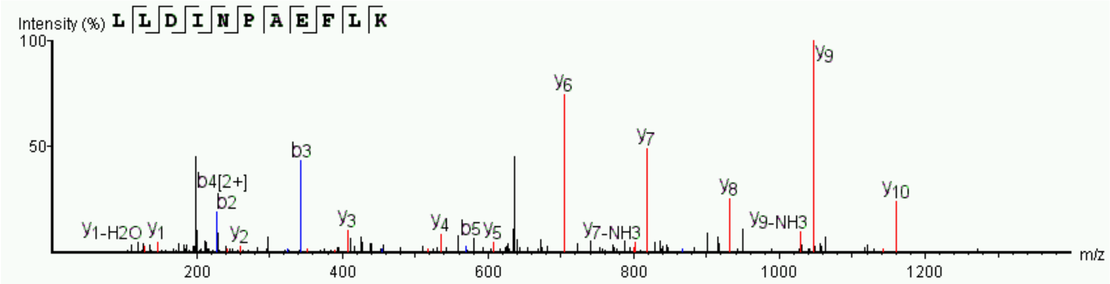

gi|571542628

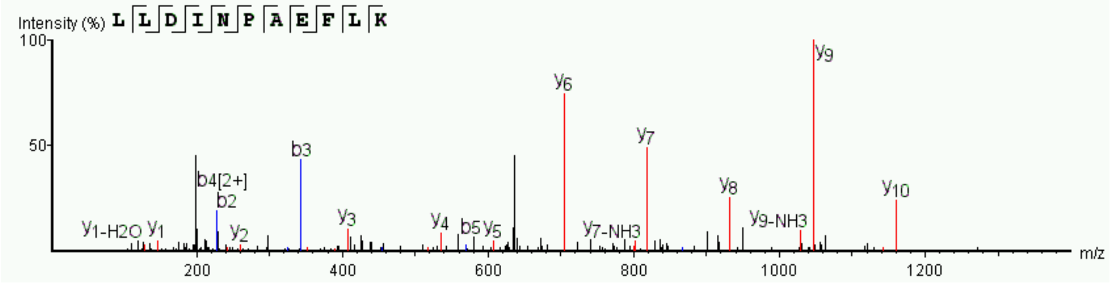

gi|571542631

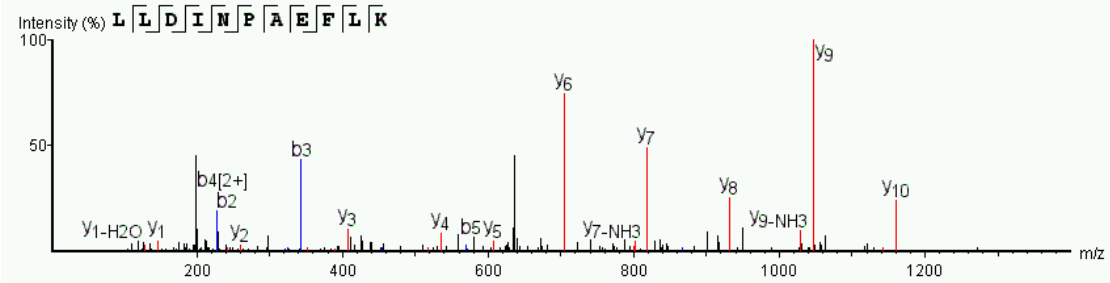

gi|571542635

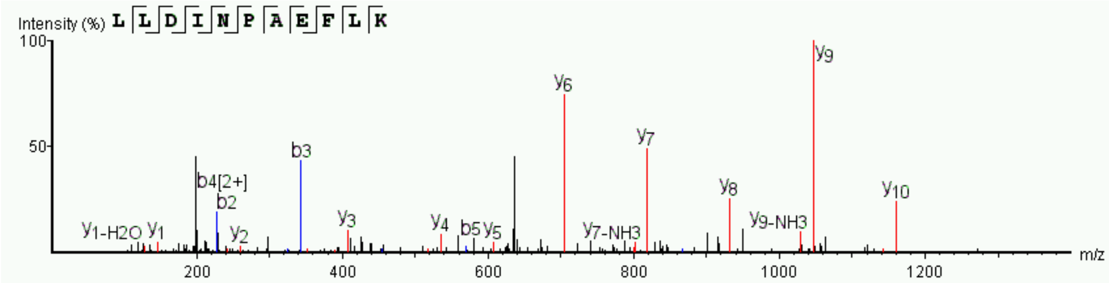

gi|571542638

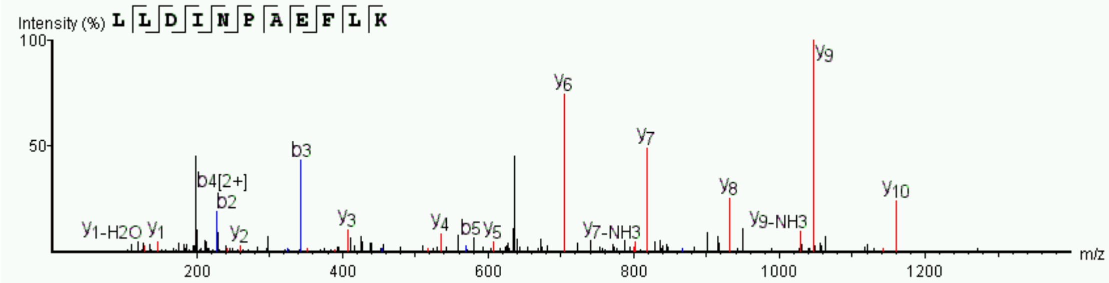

gi|571542641

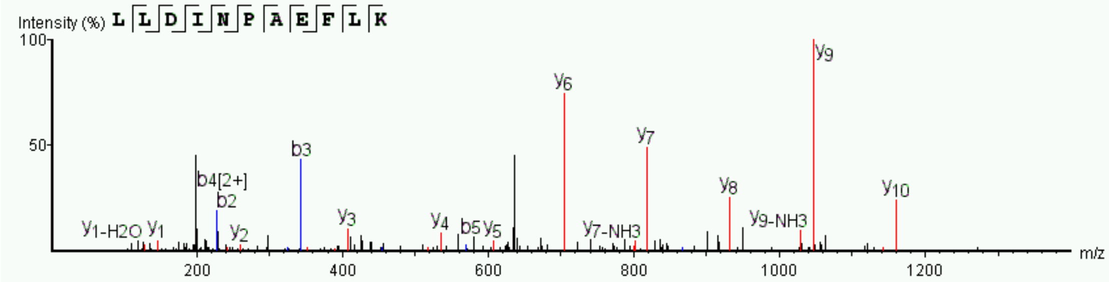

gi|571542577

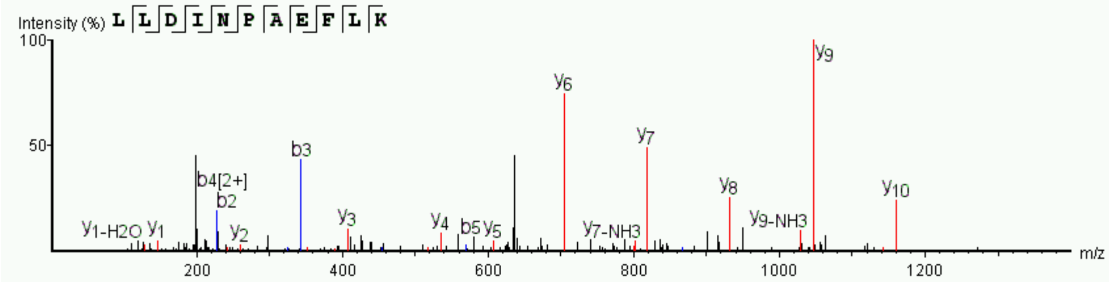

gi|571542645

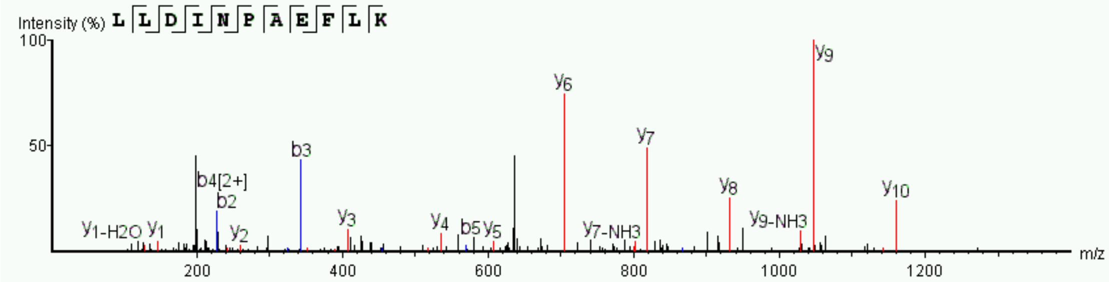

gi|571542581

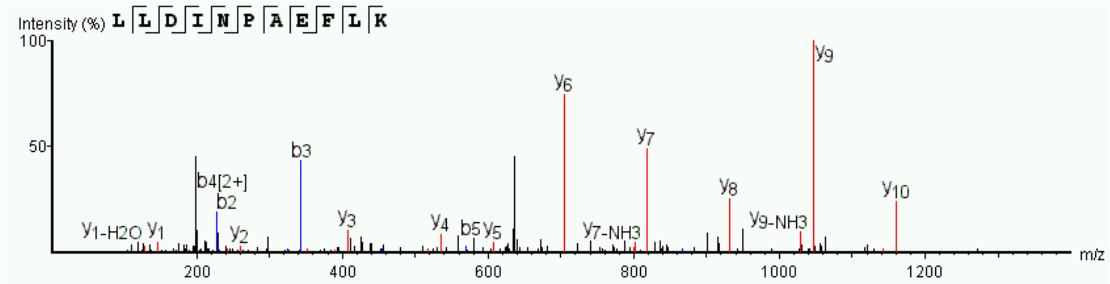

gi|571542585

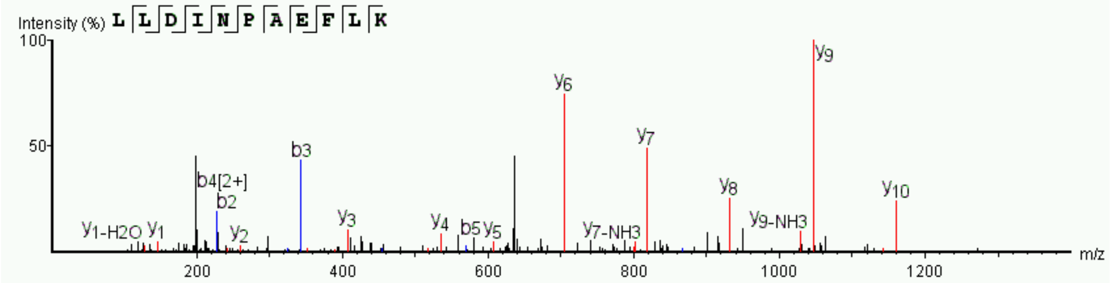

gi|571542589

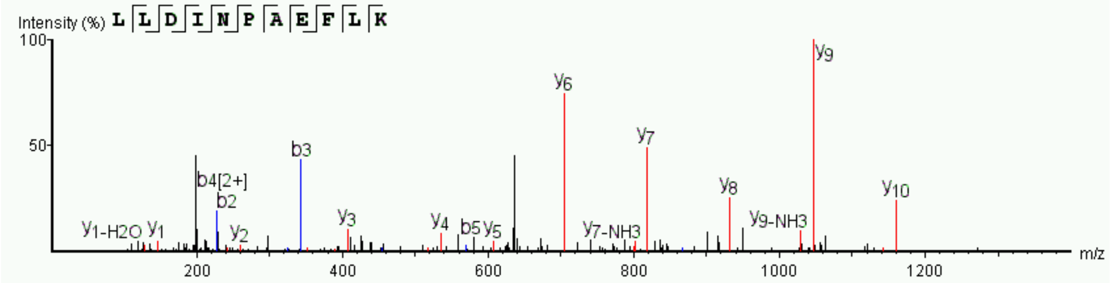

gi|571542593

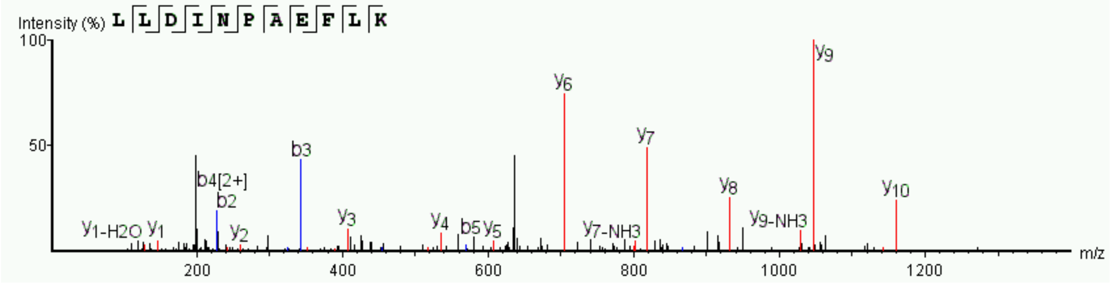

gi|571542597

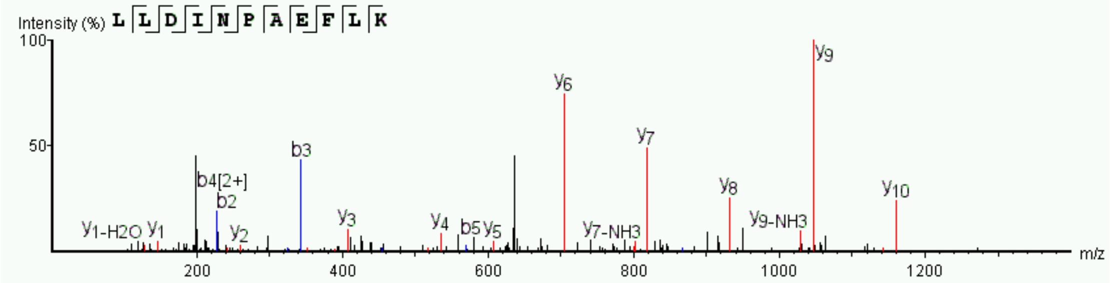

gi|571542600

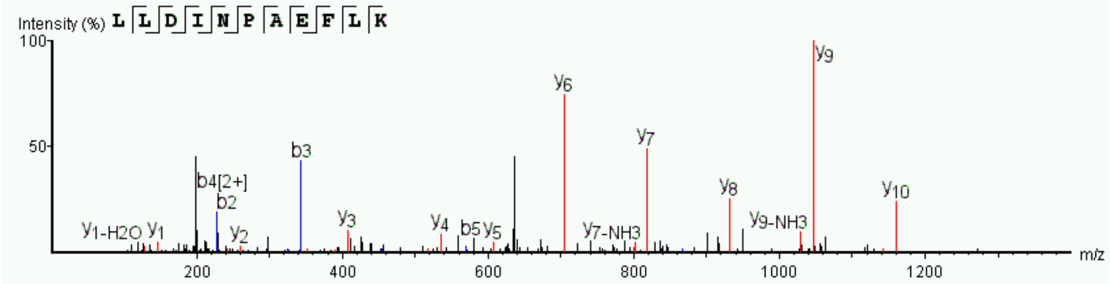

gi|571542604

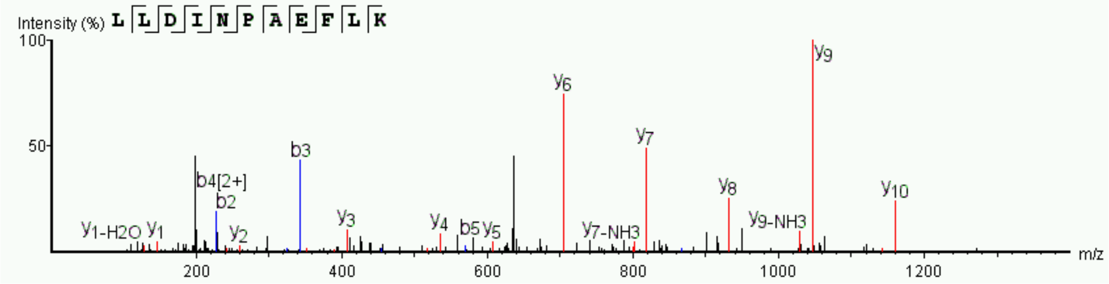

gi|571571722

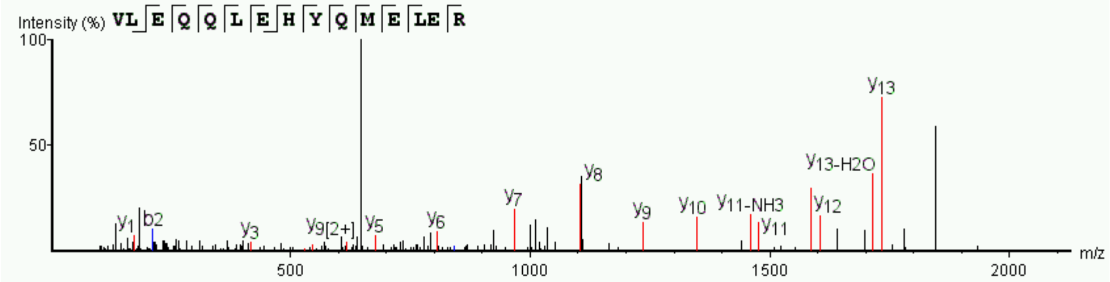

gi|571571726

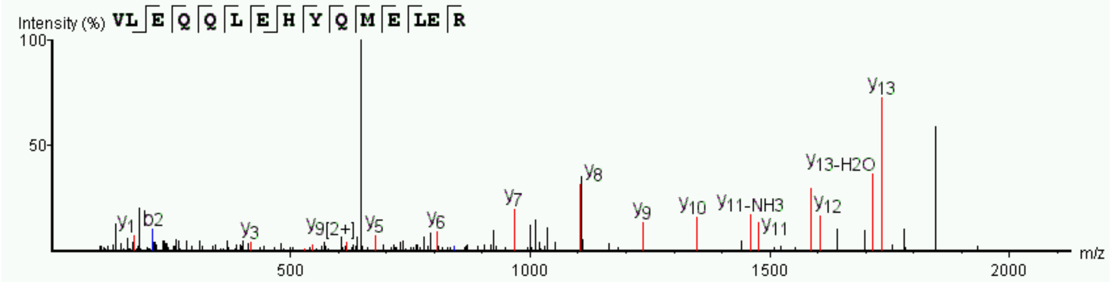

gi|571571730

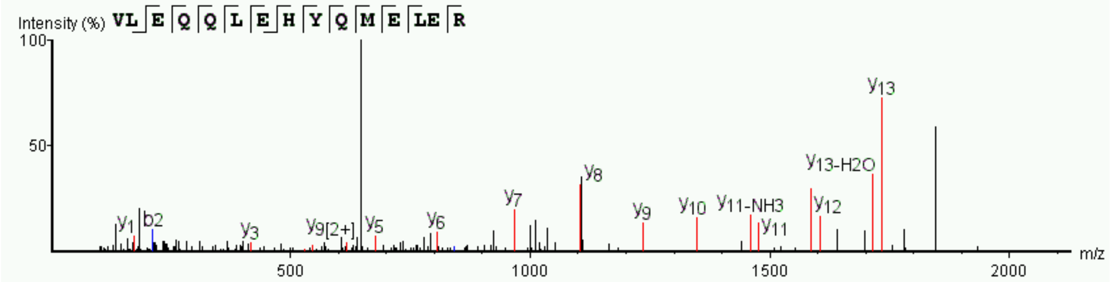

gi|571571733

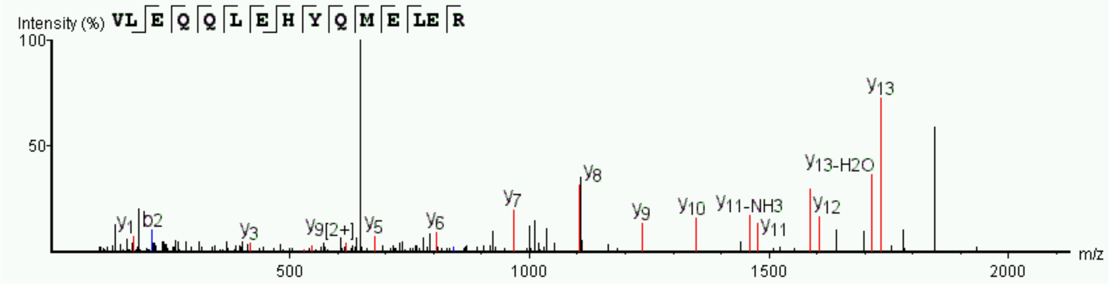

gi|571560288

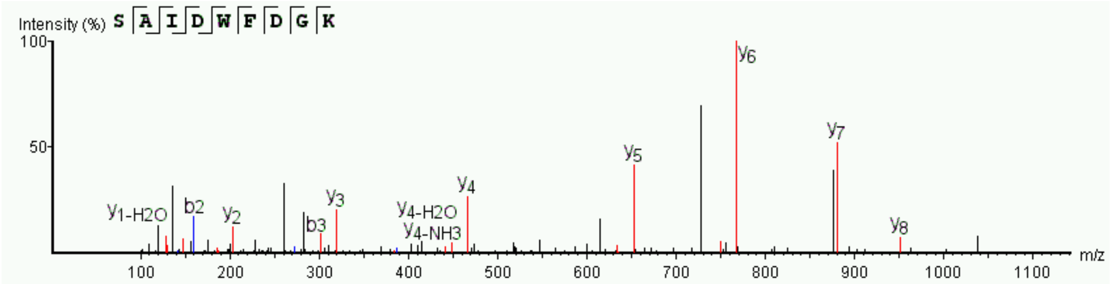

gi|571560291

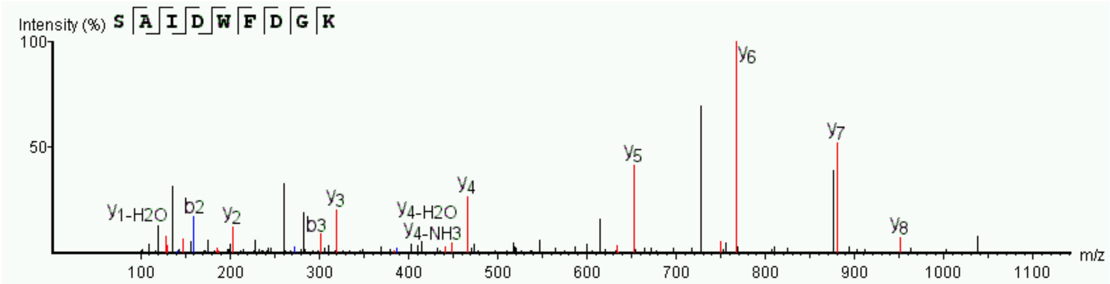

gi|571560294

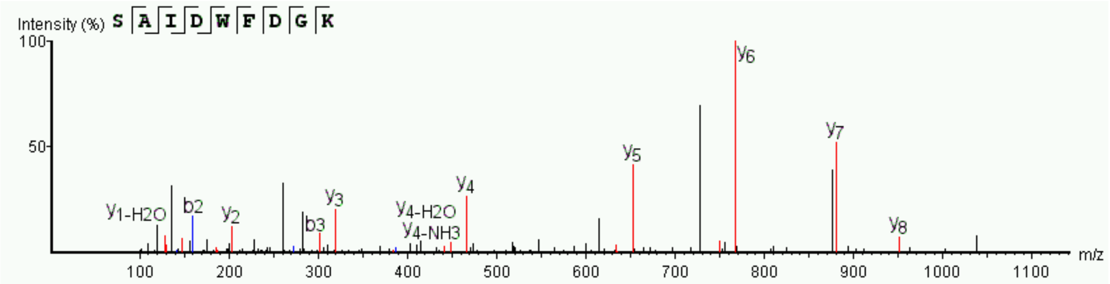

gi|571512336

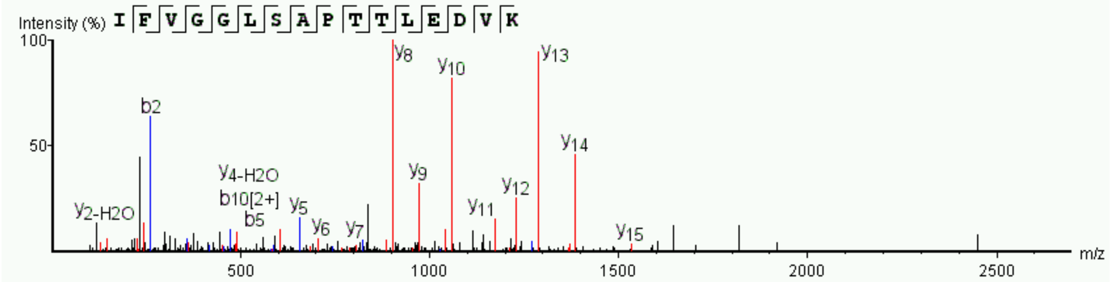

gi|571512339

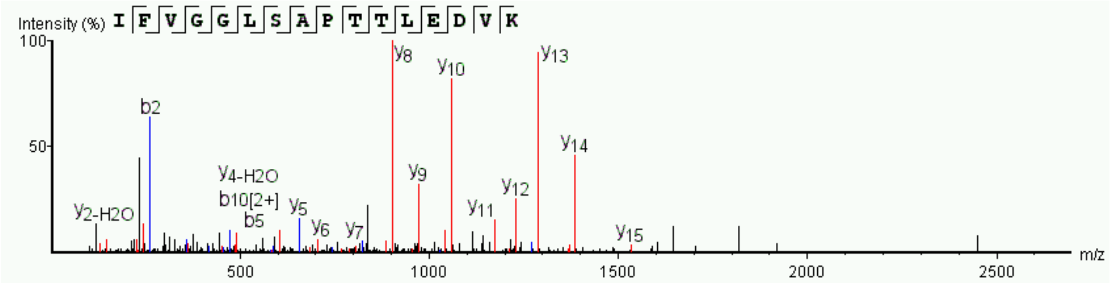

gi|571512342

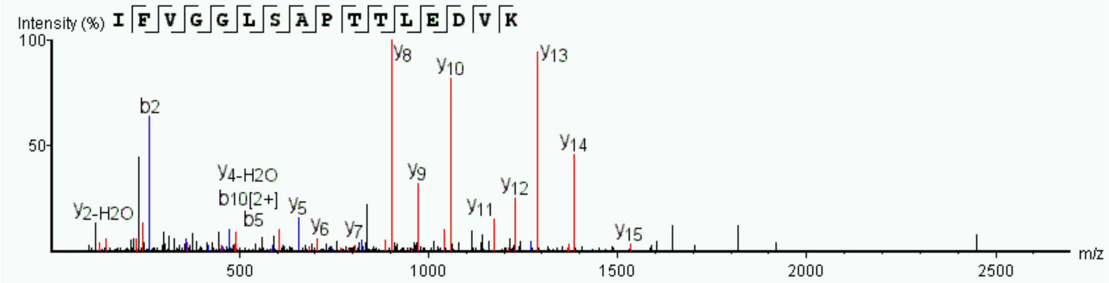

gi|571575626

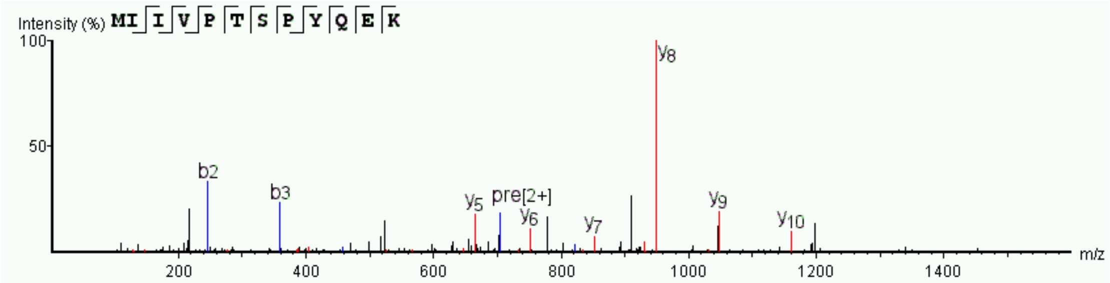

gi|571575628

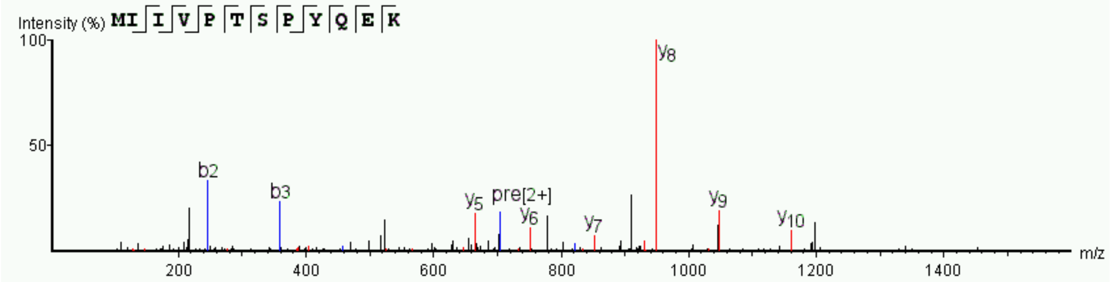

gi|571575630

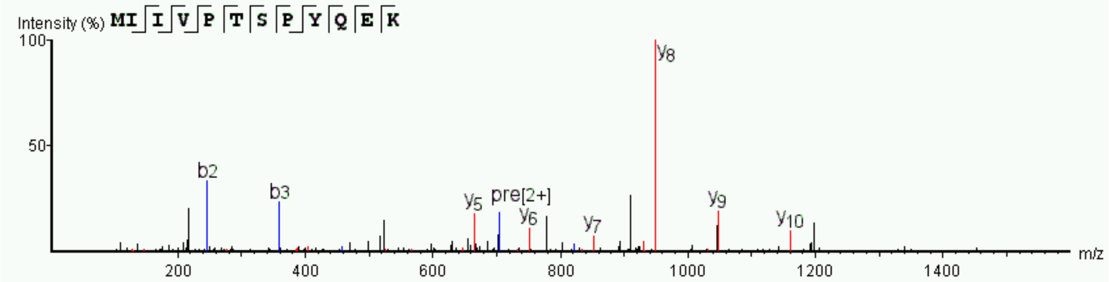

gi|571568001

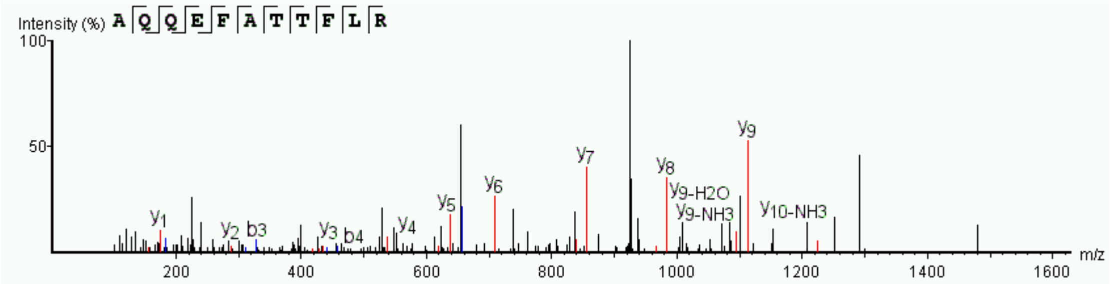

gi|328779563

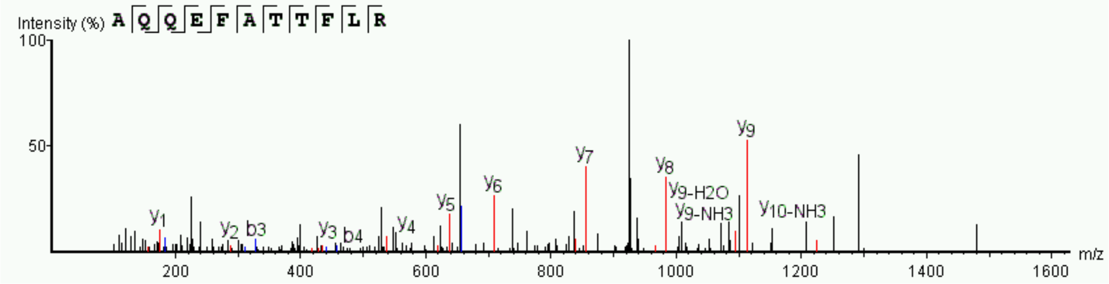

gi|571579476

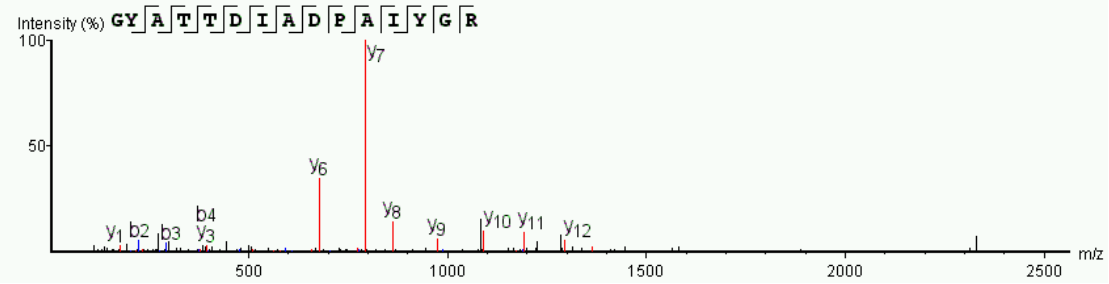

gi|571558143

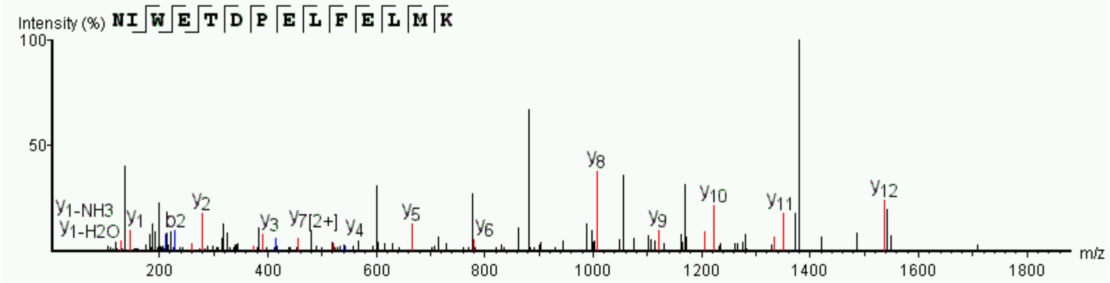

gi|571558147

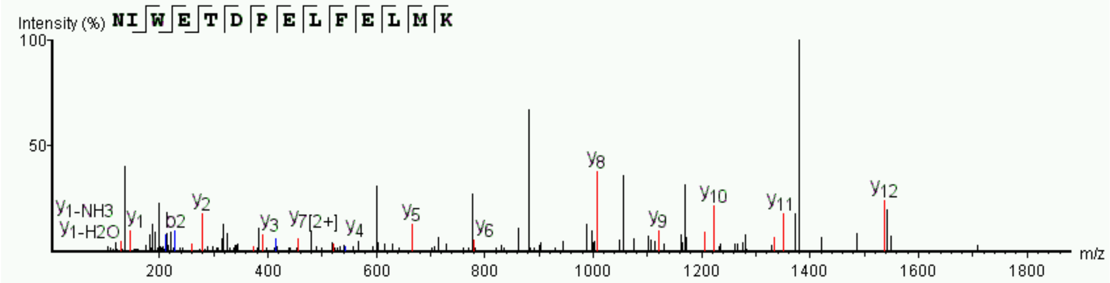

gi|571558150

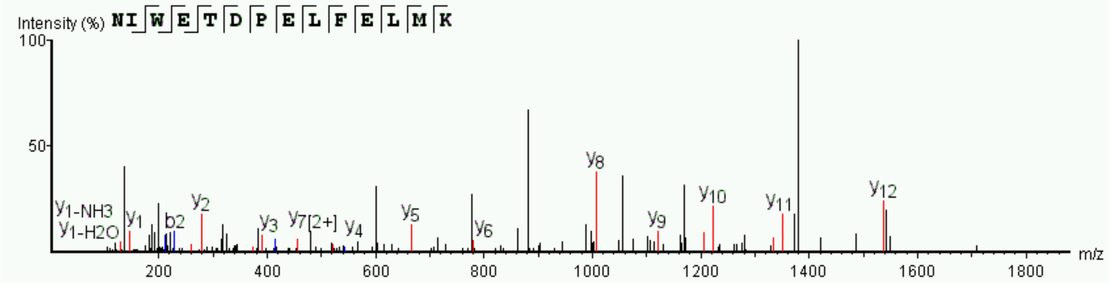

gi|571560076

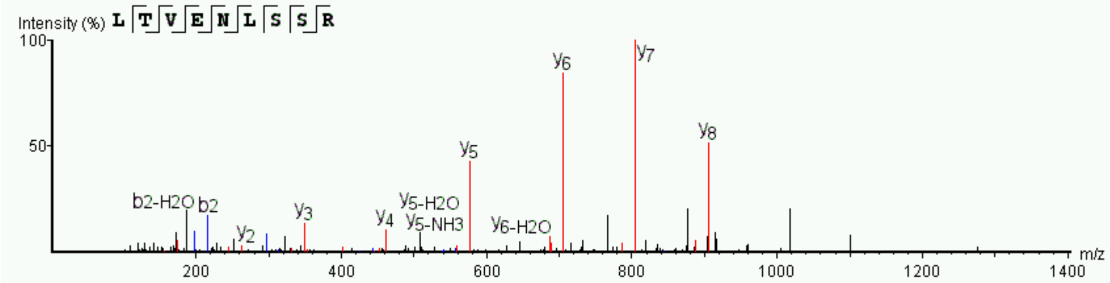

gi|571560128

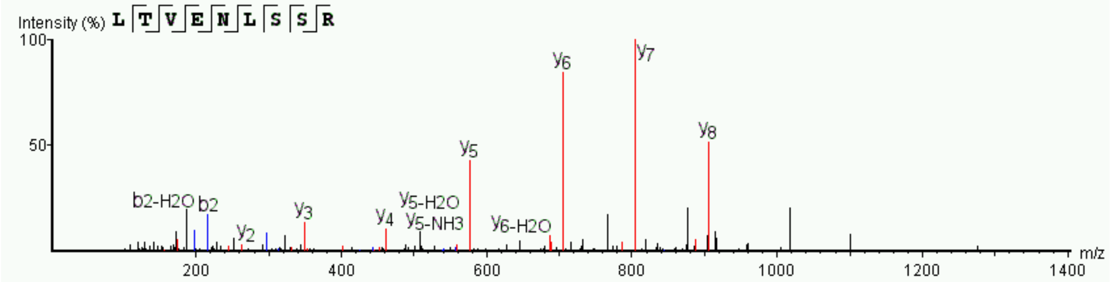

gi|571560154

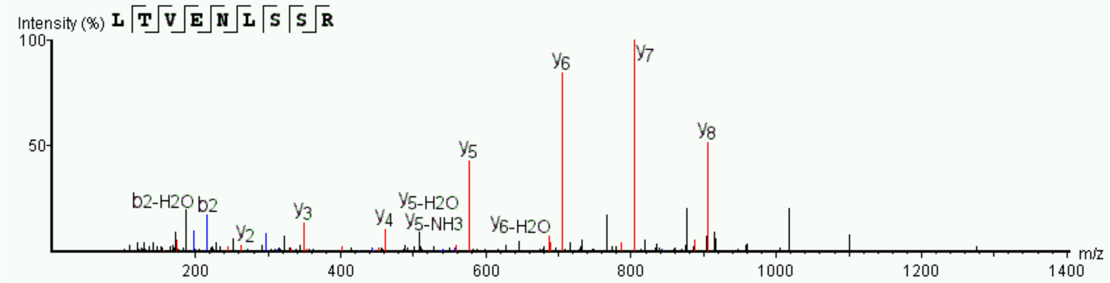

gi|571538301

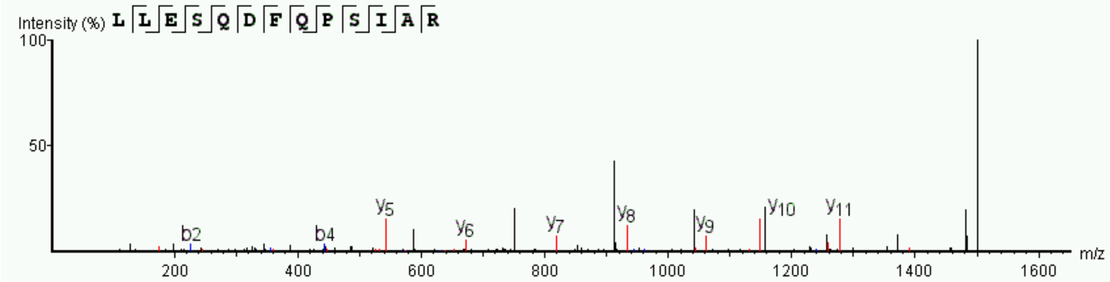

gi|571538305

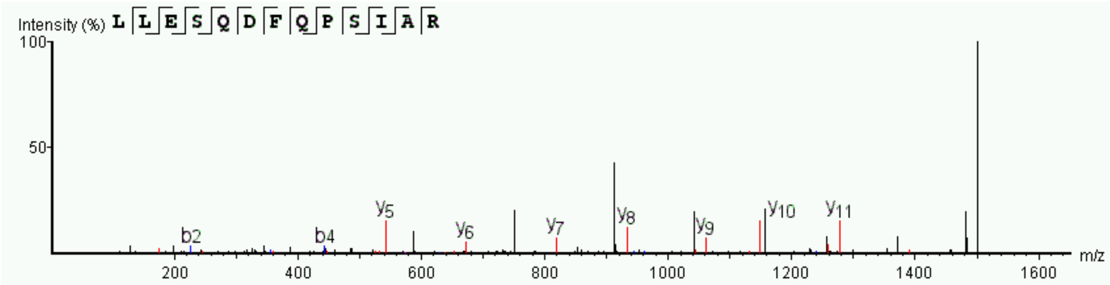

gi|571538309

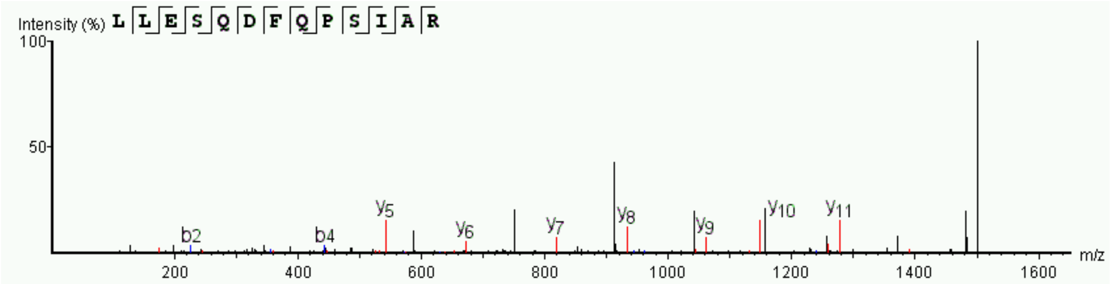

gi|571538313

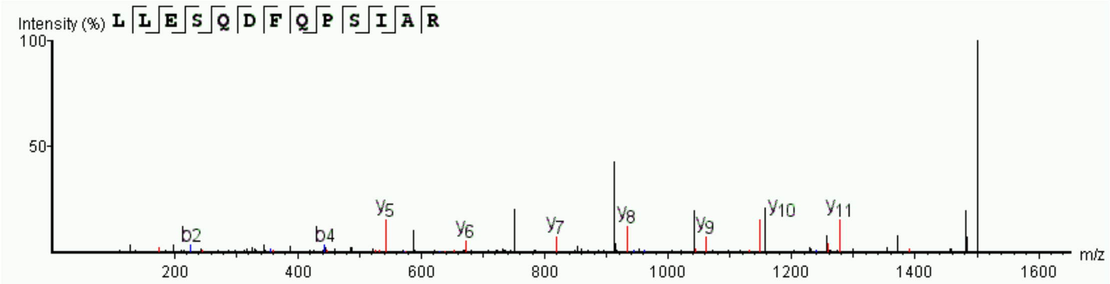

gi|328778947

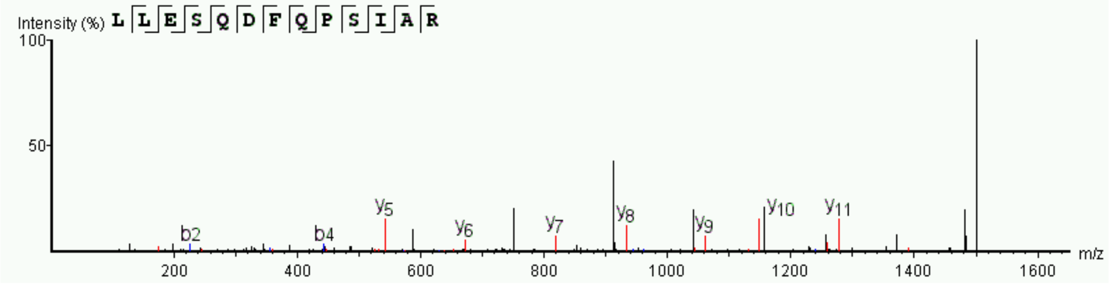

gi|571577865

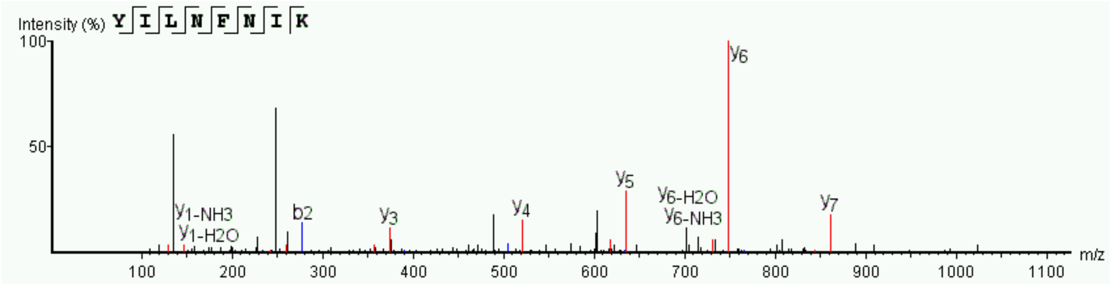

gi|571577867

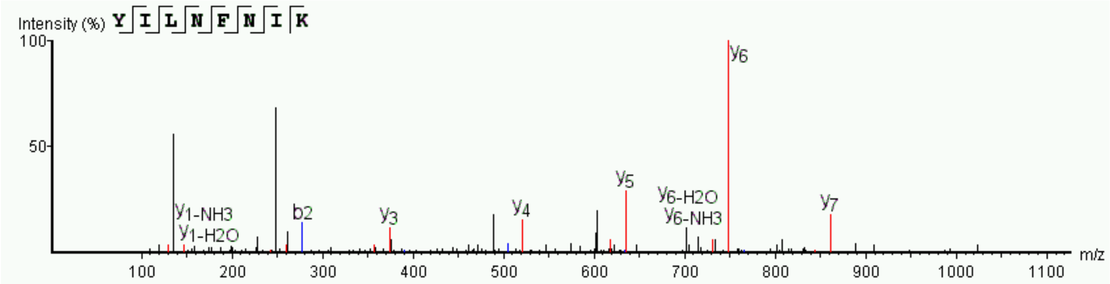

gi|571577869

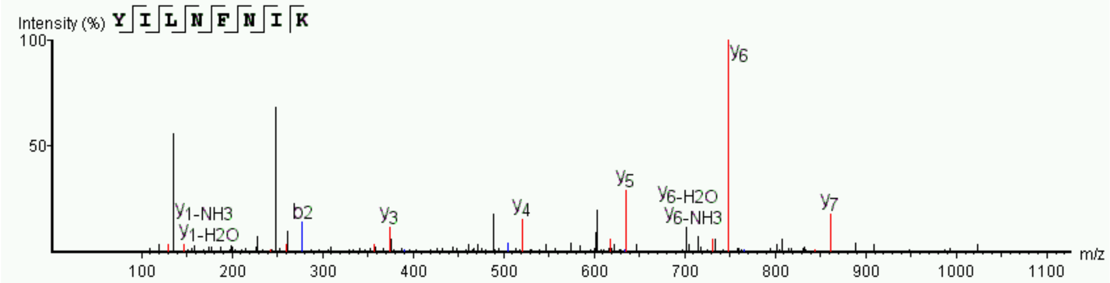

gi|571577871

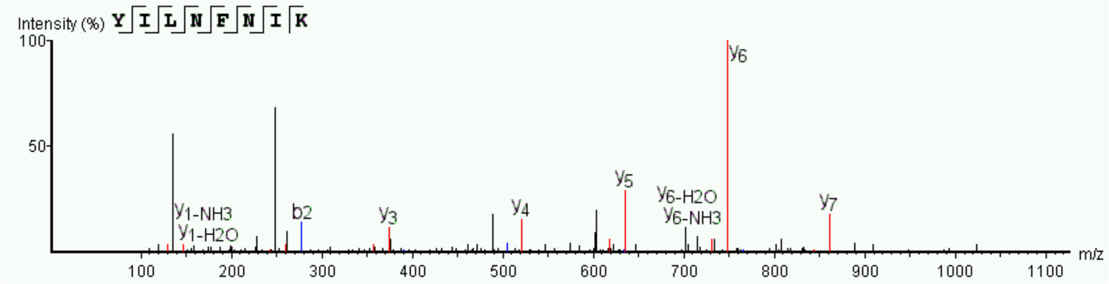

gi|571506280

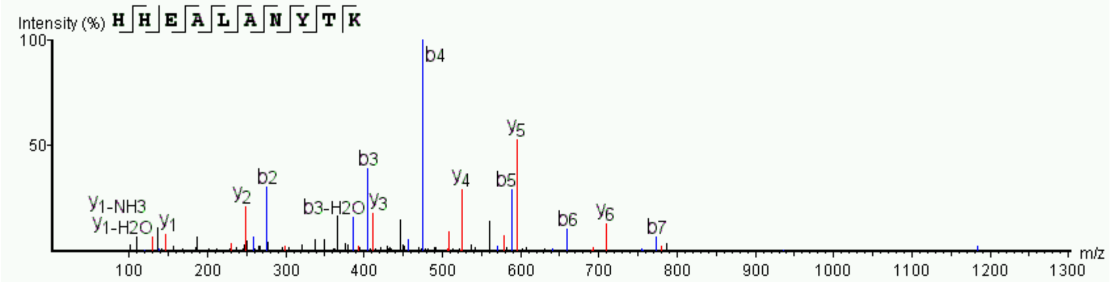

gi|571506283

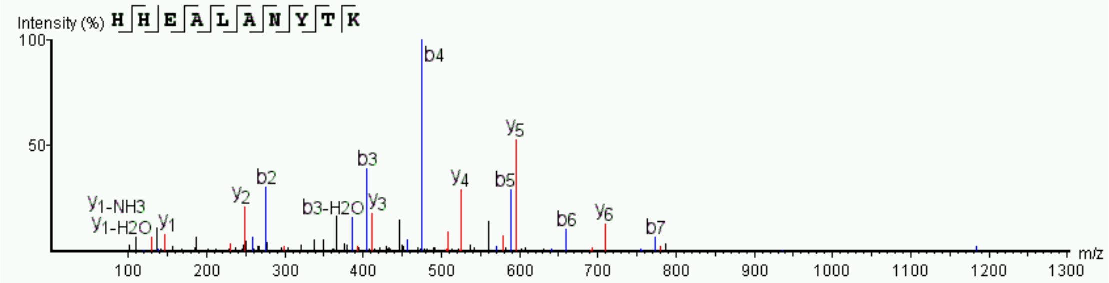

gi|110758613

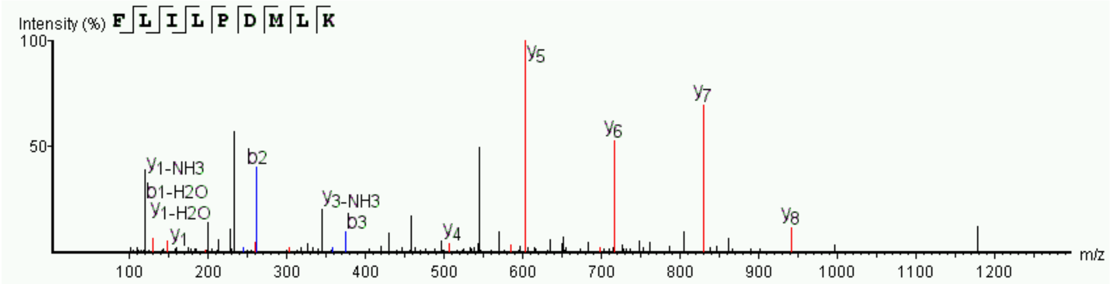

gi|571542313

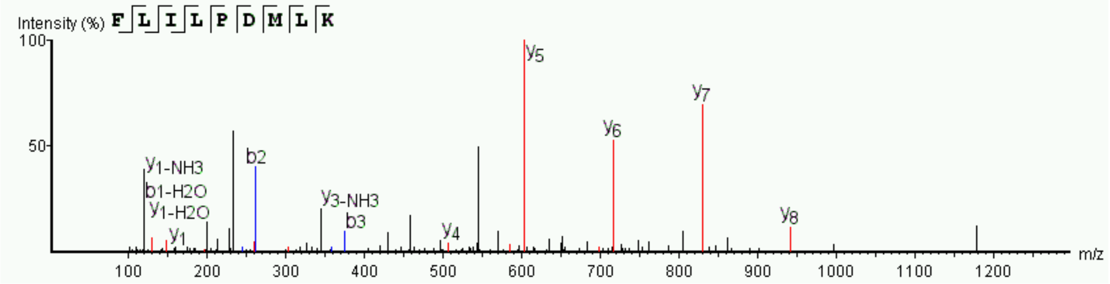

gi|48102954

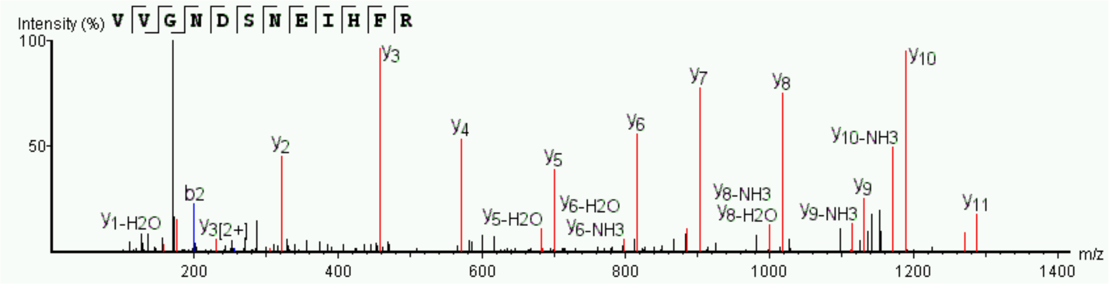

gi|571532415

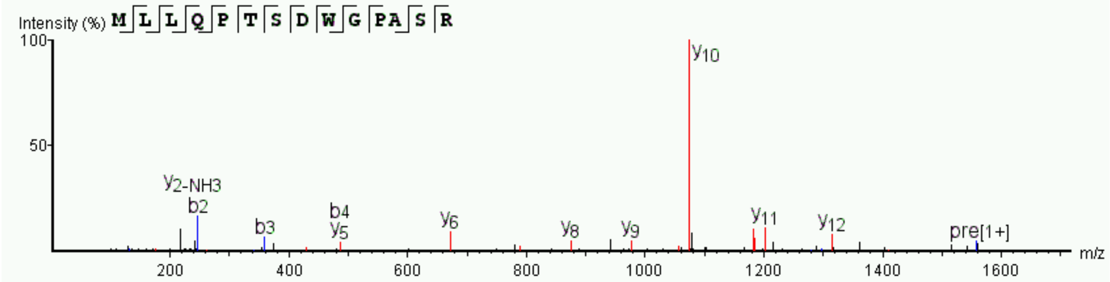

gi|571532420

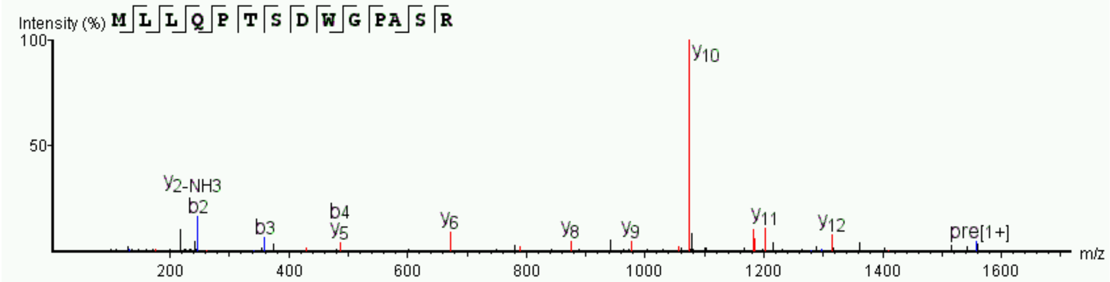

gi|571532423

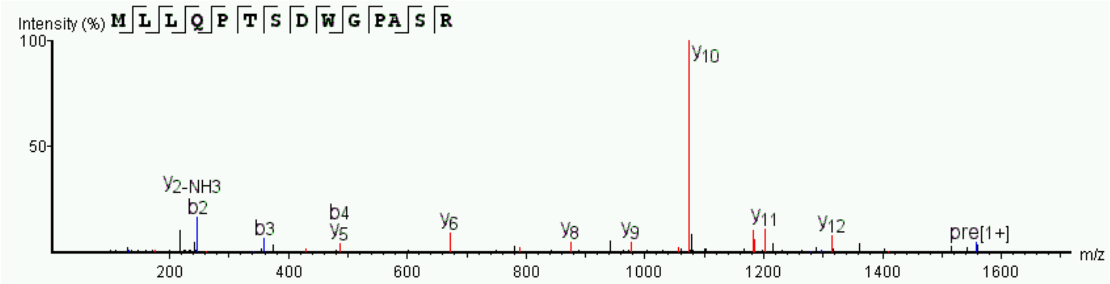

gi|571532426

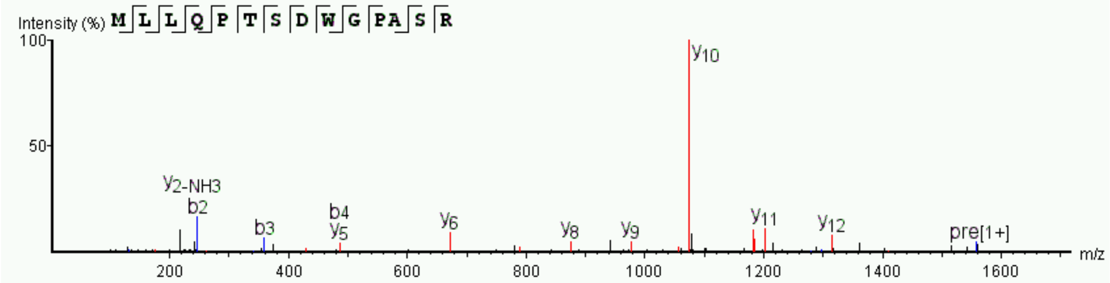

gi|571532429

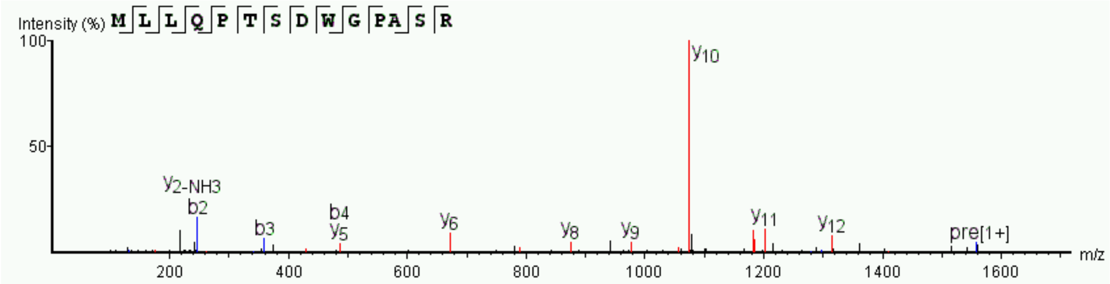

gi|571532432

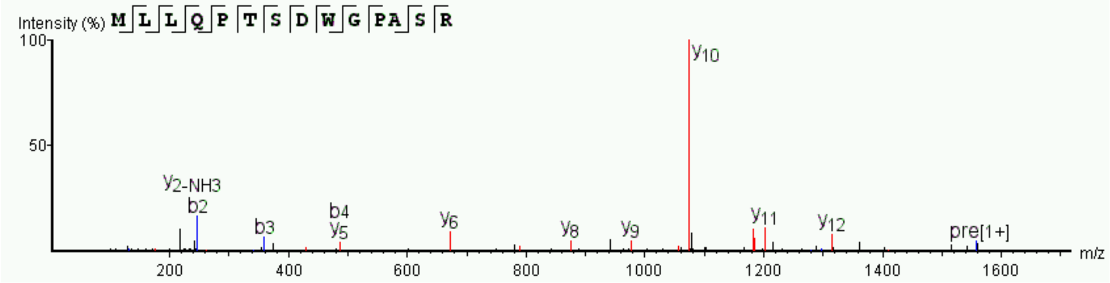

gi|571532435

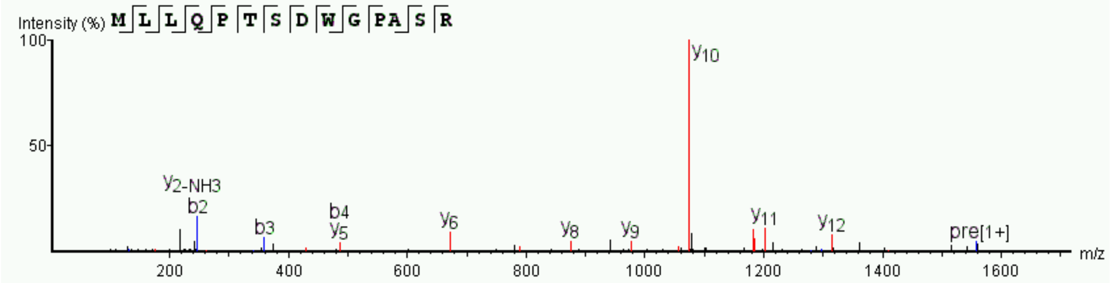

gi|328776203

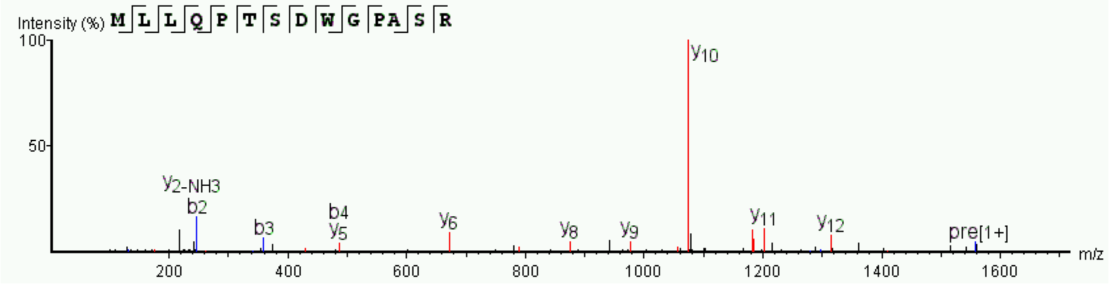

gi|571508459

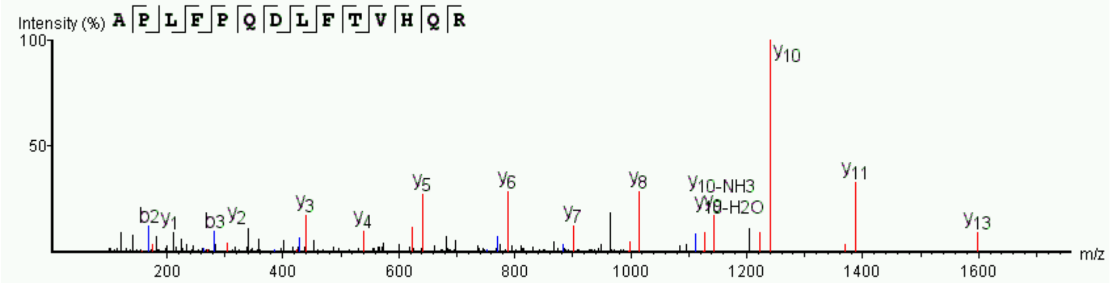

gi|571508466

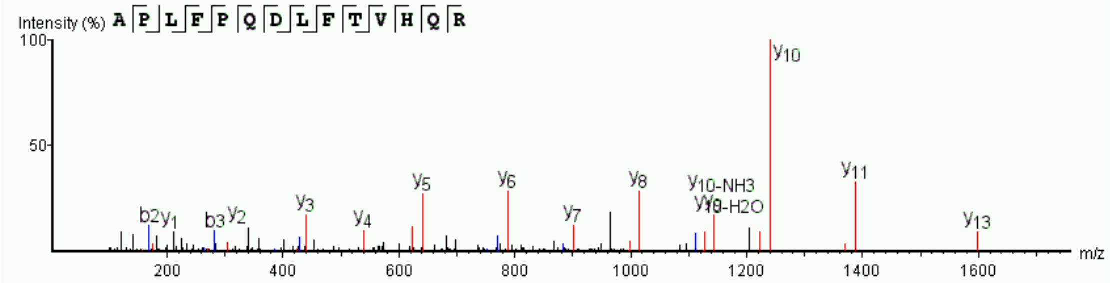

gi|571508469

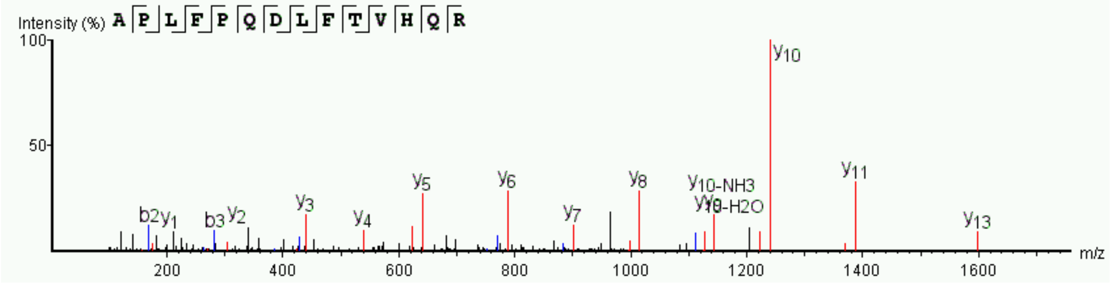

gi|571508472

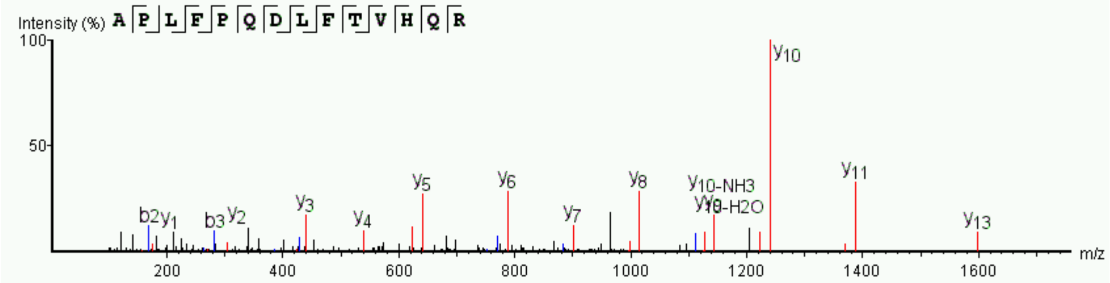

gi|328792320

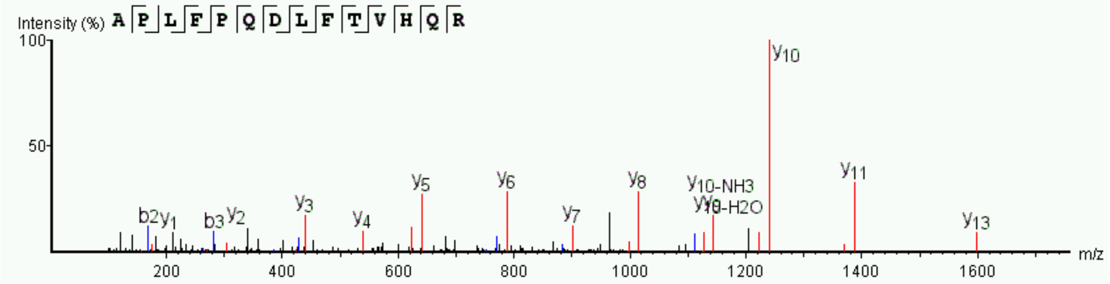

gi|571515131

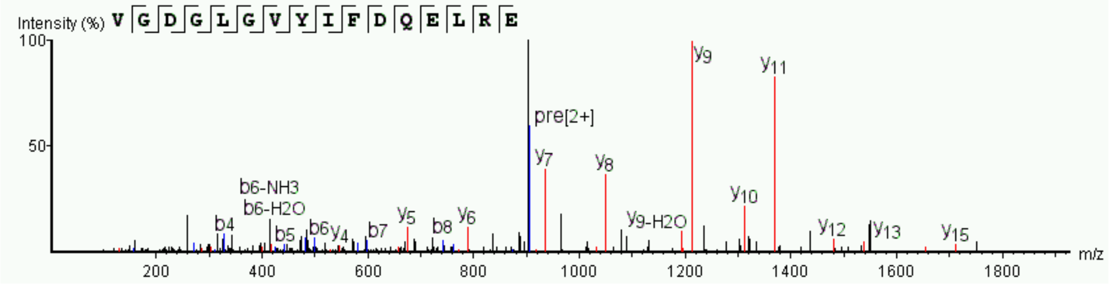

gi|571542358

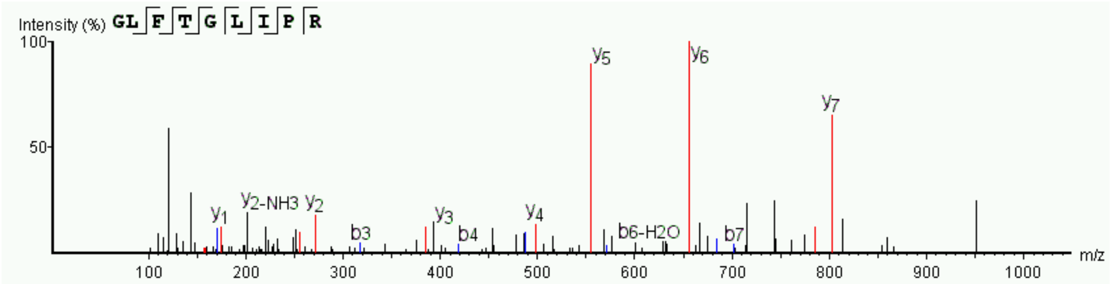

gi|571542362

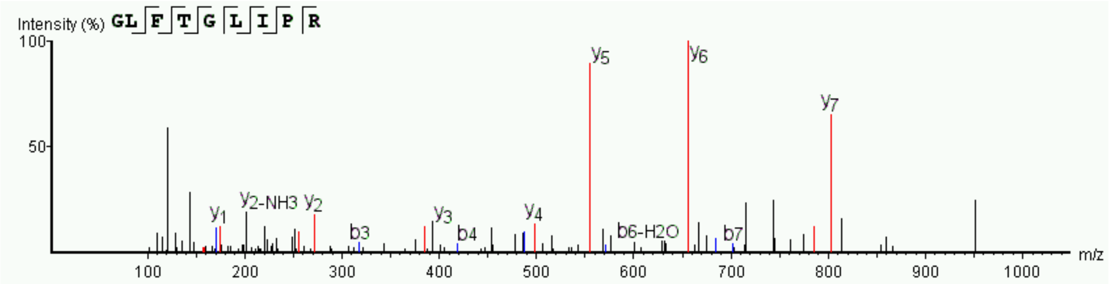

gi|571509816

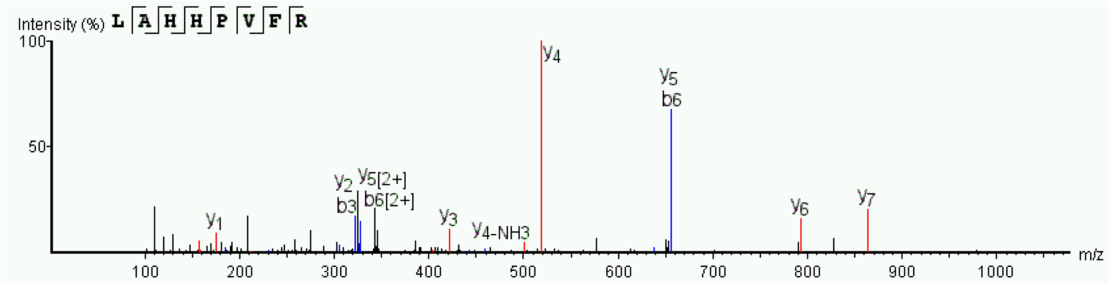

gi|571558001

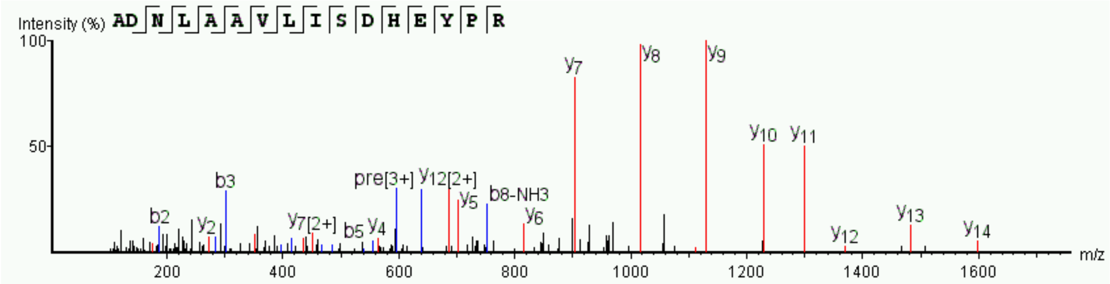

gi|571558005

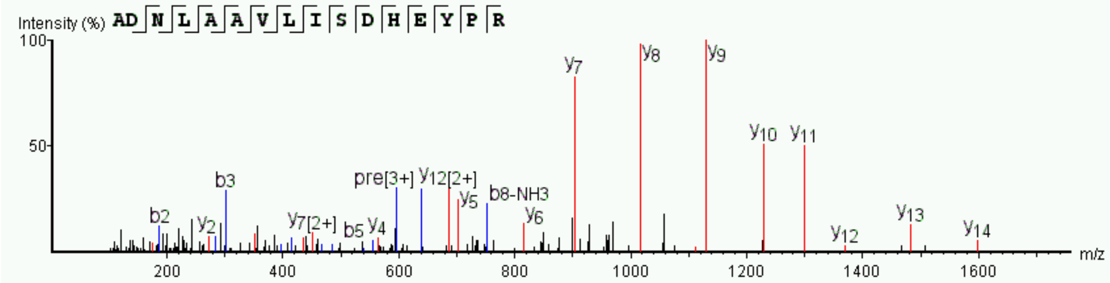

gi|328781619

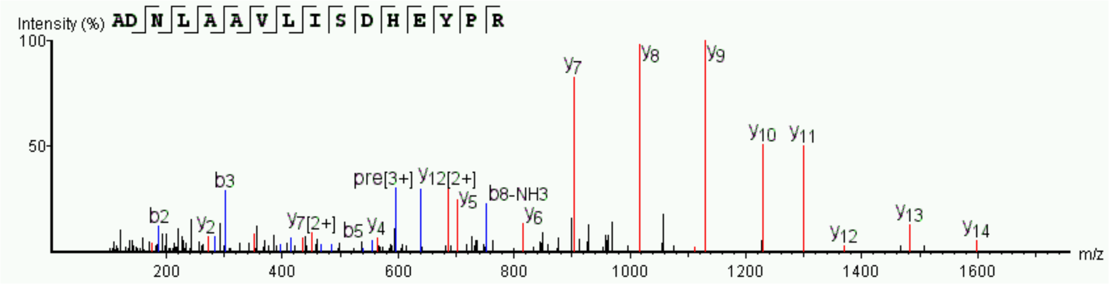

gi|66525345

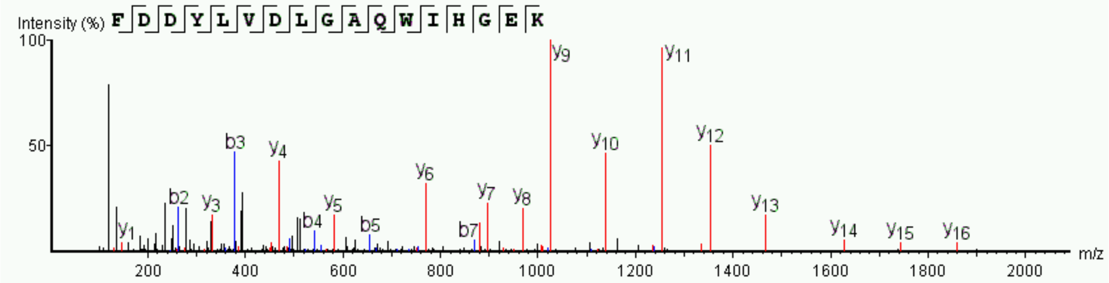

gi|571552849

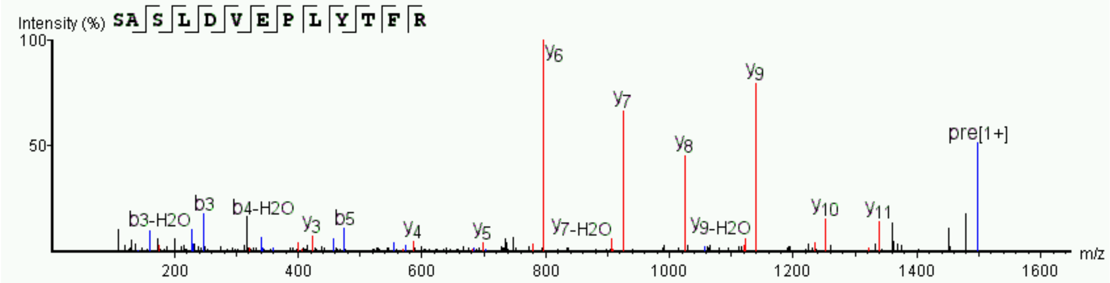

gi|328782716

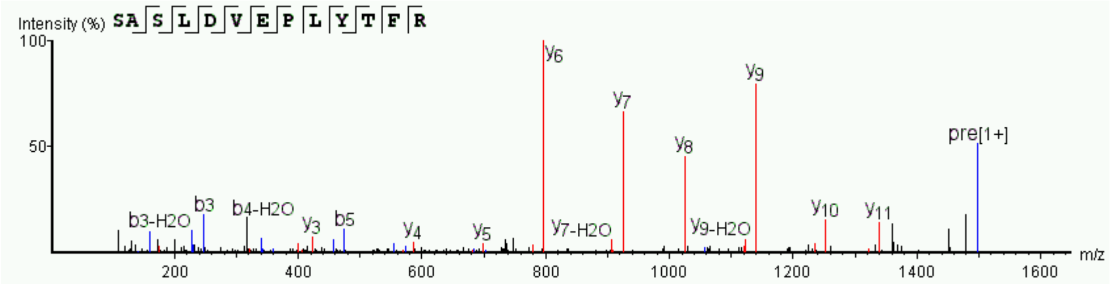

gi|571552867

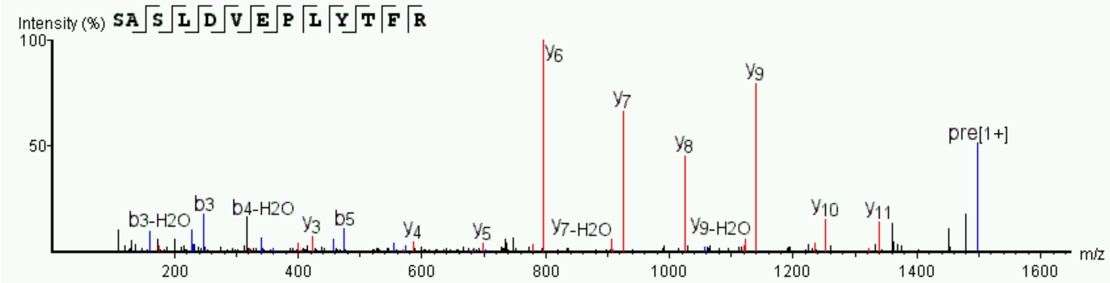

gi|571552871

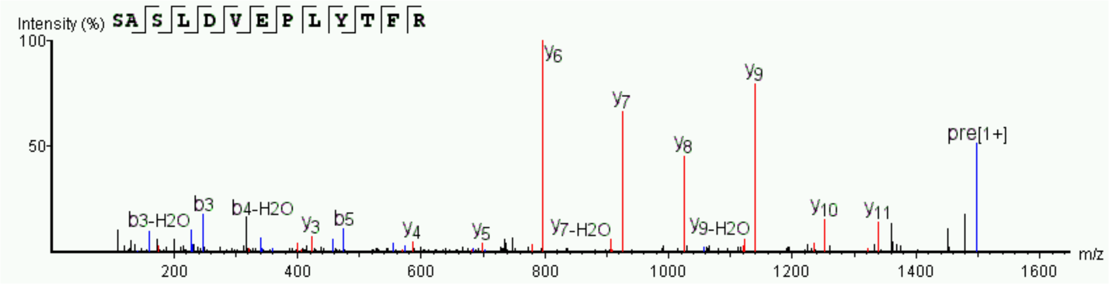

gi|571571878

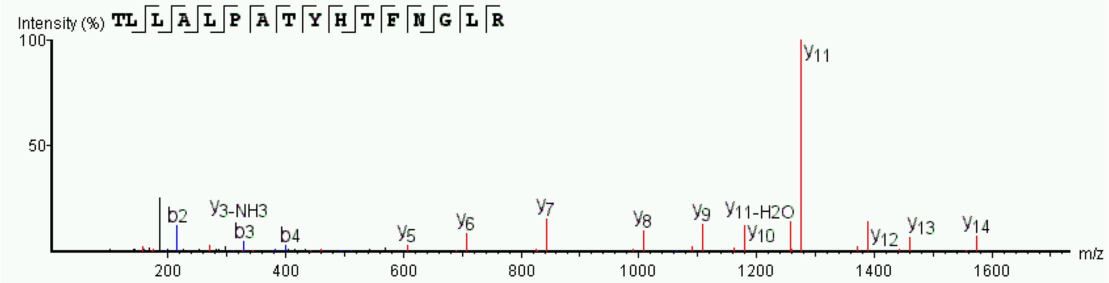

gi|66513846

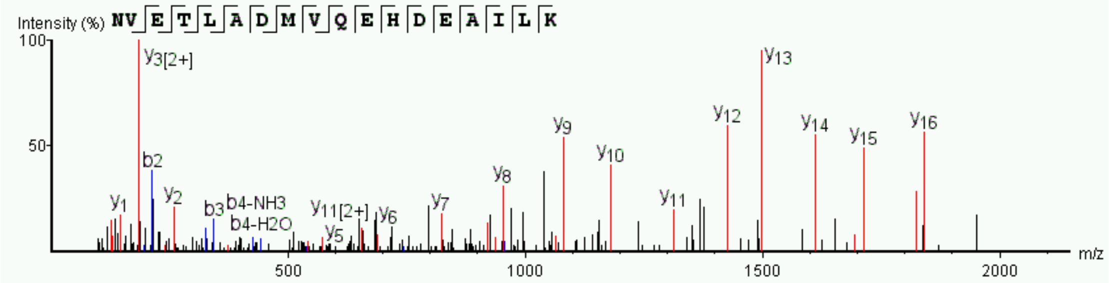

gi|66546725

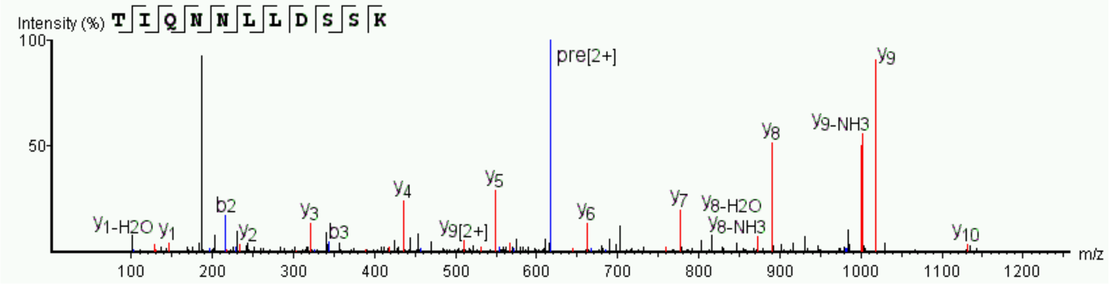

gi|571544247

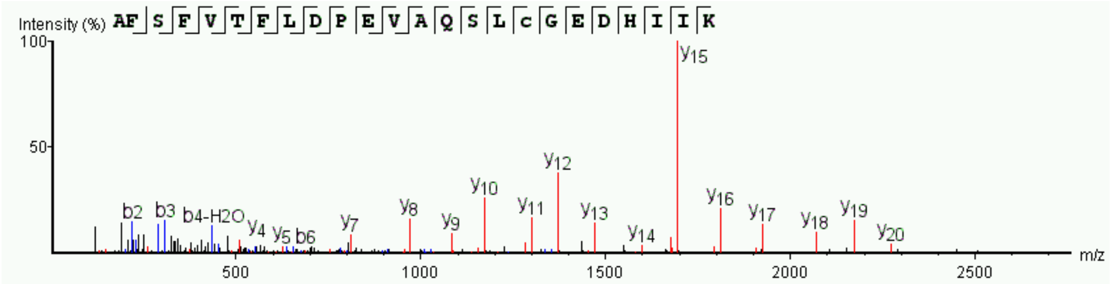

gi|571544251

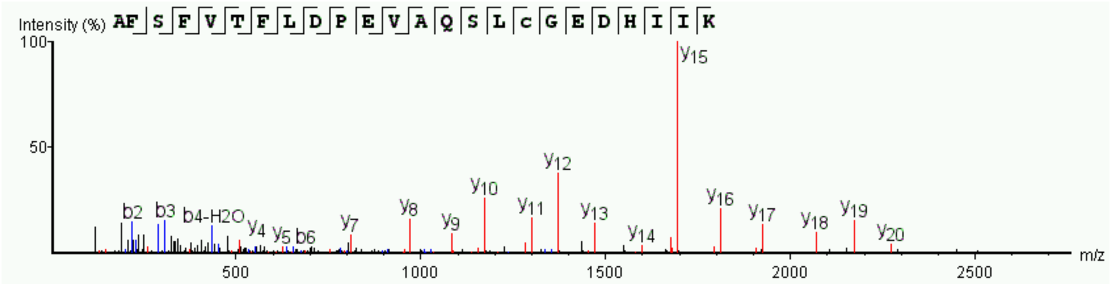

gi|328783809

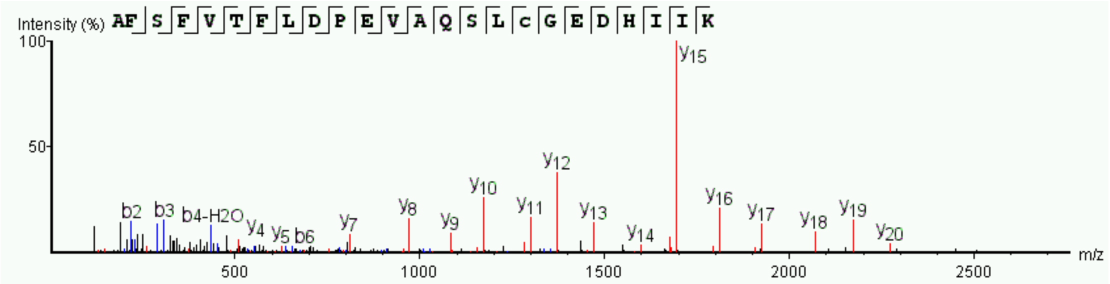

gi|571520111

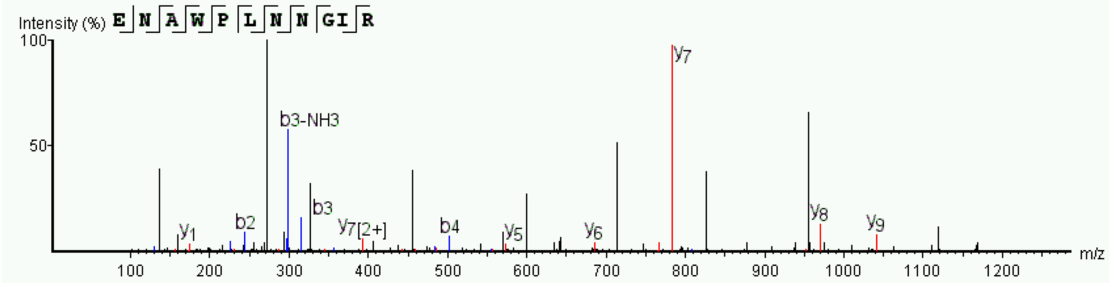

gi|571520119

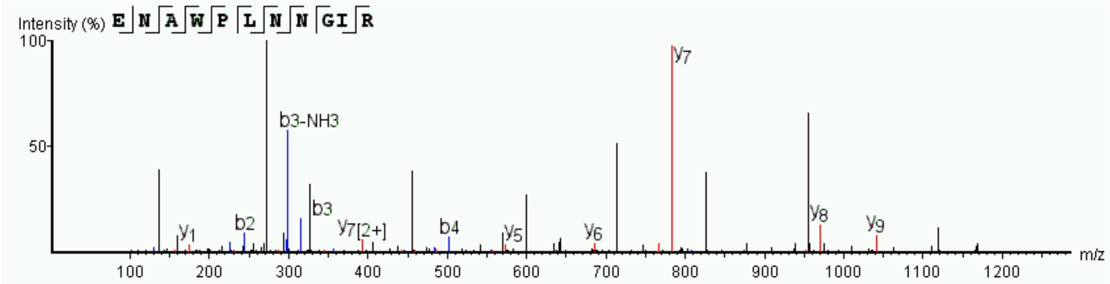

gi|328788626

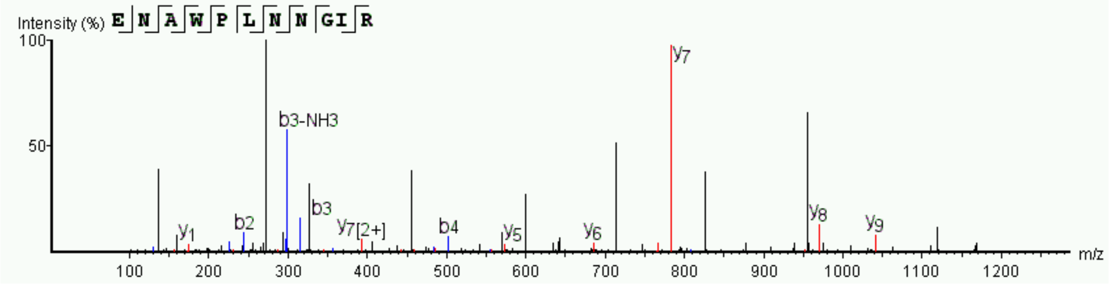

gi|571565058

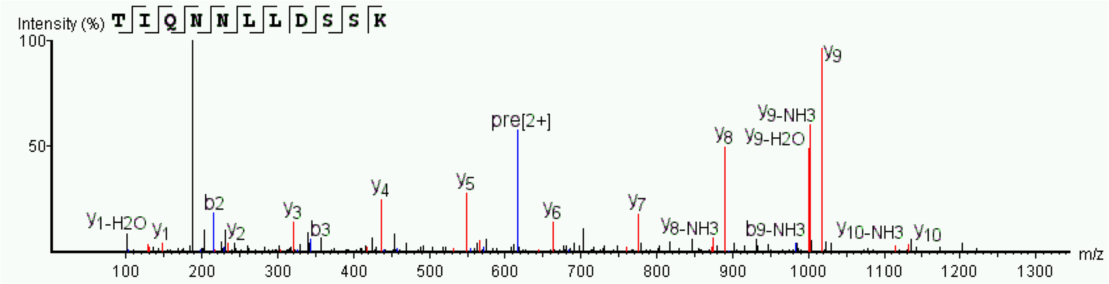

gi|328784195

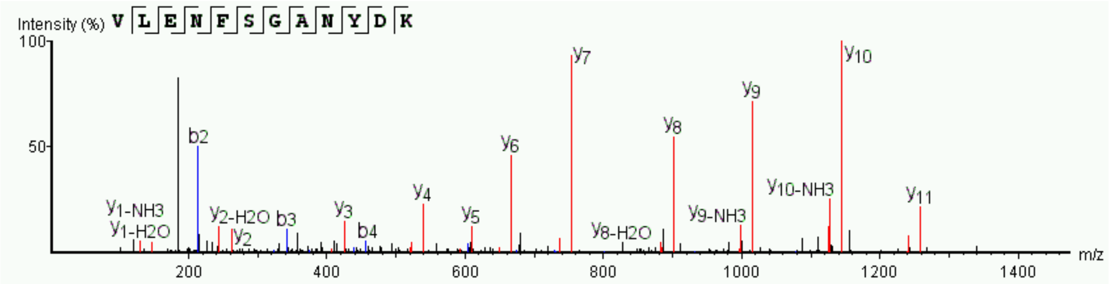

gi|66556835

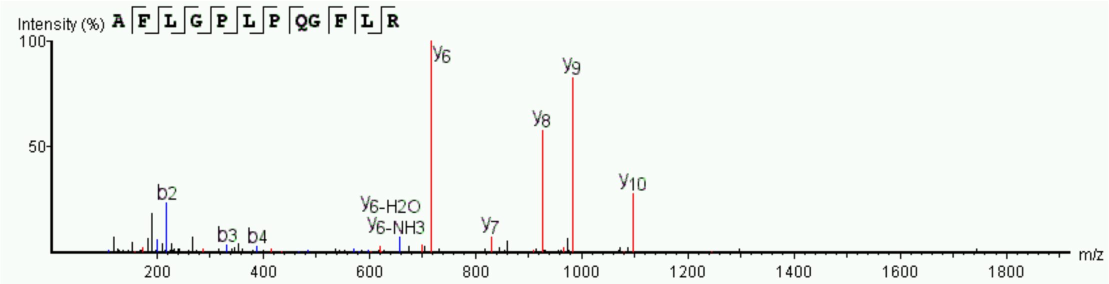

gi|328775950

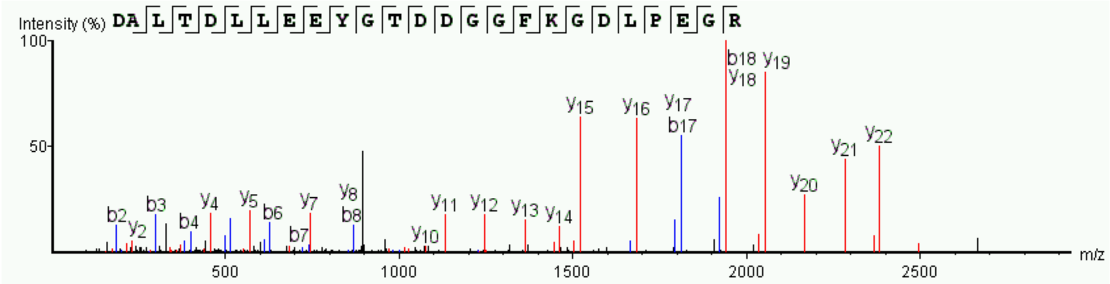

gi|571537241

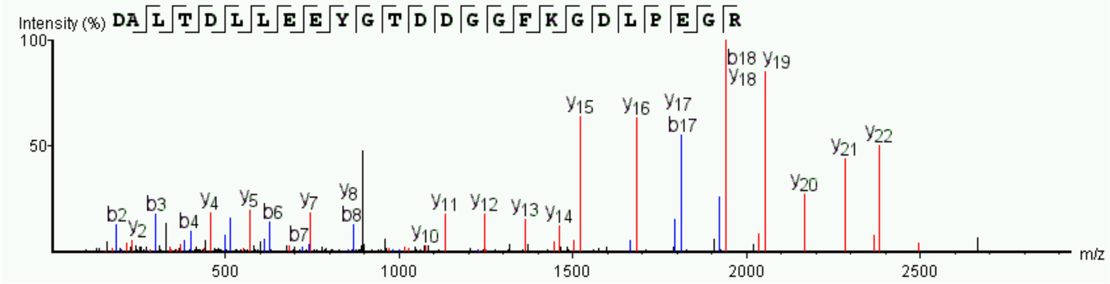

gi|571537245

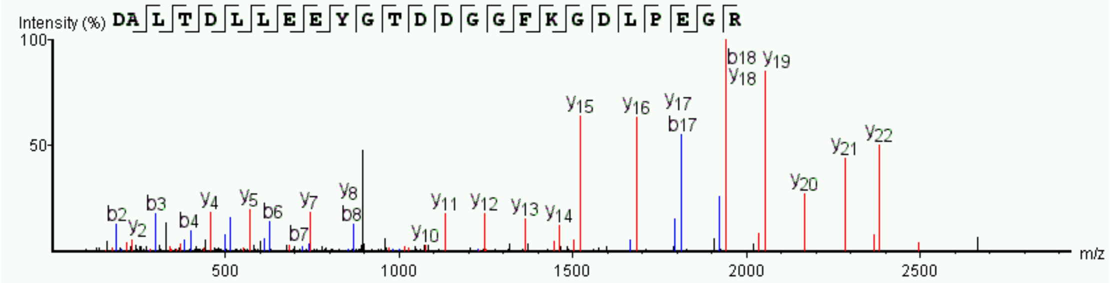

gi|571537249

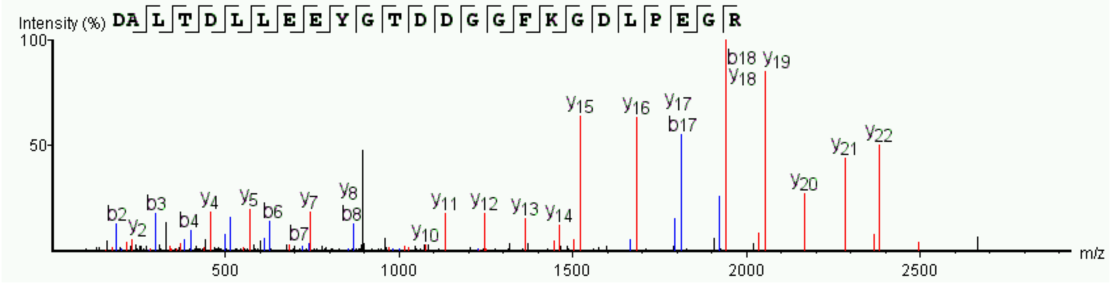

gi|571537253

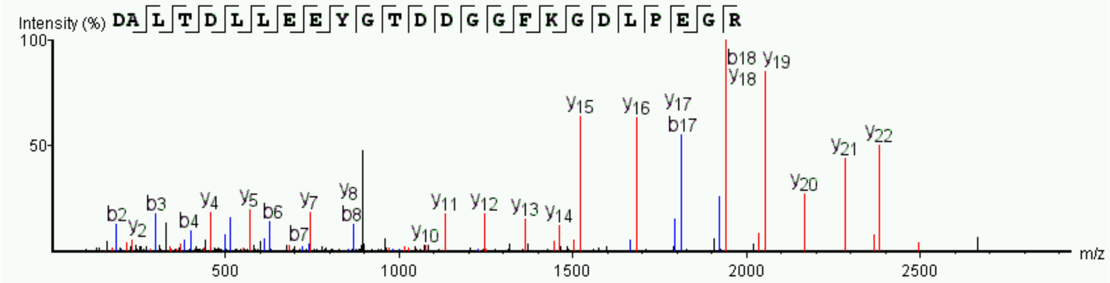

gi|571537256

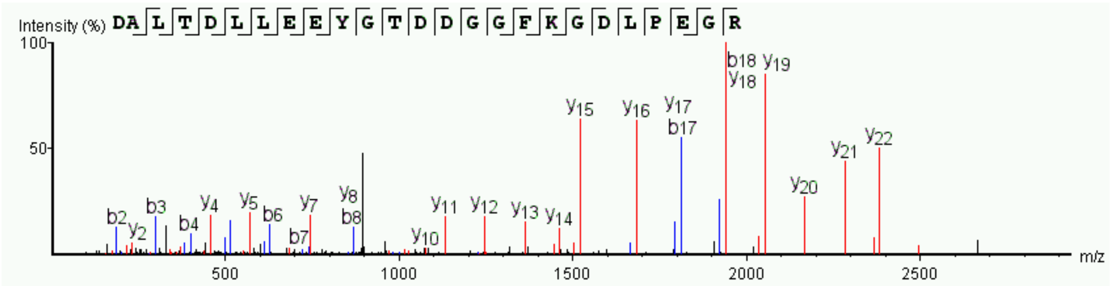

gi|571537262

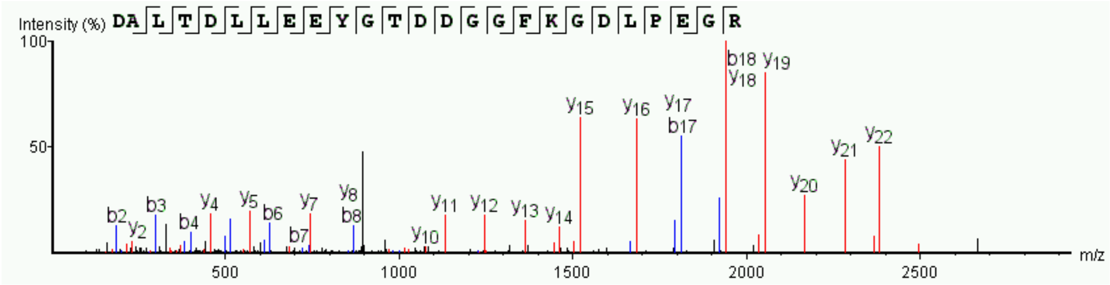

gi|571537266

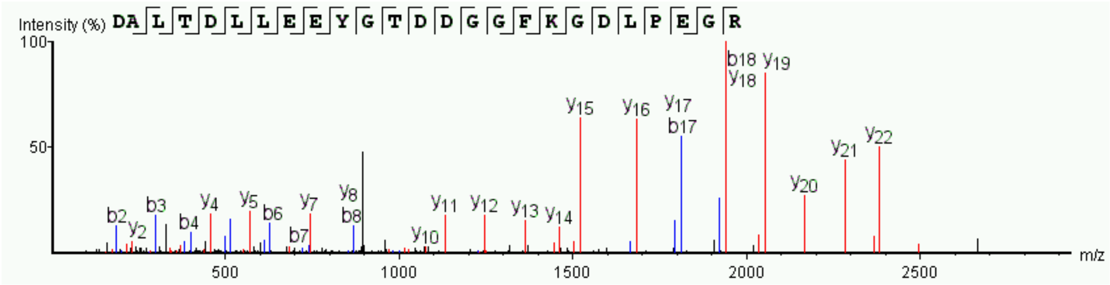

gi|66515987

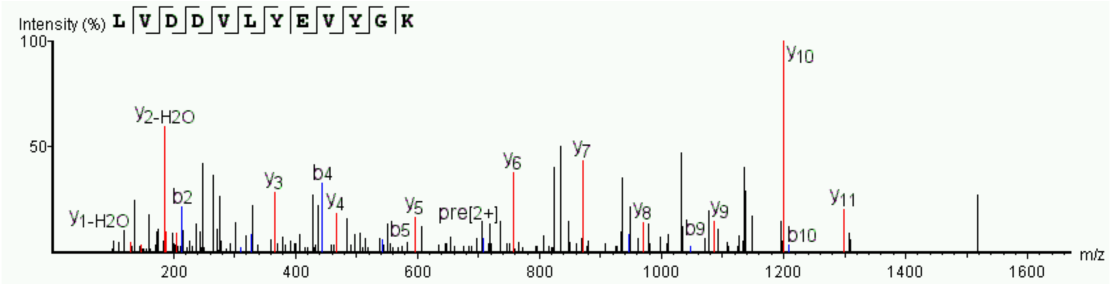

gi|571529348

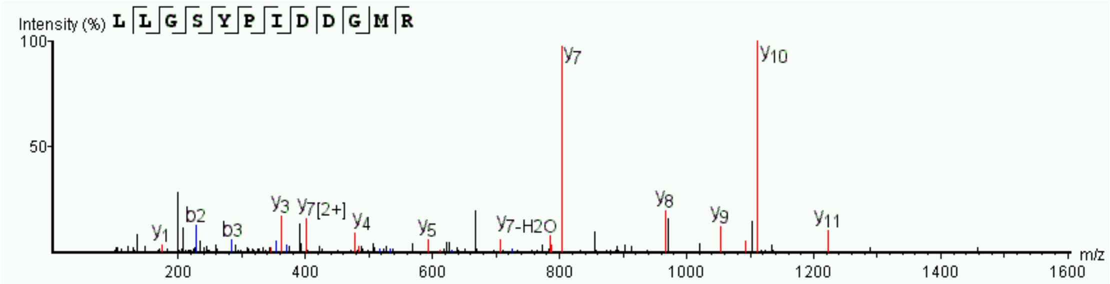

gi|571529351

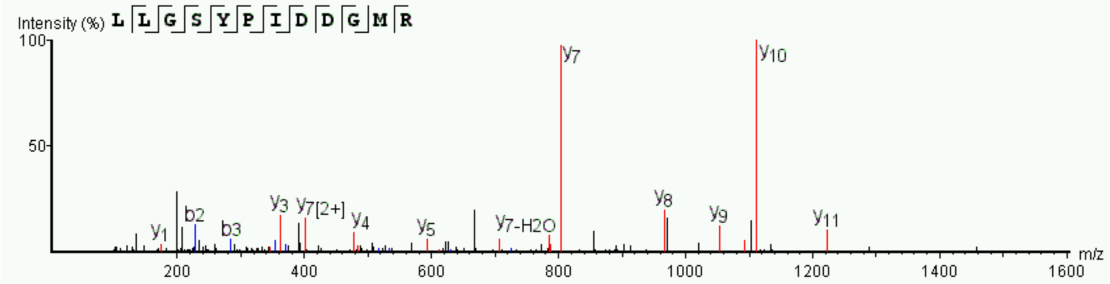

gi|571544690

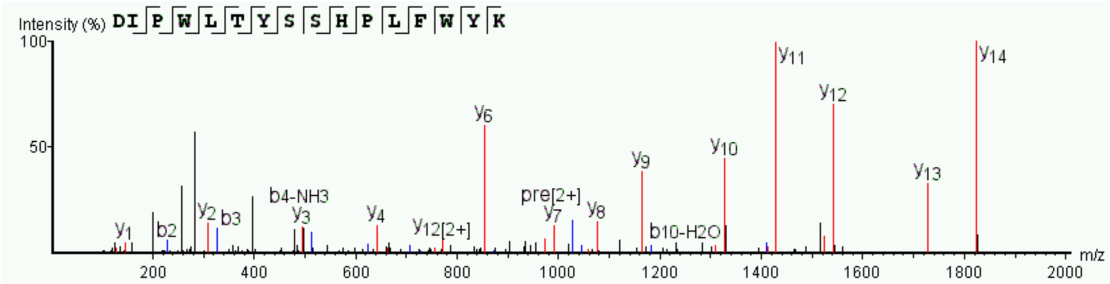

gi|571544693

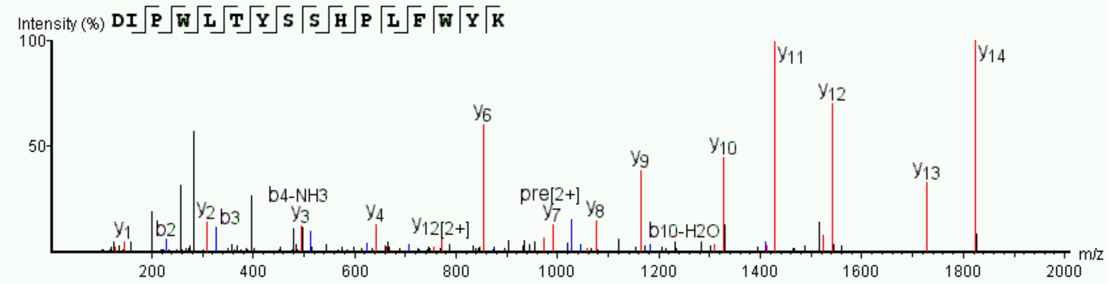

gi|571503359

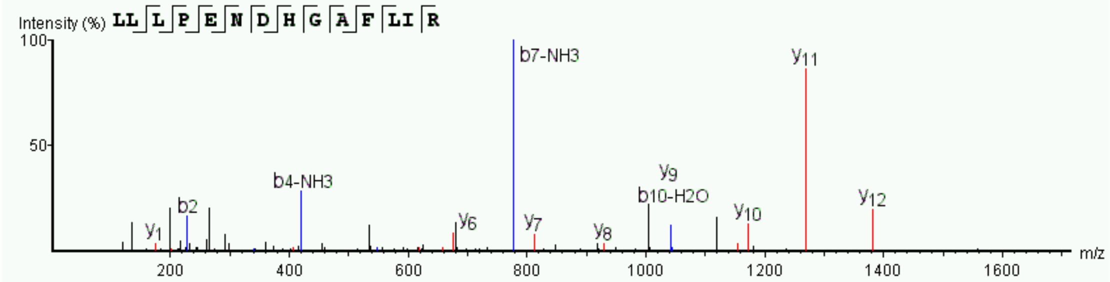

gi|571503362

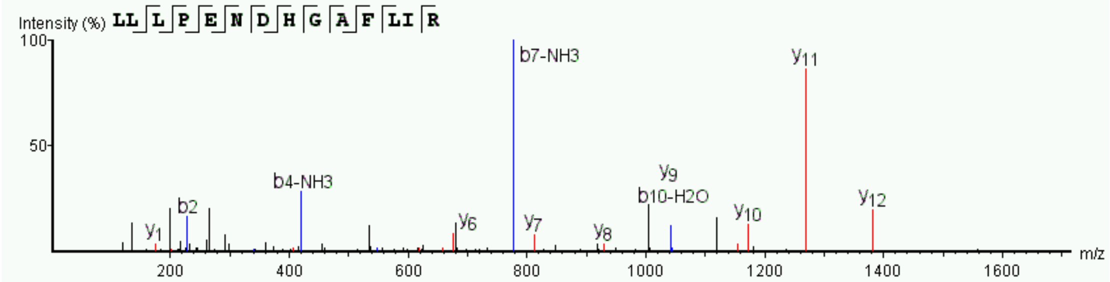

gi|571503366

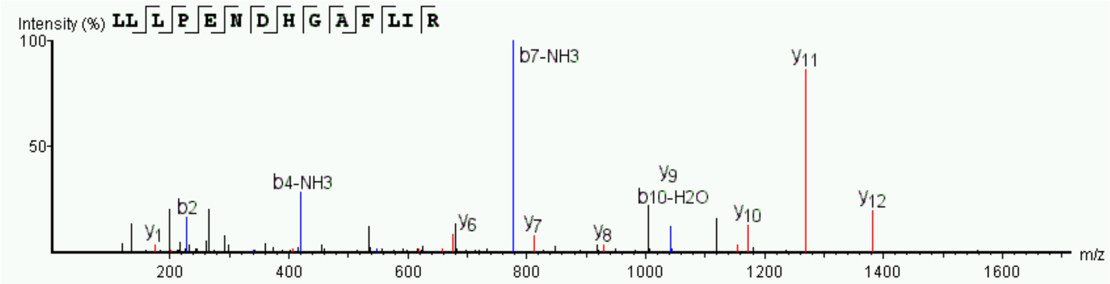

gi|571503369

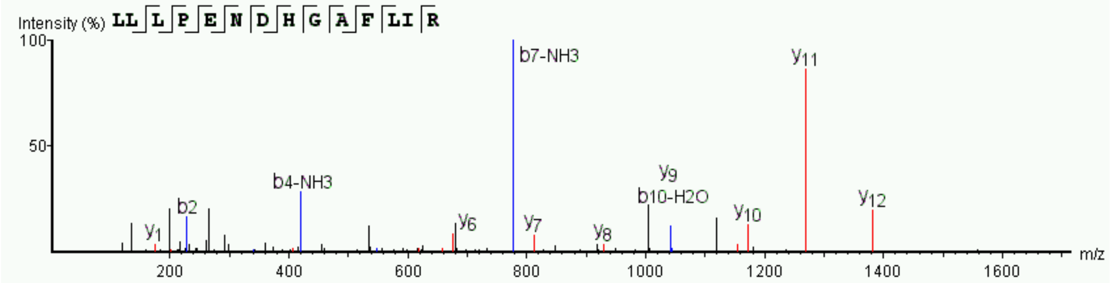

gi|571503372

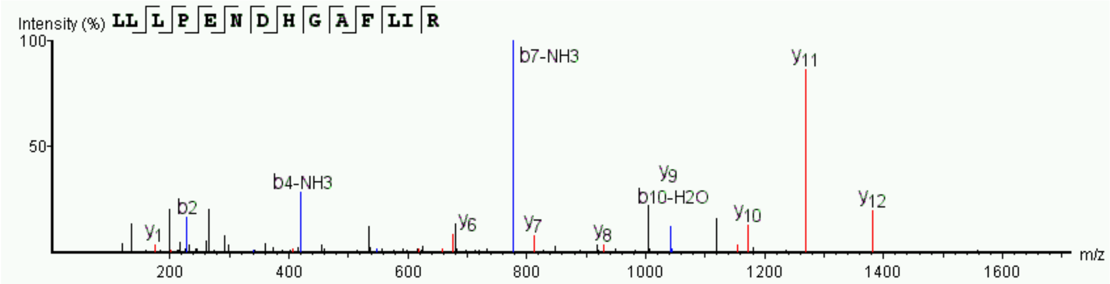

gi|571503376

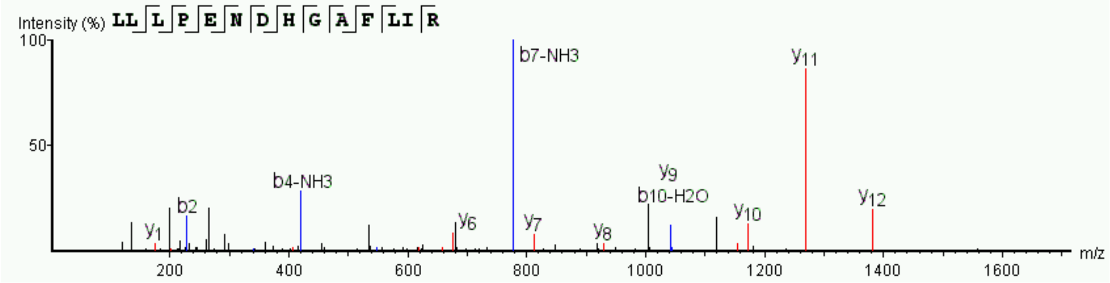

gi|571503379

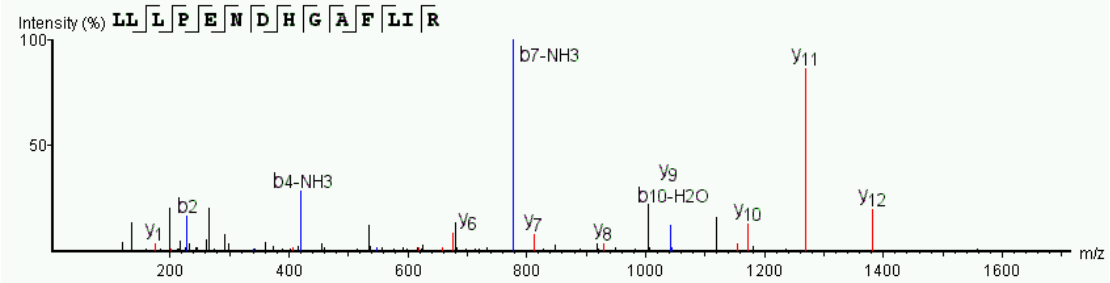

gi|328788805

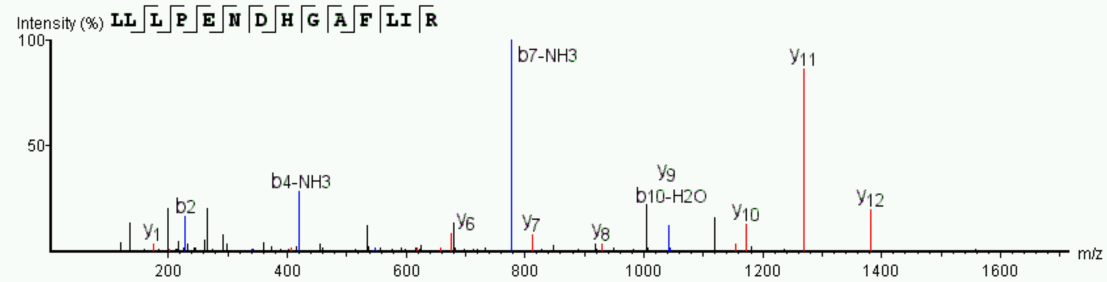

gi|571571820

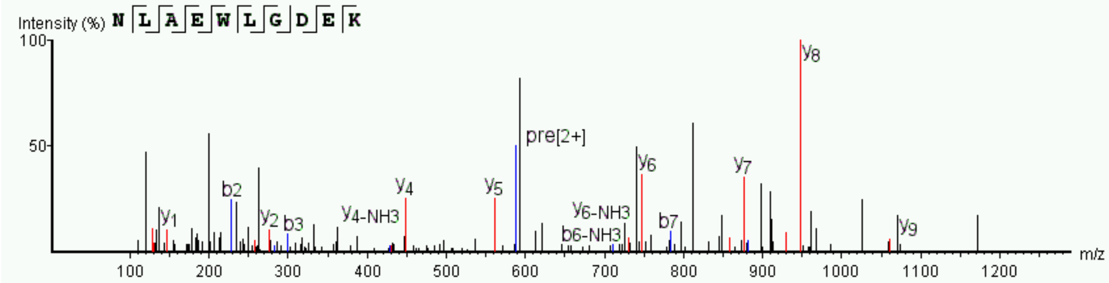

gi|571571824

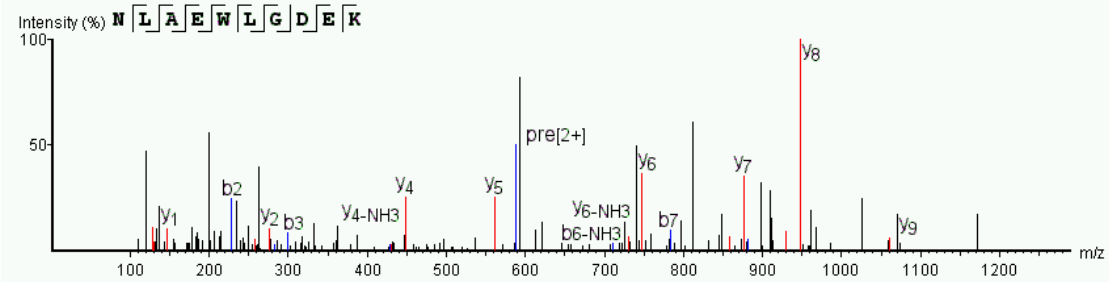

gi|571571828

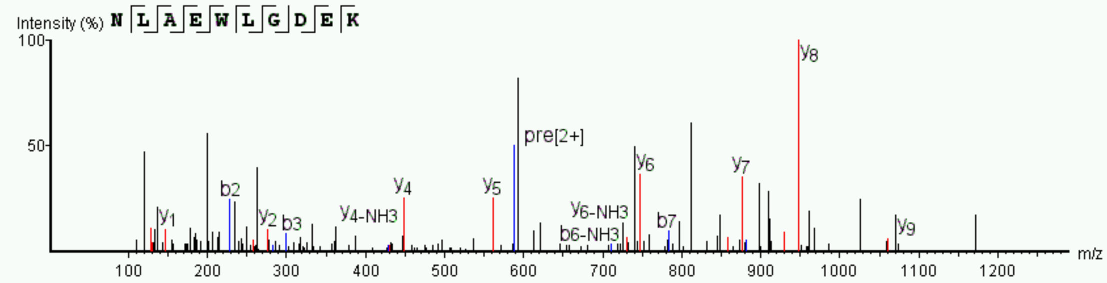

gi|571523648

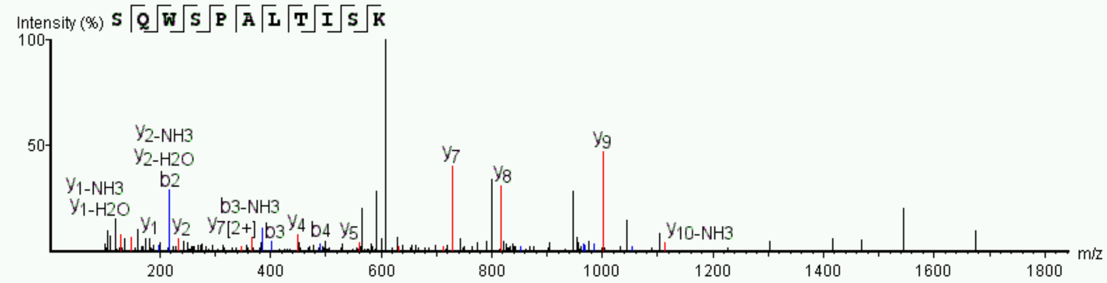

gi|110765112

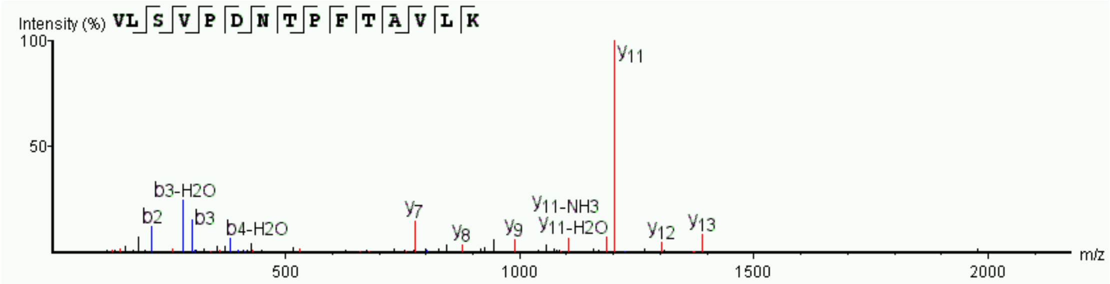

gi|571538017

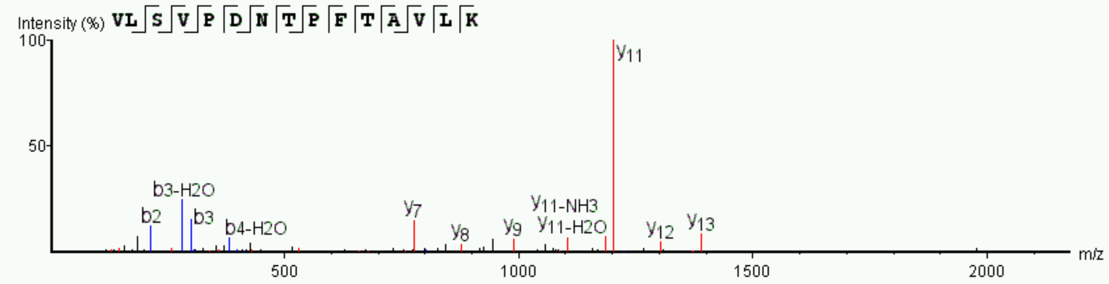

gi|571558837

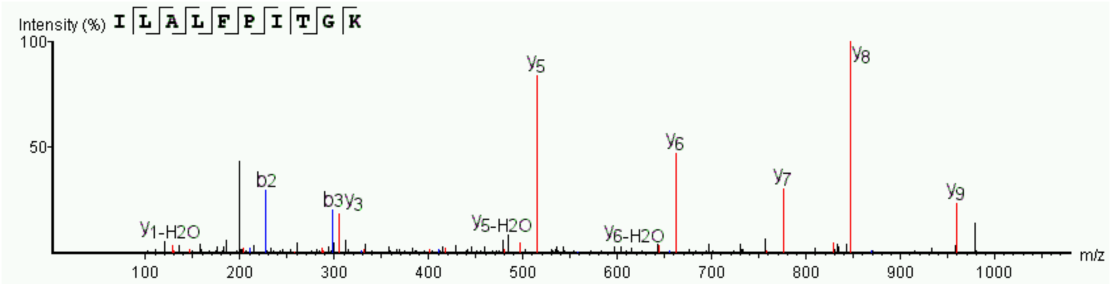

gi|328784430

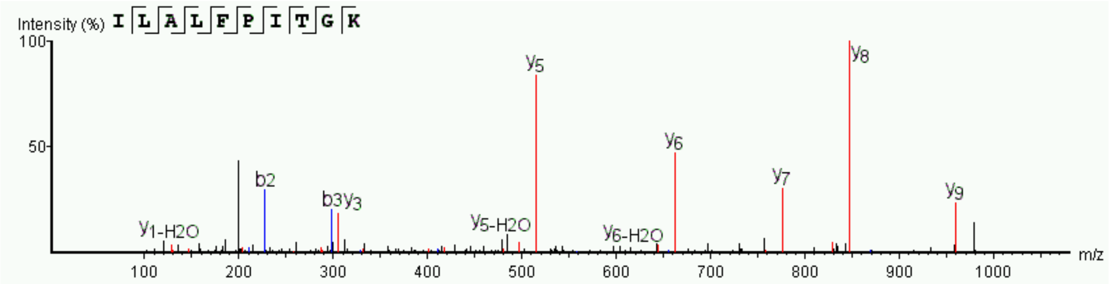

gi|66524379

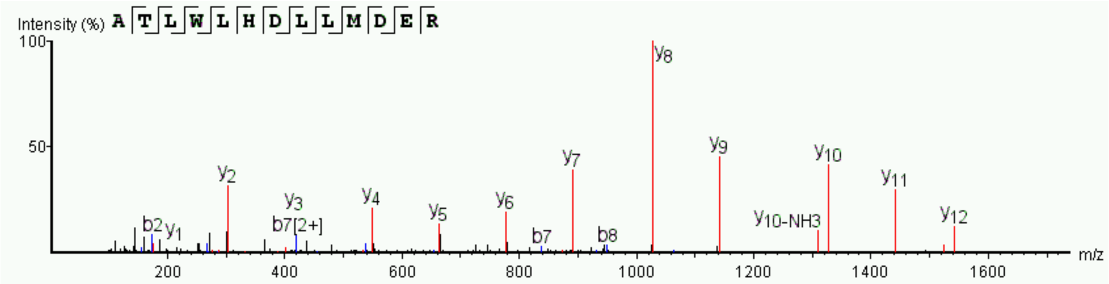

gi|328783062

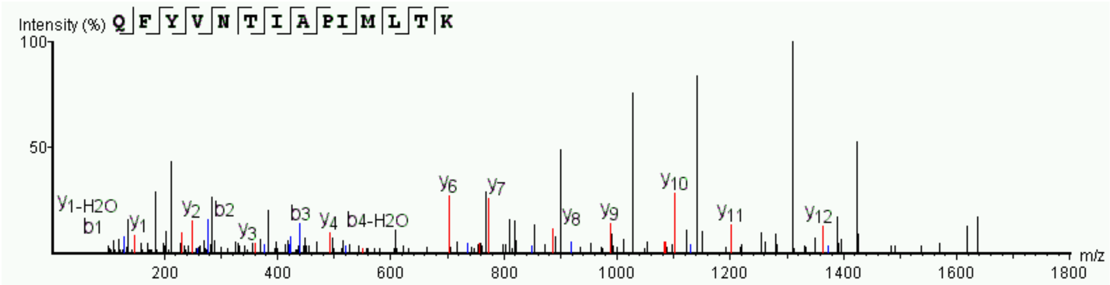

gi|571502248

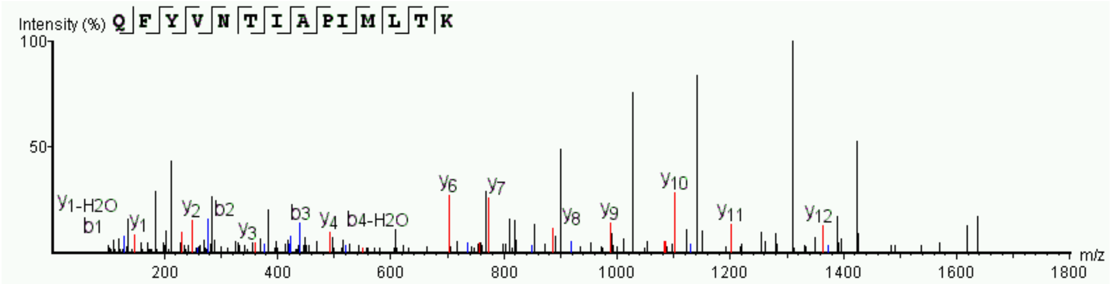

gi|571502252

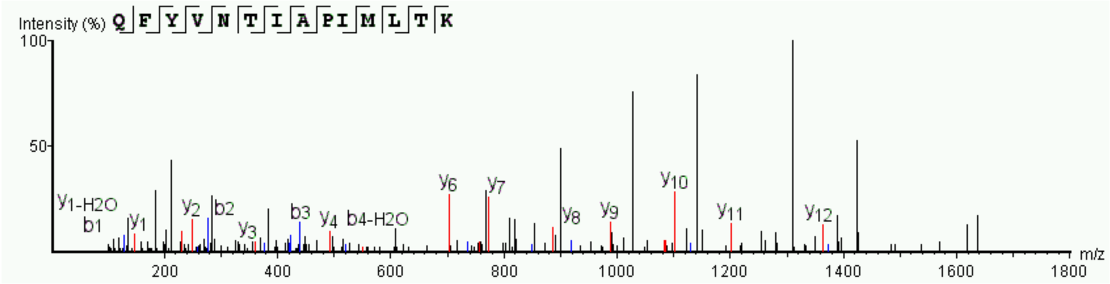

gi|571568695

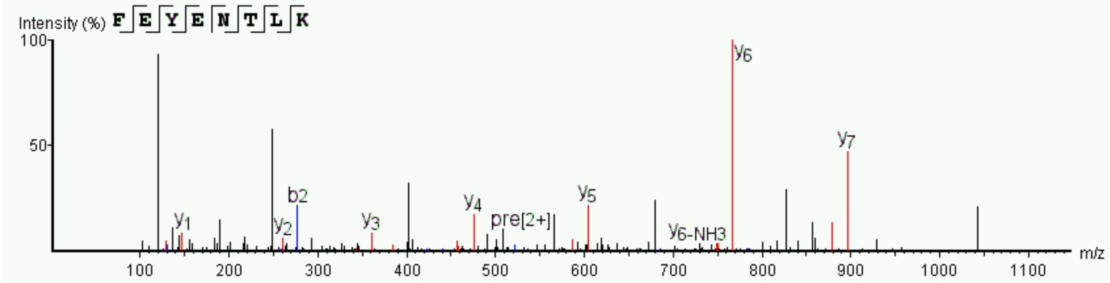

gi|571568698

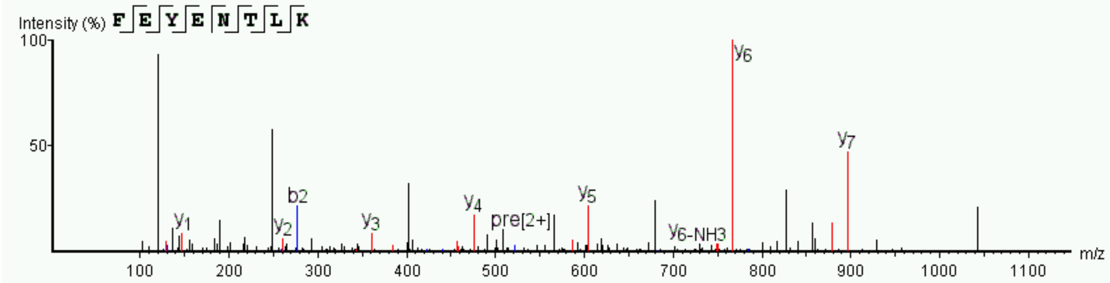

gi|328789366

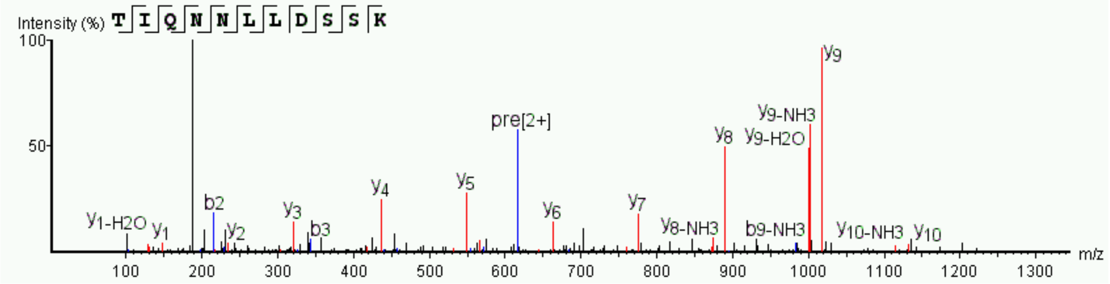

gi|571557248

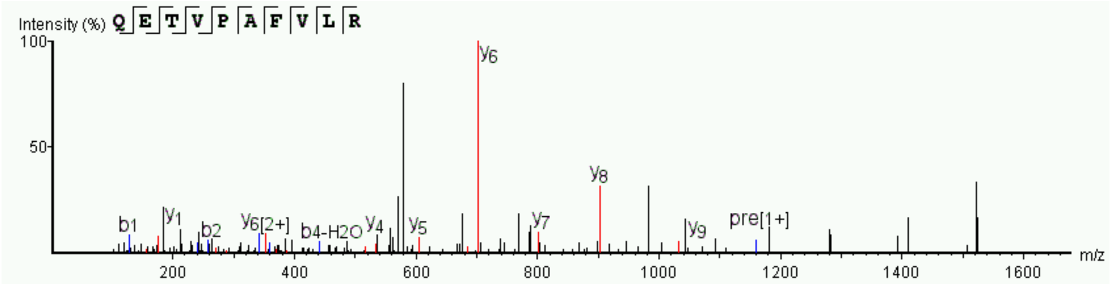

gi|110760649

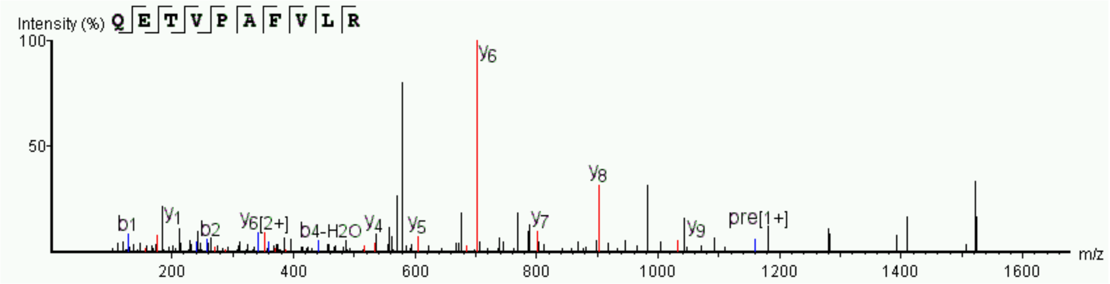

gi|571570476

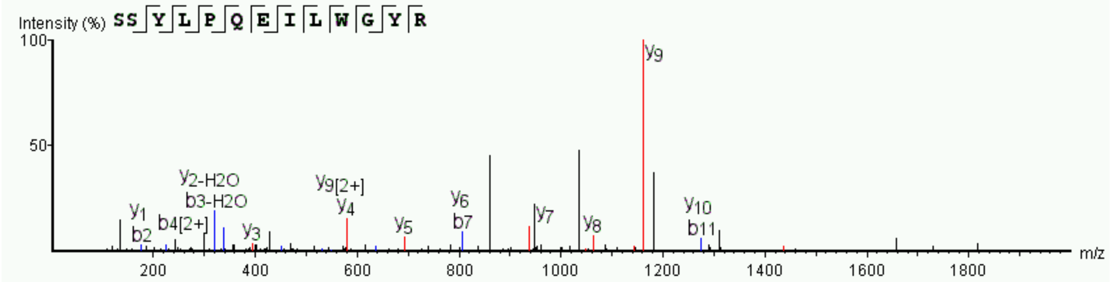

gi|571570525

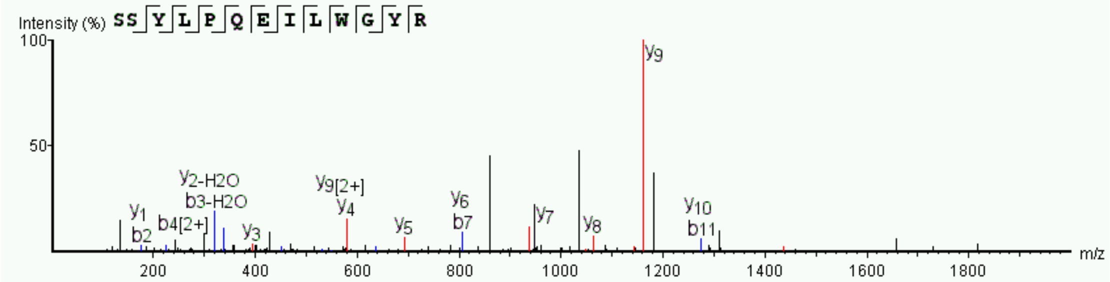

gi|571570530

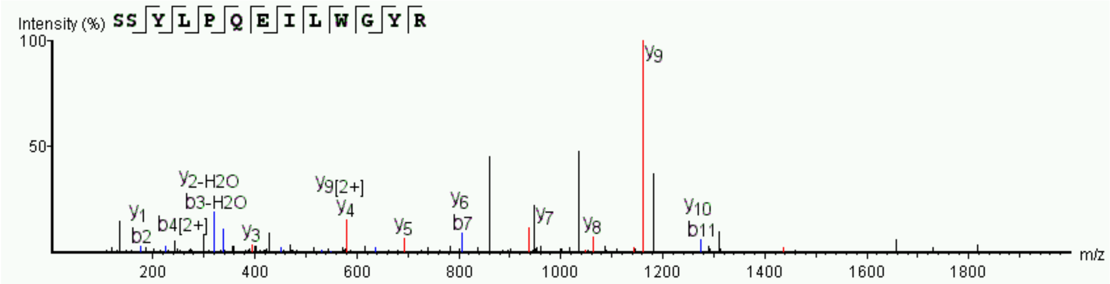

gi|571570534

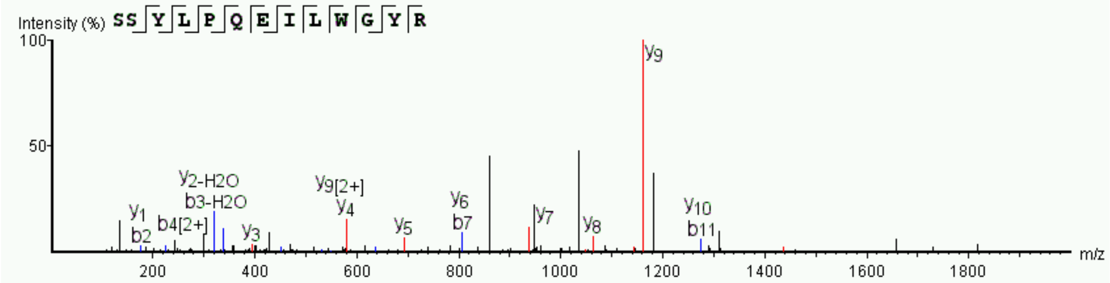

gi|328782779

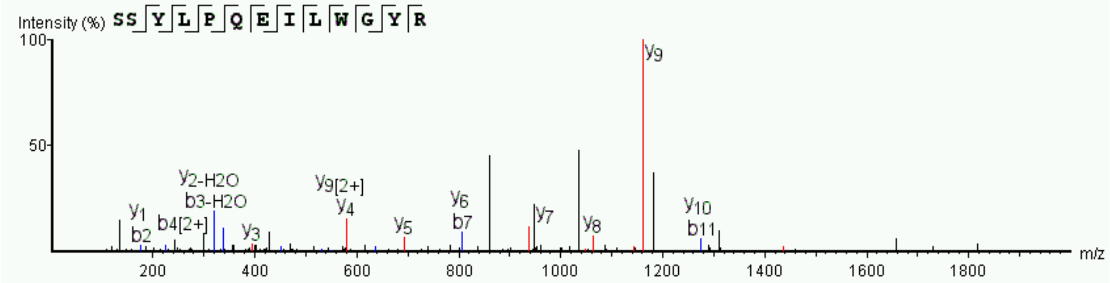

gi|571570487

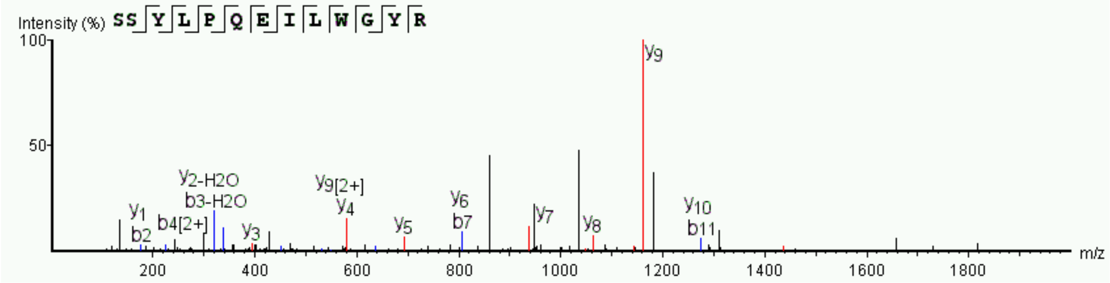

gi|571570493

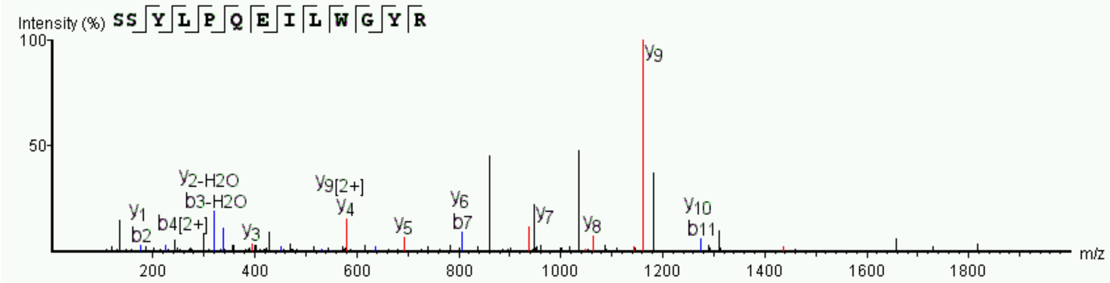

gi|571570496

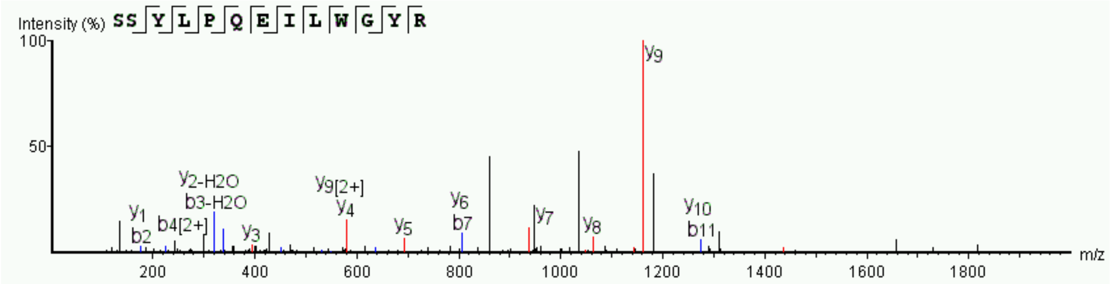

gi|571570501

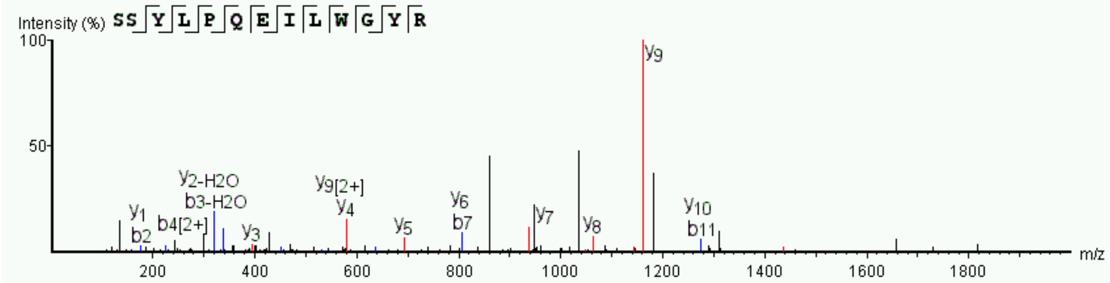

gi|571570507

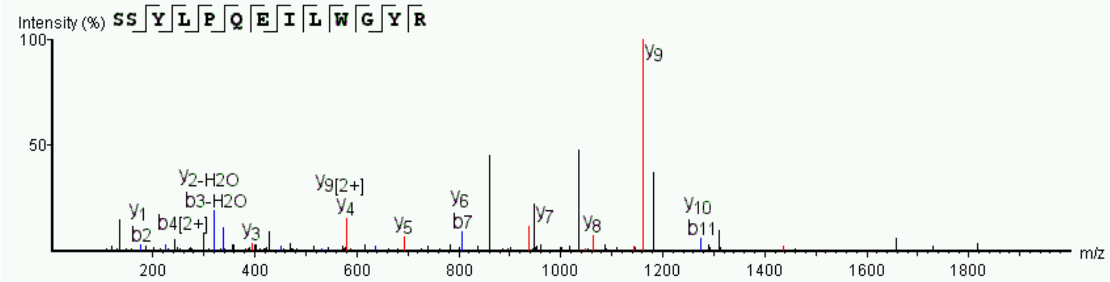

gi|571570511

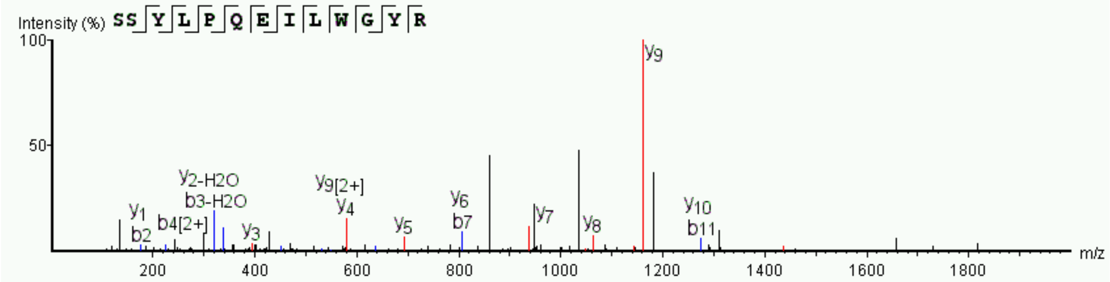

gi|571570515

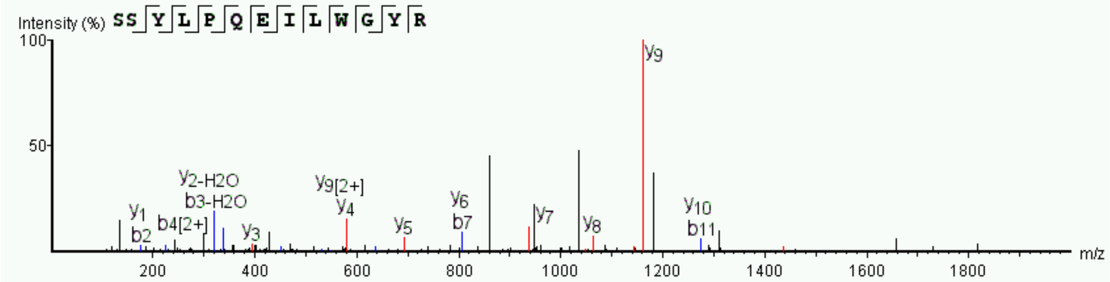

gi|571570521

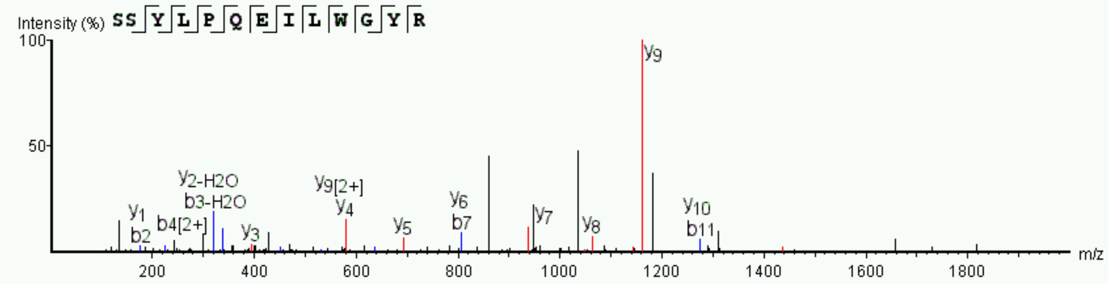

gi|571525006

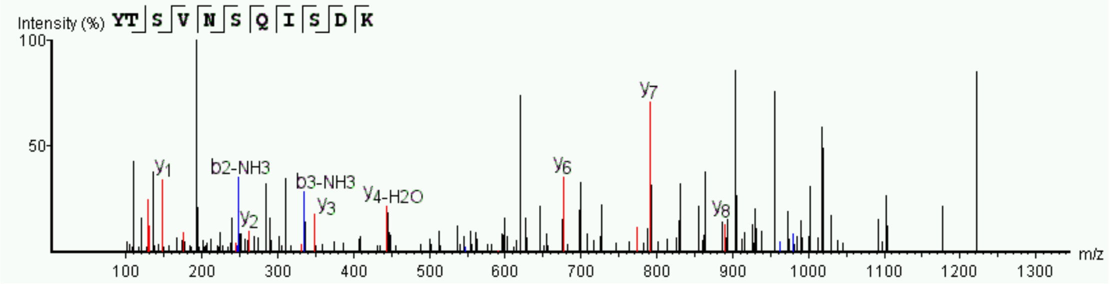

gi|571551216

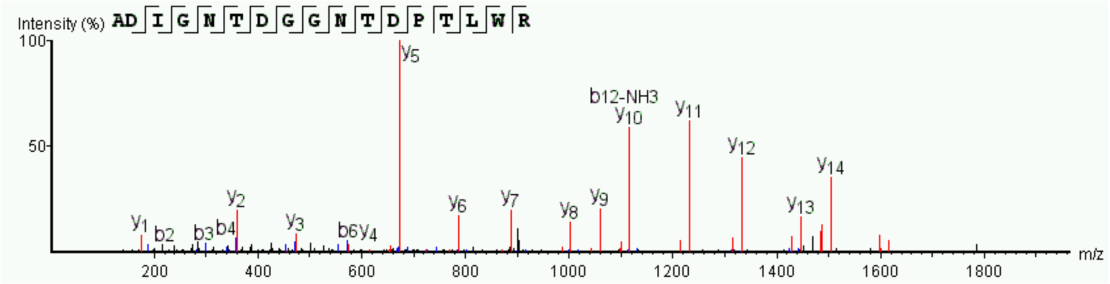

gi|571566049

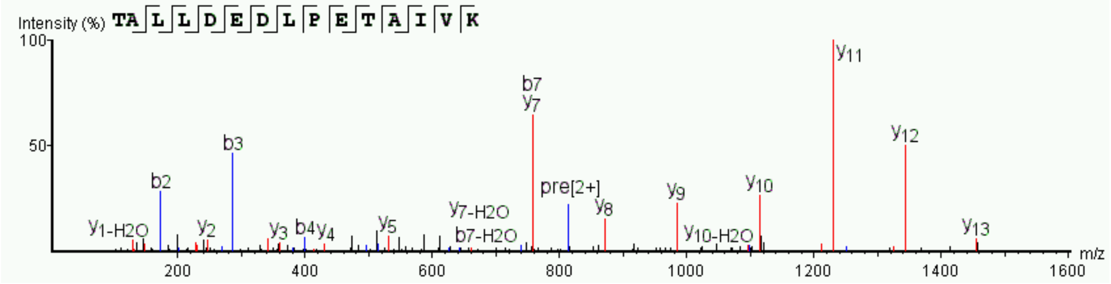

gi|328776180

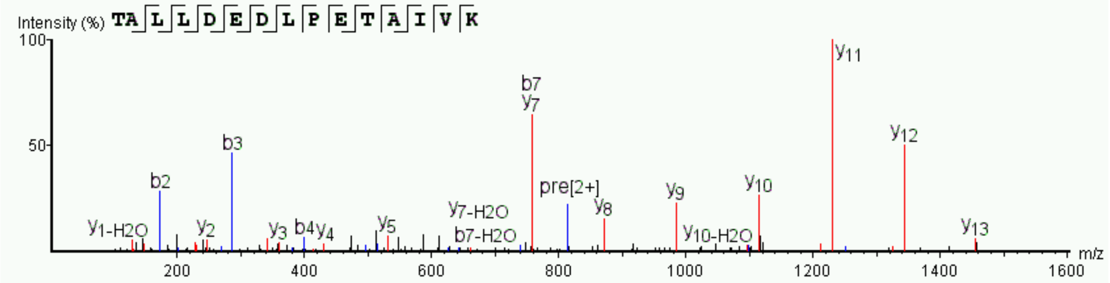

gi|571518324

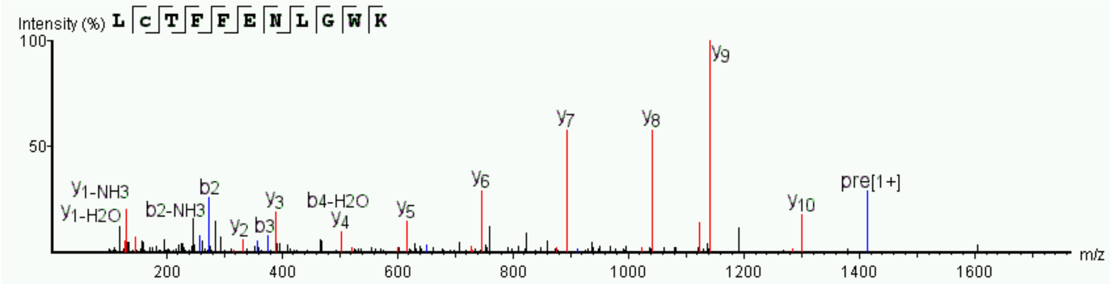

gi|571518327

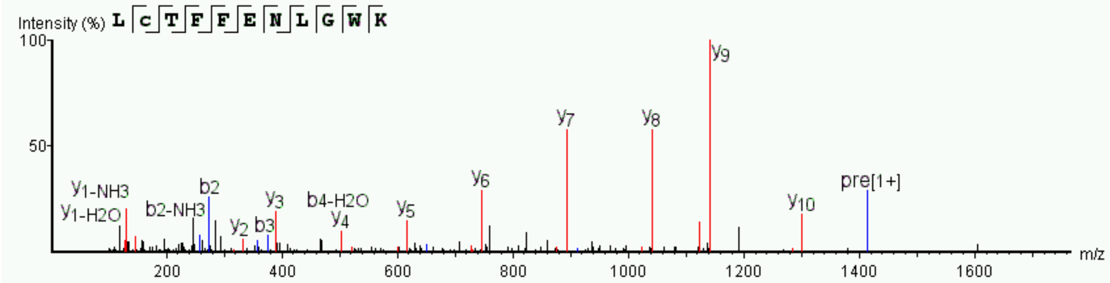

gi|571518333

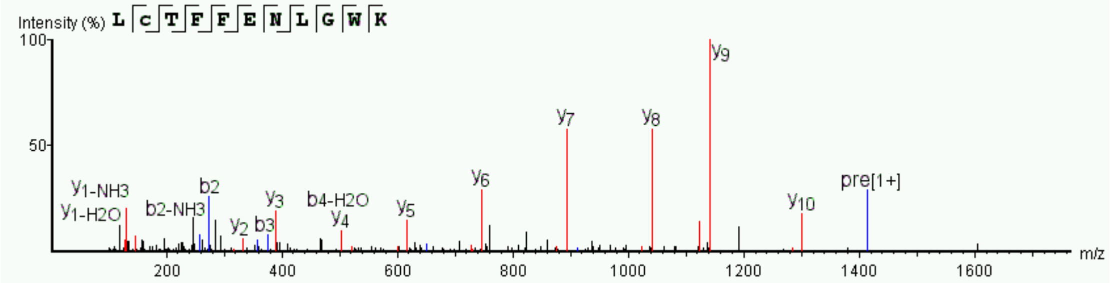

gi|328780255

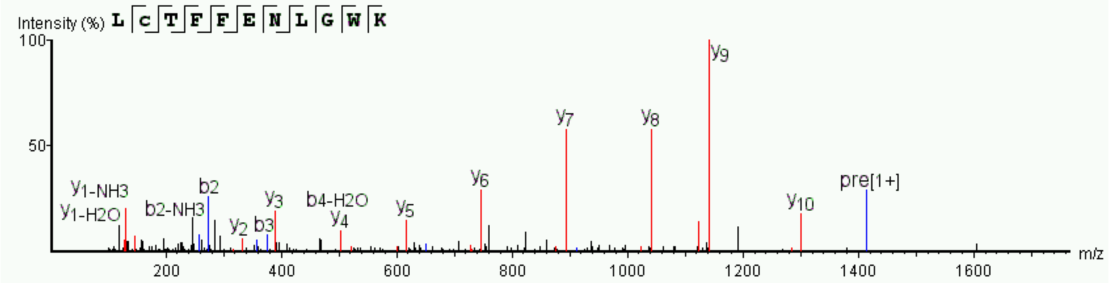

gi|571543658

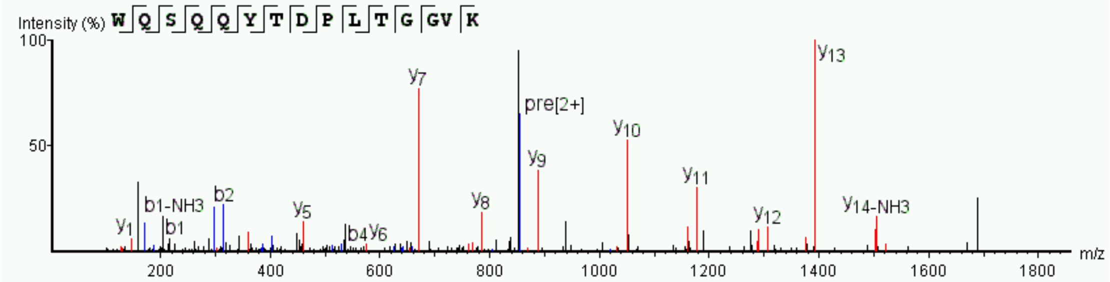

gi|571543688

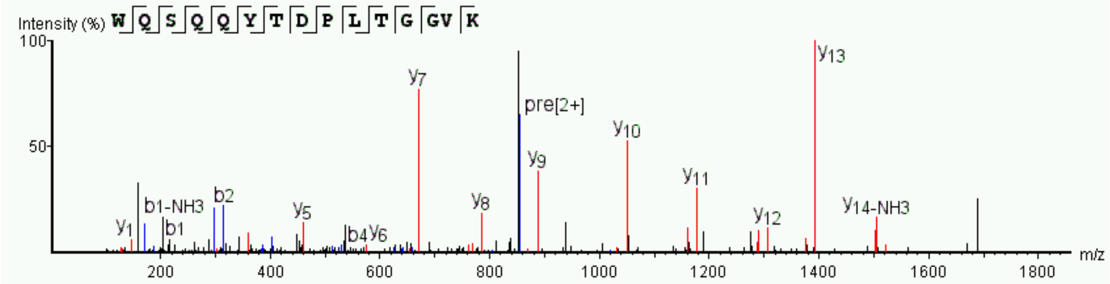

gi|571543693

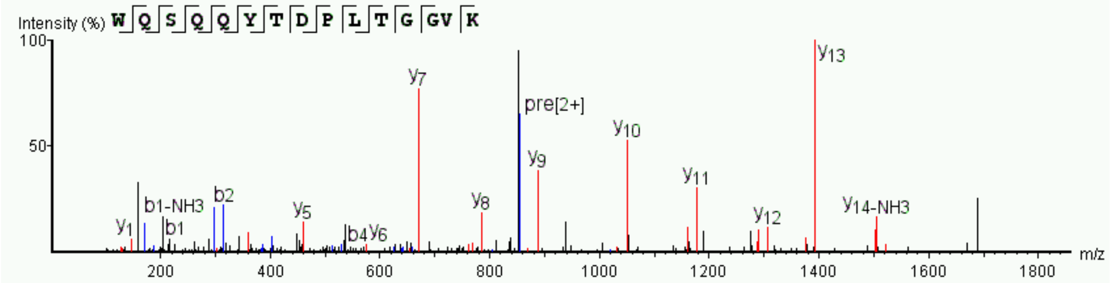

gi|571543661

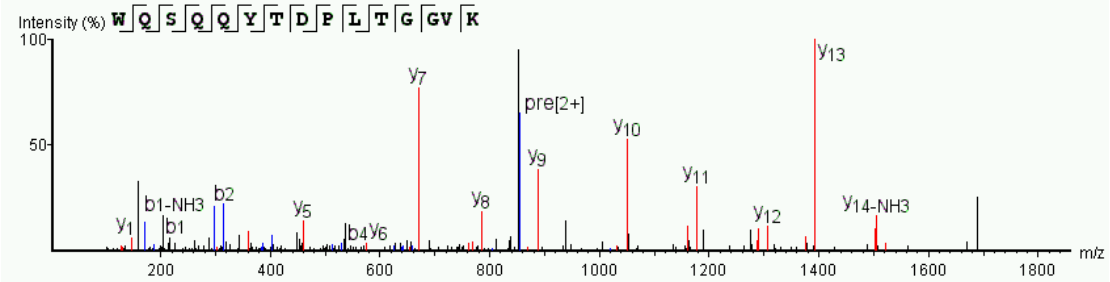

gi|571543664

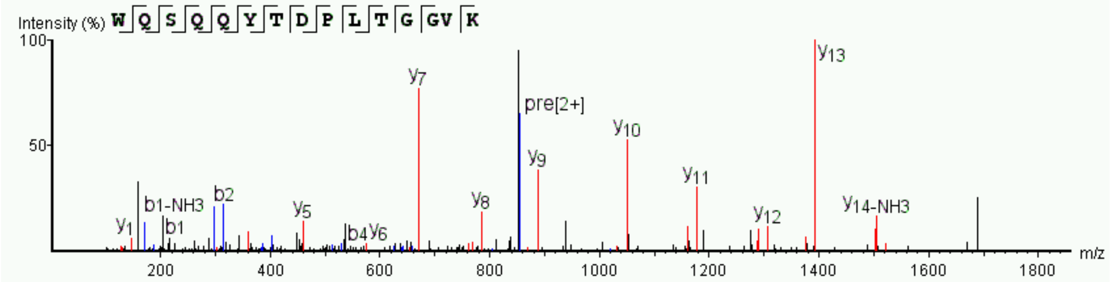

gi|571543668

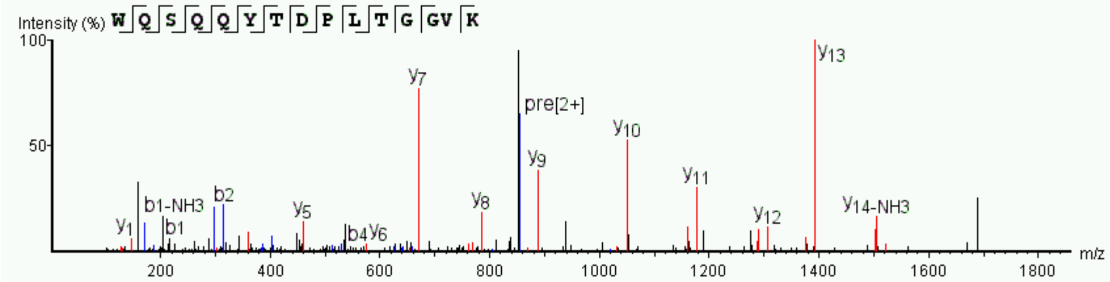

gi|571543672

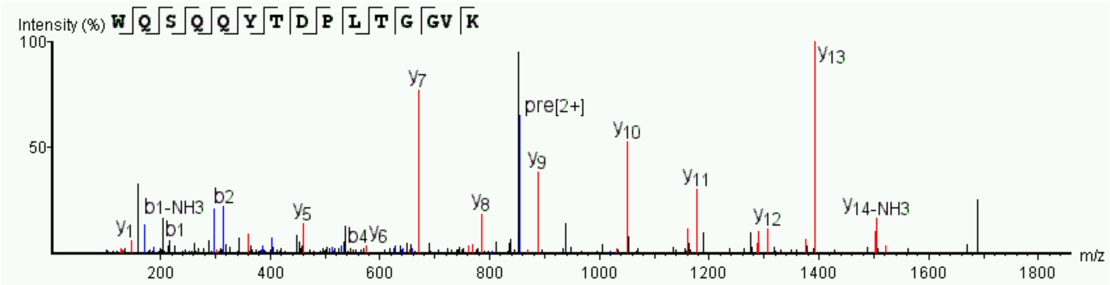

gi|571543676

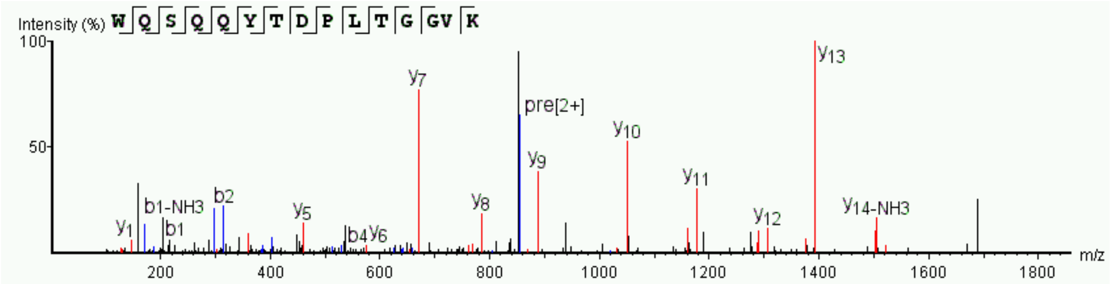

gi|571543679

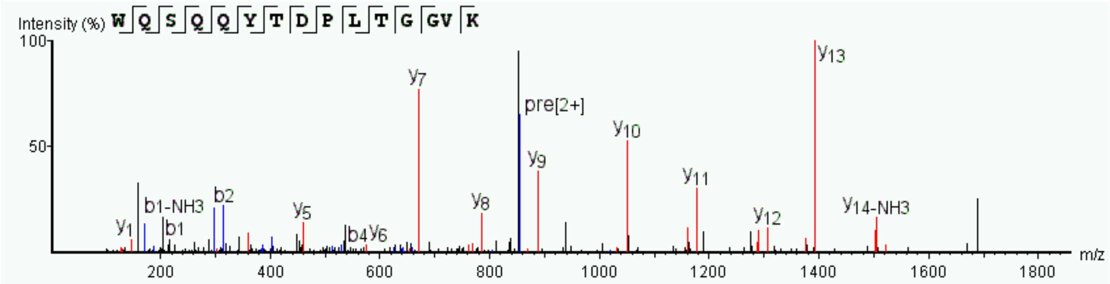

gi|571543682

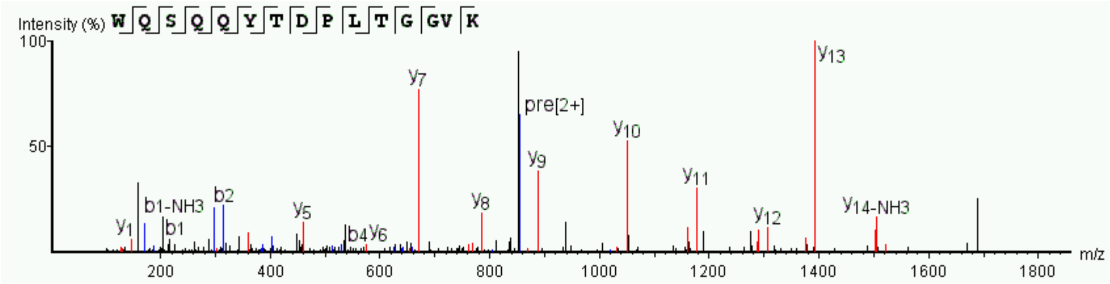

gi|571543685

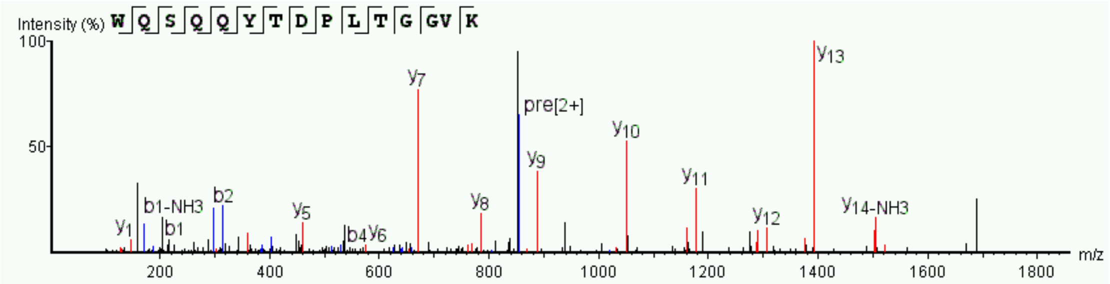

gi|571575403

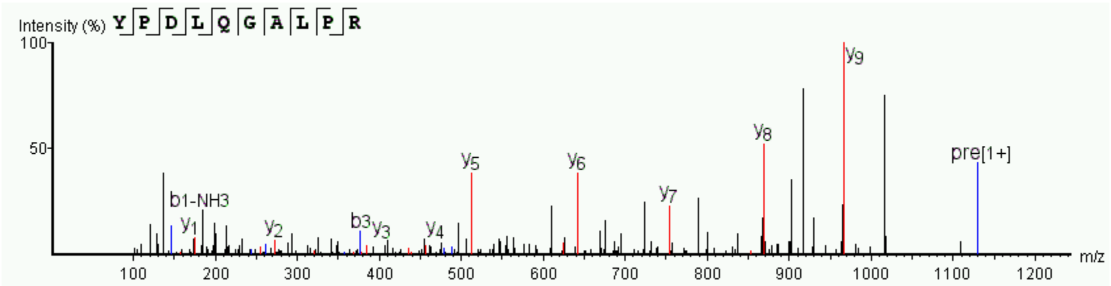

gi|571575406

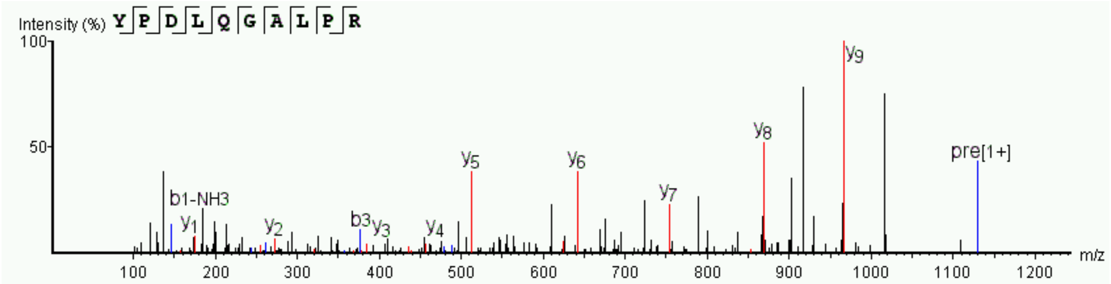

gi|571575408

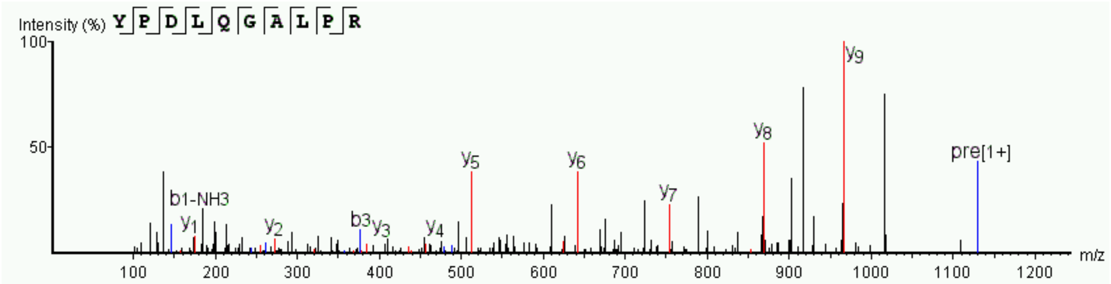

gi|328775931

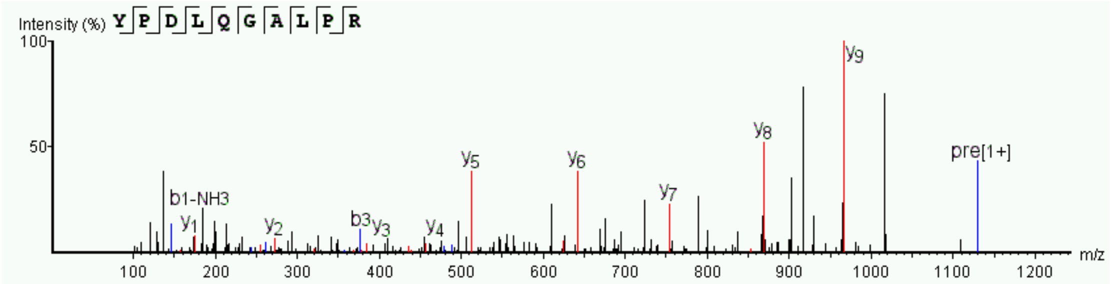

gi|571531426

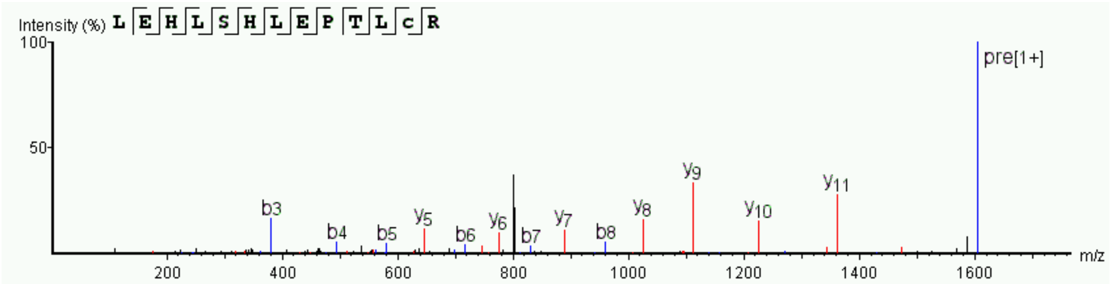

gi|66509585

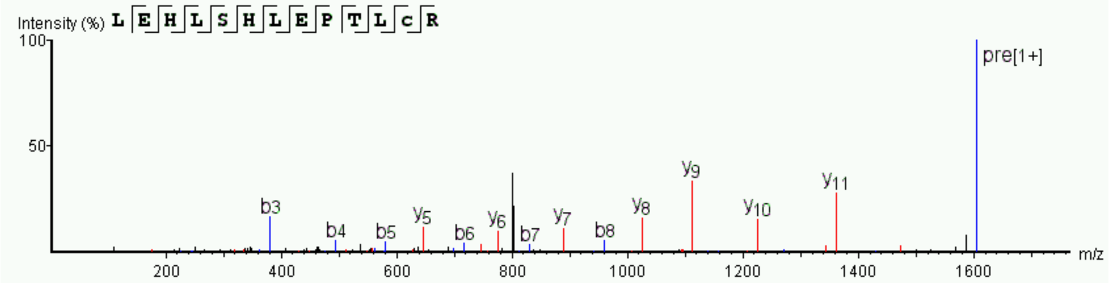

gi|571502919

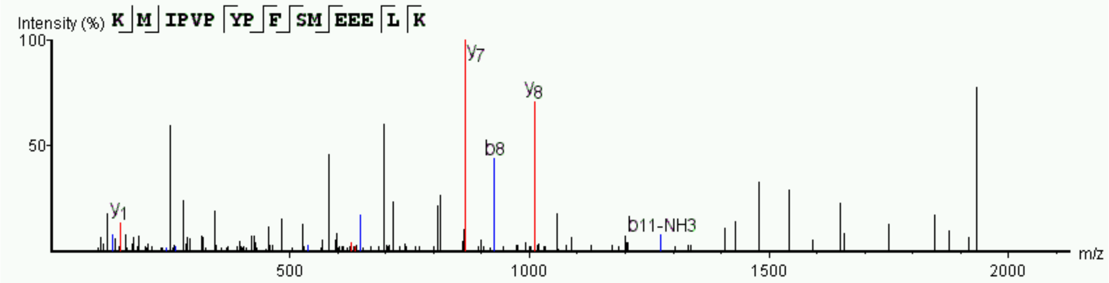

gi|571545145

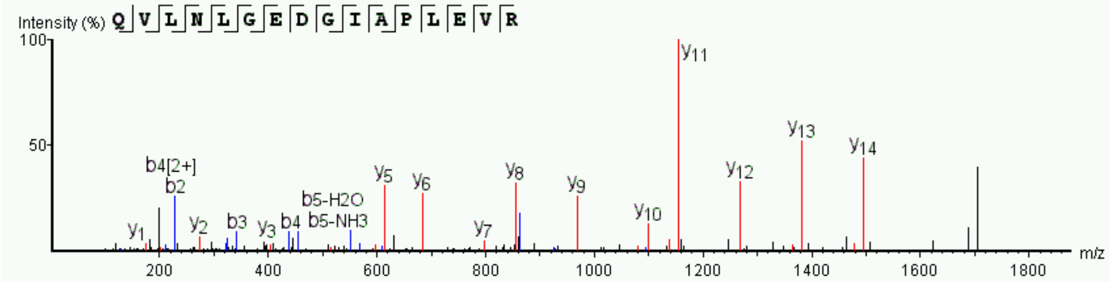

gi|571545149

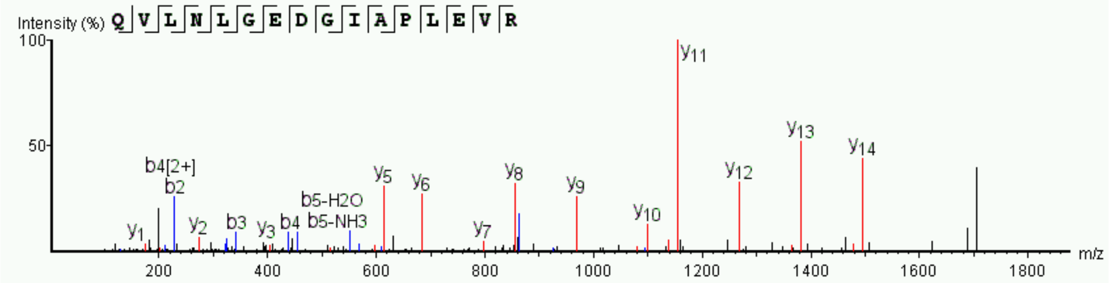

gi|571520595

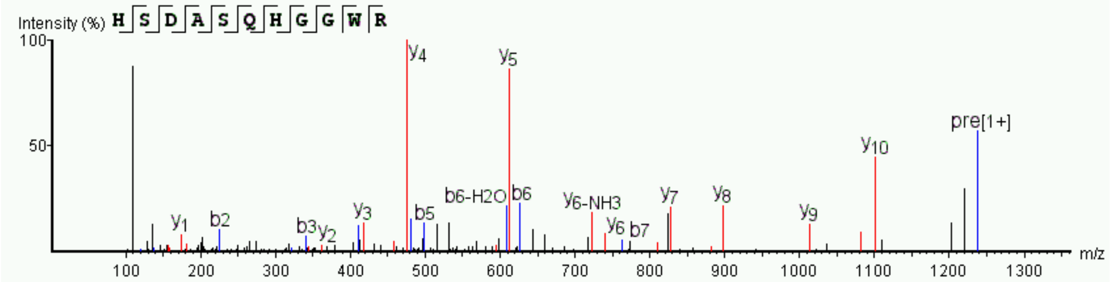

gi|571520632

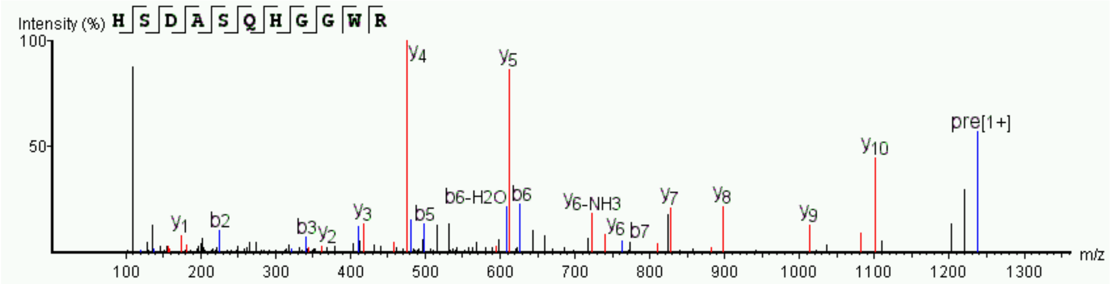

gi|571520635

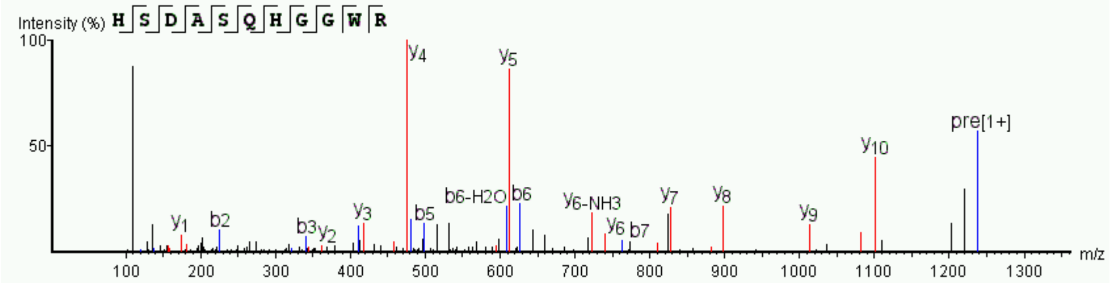

gi|571520638

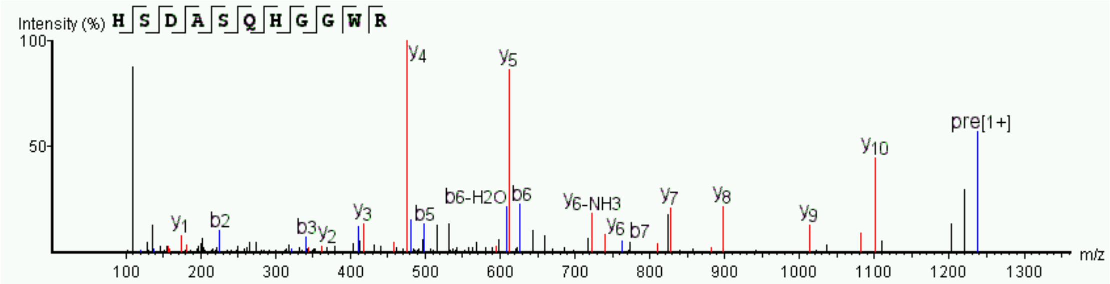

gi|571520599

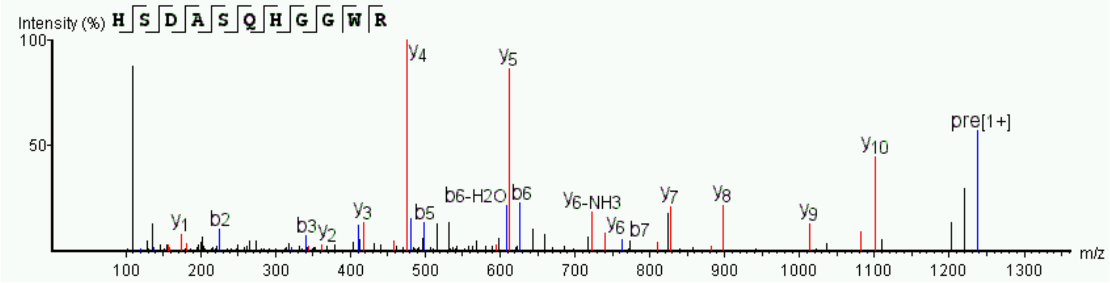

gi|571520603

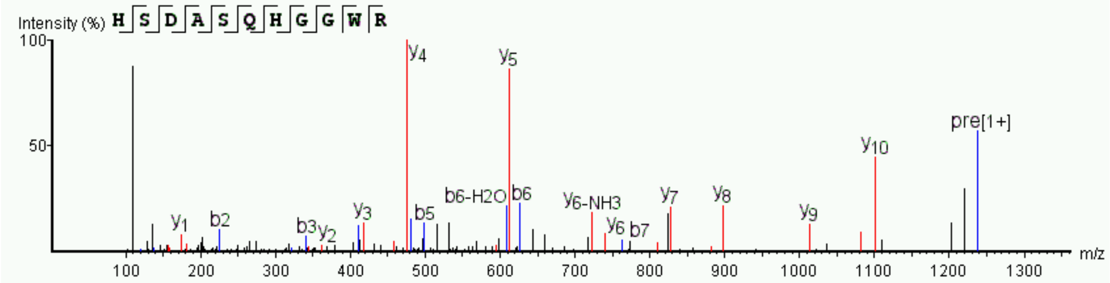

gi|571520607

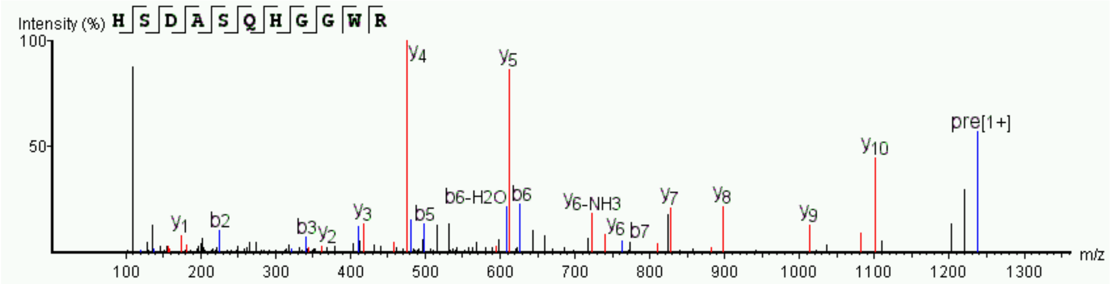

gi|571520613

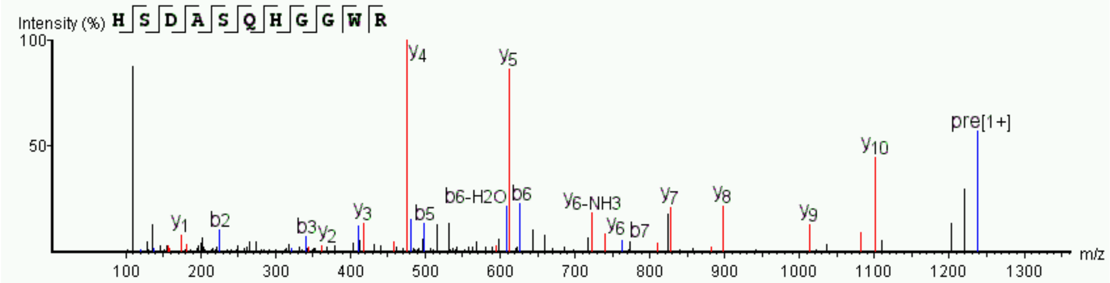

gi|571520616

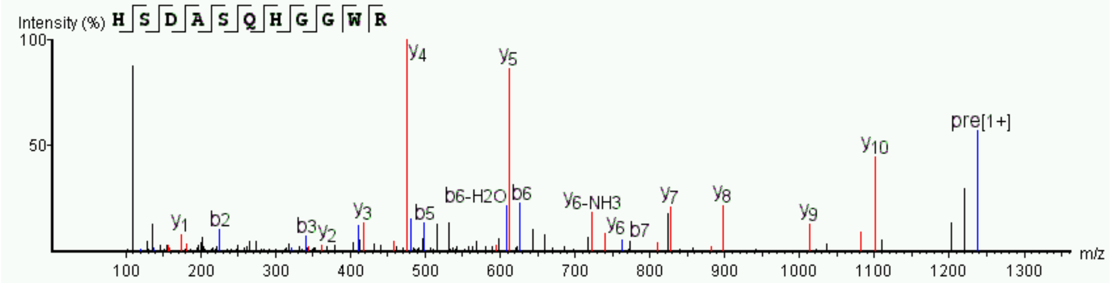

gi|571520620

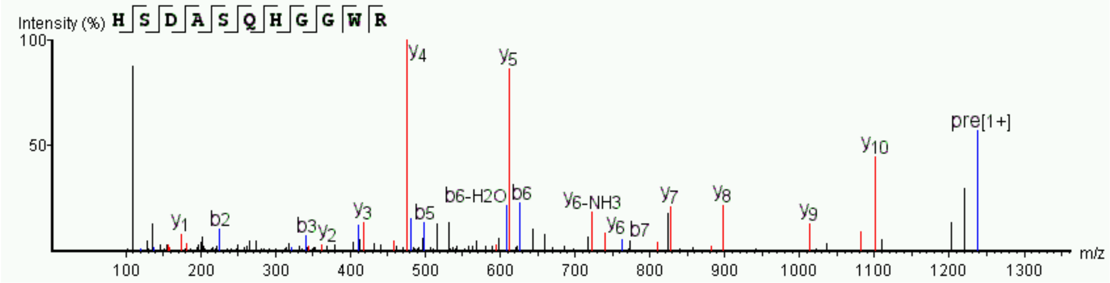

gi|571520624

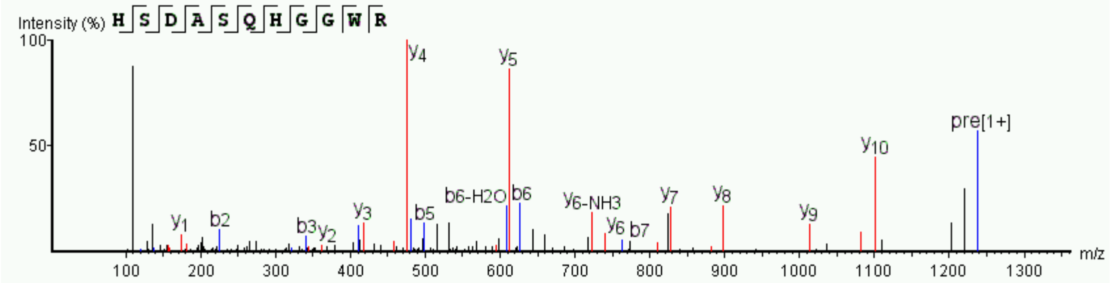

gi|571520628

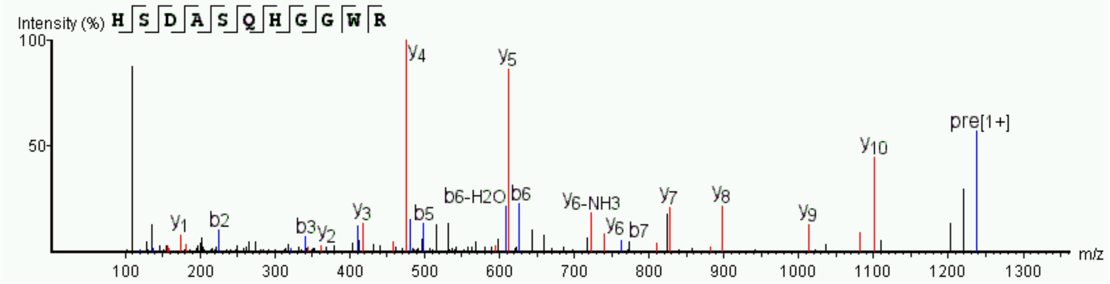

gi|328789160

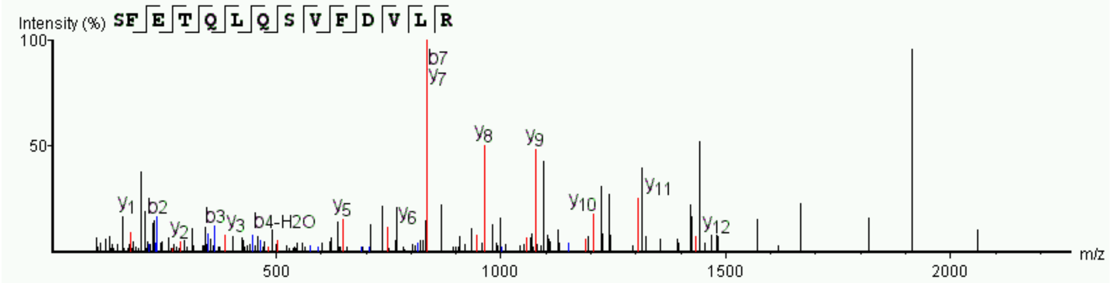

gi|571503499

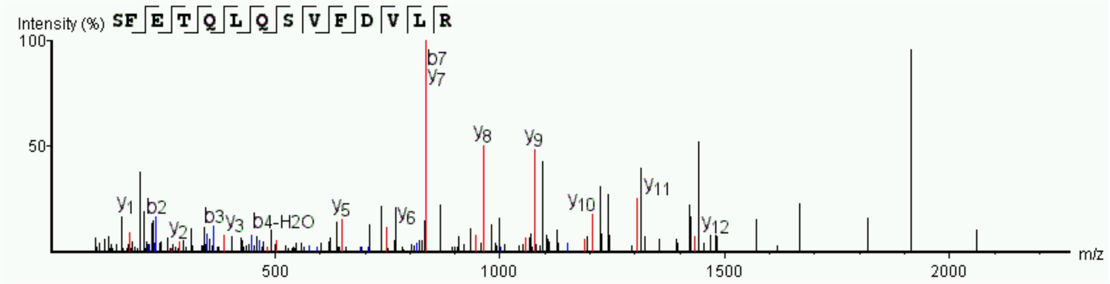

gi|571577919

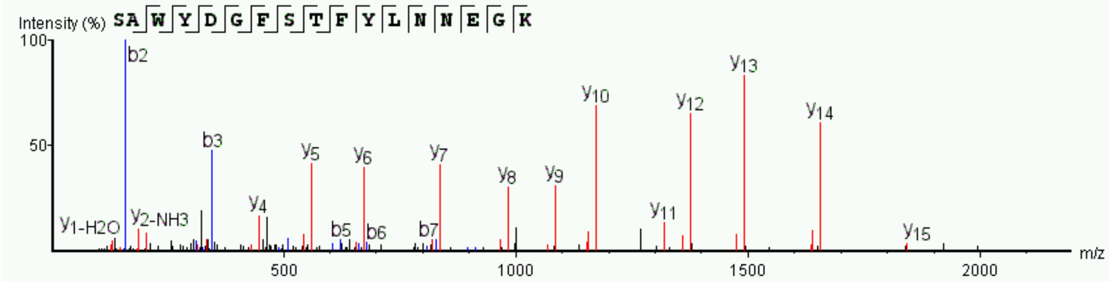

gi|571577921

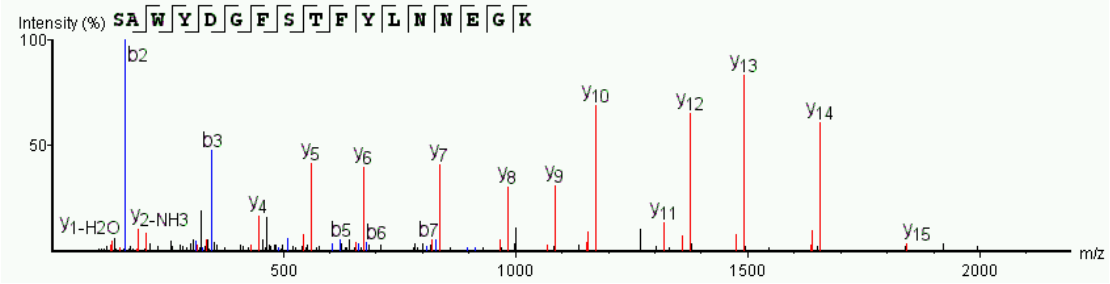

gi|571577923

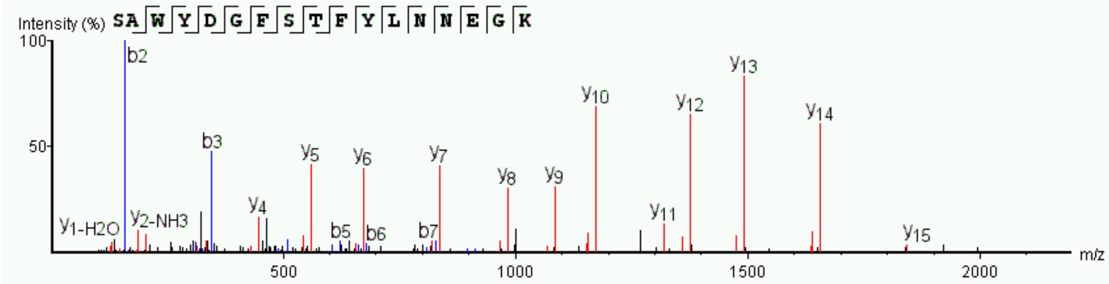

gi|571577925

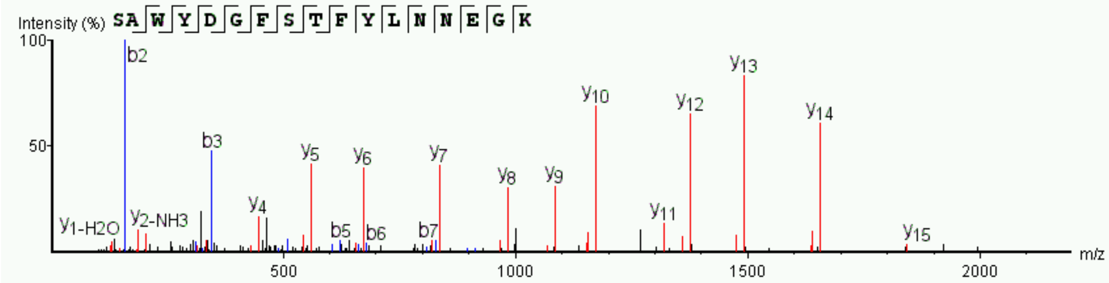

gi|571577927

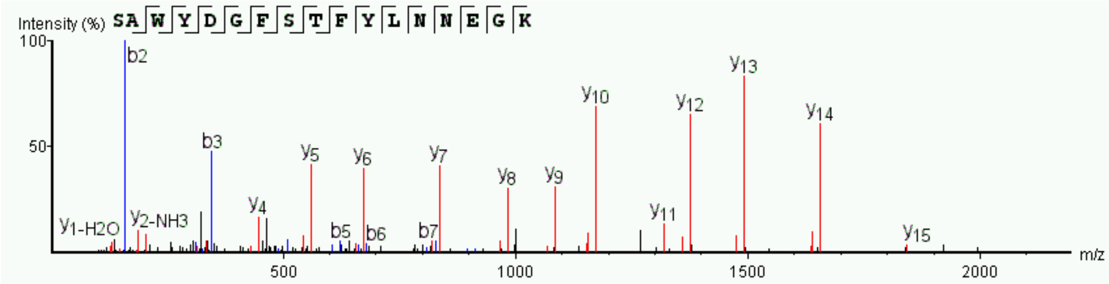

gi|571577929

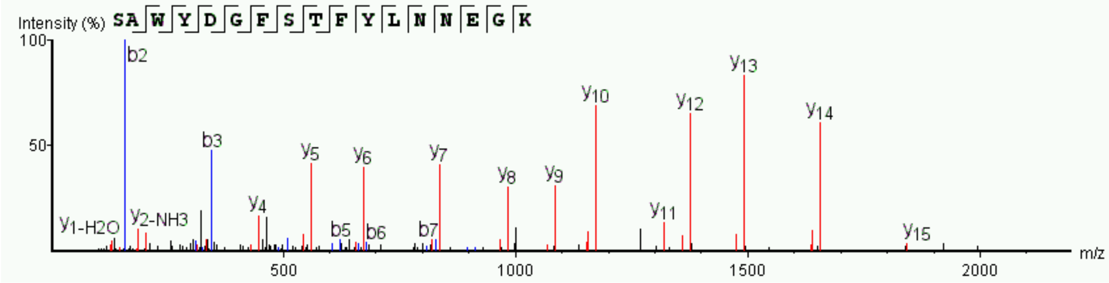

gi|571577931

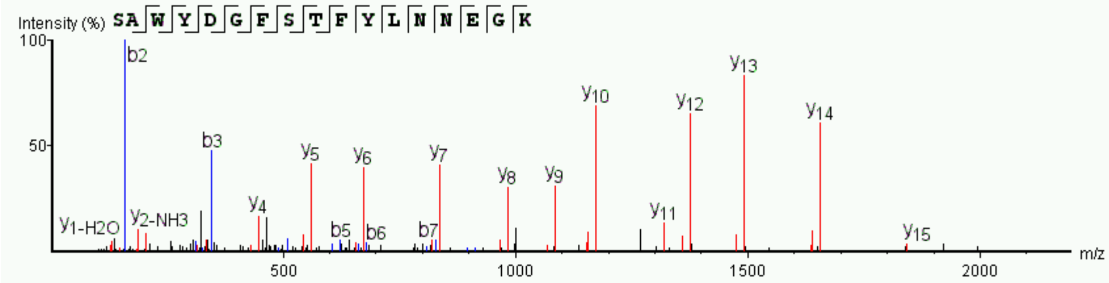

gi|571577933

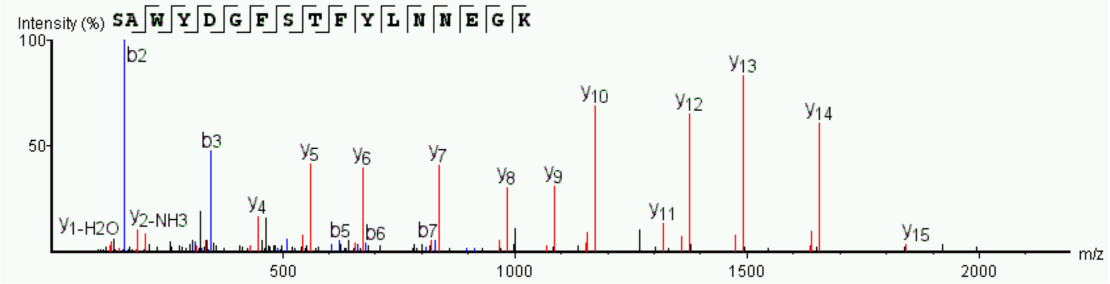

gi|328784942

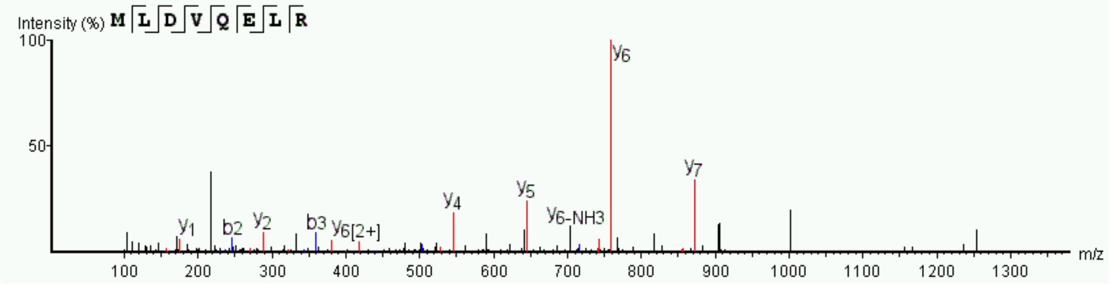

gi|328787733

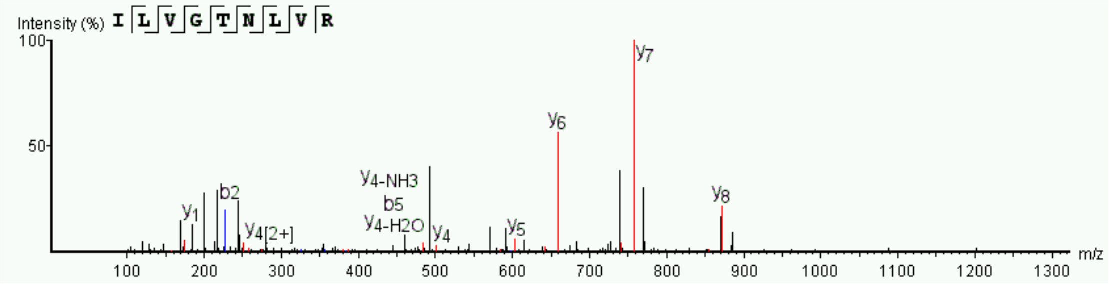

gi|571501581

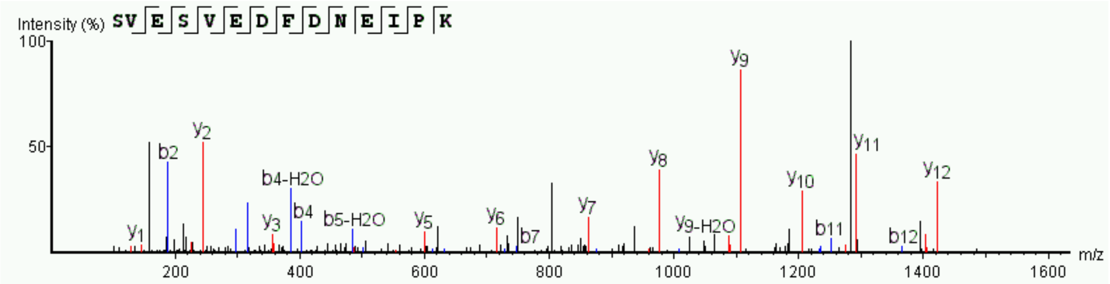

gi|571503771

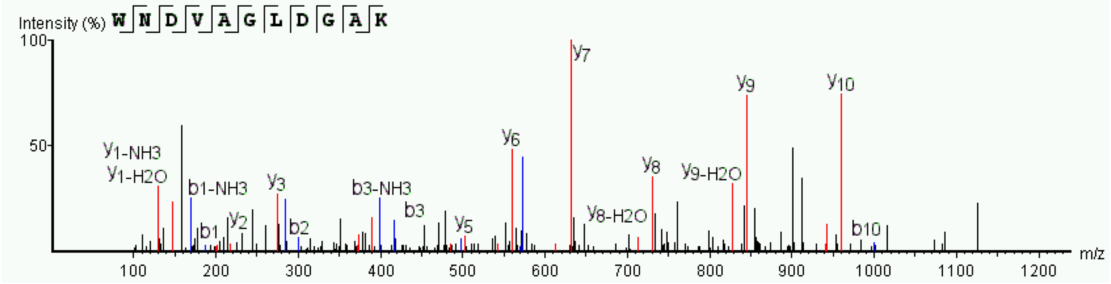

gi|66564933

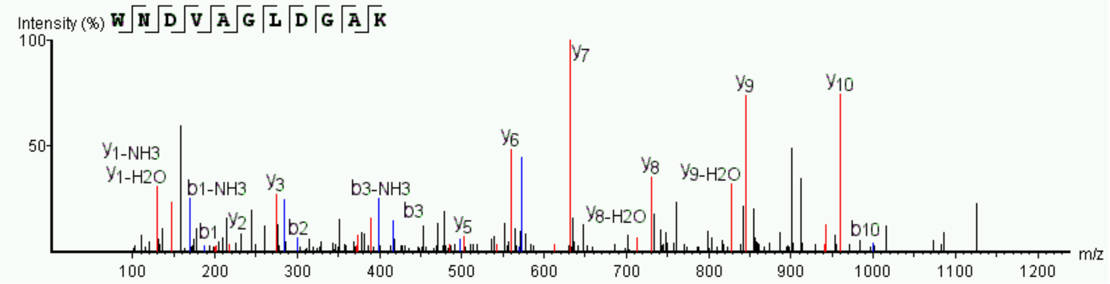

gi|571516617

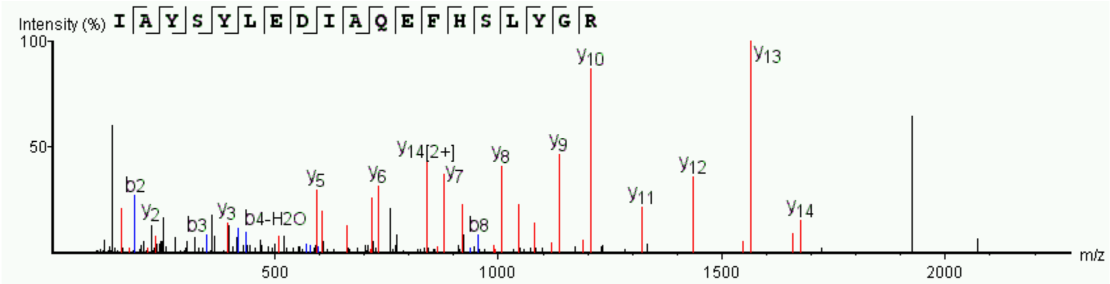

gi|66513815

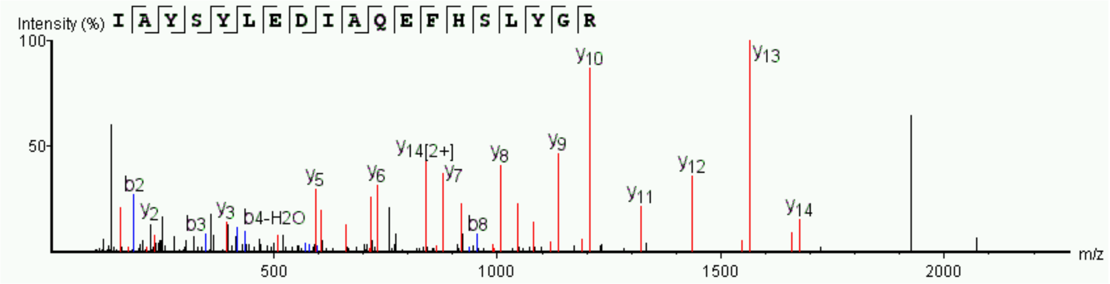

gi|66524947

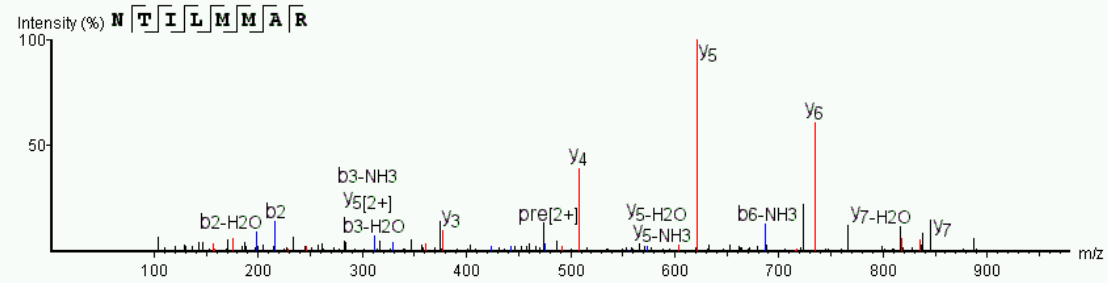

gi|571533194

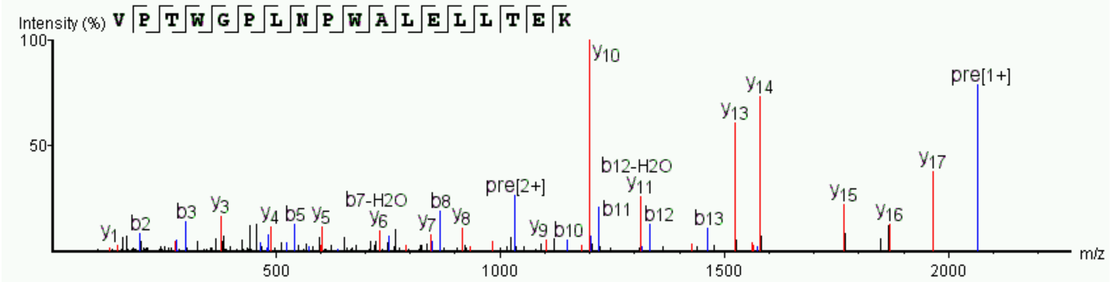

gi|571533201

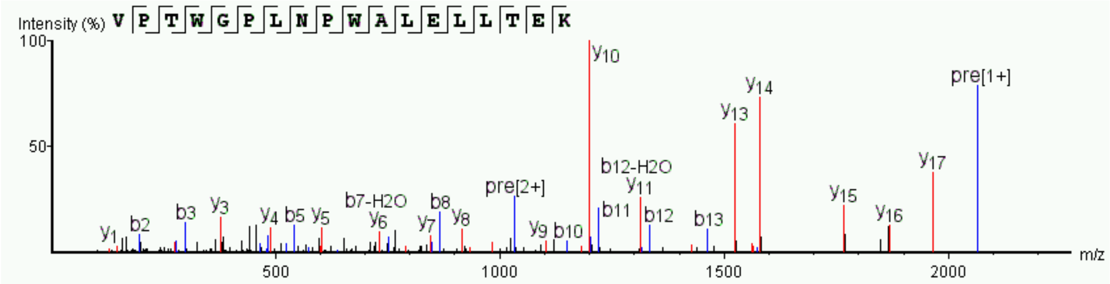

gi|571533197

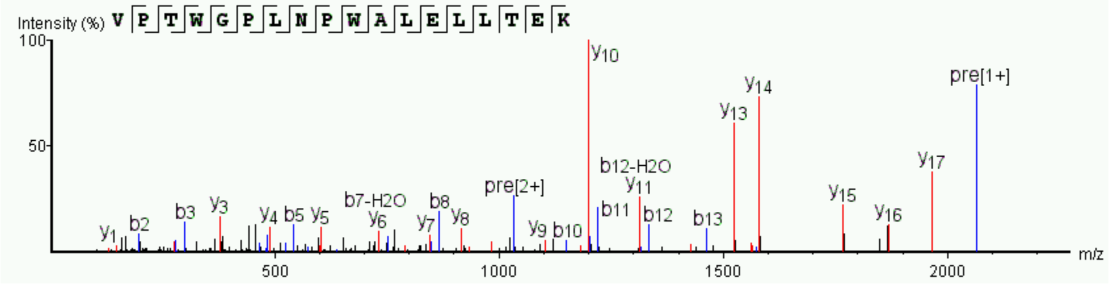

gi|335892852

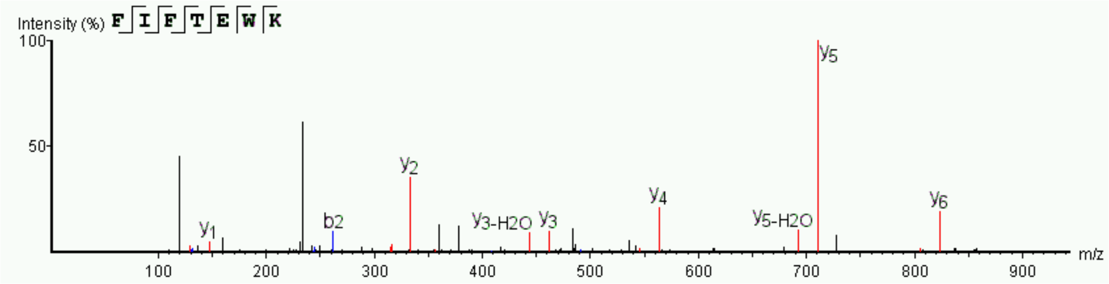

gi|336088626

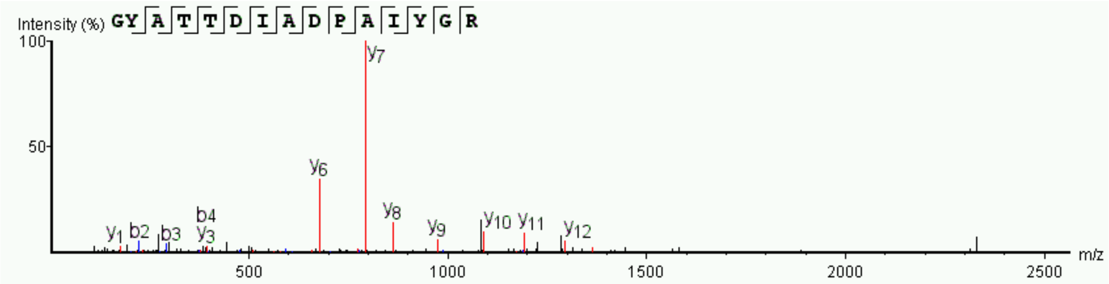

gi|296010815

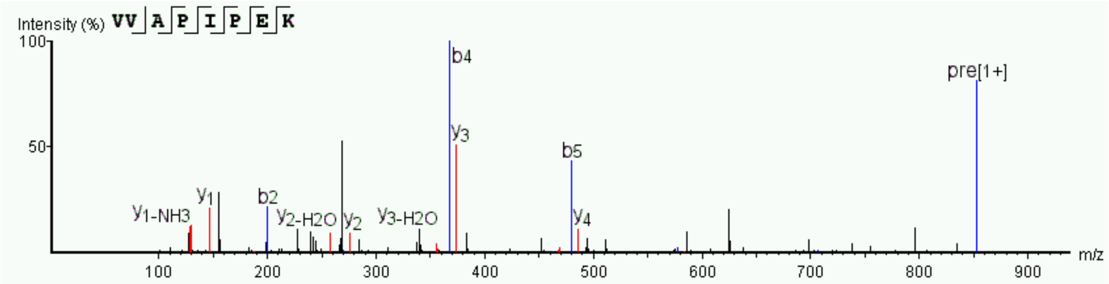

gi|507418856

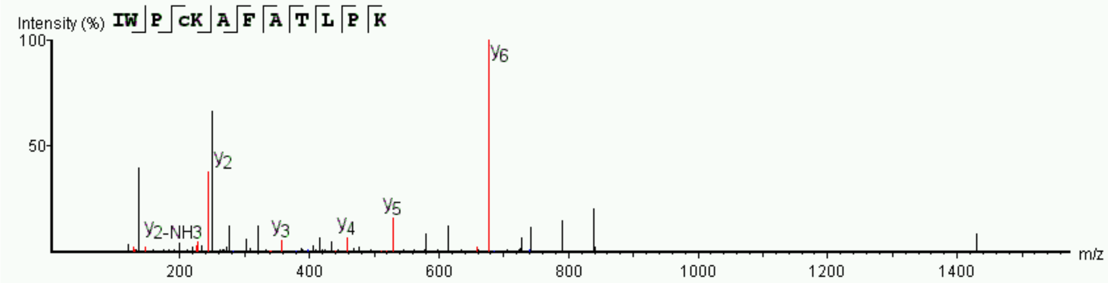

gi|289577262

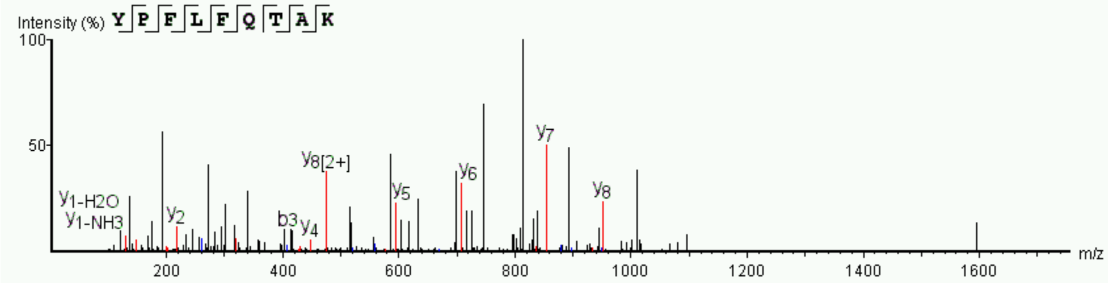

gi|162952028

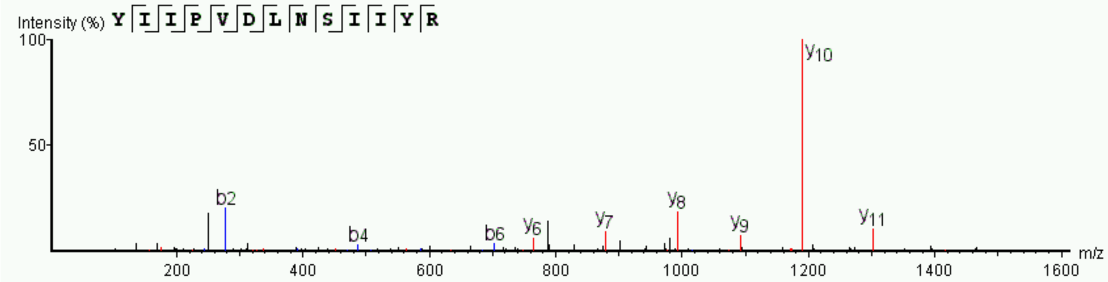

gi|571536261

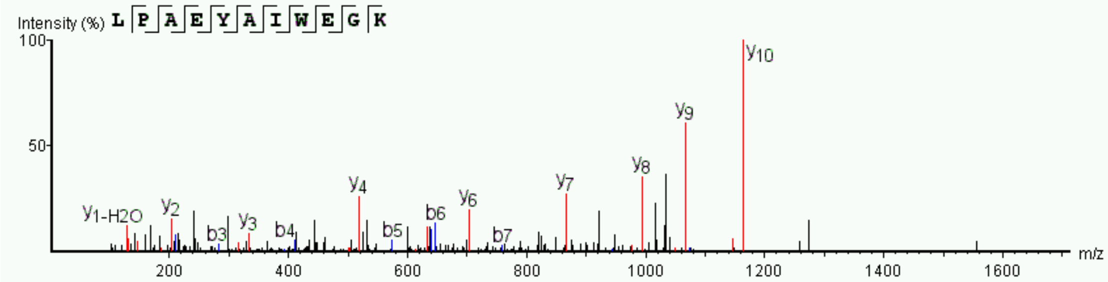

gi|571576054

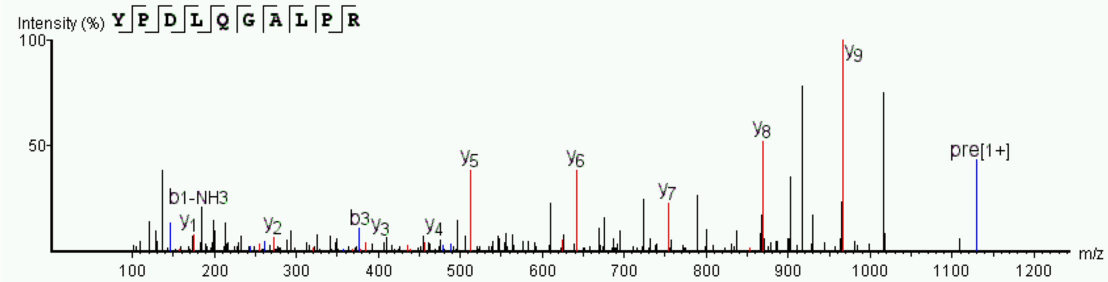

gi|571566027

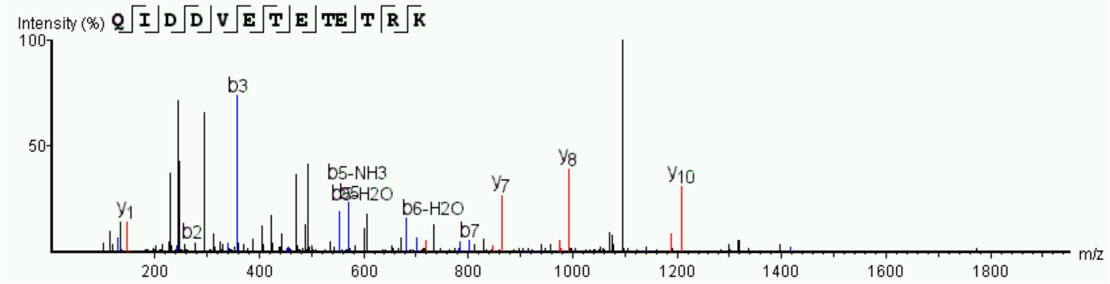

gi|571501581

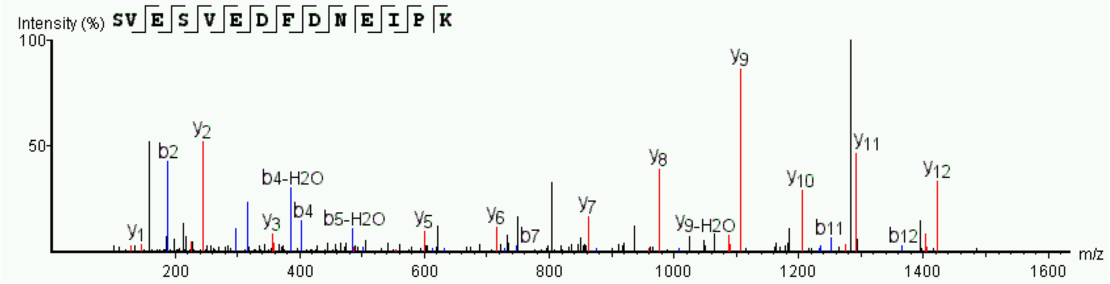

gi|328786521

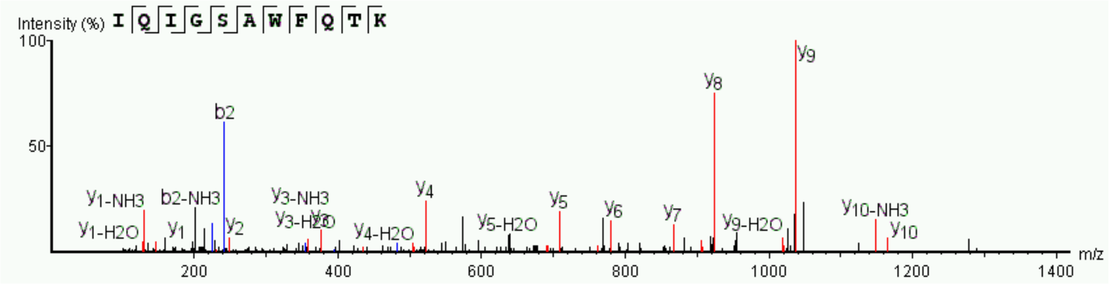

gi|571559017

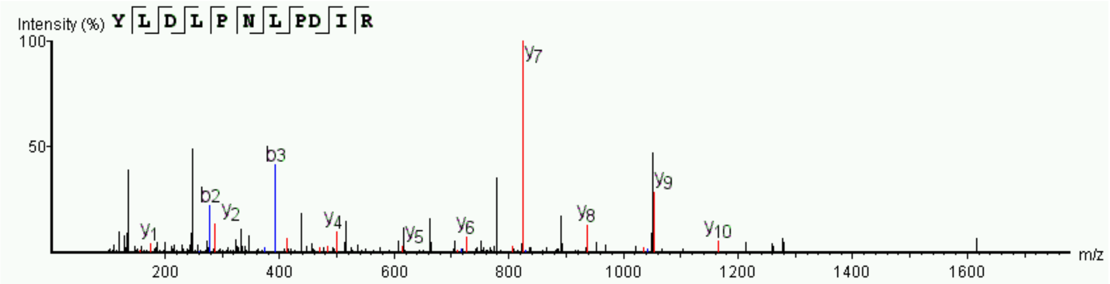

gi|571528259

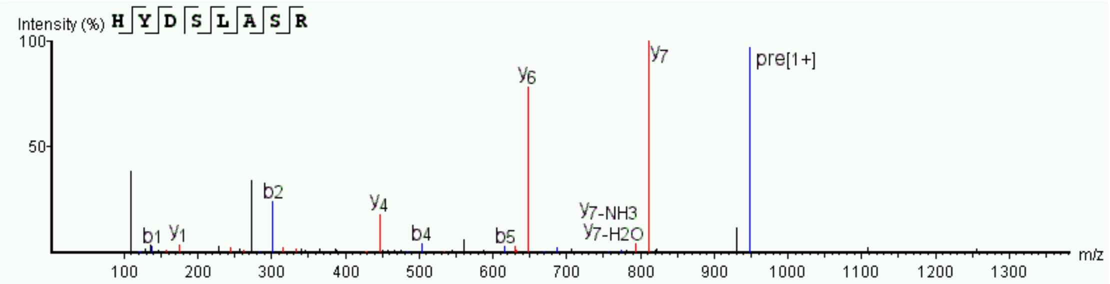

gi|571520744

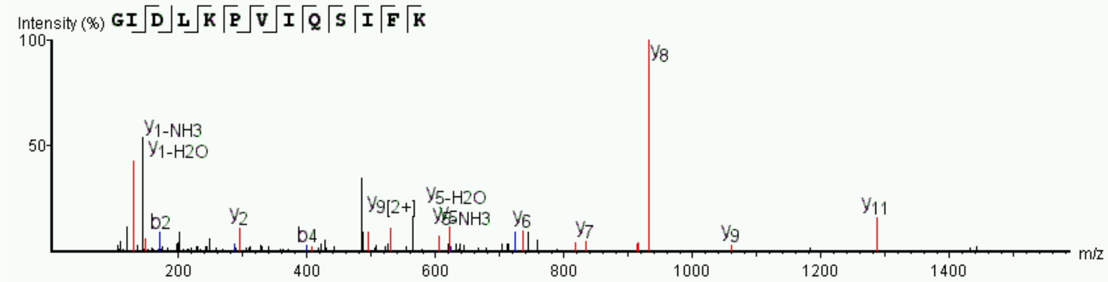

gi|328784535

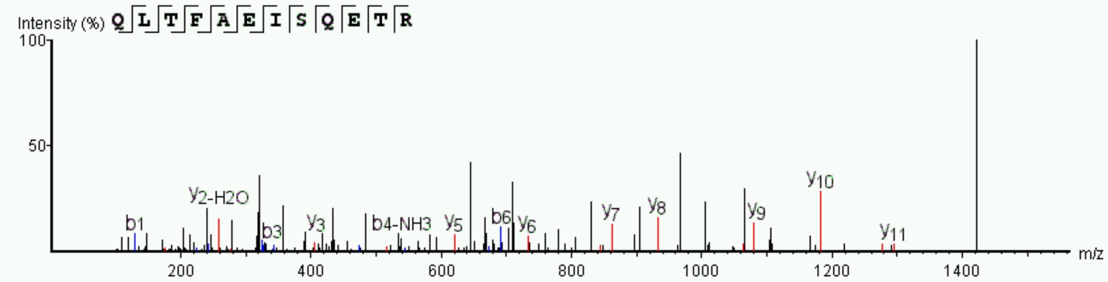

gi|571563070

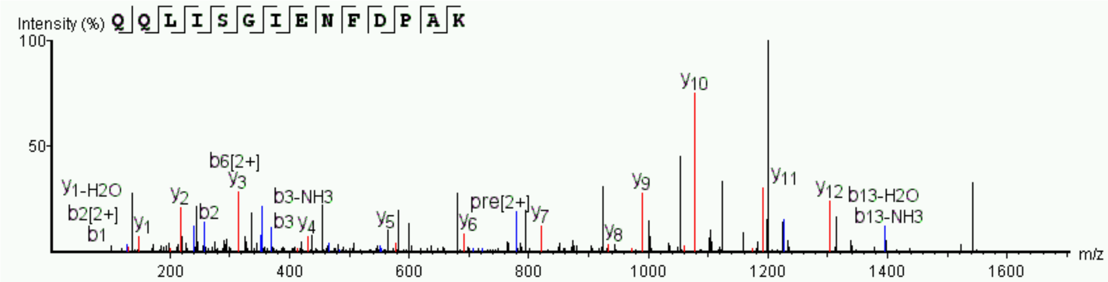

gi|66565869

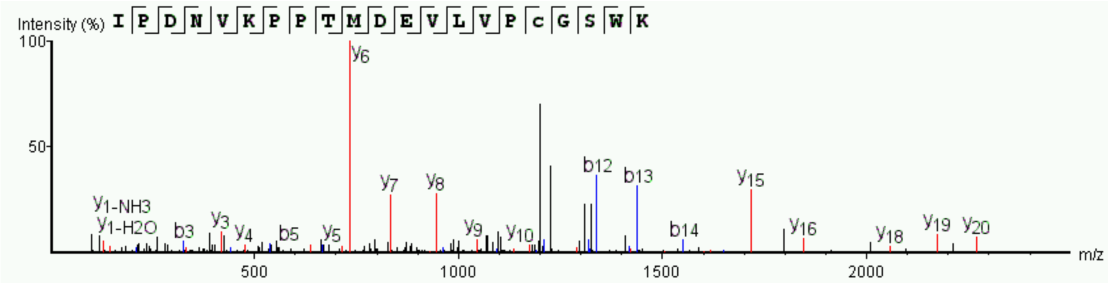

gi|571561759

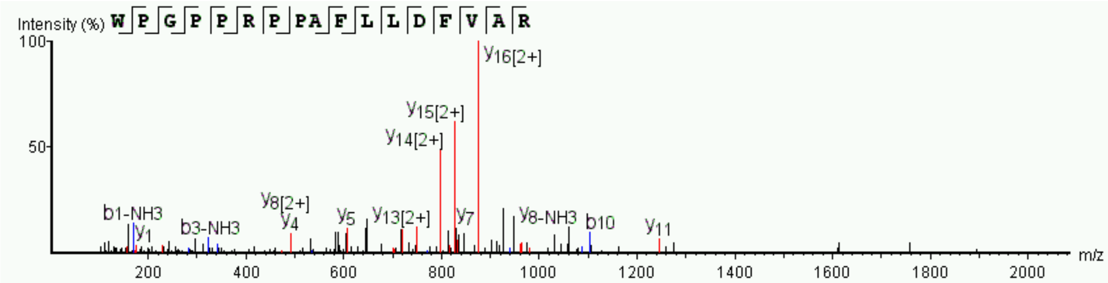

gi|328789486

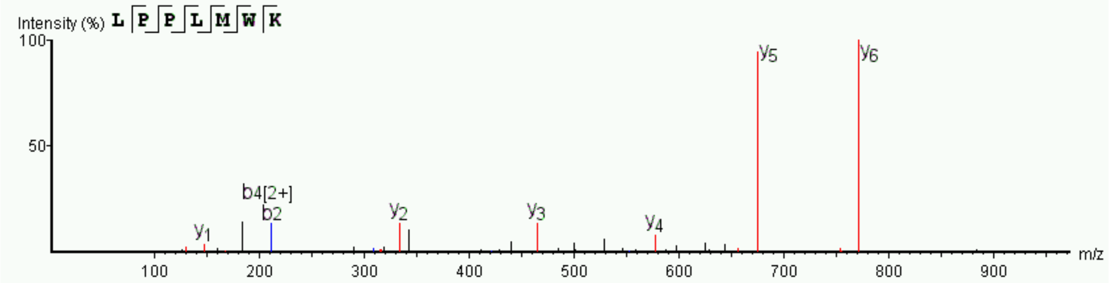

gi|66506397

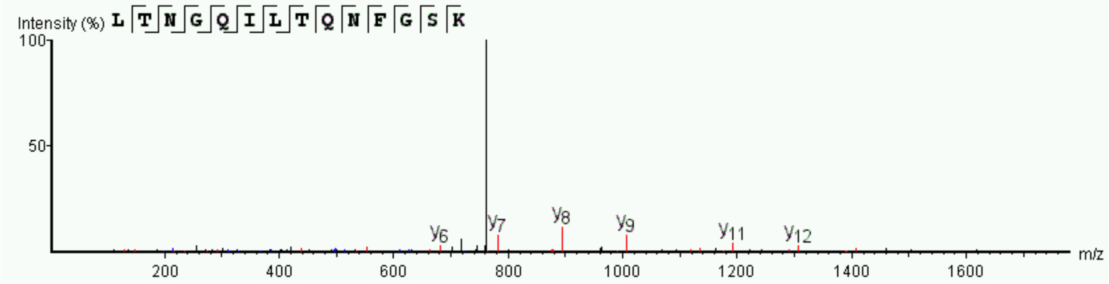

gi|328780745

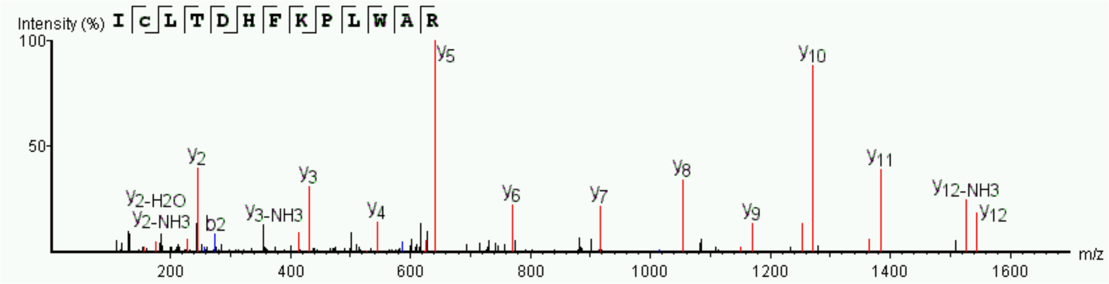

gi|571533174

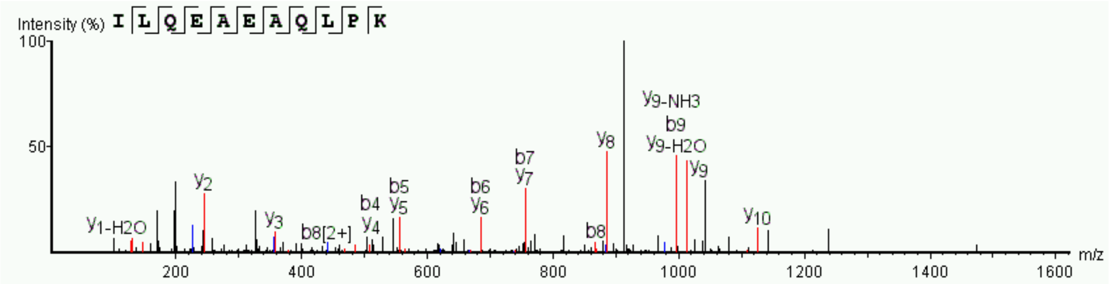

gi|571513874

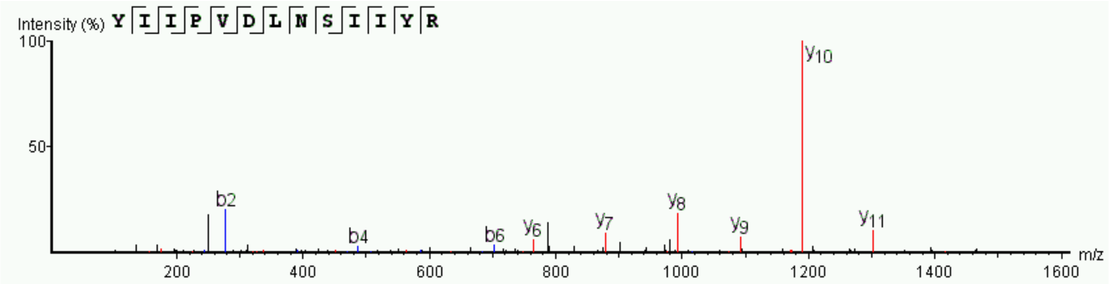

gi|571562624

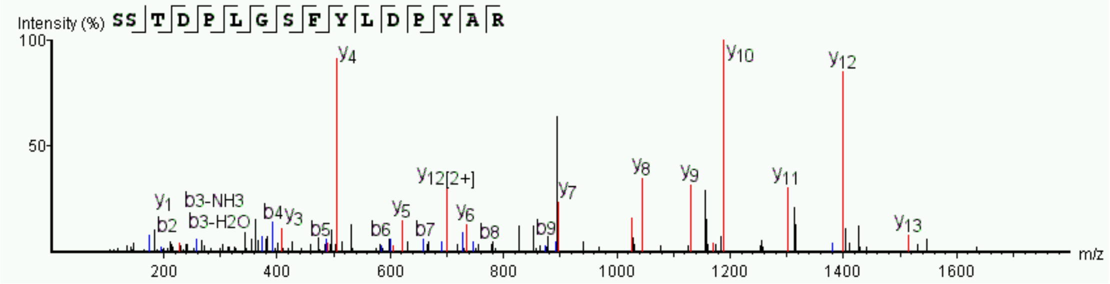

gi|571526645

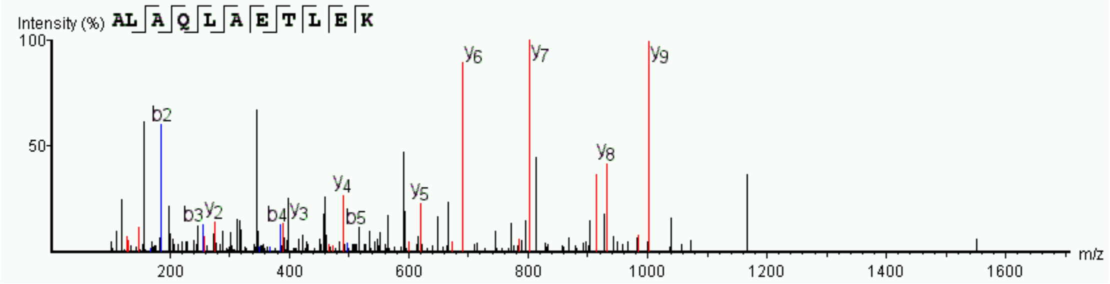

gi|571538400

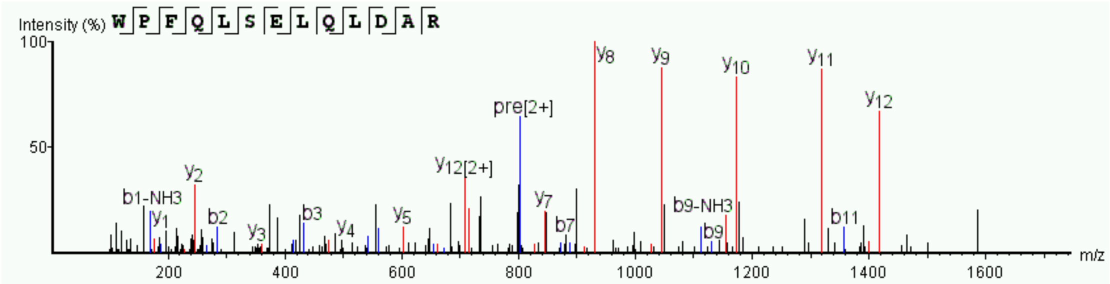

gi|571538396

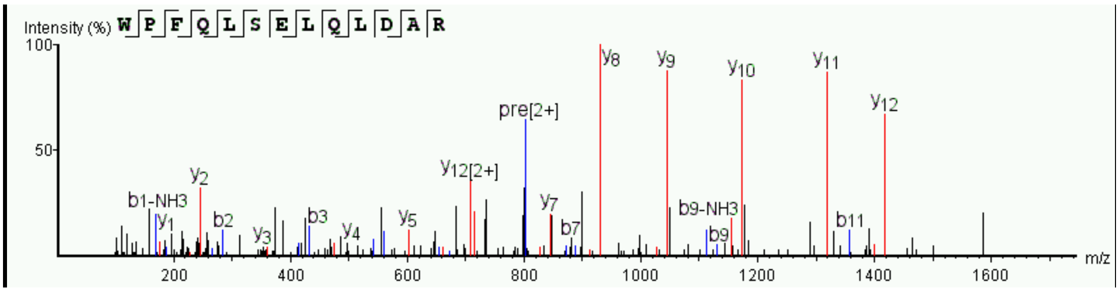

gi|571538392

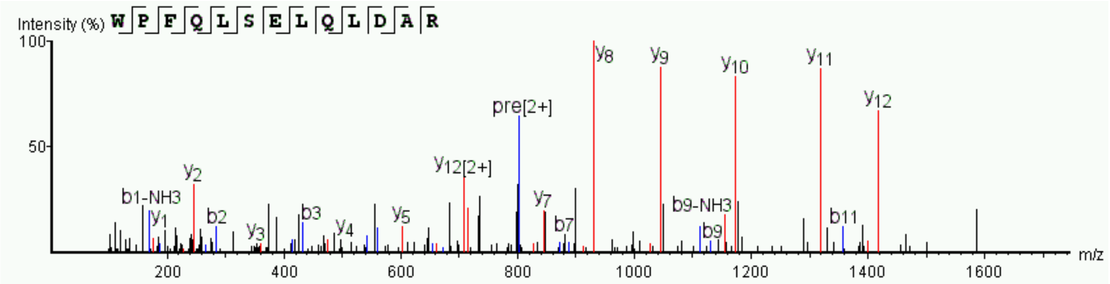

gi|571575573

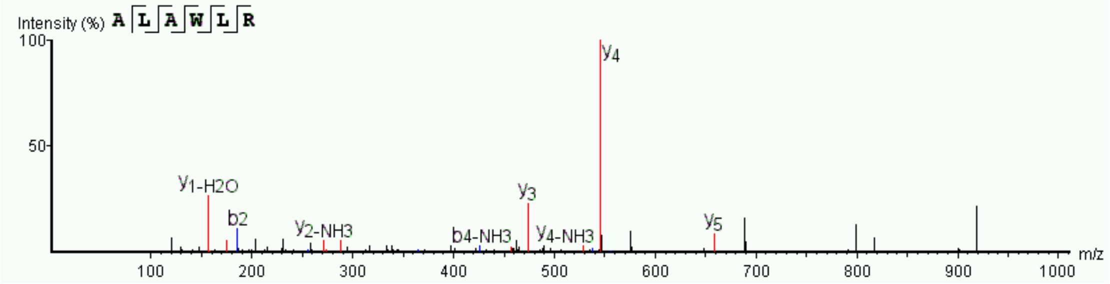

gi|571566017

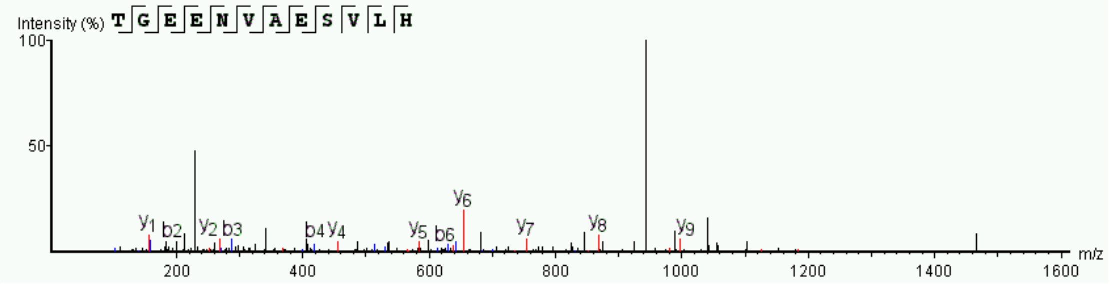

gi|110767674

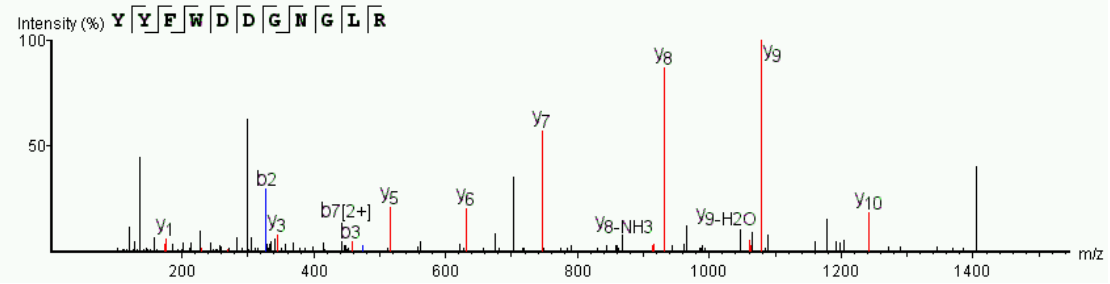

gi|571561389

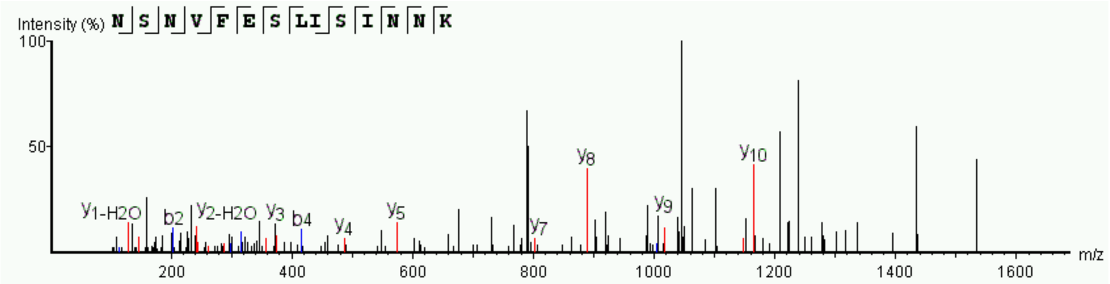

gi|571526744

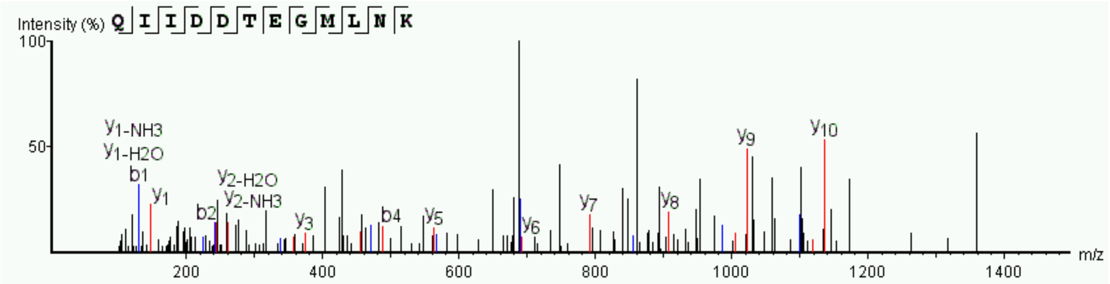

gi|328781704

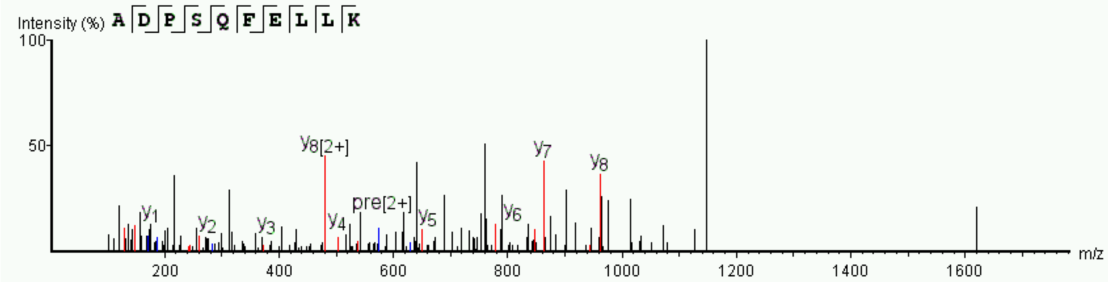

gi|66515508

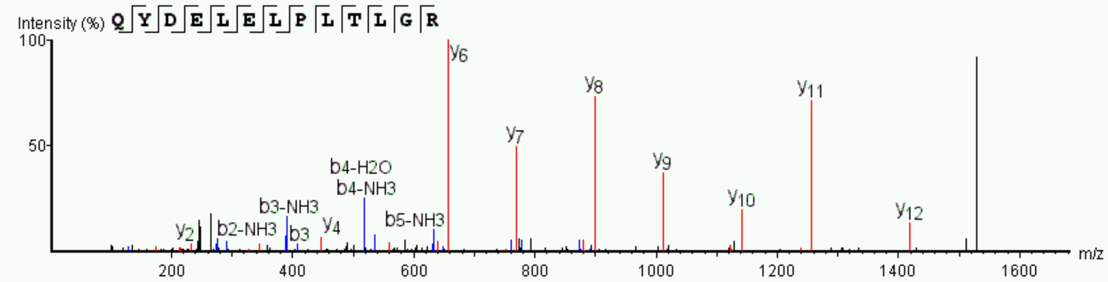

gi|48104663

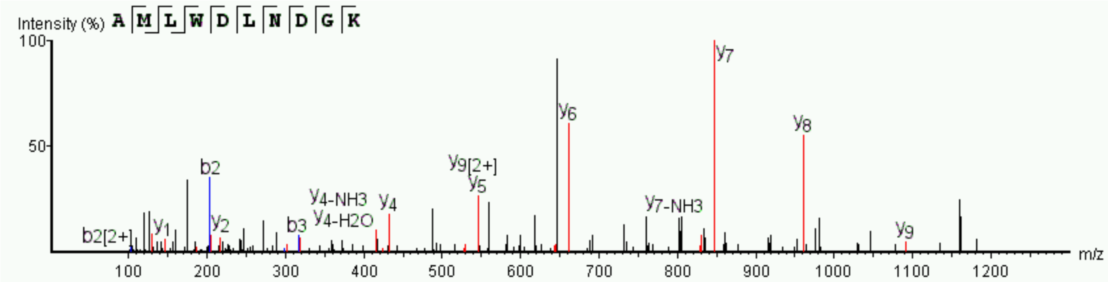

gi|66499475

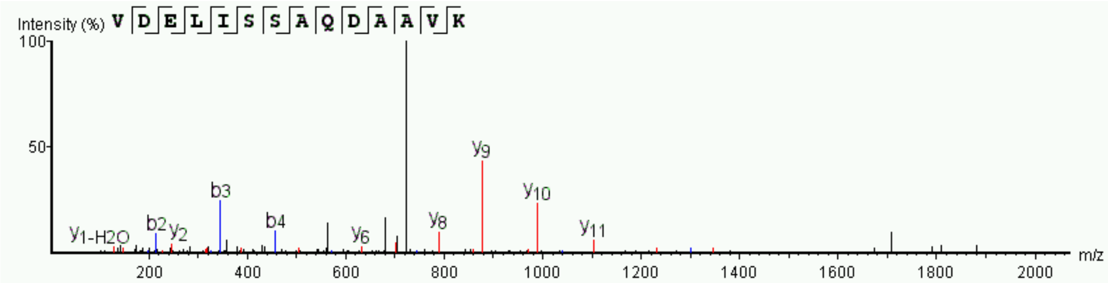

gi|66554913

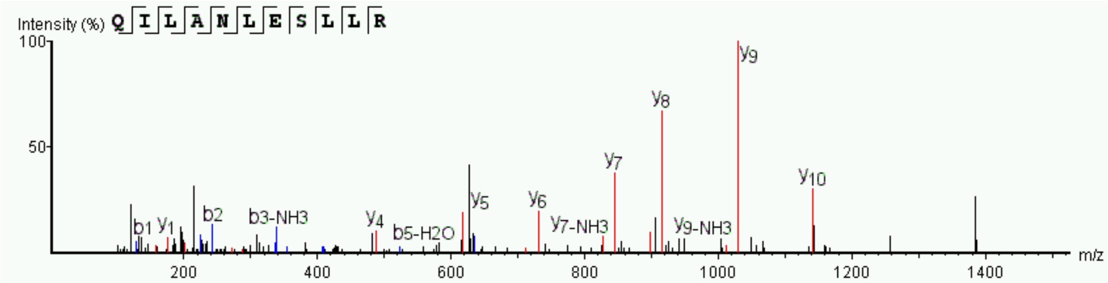

gi|328789575

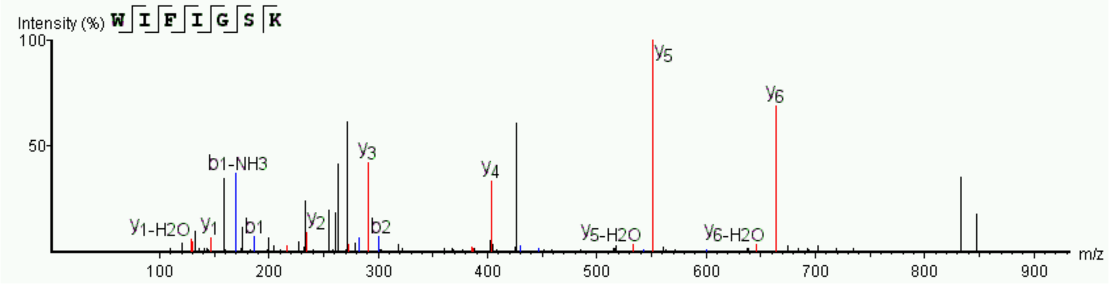

gi|571532691

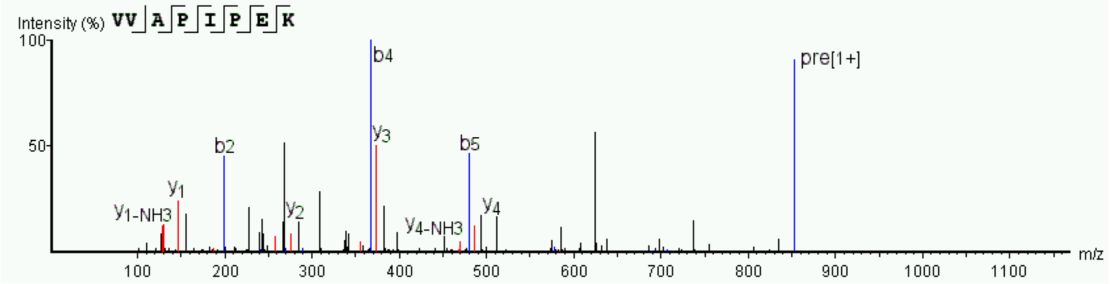

gi|571510049

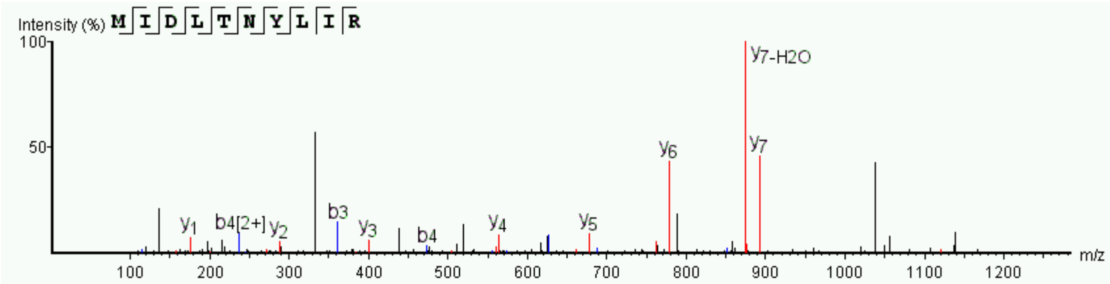

gi|571510045

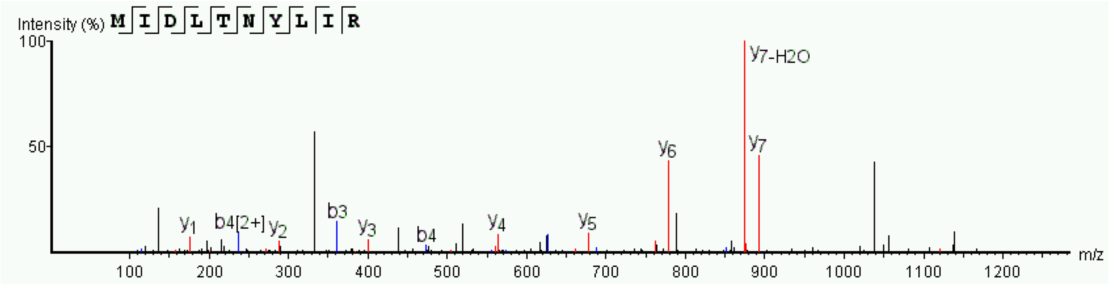

gi|328783051

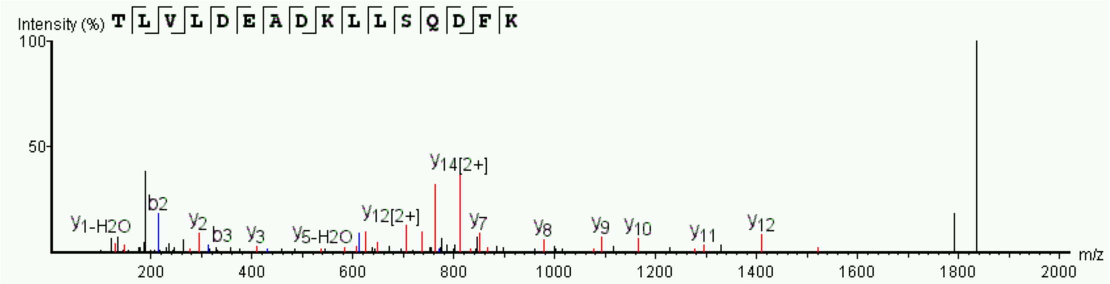

gi|571552022

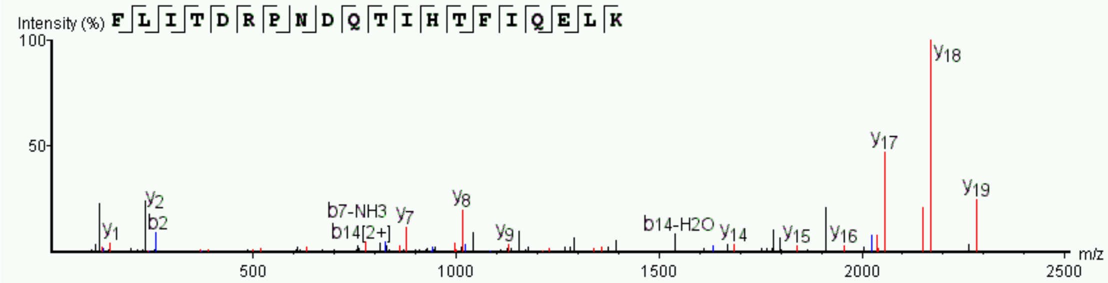

gi|328784752

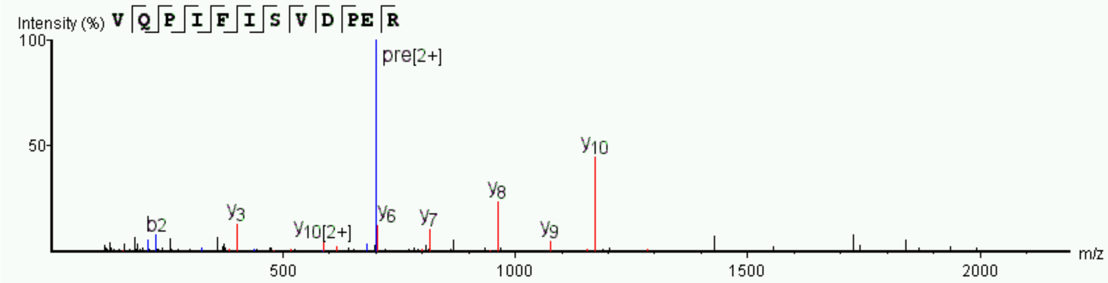

gi|571537013

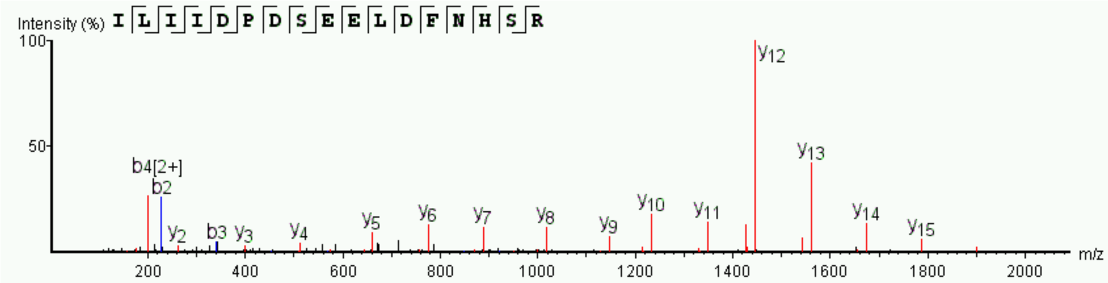

gi|571530518

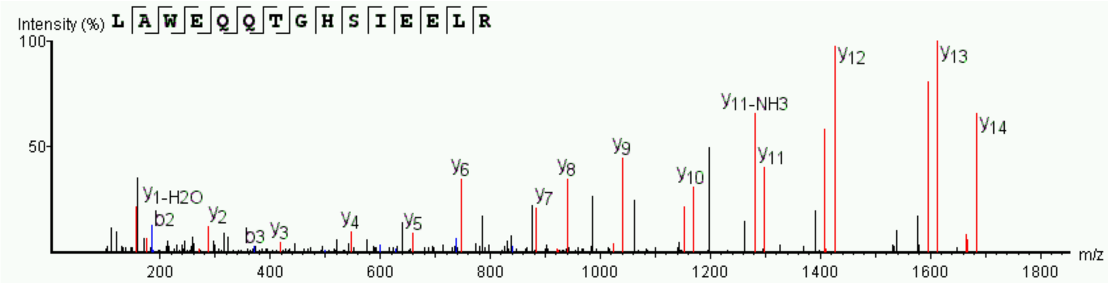

gi|48103834

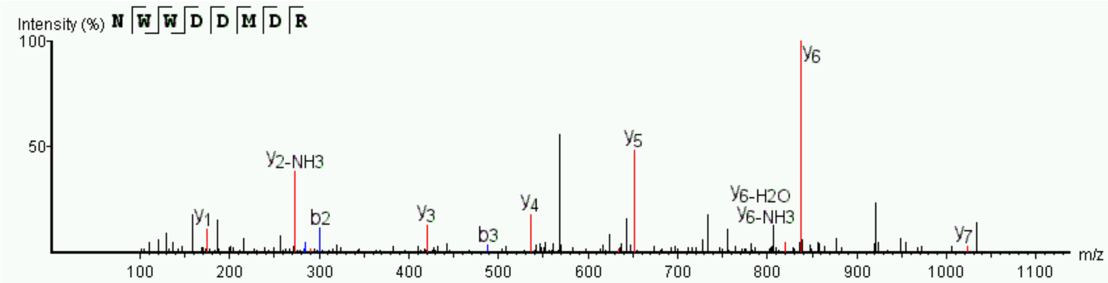

gi|110768098

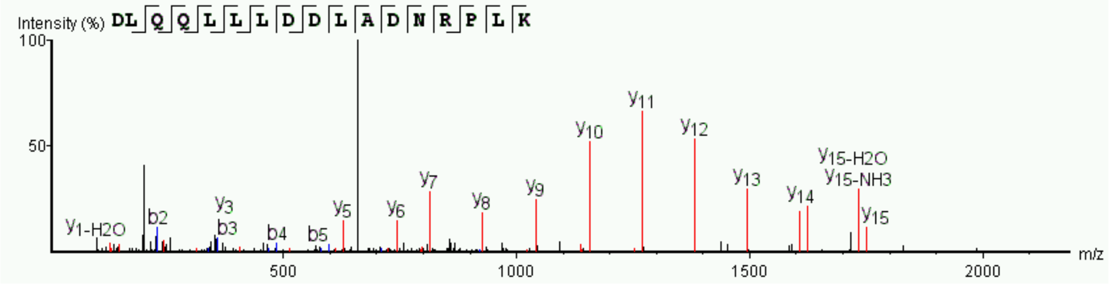

gi|571528227

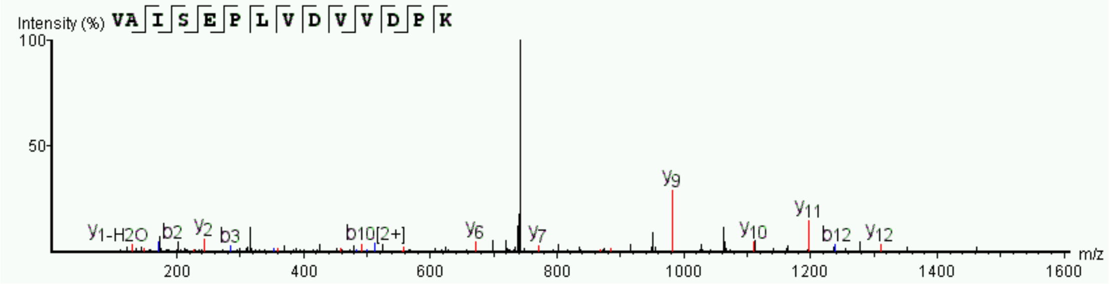

gi|66524764

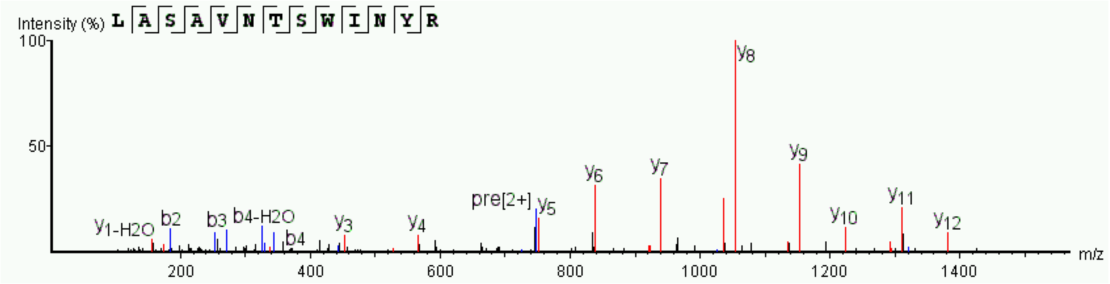

gi|48095935

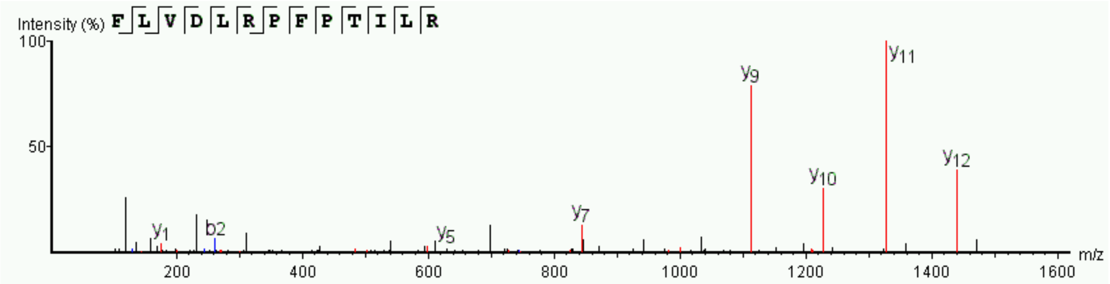

-

gi|571577775

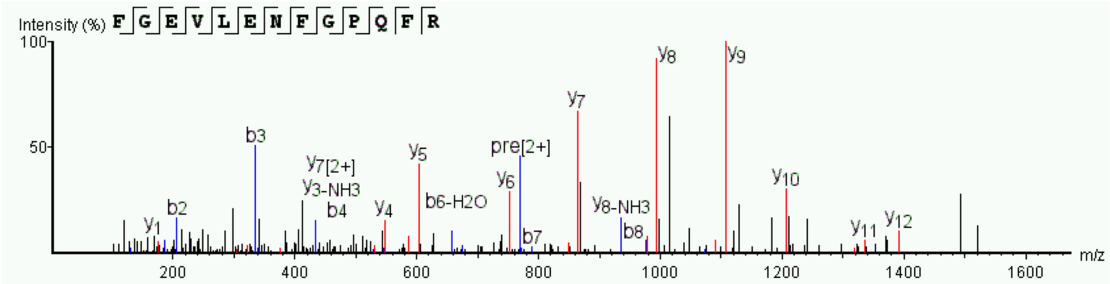

gi|66546020

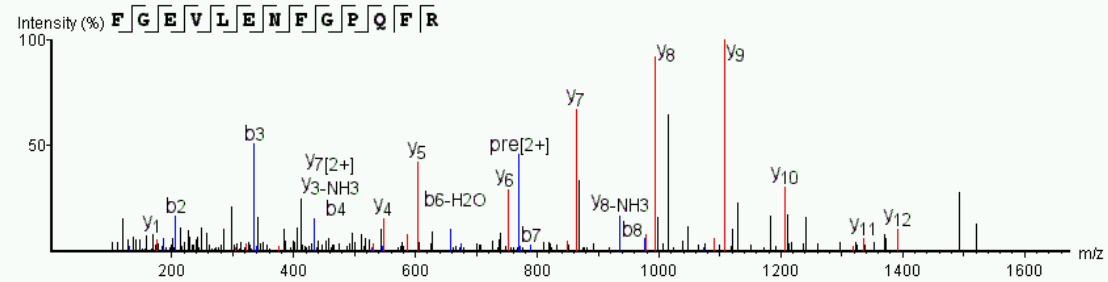

gi|66542158

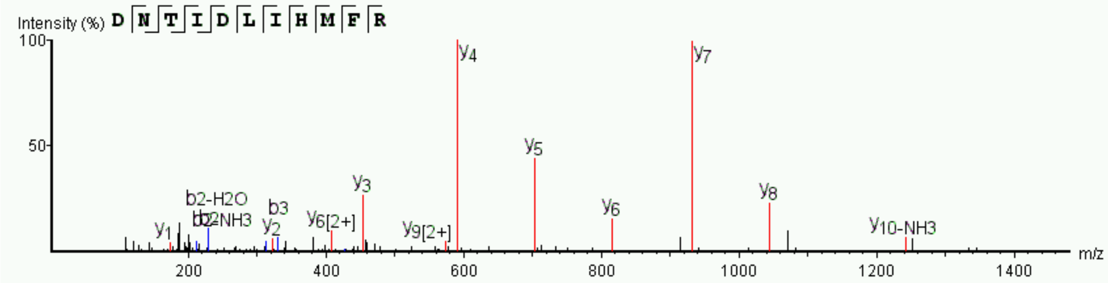

gi|328776594

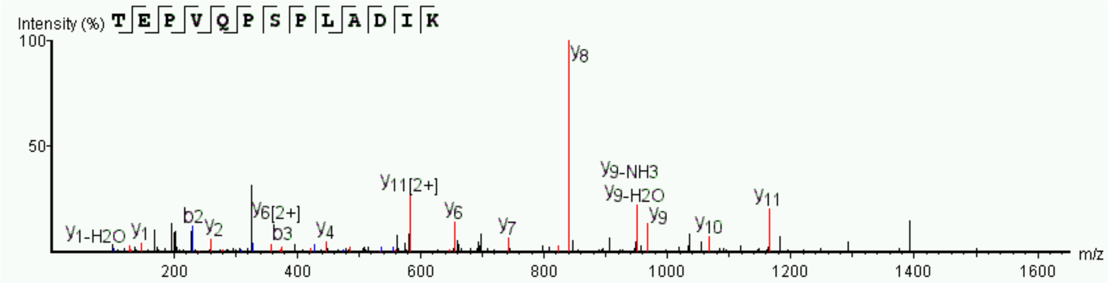

gi|571514502

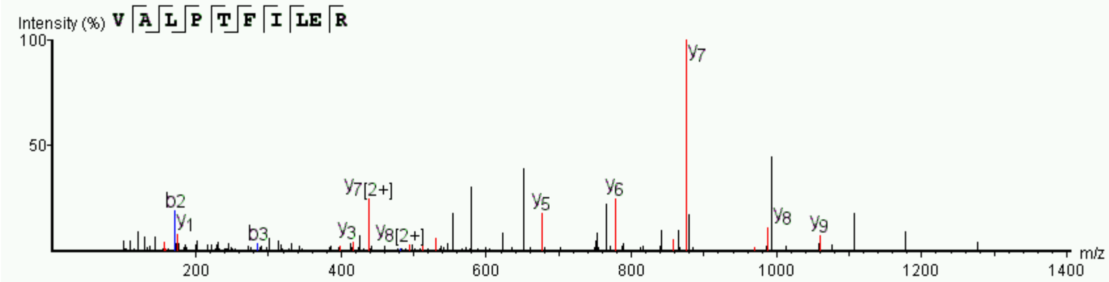

gi|571514506

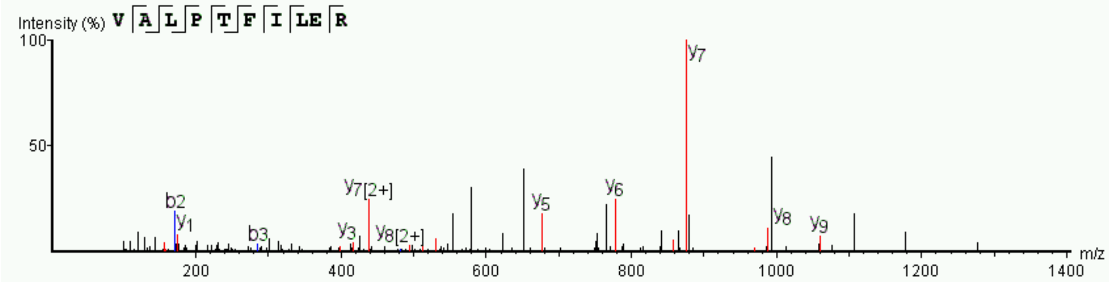

gi|571514510

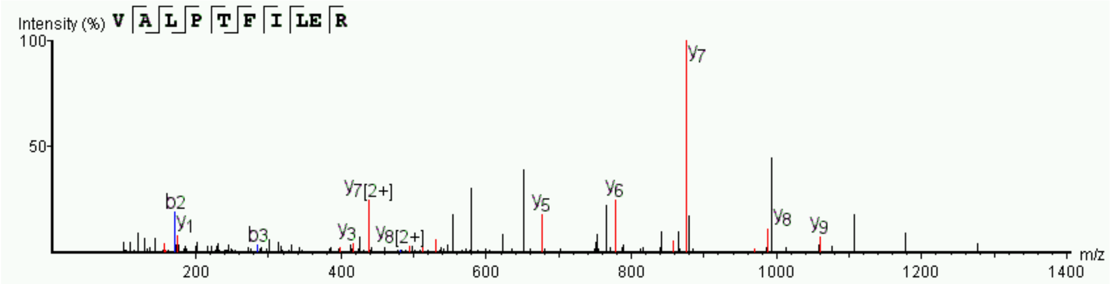

gi|571514498

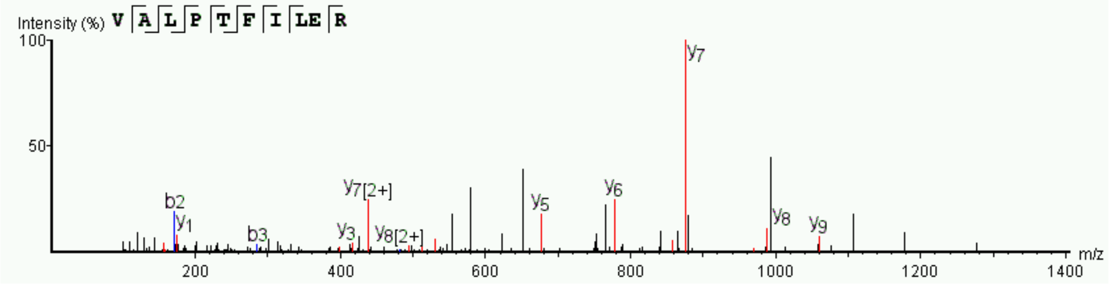

gi|571540960

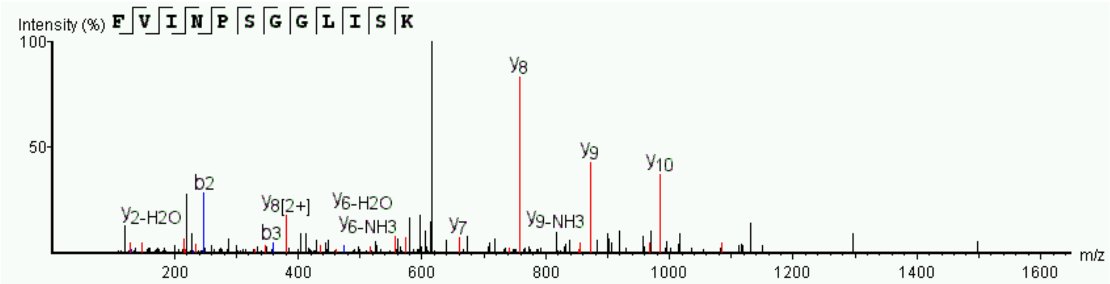

gi|571540964

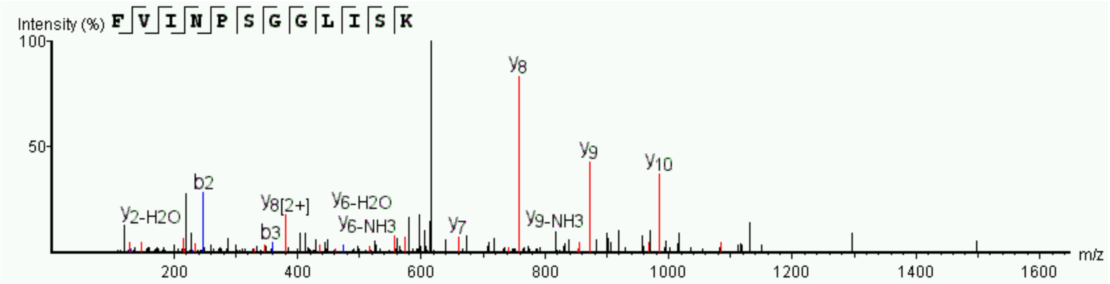

gi|571579236

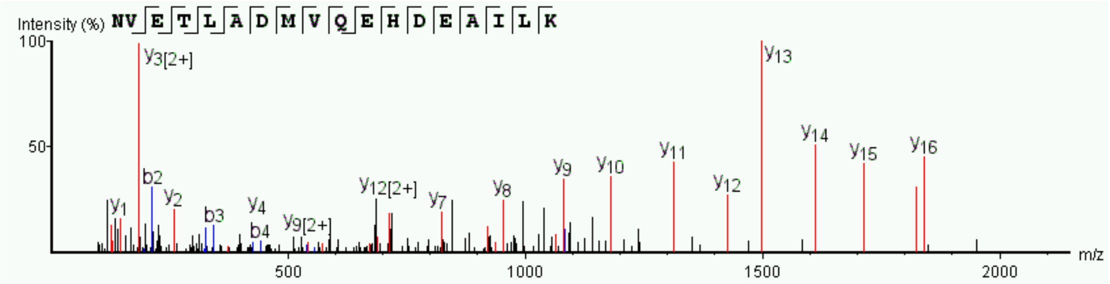

gi|48097576

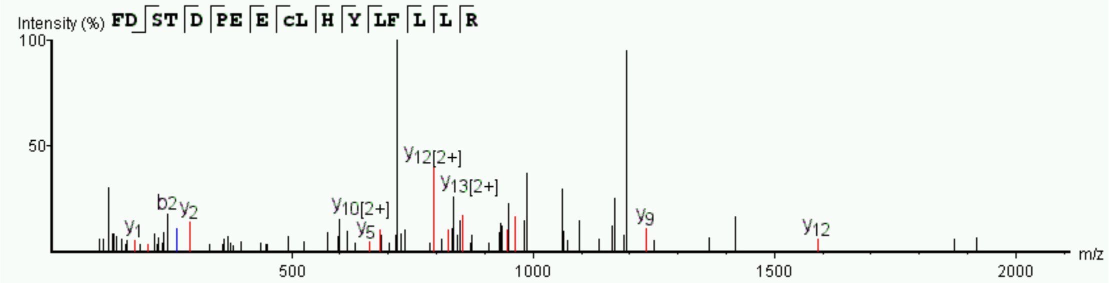

gi|66543732

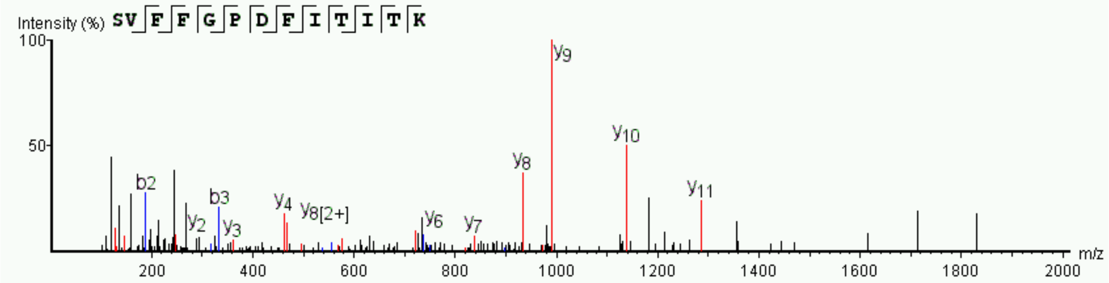

gi|571517320

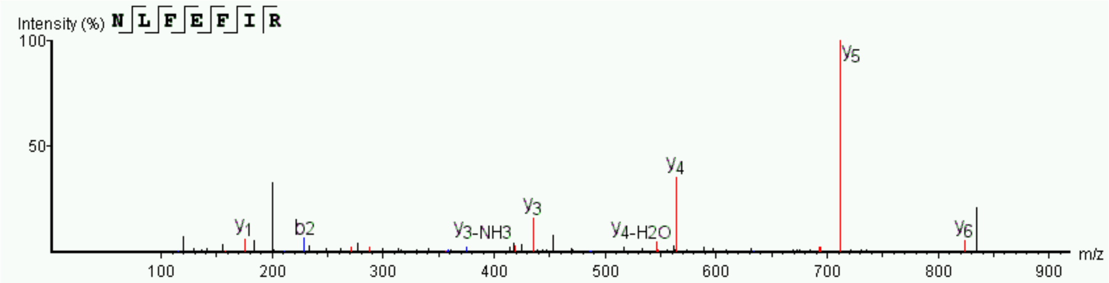

gi|571546530

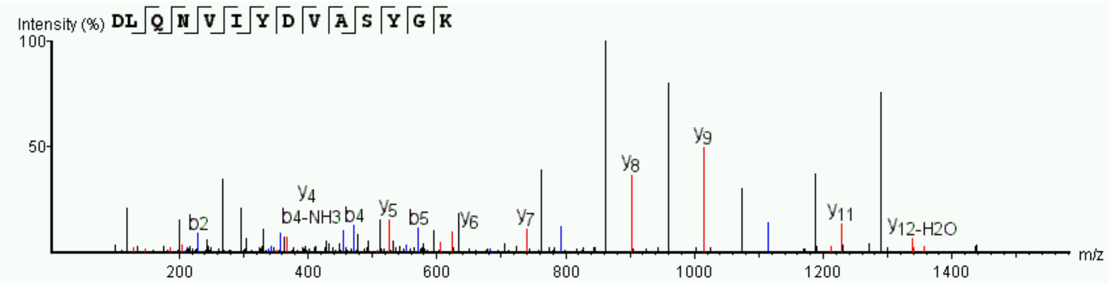

gi|48094341

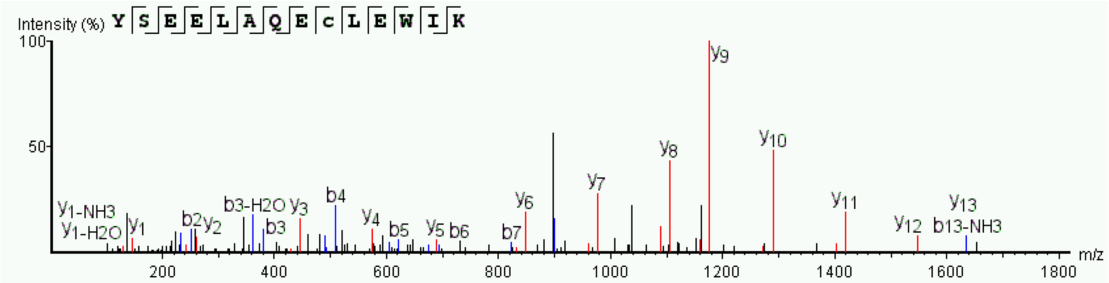

gi|571572803

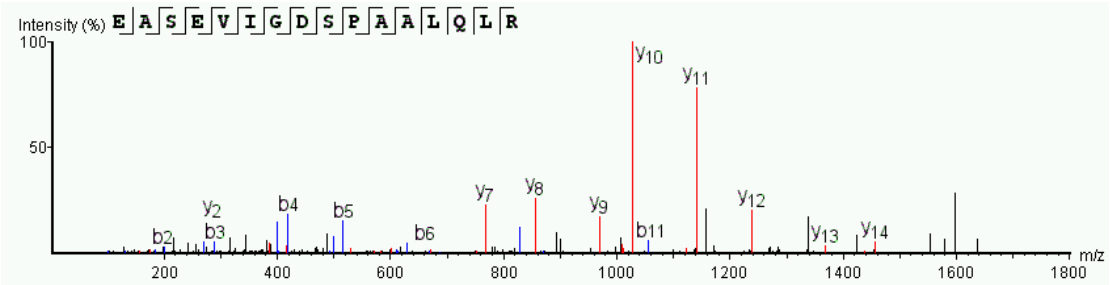

gi|110763030

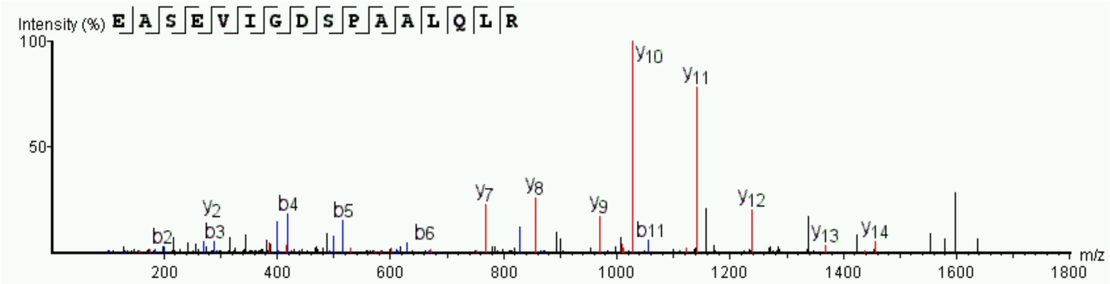

gi|571572787

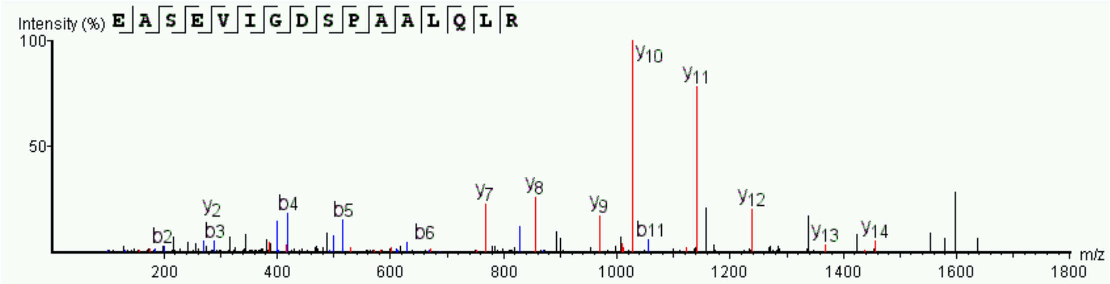

gi|571572789

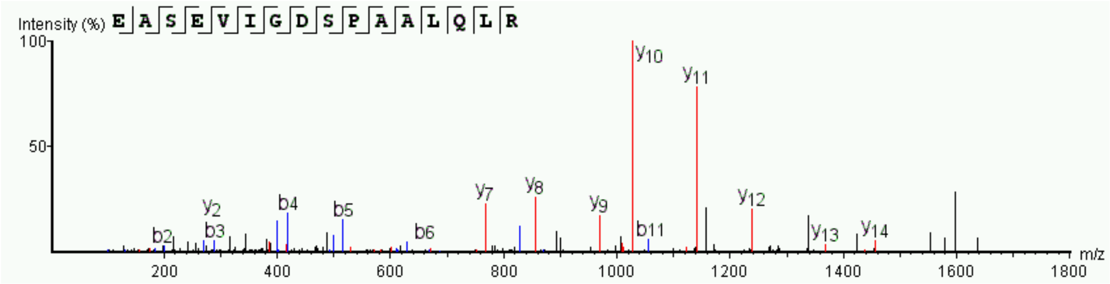

gi|571572791

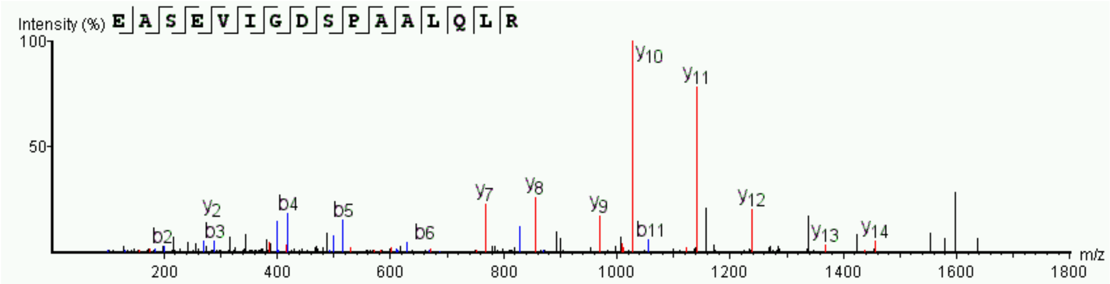

gi|571572793

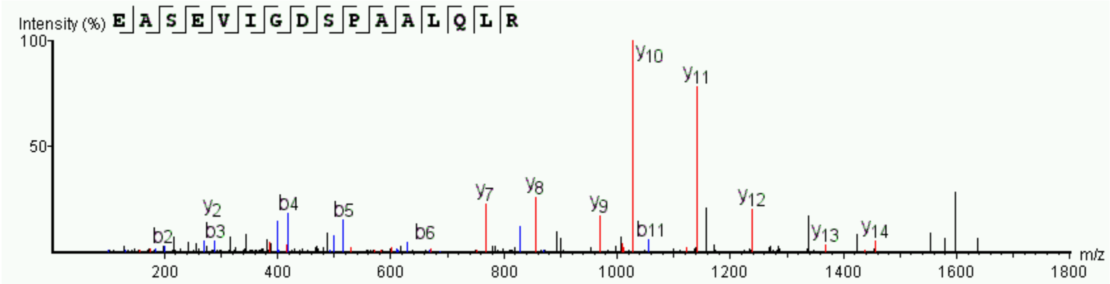

gi|571572796

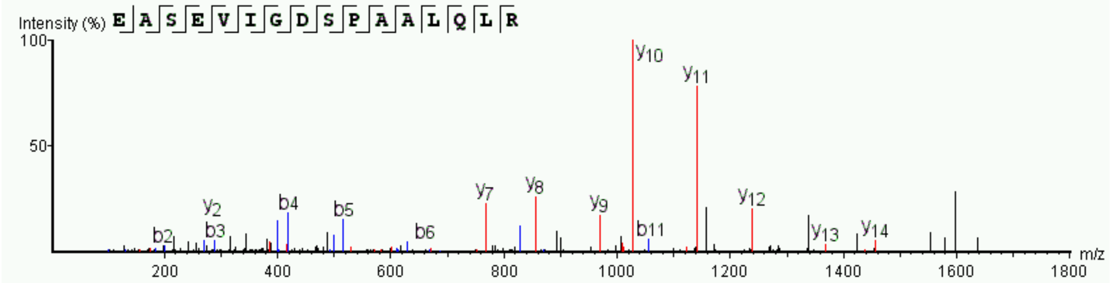

gi|571572798

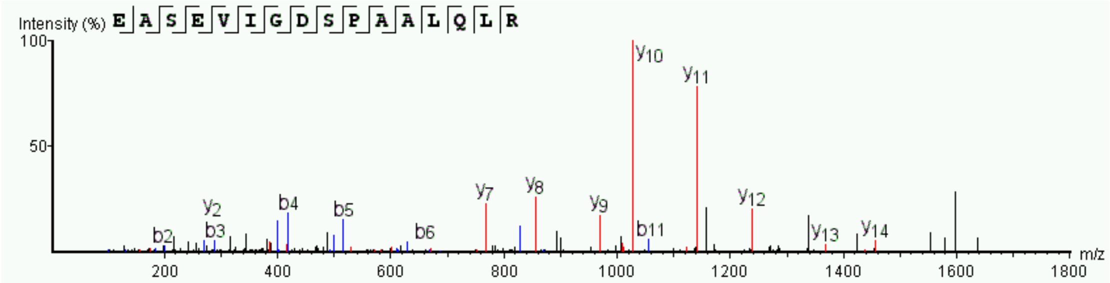

gi|571572801

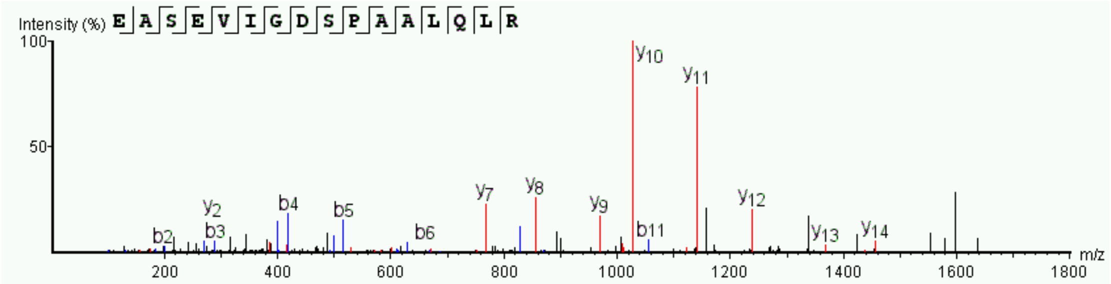

gi|66566113

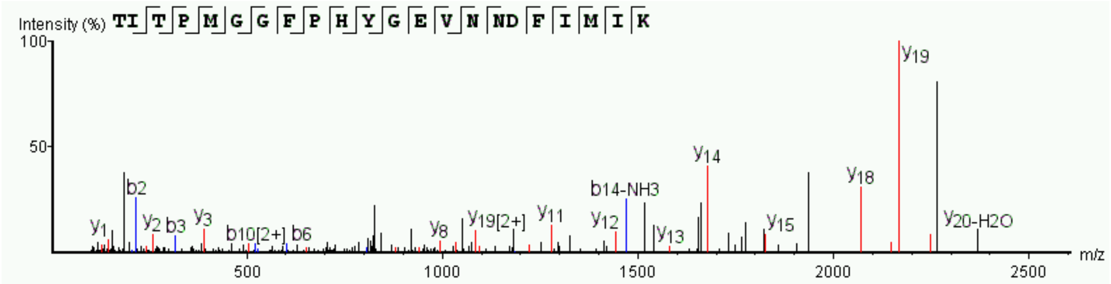

gi|66552169

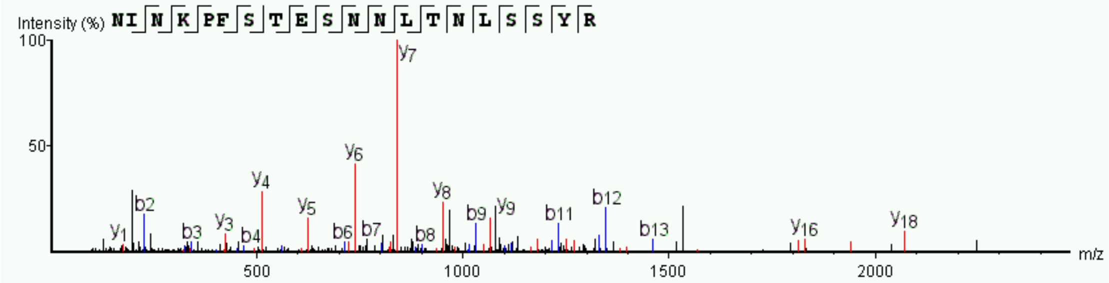

gi|110762813

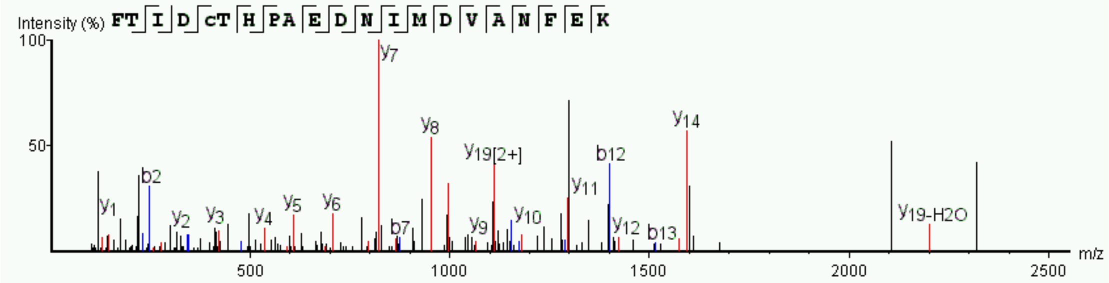

gi|571573543

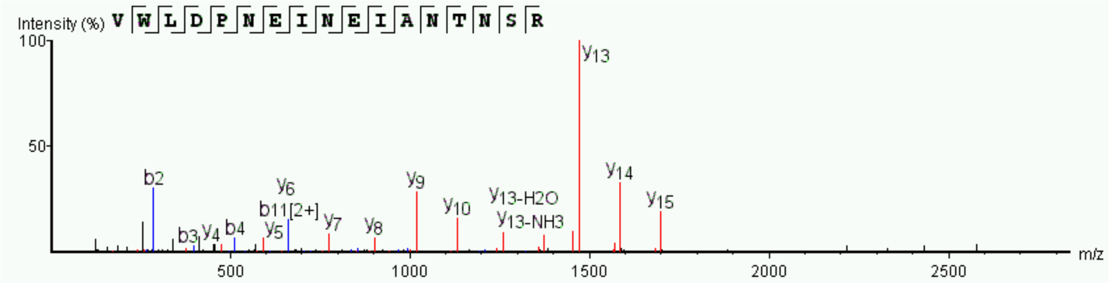

gi|66565444

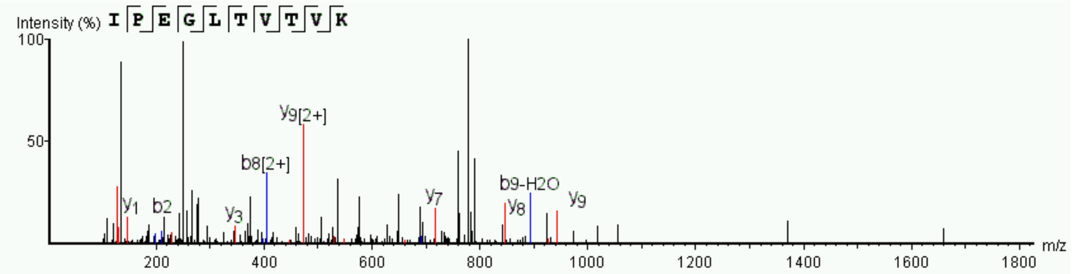

gi|571548748

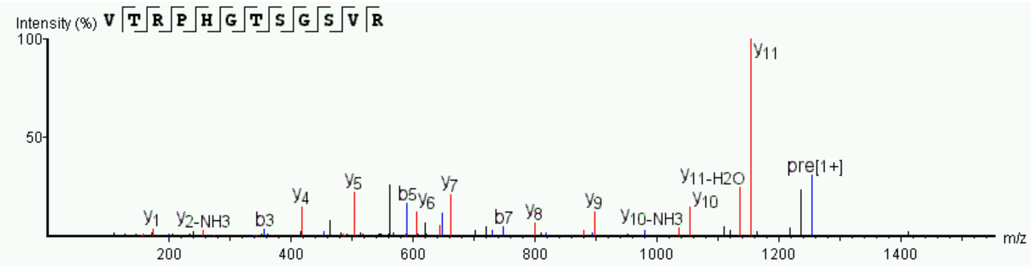

gi|571548754

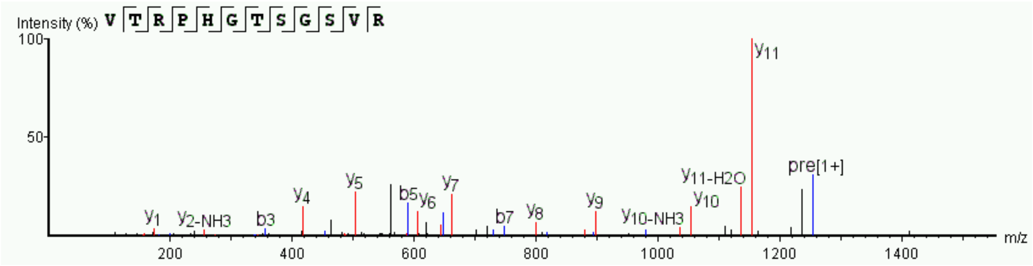

gi|571548751

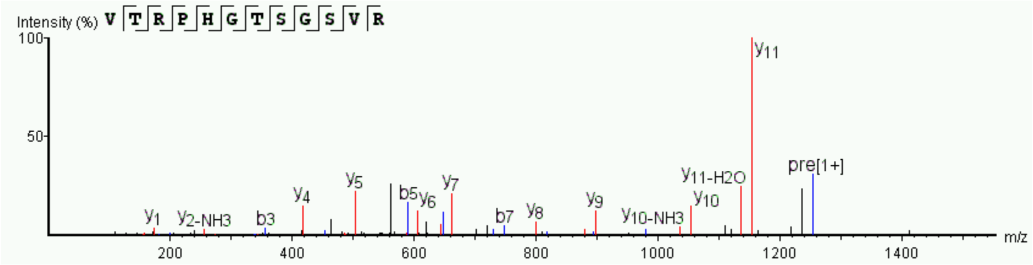

gi|571500086

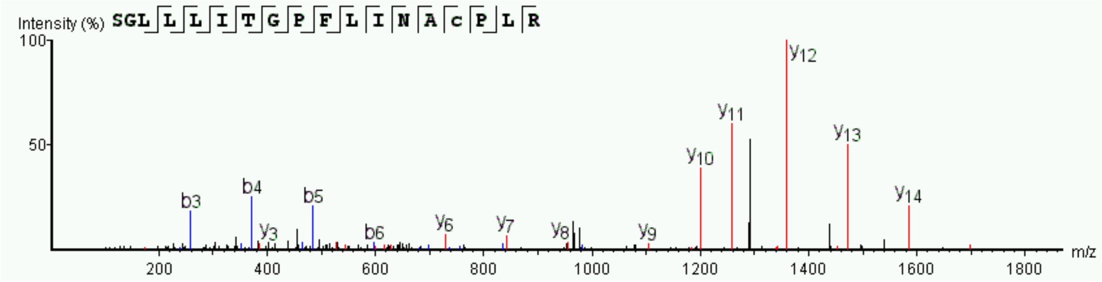

gi|571572819

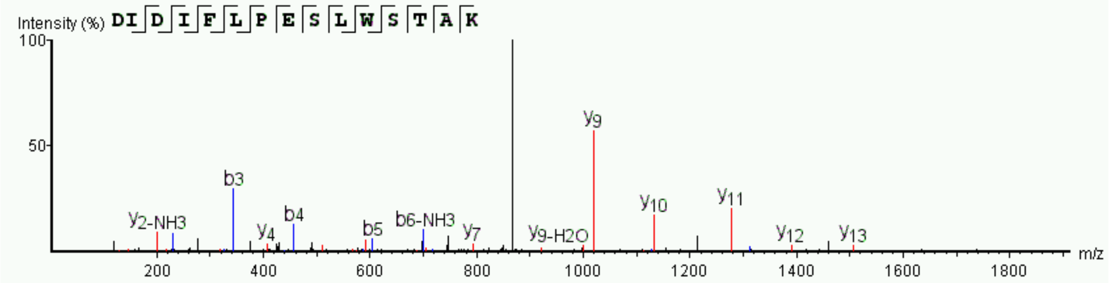

gi|66505792

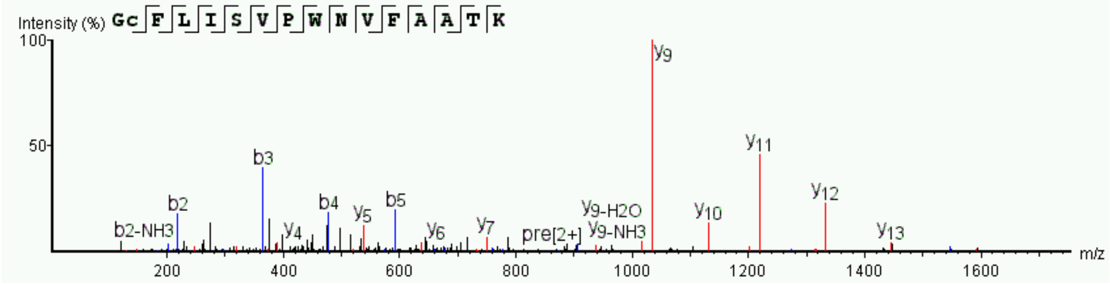

gi|571519817

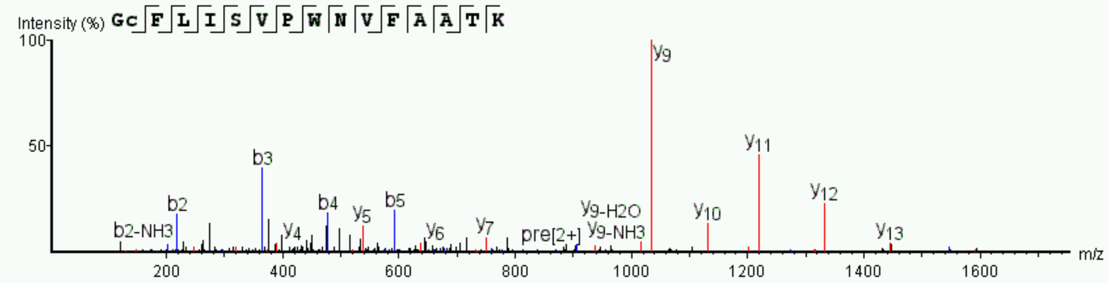

gi|571561861

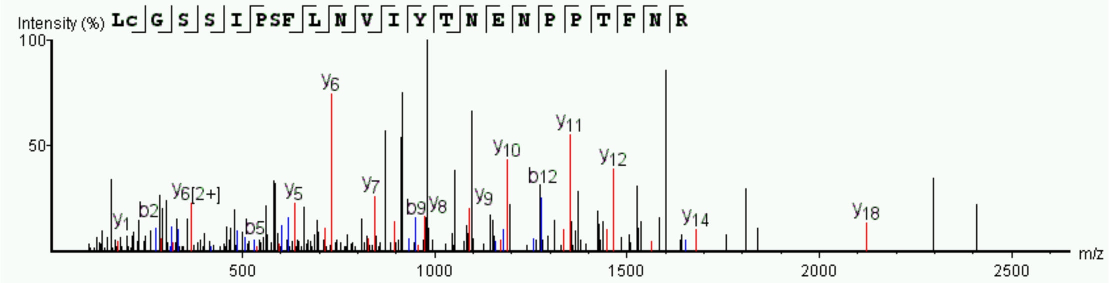

gi|48096310

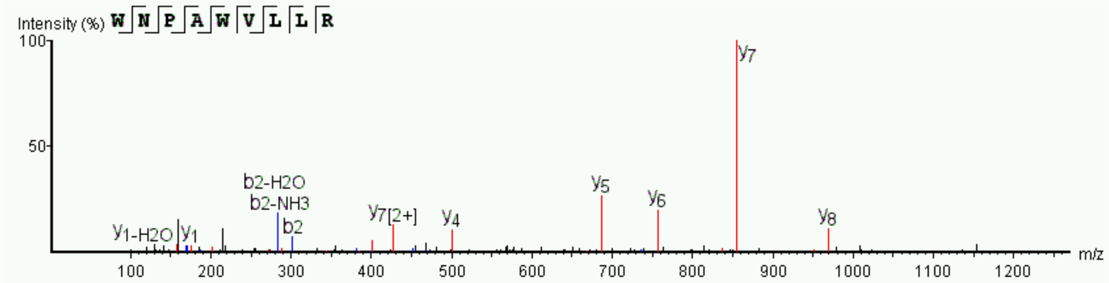

gi|66517653

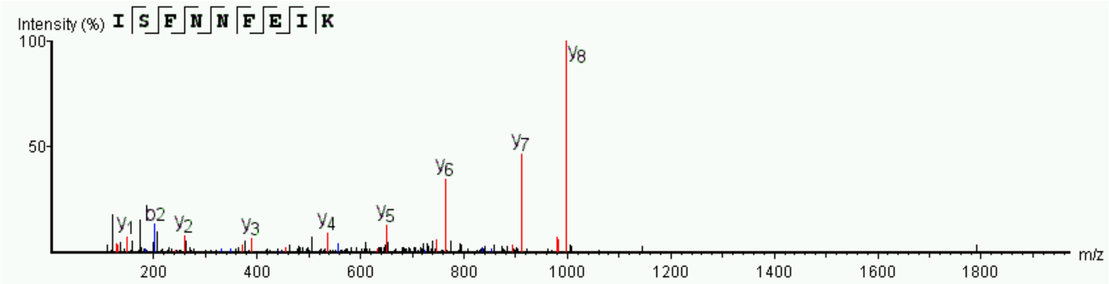

gi|110758776

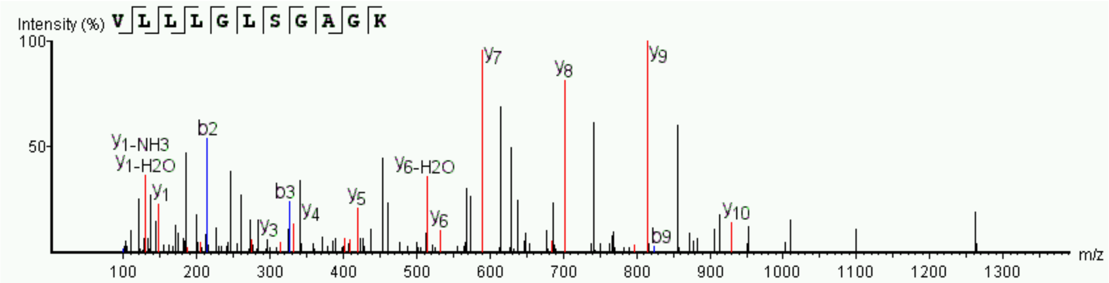

gi|571522855

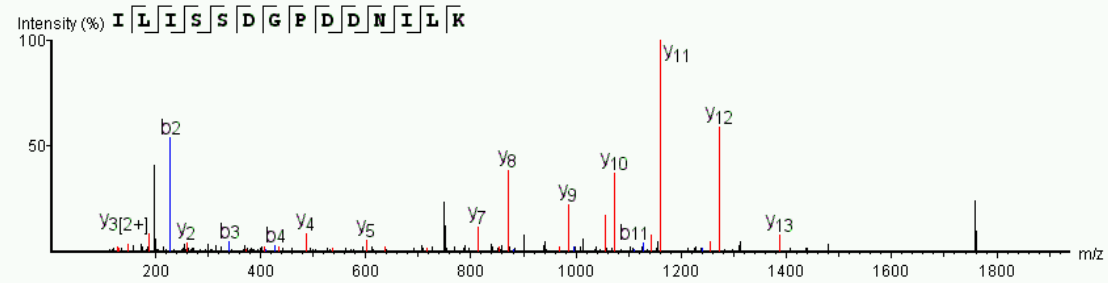

gi|571532910

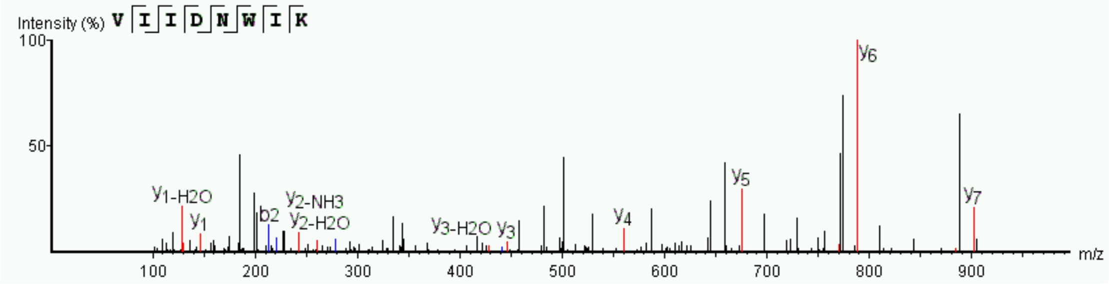

gi|110765976

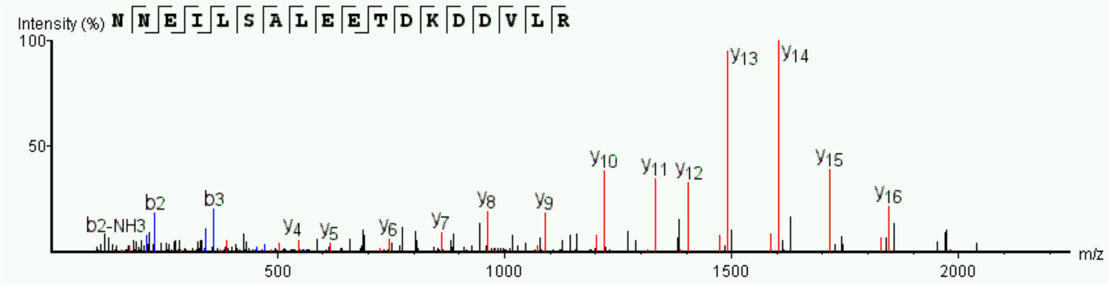

gi|571563087

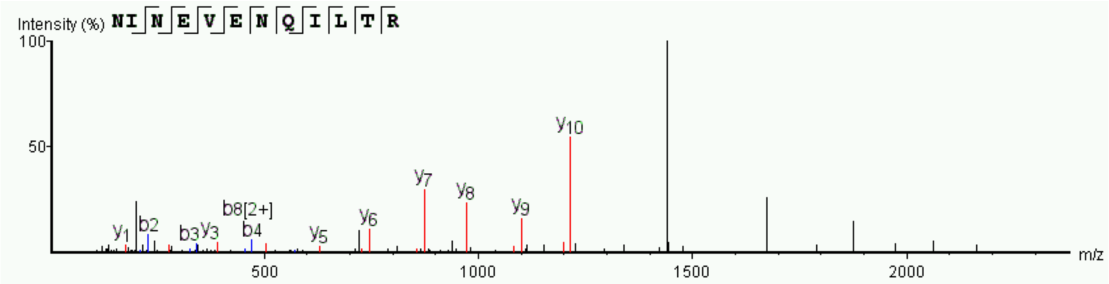

gi|571570202

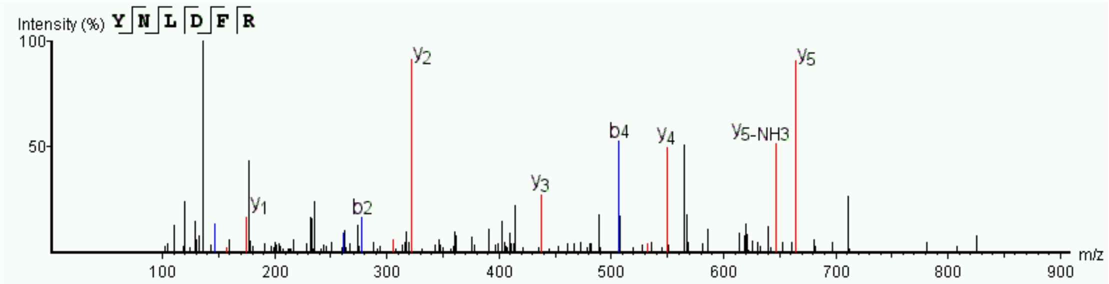

gi|66564768

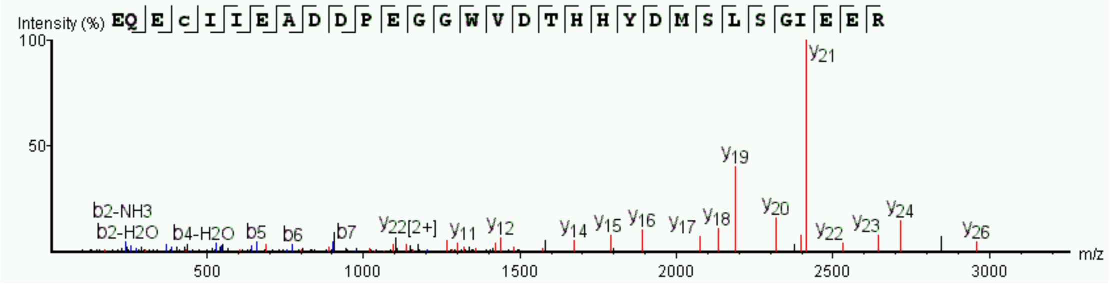

gi|571538682

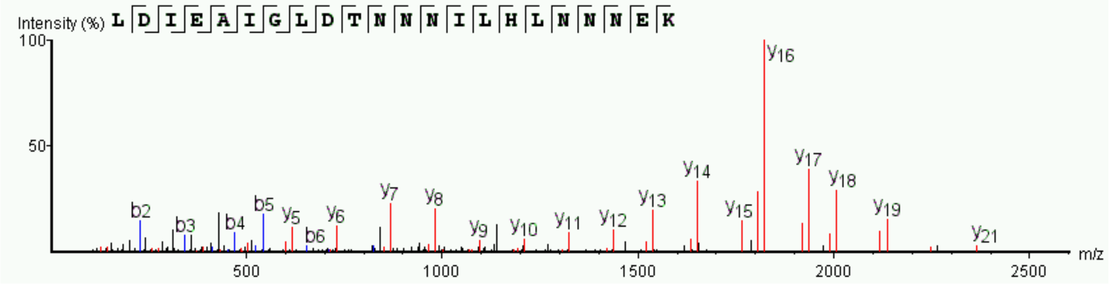

gi|328793281

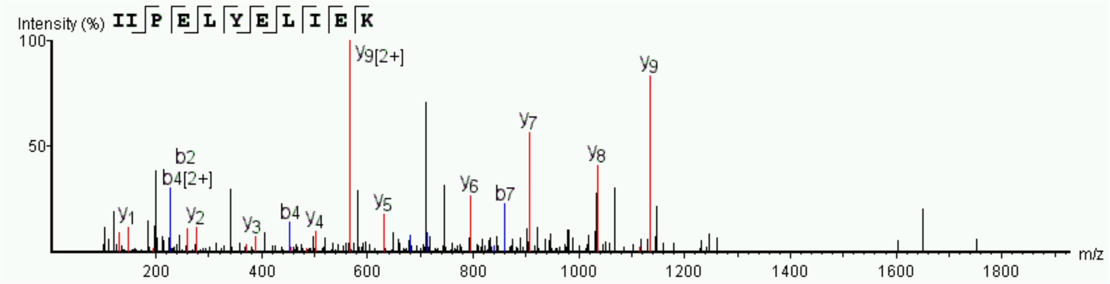

gi|110748994

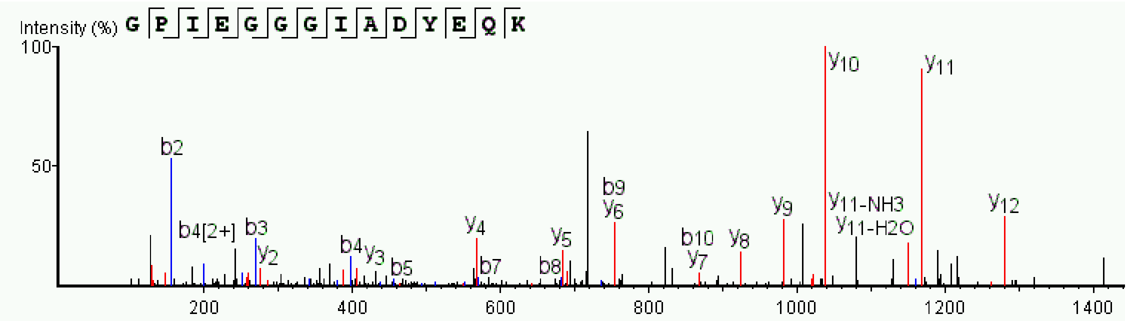

gi|571501620

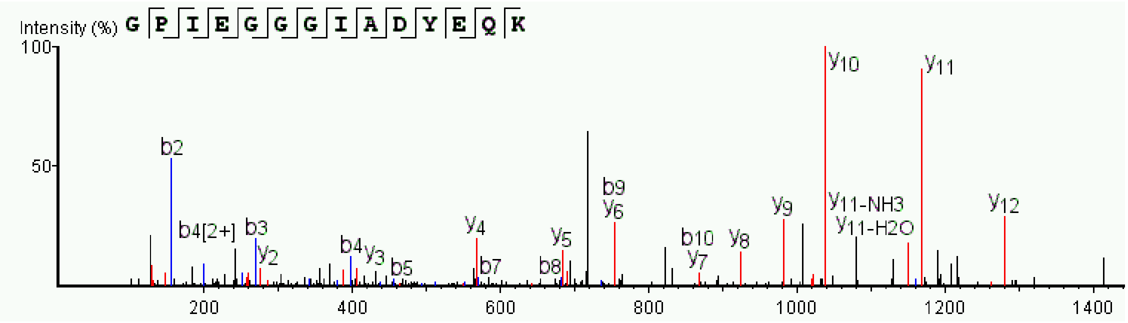

gi|66512450

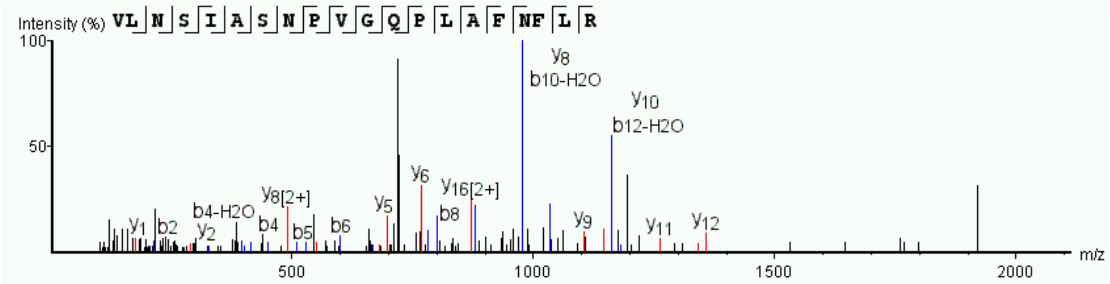

gi|571539854

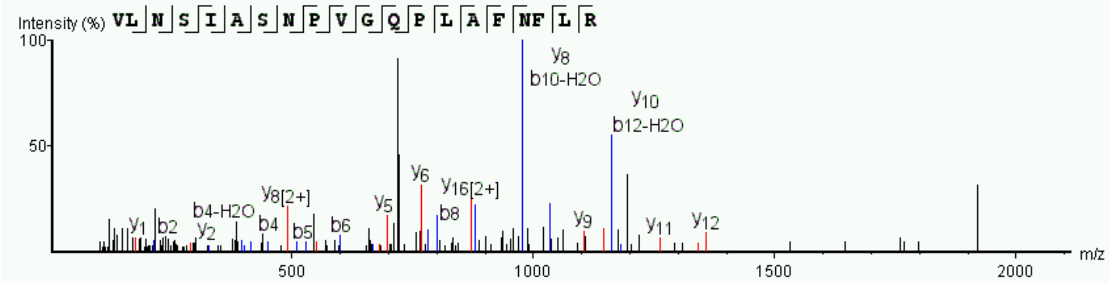

gi|571539848

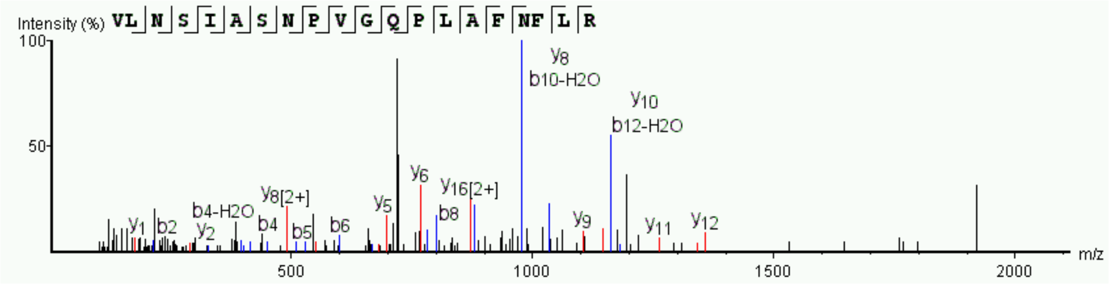

gi|571529433

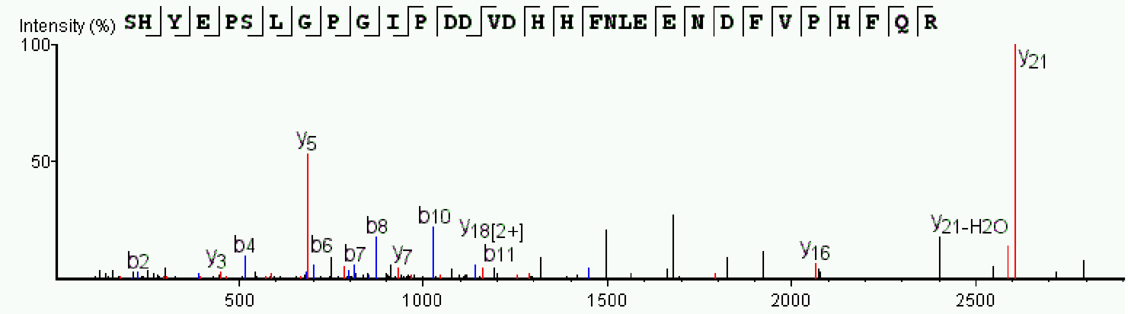

gi|571529436

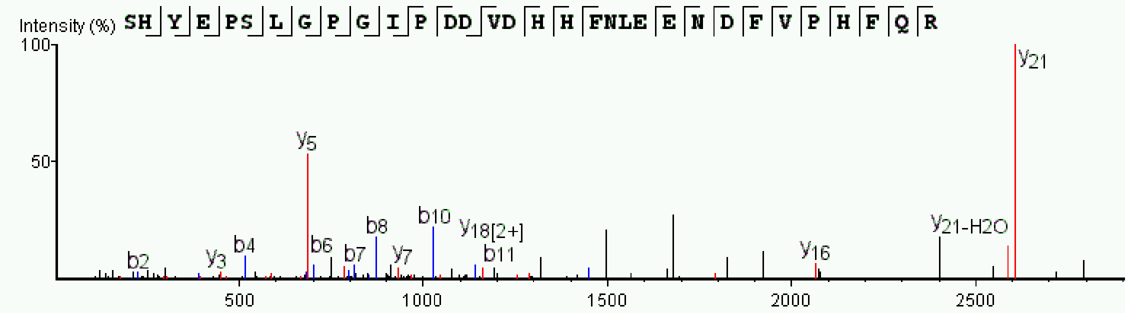

gi|571529409

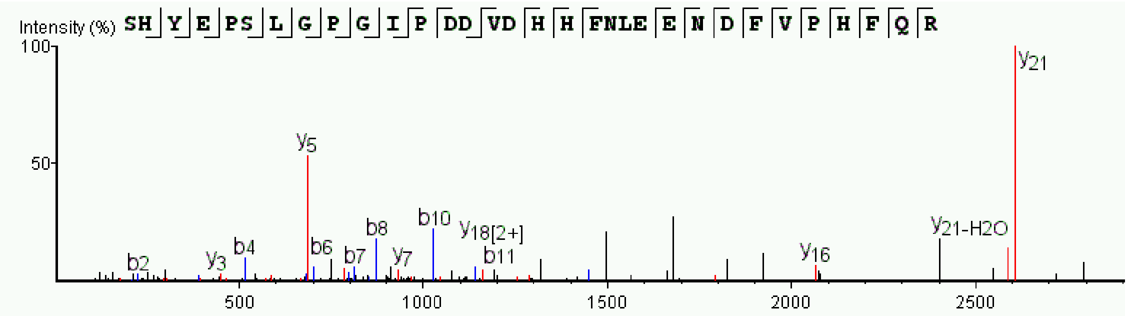

gi|571529406

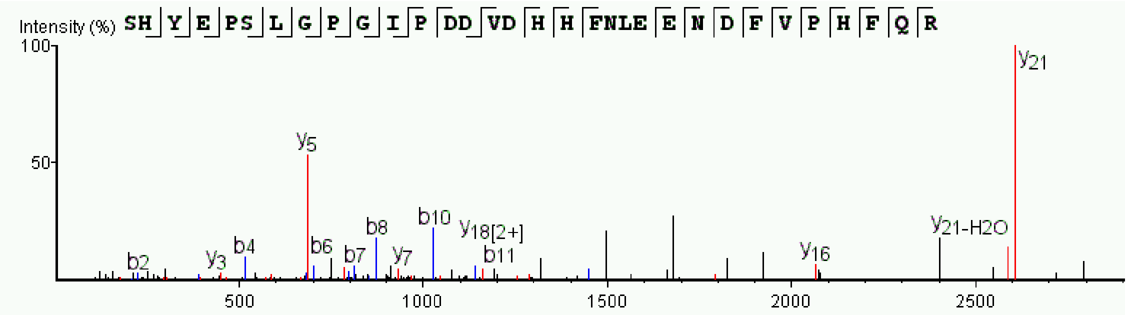

gi|571529412

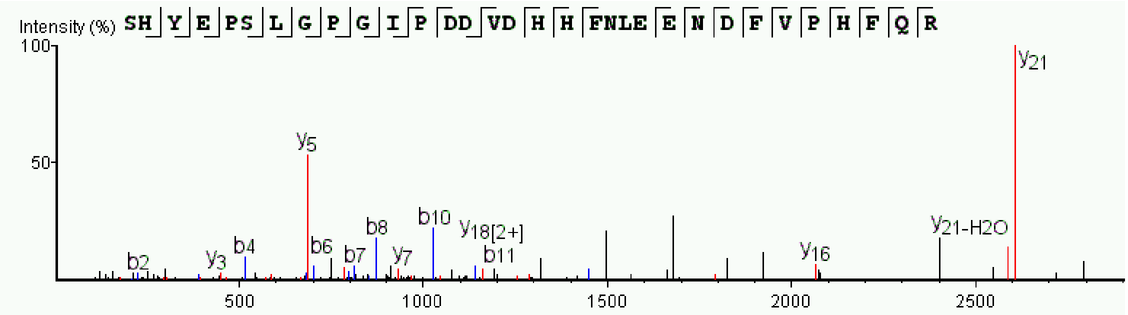

gi|571529417

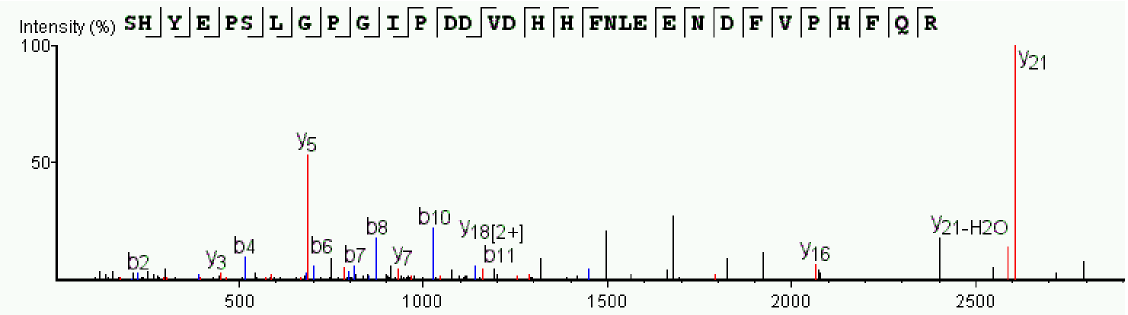

gi|571529421

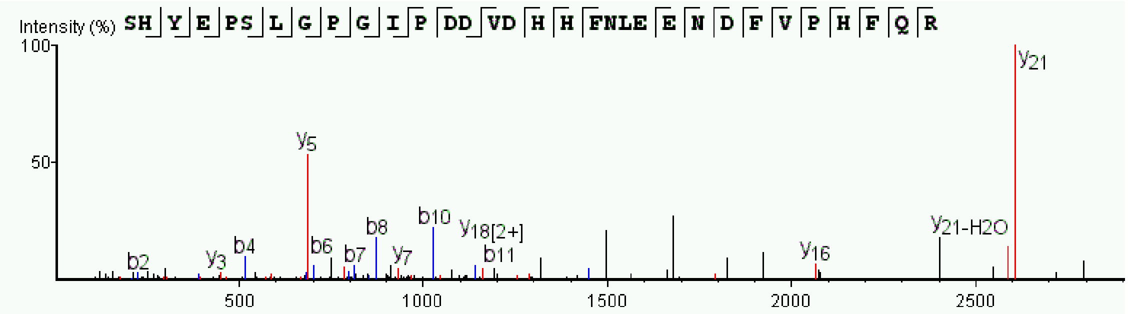

gi|571529425

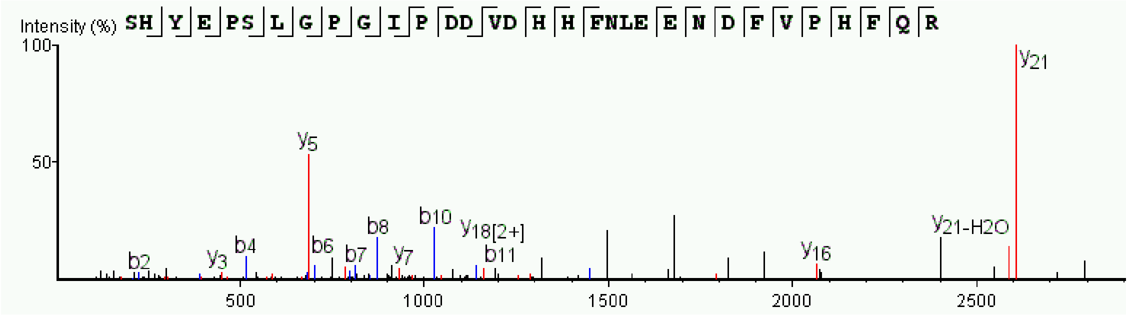

gi|571529429

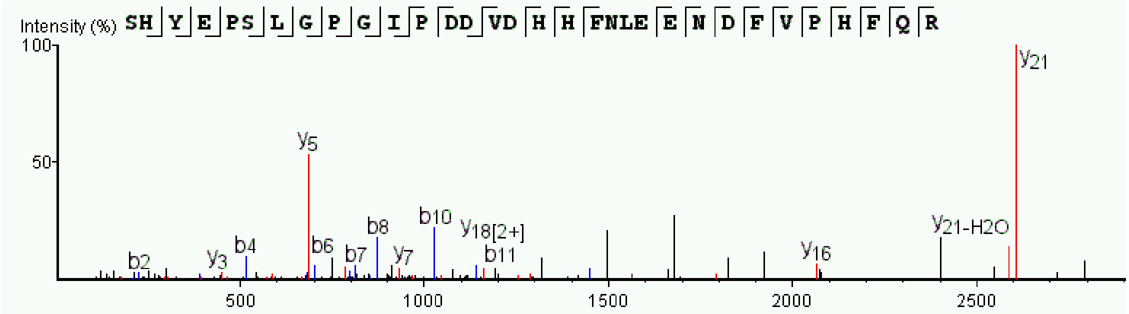

gi|328788208

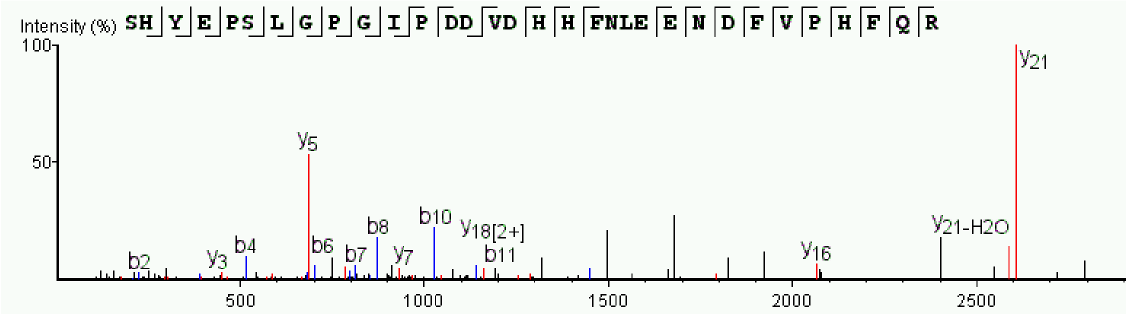

gi|110760120

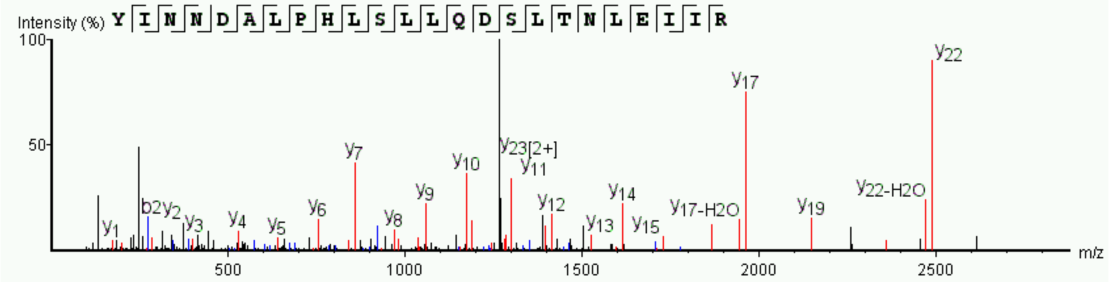

gi|571502990

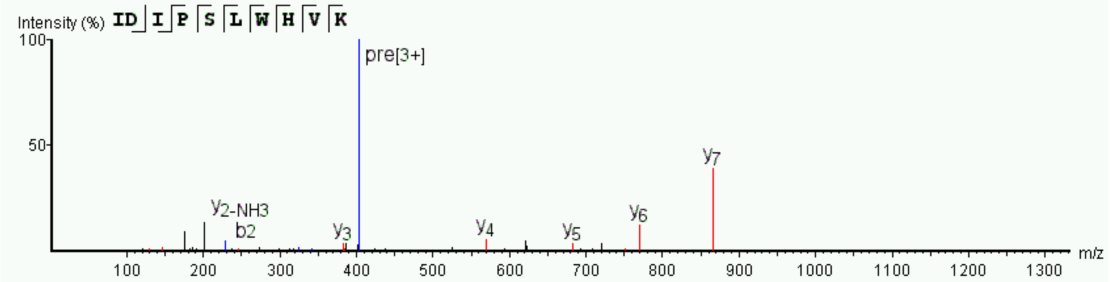

gi|66555021

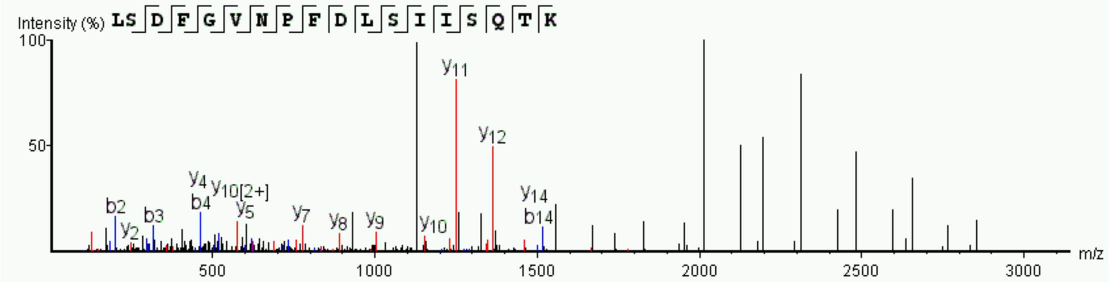

gi|328781481

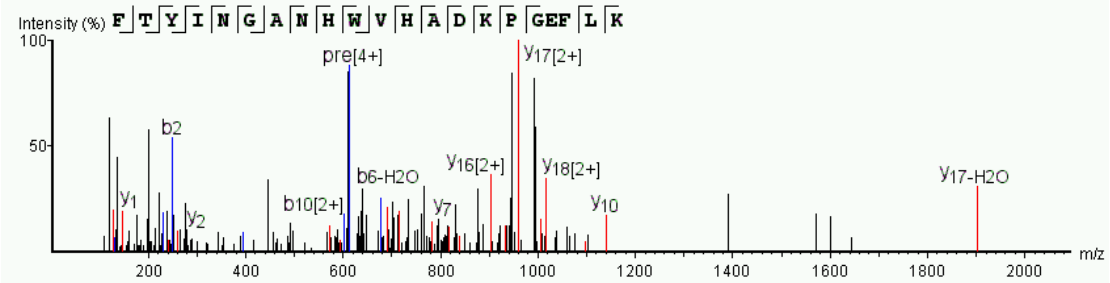

gi|571500982

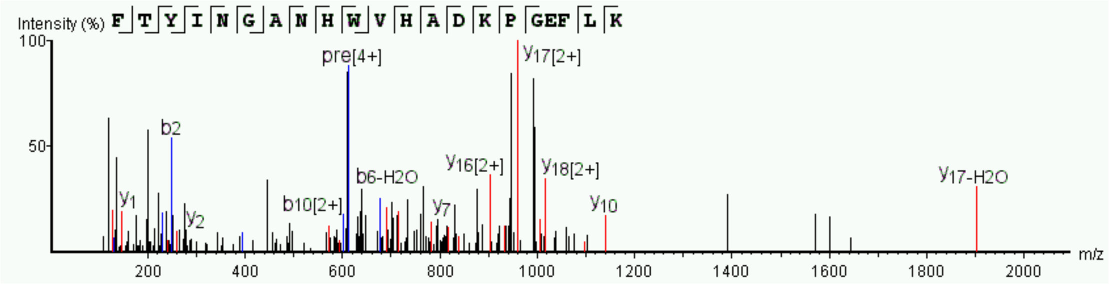

gi|48124643

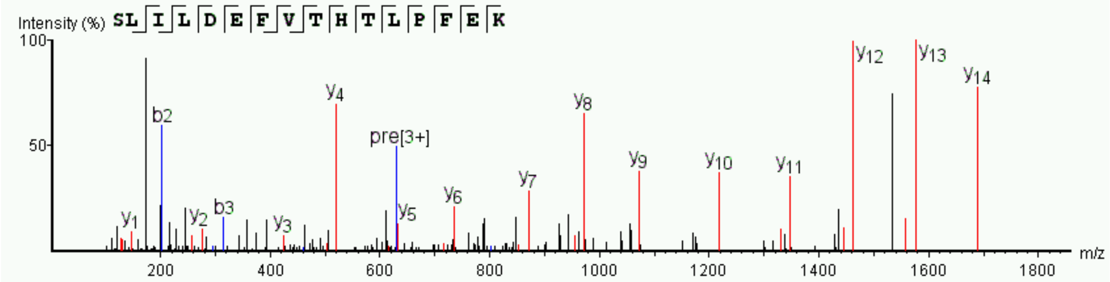

gi|571522066

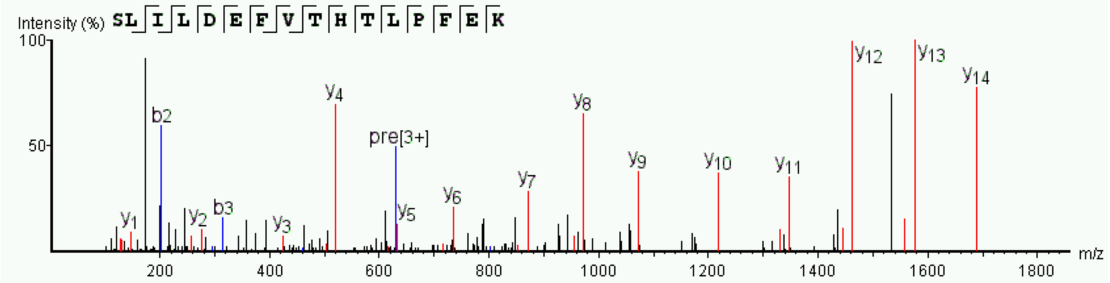

gi|571530387

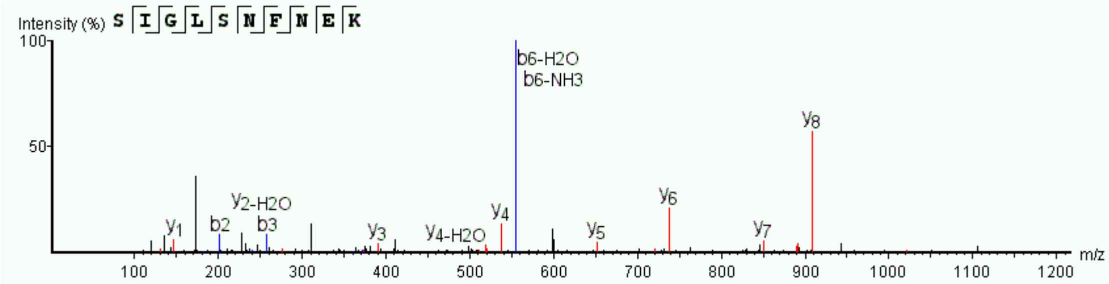

gi|571530383

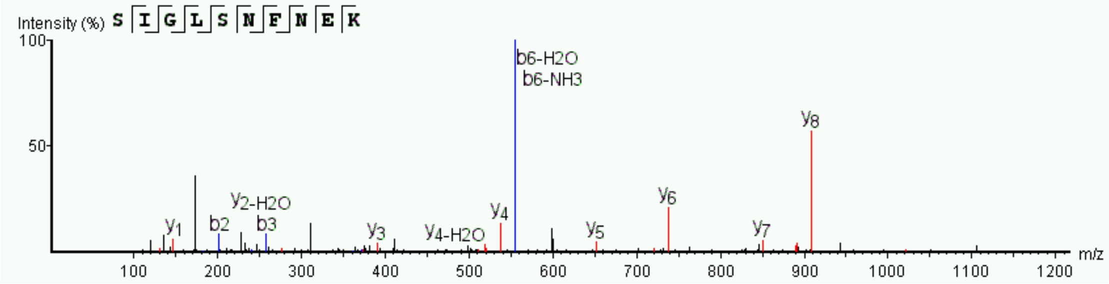

gi|571559072

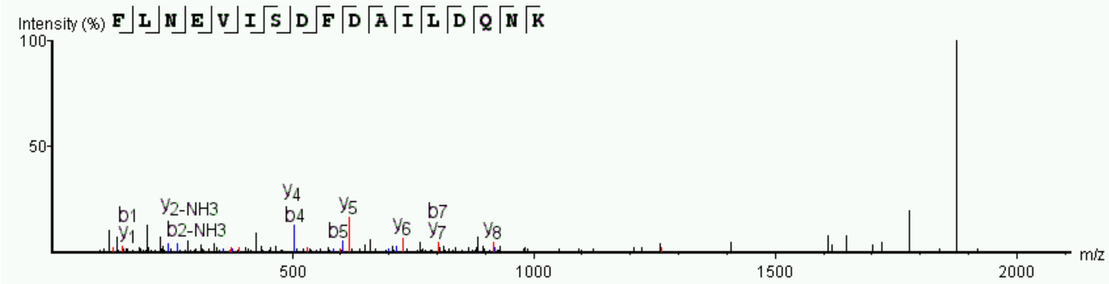

gi|571559068

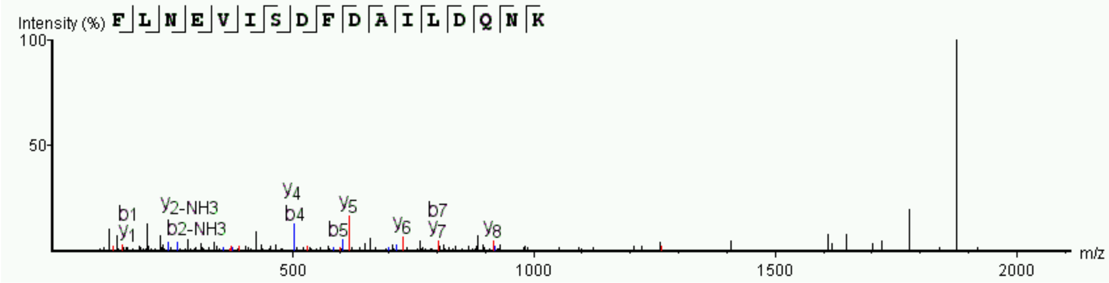

gi|571559065

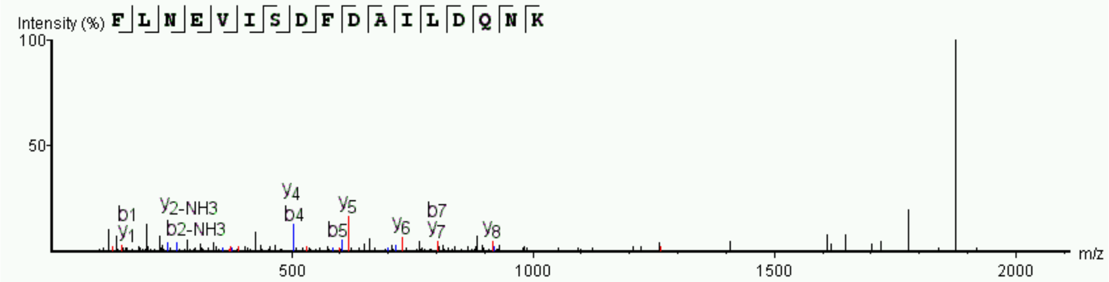

gi|328782357

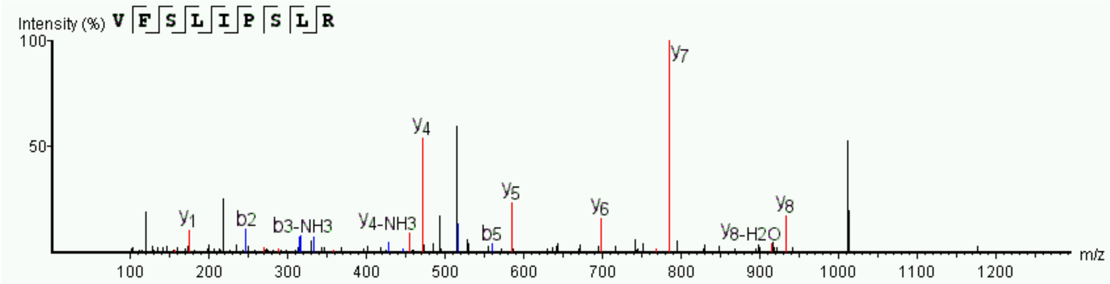

gi|571539002

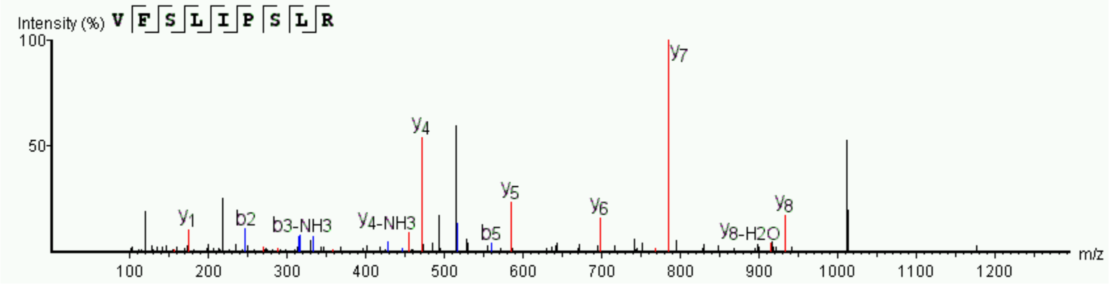

gi|571574750

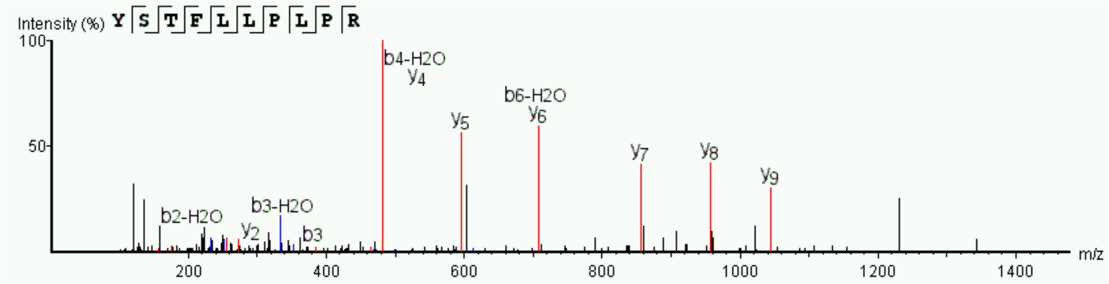

gi|66549073

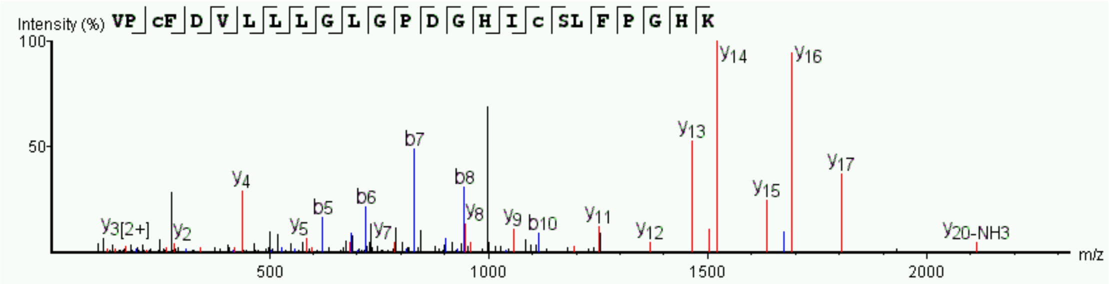

Supplement: Supplemental Data [file supp_RA117.000159_4755_1_supp_19945_wyx65z.pdf]
